# Exploring the plasma proteome linked to corpus luteum presence and conception mode across pregnancy stages and postpartum

**DOI:** 10.1007/s10815-025-03632-0

**Published:** 2025-09-20

**Authors:** Dhanya Ramachandran, Robin Tarek Dewender, Bianca Schröder-Heurich, Wiebke Froböse, Florian Avdulahu, Katja Richter, Valerie L. Baker, Virginia D. Winn, Andreas Pich, Frauke von Versen-Höynck

**Affiliations:** 1 Gynecology Research Unit, Hannover Medical School, Carl-Neuberg-Strasse 1, 30625 Hannover, Germany; 2 Core Unit Proteomics, Institute of Toxicology, Hannover Medical School, Carl-Neuberg-Straße 1, 30625 Hannover, Germany; 3 Department of Gynecology and Obstetrics, Division of Reproductive Endocrinology and Infertility, Johns Hopkins University School of Medicine, Lutherville, MD 21093 USA; 4 Department of Obstetrics and Gynecology, Stanford University Medical Center, Stanford, CA 94035 USA; 5 Department of Obstetrics and Gynecology, Hannover Medical School, Carl-Neuberg-Strasse 1, 30625 Hannover, Germany

**Keywords:** *Corpus luteum*, Frozen embryo transfer, In vitro fertilization, Mode of conception, Proteome, Unassisted conception

## Abstract

**Purpose:**

Observational data suggest that women conceiving without a *corpus luteum* are at higher risk of developing preeclampsia. While the underlying mechanisms remain unclear, the absence of *corpus luteum*-derived secretory products may be a contributing factor. This study investigates whether the plasma proteome differs between women who conceive with or without a *corpus luteum* and examines the relationship with mode of conception.

**Methods:**

Plasma samples from 12 participants were collected at three time points: first trimester, third trimester, and postpartum. The cohort included women who conceived unassisted (UC) after infertility, via artificial cycle frozen embryo transfer (AC FET), or natural cycle frozen embryo transfer (NC FET). A total of 36 plasma protein samples were analyzed using mass spectrometry-based proteomics to compare the proteome of women who conceived with and without a *corpus luteum*, across different conception methods and pregnancy stages.

**Results:**

In total, 528 proteins were quantified. No differentially expressed plasma proteins were identified between women with and without a *corpus luteum*. However, 15 proteins showed differential expression between UC and FET at all time points, with Bonferroni-corrected *p* < 9.47 × 10^−5^ and FC ≥ |2|. Several altered proteins, including PAPPA and ANG, were linked to preeclampsia. SERPINA7 was differentially detected when comparing time points within the unassisted conception method. No significant differences were detected between AC FET and NC FET.

**Conclusion:**

This pilot study revealed a unique proteomic signature associated with the mode of conception. The findings suggest biologically plausible candidate proteins for further testing. Validation in larger cohorts or with alternative proteome analysis technologies is needed.

**Supplementary Information:**

The online version contains supplementary material available at 10.1007/s10815-025-03632-0.

## Introduction

Pregnancies resulting from in vitro fertilization (IVF) have a higher risk of preeclampsia compared to those conceived naturally [1–4]. This risk is particularly elevated in pregnancies achieved using cryopreserved embryos, not only when compared to unassisted conceptions (UC) but also to those involving the transfer of “fresh” embryos during conventional IVF cycles [5, 6]. Recent observational studies have suggested that the absence of a *corpus luteum* (CL), such as in artificial cycle (AC, also called programmed) frozen embryo transfer (FET) protocols, is associated with an increased risk of developing preeclampsia [7–12]. The AC FET method differs from the natural cycle frozen embryo transfer (NC FET) in that the endometrium is prepared for the embryo implantation by administering steroid hormones, and occurs without a CL, whereas in NC FET, the natural menstrual cycle of a women is utilized and a CL is present [13, 14].

Studies examining cardiovascular physiology and circulating factors during pregnancy reveal significant differences between UC and pregnancies achieved through IVF [15–17]. These differences vary depending on the treatment protocol used. In patients without a CL, impaired cardiovascular and renal adaptation has been observed during the first trimester, including reduced aortic compliance and diminished cardiac output [12, 15]. Additionally, hormone levels have been found to differ significantly based on the presence and number of CL [12]. This is especially true for molecules primarily secreted by the CL, such as relaxin, which remains undetectable throughout pregnancy in AC FET cycles, but is markedly higher in conceptions involving multiple CL after conventional IVF [16–18], suggesting that the availability and optimal levels of CL products are essential for the establishment, maintenance, and health of a pregnancy. Abnormalities in these products can affect every stage, from successful conception to the development of pregnancy complications that may only become evident in the second half of pregnancy. However, our understanding of CL factors is limited, with most knowledge derived from studies focusing on single or a few specific molecules. Therefore, this study aimed to compare the plasma proteomes of pregnant and postpartum women who conceived with and without a CL and examine its association with the mode of conception.

## Materials and methods

### Patient selection, sample collection, and processing

The patients who provided data and plasma for this study were participants in the prospective cohort study POFI (Pregnancy Outcomes Following Infertility) [12] and its embedded POFIECH study (Pregnancy Outcomes Following Infertility and Endothelial Cell Health) [19, 20] conducted between 2014 and 2018 at an academic fertility clinic. All patients gave written informed consent for the collection of biological samples and the use of their demographic and clinical data. The subset of patients included in this analysis had a live birth after UC or FET and sufficient plasma volume and samples from all study time points available. Exclusion criteria were age > 50 years, BMI > 30 kg/m^2^, latex allergy, known vascular disease (i.e., chronic hypertension, lupus erythematosus, and rheumatoid disease), steroid or heparin intake, donor egg transfer, and multiple or surrogate pregnancies. The participants were divided into groups based on the number of *corpora lutea* and method of conception: 1 CL – UC after infertility (*N* = 4) and NC FET (*N* = 4), and 0 CL – AC FET (*N* = 4). Plasma samples were collected across three time points: during the first or third trimester (T1 and T2, respectively), and postpartum (T3), with data available from the same four participants at each time point within each group.

Blood was drawn in the morning following an overnight fast and avoidance of caffeinated products, alcohol, and pain medications during the preceding 24 h. Blood samples were placed on ice immediately and double-spun at 800 g for 10 min at 4 °C. Plasma was stored at − 80 °C until further use, and all processing was completed within 60 min of blood collection.

### Experimental design and statistical rationale

#### Design

*Comparison groups*: To identify proteomic changes associated with the presence of a CL, we compared participants with and without a CL across the three time points T1, T2, and T3. Additionally, to assess proteomic changes related to different modes of conception, we compared UC, NC FET, and AC FET at T1, T2, and T3. We also compared the three time points within each mode of conception.

### Mass spectrometry-based proteome analysis

*Sample preparation and digestion*: To improve the detection of low-abundance proteins and to reduce the abundance of highly abundant proteins in the analyzed plasma samples, the samples were prepared using the ProteoMinerTM protein Small-Capacity enrichment kit (BioRad) that is recommended for 10–20 mg of protein. The columns were prepared as per manufacturer’s instructions. The thawed plasma samples were centrifuged for 10 min at 16,100 × g and 175 μl of plasma (ca. 12 mg) and 25 μL of wash buffer were loaded onto the affinity column and left to bind for 2 h at room temperature, during which the columns were rotated. Two hundred microliters of washing buffer was applied, incubated for 5 min, followed by centrifugation at 1000 × g for 1 min. This was performed a total of three times. The final washing step was performed with 200 μL of water, incubated for 1 min, and subsequent centrifugation at 1000 × g for 1 min. For elution, 60 μL of elution buffer was added to the column, incubated for 15 min on the column during which the column bed was agitated by shaking or vortexing, and then centrifuged at 1000 × g for 1 min. This step was repeated twice, and the liquid was collected in a fresh collection tube. Ten microliters of the eluate was taken for protein determination, and the rest of the sample was dried in a vacuum concentrator and then stored at − 20 °C.

Equalized protein samples were resolved in 300 μl of deionized water. Protein concentrations were determined in triplicates with the Bio-Rad DC protein assay according to the manufacturer’s instructions. The digestion in solution was performed using the filter-aided sample preparation (FASP) method developed by Wisniewski et al. (2009) [21].

Dithiothreitol was added to 100 μg of equalized plasma protein (~ 100 μl) to reach a final concentration of 15 mM and incubated for 1 h at 37 °C. The sample was then cooled for about 5 min to room temperature and mixed 1:1 with 500 μL urea buffer (8 M urea, 100 mM Tris–HCl, pH 8.5), followed by transfer to Amicon 10 K filters (in 450 μL portions) with subsequent centrifugation at 14,000 × g for 15 min. Again, 200 μL of urea buffer was added and centrifuged at 14,000 × g for 15 min. For alkylation, 100 μL of the urea buffer containing 0.05 M iodoacetamide (IAA) was added and shaken for 1 min at 600 rpm. The mixture was then incubated in the dark for 20 min without shaking and subsequently centrifuged (14,000 × g, 15 min). After adding 150 μL of the urea buffer, another centrifugation (14,000 × g, 15 min) was performed. Then, 150 μL of the 50 mM ammonium bicarbonate buffer, pH 7.4 (ABC buffer) was added and centrifuged (14,000 × g, 15 min). This step was repeated two more times. Next, the denatured proteins were digested in 100 μL ABC buffer with 1 μg Lys-C for 4 h at 37 °C in a thermomixer. One microgram of trypsin was added to 100 μL ABC buffer and incubated at 37 °C overnight. After digestion, the mixture was centrifuged again the following day, and the solution containing the peptides was collected in a fresh collection vessel. The mixture was then washed twice with 100 μL ABC buffer. Finally, 100 μL of 10% ACN/ABC buffer (acetonitrile/ammonium bicarbonate buffer) was added and centrifuged again, so that 500 μL of the digested plasma was now available. This was dried in the concentrator plus vacuum centrifuge until no moisture was present. The dried samples were stored at − 20 °C until further use.

After digestion, the samples were fractionated using the Pierce™ High pH Reversed-Phase Peptide Fractionation Kit (Thermo Fisher Scientific). Seven fractions were prepared from each sample. The dried, digested enriched plasma proteins were dissolved in 300 μL of 0.1% trifluoroacetic acid (TFA). The spin columns from the kit were placed in 2.0 mL tubes and centrifuged at 5000 × g for 1 min. 300 μL of ACN was added to wash the column and centrifuged again at 5000 × g for 1 min. This step was repeated once more. Next, 300 μL of 0.1% TFA was added and centrifuged at 5000 × g for 1 min, with one more repetition. The flow-throughs were discarded. Next, the sample was loaded on to the spin column and centrifuged at 3000 × g for 1 min. For washing, 300 μL of water was added and centrifuged at 3000 × g for 1 min. Afterwards, 300 μl of elution solution, each consisting of different percentages of ACN in 0.1% triethylamine (with 5, 7.5, 10, 12.5, 15, 17.5, or 50% ACN in 0.1% triethylamine in the seven individual fractions), were added and fractions eluted by centrifugation at 3000 × g for 2 min. The eluates after loading the sample and from the washing step were dried in a vacuum concentrator and stored together at − 20 °C. Additionally, a small aliquot (2.5 µL) from the neat plasma was used for an in-solution digest under similar conditions as described above to recover proteins from the untreated plasma. For all analyses, only one technical replicate was generated because the technical variability was clearly lower than the biological variability.

Peptide samples were separated with a nano-flow ultra-high pressure liquid chromatography system (RSLC, Thermo Scientific) equipped with a trapping column (3 µm C18 particle, 2 cm length, 75 µm ID, Acclaim PepMap, Thermo Scientific) and a 50 cm long separation column (2 µm C18 particle, 75 µm ID, Acclaim PepMap, Thermo Scientific). Peptide mixtures were injected, enriched, and desalted on the trapping column at a flow rate of 6 µL/min with 0.1% Trifluoracetic acid (TFA) for 5 min. The trapping column was switched online with the separating column and peptides were eluted with a multi-step binary gradient: linear gradient of buffer B (80% ACN, 0.1% formic acid) in buffer A (0.1% formic acid) from 4 to 25% in 30 min, 25 to 50% in 10 min, 50 to 90% in 5 min, and 10 min at 90% B. The column was reconditioned to 4% B in 15 min. The flow rate was 500 nL/min, and the column temperature was set to 35 °C. The RSLC system was coupled online via a Nano Spray Flex Ion Source II (Thermo Scientific) to an Orbitrap Fusion Lumos Tribrid mass spectrometer. Metal-coated fused-silica emitters (SilicaTip, 10 µm i.d., New Objectives) and a voltage of 2.1 kV were used for the electrospray. Overview scans were acquired at a resolution of 60 k in a mass range of m/z 375–1500 in the Orbitrap analyzer and stored in profile mode. For MS2 analysis, selected peptides were fragmented with high-energy collisional dissociation (HCD) and an energy of 35 eV. Fragment ion mass spectra were recorded in the Orbitrap with a resolution of 15 k at normal scan rate and stored as centroid m/z value and intensity pairs. Active exclusion was activated so that ions fragmented were once excluded from further fragmentation for 60 s within a mass window of 5 ppm of the specific m/z value.

The LC–MS system was checked for performance on a weekly basis using standard peptide mixtures with high and low complexity. All samples were analyzed randomly to avoid artifacts by analysis in different batches. The samples were normalized based on their protein content by normalizing protein intensities of each sample to the median value. Due to the low number of tested samples, an influence of the plasma storage duration was not tested. All samples were stored at − 80 °C.

Raw data were processed using the MaxQuant software package (version 1.6.17.0) [22] with the implemented Andromeda search engine [23]. The human protein entries from the Uniprot database were taken as the reference proteome database containing 42,481 entries (downloaded in January 2023). Proteins with a false discovery rate (FDR) of < 0.01 on the peptide and protein level were noted. 

*Bioinformatic analysis*: All MS data were evaluated using the Perseus program (version 1.6.14.0). The Label-Free Quantification (LFQ) intensities were taken, and all protein groups were filtered out that counted for the classification as reverse hits and potential contaminants and were therefore not used for further processing. The log_2_-intensities were calculated, and out of the 2535 groups identified, only those that could be quantified in at least 70% of all 36 samples were included for further analysis, and missing values were imputed using general settings (width 0.3, downshift 1.8). The values were normalized by subtracting the median intensity. Missing data were imputed using FactoMineR and missMDA packages in R version 4.1.3 [24, 25]. A total of 528 proteins were measured across all 36 samples.

### Statistical analysis

Proteins detected with unpaired *t*-test *p*-values below the Bonferroni correction threshold (0.05/528 = 9.46 × 10^-5^) and with a fold change (FC) of ≥|2| were considered significant. Volcano plots, heatmaps, and individual graphs were generated in GraphPad Prism v10. Unpaired t-tests between two groups or two-way ANOVA (with time and conception as variables) were used to report *p*-values. *p*-values < 0.05 were considered significant and indicated by an appropriate asterisk (*p*-value: * < 0.05, ** < 0.01, *** < 0.001, or **** < 0.0001).

### Pathway and gene set enrichment analysis

All proteins at *p* < 0.05 in each comparison were uploaded into STRING-DB v12 (www.string-db.org) and analyzed for functional enrichment in Gene Ontology (Biological Process, Molecular Function, Cellular Component), local network clustering, KEGG and Reactome pathways, and Wikipathways.

## Results

### Demographic characteristics

**Fig. 1:**
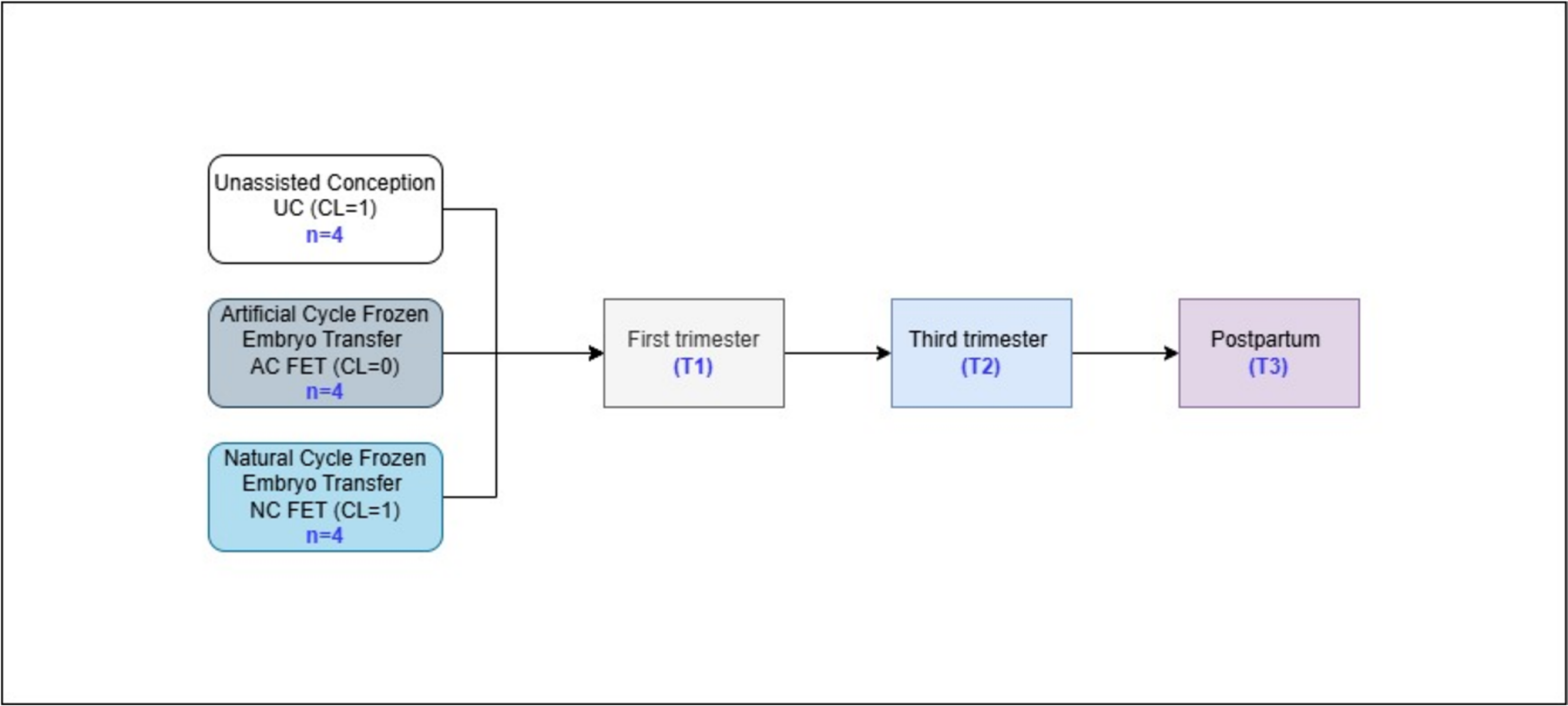
Project workflow. Sample numbers and collection time points are indicated in blue.

The primary outcomes of interest were the impact of CL number and the mode of conception on the maternal proteome profile. Both UC and NC FET had 1 CL, whereas patients with AC FET had no CL. Primary analyses involved the comparison between CL = 1 (UC + NC FET) versus CL = 0 (AC FET), UC and NC FET, UC and AC FET, or AC FET vs NC FET at the end of the first trimester (T1), at the end of the third trimester (T2), and postpartum (T3) (Fig. 1). This was followed by a comparison between the different time points within conception groups (T1 vs T2, T2 vs T3, and T1 vs T3). Demographic and clinical characteristics of the participants are summarized in Supplementary Table 1, with study groups and collection time points detailed in Fig. 1.

### Differential expression and gene set enrichment analysis

Plasma proteomics of the collected blood samples was conducted using the ProteoMiner Kit that reduces the abundance of highly-abundant proteins and increases the abundance of proteins with lower abundance. Untreated plasma was used as a control, and the number of proteins quantified in both setups was taken from the proteome analysis of untreated plasma. Overall, 528 proteins could be quantified and were included in the bioinformatic analysis.

**Fig. 2:**
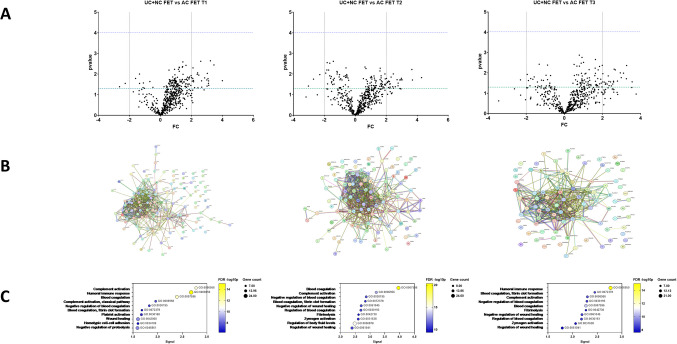
Comparison of **A** unassisted (UC) and natural cycle (NC) frozen embryo transfer (FET) vs artificial cycle FET (AC FET) conceptions. The green line indicates *p* = 0.05 whereas the purple line represents the multiple testing threshold (*p* = 0.05/528 = 9.46 × 10^-5^). **B** STRING interaction network and **C** pathway enrichment analysis of proteins differentially detected in UC + NC FET compared to AC FET at *p* < 0.05 across T1, T2, and T3. The *Y*-axis displays the top ten highest signal String Biological Process (GO, Gene ontology) categories, with the enrichment signal on the *X*-axis. Bubbles are colored according to FDR -log10 *p*-values, and their sizes correspond to the number of genes.

Samples were compared across time points T1, T2, and T3 between UC and NC FET (CL = 0) versus AC FET (CL = 1) (Fig. 2A). Although we did not identify differential proteins above the multiple testing correction threshold, all proteins at *p* < 0.05 were submitted to STRING-DB for enrichment analysis, with interaction network graphs (Fig. 2B) and top ten biological processes at T1, T2, and T3 shown in Fig. 2C. The gene ontology: biological processes (GO: BP) terms Complement activation (T1, FDR *p*-value 2.66 × 10^-12^), Blood coagulation (T2, FDR *p*-value 4.27 × 10^-21^), and Humoral immune response (T2, FDR *p*-value 8.92 × 10^-16^) were the top categories identified (full list in Supplementary Table 3).

**Fig. 3:**
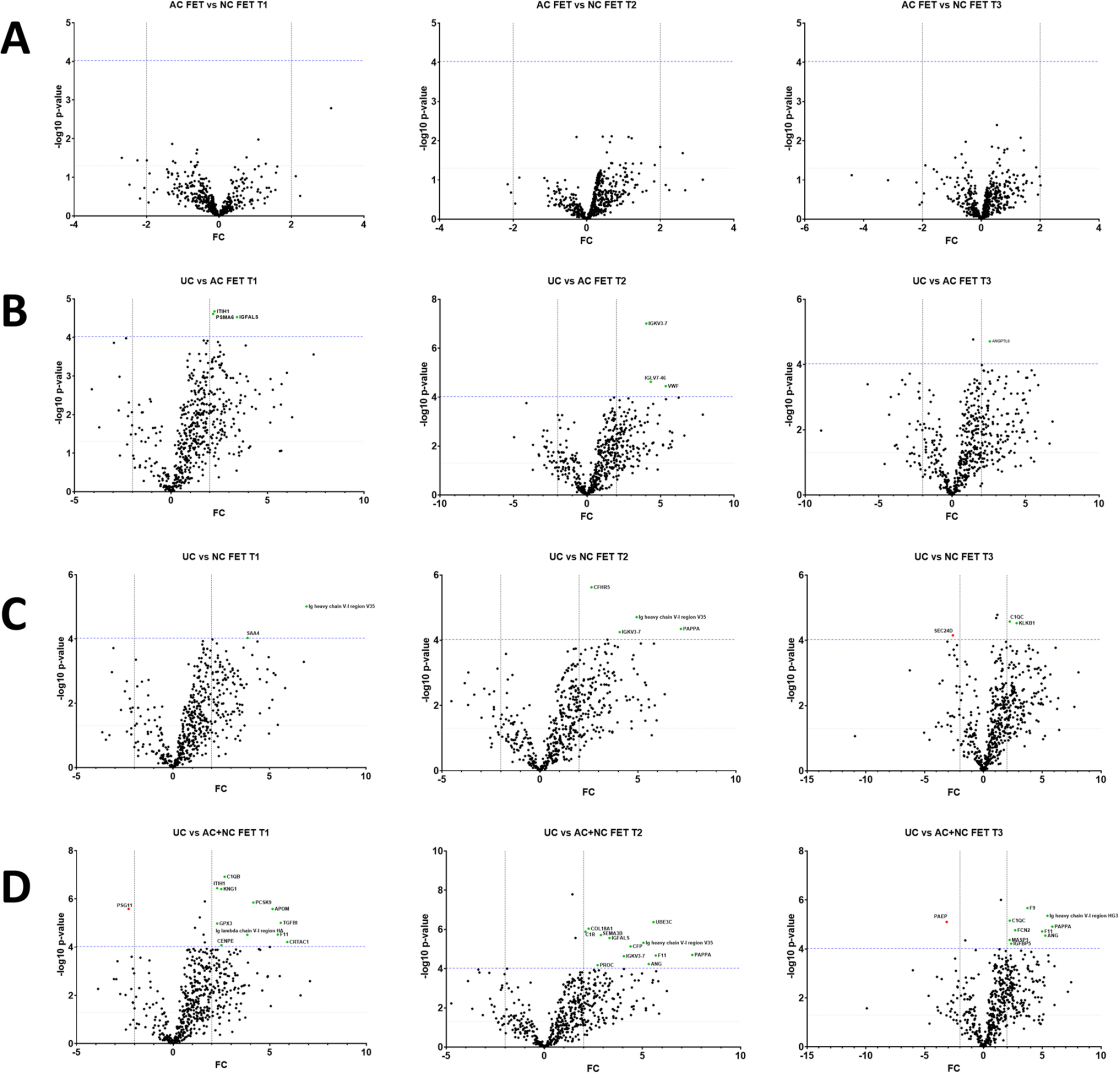
Comparisons of **A** AC FET versus NC FET, **B** UC versus AC FET, **C** UC versus NC FET, and **D** UC versus both AC and NC FET across time points T1, T2, and T3. Proteins with a Bonferroni corrected *p* < 9.47 × 10^-5^ and a FC ≥|2| are labelled. Proteins with higher expression in the first group are shown in green, while those with reduced expression in the first group are indicated in red

We also tested for differentially detected proteins within the groups AC FET and NC FET (Fig. 3A), between UC and AC FET (Fig. 3B) and between UC and NC FET (Fig. 3C). Comparing UC versus both FET methods revealed that 11 proteins were decreased in NC + AC FET compared to UC at T1 (C1QB, ITIH1, KNG1, PCSK9, APOM, TGFB1, GPX3, F11, CRTAC1, CENPE, and Ig lambda chain V-I region HA) and 1 protein was increased (PSG11) in NC + AC FET compared to UC at T1. At T2, 12 proteins were reduced (UBE3C, PAPPA, F11, ANG, CFP, IGKV3-7, IGFALS, IGKV3-7, PROC, COL18A, SEMA3B, C1R, and Ig heavy chain V-I region V35), and at T3, 9 proteins were reduced (F9, PAPPA, F11, ANG, FCN2, MASP1, IGFBP5, C1QC, and Ig heavy chain V-I region HG3) and one protein PAEP was elevated in NC + AC FET as compared to UC (Fig. 3 D2E).

**Fig. 4:**
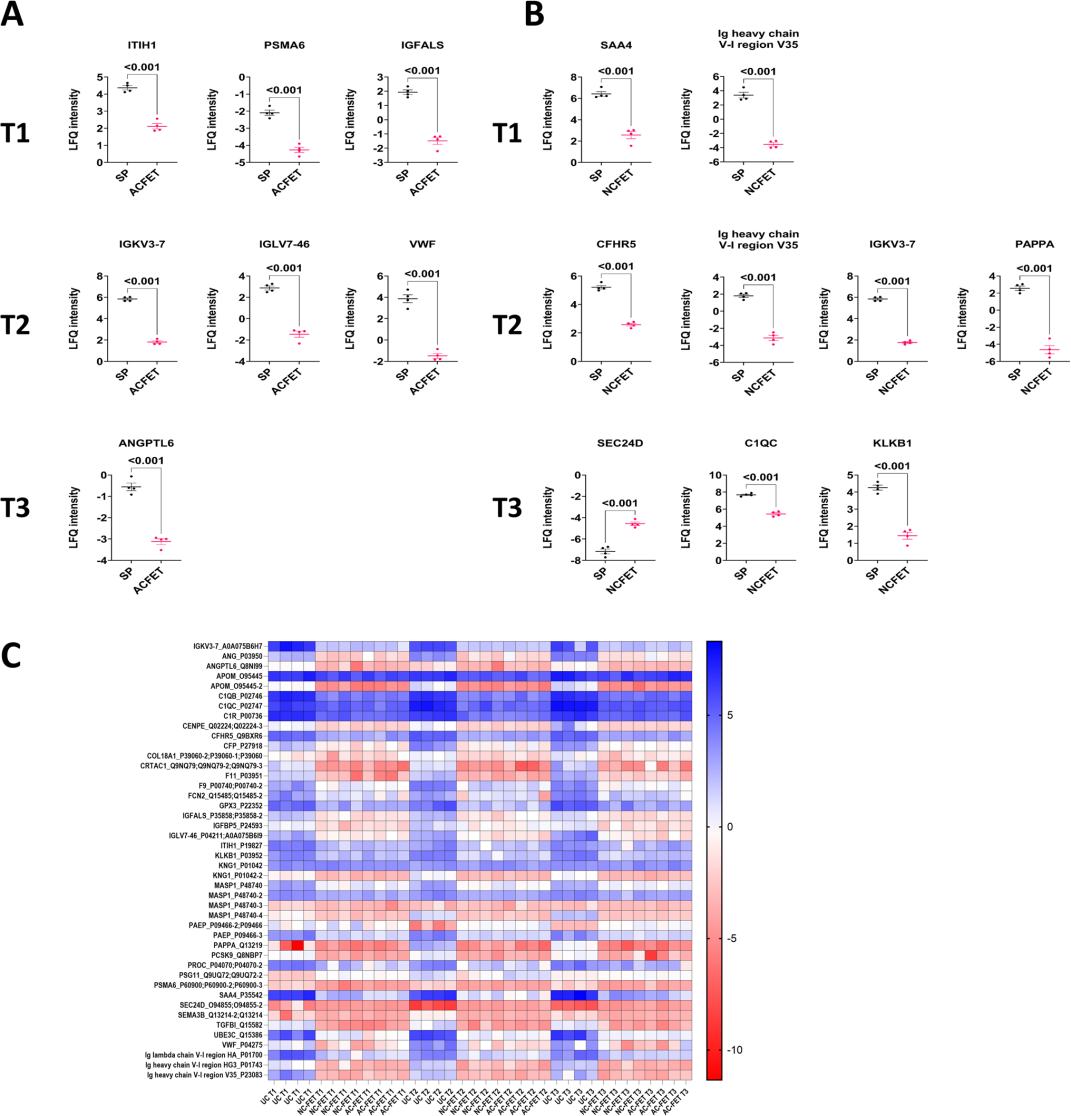
Individual graphs of proteins below the Bonferroni correction threshold. (**A**) UC versus AC FET and (**B**) UC versus NC FET at T1, T2, T3. (**C**) Heatmap of LFQ intensities for all proteins (names and majority protein IDs) with a Bonferroni corrected *p* < 9.47 × 10^-5^ and FC≥|2| across all comparison groups

In differential analysis, none of the proteins were expressed differentially between AC FET and NC FET at T1, T2, or T3 above the Bonferroni multiple-testing correction threshold (Fig. 3A, Supplementary Table 2), whereas the intensities for ITIH1, PSMA6, IGFALS were lower in the plasma of women in the first trimester in AC FET versus UC (*p* < 0.001) (Figs. 3B and 4A). Similarly, IGKV3-7, IGLV7-46, and VWF were lower at T2, and ANGPTL6 was lower at T3 in women undergoing AC FET in comparison to UC (*p* < 0.001) (Figs. 3B and 4A). Comparing NC FET with UC, Ig heavy chain V-I region V35 and SAA4 were significantly lower in NC FET at T1. CFHR5, Ig heavy chain V-I region V35, IGKV3-7, and PAPPA were lower at T2 in NC FET, and C1QC and KLKB1 were lower at T3 in NC FET, with SEC24D levels being higher in NC FET as compared to the UC group (*p* < 0.001) (Figs. 3C and 4B). LFQ intensities for all proteins that were detected at *p* < 9.47 × 10^-5^ and FC ≥|2| in group comparisons were visualized in a heatmap (Fig. 4C).

**Fig. 5:**
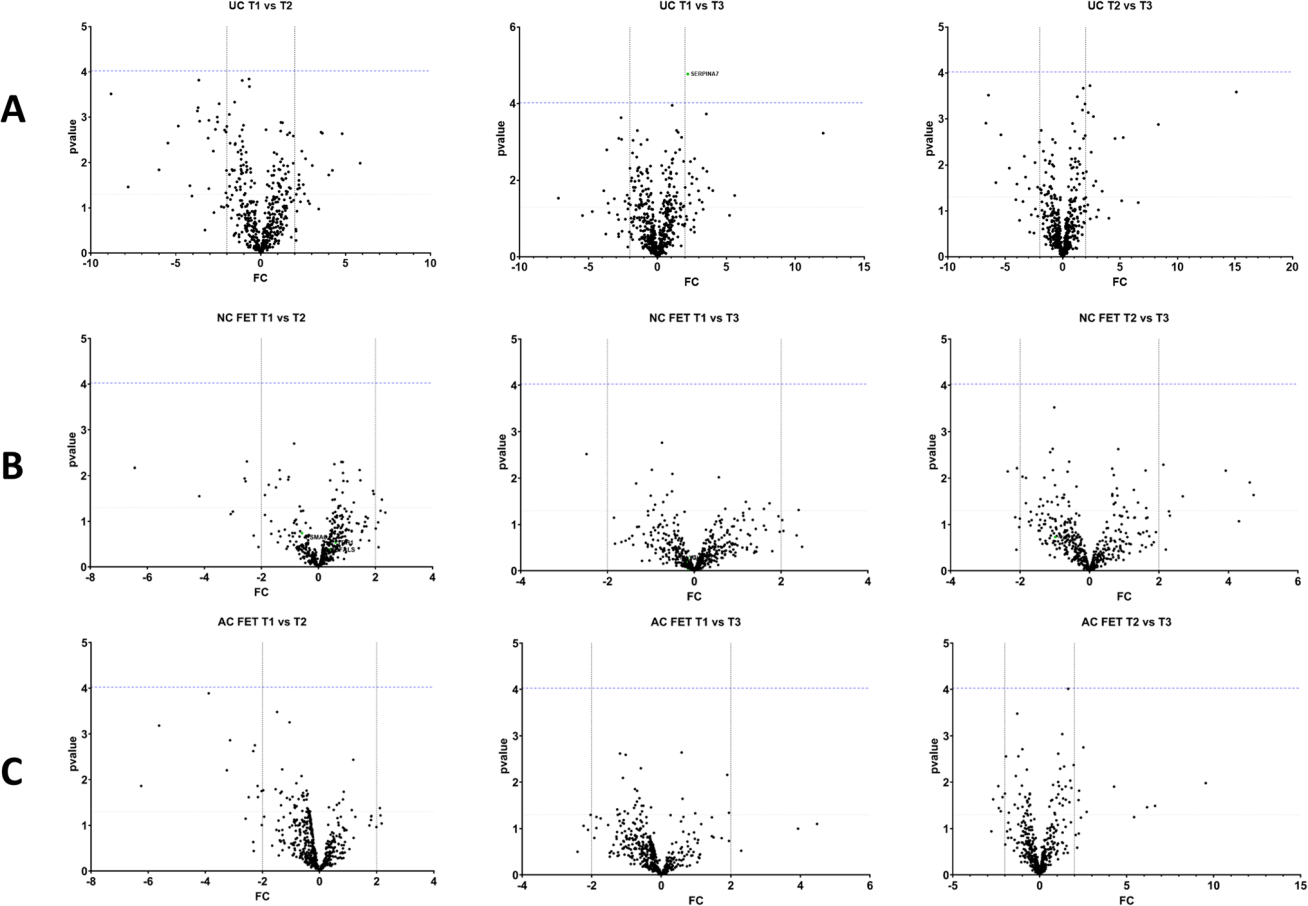

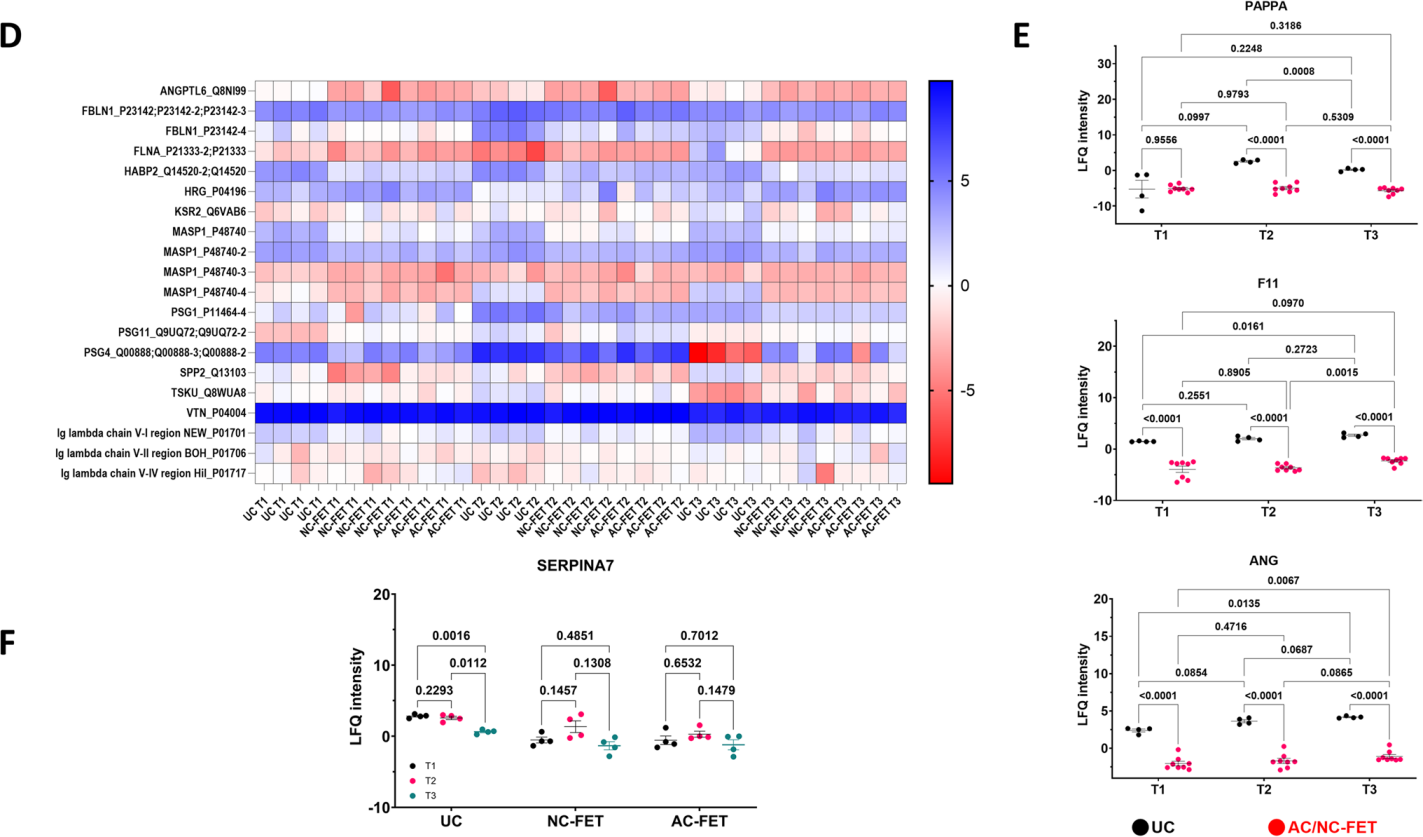
Comparison of (**A**) UC, (**B**) NC FET, (**C**) AC FET between the time points T1, T2, and T3. Proteins with a Bonferroni corrected *p* < 9.47 × 10^-5^ and FC≥|2| are labelled. Proteins with a higher expression in the first group are shown in green, while those with reduced expression in the first group are indicated in red. (**D**) Heatmap displaying LFQ intensities for all proteins (names and majority protein IDs) with a *p* < 0.05 that are common across all time point comparisons within groups. (**E**) LFQ intensities of three proteins that were significant across T1, T2, and T3 in the comparison of UC versus AC/NC FET, with p-values indicated following two-way ANOVA and post-hoc tests. (**F**) LFQ intensities of SERPINA7, the only protein withstanding multiple testing correction within the time points analysis, comparing UC, NC FET and AC FET across T1 (black dots) T2 (red dots) and T3 (green dots). *P*-values are indicated following two-way ANOVA and post-hoc tests

In time point analysis within UC (Fig. 5A), NC FET (Fig. 5B), and AC FET (Fig. 5C), only one protein, SERPINA7, was elevated in T1 over T3 in UC (*p* = 0.0022) (Fig. 5A and F). While many proteins were specific to distinct time point analysis between conception groups, 132 proteins were common between all the time points T1, T2, and T3 when comparing UC with AC FET and 128 protein were common when comparing UC with NC FET, respectively, at *p* < 0.05 (SupplementaryFigs. 1B, 1 C, 1D, Supplementary Table 2). There were no common proteins between T1, T2, and T3 while comparing AC FET and NC FET (SupplementaryFig. 1). Intensities for all proteins that were detected at *p* < 9.47 × 10^-5^ and FC ≥|2| either in time point or group comparisons were visualized in a heatmap (Fig. 5D) or as a Venn diagram (Supplementary Fig. 2). Three proteins, PAPPA, F11, and ANG, were decreased in AC FET and NC FET as compared to UC at all three time points (Fig. 5E). SERPINA7 was differentially detected while comparing time points within the same conception method, UC (*p* < 9.47 × 10^-5^ and FC ≥|2|) (Fig. 5F).

**Fig. 6:**
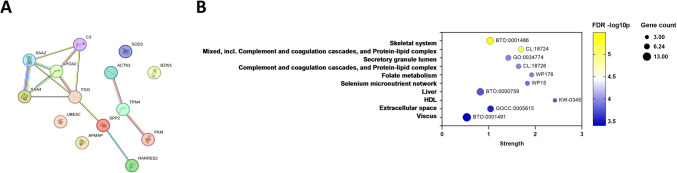
STRING-DB (**A**) interaction network and (**B**) bubble plot illustrating the enrichment analysis of proteins differentially detected in AC FET versus NC FET at an FDR < 0.0005 at T1. The Y-axis represents the differentially detected STRING categories, while the X-axis indicates the strength of the enrichment. Bubbles are colored according to FDR -log10 *P* values, with lower *p*-values shown in yellow and higher *p*-values in deep blue. The size of the bubbles corresponds to the number of genes represented. GO=GO Component, CL=STRING clusters, Reactome=HSA, WP=WikiPathways, DOID=Diseases, BTO=Tissues, GOCC=Compartments, KW= UniProt keyword, SM=SMART

Following gene-set enrichment analysis in STRING-DB, multiple pathways were identified comparing conception methods and time points (Supplementary Figs. 3 and 4, Supplementary Table 3). For instance, comparing AC FET and NC FET at T1, a gene interaction network was visualized (Fig. 6A) and genes were enriched in complement and coagulation cascades as well as protein-lipid complexes (STRING clusters CL: 18,724) with an FDR of 6.44 × 10E-6 (Fig. 6B, Supplementary Table 3).

## Discussion

In our mass-spectrometry-based approach to explore the plasma proteome in pregnancies with and without a CL, and across different modes of conception and at various stages of pregnancy, we detected 2535 proteins across samples, of which 528 proteins could be quantified in all samples. We observed significant differences between UC compared to both FET protocols (AC and NC) at all three time points. However, contrary to our expectations, no significant differences were detected between CL = 1 and CL = 0 or between AC FET and NC FET. This may be attributed to the methodology used, which covers only a part of the complete plasma proteome and prevents the detection of subtle differences.

The mode of conception has been linked to pregnancy health and adverse pregnancy outcomes. Patients undergoing AC FET face an increased risk of preeclampsia compared to those undergoing NC FET [26]. This increased risk may be related to the absence of the CL in AC FET cycles, which produces essential hormones such as estrogen and progesterone during early pregnancy. While these hormones are supplemented during AC FET, other CL products, including the vasoactive peptide hormone relaxin, are not [1]. The secretory products of the CL have been minimally studied in humans. Therefore, the aim of our study was to investigate whether the maternal plasma proteome profile differs based on the presence of a CL and the mode of conception.

Using mass spectrometry-based proteomics, we identified differences in maternal circulating proteins between UC and FETs. Moreover, the proteome profile varied across the time points studied: the first trimester, third trimester, and postpartum. Our study included UC and conceptions through NC FET, both occurring in the presence of a CL. In contrast, conceptions following an AC FET lacked a CL. The CL persists until the end of pregnancy and secretes multiple products, including sex steroids, relaxin, pro-renin, and vascular endothelial growth factor into the maternal circulation. Our own work in the field of assisted reproduction suggests that the increased risk of preeclampsia as observed in FET in the absence of a CL [12] might be at least partially driven by a lack of CL hormones. Our previous data showed absent relaxin and low pro-renin in the maternal circulation in early pregnancy in conceptions lacking a CL [17]. Thus, the physiologic maternal milieu is not fully replicated in these patients. Optimal levels of CL products are crucial for normal cardiovascular adaptation to pregnancy, and alterations of CL products early in pregnancy might impact vascular health and pregnancy outcome as observed in AC FET [20].

The identification of key hormonal molecules essential for endometrial development has driven the supplementation strategies used in current IVF practice. With the advent of discovery-based “omics” technologies, it is now possible to explore variations in the maternal proteome across different stages of pregnancy and modes of conception, while delving deeper into potential alterations in the endocrine milieu associated with ART.

Some of the differentially expressed proteins between the conception modes identified in this work, such as ITIH1, PSMA6, IGFALS, and IGLV7-46, have not been studied in the context of human pregnancy. However, VWF [27, 28], SAA4 (Serum Amyloid A4) [29, 30], and ANG (angiogenin) [31] have been investigated in relation to pregnancy, placental development [32], and pregnancy complications [33]. Apolipoprotein M (APOM) has been reported to be a diagnostic marker for preeclampsia [34], whereas serum peptides derived from kininogen-1 (KNG1) were part of a panel able to differentially diagnose preeclamptic pregnancies [35].

Additionally, SAA4 [36, 37], ANG [38], CFHR5 [39, 40], IGKV3-7 [41], and PAPPA [42–44] have been suggested to play a role in the context of preeclampsia. SAA4 mRNA and protein are expressed in first-trimester and term trophoblast cells [45]. Notably, SAA4 mRNA levels are upregulated during weeks 10 and 12 of pregnancy, suggesting a potential role in trophoblast invasion. In our study, SAA4 was significantly lower in NC FET compared to UC in the first trimester. Similarly, C1q, the recognition molecule of the classical complement pathway, has been implicated in pregnancy outcomes. Deficiency of C1q is associated with impaired trophoblast invasion and pregnancy failure [46]. Agostinis et al. reported reduced levels of C1q in women with preeclampsia [47]. In women who conceived via assisted reproductive technology (ART), C1q levels showed a trend comparable to preeclamptic patients, even though these women did not exhibit preeclampsia-like symptoms during pregnancy. The authors suggest that this may be due to immunological dysfunction at the maternal–fetal interface in ART pregnancies, supported by findings of C1q deposition in the placentas of oocyte donation pregnancies, similar to preeclampsia cases. The glycoprotein PAPPA (Pregnancy-associated plasma protein A), produced by placental syncytiotrophoblasts, is linked to preeclampsia, with low maternal serum levels indicating a higher risk [48]. In our cohort, UC conceptions showed lower PAPPA LFQ intensities postpartum compared to the third trimester. However, in the FET group, PAPPA levels remained consistent throughout pregnancy. Despite this, PAPPA levels in the FET group were lower than those in the UC group during both the third trimester and postpartum. However, whether SAA4, C1q, and PAPPA regulation is influenced by the mode of conception remains speculative at this point.

While our current study primarily focuses on identifying differentially expressed proteins in the context of corpus luteum (CL) status, we recognize the importance of understanding the functional roles and mechanisms of these proteins. It is plausible that CL-derived peptide hormones influence the expression and activity of the proteins in trophoblast and endothelial cells. Future studies could investigate how these hormones modulate the protein signatures in these cell types, potentially revealing novel regulatory mechanisms involved in CL function and preeclampsia pathogenesis. Understanding the protein–protein interactions and signaling pathways involving PAPPA, SAA4, and ANG could provide insights into their functional roles. PAPPA is a metalloproteinase reported to cleave insulin-like growth factor-binding proteins (IGFBPs) [49] and influence trophoblast invasion and placental development [50]. Investigating similar interactions for SAA4 and ANG may uncover new regulatory networks in CL function and preeclampsia. SAA4 has been implicated in inflammation and oxidative stress, which are key factors in preeclampsia [51]. Investigating the role of SAA4 in mediating these processes in trophoblast and endothelial cells could provide valuable insights into the pathogenesis of the disease. ANG, secreted by trophoblasts [52], is involved in angiogenesis—the formation of new blood vessels. Irregularities in the levels of ANG and can lead to erratic blood vessel formation and endothelial dysfunction, both of which are highly relevant to preeclampsia development [53]. A recent study has shown that inhibitors of angiogenin may contribute to the pathophysiology of preeclampsia [54]. A direct mechanistic cause or the role of any hormones or CL-derived products remains to be investigated.

SERPINA7, also known as Thyroxine-binding globulin or TBG, is essential for thyroid hormone transport and metabolism [55]. TBG levels are known to be elevated during pregnancy, as also seen in our unassisted conception group, between first trimester and postpartum time points, with similar trends in both the NC FET and AC FET groups. Thyroid hormones are needed for CL function and maintenance, with thyroid hormone receptors found on the surface of the CL [56]. Increased SERPINA7 levels may result in a decrease in free thyroid hormone in the blood, causing thyroid-stimulating hormone (TSH) levels to increase. While there are conflicting reports regarding the association between TSH levels and preeclampsia risk [57], fluctuations in TSH levels can impact the release of follicle-stimulating hormone (FSH) and luteinizing hormone (LH), both necessary for CL function.

Gene set enrichment analysis revealed several pathways affected by the different conception methods, with the activation of the complement and coagulation cascades being particularly noteworthy. In our cohort, this activation was observed, for instance, across all time points when comparing pregnancies with and without a CL and during the first trimester in AC FET compared to NC FET. Similarly, Youssef et al. reported complement and coagulation cascade activation in early-onset severe preeclampsia using LC–MS/MS proteomics analysis of serum samples from 14 cases and 6 controls [58]. These pathways have previously been reported to be associated with preeclampsia and preterm birth [59–61]. These findings open new avenues for exploring potential diagnostic and therapeutic targets for preeclampsia within the complement and coagulation pathways, not only in UC pregnancy but also in ART conceptions.

A study investigating the maternal proteome in preeclampsia via LC–MS/MS approaches reported on 17 differentially expressed proteins in the serum [58], from which our study also identified VWF, PAPPA, FCN3, and ITIH1 as candidates associated with the mode of conception.

In contrast to MS-based approaches, a recent study utilized a panel of 5000 proteins to identify plasma biomarkers in late-onset preeclampsia in a larger cohort of normal and preeclamptic pregnancies [62]. One of the proteins identified in the study, HTRA1, a serine protease, was differentially detected at T1 and T2 in UC in our study. Similarly, another recent longitudinal cohort study investigating 1006 proteins for plasma biomarkers of preeclampsia reported differentially expressed proteins at different gestational time points [63], but we did not find any candidates directly overlapping with our current study.

To our knowledge, ours is the first study to explore the plasma proteome between pregnancies with and without a CL, across different modes of conception and at various stages of pregnancy. However, we acknowledge the limitations of this pilot study. The sample size was small, and the proteins detected differentially will need to be confirmed in a larger patient cohort. The small sample numbers in the current study may have limited the ability to detect true biological effects due to insufficient statistical power, and these results or lack of findings must be treated with caution.

While we did not find strongly changed individual proteins, the enrichment analysis identified subtle biological changes in related genes belonging to specific gene-sets or pathways. This suggests that although individual protein effects may be too subtle to be identified by this small sample set, comparing the presence and absence of a CL, there may be consistent significantly impacted pathways that can throw light on the underlying biology.

The identified pathways of complement activation, humoral immune response, and blood coagulation indicate an impact of CL or CL-derived products on multiple proteins belonging to these pathways. One of the proteins belonging to the complement pathway, the Human C4b-binding protein (C4BP), was reported to be expressed in the CL [64]. While the CL itself is also known to contain immune cells [65], CL-secreted hormones such as progesterone can also affect cytokine secretion and humoral immunity [66]. Estradiol, another CL secreted hormone, is reported to transcriptionally activate multiple coagulation genes via estrogen response elements present in their promoter regions, thereby also increasing the risk of clotting during pregnancy [67]. This may partly explain our findings of strong pathway changes while comparing pregnancies with and without a CL.

Although we find significant associations in our gene-set and pathway enrichment analyses, we would like to note that all proteins below *p* < 0.05 were included here, and that replication in a larger cohort size may be warranted.

Additionally, the mass spectrometry-based methodology does not cover all plasma proteins, suggesting that alternative technologies with a higher proteome coverage should lead to the identification of more responsive proteins. Using a newer MS instrument, such as the Orbitrap Astral (Thermo Scientific) or the timsTOF Pro 2 with PASEF technology (Bruker), may lead to a higher proteome coverage with increased sensitivity. Alternatively, Proximity Extension Assays (PEA, Olink, Thermo Fischer Scientific), having a much higher sensitivity due to the combination of antibody-based protein detection and PCR amplification, may be used; however, this technique only allows for targeted analysis and misses unexpected proteins [68].

We were also unable to determine whether the observed differences were related to selection bias or pre-existing factors independent of the mode of conception and CL status. While we aimed to match participants across the three groups, the availability of plasma samples at all three time points was limited. Consequently, factors such as the cause for infertility or the use of aspirin (acetylsalicylic acid) and prednisone in FET cycles may have influenced our findings. These medications are known to exert diverse and tissue-specific effects on the proteome.

Aspirin acts not only through its well-established inhibition of cyclooxygenases (COX-1 and COX-2), but also via covalent acetylation of a broad range of non-COX proteins, particularly lysine residues. This post-translational modification can alter protein function, stability, subcellular localization, and interaction networks. Proteomic analyses have identified several aspirin-sensitive targets, including metabolic enzymes (e.g., GAPDH), chaperones (e.g., heat shock proteins), structural proteins (e.g., tubulin), and mitochondrial components—many of which play important roles in reproductive physiology.

Prednisone, a synthetic glucocorticoid, exerts its effects primarily through genomic mechanisms mediated by the glucocorticoid receptor. These genomic changes, however, translate into downstream proteomic remodeling across various tissues. Prednisone has been shown to suppress pro-inflammatory proteins in immune cells, enhance hepatic gluconeogenesis, promote muscle catabolism, and modulate extracellular matrix and transport proteins in the kidney and bone. Although clinical data on the specific effects of these agents in IVF settings remain limited, mechanistic studies suggest potentially significant modulation of molecular pathways involved in implantation, placentation, and early embryonic development.

Importantly, aspirin use is common in ART cycles in the USA but not in unassisted conceptions. This represents a systematic difference that is now nearly impossible to control for in studies comparing different modes of conception and should be considered a potential confounding factor in the interpretation of proteomic findings.

Additionally, it is unclear whether the differentially expressed proteins are secreted by the CL, by other secretory tissues such as the placenta, or if their expression is a downstream consequence. The analysis of CL tissue, particularly through single-cell sequencing, could provide further important insights.

## Conclusion

Understanding of the maternal proteome in relation to pregnancy, CL number, and mode of conception has been largely restricted to studies focused on individual molecules. This study provides the first comprehensive description of the maternal plasma proteome during pregnancy and postpartum, highlighting a unique proteomic signature associated with different modes of conception. We have identified biologically plausible candidate proteins, enriched gene sets, and pathways for further investigation. Future studies involving larger cohorts and methodologies capable of detecting smaller proteins and peptides are necessary.

## Supplementary Information

Below is the link to the electronic supplementary material.

ESM 1 (21.0 KB DOCX)

ESM 2 (5.01 MB PDF)

ESM 3 (30.3 MB PDF)

ESM 4 (3.28 MB TIF)

ESM 5 (3.78 MB TIF)

ESM 6 (14.8 MB TIF)

ESM 7 (11.5 MB TIF)

ESM 8 (9.31 MB TIF)

ESM 9 (4.75 MB TIF)

## Data Availability

The data that support the findings of this study are available from the corresponding author, Frauke von Versen-Höynck, upon reasonable request. The MS proteomics data have been deposited to the ProteomeXchange Consortium via the PRIDE partner repository (https://www.ebi.ac.uk/pride/) with the dataset identifier PXD067974.
