# Supplementary material for: Exploring the plasma proteome linked to corpus luteum presence and conception mode across pregnancy stages and postpartum
Source: J Assist Reprod Genet. 2025 Sep 20;42(10):3275–89. doi: 10.1007/s10815-025-03632-0 (PMC12602745; doi:10.1007/s10815-025-03632-0)
Supplement: Supplementary file 1 — (21.0 KB DOCX) [file 10815_2025_3632_MOESM1_ESM.docx]

**Supplementary Table 1:** Demographic characteristics of the study population.

|  | **Spontaneous** | **NC FET** | **AC FET** |
| --- | --- | --- | --- |
| Participant age, (years)* | 38.4±2.8 | 33.5±1.8 | 37.1±2.3 |
| Participant race‡  White  Asian  Other | 2 (50)  2 (50)  0 | 0  3 (75)  1 (25) | 3 (75)  0  1 (25) |
| BMI* | 22.1±3.5 | 21.8±2.3 | 22.2±2.5 |
| Gravida† | 0.5 (0-4) | 0.5 (0-1.75) | 0.5 (0-1.75) |
| Para† | 0 (0-1.5) | 0 | 0 (0-0.75) |
| SBP before pregnancy* | 114.8±14.7 | 106.5±11.1 | 114.3±15.6 |
| DBP before pregnancy* | 76.5±8.5 | 68.3±8.5 | 67.6±10.3 |
| Smoking ‡ | 1 (25) | 0 | 0 |
| Gestational age at T1 (days)† | 90.5  (82.8-96.8) | 87  (82-93.5) | 83  (82-88.5) |
| Gestational age at T2 (days)† | 252  (243-252) | 256.5  (244.8-258.5) | 253  (250.3-258) |
| Days postpartum at T3 (days)† | 63.5  (50.8-76.3) | 48  (43-67.3) | 62.5  (52.5-74) |
| Gestational age at delivery (days)† | 273.5  (267.5-284) | 280.5  (264.5-285.3) | 276.5  (265.8-280.5) |
| **Reasons for infertility** |  |  |  |
| Age | 0 | 0 | 0 |
| Diminished ovarian reserve | 2 (50) | 0 | 2 (50) |
| Male factor | 0 | 2 (50) | 2 (50) |
| PCOS | 1 (25) | 0 | 0 |
| Other ovulatory disorder | 1 (25) | 0 | 0 |
| Tubal | 0 | 0 | 0 |
| Uterine | 1 (25) | 1 (25) | 0 |
| Endometriosis | 0 | 1 (25) | 0 |
| Recurrent pregnancy loss | 1 (25) | 0 | 1 (25) |
| Single gene disorder | 0 | 0 | 1 (25) |
| Same sex partner | 0 | 1 (25) | 0 |
| Unexplained | 0 | 1 (25) | 0 |
| **Medication used** |  |  |  |
| Ovulation trigger (hCG)‡ | 0 | 2 (50) | 0 |
| Estradiol‡ | 0 | 0 | 4 |
| Progesteron (i.m.)‡ | 1 (25) | 0 | 2 (50) |
| Progesteron (vaginal)‡ | 1 (25) | 3 (25) | 4 (100) |
| Aspirin‡ | 0 | 3 (75) | 4 (100) |
| Prednisone‡ | 0 | 1 (25) | 3 (75) |
| Levothyroxine‡ | 1 (25) | 0 | 0 |

BMI; body-mass index, SBP; systolic blood pressure, DBP; diastolic blood pressure, T1; first trimester; T2; third trimester, T3; postpartum, PCOS; Polycystic ovarian syndrome, hCG; human chorionic gonatropin

*Mean value ± SD are reported

†Median value (interquartile range) are shown

‡Number (% of total) are shown
