# Supplementary material for: Exploring the plasma proteome linked to corpus luteum presence and conception mode across pregnancy stages and postpartum
Source: J Assist Reprod Genet. 2025 Sep 20;42(10):3275–89. doi: 10.1007/s10815-025-03632-0 (PMC12602745; doi:10.1007/s10815-025-03632-0)
Supplement: Supplementary file 2 — (5.01 MB PDF) [file 10815_2025_3632_MOESM2_ESM.pdf]

Supplemenary Table 2A: group comparison

Test	FC	pvalue	Protein IDs	Protein names	Gene names
AC FET vs NC FET T1	-0,587687	0,04066	P01024;O95568	Complement C3	C3
AC FET vs NC FET T1	-0,774132	0,03997	P0DP03;P01768	Ig heavy chain V-III region CAM	Ig heavy chain V-III region CAM
AC FET vs NC FET T1	-0,614617	0,04842	P02652	Apolipoprotein A-II	APOA2
AC FET vs NC FET T1	-0,602081	0,01942	P02679;P02679-2	Fibrinogen gamma chain	FGG
AC FET vs NC FET T1	-0,621685	0,02415	P08294	Extracellular superoxide dismutase [Cu-Zn]	SOD3
AC FET vs NC FET T1	-2,252048	0,03648	P0DJI9;P0DJI9-2	Serum amyloid A-2 protein	SAA2
AC FET vs NC FET T1	-2,683634	0,03142	P12814;P12814-3;P12814-2	Alpha-actinin-1	ACTN1
AC FET vs NC FET T1	1,292398	0,04473	P14618;P14618-2	Pyruvate kinase PKM	PKM
AC FET vs NC FET T1	-1,292011	0,01371	P35542	Serum amyloid A-4 protein	SAA4
AC FET vs NC FET T1	-1,992601	0,03661	P67936	Tropomyosin alpha-4 chain	TPM4
AC FET vs NC FET T1	3,0954244	0,001633	Q13103	Secreted phosphoprotein 24	SPP2
AC FET vs NC FET T1	-1,268653	0,03856	Q15386;Q15386-2	Ubiquitin-protein ligase E3C	UBE3C
AC FET vs NC FET T1	-1,20714	0,0422	Q7L1Q6-2;Q7L1Q6;Q7L1Q6-3	Basic leucine zipper and W2 domain-containing protein 1	BZW1
AC FET vs NC FET T1	1,0870426	0,01056	Q99969	Retinoic acid receptor responder protein 2	RARRES2
AC FET vs NC FET T1	0,7612188	0,03058	Q9HDC9;Q9HDC9-2	Adipocyte plasma membrane-associated protein	APMAP
AC FET vs NC FET T2	-0,278397	0,008013	P00734	Prothrombin	F2
AC FET vs NC FET T2	1,2546225	0,03842	P01594;P01593	Ig kappa chain V-I region AU	Ig kappa chain V-I region AU;Ig kappa chain V-I region AG
AC FET vs NC FET T2	0,4350814	0,007847	P01715	Ig lambda chain V-IV region Bau	Ig lambda chain V-IV region Bau
AC FET vs NC FET T2	0,6920483	0,0375	P02649;CON__Q03247	Apolipoprotein E	APOE
AC FET vs NC FET T2	0,6781847	0,007671	P06396;P06396-4;P06396-3	Gelsolin	GSN
AC FET vs NC FET T2	1,0584051	0,0374	P08294	Extracellular superoxide dismutase [Cu-Zn]	SOD3
AC FET vs NC FET T2	0,5465033	0,01976	P19823	Inter-alpha-trypsin inhibitor heavy chain H2	ITIH2
AC FET vs NC FET T2	1,849262	0,0416	P25311	Zinc-alpha-2-glycoprotein	AZGP1
AC FET vs NC FET T2	1,0062354	0,049	P35858;P35858-2	Insulin-like growth factor-binding protein complex acid labile subunit	IGFALS
AC FET vs NC FET T2	0,9075439	0,0417	P55058;P55058-4;P55058-3;P55058-2	Phospholipid transfer protein	PLTP
AC FET vs NC FET T2	0,6646713	0,03692	P61769	Beta-2-microglobulin	B2M
AC FET vs NC FET T2	1,222584	0,008643	Q4LDE5;Q4LDE5-4;Q4LDE5-3;Q4LDE5-2	Sushi, von Willebrand factor type A, EGF and pentraxin domain-containing protein 1	SVEP1
AC FET vs NC FET T2	2,6112086	0,02067	Q8IY18	Structural maintenance of chromosomes protein 5	SMC5
AC FET vs NC FET T2	1,480107	0,03755	Q99996-3;Q99996;Q99996-6;Q99996-5;Q99996-1;Q99996-4	A-kinase anchor protein 9	AKAP9
AC FET vs NC FET T2	0,6322267	0,01083	Q9BXR6	Complement factor H-related protein 5	CFHR5
AC FET vs NC FET T2	1,1372192	0,007986	Q9HDC9;Q9HDC9-2	Adipocyte plasma membrane-associated protein	APMAP
AC FET vs NC FET T2	1,9972832	0,01436	Q9UQ72;Q9UQ72-2	Pregnancy-specific beta-1-glycoprotein 11	PSG11
AC FET vs NC FET T3	-0,667342	0,02197	A0A075B6I0	.	IGLV8-61
AC FET vs NC FET T3	-1,90013	0,04228	A0A0A0MS15	.	IGHV3-49
AC FET vs NC FET T3	-0,626885	0,04866	A0A0B4J1U7	.	IGHV6-1
AC FET vs NC FET T3	0,5808974	0,01522	P00709	Alpha-lactalbumin	LALBA
AC FET vs NC FET T3	1,3397057	0,008378	P00746	Complement factor D	CFD
AC FET vs NC FET T3	0,885047	0,01612	P01594;P01593	Ig kappa chain V-I region AU	Ig kappa chain V-I region AU;Ig kappa chain V-I region AG
AC FET vs NC FET T3	-0,883593	0,02684	P0DP03;P01768	Ig heavy chain V-III region CAM	Ig heavy chain V-III region CAM
AC FET vs NC FET T3	0,6324713	0,04299	P02774-3;P02774;P02774-2	Vitamin D-binding protein	GC
AC FET vs NC FET T3	0,7894065	0,01714	P03952;P20718	Plasma kallikrein	KLKB1
AC FET vs NC FET T3	-0,532557	0,03595	P04180	Phosphatidylcholine-sterol acyltransferase	LCAT
AC FET vs NC FET T3	0,6139686	0,02934	P06396;P06396-4;P06396-3	Gelsolin	GSN
AC FET vs NC FET T3	1,4498351	0,01761	P18065	Insulin-like growth factor-binding protein 2	IGFBP2
AC FET vs NC FET T3	0,5306225	0,003959	P35555	Fibrillin-1	FBN1
AC FET vs NC FET T3	0,548268	0,03644	Q15293;Q15293-2	Reticulocalbin-1	RCN1
AC FET vs NC FET T3	0,3621158	0,0141	Q8IV50-2;Q8IV50	LysM and putative peptidoglycan-binding domain-containing protein 2	LYSMD2
AC FET vs NC FET T3	1,8654272	0,04759	Q8IYW2	Cilia- and flagella-associated protein 46	CFAP46
AC FET vs NC FET T3	0,7759815	0,01993	Q8TDL5;Q8TDL5-2	BPI fold-containing family B member 1	BPIFB1
AC FET vs NC FET T3	-0,529814	0,01061	Q96KN2	Beta-Ala-His dipeptidase	CNDP1
UC vs AC FET T1	5,1389663	1,61E-02	A0A075B6H7;A0A0C4DH55	.	IGKV3-7
UC vs AC FET T1	3,2133588	0,001125	A0A075B6J9	.	IGLV2-18
UC vs AC FET T1	1,7694744	0,02642	A0A087WSY6	.	IGKV3D-15
UC vs AC FET T1	2,3598646	0,04033	A0A0A0MS15	.	IGHV3-49
UC vs AC FET T1	0,8539554	0,000398	A0A0B4J1V0	.	IGHV3-15
UC vs AC FET T1	3,5780356	0,0008772	A0A0B4J1X5	.	IGHV3-74
UC vs AC FET T1	4,5828817	0,002945	A0A0B4J1Y9	.	IGHV3-72
UC vs AC FET T1	2,5416463	0,002172	A0A0C4DH25	.	IGKV3D-20
UC vs AC FET T1	5,7225763	0,001621	A0A0C4DH35	.	IGHV3-35
UC vs AC FET T1	-2,028394	0,008697	A0A0C4DH38	.	IGHV5-51
UC vs AC FET T1	3,3354197	3,55E-02	A0A0J9YX35	.	.
UC vs AC FET T1	-2,959992	0,0001381	A4FU69-3;A4FU69;A4FU69-2;A4FU69-4;A4FU69-6	EF-hand calcium-binding domain-containing protein 5	EFCAB5
UC vs AC FET T1	2,7305213	0,0002786	A8K2U0;A8K2U0-2	Alpha-2-macroglobulin-like protein 1	A2ML1
UC vs AC FET T1	0,9586608	0,0002676	O00391;O00391-2	Sulfhydryl oxidase 1	QSOX1
UC vs AC FET T1	1,7951682	0,001403	O14791-2;O14791;O14791-3	Apolipoprotein L1	APOL1
UC vs AC FET T1	3,8382768	0,003366	O15016;O15016-2;O15016-3	Tripartite motif-containing protein 66	TRIM66
UC vs AC FET T1	-0,93413	0,02233	O15084;O15084-4;O15084-1;O15084-2	Serine/threonine-protein phosphatase 6 regulatory ankyrin repeat subunit A	ANKRD28
UC vs AC FET T1	1,8160147	0,0002715	O43399;O43399-5;O43399-7;O43399-2;O43399-4;O43399-3	Tumor protein D54	TPD52L2
UC vs AC FET T1	1,8165163	0,0001413	O43861-2;O43861	Probable phospholipid-transporting ATPase IIB	ATP9B
UC vs AC FET T1	1,2409928	0,04934	O43866	CD5 antigen-like	CD5L
UC vs AC FET T1	3,9031031	0,001026	O75636;O75636-2	Ficolin-3	FCN3
UC vs AC FET T1	7,3832148	0,0002771	O75882;O75882-2;O75882-3	Attractin	ATRN
UC vs AC FET T1	1,6381485	0,001077	O76076	WNT1-inducible-signaling pathway protein 2	WISP2
UC vs AC FET T1	0,7861288	0,03508	O95428-6;O95428;O95428-5;O95428-4;O95428-2;O95428-3	Papilin	PAPLN
UC vs AC FET T1	5,3293923	6,38E-03	O95445-2	Apolipoprotein M	APOM
UC vs AC FET T1	1,0665433	0,01835	O95967	EGF-containing fibulin-like extracellular matrix protein 2	EFEMP2
UC vs AC FET T1	0,9954674	0,02881	P00450	Ceruloplasmin	CP

UC vs AC FET T1	1,3543333	0,009462	P00451	Coagulation factor VIII	F8
UC vs AC FET T1	1,7326376	0,003536	P00734	Prothrombin	F2
UC vs AC FET T1	1,3393638	0,03069	P00739;P00739-2	Haptoglobin-related protein	HPR
UC vs AC FET T1	2,8950999	0,0009816	P00740;P00740-2	Coagulation factor IX	F9
UC vs AC FET T1	2,8471334	5,97E-03	P00742	Coagulation factor X	F10
UC vs AC FET T1	1,6687739	0,0009955	P00747;Q02325	Plasminogen	PLG
UC vs AC FET T1	2,060703	0,03761	P00751;P00751-2	Complement factor B	CFB
UC vs AC FET T1	1,2471049	0,002685	P01011;P01011-2;P01011-3	Alpha-1-antichymotrypsin	SERPINA3
UC vs AC FET T1	2,2127626	0,007681	P01031	Complement C5	C5
UC vs AC FET T1	1,8960205	0,0008221	P01034	Cystatin-C	CST3
UC vs AC FET T1	-1,169829	0,03996	P01040	Cystatin-A	CSTA
UC vs AC FET T1	2,5473589	1,71E-02	P01042-2	Kininogen-1	KNG1
UC vs AC FET T1	2,1806964	0,006185	P01344-3;P01344;P01344-2	Insulin-like growth factor II	IGF2
UC vs AC FET T1	0,6616998	0,00775	P04432;P01597	Ig kappa chain V-I region Daudi	Ig kappa chain V-I region Daudi;Ig kappa chain V-I region DEE
UC vs AC FET T1	3,5976173	0,004264	P01599	Ig kappa chain V-I region Gal	Ig kappa chain V-I region Gal
UC vs AC FET T1	1,1035087	0,01176	P01619	Ig kappa chain V-III region B6	Ig kappa chain V-III region B6
UC vs AC FET T1	2,1719753	0,018	P01624	Ig kappa chain V-III region POM	Ig kappa chain V-III region POM
UC vs AC FET T1	3,8678501	0,0001619	P01700	Ig lambda chain V-I region HA	Ig lambda chain V-I region HA
UC vs AC FET T1	1,5148851	0,00147	P01701	Ig lambda chain V-I region NEW	Ig lambda chain V-I region NEW
UC vs AC FET T1	-1,532885	0,04926	P01714	Ig lambda chain V-III region SH	Ig lambda chain V-III region SH
UC vs AC FET T1	-1,045522	0,004598	P01715	Ig lambda chain V-IV region Bau	Ig lambda chain V-IV region Bau
UC vs AC FET T1	0,6996372	0,0297	P01742	Ig heavy chain V-I region EU	Ig heavy chain V-I region EU
UC vs AC FET T1	1,5246593	0,01021	P01766	Ig heavy chain V-III region BRO	Ig heavy chain V-III region BRO
UC vs AC FET T1	5,2717114	0,002279	P0DP03;P01768	Ig heavy chain V-III region CAM	Ig heavy chain V-III region CAM
UC vs AC FET T1	2,388274	0,01311	P01859	Ig gamma-2 chain C region	IGHG2
UC vs AC FET T1	1,8547723	0,0125	P01860	Ig gamma-3 chain C region	IGHG3
UC vs AC FET T1	-4,105843	0,002209	P01861	Ig gamma-4 chain C region	IGHG4
UC vs AC FET T1	0,8614914	0,0005825	P01871;P01871-2	Ig mu chain C region	IGHM
UC vs AC FET T1	1,6850221	0,02189	P01876	Ig alpha-1 chain C region	IGHA1
UC vs AC FET T1	1,3028023	0,008136	P02647;Q9HB71-2	Apolipoprotein A-I	APOA1
UC vs AC FET T1	1,6688116	0,008942	P02649;CON__Q03247	Apolipoprotein E	APOE
UC vs AC FET T1	1,3750021	0,009041	P02656	Apolipoprotein C-III	APOC3
UC vs AC FET T1	1,2003715	0,009203	P02671;P02671-2;REV__Q9UKV0-4;REV__Q9UKV0-2;REV__Q9UKV0;REV__Q9UKV0-5;REV__Q9UKV0-7;Q14314	Fibrinogen alpha chain	FGA
UC vs AC FET T1	1,7140033	0,005392	P02675	Fibrinogen beta chain	FGB
UC vs AC FET T1	0,5039008	0,03598	P02679;P02679-2	Fibrinogen gamma chain	FGG
UC vs AC FET T1	0,7609899	0,03075	P02743	Serum amyloid P-component	APCS
UC vs AC FET T1	2,580271	1,40E-02	P02746	Complement C1q subcomponent subunit B	C1QB
UC vs AC FET T1	1,3725531	0,005655	P02747	Complement C1q subcomponent subunit C	C1QC
UC vs AC FET T1	0,8443139	0,0437	P02748;REV__Q4AC99	Complement component C9	C9
UC vs AC FET T1	0,6381514	0,007164	P02749;CON__P17690	Beta-2-glycoprotein 1	APOH
UC vs AC FET T1	-0,548842	0,04766	P02760	Protein AMBP	AMBP
UC vs AC FET T1	1,4173553	0,001803	P02765	Alpha-2-HS-glycoprotein	AHSG
UC vs AC FET T1	2,0668685	0,000649	P02766	Transthyretin	TTR
UC vs AC FET T1	1,5418851	0,002172	P02774-3;P02774;P02774-2	Vitamin D-binding protein	GC
UC vs AC FET T1	2,5157453	0,003983	P02775	Platelet basic protein	PPBP
UC vs AC FET T1	2,6340196	0,02373	P02776	Platelet factor 4	PF4
UC vs AC FET T1	1,0335958	0,01883	P02790	Hemopexin	HPX
UC vs AC FET T1	3,9813311	0,001367	P03950	Angiogenin	ANG
UC vs AC FET T1	5,6900561	0,005517	P03951	Coagulation factor XI	F11
UC vs AC FET T1	1,6409686	0,00504	P03952;P20718	Plasma kallikrein	KLKB1
UC vs AC FET T1	0,3747709	0,04974	P04004	Vitronectin	VTN
UC vs AC FET T1	1,9168591	0,0004588	P04040	Catalase	CAT
UC vs AC FET T1	3,572345	0,005001	P04070;P04070-2	Vitamin K-dependent protein C	PROC
UC vs AC FET T1	1,0157263	0,005677	P04114	Apolipoprotein B-100	APOB
UC vs AC FET T1	1,5889342	0,005241	P04180	Phosphatidylcholine-sterol acyltransferase	LCAT
UC vs AC FET T1	2,4257085	0,02964	P04275	von Willebrand factor	VWF
UC vs AC FET T1	2,7425459	0,01604	P04406;P04406-2;O14556	Glyceraldehyde-3-phosphate dehydrogenase	GAPDH
UC vs AC FET T1	1,4741457	0,03719	P04433;A0A0A0MRZ8	Ig kappa chain V-III region VG	IGKV3D-11
UC vs AC FET T1	2,5154956	0,0003091	P05023-2;P05023-4;P05023;P05023-3	Sodium/potassium-transporting ATPase subunit alpha-1	ATP1A1
UC vs AC FET T1	0,9333036	0,02094	P05090	Apolipoprotein D	APOD
UC vs AC FET T1	3,0030487	0,004147	P05155-2;P05155;P05155-3	Plasma protease C1 inhibitor	SERPING1
UC vs AC FET T1	1,0813658	0,01926	P05156;CON__Q32PI4	Complement factor I	CFI
UC vs AC FET T1	1,6910727	0,008228	P05160	Coagulation factor XIII B chain	F13B
UC vs AC FET T1	1,3675295	0,01769	P05164-3;P05164;P05164-2	Myeloperoxidase	MPO
UC vs AC FET T1	3,3921697	0,006113	P05543	Thyroxine-binding globulin	SERPINA7
UC vs AC FET T1	1,5692503	0,01385	P05546	Heparin cofactor 2	SERPIND1
UC vs AC FET T1	2,0236631	0,002451	P06331	Ig heavy chain V-II region ARH-77	Ig heavy chain V-II region ARH-77
UC vs AC FET T1	0,9771035	0,02912	P06396;P06396-4;P06396-3	Gelsolin	GSN
UC vs AC FET T1	-1,501265	0,02509	P06702	Protein S100-A9	S100A9
UC vs AC FET T1	0,9096054	0,001291	P06850	Corticoliberin	CRH
UC vs AC FET T1	0,7895982	0,02344	P07225	Vitamin K-dependent protein S	PROS1
UC vs AC FET T1	2,1983774	0,003011	P07237	Protein disulfide-isomerase	P4HB
UC vs AC FET T1	1,1501246	0,0009677	P07333;P07333-2	Macrophage colony-stimulating factor 1 receptor	CSF1R
UC vs AC FET T1	2,7844627	0,008781	P07357	Complement component C8 alpha chain	C8A
UC vs AC FET T1	2,5267007	0,0001599	P07358	Complement component C8 beta chain	C8B
UC vs AC FET T1	0,9933824	0,001096	P07998	Ribonuclease pancreatic	RNASE1
UC vs AC FET T1	1,3564384	0,01596	P08253;P08253-3;P08253-2	72 kDa type IV collagenase	MMP2
UC vs AC FET T1	2,6724213	0,0007131	P08294	Extracellular superoxide dismutase [Cu-Zn]	SOD3
UC vs AC FET T1	1,2850344	0,04959	P08493-2;P08493	Matrix Gla protein	MGP
UC vs AC FET T1	3,4703391	0,008789	P08567	Pleckstrin	PLEK
UC vs AC FET T1	1,7226913	0,008063	P08603;P08603-2	Complement factor H	CFH
UC vs AC FET T1	1,5124776	0,007563	P08697;P08697-2	Alpha-2-antiplasmin	SERPINF2
UC vs AC FET T1	2,7692158	0,000767	P08709-2;P08709	Coagulation factor VII	F7
UC vs AC FET T1	1,4504879	0,0002682	P09382	Galectin-1	LGALS1
UC vs AC FET T1	2,4333856	0,00032	P09466-3	Glycodelin	PAEP
UC vs AC FET T1	2,1112635	0,04118	P09486	SPARC	SPARC
UC vs AC FET T1	1,479881	0,004231	P09871	Complement C1s subcomponent	C1S

UC vs AC FET T1	-1,837749	0,03206	P0C0L4;P0C0L4-2	Complement C4-A	C4A
UC vs AC FET T1	1,7607496	0,001102	P0C0L5	Complement C4-B	C4B
UC vs AC FET T1	3,54791	0,02637	P0DJI8	Serum amyloid A-1 protein	SAA1
UC vs AC FET T1	3,7234568	0,0498	P0DML3;P0DML2;P0DML3-2;P0DML3-3;P01241-2;P01241;P01241-5	Chorionic somatomammotropin hormone 2	CSH2;CSH1
UC vs AC FET T1	3,1298301	0,02102	P0DOX2	.	.
UC vs AC FET T1	1,6759092	0,01653	P0DOX3	.	.
UC vs AC FET T1	3,6156426	0,003136	P0DOX4	.	.
UC vs AC FET T1	1,1760995	0,0429	P0DOX5;P01857	Ig gamma-1 chain C region	IGHG1
UC vs AC FET T1	1,1766271	0,03231	P0DOX6	.	.
UC vs AC FET T1	1,5789044	0,009301	P0DOX7	.	.
UC vs AC FET T1	2,3193271	0,0004163	P0DOX8	.	.
UC vs AC FET T1	1,8586562	0,00109	P0DOY3	.	.
UC vs AC FET T1	1,2597928	0,04329	P10321	HLA class I histocompatibility antigen, Cw-7 alpha chain	HLA-C
UC vs AC FET T1	2,0561268	0,008902	P10643	Complement component C7	C7
UC vs AC FET T1	1,2336294	0,01687	P10645	Chromogranin-A	CHGA
UC vs AC FET T1	2,6696082	0,02693	P10720	Platelet factor 4 variant	PF4V1
UC vs AC FET T1	1,6074722	0,002069	P11021	78 kDa glucose-regulated protein	HSPA5
UC vs AC FET T1	1,8693525	0,02515	P11226	Mannose-binding protein C	MBL2
UC vs AC FET T1	1,7790015	0,02131	P11597;P11597-2	Cholesteryl ester transfer protein	CETP
UC vs AC FET T1	2,6317813	0,008323	P12259	Coagulation factor V	F5
UC vs AC FET T1	2,867152	0,004563	P13497;P13497-5;P13497-2;P13497-6;P13497-4;P13497-3	Bone morphogenetic protein 1	BMP1
UC vs AC FET T1	1,420605	0,009723	P13667	Protein disulfide-isomerase A4	PDIA4
UC vs AC FET T1	-2,186335	0,004862	P13798	Acylamino-acid-releasing enzyme	APEH
UC vs AC FET T1	1,7081462	0,002343	P14209;P14209-2;P14209-3	CD99 antigen	CD99
UC vs AC FET T1	1,4406402	0,02004	P14543;P14543-2	Nidogen-1	NID1
UC vs AC FET T1	2,3967416	0,002237	P14625;Q58FF3	Endoplasmin	HSP90B1
UC vs AC FET T1	1,2723706	0,002997	P15907	Beta-galactoside alpha-2,6-sialyltransferase 1	ST6GAL1
UC vs AC FET T1	-0,828521	0,02195	P16035	Metalloproteinase inhibitor 2	TIMP2
UC vs AC FET T1	4,3672626	0,002289	P17936;P17936-2	Insulin-like growth factor-binding protein 3	IGFBP3
UC vs AC FET T1	2,7519589	0,007227	P18206;P18206-2;P18206-3	Vinculin	VCL
UC vs AC FET T1	0,894315	0,004642	P18428	Lipopolysaccharide-binding protein	LBP
UC vs AC FET T1	1,4225862	0,002626	P19823	Inter-alpha-trypsin inhibitor heavy chain H2	ITIH2
UC vs AC FET T1	2,251159	2,11E-05	P19827	Inter-alpha-trypsin inhibitor heavy chain H1	ITIH1
UC vs AC FET T1	1,6730802	0,03653	P20851-2;P20851;REV__Q7Z7B0-3;REV__Q7Z7B0-2;REV__Q7Z7B0	C4b-binding protein beta chain	C4BPB
UC vs AC FET T1	1,8173461	0,001723	P21333-2;P21333	Filamin-A	FLNA
UC vs AC FET T1	2,1240323	0,0004381	P22352	Glutathione peroxidase 3	GPX3
UC vs AC FET T1	2,8721969	0,03817	P22792	Carboxypeptidase N subunit 2	CPN2
UC vs AC FET T1	6,27206	1,17E-02	P23083	Ig heavy chain V-I region V35	Ig heavy chain V-I region V35
UC vs AC FET T1	0,8317697	0,01248	P23142;P23142-2;P23142-3	Fibulin-1	FBLN1
UC vs AC FET T1	2,1288004	0,0004139	P24593	Insulin-like growth factor-binding protein 5	IGFBP5
UC vs AC FET T1	0,7276588	0,01898	P24844	Myosin regulatory light polypeptide 9	MYL9
UC vs AC FET T1	2,7800569	0,02924	P26927	Hepatocyte growth factor-like protein	MST1
UC vs AC FET T1	0,4956348	0,006658	P27169	Serum paraoxonase/arylesterase 1	PON1
UC vs AC FET T1	2,8599246	0,0009171	P27918	Properdin	CFP
UC vs AC FET T1	2,2674201	0,002926	P28370-2;P28370	Probable global transcription activator SNF2L1	SMARCA1
UC vs AC FET T1	1,7077544	0,002706	P29622	Kallistatin	SERPINA4
UC vs AC FET T1	-3,719256	0,02146	P31025;Q5VSP4	Lipocalin-1	LCN1
UC vs AC FET T1	-1,653027	0,005565	P31151;Q86SG5	Protein S100-A7	S100A7
UC vs AC FET T1	1,7296699	0,001134	P34096	Ribonuclease 4	RNASE4
UC vs AC FET T1	3,7839305	0,009366	P35443	Thrombospondin-4	THBS4
UC vs AC FET T1	5,1510327	1,20E-03	P35542	Serum amyloid A-4 protein	SAA4
UC vs AC FET T1	3,41699	2,97E-05	P35858;P35858-2	Insulin-like growth factor-binding protein complex acid labile subunit	IGFALS
UC vs AC FET T1	1,0834186	0,001181	P36955;CON__Q95121	Pigment epithelium-derived factor	SERPINF1
UC vs AC FET T1	0,7222197	0,03117	P43652	Afamin	AFM
UC vs AC FET T1	2,4623765	0,02923	P48059;P48059-4;P48059-2;P48059-5;P48059-3;Q7Z4I7-4;Q7Z4I7-3;Q7Z4I7-2;P0CW19-2	LIM and senescent cell antigen-like-containing domain protein 1	LIMS1
UC vs AC FET T1	1,1565242	0,04784	P48307-2;P48307	Tissue factor pathway inhibitor 2	TFPI2
UC vs AC FET T1	2,4956892	0,0005153	P48740	Mannan-binding lectin serine protease 1	MASP1
UC vs AC FET T1	1,0522134	0,0258	P48740-2	Mannan-binding lectin serine protease 1	MASP1
UC vs AC FET T1	1,7613919	0,02173	P48740-3	Mannan-binding lectin serine protease 1	MASP1
UC vs AC FET T1	2,1460711	0,0003677	P48740-4	Mannan-binding lectin serine protease 1	MASP1
UC vs AC FET T1	2,6233642	0,0004725	P49454	Centromere protein F	CENPF
UC vs AC FET T1	-1,467357	0,008736	P49767	Vascular endothelial growth factor C	VEGFC
UC vs AC FET T1	0,3422081	0,02615	P49908	Selenoprotein P	SEPP1
UC vs AC FET T1	2,5333741	0,0006082	P49913	Cathelicidin antimicrobial peptide	CAMP
UC vs AC FET T1	1,2707336	0,001571	P51884;CON__Q05443	Lumican	LUM
UC vs AC FET T1	1,6927299	0,0001198	P54132	Bloom syndrome protein	BLM
UC vs AC FET T1	1,8391521	0,008711	P55056	Apolipoprotein C-IV	APOC4
UC vs AC FET T1	1,3754046	0,003951	P55058;P55058-4;P55058-3;P55058-2	Phospholipid transfer protein	PLTP
UC vs AC FET T1	1,6590336	0,01006	P55103	Inhibin beta C chain	INHBC
UC vs AC FET T1	-1,512526	0,008832	P55285-2;P55285	Cadherin-6	CDH6
UC vs AC FET T1	1,4131806	0,04523	P55287-2;P55287	Cadherin-11	CDH11
UC vs AC FET T1	0,9656445	0,02616	P55774	C-C motif chemokine 18	CCL18
UC vs AC FET T1	2,054904	0,005316	P57077;P57077-1	MAP3K7 C-terminal-like protein	MAP3K7CL
UC vs AC FET T1	0,6740262	0,009766	P59666;P59665	Neutrophil defensin 3	DEFA3;DEFA1
UC vs AC FET T1	2,7661464	0,03396	P60709	Actin, cytoplasmic 1	ACTB
UC vs AC FET T1	2,1784753	2,45E-05	P60900;P60900-2;P60900-3	Proteasome subunit alpha type-6	PSMA6
UC vs AC FET T1	2,4299948	0,000131	P61224-3;P61224;P61224-2;P61224-4;A6NIZ1;P62834	Ras-related protein Rap-1b	RAP1B;RAP1A
UC vs AC FET T1	3,0372998	0,007256	P61626	Lysozyme C	LYZ
UC vs AC FET T1	-2,669675	0,001044	P61769	Beta-2-microglobulin	B2M
UC vs AC FET T1	1,7898346	0,01541	P62258;P62258-2	14-3-3 protein epsilon	YWHAE
UC vs AC FET T1	-2,08007	0,03285	P62979;P62987;P0CG47;P0CG48	Ubiquitin-40S ribosomal protein S27a	RPS27A;UBA52;UBB;UBC
UC vs AC FET T1	2,589618	0,04191	P63104;P63104-2	14-3-3 protein zeta/delta	YWHAZ
UC vs AC FET T1	2,2666069	0,02124	P67936	Tropomyosin alpha-4 chain	TPM4
UC vs AC FET T1	2,2148149	0,01528	P69905	Hemoglobin subunit alpha	HBA1

UC vs AC FET T1	1,5993369	0,003841	P80108;P80108-2	Phosphatidylinositol-glycan-specific phospholipase D	GPLD1
UC vs AC FET T1	3,5281599	2,47E-02	P80748	Ig lambda chain V-III region LOI	Ig lambda chain V-III region LOI
UC vs AC FET T1	0,6862733	0,02569	P98095-2;P98095	Fibulin-2	FBLN2
UC vs AC FET T1	4,3904372	0,007104	Q00526;P11802-2;Q00535-2;Q00535;P11802;Q14004-2;Q14004	Cyclin-dependent kinase 3	CDK3
UC vs AC FET T1	-0,572131	0,02835	Q00887-2	Pregnancy-specific beta-1-glycoprotein 9	PSG9
UC vs AC FET T1	1,4329594	0,009028	Q02108-2;Q02108	Guanylate cyclase soluble subunit alpha-3	GUCY1A3
UC vs AC FET T1	2,5530605	2,52E-02	Q02224;Q02224-3	Centromere-associated protein E	CENPE
UC vs AC FET T1	2,4026208	0,007488	Q02818	Nucleobindin-1	NUCB1
UC vs AC FET T1	1,9595774	0,002923	Q02985-2;Q02985	Complement factor H-related protein 3	CFHR3
UC vs AC FET T1	1,3312026	0,00136	Q03001;Q03001-9;Q03001-13;Q03001-10	Dystonin	DST
UC vs AC FET T1	1,9825532	0,005714	Q03591	Complement factor H-related protein 1	CFHR1
UC vs AC FET T1	1,065594	0,01341	Q04756	Hepatocyte growth factor activator	HGFAC
UC vs AC FET T1	2,2300055	0,0003303	Q06033-2;Q06033	Inter-alpha-trypsin inhibitor heavy chain H3	ITIH3
UC vs AC FET T1	1,8101446	0,01721	Q07065	Cytoskeleton-associated protein 4	CKAP4
UC vs AC FET T1	2,1583636	0,03349	Q07954;Q07954-2	Prolow-density lipoprotein receptor-related protein 1	LRP1
UC vs AC FET T1	0,5741312	0,0176	Q08174;Q08174-2	Protocadherin-1	PCDH1
UC vs AC FET T1	1,276612	0,02002	Q08380	Galectin-3-binding protein	LGALS3BP
UC vs AC FET T1	2,151927	0,0006365	Q0VAK6;Q0VAK6-2	Leiomodin-3	LMOD3
UC vs AC FET T1	1,1418672	0,00536	Q12794-2;Q12794;Q12794-7;Q12794-4;Q12794-3;Q12794-6;Q12794-5	Hyaluronidase-1	HYAL1
UC vs AC FET T1	1,4705584	0,009951	Q12805-2;Q12805-4;Q12805-3;Q12805	EGF-containing fibulin-like extracellular matrix protein 1	EFEMP1
UC vs AC FET T1	1,2773311	0,01047	Q13103	Secreted phosphoprotein 24	SPP2
UC vs AC FET T1	1,5879321	0,003943	Q13418-2;Q13418;Q13418-3	Integrin-linked protein kinase	ILK
UC vs AC FET T1	3,0904328	0,001129	Q13488-2	V-type proton ATPase 116 kDa subunit a isoform 3	TCIRG1
UC vs AC FET T1	-2,019591	0,019	Q13790	Apolipoprotein F	APOF
UC vs AC FET T1	1,610235	0,03932	Q14126	Desmoglein-2	DSG2
UC vs AC FET T1	0,7588204	0,009601	Q14213	Interleukin-27 subunit beta	EBI3
UC vs AC FET T1	1,0315273	0,04093	Q14515;Q14515-2	SPARC-like protein 1	SPARCL1
UC vs AC FET T1	2,5219982	4,68E-02	Q14520-2;Q14520	Hyaluronan-binding protein 2	HABP2
UC vs AC FET T1	1,3771672	0,0119	Q14623	Indian hedgehog protein	IHH
UC vs AC FET T1	1,0733144	0,0008504	Q14624;Q14624-3;Q14624-4	Inter-alpha-trypsin inhibitor heavy chain H4	ITIH4
UC vs AC FET T1	2,1023225	0,002673	Q14697;Q14697-2	Neutral alpha-glucosidase AB	GANAB
UC vs AC FET T1	1,2572761	0,04352	Q15022	Polycomb protein SUZ12	SUZ12
UC vs AC FET T1	0,5722678	0,01019	Q15113	Procollagen C-endopeptidase enhancer 1	PCOLCE
UC vs AC FET T1	0,7160069	0,006454	Q15293;Q15293-2	Reticulocalbin-1	RCN1
UC vs AC FET T1	3,2211363	0,01288	Q15485;Q15485-2	Ficolin-2	FCN2
UC vs AC FET T1	2,2687542	0,006129	Q15848	Adiponectin	ADIPOQ
UC vs AC FET T1	1,8466057	0,02955	Q16594	Transcription initiation factor TFIID subunit 9	TAF9
UC vs AC FET T1	1,1762436	0,02378	Q16769;Q16769-2	Glutaminyl-peptide cyclotransferase	QPCT
UC vs AC FET T1	0,8002481	0,03609	Q2TAC6;Q2TAC6-3;Q2TAC6-2	Kinesin-like protein KIF19	KIF19
UC vs AC FET T1	0,9558105	0,0009626	Q5CZC0;Q5CZC0-2	Fibrous sheath-interacting protein 2	FSIP2
UC vs AC FET T1	2,5519327	0,0002377	Q5HYK7-3;Q5HYK7-2;Q5HYK7;Q5HYK7-5;Q5HYK7-4	SH3 domain-containing protein 19	SH3D19
UC vs AC FET T1	2,789639	0,01959	Q5JPF3;Q5JPF3-2;A6QL64-3;Q5JPF3-3	Ankyrin repeat domain-containing protein 36C	ANKRD36C;ANKRD36
UC vs AC FET T1	0,8373748	0,006182	Q5T0U0;Q5T0U0-2	Coiled-coil domain-containing protein 122	CCDC122
UC vs AC FET T1	0,5178201	0,01206	Q5VT25-3;Q5VT25-4;Q5VT25-5;Q5VT25;Q5VT25-2;Q5VT25-6	Serine/threonine-protein kinase MRCK alpha	CDC42BPA
UC vs AC FET T1	2,1134881	0,001077	Q6EEV6	Small ubiquitin-related modifier 4	SUMO4
UC vs AC FET T1	3,1146313	0,005128	Q6ISB3;Q6ISB3-2	Grainyhead-like protein 2 homolog	GRHL2
UC vs AC FET T1	2,6180837	0,03308	Q6P387-2;Q6P387	Uncharacterized protein C16orf46	C16orf46
UC vs AC FET T1	3,2413995	0,01308	Q6Q788	Apolipoprotein A-V	APOA5
UC vs AC FET T1	1,4582316	0,002376	Q6UVK1	Chondroitin sulfate proteoglycan 4	CSPG4
UC vs AC FET T1	0,9731022	0,03503	Q6UWP8;Q6UWP8-2	Suprabasin	SBSN
UC vs AC FET T1	1,3426703	0,02036	Q6UY14-3;Q6UY14;Q6UY14-2	ADAMTS-like protein 4	ADAMTSL4
UC vs AC FET T1	2,7233012	0,006174	Q76LX8;Q76LX8-2;Q76LX8-3;Q76LX8-4	A disintegrin and metalloproteinase with thrombospondin motifs 13	ADAMTS13
UC vs AC FET T1	1,3113197	0,03687	Q7Z478	ATP-dependent RNA helicase DHX29	DHX29
UC vs AC FET T1	1,0247136	0,02003	Q86U17	Serpin A11	SERPINA11
UC vs AC FET T1	2,2369463	0,006468	Q86UQ4;Q86UQ4-4;Q86UQ4-3;Q86UQ4-6;Q86UQ4-7;Q86UQ4-5	ATP-binding cassette sub-family A member 13	ABCA13
UC vs AC FET T1	0,8973983	0,008776	Q8IV50-2;Q8IV50	LysM and putative peptidoglycan-binding domain-containing protein 2	LYSMD2
UC vs AC FET T1	1,0616848	0,002468	Q8IVL1-11;Q8IVL1-4	Neuron navigator 2	NAV2
UC vs AC FET T1	2,6013998	0,009641	Q8IYI0;Q8IYI0-2	Uncharacterized protein C20orf196	C20orf196
UC vs AC FET T1	1,748167	0,002999	Q8IZK6-2;Q8IZK6	Mucolipin-2	MCOLN2
UC vs AC FET T1	2,7169812	0,002715	Q8N7Z5;Q8WY50	Putative ankyrin repeat domain-containing protein 31	ANKRD31
UC vs AC FET T1	0,5960707	0,007754	Q8N8A2-4;Q8N8A2;Q8N8A2-2;Q8N8A2-3;Q8N8A2-5	Serine/threonine-protein phosphatase 6 regulatory ankyrin repeat subunit B	ANKRD44
UC vs AC FET T1	1,2398024	0,004553	Q8NB25-2;Q8NB25-3;Q8NB25	Protein FAM184A	FAM184A
UC vs AC FET T1	4,1915739	6,31E-03	Q8NBP7	Proprotein convertase subtilisin/kexin type 9	PCSK9
UC vs AC FET T1	-2,330242	0,000105	Q8ND83-3;Q8ND83-2;Q8ND83-4	SLAIN motif-containing protein 1	SLAIN1
UC vs AC FET T1	1,6964781	0,0007912	Q8NDV7-6;Q8NDV7;Q8NDV7-2;Q8NDV7-5;Q8NDV7-4;Q8NDV7-3	Trinucleotide repeat-containing gene 6A protein	TNRC6A
UC vs AC FET T1	3,2575261	1,73E-02	Q8NI99	Angiopoietin-related protein 6	ANGPTL6
UC vs AC FET T1	1,889589	0,0001223	Q8TE73	Dynein heavy chain 5, axonemal	DNAH5
UC vs AC FET T1	2,5842276	0,0005256	Q8WWZ8;Q8WWZ8-2	Oncoprotein-induced transcript 3 protein	OIT3
UC vs AC FET T1	1,5105512	0,0004064	Q92496;Q92496-2;Q92496-3	Complement factor H-related protein 4	CFHR4
UC vs AC FET T1	2,0173445	0,008031	Q92626;Q92626-2	Peroxidasin homolog	PXDN
UC vs AC FET T1	0,4363615	0,03079	Q96CM8-3;Q96CM8-4;Q96CM8;Q96CM8-2	Acyl-CoA synthetase family member 2, mitochondrial	ACSF2

UC vs AC FET T1	1,2595705	0,04361	Q96IY4;Q96IY4-2;CON__Q2KIG3	Carboxypeptidase B2	CPB2
UC vs AC FET T1	1,2155064	0,002396	Q96JB1-2;Q96JB1	Dynein heavy chain 8, axonemal	DNAH8
UC vs AC FET T1	2,1600974	0,001023	Q96KN2	Beta-Ala-His dipeptidase	CNDP1
UC vs AC FET T1	2,1256406	0,008233	Q96PD5;Q96PD5-2	N-acetylmuramoyl-L-alanine amidase	PGLYRP2
UC vs AC FET T1	-0,767876	0,03056	Q96QR1	Secretoglobin family 3A member 1	SCGB3A1
UC vs AC FET T1	3,4098761	0,001494	Q96RL1-3;Q96RL1-4	BRCA1-A complex subunit RAP80	UIMC1
UC vs AC FET T1	1,1460498	0,016	Q99574	Neuroserpin	SERPINI1
UC vs AC FET T1	1,3601797	0,01221	Q99969	Retinoic acid receptor responder protein 2	RARRES2
UC vs AC FET T1	-2,711569	0,007788	Q99996-3;Q99996;Q99996-6;Q99996-5;Q99996-1;Q99996-4	A-kinase anchor protein 9	AKAP9
UC vs AC FET T1	0,5717969	0,04534	Q9BRK5-6;Q9BRK5;Q9BRK5-4;Q9BRK5-3;Q9BRK5-2	45 kDa calcium-binding protein	SDF4
UC vs AC FET T1	1,8431432	0,005375	Q9BWP8-8;Q9BWP8-7;Q9BWP8-6;Q9BWP8-5;Q9BWP8-4;Q9BWP8-3;Q9BWP8-2;Q9BWP8-9;Q9BWP8;Q9BWP8-10	Collectin-11	COLEC11
UC vs AC FET T1	1,9617503	0,009333	Q9BXR6	Complement factor H-related protein 5	CFHR5
UC vs AC FET T1	1,071785	0,004257	Q9H6X2-5;Q9H6X2;Q9H6X2-4;Q9H6X2-6;Q9H6X2-2;Q9H6X2-3	Anthrax toxin receptor 1	ANTXR1
UC vs AC FET T1	-0,479312	0,02033	Q9HCL0-2;Q9HCL0	Protocadherin-18	PCDH18
UC vs AC FET T1	1,1931927	0,0006946	Q9HDC9;Q9HDC9-2	Adipocyte plasma membrane-associated protein	APMAP
UC vs AC FET T1	0,8121534	0,04401	Q9NPG4;O14917-2	Protocadherin-12	PCDH12
UC vs AC FET T1	6,0013885	0,0008282	Q9NQ79;Q9NQ79-2;Q9NQ79-3	Cartilage acidic protein 1	CRTAC1
UC vs AC FET T1	1,5161117	0,03685	Q9NY15;Q9NY15-2	Stabilin-1	STAB1
UC vs AC FET T1	-1,643362	0,02212	Q9NZT1	Calmodulin-like protein 5	CALML5
UC vs AC FET T1	2,8627532	0,00112	Q9UHG3	Prenylcysteine oxidase 1	PCYOX1
UC vs AC FET T1	-1,09291	0,00399	Q9UJJ9	N-acetylglucosamine-1-phosphotransferase subunit gamma	GNPTG
UC vs AC FET T1	2,7101514	0,005407	Q9UK55	Protein Z-dependent protease inhibitor	SERPINA10
UC vs AC FET T1	-2,226067	1,10E-02	Q9UQ72;Q9UQ72-2	Pregnancy-specific beta-1-glycoprotein 11	PSG11
UC vs AC FET T1	0,6607212	0,005582	Q9Y4B5	Microtubule cross-linking factor 1	MTCL1
UC vs AC FET T1	1,1110631	0,01706	Q9Y4L1	Hypoxia up-regulated protein 1	HYOU1
UC vs AC FET T2	4,0303423	9,80E-08	A0A075B6H7;A0A0C4DH55	.	IGKV3-7
UC vs AC FET T2	1,623387	0,0237	A0A075B6I0	.	IGLV8-61
UC vs AC FET T2	4,1669268	0,0002767	A0A075B6J9	.	IGLV2-18
UC vs AC FET T2	1,4608738	0,009154	A0A0C4DH68;A0A075B6R9	.	IGKV2-24;IGKV2D-24
UC vs AC FET T2	0,8064064	0,01084	A0A0B4J1V0	.	IGHV3-15
UC vs AC FET T2	2,6723559	0,01565	A0A0B4J1X5	.	IGHV3-74
UC vs AC FET T2	4,0108617	0,01001	A0A0B4J1Y9	.	IGHV3-72
UC vs AC FET T2	1,4840267	0,002294	A0A0C4DH25	.	IGKV3D-20
UC vs AC FET T2	-1,47241	0,03982	A0A0C4DH38	.	IGHV5-51
UC vs AC FET T2	1,7493222	0,01194	A0A0C4DH67;A0A0C4DH69	.	IGKV1-8;IGKV1-9
UC vs AC FET T2	4,5165059	7,20E-03	A0A0J9YX35	.	.
UC vs AC FET T2	-1,726404	0,01793	A0A1B0GTC6	.	.
UC vs AC FET T2	-3,674604	0,008932	A4FU69-3;A4FU69;A4FU69-2;A4FU69-4;A4FU69-6	EF-hand calcium-binding domain-containing protein 5	EFCAB5
UC vs AC FET T2	3,931147	0,001112	A8K2U0;A8K2U0-2	Alpha-2-macroglobulin-like protein 1	A2ML1
UC vs AC FET T2	2,9348466	0,01072	O00187;O00187-2	Mannan-binding lectin serine protease 2	MASP2
UC vs AC FET T2	-2,285912	0,0178	O00217	NADH dehydrogenase [ubiquinone] iron-sulfur protein 8, mitochondrial	NDUFS8
UC vs AC FET T2	-2,651681	0,02688	O00602	Ficolin-1	FCN1
UC vs AC FET T2	1,7859657	0,0007307	O14791-2;O14791;O14791-3	Apolipoprotein L1	APOL1
UC vs AC FET T2	5,6563101	8,47E-03	O15016;O15016-2;O15016-3	Tripartite motif-containing protein 66	TRIM66
UC vs AC FET T2	2,377331	0,002505	O43184-3;O43184-4;O43184-2;O43184	Disintegrin and metalloproteinase domain-containing protein 12	ADAM12
UC vs AC FET T2	1,8306377	0,03786	O43399;O43399-5;O43399-7;O43399-2;O43399-4;O43399-3	Tumor protein D54	TPD52L2
UC vs AC FET T2	1,6964483	0,004848	O43861-2;O43861	Probable phospholipid-transporting ATPase IIB	ATP9B
UC vs AC FET T2	3,591841	0,0009781	O75636;O75636-2	Ficolin-3	FCN3
UC vs AC FET T2	5,7944376	0,002807	O75882;O75882-2;O75882-3	Attractin	ATRN
UC vs AC FET T2	2,4218242	0,02212	O76076	WNT1-inducible-signaling pathway protein 2	WISP2
UC vs AC FET T2	-4,1229	0,0001746	O94855;O94855-2	Protein transport protein Sec24D	SEC24D
UC vs AC FET T2	1,3299291	0,007644	O95445	Apolipoprotein M	APOM
UC vs AC FET T2	5,7368019	1,12E-02	O95445-2	Apolipoprotein M	APOM
UC vs AC FET T2	1,8597055	0,04884	O95967	EGF-containing fibulin-like extracellular matrix protein 2	EFEMP2
UC vs AC FET T2	1,3020532	0,0246	P00450	Ceruloplasmin	CP
UC vs AC FET T2	1,030268	0,02825	P00488	Coagulation factor XIII A chain	F13A1
UC vs AC FET T2	3,6935547	0,001432	P00709	Alpha-lactalbumin	LALBA
UC vs AC FET T2	0,9719093	0,01001	P00734	Prothrombin	F2
UC vs AC FET T2	2,2180855	0,0001334	P00736	Complement C1r subcomponent	C1R
UC vs AC FET T2	1,1431921	0,03203	P00738;P00738-2	Haptoglobin	HP
UC vs AC FET T2	2,1269629	0,002927	P00739;P00739-2	Haptoglobin-related protein	HPR
UC vs AC FET T2	3,2655313	0,0007253	P00740;P00740-2	Coagulation factor IX	F9
UC vs AC FET T2	2,8914945	0,002217	P00742	Coagulation factor X	F10
UC vs AC FET T2	2,4078265	0,01121	P00746	Complement factor D	CFD
UC vs AC FET T2	2,1065881	2,57E-02	P00747;Q02325	Plasminogen	PLG
UC vs AC FET T2	2,0220521	0,003278	P00751;P00751-2	Complement factor B	CFB
UC vs AC FET T2	-1,816472	0,008731	P01009;P01009-2;P01009-3;P20848	Alpha-1-antitrypsin	SERPINA1
UC vs AC FET T2	2,5268533	0,0004021	P01031	Complement C5	C5
UC vs AC FET T2	-2,706145	0,002348	P01040	Cystatin-A	CSTA
UC vs AC FET T2	3,2774322	0,0003547	P01042-2	Kininogen-1	KNG1
UC vs AC FET T2	1,2608984	0,0087	P01591	Immunoglobulin J chain	IGJ
UC vs AC FET T2	-1,584771	0,04616	P01594;P01593	Ig kappa chain V-I region AU	Ig kappa chain V-I region AU;Ig kappa chain V-I region AG
UC vs AC FET T2	2,1478202	0,004989	P01619	Ig kappa chain V-III region B6	Ig kappa chain V-III region B6
UC vs AC FET T2	2,984905	1,64E-02	P01700	Ig lambda chain V-I region HA	Ig lambda chain V-I region HA
UC vs AC FET T2	1,3677635	0,0009914	P01701	Ig lambda chain V-I region NEW	Ig lambda chain V-I region NEW
UC vs AC FET T2	-0,660502	0,04641	P01706	Ig lambda chain V-II region BOH	Ig lambda chain V-II region BOH

UC vs AC FET T2	2,4924453	0,003243	P01709	Ig lambda chain V-II region MGC	Ig lambda chain V-II region MGC
UC vs AC FET T2	-1,912606	0,01845	P01715	Ig lambda chain V-IV region Bau	Ig lambda chain V-IV region Bau
UC vs AC FET T2	-1,385208	0,01132	P01717	Ig lambda chain V-IV region Hil	Ig lambda chain V-IV region Hil
UC vs AC FET T2	4,8141919	0,001216	P01743	Ig heavy chain V-I region HG3	Ig heavy chain V-I region HG3
UC vs AC FET T2	-2,788304	0,0119	P01764	Ig heavy chain V-III region 23	IGHV3-23
UC vs AC FET T2	3,8557728	0,01248	P0DP03;P01768	Ig heavy chain V-III region CAM	Ig heavy chain V-III region CAM
UC vs AC FET T2	-1,883466	0,0008569	P01834	Ig kappa chain C region	IGKC
UC vs AC FET T2	2,5665658	0,04051	P01860	Ig gamma-3 chain C region	IGHG3
UC vs AC FET T2	-2,43064	0,007081	P01861	Ig gamma-4 chain C region	IGHG4
UC vs AC FET T2	1,553745	0,005483	P01871;P01871-2	Ig mu chain C region	IGHM
UC vs AC FET T2	1,8990977	0,001642	P01876	Ig alpha-1 chain C region	IGHA1
UC vs AC FET T2	1,2333496	0,004102	P02647;Q9HB71-2	Apolipoprotein A-I	APOA1
UC vs AC FET T2	1,0863211	0,008662	P02649;CON__Q03247	Apolipoprotein E	APOE
UC vs AC FET T2	1,8790491	0,0004163	P02671;P02671-2;REV__Q9UKV0-4;REV__Q9UKV0-2;REV__Q9UKV0;REV__Q9UKV0-5;REV__Q9UKV0-7;Q14314	Fibrinogen alpha chain	FGA
UC vs AC FET T2	1,9849312	7,97E-03	P02675	Fibrinogen beta chain	FGB
UC vs AC FET T2	1,1402471	0,0005795	P02679;P02679-2	Fibrinogen gamma chain	FGG
UC vs AC FET T2	0,8915484	5,63E-03	P02743	Serum amyloid P-component	APCS
UC vs AC FET T2	-1,276759	0,001996	P02745	Complement C1q subcomponent subunit A	C1QA
UC vs AC FET T2	2,4940016	0,002562	P02746	Complement C1q subcomponent subunit B	C1QB
UC vs AC FET T2	0,7793391	0,02096	P02748;REV__Q4AC99	Complement component C9	C9
UC vs AC FET T2	2,3473399	0,001816	P02751-1;P02751-8;P02751-3;P02751;P02751-14;P02751-7;P02751-17;P02751-9;P02751-6;P02751-4;P02751-12;P02751-16;P02751-2	Fibronectin	FN1
UC vs AC FET T2	-1,46388	0,03401	P02760	Protein AMBP	AMBP
UC vs AC FET T2	1,7071474	0,0003725	P02765	Alpha-2-HS-glycoprotein	AHSG
UC vs AC FET T2	1,4743955	0,01864	P02766	Transthyretin	TTR
UC vs AC FET T2	1,1876791	0,01148	P02774-3;P02774;P02774-2	Vitamin D-binding protein	GC
UC vs AC FET T2	4,0895201	0,02132	P02776	Platelet factor 4	PF4
UC vs AC FET T2	2,7935305	0,005838	P02788;P02788-2	Lactotransferrin	LTF
UC vs AC FET T2	1,3479502	0,001119	P02790	Hemopexin	HPX
UC vs AC FET T2	4,9331214	0,0005494	P03950	Angiogenin	ANG
UC vs AC FET T2	5,7963707	6,17E-03	P03951	Coagulation factor XI	F11
UC vs AC FET T2	2,5214183	0,0009913	P03952;P20718	Plasma kallikrein	KLKB1
UC vs AC FET T2	-0,705026	0,01944	P03973	Antileukoproteinase	SLPI
UC vs AC FET T2	2,4648031	0,006349	P04040	Catalase	CAT
UC vs AC FET T2	2,9779708	0,003716	P04070;P04070-2	Vitamin K-dependent protein C	PROC
UC vs AC FET T2	0,7945631	0,01509	P04114	Apolipoprotein B-100	APOB
UC vs AC FET T2	1,8564246	0,0005537	P04180	Phosphatidylcholine-sterol acyltransferase	LCAT
UC vs AC FET T2	4,336777	2,36E-05	P04211;A0A075B6I9	Ig lambda chain V region 4A	IGLV7-46
UC vs AC FET T2	5,3530991	3,55E-05	P04275	von Willebrand factor	VWF
UC vs AC FET T2	2,5401866	0,002653	P04433;A0A0A0MRZ8	Ig kappa chain V-III region VG	IGKV3D-11
UC vs AC FET T2	3,1559875	0,01308	P05023-2;P05023-4;P05023;P05023-3	Sodium/potassium-transporting ATPase subunit alpha-1	ATP1A1
UC vs AC FET T2	0,9409139	0,01875	P05090	Apolipoprotein D	APOD
UC vs AC FET T2	3,2422149	0,001622	P05155-2;P05155;P05155-3	Plasma protease C1 inhibitor	SERPING1
UC vs AC FET T2	1,1871064	0,01138	P05156;CON__Q32PI4	Complement factor I	CFI
UC vs AC FET T2	1,8663499	0,001974	P05160	Coagulation factor XIII B chain	F13B
UC vs AC FET T2	2,2794197	0,003369	P05543	Thyroxine-binding globulin	SERPINA7
UC vs AC FET T2	1,9946472	0,005107	P06331	Ig heavy chain V-II region ARH-77	Ig heavy chain V-II region ARH-77
UC vs AC FET T2	-2,39146	0,04664	P06702	Protein S100-A9	S100A9
UC vs AC FET T2	1,1805904	0,0005198	P06727;Q9BT92	Apolipoprotein A-IV	APOA4
UC vs AC FET T2	0,8809135	0,002899	P07225	Vitamin K-dependent protein S	PROS1
UC vs AC FET T2	2,3449757	0,002178	P07237	Protein disulfide-isomerase	P4HB
UC vs AC FET T2	0,9638035	0,04878	P07307-3;P07307-2;P07307	Asialoglycoprotein receptor 2	ASGR2
UC vs AC FET T2	0,8836388	0,0147	P07333;P07333-2	Macrophage colony-stimulating factor 1 receptor	CSF1R
UC vs AC FET T2	2,9392245	0,00141	P07357	Complement component C8 alpha chain	C8A
UC vs AC FET T2	3,0898836	0,0001616	P07358	Complement component C8 beta chain	C8B
UC vs AC FET T2	2,6894444	0,0009011	P07437;Q9BUF5	Tubulin beta chain	TUBB
UC vs AC FET T2	3,1388536	0,02639	P07996;P07996-2	Thrombospondin-1	THBS1
UC vs AC FET T2	2,2726877	0,001908	P08294	Extracellular superoxide dismutase [Cu-Zn]	SOD3
UC vs AC FET T2	4,5007959	0,0001892	P08493-2;P08493	Matrix Gla protein	MGP
UC vs AC FET T2	1,7294619	0,00403	P08603;P08603-2	Complement factor H	CFH
UC vs AC FET T2	5,6387658	0,002486	P08670	Vimentin	VIM
UC vs AC FET T2	0,7723048	0,005951	P08697;P08697-2	Alpha-2-antiplasmin	SERPINF2
UC vs AC FET T2	2,2453949	0,002826	P08709-2;P08709	Coagulation factor VII	F7
UC vs AC FET T2	2,3092115	0,004645	P08833	Insulin-like growth factor-binding protein 1	IGFBP1
UC vs AC FET T2	-4,959848	0,004333	P09466-2;P09466	Glycodelin	PAEP
UC vs AC FET T2	2,7109287	2,74E-02	P09466-3	Glycodelin	PAEP
UC vs AC FET T2	3,5777395	0,02444	P09486	SPARC	SPARC
UC vs AC FET T2	1,4005358	1,60E-02	P09871	Complement C1s subcomponent	C1S
UC vs AC FET T2	-2,251889	0,04072	P0C0L4;P0C0L4-2	Complement C4-A	C4A
UC vs AC FET T2	1,7794802	0,002328	P0C0L5	Complement C4-B	C4B
UC vs AC FET T2	2,3347805	0,0006016	P0DML3;P0DML2;P0DML3-2;P0DML3-3;P01241-2;P01241;P01241-5	Chorionic somatomammotropin hormone 2	CSH2;CSH1
UC vs AC FET T2	-2,727902	0,00624	P0DN87;P0DN86;P0DN86-2;Q6NT52;A6NKQ9-2;A6NKQ9	Choriogonadotropin subunit beta variant 2	CGB2;CGB1
UC vs AC FET T2	2,7154677	0,006302	P0DOX2	.	.
UC vs AC FET T2	3,0521209	0,0008055	P0DOX4	.	.
UC vs AC FET T2	1,2844627	0,005986	P0DOX5;P01857	Ig gamma-1 chain C region	IGHG1
UC vs AC FET T2	1,9851107	0,001381	P0DOX6	.	.
UC vs AC FET T2	1,7310989	0,0002908	P0DOX7	.	.
UC vs AC FET T2	2,0677769	0,006173	P0DOX8	.	.
UC vs AC FET T2	1,467365	0,01294	P0DOY3	.	.
UC vs AC FET T2	1,2527993	0,02639	P10643	Complement component C7	C7
UC vs AC FET T2	2,2716247	0,0273	P10645	Chromogranin-A	CHGA
UC vs AC FET T2	0,2710321	0,04825	P10909-6	Clusterin	CLU

UC vs AC FET T2	1,8164613	0,0009163	P11464-4	Pregnancy-specific beta-1-glycoprotein 1	PSG1
UC vs AC FET T2	1,7254155	0,01837	P11465	Pregnancy-specific beta-1-glycoprotein 2	PSG2
UC vs AC FET T2	2,3984849	0,009803	P11597;P11597-2	Cholesteryl ester transfer protein	CETP
UC vs AC FET T2	3,0513661	0,000763	P12259	Coagulation factor V	F5
UC vs AC FET T2	-1,820968	0,0018	P12273	Prolactin-inducible protein	PIP
UC vs AC FET T2	-0,986969	0,03618	P12532;P12532-2	Creatine kinase U-type, mitochondrial	CKMT1A
UC vs AC FET T2	4,749614	2,35E-02	P13497;P13497-5;P13497-2;P13497-6;P13497-4;P13497-3	Bone morphogenetic protein 1	BMP1
UC vs AC FET T2	4,4452317	0,007745	P13727;P13727-2	Bone marrow proteoglycan	PRG2
UC vs AC FET T2	-2,087011	0,04697	P13798	Acylamino-acid-releasing enzyme	APEH
UC vs AC FET T2	2,0053741	0,04111	P14209;P14209-2;P14209-3	CD99 antigen	CD99
UC vs AC FET T2	1,8406643	0,0001031	P15907	Beta-galactoside alpha-2,6-sialyltransferase 1	ST6GAL1
UC vs AC FET T2	3,4699072	1,87E-02	P16885	1-phosphatidylinositol 4,5-bisphosphate phosphodiesterase gamma-2	PLCG2
UC vs AC FET T2	4,2240143	0,01584	P17936;P17936-2	Insulin-like growth factor-binding protein 3	IGFBP3
UC vs AC FET T2	2,1508687	0,0241	P19652	Alpha-1-acid glycoprotein 2	ORM2
UC vs AC FET T2	1,4897783	0,01373	P19827	Inter-alpha-trypsin inhibitor heavy chain H1	ITIH1
UC vs AC FET T2	-1,658432	0,04003	P21333-2;P21333	Filamin-A	FLNA
UC vs AC FET T2	2,9302452	0,0014	P22352	Glutathione peroxidase 3	GPX3
UC vs AC FET T2	1,9259574	0,02091	P22792	Carboxypeptidase N subunit 2	CPN2
UC vs AC FET T2	5,1667467	8,68E-03	P23083	Ig heavy chain V-I region V35	Ig heavy chain V-I region V35
UC vs AC FET T2	2,5240896	0,001298	P23142-4	Fibulin-1	FBLN1
UC vs AC FET T2	-1,679636	0,01803	P23280-3;P23280	Carbonic anhydrase 6	CA6
UC vs AC FET T2	1,8300703	0,006303	P24593	Insulin-like growth factor-binding protein 5	IGFBP5
UC vs AC FET T2	-1,785675	0,04154	P25311	Zinc-alpha-2-glycoprotein	AZGP1
UC vs AC FET T2	4,471864	0,007643	P26927	Hepatocyte growth factor-like protein	MST1
UC vs AC FET T2	4,107342	0,0005284	P27918	Properdin	CFP
UC vs AC FET T2	2,2756607	0,001306	P28370-2;P28370	Probable global transcription activator SNF2L1	SMARCA1
UC vs AC FET T2	1,5070055	0,006691	P29622	Kallistatin	SERPINA4
UC vs AC FET T2	-0,868934	0,00108	P30101	Protein disulfide-isomerase A3	PDIA3
UC vs AC FET T2	-2,890497	0,01047	P31025;Q5VSP4	Lipocalin-1	LCN1
UC vs AC FET T2	-2,628507	0,01696	P31151;Q86SG5	Protein S100-A7	S100A7
UC vs AC FET T2	1,2800438	0,001674	P33151;P33151-2	Cadherin-5	CDH5
UC vs AC FET T2	1,6865734	0,01281	P34096	Ribonuclease 4	RNASE4
UC vs AC FET T2	1,8371236	0,03183	P35443	Thrombospondin-4	THBS4
UC vs AC FET T2	6,2332366	0,0001044	P35542	Serum amyloid A-4 protein	SAA4
UC vs AC FET T2	2,888778	0,03301	P35556	Fibrillin-2	FBN2
UC vs AC FET T2	2,8177922	0,0001125	P35858;P35858-2	Insulin-like growth factor-binding protein complex acid labile subunit	IGFALS
UC vs AC FET T2	1,6572225	0,02393	P36955;CON__Q95121	Pigment epithelium-derived factor	SERPINF1
UC vs AC FET T2	1,7581456	0,009147	P36980-2;P36980	Complement factor H-related protein 2	CFHR2
UC vs AC FET T2	2,0447911	0,001985	P39060-2;P39060-1;P39060	Collagen alpha-1(XVIII) chain	COL18A1
UC vs AC FET T2	2,6451175	0,000601	P48740	Mannan-binding lectin serine protease 1	MASP1
UC vs AC FET T2	0,8376305	0,02048	P48740-2	Mannan-binding lectin serine protease 1	MASP1
UC vs AC FET T2	3,8567145	7,92E-03	P48740-4	Mannan-binding lectin serine protease 1	MASP1
UC vs AC FET T2	-1,416993	0,03293	P49619-3;P49619-2;P49619	Diacylglycerol kinase gamma	DGKG
UC vs AC FET T2	-1,003186	0,02301	P49767	Vascular endothelial growth factor C	VEGFC
UC vs AC FET T2	0,7875865	0,01715	P49908	Selenoprotein P	SEPP1
UC vs AC FET T2	1,6475036	0,04302	P49913	Cathelicidin antimicrobial peptide	CAMP
UC vs AC FET T2	0,9516413	0,01629	P51884;CON__Q05443	Lumican	LUM
UC vs AC FET T2	1,3421301	0,006255	P54132	Bloom syndrome protein	BLM
UC vs AC FET T2	1,9058349	0,02406	P55056	Apolipoprotein C-IV	APOC4
UC vs AC FET T2	1,8506333	0,008818	P55103	Inhibin beta C chain	INHBC
UC vs AC FET T2	-0,546382	0,04842	P55285-2;P55285	Cadherin-6	CDH6
UC vs AC FET T2	-0,894714	0,04325	P55774	C-C motif chemokine 18	CCL18
UC vs AC FET T2	2,6494828	0,00186	P57077;P57077-1	MAP3K7 C-terminal-like protein	MAP3K7CL
UC vs AC FET T2	1,6187964	0,01819	P58166	Inhibin beta E chain	INHBE
UC vs AC FET T2	3,031625	0,001889	P60709	Actin, cytoplasmic 1	ACTB
UC vs AC FET T2	1,91037	0,0005227	P60900;P60900-2;P60900-3	Proteasome subunit alpha type-6	PSMA6
UC vs AC FET T2	4,1282937	0,001024	P61626	Lysozyme C	LYZ
UC vs AC FET T2	-3,013116	0,004006	P61769	Beta-2-microglobulin	B2M
UC vs AC FET T2	-0,864554	0,0433	P61981	14-3-3 protein gamma	YWHAG
UC vs AC FET T2	-1,502251	0,03891	P62979;P62987;P0CG47;P0CG48	Ubiquitin-40S ribosomal protein S27a	RPS27A;UBA52;UBB;UBC
UC vs AC FET T2	1,2581398	0,0008045	P63104;P63104-2	14-3-3 protein zeta/delta	YWHAZ
UC vs AC FET T2	-1,693896	0,0005454	P67936	Tropomyosin alpha-4 chain	TPM4
UC vs AC FET T2	-3,283279	0,0218	P68363;P68363-2	Tubulin alpha-1B chain	TUBA1B
UC vs AC FET T2	1,7078798	0,0008633	P80108;P80108-2	Phosphatidylinositol-glycan-specific phospholipase D	GPLD1
UC vs AC FET T2	3,4197953	3,52E-02	P80748	Ig lambda chain V-III region LOI	Ig lambda chain V-III region LOI
UC vs AC FET T2	-1,959331	0,01681	Q00889-2;Q00889	Pregnancy-specific beta-1-glycoprotein 6	PSG6
UC vs AC FET T2	2,2184723	0,0002593	Q02224;Q02224-3	Centromere-associated protein E	CENPE
UC vs AC FET T2	-2,530849	0,04132	Q02487-2;Q02487	Desmocollin-2	DSC2
UC vs AC FET T2	2,5764008	0,002185	Q02985-2;Q02985	Complement factor H-related protein 3	CFHR3
UC vs AC FET T2	1,290611	0,03756	Q03001;Q03001-9;Q03001-13;Q03001-10	Dystonin	DST
UC vs AC FET T2	2,8725712	0,0003209	Q03591	Complement factor H-related protein 1	CFHR1
UC vs AC FET T2	0,9924066	0,01537	Q04756	Hepatocyte growth factor activator	HGFAC
UC vs AC FET T2	3,313432	0,0006076	Q06033-2;Q06033	Inter-alpha-trypsin inhibitor heavy chain H3	ITIH3
UC vs AC FET T2	1,8077684	0,0135	Q06190	Serine/threonine-protein phosphatase 2A regulatory subunit B subunit alpha	PPP2R3A
UC vs AC FET T2	2,2670879	0,04432	Q07954;Q07954-2	Prolow-density lipoprotein receptor-related protein 1	LRP1
UC vs AC FET T2	0,7952292	0,04389	Q08380	Galectin-3-binding protein	LGALS3BP
UC vs AC FET T2	1,7410745	0,005211	Q0VAK6;Q0VAK6-2	Leiomodin-3	LMOD3
UC vs AC FET T2	1,4452245	0,03728	Q12805-2;Q12805-4;Q12805-3;Q12805	EGF-containing fibulin-like extracellular matrix protein 1	EFEMP1
UC vs AC FET T2	2,0852835	0,0192	Q13103	Secreted phosphoprotein 24	SPP2
UC vs AC FET T2	2,7394437	0,01925	Q13201;Q13201-2	Multimerin-1	MMRN1
UC vs AC FET T2	2,7614788	3,32E-02	Q13214-2;Q13214	Semaphorin-3B	SEMA3B
UC vs AC FET T2	7,8780509	0,0005185	Q13219	Pappalysin-1	PAPPA
UC vs AC FET T2	1,4193013	0,005982	Q13418-2;Q13418;Q13418-3	Integrin-linked protein kinase	ILK
UC vs AC FET T2	5,1725293	0,009402	Q13488-2	V-type proton ATPase 116 kDa subunit a isoform 3	TCIRG1
UC vs AC FET T2	2,7737447	0,0004393	Q13635-2	.	.
UC vs AC FET T2	1,582814	0,002219	Q14520-2;Q14520	Hyaluronan-binding protein 2	HABP2
UC vs AC FET T2	2,0137994	0,01266	Q15022	Polycomb protein SUZ12	SUZ12
UC vs AC FET T2	5,553957	1,22E-02	Q15386;Q15386-2	Ubiquitin-protein ligase E3C	UBE3C
UC vs AC FET T2	5,3692822	0,0001213	Q15582	Transforming growth factor-beta-induced protein ig-h3	TGFBI
UC vs AC FET T2	-0,975858	0,03157	Q16557	Pregnancy-specific beta-1-glycoprotein 3	PSG3

UC vs AC FET T2	1,5619195	0,02059	Q16610-4;Q16610;Q16610-2	Extracellular matrix protein 1	ECM1
UC vs AC FET T2	2,3458811	0,009677	Q16769;Q16769-2	Glutaminyl-peptide cyclotransferase	QPCT
UC vs AC FET T2	2,0429328	9,68E-03	Q4L180-3;Q4L180-7;Q4L180-5;Q4L180-2;Q4L180;Q4L180-6	Filamin A-interacting protein 1-like	FILIP1L
UC vs AC FET T2	1,8985283	0,0007853	Q4LDE5;Q4LDE5-4;Q4LDE5-3;Q4LDE5-2	Sushi, von Willebrand factor type A, EGF and pentraxin domain-containing protein 1	SVEP1
UC vs AC FET T2	2,2460944	0,04304	Q5JPF3;Q5JPF3-2;A6QL64-3;Q5JPF3-3	Ankyrin repeat domain-containing protein 36C	ANKRD36C;ANKRD36
UC vs AC FET T2	0,5824321	0,01345	Q5T0U0;Q5T0U0-2	Coiled-coil domain-containing protein 122	CCDC122
UC vs AC FET T2	3,4447553	0,002689	Q6P1M0-2	Long-chain fatty acid transport protein 4	SLC27A4
UC vs AC FET T2	2,1906326	0,004873	Q6Q788	Apolipoprotein A-V	APOA5
UC vs AC FET T2	1,6012027	0,0004306	Q6UVK1	Chondroitin sulfate proteoglycan 4	CSPG4
UC vs AC FET T2	3,8669548	0,002721	Q6UXH9-2;Q6UXH9-3;Q6UXH9	Inactive serine protease PAMR1	PAMR1
UC vs AC FET T2	3,5508791	4,02E-02	Q6UY14-3;Q6UY14;Q6UY14-2	ADAMTS-like protein 4	ADAMTSL4
UC vs AC FET T2	1,7780163	0,005923	Q76LX8;Q76LX8-2;Q76LX8-3;Q76LX8-4	A disintegrin and metalloproteinase with thrombospondin motifs 13	ADAMTS13
UC vs AC FET T2	-1,41593	0,006027	Q7L1Q6-2;Q7L1Q6;Q7L1Q6-3	Basic leucine zipper and W2 domain-containing protein 1	BZW1
UC vs AC FET T2	-2,943925	0,002286	Q7LBC6-3	Lysine-specific demethylase 3B	KDM3B
UC vs AC FET T2	-0,801313	0,02504	Q7Z572	Spermatogenesis-associated protein 21	SPATA21
UC vs AC FET T2	1,3642547	0,04626	Q86U17	Serpin A11	SERPINA11
UC vs AC FET T2	1,903113	0,01884	Q86UQ4;Q86UQ4-4;Q86UQ4-3;Q86UQ4-6;Q86UQ4-7;Q86UQ4-5	ATP-binding cassette sub-family A member 13	ABCA13
UC vs AC FET T2	1,4221378	0,005433	Q8IVL1-11;Q8IVL1-4	Neuron navigator 2	NAV2
UC vs AC FET T2	0,754099	0,007459	Q8IZK6-2;Q8IZK6	Mucolipin-2	MCOLN2
UC vs AC FET T2	-1,984927	0,0005396	Q8IZP9-9;Q8IZP9-10;Q8IZP9-8;Q8IZP9-7;Q8IZP9-5;Q8IZP9-6;Q8IZP9-3;Q8IZP9-4;Q8IZP9-2;Q8IZP9	G-protein coupled receptor 64	GPR64
UC vs AC FET T2	3,4198889	0,01162	Q8N7Z5;Q8WY50	Putative ankyrin repeat domain-containing protein 31	ANKRD31
UC vs AC FET T2	-1,593526	0,01826	Q8N8A2-4;Q8N8A2;Q8N8A2-2;Q8N8A2-3;Q8N8A2-5	Serine/threonine-protein phosphatase 6 regulatory ankyrin repeat subunit B	ANKRD44
UC vs AC FET T2	4,248429	1,47E-02	Q8NBP7	Proprotein convertase subtilisin/kexin type 9	PCSK9
UC vs AC FET T2	-2,760787	0,01081	Q8ND83-3;Q8ND83-2;Q8ND83-4	SLAIN motif-containing protein 1	SLAIN1
UC vs AC FET T2	1,5951841	0,0001293	Q8NDV7-6;Q8NDV7;Q8NDV7-2;Q8NDV7-5;Q8NDV7-4;Q8NDV7-3	Trinucleotide repeat-containing gene 6A protein	TNRC6A
UC vs AC FET T2	2,735602	0,00936	Q8NGK2	Olfactory receptor 52B4	OR52B4
UC vs AC FET T2	1,4889302	0,01153	Q8NI99	Angiopoietin-related protein 6	ANGPTL6
UC vs AC FET T2	-0,535787	0,04762	Q8TDL5;Q8TDL5-2	BPI fold-containing family B member 1	BPIFB1
UC vs AC FET T2	2,1805502	0,006956	Q8TE73	Dynein heavy chain 5, axonemal	DNAH5
UC vs AC FET T2	1,1119378	0,01851	Q8WUA8	Tsukushin	TSKU
UC vs AC FET T2	2,0625175	0,01124	Q8WWZ8;Q8WWZ8-2	Oncoprotein-induced transcript 3 protein	OIT3
UC vs AC FET T2	3,5251903	0,01295	Q8WZ42-5	Titin	TTN
UC vs AC FET T2	1,2306974	0,01723	Q92496;Q92496-2;Q92496-3	Complement factor H-related protein 4	CFHR4
UC vs AC FET T2	1,6101651	0,005441	Q92743	Serine protease HTRA1	HTRA1
UC vs AC FET T2	1,8899162	0,02705	Q92954-3;Q92954-6;Q92954;Q92954-4;Q92954-2;Q92954-5	Proteoglycan 4	PRG4
UC vs AC FET T2	-0,991221	0,02344	Q96CM8-3;Q96CM8-4;Q96CM8;Q96CM8-2	Acyl-CoA synthetase family member 2, mitochondrial	ACSF2
UC vs AC FET T2	3,126056	0,009783	Q96IY4;Q96IY4-2;CON__Q2KIG3	Carboxypeptidase B2	CPB2
UC vs AC FET T2	0,9213372	0,02378	Q96JB1-2;Q96JB1	Dynein heavy chain 8, axonemal	DNAH8
UC vs AC FET T2	1,8859031	0,006172	Q96KN2	Beta-Ala-His dipeptidase	CNDP1
UC vs AC FET T2	3,1485677	0,01051	Q96PD5;Q96PD5-2	N-acetylmuramoyl-L-alanine amidase	PGLYRP2
UC vs AC FET T2	-2,385878	0,0247	Q96QR1	Secretoglobin family 3A member 1	SCGB3A1
UC vs AC FET T2	4,0922808	2,17E-02	Q96RL1-3;Q96RL1-4	BRCA1-A complex subunit RAP80	UIMC1
UC vs AC FET T2	0,7303585	0,01045	Q99574	Neuroserpin	SERPINI1
UC vs AC FET T2	1,4793308	0,0007273	Q99969	Retinoic acid receptor responder protein 2	RARRES2
UC vs AC FET T2	-1,819998	0,02801	Q99996-3;Q99996;Q99996-6;Q99996-5;Q99996-1;Q99996-4	A-kinase anchor protein 9	AKAP9
UC vs AC FET T2	-0,781229	0,03644	Q9BQE3	Tubulin alpha-1C chain	TUBA1C
UC vs AC FET T2	1,9973881	3,66E-02	Q9BXR6	Complement factor H-related protein 5	CFHR5
UC vs AC FET T2	-0,884557	0,04181	Q9BY76-3;Q9BY76;Q9BY76-2	Angiopoietin-related protein 4	ANGPTL4
UC vs AC FET T2	1,2813779	0,007488	Q9BZR9	Probable E3 ubiquitin-protein ligase TRIM8	TRIM8
UC vs AC FET T2	1,6784317	0,004565	Q9HDC9;Q9HDC9-2	Adipocyte plasma membrane-associated protein	APMAP
UC vs AC FET T2	-0,757644	0,03257	Q9NPH2-2;Q9NPH2-3;Q9NPH2	Inositol-3-phosphate synthase 1	ISYNA1
UC vs AC FET T2	6,6229908	0,003714	Q9NQ79;Q9NQ79-2;Q9NQ79-3	Cartilage acidic protein 1	CRTAC1
UC vs AC FET T2	-1,105904	0,03184	Q9NRA1;Q9NRA1-3;Q9NRA1-2;Q9NRA1-4	Platelet-derived growth factor C	PDGFC
UC vs AC FET T2	1,1447257	0,03884	Q9NY15;Q9NY15-2	Stabilin-1	STAB1
UC vs AC FET T2	-3,257993	0,02487	Q9NZT1	Calmodulin-like protein 5	CALML5
UC vs AC FET T2	3,646579	0,001418	Q9UHG3	Prenylcysteine oxidase 1	PCYOX1
UC vs AC FET T2	2,4369462	0,009864	Q9UK55	Protein Z-dependent protease inhibitor	SERPINA10
UC vs AC FET T2	1,1354135	0,00204	Q9Y4C2-2;Q9Y4C2	TRPM8 channel-associated factor 1	TCAF1
UC vs AC FET T3	3,0377158	0,04724	A0A075B6H7;A0A0C4DH55	.	IGKV3-7
UC vs AC FET T3	2,5877884	1,29E-02	A0A075B6I0	.	IGLV8-61
UC vs AC FET T3	3,6432261	0,01134	A0A075B6J9	.	IGLV2-18
UC vs AC FET T3	1,693516	0,03504	A0A075B6P5;P01615	Ig kappa chain V-II region FR	IGKV2D-28
UC vs AC FET T3	1,7814027	0,009523	A0A0A0MS15	.	IGHV3-49
UC vs AC FET T3	0,8853906	0,003331	A0A0B4J1V0	.	IGHV3-15
UC vs AC FET T3	1,5956321	0,03579	A0A0B4J1X5	.	IGHV3-74
UC vs AC FET T3	4,183794	0,007588	A0A0B4J1Y9	.	IGHV3-72
UC vs AC FET T3	1,3311015	0,003945	A0A0C4DH33	.	IGHV1-24
UC vs AC FET T3	3,4612028	0,024	A0A0C4DH35	.	IGHV3-35
UC vs AC FET T3	-2,170336	0,006923	A0A0C4DH38	.	IGHV5-51

UC vs AC FET T3	1,6523865	0,004306	A0A0C4DH67;A0A0C4DH69	.	IGKV1-8;IGKV1-9
UC vs AC FET T3	3,7593628	0,006849	A0A0J9YX35	.	.
UC vs AC FET T3	-5,748448	0,0004034	A4FU69-3;A4FU69;A4FU69-2;A4FU69-4;A4FU69-6	EF-hand calcium-binding domain-containing protein 5	EFCAB5
UC vs AC FET T3	2,1361635	0,0001526	A8K2U0;A8K2U0-2	Alpha-2-macroglobulin-like protein 1	A2ML1
UC vs AC FET T3	3,8408926	0,006695	O00187;O00187-2	Mannan-binding lectin serine protease 2	MASP2
UC vs AC FET T3	-3,707067	0,03774	O00217	NADH dehydrogenase [ubiquinone] iron-sulfur protein 8, mitochondrial	NDUFS8
UC vs AC FET T3	-1,795495	0,04078	O00602	Ficolin-1	FCN1
UC vs AC FET T3	1,5136912	0,02135	O14791-2;O14791;O14791-3	Apolipoprotein L1	APOL1
UC vs AC FET T3	4,7697361	0,01209	O43184-3;O43184-4;O43184-2;O43184	Disintegrin and metalloproteinase domain-containing protein 12	ADAM12
UC vs AC FET T3	2,0050236	0,0115	O43399;O43399-5;O43399-7;O43399-2;O43399-4;O43399-3	Tumor protein D54	TPD52L2
UC vs AC FET T3	2,1627055	0,01429	O43861-2;O43861	Probable phospholipid-transporting ATPase IIB	ATP9B
UC vs AC FET T3	3,0990369	0,006666	O75636;O75636-2	Ficolin-3	FCN3
UC vs AC FET T3	5,8608584	0,0004271	O75882;O75882-2;O75882-3	Attractin	ATRN
UC vs AC FET T3	1,7676437	0,002493	O76076	WNT1-inducible-signaling pathway protein 2	WISP2
UC vs AC FET T3	-2,882843	0,0001903	O94855;O94855-2	Protein transport protein Sec24D	SEC24D
UC vs AC FET T3	1,2964536	0,003986	O95428-6;O95428;O95428-5;O95428-4;O95428-2;O95428-3	Papilin	PAPLN
UC vs AC FET T3	5,7260967	1,82E-02	O95445-2	Apolipoprotein M	APOM
UC vs AC FET T3	1,0877516	0,006055	P00450	Ceruloplasmin	CP
UC vs AC FET T3	1,163379	0,007175	P00488	Coagulation factor XIII A chain	F13A1
UC vs AC FET T3	4,1090475	0,01149	P00709	Alpha-lactalbumin	LALBA
UC vs AC FET T3	0,8321183	0,007575	P00734	Prothrombin	F2
UC vs AC FET T3	1,5489929	0,001113	P00736	Complement C1r subcomponent	C1R
UC vs AC FET T3	1,5391028	0,03926	P00739;P00739-2	Haptoglobin-related protein	HPR
UC vs AC FET T3	3,6257732	2,22E-02	P00740;P00740-2	Coagulation factor IX	F9
UC vs AC FET T3	2,1763275	0,004428	P00742	Coagulation factor X	F10
UC vs AC FET T3	1,7167075	0,0004871	P00747;Q02325	Plasminogen	PLG
UC vs AC FET T3	2,1561301	0,009609	P00748	Coagulation factor XII	F12
UC vs AC FET T3	2,2522008	0,0234	P00751;P00751-2	Complement factor B	CFB
UC vs AC FET T3	-1,046523	0,01457	P01009;P01009-2;P01009-3;P20848	Alpha-1-antitrypsin	SERPINA1
UC vs AC FET T3	0,6607473	0,02778	P01024;O95568	Complement C3	C3
UC vs AC FET T3	1,3951471	0,01721	P01031	Complement C5	C5
UC vs AC FET T3	-2,28671	0,000681	P01040	Cystatin-A	CSTA
UC vs AC FET T3	0,9232838	0,02556	P01591	Immunoglobulin J chain	IGJ
UC vs AC FET T3	3,5894081	0,0005306	P01599	Ig kappa chain V-I region Gal	Ig kappa chain V-I region Gal
UC vs AC FET T3	2,2589242	0,0005583	P01619	Ig kappa chain V-III region B6	Ig kappa chain V-III region B6
UC vs AC FET T3	2,8139398	0,003989	P01700	Ig lambda chain V-I region HA	Ig lambda chain V-I region HA
UC vs AC FET T3	2,4862497	0,004727	P01701	Ig lambda chain V-I region NEW	Ig lambda chain V-I region NEW
UC vs AC FET T3	2,1267512	0,006791	P01709	Ig lambda chain V-II region MGC	Ig lambda chain V-II region MGC
UC vs AC FET T3	-2,432538	0,009957	P01715	Ig lambda chain V-IV region Bau	Ig lambda chain V-IV region Bau
UC vs AC FET T3	5,6131338	2,11E-04	P01743	Ig heavy chain V-I region HG3	Ig heavy chain V-I region HG3
UC vs AC FET T3	-2,218075	0,001091	P01764	Ig heavy chain V-III region 23	IGHV3-23
UC vs AC FET T3	4,3360735	0,003236	P0DP03;P01768	Ig heavy chain V-III region CAM	Ig heavy chain V-III region CAM
UC vs AC FET T3	5,2863977	0,0006261	P01782;P0DP04	Ig heavy chain V-III region DOB	Ig heavy chain V-III region DOB
UC vs AC FET T3	-2,217922	0,0003759	P01834	Ig kappa chain C region	IGKC
UC vs AC FET T3	1,3219736	0,01864	P01859	Ig gamma-2 chain C region	IGHG2
UC vs AC FET T3	1,4176786	0,01396	P01860	Ig gamma-3 chain C region	IGHG3
UC vs AC FET T3	-1,779301	0,0488	P01861	Ig gamma-4 chain C region	IGHG4
UC vs AC FET T3	1,4768283	0,004598	P01871;P01871-2	Ig mu chain C region	IGHM
UC vs AC FET T3	1,7263138	0,0005263	P01876	Ig alpha-1 chain C region	IGHA1
UC vs AC FET T3	0,9901445	0,001038	P02647;Q9HB71-2	Apolipoprotein A-I	APOA1
UC vs AC FET T3	1,3736327	0,01097	P02649;CON__Q03247	Apolipoprotein E	APOE
UC vs AC FET T3	0,5735018	0,001378	P02654	Apolipoprotein C-I	APOC1
UC vs AC FET T3	-1,050679	0,04176	P02655	Apolipoprotein C-II	APOC2
UC vs AC FET T3	1,8276403	0,002978	P02671;P02671-2;REV__Q9UKV0-4;REV__Q9UKV0-2;REV__Q9UKV0;REV__Q9UKV0-5;REV__Q9UKV0-7;Q14314	Fibrinogen alpha chain	FGA
UC vs AC FET T3	2,0081384	0,002131	P02675	Fibrinogen beta chain	FGB
UC vs AC FET T3	1,1342623	0,002094	P02679;P02679-2	Fibrinogen gamma chain	FGG
UC vs AC FET T3	1,0939157	0,0494	P02743	Serum amyloid P-component	APCS
UC vs AC FET T3	1,8238685	0,000548	P02746	Complement C1q subcomponent subunit B	C1QB
UC vs AC FET T3	2,2435453	4,40E-02	P02747	Complement C1q subcomponent subunit C	C1QC
UC vs AC FET T3	0,9958045	0,01159	P02753	Retinol-binding protein 4	RBP4
UC vs AC FET T3	-1,036603	0,0117	P02760	Protein AMBP	AMBP
UC vs AC FET T3	1,135155	0,01127	P02766	Transthyretin	TTR
UC vs AC FET T3	1,7540739	0,001255	P02774-3;P02774;P02774-2	Vitamin D-binding protein	GC
UC vs AC FET T3	4,0148962	0,0007776	P02775	Platelet basic protein	PPBP
UC vs AC FET T3	5,1176322	0,0002528	P02776	Platelet factor 4	PF4
UC vs AC FET T3	1,4934099	0,00178	P02790	Hemopexin	HPX
UC vs AC FET T3	4,8393657	0,0007586	P03950	Angiogenin	ANG
UC vs AC FET T3	5,0415704	0,0002551	P03951	Coagulation factor XI	F11
UC vs AC FET T3	2,0270913	0,0001034	P03952;P20718	Plasma kallikrein	KLKB1
UC vs AC FET T3	-1,342071	0,04914	P03973	Antileukoproteinase	SLPI
UC vs AC FET T3	1,9224424	0,03671	P04040	Catalase	CAT
UC vs AC FET T3	0,87374	0,01111	P04114	Apolipoprotein B-100	APOB
UC vs AC FET T3	1,3124125	0,006443	P04180	Phosphatidylcholine-sterol acyltransferase	LCAT
UC vs AC FET T3	-1,806834	0,008737	P04196	Histidine-rich glycoprotein	HRG
UC vs AC FET T3	5,4084282	0,0001503	P04211;A0A075B6I9	Ig lambda chain V region 4A	IGLV7-46
UC vs AC FET T3	1,2760665	0,02826	P04217	Alpha-1B-glycoprotein	A1BG
UC vs AC FET T3	4,2844169	0,02672	P04275	von Willebrand factor	VWF
UC vs AC FET T3	1,6138222	0,007745	P04433;A0A0A0MRZ8	Ig kappa chain V-III region VG	IGKV3D-11
UC vs AC FET T3	-0,811324	0,02065	P05019-3;P05019-2;P05019-4;P05019	Insulin-like growth factor I	IGF1
UC vs AC FET T3	3,7988104	0,003202	P05023-2;P05023-4;P05023;P05023-3	Sodium/potassium-transporting ATPase subunit alpha-1	ATP1A1
UC vs AC FET T3	1,6965034	0,004611	P05160	Coagulation factor XIII B chain	F13B
UC vs AC FET T3	1,6617744	0,007708	P05546	Heparin cofactor 2	SERPIND1

UC vs AC FET T3	3,3802173	0,003612	P06331	Ig heavy chain V-II region ARH-77	Ig heavy chain V-II region ARH-77
UC vs AC FET T3	2,7665265	0,005805	P06753-5;P06753-2;P06753-4;P06753-3;P06753-6;P06753-7	Tropomyosin alpha-3 chain	TPM3
UC vs AC FET T3	0,6506623	0,01475	P06850	Corticoliberin	CRH
UC vs AC FET T3	1,0403788	0,004393	P07225	Vitamin K-dependent protein S	PROS1
UC vs AC FET T3	1,4388025	0,01439	P07237	Protein disulfide-isomerase	P4HB
UC vs AC FET T3	0,943449	0,001686	P07333;P07333-2	Macrophage colony-stimulating factor 1 receptor	CSF1R
UC vs AC FET T3	2,0422618	0,02533	P07357	Complement component C8 alpha chain	C8A
UC vs AC FET T3	2,3037198	0,0134	P07358	Complement component C8 beta chain	C8B
UC vs AC FET T3	3,1203992	0,02583	P07996;P07996-2	Thrombospondin-1	THBS1
UC vs AC FET T3	1,8699367	0,004131	P08294	Extracellular superoxide dismutase [Cu-Zn]	SOD3
UC vs AC FET T3	4,4487088	7,77E-04	P08493-2;P08493	Matrix Gla protein	MGP
UC vs AC FET T3	2,5516428	0,02688	P08567	Pleckstrin	PLEK
UC vs AC FET T3	1,1640795	0,02474	P08571	Monocyte differentiation antigen CD14	CD14
UC vs AC FET T3	1,6845653	3,91E-02	P08603;P08603-2	Complement factor H	CFH
UC vs AC FET T3	0,7697656	0,04946	P08697;P08697-2	Alpha-2-antiplasmin	SERPINF2
UC vs AC FET T3	2,4153202	0,00192	P08709-2;P08709	Coagulation factor VII	F7
UC vs AC FET T3	2,3764491	3,19E-02	P09382	Galectin-1	LGALS1
UC vs AC FET T3	-3,187596	0,000407	P09466-2;P09466	Glycodelin	PAEP
UC vs AC FET T3	2,1101427	0,009566	P09466-3	Glycodelin	PAEP
UC vs AC FET T3	3,7298892	0,003211	P09486	SPARC	SPARC
UC vs AC FET T3	1,1968863	0,0004055	P09871	Complement C1s subcomponent	C1S
UC vs AC FET T3	-2,604058	0,03453	P0C0L4;P0C0L4-2	Complement C4-A	C4A
UC vs AC FET T3	1,5146954	0,0003691	P0C0L5	Complement C4-B	C4B
UC vs AC FET T3	1,2026637	0,02737	P0DJI8	Serum amyloid A-1 protein	SAA1
UC vs AC FET T3	-1,591672	0,003177	P0DN87;P0DN86;P0DN86-2;Q6NT52;A6NKQ9-2;A6NKQ9	Choriogonadotropin subunit beta variant 2	CGB2;CGB1
UC vs AC FET T3	3,1002982	0,002372	P0DOX2	.	.
UC vs AC FET T3	2,6943545	0,04687	P0DOX4	.	.
UC vs AC FET T3	1,5308607	0,0002844	P0DOX5;P01857	Ig gamma-1 chain C region	IGHG1
UC vs AC FET T3	1,2997038	0,003592	P0DOX7	.	.
UC vs AC FET T3	1,847523	0,0003776	P0DOX8	.	.
UC vs AC FET T3	3,1364371	0,0235	P10720	Platelet factor 4 variant	PF4V1
UC vs AC FET T3	1,5509533	0,03159	P11226	Mannose-binding protein C	MBL2
UC vs AC FET T3	1,4340091	1,70E-05	P11464-4	Pregnancy-specific beta-1-glycoprotein 1	PSG1
UC vs AC FET T3	2,4411339	0,007575	P11465	Pregnancy-specific beta-1-glycoprotein 2	PSG2
UC vs AC FET T3	2,593281	0,0003866	P12259	Coagulation factor V	F5
UC vs AC FET T3	-1,774047	0,03325	P12273	Prolactin-inducible protein	PIP
UC vs AC FET T3	4,8126908	0,001223	P12814;P12814-3;P12814-2	Alpha-actinin-1	ACTN1
UC vs AC FET T3	1,9551405	0,04986	P13497;P13497-5;P13497-2;P13497-6;P13497-4;P13497-3	Bone morphogenetic protein 1	BMP1
UC vs AC FET T3	1,8105365	0,0009268	P13667	Protein disulfide-isomerase A4	PDIA4
UC vs AC FET T3	3,5166137	1,13E-03	P13727;P13727-2	Bone marrow proteoglycan	PRG2
UC vs AC FET T3	2,0461561	0,01366	P15907	Beta-galactoside alpha-2,6-sialyltransferase 1	ST6GAL1
UC vs AC FET T3	-1,556513	0,009093	P16035	Metalloproteinase inhibitor 2	TIMP2
UC vs AC FET T3	3,6656732	0,0003842	P16885	1-phosphatidylinositol 4,5-bisphosphate phosphodiesterase gamma-2	PLCG2
UC vs AC FET T3	3,9908983	0,04441	P18206;P18206-2;P18206-3	Vinculin	VCL
UC vs AC FET T3	0,7426059	0,007348	P19823	Inter-alpha-trypsin inhibitor heavy chain H2	ITIH2
UC vs AC FET T3	0,9237225	0,04388	P19827	Inter-alpha-trypsin inhibitor heavy chain H1	ITIH1
UC vs AC FET T3	4,8137422	0,009718	P21333-2;P21333	Filamin-A	FLNA
UC vs AC FET T3	2,7204278	0,0001525	P22352	Glutathione peroxidase 3	GPX3
UC vs AC FET T3	5,0200488	0,001579	P23083	Ig heavy chain V-I region V35	Ig heavy chain V-I region V35
UC vs AC FET T3	2,8237102	0,0008089	P23142-4	Fibulin-1	FBLN1
UC vs AC FET T3	-2,024907	0,01018	P23280-3;P23280	Carbonic anhydrase 6	CA6
UC vs AC FET T3	2,4302244	0,002575	P24593	Insulin-like growth factor-binding protein 5	IGFBP5
UC vs AC FET T3	4,7525792	0,005138	P26927	Hepatocyte growth factor-like protein	MST1
UC vs AC FET T3	1,3092178	0,0446	P28370-2;P28370	Probable global transcription activator SNF2L1	SMARCA1
UC vs AC FET T3	1,4642831	0,03475	P30043	Flavin reductase (NADPH)	BLVRB
UC vs AC FET T3	-4,283841	0,003455	P31025;Q5VSP4	Lipocalin-1	LCN1
UC vs AC FET T3	-2,891507	0,006594	P31151;Q86SG5	Protein S100-A7	S100A7
UC vs AC FET T3	1,8116734	0,001932	P35443	Thrombospondin-4	THBS4
UC vs AC FET T3	1,0241916	0,002456	P35555	Fibrillin-1	FBN1
UC vs AC FET T3	1,3667979	0,0007533	P35556	Fibrillin-2	FBN2
UC vs AC FET T3	6,6294705	2,64E-02	P35579;P35579-2	Myosin-9	MYH9
UC vs AC FET T3	2,5531294	0,0003309	P35858;P35858-2	Insulin-like growth factor-binding protein complex acid labile subunit	IGFALS
UC vs AC FET T3	1,6481181	0,009635	P39060-2;P39060-1;P39060	Collagen alpha-1(XVIII) chain	COL18A1
UC vs AC FET T3	3,1068079	0,01607	P48059;P48059-4;P48059-2;P48059-5;P48059-3;Q7Z4I7-4;Q7Z4I7-3;Q7Z4I7-2;P0CW19-2	LIM and senescent cell antigen-like-containing domain protein 1	LIMS1
UC vs AC FET T3	1,2809386	0,0007767	P48307-2;P48307	Tissue factor pathway inhibitor 2	TFPI2
UC vs AC FET T3	2,1706617	0,0001552	P48740	Mannan-binding lectin serine protease 1	MASP1
UC vs AC FET T3	0,8615286	0,000656	P48740-2	Mannan-binding lectin serine protease 1	MASP1
UC vs AC FET T3	4,4975239	1,65E-04	P48740-4	Mannan-binding lectin serine protease 1	MASP1
UC vs AC FET T3	-1,302802	0,009383	P49767	Vascular endothelial growth factor C	VEGFC
UC vs AC FET T3	0,6946862	0,04498	P49908	Selenoprotein P	SEPP1
UC vs AC FET T3	1,7656817	0,005453	P49913	Cathelicidin antimicrobial peptide	CAMP
UC vs AC FET T3	2,1058661	0,003901	P54132	Bloom syndrome protein	BLM
UC vs AC FET T3	1,1665766	0,0465	P55056	Apolipoprotein C-IV	APOC4
UC vs AC FET T3	-0,791329	0,01428	P55058;P55058-4;P55058-3;P55058-2	Phospholipid transfer protein	PLTP
UC vs AC FET T3	1,6652439	0,0006386	P55103	Inhibin beta C chain	INHBC
UC vs AC FET T3	3,010348	0,03931	P60660;P60660-2;P14649	Myosin light polypeptide 6	MYL6
UC vs AC FET T3	4,5068905	0,003208	P60709	Actin, cytoplasmic 1	ACTB
UC vs AC FET T3	1,7418934	0,01271	P60900;P60900-2;P60900-3	Proteasome subunit alpha type-6	PSMA6
UC vs AC FET T3	3,2960134	0,03736	P61224-3;P61224;P61224-2;P61224-4;A6NIZ1;P62834	Ras-related protein Rap-1b	RAP1B;RAP1A
UC vs AC FET T3	4,9013746	0,01183	P61626	Lysozyme C	LYZ
UC vs AC FET T3	1,0339319	0,0166	P62736;P63267;P63267-2	Actin, aortic smooth muscle	ACTA2;ACTG2
UC vs AC FET T3	-1,337559	0,02173	P62979;P62987;P0CG47;P0CG48	Ubiquitin-40S ribosomal protein S27a	RPS27A;UBA52;UBB;UBC
UC vs AC FET T3	3,7352318	0,01552	P63104;P63104-2	14-3-3 protein zeta/delta	YWHAZ
UC vs AC FET T3	3,3349091	0,01496	P67936	Tropomyosin alpha-4 chain	TPM4

UC vs AC FET T3	2,6545942	0,01487	P69905	Hemoglobin subunit alpha	HBA1
UC vs AC FET T3	2,7356718	0,03271	P80748	Ig lambda chain V-III region LOI	Ig lambda chain V-III region LOI
UC vs AC FET T3	4,1495387	0,005556	Q00526;P11802-2;Q00535-2;Q00535;P11802;Q14004-2;Q14004	Cyclin-dependent kinase 3	CDK3
UC vs AC FET T3	-0,461176	0,03849	Q00887-2	Pregnancy-specific beta-1-glycoprotein 9	PSG9
UC vs AC FET T3	-8,909658	0,01056	Q00888;Q00888-3;Q00888-2	Pregnancy-specific beta-1-glycoprotein 4	PSG4
UC vs AC FET T3	-3,88959	0,03107	Q00889-2;Q00889	Pregnancy-specific beta-1-glycoprotein 6	PSG6
UC vs AC FET T3	1,5575606	0,005113	Q02108-2;Q02108	Guanylate cyclase soluble subunit alpha-3	GUCY1A3
UC vs AC FET T3	3,7782623	0,01986	Q02224;Q02224-3	Centromere-associated protein E	CENPE
UC vs AC FET T3	-3,397175	0,0002985	Q02487-2;Q02487	Desmocollin-2	DSC2
UC vs AC FET T3	2,9335869	0,0006734	Q02985-2;Q02985	Complement factor H-related protein 3	CFHR3
UC vs AC FET T3	2,0608423	0,005646	Q03591	Complement factor H-related protein 1	CFHR1
UC vs AC FET T3	0,7145312	0,02334	Q04756	Hepatocyte growth factor activator	HGFAC
UC vs AC FET T3	1,7547586	0,007496	Q06033-2;Q06033	Inter-alpha-trypsin inhibitor heavy chain H3	ITIH3
UC vs AC FET T3	0,9602807	0,009231	Q12805-2;Q12805-4;Q12805-3;Q12805	EGF-containing fibulin-like extracellular matrix protein 1	EFEMP1
UC vs AC FET T3	1,4998563	0,03412	Q12805-5	EGF-containing fibulin-like extracellular matrix protein 1	EFEMP1
UC vs AC FET T3	1,6898518	0,0003089	Q13093	Platelet-activating factor acetylhydrolase	PLA2G7
UC vs AC FET T3	2,462198	0,00129	Q13103	Secreted phosphoprotein 24	SPP2
UC vs AC FET T3	1,6007886	0,009934	Q13214-2;Q13214	Semaphorin-3B	SEMA3B
UC vs AC FET T3	2,2911881	0,006876	Q13418-2;Q13418;Q13418-3	Integrin-linked protein kinase	ILK
UC vs AC FET T3	4,6654686	5,94E-03	Q13488-2	V-type proton ATPase 116 kDa subunit a isoform 3	TCIRG1
UC vs AC FET T3	2,3209098	8,47E-03	Q13635-2	.	.
UC vs AC FET T3	2,4414418	0,0001704	Q14520-2;Q14520	Hyaluronan-binding protein 2	HABP2
UC vs AC FET T3	0,8204849	0,003636	Q14624;Q14624-3;Q14624-4	Inter-alpha-trypsin inhibitor heavy chain H4	ITIH4
UC vs AC FET T3	1,3227239	0,001837	Q14697;Q14697-2	Neutral alpha-glucosidase AB	GANAB
UC vs AC FET T3	4,4038293	0,002383	Q14766;Q14766-4;Q14766-3;Q14766-2;Q14766-5	Latent-transforming growth factor beta-binding protein 1	LTBP1
UC vs AC FET T3	2,3127516	0,0034	Q15022	Polycomb protein SUZ12	SUZ12
UC vs AC FET T3	5,1531546	0,002992	Q15386;Q15386-2	Ubiquitin-protein ligase E3C	UBE3C
UC vs AC FET T3	2,7762878	0,006618	Q15485;Q15485-2	Ficolin-2	FCN2
UC vs AC FET T3	4,9131229	0,001167	Q15582	Transforming growth factor-beta-induced protein ig-h3	TGFBI
UC vs AC FET T3	3,6888034	0,0002312	Q15848	Adiponectin	ADIPOQ
UC vs AC FET T3	1,4230316	0,003791	Q16610-4;Q16610;Q16610-2	Extracellular matrix protein 1	ECM1
UC vs AC FET T3	0,7500138	0,009931	Q5CZC0;Q5CZC0-2	Fibrous sheath-interacting protein 2	FSIP2
UC vs AC FET T3	2,4575098	0,001823	Q5HYK7-3;Q5HYK7-2;Q5HYK7;Q5HYK7-5;Q5HYK7-4	SH3 domain-containing protein 19	SH3D19
UC vs AC FET T3	2,3801117	0,001839	Q6EEV6	Small ubiquitin-related modifier 4	SUMO4
UC vs AC FET T3	3,8275867	1,57E-02	Q6P1M0-2	Long-chain fatty acid transport protein 4	SLC27A4
UC vs AC FET T3	4,5965895	0,01741	Q6P387-2;Q6P387	Uncharacterized protein C16orf46	C16orf46
UC vs AC FET T3	1,7921892	0,02433	Q6UY14-3;Q6UY14;Q6UY14-2	ADAMTS-like protein 4	ADAMTSL4
UC vs AC FET T3	-1,54232	0,000611	Q6ZS30-1;Q6ZS30	Neurobeachin-like protein 1	NBEAL1
UC vs AC FET T3	1,1218693	0,002896	Q76LX8;Q76LX8-2;Q76LX8-3;Q76LX8-4	A disintegrin and metalloproteinase with thrombospondin motifs 13	ADAMTS13
UC vs AC FET T3	0,5565495	0,009042	Q7Z478	ATP-dependent RNA helicase DHX29	DHX29
UC vs AC FET T3	-1,206092	0,02137	Q7Z572	Spermatogenesis-associated protein 21	SPATA21
UC vs AC FET T3	-1,182624	0,0415	Q7Z5L0	Vitelline membrane outer layer protein 1 homolog	VMO1
UC vs AC FET T3	1,600698	0,003222	Q86U17	Serpin A11	SERPINA11
UC vs AC FET T3	-1,268562	0,0008695	Q86UD1	Out at first protein homolog	OAF
UC vs AC FET T3	4,3943818	0,01119	Q86UX7-2;Q86UX7	Fermitin family homolog 3	FERMT3
UC vs AC FET T3	0,3431509	0,01524	Q8IV50-2;Q8IV50	LysM and putative peptidoglycan-binding domain-containing protein 2	LYSMD2
UC vs AC FET T3	-3,042464	0,01636	Q8IZP9-9;Q8IZP9-10;Q8IZP9-8;Q8IZP9-7;Q8IZP9-5;Q8IZP9-6;Q8IZP9-3;Q8IZP9-4;Q8IZP9-2;Q8IZP9	G-protein coupled receptor 64	GPR64
UC vs AC FET T3	-1,904475	0,006648	Q8N8A2-4;Q8N8A2;Q8N8A2-2;Q8N8A2-3;Q8N8A2-5	Serine/threonine-protein phosphatase 6 regulatory ankyrin repeat subunit B	ANKRD44
UC vs AC FET T3	-3,786447	0,02902	Q8NBJ4-2;Q8NBJ4	Golgi membrane protein 1	GOLM1
UC vs AC FET T3	5,1192101	0,02265	Q8NBP7	Proprotein convertase subtilisin/kexin type 9	PCSK9
UC vs AC FET T3	-4,169212	0,0009914	Q8ND83-3;Q8ND83-2;Q8ND83-4	SLAIN motif-containing protein 1	SLAIN1
UC vs AC FET T3	1,3509516	1,22E-02	Q8NDV7-6;Q8NDV7;Q8NDV7-2;Q8NDV7-5;Q8NDV7-4;Q8NDV7-3	Trinucleotide repeat-containing gene 6A protein	TNRC6A
UC vs AC FET T3	3,0831561	0,01434	Q8NFQ6	BPI fold-containing family C protein	BPIFC
UC vs AC FET T3	2,0727068	0,03	Q8NGK2	Olfactory receptor 52B4	OR52B4
UC vs AC FET T3	2,5648808	1,95E-05	Q8NI99	Angiopoietin-related protein 6	ANGPTL6
UC vs AC FET T3	-1,867383	0,01964	Q8TDL5;Q8TDL5-2	BPI fold-containing family B member 1	BPIFB1
UC vs AC FET T3	2,4298111	0,002181	Q8TE73	Dynein heavy chain 5, axonemal	DNAH5
UC vs AC FET T3	-2,329518	0,00548	Q8WUA8	Tsukushin	TSKU
UC vs AC FET T3	2,0488998	0,01413	Q8WWZ8;Q8WWZ8-2	Oncoprotein-induced transcript 3 protein	OIT3
UC vs AC FET T3	3,2928564	0,02	Q8WZ42-5	Titin	TTN
UC vs AC FET T3	-0,882148	0,004739	Q92598-2;Q92598-3;Q92598;Q92598-4	Heat shock protein 105 kDa	HSPH1
UC vs AC FET T3	1,3729892	0,01137	Q92626;Q92626-2	Peroxidasin homolog	PXDN
UC vs AC FET T3	1,1797989	0,005523	Q92743	Serine protease HTRA1	HTRA1
UC vs AC FET T3	3,1129736	0,0003008	Q92954-3;Q92954-6;Q92954;Q92954-4;Q92954-2;Q92954-5	Proteoglycan 4	PRG4
UC vs AC FET T3	-1,459642	0,001395	Q96CM8-3;Q96CM8-4;Q96CM8;Q96CM8-2	Acyl-CoA synthetase family member 2, mitochondrial	ACSF2
UC vs AC FET T3	1,024062	0,001382	Q96JB1-2;Q96JB1	Dynein heavy chain 8, axonemal	DNAH8
UC vs AC FET T3	1,0341103	0,006467	Q96KN2	Beta-Ala-His dipeptidase	CNDP1
UC vs AC FET T3	-3,628648	0,0008113	Q96QR1	Secretoglobin family 3A member 1	SCGB3A1
UC vs AC FET T3	2,9839404	0,04031	Q96RL1-3;Q96RL1-4	BRCA1-A complex subunit RAP80	UIMC1
UC vs AC FET T3	0,7149257	0,003274	Q99574	Neuroserpin	SERPINI1

UC vs AC FET T3	1,026613	0,02963	Q9BWP8-8;Q9BWP8-7;Q9BWP8-6;Q9BWP8-5;Q9BWP8-4;Q9BWP8-3;Q9BWP8-2;Q9BWP8-9;Q9BWP8;Q9BWP8-10	Collectin-11	COLEC11
UC vs AC FET T3	2,0471556	0,0007943	Q9BXR6	Complement factor H-related protein 5	CFHR5
UC vs AC FET T3	-1,046544	0,03644	Q9BY76-3;Q9BY76;Q9BY76-2	Angiopoietin-related protein 4	ANGPTL4
UC vs AC FET T3	1,8899083	0,0003832	Q9H6X2-5;Q9H6X2;Q9H6X2-4;Q9H6X2-6;Q9H6X2-2;Q9H6X2-3	Anthrax toxin receptor 1	ANTXR1
UC vs AC FET T3	-1,769738	0,02463	Q9HCL0-2;Q9HCL0	Protocadherin-18	PCDH18
UC vs AC FET T3	1,4950788	0,01051	Q9HDC9;Q9HDC9-2	Adipocyte plasma membrane-associated protein	APMAP
UC vs AC FET T3	-0,868416	0,02746	Q9NPH2-2;Q9NPH2-3;Q9NPH2	Inositol-3-phosphate synthase 1	ISYNA1
UC vs AC FET T3	5,6403873	0,01145	Q9NQ79;Q9NQ79-2;Q9NQ79-3	Cartilage acidic protein 1	CRTAC1
UC vs AC FET T3	-1,338117	0,02465	Q9NRA1;Q9NRA1-3;Q9NRA1-2;Q9NRA1-4	Platelet-derived growth factor C	PDGFC
UC vs AC FET T3	1,9734241	0,004283	Q9NY15;Q9NY15-2	Stabilin-1	STAB1
UC vs AC FET T3	-0,8369	0,00815	Q9P1A2-3	.	.
UC vs AC FET T3	1,090234	0,0005949	Q9UBX5	Fibulin-5	FBLN5
UC vs AC FET T3	3,203378	0,0002409	Q9UHG3	Prenylcysteine oxidase 1	PCYOX1
UC vs AC FET T3	-2,207936	0,001042	Q9UJJ9	N-acetylglucosamine-1-phosphotransferase subunit gamma	GNPTG
UC vs AC FET T3	2,5145156	0,02577	Q9UK55	Protein Z-dependent protease inhibitor	SERPINA10
UC vs AC FET T3	-0,717979	0,01616	Q9UQ72;Q9UQ72-2	Pregnancy-specific beta-1-glycoprotein 11	PSG11
UC vs AC FET T3	-1,23222	0,0288	Q9Y287-2;Q9Y287	Integral membrane protein 2B	ITM2B
UC vs AC FET T3	6,8334437	0,005612	Q9Y490	Talin-1	TLN1
UC vs AC FET T3	1,6355367	0,01267	Q9Y4C2-2;Q9Y4C2	TRPM8 channel-associated factor 1	TCAF1
UC vs AC FET T3	-0,572647	0,04183	Q9Y6Z7	Collectin-10	COLEC10
UC vs NC FET T1	3,1758724	0,001174	A0A075B6J9	.	IGLV2-18
UC vs NC FET T1	0,9372287	0,0486	A0A075B6P5;P01615	Ig kappa chain V-II region FR	IGKV2D-28
UC vs NC FET T1	1,8577766	0,007357	A0A0C4DH68;A0A075B6R9	.	IGKV2-24;IGKV2D-24
UC vs NC FET T1	1,7278493	0,03044	A0A087WSY6	.	IGKV3D-15
UC vs NC FET T1	1,3401595	0,001006	A0A0A0MS15	.	IGHV3-49
UC vs NC FET T1	0,6074302	0,013	A0A0B4J1V0	.	IGHV3-15
UC vs NC FET T1	2,983036	0,001042	A0A0B4J1X5	.	IGHV3-74
UC vs NC FET T1	5,2112228	0,001155	A0A0B4J1Y9	.	IGHV3-72
UC vs NC FET T1	2,7492706	0,001833	A0A0C4DH25	.	IGKV3D-20
UC vs NC FET T1	5,3071541	0,001319	A0A0C4DH35	.	IGHV3-35
UC vs NC FET T1	-1,809334	0,01379	A0A0C4DH38	.	IGHV5-51
UC vs NC FET T1	2,9852416	0,004941	A0A0J9YX35	.	.
UC vs NC FET T1	-3,167406	0,001079	A4FU69-3;A4FU69;A4FU69-2;A4FU69-4;A4FU69-6	EF-hand calcium-binding domain-containing protein 5	EFCAB5
UC vs NC FET T1	2,7603193	3,51E-02	A8K2U0;A8K2U0-2	Alpha-2-macroglobulin-like protein 1	A2ML1
UC vs NC FET T1	3,116765	0,001465	O00187;O00187-2	Mannan-binding lectin serine protease 2	MASP2
UC vs NC FET T1	1,4009671	0,0009219	O00391;O00391-2	Sulfhydryl oxidase 1	QSOX1
UC vs NC FET T1	1,6683326	0,00381	O14791-2;O14791;O14791-3	Apolipoprotein L1	APOL1
UC vs NC FET T1	3,5699595	0,002722	O15016;O15016-2;O15016-3	Tripartite motif-containing protein 66	TRIM66
UC vs NC FET T1	1,6908418	0,001458	O43399;O43399-5;O43399-7;O43399-2;O43399-4;O43399-3	Tumor protein D54	TPD52L2
UC vs NC FET T1	1,6949857	0,0003084	O43861-2;O43861	Probable phospholipid-transporting ATPase IIB	ATP9B
UC vs NC FET T1	1,0215902	0,02928	O43866	CD5 antigen-like	CD5L
UC vs NC FET T1	2,6048989	0,01013	O75636;O75636-2	Ficolin-3	FCN3
UC vs NC FET T1	6,7744932	0,0005149	O75882;O75882-2;O75882-3	Attractin	ATRN
UC vs NC FET T1	1,3643074	0,02138	O76076	WNT1-inducible-signaling pathway protein 2	WISP2
UC vs NC FET T1	0,7456031	0,04566	O95428-6;O95428;O95428-5;O95428-4;O95428-2;O95428-3	Papilin	PAPLN
UC vs NC FET T1	0,8476119	0,045	O95445	Apolipoprotein M	APOM
UC vs NC FET T1	4,9547081	0,0003686	O95445-2	Apolipoprotein M	APOM
UC vs NC FET T1	0,8976239	0,02842	O95967	EGF-containing fibulin-like extracellular matrix protein 2	EFEMP2
UC vs NC FET T1	0,9700747	0,01874	P00450	Ceruloplasmin	CP
UC vs NC FET T1	1,546175	0,0001165	P00734	Prothrombin	F2
UC vs NC FET T1	1,7997012	0,002578	P00736	Complement C1r subcomponent	C1R
UC vs NC FET T1	2,7189007	0,001322	P00740;P00740-2	Coagulation factor IX	F9
UC vs NC FET T1	3,1821322	2,56E-03	P00742	Coagulation factor X	F10
UC vs NC FET T1	1,6291746	0,03496	P00746	Complement factor D	CFD
UC vs NC FET T1	1,4583812	0,0005184	P00747;Q02325	Plasminogen	PLG
UC vs NC FET T1	1,063982	0,005528	P00748	Coagulation factor XII	F12
UC vs NC FET T1	1,6916995	0,00461	P00751;P00751-2	Complement factor B	CFB
UC vs NC FET T1	0,9557433	0,02055	P01011;P01011-2;P01011-3	Alpha-1-antichymotrypsin	SERPINA3
UC vs NC FET T1	1,3539835	0,0003966	P01034	Cystatin-C	CST3
UC vs NC FET T1	-1,285367	0,005688	P01040	Cystatin-A	CSTA
UC vs NC FET T1	2,4296174	0,0007391	P01042-2	Kininogen-1	KNG1
UC vs NC FET T1	1,3773327	0,01214	P01344-3;P01344;P01344-2	Insulin-like growth factor II	IGF2
UC vs NC FET T1	1,1917919	0,03871	P01594;P01593	Ig kappa chain V-I region AU	Ig kappa chain V-I region AU;Ig kappa chain V-I region AG
UC vs NC FET T1	3,4937358	0,002639	P01599	Ig kappa chain V-I region Gal	Ig kappa chain V-I region Gal
UC vs NC FET T1	2,2826057	0,01223	P01624	Ig kappa chain V-III region POM	Ig kappa chain V-III region POM
UC vs NC FET T1	3,8081388	0,002559	P01700	Ig lambda chain V-I region HA	Ig lambda chain V-I region HA
UC vs NC FET T1	1,4064062	0,02286	P01701	Ig lambda chain V-I region NEW	Ig lambda chain V-I region NEW
UC vs NC FET T1	-1,448023	0,04414	P01715	Ig lambda chain V-IV region Bau	Ig lambda chain V-IV region Bau
UC vs NC FET T1	3,3164531	0,01026	P01743	Ig heavy chain V-I region HG3	Ig heavy chain V-I region HG3
UC vs NC FET T1	4,497579	0,00178	P0DP03;P01768	Ig heavy chain V-III region CAM	Ig heavy chain V-III region CAM
UC vs NC FET T1	-1,879879	0,04326	P01833	Polymeric immunoglobulin receptor	PIGR
UC vs NC FET T1	1,5179224	0,0001492	P01859	Ig gamma-2 chain C region	IGHG2
UC vs NC FET T1	1,6295838	0,02806	P01860	Ig gamma-3 chain C region	IGHG3
UC vs NC FET T1	1,450634	0,002987	P01876	Ig alpha-1 chain C region	IGHA1
UC vs NC FET T1	0,8040638	0,0006093	P02647;Q9HB71-2	Apolipoprotein A-I	APOA1
UC vs NC FET T1	1,9038143	0,0003849	P02649;CON__Q03247	Apolipoprotein E	APOE
UC vs NC FET T1	-0,692648	0,02687	P02652	Apolipoprotein A-II	APOA2

UC vs NC FET T1	0,8173914	0,01182	P02656	Apolipoprotein C-III	APOC3
UC vs NC FET T1	0,998652	0,01827	P02675	Fibrinogen beta chain	FGB
UC vs NC FET T1	0,6313343	0,01355	P02743	Serum amyloid P-component	APCS
UC vs NC FET T1	2,7493176	0,0002411	P02746	Complement C1q subcomponent subunit B	C1QB
UC vs NC FET T1	1,0736704	0,003029	P02747	Complement C1q subcomponent subunit C	C1QC
UC vs NC FET T1	0,9986124	0,03808	P02750	Leucine-rich alpha-2-glycoprotein	LRG1
UC vs NC FET T1	-0,941612	0,03355	P02763	Alpha-1-acid glycoprotein 1	ORM1
UC vs NC FET T1	1,2538061	0,0007033	P02765	Alpha-2-HS-glycoprotein	AHSG
UC vs NC FET T1	1,3072071	0,02758	P02766	Transthyretin	TTR
UC vs NC FET T1	1,572125	0,02287	P02774-3;P02774;P02774-2	Vitamin D-binding protein	GC
UC vs NC FET T1	2,846458	0,02259	P02776	Platelet factor 4	PF4
UC vs NC FET T1	3,4460783	0,02254	P02788;P02788-2	Lactotransferrin	LTF
UC vs NC FET T1	1,4784284	0,0003963	P02790	Hemopexin	HPX
UC vs NC FET T1	4,8917241	8,79E-04	P03950	Angiogenin	ANG
UC vs NC FET T1	5,1450119	0,007781	P03951	Coagulation factor XI	F11
UC vs NC FET T1	1,9441161	0,0002303	P03952;P20718	Plasma kallikrein	KLKB1
UC vs NC FET T1	1,7951583	0,0007991	P04040	Catalase	CAT
UC vs NC FET T1	2,3463602	0,02329	P04070;P04070-2	Vitamin K-dependent protein C	PROC
UC vs NC FET T1	0,9334164	0,006433	P04114	Apolipoprotein B-100	APOB
UC vs NC FET T1	1,1141195	0,009207	P04180	Phosphatidylcholine-sterol acyltransferase	LCAT
UC vs NC FET T1	-1,296541	0,01562	P04196	Histidine-rich glycoprotein	HRG
UC vs NC FET T1	3,3493538	0,01429	P04275	von Willebrand factor	VWF
UC vs NC FET T1	2,0086073	0,04329	P04406;P04406-2;O14556	Glyceraldehyde-3-phosphate dehydrogenase	GAPDH
UC vs NC FET T1	1,4299245	0,04109	P04433;A0A0A0MRZ8	Ig kappa chain V-III region VG	IGKV3D-11
UC vs NC FET T1	1,0815406	0,01298	P05090	Apolipoprotein D	APOD
UC vs NC FET T1	1,6148434	0,008097	P05154	Plasma serine protease inhibitor	SERPINA5
UC vs NC FET T1	3,6514835	0,0001871	P05155-2;P05155;P05155-3	Plasma protease C1 inhibitor	SERPING1
UC vs NC FET T1	0,9826989	0,0492	P05156;CON__Q32PI4	Complement factor I	CFI
UC vs NC FET T1	1,2297721	0,01454	P05160	Coagulation factor XIII B chain	F13B
UC vs NC FET T1	0,8658293	0,01502	P05164-3;P05164;P05164-2	Myeloperoxidase	MPO
UC vs NC FET T1	3,3583417	0,001432	P05543	Thyroxine-binding globulin	SERPINA7
UC vs NC FET T1	1,4367685	0,03354	P05546	Heparin cofactor 2	SERPIND1
UC vs NC FET T1	2,5727171	0,0007288	P06331	Ig heavy chain V-II region ARH-77	Ig heavy chain V-II region ARH-77
UC vs NC FET T1	1,2775745	0,0007212	P06396;P06396-4;P06396-3	Gelsolin	GSN
UC vs NC FET T1	-1,248878	0,03371	P06702	Protein S100-A9	S100A9
UC vs NC FET T1	0,6686993	0,01523	P06727;Q9BT92	Apolipoprotein A-IV	APOA4
UC vs NC FET T1	0,7609491	0,0195	P06850	Corticoliberin	CRH
UC vs NC FET T1	2,2661619	0,01996	P07237	Protein disulfide-isomerase	P4HB
UC vs NC FET T1	0,9898856	0,01924	P07307-3;P07307-2;P07307	Asialoglycoprotein receptor 2	ASGR2
UC vs NC FET T1	1,0099761	0,006668	P07333;P07333-2	Macrophage colony-stimulating factor 1 receptor	CSF1R
UC vs NC FET T1	2,3371902	1,63E-02	P07357	Complement component C8 alpha chain	C8A
UC vs NC FET T1	2,669765	0,0008839	P07358	Complement component C8 beta chain	C8B
UC vs NC FET T1	1,45713	0,01847	P07360	Complement component C8 gamma chain	C8G
UC vs NC FET T1	0,8410833	0,02314	P07998	Ribonuclease pancreatic	RNASE1
UC vs NC FET T1	2,1180396	0,003297	P08253;P08253-3;P08253-2	72 kDa type IV collagenase	MMP2
UC vs NC FET T1	2,0507364	0,001095	P08294	Extracellular superoxide dismutase [Cu-Zn]	SOD3
UC vs NC FET T1	2,9627826	0,03327	P08519	Apolipoprotein(a)	LPA
UC vs NC FET T1	3,2472532	0,006248	P08567	Pleckstrin	PLEK
UC vs NC FET T1	1,5840082	0,0001958	P08603;P08603-2	Complement factor H	CFH
UC vs NC FET T1	1,113596	0,00832	P08697;P08697-2	Alpha-2-antiplasmin	SERPINF2
UC vs NC FET T1	3,0151758	0,02176	P08709-2;P08709	Coagulation factor VII	F7
UC vs NC FET T1	1,3098114	0,005044	P09382	Galectin-1	LGALS1
UC vs NC FET T1	2,3218542	0,000389	P09466-3	Glycodelin	PAEP
UC vs NC FET T1	1,2738299	0,001602	P09871	Complement C1s subcomponent	C1S
UC vs NC FET T1	-2,492459	0,004239	P0C0L4;P0C0L4-2	Complement C4-A	C4A
UC vs NC FET T1	1,1033626	0,00139	P0C0L5	Complement C4-B	C4B
UC vs NC FET T1	4,9850908	0,01403	P0DML3;P0DML2;P0DML3-2;P0DML3-3;P01241-2;P01241;P01241-5	Chorionic somatomammotropin hormone 2	CSH2;CSH1
UC vs NC FET T1	2,0929856	0,00186	P0DOX2	.	.
UC vs NC FET T1	2,1153035	0,009269	P0DOX3	.	.
UC vs NC FET T1	3,6739033	0,003329	P0DOX4	.	.
UC vs NC FET T1	1,0794244	0,003056	P0DOX5;P01857	Ig gamma-1 chain C region	IGHG1
UC vs NC FET T1	0,6537124	0,02828	P0DOX6	.	.
UC vs NC FET T1	1,5854187	0,000212	P0DOX7	.	.
UC vs NC FET T1	2,2691979	0,0001382	P0DOX8	.	.
UC vs NC FET T1	1,4010267	2,98E-02	P0DOY3	.	.
UC vs NC FET T1	2,1911373	0,03656	P10643	Complement component C7	C7
UC vs NC FET T1	1,1463609	0,0217	P10645	Chromogranin-A	CHGA
UC vs NC FET T1	2,6246407	0,0257	P10720	Platelet factor 4 variant	PF4V1
UC vs NC FET T1	2,3022496	0,00643	P11021	78 kDa glucose-regulated protein	HSPA5
UC vs NC FET T1	2,3048244	0,002418	P11597;P11597-2	Cholesteryl ester transfer protein	CETP
UC vs NC FET T1	2,8440399	0,001577	P12259	Coagulation factor V	F5
UC vs NC FET T1	-2,239055	0,007195	P12273	Prolactin-inducible protein	PIP
UC vs NC FET T1	3,4338323	0,01716	P13497;P13497-5;P13497-2;P13497-6;P13497-4;P13497-3	Bone morphogenetic protein 1	BMP1
UC vs NC FET T1	1,4713495	0,00923	P13667	Protein disulfide-isomerase A4	PDIA4
UC vs NC FET T1	2,0731936	0,01683	P14625;Q58FF3	Endoplasmin	HSP90B1
UC vs NC FET T1	1,1294761	0,01722	P15907	Beta-galactoside alpha-2,6-sialyltransferase 1	ST6GAL1
UC vs NC FET T1	4,8497936	0,002212	P17936;P17936-2	Insulin-like growth factor-binding protein 3	IGFBP3
UC vs NC FET T1	2,3216434	0,001042	P19652	Alpha-1-acid glycoprotein 2	ORM2
UC vs NC FET T1	1,7312446	0,0006591	P19823	Inter-alpha-trypsin inhibitor heavy chain H2	ITIH2
UC vs NC FET T1	2,3017187	0,001889	P19827	Inter-alpha-trypsin inhibitor heavy chain H1	ITIH1
UC vs NC FET T1	0,8219509	0,02451	P20851-2;P20851;REV__Q7Z7B0-3;REV__Q7Z7B0-2;REV__Q7Z7B0	C4b-binding protein beta chain	C4BPB
UC vs NC FET T1	1,6377558	0,03312	P21333-2;P21333	Filamin-A	FLNA
UC vs NC FET T1	2,4291773	0,0055	P22352	Glutathione peroxidase 3	GPX3
UC vs NC FET T1	1,7244744	0,03585	P22792	Carboxypeptidase N subunit 2	CPN2
UC vs NC FET T1	0,9123012	0,03213	P22891;P22891-2	Vitamin K-dependent protein Z	PROZ
UC vs NC FET T1	6,9135922	9,76E-06	P23083	Ig heavy chain V-I region V35	Ig heavy chain V-I region V35
UC vs NC FET T1	0,7786326	0,01888	P23142;P23142-2;P23142-3	Fibulin-1	FBLN1
UC vs NC FET T1	-1,683619	0,02527	P23280-3;P23280	Carbonic anhydrase 6	CA6

UC vs NC FET T1	2,6214598	0,004942	P24593	Insulin-like growth factor-binding protein 5	IGFBP5
UC vs NC FET T1	3,0886621	0,01826	P26927	Hepatocyte growth factor-like protein	MST1
UC vs NC FET T1	2,2045689	0,01091	P27797	Calreticulin	CALR
UC vs NC FET T1	3,0832286	0,0009302	P27918	Properdin	CFP
UC vs NC FET T1	2,398418	0,003997	P28370-2;P28370	Probable global transcription activator SNF2L1	SMARCA1
UC vs NC FET T1	1,3523269	0,001467	P29622	Kallistatin	SERPINA4
UC vs NC FET T1	-1,853307	0,002982	P31151;Q86SG5	Protein S100-A7	S100A7
UC vs NC FET T1	1,1082106	0,01961	P33151;P33151-2	Cadherin-5	CDH5
UC vs NC FET T1	1,7313913	0,001103	P34096	Ribonuclease 4	RNASE4
UC vs NC FET T1	3,1606016	0,01833	P35443	Thrombospondin-4	THBS4
UC vs NC FET T1	3,8590217	9,30E-05	P35542	Serum amyloid A-4 protein	SAA4
UC vs NC FET T1	1,0680729	0,002589	P35555	Fibrillin-1	FBN1
UC vs NC FET T1	3,0830698	0,0008269	P35858;P35858-2	Insulin-like growth factor-binding protein complex acid labile subunit	IGFALS
UC vs NC FET T1	2,4621392	0,02771	P48059;P48059-4;P48059-2;P48059-5;P48059-3;Q7Z4I7-4;Q7Z4I7-3;Q7Z4I7-2;P0CW19-2	LIM and senescent cell antigen-like-containing domain protein 1	LIMS1
UC vs NC FET T1	2,8050699	0,0004485	P48740	Mannan-binding lectin serine protease 1	MASP1
UC vs NC FET T1	1,0865531	2,76E-02	P48740-2	Mannan-binding lectin serine protease 1	MASP1
UC vs NC FET T1	0,9835548	0,009372	P48740-3	Mannan-binding lectin serine protease 1	MASP1
UC vs NC FET T1	2,040529	0,001223	P48740-4	Mannan-binding lectin serine protease 1	MASP1
UC vs NC FET T1	2,575279	0,02199	P49454	Centromere protein F	CENPF
UC vs NC FET T1	2,1727458	0,03824	P49619-3;P49619-2;P49619	Diacylglycerol kinase gamma	DGKG
UC vs NC FET T1	0,8817229	0,04821	P49747;P49747-2	Cartilage oligomeric matrix protein	COMP
UC vs NC FET T1	-1,922677	0,0004405	P49767	Vascular endothelial growth factor C	VEGFC
UC vs NC FET T1	0,5072174	0,0442	P49908	Selenoprotein P	SEPP1
UC vs NC FET T1	2,2320509	0,001062	P49913	Cathelicidin antimicrobial peptide	CAMP
UC vs NC FET T1	1,5609763	0,002142	P54132	Bloom syndrome protein	BLM
UC vs NC FET T1	1,6561003	0,006554	P55056	Apolipoprotein C-IV	APOC4
UC vs NC FET T1	1,5206766	0,03421	P55058;P55058-4;P55058-3;P55058-2	Phospholipid transfer protein	PLTP
UC vs NC FET T1	1,6676978	0,0005756	P55103	Inhibin beta C chain	INHBC
UC vs NC FET T1	1,9395022	0,005183	P57077;P57077-1	MAP3K7 C-terminal-like protein	MAP3K7CL
UC vs NC FET T1	2,6774105	0,001678	P60900;P60900-2;P60900-3	Proteasome subunit alpha type-6	PSMA6
UC vs NC FET T1	2,3239458	0,0003257	P61224-3;P61224;P61224-2;P61224-4;A6NIZ1;P62834	Ras-related protein Rap-1b	RAP1B;RAP1A
UC vs NC FET T1	-3,089742	0,0001917	P61769	Beta-2-microglobulin	B2M
UC vs NC FET T1	1,8550519	0,01106	P62258;P62258-2	14-3-3 protein epsilon	YWHAE
UC vs NC FET T1	-2,087114	0,01362	P62979;P62987;P0CG47;P0CG48	Ubiquitin-40S ribosomal protein S27a	RPS27A;UBA52;UBB;UBC
UC vs NC FET T1	3,1076612	0,002276	P69905	Hemoglobin subunit alpha	HBA1
UC vs NC FET T1	1,2375021	0,008853	P80108;P80108-2	Phosphatidylinositol-glycan-specific phospholipase D	GPLD1
UC vs NC FET T1	3,7023697	4,91E-02	P80748	Ig lambda chain V-III region LOI	Ig lambda chain V-III region LOI
UC vs NC FET T1	3,4579002	0,01096	Q00526;P11802-2;Q00535-2;Q00535;P11802;Q14004-2;Q14004	Cyclin-dependent kinase 3	CDK3
UC vs NC FET T1	-1,610813	0,002131	Q00889-2;Q00889	Pregnancy-specific beta-1-glycoprotein 6	PSG6
UC vs NC FET T1	1,2995541	0,01223	Q02108-2;Q02108	Guanylate cyclase soluble subunit alpha-3	GUCY1A3
UC vs NC FET T1	2,4386085	0,0006389	Q02224;Q02224-3	Centromere-associated protein E	CENPE
UC vs NC FET T1	1,5074041	0,02493	Q02818	Nucleobindin-1	NUCB1
UC vs NC FET T1	1,8422407	0,003096	Q02985-2;Q02985	Complement factor H-related protein 3	CFHR3
UC vs NC FET T1	1,1998746	0,004023	Q03001;Q03001-9;Q03001-13;Q03001-10	Dystonin	DST
UC vs NC FET T1	2,5343657	0,0103	Q03591	Complement factor H-related protein 1	CFHR1
UC vs NC FET T1	1,9524517	0,03661	Q06033-2;Q06033	Inter-alpha-trypsin inhibitor heavy chain H3	ITIH3
UC vs NC FET T1	1,8108641	0,03873	Q07065	Cytoskeleton-associated protein 4	CKAP4
UC vs NC FET T1	2,3487997	0,009423	Q07954;Q07954-2	Prolow-density lipoprotein receptor-related protein 1	LRP1
UC vs NC FET T1	1,3961444	0,01552	Q08830	Fibrinogen-like protein 1	FGL1
UC vs NC FET T1	1,4107912	0,01258	Q0VAK6;Q0VAK6-2	Leiomodin-3	LMOD3
UC vs NC FET T1	0,9839237	0,009499	Q12794-2;Q12794;Q12794-7;Q12794-4;Q12794-3;Q12794-6;Q12794-5	Hyaluronidase-1	HYAL1
UC vs NC FET T1	1,4299793	0,02214	Q12805-2;Q12805-4;Q12805-3;Q12805	EGF-containing fibulin-like extracellular matrix protein 1	EFEMP1
UC vs NC FET T1	1,5461576	0,008589	Q12805-5	EGF-containing fibulin-like extracellular matrix protein 1	EFEMP1
UC vs NC FET T1	4,3727555	0,0001201	Q13103	Secreted phosphoprotein 24	SPP2
UC vs NC FET T1	1,4545495	0,0057	Q13418-2;Q13418;Q13418-3	Integrin-linked protein kinase	ILK
UC vs NC FET T1	3,357626	0,000966	Q13488-2	V-type proton ATPase 116 kDa subunit a isoform 3	TCIRG1
UC vs NC FET T1	-2,158929	0,01854	Q13790	Apolipoprotein F	APOF
UC vs NC FET T1	1,1260936	0,04948	Q14161-7;Q14161-9;Q14161-8;Q14161-5;Q14161;Q14161-2;Q14161-11;Q14161-6;Q14161-10;Q14161-4;Q14161-3	ARF GTPase-activating protein GIT2	GIT2
UC vs NC FET T1	0,6074067	0,03567	Q14213	Interleukin-27 subunit beta	EBI3
UC vs NC FET T1	2,794445	4,39E-02	Q14520-2;Q14520	Hyaluronan-binding protein 2	HABP2
UC vs NC FET T1	0,9730202	0,03638	Q14623	Indian hedgehog protein	IHH
UC vs NC FET T1	1,114325	0,0007043	Q14624;Q14624-3;Q14624-4	Inter-alpha-trypsin inhibitor heavy chain H4	ITIH4
UC vs NC FET T1	1,3467801	0,04407	Q14697;Q14697-2	Neutral alpha-glucosidase AB	GANAB
UC vs NC FET T1	1,2505717	0,01243	Q15113	Procollagen C-endopeptidase enhancer 1	PCOLCE
UC vs NC FET T1	0,8608699	0,00888	Q15166	Serum paraoxonase/lactonase 3	PON3
UC vs NC FET T1	4,3685359	0,0008467	Q15386;Q15386-2	Ubiquitin-protein ligase E3C	UBE3C
UC vs NC FET T1	2,5601039	0,001088	Q15485;Q15485-2	Ficolin-2	FCN2
UC vs NC FET T1	5,4262218	4,69E-02	Q15582	Transforming growth factor-beta-induced protein ig-h3	TGFBI
UC vs NC FET T1	3,5855999	0,01123	Q15848	Adiponectin	ADIPOQ
UC vs NC FET T1	1,7006904	0,03362	Q16594	Transcription initiation factor TFIID subunit 9	TAF9
UC vs NC FET T1	1,3317108	0,01023	Q16769;Q16769-2	Glutaminyl-peptide cyclotransferase	QPCT
UC vs NC FET T1	1,014453	0,003644	Q2TAC6;Q2TAC6-3;Q2TAC6-2	Kinesin-like protein KIF19	KIF19
UC vs NC FET T1	-2,694827	0,01613	Q4LDE5;Q4LDE5-4;Q4LDE5-3;Q4LDE5-2	Sushi, von Willebrand factor type A, EGF and pentraxin domain-containing protein 1	SVEP1
UC vs NC FET T1	0,806612	0,01856	Q5CZC0;Q5CZC0-2	Fibrous sheath-interacting protein 2	FSIP2
UC vs NC FET T1	2,1918404	0,0005086	Q5HYK7-3;Q5HYK7-2;Q5HYK7-5;Q5HYK7-4	SH3 domain-containing protein 19	SH3D19

UC vs NC FET T1	2,7315444	0,01819	Q5JPF3;Q5JPF3-2;A6QL64-3;Q5JPF3-3	Ankyrin repeat domain-containing protein 36C	ANKRD36C;ANKRD36
UC vs NC FET T1	0,7230314	0,01822	Q5T0U0;Q5T0U0-2	Coiled-coil domain-containing protein 122	CCDC122
UC vs NC FET T1	2,2831312	0,01135	Q6EEV6	Small ubiquitin-related modifier 4	SUMO4
UC vs NC FET T1	3,2913316	0,004219	Q6ISB3;Q6ISB3-2	Grainyhead-like protein 2 homolog	GRHL2
UC vs NC FET T1	2,9149081	0,0005996	Q6P387-2;Q6P387	Uncharacterized protein C16orf46	C16orf46
UC vs NC FET T1	4,5021329	0,02055	Q6Q788	Apolipoprotein A-V	APOA5
UC vs NC FET T1	1,5192966	0,003526	Q6UVK1	Chondroitin sulfate proteoglycan 4	CSPG4
UC vs NC FET T1	1,2773199	0,02556	Q6UY14-3;Q6UY14;Q6UY14-2	ADAMTS-like protein 4	ADAMTSL4
UC vs NC FET T1	0,7150782	0,04673	Q86U17	Serpin A11	SERPINA11
UC vs NC FET T1	2,1046529	0,007602	Q86UQ4;Q86UQ4-4;Q86UQ4-3;Q86UQ4-6;Q86UQ4-7;Q86UQ4-5	ATP-binding cassette sub-family A member 13	ABCA13
UC vs NC FET T1	0,5692099	0,01617	Q8IV50-2;Q8IV50	LysM and putative peptidoglycan-binding domain-containing protein 2	LYSMD2
UC vs NC FET T1	0,9262717	0,005645	Q8IVL1-11;Q8IVL1-4	Neuron navigator 2	NAV2
UC vs NC FET T1	2,0402481	0,0001037	Q8IYI0;Q8IYI0-2	Uncharacterized protein C20orf196	C20orf196
UC vs NC FET T1	1,60853	0,003463	Q8IZK6-2;Q8IZK6	Mucolipin-2	MCOLN2
UC vs NC FET T1	2,5929338	0,001892	Q8N7Z5;Q8WY50	Putative ankyrin repeat domain-containing protein 31	ANKRD31
UC vs NC FET T1	0,4373273	0,0333	Q8N8A2-4;Q8N8A2;Q8N8A2-2;Q8N8A2-3;Q8N8A2-5	Serine/threonine-protein phosphatase 6 regulatory ankyrin repeat subunit B	ANKRD44
UC vs NC FET T1	1,1017585	0,007947	Q8NB25-2;Q8NB25-3;Q8NB25	Protein FAM184A	FAM184A
UC vs NC FET T1	4,1047179	0,0003159	Q8NBP7	Proprotein convertase subtilisin/kexin type 9	PCSK9
UC vs NC FET T1	-1,965119	0,04926	Q8ND83-3;Q8ND83-2;Q8ND83-4	SLAIN motif-containing protein 1	SLAIN1
UC vs NC FET T1	1,5778246	0,0002646	Q8NDV7-6;Q8NDV7;Q8NDV7-2;Q8NDV7-5;Q8NDV7-4;Q8NDV7-3	Trinucleotide repeat-containing gene 6A protein	TNRC6A
UC vs NC FET T1	3,5827236	0,01888	Q8NI99	Angiopoietin-related protein 6	ANGPTL6
UC vs NC FET T1	1,7744667	0,0002029	Q8TE73	Dynein heavy chain 5, axonemal	DNAH5
UC vs NC FET T1	0,5362207	0,02074	Q8WWA0	Intelectin-1	ITLN1
UC vs NC FET T1	2,3683755	0,00233	Q8WWZ8;Q8WWZ8-2	Oncoprotein-induced transcript 3 protein	OIT3
UC vs NC FET T1	1,8668063	0,007608	Q92626;Q92626-2	Peroxidasin homolog	PXDN
UC vs NC FET T1	1,3828712	0,004336	Q96IY4;Q96IY4-2;CON__Q2KIG3	Carboxypeptidase B2	CPB2
UC vs NC FET T1	1,0439554	0,006561	Q96JB1-2;Q96JB1	Dynein heavy chain 8, axonemal	DNAH8
UC vs NC FET T1	1,8205719	0,0006857	Q96KN2	Beta-Ala-His dipeptidase	CNDP1
UC vs NC FET T1	2,7281919	0,001865	Q96PD5;Q96PD5-2	N-acetylmuramoyl-L-alanine amidase	PGLYRP2
UC vs NC FET T1	-0,960821	0,01342	Q96QR1	Secretoglobin family 3A member 1	SCGB3A1
UC vs NC FET T1	2,7936622	0,003653	Q96RL1-3;Q96RL1-4	BRCA1-A complex subunit RAP80	UIMC1
UC vs NC FET T1	1,0079871	0,02416	Q99574	Neuroserpin	SERPINI1
UC vs NC FET T1	2,4472222	0,0005248	Q99969	Retinoic acid receptor responder protein 2	RARRES2
UC vs NC FET T1	-2,21246	0,01453	Q99996-3;Q99996;Q99996-6;Q99996-5;Q99996-1;Q99996-4	A-kinase anchor protein 9	AKAP9
UC vs NC FET T1	1,8327556	0,006286	Q9BWP8-8;Q9BWP8-7;Q9BWP8-6;Q9BWP8-5;Q9BWP8-4;Q9BWP8-3;Q9BWP8-2;Q9BWP8-9;Q9BWP8;Q9BWP8-10	Collectin-11	COLEC11
UC vs NC FET T1	0,9265504	0,02315	Q9H6X2-5;Q9H6X2;Q9H6X2-4;Q9H6X2-6;Q9H6X2-2;Q9H6X2-3	Anthrax toxin receptor 1	ANTXR1
UC vs NC FET T1	-0,563946	0,01272	Q9HCL0-2;Q9HCL0	Protocadherin-18	PCDH18
UC vs NC FET T1	1,9544115	0,0007345	Q9HDC9;Q9HDC9-2	Adipocyte plasma membrane-associated protein	APMAP
UC vs NC FET T1	1,4167553	0,002063	Q9NPG4;O14917-2	Protocadherin-12	PCDH12
UC vs NC FET T1	5,8074966	3,37E-03	Q9NQ79;Q9NQ79-2;Q9NQ79-3	Cartilage acidic protein 1	CRTAC1
UC vs NC FET T1	-2,395708	0,002066	Q9NZT1	Calmodulin-like protein 5	CALML5
UC vs NC FET T1	2,5605812	0,00265	Q9UHG3	Prenylcysteine oxidase 1	PCYOX1
UC vs NC FET T1	-1,267562	0,001882	Q9UJJ9	N-acetylglucosamine-1-phosphotransferase subunit gamma	GNPTG
UC vs NC FET T1	3,4930272	0,001784	Q9UK55	Protein Z-dependent protease inhibitor	SERPINA10
UC vs NC FET T1	0,5090821	0,04983	Q9Y4B5	Microtubule cross-linking factor 1	MTCL1
UC vs NC FET T1	1,3309443	1,61E-03	Q9Y4L1	Hypoxia up-regulated protein 1	HYOU1
UC vs NC FET T2	4,0716472	5,61E-05	A0A075B6H7;A0A0C4DH55	.	IGKV3-7
UC vs NC FET T2	1,4718122	0,0226	A0A075B6I0	.	IGLV8-61
UC vs NC FET T2	3,7113367	0,001023	A0A075B6J9	.	IGLV2-18
UC vs NC FET T2	1,7027402	0,005412	A0A0C4DH68;A0A075B6R9	.	IGKV2-24;IGKV2D-24
UC vs NC FET T2	1,0305437	0,01002	A0A0B4J1V0	.	IGHV3-15
UC vs NC FET T2	2,5826507	0,00248	A0A0B4J1X5	.	IGHV3-74
UC vs NC FET T2	3,5175848	0,01656	A0A0B4J1Y9	.	IGHV3-72
UC vs NC FET T2	1,6317603	0,0007837	A0A0C4DH25	.	IGKV3D-20
UC vs NC FET T2	5,1513625	0,0001258	A0A0C4DH35	.	IGHV3-35
UC vs NC FET T2	4,5824059	6,64E-03	A0A0J9YX35	.	.
UC vs NC FET T2	-1,36608	0,03799	A0A1B0GTC6	.	.
UC vs NC FET T2	-3,667329	0,009627	A4FU69-3;A4FU69;A4FU69-2;A4FU69-4;A4FU69-6	EF-hand calcium-binding domain-containing protein 5	EFCAB5
UC vs NC FET T2	3,5693924	0,003844	A8K2U0;A8K2U0-2	Alpha-2-macroglobulin-like protein 1	A2ML1
UC vs NC FET T2	3,3107147	0,001028	O00187;O00187-2	Mannan-binding lectin serine protease 2	MASP2
UC vs NC FET T2	-1,852983	0,03767	O00217	NADH dehydrogenase [ubiquinone] iron-sulfur protein 8, mitochondrial	NDUFS8
UC vs NC FET T2	1,1111727	0,02221	O00391;O00391-2	Sulfhydryl oxidase 1	QSOX1
UC vs NC FET T2	-2,257474	0,04318	O00602	Ficolin-1	FCN1
UC vs NC FET T2	1,8333564	0,0006247	O14791-2;O14791;O14791-3	Apolipoprotein L1	APOL1
UC vs NC FET T2	5,4172663	2,36E-03	O15016;O15016-2;O15016-3	Tripartite motif-containing protein 66	TRIM66
UC vs NC FET T2	3,4208632	0,001242	O43184-3;O43184-4;O43184-2;O43184	Disintegrin and metalloproteinase domain-containing protein 12	ADAM12
UC vs NC FET T2	1,9766386	0,02907	O43399;O43399-5;O43399-7;O43399-2;O43399-4;O43399-3	Tumor protein D54	TPD52L2
UC vs NC FET T2	1,828876	0,002495	O43861-2;O43861	Probable phospholipid-transporting ATPase IIB	ATP9B

UC vs NC FET T2	3,4488883	0,004212	O75636;O75636-2	Ficolin-3	FCN3
UC vs NC FET T2	5,8188484	0,0001268	O75882;O75882-2;O75882-3	Attractin	ATRN
UC vs NC FET T2	2,2018523	0,01776	O76076	WNT1-inducible-signaling pathway protein 2	WISP2
UC vs NC FET T2	-3,654651	0,0009733	O94855;O94855-2	Protein transport protein Sec24D	SEC24D
UC vs NC FET T2	1,1145335	0,009745	O95428-6;O95428;O95428-5;O95428-4;O95428-2;O95428-3	Papilin	PAPLN
UC vs NC FET T2	1,1910267	0,00137	O95445	Apolipoprotein M	APOM
UC vs NC FET T2	5,955465	2,86E-02	O95445-2	Apolipoprotein M	APOM
UC vs NC FET T2	1,1638019	0,03992	O95967	EGF-containing fibulin-like extracellular matrix protein 2	EFEMP2
UC vs NC FET T2	1,4933376	0,01344	P00450	Ceruloplasmin	CP
UC vs NC FET T2	1,5926794	0,04985	P00451	Coagulation factor VIII	F8
UC vs NC FET T2	1,1621623	0,02185	P00488	Coagulation factor XIII A chain	F13A1
UC vs NC FET T2	3,805992	0,001201	P00709	Alpha-lactalbumin	LALBA
UC vs NC FET T2	0,6935124	0,02808	P00734	Prothrombin	F2
UC vs NC FET T2	1,969202	0,003297	P00736	Complement C1r subcomponent	C1R
UC vs NC FET T2	1,8463473	0,001591	P00739;P00739-2	Haptoglobin-related protein	HPR
UC vs NC FET T2	3,0504475	0,007021	P00740;P00740-2	Coagulation factor IX	F9
UC vs NC FET T2	2,1515784	0,004539	P00746	Complement factor D	CFD
UC vs NC FET T2	1,8261681	0,003409	P00748	Coagulation factor XII	F12
UC vs NC FET T2	1,9462509	0,002977	P00751;P00751-2	Complement factor B	CFB
UC vs NC FET T2	-0,931959	0,03445	P01008	Antithrombin-III	SERPINC1
UC vs NC FET T2	1,8705277	0,02255	P01031	Complement C5	C5
UC vs NC FET T2	-1,587643	0,02915	P01040	Cystatin-A	CSTA
UC vs NC FET T2	3,3882455	0,0002608	P01042-2	Kininogen-1	KNG1
UC vs NC FET T2	0,9013281	0,007197	P01591	Immunoglobulin J chain	IGJ
UC vs NC FET T2	1,6765895	0,03983	P01619	Ig kappa chain V-III region B6	Ig kappa chain V-III region B6
UC vs NC FET T2	1,5515467	0,04626	P01624	Ig kappa chain V-III region POM	Ig kappa chain V-III region POM
UC vs NC FET T2	2,6380639	0,0007323	P01700	Ig lambda chain V-I region HA	Ig lambda chain V-I region HA
UC vs NC FET T2	1,1752292	0,01675	P01701	Ig lambda chain V-I region NEW	Ig lambda chain V-I region NEW
UC vs NC FET T2	1,7840955	0,006714	P01709	Ig lambda chain V-II region MGC	Ig lambda chain V-II region MGC
UC vs NC FET T2	-1,477524	0,03555	P01715	Ig lambda chain V-IV region Bau	Ig lambda chain V-IV region Bau
UC vs NC FET T2	-1,687623	0,01165	P01717	Ig lambda chain V-IV region Hil	Ig lambda chain V-IV region Hil
UC vs NC FET T2	4,7979751	0,000741	P01743	Ig heavy chain V-I region HG3	Ig heavy chain V-I region HG3
UC vs NC FET T2	-2,38479	0,0214	P01764	Ig heavy chain V-III region 23	IGHV3-23
UC vs NC FET T2	3,5549828	0,01544	P0DP03;P01768	Ig heavy chain V-III region CAM	Ig heavy chain V-III region CAM
UC vs NC FET T2	-2,168764	0,0007396	P01834	Ig kappa chain C region	IGKC
UC vs NC FET T2	2,4882693	0,005737	P01860	Ig gamma-3 chain C region	IGHG3
UC vs NC FET T2	1,3046856	0,005582	P01871;P01871-2	Ig mu chain C region	IGHM
UC vs NC FET T2	1,9556174	0,004115	P01876	Ig alpha-1 chain C region	IGHA1
UC vs NC FET T2	1,2358408	0,003577	P02647;Q9HB71-2	Apolipoprotein A-I	APOA1
UC vs NC FET T2	1,7783694	0,0006132	P02649;CON__Q03247	Apolipoprotein E	APOE
UC vs NC FET T2	1,6917486	0,001829	P02671;P02671-2;REV__Q9UKV0-4;REV__Q9UKV0-2;REV__Q9UKV0;REV__Q9UKV0-5;REV__Q9UKV0-7;Q14314	Fibrinogen alpha chain	FGA
UC vs NC FET T2	1,8270469	0,00363	P02675	Fibrinogen beta chain	FGB
UC vs NC FET T2	0,9044704	0,002493	P02679;P02679-2	Fibrinogen gamma chain	FGG
UC vs NC FET T2	0,7387991	0,03493	P02743	Serum amyloid P-component	APCS
UC vs NC FET T2	-1,402282	0,00116	P02745	Complement C1q subcomponent subunit A	C1QA
UC vs NC FET T2	2,6372728	0,000173	P02746	Complement C1q subcomponent subunit B	C1QB
UC vs NC FET T2	2,5030007	0,004016	P02747	Complement C1q subcomponent subunit C	C1QC
UC vs NC FET T2	1,5632129	0,04313	P02749;CON__P17690	Beta-2-glycoprotein 1	APOH
UC vs NC FET T2	2,1016746	2,09E-02	P02765	Alpha-2-HS-glycoprotein	AHSG
UC vs NC FET T2	1,6022005	0,01382	P02766	Transthyretin	TTR
UC vs NC FET T2	1,6196885	0,0005859	P02774-3;P02774;P02774-2	Vitamin D-binding protein	GC
UC vs NC FET T2	2,9198273	0,04709	P02775	Platelet basic protein	PPBP
UC vs NC FET T2	5,0043464	0,006624	P02776	Platelet factor 4	PF4
UC vs NC FET T2	3,2988119	0,01028	P02788;P02788-2	Lactotransferrin	LTF
UC vs NC FET T2	1,5375929	0,006189	P02790	Hemopexin	HPX
UC vs NC FET T2	5,5535523	6,47E-03	P03951	Coagulation factor XI	F11
UC vs NC FET T2	3,1232038	0,002294	P03952;P20718	Plasma kallikrein	KLKB1
UC vs NC FET T2	-0,821073	0,01226	P03973	Antileukoproteinase	SLPI
UC vs NC FET T2	2,595636	0,004494	P04040	Catalase	CAT
UC vs NC FET T2	2,4516578	0,006296	P04070;P04070-2	Vitamin K-dependent protein C	PROC
UC vs NC FET T2	1,1507192	0,001479	P04114	Apolipoprotein B-100	APOB
UC vs NC FET T2	1,5888739	0,004305	P04180	Phosphatidylcholine-sterol acyltransferase	LCAT
UC vs NC FET T2	3,7707939	0,000943	P04211;A0A075B6I9	Ig lambda chain V region 4A	IGLV7-46
UC vs NC FET T2	4,753777	0,005547	P04275	von Willebrand factor	VWF
UC vs NC FET T2	2,5648535	0,001521	P04433;A0A0A0MRZ8	Ig kappa chain V-III region VG	IGKV3D-11
UC vs NC FET T2	4,1495249	0,001633	P05023-2;P05023-4;P05023;P05023-3	Sodium/potassium-transporting ATPase subunit alpha-1	ATP1A1
UC vs NC FET T2	1,2775331	0,0009912	P05090	Apolipoprotein D	APOD
UC vs NC FET T2	3,0906444	0,0005804	P05155-2;P05155;P05155-3	Plasma protease C1 inhibitor	SERPING1
UC vs NC FET T2	2,5968165	0,004073	P05160	Coagulation factor XIII B chain	F13B
UC vs NC FET T2	1,7541733	0,009176	P05546	Heparin cofactor 2	SERPIND1
UC vs NC FET T2	2,6524826	0,002779	P06331	Ig heavy chain V-II region ARH-77	Ig heavy chain V-II region ARH-77
UC vs NC FET T2	1,4181929	0,0002207	P06727;Q9BT92	Apolipoprotein A-IV	APOA4
UC vs NC FET T2	-1,460747	0,02098	P06753-5;P06753-2;P06753-4;P06753-3;P06753-6;P06753-7	Tropomyosin alpha-3 chain	TPM3
UC vs NC FET T2	1,1363997	0,003957	P07225	Vitamin K-dependent protein S	PROS1
UC vs NC FET T2	2,2441002	0,001576	P07237	Protein disulfide-isomerase	P4HB
UC vs NC FET T2	1,119722	0,02774	P07307-3;P07307-2;P07307	Asialoglycoprotein receptor 2	ASGR2
UC vs NC FET T2	1,0916423	0,005142	P07333;P07333-2	Macrophage colony-stimulating factor 1 receptor	CSF1R
UC vs NC FET T2	3,3291554	0,0001301	P07357	Complement component C8 alpha chain	C8A
UC vs NC FET T2	3,4921837	0,0001263	P07358	Complement component C8 beta chain	C8B
UC vs NC FET T2	3,486757	0,0009128	P07437;Q9BUF5	Tubulin beta chain	TUBB
UC vs NC FET T2	2,6809422	0,00203	P07900;P07900-2;Q14568;Q58FF6;Q58FG1	Heat shock protein HSP 90-alpha	HSP90AA1
UC vs NC FET T2	2,338614	0,01812	P08253;P08253-3;P08253-2	72 kDa type IV collagenase	MMP2
UC vs NC FET T2	3,3310928	0,0002318	P08294	Extracellular superoxide dismutase [Cu-Zn]	SOD3
UC vs NC FET T2	4,7207771	0,0003841	P08493-2;P08493	Matrix Gla protein	MGP
UC vs NC FET T2	1,7331214	0,002758	P08603;P08603-2	Complement factor H	CFH

UC vs NC FET T2	5,3802228	0,02935	P08670	Vimentin	VIM
UC vs NC FET T2	0,7864561	0,02863	P08697;P08697-2	Alpha-2-antiplasmin	SERPINF2
UC vs NC FET T2	2,1887293	0,002523	P08709-2;P08709	Coagulation factor VII	F7
UC vs NC FET T2	2,845179	0,003089	P08833	Insulin-like growth factor-binding protein 1	IGFBP1
UC vs NC FET T2	-4,517772	0,007359	P09466-2;P09466	Glycodelin	PAEP
UC vs NC FET T2	2,8029379	1,62E-02	P09466-3	Glycodelin	PAEP
UC vs NC FET T2	1,4586387	0,0007067	P09871	Complement C1s subcomponent	C1S
UC vs NC FET T2	1,6649742	0,002499	P0C0L5	Complement C4-B	C4B
UC vs NC FET T2	3,3977852	0,002299	P0DML3;P0DML2;P0DML3-2;P0DML3-3;P01241-2;P01241;P01241-5	Chorionic somatomammotropin hormone 2	CSH2;CSH1
UC vs NC FET T2	-2,350021	0,01116	P0DN87;P0DN86;P0DN86-2;Q6NT52;A6NKQ9-2;A6NKQ9	Choriogonadotropin subunit beta variant 2	CGB2;CGB1
UC vs NC FET T2	2,4640031	0,0229	P0DOX2	.	.
UC vs NC FET T2	3,4632507	0,0004905	P0DOX4	.	.
UC vs NC FET T2	1,1986589	0,01266	P0DOX5;P01857	Ig gamma-1 chain C region	IGHG1
UC vs NC FET T2	2,2425165	0,0005233	P0DOX6	.	.
UC vs NC FET T2	1,9223351	0,0005439	P0DOX7	.	.
UC vs NC FET T2	1,8374391	0,005599	P0DOX8	.	.
UC vs NC FET T2	1,3222132	0,04381	P0DOY3	.	.
UC vs NC FET T2	1,8691082	0,01092	P11464-4	Pregnancy-specific beta-1-glycoprotein 1	PSG1
UC vs NC FET T2	2,2347164	0,00172	P11465	Pregnancy-specific beta-1-glycoprotein 2	PSG2
UC vs NC FET T2	3,0447669	0,01943	P11597;P11597-2	Cholesteryl ester transfer protein	CETP
UC vs NC FET T2	2,8481998	0,001896	P12259	Coagulation factor V	F5
UC vs NC FET T2	-2,002566	0,02559	P12273	Prolactin-inducible protein	PIP
UC vs NC FET T2	-1,158843	0,01993	P12532;P12532-2	Creatine kinase U-type, mitochondrial	CKMT1A
UC vs NC FET T2	6,3696716	0,004505	P13497;P13497-5;P13497-2;P13497-6;P13497-4;P13497-3	Bone morphogenetic protein 1	BMP1
UC vs NC FET T2	4,3859067	0,005246	P13727;P13727-2	Bone marrow proteoglycan	PRG2
UC vs NC FET T2	-2,341623	0,003671	P13798	Acylamino-acid-releasing enzyme	APEH
UC vs NC FET T2	0,5665135	0,03294	P14061	Estradiol 17-beta-dehydrogenase 1	HSD17B1
UC vs NC FET T2	1,970749	0,01071	P14209;P14209-2;P14209-3	CD99 antigen	CD99
UC vs NC FET T2	1,6465207	0,03504	P14543;P14543-2	Nidogen-1	NID1
UC vs NC FET T2	3,5618396	1,53E-02	P16885	1-phosphatidylinositol 4,5-bisphosphate phosphodiesterase gamma-2	PLCG2
UC vs NC FET T2	1,9512568	0,01263	P19827	Inter-alpha-trypsin inhibitor heavy chain H1	ITIH1
UC vs NC FET T2	-1,948407	0,01912	P21333-2;P21333	Filamin-A	FLNA
UC vs NC FET T2	3,027287	0,0008419	P22352	Glutathione peroxidase 3	GPX3
UC vs NC FET T2	0,8232627	0,02455	P22692	Insulin-like growth factor-binding protein 4	IGFBP4
UC vs NC FET T2	4,9383151	1,95E-05	P23083	Ig heavy chain V-I region V35	Ig heavy chain V-I region V35
UC vs NC FET T2	0,8563042	0,03633	P23142;P23142-2;P23142-3	Fibulin-1	FBLN1
UC vs NC FET T2	3,44452	9,58E-05	P23142-4	Fibulin-1	FBLN1
UC vs NC FET T2	-1,366296	0,03379	P23280-3;P23280	Carbonic anhydrase 6	CA6
UC vs NC FET T2	1,9491658	0,005273	P24593	Insulin-like growth factor-binding protein 5	IGFBP5
UC vs NC FET T2	4,4433246	0,006971	P26927	Hepatocyte growth factor-like protein	MST1
UC vs NC FET T2	1,8765799	0,006691	P28370-2;P28370	Probable global transcription activator SNF2L1	SMARCA1
UC vs NC FET T2	1,0547277	0,01708	P30041	Peroxiredoxin-6	PRDX6
UC vs NC FET T2	-0,532871	0,009794	P30101	Protein disulfide-isomerase A3	PDIA3
UC vs NC FET T2	-3,836978	0,002061	P31025;Q5VSP4	Lipocalin-1	LCN1
UC vs NC FET T2	-1,684572	0,004161	P31151;Q86SG5	Protein S100-A7	S100A7
UC vs NC FET T2	1,8164201	0,01443	P33151;P33151-2	Cadherin-5	CDH5
UC vs NC FET T2	2,0266945	0,003998	P34096	Ribonuclease 4	RNASE4
UC vs NC FET T2	1,8617658	0,0144	P35443	Thrombospondin-4	THBS4
UC vs NC FET T2	5,1595149	1,43E-02	P35542	Serum amyloid A-4 protein	SAA4
UC vs NC FET T2	1,7418705	0,0373	P35555	Fibrillin-1	FBN1
UC vs NC FET T2	3,2913373	0,002281	P35556	Fibrillin-2	FBN2
UC vs NC FET T2	3,8240275	0,0001489	P35858;P35858-2	Insulin-like growth factor-binding protein complex acid labile subunit	IGFALS
UC vs NC FET T2	1,4304663	0,02227	P36980-2;P36980	Complement factor H-related protein 2	CFHR2
UC vs NC FET T2	2,4713156	1,86E-02	P39060-2;P39060-1;P39060	Collagen alpha-1(XVIII) chain	COL18A1
UC vs NC FET T2	1,2483673	0,0175	P43652	Afamin	AFM
UC vs NC FET T2	2,9123774	0,0002832	P48740	Mannan-binding lectin serine protease 1	MASP1
UC vs NC FET T2	1,038063	0,01394	P48740-2	Mannan-binding lectin serine protease 1	MASP1
UC vs NC FET T2	3,9175271	6,97E-03	P48740-4	Mannan-binding lectin serine protease 1	MASP1
UC vs NC FET T2	2,03252	0,02597	P49454	Centromere protein F	CENPF
UC vs NC FET T2	1,4220233	0,0008804	P49913	Cathelicidin antimicrobial peptide	CAMP
UC vs NC FET T2	1,4836516	0,009338	P51884;CON__Q05443	Lumican	LUM
UC vs NC FET T2	1,5135099	0,002976	P54132	Bloom syndrome protein	BLM
UC vs NC FET T2	1,8947368	0,008153	P55056	Apolipoprotein C-IV	APOC4
UC vs NC FET T2	2,6964914	0,01258	P55103	Inhibin beta C chain	INHBC
UC vs NC FET T2	1,0534972	0,0157	P55287-2;P55287	Cadherin-11	CDH11
UC vs NC FET T2	2,7528267	0,001381	P57077;P57077-1	MAP3K7 C-terminal-like protein	MAP3K7CL
UC vs NC FET T2	0,5077926	0,02162	P59666;P59665	Neutrophil defensin 3	DEFA3;DEFA1
UC vs NC FET T2	2,2359314	0,006957	P60709	Actin, cytoplasmic 1	ACTB
UC vs NC FET T2	2,0928935	0,0002635	P60900;P60900-2;P60900-3	Proteasome subunit alpha type-6	PSMA6
UC vs NC FET T2	4,7762467	0,0002861	P61626	Lysozyme C	LYZ
UC vs NC FET T2	-2,348445	0,01312	P61769	Beta-2-microglobulin	B2M
UC vs NC FET T2	1,0976577	0,005586	P63104;P63104-2	14-3-3 protein zeta/delta	YWHAZ
UC vs NC FET T2	-2,981623	0,02365	P68363;P68363-2	Tubulin alpha-1B chain	TUBA1B
UC vs NC FET T2	1,4536252	0,01079	P80108;P80108-2	Phosphatidylinositol-glycan-specific phospholipase D	GPLD1
UC vs NC FET T2	3,6604876	0,0002625	P80748	Ig lambda chain V-III region LOI	Ig lambda chain V-III region LOI
UC vs NC FET T2	1,8719296	0,008049	Q00888;Q00888-3;Q00888-2	Pregnancy-specific beta-1-glycoprotein 4	PSG4
UC vs NC FET T2	2,2038176	0,04668	Q02108-2;Q02108	Guanylate cyclase soluble subunit alpha-3	GUCY1A3
UC vs NC FET T2	2,317216	0,0001943	Q02224;Q02224-3	Centromere-associated protein E	CENPE
UC vs NC FET T2	2,6886799	0,001502	Q02985-2;Q02985	Complement factor H-related protein 3	CFHR3
UC vs NC FET T2	1,4632636	0,02344	Q03001;Q03001-9;Q03001-13;Q03001-10	Dystonin	DST
UC vs NC FET T2	3,2503653	0,006077	Q03591	Complement factor H-related protein 1	CFHR1
UC vs NC FET T2	2,7635999	0,02148	Q06033-2;Q06033	Inter-alpha-trypsin inhibitor heavy chain H3	ITIH3
UC vs NC FET T2	2,040996	0,007651	Q06190	Serine/threonine-protein phosphatase 2A regulatory subunit B subunit alpha	PPP2R3A
UC vs NC FET T2	2,0423193	0,01375	Q12805-2;Q12805-4;Q12805-3;Q12805	EGF-containing fibulin-like extracellular matrix protein 1	EFEMP1
UC vs NC FET T2	1,5477605	0,02213	Q12805-5	EGF-containing fibulin-like extracellular matrix protein 1	EFEMP1
UC vs NC FET T2	2,5632151	0,009771	Q13103	Secreted phosphoprotein 24	SPP2
UC vs NC FET T2	3,1389261	0,01634	Q13201;Q13201-2	Multimerin-1	MMRN1

UC vs NC FET T2	3,0050611	0,0001612	Q13214-2;Q13214	Semaphorin-3B	SEMA3B
UC vs NC FET T2	7,1983225	4,47E-05	Q13219	Pappalysin-1	PAPPA
UC vs NC FET T2	1,5961444	0,00296	Q13418-2;Q13418;Q13418-3	Integrin-linked protein kinase	ILK
UC vs NC FET T2	5,1806676	0,009778	Q13488-2	V-type proton ATPase 116 kDa subunit a isoform 3	TCIRG1
UC vs NC FET T2	2,9245018	0,0002194	Q13635-2	.	.
UC vs NC FET T2	0,7086104	0,02909	Q14161-7;Q14161-9;Q14161-8;Q14161-5;Q14161;Q14161-2;Q14161-11;Q14161-6;Q14161-10;Q14161-4;Q14161-3	ARF GTPase-activating protein GIT2	GIT2
UC vs NC FET T2	1,4671102	0,01157	Q14515;Q14515-2	SPARC-like protein 1	SPARCL1
UC vs NC FET T2	2,1179686	0,002358	Q14520-2;Q14520	Hyaluronan-binding protein 2	HABP2
UC vs NC FET T2	3,0009725	0,001189	Q15022	Polycomb protein SUZ12	SUZ12
UC vs NC FET T2	1,7010551	0,0247	Q15166	Serum paraoxonase/lactonase 3	PON3
UC vs NC FET T2	5,5729319	1,13E-02	Q15386;Q15386-2	Ubiquitin-protein ligase E3C	UBE3C
UC vs NC FET T2	2,9318333	0,0193	Q15485;Q15485-2	Ficolin-2	FCN2
UC vs NC FET T2	4,9806446	0,0011	Q15582	Transforming growth factor-beta-induced protein ig-h3	TGFBI
UC vs NC FET T2	5,3635593	0,00198	Q15848	Adiponectin	ADIPOQ
UC vs NC FET T2	2,1445079	0,02648	Q16610-4;Q16610;Q16610-2	Extracellular matrix protein 1	ECM1
UC vs NC FET T2	2,498823	0,005884	Q16769;Q16769-2	Glutaminyl-peptide cyclotransferase	QPCT
UC vs NC FET T2	2,2684464	7,93E-03	Q4L180-3;Q4L180-7;Q4L180-5;Q4L180-2;Q4L180;Q4L180-6	Filamin A-interacting protein 1-like	FILIP1L
UC vs NC FET T2	3,1211123	4,63E-02	Q4LDE5;Q4LDE5-4;Q4LDE5-3;Q4LDE5-2	Sushi, von Willebrand factor type A, EGF and pentraxin domain-containing protein 1	SVEP1
UC vs NC FET T2	2,8176754	3,37E-03	Q5JPF3;Q5JPF3-2;A6QL64-3;Q5JPF3-3	Ankyrin repeat domain-containing protein 36C	ANKRD36C;ANKRD36
UC vs NC FET T2	0,8170505	0,003759	Q5T0U0;Q5T0U0-2	Coiled-coil domain-containing protein 122	CCDC122
UC vs NC FET T2	3,5140873	0,002392	Q6P1M0-2	Long-chain fatty acid transport protein 4	SLC27A4
UC vs NC FET T2	3,0214562	0,007803	Q6Q788	Apolipoprotein A-V	APOA5
UC vs NC FET T2	1,729181	0,0001843	Q6UVK1	Chondroitin sulfate proteoglycan 4	CSPG4
UC vs NC FET T2	3,8495551	0,001749	Q6UXH9-2;Q6UXH9-3;Q6UXH9	Inactive serine protease PAMR1	PAMR1
UC vs NC FET T2	3,9602714	3,31E-02	Q6UY14-3;Q6UY14;Q6UY14-2	ADAMTS-like protein 4	ADAMTSL4
UC vs NC FET T2	1,8637242	0,01508	Q76LX8;Q76LX8-2;Q76LX8-3;Q76LX8-4	A disintegrin and metalloproteinase with thrombospondin motifs 13	ADAMTS13
UC vs NC FET T2	-2,579796	0,00452	Q7LBC6-3	Lysine-specific demethylase 3B	KDM3B
UC vs NC FET T2	1,4185459	0,0008607	Q7Z478	ATP-dependent RNA helicase DHX29	DHX29
UC vs NC FET T2	3,1790065	0,01186	Q7Z7A1-5;Q7Z7A1;Q7Z7A1-2;Q7Z7A1-3;Q7Z7A1-4	Centriolin	CNTRL
UC vs NC FET T2	2,0663511	0,01368	Q86UQ4;Q86UQ4-4;Q86UQ4-3;Q86UQ4-6;Q86UQ4-7;Q86UQ4-5	ATP-binding cassette sub-family A member 13	ABCA13
UC vs NC FET T2	1,3335636	0,02087	Q8IV50-2;Q8IV50	LysM and putative peptidoglycan-binding domain-containing protein 2	LYSMD2
UC vs NC FET T2	1,6130175	0,00177	Q8IVL1-11;Q8IVL1-4	Neuron navigator 2	NAV2
UC vs NC FET T2	1,7622575	0,03269	Q8IYW2	Cilia- and flagella-associated protein 46	CFAP46
UC vs NC FET T2	0,9610652	0,002367	Q8IZK6-2;Q8IZK6	Mucolipin-2	MCOLN2
UC vs NC FET T2	-1,64171	0,001596	Q8IZP9-9;Q8IZP9-10;Q8IZP9-8;Q8IZP9-7;Q8IZP9-5;Q8IZP9-6;Q8IZP9-3;Q8IZP9-4;Q8IZP9-2;Q8IZP9	G-protein coupled receptor 64	GPR64
UC vs NC FET T2	3,557531	0,009799	Q8N7Z5;Q8WY50	Putative ankyrin repeat domain-containing protein 31	ANKRD31
UC vs NC FET T2	-1,301451	0,03207	Q8N8A2-4;Q8N8A2;Q8N8A2-2;Q8N8A2-3;Q8N8A2-5	Serine/threonine-protein phosphatase 6 regulatory ankyrin repeat subunit B	ANKRD44
UC vs NC FET T2	4,6703466	3,06E-02	Q8NBP7	Proprotein convertase subtilisin/kexin type 9	PCSK9
UC vs NC FET T2	-2,84288	0,003047	Q8ND83-3;Q8ND83-2;Q8ND83-4	SLAIN motif-containing protein 1	SLAIN1
UC vs NC FET T2	2,4636783	0,001588	Q8NGK2	Olfactory receptor 52B4	OR52B4
UC vs NC FET T2	2,4779488	0,0146	Q8NI99	Angiopoietin-related protein 6	ANGPTL6
UC vs NC FET T2	2,2872821	0,004693	Q8TE73	Dynein heavy chain 5, axonemal	DNAH5
UC vs NC FET T2	1,6882205	0,006458	Q8WUA8	Tsukushin	TSKU
UC vs NC FET T2	2,0449383	0,01017	Q8WWZ8;Q8WWZ8-2	Oncoprotein-induced transcript 3 protein	OIT3
UC vs NC FET T2	3,6140721	0,01171	Q8WZ42-5	Titin	TTN
UC vs NC FET T2	1,4496645	0,01071	Q92496;Q92496-2;Q92496-3	Complement factor H-related protein 4	CFHR4
UC vs NC FET T2	2,0417562	0,000405	Q92743	Serine protease HTRA1	HTRA1
UC vs NC FET T2	3,2642503	0,009642	Q92954-3;Q92954-6;Q92954;Q92954-4;Q92954-2;Q92954-5	Proteoglycan 4	PRG4
UC vs NC FET T2	2,6759906	3,97E-02	Q96IY4;Q96IY4-2;CON__Q2KIG3	Carboxypeptidase B2	CPB2
UC vs NC FET T2	1,1365721	0,001603	Q96JB1-2;Q96JB1	Dynein heavy chain 8, axonemal	DNAH8
UC vs NC FET T2	1,0907197	0,01901	Q96KN2	Beta-Ala-His dipeptidase	CNDP1
UC vs NC FET T2	3,5352149	0,001452	Q96PD5;Q96PD5-2	N-acetylmuramoyl-L-alanine amidase	PGLYRP2
UC vs NC FET T2	-1,957936	0,0467	Q96QR1	Secretoglobin family 3A member 1	SCGB3A1
UC vs NC FET T2	3,9787449	0,0001614	Q96RL1-3;Q96RL1-4	BRCA1-A complex subunit RAP80	UIMC1
UC vs NC FET T2	0,9318253	0,003215	Q99574	Neuroserpin	SERPINI1
UC vs NC FET T2	1,6954498	0,0005332	Q99969	Retinoic acid receptor responder protein 2	RARRES2
UC vs NC FET T2	2,6296148	2,38E-06	Q9BXR6	Complement factor H-related protein 5	CFHR5
UC vs NC FET T2	1,5964596	0,002744	Q9BZR9	Probable E3 ubiquitin-protein ligase TRIM8	TRIM8
UC vs NC FET T2	-1,734799	0,0002616	Q9HCL0-2;Q9HCL0	Protocadherin-18	PCDH18
UC vs NC FET T2	2,8156509	0,000376	Q9HDC9;Q9HDC9-2	Adipocyte plasma membrane-associated protein	APMAP
UC vs NC FET T2	5,8627249	9,95E-03	Q9NQ79;Q9NQ79-2;Q9NQ79-3	Cartilage acidic protein 1	CRTAC1
UC vs NC FET T2	1,8293968	0,0001798	Q9NY15;Q9NY15-2	Stabilin-1	STAB1
UC vs NC FET T2	-1,733434	0,02646	Q9NZI8-2;Q9NZI8	Insulin-like growth factor 2 mRNA-binding protein 1	IGF2BP1
UC vs NC FET T2	-3,354354	0,004656	Q9NZT1	Calmodulin-like protein 5	CALML5
UC vs NC FET T2	3,2687507	0,0007722	Q9UHG3	Prenylcysteine oxidase 1	PCYOX1
UC vs NC FET T2	2,6315527	0,0116	Q9UK55	Protein Z-dependent protease inhibitor	SERPINA10
UC vs NC FET T2	1,9327845	0,01373	Q9UQ72;Q9UQ72-2	Pregnancy-specific beta-1-glycoprotein 11	PSG11
UC vs NC FET T2	0,8240063	0,04972	Q9Y4B5	Microtubule cross-linking factor 1	MTCL1
UC vs NC FET T2	1,3640631	0,0008211	Q9Y4C2-2;Q9Y4C2	TRPM8 channel-associated factor 1	TCAF1
UC vs NC FET T3	1,9204466	0,0001111	A0A075B6I0	.	IGLV8-61
UC vs NC FET T3	2,5403775	0,00855	A0A075B6I4	.	IGLV10-54
UC vs NC FET T3	3,4205923	0,01066	A0A075B6J9	.	IGLV2-18

UC vs NC FET T3	1,561759	0,04034	A0A075B6P5;P01615	Ig kappa chain V-II region FR	IGKV2D-28
UC vs NC FET T3	2,0482302	0,005692	A0A0C4DH68;A0A075B6R9	.	IGKV2-24;IGKV2D-24
UC vs NC FET T3	1,9932885	0,01442	A0A0B4J1X5	.	IGHV3-74
UC vs NC FET T3	4,2135382	0,009137	A0A0B4J1Y9	.	IGHV3-72
UC vs NC FET T3	1,6103914	0,002925	A0A0C4DH33	.	IGHV1-24
UC vs NC FET T3	3,3079242	0,03244	A0A0C4DH35	.	IGHV3-35
UC vs NC FET T3	-2,263726	0,003966	A0A0C4DH38	.	IGHV5-51
UC vs NC FET T3	1,5582266	0,02316	A0A0C4DH67;A0A0C4DH69	.	IGKV1-8;IGKV1-9
UC vs NC FET T3	4,6640688	0,001029	A0A0J9YX35	.	.
UC vs NC FET T3	-6,26304	0,0008326	A4FU69-3;A4FU69;A4FU69-2;A4FU69-4;A4FU69-6	EF-hand calcium-binding domain-containing protein 5	EFCAB5
UC vs NC FET T3	2,8542172	0,0002155	A8K2U0;A8K2U0-2	Alpha-2-macroglobulin-like protein 1	A2ML1
UC vs NC FET T3	1,0638795	0,008979	M0R2J8	.	DCDC1
UC vs NC FET T3	3,0141859	0,0005151	O00187;O00187-2	Mannan-binding lectin serine protease 2	MASP2
UC vs NC FET T3	-3,55312	0,04362	O00217	NADH dehydrogenase [ubiquinone] iron-sulfur protein 8, mitochondrial	NDUFS8
UC vs NC FET T3	1,1854072	0,04616	O14791-2;O14791;O14791-3	Apolipoprotein L1	APOL1
UC vs NC FET T3	1,9929611	0,04351	P19105;O14950	Myosin regulatory light chain 12A	MYL12A;MYL12B
UC vs NC FET T3	3,8862112	0,0001789	O15016;O15016-2;O15016-3	Tripartite motif-containing protein 66	TRIM66
UC vs NC FET T3	6,2611873	0,01263	O43184-3;O43184-4;O43184-2;O43184	Disintegrin and metalloproteinase domain-containing protein 12	ADAM12
UC vs NC FET T3	2,1287429	0,01152	O43399;O43399-5;O43399-7;O43399-2;O43399-4;O43399-3	Tumor protein D54	TPD52L2
UC vs NC FET T3	2,2492538	0,0142	O43861-2;O43861	Probable phospholipid-transporting ATPase IIB	ATP9B
UC vs NC FET T3	1,9853355	0,009199	Q99880;Q99879;Q99877;Q93079;Q8N257;Q5QNW6;Q16778;P62807;P58876;P57053;P33778;P23527;P06899;O60814;Q5QNW6-2	Histone H2B type 1-L	HIST1H2BL;HIST1H2BM;HIST1H2BN;HIST1H2BH;HIST3H2BB;HIST2H2BF;HIST2H2BE;HIST1H2BC;HIST1H2BD;H2BFS;HIST1H2BB;HIST1H2BO;HIST1H2BJ;HIST1H2BK
UC vs NC FET T3	3,3261447	0,003447	O75636;O75636-2	Ficolin-3	FCN3
UC vs NC FET T3	5,2201972	0,001371	O75882;O75882-2;O75882-3	Attractin	ATRN
UC vs NC FET T3	1,6063463	0,0005153	O76076	WNT1-inducible-signaling pathway protein 2	WISP2
UC vs NC FET T3	-2,597672	7,03E-05	O94855;O94855-2	Protein transport protein Sec24D	SEC24D
UC vs NC FET T3	1,6072713	0,00212	O95428-6;O95428;O95428-5;O95428-4;O95428-2;O95428-3	Papilin	PAPLN
UC vs NC FET T3	6,357223	5,92E-03	O95445-2	Apolipoprotein M	APOM
UC vs NC FET T3	1,1855488	0,003388	P00450	Ceruloplasmin	CP
UC vs NC FET T3	1,0489836	0,002713	P00488	Coagulation factor XIII A chain	F13A1
UC vs NC FET T3	4,6899449	0,008481	P00709	Alpha-lactalbumin	LALBA
UC vs NC FET T3	0,6217909	0,02487	P00734	Prothrombin	F2
UC vs NC FET T3	1,4356294	0,0003457	P00736	Complement C1r subcomponent	C1R
UC vs NC FET T3	1,6138687	0,007086	P00739;P00739-2	Haptoglobin-related protein	HPR
UC vs NC FET T3	3,8446522	0,000143	P00740;P00740-2	Coagulation factor IX	F9
UC vs NC FET T3	2,4392133	0,002024	P00742	Coagulation factor X	F10
UC vs NC FET T3	1,2013102	0,009971	P00746	Complement factor D	CFD
UC vs NC FET T3	1,6818228	0,009614	P00748	Coagulation factor XII	F12
UC vs NC FET T3	2,8341541	0,004954	P00751;P00751-2	Complement factor B	CFB
UC vs NC FET T3	0,9677553	0,0469	P01023	Alpha-2-macroglobulin	A2M
UC vs NC FET T3	1,602416	0,005459	P01031	Complement C5	C5
UC vs NC FET T3	-2,539686	0,0001928	P01040	Cystatin-A	CSTA
UC vs NC FET T3	3,1780777	1,07E-02	P01042-2	Kininogen-1	KNG1
UC vs NC FET T3	0,9908412	0,01226	P01344-3;P01344;P01344-2	Insulin-like growth factor II	IGF2
UC vs NC FET T3	4,0020698	0,0003696	P01599	Ig kappa chain V-I region Gal	Ig kappa chain V-I region Gal
UC vs NC FET T3	2,0163579	0,005861	P01619	Ig kappa chain V-III region B6	Ig kappa chain V-III region B6
UC vs NC FET T3	1,327354	0,03575	P01624	Ig kappa chain V-III region POM	Ig kappa chain V-III region POM
UC vs NC FET T3	2,4380331	0,00253	P01700	Ig lambda chain V-I region HA	Ig lambda chain V-I region HA
UC vs NC FET T3	1,4323869	0,02679	P01701	Ig lambda chain V-I region NEW	Ig lambda chain V-I region NEW
UC vs NC FET T3	1,6453214	0,01145	P01709	Ig lambda chain V-II region MGC	Ig lambda chain V-II region MGC
UC vs NC FET T3	-2,042971	0,01915	P01715	Ig lambda chain V-IV region Bau	Ig lambda chain V-IV region Bau
UC vs NC FET T3	5,2612882	0,0006188	P01743	Ig heavy chain V-I region HG3	Ig heavy chain V-I region HG3
UC vs NC FET T3	-2,029768	0,002636	P01764	Ig heavy chain V-III region 23	IGHV3-23
UC vs NC FET T3	3,4524801	0,007735	P0DP03;P01768	Ig heavy chain V-III region CAM	Ig heavy chain V-III region CAM
UC vs NC FET T3	5,006731	0,00339	P01782;P0DP04	Ig heavy chain V-III region DOB	Ig heavy chain V-III region DOB
UC vs NC FET T3	-2,471989	0,0002888	P01834	Ig kappa chain C region	IGKC
UC vs NC FET T3	1,2561502	0,04161	P01859	Ig gamma-2 chain C region	IGHG2
UC vs NC FET T3	1,7569013	0,01085	P01860	Ig gamma-3 chain C region	IGHG3
UC vs NC FET T3	1,1866407	0,009176	P01871;P01871-2	Ig mu chain C region	IGHM
UC vs NC FET T3	1,6097093	0,0007486	P01876	Ig alpha-1 chain C region	IGHA1
UC vs NC FET T3	0,6670847	0,002962	P02647;Q9HB71-2	Apolipoprotein A-I	APOA1
UC vs NC FET T3	1,1516538	0,003033	P02649;CON__Q03247	Apolipoprotein E	APOE
UC vs NC FET T3	1,5452948	0,0008704	P02671;P02671-2;REV__Q9UKV0-4;REV__Q9UKV0-2;REV__Q9UKV0;REV__Q9UKV0-5;REV__Q9UKV0-7;Q14314	Fibrinogen alpha chain	FGA
UC vs NC FET T3	1,618413	0,0008259	P02675	Fibrinogen beta chain	FGB
UC vs NC FET T3	0,7839484	0,004069	P02679;P02679-2	Fibrinogen gamma chain	FGG
UC vs NC FET T3	2,2401147	2,29E-02	P02746	Complement C1q subcomponent subunit B	C1QB
UC vs NC FET T3	2,2343369	2,65E-05	P02747	Complement C1q subcomponent subunit C	C1QC
UC vs NC FET T3	1,8306928	0,02702	P02750	Leucine-rich alpha-2-glycoprotein	LRG1
UC vs NC FET T3	1,8276124	0,00692	P02751-1;P02751-8;P02751-3;P02751;P02751-14;P02751-7;P02751-17;P02751-9;P02751-6;P02751-4;P02751-12;P02751-16;P02751-2	Fibronectin	FN1
UC vs NC FET T3	1,0545988	0,01098	P02753	Retinol-binding protein 4	RBP4
UC vs NC FET T3	-1,062254	0,01588	P02760	Protein AMBP	AMBP
UC vs NC FET T3	1,6168461	0,003669	P02766	Transthyretin	TTR
UC vs NC FET T3	2,3865452	0,0004731	P02774-3;P02774;P02774-2	Vitamin D-binding protein	GC
UC vs NC FET T3	3,850319	0,0008339	P02775	Platelet basic protein	PPBP

UC vs NC FET T3	4,3729591	0,0007518	P02776	Platelet factor 4	PF4
UC vs NC FET T3	1,2911925	0,04932	P02787;CON__Q2HJF0	Serotransferrin	TF
UC vs NC FET T3	1,4289265	0,003126	P02790	Hemopexin	HPX
UC vs NC FET T3	5,6858153	1,11E-03	P03950	Angiogenin	ANG
UC vs NC FET T3	4,9317508	2,13E-03	P03951	Coagulation factor XI	F11
UC vs NC FET T3	2,8164978	2,97E-05	P03952;P20718	Plasma kallikrein	KLKB1
UC vs NC FET T3	-0,562778	0,02982	P04004	Vitronectin	VTN
UC vs NC FET T3	3,0552955	0,006759	P04040	Catalase	CAT
UC vs NC FET T3	1,4191899	0,0372	P04070;P04070-2	Vitamin K-dependent protein C	PROC
UC vs NC FET T3	0,7798557	0,03227	P04180	Phosphatidylcholine-sterol acyltransferase	LCAT
UC vs NC FET T3	4,6157625	0,0002378	P04211;A0A075B6I9	Ig lambda chain V region 4A	IGLV7-46
UC vs NC FET T3	2,5247502	0,02303	P04217	Alpha-1B-glycoprotein	A1BG
UC vs NC FET T3	4,9207234	0,00837	P04275	von Willebrand factor	VWF
UC vs NC FET T3	2,7242243	0,0006463	P04406;P04406-2;O14556	Glyceraldehyde-3-phosphate dehydrogenase	GAPDH
UC vs NC FET T3	-0,634553	0,039	P05019-3;P05019-2;P05019-4;P05019	Insulin-like growth factor I	IGF1
UC vs NC FET T3	4,4889052	0,004221	P05023-2;P05023-4;P05023;P05023-3	Sodium/potassium-transporting ATPase subunit alpha-1	ATP1A1
UC vs NC FET T3	1,0440612	0,001482	P05090	Apolipoprotein D	APOD
UC vs NC FET T3	-1,178319	0,001282	P05109	Protein S100-A8	S100A8
UC vs NC FET T3	3,7529302	0,000621	P05155-2;P05155;P05155-3	Plasma protease C1 inhibitor	SERPING1
UC vs NC FET T3	1,9185138	1,56E-02	P05160	Coagulation factor XIII B chain	F13B
UC vs NC FET T3	0,6581584	0,01465	P05164-3;P05164;P05164-2	Myeloperoxidase	MPO
UC vs NC FET T3	1,9720351	0,0243	P05543	Thyroxine-binding globulin	SERPINA7
UC vs NC FET T3	2,5410109	0,000728	P05546	Heparin cofactor 2	SERPIND1
UC vs NC FET T3	2,746417	0,004045	P06331	Ig heavy chain V-II region ARH-77	Ig heavy chain V-II region ARH-77
UC vs NC FET T3	0,7935286	0,01349	P06396;P06396-4;P06396-3	Gelsolin	GSN
UC vs NC FET T3	0,6898713	0,02992	P06727;Q9BT92	Apolipoprotein A-IV	APOA4
UC vs NC FET T3	1,0384123	0,03025	P06733	Alpha-enolase	ENO1
UC vs NC FET T3	2,4982395	0,04387	P06753-5;P06753-2;P06753-4;P06753-3;P06753-6;P06753-7	Tropomyosin alpha-3 chain	TPM3
UC vs NC FET T3	0,8278583	0,0001504	P06850	Corticoliberin	CRH
UC vs NC FET T3	0,905055	0,007723	P07225	Vitamin K-dependent protein S	PROS1
UC vs NC FET T3	3,4238968	0,02073	P07237	Protein disulfide-isomerase	P4HB
UC vs NC FET T3	1,0792656	2,07E-05	P07333;P07333-2	Macrophage colony-stimulating factor 1 receptor	CSF1R
UC vs NC FET T3	2,6372223	0,003197	P07357	Complement component C8 alpha chain	C8A
UC vs NC FET T3	2,6310692	0,006145	P07358	Complement component C8 beta chain	C8B
UC vs NC FET T3	1,4742676	0,0004186	P07900;P07900-2;Q14568;Q58FF6;Q58FG1	Heat shock protein HSP 90-alpha	HSP90AA1
UC vs NC FET T3	2,0074615	0,03885	P07996;P07996-2	Thrombospondin-1	THBS1
UC vs NC FET T3	1,3178415	0,03427	P08253;P08253-3;P08253-2	72 kDa type IV collagenase	MMP2
UC vs NC FET T3	2,7948065	0,0002571	P08294	Extracellular superoxide dismutase [Cu-Zn]	SOD3
UC vs NC FET T3	4,5536777	7,52E-04	P08493-2;P08493	Matrix Gla protein	MGP
UC vs NC FET T3	4,513207	0,02831	P08514;P08514-3;P08514-2	Integrin alpha-IIb	ITGA2B
UC vs NC FET T3	2,8979096	0,01988	P08567	Pleckstrin	PLEK
UC vs NC FET T3	1,4952302	0,001283	P08603;P08603-2	Complement factor H	CFH
UC vs NC FET T3	2,2169137	0,0005585	P08709-2;P08709	Coagulation factor VII	F7
UC vs NC FET T3	-3,068335	0,0001099	P09466-2;P09466	Glycodelin	PAEP
UC vs NC FET T3	2,3417628	0,004854	P09466-3	Glycodelin	PAEP
UC vs NC FET T3	2,1169224	0,02474	P09486	SPARC	SPARC
UC vs NC FET T3	0,8170476	0,0189	P09871	Complement C1s subcomponent	C1S
UC vs NC FET T3	-2,828537	0,02108	P0C0L4;P0C0L4-2	Complement C4-A	C4A
UC vs NC FET T3	1,0225029	0,009717	P0C0L5	Complement C4-B	C4B
UC vs NC FET T3	-2,653679	0,02054	P0DJI9;P0DJI9-2	Serum amyloid A-2 protein	SAA2
UC vs NC FET T3	-1,415568	0,0005474	P0DN87;P0DN86;P0DN86-2;Q6NT52;A6NKQ9-2;A6NKQ9	Choriogonadotropin subunit beta variant 2	CGB2;CGB1
UC vs NC FET T3	2,9719518	0,0374	P0DOX4	.	.
UC vs NC FET T3	1,2726579	0,0003661	P0DOX5;P01857	Ig gamma-1 chain C region	IGHG1
UC vs NC FET T3	1,4137526	0,0002768	P0DOX7	.	.
UC vs NC FET T3	1,7445846	0,002308	P0DOX8	.	.
UC vs NC FET T3	1,7645597	0,007656	P10643	Complement component C7	C7
UC vs NC FET T3	3,4539765	0,01924	P10720	Platelet factor 4 variant	PF4V1
UC vs NC FET T3	2,7686199	0,001379	P11021	78 kDa glucose-regulated protein	HSPA5
UC vs NC FET T3	1,4778847	9,49E-03	P11464-4	Pregnancy-specific beta-1-glycoprotein 1	PSG1
UC vs NC FET T3	2,1134003	7,23E-03	P11465	Pregnancy-specific beta-1-glycoprotein 2	PSG2
UC vs NC FET T3	1,8905949	4,47E-02	P11597;P11597-2	Cholesteryl ester transfer protein	CETP
UC vs NC FET T3	1,9581184	0,004497	P12259	Coagulation factor V	F5
UC vs NC FET T3	-1,082917	0,04785	P12532;P12532-2	Creatine kinase U-type, mitochondrial	CKMT1A
UC vs NC FET T3	5,3800505	0,005343	P12814;P12814-3;P12814-2	Alpha-actinin-1	ACTN1
UC vs NC FET T3	1,9679139	0,0001686	P13667	Protein disulfide-isomerase A4	PDIA4
UC vs NC FET T3	3,6753944	7,58E-03	P13727;P13727-2	Bone marrow proteoglycan	PRG2
UC vs NC FET T3	-2,204305	0,0006113	P13798	Acylamino-acid-releasing enzyme	APEH
UC vs NC FET T3	0,982892	0,0004854	P14061	Estradiol 17-beta-dehydrogenase 1	HSD17B1
UC vs NC FET T3	1,289247	0,02442	P14209;P14209-2;P14209-3	CD99 antigen	CD99
UC vs NC FET T3	2,1644761	0,02138	P14618;P14618-2	Pyruvate kinase PKM	PKM
UC vs NC FET T3	2,2642234	0,01481	P15907	Beta-galactoside alpha-2,6-sialyltransferase 1	ST6GAL1
UC vs NC FET T3	-1,818277	0,00422	P16035	Metalloproteinase inhibitor 2	TIMP2
UC vs NC FET T3	3,8530625	0,0006259	P16885	1-phosphatidylinositol 4,5-bisphosphate phosphodiesterase gamma-2	PLCG2
UC vs NC FET T3	4,6758676	0,03065	P17936;P17936-2	Insulin-like growth factor-binding protein 3	IGFBP3
UC vs NC FET T3	1,1259847	0,000432	P19823	Inter-alpha-trypsin inhibitor heavy chain H2	ITIH2
UC vs NC FET T3	5,0515078	0,009249	P21333-2;P21333	Filamin-A	FLNA
UC vs NC FET T3	2,1889048	0,01142	P22352	Glutathione peroxidase 3	GPX3
UC vs NC FET T3	0,8191009	0,005267	P22692	Insulin-like growth factor-binding protein 4	IGFBP4
UC vs NC FET T3	5,1137263	0,001461	P23083	Ig heavy chain V-I region V35	Ig heavy chain V-I region V35
UC vs NC FET T3	3,3571153	0,0009442	P23142-4	Fibulin-1	FBLN1
UC vs NC FET T3	-1,219541	0,03923	P23280-3;P23280	Carbonic anhydrase 6	CA6
UC vs NC FET T3	2,2814026	0,0193	P24593	Insulin-like growth factor-binding protein 5	IGFBP5
UC vs NC FET T3	4,3116627	0,004172	P26927	Hepatocyte growth factor-like protein	MST1
UC vs NC FET T3	1,4422394	0,03741	P28370-2;P28370	Probable global transcription activator SNF2L1	SMARCA1
UC vs NC FET T3	1,2152213	0,006428	P30043	Flavin reductase (NADPH)	BLVRB
UC vs NC FET T3	-2,252538	0,0003828	P31151;Q86SG5	Protein S100-A7	S100A7
UC vs NC FET T3	1,8462045	2,60E-02	P34096	Ribonuclease 4	RNASE4
UC vs NC FET T3	1,3675256	0,009547	P35443	Thrombospondin-4	THBS4

UC vs NC FET T3	4,1962876	0,0001963	P35542	Serum amyloid A-4 protein	SAA4
UC vs NC FET T3	1,5548141	0,0001903	P35555	Fibrillin-1	FBN1
UC vs NC FET T3	7,7282905	1,09E-02	P35579;P35579-2	Myosin-9	MYH9
UC vs NC FET T3	2,5912123	0,0005652	P35858;P35858-2	Insulin-like growth factor-binding protein complex acid labile subunit	IGFALS
UC vs NC FET T3	1,4487205	0,02766	P36980-2;P36980	Complement factor H-related protein 2	CFHR2
UC vs NC FET T3	2,3020659	0,009856	P39060-2;P39060-1;P39060	Collagen alpha-1(XVIII) chain	COL18A1
UC vs NC FET T3	-1,705725	0,02345	P46013;P46013-2	Antigen KI-67	MKI67
UC vs NC FET T3	3,3807453	0,01405	P48059;P48059-4;P48059-2;P48059-5;P48059-3;Q7Z4I7-4;Q7Z4I7-3;Q7Z4I7-2;P0CW19-2	LIM and senescent cell antigen-like-containing domain protein 1	LIMS1
UC vs NC FET T3	1,4858419	1,24E-02	P48307-2;P48307	Tissue factor pathway inhibitor 2	TFPI2
UC vs NC FET T3	1,1230679	0,0359	P48740-2	Mannan-binding lectin serine protease 1	MASP1
UC vs NC FET T3	4,6942541	6,94E-04	P48740-4	Mannan-binding lectin serine protease 1	MASP1
UC vs NC FET T3	3,3360437	0,008766	P49454	Centromere protein F	CENPF
UC vs NC FET T3	-1,050539	0,01955	P49767	Vascular endothelial growth factor C	VEGFC
UC vs NC FET T3	0,7682452	0,008539	P49908	Selenoprotein P	SEPP1
UC vs NC FET T3	2,2773084	0,004815	P54132	Bloom syndrome protein	BLM
UC vs NC FET T3	1,234046	0,02983	P55056	Apolipoprotein C-IV	APOC4
UC vs NC FET T3	2,1464839	0,03886	P55103	Inhibin beta C chain	INHBC
UC vs NC FET T3	0,808147	0,006823	P55287-2;P55287	Cadherin-11	CDH11
UC vs NC FET T3	3,5983203	0,000217	P57077;P57077-1	MAP3K7 C-terminal-like protein	MAP3K7CL
UC vs NC FET T3	4,8622789	0,0008073	P60709	Actin, cytoplasmic 1	ACTB
UC vs NC FET T3	2,3751641	0,002383	P60900;P60900-2;P60900-3	Proteasome subunit alpha type-6	PSMA6
UC vs NC FET T3	3,5106231	0,03253	P61224-3;P61224;P61224-2;P61224-4;A6NIZ1;P62834	Ras-related protein Rap-1b	RAP1B;RAP1A
UC vs NC FET T3	2,6982098	0,006766	P61626	Lysozyme C	LYZ
UC vs NC FET T3	0,9626385	0,02803	P62736;P63267;P63267-2	Actin, aortic smooth muscle	ACTA2;ACTG2
UC vs NC FET T3	3,4293443	0,003138	P63104;P63104-2	14-3-3 protein zeta/delta	YWHAZ
UC vs NC FET T3	3,2807562	0,01803	P68363;P68363-2	Tubulin alpha-1B chain	TUBA1B
UC vs NC FET T3	2,2869115	0,04677	P68871;P02100	Hemoglobin subunit beta	HBB
UC vs NC FET T3	4,0850458	0,008324	P69905	Hemoglobin subunit alpha	HBA1
UC vs NC FET T3	1,0601854	0,0004394	P80108;P80108-2	Phosphatidylinositol-glycan-specific phospholipase D	GPLD1
UC vs NC FET T3	2,87675	0,02661	P80748	Ig lambda chain V-III region LOI	Ig lambda chain V-III region LOI
UC vs NC FET T3	4,3550478	0,005237	Q00526;P11802-2;Q00535-2;Q00535;P11802;Q14004-2;Q14004	Cyclin-dependent kinase 3	CDK3
UC vs NC FET T3	-0,371364	0,01767	Q00887-2	Pregnancy-specific beta-1-glycoprotein 9	PSG9
UC vs NC FET T3	-3,823289	0,03318	Q00889-2;Q00889	Pregnancy-specific beta-1-glycoprotein 6	PSG6
UC vs NC FET T3	1,7383358	0,005961	Q02108-2;Q02108	Guanylate cyclase soluble subunit alpha-3	GUCY1A3
UC vs NC FET T3	3,9798349	0,01779	Q02224;Q02224-3	Centromere-associated protein E	CENPE
UC vs NC FET T3	-2,896674	0,0002803	Q02487-2;Q02487	Desmocollin-2	DSC2
UC vs NC FET T3	3,0800086	0,001133	Q02985-2;Q02985	Complement factor H-related protein 3	CFHR3
UC vs NC FET T3	1,5640632	0,04485	Q03001;Q03001-9;Q03001-13;Q03001-10	Dystonin	DST
UC vs NC FET T3	2,5939279	0,002768	Q03591	Complement factor H-related protein 1	CFHR1
UC vs NC FET T3	2,9472852	0,01187	Q06033-2;Q06033	Inter-alpha-trypsin inhibitor heavy chain H3	ITIH3
UC vs NC FET T3	2,0481374	0,04549	Q07954;Q07954-2	Prolow-density lipoprotein receptor-related protein 1	LRP1
UC vs NC FET T3	0,5779667	0,003358	Q08174;Q08174-2	Protocadherin-1	PCDH1
UC vs NC FET T3	1,4496191	0,008951	Q0VAK6;Q0VAK6-2	Leiomodin-3	LMOD3
UC vs NC FET T3	1,0289564	0,005149	Q12805-2;Q12805-4;Q12805-3;Q12805	EGF-containing fibulin-like extracellular matrix protein 1	EFEMP1
UC vs NC FET T3	2,2930461	0,0001511	Q12805-5	EGF-containing fibulin-like extracellular matrix protein 1	EFEMP1
UC vs NC FET T3	1,6839354	0,00264	Q13093	Platelet-activating factor acetylhydrolase	PLA2G7
UC vs NC FET T3	2,9677277	1,14E-02	Q13103	Secreted phosphoprotein 24	SPP2
UC vs NC FET T3	1,843709	0,009233	Q13214-2;Q13214	Semaphorin-3B	SEMA3B
UC vs NC FET T3	6,1342779	0,0001691	Q13219	Pappalysin-1	PAPPA
UC vs NC FET T3	0,9140771	0,03169	Q13361-2;Q13361	Microfibrillar-associated protein 5	MFAP5
UC vs NC FET T3	2,4930415	0,007257	Q13418-2;Q13418;Q13418-3	Integrin-linked protein kinase	ILK
UC vs NC FET T3	3,2516667	0,02732	Q13464	Rho-associated protein kinase 1	ROCK1
UC vs NC FET T3	4,8467559	9,00E-03	Q13488-2	V-type proton ATPase 116 kDa subunit a isoform 3	TCIRG1
UC vs NC FET T3	2,5139664	6,89E-03	Q13635-2	.	.
UC vs NC FET T3	2,4151144	0,001327	Q14520-2;Q14520	Hyaluronan-binding protein 2	HABP2
UC vs NC FET T3	1,1059732	0,009458	Q14624;Q14624-3;Q14624-4	Inter-alpha-trypsin inhibitor heavy chain H4	ITIH4
UC vs NC FET T3	1,8726634	0,005542	Q14697;Q14697-2	Neutral alpha-glucosidase AB	GANAB
UC vs NC FET T3	4,1204795	0,00566	Q14766;Q14766-4;Q14766-3;Q14766-2;Q14766-5	Latent-transforming growth factor beta-binding protein 1	LTBP1
UC vs NC FET T3	2,5391216	0,004127	Q15022	Polycomb protein SUZ12	SUZ12
UC vs NC FET T3	0,6800885	0,02628	Q15113	Procollagen C-endopeptidase enhancer 1	PCOLCE
UC vs NC FET T3	5,2340428	0,002925	Q15386;Q15386-2	Ubiquitin-protein ligase E3C	UBE3C
UC vs NC FET T3	2,611269	0,003664	Q15485;Q15485-2	Ficolin-2	FCN2
UC vs NC FET T3	4,6614537	0,003808	Q15582	Transforming growth factor-beta-induced protein ig-h3	TGFBI
UC vs NC FET T3	5,7038541	0,007476	Q15848	Adiponectin	ADIPOQ
UC vs NC FET T3	-0,672394	0,001563	Q16557	Pregnancy-specific beta-1-glycoprotein 3	PSG3
UC vs NC FET T3	1,519031	0,03405	Q16610-4;Q16610;Q16610-2	Extracellular matrix protein 1	ECM1
UC vs NC FET T3	-2,786815	0,04227	Q2KHM9	Uncharacterized protein KIAA0753	KIAA0753
UC vs NC FET T3	2,4968156	0,003949	Q5HYK7-3;Q5HYK7-2;Q5HYK7;Q5HYK7-5;Q5HYK7-4	SH3 domain-containing protein 19	SH3D19
UC vs NC FET T3	2,3348674	0,04783	Q5JPF3;Q5JPF3-2;A6QL64-3;Q5JPF3-3	Ankyrin repeat domain-containing protein 36C	ANKRD36C;ANKRD36
UC vs NC FET T3	1,8409457	0,04982	Q5T5C0;Q5T5C0-2;Q5T5C0-3	Syntaxin-binding protein 5	STXBP5
UC vs NC FET T3	2,5948201	0,002632	Q6EEV6	Small ubiquitin-related modifier 4	SUMO4
UC vs NC FET T3	4,0122448	0,001374	Q6P387-2;Q6P387	Uncharacterized protein C16orf46	C16orf46
UC vs NC FET T3	-0,470063	0,01149	Q6P3W6	Neuroblastoma breakpoint family member 10	NBPF10
UC vs NC FET T3	2,4243588	0,003415	Q6Q788	Apolipoprotein A-V	APOA5
UC vs NC FET T3	2,1318928	7,98E-03	Q6UVK1	Chondroitin sulfate proteoglycan 4	CSPG4
UC vs NC FET T3	2,8260935	0,01095	Q6UY14-3;Q6UY14;Q6UY14-2	ADAMTS-like protein 4	ADAMTSL4
UC vs NC FET T3	2,7315127	0,01086	Q6VAB6;Q6VAB6-2	Kinase suppressor of Ras 2	KSR2
UC vs NC FET T3	-1,547983	0,0004496	Q6ZS30-1;Q6ZS30	Neurobeachin-like protein 1	NBEAL1
UC vs NC FET T3	-2,301563	0,002539	Q7L1Q6-2;Q7L1Q6;Q7L1Q6-3	Basic leucine zipper and W2 domain-containing protein 1	BZW1
UC vs NC FET T3	-1,088337	0,03345	Q7Z572	Spermatogenesis-associated protein 21	SPATA21
UC vs NC FET T3	-1,272158	0,03703	Q7Z5L0	Vitelline membrane outer layer protein 1 homolog	VMO1

UC vs NC FET T3	1,3690393	1,95E-02	Q86U17	Serpin A11	SERPINA11
UC vs NC FET T3	4,9016828	0,00635	Q86UX7-2;Q86UX7	Fermitin family homolog 3	FERMT3
UC vs NC FET T3	0,7052668	0,0002281	Q8IV50-2;Q8IV50	LysM and putative peptidoglycan-binding domain-containing protein 2	LYSMD2
UC vs NC FET T3	-3,038091	0,0167	Q8IZP9-9;Q8IZP9-10;Q8IZP9-8;Q8IZP9-7;Q8IZP9-5;Q8IZP9-6;Q8IZP9-3;Q8IZP9-4;Q8IZP9-2;Q8IZP9	G-protein coupled receptor 64	GPR64
UC vs NC FET T3	-1,914556	0,006781	Q8N8A2-4;Q8N8A2;Q8N8A2-2;Q8N8A2-3;Q8N8A2-5	Serine/threonine-protein phosphatase 6 regulatory ankyrin repeat subunit B	ANKRD44
UC vs NC FET T3	-4,040371	0,04441	Q8NBJ4-2;Q8NBJ4	Golgi membrane protein 1	GOLM1
UC vs NC FET T3	3,169312	0,01164	Q8NBP7	Proprotein convertase subtilisin/kexin type 9	PCSK9
UC vs NC FET T3	-3,731359	0,002116	Q8ND83-3;Q8ND83-2;Q8ND83-4	SLAIN motif-containing protein 1	SLAIN1
UC vs NC FET T3	1,3388389	1,85E-02	Q8NDV7-6;Q8NDV7;Q8NDV7-2;Q8NDV7-5;Q8NDV7-4;Q8NDV7-3	Trinucleotide repeat-containing gene 6A protein	TNRC6A
UC vs NC FET T3	2,6674171	0,01612	Q8NFQ6	BPI fold-containing family C protein	BPIFC
UC vs NC FET T3	2,1432492	0,02787	Q8NGK2	Olfactory receptor 52B4	OR52B4
UC vs NC FET T3	2,4730463	0,0002093	Q8NI99	Angiopoietin-related protein 6	ANGPTL6
UC vs NC FET T3	2,5061561	0,0029	Q8TE73	Dynein heavy chain 5, axonemal	DNAH5
UC vs NC FET T3	-2,489371	0,02247	Q8WUA8	Tsukushin	TSKU
UC vs NC FET T3	1,8096832	0,01615	Q8WWZ8;Q8WWZ8-2	Oncoprotein-induced transcript 3 protein	OIT3
UC vs NC FET T3	3,4375233	0,01839	Q8WZ42-5	Titin	TTN
UC vs NC FET T3	-0,761703	0,003501	Q92598-2;Q92598-3;Q92598;Q92598-4	Heat shock protein 105 kDa	HSPH1
UC vs NC FET T3	1,6922336	0,008162	Q92626;Q92626-2	Peroxidasin homolog	PXDN
UC vs NC FET T3	1,0700429	0,003103	Q92743	Serine protease HTRA1	HTRA1
UC vs NC FET T3	3,1739893	0,008472	Q92954-3;Q92954-6;Q92954;Q92954-4;Q92954-2;Q92954-5	Proteoglycan 4	PRG4
UC vs NC FET T3	-1,398474	0,002273	Q96CM8-3;Q96CM8-4;Q96CM8;Q96CM8-2	Acyl-CoA synthetase family member 2, mitochondrial	ACSF2
UC vs NC FET T3	2,4570265	0,004356	Q96IY4;Q96IY4-2;CON__Q2KIG3	Carboxypeptidase B2	CPB2
UC vs NC FET T3	1,1770595	1,65E-05	Q96JB1-2;Q96JB1	Dynein heavy chain 8, axonemal	DNAH8
UC vs NC FET T3	3,1689291	0,02744	Q96PD5;Q96PD5-2	N-acetylmuramoyl-L-alanine amidase	PGLYRP2
UC vs NC FET T3	-3,48079	0,001704	Q96QR1	Secretoglobin family 3A member 1	SCGB3A1
UC vs NC FET T3	3,8506207	0,01687	Q96RL1-3;Q96RL1-4	BRCA1-A complex subunit RAP80	UIMC1
UC vs NC FET T3	-3,05623	0,03887	Q9BQS8;Q9BQS8-4;Q9BQS8-3;Q9BQS8-2	FYVE and coiled-coil domain-containing protein 1	FYCO1
UC vs NC FET T3	0,366485	0,01567	Q9BXP8;Q9BXP8-2	Pappalysin-2	PAPPA2
UC vs NC FET T3	2,0911818	2,70E-02	Q9BXR6	Complement factor H-related protein 5	CFHR5
UC vs NC FET T3	2,1390251	0,0006976	Q9H6X2-5;Q9H6X2;Q9H6X2-4;Q9H6X2-6;Q9H6X2-2;Q9H6X2-3	Anthrax toxin receptor 1	ANTXR1
UC vs NC FET T3	1,716785	0,01244	Q9HDC9;Q9HDC9-2	Adipocyte plasma membrane-associated protein	APMAP
UC vs NC FET T3	2,4576926	0,001285	Q9NY15;Q9NY15-2	Stabilin-1	STAB1
UC vs NC FET T3	-2,059936	0,01277	Q9NZT1	Calmodulin-like protein 5	CALML5
UC vs NC FET T3	2,4335213	0,003711	Q9UHG3	Prenylcysteine oxidase 1	PCYOX1
UC vs NC FET T3	-2,251465	0,0001405	Q9UJJ9	N-acetylglucosamine-1-phosphotransferase subunit gamma	GNPTG
UC vs NC FET T3	3,180285	0,02494	Q9UK55	Protein Z-dependent protease inhibitor	SERPINA10
UC vs NC FET T3	1,9133107	0,002055	Q9UM47	Neurogenic locus notch homolog protein 3	NOTCH3
UC vs NC FET T3	-0,598844	0,005115	Q9UQ72;Q9UQ72-2	Pregnancy-specific beta-1-glycoprotein 11	PSG11
UC vs NC FET T3	8,073518	0,000955	Q9Y490	Talin-1	TLN1
UC vs NC FET T3	1,8559226	0,01175	Q9Y4C2-2;Q9Y4C2	TRPM8 channel-associated factor 1	TCAF1
UC vs NC FET T3	1,0036935	0,04273	Q9Y5C1	Angiopoietin-related protein 3	ANGPTL3
UC vs NC FET T3	0,399522	0,04158	Q9Y6B6;Q9NR31	GTP-binding protein SAR1b	SAR1B;SAR1A
UC vs AC+NC FET T1	5,053929	2,78E-02	A0A075B6H7;A0A0C4DH55	.	IGKV3-7
UC vs AC+NC FET T1	3,1946156	0,001198	A0A075B6J9	.	IGLV2-18
UC vs AC+NC FET T1	0,7370807	0,02242	A0A075B6P5;P01615	Ig kappa chain V-II region FR	IGKV2D-28
UC vs AC+NC FET T1	1,3105947	0,01753	A0A0C4DH68;A0A075B6R9	.	IGKV2-24;IGKV2D-24
UC vs AC+NC FET T1	1,7486618	0,02977	A0A087WSY6	.	IGKV3D-15
UC vs AC+NC FET T1	1,8500121	0,002092	A0A0A0MS15	.	IGHV3-49
UC vs AC+NC FET T1	0,7306928	0,0001345	A0A0B4J1V0	.	IGHV3-15
UC vs AC+NC FET T1	3,2805358	2,33E-02	A0A0B4J1X5	.	IGHV3-74
UC vs AC+NC FET T1	4,8970522	1,61E-03	A0A0B4J1Y9	.	IGHV3-72
UC vs AC+NC FET T1	2,6454584	0,0008297	A0A0C4DH25	.	IGKV3D-20
UC vs AC+NC FET T1	5,5148652	0,001596	A0A0C4DH35	.	IGHV3-35
UC vs AC+NC FET T1	-1,918864	0,0115	A0A0C4DH38	.	IGHV5-51
UC vs AC+NC FET T1	3,1603306	5,69E-03	A0A0J9YX35	.	.
UC vs AC+NC FET T1	-3,063699	2,12E-03	A4FU69-3;A4FU69;A4FU69-2;A4FU69-4;A4FU69-6	EF-hand calcium-binding domain-containing protein 5	EFCAB5
UC vs AC+NC FET T1	2,7454203	3,12E-03	A8K2U0;A8K2U0-2	Alpha-2-macroglobulin-like protein 1	A2ML1
UC vs AC+NC FET T1	3,3268152	1,97E-04	O00187;O00187-2	Mannan-binding lectin serine protease 2	MASP2
UC vs AC+NC FET T1	1,179814	6,27E-03	O00391;O00391-2	Sulfhydryl oxidase 1	QSOX1
UC vs AC+NC FET T1	1,7317504	2,38E-03	O14791-2;O14791;O14791-3	Apolipoprotein L1	APOL1
UC vs AC+NC FET T1	3,7041181	0,003788	O15016;O15016-2;O15016-3	Tripartite motif-containing protein 66	TRIM66
UC vs AC+NC FET T1	-0,806109	0,006763	O15084;O15084-4;O15084-1;O15084-2	Serine/threonine-protein phosphatase 6 regulatory ankyrin repeat subunit A	ANKRD28
UC vs AC+NC FET T1	1,7534282	1,87E-04	O43399;O43399-5;O43399-7;O43399-2;O43399-4;O43399-3	Tumor protein D54	TPD52L2
UC vs AC+NC FET T1	1,1312915	0,00287	O43866	CD5 antigen-like	CD5L
UC vs AC+NC FET T1	1,0405358	0,03113	Q99880;Q99879;Q99877;Q93079;Q8N257;Q5QNW6;Q16778;P62807;P58876;P57053;P33778;P23527;P06899;O60814;Q5QNW6-2	Histone H2B type 1-L	HIST1H2BL;HIST1H2BM;HIST1H2BN;HIST1H2BH;HIST3H2BB;HIST2H2BF;HIST2H2BE;HIST1H2BC;HIST1H2BD;H2BFS;HIST1H2BB;HIST1H2BO;HIST1H2BJ;HIST1H2BK
UC vs AC+NC FET T1	3,254001	3,37E-02	O75636;O75636-2	Ficolin-3	FCN3
UC vs AC+NC FET T1	7,078854	2,57E-03	O75882;O75882-2;O75882-3	Attractin	ATRN

UC vs AC+NC FET T1	0,7658659	0,03757	O95428-6;O95428;O95428-5;O95428-4;O95428-2;O95428-3	Papilin	PAPLN
UC vs AC+NC FET T1	0,786193	0,02427	O95445	Apolipoprotein M	APOM
UC vs AC+NC FET T1	5,1420502	2,65E-06	O95445-2	Apolipoprotein M	APOM
UC vs AC+NC FET T1	0,9820836	0,01543	O95967	EGF-containing fibulin-like extracellular matrix protein 2	EFEMP2
UC vs AC+NC FET T1	0,982771	0,004534	P00450	Ceruloplasmin	CP
UC vs AC+NC FET T1	1,3701235	0,001174	P00451	Coagulation factor VIII	F8
UC vs AC+NC FET T1	1,6394063	1,28E-06	P00734	Prothrombin	F2
UC vs AC+NC FET T1	1,8976353	2,65E-04	P00736	Complement C1r subcomponent	C1R
UC vs AC+NC FET T1	1,1974689	0,03553	P00739;P00739-2	Haptoglobin-related protein	HPR
UC vs AC+NC FET T1	2,8070003	0,0003188	P00740;P00740-2	Coagulation factor IX	F9
UC vs AC+NC FET T1	3,0146328	6,18E-04	P00742	Coagulation factor X	F10
UC vs AC+NC FET T1	1,9250002	0,01213	P00746	Complement factor D	CFD
UC vs AC+NC FET T1	1,5635775	9,99E-03	P00747;Q02325	Plasminogen	PLG
UC vs AC+NC FET T1	1,1021575	0,004089	P00748	Coagulation factor XII	F12
UC vs AC+NC FET T1	1,8762013	0,0006707	P00751;P00751-2	Complement factor B	CFB
UC vs AC+NC FET T1	1,1014241	0,001076	P01011;P01011-2;P01011-3	Alpha-1-antichymotrypsin	SERPINA3
UC vs AC+NC FET T1	1,6500348	0,002254	P01031	Complement C5	C5
UC vs AC+NC FET T1	1,625002	9,13E-03	P01034	Cystatin-C	CST3
UC vs AC+NC FET T1	-1,227598	0,0003565	P01040	Cystatin-A	CSTA
UC vs AC+NC FET T1	2,4884882	3,91E-07	P01042-2	Kininogen-1	KNG1
UC vs AC+NC FET T1	1,7790145	0,00248	P01344-3;P01344;P01344-2	Insulin-like growth factor II	IGF2
UC vs AC+NC FET T1	0,5410465	0,04188	P01591	Immunoglobulin J chain	IGJ
UC vs AC+NC FET T1	0,6969989	0,02648	P01594;P01593	Ig kappa chain V-I region AU	Ig kappa chain V-I region AU;Ig kappa chain V-I region AG
UC vs AC+NC FET T1	0,5848253	0,008074	P04432;P01597	Ig kappa chain V-I region Daudi	Ig kappa chain V-I region Daudi;Ig kappa chain V-I region DEE
UC vs AC+NC FET T1	3,5456766	0,004224	P01599	Ig kappa chain V-I region Gal	Ig kappa chain V-I region Gal
UC vs AC+NC FET T1	0,9890927	0,02619	P01619	Ig kappa chain V-III region B6	Ig kappa chain V-III region B6
UC vs AC+NC FET T1	2,2272905	0,01633	P01624	Ig kappa chain V-III region POM	Ig kappa chain V-III region POM
UC vs AC+NC FET T1	3,8379945	3,09E-05	P01700	Ig lambda chain V-I region HA	Ig lambda chain V-I region HA
UC vs AC+NC FET T1	-1,246772	0,001193	P01715	Ig lambda chain V-IV region Bau	Ig lambda chain V-IV region Bau
UC vs AC+NC FET T1	0,6190997	0,0417	P01742	Ig heavy chain V-I region EU	Ig heavy chain V-I region EU
UC vs AC+NC FET T1	3,6739257	8,20E-03	P01743	Ig heavy chain V-I region HG3	Ig heavy chain V-I region HG3
UC vs AC+NC FET T1	1,6034152	0,008596	P01766	Ig heavy chain V-III region BRO	Ig heavy chain V-III region BRO
UC vs AC+NC FET T1	4,8846452	0,001715	P0DP03;P01768	Ig heavy chain V-III region CAM	Ig heavy chain V-III region CAM
UC vs AC+NC FET T1	-2,076664	0,02046	P01833	Polymeric immunoglobulin receptor	PIGR
UC vs AC+NC FET T1	1,9530982	0,0001417	P01859	Ig gamma-2 chain C region	IGHG2
UC vs AC+NC FET T1	1,7421781	0,004835	P01860	Ig gamma-3 chain C region	IGHG3
UC vs AC+NC FET T1	-3,884955	5,42E-03	P01861	Ig gamma-4 chain C region	IGHG4
UC vs AC+NC FET T1	0,8234457	0,0005545	P01871;P01871-2	Ig mu chain C region	IGHM
UC vs AC+NC FET T1	1,5678281	0,0007527	P01876	Ig alpha-1 chain C region	IGHA1
UC vs AC+NC FET T1	1,0534331	0,0001183	P02647;Q9HB71-2	Apolipoprotein A-I	APOA1
UC vs AC+NC FET T1	1,7863129	5,78E-03	P02649;CON__Q03247	Apolipoprotein E	APOE
UC vs AC+NC FET T1	1,0961968	0,000374	P02656	Apolipoprotein C-III	APOC3
UC vs AC+NC FET T1	0,8654603	0,001822	P02671;P02671-2;REV__Q9UKV0-4;REV__Q9UKV0-2;REV__Q9UKV0;REV__Q9UKV0-5;REV__Q9UKV0-7;Q14314	Fibrinogen alpha chain	FGA
UC vs AC+NC FET T1	1,3563277	0,0002748	P02675	Fibrinogen beta chain	FGB
UC vs AC+NC FET T1	0,6961621	0,001657	P02743	Serum amyloid P-component	APCS
UC vs AC+NC FET T1	2,6647943	1,22E-07	P02746	Complement C1q subcomponent subunit B	C1QB
UC vs AC+NC FET T1	1,2231117	0,0003037	P02747	Complement C1q subcomponent subunit C	C1QC
UC vs AC+NC FET T1	0,9012769	0,01639	P02749;CON__P17690	Beta-2-glycoprotein 1	APOH
UC vs AC+NC FET T1	0,9596542	0,03086	P02750	Leucine-rich alpha-2-glycoprotein	LRG1
UC vs AC+NC FET T1	-0,592472	0,03525	P02760	Protein AMBP	AMBP
UC vs AC+NC FET T1	1,3355807	0,0004831	P02765	Alpha-2-HS-glycoprotein	AHSG
UC vs AC+NC FET T1	1,6870378	0,000684	P02766	Transthyretin	TTR
UC vs AC+NC FET T1	1,557005	0,0001605	P02774-3;P02774;P02774-2	Vitamin D-binding protein	GC
UC vs AC+NC FET T1	2,0897259	0,007118	P02775	Platelet basic protein	PPBP
UC vs AC+NC FET T1	2,7402388	0,000761	P02776	Platelet factor 4	PF4
UC vs AC+NC FET T1	2,3863456	0,007446	P02788;P02788-2	Lactotransferrin	LTF
UC vs AC+NC FET T1	4,4365276	1,22E-04	P03950	Angiogenin	ANG
UC vs AC+NC FET T1	5,417534	3,03E-05	P03951	Coagulation factor XI	F11
UC vs AC+NC FET T1	0,7393478	0,0491	P04003;CON__Q28065	C4b-binding protein alpha chain	C4BPA
UC vs AC+NC FET T1	0,3270994	0,0177	P04004	Vitronectin	VTN
UC vs AC+NC FET T1	1,8560087	0,0004617	P04040	Catalase	CAT
UC vs AC+NC FET T1	2,9593526	0,0002055	P04070;P04070-2	Vitamin K-dependent protein C	PROC
UC vs AC+NC FET T1	0,9745713	0,0003456	P04114	Apolipoprotein B-100	APOB
UC vs AC+NC FET T1	1,3515269	0,0001534	P04180	Phosphatidylcholine-sterol acyltransferase	LCAT
UC vs AC+NC FET T1	-1,042884	0,02375	P04196	Histidine-rich glycoprotein	HRG
UC vs AC+NC FET T1	3,5057557	2,19E-02	P04211;A0A075B6I9	Ig lambda chain V region 4A	IGLV7-46
UC vs AC+NC FET T1	2,8875312	0,001601	P04275	von Willebrand factor	VWF
UC vs AC+NC FET T1	2,3755766	0,02589	P04406;P04406-2;O14556	Glyceraldehyde-3-phosphate dehydrogenase	GAPDH
UC vs AC+NC FET T1	1,4520351	0,04115	P04433;A0A0A0MRZ8	Ig kappa chain V-III region VG	IGKV3D-11
UC vs AC+NC FET T1	2,9926375	0,01313	P05023-2;P05023-4;P05023;P05023-3	Sodium/potassium-transporting ATPase subunit alpha-1	ATP1A1
UC vs AC+NC FET T1	1,0074221	0,0002496	P05090	Apolipoprotein D	APOD
UC vs AC+NC FET T1	1,25638	0,01867	P05154	Plasma serine protease inhibitor	SERPINA5
UC vs AC+NC FET T1	3,3272661	1,47E-03	P05155-2;P05155;P05155-3	Plasma protease C1 inhibitor	SERPING1
UC vs AC+NC FET T1	1,0320324	0,016	P05156;CON__Q32PI4	Complement factor I	CFI
UC vs AC+NC FET T1	1,4604224	0,0003408	P05160	Coagulation factor XIII B chain	F13B
UC vs AC+NC FET T1	1,1166794	0,001044	P05164-3;P05164;P05164-2	Myeloperoxidase	MPO
UC vs AC+NC FET T1	3,3752557	5,21E-03	P05543	Thyroxine-binding globulin	SERPINA7
UC vs AC+NC FET T1	1,5030094	0,0006072	P05546	Heparin cofactor 2	SERPIND1
UC vs AC+NC FET T1	2,2981901	0,0001296	P06331	Ig heavy chain V-II region ARH-77	Ig heavy chain V-II region ARH-77
UC vs AC+NC FET T1	1,127339	0,0001398	P06396;P06396-4;P06396-3	Gelsolin	GSN
UC vs AC+NC FET T1	-1,375071	0,02262	P06702	Protein S100-A9	S100A9
UC vs AC+NC FET T1	0,4857417	0,04626	P06727;Q9BT92	Apolipoprotein A-IV	APOA4
UC vs AC+NC FET T1	0,8352773	0,0001288	P06850	Corticoliberin	CRH
UC vs AC+NC FET T1	0,6493009	0,006554	P07225	Vitamin K-dependent protein S	PROS1

UC vs AC+NC FET T1	2,2322696	0,0002824	P07237	Protein disulfide-isomerase	P4HB
UC vs AC+NC FET T1	0,7651231	0,01501	P07307-3;P07307-2;P07307	Asialoglycoprotein receptor 2	ASGR2
UC vs AC+NC FET T1	1,0800504	7,03E-03	P07333;P07333-2	Macrophage colony-stimulating factor 1 receptor	CSF1R
UC vs AC+NC FET T1	2,5608264	9,34E-03	P07357	Complement component C8 alpha chain	C8A
UC vs AC+NC FET T1	2,5982329	3,33E-02	P07358	Complement component C8 beta chain	C8B
UC vs AC+NC FET T1	1,7355641	0,005148	P07360	Complement component C8 gamma chain	C8G
UC vs AC+NC FET T1	0,9172329	0,0005883	P07998	Ribonuclease pancreatic	RNASE1
UC vs AC+NC FET T1	1,737239	0,0001179	P08253;P08253-3;P08253-2	72 kDa type IV collagenase	MMP2
UC vs AC+NC FET T1	2,3615789	0,0005227	P08294	Extracellular superoxide dismutase [Cu-Zn]	SOD3
UC vs AC+NC FET T1	2,4258639	0,01242	P08519	Apolipoprotein(a)	LPA
UC vs AC+NC FET T1	3,3587962	0,007862	P08567	Pleckstrin	PLEK
UC vs AC+NC FET T1	1,6533498	5,90E-03	P08603;P08603-2	Complement factor H	CFH
UC vs AC+NC FET T1	1,3130368	0,001192	P08697;P08697-2	Alpha-2-antiplasmin	SERPINF2
UC vs AC+NC FET T1	0,6271244	0,009007	P08833	Insulin-like growth factor-binding protein 1	IGFBP1
UC vs AC+NC FET T1	2,3776199	0,0005444	P09466-3	Glycodelin	PAEP
UC vs AC+NC FET T1	2,5602895	0,006229	P09486	SPARC	SPARC
UC vs AC+NC FET T1	1,3768555	5,88E-06	P09871	Complement C1s subcomponent	C1S
UC vs AC+NC FET T1	-2,165104	0,005463	P0C0L4;P0C0L4-2	Complement C4-A	C4A
UC vs AC+NC FET T1	1,4320561	0,0001288	P0C0L5	Complement C4-B	C4B
UC vs AC+NC FET T1	2,3129336	0,03536	P0DJI8	Serum amyloid A-1 protein	SAA1
UC vs AC+NC FET T1	4,3542738	0,001213	P0DML3;P0DML2;P0DML3-2;P0DML3-3;P01241-2;P01241;P01241-5	Chorionic somatomammotropin hormone 2	CSH2;CSH1
UC vs AC+NC FET T1	2,6114079	0,000421	P0DOX2	.	.
UC vs AC+NC FET T1	1,8956063	0,008653	P0DOX3	.	.
UC vs AC+NC FET T1	3,644773	0,003945	P0DOX4	.	.
UC vs AC+NC FET T1	1,127762	0,0004802	P0DOX5;P01857	Ig gamma-1 chain C region	IGHG1
UC vs AC+NC FET T1	0,9151698	0,003208	P0DOX6	.	.
UC vs AC+NC FET T1	1,6298414	1,74E-03	P0DOY3	.	.
UC vs AC+NC FET T1	2,1236321	0,000574	P10643	Complement component C7	C7
UC vs AC+NC FET T1	1,1899952	0,01903	P10645	Chromogranin-A	CHGA
UC vs AC+NC FET T1	2,6471245	0,02795	P10720	Platelet factor 4 variant	PF4V1
UC vs AC+NC FET T1	1,9548609	0,0001421	P11021	78 kDa glucose-regulated protein	HSPA5
UC vs AC+NC FET T1	1,6278494	0,03185	P11226	Mannose-binding protein C	MBL2
UC vs AC+NC FET T1	1,3683137	0,005479	P11465	Pregnancy-specific beta-1-glycoprotein 2	PSG2
UC vs AC+NC FET T1	2,0419129	0,0001282	P11597;P11597-2	Cholesteryl ester transfer protein	CETP
UC vs AC+NC FET T1	2,7379106	9,22E-03	P12259	Coagulation factor V	F5
UC vs AC+NC FET T1	3,1504922	0,0001725	P13497;P13497-5;P13497-2;P13497-6;P13497-4;P13497-3	Bone morphogenetic protein 1	BMP1
UC vs AC+NC FET T1	1,4459773	0,009821	P13667	Protein disulfide-isomerase A4	PDIA4
UC vs AC+NC FET T1	1,0876764	0,02487	P13671	Complement component C6	C6
UC vs AC+NC FET T1	-2,740346	0,00882	P13798	Acylamino-acid-releasing enzyme	APEH
UC vs AC+NC FET T1	1,5335108	0,0006718	P14209;P14209-2;P14209-3	CD99 antigen	CD99
UC vs AC+NC FET T1	1,4365987	0,005966	P14543;P14543-2	Nidogen-1	NID1
UC vs AC+NC FET T1	2,2349676	0,0009212	P14625;Q58FF3	Endoplasmin	HSP90B1
UC vs AC+NC FET T1	1,2009233	0,002591	P15907	Beta-galactoside alpha-2,6-sialyltransferase 1	ST6GAL1
UC vs AC+NC FET T1	4,6085281	6,31E-03	P17936;P17936-2	Insulin-like growth factor-binding protein 3	IGFBP3
UC vs AC+NC FET T1	2,3844685	0,005898	P18206;P18206-2;P18206-3	Vinculin	VCL
UC vs AC+NC FET T1	0,9553915	0,003111	P18428	Lipopolysaccharide-binding protein	LBP
UC vs AC+NC FET T1	2,7848433	0,007147	P19652	Alpha-1-acid glycoprotein 2	ORM2
UC vs AC+NC FET T1	1,5769154	4,17E-02	P19823	Inter-alpha-trypsin inhibitor heavy chain H2	ITIH2
UC vs AC+NC FET T1	2,2764388	3,63E-07	P19827	Inter-alpha-trypsin inhibitor heavy chain H1	ITIH1
UC vs AC+NC FET T1	1,2475156	0,005603	P20851-2;P20851;REV__Q7Z7B0-3;REV__Q7Z7B0-2;REV__Q7Z7B0	C4b-binding protein beta chain	C4BPB
UC vs AC+NC FET T1	1,727551	0,0007972	P21333-2;P21333	Filamin-A	FLNA
UC vs AC+NC FET T1	2,2766048	1,06E-05	P22352	Glutathione peroxidase 3	GPX3
UC vs AC+NC FET T1	2,2983357	0,002956	P22792	Carboxypeptidase N subunit 2	CPN2
UC vs AC+NC FET T1	0,787493	0,03467	P22891;P22891-2	Vitamin K-dependent protein Z	PROZ
UC vs AC+NC FET T1	6,5928261	1,00E-02	P23083	Ig heavy chain V-I region V35	Ig heavy chain V-I region V35
UC vs AC+NC FET T1	0,8052012	0,01508	P23142;P23142-2;P23142-3	Fibulin-1	FBLN1
UC vs AC+NC FET T1	-1,504914	0,03692	P23280-3;P23280	Carbonic anhydrase 6	CA6
UC vs AC+NC FET T1	2,3751301	6,10E-03	P24593	Insulin-like growth factor-binding protein 5	IGFBP5
UC vs AC+NC FET T1	2,9343595	0,02435	P26927	Hepatocyte growth factor-like protein	MST1
UC vs AC+NC FET T1	0,6532427	0,01013	P27169	Serum paraoxonase/arylesterase 1	PON1
UC vs AC+NC FET T1	1,9609721	0,0007913	P27797	Calreticulin	CALR
UC vs AC+NC FET T1	2,9715766	6,28E-03	P27918	Properdin	CFP
UC vs AC+NC FET T1	2,332919	0,004104	P28370-2;P28370	Probable global transcription activator SNF2L1	SMARCA1
UC vs AC+NC FET T1	1,824319	0,01516	P30041	Peroxiredoxin-6	PRDX6
UC vs AC+NC FET T1	-2,94031	0,002137	P31025;Q5VSP4	Lipocalin-1	LCN1
UC vs AC+NC FET T1	-1,753167	0,005013	P31151;Q86SG5	Protein S100-A7	S100A7
UC vs AC+NC FET T1	0,8669158	0,03748	P33151;P33151-2	Cadherin-5	CDH5
UC vs AC+NC FET T1	1,7305306	0,0006195	P34096	Ribonuclease 4	RNASE4
UC vs AC+NC FET T1	3,4722661	0,0001479	P35443	Thrombospondin-4	THBS4
UC vs AC+NC FET T1	4,5050272	1,30E-04	P35542	Serum amyloid A-4 protein	SAA4
UC vs AC+NC FET T1	0,7427193	0,01077	P35555	Fibrillin-1	FBN1
UC vs AC+NC FET T1	3,2500299	1,26E-04	P35858;P35858-2	Insulin-like growth factor-binding protein complex acid labile subunit	IGFALS
UC vs AC+NC FET T1	1,1166838	0,001678	P36955;CON__Q95121	Pigment epithelium-derived factor	SERPINF1
UC vs AC+NC FET T1	1,8724887	0,03498	P36980-2;P36980	Complement factor H-related protein 2	CFHR2
UC vs AC+NC FET T1	1,2638991	0,03028	P39060-2;P39060-1;P39060	Collagen alpha-1(XVIII) chain	COL18A1
UC vs AC+NC FET T1	0,7672824	0,007991	P43652	Afamin	AFM
UC vs AC+NC FET T1	2,4622578	0,03005	P48059;P48059-4;P48059-2;P48059-5;P48059-3;Q7Z4I7-4;Q7Z4I7-3;Q7Z4I7-2;P0CW19-2	LIM and senescent cell antigen-like-containing domain protein 1	LIMS1
UC vs AC+NC FET T1	2,6503795	1,31E-03	P48740	Mannan-binding lectin serine protease 1	MASP1
UC vs AC+NC FET T1	1,0693833	4,19E-02	P48740-2	Mannan-binding lectin serine protease 1	MASP1
UC vs AC+NC FET T1	1,3724734	0,001276	P48740-3	Mannan-binding lectin serine protease 1	MASP1
UC vs AC+NC FET T1	2,0933001	0,000241	P48740-4	Mannan-binding lectin serine protease 1	MASP1
UC vs AC+NC FET T1	2,0462087	0,04865	P49619-3;P49619-2;P49619	Diacylglycerol kinase gamma	DGKG
UC vs AC+NC FET T1	0,6349045	0,0128	P49747;P49747-2	Cartilage oligomeric matrix protein	COMP
UC vs AC+NC FET T1	-1,695017	0,0002748	P49767	Vascular endothelial growth factor C	VEGFC
UC vs AC+NC FET T1	0,4247128	0,004435	P49908	Selenoprotein P	SEPP1
UC vs AC+NC FET T1	2,3827125	0,0006607	P49913	Cathelicidin antimicrobial peptide	CAMP

UC vs AC+NC FET T1	0,9959298	0,003994	P51884;CON__Q05443	Lumican	LUM
UC vs AC+NC FET T1	1,6268531	9,44E-04	P54132	Bloom syndrome protein	BLM
UC vs AC+NC FET T1	1,7476262	4,26E-02	P55056	Apolipoprotein C-IV	APOC4
UC vs AC+NC FET T1	1,4480406	0,0003627	P55058;P55058-4;P55058-3;P55058-2	Phospholipid transfer protein	PLTP
UC vs AC+NC FET T1	1,6633657	0,000167	P55103	Inhibin beta C chain	INHBC
UC vs AC+NC FET T1	-1,170302	0,004752	P55285-2;P55285	Cadherin-6	CDH6
UC vs AC+NC FET T1	1,9972031	0,00614	P57077;P57077-1	MAP3K7 C-terminal-like protein	MAP3K7CL
UC vs AC+NC FET T1	0,5965183	0,001357	P59666;P59665	Neutrophil defensin 3	DEFA3;DEFA1
UC vs AC+NC FET T1	2,4279429	1,22E-03	P60900;P60900-2;P60900-3	Proteasome subunit alpha type-6	PSMA6
UC vs AC+NC FET T1	2,0796035	0,007985	P61626	Lysozyme C	LYZ
UC vs AC+NC FET T1	-2,879709	0,0003854	P61769	Beta-2-microglobulin	B2M
UC vs AC+NC FET T1	1,8224433	0,01127	P62258;P62258-2	14-3-3 protein epsilon	YWHAE
UC vs AC+NC FET T1	-2,083592	0,0007825	P62979;P62987;P0CG47;P0CG48	Ubiquitin-40S ribosomal protein S27a	RPS27A;UBA52;UBB;UBC
UC vs AC+NC FET T1	2,6612381	0,003305	P69905	Hemoglobin subunit alpha	HBA1
UC vs AC+NC FET T1	1,4184195	3,31E-02	P80108;P80108-2	Phosphatidylinositol-glycan-specific phospholipase D	GPLD1
UC vs AC+NC FET T1	3,6152648	8,49E-03	P80748	Ig lambda chain V-III region LOI	Ig lambda chain V-III region LOI
UC vs AC+NC FET T1	0,6366918	0,01265	P98095-2;P98095	Fibulin-2	FBLN2
UC vs AC+NC FET T1	3,9241687	0,003917	Q00526;P11802-2;Q00535-2;Q00535;P11802;Q14004-2;Q14004	Cyclin-dependent kinase 3	CDK3
UC vs AC+NC FET T1	0,9265334	0,04071	Q00610-2;Q00610	Clathrin heavy chain 1	CLTC
UC vs AC+NC FET T1	1,0846831	0,01942	Q00888;Q00888-3;Q00888-2	Pregnancy-specific beta-1-glycoprotein 4	PSG4
UC vs AC+NC FET T1	1,3662567	0,01056	Q02108-2;Q02108	Guanylate cyclase soluble subunit alpha-3	GUCY1A3
UC vs AC+NC FET T1	2,4958345	8,34E-05	Q02224;Q02224-3	Centromere-associated protein E	CENPE
UC vs AC+NC FET T1	1,9550124	0,003568	Q02818	Nucleobindin-1	NUCB1
UC vs AC+NC FET T1	1,900909	0,003532	Q02985-2;Q02985	Complement factor H-related protein 3	CFHR3
UC vs AC+NC FET T1	1,2655386	2,60E-03	Q03001;Q03001-9;Q03001-13;Q03001-10	Dystonin	DST
UC vs AC+NC FET T1	2,2584594	0,000475	Q03591	Complement factor H-related protein 1	CFHR1
UC vs AC+NC FET T1	1,0284384	0,01633	Q04756	Hepatocyte growth factor activator	HGFAC
UC vs AC+NC FET T1	2,0912286	0,0002633	Q06033-2;Q06033	Inter-alpha-trypsin inhibitor heavy chain H3	ITIH3
UC vs AC+NC FET T1	1,8105044	0,0007217	Q07065	Cytoskeleton-associated protein 4	CKAP4
UC vs AC+NC FET T1	2,2535817	0,002466	Q07954;Q07954-2	Prolow-density lipoprotein receptor-related protein 1	LRP1
UC vs AC+NC FET T1	0,4922828	0,02872	Q08174;Q08174-2	Protocadherin-1	PCDH1
UC vs AC+NC FET T1	1,0824596	0,0194	Q08380	Galectin-3-binding protein	LGALS3BP
UC vs AC+NC FET T1	1,3711308	0,003125	Q08830	Fibrinogen-like protein 1	FGL1
UC vs AC+NC FET T1	1,7813591	4,15E-02	Q0VAK6;Q0VAK6-2	Leiomodin-3	LMOD3
UC vs AC+NC FET T1	1,0628955	0,007211	Q12794-2;Q12794;Q12794-7;Q12794-4;Q12794-3;Q12794-6;Q12794-5	Hyaluronidase-1	HYAL1
UC vs AC+NC FET T1	1,4502689	0,0005759	Q12805-2;Q12805-4;Q12805-3;Q12805	EGF-containing fibulin-like extracellular matrix protein 1	EFEMP1
UC vs AC+NC FET T1	2,8250433	0,001555	Q13103	Secreted phosphoprotein 24	SPP2
UC vs AC+NC FET T1	1,5212408	0,004265	Q13418-2;Q13418;Q13418-3	Integrin-linked protein kinase	ILK
UC vs AC+NC FET T1	3,2240294	0,001471	Q13488-2	V-type proton ATPase 116 kDa subunit a isoform 3	TCIRG1
UC vs AC+NC FET T1	-2,08926	0,01952	Q13790	Apolipoprotein F	APOF
UC vs AC+NC FET T1	1,3044001	0,01373	Q14126	Desmoglein-2	DSG2
UC vs AC+NC FET T1	0,6831136	0,01198	Q14213	Interleukin-27 subunit beta	EBI3
UC vs AC+NC FET T1	0,77218	0,0443	Q14515;Q14515-2	SPARC-like protein 1	SPARCL1
UC vs AC+NC FET T1	2,6582216	4,98E-03	Q14520-2;Q14520	Hyaluronan-binding protein 2	HABP2
UC vs AC+NC FET T1	1,1750937	0,01569	Q14623	Indian hedgehog protein	IHH
UC vs AC+NC FET T1	1,0938197	0,0008908	Q14624;Q14624-3;Q14624-4	Inter-alpha-trypsin inhibitor heavy chain H4	ITIH4
UC vs AC+NC FET T1	1,7245513	0,002666	Q14697;Q14697-2	Neutral alpha-glucosidase AB	GANAB
UC vs AC+NC FET T1	1,1914387	0,04977	Q15022	Polycomb protein SUZ12	SUZ12
UC vs AC+NC FET T1	0,9114197	0,002017	Q15113	Procollagen C-endopeptidase enhancer 1	PCOLCE
UC vs AC+NC FET T1	0,6951209	0,02262	Q15166	Serum paraoxonase/lactonase 3	PON3
UC vs AC+NC FET T1	0,643982	0,004286	Q15293;Q15293-2	Reticulocalbin-1	RCN1
UC vs AC+NC FET T1	5,0028622	9,87E-05	Q15386;Q15386-2	Ubiquitin-protein ligase E3C	UBE3C
UC vs AC+NC FET T1	2,8906201	0,0001553	Q15485;Q15485-2	Ficolin-2	FCN2
UC vs AC+NC FET T1	5,5671395	9,85E-06	Q15582	Transforming growth factor-beta-induced protein ig-h3	TGFBI
UC vs AC+NC FET T1	2,9271771	0,0004854	Q15848	Adiponectin	ADIPOQ
UC vs AC+NC FET T1	1,773648	0,03318	Q16594	Transcription initiation factor TFIID subunit 9	TAF9
UC vs AC+NC FET T1	1,2539772	0,006268	Q16769;Q16769-2	Glutaminyl-peptide cyclotransferase	QPCT
UC vs AC+NC FET T1	0,9073506	0,003114	Q2TAC6;Q2TAC6-3;Q2TAC6-2	Kinesin-like protein KIF19	KIF19
UC vs AC+NC FET T1	-1,997356	0,03445	Q4LDE5;Q4LDE5-4;Q4LDE5-3;Q4LDE5-2	Sushi, von Willebrand factor type A, EGF and pentraxin domain-containing protein 1	SVEP1
UC vs AC+NC FET T1	0,8812113	0,0001623	Q5CZC0;Q5CZC0-2	Fibrous sheath-interacting protein 2	FSIP2
UC vs AC+NC FET T1	2,3718865	0,0003511	Q5HYK7-3;Q5HYK7-2;Q5HYK7;Q5HYK7-5;Q5HYK7-4	SH3 domain-containing protein 19	SH3D19
UC vs AC+NC FET T1	2,7605917	0,01903	Q5JPF3;Q5JPF3-2;A6QL64-3;Q5JPF3-3	Ankyrin repeat domain-containing protein 36C	ANKRD36C;ANKRD36
UC vs AC+NC FET T1	0,7802031	0,000129	Q5T0U0;Q5T0U0-2	Coiled-coil domain-containing protein 122	CCDC122
UC vs AC+NC FET T1	0,439817	0,005779	Q5VT25-3;Q5VT25-4;Q5VT25-5;Q5VT25;Q5VT25-2;Q5VT25-6	Serine/threonine-protein kinase MRCK alpha	CDC42BPA
UC vs AC+NC FET T1	2,1983096	0,0001304	Q6EEV6	Small ubiquitin-related modifier 4	SUMO4
UC vs AC+NC FET T1	3,2029814	0,00478	Q6ISB3;Q6ISB3-2	Grainyhead-like protein 2 homolog	GRHL2
UC vs AC+NC FET T1	2,7664959	0,0004074	Q6P387-2;Q6P387	Uncharacterized protein C16orf46	C16orf46
UC vs AC+NC FET T1	3,8717662	0,0004435	Q6Q788	Apolipoprotein A-V	APOA5
UC vs AC+NC FET T1	1,4887641	4,24E-02	Q6UVK1	Chondroitin sulfate proteoglycan 4	CSPG4
UC vs AC+NC FET T1	1,3099951	0,02196	Q6UY14-3;Q6UY14;Q6UY14-2	ADAMTS-like protein 4	ADAMTSL4
UC vs AC+NC FET T1	-1,210094	0,0321	Q6VAB6;Q6VAB6-2	Kinase suppressor of Ras 2	KSR2
UC vs AC+NC FET T1	2,2503766	0,0007452	Q76LX8;Q76LX8-2;Q76LX8-3;Q76LX8-4	A disintegrin and metalloproteinase with thrombospondin motifs 13	ADAMTS13
UC vs AC+NC FET T1	1,027428	0,006481	Q7Z478	ATP-dependent RNA helicase DHX29	DHX29
UC vs AC+NC FET T1	0,8698959	0,01431	Q86U17	Serpin A11	SERPINA11
UC vs AC+NC FET T1	2,1707996	0,005027	Q86UQ4;Q86UQ4-4;Q86UQ4-3;Q86UQ4-6;Q86UQ4-7;Q86UQ4-5	ATP-binding cassette sub-family A member 13	ABCA13
UC vs AC+NC FET T1	0,7333041	0,0003018	Q8IV50-2;Q8IV50	LysM and putative peptidoglycan-binding domain-containing protein 2	LYSMD2

UC vs AC+NC FET T1	0,9939782	8,12E-03	Q8IVL1-11;Q8IVL1-4	Neuron navigator 2	NAV2
UC vs AC+NC FET T1	2,3208239	2,79E-02	Q8IYI0;Q8IYI0-2	Uncharacterized protein C20orf196	C20orf196
UC vs AC+NC FET T1	1,6783485	0,004063	Q8IZK6-2;Q8IZK6	Mucolipin-2	MCOLN2
UC vs AC+NC FET T1	2,6549575	0,002873	Q8N7Z5;Q8WY50	Putative ankyrin repeat domain-containing protein 31	ANKRD31
UC vs AC+NC FET T1	0,516699	0,007508	Q8N8A2-4;Q8N8A2;Q8N8A2-2;Q8N8A2-3;Q8N8A2-5	Serine/threonine-protein phosphatase 6 regulatory ankyrin repeat subunit B	ANKRD44
UC vs AC+NC FET T1	1,1707805	0,005702	Q8NB25-2;Q8NB25-3;Q8NB25	Protein FAM184A	FAM184A
UC vs AC+NC FET T1	4,1481459	1,42E-06	Q8NBP7	Proprotein convertase subtilisin/kexin type 9	PCSK9
UC vs AC+NC FET T1	-2,147681	0,0002519	Q8ND83-3;Q8ND83-2;Q8ND83-4	SLAIN motif-containing protein 1	SLAIN1
UC vs AC+NC FET T1	1,6371514	6,37E-05	Q8NDV7-6;Q8NDV7;Q8NDV7-2;Q8NDV7-5;Q8NDV7-4;Q8NDV7-3	Trinucleotide repeat-containing gene 6A protein	TNRC6A
UC vs AC+NC FET T1	3,4201249	4,61E-02	Q8NI99	Angiopoietin-related protein 6	ANGPTL6
UC vs AC+NC FET T1	1,8320279	4,47E-02	Q8TE73	Dynein heavy chain 5, axonemal	DNAH5
UC vs AC+NC FET T1	0,9175169	0,0218	Q8WWA0	Intelectin-1	ITLN1
UC vs AC+NC FET T1	2,4763016	0,001202	Q8WWZ8;Q8WWZ8-2	Oncoprotein-induced transcript 3 protein	OIT3
UC vs AC+NC FET T1	1,2859553	0,002982	Q92496;Q92496-2;Q92496-3	Complement factor H-related protein 4	CFHR4
UC vs AC+NC FET T1	1,9420754	0,007616	Q92626;Q92626-2	Peroxidasin homolog	PXDN
UC vs AC+NC FET T1	0,3562452	0,04188	Q96CM8-3;Q96CM8-4;Q96CM8;Q96CM8-2	Acyl-CoA synthetase family member 2, mitochondrial	ACSF2
UC vs AC+NC FET T1	1,3212208	0,0005145	Q96IY4;Q96IY4-2;CON__Q2KIG3	Carboxypeptidase B2	CPB2
UC vs AC+NC FET T1	1,1297309	1,59E-05	Q96JB1-2;Q96JB1	Dynein heavy chain 8, axonemal	DNAH8
UC vs AC+NC FET T1	1,9903346	0,0001843	Q96KN2	Beta-Ala-His dipeptidase	CNDP1
UC vs AC+NC FET T1	2,4269162	0,001746	Q96PD5;Q96PD5-2	N-acetylmuramoyl-L-alanine amidase	PGLYRP2
UC vs AC+NC FET T1	-0,864349	0,02033	Q96QR1	Secretoglobin family 3A member 1	SCGB3A1
UC vs AC+NC FET T1	3,1017692	0,002014	Q96RL1-3;Q96RL1-4	BRCA1-A complex subunit RAP80	UIMC1
UC vs AC+NC FET T1	1,0770185	0,02113	Q99574	Neuroserpin	SERPINI1
UC vs AC+NC FET T1	1,9037009	0,001019	Q99969	Retinoic acid receptor responder protein 2	RARRES2
UC vs AC+NC FET T1	-2,462014	0,009686	Q99996-3;Q99996;Q99996-6;Q99996-5;Q99996-1;Q99996-4	A-kinase anchor protein 9	AKAP9
UC vs AC+NC FET T1	0,7154097	0,03741	Q9BRK5-6;Q9BRK5;Q9BRK5-4;Q9BRK5-3;Q9BRK5-2	45 kDa calcium-binding protein	SDF4
UC vs AC+NC FET T1	1,8379494	1,93E-02	Q9BWP8-8;Q9BWP8-7;Q9BWP8-6;Q9BWP8-5;Q9BWP8-4;Q9BWP8-3;Q9BWP8-2;Q9BWP8-9;Q9BWP8;Q9BWP8-10	Collectin-11	COLEC11
UC vs AC+NC FET T1	2,0904835	4,33E-03	Q9BXR6	Complement factor H-related protein 5	CFHR5
UC vs AC+NC FET T1	0,9991677	0,004249	Q9H6X2-5;Q9H6X2;Q9H6X2-4;Q9H6X2-6;Q9H6X2-2;Q9H6X2-3	Anthrax toxin receptor 1	ANTXR1
UC vs AC+NC FET T1	-0,521629	0,01373	Q9HCL0-2;Q9HCL0	Protocadherin-18	PCDH18
UC vs AC+NC FET T1	1,5738021	3,06E-05	Q9HDC9;Q9HDC9-2	Adipocyte plasma membrane-associated protein	APMAP
UC vs AC+NC FET T1	1,1144544	0,002297	Q9NPG4;O14917-2	Protocadherin-12	PCDH12
UC vs AC+NC FET T1	5,9044426	6,18E-05	Q9NQ79;Q9NQ79-2;Q9NQ79-3	Cartilage acidic protein 1	CRTAC1
UC vs AC+NC FET T1	-2,019535	0,001228	Q9NZT1	Calmodulin-like protein 5	CALML5
UC vs AC+NC FET T1	2,7116672	1,33E-03	Q9UHG3	Prenylcysteine oxidase 1	PCYOX1
UC vs AC+NC FET T1	-1,180236	0,002709	Q9UJJ9	N-acetylglucosamine-1-phosphotransferase subunit gamma	GNPTG
UC vs AC+NC FET T1	3,1015893	0,00253	Q9UK55	Protein Z-dependent protease inhibitor	SERPINA10
UC vs AC+NC FET T1	0,5604568	0,04571	Q9UNW1;Q9UNW1-4;Q9UNW1-3;Q9UNW1-2	Multiple inositol polyphosphate phosphatase 1	MINPP1
UC vs AC+NC FET T1	-2,306842	2,61E-06	Q9UQ72;Q9UQ72-2	Pregnancy-specific beta-1-glycoprotein 11	PSG11
UC vs AC+NC FET T1	0,5849016	0,0007231	Q9Y4B5	Microtubule cross-linking factor 1	MTCL1
UC vs AC+NC FET T1	1,2210037	1,18E-02	Q9Y4L1	Hypoxia up-regulated protein 1	HYOU1
UC vs AC+NC FET T2	4,0509948	2,31E-05	A0A075B6H7;A0A0C4DH55	.	IGKV3-7
UC vs AC+NC FET T2	1,5475996	0,02161	A0A075B6I0	.	IGLV8-61
UC vs AC+NC FET T2	3,9391317	2,43E-04	A0A075B6J9	.	IGLV2-18
UC vs AC+NC FET T2	1,0825728	0,04708	A0A075B6P5;P01615	Ig kappa chain V-II region FR	IGKV2D-28
UC vs AC+NC FET T2	1,581807	0,006059	A0A0C4DH68;A0A075B6R9	.	IGKV2-24;IGKV2D-24
UC vs AC+NC FET T2	0,9184751	0,003893	A0A0B4J1V0	.	IGHV3-15
UC vs AC+NC FET T2	3,7642232	0,008951	A0A0B4J1Y9	.	IGHV3-72
UC vs AC+NC FET T2	1,5578935	0,0017	A0A0C4DH25	.	IGKV3D-20
UC vs AC+NC FET T2	4,9788088	0,0001758	A0A0C4DH35	.	IGHV3-35
UC vs AC+NC FET T2	-1,369342	0,04193	A0A0C4DH38	.	IGHV5-51
UC vs AC+NC FET T2	1,3678272	0,02461	A0A0C4DH67;A0A0C4DH69	.	IGKV1-8;IGKV1-9
UC vs AC+NC FET T2	4,5494559	8,25E-03	A0A0J9YX35	.	.
UC vs AC+NC FET T2	-1,546242	0,0265	A0A1B0GTC6	.	.
UC vs AC+NC FET T2	-3,670966	0,01114	A4FU69-3;A4FU69;A4FU69-2;A4FU69-4;A4FU69-6	EF-hand calcium-binding domain-containing protein 5	EFCAB5
UC vs AC+NC FET T2	3,7502697	0,002125	A8K2U0;A8K2U0-2	Alpha-2-macroglobulin-like protein 1	A2ML1
UC vs AC+NC FET T2	3,1227807	0,0001699	O00187;O00187-2	Mannan-binding lectin serine protease 2	MASP2
UC vs AC+NC FET T2	1,3482285	0,03272	O00204-2;O00204	Sulfotransferase family cytosolic 2B member 1	SULT2B1
UC vs AC+NC FET T2	-2,069448	0,0265	O00217	NADH dehydrogenase [ubiquinone] iron-sulfur protein 8, mitochondrial	NDUFS8
UC vs AC+NC FET T2	0,7504073	0,02782	O00391;O00391-2	Sulfhydryl oxidase 1	QSOX1
UC vs AC+NC FET T2	-2,454577	0,03428	O00602	Ficolin-1	FCN1
UC vs AC+NC FET T2	1,809661	7,99E-03	O14791-2;O14791;O14791-3	Apolipoprotein L1	APOL1
UC vs AC+NC FET T2	5,5367882	3,83E-04	O15016;O15016-2;O15016-3	Tripartite motif-containing protein 66	TRIM66
UC vs AC+NC FET T2	2,8990971	1,14E-02	O43184-3;O43184-4;O43184-2;O43184	Disintegrin and metalloproteinase domain-containing protein 12	ADAM12
UC vs AC+NC FET T2	1,9036381	0,03449	O43399;O43399-5;O43399-7;O43399-2;O43399-4;O43399-3	Tumor protein D54	TPD52L2
UC vs AC+NC FET T2	1,7626622	0,004437	O43861-2;O43861	Probable phospholipid-transporting ATPase IIB	ATP9B
UC vs AC+NC FET T2	3,5203646	3,20E-02	O75636;O75636-2	Ficolin-3	FCN3

UC vs AC+NC FET T2	5,806643	4,37E-04	O75882;O75882-2;O75882-3	Attractin	ATRN
UC vs AC+NC FET T2	2,3118383	0,007585	O76076	WNT1-inducible-signaling pathway protein 2	WISP2
UC vs AC+NC FET T2	-3,888775	0,0004299	O94855;O94855-2	Protein transport protein Sec24D	SEC24D
UC vs AC+NC FET T2	0,9314202	0,01754	O95428-6;O95428;O95428-5;O95428-4;O95428-2;O95428-3	Papilin	PAPLN
UC vs AC+NC FET T2	1,2604779	0,0004632	O95445	Apolipoprotein M	APOM
UC vs AC+NC FET T2	5,8461334	1,97E-02	O95445-2	Apolipoprotein M	APOM
UC vs AC+NC FET T2	1,5117537	0,003995	O95967	EGF-containing fibulin-like extracellular matrix protein 2	EFEMP2
UC vs AC+NC FET T2	1,3976954	0,01985	P00450	Ceruloplasmin	CP
UC vs AC+NC FET T2	1,47522	0,04061	P00451	Coagulation factor VIII	F8
UC vs AC+NC FET T2	1,0962151	0,01891	P00488	Coagulation factor XIII A chain	F13A1
UC vs AC+NC FET T2	3,7497734	0,001905	P00709	Alpha-lactalbumin	LALBA
UC vs AC+NC FET T2	0,8327109	0,01491	P00734	Prothrombin	F2
UC vs AC+NC FET T2	2,0936438	1,30E-06	P00736	Complement C1r subcomponent	C1R
UC vs AC+NC FET T2	0,9325353	0,04763	P00738;P00738-2	Haptoglobin	HP
UC vs AC+NC FET T2	3,1579894	4,80E-03	P00740;P00740-2	Coagulation factor IX	F9
UC vs AC+NC FET T2	3,2952992	7,03E-04	P00742	Coagulation factor X	F10
UC vs AC+NC FET T2	2,2797024	0,001875	P00746	Complement factor D	CFD
UC vs AC+NC FET T2	2,1299502	6,15E-03	P00747;Q02325	Plasminogen	PLG
UC vs AC+NC FET T2	1,886072	1,18E-02	P00748	Coagulation factor XII	F12
UC vs AC+NC FET T2	1,9841515	0,003293	P00751;P00751-2	Complement factor B	CFB
UC vs AC+NC FET T2	-1,101943	0,007074	P01008	Antithrombin-III	SERPINC1
UC vs AC+NC FET T2	-1,295421	0,003907	P01009;P01009-2;P01009-3;P20848	Alpha-1-antitrypsin	SERPINA1
UC vs AC+NC FET T2	-2,146894	0,0007159	P01040	Cystatin-A	CSTA
UC vs AC+NC FET T2	3,3328388	0,000549	P01042-2	Kininogen-1	KNG1
UC vs AC+NC FET T2	1,0811132	0,0007949	P01591	Immunoglobulin J chain	IGJ
UC vs AC+NC FET T2	1,9122049	0,006269	P01619	Ig kappa chain V-III region B6	Ig kappa chain V-III region B6
UC vs AC+NC FET T2	2,8114845	1,38E-03	P01700	Ig lambda chain V-I region HA	Ig lambda chain V-I region HA
UC vs AC+NC FET T2	1,2714963	0,0005397	P01701	Ig lambda chain V-I region NEW	Ig lambda chain V-I region NEW
UC vs AC+NC FET T2	2,1382704	0,001659	P01709	Ig lambda chain V-II region MGC	Ig lambda chain V-II region MGC
UC vs AC+NC FET T2	-1,695065	0,02399	P01715	Ig lambda chain V-IV region Bau	Ig lambda chain V-IV region Bau
UC vs AC+NC FET T2	-1,536415	0,005641	P01717	Ig lambda chain V-IV region Hil	Ig lambda chain V-IV region Hil
UC vs AC+NC FET T2	4,8060835	3,99E-04	P01743	Ig heavy chain V-I region HG3	Ig heavy chain V-I region HG3
UC vs AC+NC FET T2	-2,586547	0,01631	P01764	Ig heavy chain V-III region 23	IGHV3-23
UC vs AC+NC FET T2	3,7053778	0,01462	P0DP03;P01768	Ig heavy chain V-III region CAM	Ig heavy chain V-III region CAM
UC vs AC+NC FET T2	-2,026115	0,0001906	P01834	Ig kappa chain C region	IGKC
UC vs AC+NC FET T2	2,5274175	0,003189	P01860	Ig gamma-3 chain C region	IGHG3
UC vs AC+NC FET T2	-1,923514	0,01191	P01861	Ig gamma-4 chain C region	IGHG4
UC vs AC+NC FET T2	1,4292153	0,000447	P01871;P01871-2	Ig mu chain C region	IGHM
UC vs AC+NC FET T2	1,9273576	0,0001541	P01876	Ig alpha-1 chain C region	IGHA1
UC vs AC+NC FET T2	1,2345952	0,002034	P02647;Q9HB71-2	Apolipoprotein A-I	APOA1
UC vs AC+NC FET T2	1,4323453	4,37E-02	P02649;CON__Q03247	Apolipoprotein E	APOE
UC vs AC+NC FET T2	1,7853988	3,74E-04	P02671;P02671-2;REV__Q9UKV0-4;REV__Q9UKV0-2;REV__Q9UKV0;REV__Q9UKV0-5;REV__Q9UKV0-7;Q14314	Fibrinogen alpha chain	FGA
UC vs AC+NC FET T2	1,905989	3,04E-04	P02675	Fibrinogen beta chain	FGB
UC vs AC+NC FET T2	1,0223588	1,65E-03	P02679;P02679-2	Fibrinogen gamma chain	FGG
UC vs AC+NC FET T2	-1,339521	0,000902	P02745	Complement C1q subcomponent subunit A	C1QA
UC vs AC+NC FET T2	2,5656372	3,54E-04	P02746	Complement C1q subcomponent subunit B	C1QB
UC vs AC+NC FET T2	2,4547869	7,78E-03	P02747	Complement C1q subcomponent subunit C	C1QC
UC vs AC+NC FET T2	0,7398123	0,0228	P02748;REV__Q4AC99	Complement component C9	C9
UC vs AC+NC FET T2	1,9477099	0,04325	P02750	Leucine-rich alpha-2-glycoprotein	LRG1
UC vs AC+NC FET T2	1,9132229	0,000898	P02751-1;P02751-8;P02751-3;P02751;P02751-14;P02751-7;P02751-17;P02751-9;P02751-6;P02751-4;P02751-12;P02751-16;P02751-2	Fibronectin	FN1
UC vs AC+NC FET T2	1,904411	1,22E-03	P02765	Alpha-2-HS-glycoprotein	AHSG
UC vs AC+NC FET T2	1,538298	0,01728	P02766	Transthyretin	TTR
UC vs AC+NC FET T2	1,4036838	3,32E-02	P02774-3;P02774;P02774-2	Vitamin D-binding protein	GC
UC vs AC+NC FET T2	4,5469332	0,01167	P02776	Platelet factor 4	PF4
UC vs AC+NC FET T2	3,0461712	0,0002114	P02788;P02788-2	Lactotransferrin	LTF
UC vs AC+NC FET T2	1,4427716	0,0003287	P02790	Hemopexin	HPX
UC vs AC+NC FET T2	5,3201848	5,80E-05	P03950	Angiogenin	ANG
UC vs AC+NC FET T2	5,6749615	2,13E-05	P03951	Coagulation factor XI	F11
UC vs AC+NC FET T2	2,822311	2,95E-03	P03952;P20718	Plasma kallikrein	KLKB1
UC vs AC+NC FET T2	-0,76305	0,01258	P03973	Antileukoproteinase	SLPI
UC vs AC+NC FET T2	0,5712699	0,0159	P04003;CON__Q28065	C4b-binding protein alpha chain	C4BPA
UC vs AC+NC FET T2	2,5302196	0,006356	P04040	Catalase	CAT
UC vs AC+NC FET T2	2,7148143	6,62E-05	P04070;P04070-2	Vitamin K-dependent protein C	PROC
UC vs AC+NC FET T2	0,9726411	0,0006419	P04114	Apolipoprotein B-100	APOB
UC vs AC+NC FET T2	1,7226492	0,0002982	P04180	Phosphatidylcholine-sterol acyltransferase	LCAT
UC vs AC+NC FET T2	4,0537854	1,04E-04	P04211;A0A075B6I9	Ig lambda chain V region 4A	IGLV7-46
UC vs AC+NC FET T2	1,3365992	0,01442	P04217	Alpha-1B-glycoprotein	A1BG
UC vs AC+NC FET T2	5,0534381	3,27E-03	P04275	von Willebrand factor	VWF
UC vs AC+NC FET T2	2,5525201	0,002611	P04433;A0A0A0MRZ8	Ig kappa chain V-III region VG	IGKV3D-11
UC vs AC+NC FET T2	3,6527562	3,34E-02	P05023-2;P05023-4;P05023;P05023-3	Sodium/potassium-transporting ATPase subunit alpha-1	ATP1A1
UC vs AC+NC FET T2	1,1092235	0,0009077	P05090	Apolipoprotein D	APOD
UC vs AC+NC FET T2	3,1664296	3,21E-04	P05155-2;P05155;P05155-3	Plasma protease C1 inhibitor	SERPING1
UC vs AC+NC FET T2	1,029806	0,02629	P05156;CON__Q32PI4	Complement factor I	CFI
UC vs AC+NC FET T2	2,2315832	4,51E-02	P05160	Coagulation factor XIII B chain	F13B
UC vs AC+NC FET T2	1,7527047	0,005419	P05543	Thyroxine-binding globulin	SERPINA7
UC vs AC+NC FET T2	1,91842	1,29E-02	P05546	Heparin cofactor 2	SERPIND1
UC vs AC+NC FET T2	2,3235649	0,001122	P06331	Ig heavy chain V-II region ARH-77	Ig heavy chain V-II region ARH-77
UC vs AC+NC FET T2	1,2993916	0,000203	P06727;Q9BT92	Apolipoprotein A-IV	APOA4
UC vs AC+NC FET T2	1,0086566	2,72E-02	P07225	Vitamin K-dependent protein S	PROS1
UC vs AC+NC FET T2	2,294538	0,001009	P07237	Protein disulfide-isomerase	P4HB
UC vs AC+NC FET T2	1,0417628	0,03462	P07307-3;P07307-2;P07307	Asialoglycoprotein receptor 2	ASGR2

UC vs AC+NC FET T2	0,9876405	0,009287	P07333;P07333-2	Macrophage colony-stimulating factor 1 receptor	CSF1R
UC vs AC+NC FET T2	3,13419	1,52E-03	P07357	Complement component C8 alpha chain	C8A
UC vs AC+NC FET T2	3,2910336	1,20E-03	P07358	Complement component C8 beta chain	C8B
UC vs AC+NC FET T2	3,0881007	1,64E-03	P07437;Q9BUF5	Tubulin beta chain	TUBB
UC vs AC+NC FET T2	2,0701022	0,002379	P07900;P07900-2;Q14568;Q58FF6;Q58FG1	Heat shock protein HSP 90-alpha	HSP90AA1
UC vs AC+NC FET T2	2,782662	0,03913	P07996;P07996-2	Thrombospondin-1	THBS1
UC vs AC+NC FET T2	1,7744681	0,0095	P08253;P08253-3;P08253-2	72 kDa type IV collagenase	MMP2
UC vs AC+NC FET T2	4,6107865	0,0003999	P08493-2;P08493	Matrix Gla protein	MGP
UC vs AC+NC FET T2	1,7312917	0,004071	P08603;P08603-2	Complement factor H	CFH
UC vs AC+NC FET T2	5,5094943	0,0005151	P08670	Vimentin	VIM
UC vs AC+NC FET T2	0,7793804	0,003906	P08697;P08697-2	Alpha-2-antiplasmin	SERPINF2
UC vs AC+NC FET T2	2,2170621	0,002027	P08709-2;P08709	Coagulation factor VII	F7
UC vs AC+NC FET T2	2,5771953	0,001845	P08833	Insulin-like growth factor-binding protein 1	IGFBP1
UC vs AC+NC FET T2	-4,73881	0,005928	P09466-2;P09466	Glycodelin	PAEP
UC vs AC+NC FET T2	2,7569333	2,88E-02	P09466-3	Glycodelin	PAEP
UC vs AC+NC FET T2	3,1629418	0,03248	P09486	SPARC	SPARC
UC vs AC+NC FET T2	1,4295872	1,62E-08	P09871	Complement C1s subcomponent	C1S
UC vs AC+NC FET T2	1,7222272	0,002337	P0C0L5	Complement C4-B	C4B
UC vs AC+NC FET T2	2,8662828	1,30E-02	P0DML3;P0DML2;P0DML3-2;P0DML3-3;P01241-2;P01241-5	Chorionic somatomammotropin hormone 2	CSH2;CSH1
UC vs AC+NC FET T2	-2,538961	0,008547	P0DN87;P0DN86;P0DN86-2;Q6NT52;A6NKQ9-2;A6NKQ9	Choriogonadotropin subunit beta variant 2	CGB2;CGB1
UC vs AC+NC FET T2	2,5897354	0,0001401	P0DOX2	.	.
UC vs AC+NC FET T2	3,2576858	0,0007766	P0DOX4	.	.
UC vs AC+NC FET T2	1,2415608	0,005709	P0DOX5;P01857	Ig gamma-1 chain C region	IGHG1
UC vs AC+NC FET T2	2,1138136	0,001095	P0DOX6	.	.
UC vs AC+NC FET T2	1,826717	2,08E-04	P0DOX7	.	.
UC vs AC+NC FET T2	1,3947891	0,009519	P0DOY3	.	.
UC vs AC+NC FET T2	1,7453281	0,01511	P10643	Complement component C7	C7
UC vs AC+NC FET T2	2,0460686	0,04105	P10645	Chromogranin-A	CHGA
UC vs AC+NC FET T2	0,2424217	0,01888	P10909-6	Clusterin	CLU
UC vs AC+NC FET T2	1,8427848	3,63E-02	P11464-4	Pregnancy-specific beta-1-glycoprotein 1	PSG1
UC vs AC+NC FET T2	1,9800659	0,0008285	P11465	Pregnancy-specific beta-1-glycoprotein 2	PSG2
UC vs AC+NC FET T2	2,7216259	0,0002618	P11597;P11597-2	Cholesteryl ester transfer protein	CETP
UC vs AC+NC FET T2	2,949783	3,82E-03	P12259	Coagulation factor V	F5
UC vs AC+NC FET T2	-1,911767	0,0005702	P12273	Prolactin-inducible protein	PIP
UC vs AC+NC FET T2	-1,072906	0,02715	P12532;P12532-2	Creatine kinase U-type, mitochondrial	CKMT1A
UC vs AC+NC FET T2	5,5596428	7,24E-03	P13497;P13497-5;P13497-2;P13497-6;P13497-4;P13497-3	Bone morphogenetic protein 1	BMP1
UC vs AC+NC FET T2	4,4155692	2,34E-02	P13727;P13727-2	Bone marrow proteoglycan	PRG2
UC vs AC+NC FET T2	-2,214317	0,001193	P13798	Acylamino-acid-releasing enzyme	APEH
UC vs AC+NC FET T2	1,9880615	0,008234	P14209;P14209-2;P14209-3	CD99 antigen	CD99
UC vs AC+NC FET T2	1,4254886	0,04764	P14543;P14543-2	Nidogen-1	NID1
UC vs AC+NC FET T2	1,9469383	4,29E-02	P15907	Beta-galactoside alpha-2,6-sialyltransferase 1	ST6GAL1
UC vs AC+NC FET T2	3,5158734	2,71E-02	P16885	1-phosphatidylinositol 4,5-bisphosphate phosphodiesterase gamma-2	PLCG2
UC vs AC+NC FET T2	3,9598724	3,70E-02	P17936;P17936-2	Insulin-like growth factor-binding protein 3	IGFBP3
UC vs AC+NC FET T2	1,9751197	0,002644	P19652	Alpha-1-acid glycoprotein 2	ORM2
UC vs AC+NC FET T2	1,7205175	0,005023	P19827	Inter-alpha-trypsin inhibitor heavy chain H1	ITIH1
UC vs AC+NC FET T2	-1,803419	0,02762	P21333-2;P21333	Filamin-A	FLNA
UC vs AC+NC FET T2	2,9787661	0,0007418	P22352	Glutathione peroxidase 3	GPX3
UC vs AC+NC FET T2	0,6781768	0,04459	P22692	Insulin-like growth factor-binding protein 4	IGFBP4
UC vs AC+NC FET T2	1,7042056	0,003084	P22792	Carboxypeptidase N subunit 2	CPN2
UC vs AC+NC FET T2	5,0525309	4,79E-06	P23083	Ig heavy chain V-I region V35	Ig heavy chain V-I region V35
UC vs AC+NC FET T2	2,9843048	3,24E-02	P23142-4	Fibulin-1	FBLN1
UC vs AC+NC FET T2	-1,522966	0,02586	P23280-3;P23280	Carbonic anhydrase 6	CA6
UC vs AC+NC FET T2	1,889618	0,0005528	P24593	Insulin-like growth factor-binding protein 5	IGFBP5
UC vs AC+NC FET T2	4,4575943	0,008855	P26927	Hepatocyte growth factor-like protein	MST1
UC vs AC+NC FET T2	4,394441	7,33E-06	P27918	Properdin	CFP
UC vs AC+NC FET T2	1,7497696	0,004818	P29622	Kallistatin	SERPINA4
UC vs AC+NC FET T2	1,0350662	0,00209	P30041	Peroxiredoxin-6	PRDX6
UC vs AC+NC FET T2	-0,700902	0,0005172	P30101	Protein disulfide-isomerase A3	PDIA3
UC vs AC+NC FET T2	-3,363738	0,0001122	P31025;Q5VSP4	Lipocalin-1	LCN1
UC vs AC+NC FET T2	-2,156539	0,0009354	P31151;Q86SG5	Protein S100-A7	S100A7
UC vs AC+NC FET T2	-1,776866	0,04869	P31944	Caspase-14	CASP14
UC vs AC+NC FET T2	1,548232	0,0004102	P33151;P33151-2	Cadherin-5	CDH5
UC vs AC+NC FET T2	1,856634	0,002006	P34096	Ribonuclease 4	RNASE4
UC vs AC+NC FET T2	1,8494447	0,008822	P35443	Thrombospondin-4	THBS4
UC vs AC+NC FET T2	5,6963757	1,35E-04	P35542	Serum amyloid A-4 protein	SAA4
UC vs AC+NC FET T2	1,5426654	0,02903	P35555	Fibrillin-1	FBN1
UC vs AC+NC FET T2	3,0900576	0,0005651	P35556	Fibrillin-2	FBN2
UC vs AC+NC FET T2	3,3209099	2,73E-06	P35858;P35858-2	Insulin-like growth factor-binding protein complex acid labile subunit	IGFALS
UC vs AC+NC FET T2	1,8860093	0,01621	P36955;CON__Q95121	Pigment epithelium-derived factor	SERPINF1
UC vs AC+NC FET T2	1,5943059	0,004546	P36980-2;P36980	Complement factor H-related protein 2	CFHR2
UC vs AC+NC FET T2	2,2580534	9,14E-07	P39060-2;P39060-1;P39060	Collagen alpha-1(XVIII) chain	COL18A1
UC vs AC+NC FET T2	0,9167982	0,01033	P43652	Afamin	AFM
UC vs AC+NC FET T2	-1,684921	0,04077	P46013;P46013-2	Antigen KI-67	MKI67
UC vs AC+NC FET T2	2,7787474	0,0001217	P48740	Mannan-binding lectin serine protease 1	MASP1
UC vs AC+NC FET T2	0,9378468	0,01474	P48740-2	Mannan-binding lectin serine protease 1	MASP1
UC vs AC+NC FET T2	3,8871208	5,74E-03	P48740-4	Mannan-binding lectin serine protease 1	MASP1
UC vs AC+NC FET T2	2,2061343	0,004465	P49454	Centromere protein F	CENPF
UC vs AC+NC FET T2	-1,264077	0,04527	P49619-3;P49619-2;P49619	Diacylglycerol kinase gamma	DGKG
UC vs AC+NC FET T2	-1,173657	0,01895	P49767	Vascular endothelial growth factor C	VEGFC
UC vs AC+NC FET T2	0,8252896	0,01081	P49908	Selenoprotein P	SEPP1
UC vs AC+NC FET T2	1,5347635	0,0007588	P49913	Cathelicidin antimicrobial peptide	CAMP
UC vs AC+NC FET T2	1,2176465	0,0006966	P51884;CON__Q05443	Lumican	LUM
UC vs AC+NC FET T2	1,42782	0,00525	P54132	Bloom syndrome protein	BLM
UC vs AC+NC FET T2	1,9002858	0,0004274	P55056	Apolipoprotein C-IV	APOC4
UC vs AC+NC FET T2	2,2735624	0,001889	P55103	Inhibin beta C chain	INHBC
UC vs AC+NC FET T2	0,8356026	0,03221	P55287-2;P55287	Cadherin-11	CDH11
UC vs AC+NC FET T2	-1,024316	0,01754	P55774	C-C motif chemokine 18	CCL18
UC vs AC+NC FET T2	2,7011547	0,00231	P57077;P57077-1	MAP3K7 C-terminal-like protein	MAP3K7CL

UC vs AC+NC FET T2	1,5945404	0,01001	P58166	Inhibin beta E chain	INHBE
UC vs AC+NC FET T2	0,3742899	0,03737	P59666;P59665	Neutrophil defensin 3	DEFA3;DEFA1
UC vs AC+NC FET T2	2,6337782	0,002699	P60709	Actin, cytoplasmic 1	ACTB
UC vs AC+NC FET T2	2,0016317	0,0004804	P60900;P60900-2;P60900-3	Proteasome subunit alpha type-6	PSMA6
UC vs AC+NC FET T2	4,4522702	0,0001994	P61626	Lysozyme C	LYZ
UC vs AC+NC FET T2	-2,68078	0,006978	P61769	Beta-2-microglobulin	B2M
UC vs AC+NC FET T2	-1,157658	0,03731	P62979;P62987;P0CG47;P0CG48	Ubiquitin-40S ribosomal protein S27a	RPS27A;UBA52;UBB;UBC
UC vs AC+NC FET T2	1,1778988	0,0004871	P63104;P63104-2	14-3-3 protein zeta/delta	YWHAZ
UC vs AC+NC FET T2	-1,655814	0,001493	P67936	Tropomyosin alpha-4 chain	TPM4
UC vs AC+NC FET T2	-3,132451	0,02347	P68363;P68363-2	Tubulin alpha-1B chain	TUBA1B
UC vs AC+NC FET T2	1,5807525	1,17E-02	P80108;P80108-2	Phosphatidylinositol-glycan-specific phospholipase D	GPLD1
UC vs AC+NC FET T2	3,5401414	3,54E-03	P80748	Ig lambda chain V-III region LOI	Ig lambda chain V-III region LOI
UC vs AC+NC FET T2	1,3238186	0,004512	Q00888;Q00888-3;Q00888-2	Pregnancy-specific beta-1-glycoprotein 4	PSG4
UC vs AC+NC FET T2	-1,569559	0,02756	Q00889-2;Q00889	Pregnancy-specific beta-1-glycoprotein 6	PSG6
UC vs AC+NC FET T2	2,2678442	0,0003139	Q02224;Q02224-3	Centromere-associated protein E	CENPE
UC vs AC+NC FET T2	2,6325403	0,002533	Q02985-2;Q02985	Complement factor H-related protein 3	CFHR3
UC vs AC+NC FET T2	1,3769373	0,03121	Q03001;Q03001-9;Q03001-13;Q03001-10	Dystonin	DST
UC vs AC+NC FET T2	3,0385159	4,19E-02	Q06033-2;Q06033	Inter-alpha-trypsin inhibitor heavy chain H3	ITIH3
UC vs AC+NC FET T2	1,9243822	0,01095	Q06190	Serine/threonine-protein phosphatase 2A regulatory subunit B subunit alpha	PPP2R3A
UC vs AC+NC FET T2	1,9075877	0,02336	Q07954;Q07954-2	Prolow-density lipoprotein receptor-related protein 1	LRP1
UC vs AC+NC FET T2	1,7285211	0,003699	Q0VAK6;Q0VAK6-2	Leiomodin-3	LMOD3
UC vs AC+NC FET T2	1,7437719	0,0009394	Q12805-2;Q12805-4;Q12805-3;Q12805	EGF-containing fibulin-like extracellular matrix protein 1	EFEMP1
UC vs AC+NC FET T2	2,3242493	0,01304	Q13103	Secreted phosphoprotein 24	SPP2
UC vs AC+NC FET T2	2,9391849	0,01843	Q13201;Q13201-2	Multimerin-1	MMRN1
UC vs AC+NC FET T2	2,8832699	1,93E-06	Q13214-2;Q13214	Semaphorin-3B	SEMA3B
UC vs AC+NC FET T2	7,5381867	1,99E-05	Q13219	Pappalysin-1	PAPPA
UC vs AC+NC FET T2	1,5077229	0,005166	Q13418-2;Q13418;Q13418-3	Integrin-linked protein kinase	ILK
UC vs AC+NC FET T2	5,1765985	0,01045	Q13488-2	V-type proton ATPase 116 kDa subunit a isoform 3	TCIRG1
UC vs AC+NC FET T2	2,8491232	0,0005272	Q13635-2	.	.
UC vs AC+NC FET T2	0,6552622	0,03535	Q14161-7;Q14161-9;Q14161-8;Q14161-5;Q14161;Q14161-2;Q14161-11;Q14161-6;Q14161-10;Q14161-4;Q14161-3	ARF GTPase-activating protein GIT2	GIT2
UC vs AC+NC FET T2	1,8359431	0,007217	Q14515;Q14515-2	SPARC-like protein 1	SPARCL1
UC vs AC+NC FET T2	1,8503913	0,0003535	Q14520-2;Q14520	Hyaluronan-binding protein 2	HABP2
UC vs AC+NC FET T2	2,5073859	0,001394	Q15022	Polycomb protein SUZ12	SUZ12
UC vs AC+NC FET T2	1,2756451	0,009164	Q15113	Procollagen C-endopeptidase enhancer 1	PCOLCE
UC vs AC+NC FET T2	1,4786421	0,01893	Q15166	Serum paraoxonase/lactonase 3	PON3
UC vs AC+NC FET T2	5,5634444	4,22E-07	Q15386;Q15386-2	Ubiquitin-protein ligase E3C	UBE3C
UC vs AC+NC FET T2	3,037192	0,002394	Q15485;Q15485-2	Ficolin-2	FCN2
UC vs AC+NC FET T2	5,1749634	1,32E-02	Q15582	Transforming growth factor-beta-induced protein ig-h3	TGFBI
UC vs AC+NC FET T2	4,7112309	4,38E-03	Q15848	Adiponectin	ADIPOQ
UC vs AC+NC FET T2	-0,780603	0,007249	Q16557	Pregnancy-specific beta-1-glycoprotein 3	PSG3
UC vs AC+NC FET T2	1,8532137	0,007618	Q16610-4;Q16610;Q16610-2	Extracellular matrix protein 1	ECM1
UC vs AC+NC FET T2	2,422352	0,008427	Q16769;Q16769-2	Glutaminyl-peptide cyclotransferase	QPCT
UC vs AC+NC FET T2	2,1556896	1,11E-03	Q4L180-3;Q4L180-7;Q4L180-5;Q4L180-2;Q4L180;Q4L180-6	Filamin A-interacting protein 1-like	FILIP1L
UC vs AC+NC FET T2	2,5098203	1,75E-02	Q4LDE5;Q4LDE5-4;Q4LDE5-3;Q4LDE5-2	Sushi, von Willebrand factor type A, EGF and pentraxin domain-containing protein 1	SVEP1
UC vs AC+NC FET T2	2,0440271	0,005347	Q5HYK7-3;Q5HYK7-2;Q5HYK7;Q5HYK7-5;Q5HYK7-4	SH3 domain-containing protein 19	SH3D19
UC vs AC+NC FET T2	2,5318849	0,0001167	Q5JPF3;Q5JPF3-2;A6QL64-3;Q5JPF3-3	Ankyrin repeat domain-containing protein 36C	ANKRD36C;ANKRD36
UC vs AC+NC FET T2	0,6997413	0,005314	Q5T0U0;Q5T0U0-2	Coiled-coil domain-containing protein 122	CCDC122
UC vs AC+NC FET T2	3,4794213	0,003306	Q6P1M0-2	Long-chain fatty acid transport protein 4	SLC27A4
UC vs AC+NC FET T2	2,6060444	0,0007181	Q6Q788	Apolipoprotein A-V	APOA5
UC vs AC+NC FET T2	3,8582549	0,002907	Q6UXH9-2;Q6UXH9-3;Q6UXH9	Inactive serine protease PAMR1	PAMR1
UC vs AC+NC FET T2	3,7555753	9,01E-03	Q6UY14-3;Q6UY14;Q6UY14-2	ADAMTS-like protein 4	ADAMTSL4
UC vs AC+NC FET T2	1,8208703	0,0039	Q76LX8;Q76LX8-2;Q76LX8-3;Q76LX8-4	A disintegrin and metalloproteinase with thrombospondin motifs 13	ADAMTS13
UC vs AC+NC FET T2	-2,76186	0,003424	Q7LBC6-3	Lysine-specific demethylase 3B	KDM3B
UC vs AC+NC FET T2	1,3627172	0,0008366	Q7Z478	ATP-dependent RNA helicase DHX29	DHX29
UC vs AC+NC FET T2	-0,648538	0,0476	Q7Z572	Spermatogenesis-associated protein 21	SPATA21
UC vs AC+NC FET T2	2,5673744	0,02498	Q7Z7A1-5;Q7Z7A1;Q7Z7A1-2;Q7Z7A1-3;Q7Z7A1-4	Centriolin	CNTRL
UC vs AC+NC FET T2	1,984732	0,01816	Q86UQ4;Q86UQ4-4;Q86UQ4-3;Q86UQ4-6;Q86UQ4-7;Q86UQ4-5	ATP-binding cassette sub-family A member 13	ABCA13
UC vs AC+NC FET T2	0,9116645	0,04138	Q8IV50-2;Q8IV50	LysM and putative peptidoglycan-binding domain-containing protein 2	LYSMD2
UC vs AC+NC FET T2	1,5175777	0,003657	Q8IVL1-11;Q8IVL1-4	Neuron navigator 2	NAV2
UC vs AC+NC FET T2	1,1397914	0,0225	Q8IYW2	Cilia- and flagella-associated protein 46	CFAP46
UC vs AC+NC FET T2	0,8575821	0,003534	Q8IZK6-2;Q8IZK6	Mucolipin-2	MCOLN2
UC vs AC+NC FET T2	-1,813318	0,0009941	Q8IZP9-9;Q8IZP9-10;Q8IZP9-8;Q8IZP9-7;Q8IZP9-5;Q8IZP9-6;Q8IZP9-3;Q8IZP9-4;Q8IZP9-2;Q8IZP9	G-protein coupled receptor 64	GPR64
UC vs AC+NC FET T2	3,48871	0,01153	Q8N7Z5;Q8WY50	Putative ankyrin repeat domain-containing protein 31	ANKRD31
UC vs AC+NC FET T2	-1,447489	0,0249	Q8N8A2-4;Q8N8A2;Q8N8A2-2;Q8N8A2-3;Q8N8A2-5	Serine/threonine-protein phosphatase 6 regulatory ankyrin repeat subunit B	ANKRD44
UC vs AC+NC FET T2	-2,037308	0,02693	Q8NBJ4-2;Q8NBJ4	Golgi membrane protein 1	GOLM1
UC vs AC+NC FET T2	4,4593878	9,17E-04	Q8NBP7	Proprotein convertase subtilisin/kexin type 9	PCSK9
UC vs AC+NC FET T2	-2,801834	0,0001637	Q8ND83-3;Q8ND83-2;Q8ND83-4	SLAIN motif-containing protein 1	SLAIN1
UC vs AC+NC FET T2	1,6575624	3,34E-02	Q8NDV7-6;Q8NDV7;Q8NDV7-2;Q8NDV7-5;Q8NDV7-4;Q8NDV7-3	Trinucleotide repeat-containing gene 6A protein	TNRC6A

UC vs AC+NC FET T2	2,5996401	0,0005542	Q8NGK2	Olfactory receptor 52B4	OR52B4
UC vs AC+NC FET T2	1,9834395	0,002512	Q8NI99	Angiopoietin-related protein 6	ANGPTL6
UC vs AC+NC FET T2	2,2339162	0,006836	Q8TE73	Dynein heavy chain 5, axonemal	DNAH5
UC vs AC+NC FET T2	1,4000791	0,0005311	Q8WUA8	Tsukushin	TSKU
UC vs AC+NC FET T2	2,0537279	0,01099	Q8WWZ8;Q8WWZ8-2	Oncoprotein-induced transcript 3 protein	OIT3
UC vs AC+NC FET T2	3,5696312	0,01324	Q8WZ42-5	Titin	TTN
UC vs AC+NC FET T2	1,3401809	0,0116	Q92496;Q92496-2;Q92496-3	Complement factor H-related protein 4	CFHR4
UC vs AC+NC FET T2	1,8259606	0,0001549	Q92743	Serine protease HTRA1	HTRA1
UC vs AC+NC FET T2	2,5770833	0,005709	Q92954-3;Q92954-6;Q92954;Q92954-4;Q92954-2;Q92954-5	Proteoglycan 4	PRG4
UC vs AC+NC FET T2	-0,843642	0,0383	Q96CM8-3;Q96CM8-4;Q96CM8;Q96CM8-2	Acyl-CoA synthetase family member 2, mitochondrial	ACSF2
UC vs AC+NC FET T2	2,9010233	1,14E-02	Q96IY4;Q96IY4-2;CON__Q2KIG3	Carboxypeptidase B2	CPB2
UC vs AC+NC FET T2	1,0289547	0,001474	Q96JB1-2;Q96JB1	Dynein heavy chain 8, axonemal	DNAH8
UC vs AC+NC FET T2	1,4883114	0,002674	Q96KN2	Beta-Ala-His dipeptidase	CNDP1
UC vs AC+NC FET T2	3,3418913	0,0003207	Q96PD5;Q96PD5-2	N-acetylmuramoyl-L-alanine amidase	PGLYRP2
UC vs AC+NC FET T2	-2,171907	0,03451	Q96QR1	Secretoglobin family 3A member 1	SCGB3A1
UC vs AC+NC FET T2	4,0355129	1,20E-03	Q96RL1-3;Q96RL1-4	BRCA1-A complex subunit RAP80	UIMC1
UC vs AC+NC FET T2	0,8310919	0,005135	Q99574	Neuroserpin	SERPINI1
UC vs AC+NC FET T2	1,5873903	2,72E-06	Q99969	Retinoic acid receptor responder protein 2	RARRES2
UC vs AC+NC FET T2	0,7501894	0,03011	Q9BWP8-8;Q9BWP8-7;Q9BWP8-6;Q9BWP8-5;Q9BWP8-4;Q9BWP8-3;Q9BWP8-2;Q9BWP8-9;Q9BWP8;Q9BWP8-10	Collectin-11	COLEC11
UC vs AC+NC FET T2	2,3135015	1,65E-04	Q9BXR6	Complement factor H-related protein 5	CFHR5
UC vs AC+NC FET T2	1,4389187	0,00491	Q9BZR9	Probable E3 ubiquitin-protein ligase TRIM8	TRIM8
UC vs AC+NC FET T2	-1,895889	0,0001004	Q9HCL0-2;Q9HCL0	Protocadherin-18	PCDH18
UC vs AC+NC FET T2	2,2470413	0,0005695	Q9HDC9;Q9HDC9-2	Adipocyte plasma membrane-associated protein	APMAP
UC vs AC+NC FET T2	6,2428579	1,39E-03	Q9NQ79;Q9NQ79-2;Q9NQ79-3	Cartilage acidic protein 1	CRTAC1
UC vs AC+NC FET T2	1,4870612	0,0003537	Q9NY15;Q9NY15-2	Stabilin-1	STAB1
UC vs AC+NC FET T2	-1,528492	0,03375	Q9NZI8-2;Q9NZI8	Insulin-like growth factor 2 mRNA-binding protein 1	IGF2BP1
UC vs AC+NC FET T2	-3,306174	0,0001578	Q9NZT1	Calmodulin-like protein 5	CALML5
UC vs AC+NC FET T2	3,4576648	4,43E-04	Q9UHG3	Prenylcysteine oxidase 1	PCYOX1
UC vs AC+NC FET T2	2,5342494	0,0006655	Q9UK55	Protein Z-dependent protease inhibitor	SERPINA10
UC vs AC+NC FET T2	1,2497383	0,001115	Q9Y4C2-2;Q9Y4C2	TRPM8 channel-associated factor 1	TCAF1
UC vs AC+NC FET T3	2,2541175	1,14E-03	A0A075B6I0	.	IGLV8-61
UC vs AC+NC FET T3	1,7780732	0,03561	A0A075B6I4	.	IGLV10-54
UC vs AC+NC FET T3	3,5319092	0,0124	A0A075B6J9	.	IGLV2-18
UC vs AC+NC FET T3	1,6276375	0,0392	A0A075B6P5;P01615	Ig kappa chain V-II region FR	IGKV2D-28
UC vs AC+NC FET T3	1,6240035	0,01095	A0A0C4DH68;A0A075B6R9	.	IGKV2-24;IGKV2D-24
UC vs AC+NC FET T3	1,0098931	0,00509	A0A0B4J1V0	.	IGHV3-15
UC vs AC+NC FET T3	1,7944603	0,02012	A0A0B4J1X5	.	IGHV3-74
UC vs AC+NC FET T3	4,1986661	0,006259	A0A0B4J1Y9	.	IGHV3-72
UC vs AC+NC FET T3	1,4707464	0,002652	A0A0C4DH33	.	IGHV1-24
UC vs AC+NC FET T3	3,3845635	0,02936	A0A0C4DH35	.	IGHV3-35
UC vs AC+NC FET T3	-2,217031	0,003426	A0A0C4DH38	.	IGHV5-51
UC vs AC+NC FET T3	1,6053065	0,001837	A0A0C4DH67;A0A0C4DH69	.	IGKV1-8;IGKV1-9
UC vs AC+NC FET T3	4,2117158	0,002155	A0A0J9YX35	.	.
UC vs AC+NC FET T3	-6,005744	0,0007404	A4FU69-3;A4FU69;A4FU69-2;A4FU69-4;A4FU69-6	EF-hand calcium-binding domain-containing protein 5	EFCAB5
UC vs AC+NC FET T3	2,4951903	9,15E-03	A8K2U0;A8K2U0-2	Alpha-2-macroglobulin-like protein 1	A2ML1
UC vs AC+NC FET T3	1,4606832	0,004715	M0R2J8	.	DCDC1
UC vs AC+NC FET T3	3,4275392	1,58E-02	O00187;O00187-2	Mannan-binding lectin serine protease 2	MASP2
UC vs AC+NC FET T3	-3,630093	0,04146	O00217	NADH dehydrogenase [ubiquinone] iron-sulfur protein 8, mitochondrial	NDUFS8
UC vs AC+NC FET T3	0,478422	0,01366	O00391;O00391-2	Sulfhydryl oxidase 1	QSOX1
UC vs AC+NC FET T3	1,3495492	0,03255	O14791-2;O14791;O14791-3	Apolipoprotein L1	APOL1
UC vs AC+NC FET T3	4,0642534	0,0001325	O15016;O15016-2;O15016-3	Tripartite motif-containing protein 66	TRIM66
UC vs AC+NC FET T3	5,5154617	0,0001816	O43184-3;O43184-4;O43184-2;O43184	Disintegrin and metalloproteinase domain-containing protein 12	ADAM12
UC vs AC+NC FET T3	2,0668833	0,01212	O43399;O43399-5;O43399-7;O43399-2;O43399-4;O43399-3	Tumor protein D54	TPD52L2
UC vs AC+NC FET T3	2,2059796	0,01475	O43861-2;O43861	Probable phospholipid-transporting ATPase IIB	ATP9B
UC vs AC+NC FET T3	2,0839559	0,003902	Q99880;Q99879;Q99877;Q93079;Q8N257;Q5QNW6;Q16778;P62807;P58876;P57053;P33778;P23527;P06899;O60814;Q5QNW6-2	Histone H2B type 1-L	HIST1H2BL;HIST1H2BM;HIST1H2BN;HIST1H2BH;HIST3H2BB;HIST2H2BF;HIST2H2BE;HIST1H2BC;HIST1H2BD;H2BFS;HIST1H2BB;HIST1H2BO;HIST1H2BJ;HIST1H2BK
UC vs AC+NC FET T3	3,2125908	1,25E-02	O75636;O75636-2	Ficolin-3	FCN3
UC vs AC+NC FET T3	5,5405278	0,0003253	O75882;O75882-2;O75882-3	Attractin	ATRN
UC vs AC+NC FET T3	1,686995	2,18E-03	O76076	WNT1-inducible-signaling pathway protein 2	WISP2
UC vs AC+NC FET T3	-2,740257	1,34E-02	O94855;O94855-2	Protein transport protein Sec24D	SEC24D
UC vs AC+NC FET T3	1,4518624	0,00181	O95428-6;O95428;O95428-5;O95428-4;O95428-2;O95428-3	Papilin	PAPLN
UC vs AC+NC FET T3	6,0416598	5,05E-04	O95445-2	Apolipoprotein M	APOM
UC vs AC+NC FET T3	1,1366502	0,005645	P00450	Ceruloplasmin	CP
UC vs AC+NC FET T3	1,1061813	0,0005852	P00488	Coagulation factor XIII A chain	F13A1
UC vs AC+NC FET T3	4,3994962	0,009502	P00709	Alpha-lactalbumin	LALBA
UC vs AC+NC FET T3	0,7269546	0,01397	P00734	Prothrombin	F2
UC vs AC+NC FET T3	1,4923111	3,62E-02	P00736	Complement C1r subcomponent	C1R
UC vs AC+NC FET T3	1,5764858	0,0005011	P00739;P00739-2	Haptoglobin-related protein	HPR
UC vs AC+NC FET T3	3,7352127	2,14E-06	P00740;P00740-2	Coagulation factor IX	F9
UC vs AC+NC FET T3	2,3077704	0,0001856	P00742	Coagulation factor X	F10
UC vs AC+NC FET T3	1,6918217	5,78E-03	P00747;Q02325	Plasminogen	PLG
UC vs AC+NC FET T3	1,9189764	0,0002288	P00748	Coagulation factor XII	F12
UC vs AC+NC FET T3	2,5431775	0,001555	P00751;P00751-2	Complement factor B	CFB
UC vs AC+NC FET T3	-0,96891	0,04952	P01008	Antithrombin-III	SERPINC1

UC vs AC+NC FET T3	-0,843058	0,02896	P01009;P01009-2;P01009-3;P20848	Alpha-1-antitrypsin	SERPINA1
UC vs AC+NC FET T3	0,5436014	0,04957	P01024;O95568	Complement C3	C3
UC vs AC+NC FET T3	1,4987816	0,0009369	P01031	Complement C5	C5
UC vs AC+NC FET T3	-2,413198	3,93E-02	P01040	Cystatin-A	CSTA
UC vs AC+NC FET T3	3,1985273	1,18E-04	P01042-2	Kininogen-1	KNG1
UC vs AC+NC FET T3	0,9074791	0,02623	P01344-3;P01344;P01344-2	Insulin-like growth factor II	IGF2
UC vs AC+NC FET T3	0,8259517	0,005255	P01591	Immunoglobulin J chain	IGJ
UC vs AC+NC FET T3	3,7957389	0,0004921	P01599	Ig kappa chain V-I region Gal	Ig kappa chain V-I region Gal
UC vs AC+NC FET T3	2,1376411	2,65E-02	P01619	Ig kappa chain V-III region B6	Ig kappa chain V-III region B6
UC vs AC+NC FET T3	2,6259865	0,002867	P01700	Ig lambda chain V-I region HA	Ig lambda chain V-I region HA
UC vs AC+NC FET T3	1,9593183	0,0003471	P01701	Ig lambda chain V-I region NEW	Ig lambda chain V-I region NEW
UC vs AC+NC FET T3	1,8860363	0,005216	P01709	Ig lambda chain V-II region MGC	Ig lambda chain V-II region MGC
UC vs AC+NC FET T3	-2,237755	0,01025	P01715	Ig lambda chain V-IV region Bau	Ig lambda chain V-IV region Bau
UC vs AC+NC FET T3	5,437211	4,43E-06	P01743	Ig heavy chain V-I region HG3	Ig heavy chain V-I region HG3
UC vs AC+NC FET T3	-2,123922	0,002024	P01764	Ig heavy chain V-III region 23	IGHV3-23
UC vs AC+NC FET T3	3,8942768	0,004742	P0DP03;P01768	Ig heavy chain V-III region CAM	Ig heavy chain V-III region CAM
UC vs AC+NC FET T3	5,1465644	0,001441	P01782;P0DP04	Ig heavy chain V-III region DOB	Ig heavy chain V-III region DOB
UC vs AC+NC FET T3	-2,344956	7,84E-04	P01834	Ig kappa chain C region	IGKC
UC vs AC+NC FET T3	1,2890619	0,008266	P01859	Ig gamma-2 chain C region	IGHG2
UC vs AC+NC FET T3	1,5872899	0,007232	P01860	Ig gamma-3 chain C region	IGHG3
UC vs AC+NC FET T3	1,3317345	0,0002409	P01871;P01871-2	Ig mu chain C region	IGHM
UC vs AC+NC FET T3	1,6680115	0,0006568	P01876	Ig alpha-1 chain C region	IGHA1
UC vs AC+NC FET T3	0,8286146	2,74E-02	P02647;Q9HB71-2	Apolipoprotein A-I	APOA1
UC vs AC+NC FET T3	1,2626432	0,0009011	P02649;CON__Q03247	Apolipoprotein E	APOE
UC vs AC+NC FET T3	0,3524359	0,04214	P02654	Apolipoprotein C-I	APOC1
UC vs AC+NC FET T3	1,6864675	4,31E-03	P02671;P02671-2;REV__Q9UKV0-4;REV__Q9UKV0-2;REV__Q9UKV0;REV__Q9UKV0-5;REV__Q9UKV0-7;Q14314	Fibrinogen alpha chain	FGA
UC vs AC+NC FET T3	1,8132757	1,52E-02	P02675	Fibrinogen beta chain	FGB
UC vs AC+NC FET T3	0,9591054	0,0006321	P02679;P02679-2	Fibrinogen gamma chain	FGG
UC vs AC+NC FET T3	2,0319916	4,63E-03	P02746	Complement C1q subcomponent subunit B	C1QB
UC vs AC+NC FET T3	2,2389411	7,00E-06	P02747	Complement C1q subcomponent subunit C	C1QC
UC vs AC+NC FET T3	2,0098323	0,0123	P02750	Leucine-rich alpha-2-glycoprotein	LRG1
UC vs AC+NC FET T3	2,0137364	0,001184	P02751-1;P02751-8;P02751-3;P02751;P02751-14;P02751-7;P02751-17;P02751-9;P02751-6;P02751-4;P02751-12;P02751-16;P02751-2	Fibronectin	FN1
UC vs AC+NC FET T3	1,0252017	0,005119	P02753	Retinol-binding protein 4	RBP4
UC vs AC+NC FET T3	-1,049429	0,009795	P02760	Protein AMBP	AMBP
UC vs AC+NC FET T3	1,3760005	0,002617	P02766	Transthyretin	TTR
UC vs AC+NC FET T3	2,0703095	0,0002964	P02774-3;P02774;P02774-2	Vitamin D-binding protein	GC
UC vs AC+NC FET T3	3,9326076	0,0008102	P02775	Platelet basic protein	PPBP
UC vs AC+NC FET T3	1,4611682	0,0009774	P02790	Hemopexin	HPX
UC vs AC+NC FET T3	5,2625905	2,84E-05	P03950	Angiogenin	ANG
UC vs AC+NC FET T3	4,9866606	1,93E-05	P03951	Coagulation factor XI	F11
UC vs AC+NC FET T3	2,4217945	5,49E-04	P03952;P20718	Plasma kallikrein	KLKB1
UC vs AC+NC FET T3	0,5834764	0,02626	P04003;CON__Q28065	C4b-binding protein alpha chain	C4BPA
UC vs AC+NC FET T3	2,4888689	0,01193	P04040	Catalase	CAT
UC vs AC+NC FET T3	1,904251	0,009058	P04070;P04070-2	Vitamin K-dependent protein C	PROC
UC vs AC+NC FET T3	0,6648027	0,02939	P04114	Apolipoprotein B-100	APOB
UC vs AC+NC FET T3	1,0461341	0,009103	P04180	Phosphatidylcholine-sterol acyltransferase	LCAT
UC vs AC+NC FET T3	-1,608952	0,007062	P04196	Histidine-rich glycoprotein	HRG
UC vs AC+NC FET T3	1,9004084	0,006487	P04217	Alpha-1B-glycoprotein	A1BG
UC vs AC+NC FET T3	4,6025702	0,002167	P04275	von Willebrand factor	VWF
UC vs AC+NC FET T3	2,4562998	0,003389	P04406;P04406-2;O14556	Glyceraldehyde-3-phosphate dehydrogenase	GAPDH
UC vs AC+NC FET T3	1,4926852	0,004437	P04433;A0A0A0MRZ8	Ig kappa chain V-III region VG	IGKV3D-11
UC vs AC+NC FET T3	-0,722939	0,02386	P05019-3;P05019-2;P05019-4;P05019	Insulin-like growth factor I	IGF1
UC vs AC+NC FET T3	4,1438578	0,001652	P05023-2;P05023-4;P05023;P05023-3	Sodium/potassium-transporting ATPase subunit alpha-1	ATP1A1
UC vs AC+NC FET T3	0,9599825	0,001594	P05090	Apolipoprotein D	APOD
UC vs AC+NC FET T3	-1,362869	0,01128	P05109	Protein S100-A8	S100A8
UC vs AC+NC FET T3	3,4289819	5,23E-03	P05155-2;P05155;P05155-3	Plasma protease C1 inhibitor	SERPING1
UC vs AC+NC FET T3	1,8075086	2,48E-03	P05160	Coagulation factor XIII B chain	F13B
UC vs AC+NC FET T3	0,6329353	0,009326	P05164-3;P05164;P05164-2	Myeloperoxidase	MPO
UC vs AC+NC FET T3	1,901768	0,001587	P05543	Thyroxine-binding globulin	SERPINA7
UC vs AC+NC FET T3	2,1013926	0,0005276	P05546	Heparin cofactor 2	SERPIND1
UC vs AC+NC FET T3	3,0633172	3,84E-02	P06331	Ig heavy chain V-II region ARH-77	Ig heavy chain V-II region ARH-77
UC vs AC+NC FET T3	0,4865443	0,01528	P06396;P06396-4;P06396-3	Gelsolin	GSN
UC vs AC+NC FET T3	1,189242	0,04915	P06681;P06681-3;P06681-2	Complement C2	C2
UC vs AC+NC FET T3	0,578621	0,04825	P06727;Q9BT92	Apolipoprotein A-IV	APOA4
UC vs AC+NC FET T3	2,632383	0,004099	P06753-5;P06753-2;P06753-4;P06753-3;P06753-6;P06753-7	Tropomyosin alpha-3 chain	TPM3
UC vs AC+NC FET T3	0,9727169	0,006733	P07225	Vitamin K-dependent protein S	PROS1
UC vs AC+NC FET T3	2,4313496	0,002853	P07237	Protein disulfide-isomerase	P4HB
UC vs AC+NC FET T3	1,0113573	2,47E-03	P07333;P07333-2	Macrophage colony-stimulating factor 1 receptor	CSF1R
UC vs AC+NC FET T3	2,3397421	0,002265	P07357	Complement component C8 alpha chain	C8A
UC vs AC+NC FET T3	2,4673945	0,01007	P07358	Complement component C8 beta chain	C8B
UC vs AC+NC FET T3	1,0816084	0,007768	P07900;P07900-2;Q14568;Q58FF6;Q58FG1	Heat shock protein HSP 90-alpha	HSP90AA1
UC vs AC+NC FET T3	2,5639304	0,01291	P07996;P07996-2	Thrombospondin-1	THBS1
UC vs AC+NC FET T3	0,8580288	0,02288	P08253;P08253-3;P08253-2	72 kDa type IV collagenase	MMP2
UC vs AC+NC FET T3	2,3323716	0,0002547	P08294	Extracellular superoxide dismutase [Cu-Zn]	SOD3
UC vs AC+NC FET T3	4,5011932	1,97E-03	P08493-2;P08493	Matrix Gla protein	MGP
UC vs AC+NC FET T3	2,7247762	0,02335	P08567	Pleckstrin	PLEK
UC vs AC+NC FET T3	1,5898978	7,08E-03	P08603;P08603-2	Complement factor H	CFH
UC vs AC+NC FET T3	2,3161169	0,0009444	P08709-2;P08709	Coagulation factor VII	F7
UC vs AC+NC FET T3	2,4930067	2,14E-02	P09382	Galectin-1	LGALS1

UC vs AC+NC FET T3	-3,127965	7,87E-06	P09466-2;P09466	Glycodelin	PAEP
UC vs AC+NC FET T3	2,2259527	0,007627	P09466-3	Glycodelin	PAEP
UC vs AC+NC FET T3	2,9234058	0,005345	P09486	SPARC	SPARC
UC vs AC+NC FET T3	1,0069669	0,0004285	P09871	Complement C1s subcomponent	C1S
UC vs AC+NC FET T3	-2,716298	0,02814	P0C0L4;P0C0L4-2	Complement C4-A	C4A
UC vs AC+NC FET T3	1,2685992	3,05E-02	P0C0L5	Complement C4-B	C4B
UC vs AC+NC FET T3	-1,50362	1,47E-03	P0DN87;P0DN86;P0DN86-2;Q6NT52;A6NKQ9-2;A6NKQ9	Choriogonadotropin subunit beta variant 2	CGB2;CGB1
UC vs AC+NC FET T3	2,6317953	0,0007013	P0DOX2	.	.
UC vs AC+NC FET T3	2,8331531	0,04184	P0DOX4	.	.
UC vs AC+NC FET T3	1,3567282	0,0001034	P0DOX7	.	.
UC vs AC+NC FET T3	1,7960538	1,61E-02	P0DOX8	.	.
UC vs AC+NC FET T3	1,6664723	0,002433	P10643	Complement component C7	C7
UC vs AC+NC FET T3	3,2952068	0,02143	P10720	Platelet factor 4 variant	PF4V1
UC vs AC+NC FET T3	2,0423255	0,001294	P11021	78 kDa glucose-regulated protein	HSPA5
UC vs AC+NC FET T3	1,4321003	0,03687	P11226	Mannose-binding protein C	MBL2
UC vs AC+NC FET T3	1,4559469	3,14E-03	P11464-4	Pregnancy-specific beta-1-glycoprotein 1	PSG1
UC vs AC+NC FET T3	2,2772671	3,88E-03	P11465	Pregnancy-specific beta-1-glycoprotein 2	PSG2
UC vs AC+NC FET T3	1,8748718	0,0004337	P11597;P11597-2	Cholesteryl ester transfer protein	CETP
UC vs AC+NC FET T3	2,2756997	8,16E-03	P12259	Coagulation factor V	F5
UC vs AC+NC FET T3	-1,598975	0,04502	P12273	Prolactin-inducible protein	PIP
UC vs AC+NC FET T3	-0,881206	0,007842	P12532;P12532-2	Creatine kinase U-type, mitochondrial	CKMT1A
UC vs AC+NC FET T3	5,0963707	4,66E-02	P12814;P12814-3;P12814-2	Alpha-actinin-1	ACTN1
UC vs AC+NC FET T3	3,596004	4,47E-03	P13727;P13727-2	Bone marrow proteoglycan	PRG2
UC vs AC+NC FET T3	-2,192293	0,003692	P13798	Acylamino-acid-releasing enzyme	APEH
UC vs AC+NC FET T3	0,814431	0,0007892	P14061	Estradiol 17-beta-dehydrogenase 1	HSD17B1
UC vs AC+NC FET T3	1,2589551	0,01196	P14209;P14209-2;P14209-3	CD99 antigen	CD99
UC vs AC+NC FET T3	1,7561245	0,02797	P14618;P14618-2	Pyruvate kinase PKM	PKM
UC vs AC+NC FET T3	2,1551897	0,01489	P15907	Beta-galactoside alpha-2,6-sialyltransferase 1	ST6GAL1
UC vs AC+NC FET T3	-1,687395	0,003976	P16035	Metalloproteinase inhibitor 2	TIMP2
UC vs AC+NC FET T3	3,7593679	0,0006019	P16885	1-phosphatidylinositol 4,5-bisphosphate phosphodiesterase gamma-2	PLCG2
UC vs AC+NC FET T3	4,2821015	0,000214	P17936;P17936-2	Insulin-like growth factor-binding protein 3	IGFBP3
UC vs AC+NC FET T3	0,9342953	0,0004957	P19823	Inter-alpha-trypsin inhibitor heavy chain H2	ITIH2
UC vs AC+NC FET T3	1,3932308	0,01246	P19827	Inter-alpha-trypsin inhibitor heavy chain H1	ITIH1
UC vs AC+NC FET T3	-1,159598	0,008846	P20929-3;P20929-2;P20929-4;P20929	Nebulin	NEB
UC vs AC+NC FET T3	4,932625	0,009697	P21333-2;P21333	Filamin-A	FLNA
UC vs AC+NC FET T3	2,4546663	1,72E-02	P22352	Glutathione peroxidase 3	GPX3
UC vs AC+NC FET T3	0,727193	0,005971	P22692	Insulin-like growth factor-binding protein 4	IGFBP4
UC vs AC+NC FET T3	5,0668876	0,002247	P23083	Ig heavy chain V-I region V35	Ig heavy chain V-I region V35
UC vs AC+NC FET T3	3,0904127	3,26E-03	P23142-4	Fibulin-1	FBLN1
UC vs AC+NC FET T3	-1,622224	0,01139	P23280-3;P23280	Carbonic anhydrase 6	CA6
UC vs AC+NC FET T3	2,3558135	6,07E-05	P24593	Insulin-like growth factor-binding protein 5	IGFBP5
UC vs AC+NC FET T3	0,6117204	0,04646	P24844	Myosin regulatory light polypeptide 9	MYL9
UC vs AC+NC FET T3	4,5321209	0,0004224	P26927	Hepatocyte growth factor-like protein	MST1
UC vs AC+NC FET T3	3,7310468	2,71E-02	P27918	Properdin	CFP
UC vs AC+NC FET T3	1,3757286	0,04156	P28370-2;P28370	Probable global transcription activator SNF2L1	SMARCA1
UC vs AC+NC FET T3	1,3397522	0,00219	P30043	Flavin reductase (NADPH)	BLVRB
UC vs AC+NC FET T3	-4,666694	8,21E-03	P31025;Q5VSP4	Lipocalin-1	LCN1
UC vs AC+NC FET T3	-2,572022	1,54E-02	P31151;Q86SG5	Protein S100-A7	S100A7
UC vs AC+NC FET T3	1,761347	0,008752	P34096	Ribonuclease 4	RNASE4
UC vs AC+NC FET T3	1,5895995	0,002672	P35443	Thrombospondin-4	THBS4
UC vs AC+NC FET T3	4,778608	1,87E-03	P35542	Serum amyloid A-4 protein	SAA4
UC vs AC+NC FET T3	1,2895029	0,000189	P35555	Fibrillin-1	FBN1
UC vs AC+NC FET T3	2,0456382	0,00704	P35556	Fibrillin-2	FBN2
UC vs AC+NC FET T3	7,1788805	5,61E-03	P35579;P35579-2	Myosin-9	MYH9
UC vs AC+NC FET T3	2,5721709	0,0005509	P35858;P35858-2	Insulin-like growth factor-binding protein complex acid labile subunit	IGFALS
UC vs AC+NC FET T3	1,34542	0,0326	P36980-2;P36980	Complement factor H-related protein 2	CFHR2
UC vs AC+NC FET T3	1,975092	0,002981	P39060-2;P39060-1;P39060	Collagen alpha-1(XVIII) chain	COL18A1
UC vs AC+NC FET T3	-1,406589	0,03681	P46013;P46013-2	Antigen KI-67	MKI67
UC vs AC+NC FET T3	3,2437766	0,01537	P48059;P48059-4;P48059-2;P48059-5;P48059-3;Q7Z4I7-4;Q7Z4I7-3;Q7Z4I7-2;P0CW19-2	LIM and senescent cell antigen-like-containing domain protein 1	LIMS1
UC vs AC+NC FET T3	1,3833903	1,48E-03	P48307-2;P48307	Tissue factor pathway inhibitor 2	TFPI2
UC vs AC+NC FET T3	2,2236372	4,30E-05	P48740	Mannan-binding lectin serine protease 1	MASP1
UC vs AC+NC FET T3	0,9922982	0,0005937	P48740-2	Mannan-binding lectin serine protease 1	MASP1
UC vs AC+NC FET T3	4,595889	3,33E-04	P48740-4	Mannan-binding lectin serine protease 1	MASP1
UC vs AC+NC FET T3	3,0323147	0,001973	P49454	Centromere protein F	CENPF
UC vs AC+NC FET T3	-1,176671	0,01156	P49767	Vascular endothelial growth factor C	VEGFC
UC vs AC+NC FET T3	0,7314657	0,008182	P49908	Selenoprotein P	SEPP1
UC vs AC+NC FET T3	1,3526533	0,01381	P49913	Cathelicidin antimicrobial peptide	CAMP
UC vs AC+NC FET T3	2,1915872	0,004854	P54132	Bloom syndrome protein	BLM
UC vs AC+NC FET T3	1,2003113	0,02752	P55056	Apolipoprotein C-IV	APOC4
UC vs AC+NC FET T3	1,9058639	0,0006201	P55103	Inhibin beta C chain	INHBC
UC vs AC+NC FET T3	3,5153952	0,0001721	P57077;P57077-1	MAP3K7 C-terminal-like protein	MAP3K7CL
UC vs AC+NC FET T3	4,6845847	0,0004441	P60709	Actin, cytoplasmic 1	ACTB
UC vs AC+NC FET T3	2,0585287	0,0007266	P60900;P60900-2;P60900-3	Proteasome subunit alpha type-6	PSMA6
UC vs AC+NC FET T3	3,4033182	0,03506	P61224-3;P61224;P61224-2;P61224-4;A6NIZ1;P62834	Ras-related protein Rap-1b	RAP1B;RAP1A
UC vs AC+NC FET T3	3,7997922	0,000677	P61626	Lysozyme C	LYZ
UC vs AC+NC FET T3	0,9982852	0,01742	P62736;P63267;P63267-2	Actin, aortic smooth muscle	ACTA2;ACTG2
UC vs AC+NC FET T3	-1,037977	0,02879	P62979;P62987;P0CG47;P0CG48	Ubiquitin-40S ribosomal protein S27a	RPS27A;UBA52;UBB;UBC
UC vs AC+NC FET T3	3,582288	0,00147	P63104;P63104-2	14-3-3 protein zeta/delta	YWHAZ
UC vs AC+NC FET T3	2,580437	0,02735	P67936	Tropomyosin alpha-4 chain	TPM4
UC vs AC+NC FET T3	2,7006472	0,02526	P68363;P68363-2	Tubulin alpha-1B chain	TUBA1B
UC vs AC+NC FET T3	1,6500582	0,01614	P68871;P02100	Hemoglobin subunit beta	HBB
UC vs AC+NC FET T3	3,36982	0,0002629	P69905	Hemoglobin subunit alpha	HBA1
UC vs AC+NC FET T3	1,2354451	3,17E-02	P80108;P80108-2	Phosphatidylinositol-glycan-specific phospholipase D	GPLD1
UC vs AC+NC FET T3	2,8062109	0,03109	P80748	Ig lambda chain V-III region LOI	Ig lambda chain V-III region LOI
UC vs AC+NC FET T3	4,2522933	0,005568	Q00526;P11802-2;Q00535-2;Q00535;P11802;Q14004-2;Q14004	Cyclin-dependent kinase 3	CDK3

UC vs AC+NC FET T3	-0,41627	0,001095	Q00887-2	Pregnancy-specific beta-1-glycoprotein 9	PSG9
UC vs AC+NC FET T3	-9,918588	2,65E-02	Q00888;Q00888-3;Q00888-2	Pregnancy-specific beta-1-glycoprotein 4	PSG4
UC vs AC+NC FET T3	-3,85644	0,03283	Q00889-2;Q00889	Pregnancy-specific beta-1-glycoprotein 6	PSG6
UC vs AC+NC FET T3	1,6479482	0,006086	Q02108-2;Q02108	Guanylate cyclase soluble subunit alpha-3	GUCY1A3
UC vs AC+NC FET T3	3,8790486	0,01894	Q02224;Q02224-3	Centromere-associated protein E	CENPE
UC vs AC+NC FET T3	3,0067978	0,001062	Q02985-2;Q02985	Complement factor H-related protein 3	CFHR3
UC vs AC+NC FET T3	1,5129832	0,0484	Q03001;Q03001-9;Q03001-13;Q03001-10	Dystonin	DST
UC vs AC+NC FET T3	0,6284617	0,0451	Q04756	Hepatocyte growth factor activator	HGFAC
UC vs AC+NC FET T3	2,3510219	0,001471	Q06033-2;Q06033	Inter-alpha-trypsin inhibitor heavy chain H3	ITIH3
UC vs AC+NC FET T3	1,5133092	0,01523	Q07954;Q07954-2	Prolow-density lipoprotein receptor-related protein 1	LRP1
UC vs AC+NC FET T3	0,4415558	0,0121	Q08174;Q08174-2	Protocadherin-1	PCDH1
UC vs AC+NC FET T3	1,3484215	0,0008056	Q0VAK6;Q0VAK6-2	Leiomodin-3	LMOD3
UC vs AC+NC FET T3	0,9946185	3,23E-02	Q12805-2;Q12805-4;Q12805-3;Q12805	EGF-containing fibulin-like extracellular matrix protein 1	EFEMP1
UC vs AC+NC FET T3	1,8964512	0,0001212	Q12805-5	EGF-containing fibulin-like extracellular matrix protein 1	EFEMP1
UC vs AC+NC FET T3	1,6868936	4,21E-03	Q13093	Platelet-activating factor acetylhydrolase	PLA2G7
UC vs AC+NC FET T3	2,7149628	4,88E-04	Q13103	Secreted phosphoprotein 24	SPP2
UC vs AC+NC FET T3	1,7222488	0,01006	Q13214-2;Q13214	Semaphorin-3B	SEMA3B
UC vs AC+NC FET T3	5,854893	1,22E-05	Q13219	Pappalysin-1	PAPPA
UC vs AC+NC FET T3	0,7918739	0,04458	Q13361-2;Q13361	Microfibrillar-associated protein 5	MFAP5
UC vs AC+NC FET T3	2,3921148	0,007597	Q13418-2;Q13418;Q13418-3	Integrin-linked protein kinase	ILK
UC vs AC+NC FET T3	2,9699611	0,0388	Q13464	Rho-associated protein kinase 1	ROCK1
UC vs AC+NC FET T3	4,7561122	1,46E-02	Q13488-2	V-type proton ATPase 116 kDa subunit a isoform 3	TCIRG1
UC vs AC+NC FET T3	2,4174381	8,03E-04	Q13635-2	.	.
UC vs AC+NC FET T3	2,4282781	4,94E-03	Q14520-2;Q14520	Hyaluronan-binding protein 2	HABP2
UC vs AC+NC FET T3	0,9632291	0,0001772	Q14624;Q14624-3;Q14624-4	Inter-alpha-trypsin inhibitor heavy chain H4	ITIH4
UC vs AC+NC FET T3	1,5976936	0,0002572	Q14697;Q14697-2	Neutral alpha-glucosidase AB	GANAB
UC vs AC+NC FET T3	4,2621544	0,002136	Q14766;Q14766-4;Q14766-3;Q14766-2;Q14766-5	Latent-transforming growth factor beta-binding protein 1	LTBP1
UC vs AC+NC FET T3	2,4259366	0,004157	Q15022	Polycomb protein SUZ12	SUZ12
UC vs AC+NC FET T3	0,6299313	0,006957	Q15113	Procollagen C-endopeptidase enhancer 1	PCOLCE
UC vs AC+NC FET T3	5,1935987	0,003123	Q15386;Q15386-2	Ubiquitin-protein ligase E3C	UBE3C
UC vs AC+NC FET T3	2,6937784	1,72E-05	Q15485;Q15485-2	Ficolin-2	FCN2
UC vs AC+NC FET T3	4,7872883	1,89E-03	Q15582	Transforming growth factor-beta-induced protein ig-h3	TGFBI
UC vs AC+NC FET T3	1,4710313	0,0005517	Q16610-4;Q16610;Q16610-2	Extracellular matrix protein 1	ECM1
UC vs AC+NC FET T3	-2,806515	0,03523	Q2KHM9	Uncharacterized protein KIAA0753	KIAA0753
UC vs AC+NC FET T3	0,8476356	3,00E-02	Q5CZC0;Q5CZC0-2	Fibrous sheath-interacting protein 2	FSIP2
UC vs AC+NC FET T3	2,4771627	3,63E-03	Q5HYK7-3;Q5HYK7-2;Q5HYK7;Q5HYK7-5;Q5HYK7-4	SH3 domain-containing protein 19	SH3D19
UC vs AC+NC FET T3	2,1552504	0,007268	Q5JPF3;Q5JPF3-2;A6QL64-3;Q5JPF3-3	Ankyrin repeat domain-containing protein 36C	ANKRD36C;ANKRD36
UC vs AC+NC FET T3	1,4267638	0,02231	Q5T5C0;Q5T5C0-2;Q5T5C0-3	Syntaxin-binding protein 5	STXBP5
UC vs AC+NC FET T3	2,4874659	0,002527	Q6EEV6	Small ubiquitin-related modifier 4	SUMO4
UC vs AC+NC FET T3	2,2872103	0,0007954	Q6Q788	Apolipoprotein A-V	APOA5
UC vs AC+NC FET T3	2,1186351	6,91E-04	Q6UVK1	Chondroitin sulfate proteoglycan 4	CSPG4
UC vs AC+NC FET T3	2,3091413	0,006172	Q6UY14-3;Q6UY14;Q6UY14-2	ADAMTS-like protein 4	ADAMTSL4
UC vs AC+NC FET T3	2,2904441	0,002502	Q6VAB6;Q6VAB6-2	Kinase suppressor of Ras 2	KSR2
UC vs AC+NC FET T3	-1,545152	4,46E-05	Q6ZS30-1;Q6ZS30	Neurobeachin-like protein 1	NBEAL1
UC vs AC+NC FET T3	1,0361804	0,007529	Q76LX8;Q76LX8-2;Q76LX8-3;Q76LX8-4	A disintegrin and metalloproteinase with thrombospondin motifs 13	ADAMTS13
UC vs AC+NC FET T3	-1,547277	0,006922	Q7L1Q6-2;Q7L1Q6;Q7L1Q6-3	Basic leucine zipper and W2 domain-containing protein 1	BZW1
UC vs AC+NC FET T3	-1,147215	0,02872	Q7Z572	Spermatogenesis-associated protein 21	SPATA21
UC vs AC+NC FET T3	-1,227391	0,04046	Q7Z5L0	Vitelline membrane outer layer protein 1 homolog	VMO1
UC vs AC+NC FET T3	1,4848686	9,91E-07	Q86U17	Serpin A11	SERPINA11
UC vs AC+NC FET T3	-1,021451	0,001547	Q86UD1	Out at first protein homolog	OAF
UC vs AC+NC FET T3	4,6480323	0,009366	Q86UX7-2;Q86UX7	Fermitin family homolog 3	FERMT3
UC vs AC+NC FET T3	0,5242089	0,0003721	Q8IV50-2;Q8IV50	LysM and putative peptidoglycan-binding domain-containing protein 2	LYSMD2
UC vs AC+NC FET T3	-3,040277	0,0175	Q8IZP9-9;Q8IZP9-10;Q8IZP9-8;Q8IZP9-7;Q8IZP9-5;Q8IZP9-6;Q8IZP9-3;Q8IZP9-4;Q8IZP9-2;Q8IZP9	G-protein coupled receptor 64	GPR64
UC vs AC+NC FET T3	-1,909515	0,008063	Q8N8A2-4;Q8N8A2;Q8N8A2-2;Q8N8A2-3;Q8N8A2-5	Serine/threonine-protein phosphatase 6 regulatory ankyrin repeat subunit B	ANKRD44
UC vs AC+NC FET T3	-3,913409	0,02357	Q8NBJ4-2;Q8NBJ4	Golgi membrane protein 1	GOLM1
UC vs AC+NC FET T3	4,1442611	0,0008234	Q8NBP7	Proprotein convertase subtilisin/kexin type 9	PCSK9
UC vs AC+NC FET T3	-3,950286	0,001379	Q8ND83-3;Q8ND83-2;Q8ND83-4	SLAIN motif-containing protein 1	SLAIN1
UC vs AC+NC FET T3	1,3448953	1,36E-02	Q8NDV7-6;Q8NDV7;Q8NDV7-2;Q8NDV7-5;Q8NDV7-4;Q8NDV7-3	Trinucleotide repeat-containing gene 6A protein	TNRC6A
UC vs AC+NC FET T3	2,8752866	0,0155	Q8NFQ6	BPI fold-containing family C protein	BPIFC
UC vs AC+NC FET T3	2,107978	0,02756	Q8NGK2	Olfactory receptor 52B4	OR52B4
UC vs AC+NC FET T3	2,5189635	3,95E-03	Q8NI99	Angiopoietin-related protein 6	ANGPTL6
UC vs AC+NC FET T3	-1,479392	0,03746	Q8TDL5;Q8TDL5-2	BPI fold-containing family B member 1	BPIFB1
UC vs AC+NC FET T3	2,4679836	0,002846	Q8TE73	Dynein heavy chain 5, axonemal	DNAH5
UC vs AC+NC FET T3	-2,409444	0,0002478	Q8WUA8	Tsukushin	TSKU
UC vs AC+NC FET T3	1,9292915	0,0003174	Q8WWZ8;Q8WWZ8-2	Oncoprotein-induced transcript 3 protein	OIT3
UC vs AC+NC FET T3	3,3651899	0,01938	Q8WZ42-5	Titin	TTN
UC vs AC+NC FET T3	-0,821926	0,001595	Q92598-2;Q92598-3;Q92598;Q92598-4	Heat shock protein 105 kDa	HSPH1
UC vs AC+NC FET T3	1,5326114	0,0091	Q92626;Q92626-2	Peroxidasin homolog	PXDN
UC vs AC+NC FET T3	1,1249209	0,0004977	Q92743	Serine protease HTRA1	HTRA1
UC vs AC+NC FET T3	3,1434815	1,71E-02	Q92954-3;Q92954-6;Q92954;Q92954-4;Q92954-2;Q92954-5	Proteoglycan 4	PRG4
UC vs AC+NC FET T3	-1,429058	0,002354	Q96CM8-3;Q96CM8-4;Q96CM8;Q96CM8-2	Acyl-CoA synthetase family member 2, mitochondrial	ACSF2
UC vs AC+NC FET T3	2,2634047	0,00334	Q96IY4;Q96IY4-2;CON__Q2KIG3	Carboxypeptidase B2	CPB2
UC vs AC+NC FET T3	1,1005608	2,02E-03	Q96JB1-2;Q96JB1	Dynein heavy chain 8, axonemal	DNAH8
UC vs AC+NC FET T3	0,7692033	0,01759	Q96KN2	Beta-Ala-His dipeptidase	CNDP1

UC vs AC+NC FET T3	2,5445846	0,02534	Q96PD5;Q96PD5-2	N-acetylmuramoyl-L-alanine amidase	PGLYRP2
UC vs AC+NC FET T3	-3,554719	0,001698	Q96QR1	Secretoglobin family 3A member 1	SCGB3A1
UC vs AC+NC FET T3	3,4172806	0,02616	Q96RL1-3;Q96RL1-4	BRCA1-A complex subunit RAP80	UIMC1
UC vs AC+NC FET T3	0,758154	7,51E-03	Q99574	Neuroserpin	SERPINI1
UC vs AC+NC FET T3	-2,814819	0,03527	Q9BQS8;Q9BQS8-4;Q9BQS8-3;Q9BQS8-2	FYVE and coiled-coil domain-containing protein 1	FYCO1
UC vs AC+NC FET T3	0,8617922	0,03144	Q9BWP8-8;Q9BWP8-7;Q9BWP8-6;Q9BWP8-5;Q9BWP8-4;Q9BWP8-3;Q9BWP8-2;Q9BWP8-9;Q9BWP8;Q9BWP8-10	Collectin-11	COLEC11
UC vs AC+NC FET T3	2,0691687	1,66E-03	Q9BXR6	Complement factor H-related protein 5	CFHR5
UC vs AC+NC FET T3	2,0144667	0,000439	Q9H6X2-5;Q9H6X2;Q9H6X2-4;Q9H6X2-6;Q9H6X2-2;Q9H6X2-3	Anthrax toxin receptor 1	ANTXR1
UC vs AC+NC FET T3	-1,452293	0,038	Q9HCL0-2;Q9HCL0	Protocadherin-18	PCDH18
UC vs AC+NC FET T3	1,6059319	0,001845	Q9HDC9;Q9HDC9-2	Adipocyte plasma membrane-associated protein	APMAP
UC vs AC+NC FET T3	-0,763073	0,03425	Q9NPH2-2;Q9NPH2-3;Q9NPH2	Inositol-3-phosphate synthase 1	ISYNA1
UC vs AC+NC FET T3	6,0399345	1,95E-02	Q9NQ79;Q9NQ79-2;Q9NQ79-3	Cartilage acidic protein 1	CRTAC1
UC vs AC+NC FET T3	-1,20862	0,0363	Q9NRA1;Q9NRA1-3;Q9NRA1-2;Q9NRA1-4	Platelet-derived growth factor C	PDGFC
UC vs AC+NC FET T3	2,2155583	0,001802	Q9NY15;Q9NY15-2	Stabilin-1	STAB1
UC vs AC+NC FET T3	-2,564228	0,007665	Q9NZT1	Calmodulin-like protein 5	CALML5
UC vs AC+NC FET T3	0,9719096	0,001654	Q9UBX5	Fibulin-5	FBLN5
UC vs AC+NC FET T3	2,8184496	2,92E-03	Q9UHG3	Prenylcysteine oxidase 1	PCYOX1
UC vs AC+NC FET T3	-2,2297	4,86E-02	Q9UJJ9	N-acetylglucosamine-1-phosphotransferase subunit gamma	GNPTG
UC vs AC+NC FET T3	2,8474003	0,0007249	Q9UK55	Protein Z-dependent protease inhibitor	SERPINA10
UC vs AC+NC FET T3	1,4297412	0,004417	Q9UM47	Neurogenic locus notch homolog protein 3	NOTCH3
UC vs AC+NC FET T3	-0,658412	0,0001113	Q9UQ72;Q9UQ72-2	Pregnancy-specific beta-1-glycoprotein 11	PSG11
UC vs AC+NC FET T3	-1,134125	0,04006	Q9Y287-2;Q9Y287	Integral membrane protein 2B	ITM2B
UC vs AC+NC FET T3	7,4534808	0,002279	Q9Y490	Talin-1	TLN1
UC vs AC+NC FET T3	1,7457296	0,01291	Q9Y4C2-2;Q9Y4C2	TRPM8 channel-associated factor 1	TCAF1
UC vs AC+NC FET T3	0,7903255	0,02514	Q9Y5C1	Angiopoietin-related protein 3	ANGPTL3

Supplemenary Table 2A: time point comparison

Test	FC	pvalue	Protein IDs	Protein names	Gene names
UC T1 vs T2	1,077518	0,02993	A0A075B6H7;A0A0C4DH55	.	IGKV3-7
UC T1 vs T2	0,7854376	0,006011	A0A0A0MS15	.	IGHV3-49
UC T1 vs T2	-1,402308	0,02657	A0A0C4DH67;A0A0C4DH69	.	IGKV1-8;IGKV1-9
UC T1 vs T2	-1,33976	0,005861	A0A0J9YX35	.	.
UC T1 vs T2	2,2874228	0,03645	O00602	Ficolin-1	FCN1
UC T1 vs T2	-0,601921	0,0136	O14791-2;O14791;O14791-3	Apolipoprotein L1	APOL1
UC T1 vs T2	-1,836001	0,0335	O15016;O15016-2;O15016-3	Tripartite motif-containing protein 66	TRIM66
UC T1 vs T2	-5,4673	3,72E-03	O43184-3;O43184-4;O43184-2;O43184	Disintegrin and metalloproteinase domain-containing protein 12	ADAM12
UC T1 vs T2	0,939981	0,006504	O43866	CD5 antigen-like	CD5L
UC T1 vs T2	4,217356	0,01483	O94855;O94855-2	Protein transport protein Sec24D	SEC24D
UC T1 vs T2	-3,056739	0,0376	P00709	Alpha-lactalbumin	LALBA
UC T1 vs T2	0,7582536	0,01908	P00734	Prothrombin	F2
UC T1 vs T2	0,3813968	0,03076	P00736	Complement C1r subcomponent	C1R
UC T1 vs T2	-1,245115	0,02314	P00740;P00740-2	Coagulation factor IX	F9
UC T1 vs T2	0,4974837	0,03328	P00742	Coagulation factor X	F10
UC T1 vs T2	-0,592787	0,01644	P00747;Q02325	Plasminogen	PLG
UC T1 vs T2	-0,798874	0,0145	P00748	Coagulation factor XII	F12
UC T1 vs T2	1,6324539	0,01614	P01011;P01011-2;P01011-3	Alpha-1-antichymotrypsin	SERPINA3
UC T1 vs T2	1,2994209	0,02373	P01034	Cystatin-C	CST3
UC T1 vs T2	-0,916175	0,04804	P01042-2	Kininogen-1	KNG1
UC T1 vs T2	1,7516294	0,03723	P01594;P01593	Ig kappa chain V-I region AU	.
UC T1 vs T2	1,5742636	0,04437	P01624	Ig kappa chain V-III region POM	.
UC T1 vs T2	1,1344357	0,03286	P01700	Ig lambda chain V-I region HA	.
UC T1 vs T2	-1,198688	0,03955	P01709	Ig lambda chain V-II region MGC	.
UC T1 vs T2	-1,172213	0,01182	P01743	Ig heavy chain V-I region HG3	.
UC T1 vs T2	2,2516527	0,01848	P01764	Ig heavy chain V-III region 23	IGHV3-23
UC T1 vs T2	-2,047822	0,04667	P01833	Polymeric immunoglobulin receptor	PIGR
UC T1 vs T2	1,4950166	0,04299	P01859	Ig gamma-2 chain C region	IGHG2
UC T1 vs T2	-1,095335	1,89E-03	P02671;P02671-2;REV__Q9UKV0-4;REV__Q9UKV0-2;REV__Q9UKV0;REV__Q9UKV0-5;REV__Q9UKV0-7;Q14314	Fibrinogen alpha chain	FGA
UC T1 vs T2	-0,667706	0,00021	P02675	Fibrinogen beta chain	FGB
UC T1 vs T2	-1,091564	0,000154	P02679;P02679-2	Fibrinogen gamma chain	FGG
UC T1 vs T2	1,2771764	0,001319	P02745	Complement C1q subcomponent subunit A	C1QA
UC T1 vs T2	-1,049796	0,004476	P02747	Complement C1q subcomponent subunit C	C1QC
UC T1 vs T2	-2,128999	0,001917	P02751-1;P02751-8;P02751-3;P02751;P02751-14;P02751-7;P02751-17;P02751-9;P02751-6;P02751-4;P02751-12;P02751-16;P02751-2	Fibronectin	FN1
UC T1 vs T2	-1,240314	0,002607	P03950	Angiogenin	ANG

UC T1 vs T2	1,9198027	0,002596	P04196	Histidine-rich glycoprotein	HRG
UC T1 vs T2	-1,083742	0,003403	P04217	Alpha-1B-glycoprotein	A1BG
UC T1 vs T2	-3,056426	0,001169	P04275	von Willebrand factor	VWF
UC T1 vs T2	2,2420964	0,03102	P04406;P04406-2;O14556	Glyceraldehyde-3-phosphate dehydrogenase	GAPDH
UC T1 vs T2	-1,137267	0,01539	P05023-2;P05023-4;P05023;P05023-3	Sodium/potassium-transporting ATPase subunit alpha-1	ATP1A1
UC T1 vs T2	0,651021	0,01926	P05090	Apolipoprotein D	APOD
UC T1 vs T2	0,4306307	0,01547	P05155-2;P05155;P05155-3	Plasma protease C1 inhibitor	SERPING1
UC T1 vs T2	-0,854718	0,009875	P05160	Coagulation factor XIII B chain	F13B
UC T1 vs T2	1,344285	0,0116	P06396;P06396-4;P06396-3	Gelsolin	GSN
UC T1 vs T2	-0,589316	0,0349	P06681;P06681-3;P06681-2	Complement C2	C2
UC T1 vs T2	-0,861942	0,005918	P06727;Q9BT92	Apolipoprotein A-IV	APOA4
UC T1 vs T2	2,1732101	0,03838	P06733	Alpha-enolase	ENO1
UC T1 vs T2	1,1544776	0,03426	P07360	Complement component C8 gamma chain	C8G
UC T1 vs T2	-3,729559	0,000727	P08493-2;P08493	Matrix Gla protein	MGP
UC T1 vs T2	2,5044372	0,03076	P08567	Pleckstrin	PLEK
UC T1 vs T2	-1,84347	0,01832	P08833	Insulin-like growth factor-binding protein 1	IGFBP1
UC T1 vs T2	4,7941732	0,002305	P09466-2;P09466	Glycodelin	PAEP
UC T1 vs T2	0,311028	0,001887	P09871	Complement C1s subcomponent	C1S
UC T1 vs T2	-4,853685	0,001564	P0DML3;P0DML2;P0DML3-2;P0DML3-3;P01241-2;P01241;P01241-5	Chorionic somatomammotropin hormone 2	CSH2;CSH1
UC T1 vs T2	2,6360229	0,00822	P0DN87;P0DN86;P0DN86-2;Q6NT52;A6NKQ9-2;A6NKQ9	Choriogonadotropin subunit beta variant 2	CGB2;CGB1
UC T1 vs T2	-1,49717	0,003791	P0DOX6	.	.
UC T1 vs T2	0,7301736	0,02814	P0DOX8	.	.
UC T1 vs T2	1,2384381	0,02259	P0DOY3	.	.
UC T1 vs T2	-3,686028	0,000614	P11464-4	Pregnancy-specific beta-1-glycoprotein 1	PSG1
UC T1 vs T2	-2,561501	0,000993	P11465	Pregnancy-specific beta-1-glycoprotein 2	PSG2
UC T1 vs T2	-4,171221	0,0325	P13727;P13727-2	Bone marrow proteoglycan	PRG2
UC T1 vs T2	-0,858961	0,01629	P15907	Beta-galactoside alpha-2,6-sialyltransferase 1	ST6GAL1
UC T1 vs T2	1,0596786	0,01408	P19652	Alpha-1-acid glycoprotein 2	ORM2
UC T1 vs T2	1,1645474	0,009636	P19823	Inter-alpha-trypsin inhibitor heavy chain H2	ITIH2
UC T1 vs T2	1,2739592	0,02361	P20851-2;P20851;REV__Q7Z7B0-3;REV__Q7Z7B0-2;REV__Q7Z7B0	C4b-binding protein beta chain	C4BPB
UC T1 vs T2	3,5513716	0,00214	P21333-2;P21333	Filamin-A	FLNA
UC T1 vs T2	-1,017351	0,01128	P22692	Insulin-like growth factor-binding protein 4	IGFBP4
UC T1 vs T2	0,6603775	0,03704	P22792	Carboxypeptidase N subunit 2	CPN2
UC T1 vs T2	1,5801044	0,01193	P23083	Ig heavy chain V-I region V35	.
UC T1 vs T2	-0,927852	0,02927	P23142;P23142-2;P23142-3	Fibulin-1	FBLN1
UC T1 vs T2	-3,599879	0,001224	P23142-4	Fibulin-1	FBLN1
UC T1 vs T2	-1,069108	0,01708	P24593	Insulin-like growth factor-binding protein 5	IGFBP5
UC T1 vs T2	-1,801742	0,003736	P27918	Properdin	CFP
UC T1 vs T2	0,7964953	0,01496	P30041	Peroxiredoxin-6	PRDX6
UC T1 vs T2	0,7665786	0,01195	P30101	Protein disulfide-isomerase A3	PDIA3
UC T1 vs T2	1,1626496	0,03553	P31025;Q5VSP4	Lipocalin-1	LCN1
UC T1 vs T2	-5,991762	0,01448	P35556	Fibrillin-2	FBN2
UC T1 vs T2	-1,145679	0,01766	P39060-2;P39060-1;P39060	Collagen alpha-1(XVIII) chain	COL18A1
UC T1 vs T2	-1,15693	0,008453	P43251-4;P43251;P43251-3;P43251-2	Biotinidase	BTD
UC T1 vs T2	-0,538465	0,0253	P43652	Afamin	AFM
UC T1 vs T2	2,2472876	0,02472	P46013;P46013-2	Antigen KI-67	MKI67
UC T1 vs T2	-1,8494	0,000868	P48740-4	Mannan-binding lectin serine protease 1	MASP1
UC T1 vs T2	3,0362107	0,01163	P49619-3;P49619-2;P49619	Diacylglycerol kinase gamma	DGKG
UC T1 vs T2	-1,163102	0,01409	P49767	Vascular endothelial growth factor C	VEGFC
UC T1 vs T2	1,0166497	0,02839	P49913	Cathelicidin antimicrobial peptide	CAMP
UC T1 vs T2	-1,1839	0,005352	P55285-2;P55285	Cadherin-6	CDH6
UC T1 vs T2	1,5694242	0,002428	P55774	C-C motif chemokine 18	CCL18
UC T1 vs T2	-2,019666	0,01496	P61626	Lysozyme C	LYZ
UC T1 vs T2	1,6762718	0,03557	P62258;P62258-2	14-3-3 protein epsilon	YWHAE
UC T1 vs T2	2,5717134	0,0489	P62736;P63267;P63267-2	Actin, aortic smooth muscle	ACTA2;ACTG2
UC T1 vs T2	-1,202179	0,03424	P62979;P62987;P0CG47;P0CG48	Ubiquitin-40S ribosomal protein S27a	RPS27A;UBA52;UBB;UBC
UC T1 vs T2	3,624873	0,002254	P67936	Tropomyosin alpha-4 chain	TPM4
UC T1 vs T2	5,849669	0,01033	P68363;P68363-2	Tubulin alpha-1B chain	TUBA1B
UC T1 vs T2	-0,690278	0,000143	P80108;P80108-2	Phosphatidylinositol-glycan-specific phospholipase D	GPLD1
UC T1 vs T2	-2,450885	0,000502	Q00887	Pregnancy-specific beta-1-glycoprotein 9	PSG9
UC T1 vs T2	-3,096928	2,89E-03	Q00888;Q00888-3;Q00888-2	Pregnancy-specific beta-1-glycoprotein 4	PSG4
UC T1 vs T2	-1,488904	0,03561	Q00889-2;Q00889	Pregnancy-specific beta-1-glycoprotein 6	PSG6
UC T1 vs T2	1,2539487	0,008305	Q04756	Hepatocyte growth factor activator	HGFAC
UC T1 vs T2	-1,133962	0,006497	Q06033-2;Q06033	Inter-alpha-trypsin inhibitor heavy chain H3	ITIH3
UC T1 vs T2	-1,697572	0,01423	Q06190	Serine/threonine-protein phosphatase 2A regulatory subunit B subunit alpha	PPP2R3A
UC T1 vs T2	-7,807791	0,03448	Q13219	Pappalysin-1	PAPPA
UC T1 vs T2	1,0793115	0,04365	Q14623	Indian hedgehog protein	IHH
UC T1 vs T2	-1,422043	0,04067	Q15022	Polycomb protein SUZ12	SUZ12
UC T1 vs T2	1,0760293	0,03277	Q15166	Serum paraoxonase/lactonase 3	PON3
UC T1 vs T2	-1,282384	0,02249	Q15848	Adiponectin	ADIPOQ
UC T1 vs T2	-1,515182	4,00E-03	Q16557	Pregnancy-specific beta-1-glycoprotein 3	PSG3
UC T1 vs T2	-1,646776	0,01504	Q16610-4;Q16610;Q16610-2	Extracellular matrix protein 1	ECM1

UC T1 vs T2	-2,043282	0,002119	Q4L180-3;Q4L180-7;Q4L180-5;Q4L180-2;Q4L180;Q4L180-6	Filamin A-interacting protein 1-like	FILIP1L
UC T1 vs T2	-8,813947	0,000305	Q4LDE5;Q4LDE5-4;Q4LDE5-3;Q4LDE5-2	Sushi, von Willebrand factor type A, EGF and pentraxin domain-containing protein 1	SVEP1
UC T1 vs T2	1,5159521	0,04961	Q6P387-2;Q6P387	Uncharacterized protein C16orf46	C16orf46
UC T1 vs T2	-2,792464	0,005585	Q6UXH9-2;Q6UXH9-3;Q6UXH9	Inactive serine protease PAMR1	PAMR1
UC T1 vs T2	-2,541782	0,00127	Q6UY14-3;Q6UY14;Q6UY14-2	ADAMTS-like protein 4	ADAMTSL4
UC T1 vs T2	-1,549604	0,01479	Q6VAB6;Q6VAB6-2	Kinase suppressor of Ras 2	KSR2
UC T1 vs T2	1,3222766	0,02425	Q76LX8;Q76LX8-2;Q76LX8-3;Q76LX8-4	A disintegrin and metalloproteinase with thrombospondin motifs 13	ADAMTS13
UC T1 vs T2	4,0058846	0,01884	Q7LBC6-3	Lysine-specific demethylase 3B	KDM3B
UC T1 vs T2	1,7008244	0,002196	Q8IZP9-9;Q8IZP9-10;Q8IZP9-8;Q8IZP9-7;Q8IZP9-5;Q8IZP9-6;Q8IZP9-3;Q8IZP9-4;Q8IZP9-2;Q8IZP9	G-protein coupled receptor 64	GPR64
UC T1 vs T2	1,9120162	0,01036	Q8N8A2-4;Q8N8A2;Q8N8A2-2;Q8N8A2-3;Q8N8A2-5	Serine/threonine-protein phosphatase 6 regulatory ankyrin repeat subunit B	ANKRD44
UC T1 vs T2	1,4763618	0,02243	Q8NBJ4-2;Q8NBJ4	Golgi membrane protein 1	GOLM1
UC T1 vs T2	1,4532247	0,01782	Q8NI99	Angiopoietin-related protein 6	ANGPTL6
UC T1 vs T2	-1,537695	0,000463	Q8WUA8	Tsukushin	TSKU
UC T1 vs T2	-2,667848	0,001852	Q92743	Serine protease HTRA1	HTRA1
UC T1 vs T2	1,1287504	0,01447	Q96CM8-3;Q96CM8-4;Q96CM8;Q96CM8-2	Acyl-CoA synthetase family member 2, mitochondrial	ACSF2
UC T1 vs T2	-1,015321	0,001504	Q96IY4;Q96IY4-2;CON__Q2KIG3	Carboxypeptidase B2	CPB2
UC T1 vs T2	1,170239	0,002019	Q9BWP8-8;Q9BWP8-7;Q9BWP8-6;Q9BWP8-5;Q9BWP8-4;Q9BWP8-3;Q9BWP8-2;Q9BWP8-9;Q9BWP8;Q9BWP8-10	Collectin-11	COLEC11
UC T1 vs T2	-1,99703	0,001597	Q9BZR9	Probable E3 ubiquitin-protein ligase TRIM8	TRIM8
UC T1 vs T2	1,189278	0,001299	Q9HCL0-2;Q9HCL0	Protocadherin-18	PCDH18
UC T1 vs T2	2,4186091	0,005602	Q9NZI8-2;Q9NZI8	Insulin-like growth factor 2 mRNA-binding protein 1	IGF2BP1
UC T1 vs T2	-0,827489	0,01211	Q9UBX5	Fibulin-5	FBLN5
UC T1 vs T2	-0,367183	0,02846	Q9UHG3	Prenylcysteine oxidase 1	PCYOX1
UC T1 vs T2	-3,647025	0,000151	Q9UQ72;Q9UQ72-2	Pregnancy-specific beta-1-glycoprotein 11	PSG11
UC T1 vs T3	-1,991296	0,004849	A0A075B6I0	.	IGLV8-61
UC T1 vs T3	0,5102079	0,03316	A0A0A0MS15	.	IGHV3-49
UC T1 vs T3	-1,812025	0,001934	A0A0C4DH67;A0A0C4DH69	.	IGKV1-8;IGKV1-9
UC T1 vs T3	3,5817062	0,005879	A4FU69-3;A4FU69;A4FU69-2;A4FU69-4;A4FU69-6	EF-hand calcium-binding domain-containing protein 5	EFCAB5
UC T1 vs T3	3,0380311	0,03264	A6NM62	Leucine-rich repeat-containing protein 53	LRRC53
UC T1 vs T3	-0,640073	0,03477	M0R2J8	.	DCDC1
UC T1 vs T3	1,0642493	0,00011	O00391;O00391-2	Sulfhydryl oxidase 1	QSOX1
UC T1 vs T3	-3,580859	4,00E-02	O43184-3;O43184-4;O43184-2;O43184	Disintegrin and metalloproteinase domain-containing protein 12	ADAM12
UC T1 vs T3	-1,197391	0,03242	Q99880;Q99879;Q99877;Q93079;Q8N257;Q5QNW6;Q16778;P62807;P58876;P57053;P33778;P23527;P06899;O60814;Q5QNW6-2	Histone H2B type 1-L	HIST1H2BL;HIST1H2BM;HIST1H2BN;HIST1H2BH;HIST3H2BB;HIST2H2BF;HIST2H2BE;HIST1H2BC;HIST1H2BD;H2BFS;HIST1H2BB;HIST1H2BO;HIST1H2BJ;HIST1H2BK
UC T1 vs T3	3,2329273	0,03732	O94855;O94855-2	Protein transport protein Sec24D	SEC24D
UC T1 vs T3	-0,817712	0,04116	O95428-6;O95428;O95428-5;O95428-4;O95428-2;O95428-3	Papilin	PAPLN
UC T1 vs T3	-1,143102	0,01407	O95445-2	Apolipoprotein M	APOM
UC T1 vs T3	-3,896882	0,01873	P00709	Alpha-lactalbumin	LALBA
UC T1 vs T3	0,8428681	0,008672	P00734	Prothrombin	F2
UC T1 vs T3	0,8189032	0,03661	P00742	Coagulation factor X	F10
UC T1 vs T3	1,3494284	0,02625	P01011;P01011-2;P01011-3	Alpha-1-antichymotrypsin	SERPINA3
UC T1 vs T3	2,4909117	0,01223	P01019	Angiotensinogen	AGT
UC T1 vs T3	1,49733	0,00056	P01034	Cystatin-C	CST3
UC T1 vs T3	1,3757784	0,006682	P01040	Cystatin-A	CSTA
UC T1 vs T3	-0,699611	0,008743	P01042-2	Kininogen-1	KNG1
UC T1 vs T3	-0,706115	0,006431	P01591	Immunoglobulin J chain	IGJ
UC T1 vs T3	-1,339088	0,004611	P01619	Ig kappa chain V-III region B6	.
UC T1 vs T3	-0,74828	0,000872	P01701	Ig lambda chain V-I region NEW	.
UC T1 vs T3	-1,611917	0,01536	P01709	Ig lambda chain V-II region MGC	.
UC T1 vs T3	0,6811447	0,03652	P01742	Ig heavy chain V-I region EU	.
UC T1 vs T3	-1,421248	0,00494	P01743	Ig heavy chain V-I region HG3	.
UC T1 vs T3	1,9003789	0,003205	P01764	Ig heavy chain V-III region 23	IGHV3-23

UC T1 vs T3	2,5272663	8,26E-03	P01834	Ig kappa chain C region	IGKC
UC T1 vs T3	0,9538	0,03209	P01859	Ig gamma-2 chain C region	IGHG2
UC T1 vs T3	-2,197341	0,02442	P01861	Ig gamma-4 chain C region	IGHG4
UC T1 vs T3	-0,655857	0,01673	P01871;P01871-2	Ig mu chain C region	IGHM
UC T1 vs T3	0,2528188	0,02142	P02647;Q9HB71-2	Apolipoprotein A-I	APOA1
UC T1 vs T3	-1,036418	1,88E-02	P02654	Apolipoprotein C-I	APOC1
UC T1 vs T3	1,1861432	0,0264	P02655	Apolipoprotein C-II	APOC2
UC T1 vs T3	-0,451838	0,0105	P02671;P02671-2;REV__Q9UKV0-4;REV__Q9UKV0-2;REV__Q9UKV0;REV__Q9UKV0-5;REV__Q9UKV0-7;Q14314	Fibrinogen alpha chain	FGA
UC T1 vs T3	-1,455328	0,000502	P02747	Complement C1q subcomponent subunit C	C1QC
UC T1 vs T3	-2,605565	8,56E-04	P02751-1;P02751-8;P02751-3;P02751;P02751-14;P02751-7;P02751-17;P02751-9;P02751-6;P02751-4;P02751-12;P02751-16;P02751-2	Fibronectin	FN1
UC T1 vs T3	-0,841571	0,01735	P02753	Retinol-binding protein 4	RBP4
UC T1 vs T3	-1,21214	0,0433	P02763	Alpha-1-acid glycoprotein 1	ORM1
UC T1 vs T3	-1,561125	0,04528	P02775	Platelet basic protein	PPBP
UC T1 vs T3	-1,74505	0,01524	P02776	Platelet factor 4	PF4
UC T1 vs T3	-1,317738	0,04828	P02787;CON__Q2HJF0	Serotransferrin	TF
UC T1 vs T3	-1,772011	0,000904	P03950	Angiogenin	ANG
UC T1 vs T3	-1,214591	0,006223	P03951	Coagulation factor XI	F11
UC T1 vs T3	1,1167624	0,002749	P04004	Vitronectin	VTN
UC T1 vs T3	-1,090026	0,04696	P04217	Alpha-1B-glycoprotein	A1BG
UC T1 vs T3	3,5428979	0,000186	P04278;P04278-5;P04278-4;P04278-2;P04278-3	Sex hormone-binding globulin	SHBG
UC T1 vs T3	0,8614496	0,01455	P05019-3;P05019-2;P05019-4;P05019	Insulin-like growth factor I	IGF1
UC T1 vs T3	-2,052523	0,02966	P05023-2;P05023-4;P05023;P05023-3	Sodium/potassium-transporting ATPase subunit alpha-1	ATP1A1
UC T1 vs T3	0,7804524	0,04265	P05109	Protein S100-A8	S100A8
UC T1 vs T3	1,0094235	0,04414	P05154	Plasma serine protease inhibitor	SERPINA5
UC T1 vs T3	2,1996987	1,68E-05	P05543	Thyroxine-binding globulin	SERPINA7
UC T1 vs T3	-0,764914	0,02308	P06727;Q9BT92	Apolipoprotein A-IV	APOA4
UC T1 vs T3	-0,651414	0,02698	P07225	Vitamin K-dependent protein S	PROS1
UC T1 vs T3	0,6759665	0,03869	P07307-3;P07307-2;P07307	Asialoglycoprotein receptor 2	ASGR2
UC T1 vs T3	1,4205649	0,02085	P07360	Complement component C8 gamma chain	C8G
UC T1 vs T3	1,8428457	0,04949	P08185	Corticosteroid-binding globulin	SERPINA6
UC T1 vs T3	0,5939429	0,001742	P08253;P08253-3;P08253-2	72 kDa type IV collagenase	MMP2
UC T1 vs T3	-3,676386	0,001602	P08493-2;P08493	Matrix Gla protein	MGP
UC T1 vs T3	-1,200487	0,001161	P09382	Galectin-1	LGALS1
UC T1 vs T3	3,3044615	0,004835	P09466-2;P09466	Glycodelin	PAEP
UC T1 vs T3	1,6952625	4,04E-02	P0DN87;P0DN86;P0DN86-2;Q6NT52;A6NKQ9-2;A6NKQ9	Choriogonadotropin subunit beta variant 2	CGB2;CGB1
UC T1 vs T3	0,8776295	0,01286	P0DOY3	.	.
UC T1 vs T3	1,0687125	0,003567	P10643	Complement component C7	C7
UC T1 vs T3	-0,698234	0,03364	P11021	78 kDa glucose-regulated protein	HSPA5
UC T1 vs T3	-1,897128	0,01039	P11464-4	Pregnancy-specific beta-1-glycoprotein 1	PSG1
UC T1 vs T3	2,3686331	0,03975	P14314-2;P14314	Glucosidase 2 subunit beta	PRKCSH
UC T1 vs T3	-2,09065	0,04047	P16885	1-phosphatidylinositol 4,5-bisphosphate phosphodiesterase gamma-2	PLCG2
UC T1 vs T3	1,3091395	0,001903	P18428	Lipopolysaccharide-binding protein	LBP
UC T1 vs T3	0,8370554	0,004569	P19823	Inter-alpha-trypsin inhibitor heavy chain H2	ITIH2
UC T1 vs T3	0,7543542	0,02095	P19827	Inter-alpha-trypsin inhibitor heavy chain H1	ITIH1
UC T1 vs T3	2,3021686	0,02393	P20742;P20742-2	Pregnancy zone protein	PZP
UC T1 vs T3	-3,130301	0,03035	P21333-2;P21333	Filamin-A	FLNA
UC T1 vs T3	-0,594728	0,04857	P22352	Glutathione peroxidase 3	GPX3
UC T1 vs T3	-0,783474	0,005774	P22692	Insulin-like growth factor-binding protein 4	IGFBP4
UC T1 vs T3	-1,384918	0,04891	P23142-4	Fibulin-1	FBLN1
UC T1 vs T3	-1,234405	0,02453	P25311	Zinc-alpha-2-glycoprotein	AZGP1
UC T1 vs T3	0,6040566	0,005412	P27169	Serum paraoxonase/arylesterase 1	PON1
UC T1 vs T3	1,920151	0,04681	P27797	Calreticulin	CALR
UC T1 vs T3	-1,391093	0,007656	P27918	Properdin	CFP
UC T1 vs T3	2,3020055	0,006664	P31025;Q5VSP4	Lipocalin-1	LCN1
UC T1 vs T3	-0,953336	0,04195	P35542	Serum amyloid A-4 protein	SAA4
UC T1 vs T3	-0,7668	0,00513	P35555	Fibrillin-1	FBN1
UC T1 vs T3	-7,185594	0,02935	P35579;P35579-2	Myosin-9	MYH9
UC T1 vs T3	0,8877423	0,03363	P35858;P35858-2	Insulin-like growth factor-binding protein complex acid labile subunit	IGFALS
UC T1 vs T3	0,4861152	0,03585	P36955;CON__Q95121	Pigment epithelium-derived factor	SERPINF1
UC T1 vs T3	-1,144449	0,04254	P39060-2;P39060-1;P39060	Collagen alpha-1(XVIII) chain	COL18A1
UC T1 vs T3	-1,162613	0,03694	P43251-4;P43251;P43251-3;P43251-2	Biotinidase	BTD
UC T1 vs T3	2,8438062	0,009213	P46013;P46013-2	Antigen KI-67	MKI67
UC T1 vs T3	-2,637042	0,000232	P48740-4	Mannan-binding lectin serine protease 1	MASP1
UC T1 vs T3	4,0088953	0,01856	P49619-3;P49619-2;P49619	Diacylglycerol kinase gamma	DGKG
UC T1 vs T3	1,0648477	0,002895	P55058;P55058-4;P55058-3;P55058-2	Phospholipid transfer protein	PLTP
UC T1 vs T3	-1,50819	0,002476	P55285-2;P55285	Cadherin-6	CDH6

UC T1 vs T3	-1,63534	0,01064	P57077;P57077-1	MAP3K7 C-terminal-like protein	MAP3K7CL
UC T1 vs T3	-1,252037	0,03119	P61626	Lysozyme C	LYZ
UC T1 vs T3	-1,338656	0,01023	P62979;P62987;P0CG47;P0CG48	Ubiquitin-40S ribosomal protein S27a	RPS27A;UBA52;UBB;UBC
UC T1 vs T3	12,017132	0,000588	Q00888;Q00888-3;Q00888-2	Pregnancy-specific beta-1-glycoprotein 4	PSG4
UC T1 vs T3	-1,218286	0,0232	Q02985-2;Q02985	Complement factor H-related protein 3	CFHR3
UC T1 vs T3	1,6089122	0,001581	Q08830	Fibrinogen-like protein 1	FGL1
UC T1 vs T3	-0,344533	0,03808	Q0VAK6;Q0VAK6-2	Leiomodin-3	LMOD3
UC T1 vs T3	2,0074208	0,01536	Q12794-2;Q12794;Q12794-7;Q12794-4;Q12794-3;Q12794-6;Q12794-5	Hyaluronidase-1	HYAL1
UC T1 vs T3	-0,502288	0,004263	Q12805-5	EGF-containing fibulin-like extracellular matrix protein 1	EFEMP1
UC T1 vs T3	-1,077173	0,01939	Q13103	Secreted phosphoprotein 24	SPP2
UC T1 vs T3	-1,863318	0,008659	Q13488-2	V-type proton ATPase 116 kDa subunit a isoform 3	TCIRG1
UC T1 vs T3	0,6671426	0,02598	Q14520-2;Q14520	Hyaluronan-binding protein 2	HABP2
UC T1 vs T3	-2,357007	0,03531	Q14766;Q14766-4;Q14766-3;Q14766-2;Q14766-5	Latent-transforming growth factor beta-binding protein 1	LTBP1
UC T1 vs T3	-1,334386	0,04371	Q15022	Polycomb protein SUZ12	SUZ12
UC T1 vs T3	-1,17398	0,04143	Q15848	Adiponectin	ADIPOQ
UC T1 vs T3	-1,451504	0,005654	Q16610-4;Q16610;Q16610-2	Extracellular matrix protein 1	ECM1
UC T1 vs T3	5,5960625	0,025	Q2KHM9	Uncharacterized protein KIAA0753	KIAA0753
UC T1 vs T3	0,4023683	0,006557	Q5VT25-3;Q5VT25-4;Q5VT25-5;Q5VT25;Q5VT25-2;Q5VT25-6	Serine/threonine-protein kinase MRCK alpha	CDC42BPA
UC T1 vs T3	-0,51266	0,02397	Q6UVK1	Chondroitin sulfate proteoglycan 4	CSPG4
UC T1 vs T3	-2,801931	0,000805	Q6VAB6;Q6VAB6-2	Kinase suppressor of Ras 2	KSR2
UC T1 vs T3	0,9987772	0,04177	Q6ZS30-1;Q6ZS30	Neurobeachin-like protein 1	NBEAL1
UC T1 vs T3	0,9577601	0,006728	Q76LX8;Q76LX8-2;Q76LX8-3;Q76LX8-4	A disintegrin and metalloproteinase with thrombospondin motifs 13	ADAMTS13
UC T1 vs T3	1,3640176	0,03921	Q7Z572	Spermatogenesis-associated protein 21	SPATA21
UC T1 vs T3	1,3825376	0,0005	Q86UD1	Out at first protein homolog	OAF
UC T1 vs T3	0,3060765	0,004127	Q8IYW2	Cilia- and flagella-associated protein 46	CFAP46
UC T1 vs T3	1,2062662	0,01483	Q8IZK6-2;Q8IZK6	Mucolipin-2	MCOLN2
UC T1 vs T3	2,9740178	0,01885	Q8IZP9-9;Q8IZP9-10;Q8IZP9-8;Q8IZP9-7;Q8IZP9-5;Q8IZP9-6;Q8IZP9-3;Q8IZP9-4;Q8IZP9-2;Q8IZP9	G-protein coupled receptor 64	GPR64
UC T1 vs T3	2,3892078	0,003243	Q8N8A2-4;Q8N8A2;Q8N8A2-2;Q8N8A2-3;Q8N8A2-5	Serine/threonine-protein phosphatase 6 regulatory ankyrin repeat subunit B	ANKRD44
UC T1 vs T3	1,6803541	0,04196	Q8ND83-3;Q8ND83-2;Q8ND83-4	SLAIN motif-containing protein 1	SLAIN1
UC T1 vs T3	-1,966148	0,04391	Q8NFQ6	BPI fold-containing family C protein	BPIFC
UC T1 vs T3	0,4691656	0,04735	Q8NI99	Angiopoietin-related protein 6	ANGPTL6
UC T1 vs T3	3,7292616	1,59E-02	Q8WUA8	Tsukushin	TSKU
UC T1 vs T3	0,6164209	0,009539	Q92598-2;Q92598-3;Q92598;Q92598-4	Heat shock protein 105 kDa	HSPH1
UC T1 vs T3	1,7432161	0,000755	Q96CM8-3;Q96CM8-4;Q96CM8;Q96CM8-2	Acyl-CoA synthetase family member 2, mitochondrial	ACSF2
UC T1 vs T3	0,8644955	0,02456	Q96KN2	Beta-Ala-His dipeptidase	CNDP1
UC T1 vs T3	2,6865256	0,002727	Q96QR1	Secretoglobin family 3A member 1	SCGB3A1
UC T1 vs T3	0,8550637	0,03687	Q9BWP8-8;Q9BWP8-7;Q9BWP8-6;Q9BWP8-5;Q9BWP8-4;Q9BWP8-3;Q9BWP8-2;Q9BWP8-9;Q9BWP8;Q9BWP8-10	Collectin-11	COLEC11
UC T1 vs T3	-1,097998	0,006847	Q9H6X2-5;Q9H6X2;Q9H6X2-4;Q9H6X2-6;Q9H6X2-2;Q9H6X2-3	Anthrax toxin receptor 1	ANTXR1
UC T1 vs T3	-0,823706	0,03999	Q9HDC9;Q9HDC9-2	Adipocyte plasma membrane-associated protein	APMAP
UC T1 vs T3	0,9606473	0,00611	Q9NPG4;O14917-2	Protocadherin-12	PCDH12
UC T1 vs T3	-0,463453	0,02203	Q9UBX5	Fibulin-5	FBLN5
UC T1 vs T3	1,0159553	0,009048	Q9UJJ9	N-acetylglucosamine-1-phosphotransferase subunit gamma	GNPTG
UC T1 vs T3	-1,694498	1,86E-02	Q9UQ72;Q9UQ72-2	Pregnancy-specific beta-1-glycoprotein 11	PSG11
UC T1 vs T3	1,273834	0,03354	Q9Y287-2;Q9Y287	Integral membrane protein 2B	ITM2B
UC T1 vs T3	0,4160392	0,04066	Q9Y6Z7	Collectin-10	COLEC10
UC T2 vs T3	2,8877704	0,02256	A4FU69-3;A4FU69;A4FU69-2;A4FU69-4;A4FU69-6	EF-hand calcium-binding domain-containing protein 5	EFCAB5
UC T2 vs T3	0,9210646	0,01124	O00391;O00391-2	Sulfhydryl oxidase 1	QSOX1

UC T2 vs T3	1,4525855	0,02628	O14791-2;O14791;O14791-3	Apolipoprotein L1	APOL1
UC T2 vs T3	1,2972887	0,01553	O15016;O15016-2;O15016-3	Tripartite motif-containing protein 66	TRIM66
UC T2 vs T3	0,4993699	0,02965	P00747;Q02325	Plasminogen	PLG
UC T2 vs T3	0,7488339	0,02051	P00748	Coagulation factor XII	F12
UC T2 vs T3	-0,902041	0,006042	P01701	Ig lambda chain V-I region NEW	.
UC T2 vs T3	-1,16942	0,009308	P01706	Ig lambda chain V-II region BOH	.
UC T2 vs T3	-1,680432	0,005028	P01717	Ig lambda chain V-IV region Hil	.
UC T2 vs T3	0,6434972	0,004307	P02671;P02671-2;REV__Q9UKV0-4;REV__Q9UKV0-2;REV__Q9UKV0;REV__Q9UKV0-5;REV__Q9UKV0-7;Q14314	Fibrinogen alpha chain	FGA
UC T2 vs T3	0,6803253	0,01005	P02675	Fibrinogen beta chain	FGB
UC T2 vs T3	0,7758415	0,007438	P02679;P02679-2	Fibrinogen gamma chain	FGG
UC T2 vs T3	-0,717128	0,04485	P02753	Retinol-binding protein 4	RBP4
UC T2 vs T3	-0,678524	0,04728	P03951	Coagulation factor XI	F11
UC T2 vs T3	-0,878154	0,00278	P04003;CON__Q28065	C4b-binding protein alpha chain	C4BPA
UC T2 vs T3	1,0496461	0,00185	P04004	Vitronectin	VTN
UC T2 vs T3	-1,462357	0,01452	P04196	Histidine-rich glycoprotein	HRG
UC T2 vs T3	2,6676481	0,000882	P04278;P04278-5;P04278-4;P04278-2;P04278-3	Sex hormone-binding globulin	SHBG
UC T2 vs T3	-2,033742	0,003191	P04406;P04406-2;O14556	Glyceraldehyde-3-phosphate dehydrogenase	GAPDH
UC T2 vs T3	-0,780627	0,01063	P05090	Apolipoprotein D	APOD
UC T2 vs T3	0,7849019	0,01178	P05160	Coagulation factor XIII B chain	F13B
UC T2 vs T3	1,9398997	0,00047	P05543	Thyroxine-binding globulin	SERPINA7
UC T2 vs T3	-1,065241	0,02917	P06396;P06396-4;P06396-3	Gelsolin	GSN
UC T2 vs T3	-1,363755	0,04307	P06733	Alpha-enolase	ENO1
UC T2 vs T3	-2,411378	0,008875	P06753-5;P06753-2;P06753-4;P06753-3;P06753-6;P06753-7	Tropomyosin alpha-3 chain	TPM3
UC T2 vs T3	-0,900117	0,009812	P07225	Vitamin K-dependent protein S	PROS1
UC T2 vs T3	1,2246425	0,02013	P07307-3;P07307-2;P07307	Asialoglycoprotein receptor 2	ASGR2
UC T2 vs T3	-1,237699	0,04112	P07357	Complement component C8 alpha chain	C8A
UC T2 vs T3	1,7888998	0,000214	P11464-4	Pregnancy-specific beta-1-glycoprotein 1	PSG1
UC T2 vs T3	0,9957618	0,03091	P11465	Pregnancy-specific beta-1-glycoprotein 2	PSG2
UC T2 vs T3	-4,646316	0,01172	P12814;P12814-3;P12814-2	Alpha-actinin-1	ACTN1
UC T2 vs T3	4,5518239	0,002653	P13497;P13497-5;P13497-2;P13497-6;P13497-4;P13497-3	Bone morphogenetic protein 1	BMP1
UC T2 vs T3	1,2693408	0,000327	P13727;P13727-2	Bone marrow proteoglycan	PRG2
UC T2 vs T3	2,0572922	0,014	P14543;P14543-2	Nidogen-1	NID1
UC T2 vs T3	-3,340103	0,006474	P14618;P14618-2	Pyruvate kinase PKM	PKM
UC T2 vs T3	-0,961722	0,03268	P17936;P17936-2	Insulin-like growth factor-binding protein 3	IGFBP3
UC T2 vs T3	-2,186653	0,03037	P18065	Insulin-like growth factor-binding protein 2	IGFBP2
UC T2 vs T3	1,0900252	0,008529	P18428	Lipopolysaccharide-binding protein	LBP
UC T2 vs T3	2,6823828	0,02817	P20742;P20742-2	Pregnancy zone protein	PZP
UC T2 vs T3	-1,020664	0,04815	P20851-2;P20851;REV__Q7Z7B0-3;REV__Q7Z7B0-2;REV__Q7Z7B0	C4b-binding protein beta chain	C4BPB
UC T2 vs T3	-6,681673	0,001232	P21333-2;P21333	Filamin-A	FLNA
UC T2 vs T3	1,4812496	0,004382	P23142;P23142-2;P23142-3	Fibulin-1	FBLN1
UC T2 vs T3	2,2149608	0,000723	P23142-4	Fibulin-1	FBLN1
UC T2 vs T3	-1,874158	0,03491	P25311	Zinc-alpha-2-glycoprotein	AZGP1
UC T2 vs T3	-1,125026	0,03003	P35542	Serum amyloid A-4 protein	SAA4
UC T2 vs T3	2,4813128	0,005279	P35556	Fibrillin-2	FBN2
UC T2 vs T3	-6,46157	0,000303	P35579;P35579-2	Myosin-9	MYH9
UC T2 vs T3	1,2338393	0,01133	P35858;P35858-2	Insulin-like growth factor-binding protein complex acid labile subunit	IGFALS
UC T2 vs T3	-0,787642	0,01985	P48740-4	Mannan-binding lectin serine protease 1	MASP1
UC T2 vs T3	-1,870565	0,001767	P55774	C-C motif chemokine 18	CCL18
UC T2 vs T3	-1,871139	0,04086	P60709	Actin, cytoplasmic 1	ACTB
UC T2 vs T3	-2,916832	0,03276	P62736;P63267;P63267-2	Actin, aortic smooth muscle	ACTA2;ACTG2
UC T2 vs T3	-1,97229	0,03311	P63104;P63104-2	14-3-3 protein zeta/delta	YWHAZ
UC T2 vs T3	-3,236149	0,01837	P67936	Tropomyosin alpha-4 chain	TPM4
UC T2 vs T3	-5,377861	0,002203	P68363;P68363-2	Tubulin alpha-1B chain	TUBA1B
UC T2 vs T3	0,8569853	0,001248	P80108;P80108-2	Phosphatidylinositol-glycan-specific phospholipase D	GPLD1
UC T2 vs T3	15,11406	0,000259	Q00888;Q00888-3;Q00888-2	Pregnancy-specific beta-1-glycoprotein 4	PSG4
UC T2 vs T3	3,4315477	0,03725	Q00889-2;Q00889	Pregnancy-specific beta-1-glycoprotein 6	PSG6
UC T2 vs T3	-0,945341	0,01795	Q04756	Hepatocyte growth factor activator	HGFAC
UC T2 vs T3	1,7719965	0,01505	Q06190	Serine/threonine-protein phosphatase 2A regulatory subunit B subunit alpha	PPP2R3A
UC T2 vs T3	1,4830325	0,009692	Q08380	Galectin-3-binding protein	LGALS3BP
UC T2 vs T3	1,1891449	0,03485	Q13214-2;Q13214	Semaphorin-3B	SEMA3B
UC T2 vs T3	2,3701761	0,000188	Q13219	Pappalysin-1	PAPPA
UC T2 vs T3	0,7758458	0,03523	Q14520-2;Q14520	Hyaluronan-binding protein 2	HABP2
UC T2 vs T3	-0,651191	0,003265	Q15113	Procollagen C-endopeptidase enhancer 1	PCOLCE
UC T2 vs T3	-1,111316	0,008297	Q15485;Q15485-2	Ficolin-2	FCN2
UC T2 vs T3	1,3650562	8,39E-03	Q16557	Pregnancy-specific beta-1-glycoprotein 3	PSG3
UC T2 vs T3	8,3237083	0,001314	Q4LDE5;Q4LDE5-4;Q4LDE5-3;Q4LDE5-2	Sushi, von Willebrand factor type A, EGF and pentraxin domain-containing protein 1	SVEP1
UC T2 vs T3	-1,544629	0,04491	Q6P387-2;Q6P387	Uncharacterized protein C16orf46	C16orf46

UC T2 vs T3	1,8492439	0,01954	Q6UY14-3;Q6UY14;Q6UY14-2	ADAMTS-like protein 4	ADAMTSL4
UC T2 vs T3	-1,252326	0,03756	Q6VAB6;Q6VAB6-2	Kinase suppressor of Ras 2	KSR2
UC T2 vs T3	-3,98736	0,0261	Q86UX7-2;Q86UX7	Fermitin family homolog 3	FERMT3
UC T2 vs T3	-0,984059	0,0481	Q8NI99	Angiopoietin-related protein 6	ANGPTL6
UC T2 vs T3	5,266957	2,53E-03	Q8WUA8	Tsukushin	TSKU
UC T2 vs T3	1,7172087	0,000637	Q92743	Serine protease HTRA1	HTRA1
UC T2 vs T3	1,0856397	0,00727	Q99969	Retinoic acid receptor responder protein 2	RARRES2
UC T2 vs T3	1,8119333	0,002654	Q9BZR9	Probable E3 ubiquitin-protein ligase TRIM8	TRIM8
UC T2 vs T3	-1,627979	0,02779	Q9NZI8-2;Q9NZI8	Insulin-like growth factor 2 mRNA-binding protein 1	IGF2BP1
UC T2 vs T3	1,9525268	0,002302	Q9UQ72;Q9UQ72-2	Pregnancy-specific beta-1-glycoprotein 11	PSG11
UC T2 vs T3	-5,822768	0,02426	Q9Y490	Talin-1	TLN1
UC T2 vs T3	-1,515867	0,00787	Q9Y5C1	Angiopoietin-related protein 3	ANGPTL3
NCFET T1 vs T2	-4,175199	0,02834	O43184-3;O43184-4;O43184-2;O43184	Disintegrin and metalloproteinase domain-containing protein 12	ADAM12
NCFET T1 vs T2	1,0144553	1,33E-02	P00742	Coagulation factor X	F10
NCFET T1 vs T2	1,9426644	0,02526	P01721	Ig lambda chain V-VI region AR	.
NCFET T1 vs T2	0,4134045	0,01274	P02647;Q9HB71-2	Apolipoprotein A-I	APOA1
NCFET T1 vs T2	0,6381931	0,02039	P02652	Apolipoprotein A-II	APOA2
NCFET T1 vs T2	0,8046799	0,005098	P02765	Alpha-2-HS-glycoprotein	AHSG
NCFET T1 vs T2	0,8470135	0,02355	P05090	Apolipoprotein D	APOD
NCFET T1 vs T2	0,5749369	0,03309	P05109	Protein S100-A8	S100A8
NCFET T1 vs T2	0,8543525	0,005131	P06396;P06396-4;P06396-3	Gelsolin	GSN
NCFET T1 vs T2	0,8760996	0,01306	P07225	Vitamin K-dependent protein S	PROS1
NCFET T1 vs T2	1,4459405	0,007555	P07357	Complement component C8 alpha chain	C8A
NCFET T1 vs T2	2,2259943	0,03347	P07900;P07900-2;Q14568;Q58FF6;Q58FG1	Heat shock protein HSP 90-alpha	HSP90AA1
NCFET T1 vs T2	0,8617472	0,008771	P08294	Extracellular superoxide dismutase [Cu-Zn]	SOD3
NCFET T1 vs T2	0,4958367	0,03385	P09871	Complement C1s subcomponent	C1S
NCFET T1 vs T2	-6,440991	0,006785	P0DML3;P0DML2;P0DML3-2;P0DML3-3;P01241-2;P01241;P01241-5	Chorionic somatomammotropin hormone 2	CSH2;CSH1
NCFET T1 vs T2	1,2420233	0,03861	P11226	Mannose-binding protein C	MBL2
NCFET T1 vs T2	-1,873167	0,02667	P11465	Pregnancy-specific beta-1-glycoprotein 2	PSG2
NCFET T1 vs T2	-0,85018	0,002008	P23142;P23142-2;P23142-3	Fibulin-1	FBLN1
NCFET T1 vs T2	-1,361646	0,007637	P23142-4	Fibulin-1	FBLN1
NCFET T1 vs T2	-1,741402	0,01588	P24593	Insulin-like growth factor-binding protein 5	IGFBP5
NCFET T1 vs T2	1,4721832	1,27E-02	P35542	Serum amyloid A-4 protein	SAA4
NCFET T1 vs T2	0,7067822	0,04661	P55290;P55290-4;P55290-5;P55290-3;P55290-2	Cadherin-13	CDH13
NCFET T1 vs T2	-2,507796	0,004979	Q00887	Pregnancy-specific beta-1-glycoprotein 9	PSG9
NCFET T1 vs T2	-1,341199	0,01196	Q00887-2	Pregnancy-specific beta-1-glycoprotein 9	PSG9
NCFET T1 vs T2	-2,585357	0,01156	Q00888;Q00888-3;Q00888-2	Pregnancy-specific beta-1-glycoprotein 4	PSG4
NCFET T1 vs T2	-1,057877	0,01227	Q00889-2;Q00889	Pregnancy-specific beta-1-glycoprotein 6	PSG6
NCFET T1 vs T2	-1,487394	0,01821	Q13103	Secreted phosphoprotein 24	SPP2
NCFET T1 vs T2	0,5601597	0,005702	Q14624;Q14624-3;Q14624-4	Inter-alpha-trypsin inhibitor heavy chain H4	ITIH4
NCFET T1 vs T2	1,9162145	0,02169	Q15166	Serum paraoxonase/lactonase 3	PON3
NCFET T1 vs T2	-2,553947	0,01337	Q16557	Pregnancy-specific beta-1-glycoprotein 3	PSG3
NCFET T1 vs T2	-0,656295	0,0471	Q92743	Serine protease HTRA1	HTRA1
NCFET T1 vs T2	-1,040182	0,01069	Q99969	Retinoic acid receptor responder protein 2	RARRES2
NCFET T1 vs T2	0,7670794	0,04551	Q9HDC9;Q9HDC9-2	Adipocyte plasma membrane-associated protein	APMAP
NCFET T1 vs T3	-0,714483	0,03256	A0A0B4J1U7	.	IGHV6-1
NCFET T1 vs T3	-0,626631	0,02297	P02649;CON__Q03247	Apolipoprotein E	APOE
NCFET T1 vs T3	0,5664055	0,009586	P02679;P02679-2	Fibrinogen gamma chain	FGG
NCFET T1 vs T3	-0,512575	0,01926	P02745	Complement C1q subcomponent subunit A	C1QA
NCFET T1 vs T3	0,7148659	0,04558	P02748;REV__Q4AC99	Complement component C9	C9
NCFET T1 vs T3	-0,97792	0,006617	P03950	Angiogenin	ANG
NCFET T1 vs T3	-0,743742	0,001738	P06727;Q9BT92	Apolipoprotein A-IV	APOA4
NCFET T1 vs T3	-0,503399	0,008105	P33151;P33151-2	Cadherin-5	CDH5
NCFET T1 vs T3	-1,339208	0,01303	P49747;P49747-2	Cartilage oligomeric matrix protein	COMP
NCFET T1 vs T3	-1,002797	0,02395	P55285-2;P55285	Cadherin-6	CDH6
NCFET T1 vs T3	1,6046116	0,04587	P60709	Actin, cytoplasmic 1	ACTB
NCFET T1 vs T3	1,2696702	0,04557	Q08830	Fibrinogen-like protein 1	FGL1
NCFET T1 vs T3	-2,482201	0,003054	Q13103	Secreted phosphoprotein 24	SPP2
NCFET T1 vs T3	-0,902059	0,03719	Q15113	Procollagen C-endopeptidase enhancer 1	PCOLCE
NCFET T1 vs T3	2,4041366	0,04848	Q8IYW2	Cilia- and flagella-associated protein 46	CFAP46
NCFET T1 vs T3	1,7327424	3,48E-02	Q96RL1-3;Q96RL1-4	BRCA1-A complex subunit RAP80	UIMC1
NCFET T1 vs T3	-0,939654	0,04792	Q99969	Retinoic acid receptor responder protein 2	RARRES2
NCFET T1 vs T3	1,1877267	0,0323	Q9UGM5;Q9UGM5-2	Fetuin-B	FETUB
NCFET T2 vs T3	0,8046362	0,0238	O14791-2;O14791;O14791-3	Apolipoprotein L1	APOL1
NCFET T2 vs T3	4,7267653	0,02311	O43184-3;O43184-4;O43184-2;O43184	Disintegrin and metalloproteinase domain-containing protein 12	ADAM12
NCFET T2 vs T3	-0,938471	0,02526	P00742	Coagulation factor X	F10
NCFET T2 vs T3	-0,781705	0,02656	P00746	Complement factor D	CFD
NCFET T2 vs T3	-1,83112	0,03457	P01721	Ig lambda chain V-VI region AR	.
NCFET T2 vs T3	0,6553195	0,006231	P02679;P02679-2	Fibrinogen gamma chain	FGG
NCFET T2 vs T3	-0,375299	0,01519	P02745	Complement C1q subcomponent subunit A	C1QA
NCFET T2 vs T3	-0,585639	4,48E-03	P02765	Alpha-2-HS-glycoprotein	AHSG
NCFET T2 vs T3	0,6566269	0,0241	P02774-3;P02774;P02774-2	Vitamin D-binding protein	GC
NCFET T2 vs T3	-1,300326	0,009766	P03951	Coagulation factor XI	F11
NCFET T2 vs T3	0,6588186	0,02214	P03973	Antileukoproteinase	SLPI

NCFET T2 vs T3	-0,910791	0,03742	P04003;CON__Q28065	C4b-binding protein alpha chain	C4BPA
NCFET T2 vs T3	1,6332114	0,04404	P04278;P04278-5;P04278-4;P04278-2;P04278-3	Sex hormone-binding globulin	SHBG
NCFET T2 vs T3	-1,014099	0,000302	P05090	Apolipoprotein D	APOD
NCFET T2 vs T3	-0,599139	0,03228	P05109	Protein S100-A8	S100A8
NCFET T2 vs T3	2,685945	0,02463	P05543	Thyroxine-binding globulin	SERPINA7
NCFET T2 vs T3	-1,059354	0,002358	P06396;P06396-4;P06396-3	Gelsolin	GSN
NCFET T2 vs T3	-0,631294	0,007254	P06727;Q9BT92	Apolipoprotein A-IV	APOA4
NCFET T2 vs T3	-1,131462	0,002789	P07225	Vitamin K-dependent protein S	PROS1
NCFET T2 vs T3	-1,929632	0,009295	P07357	Complement component C8 alpha chain	C8A
NCFET T2 vs T3	-1,410943	0,02838	P07900;P07900-2;Q14568;Q58FF6;Q58FG1	Heat shock protein HSP 90-alpha	HSP90AA1
NCFET T2 vs T3	-0,74554	0,0265	P09871	Complement C1s subcomponent	C1S
NCFET T2 vs T3	4,61546	0,01238	P0DML3;P0DML2;P0DML3-2;P0DML3-3;P01241-2;P01241;P01241-5	Chorionic somatomammotropin hormone 2	CSH2;CSH1
NCFET T2 vs T3	-0,584968	0,03973	P0DOX7	.	.
NCFET T2 vs T3	-1,335434	0,03896	P0DOY3	.	.
NCFET T2 vs T3	1,3976764	3,35E-02	P11464-4	Pregnancy-specific beta-1-glycoprotein 1	PSG1
NCFET T2 vs T3	-0,730621	0,0228	P13671	Complement component C6	C6
NCFET T2 vs T3	0,6989815	0,03621	P18428	Lipopolysaccharide-binding protein	LBP
NCFET T2 vs T3	-1,449433	0,0408	P22352	Glutathione peroxidase 3	GPX3
NCFET T2 vs T3	0,8290465	0,002389	P23142;P23142-2;P23142-3	Fibulin-1	FBLN1
NCFET T2 vs T3	2,1275561	0,005193	P23142-4	Fibulin-1	FBLN1
NCFET T2 vs T3	-2,088253	6,13E-03	P35542	Serum amyloid A-4 protein	SAA4
NCFET T2 vs T3	0,6993682	0,008586	P48307-2;P48307	Tissue factor pathway inhibitor 2	TFPI2
NCFET T2 vs T3	-1,08462	0,03273	P49747;P49747-2	Cartilage oligomeric matrix protein	COMP
NCFET T2 vs T3	-1,207863	0,03176	P51884;CON__Q05443	Lumican	LUM
NCFET T2 vs T3	-1,052629	0,006703	P55290;P55290-4;P55290-5;P55290-3;P55290-2	Cadherin-13	CDH13
NCFET T2 vs T3	0,7880451	0,01662	Q00889-2;Q00889	Pregnancy-specific beta-1-glycoprotein 6	PSG6
NCFET T2 vs T3	0,9967029	0,0423	Q08380	Galectin-3-binding protein	LGALS3BP
NCFET T2 vs T3	-0,994807	0,01418	Q13103	Secreted phosphoprotein 24	SPP2
NCFET T2 vs T3	-2,353837	0,007163	Q15166	Serum paraoxonase/lactonase 3	PON3
NCFET T2 vs T3	1,2780102	0,01791	Q16557	Pregnancy-specific beta-1-glycoprotein 3	PSG3
NCFET T2 vs T3	3,9227177	0,006896	Q4LDE5;Q4LDE5-4;Q4LDE5-3;Q4LDE5-2	Sushi, von Willebrand factor type A, EGF and pentraxin domain-containing protein 1	SVEP1
NCFET T2 vs T3	0,7454954	0,03351	Q92743	Serine protease HTRA1	HTRA1
NCFET T2 vs T3	-1,828412	0,009888	Q9HDC9;Q9HDC9-2	Adipocyte plasma membrane-associated protein	APMAP
NCFET T2 vs T3	1,6157978	0,006845	Q9UGM5;Q9UGM5-2	Fetuin-B	FETUB
ACFET T1 vs T2	0,6857395	0,03271	A0A075B6P5;P01615	Ig kappa chain V-II region FR	IGKV2D-28
ACFET T1 vs T2	-0,558008	0,02293	O00602	Ficolin-1	FCN1
ACFET T1 vs T2	-3,879164	0,00013	O43184-3;O43184-4;O43184-2;O43184	Disintegrin and metalloproteinase domain-containing protein 12	ADAM12
ACFET T1 vs T2	0,6039128	0,03562	P00736	Complement C1r subcomponent	C1R
ACFET T1 vs T2	-0,874683	0,03612	P00740;P00740-2	Coagulation factor IX	F9
ACFET T1 vs T2	-0,411931	0,02055	P00915	Carbonic anhydrase 1	CA1
ACFET T1 vs T2	-1,538566	0,01629	P01009;P01009-2;P01009-3;P20848	Alpha-1-antitrypsin	SERPINA1
ACFET T1 vs T2	-0,800392	0,03806	P01034	Cystatin-C	CST3
ACFET T1 vs T2	0,820962	0,02719	P01780	Ig heavy chain V-III region JON	.
ACFET T1 vs T2	-0,870072	0,0405	P02656	Apolipoprotein C-III	APOC3
ACFET T1 vs T2	-0,541104	0,02796	P03973	Antileukoproteinase	SLPI
ACFET T1 vs T2	-0,453592	0,02739	P04004	Vitronectin	VTN
ACFET T1 vs T2	2,1125202	0,04187	P04196	Histidine-rich glycoprotein	HRG
ACFET T1 vs T2	-0,817778	0,03834	P08185	Corticosteroid-binding globulin	SERPINA6
ACFET T1 vs T2	-0,327608	0,0498	P08571	Monocyte differentiation antigen CD14	CD14
ACFET T1 vs T2	-1,03796	0,02723	P09466-2;P09466	Glycodelin	PAEP
ACFET T1 vs T2	-6,242362	0,0138	P0DML3;P0DML2;P0DML3-2;P0DML3-3;P01241-2;P01241;P01241-5	Chorionic somatomammotropin hormone 2	CSH2;CSH1
ACFET T1 vs T2	0,847147	1,85E-02	P0DOY3	.	.
ACFET T1 vs T2	-2,479422	0,02434	P11464-4	Pregnancy-specific beta-1-glycoprotein 1	PSG1
ACFET T1 vs T2	-2,02633	0,01787	P11465	Pregnancy-specific beta-1-glycoprotein 2	PSG2
ACFET T1 vs T2	-2,168007	0,01384	P13497;P13497-5;P13497-2;P13497-6;P13497-4;P13497-3	Bone morphogenetic protein 1	BMP1
ACFET T1 vs T2	-1,347718	0,03634	P14543;P14543-2	Nidogen-1	NID1
ACFET T1 vs T2	-0,610636	0,0222	P18428	Lipopolysaccharide-binding protein	LBP
ACFET T1 vs T2	-0,376444	0,04946	P19320;P19320-3;P19320-2	Vascular cell adhesion protein 1	VCAM1
ACFET T1 vs T2	-0,809616	0,01205	P20742;P20742-2	Pregnancy zone protein	PZP
ACFET T1 vs T2	-0,589047	0,02363	P22692	Insulin-like growth factor-binding protein 4	IGFBP4
ACFET T1 vs T2	-1,306791	0,005989	P23142;P23142-2;P23142-3	Fibulin-1	FBLN1
ACFET T1 vs T2	-2,319813	0,002369	P23142-4	Fibulin-1	FBLN1
ACFET T1 vs T2	-0,60018	0,04824	P23280-3;P23280	Carbonic anhydrase 6	CA6
ACFET T1 vs T2	-1,367838	0,01983	P24593	Insulin-like growth factor-binding protein 5	IGFBP5
ACFET T1 vs T2	-0,628545	0,008373	P24844	Myosin regulatory light polypeptide 9	MYL9
ACFET T1 vs T2	0,327589	0,04034	P27169	Serum paraoxonase/arylesterase 1	PON1
ACFET T1 vs T2	-0,945295	0,02385	P35858;P35858-2	Insulin-like growth factor-binding protein complex acid labile subunit	IGFALS
ACFET T1 vs T2	-0,860365	0,04147	P43251-4;P43251;P43251-3;P43251-2	Biotinidase	BTD

ACFET T1 vs T2	0,4968839	0,04582	P49908	Selenoprotein P	SEPP1
ACFET T1 vs T2	-0,440735	0,02244	P58166	Inhibin beta E chain	INHBE
ACFET T1 vs T2	-0,733205	0,02665	P61769	Beta-2-microglobulin	B2M
ACFET T1 vs T2	-0,410809	0,0432	P63261	Actin, cytoplasmic 2	ACTG1
ACFET T1 vs T2	-2,154022	0,02414	Q00887	Pregnancy-specific beta-1-glycoprotein 9	PSG9
ACFET T1 vs T2	-3,130228	0,001374	Q00888;Q00888-3;Q00888-2	Pregnancy-specific beta-1-glycoprotein 4	PSG4
ACFET T1 vs T2	-3,243842	0,00626	Q00889-2;Q00889	Pregnancy-specific beta-1-glycoprotein 6	PSG6
ACFET T1 vs T2	-1,968694	0,01716	Q02818	Nucleobindin-1	NUCB1
ACFET T1 vs T2	1,1807613	0,003676	Q04756	Hepatocyte growth factor activator	HGFAC
ACFET T1 vs T2	-1,138887	0,02526	Q08380	Galectin-3-binding protein	LGALS3BP
ACFET T1 vs T2	1,1300988	0,04627	Q13103	Secreted phosphoprotein 24	SPP2
ACFET T1 vs T2	-1,047887	5,56E-04	Q14520-2;Q14520	Hyaluronan-binding protein 2	HABP2
ACFET T1 vs T2	-2,26216	0,001771	Q16557	Pregnancy-specific beta-1-glycoprotein 3	PSG3
ACFET T1 vs T2	-5,615534	0,000654	Q4LDE5;Q4LDE5-4;Q4LDE5-3;Q4LDE5-2	Sushi, von Willebrand factor type A, EGF and pentraxin domain-containing protein 1	SVEP1
ACFET T1 vs T2	-0,4544	0,01742	Q8TDL5;Q8TDL5-2	BPI fold-containing family B member 1	BPIFB1
ACFET T1 vs T2	-1,322079	0,01836	Q92743	Serine protease HTRA1	HTRA1
ACFET T1 vs T2	-0,574041	0,01959	Q9BRK5-6;Q9BRK5;Q9BRK5-4;Q9BRK5-3;Q9BRK5-2	45 kDa calcium-binding protein	SDF4
ACFET T1 vs T2	-0,408247	0,04432	Q9BY76-3;Q9BY76;Q9BY76-2	Angiopoietin-related protein 4	ANGPTL4
ACFET T1 vs T2	-0,4308	0,04218	Q9NRA1;Q9NRA1-3;Q9NRA1-2;Q9NRA1-4	Platelet-derived growth factor C	PDGFC
ACFET T1 vs T2	-0,379268	0,04742	Q9UNW1;Q9UNW1-4;Q9UNW1-3;Q9UNW1-2	Multiple inositol polyphosphate phosphatase 1	MINPP1
ACFET T1 vs T2	-1,485456	3,32E-04	Q9UQ72;Q9UQ72-2	Pregnancy-specific beta-1-glycoprotein 11	PSG11
ACFET T1 vs T3	1,9469683	0,04559	O00204-2;O00204	Sulfotransferase family cytosolic 2B member 1	SULT2B1
ACFET T1 vs T3	-0,746398	3,11E-02	O95445-2	Apolipoprotein M	APOM
ACFET T1 vs T3	-0,626694	0,02224	P00709	Alpha-lactalbumin	LALBA
ACFET T1 vs T3	-0,697973	0,02623	P01009;P01009-2;P01009-3;P20848	Alpha-1-antitrypsin	SERPINA1
ACFET T1 vs T3	-0,584378	0,005032	P02749;CON__P17690	Beta-2-glycoprotein 1	APOH
ACFET T1 vs T3	-0,292119	0,03485	P02753	Retinol-binding protein 4	RBP4
ACFET T1 vs T3	-0,781693	0,03577	P02766	Transthyretin	TTR
ACFET T1 vs T3	0,6135819	0,02284	P04004	Vitronectin	VTN
ACFET T1 vs T3	-0,560161	0,03209	P04196	Histidine-rich glycoprotein	HRG
ACFET T1 vs T3	1,8977611	0,006992	P04278;P04278-5;P04278-4;P04278-2;P04278-3	Sex hormone-binding globulin	SHBG
ACFET T1 vs T3	-0,600328	0,04928	P06727;Q9BT92	Apolipoprotein A-IV	APOA4
ACFET T1 vs T3	-1,263662	0,03872	P07237	Protein disulfide-isomerase	P4HB
ACFET T1 vs T3	-0,958984	0,04732	P08294	Extracellular superoxide dismutase [Cu-Zn]	SOD3
ACFET T1 vs T3	-0,512712	0,03211	P08493-2;P08493	Matrix Gla protein	MGP
ACFET T1 vs T3	0,978874	0,0468	P12532;P12532-2	Creatine kinase U-type, mitochondrial	CKMT1A
ACFET T1 vs T3	-1,050585	0,04234	P35579;P35579-2	Myosin-9	MYH9
ACFET T1 vs T3	-1,022207	0,002561	P49747;P49747-2	Cartilage oligomeric matrix protein	COMP
ACFET T1 vs T3	-1,185396	0,002414	P51884;CON__Q05443	Lumican	LUM
ACFET T1 vs T3	-1,101886	0,008118	P55058;P55058-4;P55058-3;P55058-2	Phospholipid transfer protein	PLTP
ACFET T1 vs T3	0,5865862	0,002285	Q14520-2;Q14520	Hyaluronan-binding protein 2	HABP2
ACFET T1 vs T3	-0,693515	0,01543	Q15293;Q15293-2	Reticulocalbin-1	RCN1
ACFET T1 vs T3	-0,755786	0,01417	Q8TDL5;Q8TDL5-2	BPI fold-containing family B member 1	BPIFB1
ACFET T1 vs T3	-0,551745	0,04259	Q9NY15;Q9NY15-2	Stabilin-1	STAB1
ACFET T1 vs T3	-1,193307	0,04816	Q9Y5C1	Angiopoietin-related protein 3	ANGPTL3
ACFET T2 vs T3	-0,563584	0,0176	A0A075B6P5;P01615	Ig kappa chain V-II region FR	IGKV2D-28
ACFET T2 vs T3	1,351145	0,0226	A0A0A0MS15	.	IGHV3-49
ACFET T2 vs T3	-0,718902	0,02517	A0A0C4DH72	.	IGKV1-6
ACFET T2 vs T3	1,0649946	0,009925	O00391;O00391-2	Sulfhydryl oxidase 1	QSOX1
ACFET T2 vs T3	1,180311	0,00514	O14791-2;O14791;O14791-3	Apolipoprotein L1	APOL1
ACFET T2 vs T3	4,2788464	0,0125	O43184-3;O43184-4;O43184-2;O43184	Disintegrin and metalloproteinase domain-containing protein 12	ADAM12
ACFET T2 vs T3	-1,369925	0,007368	O95445	Apolipoprotein M	APOM
ACFET T2 vs T3	-0,833044	0,02099	P00736	Complement C1r subcomponent	C1R
ACFET T2 vs T3	-0,72664	0,04067	P00738;P00738-2	Haptoglobin	HP
ACFET T2 vs T3	0,8559687	0,0457	P00740;P00740-2	Coagulation factor IX	F9
ACFET T2 vs T3	-2,377659	0,0122	P00746	Complement factor D	CFD
ACFET T2 vs T3	-0,51368	0,0494	P01700	Ig lambda chain V-I region HA	.
ACFET T2 vs T3	0,7698567	0,0115	P02679;P02679-2	Fibrinogen gamma chain	FGG
ACFET T2 vs T3	-0,56856	1,85E-02	P02747	Complement C1q subcomponent subunit C	C1QC
ACFET T2 vs T3	-0,988889	0,03394	P02749;CON__P17690	Beta-2-glycoprotein 1	APOH
ACFET T2 vs T3	-1,433325	3,04E-02	P03951	Coagulation factor XI	F11
ACFET T2 vs T3	-0,821104	0,04685	P04003;CON__Q28065	C4b-binding protein alpha chain	C4BPA
ACFET T2 vs T3	1,0671737	0,002429	P04004	Vitronectin	VTN
ACFET T2 vs T3	-2,672681	0,0235	P04196	Histidine-rich glycoprotein	HRG
ACFET T2 vs T3	1,9522765	0,004291	P04278;P04278-5;P04278-4;P04278-2;P04278-3	Sex hormone-binding globulin	SHBG
ACFET T2 vs T3	-1,150369	0,01811	P05156;CON__Q32PI4	Complement factor I	CFI
ACFET T2 vs T3	-0,995138	0,001946	P06396;P06396-4;P06396-3	Gelsolin	GSN
ACFET T2 vs T3	2,233897	0,02444	P06681;P06681-3;P06681-2	Complement C2	C2

ACFET T2 vs T3	-0,616192	0,02283	P06727;Q9BT92	Apolipoprotein A-IV	APOA4
ACFET T2 vs T3	-0,740652	0,005384	P07225	Vitamin K-dependent protein S	PROS1
ACFET T2 vs T3	-1,248837	0,03647	P07237	Protein disulfide-isomerase	P4HB
ACFET T2 vs T3	-2,134662	0,02104	P07357	Complement component C8 alpha chain	C8A
ACFET T2 vs T3	1,1120908	0,01213	P08185	Corticosteroid-binding globulin	SERPINA6
ACFET T2 vs T3	6,1711745	0,03469	P0DML3;P0DML2;P0DML3-2;P0DML3-3;P01241-2;P01241;P01241-5	Chorionic somatomammotropin hormone 2	CSH2;CSH1
ACFET T2 vs T3	-1,291778	0,000335	P0DOY3	.	.
ACFET T2 vs T3	1,4064476	4,62E-03	P11464-4	Pregnancy-specific beta-1-glycoprotein 1	PSG1
ACFET T2 vs T3	1,7114802	2,70E-02	P11465	Pregnancy-specific beta-1-glycoprotein 2	PSG2
ACFET T2 vs T3	1,1474268	0,02875	P12532;P12532-2	Creatine kinase U-type, mitochondrial	CKMT1A
ACFET T2 vs T3	1,7573503	0,009279	P13497;P13497-5;P13497-2;P13497-6;P13497-4;P13497-3	Bone morphogenetic protein 1	BMP1
ACFET T2 vs T3	-1,77376	0,04544	P14625;Q58FF3	Endoplasmin	HSP90B1
ACFET T2 vs T3	-1,939065	0,002772	P18065	Insulin-like growth factor-binding protein 2	IGFBP2
ACFET T2 vs T3	2,2666657	0,01533	P20742;P20742-2	Pregnancy zone protein	PZP
ACFET T2 vs T3	1,0672605	0,0183	P23142;P23142-2;P23142-3	Fibulin-1	FBLN1
ACFET T2 vs T3	2,5145814	0,001778	P23142-4	Fibulin-1	FBLN1
ACFET T2 vs T3	1,3168458	0,03165	P24593	Insulin-like growth factor-binding protein 5	IGFBP5
ACFET T2 vs T3	-0,701523	0,02739	P33151;P33151-2	Cadherin-5	CDH5
ACFET T2 vs T3	-1,997334	0,01771	P35542	Serum amyloid A-4 protein	SAA4
ACFET T2 vs T3	0,9691765	0,02169	P35858;P35858-2	Insulin-like growth factor-binding protein complex acid labile subunit	IGFALS
ACFET T2 vs T3	-0,614809	0,03257	P49747;P49747-2	Cartilage oligomeric matrix protein	COMP
ACFET T2 vs T3	-0,717499	0,0478	P51884;CON__Q05443	Lumican	LUM
ACFET T2 vs T3	-0,705218	0,02618	P55058;P55058-4;P55058-3;P55058-2	Phospholipid transfer protein	PLTP
ACFET T2 vs T3	0,6602212	0,02507	P58166	Inhibin beta E chain	INHBE
ACFET T2 vs T3	0,5598104	0,03364	P80108;P80108-2	Phosphatidylinositol-glycan-specific phospholipase D	GPLD1
ACFET T2 vs T3	6,6331249	0,0323	Q00887	Pregnancy-specific beta-1-glycoprotein 9	PSG9
ACFET T2 vs T3	1,5012885	0,02401	Q00889-2;Q00889	Pregnancy-specific beta-1-glycoprotein 6	PSG6
ACFET T2 vs T3	-1,223216	0,002765	Q04756	Hepatocyte growth factor activator	HGFAC
ACFET T2 vs T3	-2,23694	0,04233	Q07954;Q07954-2	Prolow-density lipoprotein receptor-related protein 1	LRP1
ACFET T2 vs T3	1,4910324	0,01242	Q08380	Galectin-3-binding protein	LGALS3BP
ACFET T2 vs T3	1,6344736	9,73E-05	Q14520-2;Q14520	Hyaluronan-binding protein 2	HABP2
ACFET T2 vs T3	-2,34765	0,03616	Q15166	Serum paraoxonase/lactonase 3	PON3
ACFET T2 vs T3	-0,547687	0,03072	Q15293;Q15293-2	Reticulocalbin-1	RCN1
ACFET T2 vs T3	2,699654	0,04353	Q16557	Pregnancy-specific beta-1-glycoprotein 3	PSG3
ACFET T2 vs T3	9,5498958	0,01049	Q4LDE5;Q4LDE5-4;Q4LDE5-3;Q4LDE5-2	Sushi, von Willebrand factor type A, EGF and pentraxin domain-containing protein 1	SVEP1
ACFET T2 vs T3	-1,020664	0,01191	Q76LX8;Q76LX8-2;Q76LX8-3;Q76LX8-4	A disintegrin and metalloproteinase with thrombospondin motifs 13	ADAMTS13
ACFET T2 vs T3	1,8255016	0,01335	Q8WUA8	Tsukushin	TSKU
ACFET T2 vs T3	1,2868425	0,0143	Q92743	Serine protease HTRA1	HTRA1
ACFET T2 vs T3	0,7213968	0,02416	Q92954-3;Q92954-6;Q92954;Q92954-4;Q92954-2;Q92954-5	Proteoglycan 4	PRG4
ACFET T2 vs T3	0,881067	0,04925	Q9UGM5;Q9UGM5-2	Fetuin-B	FETUB
ACFET T2 vs T3	1,2990465	0,000916	Q9UQ72;Q9UQ72-2	Pregnancy-specific beta-1-glycoprotein 11	PSG11
