# Supplementary material for: Exploring the plasma proteome linked to corpus luteum presence and conception mode across pregnancy stages and postpartum
Source: J Assist Reprod Genet. 2025 Sep 20;42(10):3275–89. doi: 10.1007/s10815-025-03632-0 (PMC12602745; doi:10.1007/s10815-025-03632-0)
Supplement: Supplementary file 3 — (30.3 MB PDF) [file 10815_2025_3632_MOESM3_ESM.pdf]

UC+NCFET vs ACFET T1

#category	term ID	term description	observed gene count	background gene count	strength	false discovery rate	matching proteins in your network (labels)
GO Process	GO:0006959	Humoral immune response	24	268	1.14	8.31e-16	ST6GAL1,EBI3,C5,C3,LYZ,FCN3,PPBP,MASP1,CAMP,FGB,F2,C8A,CFHR4,CFHR3,C1QC,GAPDH,MASP2,C1S,C4B,C1R,IGHV3-15,CCL18,KNG1,FGA
GO Process	GO:0006950	Response to stress	64	3358	0.47	8.35e-14	VCL,F9,TFPI2,C5,PROC,PLEK,CAT,C3,PXDN,LYZ,ATRN,HYAL1,FCN3,AHSG,CRH,SAA4,MYL9,CSF1R,IGF2BP1,PPBP,MASP1,CAMP,HSP90B1,PCSK9,SHLD1,FGB,DST,F2,PLG,STAB1,APOL1,SERPINF2,FGG,TUBB,THBS4,BLM,C8A,F13B,MCOLN2,ADAMTS13,C1QC,HLA-C,TAF9,SOD3,TNRC6A,ILK,GAPDH,RNASE1,CST3,MASP2,SAA1,F11,C1S,CDK3,C4B,UIMC1,C1R,SERPINA3,RNASE4,IGHV3-15,CCL18,KNG1,ACTB,FGA
GO Process	GO:0007596	Blood coagulation	18	173	1.21	1.55e-12	VCL,F9,TFPI2,PROC,PLEK,MYL9,FGB,F2,PLG,FGG,F13B,ADAMTS13,ILK,SAA1,F11,KNG1,ACTB,FGA
GO Process	GO:0006956	Complement activation	13	60	1.53	2.66e-12	C5,C3,FCN3,MASP1,C8A,CFHR4,CFHR3,C1QC,MASP2,C1S,C4B,C1R,IGHV3-15
GO Process	GO:0006952	Defense response	38	1394	0.63	3.77e-11	C5,C3,LYZ,ATRN,HYAL1,FCN3,AHSG,CRH,SAA4,CSF1R,PPBP,MASP1,CAMP,FGB,F2,STAB1,APOL1,SERPINF2,TUBB,C8A,MCOLN2,ADAMTS13,C1QC,HLA-C,GAPDH,RNASE1,CST3,MASP2,SAA1,C1S,C4B,C1R,SERPINA3,RNASE4,IGHV3-15,CCL18,KNG1,FGA
GO Process	GO:0006955	Immune response	35	1321	0.61	1.07e-09	ST6GAL1,LGALS1,EBI3,C5,C3,PXDN,LYZ,PSG9,FCN3,CSF1R,PPBP,MASP1,CAMP,SHLD1,FGB,F2,APOL1,TUBB,C8A,CFHR4,CFHR3,MCOLN2,ADAMTS13,C1QC,HLA-C,GAPDH,RAPGEF4,MASP2,C1S,C4B,C1R,IGHV3-15,CCL18,KNG1,FGA
GO Process	GO:0042060	Wound healing	19	336	0.94	1.99e-09	VCL,F9,TFPI2,PROC,PLEK,MYL9,FGB,DST,F2,PLG,FGG,F13B,ADAMTS13,ILK,SAA1,F11,KNG1,ACTB,FGA
GO Process	GO:0045861	Negative regulation of proteolysis	19	339	0.94	1.99e-09	TFPI2,C5,C3,TIMP2,LCN1,ITIH1,AHSG,SERPINI1,A2ML1,F2,SERPINF2,TAF9,GAPDH,CST3,ITIH3,C4B,SERPINA3,SERPINA4,KNG1
GO Process	GO:0051346	Negative regulation of hydrolase activity	19	354	0.92	3.68e-09	TFPI2,C5,APOC3,APOA1,C3,TIMP2,LCN1,ITIH1,AHSG,SERPINI1,A2ML1,SERPINF2,GAPDH,CST3,ITIH3,C4B,SERPINA3,SERPINA4,KNG1
GO Process	GO:0050878	Regulation of body fluid levels	19	371	0.9	7.45e-09	VCL,F9,TFPI2,PROC,PLEK,MYL9,FGB,F2,PLG,SERPINF2,FGG,F13B,ADAMTS13,ILK,SAA1,F11,KNG1,ACTB,FGA
GO Process	GO:0098542	Defense response to other organism	29	989	0.66	8.15e-09	C5,C3,LYZ,FCN3,CSF1R,PPBP,MASP1,CAMP,FGB,F2,STAB1,APOL1,TUBB,C8A,MCOLN2,ADAMTS13,C1QC,HLA-C,GAPDH,RNASE1,MASP2,C1S,C4B,C1R,RNASE4,IGHV3-15,CCL18,KNG1,FGA
GO Process	GO:0050896	Response to stimulus	88	7835	0.24	1.91e-08	ST6GAL1,VCL,LGALS1,F9,EBI3,TFPI2,C5,APOC3,GPLD1,PROC,PLEK,APOA1,TTR,CAT,C3,PXDN,LYZ,TIMP2,ATRN,LCN1,LCAT,CDH6,HYAL1,PSG9,FCN3,AHSG,CRH,SAA4,MYL9,CSF1R,IGF2BP1,IHH,PPBP,MASP1,CAMP,GUCY1A1,HSP90B1,PCSK9,SHLD1,FGB,DST,F2,PLG,STAB1,APOL1,SERPINF2,FGG,TUBB,THBS4,BLM,AKAP9,C8A,F13B,CFHR4,CFHR3,MCOLN2,ADAMTS13,CCN5,C1QC,APOM,HLA-C,TAF9,SOD3,TNRC6A,ILK,GAPDH,RAPGEF4,RNASE1,CST3,MASP2,SAA1,F11,C1S,NUCB1,IGF2,CDK3,C4B,TGFBI,IGFALS,UIMC1,C1R,SERPINA3,RNASE4,IGHV3-15,CCL18,KNG1,ACTB,FGA
GO Process	GO:0006958	Complement activation, classical pathway	9	40	1.54	2.08e-08	C5,C3,C8A,C1QC,MASP2,C1S,C4B,C1R,IGHV3-15
GO Process	GO:0002376	Immune system process	41	2121	0.48	5.58e-08	ST6GAL1,LGALS1,EBI3,C5,GPLD1,PLEK,C3,PXDN,LYZ,PSG9,FCN3,CSF1R,PPBP,MASP1,CAMP,SHLD1,FGB,F2,PLG,APOL1,TUBB,C8A,CFHR4,CFHR3,MCOLN2,ADAMTS13,C1QC,HLA-C,CD99,ACTN1,GAPDH,RAPGEF4,MASP2,SAA1,C1S,C4B,C1R,IGHV3-15,CCL18,KNG1,FGA
GO Process	GO:0030195	Negative regulation of blood coagulation	9	46	1.48	5.58e-08	PROC,FGB,F2,PLG,SERPINF2,FGG,F11,KNG1,FGA
GO Process	GO:0030168	Platelet activation	11	97	1.25	6.98e-08	VCL,PLEK,MYL9,FGB,F2,FGG,ADAMTS13,ILK,SAA1,ACTB,FGA
GO Process	GO:0002250	Adaptive immune response	17	359	0.87	1.86e-07	EBI3,C5,C3,PSG9,SHLD1,FGB,C8A,MCOLN2,C1QC,HLA-C,RAPGEF4,MASP2,C1S,C4B,C1R,IGHV3-15,FGA
GO Process	GO:0002252	Immune effector process	17	375	0.85	3.37e-07	LGALS1,C5,C3,FCN3,MASP1,SHLD1,F2,TUBB,C8A,CFHR4,CFHR3,C1QC,MASP2,C1S,C4B,C1R,IGHV3-15
GO Process	GO:0034109	Homotypic cell-cell adhesion	9	60	1.37	3.37e-07	VCL,PLEK,MYL9,FGB,FGG,CD99,ILK,ACTB,FGA
GO Process	GO:0072378	Blood coagulation, fibrin clot formation	7	24	1.66	4.85e-07	F9,FGB,F2,FGG,F13B,F11,FGA
GO Process	GO:0052547	Regulation of peptidase activity	18	446	0.8	5.39e-07	TFPI2,C5,C3,TIMP2,LCN1,ITIH1,AHSG,SERPINI1,A2ML1,ANTXR1,SERPINF2,GAPDH,CST3,ITIH3,C4B,SERPINA3,SERPINA4,KNG1
GO Process	GO:0070527	Platelet aggregation	8	43	1.46	6.00e-07	VCL,PLEK,MYL9,FGB,FGG,ILK,ACTB,FGA
GO Process	GO:0016064	Immunoglobulin mediated immune response	10	95	1.21	7.05e-07	C5,C3,SHLD1,C8A,C1QC,MASP2,C1S,C4B,C1R,IGHV3-15
GO Process	GO:0051707	Response to other organism	30	1328	0.54	7.34e-07	C5,C3,LYZ,HYAL1,FCN3,CSF1R,PPBP,MASP1,CAMP,FGB,F2,STAB1,APOL1,TUBB,C8A,MCOLN2,ADAMTS13,C1QC,HLA-C,GAPDH,RNASE1,MASP2,C1S,C4B,C1R,RNASE4,IGHV3-15,CCL18,KNG1,FGA
GO Process	GO:0002460	Adaptive immune response based on somatic recombination of immune receptors built from immunoglobulin superfamily domains	12	169	1.04	8.51e-07	EBI3,C5,C3,PSG9,SHLD1,C8A,C1QC,MASP2,C1S,C4B,C1R,IGHV3-15
GO Process	GO:0006954	Inflammatory response	19	538	0.74	1.05e-06	C5,C3,LYZ,ATRN,HYAL1,AHSG,CRH,SAA4,CSF1R,PPBP,CAMP,F2,STAB1,SERPINF2,SAA1,C4B,SERPINA3,CCL18,KNG1
GO Process	GO:0030162	Regulation of proteolysis	22	739	0.66	1.08e-06	TFPI2,C5,GPLD1,C3,TIMP2,LCN1,ITIH1,AHSG,SERPINI1,A2ML1,ANTXR1,SH3D19,F2,SERPINF2,TAF9,GAPDH,CST3,ITIH3,C4B,SERPINA3,SERPINA4,KNG1
GO Process	GO:0002253	Activation of immune response	14	271	0.9	1.45e-06	C5,GPLD1,C3,FCN3,MASP1,C8A,CFHR4,CFHR3,C1QC,MASP2,C1S,C4B,C1R,IGHV3-15
GO Process	GO:0045087	Innate immune response	22	754	0.66	1.45e-06	C5,C3,FCN3,CSF1R,MASP1,CAMP,FGB,APOL1,TUBB,C8A,MCOLN2,ADAMTS13,C1QC,HLA-C,GAPDH,MASP2,C1S,C4B,C1R,IGHV3-15,CCL18,FGA
GO Process	GO:0051239	Regulation of multicellular organismal process	44	2749	0.39	1.45e-06	VCL,EBI3,C5,APOC3,MGP,GPLD1,PROC,APOA1,C3,ATRN,LCAT,HYAL1,LUM,PSG9,AHSG,CRH,MYL9,CSF1R,IGF2BP1,IHH,CAMP,GUCY1A1,PCSK9,SHLD1,FGB,F2,PLG,STAB1,SERPINF2,FGG,THBS4,AKAP9,MCOLN2,C1QC,APOM,GAPDH,CST3,SAA1,F11,IGF2,PAEP,KNG1,ACTB,FGA
GO Process	GO:0044419	Biological process involved in interspecies interaction between organisms	31	1490	0.51	1.74e-06	C5,C3,LYZ,HYAL1,FCN3,CSF1R,PPBP,MASP1,CAMP,FGB,F2,PLG,STAB1,APOL1,TUBB,C8A,MCOLN2,ADAMTS13,C1QC,HLA-C,GAPDH,RNASE1,MASP2,C1S,C4B,C1R,RNASE4,IGHV3-15,CCL18,KNG1,FGA
GO Process	GO:0051336	Regulation of hydrolase activity	25	1011	0.58	2.62e-06	TFPI2,C5,APOC3,GPLD1,PLEK,APOA1,C3,TIMP2,LCN1,ITIH1,AHSG,SERPINI1,A2ML1,HSP90B1,ANTXR1,SERPINF2,GAPDH,RAPGEF4,CST3,ITIH3,C4B,SERPINA3,SERPINA4,CCL18,KNG1
GO Process	GO:0042730	Fibrinolysis	6	19	1.69	3.02e-06	FGB,F2,PLG,SERPINF2,FGG,FGA
GO Process	GO:0002684	Positive regulation of immune system process	23	874	0.61	3.51e-06	LGALS1,EBI3,C5,GPLD1,C3,FCN3,CSF1R,IHH,MASP1,SHLD1,THBS4,C8A,CFHR4,CFHR3,C1QC,CD99,MASP2,C1S,IGF2,C4B,C1R,IGHV3-15,ACTB
GO Process	GO:0002449	Lymphocyte mediated immunity	11	159	1.03	3.71e-06	C5,C3,SHLD1,TUBB,C8A,C1QC,MASP2,C1S,C4B,C1R,IGHV3-15
GO Process	GO:0007155	Cell adhesion	24	965	0.59	4.47e-06	VCL,LGALS1,PLEK,PXDN,ATRN,CDH6,MYL9,IHH,ANTXR1,FGB,DST,STAB1,PSG11,FGG,THBS4,ADAMTS13,CCN5,CD99,ACTN1,ILK,TGFBI,IGFALS,ACTB,FGA

GO Process	GO:0002443	Leukocyte mediated immunity	12	211	0.95	6.00e-06	C5,C3,SHLD1,F2,TUBB,C8A,C1QC,MASP2,C1S,C4B,C1R,IGHV3-15
GO Process	GO:0048519	Negative regulation of biological process	63	5313	0.26	1.51e-05	ST6GAL1,VCL,TFPI2,C5,APOC3,GPLD1,PROC,PLEK,APOA1,CAT,C3,PXDN,TIMP2,LCN1,HYAL1,PSG9,FCN3,ITIH1,AHSG,CRH,CSF1R,IGF2BP1,IHH,SERPINI1,MASP1,GUCY1A1,A2ML1,HSP90B1,ANTXR1,PCSK9,SHLD1,FGB,F2,PLG,STAB1,SERPINF2,FGG,THBS4,BLM,MCOLN2,CCN5,C1QC,APOM,TAF9,TNRC6A,ILK,GAPDH,CST3,SAA1,F11,IGF2,CDK3,LMOD3,ITIH3,C4B,TGFBI,PAEP,UIMC1,SERPINA3,SERPINA4,KNG1,ACTB,FGA
GO Process	GO:0043086	Negative regulation of catalytic activity	20	771	0.6	4.08e-05	TFPI2,C5,APOC3,APOA1,C3,TIMP2,LCN1,ITIH1,AHSG,SERPINI1,A2ML1,SERPINF2,AKAP9,GAPDH,CST3,ITIH3,C4B,SERPINA3,SERPINA4,KNG1
GO Process	GO:0048584	Positive regulation of response to stimulus	35	2131	0.41	4.31e-05	LGALS1,C5,GPLD1,APOA1,CAT,C3,FCN3,CSF1R,IHH,MASP1,GUCY1A1,SHLD1,FGB,F2,PLG,SERPINF2,FGG,THBS4,C8A,CFHR4,CFHR3,C1QC,TAF9,ILK,GAPDH,MASP2,C1S,IGF2,C4B,UIMC1,C1R,IGHV3-15,CCL18,ACTB,FGA
GO Process	GO:1903028	Positive regulation of opsonization	5	16	1.69	4.96e-05	C3,FCN3,MASP1,MASP2,C4B
GO Process	GO:0031638	Zymogen activation	7	59	1.26	5.57e-05	F9,FGB,F2,FGG,F11,C1R,FGA
GO Process	GO:0051241	Negative regulation of multicellular organismal process	23	1035	0.54	5.99e-05	APOC3,PROC,APOA1,PSG9,AHSG,CRH,IHH,GUCY1A1,PCSK9,FGB,F2,PLG,STAB1,SERPINF2,FGG,THBS4,C1QC,APOM,CST3,F11,PAEP,KNG1,FGA
GO Process	GO:0065007	Biological regulation	106	12385	0.12	8.01e-05	ST6GAL1,VCL,LGALS1,TPD52L2,F9,EBI3,TFPI2,C5,APOC3,MGP,GPLD1,PROC,PLEK,APOA1,TTR,CAT,C3,PXDN,LYZ,TIMP2,ATRN,LCN1,LCAT,CDH6,CENPE,HYAL1,LUM,PSG9,FCN3,ITIH1,AHSG,ANKRD31,CRH,MYL9,CSF1R,IGF2BP1,IHH,SERPINI1,PPBP,MASP1,CAMP,GUCY1A1,A2ML1,HSP90B1,ANTXR1,SH3D19,PCSK9,SHLD1,FGB,DST,F2,PLG,STAB1,SERPINF2,OIT3,FGG,TUBB,THBS4,BLM,AKAP9,CNDP1,MTCL1,C8A,F13B,CFHR4,CFHR3,MCOLN2,ADAMTS13,CCN5,C1QC,APOM,TAF9,CD99,SOD3,ACTN1,TNRC6A,NAV2,ILK,GAPDH,RAPGEF4,CST3,MASP2,SAA1,F11,C1S,NUCB1,IGF2,ATP9B,SLAIN1,CDK3,ABCA13,LMOD3,ITIH3,C4B,TGFBI,IGFALS,PAEP,UIMC1,C1R,SERPINA3,SERPINA4,IGHV3-15,CCL18,KNG1,ACTB,FGA
GO Process	GO:0044092	Negative regulation of molecular function	24	1143	0.51	8.13e-05	TFPI2,C5,APOC3,APOA1,CAT,C3,PXDN,TIMP2,LCN1,ITIH1,AHSG,SERPINI1,A2ML1,PCSK9,SERPINF2,AKAP9,GAPDH,CST3,ITIH3,C4B,SERPINA3,SERPINA4,KNG1,ACTB
GO Process	GO:0006953	Acute-phase response	6	42	1.35	0.00014	AHSG,SAA4,F2,SERPINF2,SAA1,SERPINA3
GO Process	GO:0009605	Response to external stimulus	36	2355	0.37	0.00014	C5,APOA1,CAT,C3,LYZ,HYAL1,FCN3,CSF1R,IHH,PPBP,MASP1,CAMP,PCSK9,FGB,F2,STAB1,APOL1,TUBB,C8A,MCOLN2,ADAMTS13,C1QC,HLA-C,TNRC6A,GAPDH,RNASE1,MASP2,SAA1,C1S,C4B,C1R,RNASE4,IGHV3-15,CCL18,KNG1,FGA
GO Process	GO:0048518	Positive regulation of biological process	67	6207	0.22	0.00014	ST6GAL1,LGALS1,EBI3,C5,GPLD1,PROC,PLEK,APOA1,CAT,C3,CENPE,HYAL1,LUM,PSG9,FCN3,AHSG,ANKRD31,CRH,CSF1R,IGF2BP1,IHH,SERPINI1,PPBP,MASP1,CAMP,GUCY1A1,ANTXR1,SH3D19,PCSK9,SHLD1,FGB,F2,PLG,SERPINF2,FGG,THBS4,BLM,AKAP9,MTCL1,C8A,CFHR4,CFHR3,MCOLN2,C1QC,TAF9,CD99,ACTN1,TNRC6A,ILK,GAPDH,MASP2,SAA1,F11,C1S,IGF2,SLAIN1,ABCA13,LMOD3,C4B,PAEP,UIMC1,C1R,IGHV3-15,CCL18,KNG1,ACTB,FGA
GO Process	GO:0019730	Antimicrobial humoral response	9	149	0.97	0.00019	LYZ,PPBP,CAMP,FGB,F2,GAPDH,CCL18,KNG1,FGA
GO Process	GO:0030155	Regulation of cell adhesion	19	784	0.58	0.00019	ST6GAL1,VCL,LGALS1,EBI3,APOA1,HYAL1,IHH,SERPINI1,FGB,PLG,SERPINF2,FGG,ILK,SAA1,IGF2,TGFBI,KNG1,ACTB,FGA
GO Process	GO:0050766	Positive regulation of phagocytosis	7	75	1.16	0.00021	APOA1,C3,FCN3,AHSG,MASP1,MASP2,C4B
GO Process	GO:0051246	Regulation of protein metabolic process	38	2622	0.35	0.00021	TFPI2,C5,GPLD1,APOA1,C3,TIMP2,LCN1,CENPE,ITIH1,AHSG,CSF1R,IGF2BP1,SERPINI1,CAMP,A2ML1,HSP90B1,ANTXR1,SH3D19,PCSK9,F2,SERPINF2,THBS4,BLM,AKAP9,TAF9,TNRC6A,ILK,GAPDH,CST3,IGF2,ITIH3,C4B,PAEP,SERPINA3,SERPINA4,KNG1,ACTB,FGA
GO Process	GO:0051248	Negative regulation of protein metabolic process	22	1038	0.52	0.00021	TFPI2,C5,C3,TIMP2,LCN1,ITIH1,AHSG,IGF2BP1,SERPINI1,A2ML1,F2,SERPINF2,TAF9,TNRC6A,GAPDH,CST3,ITIH3,C4B,SERPINA3,SERPINA4,KNG1,FGA
GO Process	GO:0016485	Protein processing	10	202	0.89	0.00024	F9,IHH,PCSK9,FGB,F2,FGG,ADAMTS13,F11,C1R,FGA
GO Process	GO:0032101	Regulation of response to external stimulus	21	964	0.53	0.00024	ST6GAL1,LGALS1,C5,PROC,APOA1,C3,FCN3,AHSG,CSF1R,MASP1,FGB,F2,PLG,SERPINF2,FGG,THBS4,MASP2,SAA1,F11,KNG1,FGA
GO Process	GO:0048583	Regulation of response to stimulus	49	3931	0.29	0.00024	ST6GAL1,LGALS1,C5,APOC3,GPLD1,PROC,PLEK,APOA1,CAT,C3,PXDN,PSG9,FCN3,AHSG,CRH,CSF1R,IHH,MASP1,GUCY1A1,PCSK9,SHLD1,FGB,F2,PLG,SERPINF2,FGG,THBS4,AKAP9,C8A,CFHR4,CFHR3,C1QC,TAF9,ILK,GAPDH,MASP2,SAA1,F11,C1S,IGF2,CDK3,C4B,UIMC1,C1R,IGHV3-15,CCL18,KNG1,ACTB,FGA
GO Process	GO:0050778	Positive regulation of immune response	15	502	0.67	0.00024	C5,GPLD1,C3,FCN3,MASP1,SHLD1,C8A,CFHR4,CFHR3,C1QC,MASP2,C1S,C4B,C1R,IGHV3-15
GO Process	GO:0006508	Proteolysis	24	1247	0.47	0.00031	F9,PROC,C3,LCN1,FCN3,IHH,MASP1,HSP90B1,PCSK9,FGB,F2,PLG,UBE3C,FGG,CNDP1,ADAMTS13,TAF9,MASP2,F11,C1S,PCYOX1,UIMC1,C1R,FGA
GO Process	GO:0032102	Negative regulation of response to external stimulus	13	387	0.72	0.00035	ST6GAL1,C5,PROC,APOA1,FGB,F2,PLG,SERPINF2,FGG,SAA1,F11,KNG1,FGA
GO Process	GO:0031639	Plasminogen activation	4	11	1.75	0.00040	FGB,FGG,F11,FGA
GO Process	GO:0001906	Cell killing	8	129	0.98	0.00054	C3,LYZ,PPBP,F2,TUBB,GAPDH,CCL18,KNG1
GO Process	GO:1900024	Regulation of substrate adhesion-dependent cell spreading	6	61	1.18	0.00084	ST6GAL1,APOA1,FGB,FGG,ILK,FGA
GO Process	GO:0002682	Regulation of immune system process	25	1438	0.43	0.0010	LGALS1,EBI3,C5,GPLD1,APOA1,C3,PSG9,FCN3,CSF1R,IHH,MASP1,SHLD1,THBS4,C8A,CFHR4,CFHR3,C1QC,CD99,MASP2,C1S,IGF2,C4B,C1R,IGHV3-15,ACTB
GO Process	GO:0065008	Regulation of biological quality	45	3654	0.28	0.0010	VCL,F9,TFPI2,APOC3,GPLD1,PROC,PLEK,APOA1,TTR,LYZ,LCN1,LCAT,CRH,MYL9,CSF1R,IGF2BP1,IHH,GUCY1A1,HSP90B1,PCSK9,FGB,F2,PLG,SERPINF2,OIT3,FGG,TUBB,AKAP9,F13B,ADAMTS13,APOM,TAF9,SOD3,NAV2,ILK,GAPDH,RAPGEF4,SAA1,F11,ATP9B,LMOD3,SERPINA3,KNG1,ACTB,FGA
GO Process	GO:0031589	Cell-substrate adhesion	9	195	0.85	0.0012	VCL,ATRN,ANTXR1,FGB,FGG,ADAMTS13,ACTN1,ILK,FGA
GO Process	GO:0034375	High-density lipoprotein particle remodeling	4	16	1.59	0.0013	APOC3,APOA1,LCAT,APOM
GO Process	GO:0043691	Reverse cholesterol transport	4	18	1.54	0.0019	APOC3,APOA1,LCAT,APOM
GO Process	GO:0051917	Regulation of fibrinolysis	4	18	1.54	0.0019	F2,PLG,SERPINF2,F11
GO Process	GO:0098609	Cell-cell adhesion	14	542	0.6	0.0023	VCL,LGALS1,PLEK,CDH6,MYL9,IHH,FGB,PSG11,FGG,THBS4,CD99,ILK,ACTB,FGA
GO Process	GO:0050790	Regulation of catalytic activity	33	2370	0.33	0.0024	TFPI2,C5,APOC3,GPLD1,PLEK,APOA1,C3,TIMP2,LCN1,CENPE,ITIH1,AHSG,CSF1R,SERPINI1,A2ML1,HSP90B1,ANTXR1,F2,SERPINF2,BLM,AKAP9,GAPDH,RAPGEF4,CST3,NUCB1,IGF2,ITIH3,C4B,SERPINA3,SERPINA4,CCL18,KNG1,ACTB
GO Process	GO:0050789	Regulation of biological process	98	11655	0.12	0.0026	ST6GAL1,VCL,LGALS1,TPD52L2,EBI3,TFPI2,C5,APOC3,MGP,GPLD1,PROC,PLEK,APOA1,TTR,CAT,C3,PXDN,TIMP2,ATRN,LCN1,LCAT,CDH6,CENPE,HYAL1,LUM,PSG9,FCN3,ITIH1,AHSG,ANKRD31,CRH,MYL9,CSF1R,IGF2BP1,IHH,SERPINI1,PPBP,MASP1,CAMP,GUCY1A1,A2ML1,HSP90B1,ANTXR1,SH3D19,PCSK9,SHLD1,FGB,DST,F2,PLG,STAB1,SERPINF2,FGG,TUBB,THBS4,BLM,AKAP9,MTCL1,C8A,CFHR4,CFHR3,MCOLN2,ADAMTS13,CCN5,C1QC,APOM,TAF9,CD99,ACTN1,TNRC6A,ILK,GAPDH,RAPGEF4,CST3,MASP2,SAA1,F11,C1S,NUCB1,IGF2,SLAIN1,CDK3,ABCA13,LMOD3,ITIH3,C4B,TGFBI,IGFALS,PAEP,UIMC1,C1R,SERPINA3,SERPINA4,IGHV3-15,CCL18,KNG1,ACTB,FGA
GO Process	GO:0060627	Regulation of vesicle-mediated transport	14	551	0.6	0.0026	APOC3,APOA1,C3,FCN3,AHSG,MASP1,PCSK9,FGB,FGG,RAPGEF4,MASP2,ABCA13,C4B,FGA
GO Process	GO:1900026	Positive regulation of substrate adhesion-dependent cell spreading	5	45	1.24	0.0027	APOA1,FGB,FGG,ILK,FGA

GO Process	GO:0045785	Positive regulation of cell adhesion	13	485	0.62	0.0031	LGALS1,EBI3,APOA1,HYAL1,IHH,FGB,SERPINF2,FGG,ILK,SAA1,IGF2,ACTB,FGA
GO Process	GO:0042157	Lipoprotein metabolic process	7	123	0.95	0.0033	APOC3,GPLD1,APOA1,LCAT,PCSK9,APOL1,APOM
GO Process	GO:0051050	Positive regulation of transport	18	915	0.48	0.0044	GPLD1,APOA1,C3,FCN3,AHSG,CRH,MASP1,PCSK9,FGB,F2,FGG,AKAP9,MTCL1,MASP2,ABCA13,C4B,ACTB,FGA
GO Process	GO:0034114	Regulation of heterotypic cell-cell adhesion	4	24	1.41	0.0045	APOA1,FGB,FGG,FGA
GO Process	GO:0042742	Defense response to bacterium	10	306	0.7	0.0057	LYZ,PPBP,CAMP,FGB,F2,STAB1,RNASE1,RNASE4,IGHV3-15,FGA
GO Process	GO:0007160	Cell-matrix adhesion	7	136	0.9	0.0058	VCL,FGB,FGG,ADAMTS13,ACTN1,ILK,FGA
GO Process	GO:0097746	Blood vessel diameter maintenance	7	140	0.89	0.0068	GUCY1A1,FGB,SERPINF2,FGG,SOD3,KNG1,FGA
GO Process	GO:0002752	Cell surface pattern recognition receptor signaling pathway	3	9	1.71	0.0079	FCN3,MASP1,MASP2
GO Process	GO:0051873	Killing by host of symbiont cells	4	29	1.33	0.0083	CAMP,F2,APOL1,GAPDH
GO Process	GO:0001867	Complement activation, lectin pathway	3	10	1.67	0.0098	FCN3,MASP1,MASP2
GO Process	GO:0051702	Biological process involved in interaction with symbiont	6	108	0.94	0.0128	CSF1R,CAMP,F2,PLG,APOL1,GAPDH
GO Process	GO:0001817	Regulation of cytokine production	15	739	0.5	0.0133	EBI3,C5,APOA1,C3,LUM,PSG9,CSF1R,IGF2BP1,CAMP,F2,SERPINF2,MCOLN2,GAPDH,SAA1,PAEP
GO Process	GO:0051240	Positive regulation of multicellular organismal process	23	1505	0.37	0.0133	EBI3,C5,GPLD1,C3,HYAL1,LUM,CRH,CSF1R,IHH,CAMP,SHLD1,FGB,F2,PLG,SERPINF2,FGG,MCOLN2,GAPDH,SAA1,IGF2,PAEP,ACTB,FGA
GO Process	GO:0065009	Regulation of molecular function	37	3085	0.27	0.0133	TFPI2,C5,APOC3,GPLD1,PLEK,APOA1,CAT,C3,PXDN,TIMP2,LCN1,CENPE,ITIH1,AHSG,CRH,CSF1R,SERPINI1,A2ML1,HSP90B1,ANTXR1,PCSK9,F2,SERPINF2,BLM,AKAP9,GAPDH,RAPGEF4,CST3,NUCB1,IGF2,ITIH3,C4B,SERPINA3,SERPINA4,CCL18,KNG1,ACTB
GO Process	GO:0010810	Regulation of cell-substrate adhesion	8	217	0.76	0.0136	ST6GAL1,VCL,APOA1,FGB,PLG,FGG,ILK,FGA
GO Process	GO:0051838	Cytolysis by host of symbiont cells	3	12	1.59	0.0146	CAMP,F2,APOL1
GO Process	GO:0061844	Antimicrobial humoral immune response mediated by antimicrobial peptide	6	113	0.92	0.0154	PPBP,CAMP,F2,GAPDH,CCL18,KNG1
GO Process	GO:0050776	Regulation of immune response	16	844	0.47	0.0160	C5,GPLD1,C3,PSG9,FCN3,MASP1,SHLD1,C8A,CFHR4,CFHR3,C1QC,MASP2,C1S,C4B,C1R,IGHV3-15
GO Process	GO:0031640	Killing of cells of another organism	5	71	1.04	0.0164	LYZ,PPBP,GAPDH,CCL18,KNG1
GO Process	GO:0051918	Negative regulation of fibrinolysis	3	13	1.55	0.0172	F2,PLG,SERPINF2
GO Process	GO:0010896	Regulation of triglyceride catabolic process	3	14	1.52	0.0207	APOC3,GPLD1,APOA1
GO Process	GO:0001775	Cell activation	14	693	0.5	0.0223	VCL,LGALS1,EBI3,PLEK,MYL9,CAMP,FGB,F2,FGG,ADAMTS13,ILK,SAA1,ACTB,FGA
GO Process	GO:0090207	Regulation of triglyceride metabolic process	4	40	1.19	0.0223	APOC3,GPLD1,APOA1,C3
GO Process	GO:0010712	Regulation of collagen metabolic process	4	41	1.18	0.0241	IHH,F2,SERPINF2,CST3
GO Process	GO:0034116	Positive regulation of heterotypic cell-cell adhesion	3	15	1.49	0.0241	FGB,FGG,FGA
GO Process	GO:0070555	Response to interleukin-1	6	125	0.87	0.0243	HYAL1,CAMP,FGB,FGG,TAF9,CCL18
GO Process	GO:0080134	Regulation of response to stress	21	1373	0.38	0.0246	LGALS1,PROC,APOA1,C3,FCN3,AHSG,MASP1,SHLD1,FGB,F2,PLG,SERPINF2,FGG,TAF9,MASP2,SAA1,F11,UIMC1,KNG1,ACTB,FGA
GO Process	GO:0022604	Regulation of cell morphogenesis	9	311	0.65	0.0264	ST6GAL1,APOA1,CSF1R,SH3D19,FGB,F2,FGG,ILK,FGA
GO Process	GO:0006957	Complement activation, alternative pathway	3	16	1.46	0.0272	C5,C3,C8A
GO Process	GO:0022409	Positive regulation of cell-cell adhesion	9	322	0.64	0.0329	LGALS1,EBI3,IHH,FGB,SERPINF2,FGG,IGF2,ACTB,FGA
GO Process	GO:0090136	Epithelial cell-cell adhesion	3	18	1.41	0.0359	VCL,IHH,THBS4
GO Process	GO:0022407	Regulation of cell-cell adhesion	11	480	0.55	0.0365	LGALS1,EBI3,APOA1,IHH,FGB,PLG,SERPINF2,FGG,IGF2,ACTB,FGA
GO Process	GO:0022603	Regulation of anatomical structure morphogenesis	16	920	0.43	0.0365	ST6GAL1,C5,APOA1,C3,HYAL1,CSF1R,CAMP,SH3D19,FGB,F2,STAB1,FGG,THBS4,ILK,CST3,FGA
GO Process	GO:0001819	Positive regulation of cytokine production	11	482	0.55	0.0375	EBI3,C5,C3,LUM,CSF1R,CAMP,SERPINF2,MCOLN2,GAPDH,SAA1,PAEP
GO Process	GO:0042632	Cholesterol homeostasis	5	89	0.94	0.0386	APOC3,APOA1,LCAT,PCSK9,APOM
GO Process	GO:0097435	Supramolecular fiber organization	12	570	0.51	0.0402	PXDN,LUM,MYL9,HSP90B1,DST,SERPINF2,AKAP9,ACTN1,CST3,SLAIN1,LMOD3,TPM4
GO Process	GO:0007010	Cytoskeleton organization	19	1229	0.38	0.0405	PLEK,DNAH5,CENPE,MYL9,HSP90B1,ANTXR1,SH3D19,DST,DNAH8,TUBB,AKAP9,MTCL1,ACTN1,GAPDH,SLAIN1,FSIP2,LMOD3,TPM4,ACTB
GO Process	GO:0009617	Response to bacterium	13	663	0.48	0.0437	C3,LYZ,PPBP,CAMP,FGB,F2,STAB1,ADAMTS13,RNASE1,C4B,RNASE4,IGHV3-15,FGA
GO Process	GO:0048522	Positive regulation of cellular process	54	5584	0.18	0.0492	ST6GAL1,LGALS1,EBI3,GPLD1,PROC,PLEK,APOA1,CAT,C3,CENPE,HYAL1,LUM,FCN3,ANKRD31,CRH,CSF1R,IGF2BP1,IHH,SERPINI1,PPBP,MASP1,CAMP,GUCY1A1,SH3D19,PCSK9,SHLD1,FGB,F2,PLG,SERPINF2,FGG,THBS4,BLM,AKAP9,MTCL1,TAF9,CD99,ACTN1,TNRC6A,ILK,GAPDH,MASP2,SAA1,IGF2,SLAIN1,ABCA13,LMOD3,C4B,UIMC1,IGHV3-15,CCL18,KNG1,ACTB,FGA
GO Function	GO:0004866	Endopeptidase inhibitor activity	17	177	1.17	3.50e-11	TFPI2,C5,C3,TIMP2,LCN1,ITIH1,AHSG,SERPINI1,A2ML1,SERPINF2,GAPDH,CST3,ITIH3,C4B,SERPINA3,SERPINA4,KNG1
GO Function	GO:0004857	Enzyme inhibitor activity	18	396	0.85	1.58e-07	TFPI2,C5,APOC3,C3,TIMP2,LCN1,ITIH1,AHSG,SERPINI1,A2ML1,SERPINF2,GAPDH,CST3,ITIH3,C4B,SERPINA3,SERPINA4,KNG1
GO Function	GO:0005509	Calcium ion binding	22	717	0.68	1.39e-06	F9,MGP,PROC,CDH6,ITIH1,MYL9,IHH,MASP1,HSP90B1,DST,F2,STAB1,OIT3,THBS4,CRTAC1,ADAMTS13,ACTN1,MASP2,C1S,NUCB1,C1R,TPM4
GO Function	GO:0005102	Signaling receptor binding	31	1499	0.51	4.31e-06	EBI3,C5,APOC3,APOA1,TTR,C3,PXDN,LCN1,CRH,IHH,PPBP,HSP90B1,PCSK9,FGB,DST,F2,PLG,FGG,TUBB,THBS4,AKAP9,ADAMTS13,CCN5,ACTN1,SAA1,IGF2,TGFBI,IGHV3-15,CCL18,KNG1,FGA
GO Function	GO:0005515	Protein binding	76	7242	0.21	7.45e-05	ST6GAL1,VCL,LGALS1,TPD52L2,EBI3,C5,APOC3,PLEK,APOA1,TTR,CAT,C3,PXDN,LYZ,TIMP2,LCN1,LCAT,CDH6,DNAH5,CENPE,PSG9,FCN3,CRH,MYL9,CSF1R,IHH,PPBP,MASP1,A2ML1,HSP90B1,ANTXR1,SH3D19,PCSK9,FGB,DST,F2,PLG,SERPINF2,DNAH8,FGG,TUBB,THBS4,BLM,AKAP9,MTCL1,C8A,CFHR4,CFHR3,MCOLN2,ADAMTS13,CCN5,HLA-C,TAF9,ACTN1,ILK,GAPDH,RAPGEF4,CST3,MASP2,SAA1,F11,C1S,NUCB1,IGF2,LMOD3,C4B,TGFBI,IGFALS,UIMC1,C1R,IGHV3-15,CCL18,KNG1,TPM4,ACTB,FGA
GO Function	GO:0098772	Molecular function regulator activity	33	1960	0.42	0.00013	EBI3,TFPI2,C5,APOC3,GPLD1,APOA1,TTR,C3,PXDN,TIMP2,LCN1,ITIH1,AHSG,CRH,SERPINI1,PPBP,A2ML1,PCSK9,F2,SERPINF2,THBS4,AKAP9,GAPDH,RAPGEF4,CST3,NUCB1,IGF2,ITIH3,C4B,SERPINA3,SERPINA4,CCL18,KNG1
GO Function	GO:0004252	Serine-type endopeptidase activity	10	176	0.95	0.00015	F9,PROC,MASP1,PCSK9,F2,PLG,MASP2,F11,C1S,C1R
GO Function	GO:0005201	Extracellular matrix structural constituent	9	131	1.03	0.00015	TFPI2,MGP,PXDN,LUM,FGB,OIT3,FGG,TGFBI,FGA
GO Function	GO:0004867	Serine-type endopeptidase inhibitor activity	8	98	1.1	0.00017	TFPI2,ITIH1,SERPINI1,A2ML1,SERPINF2,ITIH3,SERPINA3,SERPINA4
GO Function	GO:0001848	Complement binding	5	26	1.47	0.00049	C8A,CFHR4,CFHR3,MASP2,C4B
GO Function	GO:0005539	Glycosaminoglycan binding	10	245	0.8	0.0018	ITIH1,F2,STAB1,THBS4,CCN5,SOD3,NAV2,SAA1,F11,KNG1
GO Function	GO:0005488	Binding	105	12838	0.1	0.0031	ST6GAL1,VCL,LGALS1,TPD52L2,F9,EBI3,C5,APOC3,MGP,PROC,PLEK,APOA1,TTR,CAT,C3,PXDN,LYZ,TIMP2,ATRN,LCN1,LCAT,CDH6,DNAH5,CENPE,LUM,PSG9,FCN3,ITIH1,CRH,MYL9,CSF1R,IGF2BP1,IHH,PPBP,MASP1,CAMP,GUCY1A1,A2ML1,HSP90B1,ANTXR1,SH3D19,PCSK9,FGB,DST,F2,PLG,STAB1,APOL1,SERPINF2,DNAH8,OIT3,FGG,TUBB,THBS4,GANAB,BLM,AKAP9,CNDP1,MTCL1,C8A,CFHR4,CFHR3,CRTAC1,MCOLN2,ADAMTS13,CCN5,APOM,HLA-C,TAF9,SOD3,ACTN1,TNRC6A,NAV2,ILK,GAPDH,RAPGEF4,BPIFC,RNASE1,CST3,MASP2,SAA1,F11,C1S,NUCB1,PCYOX1,IGF2,ATP9B,CDK3,ABCA13,LMOD3,C4B,TGFBI,IGFALS,PAEP,UIMC1,C1R,SERPINA3,RNASE4,IGHV3-15,CCL18,KNG1,TPM4,ACTB,TRIM66,FGA

GO Function	GO:0030234	Enzyme regulator activity	22	1239	0.44	0.0043	TFPI2,C5,APOC3,APOA1,C3,TIMP2,LCN1,ITIH1,AHSG,SERPINI1,A2ML1,SERPINF2,GAPDH,RAPGEF4,CST3,NUCB1,IGF2,ITIH3,C4B,SERPINA3,SERPINA4,KNG1
GO Function	GO:0008201	Heparin binding	8	173	0.86	0.0057	F2,THBS4,CCN5,SOD3,NAV2,SAA1,F11,KNG1
GO Function	GO:0005198	Structural molecule activity	16	776	0.5	0.0116	VCL,TFPI2,MGP,PXDN,LUM,MYL9,FGB,DST,OIT3,FGG,TUBB,ACTN1,TGFBI,TPM4,ACTB,FGA
GO Function	GO:0070325	Lipoprotein particle receptor binding	4	30	1.32	0.0151	APOC3,APOA1,HSP90B1,PCSK9
GO Function	GO:0005178	Integrin binding	7	159	0.83	0.0219	DST,THBS4,ADAMTS13,CCN5,ACTN1,IGF2,TGFBI
GO Function	GO:0004175	Endopeptidase activity	11	437	0.59	0.0315	F9,PROC,MASP1,PCSK9,F2,PLG,ADAMTS13,MASP2,F11,C1S,C1R
GO Function	GO:0008233	Peptidase activity	13	617	0.51	0.0422	F9,PROC,IHH,MASP1,PCSK9,F2,PLG,CNDP1,ADAMTS13,MASP2,F11,C1S,C1R
GO Component	GO:0005615	Extracellular space	86	3247	0.61	5.27e-34	VCL,LGALS1,F9,EBI3,TFPI2,C5,APOC3,MGP,GPLD1,PROC,APOA1,TTR,CAT,C3,PXDN,LYZ,TIMP2,ATRN,LCN1,LCAT,HYAL1,LUM,FCN3,ITIH1,AHSG,CRH,SAA4,IHH,SERPINI1,PPBP,MASP1,CAMP,A2ML1,HSP90B1,PCSK9,FGB,F2,PLG,APOL1,SERPINF2,OIT3,FGG,TUBB,THBS4,GANAB,FAM184A,MTCL1,C8A,F13B,CFHR4,CFHR3,CRTAC1,ADAMTS13,CCN5,C1QC,APOM,HLA-C,SOD3,ACTN1,GAPDH,BPIFC,RNASE1,CST3,MASP2,SAA1,F11,C1S,NUCB1,PCYOX1,IGF2,ITIH3,C4B,TGFBI,IGFALS,PAEP,SBSN,C1R,SERPINA3,SERPINA4,RNASE4,IGHV3-15,CCL18,KNG1,TPM4,ACTB,FGA
GO Component	GO:0005576	Extracellular region	93	4175	0.54	1.29e-32	ST6GAL1,VCL,LGALS1,F9,EBI3,TFPI2,C5,APOC3,MGP,GPLD1,PROC,PLEK,APOA1,TTR,CAT,C3,PXDN,LYZ,TIMP2,ATRN,LCN1,LCAT,DNAH5,CENPE,HYAL1,LUM,PSG9,FCN3,ITIH1,AHSG,CRH,SAA4,IHH,SERPINI1,PPBP,MASP1,CAMP,A2ML1,HSP90B1,PCSK9,FGB,F2,PLG,APOL1,PSG11,SERPINF2,OIT3,FGG,TUBB,THBS4,GANAB,FAM184A,CNDP1,MTCL1,C8A,F13B,CFHR4,CFHR3,CRTAC1,ADAMTS13,CCN5,C1QC,APOM,HLA-C,SOD3,ACTN1,GAPDH,BPIFC,RNASE1,CST3,MASP2,SAA1,F11,C1S,NUCB1,PCYOX1,IGF2,ITIH3,C4B,TGFBI,IGFALS,PAEP,SBSN,C1R,SERPINA3,SERPINA4,RNASE4,IGHV3-15,CCL18,KNG1,TPM4,ACTB,FGA
GO Component	GO:0070062	Extracellular exosome	58	2096	0.63	1.57e-20	VCL,LGALS1,F9,C5,APOC3,MGP,GPLD1,APOA1,TTR,CAT,C3,PXDN,LYZ,ATRN,LCAT,HYAL1,LUM,ITIH1,AHSG,SAA4,SERPINI1,CAMP,A2ML1,HSP90B1,FGB,F2,PLG,SERPINF2,FGG,TUBB,THBS4,GANAB,C8A,CFHR3,CRTAC1,HLA-C,SOD3,ACTN1,GAPDH,RNASE1,CST3,MASP2,SAA1,F11,NUCB1,PCYOX1,ITIH3,C4B,TGFBI,IGFALS,SBSN,C1R,SERPINA3,SERPINA4,KNG1,TPM4,ACTB,FGA
GO Component	GO:0072562	Blood microparticle	21	118	1.44	1.71e-20	APOA1,C3,FCN3,ITIH1,AHSG,FGB,F2,PLG,APOL1,SERPINF2,FGG,C8A,CFHR3,C1QC,C1S,C4B,C1R,SERPINA3,KNG1,ACTB,FGA
GO Component	GO:0031012	Extracellular matrix	33	552	0.97	8.13e-20	LGALS1,F9,TFPI2,APOC3,MGP,GPLD1,APOA1,PXDN,TIMP2,ATRN,LUM,FCN3,ITIH1,AHSG,IHH,HSP90B1,FGB,DST,F2,PLG,SERPINF2,FGG,THBS4,ADAMTS13,CCN5,C1QC,SOD3,NAV2,TGFBI,IGFALS,SERPINA3,KNG1,FGA
GO Component	GO:0062023	Collagen-containing extracellular matrix	27	407	1.01	5.60e-17	LGALS1,F9,APOC3,MGP,APOA1,PXDN,TIMP2,ATRN,LUM,FCN3,ITIH1,AHSG,HSP90B1,FGB,DST,F2,PLG,SERPINF2,FGG,THBS4,C1QC,SOD3,NAV2,TGFBI,SERPINA3,KNG1,FGA
GO Component	GO:0034774	Secretory granule lumen	23	321	1.05	6.29e-15	VCL,APOA1,TTR,CAT,C3,LYZ,TIMP2,AHSG,SERPINI1,PPBP,CAMP,FGB,PLG,SERPINF2,FGG,TUBB,ACTN1,IGF2,ITIH3,SERPINA3,SERPINA4,KNG1,FGA
GO Component	GO:0031982	Vesicle	68	3957	0.43	1.55e-14	VCL,LGALS1,F9,C5,APOC3,MGP,GPLD1,APOA1,TTR,CAT,C3,PXDN,LYZ,TIMP2,ATRN,LCAT,HYAL1,LUM,ITIH1,AHSG,SAA4,SERPINI1,PPBP,CAMP,A2ML1,HSP90B1,ANTXR1,PCSK9,FGB,DST,F2,PLG,STAB1,SERPINF2,FGG,TUBB,THBS4,GANAB,C8A,CFHR3,CRTAC1,MCOLN2,HLA-C,SOD3,ACTN1,GAPDH,RNASE1,CST3,MASP2,SAA1,F11,NUCB1,PCYOX1,IGF2,ATP9B,ABCA13,ITIH3,C4B,TGFBI,IGFALS,SBSN,C1R,SERPINA3,SERPINA4,KNG1,TPM4,ACTB,FGA
GO Component	GO:0031093	Platelet alpha granule lumen	11	66	1.41	3.06e-10	AHSG,PPBP,FGB,PLG,SERPINF2,FGG,ACTN1,IGF2,SERPINA3,KNG1,FGA
GO Component	GO:0005788	Endoplasmic reticulum lumen	18	312	0.95	4.89e-10	LGALS1,F9,EBI3,PROC,APOA1,C3,AHSG,HSP90B1,PCSK9,F2,APOL1,FGG,GANAB,ADAMTS13,CST3,NUCB1,KNG1,FGA
GO Component	GO:1905370	Serine-type endopeptidase complex	8	26	1.68	4.31e-09	F9,PROC,C3,FCN3,MASP1,F2,MASP2,F11
GO Component	GO:0030141	Secretory granule	26	873	0.66	8.44e-09	VCL,APOA1,TTR,CAT,C3,LYZ,TIMP2,AHSG,SERPINI1,PPBP,CAMP,FGB,PLG,SERPINF2,FGG,TUBB,HLA-C,ACTN1,CST3,IGF2,ABCA13,ITIH3,SERPINA3,SERPINA4,KNG1,FGA
GO Component	GO:0034358	Plasma lipoprotein particle	8	38	1.51	4.71e-08	APOC3,APOA1,LCAT,SAA4,APOL1,APOM,SAA1,PCYOX1
GO Component	GO:0034364	High-density lipoprotein particle	7	29	1.57	2.29e-07	APOC3,APOA1,LCAT,SAA4,APOL1,APOM,SAA1
GO Component	GO:1905368	Peptidase complex	10	138	1.05	3.09e-06	F9,PROC,C3,FCN3,MASP1,F2,UBE3C,TAF9,MASP2,F11
GO Component	GO:1905369	Endopeptidase complex	9	104	1.13	3.52e-06	F9,PROC,C3,FCN3,MASP1,F2,UBE3C,MASP2,F11
GO Component	GO:0071944	Cell periphery	68	6015	0.24	4.82e-06	VCL,LGALS1,F9,EBI3,TFPI2,C5,APOC3,MGP,GPLD1,PLEK,APOA1,CAT,C3,PXDN,TIMP2,ATRN,CDH6,LUM,PSG9,FCN3,ITIH1,AHSG,MYL9,CSF1R,IHH,MASP1,HSP90B1,ANTXR1,SH3D19,PCSK9,FGB,DST,F2,PLG,STAB1,SERPINF2,FGG,THBS4,AKAP9,MTCL1,C8A,MCOLN2,ADAMTS13,CCN5,C1QC,HLA-C,CD99,SOD3,ACTN1,NAV2,ILK,GAPDH,RAPGEF4,CST3,MASP2,F11,PCYOX1,ATP9B,ABCA13,C4B,TGFBI,IGFALS,SERPINA3,IGHV3-15,KNG1,TPM4,ACTB,FGA
GO Component	GO:0034361	Very-low-density lipoprotein particle	5	20	1.59	3.37e-05	APOC3,APOA1,APOL1,APOM,PCYOX1
GO Component	GO:0031410	Cytoplasmic vesicle	37	2482	0.36	4.59e-05	VCL,APOC3,APOA1,TTR,CAT,C3,LYZ,TIMP2,HYAL1,AHSG,SERPINI1,PPBP,CAMP,HSP90B1,ANTXR1,PCSK9,FGB,DST,PLG,STAB1,SERPINF2,FGG,TUBB,GANAB,MCOLN2,HLA-C,ACTN1,CST3,SAA1,IGF2,ATP9B,ABCA13,ITIH3,SERPINA3,SERPINA4,KNG1,FGA
GO Component	GO:0005577	Fibrinogen complex	4	8	1.89	4.74e-05	FGB,SERPINF2,FGG,FGA
GO Component	GO:0009986	Cell surface	20	894	0.54	8.65e-05	EBI3,C3,PXDN,PSG9,FCN3,CSF1R,MASP1,ANTXR1,PCSK9,FGB,PLG,PSG11,SERPINF2,OIT3,FGG,ADAMTS13,HLA-C,MASP2,IGHV3-15,FGA
GO Component	GO:0005602	Complement component C1 complex	3	5	1.97	0.00082	C1QC,C1S,C1R
GO Component	GO:0032991	Protein-containing complex	58	5506	0.21	0.00096	VCL,LGALS1,F9,EBI3,C5,APOC3,PROC,APOA1,TTR,CAT,C3,LCAT,CDH6,DNAH5,LUM,PSG9,FCN3,SAA4,MYL9,CSF1R,IGF2BP1,MASP1,GUCY1A1,HSP90B1,PCSK9,FGB,F2,UBE3C,APOL1,SERPINF2,DNAH8,FGG,TUBB,GANAB,BLM,AKAP9,C8A,F13B,C1QC,APOM,HLA-C,TAF9,TNRC6A,GAPDH,MASP2,SAA1,F11,C1S,PCYOX1,CDK3,C4B,IGFALS,UIMC1,C1R,IGHV3-15,TPM4,ACTB,FGA
GO Component	GO:0012505	Endomembrane system	52	4721	0.23	0.0010	ST6GAL1,VCL,LGALS1,F9,EBI3,APOC3,PROC,APOA1,TTR,CAT,C3,PXDN,LYZ,TIMP2,LUM,AHSG,SERPINI1,PPBP,CAMP,HSP90B1,ANTXR1,PCSK9,FGB,DST,F2,PLG,APOL1,SERPINF2,OIT3,FGG,TUBB,THBS4,GANAB,AKAP9,MCOLN2,ADAMTS13,HLA-C,SOD3,ACTN1,TNRC6A,GAPDH,CST3,NUCB1,IGF2,ATP9B,ABCA13,ITIH3,TGFBI,SERPINA3,SERPINA4,KNG1,FGA
GO Component	GO:0034366	Spherical high-density lipoprotein particle	3	8	1.76	0.0022	APOC3,APOA1,APOM
GO Component	GO:1904724	Tertiary granule lumen	5	55	1.15	0.0022	LYZ,TIMP2,PPBP,CAMP,CST3
GO Component	GO:0009897	External side of plasma membrane	11	388	0.64	0.0027	EBI3,FCN3,MASP1,ANTXR1,PCSK9,FGB,PLG,FGG,MASP2,IGHV3-15,FGA
GO Component	GO:0005775	Vacuolar lumen	7	175	0.79	0.0086	TTR,C3,LYZ,HYAL1,LUM,TUBB,SERPINA3
GO Component	GO:0035578	Azurophil granule lumen	5	91	0.93	0.0186	TTR,C3,LYZ,TUBB,SERPINA3
GO Component	GO:0005604	Basement membrane	5	98	0.9	0.0252	PXDN,ATRN,DST,THBS4,TGFBI
GO Component	GO:0042582	Azurophil granule	6	154	0.78	0.0271	TTR,C3,LYZ,TUBB,ABCA13,SERPINA3
GO Component	GO:0071682	Endocytic vesicle lumen	3	23	1.31	0.0271	APOA1,HSP90B1,SAA1

GO Component	GO:0098552	Side of membrane	12	611	0.48	0.0291	EBI3,FCN3,MASP1,ANTXR1,PCSK9,FGB,PLG,FGG,HLA-C,MASP2,IGHV3-15,FGA
GO Component	GO:0005796	Golgi lumen	5	106	0.86	0.0317	F9,PROC,LUM,F2,SOD3
GO Component	GO:0035580	Specific granule lumen	4	62	1.0	0.0356	VCL,LYZ,TIMP2,CAMP
GO Component	GO:0030016	Myofibril	7	237	0.66	0.0411	VCL,MYL9,DST,ACTN1,ILK,LMOD3,TPM4
GO Component	GO:0005783	Endoplasmic reticulum	25	2021	0.28	0.0455	ST6GAL1,LGALS1,F9,EBI3,PROC,APOA1,CAT,C3,PXDN,AHSG,HSP90B1,PCSK9,FGB,DST,F2,APOL1,FGG,THBS4,GANAB,ADAMTS13,HLA-C,CST3,NUCB1,KNG1,FGA
GO Component	GO:0005764	Lysosome	13	746	0.43	0.0463	GPLD1,TTR,CAT,C3,LYZ,HYAL1,LUM,PCSK9,TUBB,MCOLN2,PCYOX1,ABCA13,SERPINA3
GO Component	GO:0070013	Intracellular organelle lumen	53	5660	0.16	0.0463	VCL,LGALS1,F9,EBI3,PROC,APOA1,TTR,CAT,C3,LYZ,TIMP2,CDH6,HYAL1,LUM,AHSG,CSF1R,IGF2BP1,SERPINI1,PPBP,MASP1,CAMP,HSP90B1,SH3D19,PCSK9,FGB,DST,F2,PLG,APOL1,SERPINF2,FGG,TUBB,GANAB,BLM,ADAMTS13,TAF9,SOD3,ACTN1,TNRC6A,NAV2,ILK,CST3,SAA1,NUCB1,IGF2,ITIH3,UIMC1,SERPINA3,SERPINA4,KNG1,ACTB,TRIM66,FGA
STRING clusters	CL:18726	Complement and coagulation cascades, and Protein-lipid complex	36	161	1.54	3.60e-39	F9,C5,APOC3,PROC,APOA1,C3,LCAT,FCN3,ITIH1,AHSG,SAA4,MASP1,PCSK9,FGB,F2,PLG,APOL1,SERPINF2,FGG,C8A,F13B,CFHR4,CFHR3,ADAMTS13,C1QC,APOM,MASP2,SAA1,F11,C1S,PCYOX1,ITIH3,C4B,C1R,SERPINA4,FGA
STRING clusters	CL:18723	Mixed, incl. Complement and coagulation cascades, and Protein-lipid complex	37	196	1.47	1.39e-38	F9,C5,APOC3,PROC,APOA1,C3,LCAT,FCN3,ITIH1,AHSG,SAA4,MASP1,PCSK9,FGB,F2,PLG,STAB1,APOL1,SERPINF2,FGG,C8A,F13B,CFHR4,CFHR3,ADAMTS13,C1QC,APOM,MASP2,SAA1,F11,C1S,PCYOX1,ITIH3,C4B,C1R,SERPINA4,FGA
STRING clusters	CL:18728	Complement and coagulation cascades, and Positive regulation of opsonization	27	109	1.58	3.87e-30	F9,C5,PROC,C3,FCN3,ITIH1,AHSG,MASP1,FGB,F2,PLG,SERPINF2,FGG,C8A,F13B,CFHR4,CFHR3,ADAMTS13,C1QC,MASP2,F11,C1S,ITIH3,C4B,C1R,SERPINA4,FGA
STRING clusters	CL:18730	Hemostasis, and Dissolution of Fibrin Clot	15	55	1.63	1.88e-16	F9,PROC,ITIH1,AHSG,FGB,F2,PLG,SERPINF2,FGG,F13B,ADAMTS13,F11,ITIH3,SERPINA4,FGA
STRING clusters	CL:18731	Hemostasis, and Dissolution of Fibrin Clot	14	50	1.64	1.96e-15	F9,PROC,ITIH1,AHSG,FGB,F2,PLG,SERPINF2,FGG,F13B,ADAMTS13,F11,ITIH3,FGA
STRING clusters	CL:18846	Complement cascade	12	50	1.57	2.35e-12	C5,C3,FCN3,MASP1,C8A,CFHR4,CFHR3,C1QC,MASP2,C1S,C4B,C1R
STRING clusters	CL:18733	Mixed, incl. COVID-19, thrombosis and anticoagulation, and Inter-alpha-trypsin inhibitor heavy chain C-terminus	9	21	1.82	7.96e-11	ITIH1,AHSG,FGB,PLG,SERPINF2,FGG,F13B,ITIH3,FGA
STRING clusters	CL:18848	Complement cascade	10	40	1.59	2.79e-10	C5,C3,FCN3,MASP1,C8A,C1QC,MASP2,C1S,C4B,C1R
STRING clusters	CL:18851	Initial triggering of complement, and Negative regulation of complement activation	8	24	1.71	7.38e-09	C3,FCN3,MASP1,C1QC,MASP2,C1S,C4B,C1R
STRING clusters	CL:18960	High-density lipoprotein particle	8	26	1.68	1.17e-08	APOC3,APOA1,LCAT,SAA4,APOL1,APOM,SAA1,PCYOX1
STRING clusters	CL:18956	Lipoprotein particle, and Assembly of active LPL and LIPC lipase complexes	9	46	1.48	2.06e-08	APOC3,APOA1,LCAT,SAA4,PCSK9,APOL1,APOM,SAA1,PCYOX1
STRING clusters	CL:18734	COVID-19, thrombosis and anticoagulation, and Negative regulation of fibrinolysis	6	11	1.93	1.82e-07	FGB,PLG,SERPINF2,FGG,F13B,FGA
STRING clusters	CL:18852	Creation of C4 and C2 activators	6	15	1.79	7.41e-07	FCN3,MASP1,C1QC,MASP2,C1S,C1R
STRING clusters	CL:18962	High-density lipoprotein particle	6	21	1.65	3.72e-06	APOC3,APOA1,LCAT,APOL1,APOM,PCYOX1
STRING clusters	CL:18784	Formation of Fibrin Clot (Clotting Cascade)	5	18	1.63	6.66e-05	F9,PROC,F2,ADAMTS13,F11
STRING clusters	CL:18737	Fibrinogen, and Thrombophilia	4	6	2.01	6.81e-05	FGB,PLG,FGG,FGA
STRING clusters	CL:18786	Hemophilia B, and Blood coagulation, common pathway	4	12	1.71	0.00052	F9,F2,ADAMTS13,F11
STRING clusters	CL:18966	High-density lipoprotein particle	4	16	1.59	0.0013	APOC3,APOA1,LCAT,APOM
STRING clusters	CL:18854	Cell surface pattern recognition receptor signaling pathway	3	6	1.89	0.0037	FCN3,MASP1,MASP2
STRING clusters	CL:18869	Complement component C1 complex	3	6	1.89	0.0037	C1QC,C1S,C1R
STRING clusters	CL:19115	Mixed, incl. Cystatin superfamily, and Specific granule lumen	5	60	1.11	0.0099	LGALS1,LYZ,LCN1,CAMP,CST3
STRING clusters	CL:18755	Mixed, incl. Inter-alpha-trypsin inhibitor heavy chain C-terminus, and Alpha-1-acid glycoprotein	3	10	1.67	0.0111	ITIH1,AHSG,ITIH3
STRING clusters	CL:18970	Triglyceride-rich lipoprotein particle remodeling, and Spherical high-density lipoprotein particle	3	11	1.63	0.0135	APOC3,APOA1,LCAT
KEGG	hsa04610	Complement and coagulation cascades	21	82	1.6	1.37e-23	F9,C5,PROC,C3,FGB,F2,PLG,SERPINF2,FGG,C8A,F13B,CFHR4,CFHR3,C1QC,MASP2,F11,C1S,C4B,C1R,KNG1,FGA
KEGG	hsa05150	Staphylococcus aureus infection	10	86	1.26	9.67e-08	C5,C3,CAMP,PLG,FGG,C1QC,MASP2,C1S,C4B,C1R
KEGG	hsa05322	Systemic lupus erythematosus	8	94	1.12	3.37e-05	C5,C3,C8A,C1QC,ACTN1,C1S,C4B,C1R
KEGG	hsa05133	Pertussis	6	73	1.11	0.00098	C5,C3,C1QC,C1S,C4B,C1R
KEGG	hsa04670	Leukocyte transendothelial migration	6	111	0.92	0.0072	VCL,MYL9,CD99,ACTN1,RAPGEF4,ACTB
KEGG	hsa04611	Platelet activation	6	122	0.88	0.0098	GUCY1A1,FGB,F2,FGG,ACTB,FGA
KEGG	hsa04145	Phagosome	6	141	0.82	0.0164	C3,TUBB,THBS4,HLA-C,C1R,ACTB
KEGG	hsa04979	Cholesterol metabolism	4	48	1.11	0.0164	APOC3,APOA1,LCAT,PCSK9
Reactome	HSA-381426	Regulation of Insulin-like Growth Factor (IGF) transport and uptake by Insulin-like Growth Factor Binding Proteins (IGFBPs)	17	124	1.33	7.12e-14	LGALS1,PROC,APOA1,C3,AHSG,HSP90B1,PCSK9,F2,PLG,APOL1,FGG,CST3,NUCB1,IGF2,IGFALS,KNG1,FGA
Reactome	HSA-109582	Hemostasis	28	607	0.85	4.91e-13	VCL,F9,PROC,PLEK,APOA1,CENPE,PSG9,AHSG,PPBP,GUCY1A1,FGB,F2,PLG,PSG11,SERPINF2,FGG,F13B,CD99,ACTN1,RAPGEF4,F11,IGF2,ITIH3,SERPINA3,SERPINA4,KNG1,ACTB,FGA
Reactome	HSA-114608	Platelet degranulation	16	126	1.29	6.40e-13	VCL,PLEK,APOA1,AHSG,PPBP,FGB,PLG,SERPINF2,FGG,ACTN1,IGF2,ITIH3,SERPINA3,SERPINA4,KNG1,FGA
Reactome	HSA-166658	Complement cascade	12	59	1.5	1.19e-11	C5,C3,FCN3,F2,C8A,CFHR4,CFHR3,C1QC,MASP2,C1S,C4B,C1R
Reactome	HSA-76002	Platelet activation, signaling and aggregation	18	260	1.03	7.99e-11	VCL,PLEK,APOA1,AHSG,PPBP,FGB,F2,PLG,SERPINF2,FGG,ACTN1,RAPGEF4,IGF2,ITIH3,SERPINA3,SERPINA4,KNG1,FGA
Reactome	HSA-168249	Innate Immune System	31	1041	0.66	2.62e-10	VCL,C5,TTR,CAT,C3,LYZ,TIMP2,FCN3,AHSG,PPBP,CAMP,HSP90B1,FGB,F2,FGG,TUBB,C8A,CFHR4,CFHR3,C1QC,HLA-C,CST3,MASP2,SAA1,C1S,ABCA13,C4B,C1R,SERPINA3,ACTB,FGA
Reactome	HSA-8957275	Post-translational protein phosphorylation	13	107	1.28	2.62e-10	LGALS1,PROC,APOA1,C3,AHSG,HSP90B1,PCSK9,APOL1,FGG,CST3,NUCB1,KNG1,FGA
Reactome	HSA-977606	Regulation of Complement cascade	10	49	1.5	9.92e-10	C5,C3,F2,C8A,CFHR4,CFHR3,C1QC,C1S,C4B,C1R
Reactome	HSA-140877	Formation of Fibrin Clot (Clotting Cascade)	9	39	1.55	4.23e-09	F9,PROC,FGB,F2,FGG,F13B,F11,KNG1,FGA
Reactome	HSA-166663	Initial triggering of complement	7	24	1.66	1.80e-07	C3,FCN3,C1QC,MASP2,C1S,C4B,C1R
Reactome	HSA-168256	Immune System	37	1979	0.46	3.89e-07	VCL,EBI3,C5,TTR,CAT,C3,LYZ,TIMP2,CENPE,FCN3,AHSG,CSF1R,PPBP,CAMP,HSP90B1,FGB,F2,UBE3C,FGG,TUBB,C8A,CFHR4,CFHR3,C1QC,HLA-C,CD99,RAPGEF4,CST3,MASP2,SAA1,C1S,ABCA13,C4B,C1R,SERPINA3,ACTB,FGA
Reactome	HSA-140875	Common Pathway of Fibrin Clot Formation	6	22	1.63	3.62e-06	PROC,FGB,F2,FGG,F13B,FGA
Reactome	HSA-166786	Creation of C4 and C2 activators	5	16	1.69	3.05e-05	FCN3,C1QC,MASP2,C1S,C1R
Reactome	HSA-140837	Intrinsic Pathway of Fibrin Clot Formation	5	23	1.53	0.00013	F9,PROC,F2,F11,KNG1
Reactome	HSA-977225	Amyloid fiber formation	7	79	1.14	0.00019	APOA1,TTR,LYZ,CST3,SAA1,TGFBI,FGA
Reactome	HSA-6798695	Neutrophil degranulation	14	476	0.66	0.00043	VCL,TTR,CAT,C3,LYZ,TIMP2,AHSG,PPBP,CAMP,TUBB,HLA-C,CST3,ABCA13,SERPINA3
Reactome	HSA-6802948	Signaling by high-kinase activity BRAF mutants	5	36	1.33	0.00079	VCL,FGB,FGG,ACTB,FGA

Reactome	HSA-6802952	Signaling by BRAF and RAF1 fusions	6	65	1.16	0.00079	VCL,FGB,FGG,AKAP9,ACTB,FGA
Reactome	HSA-76009	Platelet Aggregation (Plug Formation)	5	39	1.3	0.0010	FGB,F2,FGG,RAPGEF4,FGA
Reactome	HSA-5674135	MAP2K and MAPK activation	5	40	1.29	0.0011	VCL,FGB,FGG,ACTB,FGA
Reactome	HSA-9656223	Signaling by RAF1 mutants	5	41	1.28	0.0011	VCL,FGB,FGG,ACTB,FGA
Reactome	HSA-392499	Metabolism of proteins	29	1917	0.37	0.0012	ST6GAL1,LGALS1,F9,GPLD1,PROC,APOA1,TTR,C3,LYZ,AHSG,HSP90B1,PCSK9,F2,PLG,APOL1,FGG,GANAB,BLM,ADAMTS13,CST3,SAA1,NUCB1,IGF2,TGFBI,IGFALS,UIMC1,KNG1,ACTB,FGA
Reactome	HSA-2173782	Binding and Uptake of Ligands by Scavenger Receptors	5	43	1.26	0.0013	APOA1,HSP90B1,STAB1,APOL1,SAA1
Reactome	HSA-6802946	Signaling by moderate kinase activity BRAF mutants	5	45	1.24	0.0015	VCL,FGB,FGG,ACTB,FGA
Reactome	HSA-6802955	Paradoxical activation of RAF signaling by kinase inactive BRAF	5	45	1.24	0.0015	VCL,FGB,FGG,ACTB,FGA
Reactome	HSA-9649948	Signaling downstream of RAS mutants	5	45	1.24	0.0015	VCL,FGB,FGG,ACTB,FGA
Reactome	HSA-1474244	Extracellular matrix organization	10	300	0.71	0.0023	TTR,PXDN,TIMP2,LUM,FGB,DST,PLG,FGG,ACTN1,FGA
Reactome	HSA-174577	Activation of C3 and C5	3	7	1.82	0.0023	C5,C3,C4B
Reactome	HSA-354192	Integrin signaling	4	27	1.36	0.0032	FGB,FGG,RAPGEF4,FGA
Reactome	HSA-159763	Transport of gamma-carboxylated protein precursors from the endoplasmic reticulum to the Golgi apparatus	3	9	1.71	0.0037	F9,PROC,F2
Reactome	HSA-173623	Classical antibody-mediated complement activation	3	9	1.71	0.0037	C1QC,C1S,C1R
Reactome	HSA-159740	Gamma-carboxylation of protein precursors	3	10	1.67	0.0046	F9,PROC,F2
Reactome	HSA-159782	Removal of aminoterminal propeptides from gamma-carboxylated proteins	3	10	1.67	0.0046	F9,PROC,F2
Reactome	HSA-8964058	HDL remodeling	3	10	1.67	0.0046	APOC3,APOA1,LCAT
Reactome	HSA-1643685	Disease	25	1702	0.36	0.0052	ST6GAL1,VCL,F9,APOA1,TTR,C3,HYAL1,LUM,IHH,ANTXR1,FGB,F2,FGG,TUBB,GANAB,BLM,AKAP9,ADAMTS13,HLA-C,TAF9,MASP2,F11,TPM4,ACTB,FGA
Reactome	HSA-597592	Post-translational protein modification	22	1405	0.39	0.0058	ST6GAL1,LGALS1,F9,GPLD1,PROC,APOA1,C3,AHSG,HSP90B1,PCSK9,F2,APOL1,FGG,GANAB,BLM,ADAMTS13,CST3,NUCB1,UIMC1,KNG1,ACTB,FGA
Reactome	HSA-354194	GRB2:SOS provides linkage to MAPK signaling for Integrins	3	15	1.49	0.0109	FGB,FGG,FGA
Reactome	HSA-372708	p130Cas linkage to MAPK signaling for integrins	3	15	1.49	0.0109	FGB,FGG,FGA
Reactome	HSA-445355	Smooth Muscle Contraction	4	43	1.16	0.0123	VCL,MYL9,GUCY1A1,TPM4
Reactome	HSA-9651496	Defects of contact activation system (CAS) and kallikrein/kinin system (KKS)	3	16	1.46	0.0123	F9,F2,F11
Reactome	HSA-975634	Retinoid metabolism and transport	4	44	1.15	0.0125	APOC3,APOA1,TTR,APOM
Reactome	HSA-5602498	MyD88 deficiency (TLR2/4)	3	17	1.44	0.0131	FGB,FGG,FGA
Reactome	HSA-446353	Cell-extracellular matrix interactions	3	18	1.41	0.0149	ACTN1,ILK,ACTB
Reactome	HSA-5603041	IRAK4 deficiency (TLR2/4)	3	18	1.41	0.0149	FGB,FGG,FGA
Reactome	HSA-446728	Cell junction organization	5	92	0.93	0.0180	CDH6,DST,ACTN1,ILK,ACTB
Reactome	HSA-5686938	Regulation of TLR by endogenous ligand	3	20	1.37	0.0182	FGB,FGG,FGA
Reactome	HSA-2855086	Ficolins bind to repetitive carbohydrate structures on the target cell surface	2	4	1.89	0.0259	FCN3,MASP2
Reactome	HSA-3000471	Scavenging by Class B Receptors	2	6	1.71	0.0470	APOA1,SAA1
Reactome	HSA-9673221	Defective F9 activation	2	6	1.71	0.0470	F9,F11
WikiPathways	WP558	Complement and coagulation cascades	15	58	1.6	4.45e-16	F9,PROC,C3,MASP1,FGB,F2,PLG,SERPINF2,F13B,C1QC,MASP2,C1S,C4B,C1R,KNG1
WikiPathways	WP272	Blood clotting cascade	9	22	1.8	8.87e-11	F9,FGB,F2,PLG,SERPINF2,FGG,F13B,F11,FGA
WikiPathways	WP2806	Complement system	13	96	1.32	8.87e-11	C5,APOA1,C3,MASP1,FGB,PLG,FGG,C8A,CFHR4,MASP2,F11,C1S,FGA
WikiPathways	WP545	Complement activation	9	22	1.8	8.87e-11	C5,C3,MASP1,C8A,C1QC,MASP2,C1S,C4B,C1R
WikiPathways	WP176	Folate metabolism	11	67	1.41	4.05e-10	APOA1,CAT,SAA4,FGB,F2,PLG,FGG,SOD3,SAA1,SERPINA3,FGA
WikiPathways	WP15	Selenium micronutrient network	11	84	1.31	3.10e-09	APOA1,CAT,SAA4,FGB,F2,PLG,FGG,SOD3,SAA1,SERPINA3,FGA
WikiPathways	WP4927	COVID-19, thrombosis and anticoagulation	6	7	2.12	1.13e-08	FGB,F2,PLG,FGG,F13B,FGA
WikiPathways	WP5090	Complement system in neuronal development and plasticity	10	105	1.17	3.35e-07	C5,C3,FCN3,MASP1,C8A,C1QC,MASP2,C1S,C4B,C1R
WikiPathways	WP5115	Network map of SARS-CoV-2 signaling pathway	12	218	0.93	2.57e-06	APOA1,FGB,APOL1,FGG,C8A,APOM,SAA1,C1S,ITIH3,C1R,ACTB,FGA
WikiPathways	WP1533	Vitamin B12 metabolism	7	50	1.34	5.93e-06	APOA1,SAA4,F2,PLG,SOD3,SAA1,SERPINA3
WikiPathways	WP3941	Oxidative damage response	5	39	1.3	0.00063	C5,C1QC,C1S,C4B,C1R
WikiPathways	WP2328	Allograft rejection	6	88	1.02	0.0021	C5,C3,C8A,C1QC,HLA-C,C4B
WikiPathways	WP3601	Lipid particles composition	3	10	1.67	0.0042	APOC3,APOA1,LCAT
WikiPathways	WP5186	Vitamin K metabolism and activation of dependent proteins	3	12	1.59	0.0062	F9,PROC,F2
WikiPathways	WP5323	Fatty Acids and Lipoproteins Transport in Hepatocytes	10	380	0.61	0.0109	APOC3,APOA1,LCAT,SAA4,PCSK9,APOL1,APOM,SAA1,PCYOX1,ATP9B
WikiPathways	WP4136	Fibrin complement receptor 3 signaling pathway	4	43	1.16	0.0113	FGB,PLG,FGG,FGA
WikiPathways	WP5109	Familial hyperlipidemia type 2	3	16	1.46	0.0113	APOA1,LCAT,PCSK9
WikiPathways	WP4493	Cells and molecules involved in local acute inflammatory response	3	17	1.44	0.0117	C5,C3,KNG1
WikiPathways	WP4522	Metabolic pathway of LDL, HDL and TG, including diseases	3	17	1.44	0.0117	APOA1,LCAT,PCSK9
WikiPathways	WP430	Statin inhibition of cholesterol production	3	29	1.21	0.0434	APOC3,APOA1,LCAT
Monarch	EFO:0007937	Blood protein measurement	45	1810	0.59	9.11e-12	ST6GAL1,EBI3,C5,APOC3,PROC,CAT,C3,PXDN,LYZ,TIMP2,LCAT,PSG9,ITIH1,AHSG,SAA4,ANKRD44,CSF1R,MASP1,HSP90B1,ANTXR1,F2,PLG,APOL1,SERPINF2,CNDP1,F13B,CFHR4,CRTAC1,ADAMTS13,CCN5,C1QC,APOM,SOD3,CST3,SAA1,F11,C1S,PCYOX1,C4B,TGFBI,C1R,SERPINA4,RNASE4,CCL18,KNG1
Monarch	HP:0000225	Gingival bleeding	9	45	1.49	3.47e-07	CAT,FGB,F2,SERPINF2,FGG,F13B,C1S,C1R,FGA
Monarch	HP:0100659	Abnormal cerebral vascular morphology	18	381	0.86	3.58e-07	F9,PROC,TTR,GUCY1A1,ANTXR1,PCSK9,FGB,F2,SERPINF2,FGG,GANAB,F13B,CRTAC1,ADAMTS13,CST3,KNG1,ACTB,FGA
Monarch	HP:0005339	Abnormality of complement system	7	24	1.66	2.64e-06	C5,C3,C8A,C1QC,MASP2,C1S,C4B
Monarch	HP:0000118	Phenotypic abnormality	65	5129	0.29	3.77e-06	ST6GAL1,VCL,F9,C5,APOC3,MGP,PROC,APOA1,TTR,CAT,C3,PXDN,LYZ,LCAT,DNAH5,CENPE,HYAL1,FCN3,AHSG,CRH,CSF1R,IHH,SERPINI1,MASP1,GUCY1A1,A2ML1,ANTXR1,PCSK9,FGB,DST,F2,PLG,STAB1,SERPINF2,DNAH8,FGG,TUBB,THBS4,GANAB,BLM,AKAP9,C8A,F13B,CFHR4,CRTAC1,ADAMTS13,C1QC,ACTN1,NAV2,CST3,MASP2,F11,C1S,IGF2,FSIP2,ABCA13,LMOD3,ITIH3,C4B,TGFBI,IGFALS,C1R,KNG1,ACTB,FGA
Monarch	HP:0001928	Abnormality of coagulation	12	170	1.04	4.35e-06	F9,PROC,A2ML1,FGB,F2,PLG,SERPINF2,FGG,F13B,F11,KNG1,FGA
Monarch	HP:0003256	Abnormality of the coagulation cascade	10	105	1.17	5.99e-06	F9,PROC,FGB,F2,PLG,FGG,F13B,F11,KNG1,FGA
Monarch	HP:0011025	Abnormal cardiovascular system physiology	27	1169	0.55	1.17e-05	VCL,F9,MGP,PROC,APOA1,TTR,LYZ,LCAT,GUCY1A1,A2ML1,PCSK9,FGB,DST,F2,SERPINF2,FGG,TUBB,GANAB,AKAP9,F13B,CRTAC1,ADAMTS13,CST3,F11,KNG1,ACTB,FGA
Monarch	HP:0025015	Abnormal vascular morphology	25	1027	0.58	1.50e-05	F9,MGP,PROC,TTR,CAT,LCAT,DNAH5,MASP1,GUCY1A1,A2ML1,ANTXR1,PCSK9,FGB,F2,SERPINF2,FGG,GANAB,F13B,CRTAC1,ADAMTS13,CST3,IGF2,KNG1,ACTB,FGA

Monarch	HP:0004431	Complement deficiency	6	21	1.65	1.98e-05	C5,C3,C8A,C1QC,MASP2,C4B
Monarch	EFO:0004747	Protein measurement	68	5856	0.26	2.28e-05	ST6GAL1,EBI3,C5,APOC3,GPLD1,PROC,APOA1,CAT,C3,PXDN,LYZ,TIMP2,LCAT,DNAH5,PSG9,ITIH1,AHSG,SAA4,ANKRD44,CSF1R,IGF2BP1,MASP1,HSP90B1,ANTXR1,PCSK9,FGB,F2,PLG,UBE3C,STAB1,APOL1,SERPINF2,DNAH8,OIT3,FGG,THBS4,CNDP1,F13B,CFHR4,CRTAC1,ADAMTS13,CCN5,C1QC,APOM,HLA-C,SOD3,ACTN1,TNRC6A,NAV2,RAPGEF4,CST3,SAA1,F11,C1S,PCYOX1,ABCA13,ITIH3,C4B,TGFBI,IGFALS,C1R,SERPINA4,RNASE4,CCL18,KNG1,ACTB,TRIM66,FGA
Monarch	HP:0002597	Abnormality of the vasculature	32	1673	0.47	2.28e-05	F9,MGP,PROC,APOA1,TTR,CAT,LYZ,LCAT,DNAH5,MASP1,GUCY1A1,A2ML1,ANTXR1,PCSK9,FGB,F2,PLG,SERPINF2,FGG,GANAB,BLM,F13B,CRTAC1,ADAMTS13,CST3,C1S,IGF2,TGFBI,C1R,KNG1,ACTB,FGA
Monarch	HP:0002725	Systemic lupus erythematosus	6	26	1.55	4.55e-05	C3,C8A,C1QC,MASP2,C1S,C1R
Monarch	HP:0000421	Epistaxis	8	78	1.2	6.91e-05	F9,FGB,F2,FGG,F13B,ACTN1,F11,FGA
Monarch	HP:0001892	Abnormal bleeding	15	411	0.75	7.95e-05	F9,PROC,CAT,A2ML1,FGB,F2,SERPINF2,FGG,F13B,ACTN1,CST3,F11,C1S,C1R,FGA
Monarch	HP:0003216	Generalized amyloid deposition	4	5	2.09	0.00015	APOA1,LYZ,CST3,FGA
Monarch	HP:0002715	Abnormality of the immune system	30	1682	0.44	0.00020	VCL,C5,MGP,APOA1,CAT,C3,LYZ,LCAT,DNAH5,HYAL1,FCN3,A2ML1,FGB,F2,PLG,FGG,GANAB,BLM,C8A,F13B,CFHR4,C1QC,NAV2,MASP2,C1S,IGF2,C4B,C1R,ACTB,FGA
Monarch	HP:0000080	Abnormality of reproductive system physiology	18	659	0.63	0.00022	F9,TTR,DNAH5,AHSG,GUCY1A1,A2ML1,ANTXR1,FGB,F2,DNAH8,FGG,GANAB,BLM,F13B,F11,IGF2,FSIP2,FGA
Monarch	HP:0004936	Venous thrombosis	7	65	1.22	0.00025	F9,PROC,FGB,F2,FGG,KNG1,FGA
Monarch	HP:0005261	Joint hemorrhage	5	19	1.61	0.00025	F9,F2,SERPINF2,F13B,F11
Monarch	HP:0001626	Abnormality of the cardiovascular system	37	2438	0.37	0.00026	VCL,F9,MGP,PROC,APOA1,TTR,CAT,LYZ,LCAT,DNAH5,MASP1,GUCY1A1,A2ML1,ANTXR1,PCSK9,FGB,DST,F2,PLG,SERPINF2,FGG,TUBB,GANAB,BLM,AKAP9,F13B,CRTAC1,ADAMTS13,CST3,F11,C1S,IGF2,TGFBI,C1R,KNG1,ACTB,FGA
Monarch	HP:0000168	Abnormality of the gingiva	10	190	0.91	0.00036	CAT,FGB,F2,PLG,SERPINF2,FGG,F13B,C1S,C1R,FGA
Monarch	HP:0000951	Abnormality of the skin	31	1888	0.41	0.00054	VCL,F9,C5,MGP,PROC,APOA1,CAT,LYZ,CENPE,HYAL1,FCN3,AHSG,MASP1,GUCY1A1,A2ML1,ANTXR1,PCSK9,DST,F2,PLG,SERPINF2,TUBB,BLM,F13B,CFHR4,ADAMTS13,C1S,IGF2,C1R,ACTB,FGA
Monarch	HP:0011034	Amyloidosis	5	25	1.49	0.00063	APOA1,TTR,LYZ,CST3,FGA
Monarch	HP:0000790	Hematuria	8	118	1.02	0.00074	F9,APOA1,LYZ,F2,SERPINF2,GANAB,ADAMTS13,FGA
Monarch	HP:0012233	Intramuscular hematoma	4	10	1.79	0.00074	F9,F2,SERPINF2,F13B
Monarch	HP:0012649	Increased inflammatory response	21	981	0.52	0.00074	C5,MGP,APOA1,CAT,C3,LYZ,DNAH5,HYAL1,FCN3,PLG,GANAB,BLM,F13B,CFHR4,C1QC,NAV2,MASP2,C1S,C4B,C1R,FGA
Monarch	HP:0400008	Menometrorrhagia	4	10	1.79	0.00074	F9,FGB,FGG,FGA
Monarch	HP:0000119	Abnormality of the genitourinary system	33	2189	0.37	0.0011	F9,APOA1,TTR,C3,LYZ,LCAT,DNAH5,AHSG,CRH,MASP1,GUCY1A1,A2ML1,ANTXR1,PCSK9,FGB,F2,PLG,SERPINF2,DNAH8,FGG,TUBB,GANAB,BLM,F13B,ADAMTS13,C1QC,F11,IGF2,FSIP2,LMOD3,C1R,ACTB,FGA
Monarch	HP:0002170	Intracranial hemorrhage	8	130	0.98	0.0011	F9,FGB,F2,SERPINF2,FGG,F13B,CST3,FGA
Monarch	EFO:0004634	Coagulation factor measurement	8	132	0.97	0.0012	PROC,FGB,FGG,CFHR4,ADAMTS13,F11,KNG1,FGA
Monarch	HP:0001871	Abnormality of blood and blood-forming tissues	23	1214	0.47	0.0012	VCL,F9,PROC,APOA1,CAT,LCAT,GUCY1A1,A2ML1,FGB,F2,PLG,SERPINF2,FGG,BLM,F13B,ADAMTS13,ACTN1,CST3,F11,C1S,C1R,KNG1,FGA
Monarch	HP:0010978	Abnormality of immune system physiology	24	1347	0.44	0.0019	C5,MGP,APOA1,CAT,C3,LYZ,DNAH5,HYAL1,FCN3,PLG,GANAB,BLM,C8A,F13B,CFHR4,C1QC,NAV2,MASP2,C1S,IGF2,C4B,C1R,ACTB,FGA
Monarch	HP:0010990	Abnormality of the common coagulation pathway	5	35	1.35	0.0019	FGB,PLG,FGG,F13B,FGA
Monarch	HP:0011029	Internal hemorrhage	10	244	0.8	0.0019	F9,PROC,FGB,F2,SERPINF2,FGG,F13B,CST3,F11,FGA
Monarch	HP:0011830	Abnormal oral mucosa morphology	11	305	0.75	0.0019	CAT,LYZ,FGB,F2,PLG,SERPINF2,FGG,F13B,C1S,C1R,FGA
Monarch	HP:0006298	Prolonged bleeding after dental extraction	4	16	1.59	0.0022	F9,F2,F13B,F11
Monarch	HP:0012223	Splenic rupture	3	4	2.07	0.0026	FGB,FGG,FGA
Monarch	HP:0030680	Abnormality of cardiovascular system morphology	27	1680	0.4	0.0026	VCL,F9,MGP,PROC,TTR,CAT,LCAT,DNAH5,MASP1,GUCY1A1,A2ML1,ANTXR1,PCSK9,FGB,F2,SERPINF2,FGG,TUBB,GANAB,F13B,CRTAC1,ADAMTS13,CST3,IGF2,KNG1,ACTB,FGA
Monarch	HP:0011884	Abnormal umbilical stump bleeding	3	5	1.97	0.0039	F2,SERPINF2,F13B
Monarch	HP:0030012	Abnormal female reproductive system physiology	10	273	0.75	0.0039	F9,ANTXR1,FGB,F2,FGG,BLM,F13B,F11,IGF2,FGA
Monarch	HP:0001574	Abnormality of the integument	32	2266	0.34	0.0040	VCL,F9,C5,MGP,PROC,APOA1,CAT,LYZ,CENPE,HYAL1,FCN3,AHSG,IHH,MASP1,GUCY1A1,A2ML1,ANTXR1,PCSK9,DST,F2,PLG,SERPINF2,TUBB,BLM,F13B,CFHR4,ADAMTS13,C1S,IGF2,C1R,ACTB,FGA
Monarch	HP:0001939	Abnormality of metabolism/homeostasis	31	2168	0.35	0.0044	VCL,F9,APOC3,APOA1,TTR,CAT,LYZ,LCAT,CSF1R,A2ML1,ANTXR1,PCSK9,FGB,DST,F2,PLG,SERPINF2,FGG,TUBB,GANAB,BLM,AKAP9,ADAMTS13,CST3,IGF2,LMOD3,TGFBI,IGFALS,KNG1,ACTB,FGA
Monarch	HP:0025031	Abnormality of the digestive system	33	2389	0.33	0.0044	F9,C5,APOA1,TTR,LYZ,LCAT,DNAH5,FCN3,MASP1,GUCY1A1,A2ML1,ANTXR1,PCSK9,FGB,DST,F2,PLG,FGG,TUBB,GANAB,BLM,F13B,ADAMTS13,NAV2,MASP2,F11,C1S,IGF2,LMOD3,C4B,C1R,ACTB,FGA
Monarch	HP:0000077	Abnormality of the kidney	20	1077	0.46	0.0046	F9,APOA1,TTR,C3,LYZ,LCAT,MASP1,ANTXR1,PCSK9,F2,PLG,SERPINF2,GANAB,BLM,ADAMTS13,C1QC,IGF2,C1R,ACTB,FGA
Monarch	HP:0000078	Abnormality of the genital system	23	1358	0.42	0.0046	F9,TTR,DNAH5,AHSG,MASP1,GUCY1A1,A2ML1,ANTXR1,FGB,F2,PLG,DNAH8,FGG,TUBB,GANAB,BLM,F13B,F11,IGF2,FSIP2,LMOD3,ACTB,FGA
Monarch	HP:0000140	Abnormality of the menstrual cycle	9	228	0.79	0.0046	F9,ANTXR1,FGB,F2,FGG,F13B,F11,IGF2,FGA
Monarch	HP:0000707	Abnormality of the nervous system	42	3471	0.27	0.0046	ST6GAL1,F9,MGP,PROC,APOA1,TTR,CAT,DNAH5,CENPE,AHSG,CRH,CSF1R,SERPINI1,MASP1,GUCY1A1,A2ML1,ANTXR1,PCSK9,FGB,DST,F2,PLG,STAB1,SERPINF2,FGG,TUBB,GANAB,BLM,AKAP9,C8A,F13B,CRTAC1,ADAMTS13,CST3,IGF2,LMOD3,ITIH3,C4B,TGFBI,KNG1,ACTB,FGA
Monarch	HP:0005268	Miscarriage	4	21	1.47	0.0046	MGP,FGB,FGG,FGA
Monarch	HP:0011420	Age of death	4	21	1.47	0.0046	MGP,FGB,FGG,FGA
Monarch	HP:0012211	Abnormal renal physiology	14	560	0.59	0.0046	F9,APOA1,TTR,C3,LYZ,LCAT,F2,PLG,SERPINF2,GANAB,ADAMTS13,C1QC,C1R,FGA
Monarch	HP:0003645	Prolonged partial thromboplastin time	4	24	1.41	0.0057	F9,F2,F11,KNG1
Monarch	HP:0010935	Abnormality of the upper urinary tract	20	1101	0.45	0.0057	F9,APOA1,TTR,C3,LYZ,LCAT,MASP1,ANTXR1,PCSK9,F2,PLG,SERPINF2,GANAB,BLM,ADAMTS13,C1QC,IGF2,C1R,ACTB,FGA
Monarch	HP:0001000	Abnormality of skin pigmentation	12	439	0.63	0.0064	PROC,CAT,A2ML1,ANTXR1,DST,F2,TUBB,BLM,C1S,IGF2,C1R,ACTB
Monarch	HP:0000969	Edema	12	448	0.62	0.0076	APOA1,LYZ,A2ML1,ANTXR1,FGB,F2,FGG,TUBB,LMOD3,TGFBI,ACTB,FGA
Monarch	HP:0000123	Nephritis	5	59	1.12	0.0104	C3,PLG,GANAB,C1QC,C1R
Monarch	HP:0001342	Cerebral hemorrhage	5	61	1.1	0.0119	FGB,FGG,F13B,CST3,FGA
Monarch	HP:0002239	Gastrointestinal hemorrhage	6	111	0.92	0.0193	F9,FGB,F2,FGG,F11,FGA
Monarch	HP:0000234	Abnormality of the head	35	2865	0.28	0.0195	F9,MGP,PROC,CAT,LYZ,DNAH5,CENPE,HYAL1,AHSG,CSF1R,IHH,MASP1,A2ML1,ANTXR1,PCSK9,FGB,DST,F2,PLG,SERPINF2,FGG,TUBB,BLM,F13B,CRTAC1,ACTN1,F11,C1S,IGF2,ABCA13,LMOD3,IGFALS,C1R,ACTB,FGA
Monarch	HP:0000704	Periodontitis	4	35	1.25	0.0195	CAT,PLG,C1S,C1R
Monarch	HP:0001386	Joint swelling	4	35	1.25	0.0195	FGB,F2,FGG,FGA
Monarch	HP:0001934	Persistent bleeding after trauma	3	12	1.59	0.0195	F9,SERPINF2,F13B
Monarch	HP:0000271	Abnormality of the face	33	2641	0.29	0.0206	F9,MGP,PROC,CAT,LYZ,DNAH5,CENPE,HYAL1,AHSG,CSF1R,MASP1,A2ML1,ANTXR1,PCSK9,FGB,DST,F2,PLG,SERPINF2,FGG,TUBB,BLM,F13B,CRTAC1,ACTN1,F11,C1S,IGF2,ABCA13,LMOD3,C1R,ACTB,FGA

Monarch	HP:0002011	Morphological central nervous system abnormality	31	2416	0.3	0.0212	F9,MGP,PROC,TTR,DNAH5,CENPE,AHSG,CSF1R,SERPINI1,MASP1,GUCY1A1,ANTXR1,PCSK9,FGB,F2,PLG,SERPINF2,FGG,TUBB,GANAB,BLM,C8A,F13B,CRTAC1,ADAMTS13,CST3,IGF2,C4B,KNG1,ACTB,FGA
Monarch	HP:0011121	Abnormality of skin morphology	24	1648	0.35	0.0226	VCL,F9,MGP,PROC,APOA1,CAT,CENPE,AHSG,MASP1,GUCY1A1,A2ML1,ANTXR1,PCSK9,DST,F2,SERPINF2,TUBB,BLM,F13B,ADAMTS13,C1S,IGF2,C1R,ACTB
Monarch	HP:0012639	Abnormal nervous system morphology	32	2546	0.29	0.0229	F9,MGP,PROC,TTR,DNAH5,CENPE,AHSG,CSF1R,SERPINI1,MASP1,GUCY1A1,ANTXR1,PCSK9,FGB,F2,PLG,SERPINF2,FGG,TUBB,GANAB,BLM,C8A,F13B,CRTAC1,ADAMTS13,CST3,IGF2,LMOD3,C4B,KNG1,ACTB,FGA
Monarch	HP:0005368	Abnormality of humoral immunity	8	234	0.72	0.0264	C5,C3,BLM,C8A,C1QC,MASP2,C1S,C4B
Monarch	HP:0012874	Abnormal male reproductive system physiology	8	234	0.72	0.0264	TTR,DNAH5,GUCY1A1,ANTXR1,F2,DNAH8,GANAB,BLM
Monarch	EFO:0004310	Partial thromboplastin time	3	15	1.49	0.0294	F11,C1S,KNG1
Monarch	HP:0001297	Stroke	8	249	0.7	0.0373	TTR,GUCY1A1,F2,CRTAC1,ADAMTS13,CST3,KNG1,ACTB
Monarch	HP:0010979	Abnormality of lipoprotein cholesterol concentration	4	47	1.12	0.0462	APOC3,APOA1,LCAT,PCSK9
DISEASES	DOID:1247	Blood coagulation disease	13	89	1.36	4.34e-10	F9,PROC,FGB,F2,PLG,SERPINF2,FGG,F13B,ADAMTS13,F11,C4B,KNG1,FGA
DISEASES	DOID:9120	Amyloidosis	12	75	1.39	7.63e-10	APOC3,APOA1,TTR,C3,LYZ,SAA4,CST3,SAA1,TGFBI,SERPINA3,ACTB,FGA
DISEASES	DOID:74	Hematopoietic system disease	19	473	0.79	5.40e-07	F9,PROC,CAMP,FGB,F2,PLG,SERPINF2,FGG,F13B,ADAMTS13,CD99,SAA1,F11,C4B,SERPINA3,IGHV3-15,KNG1,ACTB,FGA
DISEASES	DOID:7	Disease of anatomical entity	63	4798	0.31	9.25e-07	VCL,LGALS1,F9,C5,MGP,PROC,APOA1,TTR,C3,PXDN,LCAT,DNAH5,CENPE,LUM,AHSG,CRH,SAA4,ANKRD44,CSF1R,IHH,SERPINI1,CAMP,A2ML1,ANTXR1,SH3D19,PCSK9,FGB,DST,F2,PLG,APOL1,SERPINF2,FGG,TUBB,GANAB,BLM,AKAP9,CNDP1,F13B,CFHR4,CFHR3,ADAMTS13,C1QC,HLA-C,CD99,TNRC6A,GAPDH,CST3,SAA1,F11,C1S,NUCB1,IGF2,LMOD3,C4B,TGFBI,C1R,SERPINA3,IGHV3-15,KNG1,ACTB,TRIM66,FGA
DISEASES	DOID:4	Disease	74	6291	0.26	9.61e-07	VCL,LGALS1,F9,C5,APOC3,MGP,PROC,APOA1,TTR,CAT,C3,PXDN,LYZ,LCAT,CDH6,DNAH5,CENPE,HYAL1,LUM,AHSG,CRH,SAA4,ANKRD44,CSF1R,IHH,SERPINI1,MASP1,CAMP,A2ML1,ANTXR1,SH3D19,PCSK9,FGB,DST,F2,PLG,APOL1,SERPINF2,DNAH8,FGG,TUBB,GANAB,BLM,AKAP9,CNDP1,F13B,CFHR4,CFHR3,MCOLN2,ADAMTS13,C1QC,HLA-C,CD99,TNRC6A,GAPDH,CST3,SAA1,F11,C1S,NUCB1,PCYOX1,IGF2,ABCA13,LMOD3,C4B,TGFBI,C1R,SERPINA3,IGHV3-15,KNG1,TPM4,ACTB,TRIM66,FGA
DISEASES	DOID:0050636	Familial visceral amyloidosis	6	21	1.65	1.25e-05	APOC3,APOA1,TTR,LYZ,ACTB,FGA
DISEASES	DOID:2452	Thrombophilia	6	21	1.65	1.25e-05	F9,PROC,F2,PLG,ADAMTS13,C4B
DISEASES	DOID:409	Liver disease	9	97	1.16	1.53e-05	TTR,AHSG,SAA4,F2,GANAB,GAPDH,SAA1,IGHV3-15,ACTB
DISEASES	DOID:557	Kidney disease	13	275	0.87	2.17e-05	C5,TTR,C3,SAA4,F2,APOL1,GANAB,CFHR3,ADAMTS13,CST3,SAA1,IGF2,C4B
DISEASES	DOID:0050739	Autosomal genetic disease	42	2802	0.37	3.39e-05	F9,APOC3,MGP,PROC,APOA1,TTR,C3,LYZ,CDH6,DNAH5,ANKRD44,CSF1R,IHH,SERPINI1,MASP1,CAMP,ANTXR1,PCSK9,FGB,DST,F2,PLG,SERPINF2,FGG,GANAB,BLM,AKAP9,F13B,CFHR4,CFHR3,CST3,SAA1,F11,C1S,PCYOX1,IGF2,LMOD3,TGFBI,SERPINA3,KNG1,ACTB,FGA
DISEASES	DOID:0050736	Autosomal dominant disease	27	1386	0.48	9.88e-05	F9,APOC3,PROC,APOA1,TTR,C3,LYZ,CDH6,ANKRD44,CSF1R,IHH,SERPINI1,F2,GANAB,AKAP9,F13B,CFHR4,CFHR3,CST3,SAA1,C1S,IGF2,TGFBI,SERPINA3,KNG1,ACTB,FGA
DISEASES	DOID:630	Genetic disease	49	3778	0.3	0.00013	F9,APOC3,MGP,PROC,APOA1,TTR,CAT,C3,LYZ,LCAT,CDH6,DNAH5,HYAL1,ANKRD44,CSF1R,IHH,SERPINI1,MASP1,CAMP,A2ML1,ANTXR1,PCSK9,FGB,DST,F2,PLG,SERPINF2,DNAH8,FGG,GANAB,BLM,AKAP9,CNDP1,F13B,CFHR4,CFHR3,MCOLN2,CST3,SAA1,F11,C1S,PCYOX1,IGF2,LMOD3,TGFBI,SERPINA3,KNG1,ACTB,FGA
DISEASES	DOID:0014667	Disease of metabolism	23	1076	0.52	0.00016	F9,APOC3,MGP,APOA1,TTR,CAT,C3,LYZ,LCAT,HYAL1,AHSG,SAA4,PCSK9,F2,CNDP1,MCOLN2,CST3,SAA1,PCYOX1,TGFBI,SERPINA3,ACTB,FGA
DISEASES	DOID:2914	Immune system disease	18	675	0.62	0.00016	C5,TTR,C3,PXDN,SAA4,A2ML1,DST,F2,PLG,CFHR3,ADAMTS13,HLA-C,CD99,SAA1,C4B,IGHV3-15,KNG1,ACTB
DISEASES	DOID:0050177	Monogenic disease	44	3266	0.32	0.00019	F9,APOC3,MGP,PROC,APOA1,TTR,C3,LYZ,CDH6,DNAH5,ANKRD44,CSF1R,IHH,SERPINI1,MASP1,CAMP,A2ML1,ANTXR1,PCSK9,FGB,DST,F2,PLG,SERPINF2,DNAH8,FGG,GANAB,BLM,AKAP9,F13B,CFHR4,CFHR3,CST3,SAA1,F11,C1S,PCYOX1,IGF2,LMOD3,TGFBI,SERPINA3,KNG1,ACTB,FGA
DISEASES	DOID:626	Complement deficiency	5	23	1.53	0.00024	C5,C3,CFHR3,ADAMTS13,KNG1
DISEASES	DOID:65	Connective tissue disease	18	774	0.56	0.00073	F9,C5,MGP,TTR,CSF1R,IHH,A2ML1,ANTXR1,DST,PLG,HLA-C,CD99,SAA1,C1S,IGF2,C4B,C1R,SERPINA3
DISEASES	DOID:2236	Congenital afibrinogenemia	3	3	2.19	0.0012	FGB,FGG,FGA
DISEASES	DOID:612	Primary immunodeficiency disease	13	470	0.63	0.0030	C5,TTR,C3,PXDN,A2ML1,DST,F2,CFHR3,ADAMTS13,HLA-C,SAA1,C4B,KNG1
DISEASES	DOID:10871	Age related macular degeneration	5	44	1.25	0.0032	C3,F13B,CFHR4,CFHR3,CST3
DISEASES	DOID:16	Integumentary system disease	14	575	0.58	0.0049	APOA1,C3,AHSG,CAMP,A2ML1,ANTXR1,DST,TUBB,BLM,HLA-C,CST3,C1S,KNG1,ACTB
DISEASES	DOID:37	Skin disease	13	518	0.59	0.0068	APOA1,C3,CAMP,A2ML1,ANTXR1,DST,TUBB,BLM,HLA-C,CST3,C1S,KNG1,ACTB
DISEASES	DOID:655	Inherited metabolic disorder	18	949	0.47	0.0076	F9,APOC3,APOA1,TTR,CAT,C3,LYZ,LCAT,HYAL1,PCSK9,CNDP1,MCOLN2,CST3,SAA1,PCYOX1,SERPINA3,ACTB,FGA
DISEASES	DOID:0080301	Atypical hemolytic-uremic syndrome	3	11	1.63	0.0146	C3,CFHR3,ADAMTS13
DISEASES	DOID:1387	Hypolipoproteinemia	3	11	1.63	0.0146	APOA1,LCAT,PCSK9
DISEASES	DOID:1287	Cardiovascular system disease	12	493	0.58	0.0152	VCL,APOA1,TTR,DNAH5,PCSK9,F2,PLG,AKAP9,ADAMTS13,HLA-C,CD99,SAA1
DISEASES	DOID:863	Nervous system disease	30	2275	0.31	0.0158	LGALS1,PROC,APOA1,TTR,C3,LCAT,CENPE,LUM,ANKRD44,CSF1R,SERPINI1,A2ML1,SH3D19,PCSK9,DST,F2,PLG,CNDP1,F13B,CFHR4,CFHR3,C1QC,CD99,TNRC6A,CST3,IGF2,TGFBI,SERPINA3,ACTB,TRIM66
DISEASES	DOID:2213	Hemorrhagic disease	3	12	1.59	0.0159	F2,SERPINF2,ADAMTS13
DISEASES	DOID:2921	Glomerulonephritis	4	36	1.24	0.0171	C3,APOL1,CFHR3,C4B
DISEASES	DOID:12554	Hemolytic-uremic syndrome	3	14	1.52	0.0221	C3,CFHR3,ADAMTS13
DISEASES	DOID:17	Musculoskeletal system disease	19	1154	0.41	0.0221	F9,C5,MGP,TTR,CSF1R,IHH,A2ML1,ANTXR1,DST,PLG,HLA-C,CD99,SAA1,C1S,IGF2,LMOD3,C4B,C1R,SERPINA3
DISEASES	DOID:75	Lymphatic system disease	7	174	0.8	0.0221	SAA4,F2,PLG,CD99,SAA1,IGHV3-15,ACTB
DISEASES	DOID:0050828	Artery disease	6	130	0.85	0.0301	APOA1,PCSK9,F2,PLG,ADAMTS13,SAA1
DISEASES	DOID:1391	Norum disease	2	2	2.19	0.0301	APOA1,LCAT
DISEASES	DOID:178	Vascular disease	8	249	0.7	0.0301	APOA1,PCSK9,F2,PLG,ADAMTS13,HLA-C,CD99,SAA1
DISEASES	DOID:2529	Splenic disease	3	17	1.44	0.0301	SAA4,F2,SAA1
DISEASES	DOID:417	Autoimmune disease	9	317	0.64	0.0301	C5,TTR,PXDN,A2ML1,DST,F2,HLA-C,SAA1,C4B
DISEASES	DOID:0050639	Primary cutaneous amyloidosis	3	19	1.39	0.0386	C3,CST3,ACTB
DISEASES	DOID:4734	Calciphylaxis	2	3	2.01	0.0420	MGP,AHSG
DISEASES	DOID:170	Endocrine gland cancer	5	93	0.92	0.0429	CD99,GAPDH,IGF2,IGHV3-15,ACTB
DISEASES	DOID:0050737	Autosomal recessive disease	24	1785	0.32	0.0455	MGP,DNAH5,IHH,MASP1,CAMP,ANTXR1,PCSK9,FGB,DST,F2,PLG,SERPINF2,FGG,BLM,F13B,SAA1,F11,C1S,PCYOX1,IGF2,LMOD3,TGFBI,KNG1,FGA
DISEASES	DOID:3571	Liver cancer	3	21	1.35	0.0460	GAPDH,IGHV3-15,ACTB
DISEASES	DOID:865	Vasculitis	3	21	1.35	0.0460	F2,HLA-C,SAA1

TISSUES	BTO:0000759	Liver	60	2125	0.64	2.21e-21	ST6GAL1,VCL,LGALS1,TPD52L2,F9,APOC3,GPLD1,PROC,APOA1,TTR,CAT,C3,PXDN,ATRN,LUM,FCN3,ITIH1,AHSG,SAA4,CSF1R,PPBP,MASP1,HSP90B1,FGB,DST,F2,PLG,STAB1,APOL1,SERPINF2,FGG,TUBB,GANAB,AKAP9,C8A,F13B,CFHR4,CFHR3,ADAMTS13,APOM,HLA-C,CD99,ACTN1,GAPDH,MASP2,SAA1,F11,C1S,NUCB1,PCYOX1,IGF2,ITIH3,C4B,TGFBI,IGFALS,C1R,SERPINA3,KNG1,ACTB,FGA
TISSUES	BTO:0000392	Plasma cell	22	171	1.3	1.89e-18	C5,TTR,C3,LUM,FCN3,AHSG,SAA4,CSF1R,IHH,MASP1,FGB,F2,APOL1,SERPINF2,ADAMTS13,SOD3,C1S,C4B,C1R,SERPINA3,KNG1,FGA
TISSUES	BTO:0004850	Bone marrow cell	23	198	1.26	1.89e-18	C5,TTR,C3,LUM,FCN3,AHSG,SAA4,CSF1R,IHH,MASP1,FGB,F2,APOL1,SERPINF2,ADAMTS13,SOD3,F11,C1S,C4B,C1R,SERPINA3,KNG1,FGA
TISSUES	BTO:0000345	Digestive gland	63	2881	0.53	1.33e-17	ST6GAL1,VCL,LGALS1,TPD52L2,F9,APOC3,GPLD1,PROC,APOA1,TTR,CAT,C3,PXDN,ATRN,HYAL1,LUM,FCN3,ITIH1,AHSG,SAA4,CSF1R,PPBP,MASP1,HSP90B1,FGB,DST,F2,PLG,STAB1,APOL1,SERPINF2,FGG,TUBB,GANAB,AKAP9,C8A,F13B,CFHR4,CFHR3,ADAMTS13,APOM,HLA-C,CD99,ACTN1,GAPDH,RNASE1,MASP2,SAA1,F11,C1S,NUCB1,PCYOX1,IGF2,ITIH3,C4B,TGFBI,IGFALS,C1R,SERPINA3,RNASE4,KNG1,ACTB,FGA
TISSUES	BTO:0001486	Skeletal system	41	1307	0.69	4.44e-15	C5,APOC3,APOA1,TTR,C3,PXDN,ATRN,HYAL1,LUM,FCN3,AHSG,SAA4,CSF1R,IHH,MASP1,CAMP,HSP90B1,FGB,F2,UBE3C,APOL1,SERPINF2,FGG,GANAB,CRTAC1,ADAMTS13,CCN5,SOD3,GAPDH,CST3,F11,C1S,PCYOX1,IGF2,LMOD3,C4B,C1R,SERPINA3,KNG1,ACTB,FGA
TISSUES	BTO:0000141	Bone marrow	26	528	0.88	5.19e-13	C5,TTR,C3,PXDN,LUM,FCN3,AHSG,SAA4,CSF1R,IHH,MASP1,CAMP,FGB,F2,APOL1,SERPINF2,GANAB,ADAMTS13,SOD3,F11,C1S,C4B,C1R,SERPINA3,KNG1,FGA
TISSUES	BTO:0001491	Viscus	75	5378	0.34	2.82e-11	ST6GAL1,VCL,LGALS1,TPD52L2,F9,APOC3,GPLD1,PROC,PLEK,APOA1,TTR,CAT,C3,PXDN,LYZ,ATRN,CDH6,LUM,FCN3,ITIH1,AHSG,SAA4,CSF1R,PPBP,MASP1,GUCY1A1,HSP90B1,ANTXR1,SH3D19,FGB,DST,F2,PLG,STAB1,APOL1,SERPINF2,FGG,TUBB,GANAB,AKAP9,C8A,F13B,CFHR4,CFHR3,ADAMTS13,APOM,HLA-C,CD99,SOD3,ACTN1,TNRC6A,ILK,GAPDH,RNASE1,CST3,MASP2,SAA1,F11,C1S,NUCB1,PCYOX1,IGF2,ITIH3,C4B,TGFBI,IGFALS,C1R,SERPINA3,SERPINA4,RNASE4,CCL18,KNG1,TPM4,ACTB,FGA
TISSUES	BTO:0000522	Gland	83	7004	0.26	3.06e-09	ST6GAL1,VCL,LGALS1,TPD52L2,F9,C5,APOC3,GPLD1,PROC,APOA1,TTR,CAT,C3,PXDN,LYZ,ATRN,LCN1,CDH6,HYAL1,LUM,FCN3,ITIH1,AHSG,CRH,SAA4,CSF1R,IHH,PPBP,MASP1,CAMP,GUCY1A1,HSP90B1,ANTXR1,SH3D19,FGB,DST,F2,PLG,STAB1,APOL1,SERPINF2,FGG,TUBB,GANAB,AKAP9,C8A,F13B,CFHR4,CFHR3,ADAMTS13,CCN5,APOM,HLA-C,CD99,SOD3,ACTN1,TNRC6A,GAPDH,RAPGEF4,RNASE1,CST3,MASP2,SAA1,F11,C1S,NUCB1,PCYOX1,IGF2,ATP9B,FSIP2,ITIH3,C4B,TGFBI,IGFALS,UIMC1,C1R,SERPINA3,RNASE4,CCL18,KNG1,TPM4,ACTB,FGA
TISSUES	BTO:0001488	Endocrine gland	78	6403	0.28	7.21e-09	ST6GAL1,VCL,LGALS1,TPD52L2,F9,APOC3,GPLD1,PROC,APOA1,TTR,CAT,C3,PXDN,ATRN,LCN1,HYAL1,LUM,FCN3,ITIH1,AHSG,CRH,SAA4,CSF1R,IHH,PPBP,MASP1,CAMP,HSP90B1,ANTXR1,SH3D19,FGB,DST,F2,PLG,STAB1,APOL1,SERPINF2,FGG,TUBB,GANAB,AKAP9,C8A,F13B,CFHR4,CFHR3,ADAMTS13,CCN5,APOM,HLA-C,CD99,SOD3,ACTN1,GAPDH,RAPGEF4,RNASE1,CST3,MASP2,SAA1,F11,C1S,NUCB1,PCYOX1,IGF2,ATP9B,FSIP2,ITIH3,C4B,TGFBI,IGFALS,UIMC1,C1R,SERPINA3,RNASE4,CCL18,KNG1,TPM4,ACTB,FGA
TISSUES	BTO:0000132	Blood platelet	16	363	0.83	6.68e-07	VCL,LGALS1,APOA1,TTR,CAT,C3,AHSG,PPBP,HSP90B1,FGB,FGG,ACTN1,GAPDH,TPM4,ACTB,FGA
TISSUES	BTO:0001489	Whole body	113	13099	0.13	1.05e-06	ST6GAL1,VCL,LGALS1,TPD52L2,F9,EBI3,TFPI2,C5,APOC3,MGP,GPLD1,PROC,PLEK,APOA1,TTR,CAT,C3,PXDN,LYZ,TIMP2,ATRN,LCN1,LCAT,CDH6,DNAH5,CENPE,HYAL1,LUM,PSG9,FCN3,ITIH1,AHSG,CRH,SAA4,MYL9,CSF1R,IHH,SERPINI1,PPBP,MASP1,CAMP,GUCY1A1,A2ML1,HSP90B1,ANTXR1,SH3D19,PCSK9,FGB,DST,F2,PLG,UBE3C,STAB1,APOL1,PSG11,SERPINF2,DNAH8,FGG,TUBB,THBS4,GANAB,FAM184A,AKAP9,CNDP1,MTCL1,C8A,F13B,CFHR4,CFHR3,CRTAC1,ADAMTS13,CCN5,C1QC,APOM,HLA-C,CD99,SOD3,ACTN1,TNRC6A,NAV2,ILK,GAPDH,RAPGEF4,RNASE1,CST3,MAP3K7CL,MASP2,SAA1,F11,C1S,NUCB1,PCYOX1,IGF2,ATP9B,SLAIN1,FSIP2,LMOD3,ITIH3,C4B,TGFBI,IGFALS,PAEP,UIMC1,SBSN,C1R,SERPINA3,SERPINA4,RNASE4,CCL18,KNG1,TPM4,ACTB,FGA
TISSUES	BTO:0000449	Fetus	28	1318	0.52	4.54e-06	LGALS1,EBI3,TFPI2,TIMP2,LUM,PSG9,CRH,MYL9,CSF1R,HSP90B1,ANTXR1,DST,APOL1,PSG11,TUBB,GANAB,HLA-C,ACTN1,ILK,GAPDH,RNASE1,CST3,NUCB1,PCYOX1,IGF2,TGFBI,TPM4,ACTB
TISSUES	BTO:0001078	Placenta	27	1244	0.53	4.75e-06	LGALS1,EBI3,TFPI2,TIMP2,LUM,PSG9,CRH,MYL9,CSF1R,HSP90B1,ANTXR1,DST,APOL1,PSG11,GANAB,HLA-C,ACTN1,ILK,GAPDH,RNASE1,CST3,NUCB1,PCYOX1,IGF2,TGFBI,TPM4,ACTB
TISSUES	BTO:0000089	Blood	33	1824	0.45	6.38e-06	VCL,LGALS1,TPD52L2,PROC,PLEK,APOA1,TTR,CAT,C3,PSG9,AHSG,PPBP,HSP90B1,FGB,F2,FGG,AKAP9,C8A,C1QC,APOM,HLA-C,CD99,SOD3,ACTN1,GAPDH,CST3,C1S,NUCB1,C4B,CCL18,TPM4,ACTB,FGA
TISSUES	BTO:0000237	Cerebrospinal fluid	7	45	1.38	6.38e-06	APOC3,TTR,AHSG,F2,CST3,IGF2,KNG1
TISSUES	BTO:0000570	Hematopoietic system	42	2755	0.37	6.38e-06	ST6GAL1,VCL,LGALS1,TPD52L2,APOC3,GPLD1,PROC,PLEK,APOA1,TTR,CAT,C3,ATRN,PSG9,ITIH1,AHSG,PPBP,HSP90B1,FGB,F2,FGG,GANAB,AKAP9,C8A,C1QC,APOM,HLA-C,CD99,SOD3,ACTN1,GAPDH,CST3,C1S,NUCB1,IGF2,C4B,TGFBI,CCL18,KNG1,TPM4,ACTB,FGA
TISSUES	BTO:0001239	Serum	6	25	1.57	6.38e-06	GPLD1,APOA1,C3,ATRN,ITIH1,KNG1
TISSUES	BTO:0000284	Organism form	38	2542	0.37	4.97e-05	ST6GAL1,VCL,LGALS1,TPD52L2,EBI3,TFPI2,TIMP2,LUM,PSG9,CRH,MYL9,CSF1R,CAMP,HSP90B1,ANTXR1,DST,UBE3C,APOL1,PSG11,TUBB,GANAB,AKAP9,APOM,HLA-C,CD99,ACTN1,NAV2,ILK,GAPDH,RNASE1,CST3,NUCB1,PCYOX1,IGF2,TGFBI,UIMC1,TPM4,ACTB
TISSUES	BTO:0000511	Gastrointestinal tract	31	1816	0.42	4.97e-05	VCL,LGALS1,PROC,APOA1,TTR,CAT,C3,LYZ,LUM,AHSG,HSP90B1,SH3D19,DST,TUBB,GANAB,AKAP9,HLA-C,CD99,SOD3,ACTN1,TNRC6A,ILK,GAPDH,CST3,C1S,IGF2,TGFBI,C1R,SERPINA4,TPM4,ACTB
TISSUES	BTO:0000255	Brain cell line	10	188	0.92	6.30e-05	VCL,LGALS1,APOA1,TTR,FGB,TUBB,GAPDH,TPM4,ACTB,TRIM66
TISSUES	BTO:0003914	Interstitial cell of Cajal	10	193	0.91	7.58e-05	LGALS1,APOA1,TTR,CAT,AHSG,TUBB,GANAB,GAPDH,TPM4,ACTB
TISSUES	BTO:0000203	Respiratory system	29	1707	0.42	0.00012	ST6GAL1,LGALS1,C3,PXDN,LYZ,ATRN,LCN1,DNAH5,LUM,FCN3,HSP90B1,SH3D19,TUBB,GANAB,AKAP9,CCN5,HLA-C,ACTN1,GAPDH,RAPGEF4,MASP2,C1S,NUCB1,PCYOX1,C4B,TGFBI,CCL18,KNG1,ACTB
TISSUES	BTO:0003099	Internal female genital organ	39	2804	0.33	0.00016	VCL,LGALS1,TPD52L2,EBI3,TFPI2,MGP,C3,TIMP2,LUM,PSG9,CRH,MYL9,CSF1R,MASP1,A2ML1,HSP90B1,ANTXR1,DST,APOL1,PSG11,TUBB,GANAB,CCN5,HLA-C,CD99,ACTN1,ILK,GAPDH,RNASE1,CST3,C1S,NUCB1,PCYOX1,IGF2,TGFBI,PAEP,UIMC1,TPM4,ACTB

TISSUES	BTO:0000174	Embryonic structure	35	2369	0.36	0.00017	ST6GAL1,VCL,LGALS1,TPD52L2,EBI3,TFPI2,TIMP2,LUM,PSG9,CRH,MYL9,CSF1R,HSP90B1,ANTXR1,DST,UBE3C,APOL1,PSG11,TUBB,GANAB,AKAP9,APOM,HLA-C,ACTN1,ILK,GAPDH,RNASE1,CST3,NUCB1,PCYOX1,IGF2,TGFBI,UIMC1,TPM4,ACTB
TISSUES	BTO:0000042	Animal	117	15148	0.08	0.00048	ST6GAL1,VCL,LGALS1,TPD52L2,F9,EBI3,TFPI2,C5,APOC3,MGP,GPLD1,PROC,PLEK,APOA1,TTR,CAT,C3,PXDN,LYZ,TIMP2,ATRN,LCN1,LCAT,CDH6,DNAH5,CENPE,HYAL1,LUM,PSG9,FCN3,ITIH1,AHSG,CRH,SAA4,MYL9,CSF1R,IGF2BP1,IHH,SERPINI1,PPBP,MASP1,CAMP,GUCY1A1,A2ML1,HSP90B1,ANTXR1,SH3D19,PCSK9,SHLD1,FGB,DST,F2,PLG,UBE3C,STAB1,APOL1,PSG11,SERPINF2,DNAH8,FGG,TUBB,THBS4,GANAB,FAM184A,AKAP9,CNDP1,MTCL1,C8A,F13B,CFHR4,CFHR3,CRTAC1,ADAMTS13,CCN5,C1QC,APOM,HLA-C,CD99,SOD3,ACTN1,TNRC6A,NAV2,ILK,GAPDH,RAPGEF4,RNASE1,CST3,MAP3K7CL,MASP2,SAA1,F11,C1S,NUCB1,PCYOX1,IGF2,ATP9B,SLAIN1,FSIP2,ABCA13,LMOD3,ITIH3,C4B,TGFBI,IGFALS,PAEP,UIMC1,SBSN,C1R,SERPINA3,SERPINA4,RNASE4,CCL18,KNG1,TPM4,ACTB,TRIM66,FGA
TISSUES	BTO:0000007	HEK-293 cell	3	5	1.97	0.0011	PXDN,GAPDH,ACTB
TISSUES	BTO:0000088	Cardiovascular system	20	1057	0.47	0.0012	VCL,TFPI2,MGP,APOA1,C3,TIMP2,HYAL1,LUM,MYL9,HSP90B1,F2,THBS4,F13B,GAPDH,PCYOX1,LMOD3,TGFBI,CCL18,ACTB,FGA
TISSUES	BTO:0000917	Needle	3	7	1.82	0.0021	PXDN,GAPDH,ACTB
TISSUES	BTO:0001419	Urine	4	28	1.35	0.0034	LYZ,F2,RNASE1,MASP2
TISSUES	BTO:0000102	Blood clot	3	11	1.63	0.0058	F2,PLG,ADAMTS13
TISSUES	BTO:0000166	Cecum	4	35	1.25	0.0070	C3,GAPDH,CST3,ACTB
TISSUES	BTO:0004078	Non-small cell lung adenocarcinoma cell line	3	12	1.59	0.0070	PCSK9,GAPDH,ACTB
TISSUES	BTO:0000421	Connective tissue	17	954	0.44	0.0094	LGALS1,TFPI2,APOA1,TTR,CAT,LUM,AHSG,IHH,HSP90B1,TUBB,THBS4,GANAB,CRTAC1,GAPDH,CST3,TPM4,ACTB
TISSUES	BTO:0000648	Intestine	21	1349	0.38	0.0095	VCL,PROC,C3,LYZ,LUM,HSP90B1,SH3D19,DST,GANAB,HLA-C,SOD3,ACTN1,ILK,GAPDH,CST3,C1S,IGF2,TGFBI,C1R,SERPINA4,ACTB
TISSUES	BTO:0001279	Spinal cord	8	233	0.73	0.0095	APOC3,TTR,AHSG,F2,CST3,IGF2,KNG1,ACTB
TISSUES	BTO:0000988	Pancreas	13	626	0.51	0.0133	APOC3,GPLD1,TTR,HSP90B1,APOL1,HLA-C,CD99,GAPDH,RNASE1,IGF2,TGFBI,SERPINA3,ACTB
TISSUES	BTO:0005692	Column	2	2	2.19	0.0134	GAPDH,ACTB
TISSUES	BTO:0000067	Kidney cell line	4	44	1.15	0.0135	ST6GAL1,PXDN,GAPDH,ACTB
TISSUES	BTO:0000763	Lung	21	1395	0.37	0.0135	LGALS1,C3,PXDN,LCN1,DNAH5,LUM,FCN3,HSP90B1,TUBB,AKAP9,CCN5,HLA-C,ACTN1,GAPDH,MASP2,C1S,NUCB1,C4B,TGFBI,CCL18,ACTB
TISSUES	BTO:0005739	Transfer cell	2	3	2.01	0.0208	PXDN,ACTB
TISSUES	BTO:0002854	Corn silk	2	4	1.89	0.0303	MASP1,MASP2
TISSUES	BTO:0001090	Mouth	11	532	0.51	0.0370	C5,LYZ,LCN1,CENPE,A2ML1,DST,GANAB,ACTN1,SBSN,TPM4,ACTB
TISSUES	BTO:0001702	Left atrium	4	60	1.01	0.0370	VCL,APOA1,C3,GAPDH
TISSUES	BTO:0001703	Right atrium	4	61	1.01	0.0382	VCL,APOA1,C3,GAPDH
TISSUES	BTO:0000562	Heart	13	738	0.44	0.0472	VCL,APOA1,C3,HYAL1,LUM,F2,THBS4,F13B,GAPDH,PCYOX1,LMOD3,ACTB,FGA
TISSUES	BTO:0000567	HeLa cell	2	6	1.71	0.0482	GAPDH,ACTB
TISSUES	BTO:0002181	HEK-293T cell	2	6	1.71	0.0482	GAPDH,ACTB
COMPARTMENTS	GOCC:0005576	Extracellular region	77	2079	0.76	1.21e-38	ST6GAL1,VCL,LGALS1,F9,EBI3,TFPI2,C5,APOC3,MGP,GPLD1,PROC,APOA1,TTR,C3,PXDN,LYZ,TIMP2,LCN1,LCAT,CENPE,HYAL1,LUM,FCN3,ITIH1,AHSG,CRH,SAA4,SERPINI1,PPBP,MASP1,CAMP,A2ML1,HSP90B1,PCSK9,FGB,DST,F2,PLG,APOL1,SERPINF2,FGG,TUBB,THBS4,C8A,F13B,CFHR4,CFHR3,CRTAC1,ADAMTS13,CCN5,C1QC,APOM,HLA-C,SOD3,GAPDH,RNASE1,CST3,MASP2,SAA1,F11,C1S,NUCB1,PCYOX1,IGF2,ITIH3,C4B,TGFBI,IGFALS,PAEP,C1R,SERPINA3,SERPINA4,RNASE4,CCL18,KNG1,ACTB,FGA
COMPARTMENTS	GOCC:0005615	Extracellular space	58	1027	0.94	3.47e-36	VCL,F9,EBI3,C5,APOC3,MGP,GPLD1,PROC,APOA1,TTR,C3,PXDN,LYZ,LCN1,LCAT,HYAL1,LUM,FCN3,ITIH1,AHSG,CRH,SAA4,SERPINI1,MASP1,CAMP,A2ML1,HSP90B1,PCSK9,FGB,F2,PLG,APOL1,SERPINF2,FGG,THBS4,C8A,CFHR3,CCN5,C1QC,APOM,HLA-C,SOD3,GAPDH,CST3,MASP2,SAA1,F11,C1S,PCYOX1,IGF2,C4B,TGFBI,IGFALS,C1R,SERPINA3,KNG1,ACTB,FGA
COMPARTMENTS	GOCC:0072562	Blood microparticle	21	118	1.44	2.56e-20	APOA1,C3,FCN3,ITIH1,AHSG,FGB,F2,PLG,APOL1,SERPINF2,FGG,C8A,CFHR3,C1QC,C1S,C4B,C1R,SERPINA3,KNG1,ACTB,FGA
COMPARTMENTS	GOCC:0043230	Extracellular organelle	31	524	0.96	5.51e-18	VCL,F9,C5,MGP,APOA1,TTR,C3,PXDN,LUM,AHSG,CAMP,HSP90B1,FGB,F2,PLG,SERPINF2,FGG,HLA-C,GAPDH,RNASE1,CST3,MASP2,SAA1,F11,C4B,TGFBI,C1R,SERPINA3,KNG1,ACTB,FGA
COMPARTMENTS	GOCC:0065010	Extracellular membrane-bounded organelle	29	473	0.98	3.77e-17	VCL,F9,MGP,APOA1,TTR,C3,PXDN,LUM,AHSG,CAMP,HSP90B1,FGB,F2,PLG,SERPINF2,FGG,HLA-C,GAPDH,RNASE1,CST3,SAA1,F11,C4B,TGFBI,C1R,SERPINA3,KNG1,ACTB,FGA
COMPARTMENTS	GOCC:1903561	Extracellular vesicle	29	500	0.95	1.31e-16	VCL,F9,MGP,APOA1,TTR,C3,PXDN,LUM,AHSG,CAMP,HSP90B1,FGB,F2,PLG,SERPINF2,FGG,HLA-C,GAPDH,RNASE1,CST3,SAA1,F11,C4B,TGFBI,C1R,SERPINA3,KNG1,ACTB,FGA
COMPARTMENTS	GOCC:0060205	Cytoplasmic vesicle lumen	22	245	1.14	6.77e-16	VCL,APOA1,TTR,C3,LYZ,TIMP2,AHSG,SERPINI1,PPBP,CAMP,FGB,PLG,SERPINF2,FGG,TUBB,ACTN1,IGF2,ITIH3,SERPINA3,SERPINA4,KNG1,FGA
COMPARTMENTS	GOCC:0034774	Secretory granule lumen	21	241	1.13	5.89e-15	VCL,APOA1,TTR,C3,LYZ,TIMP2,AHSG,PPBP,CAMP,FGB,PLG,SERPINF2,FGG,TUBB,ACTN1,IGF2,ITIH3,SERPINA3,SERPINA4,KNG1,FGA
COMPARTMENTS	GOCC:0031982	Vesicle	46	2125	0.53	1.14e-11	VCL,F9,MGP,APOA1,TTR,CAT,C3,PXDN,LYZ,TIMP2,HYAL1,LUM,ITIH1,AHSG,SERPINI1,PPBP,CAMP,HSP90B1,ANTXR1,PCSK9,FGB,DST,F2,PLG,STAB1,SERPINF2,FGG,TUBB,HLA-C,ACTN1,GAPDH,RNASE1,CST3,SAA1,F11,IGF2,ABCA13,ITIH3,C4B,TGFBI,C1R,SERPINA3,SERPINA4,KNG1,ACTB,FGA
COMPARTMENTS	GOCC:0031093	Platelet alpha granule lumen	11	66	1.41	4.69e-10	AHSG,PPBP,FGB,PLG,SERPINF2,FGG,ACTN1,IGF2,SERPINA3,KNG1,FGA
COMPARTMENTS	GOCC:0070062	Extracellular exosome	20	428	0.86	1.66e-09	VCL,MGP,APOA1,TTR,C3,PXDN,AHSG,CAMP,HSP90B1,FGB,PLG,FGG,HLA-C,GAPDH,CST3,SAA1,TGFBI,KNG1,ACTB,FGA
COMPARTMENTS	GOCC:0030141	Secretory granule	24	719	0.71	8.58e-09	VCL,APOA1,TTR,CAT,C3,LYZ,TIMP2,AHSG,PPBP,CAMP,FGB,PLG,SERPINF2,FGG,TUBB,ACTN1,CST3,IGF2,ABCA13,ITIH3,SERPINA3,SERPINA4,KNG1,FGA
COMPARTMENTS	GOCC:0034358	Plasma lipoprotein particle	9	48	1.46	1.45e-08	APOC3,APOA1,LCAT,SAA4,PCSK9,APOL1,APOM,SAA1,PCYOX1
COMPARTMENTS	GOCC:1905286	Serine-type peptidase complex	6	11	1.93	8.68e-08	F9,C3,FCN3,MASP1,MASP2,F11
COMPARTMENTS	GOCC:0005788	Endoplasmic reticulum lumen	12	172	1.03	2.79e-07	F9,APOA1,C3,AHSG,HSP90B1,PCSK9,APOL1,FGG,GANAB,NUCB1,KNG1,FGA
COMPARTMENTS	GOCC:0034364	High-density lipoprotein particle	7	30	1.56	3.60e-07	APOC3,APOA1,LCAT,SAA4,APOL1,APOM,SAA1
COMPARTMENTS	GOCC:1905370	Serine-type endopeptidase complex	5	7	2.04	7.96e-07	C3,FCN3,MASP1,MASP2,F11
COMPARTMENTS	GOCC:0005577	Fibrinogen complex	6	20	1.67	1.27e-06	FGB,F2,PLG,SERPINF2,FGG,FGA
COMPARTMENTS	GOCC:0031410	Cytoplasmic vesicle	33	1738	0.47	1.32e-06	VCL,APOA1,TTR,CAT,C3,LYZ,TIMP2,HYAL1,AHSG,SERPINI1,PPBP,CAMP,HSP90B1,ANTXR1,PCSK9,FGB,DST,PLG,STAB1,SERPINF2,FGG,TUBB,HLA-C,ACTN1,CST3,SAA1,IGF2,ABCA13,ITIH3,SERPINA3,SERPINA4,KNG1,FGA
COMPARTMENTS	GOCC:0062167	Complement component C1q complex	5	10	1.89	2.52e-06	C3,C1QC,C1S,C4B,C1R

COMPARTMENTS	GOCC:0110165	Cellular anatomical entity	115	14060	0.1	1.15e-05	ST6GAL1,VCL,LGALS1,TPD52L2,F9,EBI3,TFPI2,C5,APOC3,MGP,GPLD1,PROC,PLEK,APOA1,TTR,CAT,C3,PXDN,LYZ,TIMP2,ATRN,LCN1,LCAT,CDH6,DNAH5,CENPE,HYAL1,LUM,PSG9,FCN3,ITIH1,AHSG,CRH,SAA4,MYL9,CSF1R,IGF2BP1,IHH,SERPINI1,PPBP,MASP1,CAMP,GUCY1A1,A2ML1,HSP90B1,ANTXR1,SH3D19,PCSK9,SHLD1,FGB,DST,F2,PLG,STAB1,APOL1,SERPINF2,DNAH8,FGG,TUBB,THBS4,GANAB,BLM,AKAP9,CNDP1,MTCL1,C8A,F13B,CFHR4,CFHR3,CRTAC1,MCOLN2,ADAMTS13,CCN5,C1QC,APOM,HLA-C,TAF9,CD99,SOD3,ACTN1,TNRC6A,NAV2,ILK,GAPDH,RAPGEF4,RNASE1,CST3,MAP3K7CL,MASP2,SAA1,F11,C1S,NUCB1,PCYOX1,IGF2,ATP9B,FSIP2,ABCA13,LMOD3,ITIH3,C4B,TGFBI,IGFALS,PAEP,UIMC1,C1R,SERPINA3,SERPINA4,RNASE4,CCL18,KNG1,TPM4,ACTB,TRIM66,FGA
COMPARTMENTS	GOCC:0031012	Extracellular matrix	12	259	0.86	1.46e-05	TFPI2,GPLD1,PXDN,TIMP2,LUM,HSP90B1,DST,PLG,THBS4,ADAMTS13,SOD3,TGFBI
COMPARTMENTS	GOCC:1905368	Peptidase complex	9	136	1.01	3.15e-05	F9,C3,FCN3,MASP1,F2,UBE3C,TAF9,MASP2,F11
COMPARTMENTS	GOCC:0034361	Very-low-density lipoprotein particle	5	20	1.59	3.66e-05	APOC3,APOA1,APOL1,APOM,PCYOX1
COMPARTMENTS	GOCC:0070013	Intracellular organelle lumen	41	2902	0.34	4.15e-05	VCL,F9,PROC,APOA1,TTR,CAT,C3,LYZ,TIMP2,HYAL1,LUM,AHSG,SERPINI1,PPBP,CAMP,HSP90B1,PCSK9,FGB,F2,PLG,APOL1,SERPINF2,FGG,TUBB,GANAB,BLM,TAF9,SOD3,ACTN1,GAPDH,CST3,SAA1,NUCB1,IGF2,ITIH3,UIMC1,SERPINA3,SERPINA4,KNG1,ACTB,FGA
COMPARTMENTS	GOCC:0012505	Endomembrane system	43	3156	0.32	4.75e-05	ST6GAL1,VCL,F9,PROC,APOA1,TTR,CAT,C3,PXDN,LYZ,TIMP2,LUM,AHSG,PPBP,CAMP,HSP90B1,ANTXR1,PCSK9,FGB,F2,PLG,APOL1,SERPINF2,FGG,TUBB,THBS4,GANAB,AKAP9,HLA-C,SOD3,ACTN1,TNRC6A,GAPDH,CST3,NUCB1,IGF2,ATP9B,ABCA13,ITIH3,SERPINA3,SERPINA4,KNG1,FGA
COMPARTMENTS	GOCC:0030312	External encapsulating structure	11	259	0.82	8.59e-05	TFPI2,GPLD1,PXDN,TIMP2,LUM,HSP90B1,DST,PLG,THBS4,ADAMTS13,SOD3
COMPARTMENTS	GOCC:0034366	Spherical high-density lipoprotein particle	4	11	1.75	0.00013	APOC3,APOA1,LCAT,APOM
COMPARTMENTS	GOCC:0032991	Protein-containing complex	59	5325	0.24	0.00015	VCL,LGALS1,F9,C5,APOC3,APOA1,CAT,C3,PXDN,LCAT,CDH6,DNAH5,LUM,PSG9,FCN3,SAA4,MYL9,CSF1R,IGF2BP1,MASP1,GUCY1A1,HSP90B1,PCSK9,FGB,DST,F2,PLG,UBE3C,APOL1,SERPINF2,DNAH8,FGG,GANAB,BLM,AKAP9,C8A,ADAMTS13,C1QC,APOM,HLA-C,TAF9,TNRC6A,GAPDH,CST3,MASP2,SAA1,F11,C1S,PCYOX1,IGF2,CDK3,C4B,IGFALS,UIMC1,C1R,IGHV3-15,TPM4,ACTB,FGA
COMPARTMENTS	GOCC:0062023	Collagen-containing extracellular matrix	9	198	0.85	0.00045	PXDN,TIMP2,LUM,HSP90B1,DST,PLG,THBS4,SOD3,TGFBI
COMPARTMENTS	GOCC:0005602	Complement component C1 complex	3	5	1.97	0.00081	C1QC,C1S,C1R
COMPARTMENTS	GOCC:0034365	Discoidal high-density lipoprotein particle	3	6	1.89	0.0012	APOA1,LCAT,APOM
COMPARTMENTS	GOCC:1905369	Endopeptidase complex	6	90	1.01	0.0019	C3,FCN3,MASP1,UBE3C,MASP2,F11
COMPARTMENTS	GOCC:1904724	Tertiary granule lumen	5	55	1.15	0.0022	LYZ,TIMP2,PPBP,CAMP,CST3
COMPARTMENTS	GOCC:0045298	Tubulin complex	3	10	1.67	0.0037	PXDN,GAPDH,ACTB
COMPARTMENTS	GOCC:0005579	Membrane attack complex	3	12	1.59	0.0056	C5,C3,C8A
COMPARTMENTS	GOCC:0005775	Vacuolar lumen	7	168	0.81	0.0068	TTR,C3,LYZ,HYAL1,LUM,TUBB,SERPINA3
COMPARTMENTS	GOCC:0009986	Cell surface	11	438	0.59	0.0073	PXDN,CSF1R,ANTXR1,PCSK9,FGB,PLG,SERPINF2,FGG,ADAMTS13,HLA-C,FGA
COMPARTMENTS	GOCC:0097179	Protease inhibitor complex	3	14	1.52	0.0078	PXDN,GAPDH,ACTB
COMPARTMENTS	GOCC:0099512	Supramolecular fiber	12	555	0.53	0.0133	VCL,CENPE,LUM,MYL9,DST,TUBB,ACTN1,GAPDH,SAA1,LMOD3,TPM4,ACTB
COMPARTMENTS	GOCC:0035578	Azurophil granule lumen	5	91	0.93	0.0169	TTR,C3,LYZ,TUBB,SERPINA3
COMPARTMENTS	GOCC:0099080	Supramolecular complex	15	844	0.44	0.0173	VCL,CENPE,LUM,MYL9,IGF2BP1,DST,F2,TUBB,ACTN1,TNRC6A,GAPDH,SAA1,LMOD3,TPM4,ACTB
COMPARTMENTS	GOCC:0140224	SLAC complex	2	3	2.01	0.0173	GAPDH,ACTB
COMPARTMENTS	GOCC:0034362	Low-density lipoprotein particle	3	22	1.33	0.0223	APOA1,PCSK9,APOM
COMPARTMENTS	GOCC:0042582	Azurophil granule	6	154	0.78	0.0239	TTR,C3,LYZ,TUBB,ABCA13,SERPINA3
COMPARTMENTS	GOCC:0043292	Contractile fiber	6	154	0.78	0.0239	VCL,MYL9,DST,ACTN1,LMOD3,TPM4
COMPARTMENTS	GOCC:0071682	Endocytic vesicle lumen	3	23	1.31	0.0239	APOA1,HSP90B1,SAA1
COMPARTMENTS	GOCC:0005796	Golgi lumen	5	102	0.88	0.0241	F9,PROC,LUM,F2,SOD3
COMPARTMENTS	GOCC:0009897	External side of plasma membrane	5	102	0.88	0.0241	ANTXR1,FGB,PLG,FGG,FGA
COMPARTMENTS	GOCC:0035580	Specific granule lumen	4	62	1.0	0.0316	VCL,LYZ,TIMP2,CAMP
UniProt Keywords	KW-0964	Secreted	74	1839	0.8	1.68e-39	ST6GAL1,LGALS1,F9,EBI3,TFPI2,C5,APOC3,MGP,GPLD1,PROC,APOA1,TTR,C3,PXDN,LYZ,TIMP2,ATRN,LCN1,LCAT,HYAL1,LUM,PSG9,FCN3,ITIH1,AHSG,CRH,SAA4,IHH,SERPINI1,PPBP,CAMP,A2ML1,PCSK9,FGB,F2,PLG,APOL1,PSG11,SERPINF2,FGG,THBS4,CNDP1,C8A,F13B,CFHR4,CFHR3,CRTAC1,ADAMTS13,CCN5,C1QC,APOM,SOD3,BPIFC,RNASE1,CST3,MASP2,SAA1,F11,NUCB1,IGF2,ITIH3,C4B,TGFBI,IGFALS,PAEP,SBSN,C1R,SERPINA3,SERPINA4,RNASE4,IGHV3-15,CCL18,KNG1,FGA
UniProt Keywords	KW-0732	Signal	83	3277	0.59	1.61e-31	F9,EBI3,TFPI2,C5,APOC3,MGP,GPLD1,PROC,APOA1,TTR,C3,PXDN,LYZ,TIMP2,ATRN,LCN1,LCAT,CDH6,HYAL1,LUM,PSG9,FCN3,ITIH1,AHSG,CRH,SAA4,CSF1R,IHH,SERPINI1,PPBP,CAMP,A2ML1,HSP90B1,ANTXR1,PCSK9,FGB,F2,PLG,STAB1,APOL1,PSG11,SERPINF2,OIT3,FGG,THBS4,GANAB,CNDP1,C8A,F13B,CFHR4,CFHR3,CRTAC1,ADAMTS13,CCN5,C1QC,APOM,HLA-C,CD99,SOD3,BPIFC,RNASE1,CST3,MASP2,SAA1,F11,C1S,NUCB1,PCYOX1,IGF2,ITIH3,C4B,TGFBI,IGFALS,PAEP,SBSN,C1R,SERPINA3,SERPINA4,RNASE4,IGHV3-15,CCL18,KNG1,FGA
UniProt Keywords	KW-1015	Disulfide bond	64	3338	0.47	1.76e-15	ST6GAL1,F9,TFPI2,C5,MGP,PROC,C3,PXDN,LYZ,TIMP2,ATRN,LCN1,LCAT,HYAL1,LUM,PSG9,FCN3,ITIH1,AHSG,CSF1R,PPBP,MASP1,CAMP,A2ML1,HSP90B1,ANTXR1,PCSK9,FGB,F2,PLG,STAB1,PSG11,SERPINF2,OIT3,FGG,THBS4,GANAB,C8A,F13B,CFHR4,CFHR3,CRTAC1,MCOLN2,ADAMTS13,C1QC,APOM,HLA-C,SOD3,BPIFC,RNASE1,CST3,MASP2,F11,C1S,IGF2,C4B,TGFBI,PAEP,C1R,RNASE4,IGHV3-15,CCL18,KNG1,FGA
UniProt Keywords	KW-0094	Blood coagulation	12	46	1.61	3.28e-13	F9,TFPI2,PROC,FGB,F2,PLG,FGG,F13B,ADAMTS13,F11,KNG1,FGA
UniProt Keywords	KW-0325	Glycoprotein	69	4386	0.39	6.16e-13	ST6GAL1,F9,EBI3,TFPI2,C5,APOC3,GPLD1,PROC,APOA1,TTR,C3,PXDN,ATRN,LCAT,CDH6,HYAL1,LUM,PSG9,FCN3,ITIH1,AHSG,SAA4,CSF1R,IHH,SERPINI1,A2ML1,HSP90B1,ANTXR1,PCSK9,FGB,F2,PLG,STAB1,APOL1,PSG11,SERPINF2,OIT3,FGG,THBS4,GANAB,CNDP1,C8A,F13B,CFHR4,CFHR3,CRTAC1,ADAMTS13,C1QC,APOM,HLA-C,CD99,SOD3,BPIFC,RNASE1,CST3,F11,C1S,NUCB1,PCYOX1,IGF2,ITIH3,C4B,IGFALS,PAEP,C1R,SERPINA3,SERPINA4,KNG1,FGA
UniProt Keywords	KW-0180	Complement pathway	8	30	1.62	9.07e-09	C5,C3,C8A,C1QC,MASP2,C1S,C4B,C1R
UniProt Keywords	KW-0646	Protease inhibitor	11	117	1.16	5.21e-08	TFPI2,TIMP2,ITIH1,SERPINI1,A2ML1,SERPINF2,CST3,ITIH3,SERPINA3,SERPINA4,KNG1
UniProt Keywords	KW-0034	Amyloid	7	24	1.66	6.47e-08	APOA1,TTR,LYZ,CST3,SAA1,TGFBI,FGA
UniProt Keywords	KW-0301	Gamma-carboxyglutamic acid	6	16	1.76	2.69e-07	F9,MGP,PROC,TTR,F2,TGFBI
UniProt Keywords	KW-1008	Amyloidosis	7	31	1.54	2.69e-07	APOA1,TTR,LYZ,CST3,SAA1,TGFBI,FGA
UniProt Keywords	KW-0165	Cleavage on pair of basic residues	14	280	0.89	3.49e-07	F9,C5,PROC,C3,CDH6,CRH,PPBP,CAMP,F2,PLG,C8A,ADAMTS13,IGF2,C4B
UniProt Keywords	KW-0245	EGF-like domain	13	232	0.94	3.49e-07	F9,PROC,ATRN,HYAL1,MASP1,STAB1,OIT3,THBS4,C8A,CRTAC1,MASP2,C1S,C1R
UniProt Keywords	KW-0399	Innate immunity	14	329	0.82	2.11e-06	C5,C3,FCN3,CSF1R,FGB,C8A,MCOLN2,C1QC,HLA-C,MASP2,C1S,C4B,C1R,FGA
UniProt Keywords	KW-0106	Calcium	22	886	0.59	3.08e-06	F9,PROC,PXDN,CDH6,FCN3,MYL9,IHH,HSP90B1,PCSK9,DST,F2,OIT3,FGG,THBS4,MCOLN2,ADAMTS13,ACTN1,MASP2,C1S,NUCB1,TPM4,FGA

UniProt Keywords	KW-0722	Serine protease inhibitor	8	80	1.19	3.99e-06	TFPI2,ITIH1,SERPINI1,A2ML1,SERPINF2,ITIH3,SERPINA3,SERPINA4
UniProt Keywords	KW-0768	Sushi	7	57	1.28	6.79e-06	MASP1,F13B,CFHR4,CFHR3,MASP2,C1S,C1R
UniProt Keywords	KW-0345	HDL	5	16	1.69	6.97e-06	APOA1,SAA4,APOL1,APOM,SAA1
UniProt Keywords	KW-0011	Acute phase	5	19	1.61	1.36e-05	SAA4,F2,SERPINF2,SAA1,SERPINA3
UniProt Keywords	KW-0720	Serine protease	9	136	1.01	1.36e-05	F9,PROC,PCSK9,F2,PLG,MASP2,F11,C1S,C1R
UniProt Keywords	KW-0379	Hydroxylation	9	143	0.99	1.90e-05	F9,PROC,FCN3,C1QC,MASP2,C1S,C1R,KNG1,FGA
UniProt Keywords	KW-0792	Thrombophilia	4	10	1.79	4.67e-05	F9,PROC,F2,PLG
UniProt Keywords	KW-0391	Immunity	15	537	0.64	7.50e-05	C5,C3,FCN3,CSF1R,FGB,C8A,MCOLN2,C1QC,HLA-C,MASP2,C1S,C4B,C1R,IGHV3-15,FGA
UniProt Keywords	KW-0765	Sulfation	6	57	1.21	8.66e-05	F9,LUM,PCSK9,SERPINF2,FGG,C4B
UniProt Keywords	KW-0395	Inflammatory response	8	163	0.88	0.00039	C5,C3,ATRN,CSF1R,STAB1,C4B,CCL18,KNG1
UniProt Keywords	KW-0677	Repeat	52	4794	0.23	0.00075	VCL,F9,EBI3,TFPI2,GPLD1,PROC,PLEK,APOA1,PXDN,ATRN,CDH6,DNAH5,LUM,PSG9,FCN3,AHSG,ANKRD31,MYL9,ANKRD44,CSF1R,IGF2BP1,MASP1,SH3D19,DST,F2,PLG,STAB1,PSG11,DNAH8,THBS4,C8A,F13B,CFHR4,CFHR3,CRTAC1,ADAMTS13,C1QC,ACTN1,ILK,RAPGEF4,MASP2,F11,C1S,NUCB1,FSIP2,ABCA13,TGFBI,IGFALS,UIMC1,C1R,KNG1,TRIM66
UniProt Keywords	KW-0882	Thioester bond	3	8	1.76	0.0010	C3,A2ML1,C4B
UniProt Keywords	KW-0179	Complement alternate pathway	3	12	1.59	0.0026	C5,C3,C8A
UniProt Keywords	KW-0645	Protease	12	516	0.56	0.0035	F9,PROC,IHH,PCSK9,F2,PLG,CNDP1,ADAMTS13,MASP2,F11,C1S,C1R
UniProt Keywords	KW-0497	Mitogen	4	39	1.2	0.0037	CAT,PPBP,THBS4,IGF2
UniProt Keywords	KW-0797	Tissue remodeling	2	4	1.89	0.0130	PLG,THBS4
UniProt Keywords	KW-0153	Cholesterol metabolism	4	65	0.98	0.0211	APOA1,LCAT,PCSK9,APOL1
UniProt Keywords	KW-0873	Pyrrolidone carboxylic acid	4	67	0.97	0.0228	LUM,FGB,RNASE4,KNG1
UniProt Keywords	KW-0473	Membrane attack complex	2	7	1.65	0.0281	C5,C8A
UniProt Keywords	KW-0558	Oxidation	3	32	1.16	0.0281	APOA1,GAPDH,ACTB
UniProt Keywords	KW-0376	Hydrogen peroxide	2	9	1.54	0.0392	CAT,PXDN
UniProt Keywords	KW-1068	Hemolytic uremic syndrome	2	10	1.49	0.0456	C3,CFHR3
UniProt Keywords	KW-0865	Zymogen	6	210	0.65	0.0466	F9,PROC,PCSK9,F2,PLG,ADAMTS13
InterPro	IPR001881	EGF-like calcium-binding domain	10	124	1.1	0.00011	F9,PROC,MASP1,STAB1,OIT3,THBS4,CRTAC1,MASP2,C1S,C1R
InterPro	IPR018097	EGF-like calcium-binding, conserved site	9	99	1.15	0.00011	F9,PROC,MASP1,OIT3,THBS4,CRTAC1,MASP2,C1S,C1R
InterPro	IPR000436	Sushi/SCR/CCP domain	7	56	1.29	0.00037	MASP1,F13B,CFHR4,CFHR3,MASP2,C1S,C1R
InterPro	IPR033116	Serine proteases, trypsin family, serine active site	8	92	1.13	0.00037	F9,PROC,F2,PLG,MASP2,F11,C1S,C1R
InterPro	IPR035976	Sushi/SCR/CCP superfamily	7	58	1.27	0.00037	MASP1,F13B,CFHR4,CFHR3,MASP2,C1S,C1R
InterPro	IPR001314	Peptidase S1A, chymotrypsin family	8	107	1.06	0.00091	F9,PROC,F2,PLG,MASP2,F11,C1S,C1R
InterPro	IPR040839	Macroglobulin domain MG4	4	8	1.89	0.00091	C5,C3,A2ML1,C4B
InterPro	IPR001254	Serine proteases, trypsin domain	8	115	1.03	0.00099	F9,PROC,F2,PLG,MASP2,F11,C1S,C1R
InterPro	IPR001599	Alpha-2-macroglobulin	4	9	1.84	0.00099	C5,C3,A2ML1,C4B
InterPro	IPR002890	Macroglobulin domain	4	9	1.84	0.00099	C5,C3,A2ML1,C4B
InterPro	IPR009003	Peptidase S1, PA clan	8	123	1.0	0.00099	F9,PROC,F2,PLG,MASP2,F11,C1S,C1R
InterPro	IPR009048	Alpha-macroglobulin, receptor-binding	4	9	1.84	0.00099	C5,C3,A2ML1,C4B
InterPro	IPR011625	Alpha-2-macroglobulin, bait region domain	4	9	1.84	0.00099	C5,C3,A2ML1,C4B
InterPro	IPR011626	Alpha-macroglobulin-like, TED domain	4	9	1.84	0.00099	C5,C3,A2ML1,C4B
InterPro	IPR035914	Spermadhesin, CUB domain superfamily	6	54	1.24	0.00099	ATRN,MASP1,ADAMTS13,MASP2,C1S,C1R
InterPro	IPR036595	Alpha-macroglobulin, receptor-binding domain superfamily	4	9	1.84	0.00099	C5,C3,A2ML1,C4B
InterPro	IPR041555	Macroglobulin domain MG3	4	9	1.84	0.00099	C5,C3,A2ML1,C4B
InterPro	IPR043504	Peptidase S1, PA clan, chymotrypsin-like fold	8	116	1.03	0.00099	F9,PROC,F2,PLG,MASP2,F11,C1S,C1R
InterPro	IPR000294	Gamma-carboxyglutamic acid-rich (GLA) domain	4	15	1.62	0.0019	F9,MGP,PROC,F2
InterPro	IPR008930	Terpenoid cyclases/protein prenyltransferase alpha-alpha toroid	4	15	1.62	0.0019	C5,C3,A2ML1,C4B
InterPro	IPR012290	Fibrinogen, alpha/beta/gamma chain, coiled coil domain	3	3	2.19	0.0019	FGB,FGG,FGA
InterPro	IPR024175	Peptidase S1A, complement C1r/C1S/mannan-binding	3	3	2.19	0.0019	MASP2,C1S,C1R
InterPro	IPR035972	Gamma-carboxyglutamic acid-rich (GLA) domain superfamily	4	15	1.62	0.0019	F9,MGP,PROC,F2
InterPro	IPR037579	Fibrinogen	3	3	2.19	0.0019	FGB,FGG,FGA
InterPro	IPR001840	Anaphylatoxin, complement system domain	3	4	2.07	0.0025	C5,C3,C4B
InterPro	IPR018081	Anaphylatoxin, complement system	3	4	2.07	0.0025	C5,C3,C4B
InterPro	IPR020837	Fibrinogen, conserved site	4	19	1.51	0.0034	FCN3,FGB,FGG,FGA
InterPro	IPR000020	Anaphylatoxin/fibulin	3	6	1.89	0.0054	C5,C3,C4B
InterPro	IPR001134	Netrin domain	4	23	1.43	0.0062	C5,C3,TIMP2,C4B
InterPro	IPR046350	Cystatin superfamily	4	23	1.43	0.0062	AHSG,CAMP,CST3,KNG1
InterPro	IPR000742	EGF-like domain	9	236	0.77	0.0067	F9,PROC,ATRN,MASP1,STAB1,OIT3,THBS4,MASP2,C1R
InterPro	IPR000859	CUB domain	5	51	1.18	0.0067	ATRN,MASP1,MASP2,C1S,C1R
InterPro	IPR008993	Tissue inhibitor of metalloproteinases-like, OB-fold	4	24	1.41	0.0067	C5,C3,TIMP2,C4B
InterPro	IPR014716	Fibrinogen, alpha/beta/gamma chain, C-terminal globular, subdomain 1	4	26	1.38	0.0082	FCN3,FGB,FGG,FGA
InterPro	IPR019742	Alpha-2-macroglobulin, conserved site	3	8	1.76	0.0084	C3,A2ML1,C4B
InterPro	IPR047565	Alpha-macroglobulin-like, thiol-ester bond-forming region	3	8	1.76	0.0084	C3,A2ML1,C4B
InterPro	IPR002181	Fibrinogen, alpha/beta/gamma chain, C-terminal globular domain	4	32	1.29	0.0157	FCN3,FGB,FGG,FGA
InterPro	IPR023795	Serpin, conserved site	4	32	1.29	0.0157	SERPINI1,SERPINF2,SERPINA3,SERPINA4
InterPro	IPR036056	Fibrinogen-like, C-terminal	4	32	1.29	0.0157	FCN3,FGB,FGG,FGA
InterPro	IPR042185	Serpin superfamily, domain 2	4	35	1.25	0.0199	SERPINI1,SERPINF2,SERPINA3,SERPINA4
InterPro	IPR000215	Serpin family	4	36	1.24	0.0215	SERPINI1,SERPINF2,SERPINA3,SERPINA4
InterPro	IPR023796	Serpin domain	4	36	1.24	0.0215	SERPINI1,SERPINF2,SERPINA3,SERPINA4
InterPro	IPR036186	Serpin superfamily	4	36	1.24	0.0215	SERPINI1,SERPINF2,SERPINA3,SERPINA4
InterPro	IPR042178	Serpin superfamily, domain 1	4	36	1.24	0.0215	SERPINI1,SERPINF2,SERPINA3,SERPINA4
InterPro	IPR041425	Complement C3/4/5, macroglobulin domain MG1	2	2	2.19	0.0387	C5,C3
InterPro	IPR000010	Cystatin domain	3	17	1.44	0.0422	AHSG,CST3,KNG1
InterPro	IPR018933	Netrin module, non-TIMP type	3	17	1.44	0.0422	C5,C3,C4B
SMART	SM00020	Trypsin-like serine protease	9	118	1.07	0.00011	F9,PROC,MASP1,F2,PLG,MASP2,F11,C1S,C1R
SMART	SM00032	Domain abundant in complement control proteins	7	56	1.29	0.00011	MASP1,F13B,CFHR4,CFHR3,MASP2,C1S,C1R
SMART	SM00179	Calcium-binding EGF-like domain	8	101	1.09	0.00014	F9,PROC,MASP1,OIT3,THBS4,MASP2,C1S,C1R
SMART	SM01359	Alpha-2-Macroglobulin	4	9	1.84	0.00023	C5,C3,A2ML1,C4B
SMART	SM01360	Alpha-2-macroglobulin family	4	9	1.84	0.00023	C5,C3,A2ML1,C4B
SMART	SM01361	A-macroglobulin receptor	4	9	1.84	0.00023	C5,C3,A2ML1,C4B
SMART	SM00069	Domain containing Gla (gamma-carboxyglutamate) residues.	4	15	1.62	0.00059	F9,MGP,PROC,F2
SMART	SM01212	Fibrinogen alpha/beta chain family	3	3	2.19	0.00059	FGB,FGG,FGA
SMART	SM00104	Anaphylatoxin homologous domain	3	6	1.89	0.0019	C5,C3,C4B

SMART	SM00042	Domain first found in C1r, C1s, uEGF, and bone morphogenetic protein.	5	50	1.19	0.0022	ATRN,MASP1,MASP2,C1S,C1R
SMART	SM01419	Alpha-macro-globulin thiol-ester bond-forming region	3	8	1.76	0.0030	C3,A2ML1,C4B
SMART	SM00186	Fibrinogen-related domains (FReDs)	4	29	1.33	0.0039	FCN3,FGB,FGG,FGA
SMART	SM00093	SERine Proteinase INhibitors	4	35	1.25	0.0070	SERPINI1,SERPINF2,SERPINA3,SERPINA4
SMART	SM00643	Netrin C-terminal Domain	3	15	1.49	0.0114	C5,C3,C4B
SMART	SM00043	Cystatin-like domain	3	17	1.44	0.0148	AHSG,CST3,KNG1
SMART	SM00181	Epidermal growth factor-like domain.	7	209	0.72	0.0251	F9,PROC,ATRN,HYAL1,STAB1,OIT3,THBS4
SMART	SM00197	Serum amyloid A proteins	2	4	1.89	0.0290	SAA4,SAA1
SMART	SM00554	Four repeated domains in the Fasciclin I family of proteins, present in many other contexts.	2	4	1.89	0.0290	STAB1,TGFBI

UC+NCFET vs ACFET T2

#category	term ID	term description	observed gene count	background gene count	strength	false discovery rate	matching proteins in your network (labels)
GO Process	GO:0007596	Blood coagulation	23	173	1.42	4.27e-21	CPB2,SERPIND1,F9,TFPI2,PROC,F12,VWF,DGKG,SERPING1,FGB,F2,PLG,FGG,FN1,F5,F11,SERPINA1,ACTG1,PLCG2,KNG1,HBB,ACTB,FGA
GO Process	GO:0050878	Regulation of body fluid levels	24	371	1.11	6.17e-16	CPB2,SERPIND1,F9,TFPI2,PROC,F12,THBS1,VWF,DGKG,SERPING1,FGB,F2,PLG,FGG,FN1,F5,F11,SERPINA1,ACTG1,PLCG2,KNG1,HBB,ACTB,FGA
GO Process	GO:0006956	Complement activation	13	60	1.63	1.09e-13	C5,FCN3,SERPING1,MASP1,CFHR3,CFH,FCN1,C1S,C4A,C4B,CFB,C1R,IGHV3-72
GO Process	GO:0006959	Humoral immune response	19	268	1.15	5.77e-13	C5,FCN3,SERPING1,MASP1,FGB,F2,CFHR3,CFH,FCN1,C1S,C4A,C4B,CFB,JCHAIN,C1R,IGHV3-72,KNG1,B2M,FGA
GO Process	GO:0006950	Response to stress	53	3358	0.49	6.57e-13	CPB2,SERPIND1,CHGA,F9,TFPI2,C5,PROC,BPIFB1,F12,APCS,THBS1,VWF,DGKG,AMBP,TCIRG1,FCN3,SAA4,SERPING1,DSC2,MASP1,PDIA3,LALBA,ANGPTL4,FGB,F2,PLG,NDUFS8,FGG,FN1,HP,CFH,F5,FCN1,HLA-C,F11,C1S,C4A,MST1,C4B,SERPINA1,CFB,UIMC1,JCHAIN,HPR,C1R,ACTG1,IGHV3-72,PLCG2,KNG1,HBB,ACTB,B2M,FGA
GO Process	GO:0030195	Negative regulation of blood coagulation	11	46	1.67	7.40e-12	CPB2,PROC,F12,SERPING1,FGB,F2,PLG,FGG,F11,KNG1,FGA
GO Process	GO:0061045	Negative regulation of wound healing	12	69	1.53	9.68e-12	CPB2,PROC,F12,APCS,SERPING1,FGB,F2,PLG,FGG,F11,KNG1,FGA
GO Process	GO:0030193	Regulation of blood coagulation	12	70	1.53	1.04e-11	CPB2,PROC,F12,THBS1,SERPING1,FGB,F2,PLG,FGG,F11,KNG1,FGA
GO Process	GO:0061041	Regulation of wound healing	14	130	1.33	1.55e-11	CPB2,PROC,F12,APCS,THBS1,SERPING1,FGB,F2,PLG,FGG,F11,ACTG1,KNG1,FGA
GO Process	GO:0006952	Defense response	33	1394	0.67	3.19e-11	CHGA,C5,BPIFB1,F12,APCS,THBS1,TCIRG1,FCN3,SAA4,SERPING1,MASP1,LALBA,FGB,F2,FN1,HP,CFH,FCN1,HLA-C,C1S,C4A,C4B,SERPINA1,CFB,JCHAIN,HPR,C1R,ACTG1,IGHV3-72,PLCG2,KNG1,B2M,FGA
GO Process	GO:0031638	Zymogen activation	11	59	1.56	4.26e-11	F9,F12,FGB,F2,FGG,HP,F5,F11,HPR,C1R,FGA
GO Process	GO:0072378	Blood coagulation, fibrin clot formation	9	24	1.87	4.26e-11	F9,F12,FGB,F2,FGG,FN1,F5,F11,FGA
GO Process	GO:0098542	Defense response to other organism	27	989	0.73	3.67e-10	CHGA,C5,BPIFB1,F12,APCS,FCN3,SERPING1,MASP1,LALBA,FGB,F2,HP,CFH,FCN1,HLA-C,C1S,C4A,C4B,CFB,JCHAIN,C1R,ACTG1,IGHV3-72,PLCG2,KNG1,B2M,FGA
GO Process	GO:0042730	Fibrinolysis	8	19	1.92	4.10e-10	CPB2,F12,SERPING1,FGB,F2,PLG,FGG,FGA
GO Process	GO:0002252	Immune effector process	18	375	0.98	6.50e-10	CHGA,C5,TCIRG1,FCN3,SERPING1,MASP1,F2,CFHR3,CFH,FCN1,C1S,C4A,C4B,CFB,C1R,IGHV3-72,PLCG2,B2M
GO Process	GO:0045087	Innate immune response	23	754	0.78	2.73e-09	CHGA,C5,BPIFB1,F12,APCS,FCN3,SERPING1,MASP1,FGB,CFH,FCN1,HLA-C,C1S,C4A,C4B,CFB,JCHAIN,C1R,ACTG1,IGHV3-72,PLCG2,B2M,FGA
GO Process	GO:0051346	Negative regulation of hydrolase activity	17	354	0.98	2.73e-09	SERPIND1,TFPI2,C5,APCS,THBS1,CSTA,AMBP,SERPING1,A2ML1,SLC27A4,ANGPTL4,SERPINA7,C4A,ITIH3,C4B,SERPINA1,KNG1
GO Process	GO:0030168	Platelet activation	11	97	1.35	3.86e-09	VWF,DGKG,FGB,F2,FGG,FN1,ACTG1,PLCG2,HBB,ACTB,FGA
GO Process	GO:0002253	Activation of immune response	15	271	1.04	7.60e-09	C5,GPLD1,FCN3,SERPING1,MASP1,CFHR3,CFH,FCN1,C1S,C4A,C4B,CFB,C1R,IGHV3-72,PLCG2
GO Process	GO:0045861	Negative regulation of proteolysis	16	339	0.97	1.42e-08	CPB2,SERPIND1,TFPI2,C5,THBS1,CSTA,AMBP,SERPING1,A2ML1,F2,SERPINA7,C4A,ITIH3,C4B,SERPINA1,KNG1
GO Process	GO:0010951	Negative regulation of endopeptidase activity	14	240	1.06	1.86e-08	SERPIND1,TFPI2,C5,THBS1,CSTA,AMBP,SERPING1,A2ML1,SERPINA7,C4A,ITIH3,C4B,SERPINA1,KNG1
GO Process	GO:0006955	Immune response	28	1321	0.62	3.17e-08	CHGA,C5,BPIFB1,F12,APCS,THBS1,TCIRG1,FCN3,SERPING1,MASP1,FGB,F2,CFHR3,CFH,FCN1,HLA-C,C1S,C4A,C4B,CFB,JCHAIN,C1R,ACTG1,IGHV3-72,PLCG2,KNG1,B2M,FGA
GO Process	GO:0006508	Proteolysis	27	1247	0.63	4.63e-08	CPB2,F9,PROC,F12,FCN3,MASP1,FGB,F2,PLG,UBE3C,PAPPA,FGG,HP,CNDP1,CFH,F5,FCN1,F11,C1S,PCYOX1,MST1,CFB,UIMC1,HPR,C1R,PAMR1,FGA
GO Process	GO:0044419	Biological process involved in interspecies interaction between organisms	29	1490	0.58	9.10e-08	CHGA,C5,BPIFB1,F12,APCS,FCN3,SERPING1,MASP1,LALBA,FGB,F2,PLG,FN1,HP,CFH,FCN1,HLA-C,C1S,C4A,C4B,CFB,JCHAIN,C1R,ACTG1,IGHV3-72,PLCG2,KNG1,B2M,FGA
GO Process	GO:1903028	Positive regulation of opsonization	6	16	1.87	3.91e-07	FCN3,MASP1,FCN1,C4A,C4B,CFB
GO Process	GO:0050896	Response to stimulus	70	7835	0.25	3.96e-07	CPB2,SERPIND1,CHGA,F9,TFPI2,C5,GPLD1,PROC,BPIFB1,F12,APCS,THBS1,VWF,LCAT,DGKG,AMBP,TCIRG1,FCN3,SAA4,SERPING1,DSC2,PSG6,AZGP1,SCGB3A1,MASP1,PDIA3,SLC27A4,LALBA,ANGPTL4,FGB,F2,PLG,NDUFS8,PAPPA,TUBA1B,FGG,FN1,HP,AKAP9,CFHR3,CFH,F5,FCN1,ASPN,APOM,HLA-C,CA6,ADGRG2,YWHAZ,F11,C1S,C4A,MST1,C4B,SERPINA1,TGFBI,CFB,UIMC1,PDGFC,JCHAIN,HPR,C1R,ACTG1,IGHV3-72,PLCG2,KNG1,HBB,ACTB,B2M,FGA
GO Process	GO:0030162	Regulation of proteolysis	20	739	0.73	4.08e-07	CPB2,SERPIND1,TFPI2,C5,GPLD1,F12,THBS1,CSTA,AMBP,SERPING1,A2ML1,SH3D19,F2,SERPINA7,FN1,C4A,ITIH3,C4B,SERPINA1,KNG1
GO Process	GO:0034109	Homotypic cell-cell adhesion	8	60	1.42	6.50e-07	DSC2,FGB,FGG,FN1,ACTG1,HBB,ACTB,FGA
GO Process	GO:0051917	Regulation of fibrinolysis	6	18	1.82	6.50e-07	CPB2,F12,THBS1,F2,PLG,F11
GO Process	GO:0002376	Immune system process	33	2121	0.49	8.35e-07	CHGA,C5,GPLD1,BPIFB1,F12,APCS,THBS1,TCIRG1,FCN3,SERPING1,AZGP1,MASP1,PDIA3,FGB,F2,PLG,HP,CFHR3,CFH,FCN1,HLA-C,C1S,C4A,C4B,CFB,JCHAIN,C1R,ACTG1,IGHV3-72,PLCG2,KNG1,B2M,FGA
GO Process	GO:0006958	Complement activation, classical pathway	7	40	1.54	1.21e-06	C5,SERPING1,C1S,C4A,C4B,C1R,IGHV3-72
GO Process	GO:0070527	Platelet aggregation	7	43	1.51	1.85e-06	FGB,FGG,FN1,ACTG1,HBB,ACTB,FGA
GO Process	GO:0050778	Positive regulation of immune response	16	502	0.8	2.13e-06	C5,GPLD1,FCN3,SERPING1,MASP1,CFHR3,CFH,FCN1,C1S,C4A,C4B,CFB,C1R,IGHV3-72,PLCG2,B2M
GO Process	GO:0052547	Regulation of peptidase activity	15	446	0.82	3.25e-06	SERPIND1,TFPI2,C5,THBS1,CSTA,AMBP,SERPING1,A2ML1,SERPINA7,FN1,C4A,ITIH3,C4B,SERPINA1,KNG1
GO Process	GO:0048584	Positive regulation of response to stimulus	32	2131	0.47	3.27e-06	CPB2,CHGA,C5,GPLD1,F12,THBS1,VWF,FCN3,SERPING1,MASP1,PDIA3,FGB,F2,PLG,FGG,FN1,CFHR3,CFH,FCN1,C1S,C4A,C4B,CFB,UIMC1,PDGFC,C1R,ACTG1,IGHV3-72,PLCG2,ACTB,B2M,FGA
GO Process	GO:0043086	Negative regulation of catalytic activity	19	771	0.69	3.87e-06	SERPIND1,TFPI2,C5,APCS,THBS1,CSTA,AMBP,SERPING1,A2ML1,SLC27A4,ANGPTL4,SERPINA7,HP,AKAP9,C4A,ITIH3,C4B,SERPINA1,KNG1
GO Process	GO:0032102	Negative regulation of response to external stimulus	14	387	0.85	4.04e-06	CPB2,C5,PROC,F12,APCS,THBS1,SERPING1,FGB,F2,PLG,FGG,F11,KNG1,FGA
GO Process	GO:0002526	Acute inflammatory response	8	80	1.29	4.11e-06	F12,APCS,SAA4,F2,FN1,HP,SERPINA1,HPR
GO Process	GO:0002443	Leukocyte mediated immunity	11	211	1.01	4.47e-06	CHGA,C5,TCIRG1,SERPING1,F2,C1S,C4A,C4B,C1R,IGHV3-72,B2M
GO Process	GO:0002250	Adaptive immune response	13	359	0.85	1.28e-05	C5,TCIRG1,SERPING1,FGB,HLA-C,C1S,C4A,C4B,JCHAIN,C1R,IGHV3-72,B2M,FGA

GO Process	GO:0048583	Regulation of response to stimulus	44	3931	0.34	1.28e-05	CPB2,CHGA,C5,GPLD1,PROC,BPIFB1,F12,APCS,THBS1,VWF,DGKG,AMBP,FCN3,SERPING1,MASP1,PDIA3,SLC27A4,FGB,F2,PLG,FGG,FN1,AKAP9,CFHR3,CFH,FCN1,ASPN,YWHAZ,F11,C1S,C4A,MST1,C4B,CFB,UIMC1,PDGFC,C1R,ACTG1,IGHV3-72,PLCG2,KNG1,ACTB,B2M,FGA
GO Process	GO:0016064	Immunoglobulin mediated immune response	8	95	1.22	1.31e-05	C5,TCIRG1,SERPING1,C1S,C4A,C4B,C1R,IGHV3-72
GO Process	GO:0032101	Regulation of response to external stimulus	20	964	0.61	2.12e-05	CPB2,C5,PROC,F12,APCS,THBS1,FCN3,SERPING1,MASP1,FGB,F2,PLG,FGG,CFH,FCN1,F11,MST1,PLCG2,KNG1,FGA
GO Process	GO:0009605	Response to external stimulus	32	2355	0.43	2.82e-05	SERPIND1,CHGA,C5,BPIFB1,F12,APCS,THBS1,FCN3,SERPING1,DSC2,MASP1,SLC27A4,LALBA,FGB,F2,HP,CFH,F5,FCN1,HLA-C,C1S,C4A,C4B,CFB,JCHAIN,C1R,ACTG1,IGHV3-72,PLCG2,KNG1,B2M,FGA
GO Process	GO:0006953	Acute-phase response	6	42	1.45	3.56e-05	APCS,SAA4,F2,FN1,HP,SERPINA1
GO Process	GO:0002449	Lymphocyte mediated immunity	9	159	1.05	4.29e-05	C5,TCIRG1,SERPING1,C1S,C4A,C4B,C1R,IGHV3-72,B2M
GO Process	GO:0050766	Positive regulation of phagocytosis	7	75	1.26	4.43e-05	FCN3,MASP1,FCN1,C4A,C4B,CFB,PLCG2
GO Process	GO:0002682	Regulation of immune system process	24	1438	0.52	4.44e-05	C5,GPLD1,BPIFB1,APCS,THBS1,AMBP,FCN3,SERPING1,PSG6,MASP1,FN1,CFHR3,CFH,FCN1,C1S,C4A,MST1,C4B,CFB,C1R,IGHV3-72,PLCG2,ACTB,B2M
GO Process	GO:0044092	Negative regulation of molecular function	21	1143	0.56	6.28e-05	SERPIND1,TFPI2,C5,APCS,THBS1,CSTA,AMBP,SERPING1,A2ML1,SLC27A4,ANGPTL4,SERPINA7,HP,AKAP9,C4A,ITIH3,C4B,SERPINA1,KNG1,ACTB,B2M
GO Process	GO:0050776	Regulation of immune response	18	844	0.62	6.28e-05	C5,GPLD1,BPIFB1,AMBP,FCN3,SERPING1,MASP1,CFHR3,CFH,FCN1,C1S,C4A,C4B,CFB,C1R,IGHV3-72,PLCG2,B2M
GO Process	GO:0002460	Adaptive immune response based on somatic recombination of immune receptors built from immunoglobulin superfamily domains	9	169	1.02	6.43e-05	C5,TCIRG1,SERPING1,C1S,C4A,C4B,C1R,IGHV3-72,B2M
GO Process	GO:0002684	Positive regulation of immune system process	18	874	0.61	9.93e-05	C5,GPLD1,THBS1,FCN3,SERPING1,MASP1,CFHR3,CFH,FCN1,C1S,C4A,C4B,CFB,C1R,IGHV3-72,PLCG2,ACTB,B2M
GO Process	GO:0030194	Positive regulation of blood coagulation	5	28	1.55	0.00013	CPB2,F12,THBS1,F2,PLG
GO Process	GO:0006954	Inflammatory response	14	538	0.71	0.00015	C5,F12,APCS,THBS1,TCIRG1,SAA4,F2,FN1,HP,C4A,C4B,SERPINA1,HPR,KNG1
GO Process	GO:0031639	Plasminogen activation	4	11	1.86	0.00016	FGB,FGG,F11,FGA
GO Process	GO:0051336	Regulation of hydrolase activity	19	1011	0.57	0.00017	SERPIND1,TFPI2,C5,GPLD1,APCS,THBS1,CSTA,AMBP,SERPING1,A2ML1,SLC27A4,ANGPTL4,SERPINA7,FN1,C4A,ITIH3,C4B,SERPINA1,KNG1
GO Process	GO:0090303	Positive regulation of wound healing	6	60	1.29	0.00020	CPB2,F12,THBS1,F2,PLG,ACTG1
GO Process	GO:0051241	Negative regulation of multicellular organismal process	19	1035	0.56	0.00023	CPB2,SPARC,PROC,F12,APCS,THBS1,SERPING1,FGB,F2,PLG,FGG,FN1,ASPN,APOM,F11,PAEP,KNG1,B2M,FGA
GO Process	GO:0051248	Negative regulation of protein metabolic process	19	1038	0.56	0.00023	CPB2,SERPIND1,TFPI2,C5,APCS,THBS1,CSTA,AMBP,SERPING1,A2ML1,F2,SERPINA7,C4A,ITIH3,C4B,SERPINA1,KNG1,ITM2B,FGA
GO Process	GO:0051918	Negative regulation of fibrinolysis	4	13	1.78	0.00026	CPB2,THBS1,F2,PLG
GO Process	GO:0080134	Regulation of response to stress	22	1373	0.5	0.00026	CPB2,PROC,F12,APCS,THBS1,AMBP,FCN3,SERPING1,MASP1,FGB,F2,PLG,FGG,CFH,FCN1,F11,UIMC1,ACTG1,PLCG2,KNG1,ACTB,FGA
GO Process	GO:0065008	Regulation of biological quality	39	3654	0.32	0.00029	CPB2,SERPIND1,CHGA,F9,TFPI2,GPLD1,PROC,F12,THBS1,VWF,LCAT,DGKG,TCIRG1,SERPING1,DSC2,AZGP1,ANGPTL4,FGB,F2,PLG,SERPINA7,FGG,FN1,AKAP9,F5,APOM,YWHAZ,F11,SERPINA1,PDGFC,JCHAIN,ACTG1,PLCG2,NEB,KNG1,HBB,ACTB,B2M,FGA
GO Process	GO:0048519	Negative regulation of biological process	49	5313	0.26	0.00043	CPB2,SERPIND1,CHGA,TFPI2,C5,GPLD1,SPARC,PROC,BPIFB1,F12,APCS,THBS1,CSTA,DGKG,AMBP,FCN3,SERPING1,AZGP1,SCGB3A1,MASP1,A2ML1,SLC27A4,ANGPTL4,FGB,F2,PLG,SERPINA7,FGG,FN1,HP,FCN1,ASPN,APOM,YWHAZ,F11,C4A,MST1,ITIH3,C4B,SERPINA1,TGFBI,PAEP,UIMC1,PLCG2,KNG1,ACTB,ITM2B,B2M,FGA
GO Process	GO:0010035	Response to inorganic substance	13	532	0.68	0.00063	GPLD1,THBS1,LCAT,TCIRG1,FGB,FGG,HP,ASPN,ACTG1,PLCG2,HBB,B2M,FGA
GO Process	GO:0051239	Regulation of multicellular organismal process	32	2749	0.36	0.00063	CPB2,CHGA,C5,MGP,GPLD1,SPARC,PROC,F12,APCS,THBS1,LCAT,SERPING1,DSC2,ANGPTL4,FGB,F2,PLG,FGG,FN1,AKAP9,FCN1,ASPN,APOM,F11,MST1,PAEP,PDGFC,PLCG2,KNG1,ACTB,B2M,FGA
GO Process	GO:0051919	Positive regulation of fibrinolysis	3	4	2.17	0.00074	F12,PLG,F11
GO Process	GO:0032501	Multicellular organismal process	55	6490	0.22	0.00084	CPB2,SERPIND1,CHGA,F9,TFPI2,C5,MGP,GPLD1,PROC,F12,THBS1,VWF,LCAT,DGKG,AMBP,TCIRG1,SERPING1,SEC24D,DSC2,PSG6,AZGP1,SLC27A4,ANGPTL4,FGB,F2,PLG,PSG11,PAPPA,FGG,FN1,AKAP9,PCDH18,F5,CRTAC1,SMARCA1,ASPN,APOM,CA6,ADGRG2,YWHAZ,F11,MST1,SERPINA1,TGFBI,PDGFC,JCHAIN,ACTG1,PLCG2,NEB,KNG1,HBB,ACTB,ITM2B,B2M,FGA
GO Process	GO:0072377	Blood coagulation, common pathway	3	5	2.07	0.0012	F2,F5,FGA
GO Process	GO:0019538	Protein metabolic process	39	3910	0.29	0.0014	CPB2,F9,GPLD1,PROC,F12,THBS1,LCAT,CSTA,AMBP,TCIRG1,FCN3,MASP1,FGB,F2,PLG,UBE3C,KDM3B,PAPPA,FGG,FN1,HP,CNDP1,CFH,F5,FCN1,APOM,YWHAZ,F11,C1S,PCYOX1,MST1,CFB,UIMC1,HPR,C1R,PAMR1,ACTB,B2M,FGA
GO Process	GO:2000351	Regulation of endothelial cell apoptotic process	5	51	1.29	0.0015	THBS1,ANGPTL4,FGB,FGG,FGA
GO Process	GO:0051246	Regulation of protein metabolic process	30	2622	0.35	0.0019	CPB2,SERPIND1,TFPI2,C5,GPLD1,F12,APCS,THBS1,CSTA,AMBP,CENPE,SERPING1,A2ML1,SH3D19,F2,SERPINA7,FN1,AKAP9,C4A,MST1,ITIH3,C4B,SERPINA1,PAEP,PDGFC,PLCG2,KNG1,ACTB,ITM2B,FGA
GO Process	GO:0001775	Cell activation	14	693	0.6	0.0020	CHGA,VWF,DGKG,TCIRG1,FGB,F2,FGG,FN1,ACTG1,PLCG2,HBB,ACTB,B2M,FGA
GO Process	GO:0051592	Response to calcium ion	7	149	0.97	0.0023	GPLD1,THBS1,FGB,FGG,ACTG1,PLCG2,FGA
GO Process	GO:0051050	Positive regulation of transport	16	915	0.54	0.0026	CPB2,GPLD1,FCN3,MASP1,FGB,F2,FGG,AKAP9,FCN1,C4A,C4B,CFB,PLCG2,ACTB,B2M,FGA
GO Process	GO:1904036	Negative regulation of epithelial cell apoptotic process	5	59	1.22	0.0028	ANGPTL4,FGB,FGG,MST1,FGA
GO Process	GO:0010038	Response to metal ion	10	362	0.74	0.0029	GPLD1,THBS1,LCAT,TCIRG1,FGB,FGG,ACTG1,PLCG2,B2M,FGA
GO Process	GO:1904035	Regulation of epithelial cell apoptotic process	6	106	1.05	0.0034	THBS1,ANGPTL4,FGB,FGG,MST1,FGA
GO Process	GO:0002752	Cell surface pattern recognition receptor signaling pathway	3	9	1.82	0.0041	FCN3,MASP1,FCN1
GO Process	GO:2000352	Negative regulation of endothelial cell apoptotic process	4	31	1.41	0.0043	ANGPTL4,FGB,FGG,FGA
GO Process	GO:0002697	Regulation of immune effector process	10	383	0.71	0.0045	FCN3,SERPING1,MASP1,CFH,FCN1,C4A,C4B,CFB,PLCG2,B2M
GO Process	GO:0002220	Innate immune response activating cell surface receptor signaling pathway	4	32	1.39	0.0046	FCN3,MASP1,FCN1,PLCG2
GO Process	GO:0007155	Cell adhesion	16	965	0.51	0.0046	THBS1,VWF,CSTA,AMBP,DSC2,AZGP1,FGB,PSG11,FGG,FN1,PCDH18,TGFBI,ACTG1,HBB,ACTB,FGA
GO Process	GO:0042742	Defense response to bacterium	9	306	0.76	0.0046	CHGA,LALBA,FGB,F2,HP,JCHAIN,IGHV3-72,B2M,FGA
GO Process	GO:0001867	Complement activation, lectin pathway	3	10	1.77	0.0050	FCN3,MASP1,FCN1
GO Process	GO:0001895	Retina homeostasis	5	78	1.1	0.0089	AZGP1,JCHAIN,ACTG1,ACTB,B2M
GO Process	GO:0051240	Positive regulation of multicellular organismal process	20	1505	0.42	0.0092	CPB2,CHGA,C5,GPLD1,F12,THBS1,ANGPTL4,FGB,F2,PLG,FGG,FN1,FCN1,MST1,PAEP,PDGFC,PLCG2,ACTB,B2M,FGA
GO Process	GO:0007597	Blood coagulation, intrinsic pathway	3	13	1.66	0.0093	F9,F12,F11
GO Process	GO:0002699	Positive regulation of immune effector process	8	264	0.78	0.0098	FCN3,MASP1,FCN1,C4A,C4B,CFB,PLCG2,B2M

GO Process	GO:0048518	Positive regulation of biological process	50	6207	0.2	0.0129	CPB2,CHGA,C5,GPLD1,SPARC,PROC,F12,THBS1,VWF,CENPE,TCIRG1,FCN3,SERPING1,SCGB3A1,MASP1,PDIA3,SLC27A4,ANGPTL4,SH3D19,FGB,F2,PLG,FGG,FN1,HP,AKAP9,CFHR3,CFH,SMARCA1,FCN1,F11,C1S,C4A,MST1,C4B,CFB,PAEP,UIMC1,PDGFC,JCHAIN,HPR,C1R,ACTG1,IGHV3-72,PLCG2,KNG1,HBB,ACTB,B2M,FGA
GO Process	GO:0034116	Positive regulation of heterotypic cell-cell adhesion	3	15	1.6	0.0131	FGB,FGG,FGA
GO Process	GO:0050790	Regulation of catalytic activity	26	2370	0.33	0.0145	SERPIND1,TFPI2,C5,GPLD1,APCS,THBS1,CSTA,AMBP,CENPE,SERPING1,A2ML1,SLC27A4,ANGPTL4,F2,SERPINA7,FN1,HP,AKAP9,C4A,MST1,ITIH3,C4B,SERPINA1,PDGFC,KNG1,ACTB
GO Process	GO:0098609	Cell-cell adhesion	11	542	0.6	0.0146	CSTA,DSC2,FGB,PSG11,FGG,FN1,PCDH18,ACTG1,HBB,ACTB,FGA
GO Process	GO:0006957	Complement activation, alternative pathway	3	16	1.57	0.0151	C5,CFH,CFB
GO Process	GO:0060627	Regulation of vesicle-mediated transport	11	551	0.59	0.0165	FCN3,MASP1,FGB,FGG,FCN1,C4A,C4B,CFB,PLCG2,B2M,FGA
GO Process	GO:1902041	Regulation of extrinsic apoptotic signaling pathway via death domain receptors	4	47	1.22	0.0165	THBS1,FGB,FGG,FGA
GO Process	GO:0019730	Antimicrobial humoral response	6	149	0.9	0.0175	FGB,F2,JCHAIN,KNG1,B2M,FGA
GO Process	GO:0010810	Regulation of cell-substrate adhesion	7	217	0.8	0.0181	THBS1,FGB,PLG,FGG,FN1,ACTG1,FGA
GO Process	GO:0009617	Response to bacterium	12	663	0.55	0.0192	CHGA,LALBA,FGB,F2,HP,C4B,CFB,JCHAIN,IGHV3-72,PLCG2,B2M,FGA
GO Process	GO:0002483	Antigen processing and presentation of endogenous peptide antigen	3	18	1.52	0.0195	AZGP1,HLA-C,B2M
GO Process	GO:0010755	Regulation of plasminogen activation	3	18	1.52	0.0195	CPB2,F12,THBS1
GO Process	GO:0048585	Negative regulation of response to stimulus	20	1612	0.39	0.0204	CPB2,C5,PROC,BPIFB1,F12,APCS,THBS1,DGKG,AMBP,SERPING1,MASP1,SLC27A4,FGB,F2,PLG,FGG,ASPN,F11,KNG1,FGA
GO Process	GO:1901564	Organonitrogen compound metabolic process	42	4981	0.22	0.0245	CPB2,F9,GPLD1,PROC,F12,THBS1,LCAT,CSTA,AMBP,TCIRG1,FCN3,MASP1,FGB,F2,PLG,UBE3C,NDUFS8,KDM3B,PAPPA,FGG,FN1,HP,CNDP1,CFH,F5,FCN1,APOM,YWHAZ,F11,C1S,PCYOX1,ACSF2,MST1,ITIH3,CFB,UIMC1,HPR,C1R,PAMR1,ACTB,B2M,FGA
GO Process	GO:0030449	Regulation of complement activation	3	22	1.43	0.0322	SERPING1,MASP1,CFH
GO Process	GO:0046597	Negative regulation of viral entry into host cell	3	23	1.41	0.0360	APCS,FCN3,FCN1
GO Process	GO:0048002	Antigen processing and presentation of peptide antigen	4	60	1.12	0.0369	AZGP1,PDIA3,HLA-C,B2M
GO Process	GO:0050789	Regulation of biological process	76	11655	0.11	0.0380	CPB2,SERPIND1,CHGA,TFPI2,C5,MGP,GPLD1,SPARC,PROC,BPIFB1,F12,APCS,THBS1,VWF,LCAT,CSTA,DGKG,AMBP,CENPE,TCIRG1,FCN3,SERPING1,DSC2,PSG6,AZGP1,SCGB3A1,MASP1,A2ML1,PDIA3,SLC27A4,LALBA,ANGPTL4,SH3D19,FGB,F2,PLG,KDM3B,SERPINA7,PAPPA,FGG,FN1,HP,AKAP9,CFHR3,CFH,SMARCA1,FCN1,ASPN,APOM,ADGRG2,YWHAZ,F11,C1S,C4A,MST1,ITIH3,C4B,SERPINA1,TGFBI,CFB,PAEP,UIMC1,PDGFC,JCHAIN,HPR,C1R,ACTG1,IGHV3-72,PLCG2,NEB,KNG1,HBB,ACTB,ITM2B,B2M,FGA
GO Process	GO:0150111	Regulation of transepithelial transport	2	4	1.99	0.0437	ACTG1,ACTB
GO Process	GO:1900738	Positive regulation of phospholipase C-activating G protein-coupled receptor signaling pathway	2	4	1.99	0.0437	CHGA,F2
GO Process	GO:0019731	Antibacterial humoral response	4	64	1.09	0.0446	FGB,JCHAIN,B2M,FGA
GO Process	GO:0065007	Biological regulation	79	12385	0.1	0.0459	CPB2,SERPIND1,CHGA,F9,TFPI2,C5,MGP,GPLD1,SPARC,PROC,BPIFB1,F12,APCS,THBS1,VWF,LCAT,CSTA,DGKG,AMBP,CENPE,TCIRG1,FCN3,SERPING1,DSC2,PSG6,AZGP1,SCGB3A1,MASP1,A2ML1,PDIA3,SLC27A4,LALBA,ANGPTL4,SH3D19,FGB,F2,PLG,KDM3B,SERPINA7,PAPPA,FGG,FN1,HP,AKAP9,CNDP1,CFHR3,CFH,F5,SMARCA1,FCN1,ASPN,APOM,ADGRG2,YWHAZ,F11,C1S,C4A,MST1,ITIH3,C4B,SERPINA1,TGFBI,CFB,PAEP,UIMC1,PDGFC,JCHAIN,HPR,C1R,ACTG1,IGHV3-72,PLCG2,NEB,KNG1,HBB,ACTB,ITM2B,B2M,FGA
GO Process	GO:0006910	Phagocytosis, recognition	3	26	1.36	0.0465	FCN3,FCN1,IGHV3-72
GO Process	GO:0090276	Regulation of peptide hormone secretion	6	185	0.81	0.0466	CHGA,GPLD1,TCIRG1,FGB,FGG,FGA
GO Function	GO:0004252	Serine-type endopeptidase activity	14	176	1.2	3.67e-09	F9,PROC,F12,MASP1,F2,PLG,HP,F11,C1S,MST1,CFB,HPR,C1R,PAMR1
GO Function	GO:0004866	Endopeptidase inhibitor activity	13	177	1.16	1.64e-08	SERPIND1,TFPI2,C5,CSTA,AMBP,SERPING1,A2ML1,SERPINA7,C4A,ITIH3,C4B,SERPINA1,KNG1
GO Function	GO:0061134	Peptidase regulator activity	14	227	1.08	1.85e-08	SERPIND1,TFPI2,C5,CSTA,AMBP,SERPING1,A2ML1,SERPINA7,FN1,C4A,ITIH3,C4B,SERPINA1,KNG1
GO Function	GO:0004175	Endopeptidase activity	16	437	0.86	6.18e-07	F9,PROC,F12,MASP1,PDIA3,F2,PLG,PAPPA,HP,F11,C1S,MST1,CFB,HPR,C1R,PAMR1
GO Function	GO:0008233	Peptidase activity	18	617	0.76	1.47e-06	CPB2,F9,PROC,F12,MASP1,PDIA3,F2,PLG,PAPPA,HP,CNDP1,F11,C1S,MST1,CFB,HPR,C1R,PAMR1
GO Function	GO:0005509	Calcium ion binding	19	717	0.72	2.09e-06	F9,MGP,SPARC,PROC,F12,APCS,THBS1,DGKG,DSC2,MASP1,LALBA,F2,SPATA21,PCDH18,CRTAC1,ASPN,C1S,C1R,PAMR1
GO Function	GO:0001848	Complement binding	6	26	1.66	5.21e-06	APCS,CFHR3,CFH,C4A,C4B,CFB
GO Function	GO:0004857	Enzyme inhibitor activity	14	396	0.84	7.70e-06	SERPIND1,TFPI2,C5,CSTA,AMBP,SERPING1,A2ML1,ANGPTL4,SERPINA7,C4A,ITIH3,C4B,SERPINA1,KNG1
GO Function	GO:0004867	Serine-type endopeptidase inhibitor activity	8	98	1.21	2.43e-05	SERPIND1,TFPI2,AMBP,SERPING1,A2ML1,SERPINA7,ITIH3,SERPINA1
GO Function	GO:0005201	Extracellular matrix structural constituent	8	131	1.08	0.00018	TFPI2,MGP,THBS1,FGB,FGG,FN1,TGFBI,FGA
GO Function	GO:0005518	Collagen binding	6	66	1.25	0.00056	SPARC,THBS1,VWF,FN1,ASPN,TGFBI
GO Function	GO:0003823	Antigen binding	6	71	1.22	0.00078	FCN3,PLG,FCN1,HLA-C,JCHAIN,IGHV3-72
GO Function	GO:0001846	Opsonin binding	4	21	1.57	0.0021	APCS,CFHR3,CFH,C4A
GO Function	GO:0005488	Binding	84	12838	0.11	0.0066	CPB2,SERPIND1,F9,C5,MGP,SPARC,PROC,BPIFB1,F12,APCS,THBS1,VWF,LCAT,CSTA,DGKG,AMBP,CENPE,TCIRG1,FCN3,SEC24D,DSC2,PSG6,SCGB3A1,MASP1,A2ML1,PDIA3,SLC27A4,LALBA,ANGPTL4,SH3D19,FGB,F2,PLG,NDUFS8,KDM3B,PAPPA,SPATA21,TUBA1B,FGG,FN1,HP,AKAP9,CNDP1,PCDH18,CFHR3,CFH,F5,CRTAC1,SMARCA1,FCN1,ASPN,APOM,HLA-C,CA6,YWHAZ,F11,C1S,PCYOX1,C4A,ACSF2,MST1,C4B,SERPINA1,TGFBI,CFB,PAEP,UIMC1,PDGFC,JCHAIN,HPR,TUBA1C,C1R,ACTG1,IGHV3-72,PLCG2,PAMR1,NEB,KNG1,HBB,ACTB,TRIM66,ITM2B,B2M,FGA
GO Function	GO:0030492	Hemoglobin binding	3	9	1.82	0.0069	HP,HPR,HBB
GO Function	GO:0008201	Heparin binding	7	173	0.9	0.0089	SERPIND1,THBS1,F2,FN1,CFH,F11,KNG1
GO Function	GO:0005539	Glycosaminoglycan binding	8	245	0.81	0.0101	SERPIND1,THBS1,AMBP,F2,FN1,CFH,F11,KNG1
GO Function	GO:0005515	Protein binding	56	7242	0.18	0.0165	C5,F12,APCS,THBS1,VWF,LCAT,CSTA,AMBP,CENPE,TCIRG1,FCN3,SEC24D,DSC2,PSG6,SCGB3A1,MASP1,A2ML1,PDIA3,ANGPTL4,SH3D19,FGB,F2,PLG,TUBA1B,FGG,FN1,HP,AKAP9,CFHR3,CFH,SMARCA1,FCN1,HLA-C,YWHAZ,F11,C1S,C4A,MST1,C4B,SERPINA1,TGFBI,CFB,UIMC1,PDGFC,JCHAIN,HPR,C1R,ACTG1,IGHV3-72,PLCG2,NEB,KNG1,HBB,ACTB,B2M,FGA
GO Function	GO:1901681	Sulfur compound binding	8	272	0.76	0.0188	SERPIND1,THBS1,AMBP,F2,FN1,CFH,F11,KNG1
GO Function	GO:0005198	Structural molecule activity	13	776	0.52	0.0345	TFPI2,MGP,THBS1,FGB,TUBA1B,FGG,FN1,TGFBI,TUBA1C,ACTG1,NEB,ACTB,FGA
GO Function	GO:0005102	Signaling receptor binding	19	1499	0.4	0.0373	C5,THBS1,VWF,SCGB3A1,ANGPTL4,FGB,F2,PLG,FGG,FN1,AKAP9,FCN1,MST1,TGFBI,PDGFC,JCHAIN,IGHV3-72,KNG1,FGA
GO Function	GO:0019862	IgA binding	2	3	2.12	0.0474	AMBP,JCHAIN

GO Component	GO:0005615	Extracellular space	74	3247	0.65	3.02e-33	CPB2,SERPIND1,CHGA,F9,TFPI2,C5,MGP,GPLD1,SPARC,PROC,BPIFB1,F12,APCS,THBS1,VWF,LCAT,CSTA,AMBP,FCN3,SAA4,SERPING1,DSC2,AZGP1,SCGB3A1,MASP1,A2ML1,PDIA3,LALBA,ANGPTL4,FGB,F2,PLG,SERPINA7,PAPPA,FGG,FN1,HP,CFHR3,CFH,F5,CRTAC1,FCN1,ASPN,APOM,HLA-C,CA6,ADGRG2,YWHAZ,F11,C1S,PCYOX1,C4A,MST1,ITIH3,C4B,SERPINA1,TGFBI,CFB,PAEP,PDGFC,SBSN,JCHAIN,HPR,C1R,ACTG1,IGHV3-72,PLCG2,NEB,KNG1,HBB,ACTB,ITM2B,B2M,FGA
GO Component	GO:0005576	Extracellular region	79	4175	0.57	1.49e-31	CPB2,SERPIND1,CHGA,F9,TFPI2,C5,MGP,GPLD1,SPARC,PROC,BPIFB1,F12,APCS,THBS1,VWF,LCAT,CSTA,AMBP,CENPE,FCN3,SAA4,SERPING1,DSC2,PSG6,AZGP1,SCGB3A1,MASP1,A2ML1,PDIA3,LALBA,ANGPTL4,FGB,F2,PLG,PSG11,SERPINA7,PAPPA,FGG,FN1,HP,CNDP1,CFHR3,CFH,F5,CRTAC1,FCN1,ASPN,APOM,HLA-C,CA6,ADGRG2,YWHAZ,F11,C1S,PCYOX1,C4A,MST1,ITIH3,C4B,SERPINA1,TGFBI,CFB,PAEP,PDGFC,SBSN,JCHAIN,HPR,C1R,ACTG1,IGHV3-72,PLCG2,PAMR1,NEB,KNG1,HBB,ACTB,ITM2B,B2M,FGA
GO Component	GO:0072562	Blood microparticle	26	118	1.64	7.27e-31	APCS,AMBP,FCN3,SERPING1,ANGPTL4,FGB,F2,PLG,FGG,FN1,HP,CFHR3,CFH,YWHAZ,C1S,C4A,C4B,CFB,JCHAIN,HPR,C1R,ACTG1,KNG1,HBB,ACTB,FGA
GO Component	GO:1903561	Extracellular vesicle	57	2120	0.72	1.86e-26	CPB2,SERPIND1,F9,C5,MGP,GPLD1,BPIFB1,F12,APCS,THBS1,VWF,LCAT,AMBP,SAA4,SERPING1,DSC2,AZGP1,SCGB3A1,A2ML1,PDIA3,FGB,F2,PLG,SERPINA7,FGG,FN1,HP,CFHR3,CFH,F5,CRTAC1,HLA-C,CA6,ADGRG2,YWHAZ,F11,PCYOX1,C4A,ITIH3,C4B,SERPINA1,TGFBI,CFB,PDGFC,SBSN,JCHAIN,HPR,C1R,ACTG1,PLCG2,NEB,KNG1,HBB,ACTB,ITM2B,B2M,FGA
GO Component	GO:0070062	Extracellular exosome	56	2096	0.72	6.33e-26	CPB2,SERPIND1,F9,C5,MGP,GPLD1,BPIFB1,F12,APCS,THBS1,VWF,LCAT,AMBP,SAA4,SERPING1,DSC2,AZGP1,SCGB3A1,A2ML1,PDIA3,FGB,F2,PLG,SERPINA7,FGG,FN1,HP,CFHR3,CFH,CRTAC1,HLA-C,CA6,ADGRG2,YWHAZ,F11,PCYOX1,C4A,ITIH3,C4B,SERPINA1,TGFBI,CFB,PDGFC,SBSN,JCHAIN,HPR,C1R,ACTG1,PLCG2,NEB,KNG1,HBB,ACTB,ITM2B,B2M,FGA
GO Component	GO:0031982	Vesicle	63	3957	0.5	4.16e-18	CPB2,SERPIND1,CHGA,F9,C5,MGP,GPLD1,SPARC,BPIFB1,F12,APCS,THBS1,VWF,LCAT,AMBP,TCIRG1,SAA4,SERPING1,SEC24D,DSC2,AZGP1,SCGB3A1,A2ML1,PDIA3,FGB,F2,PLG,SERPINA7,FGG,FN1,HP,CFHR3,CFH,F5,CRTAC1,FCN1,HLA-C,CA6,ADGRG2,YWHAZ,F11,PCYOX1,C4A,ITIH3,C4B,SERPINA1,TGFBI,CFB,PDGFC,SBSN,JCHAIN,HPR,TUBA1C,C1R,ACTG1,PLCG2,NEB,KNG1,HBB,ACTB,ITM2B,B2M,FGA
GO Component	GO:0062023	Collagen-containing extracellular matrix	24	407	1.07	2.69e-16	F9,MGP,SPARC,F12,APCS,THBS1,VWF,AMBP,FCN3,SERPING1,AZGP1,ANGPTL4,FGB,F2,PLG,FGG,FN1,FCN1,ASPN,MST1,SERPINA1,TGFBI,KNG1,FGA
GO Component	GO:0031012	Extracellular matrix	26	552	0.97	1.37e-15	F9,TFPI2,MGP,GPLD1,SPARC,F12,APCS,THBS1,VWF,AMBP,FCN3,SERPING1,AZGP1,ANGPTL4,FGB,F2,PLG,FGG,FN1,FCN1,ASPN,MST1,SERPINA1,TGFBI,KNG1,FGA
GO Component	GO:1905370	Serine-type endopeptidase complex	10	26	1.88	2.65e-13	F9,PROC,F12,FCN3,MASP1,F2,CFH,F5,FCN1,F11
GO Component	GO:0031093	Platelet alpha granule lumen	12	66	1.55	6.73e-13	SPARC,THBS1,VWF,SERPING1,FGB,PLG,FGG,FN1,F5,SERPINA1,KNG1,FGA
GO Component	GO:0005788	Endoplasmic reticulum lumen	16	312	1.0	1.10e-09	SERPIND1,F9,PROC,THBS1,SERPING1,PDIA3,F2,FGG,FN1,F5,C4A,SERPINA1,PDGFC,KNG1,B2M,FGA
GO Component	GO:0034774	Secretory granule lumen	16	321	0.99	1.55e-09	SPARC,THBS1,VWF,SERPING1,FGB,PLG,FGG,FN1,HP,F5,FCN1,ITIH3,SERPINA1,KNG1,B2M,FGA
GO Component	GO:1905369	Endopeptidase complex	11	104	1.32	1.63e-09	F9,PROC,F12,FCN3,MASP1,F2,UBE3C,CFH,F5,FCN1,F11
GO Component	GO:0005577	Fibrinogen complex	5	8	2.09	3.45e-07	THBS1,FGB,FGG,FN1,FGA
GO Component	GO:0030141	Secretory granule	20	873	0.65	1.53e-06	CHGA,SPARC,THBS1,VWF,TCIRG1,SERPING1,FGB,PLG,FGG,FN1,HP,F5,FCN1,HLA-C,ITIH3,SERPINA1,KNG1,HBB,B2M,FGA
GO Component	GO:0009986	Cell surface	19	894	0.62	1.12e-05	SPARC,THBS1,AMBP,FCN3,PSG6,AZGP1,MASP1,PDIA3,FGB,PLG,PSG11,FGG,FCN1,HLA-C,ADGRG2,PDGFC,IGHV3-72,B2M,FGA
GO Component	GO:0034358	Plasma lipoprotein particle	5	38	1.41	0.00019	LCAT,SAA4,APOM,PCYOX1,HPR
GO Component	GO:0071944	Cell periphery	53	6015	0.24	0.00019	F9,TFPI2,C5,MGP,GPLD1,SPARC,F12,APCS,THBS1,VWF,CSTA,DGKG,AMBP,TCIRG1,FCN3,SERPING1,DSC2,PSG6,AZGP1,LRRC53,MASP1,SLC27A4,ANGPTL4,SH3D19,FGB,F2,PLG,FGG,FN1,AKAP9,PCDH18,F5,FCN1,ASPN,HLA-C,ADGRG2,F11,PCYOX1,C4A,MST1,C4B,SERPINA1,TGFBI,CFB,PDGFC,ACTG1,IGHV3-72,PLCG2,KNG1,ACTB,ITM2B,B2M,FGA
GO Component	GO:0005601	Classical-complement-pathway C3/C5 convertase complex	3	4	2.17	0.00029	C4A,C4B,CFB
GO Component	GO:0009897	External side of plasma membrane	11	388	0.75	0.00036	THBS1,FCN3,AZGP1,MASP1,FGB,PLG,FGG,FCN1,IGHV3-72,B2M,FGA
GO Component	GO:0034364	High-density lipoprotein particle	4	29	1.43	0.0014	LCAT,SAA4,APOM,HPR
GO Component	GO:0098552	Side of membrane	12	611	0.59	0.0044	THBS1,FCN3,AZGP1,MASP1,FGB,PLG,FGG,FCN1,HLA-C,IGHV3-72,B2M,FGA
GO Component	GO:0097708	Intracellular vesicle	27	2484	0.33	0.0054	CHGA,SPARC,THBS1,VWF,TCIRG1,SERPING1,SEC24D,DSC2,PDIA3,FGB,PLG,FGG,FN1,HP,F5,FCN1,HLA-C,YWHAZ,ITIH3,SERPINA1,ACTG1,PLCG2,KNG1,HBB,ITM2B,B2M,FGA
GO Component	GO:0030134	COPII-coated ER to Golgi transport vesicle	5	90	1.04	0.0070	SEC24D,F5,HLA-C,SERPINA1,B2M
GO Component	GO:0005783	Endoplasmic reticulum	23	2021	0.35	0.0100	SERPIND1,F9,PROC,F12,THBS1,VWF,AMBP,SERPING1,SEC24D,PDIA3,SLC27A4,FGB,F2,FGG,FN1,F5,HLA-C,C4A,SERPINA1,PDGFC,KNG1,B2M,FGA
GO Component	GO:0032991	Protein-containing complex	45	5506	0.21	0.0116	F9,C5,PROC,F12,THBS1,LCAT,CSTA,TCIRG1,FCN3,SAA4,SEC24D,MASP1,PDIA3,LALBA,FGB,F2,UBE3C,NDUFS8,KDM3B,FGG,FN1,HP,AKAP9,CFH,F5,SMARCA1,FCN1,APOM,HLA-C,F11,C1S,PCYOX1,C4A,C4B,CFB,UIMC1,JCHAIN,HPR,C1R,ACTG1,IGHV3-72,HBB,ACTB,B2M,FGA
GO Component	GO:0031410	Cytoplasmic vesicle	26	2482	0.31	0.0122	CHGA,SPARC,THBS1,VWF,TCIRG1,SERPING1,SEC24D,DSC2,PDIA3,FGB,PLG,FGG,FN1,HP,F5,FCN1,HLA-C,YWHAZ,ITIH3,SERPINA1,ACTG1,KNG1,HBB,ITM2B,B2M,FGA
GO Component	GO:0071682	Endocytic vesicle lumen	3	23	1.41	0.0157	SPARC,HP,HBB
GO Component	GO:0030139	Endocytic vesicle	8	338	0.67	0.0189	SPARC,TCIRG1,PDIA3,HP,HLA-C,ACTG1,HBB,B2M
GO Component	GO:0098871	Postsynaptic actin cytoskeleton	2	4	1.99	0.0189	ACTG1,ACTB
GO Component	GO:0005602	Complement component C1 complex	2	5	1.9	0.0254	C1S,C1R
GO Component	GO:0097433	Dense body	2	5	1.9	0.0254	ACTG1,ACTB
GO Component	GO:0045335	Phagocytic vesicle	5	139	0.85	0.0382	TCIRG1,PDIA3,HLA-C,ACTG1,B2M
GO Component	GO:0005886	Plasma membrane	43	5544	0.18	0.0486	F9,C5,SPARC,F12,THBS1,CSTA,DGKG,AMBP,TCIRG1,FCN3,DSC2,PSG6,AZGP1,LRRC53,MASP1,SLC27A4,SH3D19,FGB,F2,PLG,FGG,FN1,AKAP9,PCDH18,F5,FCN1,HLA-C,ADGRG2,F11,PCYOX1,C4A,C4B,TGFBI,CFB,PDGFC,ACTG1,IGHV3-72,PLCG2,KNG1,ACTB,ITM2B,B2M,FGA
GO Component	GO:0034366	Spherical high-density lipoprotein particle	2	8	1.69	0.0493	APOM,HPR
GO Component	GO:0042612	MHC class I protein complex	2	8	1.69	0.0493	HLA-C,B2M
STRING clusters	CL:18726	Complement and coagulation cascades, and Protein-lipid complex	37	161	1.66	2.70e-45	CPB2,SERPIND1,F9,C5,PROC,F12,APCS,VWF,LCAT,AMBP,FCN3,SAA4,SERPING1,MASP1,ANGPTL4,FGB,F2,PLG,SERPINA7,FGG,HP,CFHR3,CFH,F5,FCN1,APOM,F11,C1S,PCYOX1,C4A,ITIH3,C4B,SERPINA1,CFB,HPR,C1R,FGA
STRING clusters	CL:18728	Complement and coagulation cascades, and Positive regulation of opsonization	31	109	1.75	2.55e-40	CPB2,SERPIND1,F9,C5,PROC,F12,APCS,VWF,AMBP,FCN3,SERPING1,MASP1,FGB,F2,PLG,SERPINA7,FGG,HP,CFHR3,CFH,F5,FCN1,F11,C1S,C4A,ITIH3,C4B,SERPINA1,CFB,C1R,FGA

STRING clusters	CL:18731	Hemostasis, and Dissolution of Fibrin Clot	17	50	1.83	3.90e-22	CPB2,SERPIND1,F9,PROC,F12,VWF,AMBP,FGB,F2,PLG,FGG,HP,F5,F11,ITIH3,SERPINA1,FGA
STRING clusters	CL:18846	Complement cascade	13	50	1.71	2.68e-15	C5,APCS,FCN3,SERPING1,MASP1,CFHR3,CFH,FCN1,C1S,C4A,C4B,CFB,C1R
STRING clusters	CL:18848	Complement cascade	11	40	1.73	5.71e-13	C5,FCN3,SERPING1,MASP1,CFH,FCN1,C1S,C4A,C4B,CFB,C1R
STRING clusters	CL:18732	Mixed, incl. COVID-19, thrombosis and anticoagulation, and Scavenging of heme from plasma	10	26	1.88	6.46e-13	CPB2,SERPIND1,AMBP,FGB,PLG,FGG,HP,ITIH3,SERPINA1,FGA
STRING clusters	CL:18849	Initial triggering of complement, and Regulation of complement activation	10	31	1.8	2.56e-12	FCN3,SERPING1,MASP1,CFH,FCN1,C1S,C4A,C4B,CFB,C1R
STRING clusters	CL:18733	Mixed, incl. COVID-19, thrombosis and anticoagulation, and Inter-alpha-trypsin inhibitor heavy chain C-terminus	9	21	1.93	6.83e-12	CPB2,SERPIND1,AMBP,FGB,PLG,FGG,ITIH3,SERPINA1,FGA
STRING clusters	CL:18851	Initial triggering of complement, and Negative regulation of complement activation	8	24	1.82	9.90e-10	FCN3,SERPING1,MASP1,FCN1,C1S,C4A,C4B,C1R
STRING clusters	CL:18784	Formation of Fibrin Clot (Clotting Cascade)	7	18	1.88	9.22e-09	F9,PROC,F12,VWF,F2,F5,F11
STRING clusters	CL:18734	COVID-19, thrombosis and anticoagulation, and Negative regulation of fibrinolysis	6	11	2.03	4.61e-08	CPB2,SERPIND1,FGB,PLG,FGG,FGA
STRING clusters	CL:18852	Creation of C4 and C2 activators	6	15	1.9	1.85e-07	FCN3,SERPING1,MASP1,FCN1,C1S,C1R
STRING clusters	CL:18737	Fibrinogen, and Thrombophilia	5	6	2.22	3.23e-07	SERPIND1,FGB,PLG,FGG,FGA
STRING clusters	CL:18786	Hemophilia B, and Blood coagulation, common pathway	5	12	1.91	4.01e-06	F9,VWF,F2,F5,F11
STRING clusters	CL:18957	Lipoprotein particle	6	36	1.52	1.41e-05	LCAT,SAA4,ANGPTL4,APOM,PCYOX1,HPR
STRING clusters	CL:18960	High-density lipoprotein particle	5	26	1.58	9.05e-05	LCAT,SAA4,APOM,PCYOX1,HPR
STRING clusters	CL:18962	High-density lipoprotein particle	4	21	1.57	0.0014	LCAT,APOM,PCYOX1,HPR
STRING clusters	CL:18854	Cell surface pattern recognition receptor signaling pathway	3	6	1.99	0.0019	FCN3,MASP1,FCN1
STRING clusters	CL:18869	Complement component C1 complex	3	6	1.99	0.0019	SERPING1,C1S,C1R
STRING clusters	CL:18755	Mixed, incl. Inter-alpha-trypsin inhibitor heavy chain C-terminus, and Alpha-1-acid glycoprotein	3	10	1.77	0.0058	AMBP,ITIH3,SERPINA1
KEGG	hsa04610	Complement and coagulation cascades	24	82	1.76	3.67e-31	CPB2,SERPIND1,F9,C5,PROC,F12,VWF,SERPING1,FGB,F2,PLG,FGG,CFHR3,CFH,F5,F11,C1S,C4A,C4B,SERPINA1,CFB,C1R,KNG1,FGA
KEGG	hsa05150	Staphylococcus aureus infection	9	86	1.31	2.04e-07	C5,PLG,FGG,CFH,C1S,C4A,C4B,CFB,C1R
KEGG	hsa04611	Platelet activation	8	122	1.11	3.49e-05	VWF,FGB,F2,FGG,ACTG1,PLCG2,ACTB,FGA
KEGG	hsa04145	Phagosome	8	141	1.05	7.42e-05	THBS1,TCIRG1,TUBA1B,HLA-C,TUBA1C,C1R,ACTG1,ACTB
KEGG	hsa05133	Pertussis	6	73	1.21	0.00020	C5,SERPING1,C1S,C4A,C4B,C1R
KEGG	hsa05110	Vibrio cholerae infection	4	47	1.22	0.0071	TCIRG1,ACTG1,PLCG2,ACTB
KEGG	hsa05322	Systemic lupus erythematosus	5	94	1.02	0.0071	C5,C1S,C4A,C4B,C1R
KEGG	hsa05130	Pathogenic Escherichia coli infection	6	187	0.8	0.0186	SEC24D,F2,TUBA1B,TUBA1C,ACTG1,ACTB
KEGG	hsa04510	Focal adhesion	6	195	0.78	0.0205	THBS1,VWF,FN1,PDGFC,ACTG1,ACTB
KEGG	hsa04810	Regulation of actin cytoskeleton	6	209	0.75	0.0262	F2,FN1,PDGFC,ACTG1,KNG1,ACTB
KEGG	hsa05143	African trypanosomiasis	3	36	1.22	0.0306	HPR,KNG1,HBB
Reactome	HSA-109582	Hemostasis	30	607	0.99	6.95e-18	SERPIND1,F9,SPARC,PROC,F12,THBS1,VWF,DGKG,CENPE,SERPING1,PSG6,FGB,F2,PLG,PSG11,TUBA1B,FGG,FN1,F5,YWHAZ,F11,ITIH3,SERPINA1,JCHAIN,TUBA1C,PLCG2,KNG1,HBB,ACTB,FGA
Reactome	HSA-140877	Formation of Fibrin Clot (Clotting Cascade)	13	39	1.82	3.82e-16	SERPIND1,F9,PROC,F12,VWF,SERPING1,FGB,F2,FGG,F5,F11,KNG1,FGA
Reactome	HSA-166658	Complement cascade	13	59	1.64	2.62e-14	CPB2,C5,FCN3,SERPING1,F2,CFHR3,CFH,FCN1,C1S,C4A,C4B,CFB,C1R
Reactome	HSA-977606	Regulation of Complement cascade	11	49	1.65	4.94e-12	CPB2,C5,SERPING1,F2,CFHR3,CFH,C1S,C4A,C4B,CFB,C1R
Reactome	HSA-140837	Intrinsic Pathway of Fibrin Clot Formation	9	23	1.89	1.72e-11	SERPIND1,F9,PROC,F12,VWF,SERPING1,F2,F11,KNG1
Reactome	HSA-76002	Platelet activation, signaling and aggregation	17	260	1.11	1.72e-11	SPARC,THBS1,VWF,DGKG,SERPING1,FGB,F2,PLG,FGG,FN1,F5,YWHAZ,ITIH3,SERPINA1,PLCG2,KNG1,FGA
Reactome	HSA-114608	Platelet degranulation	13	126	1.31	7.84e-11	SPARC,THBS1,VWF,SERPING1,FGB,PLG,FGG,FN1,F5,ITIH3,SERPINA1,KNG1,FGA
Reactome	HSA-168249	Innate Immune System	27	1041	0.71	4.60e-10	CPB2,CHGA,C5,BPIFB1,TCIRG1,FCN3,SERPING1,FGB,F2,FGG,HP,CFHR3,CFH,FCN1,HLA-C,C1S,C4A,C4B,SERPINA1,CFB,C1R,ACTG1,PLCG2,HBB,ACTB,B2M,FGA
Reactome	HSA-381426	Regulation of Insulin-like Growth Factor (IGF) transport and uptake by Insulin-like Growth Factor Binding Proteins (IGFBPs)	12	124	1.28	9.67e-10	SERPIND1,PROC,F2,PLG,PAPPA,FGG,FN1,F5,C4A,SERPINA1,KNG1,FGA
Reactome	HSA-168256	Immune System	35	1979	0.54	4.11e-09	CPB2,CHGA,C5,BPIFB1,CENPE,TCIRG1,FCN3,SERPING1,SEC24D,PDIA3,FGB,F2,UBE3C,TUBA1B,FGG,FN1,HP,CFHR3,CFH,FCN1,HLA-C,YWHAZ,C1S,C4A,C4B,SERPINA1,CFB,TUBA1C,C1R,ACTG1,PLCG2,HBB,ACTB,B2M,FGA
Reactome	HSA-140875	Common Pathway of Fibrin Clot Formation	7	22	1.8	1.84e-08	SERPIND1,PROC,FGB,F2,FGG,F5,FGA
Reactome	HSA-166663	Initial triggering of complement	7	24	1.76	2.83e-08	FCN3,FCN1,C1S,C4A,C4B,CFB,C1R
Reactome	HSA-9651496	Defects of contact activation system (CAS) and kallikrein/kinin system (KKS)	6	16	1.87	1.63e-07	F9,F12,VWF,SERPING1,F2,F11
Reactome	HSA-6802948	Signaling by high-kinase activity BRAF mutants	7	36	1.58	2.68e-07	VWF,FGB,FGG,FN1,ACTG1,ACTB,FGA
Reactome	HSA-6802952	Signaling by BRAF and RAF1 fusions	8	65	1.38	4.39e-07	VWF,FGB,FGG,FN1,AKAP9,ACTG1,ACTB,FGA
Reactome	HSA-5674135	MAP2K and MAPK activation	7	40	1.54	4.58e-07	VWF,FGB,FGG,FN1,ACTG1,ACTB,FGA
Reactome	HSA-9656223	Signaling by RAF1 mutants	7	41	1.53	5.06e-07	VWF,FGB,FGG,FN1,ACTG1,ACTB,FGA
Reactome	HSA-6802946	Signaling by moderate kinase activity BRAF mutants	7	45	1.49	8.26e-07	VWF,FGB,FGG,FN1,ACTG1,ACTB,FGA
Reactome	HSA-6802955	Paradoxical activation of RAF signaling by kinase inactive BRAF	7	45	1.49	8.26e-07	VWF,FGB,FGG,FN1,ACTG1,ACTB,FGA
Reactome	HSA-8957275	Post-translational protein phosphorylation	9	107	1.22	8.26e-07	SERPIND1,PROC,FGG,FN1,F5,C4A,SERPINA1,KNG1,FGA
Reactome	HSA-9649948	Signaling downstream of RAS mutants	7	45	1.49	8.26e-07	VWF,FGB,FGG,FN1,ACTG1,ACTB,FGA
Reactome	HSA-2168880	Scavenging of heme from plasma	5	15	1.82	3.81e-06	AMBP,HP,JCHAIN,HPR,HBB
Reactome	HSA-354194	GRB2:SOS provides linkage to MAPK signaling for Integrins	5	15	1.82	3.81e-06	VWF,FGB,FGG,FN1,FGA
Reactome	HSA-372708	p130Cas linkage to MAPK signaling for integrins	5	15	1.82	3.81e-06	VWF,FGB,FGG,FN1,FGA
Reactome	HSA-76009	Platelet Aggregation (Plug Formation)	6	39	1.48	7.80e-06	VWF,FGB,F2,FGG,FN1,FGA
Reactome	HSA-2173782	Binding and Uptake of Ligands by Scavenger Receptors	6	43	1.44	1.27e-05	SPARC,AMBP,HP,JCHAIN,HPR,HBB
Reactome	HSA-174577	Activation of C3 and C5	4	7	2.05	1.45e-05	C5,C4A,C4B,CFB
Reactome	HSA-5653656	Vesicle-mediated transport	16	666	0.68	2.15e-05	SPARC,AMBP,CENPE,SEC24D,SH3D19,TUBA1B,HP,F5,YWHAZ,SERPINA1,JCHAIN,HPR,TUBA1C,ACTG1,HBB,ACTB
Reactome	HSA-166786	Creation of C4 and C2 activators	4	16	1.69	0.00018	FCN3,FCN1,C1S,C1R
Reactome	HSA-392499	Metabolism of proteins	26	1917	0.43	0.00018	CPB2,SERPIND1,F9,GPLD1,PROC,APCS,THBS1,SEC24D,PDIA3,F2,PLG,PAPPA,TUBA1B,FGG,FN1,F5,C4A,SERPINA1,TGFBI,UIMC1,TUBA1C,KNG1,ACTB,ITM2B,B2M,FGA
Reactome	HSA-216083	Integrin cell surface interactions	6	85	1.14	0.00043	THBS1,VWF,FGB,FGG,FN1,FGA
Reactome	HSA-1236974	ER-Phagosome pathway	6	89	1.12	0.00053	PDIA3,FGB,FGG,HLA-C,B2M,FGA
Reactome	HSA-9662001	Defective factor VIII causes hemophilia A	3	7	1.93	0.00088	F9,VWF,F2
Reactome	HSA-983170	Antigen Presentation: Folding, assembly and peptide loading of class I MHC	4	29	1.43	0.0013	SEC24D,PDIA3,HLA-C,B2M
Reactome	HSA-1474244	Extracellular matrix organization	9	300	0.77	0.0015	SPARC,THBS1,VWF,FGB,PLG,FGG,FN1,ASPN,FGA

Reactome	HSA-159763	Transport of gamma-carboxylated protein precursors from the endoplasmic reticulum to the Golgi apparatus	3	9	1.82	0.0015	F9,PROC,F2
Reactome	HSA-5626467	RHO GTPases activate IQGAPs	4	31	1.41	0.0015	TUBA1B,TUBA1C,ACTG1,ACTB
Reactome	HSA-159740	Gamma-carboxylation of protein precursors	3	10	1.77	0.0018	F9,PROC,F2
Reactome	HSA-159782	Removal of aminoterminal propeptides from gamma-carboxylated proteins	3	10	1.77	0.0018	F9,PROC,F2
Reactome	HSA-1445148	Translocation of SLC2A4 (GLUT4) to the plasma membrane	5	71	1.14	0.0020	TUBA1B,YWHAZ,TUBA1C,ACTG1,ACTB
Reactome	HSA-1643685	Disease	22	1702	0.41	0.0020	F9,F12,THBS1,VWF,SERPING1,SEC24D,SLC27A4,FGB,F2,TUBA1B,FGG,FN1,AKAP9,HLA-C,YWHAZ,F11,TUBA1C,ACTG1,PLCG2,ACTB,B2M,FGA
Reactome	HSA-977225	Amyloid fiber formation	5	79	1.1	0.0031	APCS,TGFBI,ITM2B,B2M,FGA
Reactome	HSA-597592	Post-translational protein modification	19	1405	0.43	0.0037	SERPIND1,F9,GPLD1,PROC,THBS1,SEC24D,PDIA3,F2,TUBA1B,FGG,FN1,F5,C4A,SERPINA1,UIMC1,TUBA1C,KNG1,ACTB,FGA
Reactome	HSA-202733	Cell surface interactions at the vascular wall	6	139	0.93	0.0042	PROC,PSG6,F2,PSG11,FN1,JCHAIN
Reactome	HSA-1280218	Adaptive Immune System	13	758	0.53	0.0059	CENPE,SEC24D,PDIA3,FGB,UBE3C,TUBA1B,FGG,HLA-C,YWHAZ,TUBA1C,PLCG2,B2M,FGA
Reactome	HSA-190828	Gap junction trafficking	4	48	1.22	0.0059	TUBA1B,TUBA1C,ACTG1,ACTB
Reactome	HSA-437239	Recycling pathway of L1	4	48	1.22	0.0059	TUBA1B,TUBA1C,ACTG1,ACTB
Reactome	HSA-5602498	MyD88 deficiency (TLR2/4)	3	17	1.54	0.0059	FGB,FGG,FGA
Reactome	HSA-5603041	IRAK4 deficiency (TLR2/4)	3	18	1.52	0.0063	FGB,FGG,FGA
Reactome	HSA-5686938	Regulation of TLR by endogenous ligand	3	20	1.47	0.0081	FGB,FGG,FGA
Reactome	HSA-9657688	Defective factor XII causes hereditary angioedema	2	3	2.12	0.0097	F12,F2
Reactome	HSA-9657689	Defective SERPING1 causes hereditary angioedema	2	3	2.12	0.0097	F12,SERPING1
Reactome	HSA-9672391	Defective F8 cleavage by thrombin	2	3	2.12	0.0097	VWF,F2
Reactome	HSA-2855086	Ficolins bind to repetitive carbohydrate structures on the target cell surface	2	4	1.99	0.0138	FCN3,FCN1
Reactome	HSA-9673221	Defective F9 activation	2	6	1.82	0.0238	F9,F11
Reactome	HSA-389958	Cooperation of Prefoldin and TriC/CCT in actin and tubulin folding	3	32	1.27	0.0244	TUBA1B,TUBA1C,ACTB
Reactome	HSA-983169	Class I MHC mediated antigen processing & presentation	8	376	0.62	0.0244	SEC24D,PDIA3,FGB,UBE3C,FGG,HLA-C,B2M,FGA
Reactome	HSA-5694530	Cargo concentration in the ER	3	33	1.25	0.0258	SEC24D,F5,SERPINA1
Reactome	HSA-5663220	RHO GTPases Activate Formins	5	139	0.85	0.0264	CENPE,TUBA1B,TUBA1C,ACTG1,ACTB
Reactome	HSA-2172127	DAP12 interactions	3	39	1.18	0.0389	HLA-C,PLCG2,B2M
Reactome	HSA-199977	ER to Golgi Anterograde Transport	5	154	0.81	0.0394	SEC24D,TUBA1B,F5,SERPINA1,TUBA1C
Reactome	HSA-983231	Factors involved in megakaryocyte development and platelet production	5	155	0.8	0.0400	CENPE,TUBA1B,TUBA1C,HBB,ACTB
Reactome	HSA-173623	Classical antibody-mediated complement activation	2	9	1.64	0.0405	C1S,C1R
Reactome	HSA-199991	Membrane Trafficking	10	626	0.5	0.0411	CENPE,SEC24D,SH3D19,TUBA1B,F5,YWHAZ,SERPINA1,TUBA1C,ACTG1,ACTB
Reactome	HSA-9006934	Signaling by Receptor Tyrosine Kinases	9	521	0.53	0.0414	SPARC,THBS1,TCIRG1,PLG,FN1,MST1,PDGFC,ACTG1,ACTB
WikiPathways	WP558	Complement and coagulation cascades	19	58	1.81	1.19e-24	CPB2,SERPIND1,F9,PROC,F12,VWF,SERPING1,MASP1,FGB,F2,PLG,CFH,F5,C1S,C4B,SERPINA1,CFB,C1R,KNG1
WikiPathways	WP2806	Complement system	16	96	1.52	1.28e-16	C5,F12,APCS,THBS1,SERPING1,MASP1,FGB,PLG,FGG,CFH,FCN1,F11,C1S,C4A,CFB,FGA
WikiPathways	WP272	Blood clotting cascade	10	22	1.95	1.04e-13	F9,F12,VWF,FGB,F2,PLG,FGG,F5,F11,FGA
WikiPathways	WP5115	Network map of SARS-CoV-2 signaling pathway	14	218	1.1	2.20e-09	FGB,FGG,FN1,HP,CFH,APOM,C1S,ITIH3,CFB,C1R,ACTG1,HBB,ACTB,FGA
WikiPathways	WP5090	Complement system in neuronal development and plasticity	11	105	1.31	2.46e-09	C5,FCN3,SERPING1,MASP1,CFH,FCN1,C1S,C4A,C4B,CFB,C1R
WikiPathways	WP545	Complement activation	7	22	1.8	1.26e-08	C5,MASP1,C1S,C4A,C4B,CFB,C1R
WikiPathways	WP4927	COVID-19, thrombosis and anticoagulation	5	7	2.15	2.55e-07	FGB,F2,PLG,FGG,FGA
WikiPathways	WP176	Folate metabolism	7	67	1.31	9.03e-06	SAA4,FGB,F2,PLG,FGG,HBB,FGA
WikiPathways	WP15	Selenium micronutrient network	7	84	1.22	3.37e-05	SAA4,FGB,F2,PLG,FGG,HBB,FGA
WikiPathways	WP2272	Pathogenic Escherichia coli infection	5	55	1.25	0.0010	TUBA1B,YWHAZ,TUBA1C,ACTG1,ACTB
WikiPathways	WP5089	Kinin-Kallikrein pathway	3	7	1.93	0.0010	F12,SERPING1,KNG1
WikiPathways	WP5186	Vitamin K metabolism and activation of dependent proteins	3	12	1.69	0.0035	F9,PROC,F2
WikiPathways	WP3941	Oxidative damage response	4	39	1.31	0.0039	C5,C1S,C4B,C1R
WikiPathways	WP4136	Fibrin complement receptor 3 signaling pathway	4	43	1.26	0.0052	FGB,PLG,FGG,FGA
WikiPathways	WP2328	Allograft rejection	5	88	1.05	0.0057	C5,TUBA1B,HLA-C,C4A,C4B
WikiPathways	WP1533	Vitamin B12 metabolism	4	50	1.2	0.0078	SAA4,F2,PLG,HBB
WikiPathways	WP4969	RAS and bradykinin pathways in COVID-19	3	28	1.32	0.0233	F12,SERPING1,KNG1
WikiPathways	WP306	Focal adhesion	6	195	0.78	0.0238	THBS1,VWF,FN1,PDGFC,ACTG1,ACTB
Monarch	EFO:0007937	Blood protein measurement	38	1810	0.62	1.09e-10	CPB2,CHGA,C5,PROC,BPIFB1,F12,VWF,LCAT,AMBP,SAA4,SERPING1,DSC2,ANKRD44,MASP1,F2,PLG,PAPPA,FN1,CNDP1,CFH,F5,CRTAC1,FCN1,ASPN,APOM,CA6,F11,C1S,PCYOX1,MST1,C4B,SERPINA1,TGFBI,CFB,HPR,C1R,PLCG2,KNG1
Monarch	HP:0001928	Abnormality of coagulation	14	170	1.21	2.92e-09	F9,PROC,F12,VWF,A2ML1,FGB,F2,PLG,FGG,F5,F11,KNG1,HBB,FGA
Monarch	HP:0003256	Abnormality of the coagulation cascade	12	105	1.35	2.92e-09	F9,PROC,F12,VWF,FGB,F2,PLG,FGG,F5,F11,KNG1,FGA
Monarch	HP:0002597	Abnormality of the vasculature	34	1673	0.6	2.95e-09	SERPIND1,F9,MGP,SPARC,PROC,F12,VWF,LCAT,CSTA,TCIRG1,SERPING1,SEC24D,MASP1,A2ML1,FGB,F2,PLG,KDM3B,FGG,FN1,F5,CRTAC1,C1S,C4A,SERPINA1,TGFBI,C1R,ACTG1,PLCG2,KNG1,HBB,ACTB,B2M,FGA
Monarch	HP:0001871	Abnormality of blood and blood-forming tissues	29	1214	0.67	3.97e-09	SERPIND1,F9,SPARC,PROC,F12,VWF,LCAT,TCIRG1,SEC24D,A2ML1,SLC27A4,FGB,F2,PLG,NDUFS8,FGG,FN1,CFH,F5,F11,C1S,C4A,SERPINA1,C1R,PLCG2,KNG1,HBB,B2M,FGA
Monarch	HP:0001892	Abnormal bleeding	18	411	0.94	8.76e-09	F9,SPARC,PROC,F12,VWF,TCIRG1,SEC24D,A2ML1,FGB,F2,FGG,FN1,F5,F11,C1S,C4A,C1R,FGA
Monarch	HP:0001977	Abnormal thrombosis	11	94	1.36	8.76e-09	SERPIND1,F9,PROC,F12,FGB,F2,FGG,F5,KNG1,HBB,FGA
Monarch	HP:0004936	Venous thrombosis	10	65	1.48	8.76e-09	SERPIND1,F9,PROC,FGB,F2,FGG,F5,KNG1,HBB,FGA
Monarch	HP:0001626	Abnormality of the cardiovascular system	39	2438	0.5	2.38e-08	SERPIND1,F9,MGP,SPARC,PROC,F12,VWF,LCAT,CSTA,TCIRG1,SERPING1,SEC24D,DSC2,MASP1,A2ML1,FGB,F2,PLG,NDUFS8,KDM3B,FGG,FN1,AKAP9,F5,CRTAC1,F11,C1S,C4A,SERPINA1,TGFBI,C1R,ACTG1,PLCG2,NEB,KNG1,HBB,ACTB,B2M,FGA
Monarch	HP:0025015	Abnormal vascular morphology	25	1027	0.68	6.62e-08	SERPIND1,F9,MGP,SPARC,PROC,F12,VWF,LCAT,TCIRG1,SEC24D,MASP1,A2ML1,FGB,F2,KDM3B,FGG,FN1,F5,CRTAC1,C4A,ACTG1,KNG1,HBB,ACTB,FGA
Monarch	HP:0011025	Abnormal cardiovascular system physiology	26	1169	0.64	1.57e-07	F9,MGP,SPARC,PROC,VWF,LCAT,TCIRG1,SERPING1,DSC2,A2ML1,FGB,F2,FGG,FN1,AKAP9,F5,CRTAC1,F11,SERPINA1,ACTG1,PLCG2,KNG1,HBB,ACTB,B2M,FGA
Monarch	HP:0030680	Abnormality of cardiovascular system morphology	30	1680	0.55	7.26e-07	SERPIND1,F9,MGP,SPARC,PROC,F12,VWF,LCAT,TCIRG1,SEC24D,DSC2,MASP1,A2ML1,FGB,F2,NDUFS8,KDM3B,FGG,FN1,F5,CRTAC1,C4A,SERPINA1,ACTG1,NEB,KNG1,HBB,ACTB,B2M,FGA
Monarch	EFO:0004634	Coagulation factor measurement	10	132	1.17	2.45e-06	CPB2,PROC,VWF,FGB,FGG,CFH,F5,F11,KNG1,FGA

Monarch	EFO:0004747	Protein measurement	58	5856	0.29	3.94e-06	CPB2,CHGA,C5,GPLD1,PROC,BPIFB1,F12,APCS,VWF,LCAT,DGKG,AMBP,TCIRG1,SAA4,SERPING1,DSC2,ANKRD44,MASP1,ANGPTL4,FGB,F2,PLG,UBE3C,PAPPA,FGG,FN1,HP,CNDP1,CFH,F5,CRTAC1,FCN1,ASPN,APOM,HLA-C,CA6,YWHAZ,F11,C1S,PCYOX1,MST1,ITIH3,C4B,SERPINA1,TGFBI,CFB,PDGFC,HPR,TUBA1C,C1R,ACTG1,PLCG2,NEB,KNG1,HBB,ACTB,TRIM66,FGA
Monarch	HP:0000225	Gingival bleeding	7	45	1.49	5.87e-06	FGB,F2,FGG,F5,C1S,C1R,FGA
Monarch	HP:0003645	Prolonged partial thromboplastin time	6	24	1.69	5.87e-06	F9,F12,F2,F5,F11,KNG1
Monarch	HP:0011276	Vascular skin abnormality	15	433	0.83	5.87e-06	PROC,F12,VWF,CSTA,TCIRG1,SERPING1,SEC24D,MASP1,F2,F5,C1S,C4A,SERPINA1,C1R,PLCG2
Monarch	HP:0000951	Abnormality of the skin	30	1888	0.5	7.17e-06	F9,C5,MGP,SPARC,PROC,F12,VWF,CSTA,CENPE,TCIRG1,FCN3,SERPING1,SEC24D,DSC2,MASP1,A2ML1,SLC27A4,F2,PLG,F5,C1S,C4A,SERPINA1,C1R,PLCG2,NEB,HBB,ACTB,B2M,FGA
Monarch	HP:0000421	Epistaxis	8	78	1.31	7.53e-06	F9,VWF,FGB,F2,FGG,F5,F11,FGA
Monarch	HP:0100659	Abnormal cerebral vascular morphology	14	381	0.86	7.53e-06	F9,SPARC,PROC,FGB,F2,FGG,FN1,F5,CRTAC1,ACTG1,KNG1,HBB,ACTB,FGA
Monarch	EFO:0004310	Partial thromboplastin time	5	15	1.82	2.52e-05	F12,F5,F11,C1S,KNG1
Monarch	HP:0001574	Abnormality of the integument	32	2266	0.44	2.62e-05	F9,C5,MGP,SPARC,PROC,F12,VWF,CSTA,CENPE,TCIRG1,FCN3,SERPING1,SEC24D,DSC2,MASP1,A2ML1,SLC27A4,F2,PLG,NDUFS8,F5,C1S,C4A,SERPINA1,C1R,ACTG1,PLCG2,NEB,HBB,ACTB,B2M,FGA
Monarch	HP:0006298	Prolonged bleeding after dental extraction	5	16	1.79	3.01e-05	F9,VWF,F2,F5,F11
Monarch	HP:0011029	Internal hemorrhage	11	244	0.95	3.42e-05	F9,SPARC,PROC,VWF,FGB,F2,FGG,FN1,F5,F11,FGA
Monarch	HP:0005542	Prolonged whole-blood clotting time	4	5	2.2	3.69e-05	F9,F12,VWF,F5
Monarch	HP:0000969	Edema	14	448	0.79	3.88e-05	F12,SERPING1,A2ML1,FGB,F2,FGG,FN1,SERPINA1,TGFBI,ACTG1,PLCG2,NEB,ACTB,FGA
Monarch	HP:0005261	Joint hemorrhage	5	19	1.71	5.29e-05	F9,VWF,F2,F5,F11
Monarch	HP:0002625	Deep venous thrombosis	5	20	1.69	6.35e-05	SERPIND1,F9,PROC,F2,F5
Monarch	HP:0002239	Gastrointestinal hemorrhage	8	111	1.15	6.56e-05	F9,VWF,FGB,F2,FGG,F5,F11,FGA
Monarch	HP:0011121	Abnormality of skin morphology	26	1648	0.49	6.56e-05	F9,MGP,SPARC,PROC,F12,VWF,CSTA,CENPE,TCIRG1,SERPING1,SEC24D,DSC2,MASP1,A2ML1,SLC27A4,F2,F5,C1S,C4A,SERPINA1,C1R,PLCG2,NEB,HBB,ACTB,B2M
Monarch	HP:0011354	Generalized abnormality of skin	18	821	0.64	7.17e-05	F9,PROC,F12,VWF,CSTA,CENPE,TCIRG1,SERPING1,SEC24D,MASP1,F2,F5,C1S,C4A,SERPINA1,C1R,PLCG2,HBB
Monarch	HP:0000118	Phenotypic abnormality	50	5129	0.28	0.00010	SERPIND1,F9,C5,MGP,SPARC,PROC,F12,VWF,LCAT,CSTA,CENPE,TCIRG1,FCN3,SERPING1,SEC24D,DSC2,MASP1,A2ML1,SLC27A4,FGB,F2,PLG,NDUFS8,KDM3B,PAPPA,FGG,FN1,AKAP9,CFH,F5,CRTAC1,ADGRG2,F11,C1S,C4A,ITIH3,C4B,SERPINA1,TGFBI,C1R,ACTG1,PLCG2,PAMR1,NEB,KNG1,HBB,ACTB,ITM2B,B2M,FGA
Monarch	HP:0005339	Abnormality of complement system	5	24	1.61	0.00011	C5,CFH,C1S,C4A,C4B
Monarch	HP:0002170	Intracranial hemorrhage	8	130	1.08	0.00017	F9,SPARC,FGB,F2,FGG,FN1,F5,FGA
Monarch	HP:0400008	Menometrorrhagia	4	10	1.9	0.00019	F9,FGB,FGG,FGA
Monarch	HP:0000168	Abnormality of the gingiva	9	190	0.97	0.00023	TCIRG1,FGB,F2,PLG,FGG,F5,C1S,C1R,FGA
Monarch	HP:0001933	Subcutaneous hemorrhage	9	189	0.97	0.00023	PROC,VWF,TCIRG1,SEC24D,F2,F5,C1S,C4A,C1R
Monarch	HP:0010989	Abnormality of the intrinsic pathway	5	29	1.53	0.00023	F9,F12,VWF,F11,KNG1
Monarch	HP:0012211	Abnormal renal physiology	14	560	0.69	0.00033	F9,LCAT,F2,PLG,NDUFS8,FN1,CFH,F5,C4A,SERPINA1,C1R,HBB,B2M,FGA
Monarch	HP:0030163	Abnormal vascular physiology	11	340	0.8	0.00046	MGP,PROC,TCIRG1,F2,F5,CRTAC1,SERPINA1,ACTG1,KNG1,HBB,ACTB
Monarch	HP:0001386	Joint swelling	5	35	1.45	0.00047	FGB,F2,FGG,SERPINA1,FGA
Monarch	HP:0010990	Abnormality of the common coagulation pathway	5	35	1.45	0.00047	FGB,PLG,FGG,F5,FGA
Monarch	HP:0002715	Abnormality of the immune system	24	1682	0.45	0.00081	C5,MGP,LCAT,CSTA,TCIRG1,FCN3,SERPING1,A2ML1,SLC27A4,FGB,F2,PLG,FGG,CFH,C1S,C4A,C4B,SERPINA1,C1R,PLCG2,HBB,ACTB,B2M,FGA
Monarch	HP:0000140	Abnormality of the menstrual cycle	9	228	0.89	0.00083	F9,VWF,FGB,F2,FGG,F5,F11,SERPINA1,FGA
Monarch	HP:0000790	Hematuria	7	118	1.07	0.00084	F9,F2,FN1,CFH,F5,HBB,FGA
Monarch	HP:0000271	Abnormality of the face	31	2641	0.36	0.0011	F9,MGP,SPARC,PROC,F12,VWF,CENPE,TCIRG1,SERPING1,SEC24D,MASP1,A2ML1,FGB,F2,PLG,KDM3B,PAPPA,FGG,F5,CRTAC1,F11,C1S,SERPINA1,C1R,ACTG1,PLCG2,NEB,HBB,ACTB,B2M,FGA
Monarch	HP:0012223	Splenic rupture	3	4	2.17	0.0011	FGB,FGG,FGA
Monarch	HP:0100724	Hypercoagulability	4	19	1.62	0.0012	F9,PROC,F5,HBB
Monarch	HP:0000978	Bruising susceptibility	7	130	1.03	0.0013	VWF,TCIRG1,SEC24D,F2,F5,C1S,C1R
Monarch	HP:0011024	Abnormality of the gastrointestinal tract	21	1384	0.48	0.0013	F9,F12,VWF,TCIRG1,FCN3,SERPING1,FGB,F2,PLG,KDM3B,FGG,F5,F11,SERPINA1,C1R,PLCG2,NEB,HBB,ACTB,B2M,FGA
Monarch	HP:0012719	Functional abnormality of the gastrointestinal tract	16	840	0.57	0.0013	F9,VWF,TCIRG1,FCN3,SERPING1,FGB,F2,FGG,F5,F11,SERPINA1,PLCG2,NEB,ACTB,B2M,FGA
Monarch	HP:0025031	Abnormality of the digestive system	29	2389	0.38	0.0013	F9,C5,F12,VWF,LCAT,TCIRG1,FCN3,SERPING1,MASP1,A2ML1,FGB,F2,PLG,NDUFS8,KDM3B,FGG,F5,F11,C1S,C4B,SERPINA1,C1R,ACTG1,PLCG2,NEB,HBB,ACTB,B2M,FGA
Monarch	HP:0004431	Complement deficiency	4	21	1.57	0.0015	C5,CFH,C4A,C4B
Monarch	HP:0005268	Miscarriage	4	21	1.57	0.0015	MGP,FGB,FGG,FGA
Monarch	HP:0011420	Age of death	4	21	1.57	0.0015	MGP,FGB,FGG,FGA
Monarch	HP:0000077	Abnormality of the kidney	18	1077	0.52	0.0016	F9,LCAT,MASP1,F2,PLG,NDUFS8,FN1,CFH,F5,ADGRG2,C4A,SERPINA1,C1R,ACTG1,HBB,ACTB,B2M,FGA
Monarch	HP:0000234	Abnormality of the head	32	2865	0.34	0.0016	F9,MGP,SPARC,PROC,F12,VWF,CENPE,TCIRG1,SERPING1,SEC24D,MASP1,A2ML1,FGB,F2,PLG,NDUFS8,KDM3B,PAPPA,FGG,F5,CRTAC1,F11,C1S,SERPINA1,C1R,ACTG1,PLCG2,NEB,HBB,ACTB,B2M,FGA
Monarch	HP:0010935	Abnormality of the upper urinary tract	18	1101	0.51	0.0020	F9,LCAT,MASP1,F2,PLG,NDUFS8,FN1,CFH,F5,ADGRG2,C4A,SERPINA1,C1R,ACTG1,HBB,ACTB,B2M,FGA
Monarch	HP:0000366	Abnormality of the nose	21	1470	0.45	0.0025	F9,MGP,VWF,CENPE,TCIRG1,MASP1,FGB,F2,PLG,KDM3B,FGG,F5,F11,SERPINA1,ACTG1,PLCG2,NEB,HBB,ACTB,B2M,FGA
Monarch	HP:0001367	Abnormal joint morphology	16	905	0.54	0.0025	F9,VWF,MASP1,A2ML1,FGB,F2,FGG,FN1,F5,F11,SERPINA1,ACTG1,NEB,HBB,ACTB,FGA
Monarch	HP:0002725	Systemic lupus erythematosus	4	26	1.48	0.0026	SERPING1,C1S,C4A,C1R
Monarch	EFO:0008390	Prothrombin time measurement	3	8	1.87	0.0033	F2,FGG,F5
Monarch	HP:0100665	Angioedema	3	8	1.87	0.0033	F12,SERPING1,PLCG2
Monarch	HP:0025032	Abnormality of digestive system physiology	23	1763	0.41	0.0036	F9,C5,F12,VWF,TCIRG1,FCN3,SERPING1,A2ML1,FGB,F2,NDUFS8,KDM3B,FGG,F5,F11,SERPINA1,ACTG1,PLCG2,NEB,HBB,ACTB,B2M,FGA
Monarch	HP:0000163	Abnormal oral cavity morphology	24	1901	0.4	0.0038	MGP,SPARC,CENPE,TCIRG1,SERPING1,SEC24D,MASP1,A2ML1,FGB,F2,PLG,KDM3B,PAPPA,FGG,F5,CRTAC1,C1S,C1R,ACTG1,NEB,HBB,ACTB,B2M,FGA
Monarch	HP:0001939	Abnormality of metabolism/homeostasis	26	2168	0.37	0.0038	F9,F12,LCAT,TCIRG1,SERPING1,A2ML1,FGB,F2,PLG,NDUFS8,FGG,FN1,AKAP9,CFH,F5,SERPINA1,TGFBI,ACTG1,PLCG2,NEB,KNG1,HBB,ACTB,ITM2B,B2M,FGA
Monarch	EFO:0003907	Deep vein thrombosis	3	9	1.82	0.0041	F2,F5,KNG1
Monarch	HP:0005368	Abnormality of humoral immunity	8	234	0.83	0.0047	C5,CFH,C1S,C4A,C4B,SERPINA1,PLCG2,B2M
Monarch	HP:0012649	Increased inflammatory response	16	981	0.51	0.0056	C5,MGP,CSTA,TCIRG1,FCN3,PLG,CFH,C1S,C4A,C4B,SERPINA1,C1R,PLCG2,HBB,B2M,FGA
Monarch	HP:0000704	Periodontitis	4	35	1.35	0.0064	TCIRG1,PLG,C1S,C1R
Monarch	HP:0002960	Autoimmunity	6	122	0.99	0.0068	TCIRG1,SERPING1,C1S,C4A,C1R,PLCG2
Monarch	HP:0033353	Abnormal blood vessel morphology	14	792	0.54	0.0074	SERPIND1,MGP,PROC,F12,VWF,LCAT,TCIRG1,SEC24D,MASP1,A2ML1,KDM3B,FN1,C4A,ACTB
Monarch	HP:0001934	Persistent bleeding after trauma	3	12	1.69	0.0075	F9,VWF,F5
Monarch	HP:0000132	Menorrhagia	4	38	1.32	0.0081	VWF,F2,F5,F11
Monarch	HP:0000119	Abnormality of the genitourinary system	25	2189	0.35	0.0109	F9,VWF,LCAT,MASP1,A2ML1,FGB,F2,PLG,NDUFS8,KDM3B,FGG,FN1,CFH,F5,ADGRG2,F11,C4A,SERPINA1,C1R,ACTG1,NEB,HBB,ACTB,B2M,FGA

Monarch	HP:0003010	Prolonged bleeding time	4	42	1.27	0.0114	F9,VWF,F2,F5
Monarch	HP:0001000	Abnormality of skin pigmentation	10	439	0.65	0.0121	PROC,CSTA,A2ML1,F2,C1S,SERPINA1,C1R,PLCG2,HBB,ACTB
Monarch	HP:0001787	Abnormal delivery	4	43	1.26	0.0121	F9,F2,KDM3B,NEB
Monarch	EFO:0003843	Pain	9	356	0.7	0.0126	C5,F12,TCIRG1,SERPING1,F2,SERPINA1,PLCG2,HBB,B2M
Monarch	HP:0002659	Increased susceptibility to fractures	7	205	0.83	0.0129	SPARC,TCIRG1,SEC24D,FN1,NEB,HBB,B2M
Monarch	HP:0011355	Localized skin lesion	13	738	0.54	0.0131	MGP,PROC,F12,CSTA,TCIRG1,SEC24D,MASP1,A2ML1,C1S,C1R,HBB,ACTB,B2M
Monarch	HP:0012639	Abnormal nervous system morphology	27	2546	0.32	0.0170	F9,MGP,SPARC,PROC,CENPE,TCIRG1,SERPING1,SEC24D,MASP1,FGB,F2,PLG,NDUFS8,KDM3B,FGG,FN1,F5,CRTAC1,C4B,ACTG1,NEB,KNG1,HBB,ACTB,ITM2B,B2M,FGA
Monarch	HP:0000080	Abnormality of reproductive system physiology	12	659	0.55	0.0178	F9,VWF,A2ML1,FGB,F2,FGG,F5,ADGRG2,F11,SERPINA1,HBB,FGA
Monarch	HP:0000707	Abnormality of the nervous system	33	3471	0.27	0.0185	F9,MGP,SPARC,PROC,CENPE,TCIRG1,SERPING1,SEC24D,MASP1,A2ML1,FGB,F2,PLG,NDUFS8,KDM3B,FGG,FN1,AKAP9,F5,CRTAC1,ITIH3,C4B,SERPINA1,TGFBI,ACTG1,PAMR1,NEB,KNG1,HBB,ACTB,ITM2B,B2M,FGA
Monarch	HP:0004846	Prolonged bleeding after surgery	3	18	1.52	0.0185	F9,VWF,F5
Monarch	HP:0005225	Intestinal edema	2	2	2.29	0.0185	F12,SERPING1
Monarch	HP:0010978	Abnormality of immune system physiology	18	1347	0.42	0.0185	C5,MGP,CSTA,TCIRG1,FCN3,SERPING1,PLG,CFH,C1S,C4A,C4B,SERPINA1,C1R,PLCG2,HBB,ACTB,B2M,FGA
Monarch	HP:0011855	Pharyngeal edema	2	2	2.29	0.0185	F12,SERPING1
Monarch	HP:0011971	Dermatographic urticaria	2	2	2.29	0.0185	SERPING1,PLCG2
Monarch	HP:0011842	Abnormal skeletal morphology	27	2573	0.32	0.0187	F9,MGP,SPARC,VWF,CENPE,TCIRG1,SEC24D,MASP1,A2ML1,FGB,F2,PLG,NDUFS8,KDM3B,FGG,FN1,F5,F11,C1S,SERPINA1,C1R,ACTG1,NEB,HBB,ACTB,B2M,FGA
Monarch	HP:0006487	Bowing of the long bones	7	224	0.79	0.0197	SPARC,TCIRG1,SEC24D,FN1,NEB,HBB,B2M
Monarch	HP:0025142	Constitutional symptom	14	906	0.48	0.0240	PROC,F12,TCIRG1,SERPING1,DSC2,F2,AKAP9,C1S,SERPINA1,TGFBI,PLCG2,NEB,HBB,B2M
Monarch	HP:0033151	Abnormal pharynx morphology	6	162	0.86	0.0240	MGP,F12,TCIRG1,SERPING1,PLG,B2M
Monarch	HP:0006323	Premature loss of primary teeth	3	21	1.45	0.0262	TCIRG1,C1S,C1R
Monarch	HP:0000079	Abnormality of the urinary system	20	1657	0.38	0.0264	F9,LCAT,MASP1,F2,PLG,NDUFS8,KDM3B,FN1,CFH,F5,ADGRG2,C4A,SERPINA1,C1R,ACTG1,NEB,HBB,ACTB,B2M,FGA
Monarch	HP:0012337	Abnormal homeostasis	17	1275	0.42	0.0266	F12,TCIRG1,SERPING1,A2ML1,FGB,F2,NDUFS8,FGG,FN1,SERPINA1,TGFBI,ACTG1,PLCG2,NEB,HBB,ACTB,FGA
Monarch	HP:0011898	Abnormality of circulating fibrinogen	3	22	1.43	0.0286	FGB,FGG,FGA
Monarch	HP:0011900	Hypofibrinogenemia	3	22	1.43	0.0286	FGB,FGG,FGA
Monarch	HP:0008065	Aplasia/Hypoplasia of the skin	6	170	0.84	0.0287	MGP,PROC,TCIRG1,SEC24D,C1S,C1R
Monarch	HP:0000123	Nephritis	4	59	1.13	0.0300	PLG,CFH,C4A,C1R
Monarch	HP:0002027	Abdominal pain	6	172	0.84	0.0300	F12,TCIRG1,SERPING1,F2,SERPINA1,HBB
Monarch	HP:0011031	Abnormality of iron homeostasis	3	23	1.41	0.0306	F2,SERPINA1,HBB
Monarch	HP:0000940	Abnormal diaphysis morphology	7	249	0.74	0.0326	SPARC,TCIRG1,SEC24D,FN1,NEB,HBB,B2M
Monarch	HP:0001297	Stroke	7	249	0.74	0.0326	F2,F5,CRTAC1,ACTG1,KNG1,HBB,ACTB
Monarch	HP:0001342	Cerebral hemorrhage	4	61	1.11	0.0328	FGB,FGG,FN1,FGA
Monarch	EFO:0008291	Stem Cell Factor measurement	3	24	1.39	0.0332	LCAT,F5,PLCG2
Monarch	EFO:0004503	Hematological measurement	39	4592	0.22	0.0337	CPB2,GPLD1,PROC,F12,APCS,VWF,LCAT,DGKG,TCIRG1,SERPING1,AZGP1,ANGPTL4,FGB,F2,PLG,PAPPA,FGG,FN1,AKAP9,CFH,F5,HLA-C,MAP3K7CL,F11,C1S,ITIH3,C4B,SERPINA1,CFB,PDGFC,HPR,TUBA1C,PLCG2,NEB,KNG1,HBB,ACTB,TRIM66,FGA
Monarch	HP:0000282	Facial edema	4	62	1.1	0.0339	F12,SERPING1,ACTG1,ACTB
Monarch	EFO:0004694	Factor XI measurement	2	4	1.99	0.0359	F11,KNG1
Monarch	HP:0002011	Morphological central nervous system abnormality	25	2416	0.31	0.0359	F9,MGP,SPARC,PROC,CENPE,TCIRG1,SEC24D,MASP1,FGB,F2,PLG,NDUFS8,KDM3B,FGG,FN1,F5,CRTAC1,C4B,ACTG1,KNG1,HBB,ACTB,ITM2B,B2M,FGA
Monarch	HP:0011034	Amyloidosis	3	25	1.37	0.0359	ITM2B,B2M,FGA
Monarch	HP:0012541	Cephalohematoma	2	4	1.99	0.0359	F9,F2
Monarch	HP:0031368	Intestinal perforation	2	4	1.99	0.0359	C1R,B2M
Monarch	HP:0001627	Abnormal heart morphology	16	1206	0.42	0.0363	MGP,VWF,TCIRG1,SEC24D,DSC2,MASP1,A2ML1,F2,NDUFS8,KDM3B,FN1,SERPINA1,NEB,HBB,ACTB,B2M
Monarch	HP:0011843	Abnormal musculoskeletal physiology	17	1330	0.4	0.0363	SPARC,CENPE,TCIRG1,SEC24D,MASP1,A2ML1,F2,KDM3B,FN1,C1S,SERPINA1,C1R,ACTG1,NEB,HBB,ACTB,B2M
Monarch	HP:0033127	Abnormality of the musculoskeletal system	30	3173	0.27	0.0363	F9,MGP,SPARC,VWF,CENPE,TCIRG1,SEC24D,MASP1,A2ML1,FGB,F2,PLG,NDUFS8,KDM3B,FGG,FN1,F5,F11,C1S,SERPINA1,TGFBI,C1R,ACTG1,PLCG2,NEB,HBB,ACTB,ITM2B,B2M,FGA
Monarch	HP:0032101	Unusual infection	12	748	0.5	0.0400	C5,MGP,TCIRG1,FCN3,PLG,CFH,C4B,SERPINA1,C1R,PLCG2,HBB,B2M
Monarch	HP:0012531	Pain	10	539	0.56	0.0408	F12,TCIRG1,SERPING1,F2,C1S,SERPINA1,TGFBI,PLCG2,HBB,B2M
Monarch	HP:0000078	Abnormality of the genital system	17	1358	0.39	0.0440	F9,VWF,MASP1,A2ML1,FGB,F2,PLG,KDM3B,FGG,F5,ADGRG2,F11,SERPINA1,NEB,HBB,ACTB,FGA
Monarch	HP:0002829	Arthralgia	5	124	0.9	0.0440	F2,C1S,SERPINA1,PLCG2,HBB
Monarch	HP:0000235	Abnormality of the fontanelles or cranial sutures	8	355	0.65	0.0443	SPARC,CENPE,TCIRG1,SEC24D,MASP1,ACTG1,NEB,ACTB
Monarch	HP:0006308	Atrophy of alveolar ridges	2	5	1.9	0.0451	C1S,C1R
Monarch	HP:0030502	Retinoschisis	2	5	1.9	0.0451	ACTG1,ACTB
Monarch	HP:0100308	Cerebral cortical hemiatrophy	2	5	1.9	0.0451	ACTG1,ACTB
DISEASES	DOID:1247	Blood coagulation disease	16	89	1.55	5.12e-16	SERPIND1,F9,PROC,F12,VWF,FGB,F2,PLG,FGG,HP,CFH,F5,F11,C4B,KNG1,FGA
DISEASES	DOID:2452	Thrombophilia	10	21	1.97	6.35e-13	SERPIND1,F9,PROC,VWF,F2,PLG,HP,CFH,F5,C4B
DISEASES	DOID:7	Disease of anatomical entity	61	4798	0.4	1.50e-11	SERPIND1,CHGA,F9,C5,MGP,SPARC,PROC,F12,APCS,VWF,LCAT,CSTA,CENPE,TCIRG1,SAA4,SERPING1,SEC24D,DSC2,ANKRD44,SCGB3A1,A2ML1,SLC27A4,LALBA,SH3D19,FGB,F2,PLG,SERPINA7,PAPPA,FGG,FN1,HP,AKAP9,CNDP1,CFHR3,CFH,F5,ASPN,HLA-C,ADGRG2,YWHAZ,F11,C1S,C4A,MST1,C4B,SERPINA1,TGFBI,CFB,JCHAIN,C1R,ACTG1,PLCG2,NEB,KNG1,HBB,ACTB,TRIM66,ITM2B,B2M,FGA
DISEASES	DOID:9120	Amyloidosis	12	75	1.5	2.08e-11	APCS,SAA4,AZGP1,FN1,SERPINA1,TGFBI,ACTG1,HBB,ACTB,ITM2B,B2M,FGA
DISEASES	DOID:4	Disease	68	6291	0.33	1.72e-10	SERPIND1,CHGA,F9,C5,MGP,SPARC,PROC,F12,APCS,VWF,LCAT,CSTA,CENPE,TCIRG1,SAA4,SERPING1,SEC24D,DSC2,ANKRD44,AZGP1,SCGB3A1,MASP1,A2ML1,SLC27A4,LALBA,SH3D19,FGB,F2,PLG,NDUFS8,SERPINA7,PAPPA,FGG,FN1,HP,AKAP9,CNDP1,CFHR3,CFH,F5,ASPN,HLA-C,CA6,ADGRG2,YWHAZ,F11,C1S,PCYOX1,C4A,MST1,C4B,SERPINA1,TGFBI,CFB,PDGFC,JCHAIN,HPR,C1R,ACTG1,PLCG2,NEB,KNG1,HBB,ACTB,TRIM66,ITM2B,B2M,FGA
DISEASES	DOID:74	Hematopoietic system disease	20	473	0.92	3.73e-10	SERPIND1,F9,PROC,F12,VWF,TCIRG1,FGB,F2,PLG,FGG,HP,CFH,F5,F11,C4B,KNG1,HBB,ACTB,B2M,FGA
DISEASES	DOID:2914	Immune system disease	21	675	0.79	2.14e-08	C5,APCS,SAA4,SERPING1,A2ML1,LALBA,F2,PLG,FN1,HP,CFHR3,CFH,HLA-C,C4A,C4B,CFB,JCHAIN,PLCG2,KNG1,ACTB,B2M
DISEASES	DOID:0050636	Familial visceral amyloidosis	7	21	1.82	4.22e-08	APCS,AZGP1,SERPINA1,HBB,ACTB,B2M,FGA
DISEASES	DOID:0050736	Autosomal dominant disease	28	1386	0.6	1.04e-07	SERPIND1,F9,PROC,F12,APCS,VWF,SERPING1,ANKRD44,AZGP1,F2,HP,AKAP9,CFHR3,CFH,F5,C1S,SERPINA1,TGFBI,CFB,JCHAIN,ACTG1,PLCG2,KNG1,HBB,ACTB,ITM2B,B2M,FGA

DISEASES	DOID:630	Genetic disease	45	3778	0.37	1.92e-06	SERPIND1,F9,MGP,PROC,F12,APCS,VWF,LCAT,TCIRG1,SERPING1,SEC24D,ANKRD44,AZGP1,MASP1,A2ML1,FGB,F2,PLG,NDUFS8,PAPPA,FGG,HP,AKAP9,CNDP1,CFHR3,CFH,F5,ADGRG2,F11,C1S,PCYOX1,MST1,SERPINA1,TGFBI,CFB,JCHAIN,ACTG1,PLCG2,NEB,KNG1,HBB,ACTB,ITM2B,B2M,FGA
DISEASES	DOID:626	Complement deficiency	6	23	1.71	2.58e-06	C5,SERPING1,CFHR3,CFH,CFB,KNG1
DISEASES	DOID:0050177	Monogenic disease	41	3266	0.39	2.71e-06	SERPIND1,F9,MGP,PROC,F12,APCS,VWF,TCIRG1,SERPING1,SEC24D,ANKRD44,AZGP1,MASP1,A2ML1,FGB,F2,PLG,FGG,HP,AKAP9,CFHR3,CFH,F5,ADGRG2,F11,C1S,PCYOX1,MST1,SERPINA1,TGFBI,CFB,JCHAIN,ACTG1,PLCG2,NEB,KNG1,HBB,ACTB,ITM2B,B2M,FGA
DISEASES	DOID:0050739	Autosomal genetic disease	37	2802	0.42	5.40e-06	SERPIND1,F9,MGP,PROC,F12,APCS,VWF,TCIRG1,SERPING1,ANKRD44,AZGP1,MASP1,FGB,F2,PLG,FGG,HP,AKAP9,CFHR3,CFH,F5,F11,C1S,PCYOX1,SERPINA1,TGFBI,CFB,JCHAIN,ACTG1,PLCG2,NEB,KNG1,HBB,ACTB,ITM2B,B2M,FGA
DISEASES	DOID:14735	Hereditary angioedema	4	6	2.12	4.13e-05	F12,SERPING1,C1S,KNG1
DISEASES	DOID:557	Kidney disease	11	275	0.9	6.14e-05	C5,APCS,SAA4,F2,FN1,HP,CFHR3,CFH,C4B,CFB,B2M
DISEASES	DOID:37	Skin disease	14	518	0.73	0.00012	F12,APCS,CSTA,SERPING1,DSC2,A2ML1,SLC27A4,HP,HLA-C,C1S,SERPINA1,ACTG1,KNG1,ACTB
DISEASES	DOID:612	Primary immunodeficiency disease	13	470	0.74	0.00024	C5,SERPING1,A2ML1,F2,HP,CFHR3,CFH,HLA-C,C4A,C4B,CFB,PLCG2,KNG1
DISEASES	DOID:0014667	Disease of metabolism	19	1076	0.54	0.00047	F9,MGP,APCS,LCAT,SAA4,AZGP1,F2,NDUFS8,FN1,CNDP1,PCYOX1,SERPINA1,TGFBI,ACTG1,HBB,ACTB,ITM2B,B2M,FGA
DISEASES	DOID:2236	Congenital afibrinogenemia	3	3	2.29	0.00052	FGB,FGG,FGA
DISEASES	DOID:10772	Thrombotic thrombocytopenic purpura	3	4	2.17	0.00086	VWF,HP,CFH
DISEASES	DOID:0050639	Primary cutaneous amyloidosis	4	19	1.62	0.00096	APCS,SERPINA1,ACTG1,ACTB
DISEASES	DOID:17	Musculoskeletal system disease	19	1154	0.51	0.0010	F9,C5,MGP,SPARC,F12,APCS,TCIRG1,SEC24D,A2ML1,PLG,ASPN,HLA-C,C1S,C4A,C4B,JCHAIN,C1R,NEB,B2M
DISEASES	DOID:65	Connective tissue disease	15	774	0.58	0.0017	F9,C5,MGP,SPARC,TCIRG1,SEC24D,A2ML1,PLG,ASPN,HLA-C,C1S,C4A,C4B,JCHAIN,C1R
DISEASES	DOID:0080301	Atypical hemolytic-uremic syndrome	3	11	1.73	0.0074	CFHR3,CFH,CFB
DISEASES	DOID:2921	Glomerulonephritis	4	36	1.34	0.0080	CFHR3,CFH,C4B,CFB
DISEASES	DOID:12554	Hemolytic-uremic syndrome	3	14	1.63	0.0128	CFHR3,CFH,CFB
DISEASES	DOID:0050117	Disease by infectious agent	9	368	0.68	0.0184	APCS,F2,PLG,HLA-C,CA6,YWHAZ,C4A,PDGFC,HPR
DISEASES	DOID:0060229	Baraitser-Winter syndrome	2	2	2.29	0.0216	ACTG1,ACTB
DISEASES	DOID:409	Liver disease	5	97	1.01	0.0234	APCS,SAA4,F2,SERPINA1,ACTB
DISEASES	DOID:0060903	Thrombosis	3	21	1.45	0.0310	VWF,F2,PLG
DISEASES	DOID:1287	Cardiovascular system disease	10	493	0.6	0.0310	CHGA,APCS,VWF,DSC2,F2,PLG,PAPPA,AKAP9,HLA-C,C4A
DISEASES	DOID:10881	Hand, foot and mouth disease	2	3	2.12	0.0319	CA6,C4A
DISEASES	DOID:2216	Factor V deficiency	2	3	2.12	0.0319	F2,F5
DISEASES	DOID:2231	Factor XII deficiency	2	3	2.12	0.0319	F12,F11
DISEASES	DOID:3118	Hepatobiliary disease	6	168	0.85	0.0319	APCS,SAA4,F2,MST1,SERPINA1,ACTB
DISEASES	DOID:655	Inherited metabolic disorder	14	949	0.46	0.0387	F9,APCS,LCAT,AZGP1,NDUFS8,CNDP1,PCYOX1,SERPINA1,ACTG1,HBB,ACTB,ITM2B,B2M,FGA
DISEASES	DOID:0060002	C1 inhibitor deficiency	2	4	1.99	0.0419	SERPING1,KNG1
DISEASES	DOID:0070027	CST3-related cerebral amyloid angiopathy	2	4	1.99	0.0419	APCS,ITM2B
DISEASES	DOID:10128	Venous insufficiency	2	4	1.99	0.0419	F2,C4A
DISEASES	DOID:12134	Factor VIII deficiency	2	4	1.99	0.0419	F9,VWF
DISEASES	DOID:3756	Protein C deficiency	2	4	1.99	0.0419	PROC,F2
TISSUES	BTO:0004850	Bone marrow cell	31	198	1.49	9.11e-33	CPB2,C5,APCS,THBS1,VWF,AMBP,FCN3,SAA4,SERPING1,DSC2,AZGP1,MASP1,FGB,F2,FN1,HP,CFH,F5,F11,C1S,C4A,MST1,C4B,SERPINA1,CFB,JCHAIN,C1R,PAMR1,KNG1,B2M,FGA
TISSUES	BTO:0000141	Bone marrow	37	528	1.14	1.73e-28	CPB2,SERPIND1,C5,APCS,THBS1,VWF,CSTA,AMBP,FCN3,SAA4,SERPING1,DSC2,AZGP1,MASP1,FGB,F2,KDM3B,FN1,HP,CFH,F5,YWHAZ,F11,C1S,C4A,MST1,C4B,SERPINA1,CFB,JCHAIN,TUBA1C,C1R,PAMR1,KNG1,HBB,B2M,FGA
TISSUES	BTO:0000392	Plasma cell	27	171	1.49	1.73e-28	C5,APCS,THBS1,VWF,AMBP,FCN3,SAA4,SERPING1,DSC2,AZGP1,MASP1,FGB,F2,FN1,HP,CFH,C1S,C4A,C4B,SERPINA1,CFB,JCHAIN,C1R,PAMR1,KNG1,B2M,FGA
TISSUES	BTO:0001486	Skeletal system	49	1307	0.87	7.62e-28	CPB2,SERPIND1,CHGA,C5,SPARC,APCS,THBS1,VWF,CSTA,AMBP,FCN3,SAA4,SERPING1,DSC2,AZGP1,MASP1,FGB,F2,UBE3C,KDM3B,SERPINA7,TUBA1B,FGG,FN1,HP,CFH,F5,CRTAC1,ASPN,YWHAZ,F11,C1S,PCYOX1,C4A,MST1,C4B,SERPINA1,CFB,JCHAIN,TUBA1C,C1R,ACTG1,PAMR1,NEB,KNG1,HBB,ACTB,B2M,FGA
TISSUES	BTO:0000759	Liver	52	2125	0.68	1.72e-21	CPB2,SERPIND1,CHGA,F9,GPLD1,PROC,F12,APCS,THBS1,VWF,AMBP,FCN3,SAA4,SERPING1,AZGP1,MASP1,PDIA3,FGB,F2,PLG,SERPINA7,TUBA1B,FGG,FN1,HP,AKAP9,PCDH18,CFHR3,CFH,SMARCA1,APOM,HLA-C,YWHAZ,F11,C1S,PCYOX1,C4A,MST1,ITIH3,C4B,SERPINA1,TGFBI,CFB,JCHAIN,TUBA1C,C1R,ACTG1,KNG1,HBB,ACTB,B2M,FGA
TISSUES	BTO:0000345	Digestive gland	58	2881	0.6	9.42e-21	CPB2,SERPIND1,CHGA,F9,GPLD1,SPARC,PROC,F12,APCS,THBS1,VWF,AMBP,TCIRG1,FCN3,SAA4,SERPING1,SEC24D,AZGP1,MASP1,PDIA3,ANGPTL4,FGB,F2,PLG,NDUFS8,SERPINA7,TUBA1B,FGG,FN1,HP,AKAP9,PCDH18,CFHR3,CFH,SMARCA1,APOM,HLA-C,YWHAZ,F11,C1S,PCYOX1,C4A,MST1,ITIH3,C4B,SERPINA1,TGFBI,CFB,JCHAIN,TUBA1C,C1R,ACTG1,KNG1,HBB,ACTB,ITM2B,B2M,FGA
TISSUES	BTO:0001491	Viscus	64	5378	0.37	8.76e-12	CPB2,SERPIND1,CHGA,F9,GPLD1,SPARC,PROC,F12,APCS,THBS1,VWF,CSTA,AMBP,TCIRG1,FCN3,SAA4,SERPING1,SEC24D,DSC2,AZGP1,MASP1,PDIA3,LALBA,ANGPTL4,SH3D19,FGB,F2,PLG,SERPINA7,TUBA1B,FGG,FN1,HP,AKAP9,PCDH18,CFHR3,CFH,SMARCA1,ASPN,APOM,HLA-C,YWHAZ,F11,C1S,PCYOX1,C4A,ACSF2,MST1,ITIH3,C4B,SERPINA1,TGFBI,CFB,JCHAIN,TUBA1C,C1R,ACTG1,PLCG2,KNG1,HBB,ACTB,ITM2B,B2M,FGA
TISSUES	BTO:0000237	Cerebrospinal fluid	9	45	1.6	1.98e-09	CHGA,SERPING1,F2,SERPINA7,HP,CFH,C4A,SERPINA1,KNG1
TISSUES	BTO:0001489	Whole body	94	13099	0.15	1.12e-08	CPB2,SERPIND1,CHGA,F9,TFPI2,C5,MGP,GPLD1,SPARC,PROC,BPIFB1,F12,APCS,THBS1,VWF,LCAT,CSTA,DGKG,AMBP,CENPE,TCIRG1,FCN3,SAA4,SERPING1,SEC24D,DSC2,PSG6,AZGP1,SCGB3A1,MASP1,A2ML1,PDIA3,SLC27A4,LALBA,ANGPTL4,SH3D19,FGB,F2,PLG,UBE3C,NDUFS8,PSG11,KDM3B,SERPINA7,PAPPA,TUBA1B,FGG,ISYNA1,FN1,HP,AKAP9,CNDP1,PCDH18,CFHR3,CFH,F5,CRTAC1,SMARCA1,FCN1,ASPN,APOM,HLA-C,CA6,ADGRG2,YWHAZ,MAP3K7CL,F11,C1S,PCYOX1,C4A,ACSF2,MST1,ITIH3,C4B,SERPINA1,TGFBI,CFB,PAEP,UIMC1,PDGFC,SBSN,JCHAIN,TUBA1C,C1R,ACTG1,PLCG2,PAMR1,NEB,KNG1,HBB,ACTB,ITM2B,B2M,FGA
TISSUES	BTO:0001488	Endocrine gland	64	6403	0.29	2.75e-08	CPB2,SERPIND1,CHGA,F9,GPLD1,SPARC,PROC,F12,APCS,THBS1,VWF,CSTA,AMBP,TCIRG1,FCN3,SAA4,SERPING1,SEC24D,AZGP1,MASP1,PDIA3,ANGPTL4,SH3D19,FGB,F2,PLG,NDUFS8,SERPINA7,TUBA1B,FGG,ISYNA1,FN1,HP,AKAP9,PCDH18,CFHR3,CFH,SMARCA1,APOM,HLA-C,YWHAZ,F11,C1S,PCYOX1,C4A,ACSF2,MST1,ITIH3,C4B,SERPINA1,TGFBI,CFB,UIMC1,PDGFC,JCHAIN,TUBA1C,C1R,ACTG1,KNG1,HBB,ACTB,ITM2B,B2M,FGA

TISSUES	BTO:0000522	Gland	67	7004	0.28	3.43e-08	CPB2,SERPIND1,CHGA,F9,C5,GPLD1,SPARC,PROC,BPIFB1,F12,APCS,THBS1,VWF,CSTA,AMBP,TCIRG1,FCN3,SAA4,SERPING1,SEC24D,AZGP1,MASP1,PDIA3,ANGPTL4,SH3D19,FGB,F2,PLG,NDUFS8,SERPINA7,TUBA1B,FGG,ISYNA1,FN1,HP,AKAP9,PCDH18,CFHR3,CFH,SMARCA1,APOM,HLA-C,CA6,YWHAZ,F11,C1S,PCYOX1,C4A,ACSF2,MST1,ITIH3,C4B,SERPINA1,TGFBI,CFB,UIMC1,PDGFC,JCHAIN,TUBA1C,C1R,ACTG1,KNG1,HBB,ACTB,ITM2B,B2M,FGA
TISSUES	BTO:0000203	Respiratory system	30	1707	0.54	1.80e-07	SPARC,BPIFB1,THBS1,VWF,TCIRG1,FCN3,SERPING1,PDIA3,SH3D19,TUBA1B,FN1,AKAP9,PCDH18,CFH,SMARCA1,FCN1,HLA-C,C1S,PCYOX1,C4B,SERPINA1,TGFBI,PDGFC,JCHAIN,TUBA1C,ACTG1,KNG1,HBB,ACTB,B2M
TISSUES	BTO:0001078	Placenta	24	1244	0.58	2.12e-06	TFPI2,SPARC,THBS1,VWF,SERPING1,DSC2,PSG6,PDIA3,ANGPTL4,PSG11,PAPPA,TUBA1B,FN1,F5,HLA-C,YWHAZ,PCYOX1,SERPINA1,TGFBI,TUBA1C,ACTG1,HBB,ACTB,B2M
TISSUES	BTO:0000088	Cardiovascular system	22	1057	0.61	2.43e-06	TFPI2,MGP,SPARC,THBS1,VWF,SERPING1,PDIA3,SLC27A4,ANGPTL4,F2,TUBA1B,FN1,PCDH18,ASPN,PCYOX1,SERPINA1,TGFBI,ACTG1,HBB,ACTB,B2M,FGA
TISSUES	BTO:0000089	Blood	29	1824	0.5	2.85e-06	PROC,F12,THBS1,VWF,SERPING1,AZGP1,PDIA3,FGB,F2,TUBA1B,FGG,FN1,HP,AKAP9,PCDH18,F5,APOM,HLA-C,YWHAZ,C1S,C4B,SERPINA1,CFB,ACTG1,PLCG2,HBB,ACTB,B2M,FGA
TISSUES	BTO:0000763	Lung	25	1395	0.55	3.45e-06	SPARC,BPIFB1,THBS1,VWF,TCIRG1,FCN3,SERPING1,PDIA3,TUBA1B,FN1,AKAP9,PCDH18,SMARCA1,FCN1,HLA-C,C1S,C4B,SERPINA1,TGFBI,PDGFC,TUBA1C,ACTG1,HBB,ACTB,B2M
TISSUES	BTO:0000570	Hematopoietic system	36	2755	0.41	4.43e-06	GPLD1,PROC,F12,THBS1,VWF,SERPING1,AZGP1,PDIA3,ANGPTL4,FGB,F2,PAPPA,TUBA1B,FGG,FN1,HP,AKAP9,PCDH18,F5,APOM,HLA-C,YWHAZ,C1S,C4B,SERPINA1,TGFBI,CFB,JCHAIN,TUBA1C,ACTG1,PLCG2,KNG1,HBB,ACTB,B2M,FGA
TISSUES	BTO:0001279	Spinal cord	11	233	0.97	4.96e-06	CHGA,SERPING1,F2,SERPINA7,HP,PCDH18,CFH,C4A,SERPINA1,KNG1,ACTB
TISSUES	BTO:0001307	Stomach	12	312	0.88	9.62e-06	CHGA,SPARC,PDIA3,LALBA,TUBA1B,FN1,AKAP9,HLA-C,TUBA1C,ACTG1,ACTB,B2M
TISSUES	BTO:0003099	Internal female genital organ	35	2804	0.39	1.94e-05	TFPI2,MGP,SPARC,THBS1,VWF,SERPING1,DSC2,PSG6,MASP1,A2ML1,PDIA3,ANGPTL4,NDUFS8,PSG11,PAPPA,TUBA1B,ISYNA1,FN1,PCDH18,F5,HLA-C,YWHAZ,C1S,PCYOX1,SERPINA1,TGFBI,PAEP,UIMC1,PDGFC,TUBA1C,ACTG1,HBB,ACTB,ITM2B,B2M
TISSUES	BTO:0000174	Embryonic structure	31	2369	0.41	4.56e-05	TFPI2,SPARC,THBS1,VWF,SERPING1,DSC2,PSG6,PDIA3,ANGPTL4,UBE3C,PSG11,PAPPA,TUBA1B,ISYNA1,FN1,AKAP9,PCDH18,F5,ASPN,APOM,HLA-C,YWHAZ,PCYOX1,SERPINA1,TGFBI,UIMC1,TUBA1C,ACTG1,HBB,ACTB,B2M
TISSUES	BTO:0001419	Urine	5	28	1.55	6.12e-05	AMBP,F2,FN1,SERPINA1,B2M
TISSUES	BTO:0001202	Saliva	6	57	1.32	7.17e-05	THBS1,DSC2,AZGP1,HP,C4B,JCHAIN
TISSUES	BTO:0000042	Animal	95	15148	0.09	8.13e-05	CPB2,SERPIND1,CHGA,F9,TFPI2,C5,MGP,GPLD1,SPARC,PROC,BPIFB1,F12,APCS,THBS1,VWF,LCAT,CSTA,DGKG,AMBP,CENPE,TCIRG1,FCN3,SAA4,SERPING1,SEC24D,DSC2,PSG6,AZGP1,SCGB3A1,MASP1,A2ML1,PDIA3,SLC27A4,LALBA,ANGPTL4,SH3D19,FGB,F2,PLG,UBE3C,NDUFS8,PSG11,KDM3B,SERPINA7,PAPPA,TUBA1B,FGG,ISYNA1,FN1,HP,AKAP9,CNDP1,PCDH18,CFHR3,CFH,F5,CRTAC1,SMARCA1,FCN1,ASPN,APOM,HLA-C,CA6,ADGRG2,YWHAZ,MAP3K7CL,F11,C1S,PCYOX1,C4A,ACSF2,MST1,ITIH3,C4B,SERPINA1,TGFBI,CFB,PAEP,UIMC1,PDGFC,SBSN,JCHAIN,TUBA1C,C1R,ACTG1,PLCG2,PAMR1,NEB,KNG1,HBB,ACTB,TRIM66,ITM2B,B2M,FGA
TISSUES	BTO:0000421	Connective tissue	18	954	0.57	0.00013	TFPI2,SPARC,THBS1,PDIA3,ANGPTL4,PAPPA,TUBA1B,FN1,F5,CRTAC1,ASPN,YWHAZ,ACSF2,SERPINA1,TUBA1C,ACTG1,ACTB,B2M
TISSUES	BTO:0000020	Abdomen	7	113	1.09	0.00020	F2,TUBA1B,SERPINA1,ACTG1,KNG1,ACTB,B2M
TISSUES	BTO:0000132	Blood platelet	11	363	0.78	0.00023	THBS1,VWF,PDIA3,FGB,FGG,F5,YWHAZ,SERPINA1,ACTG1,ACTB,FGA
TISSUES	BTO:0000511	Gastrointestinal tract	25	1816	0.43	0.00025	CHGA,SPARC,PROC,SERPING1,AZGP1,PDIA3,LALBA,SH3D19,TUBA1B,FN1,AKAP9,PCDH18,ASPN,HLA-C,YWHAZ,C1S,SERPINA1,TGFBI,CFB,JCHAIN,TUBA1C,C1R,ACTG1,ACTB,B2M
TISSUES	BTO:0000988	Pancreas	14	626	0.64	0.00029	CHGA,GPLD1,SPARC,TCIRG1,SEC24D,PDIA3,ANGPTL4,TUBA1B,FN1,HLA-C,TGFBI,ACTG1,ACTB,B2M
TISSUES	BTO:0000562	Heart	15	738	0.6	0.00038	THBS1,VWF,SERPING1,PDIA3,SLC27A4,F2,TUBA1B,FN1,PCDH18,ASPN,PCYOX1,SERPINA1,HBB,ACTB,FGA
TISSUES	BTO:0000058	Alimentary canal	26	2021	0.4	0.00047	CHGA,SPARC,PROC,THBS1,SERPING1,AZGP1,PDIA3,LALBA,SH3D19,TUBA1B,FN1,AKAP9,PCDH18,ASPN,HLA-C,YWHAZ,C1S,SERPINA1,TGFBI,CFB,JCHAIN,TUBA1C,C1R,ACTG1,ACTB,B2M
TISSUES	BTO:0001239	Serum	4	25	1.5	0.00084	GPLD1,PAPPA,HP,KNG1
TISSUES	BTO:0000648	Intestine	20	1349	0.47	0.00087	PROC,AZGP1,PDIA3,SH3D19,TUBA1B,FN1,PCDH18,ASPN,HLA-C,YWHAZ,C1S,SERPINA1,TGFBI,CFB,JCHAIN,TUBA1C,C1R,ACTG1,ACTB,B2M
TISSUES	BTO:0000091	Ascites	5	61	1.21	0.0012	TUBA1B,SERPINA1,KNG1,ACTB,B2M
TISSUES	BTO:0001090	Mouth	12	532	0.65	0.0012	C5,BPIFB1,CSTA,CENPE,A2ML1,FN1,CA6,YWHAZ,SBSN,JCHAIN,ACTB,B2M
TISSUES	BTO:0000140	Bone	7	165	0.92	0.0015	SPARC,THBS1,TUBA1B,FN1,ACTG1,ACTB,B2M
TISSUES	BTO:0000102	Blood clot	3	11	1.73	0.0025	VWF,F2,PLG
TISSUES	BTO:0001424	Uterus	17	1117	0.48	0.0027	MGP,SPARC,THBS1,SERPING1,A2ML1,NDUFS8,TUBA1B,ISYNA1,FN1,YWHAZ,C1S,PAEP,UIMC1,PDGFC,ACTG1,ACTB,ITM2B
TISSUES	BTO:0001085	Vascular system	10	420	0.67	0.0034	TFPI2,MGP,THBS1,VWF,ANGPTL4,F2,FN1,TGFBI,ACTB,B2M
TISSUES	BTO:0003914	Interstitial cell of Cajal	7	193	0.85	0.0037	PDIA3,TUBA1B,YWHAZ,SERPINA1,TUBA1C,ACTG1,ACTB
TISSUES	BTO:0000121	Bile	3	20	1.47	0.0104	AZGP1,SERPINA1,JCHAIN
TISSUES	BTO:0000084	Vermiform appendix	3	22	1.43	0.0131	JCHAIN,ACTB,B2M
TISSUES	BTO:0001499	Tear	3	23	1.41	0.0145	JCHAIN,HBB,B2M
TISSUES	BTO:0000634	Integument	23	2112	0.33	0.0158	CHGA,SPARC,BPIFB1,THBS1,VWF,CSTA,DSC2,AZGP1,A2ML1,PDIA3,ANGPTL4,TUBA1B,FN1,AKAP9,YWHAZ,TGFBI,PAEP,PDGFC,TUBA1C,C1R,ACTG1,ACTB,B2M
TISSUES	BTO:0000255	Brain cell line	6	188	0.8	0.0212	PDIA3,FGB,TUBA1B,YWHAZ,ACTB,TRIM66
TISSUES	BTO:0001493	Trunk	14	1014	0.43	0.0290	CHGA,AZGP1,ANGPTL4,FGB,F2,TUBA1B,FN1,HP,HLA-C,SERPINA1,ACTG1,KNG1,ACTB,B2M
TISSUES	BTO:0000394	Aorta endothelium	3	33	1.25	0.0349	THBS1,ANGPTL4,FN1
TISSUES	BTO:0000765	Exocrine gland	5	150	0.82	0.0486	C5,BPIFB1,MASP1,CA6,B2M
COMPARTMENTS	GOCC:0005576	Extracellular region	70	2079	0.82	2.83e-41	CPB2,SERPIND1,CHGA,F9,TFPI2,C5,MGP,GPLD1,SPARC,PROC,BPIFB1,F12,APCS,THBS1,VWF,LCAT,CSTA,AMBP,CENPE,FCN3,SAA4,SERPING1,PSG6,AZGP1,SCGB3A1,MASP1,A2ML1,PDIA3,LALBA,ANGPTL4,FGB,F2,PLG,SERPINA7,PAPPA,FGG,FN1,HP,CFHR3,CFH,F5,CRTAC1,FCN1,ASPN,APOM,HLA-C,CA6,YWHAZ,F11,C1S,PCYOX1,C4A,MST1,ITIH3,C4B,SERPINA1,TGFBI,CFB,PAEP,JCHAIN,HPR,C1R,ACTG1,PLCG2,KNG1,HBB,ACTB,ITM2B,B2M,FGA

COMPARTMENTS	GOCC:0005615	Extracellular space	56	1027	1.03	3.05e-41	CPB2,SERPIND1,F9,C5,MGP,GPLD1,PROC,F12,APCS,THBS1,VWF,LCAT,CSTA,AMBP,FCN3,SAA4,SERPING1,AZGP1,SCGB3A1,MASP1,A2ML1,PDIA3,ANGPTL4,FGB,F2,PLG,PAPPA,FGG,FN1,HP,CFHR3,CFH,APOM,HLA-C,CA6,YWHAZ,F11,C1S,PCYOX1,C4A,MST1,C4B,SERPINA1,TGFBI,CFB,JCHAIN,HPR,C1R,ACTG1,PLCG2,KNG1,HBB,ACTB,ITM2B,B2M,FGA
COMPARTMENTS	GOCC:0072562	Blood microparticle	26	118	1.64	8.17e-31	APCS,AMBP,FCN3,SERPING1,ANGPTL4,FGB,F2,PLG,FGG,FN1,HP,CFHR3,CFH,YWHAZ,C1S,C4A,C4B,CFB,JCHAIN,HPR,C1R,ACTG1,KNG1,HBB,ACTB,FGA
COMPARTMENTS	GOCC:0043230	Extracellular organelle	31	524	1.07	1.95e-21	CPB2,F9,C5,MGP,APCS,THBS1,VWF,AMBP,SERPING1,AZGP1,FGB,F2,PLG,FGG,FN1,HP,CFH,HLA-C,YWHAZ,F11,C4A,C4B,SERPINA1,TGFBI,C1R,ACTG1,KNG1,HBB,ACTB,B2M,FGA
COMPARTMENTS	GOCC:0065010	Extracellular membrane-bounded organelle	30	473	1.1	1.95e-21	CPB2,F9,MGP,APCS,THBS1,VWF,AMBP,SERPING1,AZGP1,FGB,F2,PLG,FGG,FN1,HP,CFH,HLA-C,YWHAZ,F11,C4A,C4B,SERPINA1,TGFBI,C1R,ACTG1,KNG1,HBB,ACTB,B2M,FGA
COMPARTMENTS	GOCC:1903561	Extracellular vesicle	30	500	1.07	5.93e-21	CPB2,F9,MGP,APCS,THBS1,VWF,AMBP,SERPING1,AZGP1,FGB,F2,PLG,FGG,FN1,HP,CFH,HLA-C,YWHAZ,F11,C4A,C4B,SERPINA1,TGFBI,C1R,ACTG1,KNG1,HBB,ACTB,B2M,FGA
COMPARTMENTS	GOCC:0070062	Extracellular exosome	22	428	1.01	1.98e-13	MGP,THBS1,VWF,AMBP,AZGP1,FGB,PLG,FGG,FN1,HP,CFH,HLA-C,YWHAZ,C4A,SERPINA1,TGFBI,ACTG1,KNG1,HBB,ACTB,B2M,FGA
COMPARTMENTS	GOCC:0031093	Platelet alpha granule lumen	12	66	1.55	1.32e-12	SPARC,THBS1,VWF,SERPING1,FGB,PLG,FGG,FN1,F5,SERPINA1,KNG1,FGA
COMPARTMENTS	GOCC:0031982	Vesicle	41	2125	0.58	2.11e-12	CPB2,CHGA,F9,MGP,SPARC,APCS,THBS1,VWF,AMBP,TCIRG1,SERPING1,SEC24D,DSC2,AZGP1,PDIA3,FGB,F2,PLG,FGG,FN1,HP,CFH,F5,FCN1,HLA-C,YWHAZ,F11,C4A,ITIH3,C4B,SERPINA1,TGFBI,C1R,ACTG1,PLCG2,KNG1,HBB,ACTB,ITM2B,B2M,FGA
COMPARTMENTS	GOCC:0034774	Secretory granule lumen	16	241	1.12	4.34e-11	SPARC,THBS1,VWF,SERPING1,FGB,PLG,FGG,FN1,HP,F5,FCN1,ITIH3,SERPINA1,KNG1,B2M,FGA
COMPARTMENTS	GOCC:0005577	Fibrinogen complex	8	20	1.9	1.50e-10	THBS1,VWF,FGB,F2,PLG,FGG,FN1,FGA
COMPARTMENTS	GOCC:0005788	Endoplasmic reticulum lumen	11	172	1.1	3.31e-07	F9,THBS1,PDIA3,FGG,FN1,F5,C4A,SERPINA1,KNG1,B2M,FGA
COMPARTMENTS	GOCC:0030141	Secretory granule	19	719	0.72	6.38e-07	CHGA,SPARC,THBS1,VWF,TCIRG1,SERPING1,FGB,PLG,FGG,FN1,HP,F5,FCN1,ITIH3,SERPINA1,KNG1,HBB,B2M,FGA
COMPARTMENTS	GOCC:0062167	Complement component C1q complex	5	10	1.99	1.16e-06	SERPING1,C1S,C4A,C4B,C1R
COMPARTMENTS	GOCC:1905286	Serine-type peptidase complex	5	11	1.95	1.59e-06	F9,FCN3,MASP1,FCN1,F11
COMPARTMENTS	GOCC:1905370	Serine-type endopeptidase complex	4	7	2.05	2.26e-05	FCN3,MASP1,FCN1,F11
COMPARTMENTS	GOCC:0031012	Extracellular matrix	10	259	0.88	0.00012	CPB2,TFPI2,GPLD1,SPARC,THBS1,VWF,PLG,FN1,ASPN,TGFBI
COMPARTMENTS	GOCC:0110165	Cellular anatomical entity	91	14060	0.11	0.00017	CPB2,SERPIND1,CHGA,F9,TFPI2,C5,MGP,GPLD1,SPARC,PROC,BPIFB1,F12,APCS,THBS1,VWF,LCAT,CSTA,DGKG,AMBP,CENPE,TCIRG1,FCN3,SAA4,SERPING1,SEC24D,DSC2,PSG6,AZGP1,SCGB3A1,MASP1,A2ML1,PDIA3,SLC27A4,LALBA,ANGPTL4,SH3D19,FGB,F2,PLG,NDUFS8,KDM3B,SERPINA7,PAPPA,TUBA1B,FGG,ISYNA1,FN1,HP,AKAP9,CNDP1,CFHR3,CFH,F5,CRTAC1,SMARCA1,FCN1,ASPN,APOM,HLA-C,CA6,ADGRG2,YWHAZ,MAP3K7CL,F11,C1S,PCYOX1,C4A,ACSF2,MST1,ITIH3,C4B,SERPINA1,TGFBI,CFB,PAEP,UIMC1,PDGFC,JCHAIN,HPR,TUBA1C,C1R,ACTG1,PLCG2,NEB,KNG1,HBB,ACTB,TRIM66,ITM2B,B2M,FGA
COMPARTMENTS	GOCC:0005601	Classical-complement-pathway C3/C5 convertase complex	3	4	2.17	0.00043	C4A,C4B,CFB
COMPARTMENTS	GOCC:0031410	Cytoplasmic vesicle	24	1738	0.43	0.00051	CHGA,SPARC,THBS1,VWF,TCIRG1,SERPING1,SEC24D,DSC2,PDIA3,FGB,PLG,FGG,FN1,HP,F5,FCN1,HLA-C,ITIH3,SERPINA1,KNG1,HBB,ITM2B,B2M,FGA
COMPARTMENTS	GOCC:0034358	Plasma lipoprotein particle	5	48	1.31	0.00062	LCAT,SAA4,APOM,PCYOX1,HPR
COMPARTMENTS	GOCC:1905368	Peptidase complex	7	136	1.01	0.00066	F9,FCN3,MASP1,F2,UBE3C,FCN1,F11
COMPARTMENTS	GOCC:0030312	External encapsulating structure	9	259	0.84	0.00069	TFPI2,GPLD1,SPARC,THBS1,VWF,PLG,PAPPA,FN1,ASPN
COMPARTMENTS	GOCC:0009897	External side of plasma membrane	6	102	1.06	0.0013	THBS1,FGB,PLG,FGG,FCN1,FGA
COMPARTMENTS	GOCC:0030134	COPII-coated ER to Golgi transport vesicle	5	62	1.2	0.0016	SEC24D,F5,HLA-C,SERPINA1,B2M
COMPARTMENTS	GOCC:0034364	High-density lipoprotein particle	4	30	1.42	0.0018	LCAT,SAA4,APOM,HPR
COMPARTMENTS	GOCC:0034366	Spherical high-density lipoprotein particle	3	11	1.73	0.0030	LCAT,APOM,HPR
COMPARTMENTS	GOCC:0005798	Golgi-associated vesicle	6	128	0.97	0.0040	SEC24D,F5,HLA-C,SERPINA1,ITM2B,B2M
COMPARTMENTS	GOCC:0009986	Cell surface	10	438	0.65	0.0057	SPARC,THBS1,PDIA3,FGB,PLG,FGG,FCN1,HLA-C,PDGFC,FGA
COMPARTMENTS	GOCC:1905369	Endopeptidase complex	5	90	1.04	0.0075	FCN3,MASP1,UBE3C,FCN1,F11
COMPARTMENTS	GOCC:0005783	Endoplasmic reticulum	16	1095	0.46	0.0081	F9,PROC,THBS1,VWF,SEC24D,PDIA3,SLC27A4,FGG,FN1,F5,HLA-C,C4A,SERPINA1,KNG1,B2M,FGA
COMPARTMENTS	GOCC:1904090	Peptidase inhibitor complex	3	17	1.54	0.0081	CSTA,SERPINA1,ACTB
COMPARTMENTS	GOCC:0097433	Dense body	2	3	2.12	0.0144	ACTG1,ACTB
COMPARTMENTS	GOCC:0071682	Endocytic vesicle lumen	3	23	1.41	0.0168	SPARC,HP,HBB
COMPARTMENTS	GOCC:0005833	Hemoglobin complex	3	24	1.39	0.0183	F2,HP,HBB
COMPARTMENTS	GOCC:0012505	Endomembrane system	30	3156	0.27	0.0191	CHGA,F9,SPARC,PROC,THBS1,VWF,TCIRG1,SERPING1,SEC24D,PDIA3,SLC27A4,LALBA,FGB,F2,PLG,FGG,FN1,HP,AKAP9,F5,FCN1,HLA-C,C4A,ITIH3,SERPINA1,KNG1,HBB,ITM2B,B2M,FGA
COMPARTMENTS	GOCC:0030139	Endocytic vesicle	7	260	0.72	0.0216	SPARC,TCIRG1,PDIA3,HP,HLA-C,HBB,B2M
COMPARTMENTS	GOCC:0032991	Protein-containing complex	43	5325	0.2	0.0222	CPB2,F9,C5,THBS1,VWF,LCAT,CSTA,FCN3,SAA4,SERPING1,SEC24D,MASP1,PDIA3,FGB,F2,PLG,UBE3C,NDUFS8,PAPPA,FGG,FN1,HP,AKAP9,SMARCA1,FCN1,APOM,HLA-C,F11,C1S,PCYOX1,C4A,C4B,SERPINA1,CFB,UIMC1,JCHAIN,HPR,C1R,ACTG1,HBB,ACTB,B2M,FGA
COMPARTMENTS	GOCC:0098552	Side of membrane	7	264	0.72	0.0226	THBS1,FGB,PLG,FGG,FCN1,HLA-C,FGA
COMPARTMENTS	GOCC:0005602	Complement component C1 complex	2	5	1.9	0.0255	C1S,C1R
COMPARTMENTS	GOCC:0062023	Collagen-containing extracellular matrix	6	198	0.78	0.0284	SPARC,THBS1,VWF,PLG,FN1,TGFBI
COMPARTMENTS	GOCC:0031232	Extrinsic component of external side of plasma membrane	2	6	1.82	0.0326	PLG,FCN1
COMPARTMENTS	GOCC:0034365	Discoidal high-density lipoprotein particle	2	6	1.82	0.0326	LCAT,APOM
COMPARTMENTS	GOCC:0030135	Coated vesicle	6	223	0.72	0.0488	VWF,SEC24D,F5,HLA-C,SERPINA1,B2M
COMPARTMENTS	GOCC:0031838	Haptoglobin-hemoglobin complex	2	8	1.69	0.0488	HP,HBB
COMPARTMENTS	GOCC:0042612	MHC class I protein complex	2	8	1.69	0.0488	HLA-C,B2M
COMPARTMENTS	GOCC:0070013	Intracellular organelle lumen	27	2902	0.26	0.0488	F9,SPARC,PROC,THBS1,VWF,SERPING1,PDIA3,LALBA,FGB,F2,PLG,NDUFS8,FGG,FN1,HP,F5,SMARCA1,FCN1,C4A,ITIH3,SERPINA1,UIMC1,KNG1,HBB,ACTB,B2M,FGA
UniProt Keywords	KW-0964	Secreted	63	1839	0.83	2.03e-36	CPB2,CHGA,F9,TFPI2,C5,MGP,GPLD1,SPARC,PROC,BPIFB1,F12,APCS,THBS1,VWF,LCAT,AMBP,FCN3,SAA4,SERPING1,PSG6,AZGP1,SCGB3A1,A2ML1,LALBA,ANGPTL4,FGB,F2,PLG,PSG11,SERPINA7,PAPPA,FGG,FN1,HP,CNDP1,CFHR3,CFH,F5,CRTAC1,FCN1,ASPN,APOM,CA6,F11,C4A,MST1,ITIH3,C4B,SERPINA1,TGFBI,CFB,PAEP,PDGFC,SBSN,JCHAIN,HPR,C1R,IGHV3-72,PAMR1,KNG1,ITM2B,B2M,FGA

UniProt Keywords	KW-0732	Signal	70	3277	0.62	2.30e-29	CPB2,SERPIND1,CHGA,F9,TFPI2,C5,MGP,GPLD1,SPARC,PROC,BPIFB1,F12,APCS,THBS1,VWF,LCAT,AMBP,FCN3,SAA4,SERPING1,DSC2,PSG6,AZGP1,SCGB3A1,A2ML1,PDIA3,LALBA,ANGPTL4,FGB,F2,PLG,PSG11,SERPINA7,PAPPA,FGG,FN1,HP,CNDP1,PCDH18,CFHR3,CFH,F5,CRTAC1,FCN1,ASPN,APOM,HLA-C,CA6,ADGRG2,F11,C1S,PCYOX1,C4A,MST1,ITIH3,C4B,SERPINA1,TGFBI,CFB,PAEP,PDGFC,SBSN,JCHAIN,HPR,C1R,IGHV3-72,PAMR1,KNG1,B2M,FGA
UniProt Keywords	KW-0094	Blood coagulation	17	46	1.86	4.08e-23	CPB2,SERPIND1,F9,TFPI2,PROC,F12,VWF,SERPING1,FGB,F2,PLG,FGG,F5,F11,SERPINA1,KNG1,FGA
UniProt Keywords	KW-1015	Disulfide bond	60	3338	0.55	9.84e-20	CPB2,CHGA,F9,TFPI2,C5,MGP,SPARC,PROC,BPIFB1,F12,APCS,THBS1,VWF,LCAT,AMBP,FCN3,SERPING1,PSG6,AZGP1,SCGB3A1,MASP1,A2ML1,PDIA3,LALBA,ANGPTL4,FGB,F2,PLG,PSG11,PAPPA,FGG,FN1,HP,CFHR3,CFH,F5,CRTAC1,FCN1,ASPN,APOM,HLA-C,CA6,F11,C1S,C4A,MST1,C4B,TGFBI,CFB,PAEP,PDGFC,JCHAIN,HPR,C1R,IGHV3-72,PAMR1,KNG1,ITM2B,B2M,FGA
UniProt Keywords	KW-0325	Glycoprotein	65	4386	0.47	1.11e-17	CPB2,SERPIND1,CHGA,F9,TFPI2,C5,GPLD1,SPARC,PROC,BPIFB1,F12,APCS,THBS1,VWF,LCAT,AMBP,FCN3,SAA4,SERPING1,DSC2,PSG6,AZGP1,A2ML1,LALBA,ANGPTL4,FGB,F2,PLG,PSG11,SERPINA7,PAPPA,FGG,FN1,HP,CNDP1,PCDH18,CFHR3,CFH,F5,CRTAC1,FCN1,ASPN,APOM,HLA-C,CA6,ADGRG2,F11,C1S,PCYOX1,C4A,MST1,ITIH3,C4B,SERPINA1,CFB,PAEP,PDGFC,JCHAIN,C1R,PAMR1,KNG1,HBB,ITM2B,B2M,FGA
UniProt Keywords	KW-0768	Sushi	10	57	1.54	1.28e-10	MASP1,PAPPA,HP,CFHR3,CFH,C1S,CFB,HPR,C1R,PAMR1
UniProt Keywords	KW-0765	Sulfation	9	57	1.49	3.63e-09	SERPIND1,CHGA,F9,FGG,FN1,CFH,F5,C4A,C4B
UniProt Keywords	KW-0792	Thrombophilia	6	10	2.07	8.19e-09	SERPIND1,F9,PROC,F2,PLG,F5
UniProt Keywords	KW-0165	Cleavage on pair of basic residues	14	280	0.99	1.76e-08	CHGA,F9,C5,PROC,VWF,AMBP,DSC2,F2,PLG,C4A,C4B,CFB,PDGFC,ITM2B
UniProt Keywords	KW-0399	Innate immunity	14	329	0.92	1.19e-07	C5,BPIFB1,FCN3,SERPING1,FGB,CFH,FCN1,HLA-C,C1S,C4A,C4B,CFB,C1R,FGA
UniProt Keywords	KW-0391	Immunity	17	537	0.79	1.34e-07	C5,BPIFB1,FCN3,SERPING1,FGB,HP,CFH,FCN1,HLA-C,C1S,C4A,C4B,CFB,C1R,IGHV3-72,B2M,FGA
UniProt Keywords	KW-0646	Protease inhibitor	9	117	1.18	7.75e-07	SERPIND1,TFPI2,CSTA,AMBP,SERPING1,A2ML1,ITIH3,SERPINA1,KNG1
UniProt Keywords	KW-0106	Calcium	20	886	0.65	1.05e-06	CHGA,F9,SPARC,PROC,APCS,THBS1,DGKG,FCN3,DSC2,LALBA,F2,SPATA21,FGG,PCDH18,F5,FCN1,ASPN,C1S,PLCG2,FGA
UniProt Keywords	KW-0180	Complement pathway	6	30	1.6	1.10e-06	C5,SERPING1,C1S,C4A,C4B,C1R
UniProt Keywords	KW-0720	Serine protease	9	136	1.12	2.14e-06	F9,PROC,F12,F2,PLG,F11,C1S,CFB,C1R
UniProt Keywords	KW-0301	Gamma-carboxyglutamic acid	5	16	1.79	2.24e-06	F9,MGP,PROC,F2,TGFBI
UniProt Keywords	KW-0011	Acute phase	5	19	1.71	4.37e-06	SAA4,F2,FN1,HP,SERPINA1
UniProt Keywords	KW-0280	Fibrinolysis	4	8	1.99	1.01e-05	CPB2,F12,SERPING1,PLG
UniProt Keywords	KW-0722	Serine protease inhibitor	7	80	1.24	1.01e-05	SERPIND1,TFPI2,AMBP,SERPING1,A2ML1,ITIH3,SERPINA1
UniProt Keywords	KW-0034	Amyloid	5	24	1.61	1.03e-05	APCS,TGFBI,ITM2B,B2M,FGA
UniProt Keywords	KW-0721	Serine protease homolog	4	9	1.94	1.29e-05	HP,MST1,HPR,PAMR1
UniProt Keywords	KW-0865	Zymogen	9	210	0.93	4.89e-05	CPB2,F9,PROC,F12,F2,PLG,PAPPA,F5,CFB
UniProt Keywords	KW-0873	Pyrrolidone carboxylic acid	6	67	1.25	5.17e-05	AZGP1,FGB,FN1,JCHAIN,KNG1,B2M
UniProt Keywords	KW-0677	Repeat	46	4794	0.28	5.60e-05	SERPIND1,F9,TFPI2,GPLD1,PROC,F12,THBS1,VWF,DGKG,AMBP,FCN3,SERPING1,DSC2,ANKRD44,PSG6,LRRC53,MASP1,PDIA3,SH3D19,F2,PLG,NDUFS8,PSG11,PAPPA,FN1,HP,PCDH18,CFHR3,CFH,F5,CRTAC1,SMARCA1,FCN1,ASPN,F11,C1S,MST1,TGFBI,CFB,UIMC1,C1R,PLCG2,PAMR1,NEB,KNG1,TRIM66
UniProt Keywords	KW-0420	Kringle	4	16	1.69	7.21e-05	F12,F2,PLG,MST1
UniProt Keywords	KW-0245	EGF-like domain	9	232	0.88	9.14e-05	F9,PROC,F12,THBS1,MASP1,CRTAC1,C1S,C1R,PAMR1
UniProt Keywords	KW-0272	Extracellular matrix	9	267	0.82	0.00026	SPARC,THBS1,VWF,ANGPTL4,FN1,CRTAC1,ASPN,SERPINA1,TGFBI
UniProt Keywords	KW-0379	Hydroxylation	7	143	0.98	0.00026	F9,PROC,FCN3,C1S,C1R,KNG1,FGA
UniProt Keywords	KW-0645	Protease	12	516	0.66	0.00032	CPB2,F9,PROC,F12,F2,PLG,PAPPA,CNDP1,F11,C1S,CFB,C1R
UniProt Keywords	KW-0882	Thioester bond	3	8	1.87	0.00043	A2ML1,C4A,C4B
UniProt Keywords	KW-1008	Amyloidosis	4	31	1.41	0.00060	TGFBI,ITM2B,B2M,FGA
UniProt Keywords	KW-0558	Oxidation	4	32	1.39	0.00065	CHGA,FN1,ACTG1,ACTB
UniProt Keywords	KW-1068	Hemolytic uremic syndrome	3	10	1.77	0.00068	CFHR3,CFH,CFB
UniProt Keywords	KW-0179	Complement alternate pathway	3	12	1.69	0.0010	C5,CFH,CFB
UniProt Keywords	KW-0971	Glycation	3	12	1.69	0.0010	CFB,HBB,B2M
UniProt Keywords	KW-0351	Hemoglobin-binding	2	2	2.29	0.0027	HP,HPR
UniProt Keywords	KW-0490	MHC I	2	8	1.69	0.0196	HLA-C,B2M
UniProt Keywords	KW-0358	Heparin-binding	4	88	0.95	0.0210	SERPIND1,THBS1,FN1,F11
UniProt Keywords	KW-0838	Vasoactive	2	12	1.52	0.0371	KNG1,HBB
InterPro	IPR001314	Peptidase S1A, chymotrypsin family	13	107	1.38	2.51e-10	F9,PROC,F12,F2,PLG,HP,F11,C1S,MST1,CFB,HPR,C1R,PAMR1
InterPro	IPR001254	Serine proteases, trypsin domain	13	115	1.35	2.94e-10	F9,PROC,F12,F2,PLG,HP,F11,C1S,MST1,CFB,HPR,C1R,PAMR1
InterPro	IPR009003	Peptidase S1, PA clan	13	123	1.32	4.33e-10	F9,PROC,F12,F2,PLG,HP,F11,C1S,MST1,CFB,HPR,C1R,PAMR1
InterPro	IPR035976	Sushi/SCR/CCP superfamily	10	58	1.53	2.79e-09	MASP1,PAPPA,HP,CFHR3,CFH,C1S,CFB,HPR,C1R,PAMR1
InterPro	IPR043504	Peptidase S1, PA clan, chymotrypsin-like fold	12	116	1.31	2.94e-09	F9,PROC,F12,F2,PLG,HP,F11,C1S,MST1,HPR,C1R,PAMR1
InterPro	IPR000436	Sushi/SCR/CCP domain	9	56	1.5	4.49e-08	MASP1,PAPPA,HP,CFHR3,CFH,C1S,CFB,C1R,PAMR1
InterPro	IPR020837	Fibrinogen, conserved site	6	19	1.79	2.16e-06	FCN3,ANGPTL4,FGB,FGG,FCN1,FGA
InterPro	IPR033116	Serine proteases, trypsin family, serine active site	9	92	1.28	2.16e-06	F9,PROC,F12,F2,PLG,F11,C1S,CFB,C1R
InterPro	IPR014716	Fibrinogen, alpha/beta/gamma chain, C-terminal globular, subdomain 1	6	26	1.66	9.27e-06	FCN3,ANGPTL4,FGB,FGG,FCN1,FGA
InterPro	IPR001881	EGF-like calcium-binding domain	9	124	1.16	1.73e-05	F9,PROC,F12,THBS1,MASP1,CRTAC1,C1S,C1R,PAMR1
InterPro	IPR002181	Fibrinogen, alpha/beta/gamma chain, C-terminal globular domain	6	32	1.57	2.26e-05	FCN3,ANGPTL4,FGB,FGG,FCN1,FGA
InterPro	IPR036056	Fibrinogen-like, C-terminal	6	32	1.57	2.26e-05	FCN3,ANGPTL4,FGB,FGG,FCN1,FGA
InterPro	IPR040839	Macroglobulin domain MG4	4	8	1.99	0.00016	C5,A2ML1,C4A,C4B
InterPro	IPR001599	Alpha-2-macroglobulin	4	9	1.94	0.00022	C5,A2ML1,C4A,C4B
InterPro	IPR002890	Macroglobulin domain	4	9	1.94	0.00022	C5,A2ML1,C4A,C4B
InterPro	IPR009048	Alpha-macroglobulin, receptor-binding	4	9	1.94	0.00022	C5,A2ML1,C4A,C4B
InterPro	IPR011625	Alpha-2-macroglobulin, bait region domain	4	9	1.94	0.00022	C5,A2ML1,C4A,C4B
InterPro	IPR011626	Alpha-macroglobulin-like, TED domain	4	9	1.94	0.00022	C5,A2ML1,C4A,C4B
InterPro	IPR013806	Kringle-like fold	5	26	1.58	0.00022	F12,F2,PLG,FN1,MST1
InterPro	IPR036595	Alpha-macroglobulin, receptor-binding domain superfamily	4	9	1.94	0.00022	C5,A2ML1,C4A,C4B
InterPro	IPR041555	Macroglobulin domain MG3	4	9	1.94	0.00022	C5,A2ML1,C4A,C4B
InterPro	IPR018114	Serine proteases, trypsin family, histidine active site	7	100	1.14	0.00038	F9,PROC,F12,F2,PLG,F11,CFB
InterPro	IPR000294	Gamma-carboxyglutamic acid-rich (GLA) domain	4	15	1.72	0.00070	F9,MGP,PROC,F2
InterPro	IPR008930	Terpenoid cyclases/protein prenyltransferase alpha-alpha toroid	4	15	1.72	0.00070	C5,A2ML1,C4A,C4B
InterPro	IPR012290	Fibrinogen, alpha/beta/gamma chain, coiled coil domain	3	3	2.29	0.00070	FGB,FGG,FGA
InterPro	IPR035972	Gamma-carboxyglutamic acid-rich (GLA) domain superfamily	4	15	1.72	0.00070	F9,MGP,PROC,F2
InterPro	IPR037579	Fibrinogen	3	3	2.29	0.00070	FGB,FGG,FGA
InterPro	IPR000001	Kringle	4	16	1.69	0.00072	F12,F2,PLG,MST1

InterPro	IPR018056	Kringle, conserved site	4	16	1.69	0.00072	F12,F2,PLG,MST1
InterPro	IPR038178	Kringle superfamily	4	16	1.69	0.00072	F12,F2,PLG,MST1
InterPro	IPR001840	Anaphylatoxin, complement system domain	3	4	2.17	0.00100	C5,C4A,C4B
InterPro	IPR018081	Anaphylatoxin, complement system	3	4	2.17	0.00100	C5,C4A,C4B
InterPro	IPR000859	CUB domain	5	51	1.29	0.0020	MASP1,C1S,PDGFC,C1R,PAMR1
InterPro	IPR000020	Anaphylatoxin/fibulin	3	6	1.99	0.0022	C5,C4A,C4B
InterPro	IPR003609	PAN/Apple domain	3	6	1.99	0.0022	PLG,F11,MST1
InterPro	IPR035914	Spermadhesin, CUB domain superfamily	5	54	1.26	0.0024	MASP1,C1S,PDGFC,C1R,PAMR1
InterPro	IPR018097	EGF-like calcium-binding, conserved site	6	99	1.08	0.0030	F9,PROC,MASP1,CRTAC1,C1S,C1R
InterPro	IPR019742	Alpha-2-macroglobulin, conserved site	3	8	1.87	0.0038	A2ML1,C4A,C4B
InterPro	IPR047565	Alpha-macroglobulin-like, thiol-ester bond-forming region	3	8	1.87	0.0038	A2ML1,C4A,C4B
InterPro	IPR023795	Serpin, conserved site	4	32	1.39	0.0057	SERPIND1,SERPING1,SERPINA7,SERPINA1
InterPro	IPR042185	Serpin superfamily, domain 2	4	35	1.35	0.0077	SERPIND1,SERPING1,SERPINA7,SERPINA1
InterPro	IPR000215	Serpin family	4	36	1.34	0.0083	SERPIND1,SERPING1,SERPINA7,SERPINA1
InterPro	IPR023796	Serpin domain	4	36	1.34	0.0083	SERPIND1,SERPING1,SERPINA7,SERPINA1
InterPro	IPR036186	Serpin superfamily	4	36	1.34	0.0083	SERPIND1,SERPING1,SERPINA7,SERPINA1
InterPro	IPR042178	Serpin superfamily, domain 1	4	36	1.34	0.0083	SERPIND1,SERPING1,SERPINA7,SERPINA1
InterPro	IPR022272	Lipocalin family conserved site	3	13	1.66	0.0104	AMBP,SLC27A4,PAEP
InterPro	IPR018933	Netrin module, non-TIMP type	3	17	1.54	0.0205	C5,C4A,C4B
InterPro	IPR000742	EGF-like domain	7	236	0.77	0.0350	F9,PROC,F12,THBS1,MASP1,C1R,PAMR1
InterPro	IPR024175	Peptidase S1A, complement C1r/C1S/mannan-binding	2	3	2.12	0.0367	C1S,C1R
InterPro	IPR001134	Netrin domain	3	23	1.41	0.0429	C5,C4A,C4B
InterPro	IPR008993	Tissue inhibitor of metalloproteinases-like, OB-fold	3	24	1.39	0.0472	C5,C4A,C4B
SMART	SM00020	Trypsin-like serine protease	14	118	1.37	3.78e-12	F9,PROC,F12,MASP1,F2,PLG,HP,F11,C1S,MST1,CFB,HPR,C1R,PAMR1
SMART	SM00032	Domain abundant in complement control proteins	9	56	1.5	1.54e-08	MASP1,PAPPA,HP,CFHR3,CFH,C1S,CFB,C1R,PAMR1
SMART	SM00186	Fibrinogen-related domains (FReDs)	6	29	1.61	5.63e-06	FCN3,ANGPTL4,FGB,FGG,FCN1,FGA
SMART	SM01359	Alpha-2-Macroglobulin	4	9	1.94	8.68e-05	C5,A2ML1,C4A,C4B
SMART	SM01360	Alpha-2-macroglobulin family	4	9	1.94	8.68e-05	C5,A2ML1,C4A,C4B
SMART	SM01361	A-macroglobulin receptor	4	9	1.94	8.68e-05	C5,A2ML1,C4A,C4B
SMART	SM00069	Domain containing Gla (gamma-carboxyglutamate) residues.	4	15	1.72	0.00026	F9,MGP,PROC,F2
SMART	SM00130	Kringle domain	4	16	1.69	0.00026	F12,F2,PLG,MST1
SMART	SM01212	Fibrinogen alpha/beta chain family	3	3	2.29	0.00026	FGB,FGG,FGA
SMART	SM00042	Domain first found in C1r, C1s, uEGF, and bone morphogenetic protein.	5	50	1.29	0.00069	MASP1,C1S,PDGFC,C1R,PAMR1
SMART	SM00104	Anaphylatoxin homologous domain	3	6	1.99	0.00077	C5,C4A,C4B
SMART	SM01419	Alpha-macro-globulin thiol-ester bond-forming region	3	8	1.87	0.0014	A2ML1,C4A,C4B
SMART	SM00093	SERine Proteinase INhibitors	4	35	1.35	0.0028	SERPIND1,SERPING1,SERPINA7,SERPINA1
SMART	SM00643	Netrin C-terminal Domain	3	15	1.6	0.0057	C5,C4A,C4B
SMART	SM00179	Calcium-binding EGF-like domain	5	101	0.99	0.0110	F9,PROC,MASP1,C1S,C1R
SMART	SM00058	Fibronectin type 1 domain	2	4	1.99	0.0192	F12,FN1
SMART	SM00473	Divergent subfamily of APPLE domains	2	5	1.9	0.0252	PLG,MST1

UC+NCFET vs ACFET T3

#category	term ID	term description	observed gene count	background gene count	strength	false discovery rate	matching proteins in your network (labels)
GO Process	GO:0006959	Humoral immune response	21	268	1.24	8.92e-16	HRG,C3,CFHR5,LYZ,PPBP,PF4,MASP1,CAMP,FGB,F2,CFHR3,CFH,S100A7,C1QC,MASP2,C1S,C4B,C1R,KNG1,B2M,FGA
GO Process	GO:0007596	Blood coagulation	15	173	1.29	3.59e-11	F9,HRG,PF4,FGB,F2,PLG,FGG,F5,F7,PROS1,F11,SERPINA1,PLCG2,KNG1,FGA
GO Process	GO:0006952	Defense response	31	1394	0.7	8.68e-11	HRG,C3,BPIFB1,APCS,CFHR5,LYZ,ATRN,TCIRG1,AHSG,SAA4,PPBP,PF4,MASP1,CAMP,FGB,F2,PRG2,APOL1,CFH,S100A7,C1QC,F7,MASP2,C1S,C4B,SERPINA1,C1R,PLCG2,KNG1,B2M,FGA
GO Process	GO:0006956	Complement activation	10	60	1.57	1.49e-09	C3,CFHR5,MASP1,CFHR3,CFH,C1QC,MASP2,C1S,C4B,C1R
GO Process	GO:0072378	Blood coagulation, fibrin clot formation	8	24	1.87	2.39e-09	F9,FGB,F2,FGG,F5,F7,F11,FGA
GO Process	GO:0098542	Defense response to other organism	25	989	0.75	2.39e-09	HRG,C3,BPIFB1,APCS,CFHR5,LYZ,PPBP,PF4,MASP1,CAMP,FGB,F2,PRG2,APOL1,CFH,S100A7,C1QC,MASP2,C1S,C4B,C1R,PLCG2,KNG1,B2M,FGA
GO Process	GO:0006950	Response to stress	44	3358	0.47	2.78e-09	F9,HRG,C3,BPIFB1,APCS,CFHR5,LYZ,ATRN,TCIRG1,AHSG,SAA4,DSC2,PPBP,PF4,MASP1,CAMP,FGB,F2,PLG,PRG2,APOL1,FGG,THBS4,CFH,F5,S100A7,C1QC,F7,GPX3,PROS1,TNRC6A,MASP2,F11,C1S,CDK3,C4B,SERPINA1,C1R,VEGFC,PLCG2,HSPH1,KNG1,B2M,FGA
GO Process	GO:0061045	Negative regulation of wound healing	10	69	1.51	2.86e-09	HRG,APCS,FGB,F2,PLG,FGG,PROS1,F11,KNG1,FGA
GO Process	GO:0030193	Regulation of blood coagulation	10	70	1.5	2.99e-09	HRG,FGB,F2,PLG,FGG,F7,PROS1,F11,KNG1,FGA
GO Process	GO:0030195	Negative regulation of blood coagulation	9	46	1.64	2.99e-09	HRG,FGB,F2,PLG,FGG,PROS1,F11,KNG1,FGA
GO Process	GO:0006955	Immune response	27	1321	0.66	1.18e-08	HRG,C3,BPIFB1,APCS,CFHR5,LYZ,TCIRG1,PPBP,PF4,MASP1,CAMP,FGB,F2,PRG2,APOL1,CFHR3,CFH,S100A7,C1QC,MASP2,C1S,C4B,C1R,PLCG2,KNG1,B2M,FGA
GO Process	GO:0042730	Fibrinolysis	7	19	1.92	1.30e-08	HRG,FGB,F2,PLG,FGG,PROS1,FGA
GO Process	GO:0031638	Zymogen activation	9	59	1.53	1.33e-08	F9,FGB,F2,FGG,F5,F7,F11,C1R,FGA
GO Process	GO:0061041	Regulation of wound healing	11	130	1.28	2.32e-08	HRG,APCS,FGB,F2,PLG,FGG,F7,PROS1,F11,KNG1,FGA
GO Process	GO:0051707	Response to other organism	26	1328	0.64	6.31e-08	HRG,APOB,C3,BPIFB1,APCS,CFHR5,LYZ,PPBP,PF4,MASP1,CAMP,FGB,F2,PRG2,APOL1,CFH,S100A7,C1QC,MASP2,C1S,C4B,C1R,PLCG2,KNG1,B2M,FGA
GO Process	GO:0019730	Antimicrobial humoral response	11	149	1.22	7.72e-08	HRG,LYZ,PPBP,PF4,CAMP,FGB,F2,S100A7,KNG1,B2M,FGA
GO Process	GO:0044419	Biological process involved in interspecies interaction between organisms	27	1490	0.61	1.14e-07	HRG,APOB,C3,BPIFB1,APCS,CFHR5,LYZ,PPBP,PF4,MASP1,CAMP,FGB,F2,PLG,PRG2,APOL1,CFH,S100A7,C1QC,MASP2,C1S,C4B,C1R,PLCG2,KNG1,B2M,FGA
GO Process	GO:0032101	Regulation of response to external stimulus	22	964	0.71	1.20e-07	HRG,APOA1,C3,APCS,AHSG,PLA2G7,MASP1,FGB,F2,PLG,FGG,THBS4,CFH,S100A7,F7,PROS1,MASP2,F11,VEGFC,PLCG2,KNG1,FGA
GO Process	GO:0002684	Positive regulation of immune system process	21	874	0.73	1.31e-07	GPLD1,HRG,C3,CFHR5,PLA2G7,PF4,MASP1,THBS4,CFHR3,CFH,S100A7,C1QC,F7,MASP2,C1S,C4B,C1R,VEGFC,PLCG2,HSPH1,B2M
GO Process	GO:0002682	Regulation of immune system process	26	1438	0.61	2.50e-07	GPLD1,HRG,APOA1,C3,BPIFB1,APCS,CFHR5,PLA2G7,PF4,MASP1,PSG1,PRG2,THBS4,CFHR3,CFH,S100A7,C1QC,F7,MASP2,C1S,C4B,C1R,VEGFC,PLCG2,HSPH1,B2M
GO Process	GO:0009605	Response to external stimulus	33	2355	0.5	3.71e-07	HRG,APOB,APOA1,C3,BPIFB1,APCS,CFHR5,LYZ,DSC2,PPBP,PF4,MASP1,CAMP,SLC27A4,FGB,F2,PRG2,APOL1,CFH,F5,S100A7,C1QC,F7,TNRC6A,MASP2,C1S,C4B,C1R,VEGFC,PLCG2,KNG1,B2M,FGA
GO Process	GO:0002252	Immune effector process	14	375	0.92	7.65e-07	C3,CFHR5,TCIRG1,MASP1,F2,CFHR3,CFH,C1QC,MASP2,C1S,C4B,C1R,PLCG2,B2M
GO Process	GO:0002253	Activation of immune response	12	271	1.0	1.92e-06	GPLD1,C3,CFHR5,MASP1,CFHR3,CFH,C1QC,MASP2,C1S,C4B,C1R,PLCG2
GO Process	GO:0045087	Innate immune response	18	754	0.73	2.77e-06	C3,BPIFB1,APCS,CFHR5,MASP1,CAMP,FGB,APOL1,CFH,S100A7,C1QC,MASP2,C1S,C4B,C1R,PLCG2,B2M,FGA
GO Process	GO:0051838	Cytolysis by host of symbiont cells	5	12	1.97	3.70e-06	HRG,CFHR5,CAMP,F2,APOL1

GO Process	GO:0051873	Killing by host of symbiont cells	6	29	1.67	3.73e-06	HRG,CFHR5,PF4,CAMP,F2,APOL1
GO Process	GO:0006954	Inflammatory response	15	538	0.8	7.60e-06	C3,APCS,LYZ,ATRN,TCIRG1,AHSG,SAA4,PPBP,PF4,CAMP,F2,F7,C4B,SERPINA1,KNG1
GO Process	GO:0050896	Response to stimulus	61	7835	0.24	8.01e-06	F9,GPLD1,HRG,APOB,APOA1,C3,BPIFB1,APCS,CFHR5,LYZ,ATRN,LCAT,TCIRG1,AHSG,SAA4,DSC2,SCGB3A1,PPBP,PF4,MASP1,CAMP,SLC27A4,FGB,F2,PLG,PSG1,PRG2,CSPG4,APOL1,FGG,THBS4,AKAP9,CFHR3,CFH,F5,S100A7,CCN5,C1QC,F7,CA6,ADGRG2,GPX3,PROS1,TNRC6A,MASP2,F11,C1S,LTBP1,CDK3,C4B,SERPINA1,TGFBI,IGFALS,PDGFC,C1R,VEGFC,PLCG2,HSPH1,KNG1,B2M,FGA
GO Process	GO:0002376	Immune system process	29	2121	0.49	8.23e-06	GPLD1,HRG,C3,BPIFB1,APCS,CFHR5,LYZ,TCIRG1,PPBP,PF4,MASP1,CAMP,FGB,F2,PLG,PRG2,APOL1,CFHR3,CFH,S100A7,C1QC,MASP2,C1S,C4B,C1R,PLCG2,KNG1,B2M,FGA
GO Process	GO:0051241	Negative regulation of multicellular organismal process	20	1035	0.64	9.92e-06	SPARC,HRG,APOA1,APCS,AHSG,PF4,FGB,F2,PLG,PRG2,FGG,THBS4,C1QC,PROS1,F11,PAEP,APOC1,KNG1,B2M,FGA
GO Process	GO:0016485	Protein processing	10	202	1.04	1.22e-05	F9,PLA2G7,FGB,F2,FGG,F5,F7,F11,C1R,FGA
GO Process	GO:0051239	Regulation of multicellular organismal process	33	2749	0.43	1.31e-05	MGP,GPLD1,SPARC,HRG,APOA1,C3,APCS,ATRN,LCAT,AHSG,DSC2,PF4,CAMP,FGB,F2,PLG,PRG2,FGG,THBS4,AKAP9,C1QC,F7,PROS1,F11,LTBP1,PAEP,PDGFC,APOC1,VEGFC,PLCG2,KNG1,B2M,FGA
GO Process	GO:0051702	Biological process involved in interaction with symbiont	8	108	1.22	1.59e-05	HRG,APCS,CFHR5,PF4,CAMP,F2,PLG,APOL1
GO Process	GO:0006958	Complement activation, classical pathway	6	40	1.53	1.73e-05	C3,C1QC,MASP2,C1S,C4B,C1R
GO Process	GO:0050778	Positive regulation of immune response	14	502	0.8	1.98e-05	GPLD1,HRG,C3,CFHR5,MASP1,CFHR3,CFH,C1QC,MASP2,C1S,C4B,C1R,PLCG2,B2M
GO Process	GO:0061844	Antimicrobial humoral immune response mediated by antimicrobial peptide	8	113	1.2	2.07e-05	HRG,PPBP,PF4,CAMP,F2,S100A7,KNG1,B2M
GO Process	GO:0050766	Positive regulation of phagocytosis	7	75	1.32	2.39e-05	APOA1,C3,AHSG,MASP1,MASP2,C4B,PLCG2
GO Process	GO:0051346	Negative regulation of hydrolase activity	12	354	0.88	2.39e-05	HRG,APOA1,C3,APCS,AHSG,SLC27A4,SERPINA11,PROS1,C4B,SERPINA1,APOC1,KNG1
GO Process	GO:0048583	Regulation of response to stimulus	39	3931	0.35	6.19e-05	GPLD1,HRG,APOA1,C3,BPIFB1,APCS,CFHR5,AHSG,PLA2G7,PF4,MASP1,SLC27A4,FGB,F2,PLG,CSPG4,FGG,THBS4,FBLN5,AKAP9,CFHR3,CFH,S100A7,C1QC,F7,PROS1,MASP2,F11,C1S,LTBP1,CDK3,C4B,PDGFC,C1R,VEGFC,PLCG2,KNG1,B2M,FGA
GO Process	GO:0016064	Immunoglobulin mediated immune response	7	95	1.22	0.00010	C3,TCIRG1,C1QC,MASP2,C1S,C4B,C1R
GO Process	GO:0030168	Platelet activation	7	97	1.21	0.00011	HRG,PF4,FGB,F2,FGG,PLCG2,FGA
GO Process	GO:0034369	Plasma lipoprotein particle remodeling	5	29	1.59	0.00011	APOB,APOA1,LCAT,PLA2G7,APOC1
GO Process	GO:0048584	Positive regulation of response to stimulus	27	2131	0.45	0.00011	GPLD1,HRG,APOA1,C3,CFHR5,PLA2G7,MASP1,FGB,F2,PLG,CSPG4,FGG,THBS4,CFHR3,CFH,S100A7,C1QC,F7,MASP2,C1S,C4B,PDGFC,C1R,VEGFC,PLCG2,B2M,FGA
GO Process	GO:0031639	Plasminogen activation	4	11	1.91	0.00012	FGB,FGG,F11,FGA
GO Process	GO:0006508	Proteolysis	20	1247	0.56	0.00013	F9,C3,PLA2G7,HABP2,MASP1,FGB,F2,PLG,UBE3C,FGG,CNDP1,CFH,F5,F7,MASP2,F11,C1S,PCYOX1,C1R,FGA
GO Process	GO:0002443	Leukocyte mediated immunity	9	211	0.98	0.00014	C3,TCIRG1,F2,C1QC,MASP2,C1S,C4B,C1R,B2M
GO Process	GO:0002449	Lymphocyte mediated immunity	8	159	1.05	0.00018	C3,TCIRG1,C1QC,MASP2,C1S,C4B,C1R,B2M
GO Process	GO:0032103	Positive regulation of response to external stimulus	12	453	0.77	0.00023	HRG,C3,PLA2G7,MASP1,F2,PLG,THBS4,S100A7,F7,MASP2,VEGFC,PLCG2
GO Process	GO:0002460	Adaptive immune response based on somatic recombination of immune receptors built from immunoglobulin superfamily domains	8	169	1.03	0.00028	C3,TCIRG1,C1QC,MASP2,C1S,C4B,C1R,B2M
GO Process	GO:0032102	Negative regulation of response to external stimulus	11	387	0.8	0.00034	HRG,APOA1,APCS,FGB,F2,PLG,FGG,PROS1,F11,KNG1,FGA
GO Process	GO:0009617	Response to bacterium	14	663	0.67	0.00036	APOB,C3,LYZ,PPBP,PF4,CAMP,FGB,F2,PRG2,S100A7,C4B,PLCG2,B2M,FGA
GO Process	GO:1903028	Positive regulation of opsonization	4	16	1.75	0.00037	C3,MASP1,MASP2,C4B
GO Process	GO:0006953	Acute-phase response	5	42	1.43	0.00044	APCS,AHSG,SAA4,F2,SERPINA1
GO Process	GO:0002526	Acute inflammatory response	6	80	1.23	0.00048	APCS,AHSG,SAA4,F2,F7,SERPINA1
GO Process	GO:0080134	Regulation of response to stress	20	1373	0.51	0.00049	HRG,APOA1,C3,APCS,AHSG,PLA2G7,MASP1,FGB,F2,PLG,FGG,FBLN5,CFH,F7,PROS1,MASP2,F11,PLCG2,KNG1,FGA
GO Process	GO:0001906	Cell killing	7	129	1.08	0.00050	C3,LYZ,PPBP,PF4,F2,KNG1,B2M
GO Process	GO:0051917	Regulation of fibrinolysis	4	18	1.7	0.00051	HRG,F2,PLG,F11
GO Process	GO:0072377	Blood coagulation, common pathway	3	5	2.13	0.00091	F2,F5,FGA
GO Process	GO:0050776	Regulation of immune response	15	844	0.6	0.0010	GPLD1,HRG,C3,BPIFB1,CFHR5,MASP1,CFHR3,CFH,C1QC,MASP2,C1S,C4B,C1R,PLCG2,B2M
GO Process	GO:0002250	Adaptive immune response	10	359	0.79	0.0011	C3,TCIRG1,FGB,C1QC,MASP2,C1S,C4B,C1R,B2M,FGA
GO Process	GO:0034114	Regulation of heterotypic cell-cell adhesion	4	24	1.57	0.0013	APOA1,FGB,FGG,FGA
GO Process	GO:0060627	Regulation of vesicle-mediated transport	12	551	0.69	0.0013	APOA1,C3,AHSG,MASP1,FGB,FGG,MASP2,C4B,APOC1,PLCG2,B2M,FGA
GO Process	GO:0042742	Defense response to bacterium	9	306	0.82	0.0020	LYZ,PPBP,CAMP,FGB,F2,PRG2,S100A7,B2M,FGA
GO Process	GO:0030194	Positive regulation of blood coagulation	4	28	1.5	0.0021	HRG,F2,PLG,F7
GO Process	GO:0051050	Positive regulation of transport	15	915	0.56	0.0024	GPLD1,APOA1,C3,AHSG,MASP1,FGB,F2,FGG,AKAP9,MASP2,C4B,VEGFC,PLCG2,B2M,FGA
GO Process	GO:0042157	Lipoprotein metabolic process	6	123	1.04	0.0040	GPLD1,APOB,APOA1,LCAT,APOL1,APOC1
GO Process	GO:0045861	Negative regulation of proteolysis	9	339	0.77	0.0040	HRG,C3,AHSG,F2,SERPINA11,PROS1,C4B,SERPINA1,KNG1
GO Process	GO:0043086	Negative regulation of catalytic activity	13	771	0.58	0.0067	HRG,APOA1,C3,APCS,AHSG,SLC27A4,SERPINA11,AKAP9,PROS1,C4B,SERPINA1,APOC1,KNG1
GO Process	GO:0007597	Blood coagulation, intrinsic pathway	3	13	1.71	0.0073	F9,F7,F11
GO Process	GO:0051918	Negative regulation of fibrinolysis	3	13	1.71	0.0073	HRG,F2,PLG
GO Process	GO:0002697	Regulation of immune effector process	9	383	0.72	0.0097	APOA1,C3,MASP1,PRG2,CFH,MASP2,C4B,PLCG2,B2M
GO Process	GO:0032501	Multicellular organismal process	47	6490	0.21	0.0097	F9,MGP,GPLD1,HRG,APOB,APOA1,C3,ANGPTL6,LYZ,ATRN,LCAT,DNAH5,TCIRG1,AHSG,PLA2G7,SEC24D,DSC2,PF4,SLC27A4,FGB,F2,PLG,PSG1,CSPG4,FGG,THBS4,AKAP9,PCDH18,F5,S100A7,F7,CA6,ADGRG2,PROS1,F11,PSG2,SERPINA1,TGFBI,PDGFC,MYL6,APOC1,VEGFC,PLCG2,KNG1,TPM4,B2M,FGA
GO Process	GO:0034116	Positive regulation of heterotypic cell-cell adhesion	3	15	1.65	0.0102	FGB,FGG,FGA
GO Process	GO:1900026	Positive regulation of substrate adhesion-dependent cell spreading	4	45	1.3	0.0102	APOA1,FGB,FGG,FGA
GO Process	GO:0006957	Complement activation, alternative pathway	3	16	1.62	0.0118	C3,CFHR5,CFH
GO Process	GO:0034375	High-density lipoprotein particle remodeling	3	16	1.62	0.0118	APOA1,LCAT,APOC1
GO Process	GO:0050829	Defense response to Gram-negative bacterium	5	97	1.06	0.0136	LYZ,CAMP,F2,S100A7,B2M
GO Process	GO:0002690	Positive regulation of leukocyte chemotaxis	5	99	1.05	0.0148	PLA2G7,THBS4,S100A7,F7,VEGFC
GO Process	GO:0048518	Positive regulation of biological process	45	6207	0.21	0.0153	GPLD1,SPARC,HRG,APOB,APOA1,C3,CFHR5,TCIRG1,AHSG,PLA2G7,SCGB3A1,PPBP,PF4,MASP1,CAMP,SLC27A4,FGB,F2,PLG,PRG2,PSG1,CSPG4,FGG,THBS4,AKAP9,CFHR3,CFH,S100A7,C1QC,F7,TNRC6A,MASP2,F11,C1S,SLAIN1,C4B,PAEP,PDGFC,C1R,APOC1,VEGFC,PLCG2,HSPH1,KNG1,B2M,FGA
GO Process	GO:0051258	Protein polymerization	5	101	1.04	0.0159	FGB,FGG,AKAP9,SLAIN1,FGA
GO Process	GO:0002237	Response to molecule of bacterial origin	8	333	0.73	0.0198	APOB,PPBP,PF4,CAMP,S100A7,C4B,PLCG2,B2M
GO Process	GO:0030335	Positive regulation of cell migration	10	529	0.63	0.0198	GPLD1,SPARC,PLA2G7,PLG,THBS4,S100A7,F7,PDGFC,VEGFC,PLCG2

GO Process	GO:0044092	Negative regulation of molecular function	15	1143	0.47	0.0233	HRG,APOA1,C3,APCS,CFHR5,AHSG,SLC27A4,SERPINA11,AKAP9,PROS1,C4B,SERPINA1,APOC1,KNG1,B2M
GO Process	GO:0030449	Regulation of complement activation	3	22	1.48	0.0253	C3,MASP1,CFH
GO Process	GO:0034109	Homotypic cell-cell adhesion	4	60	1.17	0.0260	DSC2,FGB,FGG,FGA
GO Process	GO:0007598	Blood coagulation, extrinsic pathway	2	3	2.17	0.0262	F5,F7
GO Process	GO:0034377	Plasma lipoprotein particle assembly	3	23	1.47	0.0271	APOB,APOA1,APOC1
GO Process	GO:0008203	Cholesterol metabolic process	5	119	0.97	0.0301	APOB,APOA1,LCAT,APOL1,APOC1
GO Process	GO:0034381	Plasma lipoprotein particle clearance	3	24	1.45	0.0301	APOB,APOA1,APOC1
GO Process	GO:0019731	Antibacterial humoral response	4	64	1.15	0.0308	CAMP,FGB,B2M,FGA
GO Process	GO:0010038	Response to metal ion	8	362	0.69	0.0309	GPLD1,LCAT,TCIRG1,FGB,FGG,PLCG2,B2M,FGA
GO Process	GO:0030301	Cholesterol transport	4	65	1.14	0.0318	APOB,APOA1,LCAT,APOC1
GO Process	GO:0048585	Negative regulation of response to stimulus	18	1612	0.4	0.0318	HRG,APOA1,BPIFB1,APCS,AHSG,PF4,MASP1,SLC27A4,FGB,F2,PLG,FGG,PROS1,F11,LTBP1,CDK3,KNG1,FGA
GO Process	GO:0010873	Positive regulation of cholesterol esterification	2	4	2.05	0.0355	APOA1,APOC1
GO Process	GO:0051919	Positive regulation of fibrinolysis	2	4	2.05	0.0355	PLG,F11
GO Process	GO:0010811	Positive regulation of cell-substrate adhesion	5	128	0.94	0.0382	HRG,APOA1,FGB,FGG,FGA
GO Process	GO:0033344	Cholesterol efflux	3	27	1.4	0.0382	APOB,APOA1,APOC1
GO Process	GO:0051240	Positive regulation of multicellular organismal process	17	1505	0.4	0.0400	GPLD1,HRG,C3,PF4,CAMP,FGB,F2,PLG,PRG2,FGG,F7,PAEP,PDGFC,VEGFC,PLCG2,B2M,FGA
GO Process	GO:0045765	Regulation of angiogenesis	7	288	0.74	0.0401	SPARC,HRG,C3,PF4,CAMP,THBS4,VEGFC
GO Process	GO:0031640	Killing of cells of another organism	4	71	1.1	0.0402	LYZ,PPBP,PF4,KNG1
GO Process	GO:0046470	Phosphatidylcholine metabolic process	4	71	1.1	0.0402	GPLD1,APOA1,LCAT,PLA2G7
GO Process	GO:0050714	Positive regulation of protein secretion	5	131	0.93	0.0402	GPLD1,FGB,FGG,VEGFC,FGA
GO Process	GO:1902042	Negative regulation of extrinsic apoptotic signaling pathway via death domain receptors	3	28	1.38	0.0402	FGB,FGG,FGA
GO Process	GO:0007155	Cell adhesion	13	965	0.48	0.0414	ATRN,HABP2,DSC2,FGB,FGG,THBS4,FBLN5,PCDH18,CCN5,PSG2,TGFBI,IGFALS,FGA
GO Function	GO:0005509	Calcium ion binding	21	717	0.82	3.56e-08	F9,MGP,SPARC,APCS,HABP2,DSC2,MASP1,F2,SPATA21,THBS4,FBLN5,PCDH18,S100A7,F7,PROS1,MASP2,C1S,LTBP1,C1R,MYL6,TPM4
GO Function	GO:0001848	Complement binding	6	26	1.71	1.33e-05	APCS,CFHR5,CFHR3,CFH,MASP2,C4B
GO Function	GO:0004252	Serine-type endopeptidase activity	10	176	1.1	1.42e-05	F9,HABP2,MASP1,F2,PLG,F7,MASP2,F11,C1S,C1R
GO Function	GO:0008201	Heparin binding	10	173	1.11	1.42e-05	HRG,APOB,PF4,F2,PRG2,THBS4,CFH,CCN5,F11,KNG1
GO Function	GO:0005539	Glycosaminoglycan binding	11	245	1.0	1.80e-05	HRG,APOB,HABP2,PF4,F2,PRG2,THBS4,CFH,CCN5,F11,KNG1
GO Function	GO:0001846	Opsonin binding	5	21	1.73	5.84e-05	APCS,CFHR5,CFHR3,CFH,MASP2
GO Function	GO:0005102	Signaling receptor binding	23	1499	0.54	7.37e-05	HRG,APOB,APOA1,C3,ANGPTL6,SCGB3A1,PPBP,PF4,FGB,F2,PLG,FGG,THBS4,FBLN5,AKAP9,S100A7,CCN5,F7,TGFBI,PDGFC,VEGFC,KNG1,FGA
GO Function	GO:0004866	Endopeptidase inhibitor activity	8	177	1.01	0.00076	HRG,C3,AHSG,SERPINA11,PROS1,C4B,SERPINA1,KNG1
GO Function	GO:0005201	Extracellular matrix structural constituent	7	131	1.08	0.0010	MGP,FGB,PRG2,FGG,LTBP1,TGFBI,FGA
GO Function	GO:0005488	Binding	76	12838	0.12	0.0023	F9,MGP,SPARC,HRG,APOB,APOA1,C3,ANGPTL6,BPIFB1,APCS,CFHR5,LYZ,ATRN,LCAT,DNAH5,TCIRG1,PLA2G7,HABP2,SEC24D,DSC2,SCGB3A1,PPBP,PF4,MASP1,CAMP,SLC27A4,FGB,F2,PLG,PSG1,PRG2,CSPG4,APOL1,SPATA21,FGG,THBS4,FBLN5,AKAP9,CNDP1,PCDH18,CFHR3,CFH,F5,S100A7,CCN5,F7,CA6,GPX3,PROS1,TNRC6A,BPIFC,MASP2,F11,C1S,LTBP1,PCYOX1,NBEAL1,ACSF2,CDK3,C4B,SERPINA1,TGFBI,IGFALS,PAEP,PDGFC,C1R,MYL6,APOC1,VEGFC,PLCG2,HSPH1,KNG1,TPM4,TRIM66,B2M,FGA
GO Function	GO:0001851	Complement component C3b binding	3	11	1.79	0.0085	CFHR5,CFHR3,CFH
GO Function	GO:0004857	Enzyme inhibitor activity	9	396	0.71	0.0205	HRG,C3,AHSG,SERPINA11,PROS1,C4B,SERPINA1,APOC1,KNG1
GO Function	GO:0005515	Protein binding	50	7242	0.19	0.0259	HRG,APOB,APOA1,C3,ANGPTL6,APCS,CFHR5,LYZ,LCAT,DNAH5,TCIRG1,SEC24D,DSC2,SCGB3A1,PPBP,PF4,MASP1,FGB,F2,PLG,PSG1,CSPG4,FGG,THBS4,FBLN5,AKAP9,CFHR3,CFH,S100A7,CCN5,F7,GPX3,MASP2,F11,C1S,LTBP1,NBEAL1,C4B,SERPINA1,TGFBI,IGFALS,PDGFC,C1R,VEGFC,PLCG2,HSPH1,KNG1,TPM4,B2M,FGA
GO Function	GO:0008233	Peptidase activity	11	617	0.6	0.0259	F9,HABP2,MASP1,F2,PLG,CNDP1,F7,MASP2,F11,C1S,C1R
GO Component	GO:0005615	Extracellular space	67	3247	0.66	2.87e-31	F9,MGP,GPLD1,SPARC,HRG,APOB,APOA1,C3,ANGPTL6,BPIFB1,APCS,CFHR5,LYZ,ATRN,LCAT,AHSG,PLA2G7,HABP2,SAA4,DSC2,SCGB3A1,PPBP,PF4,MASP1,CAMP,FGB,F2,PLG,PRG2,CSPG4,APOL1,SERPINA11,FGG,THBS4,FBLN5,CFHR3,CFH,F5,S100A7,CCN5,C1QC,F7,CA6,ADGRG2,GPX3,PROS1,BPIFC,MASP2,F11,C1S,PCYOX1,C4B,SERPINA1,TGFBI,IGFALS,PAEP,PDGFC,C1R,MYL6,APOC1,VEGFC,PLCG2,HSPH1,KNG1,TPM4,B2M,FGA
GO Component	GO:0005576	Extracellular region	72	4175	0.59	1.65e-30	F9,MGP,GPLD1,SPARC,HRG,APOB,APOA1,C3,ANGPTL6,BPIFB1,APCS,CFHR5,LYZ,ATRN,LCAT,DNAH5,AHSG,PLA2G7,HABP2,SAA4,DSC2,SCGB3A1,PPBP,PF4,MASP1,CAMP,FGB,F2,PLG,PSG1,PRG2,CSPG4,APOL1,SERPINA11,FGG,THBS4,FBLN5,CNDP1,CFHR3,CFH,F5,S100A7,CCN5,C1QC,F7,CA6,ADGRG2,GPX3,PROS1,BPIFC,MASP2,F11,C1S,PSG2,LTBP1,PCYOX1,C4B,SERPINA1,TGFBI,IGFALS,PAEP,PDGFC,C1R,MYL6,APOC1,VEGFC,PLCG2,HSPH1,KNG1,TPM4,B2M,FGA
GO Component	GO:1903561	Extracellular vesicle	49	2120	0.71	7.37e-22	F9,MGP,GPLD1,HRG,APOB,APOA1,C3,ANGPTL6,BPIFB1,APCS,LYZ,ATRN,LCAT,AHSG,SAA4,DSC2,SCGB3A1,CAMP,FGB,F2,PLG,PRG2,CSPG4,FGG,THBS4,FBLN5,CFHR3,CFH,F5,CA6,ADGRG2,GPX3,PROS1,MASP2,F11,PCYOX1,C4B,SERPINA1,TGFBI,IGFALS,PDGFC,C1R,MYL6,PLCG2,HSPH1,KNG1,TPM4,B2M,FGA
GO Component	GO:0070062	Extracellular exosome	48	2096	0.71	2.26e-21	F9,MGP,GPLD1,HRG,APOB,APOA1,C3,ANGPTL6,BPIFB1,APCS,LYZ,ATRN,LCAT,AHSG,SAA4,DSC2,SCGB3A1,CAMP,FGB,F2,PLG,PRG2,CSPG4,FGG,THBS4,FBLN5,CFHR3,CFH,CA6,ADGRG2,GPX3,PROS1,MASP2,F11,PCYOX1,C4B,SERPINA1,TGFBI,IGFALS,PDGFC,C1R,MYL6,PLCG2,HSPH1,KNG1,TPM4,B2M,FGA
GO Component	GO:0072562	Blood microparticle	19	118	1.56	4.44e-21	HRG,APOA1,C3,APCS,AHSG,FGB,F2,PLG,APOL1,FGG,CFHR3,CFH,C1QC,PROS1,C1S,C4B,C1R,KNG1,FGA
GO Component	GO:0031012	Extracellular matrix	29	552	1.07	1.42e-20	F9,MGP,GPLD1,SPARC,HRG,APOA1,ANGPTL6,APCS,ATRN,AHSG,PF4,FGB,F2,PLG,PRG2,CSPG4,FGG,THBS4,FBLN5,S100A7,CCN5,C1QC,F7,LTBP1,SERPINA1,TGFBI,IGFALS,KNG1,FGA
GO Component	GO:0062023	Collagen-containing extracellular matrix	26	407	1.16	2.84e-20	F9,MGP,SPARC,HRG,APOA1,ANGPTL6,APCS,ATRN,AHSG,PF4,FGB,F2,PLG,PRG2,CSPG4,FGG,THBS4,FBLN5,S100A7,C1QC,F7,LTBP1,SERPINA1,TGFBI,KNG1,FGA
GO Component	GO:0031982	Vesicle	57	3957	0.51	2.71e-17	F9,MGP,GPLD1,SPARC,HRG,APOB,APOA1,C3,ANGPTL6,BPIFB1,APCS,LYZ,ATRN,LCAT,TCIRG1,AHSG,SAA4,SEC24D,DSC2,SCGB3A1,PPBP,PF4,CAMP,FGB,F2,PLG,PRG2,CSPG4,FGG,THBS4,FBLN5,CFHR3,CFH,F5,S100A7,F7,CA6,ADGRG2,GPX3,PROS1,MASP2,F11,PCYOX1,C4B,SERPINA1,TGFBI,IGFALS,PDGFC,C1R,MYL6,VEGFC,PLCG2,HSPH1,KNG1,TPM4,B2M,FGA
GO Component	GO:0031093	Platelet alpha granule lumen	14	66	1.68	8.15e-17	SPARC,HRG,AHSG,PPBP,PF4,FGB,PLG,FGG,F5,PROS1,SERPINA1,VEGFC,KNG1,FGA
GO Component	GO:0031983	Vesicle lumen	21	326	1.16	3.91e-16	SPARC,HRG,APOB,APOA1,C3,LYZ,AHSG,PPBP,PF4,CAMP,FGB,PLG,FGG,F5,S100A7,PROS1,SERPINA1,VEGFC,KNG1,B2M,FGA
GO Component	GO:0034774	Secretory granule lumen	20	321	1.14	4.44e-15	SPARC,HRG,APOA1,C3,LYZ,AHSG,PPBP,PF4,CAMP,FGB,PLG,FGG,F5,S100A7,PROS1,SERPINA1,VEGFC,KNG1,B2M,FGA
GO Component	GO:1905370	Serine-type endopeptidase complex	9	26	1.89	3.46e-12	F9,C3,MASP1,F2,CFH,F5,F7,MASP2,F11

GO Component	GO:0005788	Endoplasmic reticulum lumen	16	312	1.06	1.20e-10	F9,APOB,APOA1,C3,AHSG,F2,APOL1,FGG,F5,F7,LTBP1,SERPINA1,PDGFC,KNG1,B2M,FGA
GO Component	GO:0030141	Secretory granule	23	873	0.77	4.99e-10	SPARC,HRG,APOA1,C3,ANGPTL6,LYZ,TCIRG1,AHSG,PPBP,PF4,CAMP,FGB,PLG,PRG2,FGG,F5,S100A7,PROS1,SERPINA1,VEGFC,KNG1,B2M,FGA
GO Component	GO:0034358	Plasma lipoprotein particle	8	38	1.67	2.45e-09	APOB,APOA1,LCAT,PLA2G7,SAA4,APOL1,PCYOX1,APOC1
GO Component	GO:1905369	Endopeptidase complex	10	104	1.33	7.13e-09	F9,C3,MASP1,F2,UBE3C,CFH,F5,F7,MASP2,F11
GO Component	GO:0034364	High-density lipoprotein particle	7	29	1.73	1.60e-08	APOB,APOA1,LCAT,PLA2G7,SAA4,APOL1,APOC1
GO Component	GO:0034361	Very-low-density lipoprotein particle	5	20	1.75	5.62e-06	APOB,APOA1,APOL1,PCYOX1,APOC1
GO Component	GO:0071944	Cell periphery	49	6015	0.26	6.26e-05	F9,MGP,GPLD1,SPARC,HRG,APOB,APOA1,C3,ANGPTL6,APCS,ATRN,TCIRG1,AHSG,DSC2,PF4,MASP1,SLC27A4,FGB,F2,PLG,PSG1,PRG2,CSPG4,FGG,THBS4,FBLN5,AKAP9,PCDH18,F5,S100A7,CCN5,C1QC,F7,ADGRG2,PROS1,MASP2,F11,LTBP1,PCYOX1,C4B,SERPINA1,TGFBI,IGFALS,PDGFC,PLCG2,KNG1,TPM4,B2M,FGA
GO Component	GO:0097708	Intracellular vesicle	28	2484	0.4	0.00015	SPARC,HRG,APOB,APOA1,C3,ANGPTL6,LYZ,TCIRG1,AHSG,SEC24D,DSC2,PPBP,PF4,CAMP,FGB,PLG,PRG2,FGG,F5,S100A7,PROS1,SERPINA1,VEGFC,PLCG2,HSPH1,KNG1,B2M,FGA
GO Component	GO:0005602	Complement component C1 complex	3	5	2.13	0.00030	C1QC,C1S,C1R
GO Component	GO:0071682	Endocytic vesicle lumen	4	23	1.59	0.00037	SPARC,APOB,APOA1,HSPH1
GO Component	GO:0031410	Cytoplasmic vesicle	27	2482	0.39	0.00043	SPARC,HRG,APOB,APOA1,C3,ANGPTL6,LYZ,TCIRG1,AHSG,SEC24D,DSC2,PPBP,PF4,CAMP,FGB,PLG,PRG2,FGG,F5,S100A7,PROS1,SERPINA1,VEGFC,HSPH1,KNG1,B2M,FGA
GO Component	GO:0009986	Cell surface	15	894	0.57	0.00061	SPARC,HRG,C3,MASP1,FGB,PLG,PSG1,CSPG4,FGG,ADGRG2,MASP2,PSG2,PDGFC,B2M,FGA
GO Component	GO:0005577	Fibrinogen complex	3	8	1.92	0.00078	FGB,FGG,FGA
GO Component	GO:0005783	Endoplasmic reticulum	23	2021	0.41	0.0012	F9,APOB,APOA1,C3,AHSG,SEC24D,SLC27A4,FGB,F2,APOL1,FGG,THBS4,F5,S100A7,F7,PROS1,LTBP1,SERPINA1,PDGFC,APOC1,KNG1,B2M,FGA
GO Component	GO:0042627	Chylomicron	3	13	1.71	0.0025	APOB,APOA1,APOC1
GO Component	GO:0012505	Endomembrane system	38	4721	0.26	0.0031	F9,SPARC,HRG,APOB,APOA1,C3,ANGPTL6,LYZ,TCIRG1,AHSG,SEC24D,PPBP,PF4,CAMP,SLC27A4,FGB,F2,PLG,PRG2,CSPG4,APOL1,FGG,THBS4,AKAP9,F5,S100A7,F7,PROS1,TNRC6A,LTBP1,SERPINA1,TGFBI,PDGFC,APOC1,VEGFC,KNG1,B2M,FGA
GO Component	GO:0005796	Golgi lumen	5	106	1.02	0.0070	F9,F2,CSPG4,F7,PROS1
GO Component	GO:1904724	Tertiary granule lumen	4	55	1.21	0.0070	LYZ,PPBP,CAMP,B2M
GO Component	GO:0030134	COPII-coated ER to Golgi transport vesicle	4	90	1.0	0.0400	SEC24D,F5,SERPINA1,B2M
GO Component	GO:0070820	Tertiary granule	5	164	0.83	0.0451	LYZ,TCIRG1,PPBP,CAMP,B2M
STRING clusters	CL:18726	Complement and coagulation cascades, and Protein-lipid complex	31	161	1.63	5.03e-37	F9,HRG,APOA1,C3,APCS,CFHR5,LCAT,AHSG,HABP2,SAA4,MASP1,FGB,F2,PLG,APOL1,SERPINA11,FGG,CFHR3,CFH,F5,C1QC,F7,MASP2,F11,C1S,PCYOX1,C4B,SERPINA1,C1R,APOC1,FGA
STRING clusters	CL:18723	Mixed, incl. Complement and coagulation cascades, and Protein-lipid complex	32	196	1.56	8.57e-37	F9,HRG,APOA1,C3,APCS,CFHR5,LCAT,AHSG,HABP2,SAA4,MASP1,FGB,F2,PLG,APOL1,SERPINA11,FGG,CFHR3,CFH,F5,C1QC,F7,PROS1,MASP2,F11,C1S,PCYOX1,C4B,SERPINA1,C1R,APOC1,FGA
STRING clusters	CL:18728	Complement and coagulation cascades, and Positive regulation of opsonization	25	109	1.71	2.15e-31	F9,HRG,C3,APCS,CFHR5,AHSG,HABP2,MASP1,FGB,F2,PLG,SERPINA11,FGG,CFHR3,CFH,F5,C1QC,F7,MASP2,F11,C1S,C4B,SERPINA1,C1R,FGA
STRING clusters	CL:18731	Hemostasis, and Dissolution of Fibrin Clot	13	50	1.76	6.04e-16	F9,HRG,AHSG,HABP2,FGB,F2,PLG,FGG,F5,F7,F11,SERPINA1,FGA
STRING clusters	CL:18846	Complement cascade	11	50	1.69	1.25e-12	C3,APCS,CFHR5,MASP1,CFHR3,CFH,C1QC,MASP2,C1S,C4B,C1R
STRING clusters	CL:18733	Mixed, incl. COVID-19, thrombosis and anticoagulation, and Inter-alpha-trypsin inhibitor heavy chain C-terminus	8	21	1.93	2.01e-10	HRG,AHSG,HABP2,FGB,PLG,FGG,SERPINA1,FGA
STRING clusters	CL:18849	Initial triggering of complement, and Regulation of complement activation	8	31	1.76	2.32e-09	C3,MASP1,CFH,C1QC,MASP2,C1S,C4B,C1R
STRING clusters	CL:18851	Initial triggering of complement, and Negative regulation of complement activation	7	24	1.81	2.13e-08	C3,MASP1,C1QC,MASP2,C1S,C4B,C1R
STRING clusters	CL:18734	COVID-19, thrombosis and anticoagulation, and Negative regulation of fibrinolysis	6	11	2.09	2.36e-08	HRG,HABP2,FGB,PLG,FGG,FGA
STRING clusters	CL:18737	Fibrinogen, and Thrombophilia	5	6	2.27	2.01e-07	HRG,FGB,PLG,FGG,FGA
STRING clusters	CL:18960	High-density lipoprotein particle	6	26	1.71	1.45e-06	APOA1,LCAT,SAA4,APOL1,PCYOX1,APOC1
STRING clusters	CL:18786	Hemophilia B, and Blood coagulation, common pathway	5	12	1.97	2.34e-06	F9,F2,F5,F7,F11
STRING clusters	CL:18852	Creation of C4 and C2 activators	5	15	1.87	5.49e-06	MASP1,C1QC,MASP2,C1S,C1R
STRING clusters	CL:18962	High-density lipoprotein particle	5	21	1.73	1.97e-05	APOA1,LCAT,APOL1,PCYOX1,APOC1
STRING clusters	CL:18788	Extrinsic Pathway of Fibrin Clot Formation, and Factor V deficiency	3	6	2.05	0.0013	F2,F5,F7
STRING clusters	CL:18869	Complement component C1 complex	3	6	2.05	0.0013	C1QC,C1S,C1R
STRING clusters	CL:18970	Triglyceride-rich lipoprotein particle remodeling, and Spherical high-density lipoprotein particle	3	11	1.79	0.0051	APOA1,LCAT,APOC1
KEGG	hsa04610	Complement and coagulation cascades	21	82	1.76	3.22e-27	F9,C3,CFHR5,FGB,F2,PLG,FGG,CFHR3,CFH,F5,C1QC,F7,PROS1,MASP2,F11,C1S,C4B,SERPINA1,C1R,KNG1,FGA
KEGG	hsa05150	Staphylococcus aureus infection	10	86	1.42	2.48e-09	C3,CAMP,PLG,FGG,CFH,C1QC,MASP2,C1S,C4B,C1R
KEGG	hsa05133	Pertussis	5	73	1.19	0.0028	C3,C1QC,C1S,C4B,C1R
KEGG	hsa04979	Cholesterol metabolism	4	48	1.27	0.0067	APOB,APOA1,LCAT,APOC1
KEGG	hsa05322	Systemic lupus erythematosus	5	94	1.08	0.0067	C3,C1QC,C1S,C4B,C1R
KEGG	hsa04611	Platelet activation	5	122	0.96	0.0144	FGB,F2,FGG,PLCG2,FGA
KEGG	hsa05143	African trypanosomiasis	3	36	1.27	0.0332	APOA1,APOL1,KNG1
KEGG	hsa04975	Fat digestion and absorption	3	41	1.21	0.0414	APOB,APOA1,SLC27A4
Reactome	HSA-114608	Platelet degranulation	15	126	1.43	1.35e-13	SPARC,HRG,APOA1,AHSG,PPBP,PF4,FGB,PLG,FGG,F5,PROS1,SERPINA1,VEGFC,KNG1,FGA
Reactome	HSA-140877	Formation of Fibrin Clot (Clotting Cascade)	11	39	1.8	1.74e-13	F9,PF4,FGB,F2,FGG,F5,F7,PROS1,F11,KNG1,FGA
Reactome	HSA-381426	Regulation of Insulin-like Growth Factor (IGF) transport and uptake by Insulin-like Growth Factor Binding Proteins (IGFBPs)	14	124	1.4	7.78e-13	APOB,APOA1,C3,AHSG,F2,PLG,APOL1,FGG,F5,LTBP1,SERPINA1,IGFALS,KNG1,FGA
Reactome	HSA-109582	Hemostasis	23	607	0.93	1.42e-12	F9,SPARC,HRG,APOB,APOA1,AHSG,PPBP,PF4,FGB,F2,PLG,PSG1,FGG,F5,F7,PROS1,F11,PSG2,SERPINA1,VEGFC,PLCG2,KNG1,FGA
Reactome	HSA-76002	Platelet activation, signaling and aggregation	17	260	1.17	1.78e-12	SPARC,HRG,APOA1,AHSG,PPBP,PF4,FGB,F2,PLG,FGG,F5,PROS1,SERPINA1,VEGFC,PLCG2,KNG1,FGA
Reactome	HSA-166658	Complement cascade	11	59	1.62	4.02e-12	C3,CFHR5,F2,CFHR3,CFH,C1QC,PROS1,MASP2,C1S,C4B,C1R
Reactome	HSA-977606	Regulation of Complement cascade	10	49	1.66	2.68e-11	C3,CFHR5,F2,CFHR3,CFH,C1QC,PROS1,C1S,C4B,C1R
Reactome	HSA-168249	Innate Immune System	26	1041	0.75	1.17e-10	APOB,C3,BPIFB1,CFHR5,LYZ,TCIRG1,AHSG,PPBP,CAMP,FGB,F2,PRG2,FGG,CFHR3,CFH,S100A7,C1QC,PROS1,MASP2,C1S,C4B,SERPINA1,C1R,PLCG2,B2M,FGA
Reactome	HSA-8957275	Post-translational protein phosphorylation	11	107	1.36	1.05e-09	APOB,APOA1,C3,AHSG,APOL1,FGG,F5,LTBP1,SERPINA1,KNG1,FGA
Reactome	HSA-140875	Common Pathway of Fibrin Clot Formation	7	22	1.85	8.07e-09	PF4,FGB,F2,FGG,F5,PROS1,FGA
Reactome	HSA-166663	Initial triggering of complement	6	24	1.75	6.75e-07	C3,C1QC,MASP2,C1S,C4B,C1R
Reactome	HSA-168256	Immune System	28	1979	0.5	3.68e-06	APOB,C3,BPIFB1,CFHR5,LYZ,TCIRG1,AHSG,SEC24D,PPBP,CAMP,FGB,F2,UBE3C,PRG2,FGG,CFHR3,CFH,S100A7,C1QC,PROS1,MASP2,C1S,C4B,SERPINA1,C1R,PLCG2,B2M,FGA
Reactome	HSA-140837	Intrinsic Pathway of Fibrin Clot Formation	5	23	1.69	2.29e-05	F9,F2,PROS1,F11,KNG1

Reactome	HSA-159763	Transport of gamma-carboxylated protein precursors from the endoplasmic reticulum to the Golgi apparatus	4	9	2.0	3.85e-05	F9,F2,F7,PROS1
Reactome	HSA-159740	Gamma-carboxylation of protein precursors	4	10	1.95	5.04e-05	F9,F2,F7,PROS1
Reactome	HSA-159782	Removal of aminoterminal propeptides from gamma-carboxylated proteins	4	10	1.95	5.04e-05	F9,F2,F7,PROS1
Reactome	HSA-166786	Creation of C4 and C2 activators	4	16	1.75	0.00020	C1QC,MASP2,C1S,C1R
Reactome	HSA-2173782	Binding and Uptake of Ligands by Scavenger Receptors	5	43	1.42	0.00025	SPARC,APOB,APOA1,APOL1,HSPH1
Reactome	HSA-977225	Amyloid fiber formation	6	79	1.23	0.00025	APOA1,APCS,LYZ,TGFBI,B2M,FGA
Reactome	HSA-392499	Metabolism of proteins	24	1917	0.45	0.00027	F9,GPLD1,APOB,APOA1,C3,APCS,LYZ,AHSG,PLA2G7,SEC24D,F2,PLG,APOL1,FGG,F5,F7,PROS1,LTBP1,SERPINA1,TGFBI,IGFALS,KNG1,B2M,FGA
Reactome	HSA-5686938	Regulation of TLR by endogenous ligand	4	20	1.65	0.00036	APOB,FGB,FGG,FGA
Reactome	HSA-173623	Classical antibody-mediated complement activation	3	9	1.87	0.0017	C1QC,C1S,C1R
Reactome	HSA-76009	Platelet Aggregation (Plug Formation)	4	39	1.36	0.0036	FGB,F2,FGG,FGA
Reactome	HSA-202733	Cell surface interactions at the vascular wall	6	139	0.99	0.0041	APOB,PF4,F2,PSG1,PROS1,PSG2
Reactome	HSA-6798695	Neutrophil degranulation	10	476	0.67	0.0048	C3,LYZ,TCIRG1,AHSG,PPBP,CAMP,PRG2,S100A7,SERPINA1,B2M
Reactome	HSA-354194	GRB2:SOS provides linkage to MAPK signaling for Integrins	3	15	1.65	0.0052	FGB,FGG,FGA
Reactome	HSA-372708	p130Cas linkage to MAPK signaling for integrins	3	15	1.65	0.0052	FGB,FGG,FGA
Reactome	HSA-9651496	Defects of contact activation system (CAS) and kallikrein/kinin system (KKS)	3	16	1.62	0.0058	F9,F2,F11
Reactome	HSA-5602498	MyD88 deficiency (TLR2/4)	3	17	1.6	0.0063	FGB,FGG,FGA
Reactome	HSA-5603041	IRAK4 deficiency (TLR2/4)	3	18	1.57	0.0072	FGB,FGG,FGA
Reactome	HSA-8963898	Plasma lipoprotein assembly	3	19	1.55	0.0080	APOB,APOA1,APOC1
Reactome	HSA-597592	Post-translational protein modification	17	1405	0.43	0.0097	F9,GPLD1,APOB,APOA1,C3,AHSG,SEC24D,F2,APOL1,FGG,F5,F7,PROS1,LTBP1,SERPINA1,KNG1,FGA
Reactome	HSA-6802952	Signaling by BRAF and RAF1 fusions	4	65	1.14	0.0156	FGB,FGG,AKAP9,FGA
Reactome	HSA-3000497	Scavenging by Class H Receptors	2	4	2.05	0.0174	SPARC,APOB
Reactome	HSA-140834	Extrinsic Pathway of Fibrin Clot Formation	2	5	1.95	0.0231	F9,F7
Reactome	HSA-174824	Plasma lipoprotein assembly, remodeling, and clearance	4	75	1.08	0.0231	APOB,APOA1,LCAT,APOC1
Reactome	HSA-8866423	VLDL assembly	2	5	1.95	0.0231	APOB,APOC1
Reactome	HSA-1474244	Extracellular matrix organization	7	300	0.72	0.0233	SPARC,FGB,PLG,FGG,FBLN5,LTBP1,FGA
Reactome	HSA-3000471	Scavenging by Class B Receptors	2	6	1.87	0.0267	APOB,APOA1
Reactome	HSA-3000484	Scavenging by Class F Receptors	2	6	1.87	0.0267	APOB,HSPH1
Reactome	HSA-5694530	Cargo concentration in the ER	3	33	1.31	0.0267	SEC24D,F5,SERPINA1
Reactome	HSA-8963899	Plasma lipoprotein remodeling	3	34	1.3	0.0267	APOB,APOA1,LCAT
Reactome	HSA-8964046	VLDL clearance	2	6	1.87	0.0267	APOB,APOC1
Reactome	HSA-9673221	Defective F9 activation	2	6	1.87	0.0267	F9,F11
Reactome	HSA-174577	Activation of C3 and C5	2	7	1.81	0.0297	C3,C4B
Reactome	HSA-6802948	Signaling by high-kinase activity BRAF mutants	3	36	1.27	0.0297	FGB,FGG,FGA
Reactome	HSA-6803157	Antimicrobial peptides	4	87	1.01	0.0297	BPIFB1,LYZ,CAMP,S100A7
Reactome	HSA-9662001	Defective factor VIII causes hemophilia A	2	7	1.81	0.0297	F9,F2
Reactome	HSA-8964043	Plasma lipoprotein clearance	3	37	1.26	0.0299	APOB,APOA1,APOC1
Reactome	HSA-1236974	ER-Phagosome pathway	4	89	1.0	0.0310	FGB,FGG,B2M,FGA
Reactome	HSA-168898	Toll-like Receptor Cascades	5	161	0.84	0.0338	APOB,FGB,FGG,PLCG2,FGA
Reactome	HSA-5674135	MAP2K and MAPK activation	3	40	1.23	0.0351	FGB,FGG,FGA
Reactome	HSA-9656223	Signaling by RAF1 mutants	3	41	1.21	0.0369	FGB,FGG,FGA
Reactome	HSA-6802946	Signaling by moderate kinase activity BRAF mutants	3	45	1.17	0.0454	FGB,FGG,FGA
Reactome	HSA-6802955	Paradoxical activation of RAF signaling by kinase inactive BRAF	3	45	1.17	0.0454	FGB,FGG,FGA
Reactome	HSA-8963888	Chylomicron assembly	2	10	1.65	0.0454	APOB,APOA1
Reactome	HSA-8963901	Chylomicron remodeling	2	10	1.65	0.0454	APOB,APOA1
Reactome	HSA-8964058	HDL remodeling	2	10	1.65	0.0454	APOA1,LCAT
Reactome	HSA-9649948	Signaling downstream of RAS mutants	3	45	1.17	0.0454	FGB,FGG,FGA
WikiPathways	WP558	Complement and coagulation cascades	17	58	1.82	3.82e-22	F9,C3,MASP1,FGB,F2,PLG,CFH,F5,C1QC,F7,PROS1,MASP2,C1S,C4B,SERPINA1,C1R,KNG1
WikiPathways	WP2806	Complement system	13	96	1.48	6.85e-13	APOA1,C3,APCS,MASP1,FGB,PLG,FGG,CFH,PROS1,MASP2,F11,C1S,FGA
WikiPathways	WP272	Blood clotting cascade	9	22	1.96	2.17e-12	F9,FGB,F2,PLG,FGG,F5,F7,F11,FGA
WikiPathways	WP176	Folate metabolism	10	67	1.52	3.00e-10	APOB,APOA1,SAA4,FGB,F2,PLG,FGG,F7,GPX3,FGA
WikiPathways	WP15	Selenium micronutrient network	10	84	1.43	1.86e-09	APOB,APOA1,SAA4,FGB,F2,PLG,FGG,F7,GPX3,FGA
WikiPathways	WP545	Complement activation	7	22	1.85	5.06e-09	C3,MASP1,C1QC,MASP2,C1S,C4B,C1R
WikiPathways	WP4927	COVID-19, thrombosis and anticoagulation	5	7	2.2	1.34e-07	FGB,F2,PLG,FGG,FGA
WikiPathways	WP5090	Complement system in neuronal development and plasticity	9	105	1.28	1.94e-07	C3,MASP1,CFH,C1QC,PROS1,MASP2,C1S,C4B,C1R
WikiPathways	WP5115	Network map of SARS-CoV-2 signaling pathway	11	218	1.05	4.98e-07	HRG,APOA1,PF4,FGB,APOL1,FGG,CFH,C1S,C1R,APOC1,FGA
WikiPathways	WP1533	Vitamin B12 metabolism	6	50	1.43	1.38e-05	APOB,APOA1,SAA4,F2,PLG,F7
WikiPathways	WP5186	Vitamin K metabolism and activation of dependent proteins	4	12	1.87	4.53e-05	F9,F2,F7,PROS1
WikiPathways	WP430	Statin inhibition of cholesterol production	4	29	1.49	0.00088	APOB,APOA1,LCAT,APOC1
WikiPathways	WP3601	Lipid particles composition	3	10	1.83	0.0014	APOB,APOA1,LCAT
WikiPathways	WP5304	Cholesterol metabolism	5	72	1.19	0.0014	APOB,APOA1,LCAT,SLC27A4,APOC1
WikiPathways	WP3941	Oxidative damage response	4	39	1.36	0.0021	C1QC,C1S,C4B,C1R
WikiPathways	WP4136	Fibrin complement receptor 3 signaling pathway	4	43	1.32	0.0027	FGB,PLG,FGG,FGA
WikiPathways	WP5323	Fatty Acids and Lipoproteins Transport in Hepatocytes	9	380	0.72	0.0027	APOB,APOA1,LCAT,PLA2G7,SAA4,SLC27A4,APOL1,PCYOX1,APOC1
WikiPathways	WP5109	Familial hyperlipidemia type 2	3	16	1.62	0.0034	APOB,APOA1,LCAT
WikiPathways	WP4522	Metabolic pathway of LDL, HDL and TG, including diseases	3	17	1.6	0.0038	APOB,APOA1,LCAT
WikiPathways	WP5333	Cholesterol metabolism	3	36	1.27	0.0270	APOB,APOA1,SLC27A4
Monarch	EFO:0007937	Blood protein measurement	40	1810	0.69	1.15e-14	HRG,APOB,C3,BPIFB1,CFHR5,LYZ,LCAT,AHSG,HABP2,SAA4,DSC2,ANKRD44,MASP1,F2,PLG,PSG1,PRG2,APOL1,OAF,SERPINA11,CNDP1,CFH,F5,CCN5,C1QC,F7,CA6,F11,C1S,PSG2,PCYOX1,NBEAL1,C4B,SERPINA1,TGFBI,C1R,APOC1,VEGFC,PLCG2,KNG1
Monarch	HP:0003256	Abnormality of the coagulation cascade	11	105	1.37	2.32e-08	F9,FGB,F2,PLG,FGG,F5,F7,PROS1,F11,KNG1,FGA
Monarch	EFO:0004747	Protein measurement	57	5856	0.34	5.45e-08	GPLD1,HRG,APOB,APOA1,C3,ANGPTL6,BPIFB1,APCS,CFHR5,LYZ,LCAT,DNAH5,TCIRG1,AHSG,PLA2G7,HABP2,SAA4,DSC2,ANKRD44,MASP1,FGB,F2,PLG,PSG1,UBE3C,PRG2,APOL1,OAF,SERPINA11,FGG,THBS4,CNDP1,CFH,F5,CCN5,C1QC,F7,CA6,TNRC6A,F11,C1S,PSG2,LTBP1,PCYOX1,NBEAL1,C4B,SERPINA1,TGFBI,IGFALS,PDGFC,C1R,APOC1,VEGFC,PLCG2,KNG1,TRIM66,FGA

Monarch	HP:0001871	Abnormality of blood and blood-forming tissues	26	1214	0.68	5.45e-08	F9,SPARC,HRG,APOB,APOA1,ANGPTL6,LCAT,TCIRG1,SEC24D,SLC27A4,FGB,F2,PLG,FGG,CFH,F5,F7,PROS1,F11,C1S,SERPINA1,C1R,PLCG2,KNG1,B2M,FGA
Monarch	HP:0002597	Abnormality of the vasculature	30	1673	0.6	5.45e-08	F9,MGP,SPARC,APOB,APOA1,ANGPTL6,LYZ,LCAT,DNAH5,TCIRG1,HABP2,SEC24D,MASP1,FGB,F2,PLG,FGG,FBLN5,F5,F7,PROS1,C1S,SERPINA1,TGFBI,C1R,VEGFC,PLCG2,KNG1,B2M,FGA
Monarch	HP:0001928	Abnormality of coagulation	12	170	1.2	5.79e-08	F9,HRG,FGB,F2,PLG,FGG,F5,F7,PROS1,F11,KNG1,FGA
Monarch	HP:0000225	Gingival bleeding	8	45	1.6	1.45e-07	FGB,F2,FGG,F5,F7,C1S,C1R,FGA
Monarch	HP:0011025	Abnormal cardiovascular system physiology	24	1169	0.66	3.67e-07	F9,MGP,SPARC,APOB,APOA1,ANGPTL6,LYZ,LCAT,TCIRG1,DSC2,FGB,F2,FGG,FBLN5,AKAP9,F5,F7,PROS1,F11,SERPINA1,PLCG2,KNG1,B2M,FGA
Monarch	HP:0001892	Abnormal bleeding	15	411	0.91	7.94e-07	F9,SPARC,ANGPTL6,TCIRG1,SEC24D,FGB,F2,FGG,F5,F7,PROS1,F11,C1S,C1R,FGA
Monarch	HP:0002170	Intracranial hemorrhage	10	130	1.24	7.94e-07	F9,SPARC,ANGPTL6,FGB,F2,FGG,F5,F7,PROS1,FGA
Monarch	HP:0001977	Abnormal thrombosis	9	94	1.33	8.86e-07	F9,HRG,FGB,F2,FGG,F5,PROS1,KNG1,FGA
Monarch	HP:0004936	Venous thrombosis	8	65	1.44	1.19e-06	F9,FGB,F2,FGG,F5,PROS1,KNG1,FGA
Monarch	HP:0001626	Abnormality of the cardiovascular system	33	2438	0.48	1.82e-06	F9,MGP,SPARC,APOB,APOA1,ANGPTL6,LYZ,LCAT,DNAH5,TCIRG1,HABP2,SEC24D,DSC2,MASP1,FGB,F2,PLG,FGG,FBLN5,AKAP9,F5,F7,PROS1,F11,C1S,SERPINA1,TGFBI,C1R,VEGFC,PLCG2,KNG1,B2M,FGA
Monarch	HP:0005339	Abnormality of complement system	6	24	1.75	3.09e-06	C3,CFH,C1QC,MASP2,C1S,C4B
Monarch	HP:0000421	Epistaxis	8	78	1.36	3.57e-06	F9,FGB,F2,FGG,F5,F7,F11,FGA
Monarch	HP:0012211	Abnormal renal physiology	16	560	0.81	3.57e-06	F9,APOA1,C3,CFHR5,LYZ,LCAT,F2,PLG,FBLN5,CFH,F5,C1QC,SERPINA1,C1R,B2M,FGA
Monarch	HP:0025015	Abnormal vascular morphology	21	1027	0.66	3.57e-06	F9,MGP,SPARC,APOB,ANGPTL6,LCAT,DNAH5,TCIRG1,HABP2,SEC24D,MASP1,FGB,F2,FGG,FBLN5,F5,F7,PROS1,VEGFC,KNG1,FGA
Monarch	HP:0012649	Increased inflammatory response	20	981	0.66	7.85e-06	MGP,APOA1,C3,CFHR5,LYZ,DNAH5,TCIRG1,PLG,FBLN5,CFH,C1QC,MASP2,C1S,C4B,SERPINA1,C1R,VEGFC,PLCG2,B2M,FGA
Monarch	EFO:0004634	Coagulation factor measurement	9	132	1.18	7.92e-06	CFHR5,FGB,FGG,CFH,F5,F7,F11,KNG1,FGA
Monarch	HP:0002715	Abnormality of the immune system	26	1682	0.54	7.92e-06	MGP,APOA1,C3,CFHR5,LYZ,LCAT,DNAH5,TCIRG1,HABP2,SLC27A4,FGB,F2,PLG,FGG,FBLN5,CFH,C1QC,MASP2,C1S,C4B,SERPINA1,C1R,VEGFC,PLCG2,B2M,FGA
Monarch	HP:0000123	Nephritis	7	59	1.42	9.43e-06	C3,CFHR5,PLG,FBLN5,CFH,C1QC,C1R
Monarch	HP:0011029	Internal hemorrhage	11	244	1.0	9.43e-06	F9,SPARC,ANGPTL6,FGB,F2,FGG,F5,F7,PROS1,F11,FGA
Monarch	HP:0000168	Abnormality of the gingiva	10	190	1.07	1.03e-05	TCIRG1,FGB,F2,PLG,FGG,F5,F7,C1S,C1R,FGA
Monarch	EFO:0004310	Partial thromboplastin time	5	15	1.87	1.11e-05	HRG,F5,F11,C1S,KNG1
Monarch	HP:0000077	Abnormality of the kidney	20	1077	0.62	2.39e-05	F9,APOB,APOA1,C3,CFHR5,LYZ,LCAT,HABP2,MASP1,F2,PLG,FBLN5,CFH,F5,C1QC,ADGRG2,SERPINA1,C1R,B2M,FGA
Monarch	HP:0002239	Gastrointestinal hemorrhage	8	111	1.21	2.53e-05	F9,FGB,F2,FGG,F5,F7,F11,FGA
Monarch	HP:0005261	Joint hemorrhage	5	19	1.77	2.59e-05	F9,F2,F5,F7,F11
Monarch	HP:0010935	Abnormality of the upper urinary tract	20	1101	0.61	3.07e-05	F9,APOB,APOA1,C3,CFHR5,LYZ,LCAT,HABP2,MASP1,F2,PLG,FBLN5,CFH,F5,C1QC,ADGRG2,SERPINA1,C1R,B2M,FGA
Monarch	HP:0000790	Hematuria	8	118	1.18	3.57e-05	F9,APOA1,CFHR5,LYZ,F2,CFH,F5,FGA
Monarch	HP:0004431	Complement deficiency	5	21	1.73	3.57e-05	C3,CFH,C1QC,MASP2,C4B
Monarch	HP:0011890	Prolonged bleeding following procedure	5	23	1.69	5.05e-05	F9,F2,F5,F7,F11
Monarch	HP:0000118	Phenotypic abnormality	46	5129	0.3	5.37e-05	F9,MGP,SPARC,HRG,APOB,APOA1,C3,ANGPTL6,CFHR5,LYZ,LCAT,DNAH5,TCIRG1,AHSG,PLA2G7,HABP2,SEC24D,DSC2,MASP1,SLC27A4,FGB,F2,PLG,FGG,THBS4,FBLN5,AKAP9,CFH,F5,C1QC,F7,ADGRG2,PROS1,MASP2,F11,C1S,C4B,SERPINA1,TGFBI,IGFALS,C1R,VEGFC,PLCG2,KNG1,B2M,FGA
Monarch	HP:0011830	Abnormal oral mucosa morphology	11	305	0.91	5.37e-05	LYZ,TCIRG1,FGB,F2,PLG,FGG,F5,F7,C1S,C1R,FGA
Monarch	EFO:0008390	Prothrombin time measurement	4	8	2.05	5.55e-05	F2,FGG,F5,F7
Monarch	HP:0003645	Prolonged partial thromboplastin time	5	24	1.67	5.55e-05	F9,F2,F5,F11,KNG1
Monarch	HP:0100659	Abnormal cerebral vascular morphology	12	381	0.85	5.55e-05	F9,SPARC,APOB,ANGPTL6,FGB,F2,FGG,F5,F7,PROS1,KNG1,FGA
Monarch	HP:0002725	Systemic lupus erythematosus	5	26	1.63	7.34e-05	C3,C1QC,MASP2,C1S,C1R
Monarch	HP:0030680	Abnormality of cardiovascular system morphology	24	1680	0.5	7.35e-05	F9,MGP,SPARC,APOB,ANGPTL6,LCAT,DNAH5,TCIRG1,HABP2,SEC24D,DSC2,MASP1,FGB,F2,FGG,FBLN5,F5,F7,PROS1,SERPINA1,VEGFC,KNG1,B2M,FGA
Monarch	HP:0400008	Menometrorrhagia	4	10	1.95	0.00010	F9,FGB,FGG,FGA
Monarch	HP:0000951	Abnormality of the skin	25	1888	0.47	0.00015	F9,MGP,SPARC,APOB,APOA1,LYZ,TCIRG1,AHSG,SEC24D,DSC2,MASP1,SLC27A4,F2,PLG,FBLN5,F5,F7,PROS1,C1S,SERPINA1,C1R,VEGFC,PLCG2,B2M,FGA
Monarch	HP:0000119	Abnormality of the genitourinary system	27	2189	0.44	0.00018	F9,APOB,APOA1,C3,CFHR5,LYZ,LCAT,DNAH5,AHSG,HABP2,MASP1,FGB,F2,PLG,FGG,FBLN5,CFH,F5,C1QC,F7,ADGRG2,F11,SERPINA1,C1R,VEGFC,B2M,FGA
Monarch	HP:0001386	Joint swelling	5	35	1.5	0.00024	FGB,F2,FGG,SERPINA1,FGA
Monarch	HP:0010990	Abnormality of the common coagulation pathway	5	35	1.5	0.00024	FGB,PLG,FGG,F5,FGA
Monarch	HP:0000140	Abnormality of the menstrual cycle	9	228	0.95	0.00028	F9,FGB,F2,FGG,F5,F7,F11,SERPINA1,FGA
Monarch	HP:0005368	Abnormality of humoral immunity	9	234	0.93	0.00034	C3,CFH,C1QC,MASP2,C1S,C4B,SERPINA1,PLCG2,B2M
Monarch	HP:0002960	Autoimmunity	7	122	1.11	0.00042	C3,TCIRG1,C1QC,MASP2,C1S,C1R,PLCG2
Monarch	HP:0006298	Prolonged bleeding after dental extraction	4	16	1.75	0.00042	F9,F2,F5,F11
Monarch	HP:0010978	Abnormality of immune system physiology	20	1347	0.52	0.00042	MGP,APOA1,C3,CFHR5,LYZ,DNAH5,TCIRG1,PLG,FBLN5,CFH,C1QC,MASP2,C1S,C4B,SERPINA1,C1R,VEGFC,PLCG2,B2M,FGA
Monarch	HP:0000978	Bruising susceptibility	7	130	1.08	0.00061	TCIRG1,SEC24D,F2,F5,F7,C1S,C1R
Monarch	HP:0001933	Subcutaneous hemorrhage	8	189	0.98	0.00063	TCIRG1,SEC24D,F2,F5,F7,PROS1,C1S,C1R
Monarch	HP:0011121	Abnormality of skin morphology	22	1648	0.48	0.00063	F9,MGP,SPARC,APOB,APOA1,TCIRG1,AHSG,SEC24D,DSC2,MASP1,SLC27A4,F2,FBLN5,F5,F7,PROS1,C1S,SERPINA1,C1R,VEGFC,PLCG2,B2M
Monarch	HP:0012223	Splenic rupture	3	4	2.23	0.00065	FGB,FGG,FGA
Monarch	HP:0100724	Hypercoagulability	4	19	1.67	0.00066	F9,HRG,F5,PROS1
Monarch	HP:0002625	Deep venous thrombosis	4	20	1.65	0.00078	F9,F2,F5,PROS1
Monarch	HP:0003216	Generalized amyloid deposition	3	5	2.13	0.00089	APOA1,LYZ,FGA
Monarch	HP:0005268	Miscarriage	4	21	1.63	0.00089	MGP,FGB,FGG,FGA
Monarch	HP:0011276	Vascular skin abnormality	11	433	0.75	0.00089	TCIRG1,SEC24D,MASP1,F2,F5,F7,PROS1,C1S,SERPINA1,C1R,PLCG2
Monarch	HP:0011420	Age of death	4	21	1.63	0.00089	MGP,FGB,FGG,FGA
Monarch	HP:0032101	Unusual infection	14	748	0.62	0.0011	MGP,C3,DNAH5,TCIRG1,PLG,FBLN5,CFH,C1QC,MASP2,C4B,SERPINA1,C1R,PLCG2,B2M
Monarch	HP:0011034	Amyloidosis	4	25	1.55	0.0014	APOA1,LYZ,B2M,FGA
Monarch	EFO:0003907	Deep vein thrombosis	3	9	1.87	0.0031	F2,F5,KNG1
Monarch	HP:0000100	Nephrotic syndrome	6	117	1.06	0.0031	APOA1,C3,LYZ,CFH,SERPINA1,FGA
Monarch	HP:0002719	Recurrent infections	13	721	0.61	0.0034	MGP,C3,DNAH5,TCIRG1,PLG,FBLN5,CFH,C1QC,MASP2,SERPINA1,C1R,PLCG2,B2M
Monarch	HP:0011891	Post-partum hemorrhage	3	10	1.83	0.0038	F2,F5,F7
Monarch	HP:0012233	Intramuscular hematoma	3	10	1.83	0.0038	F9,F2,F7
Monarch	HP:0000704	Periodontitis	4	35	1.41	0.0042	TCIRG1,PLG,C1S,C1R
Monarch	HP:0000099	Glomerulonephritis	4	37	1.38	0.0051	C3,CFHR5,CFH,C1QC
Monarch	HP:0025031	Abnormality of the digestive system	25	2389	0.37	0.0053	F9,APOB,APOA1,LYZ,LCAT,DNAH5,TCIRG1,HABP2,MASP1,FGB,F2,PLG,FGG,FBLN5,F5,F7,MASP2,F11,C1S,C4B,SERPINA1,C1R,PLCG2,B2M,FGA
Monarch	HP:0000132	Menorrhagia	4	38	1.37	0.0055	F2,F5,F7,F11
Monarch	HP:0000969	Edema	10	448	0.7	0.0055	APOA1,LYZ,FGB,F2,FGG,SERPINA1,TGFBI,VEGFC,PLCG2,FGA
Monarch	HP:0011024	Abnormality of the gastrointestinal tract	18	1384	0.46	0.0055	F9,DNAH5,TCIRG1,HABP2,FGB,F2,PLG,FGG,FBLN5,F5,F7,MASP2,F11,SERPINA1,C1R,PLCG2,B2M,FGA

Monarch	HP:0000080	Abnormality of reproductive system physiology	12	659	0.61	0.0059	F9,DNAH5,AHSG,FGB,F2,FGG,F5,F7,ADGRG2,F11,SERPINA1,FGA
Monarch	HP:0025408	Abnormal spleen morphology	10	475	0.67	0.0083	APOA1,LYZ,LCAT,DNAH5,TCIRG1,FGB,F2,FGG,SERPINA1,FGA
Monarch	HP:0100763	Abnormality of the lymphatic system	11	576	0.63	0.0083	APOA1,LYZ,LCAT,DNAH5,TCIRG1,HABP2,FGB,F2,FGG,SERPINA1,FGA
Monarch	HP:0001939	Abnormality of metabolism/homeostasis	23	2168	0.38	0.0088	F9,APOB,APOA1,CFHR5,LYZ,LCAT,TCIRG1,PLA2G7,FGB,F2,PLG,FGG,AKAP9,CFH,F5,SERPINA1,TGFBI,IGFALS,VEGFC,PLCG2,KNG1,B2M,FGA
Monarch	HP:0032263	Increased blood pressure	8	303	0.77	0.0102	MGP,APOB,APOA1,ANGPTL6,LYZ,F2,SERPINA1,FGA
Monarch	HP:0011354	Generalized abnormality of skin	13	821	0.55	0.0107	F9,TCIRG1,SEC24D,MASP1,F2,FBLN5,F5,F7,PROS1,C1S,SERPINA1,C1R,PLCG2
Monarch	HP:0002097	Emphysema	4	50	1.25	0.0131	MGP,FBLN5,SERPINA1,B2M
Monarch	HP:0012719	Functional abnormality of the gastrointestinal tract	13	840	0.54	0.0131	F9,TCIRG1,FGB,F2,FGG,F5,F7,MASP2,F11,SERPINA1,PLCG2,B2M,FGA
Monarch	HP:0000152	Abnormality of head or neck	27	2882	0.32	0.0135	F9,MGP,SPARC,APOB,LYZ,DNAH5,TCIRG1,AHSG,HABP2,SEC24D,MASP1,FGB,F2,PLG,FGG,FBLN5,F5,F7,F11,C1S,SERPINA1,IGFALS,C1R,VEGFC,PLCG2,B2M,FGA
Monarch	HP:0004846	Prolonged bleeding after surgery	3	18	1.57	0.0138	F9,F5,F7
Monarch	EFO:0004983	Complement C3 measurement	2	2	2.35	0.0150	C3,CFH
Monarch	EFO:0008155	Histidine-rich glycoprotein measurement	2	2	2.35	0.0150	HRG,KNG1
Monarch	HP:0008065	Aplasia/Hypoplasia of the skin	6	170	0.9	0.0170	MGP,TCIRG1,SEC24D,PROS1,C1S,C1R
Monarch	EFO:0006794	Cerebrospinal fluid biomarker measurement	4	55	1.21	0.0172	HRG,CFHR5,PLA2G7,C1R
Monarch	HP:0006323	Premature loss of primary teeth	3	21	1.5	0.0195	TCIRG1,C1S,C1R
Monarch	HP:0030163	Abnormal vascular physiology	8	340	0.72	0.0195	MGP,ANGPTL6,TCIRG1,F2,F5,PROS1,SERPINA1,KNG1
Monarch	HP:0000271	Abnormality of the face	25	2641	0.33	0.0208	F9,MGP,SPARC,APOB,LYZ,DNAH5,TCIRG1,AHSG,SEC24D,MASP1,FGB,F2,PLG,FGG,FBLN5,F5,F7,F11,C1S,SERPINA1,C1R,VEGFC,PLCG2,B2M,FGA
Monarch	HP:0011898	Abnormality of circulating fibrinogen	3	22	1.48	0.0216	FGB,FGG,FGA
Monarch	HP:0011900	Hypofibrinogenemia	3	22	1.48	0.0216	FGB,FGG,FGA
Monarch	EFO:0004503	Hematological measurement	36	4592	0.24	0.0220	GPLD1,HRG,APOB,APCS,LYZ,ATRN,LCAT,TCIRG1,HABP2,FGB,F2,PLG,CSPG4,FGG,AKAP9,CFH,F5,F7,TNRC6A,MAP3K7CL,MASP2,F11,C1S,LTBP1,NBEAL1,C4B,SERPINA1,IGFALS,PDGFC,APOC1,VEGFC,PLCG2,KNG1,TPM4,TRIM66,FGA
Monarch	EFO:0000618	Nervous system disease	8	350	0.71	0.0223	C3,CFHR5,CFH,F5,NBEAL1,APOC1,PLCG2,KNG1
Monarch	HP:0001342	Cerebral hemorrhage	4	61	1.17	0.0229	FGB,FGG,PROS1,FGA
Monarch	HP:0033127	Abnormality of the musculoskeletal system	28	3173	0.3	0.0232	F9,MGP,SPARC,APOB,APOA1,DNAH5,TCIRG1,AHSG,HABP2,SEC24D,MASP1,FGB,F2,PLG,FGG,FBLN5,F5,F7,F11,C1S,SERPINA1,TGFBI,IGFALS,C1R,VEGFC,PLCG2,B2M,FGA
Monarch	EFO:0008291	Stem Cell Factor measurement	3	24	1.45	0.0258	LCAT,F5,PLCG2
Monarch	HP:0000234	Abnormality of the head	26	2865	0.31	0.0270	F9,MGP,SPARC,APOB,LYZ,DNAH5,TCIRG1,AHSG,SEC24D,MASP1,FGB,F2,PLG,FGG,FBLN5,F5,F7,F11,C1S,SERPINA1,IGFALS,C1R,VEGFC,PLCG2,B2M,FGA
Monarch	HP:0000924	Abnormality of the skeletal system	25	2708	0.32	0.0283	F9,MGP,SPARC,APOB,DNAH5,TCIRG1,AHSG,HABP2,SEC24D,MASP1,FGB,F2,PLG,FGG,FBLN5,F5,F7,F11,C1S,SERPINA1,IGFALS,C1R,VEGFC,B2M,FGA
Monarch	HP:0000366	Abnormality of the nose	17	1470	0.41	0.0284	F9,MGP,DNAH5,TCIRG1,AHSG,MASP1,FGB,F2,PLG,FGG,F5,F7,F11,SERPINA1,PLCG2,B2M,FGA
Monarch	HP:0002829	Arthralgia	5	124	0.96	0.0297	APOB,F2,C1S,SERPINA1,PLCG2
Monarch	EFO:0004694	Factor XI measurement	2	4	2.05	0.0303	F11,KNG1
Monarch	HP:0002012	Abnormality of the abdominal organs	14	1068	0.47	0.0303	APOB,APOA1,LYZ,LCAT,DNAH5,TCIRG1,FGB,F2,FGG,C1S,C4B,SERPINA1,B2M,FGA
Monarch	HP:0002205	Recurrent respiratory infections	9	472	0.63	0.0303	MGP,DNAH5,TCIRG1,PLG,FBLN5,MASP2,SERPINA1,PLCG2,B2M
Monarch	HP:0008151	Prolonged prothrombin time	3	26	1.41	0.0303	F2,F5,F7
Monarch	HP:0012541	Cephalohematoma	2	4	2.05	0.0303	F9,F2
Monarch	HP:0031368	Intestinal perforation	2	4	2.05	0.0303	C1R,B2M
Monarch	HP:0011842	Abnormal skeletal morphology	24	2573	0.32	0.0308	F9,MGP,SPARC,APOB,DNAH5,TCIRG1,AHSG,SEC24D,MASP1,FGB,F2,PLG,FGG,FBLN5,F5,F7,F11,C1S,SERPINA1,IGFALS,C1R,VEGFC,B2M,FGA
Monarch	HP:0002011	Morphological central nervous system abnormality	23	2416	0.33	0.0316	F9,MGP,SPARC,APOB,ANGPTL6,DNAH5,TCIRG1,AHSG,HABP2,SEC24D,MASP1,FGB,F2,PLG,FGG,FBLN5,F5,F7,PROS1,C4B,KNG1,B2M,FGA
Monarch	HP:0010981	Hypolipoproteinemia	3	27	1.4	0.0316	APOB,APOA1,LCAT
Monarch	HP:0000078	Abnormality of the genital system	16	1358	0.42	0.0323	F9,DNAH5,AHSG,MASP1,FGB,F2,PLG,FGG,FBLN5,F5,F7,ADGRG2,F11,SERPINA1,VEGFC,FGA
Monarch	HP:0002659	Increased susceptibility to fractures	6	205	0.82	0.0347	SPARC,TCIRG1,HABP2,SEC24D,FBLN5,B2M
Monarch	HP:0410042	Abnormal liver morphology	11	715	0.54	0.0354	APOB,APOA1,LYZ,LCAT,TCIRG1,F2,C1S,C4B,SERPINA1,B2M,FGA
Monarch	HP:0000370	Abnormality of the middle ear	9	490	0.61	0.0358	MGP,SPARC,DNAH5,TCIRG1,SEC24D,MASP1,PLG,PLCG2,B2M
Monarch	HP:0010989	Abnormality of the intrinsic pathway	3	29	1.36	0.0363	F9,F11,KNG1
Monarch	HP:0005542	Prolonged whole-blood clotting time	2	5	1.95	0.0377	F9,F5
Monarch	HP:0006308	Atrophy of alveolar ridges	2	5	1.95	0.0377	C1S,C1R
Monarch	HP:0011109	Chronic sinusitis	4	75	1.08	0.0390	MGP,DNAH5,SERPINA1,B2M
Monarch	EFO:0008300	TNF-related apoptosis-inducing ligand measurement	3	32	1.32	0.0457	CFH,SERPINA1,KNG1
Monarch	HP:0000793	Membranoproliferative glomerulonephritis	2	6	1.87	0.0486	C3,C1QC
Monarch	HP:0030780	Abnormality of the protein C anticoagulant pathway	2	6	1.87	0.0486	F5,PROS1
Monarch	HP:0100279	Ulcerative colitis	2	6	1.87	0.0486	MASP2,PLCG2
DISEASES	DOID:1247	Blood coagulation disease	15	89	1.58	2.41e-15	F9,HRG,PF4,FGB,F2,PLG,FGG,CFH,F5,F7,PROS1,F11,C4B,KNG1,FGA
DISEASES	DOID:2452	Thrombophilia	8	21	1.93	1.01e-09	F9,HRG,F2,PLG,CFH,F5,PROS1,C4B
DISEASES	DOID:74	Hematopoietic system disease	18	473	0.93	6.97e-09	F9,HRG,TCIRG1,PF4,CAMP,FGB,F2,PLG,FGG,CFH,F5,F7,PROS1,F11,C4B,KNG1,B2M,FGA
DISEASES	DOID:4	Disease	58	6291	0.31	8.55e-08	F9,MGP,SPARC,HRG,APOB,APOA1,C3,APCS,CFHR5,LYZ,LCAT,DNAH5,TCIRG1,AHSG,PLA2G7,SAA4,SEC24D,DSC2,ANKRD44,SCGB3A1,PF4,MASP1,CAMP,SLC27A4,FGB,F2,PLG,PRG2,APOL1,FGG,FBLN5,AKAP9,CNDP1,CFHR3,CFH,F5,C1QC,F7,CA6,ADGRG2,GPX3,PROS1,TNRC6A,F11,C1S,PCYOX1,C4B,SERPINA1,TGFBI,PDGFC,C1R,VEGFC,PLCG2,KNG1,TPM4,TRIM66,B2M,FGA
DISEASES	DOID:7	Disease of anatomical entity	50	4798	0.37	8.55e-08	F9,MGP,SPARC,HRG,APOB,APOA1,C3,APCS,CFHR5,LCAT,DNAH5,TCIRG1,AHSG,PLA2G7,SAA4,SEC24D,DSC2,ANKRD44,SCGB3A1,PF4,CAMP,SLC27A4,FGB,F2,PLG,APOL1,FGG,FBLN5,AKAP9,CNDP1,CFHR3,CFH,F5,C1QC,F7,ADGRG2,PROS1,TNRC6A,F11,C1S,C4B,SERPINA1,TGFBI,C1R,VEGFC,PLCG2,KNG1,TRIM66,B2M,FGA
DISEASES	DOID:9120	Amyloidosis	9	75	1.43	9.61e-08	APOA1,C3,APCS,LYZ,SAA4,SERPINA1,TGFBI,B2M,FGA
DISEASES	DOID:0050636	Familial visceral amyloidosis	6	21	1.81	1.17e-06	APOA1,APCS,LYZ,SERPINA1,B2M,FGA
DISEASES	DOID:2921	Glomerulonephritis	6	36	1.57	1.72e-05	C3,CFHR5,APOL1,CFHR3,CFH,C4B
DISEASES	DOID:0050177	Monogenic disease	36	3266	0.39	3.07e-05	F9,MGP,HRG,APOB,APOA1,C3,APCS,CFHR5,LYZ,DNAH5,TCIRG1,SEC24D,ANKRD44,MASP1,CAMP,FGB,F2,PLG,FGG,FBLN5,AKAP9,CFHR3,CFH,F5,F7,ADGRG2,PROS1,F11,C1S,PCYOX1,SERPINA1,TGFBI,PLCG2,KNG1,B2M,FGA
DISEASES	DOID:0050739	Autosomal genetic disease	33	2802	0.42	3.07e-05	F9,MGP,HRG,APOB,APOA1,C3,APCS,CFHR5,LYZ,DNAH5,TCIRG1,ANKRD44,MASP1,CAMP,FGB,F2,PLG,FGG,FBLN5,AKAP9,CFHR3,CFH,F5,F7,F11,C1S,PCYOX1,SERPINA1,TGFBI,PLCG2,KNG1,B2M,FGA
DISEASES	DOID:626	Complement deficiency	5	23	1.69	5.38e-05	C3,CFHR5,CFHR3,CFH,KNG1
DISEASES	DOID:630	Genetic disease	38	3778	0.35	8.43e-05	F9,MGP,HRG,APOB,APOA1,C3,APCS,CFHR5,LYZ,LCAT,DNAH5,TCIRG1,SEC24D,ANKRD44,MASP1,CAMP,FGB,F2,PLG,FGG,FBLN5,AKAP9,CNDP1,CFHR3,CFH,F5,F7,ADGRG2,PROS1,F11,C1S,PCYOX1,SERPINA1,TGFBI,PLCG2,KNG1,B2M,FGA

DISEASES	DOID:0014667	Disease of metabolism	19	1076	0.6	8.59e-05	F9,MGP,APOB,APOA1,C3,APCS,LYZ,LCAT,AHSG,SAA4,F2,CNDP1,GPX3,PROS1,PCYOX1,SERPINA1,TGFBI,B2M,FGA
DISEASES	DOID:0080301	Atypical hemolytic-uremic syndrome	4	11	1.91	0.00015	C3,CFHR5,CFHR3,CFH
DISEASES	DOID:557	Kidney disease	10	275	0.91	0.00016	C3,APCS,CFHR5,SAA4,F2,APOL1,CFHR3,CFH,C4B,B2M
DISEASES	DOID:0050736	Autosomal dominant disease	21	1386	0.53	0.00018	F9,HRG,APOA1,C3,APCS,CFHR5,LYZ,ANKRD44,F2,FBLN5,AKAP9,CFHR3,CFH,F5,C1S,SERPINA1,TGFBI,PLCG2,KNG1,B2M,FGA
DISEASES	DOID:12554	Hemolytic-uremic syndrome	4	14	1.81	0.00027	C3,CFHR5,CFHR3,CFH
DISEASES	DOID:2236	Congenital afibrinogenemia	3	3	2.35	0.00041	FGB,FGG,FGA
DISEASES	DOID:10871	Age related macular degeneration	5	44	1.41	0.00058	C3,CFHR5,FBLN5,CFHR3,CFH
DISEASES	DOID:2920	Membranoproliferative glomerulonephritis	3	4	2.23	0.00064	C3,CFHR5,CFH
DISEASES	DOID:0060903	Thrombosis	4	21	1.63	0.00086	PF4,F2,PLG,F7
DISEASES	DOID:11249	Vitamin K deficiency bleeding	3	5	2.13	0.00086	MGP,F2,PROS1
DISEASES	DOID:12259	Hemophilia B	3	5	2.13	0.00086	F9,F7,F11
DISEASES	DOID:2229	Factor XI deficiency	3	5	2.13	0.00086	F9,F7,F11
DISEASES	DOID:2451	Protein S deficiency	3	5	2.13	0.00086	F2,PROS1,C4B
DISEASES	DOID:0050117	Disease by infectious agent	10	368	0.78	0.0011	C3,APCS,AHSG,F2,PLG,PRG2,APOL1,C1QC,CA6,PDGFC
DISEASES	DOID:2914	Immune system disease	13	675	0.63	0.0016	C3,APCS,CFHR5,SAA4,F2,PLG,CFHR3,CFH,C4B,VEGFC,PLCG2,KNG1,B2M
DISEASES	DOID:0050637	Finnish type amyloidosis	3	9	1.87	0.0026	C3,APCS,SERPINA1
DISEASES	DOID:11949	Creutzfeldt-Jakob disease	3	9	1.87	0.0026	C3,APCS,C1QC
DISEASES	DOID:1387	Hypolipoproteinemia	3	11	1.79	0.0040	APOB,APOA1,LCAT
DISEASES	DOID:16	Integumentary system disease	11	575	0.63	0.0074	APOA1,C3,APCS,AHSG,DSC2,CAMP,SLC27A4,FBLN5,C1S,SERPINA1,KNG1
DISEASES	DOID:5113	Nutritional deficiency disease	4	43	1.32	0.0074	MGP,F2,GPX3,PROS1
DISEASES	DOID:1287	Cardiovascular system disease	10	493	0.66	0.0097	APOB,APOA1,APCS,DNAH5,DSC2,PF4,F2,PLG,AKAP9,F7
DISEASES	DOID:409	Liver disease	5	97	1.06	0.0108	APCS,AHSG,SAA4,F2,SERPINA1
DISEASES	DOID:9799	Eye degenerative disease	7	236	0.82	0.0121	APOB,C3,CFHR5,FBLN5,CFHR3,CFH,TGFBI
DISEASES	DOID:1391	Norum disease	2	2	2.35	0.0131	APOA1,LCAT
DISEASES	DOID:37	Skin disease	10	518	0.64	0.0131	APOA1,C3,APCS,DSC2,CAMP,SLC27A4,FBLN5,C1S,SERPINA1,KNG1
DISEASES	DOID:0050639	Primary cutaneous amyloidosis	3	19	1.55	0.0132	C3,APCS,SERPINA1
DISEASES	DOID:2215	Factor VII deficiency	2	3	2.17	0.0202	F2,F7
DISEASES	DOID:2216	Factor V deficiency	2	3	2.17	0.0202	F2,F5
DISEASES	DOID:2222	Factor X deficiency	2	3	2.17	0.0202	F2,F7
DISEASES	DOID:4734	Calciphylaxis	2	3	2.17	0.0202	MGP,AHSG
DISEASES	DOID:801	Hemarthrosis	2	3	2.17	0.0202	F9,F7
DISEASES	DOID:0050737	Autosomal recessive disease	19	1785	0.38	0.0272	MGP,APOB,DNAH5,TCIRG1,MASP1,CAMP,FGB,F2,PLG,FGG,FBLN5,F5,F7,F11,C1S,PCYOX1,TGFBI,KNG1,FGA
DISEASES	DOID:12134	Factor VIII deficiency	2	4	2.05	0.0272	F9,F7
DISEASES	DOID:1388	Tangier disease	2	4	2.05	0.0272	APOA1,LCAT
DISEASES	DOID:2569	Retinal drusen	2	4	2.05	0.0272	FBLN5,CFH
DISEASES	DOID:0111902	Thrombophilia due to activated protein C resistance	2	5	1.95	0.0351	F2,F5
DISEASES	DOID:1237	Corneal degeneration	2	5	1.95	0.0351	APOB,TGFBI
DISEASES	DOID:14735	Hereditary angioedema	2	6	1.87	0.0450	C1S,KNG1
DISEASES	DOID:1884	Viral hepatitis	2	6	1.87	0.0450	AHSG,F2
TISSUES	BTO:0004850	Bone marrow cell	22	198	1.4	1.37e-20	APOB,C3,APCS,AHSG,SAA4,DSC2,MASP1,FGB,F2,CSPG4,APOL1,CFH,F5,PROS1,F11,C1S,C4B,SERPINA1,C1R,KNG1,B2M,FGA
TISSUES	BTO:0000392	Plasma cell	20	171	1.42	3.77e-19	APOB,C3,APCS,AHSG,SAA4,DSC2,MASP1,FGB,F2,CSPG4,APOL1,CFH,PROS1,C1S,C4B,SERPINA1,C1R,KNG1,B2M,FGA
TISSUES	BTO:0000141	Bone marrow	25	528	1.03	7.98e-16	APOB,C3,APCS,AHSG,SAA4,DSC2,MASP1,CAMP,FGB,F2,PRG2,CSPG4,APOL1,CFH,F5,PROS1,F11,C1S,C4B,SERPINA1,C1R,HSPH1,KNG1,B2M,FGA
TISSUES	BTO:0001486	Skeletal system	32	1307	0.74	3.88e-13	SPARC,APOB,APOA1,C3,APCS,ATRN,AHSG,SAA4,DSC2,MASP1,CAMP,FGB,F2,UBE3C,PRG2,CSPG4,APOL1,FGG,CFH,F5,CCN5,PROS1,F11,C1S,PCYOX1,C4B,SERPINA1,C1R,HSPH1,KNG1,B2M,FGA
TISSUES	BTO:0000759	Liver	39	2125	0.61	7.70e-13	F9,GPLD1,HRG,APOB,APOA1,C3,APCS,CFHR5,ATRN,AHSG,HABP2,SAA4,PPBP,MASP1,FGB,F2,PLG,APOL1,FGG,AKAP9,PCDH18,CFHR3,CFH,F7,PROS1,MASP2,F11,C1S,PCYOX1,C4B,SERPINA1,TGFBI,IGFALS,C1R,MYL6,APOC1,KNG1,B2M,FGA
TISSUES	BTO:0000345	Digestive gland	43	2881	0.52	1.55e-11	F9,GPLD1,SPARC,HRG,APOB,APOA1,C3,APCS,CFHR5,ATRN,TCIRG1,AHSG,HABP2,SAA4,SEC24D,PPBP,MASP1,FGB,F2,PLG,APOL1,FGG,AKAP9,PCDH18,CFHR3,CFH,F7,GPX3,PROS1,MASP2,F11,C1S,PCYOX1,C4B,SERPINA1,TGFBI,IGFALS,C1R,MYL6,APOC1,KNG1,B2M,FGA
TISSUES	BTO:0001491	Viscus	53	5378	0.34	3.99e-08	F9,GPLD1,SPARC,HRG,APOB,APOA1,C3,APCS,CFHR5,LYZ,ATRN,TCIRG1,AHSG,PLA2G7,HABP2,SAA4,SEC24D,DSC2,PPBP,MASP1,FGB,F2,PLG,APOL1,FGG,FBLN5,AKAP9,PCDH18,CFHR3,CFH,F7,GPX3,PROS1,TNRC6A,MASP2,F11,C1S,PCYOX1,ACSF2,C4B,SERPINA1,TGFBI,IGFALS,C1R,MYL6,APOC1,VEGFC,PLCG2,HSPH1,KNG1,TPM4,B2M,FGA
TISSUES	BTO:0000203	Respiratory system	27	1707	0.55	1.20e-06	SPARC,C3,BPIFB1,LYZ,ATRN,DNAH5,TCIRG1,FBLN5,AKAP9,PCDH18,CFH,CCN5,GPX3,PROS1,MASP2,C1S,LTBP1,PCYOX1,C4B,SERPINA1,TGFBI,PDGFC,MYL6,APOC1,VEGFC,KNG1,B2M
TISSUES	BTO:0001239	Serum	6	25	1.73	1.20e-06	GPLD1,APOA1,C3,ATRN,PRG2,KNG1
TISSUES	BTO:0001419	Urine	6	28	1.68	1.92e-06	LYZ,F2,FBLN5,MASP2,SERPINA1,B2M
TISSUES	BTO:0000522	Gland	56	7004	0.25	1.87e-05	F9,GPLD1,SPARC,HRG,APOB,APOA1,C3,BPIFB1,APCS,CFHR5,LYZ,ATRN,TCIRG1,AHSG,PLA2G7,HABP2,SAA4,SEC24D,PPBP,MASP1,CAMP,FGB,F2,PLG,APOL1,FGG,FBLN5,AKAP9,PCDH18,CFHR3,CFH,CCN5,F7,CA6,GPX3,PROS1,TNRC6A,MASP2,F11,C1S,LTBP1,PCYOX1,ACSF2,C4B,SERPINA1,TGFBI,IGFALS,PDGFC,C1R,MYL6,APOC1,HSPH1,KNG1,TPM4,B2M,FGA
TISSUES	BTO:0001489	Whole body	80	13099	0.14	1.87e-05	F9,MGP,GPLD1,SPARC,HRG,APOB,APOA1,C3,BPIFB1,APCS,CFHR5,LYZ,ATRN,LCAT,DNAH5,TCIRG1,AHSG,PLA2G7,HABP2,SAA4,SEC24D,DSC2,SCGB3A1,PPBP,PF4,MASP1,CAMP,SLC27A4,FGB,F2,PLG,PSG1,UBE3C,PRG2,CSPG4,APOL1,FGG,THBS4,FBLN5,AKAP9,CNDP1,PCDH18,CFHR3,CFH,F5,S100A7,CCN5,C1QC,F7,CA6,ADGRG2,GPX3,PROS1,TNRC6A,MAP3K7CL,MASP2,F11,C1S,PSG2,LTBP1,PCYOX1,NBEAL1,SLAIN1,ACSF2,C4B,SERPINA1,TGFBI,IGFALS,PAEP,PDGFC,C1R,MYL6,APOC1,VEGFC,PLCG2,HSPH1,KNG1,TPM4,B2M,FGA
TISSUES	BTO:0000089	Blood	25	1824	0.49	5.23e-05	APOB,APOA1,C3,AHSG,PLA2G7,PPBP,PF4,FGB,F2,FGG,AKAP9,PCDH18,F5,C1QC,GPX3,C1S,LTBP1,C4B,SERPINA1,MYL6,PLCG2,HSPH1,TPM4,B2M,FGA
TISSUES	BTO:0001488	Endocrine gland	52	6403	0.26	5.23e-05	F9,GPLD1,SPARC,HRG,APOB,APOA1,C3,APCS,CFHR5,ATRN,TCIRG1,AHSG,PLA2G7,HABP2,SAA4,SEC24D,PPBP,MASP1,CAMP,FGB,F2,PLG,APOL1,FGG,FBLN5,AKAP9,PCDH18,CFHR3,CFH,CCN5,F7,GPX3,PROS1,MASP2,F11,C1S,LTBP1,PCYOX1,ACSF2,C4B,SERPINA1,TGFBI,IGFALS,PDGFC,C1R,MYL6,APOC1,HSPH1,KNG1,TPM4,B2M,FGA
TISSUES	BTO:0000570	Hematopoietic system	31	2755	0.4	8.32e-05	GPLD1,APOB,APOA1,C3,ATRN,AHSG,PLA2G7,PPBP,PF4,FGB,F2,PRG2,FGG,AKAP9,PCDH18,F5,C1QC,GPX3,C1S,LTBP1,C4B,SERPINA1,TGFBI,MYL6,VEGFC,PLCG2,HSPH1,KNG1,TPM4,B2M,FGA
TISSUES	BTO:0000132	Blood platelet	11	363	0.83	0.00013	APOA1,C3,AHSG,PPBP,FGB,FGG,F5,LTBP1,SERPINA1,TPM4,FGA
TISSUES	BTO:0000237	Cerebrospinal fluid	5	45	1.4	0.00040	AHSG,F2,CFH,SERPINA1,KNG1
TISSUES	BTO:0000763	Lung	20	1395	0.51	0.00040	SPARC,C3,BPIFB1,DNAH5,TCIRG1,FBLN5,AKAP9,PCDH18,CCN5,GPX3,MASP2,C1S,LTBP1,C4B,SERPINA1,TGFBI,PDGFC,MYL6,APOC1,B2M

TISSUES	BTO:0000042	Animal	83	15148	0.09	0.0013	F9,MGP,GPLD1,SPARC,HRG,APOB,APOA1,C3,ANGPTL6,BPIFB1,APCS,CFHR5,LYZ,ATRN,LCAT,DNAH5,TCIRG1,AHSG,PLA2G7,HABP2,SAA4,SEC24D,DSC2,SCGB3A1,PPBP,PF4,MASP1,CAMP,SLC27A4,FGB,F2,PLG,PSG1,UBE3C,PRG2,CSPG4,APOL1,OAF,FGG,THBS4,FBLN5,AKAP9,CNDP1,PCDH18,CFHR3,CFH,F5,S100A7,CCN5,C1QC,F7,CA6,ADGRG2,GPX3,PROS1,TNRC6A,MAP3K7CL,MASP2,F11,C1S,PSG2,LTBP1,PCYOX1,NBEAL1,SLAIN1,ACSF2,C4B,SERPINA1,TGFBI,IGFALS,PAEP,PDGFC,C1R,MYL6,APOC1,VEGFC,PLCG2,HSPH1,KNG1,TPM4,TRIM66,B2M,FGA
TISSUES	BTO:0000088	Cardiovascular system	16	1057	0.53	0.0018	MGP,SPARC,APOB,APOA1,C3,SLC27A4,F2,THBS4,PCDH18,GPX3,PCYOX1,SERPINA1,TGFBI,VEGFC,B2M,FGA
TISSUES	BTO:0001279	Spinal cord	7	233	0.83	0.0095	AHSG,F2,CSPG4,PCDH18,CFH,SERPINA1,KNG1
TISSUES	BTO:0000574	Hematopoietic cell	14	1019	0.49	0.0165	APOB,APOA1,PPBP,PF4,AKAP9,PCDH18,C1QC,GPX3,C1S,SERPINA1,MYL6,PLCG2,HSPH1,B2M
TISSUES	BTO:0000751	Leukocyte	13	924	0.5	0.0221	APOB,PPBP,PF4,AKAP9,PCDH18,C1QC,GPX3,C1S,SERPINA1,MYL6,PLCG2,HSPH1,B2M
TISSUES	BTO:0002854	Corn silk	2	4	2.05	0.0251	MASP1,MASP2
TISSUES	BTO:0005288	CL-48 cell	6	200	0.83	0.0264	AHSG,MASP1,SERPINA11,FGG,GPX3,SERPINA1
TISSUES	BTO:0001078	Placenta	15	1244	0.43	0.0327	SPARC,DSC2,PSG1,PRG2,APOL1,FBLN5,F5,GPX3,PSG2,PCYOX1,SERPINA1,TGFBI,MYL6,TPM4,B2M
TISSUES	BTO:0000562	Heart	11	738	0.52	0.0352	APOA1,C3,SLC27A4,F2,THBS4,PCDH18,GPX3,PCYOX1,SERPINA1,VEGFC,FGA
TISSUES	BTO:0000765	Exocrine gland	5	150	0.87	0.0458	BPIFB1,LYZ,MASP1,CA6,B2M
COMPARTMENTS	GOCC:0005576	Extracellular region	59	2079	0.8	6.83e-33	F9,MGP,GPLD1,SPARC,HRG,APOB,APOA1,C3,ANGPTL6,BPIFB1,APCS,CFHR5,LYZ,LCAT,AHSG,PLA2G7,HABP2,SAA4,SCGB3A1,PPBP,PF4,MASP1,CAMP,FGB,F2,PLG,PRG2,CSPG4,APOL1,FGG,THBS4,FBLN5,CFHR3,CFH,F5,S100A7,CCN5,C1QC,F7,CA6,GPX3,PROS1,MASP2,F11,C1S,LTBP1,PCYOX1,C4B,SERPINA1,TGFBI,IGFALS,PAEP,C1R,APOC1,VEGFC,PLCG2,KNG1,B2M,FGA
COMPARTMENTS	GOCC:0005615	Extracellular space	46	1027	1.0	7.23e-32	F9,MGP,GPLD1,HRG,APOB,APOA1,C3,APCS,LYZ,LCAT,AHSG,PLA2G7,SAA4,SCGB3A1,MASP1,CAMP,FGB,F2,PLG,CSPG4,APOL1,FGG,THBS4,CFHR3,CFH,S100A7,CCN5,C1QC,F7,CA6,GPX3,PROS1,MASP2,F11,C1S,PCYOX1,C4B,SERPINA1,TGFBI,IGFALS,C1R,APOC1,PLCG2,KNG1,B2M,FGA
COMPARTMENTS	GOCC:0072562	Blood microparticle	19	118	1.56	1.16e-20	HRG,APOA1,C3,APCS,AHSG,FGB,F2,PLG,APOL1,FGG,CFHR3,CFH,C1QC,PROS1,C1S,C4B,C1R,KNG1,FGA
COMPARTMENTS	GOCC:0034774	Secretory granule lumen	20	241	1.27	9.72e-17	SPARC,HRG,APOA1,C3,LYZ,AHSG,PPBP,PF4,CAMP,FGB,PLG,FGG,F5,S100A7,PROS1,SERPINA1,VEGFC,KNG1,B2M,FGA
COMPARTMENTS	GOCC:0031093	Platelet alpha granule lumen	14	66	1.68	1.57e-16	SPARC,HRG,AHSG,PPBP,PF4,FGB,PLG,FGG,F5,PROS1,SERPINA1,VEGFC,KNG1,FGA
COMPARTMENTS	GOCC:0043230	Extracellular organelle	25	524	1.03	2.39e-16	F9,MGP,APOB,APOA1,C3,APCS,AHSG,CAMP,FGB,F2,PLG,CSPG4,FGG,CFH,GPX3,PROS1,MASP2,F11,C4B,SERPINA1,TGFBI,C1R,KNG1,B2M,FGA
COMPARTMENTS	GOCC:0065010	Extracellular membrane-bounded organelle	24	473	1.06	3.12e-16	F9,MGP,APOB,APOA1,C3,APCS,AHSG,CAMP,FGB,F2,PLG,CSPG4,FGG,CFH,GPX3,PROS1,F11,C4B,SERPINA1,TGFBI,C1R,KNG1,B2M,FGA
COMPARTMENTS	GOCC:1903561	Extracellular vesicle	24	500	1.03	9.44e-16	F9,MGP,APOB,APOA1,C3,APCS,AHSG,CAMP,FGB,F2,PLG,CSPG4,FGG,CFH,GPX3,PROS1,F11,C4B,SERPINA1,TGFBI,C1R,KNG1,B2M,FGA
COMPARTMENTS	GOCC:0031982	Vesicle	38	2125	0.6	1.95e-12	F9,MGP,SPARC,HRG,APOB,APOA1,C3,APCS,LYZ,TCIRG1,AHSG,SEC24D,DSC2,PPBP,PF4,CAMP,FGB,F2,PLG,PRG2,CSPG4,FGG,CFH,F5,S100A7,GPX3,PROS1,F11,C4B,SERPINA1,TGFBI,C1R,VEGFC,PLCG2,HSPH1,KNG1,B2M,FGA
COMPARTMENTS	GOCC:0030141	Secretory granule	22	719	0.84	1.58e-10	SPARC,HRG,APOA1,C3,LYZ,TCIRG1,AHSG,PPBP,PF4,CAMP,FGB,PLG,PRG2,FGG,F5,S100A7,PROS1,SERPINA1,VEGFC,KNG1,B2M,FGA
COMPARTMENTS	GOCC:0005788	Endoplasmic reticulum lumen	13	172	1.23	2.92e-10	F9,APOB,APOA1,C3,AHSG,APOL1,FGG,F5,F7,SERPINA1,KNG1,B2M,FGA
COMPARTMENTS	GOCC:0070062	Extracellular exosome	17	428	0.95	1.45e-09	MGP,APOB,APOA1,C3,AHSG,CAMP,FGB,PLG,CSPG4,FGG,CFH,GPX3,SERPINA1,TGFBI,KNG1,B2M,FGA
COMPARTMENTS	GOCC:1905286	Serine-type peptidase complex	6	11	2.09	1.05e-08	F9,C3,MASP1,F7,MASP2,F11
COMPARTMENTS	GOCC:0034358	Plasma lipoprotein particle	8	48	1.57	1.69e-08	APOB,APOA1,LCAT,PLA2G7,SAA4,APOL1,PCYOX1,APOC1
COMPARTMENTS	GOCC:0034364	High-density lipoprotein particle	7	30	1.72	2.73e-08	APOB,APOA1,LCAT,PLA2G7,SAA4,APOL1,APOC1
COMPARTMENTS	GOCC:0005577	Fibrinogen complex	6	20	1.83	1.46e-07	FGB,F2,PLG,FGG,F7,FGA
COMPARTMENTS	GOCC:0062167	Complement component C1q complex	5	10	2.05	4.50e-07	C3,C1QC,C1S,C4B,C1R
COMPARTMENTS	GOCC:0031410	Cytoplasmic vesicle	26	1738	0.52	2.52e-06	SPARC,HRG,APOB,APOA1,C3,LYZ,TCIRG1,AHSG,SEC24D,DSC2,PPBP,PF4,CAMP,FGB,PLG,PRG2,FGG,F5,S100A7,PROS1,SERPINA1,VEGFC,HSPH1,KNG1,B2M,FGA
COMPARTMENTS	GOCC:0034361	Very-low-density lipoprotein particle	5	20	1.75	6.80e-06	APOB,APOA1,APOL1,PCYOX1,APOC1
COMPARTMENTS	GOCC:1905370	Serine-type endopeptidase complex	4	7	2.11	9.88e-06	C3,MASP1,MASP2,F11
COMPARTMENTS	GOCC:1905368	Peptidase complex	8	136	1.12	2.02e-05	F9,C3,MASP1,F2,UBE3C,F7,MASP2,F11
COMPARTMENTS	GOCC:0012505	Endomembrane system	33	3156	0.37	7.63e-05	F9,SPARC,HRG,APOB,APOA1,C3,LYZ,TCIRG1,AHSG,SEC24D,PPBP,PF4,CAMP,SLC27A4,FGB,F2,PLG,PRG2,CSPG4,APOL1,FGG,THBS4,AKAP9,F5,S100A7,F7,PROS1,TNRC6A,SERPINA1,VEGFC,KNG1,B2M,FGA
COMPARTMENTS	GOCC:0005602	Complement component C1 complex	3	5	2.13	0.00035	C1QC,C1S,C1R
COMPARTMENTS	GOCC:0071682	Endocytic vesicle lumen	4	23	1.59	0.00043	SPARC,APOB,APOA1,HSPH1
COMPARTMENTS	GOCC:0005783	Endoplasmic reticulum	17	1095	0.54	0.00048	F9,APOB,APOA1,C3,AHSG,SEC24D,SLC27A4,APOL1,FGG,THBS4,F5,S100A7,F7,SERPINA1,KNG1,B2M,FGA
COMPARTMENTS	GOCC:0070013	Intracellular organelle lumen	28	2902	0.33	0.0029	F9,SPARC,HRG,APOB,APOA1,C3,LYZ,AHSG,PPBP,PF4,CAMP,FGB,F2,PLG,PRG2,CSPG4,APOL1,FGG,F5,S100A7,F7,PROS1,SERPINA1,VEGFC,HSPH1,KNG1,B2M,FGA
COMPARTMENTS	GOCC:0042627	Chylomicron	3	14	1.68	0.0034	APOB,APOA1,APOC1
COMPARTMENTS	GOCC:1905369	Endopeptidase complex	5	90	1.09	0.0040	C3,MASP1,UBE3C,MASP2,F11
COMPARTMENTS	GOCC:0005796	Golgi lumen	5	102	1.04	0.0068	F9,F2,CSPG4,F7,PROS1
COMPARTMENTS	GOCC:1904724	Tertiary granule lumen	4	55	1.21	0.0079	LYZ,PPBP,CAMP,B2M
COMPARTMENTS	GOCC:0030312	External encapsulating structure	7	259	0.78	0.0102	GPLD1,SPARC,HRG,PLG,THBS4,FBLN5,LTBP1
COMPARTMENTS	GOCC:0031012	Extracellular matrix	7	259	0.78	0.0102	GPLD1,SPARC,PLG,THBS4,FBLN5,LTBP1,TGFBI
COMPARTMENTS	GOCC:0034362	Low-density lipoprotein particle	3	22	1.48	0.0102	APOB,APOA1,PLA2G7
COMPARTMENTS	GOCC:0030134	COPII-coated ER to Golgi transport vesicle	4	62	1.16	0.0112	SEC24D,F5,SERPINA1,B2M
COMPARTMENTS	GOCC:0062023	Collagen-containing extracellular matrix	6	198	0.83	0.0153	SPARC,PLG,THBS4,FBLN5,LTBP1,TGFBI
COMPARTMENTS	GOCC:0032991	Protein-containing complex	39	5325	0.21	0.0189	F9,APOB,APOA1,C3,LCAT,DNAH5,PLA2G7,SAA4,SEC24D,MASP1,FGB,F2,PLG,UBE3C,APOL1,FGG,AKAP9,S100A7,C1QC,F7,GPX3,TNRC6A,MASP2,F11,C1S,LTBP1,PCYOX1,CDK3,C4B,SERPINA1,IGFALS,C1R,MYL6,APOC1,VEGFC,HSPH1,TPM4,B2M,FGA
COMPARTMENTS	GOCC:0034365	Discoidal high-density lipoprotein particle	2	6	1.87	0.0263	APOA1,LCAT
COMPARTMENTS	GOCC:0110165	Cellular anatomical entity	76	14060	0.08	0.0357	F9,MGP,GPLD1,SPARC,HRG,APOB,APOA1,C3,ANGPTL6,BPIFB1,APCS,CFHR5,LYZ,ATRN,LCAT,DNAH5,TCIRG1,AHSG,PLA2G7,HABP2,SAA4,SEC24D,DSC2,SCGB3A1,PPBP,PF4,MASP1,CAMP,SLC27A4,FGB,F2,PLG,PRG2,CSPG4,APOL1,FGG,THBS4,FBLN5,AKAP9,CNDP1,CFHR3,CFH,F5,S100A7,CCN5,C1QC,F7,CA6,ADGRG2,GPX3,PROS1,TNRC6A,MAP3K7CL,MASP2,F11,C1S,LTBP1,PCYOX1,ACSF2,C4B,SERPINA1,TGFBI,IGFALS,PAEP,PDGFC,C1R,MYL6,APOC1,VEGFC,PLCG2,HSPH1,KNG1,TPM4,TRIM66,B2M,FGA
COMPARTMENTS	GOCC:0009986	Cell surface	8	438	0.61	0.0391	SPARC,HRG,FGB,PLG,CSPG4,FGG,PDGFC,FGA
COMPARTMENTS	GOCC:0034363	Intermediate-density lipoprotein particle	2	8	1.75	0.0396	APOB,APOA1
COMPARTMENTS	GOCC:0070820	Tertiary granule	5	164	0.83	0.0428	LYZ,TCIRG1,PPBP,CAMP,B2M
COMPARTMENTS	GOCC:0005775	Vacuolar lumen	5	168	0.82	0.0464	APOB,C3,LYZ,CSPG4,S100A7
COMPARTMENTS	GOCC:0062136	Low-density lipoprotein receptor complex	2	9	1.7	0.0464	APOB,APOA1

UniProt Keywords	KW-0964	Secreted	61	1839	0.87	5.85e-39	F9,MGP,GPLD1,SPARC,HRG,APOB,APOA1,C3,ANGPTL6,BPIFB1,APCS,CFHR5,LYZ,ATRN,LCAT,AHSG,PLA2G7,HABP2,SAA4,SCGB3A1,PPBP,PF4,CAMP,FGB,F2,PLG,PSG1,PRG2,APOL1,SERPINA11,FGG,THBS4,FBLN5,CNDP1,CFHR3,CFH,F5,S100A7,CCN5,C1QC,F7,CA6,GPX3,PROS1,BPIFC,MASP2,F11,PSG2,LTBP1,C4B,SERPINA1,TGFBI,IGFALS,PAEP,PDGFC,C1R,APOC1,VEGFC,KNG1,B2M,FGA
UniProt Keywords	KW-0732	Signal	67	3277	0.66	8.34e-32	F9,MGP,GPLD1,SPARC,HRG,APOB,APOA1,C3,ANGPTL6,BPIFB1,APCS,CFHR5,LYZ,ATRN,LCAT,AHSG,PLA2G7,HABP2,SAA4,DSC2,SCGB3A1,PPBP,PF4,CAMP,FGB,F2,PLG,PSG1,PRG2,CSPG4,APOL1,OAF,SERPINA11,FGG,THBS4,FBLN5,CNDP1,PCDH18,CFHR3,CFH,F5,CCN5,C1QC,F7,CA6,ADGRG2,GPX3,PROS1,BPIFC,MASP2,F11,C1S,PSG2,LTBP1,PCYOX1,C4B,SERPINA1,TGFBI,IGFALS,PAEP,PDGFC,C1R,APOC1,VEGFC,KNG1,B2M,FGA
UniProt Keywords	KW-0094	Blood coagulation	13	46	1.8	8.12e-17	F9,HRG,FGB,F2,PLG,FGG,F5,F7,PROS1,F11,SERPINA1,KNG1,FGA
UniProt Keywords	KW-1015	Disulfide bond	52	3338	0.54	1.26e-16	F9,MGP,SPARC,HRG,APOB,C3,ANGPTL6,BPIFB1,APCS,CFHR5,LYZ,ATRN,LCAT,AHSG,HABP2,SCGB3A1,PPBP,PF4,MASP1,CAMP,FGB,F2,PLG,PSG1,PRG2,CSPG4,FGG,THBS4,FBLN5,CFHR3,CFH,F5,S100A7,C1QC,F7,CA6,PROS1,BPIFC,MASP2,F11,C1S,PSG2,LTBP1,C4B,TGFBI,PAEP,PDGFC,C1R,VEGFC,KNG1,B2M,FGA
UniProt Keywords	KW-0325	Glycoprotein	55	4386	0.45	8.83e-14	F9,GPLD1,SPARC,HRG,APOB,APOA1,C3,ANGPTL6,BPIFB1,APCS,CFHR5,ATRN,LCAT,AHSG,PLA2G7,HABP2,SAA4,DSC2,FGB,F2,PLG,PSG1,PRG2,CSPG4,APOL1,SERPINA11,FGG,THBS4,FBLN5,CNDP1,PCDH18,CFHR3,CFH,F5,C1QC,F7,CA6,ADGRG2,PROS1,BPIFC,F11,C1S,PSG2,LTBP1,PCYOX1,C4B,SERPINA1,IGFALS,PAEP,PDGFC,C1R,VEGFC,KNG1,B2M,FGA
UniProt Keywords	KW-0165	Cleavage on pair of basic residues	14	280	1.05	4.33e-09	F9,HRG,C3,HABP2,DSC2,PPBP,CAMP,F2,PLG,F7,PROS1,C4B,PDGFC,VEGFC
UniProt Keywords	KW-0792	Thrombophilia	6	10	2.13	4.33e-09	F9,HRG,F2,PLG,F5,PROS1
UniProt Keywords	KW-0301	Gamma-carboxyglutamic acid	6	16	1.92	3.42e-08	F9,MGP,F2,F7,PROS1,TGFBI
UniProt Keywords	KW-0245	EGF-like domain	12	232	1.06	5.69e-08	F9,ATRN,HABP2,MASP1,THBS4,FBLN5,F7,PROS1,MASP2,C1S,LTBP1,C1R
UniProt Keywords	KW-0379	Hydroxylation	10	143	1.19	9.35e-08	F9,C1QC,F7,PROS1,MASP2,C1S,LTBP1,C1R,KNG1,FGA
UniProt Keywords	KW-0034	Amyloid	6	24	1.75	1.99e-07	APOA1,APCS,LYZ,TGFBI,B2M,FGA
UniProt Keywords	KW-0180	Complement pathway	6	30	1.65	5.89e-07	C3,C1QC,MASP2,C1S,C4B,C1R
UniProt Keywords	KW-0106	Calcium	19	886	0.68	6.26e-07	F9,SPARC,APCS,DSC2,F2,SPATA21,FGG,THBS4,FBLN5,PCDH18,F5,S100A7,F7,PROS1,MASP2,C1S,PLCG2,TPM4,FGA
UniProt Keywords	KW-0768	Sushi	7	57	1.44	6.26e-07	CFHR5,MASP1,CFHR3,CFH,MASP2,C1S,C1R
UniProt Keywords	KW-0720	Serine protease	9	136	1.17	6.97e-07	F9,HABP2,F2,PLG,F7,MASP2,F11,C1S,C1R
UniProt Keywords	KW-1068	Hemolytic uremic syndrome	4	10	1.95	1.40e-05	C3,CFHR5,CFHR3,CFH
UniProt Keywords	KW-1008	Amyloidosis	5	31	1.56	1.96e-05	APOA1,LYZ,TGFBI,B2M,FGA
UniProt Keywords	KW-0399	Innate immunity	10	329	0.83	9.33e-05	C3,BPIFB1,FGB,CFH,C1QC,MASP2,C1S,C4B,C1R,FGA
UniProt Keywords	KW-0797	Tissue remodeling	3	4	2.23	9.87e-05	PLG,CSPG4,THBS4
UniProt Keywords	KW-0391	Immunity	12	537	0.7	0.00018	C3,BPIFB1,FGB,PRG2,CFH,C1QC,MASP2,C1S,C4B,C1R,B2M,FGA
UniProt Keywords	KW-0765	Sulfation	5	57	1.29	0.00025	F9,FGG,CFH,F5,C4B
UniProt Keywords	KW-0280	Fibrinolysis	3	8	1.92	0.00039	HRG,PLG,PROS1
UniProt Keywords	KW-0677	Repeat	40	4794	0.27	0.00039	F9,GPLD1,HRG,APOA1,CFHR5,ATRN,DNAH5,AHSG,HABP2,DSC2,ANKRD44,MASP1,F2,PLG,PSG1,CSPG4,THBS4,FBLN5,PCDH18,CFHR3,CFH,F5,S100A7,C1QC,F7,PROS1,MASP2,F11,C1S,PSG2,LTBP1,NBEAL1,TGFBI,IGFALS,C1R,MYL6,VEGFC,PLCG2,KNG1,TRIM66
UniProt Keywords	KW-0497	Mitogen	4	39	1.36	0.0011	PPBP,THBS4,PDGFC,VEGFC
UniProt Keywords	KW-0358	Heparin-binding	5	88	1.1	0.0015	HRG,APOB,PF4,PRG2,F11
UniProt Keywords	KW-0345	HDL	3	16	1.62	0.0019	APOA1,SAA4,APOL1
UniProt Keywords	KW-0420	Kringle	3	16	1.62	0.0019	HABP2,F2,PLG
UniProt Keywords	KW-0913	Age-related macular degeneration	3	16	1.62	0.0019	C3,FBLN5,CFH
UniProt Keywords	KW-0645	Protease	10	516	0.64	0.0026	F9,HABP2,F2,PLG,CNDP1,F7,MASP2,F11,C1S,C1R
UniProt Keywords	KW-0011	Acute phase	3	19	1.55	0.0027	SAA4,F2,SERPINA1
UniProt Keywords	KW-0445	Lipid transport	5	110	1.01	0.0034	APOB,APOA1,SLC27A4,APOL1,APOC1
UniProt Keywords	KW-0153	Cholesterol metabolism	4	65	1.14	0.0052	APOB,APOA1,LCAT,APOL1
UniProt Keywords	KW-0443	Lipid metabolism	9	485	0.62	0.0069	APOB,APOA1,C3,LCAT,PLA2G7,SLC27A4,APOL1,ACSF2,PLCG2
UniProt Keywords	KW-0865	Zymogen	6	210	0.81	0.0078	F9,F2,PLG,F5,F7,PROS1
UniProt Keywords	KW-0065	Atherosclerosis	2	8	1.75	0.0156	APOB,APOA1
UniProt Keywords	KW-0882	Thioester bond	2	8	1.75	0.0156	C3,C4B
UniProt Keywords	KW-0929	Antimicrobial	4	93	0.98	0.0159	LYZ,PPBP,CAMP,PRG2
UniProt Keywords	KW-0272	Extracellular matrix	6	267	0.7	0.0225	SPARC,THBS4,FBLN5,LTBP1,SERPINA1,TGFBI
UniProt Keywords	KW-0179	Complement alternate pathway	2	12	1.57	0.0275	C3,CFH
UniProt Keywords	KW-0971	Glycation	2	12	1.57	0.0275	APOA1,B2M
UniProt Keywords	KW-0339	Growth factor	4	129	0.84	0.0449	PPBP,THBS4,PDGFC,VEGFC
UniProt Keywords	KW-0037	Angiogenesis	4	131	0.83	0.0464	HRG,ANGPTL6,CSPG4,VEGFC
UniProt Keywords	KW-0248	Ehlers-Danlos syndrome	2	17	1.42	0.0465	C1S,C1R
UniProt Keywords	KW-0186	Copper	3	65	1.01	0.0494	SPARC,HRG,F5
InterPro	IPR001881	EGF-like calcium-binding domain	11	124	1.3	1.45e-07	F9,HABP2,MASP1,THBS4,FBLN5,F7,PROS1,MASP2,C1S,LTBP1,C1R
InterPro	IPR018097	EGF-like calcium-binding, conserved site	10	99	1.35	1.91e-07	F9,MASP1,THBS4,FBLN5,F7,PROS1,MASP2,C1S,LTBP1,C1R
InterPro	IPR033116	Serine proteases, trypsin family, serine active site	9	92	1.34	1.60e-06	F9,HABP2,F2,PLG,F7,MASP2,F11,C1S,C1R
InterPro	IPR001314	Peptidase S1A, chymotrypsin family	9	107	1.27	4.16e-06	F9,HABP2,F2,PLG,F7,MASP2,F11,C1S,C1R
InterPro	IPR001254	Serine proteases, trypsin domain	9	115	1.24	6.02e-06	F9,HABP2,F2,PLG,F7,MASP2,F11,C1S,C1R
InterPro	IPR043504	Peptidase S1, PA clan, chymotrypsin-like fold	9	116	1.24	6.02e-06	F9,HABP2,F2,PLG,F7,MASP2,F11,C1S,C1R
InterPro	IPR009003	Peptidase S1, PA clan	9	123	1.21	7.47e-06	F9,HABP2,F2,PLG,F7,MASP2,F11,C1S,C1R
InterPro	IPR000436	Sushi/SCR/CCP domain	7	56	1.45	1.10e-05	CFHR5,MASP1,CFHR3,CFH,MASP2,C1S,C1R
InterPro	IPR000742	EGF-like domain	11	236	1.02	1.10e-05	F9,ATRN,HABP2,MASP1,THBS4,FBLN5,F7,PROS1,MASP2,LTBP1,C1R
InterPro	IPR035976	Sushi/SCR/CCP superfamily	7	58	1.43	1.10e-05	CFHR5,MASP1,CFHR3,CFH,MASP2,C1S,C1R
InterPro	IPR000294	Gamma-carboxyglutamic acid-rich (GLA) domain	5	15	1.87	1.49e-05	F9,MGP,F2,F7,PROS1
InterPro	IPR035972	Gamma-carboxyglutamic acid-rich (GLA) domain superfamily	5	15	1.87	1.49e-05	F9,MGP,F2,F7,PROS1
InterPro	IPR000859	CUB domain	6	51	1.42	0.00011	ATRN,MASP1,MASP2,C1S,PDGFC,C1R
InterPro	IPR035914	Spermadhesin, CUB domain superfamily	6	54	1.4	0.00014	ATRN,MASP1,MASP2,C1S,PDGFC,C1R
InterPro	IPR000152	EGF-type aspartate/asparagine hydroxylation site	7	98	1.2	0.00021	F9,FBLN5,F7,PROS1,MASP2,C1S,LTBP1
InterPro	IPR012290	Fibrinogen, alpha/beta/gamma chain, coiled coil domain	3	3	2.35	0.00076	FGB,FGG,FGA
InterPro	IPR024175	Peptidase S1A, complement C1r/C1S/mannan-binding	3	3	2.35	0.00076	MASP2,C1S,C1R
InterPro	IPR037579	Fibrinogen	3	3	2.35	0.00076	FGB,FGG,FGA
InterPro	IPR020837	Fibrinogen, conserved site	4	19	1.67	0.0011	ANGPTL6,FGB,FGG,FGA
InterPro	IPR046350	Cystatin superfamily	4	23	1.59	0.0021	HRG,AHSG,CAMP,KNG1
InterPro	IPR018114	Serine proteases, trypsin family, histidine active site	6	100	1.13	0.0027	F9,HABP2,F2,PLG,F7,F11
InterPro	IPR014716	Fibrinogen, alpha/beta/gamma chain, C-terminal globular, subdomain 1	4	26	1.54	0.0030	ANGPTL6,FGB,FGG,FGA
InterPro	IPR002181	Fibrinogen, alpha/beta/gamma chain, C-terminal globular domain	4	32	1.45	0.0060	ANGPTL6,FGB,FGG,FGA
InterPro	IPR036056	Fibrinogen-like, C-terminal	4	32	1.45	0.0060	ANGPTL6,FGB,FGG,FGA

InterPro	IPR017857	Coagulation factor-like, Gla domain superfamily	3	12	1.75	0.0107	F9,F7,PROS1
InterPro	IPR000001	Kringle	3	16	1.62	0.0216	HABP2,F2,PLG
InterPro	IPR018056	Kringle, conserved site	3	16	1.62	0.0216	HABP2,F2,PLG
InterPro	IPR038178	Kringle superfamily	3	16	1.62	0.0216	HABP2,F2,PLG
InterPro	IPR000010	Cystatin domain	3	17	1.6	0.0227	HRG,AHSG,KNG1
SMART	SM00179	Calcium-binding EGF-like domain	10	101	1.35	5.25e-08	F9,MASP1,THBS4,FBLN5,F7,PROS1,MASP2,C1S,LTBP1,C1R
SMART	SM00020	Trypsin-like serine protease	10	118	1.28	1.09e-07	F9,HABP2,MASP1,F2,PLG,F7,MASP2,F11,C1S,C1R
SMART	SM00032	Domain abundant in complement control proteins	7	56	1.45	3.34e-06	CFHR5,MASP1,CFHR3,CFH,MASP2,C1S,C1R
SMART	SM00069	Domain containing Gla (gamma-carboxyglutamate) residues.	5	15	1.87	4.68e-06	F9,MGP,F2,F7,PROS1
SMART	SM00042	Domain first found in C1r, C1s, uEGF, and bone morphogenetic protein.	6	50	1.43	2.90e-05	ATRN,MASP1,MASP2,C1S,PDGFC,C1R
SMART	SM01212	Fibrinogen alpha/beta chain family	3	3	2.35	0.00023	FGB,FGG,FGA
SMART	SM00186	Fibrinogen-related domains (FReDs)	4	29	1.49	0.0016	ANGPTL6,FGB,FGG,FGA
SMART	SM00181	Epidermal growth factor-like domain.	7	209	0.87	0.0052	F9,ATRN,HABP2,THBS4,F7,PROS1,LTBP1
SMART	SM00130	Kringle domain	3	16	1.62	0.0071	HABP2,F2,PLG
SMART	SM00043	Cystatin-like domain	3	17	1.6	0.0075	HRG,AHSG,KNG1
SMART	SM00104	Anaphylatoxin homologous domain	2	6	1.87	0.0402	C3,C4B

AC vs NCFET T1

#category	term ID	term description	observed gene count	background gene count	strength	false discovery rate	matching proteins in your network (labels)
GO Component	GO:0034774	Secretory granule lumen	6	321	1.42	0.00011	SPP2,C3,PKM,FGG,ACTN1,RARRES2
GO Component	GO:0034364	High-density lipoprotein particle	3	29	2.16	0.00072	SAA4,APOA2,SAA2
GO Component	GO:0031982	Vesicle	11	3957	0.59	0.00088	SPP2,C3,SAA4,PKM,FGG,APOA2,SOD3,ACTN1,RARRES2,SAA2,TPM4
GO Component	GO:0070062	Extracellular exosome	9	2096	0.78	0.00088	C3,SAA4,PKM,FGG,APOA2,SOD3,ACTN1,SAA2,TPM4
GO Component	GO:0005576	Extracellular region	11	4175	0.57	0.0011	SPP2,C3,SAA4,PKM,FGG,APOA2,SOD3,ACTN1,RARRES2,SAA2,TPM4
GO Component	GO:0005615	Extracellular space	10	3247	0.64	0.0011	C3,SAA4,PKM,FGG,APOA2,SOD3,ACTN1,RARRES2,SAA2,TPM4
GO Component	GO:0062023	Collagen-containing extracellular matrix	5	407	1.24	0.0011	SPP2,PKM,FGG,SOD3,RARRES2
GO Component	GO:0005788	Endoplasmic reticulum lumen	4	312	1.26	0.0057	SPP2,C3,FGG,APOA2
GO Component	GO:0031089	Platelet dense granule lumen	2	14	2.3	0.0057	SPP2,RARRES2
GO Component	GO:0072562	Blood microparticle	3	118	1.55	0.0073	C3,FGG,APOA2
STRING clusters	CL:18724	Mixed, incl. Complement and coagulation cascades, and Protein-lipid complex	6	172	1.69	6.44e-06	SPP2,C3,SAA4,FGG,APOA2,SAA2
STRING clusters	CL:18726	Complement and coagulation cascades, and Protein-lipid complex	5	161	1.64	0.00011	C3,SAA4,FGG,APOA2,SAA2
STRING clusters	CL:18960	High-density lipoprotein particle	3	26	2.21	0.00094	SAA4,APOA2,SAA2
Reactome	HSA-114608	Platelet degranulation	4	126	1.65	0.0021	SPP2,FGG,ACTN1,RARRES2
Reactome	HSA-381426	Regulation of Insulin-like Growth Factor (IGF) transport and uptake by Insulin-like Growth Factor Binding Proteins (IGFBPs)	4	124	1.66	0.0021	SPP2,C3,FGG,APOA2
Reactome	HSA-8957275	Post-translational protein phosphorylation	4	107	1.72	0.0021	SPP2,C3,FGG,APOA2
WikiPathways	WP176	Folate metabolism	4	67	1.92	0.00012	SAA4,FGG,SOD3,SAA2
WikiPathways	WP15	Selenium micronutrient network	4	84	1.83	0.00014	SAA4,FGG,SOD3,SAA2
WikiPathways	WP1533	Vitamin B12 metabolism	3	50	1.93	0.0017	SAA4,SOD3,SAA2
DISEASES	DOID:9120	Amyloidosis	4	75	1.88	0.0011	C3,SAA4,APOA2,SAA2
TISSUES	BTO:0001486	Skeletal system	10	1307	1.03	3.25e-06	SPP2,APMAP,C3,SAA4,UBE3C,PKM,FGG,APOA2,SOD3,BZW1
TISSUES	BTO:0000759	Liver	10	2125	0.82	0.00018	SPP2,APMAP,C3,SAA4,PKM,FGG,APOA2,ACTN1,BZW1,SAA2
TISSUES	BTO:0001491	Viscus	13	5378	0.53	0.00040	SPP2,APMAP,C3,SAA4,PKM,FGG,APOA2,SOD3,ACTN1,BZW1,RARRES2,SAA2, TPM4
TISSUES	BTO:0000141	Bone marrow	6	528	1.2	0.00058	APMAP,C3,SAA4,APOA2,SOD3,BZW1
TISSUES	BTO:0000132	Blood platelet	5	363	1.29	0.0016	C3,PKM,FGG,ACTN1,TPM4
TISSUES	BTO:0001488	Endocrine gland	13	6403	0.46	0.0016	SPP2,APMAP,C3,SAA4,PKM,FGG,APOA2,SOD3,ACTN1,BZW1,RARRES2,SAA2, TPM4
TISSUES	BTO:0000392	Plasma cell	4	171	1.52	0.0017	C3,SAA4,APOA2,SOD3
TISSUES	BTO:0001239	Serum	2	25	2.05	0.0329	C3,APOA2
COMPARTMENTS	GOCC:0005615	Extracellular space	8	1027	1.04	0.00029	C3,SAA4,PKM,FGG,APOA2,SOD3,RARRES2,SAA2
COMPARTMENTS	GOCC:0034774	Secretory granule lumen	5	241	1.47	0.00061	SPP2,C3,FGG,ACTN1,RARRES2
COMPARTMENTS	GOCC:0034364	High-density lipoprotein particle	3	30	2.15	0.00071	SAA4,APOA2,SAA2
COMPARTMENTS	GOCC:0005576	Extracellular region	9	2079	0.78	0.00077	SPP2,C3,SAA4,PKM,FGG,APOA2,SOD3,RARRES2,SAA2
COMPARTMENTS	GOCC:0030141	Secretory granule	6	719	1.07	0.0019	SPP2,C3,PKM,FGG,ACTN1,RARRES2
COMPARTMENTS	GOCC:0065010	Extracellular membrane-bounded organelle	5	473	1.17	0.0026	C3,PKM,FGG,APOA2,SAA2
COMPARTMENTS	GOCC:1903561	Extracellular vesicle	5	500	1.15	0.0032	C3,PKM,FGG,APOA2,SAA2
COMPARTMENTS	GOCC:0031982	Vesicle	8	2125	0.72	0.0047	SPP2,C3,PKM,FGG,APOA2,ACTN1,RARRES2,SAA2
COMPARTMENTS	GOCC:0031089	Platelet dense granule lumen	2	14	2.3	0.0080	SPP2,RARRES2
COMPARTMENTS	GOCC:0072562	Blood microparticle	3	118	1.55	0.0106	C3,FGG,APOA2
COMPARTMENTS	GOCC:0070062	Extracellular exosome	4	428	1.12	0.0232	C3,PKM,FGG,APOA2
COMPARTMENTS	GOCC:0005788	Endoplasmic reticulum lumen	3	172	1.39	0.0268	SPP2,C3,FGG
COMPARTMENTS	GOCC:0062023	Collagen-containing extracellular matrix	3	198	1.33	0.0383	PKM,SOD3,RARRES2
COMPARTMENTS	GOCC:0001725	Stress fiber	2	39	1.86	0.0395	ACTN1,TPM4
UniProt Keywords	KW-0345	HDL	3	16	2.42	0.00018	SAA4,APOA2,SAA2
UniProt Keywords	KW-0964	Secreted	8	1839	0.79	0.0035	SPP2,C3,SAA4,FGG,APOA2,SOD3,RARRES2,SAA2
UniProt Keywords	KW-0011	Acute phase	2	19	2.17	0.0219	SAA4,SAA2
SMART	SM00197	Serum amyloid A proteins	2	4	2.85	0.0058	SAA4,SAA2

AC vs NCFET T2

#category	term ID	term description	observed gene count	background gene count	strength	false discovery rate	matching proteins in your network (labels)
GO Component	GO:0005576	Extracellular region	12	4175	0.58	0.0036	APOE,CFHR5,AZGP1,F2,PSG11,ITIH2,GSN,SVEP1,SOD3,IGFALS,PLTP,B2M
GO Component	GO:0005615	Extracellular space	11	3247	0.65	0.0036	APOE,CFHR5,AZGP1,F2,ITIH2,GSN,SVEP1,SOD3,IGFALS,PLTP,B2M
GO Component	GO:0031012	Extracellular matrix	6	552	1.15	0.0036	APOE,AZGP1,F2,ITIH2,SOD3,IGFALS
GO Component	GO:0062023	Collagen-containing extracellular matrix	5	407	1.21	0.0036	APOE,AZGP1,F2,ITIH2,SOD3
GO Component	GO:0072562	Blood microparticle	4	118	1.65	0.0036	APOE,F2,ITIH2,GSN
GO Component	GO:0005788	Endoplasmic reticulum lumen	4	312	1.23	0.0157	APOE,F2,ITIH2,B2M
GO Component	GO:0070062	Extracellular exosome	8	2096	0.7	0.0157	APOE,AZGP1,F2,ITIH2,GSN,SOD3,IGFALS,B2M
GO Component	GO:0034364	High-density lipoprotein particle	2	29	1.96	0.0423	APOE,PLTP
STRING clusters	CL:18726	Complement and coagulation cascades, and Protein-lipid complex	5	161	1.61	0.00051	APOE,CFHR5,F2,ITIH2,PLTP
Reactome	HSA-381426	Regulation of Insulin-like Growth Factor (IGF) transport and uptake by Insulin-like Growth Factor Binding Proteins (IGFBPs)	4	124	1.63	0.0050	APOE,F2,ITIH2,IGFALS
Reactome	HSA-8964058	HDL remodeling	2	10	2.42	0.0347	APOE,PLTP
Reactome	HSA-977225	Amyloid fiber formation	3	79	1.7	0.0347	APOE,GSN,B2M
WikiPathways	WP1533	Vitamin B12 metabolism	3	50	1.9	0.0064	APOE,F2,SOD3

WikiPathways	WP5110	Familial hyperlipidemia type 3	2	13	2.31	0.0220	APOE,PLTP
Monarch	HP:0030843	Cardiac amyloidosis	2	2	3.12	0.0395	GSN,B2M
DISEASES	DOID:9120	Amyloidosis	4	75	1.85	0.0014	APOE,AZGP1,GSN,B2M
DISEASES	DOID:0050636	Familial visceral amyloidosis	3	21	2.27	0.0016	APOE,AZGP1,B2M
DISEASES	DOID:0050637	Finnish type amyloidosis	2	9	2.47	0.0453	APOE,GSN
DISEASES	DOID:0050736	Autosomal dominant disease	7	1386	0.82	0.0453	APOE,CFHR5,AZGP1,F2,AKAP9,GSN,B2M
TISSUES	BTO:0000759	Liver	12	2125	0.87	2.06e-06	APMAP,APOE,CFHR5,AZGP1,F2,AKAP9,ITIH2,SMC5,GSN,IGFALS,PLTP,B2M
TISSUES	BTO:0004850	Bone marrow cell	6	198	1.6	6.41e-06	AZGP1,F2,ITIH2,SOD3,PLTP,B2M
TISSUES	BTO:0000141	Bone marrow	7	528	1.24	3.37e-05	APMAP,AZGP1,F2,ITIH2,SOD3,PLTP,B2M
TISSUES	BTO:0000392	Plasma cell	5	171	1.58	7.26e-05	AZGP1,F2,ITIH2,SOD3,B2M
TISSUES	BTO:0001486	Skeletal system	8	1307	0.91	0.00063	APMAP,APOE,AZGP1,F2,ITIH2,SOD3,PLTP,B2M
TISSUES	BTO:0001488	Endocrine gland	14	6403	0.46	0.00063	APMAP,APOE,CFHR5,AZGP1,F2,AKAP9,ITIH2,SMC5,GSN,SVEP1,SOD3,IGFALS,PLTP,B2M
TISSUES	BTO:0001491	Viscus	13	5378	0.5	0.00084	APMAP,APOE,CFHR5,AZGP1,F2,AKAP9,ITIH2,SMC5,GSN,SOD3,IGFALS,PLTP,B2M
TISSUES	BTO:0000817	Mammary gland	4	198	1.42	0.0032	AZGP1,F2,SMC5,SVEP1
TISSUES	BTO:0000431	Excretory gland	7	1385	0.82	0.0074	APOE,AZGP1,F2,SMC5,GSN,SVEP1,B2M
TISSUES	BTO:0001493	Trunk	6	1014	0.89	0.0128	APOE,AZGP1,F2,SMC5,SVEP1,B2M
TISSUES	BTO:0001368	Thorax	5	640	1.01	0.0146	AZGP1,F2,SMC5,SVEP1,B2M
TISSUES	BTO:0000089	Blood	7	1824	0.7	0.0314	APOE,AZGP1,F2,AKAP9,GSN,SOD3,B2M
TISSUES	BTO:0001085	Vascular system	4	420	1.1	0.0352	APOE,F2,PLTP,B2M
TISSUES	BTO:0001419	Urine	2	28	1.97	0.0352	F2,B2M
COMPARTMENTS	GOCC:0005615	Extracellular space	9	1027	1.06	2.55e-05	APOE,AZGP1,F2,ITIH2,GSN,SOD3,IGFALS,PLTP,B2M
COMPARTMENTS	GOCC:0005576	Extracellular region	10	2079	0.8	0.00037	APOE,CFHR5,AZGP1,F2,ITIH2,GSN,SOD3,IGFALS,PLTP,B2M
COMPARTMENTS	GOCC:0072562	Blood microparticle	4	118	1.65	0.0014	APOE,F2,ITIH2,GSN
COMPARTMENTS	GOCC:0065010	Extracellular membrane-bounded organelle	5	473	1.14	0.0116	APOE,AZGP1,F2,GSN,B2M
COMPARTMENTS	GOCC:1903561	Extracellular vesicle	5	500	1.12	0.0121	APOE,AZGP1,F2,GSN,B2M
UniProt Keywords	KW-0964	Secreted	12	1839	0.93	1.06e-07	APOE,CFHR5,AZGP1,F2,PSG11,ITIH2,GSN,SVEP1,SOD3,IGFALS,PLTP,B2M
UniProt Keywords	KW-0732	Signal	12	3277	0.68	4.24e-05	APOE,CFHR5,AZGP1,F2,PSG11,ITIH2,GSN,SVEP1,SOD3,IGFALS,PLTP,B2M
UniProt Keywords	KW-0971	Glycation	3	12	2.52	4.24e-05	APOE,SOD3,B2M
UniProt Keywords	KW-1008	Amyloidosis	3	31	2.1	0.00035	APOE,GSN,B2M
UniProt Keywords	KW-0325	Glycoprotein	12	4386	0.56	0.00046	APMAP,APOE,CFHR5,AZGP1,F2,PSG11,ITIH2,SVEP1,SOD3,IGFALS,PLTP,B2M
UniProt Keywords	KW-1015	Disulfide bond	10	3338	0.59	0.0029	CFHR5,AZGP1,F2,PSG11,ITIH2,GSN,SVEP1,SOD3,PLTP,B2M
UniProt Keywords	KW-0301	Gamma-carboxyglutamic acid	2	16	2.22	0.0079	F2,ITIH2
UniProt Keywords	KW-0034	Amyloid	2	24	2.04	0.0146	GSN,B2M

AC vs NCFET T3

#category	term ID	term description	observed gene count	background gene count	strength	false discovery rate	matching proteins in your network (labels)
COMPARTMENTS	GOCC:0005576	Extracellular region	9	2079	0.82	0.0018	IGFBP2,BPIFB1,LCAT,KLKB1,LALBA,FBN1,GSN,GC,CFD
COMPARTMENTS	GOCC:0005615	Extracellular space	7	1027	1.01	0.0018	IGFBP2,LCAT,KLKB1,FBN1,GSN,GC,CFD
UniProt Keywords	KW-0964	Secreted	10	1839	0.92	7.60e-06	IGFBP2,BPIFB1,LCAT,KLKB1,LALBA,FBN1,CNDP1,GSN,GC,CFD
UniProt Keywords	KW-0732	Signal	11	3277	0.71	5.16e-05	RCN1,IGFBP2,BPIFB1,LCAT,KLKB1,LALBA,FBN1,CNDP1,GSN,GC,CFD
UniProt Keywords	KW-1015	Disulfide bond	9	3338	0.61	0.0096	IGFBP2,BPIFB1,LCAT,KLKB1,LALBA,FBN1,GSN,GC,CFD
UniProt Keywords	KW-1212	Corneal dystrophy	2	20	2.18	0.0155	LCAT,GSN

UC vs ACFET T1

#category	term ID	term description	observed gene count	background gene count	strength	false discovery rate	matching proteins in your network (labels)
GO Process	GO:0006959	Humoral immune response	45	268	1.12	2.44e-30	ST6GAL1,EBI3,C5,PF4V1,C4BPB,CFP,CFHR5,LYZ,C9,FCN3,SERPING1,FCN2,PPBP,PF4,MASP1,CAMP,FGB,F2,C1QB,CFHR1,C7,ANG,C8A,CFHR4,CFHR3,CFH,S100A7,S100A9,C8B,MBL2,C1QC,CFI,GAPDH,C1S,C4A,COLEC11,C4B,CFB,RARRES2,IGHV3-15,CCL18,IGHV3-72,KNG1,B2M,FGA
GO Process	GO:0006950	Response to stress	126	3358	0.47	4.06e-29	CPB2,APOH,VCL,SERPIND1,CHGA,LBP,F9,MMP2,TFPI2,C5,MPO,PF4V1,PROC,PLEK,CAT,LRP1,C4BPB,CFP,APOE,PXDN,APCS,CFHR5,LYZ,VWF,SERPINA10,LGALS3BP,ATRN,C9,YWHAE,KLKB1,AMBP,TCIRG1,HPX,HYAL1,ITIH4,FCN3,AHSG,CRH,SAA4,SERPING1,MYL9,CSF1R,FCN2,PGLYRP2,PPBP,PF4,MASP1,CAMP,HSP90B1,PCSK9,SHLD1,FGB,DST,F2,PLG,STAB1,C1QB,CFHR1,APOL1,SUMO4,SERPINF2,C7,HBA1,HSPA5,P4HB,FBLN1,ANG,FGG,THBS4,APOD,BLM,F8,C8A,F13B,CFH,F5,CD5L,S100A7,S100A9,FLNA,MCOLN2,C8B,ADAMTS13,GSN,MBL2,C1QC,F10,F7,HLA-C,TAF9,SOD3,GPX3,PROS1,CFI,TNRC6A,ILK,GAPDH,RNASE1,CST3,SAA1,F11,C1S,ADIPOQ,C4A,CDK3,COLEC11,MST1,C4B,CFB,RARRES2,HGFAC,UIMC1,SELENOP,HPR,SERPINA3,RNASE4,IGHV3-15,CCL18,IGHV3-72,VEGFC,HYOU1,KNG1,ACTB,B2M,FGA,PDIA4
GO Process	GO:0006956	Complement activation	27	60	1.55	6.66e-27	C5,C4BPB,CFP,CFHR5,C9,FCN3,SERPING1,FCN2,MASP1,C1QB,CFHR1,C7,C8A,CFHR4,CFHR3,CFH,C8B,MBL2,C1QC,CFI,C1S,C4A,COLEC11,C4B,CFB,IGHV3-15,IGHV3-72
GO Process	GO:0007596	Blood coagulation	36	173	1.21	6.66e-27	CPB2,APOH,VCL,SERPIND1,F9,TFPI2,PF4V1,PROC,PLEK,C4BPB,VWF,SERPINA10,KLKB1,SERPING1,MYL9,PF4,FGB,F2,PLG,FBLN1,FGG,F8,F13B,F5,FLNA,ADAMTS13,F10,F7,PROS1,ILK,SAA1,F11,HGFAC,KNG1,ACTB,FGA
GO Process	GO:0006952	Defense response	74	1394	0.62	8.61e-23	CHGA,LBP,C5,MPO,PF4V1,LRP1,C4BPB,CFP,APCS,CFHR5,LYZ,LGALS3BP,ATRN,C9,KLKB1,TCIRG1,HPX,HYAL1,ITIH4,FCN3,AHSG,CRH,SAA4,SERPING1,CSF1R,FCN2,PGLYRP2,PPBP,PF4,MASP1,CAMP,FGB,F2,STAB1,C1QB,CFHR1,APOL1,SERPINF2,C7,ANG,F8,C8A,CFH,CD5L,S100A7,S100A9,MCOLN2,C8B,ADAMTS13,GSN,MBL2,C1QC,F7,HLA-C,CFI,GAPDH,RNASE1,CST3,SAA1,C1S,C4A,COLEC11,C4B,CFB,RARRES2,HPR,SERPINA3,RNASE4,IGHV3-15,CCL18,IGHV3-72,KNG1,B2M,FGA
GO Process	GO:0050878	Regulation of body fluid levels	39	371	0.92	7.31e-20	CPB2,APOH,VCL,SERPIND1,F9,TFPI2,VTN,PF4V1,PROC,PLEK,C4BPB,APOE,VWF,SERPINA10,KLKB1,SERPING1,MYL9,PF4,FGB,F2,PLG,SERPINF2,FBLN1,FGG,F8,F13B,F5,FLNA,ADAMTS13,F10,F7,PROS1,ILK,SAA1,F11,HGFAC,KNG1,ACTB,FGA
GO Process	GO:0042060	Wound healing	37	336	0.94	2.18e-19	CPB2,APOH,VCL,SERPIND1,F9,TFPI2,PF4V1,PROC,PLEK,C4BPB,VWF,SERPINA10,KLKB1,SERPING1,MYL9,PF4,FGB,DST,F2,PLG,FBLN1,FGG,F8,F13B,F5,FLNA,ADAMTS13,F10,F7,PROS1,ILK,SAA1,F11,HGFAC,KNG1,ACTB,FGA
GO Process	GO:0010466	Negative regulation of peptidase activity	32	249	1.01	2.66e-18	SPP2,SERPIND1,TFPI2,C5,VTN,LRP1,SERPINF1,SERPINA10,TIMP2,LCN1,CSTA,AMBP,ITIH4,ITIH1,AHSG,SERPING1,SERPINI1,A2ML1,SERPINF2,SERPINA7,SERPINA11,PAPLN,ITIH2,PROS1,GAPDH,CST3,C4A,ITIH3,C4B,SERPINA3,SERPINA4,KNG1
GO Process	GO:0010951	Negative regulation of endopeptidase activity	31	240	1.01	9.35e-18	SPP2,SERPIND1,TFPI2,C5,VTN,SERPINF1,SERPINA10,TIMP2,LCN1,CSTA,AMBP,ITIH4,ITIH1,AHSG,SERPING1,SERPINI1,A2ML1,SERPINF2,SERPINA7,SERPINA11,PAPLN,ITIH2,PROS1,GAPDH,CST3,C4A,ITIH3,C4B,SERPINA3,SERPINA4,KNG1

GO Process	GO:0098542	Defense response to other organism	56	989	0.65	1.19e-17	CHGA,LBP,C5,MPO,PF4V1,C4BPB,CFP,APCS,CFHR5,LYZ,C9,HPX,FCN3,SERPING1,CSF1R,FCN2,PGLYRP2,PPBP,PF4,MASP1,CAMP,FGB,F2,STAB1,C1QB,CFHR1,APOL1,C7,ANG,C8A,CFH,S100A7,S100A9,MCOLN2,C8B,ADAMTS13,GSN,MBL2,C1QC,HLA-C,CFI,GAPDH,RNASE1,C1S,C4A,COLEC11,C4B,CFB,RARRES2,RNASE4,IGHV3-15,CCL18,IGHV3-72,KNG1,B2M,FGA
GO Process	GO:0009611	Response to wounding	39	444	0.84	1.58e-17	CPB2,APOH,VCL,SERPIND1,F9,MMP2,TFPI2,PF4V1,PROC,PLEK,C4BPB,VWF,SERPINA10,KLKB1,SERPING1,MYL9,PF4,FGB,DST,F2,PLG,FBLN1,FGG,APOD,F8,F13B,F5,FLNA,ADAMTS13,F10,F7,PROS1,ILK,SAA1,F11,HGFAC,KNG1,ACTB,FGA
GO Process	GO:0045861	Negative regulation of proteolysis	35	339	0.91	1.58e-17	SPP2,CPB2,SERPIND1,TFPI2,C5,VTN,LRP1,SERPINF1,SERPINA10,TIMP2,LCN1,CSTA,AMBP,ITIH4,ITIH1,AHSG,SERPING1,SERPINI1,A2ML1,F2,SERPINF2,SERPINA7,SERPINA11,PAPLN,ITIH2,TAF9,PROS1,GAPDH,CST3,C4A,ITIH3,C4B,SERPINA3,SERPINA4,KNG1
GO Process	GO:0006955	Immune response	64	1321	0.58	2.09e-17	ST6GAL1,LGALS1,CHGA,LBP,EBI3,C5,VTN,PF4V1,LRP1,C4BPB,CFP,PXDN,APCS,CFHR5,LYZ,C9,TCIRG1,HPX,PSG9,FCN3,SERPING1,CSF1R,FCN2,PGLYRP2,PPBP,PF4,MASP1,CAMP,SHLD1,FGB,F2,C1QB,CFHR1,APOL1,C7,ANG,C8A,CFHR4,CFHR3,CFH,S100A7,S100A9,MCOLN2,C8B,ADAMTS13,GSN,MBL2,C1QC,HLA-C,CFI,GAPDH,RAPGEF4,C1S,C4A,COLEC11,C4B,CFB,RARRES2,IGHV3-15,CCL18,IGHV3-72,KNG1,B2M,FGA
GO Process	GO:0002252	Immune effector process	36	375	0.88	3.17e-17	LGALS1,CHGA,LBP,C5,MPO,LRP1,C4BPB,CFP,CFHR5,C9,TCIRG1,FCN3,SERPING1,FCN2,MASP1,SHLD1,F2,C1QB,CFHR1,C7,C8A,CFHR4,CFHR3,CFH,C8B,MBL2,C1QC,CFI,C1S,C4A,COLEC11,C4B,CFB,IGHV3-15,IGHV3-72,B2M
GO Process	GO:0051346	Negative regulation of hydrolase activity	35	354	0.89	4.36e-17	SPP2,SERPIND1,TFPI2,C5,VTN,APOC3,APOA1,LRP1,SERPINF1,APCS,SERPINA10,TIMP2,LCN1,CSTA,AMBP,ITIH4,ITIH1,AHSG,SERPING1,SERPINI1,A2ML1,SERPINF2,SERPINA7,SERPINA11,PAPLN,ITIH2,PROS1,GAPDH,CST3,C4A,ITIH3,C4B,SERPINA3,SERPINA4,KNG1
GO Process	GO:0072378	Blood coagulation, fibrin clot formation	15	24	1.69	3.25e-16	APOH,F9,KLKB1,FGB,F2,FBLN1,FGG,F8,F13B,F5,FLNA,F10,F7,F11,FGA
GO Process	GO:0052547	Regulation of peptidase activity	37	446	0.82	7.02e-16	SPP2,SERPIND1,TFPI2,PCOLCE,C5,VTN,LRP1,SERPINF1,SERPINA10,TIMP2,LCN1,CSTA,AMBP,ITIH4,ITIH1,AHSG,SERPING1,SERPINI1,A2ML1,ANTXR1,SERPINF2,SERPINA7,FBLN1,SERPINA11,PAPLN,ITIH2,S100A9,GSN,PROS1,GAPDH,CST3,C4A,ITIH3,C4B,SERPINA3,SERPINA4,KNG1
GO Process	GO:0006958	Complement activation, classical pathway	16	40	1.5	4.55e-15	C5,C4BPB,C9,SERPING1,C1QB,C7,C8A,C8B,MBL2,C1QC,CFI,C1S,C4A,C4B,IGHV3-15,IGHV3-72
GO Process	GO:0050896	Response to stimulus	167	7835	0.23	6.67e-15	ST6GAL1,CPB2,APOH,VCL,SERPIND1,LGALS1,CHGA,LBP,F9,MMP2,EBI3,PON1,TFPI2,C5,MPO,VTN,PF4V1,APOC3,GPLD1,APOB,IGFBP5,PROC,PLEK,APOA1,TTR,CAT,LRP1,C4BPB,CFP,APOE,PXDN,SERPINF1,APCS,CFHR5,LYZ,VWF,SERPINA10,TIMP2,LGALS3BP,ATRN,C9,LCN1,LCAT,YWHAE,KLKB1,CDH6,AMBP,TCIRG1,HPX,HYAL1,ITIH4,PSG9,FCN3,AHSG,CRH,SAA4,SERPING1,MYL9,CSF1R,FCN2,PGLYRP2,SCGB3A1,IHH,PPBP,PF4,MASP1,CAMP,GUCY1A1,HSP90B1,PCSK9,BMP1,SHLD1,FGB,DST,F2,INHBC,PLG,CSPG4,STAB1,C1QB,CFHR1,APOL1,SUMO4,SERPINF2,C7,HBA1,HSPA5,P4HB,FBLN1,ANG,FGG,LIMS1,THBS4,APOD,BLM,AKAP9,F8,C8A,CENPF,F13B,CFHR4,CFHR3,CFH,F5,CD5L,S100A7,S100A9,FLNA,MCOLN2,C8B,ADAMTS13,CCN5,GSN,MBL2,C1QC,F10,F7,APOM,HLA-C,CALML5,TAF9,IGFBP3,SOD3,GPX3,PROS1,EFEMP1,CFI,TNRC6A,YWHAZ,ILK,GAPDH,RAPGEF4,RNASE1,CST3,SAA1,F11,C1S,NUCB1,ADIPOQ,IGF2,C4A,CDK3,COLEC11,MST1,SPARCL1,C4B,CFB,IGFALS,RARRES2,HGFAC,UIMC1,SELENOP,HPR,ATP1A1,SERPINA3,RNASE4,IGHV3-15,CCL18,IGHV3-72,VEGFC,HYOU1,KNG1,ACTB,B2M,FGA,PDIA4,SDF4
GO Process	GO:0045087	Innate immune response	45	754	0.67	1.12e-14	CHGA,LBP,C5,C4BPB,CFP,APCS,CFHR5,C9,HPX,FCN3,SERPING1,CSF1R,FCN2,PGLYRP2,MASP1,CAMP,FGB,C1QB,APOL1,C7,ANG,C8A,CFH,S100A7,S100A9,MCOLN2,C8B,ADAMTS13,GSN,MBL2,C1QC,HLA-C,CFI,GAPDH,C1S,C4A,COLEC11,C4B,CFB,RARRES2,IGHV3-15,CCL18,IGHV3-72,B2M,FGA
GO Process	GO:0030195	Negative regulation of blood coagulation	16	46	1.44	2.35e-14	CPB2,APOH,VTN,PROC,APOE,KLKB1,SERPING1,FGB,F2,PLG,SERPINF2,FGG,PROS1,F11,KNG1,FGA
GO Process	GO:0030162	Regulation of proteolysis	44	739	0.67	2.60e-14	SPP2,CPB2,SERPIND1,TFPI2,PCOLCE,C5,VTN,GPLD1,LRP1,APOE,SERPINF1,SERPINA10,TIMP2,LCN1,CSTA,KLKB1,AMBP,ITIH4,ITIH1,AHSG,SERPING1,SERPINI1,A2ML1,ANTXR1,SH3D19,F2,SERPINF2,SERPINA7,FBLN1,SERPINA11,PAPLN,ITIH2,S100A9,GSN,TAF9,PROS1,GAPDH,CST3,C4A,ITIH3,C4B,SERPINA3,SERPINA4,KNG1
GO Process	GO:0051707	Response to other organism	59	1328	0.54	2.60e-14	CHGA,LBP,C5,MPO,PF4V1,APOB,C4BPB,CFP,APCS,CFHR5,LYZ,C9,HPX,HYAL1,FCN3,SERPING1,CSF1R,FCN2,PGLYRP2,PPBP,PF4,MASP1,CAMP,FGB,F2,STAB1,C1QB,CFHR1,APOL1,C7,ANG,C8A,CFH,S100A7,S100A9,MCOLN2,C8B,ADAMTS13,GSN,MBL2,C1QC,HLA-C,CFI,GAPDH,RNASE1,C1S,ADIPOQ,C4A,COLEC11,C4B,CFB,RARRES2,RNASE4,IGHV3-15,CCL18,IGHV3-72,KNG1,B2M,FGA
GO Process	GO:0009607	Response to biotic stimulus	60	1375	0.54	2.66e-14	CHGA,LBP,C5,MPO,PF4V1,APOB,C4BPB,CFP,APCS,CFHR5,LYZ,C9,HPX,HYAL1,FCN3,SERPING1,CSF1R,FCN2,PGLYRP2,PPBP,PF4,MASP1,CAMP,FGB,F2,STAB1,C1QB,CFHR1,APOL1,C7,HSPA5,ANG,C8A,CFH,S100A7,S100A9,MCOLN2,C8B,ADAMTS13,GSN,MBL2,C1QC,HLA-C,CFI,GAPDH,RNASE1,C1S,ADIPOQ,C4A,COLEC11,C4B,CFB,RARRES2,RNASE4,IGHV3-15,CCL18,IGHV3-72,KNG1,B2M,FGA
GO Process	GO:0002253	Activation of immune response	28	271	0.91	5.66e-14	C5,GPLD1,C4BPB,CFP,CFHR5,C9,FCN3,SERPING1,FCN2,MASP1,C1QB,CFHR1,C7,C8A,CFHR4,CFHR3,CFH,C8B,MBL2,C1QC,CFI,C1S,C4A,COLEC11,C4B,CFB,IGHV3-15,IGHV3-72
GO Process	GO:0052548	Regulation of endopeptidase activity	33	414	0.8	1.08e-13	SPP2,SERPIND1,TFPI2,C5,VTN,SERPINF1,SERPINA10,TIMP2,LCN1,CSTA,AMBP,ITIH4,ITIH1,AHSG,SERPING1,SERPINI1,A2ML1,SERPINF2,SERPINA7,SERPINA11,PAPLN,ITIH2,S100A9,GSN,PROS1,GAPDH,CST3,C4A,ITIH3,C4B,SERPINA3,SERPINA4,KNG1
GO Process	GO:0044419	Biological process involved in interspecies interaction between organisms	61	1490	0.51	2.06e-13	CHGA,LBP,C5,MPO,PF4V1,APOB,C4BPB,CFP,APOE,APCS,CFHR5,LYZ,C9,HPX,HYAL1,FCN3,SERPING1,CSF1R,FCN2,PGLYRP2,PPBP,PF4,MASP1,CAMP,FGB,F2,PLG,STAB1,C1QB,CFHR1,APOL1,C7,ANG,C8A,CFH,S100A7,S100A9,MCOLN2,C8B,ADAMTS13,GSN,MBL2,C1QC,HLA-C,CFI,GAPDH,RNASE1,C1S,ADIPOQ,C4A,COLEC11,C4B,CFB,RARRES2,RNASE4,IGHV3-15,CCL18,IGHV3-72,KNG1,B2M,FGA
GO Process	GO:0061045	Negative regulation of wound healing	17	69	1.29	2.22e-13	CPB2,APOH,VTN,PROC,APOE,APCS,KLKB1,SERPING1,FGB,F2,PLG,SERPINF2,FGG,PROS1,F11,KNG1,FGA
GO Process	GO:0030193	Regulation of blood coagulation	17	70	1.28	2.65e-13	CPB2,APOH,VTN,PROC,APOE,KLKB1,SERPING1,FGB,F2,PLG,SERPINF2,FGG,F7,PROS1,F11,KNG1,FGA
GO Process	GO:0051336	Regulation of hydrolase activity	49	1011	0.58	5.65e-13	SPP2,APOH,SERPIND1,TFPI2,PCOLCE,C5,VTN,APOC3,GPLD1,PLEK,APOA1,LRP1,SERPINF1,APCS,SERPINA10,TIMP2,LCN1,CSTA,AMBP,ITIH4,ITIH1,AHSG,SERPING1,SERPINI1,A2ML1,HSP90B1,ANTXR1,SERPINF2,SERPINA7,FBLN1,SERPINA11,ANG,LIMS1,PAPLN,ITIH2,S100A9,GSN,PROS1,GAPDH,RAPGEF4,CST3,C4A,ITIH3,C4B,APOA5,SERPINA3,SERPINA4,CCL18,KNG1

GO Process	GO:0002684	Positive regulation of immune system process	45	874	0.61	1.18e-12	LGALS1,LBP,EBI3,C5,GPLD1,C4BPB,CFP,CFHR5,C9,HPX,FCN3,SERPING1,CSF1R,FCN2,IHH,PF4,MASP1,SHLD1,C1QB,CFHR1,C7,THBS4,C8A,CFHR4,CFHR3,CFH,S100A7,C8B,MBL2,C1QC,F7,CD99,CFI,C1S,IGF2,C4A,COLEC11,C4B,CFB,RARRES2,IGHV3-15,IGHV3-72,VEGFC,ACTB,B2M
GO Process	GO:0016064	Immunoglobulin mediated immune response	18	95	1.17	1.41e-12	C5,C4BPB,C9,TCIRG1,SERPING1,SHLD1,C1QB,C7,C8A,C8B,MBL2,C1QC,CFI,C1S,C4A,C4B,IGHV3-15,IGHV3-72
GO Process	GO:0002376	Immune system process	72	2121	0.43	2.45e-12	ST6GAL1,LGALS1,CHGA,LBP,EBI3,C5,MPO,VTN,PF4V1,GPLD1,PLEK,LRP1,C4BPB,CFP,PXDN,APCS,CFHR5,LYZ,C9,TCIRG1,HPX,PSG9,FCN3,SERPING1,CSF1R,FCN2,PGLYRP2,PPBP,PF4,MASP1,CAMP,SHLD1,FGB,F2,PLG,C1QB,CFHR1,APOL1,C7,ANG,C8A,CFHR4,CFHR3,CFH,CD5L,S100A7,S100A9,FLNA,MCOLN2,C8B,ADAMTS13,GSN,MBL2,C1QC,HLA-C,CD99,CFI,GAPDH,RAPGEF4,SAA1,C1S,C4A,COLEC11,C4B,CFB,RARRES2,IGHV3-15,CCL18,IGHV3-72,KNG1,B2M,FGA
GO Process	GO:0048583	Regulation of response to stimulus	104	3931	0.32	2.87e-12	ST6GAL1,CPB2,APOH,LGALS1,CHGA,LBP,C5,VTN,APOC3,GPLD1,IGFBP5,PROC,PLEK,APOA1,CAT,LRP1,C4BPB,CFP,APOE,PXDN,SERPINF1,APCS,CFHR5,VWF,PSMA6,C9,NID1,KLKB1,AMBP,HPX,PSG9,FCN3,AHSG,CRH,SERPING1,CSF1R,FCN2,PGLYRP2,IHH,PF4,MASP1,GUCY1A1,PCSK9,SHLD1,FGB,F2,INHBC,PLG,CSPG4,C1QB,CFHR1,SUMO4,SERPINF2,C7,HSPA5,P4HB,FBLN1,FGG,LIMS1,THBS4,APOD,AKAP9,C8A,CFHR4,CFHR3,CFH,CD5L,S100A7,S100A9,FLNA,C8B,MBL2,C1QC,F10,F7,TAF9,IGFBP3,PROS1,CFI,YWHAZ,ILK,GAPDH,SAA1,F11,C1S,ADIPOQ,IGF2,C4A,CDK3,COLEC11,MST1,C4B,CFB,RARRES2,UIMC1,IGHV3-15,CCL18,IGHV3-72,VEGFC,HYOU1,KNG1,ACTB,B2M,FGA
GO Process	GO:0048584	Positive regulation of response to stimulus	72	2131	0.43	2.89e-12	CPB2,APOH,LGALS1,CHGA,LBP,C5,VTN,GPLD1,IGFBP5,APOA1,CAT,C4BPB,CFP,APOE,CFHR5,VWF,C9,NID1,HPX,FCN3,SERPING1,CSF1R,FCN2,IHH,MASP1,GUCY1A1,SHLD1,FGB,F2,INHBC,PLG,CSPG4,C1QB,CFHR1,SERPINF2,C7,FGG,LIMS1,THBS4,C8A,CFHR4,CFHR3,CFH,S100A7,S100A9,FLNA,C8B,MBL2,C1QC,F10,F7,TAF9,IGFBP3,CFI,ILK,GAPDH,C1S,ADIPOQ,IGF2,C4A,COLEC11,C4B,CFB,RARRES2,UIMC1,IGHV3-15,CCL18,IGHV3-72,VEGFC,ACTB,B2M,FGA
GO Process	GO:0031638	Zymogen activation	15	59	1.3	6.11e-12	APOH,F9,KLKB1,FGB,F2,FGG,F8,F5,CD5L,F10,F7,F11,HGFAC,HPR,FGA
GO Process	GO:0002460	Adaptive immune response based on somatic recombination of immune receptors built from immunoglobulin superfamily domains	21	169	0.99	1.09e-11	EBI3,C5,C4BPB,C9,TCIRG1,PSG9,SERPING1,SHLD1,C1QB,C7,C8A,C8B,MBL2,C1QC,CFI,C1S,C4A,C4B,IGHV3-15,IGHV3-72,B2M
GO Process	GO:0006954	Inflammatory response	34	538	0.7	1.49e-11	LBP,C5,PF4V1,LRP1,APCS,LYZ,ATRN,KLKB1,TCIRG1,HYAL1,ITIH4,AHSG,CRH,SAA4,CSF1R,PPBP,PF4,CAMP,F2,STAB1,SERPINF2,F8,CD5L,S100A9,MBL2,F7,SAA1,C4A,C4B,RARRES2,HPR,SERPINA3,CCL18,KNG1
GO Process	GO:0051248	Negative regulation of protein metabolic process	47	1038	0.55	2.01e-11	SPP2,CPB2,SERPIND1,TFPI2,C5,VTN,IGFBP5,LRP1,APOE,SERPINF1,APCS,SERPINA10,TIMP2,LCN1,YWHAE,CSTA,AMBP,ITIH4,ITIH1,AHSG,SERPING1,SERPINI1,A2ML1,F2,SERPINF2,SERPINA7,FBLN1,SERPINA11,ANG,APOD,PAPLN,ITIH2,FLNA,TAF9,IGFBP3,PROS1,TNRC6A,GAPDH,CST3,ADIPOQ,C4A,ITIH3,C4B,SERPINA3,SERPINA4,KNG1,FGA
GO Process	GO:0032101	Regulation of response to external stimulus	45	964	0.57	2.64e-11	ST6GAL1,CPB2,APOH,LGALS1,LBP,C5,VTN,PROC,APOA1,APOE,SERPINF1,APCS,PSMA6,KLKB1,HPX,FCN3,AHSG,SERPING1,CSF1R,FCN2,PGLYRP2,MASP1,FGB,F2,PLG,SERPINF2,FGG,THBS4,CFH,S100A7,S100A9,FLNA,MBL2,F7,PROS1,CFI,SAA1,F11,ADIPOQ,COLEC11,MST1,RARRES2,VEGFC,KNG1,FGA
GO Process	GO:0048518	Positive regulation of biological process	136	6207	0.24	2.79e-11	ST6GAL1,CPB2,CETP,APOH,LGALS1,CHGA,LBP,MMP2,EBI3,PON1,PCOLCE,C5,VTN,GPLD1,SPARC,APOB,IGFBP5,PROC,PLEK,APOA1,CAT,LRP1,C4BPB,CFP,DHX29,APOE,SERPINF1,CFHR5,VWF,C9,NID1,YWHAE,KLKB1,CENPE,TCIRG1,HPX,HYAL1,LUM,PSG9,FCN3,AHSG,ANKRD31,CRH,SERPING1,CSF1R,FCN2,SCGB3A1,IHH,SERPINI1,PPBP,PF4,MASP1,CAMP,GUCY1A1,ANTXR1,SH3D19,PCSK9,BMP1,SHLD1,FGB,F2,INHBC,PLG,EFEMP2,CSPG4,C1QB,CFHR1,SUZ12,SERPINF2,C7,HBA1,HSPA5,P4HB,FBLN1,ANG,FGG,LIMS1,THBS4,BLM,AKAP9,MTCL1,C8A,CFHR4,CFHR3,CFH,CD5L,S100A7,S100A9,ADAMTSL4,FLNA,MCOLN2,SMARCA1,C8B,GSN,MBL2,C1QC,F10,F7,TAF9,IGFBP3,CD99,CFI,TNRC6A,ILK,GAPDH,FBLN2,SAA1,F11,C1S,ADIPOQ,IGF2,C4A,SLAIN1,ABCA13,COLEC11,MST1,LMOD3,C4B,CFB,PLTP,PAEP,RARRES2,UIMC1,HPR,APOA5,ATP1A1,APOC4,IGHV3-15,CCL18,IGHV3-72,VEGFC,KNG1,ACTB,GRHL2,B2M,FGA
GO Process	GO:0050778	Positive regulation of immune response	32	502	0.7	6.60e-11	LBP,C5,GPLD1,C4BPB,CFP,CFHR5,C9,HPX,FCN3,SERPING1,FCN2,MASP1,SHLD1,C1QB,CFHR1,C7,C8A,CFHR4,CFHR3,CFH,C8B,MBL2,C1QC,CFI,C1S,C4A,COLEC11,C4B,CFB,IGHV3-15,IGHV3-72,B2M
GO Process	GO:0002682	Regulation of immune system process	55	1438	0.48	7.58e-11	LGALS1,LBP,EBI3,C5,GPLD1,APOA1,C4BPB,CFP,APOE,APCS,CFHR5,C9,AMBP,HPX,PSG9,FCN3,SERPING1,CSF1R,FCN2,PGLYRP2,IHH,PF4,MASP1,SHLD1,C1QB,CFHR1,C7,THBS4,APOD,C8A,CFHR4,CFHR3,CFH,CD5L,S100A7,C8B,MBL2,C1QC,F7,CD99,CFI,C1S,ADIPOQ,IGF2,C4A,COLEC11,MST1,C4B,CFB,RARRES2,IGHV3-15,IGHV3-72,VEGFC,ACTB,B2M
GO Process	GO:0009605	Response to external stimulus	73	2355	0.39	1.02e-10	SERPIND1,CHGA,LBP,MMP2,PON1,C5,MPO,PF4V1,APOB,APOA1,CAT,C4BPB,CFP,APOE,APCS,CFHR5,LYZ,C9,HPX,HYAL1,FCN3,SERPING1,CSF1R,FCN2,PGLYRP2,IHH,PPBP,PF4,MASP1,CAMP,PCSK9,FGB,F2,STAB1,C1QB,CFHR1,APOL1,C7,HSPA5,ANG,C8A,CFH,F5,S100A7,S100A9,MCOLN2,C8B,ADAMTS13,GSN,MBL2,C1QC,F7,HLA-C,CFI,TNRC6A,GAPDH,RNASE1,SAA1,C1S,ADIPOQ,C4A,COLEC11,C4B,CFB,RARRES2,RNASE4,IGHV3-15,CCL18,IGHV3-72,VEGFC,KNG1,B2M,FGA
GO Process	GO:0061041	Regulation of wound healing	18	130	1.04	1.28e-10	CPB2,APOH,VTN,PROC,APOE,APCS,KLKB1,SERPING1,FGB,F2,PLG,SERPINF2,FGG,F7,PROS1,F11,KNG1,FGA
GO Process	GO:0044092	Negative regulation of molecular function	48	1143	0.52	1.30e-10	SPP2,SERPIND1,TFPI2,C5,VTN,APOC3,APOA1,CAT,LRP1,APOE,PXDN,SERPINF1,APCS,CFHR5,SERPINA10,TIMP2,LCN1,YWHAE,CSTA,AMBP,ITIH4,ITIH1,AHSG,SERPING1,SERPINI1,A2ML1,PCSK9,CFHR1,SUMO4,SERPINF2,SERPINA7,SERPINA11,PAPLN,AKAP9,ITIH2,FLNA,PROS1,GAPDH,CST3,ADIPOQ,C4A,ITIH3,C4B,SERPINA3,SERPINA4,KNG1,ACTB,B2M
GO Process	GO:1903027	Regulation of opsonization	10	18	1.64	2.32e-10	C4BPB,CFP,FCN3,FCN2,MASP1,MBL2,C4A,COLEC11,C4B,CFB
GO Process	GO:0051241	Negative regulation of multicellular organismal process	45	1035	0.53	2.46e-10	CPB2,APOH,LBP,MMP2,VTN,APOC3,SPARC,IGFBP5,PROC,APOA1,APOE,SERPINF1,APCS,KLKB1,PSG9,AHSG,CRH,SERPING1,PGLYRP2,IHH,PF4,GUCY1A1,PCSK9,FGB,F2,PLG,STAB1,SERPINF2,FBLN1,FGG,THBS4,APOD,C1QC,APOM,PROS1,EFEMP1,CST3,F11,ADIPOQ,PAEP,ATP1A1,KNG1,GRHL2,B2M,FGA
GO Process	GO:0002449	Lymphocyte mediated immunity	19	159	0.97	2.84e-10	C5,C4BPB,C9,TCIRG1,SERPING1,SHLD1,C1QB,C7,C8A,C8B,MBL2,C1QC,CFI,C1S,C4A,C4B,IGHV3-15,IGHV3-72,B2M
GO Process	GO:0042730	Fibrinolysis	10	19	1.62	3.33e-10	CPB2,KLKB1,SERPING1,FGB,F2,PLG,SERPINF2,FGG,PROS1,FGA
GO Process	GO:1903034	Regulation of response to wounding	19	162	0.97	3.72e-10	CPB2,APOH,VTN,PROC,APOE,APCS,KLKB1,SERPING1,FGB,F2,PLG,SERPINF2,FGG,FLNA,F7,PROS1,F11,KNG1,FGA
GO Process	GO:0002443	Leukocyte mediated immunity	21	211	0.89	4.29e-10	CHGA,C5,C4BPB,C9,TCIRG1,SERPING1,SHLD1,F2,C1QB,C7,C8A,C8B,MBL2,C1QC,CFI,C1S,C4A,C4B,IGHV3-15,IGHV3-72,B2M

GO Process	GO:0065007	Biological regulation	208	12385	0.12	4.73e-10	SPP2,ST6GAL1,CPB2,CETP,APOH,VCL,SERPIND1,LGALS1,CHGA,TPD52L2,LBP,F9,MMP2,EBI3,PON1,TFPI2,PCOLCE,C5,MPO,VTN,AFM,PF4V1,APOC3,MGP,GPLD1,SPARC,APOB,IGFBP5,PROC,PLEK,APOA1,TTR,CAT,LRP1,C4BPB,CFP,DHX29,APOE,PXDN,SERPINF1,APCS,CFHR5,LYZ,VWF,PSMA6,DSG2,SERPINA10,TIMP2,LGALS3BP,ATRN,C9,LCN1,LCAT,NID1,YWHAE,CSTA,CP,KLKB1,CDH6,AMBP,CENPE,TCIRG1,HPX,HYAL1,ITIH4,LUM,CDH11,PSG9,FCN3,ITIH1,AHSG,ANKRD31,CRH,SERPING1,MYL9,CSF1R,FCN2,PGLYRP2,SCGB3A1,IHH,SERPINI1,PPBP,PF4,MASP1,CAMP,GUCY1A1,A2ML1,HSP90B1,ANTXR1,SH3D19,PCSK9,BMP1,SHLD1,FGB,DST,F2,INHBC,PLG,EFEMP2,CSPG4,STAB1,C1QB,CFHR1,SUZ12,SUMO4,CPN2,SERPINF2,C7,HBA1,HSPA5,P4HB,SERPINA7,FBLN1,OIT3,SERPINA11,ANG,FGG,LIMS1,THBS4,APOD,PAPLN,BLM,AKAP9,ITIH2,CNDP1,MTCL1,F8,C8A,CENPF,F13B,CFHR4,CFHR3,CFH,QSOX1,F5,CD5L,S100A7,S100A9,ADAMTSL4,FLNA,MCOLN2,SMARCA1,C8B,ADAMTS13,CCN5,GSN,MBL2,C1QC,F10,F7,APOM,CALML5,TAF9,IGFBP3,CD99,SOD3,PROS1,EFEMP1,CFI,TNRC6A,YWHAZ,NAV2,ILK,GAPDH,RAPGEF4,CST3,FBLN2,SAA1,F11,C1S,NUCB1,ADIPOQ,IGF2,C4A,ATP9B,SLAIN1,CDK3,ABCA13,COLEC11,MST1,LMOD3,SPARCL1,ITIH3,C4B,CFB,IGFALS,PLTP,PAEP,RARRES2,HGFAC,UIMC1,SELENOP,HPR,APOA5,ATP1A1,SERPINA3,SERPINA4,APOC4,IGHV3-15,CCL18,IGHV3-72,VEGFC,HYOU1,KNG1,ACTB,GRHL2,B2M,FGA
GO Process	GO:0043086	Negative regulation of catalytic activity	38	771	0.59	4.87e-10	SPP2,SERPIND1,TFPI2,C5,VTN,APOC3,APOA1,LRP1,APOE,SERPINF1,APCS,SERPINA10,TIMP2,LCN1,CSTA,AMBP,ITIH4,ITIH1,AHSG,SERPING1,SERPINI1,A2ML1,SERPINF2,SERPINA7,SERPINA11,PAPLN,AKAP9,ITIH2,PROS1,GAPDH,CST3,ADIPOQ,C4A,ITIH3,C4B,SERPINA3,SERPINA4,KNG1
GO Process	GO:0051246	Regulation of protein metabolic process	76	2622	0.36	6.41e-10	SPP2,CPB2,SERPIND1,TFPI2,PCOLCE,C5,VTN,GPLD1,IGFBP5,APOA1,LRP1,C4BPB,DHX29,APOE,SERPINF1,APCS,SERPINA10,TIMP2,LCN1,YWHAE,CSTA,KLKB1,AMBP,CENPE,HPX,ITIH4,ITIH1,AHSG,SERPING1,CSF1R,SERPINI1,CAMP,A2ML1,HSP90B1,ANTXR1,SH3D19,PCSK9,F2,INHBC,CSPG4,SERPINF2,HSPA5,SERPINA7,FBLN1,SERPINA11,ANG,THBS4,APOD,PAPLN,BLM,AKAP9,ITIH2,S100A9,FLNA,GSN,TAF9,IGFBP3,PROS1,TNRC6A,ILK,GAPDH,CST3,ADIPOQ,IGF2,C4A,MST1,ITIH3,C4B,PAEP,RARRES2,SERPINA3,SERPINA4,VEGFC,KNG1,ACTB,FGA
GO Process	GO:0002250	Adaptive immune response	26	359	0.76	7.49e-10	EBI3,C5,C4BPB,C9,TCIRG1,PSG9,SERPING1,SHLD1,FGB,C1QB,C7,C8A,MCOLN2,C8B,MBL2,C1QC,HLA-C,CFI,RAPGEF4,C1S,C4A,C4B,IGHV3-15,IGHV3-72,B2M,FGA
GO Process	GO:0030168	Platelet activation	15	97	1.09	2.38e-09	VCL,PF4V1,PLEK,VWF,MYL9,PF4,FGB,F2,FGG,FLNA,ADAMTS13,ILK,SAA1,ACTB,FGA
GO Process	GO:0006957	Complement activation, alternative pathway	9	16	1.65	2.57e-09	C5,CFP,CFHR5,C9,C7,C8A,CFH,C8B,CFB
GO Process	GO:1903028	Positive regulation of opsonization	9	16	1.65	2.57e-09	CFP,FCN3,FCN2,MASP1,MBL2,C4A,COLEC11,C4B,CFB
GO Process	GO:0002526	Acute inflammatory response	14	80	1.14	2.59e-09	LBP,APCS,KLKB1,ITIH4,AHSG,SAA4,F2,SERPINF2,F8,MBL2,F7,SAA1,HPR,SERPINA3
GO Process	GO:0042742	Defense response to bacterium	23	306	0.77	6.62e-09	CHGA,LBP,MPO,CFP,LYZ,FCN2,PGLYRP2,PPBP,CAMP,FGB,F2,STAB1,ANG,S100A7,S100A9,MBL2,RNASE1,RARRES2,RNASE4,IGHV3-15,IGHV3-72,B2M,FGA
GO Process	GO:0019730	Antimicrobial humoral response	17	149	0.95	7.30e-09	PF4V1,LYZ,PPBP,PF4,CAMP,FGB,F2,ANG,S100A7,S100A9,GAPDH,COLEC11,RARRES2,CCL18,KNG1,B2M,FGA
GO Process	GO:0034369	Plasma lipoprotein particle remodeling	10	29	1.43	7.82e-09	CETP,MPO,APOC3,APOB,APOA1,APOE,LCAT,APOM,PLTP,APOA5
GO Process	GO:0006953	Acute-phase response	11	42	1.31	9.50e-09	LBP,APCS,ITIH4,AHSG,SAA4,F2,SERPINF2,F8,MBL2,SAA1,SERPINA3
GO Process	GO:0016485	Protein processing	19	202	0.87	1.02e-08	APOH,F9,KLKB1,IHH,PCSK9,BMP1,FGB,F2,FGG,F8,F5,CD5L,ADAMTS13,F10,F7,F11,HGFAC,HPR,FGA
GO Process	GO:0048519	Negative regulation of biological process	116	5313	0.24	1.11e-08	SPP2,ST6GAL1,CPB2,CETP,APOH,VCL,SERPIND1,CHGA,LBP,MMP2,TFPI2,C5,MPO,VTN,APOC3,GPLD1,SPARC,IGFBP5,PROC,PLEK,APOA1,CAT,LRP1,C4BPB,APOE,PXDN,SERPINF1,APCS,SERPINA10,TIMP2,LCN1,YWHAE,CSTA,KLKB1,AMBP,HYAL1,ITIH4,PSG9,FCN3,ITIH1,AHSG,CRH,SERPING1,CSF1R,PGLYRP2,SCGB3A1,IHH,SERPINI1,PF4,MASP1,GUCY1A1,A2ML1,HSP90B1,ANTXR1,PCSK9,SHLD1,FGB,F2,PLG,EFEMP2,STAB1,SUZ12,SUMO4,SERPINF2,HSPA5,SERPINA7,FBLN1,SERPINA11,ANG,FGG,LIMS1,THBS4,APOD,PAPLN,BLM,ITIH2,CENPF,QSOX1,FLNA,MCOLN2,CCN5,GSN,MBL2,C1QC,APOM,TAF9,IGFBP3,PROS1,EFEMP1,TNRC6A,YWHAZ,ILK,GAPDH,CST3,SAA1,F11,ADIPOQ,IGF2,C4A,CDK3,MST1,LMOD3,ITIH3,C4B,PAEP,UIMC1,ATP1A1,SERPINA3,SERPINA4,VEGFC,HYOU1,KNG1,ACTB,GRHL2,B2M,FGA
GO Process	GO:0050776	Regulation of immune response	37	844	0.54	2.01e-08	LBP,C5,GPLD1,C4BPB,CFP,APOE,CFHR5,C9,AMBP,HPX,PSG9,FCN3,SERPING1,FCN2,PGLYRP2,MASP1,SHLD1,C1QB,CFHR1,C7,C8A,CFHR4,CFHR3,CFH,CD5L,C8B,MBL2,C1QC,CFI,C1S,C4A,COLEC11,C4B,CFB,IGHV3-15,IGHV3-72,B2M
GO Process	GO:0007155	Cell adhesion	39	965	0.5	5.74e-08	VCL,LGALS1,VTN,PCDH12,PLEK,PXDN,VWF,DSG2,LGALS3BP,ATRN,NID1,CSTA,CDH6,AMBP,CDH11,HABP2,MYL9,PCDH1,IHH,ANTXR1,FGB,DST,STAB1,PSG11,FGG,LIMS1,THBS4,PCDH18,S100A9,FLNA,ADAMTS13,CCN5,CD99,ILK,SPARCL1,IGFALS,ACTB,GRHL2,FGA
GO Process	GO:0051239	Regulation of multicellular organismal process	73	2749	0.32	9.01e-08	CPB2,APOH,VCL,CHGA,LBP,MMP2,EBI3,C5,VTN,APOC3,MGP,GPLD1,SPARC,IGFBP5,PROC,APOA1,LRP1,APOE,SERPINF1,APCS,DSG2,ATRN,LCAT,YWHAE,KLKB1,HYAL1,LUM,PSG9,AHSG,CRH,SERPING1,MYL9,CSF1R,PGLYRP2,IHH,PF4,CAMP,GUCY1A1,PCSK9,BMP1,SHLD1,FGB,F2,PLG,STAB1,SERPINF2,FBLN1,FGG,THBS4,APOD,AKAP9,MCOLN2,C1QC,F7,APOM,PROS1,EFEMP1,GAPDH,CST3,SAA1,F11,ADIPOQ,IGF2,MST1,PAEP,APOA5,ATP1A1,VEGFC,KNG1,ACTB,GRHL2,B2M,FGA
GO Process	GO:0097006	Regulation of plasma lipoprotein particle levels	11	54	1.21	9.01e-08	CETP,MPO,APOC3,APOB,APOA1,APOE,LCAT,APOM,ADIPOQ,PLTP,APOA5
GO Process	GO:0050790	Regulation of catalytic activity	66	2370	0.34	1.24e-07	SPP2,APOH,SERPIND1,TFPI2,PCOLCE,C5,VTN,APOC3,GPLD1,PLEK,APOA1,LRP1,APOE,SERPINF1,APCS,SERPINA10,TIMP2,LCN1,CSTA,AMBP,CENPE,ITIH4,ITIH1,AHSG,SERPING1,CSF1R,SERPINI1,A2ML1,HSP90B1,ANTXR1,F2,SUZ12,CPN2,SERPINF2,SERPINA7,FBLN1,SERPINA11,ANG,LIMS1,PAPLN,BLM,AKAP9,ITIH2,S100A9,GSN,CALML5,IGFBP3,PROS1,GAPDH,RAPGEF4,CST3,NUCB1,ADIPOQ,IGF2,C4A,MST1,ITIH3,C4B,APOA5,SERPINA3,SERPINA4,CCL18,VEGFC,KNG1,ACTB,GRHL2
GO Process	GO:0051917	Regulation of fibrinolysis	8	18	1.54	1.29e-07	CPB2,APOH,VTN,KLKB1,F2,PLG,SERPINF2,F11

GO Process	GO:0050789	Regulation of biological process	194	11655	0.12	1.32e-07	SPP2,ST6GAL1,CPB2,CETP,APOH,VCL,SERPIND1,LGALS1,CHGA,TPD52L2,LBP,MMP2,EBI3,PON1,TFPI2,PCOLCE,C5,MPO,VTN,PF4V1,APOC3,MGP,GPLD1,SPARC,APOB,IGFBP5,PROC,PLEK,APOA1,TTR,CAT,LRP1,C4BPB,CFP,DHX29,APOE,PXDN,SERPINF1,APCS,CFHR5,VWF,PSMA6,DSG2,SERPINA10,TIMP2,LGALS3BP,ATRN,C9,LCN1,LCAT,NID1,YWHAE,CSTA,KLKB1,CDH6,AMBP,CENPE,TCIRG1,HPX,HYAL1,ITIH4,LUM,CDH11,PSG9,FCN3,ITIH1,AHSG,ANKRD31,CRH,SERPING1,MYL9,CSF1R,FCN2,PGLYRP2,SCGB3A1,IHH,SERPINI1,PPBP,PF4,MASP1,CAMP,GUCY1A1,A2ML1,HSP90B1,ANTXR1,SH3D19,PCSK9,BMP1,SHLD1,FGB,DST,F2,INHBC,PLG,EFEMP2,CSPG4,STAB1,C1QB,CFHR1,SUZ12,SUMO4,SERPINF2,C7,HBA1,HSPA5,P4HB,SERPINA7,FBLN1,SERPINA11,ANG,FGG,LIMS1,THBS4,APOD,PAPLN,BLM,AKAP9,ITIH2,MTCL1,C8A,CENPF,CFHR4,CFHR3,CFH,QSOX1,CD5L,S100A7,S100A9,ADAMTSL4,FLNA,MCOLN2,SMARCA1,C8B,ADAMTS13,CCN5,GSN,MBL2,C1QC,F10,F7,APOM,CALML5,TAF9,IGFBP3,CD99,PROS1,EFEMP1,CFI,TNRC6A,YWHAZ,ILK,GAPDH,RAPGEF4,CST3,FBLN2,SAA1,F11,C1S,NUCB1,ADIPOQ,IGF2,C4A,SLAIN1,CDK3,ABCA13,COLEC11,MST1,LMOD3,SPARCL1,ITIH3,C4B,CFB,IGFALS,PLTP,PAEP,RARRES2,UIMC1,SELENOP,HPR,APOA5,ATP1A1,SERPINA3,SERPINA4,APOC4,IGHV3-15,CCL18,IGHV3-72,VEGFC,HYOU1,KNG1,ACTB,GRHL2,B2M,FGA
GO Process	GO:0065009	Regulation of molecular function	78	3085	0.3	1.58e-07	SPP2,APOH,SERPIND1,PON1,TFPI2,PCOLCE,C5,VTN,APOC3,GPLD1,PLEK,APOA1,CAT,LRP1,APOE,PXDN,SERPINF1,APCS,CFHR5,PSMA6,SERPINA10,TIMP2,LCN1,YWHAE,CSTA,AMBP,CENPE,ITIH4,ITIH1,AHSG,CRH,SERPING1,CSF1R,SERPINI1,A2ML1,HSP90B1,ANTXR1,PCSK9,F2,CFHR1,SUZ12,SUMO4,CPN2,SERPINF2,SERPINA7,FBLN1,SERPINA11,ANG,LIMS1,PAPLN,BLM,AKAP9,ITIH2,S100A9,FLNA,GSN,CALML5,IGFBP3,PROS1,GAPDH,RAPGEF4,CST3,NUCB1,ADIPOQ,IGF2,C4A,MST1,ITIH3,C4B,APOA5,SERPINA3,SERPINA4,CCL18,VEGFC,KNG1,ACTB,GRHL2,B2M
GO Process	GO:0009617	Response to bacterium	31	663	0.57	1.59e-07	CHGA,LBP,MPO,PF4V1,APOB,C4BPB,CFP,LYZ,FCN2,PGLYRP2,PPBP,PF4,CAMP,FGB,F2,STAB1,ANG,S100A7,S100A9,ADAMTS13,MBL2,RNASE1,ADIPOQ,C4B,CFB,RARRES2,RNASE4,IGHV3-15,IGHV3-72,B2M,FGA
GO Process	GO:0006508	Proteolysis	44	1247	0.44	2.00e-07	CPB2,APOH,F9,MMP2,PCOLCE,PROC,PSMA6,LCN1,KLKB1,FCN3,HABP2,FCN2,IHH,MASP1,APEH,HSP90B1,PCSK9,BMP1,FGB,F2,PLG,HSPA5,FGG,CNDP1,F8,CFH,F5,CD5L,ADAMTS13,MBL2,F10,F7,TAF9,CFI,F11,C1S,PCYOX1,COLEC11,MST1,CFB,HGFAC,UIMC1,HPR,FGA
GO Process	GO:0065008	Regulation of biological quality	87	3654	0.27	2.00e-07	CPB2,CETP,APOH,VCL,SERPIND1,CHGA,F9,MMP2,TFPI2,VTN,AFM,PF4V1,APOC3,GPLD1,APOB,IGFBP5,PROC,PLEK,APOA1,TTR,C4BPB,APOE,SERPINF1,LYZ,VWF,DSG2,SERPINA10,LCN1,LCAT,YWHAE,CP,KLKB1,TCIRG1,HPX,CRH,SERPING1,MYL9,CSF1R,IHH,PF4,GUCY1A1,HSP90B1,PCSK9,FGB,F2,PLG,CPN2,SERPINF2,SERPINA7,FBLN1,OIT3,ANG,FGG,AKAP9,F8,F13B,F5,S100A9,FLNA,ADAMTS13,GSN,F10,F7,APOM,TAF9,SOD3,PROS1,YWHAZ,NAV2,ILK,GAPDH,RAPGEF4,SAA1,F11,ADIPOQ,ATP9B,LMOD3,HGFAC,APOA5,ATP1A1,SERPINA3,APOC4,VEGFC,KNG1,ACTB,B2M,FGA
GO Process	GO:0034109	Homotypic cell-cell adhesion	11	60	1.16	2.16e-07	VCL,PLEK,DSG2,MYL9,FGB,FGG,FLNA,CD99,ILK,ACTB,FGA
GO Process	GO:0032102	Negative regulation of response to external stimulus	23	387	0.67	3.83e-07	ST6GAL1,CPB2,APOH,C5,VTN,PROC,APOA1,APOE,SERPINF1,APCS,KLKB1,SERPING1,FGB,F2,PLG,SERPINF2,FGG,PROS1,SAA1,F11,ADIPOQ,KNG1,FGA
GO Process	GO:0032374	Regulation of cholesterol transport	11	64	1.13	3.83e-07	CETP,PON1,APOC3,APOA1,LRP1,APOE,PCSK9,ADIPOQ,ABCA13,PLTP,APOA5
GO Process	GO:0007597	Blood coagulation, intrinsic pathway	7	13	1.63	4.87e-07	APOH,F9,KLKB1,F8,FLNA,F7,F11
GO Process	GO:0051050	Positive regulation of transport	36	915	0.49	4.95e-07	CPB2,CETP,PON1,VTN,GPLD1,APOA1,LRP1,CFP,APOE,YWHAE,FCN3,AHSG,CRH,FCN2,MASP1,PCSK9,FGB,F2,ANG,FGG,AKAP9,MTCL1,FLNA,MBL2,ADIPOQ,C4A,ABCA13,COLEC11,C4B,CFB,PLTP,APOA5,VEGFC,ACTB,B2M,FGA
GO Process	GO:0080134	Regulation of response to stress	45	1373	0.41	1.03e-06	CPB2,APOH,LGALS1,LBP,VTN,PROC,APOA1,APOE,SERPINF1,APCS,PSMA6,KLKB1,AMBP,HPX,FCN3,AHSG,SERPING1,FCN2,PGLYRP2,MASP1,SHLD1,FGB,F2,PLG,SERPINF2,HSPA5,P4HB,FGG,CFH,S100A9,FLNA,MBL2,F7,TAF9,PROS1,CFI,SAA1,F11,ADIPOQ,COLEC11,UIMC1,HYOU1,KNG1,ACTB,FGA
GO Process	GO:0010810	Regulation of cell-substrate adhesion	17	217	0.79	1.09e-06	ST6GAL1,VCL,VTN,APOA1,NID1,FGB,PLG,EFEMP2,P4HB,FBLN1,FGG,LIMS1,APOD,FLNA,ILK,FBLN2,FGA
GO Process	GO:0034375	High-density lipoprotein particle remodeling	7	16	1.54	1.42e-06	CETP,APOC3,APOA1,APOE,LCAT,APOM,PLTP
GO Process	GO:0050766	Positive regulation of phagocytosis	11	75	1.06	1.56e-06	APOA1,CFP,FCN3,AHSG,FCN2,MASP1,MBL2,C4A,COLEC11,C4B,CFB
GO Process	GO:0051873	Killing by host of symbiont cells	8	29	1.34	2.29e-06	CFHR5,PF4,CAMP,F2,CFHR1,APOL1,MBL2,GAPDH
GO Process	GO:0070527	Platelet aggregation	9	43	1.22	2.29e-06	VCL,PLEK,MYL9,FGB,FGG,FLNA,ILK,ACTB,FGA
GO Process	GO:1900024	Regulation of substrate adhesion-dependent cell spreading	10	61	1.11	2.79e-06	ST6GAL1,APOA1,FGB,P4HB,FBLN1,FGG,LIMS1,FLNA,ILK,FGA
GO Process	GO:0050764	Regulation of phagocytosis	12	104	0.96	3.67e-06	APOA1,CFP,FCN3,AHSG,FCN2,MASP1,MBL2,ADIPOQ,C4A,COLEC11,C4B,CFB
GO Process	GO:0010811	Positive regulation of cell-substrate adhesion	13	128	0.9	3.82e-06	VTN,APOA1,NID1,FGB,EFEMP2,P4HB,FBLN1,FGG,LIMS1,FLNA,ILK,FBLN2,FGA
GO Process	GO:0051702	Biological process involved in interaction with symbiont	12	108	0.94	5.29e-06	APOE,APCS,CFHR5,CSF1R,PF4,CAMP,F2,PLG,CFHR1,APOL1,MBL2,GAPDH
GO Process	GO:0031639	Plasminogen activation	6	11	1.63	5.73e-06	APOH,KLKB1,FGB,FGG,F11,FGA
GO Process	GO:0061844	Antimicrobial humoral immune response mediated by antimicrobial peptide	12	113	0.92	8.21e-06	PF4V1,PPBP,PF4,CAMP,F2,ANG,S100A7,S100A9,GAPDH,CCL18,KNG1,B2M
GO Process	GO:1905952	Regulation of lipid localization	14	167	0.82	9.94e-06	CETP,PON1,APOC3,APOB,APOA1,LRP1,APOE,CRH,PCSK9,ADIPOQ,ABCA13,PLTP,APOA5,APOC4
GO Process	GO:0051918	Negative regulation of fibrinolysis	6	13	1.56	1.20e-05	CPB2,APOH,VTN,F2,PLG,SERPINF2
GO Process	GO:0032376	Positive regulation of cholesterol transport	8	38	1.22	1.29e-05	CETP,PON1,APOA1,LRP1,APOE,ADIPOQ,ABCA13,PLTP
GO Process	GO:0030155	Regulation of cell adhesion	30	784	0.48	1.75e-05	ST6GAL1,VCL,LGALS1,MMP2,EBI3,VTN,APOA1,NID1,HYAL1,IHH,SERPINI1,FGB,PLG,EFEMP2,SERPINF2,P4HB,FBLN1,FGG,LIMS1,APOD,FLNA,ILK,FBLN2,SAA1,ADIPOQ,IGF2,KNG1,ACTB,B2M,FGA
GO Process	GO:0032103	Positive regulation of response to external stimulus	22	453	0.58	2.09e-05	CPB2,APOH,LGALS1,LBP,VTN,HPX,FCN3,CSF1R,FCN2,MASP1,F2,PLG,SERPINF2,THBS4,S100A7,S100A9,FLNA,MBL2,F7,COLEC11,RARRES2,VEGFC
GO Process	GO:0032501	Multicellular organismal process	122	6490	0.17	2.46e-05	RCN1,SPP2,CPB2,CETP,APOH,VCL,SERPIND1,LGALS1,CHGA,F9,MMP2,TFPI2,C5,MPO,VTN,PF4V1,APOC3,MGP,GPLD1,PCDH12,APOB,IGFBP5,PROC,PLEK,APOA1,CAT,LRP1,C4BPB,APOE,PXDN,ANGPTL6,SERPINF1,LYZ,VWF,DSG2,SERPINA10,ATRN,LCN1,LCAT,NID1,YWHAE,KLKB1,DNAH5,AMBP,TCIRG1,HYAL1,LUM,CDH11,PSG9,AHSG,CRH,SERPING1,MYL9,CSF1R,PCDH1,IHH,SERPINI1,PF4,GUCY1A1,ANTXR1,PCSK9,BMP1,SHLD1,FGB,F2,PLG,EFEMP2,CSPG4,SUZ12,PSG11,SERPINF2,HSPA5,FBLN1,ANG,FGG,THBS4,APOD,AKAP9,F8,PCDH18,CENPF,F13B,F5,S100A7,S100A9,FLNA,CRTAC1,SMARCA1,ADAMTS13,GSN,F10,F7,APOM,IGFBP3,SOD3,PROS1,EFEMP1,YWHAZ,NAV2,ILK,SAA1,F11,ADIPOQ,IGF2,FSIP2,MST1,LMOD3,PLTP,RARRES2,HGFAC,SELENOP,APOA5,ATP1A1,SERPINA3,VEGFC,KNG1,TPM4,ACTB,GRHL2,B2M,FGA,SDF4
GO Process	GO:0002697	Regulation of immune effector process	20	383	0.61	2.58e-05	LBP,APOA1,C4BPB,CFP,HPX,FCN3,SERPING1,FCN2,PGLYRP2,MASP1,SHLD1,CFH,CD5L,MBL2,CFI,C4A,COLEC11,C4B,CFB,B2M
GO Process	GO:0098609	Cell-cell adhesion	24	542	0.54	2.77e-05	VCL,LGALS1,PCDH12,PLEK,DSG2,CSTA,CDH6,CDH11,MYL9,PCDH1,IHH,FGB,PSG11,FGG,LIMS1,THBS4,PCDH18,S100A9,FLNA,CD99,ILK,SPARCL1,ACTB,FGA
GO Process	GO:0030194	Positive regulation of blood coagulation	7	28	1.29	2.88e-05	CPB2,APOH,VTN,F2,PLG,SERPINF2,F7

GO Process	GO:0050830	Defense response to Gram-positive bacterium	12	130	0.86	3.03e-05	CHGA,LBP,LYZ,FCN2,PGLYRP2,CAMP,ANG,MBL2,RNASE1,RARRES2,RNASE4,B2M
GO Process	GO:0060627	Regulation of vesicle-mediated transport	24	551	0.54	3.58e-05	VTN,APOC3,APOA1,LRP1,CFP,APOE,FCN3,AHSG,FCN2,MASP1,PCSK9,FGB,FGG,MBL2,RAPGEF4,ADIPOQ,C4A,ABCA13,COLEC11,C4B,CFB,APOA5,B2M,FGA
GO Process	GO:1900026	Positive regulation of substrate adhesion-dependent cell spreading	8	45	1.15	3.72e-05	APOA1,FGB,P4HB,FGG,LIMS1,FLNA,ILK,FGA
GO Process	GO:0009892	Negative regulation of metabolic process	69	2982	0.26	4.77e-05	SPP2,CPB2,SERPIND1,LBP,TFPI2,C5,VTN,APOC3,GPLD1,IGFBP5,PLEK,APOA1,LRP1,APOE,SERPINF1,APCS,SERPINA10,TIMP2,LCN1,YWHAE,CSTA,AMBP,ITIH4,PSG9,FCN3,ITIH1,AHSG,SERPING1,PGLYRP2,SERPINI1,PF4,A2ML1,PCSK9,SHLD1,F2,SUZ12,SUMO4,SERPINF2,SERPINA7,FBLN1,SERPINA11,ANG,LIMS1,APOD,PAPLN,BLM,ITIH2,CENPF,QSOX1,FLNA,TAF9,IGFBP3,PROS1,TNRC6A,YWHAZ,GAPDH,CST3,ADIPOQ,IGF2,C4A,MST1,ITIH3,C4B,UIMC1,ATP1A1,SERPINA3,SERPINA4,KNG1,FGA
GO Process	GO:1905954	Positive regulation of lipid localization	11	111	0.89	4.86e-05	CETP,PON1,APOB,APOA1,LRP1,APOE,CRH,ADIPOQ,ABCA13,PLTP,APOC4
GO Process	GO:0043691	Reverse cholesterol transport	6	18	1.42	4.94e-05	CETP,APOC3,APOA1,APOE,LCAT,APOM
GO Process	GO:0006869	Lipid transport	18	329	0.63	5.18e-05	CETP,APOH,LBP,APOC3,APOB,APOA1,APOE,LCAT,APOL1,APOD,CFHR4,APOM,APOF,ATP9B,ABCA13,PLTP,APOA5,APOC4
GO Process	GO:0032368	Regulation of lipid transport	12	139	0.83	5.55e-05	CETP,PON1,APOC3,APOA1,LRP1,APOE,CRH,PCSK9,ADIPOQ,ABCA13,PLTP,APOA5
GO Process	GO:0045785	Positive regulation of cell adhesion	22	485	0.55	5.63e-05	LGALS1,EBI3,VTN,APOA1,NID1,HYAL1,IHH,FGB,EFEMP2,SERPINF2,P4HB,FBLN1,FGG,LIMS1,FLNA,ILK,FBLN2,SAA1,IGF2,ACTB,B2M,FGA
GO Process	GO:0002752	Cell surface pattern recognition receptor signaling pathway	5	9	1.64	6.39e-05	FCN3,FCN2,MASP1,MBL2,COLEC11
GO Process	GO:0034372	Very-low-density lipoprotein particle remodeling	5	9	1.64	6.39e-05	CETP,APOA1,APOE,LCAT,APOA5
GO Process	GO:0098869	Cellular oxidant detoxification	10	91	0.94	6.56e-05	MPO,CAT,APOE,PXDN,AMBP,HBA1,S100A9,APOM,SOD3,GPX3
GO Process	GO:0008203	Cholesterol metabolic process	11	119	0.86	8.67e-05	CETP,PON1,APOB,APOA1,CAT,APOE,LCAT,PCSK9,APOL1,APOF,APOA5
GO Process	GO:0001867	Complement activation, lectin pathway	5	10	1.6	9.21e-05	FCN3,FCN2,MASP1,MBL2,COLEC11
GO Process	GO:0030198	Extracellular matrix organization	16	278	0.66	0.00012	MMP2,VTN,PXDN,NID1,LUM,IHH,BMP1,PLG,EFEMP2,SERPINF2,FBLN1,PAPLN,QSOX1,ADAMTSL4,ADAMTS13,FBLN2
GO Process	GO:0030449	Regulation of complement activation	6	22	1.33	0.00012	C4BPB,SERPING1,MASP1,CFH,CD5L,CFI
GO Process	GO:0010874	Regulation of cholesterol efflux	7	37	1.17	0.00013	CETP,PON1,APOA1,LRP1,APOE,ADIPOQ,PLTP
GO Process	GO:0019538	Protein metabolic process	82	3910	0.22	0.00013	ST6GAL1,CPB2,GNPTG,APOH,F9,MMP2,PCOLCE,APOC3,GPLD1,APOB,PROC,APOA1,CAT,DHX29,APOE,PSMA6,LCN1,LCAT,CSTA,KLKB1,AMBP,TCIRG1,HPX,HYAL1,FCN3,HABP2,CSF1R,FCN2,IHH,MASP1,APEH,HSP90B1,PCSK9,BMP1,FGB,F2,PLG,SUZ12,APOL1,SUMO4,HSPA5,P4HB,FBLN1,FGG,GANAB,QPCT,CNDP1,F8,CDC42BPA,F13B,CFH,F5,CD5L,S100A9,FLNA,ADAMTS13,GSN,MBL2,F10,F7,APOM,TAF9,IGFBP3,EFEMP1,CFI,YWHAZ,ILK,GAPDH,F11,C1S,PCYOX1,CDK3,COLEC11,MST1,CFB,HGFAC,UIMC1,HPR,APOA5,ACTB,B2M,FGA
GO Process	GO:1901700	Response to oxygen-containing compound	43	1547	0.34	0.00014	ST6GAL1,CPB2,LBP,MMP2,PON1,MPO,PF4V1,GPLD1,APOB,IGFBP5,CAT,LRP1,APOE,SERPINF1,AMBP,HYAL1,IHH,PPBP,PF4,CAMP,HSP90B1,PCSK9,HBA1,HSPA5,APOD,BLM,AKAP9,F5,S100A7,S100A9,FLNA,ADAMTS13,F7,TAF9,SOD3,GPX3,ADIPOQ,IGF2,RARRES2,ATP1A1,ACTB,B2M,SDF4
GO Process	GO:0019835	Cytolysis	6	23	1.31	0.00015	C5,LYZ,C9,C7,C8A,C8B
GO Process	GO:0034377	Plasma lipoprotein particle assembly	6	23	1.31	0.00015	APOC3,APOB,APOA1,APOE,APOM,APOA5
GO Process	GO:0034381	Plasma lipoprotein particle clearance	6	24	1.29	0.00017	APOC3,APOB,APOA1,APOE,APOM,ADIPOQ
GO Process	GO:0051838	Cytolysis by host of symbiont cells	5	12	1.52	0.00017	CFHR5,CAMP,F2,CFHR1,APOL1
GO Process	GO:0050793	Regulation of developmental process	59	2492	0.27	0.00018	ST6GAL1,CETP,APOH,VCL,C5,MGP,SPARC,APOB,IGFBP5,PROC,APOA1,APOE,SERPINF1,APCS,ATRN,NID1,TCIRG1,HYAL1,PSG9,AHSG,CSF1R,PGLYRP2,SCGB3A1,IHH,PF4,CAMP,SH3D19,BMP1,SHLD1,FGB,F2,EFEMP2,STAB1,SUZ12,SERPINF2,P4HB,FBLN1,FGG,LIMS1,THBS4,CENPF,FLNA,C1QC,IGFBP3,EFEMP1,YWHAZ,ILK,CST3,ADIPOQ,IGF2,MST1,LMOD3,PAEP,RARRES2,VEGFC,ACTB,GRHL2,B2M,FGA
GO Process	GO:0015918	Sterol transport	9	82	0.94	0.00021	CETP,APOC3,APOB,APOA1,APOE,LCAT,APOM,PLTP,APOA5
GO Process	GO:0010605	Negative regulation of macromolecule metabolic process	63	2760	0.25	0.00024	SPP2,CPB2,SERPIND1,LBP,TFPI2,C5,VTN,IGFBP5,APOA1,LRP1,APOE,SERPINF1,APCS,SERPINA10,TIMP2,LCN1,YWHAE,CSTA,AMBP,ITIH4,PSG9,FCN3,ITIH1,AHSG,SERPING1,PGLYRP2,SERPINI1,PF4,A2ML1,PCSK9,SHLD1,F2,SUZ12,SUMO4,SERPINF2,SERPINA7,FBLN1,SERPINA11,ANG,LIMS1,APOD,PAPLN,BLM,ITIH2,CENPF,FLNA,TAF9,IGFBP3,PROS1,TNRC6A,YWHAZ,GAPDH,CST3,ADIPOQ,IGF2,C4A,ITIH3,C4B,UIMC1,SERPINA3,SERPINA4,KNG1,FGA
GO Process	GO:0010875	Positive regulation of cholesterol efflux	6	26	1.26	0.00025	PON1,APOA1,LRP1,APOE,ADIPOQ,PLTP
GO Process	GO:0048870	Cell motility	33	1061	0.39	0.00026	CHGA,LBP,MMP2,C5,VTN,PF4V1,GPLD1,APOB,APOA1,ATRN,YWHAE,DNAH5,PPBP,PF4,DST,PLG,CSPG4,DNAH8,ANG,THBS4,CDC42BPA,S100A9,FLNA,MCOLN2,CD99,ILK,SAA1,FSIP2,MST1,PLTP,CCL18,VEGFC,ACTB
GO Process	GO:0051172	Negative regulation of nitrogen compound metabolic process	57	2403	0.27	0.00026	SPP2,CPB2,SERPIND1,TFPI2,C5,VTN,IGFBP5,LRP1,APOE,SERPINF1,APCS,SERPINA10,TIMP2,LCN1,YWHAE,CSTA,AMBP,ITIH4,FCN3,ITIH1,AHSG,SERPING1,SERPINI1,A2ML1,SHLD1,F2,SUZ12,SUMO4,SERPINF2,SERPINA7,FBLN1,SERPINA11,ANG,LIMS1,APOD,PAPLN,BLM,ITIH2,CENPF,FLNA,TAF9,IGFBP3,PROS1,TNRC6A,YWHAZ,GAPDH,CST3,ADIPOQ,IGF2,C4A,ITIH3,C4B,UIMC1,SERPINA3,SERPINA4,KNG1,FGA
GO Process	GO:0072377	Blood coagulation, common pathway	4	5	1.8	0.00028	F2,F5,F10,FGA
GO Process	GO:0033344	Cholesterol efflux	6	27	1.24	0.00029	APOC3,APOB,APOA1,APOE,APOM,APOA5
GO Process	GO:0048522	Positive regulation of cellular process	105	5584	0.17	0.00029	ST6GAL1,CPB2,LGALS1,CHGA,LBP,MMP2,EBI3,VTN,GPLD1,SPARC,APOB,IGFBP5,PROC,PLEK,APOA1,CAT,LRP1,CFP,DHX29,APOE,SERPINF1,VWF,NID1,YWHAE,CENPE,TCIRG1,HPX,HYAL1,LUM,FCN3,ANKRD31,CRH,CSF1R,FCN2,SCGB3A1,IHH,SERPINI1,PPBP,PF4,MASP1,CAMP,GUCY1A1,SH3D19,PCSK9,SHLD1,FGB,F2,INHBC,PLG,EFEMP2,CSPG4,SUZ12,SERPINF2,HBA1,HSPA5,P4HB,FBLN1,ANG,FGG,LIMS1,THBS4,BLM,AKAP9,MTCL1,CD5L,S100A7,S100A9,ADAMTSL4,FLNA,SMARCA1,GSN,MBL2,F10,F7,TAF9,IGFBP3,CD99,TNRC6A,ILK,GAPDH,FBLN2,SAA1,ADIPOQ,IGF2,C4A,SLAIN1,ABCA13,COLEC11,MST1,LMOD3,C4B,CFB,RARRES2,UIMC1,HPR,APOA5,IGHV3-15,CCL18,IGHV3-72,VEGFC,KNG1,ACTB,GRHL2,B2M,FGA
GO Process	GO:0030301	Cholesterol transport	8	65	0.99	0.00034	CETP,APOC3,APOB,APOA1,APOE,LCAT,APOM,APOA5
GO Process	GO:0032370	Positive regulation of lipid transport	9	89	0.9	0.00037	CETP,PON1,APOA1,LRP1,APOE,CRH,ADIPOQ,ABCA13,PLTP
GO Process	GO:0042632	Cholesterol homeostasis	9	89	0.9	0.00037	CETP,APOC3,APOB,APOA1,APOE,LCAT,PCSK9,APOM,APOA5
GO Process	GO:0002920	Regulation of humoral immune response	7	46	1.08	0.00039	C4BPB,HPX,SERPING1,MASP1,CFH,CD5L,CFI
GO Process	GO:0006641	Triglyceride metabolic process	8	68	0.97	0.00045	CETP,APOH,APOC3,APOB,CAT,APOE,PCSK9,APOA5
GO Process	GO:0006639	Acylglycerol metabolic process	9	95	0.87	0.00059	CETP,APOH,APOC3,APOB,CAT,APOE,PCSK9,ANG,APOA5
GO Process	GO:0042157	Lipoprotein metabolic process	10	123	0.81	0.00063	APOC3,GPLD1,APOB,APOA1,APOE,LCAT,PCSK9,APOL1,APOM,APOA5
GO Process	GO:1903036	Positive regulation of response to wounding	8	72	0.94	0.00065	CPB2,APOH,VTN,F2,PLG,SERPINF2,FLNA,F7
GO Process	GO:0050829	Defense response to Gram-negative bacterium	9	97	0.86	0.00067	CHGA,LBP,LYZ,FCN2,CAMP,F2,S100A7,RARRES2,B2M
GO Process	GO:0006979	Response to oxidative stress	17	368	0.56	0.00070	MMP2,MPO,CAT,APOE,PXDN,ATRN,AMBP,HYAL1,SUMO4,HBA1,APOD,S100A7,MBL2,SOD3,GPX3,ADIPOQ,SELENOP
GO Process	GO:0048662	Negative regulation of smooth muscle cell proliferation	7	53	1.02	0.00086	IGFBP5,APOE,EFEMP2,ANG,APOD,IGFBP3,ADIPOQ

GO Process	GO:0030334	Regulation of cell migration	29	927	0.39	0.00089	APOH,VCL,LBP,MMP2,C5,VTN,GPLD1,SPARC,IGFBP5,LRP1,APOE,SERPINF1,HYAL1,CSF1R,PLG,HSPA5,FBLN1,THBS4,APOD,S100A7,FLNA,F10,F7,IGFBP3,CD99,ADIPOQ,MST1,RARRES2,VEGFC
GO Process	GO:0048771	Tissue remodeling	9	101	0.85	0.00089	SPP2,MMP2,IGFBP5,TCIRG1,IHH,PLG,CSPG4,THBS4,FLNA
GO Process	GO:0001906	Cell killing	10	129	0.79	0.00090	CHGA,LYZ,C9,PPBP,PF4,F2,GAPDH,CCL18,KNG1,B2M
GO Process	GO:0031347	Regulation of defense response	23	638	0.45	0.00091	LGALS1,LBP,PROC,APOA1,APOE,SERPINF1,APCS,PSMA6,KLKB1,HPX,FCN3,AHSG,SERPING1,FCN2,PGLYRP2,MASP1,CFH,S100A9,MBL2,CFI,SAA1,ADIPOQ,COLEC11
GO Process	GO:0008228	Opsonization	4	9	1.54	0.0013	LBP,FCN2,MBL2,C4B
GO Process	GO:0034097	Response to cytokine	26	804	0.41	0.0015	MMP2,EBI3,PF4V1,APOB,TIMP2,TCIRG1,HPX,HYAL1,ITIH4,CSF1R,PPBP,PF4,CAMP,FGB,HSPA5,P4HB,FGG,LIMS1,ADAMTS13,GSN,TAF9,ILK,GAPDH,ADIPOQ,CCL18,VEGFC
GO Process	GO:0070328	Triglyceride homeostasis	6	38	1.09	0.0015	CETP,APOC3,APOA1,APOE,APOA5,APOC4
GO Process	GO:0048585	Negative regulation of response to stimulus	41	1612	0.3	0.0016	ST6GAL1,CPB2,APOH,C5,VTN,IGFBP5,PROC,PLEK,APOA1,LRP1,C4BPB,APOE,PXDN,SERPINF1,APCS,KLKB1,AMBP,AHSG,SERPING1,PGLYRP2,PF4,MASP1,SHLD1,FGB,F2,PLG,SERPINF2,HSPA5,FBLN1,FGG,APOD,TAF9,IGFBP3,PROS1,SAA1,F11,ADIPOQ,CDK3,HYOU1,KNG1,FGA
GO Process	GO:0051240	Positive regulation of multicellular organismal process	39	1505	0.31	0.0017	CPB2,APOH,CHGA,LBP,EBI3,C5,VTN,GPLD1,LRP1,APOE,SERPINF1,HYAL1,LUM,CRH,CSF1R,IHH,PF4,CAMP,BMP1,SHLD1,FGB,F2,PLG,SERPINF2,FGG,MCOLN2,F7,GAPDH,SAA1,ADIPOQ,IGF2,MST1,PAEP,APOA5,ATP1A1,VEGFC,ACTB,B2M,FGA
GO Process	GO:0030212	Hyaluronan metabolic process	5	23	1.23	0.0019	HYAL1,ITIH4,ITIH1,ITIH2,ITIH3
GO Process	GO:1901564	Organonitrogen compound metabolic process	93	4981	0.17	0.0019	ST6GAL1,CPB2,CETP,GNPTG,APOH,F9,MMP2,PON1,PCOLCE,APOC3,GPLD1,APOB,PROC,APOA1,TTR,CAT,DHX29,APOE,PXDN,PSMA6,LCN1,LCAT,CSTA,KLKB1,AMBP,TCIRG1,HPX,HYAL1,ITIH4,FCN3,ITIH1,HABP2,CSF1R,FCN2,PGLYRP2,IHH,MASP1,APEH,GUCY1A1,HSP90B1,PCSK9,BMP1,FGB,F2,PLG,SUZ12,APOL1,SUMO4,HSPA5,P4HB,FBLN1,FGG,GANAB,QPCT,ITIH2,CNDP1,F8,CDC42BPA,F13B,CFH,F5,CD5L,S100A9,FLNA,ADAMTS13,GSN,MBL2,F10,F7,APOM,TAF9,IGFBP3,EFEMP1,CFI,YWHAZ,ILK,GAPDH,F11,C1S,PCYOX1,ACSF2,CDK3,COLEC11,MST1,ITIH3,CFB,HGFAC,UIMC1,HPR,APOA5,ACTB,B2M,FGA
GO Process	GO:0008015	Blood circulation	17	403	0.52	0.0020	CHGA,MMP2,APOE,SERPING1,GUCY1A1,FGB,SERPINF2,FGG,F5,GSN,SOD3,NAV2,ADIPOQ,ATP1A1,VEGFC,KNG1,FGA
GO Process	GO:0032879	Regulation of localization	49	2103	0.26	0.0020	CPB2,CETP,VCL,CHGA,PON1,VTN,APOC3,GPLD1,APOB,APOA1,LRP1,CFP,APOE,YWHAE,TCIRG1,FCN3,AHSG,CRH,FCN2,MASP1,PCSK9,FGB,F2,SUMO4,ANG,FGG,APOD,AKAP9,MTCL1,FLNA,MCOLN2,GSN,MBL2,RAPGEF4,SAA1,ADIPOQ,C4A,ABCA13,COLEC11,C4B,CFB,PLTP,APOA5,ATP1A1,APOC4,VEGFC,ACTB,B2M,FGA
GO Process	GO:0050832	Defense response to fungus	7	62	0.95	0.0020	CHGA,MPO,CAMP,ANG,S100A9,GAPDH,RARRES2
GO Process	GO:0034114	Regulation of heterotypic cell-cell adhesion	5	24	1.22	0.0022	APOA1,FGB,FGG,ADIPOQ,FGA
GO Process	GO:0040012	Regulation of locomotion	30	1032	0.36	0.0022	ST6GAL1,APOH,VCL,LBP,MMP2,C5,VTN,GPLD1,SPARC,IGFBP5,LRP1,APOE,SERPINF1,HYAL1,CSF1R,PLG,HSPA5,FBLN1,THBS4,APOD,S100A7,FLNA,F10,F7,IGFBP3,CD99,ADIPOQ,MST1,RARRES2,VEGFC
GO Process	GO:0051049	Regulation of transport	43	1763	0.28	0.0024	CPB2,CETP,CHGA,PON1,VTN,APOC3,GPLD1,APOA1,LRP1,CFP,APOE,YWHAE,TCIRG1,FCN3,AHSG,CRH,FCN2,MASP1,PCSK9,FGB,F2,ANG,FGG,APOD,AKAP9,MTCL1,FLNA,MBL2,RAPGEF4,SAA1,ADIPOQ,C4A,ABCA13,COLEC11,C4B,CFB,PLTP,APOA5,ATP1A1,VEGFC,ACTB,B2M,FGA
GO Process	GO:0042744	Hydrogen peroxide catabolic process	5	25	1.2	0.0026	MPO,CAT,PXDN,HBA1,GPX3
GO Process	GO:0001568	Blood vessel development	19	505	0.47	0.0028	MMP2,GPLD1,APOB,LRP1,APOE,PXDN,ANGPTL6,IHH,ANTXR1,PLG,EFEMP2,CSPG4,SERPINF2,ANG,APOD,S100A7,FLNA,YWHAZ,VEGFC
GO Process	GO:0010033	Response to organic substance	58	2692	0.23	0.0028	ST6GAL1,CPB2,LBP,MMP2,EBI3,PON1,MPO,PF4V1,GPLD1,APOB,IGFBP5,CAT,LRP1,SERPINF1,TIMP2,LCAT,TCIRG1,HPX,HYAL1,ITIH4,PSG9,CSF1R,IHH,PPBP,PF4,CAMP,HSP90B1,PCSK9,FGB,HSPA5,P4HB,ANG,FGG,LIMS1,THBS4,BLM,AKAP9,F5,S100A7,S100A9,FLNA,ADAMTS13,GSN,F7,TAF9,GPX3,ILK,GAPDH,ADIPOQ,IGF2,C4B,RARRES2,ATP1A1,CCL18,VEGFC,ACTB,B2M,SDF4
GO Process	GO:0001775	Cell activation	23	693	0.42	0.0029	VCL,LGALS1,CHGA,LBP,EBI3,PF4V1,PLEK,LRP1,VWF,TCIRG1,MYL9,PF4,CAMP,FGB,F2,FGG,FLNA,ADAMTS13,ILK,SAA1,ACTB,B2M,FGA
GO Process	GO:0033700	Phospholipid efflux	4	12	1.42	0.0029	APOC3,APOA1,APOE,APOA5
GO Process	GO:0034380	High-density lipoprotein particle assembly	4	12	1.42	0.0029	APOA1,APOE,APOM,APOA5
GO Process	GO:0044403	Biological process involved in symbiotic interaction	13	257	0.6	0.0029	APOE,APCS,CFHR5,CSF1R,PGLYRP2,PF4,CAMP,F2,PLG,CFHR1,APOL1,MBL2,GAPDH
GO Process	GO:0048514	Blood vessel morphogenesis	17	419	0.5	0.0029	MMP2,GPLD1,APOB,LRP1,APOE,PXDN,ANGPTL6,IHH,EFEMP2,CSPG4,SERPINF2,ANG,APOD,S100A7,FLNA,YWHAZ,VEGFC
GO Process	GO:1990266	Neutrophil migration	8	92	0.84	0.0029	LBP,PF4V1,PPBP,PF4,S100A9,MCOLN2,SAA1,CCL18
GO Process	GO:0008202	Steroid metabolic process	13	258	0.6	0.0030	CETP,PON1,APOB,APOA1,CAT,APOE,LCAT,CRH,PCSK9,APOL1,APOF,GC,APOA5
GO Process	GO:0002699	Positive regulation of immune effector process	13	264	0.59	0.0037	LBP,CFP,HPX,FCN3,FCN2,MASP1,SHLD1,MBL2,C4A,COLEC11,C4B,CFB,B2M
GO Process	GO:0009636	Response to toxic substance	12	229	0.62	0.0041	PON1,MPO,CAT,APOE,PXDN,AMBP,HBA1,S100A9,ADAMTS13,APOM,SOD3,GPX3
GO Process	GO:0031640	Killing of cells of another organism	7	71	0.89	0.0041	CHGA,LYZ,PPBP,PF4,GAPDH,CCL18,KNG1
GO Process	GO:0070887	Cellular response to chemical stimulus	56	2609	0.23	0.0042	CPB2,CHGA,LBP,MMP2,EBI3,C5,MPO,PF4V1,GPLD1,APOB,IGFBP5,CAT,LRP1,APOE,PXDN,SERPINF1,AMBP,TCIRG1,HPX,HYAL1,PSG9,CSF1R,PPBP,PF4,CAMP,HSP90B1,PCSK9,FGB,SUMO4,HBA1,HSPA5,P4HB,FGG,LIMS1,BLM,AKAP9,S100A9,FLNA,ADAMTS13,GSN,APOM,SOD3,GPX3,ILK,GAPDH,SAA1,ADIPOQ,IGF2,MST1,RARRES2,ATP1A1,CCL18,VEGFC,HYOU1,ACTB,B2M
GO Process	GO:0002688	Regulation of leukocyte chemotaxis	9	128	0.74	0.0043	LBP,C5,CSF1R,THBS4,S100A7,F7,MST1,RARRES2,VEGFC
GO Process	GO:0031589	Cell-substrate adhesion	11	195	0.65	0.0044	VCL,VTN,VWF,ATRN,NID1,ANTXR1,FGB,FGG,ADAMTS13,ILK,FGA
GO Process	GO:0055088	Lipid homeostasis	10	161	0.69	0.0045	CETP,APOC3,APOB,APOA1,APOE,LCAT,PCSK9,APOM,APOA5,APOC4
GO Process	GO:0010896	Regulation of triglyceride catabolic process	4	14	1.35	0.0046	APOC3,GPLD1,APOA1,APOA5
GO Process	GO:0022604	Regulation of cell morphogenesis	14	311	0.55	0.0047	ST6GAL1,SPARC,APOA1,CSF1R,SH3D19,FGB,F2,P4HB,FBLN1,FGG,LIMS1,FLNA,ILK,FGA
GO Process	GO:0051919	Positive regulation of fibrinolysis	3	4	1.77	0.0047	KLKB1,PLG,F11
GO Process	GO:0051258	Protein polymerization	8	101	0.8	0.0049	VTN,FGB,ANG,FGG,AKAP9,GSN,SLAIN1,FGA
GO Process	GO:0010035	Response to inorganic substance	19	532	0.45	0.0050	MMP2,PON1,MPO,GPLD1,APOB,CAT,SERPINF1,LCAT,TCIRG1,FGB,HBA1,HSPA5,FGG,BLM,ADAMTS13,SOD3,SELENOP,B2M,FGA
GO Process	GO:0046486	Glycerolipid metabolic process	15	357	0.52	0.0053	CETP,APOH,PON1,APOC3,GPLD1,APOB,PLEK,APOA1,CAT,APOE,LCAT,CSF1R,PCSK9,ANG,APOA5
GO Process	GO:0003013	Circulatory system process	18	493	0.46	0.0059	CHGA,MMP2,LRP1,APOE,SERPING1,GUCY1A1,FGB,SERPINF2,FGG,F5,GSN,SOD3,NAV2,ADIPOQ,ATP1A1,VEGFC,KNG1,FGA
GO Process	GO:0000302	Response to reactive oxygen species	10	169	0.67	0.0062	MMP2,MPO,CAT,APOE,AMBP,HYAL1,HBA1,APOD,S100A7,SOD3
GO Process	GO:0034374	Low-density lipoprotein particle remodeling	4	16	1.29	0.0068	CETP,MPO,APOB,APOE
GO Process	GO:0097529	Myeloid leukocyte migration	9	138	0.71	0.0069	CHGA,LBP,PF4V1,PPBP,PF4,S100A9,MCOLN2,SAA1,CCL18
GO Process	GO:0001503	Ossification	13	287	0.55	0.0075	MMP2,MGP,GPLD1,IGFBP5,CAT,TCIRG1,CDH11,AHSG,IHH,BMP1,IGFBP3,IGF2,TPM4
GO Process	GO:0030593	Neutrophil chemotaxis	7	80	0.84	0.0075	LBP,PF4V1,PPBP,PF4,S100A9,SAA1,CCL18
GO Process	GO:0097746	Blood vessel diameter maintenance	9	140	0.7	0.0075	MMP2,APOE,GUCY1A1,FGB,SERPINF2,FGG,SOD3,KNG1,FGA
GO Process	GO:0060284	Regulation of cell development	18	506	0.45	0.0077	VCL,PROC,APOA1,SERPINF1,FGB,F2,P4HB,FBLN1,FGG,LIMS1,FLNA,ILK,ADIPOQ,LMOD3,PAEP,VEGFC,B2M,FGA

GO Process	GO:0042221	Response to chemical	76	4010	0.17	0.0082	ST6GAL1,CPB2,SERPIND1,CHGA,LBP,MMP2,EBI3,PON1,C5,MPO,PF4V1,GPLD1,APOB,IGFBP5,APOA1,CAT,LRP1,APOE,PXDN,SERPINF1,TIMP2,LCAT,AMBP,TCIRG1,HPX,HYAL1,ITIH4,PSG9,CSF1R,IHH,PPBP,PF4,CAMP,HSP90B1,PCSK9,FGB,SUMO4,HBA1,HSPA5,P4HB,ANG,FGG,LIMS1,THBS4,APOD,BLM,AKAP9,CENPF,F5,S100A7,S100A9,FLNA,ADAMTS13,GSN,F7,APOM,TAF9,SOD3,GPX3,ILK,GAPDH,SAA1,ADIPOQ,IGF2,MST1,C4B,RARRES2,SELENOP,ATP1A1,CCL18,VEGFC,HYOU1,ACTB,B2M,FGA,SDF4
GO Process	GO:0048660	Regulation of smooth muscle cell proliferation	9	142	0.7	0.0082	MMP2,IGFBP5,APOE,EFEMP2,SERPINF2,ANG,APOD,IGFBP3,ADIPOQ
GO Process	GO:1903053	Regulation of extracellular matrix organization	6	56	0.93	0.0082	CPB2,LRP1,NID1,ANTXR1,EFEMP2,CST3
GO Process	GO:0045595	Regulation of cell differentiation	38	1582	0.28	0.0084	CETP,VCL,APOB,IGFBP5,PROC,APOA1,SERPINF1,APCS,NID1,TCIRG1,PSG9,PGLYRP2,IHH,PF4,FGB,F2,EFEMP2,SUZ12,SERPINF2,P4HB,FBLN1,FGG,LIMS1,FLNA,C1QC,IGFBP3,EFEMP1,ILK,ADIPOQ,IGF2,LMOD3,PAEP,RARRES2,VEGFC,ACTB,GRHL2,B2M,FGA
GO Process	GO:0051004	Regulation of lipoprotein lipase activity	4	18	1.24	0.0095	APOH,APOC3,APOA1,APOA5
GO Process	GO:0071345	Cellular response to cytokine stimulus	22	711	0.39	0.0095	MMP2,EBI3,PF4V1,APOB,TCIRG1,HPX,HYAL1,CSF1R,PPBP,PF4,CAMP,FGB,HSPA5,P4HB,FGG,LIMS1,ADAMTS13,GSN,ILK,GAPDH,CCL18,VEGFC
GO Process	GO:0010901	Regulation of very-low-density lipoprotein particle remodeling	3	6	1.6	0.0099	APOC3,APOA1,APOA5
GO Process	GO:0035239	Tube morphogenesis	21	669	0.39	0.0110	MMP2,GPLD1,APOB,LRP1,APOE,PXDN,ANGPTL6,CSF1R,IHH,EFEMP2,CSPG4,SERPINF2,ANG,APOD,S100A7,FLNA,YWHAZ,ILK,MST1,VEGFC,GRHL2
GO Process	GO:0043903	Regulation of biological process involved in symbiotic interaction	6	60	0.9	0.0111	LGALS1,APCS,FCN3,P4HB,FBLN1,GSN
GO Process	GO:0035295	Tube development	25	880	0.35	0.0118	MMP2,GPLD1,APOB,IGFBP5,CAT,LRP1,APOE,PXDN,ANGPTL6,CRH,CSF1R,IHH,EFEMP2,CSPG4,SERPINF2,ANG,APOD,S100A7,FLNA,YWHAZ,ILK,MST1,RARRES2,VEGFC,GRHL2
GO Process	GO:0051093	Negative regulation of developmental process	26	933	0.34	0.0118	CETP,APOH,SPARC,IGFBP5,APOE,SERPINF1,APCS,AHSG,PGLYRP2,IHH,PF4,F2,STAB1,SUZ12,FBLN1,THBS4,C1QC,EFEMP1,CST3,ADIPOQ,IGF2,PAEP,VEGFC,ACTB,GRHL2,B2M
GO Process	GO:0010720	Positive regulation of cell development	13	304	0.53	0.0121	PROC,APOA1,SERPINF1,FGB,P4HB,FGG,LIMS1,FLNA,ILK,ADIPOQ,LMOD3,VEGFC,FGA
GO Process	GO:0030335	Positive regulation of cell migration	18	529	0.43	0.0123	LBP,MMP2,VTN,GPLD1,SPARC,IGFBP5,HYAL1,CSF1R,PLG,HSPA5,FBLN1,THBS4,S100A7,F10,F7,CD99,RARRES2,VEGFC
GO Process	GO:0034384	High-density lipoprotein particle clearance	3	7	1.53	0.0135	APOA1,APOE,APOM
GO Process	GO:0031099	Regeneration	9	154	0.66	0.0136	CPB2,MMP2,VTN,SERPINA10,IHH,PLG,APOD,F7,APOA5
GO Process	GO:0090207	Regulation of triglyceride metabolic process	5	40	0.99	0.0148	APOC3,GPLD1,APOA1,APOE,APOA5
GO Process	GO:0002685	Regulation of leukocyte migration	11	231	0.57	0.0153	LBP,C5,CSF1R,THBS4,APOD,S100A7,F7,CD99,MST1,RARRES2,VEGFC
GO Process	GO:0016477	Cell migration	25	903	0.34	0.0168	CHGA,LBP,MMP2,C5,VTN,PF4V1,GPLD1,APOA1,ATRN,YWHAE,PPBP,PF4,PLG,CSPG4,ANG,THBS4,CDC42BPA,S100A9,FLNA,MCOLN2,CD99,ILK,SAA1,CCL18,VEGFC
GO Process	GO:0032489	Regulation of Cdc42 protein signal transduction	3	8	1.47	0.0180	APOC3,APOA1,APOE
GO Process	GO:0045088	Regulation of innate immune response	11	238	0.56	0.0193	LBP,APOE,HPX,FCN3,SERPING1,FCN2,MASP1,CFH,MBL2,CFI,COLEC11
GO Process	GO:0051235	Maintenance of location	9	166	0.63	0.0223	APOA1,APOE,HSP90B1,HSPA5,AKAP9,S100A7,S100A9,FLNA,GSN
GO Process	GO:0002690	Positive regulation of leukocyte chemotaxis	7	99	0.75	0.0225	LBP,CSF1R,THBS4,S100A7,F7,RARRES2,VEGFC
GO Process	GO:0015914	Phospholipid transport	7	99	0.75	0.0225	CETP,APOC3,APOA1,APOE,ATP9B,PLTP,APOA5
GO Process	GO:0045807	Positive regulation of endocytosis	7	99	0.75	0.0225	VTN,LRP1,APOE,PCSK9,ABCA13,APOA5,B2M
GO Process	GO:0050727	Regulation of inflammatory response	14	371	0.47	0.0225	LGALS1,LBP,PROC,APOA1,APOE,SERPINF1,APCS,PSMA6,KLKB1,AHSG,PGLYRP2,S100A9,SAA1,ADIPOQ
GO Process	GO:0010640	Regulation of platelet-derived growth factor receptor signaling pathway	4	24	1.12	0.0228	LRP1,APOD,F7,ADIPOQ
GO Process	GO:0090075	Relaxation of muscle	4	24	1.12	0.0228	CHGA,GUCY1A1,GSN,ATP1A1
GO Process	GO:0051006	Positive regulation of lipoprotein lipase activity	3	9	1.42	0.0229	APOH,APOA1,APOA5
GO Process	GO:0046596	Regulation of viral entry into host cell	5	45	0.94	0.0230	LGALS1,APCS,FCN3,P4HB,GSN
GO Process	GO:0046470	Phosphatidylcholine metabolic process	6	71	0.82	0.0237	CETP,PON1,GPLD1,APOA1,LCAT,APOA5
GO Process	GO:0007565	Female pregnancy	9	170	0.62	0.0253	MMP2,IGFBP5,AMBP,PSG9,CRH,IHH,PSG11,FBLN1,MST1
GO Process	GO:0051043	Regulation of membrane protein ectodomain proteolysis	4	25	1.1	0.0256	GPLD1,APOE,TIMP2,SH3D19
GO Process	GO:0050900	Leukocyte migration	11	249	0.54	0.0263	CHGA,LBP,PF4V1,PPBP,PF4,PLG,S100A9,MCOLN2,CD99,SAA1,CCL18
GO Process	GO:0007160	Cell-matrix adhesion	8	136	0.67	0.0267	VCL,VTN,NID1,FGB,FGG,ADAMTS13,ILK,FGA
GO Process	GO:0097435	Supramolecular fiber organization	18	570	0.4	0.0267	PXDN,LUM,MYL9,HSP90B1,BMP1,DST,EFEMP2,SERPINF2,ANG,AKAP9,FLNA,GSN,KIF19,CST3,SLAIN1,LMOD3,TPM4,B2M
GO Process	GO:0006910	Phagocytosis, recognition	4	26	1.08	0.0284	FCN3,FCN2,IGHV3-15,IGHV3-72
GO Process	GO:0014912	Negative regulation of smooth muscle cell migration	4	26	1.08	0.0284	IGFBP5,LRP1,IGFBP3,ADIPOQ
GO Process	GO:0090209	Negative regulation of triglyceride metabolic process	3	10	1.37	0.0284	APOC3,GPLD1,APOE
GO Process	GO:1902533	Positive regulation of intracellular signal transduction	26	997	0.31	0.0284	LGALS1,IGFBP5,APOA1,CAT,APOE,VWF,CSF1R,GUCY1A1,FGB,F2,CSPG4,SERPINF2,FGG,LIMS1,S100A7,S100A9,FLNA,F10,F7,IGFBP3,ILK,GAPDH,ADIPOQ,IGF2,CCL18,FGA
GO Process	GO:0045216	Cell-cell junction organization	9	174	0.61	0.0286	VCL,DSG2,CDH6,CDH11,CSF1R,LIMS1,FLNA,ACTB,GRHL2
GO Process	GO:1901701	Cellular response to oxygen-containing compound	27	1057	0.3	0.0295	CPB2,LBP,MMP2,MPO,PF4V1,GPLD1,IGFBP5,CAT,LRP1,SERPINF1,AMBP,PPBP,PF4,CAMP,HSP90B1,PCSK9,HSPA5,BLM,AKAP9,FLNA,ADAMTS13,SOD3,ADIPOQ,IGF2,RARRES2,ACTB,B2M
GO Process	GO:0010941	Regulation of cell death	37	1651	0.25	0.0325	ST6GAL1,APOH,LGALS1,CHGA,MMP2,MPO,GPLD1,PROC,CAT,LRP1,APOE,SERPINF1,CSF1R,IHH,PF4,HSP90B1,PCSK9,FGB,HBA1,HSPA5,P4HB,FGG,S100A9,ADAMTSL4,FLNA,CCN5,GSN,TAF9,IGFBP3,YWHAZ,ADIPOQ,MST1,HPR,HYOU1,KNG1,ACTB,FGA
GO Process	GO:0020027	Hemoglobin metabolic process	3	11	1.33	0.0348	CAT,AMBP,HPX
GO Process	GO:0034975	Protein folding in endoplasmic reticulum	3	11	1.33	0.0348	HSP90B1,HSPA5,P4HB
GO Process	GO:1900121	Negative regulation of receptor binding	3	11	1.33	0.0348	PCSK9,ADIPOQ,B2M
GO Process	GO:0031529	Ruffle organization	4	28	1.05	0.0353	PLEK,TCIRG1,CSF1R,CSPG4
GO Process	GO:0048878	Chemical homeostasis	24	904	0.32	0.0353	CPB2,CETP,APOC3,APOB,IGFBP5,APOA1,APOE,SERPINF1,LCAT,YWHAE,CP,TCIRG1,HPX,HSP90B1,PCSK9,F2,OIT3,S100A9,APOM,ADIPOQ,APOA5,ATP1A1,APOC4,B2M
GO Process	GO:0071622	Regulation of granulocyte chemotaxis	5	52	0.88	0.0391	LBP,CSF1R,THBS4,S100A7,RARRES2
GO Process	GO:0010758	Regulation of macrophage chemotaxis	4	29	1.04	0.0394	C5,CSF1R,MST1,RARRES2
GO Process	GO:0006897	Endocytosis	15	447	0.42	0.0407	VTN,LRP1,APOE,LGALS3BP,FCN3,AHSG,FCN2,STAB1,CD5L,GSN,CFI,ATP9B,IGHV3-15,IGHV3-72,ACTB
GO Process	GO:0010543	Regulation of platelet activation	5	53	0.87	0.0418	PLEK,APOE,F2,FGG,FLNA
GO Process	GO:0019732	Antifungal humoral response	3	12	1.29	0.0418	CAMP,ANG,RARRES2
GO Process	GO:0022603	Regulation of anatomical structure morphogenesis	24	920	0.31	0.0435	ST6GAL1,APOH,C5,SPARC,APOA1,SERPINF1,HYAL1,CSF1R,PF4,CAMP,SH3D19,FGB,F2,STAB1,P4HB,FBLN1,FGG,LIMS1,THBS4,FLNA,ILK,CST3,VEGFC,FGA
GO Process	GO:0097421	Liver regeneration	4	30	1.02	0.0435	CPB2,VTN,SERPINA10,IHH
GO Process	GO:0030595	Leukocyte chemotaxis	8	149	0.63	0.0436	CHGA,LBP,PF4V1,PPBP,PF4,S100A9,SAA1,CCL18
GO Process	GO:0050920	Regulation of chemotaxis	10	227	0.54	0.0441	ST6GAL1,LBP,C5,CSF1R,THBS4,S100A7,F7,MST1,RARRES2,VEGFC
GO Process	GO:0002687	Positive regulation of leukocyte migration	8	151	0.62	0.0470	LBP,CSF1R,THBS4,S100A7,F7,CD99,RARRES2,VEGFC

GO Process	GO:0043170	Macromolecule metabolic process	97	5781	0.12	0.0471	ST6GAL1,CPB2,GNPTG,APOH,F9,MMP2,PCOLCE,APOC3,GPLD1,PCDH12,APOB,PROC,APOA1,CAT,DHX29,APOE,PSMA6,LCN1,LCAT,CSTA,KLKB1,AMBP,TCIRG1,HPX,HYAL1,ITIH4,PSG9,FCN3,ITIH1,ANKRD31,HABP2,CSF1R,FCN2,PGLYRP2,IHH,MASP1,APEH,HSP90B1,PCSK9,BMP1,SHLD1,FGB,F2,PLG,SUZ12,APOL1,SUMO4,HSPA5,P4HB,FBLN1,ANG,FGG,GANAB,QPCT,BLM,ITIH2,CNDP1,F8,CDC42BPA,CENPF,F13B,CFH,F5,CD5L,S100A9,FLNA,ADAMTS13,GSN,MBL2,F10,F7,APOM,TAF9,IGFBP3,EFEMP1,CFI,YWHAZ,ILK,GAPDH,RNASE1,F11,C1S,PCYOX1,ADIPOQ,CDK3,COLEC11,MST1,ITIH3,CFB,HGFAC,UIMC1,HPR,APOA5,RNASE4,ACTB,B2M,FGA
GO Process	GO:0035606	Peptidyl-cysteine S-trans-nitrosylation	2	2	1.9	0.0475	S100A9,GAPDH
GO Process	GO:0010743	Regulation of macrophage derived foam cell differentiation	4	31	1.01	0.0476	CETP,APOB,PF4,ADIPOQ
GO Process	GO:0051094	Positive regulation of developmental process	31	1332	0.26	0.0481	C5,APOB,PROC,APOA1,APOE,SERPINF1,NID1,HYAL1,SCGB3A1,IHH,PF4,CAMP,BMP1,SHLD1,FGB,EFEMP2,SERPINF2,P4HB,FGG,LIMS1,FLNA,IGFBP3,ILK,ADIPOQ,IGF2,MST1,LMOD3,RARRES2,VEGFC,ACTB,FGA
GO Process	GO:0010642	Negative regulation of platelet-derived growth factor receptor signaling pathway	3	13	1.26	0.0490	LRP1,APOD,ADIPOQ
GO Process	GO:2001046	Positive regulation of integrin-mediated signaling pathway	3	13	1.26	0.0490	NID1,LIMS1,FLNA
GO Process	GO:0070482	Response to oxygen levels	12	318	0.47	0.0496	MMP2,CAT,GUCY1A1,HSP90B1,P4HB,ANG,F7,SOD3,ADIPOQ,MST1,VEGFC,HYOU1
GO Function	GO:0004866	Endopeptidase inhibitor activity	30	177	1.13	1.33e-19	SPP2,SERPIND1,TFPI2,C5,SERPINF1,SERPINA10,TIMP2,LCN1,CSTA,AMBP,ITIH4,ITIH1,AHSG,SERPING1,SERPINI1,A2ML1,SERPINF2,SERPINA7,SERPINA11,PAPLN,ITIH2,PROS1,GAPDH,CST3,C4A,ITIH3,C4B,SERPINA3,SERPINA4,KNG1
GO Function	GO:0061134	Peptidase regulator activity	32	227	1.05	2.21e-19	SPP2,SERPIND1,TFPI2,PCOLCE,C5,SERPINF1,SERPINA10,TIMP2,LCN1,CSTA,AMBP,ITIH4,ITIH1,AHSG,SERPING1,SERPINI1,A2ML1,SERPINF2,SERPINA7,FBLN1,SERPINA11,PAPLN,ITIH2,PROS1,GAPDH,CST3,C4A,ITIH3,C4B,SERPINA3,SERPINA4,KNG1
GO Function	GO:0005509	Calcium ion binding	48	717	0.72	1.35e-17	RCN1,F9,PON1,MGP,SPARC,PCDH12,PROC,LRP1,APCS,DSG2,NID1,CDH6,CDH11,ITIH1,HABP2,MYL9,PCDH1,IHH,MASP1,HSP90B1,BMP1,DST,F2,EFEMP2,STAB1,HSPA5,FBLN1,OIT3,THBS4,PCDH18,S100A7,S100A9,CRTAC1,ADAMTS13,GSN,F10,F7,CALML5,PROS1,EFEMP1,EFCAB5,FBLN2,C1S,NUCB1,COLEC11,SPARCL1,TPM4,SDF4
GO Function	GO:0005539	Glycosaminoglycan binding	28	245	0.95	8.62e-15	APOH,SERPIND1,PCOLCE,MPO,VTN,PF4V1,APOB,APOE,SERPINA10,AMBP,ITIH1,HABP2,PGLYRP2,PF4,F2,EFEMP2,STAB1,ANG,THBS4,ITIH2,CFH,CCN5,SOD3,NAV2,SAA1,F11,APOA5,KNG1
GO Function	GO:0004857	Enzyme inhibitor activity	31	396	0.79	2.20e-12	SPP2,SERPIND1,TFPI2,C5,APOC3,SERPINF1,SERPINA10,TIMP2,LCN1,CSTA,AMBP,ITIH4,ITIH1,AHSG,SERPING1,SERPINI1,A2ML1,SERPINF2,SERPINA7,SERPINA11,PAPLN,ITIH2,PROS1,GAPDH,CST3,C4A,ITIH3,C4B,SERPINA3,SERPINA4,KNG1
GO Function	GO:0008201	Heparin binding	22	173	1.0	3.20e-12	APOH,SERPIND1,PCOLCE,MPO,VTN,PF4V1,APOB,APOE,SERPINA10,PF4,F2,EFEMP2,ANG,THBS4,CFH,CCN5,SOD3,NAV2,SAA1,F11,APOA5,KNG1
GO Function	GO:0004867	Serine-type endopeptidase inhibitor activity	18	98	1.16	3.25e-12	SERPIND1,TFPI2,SERPINF1,SERPINA10,AMBP,ITIH4,ITIH1,SERPING1,SERPINI1,A2ML1,SERPINF2,SERPINA7,SERPINA11,PAPLN,ITIH2,ITIH3,SERPINA3,SERPINA4
GO Function	GO:0004252	Serine-type endopeptidase activity	21	176	0.97	3.40e-11	F9,MMP2,PROC,KLKB1,HABP2,MASP1,APEH,PCSK9,BMP1,F2,PLG,CD5L,F10,F7,CFI,F11,C1S,MST1,CFB,HGFAC,HPR
GO Function	GO:0005102	Signaling receptor binding	57	1499	0.48	4.88e-11	LBP,EBI3,C5,VTN,PF4V1,APOC3,APOB,APOA1,TTR,LRP1,APOE,PXDN,ANGPTL6,VWF,LCN1,CRH,FCN2,SCGB3A1,IHH,PPBP,PF4,HSP90B1,PCSK9,BMP1,FGB,DST,F2,INHBC,PLG,HBA1,P4HB,FBLN1,ANG,FGG,THBS4,AKAP9,S100A7,S100A9,FLNA,ADAMTS13,CCN5,MBL2,F7,EFEMP1,APOF,SAA1,ADIPOQ,IGF2,MST1,RARRES2,APOA5,IGHV3-15,CCL18,IGHV3-72,VEGFC,KNG1,FGA
GO Function	GO:1901681	Sulfur compound binding	24	272	0.84	1.76e-10	APOH,SERPIND1,PCOLCE,MPO,VTN,PF4V1,APOB,LRP1,APOE,SERPINA10,AMBP,PF4,F2,EFEMP2,ANG,THBS4,CFH,CCN5,SOD3,NAV2,SAA1,F11,APOA5,KNG1
GO Function	GO:0098772	Molecular function regulator activity	65	1960	0.42	1.97e-10	SPP2,APOH,SERPIND1,EBI3,TFPI2,PCOLCE,C5,PF4V1,APOC3,GPLD1,APOA1,TTR,APOE,PXDN,SERPINF1,SERPINA10,TIMP2,LCN1,YWHAE,CSTA,AMBP,ITIH4,ITIH1,AHSG,CRH,SERPING1,SCGB3A1,SERPINI1,PPBP,PF4,A2ML1,PCSK9,BMP1,F2,INHBC,SUZ12,CPN2,SERPINF2,SERPINA7,FBLN1,SERPINA11,THBS4,PAPLN,AKAP9,ITIH2,FLNA,CALML5,IGFBP3,PROS1,EFEMP1,GAPDH,RAPGEF4,CST3,NUCB1,ADIPOQ,IGF2,C4A,ITIH3,C4B,APOA5,SERPINA3,SERPINA4,CCL18,VEGFC,KNG1
GO Function	GO:0005515	Protein binding	146	7242	0.2	1.36e-09	ST6GAL1,GNPTG,APOH,VCL,LGALS1,TPD52L2,LBP,MMP2,EBI3,PON1,C5,VTN,PF4V1,APOC3,APOB,IGFBP5,PLEK,APOA1,TTR,CAT,LRP1,DHX29,APOE,PXDN,ANGPTL6,APCS,CFHR5,LYZ,VWF,PSMA6,DSG2,TIMP2,LCN1,LCAT,NID1,YWHAE,CSTA,CP,CDH6,DNAH5,AMBP,CENPE,TCIRG1,CDH11,PSG9,FCN3,CRH,MYL9,CSF1R,FCN2,SCGB3A1,IHH,PPBP,PF4,MASP1,APEH,A2ML1,HSP90B1,ANTXR1,SH3D19,PCSK9,BMP1,FGB,DST,F2,INHBC,PLG,EFEMP2,CSPG4,C1QB,CFHR1,SUZ12,SUMO4,SERPINF2,HBA1,HSPA5,P4HB,FBLN1,DNAH8,ANG,FGG,LIMS1,THBS4,BLM,AKAP9,MTCL1,C8A,CDC42BPA,CENPF,CFHR4,CFHR3,CFH,S100A7,S100A9,ADAMTSL4,FLNA,MCOLN2,SMARCA1,ADAMTS13,CCN5,GSN,MBL2,F7,HLA-C,TAF9,IGFBP3,GPX3,KIF19,EFEMP1,YWHAZ,ILK,GAPDH,RAPGEF4,APOF,CST3,SAA1,F11,C1S,NUCB1,ADIPOQ,IGF2,C4A,COLEC11,MST1,LMOD3,C4B,CFB,IGFALS,PLTP,RARRES2,GC,UIMC1,HPR,APOA5,ATP1A1,IGHV3-15,CCL18,IGHV3-72,VEGFC,HYOU1,KNG1,TPM4,ACTB,B2M,FGA,SDF4
GO Function	GO:0001848	Complement binding	10	26	1.48	4.42e-09	APCS,CFHR5,CFHR1,C8A,CFHR4,CFHR3,CFH,C4A,C4B,CFB
GO Function	GO:0005488	Binding	208	12838	0.11	6.34e-08	RCN1,ST6GAL1,CPB2,CETP,GNPTG,APOH,VCL,SERPIND1,LGALS1,TPD52L2,LBP,F9,MMP2,EBI3,PON1,PCOLCE,C5,MPO,VTN,AFM,PF4V1,APOC3,MGP,SPARC,PCDH12,APOB,IGFBP5,PROC,PLEK,APOA1,TTR,CAT,LRP1,DHX29,APOE,PXDN,ANGPTL6,APCS,CFHR5,LYZ,VWF,PSMA6,DSG2,SERPINA10,TIMP2,ATRN,LCN1,LCAT,NID1,YWHAE,CSTA,CP,KLKB1,CDH6,DNAH5,AMBP,CENPE,TCIRG1,HPX,LUM,CDH11,PSG9,FCN3,ITIH1,CRH,HABP2,MYL9,CSF1R,PCDH1,FCN2,PGLYRP2,SCGB3A1,IHH,PPBP,PF4,MASP1,CAMP,APEH,GUCY1A1,A2ML1,HSP90B1,ANTXR1,SH3D19,PCSK9,BMP1,FGB,DST,F2,INHBC,PLG,EFEMP2,CSPG4,STAB1,C1QB,CFHR1,SUZ12,APOL1,SUMO4,SERPINF2,HBA1,HSPA5,P4HB,FBLN1,DNAH8,OIT3,ANG,FGG,LIMS1,THBS4,GANAB,QPCT,APOD,BLM,AKAP9,ITIH2,CNDP1,MTCL1,F8,C8A,PCDH18,CDC42BPA,CENPF,CFHR4,CFHR3,CFH,QSOX1,F5,S100A7,S100A9,ADAMTSL4,FLNA,CRTAC1,MCOLN2,SMARCA1,C8B,ADAMTS13,CCN5,GSN,MBL2,F10,F7,APOM,HLA-C,CKAP4,CALML5,TAF9,IGFBP3,SOD3,GPX3,KIF19,PROS1,EFEMP1,CFI,EFCAB5,TNRC6A,YWHAZ,NAV2,ILK,GAPDH,RAPGEF4,RNASE1,APOF,CST3,FBLN2,SAA1,F11,C1S,NUCB1,PCYOX1,ADIPOQ,IGF2,C4A,ATP9B,ACSF2,CDK3,ABCA13,COLEC11,MST1,LMOD3,SPARCL1,C4B,CFB,IGFALS,PLTP,PAEP,RARRES2,GC,UIMC1,SELENOP,HPR,APOA5,ATP1A1,SERPINA3,RNASE4,IGHV3-15,CCL18,IGHV3-72,VEGFC,HYOU1,KNG1,TPM4,ACTB,TRIM66,GRHL2,B2M,FGA,PDIA4,SDF4

GO Function	GO:0030234	Enzyme regulator activity	44	1239	0.45	2.45e-07	SPP2,APOH,SERPIND1,TFPI2,PCOLCE,C5,APOC3,APOA1,APOE,SERPINF1,SERPINA10,TIMP2,LCN1,CSTA,AMBP,ITIH4,ITIH1,AHSG,SERPING1,SERPINI1,A2ML1,SUZ12,CPN2,SERPINF2,SERPINA7,FBLN1,SERPINA11,PAPLN,ITIH2,CALML5,IGFBP3,PROS1,GAPDH,RAPGEF4,CST3,NUCB1,IGF2,C4A,ITIH3,C4B,APOA5,SERPINA3,SERPINA4,KNG1
GO Function	GO:0004175	Endopeptidase activity	24	437	0.64	1.09e-06	F9,MMP2,PROC,PSMA6,KLKB1,HABP2,MASP1,APEH,PCSK9,BMP1,F2,PLG,PAPLN,CD5L,ADAMTS13,F10,F7,CFI,F11,C1S,MST1,CFB,HGFAC,HPR
GO Function	GO:0070325	Lipoprotein particle receptor binding	8	30	1.32	4.51e-06	APOC3,APOB,APOA1,LRP1,APOE,HSP90B1,PCSK9,APOA5
GO Function	GO:0005201	Extracellular matrix structural constituent	13	131	0.89	7.58e-06	TFPI2,VTN,MGP,PXDN,NID1,LUM,FGB,EFEMP2,FBLN1,OIT3,FGG,FBLN2,FGA
GO Function	GO:0001846	Opsonin binding	7	21	1.42	9.05e-06	APCS,CFHR5,CFHR1,CFHR4,CFHR3,CFH,C4A
GO Function	GO:0008233	Peptidase activity	27	617	0.54	9.13e-06	CPB2,F9,MMP2,PROC,PSMA6,KLKB1,HABP2,IHH,MASP1,APEH,PCSK9,BMP1,F2,PLG,PAPLN,CNDP1,CD5L,ADAMTS13,F10,F7,CFI,F11,C1S,MST1,CFB,HGFAC,HPR
GO Function	GO:0044877	Protein-containing complex binding	38	1261	0.38	0.00018	VCL,PCOLCE,VTN,SPARC,APOA1,TTR,LRP1,DHX29,APOE,APCS,VWF,NID1,YWHAE,AMBP,LUM,PSG9,ANTXR1,PCSK9,DST,STAB1,HSPA5,P4HB,FBLN1,THBS4,C8A,CENPF,FLNA,SMARCA1,C8B,ADAMTS13,CCN5,GSN,IGF2,C4A,SPARCL1,PLTP,TPM4,B2M
GO Function	GO:0001851	Complement component C3b binding	5	11	1.55	0.00022	CFHR5,CFHR1,CFHR4,CFHR3,CFH
GO Function	GO:0016209	Antioxidant activity	9	76	0.97	0.00022	MPO,CAT,APOE,PXDN,AMBP,S100A9,APOM,SOD3,GPX3
GO Function	GO:0043169	Cation binding	88	4335	0.2	0.00027	RCN1,CPB2,CETP,F9,MMP2,PON1,MPO,AFM,MGP,SPARC,PCDH12,PROC,APOA1,CAT,LRP1,APOE,PXDN,APCS,DSG2,TIMP2,LCN1,NID1,CP,KLKB1,CDH6,HPX,CDH11,FCN3,ITIH1,HABP2,MYL9,PCDH1,FCN2,PGLYRP2,IHH,MASP1,HSP90B1,ANTXR1,BMP1,DST,F2,EFEMP2,STAB1,SUZ12,HBA1,HSPA5,FBLN1,OIT3,ANG,FGG,LIMS1,THBS4,QPCT,BLM,CNDP1,F8,PCDH18,CDC42BPA,F5,S100A7,S100A9,CRTAC1,ADAMTS13,GSN,F10,F7,CALML5,IGFBP3,SOD3,PROS1,EFEMP1,CFI,EFCAB5,FBLN2,C1S,NUCB1,ATP9B,COLEC11,SPARCL1,PLTP,UIMC1,APOA5,ATP1A1,KNG1,TPM4,TRIM66,FGA,SDF4
GO Function	GO:0005319	Lipid transporter activity	12	163	0.76	0.00038	CETP,APOB,APOA1,APOE,APOD,CFHR4,APOM,APOF,ATP9B,ABCA13,PLTP,APOC4
GO Function	GO:0097367	Carbohydrate derivative binding	55	2278	0.28	0.00040	APOH,SERPIND1,LBP,PCOLCE,MPO,VTN,PF4V1,APOB,LRP1,DHX29,APOE,SERPINA10,NID1,DNAH5,AMBP,CENPE,ITIH1,HABP2,CSF1R,FCN2,PGLYRP2,PF4,CAMP,GUCY1A1,HSP90B1,F2,EFEMP2,STAB1,HSPA5,DNAH8,ANG,THBS4,BLM,ITIH2,CDC42BPA,CFH,SMARCA1,CCN5,SOD3,KIF19,NAV2,ILK,RAPGEF4,SAA1,F11,ADIPOQ,ATP9B,ACSF2,CDK3,ABCA13,APOA5,ATP1A1,HYOU1,KNG1,ACTB
GO Function	GO:0005518	Collagen binding	8	66	0.98	0.00064	PCOLCE,VTN,SPARC,VWF,NID1,LUM,ANTXR1,SPARCL1
GO Function	GO:0042802	Identical protein binding	52	2144	0.28	0.00068	ST6GAL1,GNPTG,APOH,TPD52L2,PON1,VTN,PLEK,APOA1,TTR,CAT,APOE,APCS,CFHR5,LYZ,VWF,YWHAE,AMBP,FCN3,CSF1R,FCN2,MASP1,APEH,BMP1,DST,EFEMP2,C1QB,CFHR1,SERPINF2,FBLN1,ANG,FGG,BLM,MTCL1,CDC42BPA,CENPF,CFH,ADAMTSL4,FLNA,MCOLN2,MBL2,GPX3,YWHAZ,GAPDH,CST3,F11,C1S,ADIPOQ,COLEC11,TPM4,ACTB,B2M,SDF4
GO Function	GO:0046872	Metal ion binding	84	4250	0.19	0.0013	RCN1,CPB2,F9,MMP2,PON1,MPO,AFM,MGP,SPARC,PCDH12,PROC,CAT,LRP1,APOE,PXDN,APCS,DSG2,TIMP2,LCN1,NID1,CP,KLKB1,CDH6,HPX,CDH11,FCN3,ITIH1,HABP2,MYL9,PCDH1,FCN2,PGLYRP2,IHH,MASP1,HSP90B1,ANTXR1,BMP1,DST,F2,EFEMP2,STAB1,SUZ12,HBA1,HSPA5,FBLN1,OIT3,ANG,FGG,LIMS1,THBS4,QPCT,BLM,CNDP1,F8,PCDH18,CDC42BPA,F5,S100A7,S100A9,CRTAC1,ADAMTS13,GSN,F10,F7,CALML5,IGFBP3,SOD3,PROS1,EFEMP1,CFI,EFCAB5,FBLN2,C1S,NUCB1,ATP9B,COLEC11,SPARCL1,UIMC1,ATP1A1,KNG1,TPM4,TRIM66,FGA,SDF4
GO Function	GO:0043167	Ion binding	109	6033	0.15	0.0017	RCN1,CPB2,CETP,F9,MMP2,PON1,MPO,AFM,MGP,SPARC,PCDH12,PROC,PLEK,APOA1,CAT,LRP1,DHX29,APOE,PXDN,APCS,PSMA6,DSG2,TIMP2,LCN1,NID1,CP,KLKB1,CDH6,DNAH5,CENPE,HPX,CDH11,FCN3,ITIH1,HABP2,MYL9,CSF1R,PCDH1,FCN2,PGLYRP2,IHH,MASP1,GUCY1A1,HSP90B1,ANTXR1,BMP1,DST,F2,EFEMP2,STAB1,SUZ12,HBA1,HSPA5,FBLN1,DNAH8,OIT3,ANG,FGG,LIMS1,THBS4,QPCT,BLM,CNDP1,F8,PCDH18,CDC42BPA,QSOX1,F5,S100A7,S100A9,CRTAC1,SMARCA1,ADAMTS13,GSN,F10,F7,CALML5,IGFBP3,SOD3,KIF19,PROS1,EFEMP1,CFI,EFCAB5,NAV2,ILK,RAPGEF4,FBLN2,C1S,NUCB1,PCYOX1,ADIPOQ,ATP9B,ACSF2,CDK3,ABCA13,COLEC11,SPARCL1,PLTP,UIMC1,APOA5,ATP1A1,HYOU1,KNG1,TPM4,ACTB,TRIM66,FGA,SDF4
GO Function	GO:0050840	Extracellular matrix binding	7	56	0.99	0.0019	LGALS1,VTN,SPARC,PXDN,NID1,FBLN2,SPARCL1
GO Function	GO:0120020	Cholesterol transfer activity	5	23	1.23	0.0032	CETP,APOB,APOA1,APOE,PLTP
GO Function	GO:0050750	Low-density lipoprotein particle receptor binding	5	25	1.2	0.0043	APOB,APOE,HSP90B1,PCSK9,APOA5
GO Function	GO:0030545	Signaling receptor regulator activity	20	552	0.46	0.0048	EBI3,C5,PF4V1,APOA1,TTR,PXDN,CRH,SCGB3A1,PPBP,PF4,PCSK9,BMP1,F2,INHBC,THBS4,EFEMP1,ADIPOQ,IGF2,CCL18,VEGFC
GO Function	GO:0071813	Lipoprotein particle binding	5	28	1.15	0.0065	APOA1,APOE,PCSK9,STAB1,PLTP
GO Function	GO:0005044	Scavenger receptor activity	6	48	0.99	0.0067	VTN,LRP1,LGALS3BP,STAB1,CD5L,CFI
GO Function	GO:0005496	Steroid binding	8	101	0.8	0.0080	CETP,APOC3,APOA1,APOD,APOF,GC,APOA5,ATP1A1
GO Function	GO:0015485	Cholesterol binding	6	52	0.96	0.0096	CETP,APOC3,APOA1,APOD,APOF,APOA5
GO Function	GO:0048018	Receptor ligand activity	18	499	0.45	0.0108	EBI3,C5,PF4V1,APOA1,TTR,CRH,SCGB3A1,PPBP,PF4,BMP1,F2,INHBC,THBS4,EFEMP1,ADIPOQ,IGF2,CCL18,VEGFC
GO Function	GO:0050839	Cell adhesion molecule binding	19	560	0.43	0.0143	VCL,VTN,DHX29,VWF,DSG2,YWHAE,CDH6,CDH11,DST,HSPA5,P4HB,FBLN1,FGG,THBS4,FLNA,ADAMTS13,CCN5,YWHAZ,IGF2
GO Function	GO:0042803	Protein homodimerization activity	22	709	0.39	0.0146	ST6GAL1,GNPTG,TPD52L2,PON1,PLEK,APOA1,CAT,APOE,AMBP,CSF1R,MASP1,DST,EFEMP2,SERPINF2,ANG,BLM,MTCL1,CENPF,FLNA,ADIPOQ,TPM4,B2M
GO Function	GO:0060228	Phosphatidylcholine-sterol O-acyltransferase activator activity	3	6	1.6	0.0155	APOA1,APOE,APOA5
GO Function	GO:0034185	Apolipoprotein binding	4	19	1.22	0.0171	LRP1,LCAT,PCSK9,PLG
GO Function	GO:0043394	Proteoglycan binding	5	37	1.03	0.0171	LRP1,APOE,NID1,FCN2,CFH
GO Function	GO:0005178	Integrin binding	9	159	0.65	0.0253	VTN,VWF,DST,P4HB,FBLN1,THBS4,ADAMTS13,CCN5,IGF2
GO Function	GO:0003823	Antigen binding	6	71	0.82	0.0369	FCN3,FCN2,PLG,HLA-C,IGHV3-15,IGHV3-72
GO Function	GO:0008009	Chemokine activity	5	48	0.91	0.0460	C5,PF4V1,PPBP,PF4,CCL18

GO Component	GO:0005615	Extracellular space	187	3247	0.66	5.32e-87	CPB2,CETP,GNPTG,APOH,VCL,SERPIND1,LGALS1,CHGA,LBP,F9,MMP2,EBI3,PON1,TFPI2,PCOLCE,C5,MPO,VTN,AFM,PF4V1,APOC3,MGP,GPLD1,SPARC,PCDH12,APOB,IGFBP5,PROC,APOA1,TTR,CAT,C4BPB,CFP,APOE,PXDN,ANGPTL6,SERPINF1,APCS,CFHR5,LYZ,VWF,PSMA6,DSG2,SERPINA10,TIMP2,LGALS3BP,ATRN,C9,LCN1,LCAT,NID1,YWHAE,CSTA,CP,KLKB1,AMBP,HPX,HYAL1,ITIH4,LUM,CDH11,FCN3,ITIH1,AHSG,CRH,HABP2,SAA4,SERPING1,FCN2,PGLYRP2,SCGB3A1,IHH,SERPINI1,PPBP,PF4,MASP1,CAMP,APEH,A2ML1,HSP90B1,PCSK9,BMP1,FGB,F2,INHBC,PLG,EFEMP2,CSPG4,C1QB,CFHR1,APOL1,CPN2,SERPINF2,C7,HBA1,HSPA5,P4HB,SERPINA7,FBLN1,OIT3,SERPINA11,ANG,FGG,THBS4,GANAB,FAM184A,QPCT,APOD,ITIH2,MTCL1,F8,C8A,CDC42BPA,F13B,CFHR4,CFHR3,CFH,QSOX1,F5,CD5L,S100A7,S100A9,FLNA,CRTAC1,C8B,ADAMTS13,CCN5,GSN,MBL2,C1QC,F10,F7,APOM,HLA-C,CKAP4,IGFBP3,SOD3,GPX3,PROS1,EFEMP1,CFI,YWHAZ,GAPDH,RNASE1,APOF,CST3,SAA1,F11,C1S,NUCB1,PCYOX1,ADIPOQ,IGF2,C4A,COLEC11,MST1,SPARCL1,ITIH3,C4B,CFB,IGFALS,PLTP,PAEP,RARRES2,GC,HGFAC,SELENOP,SBSN,HPR,APOA5,ATP1A1,SERPINA3,SERPINA4,RNASE4,APOC4,IGHV3-15,CCL18,IGHV3-72,VEGFC,HYOU1,KNG1,TPM4,ACTB,B2M,FGA,PDIA4,SDF4
GO Component	GO:0005576	Extracellular region	199	4175	0.57	6.18e-82	SPP2,ST6GAL1,CPB2,CETP,GNPTG,APOH,VCL,SERPIND1,LGALS1,CHGA,LBP,F9,MMP2,EBI3,PON1,TFPI2,PCOLCE,C5,MPO,VTN,AFM,PF4V1,APOC3,MGP,GPLD1,SPARC,PCDH12,APOB,IGFBP5,PROC,PLEK,APOA1,TTR,CAT,C4BPB,CFP,APOE,PXDN,ANGPTL6,SERPINF1,APCS,CFHR5,LYZ,VWF,PSMA6,DSG2,SERPINA10,TIMP2,LGALS3BP,ATRN,C9,LCN1,LCAT,NID1,YWHAE,CSTA,CP,KLKB1,DNAH5,AMBP,CENPE,HPX,HYAL1,ITIH4,LUM,CDH11,PSG9,FCN3,ITIH1,AHSG,CRH,HABP2,SAA4,SERPING1,FCN2,PGLYRP2,SCGB3A1,IHH,SERPINI1,PPBP,PF4,MASP1,CAMP,APEH,A2ML1,HSP90B1,PCSK9,BMP1,FGB,F2,INHBC,PLG,EFEMP2,CSPG4,C1QB,CFHR1,APOL1,PSG11,CPN2,SERPINF2,C7,HBA1,HSPA5,P4HB,SERPINA7,FBLN1,OIT3,SERPINA11,ANG,FGG,THBS4,GANAB,FAM184A,QPCT,APOD,PAPLN,ITIH2,CNDP1,MTCL1,F8,C8A,CDC42BPA,F13B,CFHR4,CFHR3,CFH,QSOX1,F5,CD5L,S100A7,S100A9,ADAMTSL4,FLNA,CRTAC1,C8B,ADAMTS13,CCN5,GSN,MBL2,C1QC,F10,F7,APOM,HLA-C,CKAP4,CALML5,IGFBP3,SOD3,GPX3,PROS1,EFEMP1,CFI,YWHAZ,GAPDH,RNASE1,APOF,CST3,FBLN2,SAA1,F11,C1S,NUCB1,PCYOX1,ADIPOQ,IGF2,C4A,COLEC11,MST1,SPARCL1,ITIH3,C4B,CFB,IGFALS,PLTP,PAEP,RARRES2,GC,HGFAC,SELENOP,SBSN,HPR,APOA5,ATP1A1,SERPINA3,SERPINA4,RNASE4,APOC4,IGHV3-15,CCL18,IGHV3-72,VEGFC,HYOU1,KNG1,TPM4,ACTB,B2M,FGA,PDIA4,SDF4
GO Component	GO:1903561	Extracellular vesicle	124	2120	0.66	4.33e-49	CPB2,CETP,GNPTG,APOH,VCL,SERPIND1,LGALS1,LBP,F9,PON1,PCOLCE,C5,MPO,VTN,AFM,APOC3,MGP,GPLD1,PCDH12,APOB,APOA1,TTR,CAT,APOE,PXDN,ANGPTL6,SERPINF1,APCS,LYZ,VWF,PSMA6,DSG2,SERPINA10,LGALS3BP,ATRN,C9,LCAT,NID1,YWHAE,CP,KLKB1,AMBP,HPX,HYAL1,ITIH4,LUM,CDH11,ITIH1,AHSG,SAA4,SERPING1,FCN2,PGLYRP2,SCGB3A1,SERPINI1,CAMP,APEH,A2ML1,HSP90B1,FGB,F2,PLG,EFEMP2,CSPG4,CPN2,SERPINF2,C7,HBA1,HSPA5,P4HB,SERPINA7,FBLN1,FGG,THBS4,GANAB,QPCT,APOD,ITIH2,C8A,CDC42BPA,CFHR3,CFH,QSOX1,F5,S100A9,FLNA,CRTAC1,C8B,GSN,HLA-C,CKAP4,SOD3,GPX3,PROS1,EFEMP1,CFI,YWHAZ,GAPDH,RNASE1,CST3,FBLN2,SAA1,F11,NUCB1,PCYOX1,C4A,ITIH3,C4B,CFB,IGFALS,GC,SELENOP,SBSN,HPR,ATP1A1,SERPINA3,SERPINA4,HYOU1,KNG1,TPM4,ACTB,B2M,FGA,SDF4
GO Component	GO:0070062	Extracellular exosome	122	2096	0.66	3.91e-48	CPB2,CETP,GNPTG,APOH,VCL,SERPIND1,LGALS1,LBP,F9,PON1,PCOLCE,C5,MPO,VTN,AFM,APOC3,MGP,GPLD1,PCDH12,APOB,APOA1,TTR,CAT,APOE,PXDN,ANGPTL6,SERPINF1,APCS,LYZ,VWF,PSMA6,DSG2,SERPINA10,LGALS3BP,ATRN,C9,LCAT,NID1,YWHAE,CP,KLKB1,AMBP,HPX,HYAL1,ITIH4,LUM,CDH11,ITIH1,AHSG,SAA4,SERPING1,FCN2,PGLYRP2,SCGB3A1,SERPINI1,CAMP,APEH,A2ML1,HSP90B1,FGB,F2,PLG,EFEMP2,CSPG4,CPN2,SERPINF2,C7,HBA1,HSPA5,P4HB,SERPINA7,FBLN1,FGG,THBS4,GANAB,QPCT,APOD,ITIH2,C8A,CDC42BPA,CFHR3,CFH,QSOX1,S100A9,FLNA,CRTAC1,C8B,GSN,HLA-C,CKAP4,SOD3,GPX3,PROS1,EFEMP1,CFI,YWHAZ,GAPDH,RNASE1,CST3,SAA1,F11,NUCB1,PCYOX1,C4A,ITIH3,C4B,CFB,IGFALS,GC,SELENOP,SBSN,HPR,ATP1A1,SERPINA3,SERPINA4,HYOU1,KNG1,TPM4,ACTB,B2M,FGA,SDF4
GO Component	GO:0072562	Blood microparticle	46	118	1.49	4.64e-47	PON1,VTN,AFM,APOA1,APOE,APCS,LGALS3BP,C9,CP,AMBP,HPX,ITIH4,FCN3,ITIH1,AHSG,SERPING1,FCN2,FGB,F2,PLG,C1QB,CFHR1,APOL1,CPN2,SERPINF2,HBA1,FGG,ITIH2,C8A,CFHR3,CFH,CD5L,GSN,C1QC,PROS1,YWHAZ,C1S,C4A,C4B,CFB,GC,HPR,SERPINA3,KNG1,ACTB,FGA
GO Component	GO:0031012	Extracellular matrix	69	552	0.99	2.04e-43	SPP2,APOH,LGALS1,F9,MMP2,TFPI2,VTN,APOC3,MGP,GPLD1,SPARC,APOA1,APOE,PXDN,ANGPTL6,SERPINF1,APCS,VWF,TIMP2,LGALS3BP,ATRN,NID1,AMBP,HPX,ITIH4,LUM,FCN3,ITIH1,AHSG,SERPING1,FCN2,IHH,PF4,HSP90B1,FGB,DST,F2,PLG,EFEMP2,CSPG4,C1QB,CPN2,SERPINF2,FBLN1,ANG,FGG,THBS4,PAPLN,ITIH2,S100A7,S100A9,ADAMTSL4,ADAMTS13,CCN5,MBL2,C1QC,F7,SOD3,EFEMP1,NAV2,FBLN2,ADIPOQ,MST1,SPARCL1,IGFALS,RARRES2,SERPINA3,KNG1,FGA
GO Component	GO:0062023	Collagen-containing extracellular matrix	61	407	1.07	3.84e-42	SPP2,APOH,LGALS1,F9,MMP2,VTN,APOC3,MGP,SPARC,APOA1,APOE,PXDN,ANGPTL6,SERPINF1,APCS,VWF,TIMP2,LGALS3BP,ATRN,NID1,AMBP,HPX,ITIH4,LUM,FCN3,ITIH1,AHSG,SERPING1,FCN2,PF4,HSP90B1,FGB,DST,F2,PLG,EFEMP2,CSPG4,C1QB,SERPINF2,FBLN1,ANG,FGG,THBS4,ITIH2,S100A7,S100A9,ADAMTSL4,MBL2,C1QC,F7,SOD3,EFEMP1,NAV2,FBLN2,ADIPOQ,MST1,SPARCL1,RARRES2,SERPINA3,KNG1,FGA
GO Component	GO:0031982	Vesicle	151	3957	0.48	5.52e-41	SPP2,CPB2,CETP,GNPTG,APOH,VCL,SERPIND1,LGALS1,CHGA,LBP,F9,PON1,PCOLCE,C5,MPO,VTN,AFM,APOC3,MGP,GPLD1,SPARC,PCDH12,APOB,APOA1,TTR,CAT,LRP1,CFP,APOE,PXDN,ANGPTL6,SERPINF1,APCS,LYZ,VWF,PSMA6,DSG2,SERPINA10,TIMP2,LGALS3BP,ATRN,C9,LCAT,NID1,YWHAE,CP,KLKB1,AMBP,TCIRG1,HPX,HYAL1,ITIH4,LUM,CDH11,ITIH1,AHSG,SAA4,SERPING1,FCN2,PGLYRP2,SCGB3A1,SERPINI1,PPBP,PF4,CAMP,APEH,A2ML1,HSP90B1,ANTXR1,PCSK9,BMP1,FGB,DST,F2,PLG,EFEMP2,CSPG4,STAB1,CPN2,SERPINF2,C7,HBA1,HSPA5,P4HB,SERPINA7,FBLN1,ANG,FGG,THBS4,GANAB,QPCT,APOD,ITIH2,F8,C8A,CDC42BPA,CFHR3,CFH,QSOX1,F5,S100A7,S100A9,FLNA,CRTAC1,MCOLN2,C8B,GSN,F7,HLA-C,CKAP4,CALML5,SOD3,GPX3,PROS1,EFEMP1,CFI,YWHAZ,GAPDH,RNASE1,CST3,FBLN2,SAA1,F11,NUCB1,PCYOX1,IGF2,C4A,ATP9B,ABCA13,ITIH3,C4B,CFB,IGFALS,RARRES2,GC,SELENOP,SBSN,HPR,APOA5,ATP1A1,SERPINA3,SERPINA4,VEGFC,HYOU1,KNG1,TPM4,ACTB,B2M,FGA,PDIA4,SDF4
GO Component	GO:0005788	Endoplasmic reticulum lumen	45	312	1.06	9.66e-30	RCN1,SPP2,SERPIND1,LGALS1,F9,EBI3,VTN,APOB,IGFBP5,PROC,APOA1,CFP,APOE,SERPINA10,CP,AHSG,SERPING1,HSP90B1,PCSK9,F2,APOL1,HSPA5,P4HB,FGG,GANAB,ITIH2,F8,QSOX1,F5,ADAMTSL4,ADAMTS13,F10,F7,CKAP4,IGFBP3,CST3,NUCB1,C4A,SPARCL1,APOA5,HYOU1,KNG1,B2M,FGA,PDIA4

GO Component	GO:0031983	Vesicle lumen	43	326	1.02	6.37e-27	SPP2,APOH,VCL,MPO,SPARC,APOB,APOA1,TTR,CAT,CFP,LYZ,VWF,TIMP2,LGALS3BP,ITIH4,AHSG,SERPING1,SERPINI1,PPBP,PF4,CAMP,FGB,PLG,SERPINF2,FGG,QPCT,F8,QSOX1,F5,S100A7,S100A9,GSN,PROS1,IGF2,ITIH3,RARRES2,SELENOP,SERPINA3,SERPINA4,VEGFC,KNG1,B2M,FGA
GO Component	GO:0034774	Secretory granule lumen	42	321	1.01	3.72e-26	SPP2,APOH,VCL,MPO,SPARC,APOA1,TTR,CAT,CFP,LYZ,VWF,TIMP2,LGALS3BP,ITIH4,AHSG,SERPING1,SERPINI1,PPBP,PF4,CAMP,FGB,PLG,SERPINF2,FGG,QPCT,F8,QSOX1,F5,S100A7,S100A9,GSN,PROS1,IGF2,ITIH3,RARRES2,SELENOP,SERPINA3,SERPINA4,VEGFC,KNG1,B2M,FGA
GO Component	GO:0034364	High-density lipoprotein particle	17	29	1.66	4.34e-19	CETP,APOH,PON1,APOC3,APOB,APOA1,APOE,LCAT,SAA4,APOL1,APOM,APOF,SAA1,PLTP,HPR,APOA5,APOC4
GO Component	GO:0034358	Plasma lipoprotein particle	18	38	1.57	5.25e-19	CETP,APOH,PON1,APOC3,APOB,APOA1,APOE,LCAT,SAA4,APOL1,APOM,APOF,SAA1,PCYOX1,PLTP,HPR,APOA5,APOC4
GO Component	GO:1905370	Serine-type endopeptidase complex	16	26	1.69	2.91e-18	F9,PROC,KLKB1,FCN3,FCN2,MASP1,F2,F8,CFH,F5,MBL2,F10,F7,CFI,F11,COLEC11
GO Component	GO:0030141	Secretory granule	51	873	0.66	1.78e-17	SPP2,APOH,VCL,CHGA,MPO,SPARC,APOA1,TTR,CAT,CFP,ANGPTL6,LYZ,VWF,TIMP2,LGALS3BP,TCIRG1,ITIH4,AHSG,SERPING1,SERPINI1,PPBP,PF4,CAMP,APEH,FGB,PLG,SERPINF2,FGG,QPCT,F8,QSOX1,F5,S100A7,S100A9,GSN,HLA-C,CKAP4,CALML5,PROS1,CST3,IGF2,ABCA13,ITIH3,RARRES2,SELENOP,SERPINA3,SERPINA4,VEGFC,KNG1,B2M,FGA
GO Component	GO:0031093	Platelet alpha granule lumen	19	66	1.36	6.64e-17	SPARC,VWF,AHSG,SERPING1,PPBP,PF4,FGB,PLG,SERPINF2,FGG,F8,QSOX1,F5,PROS1,IGF2,SERPINA3,VEGFC,KNG1,FGA
GO Component	GO:0071944	Cell periphery	135	6015	0.25	1.38e-12	SPP2,APOH,VCL,LGALS1,F9,MMP2,EBI3,TFPI2,C5,VTN,APOC3,MGP,GPLD1,SPARC,PCDH12,APOB,PLEK,APOA1,CAT,LRP1,C4BPB,CFP,APOE,PXDN,ANGPTL6,SERPINF1,APCS,VWF,DSG2,TIMP2,LGALS3BP,ATRN,C9,NID1,CSTA,CP,KLKB1,CDH6,AMBP,TCIRG1,HPX,ITIH4,LUM,CDH11,PSG9,FCN3,ITIH1,AHSG,SERPING1,MYL9,CSF1R,PCDH1,FCN2,IHH,PF4,MASP1,HSP90B1,ANTXR1,SH3D19,PCSK9,FGB,DST,F2,PLG,EFEMP2,CSPG4,STAB1,C1QB,CPN2,SERPINF2,C7,HSPA5,P4HB,FBLN1,ANG,FGG,LIMS1,THBS4,PAPLN,AKAP9,ITIH2,MTCL1,F8,C8A,PCDH18,F5,CD5L,S100A7,S100A9,ADAMTSL4,FLNA,MCOLN2,C8B,ADAMTS13,CCN5,GSN,MBL2,C1QC,F10,F7,HLA-C,CKAP4,CD99,SOD3,PROS1,EFEMP1,NAV2,ILK,GAPDH,RAPGEF4,CST3,FBLN2,F11,PCYOX1,ADIPOQ,C4A,ATP9B,ABCA13,COLEC11,MST1,SPARCL1,C4B,CFB,IGFALS,RARRES2,ATP1A1,SERPINA3,IGHV3-15,IGHV3-72,KNG1,TPM4,ACTB,B2M,FGA,SDF4
GO Component	GO:0031410	Cytoplasmic vesicle	78	2482	0.39	1.43e-12	SPP2,APOH,VCL,CHGA,MPO,APOC3,SPARC,APOB,APOA1,TTR,CAT,LRP1,CFP,APOE,ANGPTL6,SERPINF1,LYZ,VWF,TIMP2,LGALS3BP,YWHAE,TCIRG1,HPX,HYAL1,ITIH4,AHSG,SERPING1,SERPINI1,PPBP,PF4,CAMP,APEH,HSP90B1,ANTXR1,PCSK9,FGB,DST,PLG,STAB1,SERPINF2,HBA1,HSPA5,P4HB,ANG,FGG,GANAB,QPCT,F8,QSOX1,F5,S100A7,S100A9,MCOLN2,GSN,HLA-C,CKAP4,CALML5,PROS1,YWHAZ,CST3,SAA1,IGF2,ATP9B,ABCA13,ITIH3,RARRES2,SELENOP,APOA5,ATP1A1,SERPINA3,SERPINA4,VEGFC,HYOU1,KNG1,B2M,FGA,PDIA4,SDF4
GO Component	GO:1905369	Endopeptidase complex	17	104	1.11	1.32e-11	F9,PROC,PSMA6,KLKB1,FCN3,FCN2,MASP1,F2,F8,CFH,F5,MBL2,F10,F7,CFI,F11,COLEC11
GO Component	GO:1905368	Peptidase complex	18	138	1.01	7.79e-11	F9,PROC,PSMA6,KLKB1,FCN3,FCN2,MASP1,F2,F8,CFH,F5,MBL2,F10,F7,TAF9,CFI,F11,COLEC11
GO Component	GO:0034361	Very-low-density lipoprotein particle	10	20	1.6	1.27e-10	APOH,APOC3,APOB,APOA1,APOE,APOL1,APOM,PCYOX1,APOA5,APOC4
GO Component	GO:0071682	Endocytic vesicle lumen	10	23	1.53	3.56e-10	MPO,SPARC,APOB,APOA1,APOE,HPX,HSP90B1,HBA1,SAA1,HYOU1
GO Component	GO:0012505	Endomembrane system	108	4721	0.26	1.60e-09	RCN1,SPP2,ST6GAL1,GNPTG,APOH,VCL,SERPIND1,LGALS1,CHGA,F9,EBI3,MPO,VTN,APOC3,SPARC,APOB,IGFBP5,PROC,APOA1,TTR,CAT,LRP1,CFP,APOE,PXDN,ANGPTL6,LYZ,VWF,SERPINA10,TIMP2,LGALS3BP,CP,AMBP,TCIRG1,ITIH4,LUM,AHSG,SERPING1,SERPINI1,PPBP,PF4,CAMP,APEH,HSP90B1,ANTXR1,PCSK9,BMP1,FGB,DST,F2,PLG,CSPG4,APOL1,SERPINF2,HSPA5,P4HB,OIT3,FGG,THBS4,GANAB,QPCT,APOD,AKAP9,ITIH2,F8,CENPF,QSOX1,F5,S100A7,S100A9,ADAMTSL4,FLNA,MCOLN2,ADAMTS13,GSN,F10,F7,HLA-C,CKAP4,CALML5,IGFBP3,SOD3,PROS1,TNRC6A,GAPDH,CST3,NUCB1,ADIPOQ,IGF2,C4A,ATP9B,ABCA13,SPARCL1,ITIH3,RARRES2,HGFAC,SELENOP,APOA5,ATP1A1,SERPINA3,SERPINA4,VEGFC,HYOU1,KNG1,B2M,FGA,PDIA4,SDF4
GO Component	GO:0031089	Platelet dense granule lumen	8	14	1.65	8.74e-09	SPP2,APOH,LGALS3BP,ITIH4,ITIH3,RARRES2,SELENOP,SERPINA4
GO Component	GO:0005783	Endoplasmic reticulum	61	2021	0.38	9.59e-09	RCN1,SPP2,ST6GAL1,SERPIND1,LGALS1,F9,EBI3,VTN,APOB,IGFBP5,PROC,APOA1,CAT,CFP,APOE,PXDN,VWF,SERPINA10,CP,AMBP,AHSG,SERPING1,HSP90B1,PCSK9,FGB,DST,F2,APOL1,HSPA5,P4HB,FGG,THBS4,GANAB,APOD,ITIH2,F8,QSOX1,F5,S100A7,ADAMTSL4,ADAMTS13,F10,F7,HLA-C,CKAP4,IGFBP3,PROS1,CST3,NUCB1,ADIPOQ,C4A,SPARCL1,HGFAC,APOA5,ATP1A1,HYOU1,KNG1,B2M,FGA,PDIA4,SDF4
GO Component	GO:0009986	Cell surface	36	894	0.5	9.48e-08	APOH,LBP,APMAP,EBI3,SPARC,PXDN,DSG2,AMBP,PSG9,FCN3,CSF1R,FCN2,MASP1,ANTXR1,PCSK9,FGB,PLG,CSPG4,PSG11,SERPINF2,HSPA5,P4HB,OIT3,FGG,CD5L,ADAMTS13,MBL2,F10,HLA-C,ADIPOQ,COLEC11,IGHV3-15,IGHV3-72,B2M,FGA,PDIA4
GO Component	GO:0005796	Golgi lumen	12	106	0.95	1.60e-06	F9,VTN,PROC,LUM,F2,CSPG4,F8,F10,F7,SOD3,PROS1,SDF4
GO Component	GO:0070013	Intracellular organelle lumen	112	5660	0.19	3.14e-06	RCN1,SPP2,APOH,VCL,SERPIND1,LGALS1,F9,EBI3,MPO,VTN,SPARC,APOB,IGFBP5,PROC,APOA1,TTR,CAT,CFP,APOE,LYZ,VWF,PSMA6,SERPINA10,TIMP2,LGALS3BP,CSTA,CP,CDH6,HPX,HYAL1,ITIH4,LUM,AHSG,SERPING1,CSF1R,PCDH1,SERPINI1,PPBP,PF4,MASP1,CAMP,APEH,C16orf46,HSP90B1,SH3D19,PCSK9,FGB,DST,F2,PLG,CSPG4,SUZ12,APOL1,SERPINF2,HBA1,HSPA5,P4HB,ANG,FGG,GANAB,QPCT,BLM,ITIH2,F8,CENPF,QSOX1,F5,S100A7,S100A9,ADAMTSL4,FLNA,SMARCA1,ADAMTS13,GSN,F10,F7,CKAP4,CALML5,TAF9,IGFBP3,SOD3,PROS1,TNRC6A,YWHAZ,NAV2,ILK,CST3,ANKRD28,SAA1,NUCB1,IGF2,C4A,ACSF2,SPARCL1,ITIH3,RARRES2,GC,UIMC1,SELENOP,APOA5,SERPINA3,SERPINA4,VEGFC,HYOU1,KNG1,ACTB,TRIM66,GRHL2,B2M,FGA,PDIA4,SDF4
GO Component	GO:0042627	Chylomicron	6	13	1.56	4.11e-06	APOH,APOC3,APOB,APOA1,APOE,APOA5
GO Component	GO:1904724	Tertiary granule lumen	9	55	1.11	4.64e-06	CFP,LYZ,TIMP2,PPBP,CAMP,QPCT,QSOX1,CST3,B2M
GO Component	GO:0005604	Basement membrane	11	98	0.95	5.69e-06	VTN,SPARC,PXDN,SERPINF1,ATRN,NID1,DST,EFEMP2,FBLN1,ANG,THBS4
GO Component	GO:0005579	Membrane attack complex	5	7	1.75	9.74e-06	C5,C9,C7,C8A,C8B
GO Component	GO:0034366	Spherical high-density lipoprotein particle	5	8	1.69	1.53e-05	PON1,APOC3,APOA1,APOM,HPR
GO Component	GO:0005925	Focal adhesion	20	416	0.58	2.90e-05	VCL,CAT,LRP1,YWHAE,HSP90B1,DST,CSPG4,HSPA5,P4HB,LIMS1,S100A7,FLNA,GSN,CD99,YWHAZ,ILK,HYOU1,TPM4,ACTB,B2M

GO Component	GO:0032991	Protein-containing complex	106	5506	0.18	3.39e-05	CETP,GNPTG,APOH,VCL,LGALS1,F9,EBI3,PON1,C5,VTN,APOC3,APOB,IGFBP5,PROC,APOA1,TTR,CAT,LRP1,DHX29,APOE,CFHR5,PSMA6,C9,LCAT,NID1,CSTA,KLKB1,CDH6,DNAH5,TCIRG1,LUM,CDH11,PSG9,FCN3,SAA4,MYL9,CSF1R,FCN2,MASP1,GUCY1A1,HSP90B1,PCSK9,FGB,F2,C1QB,CFHR1,SUZ12,APOL1,SUMO4,SERPINF2,C7,HBA1,HSPA5,P4HB,DNAH8,ANG,FGG,GANAB,BLM,AKAP9,F8,C8A,CENPF,F13B,CFH,F5,FLNA,SMARCA1,C8B,MBL2,C1QC,F10,F7,APOM,HLA-C,TAF9,IGFBP3,KIF19,CFI,TNRC6A,GAPDH,APOF,SAA1,F11,C1S,PCYOX1,ADIPOQ,C4A,CDK3,COLEC11,C4B,CFB,IGFALS,PLTP,UIMC1,HPR,APOA5,ATP1A1,APOC4,IGHV3-15,IGHV3-72,HYOU1,TPM4,ACTB,B2M,FGA
GO Component	GO:0035580	Specific granule lumen	8	62	1.01	0.00010	VCL,CFP,LYZ,TIMP2,CAMP,QPCT,QSOX1,B2M
GO Component	GO:0042470	Melanosome	10	109	0.86	0.00010	SERPINF1,YWHAE,HSP90B1,HSPA5,P4HB,GANAB,GSN,YWHAZ,ATP1A1,PDIA4
GO Component	GO:0043227	Membrane-bounded organelle	199	13188	0.08	0.00029	RCN1,SPP2,ST6GAL1,CPB2,CETP,GNPTG,APOH,VCL,SERPIND1,LGALS1,CHGA,LBP,F9,MMP2,EBI3,PON1,PCOLCE,C5,MPO,VTN,AFM,APOC3,MGP,GPLD1,SPARC,PCDH12,APOB,IGFBP5,PROC,APOA1,TTR,CAT,LRP1,CFP,APOE,PXDN,ANGPTL6,SERPINF1,APCS,LYZ,VWF,PSMA6,DSG2,SERPINA10,TIMP2,LGALS3BP,ATRN,C9,LCAT,NID1,YWHAE,CSTA,CP,KLKB1,CDH6,DNAH5,AMBP,CENPE,TCIRG1,HPX,HYAL1,ITIH4,LUM,CDH11,ITIH1,AHSG,ANKRD31,SAA4,SERPING1,CSF1R,PCDH1,FCN2,PGLYRP2,SCGB3A1,SERPINI1,PPBP,PF4,MASP1,CAMP,APEH,C16orf46,A2ML1,HSP90B1,ANTXR1,SH3D19,PCSK9,BMP1,FGB,DST,F2,PLG,EFEMP2,CSPG4,STAB1,SUZ12,APOL1,SUMO4,CPN2,SERPINF2,C7,HBA1,HSPA5,P4HB,SERPINA7,FBLN1,DNAH8,OIT3,ANG,FGG,THBS4,GANAB,QPCT,APOD,BLM,AKAP9,ITIH2,F8,C8A,CDC42BPA,CENPF,CFHR3,CFH,QSOX1,F5,S100A7,S100A9,ADAMTSL4,FLNA,CRTAC1,MCOLN2,SMARCA1,C8B,ADAMTS13,CCN5,GSN,F10,F7,HLA-C,CKAP4,CALML5,TAF9,IGFBP3,SOD3,GPX3,KIF19,PROS1,EFEMP1,CFI,TNRC6A,YWHAZ,NAV2,ILK,GAPDH,RNASE1,CST3,ANKRD28,MAP3K7CL,FBLN2,SAA1,F11,NUCB1,PCYOX1,ADIPOQ,IGF2,C4A,ATP9B,FSIP2,ACSF2,CDK3,ABCA13,MST1,SPARCL1,ITIH3,C4B,CFB,IGFALS,PLTP,RARRES2,GC,HGFAC,UIMC1,SELENOP,SBSN,HPR,APOA5,ATP1A1,SERPINA3,SERPINA4,VEGFC,HYOU1,KNG1,TPM4,ACTB,TRIM66,GRHL2,B2M,FGA,PDIA4,SDF4
GO Component	GO:0005577	Fibrinogen complex	4	8	1.6	0.00041	FGB,SERPINF2,FGG,FGA
GO Component	GO:0030139	Endocytic vesicle	16	338	0.57	0.00041	MPO,SPARC,APOB,APOA1,LRP1,APOE,TCIRG1,HPX,HSP90B1,STAB1,HBA1,GSN,HLA-C,SAA1,HYOU1,B2M
GO Component	GO:0009897	External side of plasma membrane	17	388	0.54	0.00055	EBI3,FCN3,FCN2,MASP1,ANTXR1,PCSK9,FGB,PLG,P4HB,FGG,MBL2,F10,COLEC11,IGHV3-15,IGHV3-72,B2M,FGA
GO Component	GO:0034362	Low-density lipoprotein particle	4	12	1.42	0.0014	APOB,APOE,APOM,APOF
GO Component	GO:0034663	Endoplasmic reticulum chaperone complex	4	12	1.42	0.0014	HSP90B1,HSPA5,P4HB,HYOU1
GO Component	GO:0005581	Collagen trimer	8	95	0.82	0.0016	LUM,FCN3,FCN2,C1QB,MBL2,C1QC,ADIPOQ,COLEC11
GO Component	GO:0043226	Organelle	205	14017	0.06	0.0019	RCN1,SPP2,ST6GAL1,CPB2,CETP,GNPTG,APOH,VCL,SERPIND1,LGALS1,CHGA,LBP,F9,MMP2,EBI3,PON1,PCOLCE,C5,MPO,VTN,AFM,APOC3,MGP,GPLD1,SPARC,PCDH12,APOB,IGFBP5,PROC,APOA1,TTR,CAT,LRP1,CFP,DHX29,APOE,PXDN,ANGPTL6,SERPINF1,APCS,LYZ,VWF,PSMA6,DSG2,SERPINA10,TIMP2,LGALS3BP,ATRN,C9,LCAT,NID1,YWHAE,CSTA,CP,KLKB1,CDH6,DNAH5,AMBP,CENPE,TCIRG1,HPX,HYAL1,ITIH4,LUM,CDH11,ITIH1,AHSG,ANKRD31,SAA4,SERPING1,MYL9,CSF1R,PCDH1,FCN2,PGLYRP2,SCGB3A1,SERPINI1,PPBP,PF4,MASP1,CAMP,APEH,C16orf46,A2ML1,HSP90B1,ANTXR1,SH3D19,PCSK9,BMP1,SHLD1,FGB,DST,F2,PLG,EFEMP2,CSPG4,STAB1,SUZ12,APOL1,SUMO4,CPN2,SERPINF2,C7,HBA1,HSPA5,P4HB,SERPINA7,FBLN1,DNAH8,OIT3,ANG,FGG,THBS4,GANAB,QPCT,APOD,BLM,AKAP9,ITIH2,MTCL1,F8,C8A,CDC42BPA,CENPF,CFHR3,CFH,QSOX1,F5,S100A7,S100A9,ADAMTSL4,FLNA,CRTAC1,MCOLN2,SMARCA1,C8B,ADAMTS13,CCN5,GSN,F10,F7,HLA-C,CKAP4,CALML5,TAF9,IGFBP3,SOD3,GPX3,KIF19,PROS1,EFEMP1,CFI,TNRC6A,YWHAZ,NAV2,ILK,GAPDH,RNASE1,CST3,ANKRD28,MAP3K7CL,FBLN2,SAA1,F11,NUCB1,PCYOX1,ADIPOQ,IGF2,C4A,ATP9B,SLAIN1,FSIP2,ACSF2,CDK3,ABCA13,MST1,LMOD3,SPARCL1,ITIH3,C4B,CFB,IGFALS,PLTP,RARRES2,GC,HGFAC,UIMC1,SELENOP,SBSN,HPR,APOA5,ATP1A1,SERPINA3,SERPINA4,VEGFC,HYOU1,KNG1,TPM4,ACTB,TRIM66,GRHL2,B2M,FGA,PDIA4,SDF4
GO Component	GO:0005601	Classical-complement-pathway C3/C5 convertase complex	3	4	1.77	0.0021	C4A,C4B,CFB
GO Component	GO:0042567	Insulin-like growth factor ternary complex	3	4	1.77	0.0021	IGFBP5,IGFBP3,IGFALS
GO Component	GO:0110165	Cellular anatomical entity	246	18293	0.03	0.0021	RCN1,SPP2,ST6GAL1,CPB2,CETP,GNPTG,APOH,VCL,SERPIND1,LGALS1,CHGA,TPD52L2,LBP,APMAP,F9,MMP2,EBI3,PON1,TFPI2,PCOLCE,C5,MPO,VTN,AFM,PF4V1,APOC3,MGP,GPLD1,SPARC,PCDH12,APOB,IGFBP5,PROC,PLEK,APOA1,TTR,CAT,LRP1,C4BPB,CFP,DHX29,APOE,PXDN,ANGPTL6,SERPINF1,APCS,CFHR5,LYZ,VWF,PSMA6,DSG2,SERPINA10,TIMP2,LGALS3BP,ATRN,C9,LCN1,LCAT,NID1,YWHAE,CSTA,CP,KLKB1,CDH6,DNAH5,AMBP,CENPE,TCIRG1,HPX,HYAL1,ITIH4,LUM,CDH11,PSG9,FCN3,ITIH1,AHSG,ANKRD31,CRH,HABP2,SAA4,SERPING1,MYL9,CSF1R,PCDH1,FCN2,PGLYRP2,SCGB3A1,IHH,SERPINI1,PPBP,PF4,MASP1,CAMP,APEH,GUCY1A1,C16orf46,A2ML1,HSP90B1,ANTXR1,SH3D19,PCSK9,BMP1,SHLD1,FGB,DST,F2,INHBC,PLG,EFEMP2,CSPG4,STAB1,C1QB,CFHR1,SUZ12,APOL1,SUMO4,PSG11,CPN2,SERPINF2,C7,HBA1,HSPA5,P4HB,SERPINA7,FBLN1,DNAH8,OIT3,SERPINA11,ANG,FGG,LIMS1,THBS4,GANAB,FAM184A,QPCT,APOD,PAPLN,BLM,AKAP9,ITIH2,CNDP1,MTCL1,F8,C8A,PCDH18,CDC42BPA,CENPF,F13B,CFHR4,CFHR3,CFH,QSOX1,F5,CD5L,S100A7,S100A9,ADAMTSL4,FLNA,CRTAC1,MCOLN2,SMARCA1,C8B,ADAMTS13,CCN5,GSN,MBL2,C1QC,F10,F7,APOM,HLA-C,CKAP4,CALML5,TAF9,IGFBP3,CD99,SOD3,GPX3,KIF19,PROS1,EFEMP1,CFI,TNRC6A,YWHAZ,NAV2,ILK,GAPDH,RAPGEF4,RNASE1,APOF,CST3,ANKRD28,MAP3K7CL,FBLN2,SAA1,F11,C1S,NUCB1,PCYOX1,ADIPOQ,IGF2,C4A,ATP9B,SLAIN1,FSIP2,ACSF2,CDK3,ABCA13,COLEC11,MST1,LMOD3,SPARCL1,ITIH3,C4B,CFB,IGFALS,PLTP,PAEP,RARRES2,GC,HGFAC,UIMC1,SELENOP,SBSN,HPR,APOA5,ATP1A1,SERPINA3,SERPINA4,RNASE4,APOC4,IGHV3-15,CCL18,IGHV3-72,VEGFC,HYOU1,KNG1,TPM4,ACTB,TRIM66,GRHL2,B2M,FGA,PDIA4,SDF4
GO Component	GO:0070820	Tertiary granule	10	164	0.68	0.0022	CFP,LYZ,TIMP2,TCIRG1,PPBP,CAMP,QPCT,QSOX1,CST3,B2M
GO Component	GO:0005602	Complement component C1 complex	3	5	1.67	0.0032	C1QB,C1QC,C1S
GO Component	GO:0034363	Intermediate-density lipoprotein particle	3	5	1.67	0.0032	APOC3,APOB,APOE
GO Component	GO:0005775	Vacuolar lumen	10	175	0.65	0.0035	MPO,APOB,TTR,LYZ,HYAL1,LUM,CSPG4,S100A7,GC,SERPINA3
GO Component	GO:1904813	ficolin-1-rich granule lumen	8	124	0.71	0.0075	VCL,CAT,TIMP2,APEH,QPCT,GSN,CALML5,CST3
GO Component	GO:0042581	Specific granule	9	159	0.65	0.0078	VCL,CFP,LYZ,TIMP2,CAMP,QPCT,QSOX1,CKAP4,B2M
GO Component	GO:0005764	Lysosome	21	746	0.35	0.0193	GNPTG,MPO,GPLD1,APOB,TTR,CAT,LRP1,LYZ,CP,TCIRG1,HYAL1,LUM,PCSK9,CSPG4,S100A7,MCOLN2,CKAP4,PCYOX1,ABCA13,GC,SERPINA3
GO Component	GO:0101002	ficolin-1-rich granule	9	185	0.58	0.0212	VCL,CAT,TIMP2,TCIRG1,APEH,QPCT,GSN,CALML5,CST3
GO Component	GO:0034365	Discoidal high-density lipoprotein particle	2	2	1.9	0.0245	APOE,APOM

GO Component	GO:0098552	Side of membrane	18	611	0.37	0.0268	EBI3,FCN3,FCN2,MASP1,ANTXR1,PCSK9,FGB,PLG,P4HB,FGG,MBL2,F10,HLA-C,COLEC11,IGHV3-15,IGHV3-72,B2M,FGA
GO Component	GO:0005773	Vacuole	22	839	0.32	0.0332	GNPTG,MPO,GPLD1,APOB,TTR,CAT,LRP1,LYZ,CP,TCIRG1,HYAL1,LUM,PCSK9,CSPG4,S100A7,MCOLN2,CKAP4,PCYOX1,ABCA13,MST1,GC,SERPINA3
GO Component	GO:0062167	Complement component C1q complex	2	3	1.72	0.0390	C1QB,C1QC
STRING clusters	CL:18723	Mixed, incl. Complement and coagulation cascades, and Protein-lipid complex	82	196	1.52	3.00e-88	SPP2,CPB2,CETP,APOH,SERPIND1,F9,PON1,C5,AFM,APOC3,PROC,APOA1,LRP1,C4BPB,CFP,APOE,APCS,CFHR5,VWF,SERPINA10,C9,LCAT,KLKB1,AMBP,HPX,FCN3,ITIH1,AHSG,HABP2,SAA4,SERPING1,FCN2,PGLYRP2,MASP1,PCSK9,FGB,F2,PLG,STAB1,C1QB,CFHR1,APOL1,CPN2,SERPINF2,C7,SERPINA7,SERPINA11,FGG,ITIH2,F8,C8A,F13B,CFHR4,CFHR3,CFH,F5,CD5L,C8B,ADAMTS13,MBL2,C1QC,F10,F7,APOM,PROS1,CFI,APOF,SAA1,F11,C1S,PCYOX1,C4A,COLEC11,ITIH3,C4B,CFB,PLTP,HPR,APOA5,SERPINA4,APOC4,FGA
STRING clusters	CL:18724	Mixed, incl. Complement and coagulation cascades, and Protein-lipid complex	79	172	1.56	1.37e-87	SPP2,CPB2,CETP,APOH,SERPIND1,F9,PON1,C5,AFM,APOC3,PROC,APOA1,LRP1,C4BPB,CFP,APOE,APCS,CFHR5,VWF,SERPINA10,C9,LCAT,KLKB1,AMBP,HPX,FCN3,ITIH1,AHSG,HABP2,SAA4,SERPING1,FCN2,PGLYRP2,MASP1,PCSK9,FGB,F2,PLG,C1QB,CFHR1,APOL1,CPN2,SERPINF2,C7,SERPINA7,SERPINA11,FGG,ITIH2,F8,C8A,F13B,CFHR4,CFHR3,CFH,F5,C8B,ADAMTS13,MBL2,C1QC,F10,F7,APOM,CFI,APOF,SAA1,F11,C1S,PCYOX1,C4A,COLEC11,ITIH3,C4B,CFB,PLTP,HPR,APOA5,SERPINA4,APOC4,FGA
STRING clusters	CL:18725	Complement and coagulation cascades, and Protein-lipid complex	78	166	1.57	3.58e-87	CPB2,CETP,APOH,SERPIND1,F9,PON1,C5,AFM,APOC3,PROC,APOA1,LRP1,C4BPB,CFP,APOE,APCS,CFHR5,VWF,SERPINA10,C9,LCAT,KLKB1,AMBP,HPX,FCN3,ITIH1,AHSG,HABP2,SAA4,SERPING1,FCN2,PGLYRP2,MASP1,PCSK9,FGB,F2,PLG,C1QB,CFHR1,APOL1,CPN2,SERPINF2,C7,SERPINA7,SERPINA11,FGG,ITIH2,F8,C8A,F13B,CFHR4,CFHR3,CFH,F5,C8B,ADAMTS13,MBL2,C1QC,F10,F7,APOM,CFI,APOF,SAA1,F11,C1S,PCYOX1,C4A,COLEC11,ITIH3,C4B,CFB,PLTP,HPR,APOA5,SERPINA4,APOC4,FGA
STRING clusters	CL:18726	Complement and coagulation cascades, and Protein-lipid complex	77	161	1.58	1.49e-86	CPB2,CETP,APOH,SERPIND1,F9,PON1,C5,AFM,APOC3,PROC,APOA1,LRP1,C4BPB,CFP,APOE,APCS,CFHR5,VWF,C9,LCAT,KLKB1,AMBP,HPX,FCN3,ITIH1,AHSG,HABP2,SAA4,SERPING1,FCN2,PGLYRP2,MASP1,PCSK9,FGB,F2,PLG,C1QB,CFHR1,APOL1,CPN2,SERPINF2,C7,SERPINA7,SERPINA11,FGG,ITIH2,F8,C8A,F13B,CFHR4,CFHR3,CFH,F5,C8B,ADAMTS13,MBL2,C1QC,F10,F7,APOM,CFI,APOF,SAA1,F11,C1S,PCYOX1,C4A,COLEC11,ITIH3,C4B,CFB,PLTP,HPR,APOA5,SERPINA4,APOC4,FGA
STRING clusters	CL:18727	Complement and coagulation cascades, and Positive regulation of opsonization	59	115	1.61	1.86e-66	CPB2,SERPIND1,F9,C5,AFM,PROC,LRP1,C4BPB,CFP,APCS,CFHR5,VWF,C9,KLKB1,AMBP,HPX,FCN3,ITIH1,AHSG,HABP2,SERPING1,FCN2,PGLYRP2,MASP1,FGB,F2,PLG,C1QB,CFHR1,CPN2,SERPINF2,C7,SERPINA7,SERPINA11,FGG,ITIH2,F8,C8A,F13B,CFHR4,CFHR3,CFH,F5,C8B,ADAMTS13,MBL2,C1QC,F10,F7,CFI,F11,C1S,C4A,COLEC11,ITIH3,C4B,CFB,SERPINA4,FGA
STRING clusters	CL:18728	Complement and coagulation cascades, and Positive regulation of opsonization	56	109	1.61	5.92e-63	CPB2,SERPIND1,F9,C5,PROC,LRP1,C4BPB,CFP,APCS,CFHR5,VWF,C9,KLKB1,AMBP,HPX,FCN3,ITIH1,AHSG,HABP2,SERPING1,FCN2,MASP1,FGB,F2,PLG,C1QB,CFHR1,SERPINF2,C7,SERPINA7,SERPINA11,FGG,ITIH2,F8,C8A,F13B,CFHR4,CFHR3,CFH,F5,C8B,ADAMTS13,MBL2,C1QC,F10,F7,CFI,F11,C1S,C4A,COLEC11,ITIH3,C4B,CFB,SERPINA4,FGA
STRING clusters	CL:18730	Hemostasis, and Dissolution of Fibrin Clot	28	55	1.6	4.32e-30	CPB2,SERPIND1,F9,PROC,LRP1,VWF,KLKB1,AMBP,HPX,ITIH1,AHSG,HABP2,FGB,F2,PLG,SERPINF2,FGG,ITIH2,F8,F13B,F5,ADAMTS13,F10,F7,F11,ITIH3,SERPINA4,FGA
STRING clusters	CL:18731	Hemostasis, and Dissolution of Fibrin Clot	27	50	1.63	1.53e-29	CPB2,SERPIND1,F9,PROC,LRP1,VWF,KLKB1,AMBP,HPX,ITIH1,AHSG,HABP2,FGB,F2,PLG,SERPINF2,FGG,ITIH2,F8,F13B,F5,ADAMTS13,F10,F7,F11,ITIH3,FGA
STRING clusters	CL:18846	Complement cascade	26	50	1.61	4.24e-28	C5,C4BPB,CFP,APCS,CFHR5,C9,FCN3,SERPING1,FCN2,MASP1,C1QB,CFHR1,C7,C8A,CFHR4,CFHR3,CFH,C8B,MBL2,C1QC,CFI,C1S,C4A,COLEC11,C4B,CFB
STRING clusters	CL:18848	Complement cascade	21	40	1.62	1.75e-22	C5,C4BPB,CFP,C9,FCN3,SERPING1,FCN2,MASP1,C1QB,C7,C8A,CFH,C8B,MBL2,C1QC,CFI,C1S,C4A,COLEC11,C4B,CFB
STRING clusters	CL:18732	Mixed, incl. COVID-19, thrombosis and anticoagulation, and Scavenging of heme from plasma	16	26	1.69	1.18e-17	CPB2,SERPIND1,LRP1,AMBP,HPX,ITIH1,AHSG,HABP2,FGB,PLG,SERPINF2,FGG,ITIH2,F13B,ITIH3,FGA
STRING clusters	CL:18960	High-density lipoprotein particle	16	26	1.69	1.18e-17	CETP,APOH,PON1,APOC3,APOA1,LCAT,SAA4,APOL1,APOM,APOF,SAA1,PCYOX1,PLTP,HPR,APOA5,APOC4
STRING clusters	CL:18956	Lipoprotein particle, and Assembly of active LPL and LIPC lipase complexes	18	46	1.49	2.38e-17	CETP,APOH,PON1,APOC3,APOA1,APOE,LCAT,SAA4,PCSK9,APOL1,APOM,APOF,SAA1,PCYOX1,PLTP,HPR,APOA5,APOC4
STRING clusters	CL:18733	Mixed, incl. COVID-19, thrombosis and anticoagulation, and Inter-alpha-trypsin inhibitor heavy chain C-terminus	14	21	1.72	9.16e-16	CPB2,SERPIND1,AMBP,ITIH1,AHSG,HABP2,FGB,PLG,SERPINF2,FGG,ITIH2,F13B,ITIH3,FGA
STRING clusters	CL:18962	High-density lipoprotein particle	14	21	1.72	9.16e-16	CETP,APOH,PON1,APOC3,APOA1,LCAT,APOL1,APOM,APOF,PCYOX1,PLTP,HPR,APOA5,APOC4
STRING clusters	CL:18849	Initial triggering of complement, and Regulation of complement activation	15	31	1.58	1.89e-15	CFP,FCN3,SERPING1,FCN2,MASP1,C1QB,CFH,MBL2,C1QC,CFI,C1S,C4A,COLEC11,C4B,CFB
STRING clusters	CL:18784	Formation of Fibrin Clot (Clotting Cascade)	11	18	1.68	6.92e-12	F9,PROC,VWF,KLKB1,F2,F8,F5,ADAMTS13,F10,F7,F11
STRING clusters	CL:18851	Initial triggering of complement, and Negative regulation of complement activation	11	24	1.56	7.40e-11	FCN3,SERPING1,FCN2,MASP1,C1QB,MBL2,C1QC,C1S,C4A,COLEC11,C4B
STRING clusters	CL:18966	High-density lipoprotein particle	10	16	1.69	7.74e-11	CETP,APOH,PON1,APOC3,APOA1,LCAT,APOM,PLTP,APOA5,APOC4
STRING clusters	CL:18734	COVID-19, thrombosis and anticoagulation, and Negative regulation of fibrinolysis	9	11	1.81	2.04e-10	CPB2,SERPIND1,HABP2,FGB,PLG,SERPINF2,FGG,F13B,FGA
STRING clusters	CL:18786	Hemophilia B, and Blood coagulation, common pathway	9	12	1.77	3.39e-10	F9,VWF,F2,F8,F5,ADAMTS13,F10,F7,F11
STRING clusters	CL:18852	Creation of C4 and C2 activators	9	15	1.67	1.40e-09	FCN3,SERPING1,FCN2,MASP1,C1QB,MBL2,C1QC,C1S,COLEC11
STRING clusters	CL:19457	Extracellular matrix organization	15	180	0.82	4.72e-06	MMP2,PCOLCE,VTN,SERPINF1,TIMP2,NID1,LUM,BMP1,DST,EFEMP2,P4HB,FBLN1,THBS4,EFEMP1,FBLN2
STRING clusters	CL:18970	Triglyceride-rich lipoprotein particle remodeling, and Spherical high-density lipoprotein particle	6	11	1.63	7.08e-06	CETP,PON1,APOC3,APOA1,LCAT,APOA5
STRING clusters	CL:18737	Fibrinogen, and Thrombophilia	5	6	1.82	2.19e-05	SERPIND1,FGB,PLG,FGG,FGA
STRING clusters	CL:18911	Membrane attack complex	5	7	1.75	3.58e-05	C5,C9,C7,C8A,C8B
STRING clusters	CL:18755	Mixed, incl. Inter-alpha-trypsin inhibitor heavy chain C-terminus, and Alpha-1-acid glycoprotein	5	10	1.6	0.00013	AMBP,ITIH1,AHSG,ITIH2,ITIH3
STRING clusters	CL:19114	Mixed, incl. S100/CaBP-9k-type, calcium binding, subdomain, and Cystatin superfamily	10	103	0.88	0.00024	LGALS1,MPO,LYZ,LGALS3BP,LCN1,CSTA,CAMP,S100A7,S100A9,CST3
STRING clusters	CL:19115	Mixed, incl. Cystatin superfamily, and Specific granule lumen	8	60	1.02	0.00031	LGALS1,MPO,LYZ,LGALS3BP,LCN1,CSTA,CAMP,CST3
STRING clusters	CL:19695	Elastic fibre formation, and Matrix metalloproteinases	8	61	1.01	0.00034	MMP2,PCOLCE,TIMP2,BMP1,EFEMP2,FBLN1,EFEMP1,FBLN2
STRING clusters	CL:18934	Hemolytic uremic syndrome	4	5	1.8	0.00040	CFHR5,CFHR1,CFHR4,CFHR3
STRING clusters	CL:18788	Extrinsic Pathway of Fibrin Clot Formation, and Factor V deficiency	4	6	1.72	0.00064	F2,F5,F10,F7
STRING clusters	CL:18854	Cell surface pattern recognition receptor signaling pathway	4	6	1.72	0.00064	FCN3,FCN2,MASP1,MBL2

STRING clusters	CL:18869	Complement component C1 complex	4	6	1.72	0.00064	SERPING1,C1QB,C1QC,C1S
STRING clusters	CL:19696	Elastic fibre formation, and Tolloid/BMP1 peptidase domain	6	34	1.14	0.0012	PCOLCE,BMP1,EFEMP2,FBLN1,EFEMP1,FBLN2
STRING clusters	CL:24268	Insulin-like growth factor-binding protein family 1-6, chordata, and Insulin-like growth factor	4	10	1.5	0.0026	IGFBP5,IGFBP3,IGF2,IGFALS
STRING clusters	CL:19699	Elastic fibre formation	4	14	1.35	0.0075	EFEMP2,FBLN1,EFEMP1,FBLN2
STRING clusters	CL:18123	Cell-extracellular matrix interactions, and Alpha-catenin/vinculin-like superfamily	4	17	1.27	0.0136	VCL,LIMS1,FLNA,ILK
STRING clusters	CL:18945	Mixed, incl. Peptide amidation, and TonB box, conserved site	3	6	1.6	0.0168	AFM,PGLYRP2,CPN2
STRING clusters	CL:1545	Photodynamic therapy-induced unfolded protein response, and IRE1-TRAF2-ASK1 complex	4	19	1.22	0.0188	HSP90B1,HSPA5,HYOU1,PDIA4
STRING clusters	CL:19118	Mixed, incl. Proteinase inhibitor I25, cystatin, conserved site, and Cysteine peptidase, asparagine active site	4	24	1.12	0.0405	LGALS1,LGALS3BP,CSTA,CST3
KEGG	hsa04610	Complement and coagulation cascades	41	82	1.6	3.72e-45	CPB2,SERPIND1,F9,C5,VTN,PROC,C4BPB,CFHR5,VWF,C9,KLKB1,SERPING1,FGB,F2,PLG,C1QB,CFHR1,SERPINF2,C7,FGG,F8,C8A,F13B,CFHR4,CFHR3,CFH,F5,C8B,MBL2,C1QC,F10,F7,PROS1,CFI,F11,C1S,C4A,C4B,CFB,KNG1,FGA
KEGG	hsa05150	Staphylococcus aureus infection	13	86	1.08	5.50e-08	C5,CAMP,PLG,C1QB,FGG,CFH,MBL2,C1QC,CFI,C1S,C4A,C4B,CFB
KEGG	hsa04979	Cholesterol metabolism	10	48	1.22	2.75e-07	CETP,APOH,APOC3,APOB,APOA1,LRP1,APOE,LCAT,PCSK9,PLTP
KEGG	hsa05133	Pertussis	9	73	0.99	6.23e-05	C5,C4BPB,SERPING1,C1QB,C1QC,CALML5,C1S,C4A,C4B
KEGG	hsa05322	Systemic lupus erythematosus	10	94	0.92	6.23e-05	C5,C9,C1QB,C7,C8A,C8B,C1QC,C1S,C4A,C4B
KEGG	hsa04918	Thyroid hormone synthesis	7	73	0.88	0.0036	TTR,HSP90B1,HSPA5,SERPINA7,GPX3,ATP1A1,PDIA4
KEGG	hsa03320	PPAR signaling pathway	6	75	0.8	0.0259	APOC3,APOA1,ILK,ADIPOQ,PLTP,APOA5
KEGG	hsa04510	Focal adhesion	9	195	0.56	0.0471	VCL,VTN,VWF,MYL9,THBS4,FLNA,ILK,VEGFC,ACTB
KEGG	hsa04611	Platelet activation	7	122	0.66	0.0471	VWF,GUCY1A1,FGB,F2,FGG,ACTB,FGA
KEGG	hsa04970	Salivary secretion	6	89	0.73	0.0471	LYZ,CAMP,GUCY1A1,CALML5,CST3,ATP1A1
KEGG	hsa05143	African trypanosomiasis	4	36	0.94	0.0472	APOA1,APOL1,HPR,KNG1
Reactome	HSA-166658	Complement cascade	29	59	1.59	2.40e-30	CPB2,C5,VTN,C4BPB,CFP,CFHR5,C9,FCN3,SERPING1,FCN2,F2,C1QB,CFHR1,CPN2,C7,C8A,CFHR4,CFHR3,CFH,C8B,MBL2,C1QC,PROS1,CFI,C1S,C4A,COLEC11,C4B,CFB
Reactome	HSA-381426	Regulation of Insulin-like Growth Factor (IGF) transport and uptake by Insulin-like Growth Factor Binding Proteins (IGFBPs)	34	124	1.33	2.69e-29	RCN1,SPP2,SERPIND1,LGALS1,MMP2,APOB,IGFBP5,PROC,APOA1,APOE,SERPINA10,CP,AHSG,HSP90B1,PCSK9,F2,PLG,APOL1,P4HB,FGG,ITIH2,QSOX1,F5,CKAP4,IGFBP3,CST3,NUCB1,IGF2,C4A,SPARCL1,IGFALS,APOA5,KNG1,FGA
Reactome	HSA-114608	Platelet degranulation	32	126	1.3	1.04e-26	SPP2,APOH,VCL,SPARC,PLEK,APOA1,VWF,LGALS3BP,ITIH4,AHSG,SERPING1,PPBP,PF4,FGB,PLG,SERPINF2,HSPA5,FGG,F8,QSOX1,F5,FLNA,PROS1,IGF2,ITIH3,RARRES2,SELENOP,SERPINA3,SERPINA4,VEGFC,KNG1,FGA
Reactome	HSA-977606	Regulation of Complement cascade	25	49	1.6	1.04e-26	CPB2,C5,VTN,C4BPB,CFP,CFHR5,C9,SERPING1,F2,C1QB,CFHR1,CPN2,C7,C8A,CFHR4,CFHR3,CFH,C8B,C1QC,PROS1,CFI,C1S,C4A,C4B,CFB
Reactome	HSA-8957275	Post-translational protein phosphorylation	29	107	1.33	5.30e-25	RCN1,SPP2,SERPIND1,LGALS1,APOB,IGFBP5,PROC,APOA1,APOE,SERPINA10,CP,AHSG,HSP90B1,PCSK9,APOL1,P4HB,FGG,ITIH2,QSOX1,F5,CKAP4,IGFBP3,CST3,NUCB1,C4A,SPARCL1,APOA5,KNG1,FGA
Reactome	HSA-109582	Hemostasis	52	607	0.83	2.06e-24	SPP2,APOH,VCL,SERPIND1,F9,PF4V1,SPARC,APOB,PROC,PLEK,APOA1,VWF,LGALS3BP,KLKB1,CENPE,ITIH4,PSG9,AHSG,SERPING1,PPBP,PF4,GUCY1A1,FGB,F2,PLG,PSG11,SERPINF2,HSPA5,FGG,F8,F13B,QSOX1,F5,FLNA,F10,F7,CD99,KIF19,PROS1,YWHAZ,RAPGEF4,F11,IGF2,ITIH3,RARRES2,SELENOP,SERPINA3,SERPINA4,VEGFC,KNG1,ACTB,FGA
Reactome	HSA-168249	Innate Immune System	63	1041	0.68	1.87e-22	CPB2,VCL,CHGA,LBP,C5,MPO,VTN,APOB,TTR,CAT,C4BPB,CFP,CFHR5,LYZ,PSMA6,TIMP2,C9,TCIRG1,FCN3,AHSG,SERPING1,FCN2,PGLYRP2,PPBP,CAMP,APEH,HSP90B1,FGB,F2,C1QB,CFHR1,CPN2,C7,FGG,QPCT,C8A,CFHR4,CFHR3,CFH,QSOX1,S100A7,S100A9,C8B,GSN,MBL2,C1QC,HLA-C,CKAP4,CALML5,PROS1,CFI,CST3,SAA1,C1S,C4A,ABCA13,COLEC11,C4B,CFB,SERPINA3,ACTB,B2M,FGA
Reactome	HSA-76002	Platelet activation, signaling and aggregation	35	260	1.03	1.21e-21	SPP2,APOH,VCL,SPARC,PLEK,APOA1,VWF,LGALS3BP,ITIH4,AHSG,SERPING1,PPBP,PF4,FGB,F2,PLG,SERPINF2,HSPA5,FGG,F8,QSOX1,F5,FLNA,PROS1,YWHAZ,RAPGEF4,IGF2,ITIH3,RARRES2,SELENOP,SERPINA3,SERPINA4,VEGFC,KNG1,FGA
Reactome	HSA-140877	Formation of Fibrin Clot (Clotting Cascade)	20	39	1.61	1.75e-21	SERPIND1,F9,PF4V1,PROC,VWF,KLKB1,SERPING1,PF4,FGB,F2,FGG,F8,F13B,F5,F10,F7,PROS1,F11,KNG1,FGA
Reactome	HSA-168256	Immune System	73	1979	0.46	1.13e-14	CPB2,VCL,CHGA,LBP,MMP2,EBI3,C5,MPO,VTN,APOB,TTR,CAT,C4BPB,CFP,CFHR5,LYZ,PSMA6,TIMP2,C9,CENPE,TCIRG1,FCN3,AHSG,SERPING1,CSF1R,FCN2,PGLYRP2,PPBP,CAMP,APEH,HSP90B1,FGB,F2,C1QB,CFHR1,CPN2,C7,HSPA5,P4HB,FGG,QPCT,C8A,CFHR4,CFHR3,CFH,QSOX1,S100A7,S100A9,FLNA,C8B,GSN,MBL2,C1QC,HLA-C,CKAP4,CALML5,CD99,PROS1,CFI,YWHAZ,RAPGEF4,CST3,SAA1,C1S,C4A,ABCA13,COLEC11,C4B,CFB,SERPINA3,ACTB,B2M,FGA
Reactome	HSA-140875	Common Pathway of Fibrin Clot Formation	13	22	1.67	3.02e-14	SERPIND1,PF4V1,PROC,PF4,FGB,F2,FGG,F8,F13B,F5,F10,PROS1,FGA
Reactome	HSA-140837	Intrinsic Pathway of Fibrin Clot Formation	12	23	1.61	1.31e-12	SERPIND1,F9,PROC,VWF,KLKB1,SERPING1,F2,F8,F10,PROS1,F11,KNG1
Reactome	HSA-2173782	Binding and Uptake of Ligands by Scavenger Receptors	14	43	1.41	1.31e-12	SPARC,APOB,APOA1,LRP1,APOE,AMBP,HPX,HSP90B1,STAB1,APOL1,SAA1,COLEC11,HPR,HYOU1
Reactome	HSA-166663	Initial triggering of complement	11	24	1.56	4.68e-11	CFP,FCN3,FCN2,C1QB,MBL2,C1QC,C1S,C4A,COLEC11,C4B,CFB
Reactome	HSA-392499	Metabolism of proteins	60	1917	0.39	8.69e-09	RCN1,SPP2,ST6GAL1,CPB2,SERPIND1,LGALS1,F9,MMP2,GPLD1,APOB,IGFBP5,PROC,APOA1,TTR,CFP,APOE,APCS,LYZ,PSMA6,SERPINA10,CP,AHSG,APEH,HSP90B1,PCSK9,F2,INHBC,PLG,SUZ12,APOL1,P4HB,FGG,GANAB,BLM,ITIH2,F8,QSOX1,F5,ADAMTSL4,ADAMTS13,GSN,F10,F7,CKAP4,IGFBP3,PROS1,CST3,ANKRD28,SAA1,NUCB1,IGF2,C4A,SPARCL1,IGFALS,UIMC1,APOA5,KNG1,ACTB,B2M,FGA
Reactome	HSA-174824	Plasma lipoprotein assembly, remodeling, and clearance	13	75	1.14	9.64e-09	CETP,APOC3,APOB,APOA1,APOE,LCAT,PCSK9,BMP1,P4HB,APOF,PLTP,APOA5,APOC4
Reactome	HSA-1474244	Extracellular matrix organization	22	300	0.76	1.64e-08	MMP2,PCOLCE,VTN,SPARC,TTR,PXDN,VWF,TIMP2,NID1,KLKB1,LUM,BMP1,FGB,DST,PLG,EFEMP2,P4HB,FBLN1,FGG,EFEMP1,FBLN2,FGA
Reactome	HSA-8963899	Plasma lipoprotein remodeling	10	34	1.37	1.64e-08	CETP,APOC3,APOB,APOA1,APOE,LCAT,P4HB,APOF,PLTP,APOA5
Reactome	HSA-9651496	Defects of contact activation system (CAS) and kallikrein/kinin system (KKS)	8	16	1.6	3.84e-08	F9,VWF,KLKB1,SERPING1,F2,F8,F10,F11
Reactome	HSA-597592	Post-translational protein modification	47	1405	0.42	1.56e-07	RCN1,SPP2,ST6GAL1,SERPIND1,LGALS1,F9,GPLD1,APOB,IGFBP5,PROC,APOA1,CFP,APOE,PSMA6,SERPINA10,CP,AHSG,HSP90B1,PCSK9,F2,SUZ12,APOL1,P4HB,FGG,GANAB,BLM,ITIH2,F8,QSOX1,F5,ADAMTSL4,ADAMTS13,F10,F7,CKAP4,IGFBP3,PROS1,CST3,ANKRD28,NUCB1,C4A,SPARCL1,UIMC1,APOA5,KNG1,ACTB,FGA
Reactome	HSA-166786	Creation of C4 and C2 activators	7	16	1.54	9.15e-07	FCN3,FCN2,C1QB,MBL2,C1QC,C1S,COLEC11
Reactome	HSA-159763	Transport of gamma-carboxylated protein precursors from the endoplasmic reticulum to the Golgi apparatus	6	9	1.72	1.58e-06	F9,PROC,F2,F10,F7,PROS1
Reactome	HSA-6798695	Neutrophil degranulation	24	476	0.6	1.92e-06	VCL,MPO,TTR,CAT,CFP,LYZ,TIMP2,TCIRG1,AHSG,PPBP,CAMP,APEH,QPCT,QSOX1,S100A7,S100A9,GSN,HLA-C,CKAP4,CALML5,CST3,ABCA13,SERPINA3,B2M
Reactome	HSA-8963898	Plasma lipoprotein assembly	7	19	1.46	2.10e-06	APOC3,APOB,APOA1,APOE,BMP1,P4HB,APOC4

Reactome	HSA-159740	Gamma-carboxylation of protein precursors	6	10	1.67	2.22e-06	F9,PROC,F2,F10,F7,PROS1
Reactome	HSA-159782	Removal of aminoterminal propeptides from gamma-carboxylated proteins	6	10	1.67	2.22e-06	F9,PROC,F2,F10,F7,PROS1
Reactome	HSA-8964058	HDL remodeling	6	10	1.67	2.22e-06	CETP,APOC3,APOA1,APOE,LCAT,PLTP
Reactome	HSA-2168880	Scavenging of heme from plasma	6	15	1.5	1.25e-05	APOA1,LRP1,AMBP,HPX,APOL1,HPR
Reactome	HSA-977225	Amyloid fiber formation	10	79	1.0	1.25e-05	APOA1,TTR,APOE,APCS,LYZ,GSN,CST3,SAA1,B2M,FGA
Reactome	HSA-174577	Activation of C3 and C5	5	7	1.75	1.51e-05	C5,CFP,C4A,C4B,CFB
Reactome	HSA-9662001	Defective factor VIII causes hemophilia A	5	7	1.75	1.51e-05	F9,VWF,F2,F8,F10
Reactome	HSA-166665	Terminal pathway of complement	5	8	1.69	2.30e-05	C5,C9,C7,C8A,C8B
Reactome	HSA-5686938	Regulation of TLR by endogenous ligand	6	20	1.37	4.32e-05	LBP,APOB,FGB,FGG,S100A9,FGA
Reactome	HSA-8963888	Chylomicron assembly	5	10	1.6	4.97e-05	APOC3,APOB,APOA1,APOE,P4HB
Reactome	HSA-8963901	Chylomicron remodeling	5	10	1.6	4.97e-05	APOC3,APOB,APOA1,APOE,APOA5
Reactome	HSA-163841	Gamma carboxylation, hypusine formation and arylsulfatase activation	7	42	1.12	0.00014	F9,PROC,F2,F8,F10,F7,PROS1
Reactome	HSA-975634	Retinoid metabolism and transport	7	44	1.1	0.00018	APOC3,APOB,APOA1,TTR,LRP1,APOE,APOM
Reactome	HSA-8964041	LDL remodeling	4	6	1.72	0.00027	CETP,APOB,P4HB,APOF
Reactome	HSA-166662	Lectin pathway of complement activation	4	7	1.65	0.00039	FCN3,FCN2,MBL2,COLEC11
Reactome	HSA-3000480	Scavenging by Class A Receptors	5	18	1.34	0.00043	APOB,APOA1,APOE,HSP90B1,COLEC11
Reactome	HSA-6802948	Signaling by high-kinase activity BRAF mutants	6	36	1.12	0.00067	VCL,VWF,FGB,FGG,ACTB,FGA
Reactome	HSA-9029569	NR1H3 & NR1H2 regulate gene expression linked to cholesterol transport and efflux	6	36	1.12	0.00067	CETP,APOE,APOD,TNRC6A,PLTP,APOC4
Reactome	HSA-9668250	Defective factor IX causes hemophilia B	4	9	1.54	0.00077	F9,F8,F10,F11
Reactome	HSA-76009	Platelet Aggregation (Plug Formation)	6	39	1.08	0.00094	VWF,FGB,F2,FGG,RAPGEF4,FGA
Reactome	HSA-5653656	Vesicle-mediated transport	23	666	0.43	0.00095	SPARC,APOB,APOA1,LRP1,APOE,YWHAE,AMBP,CENPE,HPX,HSP90B1,SH3D19,STAB1,APOL1,F8,F5,KIF19,YWHAZ,ANKRD28,SAA1,COLEC11,HPR,HYOU1,ACTB
Reactome	HSA-5674135	MAP2K and MAPK activation	6	40	1.07	0.0010	VCL,VWF,FGB,FGG,ACTB,FGA
Reactome	HSA-9656223	Signaling by RAF1 mutants	6	41	1.06	0.0011	VCL,VWF,FGB,FGG,ACTB,FGA
Reactome	HSA-6802952	Signaling by BRAF and RAF1 fusions	7	65	0.93	0.0014	VCL,VWF,FGB,FGG,AKAP9,ACTB,FGA
Reactome	HSA-446728	Cell junction organization	8	92	0.84	0.0016	CDH6,CDH11,DST,ANG,LIMS1,FLNA,ILK,ACTB
Reactome	HSA-6802946	Signaling by moderate kinase activity BRAF mutants	6	45	1.02	0.0016	VCL,VWF,FGB,FGG,ACTB,FGA
Reactome	HSA-6802955	Paradoxical activation of RAF signaling by kinase inactive BRAF	6	45	1.02	0.0016	VCL,VWF,FGB,FGG,ACTB,FGA
Reactome	HSA-9649948	Signaling downstream of RAS mutants	6	45	1.02	0.0016	VCL,VWF,FGB,FGG,ACTB,FGA
Reactome	HSA-9672383	Defective factor IX causes thrombophilia	3	3	1.9	0.0016	F9,F8,F10
Reactome	HSA-9672391	Defective F8 cleavage by thrombin	3	3	1.9	0.0016	VWF,F2,F8
Reactome	HSA-9672396	Defective cofactor function of FVIIIa variant	3	3	1.9	0.0016	F9,F8,F10
Reactome	HSA-9673202	Defective F9 variant does not activate FX	3	3	1.9	0.0016	F9,F8,F10
Reactome	HSA-354192	Integrin signaling	5	27	1.16	0.0017	VWF,FGB,FGG,RAPGEF4,FGA
Reactome	HSA-3000497	Scavenging by Class H Receptors	3	4	1.77	0.0023	SPARC,APOB,STAB1
Reactome	HSA-354194	GRB2:SOS provides linkage to MAPK signaling for Integrins	4	15	1.32	0.0028	VWF,FGB,FGG,FGA
Reactome	HSA-372708	p130Cas linkage to MAPK signaling for integrins	4	15	1.32	0.0028	VWF,FGB,FGG,FGA
Reactome	HSA-140834	Extrinsic Pathway of Fibrin Clot Formation	3	5	1.67	0.0035	F9,F10,F7
Reactome	HSA-202733	Cell surface interactions at the vascular wall	9	139	0.71	0.0035	PF4V1,APOB,PROC,PSG9,PF4,F2,PSG11,CD99,PROS1
Reactome	HSA-8866423	VLDL assembly	3	5	1.67	0.0035	APOB,P4HB,APOC4
Reactome	HSA-5602498	MyD88 deficiency (TLR2/4)	4	17	1.27	0.0040	FGB,FGG,S100A9,FGA
Reactome	HSA-446353	Cell-extracellular matrix interactions	4	18	1.24	0.0047	LIMS1,FLNA,ILK,ACTB
Reactome	HSA-5603041	IRAK4 deficiency (TLR2/4)	4	18	1.24	0.0047	FGB,FGG,S100A9,FGA
Reactome	HSA-3000471	Scavenging by Class B Receptors	3	6	1.6	0.0048	APOB,APOA1,SAA1
Reactome	HSA-2129379	Molecules associated with elastic fibres	5	37	1.03	0.0053	VTN,EFEMP2,FBLN1,EFEMP1,FBLN2
Reactome	HSA-8964043	Plasma lipoprotein clearance	5	37	1.03	0.0053	APOB,APOA1,APOE,PCSK9,APOC4
Reactome	HSA-1236974	ER-Phagosome pathway	7	89	0.79	0.0061	PSMA6,FGB,FGG,S100A9,HLA-C,B2M,FGA
Reactome	HSA-1643685	Disease	39	1702	0.26	0.0079	ST6GAL1,VCL,F9,APOA1,TTR,CFP,VWF,PSMA6,YWHAE,CP,KLKB1,HYAL1,LUM,SERPING1,IHH,ANTXR1,FGB,F2,CSPG4,SUZ12,FGG,GANAB,BLM,AKAP9,F8,S100A9,ADAMTSL4,ADAMTS13,MBL2,F10,HLA-C,TAF9,YWHAZ,F11,ATP1A1,TPM4,ACTB,B2M,FGA
Reactome	HSA-173623	Classical antibody-mediated complement activation	3	9	1.42	0.0110	C1QB,C1QC,C1S
Reactome	HSA-382551	Transport of small molecules	21	723	0.36	0.0130	CETP,APOC3,APOB,APOA1,APOE,PSMA6,LCN1,LCAT,CP,TCIRG1,PCSK9,BMP1,P4HB,APOD,MCOLN2,APOF,ATP9B,PLTP,APOA5,ATP1A1,APOC4
Reactome	HSA-430116	GP1b-IX-V activation signalling	3	12	1.29	0.0213	VWF,FLNA,YWHAZ
Reactome	HSA-9672393	Defective F8 binding to von Willebrand factor	2	2	1.9	0.0245	VWF,F8
Reactome	HSA-216083	Integrin cell surface interactions	6	85	0.75	0.0257	VTN,VWF,LUM,FGB,FGG,FGA
Reactome	HSA-6803157	Antimicrobial peptides	6	87	0.74	0.0284	CHGA,LYZ,PGLYRP2,CAMP,S100A7,S100A9
Reactome	HSA-1592389	Activation of Matrix Metalloproteinases	4	33	0.98	0.0288	MMP2,TIMP2,KLKB1,PLG
Reactome	HSA-418990	Adherens junctions interactions	4	33	0.98	0.0288	CDH6,CDH11,ANG,ACTB
Reactome	HSA-168898	Toll-like Receptor Cascades	8	161	0.59	0.0330	LBP,APOB,HSP90B1,FGB,FGG,S100A9,SAA1,FGA
Reactome	HSA-3299685	Detoxification of Reactive Oxygen Species	4	36	0.94	0.0371	CAT,P4HB,SOD3,GPX3
Reactome	HSA-9657688	Defective factor XII causes hereditary angioedema	2	3	1.72	0.0371	KLKB1,F2
Reactome	HSA-9657689	Defective SERPING1 causes hereditary angioedema	2	3	1.72	0.0371	KLKB1,SERPING1
WikiPathways	WP558	Complement and coagulation cascades	28	58	1.58	1.66e-29	CPB2,SERPIND1,F9,PROC,VWF,C9,KLKB1,SERPING1,MASP1,FGB,F2,PLG,C1QB,SERPINF2,C7,F8,F13B,CFH,F5,C1QC,F10,F7,PROS1,CFI,C1S,C4B,CFB,KNG1
WikiPathways	WP2806	Complement system	27	96	1.35	1.63e-23	C5,VTN,APOA1,CFP,APCS,C9,KLKB1,SERPING1,FCN2,MASP1,FGB,PLG,C7,FGG,C8A,CFHR4,CFH,MBL2,F10,PROS1,CFI,F11,C1S,ADIPOQ,C4A,CFB,FGA
WikiPathways	WP5090	Complement system in neuronal development and plasticity	23	105	1.24	7.25e-18	C5,VTN,C4BPB,CFP,C9,FCN3,SERPING1,FCN2,MASP1,C1QB,C7,C8A,CFH,C8B,MBL2,C1QC,PROS1,CFI,C1S,C4A,COLEC11,C4B,CFB
WikiPathways	WP272	Blood clotting cascade	15	22	1.73	2.77e-17	F9,VWF,KLKB1,FGB,F2,PLG,SERPINF2,FGG,F8,F13B,F5,F10,F7,F11,FGA
WikiPathways	WP545	Complement activation	13	22	1.67	2.48e-14	C5,CFP,C9,MASP1,C1QB,C7,C8A,C8B,C1QC,C1S,C4A,C4B,CFB
WikiPathways	WP176	Folate metabolism	16	67	1.27	6.99e-13	MPO,APOB,APOA1,CAT,SAA4,FGB,F2,PLG,HBA1,FGG,F7,SOD3,GPX3,SAA1,SERPINA3,FGA
WikiPathways	WP5115	Network map of SARS-CoV-2 signaling pathway	24	218	0.94	6.99e-13	APOH,LBP,APOA1,CFP,SERPINA10,LGALS3BP,ITIH4,PF4,FGB,APOL1,FGG,APOD,C8A,CFH,GSN,APOM,IGFBP3,CFI,SAA1,C1S,ITIH3,CFB,ACTB,FGA
WikiPathways	WP15	Selenium micronutrient network	17	84	1.2	8.22e-13	MPO,APOB,APOA1,CAT,SAA4,FGB,F2,PLG,HBA1,FGG,F7,SOD3,GPX3,SAA1,SELENOP,SERPINA3,FGA
WikiPathways	WP1533	Vitamin B12 metabolism	12	50	1.28	1.24e-09	MPO,APOB,APOA1,APOE,SAA4,F2,PLG,HBA1,F7,SOD3,SAA1,SERPINA3
WikiPathways	WP5323	Fatty Acids and Lipoproteins Transport in Hepatocytes	25	380	0.71	4.63e-09	CETP,APOH,PON1,VTN,APOC3,APOB,APOA1,LRP1,APOE,LGALS3BP,LCAT,SAA4,PCSK9,APOL1,CD5L,APOM,CFI,APOF,SAA1,PCYOX1,ADIPOQ,ATP9B,PLTP,APOA5,APOC4
WikiPathways	WP430	Statin inhibition of cholesterol production	9	29	1.39	5.57e-08	CETP,APOC3,APOB,APOA1,LRP1,APOE,LCAT,PLTP,APOA5
WikiPathways	WP4927	COVID-19, thrombosis and anticoagulation	6	7	1.83	3.79e-07	FGB,F2,PLG,FGG,F13B,FGA
WikiPathways	WP3601	Lipid particles composition	6	10	1.67	1.58e-06	CETP,APOC3,APOB,APOA1,APOE,LCAT

WikiPathways	WP5186	Vitamin K metabolism and activation of dependent proteins	6	12	1.6	3.33e-06	F9,PROC,F2,F10,F7,PROS1
WikiPathways	WP5304	Cholesterol metabolism	10	72	1.04	4.03e-06	CETP,APOH,APOC3,APOB,APOA1,LRP1,APOE,LCAT,PCSK9,PLTP
WikiPathways	WP5110	Familial hyperlipidemia type 3	6	13	1.56	4.21e-06	CETP,APOA1,LRP1,APOE,LCAT,PLTP
WikiPathways	WP5112	Familial hyperlipidemia type 5	6	15	1.5	7.77e-06	CETP,APOA1,LRP1,LCAT,PLTP,APOA5
WikiPathways	WP5109	Familial hyperlipidemia type 2	6	16	1.47	9.98e-06	CETP,APOB,APOA1,LCAT,PCSK9,PLTP
WikiPathways	WP4522	Metabolic pathway of LDL, HDL and TG, including diseases	6	17	1.44	1.27e-05	CETP,APOB,APOA1,APOE,LCAT,PCSK9
WikiPathways	WP2328	Allograft rejection	10	88	0.95	1.59e-05	C5,C9,C1QB,C7,C8A,C8B,C1QC,HLA-C,C4A,C4B
WikiPathways	WP5108	Familial hyperlipidemia type 1	6	18	1.42	1.59e-05	CETP,APOA1,LRP1,LCAT,PLTP,APOA5
WikiPathways	WP5111	Familial hyperlipidemia type 4	6	22	1.33	3.87e-05	CETP,APOA1,LRP1,LCAT,PLTP,APOA5
WikiPathways	WP3941	Oxidative damage response	5	39	1.0	0.0073	C5,C1QB,C1QC,C1S,C4B
WikiPathways	WP5089	Kinin-Kallikrein pathway	3	7	1.53	0.0073	KLKB1,SERPING1,KNG1
WikiPathways	WP3942	PPAR signaling pathway	6	67	0.85	0.0096	APOC3,APOA1,ILK,ADIPOQ,PLTP,APOA5
WikiPathways	WP4136	Fibrin complement receptor 3 signaling pathway	5	43	0.96	0.0098	LBP,FGB,PLG,FGG,FGA
WikiPathways	WP2878	PPAR-alpha pathway	4	26	1.08	0.0148	APOC3,APOA1,PLTP,APOA5
WikiPathways	WP306	Focal adhesion	9	195	0.56	0.0313	VCL,VTN,VWF,MYL9,THBS4,FLNA,ILK,VEGFC,ACTB
Monarch	EFO:0007937	Blood protein measurement	96	1810	0.62	8.09e-31	ST6GAL1,CPB2,GNPTG,APOH,CHGA,LBP,EBI3,PON1,C5,MPO,VTN,AFM,APOC3,APOB,IGFBP5,PROC,CAT,APOE,PXDN,SERPINF1,CFHR5,LYZ,VWF,DSG2,SERPINA10,TIMP2,C9,LCAT,NID1,KLKB1,AMBP,HPX,ITIH4,CDH11,PSG9,ITIH1,AHSG,HABP2,SAA4,SERPING1,ANKRD44,CSF1R,FCN2,PGLYRP2,MASP1,HSP90B1,ANTXR1,F2,PLG,APOL1,CPN2,SERPINF2,C7,SERPINA11,ANG,ITIH2,CNDP1,F13B,CFHR4,CFH,QSOX1,F5,S100A9,CRTAC1,C8B,ADAMTS13,CCN5,GSN,MBL2,C1QC,F10,F7,APOM,IGFBP3,SOD3,CST3,SAA1,F11,C1S,PCYOX1,COLEC11,MST1,SPARCL1,C4B,CFB,PLTP,GC,HGFAC,HPR,SERPINA4,RNASE4,APOC4,CCL18,VEGFC,KNG1,SDF4
Monarch	EFO:0004747	Protein measurement	141	5856	0.28	1.89e-14	RCN1,ST6GAL1,CPB2,CETP,GNPTG,APOH,CHGA,LBP,EBI3,PON1,C5,MPO,VTN,AFM,APOC3,GPLD1,APOB,IGFBP5,PROC,APOA1,CAT,LRP1,C4BPB,APOE,PXDN,ANGPTL6,SERPINF1,APCS,CFHR5,LYZ,VWF,DSG2,SERPINA10,TIMP2,C9,LCAT,NID1,CP,KLKB1,DNAH5,AMBP,TCIRG1,HPX,ITIH4,CDH11,PSG9,ITIH1,AHSG,HABP2,SAA4,SERPING1,ANKRD44,CSF1R,FCN2,PGLYRP2,MASP1,APEH,HSP90B1,ANTXR1,PCSK9,FGB,F2,INHBC,PLG,STAB1,APOL1,CPN2,SERPINF2,C7,HBA1,FBLN1,DNAH8,OIT3,SERPINA11,ANG,FGG,LIMS1,THBS4,APOD,PAPLN,ITIH2,CNDP1,CDC42BPA,CENPF,F13B,CFHR4,CFH,QSOX1,F5,S100A9,CRTAC1,C8B,ADAMTS13,CCN5,GSN,MBL2,C1QC,F10,F7,APOM,HLA-C,IGFBP3,SOD3,CFI,EFCAB5,TNRC6A,YWHAZ,NAV2,RAPGEF4,CST3,ANKRD28,SAA1,F11,C1S,PCYOX1,ADIPOQ,ABCA13,COLEC11,MST1,SPARCL1,ITIH3,C4B,CFB,IGFALS,PLTP,GC,HGFAC,SELENOP,HPR,APOA5,SERPINA4,RNASE4,APOC4,CCL18,VEGFC,KNG1,ACTB,TRIM66,GRHL2,FGA,SDF4
Monarch	HP:0005339	Abnormality of complement system	13	24	1.63	1.53e-12	C5,CFP,C9,C1QB,C7,C8A,CFH,C8B,C1QC,CFI,C1S,C4A,C4B
Monarch	HP:0025015	Abnormal vascular morphology	47	1027	0.56	1.34e-10	SERPIND1,F9,MMP2,MGP,SPARC,APOB,PROC,TTR,CAT,APOE,ANGPTL6,VWF,LCAT,NID1,DNAH5,TCIRG1,HABP2,MASP1,GUCY1A1,A2ML1,ANTXR1,PCSK9,FGB,F2,EFEMP2,SERPINF2,P4HB,FGG,GANAB,F8,F13B,F5,FLNA,CRTAC1,ADAMTS13,F10,F7,PROS1,EFEMP1,CFI,CST3,IGF2,C4A,VEGFC,KNG1,ACTB,FGA
Monarch	HP:0011025	Abnormal cardiovascular system physiology	50	1169	0.53	1.74e-10	GNPTG,VCL,F9,MMP2,MGP,SPARC,APOB,PROC,APOA1,TTR,APOE,ANGPTL6,SERPINF1,LYZ,VWF,DSG2,LCAT,CP,TCIRG1,SERPING1,GUCY1A1,A2ML1,PCSK9,BMP1,FGB,DST,F2,EFEMP2,SERPINF2,HBA1,FGG,GANAB,AKAP9,F8,F13B,F5,ADAMTSL4,FLNA,CRTAC1,ADAMTS13,GSN,F10,F7,PROS1,CST3,F11,KNG1,ACTB,B2M,FGA
Monarch	HP:0004431	Complement deficiency	11	21	1.62	2.00e-10	C5,C9,C1QB,C7,C8A,CFH,C8B,C1QC,CFI,C4A,C4B
Monarch	HP:0002597	Abnormality of the vasculature	60	1673	0.45	3.73e-10	SERPIND1,F9,MMP2,MGP,SPARC,APOB,PROC,APOA1,TTR,CAT,APOE,ANGPTL6,SERPINF1,LYZ,VWF,LCAT,NID1,CSTA,DNAH5,TCIRG1,HABP2,SERPING1,MASP1,GUCY1A1,A2ML1,ANTXR1,PCSK9,BMP1,FGB,F2,PLG,EFEMP2,SERPINF2,HBA1,P4HB,FGG,GANAB,BLM,F8,CENPF,F13B,F5,FLNA,CRTAC1,ADAMTS13,GSN,F10,F7,PROS1,EFEMP1,CFI,CST3,C1S,IGF2,C4A,VEGFC,KNG1,ACTB,B2M,FGA
Monarch	HP:0003256	Abnormality of the coagulation cascade	17	105	1.11	3.73e-10	F9,PROC,VWF,FGB,F2,PLG,FGG,F8,F13B,F5,FLNA,F10,F7,PROS1,F11,KNG1,FGA
Monarch	HP:0000118	Phenotypic abnormality	119	5129	0.26	4.31e-10	ST6GAL1,CETP,GNPTG,VCL,SERPIND1,F9,MMP2,PON1,C5,MPO,APOC3,MGP,SPARC,PCDH12,APOB,PROC,APOA1,TTR,CAT,LRP1,CFP,APOE,PXDN,ANGPTL6,SERPINF1,CFHR5,LYZ,VWF,PSMA6,DSG2,C9,LCAT,NID1,CSTA,CP,KLKB1,DNAH5,CENPE,TCIRG1,HYAL1,CDH11,FCN3,AHSG,CRH,HABP2,SERPING1,CSF1R,IHH,SERPINI1,MASP1,GUCY1A1,A2ML1,ANTXR1,PCSK9,BMP1,FGB,DST,F2,PLG,EFEMP2,STAB1,C1QB,CFHR1,SUZ12,SERPINF2,C7,HBA1,P4HB,FBLN1,DNAH8,ANG,FGG,LIMS1,THBS4,GANAB,PAPLN,BLM,AKAP9,F8,C8A,CENPF,F13B,CFHR4,CFH,F5,ADAMTSL4,FLNA,CRTAC1,C8B,ADAMTS13,GSN,C1QC,F10,F7,PROS1,EFEMP1,CFI,NAV2,CST3,F11,C1S,IGF2,C4A,FSIP2,ABCA13,COLEC11,LMOD3,ITIH3,C4B,IGFALS,GC,APOA5,ATP1A1,VEGFC,KNG1,ACTB,GRHL2,B2M,FGA
Monarch	HP:0001928	Abnormality of coagulation	20	170	0.97	4.31e-10	F9,PROC,VWF,KLKB1,A2ML1,FGB,F2,PLG,SERPINF2,FGG,F8,F13B,F5,FLNA,F10,F7,PROS1,F11,KNG1,FGA
Monarch	HP:0100659	Abnormal cerebral vascular morphology	28	381	0.76	4.31e-10	F9,SPARC,APOB,PROC,TTR,APOE,ANGPTL6,GUCY1A1,ANTXR1,PCSK9,FGB,F2,SERPINF2,FGG,GANAB,F8,F13B,F5,FLNA,CRTAC1,ADAMTS13,F10,F7,PROS1,CST3,KNG1,ACTB,FGA
Monarch	EFO:0004634	Coagulation factor measurement	18	132	1.03	5.87e-10	CPB2,PROC,CFHR5,VWF,KLKB1,FGB,FGG,CFHR4,CFH,F5,ADAMTS13,F10,F7,F11,COLEC11,HGFAC,KNG1,FGA
Monarch	HP:0005261	Joint hemorrhage	10	19	1.62	1.22e-09	F9,VWF,F2,SERPINF2,F8,F13B,F5,F10,F7,F11
Monarch	HP:0000225	Gingival bleeding	12	45	1.32	4.31e-09	CAT,FGB,F2,SERPINF2,FGG,F8,F13B,F5,F10,F7,C1S,FGA
Monarch	HP:0001892	Abnormal bleeding	27	411	0.71	8.11e-09	F9,SPARC,PROC,CAT,APOE,ANGPTL6,VWF,TCIRG1,A2ML1,FGB,F2,SERPINF2,P4HB,FGG,F8,F13B,F5,FLNA,GSN,F10,F7,PROS1,CST3,F11,C1S,C4A,FGA
Monarch	HP:0001626	Abnormality of the cardiovascular system	71	2438	0.36	1.17e-08	GNPTG,VCL,SERPIND1,F9,MMP2,MGP,SPARC,APOB,PROC,APOA1,TTR,CAT,LRP1,APOE,ANGPTL6,SERPINF1,LYZ,VWF,DSG2,LCAT,NID1,CSTA,CP,KLKB1,DNAH5,TCIRG1,HABP2,SERPING1,MASP1,GUCY1A1,A2ML1,ANTXR1,PCSK9,BMP1,FGB,DST,F2,PLG,EFEMP2,SUZ12,SERPINF2,HBA1,P4HB,FGG,GANAB,BLM,AKAP9,F8,CENPF,F13B,F5,ADAMTSL4,FLNA,CRTAC1,ADAMTS13,GSN,F10,F7,PROS1,EFEMP1,CFI,CST3,F11,C1S,IGF2,C4A,VEGFC,KNG1,ACTB,B2M,FGA
Monarch	HP:0002715	Abnormality of the immune system	55	1682	0.41	7.04e-08	VCL,MMP2,C5,MPO,MGP,APOA1,CAT,CFP,APOE,CFHR5,LYZ,PSMA6,DSG2,C9,LCAT,CSTA,DNAH5,TCIRG1,HYAL1,FCN3,HABP2,SERPING1,A2ML1,FGB,F2,PLG,EFEMP2,C1QB,CFHR1,C7,HBA1,FGG,GANAB,BLM,F8,C8A,CENPF,F13B,CFHR4,CFH,FLNA,C8B,GSN,C1QC,CFI,NAV2,C1S,IGF2,C4A,C4B,VEGFC,ACTB,GRHL2,B2M,FGA
Monarch	HP:0011890	Prolonged bleeding following procedure	9	23	1.49	9.76e-08	F9,VWF,F2,F8,F13B,F5,F10,F7,F11
Monarch	HP:0006298	Prolonged bleeding after dental extraction	8	16	1.6	2.16e-07	F9,VWF,F2,F8,F13B,F5,F10,F11
Monarch	HP:0002170	Intracranial hemorrhage	15	130	0.96	2.65e-07	F9,SPARC,ANGPTL6,FGB,F2,SERPINF2,FGG,F8,F13B,F5,F10,F7,PROS1,CST3,FGA

Monarch	HP:0001871	Abnormality of blood and blood-forming tissues	44	1214	0.46	2.98e-07	VCL,SERPIND1,F9,MPO,SPARC,APOB,PROC,APOA1,CAT,APOE,ANGPTL6,VWF,DSG2,LCAT,CP,KLKB1,TCIRG1,GUCY1A1,A2ML1,FGB,F2,PLG,SERPINF2,HBA1,P4HB,FGG,BLM,F8,F13B,CFH,F5,FLNA,ADAMTS13,GSN,F10,F7,PROS1,CST3,F11,C1S,C4A,KNG1,B2M,FGA
Monarch	HP:0000951	Abnormality of the skin	57	1888	0.38	4.33e-07	VCL,F9,MMP2,C5,MGP,SPARC,APOB,PROC,APOA1,CAT,LRP1,APOE,LYZ,VWF,PSMA6,DSG2,CSTA,CENPE,TCIRG1,HYAL1,CDH11,FCN3,AHSG,SERPING1,MASP1,GUCY1A1,A2ML1,ANTXR1,PCSK9,DST,F2,PLG,EFEMP2,SUZ12,SERPINF2,HBA1,P4HB,BLM,F8,F13B,CFHR4,F5,FLNA,ADAMTS13,GSN,F10,F7,PROS1,CFI,C1S,IGF2,C4A,VEGFC,ACTB,GRHL2,B2M,FGA
Monarch	HP:0012233	Intramuscular hematoma	7	10	1.74	4.33e-07	F9,F2,SERPINF2,F8,F13B,F10,F7
Monarch	HP:0030680	Abnormality of cardiovascular system morphology	53	1680	0.4	4.33e-07	VCL,SERPIND1,F9,MMP2,MGP,SPARC,APOB,PROC,TTR,CAT,APOE,ANGPTL6,VWF,DSG2,LCAT,NID1,DNAH5,TCIRG1,HABP2,MASP1,GUCY1A1,A2ML1,ANTXR1,PCSK9,FGB,F2,EFEMP2,SUZ12,SERPINF2,HBA1,P4HB,FGG,GANAB,F8,F13B,F5,FLNA,CRTAC1,ADAMTS13,GSN,F10,F7,PROS1,EFEMP1,CFI,CST3,IGF2,C4A,VEGFC,KNG1,ACTB,B2M,FGA
Monarch	HP:0001933	Subcutaneous hemorrhage	17	189	0.85	4.94e-07	PROC,APOE,VWF,TCIRG1,F2,SERPINF2,P4HB,F8,F13B,F5,FLNA,GSN,F10,F7,PROS1,C1S,C4A
Monarch	HP:0000790	Hematuria	14	118	0.97	5.66e-07	F9,APOA1,CFHR5,LYZ,F2,CFHR1,SERPINF2,GANAB,F8,CFH,F5,ADAMTS13,F10,FGA
Monarch	HP:0000421	Epistaxis	12	78	1.08	5.79e-07	F9,VWF,FGB,F2,FGG,F8,F13B,F5,F10,F7,F11,FGA
Monarch	HP:0003645	Prolonged partial thromboplastin time	8	24	1.42	1.86e-06	F9,KLKB1,F2,F8,F5,F10,F11,KNG1
Monarch	HP:0011029	Internal hemorrhage	18	244	0.76	2.46e-06	F9,SPARC,PROC,ANGPTL6,VWF,FGB,F2,SERPINF2,FGG,F8,F13B,F5,F10,F7,PROS1,CST3,F11,FGA
Monarch	HP:0010978	Abnormality of immune system physiology	44	1347	0.41	4.33e-06	MMP2,C5,MGP,APOA1,CAT,CFP,APOE,CFHR5,LYZ,PSMA6,C9,CSTA,DNAH5,TCIRG1,HYAL1,FCN3,SERPING1,PLG,EFEMP2,C1QB,CFHR1,C7,HBA1,GANAB,BLM,C8A,F13B,CFHR4,CFH,FLNA,C8B,GSN,C1QC,CFI,NAV2,C1S,IGF2,C4A,C4B,VEGFC,ACTB,GRHL2,B2M,FGA
Monarch	HP:0001574	Abnormality of the integument	61	2266	0.33	4.88e-06	VCL,F9,MMP2,C5,MGP,SPARC,APOB,PROC,APOA1,CAT,LRP1,APOE,LYZ,VWF,PSMA6,DSG2,CSTA,CENPE,TCIRG1,HYAL1,CDH11,FCN3,AHSG,SERPING1,IHH,MASP1,GUCY1A1,A2ML1,ANTXR1,PCSK9,BMP1,DST,F2,PLG,EFEMP2,SUZ12,SERPINF2,HBA1,P4HB,LIMS1,BLM,F8,F13B,CFHR4,F5,FLNA,ADAMTS13,GSN,F10,F7,PROS1,CFI,C1S,IGF2,C4A,COLEC11,VEGFC,ACTB,GRHL2,B2M,FGA
Monarch	HP:0000123	Nephritis	10	59	1.13	5.25e-06	CFHR5,PLG,EFEMP2,C1QB,CFHR1,GANAB,CFH,C1QC,CFI,C4A
Monarch	HP:0011276	Vascular skin abnormality	23	433	0.62	6.08e-06	PROC,APOE,VWF,CSTA,TCIRG1,SERPING1,MASP1,GUCY1A1,ANTXR1,F2,SERPINF2,P4HB,BLM,F8,F13B,F5,FLNA,GSN,F10,F7,PROS1,C1S,C4A
Monarch	HP:0004846	Prolonged bleeding after surgery	7	18	1.49	6.17e-06	F9,VWF,F8,F13B,F5,F10,F7
Monarch	HP:0011891	Post-partum hemorrhage	6	10	1.67	8.82e-06	F2,F8,F13B,F5,F10,F7
Monarch	HP:0012211	Abnormal renal physiology	26	560	0.56	8.82e-06	F9,APOA1,TTR,APOE,CFHR5,LYZ,LCAT,F2,PLG,EFEMP2,C1QB,CFHR1,SERPINF2,GANAB,F8,CFH,F5,ADAMTS13,GSN,C1QC,F10,CFI,C4A,ATP1A1,B2M,FGA
Monarch	HP:0000978	Bruising susceptibility	13	130	0.9	9.74e-06	VWF,TCIRG1,F2,SERPINF2,P4HB,F8,F13B,F5,FLNA,GSN,F10,F7,C1S
Monarch	HP:0004936	Venous thrombosis	10	65	1.08	9.88e-06	SERPIND1,F9,PROC,FGB,F2,FGG,F5,PROS1,KNG1,FGA
Monarch	HP:0010935	Abnormality of the upper urinary tract	38	1101	0.43	9.88e-06	F9,APOB,APOA1,TTR,APOE,CFHR5,LYZ,LCAT,CDH11,HABP2,MASP1,ANTXR1,PCSK9,F2,PLG,EFEMP2,C1QB,CFHR1,SERPINF2,GANAB,BLM,F8,CENPF,CFH,F5,FLNA,ADAMTS13,GSN,C1QC,F10,CFI,IGF2,C4A,COLEC11,ATP1A1,ACTB,B2M,FGA
Monarch	HP:0001939	Abnormality of metabolism/homeostasis	58	2168	0.32	1.18e-05	CETP,GNPTG,VCL,F9,MMP2,PON1,MPO,APOC3,APOB,APOA1,TTR,CAT,CFP,APOE,CFHR5,LYZ,DSG2,LCAT,CP,TCIRG1,CDH11,SERPING1,CSF1R,A2ML1,ANTXR1,PCSK9,FGB,DST,F2,PLG,CFHR1,SERPINF2,HBA1,FGG,GANAB,PAPLN,BLM,AKAP9,F8,CFH,F5,FLNA,ADAMTS13,GSN,F10,CST3,IGF2,LMOD3,IGFALS,GC,APOA5,ATP1A1,VEGFC,KNG1,ACTB,GRHL2,B2M,FGA
Monarch	HP:0000168	Abnormality of the gingiva	15	190	0.79	1.43e-05	MMP2,CAT,TCIRG1,FGB,F2,PLG,SERPINF2,FGG,F8,F13B,F5,F10,F7,C1S,FGA
Monarch	HP:0000077	Abnormality of the kidney	37	1077	0.43	1.57e-05	F9,APOB,APOA1,TTR,APOE,CFHR5,LYZ,LCAT,HABP2,MASP1,ANTXR1,PCSK9,F2,PLG,EFEMP2,C1QB,CFHR1,SERPINF2,GANAB,BLM,F8,CENPF,CFH,F5,FLNA,ADAMTS13,GSN,C1QC,F10,CFI,IGF2,C4A,COLEC11,ATP1A1,ACTB,B2M,FGA
Monarch	HP:0001934	Persistent bleeding after trauma	6	12	1.6	1.62e-05	F9,VWF,SERPINF2,F8,F13B,F5
Monarch	HP:0011121	Abnormality of skin morphology	48	1648	0.36	1.82e-05	VCL,F9,MMP2,MGP,SPARC,APOB,PROC,APOA1,CAT,LRP1,APOE,VWF,DSG2,CSTA,CENPE,TCIRG1,CDH11,AHSG,SERPING1,MASP1,GUCY1A1,A2ML1,ANTXR1,PCSK9,DST,F2,EFEMP2,SUZ12,SERPINF2,HBA1,P4HB,BLM,F8,F13B,F5,FLNA,ADAMTS13,GSN,F10,F7,PROS1,C1S,IGF2,C4A,VEGFC,ACTB,GRHL2,B2M
Monarch	HP:0003216	Generalized amyloid deposition	5	5	1.9	1.85e-05	APOA1,LYZ,GSN,CST3,FGA
Monarch	HP:0001977	Abnormal thrombosis	11	94	0.96	2.16e-05	SERPIND1,F9,PROC,FGB,F2,FGG,F8,F5,PROS1,KNG1,FGA
Monarch	HP:0000132	Menorrhagia	8	38	1.22	2.34e-05	VWF,F2,F8,F13B,F5,F10,F7,F11
Monarch	HP:0005368	Abnormality of humoral immunity	16	234	0.73	2.81e-05	C5,CFP,C9,C1QB,C7,BLM,C8A,CFH,FLNA,C8B,C1QC,CFI,C1S,C4A,C4B,B2M
Monarch	HP:0011034	Amyloidosis	7	25	1.34	2.81e-05	APOA1,TTR,LYZ,GSN,CST3,B2M,FGA
Monarch	HP:0033057	Decreased serum terminal complement component	5	6	1.82	3.03e-05	C5,C9,C7,C8A,C8B
Monarch	HP:0000707	Abnormality of the nervous system	78	3471	0.25	3.59e-05	ST6GAL1,GNPTG,F9,MMP2,PON1,MGP,SPARC,PCDH12,APOB,PROC,APOA1,TTR,CAT,LRP1,APOE,ANGPTL6,NID1,CP,DNAH5,CENPE,TCIRG1,CDH11,AHSG,CRH,HABP2,SERPING1,CSF1R,SERPINI1,MASP1,GUCY1A1,A2ML1,ANTXR1,PCSK9,BMP1,FGB,DST,F2,PLG,EFEMP2,STAB1,SUZ12,SERPINF2,HBA1,P4HB,FBLN1,ANG,FGG,GANAB,BLM,AKAP9,F8,C8A,CENPF,F13B,F5,ADAMTSL4,FLNA,CRTAC1,C8B,ADAMTS13,GSN,F10,F7,PROS1,CFI,CST3,IGF2,COLEC11,LMOD3,ITIH3,C4B,ATP1A1,VEGFC,KNG1,ACTB,GRHL2,B2M,FGA
Monarch	EFO:0004310	Partial thromboplastin time	6	15	1.5	3.82e-05	APOH,KLKB1,F5,F11,C1S,KNG1
Monarch	HP:0012649	Increased inflammatory response	34	981	0.44	3.82e-05	C5,MGP,APOA1,CAT,APOE,CFHR5,LYZ,PSMA6,CSTA,DNAH5,TCIRG1,HYAL1,FCN3,PLG,EFEMP2,C1QB,CFHR1,HBA1,GANAB,BLM,F13B,CFHR4,CFH,FLNA,GSN,C1QC,CFI,NAV2,C1S,C4A,C4B,VEGFC,B2M,FGA
Monarch	HP:0000119	Abnormality of the genitourinary system	56	2189	0.3	6.81e-05	F9,MMP2,APOB,APOA1,TTR,APOE,CFHR5,LYZ,VWF,LCAT,DNAH5,CDH11,AHSG,CRH,HABP2,MASP1,GUCY1A1,A2ML1,ANTXR1,PCSK9,FGB,F2,PLG,EFEMP2,C1QB,CFHR1,SUZ12,SERPINF2,FBLN1,DNAH8,FGG,GANAB,BLM,F8,CENPF,F13B,CFH,F5,FLNA,ADAMTS13,GSN,C1QC,F10,F7,CFI,F11,IGF2,C4A,FSIP2,COLEC11,LMOD3,ATP1A1,VEGFC,ACTB,B2M,FGA
Monarch	HP:0012639	Abnormal nervous system morphology	62	2546	0.28	7.09e-05	F9,PON1,MGP,SPARC,PCDH12,APOB,PROC,TTR,APOE,ANGPTL6,NID1,CP,DNAH5,CENPE,TCIRG1,CDH11,AHSG,HABP2,SERPING1,CSF1R,SERPINI1,MASP1,GUCY1A1,ANTXR1,PCSK9,FGB,F2,PLG,EFEMP2,SUZ12,SERPINF2,HBA1,P4HB,FBLN1,ANG,FGG,GANAB,BLM,F8,C8A,CENPF,F13B,F5,FLNA,CRTAC1,C8B,ADAMTS13,GSN,F10,F7,PROS1,CFI,CST3,IGF2,COLEC11,LMOD3,C4B,ATP1A1,KNG1,ACTB,B2M,FGA
Monarch	EFO:0008390	Prothrombin time measurement	5	8	1.69	7.28e-05	F2,FGG,F5,F10,F7
Monarch	HP:0002239	Gastrointestinal hemorrhage	11	111	0.89	8.06e-05	F9,VWF,FGB,F2,FGG,F8,F5,F10,F7,F11,FGA
Monarch	HP:0010979	Abnormality of lipoprotein cholesterol concentration	8	47	1.13	8.06e-05	CETP,APOC3,APOB,APOA1,APOE,LCAT,PCSK9,APOA5

Monarch	HP:0000152	Abnormality of head or neck	67	2882	0.26	0.00010	GNPTG,F9,MMP2,PON1,MGP,SPARC,PCDH12,APOB,PROC,CAT,LRP1,APOE,SERPINF1,LYZ,VWF,NID1,CP,DNAH5,CENPE,TCIRG1,HYAL1,CDH11,AHSG,HABP2,SERPING1,CSF1R,IHH,MASP1,A2ML1,ANTXR1,PCSK9,BMP1,FGB,DST,F2,PLG,EFEMP2,SUZ12,SERPINF2,P4HB,FBLN1,ANG,FGG,BLM,F8,CENPF,F13B,F5,ADAMTSL4,FLNA,CRTAC1,GSN,F10,F7,CFI,F11,C1S,IGF2,ABCA13,COLEC11,LMOD3,IGFALS,VEGFC,ACTB,GRHL2,B2M,FGA
Monarch	HP:0000271	Abnormality of the face	63	2641	0.27	0.00010	GNPTG,F9,MMP2,PON1,MGP,SPARC,APOB,PROC,CAT,LRP1,APOE,SERPINF1,LYZ,VWF,NID1,CP,DNAH5,CENPE,TCIRG1,HYAL1,CDH11,AHSG,SERPING1,CSF1R,MASP1,A2ML1,ANTXR1,PCSK9,BMP1,FGB,DST,F2,PLG,EFEMP2,SUZ12,SERPINF2,P4HB,FBLN1,ANG,FGG,BLM,F8,CENPF,F13B,F5,ADAMTSL4,FLNA,CRTAC1,GSN,F10,F7,CFI,F11,C1S,IGF2,ABCA13,COLEC11,LMOD3,VEGFC,ACTB,GRHL2,B2M,FGA
Monarch	HP:0030140	Oral cavity bleeding	5	9	1.64	0.00010	F2,F8,F13B,F5,F10
Monarch	HP:0033353	Abnormal blood vessel morphology	29	792	0.46	0.00010	SERPIND1,MMP2,MGP,APOB,PROC,CAT,APOE,ANGPTL6,VWF,LCAT,NID1,DNAH5,TCIRG1,MASP1,GUCY1A1,A2ML1,ANTXR1,PCSK9,EFEMP2,P4HB,GANAB,FLNA,PROS1,EFEMP1,CFI,IGF2,C4A,VEGFC,ACTB
Monarch	HP:0010980	Hyperlipoproteinemia	7	33	1.22	0.00011	CETP,APOC3,APOB,APOE,LCAT,PCSK9,APOA5
Monarch	HP:0002011	Morphological central nervous system abnormality	59	2416	0.28	0.00012	F9,PON1,MGP,SPARC,PCDH12,APOB,PROC,TTR,APOE,ANGPTL6,NID1,CP,DNAH5,CENPE,TCIRG1,CDH11,AHSG,HABP2,CSF1R,SERPINI1,MASP1,GUCY1A1,ANTXR1,PCSK9,FGB,F2,PLG,EFEMP2,SUZ12,SERPINF2,HBA1,P4HB,FBLN1,ANG,FGG,GANAB,BLM,F8,C8A,CENPF,F13B,F5,FLNA,CRTAC1,C8B,ADAMTS13,F10,F7,PROS1,CFI,CST3,IGF2,COLEC11,C4B,ATP1A1,KNG1,ACTB,B2M,FGA
Monarch	HP:0002625	Deep venous thrombosis	6	20	1.37	0.00012	SERPIND1,F9,PROC,F2,F5,PROS1
Monarch	EFO:0001365	Age-related macular degeneration	7	35	1.2	0.00014	CETP,APOE,CFHR5,C9,CFH,CFI,CFB
Monarch	EFO:0004298	Cardiovascular measurement	58	2378	0.28	0.00014	ST6GAL1,CETP,APOH,VCL,F9,PON1,MPO,VTN,APOC3,GPLD1,APOB,APOA1,LRP1,C4BPB,APOE,C9,LCAT,YWHAE,KLKB1,DNAH5,CDH11,ITIH1,HABP2,SERPINI1,HSP90B1,PCSK9,SHLD1,FGB,INHBC,PLG,STAB1,FBLN1,OIT3,LIMS1,FAM184A,CDC42BPA,CFH,QSOX1,F5,CRTAC1,ADAMTS13,GSN,EFEMP1,EFCAB5,TNRC6A,NAV2,RAPGEF4,ANKRD28,MAP3K7CL,ITIH3,PLTP,HGFAC,HPR,APOA5,APOC4,KNG1,ACTB,GRHL2
Monarch	EFO:1001492	Atrophic macular degeneration	7	35	1.2	0.00014	CETP,APOE,CFHR5,C9,CFH,CFI,CFB
Monarch	HP:0000234	Abnormality of the head	66	2865	0.26	0.00014	GNPTG,F9,MMP2,PON1,MGP,SPARC,PCDH12,APOB,PROC,CAT,LRP1,APOE,SERPINF1,LYZ,VWF,NID1,CP,DNAH5,CENPE,TCIRG1,HYAL1,CDH11,AHSG,SERPING1,CSF1R,IHH,MASP1,A2ML1,ANTXR1,PCSK9,BMP1,FGB,DST,F2,PLG,EFEMP2,SUZ12,SERPINF2,P4HB,FBLN1,ANG,FGG,BLM,F8,CENPF,F13B,F5,ADAMTSL4,FLNA,CRTAC1,GSN,F10,F7,CFI,F11,C1S,IGF2,ABCA13,COLEC11,LMOD3,IGFALS,VEGFC,ACTB,GRHL2,B2M,FGA
Monarch	HP:0001367	Abnormal joint morphology	31	905	0.43	0.00014	GNPTG,F9,MMP2,APOE,SERPINF1,VWF,HYAL1,IHH,MASP1,A2ML1,BMP1,FGB,F2,EFEMP2,SUZ12,SERPINF2,FBLN1,FGG,F8,F13B,F5,FLNA,F10,F7,CFI,F11,COLEC11,LMOD3,VEGFC,ACTB,FGA
Monarch	HP:0010990	Abnormality of the common coagulation pathway	7	35	1.2	0.00014	FGB,PLG,FGG,F13B,F5,F10,FGA
Monarch	HP:0031888	Abnormal HDL cholesterol concentration	6	21	1.35	0.00014	CETP,APOC3,APOA1,APOE,LCAT,APOA5
Monarch	HP:0000099	Glomerulonephritis	7	37	1.17	0.00017	CFHR5,C1QB,CFHR1,CFH,C1QC,CFI,C4A
Monarch	EFO:0005278	Cardiovascular disease biomarker measurement	55	2228	0.29	0.00018	ST6GAL1,CETP,APOH,F9,PON1,MPO,VTN,APOC3,APOB,APOA1,LRP1,C4BPB,APOE,C9,LCAT,YWHAE,KLKB1,DNAH5,CDH11,ITIH1,HABP2,SERPINI1,HSP90B1,PCSK9,SHLD1,FGB,PLG,STAB1,FBLN1,OIT3,LIMS1,FAM184A,CDC42BPA,CFH,QSOX1,F5,CRTAC1,ADAMTS13,GSN,EFEMP1,EFCAB5,TNRC6A,NAV2,RAPGEF4,ANKRD28,MAP3K7CL,ITIH3,PLTP,HGFAC,HPR,APOA5,APOC4,KNG1,ACTB,GRHL2
Monarch	HP:0012223	Splenic rupture	4	4	1.9	0.00023	FGB,FGG,F8,FGA
Monarch	HP:0000140	Abnormality of the menstrual cycle	14	228	0.68	0.00034	F9,VWF,ANTXR1,FGB,F2,FGG,F8,F13B,F5,F10,F7,F11,IGF2,FGA
Monarch	HP:0002725	Systemic lupus erythematosus	6	26	1.26	0.00035	SERPING1,C1QB,C8A,C1QC,C1S,C4A
Monarch	HP:0005430	Recurrent Neisserial infections	4	5	1.8	0.00040	C5,C7,C8B,CFI
Monarch	HP:0011884	Abnormal umbilical stump bleeding	4	5	1.8	0.00040	F2,SERPINF2,F13B,F10
Monarch	HP:0010981	Hypolipoproteinemia	6	27	1.24	0.00041	APOC3,APOB,APOA1,APOE,LCAT,APOA5
Monarch	HP:0011354	Generalized abnormality of skin	28	821	0.43	0.00041	F9,PROC,APOE,VWF,CSTA,CENPE,TCIRG1,CDH11,SERPING1,MASP1,GUCY1A1,ANTXR1,F2,SERPINF2,HBA1,P4HB,BLM,F8,F13B,F5,FLNA,ADAMTS13,GSN,F10,F7,PROS1,C1S,C4A
Monarch	HP:0011830	Abnormal oral mucosa morphology	16	305	0.62	0.00042	MMP2,CAT,LYZ,TCIRG1,FGB,F2,PLG,SERPINF2,FGG,F8,F13B,F5,F10,F7,C1S,FGA
Monarch	HP:0030012	Abnormal female reproductive system physiology	15	273	0.64	0.00050	F9,VWF,ANTXR1,FGB,F2,FGG,BLM,F8,F13B,F5,F10,F7,F11,IGF2,FGA
Monarch	EFO:0004614	Apolipoprotein A 1 measurement	18	396	0.55	0.00068	CETP,APOH,VTN,APOC3,APOB,APOA1,LRP1,APOE,LCAT,ITIH1,PCSK9,PLG,STAB1,CRTAC1,ANKRD28,HGFAC,HPR,APOC4
Monarch	HP:0000080	Abnormality of reproductive system physiology	24	659	0.46	0.00073	F9,TTR,APOE,VWF,DNAH5,AHSG,GUCY1A1,A2ML1,ANTXR1,FGB,F2,DNAH8,FGG,GANAB,BLM,F8,F13B,F5,F10,F7,F11,IGF2,FSIP2,FGA
Monarch	HP:0002634	Arteriosclerosis	8	70	0.95	0.00075	SERPIND1,APOB,CAT,APOE,ANGPTL6,LCAT,ANTXR1,PCSK9
Monarch	HP:0025031	Abnormality of the digestive system	55	2389	0.26	0.0012	F9,MMP2,PON1,C5,APOB,APOA1,TTR,APOE,LYZ,VWF,LCAT,CP,DNAH5,TCIRG1,CDH11,FCN3,HABP2,SERPING1,MASP1,GUCY1A1,A2ML1,ANTXR1,PCSK9,BMP1,FGB,DST,F2,PLG,EFEMP2,SUZ12,HBA1,ANG,FGG,GANAB,BLM,F8,CENPF,F13B,F5,FLNA,ADAMTS13,GSN,F10,F7,NAV2,F11,C1S,IGF2,COLEC11,LMOD3,C4B,ACTB,GRHL2,B2M,FGA
Monarch	HP:0000153	Abnormality of the mouth	48	1992	0.28	0.0016	MMP2,PON1,MGP,SPARC,CAT,APOE,SERPINF1,LYZ,NID1,CENPE,TCIRG1,HYAL1,CDH11,SERPING1,MASP1,A2ML1,ANTXR1,BMP1,FGB,DST,F2,PLG,EFEMP2,SUZ12,SERPINF2,P4HB,FBLN1,ANG,FGG,BLM,F8,CENPF,F13B,F5,FLNA,CRTAC1,GSN,F10,F7,C1S,IGF2,ABCA13,COLEC11,LMOD3,ACTB,GRHL2,B2M,FGA
Monarch	HP:0031886	Abnormal LDL cholesterol concentration	6	36	1.12	0.0016	APOC3,APOB,APOE,LCAT,PCSK9,APOA5
Monarch	HP:0030137	Prolonged bleeding following circumcision	4	9	1.54	0.0019	F2,F8,F13B,F5
Monarch	HP:0008011	Peripheral opacification of the cornea	4	10	1.5	0.0026	MMP2,APOB,APOE,PCSK9
Monarch	HP:0400008	Menometrorrhagia	4	10	1.5	0.0026	F9,FGB,FGG,FGA
Monarch	HP:0000969	Edema	18	448	0.5	0.0030	APOA1,APOE,LYZ,CDH11,SERPING1,A2ML1,ANTXR1,FGB,F2,HBA1,FGG,F8,GSN,LMOD3,VEGFC,ACTB,GRHL2,FGA
Monarch	HP:0033127	Abnormality of the musculoskeletal system	66	3173	0.21	0.0031	GNPTG,VCL,F9,MMP2,PON1,MGP,SPARC,PCDH12,APOB,APOA1,TTR,LRP1,APOE,SERPINF1,VWF,DSG2,NID1,CP,DNAH5,CENPE,TCIRG1,HYAL1,CDH11,AHSG,HABP2,CSF1R,IHH,MASP1,A2ML1,ANTXR1,PCSK9,BMP1,FGB,DST,F2,PLG,EFEMP2,SUZ12,SERPINF2,P4HB,FBLN1,ANG,FGG,BLM,F8,CENPF,F13B,F5,ADAMTSL4,FLNA,GSN,F10,F7,CFI,F11,C1S,IGF2,COLEC11,LMOD3,IGFALS,ATP1A1,VEGFC,ACTB,GRHL2,B2M,FGA
Monarch	HP:0000078	Abnormality of the genital system	36	1358	0.32	0.0033	F9,MMP2,TTR,APOE,VWF,DNAH5,CDH11,AHSG,MASP1,GUCY1A1,A2ML1,ANTXR1,FGB,F2,PLG,EFEMP2,SUZ12,FBLN1,DNAH8,FGG,GANAB,BLM,F8,F13B,F5,FLNA,F10,F7,F11,IGF2,FSIP2,COLEC11,LMOD3,VEGFC,ACTB,FGA
Monarch	HP:0002621	Atherosclerosis	7	64	0.94	0.0034	SERPIND1,APOB,APOE,ANGPTL6,LCAT,ANTXR1,PCSK9
Monarch	HP:0011277	Abnormality of the urinary system physiology	28	933	0.37	0.0034	F9,APOA1,TTR,APOE,CFHR5,LYZ,LCAT,F2,PLG,EFEMP2,C1QB,CFHR1,SERPINF2,GANAB,PAPLN,BLM,F8,CFH,F5,ADAMTS13,GSN,C1QC,F10,CFI,C4A,ATP1A1,B2M,FGA

Monarch	HP:0011355	Localized skin lesion	24	738	0.41	0.0038	MMP2,MGP,APOB,PROC,APOA1,LRP1,APOE,CSTA,TCIRG1,MASP1,A2ML1,ANTXR1,PCSK9,DST,SUZ12,P4HB,BLM,FLNA,GSN,PROS1,C1S,IGF2,ACTB,B2M
Monarch	HP:0000079	Abnormality of the urinary system	41	1657	0.29	0.0039	F9,APOB,APOA1,TTR,APOE,CFHR5,LYZ,LCAT,CDH11,CRH,HABP2,MASP1,ANTXR1,PCSK9,F2,PLG,EFEMP2,C1QB,CFHR1,SERPINF2,GANAB,PAPLN,BLM,F8,CENPF,CFH,F5,FLNA,ADAMTS13,GSN,C1QC,F10,CFI,IGF2,C4A,COLEC11,LMOD3,ATP1A1,ACTB,B2M,FGA
Monarch	HP:0000163	Abnormal oral cavity morphology	45	1901	0.27	0.0044	MMP2,MGP,SPARC,CAT,APOE,SERPINF1,LYZ,NID1,CENPE,TCIRG1,HYAL1,CDH11,SERPING1,MASP1,A2ML1,ANTXR1,BMP1,FGB,DST,F2,PLG,EFEMP2,SUZ12,SERPINF2,P4HB,FGG,BLM,F8,CENPF,F13B,F5,FLNA,CRTAC1,GSN,F10,F7,C1S,IGF2,ABCA13,COLEC11,LMOD3,ACTB,GRHL2,B2M,FGA
Monarch	HP:0000924	Abnormality of the skeletal system	58	2708	0.23	0.0047	GNPTG,F9,MMP2,MGP,SPARC,PCDH12,APOB,TTR,APOE,SERPINF1,VWF,NID1,DNAH5,CENPE,TCIRG1,HYAL1,CDH11,AHSG,HABP2,CSF1R,IHH,MASP1,A2ML1,ANTXR1,PCSK9,BMP1,FGB,DST,F2,PLG,EFEMP2,SUZ12,SERPINF2,P4HB,FBLN1,FGG,BLM,F8,CENPF,F13B,F5,ADAMTSL4,FLNA,F10,F7,CFI,F11,C1S,IGF2,COLEC11,LMOD3,IGFALS,ATP1A1,VEGFC,ACTB,GRHL2,B2M,FGA
Monarch	HP:0030163	Abnormal vascular physiology	15	340	0.54	0.0048	MGP,PROC,ANGPTL6,SERPINF1,TCIRG1,GUCY1A1,BMP1,F2,F5,FLNA,CRTAC1,ADAMTS13,PROS1,KNG1,ACTB
Monarch	EFO:0003839	Retinopathy	8	98	0.81	0.0060	CETP,APOE,CFHR5,C9,EFEMP2,CFH,CFI,CFB
Monarch	HP:0010299	Abnormal dentin morphology	5	29	1.13	0.0064	SPARC,SERPINF1,CDH11,BMP1,P4HB
Monarch	HP:0010989	Abnormality of the intrinsic pathway	5	29	1.13	0.0064	F9,VWF,F8,F11,KNG1
Monarch	EFO:0004694	Factor XI measurement	3	4	1.77	0.0067	KLKB1,F11,KNG1
Monarch	HP:0005356	Decreased serum complement factor I	3	4	1.77	0.0067	C1QB,C1QC,CFI
Monarch	HP:0005381	Recurrent meningococcal disease	3	4	1.77	0.0067	C5,C7,CFI
Monarch	HP:0012541	Cephalohematoma	3	4	1.77	0.0067	F9,F2,F8
Monarch	HP:0000235	Abnormality of the fontanelles or cranial sutures	15	355	0.52	0.0071	MMP2,SPARC,APOE,SERPINF1,CENPE,TCIRG1,CDH11,MASP1,ANTXR1,BMP1,P4HB,FLNA,COLEC11,LMOD3,ACTB
Monarch	HP:0011842	Abnormal skeletal morphology	55	2573	0.23	0.0075	GNPTG,F9,MMP2,MGP,SPARC,PCDH12,APOB,TTR,APOE,SERPINF1,VWF,NID1,DNAH5,CENPE,TCIRG1,HYAL1,CDH11,AHSG,CSF1R,IHH,MASP1,A2ML1,ANTXR1,PCSK9,BMP1,FGB,DST,F2,PLG,EFEMP2,SUZ12,SERPINF2,P4HB,FBLN1,FGG,BLM,F8,CENPF,F13B,F5,ADAMTSL4,FLNA,F10,F7,CFI,F11,C1S,IGF2,COLEC11,LMOD3,IGFALS,VEGFC,ACTB,B2M,FGA
Monarch	HP:0001547	Abnormal rib cage morphology	14	325	0.53	0.0100	GNPTG,MGP,SERPINF1,TCIRG1,IHH,A2ML1,ANTXR1,BMP1,SUZ12,P4HB,FLNA,IGF2,LMOD3,ACTB
Monarch	HP:0005542	Prolonged whole-blood clotting time	3	5	1.67	0.0100	F9,VWF,F5
Monarch	HP:0007937	Reticular pigmentary degeneration	3	5	1.67	0.0100	CFH,EFEMP1,CFI
Monarch	EFO:0004872	Inflammatory biomarker measurement	32	1226	0.31	0.0103	CETP,APOH,MPO,VTN,APOC3,APOB,PLEK,APOA1,LRP1,APOE,APCS,LYZ,LCAT,ITIH1,ANKRD44,PCSK9,PLG,EFEMP2,STAB1,CENPF,CFH,F5,CRTAC1,HLA-CKAP4,YWHAZ,ANKRD28,COLEC11,GC,HGFAC,HPR,APOC4
Monarch	HP:0030972	Abnormal systemic blood pressure	15	369	0.51	0.0103	MMP2,MGP,APOB,APOA1,TTR,ANGPTL6,LYZ,SERPING1,GUCY1A1,PCSK9,F2,GANAB,ADAMTSL4,GSN,FGA
Monarch	HP:0003272	Abnormal hip bone morphology	17	461	0.46	0.0111	GNPTG,MMP2,APOE,SERPINF1,CENPE,TCIRG1,HYAL1,IHH,MASP1,BMP1,EFEMP2,SUZ12,FBLN1,FLNA,COLEC11,LMOD3,B2M
Monarch	EFO:0006925	Lipoprotein A measurement	5	34	1.06	0.0112	CETP,APOH,APOE,PLG,APOC4
Monarch	HP:0002644	Abnormal pelvic girdle bone morphology	18	512	0.44	0.0122	GNPTG,MMP2,APOE,SERPINF1,CENPE,TCIRG1,HYAL1,IHH,MASP1,ANTXR1,BMP1,EFEMP2,SUZ12,FBLN1,FLNA,COLEC11,LMOD3,B2M
Monarch	HP:0011329	Abnormality of cranial sutures	13	292	0.55	0.0122	MMP2,SPARC,SERPINF1,CENPE,TCIRG1,CDH11,MASP1,ANTXR1,BMP1,P4HB,FLNA,COLEC11,ACTB
Monarch	HP:0001386	Joint swelling	5	35	1.05	0.0123	FGB,F2,FGG,F8,FGA
Monarch	HP:0012443	Abnormality of brain morphology	49	2254	0.23	0.0124	F9,MGP,SPARC,PCDH12,APOB,PROC,TTR,APOE,ANGPTL6,NID1,CP,DNAH5,CENPE,TCIRG1,CDH11,AHSG,CSF1R,SERPINI1,MASP1,GUCY1A1,ANTXR1,PCSK9,FGB,F2,PLG,EFEMP2,SUZ12,SERPINF2,HBA1,P4HB,FBLN1,FGG,GANAB,BLM,F8,CENPF,F13B,F5,FLNA,CRTAC1,ADAMTS13,F10,F7,PROS1,CST3,ATP1A1,KNG1,ACTB,FGA
Monarch	HP:0003233	Decreased HDL cholesterol concentration	4	18	1.24	0.0131	APOA1,APOE,LCAT,APOA5
Monarch	HP:0030780	Abnormality of the protein C anticoagulant pathway	3	6	1.6	0.0137	PROC,F5,PROS1
Monarch	HP:0011492	Abnormal corneal stroma morphology	7	86	0.81	0.0148	GNPTG,MMP2,APOB,APOE,LCAT,PCSK9,GRHL2
Monarch	HP:0100724	Hypercoagulability	4	19	1.22	0.0154	F9,PROC,F5,PROS1
Monarch	HP:0001342	Cerebral hemorrhage	6	61	0.89	0.0166	FGB,FGG,F13B,PROS1,CST3,FGA
Monarch	HP:0005262	Abnormality of the synovia	13	305	0.53	0.0172	MMP2,APOE,SERPINF1,HYAL1,MASP1,BMP1,F2,EFEMP2,FBLN1,F8,FLNA,COLEC11,LMOD3
Monarch	HP:0001000	Abnormality of skin pigmentation	16	439	0.46	0.0179	MMP2,PROC,CAT,APOE,CSTA,A2ML1,ANTXR1,DST,F2,SUZ12,BLM,FLNA,PROS1,C1S,IGF2,ACTB
Monarch	HP:0007420	Spontaneous hematomas	4	20	1.2	0.0179	F8,F13B,F5,F10
Monarch	HP:0007957	Corneal opacity	11	226	0.58	0.0179	GNPTG,MMP2,APOB,APOA1,APOE,PXDN,LCAT,PCSK9,DST,CENPF,GRHL2
Monarch	HP:0000799	Renal steatosis	3	7	1.53	0.0181	APOB,APOE,PCSK9
Monarch	HP:0004418	Thrombophlebitis	3	7	1.53	0.0181	PROC,F2,PROS1
Monarch	HP:0005305	Cerebral venous thrombosis	3	7	1.53	0.0181	PROC,F2,PROS1
Monarch	HP:0012397	Aortic atherosclerotic lesion	3	7	1.53	0.0181	APOB,APOE,PCSK9
Monarch	HP:0002960	Autoimmunity	8	122	0.71	0.0192	MMP2,TCIRG1,SERPING1,C1QB,C8A,C1QC,C1S,C4A
Monarch	HP:0011867	Abnormal iliac wing morphology	6	64	0.87	0.0196	GNPTG,APOE,TCIRG1,IHH,SUZ12,FLNA
Monarch	HP:0005268	Miscarriage	4	21	1.18	0.0197	MGP,FGB,FGG,FGA
Monarch	HP:0011420	Age of death	4	21	1.18	0.0197	MGP,FGB,FGG,FGA
Monarch	HP:0012531	Pain	18	539	0.42	0.0197	GNPTG,MMP2,PON1,APOB,CAT,TCIRG1,HABP2,SERPING1,PCSK9,F2,SERPINF2,ANG,GANAB,F8,FLNA,C1S,GRHL2,B2M
Monarch	HP:0003312	Abnormal form of the vertebral bodies	12	274	0.54	0.0210	MMP2,SPARC,SERPINF1,TCIRG1,CDH11,CSF1R,IHH,ANTXR1,BMP1,P4HB,FLNA,ACTB
Monarch	HP:0031815	Abnormal oral physiology	10	196	0.6	0.0214	PON1,SERPING1,F2,FBLN1,ANG,F8,F13B,F5,GSN,F10
Monarch	HP:0000991	Xanthomatosis	4	22	1.16	0.0217	APOB,APOA1,APOE,PCSK9
Monarch	HP:0004381	Supravalvular aortic stenosis	3	8	1.47	0.0222	APOB,PCSK9,EFEMP2
Monarch	HP:0010874	Tendon xanthomatosis	3	8	1.47	0.0222	APOB,APOE,PCSK9
Monarch	EFO:0000508	Genetic disorder	8	130	0.69	0.0258	CETP,APOE,CFHR5,C9,CFH,CFI,EFCAB5,CFB
Monarch	HP:0001681	Angina pectoris	4	24	1.12	0.0281	APOB,APOE,LCAT,PCSK9
Monarch	EFO:0003907	Deep vein thrombosis	3	9	1.42	0.0282	F2,F5,KNG1
Monarch	HP:0001084	Corneal arcus	3	9	1.42	0.0282	APOB,APOE,PCSK9
Monarch	HP:0002659	Increased susceptibility to fractures	10	205	0.58	0.0283	MMP2,SPARC,SERPINF1,TCIRG1,HABP2,BMP1,EFEMP2,P4HB,LMOD3,B2M
Monarch	HP:0008065	Aplasia/Hypoplasia of the skin	9	170	0.62	0.0311	MGP,PROC,TCIRG1,DST,P4HB,FLNA,GSN,PROS1,C1S
Monarch	HP:0000236	Abnormality of the anterior fontanelle	7	102	0.73	0.0315	MMP2,SERPINF1,TCIRG1,MASP1,ANTXR1,BMP1,FLNA
Monarch	HP:0001297	Stroke	11	249	0.54	0.0320	TTR,ANGPTL6,GUCY1A1,F2,F5,FLNA,CRTAC1,ADAMTS13,CST3,KNG1,ACTB
Monarch	HP:0011328	Abnormality of fontanelles	9	171	0.62	0.0320	MMP2,SERPINF1,TCIRG1,MASP1,ANTXR1,BMP1,FLNA,LMOD3,ACTB
Monarch	EFO:0004713	FEV/FEC ratio	20	672	0.37	0.0326	MPO,LRP1,SERPINA10,TIMP2,DNAH5,SERPING1,MYL9,ANTXR1,DST,C7,FBLN1,FAM184A,MTCL1,CFH,EFEMP1,CFI,EFCAB5,NAV2,VEGFC,HYOU1
Monarch	HP:0000095	Abnormal renal glomerulus morphology	8	136	0.67	0.0326	APOE,CFHR5,C1QB,CFHR1,CFH,C1QC,CFI,C4A
Monarch	HP:0010647	Abnormal elasticity of skin	9	172	0.62	0.0326	MGP,SPARC,CSTA,ANTXR1,EFEMP2,SUZ12,FLNA,GSN,C1S
Monarch	HP:0030875	Abnormality of pulmonary circulation	8	136	0.67	0.0326	MGP,PROC,SERPINF1,TCIRG1,BMP1,F2,FLNA,PROS1
Monarch	HP:0001114	Xanthelasma	3	10	1.37	0.0340	APOB,APOE,PCSK9

Monarch	HP:0003141	Increased LDL cholesterol concentration	4	26	1.08	0.0340	APOB,APOE,LCAT,PCSK9
Monarch	HP:0008151	Prolonged prothrombin time	4	26	1.08	0.0340	F2,F5,F10,F7
Monarch	HP:0012185	Constrictive median neuropathy	3	10	1.37	0.0340	TTR,GSN,B2M
Monarch	HP:0000822	Hypertension	11	256	0.53	0.0376	MMP2,MGP,APOB,APOA1,ANGPTL6,LYZ,GUCY1A1,PCSK9,GANAB,ADAMTSL4,FGA
Monarch	HP:0002953	Vertebral compression fracture	4	27	1.07	0.0377	MMP2,SPARC,SERPINF1,P4HB
Monarch	HP:0003563	Decreased LDL cholesterol concentration	3	11	1.33	0.0410	APOC3,APOB,APOA5
Monarch	HP:0000703	Dentinogenesis imperfecta	4	28	1.05	0.0417	SPARC,SERPINF1,BMP1,P4HB
Monarch	HP:0003110	Abnormality of urine homeostasis	19	638	0.37	0.0417	F9,APOA1,APOE,CFHR5,LYZ,LCAT,F2,CFHR1,SERPINF2,GANAB,PAPLN,F8,CFH,F5,ADAMTS13,GSN,F10,ATP1A1,FGA
Monarch	HP:0007759	Opacification of the corneal stroma	6	78	0.78	0.0417	GNPTG,MMP2,APOB,APOE,LCAT,PCSK9
Monarch	HP:0032263	Increased blood pressure	12	303	0.49	0.0417	MMP2,MGP,APOB,APOA1,ANGPTL6,LYZ,GUCY1A1,PCSK9,F2,GANAB,ADAMTSL4,FGA
Monarch	HP:0025142	Constitutional symptom	24	906	0.32	0.0420	GNPTG,MMP2,PON1,APOB,PROC,TTR,CAT,DSG2,TCIRG1,CRH,HABP2,SERPING1,PCSK9,F2,SERPINF2,ANG,GANAB,AKAP9,F8,FLNA,PROS1,C1S,GRHL2,B2M
Monarch	HP:0000979	Purpura	6	79	0.78	0.0432	PROC,APOE,F2,F13B,PROS1,C4A
Monarch	EFO:0000618	Nervous system disease	13	350	0.47	0.0434	CETP,APOE,CFHR5,TIMP2,C9,ITIH4,EFEMP2,F8,CFH,F5,CFI,CFB,KNG1
Monarch	EFO:0004732	Lipoprotein measurement	33	1426	0.26	0.0434	CETP,APOH,VTN,APOC3,APOB,APOA1,LRP1,APOE,LCAT,KLKB1,ITIH1,PCSK9,FGB,F2,INHBC,PLG,STAB1,FBLN1,OIT3,LIMS1,THBS4,APOD,CDC42BPA,CRTAC1,ADAMTS13,ANKRD28,ABCA13,PLTP,HGFAC,HPR,APOA5,APOC4,GRHL2
Monarch	HP:0000926	Platyspondyly	7	111	0.7	0.0441	SPARC,SERPINF1,TCIRG1,CSF1R,BMP1,P4HB,FLNA
Monarch	HP:0011004	Abnormal systemic arterial morphology	14	398	0.44	0.0449	SERPIND1,MMP2,APOB,CAT,APOE,ANGPTL6,LCAT,GUCY1A1,ANTXR1,PCSK9,EFEMP2,P4HB,GANAB,FLNA
Monarch	HP:0006487	Bowing of the long bones	10	224	0.55	0.0463	GNPTG,SPARC,SERPINF1,TCIRG1,IHH,BMP1,P4HB,FLNA,LMOD3,B2M
Monarch	HP:0003321	Biconcave flattened vertebrae	3	12	1.29	0.0473	SPARC,SERPINF1,P4HB
Monarch	HP:0031652	Abnormal aortic valve physiology	7	113	0.69	0.0475	GNPTG,APOB,VWF,PCSK9,EFEMP2,FLNA,ACTB
DISEASES	DOID:1247	Blood coagulation disease	25	89	1.35	1.43e-20	SERPIND1,F9,PF4V1,PROC,VWF,KLKB1,PF4,FGB,F2,PLG,SERPINF2,FGG,F8,F13B,CFH,F5,FLNA,ADAMTS13,F10,F7,PROS1,F11,C4B,KNG1,FGA
DISEASES	DOID:4	Disease	145	6291	0.26	7.96e-14	CETP,GNPTG,APOH,VCL,SERPIND1,LGALS1,CHGA,LBP,F9,MMP2,PON1,C5,MPO,AFM,PF4V1,APOC3,MGP,SPARC,PCDH12,APOB,PROC,APOA1,TTR,CAT,CFP,APOE,PXDN,SERPINF1,APCS,CFHR5,LYZ,VWF,DSG2,C9,LCAT,YWHAE,CSTA,CP,KLKB1,CDH6,DNAH5,CENPE,TCIRG1,HYAL1,ITIH4,LUM,CDH11,AHSG,CRH,SAA4,SERPING1,ANKRD44,CSF1R,PCDH1,SCGB3A1,IHH,SERPINI1,PF4,MASP1,CAMP,APEH,A2ML1,ANTXR1,SH3D19,PCSK9,BMP1,FGB,DST,F2,PLG,EFEMP2,C1QB,CFHR1,SUZ12,APOL1,SUMO4,SERPINF2,HBA1,HSPA5,P4HB,SERPINA7,DNAH8,ANG,FGG,GANAB,BLM,AKAP9,CNDP1,F8,CENPF,F13B,CFHR4,CFHR3,CFH,F5,ADAMTSL4,FLNA,MCOLN2,C8B,ADAMTS13,GSN,C1QC,F10,F7,HLA-C,IGFBP3,CD99,GPX3,PROS1,EFEMP1,CFI,TNRC6A,YWHAZ,GAPDH,CST3,ANKRD28,SAA1,F11,C1S,NUCB1,PCYOX1,ADIPOQ,IGF2,C4A,CCDC122,ABCA13,COLEC11,MST1,LMOD3,C4B,CFB,SELENOP,HPR,APOA5,ATP1A1,SERPINA3,IGHV3-15,VEGFC,KNG1,TPM4,ACTB,TRIM66,GRHL2,B2M,FGA
DISEASES	DOID:7	Disease of anatomical entity	121	4798	0.3	3.36e-13	CETP,APOH,VCL,SERPIND1,LGALS1,CHGA,LBP,F9,MMP2,C5,MPO,AFM,PF4V1,MGP,SPARC,PCDH12,APOB,PROC,APOA1,TTR,CFP,APOE,PXDN,SERPINF1,APCS,CFHR5,VWF,DSG2,C9,LCAT,YWHAE,CSTA,KLKB1,DNAH5,CENPE,TCIRG1,LUM,AHSG,CRH,SAA4,SERPING1,ANKRD44,CSF1R,PCDH1,SCGB3A1,IHH,SERPINI1,PF4,CAMP,APEH,A2ML1,ANTXR1,SH3D19,PCSK9,BMP1,FGB,DST,F2,PLG,EFEMP2,C1QB,CFHR1,SUZ12,APOL1,SUMO4,SERPINF2,HBA1,P4HB,SERPINA7,ANG,FGG,GANAB,BLM,AKAP9,CNDP1,F8,F13B,CFHR4,CFHR3,CFH,F5,ADAMTSL4,FLNA,C8B,ADAMTS13,GSN,C1QC,F10,F7,HLA-C,CD99,PROS1,EFEMP1,CFI,TNRC6A,YWHAZ,GAPDH,CST3,ANKRD28,SAA1,F11,C1S,NUCB1,ADIPOQ,IGF2,C4A,CCDC122,MST1,LMOD3,C4B,CFB,ATP1A1,SERPINA3,IGHV3-15,VEGFC,KNG1,ACTB,TRIM66,GRHL2,B2M,FGA
DISEASES	DOID:74	Hematopoietic system disease	35	473	0.77	3.36e-13	SERPIND1,F9,MPO,PF4V1,PROC,VWF,KLKB1,TCIRG1,PF4,CAMP,FGB,F2,PLG,SERPINF2,HBA1,FGG,F8,F13B,CFH,F5,FLNA,ADAMTS13,F10,F7,CD99,PROS1,SAA1,F11,C4B,SERPINA3,IGHV3-15,KNG1,ACTB,B2M,FGA
DISEASES	DOID:626	Complement deficiency	12	23	1.61	6.84e-12	C5,CFHR5,C9,KLKB1,SERPING1,CFHR1,CFHR3,CFH,ADAMTS13,CFI,CFB,KNG1
DISEASES	DOID:2452	Thrombophilia	11	21	1.62	7.53e-11	SERPIND1,F9,PROC,VWF,F2,PLG,CFH,F5,ADAMTS13,PROS1,C4B
DISEASES	DOID:2007	Degeneration of macula and posterior pole	13	46	1.35	2.19e-10	CETP,APOE,CFHR5,C9,CFHR1,F13B,CFHR4,CFHR3,CFH,EFEMP1,CFI,CST3,CFB
DISEASES	DOID:9120	Amyloidosis	15	75	1.2	2.21e-10	APOC3,APOA1,TTR,APOE,APCS,LYZ,C9,SAA4,GSN,CST3,SAA1,SERPINA3,ACTB,B2M,FGA
DISEASES	DOID:10871	Age related macular degeneration	12	44	1.33	1.81e-09	CETP,APOE,CFHR5,C9,CFHR1,F13B,CFHR4,CFHR3,CFH,CFI,CST3,CFB
DISEASES	DOID:2914	Immune system disease	34	675	0.6	7.96e-09	APOH,C5,MPO,TTR,APOE,PXDN,APCS,CFHR5,C9,KLKB1,SAA4,SERPING1,A2ML1,DST,F2,PLG,CFHR1,SUMO4,HBA1,CFHR3,CFH,ADAMTS13,HLA-C,CD99,CFI,SAA1,C4A,C4B,CFB,IGHV3-15,VEGFC,KNG1,ACTB,B2M
DISEASES	DOID:0050636	Familial visceral amyloidosis	9	21	1.53	2.88e-08	APOC3,APOA1,TTR,APOE,APCS,LYZ,ACTB,B2M,FGA
DISEASES	DOID:0050736	Autosomal dominant disease	48	1386	0.44	1.12e-07	CETP,SERPIND1,F9,APOC3,PROC,APOA1,TTR,APOE,APCS,CFHR5,LYZ,VWF,C9,YWHAE,KLKB1,CDH6,SERPING1,ANKRD44,CSF1R,IHH,SERPINI1,F2,EFEMP2,CFHR1,GANAB,AKAP9,F8,F13B,CFHR4,CFHR3,CFH,F5,FLNA,GSN,EFEMP1,CFI,CST3,SAA1,C1S,IGF2,CFB,ATP1A1,SERPINA3,KNG1,ACTB,GRHL2,B2M,FGA
DISEASES	DOID:0050739	Autosomal genetic disease	74	2802	0.32	1.42e-07	CETP,GNPTG,SERPIND1,F9,APOC3,MGP,APOB,PROC,APOA1,TTR,APOE,APCS,CFHR5,LYZ,VWF,C9,YWHAE,CP,KLKB1,CDH6,DNAH5,TCIRG1,SERPING1,ANKRD44,CSF1R,IHH,SERPINI1,MASP1,CAMP,ANTXR1,PCSK9,FGB,DST,F2,PLG,EFEMP2,CFHR1,SERPINF2,HBA1,FGG,GANAB,BLM,AKAP9,F8,F13B,CFHR4,CFHR3,CFH,F5,ADAMTSL4,FLNA,GSN,F10,F7,IGFBP3,EFEMP1,CFI,CST3,SAA1,F11,C1S,PCYOX1,IGF2,COLEC11,LMOD3,CFB,APOA5,ATP1A1,SERPINA3,KNG1,ACTB,GRHL2,B2M,FGA
DISEASES	DOID:1287	Cardiovascular system disease	27	493	0.64	1.45e-07	CETP,APOH,VCL,CHGA,MPO,APOB,APOA1,TTR,APOE,APCS,VWF,DSG2,DNAH5,PF4,PCSK9,F2,PLG,AKAP9,FLNA,ADAMTS13,F10,F7,HLA-C,CD99,SAA1,ADIPOQ,C4A
DISEASES	DOID:557	Kidney disease	20	275	0.76	3.05e-07	C5,MPO,TTR,APCS,CFHR5,SAA4,F2,CFHR1,APOL1,GANAB,CFHR3,CFH,ADAMTS13,CFI,CST3,SAA1,IGF2,C4B,CFB,B2M
DISEASES	DOID:0080301	Atypical hemolytic-uremic syndrome	7	11	1.7	3.41e-07	CFHR5,CFHR1,CFHR3,CFH,ADAMTS13,CFI,CFB
DISEASES	DOID:178	Vascular disease	19	249	0.78	3.42e-07	CETP,APOH,MPO,APOB,APOA1,APOE,VWF,PF4,PCSK9,F2,PLG,ADAMTS13,F10,F7,HLA-C,CD99,SAA1,ADIPOQ,C4A
DISEASES	DOID:0050177	Monogenic disease	80	3266	0.29	5.15e-07	CETP,GNPTG,SERPIND1,F9,APOC3,MGP,APOB,PROC,APOA1,TTR,APOE,SERPINF1,APCS,CFHR5,LYZ,VWF,C9,YWHAE,CP,KLKB1,CDH6,DNAH5,TCIRG1,SERPING1,ANKRD44,CSF1R,IHH,SERPINI1,MASP1,CAMP,A2ML1,ANTXR1,PCSK9,FGB,DST,F2,PLG,EFEMP2,CFHR1,SERPINF2,HBA1,DNAH8,FGG,GANAB,BLM,AKAP9,F8,CENPF,F13B,CFHR4,CFHR3,CFH,F5,ADAMTSL4,FLNA,GSN,F10,F7,IGFBP3,PROS1,EFEMP1,CFI,CST3,SAA1,F11,C1S,PCYOX1,IGF2,COLEC11,MST1,LMOD3,CFB,APOA5,ATP1A1,SERPINA3,KNG1,ACTB,GRHL2,B2M,FGA

DISEASES	DOID:630	Genetic disease	88	3778	0.26	6.00e-07	CETP,GNPTG,SERPIND1,F9,APOC3,MGP,APOB,PROC,APOA1,TTR,CAT,APOE,SERPINF1,APCS,CFHR5,LYZ,VWF,C9,LCAT,YWHAE,CP,KLKB1,CDH6,DNAH5,TCIRG1,HYAL1,SERPING1,ANKRD44,CSF1R,IHH,SERPINI1,MASP1,CAMP,A2ML1,ANTXR1,PCSK9,FGB,DST,F2,PLG,EFEMP2,C1QB,CFHR1,SUMO4,SERPINF2,HBA1,DNAH8,FGG,GANAB,BLM,AKAP9,CNDP1,F8,CENPF,F13B,CFHR4,CFHR3,CFH,F5,ADAMTSL4,FLNA,MCOLN2,GSN,F10,F7,IGFBP3,PROS1,EFEMP1,CFI,CST3,SAA1,F11,C1S,PCYOX1,ADIPOQ,IGF2,COLEC11,MST1,LMOD3,CFB,APOA5,ATP1A1,SERPINA3,KNG1,ACTB,GRHL2,B2M,FGA
DISEASES	DOID:612	Primary immunodeficiency disease	25	470	0.62	8.33e-07	APOH,C5,MPO,TTR,PXDN,CFHR5,C9,KLKB1,SERPING1,A2ML1,DST,F2,CFHR1,SUMO4,HBA1,CFHR3,CFH,ADAMTS13,HLA-C,CFI,SAA1,C4A,C4B,CFB,KNG1
DISEASES	DOID:12554	Hemolytic-uremic syndrome	7	14	1.6	9.33e-07	CFHR5,CFHR1,CFHR3,CFH,ADAMTS13,CFI,CFB
DISEASES	DOID:18	Urinary system disease	21	350	0.67	2.01e-06	C5,MPO,TTR,APCS,CFHR5,SAA4,F2,CFHR1,APOL1,GANAB,CFHR3,CFH,ADAMTS13,CFI,CST3,ANKRD28,SAA1,IGF2,C4B,CFB,B2M
DISEASES	DOID:0050117	Disease by infectious agent	21	368	0.65	4.38e-06	APOE,APCS,YWHAE,CP,ITIH4,AHSG,PCDH1,APEH,ANTXR1,F2,PLG,C1QB,APOL1,HSPA5,C1QC,HLA-C,YWHAZ,SAA1,C4A,CCDC122,HPR
DISEASES	DOID:0060903	Thrombosis	7	21	1.42	7.76e-06	APOH,VWF,PF4,F2,PLG,F10,F7
DISEASES	DOID:0014667	Disease of metabolism	37	1076	0.43	9.76e-06	CETP,GNPTG,F9,APOC3,MGP,APOB,APOA1,TTR,CAT,APOE,APCS,LYZ,C9,LCAT,CP,HYAL1,AHSG,SAA4,PCSK9,F2,SUMO4,HBA1,CNDP1,MCOLN2,GSN,GPX3,PROS1,CST3,SAA1,PCYOX1,ADIPOQ,SELENOP,APOA5,SERPINA3,ACTB,B2M,FGA
DISEASES	DOID:649	Prion disease	6	16	1.47	3.92e-05	APOE,APCS,YWHAE,C1QB,C1QC,YWHAZ
DISEASES	DOID:0050637	Finnish type amyloidosis	5	9	1.64	8.68e-05	APOE,APCS,C9,GSN,CST3
DISEASES	DOID:0050639	Primary cutaneous amyloidosis	6	19	1.4	8.68e-05	APOE,APCS,C9,GSN,CST3,ACTB
DISEASES	DOID:11949	Creutzfeldt-Jakob disease	5	9	1.64	8.68e-05	APOE,APCS,C1QB,C1QC,YWHAZ
DISEASES	DOID:9799	Eye degenerative disease	15	236	0.7	0.00010	CETP,APOB,APOE,CFHR5,C9,PCSK9,CFHR1,F13B,CFHR4,CFHR3,CFH,EFEMP1,CFI,CST3,CFB
DISEASES	DOID:2921	Glomerulonephritis	7	36	1.19	0.00014	MPO,CFHR5,APOL1,CFHR3,CFH,C4B,CFB
DISEASES	DOID:409	Liver disease	10	97	0.91	0.00014	TTR,APCS,AHSG,SAA4,F2,GANAB,GAPDH,SAA1,IGHV3-15,ACTB
DISEASES	DOID:65	Connective tissue disease	28	774	0.45	0.00014	F9,MMP2,C5,MGP,SPARC,TTR,SERPINF1,TCIRG1,CSF1R,IHH,A2ML1,ANTXR1,BMP1,DST,PLG,EFEMP2,P4HB,F8,FLNA,F7,HLA-C,CD99,SAA1,C1S,IGF2,C4A,C4B,SERPINA3
DISEASES	DOID:37	Skin disease	22	518	0.52	0.00018	MPO,APOA1,CFP,APOE,APCS,DSG2,C9,CSTA,KLKB1,SERPING1,CAMP,A2ML1,ANTXR1,DST,EFEMP2,BLM,GSN,HLA-C,CST3,C1S,KNG1,ACTB
DISEASES	DOID:12134	Factor VIII deficiency	4	4	1.9	0.00021	F9,VWF,F8,F7
DISEASES	DOID:1588	Thrombocytopenia	5	12	1.52	0.00021	PF4V1,VWF,PF4,F2,ADAMTS13
DISEASES	DOID:16	Integumentary system disease	23	575	0.5	0.00025	MPO,APOA1,CFP,APOE,APCS,DSG2,C9,CSTA,KLKB1,AHSG,SERPING1,CAMP,A2ML1,ANTXR1,DST,EFEMP2,BLM,GSN,HLA-C,CST3,C1S,KNG1,ACTB
DISEASES	DOID:0070028	APP-related cerebral amyloid angiopathy	4	5	1.8	0.00034	APOE,APCS,CST3,SERPINA3
DISEASES	DOID:12259	Hemophilia B	4	5	1.8	0.00034	F9,F8,F7,F11
DISEASES	DOID:2229	Factor XI deficiency	4	5	1.8	0.00034	F9,F8,F7,F11
DISEASES	DOID:1168	Familial hyperlipidemia	5	15	1.42	0.00042	APOC3,APOB,APOE,PCSK9,APOA5
DISEASES	DOID:12531	Von Willebrands disease	4	6	1.72	0.00050	F9,VWF,F8,ADAMTS13
DISEASES	DOID:14735	Hereditary angioedema	4	6	1.72	0.00050	KLKB1,SERPING1,C1S,KNG1
DISEASES	DOID:2218	Blood platelet disease	7	47	1.07	0.00050	PF4V1,VWF,PF4,F2,FLNA,ADAMTS13,F7
DISEASES	DOID:863	Nervous system disease	54	2275	0.27	0.00050	CETP,LGALS1,MPO,PCDH12,APOB,PROC,APOA1,TTR,APOE,APCS,CFHR5,VWF,C9,LCAT,YWHAE,CENPE,LUM,SERPING1,ANKRD44,CSF1R,SERPINI1,A2ML1,SH3D19,PCSK9,DST,F2,PLG,C1QB,CFHR1,SUZ12,ANG,CNDP1,F13B,CFHR4,CFHR3,CFH,ADAMTSL4,FLNA,GSN,C1QC,CD99,EFEMP1,CFI,TNRC6A,YWHAZ,CST3,IGF2,CCDC122,CFB,ATP1A1,SERPINA3,ACTB,TRIM66,GRHL2
DISEASES	DOID:655	Inherited metabolic disorder	30	949	0.4	0.00056	CETP,GNPTG,F9,APOC3,APOB,APOA1,TTR,CAT,APOE,APCS,LYZ,C9,LCAT,CP,HYAL1,PCSK9,SUMO4,HBA1,CNDP1,MCOLN2,GSN,CST3,SAA1,PCYOX1,ADIPOQ,APOA5,SERPINA3,ACTB,B2M,FGA
DISEASES	DOID:3146	Lipid metabolism disorder	8	72	0.94	0.00068	CETP,APOC3,APOB,APOA1,APOE,LCAT,PCSK9,APOA5
DISEASES	DOID:0050828	Artery disease	10	130	0.78	0.00099	CETP,APOB,APOA1,APOE,PCSK9,F2,PLG,ADAMTS13,SAA1,ADIPOQ
DISEASES	DOID:17	Musculoskeletal system disease	33	1154	0.35	0.0013	F9,MMP2,C5,MGP,SPARC,TTR,APOE,SERPINF1,APCS,TCIRG1,CSF1R,IHH,A2ML1,ANTXR1,BMP1,DST,PLG,EFEMP2,P4HB,F8,FLNA,F7,HLA-C,CD99,SAA1,C1S,IGF2,C4A,LMOD3,C4B,ATP1A1,SERPINA3,B2M
DISEASES	DOID:1936	Atherosclerosis	5	21	1.27	0.0013	CETP,APOB,APOA1,APOE,ADIPOQ
DISEASES	DOID:3118	Hepatobiliary disease	11	168	0.71	0.0014	TTR,APCS,AHSG,SAA4,F2,GANAB,GAPDH,SAA1,MST1,IGHV3-15,ACTB
DISEASES	DOID:0080001	Bone disease	20	540	0.47	0.0022	F9,MMP2,C5,SPARC,TTR,SERPINF1,TCIRG1,CSF1R,IHH,BMP1,P4HB,F8,FLNA,F7,HLA-C,CD99,SAA1,C1S,IGF2,SERPINA3
DISEASES	DOID:1387	Hypolipoproteinemia	4	11	1.46	0.0023	APOB,APOA1,LCAT,PCSK9
DISEASES	DOID:5113	Nutritional deficiency disease	6	43	1.04	0.0025	MGP,TTR,F2,GPX3,PROS1,SELENOP
DISEASES	DOID:0070029	ITM2B-related cerebral amyloid angiopathy 1	4	12	1.42	0.0028	APOE,APCS,CST3,SERPINA3
DISEASES	DOID:0111146	Acquired von Willebrand syndrome	3	3	1.9	0.0028	VWF,F8,ADAMTS13
DISEASES	DOID:170	Endocrine gland cancer	8	93	0.83	0.0028	CHGA,APOE,APCS,CD99,GAPDH,IGF2,IGHV3-15,ACTB
DISEASES	DOID:2222	Factor X deficiency	3	3	1.9	0.0028	F2,F10,F7
DISEASES	DOID:2236	Congenital afibrinogenemia	3	3	1.9	0.0028	FGB,FGG,FGA
DISEASES	DOID:801	Hemarthrosis	3	3	1.9	0.0028	F9,F8,F7
DISEASES	DOID:5614	Eye disease	24	749	0.4	0.0030	CETP,LGALS1,APOB,APOE,APCS,CFHR5,C9,LUM,SERPING1,PCSK9,PLG,CFHR1,F13B,CFHR4,CFHR3,CFH,ADAMTSL4,GSN,EFEMP1,CFI,CST3,CFB,ACTB,GRHL2
DISEASES	DOID:0070030	ITM2B-related cerebral amyloid angiopathy 2	4	13	1.38	0.0034	APOE,APCS,CST3,SERPINA3
DISEASES	DOID:0060002	C1 inhibitor deficiency	3	4	1.77	0.0043	KLKB1,SERPING1,KNG1
DISEASES	DOID:10772	Thrombotic thrombocytopenic purpura	3	4	1.77	0.0043	VWF,CFH,ADAMTS13
DISEASES	DOID:417	Autoimmune disease	14	317	0.54	0.0051	APOH,C5,MPO,TTR,PXDN,A2ML1,DST,F2,SUMO4,HBA1,HLA-C,SAA1,C4A,C4B
DISEASES	DOID:11249	Vitamin K deficiency bleeding	3	5	1.67	0.0065	MGP,F2,PROS1
DISEASES	DOID:2451	Protein S deficiency	3	5	1.67	0.0065	F2,PROS1,C4B
DISEASES	DOID:3393	Coronary artery disease	5	36	1.04	0.0094	APOB,APOA1,APOE,PCSK9,PLG
DISEASES	DOID:0050155	Sensory system disease	26	929	0.34	0.0106	CETP,LGALS1,APOB,PROC,APOE,APCS,CFHR5,C9,LUM,SERPING1,A2ML1,PCSK9,PLG,CFHR1,F13B,CFHR4,CFHR3,CFH,ADAMTSL4,GSN,EFEMP1,CFI,CST3,CFB,ACTB,GRHL2
DISEASES	DOID:10873	Kuhnt-Junius degeneration	3	7	1.53	0.0130	CETP,CFH,CFB
DISEASES	DOID:865	Vasculitis	4	21	1.18	0.0144	MPO,F2,HLA-C,SAA1
DISEASES	DOID:0060158	Acquired metabolic disease	9	162	0.64	0.0181	MGP,TTR,CP,AHSG,F2,GPX3,PROS1,ADIPOQ,SELENOP
DISEASES	DOID:381	Arthropathy	4	23	1.14	0.0191	F9,F8,F7,IGF2
DISEASES	DOID:0050737	Autosomal recessive disease	40	1785	0.25	0.0195	GNPTG,MGP,APOB,APOE,VWF,CP,KLKB1,DNAH5,TCIRG1,SERPING1,IHH,MASP1,CAMP,ANTXR1,PCSK9,FGB,DST,F2,PLG,EFEMP2,SERPINF2,HBA1,FGG,BLM,F13B,F5,FLNA,F10,F7,IGFBP3,SAA1,F11,C1S,PCYOX1,IGF2,COLEC11,LMOD3,APOA5,KNG1,FGA
DISEASES	DOID:1188	Mononeuropathy	3	9	1.42	0.0218	MPO,F2,CCDC122
DISEASES	DOID:14250	Down syndrome	3	9	1.42	0.0218	APOE,C1QB,CST3
DISEASES	DOID:0060051	Autoimmune disease of cardiovascular system	4	25	1.1	0.0242	APOH,F2,HLA-C,SAA1
DISEASES	DOID:75	Lymphatic system disease	9	174	0.61	0.0277	SAA4,F2,PLG,CD99,SAA1,IGHV3-15,VEGFC,ACTB,B2M

DISEASES	DOID:77	Gastrointestinal system disease	18	576	0.39	0.0280	CHGA,AFM,TTR,APCS,AHSG,CRH,SAA4,APEH,ANTXR1,F2,GANAB,FLNA,C8B,GAPDH,SAA1,MST1,IGHV3-15,ACTB
DISEASES	DOID:5844	Myocardial infarction	4	27	1.07	0.0303	APOB,APOE,PCSK9,PLG
DISEASES	DOID:2213	Hemorrhagic disease	3	12	1.29	0.0409	F2,SERPINF2,ADAMTS13
DISEASES	DOID:0050638	Transthyretin amyloidosis	2	2	1.9	0.0471	TTR,APCS
DISEASES	DOID:0060573	Von Willebrands disease 1	2	2	1.9	0.0471	VWF,F8
DISEASES	DOID:0060574	Von Willebrands disease 2	2	2	1.9	0.0471	VWF,F8
DISEASES	DOID:0111054	Von Willebrands disease 3	2	2	1.9	0.0471	VWF,F8
DISEASES	DOID:0111676	High molecular weight kininogen deficiency	2	2	1.9	0.0471	KLKB1,KNG1
DISEASES	DOID:10124	Corneal disease	6	85	0.75	0.0471	LGALS1,APOB,APOE,LUM,PCSK9,GRHL2
DISEASES	DOID:10230	Aortic atherosclerosis	2	2	1.9	0.0471	APOB,APOE
DISEASES	DOID:1391	Norum disease	2	2	1.9	0.0471	APOA1,LCAT
DISEASES	DOID:374	Nutrition disease	7	116	0.68	0.0471	MGP,TTR,F2,GPX3,PROS1,ADIPOQ,SELENOP
TISSUES	BTO:0004850	Bone marrow cell	65	198	1.41	4.56e-63	CPB2,CETP,APOH,PON1,C5,MPO,VTN,AFM,APOB,IGFBP5,TTR,LRP1,CFP,SERPINF1,APCS,VWF,DSG2,LGALS3BP,C9,CP,KLKB1,AMBP,HPX,ITIH4,LUM,FCN3,AHSG,SAA4,SERPING1,CSF1R,FCN2,IHH,MASP1,FGB,F2,CSPG4,CFHR1,APOL1,SERPINF2,APOD,ITIH2,F8,CFH,F5,ADAMTS13,MBL2,IGFBP3,SOD3,PROS1,CFI,F11,C1S,C4A,MST1,SPARCL1,C4B,CFB,PLTP,HGFAC,SELENOP,SERPINA3,HYOU1,KNG1,B2M,FGA
TISSUES	BTO:0000392	Plasma cell	58	171	1.43	1.09e-56	APOH,PON1,C5,MPO,VTN,APOB,IGFBP5,TTR,LRP1,CFP,SERPINF1,APCS,VWF,DSG2,LGALS3BP,C9,CP,KLKB1,AMBP,HPX,ITIH4,LUM,FCN3,AHSG,SAA4,SERPING1,CSF1R,FCN2,IHH,MASP1,FGB,F2,CSPG4,CFHR1,APOL1,SERPINF2,APOD,ITIH2,F8,CFH,ADAMTS13,MBL2,IGFBP3,SOD3,PROS1,CFI,C1S,C4A,SPARCL1,C4B,CFB,HGFAC,SELENOP,SERPINA3,HYOU1,KNG1,B2M,FGA
TISSUES	BTO:0000759	Liver	129	2125	0.68	1.93e-53	RCN1,SPP2,ST6GAL1,CPB2,CETP,APOH,VCL,SERPIND1,LGALS1,CHGA,TPD52L2,LBP,APMAP,F9,PON1,MPO,AFM,APOC3,GPLD1,APOB,PROC,APOA1,TTR,CAT,LRP1,C4BPB,APOE,PXDN,SERPINF1,APCS,CFHR5,VWF,PSMA6,SERPINA10,LGALS3BP,ATRN,C9,NID1,YWHAE,CP,KLKB1,AMBP,HPX,LUM,FCN3,ITIH1,AHSG,HABP2,SAA4,SERPING1,CSF1R,FCN2,PGLYRP2,PPBP,MASP1,APEH,HSP90B1,FGB,DST,F2,INHBC,PLG,STAB1,C1QB,CFHR1,APOL1,SERPINF2,HBA1,HSPA5,P4HB,SERPINA7,ANG,FGG,GANAB,AKAP9,ITIH2,C8A,PCDH18,F13B,CFHR4,CFHR3,CFH,CD5L,S100A9,FLNA,SMARCA1,C8B,ADAMTS13,GSN,MBL2,F10,F7,APOM,HLA-C,CKAP4,IGFBP3,CD99,PROS1,CFI,YWHAZ,GAPDH,APOF,SAA1,F11,C1S,NUCB1,PCYOX1,IGF2,C4A,COLEC11,MST1,ITIH3,C4B,CFB,IGFALS,PLTP,GC,HGFAC,APOA5,ATP1A1,SERPINA3,APOC4,HYOU1,KNG1,ACTB,B2M,FGA,PDIA4,SDF4
TISSUES	BTO:0000141	Bone marrow	76	528	1.05	4.78e-52	CPB2,CETP,APOH,SERPIND1,APMAP,PON1,C5,MPO,VTN,AFM,APOB,IGFBP5,TTR,LRP1,CFP,PXDN,SERPINF1,APCS,VWF,DSG2,LGALS3BP,C9,CSTA,CP,KLKB1,AMBP,HPX,ITIH4,LUM,FCN3,AHSG,SAA4,SERPING1,CSF1R,FCN2,IHH,MASP1,CAMP,FGB,F2,CSPG4,CFHR1,SUZ12,APOL1,SERPINF2,HBA1,LIMS1,GANAB,APOD,ITIH2,F8,CFH,QSOX1,F5,ADAMTS13,MBL2,IGFBP3,SOD3,PROS1,CFI,YWHAZ,F11,C1S,C4A,MST1,SPARCL1,C4B,CFB,PLTP,HGFAC,SELENOP,SERPINA3,HYOU1,KNG1,B2M,FGA
TISSUES	BTO:0000345	Digestive gland	139	2881	0.58	1.87e-47	RCN1,SPP2,ST6GAL1,CPB2,CETP,APOH,VCL,SERPIND1,LGALS1,CHGA,TPD52L2,LBP,APMAP,F9,PON1,MPO,AFM,APOC3,GPLD1,SPARC,APOB,PROC,APOA1,TTR,CAT,LRP1,C4BPB,DHX29,APOE,PXDN,SERPINF1,APCS,CFHR5,VWF,PSMA6,SERPINA10,LGALS3BP,ATRN,C9,NID1,YWHAE,CP,KLKB1,AMBP,TCIRG1,HPX,HYAL1,LUM,FCN3,ITIH1,AHSG,HABP2,SAA4,SERPING1,CSF1R,FCN2,PGLYRP2,PPBP,MASP1,APEH,HSP90B1,FGB,DST,F2,INHBC,PLG,STAB1,C1QB,CFHR1,APOL1,SERPINF2,HBA1,HSPA5,P4HB,SERPINA7,ANG,FGG,GANAB,QPCT,APOD,AKAP9,ITIH2,C8A,PCDH18,CDC42BPA,F13B,CFHR4,CFHR3,CFH,CD5L,S100A9,FLNA,SMARCA1,C8B,ADAMTS13,GSN,MBL2,F10,F7,APOM,HLA-C,CKAP4,IGFBP3,CD99,GPX3,PROS1,CFI,YWHAZ,GAPDH,RNASE1,APOF,SAA1,F11,C1S,NUCB1,PCYOX1,IGF2,C4A,COLEC11,MST1,ITIH3,C4B,CFB,IGFALS,PLTP,GC,HGFAC,APOA5,ATP1A1,SERPINA3,RNASE4,APOC4,HYOU1,KNG1,ACTB,B2M,FGA,PDIA4,SDF4
TISSUES	BTO:0001486	Skeletal system	100	1307	0.78	2.31e-47	SPP2,CPB2,CETP,APOH,SERPIND1,CHGA,APMAP,MMP2,PON1,C5,MPO,VTN,AFM,APOC3,SPARC,APOB,IGFBP5,APOA1,TTR,LRP1,CFP,APOE,PXDN,SERPINF1,APCS,VWF,PSMA6,DSG2,LGALS3BP,ATRN,C9,CSTA,CP,KLKB1,AMBP,HPX,HYAL1,ITIH4,LUM,FCN3,AHSG,SAA4,SERPING1,CSF1R,FCN2,IHH,MASP1,CAMP,HSP90B1,FGB,F2,CSPG4,CFHR1,SUZ12,APOL1,SERPINF2,HBA1,HSPA5,SERPINA7,FGG,LIMS1,GANAB,APOD,ITIH2,F8,CFH,QSOX1,F5,ADAMTSL4,CRTAC1,ADAMTS13,CCN5,MBL2,IGFBP3,SOD3,PROS1,CFI,YWHAZ,GAPDH,CST3,F11,C1S,PCYOX1,IGF2,C4A,MST1,LMOD3,SPARCL1,C4B,CFB,PLTP,HGFAC,SELENOP,SERPINA3,HYOU1,KNG1,ACTB,B2M,FGA,SDF4
TISSUES	BTO:0001491	Viscus	162	5378	0.38	5.62e-32	RCN1,SPP2,ST6GAL1,CPB2,CETP,APOH,VCL,SERPIND1,LGALS1,CHGA,TPD52L2,LBP,APMAP,F9,PON1,MPO,AFM,APOC3,GPLD1,SPARC,APOB,PROC,PLEK,APOA1,TTR,CAT,LRP1,C4BPB,CFP,DHX29,APOE,PXDN,SERPINF1,APCS,CFHR5,LYZ,VWF,PSMA6,DSG2,SERPINA10,LGALS3BP,ATRN,C9,NID1,YWHAE,CSTA,CP,KLKB1,CDH6,AMBP,TCIRG1,HPX,LUM,FCN3,ITIH1,AHSG,HABP2,SAA4,SERPING1,CSF1R,FCN2,PGLYRP2,PPBP,MASP1,APEH,GUCY1A1,HSP90B1,ANTXR1,SH3D19,FGB,DST,F2,INHBC,PLG,STAB1,C1QB,CFHR1,APOL1,SERPINF2,HBA1,HSPA5,P4HB,SERPINA7,FBLN1,ANG,FGG,GANAB,QPCT,APOD,AKAP9,ITIH2,F8,C8A,PCDH18,F13B,CFHR4,CFHR3,CFH,QSOX1,CD5L,S100A9,FLNA,SMARCA1,C8B,ADAMTS13,GSN,MBL2,F10,F7,APOM,HLA-C,CKAP4,CALML5,IGFBP3,CD99,SOD3,GPX3,PROS1,EFEMP1,CFI,TNRC6A,YWHAZ,ILK,GAPDH,RNASE1,APOF,CST3,SAA1,F11,C1S,NUCB1,PCYOX1,IGF2,C4A,ACSF2,COLEC11,MST1,SPARCL1,ITIH3,C4B,CFB,IGFALS,PLTP,RARRES2,GC,HGFAC,APOA5,ATP1A1,SERPINA3,SERPINA4,RNASE4,APOC4,CCL18,VEGFC,HYOU1,KNG1,TPM4,ACTB,B2M,FGA,PDIA4,SDF4
TISSUES	BTO:0001488	Endocrine gland	168	6403	0.32	2.57e-26	RCN1,SPP2,ST6GAL1,CPB2,CETP,APOH,VCL,SERPIND1,LGALS1,CHGA,TPD52L2,LBP,APMAP,F9,MMP2,PON1,MPO,VTN,AFM,APOC3,GPLD1,SPARC,APOB,PROC,APOA1,TTR,CAT,LRP1,C4BPB,DHX29,APOE,PXDN,SERPINF1,APCS,CFHR5,VWF,PSMA6,SERPINA10,LGALS3BP,ATRN,C9,LCN1,NID1,YWHAE,CSTA,CP,KLKB1,AMBP,TCIRG1,HPX,HYAL1,LUM,FCN3,ITIH1,AHSG,CRH,HABP2,SAA4,SERPING1,CSF1R,FCN2,PGLYRP2,IHH,PPBP,MASP1,CAMP,APEH,HSP90B1,ANTXR1,SH3D19,FGB,DST,F2,INHBC,PLG,STAB1,C1QB,CFHR1,APOL1,SERPINF2,HBA1,HSPA5,P4HB,SERPINA7,FBLN1,ANG,FGG,LIMS1,GANAB,QPCT,APOD,PAPLN,AKAP9,ITIH2,C8A,PCDH18,CDC42BPA,F13B,CFHR4,CFHR3,CFH,QSOX1,CD5L,S100A9,FLNA,SMARCA1,C8B,ADAMTS13,CCN5,GSN,MBL2,F10,F7,APOM,HLA-C,CKAP4,CALML5,IGFBP3,CD99,SOD3,GPX3,PROS1,EFEMP1,CFI,EFCAB5,YWHAZ,GAPDH,RAPGEF4,RNASE1,APOF,CST3,ANKRD28,FBLN2,SAA1,F11,C1S,NUCB1,PCYOX1,IGF2,C4A,ATP9B,FSIP2,ACSF2,COLEC11,MST1,ITIH3,C4B,CFB,IGFALS,PLTP,RARRES2,GC,HGFAC,UIMC1,APOA5,ATP1A1,SERPINA3,RNASE4,APOC4,CCL18,HYOU1,KNG1,TPM4,ACTB,B2M,FGA,PDIA4,SDF4

TISSUES	BTO:0000522	Gland	174	7004	0.29	4.76e-25	RCN1,SPP2,ST6GAL1,CPB2,CETP,APOH,VCL,SERPIND1,LGALS1,CHGA,TPD52L2,LBP,APMAP,F9,MMP2,PON1,C5,MPO,VTN,AFM,APOC3,GPLD1,SPARC,APOB,PROC,APOA1,TTR,CAT,LRP1,C4BPB,DHX29,APOE,PXDN,SERPINF1,APCS,CFHR5,LYZ,VWF,PSMA6,SERPINA10,LGALS3BP,ATRN,C9,LCN1,NID1,YWHAE,CSTA,CP,KLKB1,CDH6,AMBP,TCIRG1,HPX,HYAL1,LUM,FCN3,ITIH1,AHSG,CRH,HABP2,SAA4,SERPING1,CSF1R,FCN2,PGLYRP2,IHH,PPBP,MASP1,CAMP,APEH,GUCY1A1,HSP90B1,ANTXR1,SH3D19,FGB,DST,F2,INHBC,PLG,STAB1,C1QB,CFHR1,APOL1,SERPINF2,HBA1,HSPA5,P4HB,SERPINA7,FBLN1,ANG,FGG,LIMS1,GANAB,QPCT,APOD,PAPLN,AKAP9,ITIH2,F8,C8A,PCDH18,CDC42BPA,F13B,CFHR4,CFHR3,CFH,QSOX1,CD5L,S100A9,FLNA,SMARCA1,C8B,ADAMTS13,CCN5,GSN,MBL2,F10,F7,APOM,HLA-C,CKAP4,CALML5,IGFBP3,CD99,SOD3,GPX3,PROS1,EFEMP1,CFI,EFCAB5,TNRC6A,YWHAZ,GAPDH,RAPGEF4,RNASE1,APOF,CST3,ANKRD28,FBLN2,SAA1,F11,C1S,NUCB1,PCYOX1,IGF2,C4A,ATP9B,FSIP2,ACSF2,COLEC11,MST1,ITIH3,C4B,CFB,IGFALS,PLTP,RARRES2,GC,HGFAC,UIMC1,APOA5,ATP1A1,SERPINA3,RNASE4,APOC4,CCL18,HYOU1,KNG1,TPM4,ACTB,B2M,FGA,PDIA4,SDF4
TISSUES	BTO:0000237	Cerebrospinal fluid	22	45	1.59	2.24e-23	APOH,CHGA,AFM,APOC3,TTR,APOE,SERPINF1,CP,HPX,AHSG,SERPING1,F2,SERPINA7,APOD,CFH,IGFBP3,CFI,CST3,IGF2,C4A,SPARCL1,KNG1
TISSUES	BTO:0001489	Whole body	227	13099	0.14	4.87e-17	RCN1,SPP2,ST6GAL1,CPB2,CETP,GNPTG,APOH,VCL,SERPIND1,LGALS1,CHGA,TPD52L2,LBP,APMAP,F9,MMP2,EBI3,PON1,TFPI2,PCOLCE,C5,MPO,VTN,AFM,APOC3,MGP,GPLD1,SPARC,PCDH12,APOB,IGFBP5,PROC,PLEK,APOA1,TTR,CAT,LRP1,C4BPB,CFP,DHX29,APOE,PXDN,SERPINF1,APCS,CFHR5,LYZ,VWF,PSMA6,DSG2,SERPINA10,TIMP2,LGALS3BP,ATRN,C9,LCN1,LCAT,NID1,YWHAE,CSTA,CP,KLKB1,CDH6,DNAH5,AMBP,CENPE,TCIRG1,HPX,HYAL1,ITIH4,LUM,CDH11,PSG9,FCN3,ITIH1,AHSG,CRH,HABP2,SAA4,SERPING1,MYL9,CSF1R,FCN2,PGLYRP2,SCGB3A1,IHH,SERPINI1,PPBP,PF4,MASP1,CAMP,APEH,GUCY1A1,A2ML1,HSP90B1,ANTXR1,SH3D19,PCSK9,BMP1,FGB,DST,F2,INHBC,PLG,EFEMP2,CSPG4,STAB1,C1QB,CFHR1,SUZ12,APOL1,PSG11,SERPINF2,C7,HBA1,HSPA5,P4HB,SERPINA7,FBLN1,DNAH8,ANG,FGG,LIMS1,THBS4,GANAB,FAM184A,QPCT,APOD,PAPLN,AKAP9,ITIH2,CNDP1,MTCL1,F8,C8A,PCDH18,CDC42BPA,CENPF,F13B,CFHR4,CFHR3,CFH,QSOX1,F5,CD5L,S100A7,S100A9,ADAMTSL4,FLNA,CRTAC1,SMARCA1,C8B,ADAMTS13,CCN5,GSN,MBL2,C1QC,F10,F7,APOM,HLA-C,CKAP4,CALML5,IGFBP3,CD99,SOD3,GPX3,PROS1,EFEMP1,CFI,EFCAB5,TNRC6A,YWHAZ,NAV2,ILK,GAPDH,RAPGEF4,RNASE1,APOF,CST3,ANKRD28,MAP3K7CL,FBLN2,SAA1,F11,C1S,NUCB1,PCYOX1,ADIPOQ,IGF2,C4A,ATP9B,SLAIN1,FSIP2,ACSF2,COLEC11,MST1,LMOD3,SPARCL1,ITIH3,C4B,CFB,IGFALS,PLTP,PAEP,RARRES2,GC,HGFAC,UIMC1,SELENOP,SBSN,APOA5,ATP1A1,SERPINA3,SERPINA4,RNASE4,APOC4,CCL18,VEGFC,HYOU1,KNG1,TPM4,ACTB,GRHL2,B2M,FGA,PDIA4,SDF4
TISSUES	BTO:0001279	Spinal cord	26	233	0.94	4.79e-14	APOH,CHGA,AFM,APOC3,TTR,APOE,SERPINF1,CP,HPX,AHSG,SERPING1,F2,CSPG4,SERPINA7,APOD,PCDH18,CFH,FLNA,IGFBP3,CFI,CST3,IGF2,C4A,SPARCL1,KNG1,ACTB
TISSUES	BTO:0001078	Placenta	49	1244	0.49	4.01e-10	LGALS1,EBI3,TFPI2,PCOLCE,SPARC,PCDH12,IGFBP5,LRP1,VWF,TIMP2,LGALS3BP,NID1,YWHAE,LUM,PSG9,CRH,SERPING1,MYL9,CSF1R,HSP90B1,ANTXR1,BMP1,DST,EFEMP2,APOL1,PSG11,HBA1,P4HB,FBLN1,GANAB,QSOX1,F5,ADAMTSL4,HLA-C,IGFBP3,GPX3,YWHAZ,ILK,GAPDH,RNASE1,CST3,NUCB1,PCYOX1,IGF2,PLTP,ATP1A1,TPM4,ACTB,B2M
TISSUES	BTO:0000132	Blood platelet	26	363	0.75	6.13e-10	VCL,LGALS1,VTN,APOA1,TTR,CAT,VWF,C9,YWHAE,AHSG,PPBP,HSP90B1,FGB,HBA1,P4HB,FGG,LIMS1,F5,FLNA,GSN,YWHAZ,GAPDH,HYOU1,TPM4,ACTB,FGA
TISSUES	BTO:0000449	Fetus	50	1318	0.48	7.65e-10	LGALS1,EBI3,TFPI2,PCOLCE,SPARC,PCDH12,IGFBP5,LRP1,VWF,TIMP2,LGALS3BP,NID1,YWHAE,LUM,PSG9,CRH,SERPING1,MYL9,CSF1R,HSP90B1,ANTXR1,BMP1,DST,EFEMP2,APOL1,PSG11,HBA1,P4HB,FBLN1,GANAB,QSOX1,F5,ADAMTSL4,HLA-C,CKAP4,IGFBP3,GPX3,YWHAZ,ILK,GAPDH,RNASE1,CST3,NUCB1,PCYOX1,IGF2,PLTP,ATP1A1,TPM4,ACTB,B2M
TISSUES	BTO:0000042	Animal	233	15148	0.08	8.36e-10	RCN1,SPP2,ST6GAL1,CPB2,CETP,GNPTG,APOH,VCL,SERPIND1,LGALS1,CHGA,TPD52L2,LBP,APMAP,F9,MMP2,EBI3,PON1,TFPI2,PCOLCE,C5,MPO,VTN,AFM,APOC3,MGP,GPLD1,SPARC,PCDH12,APOB,IGFBP5,PROC,PLEK,APOA1,TTR,CAT,LRP1,C4BPB,CFP,DHX29,APOE,PXDN,ANGPTL6,SERPINF1,APCS,CFHR5,LYZ,VWF,PSMA6,DSG2,SERPINA10,TIMP2,LGALS3BP,ATRN,C9,LCN1,LCAT,NID1,YWHAE,CSTA,CP,KLKB1,CDH6,DNAH5,AMBP,CENPE,TCIRG1,HPX,HYAL1,ITIH4,LUM,CDH11,PSG9,FCN3,ITIH1,AHSG,CRH,HABP2,SAA4,SERPING1,MYL9,CSF1R,PCDH1,FCN2,PGLYRP2,SCGB3A1,IHH,SERPINI1,PPBP,PF4,MASP1,CAMP,APEH,GUCY1A1,A2ML1,HSP90B1,ANTXR1,SH3D19,PCSK9,BMP1,SHLD1,FGB,DST,F2,INHBC,PLG,EFEMP2,CSPG4,STAB1,C1QB,CFHR1,SUZ12,APOL1,PSG11,SERPINF2,C7,HBA1,HSPA5,P4HB,SERPINA7,FBLN1,DNAH8,ANG,FGG,LIMS1,THBS4,GANAB,FAM184A,QPCT,APOD,PAPLN,AKAP9,ITIH2,CNDP1,MTCL1,F8,C8A,PCDH18,CDC42BPA,CENPF,F13B,CFHR4,CFHR3,CFH,QSOX1,F5,CD5L,S100A7,S100A9,ADAMTSL4,FLNA,CRTAC1,SMARCA1,C8B,ADAMTS13,CCN5,GSN,MBL2,C1QC,F10,F7,APOM,HLA-C,CKAP4,CALML5,IGFBP3,CD99,SOD3,GPX3,KIF19,PROS1,EFEMP1,CFI,EFCAB5,TNRC6A,YWHAZ,NAV2,ILK,GAPDH,RAPGEF4,RNASE1,APOF,CST3,ANKRD28,MAP3K7CL,FBLN2,SAA1,F11,C1S,NUCB1,PCYOX1,ADIPOQ,IGF2,C4A,ATP9B,SLAIN1,FSIP2,ACSF2,ABCA13,COLEC11,MST1,LMOD3,SPARCL1,ITIH3,C4B,CFB,IGFALS,PLTP,PAEP,RARRES2,GC,HGFAC,UIMC1,SELENOP,SBSN,APOA5,ATP1A1,SERPINA3,SERPINA4,RNASE4,APOC4,CCL18,VEGFC,HYOU1,KNG1,TPM4,ACTB,TRIM66,GRHL2,B2M,FGA,PDIA4,SDF4
TISSUES	BTO:0000203	Respiratory system	56	1707	0.41	6.80e-09	ST6GAL1,LGALS1,MMP2,VTN,SPARC,IGFBP5,APOE,PXDN,LYZ,VWF,LGALS3BP,ATRN,LCN1,YWHAE,CP,DNAH5,TCIRG1,LUM,FCN3,SERPING1,HSP90B1,SH3D19,HBA1,HSPA5,P4HB,LIMS1,GANAB,AKAP9,PCDH18,CFH,QSOX1,S100A9,FLNA,SMARCA1,CCN5,HLA-C,CKAP4,IGFBP3,GPX3,PROS1,EFEMP1,GAPDH,RAPGEF4,C1S,NUCB1,PCYOX1,SPARCL1,C4B,ATP1A1,CCL18,VEGFC,HYOU1,KNG1,ACTB,B2M,PDIA4
TISSUES	BTO:0000570	Hematopoietic system	75	2755	0.33	8.98e-09	ST6GAL1,CETP,VCL,LGALS1,TPD52L2,PON1,MPO,VTN,APOC3,GPLD1,APOB,PROC,PLEK,APOA1,TTR,CAT,LRP1,CFP,APOE,VWF,PSMA6,LGALS3BP,ATRN,C9,YWHAE,PSG9,ITIH1,AHSG,SERPING1,PGLYRP2,PPBP,PF4,HSP90B1,FGB,F2,HBA1,P4HB,FGG,LIMS1,GANAB,AKAP9,ITIH2,C8A,PCDH18,F5,CD5L,S100A9,FLNA,GSN,C1QC,APOM,HLA-C,CKAP4,IGFBP3,CD99,SOD3,GPX3,YWHAZ,GAPDH,CST3,C1S,NUCB1,IGF2,SPARCL1,C4B,CFB,HGFAC,CCL18,VEGFC,HYOU1,KNG1,TPM4,ACTB,B2M,FGA

TISSUES	BTO:0000284	Organism form	71	2542	0.34	1.10e-08	ST6GAL1,VCL,LGALS1,TPD52L2,EBI3,TFPI2,PCOLCE,SPARC,PCDH12,IGFBP5,LRP1,CFP,DHX29,VWF,PSMA6,TIMP2,LGALS3BP,NID1,YWHAE,ITIH4,LUM,PSG9,CRH,SERPING1,MYL9,CSF1R,CAMP,HSP90B1,ANTXR1,BMP1,DST,EFEMP2,APOL1,PSG11,HBA1,HSPA5,P4HB,FBLN1,GANAB,AKAP9,PCDH18,CENPF,QSOX1,F5,ADAMTSL4,FLNA,APOM,HLA-C,CKAP4,IGFBP3,CD99,GPX3,EFEMP1,YWHAZ,NAV2,ILK,GAPDH,RNASE1,CST3,NUCB1,PCYOX1,IGF2,PLTP,UIMC1,ATP1A1,VEGFC,TPM4,ACTB,GRHL2,B2M,SDF4
TISSUES	BTO:0001239	Serum	9	25	1.45	2.66e-08	GPLD1,APOA1,LGALS3BP,ATRN,ITIH1,PGLYRP2,ITIH2,HGFAC,KNG1
TISSUES	BTO:0003099	Internal female genital organ	73	2804	0.31	1.09e-07	RCN1,APOH,VCL,LGALS1,TPD52L2,EBI3,TFPI2,PCOLCE,MGP,SPARC,PCDH12,IGFBP5,LRP1,VWF,TIMP2,LGALS3BP,NID1,YWHAE,LUM,PSG9,CRH,SERPING1,MYL9,CSF1R,MASP1,A2ML1,HSP90B1,ANTXR1,BMP1,DST,EFEMP2,SUZ12,APOL1,PSG11,HBA1,P4HB,FBLN1,GANAB,PAPLN,PCDH18,QSOX1,F5,S100A9,ADAMTSL4,FLNA,CCN5,GSN,F10,HLA-C,CKAP4,IGFBP3,CD99,GPX3,PROS1,YWHAZ,ILK,GAPDH,RNASE1,CST3,FBLN2,C1S,NUCB1,PCYOX1,IGF2,COLEC11,SPARCL1,PLTP,PAEP,UIMC1,ATP1A1,TPM4,ACTB,B2M
TISSUES	BTO:0000089	Blood	55	1824	0.38	1.66e-07	VCL,LGALS1,TPD52L2,PON1,MPO,VTN,APOB,PROC,PLEK,APOA1,TTR,CAT,LRP1,APOE,VWF,C9,YWHAE,PSG9,AHSG,SERPING1,PPBP,PF4,HSP90B1,FGB,F2,HBA1,P4HB,FGG,LIMS1,AKAP9,C8A,PCDH18,F5,S100A9,FLNA,GSN,C1QC,APOM,HLA-C,CD99,SOD3,GPX3,YWHAZ,GAPDH,CST3,C1S,NUCB1,C4B,CFB,CCL18,HYOU1,TPM4,ACTB,B2M,FGA
TISSUES	BTO:0000088	Cardiovascular system	39	1057	0.46	3.20e-07	VCL,TFPI2,PCOLCE,AFM,MGP,SPARC,APOB,APOA1,APOE,VWF,TIMP2,YWHAE,HYAL1,LUM,SERPING1,MYL9,HSP90B1,F2,HBA1,P4HB,THBS4,PCDH18,F13B,FLNA,GSN,GPX3,EFEMP1,GAPDH,PCYOX1,LMOD3,SPARCL1,PLTP,SELENOP,CCL18,VEGFC,HYOU1,ACTB,B2M,FGA
TISSUES	BTO:0000174	Embryonic structure	63	2369	0.32	9.32e-07	ST6GAL1,VCL,LGALS1,TPD52L2,EBI3,TFPI2,PCOLCE,SPARC,PCDH12,IGFBP5,LRP1,DHX29,VWF,PSMA6,TIMP2,LGALS3BP,NID1,YWHAE,LUM,PSG9,CRH,SERPING1,MYL9,CSF1R,HSP90B1,ANTXR1,BMP1,DST,EFEMP2,APOL1,PSG11,HBA1,P4HB,FBLN1,GANAB,AKAP9,PCDH18,CENPF,QSOX1,F5,ADAMTSL4,FLNA,APOM,HLA-C,CKAP4,IGFBP3,GPX3,YWHAZ,ILK,GAPDH,RNASE1,CST3,NUCB1,PCYOX1,IGF2,PLTP,UIMC1,ATP1A1,VEGFC,TPM4,ACTB,GRHL2,B2M
TISSUES	BTO:0000988	Pancreas	28	626	0.55	1.31e-06	CETP,CHGA,APOC3,GPLD1,SPARC,TTR,NID1,TCIRG1,HSP90B1,APOL1,HSPA5,P4HB,QPCT,FLNA,GSN,HLA-C,CKAP4,IGFBP3,CD99,GPX3,GAPDH,RNASE1,IGF2,ATP1A1,SERPINA3,ACTB,B2M,SDF4
TISSUES	BTO:0000421	Connective tissue	35	954	0.46	2.32e-06	SPP2,LGALS1,APMAP,MMP2,TFPI2,SPARC,APOA1,TTR,CAT,LGALS3BP,YWHAE,LUM,AHSG,IHH,HSP90B1,EFEMP2,HSPA5,P4HB,THBS4,GANAB,PAPLN,F5,CRTAC1,EFEMP1,YWHAZ,GAPDH,CST3,FBLN2,ADIPOQ,ACSF2,PLTP,TPM4,ACTB,B2M,SDF4
TISSUES	BTO:0000511	Gastrointestinal tract	49	1816	0.33	3.64e-05	VCL,LGALS1,CHGA,SPARC,APOB,PROC,APOA1,TTR,CAT,LYZ,LGALS3BP,YWHAE,KLKB1,LUM,AHSG,HABP2,SERPING1,HSP90B1,SH3D19,DST,HSPA5,P4HB,FBLN1,ANG,GANAB,AKAP9,PCDH18,QSOX1,FLNA,GSN,HLA-C,IGFBP3,CD99,SOD3,GPX3,TNRC6A,YWHAZ,ILK,GAPDH,CST3,C1S,IGF2,SPARCL1,CFB,ATP1A1,SERPINA4,TPM4,ACTB,B2M
TISSUES	BTO:0000763	Lung	40	1395	0.35	0.00011	LGALS1,MMP2,VTN,SPARC,IGFBP5,APOE,PXDN,VWF,LGALS3BP,LCN1,YWHAE,DNAH5,TCIRG1,LUM,FCN3,SERPING1,HSP90B1,HBA1,HSPA5,P4HB,AKAP9,PCDH18,QSOX1,S100A9,FLNA,SMARCA1,CCN5,HLA-C,CKAP4,GPX3,EFEMP1,GAPDH,C1S,NUCB1,SPARCL1,C4B,CCL18,ACTB,B2M,PDIA4
TISSUES	BTO:0001202	Saliva	8	57	1.04	0.00011	APOH,MPO,AFM,LGALS3BP,HPX,AHSG,CST3,C4B
TISSUES	BTO:0000255	Brain cell line	13	188	0.74	0.00012	VCL,LGALS1,APOA1,TTR,FGB,HSPA5,P4HB,YWHAZ,GAPDH,HYOU1,TPM4,ACTB,TRIM66
TISSUES	BTO:0000083	Female reproductive system	113	6111	0.16	0.00014	RCN1,ST6GAL1,GNPTG,APOH,VCL,LGALS1,TPD52L2,APMAP,MMP2,EBI3,TFPI2,PCOLCE,VTN,MGP,SPARC,PCDH12,IGFBP5,APOA1,CAT,LRP1,DHX29,APOE,PXDN,SERPINF1,VWF,PSMA6,TIMP2,LGALS3BP,ATRN,NID1,YWHAE,HPX,LUM,CDH11,PSG9,AHSG,CRH,SERPING1,MYL9,CSF1R,PGLYRP2,IHH,MASP1,CAMP,APEH,A2ML1,HSP90B1,ANTXR1,SH3D19,BMP1,FGB,DST,F2,EFEMP2,SUZ12,APOL1,PSG11,C7,HBA1,HSPA5,P4HB,FBLN1,DNAH8,LIMS1,GANAB,PAPLN,PCDH18,CDC42BPA,CFH,QSOX1,F5,S100A9,ADAMTSL4,FLNA,ADAMTS13,CCN5,GSN,F10,HLA-C,CKAP4,IGFBP3,CD99,GPX3,PROS1,EFEMP1,EFCAB5,YWHAZ,NAV2,ILK,GAPDH,RNASE1,CST3,ANKRD28,FBLN2,C1S,NUCB1,PCYOX1,IGF2,FSIP2,COLEC11,SPARCL1,PLTP,PAEP,UIMC1,ATP1A1,SERPINA3,HYOU1,TPM4,ACTB,GRHL2,B2M,PDIA4,SDF4
TISSUES	BTO:0003914	Interstitial cell of Cajal	13	193	0.72	0.00014	LGALS1,APOA1,TTR,CAT,YWHAE,AHSG,HSPA5,P4HB,GANAB,YWHAZ,GAPDH,TPM4,ACTB
TISSUES	BTO:0000058	Alimentary canal	50	2021	0.29	0.00026	VCL,LGALS1,CHGA,SPARC,APOB,PROC,APOA1,TTR,CAT,LYZ,LGALS3BP,YWHAE,KLKB1,LUM,AHSG,HABP2,SERPING1,HSP90B1,SH3D19,DST,HSPA5,P4HB,FBLN1,ANG,GANAB,AKAP9,PCDH18,QSOX1,FLNA,GSN,HLA-C,CALML5,IGFBP3,CD99,SOD3,GPX3,TNRC6A,YWHAZ,ILK,GAPDH,CST3,C1S,IGF2,SPARCL1,CFB,ATP1A1,SERPINA4,TPM4,ACTB,B2M
TISSUES	BTO:0000562	Heart	26	738	0.44	0.00026	VCL,PCOLCE,AFM,APOA1,VWF,YWHAE,HYAL1,LUM,SERPING1,F2,HBA1,THBS4,PCDH18,F13B,FLNA,GPX3,GAPDH,PCYOX1,LMOD3,SPARCL1,PLTP,SELENOP,VEGFC,HYOU1,ACTB,FGA
TISSUES	BTO:0003091	Urogenital system	125	7090	0.14	0.00028	RCN1,ST6GAL1,GNPTG,APOH,VCL,LGALS1,TPD52L2,APMAP,MMP2,EBI3,TFPI2,PCOLCE,VTN,MGP,SPARC,PCDH12,IGFBP5,APOA1,CAT,LRP1,DHX29,APOE,PXDN,SERPINF1,VWF,PSMA6,TIMP2,LGALS3BP,ATRN,NID1,YWHAE,CDH6,HPX,LUM,CDH11,PSG9,AHSG,CRH,SERPING1,MYL9,CSF1R,PGLYRP2,IHH,MASP1,CAMP,APEH,GUCY1A1,A2ML1,HSP90B1,ANTXR1,SH3D19,BMP1,FGB,DST,F2,PLG,EFEMP2,SUZ12,APOL1,PSG11,C7,HBA1,HSPA5,P4HB,FBLN1,DNAH8,LIMS1,GANAB,PAPLN,F8,PCDH18,CDC42BPA,CFH,QSOX1,F5,S100A9,ADAMTSL4,FLNA,SMARCA1,ADAMTS13,CCN5,GSN,F10,HLA-C,CKAP4,IGFBP3,CD99,GPX3,PROS1,EFEMP1,EFCAB5,TNRC6A,YWHAZ,NAV2,ILK,GAPDH,RNASE1,CST3,ANKRD28,FBLN2,C1S,NUCB1,PCYOX1,IGF2,ATP9B,FSIP2,ACSF2,COLEC11,SPARCL1,PLTP,PAEP,RARRES2,GC,UIMC1,ATP1A1,SERPINA3,VEGFC,HYOU1,KNG1,TPM4,ACTB,GRHL2,B2M,PDIA4,SDF4
TISSUES	BTO:0001253	Skin	34	1151	0.37	0.00033	ST6GAL1,LGALS1,SPARC,LRP1,APOE,PSMA6,TIMP2,LGALS3BP,LCN1,CSTA,HSP90B1,BMP1,DST,P4HB,APOD,S100A7,S100A9,FLNA,GSN,CALML5,IGFBP3,CD99,EFEMP1,YWHAZ,GAPDH,ADIPOQ,RARRES2,ATP1A1,SERPINA3,VEGFC,TPM4,ACTB,B2M,SDF4
TISSUES	BTO:0000849	Melanoma cell line	12	180	0.72	0.00034	LGALS1,MMP2,SERPINF1,YWHAE,HSP90B1,CSPG4,HSPA5,P4HB,GANAB,BLM,YWHAZ,ATP1A1

TISSUES	BTO:0000081	Reproductive system	115	6444	0.15	0.00055	RCN1,ST6GAL1,GNPTG,APOH,VCL,LGALS1,TPD52L2,APMAP,MMP2,EBI3,TFPI2,PCOLCE,VTN,MGP,SPARC,PCDH12,IGFBP5,APOA1,CAT,LRP1,DHX29,APOE,PXDN,SERPINF1,VWF,PSMA6,TIMP2,LGALS3BP,ATRN,NID1,YWHAE,HPX,LUM,CDH11,PSG9,AHSG,CRH,SERPING1,MYL9,CSF1R,PGLYRP2,IHH,MASP1,CAMP,APEH,A2ML1,HSP90B1,ANTXR1,SH3D19,BMP1,FGB,DST,F2,EFEMP2,SUZ12,APOL1,PSG11,C7,HBA1,HSPA5,P4HB,FBLN1,DNAH8,LIMS1,GANAB,PAPLN,PCDH18,CDC42BPA,CFH,QSOX1,F5,S100A9,ADAMTSL4,FLNA,ADAMTS13,CCN5,GSN,F10,HLA-C,CKAP4,IGFBP3,CD99,GPX3,PROS1,EFEMP1,EFCAB5,YWHAZ,NAV2,ILK,GAPDH,RNASE1,CST3,ANKRD28,FBLN2,C1S,NUCB1,PCYOX1,IGF2,ATP9B,FSIP2,COLEC11,SPARCL1,PLTP,PAEP,RARRES2,UIMC1,ATP1A1,SERPINA3,HYOU1,TPM4,ACTB,GRHL2,B2M,PDIA4,SDF4
TISSUES	BTO:0003092	Urinary system	35	1249	0.34	0.00066	VCL,TPD52L2,SPARC,CAT,APOE,CDH6,GUCY1A1,HSP90B1,ANTXR1,PLG,P4HB,F8,PCDH18,CFH,QSOX1,FLNA,SMARCA1,GSN,IGFBP3,CD99,GPX3,TNRC6A,GAPDH,NUCB1,IGF2,ACSF2,COLEC11,RARRES2,GC,ATP1A1,SERPINA3,VEGFC,KNG1,ACTB,B2M
TISSUES	BTO:0001244	Urinary tract	35	1258	0.34	0.00075	VCL,TPD52L2,SPARC,CAT,APOE,CDH6,GUCY1A1,HSP90B1,ANTXR1,PLG,P4HB,F8,PCDH18,CFH,QSOX1,FLNA,SMARCA1,GSN,IGFBP3,CD99,GPX3,TNRC6A,GAPDH,NUCB1,IGF2,ACSF2,COLEC11,RARRES2,GC,ATP1A1,SERPINA3,VEGFC,KNG1,ACTB,B2M
TISSUES	BTO:0001090	Mouth	20	532	0.47	0.0012	GNPTG,C5,LYZ,LCN1,CSTA,CENPE,A2ML1,DST,C7,P4HB,LIMS1,GANAB,S100A9,GSN,YWHAZ,SBSN,TPM4,ACTB,B2M,SDF4
TISSUES	BTO:0000102	Blood clot	4	11	1.46	0.0015	VWF,F2,PLG,ADAMTS13
TISSUES	BTO:0001419	Urine	5	28	1.15	0.0027	LYZ,AMBP,F2,RNASE1,B2M
TISSUES	BTO:0000227	Central nervous system	103	5825	0.14	0.0033	RCN1,GNPTG,APOH,LGALS1,CHGA,TPD52L2,APMAP,MMP2,PON1,PCOLCE,VTN,AFM,APOC3,MGP,SPARC,IGFBP5,TTR,CAT,LRP1,DHX29,APOE,SERPINF1,PSMA6,TIMP2,LGALS3BP,ATRN,LCN1,NID1,YWHAE,CP,HPX,CDH11,AHSG,CRH,SERPING1,CSF1R,SERPINI1,MASP1,GUCY1A1,HSP90B1,ANTXR1,SH3D19,PCSK9,BMP1,DST,F2,EFEMP2,CSPG4,C1QB,HBA1,HSPA5,P4HB,SERPINA7,FBLN1,GANAB,QPCT,APOD,AKAP9,CNDP1,MTCL1,F8,PCDH18,CDC42BPA,CFH,QSOX1,S100A9,FLNA,CRTAC1,GSN,C1QC,HLA-C,IGFBP3,CD99,GPX3,EFEMP1,CFI,TNRC6A,YWHAZ,NAV2,ILK,GAPDH,RAPGEF4,CST3,MAP3K7CL,C1S,NUCB1,PCYOX1,IGF2,C4A,SLAIN1,ACSF2,SPARCL1,PLTP,UIMC1,ATP1A1,SERPINA3,HYOU1,KNG1,TPM4,ACTB,B2M,FGA,SDF4
TISSUES	BTO:0000671	Kidney	29	1039	0.34	0.0037	TPD52L2,SPARC,CAT,APOE,CDH6,GUCY1A1,HSP90B1,ANTXR1,PLG,P4HB,F8,QSOX1,SMARCA1,GSN,IGFBP3,CD99,GPX3,TNRC6A,GAPDH,NUCB1,IGF2,ACSF2,COLEC11,RARRES2,GC,ATP1A1,KNG1,ACTB,B2M
TISSUES	BTO:0001388	Trachea	11	201	0.63	0.0037	ST6GAL1,LYZ,CP,P4HB,LIMS1,CFH,RAPGEF4,VEGFC,HYOU1,KNG1,B2M
TISSUES	BTO:0000431	Excretory gland	35	1385	0.3	0.0043	TPD52L2,VTN,SPARC,CAT,APOE,CDH6,HPX,AHSG,GUCY1A1,HSP90B1,ANTXR1,FGB,F2,PLG,P4HB,APOD,F8,QSOX1,SMARCA1,GSN,IGFBP3,CD99,GPX3,TNRC6A,GAPDH,NUCB1,IGF2,ACSF2,COLEC11,RARRES2,GC,ATP1A1,KNG1,ACTB,B2M
TISSUES	BTO:0001484	Nervous system	105	6016	0.14	0.0043	RCN1,GNPTG,APOH,LGALS1,CHGA,TPD52L2,APMAP,MMP2,PON1,PCOLCE,VTN,AFM,APOC3,MGP,SPARC,IGFBP5,TTR,CAT,LRP1,DHX29,APOE,SERPINF1,PSMA6,TIMP2,LGALS3BP,ATRN,LCN1,NID1,YWHAE,CP,HPX,CDH11,AHSG,CRH,SERPING1,CSF1R,SERPINI1,MASP1,GUCY1A1,HSP90B1,ANTXR1,SH3D19,PCSK9,BMP1,DST,F2,EFEMP2,CSPG4,C1QB,HBA1,HSPA5,P4HB,SERPINA7,FBLN1,THBS4,GANAB,QPCT,APOD,AKAP9,CNDP1,MTCL1,F8,PCDH18,CDC42BPA,CFH,QSOX1,S100A9,FLNA,CRTAC1,GSN,C1QC,HLA-C,IGFBP3,CD99,GPX3,EFEMP1,CFI,TNRC6A,YWHAZ,NAV2,ILK,GAPDH,RAPGEF4,CST3,MAP3K7CL,C1S,NUCB1,PCYOX1,IGF2,C4A,SLAIN1,ACSF2,SPARCL1,PLTP,UIMC1,SELENOP,ATP1A1,SERPINA3,HYOU1,KNG1,TPM4,ACTB,B2M,FGA,SDF4
TISSUES	BTO:0000007	HEK-293 cell	3	5	1.67	0.0047	PXDN,GAPDH,ACTB
TISSUES	BTO:0000634	Integument	47	2112	0.24	0.0050	ST6GAL1,VCL,LGALS1,CHGA,SPARC,LRP1,APOE,VWF,PSMA6,TIMP2,LGALS3BP,LCN1,CSTA,LUM,A2ML1,HSP90B1,ANTXR1,BMP1,DST,HSPA5,P4HB,APOD,AKAP9,S100A7,S100A9,FLNA,GSN,CALML5,IGFBP3,CD99,EFEMP1,YWHAZ,ILK,GAPDH,RNASE1,ADIPOQ,COLEC11,PLTP,PAEP,RARRES2,ATP1A1,SERPINA3,VEGFC,TPM4,ACTB,B2M,SDF4
TISSUES	BTO:0000420	Neck	12	264	0.55	0.0085	CHGA,APOE,HSPA5,P4HB,GANAB,HLA-C,GPX3,GAPDH,SAA1,ATP1A1,ACTB,B2M
TISSUES	BTO:0000121	Bile	4	20	1.2	0.0088	APOH,LGALS3BP,CP,HPX
TISSUES	BTO:0000917	Needle	3	7	1.53	0.0090	PXDN,GAPDH,ACTB
TISSUES	BTO:0000254	Female reproductive gland	60	3017	0.2	0.0098	RCN1,APOH,VCL,TPD52L2,APMAP,MMP2,VTN,SPARC,APOA1,DHX29,APOE,PSMA6,LGALS3BP,ATRN,NID1,YWHAE,HPX,AHSG,SERPING1,PGLYRP2,IHH,CAMP,HSP90B1,SH3D19,FGB,F2,HSPA5,P4HB,LIMS1,GANAB,PAPLN,PCDH18,CDC42BPA,CFH,QSOX1,FLNA,ADAMTS13,CCN5,GSN,F10,HLA-C,IGFBP3,CD99,EFCAB5,YWHAZ,GAPDH,RNASE1,CST3,ANKRD28,FBLN2,C1S,IGF2,FSIP2,COLEC11,UIMC1,ATP1A1,HYOU1,TPM4,ACTB,B2M
TISSUES	BTO:0000648	Intestine	33	1349	0.29	0.0108	VCL,APOB,PROC,LYZ,LGALS3BP,KLKB1,LUM,HABP2,HSP90B1,SH3D19,DST,HSPA5,P4HB,ANG,GANAB,PCDH18,FLNA,GSN,HLA-C,IGFBP3,SOD3,GPX3,YWHAZ,ILK,GAPDH,CST3,C1S,IGF2,CFB,ATP1A1,SERPINA4,ACTB,B2M
TISSUES	BTO:0000828	Throat	11	234	0.57	0.0109	CHGA,HSPA5,P4HB,GANAB,HLA-C,GPX3,GAPDH,SAA1,ATP1A1,ACTB,B2M
TISSUES	BTO:0005288	CL-48 cell	10	200	0.6	0.0127	SERPINA10,ITIH4,PSG9,AHSG,MASP1,SERPINA11,FGG,LIMS1,GPX3,SERPINA4
TISSUES	BTO:0000140	Bone	9	165	0.63	0.0136	SPP2,MMP2,SPARC,IHH,HSP90B1,GAPDH,ACTB,B2M,SDF4
TISSUES	BTO:0003120	Atherosclerotic plaque	3	9	1.42	0.0147	APOB,APOA1,APOE
TISSUES	BTO:0003872	Foam cell	3	9	1.42	0.0147	APOB,APOA1,APOE
TISSUES	BTO:0001085	Vascular system	15	420	0.45	0.0150	VCL,TFPI2,MGP,APOB,APOE,VWF,MYL9,F2,P4HB,EFEMP1,PLTP,CCL18,VEGFC,ACTB,B2M
TISSUES	BTO:0000672	Hindbrain	18	565	0.4	0.0152	RCN1,CHGA,PON1,VTN,APOE,PSMA6,LCN1,CRH,PCSK9,QPCT,APOD,MTCL1,C1QC,ILK,IGF2,ATP1A1,HYOU1,FGA
TISSUES	BTO:0001129	Prostate gland	16	476	0.42	0.0179	VCL,ATRN,LUM,HSP90B1,ANTXR1,P4HB,PCDH18,FLNA,GAPDH,RNASE1,NUCB1,RARRES2,ATP1A1,ACTB,B2M,SDF4
TISSUES	BTO:0000673	Metencephalon	17	528	0.4	0.0189	RCN1,CHGA,PON1,VTN,APOE,LCN1,CRH,PCSK9,QPCT,APOD,MTCL1,C1QC,ILK,IGF2,ATP1A1,HYOU1,FGA
TISSUES	BTO:0000975	Ovary	17	528	0.4	0.0189	RCN1,APOH,VCL,LGALS3BP,SERPING1,PAPLN,PCDH18,FLNA,CCN5,F10,GAPDH,IGF2,COLEC11,UIMC1,ATP1A1,ACTB,B2M
TISSUES	BTO:0000574	Hematopoietic cell	26	1019	0.3	0.0233	VCL,LGALS1,TPD52L2,PON1,MPO,APOB,PLEK,APOA1,CAT,APOE,YWHAE,PPBP,PF4,AKAP9,PCDH18,S100A9,FLNA,C1QC,CD99,GPX3,YWHAZ,GAPDH,CST3,C1S,ACTB,B2M
TISSUES	BTO:0001356	Tendon	7	112	0.69	0.0249	EFEMP2,P4HB,THBS4,PAPLN,CRTAC1,EFEMP1,CST3
TISSUES	BTO:0000020	Abdomen	7	113	0.69	0.0256	LGALS3BP,F2,GAPDH,ADIPOQ,KNG1,ACTB,B2M
TISSUES	BTO:0000484	Frontal lobe	7	113	0.69	0.0256	APOE,APOD,PCDH18,GAPDH,CST3,ACTB,SDF4
TISSUES	BTO:0004078	Non-small cell lung adenocarcinoma cell line	3	12	1.29	0.0256	PCSK9,GAPDH,ACTB
TISSUES	BTO:0005692	Column	2	2	1.9	0.0287	GAPDH,ACTB
TISSUES	BTO:0001823	Synovium	6	88	0.73	0.0364	P4HB,THBS4,PAPLN,CRTAC1,EFEMP1,CST3

TISSUES	BTO:0000232	Cerebellum	16	519	0.39	0.0368	RCN1,CHGA,VTN,APOE,LCN1,CRH,PCSK9,QPCT,APOD,MTCL1,C1QC,ILK,IGF2,ATP1A1,HYOU1,FGA
TISSUES	BTO:0000202	Sense organ	27	1124	0.28	0.0387	GNPTG,VCL,SPARC,IGFBP5,TTR,APOE,SERPINF1,LYZ,TIMP2,LCN1,CSTA,LUM,SERPING1,SERPINI1,DST,FAM184A,APOD,CFH,GSN,GPX3,YWHAZ,GAPDH,SBSN,ATP1A1,ACTB,B2M,SDF4
TISSUES	BTO:0001385	Tongue	8	161	0.59	0.0389	GNPTG,LCN1,CSTA,DST,GSN,YWHAZ,SBSN,SDF4
TISSUES	BTO:0001702	Left atrium	5	60	0.82	0.0389	VCL,APOA1,HBA1,FLNA,GAPDH
TISSUES	BTO:0000166	Cecum	4	35	0.95	0.0401	GAPDH,CST3,ACTB,B2M
TISSUES	BTO:0000091	Ascites	5	61	0.81	0.0402	LGALS3BP,GAPDH,KNG1,ACTB,B2M
TISSUES	BTO:0003445	Carotid atherosclerotic plaque	2	3	1.72	0.0425	APOB,APOE
TISSUES	BTO:0005739	Transfer cell	2	3	1.72	0.0425	PXDN,ACTB
TISSUES	BTO:0000282	Head	107	6642	0.1	0.0491	RCN1,GNPTG,VCL,LGALS1,CHGA,TPD52L2,APMAP,MMP2,PON1,PCOLCE,C5,VTN,MGP,SPARC,IGFBP5,TTR,CAT,LRP1,DHX29,APOE,SERPINF1,LYZ,PSMA6,TIMP2,LGALS3BP,ATRN,LCN1,NID1,YWHAE,CSTA,CENPE,LUM,CDH11,CRH,SERPING1,CSF1R,IHH,SERPINI1,MASP1,GUCY1A1,A2ML1,HSP90B1,ANTXR1,SH3D19,PCSK9,BMP1,DST,F2,EFEMP2,CSPG4,C1QB,C7,HBA1,HSPA5,P4HB,FBLN1,LIMS1,GANAB,FAM184A,QPCT,APOD,AKAP9,CNDP1,MTCL1,F8,PCDH18,CDC42BPA,CFH,QSOX1,S100A9,CRTAC1,GSN,C1QC,HLA-C,IGFBP3,CD99,GPX3,EFEMP1,CFI,TNRC6A,YWHAZ,NAV2,ILK,GAPDH,RAPGEF4,CST3,MAP3K7CL,C1S,NUCB1,PCYOX1,IGF2,C4A,SLAIN1,ACSF2,SPARCL1,PLTP,UIMC1,SBSN,ATP1A1,SERPINA3,HYOU1,KNG1,TPM4,ACTB,B2M,FGA,SDF4
TISSUES	BTO:0001281	Spleen	16	540	0.37	0.0491	ST6GAL1,CETP,LGALS1,PLEK,CFP,HBA1,P4HB,CD5L,FLNA,HLA-C,CKAP4,IGFBP3,C1S,CCL18,ACTB,B2M
COMPARTMENTS	GOCC:0005576	Extracellular region	171	2079	0.81	2.55e-99	SPP2,ST6GAL1,CPB2,CETP,APOH,VCL,SERPIND1,LGALS1,CHGA,LBP,F9,MMP2,EBI3,PON1,TFPI2,PCOLCE,C5,MPO,VTN,AFM,PF4V1,APOC3,MGP,GPLD1,SPARC,APOB,IGFBP5,PROC,APOA1,TTR,C4BPB,CFP,APOE,PXDN,ANGPTL6,SERPINF1,APCS,CFHR5,LYZ,VWF,SERPINA10,TIMP2,LGALS3BP,C9,LCN1,LCAT,NID1,YWHAE,CSTA,CP,KLKB1,AMBP,CENPE,HPX,HYAL1,ITIH4,LUM,CDH11,FCN3,ITIH1,AHSG,CRH,HABP2,SAA4,SERPING1,FCN2,PGLYRP2,SCGB3A1,SERPINI1,PPBP,PF4,MASP1,CAMP,A2ML1,HSP90B1,PCSK9,BMP1,FGB,DST,F2,INHBC,PLG,EFEMP2,CSPG4,C1QB,CFHR1,APOL1,CPN2,SERPINF2,C7,HBA1,HSPA5,P4HB,SERPINA7,FBLN1,ANG,FGG,THBS4,QPCT,APOD,ITIH2,F8,C8A,F13B,CFHR4,CFHR3,CFH,QSOX1,F5,CD5L,S100A7,S100A9,FLNA,CRTAC1,C8B,ADAMTS13,CCN5,GSN,MBL2,C1QC,F10,F7,APOM,HLA-C,CALML5,IGFBP3,SOD3,GPX3,PROS1,EFEMP1,CFI,YWHAZ,GAPDH,RNASE1,APOF,CST3,FBLN2,SAA1,F11,C1S,NUCB1,PCYOX1,ADIPOQ,IGF2,C4A,COLEC11,MST1,SPARCL1,ITIH3,C4B,CFB,IGFALS,PLTP,PAEP,RARRES2,GC,HGFAC,HPR,APOA5,SERPINA3,SERPINA4,RNASE4,APOC4,CCL18,VEGFC,KNG1,ACTB,B2M,FGA,PDIA4,SDF4
COMPARTMENTS	GOCC:0005615	Extracellular space	123	1027	0.97	7.73e-82	CPB2,CETP,APOH,VCL,SERPIND1,LBP,F9,MMP2,EBI3,PON1,C5,MPO,VTN,AFM,APOC3,MGP,GPLD1,APOB,IGFBP5,PROC,APOA1,TTR,CFP,APOE,PXDN,SERPINF1,APCS,LYZ,VWF,LGALS3BP,C9,LCN1,LCAT,YWHAE,CSTA,CP,KLKB1,AMBP,HPX,HYAL1,ITIH4,LUM,CDH11,FCN3,ITIH1,AHSG,CRH,SAA4,SERPING1,FCN2,SCGB3A1,SERPINI1,MASP1,CAMP,A2ML1,HSP90B1,PCSK9,FGB,F2,PLG,CSPG4,C1QB,CFHR1,APOL1,CPN2,SERPINF2,HBA1,HSPA5,FBLN1,ANG,FGG,THBS4,APOD,ITIH2,F8,C8A,CFHR3,CFH,QSOX1,CD5L,S100A7,S100A9,FLNA,CCN5,GSN,MBL2,C1QC,F7,APOM,HLA-C,IGFBP3,SOD3,GPX3,PROS1,EFEMP1,YWHAZ,GAPDH,APOF,CST3,SAA1,F11,C1S,PCYOX1,ADIPOQ,IGF2,C4A,COLEC11,MST1,C4B,CFB,IGFALS,PLTP,RARRES2,GC,HPR,APOA5,SERPINA3,APOC4,KNG1,ACTB,B2M,FGA,PDIA4
COMPARTMENTS	GOCC:0072562	Blood microparticle	46	118	1.49	1.22e-46	PON1,VTN,AFM,APOA1,APOE,APCS,LGALS3BP,C9,CP,AMBP,HPX,ITIH4,FCN3,ITIH1,AHSG,SERPING1,FCN2,FGB,F2,PLG,C1QB,CFHR1,APOL1,CPN2,SERPINF2,HBA1,FGG,ITIH2,C8A,CFHR3,CFH,CD5L,GSN,C1QC,PROS1,YWHAZ,C1S,C4A,C4B,CFB,GC,HPR,SERPINA3,KNG1,ACTB,FGA
COMPARTMENTS	GOCC:0043230	Extracellular organelle	63	524	0.98	5.56e-38	CPB2,APOH,VCL,LBP,F9,C5,MPO,VTN,AFM,MGP,APOB,APOA1,TTR,APOE,PXDN,SERPINF1,APCS,VWF,LGALS3BP,YWHAE,CP,KLKB1,AMBP,HPX,ITIH4,LUM,CDH11,AHSG,SERPING1,FCN2,CAMP,HSP90B1,FGB,F2,PLG,CSPG4,SERPINF2,HSPA5,FGG,APOD,CFH,QSOX1,S100A9,FLNA,GSN,HLA-C,GPX3,PROS1,EFEMP1,YWHAZ,GAPDH,RNASE1,CST3,SAA1,F11,C4A,C4B,GC,SERPINA3,KNG1,ACTB,B2M,FGA
COMPARTMENTS	GOCC:1903561	Extracellular vesicle	60	500	0.98	5.04e-36	CPB2,APOH,VCL,F9,MPO,VTN,AFM,MGP,APOB,APOA1,TTR,APOE,PXDN,SERPINF1,APCS,VWF,LGALS3BP,YWHAE,CP,KLKB1,AMBP,HPX,ITIH4,LUM,CDH11,AHSG,SERPING1,CAMP,HSP90B1,FGB,F2,PLG,CSPG4,SERPINF2,HSPA5,FGG,APOD,CFH,QSOX1,S100A9,FLNA,GSN,HLA-C,GPX3,PROS1,EFEMP1,YWHAZ,GAPDH,RNASE1,CST3,SAA1,F11,C4A,C4B,GC,SERPINA3,KNG1,ACTB,B2M,FGA
COMPARTMENTS	GOCC:0065010	Extracellular membrane-bounded organelle	56	473	0.97	3.62e-33	CPB2,APOH,VCL,F9,MPO,VTN,MGP,APOB,APOA1,TTR,APOE,PXDN,SERPINF1,APCS,VWF,LGALS3BP,CP,KLKB1,AMBP,HPX,ITIH4,LUM,AHSG,SERPING1,CAMP,HSP90B1,FGB,F2,PLG,CSPG4,SERPINF2,HSPA5,FGG,APOD,CFH,QSOX1,S100A9,FLNA,GSN,HLA-C,GPX3,PROS1,YWHAZ,GAPDH,RNASE1,CST3,SAA1,F11,C4A,C4B,GC,SERPINA3,KNG1,ACTB,B2M,FGA
COMPARTMENTS	GOCC:0060205	Cytoplasmic vesicle lumen	40	245	1.11	8.91e-28	SPP2,APOH,VCL,MPO,SPARC,APOA1,TTR,CFP,LYZ,VWF,TIMP2,LGALS3BP,ITIH4,AHSG,SERPING1,SERPINI1,PPBP,PF4,CAMP,FGB,PLG,SERPINF2,FGG,QPCT,F8,QSOX1,F5,S100A7,GSN,PROS1,IGF2,ITIH3,RARRES2,SELENOP,SERPINA3,SERPINA4,VEGFC,KNG1,B2M,FGA
COMPARTMENTS	GOCC:0031982	Vesicle	96	2125	0.55	3.90e-27	SPP2,CPB2,APOH,VCL,CHGA,LBP,F9,MPO,VTN,AFM,MGP,SPARC,APOB,APOA1,TTR,CAT,LRP1,CFP,APOE,PXDN,SERPINF1,APCS,LYZ,VWF,TIMP2,LGALS3BP,YWHAE,CP,KLKB1,AMBP,TCIRG1,HPX,HYAL1,ITIH4,LUM,CDH11,ITIH1,AHSG,SERPING1,SERPINI1,PPBP,PF4,CAMP,APEH,HSP90B1,ANTXR1,PCSK9,FGB,DST,F2,PLG,CSPG4,STAB1,SERPINF2,HBA1,HSPA5,FGG,QPCT,APOD,F8,CDC42BPA,CFH,QSOX1,F5,S100A7,S100A9,FLNA,GSN,HLA-C,CKAP4,CALML5,GPX3,PROS1,EFEMP1,YWHAZ,GAPDH,RNASE1,CST3,SAA1,F11,IGF2,C4A,ABCA13,ITIH3,C4B,RARRES2,GC,SELENOP,SERPINA3,SERPINA4,VEGFC,HYOU1,KNG1,ACTB,B2M,FGA
COMPARTMENTS	GOCC:0034774	Secretory granule lumen	39	241	1.11	4.71e-27	SPP2,APOH,VCL,MPO,SPARC,APOA1,TTR,CFP,LYZ,VWF,TIMP2,LGALS3BP,ITIH4,AHSG,SERPING1,PPBP,PF4,CAMP,FGB,PLG,SERPINF2,FGG,QPCT,F8,QSOX1,F5,S100A7,GSN,PROS1,IGF2,ITIH3,RARRES2,SELENOP,SERPINA3,SERPINA4,VEGFC,KNG1,B2M,FGA
COMPARTMENTS	GOCC:0070062	Extracellular exosome	45	428	0.92	2.36e-24	VCL,MPO,VTN,AFM,MGP,APOB,APOA1,TTR,APOE,PXDN,SERPINF1,VWF,LGALS3BP,YWHAE,CP,AMBP,HPX,ITIH4,CDH11,AHSG,CAMP,HSP90B1,FGB,PLG,CSPG4,HSPA5,FGG,APOD,CFH,QSOX1,S100A9,FLNA,GSN,HLA-C,GPX3,EFEMP1,YWHAZ,GAPDH,CST3,SAA1,C4A,KNG1,ACTB,B2M,FGA
COMPARTMENTS	GOCC:0034358	Plasma lipoprotein particle	20	48	1.52	3.91e-20	CETP,APOH,PON1,APOC3,APOB,APOA1,APOE,LCAT,SAA4,PCSK9,APOL1,APOM,APOF,SAA1,PCYOX1,ADIPOQ,PLTP,HPR,APOA5,APOC4
COMPARTMENTS	GOCC:0005788	Endoplasmic reticulum lumen	28	172	1.11	3.38e-19	RCN1,SPP2,F9,APOB,APOA1,APOE,AHSG,HSP90B1,PCSK9,APOL1,HSPA5,P4HB,FGG,GANAB,F8,QSOX1,F5,F7,CKAP4,IGFBP3,NUCB1,C4A,APOA5,HYOU1,KNG1,B2M,FGA,PDIA4

COMPARTMENTS	GOCC:0034364	High-density lipoprotein particle	17	30	1.65	7.56e-19	CETP,APOH,PON1,APOC3,APOB,APOA1,APOE,LCAT,SAA4,APOL1,APOM,APOF,SAA1,PLTP,HPR,APOA5,APOC4
COMPARTMENTS	GOCC:0030141	Secretory granule	47	719	0.71	1.23e-17	SPP2,APOH,VCL,CHGA,MPO,SPARC,APOA1,TTR,CAT,CFP,LYZ,VWF,TIMP2,LGALS3BP,TCIRG1,ITIH4,AHSG,SERPING1,PPBP,PF4,CAMP,APEH,FGB,PLG,SERPINF2,FGG,QPCT,F8,QSOX1,F5,S100A7,GSN,CKAP4,CALML5,PROS1,CST3,IGF2,ABCA13,ITIH3,RARRES2,SELENOP,SERPINA3,SERPINA4,VEGFC,KNG1,B2M,FGA
COMPARTMENTS	GOCC:0031093	Platelet alpha granule lumen	19	66	1.36	9.54e-17	SPARC,VWF,AHSG,SERPING1,PPBP,PF4,FGB,PLG,SERPINF2,FGG,F8,QSOX1,F5,PROS1,IGF2,SERPINA3,VEGFC,KNG1,FGA
COMPARTMENTS	GOCC:0110165	Cellular anatomical entity	231	14060	0.11	2.01e-14	RCN1,SPP2,ST6GAL1,CPB2,CETP,GNPTG,APOH,VCL,SERPIND1,LGALS1,CHGA,TPD52L2,LBP,APMAP,F9,MMP2,EBI3,PON1,TFPI2,PCOLCE,C5,MPO,VTN,AFM,PF4V1,APOC3,MGP,GPLD1,SPARC,PCDH12,APOB,IGFBP5,PROC,PLEK,APOA1,TTR,CAT,LRP1,C4BPB,CFP,DHX29,APOE,PXDN,ANGPTL6,SERPINF1,APCS,CFHR5,LYZ,VWF,PSMA6,DSG2,SERPINA10,TIMP2,LGALS3BP,ATRN,C9,LCN1,LCAT,NID1,YWHAE,CSTA,CP,KLKB1,CDH6,DNAH5,AMBP,CENPE,TCIRG1,HPX,HYAL1,ITIH4,LUM,CDH11,PSG9,FCN3,ITIH1,AHSG,CRH,HABP2,SAA4,SERPING1,MYL9,CSF1R,PCDH1,FCN2,PGLYRP2,SCGB3A1,IHH,SERPINI1,PPBP,PF4,MASP1,CAMP,APEH,GUCY1A1,C16orf46,A2ML1,HSP90B1,ANTXR1,SH3D19,PCSK9,BMP1,SHLD1,FGB,DST,F2,INHBC,PLG,EFEMP2,CSPG4,STAB1,C1QB,CFHR1,SUZ12,APOL1,CPN2,SERPINF2,C7,HBA1,HSPA5,P4HB,SERPINA7,FBLN1,DNAH8,ANG,FGG,LIMS1,THBS4,GANAB,QPCT,APOD,BLM,AKAP9,ITIH2,CNDP1,MTCL1,F8,C8A,CDC42BPA,CENPF,F13B,CFHR4,CFHR3,CFH,QSOX1,F5,CD5L,S100A7,S100A9,FLNA,CRTAC1,MCOLN2,SMARCA1,C8B,ADAMTS13,CCN5,GSN,MBL2,C1QC,F10,F7,APOM,HLA-C,CKAP4,CALML5,TAF9,IGFBP3,CD99,SOD3,GPX3,KIF19,PROS1,EFEMP1,CFI,TNRC6A,YWHAZ,NAV2,ILK,GAPDH,RAPGEF4,RNASE1,APOF,CST3,MAP3K7CL,FBLN2,SAA1,F11,C1S,NUCB1,PCYOX1,ADIPOQ,IGF2,C4A,ATP9B,FSIP2,ACSF2,ABCA13,COLEC11,MST1,LMOD3,SPARCL1,ITIH3,C4B,CFB,IGFALS,PLTP,PAEP,RARRES2,GC,HGFAC,UIMC1,SELENOP,HPR,APOA5,ATP1A1,SERPINA3,SERPINA4,RNASE4,APOC4,CCL18,VEGFC,HYOU1,KNG1,TPM4,ACTB,TRIM66,GRHL2,B2M,FGA,PDIA4,SDF4
COMPARTMENTS	GOCC:0034361	Very-low-density lipoprotein particle	11	20	1.64	6.82e-12	CETP,APOH,APOC3,APOB,APOA1,APOE,APOL1,APOM,PCYOX1,APOA5,APOC4
COMPARTMENTS	GOCC:0031410	Cytoplasmic vesicle	62	1738	0.45	1.09e-11	SPP2,APOH,VCL,CHGA,MPO,SPARC,APOB,APOA1,TTR,CAT,LRP1,CFP,APOE,LYZ,VWF,TIMP2,LGALS3BP,TCIRG1,HPX,HYAL1,ITIH4,AHSG,SERPING1,SERPINI1,PPBP,PF4,CAMP,APEH,HSP90B1,ANTXR1,PCSK9,FGB,DST,PLG,STAB1,SERPINF2,HBA1,FGG,QPCT,F8,QSOX1,F5,S100A7,GSN,HLA-C,CKAP4,CALML5,PROS1,CST3,SAA1,IGF2,ABCA13,ITIH3,RARRES2,SELENOP,SERPINA3,SERPINA4,VEGFC,HYOU1,KNG1,B2M,FGA
COMPARTMENTS	GOCC:0031012	Extracellular matrix	24	259	0.86	1.54e-11	CPB2,MMP2,TFPI2,VTN,GPLD1,SPARC,APOE,PXDN,VWF,TIMP2,NID1,LUM,HSP90B1,DST,PLG,EFEMP2,FBLN1,ANG,THBS4,ADAMTS13,SOD3,EFEMP1,FBLN2,RARRES2
COMPARTMENTS	GOCC:0070013	Intracellular organelle lumen	82	2902	0.35	7.87e-11	RCN1,SPP2,APOH,VCL,F9,MPO,SPARC,APOB,PROC,APOA1,TTR,CAT,CFP,APOE,LYZ,VWF,TIMP2,LGALS3BP,HPX,HYAL1,ITIH4,LUM,AHSG,SERPING1,SERPINI1,PPBP,PF4,CAMP,APEH,HSP90B1,PCSK9,FGB,F2,PLG,CSPG4,SUZ12,APOL1,SERPINF2,HBA1,HSPA5,P4HB,ANG,FGG,GANAB,QPCT,BLM,F8,CENPF,QSOX1,F5,S100A7,SMARCA1,GSN,F10,F7,CKAP4,CALML5,TAF9,IGFBP3,SOD3,PROS1,GAPDH,CST3,SAA1,NUCB1,IGF2,C4A,ITIH3,RARRES2,GC,UIMC1,SELENOP,APOA5,SERPINA3,SERPINA4,VEGFC,HYOU1,KNG1,ACTB,B2M,FGA,PDIA4
COMPARTMENTS	GOCC:0030312	External encapsulating structure	23	259	0.84	9.89e-11	MMP2,TFPI2,VTN,GPLD1,SPARC,APOE,PXDN,VWF,TIMP2,NID1,LUM,HSP90B1,DST,PLG,EFEMP2,FBLN1,ANG,THBS4,ADAMTS13,SOD3,EFEMP1,FBLN2,RARRES2
COMPARTMENTS	GOCC:0071682	Endocytic vesicle lumen	10	23	1.53	4.26e-10	MPO,SPARC,APOB,APOA1,APOE,HPX,HSP90B1,HBA1,SAA1,HYOU1
COMPARTMENTS	GOCC:0012505	Endomembrane system	84	3156	0.32	7.22e-10	RCN1,SPP2,ST6GAL1,APOH,VCL,CHGA,F9,MPO,SPARC,APOB,PROC,APOA1,TTR,CAT,LRP1,CFP,APOE,PXDN,LYZ,VWF,TIMP2,LGALS3BP,CP,TCIRG1,ITIH4,LUM,AHSG,SERPING1,PPBP,PF4,CAMP,APEH,HSP90B1,ANTXR1,PCSK9,FGB,F2,PLG,CSPG4,APOL1,SERPINF2,HSPA5,P4HB,FGG,THBS4,GANAB,QPCT,AKAP9,F8,CENPF,QSOX1,F5,S100A7,GSN,F10,F7,HLA-C,CKAP4,CALML5,IGFBP3,SOD3,PROS1,TNRC6A,GAPDH,CST3,NUCB1,ADIPOQ,IGF2,C4A,ATP9B,ABCA13,ITIH3,RARRES2,SELENOP,APOA5,SERPINA3,SERPINA4,VEGFC,HYOU1,KNG1,B2M,FGA,PDIA4,SDF4
COMPARTMENTS	GOCC:0031089	Platelet dense granule lumen	8	14	1.65	1.04e-08	SPP2,APOH,LGALS3BP,ITIH4,ITIH3,RARRES2,SELENOP,SERPINA4
COMPARTMENTS	GOCC:0062023	Collagen-containing extracellular matrix	18	198	0.86	1.86e-08	MMP2,VTN,SPARC,APOE,PXDN,VWF,TIMP2,NID1,LUM,HSP90B1,DST,PLG,EFEMP2,ANG,THBS4,SOD3,FBLN2,RARRES2
COMPARTMENTS	GOCC:0062167	Complement component C1q complex	7	10	1.74	5.13e-08	SERPING1,C1QB,MBL2,C1QC,C1S,C4A,C4B
COMPARTMENTS	GOCC:0005577	Fibrinogen complex	8	20	1.5	8.70e-08	VWF,FGB,F2,PLG,SERPINF2,FGG,F7,FGA
COMPARTMENTS	GOCC:0005579	Membrane attack complex	7	12	1.66	1.20e-07	C5,C9,C7,C8A,C8B,MBL2,C4A
COMPARTMENTS	GOCC:0034362	Low-density lipoprotein particle	8	22	1.46	1.48e-07	CETP,APOB,APOA1,APOE,PCSK9,APOM,APOF,ADIPOQ
COMPARTMENTS	GOCC:0034366	Spherical high-density lipoprotein particle	6	11	1.63	2.31e-06	PON1,APOC3,APOA1,LCAT,APOM,HPR
COMPARTMENTS	GOCC:1905286	Serine-type peptidase complex	6	11	1.63	2.31e-06	F9,FCN3,FCN2,MASP1,F7,F11
COMPARTMENTS	GOCC:0032991	Protein-containing complex	107	5325	0.2	3.88e-06	CPB2,CETP,GNPTG,APOH,VCL,LGALS1,F9,PON1,C5,VTN,APOC3,APOB,IGFBP5,APOA1,CAT,LRP1,DHX29,APOE,PXDN,VWF,PSMA6,C9,LCAT,NID1,CSTA,CDH6,DNAH5,LUM,CDH11,PSG9,FCN3,SAA4,SERPING1,MYL9,CSF1R,FCN2,MASP1,APEH,GUCY1A1,HSP90B1,PCSK9,FGB,DST,F2,PLG,C1QB,CFHR1,SUZ12,APOL1,SUMO4,SERPINF2,C7,HBA1,HSPA5,P4HB,DNAH8,ANG,FGG,GANAB,BLM,AKAP9,C8A,CENPF,S100A7,S100A9,FLNA,SMARCA1,C8B,ADAMTS13,MBL2,C1QC,F7,APOM,HLA-C,CALML5,TAF9,IGFBP3,GPX3,KIF19,TNRC6A,GAPDH,APOF,CST3,SAA1,F11,C1S,PCYOX1,ADIPOQ,IGF2,C4A,CDK3,C4B,CFB,IGFALS,PLTP,UIMC1,HPR,APOA5,ATP1A1,APOC4,IGHV3-15,VEGFC,HYOU1,TPM4,ACTB,B2M,FGA
COMPARTMENTS	GOCC:0009986	Cell surface	22	438	0.6	4.79e-06	APOH,LBP,APMAP,SPARC,PXDN,DSG2,CSF1R,ANTXR1,PCSK9,FGB,PLG,CSPG4,SERPINF2,P4HB,FGG,CD5L,ADAMTS13,MBL2,HLA-C,ADIPOQ,FGA,PDIA4
COMPARTMENTS	GOCC:1904724	Tertiary granule lumen	9	55	1.11	5.22e-06	CFP,LYZ,TIMP2,PPBP,CAMP,QPCT,QSOX1,CST3,B2M
COMPARTMENTS	GOCC:0042627	Chylomicron	6	14	1.53	6.24e-06	APOH,APOC3,APOB,APOA1,APOE,APOA5
COMPARTMENTS	GOCC:0005925	Focal adhesion	17	269	0.7	6.68e-06	VCL,LRP1,YWHAE,HSP90B1,DST,CSPG4,HSPA5,P4HB,LIMS1,S100A7,FLNA,GSN,YWHAZ,ILK,TPM4,ACTB,B2M
COMPARTMENTS	GOCC:0005783	Endoplasmic reticulum	36	1095	0.41	1.19e-05	RCN1,SPP2,F9,APOB,PROC,APOA1,APOE,PXDN,VWF,CP,AHSG,HSP90B1,PCSK9,APOL1,HSPA5,P4HB,FGG,THBS4,GANAB,F8,QSOX1,F5,S100A7,F7,HLA-C,CKAP4,IGFBP3,NUCB1,ADIPOQ,C4A,APOA5,HYOU1,KNG1,B2M,FGA,PDIA4
COMPARTMENTS	GOCC:0062136	Low-density lipoprotein receptor complex	5	9	1.64	2.55e-05	APOB,APOA1,LRP1,APOE,PCSK9
COMPARTMENTS	GOCC:0005796	Golgi lumen	10	102	0.89	6.52e-05	F9,PROC,LUM,F2,CSPG4,F8,F10,F7,SOD3,PROS1
COMPARTMENTS	GOCC:0030139	Endocytic vesicle	15	260	0.66	9.43e-05	MPO,SPARC,APOB,APOA1,LRP1,APOE,TCIRG1,HPX,HSP90B1,STAB1,HBA1,HLA-C,SAA1,HYOU1,B2M
COMPARTMENTS	GOCC:0005604	Basement membrane	8	61	1.01	0.00010	VTN,SPARC,PXDN,NID1,DST,EFEMP2,ANG,THBS4
COMPARTMENTS	GOCC:0035580	Specific granule lumen	8	62	1.01	0.00011	VCL,CFP,LYZ,TIMP2,CAMP,QPCT,QSOX1,B2M

COMPARTMENTS	GOCC:0070161	Anchoring junction	22	553	0.5	0.00015	VCL,LRP1,DSG2,YWHAE,PCDH1,HSP90B1,DST,CSPG4,HSPA5,P4HB,LIMS1,CDC42BPA,S100A7,FLNA,GSN,CD99,YWHAZ,ILK,TPM4,ACTB,GRHL2,B2M
COMPARTMENTS	GOCC:0034365	Discoidal high-density lipoprotein particle	4	6	1.72	0.00019	APOA1,APOE,LCAT,APOM
COMPARTMENTS	GOCC:1904090	Peptidase inhibitor complex	5	17	1.37	0.00026	VTN,PXDN,CSTA,GAPDH,ACTB
COMPARTMENTS	GOCC:1905370	Serine-type endopeptidase complex	4	7	1.65	0.00029	FCN3,FCN2,MASP1,F11
COMPARTMENTS	GOCC:0034363	Intermediate-density lipoprotein particle	4	8	1.6	0.00042	APOC3,APOB,APOA1,APOE
COMPARTMENTS	GOCC:0016942	Insulin-like growth factor binding protein complex	4	10	1.5	0.00082	IGFBP5,IGFBP3,IGF2,IGFALS
COMPARTMENTS	GOCC:0034663	Endoplasmic reticulum chaperone complex	4	11	1.46	0.0011	HSP90B1,HSPA5,P4HB,HYOU1
COMPARTMENTS	GOCC:0098797	Plasma membrane protein complex	23	682	0.42	0.0011	C5,VTN,APOB,APOA1,LRP1,APOE,VWF,C9,CDH6,CDH11,PSG9,PCSK9,DST,C7,AKAP9,C8A,FLNA,C8B,MBL2,HLA-C,C4A,ATP1A1,B2M
COMPARTMENTS	GOCC:0005601	Classical-complement-pathway C3/C5 convertase complex	3	4	1.77	0.0024	C4A,C4B,CFB
COMPARTMENTS	GOCC:0042567	Insulin-like growth factor ternary complex	3	4	1.77	0.0024	IGFBP5,IGFBP3,IGFALS
COMPARTMENTS	GOCC:0043227	Membrane-bounded organelle	146	9083	0.1	0.0025	RCN1,SPP2,ST6GAL1,CPB2,GNPTG,APOH,VCL,LGALS1,CHGA,LBP,F9,MMP2,PON1,PCOLCE,MPO,VTN,AFM,MGP,GPLD1,SPARC,APOB,PROC,APOA1,TTR,CAT,LRP1,CFP,APOE,PXDN,SERPINF1,APCS,LYZ,VWF,PSMA6,TIMP2,LGALS3BP,LCAT,YWHAE,CSTA,CP,KLKB1,DNAH5,AMBP,CENPE,TCIRG1,HPX,HYAL1,ITIH4,LUM,CDH11,ITIH1,AHSG,SERPING1,SERPINI1,PPBP,PF4,CAMP,APEH,C16orf46,HSP90B1,ANTXR1,SH3D19,PCSK9,FGB,DST,F2,PLG,CSPG4,STAB1,SUZ12,APOL1,SERPINF2,HBA1,HSPA5,P4HB,DNAH8,ANG,FGG,THBS4,GANAB,QPCT,APOD,BLM,AKAP9,F8,CDC42BPA,CENPF,CFH,QSOX1,F5,S100A7,S100A9,FLNA,SMARCA1,CCN5,GSN,F10,F7,HLA-C,CKAP4,CALML5,TAF9,IGFBP3,CD99,SOD3,GPX3,PROS1,EFEMP1,TNRC6A,YWHAZ,NAV2,ILK,GAPDH,RNASE1,CST3,SAA1,F11,NUCB1,PCYOX1,ADIPOQ,IGF2,C4A,ATP9B,FSIP2,ACSF2,ABCA13,ITIH3,C4B,PLTP,RARRES2,GC,UIMC1,SELENOP,APOA5,SERPINA3,SERPINA4,VEGFC,HYOU1,KNG1,ACTB,TRIM66,GRHL2,B2M,FGA,PDIA4,SDF4
COMPARTMENTS	GOCC:0070820	Tertiary granule	10	164	0.68	0.0025	CFP,LYZ,TIMP2,TCIRG1,PPBP,CAMP,QPCT,QSOX1,CST3,B2M
COMPARTMENTS	GOCC:0036454	Growth factor complex	5	31	1.1	0.0028	IGFBP5,IGFBP3,IGF2,IGFALS,VEGFC
COMPARTMENTS	GOCC:0005775	Vacuolar lumen	10	168	0.67	0.0029	MPO,APOB,TTR,LYZ,HYAL1,LUM,CSPG4,S100A7,GC,SERPINA3
COMPARTMENTS	GOCC:1905368	Peptidase complex	9	136	0.72	0.0030	F9,PSMA6,FCN3,FCN2,MASP1,F2,F7,TAF9,F11
COMPARTMENTS	GOCC:0005602	Complement component C1 complex	3	5	1.67	0.0034	C1QB,C1QC,C1S
COMPARTMENTS	GOCC:0042581	Specific granule	9	158	0.65	0.0084	VCL,CFP,LYZ,TIMP2,CAMP,QPCT,QSOX1,CKAP4,B2M
COMPARTMENTS	GOCC:1904813	ficolin-1-rich granule lumen	8	124	0.71	0.0084	VCL,CAT,TIMP2,APEH,QPCT,GSN,CALML5,CST3
COMPARTMENTS	GOCC:0043226	Organelle	156	10113	0.08	0.0093	RCN1,SPP2,ST6GAL1,CPB2,GNPTG,APOH,VCL,LGALS1,CHGA,LBP,F9,MMP2,PON1,PCOLCE,C5,MPO,VTN,AFM,MGP,GPLD1,SPARC,APOB,PROC,APOA1,TTR,CAT,LRP1,CFP,DHX29,APOE,PXDN,SERPINF1,APCS,LYZ,VWF,PSMA6,TIMP2,LGALS3BP,LCAT,YWHAE,CSTA,CP,KLKB1,DNAH5,AMBP,CENPE,TCIRG1,HPX,HYAL1,ITIH4,LUM,CDH11,ITIH1,AHSG,SERPING1,MYL9,FCN2,SERPINI1,PPBP,PF4,CAMP,APEH,C16orf46,HSP90B1,ANTXR1,SH3D19,PCSK9,SHLD1,FGB,DST,F2,PLG,CSPG4,STAB1,SUZ12,APOL1,SERPINF2,HBA1,HSPA5,P4HB,DNAH8,ANG,FGG,THBS4,GANAB,QPCT,APOD,BLM,AKAP9,MTCL1,F8,CDC42BPA,CENPF,CFH,QSOX1,F5,S100A7,S100A9,FLNA,SMARCA1,CCN5,GSN,F10,F7,HLA-C,CKAP4,CALML5,TAF9,IGFBP3,CD99,SOD3,GPX3,KIF19,PROS1,EFEMP1,TNRC6A,YWHAZ,NAV2,ILK,GAPDH,RNASE1,CST3,SAA1,F11,NUCB1,PCYOX1,ADIPOQ,IGF2,C4A,ATP9B,FSIP2,ACSF2,ABCA13,LMOD3,ITIH3,C4B,PLTP,RARRES2,GC,UIMC1,SELENOP,APOA5,ATP1A1,SERPINA3,SERPINA4,VEGFC,HYOU1,KNG1,TPM4,ACTB,TRIM66,GRHL2,B2M,FGA,PDIA4,SDF4
COMPARTMENTS	GOCC:0071944	Cell periphery	72	3860	0.17	0.0101	VCL,MMP2,TFPI2,C5,VTN,GPLD1,SPARC,PCDH12,APOB,PLEK,APOA1,LRP1,APOE,PXDN,VWF,DSG2,TIMP2,ATRN,C9,NID1,CSTA,CDH6,AMBP,TCIRG1,LUM,CDH11,PSG9,CSF1R,PCDH1,IHH,HSP90B1,ANTXR1,PCSK9,FGB,DST,PLG,EFEMP2,CSPG4,STAB1,C7,P4HB,FBLN1,ANG,FGG,LIMS1,THBS4,AKAP9,MTCL1,C8A,S100A9,FLNA,MCOLN2,C8B,ADAMTS13,GSN,MBL2,HLA-C,SOD3,GPX3,PROS1,EFEMP1,ILK,GAPDH,FBLN2,C4A,ABCA13,C4B,RARRES2,ATP1A1,KNG1,B2M,FGA
COMPARTMENTS	GOCC:0005764	Lysosome	18	566	0.4	0.0130	GNPTG,MPO,APOB,TTR,LRP1,LYZ,CP,TCIRG1,HYAL1,LUM,PCSK9,CSPG4,S100A7,CKAP4,PCYOX1,ABCA13,GC,SERPINA3
COMPARTMENTS	GOCC:0030054	Cell junction	27	1053	0.31	0.0148	VCL,LRP1,APOE,DSG2,YWHAE,CDH6,PCDH1,HSP90B1,DST,CSPG4,HSPA5,P4HB,LIMS1,AKAP9,CDC42BPA,S100A7,FLNA,GSN,CD99,GPX3,YWHAZ,ILK,C4A,TPM4,ACTB,GRHL2,B2M
COMPARTMENTS	GOCC:0045298	Tubulin complex	3	10	1.37	0.0148	PXDN,GAPDH,ACTB
COMPARTMENTS	GOCC:0097179	Protease inhibitor complex	3	14	1.23	0.0330	PXDN,GAPDH,ACTB
COMPARTMENTS	GOCC:0140224	SLAC complex	2	3	1.72	0.0428	GAPDH,ACTB
UniProt Keywords	KW-0964	Secreted	161	1839	0.84	1.18e-95	SPP2,ST6GAL1,CPB2,CETP,GNPTG,APOH,LGALS1,CHGA,LBP,F9,MMP2,EBI3,PON1,TFPI2,PCOLCE,C5,VTN,AFM,PF4V1,APOC3,MGP,GPLD1,SPARC,PCDH12,APOB,IGFBP5,PROC,APOA1,TTR,C4BPB,CFP,APOE,PXDN,ANGPTL6,SERPINF1,APCS,CFHR5,LYZ,VWF,SERPINA10,TIMP2,LGALS3BP,ATRN,C9,LCN1,LCAT,NID1,CP,KLKB1,AMBP,HPX,HYAL1,ITIH4,LUM,PSG9,FCN3,ITIH1,AHSG,CRH,HABP2,SAA4,SERPING1,FCN2,PGLYRP2,SCGB3A1,IHH,SERPINI1,PPBP,PF4,CAMP,A2ML1,PCSK9,BMP1,FGB,F2,INHBC,PLG,EFEMP2,C1QB,CFHR1,APOL1,PSG11,CPN2,SERPINF2,C7,SERPINA7,FBLN1,SERPINA11,ANG,FGG,THBS4,QPCT,APOD,PAPLN,ITIH2,CNDP1,F8,C8A,F13B,CFHR4,CFHR3,CFH,QSOX1,F5,CD5L,S100A7,S100A9,ADAMTSL4,CRTAC1,C8B,ADAMTS13,CCN5,GSN,MBL2,C1QC,F10,F7,APOM,IGFBP3,SOD3,GPX3,PROS1,EFEMP1,CFI,RNASE1,APOF,CST3,FBLN2,SAA1,F11,NUCB1,ADIPOQ,IGF2,C4A,COLEC11,MST1,SPARCL1,ITIH3,C4B,CFB,IGFALS,PLTP,PAEP,RARRES2,GC,HGFAC,SELENOP,SBSN,HPR,APOA5,SERPINA3,SERPINA4,RNASE4,APOC4,IGHV3-15,CCL18,IGHV3-72,VEGFC,KNG1,B2M,FGA
UniProt Keywords	KW-0732	Signal	181	3277	0.64	2.24e-80	RCN1,SPP2,CPB2,CETP,GNPTG,APOH,SERPIND1,CHGA,LBP,F9,MMP2,EBI3,PON1,TFPI2,PCOLCE,C5,MPO,VTN,AFM,PF4V1,APOC3,MGP,GPLD1,SPARC,PCDH12,APOB,IGFBP5,PROC,APOA1,TTR,LRP1,C4BPB,CFP,APOE,PXDN,ANGPTL6,SERPINF1,APCS,CFHR5,LYZ,VWF,DSG2,TIMP2,LGALS3BP,ATRN,C9,LCN1,LCAT,NID1,CP,KLKB1,CDH6,AMBP,HPX,HYAL1,ITIH4,LUM,CDH11,PSG9,FCN3,ITIH1,AHSG,CRH,HABP2,SAA4,SERPING1,CSF1R,PCDH1,FCN2,PGLYRP2,SCGB3A1,IHH,SERPINI1,PPBP,PF4,CAMP,A2ML1,HSP90B1,ANTXR1,PCSK9,BMP1,FGB,F2,INHBC,PLG,EFEMP2,CSPG4,STAB1,C1QB,CFHR1,APOL1,PSG11,CPN2,SERPINF2,C7,HSPA5,P4HB,SERPINA7,FBLN1,OIT3,SERPINA11,ANG,FGG,THBS4,GANAB,QPCT,APOD,PAPLN,ITIH2,CNDP1,F8,C8A,PCDH18,F13B,CFHR4,CFHR3,CFH,QSOX1,F5,CD5L,ADAMTSL4,CRTAC1,C8B,ADAMTS13,CCN5,GSN,MBL2,C1QC,F10,F7,APOM,HLA-C,IGFBP3,CD99,SOD3,GPX3,PROS1,EFEMP1,CFI,RNASE1,APOF,CST3,FBLN2,SAA1,F11,C1S,NUCB1,PCYOX1,ADIPOQ,IGF2,C4A,COLEC11,MST1,SPARCL1,ITIH3,C4B,CFB,IGFALS,PLTP,PAEP,RARRES2,GC,HGFAC,SELENOP,SBSN,HPR,APOA5,SERPINA3,SERPINA4,RNASE4,APOC4,IGHV3-15,CCL18,IGHV3-72,VEGFC,HYOU1,KNG1,B2M,FGA,PDIA4,SDF4

UniProt Keywords	KW-1015	Disulfide bond	143	3338	0.53	1.47e-43	SPP2,ST6GAL1,CPB2,CETP,GNPTG,APOH,CHGA,LBP,F9,MMP2,PON1,TFPI2,PCOLCE,C5,MPO,VTN,AFM,PF4V1,MGP,SPARC,APOB,IGFBP5,PROC,LRP1,C4BPB,CFP,PXDN,ANGPTL6,APCS,CFHR5,LYZ,VWF,TIMP2,LGALS3BP,ATRN,C9,LCN1,LCAT,NID1,CP,KLKB1,AMBP,HPX,HYAL1,ITIH4,LUM,PSG9,FCN3,ITIH1,AHSG,HABP2,SERPING1,CSF1R,FCN2,PGLYRP2,SCGB3A1,PPBP,PF4,MASP1,CAMP,A2ML1,HSP90B1,ANTXR1,PCSK9,BMP1,FGB,F2,INHBC,PLG,EFEMP2,CSPG4,STAB1,C1QB,CFHR1,PSG11,CPN2,SERPINF2,C7,P4HB,FBLN1,OIT3,ANG,FGG,THBS4,GANAB,QPCT,APOD,PAPLN,ITIH2,F8,C8A,F13B,CFHR4,CFHR3,CFH,QSOX1,F5,CD5L,S100A7,CRTAC1,MCOLN2,C8B,ADAMTS13,GSN,MBL2,C1QC,F10,F7,APOM,HLA-C,IGFBP3,SOD3,PROS1,EFEMP1,CFI,RNASE1,CST3,FBLN2,F11,C1S,ADIPOQ,IGF2,C4A,COLEC11,MST1,SPARCL1,C4B,CFB,PLTP,PAEP,RARRES2,GC,HGFAC,HPR,RNASE4,IGHV3-15,CCL18,IGHV3-72,VEGFC,KNG1,B2M,FGA,PDIA4
UniProt Keywords	KW-0325	Glycoprotein	156	4386	0.45	3.09e-39	RCN1,ST6GAL1,CPB2,CETP,GNPTG,APOH,SERPIND1,CHGA,LBP,APMAP,F9,MMP2,EBI3,PON1,TFPI2,PCOLCE,C5,MPO,VTN,AFM,PF4V1,APOC3,GPLD1,SPARC,PCDH12,APOB,IGFBP5,PROC,APOA1,TTR,LRP1,C4BPB,CFP,APOE,PXDN,ANGPTL6,SERPINF1,APCS,CFHR5,VWF,PSMA6,DSG2,SERPINA10,LGALS3BP,ATRN,C9,LCAT,NID1,CP,KLKB1,CDH6,AMBP,HPX,HYAL1,ITIH4,LUM,CDH11,PSG9,FCN3,ITIH1,AHSG,HABP2,SAA4,SERPING1,CSF1R,PCDH1,FCN2,PGLYRP2,IHH,SERPINI1,A2ML1,HSP90B1,ANTXR1,PCSK9,BMP1,FGB,F2,INHBC,PLG,EFEMP2,CSPG4,STAB1,C1QB,CFHR1,APOL1,PSG11,CPN2,SERPINF2,C7,SERPINA7,FBLN1,OIT3,SERPINA11,FGG,THBS4,GANAB,QPCT,APOD,ITIH2,CNDP1,F8,C8A,PCDH18,F13B,CFHR4,CFHR3,CFH,QSOX1,F5,CD5L,ADAMTSL4,CRTAC1,C8B,ADAMTS13,C1QC,F10,F7,APOM,HLA-C,IGFBP3,CD99,SOD3,PROS1,EFEMP1,CFI,RNASE1,APOF,CST3,FBLN2,F11,C1S,NUCB1,PCYOX1,ADIPOQ,IGF2,C4A,MST1,SPARCL1,ITIH3,C4B,CFB,IGFALS,PLTP,PAEP,GC,HGFAC,SELENOP,SERPINA3,SERPINA4,APOC4,VEGFC,HYOU1,KNG1,B2M,FGA,SDF4
UniProt Keywords	KW-0094	Blood coagulation	23	46	1.6	6.25e-25	CPB2,SERPIND1,F9,TFPI2,PROC,VWF,SERPINA10,KLKB1,SERPING1,FGB,F2,PLG,FGG,F8,F13B,F5,ADAMTS13,F10,F7,PROS1,F11,KNG1,FGA
UniProt Keywords	KW-0106	Calcium	55	886	0.69	3.63e-20	RCN1,CHGA,F9,MMP2,PON1,MPO,SPARC,PCDH12,PROC,LRP1,PXDN,APCS,DSG2,NID1,CDH6,CDH11,FCN3,MYL9,PCDH1,FCN2,IHH,HSP90B1,PCSK9,BMP1,DST,F2,EFEMP2,FBLN1,OIT3,FGG,THBS4,F8,PCDH18,F5,S100A7,S100A9,MCOLN2,ADAMTS13,GSN,MBL2,F10,F7,CALML5,PROS1,EFEMP1,CFI,EFCAB5,FBLN2,C1S,NUCB1,COLEC11,SPARCL1,TPM4,FGA,SDF4
UniProt Keywords	KW-0180	Complement pathway	14	30	1.57	1.13e-14	C5,C4BPB,C9,SERPING1,C1QB,C7,C8A,C8B,MBL2,C1QC,CFI,C1S,C4A,C4B
UniProt Keywords	KW-0165	Cleavage on pair of basic residues	28	280	0.9	1.85e-14	CHGA,F9,C5,PROC,VWF,DSG2,CDH6,AMBP,CDH11,CRH,HABP2,PPBP,CAMP,BMP1,F2,INHBC,PLG,C8A,ADAMTS13,F10,F7,PROS1,CFI,IGF2,C4A,C4B,CFB,VEGFC
UniProt Keywords	KW-0245	EGF-like domain	26	232	0.95	1.85e-14	F9,PROC,LRP1,ATRN,C9,NID1,HYAL1,HABP2,MASP1,BMP1,EFEMP2,STAB1,C7,FBLN1,OIT3,THBS4,C8A,CRTAC1,C8B,F10,F7,PROS1,EFEMP1,FBLN2,C1S,HGFAC
UniProt Keywords	KW-0646	Protease inhibitor	20	117	1.13	3.67e-14	SERPIND1,TFPI2,SERPINA10,TIMP2,CSTA,AMBP,ITIH4,ITIH1,SERPING1,SERPINI1,A2ML1,SERPINF2,SERPINA11,PAPLN,ITIH2,CST3,ITIH3,SERPINA3,SERPINA4,KNG1
UniProt Keywords	KW-0722	Serine protease inhibitor	16	80	1.2	3.48e-12	SERPIND1,TFPI2,SERPINA10,AMBP,ITIH4,ITIH1,SERPING1,SERPINI1,A2ML1,SERPINF2,SERPINA11,PAPLN,ITIH2,ITIH3,SERPINA3,SERPINA4
UniProt Keywords	KW-0399	Innate immunity	27	329	0.81	3.88e-12	LBP,C5,C4BPB,CFP,C9,FCN3,SERPING1,CSF1R,FCN2,FGB,C1QB,C7,C8A,CFH,S100A9,MCOLN2,C8B,MBL2,C1QC,HLA-C,CFI,C1S,C4A,COLEC11,C4B,CFB,FGA
UniProt Keywords	KW-0765	Sulfation	14	57	1.29	1.00e-11	SERPIND1,CHGA,F9,VTN,NID1,LUM,PCSK9,SERPINF2,FGG,F8,CFH,F5,C4A,C4B
UniProt Keywords	KW-0677	Repeat	113	4794	0.27	3.47e-11	RCN1,APOH,VCL,SERPIND1,F9,MMP2,EBI3,TFPI2,PCOLCE,VTN,AFM,GPLD1,PCDH12,PROC,PLEK,APOA1,LRP1,C4BPB,CFP,APOE,PXDN,CFHR5,VWF,DSG2,ATRN,NID1,CP,KLKB1,CDH6,DNAH5,AMBP,HPX,LUM,CDH11,PSG9,FCN3,AHSG,ANKRD31,HABP2,SERPING1,MYL9,ANKRD44,CSF1R,PCDH1,FCN2,MASP1,SH3D19,BMP1,DST,F2,PLG,EFEMP2,CSPG4,STAB1,C1QB,CFHR1,PSG11,CPN2,C7,P4HB,FBLN1,DNAH8,LIMS1,THBS4,PAPLN,F8,C8A,PCDH18,CENPF,F13B,CFHR4,CFHR3,CFH,F5,CD5L,S100A7,S100A9,ADAMTSL4,FLNA,CRTAC1,SMARCA1,C8B,ADAMTS13,GSN,MBL2,C1QC,F10,F7,CALML5,PROS1,EFEMP1,CFI,ILK,RAPGEF4,ANKRD28,FBLN2,F11,C1S,NUCB1,ADIPOQ,FSIP2,ABCA13,MST1,CFB,IGFALS,GC,HGFAC,UIMC1,VEGFC,KNG1,TRIM66,PDIA4,SDF4
UniProt Keywords	KW-0391	Immunity	32	537	0.67	5.18e-11	LBP,C5,C4BPB,CFP,C9,FCN3,SERPING1,CSF1R,FCN2,PGLYRP2,FGB,C1QB,C7,C8A,CFH,CD5L,S100A9,MCOLN2,C8B,MBL2,C1QC,HLA-C,CFI,C1S,C4A,COLEC11,C4B,CFB,IGHV3-15,IGHV3-72,B2M,FGA
UniProt Keywords	KW-0768	Sushi	13	57	1.25	1.37e-10	APOH,C4BPB,CFHR5,MASP1,CFHR1,C7,F13B,CFHR4,CFHR3,CFH,C1S,CFB,HPR
UniProt Keywords	KW-0301	Gamma-carboxyglutamic acid	9	16	1.65	4.23e-10	F9,MGP,PROC,TTR,F2,ITIH2,F10,F7,PROS1
UniProt Keywords	KW-0345	HDL	9	16	1.65	4.23e-10	PON1,APOA1,APOE,SAA4,APOL1,APOM,APOF,SAA1,APOA5
UniProt Keywords	KW-0179	Complement alternate pathway	8	12	1.72	2.02e-09	C5,CFP,C9,C7,C8A,CFH,C8B,CFB
UniProt Keywords	KW-0034	Amyloid	9	24	1.47	6.27e-09	APOA1,TTR,APCS,LYZ,GSN,CST3,SAA1,B2M,FGA
UniProt Keywords	KW-0792	Thrombophilia	7	10	1.74	2.38e-08	SERPIND1,F9,PROC,F2,PLG,F5,PROS1
UniProt Keywords	KW-1008	Amyloidosis	9	31	1.36	3.76e-08	APOA1,TTR,APOE,LYZ,GSN,CST3,SAA1,B2M,FGA
UniProt Keywords	KW-0720	Serine protease	14	136	0.91	1.81e-07	F9,PROC,KLKB1,HABP2,PCSK9,F2,PLG,F10,F7,CFI,F11,C1S,CFB,HGFAC
UniProt Keywords	KW-0379	Hydroxylation	14	143	0.89	3.13e-07	F9,PROC,FCN3,FCN2,C1QB,MBL2,C1QC,F10,F7,PROS1,C1S,ADIPOQ,KNG1,FGA
UniProt Keywords	KW-0011	Acute phase	7	19	1.46	6.19e-07	ITIH4,SAA4,F2,SERPINF2,F8,SAA1,SERPINA3
UniProt Keywords	KW-1068	Hemolytic uremic syndrome	6	10	1.67	6.55e-07	CFHR5,CFHR1,CFHR3,CFH,CFI,CFB
UniProt Keywords	KW-0865	Zymogen	16	210	0.78	6.82e-07	CPB2,F9,MMP2,PROC,KLKB1,PCSK9,BMP1,F2,PLG,F5,ADAMTS13,F10,F7,PROS1,CFB,HGFAC
UniProt Keywords	KW-0358	Heparin-binding	11	88	0.99	1.03e-06	APOH,SERPIND1,VTN,PF4V1,APOB,APOE,SERPINA10,PF4,SOD3,SAA1,F11
UniProt Keywords	KW-0445	Lipid transport	12	110	0.93	1.03e-06	CETP,LBP,APOC3,APOB,APOA1,APOE,APOL1,APOM,APOF,PLTP,APOA5,APOC4
UniProt Keywords	KW-0272	Extracellular matrix	17	267	0.7	2.62e-06	LGALS1,MMP2,SPARC,APOE,PXDN,VWF,LGALS3BP,NID1,LUM,BMP1,FBLN1,THBS4,ADAMTSL4,CRTAC1,EFEMP1,FBLN2,SPARCL1
UniProt Keywords	KW-0473	Membrane attack complex	5	7	1.75	4.60e-06	C5,C9,C7,C8A,C8B
UniProt Keywords	KW-0280	Fibrinolysis	5	8	1.69	7.17e-06	CPB2,KLKB1,SERPING1,PLG,PROS1
UniProt Keywords	KW-0873	Pyrrolidone carboxylic acid	9	67	1.02	8.95e-06	SERPINF1,LUM,FGB,C1QB,ANG,APOD,RNASE4,KNG1,B2M
UniProt Keywords	KW-0971	Glycation	5	12	1.52	3.12e-05	APOA1,APOE,SOD3,CFB,B2M
UniProt Keywords	KW-0395	Inflammatory response	12	163	0.76	4.15e-05	C5,ATRN,KLKB1,CSF1R,STAB1,CD5L,S100A9,C4A,C4B,RARRES2,CCL18,KNG1
UniProt Keywords	KW-0153	Cholesterol metabolism	8	65	0.99	6.40e-05	CETP,APOB,APOA1,APOE,LCAT,PCSK9,APOL1,APOF
UniProt Keywords	KW-0645	Protease	21	516	0.51	7.73e-05	CPB2,F9,MMP2,PROC,PSMA6,KLKB1,HABP2,IHH,PCSK9,BMP1,F2,PLG,CNDP1,ADAMTS13,F10,F7,CFI,F11,C1S,CFB,HGFAC
UniProt Keywords	KW-0420	Kringle	5	16	1.39	8.62e-05	HABP2,F2,PLG,MST1,HGFAC
UniProt Keywords	KW-0913	Age-related macular degeneration	5	16	1.39	8.62e-05	C9,CFH,CFI,CST3,CFB
UniProt Keywords	KW-0558	Oxidation	6	32	1.17	0.00012	CHGA,MPO,APOA1,APOE,GAPDH,ACTB
UniProt Keywords	KW-0654	Proteoglycan	7	55	1.0	0.00019	PF4V1,AMBP,LUM,ITIH1,CSPG4,ITIH2,ITIH3
UniProt Keywords	KW-0162	Chylomicron	4	9	1.54	0.00024	APOC3,APOB,APOE,APOA5

UniProt Keywords	KW-1018	Complement activation lectin pathway	3	3	1.9	0.00055	FCN3,FCN2,MBL2
UniProt Keywords	KW-0797	Tissue remodeling	3	4	1.77	0.00092	PLG,CSPG4,THBS4
UniProt Keywords	KW-0176	Collagen	7	83	0.82	0.0019	FCN3,FCN2,C1QB,MBL2,C1QC,ADIPOQ,COLEC11
UniProt Keywords	KW-0130	Cell adhesion	17	478	0.45	0.0024	VCL,VTN,PCDH12,VWF,DSG2,LGALS3BP,NID1,CSTA,CDH6,CDH11,PCDH1,DST,THBS4,PCDH18,CCN5,CD99,IGFALS
UniProt Keywords	KW-0497	Mitogen	5	39	1.0	0.0029	CAT,PPBP,THBS4,IGF2,VEGFC
UniProt Keywords	KW-0065	Atherosclerosis	3	8	1.47	0.0038	CETP,APOB,APOA1
UniProt Keywords	KW-0882	Thioester bond	3	8	1.47	0.0038	A2ML1,C4A,C4B
UniProt Keywords	KW-1065	Osteogenesis imperfecta	4	23	1.14	0.0042	SPARC,SERPINF1,BMP1,P4HB
UniProt Keywords	KW-0376	Hydrogen peroxide	3	9	1.42	0.0048	MPO,CAT,PXDN
UniProt Keywords	KW-0575	Peroxidase	4	25	1.1	0.0054	MPO,CAT,PXDN,GPX3
UniProt Keywords	KW-0850	VLDL	3	10	1.37	0.0060	APOC3,APOE,APOA5
UniProt Keywords	KW-0378	Hydrolase	36	1612	0.25	0.0087	CPB2,F9,MMP2,PON1,GPLD1,PROC,DHX29,LYZ,PSMA6,KLKB1,HYAL1,HABP2,PGLYRP2,IHH,APEH,PCSK9,BMP1,F2,PLG,HSPA5,ANG,GANAB,BLM,CNDP1,SMARCA1,ADAMTS13,F10,F7,CFI,NAV2,RNASE1,F11,C1S,CFB,HGFAC,RNASE4
UniProt Keywords	KW-0355	Hemophilia	2	2	1.9	0.0105	F9,F8
UniProt Keywords	KW-0929	Antimicrobial	6	93	0.71	0.0173	CHGA,LBP,LYZ,PPBP,CAMP,S100A9
UniProt Keywords	KW-0145	Chemotaxis	6	94	0.7	0.0179	SERPIND1,PPBP,PF4,S100A9,RARRES2,CCL18
UniProt Keywords	KW-0339	Growth factor	7	129	0.63	0.0179	PPBP,BMP1,INHBC,THBS4,EFEMP1,IGF2,VEGFC
UniProt Keywords	KW-1212	Corneal dystrophy	3	20	1.07	0.0305	LCAT,GSN,GRHL2
Pfam	PF06668	Inter-alpha-trypsin inhibitor heavy chain C-terminus	4	6	1.72	0.0110	ITIH4,ITIH1,ITIH2,ITIH3
InterPro	IPR001881	EGF-like calcium-binding domain	19	124	1.08	1.39e-10	F9,PROC,LRP1,NID1,HABP2,MASP1,BMP1,EFEMP2,STAB1,FBLN1,OIT3,THBS4,CRTAC1,F10,F7,PROS1,EFEMP1,FBLN2,C1S
InterPro	IPR018097	EGF-like calcium-binding, conserved site	17	99	1.13	3.27e-10	F9,PROC,LRP1,NID1,MASP1,BMP1,EFEMP2,FBLN1,OIT3,THBS4,CRTAC1,F10,F7,PROS1,EFEMP1,FBLN2,C1S
InterPro	IPR035976	Sushi/SCR/CCP superfamily	13	58	1.25	1.00e-08	APOH,C4BPB,CFHR5,MASP1,CFHR1,C7,F13B,CFHR4,CFHR3,CFH,C1S,CFB,HPR
InterPro	IPR000436	Sushi/SCR/CCP domain	12	56	1.23	6.51e-08	APOH,C4BPB,CFHR5,MASP1,CFHR1,C7,F13B,CFHR4,CFHR3,CFH,C1S,CFB
InterPro	IPR001314	Peptidase S1A, chymotrypsin family	15	107	1.04	6.51e-08	F9,PROC,KLKB1,HABP2,F2,PLG,F10,F7,CFI,F11,C1S,MST1,CFB,HGFAC,HPR
InterPro	IPR001254	Serine proteases, trypsin domain	15	115	1.01	1.09e-07	F9,PROC,KLKB1,HABP2,F2,PLG,F10,F7,CFI,F11,C1S,MST1,CFB,HGFAC,HPR
InterPro	IPR042185	Serpin superfamily, domain 2	10	35	1.35	1.78e-07	SERPIND1,SERPINF1,SERPINA10,SERPING1,SERPINI1,SERPINF2,SERPINA7,SERPINA11,SERPINA3,SERPINA4
InterPro	IPR000215	Serpin family	10	36	1.34	1.93e-07	SERPIND1,SERPINF1,SERPINA10,SERPING1,SERPINI1,SERPINF2,SERPINA7,SERPINA11,SERPINA3,SERPINA4
InterPro	IPR009003	Peptidase S1, PA clan	15	123	0.98	1.93e-07	F9,PROC,KLKB1,HABP2,F2,PLG,F10,F7,CFI,F11,C1S,MST1,CFB,HGFAC,HPR
InterPro	IPR023796	Serpin domain	10	36	1.34	1.93e-07	SERPIND1,SERPINF1,SERPINA10,SERPING1,SERPINI1,SERPINF2,SERPINA7,SERPINA11,SERPINA3,SERPINA4
InterPro	IPR036186	Serpin superfamily	10	36	1.34	1.93e-07	SERPIND1,SERPINF1,SERPINA10,SERPING1,SERPINI1,SERPINF2,SERPINA7,SERPINA11,SERPINA3,SERPINA4
InterPro	IPR042178	Serpin superfamily, domain 1	10	36	1.34	1.93e-07	SERPIND1,SERPINF1,SERPINA10,SERPING1,SERPINI1,SERPINF2,SERPINA7,SERPINA11,SERPINA3,SERPINA4
InterPro	IPR000742	EGF-like domain	19	236	0.8	3.22e-07	F9,PROC,LRP1,ATRN,NID1,HABP2,MASP1,BMP1,EFEMP2,STAB1,FBLN1,OIT3,THBS4,F10,F7,PROS1,EFEMP1,FBLN2,HGFAC
InterPro	IPR033116	Serine proteases, trypsin family, serine active site	13	92	1.05	3.55e-07	F9,PROC,KLKB1,HABP2,F2,PLG,F10,F7,CFI,F11,C1S,CFB,HGFAC
InterPro	IPR043504	Peptidase S1, PA clan, chymotrypsin-like fold	14	116	0.98	4.72e-07	F9,PROC,KLKB1,HABP2,F2,PLG,F10,F7,CFI,F11,C1S,MST1,HGFAC,HPR
InterPro	IPR000152	EGF-type aspartate/asparagine hydroxylation site	13	98	1.02	6.25e-07	F9,PROC,LRP1,NID1,BMP1,EFEMP2,FBLN1,F10,F7,PROS1,EFEMP1,FBLN2,C1S
InterPro	IPR000294	Gamma-carboxyglutamic acid-rich (GLA) domain	7	15	1.57	2.74e-06	F9,MGP,PROC,F2,F10,F7,PROS1
InterPro	IPR035972	Gamma-carboxyglutamic acid-rich (GLA) domain superfamily	7	15	1.57	2.74e-06	F9,MGP,PROC,F2,F10,F7,PROS1
InterPro	IPR018114	Serine proteases, trypsin family, histidine active site	12	100	0.98	6.36e-06	F9,PROC,KLKB1,HABP2,F2,PLG,F10,F7,CFI,F11,CFB,HGFAC
InterPro	IPR009030	Growth factor receptor cysteine-rich domain superfamily	13	127	0.91	8.66e-06	IGFBP5,LRP1,C9,NID1,BMP1,EFEMP2,FBLN1,C8A,CCN5,IGFBP3,PROS1,EFEMP1,FBLN2
InterPro	IPR023795	Serpin, conserved site	8	32	1.29	1.01e-05	SERPIND1,SERPINF1,SERPING1,SERPINI1,SERPINF2,SERPINA7,SERPINA3,SERPINA4
InterPro	IPR046350	Cystatin superfamily	7	23	1.38	2.32e-05	SPP2,CSTA,AHSG,CAMP,CST3,RARRES2,KNG1
InterPro	IPR000020	Anaphylatoxin/fibulin	5	6	1.82	4.03e-05	C5,FBLN1,FBLN2,C4A,C4B
InterPro	IPR036383	Thrombospondin type-1 (TSP1) repeat superfamily	9	64	1.04	9.54e-05	CFP,C9,C7,PAPLN,C8A,ADAMTSL4,C8B,ADAMTS13,CCN5
InterPro	IPR000884	Thrombospondin type-1 (TSP1) repeat	9	65	1.04	0.00010	CFP,C9,C7,PAPLN,C8A,ADAMTSL4,C8B,ADAMTS13,CCN5
InterPro	IPR020837	Fibrinogen, conserved site	6	19	1.4	0.00015	ANGPTL6,FCN3,FCN2,FGB,FGG,FGA
InterPro	IPR017857	Coagulation factor-like, Gla domain superfamily	5	12	1.52	0.00043	F9,PROC,F10,F7,PROS1
InterPro	IPR013806	Kringle-like fold	6	26	1.26	0.00065	MMP2,HABP2,F2,PLG,MST1,HGFAC
InterPro	IPR014716	Fibrinogen, alpha/beta/gamma chain, C-terminal globular, subdomain 1	6	26	1.26	0.00065	ANGPTL6,FCN3,FCN2,FGB,FGG,FGA
InterPro	IPR001862	Membrane attack complex component/perforin/complement C9	4	5	1.8	0.00069	C9,C7,C8A,C8B
InterPro	IPR012224	Peptidase S1A, coagulation factor VII/IX/X/C/Z	4	5	1.8	0.00069	F9,PROC,F10,F7
InterPro	IPR026823	Complement Clr-like EGF domain	6	27	1.24	0.00069	LRP1,NID1,EFEMP2,FBLN1,EFEMP1,FBLN2
InterPro	IPR000001	Kringle	5	16	1.39	0.0010	HABP2,F2,PLG,MST1,HGFAC
InterPro	IPR003609	PAN/Apple domain	4	6	1.72	0.0010	KLKB1,PLG,F11,MST1
InterPro	IPR010600	Inter-alpha-trypsin inhibitor heavy chain, C-terminal	4	6	1.72	0.0010	ITIH4,ITIH1,ITIH2,ITIH3
InterPro	IPR018056	Kringle, conserved site	5	16	1.39	0.0010	HABP2,F2,PLG,MST1,HGFAC
InterPro	IPR020863	Membrane attack complex component/perforin domain, conserved site	4	6	1.72	0.0010	C9,C7,C8A,C8B
InterPro	IPR038178	Kringle superfamily	5	16	1.39	0.0010	HABP2,F2,PLG,MST1,HGFAC
InterPro	IPR002181	Fibrinogen, alpha/beta/gamma chain, C-terminal globular domain	6	32	1.17	0.0013	ANGPTL6,FCN3,FCN2,FGB,FGG,FGA
InterPro	IPR036056	Fibrinogen-like, C-terminal	6	32	1.17	0.0013	ANGPTL6,FCN3,FCN2,FGB,FGG,FGA
InterPro	IPR040839	Macroglobulin domain MG4	4	8	1.6	0.0019	C5,A2ML1,C4A,C4B
InterPro	IPR001599	Alpha-2-macroglobulin	4	9	1.54	0.0027	C5,A2ML1,C4A,C4B
InterPro	IPR002890	Macroglobulin domain	4	9	1.54	0.0027	C5,A2ML1,C4A,C4B
InterPro	IPR009048	Alpha-macroglobulin, receptor-binding	4	9	1.54	0.0027	C5,A2ML1,C4A,C4B
InterPro	IPR011625	Alpha-2-macroglobulin, bait region domain	4	9	1.54	0.0027	C5,A2ML1,C4A,C4B
InterPro	IPR011626	Alpha-macroglobulin-like, TED domain	4	9	1.54	0.0027	C5,A2ML1,C4A,C4B
InterPro	IPR036595	Alpha-macroglobulin, receptor-binding domain superfamily	4	9	1.54	0.0027	C5,A2ML1,C4A,C4B
InterPro	IPR041555	Macroglobulin domain MG3	4	9	1.54	0.0027	C5,A2ML1,C4A,C4B
InterPro	IPR001134	Netrin domain	5	23	1.23	0.0032	PCOLCE,C5,TIMP2,C4A,C4B
InterPro	IPR013694	VIT domain	4	10	1.5	0.0032	ITIH4,ITIH1,ITIH2,ITIH3

InterPro	IPR023415	Low-density lipoprotein (LDL) receptor class A, conserved site	6	40	1.07	0.0032	LRP1,C9,C7,C8A,C8B,CFI
InterPro	IPR018247	EF-Hand 1, calcium-binding site	11	176	0.69	0.0035	RCN1,SPARC,MYL9,DST,S100A7,S100A9,CALML5,EFCAB5,NUCB1,SPARCL1,SDF4
InterPro	IPR008993	Tissue inhibitor of metalloproteinases-like, OB-fold	5	24	1.22	0.0037	PCOLCE,C5,TIMP2,C4A,C4B
InterPro	IPR012290	Fibrinogen, alpha/beta/gamma chain, coiled coil domain	3	3	1.9	0.0050	FGB,FGG,FGA
InterPro	IPR020864	Membrane attack complex component/perforin (MACPF) domain	4	12	1.42	0.0050	C9,C7,C8A,C8B
InterPro	IPR037579	Fibrinogen	3	3	1.9	0.0050	FGB,FGG,FGA
InterPro	IPR002172	Low-density lipoprotein (LDL) receptor class A repeat	6	47	1.0	0.0064	LRP1,C9,C7,C8A,C8B,CFI
InterPro	IPR036055	LDL receptor-like superfamily	6	47	1.0	0.0064	LRP1,C9,C7,C8A,C8B,CFI
InterPro	IPR000074	Apolipoprotein A/E	3	4	1.77	0.0080	APOA1,APOE,APOA5
InterPro	IPR001840	Anaphylatoxin, complement system domain	3	4	1.77	0.0080	C5,C4A,C4B
InterPro	IPR018081	Anaphylatoxin, complement system	3	4	1.77	0.0080	C5,C4A,C4B
InterPro	IPR008930	Terpenoid cyclases/protein prenyltransferase alpha-alpha toroid	4	15	1.32	0.0093	C5,A2ML1,C4A,C4B
InterPro	IPR002035	Von Willebrand factor, type A	7	76	0.86	0.0094	VWF,ITIH4,ITIH1,ANTXR1,ITIH2,ITIH3,CFB
InterPro	IPR011707	Multicopper oxidase, N-terminal	3	5	1.67	0.0116	CP,F8,F5
InterPro	IPR017954	Lipid-binding serum glycoprotein, conserved site	3	5	1.67	0.0116	CETP,LBP,PLTP
InterPro	IPR033138	Multicopper oxidases, conserved site	3	5	1.67	0.0116	CP,F8,F5
InterPro	IPR035914	Spermadhesin, CUB domain superfamily	6	54	0.94	0.0116	PCOLCE,ATRN,MASP1,BMP1,ADAMTS13,C1S
InterPro	IPR000010	Cystatin domain	4	17	1.27	0.0129	CSTA,AHSG,CST3,KNG1
InterPro	IPR018933	Netrin module, non-TIMP type	4	17	1.27	0.0129	PCOLCE,C5,C4A,C4B
InterPro	IPR011042	Six-bladed beta-propeller, TolB-like	5	38	1.02	0.0199	APMAP,PON1,LRP1,NID1,APEH
InterPro	IPR012674	Calycin	5	38	1.02	0.0199	LCN1,AMBP,APOD,APOM,PAEP
InterPro	IPR019742	Alpha-2-macroglobulin, conserved site	3	8	1.47	0.0297	A2ML1,C4A,C4B
InterPro	IPR023411	Ribonuclease A, active site	3	8	1.47	0.0297	ANG,RNASE1,RNASE4
InterPro	IPR047565	Alpha-macroglobulin-like, thiol-ester bond-forming region	3	8	1.47	0.0297	A2ML1,C4A,C4B
InterPro	IPR036465	Von Willebrand factor A-like domain superfamily	7	97	0.75	0.0323	VWF,ITIH4,ITIH1,ANTXR1,ITIH2,ITIH3,CFB
InterPro	IPR018073	Proteinase inhibitor I25, cystatin, conserved site	3	9	1.42	0.0372	CSTA,CST3,KNG1
InterPro	IPR001124	Lipid-binding serum glycoprotein, C-terminal	3	10	1.37	0.0473	CETP,LBP,PLTP
SMART	SM00179	Calcium-binding EGF-like domain	16	101	1.1	1.23e-09	F9,PROC,LRP1,NID1,MASP1,BMP1,EFEMP2,FBLN1,OIT3,THBS4,F10,F7,PROS1,EFEMP1,FBLN2,C1S
SMART	SM00020	Trypsin-like serine protease	16	118	1.03	5.13e-09	F9,PROC,KLKB1,HABP2,MASP1,F2,PLG,F10,F7,CFI,F11,C1S,MST1,CFB,HGFAC,HPR
SMART	SM00032	Domain abundant in complement control proteins	12	56	1.23	1.23e-08	APOH,C4BPB,CFHR5,MASP1,CFHR1,C7,F13B,CFHR4,CFHR3,CFH,C1S,CFB
SMART	SM00093	SERine Proteinase INhibitors	10	35	1.35	3.56e-08	SERPIND1,SERPINF1,SERPINA10,SERPING1,SERPINI1,SERPINF2,SERPINA7,SERPINA11,SERPINA3,SERPINA4
SMART	SM00181	Epidermal growth factor-like domain.	17	209	0.81	6.84e-07	F9,PROC,LRP1,ATRN,NID1,HYAL1,HABP2,EFEMP2,STAB1,FBLN1,OIT3,THBS4,F10,F7,PROS1,FBLN2,HGFAC
SMART	SM00069	Domain containing Gla (gamma-carboxyglutamate) residues.	7	15	1.57	8.87e-07	F9,MGP,PROC,F2,F10,F7,PROS1
SMART	SM00104	Anaphylatoxin homologous domain	5	6	1.82	1.51e-05	C5,FBLN1,FBLN2,C4A,C4B
SMART	SM00209	Thrombospondin type 1 repeats	9	64	1.04	3.27e-05	CFP,C9,C7,PAPLN,C8A,ADAMTSL4,C8B,ADAMTS13,CCN5
SMART	SM00186	Fibrinogen-related domains (FReDs)	6	29	1.21	0.00040	ANGPTL6,FCN3,FCN2,FGB,FGG,FGA
SMART	SM00130	Kringle domain	5	16	1.39	0.00042	HABP2,F2,PLG,MST1,HGFAC
SMART	SM00609	Vault protein Inter-alpha-Trypsin domain	4	7	1.65	0.00055	ITIH4,ITIH1,ITIH2,ITIH3
SMART	SM01359	Alpha-2-Macroglobulin	4	9	1.54	0.0011	C5,A2ML1,C4A,C4B
SMART	SM01360	Alpha-2-macroglobulin family	4	9	1.54	0.0011	C5,A2ML1,C4A,C4B
SMART	SM01361	A-macroglobulin receptor	4	9	1.54	0.0011	C5,A2ML1,C4A,C4B
SMART	SM00457	Membrane-attack complex / perforin	4	12	1.42	0.0021	C9,C7,C8A,C8B
SMART	SM01212	Fibrinogen alpha/beta chain family	3	3	1.9	0.0021	FGB,FGG,FGA
SMART	SM00192	Low-density lipoprotein receptor domain class A	6	48	0.99	0.0028	LRP1,C9,C7,C8A,C8B,CFI
SMART	SM00643	Netrin C-terminal Domain	4	15	1.32	0.0037	PCOLCE,C5,C4A,C4B
SMART	SM00327	Von Willebrand factor (vWF) type A domain	7	77	0.86	0.0038	VWF,ITIH4,ITIH1,ANTXR1,ITIH2,ITIH3,CFB
SMART	SM00043	Cystatin-like domain	4	17	1.27	0.0050	CSTA,AHSG,CST3,KNG1
SMART	SM00092	Pancreatic ribonuclease	3	8	1.47	0.0116	ANG,RNASE1,RNASE4
SMART	SM01419	Alpha-macro-globulin thiol-ester bond-forming region	3	8	1.47	0.0116	A2ML1,C4A,C4B
SMART	SM00001	EGF domain, unclasssified subfamily	6	70	0.83	0.0137	LRP1,ATRN,EFEMP2,STAB1,OIT3,EFEMP1
SMART	SM00328	BPI/LBP/CETP N-terminal domain	3	9	1.42	0.0137	CETP,LBP,PLTP
SMART	SM00329	BPI/LBP/CETP C-terminal domain	3	9	1.42	0.0137	CETP,LBP,PLTP
SMART	SM00042	Domain first found in C1r, C1s, uEGF, and bone morphogenetic protein.	5	50	0.9	0.0196	PCOLCE,ATRN,MASP1,BMP1,C1S
SMART	SM00223	APPLE domain	2	2	1.9	0.0281	KLKB1,F11

UC vs ACFET T2

#category	term ID	term description	observed gene count	background gene count	strength	false discovery rate	matching proteins in your network (labels)
GO Process	GO:0006959	Humoral immune response	46	268	1.16	6.28e-33	ST6GAL1,C5,LTF,CFP,CFHR5,LYZ,C9,FCN3,SERPING1,PF4,MASP1,CAMP,FGB,F2,C1QB,CFHR1,CLU,C7,ANG,SLPI,C8A,CFHR2,CFHR4,CFHR3,CFH,S100A7,S100A9,C8B,FCN1,C1QA,CFI,MASP2,C1S,C4A,C4B,CFB,RARRES2,JCHAIN,C1R,CFD,IGHV3-15,CCL18,IGHV3-72,KNG1,B2M,FGA
GO Process	GO:0006956	Complement activation	29	60	1.61	1.48e-30	C5,CFP,CFHR5,C9,FCN3,SERPING1,MASP1,C1QB,CFHR1,CLU,C7,C8A,CFHR2,CFHR4,CFHR3,CFH,C8B,FCN1,C1QA,CFI,MASP2,C1S,C4A,C4B,CFB,C1R,CFD,IGHV3-15,IGHV3-72
GO Process	GO:0006950	Response to stress	119	3358	0.48	2.80e-28	CPB2,CHGA,F9,C5,LTF,PROC,CAT,LRP1,CFP,APOE,BPIFB1,APCS,CFHR5,THBS1,LYZ,VWF,SERPINA10,LGALS3BP,ATRN,C9,KLKB1,F13A1,DGKG,AMBP,TCIRG1,HPX,FCN3,AHSG,SAA4,SERPING1,DSC2,CSF1R,PGLYRP2,PF4,MASP1,CAMP,PDIA3,LALBA,ANGPTL4,PCSK9,FGB,DST,F2,PLG,PRG2,STAB1,C1QB,CFHR1,CLU,NDUFS8,APOL1,SERPINF2,C7,P4HB,FBLN1,PSG3,ANG,FGG,TUBB,THBS4,SLPI,APOD,FN1,BLM,HP,APOA4,C8A,F13B,CFHR2,CFH,F5,S100A7,S100A9,ECM1,FLNA,MCOLN2,C8B,FCN1,ADAMTS13,C1QA,F10,F7,SOD3,GPX3,PROS1,CFI,MMRN1,TNRC6A,ILK,MASP2,F11,C1S,ORM2,C4A,MST1,C4B,SERPINA1,CFB,RARRES2,HGFAC,UIMC1,SELENOP,TSKU,JCHAIN,HPR,C1R,VIM,RNASE4,CFD,IGHV3-15,CCL18,IGHV3-72,VEGFC,PLCG2,KNG1,ACTB,TRIM8,B2M,FGA

GO Process	GO:0006952	Defense response	78	1394	0.67	7.77e-28	CHGA,C5,LTF,LRP1,CFP,BPIFB1,APCS,CFHR5,THBS1,LYZ,LGALS3BP,ATRN,C9,KLKB1,TCIRG1,HPX,FCN3,AHSG,SAA4,SERPING1,CSF1R,PGLYRP2,PF4,MASP1,CAMP,LALBA,FGB,F2,PRG2,STAB1,C1QB,CFHR1,CLU,APOL1,SERPINF2,C7,PSG3,ANG,TUBB,SLPI,FN1,HP,APOA4,C8A,CFHR2,CFH,S100A7,S100A9,ECM1,MCOLN2,C8B,FCN1,ADAMTS13,C1QA,F7,CFI,MASP2,C1S,ORM2,C4A,C4B,SERPINA1,CFB,RARRES2,JCHAIN,HPR,C1R,VIM,RNASE4,CFD,IGHV3-15,CCL18,IGHV3-72,PLCG2,KNG1,TRIM8,B2M,FGA
GO Process	GO:0098542	Defense response to other organism	62	989	0.72	1.45e-23	CHGA,C5,LTF,CFP,BPIFB1,APCS,CFHR5,LYZ,C9,HPX,FCN3,SERPING1,CSF1R,PGLYRP2,PF4,MASP1,CAMP,LALBA,FGB,F2,PRG2,STAB1,C1QB,CFHR1,CLU,APOL1,C7,ANG,TUBB,SLPI,HP,APOA4,C8A,CFHR2,CFH,S100A7,S100A9,MCOLN2,C8B,FCN1,ADAMTS13,C1QA,CFI,MASP2,C1S,C4A,C4B,CFB,RARRES2,JCHAIN,C1R,VIM,RNASE4,CFD,IGHV3-15,CCL18,IGHV3-72,PLCG2,KNG1,TRIM8,B2M,FGA
GO Process	GO:0007596	Blood coagulation	32	173	1.19	2.15e-23	CPB2,F9,PROC,VWF,SERPINA10,KLKB1,F13A1,DGKG,SERPING1,PF4,FGB,F2,PLG,FBLN1,FGG,FN1,F13B,F5,FLNA,ADAMTS13,F10,F7,PROS1,MMRN1,ILK,F11,SERPINA1,HGFAC,PLCG2,KNG1,ACTB,FGA
GO Process	GO:0045087	Innate immune response	51	754	0.76	2.85e-20	CHGA,C5,LTF,CFP,BPIFB1,APCS,CFHR5,C9,HPX,FCN3,SERPING1,CSF1R,PGLYRP2,MASP1,CAMP,FGB,C1QB,CLU,APOL1,C7,ANG,TUBB,SLPI,APOA4,C8A,CFH,S100A7,S100A9,MCOLN2,C8B,FCN1,ADAMTS13,C1QA,CFI,MASP2,C1S,C4A,C4B,CFB,RARRES2,JCHAIN,C1R,VIM,CFD,IGHV3-15,CCL18,IGHV3-72,PLCG2,TRIM8,B2M,FGA
GO Process	GO:0050896	Response to stimulus	168	7835	0.26	4.66e-20	ST6GAL1,CPB2,CHGA,F9,C5,GPLD1,LTF,APOB,IGFBP5,PROC,APOA1,TTR,CAT,LRP1,CFP,APOE,BPIFB1,SERPINF1,APCS,CFHR5,THBS1,LYZ,VWF,SERPINA10,LGALS3BP,ATRN,C9,LCN1,LCAT,KLKB1,F13A1,PPP2R3A,DGKG,CDH6,AMBP,TCIRG1,HPX,INHBE,FCN3,AHSG,IGFBP1,SAA4,SERPING1,DSC2,CSF1R,PIP,PSG6,AZGP1,PGLYRP2,SCGB3A1,PF4,MASP1,CAMP,PDIA3,SLC27A4,LALBA,ANGPTL4,PCSK9,BMP1,FGB,YWHAG,DST,F2,INHBC,PLG,PSG1,PRG2,CSPG4,STAB1,C1QB,CFHR1,CLU,NDUFS8,APOL1,SERPINF2,C7,P4HB,PAPPA,FBLN1,PSG3,ANG,TUBA1B,FGG,TUBB,THBS4,SLPI,APOD,FN1,ASGR2,BLM,HP,AKAP9,APOA4,C8A,F13B,CFHR2,CFHR4,CFHR3,CFH,F5,ADAM12,S100A7,S100A9,ECM1,FLNA,MCOLN2,C8B,FCN1,ADAMTS13,CCN5,SVEP1,C1QA,F10,F7,APOM,CA6,ADGRG2,CALML5,IGFBP3,SOD3,GPX3,PROS1,EFEMP1,CFI,MMRN1,TNRC6A,YWHAZ,ILK,MASP2,F11,C1S,ORM2,C4A,PRG4,MST1,C4B,SERPINA1,TGFBI,CFB,IGFALS,RARRES2,HGFAC,UIMC1,PDGFC,FBN2,SELENOP,TSKU,JCHAIN,HPR,C1R,ATP1A1,VIM,RNASE4,TTN,CFD,IGHV3-15,CCL18,IGHV3-72,VEGFC,SEMA3B,PLCG2,OR52B4,KNG1,ACTB,TRIM8,CDH5,B2M,FGA
GO Process	GO:0006955	Immune response	65	1321	0.62	9.86e-20	ST6GAL1,CHGA,C5,LTF,LRP1,CFP,BPIFB1,APCS,CFHR5,THBS1,LYZ,C9,TCIRG1,HPX,FCN3,SERPING1,CSF1R,PGLYRP2,PF4,MASP1,CAMP,FGB,F2,PRG2,C1QB,CFHR1,CLU,APOL1,C7,ANG,TUBB,SLPI,APOA4,C8A,CFHR2,CFHR4,CFHR3,CFH,S100A7,S100A9,MCOLN2,C8B,FCN1,ADAMTS13,C1QA,CFI,MASP2,C1S,C4A,PRG4,C4B,CFB,RARRES2,JCHAIN,C1R,VIM,CFD,IGHV3-15,CCL18,IGHV3-72,PLCG2,KNG1,TRIM8,B2M,FGA
GO Process	GO:0051707	Response to other organism	63	1328	0.6	3.08e-18	CHGA,C5,LTF,APOB,CFP,BPIFB1,APCS,CFHR5,LYZ,C9,HPX,FCN3,SERPING1,CSF1R,PGLYRP2,PF4,MASP1,CAMP,LALBA,FGB,F2,PRG2,STAB1,C1QB,CFHR1,CLU,APOL1,C7,ANG,TUBB,SLPI,HP,APOA4,C8A,CFHR2,CFH,S100A7,S100A9,MCOLN2,C8B,FCN1,ADAMTS13,C1QA,CFI,MASP2,C1S,C4A,C4B,CFB,RARRES2,JCHAIN,C1R,VIM,RNASE4,CFD,IGHV3-15,CCL18,IGHV3-72,PLCG2,KNG1,TRIM8,B2M,FGA
GO Process	GO:0002252	Immune effector process	36	375	0.91	3.30e-18	CHGA,C5,LRP1,CFP,CFHR5,C9,TCIRG1,FCN3,SERPING1,MASP1,F2,C1QB,CFHR1,CLU,C7,TUBB,C8A,CFHR2,CFHR4,CFHR3,CFH,C8B,FCN1,C1QA,CFI,MASP2,C1S,C4A,C4B,CFB,C1R,CFD,IGHV3-15,IGHV3-72,PLCG2,B2M
GO Process	GO:0044419	Biological process involved in interspecies interaction between organisms	66	1490	0.57	7.14e-18	CHGA,C5,LTF,APOB,CFP,APOE,BPIFB1,APCS,CFHR5,LYZ,C9,HPX,FCN3,SERPING1,CSF1R,PGLYRP2,PF4,MASP1,CAMP,LALBA,FGB,F2,PLG,PRG2,STAB1,C1QB,CFHR1,CLU,APOL1,C7,ANG,TUBB,SLPI,FN1,HP,APOA4,C8A,CFHR2,CFH,S100A7,S100A9,MCOLN2,C8B,FCN1,ADAMTS13,C1QA,CFI,MASP2,C1S,C4A,C4B,CFB,RARRES2,JCHAIN,C1R,VIM,RNASE4,CFD,IGHV3-15,CCL18,IGHV3-72,PLCG2,KNG1,TRIM8,B2M,FGA
GO Process	GO:0042060	Wound healing	34	336	0.93	8.79e-18	CPB2,F9,PROC,VWF,SERPINA10,KLKB1,F13A1,DGKG,SERPING1,PF4,FGB,DST,F2,PLG,FBLN1,FGG,FN1,F13B,F5,FLNA,ADAMTS13,F10,F7,PROS1,MMRN1,ILK,F11,SERPINA1,HGFAC,TSKU,PLCG2,KNG1,ACTB,FGA
GO Process	GO:0050878	Regulation of body fluid levels	35	371	0.9	1.62e-17	CPB2,F9,PROC,APOE,THBS1,VWF,SERPINA10,KLKB1,F13A1,DGKG,SERPING1,PF4,FGB,F2,PLG,SERPINF2,FBLN1,FGG,FN1,F13B,F5,FLNA,ADAMTS13,F10,F7,PROS1,MMRN1,ILK,F11,SERPINA1,HGFAC,PLCG2,KNG1,ACTB,FGA
GO Process	GO:0002253	Activation of immune response	31	271	0.99	1.68e-17	C5,GPLD1,CFP,CFHR5,C9,FCN3,SERPING1,MASP1,C1QB,CFHR1,CLU,C7,C8A,CFHR2,CFHR4,CFHR3,CFH,C8B,FCN1,C1QA,CFI,MASP2,C1S,C4A,C4B,CFB,C1R,CFD,IGHV3-15,IGHV3-72,PLCG2
GO Process	GO:0006958	Complement activation, classical pathway	17	40	1.56	5.71e-17	C5,C9,SERPING1,C1QB,CLU,C7,C8A,C8B,C1QA,CFI,MASP2,C1S,C4A,C4B,C1R,IGHV3-15,IGHV3-72
GO Process	GO:0072378	Blood coagulation, fibrin clot formation	15	24	1.72	9.67e-17	F9,KLKB1,F13A1,FGB,F2,FBLN1,FGG,FN1,F13B,F5,FLNA,F10,F7,F11,FGA
GO Process	GO:0009611	Response to wounding	36	444	0.84	3.29e-16	CPB2,F9,PROC,VWF,SERPINA10,KLKB1,F13A1,DGKG,SERPING1,PF4,FGB,DST,F2,PLG,FBLN1,FGG,APOD,FN1,APOA4,F13B,F5,FLNA,ADAMTS13,F10,F7,PROS1,MMRN1,ILK,F11,SERPINA1,HGFAC,TSKU,PLCG2,KNG1,ACTB,FGA
GO Process	GO:0002682	Regulation of immune system process	60	1438	0.55	6.49e-15	C5,GPLD1,LTF,APOA1,CFP,APOE,BPIFB1,APCS,CFHR5,THBS1,C9,AMBP,HPX,FCN3,SERPING1,CSF1R,PIP,PSG6,PGLYRP2,PF4,MASP1,PSG1,PRG2,C1QB,CFHR1,CLU,C7,PSG3,THBS4,APOD,FN1,C8A,CFHR2,CFHR4,CFHR3,CFH,S100A7,ECM1,C8B,FCN1,C1QA,F7,CD99,CFI,MASP2,C1S,ORM2,C4A,MST1,C4B,CFB,RARRES2,C1R,CFD,IGHV3-15,IGHV3-72,VEGFC,PLCG2,ACTB,B2M
GO Process	GO:0010466	Negative regulation of peptidase activity	27	249	0.96	1.40e-14	SPP2,C5,LTF,LRP1,SERPINF1,THBS1,SERPINA10,LCN1,CSTA,AMBP,ITIH1,AHSG,SERPING1,SERPINI1,A2ML1,SERPINF2,SERPINA7,SERPINA11,SLPI,ECM1,PROS1,C4A,ITIH3,C4B,SERPINA1,SERPINA4,KNG1
GO Process	GO:0051346	Negative regulation of hydrolase activity	31	354	0.87	1.40e-14	SPP2,C5,LTF,APOA1,LRP1,SERPINF1,APCS,THBS1,SERPINA10,LCN1,CSTA,AMBP,ITIH1,AHSG,SERPING1,SERPINI1,A2ML1,SLC27A4,ANGPTL4,SERPINF2,SERPINA7,SERPINA11,SLPI,ECM1,PROS1,C4A,ITIH3,C4B,SERPINA1,SERPINA4,KNG1
GO Process	GO:0048583	Regulation of response to stimulus	103	3931	0.35	4.47e-14	ST6GAL1,CPB2,CHGA,C5,GPLD1,LTF,IGFBP5,PROC,APOA1,CAT,LRP1,CFP,APOE,BPIFB1,SERPINF1,APCS,CFHR5,THBS1,VWF,PSMA6,C9,KLKB1,PPP2R3A,DGKG,AMBP,HPX,INHBE,FCN3,AHSG,IGFBP1,SERPING1,CSF1R,PGLYRP2,PF4,MASP1,PDIA3,SLC27A4,PCSK9,FGB,YWHAG,F2,INHBC,PLG,CSPG4,C1QB,CFHR1,CLU,SERPINF2,C7,P4HB,FBLN1,FGG,THBS4,APOD,FN1,AKAP9,C8A,CFHR2,CFHR4,CFHR3,CFH,S100A7,S100A9,HTRA1,ECM1,FLNA,C8B,FCN1,C1QA,F10,F7,IGFBP3,PROS1,CFI,MMRN1,YWHAZ,ILK,MASP2,F11,C1S,C4A,MST1,C4B,CFB,RARRES2,UIMC1,PDGFC,FBN2,TSKU,C1R,CFD,IGHV3-15,CCL18,IGHV3-72,VEGFC,SEMA3B,PLCG2,KNG1,ACTB,TRIM8,CDH5,B2M,FGA

GO Process	GO:0002376	Immune system process	72	2121	0.46	6.10e-14	ST6GAL1,CHGA,C5,GPLD1,LTF,LRP1,CFP,BPIFB1,APCS,CFHR5,THBS1,LYZ,C9,TCIRG1,HPX,FCN3,SERPING1,CSF1R,AZGP1,PGLYRP2,PF4,MASP1,CAMP,PDIA3,FGB,F2,PLG,PRG2,C1QB,CFHR1,CLU,APOL1,C7,ANG,TUBB,SLPI,HP,APOA4,C8A,CFHR2,CFHR4,CFHR3,CFH,S100A7,S100A9,FLNA,MCOLN2,C8B,FCN1,ADAMTS13,C1QA,CD99,CFI,MASP2,C1S,C4A,PRG4,C4B,CFB,RARRES2,JCHAIN,C1R,VIM,CFD,IGHV3-15,CCL18,IGHV3-72,PLCG2,KNG1,TRIM8,B2M,FGA
GO Process	GO:0048584	Positive regulation of response to stimulus	72	2131	0.46	7.52e-14	CPB2,CHGA,C5,GPLD1,LTF,IGFBP5,APOA1,CAT,CFP,APOE,CFHR5,THBS1,VWF,C9,PPP2R3A,HPX,INHBE,FCN3,SERPING1,CSF1R,MASP1,PDIA3,FGB,F2,INHBC,PLG,CSPG4,C1QB,CFHR1,CLU,SERPINF2,C7,FGG,THBS4,FN1,C8A,CFHR2,CFHR4,CFHR3,CFH,S100A7,S100A9,ECM1,FLNA,C8B,FCN1,C1QA,F10,F7,IGFBP3,CFI,ILK,MASP2,C1S,C4A,C4B,CFB,RARRES2,UIMC1,PDGFC,C1R,CFD,IGHV3-15,CCL18,IGHV3-72,VEGFC,PLCG2,ACTB,TRIM8,CDH5,B2M,FGA
GO Process	GO:0009605	Response to external stimulus	76	2355	0.44	8.75e-14	CHGA,C5,LTF,APOB,APOA1,CAT,CFP,APOE,BPIFB1,APCS,CFHR5,THBS1,LYZ,C9,HPX,FCN3,SERPING1,DSC2,CSF1R,PGLYRP2,PF4,MASP1,CAMP,SLC27A4,LALBA,PCSK9,FGB,F2,PRG2,STAB1,C1QB,CFHR1,CLU,APOL1,C7,ANG,TUBB,SLPI,HP,APOA4,C8A,CFHR2,CFH,F5,S100A7,S100A9,MCOLN2,C8B,FCN1,ADAMTS13,C1QA,F7,CFI,TNRC6A,MASP2,C1S,C4A,C4B,CFB,RARRES2,JCHAIN,C1R,VIM,RNASE4,TTN,CFD,IGHV3-15,CCL18,IGHV3-72,VEGFC,SEMA3B,PLCG2,KNG1,TRIM8,B2M,FGA
GO Process	GO:0045861	Negative regulation of proteolysis	29	339	0.86	2.35e-13	SPP2,CPB2,C5,LTF,LRP1,SERPINF1,THBS1,SERPINA10,LCN1,CSTA,AMBP,ITIH1,AHSG,SERPING1,SERPINI1,A2ML1,F2,SERPINF2,SERPINA7,SERPINA11,SLPI,ECM1,PROS1,C4A,ITIH3,C4B,SERPINA1,SERPINA4,KNG1
GO Process	GO:0010951	Negative regulation of endopeptidase activity	25	240	0.94	4.22e-13	SPP2,C5,LTF,SERPINF1,THBS1,SERPINA10,LCN1,CSTA,AMBP,ITIH1,AHSG,SERPING1,SERPINI1,A2ML1,SERPINF2,SERPINA7,SERPINA11,SLPI,PROS1,C4A,ITIH3,C4B,SERPINA1,SERPINA4,KNG1
GO Process	GO:0016064	Immunoglobulin mediated immune response	18	95	1.2	5.00e-13	C5,C9,TCIRG1,SERPING1,C1QB,CLU,C7,C8A,C8B,C1QA,CFI,MASP2,C1S,C4A,C4B,C1R,IGHV3-15,IGHV3-72
GO Process	GO:0061045	Negative regulation of wound healing	16	69	1.29	1.22e-12	CPB2,PROC,APOE,APCS,KLKB1,SERPING1,FGB,F2,PLG,SERPINF2,FGG,PROS1,MMRN1,F11,KNG1,FGA
GO Process	GO:0048518	Positive regulation of biological process	132	6207	0.25	1.39e-12	ST6GAL1,CPB2,CETP,CHGA,C5,GPLD1,SPARC,LTF,APOB,IGFBP5,PROC,APOA1,CAT,LRP1,CFP,APOE,SERPINF1,CFHR5,THBS1,VWF,C9,KLKB1,PPP2R3A,CENPE,TCIRG1,HPX,INHBE,LUM,FCN3,AHSG,ANKRD31,IGFBP1,SERPING1,CSF1R,PIP,SCGB3A1,SERPINI1,PF4,MASP1,CAMP,PDIA3,SLC27A4,ANGPTL4,PCSK9,BMP1,FGB,F2,INHBC,PLG,EFEMP2,PRG2,CSPG4,C1QB,CFHR1,CLU,SUZ12,SERPINF2,C7,P4HB,FBLN1,ANG,FGG,THBS4,FN1,BLM,HP,AKAP9,APOA4,C8A,CFHR2,CFHR4,CFHR3,CFH,ADAM12,S100A7,S100A9,HTRA1,ADAMTSL4,ECM1,FLNA,MCOLN2,SMARCA1,C8B,FCN1,C1QA,F10,F7,IGFBP3,CD99,CFI,MMRN1,TNRC6A,ILK,MASP2,F11,C1S,ORM2,C4A,SLAIN1,ABCA13,MST1,LMOD3,C4B,CFB,PAEP,RARRES2,TCAF1,UIMC1,PDGFC,FBN2,TSKU,JCHAIN,HPR,C1R,APOA5,ATP1A1,VIM,TTN,APOC4,CFD,IGHV3-15,CCL18,IGHV3-72,VEGFC,SEMA3B,PLCG2,KNG1,ACTB,TRIM8,CDH5,B2M,FGA
GO Process	GO:0030193	Regulation of blood coagulation	16	70	1.29	1.40e-12	CPB2,PROC,APOE,THBS1,KLKB1,SERPING1,FGB,F2,PLG,SERPINF2,FGG,F7,PROS1,F11,KNG1,FGA
GO Process	GO:0002684	Positive regulation of immune system process	43	874	0.62	1.91e-12	C5,GPLD1,CFP,CFHR5,THBS1,C9,HPX,FCN3,SERPING1,CSF1R,PF4,MASP1,C1QB,CFHR1,CLU,C7,THBS4,C8A,CFHR2,CFHR4,CFHR3,CFH,S100A7,C8B,FCN1,C1QA,F7,CD99,CFI,MASP2,C1S,C4A,C4B,CFB,RARRES2,C1R,CFD,IGHV3-15,IGHV3-72,VEGFC,PLCG2,ACTB,B2M
GO Process	GO:0050778	Positive regulation of immune response	33	502	0.74	1.99e-12	C5,GPLD1,CFP,CFHR5,C9,HPX,FCN3,SERPING1,MASP1,C1QB,CFHR1,CLU,C7,C8A,CFHR2,CFHR4,CFHR3,CFH,C8B,FCN1,C1QA,CFI,MASP2,C1S,C4A,C4B,CFB,C1R,CFD,IGHV3-15,IGHV3-72,PLCG2,B2M
GO Process	GO:0032101	Regulation of response to external stimulus	45	964	0.6	2.29e-12	ST6GAL1,CPB2,C5,LTF,PROC,APOA1,APOE,SERPINF1,APCS,THBS1,PSMA6,KLKB1,HPX,FCN3,AHSG,SERPING1,CSF1R,PGLYRP2,MASP1,FGB,F2,PLG,SERPINF2,FGG,THBS4,CFH,S100A7,S100A9,HTRA1,FLNA,FCN1,F7,PROS1,CFI,MMRN1,MASP2,F11,MST1,RARRES2,VEGFC,SEMA3B,PLCG2,KNG1,CDH5,FGA
GO Process	GO:0030195	Negative regulation of blood coagulation	14	46	1.41	2.36e-12	CPB2,PROC,APOE,KLKB1,SERPING1,FGB,F2,PLG,SERPINF2,FGG,PROS1,F11,KNG1,FGA
GO Process	GO:0065007	Biological regulation	199	12385	0.13	9.58e-12	SPP2,ST6GAL1,CPB2,CETP,CHGA,TPD52L2,F9,C5,MGP,GPLD1,SPARC,LTF,APOB,IGFBP5,PROC,APOA1,TTR,CAT,LRP1,CFP,APOE,BPIFB1,SERPINF1,APCS,CFHR5,THBS1,LYZ,VWF,PSMA6,SERPINA10,LGALS3BP,ATRN,C9,LCN1,LCAT,CSTA,CP,KLKB1,F13A1,PPP2R3A,DGKG,CDH6,AMBP,CENPE,TCIRG1,HPX,INHBE,LUM,FCN3,ITIH1,AHSG,ANKRD31,IGFBP1,SERPING1,DSC2,CSF1R,PIP,PSG6,AZGP1,PGLYRP2,SCGB3A1,SERPINI1,PF4,MASP1,CAMP,A2ML1,PDIA3,SLC27A4,LALBA,ANGPTL4,PCSK9,BMP1,FGB,YWHAG,DST,F2,INHBC,PLG,PSG1,EFEMP2,PRG2,CSPG4,STAB1,C1QB,CFHR1,CLU,SUZ12,CPN2,SERPINF2,C7,KDM3B,P4HB,SERPINA7,PAPPA,FBLN1,PSG3,OIT3,SERPINA11,ANG,FGG,TUBB,THBS4,SLPI,APOD,FN1,ASGR2,BLM,HP,AKAP9,APOA4,CNDP1,COL18A1,C8A,F13B,CFHR2,CFHR4,CFHR3,CFH,F5,ADAM12,S100A7,S100A9,HTRA1,ADAMTSL4,ECM1,FLNA,MCOLN2,SMARCA1,C8B,FCN1,ADAMTS13,CCN5,SVEP1,C1QA,F10,F7,APOM,ADGRG2,CALML5,IGFBP3,CD99,SOD3,PROS1,EFEMP1,CFI,MMRN1,TNRC6A,YWHAZ,NAV2,ILK,MASP2,F11,C1S,BZW1,ORM2,C4A,ATP9B,SLAIN1,ABCA13,MST1,LMOD3,ITIH3,C4B,SERPINA1,TGFBI,CFB,IGFALS,PAEP,RARRES2,TCAF1,HGFAC,UIMC1,PDGFC,FBN2,SELENOP,TSKU,JCHAIN,HPR,C1R,APOA5,ATP1A1,VIM,SERPINA4,TTN,APOC4,CFD,IGHV3-15,CCL18,IGHV3-72,VEGFC,SEMA3B,PLCG2,OR52B4,KNG1,ACTB,TRIM8,CDH5,B2M,FGA
GO Process	GO:0002449	Lymphocyte mediated immunity	20	159	1.03	9.86e-12	C5,C9,TCIRG1,SERPING1,C1QB,CLU,C7,TUBB,C8A,C8B,C1QA,CFI,MASP2,C1S,C4A,C4B,C1R,IGHV3-15,IGHV3-72,B2M
GO Process	GO:0002443	Leukocyte mediated immunity	22	211	0.95	1.58e-11	CHGA,C5,C9,TCIRG1,SERPING1,F2,C1QB,CLU,C7,TUBB,C8A,C8B,C1QA,CFI,MASP2,C1S,C4A,C4B,C1R,IGHV3-15,IGHV3-72,B2M
GO Process	GO:0052547	Regulation of peptidase activity	30	446	0.75	1.76e-11	SPP2,C5,LTF,LRP1,SERPINF1,THBS1,SERPINA10,LCN1,CSTA,AMBP,ITIH1,AHSG,SERPING1,SERPINI1,A2ML1,SERPINF2,SERPINA7,FBLN1,SERPINA11,SLPI,FN1,S100A9,ECM1,PROS1,C4A,ITIH3,C4B,SERPINA1,SERPINA4,KNG1
GO Process	GO:0031638	Zymogen activation	14	59	1.3	3.64e-11	F9,KLKB1,FGB,F2,FGG,HP,F5,F10,F7,F11,HGFAC,HPR,C1R,FGA
GO Process	GO:0061041	Regulation of wound healing	18	130	1.07	4.09e-11	CPB2,PROC,APOE,APCS,THBS1,KLKB1,SERPING1,FGB,F2,PLG,SERPINF2,FGG,F7,PROS1,MMRN1,F11,KNG1,FGA
GO Process	GO:0006957	Complement activation, alternative pathway	10	16	1.72	5.14e-11	C5,CFP,CFHR5,C9,C7,C8A,CFH,C8B,CFB,CFD
GO Process	GO:1903034	Regulation of response to wounding	19	162	1.0	1.21e-10	CPB2,PROC,APOE,APCS,THBS1,KLKB1,SERPING1,FGB,F2,PLG,SERPINF2,FGG,FLNA,F7,PROS1,MMRN1,F11,KNG1,FGA
GO Process	GO:0042730	Fibrinolysis	10	19	1.65	1.81e-10	CPB2,KLKB1,SERPING1,FGB,F2,PLG,SERPINF2,FGG,PROS1,FGA
GO Process	GO:0050776	Regulation of immune response	39	844	0.59	1.92e-10	C5,GPLD1,LTF,CFP,APOE,BPIFB1,CFHR5,C9,AMBP,HPX,FCN3,SERPING1,PGLYRP2,MASP1,C1QB,CFHR1,CLU,C7,C8A,CFHR2,CFHR4,CFHR3,CFH,ECM1,C8B,FCN1,C1QA,CFI,MASP2,C1S,C4A,C4B,CFB,C1R,CFD,IGHV3-15,IGHV3-72,PLCG2,B2M

GO Process	GO:0002460	Adaptive immune response based on somatic recombination of immune receptors built from immunoglobulin superfamily domains	19	169	0.98	2.28e-10	C5,C9,TCIRG1,SERPING1,C1QB,CLU,C7,C8A,C8B,C1QA,CFI,MASP2,C1S,C4A,C4B,C1R,IGHV3-15,IGHV3-72,B2M
GO Process	GO:0006954	Inflammatory response	31	538	0.69	2.68e-10	C5,LRP1,APCS,THBS1,LYZ,ATRN,KLKB1,TCIRG1,AHSG,SAA4,CSF1R,PF4,CAMP,F2,STAB1,CLU,SERPINF2,FN1,HP,S100A9,ECM1,C1QA,F7,ORM2,C4A,C4B,SERPINA1,RARRES2,HPR,CCL18,KNG1
GO Process	GO:0030162	Regulation of proteolysis	36	739	0.61	3.67e-10	SPP2,CPB2,C5,GPLD1,LTF,LRP1,APOE,SERPINF1,THBS1,SERPINA10,LCN1,CSTA,KLKB1,AMBP,ITIH1,AHSG,SERPING1,SERPINI1,A2ML1,F2,CLU,SERPINF2,SERPINA7,FBLN1,SERPINA11,SLPI,FN1,S100A9,ECM1,PROS1,C4A,ITIH3,C4B,SERPINA1,SERPINA4,KNG1
GO Process	GO:0042742	Defense response to bacterium	23	306	0.8	1.87e-09	CHGA,LTF,CFP,LYZ,PGLYRP2,CAMP,LALBA,FGB,F2,PRG2,STAB1,ANG,SLPI,HP,S100A7,S100A9,RARRES2,JCHAIN,RNASE4,IGHV3-15,IGHV3-72,B2M,FGA
GO Process	GO:0052548	Regulation of endopeptidase activity	26	414	0.73	3.30e-09	SPP2,C5,LTF,SERPINF1,THBS1,SERPINA10,LCN1,CSTA,AMBP,ITIH1,AHSG,SERPING1,SERPINI1,A2ML1,SERPINF2,SERPINA7,SERPINA11,SLPI,S100A9,PROS1,C4A,ITIH3,C4B,SERPINA1,SERPINA4,KNG1
GO Process	GO:0032102	Negative regulation of response to external stimulus	25	387	0.74	4.67e-09	ST6GAL1,CPB2,C5,LTF,PROC,APOA1,APOE,SERPINF1,APCS,THBS1,KLKB1,SERPING1,FGB,F2,PLG,SERPINF2,FGG,HTRA1,PROS1,MMRN1,F11,SEMA3B,KNG1,CDH5,FGA
GO Process	GO:0043086	Negative regulation of catalytic activity	35	771	0.58	4.82e-09	SPP2,C5,LTF,APOA1,LRP1,APOE,SERPINF1,APCS,THBS1,SERPINA10,LCN1,CSTA,AMBP,ITIH1,AHSG,SERPING1,SERPINI1,A2ML1,SLC27A4,ANGPTL4,YWHAG,SERPINF2,SERPINA7,SERPINA11,SLPI,HP,AKAP9,ECM1,PROS1,C4A,ITIH3,C4B,SERPINA1,SERPINA4,KNG1
GO Process	GO:0051248	Negative regulation of protein metabolic process	41	1038	0.52	4.84e-09	SPP2,CPB2,C5,LTF,IGFBP5,LRP1,APOE,SERPINF1,APCS,THBS1,SERPINA10,LCN1,CSTA,AMBP,ITIH1,AHSG,SERPING1,SERPINI1,A2ML1,YWHAG,F2,CLU,SERPINF2,SERPINA7,FBLN1,SERPINA11,ANG,SLPI,APOD,ECM1,FLNA,IGFBP3,PROS1,TNRC6A,C4A,ITIH3,C4B,SERPINA1,SERPINA4,KNG1,FGA
GO Process	GO:0050789	Regulation of biological process	186	11655	0.13	4.87e-09	SPP2,ST6GAL1,CPB2,CETP,CHGA,TPD52L2,C5,MGP,GPLD1,SPARC,LTF,APOB,IGFBP5,PROC,APOA1,TTR,CAT,LRP1,CFP,APOE,BPIFB1,SERPINF1,APCS,CFHR5,THBS1,VWF,PSMA6,SERPINA10,LGALS3BP,ATRN,C9,LCN1,LCAT,CSTA,KLKB1,PPP2R3A,DGKG,CDH6,AMBP,CENPE,TCIRG1,HPX,INHBE,LUM,FCN3,ITIH1,AHSG,ANKRD31,IGFBP1,SERPING1,DSC2,CSF1R,PIP,PSG6,AZGP1,PGLYRP2,SCGB3A1,SERPINI1,PF4,MASP1,CAMP,A2ML1,PDIA3,SLC27A4,LALBA,ANGPTL4,PCSK9,BMP1,FGB,YWHAG,DST,F2,INHBC,PLG,PSG1,EFEMP2,PRG2,CSPG4,STAB1,C1QB,CFHR1,CLU,SUZ12,SERPINF2,C7,KDM3B,P4HB,SERPINA7,PAPPA,FBLN1,PSG3,SERPINA11,ANG,FGG,TUBB,THBS4,SLPI,APOD,FN1,ASGR2,BLM,HP,AKAP9,APOA4,COL18A1,C8A,CFHR2,CFHR4,CFHR3,CFH,ADAM12,S100A7,S100A9,HTRA1,ADAMTSL4,ECM1,FLNA,MCOLN2,SMARCA1,C8B,FCN1,ADAMTS13,CCN5,SVEP1,C1QA,F10,F7,APOM,ADGRG2,CALML5,IGFBP3,CD99,PROS1,EFEMP1,CFI,MMRN1,TNRC6A,YWHAZ,ILK,MASP2,F11,C1S,BZW1,ORM2,C4A,SLAIN1,ABCA13,MST1,LMOD3,ITIH3,C4B,SERPINA1,TGFBI,CFB,IGFALS,PAEP,RARRES2,TCAF1,UIMC1,PDGFC,FBN2,SELENOP,TSKU,JCHAIN,HPR,C1R,APOA5,ATP1A1,VIM,SERPINA4,TTN,APOC4,CFD,IGHV3-15,CCL18,IGHV3-72,VEGFC,SEMA3B,PLCG2,OR52B4,KNG1,ACTB,TRIM8,CDH5,B2M,FGA
GO Process	GO:0044092	Negative regulation of molecular function	43	1143	0.5	6.36e-09	SPP2,C5,LTF,APOA1,CAT,LRP1,APOE,SERPINF1,APCS,CFHR5,THBS1,SERPINA10,LCN1,CSTA,AMBP,ITIH1,AHSG,SERPING1,SERPINI1,A2ML1,SLC27A4,ANGPTL4,PCSK9,YWHAG,CFHR1,SERPINF2,SERPINA7,SERPINA11,SLPI,HP,AKAP9,CFHR2,ECM1,FLNA,PROS1,C4A,ITIH3,C4B,SERPINA1,SERPINA4,KNG1,ACTB,B2M
GO Process	GO:0051246	Regulation of protein metabolic process	70	2622	0.35	7.67e-09	SPP2,CPB2,C5,GPLD1,LTF,IGFBP5,APOA1,LRP1,APOE,SERPINF1,APCS,THBS1,SERPINA10,LCN1,CSTA,KLKB1,PPP2R3A,AMBP,CENPE,HPX,INHBE,ITIH1,AHSG,SERPING1,CSF1R,SERPINI1,CAMP,A2ML1,PCSK9,YWHAG,F2,INHBC,CSPG4,CLU,SERPINF2,SERPINA7,FBLN1,SERPINA11,ANG,THBS4,SLPI,APOD,FN1,BLM,AKAP9,S100A9,ECM1,FLNA,IGFBP3,PROS1,TNRC6A,ILK,BZW1,C4A,MST1,ITIH3,C4B,SERPINA1,PAEP,RARRES2,PDGFC,VIM,SERPINA4,TTN,VEGFC,PLCG2,KNG1,ACTB,CDH5,FGA
GO Process	GO:0051241	Negative regulation of multicellular organismal process	40	1035	0.51	1.57e-08	CPB2,SPARC,LTF,IGFBP5,PROC,APOA1,APOE,SERPINF1,APCS,THBS1,KLKB1,AHSG,SERPING1,PGLYRP2,PF4,PCSK9,FGB,F2,PLG,PRG2,STAB1,SERPINF2,FBLN1,FGG,THBS4,APOD,FN1,APOA4,ECM1,APOM,PROS1,EFEMP1,F11,PAEP,TSKU,ATP1A1,SEMA3B,KNG1,B2M,FGA
GO Process	GO:0019730	Antimicrobial humoral response	16	149	0.96	2.37e-08	LTF,LYZ,PF4,CAMP,FGB,F2,ANG,SLPI,S100A7,S100A9,RARRES2,JCHAIN,CCL18,KNG1,B2M,FGA
GO Process	GO:0006508	Proteolysis	44	1247	0.47	2.51e-08	CPB2,F9,LTF,PROC,PSMA6,LCN1,KLKB1,FCN3,HABP2,PIP,MASP1,APEH,PCSK9,BMP1,FGB,F2,PLG,UBE3C,PAPPA,FGG,HP,CNDP1,CFH,F5,ADAM12,HTRA1,FCN1,ADAMTS13,F10,F7,CFI,MASP2,F11,C1S,PCYOX1,MST1,CFB,HGFAC,UIMC1,HPR,C1R,CFD,PAMR1,FGA
GO Process	GO:0051336	Regulation of hydrolase activity	39	1011	0.51	2.85e-08	SPP2,C5,GPLD1,LTF,APOA1,LRP1,SERPINF1,APCS,THBS1,SERPINA10,LCN1,CSTA,AMBP,ITIH1,AHSG,SERPING1,SERPINI1,A2ML1,SLC27A4,ANGPTL4,SERPINF2,SERPINA7,FBLN1,SERPINA11,ANG,SLPI,FN1,APOA4,S100A9,ECM1,PROS1,C4A,ITIH3,C4B,SERPINA1,APOA5,SERPINA4,CCL18,KNG1
GO Process	GO:0002250	Adaptive immune response	23	359	0.73	3.15e-08	C5,C9,TCIRG1,SERPING1,FGB,C1QB,CLU,C7,C8A,MCOLN2,C8B,C1QA,CFI,MASP2,C1S,C4A,C4B,JCHAIN,C1R,IGHV3-15,IGHV3-72,B2M,FGA
GO Process	GO:0009617	Response to bacterium	31	663	0.6	3.32e-08	CHGA,LTF,APOB,CFP,LYZ,PGLYRP2,PF4,CAMP,LALBA,FGB,F2,PRG2,STAB1,ANG,SLPI,HP,S100A7,S100A9,ADAMTS13,C4B,CFB,RARRES2,JCHAIN,VIM,RNASE4,CFD,IGHV3-15,IGHV3-72,PLCG2,B2M,FGA
GO Process	GO:1903028	Positive regulation of opsonization	8	16	1.63	4.05e-08	CFP,FCN3,MASP1,FCN1,MASP2,C4A,C4B,CFB
GO Process	GO:0065008	Regulation of biological quality	84	3654	0.29	6.37e-08	CPB2,CETP,CHGA,F9,GPLD1,LTF,APOB,IGFBP5,PROC,APOA1,TTR,APOE,SERPINF1,THBS1,LYZ,VWF,SERPINA10,LCN1,LCAT,CP,KLKB1,F13A1,DGKG,TCIRG1,HPX,SERPING1,DSC2,CSF1R,PIP,AZGP1,PF4,ANGPTL4,PCSK9,FGB,YWHAG,F2,PLG,CLU,CPN2,SERPINF2,SERPINA7,FBLN1,OIT3,ANG,FGG,TUBB,FN1,AKAP9,APOA4,F13B,F5,S100A9,FLNA,ADAMTS13,F10,F7,APOM,SOD3,PROS1,MMRN1,YWHAZ,NAV2,ILK,F11,ATP9B,LMOD3,SERPINA1,HGFAC,PDGFC,FBN2,TSKU,JCHAIN,APOA5,ATP1A1,VIM,APOC4,VEGFC,SEMA3B,PLCG2,KNG1,ACTB,CDH5,B2M,FGA
GO Process	GO:0030168	Platelet activation	13	97	1.05	1.07e-07	VWF,DGKG,PF4,FGB,F2,FGG,FN1,FLNA,ADAMTS13,ILK,PLCG2,ACTB,FGA
GO Process	GO:0002526	Acute inflammatory response	12	80	1.1	1.55e-07	APCS,KLKB1,AHSG,SAA4,F2,SERPINF2,FN1,HP,F7,ORM2,SERPINA1,HPR
GO Process	GO:0016485	Protein processing	17	202	0.85	1.75e-07	F9,KLKB1,PCSK9,BMP1,FGB,F2,FGG,HP,F5,ADAMTS13,F10,F7,F11,HGFAC,HPR,C1R,FGA
GO Process	GO:0051702	Biological process involved in interaction with symbiont	13	108	1.01	3.39e-07	LTF,APOE,APCS,CFHR5,CSF1R,PF4,CAMP,F2,PLG,CFHR1,APOL1,FN1,CFHR2
GO Process	GO:0051239	Regulation of multicellular organismal process	68	2749	0.32	3.46e-07	CPB2,CHGA,C5,MGP,GPLD1,SPARC,LTF,IGFBP5,PROC,APOA1,LRP1,APOE,SERPINF1,APCS,THBS1,ATRN,LCAT,KLKB1,LUM,AHSG,SERPING1,DSC2,CSF1R,PGLYRP2,PF4,CAMP,ANGPTL4,PCSK9,BMP1,FGB,F2,PLG,PRG2,STAB1,CLU,SERPINF2,FBLN1,FGG,THBS4,APOD,FN1,AKAP9,APOA4,ADAM12,ECM1,MCOLN2,FCN1,F7,APOM,PROS1,EFEMP1,F11,ORM2,MST1,PAEP,PDGFC,FBN2,TSKU,APOA5,ATP1A1,VEGFC,SEMA3B,PLCG2,KNG1,ACTB,CDH5,B2M,FGA

GO Process	GO:0048519	Negative regulation of biological process	105	5313	0.22	5.86e-07	SPP2,ST6GAL1,CPB2,CETP,CHGA,C5,GPLD1,SPARC,LTF,IGFBP5,PROC,APOA1,CAT,LRP1,APOE,BPIFB1,SERPINF1,APCS,THBS1,SERPINA10,LCN1,CSTA,KLKB1,PPP2R3A,DGKG,AMBP,FCN3,ITIH1,AHSG,IGFBP1,SERPING1,CSF1R,PIP,AZGP1,PGLYRP2,SCGB3A1,SERPINI1,PF4,MASP1,A2ML1,SLC27A4,ANGPTL4,PCSK9,FGB,YWHAG,F2,PLG,EFEMP2,PRG2,STAB1,CLU,SUZ12,SERPINF2,SERPINA7,FBLN1,SERPINA11,ANG,FGG,THBS4,SLPI,APOD,FN1,BLM,HP,APOA4,COL18A1,HTRA1,ECM1,FLNA,MCOLN2,FCN1,CCN5,APOM,IGFBP3,PROS1,EFEMP1,MMRN1,TNRC6A,YWHAZ,ILK,F11,C4A,MST1,LMOD3,ITIH3,C4B,SERPINA1,TGFBI,PAEP,TCAF1,UIMC1,FBN2,TSKU,ATP1A1,VIM,SERPINA4,VEGFC,SEMA3B,PLCG2,KNG1,ACTB,TRIM8,CDH5,B2M,FGA
GO Process	GO:0032501	Multicellular organismal process	120	6490	0.19	9.18e-07	SPP2,CPB2,CETP,CHGA,F9,C5,MGP,GPLD1,LTF,APOB,IGFBP5,PROC,APOA1,CAT,LRP1,APOE,ANGPTL6,SERPINF1,THBS1,LYZ,VWF,SERPINA10,ATRN,LCN1,LCAT,KLKB1,F13A1,PPP2R3A,DGKG,DNAH5,AMBP,TCIRG1,LUM,AHSG,SERPING1,SEC24D,DSC2,CSF1R,PIP,PSG6,AZGP1,SERPINI1,PF4,SLC27A4,ANGPTL4,PCSK9,BMP1,FGB,F2,PLG,PSG1,EFEMP2,CSPG4,CLU,SUZ12,SERPINF2,PAPPA,FBLN1,PSG3,ANG,FGG,THBS4,APOD,FN1,AKAP9,APOA4,COL18A1,F13B,F5,S100A7,S100A9,HTRA1,ECM1,FLNA,CRTAC1,SMARCA1,ADAMTS13,SVEP1,C1QA,F10,F7,APOM,CA6,ADGRG2,IGFBP3,SOD3,PROS1,EFEMP1,MMRN1,YWHAZ,NAV2,ILK,F11,PSG2,MST1,LMOD3,SERPINA1,TGFBI,RARRES2,HGFAC,PDGFC,FBN2,SELENOP,TSKU,JCHAIN,APOA5,ATP1A1,VIM,TTN,VEGFC,SEMA3B,PLCG2,OR52B4,KNG1,TPM4,ACTB,TRIM8,CDH5,B2M,FGA
GO Process	GO:0050766	Positive regulation of phagocytosis	11	75	1.09	9.19e-07	APOA1,CFP,FCN3,AHSG,MASP1,FCN1,MASP2,C4A,C4B,CFB,PLCG2
GO Process	GO:0006953	Acute-phase response	9	42	1.26	1.26e-06	APCS,AHSG,SAA4,F2,SERPINF2,FN1,HP,ORM2,SERPINA1
GO Process	GO:0034369	Plasma lipoprotein particle remodeling	8	29	1.37	1.61e-06	CETP,APOB,APOA1,APOE,LCAT,APOA4,APOM,APOA5
GO Process	GO:0034372	Very-low-density lipoprotein particle remodeling	6	9	1.75	1.93e-06	CETP,APOA1,APOE,LCAT,APOA4,APOA5
GO Process	GO:0043691	Reverse cholesterol transport	7	18	1.52	1.93e-06	CETP,APOA1,APOE,LCAT,CLU,APOA4,APOM
GO Process	GO:0051917	Regulation of fibrinolysis	7	18	1.52	1.93e-06	CPB2,THBS1,KLKB1,F2,PLG,SERPINF2,F11
GO Process	GO:0030301	Cholesterol transport	10	65	1.11	2.83e-06	CETP,APOB,APOA1,APOE,LCAT,CLU,APOA4,APOM,TSKU,APOA5
GO Process	GO:0051838	Cytolysis by host of symbiont cells	6	12	1.63	6.52e-06	CFHR5,CAMP,F2,CFHR1,APOL1,CFHR2
GO Process	GO:0050790	Regulation of catalytic activity	58	2370	0.32	1.03e-05	SPP2,C5,GPLD1,LTF,APOA1,LRP1,APOE,SERPINF1,APCS,THBS1,SERPINA10,LCN1,CSTA,PPP2R3A,AMBP,CENPE,ITIH1,AHSG,SERPING1,CSF1R,SERPINI1,A2ML1,SLC27A4,ANGPTL4,YWHAG,F2,CLU,SUZ12,CPN2,SERPINF2,SERPINA7,FBLN1,SERPINA11,ANG,SLPI,FN1,BLM,HP,AKAP9,APOA4,S100A9,ECM1,CALML5,IGFBP3,PROS1,C4A,MST1,ITIH3,C4B,SERPINA1,PDGFC,APOA5,SERPINA4,TTN,CCL18,VEGFC,KNG1,ACTB
GO Process	GO:0007155	Cell adhesion	33	965	0.46	1.07e-05	THBS1,VWF,LGALS3BP,ATRN,CSTA,CDH6,AMBP,HABP2,DSC2,AZGP1,FGB,DST,STAB1,FGG,THBS4,FN1,APOA4,COL18A1,ADAM12,S100A9,FLNA,ADAMTS13,CCN5,SVEP1,CD99,MMRN1,ILK,PSG2,TGFBI,IGFALS,ACTB,CDH5,FGA
GO Process	GO:0051050	Positive regulation of transport	32	915	0.47	1.07e-05	CPB2,CETP,GPLD1,APOA1,LRP1,CFP,APOE,FCN3,AHSG,MASP1,PCSK9,FGB,F2,CLU,ANG,FGG,AKAP9,FLNA,FCN1,MASP2,C4A,ABCA13,C4B,CFB,TCAF1,APOA5,TTN,VEGFC,PLCG2,ACTB,B2M,FGA
GO Process	GO:0065009	Regulation of molecular function	69	3085	0.28	1.19e-05	SPP2,C5,GPLD1,LTF,APOA1,CAT,LRP1,APOE,SERPINF1,APCS,CFHR5,THBS1,PSMA6,SERPINA10,LCN1,CSTA,PPP2R3A,AMBP,CENPE,ITIH1,AHSG,SERPING1,CSF1R,SERPINI1,A2ML1,SLC27A4,ANGPTL4,PCSK9,YWHAG,F2,CFHR1,CLU,SUZ12,CPN2,SERPINF2,SERPINA7,FBLN1,SERPINA11,ANG,SLPI,FN1,BLM,HP,AKAP9,APOA4,CFHR2,S100A9,ECM1,FLNA,CALML5,IGFBP3,PROS1,C4A,MST1,ITIH3,C4B,SERPINA1,TCAF1,PDGFC,APOA5,SERPINA4,TTN,CCL18,VEGFC,PLCG2,KNG1,ACTB,TRIM8,B2M
GO Process	GO:0019538	Protein metabolic process	81	3910	0.24	1.43e-05	ST6GAL1,CPB2,F9,GPLD1,LTF,APOB,PROC,APOA1,CAT,APOE,THBS1,PSMA6,LCN1,LCAT,CSTA,KLKB1,F13A1,PPP2R3A,AMBP,TCIRG1,HPX,FCN3,HABP2,CSF1R,PIP,MASP1,APEH,PCSK9,BMP1,FGB,F2,PLG,UBE3C,CLU,SUZ12,APOL1,KDM3B,P4HB,PAPPA,FBLN1,FGG,QPCT,FN1,HP,APOA4,CNDP1,F13B,CFH,F5,ADAM12,S100A9,HTRA1,FLNA,FCN1,ADAMTS13,F10,F7,APOM,IGFBP3,EFEMP1,CFI,YWHAZ,ILK,MASP2,F11,C1S,PCYOX1,MST1,CFB,HGFAC,UIMC1,HPR,C1R,APOA5,TTN,CFD,PAMR1,ACTB,TRIM8,B2M,FGA
GO Process	GO:0034109	Homotypic cell-cell adhesion	9	60	1.1	1.65e-05	DSC2,FGB,FGG,FN1,FLNA,CD99,ILK,ACTB,FGA
GO Process	GO:0033344	Cholesterol efflux	7	27	1.34	1.67e-05	APOB,APOA1,APOE,APOA4,APOM,TSKU,APOA5
GO Process	GO:1900024	Regulation of substrate adhesion-dependent cell spreading	9	61	1.1	1.81e-05	ST6GAL1,APOA1,FGB,P4HB,FBLN1,FGG,FLNA,ILK,FGA
GO Process	GO:0001944	Vasculature development	23	527	0.57	2.04e-05	GPLD1,APOB,LRP1,APOE,ANGPTL6,THBS1,ANGPTL4,PLG,EFEMP2,CSPG4,SERPINF2,ANG,APOD,FN1,COL18A1,S100A7,ECM1,FLNA,SVEP1,YWHAZ,TGFBI,VEGFC,CDH5
GO Process	GO:0034375	High-density lipoprotein particle remodeling	6	16	1.5	2.20e-05	CETP,APOA1,APOE,LCAT,APOA4,APOM
GO Process	GO:0080134	Regulation of response to stress	40	1373	0.39	2.25e-05	CPB2,PROC,APOA1,APOE,SERPINF1,APCS,THBS1,PSMA6,KLKB1,AMBP,HPX,FCN3,AHSG,SERPING1,PGLYRP2,MASP1,FGB,F2,PLG,CLU,SERPINF2,P4HB,FGG,CFH,S100A9,HTRA1,FLNA,FCN1,F7,PROS1,CFI,MMRN1,MASP2,F11,UIMC1,PLCG2,KNG1,ACTB,CDH5,FGA
GO Process	GO:0051873	Killing by host of symbiont cells	7	29	1.31	2.41e-05	CFHR5,PF4,CAMP,F2,CFHR1,APOL1,CFHR2
GO Process	GO:0048585	Negative regulation of response to stimulus	44	1612	0.36	2.84e-05	ST6GAL1,CPB2,C5,LTF,IGFBP5,PROC,APOA1,LRP1,APOE,BPIFB1,SERPINF1,APCS,THBS1,KLKB1,PPP2R3A,DGKG,AMBP,AHSG,IGFBP1,SERPING1,PGLYRP2,PF4,MASP1,SLC27A4,FGB,F2,PLG,CLU,SERPINF2,FBLN1,FGG,APOD,HTRA1,ECM1,IGFBP3,PROS1,MMRN1,F11,FBN2,TSKU,SEMA3B,KNG1,CDH5,FGA
GO Process	GO:0051240	Positive regulation of multicellular organismal process	42	1505	0.37	3.15e-05	CPB2,CHGA,C5,GPLD1,LTF,LRP1,APOE,SERPINF1,THBS1,LUM,CSF1R,PF4,CAMP,ANGPTL4,BMP1,FGB,F2,PLG,PRG2,CLU,SERPINF2,FGG,FN1,ADAM12,ECM1,MCOLN2,FCN1,F7,ORM2,MST1,PAEP,PDGFC,FBN2,TSKU,APOA5,ATP1A1,VEGFC,PLCG2,ACTB,CDH5,B2M,FGA
GO Process	GO:0042632	Cholesterol homeostasis	10	89	0.98	3.36e-05	CETP,APOB,APOA1,APOE,LCAT,PCSK9,APOA4,APOM,TSKU,APOA5
GO Process	GO:0048514	Blood vessel morphogenesis	20	419	0.61	3.37e-05	GPLD1,APOB,LRP1,APOE,ANGPTL6,THBS1,ANGPTL4,EFEMP2,CSPG4,SERPINF2,ANG,APOD,FN1,COL18A1,S100A7,ECM1,FLNA,YWHAZ,TGFBI,VEGFC
GO Process	GO:0001568	Blood vessel development	22	505	0.57	3.66e-05	GPLD1,APOB,LRP1,APOE,ANGPTL6,THBS1,ANGPTL4,PLG,EFEMP2,CSPG4,SERPINF2,ANG,APOD,FN1,COL18A1,S100A7,ECM1,FLNA,YWHAZ,TGFBI,VEGFC,CDH5
GO Process	GO:0030334	Regulation of cell migration	31	927	0.45	3.73e-05	C5,GPLD1,SPARC,IGFBP5,LRP1,APOE,SERPINF1,THBS1,PPP2R3A,CSF1R,PLG,FBLN1,THBS4,APOD,FN1,S100A7,ECM1,FLNA,F10,F7,IGFBP3,CD99,MST1,RARRES2,TCAF1,PDGFC,FBN2,VEGFC,SEMA3B,PLCG2,CDH5
GO Process	GO:0008203	Cholesterol metabolic process	11	119	0.89	4.93e-05	CETP,APOB,APOA1,CAT,APOE,LCAT,PCSK9,APOL1,APOA4,TSKU,APOA5
GO Process	GO:0010810	Regulation of cell-substrate adhesion	14	217	0.74	7.85e-05	ST6GAL1,APOA1,THBS1,FGB,PLG,EFEMP2,P4HB,FBLN1,FGG,APOD,FN1,FLNA,ILK,FGA
GO Process	GO:0006869	Lipid transport	17	329	0.64	9.28e-05	CETP,APOB,APOA1,APOE,LCAT,SLC27A4,CLU,APOL1,APOD,APOA4,CFHR4,APOM,ATP9B,ABCA13,TSKU,APOA5,APOC4
GO Process	GO:0031639	Plasminogen activation	5	11	1.58	0.00010	KLKB1,FGB,FGG,F11,FGA
GO Process	GO:0019835	Cytolysis	6	23	1.34	0.00011	C5,LYZ,C9,C7,C8A,C8B
GO Process	GO:0034377	Plasma lipoprotein particle assembly	6	23	1.34	0.00011	APOB,APOA1,APOE,APOA4,APOM,APOA5

GO Process	GO:0040012	Regulation of locomotion	32	1032	0.42	0.00011	ST6GAL1,C5,GPLD1,SPARC,IGFBP5,LRP1,APOE,SERPINF1,THBS1,PPP2R3A,CSF1R,PLG,FBLN1,THBS4,APOD,FN1,S100A7,ECM1,FLNA,F10,F7,IGFBP3,CD99,MST1,RARRES2,TCAF1,PDGFC,FBN2,VEGFC,SEMA3B,PLCG2,CDH5
GO Process	GO:0055088	Lipid homeostasis	12	161	0.8	0.00011	CETP,APOB,APOA1,APOE,LCAT,ANGPTL4,PCSK9,APOA4,APOM,TSKU,APOA5,APOC4
GO Process	GO:0070328	Triglyceride homeostasis	7	38	1.19	0.00011	CETP,APOA1,APOE,ANGPTL4,APOA4,APOA5,APOC4
GO Process	GO:0034380	High-density lipoprotein particle assembly	5	12	1.55	0.00014	APOA1,APOE,APOA4,APOM,APOA5
GO Process	GO:0007597	Blood coagulation, intrinsic pathway	5	13	1.51	0.00018	F9,KLKB1,FLNA,F7,F11
GO Process	GO:0051918	Negative regulation of fibrinolysis	5	13	1.51	0.00018	CPB2,THBS1,F2,PLG,SERPINF2
GO Process	GO:0050793	Regulation of developmental process	56	2492	0.28	0.00020	ST6GAL1,CETP,C5,MGP,SPARC,LTF,APOB,IGFBP5,PROC,APOA1,APOE,SERPINF1,APCS,THBS1,ATRN,DGKG,TCIRG1,AHSG,CSF1R,PGLYRP2,SCGB3A1,PF4,CAMP,ANGPTL4,BMP1,FGB,YWHAG,F2,EFEMP2,STAB1,SUZ12,SERPINF2,P4HB,FBLN1,FGG,THBS4,FN1,ADAM12,ECM1,FLNA,IGFBP3,EFEMP1,YWHAZ,ILK,MST1,LMOD3,PAEP,RARRES2,FBN2,TSKU,VEGFC,SEMA3B,ACTB,CDH5,B2M,FGA
GO Process	GO:0070527	Platelet aggregation	7	43	1.14	0.00020	FGB,FGG,FN1,FLNA,ILK,ACTB,FGA
GO Process	GO:0061844	Antimicrobial humoral immune response mediated by antimicrobial peptide	10	113	0.87	0.00021	LTF,PF4,CAMP,F2,ANG,S100A7,S100A9,CCL18,KNG1,B2M
GO Process	GO:0019731	Antibacterial humoral response	8	64	1.02	0.00022	LTF,CAMP,FGB,ANG,SLPI,JCHAIN,B2M,FGA
GO Process	GO:0032374	Regulation of cholesterol transport	8	64	1.02	0.00022	CETP,APOA1,LRP1,APOE,PCSK9,APOA4,ABCA13,APOA5
GO Process	GO:0072377	Blood coagulation, common pathway	4	5	1.83	0.00024	F2,F5,F10,FGA
GO Process	GO:1900026	Positive regulation of substrate adhesion-dependent cell spreading	7	45	1.12	0.00025	APOA1,FGB,P4HB,FGG,FLNA,ILK,FGA
GO Process	GO:0030194	Positive regulation of blood coagulation	6	28	1.26	0.00026	CPB2,THBS1,F2,PLG,SERPINF2,F7
GO Process	GO:0032103	Positive regulation of response to external stimulus	19	453	0.55	0.00033	CPB2,THBS1,HPX,FCN3,CSF1R,MASP1,F2,PLG,SERPINF2,THBS4,S100A7,S100A9,FLNA,FCN1,F7,MASP2,RARRES2,VEGFC,PLCG2
GO Process	GO:0048522	Positive regulation of cellular process	99	5584	0.18	0.00033	ST6GAL1,CPB2,CHGA,GPLD1,SPARC,LTF,APOB,IGFBP5,PROC,APOA1,CAT,LRP1,CFP,APOE,SERPINF1,THBS1,VWF,PPP2R3A,CENPE,TCIRG1,HPX,INHBE,LUM,FCN3,ANKRD31,IGFBP1,CSF1R,SCGB3A1,SERPINI1,PF4,MASP1,CAMP,PDIA3,SLC27A4,PCSK9,FGB,F2,INHBC,PLG,EFEMP2,CSPG4,CLU,SUZ12,SERPINF2,P4HB,FBLN1,ANG,FGG,THBS4,FN1,BLM,HP,AKAP9,APOA4,S100A7,S100A9,HTRA1,ADAMTSL4,ECM1,FLNA,SMARCA1,FCN1,C1QA,F10,F7,IGFBP3,CD99,MMRN1,TNRC6A,ILK,MASP2,C4A,SLAIN1,ABCA13,MST1,LMOD3,C4B,CFB,RARRES2,TCAF1,UIMC1,PDGFC,FBN2,HPR,APOA5,VIM,TTN,IGHV3-15,CCL18,IGHV3-72,VEGFC,SEMA3B,PLCG2,KNG1,ACTB,TRIM8,CDH5,B2M,FGA
GO Process	GO:0042157	Lipoprotein metabolic process	10	123	0.84	0.00039	GPLD1,APOB,APOA1,APOE,LCAT,PCSK9,APOL1,APOA4,APOM,APOA5
GO Process	GO:0044403	Biological process involved in symbiotic interaction	14	257	0.66	0.00041	LTF,APOE,APCS,CFHR5,CSF1R,PGLYRP2,PF4,CAMP,F2,PLG,CFHR1,APOL1,FN1,CFHR2
GO Process	GO:0060627	Regulation of vesicle-mediated transport	21	551	0.51	0.00041	APOA1,LRP1,CFP,APOE,FCN3,AHSG,MASP1,PCSK9,FGB,CLU,FGG,FCN1,MASP2,C4A,ABCA13,C4B,CFB,APOA5,PLCG2,B2M,FGA
GO Process	GO:0002697	Regulation of immune effector process	17	383	0.57	0.00052	APOA1,CFP,HPX,FCN3,SERPING1,PGLYRP2,MASP1,PRG2,CFH,FCN1,CFI,MASP2,C4A,C4B,CFB,PLCG2,B2M
GO Process	GO:0010811	Positive regulation of cell-substrate adhesion	10	128	0.82	0.00052	APOA1,FGB,EFEMP2,P4HB,FBLN1,FGG,FN1,FLNA,ILK,FGA
GO Process	GO:0001906	Cell killing	10	129	0.82	0.00055	CHGA,LTF,LYZ,C9,PF4,F2,TUBB,CCL18,KNG1,B2M
GO Process	GO:0035239	Tube morphogenesis	23	669	0.46	0.00069	GPLD1,APOB,LRP1,APOE,ANGPTL6,THBS1,CSF1R,ANGPTL4,EFEMP2,CSPG4,SERPINF2,ANG,APOD,FN1,COL18A1,S100A7,ECM1,FLNA,YWHAZ,ILK,MST1,TGFBI,VEGFC
GO Process	GO:1901700	Response to oxygen-containing compound	39	1547	0.33	0.00071	ST6GAL1,CPB2,GPLD1,APOB,IGFBP5,CAT,LRP1,APOE,SERPINF1,THBS1,AMBP,IGFBP1,PF4,CAMP,SLC27A4,PCSK9,YWHAG,PAPPA,SLPI,APOD,BLM,HP,AKAP9,APOA4,F5,S100A7,S100A9,FLNA,ADAMTS13,F7,SOD3,GPX3,RARRES2,PDGFC,ATP1A1,VIM,PLCG2,ACTB,B2M
GO Process	GO:0001895	Retina homeostasis	8	78	0.94	0.00073	LTF,LYZ,LCN1,PIP,AZGP1,JCHAIN,ACTB,B2M
GO Process	GO:0035295	Tube development	27	880	0.41	0.00079	GPLD1,APOB,IGFBP5,CAT,LRP1,APOE,ANGPTL6,THBS1,CSF1R,ANGPTL4,EFEMP2,CSPG4,SERPINF2,ANG,APOD,FN1,COL18A1,S100A7,ECM1,FLNA,YWHAZ,ILK,MST1,TGFBI,RARRES2,PDGFC,VEGFC
GO Process	GO:1905952	Regulation of lipid localization	11	167	0.75	0.00079	CETP,APOB,APOA1,LRP1,APOE,THBS1,PCSK9,APOA4,ABCA13,APOA5,APOC4
GO Process	GO:0001932	Regulation of protein phosphorylation	31	1108	0.37	0.00097	LTF,APOA1,LRP1,APOE,THBS1,CENPE,HPX,INHBE,CSF1R,CAMP,YWHAG,F2,INHBC,CSPG4,CLU,FBLN1,ANG,THBS4,FN1,BLM,AKAP9,IGFBP3,ILK,MST1,RARRES2,PDGFC,TTN,VEGFC,PLCG2,ACTB,CDH5
GO Process	GO:1901564	Organonitrogen compound metabolic process	89	4981	0.18	0.0010	ST6GAL1,CPB2,CETP,F9,GPLD1,LTF,APOB,PROC,APOA1,TTR,CAT,APOE,THBS1,PSMA6,LCN1,LCAT,CSTA,KLKB1,F13A1,PPP2R3A,AMBP,TCIRG1,HPX,FCN3,ITIH1,HABP2,CSF1R,PIP,PGLYRP2,MASP1,APEH,PCSK9,BMP1,FGB,F2,PLG,UBE3C,CLU,NDUFS8,SUZ12,APOL1,KDM3B,P4HB,PAPPA,FBLN1,FGG,QPCT,FN1,HP,APOA4,CNDP1,F13B,CFH,F5,ADAM12,S100A9,HTRA1,FLNA,FCN1,ADAMTS13,F10,F7,APOM,IGFBP3,EFEMP1,CFI,YWHAZ,ILK,MASP2,F11,C1S,PCYOX1,ACSF2,CKMT1A,MST1,ITIH3,CFB,HGFAC,UIMC1,HPR,C1R,APOA5,TTN,CFD,PAMR1,ACTB,TRIM8,B2M,FGA
GO Process	GO:0001525	Angiogenesis	15	325	0.59	0.0011	GPLD1,ANGPTL6,THBS1,ANGPTL4,CSPG4,ANG,APOD,FN1,COL18A1,S100A7,ECM1,FLNA,YWHAZ,TGFBI,VEGFC
GO Process	GO:0002752	Cell surface pattern recognition receptor signaling pathway	4	9	1.57	0.0011	FCN3,MASP1,FCN1,MASP2
GO Process	GO:0043903	Regulation of biological process involved in symbiotic interaction	7	60	0.99	0.0012	LTF,APCS,FCN3,P4HB,FBLN1,FCN1,TRIM8
GO Process	GO:0071634	Regulation of transforming growth factor beta production	6	39	1.11	0.0012	THBS1,LUM,SERPINF2,FBLN1,FN1,TSKU
GO Process	GO:0060284	Regulation of cell development	19	506	0.5	0.0013	LTF,PROC,APOA1,SERPINF1,FGB,F2,P4HB,FBLN1,FGG,FN1,FLNA,ILK,LMOD3,PAEP,VEGFC,SEMA3B,CDH5,B2M,FGA
GO Process	GO:0050832	Defense response to fungus	7	62	0.98	0.0014	CHGA,LTF,CAMP,ANG,S100A9,RARRES2,PLCG2
GO Process	GO:0001867	Complement activation, lectin pathway	4	10	1.53	0.0015	FCN3,MASP1,FCN1,MASP2
GO Process	GO:0030335	Positive regulation of cell migration	19	529	0.48	0.0023	GPLD1,SPARC,IGFBP5,THBS1,CSF1R,PLG,FBLN1,THBS4,FN1,S100A7,F10,F7,CD99,RARRES2,PDGFC,VEGFC,SEMA3B,PLCG2,CDH5
GO Process	GO:0010035	Response to inorganic substance	19	532	0.48	0.0024	GPLD1,APOB,CAT,SERPINF1,THBS1,LCAT,TCIRG1,FGB,FGG,BLM,HP,APOA4,ADAMTS13,SOD3,SELENOP,TTN,PLCG2,B2M,FGA
GO Process	GO:1902533	Positive regulation of intracellular signal transduction	28	997	0.38	0.0024	LTF,IGFBP5,APOA1,CAT,APOE,THBS1,VWF,CSF1R,FGB,F2,CSPG4,CLU,SERPINF2,FGG,FN1,S100A7,S100A9,ECM1,FLNA,F10,F7,IGFBP3,ILK,PDGFC,CCL18,PLCG2,TRIM8,FGA
GO Process	GO:0006639	Acylglycerol metabolic process	8	95	0.85	0.0025	CETP,APOB,CAT,APOE,DGKG,PCSK9,ANG,APOA5
GO Process	GO:0002685	Regulation of leukocyte migration	12	231	0.64	0.0026	C5,THBS1,CSF1R,THBS4,APOD,S100A7,ECM1,F7,CD99,MST1,RARRES2,VEGFC
GO Process	GO:0019732	Antifungal humoral response	4	12	1.45	0.0026	LTF,CAMP,ANG,RARRES2
GO Process	GO:0033700	Phospholipid efflux	4	12	1.45	0.0026	APOA1,APOE,APOA4,APOA5
GO Process	GO:0001817	Regulation of cytokine production	23	739	0.42	0.0028	C5,LTF,APOA1,THBS1,LUM,CSF1R,PGLYRP2,PF4,CAMP,F2,PRG2,CLU,SERPINF2,FBLN1,APOD,FN1,MCOLN2,FCN1,ORM2,PAEP,TSKU,PLCG2,B2M
GO Process	GO:0031347	Regulation of defense response	21	638	0.44	0.0028	PROC,APOA1,APOE,SERPINF1,APCS,PSMA6,KLKB1,HPX,FCN3,AHSG,SERPING1,PGLYRP2,MASP1,CFH,S100A9,HTRA1,FCN1,CFI,MASP2,PLCG2,CDH5
GO Process	GO:0050829	Defense response to Gram-negative bacterium	8	97	0.84	0.0028	CHGA,LTF,LYZ,CAMP,F2,S100A7,RARRES2,B2M

GO Process	GO:0002688	Regulation of leukocyte chemotaxis	9	128	0.77	0.0029	C5,THBS1,CSF1R,THBS4,S100A7,F7,MST1,RARRES2,VEGFC
GO Process	GO:0003013	Circulatory system process	18	493	0.49	0.0029	CHGA,LRP1,APOE,SERPING1,SLC27A4,FGB,SERPINF2,FGG,F5,SVEP1,SOD3,NAV2,ATP1A1,TTN,VEGFC,KNG1,CDH5,FGA
GO Process	GO:0072359	Circulatory system development	26	901	0.39	0.0029	GPLD1,APOB,LRP1,APOE,ANGPTL6,THBS1,DNAH5,ANGPTL4,PLG,EFEMP2,CSPG4,SERPINF2,ANG,APOD,FN1,COL18A1,S100A7,ECM1,FLNA,SVEP1,YWHAZ,ILK,TGFBI,TTN,VEGFC,CDH5
GO Process	GO:0048878	Chemical homeostasis	26	904	0.39	0.0030	CPB2,CETP,LTF,APOB,IGFBP5,APOA1,APOE,SERPINF1,LCAT,CP,TCIRG1,HPX,ANGPTL4,PCSK9,F2,OIT3,APOA4,S100A9,APOM,FBN2,TSKU,APOA5,ATP1A1,APOC4,CDH5,B2M
GO Process	GO:0045807	Positive regulation of endocytosis	8	99	0.83	0.0031	LRP1,APOE,PCSK9,CLU,ABCA13,APOA5,PLCG2,B2M
GO Process	GO:0050830	Defense response to Gram-positive bacterium	9	130	0.77	0.0031	CHGA,LTF,LYZ,PGLYRP2,CAMP,ANG,RARRES2,RNASE4,B2M
GO Process	GO:1903036	Positive regulation of response to wounding	7	72	0.91	0.0031	CPB2,THBS1,F2,PLG,SERPINF2,FLNA,F7
GO Process	GO:0042127	Regulation of cell population proliferation	39	1669	0.3	0.0032	ST6GAL1,CPB2,TPD52L2,GPLD1,SPARC,LTF,IGFBP5,APOE,SERPINF1,THBS1,TCIRG1,CSF1R,AZGP1,SCGB3A1,PF4,CAMP,F2,PLG,EFEMP2,CLU,SUZ12,SERPINF2,FBLN1,ANG,THBS4,APOD,FN1,COL18A1,HTRA1,ECM1,SMARCA1,IGFBP3,ILK,MST1,PDGFC,VEGFC,ACTB,CDH5,B2M
GO Process	GO:0010758	Regulation of macrophage chemotaxis	5	29	1.16	0.0035	C5,THBS1,CSF1R,MST1,RARRES2
GO Process	GO:0006066	Alcohol metabolic process	14	324	0.56	0.0037	CETP,APOB,APOA1,TTR,CAT,APOE,LCAT,PCSK9,APOL1,ISYNA1,APOA4,TSKU,APOA5,PLCG2
GO Process	GO:0010896	Regulation of triglyceride catabolic process	4	14	1.38	0.0038	GPLD1,APOA1,APOA4,APOA5
GO Process	GO:0022603	Regulation of anatomical structure morphogenesis	26	920	0.38	0.0038	ST6GAL1,C5,SPARC,APOA1,SERPINF1,THBS1,CSF1R,PF4,CAMP,ANGPTL4,FGB,F2,STAB1,P4HB,FBLN1,FGG,THBS4,FN1,ADAM12,ECM1,FLNA,ILK,VEGFC,SEMA3B,CDH5,FGA
GO Process	GO:0010873	Positive regulation of cholesterol esterification	3	4	1.8	0.0041	APOA1,APOA4,APOA5
GO Process	GO:0051919	Positive regulation of fibrinolysis	3	4	1.8	0.0041	KLKB1,PLG,F11
GO Process	GO:2000351	Regulation of endothelial cell apoptotic process	6	51	1.0	0.0041	THBS1,ANGPTL4,FGB,FGG,CDH5,FGA
GO Process	GO:0045765	Regulation of angiogenesis	13	288	0.58	0.0044	C5,SPARC,SERPINF1,THBS1,PF4,CAMP,ANGPTL4,STAB1,THBS4,ADAM12,ECM1,VEGFC,CDH5
GO Process	GO:2000352	Negative regulation of endothelial cell apoptotic process	5	31	1.13	0.0044	ANGPTL4,FGB,FGG,CDH5,FGA
GO Process	GO:0032368	Regulation of lipid transport	9	139	0.74	0.0047	CETP,APOA1,LRP1,APOE,THBS1,PCSK9,APOA4,ABCA13,APOA5
GO Process	GO:0048662	Negative regulation of smooth muscle cell proliferation	6	53	0.98	0.0048	IGFBP5,APOE,EFEMP2,ANG,APOD,IGFBP3
GO Process	GO:0002220	Innate immune response activating cell surface receptor signaling pathway	5	32	1.12	0.0050	FCN3,MASP1,FCN1,MASP2,PLCG2
GO Process	GO:0042592	Homeostatic process	34	1406	0.31	0.0051	CPB2,CETP,LTF,APOB,IGFBP5,APOA1,APOE,SERPINF1,LYZ,LCN1,LCAT,CP,TCIRG1,HPX,PIP,AZGP1,ANGPTL4,PCSK9,F2,PLG,OIT3,ANG,APOA4,S100A9,APOM,FBN2,TSKU,JCHAIN,APOA5,ATP1A1,APOC4,ACTB,CDH5,B2M
GO Process	GO:0048870	Cell motility	28	1061	0.35	0.0056	CHGA,C5,GPLD1,APOB,APOA1,THBS1,ATRN,DNAH5,PF4,DST,PLG,CSPG4,DNAH8,ANG,THBS4,FN1,S100A9,FLNA,MCOLN2,CD99,ILK,PSG2,MST1,CCL18,VEGFC,SEMA3B,ACTB,CDH5
GO Process	GO:0009892	Negative regulation of metabolic process	58	2982	0.22	0.0057	SPP2,CPB2,C5,GPLD1,LTF,IGFBP5,APOA1,LRP1,APOE,SERPINF1,APCS,THBS1,SERPINA10,LCN1,CSTA,AMBP,FCN3,ITIH1,AHSG,SERPING1,PGLYRP2,SERPINI1,PF4,A2ML1,SLC27A4,PCSK9,YWHAG,F2,PRG2,CLU,SUZ12,SERPINF2,SERPINA7,FBLN1,SERPINA11,ANG,SLPI,APOD,FN1,BLM,HP,ECM1,FLNA,IGFBP3,PROS1,TNRC6A,YWHAZ,C4A,MST1,ITIH3,C4B,SERPINA1,UIMC1,TSKU,ATP1A1,SERPINA4,KNG1,FGA
GO Process	GO:0008202	Steroid metabolic process	12	258	0.59	0.0061	CETP,APOB,APOA1,CAT,APOE,LCAT,PCSK9,APOL1,APOA4,GC,TSKU,APOA5
GO Process	GO:0010720	Positive regulation of cell development	13	304	0.56	0.0069	PROC,APOA1,SERPINF1,FGB,P4HB,FGG,FN1,FLNA,ILK,LMOD3,VEGFC,CDH5,FGA
GO Process	GO:1904036	Negative regulation of epithelial cell apoptotic process	6	59	0.93	0.0079	ANGPTL4,FGB,FGG,MST1,CDH5,FGA
GO Process	GO:0051004	Regulation of lipoprotein lipase activity	4	18	1.27	0.0080	APOA1,ANGPTL4,APOA4,APOA5
GO Process	GO:0050920	Regulation of chemotaxis	11	227	0.61	0.0083	ST6GAL1,C5,THBS1,CSF1R,THBS4,S100A7,F7,MST1,RARRES2,VEGFC,SEMA3B
GO Process	GO:0022604	Regulation of cell morphogenesis	13	311	0.55	0.0085	ST6GAL1,SPARC,APOA1,CSF1R,FGB,F2,P4HB,FBLN1,FGG,FN1,FLNA,ILK,FGA
GO Process	GO:0008015	Blood circulation	15	403	0.5	0.0087	CHGA,APOE,SERPING1,FGB,SERPINF2,FGG,F5,SOD3,NAV2,ATP1A1,TTN,VEGFC,KNG1,CDH5,FGA
GO Process	GO:0018149	Peptide cross-linking	5	37	1.06	0.0087	THBS1,CSTA,F13A1,FN1,F13B
GO Process	GO:0046486	Glycerolipid metabolic process	14	357	0.52	0.0087	CETP,GPLD1,APOB,APOA1,CAT,APOE,LCAT,DGKG,CSF1R,PCSK9,ANG,APOA4,APOA5,PLCG2
GO Process	GO:0051094	Positive regulation of developmental process	32	1332	0.31	0.0088	C5,LTF,APOB,PROC,APOA1,APOE,SERPINF1,THBS1,SCGB3A1,PF4,CAMP,ANGPTL4,BMP1,FGB,EFEMP2,SERPINF2,P4HB,FGG,FN1,ADAM12,ECM1,FLNA,IGFBP3,ILK,MST1,LMOD3,RARRES2,FBN2,VEGFC,ACTB,CDH5,FGA
GO Process	GO:0045595	Regulation of cell differentiation	36	1582	0.28	0.0093	CETP,LTF,APOB,IGFBP5,PROC,APOA1,SERPINF1,APCS,TCIRG1,PGLYRP2,PF4,FGB,YWHAG,F2,EFEMP2,SUZ12,SERPINF2,P4HB,FBLN1,FGG,FN1,FLNA,IGFBP3,EFEMP1,ILK,LMOD3,PAEP,RARRES2,FBN2,TSKU,VEGFC,SEMA3B,ACTB,CDH5,B2M,FGA
GO Process	GO:0032376	Positive regulation of cholesterol transport	5	38	1.05	0.0095	CETP,APOA1,LRP1,APOE,ABCA13
GO Process	GO:0009967	Positive regulation of signal transduction	35	1525	0.29	0.0098	CHGA,LTF,IGFBP5,APOA1,CAT,APOE,THBS1,VWF,PPP2R3A,HPX,INHBE,CSF1R,PDIA3,FGB,F2,INHBC,CSPG4,CLU,SERPINF2,FGG,FN1,S100A7,S100A9,ECM1,FLNA,F10,F7,IGFBP3,ILK,PDGFC,CCL18,PLCG2,TRIM8,CDH5,FGA
GO Process	GO:0010038	Response to metal ion	14	362	0.51	0.0098	GPLD1,CAT,SERPINF1,THBS1,LCAT,TCIRG1,FGB,FGG,ADAMTS13,SOD3,TTN,PLCG2,B2M,FGA
GO Process	GO:0010941	Regulation of cell death	37	1651	0.28	0.0098	ST6GAL1,CHGA,GPLD1,LTF,PROC,CAT,LRP1,APOE,SERPINF1,THBS1,CSF1R,PIP,PF4,PDIA3,SLC27A4,ANGPTL4,PCSK9,FGB,CLU,P4HB,FGG,HP,S100A9,HTRA1,ADAMTSL4,FLNA,CCN5,C1QA,IGFBP3,YWHAZ,MST1,HPR,PLCG2,KNG1,ACTB,CDH5,FGA
GO Process	GO:0007166	Cell surface receptor signaling pathway	43	2040	0.25	0.0102	C5,GPLD1,IGFBP5,APOA1,LRP1,PPP2R3A,CDH6,HPX,INHBE,FCN3,IGFBP1,CSF1R,PF4,MASP1,PDIA3,DST,F2,INHBC,CSPG4,P4HB,PAPPA,FN1,ASGR2,ADAM12,FLNA,FCN1,ADAMTS13,SVEP1,ADGRG2,EFEMP1,ILK,MASP2,RARRES2,PDGFC,VIM,CFD,IGHV3-15,CCL18,IGHV3-72,VEGFC,SEMA3B,PLCG2,CDH5
GO Process	GO:0090207	Regulation of triglyceride metabolic process	5	40	1.02	0.0115	GPLD1,APOA1,APOE,APOA4,APOA5
GO Process	GO:0034384	High-density lipoprotein particle clearance	3	7	1.56	0.0117	APOA1,APOE,APOM
GO Process	GO:0032091	Negative regulation of protein binding	7	94	0.8	0.0121	CFHR5,PCSK9,CFHR1,SLPI,CFHR2,ACTB,B2M
GO Process	GO:0048525	Negative regulation of viral process	7	94	0.8	0.0121	LTF,APCS,FCN3,FBLN1,SLPI,FCN1,TRIM8
GO Process	GO:0030449	Regulation of complement activation	4	22	1.19	0.0144	SERPING1,MASP1,CFH,CFI
GO Process	GO:0006641	Triglyceride metabolic process	6	68	0.87	0.0147	CETP,APOB,CAT,APOE,PCSK9,APOA5
GO Process	GO:0010605	Negative regulation of macromolecule metabolic process	53	2760	0.21	0.0155	SPP2,CPB2,C5,LTF,IGFBP5,APOA1,LRP1,APOE,SERPINF1,APCS,THBS1,SERPINA10,LCN1,CSTA,AMBP,FCN3,ITIH1,AHSG,SERPING1,PGLYRP2,SERPINI1,PF4,A2ML1,PCSK9,YWHAG,F2,PRG2,CLU,SUZ12,SERPINF2,SERPINA7,FBLN1,SERPINA11,ANG,SLPI,APOD,FN1,BLM,ECM1,FLNA,IGFBP3,PROS1,TNRC6A,YWHAZ,C4A,ITIH3,C4B,SERPINA1,UIMC1,TSKU,SERPINA4,KNG1,FGA

GO Process	GO:0010757	Negative regulation of plasminogen activation	3	8	1.5	0.0155	CPB2,THBS1,SERPINF2
GO Process	GO:1905920	Positive regulation of CoA-transferase activity	3	8	1.5	0.0155	APOA1,APOE,APOA4
GO Process	GO:0002690	Positive regulation of leukocyte chemotaxis	7	99	0.78	0.0157	THBS1,CSF1R,THBS4,S100A7,F7,RARRES2,VEGFC
GO Process	GO:0046597	Negative regulation of viral entry into host cell	4	23	1.17	0.0162	APCS,FCN3,FCN1,TRIM8
GO Process	GO:0051049	Regulation of transport	38	1763	0.26	0.0162	CPB2,CETP,CHGA,GPLD1,APOA1,LRP1,CFP,APOE,THBS1,TCIRG1,FCN3,AHSG,MASP1,PCSK9,FGB,F2,CLU,ANG,FGG,APOD,AKAP9,APOA4,FLNA,FCN1,MASP2,C4A,ABCA13,C4B,CFB,TCAF1,APOA5,ATP1A1,TTN,VEGFC,PLCG2,ACTB,B2M,FGA
GO Process	GO:0090208	Positive regulation of triglyceride metabolic process	4	23	1.17	0.0162	GPLD1,APOA1,APOA4,APOA5
GO Process	GO:0007565	Female pregnancy	9	170	0.65	0.0164	IGFBP5,AMBP,PSG6,PSG1,PAPPA,FBLN1,PSG3,PSG2,MST1
GO Process	GO:0048771	Tissue remodeling	7	101	0.77	0.0172	SPP2,IGFBP5,TCIRG1,PLG,CSPG4,THBS4,FLNA
GO Process	GO:0001775	Cell activation	20	693	0.39	0.0175	CHGA,LRP1,VWF,DGKG,TCIRG1,PF4,CAMP,FGB,F2,CLU,FGG,FN1,FLNA,ADAMTS13,C1QA,ILK,PLCG2,ACTB,B2M,FGA
GO Process	GO:0031640	Killing of cells of another organism	6	71	0.85	0.0175	CHGA,LTF,LYZ,PF4,CCL18,KNG1
GO Process	GO:0046470	Phosphatidylcholine metabolic process	6	71	0.85	0.0175	CETP,GPLD1,APOA1,LCAT,APOA4,APOA5
GO Process	GO:0046596	Regulation of viral entry into host cell	5	45	0.97	0.0175	APCS,FCN3,P4HB,FCN1,TRIM8
GO Process	GO:0034114	Regulation of heterotypic cell-cell adhesion	4	24	1.15	0.0181	APOA1,FGB,FGG,FGA
GO Process	GO:0034381	Plasma lipoprotein particle clearance	4	24	1.15	0.0181	APOB,APOA1,APOE,APOM
GO Process	GO:0070372	Regulation of ERK1 and ERK2 cascade	12	299	0.53	0.0189	APOE,CSF1R,FGB,SERPINF2,FBLN1,FGG,FN1,S100A7,YWHAZ,PDGFC,CCL18,FGA
GO Process	GO:0002920	Regulation of humoral immune response	5	46	0.96	0.0190	HPX,SERPING1,MASP1,CFH,CFI
GO Process	GO:0010898	Positive regulation of triglyceride catabolic process	3	9	1.45	0.0191	APOA1,APOA4,APOA5
GO Process	GO:0051006	Positive regulation of lipoprotein lipase activity	3	9	1.45	0.0191	APOA1,APOA4,APOA5
GO Process	GO:0019220	Regulation of phosphate metabolic process	32	1405	0.28	0.0195	GPLD1,LTF,APOA1,LRP1,APOE,THBS1,CENPE,HPX,INHBE,CSF1R,CAMP,YWHAG,F2,INHBC,CSPG4,CLU,FBLN1,ANG,THBS4,FN1,BLM,AKAP9,IGFBP3,ILK,MST1,RARRES2,PDGFC,TTN,VEGFC,PLCG2,ACTB,CDH5
GO Process	GO:1904035	Regulation of epithelial cell apoptotic process	7	106	0.75	0.0216	THBS1,ANGPTL4,FGB,FGG,MST1,CDH5,FGA
GO Process	GO:0006897	Endocytosis	15	447	0.45	0.0217	LRP1,APOE,THBS1,LGALS3BP,FCN3,AHSG,STAB1,ASGR2,FCN1,CFI,ATP9B,PRG4,IGHV3-15,IGHV3-72,ACTB
GO Process	GO:0051093	Negative regulation of developmental process	24	933	0.34	0.0217	CETP,SPARC,LTF,IGFBP5,APOE,SERPINF1,APCS,THBS1,AHSG,PGLYRP2,PF4,F2,STAB1,SUZ12,FBLN1,THBS4,ECM1,EFEMP1,PAEP,TSKU,VEGFC,SEMA3B,ACTB,B2M
GO Process	GO:0006910	Phagocytosis, recognition	4	26	1.11	0.0228	FCN3,FCN1,IGHV3-15,IGHV3-72
GO Process	GO:0048660	Regulation of smooth muscle cell proliferation	8	142	0.68	0.0228	IGFBP5,APOE,THBS1,EFEMP2,SERPINF2,ANG,APOD,IGFBP3
GO Process	GO:0002699	Positive regulation of immune effector process	11	264	0.55	0.0238	CFP,HPX,FCN3,MASP1,FCN1,MASP2,C4A,C4B,CFB,PLCG2,B2M
GO Process	GO:0030300	Regulation of intestinal cholesterol absorption	3	10	1.4	0.0238	APOA1,APOA4,APOA5
GO Process	GO:0010033	Response to organic substance	51	2692	0.2	0.0255	ST6GAL1,CPB2,GPLD1,APOB,IGFBP5,CAT,LRP1,SERPINF1,THBS1,LCAT,TCIRG1,HPX,IGFBP1,CSF1R,PF4,CAMP,PDIA3,SLC27A4,PCSK9,FGB,YWHAG,CLU,P4HB,PAPPA,ANG,TUBA1B,FGG,THBS4,SLPI,BLM,AKAP9,APOA4,F5,S100A7,S100A9,FLNA,ADAMTS13,F7,GPX3,ILK,C4B,RARRES2,PDGFC,ATP1A1,VIM,CCL18,VEGFC,PLCG2,ACTB,CDH5,B2M
GO Process	GO:0009966	Regulation of signal transduction	55	2978	0.19	0.0266	CHGA,LTF,IGFBP5,APOA1,CAT,LRP1,APOE,BPIFB1,THBS1,VWF,PPP2R3A,DGKG,AMBP,HPX,INHBE,AHSG,IGFBP1,CSF1R,PF4,PDIA3,SLC27A4,PCSK9,FGB,YWHAG,F2,INHBC,CSPG4,CLU,SERPINF2,P4HB,FBLN1,FGG,APOD,FN1,AKAP9,S100A7,S100A9,HTRA1,ECM1,FLNA,F10,F7,IGFBP3,YWHAZ,ILK,MST1,PDGFC,FBN2,TSKU,CCL18,VEGFC,PLCG2,TRIM8,CDH5,FGA
GO Process	GO:1905954	Positive regulation of lipid localization	7	111	0.73	0.0269	CETP,APOB,APOA1,LRP1,APOE,ABCA13,APOC4
GO Process	GO:0010647	Positive regulation of cell communication	36	1693	0.25	0.0273	CHGA,GPLD1,LTF,IGFBP5,APOA1,CAT,APOE,THBS1,VWF,PPP2R3A,HPX,INHBE,CSF1R,PDIA3,FGB,F2,INHBC,CSPG4,CLU,SERPINF2,FGG,FN1,S100A7,S100A9,ECM1,FLNA,F10,F7,IGFBP3,ILK,PDGFC,CCL18,PLCG2,TRIM8,CDH5,FGA
GO Process	GO:0002227	Innate immune response in mucosa	4	28	1.08	0.0282	LTF,BPIFB1,CAMP,APOA4
GO Process	GO:0023056	Positive regulation of signaling	36	1698	0.25	0.0286	CHGA,GPLD1,LTF,IGFBP5,APOA1,CAT,APOE,THBS1,VWF,PPP2R3A,HPX,INHBE,CSF1R,PDIA3,FGB,F2,INHBC,CSPG4,CLU,SERPINF2,FGG,FN1,S100A7,S100A9,ECM1,FLNA,F10,F7,IGFBP3,ILK,PDGFC,CCL18,PLCG2,TRIM8,CDH5,FGA
GO Process	GO:0009653	Anatomical structure morphogenesis	44	2229	0.22	0.0289	MGP,GPLD1,LTF,APOB,IGFBP5,LRP1,APOE,ANGPTL6,THBS1,ATRN,PPP2R3A,CDH6,TCIRG1,CSF1R,ANGPTL4,BMP1,EFEMP2,CSPG4,CLU,SERPINF2,ANG,APOD,FN1,COL18A1,ADAM12,S100A7,HTRA1,ECM1,FLNA,SVEP1,EFEMP1,YWHAZ,ILK,MST1,LMOD3,TGFBI,PDGFC,FBN2,TSKU,TTN,VEGFC,SEMA3B,ACTB,CDH5
GO Process	GO:0020027	Hemoglobin metabolic process	3	11	1.36	0.0289	CAT,AMBP,HPX
GO Process	GO:0030155	Regulation of cell adhesion	21	784	0.35	0.0289	ST6GAL1,APOA1,THBS1,SERPINI1,FGB,PLG,EFEMP2,SERPINF2,P4HB,FBLN1,FGG,APOD,FN1,FLNA,MMRN1,ILK,TGFBI,KNG1,ACTB,B2M,FGA
GO Process	GO:0009636	Response to toxic substance	10	229	0.57	0.0290	CAT,APOE,AMBP,HP,APOA4,S100A9,ADAMTS13,APOM,SOD3,GPX3
GO Process	GO:0071622	Regulation of granulocyte chemotaxis	5	52	0.91	0.0291	THBS1,CSF1R,THBS4,S100A7,RARRES2
GO Process	GO:0042221	Response to chemical	69	4010	0.16	0.0297	ST6GAL1,CPB2,CHGA,C5,GPLD1,APOB,IGFBP5,APOA1,CAT,LRP1,APOE,SERPINF1,THBS1,LCAT,AMBP,TCIRG1,HPX,IGFBP1,CSF1R,PIP,AZGP1,PF4,CAMP,PDIA3,SLC27A4,PCSK9,FGB,YWHAG,CLU,P4HB,PAPPA,ANG,TUBA1B,FGG,THBS4,SLPI,APOD,BLM,HP,AKAP9,APOA4,F5,S100A7,S100A9,FLNA,ADAMTS13,F7,APOM,CA6,SOD3,GPX3,ILK,MST1,C4B,RARRES2,PDGFC,SELENOP,ATP1A1,VIM,TTN,CCL18,VEGFC,SEMA3B,PLCG2,OR52B4,ACTB,CDH5,B2M,FGA
GO Process	GO:0032879	Regulation of localization	42	2103	0.23	0.0305	CPB2,CETP,CHGA,GPLD1,APOB,APOA1,LRP1,CFP,APOE,THBS1,TCIRG1,FCN3,AHSG,MASP1,PCSK9,FGB,F2,CLU,ANG,FGG,APOD,AKAP9,APOA4,FLNA,MCOLN2,FCN1,MASP2,C4A,ABCA13,C4B,CFB,TCAF1,APOA5,ATP1A1,TTN,APOC4,VEGFC,PLCG2,ACTB,TRIM8,B2M,FGA
GO Process	GO:0002687	Positive regulation of leukocyte migration	8	151	0.65	0.0309	THBS1,CSF1R,THBS4,S100A7,F7,CD99,RARRES2,VEGFC
GO Process	GO:0010543	Regulation of platelet activation	5	53	0.9	0.0309	APOE,F2,FGG,FLNA,MMRN1
GO Process	GO:0045940	Positive regulation of steroid metabolic process	4	30	1.05	0.0341	APOA1,APOE,APOA4,APOA5
GO Process	GO:0032908	Regulation of transforming growth factor beta1 production	3	12	1.33	0.0343	THBS1,LUM,TSKU
GO Process	GO:0031099	Regeneration	8	154	0.64	0.0345	CPB2,SERPINA10,IGFBP1,PLG,APOD,APOA4,F7,APOA5
GO Process	GO:0048260	Positive regulation of receptor-mediated endocytosis	5	55	0.89	0.0356	PCSK9,CLU,APOA5,PLCG2,B2M
GO Process	GO:0001894	Tissue homeostasis	10	238	0.55	0.0370	LTF,LYZ,LCN1,TCIRG1,PIP,AZGP1,JCHAIN,ACTB,CDH5,B2M
GO Process	GO:0045088	Regulation of innate immune response	10	238	0.55	0.0370	APOE,HPX,FCN3,SERPING1,MASP1,CFH,FCN1,CFI,MASP2,PLCG2

GO Process	GO:0070887	Cellular response to chemical stimulus	49	2609	0.2	0.0376	CPB2,CHGA,C5,GPLD1,APOB,IGFBP5,CAT,LRP1,APOE,SERPINF1,THBS1,AMBP,TCIRG1,HPX,IGFBP1,CSF1R,PF4,CAMP,PDIA3,SLC27A4,PCSK9,FGB,YWHAG,P4HB,TUBA1B,FGG,BLM,HP,AKAP9,APOA4,S100A9,FLNA,ADAMTS13,APOM,SOD3,GPX3,ILK,MST1,RARRES2,PDGFC,ATP1A1,VIM,CCL18,VEGFC,SEMA3B,PLCG2,ACTB,CDH5,B2M
GO Process	GO:0051172	Negative regulation of nitrogen compound metabolic process	46	2403	0.21	0.0379	SPP2,CPB2,C5,LTF,IGFBP5,LRP1,APOE,SERPINF1,APCS,THBS1,SERPINA10,LCN1,CSTA,AMBP,FCN3,ITIH1,AHSG,SERPING1,SERPINI1,A2ML1,YWHAG,F2,CLU,SUZ12,SERPINF2,SERPINA7,FBLN1,SERPINA11,ANG,SLPI,APOD,BLM,ECM1,FLNA,IGFBP3,PROS1,TNRC6A,YWHAZ,C4A,ITIH3,C4B,SERPINA1,UIMC1,SERPINA4,KNG1,FGA
GO Process	GO:1901615	Organic hydroxy compound metabolic process	15	478	0.42	0.0379	CETP,APOB,APOA1,TTR,CAT,APOE,LCAT,PCSK9,APOL1,ISYNA1,APOA4,GC,TSKU,APOA5,PLCG2
GO Process	GO:0001819	Positive regulation of cytokine production	15	482	0.42	0.0407	C5,THBS1,LUM,CSF1R,PF4,CAMP,PRG2,CLU,SERPINF2,MCOLN2,FCN1,ORM2,PAEP,PLCG2,B2M
GO Process	GO:0002544	Chronic inflammatory response	3	13	1.29	0.0407	THBS1,CAMP,S100A9
GO Process	GO:0071635	Negative regulation of transforming growth factor beta production	3	13	1.29	0.0407	FBLN1,FN1,TSKU
GO Process	GO:0001503	Ossification	11	287	0.51	0.0408	MGP,GPLD1,LTF,IGFBP5,CAT,TCIRG1,AHSG,BMP1,ECM1,IGFBP3,TPM4
GO Process	GO:0034445	Negative regulation of plasma lipoprotein oxidation	2	2	1.93	0.0408	APOA4,APOM
GO Process	GO:1902998	Positive regulation of neurofibrillary tangle assembly	2	2	1.93	0.0408	APOE,CLU
GO Process	GO:0060191	Regulation of lipase activity	6	88	0.76	0.0414	APOA1,LRP1,ANGPTL4,ANG,APOA4,APOA5
GO Process	GO:0043393	Regulation of protein binding	9	201	0.58	0.0422	LRP1,APOE,CFHR5,PCSK9,CFHR1,SLPI,CFHR2,ACTB,B2M
GO Process	GO:0050792	Regulation of viral process	8	161	0.62	0.0428	LTF,APCS,FCN3,P4HB,FBLN1,SLPI,FCN1,TRIM8
GO Process	GO:0045597	Positive regulation of cell differentiation	22	876	0.33	0.0440	LTF,APOB,PROC,APOA1,SERPINF1,PF4,FGB,EFEMP2,SERPINF2,P4HB,FGG,FN1,FLNA,IGFBP3,ILK,LMOD3,RARRES2,FBN2,VEGFC,ACTB,CDH5,FGA
GO Process	GO:0098609	Cell-cell adhesion	16	542	0.4	0.0453	CSTA,CDH6,DSC2,FGB,FGG,THBS4,FN1,APOA4,S100A9,FLNA,CD99,ILK,PSG2,ACTB,CDH5,FGA
GO Process	GO:1901701	Cellular response to oxygen-containing compound	25	1057	0.3	0.0453	CPB2,GPLD1,IGFBP5,CAT,LRP1,SERPINF1,AMBP,IGFBP1,PF4,CAMP,SLC27A4,PCSK9,YWHAG,BLM,AKAP9,APOA4,FLNA,ADAMTS13,SOD3,RARRES2,PDGFC,VIM,PLCG2,ACTB,B2M
GO Process	GO:0040013	Negative regulation of locomotion	12	340	0.47	0.0462	ST6GAL1,C5,IGFBP5,LRP1,APOE,SERPINF1,THBS1,FBLN1,APOD,IGFBP3,TCAF1,SEMA3B
GO Process	GO:0001878	Response to yeast	3	14	1.26	0.0466	CAMP,ANG,PLCG2
GO Process	GO:0042063	Gliogenesis	10	248	0.53	0.0466	LRP1,CSF1R,CSPG4,CLU,SUZ12,S100A9,C1QA,ILK,VIM,VEGFC
GO Process	GO:0035821	Modulation of process of another organism	4	34	1.0	0.0479	LTF,CAMP,SLPI,S100A9
GO Process	GO:0050708	Regulation of protein secretion	10	250	0.53	0.0491	CHGA,GPLD1,APOE,TCIRG1,FGB,ANG,FGG,TTN,VEGFC,FGA
GO Process	GO:0008285	Negative regulation of cell population proliferation	19	713	0.35	0.0494	CPB2,GPLD1,SPARC,IGFBP5,APOE,SERPINF1,THBS1,CSF1R,AZGP1,PLG,EFEMP2,FBLN1,ANG,APOD,COL18A1,IGFBP3,ILK,CDH5,B2M
GO Process	GO:0060249	Anatomical structure homeostasis	11	296	0.5	0.0494	LTF,LYZ,LCN1,TCIRG1,PIP,AZGP1,PLG,JCHAIN,ACTB,CDH5,B2M
GO Process	GO:0045766	Positive regulation of angiogenesis	8	166	0.61	0.0500	C5,THBS1,CAMP,ANGPTL4,ADAM12,ECM1,VEGFC,CDH5
GO Function	GO:0004252	Serine-type endopeptidase activity	26	176	1.1	4.63e-16	F9,LTF,PROC,KLKB1,HABP2,MASP1,APEH,PCSK9,BMP1,F2,PLG,HP,HTRA1,F10,F7,CFI,MASP2,F11,C1S,MST1,CFB,HGFAC,HPR,C1R,CFD,PAMR1
GO Function	GO:0005509	Calcium ion binding	44	717	0.72	2.05e-15	F9,MGP,SPARC,PROC,LRP1,APCS,THBS1,PPP2R3A,DGKG,CDH6,ITIH1,HABP2,DSC2,MASP1,LALBA,BMP1,DST,F2,EFEMP2,STAB1,FBLN1,OIT3,SPATA21,THBS4,S100A7,S100A9,CRTAC1,ADAMTS13,SVEP1,F10,F7,CALML5,PROS1,EFEMP1,EFCAB5,MMRN1,MASP2,C1S,FBN2,C1R,TTN,PAMR1,TPM4,CDH5
GO Function	GO:0004866	Endopeptidase inhibitor activity	24	177	1.06	1.50e-14	SPP2,C5,LTF,SERPINF1,SERPINA10,LCN1,CSTA,AMBP,ITIH1,AHSG,SERPING1,SERPINI1,A2ML1,SERPINF2,SERPINA7,SERPINA11,SLPI,PROS1,C4A,ITIH3,C4B,SERPINA1,SERPINA4,KNG1
GO Function	GO:0061134	Peptidase regulator activity	26	227	0.99	2.28e-14	SPP2,C5,LTF,SERPINF1,SERPINA10,LCN1,CSTA,AMBP,ITIH1,AHSG,SERPING1,SERPINI1,A2ML1,SERPINF2,SERPINA7,FBLN1,SERPINA11,SLPI,FN1,PROS1,C4A,ITIH3,C4B,SERPINA1,SERPINA4,KNG1
GO Function	GO:0004175	Endopeptidase activity	32	437	0.79	4.50e-13	F9,LTF,PROC,PSMA6,KLKB1,HABP2,PIP,MASP1,APEH,PDIA3,PCSK9,BMP1,F2,PLG,PAPPA,HP,ADAM12,HTRA1,ADAMTS13,F10,F7,CFI,MASP2,F11,C1S,MST1,CFB,HGFAC,HPR,C1R,CFD,PAMR1
GO Function	GO:0001848	Complement binding	12	26	1.59	5.00e-12	APCS,CFHR5,CFHR1,C8A,CFHR2,CFHR4,CFHR3,CFH,MASP2,C4A,C4B,CFB
GO Function	GO:0005539	Glycosaminoglycan binding	24	245	0.92	5.40e-12	LTF,APOB,APOE,THBS1,SERPINA10,AMBP,ITIH1,HABP2,PGLYRP2,PF4,F2,EFEMP2,PRG2,STAB1,ANG,THBS4,FN1,CFH,CCN5,SOD3,NAV2,F11,APOA5,KNG1
GO Function	GO:0005102	Signaling receptor binding	56	1499	0.5	6.85e-12	C5,APOB,APOA1,TTR,LRP1,APOE,ANGPTL6,THBS1,VWF,LCN1,INHBE,IGFBP1,SCGB3A1,PF4,ANGPTL4,PCSK9,BMP1,FGB,YWHAG,DST,F2,INHBC,PLG,CLU,P4HB,FBLN1,ANG,FGG,TUBB,THBS4,FN1,AKAP9,S100A7,S100A9,ECM1,FLNA,FCN1,ADAMTS13,CCN5,F7,EFEMP1,MST1,TGFBI,RARRES2,PDGFC,FBN2,JCHAIN,APOA5,IGHV3-15,CCL18,IGHV3-72,VEGFC,SEMA3B,KNG1,CDH5,FGA
GO Function	GO:0008233	Peptidase activity	34	617	0.67	9.10e-11	CPB2,F9,LTF,PROC,PSMA6,KLKB1,HABP2,PIP,MASP1,APEH,PDIA3,PCSK9,BMP1,F2,PLG,PAPPA,HP,CNDP1,ADAM12,HTRA1,ADAMTS13,F10,F7,CFI,MASP2,F11,C1S,MST1,CFB,HGFAC,HPR,C1R,CFD,PAMR1
GO Function	GO:0008201	Heparin binding	19	173	0.97	4.33e-10	LTF,APOB,APOE,THBS1,SERPINA10,PF4,F2,EFEMP2,PRG2,ANG,THBS4,FN1,CFH,CCN5,SOD3,NAV2,F11,APOA5,KNG1
GO Function	GO:0004857	Enzyme inhibitor activity	26	396	0.74	1.69e-09	SPP2,C5,LTF,SERPINF1,SERPINA10,LCN1,CSTA,AMBP,ITIH1,AHSG,SERPING1,SERPINI1,A2ML1,ANGPTL4,YWHAG,SERPINF2,SERPINA7,SERPINA11,SLPI,PROS1,C4A,ITIH3,C4B,SERPINA1,SERPINA4,KNG1
GO Function	GO:0001846	Opsonin binding	9	21	1.56	1.15e-08	APCS,CFHR5,CFHR1,CFHR2,CFHR4,CFHR3,CFH,MASP2,C4A
GO Function	GO:0005488	Binding	197	12838	0.11	1.15e-08	ST6GAL1,CPB2,CETP,TPD52L2,F9,C5,MGP,SPARC,LTF,APOB,IGFBP5,PROC,APOA1,TTR,CAT,LRP1,APOE,ANGPTL6,BPIFB1,APCS,CFHR5,THBS1,LYZ,VWF,PSMA6,SERPINA10,ATRN,LCN1,LCAT,CSTA,CP,KLKB1,F13A1,PPP2R3A,DGKG,CDH6,DNAH5,AMBP,CENPE,TCIRG1,HPX,INHBE,LUM,FCN3,ITIH1,IGFBP1,HABP2,SEC24D,DSC2,CSF1R,PIP,PSG6,PGLYRP2,SCGB3A1,PF4,MASP1,CAMP,APEH,A2ML1,PDIA3,SLC27A4,LALBA,ANGPTL4,PCSK9,BMP1,FGB,YWHAG,DST,F2,INHBC,PLG,PSG1,EFEMP2,PRG2,CSPG4,STAB1,C1QB,CFHR1,CLU,NDUFS8,SUZ12,APOL1,SERPINF2,KDM3B,P4HB,PAPPA,FBLN1,PSG3,DNAH8,OIT3,SPATA21,ANG,TUBA1B,FGG,TUBB,THBS4,SLPI,QPCT,APOD,FN1,ASGR2,BLM,HP,AKAP9,APOA4,CNDP1,COL18A1,C8A,CFHR2,CFHR4,CFHR3,CFH,F5,ADAM12,S100A7,S100A9,HTRA1,ADAMTSL4,ECM1,FLNA,CRTAC1,MCOLN2,SMARCA1,C8B,FCN1,ADAMTS13,CCN5,SVEP1,C1QA,F10,F7,APOM,CA6,CALML5,IGFBP3,SOD3,GPX3,PROS1,EFEMP1,CFI,EFCAB5,MMRN1,TNRC6A,YWHAZ,NAV2,ILK,MASP2,F11,C1S,PCYOX1,BZW1,C4A,ATP9B,PRG4,ACSF2,CKMT1A,ABCA13,MST1,LMOD3,C4B,SERPINA1,TGFBI,CFB,IGFALS,PAEP,RARRES2,TCAF1,GC,UIMC1,PDGFC,FBN2,SELENOP,TSKU,JCHAIN,HPR,TUBA1C,C1R,APOA5,ATP1A1,VIM,RNASE4,TTN,IGHV3-15,CCL18,IGHV3-72,VEGFC,SEMA3B,PLCG2,PAMR1,KNG1,TPM4,ACTB,TRIM66,TRIM8,CDH5,B2M,FGA
GO Function	GO:1901681	Sulfur compound binding	21	272	0.81	1.15e-08	LTF,APOB,LRP1,APOE,THBS1,SERPINA10,AMBP,PF4,F2,EFEMP2,PRG2,ANG,THBS4,FN1,CFH,CCN5,SOD3,NAV2,F11,APOA5,KNG1

GO Function	GO:0004867	Serine-type endopeptidase inhibitor activity	14	98	1.08	1.36e-08	SERPINF1,SERPINA10,AMBP,ITIH1,SERPING1,SERPINI1,A2ML1,SERPINF2,SERPINA7,SERPINA11,SLPI,ITIH3,SERPINA1,SERPINA4
GO Function	GO:0005515	Protein binding	135	7242	0.2	1.41e-08	ST6GAL1,TPD52L2,C5,APOB,IGFBP5,APOA1,TTR,CAT,LRP1,APOE,ANGPTL6,APCS,CFHR5,THBS1,LYZ,VWF,PSMA6,LCN1,LCAT,CSTA,CP,CDH6,DNAH5,AMBP,CENPE,TCIRG1,INHBE,FCN3,IGFBP1,SEC24D,DSC2,CSF1R,PIP,PSG6,SCGB3A1,PF4,MASP1,APEH,A2ML1,PDIA3,ANGPTL4,PCSK9,BMP1,FGB,YWHAG,DST,F2,INHBC,PLG,PSG1,EFEMP2,CSPG4,C1QB,CFHR1,CLU,SUZ12,SERPINF2,P4HB,FBLN1,PSG3,DNAH8,ANG,TUBA1B,FGG,TUBB,THBS4,SLPI,FN1,BLM,HP,AKAP9,APOA4,COL18A1,C8A,CFHR2,CFHR4,CFHR3,CFH,ADAM12,S100A7,S100A9,HTRA1,ADAMTSL4,ECM1,FLNA,MCOLN2,SMARCA1,FCN1,ADAMTS13,CCN5,F7,IGFBP3,GPX3,EFEMP1,YWHAZ,ILK,MASP2,F11,C1S,BZW1,C4A,MST1,LMOD3,C4B,SERPINA1,TGFBI,CFB,IGFALS,RARRES2,TCAF1,GC,UIMC1,PDGFC,FBN2,TSKU,JCHAIN,HPR,C1R,APOA5,ATP1A1,VIM,TTN,IGHV3-15,CCL18,IGHV3-72,VEGFC,SEMA3B,PLCG2,KNG1,TPM4,ACTB,TRIM8,CDH5,B2M,FGA
GO Function	GO:0098772	Molecular function regulator activity	55	1960	0.38	3.55e-07	SPP2,C5,GPLD1,LTF,APOA1,TTR,APOE,SERPINF1,SERPINA10,LCN1,CSTA,PPP2R3A,AMBP,INHBE,ITIH1,AHSG,SERPING1,SCGB3A1,SERPINI1,PF4,A2ML1,ANGPTL4,PCSK9,BMP1,YWHAG,F2,INHBC,SUZ12,CPN2,SERPINF2,SERPINA7,FBLN1,SERPINA11,THBS4,SLPI,FN1,AKAP9,APOA4,FLNA,CALML5,IGFBP3,PROS1,EFEMP1,C4A,ITIH3,C4B,SERPINA1,PDGFC,FBN2,APOA5,SERPINA4,CCL18,VEGFC,SEMA3B,KNG1
GO Function	GO:0005201	Extracellular matrix structural constituent	14	131	0.96	3.80e-07	MGP,THBS1,LUM,FGB,EFEMP2,PRG2,FBLN1,OIT3,FGG,FN1,COL18A1,TGFBI,FBN2,FGA
GO Function	GO:0001851	Complement component C3b binding	6	11	1.66	5.70e-06	CFHR5,CFHR1,CFHR2,CFHR4,CFHR3,CFH
GO Function	GO:0042802	Identical protein binding	55	2144	0.34	6.74e-06	ST6GAL1,TPD52L2,APOA1,TTR,CAT,APOE,APCS,CFHR5,THBS1,LYZ,VWF,AMBP,FCN3,CSF1R,PIP,MASP1,APEH,PDIA3,ANGPTL4,BMP1,YWHAG,DST,EFEMP2,C1QB,CFHR1,SERPINF2,FBLN1,ANG,FGG,FN1,BLM,APOA4,COL18A1,CFHR2,CFH,HTRA1,ADAMTSL4,FLNA,MCOLN2,GPX3,YWHAZ,MASP2,F11,C1S,SERPINA1,TGFBI,PDGFC,JCHAIN,C1R,VIM,TTN,TPM4,ACTB,TRIM8,B2M
GO Function	GO:0070325	Lipoprotein particle receptor binding	7	30	1.3	4.11e-05	APOB,APOA1,LRP1,APOE,PCSK9,CLU,APOA5
GO Function	GO:0030234	Enzyme regulator activity	37	1239	0.4	5.23e-05	SPP2,C5,LTF,APOA1,APOE,SERPINF1,SERPINA10,LCN1,CSTA,PPP2R3A,AMBP,ITIH1,AHSG,SERPING1,SERPINI1,A2ML1,ANGPTL4,YWHAG,SUZ12,CPN2,SERPINF2,SERPINA7,FBLN1,SERPINA11,SLPI,FN1,APOA4,CALML5,IGFBP3,PROS1,C4A,ITIH3,C4B,SERPINA1,APOA5,SERPINA4,KNG1
GO Function	GO:0016209	Antioxidant activity	9	76	1.0	0.00012	CAT,APOE,AMBP,HP,APOA4,S100A9,APOM,SOD3,GPX3
GO Function	GO:0043169	Cation binding	84	4335	0.21	0.00015	CPB2,CETP,F9,MGP,SPARC,LTF,PROC,APOA1,CAT,LRP1,APOE,APCS,THBS1,LCN1,CP,KLKB1,F13A1,PPP2R3A,DGKG,CDH6,HPX,FCN3,ITIH1,HABP2,SEC24D,DSC2,PGLYRP2,MASP1,LALBA,BMP1,DST,F2,EFEMP2,STAB1,NDUFS8,SUZ12,KDM3B,PAPPA,FBLN1,OIT3,SPATA21,ANG,FGG,THBS4,QPCT,BLM,APOA4,CNDP1,COL18A1,F5,ADAM12,S100A7,S100A9,CRTAC1,FCN1,ADAMTS13,SVEP1,F10,F7,CA6,CALML5,IGFBP3,SOD3,PROS1,EFEMP1,CFI,EFCAB5,MMRN1,MASP2,C1S,ATP9B,UIMC1,FBN2,C1R,APOA5,ATP1A1,TTN,PAMR1,KNG1,TPM4,TRIM66,TRIM8,CDH5,FGA
GO Function	GO:0005319	Lipid transporter activity	12	163	0.79	0.00018	CETP,APOB,APOA1,APOE,SLC27A4,APOD,APOA4,CFHR4,APOM,ATP9B,ABCA13,APOC4
GO Function	GO:0046872	Metal ion binding	81	4250	0.21	0.00049	CPB2,F9,MGP,SPARC,LTF,PROC,CAT,LRP1,APOE,APCS,THBS1,LCN1,CP,KLKB1,F13A1,PPP2R3A,DGKG,CDH6,HPX,FCN3,ITIH1,HABP2,SEC24D,DSC2,PGLYRP2,MASP1,LALBA,BMP1,DST,F2,EFEMP2,STAB1,NDUFS8,SUZ12,KDM3B,PAPPA,FBLN1,OIT3,SPATA21,ANG,FGG,THBS4,QPCT,BLM,APOA4,CNDP1,COL18A1,F5,ADAM12,S100A7,S100A9,CRTAC1,FCN1,ADAMTS13,SVEP1,F10,F7,CA6,CALML5,IGFBP3,SOD3,PROS1,EFEMP1,CFI,EFCAB5,MMRN1,MASP2,C1S,ATP9B,UIMC1,FBN2,C1R,ATP1A1,TTN,PAMR1,KNG1,TPM4,TRIM66,TRIM8,CDH5,FGA
GO Function	GO:0060228	Phosphatidylcholine-sterol O-acyltransferase activator activity	4	6	1.75	0.00058	APOA1,APOE,APOA4,APOA5
GO Function	GO:0044877	Protein-containing complex binding	34	1261	0.36	0.0012	SPARC,APOA1,TTR,LRP1,APOE,APCS,THBS1,VWF,AMBP,LUM,PIP,PCSK9,DST,STAB1,CLU,P4HB,FBLN1,TUBB,THBS4,FN1,C8A,FLNA,SMARCA1,C8B,ADAMTS13,CCN5,C4A,TGFBI,JCHAIN,VIM,TTN,TPM4,CDH5,B2M
GO Function	GO:0120020	Cholesterol transfer activity	5	23	1.26	0.0025	CETP,APOB,APOA1,APOE,APOA4
GO Function	GO:0050750	Low-density lipoprotein particle receptor binding	5	25	1.23	0.0033	APOB,APOE,PCSK9,CLU,APOA5
GO Function	GO:0005178	Integrin binding	10	159	0.73	0.0041	THBS1,VWF,DST,P4HB,FBLN1,THBS4,FN1,ADAMTS13,CCN5,TGFBI
GO Function	GO:0016787	Hydrolase activity	50	2347	0.26	0.0041	CPB2,APMAP,F9,GPLD1,LTF,PROC,CAT,LYZ,PSMA6,LCAT,KLKB1,HABP2,PIP,AZGP1,PGLYRP2,MASP1,APEH,PDIA3,LALBA,PCSK9,BMP1,F2,PLG,PAPPA,ANG,BLM,HP,CNDP1,ADAM12,HTRA1,SMARCA1,ADAMTS13,F10,F7,CFI,NAV2,MASP2,F11,C1S,ATP9B,MST1,CFB,HGFAC,HPR,C1R,ATP1A1,RNASE4,CFD,PLCG2,PAMR1
GO Function	GO:0071813	Lipoprotein particle binding	5	28	1.18	0.0049	APOA1,APOE,THBS1,PCSK9,STAB1
GO Function	GO:0002020	Protease binding	9	135	0.75	0.0062	VWF,CSTA,A2ML1,SERPINF2,FN1,ADAMTSL4,ECM1,SERPINA1,TTN
GO Function	GO:0070051	Fibrinogen binding	3	4	1.8	0.0064	THBS1,FBLN1,CDH5
GO Function	GO:0043167	Ion binding	100	6033	0.15	0.0065	CPB2,CETP,F9,MGP,SPARC,LTF,PROC,APOA1,CAT,LRP1,APOE,APCS,THBS1,PSMA6,LCN1,CP,KLKB1,F13A1,PPP2R3A,DGKG,CDH6,DNAH5,CENPE,HPX,FCN3,ITIH1,HABP2,SEC24D,DSC2,CSF1R,PGLYRP2,MASP1,LALBA,BMP1,DST,F2,EFEMP2,STAB1,NDUFS8,SUZ12,KDM3B,PAPPA,FBLN1,DNAH8,OIT3,SPATA21,ANG,TUBA1B,FGG,TUBB,THBS4,QPCT,BLM,APOA4,CNDP1,COL18A1,F5,ADAM12,S100A7,S100A9,CRTAC1,SMARCA1,FCN1,ADAMTS13,SVEP1,F10,F7,CA6,CALML5,IGFBP3,SOD3,PROS1,EFEMP1,CFI,EFCAB5,MMRN1,NAV2,ILK,MASP2,C1S,PCYOX1,ATP9B,ACSF2,CKMT1A,ABCA13,UIMC1,FBN2,TUBA1C,C1R,APOA5,ATP1A1,TTN,PAMR1,KNG1,TPM4,ACTB,TRIM66,TRIM8,CDH5,FGA
GO Function	GO:0005198	Structural molecule activity	23	776	0.4	0.0077	MGP,APOE,THBS1,LUM,FGB,DST,EFEMP2,PRG2,FBLN1,OIT3,TUBA1B,FGG,TUBB,FN1,COL18A1,TGFBI,FBN2,TUBA1C,VIM,TTN,TPM4,ACTB,FGA
GO Function	GO:0034987	Immunoglobulin receptor binding	4	16	1.33	0.0085	FLNA,JCHAIN,IGHV3-15,IGHV3-72
GO Function	GO:0005520	Insulin-like growth factor binding	4	17	1.3	0.0102	IGFBP5,IGFBP1,IGFBP3,IGFALS
GO Function	GO:0043394	Proteoglycan binding	5	37	1.06	0.0134	LRP1,APOE,THBS1,FN1,CFH
GO Function	GO:0034185	Apolipoprotein binding	4	19	1.25	0.0139	LRP1,LCAT,PCSK9,PLG
GO Function	GO:0005518	Collagen binding	6	66	0.89	0.0198	SPARC,THBS1,VWF,LUM,FN1,TGFBI
GO Function	GO:0031995	Insulin-like growth factor II binding	3	8	1.5	0.0239	IGFBP5,IGFBP1,IGFBP3
GO Function	GO:0019865	Immunoglobulin binding	4	23	1.17	0.0250	VWF,AMBP,PIP,JCHAIN
GO Function	GO:0003823	Antigen binding	6	71	0.85	0.0265	FCN3,PLG,FCN1,JCHAIN,IGHV3-15,IGHV3-72
GO Function	GO:0005496	Steroid binding	7	101	0.77	0.0265	CETP,APOA1,APOD,APOA4,GC,APOA5,ATP1A1
GO Function	GO:0005044	Scavenger receptor activity	5	48	0.94	0.0345	LRP1,LGALS3BP,STAB1,CFI,PRG4
GO Function	GO:0030545	Signaling receptor regulator activity	17	552	0.42	0.0345	C5,APOA1,TTR,INHBE,SCGB3A1,PF4,PCSK9,BMP1,F2,INHBC,THBS4,EFEMP1,PDGFC,FBN2,CCL18,VEGFC,SEMA3B
GO Function	GO:0031994	Insulin-like growth factor I binding	3	10	1.4	0.0345	IGFBP5,IGFBP1,IGFBP3
GO Function	GO:0042803	Protein homodimerization activity	20	709	0.38	0.0345	ST6GAL1,TPD52L2,APOA1,CAT,APOE,AMBP,CSF1R,MASP1,DST,EFEMP2,SERPINF2,ANG,BLM,APOA4,FLNA,PDGFC,JCHAIN,TPM4,TRIM8,B2M
GO Function	GO:0048018	Receptor ligand activity	16	499	0.43	0.0345	C5,APOA1,TTR,INHBE,SCGB3A1,PF4,BMP1,F2,INHBC,THBS4,EFEMP1,PDGFC,FBN2,CCL18,VEGFC,SEMA3B

GO Function	GO:0051087	Chaperone binding	7	108	0.74	0.0345	VWF,CP,FGB,PLG,CLU,FN1,ATP1A1
GO Function	GO:0097367	Carbohydrate derivative binding	45	2278	0.22	0.0354	LTF,APOB,LRP1,APOE,THBS1,SERPINA10,DGKG,DNAH5,AMBP,CENPE,ITIH1,HABP2,CSF1R,PGLYRP2,PF4,CAMP,F2,EFEMP2,PRG2,STAB1,DNAH8,ANG,TUBA1B,TUBB,THBS4,FN1,BLM,CFH,SMARCA1,FCN1,CCN5,SOD3,NAV2,ILK,F11,ATP9B,ACSF2,CKMT1A,ABCA13,TUBA1C,APOA5,ATP1A1,TTN,KNG1,ACTB
GO Function	GO:0140096	Catalytic activity, acting on a protein	45	2279	0.22	0.0354	CPB2,F9,LTF,PROC,PSMA6,KLKB1,F13A1,HABP2,CSF1R,PIP,MASP1,APEH,PDIA3,PCSK9,BMP1,F2,PLG,UBE3C,STAB1,SUZ12,KDM3B,P4HB,PAPPA,QPCT,HP,CNDP1,ADAM12,HTRA1,ADAMTS13,F10,F7,EFEMP1,CFI,ILK,MASP2,F11,C1S,MST1,CFB,HGFAC,HPR,C1R,TTN,CFD,PAMR1
GO Function	GO:0050839	Cell adhesion molecule binding	17	560	0.41	0.0362	THBS1,VWF,CDH6,DSC2,DST,P4HB,FBLN1,FGG,THBS4,FN1,FLNA,ADAMTS13,CCN5,YWHAZ,BZW1,TGFBI,CDH5
GO Function	GO:0015485	Cholesterol binding	5	52	0.91	0.0405	CETP,APOA1,APOD,APOA4,APOA5
GO Function	GO:0031210	Phosphatidylcholine binding	4	29	1.07	0.0426	CETP,APOA1,APOA4,APOA5
GO Function	GO:0038024	Cargo receptor activity	6	82	0.79	0.0429	LRP1,LGALS3BP,STAB1,ASGR2,CFI,PRG4
GO Function	GO:0004869	Cysteine-type endopeptidase inhibitor activity	5	54	0.89	0.0454	LTF,LCN1,CSTA,AHSG,KNG1
GO Function	GO:0001968	Fibronectin binding	4	30	1.05	0.0458	IGFBP5,THBS1,FBLN1,IGFBP3
GO Component	GO:0005615	Extracellular space	170	3247	0.65	3.20e-76	CPB2,CETP,CHGA,F9,C5,MGP,GPLD1,SPARC,LTF,APOB,IGFBP5,PROC,APOA1,TTR,CAT,CFP,APOE,ANGPTL6,BPIFB1,SERPINF1,APCS,CFHR5,THBS1,LYZ,VWF,PSMA6,SERPINA10,LGALS3BP,ATRN,C9,LCN1,LCAT,CSTA,CP,KLKB1,F13A1,AMBP,HPX,INHBE,LUM,FCN3,ITIH1,AHSG,IGFBP1,HABP2,SAA4,SERPING1,DSC2,PIP,AZGP1,PGLYRP2,SCGB3A1,SERPINI1,PF4,MASP1,CAMP,APEH,A2ML1,PDIA3,LALBA,ANGPTL4,PCSK9,BMP1,FGB,YWHAG,F2,INHBC,PLG,EFEMP2,PRG2,CSPG4,C1QB,CFHR1,CLU,APOL1,CPN2,SERPINF2,C7,P4HB,SERPINA7,PAPPA,FBLN1,OIT3,SERPINA11,ANG,FGG,TUBB,THBS4,SLPI,QPCT,APOD,FN1,HP,APOA4,COL18A1,C8A,F13B,CFHR2,CFHR4,CFHR3,CFH,F5,S100A7,S100A9,HTRA1,ECM1,FLNA,CRTAC1,C8B,FCN1,ADAMTS13,CCN5,SVEP1,C1QA,F10,F7,APOM,CA6,ADGRG2,IGFBP3,SOD3,GPX3,PROS1,EFEMP1,CFI,YWHAZ,MASP2,F11,C1S,PCYOX1,ORM2,C4A,PRG4,MST1,ITIH3,C4B,SERPINA1,TGFBI,CFB,IGFALS,PAEP,RARRES2,GC,HGFAC,PDGFC,FBN2,SELENOP,TSKU,JCHAIN,HPR,C1R,APOA5,ATP1A1,VIM,SERPINA4,RNASE4,TTN,APOC4,CFD,IGHV3-15,CCL18,IGHV3-72,VEGFC,SEMA3B,PLCG2,KNG1,TPM4,ACTB,B2M,FGA
GO Component	GO:0005576	Extracellular region	184	4175	0.57	1.07e-74	SPP2,ST6GAL1,CPB2,CETP,CHGA,F9,C5,MGP,GPLD1,SPARC,LTF,APOB,IGFBP5,PROC,APOA1,TTR,CAT,CFP,APOE,ANGPTL6,BPIFB1,SERPINF1,APCS,CFHR5,THBS1,LYZ,VWF,PSMA6,SERPINA10,LGALS3BP,ATRN,C9,LCN1,LCAT,CSTA,CP,KLKB1,F13A1,DNAH5,AMBP,CENPE,HPX,INHBE,LUM,FCN3,ITIH1,AHSG,IGFBP1,HABP2,SAA4,SERPING1,DSC2,PIP,PSG6,AZGP1,PGLYRP2,SCGB3A1,SERPINI1,PF4,MASP1,CAMP,APEH,A2ML1,PDIA3,LALBA,ANGPTL4,PCSK9,BMP1,FGB,YWHAG,F2,INHBC,PLG,PSG1,EFEMP2,PRG2,CSPG4,C1QB,CFHR1,CLU,APOL1,CPN2,SERPINF2,C7,P4HB,SERPINA7,PAPPA,FBLN1,PSG3,OIT3,SERPINA11,ANG,FGG,TUBB,THBS4,SLPI,QPCT,APOD,FN1,HP,APOA4,CNDP1,COL18A1,C8A,F13B,CFHR2,CFHR4,CFHR3,CFH,F5,ADAM12,S100A7,S100A9,HTRA1,ADAMTSL4,ECM1,FLNA,CRTAC1,C8B,FCN1,ADAMTS13,CCN5,SVEP1,C1QA,F10,F7,APOM,CA6,ADGRG2,CALML5,IGFBP3,SOD3,GPX3,PROS1,EFEMP1,CFI,MMRN1,YWHAZ,MASP2,F11,C1S,PSG2,PCYOX1,ORM2,C4A,PRG4,MST1,ITIH3,C4B,SERPINA1,TGFBI,CFB,IGFALS,PAEP,RARRES2,GC,HGFAC,PDGFC,FBN2,SELENOP,TSKU,JCHAIN,HPR,C1R,APOA5,ATP1A1,VIM,SERPINA4,RNASE4,TTN,APOC4,CFD,IGHV3-15,CCL18,IGHV3-72,VEGFC,SEMA3B,PLCG2,PAMR1,KNG1,TPM4,ACTB,B2M,FGA
GO Component	GO:0062023	Collagen-containing extracellular matrix	65	407	1.13	2.41e-48	SPP2,F9,MGP,SPARC,APOA1,APOE,ANGPTL6,SERPINF1,APCS,THBS1,VWF,LGALS3BP,ATRN,F13A1,AMBP,HPX,INHBE,LUM,FCN3,ITIH1,AHSG,SERPING1,AZGP1,PF4,ANGPTL4,FGB,DST,F2,PLG,EFEMP2,PRG2,CSPG4,C1QB,CLU,SERPINF2,FBLN1,ANG,FGG,THBS4,SLPI,FN1,APOA4,COL18A1,S100A7,S100A9,HTRA1,ADAMTSL4,ECM1,FCN1,C1QA,F7,SOD3,EFEMP1,MMRN1,NAV2,ORM2,PRG4,MST1,SERPINA1,TGFBI,RARRES2,FBN2,SEMA3B,KNG1,FGA
GO Component	GO:0031012	Extracellular matrix	70	552	1.03	1.73e-46	SPP2,F9,MGP,GPLD1,SPARC,APOA1,APOE,ANGPTL6,SERPINF1,APCS,THBS1,VWF,LGALS3BP,ATRN,F13A1,AMBP,HPX,INHBE,LUM,FCN3,ITIH1,AHSG,SERPING1,AZGP1,PF4,ANGPTL4,FGB,DST,F2,PLG,EFEMP2,PRG2,CSPG4,C1QB,CLU,CPN2,SERPINF2,FBLN1,ANG,FGG,THBS4,SLPI,FN1,APOA4,COL18A1,S100A7,S100A9,HTRA1,ADAMTSL4,ECM1,FCN1,ADAMTS13,CCN5,C1QA,F7,SOD3,EFEMP1,MMRN1,NAV2,ORM2,PRG4,MST1,SERPINA1,TGFBI,IGFALS,RARRES2,FBN2,SEMA3B,KNG1,FGA
GO Component	GO:0072562	Blood microparticle	44	118	1.5	1.47e-45	APOA1,APOE,APCS,LGALS3BP,C9,CP,F13A1,AMBP,HPX,FCN3,ITIH1,AHSG,SERPING1,ANGPTL4,FGB,F2,PLG,C1QB,CFHR1,CLU,APOL1,CPN2,SERPINF2,FGG,FN1,HP,APOA4,C8A,CFHR3,CFH,PROS1,YWHAZ,C1S,ORM2,C4A,C4B,CFB,GC,JCHAIN,HPR,C1R,KNG1,ACTB,FGA
GO Component	GO:1903561	Extracellular vesicle	114	2120	0.66	1.01e-44	CPB2,CETP,F9,C5,MGP,GPLD1,LTF,APOB,APOA1,TTR,CAT,APOE,ANGPTL6,BPIFB1,SERPINF1,APCS,THBS1,LYZ,VWF,PSMA6,SERPINA10,LGALS3BP,ATRN,C9,LCAT,CP,KLKB1,AMBP,HPX,LUM,ITIH1,AHSG,SAA4,SERPING1,DSC2,PIP,AZGP1,PGLYRP2,SCGB3A1,SERPINI1,CAMP,APEH,A2ML1,PDIA3,FGB,YWHAG,F2,PLG,EFEMP2,PRG2,CSPG4,CLU,CPN2,SERPINF2,C7,P4HB,SERPINA7,FBLN1,FGG,TUBB,THBS4,SLPI,QPCT,APOD,FN1,HP,APOA4,COL18A1,C8A,CFHR3,CFH,F5,S100A9,HTRA1,ECM1,FLNA,CRTAC1,C8B,CA6,ADGRG2,SOD3,GPX3,PROS1,EFEMP1,CFI,YWHAZ,MASP2,F11,PCYOX1,ORM2,C4A,ITIH3,C4B,SERPINA1,TGFBI,CFB,IGFALS,GC,PDGFC,SELENOP,JCHAIN,HPR,C1R,ATP1A1,VIM,SERPINA4,TTN,CFD,PLCG2,KNG1,TPM4,ACTB,B2M,FGA
GO Component	GO:0070062	Extracellular exosome	113	2096	0.66	1.80e-44	CPB2,CETP,F9,C5,MGP,GPLD1,LTF,APOB,APOA1,TTR,CAT,APOE,ANGPTL6,BPIFB1,SERPINF1,APCS,THBS1,LYZ,VWF,PSMA6,SERPINA10,LGALS3BP,ATRN,C9,LCAT,CP,KLKB1,AMBP,HPX,LUM,ITIH1,AHSG,SAA4,SERPING1,DSC2,PIP,AZGP1,PGLYRP2,SCGB3A1,SERPINI1,CAMP,APEH,A2ML1,PDIA3,FGB,YWHAG,F2,PLG,EFEMP2,PRG2,CSPG4,CLU,CPN2,SERPINF2,C7,P4HB,SERPINA7,FBLN1,FGG,TUBB,THBS4,SLPI,QPCT,APOD,FN1,HP,APOA4,COL18A1,C8A,CFHR3,CFH,S100A9,HTRA1,ECM1,FLNA,CRTAC1,C8B,CA6,ADGRG2,SOD3,GPX3,PROS1,EFEMP1,CFI,YWHAZ,MASP2,F11,PCYOX1,ORM2,C4A,ITIH3,C4B,SERPINA1,TGFBI,CFB,IGFALS,GC,PDGFC,SELENOP,JCHAIN,HPR,C1R,ATP1A1,VIM,SERPINA4,TTN,CFD,PLCG2,KNG1,TPM4,ACTB,B2M,FGA

GO Component	GO:0031982	Vesicle	140	3957	0.48	1.22e-37	SPP2,CPB2,CETP,CHGA,F9,C5,MGP,GPLD1,SPARC,LTF,APOB,APOA1,TTR,CAT,LRP1,CFP,APOE,ANGPTL6,BPIFB1,SERPINF1,APCS,THBS1,LYZ,VWF,PSMA6,SERPINA10,LGALS3BP,ATRN,C9,LCAT,CP,KLKB1,F13A1,AMBP,TCIRG1,HPX,LUM,ITIH1,AHSG,SAA4,SERPING1,SEC24D,DSC2,PIP,AZGP1,PGLYRP2,SCGB3A1,SERPINI1,PF4,CAMP,APEH,A2ML1,PDIA3,PCSK9,BMP1,FGB,YWHAG,DST,F2,PLG,EFEMP2,PRG2,CSPG4,STAB1,CLU,CPN2,SERPINF2,C7,P4HB,SERPINA7,FBLN1,ANG,FGG,TUBB,THBS4,SLPI,QPCT,APOD,FN1,HP,APOA4,COL18A1,C8A,CFHR3,CFH,F5,S100A7,S100A9,HTRA1,ECM1,FLNA,CRTAC1,MCOLN2,C8B,FCN1,F7,CA6,ADGRG2,CALML5,SOD3,GPX3,PROS1,EFEMP1,CFI,MMRN1,YWHAZ,MASP2,F11,PCYOX1,ORM2,C4A,ATP9B,ABCA13,ITIH3,C4B,SERPINA1,TGFBI,CFB,IGFALS,RARRES2,GC,PDGFC,SELENOP,JCHAIN,HPR,TUBA1C,C1R,APOA5,ATP1A1,VIM,SERPINA4,TTN,CFD,VEGFC,PLCG2,KNG1,TPM4,ACTB,B2M,FGA
GO Component	GO:0031983	Vesicle lumen	46	326	1.08	1.70e-31	SPP2,SPARC,LTF,APOB,APOA1,TTR,CAT,CFP,THBS1,LYZ,VWF,LGALS3BP,F13A1,AHSG,SERPING1,SERPINI1,PF4,CAMP,FGB,PLG,CLU,SERPINF2,FGG,TUBB,SLPI,QPCT,FN1,HP,F5,S100A7,S100A9,ECM1,FCN1,PROS1,MMRN1,ORM2,ITIH3,SERPINA1,RARRES2,SELENOP,SERPINA4,CFD,VEGFC,KNG1,B2M,FGA
GO Component	GO:0034774	Secretory granule lumen	45	321	1.07	1.10e-30	SPP2,SPARC,LTF,APOA1,TTR,CAT,CFP,THBS1,LYZ,VWF,LGALS3BP,F13A1,AHSG,SERPING1,SERPINI1,PF4,CAMP,FGB,PLG,CLU,SERPINF2,FGG,TUBB,SLPI,QPCT,FN1,HP,F5,S100A7,S100A9,ECM1,FCN1,PROS1,MMRN1,ORM2,ITIH3,SERPINA1,RARRES2,SELENOP,SERPINA4,CFD,VEGFC,KNG1,B2M,FGA
GO Component	GO:0005788	Endoplasmic reticulum lumen	37	312	1.0	1.31e-22	SPP2,F9,APOB,IGFBP5,PROC,APOA1,CFP,APOE,THBS1,SERPINA10,CP,AHSG,IGFBP1,SERPING1,PDIA3,PCSK9,F2,CLU,APOL1,P4HB,FGG,FN1,APOA4,COL18A1,F5,ADAMTSL4,ADAMTS13,F10,F7,IGFBP3,C4A,SERPINA1,PDGFC,APOA5,KNG1,B2M,FGA
GO Component	GO:0031093	Platelet alpha granule lumen	22	66	1.45	2.23e-21	SPARC,THBS1,VWF,F13A1,AHSG,SERPING1,PF4,FGB,PLG,CLU,SERPINF2,FGG,FN1,F5,PROS1,MMRN1,ORM2,SERPINA1,CFD,VEGFC,KNG1,FGA
GO Component	GO:0030141	Secretory granule	52	873	0.7	1.53e-19	SPP2,CHGA,SPARC,LTF,APOA1,TTR,CAT,CFP,ANGPTL6,THBS1,LYZ,VWF,LGALS3BP,F13A1,TCIRG1,AHSG,SERPING1,SERPINI1,PF4,CAMP,APEH,FGB,PLG,PRG2,CLU,SERPINF2,FGG,TUBB,SLPI,QPCT,FN1,HP,F5,S100A7,S100A9,ECM1,FCN1,CALML5,PROS1,MMRN1,ORM2,ABCA13,ITIH3,SERPINA1,RARRES2,SELENOP,SERPINA4,CFD,VEGFC,KNG1,B2M,FGA
GO Component	GO:1905370	Serine-type endopeptidase complex	14	26	1.66	1.09e-15	F9,PROC,KLKB1,FCN3,MASP1,F2,CFH,F5,FCN1,F10,F7,CFI,MASP2,F11
GO Component	GO:0034358	Plasma lipoprotein particle	14	38	1.49	6.67e-14	CETP,APOB,APOA1,APOE,LCAT,SAA4,CLU,APOL1,APOA4,APOM,PCYOX1,HPR,APOA5,APOC4
GO Component	GO:0034364	High-density lipoprotein particle	13	29	1.58	8.77e-14	CETP,APOB,APOA1,APOE,LCAT,SAA4,CLU,APOL1,APOA4,APOM,HPR,APOA5,APOC4
GO Component	GO:0071944	Cell periphery	128	6015	0.26	1.15e-12	SPP2,F9,C5,MGP,GPLD1,SPARC,LTF,APOB,APOA1,CAT,LRP1,CFP,APOE,ANGPTL6,SERPINF1,APCS,THBS1,VWF,LGALS3BP,ATRN,C9,CSTA,CP,KLKB1,F13A1,DGKG,CDH6,AMBP,TCIRG1,HPX,INHBE,LUM,FCN3,ITIH1,AHSG,SERPING1,DSC2,CSF1R,PSG6,AZGP1,PF4,MASP1,SLC27A4,ANGPTL4,PCSK9,FGB,DST,F2,PLG,PSG1,EFEMP2,PRG2,CSPG4,STAB1,C1QB,CLU,CPN2,SERPINF2,C7,P4HB,FBLN1,PSG3,ANG,FGG,THBS4,SLPI,FN1,ASGR2,AKAP9,APOA4,COL18A1,C8A,F5,ADAM12,S100A7,S100A9,HTRA1,ADAMTSL4,ECM1,FLNA,MCOLN2,C8B,FCN1,ADAMTS13,CCN5,C1QA,F10,F7,ADGRG2,CD99,SOD3,PROS1,EFEMP1,MMRN1,NAV2,ILK,MASP2,F11,PCYOX1,ORM2,C4A,ATP9B,PRG4,ABCA13,MST1,C4B,SERPINA1,TGFBI,CFB,IGFALS,RARRES2,TCAF1,PDGFC,FBN2,ATP1A1,VIM,TTN,IGHV3-15,IGHV3-72,SEMA3B,PLCG2,OR52B4,KNG1,TPM4,ACTB,CDH5,B2M,FGA
GO Component	GO:0097708	Intracellular vesicle	73	2484	0.4	7.61e-12	SPP2,CHGA,SPARC,LTF,APOB,APOA1,TTR,CAT,LRP1,CFP,APOE,ANGPTL6,SERPINF1,THBS1,LYZ,VWF,LGALS3BP,F13A1,TCIRG1,HPX,AHSG,SERPING1,SEC24D,DSC2,SERPINI1,PF4,CAMP,APEH,PDIA3,PCSK9,FGB,DST,PLG,PRG2,STAB1,CLU,SERPINF2,P4HB,ANG,FGG,TUBB,SLPI,QPCT,FN1,HP,APOA4,F5,S100A7,S100A9,ECM1,MCOLN2,FCN1,CALML5,PROS1,MMRN1,YWHAZ,ORM2,ATP9B,ABCA13,ITIH3,SERPINA1,RARRES2,SELENOP,APOA5,ATP1A1,VIM,SERPINA4,CFD,VEGFC,PLCG2,KNG1,B2M,FGA
GO Component	GO:0031410	Cytoplasmic vesicle	72	2482	0.39	2.19e-11	SPP2,CHGA,SPARC,LTF,APOB,APOA1,TTR,CAT,LRP1,CFP,APOE,ANGPTL6,SERPINF1,THBS1,LYZ,VWF,LGALS3BP,F13A1,TCIRG1,HPX,AHSG,SERPING1,SEC24D,DSC2,SERPINI1,PF4,CAMP,APEH,PDIA3,PCSK9,FGB,DST,PLG,PRG2,STAB1,CLU,SERPINF2,P4HB,ANG,FGG,TUBB,SLPI,QPCT,FN1,HP,APOA4,F5,S100A7,S100A9,ECM1,MCOLN2,FCN1,CALML5,PROS1,MMRN1,YWHAZ,ORM2,ATP9B,ABCA13,ITIH3,SERPINA1,RARRES2,SELENOP,APOA5,ATP1A1,VIM,SERPINA4,CFD,VEGFC,KNG1,B2M,FGA
GO Component	GO:1905369	Endopeptidase complex	16	104	1.11	5.41e-11	F9,PROC,PSMA6,KLKB1,FCN3,MASP1,F2,UBE3C,CFH,F5,FCN1,F10,F7,CFI,MASP2,F11
GO Component	GO:0034361	Very-low-density lipoprotein particle	9	20	1.58	1.95e-09	APOB,APOA1,APOE,APOL1,APOA4,APOM,PCYOX1,APOA5,APOC4
GO Component	GO:0012505	Endomembrane system	100	4721	0.25	1.32e-08	SPP2,ST6GAL1,CHGA,F9,SPARC,LTF,APOB,IGFBP5,PROC,APOA1,TTR,CAT,LRP1,CFP,APOE,ANGPTL6,THBS1,LYZ,VWF,SERPINA10,LGALS3BP,CP,F13A1,AMBP,TCIRG1,LUM,AHSG,IGFBP1,SERPING1,SEC24D,SERPINI1,PF4,CAMP,APEH,PDIA3,SLC27A4,LALBA,PCSK9,BMP1,FGB,DST,F2,PLG,PRG2,CSPG4,CLU,APOL1,SERPINF2,P4HB,OIT3,FGG,TUBB,THBS4,SLPI,QPCT,APOD,FN1,ASGR2,HP,AKAP9,APOA4,COL18A1,F5,S100A7,S100A9,ADAMTSL4,ECM1,FLNA,MCOLN2,FCN1,ADAMTS13,F10,F7,CALML5,IGFBP3,SOD3,PROS1,MMRN1,TNRC6A,ORM2,C4A,ATP9B,ABCA13,ITIH3,SERPINA1,TGFBI,RARRES2,HGFAC,PDGFC,SELENOP,APOA5,ATP1A1,SERPINA4,CFD,VEGFC,SEMA3B,KNG1,CDH5,B2M,FGA
GO Component	GO:0009986	Cell surface	35	894	0.52	5.48e-08	APMAP,SPARC,LTF,THBS1,AMBP,FCN3,CSF1R,PSG6,AZGP1,MASP1,PDIA3,PCSK9,FGB,PLG,PSG1,CSPG4,CLU,SERPINF2,P4HB,PSG3,OIT3,FGG,ASGR2,FCN1,ADAMTS13,F10,ADGRG2,MASP2,PSG2,PDGFC,IGHV3-15,IGHV3-72,CDH5,B2M,FGA
GO Component	GO:0031089	Platelet dense granule lumen	7	14	1.63	1.61e-07	SPP2,LGALS3BP,ECM1,ITIH3,RARRES2,SELENOP,SERPINA4
GO Component	GO:0005577	Fibrinogen complex	6	8	1.8	3.78e-07	THBS1,FGB,SERPINF2,FGG,FN1,FGA
GO Component	GO:0005783	Endoplasmic reticulum	53	2021	0.35	1.66e-06	SPP2,ST6GAL1,F9,APOB,IGFBP5,PROC,APOA1,CAT,CFP,APOE,THBS1,VWF,SERPINA10,CP,AMBP,AHSG,IGFBP1,SERPING1,SEC24D,PDIA3,SLC27A4,PCSK9,FGB,DST,F2,CLU,APOL1,P4HB,FGG,THBS4,APOD,FN1,ASGR2,APOA4,COL18A1,F5,S100A7,ADAMTSL4,ADAMTS13,F10,F7,IGFBP3,PROS1,C4A,SERPINA1,HGFAC,PDGFC,APOA5,ATP1A1,SEMA3B,KNG1,B2M,FGA
GO Component	GO:0071682	Endocytic vesicle lumen	7	23	1.41	2.32e-06	SPARC,LTF,APOB,APOA1,APOE,HPX,HP
GO Component	GO:0005604	Basement membrane	11	98	0.98	3.16e-06	SPARC,SERPINF1,ATRN,DST,EFEMP2,FBLN1,ANG,THBS4,FN1,COL18A1,TGFBI
GO Component	GO:0035580	Specific granule lumen	9	62	1.09	6.92e-06	LTF,CFP,LYZ,CAMP,SLPI,QPCT,HP,ORM2,B2M
GO Component	GO:0005579	Membrane attack complex	5	7	1.78	7.55e-06	C5,C9,C7,C8A,C8B

GO Component	GO:0070013	Intracellular organelle lumen	103	5660	0.19	2.07e-05	SPP2,F9,SPARC,LTF,APOB,IGFBP5,PROC,APOA1,TTR,CAT,CFP,APOE,THBS1,LYZ,VWF,PSMA6,SERPINA10,LGALS3BP,CSTA,CP,F13A1,CDH6,HPX,LUM,AHSG,IGFBP1,SERPING1,CSF1R,SERPINI1,PF4,MASP1,CAMP,APEH,PDIA3,LALBA,PCSK9,FGB,DST,F2,PLG,PRG2,CSPG4,CLU,NDUFS8,SUZ12,APOL1,SERPINF2,KDM3B,P4HB,ANG,FGG,TUBB,SLPI,QPCT,FN1,BLM,HP,APOA4,COL18A1,F5,ADAM12,S100A7,S100A9,ADAMTSL4,ECM1,FLNA,SMARCA1,FCN1,ADAMTS13,F10,F7,CALML5,IGFBP3,SOD3,PROS1,MMRN1,TNRC6A,YWHAZ,NAV2,ILK,ORM2,C4A,ACSF2,ITIH3,SERPINA1,RARRES2,GC,UIMC1,PDGFC,SELENOP,APOA5,VIM,SERPINA4,TTN,CFD,VEGFC,KNG1,ACTB,TRIM66,TRIM8,CDH5,B2M,FGA
GO Component	GO:0005796	Golgi lumen	10	106	0.9	4.83e-05	F9,PROC,LUM,LALBA,F2,CSPG4,F10,F7,SOD3,PROS1
GO Component	GO:0009897	External side of plasma membrane	18	388	0.59	6.52e-05	THBS1,FCN3,AZGP1,MASP1,PCSK9,FGB,PLG,P4HB,FGG,ASGR2,FCN1,F10,MASP2,IGHV3-15,IGHV3-72,CDH5,B2M,FGA
GO Component	GO:0042627	Chylomicron	5	13	1.51	6.75e-05	APOB,APOA1,APOE,APOA4,APOA5
GO Component	GO:0005602	Complement component C1 complex	4	5	1.83	9.15e-05	C1QB,C1QA,C1S,C1R
GO Component	GO:0032991	Protein-containing complex	97	5506	0.17	0.00024	CETP,F9,C5,LTF,APOB,IGFBP5,PROC,APOA1,TTR,CAT,LRP1,APOE,CFHR5,THBS1,PSMA6,C9,LCAT,CSTA,KLKB1,F13A1,PPP2R3A,CDH6,DNAH5,TCIRG1,LUM,FCN3,SAA4,SEC24D,CSF1R,MASP1,PDIA3,LALBA,PCSK9,FGB,F2,UBE3C,C1QB,CFHR1,CLU,NDUFS8,SUZ12,APOL1,SERPINF2,C7,KDM3B,P4HB,DNAH8,ANG,FGG,TUBB,FN1,BLM,HP,AKAP9,APOA4,COL18A1,C8A,F13B,CFHR2,CFH,F5,FLNA,SMARCA1,C8B,FCN1,C1QA,F10,F7,APOM,IGFBP3,CFI,MMRN1,TNRC6A,MASP2,F11,C1S,PCYOX1,C4A,C4B,CFB,IGFALS,UIMC1,JCHAIN,HPR,C1R,APOA5,ATP1A1,VIM,TTN,APOC4,IGHV3-15,IGHV3-72,TPM4,ACTB,CDH5,B2M,FGA
GO Component	GO:1904724	Tertiary granule lumen	7	55	1.03	0.00031	LTF,CFP,LYZ,CAMP,QPCT,HP,B2M
GO Component	GO:0034366	Spherical high-density lipoprotein particle	4	8	1.63	0.00033	APOA1,CLU,APOM,HPR
GO Component	GO:0043227	Membrane-bounded organelle	184	13188	0.07	0.0015	SPP2,ST6GAL1,CPB2,CETP,CHGA,F9,C5,MGP,GPLD1,SPARC,LTF,APOB,IGFBP5,PROC,APOA1,TTR,CAT,LRP1,CFP,APOE,ANGPTL6,BPIFB1,SERPINF1,APCS,THBS1,LYZ,VWF,PSMA6,SERPINA10,LGALS3BP,ATRN,C9,LCAT,CSTA,CP,KLKB1,F13A1,CDH6,DNAH5,AMBP,CENPE,TCIRG1,HPX,LUM,ITIH1,AHSG,ANKRD31,IGFBP1,SAA4,SERPING1,SEC24D,DSC2,CSF1R,PIP,AZGP1,PGLYRP2,SCGB3A1,SERPINI1,PF4,MASP1,CAMP,APEH,A2ML1,PDIA3,SLC27A4,LALBA,PCSK9,BMP1,FGB,YWHAG,DST,F2,PLG,EFEMP2,PRG2,CSPG4,STAB1,CLU,NDUFS8,SUZ12,APOL1,CPN2,SERPINF2,C7,KDM3B,P4HB,SERPINA7,FBLN1,DNAH8,OIT3,ANG,FGG,TUBB,THBS4,SLPI,QPCT,APOD,FILIP1L,FN1,ASGR2,BLM,HP,AKAP9,APOA4,COL18A1,C8A,CFHR3,CFH,F5,ADAM12,S100A7,S100A9,HTRA1,ADAMTSL4,ECM1,FLNA,CRTAC1,MCOLN2,SMARCA1,C8B,FCN1,ADAMTS13,CCN5,SVEP1,F10,F7,CA6,ADGRG2,CALML5,IGFBP3,SOD3,GPX3,PROS1,EFEMP1,CFI,MMRN1,TNRC6A,YWHAZ,NAV2,ILK,MAP3K7CL,MASP2,F11,PCYOX1,ORM2,C4A,ATP9B,ACSF2,CKMT1A,ABCA13,MST1,ITIH3,C4B,SERPINA1,TGFBI,CFB,IGFALS,RARRES2,GC,HGFAC,UIMC1,PDGFC,SELENOP,JCHAIN,HPR,TUBA1C,C1R,APOA5,ATP1A1,VIM,SERPINA4,TTN,CFD,VEGFC,SEMA3B,PLCG2,KNG1,TPM4,ACTB,TRIM66,TRIM8,CDH5,B2M,FGA
GO Component	GO:0005601	Classical-complement-pathway C3/C5 convertase complex	3	4	1.8	0.0020	C4A,C4B,CFB
GO Component	GO:0005925	Focal adhesion	16	416	0.51	0.0020	CAT,LRP1,PDIA3,YWHAG,DST,CSPG4,P4HB,S100A7,FLNA,CD99,YWHAZ,ILK,VIM,TPM4,ACTB,B2M
GO Component	GO:0042567	Insulin-like growth factor ternary complex	3	4	1.8	0.0020	IGFBP5,IGFBP3,IGFALS
GO Component	GO:1904813	ficolin-1-rich granule lumen	8	124	0.74	0.0056	CAT,APEH,PRG2,QPCT,FCN1,CALML5,SERPINA1,CFD
GO Component	GO:0030139	Endocytic vesicle	13	338	0.51	0.0086	SPARC,LTF,APOB,APOA1,LRP1,APOE,TCIRG1,HPX,PDIA3,STAB1,HP,VIM,B2M
GO Component	GO:0005775	Vacuolar lumen	9	175	0.64	0.0103	APOB,TTR,LYZ,LUM,CSPG4,TUBB,S100A7,ORM2,GC
GO Component	GO:0031232	Extrinsic component of external side of plasma membrane	3	9	1.45	0.0103	PCSK9,PLG,FCN1
GO Component	GO:0101002	ficolin-1-rich granule	9	185	0.61	0.0146	CAT,TCIRG1,APEH,PRG2,QPCT,FCN1,CALML5,SERPINA1,CFD
GO Component	GO:0043226	Organelle	188	14017	0.05	0.0185	SPP2,ST6GAL1,CPB2,CETP,CHGA,F9,C5,MGP,GPLD1,SPARC,LTF,APOB,IGFBP5,PROC,APOA1,TTR,CAT,LRP1,CFP,APOE,ANGPTL6,BPIFB1,SERPINF1,APCS,THBS1,LYZ,VWF,PSMA6,SERPINA10,LGALS3BP,ATRN,C9,LCAT,CSTA,CP,KLKB1,F13A1,DGKG,CDH6,DNAH5,AMBP,CENPE,TCIRG1,HPX,LUM,ITIH1,AHSG,ANKRD31,IGFBP1,SAA4,SERPING1,SEC24D,DSC2,CSF1R,PIP,AZGP1,PGLYRP2,SCGB3A1,SERPINI1,PF4,MASP1,CAMP,APEH,A2ML1,PDIA3,SLC27A4,LALBA,PCSK9,BMP1,FGB,YWHAG,DST,F2,PLG,EFEMP2,PRG2,CSPG4,STAB1,CLU,NDUFS8,SUZ12,APOL1,CPN2,SERPINF2,C7,KDM3B,P4HB,SERPINA7,FBLN1,DNAH8,OIT3,ANG,TUBA1B,FGG,TUBB,THBS4,SLPI,QPCT,APOD,FILIP1L,FN1,ASGR2,BLM,HP,AKAP9,APOA4,COL18A1,C8A,CFHR3,CFH,F5,ADAM12,S100A7,S100A9,HTRA1,ADAMTSL4,ECM1,FLNA,CRTAC1,MCOLN2,SMARCA1,C8B,FCN1,ADAMTS13,CCN5,SVEP1,F10,F7,CA6,ADGRG2,CALML5,IGFBP3,SOD3,GPX3,PROS1,EFEMP1,CFI,MMRN1,TNRC6A,YWHAZ,NAV2,ILK,MAP3K7CL,MASP2,F11,PCYOX1,ORM2,C4A,ATP9B,SLAIN1,ACSF2,CKMT1A,ABCA13,MST1,LMOD3,ITIH3,C4B,SERPINA1,TGFBI,CFB,IGFALS,RARRES2,GC,HGFAC,UIMC1,PDGFC,SELENOP,JCHAIN,HPR,TUBA1C,C1R,APOA5,ATP1A1,VIM,SERPINA4,TTN,CFD,VEGFC,SEMA3B,PLCG2,KNG1,TPM4,ACTB,TRIM66,TRIM8,CDH5,B2M,FGA
GO Component	GO:0034362	Low-density lipoprotein particle	3	12	1.33	0.0192	APOB,APOE,APOM
GO Component	GO:0042571	Immunoglobulin complex, circulating	3	12	1.33	0.0192	JCHAIN,IGHV3-15,IGHV3-72
GO Component	GO:0034365	Discoidal high-density lipoprotein particle	2	2	1.93	0.0232	APOE,APOM
GO Component	GO:0070820	Tertiary granule	8	164	0.62	0.0274	LTF,CFP,LYZ,TCIRG1,CAMP,QPCT,HP,B2M
GO Component	GO:0005581	Collagen trimer	6	95	0.73	0.0337	LUM,FCN3,C1QB,COL18A1,FCN1,C1QA
GO Component	GO:0062167	Complement component C1q complex	2	3	1.75	0.0368	C1QB,C1QA
STRING clusters	CL:18724	Mixed, incl. Complement and coagulation cascades, and Protein-lipid complex	78	172	1.58	1.63e-88	SPP2,CPB2,CETP,F9,C5,PROC,APOA1,LRP1,CFP,APOE,APCS,CFHR5,VWF,SERPINA10,C9,LCAT,KLKB1,F13A1,AMBP,HPX,FCN3,ITIH1,AHSG,HABP2,SAA4,SERPING1,PGLYRP2,MASP1,ANGPTL4,PCSK9,FGB,F2,PLG,C1QB,CFHR1,CLU,APOL1,CPN2,SERPINF2,C7,SERPINA7,SERPINA11,FGG,HP,APOA4,C8A,F13B,CFHR2,CFHR4,CFHR3,CFH,F5,ECM1,C8B,FCN1,ADAMTS13,C1QA,F10,F7,APOM,CFI,MASP2,F11,C1S,PCYOX1,ORM2,C4A,ITIH3,C4B,SERPINA1,CFB,HPR,C1R,APOA5,SERPINA4,APOC4,CFD,FGA
STRING clusters	CL:18723	Mixed, incl. Complement and coagulation cascades, and Protein-lipid complex	80	196	1.54	2.39e-88	SPP2,CPB2,CETP,F9,C5,PROC,APOA1,LRP1,CFP,APOE,APCS,CFHR5,VWF,SERPINA10,C9,LCAT,KLKB1,F13A1,AMBP,HPX,FCN3,ITIH1,AHSG,HABP2,SAA4,SERPING1,PGLYRP2,MASP1,ANGPTL4,PCSK9,FGB,F2,PLG,STAB1,C1QB,CFHR1,CLU,APOL1,CPN2,SERPINF2,C7,SERPINA7,SERPINA11,FGG,HP,APOA4,C8A,F13B,CFHR2,CFHR4,CFHR3,CFH,F5,ECM1,C8B,FCN1,ADAMTS13,C1QA,F10,F7,APOM,PROS1,CFI,MASP2,F11,C1S,PCYOX1,ORM2,C4A,ITIH3,C4B,SERPINA1,CFB,HPR,C1R,APOA5,SERPINA4,APOC4,CFD,FGA

STRING clusters	CL:18725	Complement and coagulation cascades, and Protein-lipid complex	76	166	1.59	9.44e-87	CPB2,CETP,F9,C5,PROC,APOA1,LRP1,CFP,APOE,APCS,CFHR5,VWF,SERPINA10,C9,LCAT,KLKB1,F13A1,AMBP,HPX,FCN3,ITIH1,AHSG,HABP2,SAA4,SERPING1,PGLYRP2,MASP1,ANGPTL4,PCSK9,FGB,F2,PLG,C1QB,CFHR1,CLU,APOL1,CPN2,SERPINF2,C7,SERPINA7,SERPINA11,FGG,HP,APOA4,C8A,F13B,CFHR2,CFHR4,CFHR3,CFH,F5,C8B,FCN1,ADAMTS13,C1QA,F10,F7,APOM,CFI,MASP2,F11,C1S,PCYOX1,ORM2,C4A,ITIH3,C4B,SERPINA1,CFB,HPR,C1R,APOA5,SERPINA4,APOC4,CFD,FGA
STRING clusters	CL:18726	Complement and coagulation cascades, and Protein-lipid complex	75	161	1.6	4.39e-86	CPB2,CETP,F9,C5,PROC,APOA1,LRP1,CFP,APOE,APCS,CFHR5,VWF,C9,LCAT,KLKB1,F13A1,AMBP,HPX,FCN3,ITIH1,AHSG,HABP2,SAA4,SERPING1,PGLYRP2,MASP1,ANGPTL4,PCSK9,FGB,F2,PLG,C1QB,CFHR1,CLU,APOL1,CPN2,SERPINF2,C7,SERPINA7,SERPINA11,FGG,HP,APOA4,C8A,F13B,CFHR2,CFHR4,CFHR3,CFH,F5,C8B,FCN1,ADAMTS13,C1QA,F10,F7,APOM,CFI,MASP2,F11,C1S,PCYOX1,ORM2,C4A,ITIH3,C4B,SERPINA1,CFB,HPR,C1R,APOA5,SERPINA4,APOC4,CFD,FGA
STRING clusters	CL:18727	Complement and coagulation cascades, and Positive regulation of opsonization	60	115	1.64	4.78e-70	CPB2,F9,C5,PROC,LRP1,CFP,APCS,CFHR5,VWF,C9,KLKB1,F13A1,AMBP,HPX,FCN3,ITIH1,AHSG,HABP2,SERPING1,PGLYRP2,MASP1,FGB,F2,PLG,C1QB,CFHR1,CPN2,SERPINF2,C7,SERPINA7,SERPINA11,FGG,HP,C8A,F13B,CFHR2,CFHR4,CFHR3,CFH,F5,C8B,FCN1,ADAMTS13,C1QA,F10,F7,CFI,MASP2,F11,C1S,ORM2,C4A,ITIH3,C4B,SERPINA1,CFB,C1R,SERPINA4,CFD,FGA
STRING clusters	CL:18728	Complement and coagulation cascades, and Positive regulation of opsonization	58	109	1.65	5.17e-68	CPB2,F9,C5,PROC,LRP1,CFP,APCS,CFHR5,VWF,C9,KLKB1,F13A1,AMBP,HPX,FCN3,ITIH1,AHSG,HABP2,SERPING1,MASP1,FGB,F2,PLG,C1QB,CFHR1,SERPINF2,C7,SERPINA7,SERPINA11,FGG,HP,C8A,F13B,CFHR2,CFHR4,CFHR3,CFH,F5,C8B,FCN1,ADAMTS13,C1QA,F10,F7,CFI,MASP2,F11,C1S,ORM2,C4A,ITIH3,C4B,SERPINA1,CFB,C1R,SERPINA4,CFD,FGA
STRING clusters	CL:18730	Hemostasis, and Dissolution of Fibrin Clot	29	55	1.65	1.71e-32	CPB2,F9,PROC,LRP1,VWF,KLKB1,F13A1,AMBP,HPX,ITIH1,AHSG,HABP2,FGB,F2,PLG,SERPINF2,FGG,HP,F13B,F5,ADAMTS13,F10,F7,F11,ORM2,ITIH3,SERPINA1,SERPINA4,FGA
STRING clusters	CL:18731	Hemostasis, and Dissolution of Fibrin Clot	28	50	1.68	6.30e-32	CPB2,F9,PROC,LRP1,VWF,KLKB1,F13A1,AMBP,HPX,ITIH1,AHSG,HABP2,FGB,F2,PLG,SERPINF2,FGG,HP,F13B,F5,ADAMTS13,F10,F7,F11,ORM2,ITIH3,SERPINA1,FGA
STRING clusters	CL:18846	Complement cascade	27	50	1.66	1.94e-30	C5,CFP,APCS,CFHR5,C9,FCN3,SERPING1,MASP1,C1QB,CFHR1,C7,C8A,CFHR2,CFHR4,CFHR3,CFH,C8B,FCN1,C1QA,CFI,MASP2,C1S,C4A,C4B,CFB,C1R,CFD
STRING clusters	CL:18848	Complement cascade	21	40	1.65	3.92e-23	C5,CFP,C9,FCN3,SERPING1,MASP1,C1QB,C7,C8A,CFH,C8B,FCN1,C1QA,CFI,MASP2,C1S,C4A,C4B,CFB,C1R,CFD
STRING clusters	CL:18732	Mixed, incl. COVID-19, thrombosis and anticoagulation, and Scavenging of heme from plasma	18	26	1.77	2.80e-21	CPB2,LRP1,F13A1,AMBP,HPX,ITIH1,AHSG,HABP2,FGB,PLG,SERPINF2,FGG,HP,F13B,ORM2,ITIH3,SERPINA1,FGA
STRING clusters	CL:18733	Mixed, incl. COVID-19, thrombosis and anticoagulation, and Inter-alpha-trypsin inhibitor heavy chain C-terminus	15	21	1.78	1.13e-17	CPB2,F13A1,AMBP,ITIH1,AHSG,HABP2,FGB,PLG,SERPINF2,FGG,F13B,ORM2,ITIH3,SERPINA1,FGA
STRING clusters	CL:18849	Initial triggering of complement, and Regulation of complement activation	16	31	1.64	2.78e-17	CFP,FCN3,SERPING1,MASP1,C1QB,CFH,FCN1,C1QA,CFI,MASP2,C1S,C4A,C4B,CFB,C1R,CFD
STRING clusters	CL:18956	Lipoprotein particle, and Assembly of active LPL and LIPC lipase complexes	15	46	1.44	9.46e-14	CETP,APOA1,APOE,LCAT,SAA4,ANGPTL4,PCSK9,CLU,APOL1,APOA4,APOM,PCYOX1,HPR,APOA5,APOC4
STRING clusters	CL:18957	Lipoprotein particle	13	36	1.48	3.00e-12	CETP,APOA1,LCAT,SAA4,ANGPTL4,CLU,APOL1,APOA4,APOM,PCYOX1,HPR,APOA5,APOC4
STRING clusters	CL:18960	High-density lipoprotein particle	12	26	1.59	3.00e-12	CETP,APOA1,LCAT,SAA4,CLU,APOL1,APOA4,APOM,PCYOX1,HPR,APOA5,APOC4
STRING clusters	CL:18962	High-density lipoprotein particle	11	21	1.65	1.23e-11	CETP,APOA1,LCAT,CLU,APOL1,APOA4,APOM,PCYOX1,HPR,APOA5,APOC4
STRING clusters	CL:18851	Initial triggering of complement, and Negative regulation of complement activation	11	24	1.59	3.64e-11	FCN3,SERPING1,MASP1,C1QB,FCN1,C1QA,MASP2,C1S,C4A,C4B,C1R
STRING clusters	CL:18784	Formation of Fibrin Clot (Clotting Cascade)	10	18	1.67	1.03e-10	F9,PROC,VWF,KLKB1,F2,F5,ADAMTS13,F10,F7,F11
STRING clusters	CL:18734	COVID-19, thrombosis and anticoagulation, and Negative regulation of fibrinolysis	9	11	1.84	1.20e-10	CPB2,F13A1,HABP2,FGB,PLG,SERPINF2,FGG,F13B,FGA
STRING clusters	CL:18852	Creation of C4 and C2 activators	9	15	1.71	8.54e-10	FCN3,SERPING1,MASP1,C1QB,FCN1,C1QA,MASP2,C1S,C1R
STRING clusters	CL:18786	Hemophilia B, and Blood coagulation, common pathway	8	12	1.75	6.87e-09	F9,VWF,F2,F5,ADAMTS13,F10,F7,F11
STRING clusters	CL:18966	High-density lipoprotein particle	8	16	1.63	3.69e-08	CETP,APOA1,LCAT,CLU,APOA4,APOM,APOA5,APOC4
STRING clusters	CL:18755	Mixed, incl. Inter-alpha-trypsin inhibitor heavy chain C-terminus, and Alpha-1-acid glycoprotein	6	10	1.71	3.18e-06	AMBP,ITIH1,AHSG,ORM2,ITIH3,SERPINA1
STRING clusters	CL:18970	Triglyceride-rich lipoprotein particle remodeling, and Spherical high-density lipoprotein particle	6	11	1.66	4.68e-06	CETP,APOA1,LCAT,CLU,APOA4,APOA5
STRING clusters	CL:18869	Complement component C1 complex	5	6	1.85	1.55e-05	SERPING1,C1QB,C1QA,C1S,C1R
STRING clusters	CL:18911	Membrane attack complex	5	7	1.78	2.49e-05	C5,C9,C7,C8A,C8B
STRING clusters	CL:24266	Insulin-like growth factor binding, and Pappalysin-1/2	6	16	1.5	2.49e-05	IGFBP5,IGFBP1,PRG2,PAPPA,IGFBP3,IGFALS
STRING clusters	CL:19457	Extracellular matrix organization	13	180	0.79	7.11e-05	SERPINF1,THBS1,LUM,BMP1,DST,EFEMP2,P4HB,FBLN1,THBS4,FN1,COL18A1,EFEMP1,FBN2
STRING clusters	CL:18934	Hemolytic uremic syndrome	4	5	1.83	0.00032	CFHR5,CFHR1,CFHR4,CFHR3
STRING clusters	CL:18737	Fibrinogen, and Thrombophilia	4	6	1.75	0.00052	FGB,PLG,FGG,FGA
STRING clusters	CL:18788	Extrinsic Pathway of Fibrin Clot Formation, and Factor V deficiency	4	6	1.75	0.00052	F2,F5,F10,F7
STRING clusters	CL:18854	Cell surface pattern recognition receptor signaling pathway	4	6	1.75	0.00052	FCN3,MASP1,FCN1,MASP2
STRING clusters	CL:35049	Transforming growth factor beta1 activation, and Protein tyrosine kinase binding	4	7	1.68	0.00074	PSG6,PSG1,PSG3,PSG2
STRING clusters	CL:24268	Insulin-like growth factor-binding protein family 1-6, chordata, and Insulin-like growth factor	4	10	1.53	0.0021	IGFBP5,IGFBP1,IGFBP3,IGFALS
STRING clusters	CL:19114	Mixed, incl. S100/CaBP-9k-type, calcium binding, subdomain, and Cystatin superfamily	8	103	0.82	0.0058	LYZ,LGALS3BP,LCN1,CSTA,CAMP,SLPI,S100A7,S100A9
STRING clusters	CL:19699	Elastic fibre formation	4	14	1.38	0.0058	EFEMP2,FBLN1,EFEMP1,FBN2
STRING clusters	CL:18757	Alpha-1-acid glycoprotein, and Protein-losing enteropathy	3	5	1.71	0.0094	AHSG,ORM2,SERPINA1
STRING clusters	CL:18774	Scavenging of heme from plasma	3	5	1.71	0.0094	LRP1,HPX,HP
STRING clusters	CL:19696	Elastic fibre formation, and Tolloid/BMP1 peptidase domain	5	34	1.09	0.0094	BMP1,EFEMP2,FBLN1,EFEMP1,FBN2
STRING clusters	CL:19115	Mixed, incl. Cystatin superfamily, and Specific granule lumen	6	60	0.93	0.0126	LYZ,LGALS3BP,LCN1,CSTA,CAMP,SLPI
STRING clusters	CL:19458	Collagen formation, and Protein complex involved in cell adhesion	8	119	0.75	0.0128	SERPINF1,THBS1,LUM,DST,P4HB,THBS4,FN1,COL18A1
STRING clusters	CL:19397	Signaling by MST1, and Campylobacteriosis	3	10	1.4	0.0403	APEH,MST1,HGFAC

KEGG	hsa04610	Complement and coagulation cascades	43	82	1.65	1.40e-49	CPB2,F9,C5,PROC,CFHR5,VWF,C9,KLKB1,F13A1,SERPING1,FGB,F2,PLG,C1QB,CFHR1,CLU,SERPINF2,C7,FGG,C8A,F13B,CFHR2,CFHR4,CFHR3,CFH,F5,C8B,C1QA,F10,F7,PROS1,CFI,MASP2,F11,C1S,C4A,C4B,SERPINA1,CFB,C1R,CFD,KNG1,FGA
KEGG	hsa05150	Staphylococcus aureus infection	15	86	1.17	1.36e-10	C5,CAMP,PLG,C1QB,FGG,CFH,C1QA,CFI,MASP2,C1S,C4A,C4B,CFB,C1R,CFD
KEGG	hsa04979	Cholesterol metabolism	9	48	1.2	2.16e-06	CETP,APOB,APOA1,LRP1,APOE,LCAT,ANGPTL4,PCSK9,APOA4
KEGG	hsa05322	Systemic lupus erythematosus	11	94	1.0	3.51e-06	C5,C9,C1QB,C7,C8A,C8B,C1QA,C1S,C4A,C4B,C1R
KEGG	hsa05133	Pertussis	9	73	1.02	3.28e-05	C5,SERPING1,C1QB,C1QA,CALML5,C1S,C4A,C4B,C1R
KEGG	hsa05020	Prion disease	12	263	0.59	0.0057	C5,PSMA6,C9,C1QB,NDUFS8,C7,TUBA1B,TUBB,C8A,C8B,C1QA,TUBA1C
KEGG	hsa04145	Phagosome	8	141	0.68	0.0180	THBS1,TCIRG1,TUBA1B,TUBB,THBS4,TUBA1C,C1R,ACTB
KEGG	hsa04510	Focal adhesion	9	195	0.59	0.0288	THBS1,VWF,THBS4,FN1,FLNA,ILK,PDGFC,VEGFC,ACTB
KEGG	hsa04611	Platelet activation	7	122	0.69	0.0304	VWF,FGB,F2,FGG,PLCG2,ACTB,FGA
KEGG	hsa05143	African trypanosomiasis	4	36	0.97	0.0402	APOA1,APOL1,HPR,KNG1
Reactome	HSA-166658	Complement cascade	30	59	1.63	8.96e-33	CPB2,C5,CFP,CFHR5,C9,FCN3,SERPING1,F2,C1QB,CFHR1,CLU,CPN2,C7,C8A,CFHR2,CFHR4,CFHR3,CFH,C8B,FCN1,C1QA,PROS1,CFI,MASP2,C1S,C4A,C4B,CFB,C1R,CFD
Reactome	HSA-977606	Regulation of Complement cascade	26	49	1.65	9.75e-29	CPB2,C5,CFP,CFHR5,C9,SERPING1,F2,C1QB,CFHR1,CLU,CPN2,C7,C8A,CFHR2,CFHR4,CFHR3,CFH,C8B,C1QA,PROS1,CFI,C1S,C4A,C4B,CFB,C1R
Reactome	HSA-114608	Platelet degranulation	32	126	1.33	1.06e-27	SPP2,SPARC,APOA1,THBS1,VWF,LGALS3BP,F13A1,AHSG,SERPING1,PF4,FGB,PLG,CLU,SERPINF2,FGG,FN1,F5,ECM1,FLNA,PROS1,MMRN1,ORM2,ITIH3,SERPINA1,RARRES2,SELENOP,SERPINA4,TTN,CFD,VEGFC,KNG1,FGA
Reactome	HSA-109582	Hemostasis	54	607	0.88	1.37e-27	SPP2,F9,SPARC,APOB,PROC,APOA1,THBS1,VWF,LGALS3BP,KLKB1,F13A1,DGKG,CENPE,AHSG,SERPING1,PSG6,PF4,FGB,F2,PLG,PSG1,CLU,SERPINF2,PSG3,TUBA1B,FGG,FN1,F13B,F5,ECM1,FLNA,F10,F7,CD99,PROS1,MMRN1,YWHAZ,F11,PSG2,ORM2,ITIH3,SERPINA1,RARRES2,SELENOP,JCHAIN,TUBA1C,SERPINA4,TTN,CFD,VEGFC,PLCG2,KNG1,ACTB,FGA
Reactome	HSA-76002	Platelet activation, signaling and aggregation	36	260	1.07	1.35e-23	SPP2,SPARC,APOA1,THBS1,VWF,LGALS3BP,F13A1,DGKG,AHSG,SERPING1,PF4,FGB,F2,PLG,CLU,SERPINF2,FGG,FN1,F5,ECM1,FLNA,PROS1,MMRN1,YWHAZ,ORM2,ITIH3,SERPINA1,RARRES2,SELENOP,SERPINA4,TTN,CFD,VEGFC,PLCG2,KNG1,FGA
Reactome	HSA-168249	Innate Immune System	60	1041	0.69	7.72e-22	CPB2,CHGA,C5,LTF,APOB,TTR,CAT,CFP,BPIFB1,CFHR5,LYZ,PSMA6,C9,TCIRG1,FCN3,AHSG,SERPING1,PGLYRP2,CAMP,APEH,FGB,F2,PRG2,C1QB,CFHR1,CLU,CPN2,C7,FGG,TUBB,SLPI,QPCT,HP,C8A,CFHR2,CFHR4,CFHR3,CFH,S100A7,S100A9,C8B,FCN1,C1QA,CALML5,PROS1,CFI,MASP2,C1S,ORM2,C4A,ABCA13,C4B,SERPINA1,CFB,C1R,CFD,PLCG2,ACTB,B2M,FGA
Reactome	HSA-381426	Regulation of Insulin-like Growth Factor (IGF) transport and uptake by Insulin-like Growth Factor Binding Proteins (IGFBPs)	26	124	1.25	1.06e-20	SPP2,APOB,IGFBP5,PROC,APOA1,APOE,SERPINA10,CP,AHSG,IGFBP1,PCSK9,F2,PLG,APOL1,P4HB,PAPPA,FGG,FN1,F5,IGFBP3,C4A,SERPINA1,IGFALS,APOA5,KNG1,FGA
Reactome	HSA-140877	Formation of Fibrin Clot (Clotting Cascade)	18	39	1.59	4.50e-19	F9,PROC,VWF,KLKB1,F13A1,SERPING1,PF4,FGB,F2,FGG,F13B,F5,F10,F7,PROS1,F11,KNG1,FGA
Reactome	HSA-168256	Immune System	75	1979	0.51	1.02e-17	CPB2,CHGA,C5,LTF,APOB,TTR,CAT,CFP,BPIFB1,CFHR5,LYZ,PSMA6,C9,F13A1,CENPE,TCIRG1,FCN3,AHSG,SERPING1,SEC24D,CSF1R,PGLYRP2,CAMP,APEH,PDIA3,FGB,F2,UBE3C,PRG2,C1QB,CFHR1,CLU,CPN2,C7,P4HB,TUBA1B,FGG,TUBB,SLPI,QPCT,FN1,HP,C8A,CFHR2,CFHR4,CFHR3,CFH,S100A7,S100A9,FLNA,C8B,FCN1,C1QA,CALML5,CD99,PROS1,CFI,YWHAZ,MASP2,C1S,ORM2,C4A,ABCA13,C4B,SERPINA1,CFB,TUBA1C,C1R,VIM,CFD,PLCG2,ACTB,TRIM8,B2M,FGA
Reactome	HSA-8957275	Post-translational protein phosphorylation	22	107	1.24	2.83e-17	SPP2,APOB,IGFBP5,PROC,APOA1,APOE,SERPINA10,CP,AHSG,IGFBP1,PCSK9,APOL1,P4HB,FGG,FN1,F5,IGFBP3,C4A,SERPINA1,APOA5,KNG1,FGA
Reactome	HSA-166663	Initial triggering of complement	12	24	1.63	9.11e-13	CFP,FCN3,C1QB,FCN1,C1QA,MASP2,C1S,C4A,C4B,CFB,C1R,CFD
Reactome	HSA-140875	Common Pathway of Fibrin Clot Formation	11	22	1.63	1.19e-11	PROC,F13A1,PF4,FGB,F2,FGG,F13B,F5,F10,PROS1,FGA
Reactome	HSA-2173782	Binding and Uptake of Ligands by Scavenger Receptors	12	43	1.37	2.25e-10	SPARC,APOB,APOA1,LRP1,APOE,AMBP,HPX,STAB1,APOL1,HP,JCHAIN,HPR
Reactome	HSA-140837	Intrinsic Pathway of Fibrin Clot Formation	10	23	1.57	4.37e-10	F9,PROC,VWF,KLKB1,SERPING1,F2,F10,PROS1,F11,KNG1
Reactome	HSA-392499	Metabolism of proteins	57	1917	0.4	1.14e-08	SPP2,ST6GAL1,CPB2,F9,GPLD1,LTF,APOB,IGFBP5,PROC,APOA1,TTR,CFP,APOE,APCS,THBS1,LYZ,PSMA6,SERPINA10,CP,INHBE,AHSG,IGFBP1,SEC24D,APEH,PDIA3,PCSK9,F2,INHBC,PLG,SUZ12,APOL1,P4HB,PAPPA,TUBA1B,FGG,FN1,ASGR2,BLM,APOA4,F5,ADAMTSL4,ADAMTS13,F10,F7,IGFBP3,PROS1,C4A,SERPINA1,TGFBI,IGFALS,UIMC1,TUBA1C,APOA5,KNG1,ACTB,B2M,FGA
Reactome	HSA-2168880	Scavenging of heme from plasma	8	15	1.65	1.75e-08	APOA1,LRP1,AMBP,HPX,APOL1,HP,JCHAIN,HPR
Reactome	HSA-1474244	Extracellular matrix organization	21	300	0.77	2.69e-08	SPARC,TTR,THBS1,VWF,KLKB1,LUM,BMP1,FGB,DST,PLG,EFEMP2,P4HB,FBLN1,FGG,FN1,COL18A1,ADAM12,HTRA1,EFEMP1,FBN2,FGA
Reactome	HSA-174824	Plasma lipoprotein assembly, remodeling, and clearance	12	75	1.13	4.83e-08	CETP,APOB,APOA1,APOE,LCAT,ANGPTL4,PCSK9,BMP1,P4HB,APOA4,APOA5,APOC4
Reactome	HSA-8963899	Plasma lipoprotein remodeling	9	34	1.35	1.59e-07	CETP,APOB,APOA1,APOE,LCAT,ANGPTL4,P4HB,APOA4,APOA5
Reactome	HSA-166786	Creation of C4 and C2 activators	7	16	1.57	6.20e-07	FCN3,C1QB,FCN1,C1QA,MASP2,C1S,C1R
Reactome	HSA-9651496	Defects of contact activation system (CAS) and kallikrein/kinin system (KKS)	7	16	1.57	6.20e-07	F9,VWF,KLKB1,SERPING1,F2,F10,F11
Reactome	HSA-166665	Terminal pathway of complement	6	8	1.8	6.32e-07	C5,C9,CLU,C7,C8A,C8B
Reactome	HSA-159763	Transport of gamma-carboxylated protein precursors from the endoplasmic reticulum to the Golgi apparatus	6	9	1.75	1.00e-06	F9,PROC,F2,F10,F7,PROS1
Reactome	HSA-597592	Post-translational protein modification	43	1405	0.41	1.09e-06	SPP2,ST6GAL1,F9,GPLD1,APOB,IGFBP5,PROC,APOA1,CFP,APOE,THBS1,PSMA6,SERPINA10,CP,AHSG,IGFBP1,SEC24D,PDIA3,PCSK9,F2,SUZ12,APOL1,P4HB,TUBA1B,FGG,FN1,ASGR2,BLM,F5,ADAMTSL4,ADAMTS13,F10,F7,IGFBP3,PROS1,C4A,SERPINA1,UIMC1,TUBA1C,APOA5,KNG1,ACTB,FGA
Reactome	HSA-8963898	Plasma lipoprotein assembly	7	19	1.49	1.25e-06	APOB,APOA1,APOE,BMP1,P4HB,APOA4,APOC4
Reactome	HSA-159740	Gamma-carboxylation of protein precursors	6	10	1.71	1.42e-06	F9,PROC,F2,F10,F7,PROS1
Reactome	HSA-159782	Removal of aminoterminal propeptides from gamma-carboxylated proteins	6	10	1.71	1.42e-06	F9,PROC,F2,F10,F7,PROS1
Reactome	HSA-6798695	Neutrophil degranulation	23	476	0.61	1.84e-06	LTF,TTR,CAT,CFP,LYZ,TCIRG1,AHSG,CAMP,APEH,PRG2,TUBB,SLPI,QPCT,HP,S100A7,S100A9,FCN1,CALML5,ORM2,ABCA13,SERPINA1,CFD,B2M
Reactome	HSA-977225	Amyloid fiber formation	10	79	1.03	6.36e-06	LTF,APOA1,TTR,APOE,APCS,LYZ,APOA4,TGFBI,B2M,FGA
Reactome	HSA-174577	Activation of C3 and C5	5	7	1.78	1.07e-05	C5,CFP,C4A,C4B,CFB
Reactome	HSA-202733	Cell surface interactions at the vascular wall	12	139	0.86	1.48e-05	APOB,PROC,PSG6,PF4,F2,PSG1,PSG3,FN1,CD99,PROS1,PSG2,JCHAIN
Reactome	HSA-8963888	Chylomicron assembly	5	10	1.63	3.72e-05	APOB,APOA1,APOE,P4HB,APOA4
Reactome	HSA-8963901	Chylomicron remodeling	5	10	1.63	3.72e-05	APOB,APOA1,APOE,APOA4,APOA5
Reactome	HSA-6803157	Antimicrobial peptides	9	87	0.94	0.00012	CHGA,LTF,BPIFB1,LYZ,PGLYRP2,CAMP,CLU,S100A7,S100A9
Reactome	HSA-975634	Retinoid metabolism and transport	7	44	1.13	0.00012	APOB,APOA1,TTR,LRP1,APOE,APOA4,APOM
Reactome	HSA-354194	GRB2:SOS provides linkage to MAPK signaling for Integrins	5	15	1.45	0.00016	VWF,FGB,FGG,FN1,FGA
Reactome	HSA-372708	p130Cas linkage to MAPK signaling for integrins	5	15	1.45	0.00016	VWF,FGB,FGG,FN1,FGA
Reactome	HSA-9662001	Defective factor VIII causes hemophilia A	4	7	1.68	0.00031	F9,VWF,F2,F10
Reactome	HSA-6802948	Signaling by high-kinase activity BRAF mutants	6	36	1.15	0.00047	VWF,FGB,FGG,FN1,ACTB,FGA
Reactome	HSA-5686938	Regulation of TLR by endogenous ligand	5	20	1.33	0.00048	APOB,FGB,FGG,S100A9,FGA

Reactome	HSA-173623	Classical antibody-mediated complement activation	4	9	1.57	0.00061	C1QB,C1QA,C1S,C1R
Reactome	HSA-216083	Integrin cell surface interactions	8	85	0.9	0.00066	THBS1,VWF,LUM,FGB,FGG,FN1,COL18A1,FGA
Reactome	HSA-76009	Platelet Aggregation (Plug Formation)	6	39	1.11	0.00066	VWF,FGB,F2,FGG,FN1,FGA
Reactome	HSA-5674135	MAP2K and MAPK activation	6	40	1.1	0.00072	VWF,FGB,FGG,FN1,ACTB,FGA
Reactome	HSA-8964058	HDL remodeling	4	10	1.53	0.00077	CETP,APOA1,APOE,LCAT
Reactome	HSA-9656223	Signaling by RAF1 mutants	6	41	1.09	0.00079	VWF,FGB,FGG,FN1,ACTB,FGA
Reactome	HSA-6802952	Signaling by BRAF and RAF1 fusions	7	65	0.96	0.00092	VWF,FGB,FGG,FN1,AKAP9,ACTB,FGA
Reactome	HSA-382551	Transport of small molecules	23	723	0.43	0.0010	CETP,APOB,APOA1,APOE,PSMA6,LCN1,LCAT,CP,TCIRG1,PIP,AZGP1,SLC27A4,ANGPTL4,PCSK9,BMP1,P4HB,APOD,APOA4,MCOLN2,ATP9B,APOA5,ATP1A1,APOC4
Reactome	HSA-1643685	Disease	40	1702	0.3	0.0011	ST6GAL1,F9,LTF,APOA1,TTR,CFP,THBS1,VWF,PSMA6,CP,KLKB1,LUM,SERPING1,SEC24D,SLC27A4,FGB,YWHAG,F2,CSPG4,SUZ12,TUBA1B,FGG,TUBB,FN1,BLM,AKAP9,S100A9,ADAMTSL4,ADAMTS13,F10,YWHAZ,MASP2,F11,TUBA1C,ATP1A1,PLCG2,TPM4,ACTB,B2M,FGA
Reactome	HSA-6802946	Signaling by moderate kinase activity BRAF mutants	6	45	1.05	0.0012	VWF,FGB,FGG,FN1,ACTB,FGA
Reactome	HSA-6802955	Paradoxical activation of RAF signaling by kinase inactive BRAF	6	45	1.05	0.0012	VWF,FGB,FGG,FN1,ACTB,FGA
Reactome	HSA-9649948	Signaling downstream of RAS mutants	6	45	1.05	0.0012	VWF,FGB,FGG,FN1,ACTB,FGA
Reactome	HSA-2855086	Ficolins bind to repetitive carbohydrate structures on the target cell surface	3	4	1.8	0.0020	FCN3,FCN1,MASP2
Reactome	HSA-3000497	Scavenging by Class H Receptors	3	4	1.8	0.0020	SPARC,APOB,STAB1
Reactome	HSA-5653656	Vesicle-mediated transport	21	666	0.43	0.0022	SPARC,APOB,APOA1,LRP1,APOE,AMBP,CENPE,HPX,SEC24D,YWHAG,STAB1,APOL1,TUBA1B,HP,F5,YWHAZ,SERPINA1,JCHAIN,HPR,TUBA1C,ACTB
Reactome	HSA-140834	Extrinsic Pathway of Fibrin Clot Formation	3	5	1.71	0.0030	F9,F10,F7
Reactome	HSA-173736	Alternative complement activation	3	5	1.71	0.0030	CFP,CFB,CFD
Reactome	HSA-8866423	VLDL assembly	3	5	1.71	0.0030	APOB,P4HB,APOC4
Reactome	HSA-5602498	MyD88 deficiency (TLR2/4)	4	17	1.3	0.0032	FGB,FGG,S100A9,FGA
Reactome	HSA-5603041	IRAK4 deficiency (TLR2/4)	4	18	1.27	0.0037	FGB,FGG,S100A9,FGA
Reactome	HSA-9029569	NR1H3 & NR1H2 regulate gene expression linked to cholesterol transport and efflux	5	36	1.07	0.0037	CETP,APOE,APOD,TNRC6A,APOC4
Reactome	HSA-1236974	ER-Phagosome pathway	7	89	0.82	0.0041	PSMA6,PDIA3,FGB,FGG,S100A9,B2M,FGA
Reactome	HSA-2129379	Molecules associated with elastic fibres	5	37	1.06	0.0041	EFEMP2,FBLN1,FN1,EFEMP1,FBN2
Reactome	HSA-6799990	Metal sequestration by antimicrobial proteins	3	6	1.63	0.0041	LTF,S100A7,S100A9
Reactome	HSA-8964041	LDL remodeling	3	6	1.63	0.0041	CETP,APOB,P4HB
Reactome	HSA-8964043	Plasma lipoprotein clearance	5	37	1.06	0.0041	APOB,APOA1,APOE,PCSK9,APOC4
Reactome	HSA-446728	Cell junction organization	7	92	0.81	0.0050	CDH6,DST,ANG,FLNA,ILK,ACTB,CDH5
Reactome	HSA-804914	Transport of fatty acids	3	8	1.5	0.0072	LCN1,SLC27A4,APOD
Reactome	HSA-9668250	Defective factor IX causes hemophilia B	3	9	1.45	0.0090	F9,F10,F11
Reactome	HSA-430116	GP1b-IX-V activation signalling	3	12	1.33	0.0178	VWF,FLNA,YWHAZ
Reactome	HSA-418990	Adherens junctions interactions	4	33	1.01	0.0236	CDH6,ANG,ACTB,CDH5
Reactome	HSA-3299685	Detoxification of Reactive Oxygen Species	4	36	0.97	0.0310	CAT,P4HB,SOD3,GPX3
Reactome	HSA-5083635	Defective B3GALTL causes PpS	4	37	0.96	0.0337	CFP,THBS1,ADAMTSL4,ADAMTS13
Reactome	HSA-9657688	Defective factor XII causes hereditary angioedema	2	3	1.75	0.0337	KLKB1,F2
Reactome	HSA-9657689	Defective SERPING1 causes hereditary angioedema	2	3	1.75	0.0337	KLKB1,SERPING1
Reactome	HSA-9672383	Defective factor IX causes thrombophilia	2	3	1.75	0.0337	F9,F10
Reactome	HSA-9672391	Defective F8 cleavage by thrombin	2	3	1.75	0.0337	VWF,F2
Reactome	HSA-9672396	Defective cofactor function of FVIIIa variant	2	3	1.75	0.0337	F9,F10
Reactome	HSA-9673202	Defective F9 variant does not activate FX	2	3	1.75	0.0337	F9,F10
Reactome	HSA-5173214	O-glycosylation of TSR domain-containing proteins	4	38	0.95	0.0342	CFP,THBS1,ADAMTSL4,ADAMTS13
Reactome	HSA-1474228	Degradation of the extracellular matrix	7	140	0.63	0.0409	KLKB1,BMP1,PLG,FN1,COL18A1,HTRA1,FBN2
Reactome	HSA-3000480	Scavenging by Class A Receptors	3	18	1.15	0.0418	APOB,APOA1,APOE
Reactome	HSA-446353	Cell-extracellular matrix interactions	3	18	1.15	0.0418	FLNA,ILK,ACTB
Reactome	HSA-1445148	Translocation of SLC2A4 (GLUT4) to the plasma membrane	5	71	0.77	0.0449	YWHAG,TUBA1B,YWHAZ,TUBA1C,ACTB
Reactome	HSA-8963889	Assembly of active LPL and LIPC lipase complexes	3	19	1.13	0.0466	ANGPTL4,APOA4,APOA5
WikiPathways	WP558	Complement and coagulation cascades	31	58	1.66	6.02e-35	CPB2,F9,PROC,VWF,C9,KLKB1,SERPING1,MASP1,FGB,F2,PLG,C1QB,CLU,SERPINF2,C7,F13B,CFH,F5,C1QA,F10,F7,PROS1,CFI,MASP2,C1S,C4B,SERPINA1,CFB,C1R,CFD,KNG1
WikiPathways	WP2806	Complement system	29	96	1.41	4.93e-27	C5,APOA1,CFP,APCS,THBS1,C9,KLKB1,F13A1,SERPING1,MASP1,FGB,PLG,C7,FGG,C8A,CFHR2,CFHR4,CFH,FCN1,F10,PROS1,CFI,MASP2,F11,C1S,C4A,CFB,CFD,FGA
WikiPathways	WP545	Complement activation	16	22	1.79	3.33e-19	C5,CFP,C9,MASP1,C1QB,C7,C8A,C8B,C1QA,MASP2,C1S,C4A,C4B,CFB,C1R,CFD
WikiPathways	WP5090	Complement system in neuronal development and plasticity	23	105	1.27	1.11e-18	C5,CFP,C9,FCN3,SERPING1,MASP1,C1QB,CLU,C7,C8A,CFH,C8B,FCN1,C1QA,PROS1,CFI,MASP2,C1S,C4A,C4B,CFB,C1R,CFD
WikiPathways	WP272	Blood clotting cascade	14	22	1.73	2.84e-16	F9,VWF,KLKB1,FGB,F2,PLG,SERPINF2,FGG,F13B,F5,F10,F7,F11,FGA
WikiPathways	WP5115	Network map of SARS-CoV-2 signaling pathway	22	218	0.93	1.21e-11	APOA1,CFP,SERPINA10,LGALS3BP,PF4,FGB,APOL1,FGG,APOD,FN1,HP,C8A,CFH,APOM,IGFBP3,CFI,C1S,ITIH3,CFB,C1R,ACTB,FGA
WikiPathways	WP4927	COVID-19, thrombosis and anticoagulation	7	7	1.93	9.74e-09	F13A1,FGB,F2,PLG,FGG,F13B,FGA
WikiPathways	WP15	Selenium micronutrient network	13	84	1.12	1.04e-08	APOB,APOA1,CAT,SAA4,FGB,F2,PLG,FGG,F7,SOD3,GPX3,SELENOP,FGA
WikiPathways	WP176	Folate metabolism	12	67	1.18	1.09e-08	APOB,APOA1,CAT,SAA4,FGB,F2,PLG,FGG,F7,SOD3,GPX3,FGA
WikiPathways	WP430	Statin inhibition of cholesterol production	8	29	1.37	6.96e-07	CETP,APOB,APOA1,LRP1,APOE,LCAT,APOA4,APOA5
WikiPathways	WP5108	Familial hyperlipidemia type 1	7	18	1.52	7.78e-07	CETP,APOA1,LRP1,LCAT,ANGPTL4,APOA4,APOA5
WikiPathways	WP2328	Allograft rejection	11	88	1.02	1.46e-06	C5,C9,C1QB,C7,TUBA1B,C8A,C8B,C1QA,C4A,C4B,VIM
WikiPathways	WP5186	Vitamin K metabolism and activation of dependent proteins	6	12	1.63	2.37e-06	F9,PROC,F2,F10,F7,PROS1
WikiPathways	WP5110	Familial hyperlipidemia type 3	6	13	1.59	3.10e-06	CETP,APOA1,LRP1,APOE,LCAT,APOA4
WikiPathways	WP5323	Fatty Acids and Lipoproteins Transport in Hepatocytes	20	380	0.65	3.10e-06	CETP,APOB,APOA1,LRP1,APOE,LGALS3BP,LCAT,SAA4,SLC27A4,PCSK9,APOL1,ASGR2,APOA4,APOM,CFI,PCYOX1,ATP9B,PRG4,APOA5,APOC4
WikiPathways	WP5112	Familial hyperlipidemia type 5	6	15	1.53	5.47e-06	CETP,APOA1,LRP1,LCAT,APOA4,APOA5
WikiPathways	WP5109	Familial hyperlipidemia type 2	6	16	1.5	7.01e-06	CETP,APOB,APOA1,LCAT,PCSK9,APOA4
WikiPathways	WP4522	Metabolic pathway of LDL, HDL and TG, including diseases	6	17	1.47	8.87e-06	CETP,APOB,APOA1,APOE,LCAT,PCSK9
WikiPathways	WP1533	Vitamin B12 metabolism	8	50	1.13	1.47e-05	APOB,APOA1,APOE,SAA4,F2,PLG,F7,SOD3
WikiPathways	WP5304	Cholesterol metabolism	9	72	1.02	1.71e-05	CETP,APOB,APOA1,LRP1,APOE,LCAT,SLC27A4,PCSK9,APOA4
WikiPathways	WP3601	Lipid particles composition	5	10	1.63	2.12e-05	CETP,APOB,APOA1,APOE,LCAT
WikiPathways	WP5111	Familial hyperlipidemia type 4	6	22	1.36	2.58e-05	CETP,APOA1,LRP1,LCAT,APOA4,APOA5
WikiPathways	WP3941	Oxidative damage response	6	39	1.11	0.00045	C5,C1QB,C1QA,C1S,C4B,C1R
WikiPathways	WP5089	Kinin-Kallikrein pathway	3	7	1.56	0.0058	KLKB1,SERPING1,KNG1
WikiPathways	WP5055	Burn wound healing	7	100	0.77	0.0082	SPARC,F13A1,AMBP,FN1,ADAM12,S100A9,VIM
WikiPathways	WP5104	Acquired partial lipodystrophy / Barraquer-Simons syndrome	3	10	1.4	0.0124	C5,CFB,CFD
WikiPathways	WP2272	Pathogenic Escherichia coli infection	5	55	0.89	0.0196	TUBA1B,TUBB,YWHAZ,TUBA1C,ACTB

WikiPathways	WP306	Focal adhesion	9	195	0.59	0.0196	THBS1,VWF,THBS4,FN1,FLNA,ILK,PDGFC,VEGFC,ACTB
WikiPathways	WP5333	Cholesterol metabolism	4	36	0.97	0.0322	APOB,APOA1,SLC27A4,APOA4
WikiPathways	WP3859	TGF-beta signaling in thyroid cells for epithelial-mesenchymal transition	3	17	1.17	0.0403	CDH6,FN1,VIM
WikiPathways	WP3942	PPAR signaling pathway	5	67	0.8	0.0403	APOA1,SLC27A4,ANGPTL4,ILK,APOA5
WikiPathways	WP4493	Cells and molecules involved in local acute inflammatory response	3	17	1.17	0.0403	C5,C7,KNG1
WikiPathways	WP2877	Vitamin D receptor pathway	8	183	0.57	0.0445	IGFBP5,IGFBP1,CAMP,S100A9,IGFBP3,ORM2,SEMA3B,KNG1
Monarch	EFO:0007937	Blood protein measurement	86	1810	0.6	5.83e-26	ST6GAL1,CPB2,CHGA,C5,LTF,APOB,IGFBP5,PROC,CAT,APOE,BPIFB1,SERPINF1,CFHR5,LYZ,VWF,SERPINA10,C9,LCAT,KLKB1,AMBP,HPX,ITIH1,AHSG,HABP2,SAA4,SERPING1,DSC2,ANKRD44,CSF1R,PGLYRP2,MASP1,YWHAG,F2,PLG,PSG1,PRG2,APOL1,CPN2,SERPINF2,C7,PAPPA,SERPINA11,ANG,FN1,ASGR2,CNDP1,COL18A1,F13B,CFHR4,CFH,F5,ADAM12,S100A9,ECM1,CRTAC1,C8B,FCN1,ADAMTS13,CCN5,SVEP1,F10,F7,APOM,CA6,IGFBP3,SOD3,F11,C1S,PSG2,PCYOX1,MST1,C4B,SERPINA1,TGFBI,CFB,GC,HGFAC,HPR,C1R,SERPINA4,RNASE4,APOC4,CCL18,VEGFC,PLCG2,KNG1
Monarch	HP:0005339	Abnormality of complement system	15	24	1.72	7.90e-16	C5,CFP,C9,C1QB,C7,C8A,CFH,C8B,C1QA,CFI,MASP2,C1S,C4A,C4B,CFD
Monarch	EFO:0004747	Protein measurement	133	5856	0.28	2.75e-14	ST6GAL1,CPB2,CETP,CHGA,C5,GPLD1,LTF,APOB,IGFBP5,PROC,APOA1,CAT,LRP1,APOE,ANGPTL6,BPIFB1,SERPINF1,APCS,CFHR5,LYZ,VWF,SERPINA10,C9,LCAT,CP,KLKB1,PPP2R3A,DGKG,DNAH5,AMBP,TCIRG1,HPX,INHBE,ITIH1,AHSG,HABP2,SAA4,SERPING1,DSC2,ANKRD44,CSF1R,PGLYRP2,MASP1,APEH,ANGPTL4,PCSK9,FGB,YWHAG,F2,INHBC,PLG,PSG1,UBE3C,PRG2,STAB1,CLU,APOL1,CPN2,SERPINF2,C7,PAPPA,FBLN1,DNAH8,OIT3,SERPINA11,ANG,FGG,THBS4,APOD,FN1,ASGR2,HP,APOA4,CNDP1,COL18A1,F13B,CFHR4,CFH,F5,ADAM12,S100A9,ECM1,CRTAC1,C8B,FCN1,ADAMTS13,CCN5,SVEP1,F10,F7,APOM,CA6,IGFBP3,SOD3,CFI,EFCAB5,TNRC6A,YWHAZ,NAV2,F11,C1S,PSG2,PCYOX1,ABCA13,MST1,ITIH3,C4B,SERPINA1,TGFBI,CFB,IGFALS,GC,HGFAC,PDGFC,FBN2,SELENOP,TSKU,HPR,TUBA1C,C1R,APOA5,SERPINA4,RNASE4,TTN,APOC4,CCL18,VEGFC,PLCG2,KNG1,ACTB,TRIM66,TRIM8,FGA
Monarch	HP:0004431	Complement deficiency	13	21	1.72	1.24e-13	C5,C9,C1QB,C7,C8A,CFH,C8B,C1QA,CFI,MASP2,C4A,C4B,CFD
Monarch	HP:0000118	Phenotypic abnormality	120	5129	0.3	4.40e-13	ST6GAL1,CETP,F9,C5,MGP,SPARC,LTF,APOB,PROC,APOA1,TTR,CAT,LRP1,CFP,APOE,ANGPTL6,SERPINF1,CFHR5,LYZ,VWF,PSMA6,C9,LCAT,CSTA,CP,KLKB1,F13A1,DNAH5,CENPE,TCIRG1,FCN3,AHSG,HABP2,SERPING1,SEC24D,DSC2,CSF1R,SERPINI1,MASP1,A2ML1,SLC27A4,PCSK9,BMP1,FGB,YWHAG,DST,F2,PLG,EFEMP2,STAB1,C1QB,CFHR1,CLU,NDUFS8,SUZ12,SERPINF2,C7,KDM3B,P4HB,PAPPA,FBLN1,DNAH8,ANG,FGG,TUBB,THBS4,FILIP1L,FN1,BLM,AKAP9,COL18A1,C8A,F13B,CFHR4,CFH,F5,HTRA1,ADAMTSL4,ECM1,FLNA,CRTAC1,C8B,ADAMTS13,SVEP1,C1QA,F10,F7,ADGRG2,PROS1,EFEMP1,CFI,NAV2,MASP2,F11,C1S,C4A,PRG4,ABCA13,LMOD3,ITIH3,C4B,SERPINA1,TGFBI,IGFALS,GC,FBN2,C1R,APOA5,ATP1A1,VIM,TTN,CFD,VEGFC,PLCG2,PAMR1,KNG1,ACTB,TRIM8,B2M,FGA
Monarch	EFO:0004634	Coagulation factor measurement	20	132	1.11	2.51e-12	CPB2,PROC,CFHR5,VWF,KLKB1,F13A1,PPP2R3A,FGB,FGG,CFHR4,CFH,F5,ADAMTS13,F10,F7,F11,HGFAC,TSKU,KNG1,FGA
Monarch	HP:0011025	Abnormal cardiovascular system physiology	49	1169	0.55	2.91e-11	F9,MGP,SPARC,APOB,PROC,APOA1,TTR,APOE,ANGPTL6,SERPINF1,LYZ,VWF,LCAT,CP,F13A1,TCIRG1,SERPING1,DSC2,A2ML1,PCSK9,BMP1,FGB,DST,F2,EFEMP2,SERPINF2,FGG,TUBB,FN1,AKAP9,F13B,F5,HTRA1,ADAMTSL4,FLNA,CRTAC1,ADAMTS13,F10,F7,PROS1,F11,SERPINA1,FBN2,TTN,PLCG2,KNG1,ACTB,B2M,FGA
Monarch	HP:0002597	Abnormality of the vasculature	59	1673	0.47	4.82e-11	F9,MGP,SPARC,APOB,PROC,APOA1,TTR,CAT,APOE,ANGPTL6,SERPINF1,LYZ,VWF,LCAT,CSTA,F13A1,DNAH5,TCIRG1,HABP2,SERPING1,SEC24D,MASP1,A2ML1,PCSK9,BMP1,FGB,F2,PLG,EFEMP2,SERPINF2,KDM3B,P4HB,FGG,FN1,BLM,COL18A1,F13B,F5,HTRA1,FLNA,CRTAC1,ADAMTS13,F10,F7,PROS1,EFEMP1,CFI,C1S,C4A,SERPINA1,TGFBI,FBN2,C1R,VEGFC,PLCG2,KNG1,ACTB,B2M,FGA
Monarch	HP:0001626	Abnormality of the cardiovascular system	73	2438	0.4	5.38e-11	F9,MGP,SPARC,APOB,PROC,APOA1,TTR,CAT,LRP1,APOE,ANGPTL6,SERPINF1,LYZ,VWF,LCAT,CSTA,CP,KLKB1,F13A1,DNAH5,TCIRG1,HABP2,SERPING1,SEC24D,DSC2,MASP1,A2ML1,PCSK9,BMP1,FGB,DST,F2,PLG,EFEMP2,NDUFS8,SUZ12,SERPINF2,KDM3B,P4HB,FGG,TUBB,FN1,BLM,AKAP9,COL18A1,F13B,F5,HTRA1,ADAMTSL4,FLNA,CRTAC1,ADAMTS13,F10,F7,PROS1,EFEMP1,CFI,F11,C1S,C4A,PRG4,SERPINA1,TGFBI,FBN2,C1R,TTN,VEGFC,PLCG2,KNG1,ACTB,TRIM8,B2M,FGA
Monarch	HP:0025015	Abnormal vascular morphology	45	1027	0.57	6.57e-11	F9,MGP,SPARC,APOB,PROC,TTR,CAT,APOE,ANGPTL6,VWF,LCAT,F13A1,DNAH5,TCIRG1,HABP2,SEC24D,MASP1,A2ML1,PCSK9,FGB,F2,EFEMP2,SERPINF2,KDM3B,P4HB,FGG,FN1,COL18A1,F13B,F5,HTRA1,FLNA,CRTAC1,ADAMTS13,F10,F7,PROS1,EFEMP1,CFI,C4A,FBN2,VEGFC,KNG1,ACTB,FGA
Monarch	HP:0003256	Abnormality of the coagulation cascade	17	105	1.14	7.60e-11	F9,PROC,VWF,F13A1,FGB,F2,PLG,FGG,F13B,F5,FLNA,F10,F7,PROS1,F11,KNG1,FGA
Monarch	HP:0001928	Abnormality of coagulation	20	170	1.0	9.65e-11	F9,PROC,VWF,KLKB1,F13A1,A2ML1,FGB,F2,PLG,SERPINF2,FGG,F13B,F5,FLNA,F10,F7,PROS1,F11,KNG1,FGA
Monarch	HP:0000225	Gingival bleeding	13	45	1.39	1.01e-10	CAT,F13A1,FGB,F2,SERPINF2,FGG,F13B,F5,F10,F7,C1S,C1R,FGA
Monarch	HP:0001892	Abnormal bleeding	28	411	0.76	2.75e-10	F9,SPARC,PROC,CAT,APOE,ANGPTL6,VWF,F13A1,TCIRG1,SEC24D,A2ML1,FGB,F2,SERPINF2,P4HB,FGG,FN1,F13B,F5,FLNA,F10,F7,PROS1,F11,C1S,C4A,C1R,FGA
Monarch	HP:0005261	Joint hemorrhage	10	19	1.65	5.27e-10	F9,VWF,F13A1,F2,SERPINF2,F13B,F5,F10,F7,F11
Monarch	HP:0100659	Abnormal cerebral vascular morphology	26	381	0.76	1.73e-09	F9,SPARC,APOB,PROC,TTR,APOE,ANGPTL6,F13A1,PCSK9,FGB,F2,SERPINF2,FGG,FN1,F13B,F5,HTRA1,FLNA,CRTAC1,ADAMTS13,F10,F7,PROS1,KNG1,ACTB,FGA
Monarch	HP:0001871	Abnormality of blood and blood-forming tissues	46	1214	0.51	2.72e-09	F9,SPARC,APOB,PROC,APOA1,CAT,APOE,ANGPTL6,VWF,LCAT,CP,KLKB1,F13A1,TCIRG1,SEC24D,A2ML1,SLC27A4,FGB,F2,PLG,NDUFS8,SERPINF2,P4HB,FGG,FN1,BLM,F13B,CFH,F5,FLNA,ADAMTS13,SVEP1,F10,F7,PROS1,F11,C1S,C4A,SERPINA1,FBN2,C1R,TTN,PLCG2,KNG1,B2M,FGA
Monarch	HP:0002715	Abnormality of the immune system	55	1682	0.44	3.75e-09	C5,MGP,APOA1,CAT,CFP,APOE,CFHR5,LYZ,PSMA6,C9,LCAT,CSTA,F13A1,DNAH5,TCIRG1,FCN3,HABP2,SERPING1,A2ML1,SLC27A4,FGB,F2,PLG,EFEMP2,C1QB,CFHR1,C7,FGG,BLM,COL18A1,C8A,F13B,CFHR4,CFH,ECM1,FLNA,C8B,SVEP1,C1QA,CFI,NAV2,MASP2,C1S,C4A,PRG4,C4B,SERPINA1,C1R,TTN,CFD,VEGFC,PLCG2,ACTB,B2M,FGA
Monarch	HP:0030680	Abnormality of cardiovascular system morphology	54	1680	0.43	1.10e-08	F9,MGP,SPARC,APOB,PROC,TTR,CAT,APOE,ANGPTL6,VWF,LCAT,F13A1,DNAH5,TCIRG1,HABP2,SEC24D,DSC2,MASP1,A2ML1,PCSK9,FGB,F2,EFEMP2,NDUFS8,SUZ12,SERPINF2,KDM3B,P4HB,FGG,TUBB,FN1,COL18A1,F13B,F5,HTRA1,FLNA,CRTAC1,ADAMTS13,F10,F7,PROS1,EFEMP1,CFI,C4A,PRG4,SERPINA1,FBN2,TTN,VEGFC,KNG1,ACTB,TRIM8,B2M,FGA
Monarch	HP:0005368	Abnormality of humoral immunity	20	234	0.86	1.30e-08	C5,CFP,C9,C1QB,C7,BLM,C8A,CFH,FLNA,C8B,C1QA,CFI,MASP2,C1S,C4A,C4B,SERPINA1,CFD,PLCG2,B2M
Monarch	HP:0001933	Subcutaneous hemorrhage	18	189	0.91	2.52e-08	PROC,APOE,VWF,F13A1,TCIRG1,SEC24D,F2,SERPINF2,P4HB,F13B,F5,FLNA,F10,F7,PROS1,C1S,C4A,C1R
Monarch	HP:0011890	Prolonged bleeding following procedure	9	23	1.52	4.30e-08	F9,VWF,F13A1,F2,F13B,F5,F10,F7,F11
Monarch	HP:0002170	Intracranial hemorrhage	15	130	0.99	8.71e-08	F9,SPARC,ANGPTL6,F13A1,FGB,F2,SERPINF2,FGG,FN1,F13B,F5,F10,F7,PROS1,FGA
Monarch	HP:0006298	Prolonged bleeding after dental extraction	8	16	1.63	9.84e-08	F9,VWF,F13A1,F2,F13B,F5,F10,F11

Monarch	HP:0010978	Abnormality of immune system physiology	45	1347	0.45	1.81e-07	C5,MGP,APOA1,CAT,CFP,APOE,CFHR5,LYZ,PSMA6,C9,CSTA,F13A1,DNAH5,TCIRG1,FCN3,SERPING1,PLG,EFEMP2,C1QB,CFHR1,C7,BLM,C8A,F13B,CFHR4,CFH,ECM1,FLNA,C8B,C1QA,CFI,NAV2,MASP2,C1S,C4A,PRG4,C4B,SERPINA1,C1R,CFD,VEGFC,PLCG2,ACTB,B2M,FGA
Monarch	HP:0000951	Abnormality of the skin	55	1888	0.39	1.87e-07	F9,C5,MGP,SPARC,APOB,PROC,APOA1,CAT,LRP1,APOE,LYZ,VWF,PSMA6,CSTA,F13A1,CENPE,TCIRG1,FCN3,AHSG,SERPING1,SEC24D,DSC2,MASP1,A2ML1,SLC27A4,PCSK9,DST,F2,PLG,EFEMP2,SUZ12,SERPINF2,P4HB,TUBB,BLM,F13B,CFHR4,F5,ECM1,FLNA,ADAMTS13,F10,F7,PROS1,CFI,C1S,C4A,SERPINA1,C1R,TTN,VEGFC,PLCG2,ACTB,B2M,FGA
Monarch	HP:0012233	Intramuscular hematoma	7	10	1.77	2.17e-07	F9,F13A1,F2,SERPINF2,F13B,F10,F7
Monarch	HP:0000421	Epistaxis	12	78	1.11	2.62e-07	F9,VWF,F13A1,FGB,F2,FGG,F13B,F5,F10,F7,F11,FGA
Monarch	HP:0012649	Increased inflammatory response	37	981	0.5	3.20e-07	C5,MGP,APOA1,CAT,APOE,CFHR5,LYZ,PSMA6,CSTA,F13A1,DNAH5,TCIRG1,FCN3,PLG,EFEMP2,C1QB,CFHR1,BLM,F13B,CFHR4,CFH,ECM1,FLNA,C1QA,CFI,NAV2,MASP2,C1S,C4A,PRG4,C4B,SERPINA1,C1R,VEGFC,PLCG2,B2M,FGA
Monarch	HP:0011276	Vascular skin abnormality	24	433	0.67	3.60e-07	PROC,APOE,VWF,CSTA,F13A1,TCIRG1,SERPING1,SEC24D,MASP1,F2,SERPINF2,P4HB,BLM,F13B,F5,FLNA,F10,F7,PROS1,C1S,C4A,SERPINA1,C1R,PLCG2
Monarch	HP:0012211	Abnormal renal physiology	27	560	0.61	5.36e-07	F9,APOA1,TTR,APOE,CFHR5,LYZ,LCAT,F2,PLG,EFEMP2,C1QB,CFHR1,NDUFS8,SERPINF2,FN1,CFH,F5,ADAMTS13,C1QA,F10,CFI,C4A,SERPINA1,C1R,ATP1A1,B2M,FGA
Monarch	HP:0000978	Bruising susceptibility	14	130	0.96	5.72e-07	VWF,F13A1,TCIRG1,SEC24D,F2,SERPINF2,P4HB,F13B,F5,FLNA,F10,F7,C1S,C1R
Monarch	HP:0001574	Abnormality of the integument	60	2266	0.35	6.93e-07	F9,C5,MGP,SPARC,APOB,PROC,APOA1,CAT,LRP1,APOE,LYZ,VWF,PSMA6,CSTA,F13A1,CENPE,TCIRG1,FCN3,AHSG,SERPING1,SEC24D,DSC2,MASP1,A2ML1,SLC27A4,PCSK9,BMP1,DST,F2,PLG,EFEMP2,NDUFS8,SUZ12,SERPINF2,P4HB,TUBB,BLM,COL18A1,F13B,CFHR4,F5,HTRA1,ECM1,FLNA,ADAMTS13,F10,F7,PROS1,CFI,C1S,C4A,SERPINA1,C1R,TTN,VEGFC,PLCG2,ACTB,TRIM8,B2M,FGA
Monarch	HP:0011029	Internal hemorrhage	18	244	0.79	7.01e-07	F9,SPARC,PROC,ANGPTL6,VWF,F13A1,FGB,F2,SERPINF2,FGG,FN1,F13B,F5,F10,F7,PROS1,F11,FGA
Monarch	HP:0000271	Abnormality of the face	66	2641	0.32	7.81e-07	F9,MGP,SPARC,LTF,APOB,PROC,CAT,LRP1,APOE,SERPINF1,LYZ,VWF,CP,F13A1,DNAH5,CENPE,TCIRG1,AHSG,SERPING1,SEC24D,CSF1R,MASP1,A2ML1,PCSK9,BMP1,FGB,YWHAG,DST,F2,PLG,EFEMP2,SUZ12,SERPINF2,KDM3B,P4HB,PAPPA,FBLN1,ANG,FGG,TUBB,FILIP1L,BLM,COL18A1,F13B,F5,ADAMTSL4,ECM1,FLNA,CRTAC1,F10,F7,CFI,F11,C1S,ABCA13,LMOD3,SERPINA1,FBN2,C1R,TTN,VEGFC,PLCG2,ACTB,TRIM8,B2M,FGA
Monarch	HP:0000168	Abnormality of the gingiva	16	190	0.85	9.28e-07	CAT,F13A1,TCIRG1,FGB,F2,PLG,SERPINF2,FGG,F13B,F5,ECM1,F10,F7,C1S,C1R,FGA
Monarch	HP:0002725	Systemic lupus erythematosus	8	26	1.42	1.39e-06	SERPING1,C1QB,C8A,C1QA,MASP2,C1S,C4A,C1R
Monarch	HP:0000790	Hematuria	13	118	0.97	1.46e-06	F9,APOA1,CFHR5,LYZ,F2,CFHR1,SERPINF2,FN1,CFH,F5,ADAMTS13,F10,FGA
Monarch	HP:0000152	Abnormality of head or neck	69	2882	0.31	1.50e-06	F9,MGP,SPARC,LTF,APOB,PROC,CAT,LRP1,APOE,SERPINF1,LYZ,VWF,CP,F13A1,DNAH5,CENPE,TCIRG1,AHSG,HABP2,SERPING1,SEC24D,CSF1R,MASP1,A2ML1,PCSK9,BMP1,FGB,YWHAG,DST,F2,PLG,EFEMP2,NDUFS8,SUZ12,SERPINF2,KDM3B,P4HB,PAPPA,FBLN1,ANG,FGG,TUBB,FILIP1L,BLM,COL18A1,F13B,F5,ADAMTSL4,ECM1,FLNA,CRTAC1,F10,F7,CFI,F11,C1S,ABCA13,LMOD3,SERPINA1,IGFALS,FBN2,C1R,TTN,VEGFC,PLCG2,ACTB,TRIM8,B2M,FGA
Monarch	HP:0000123	Nephritis	10	59	1.16	2.12e-06	CFHR5,PLG,EFEMP2,C1QB,CFHR1,CFH,C1QA,CFI,C4A,C1R
Monarch	HP:0000234	Abnormality of the head	68	2865	0.3	2.69e-06	F9,MGP,SPARC,LTF,APOB,PROC,CAT,LRP1,APOE,SERPINF1,LYZ,VWF,CP,F13A1,DNAH5,CENPE,TCIRG1,AHSG,SERPING1,SEC24D,CSF1R,MASP1,A2ML1,PCSK9,BMP1,FGB,YWHAG,DST,F2,PLG,EFEMP2,NDUFS8,SUZ12,SERPINF2,KDM3B,P4HB,PAPPA,FBLN1,ANG,FGG,TUBB,FILIP1L,BLM,COL18A1,F13B,F5,ADAMTSL4,ECM1,FLNA,CRTAC1,F10,F7,CFI,F11,C1S,ABCA13,LMOD3,SERPINA1,IGFALS,FBN2,C1R,TTN,VEGFC,PLCG2,ACTB,TRIM8,B2M,FGA
Monarch	HP:0004846	Prolonged bleeding after surgery	7	18	1.52	2.97e-06	F9,VWF,F13A1,F13B,F5,F10,F7
Monarch	HP:0000707	Abnormality of the nervous system	77	3471	0.27	3.35e-06	ST6GAL1,F9,MGP,SPARC,APOB,PROC,APOA1,TTR,CAT,LRP1,APOE,ANGPTL6,CP,F13A1,DNAH5,CENPE,TCIRG1,AHSG,HABP2,SERPING1,SEC24D,CSF1R,SERPINI1,MASP1,A2ML1,PCSK9,BMP1,FGB,YWHAG,DST,F2,PLG,EFEMP2,STAB1,NDUFS8,SUZ12,SERPINF2,KDM3B,P4HB,FBLN1,ANG,FGG,TUBB,FN1,BLM,AKAP9,COL18A1,C8A,F13B,F5,HTRA1,ADAMTSL4,ECM1,FLNA,CRTAC1,C8B,ADAMTS13,F10,F7,PROS1,CFI,LMOD3,ITIH3,C4B,SERPINA1,TGFBI,FBN2,ATP1A1,VIM,TTN,VEGFC,PAMR1,KNG1,ACTB,TRIM8,B2M,FGA
Monarch	HP:0010935	Abnormality of the upper urinary tract	37	1101	0.45	3.91e-06	F9,APOB,APOA1,TTR,APOE,CFHR5,LYZ,LCAT,HABP2,MASP1,PCSK9,F2,PLG,EFEMP2,C1QB,CFHR1,NDUFS8,SERPINF2,FN1,BLM,COL18A1,CFH,F5,FLNA,ADAMTS13,C1QA,F10,ADGRG2,CFI,C4A,SERPINA1,C1R,ATP1A1,ACTB,TRIM8,B2M,FGA
Monarch	HP:0011891	Post-partum hemorrhage	6	10	1.71	4.42e-06	F13A1,F2,F13B,F5,F10,F7
Monarch	HP:0011121	Abnormality of skin morphology	47	1648	0.38	4.84e-06	F9,MGP,SPARC,APOB,PROC,APOA1,CAT,LRP1,APOE,VWF,CSTA,F13A1,CENPE,TCIRG1,AHSG,SERPING1,SEC24D,DSC2,MASP1,A2ML1,SLC27A4,PCSK9,DST,F2,EFEMP2,SUZ12,SERPINF2,P4HB,TUBB,BLM,F13B,F5,ECM1,FLNA,ADAMTS13,F10,F7,PROS1,C1S,C4A,SERPINA1,C1R,TTN,VEGFC,PLCG2,ACTB,B2M
Monarch	HP:0000077	Abnormality of the kidney	36	1077	0.45	6.76e-06	F9,APOB,APOA1,TTR,APOE,CFHR5,LYZ,LCAT,HABP2,MASP1,PCSK9,F2,PLG,EFEMP2,C1QB,CFHR1,NDUFS8,SERPINF2,FN1,BLM,CFH,F5,FLNA,ADAMTS13,C1QA,F10,ADGRG2,CFI,C4A,SERPINA1,C1R,ATP1A1,ACTB,TRIM8,B2M,FGA
Monarch	HP:0010990	Abnormality of the common coagulation pathway	8	35	1.29	7.54e-06	F13A1,FGB,PLG,FGG,F13B,F5,F10,FGA
Monarch	HP:0001934	Persistent bleeding after trauma	6	12	1.63	9.27e-06	F9,VWF,F13A1,SERPINF2,F13B,F5
Monarch	HP:0011884	Abnormal umbilical stump bleeding	5	5	1.93	1.16e-05	F13A1,F2,SERPINF2,F13B,F10
Monarch	HP:0011830	Abnormal oral mucosa morphology	18	305	0.7	1.17e-05	LTF,CAT,LYZ,F13A1,TCIRG1,FGB,F2,PLG,SERPINF2,FGG,F13B,F5,ECM1,F10,F7,C1S,C1R,FGA
Monarch	HP:0000132	Menorrhagia	8	38	1.25	1.22e-05	VWF,F13A1,F2,F13B,F5,F10,F7,F11
Monarch	HP:0003645	Prolonged partial thromboplastin time	7	24	1.39	1.23e-05	F9,KLKB1,F2,F5,F10,F11,KNG1
Monarch	HP:0000119	Abnormality of the genitourinary system	55	2189	0.33	1.40e-05	F9,APOB,APOA1,TTR,APOE,CFHR5,LYZ,VWF,LCAT,F13A1,DNAH5,AHSG,HABP2,MASP1,A2ML1,PCSK9,FGB,F2,PLG,EFEMP2,C1QB,CFHR1,NDUFS8,SUZ12,SERPINF2,KDM3B,FBLN1,DNAH8,FGG,TUBB,FN1,BLM,COL18A1,F13B,CFH,F5,HTRA1,FLNA,ADAMTS13,C1QA,F10,F7,ADGRG2,CFI,F11,C4A,LMOD3,SERPINA1,C1R,ATP1A1,VEGFC,ACTB,TRIM8,B2M,FGA
Monarch	HP:0033057	Decreased serum terminal complement component	5	6	1.85	1.92e-05	C5,C9,C7,C8A,C8B
Monarch	HP:0012639	Abnormal nervous system morphology	60	2546	0.3	2.66e-05	F9,MGP,SPARC,APOB,PROC,TTR,APOE,ANGPTL6,CP,F13A1,DNAH5,CENPE,TCIRG1,AHSG,HABP2,SERPING1,SEC24D,CSF1R,SERPINI1,MASP1,PCSK9,FGB,YWHAG,F2,PLG,EFEMP2,NDUFS8,SUZ12,SERPINF2,KDM3B,P4HB,FBLN1,ANG,FGG,TUBB,FN1,BLM,COL18A1,C8A,F13B,F5,HTRA1,ECM1,FLNA,CRTAC1,C8B,ADAMTS13,F10,F7,PROS1,CFI,LMOD3,C4B,ATP1A1,TTN,KNG1,ACTB,TRIM8,B2M,FGA
Monarch	HP:0004936	Venous thrombosis	9	65	1.07	4.06e-05	F9,PROC,FGB,F2,FGG,F5,PROS1,KNG1,FGA

Monarch	EFO:0008390	Prothrombin time measurement	5	8	1.72	4.99e-05	F2,FGG,F5,F10,F7
Monarch	HP:0002011	Morphological central nervous system abnormality	57	2416	0.3	5.61e-05	F9,MGP,SPARC,APOB,PROC,TTR,APOE,ANGPTL6,CP,F13A1,DNAH5,CENPE,TCIRG1,AHSG,HABP2,SEC24D,CSF1R,SERPINI1,MASP1,PCSK9,FGB,YWHAG,F2,PLG,EFEMP2,NDUFS8,SUZ12,SERPINF2,KDM3B,P4HB,FBLN1,ANG,FGG,TUBB,FN1,BLM,COL18A1,C8A,F13B,F5,HTRA1,ECM1,FLNA,CRTAC1,C8B,ADAMTS13,F10,F7,PROS1,CFI,C4B,ATP1A1,KNG1,ACTB,TRIM8,B2M,FGA
Monarch	HP:0030140	Oral cavity bleeding	5	9	1.67	7.44e-05	F13A1,F2,F13B,F5,F10
Monarch	HP:0033353	Abnormal blood vessel morphology	28	792	0.48	7.44e-05	MGP,APOB,PROC,CAT,APOE,ANGPTL6,VWF,LCAT,DNAH5,TCIRG1,SEC24D,MASP1,A2ML1,PCSK9,EFEMP2,KDM3B,P4HB,FN1,COL18A1,HTRA1,FLNA,PROS1,EFEMP1,CFI,C4A,FBN2,VEGFC,ACTB
Monarch	HP:0000924	Abnormality of the skeletal system	61	2708	0.28	8.67e-05	F9,MGP,SPARC,APOB,TTR,APOE,SERPINF1,VWF,F13A1,DNAH5,CENPE,TCIRG1,AHSG,HABP2,SEC24D,CSF1R,MASP1,A2ML1,PCSK9,BMP1,FGB,YWHAG,DST,F2,PLG,EFEMP2,NDUFS8,SUZ12,SERPINF2,KDM3B,P4HB,FBLN1,FGG,TUBB,FN1,BLM,COL18A1,F13B,F5,HTRA1,ADAMTSL4,ECM1,FLNA,F10,F7,CFI,F11,C1S,PRG4,LMOD3,SERPINA1,IGFALS,FBN2,C1R,ATP1A1,TTN,VEGFC,ACTB,TRIM8,B2M,FGA
Monarch	EFO:0001365	Age-related macular degeneration	7	35	1.23	9.60e-05	CETP,APOE,CFHR5,C9,CFH,CFI,CFB
Monarch	EFO:1001492	Atrophic macular degeneration	7	35	1.23	9.60e-05	CETP,APOE,CFHR5,C9,CFH,CFI,CFB
Monarch	HP:0001367	Abnormal joint morphology	30	905	0.45	9.60e-05	F9,APOE,SERPINF1,VWF,F13A1,MASP1,A2ML1,BMP1,FGB,F2,EFEMP2,SUZ12,SERPINF2,FBLN1,FGG,FN1,F13B,F5,FLNA,F10,F7,CFI,F11,PRG4,LMOD3,SERPINA1,FBN2,VEGFC,ACTB,FGA
Monarch	HP:0000099	Glomerulonephritis	7	37	1.2	0.00011	CFHR5,C1QB,CFHR1,CFH,C1QA,CFI,C4A
Monarch	HP:0000153	Abnormality of the mouth	49	1992	0.32	0.00012	MGP,SPARC,LTF,CAT,APOE,SERPINF1,LYZ,F13A1,CENPE,TCIRG1,SERPING1,SEC24D,MASP1,A2ML1,BMP1,FGB,YWHAG,DST,F2,PLG,EFEMP2,SUZ12,SERPINF2,KDM3B,P4HB,PAPPA,FBLN1,ANG,FGG,TUBB,FILIP1L,BLM,F13B,F5,ECM1,FLNA,CRTAC1,F10,F7,C1S,ABCA13,LMOD3,FBN2,C1R,TTN,ACTB,TRIM8,B2M,FGA
Monarch	HP:0011842	Abnormal skeletal morphology	58	2573	0.28	0.00015	F9,MGP,SPARC,APOB,TTR,APOE,SERPINF1,VWF,F13A1,DNAH5,CENPE,TCIRG1,AHSG,SEC24D,CSF1R,MASP1,A2ML1,PCSK9,BMP1,FGB,YWHAG,DST,F2,PLG,EFEMP2,NDUFS8,SUZ12,SERPINF2,KDM3B,P4HB,FBLN1,FGG,TUBB,FN1,BLM,COL18A1,F13B,F5,HTRA1,ADAMTSL4,FLNA,F10,F7,CFI,F11,C1S,PRG4,LMOD3,SERPINA1,IGFALS,FBN2,C1R,TTN,VEGFC,ACTB,TRIM8,B2M,FGA
Monarch	HP:0025031	Abnormality of the digestive system	55	2389	0.29	0.00016	F9,C5,APOB,APOA1,TTR,APOE,LYZ,VWF,LCAT,CP,F13A1,DNAH5,TCIRG1,FCN3,HABP2,SERPING1,MASP1,A2ML1,PCSK9,BMP1,FGB,YWHAG,DST,F2,PLG,EFEMP2,NDUFS8,SUZ12,KDM3B,ANG,FGG,TUBB,BLM,COL18A1,F13B,F5,ECM1,FLNA,ADAMTS13,F10,F7,NAV2,MASP2,F11,C1S,LMOD3,C4B,SERPINA1,FBN2,C1R,PLCG2,ACTB,TRIM8,B2M,FGA
Monarch	HP:0033127	Abnormality of the musculoskeletal system	67	3173	0.25	0.00016	F9,MGP,SPARC,APOB,APOA1,TTR,LRP1,APOE,SERPINF1,VWF,CP,F13A1,DNAH5,CENPE,TCIRG1,AHSG,HABP2,SEC24D,CSF1R,MASP1,A2ML1,PCSK9,BMP1,FGB,YWHAG,DST,F2,PLG,EFEMP2,NDUFS8,SUZ12,SERPINF2,KDM3B,P4HB,FBLN1,ANG,FGG,TUBB,FN1,BLM,COL18A1,F13B,F5,HTRA1,ADAMTSL4,ECM1,FLNA,F10,F7,CFI,F11,C1S,PRG4,LMOD3,SERPINA1,TGFBI,IGFALS,FBN2,C1R,ATP1A1,TTN,VEGFC,PLCG2,ACTB,TRIM8,B2M,FGA
Monarch	HP:0000163	Abnormal oral cavity morphology	47	1901	0.32	0.00018	MGP,SPARC,LTF,CAT,APOE,SERPINF1,LYZ,F13A1,CENPE,TCIRG1,SERPING1,SEC24D,MASP1,A2ML1,BMP1,FGB,YWHAG,DST,F2,PLG,EFEMP2,SUZ12,SERPINF2,KDM3B,P4HB,PAPPA,FGG,TUBB,FILIP1L,BLM,F13B,F5,ECM1,FLNA,CRTAC1,F10,F7,C1S,ABCA13,LMOD3,FBN2,C1R,TTN,ACTB,TRIM8,B2M,FGA
Monarch	HP:0002239	Gastrointestinal hemorrhage	10	111	0.88	0.00023	F9,VWF,FGB,F2,FGG,F5,F10,F7,F11,FGA
Monarch	EFO:0004614	Apolipoprotein A 1 measurement	18	396	0.58	0.00028	CETP,APOB,APOA1,LRP1,APOE,LCAT,DGKG,ITIH1,ANGPTL4,PCSK9,PLG,STAB1,APOA4,COL18A1,CRTAC1,HGFAC,HPR,APOC4
Monarch	HP:0005430	Recurrent Neisserial infections	4	5	1.83	0.00031	C5,C7,C8B,CFI
Monarch	HP:0011354	Generalized abnormality of skin	27	821	0.44	0.00034	F9,PROC,APOE,VWF,CSTA,F13A1,CENPE,TCIRG1,SERPING1,SEC24D,MASP1,F2,SERPINF2,P4HB,BLM,F13B,F5,FLNA,ADAMTS13,F10,F7,PROS1,C1S,C4A,SERPINA1,C1R,PLCG2
Monarch	EFO:0004310	Partial thromboplastin time	5	15	1.45	0.00039	KLKB1,F5,F11,C1S,KNG1
Monarch	HP:0010979	Abnormality of lipoprotein cholesterol concentration	7	47	1.1	0.00041	CETP,APOB,APOA1,APOE,LCAT,PCSK9,APOA5
Monarch	HP:0002960	Autoimmunity	10	122	0.84	0.00047	TCIRG1,SERPING1,C1QB,C8A,C1QA,MASP2,C1S,C4A,C1R,PLCG2
Monarch	HP:0030163	Abnormal vascular physiology	16	340	0.6	0.00065	MGP,PROC,ANGPTL6,SERPINF1,TCIRG1,BMP1,F2,F5,HTRA1,FLNA,CRTAC1,ADAMTS13,PROS1,SERPINA1,KNG1,ACTB
Monarch	HP:0000140	Abnormality of the menstrual cycle	13	228	0.68	0.00073	F9,VWF,F13A1,FGB,F2,FGG,F13B,F5,F10,F7,F11,SERPINA1,FGA
Monarch	HP:0010980	Hyperlipoproteinemia	6	33	1.19	0.00073	CETP,APOB,APOE,LCAT,PCSK9,APOA5
Monarch	HP:0000079	Abnormality of the urinary system	41	1657	0.32	0.00082	F9,APOB,APOA1,TTR,APOE,CFHR5,LYZ,LCAT,HABP2,MASP1,PCSK9,F2,PLG,EFEMP2,C1QB,CFHR1,NDUFS8,SERPINF2,KDM3B,TUBB,FN1,BLM,COL18A1,CFH,F5,HTRA1,FLNA,ADAMTS13,C1QA,F10,ADGRG2,CFI,C4A,LMOD3,SERPINA1,C1R,ATP1A1,ACTB,TRIM8,B2M,FGA
Monarch	HP:0030012	Abnormal female reproductive system physiology	14	273	0.64	0.00098	F9,VWF,F13A1,FGB,F2,FGG,BLM,F13B,F5,F10,F7,F11,SERPINA1,FGA
Monarch	HP:0011277	Abnormality of the urinary system physiology	28	933	0.4	0.0010	F9,APOA1,TTR,APOE,CFHR5,LYZ,LCAT,F2,PLG,EFEMP2,C1QB,CFHR1,NDUFS8,SERPINF2,FN1,BLM,CFH,F5,ADAMTS13,C1QA,F10,CFI,C4A,SERPINA1,C1R,ATP1A1,B2M,FGA
Monarch	HP:0002625	Deep venous thrombosis	5	20	1.33	0.0011	F9,PROC,F2,F5,PROS1
Monarch	HP:0012443	Abnormality of brain morphology	50	2254	0.27	0.0013	F9,MGP,SPARC,APOB,PROC,TTR,APOE,ANGPTL6,CP,F13A1,DNAH5,CENPE,TCIRG1,AHSG,SEC24D,CSF1R,SERPINI1,MASP1,PCSK9,FGB,YWHAG,F2,PLG,EFEMP2,NDUFS8,SUZ12,SERPINF2,KDM3B,P4HB,FBLN1,FGG,TUBB,FN1,BLM,COL18A1,F13B,F5,HTRA1,ECM1,FLNA,CRTAC1,ADAMTS13,F10,F7,PROS1,ATP1A1,KNG1,ACTB,TRIM8,FGA
Monarch	EFO:0004574	Total cholesterol measurement	19	497	0.51	0.0014	CETP,APOB,APOA1,APOE,LCAT,PCSK9,FGB,OIT3,FN1,ASGR2,HP,APOA4,COL18A1,ADAMTS13,SERPINA1,HGFAC,HPR,APOA5,APOC4
Monarch	HP:0030137	Prolonged bleeding following circumcision	4	9	1.57	0.0014	F13A1,F2,F13B,F5
Monarch	HP:0031888	Abnormal HDL cholesterol concentration	5	21	1.3	0.0014	CETP,APOA1,APOE,LCAT,APOA5
Monarch	HP:0032101	Unusual infection	24	748	0.43	0.0016	C5,MGP,DNAH5,TCIRG1,FCN3,PLG,EFEMP2,C1QB,C7,BLM,C8A,CFH,ECM1,FLNA,C8B,C1QA,CFI,MASP2,C4B,SERPINA1,C1R,CFD,PLCG2,B2M
Monarch	HP:0001342	Cerebral hemorrhage	7	61	0.99	0.0017	F13A1,FGB,FGG,FN1,F13B,PROS1,FGA
Monarch	EFO:0004732	Lipoprotein measurement	36	1426	0.33	0.0019	CETP,APOB,APOA1,LRP1,APOE,LCAT,KLKB1,PPP2R3A,DGKG,INHBE,ITIH1,ANGPTL4,PCSK9,FGB,F2,INHBC,PLG,STAB1,FBLN1,OIT3,THBS4,APOD,FN1,ASGR2,HP,APOA4,COL18A1,CRTAC1,ADAMTS13,ABCA13,SERPINA1,HGFAC,PDGFC,HPR,APOA5,APOC4
Monarch	HP:0001939	Abnormality of metabolism/homeostasis	48	2168	0.27	0.0019	CETP,F9,APOB,APOA1,TTR,CAT,CFP,APOE,CFHR5,LYZ,LCAT,CP,TCIRG1,SERPING1,CSF1R,A2ML1,PCSK9,FGB,DST,F2,PLG,CFHR1,NDUFS8,SERPINF2,FGG,TUBB,FN1,BLM,AKAP9,CFH,F5,FLNA,ADAMTS13,F10,LMOD3,SERPINA1,TGFBI,IGFALS,GC,APOA5,ATP1A1,TTN,VEGFC,PLCG2,KNG1,ACTB,B2M,FGA
Monarch	HP:0400008	Menometrorrhagia	4	10	1.53	0.0019	F9,FGB,FGG,FGA
Monarch	HP:0011024	Abnormality of the gastrointestinal tract	35	1384	0.33	0.0024	F9,APOE,VWF,F13A1,DNAH5,TCIRG1,FCN3,HABP2,SERPING1,FGB,YWHAG,F2,PLG,EFEMP2,SUZ12,KDM3B,FGG,BLM,COL18A1,F13B,F5,ECM1,FLNA,F10,F7,MASP2,F11,LMOD3,SERPINA1,FBN2,C1R,PLCG2,ACTB,B2M,FGA
Monarch	HP:0011034	Amyloidosis	5	25	1.23	0.0026	APOA1,TTR,LYZ,B2M,FGA

Monarch	HP:0001000	Abnormality of skin pigmentation	17	439	0.52	0.0031	PROC,CAT,APOE,CSTA,A2ML1,DST,F2,SUZ12,TUBB,BLM,FLNA,PROS1,C1S,SERPINA1,C1R,PLCG2,ACTB
Monarch	HP:0003321	Biconcave flattened vertebrae	4	12	1.45	0.0033	SPARC,SERPINF1,SEC24D,P4HB
Monarch	HP:0012719	Functional abnormality of the gastrointestinal tract	25	840	0.4	0.0033	F9,VWF,F13A1,TCIRG1,FCN3,SERPING1,FGB,YWHAG,F2,FGG,BLM,F13B,F5,ECM1,FLNA,F10,F7,MASP2,F11,LMOD3,SERPINA1,PLCG2,ACTB,B2M,FGA
Monarch	HP:0011355	Localized skin lesion	23	738	0.42	0.0034	MGP,APOB,PROC,APOA1,LRP1,APOE,CSTA,TCIRG1,SEC24D,MASP1,A2ML1,PCSK9,DST,SUZ12,P4HB,BLM,ECM1,FLNA,PROS1,C1S,C1R,ACTB,B2M
Monarch	HP:0002634	Arteriosclerosis	7	70	0.93	0.0035	APOB,CAT,APOE,ANGPTL6,LCAT,PCSK9,HTRA1
Monarch	HP:0010981	Hypolipoproteinemia	5	27	1.19	0.0035	APOB,APOA1,APOE,LCAT,APOA5
Monarch	EFO:0003839	Retinopathy	8	98	0.84	0.0036	CETP,APOE,CFHR5,C9,EFEMP2,CFH,CFI,CFB
Monarch	EFO:0005278	Cardiovascular disease biomarker measurement	48	2228	0.26	0.0036	ST6GAL1,CETP,F9,APOB,APOA1,LRP1,APOE,C9,LCAT,KLKB1,DGKG,DNAH5,ITIH1,HABP2,SERPINI1,ANGPTL4,PCSK9,FGB,PLG,STAB1,FBLN1,OIT3,FILIP1L,FN1,HP,APOA4,COL18A1,CFH,F5,CRTAC1,ADAMTS13,SVEP1,EFEMP1,EFCAB5,TNRC6A,NAV2,MAP3K7CL,ITIH3,SERPINA1,HGFAC,FBN2,HPR,APOA5,TTN,APOC4,PLCG2,KNG1,ACTB
Monarch	HP:0000969	Edema	17	448	0.51	0.0037	APOA1,APOE,LYZ,SERPING1,A2ML1,FGB,F2,FGG,TUBB,FN1,LMOD3,SERPINA1,TGFBI,VEGFC,PLCG2,ACTB,FGA
Monarch	HP:0000703	Dentinogenesis imperfecta	5	28	1.18	0.0039	SPARC,SERPINF1,SEC24D,BMP1,P4HB
Monarch	EFO:0004298	Cardiovascular measurement	50	2378	0.25	0.0043	ST6GAL1,CETP,F9,GPLD1,APOB,APOA1,LRP1,APOE,C9,LCAT,KLKB1,DGKG,DNAH5,ITIH1,HABP2,SERPINI1,ANGPTL4,PCSK9,FGB,INHBC,PLG,STAB1,FBLN1,OIT3,FILIP1L,FN1,HP,APOA4,COL18A1,CFH,F5,CRTAC1,ADAMTS13,SVEP1,EFEMP1,EFCAB5,TNRC6A,NAV2,MAP3K7CL,ITIH3,SERPINA1,HGFAC,FBN2,HPR,APOA5,TTN,APOC4,PLCG2,KNG1,ACTB
Monarch	EFO:0003966	Eye disease	10	168	0.7	0.0048	CETP,APOE,CFHR5,C9,EFEMP2,CFH,CFI,CFB,FBN2,PAMR1
Monarch	HP:0002659	Increased susceptibility to fractures	11	205	0.66	0.0049	SPARC,SERPINF1,TCIRG1,HABP2,SEC24D,BMP1,EFEMP2,P4HB,FN1,LMOD3,B2M
Monarch	HP:0000080	Abnormality of reproductive system physiology	21	659	0.43	0.0050	F9,TTR,APOE,VWF,F13A1,DNAH5,AHSG,A2ML1,FGB,F2,DNAH8,FGG,BLM,F13B,F5,F10,F7,ADGRG2,F11,SERPINA1,FGA
Monarch	EFO:0004694	Factor XI measurement	3	4	1.8	0.0051	KLKB1,F11,KNG1
Monarch	HP:0000095	Abnormal renal glomerulus morphology	9	136	0.75	0.0051	APOE,CFHR5,C1QB,CFHR1,FN1,CFH,C1QA,CFI,C4A
Monarch	HP:0005356	Decreased serum complement factor I	3	4	1.8	0.0051	C1QB,C1QA,CFI
Monarch	HP:0005381	Recurrent meningococcal disease	3	4	1.8	0.0051	C5,C7,CFI
Monarch	HP:0008065	Aplasia/Hypoplasia of the skin	10	170	0.7	0.0051	MGP,PROC,TCIRG1,SEC24D,DST,P4HB,FLNA,PROS1,C1S,C1R
Monarch	HP:0011843	Abnormal musculoskeletal physiology	33	1330	0.32	0.0051	SPARC,APOE,SERPINF1,CENPE,TCIRG1,AHSG,HABP2,SEC24D,MASP1,A2ML1,BMP1,DST,F2,EFEMP2,SUZ12,SERPINF2,KDM3B,P4HB,FBLN1,FN1,COL18A1,ADAMTSL4,FLNA,C1S,PRG4,LMOD3,SERPINA1,IGFALS,FBN2,C1R,TTN,ACTB,B2M
Monarch	HP:0012223	Splenic rupture	3	4	1.8	0.0051	FGB,FGG,FGA
Monarch	HP:0040232	Delayed onset bleeding	3	4	1.8	0.0051	F9,F13A1,F13B
Monarch	HP:0000362	Otosclerosis	4	15	1.35	0.0055	SPARC,SERPINF1,SEC24D,P4HB
Monarch	HP:0002719	Recurrent infections	22	721	0.41	0.0057	C5,MGP,DNAH5,TCIRG1,FCN3,PLG,EFEMP2,C1QB,C7,BLM,CFH,ECM1,FLNA,C8B,C1QA,CFI,MASP2,SERPINA1,C1R,CFD,PLCG2,B2M
Monarch	HP:0000979	Purpura	7	79	0.87	0.0059	PROC,APOE,F13A1,F2,F13B,PROS1,C4A
Monarch	HP:0002645	Wormian bones	6	54	0.97	0.0062	SPARC,SERPINF1,SEC24D,BMP1,P4HB,FLNA
Monarch	HP:0004586	Biconcave vertebral bodies	5	33	1.11	0.0066	SPARC,SERPINF1,SEC24D,BMP1,P4HB
Monarch	HP:0000926	Platyspondyly	8	111	0.78	0.0068	SPARC,SERPINF1,TCIRG1,SEC24D,CSF1R,BMP1,P4HB,FLNA
Monarch	HP:0000078	Abnormality of the genital system	33	1358	0.31	0.0069	F9,TTR,APOE,VWF,F13A1,DNAH5,AHSG,MASP1,A2ML1,FGB,F2,PLG,EFEMP2,SUZ12,KDM3B,FBLN1,DNAH8,FGG,TUBB,BLM,F13B,F5,FLNA,F10,F7,ADGRG2,F11,LMOD3,SERPINA1,VEGFC,ACTB,TRIM8,FGA
Monarch	HP:0005692	Joint hyperflexibility	9	144	0.72	0.0069	CENPE,SEC24D,A2ML1,SUZ12,P4HB,COL18A1,FLNA,C1S,C1R
Monarch	HP:0003216	Generalized amyloid deposition	3	5	1.71	0.0071	APOA1,LYZ,FGA
Monarch	HP:0005542	Prolonged whole-blood clotting time	3	5	1.71	0.0071	F9,VWF,F5
Monarch	HP:0007937	Reticular pigmentary degeneration	3	5	1.71	0.0071	CFH,EFEMP1,CFI
Monarch	EFO:0004612	High density lipoprotein cholesterol measurement	22	740	0.4	0.0076	CETP,APOB,APOA1,LRP1,APOE,LCAT,PPP2R3A,DGKG,INHBE,ITIH1,ANGPTL4,F2,INHBC,STAB1,APOD,HP,APOA4,COL18A1,CRTAC1,PDGFC,APOA5,APOC4
Monarch	HP:0000704	Periodontitis	5	35	1.08	0.0080	CAT,TCIRG1,PLG,C1S,C1R
Monarch	HP:0001386	Joint swelling	5	35	1.08	0.0080	FGB,F2,FGG,SERPINA1,FGA
Monarch	EFO:0000618	Nervous system disease	14	350	0.53	0.0084	CETP,APOE,CFHR5,C9,EFEMP2,CLU,KDM3B,COL18A1,CFH,F5,CFI,CFB,PLCG2,KNG1
Monarch	HP:0006487	Bowing of the long bones	11	224	0.62	0.0085	SPARC,SERPINF1,TCIRG1,SEC24D,BMP1,P4HB,FN1,FLNA,LMOD3,FBN2,B2M
Monarch	HP:0031886	Abnormal LDL cholesterol concentration	5	36	1.07	0.0088	APOB,APOE,LCAT,PCSK9,APOA5
Monarch	HP:0003233	Decreased HDL cholesterol concentration	4	18	1.27	0.0089	APOA1,APOE,LCAT,APOA5
Monarch	HP:0012372	Abnormal eye morphology	44	2077	0.25	0.0089	MGP,SPARC,APOB,PROC,APOA1,TTR,APOE,SERPINF1,LCAT,CP,DNAH5,TCIRG1,SERPING1,SEC24D,CSF1R,MASP1,A2ML1,PCSK9,BMP1,YWHAG,DST,PLG,EFEMP2,NDUFS8,SUZ12,P4HB,FBLN1,TUBB,BLM,COL18A1,CFH,ADAMTSL4,FLNA,PROS1,EFEMP1,CFI,ABCA13,TGFBI,FBN2,C1R,VIM,PLCG2,ACTB,B2M
Monarch	HP:0002086	Abnormality of the respiratory system	37	1636	0.28	0.0096	MGP,APOB,PROC,CAT,APOE,SERPINF1,DNAH5,TCIRG1,FCN3,HABP2,SERPING1,DSC2,A2ML1,SLC27A4,PCSK9,BMP1,DST,F2,PLG,EFEMP2,NDUFS8,SERPINF2,ANG,BLM,F5,ECM1,FLNA,ADAMTS13,PROS1,CFI,MASP2,LMOD3,SERPINA1,FBN2,TTN,PLCG2,B2M
Monarch	HP:0030780	Abnormality of the protein C anticoagulant pathway	3	6	1.63	0.0097	PROC,F5,PROS1
Monarch	HP:0100724	Hypercoagulability	4	19	1.25	0.0103	F9,PROC,F5,PROS1
Monarch	HP:0000366	Abnormality of the nose	34	1470	0.29	0.0120	F9,MGP,APOE,VWF,F13A1,DNAH5,CENPE,TCIRG1,AHSG,MASP1,FGB,F2,PLG,EFEMP2,SUZ12,KDM3B,FGG,TUBB,BLM,COL18A1,F13B,F5,ECM1,FLNA,F10,F7,CFI,F11,SERPINA1,PLCG2,ACTB,TRIM8,B2M,FGA
Monarch	HP:0001058	Poor wound healing	4	20	1.23	0.0121	F9,F13A1,F13B,C1R
Monarch	HP:0007420	Spontaneous hematomas	4	20	1.23	0.0121	F13A1,F13B,F5,F10
Monarch	HP:0001382	Joint hypermobility	12	279	0.56	0.0127	CENPE,SEC24D,A2ML1,BMP1,EFEMP2,SUZ12,KDM3B,P4HB,COL18A1,FLNA,C1S,C1R
Monarch	HP:0000799	Renal steatosis	3	7	1.56	0.0131	APOB,APOE,PCSK9
Monarch	HP:0004418	Thrombophlebitis	3	7	1.56	0.0131	PROC,F2,PROS1
Monarch	HP:0005305	Cerebral venous thrombosis	3	7	1.56	0.0131	PROC,F2,PROS1
Monarch	HP:0012397	Aortic atherosclerotic lesion	3	7	1.56	0.0131	APOB,APOE,PCSK9
Monarch	HP:0000478	Abnormality of the eye	55	2876	0.21	0.0136	MGP,SPARC,APOB,PROC,APOA1,TTR,APOE,ANGPTL6,SERPINF1,LCAT,CSTA,CP,DNAH5,CENPE,TCIRG1,AHSG,SERPING1,SEC24D,CSF1R,SERPINI1,MASP1,A2ML1,PCSK9,BMP1,YWHAG,DST,PLG,EFEMP2,CLU,NDUFS8,SUZ12,P4HB,FBLN1,TUBB,THBS4,BLM,COL18A1,CFH,HTRA1,ADAMTSL4,FLNA,PROS1,EFEMP1,CFI,ABCA13,LMOD3,TGFBI,FBN2,C1R,VIM,TTN,PLCG2,ACTB,TRIM8,B2M
Monarch	HP:0002757	Recurrent fractures	8	127	0.73	0.0136	SPARC,SERPINF1,TCIRG1,HABP2,SEC24D,BMP1,P4HB,FN1
Monarch	HP:0005268	Miscarriage	4	21	1.21	0.0136	MGP,FGB,FGG,FGA
Monarch	HP:0011420	Age of death	4	21	1.21	0.0136	MGP,FGB,FGG,FGA
Monarch	EFO:0004535	Serum albumin measurement	8	128	0.72	0.0137	TCIRG1,SERPINF2,SERPINA11,CFH,SVEP1,SERPINA1,HGFAC,HPR
Monarch	HP:0030962	Abnormal morphology of the great vessels	18	571	0.43	0.0140	MGP,APOB,APOE,ANGPTL6,DNAH5,TCIRG1,SEC24D,MASP1,A2ML1,PCSK9,EFEMP2,KDM3B,P4HB,FN1,COL18A1,FLNA,FBN2,ACTB

Monarch	EFO:0000408	Disease	32	1373	0.29	0.0149	CETP,LRP1,APOE,CFHR5,VWF,C9,SEC24D,EFEMP2,CLU,KDM3B,P4HB,TUBB,COL18A1,CFHR4,CFH,F5,ADAM12,FLNA,EFEMP1,CFI,EFCAB5,NAV2,MAP3K7CL,BZW1,CFB,IGFALS,FBN2,TUBA1C,TTN,PLCG2,PAMR1,KNG1
Monarch	EFO:0000508	Genetic disorder	8	130	0.72	0.0149	CETP,APOE,CFHR5,C9,CFH,CFI,EFCAB5,CFB
Monarch	HP:0000991	Xanthomatosis	4	22	1.19	0.0149	APOB,APOA1,APOE,PCSK9
Monarch	HP:0004381	Supravalvular aortic stenosis	3	8	1.5	0.0160	APOB,PCSK9,EFEMP2
Monarch	HP:0010874	Tendon xanthomatosis	3	8	1.5	0.0160	APOB,APOE,PCSK9
Monarch	HP:0000940	Abnormal diaphysis morphology	11	249	0.57	0.0164	SPARC,SERPINF1,TCIRG1,SEC24D,BMP1,P4HB,FN1,FLNA,LMOD3,FBN2,B2M
Monarch	HP:0002718	Recurrent bacterial infections	9	169	0.65	0.0164	C5,DNAH5,TCIRG1,FCN3,C7,CFH,C8B,CFI,CFD
Monarch	HP:0010647	Abnormal elasticity of skin	9	172	0.65	0.0183	MGP,SPARC,CSTA,EFEMP2,SUZ12,TUBB,FLNA,C1S,C1R
Monarch	HP:0001681	Angina pectoris	4	24	1.15	0.0188	APOB,APOE,LCAT,PCSK9
Monarch	HP:0030875	Abnormality of pulmonary circulation	8	136	0.7	0.0188	MGP,PROC,SERPINF1,TCIRG1,BMP1,F2,FLNA,PROS1
Monarch	EFO:0003907	Deep vein thrombosis	3	9	1.45	0.0200	F2,F5,KNG1
Monarch	HP:0001084	Corneal arcus	3	9	1.45	0.0200	APOB,APOE,PCSK9
Monarch	HP:0009115	Aplasia/hypoplasia involving the skeleton	25	985	0.33	0.0208	MGP,APOE,SERPINF1,CENPE,TCIRG1,SEC24D,MASP1,A2ML1,BMP1,EFEMP2,SUZ12,P4HB,TUBB,FN1,BLM,FLNA,C1S,LMOD3,IGFALS,FBN2,C1R,TTN,ACTB,TRIM8,B2M
Monarch	HP:0003549	Abnormality of connective tissue	30	1294	0.29	0.0235	SPARC,LRP1,APOE,SERPINF1,CENPE,TCIRG1,AHSG,SEC24D,MASP1,BMP1,DST,EFEMP2,SUZ12,KDM3B,P4HB,TUBB,BLM,ECM1,FLNA,C1S,PRG4,LMOD3,TGFBI,FBN2,C1R,TTN,VEGFC,PLCG2,ACTB,TRIM8
Monarch	HP:0003141	Increased LDL cholesterol concentration	4	26	1.11	0.0238	APOB,APOE,LCAT,PCSK9
Monarch	HP:0008151	Prolonged prothrombin time	4	26	1.11	0.0238	F2,F5,F10,F7
Monarch	HP:0011821	Abnormal facial skeleton morphology	26	1056	0.32	0.0242	MGP,APOE,SERPINF1,DNAH5,CENPE,TCIRG1,SEC24D,A2ML1,BMP1,EFEMP2,SUZ12,P4HB,TUBB,BLM,ADAMTSL4,FLNA,CFI,C1S,LMOD3,SERPINA1,IGFALS,FBN2,C1R,TTN,ACTB,B2M
Monarch	HP:0000347	Micrognathia	19	659	0.39	0.0244	SERPINF1,CENPE,TCIRG1,SEC24D,A2ML1,BMP1,EFEMP2,SUZ12,P4HB,TUBB,BLM,FLNA,C1S,LMOD3,IGFALS,FBN2,C1R,TTN,ACTB
Monarch	HP:0001114	Xanthelasma	3	10	1.4	0.0246	APOB,APOE,PCSK9
Monarch	HP:0002980	Femoral bowing	5	49	0.94	0.0246	SPARC,SERPINF1,SEC24D,P4HB,FLNA
Monarch	HP:0009118	Aplasia/Hypoplasia of the mandible	19	660	0.39	0.0246	SERPINF1,CENPE,TCIRG1,SEC24D,A2ML1,BMP1,EFEMP2,SUZ12,P4HB,TUBB,BLM,FLNA,C1S,LMOD3,IGFALS,FBN2,C1R,TTN,ACTB
Monarch	HP:0011729	Abnormality of joint mobility	26	1063	0.32	0.0259	APOE,SERPINF1,CENPE,AHSG,SEC24D,MASP1,A2ML1,BMP1,DST,F2,EFEMP2,SUZ12,KDM3B,P4HB,FBLN1,COL18A1,ADAMTSL4,FLNA,C1S,PRG4,LMOD3,SERPINA1,FBN2,C1R,TTN,ACTB
Monarch	HP:0007759	Opacification of the corneal stroma	6	78	0.81	0.0262	APOB,APOE,LCAT,PCSK9,COL18A1,TGFBI
Monarch	EFO:0004503	Hematological measurement	77	4592	0.15	0.0283	ST6GAL1,CPB2,CETP,GPLD1,APOB,PROC,APOE,APCS,LYZ,VWF,ATRN,C9,LCAT,KLKB1,PPP2R3A,DGKG,TCIRG1,INHBE,HABP2,SERPING1,CSF1R,AZGP1,PGLYRP2,ANGPTL4,FGB,DST,F2,INHBC,PLG,EFEMP2,CSPG4,STAB1,PAPPA,FBLN1,FGG,TUBB,FN1,AKAP9,COL18A1,CFHR4,CFH,F5,ADAMTSL4,FLNA,ADAMTS13,SVEP1,F10,F7,EFCAB5,TNRC6A,NAV2,MAP3K7CL,MASP2,F11,C1S,ITIH3,C4B,SERPINA1,CFB,IGFALS,GC,HGFAC,PDGFC,FBN2,HPR,TUBA1C,APOA5,ATP1A1,VIM,VEGFC,PLCG2,KNG1,TPM4,ACTB,TRIM66,CDH5,FGA
Monarch	HP:0002644	Abnormal pelvic girdle bone morphology	16	512	0.42	0.0289	APOE,SERPINF1,CENPE,TCIRG1,SEC24D,MASP1,BMP1,EFEMP2,SUZ12,KDM3B,FBLN1,FN1,FLNA,LMOD3,TTN,B2M
Monarch	HP:0003272	Abnormal hip bone morphology	15	461	0.44	0.0289	APOE,SERPINF1,CENPE,TCIRG1,SEC24D,MASP1,BMP1,EFEMP2,SUZ12,KDM3B,FBLN1,FLNA,LMOD3,TTN,B2M
Monarch	HP:0000963	Thin skin	6	80	0.8	0.0290	PROC,SEC24D,P4HB,FLNA,PROS1,C1R
Monarch	HP:0001387	Joint stiffness	8	151	0.65	0.0318	F2,SUZ12,ADAMTSL4,FLNA,PRG4,SERPINA1,FBN2,ACTB
Monarch	HP:0010989	Abnormality of the intrinsic pathway	4	29	1.07	0.0318	F9,VWF,F11,KNG1
Monarch	HP:0030972	Abnormal systemic blood pressure	13	369	0.47	0.0323	MGP,APOB,APOA1,TTR,ANGPTL6,LYZ,SERPING1,PCSK9,F2,FN1,ADAMTSL4,SERPINA1,FGA
Monarch	EFO:0004623	Fibrinogen measurement	5	54	0.89	0.0337	PPP2R3A,FGB,FGG,HGFAC,FGA
Monarch	HP:0000592	Blue sclerae	6	83	0.79	0.0337	SPARC,SERPINF1,SEC24D,BMP1,P4HB,C1R
Monarch	HP:0002813	Abnormality of limb bone morphology	32	1459	0.27	0.0337	MGP,SPARC,APOE,SERPINF1,VWF,DNAH5,CENPE,TCIRG1,AHSG,SEC24D,CSF1R,MASP1,A2ML1,BMP1,F2,EFEMP2,SUZ12,P4HB,FBLN1,TUBB,FN1,BLM,FLNA,PRG4,LMOD3,SERPINA1,FBN2,C1R,TTN,ACTB,TRIM8,B2M
Monarch	HP:0005293	Venous insufficiency	5	54	0.89	0.0337	PROC,VWF,PROS1,EFEMP1,VEGFC
Monarch	HP:0012638	Abnormal nervous system physiology	57	3154	0.18	0.0337	ST6GAL1,MGP,SPARC,APOB,PROC,APOA1,TTR,CAT,LRP1,APOE,ANGPTL6,CP,DNAH5,CENPE,TCIRG1,AHSG,SERPING1,SEC24D,CSF1R,SERPINI1,MASP1,A2ML1,PCSK9,BMP1,YWHAG,DST,F2,EFEMP2,STAB1,NDUFS8,SUZ12,KDM3B,P4HB,FBLN1,ANG,TUBB,FN1,BLM,AKAP9,COL18A1,HTRA1,ADAMTSL4,ECM1,FLNA,ADAMTS13,LMOD3,ITIH3,SERPINA1,TGFBI,FBN2,ATP1A1,VIM,TTN,VEGFC,ACTB,TRIM8,B2M
Monarch	EFO:0003777	Heart disease	8	155	0.64	0.0359	LRP1,VWF,SEC24D,P4HB,FLNA,MAP3K7CL,FBN2,TTN
Monarch	EFO:0006794	Cerebrospinal fluid biomarker measurement	5	55	0.89	0.0359	LTF,APOE,CFHR5,C7,C1R
Monarch	HP:0001634	Mitral valve prolapse	6	86	0.77	0.0388	VWF,SEC24D,P4HB,FLNA,FBN2,TTN
Monarch	EFO:0004872	Inflammatory biomarker measurement	28	1226	0.29	0.0394	CETP,APOB,APOA1,LRP1,APOE,APCS,LYZ,LCAT,DGKG,ITIH1,ANKRD44,ANGPTL4,PCSK9,PLG,EFEMP2,STAB1,APOA4,COL18A1,CFH,F5,CRTAC1,C1QA,YWHAZ,SERPINA1,GC,HGFAC,HPR,APOC4
Monarch	HP:0009122	Aplasia/hypoplasia affecting bones of the axial skeleton	20	751	0.35	0.0394	SERPINF1,CENPE,TCIRG1,SEC24D,A2ML1,BMP1,EFEMP2,SUZ12,P4HB,TUBB,FN1,BLM,FLNA,C1S,LMOD3,IGFALS,FBN2,C1R,TTN,ACTB
Monarch	HP:0002907	Microscopic hematuria	3	13	1.29	0.0405	F2,FN1,ADAMTS13
Monarch	EFO:0008022	Angiogenin measurement	2	2	1.93	0.0410	ANG,RNASE4
Monarch	EFO:0008091	Complement C1s subcomponent measurement	2	2	1.93	0.0410	CFHR4,CFH
Monarch	EFO:0008098	Complement factor H-related protein 5 measurement	2	2	1.93	0.0410	F13B,CFH
Monarch	EFO:0008260	Plasma kallikrein measurement	2	2	1.93	0.0410	KLKB1,KNG1
Monarch	EFO:0008543	c3d:C3 ratio	2	2	1.93	0.0410	CFHR4,CFH
Monarch	HP:0001038	Warfarin-induced skin necrosis	2	2	1.93	0.0410	PROC,PROS1
Monarch	HP:0004434	Decreased serum complement C8	2	2	1.93	0.0410	C8A,C8B
Monarch	HP:0005369	Decreased serum complement factor H	2	2	1.93	0.0410	CFH,CFI
Monarch	HP:0005482	Abnormality of the alternative complement pathway	2	2	1.93	0.0410	CFP,CFH
Monarch	HP:0011971	Dermatographic urticaria	2	2	1.93	0.0410	SERPING1,PLCG2
Monarch	HP:0012531	Pain	16	539	0.4	0.0425	APOB,CAT,TCIRG1,HABP2,SERPING1,PCSK9,F2,SERPINF2,ANG,HTRA1,FLNA,C1S,SERPINA1,TGFBI,PLCG2,B2M
Monarch	HP:0033151	Abnormal pharynx morphology	8	162	0.62	0.0436	MGP,DNAH5,TCIRG1,SERPING1,PLG,BLM,CFI,B2M
Monarch	HP:0011844	Abnormal appendicular skeleton morphology	33	1557	0.25	0.0439	MGP,SPARC,APOE,SERPINF1,VWF,DNAH5,CENPE,TCIRG1,AHSG,SEC24D,CSF1R,MASP1,A2ML1,BMP1,F2,EFEMP2,SUZ12,KDM3B,P4HB,FBLN1,TUBB,FN1,BLM,FLNA,PRG4,LMOD3,SERPINA1,FBN2,C1R,TTN,ACTB,TRIM8,B2M
Monarch	HP:0000370	Abnormality of the middle ear	15	490	0.41	0.0450	MGP,SPARC,APOE,SERPINF1,DNAH5,TCIRG1,SEC24D,MASP1,PLG,P4HB,BLM,FLNA,CFI,PLCG2,B2M
Monarch	HP:0003023	Bowing of limbs due to multiple fractures	3	14	1.26	0.0458	SPARC,SERPINF1,BMP1
Monarch	HP:0002037	Inflammation of the large intestine	5	60	0.85	0.0469	F13A1,FCN3,F13B,MASP2,PLCG2
Monarch	EFO:0006925	Lipoprotein A measurement	4	34	1.0	0.0470	CETP,APOE,PLG,APOC4
Monarch	HP:0001297	Stroke	10	249	0.53	0.0470	TTR,ANGPTL6,F2,F5,HTRA1,FLNA,CRTAC1,ADAMTS13,KNG1,ACTB

DISEASES	DOID:1247	Blood coagulation disease	25	89	1.38	2.46e-21	F9,PROC,VWF,KLKB1,F13A1,PF4,FGB,F2,PLG,SERPINF2,FGG,HP,F13B,CFH,F5,FLNA,ADAMTS13,F10,F7,PROS1,MMRN1,F11,C4B,KNG1,FGA
DISEASES	DOID:4	Disease	141	6291	0.28	1.22e-15	CETP,CHGA,F9,C5,MGP,SPARC,LTF,APOB,PROC,APOA1,TTR,CAT,CFP,APOE,SERPINF1,APCS,CFHR5,LYZ,VWF,C9,LCAT,CSTA,CP,KLKB1,F13A1,CDH6,DNAH5,CENPE,TCIRG1,LUM,AHSG,SAA4,SERPING1,SEC24D,DSC2,ANKRD44,CSF1R,PIP,AZGP1,SCGB3A1,SERPINI1,PF4,MASP1,CAMP,APEH,A2ML1,SLC27A4,LALBA,PCSK9,BMP1,FGB,YWHAG,DST,F2,PLG,EFEMP2,PRG2,C1QB,CFHR1,CLU,NDUFS8,SUZ12,APOL1,SERPINF2,P4HB,SERPINA7,PAPPA,DNAH8,ANG,FGG,TUBB,SLPI,FILIP1L,FN1,BLM,HP,AKAP9,APOA4,CNDP1,COL18A1,F13B,CFHR2,CFHR4,CFHR3,CFH,F5,HTRA1,ADAMTSL4,ECM1,FLNA,MCOLN2,C8B,ADAMTS13,C1QA,F10,F7,CA6,ADGRG2,IGFBP3,CD99,GPX3,PROS1,EFEMP1,CFI,MMRN1,TNRC6A,YWHAZ,F11,C1S,PCYOX1,C4A,PRG4,CCDC122,ABCA13,MST1,LMOD3,C4B,SERPINA1,TGFBI,CFB,PDGFC,FBN2,SELENOP,TSKU,JCHAIN,HPR,C1R,APOA5,ATP1A1,VIM,TTN,CFD,IGHV3-15,VEGFC,PLCG2,KNG1,TPM4,ACTB,TRIM66,B2M,FGA
DISEASES	DOID:7	Disease of anatomical entity	120	4798	0.33	1.22e-15	CETP,CHGA,F9,C5,MGP,SPARC,LTF,APOB,PROC,APOA1,TTR,CFP,APOE,SERPINF1,APCS,CFHR5,VWF,C9,LCAT,CSTA,KLKB1,F13A1,DNAH5,CENPE,TCIRG1,LUM,AHSG,SAA4,SERPING1,SEC24D,DSC2,ANKRD44,CSF1R,PIP,SCGB3A1,SERPINI1,PF4,CAMP,APEH,A2ML1,SLC27A4,LALBA,PCSK9,BMP1,FGB,YWHAG,DST,F2,PLG,EFEMP2,C1QB,CFHR1,CLU,SUZ12,APOL1,SERPINF2,P4HB,SERPINA7,PAPPA,ANG,FGG,TUBB,SLPI,FILIP1L,FN1,BLM,HP,AKAP9,APOA4,CNDP1,COL18A1,F13B,CFHR2,CFHR4,CFHR3,CFH,F5,HTRA1,ADAMTSL4,ECM1,FLNA,C8B,ADAMTS13,C1QA,F10,F7,ADGRG2,CD99,PROS1,EFEMP1,CFI,MMRN1,TNRC6A,YWHAZ,F11,C1S,C4A,CCDC122,MST1,LMOD3,C4B,SERPINA1,TGFBI,CFB,FBN2,TSKU,JCHAIN,C1R,ATP1A1,VIM,TTN,CFD,IGHV3-15,VEGFC,PLCG2,KNG1,ACTB,TRIM66,B2M,FGA
DISEASES	DOID:2007	Degeneration of macula and posterior pole	15	46	1.44	3.33e-13	CETP,APOE,CFHR5,C9,CFHR1,FILIP1L,F13B,CFHR2,CFHR4,CFHR3,CFH,HTRA1,EFEMP1,CFI,CFB
DISEASES	DOID:626	Complement deficiency	12	23	1.64	2.47e-12	C5,CFHR5,C9,KLKB1,SERPING1,CFHR1,CFHR3,CFH,ADAMTS13,CFI,CFB,KNG1
DISEASES	DOID:10871	Age related macular degeneration	14	44	1.43	2.54e-12	CETP,APOE,CFHR5,C9,CFHR1,FILIP1L,F13B,CFHR2,CFHR4,CFHR3,CFH,HTRA1,CFI,CFB
DISEASES	DOID:9120	Amyloidosis	16	75	1.26	4.99e-12	APOA1,TTR,APOE,APCS,LYZ,C9,SAA4,AZGP1,CLU,FN1,APOA4,SERPINA1,TGFBI,ACTB,B2M,FGA
DISEASES	DOID:74	Hematopoietic system disease	31	473	0.74	2.07e-11	F9,PROC,VWF,KLKB1,F13A1,TCIRG1,PF4,CAMP,FGB,F2,PLG,SERPINF2,FGG,HP,F13B,CFH,F5,FLNA,ADAMTS13,F10,F7,CD99,PROS1,MMRN1,F11,C4B,IGHV3-15,KNG1,ACTB,B2M,FGA
DISEASES	DOID:0050636	Familial visceral amyloidosis	11	21	1.65	2.10e-11	APOA1,TTR,APOE,APCS,LYZ,AZGP1,APOA4,SERPINA1,ACTB,B2M,FGA
DISEASES	DOID:2452	Thrombophilia	11	21	1.65	2.10e-11	F9,PROC,VWF,F2,PLG,HP,CFH,F5,ADAMTS13,PROS1,C4B
DISEASES	DOID:630	Genetic disease	92	3778	0.31	3.38e-10	CETP,F9,MGP,LTF,APOB,PROC,APOA1,TTR,CAT,APOE,SERPINF1,APCS,CFHR5,LYZ,VWF,C9,LCAT,CP,KLKB1,F13A1,CDH6,DNAH5,TCIRG1,SERPING1,SEC24D,ANKRD44,CSF1R,AZGP1,SERPINI1,MASP1,CAMP,A2ML1,PCSK9,FGB,DST,F2,PLG,EFEMP2,C1QB,CFHR1,CLU,NDUFS8,SERPINF2,PAPPA,DNAH8,FGG,FILIP1L,BLM,HP,AKAP9,APOA4,CNDP1,F13B,CFHR2,CFHR4,CFHR3,CFH,F5,HTRA1,ADAMTSL4,ECM1,FLNA,MCOLN2,C1QA,F10,F7,ADGRG2,IGFBP3,PROS1,EFEMP1,CFI,F11,C1S,PCYOX1,PRG4,MST1,LMOD3,SERPINA1,TGFBI,CFB,FBN2,JCHAIN,APOA5,ATP1A1,VIM,TTN,CFD,PLCG2,KNG1,ACTB,B2M,FGA
DISEASES	DOID:0050739	Autosomal genetic disease	76	2802	0.36	5.06e-10	CETP,F9,MGP,LTF,APOB,PROC,APOA1,TTR,APOE,APCS,CFHR5,LYZ,VWF,C9,CP,KLKB1,F13A1,CDH6,DNAH5,TCIRG1,SERPING1,ANKRD44,CSF1R,AZGP1,SERPINI1,MASP1,CAMP,PCSK9,FGB,DST,F2,PLG,EFEMP2,CFHR1,CLU,SERPINF2,FGG,FILIP1L,BLM,HP,AKAP9,APOA4,F13B,CFHR2,CFHR4,CFHR3,CFH,F5,HTRA1,ADAMTSL4,FLNA,F10,F7,IGFBP3,EFEMP1,CFI,F11,C1S,PCYOX1,PRG4,LMOD3,SERPINA1,TGFBI,CFB,FBN2,JCHAIN,APOA5,ATP1A1,VIM,TTN,CFD,PLCG2,KNG1,ACTB,B2M,FGA
DISEASES	DOID:0050736	Autosomal dominant disease	50	1386	0.48	6.82e-10	CETP,F9,PROC,APOA1,TTR,APOE,APCS,CFHR5,LYZ,VWF,C9,KLKB1,CDH6,SERPING1,ANKRD44,CSF1R,AZGP1,SERPINI1,F2,EFEMP2,CFHR1,CLU,FILIP1L,HP,AKAP9,APOA4,F13B,CFHR2,CFHR4,CFHR3,CFH,F5,HTRA1,FLNA,EFEMP1,CFI,C1S,SERPINA1,TGFBI,CFB,FBN2,JCHAIN,ATP1A1,VIM,TTN,PLCG2,KNG1,ACTB,B2M,FGA
DISEASES	DOID:0050177	Monogenic disease	83	3266	0.33	7.62e-10	CETP,F9,MGP,LTF,APOB,PROC,APOA1,TTR,APOE,SERPINF1,APCS,CFHR5,LYZ,VWF,C9,CP,KLKB1,F13A1,CDH6,DNAH5,TCIRG1,SERPING1,SEC24D,ANKRD44,CSF1R,AZGP1,SERPINI1,MASP1,CAMP,A2ML1,PCSK9,FGB,DST,F2,PLG,EFEMP2,CFHR1,CLU,SERPINF2,DNAH8,FGG,FILIP1L,BLM,HP,AKAP9,APOA4,F13B,CFHR2,CFHR4,CFHR3,CFH,F5,HTRA1,ADAMTSL4,FLNA,F10,F7,ADGRG2,IGFBP3,PROS1,EFEMP1,CFI,F11,C1S,PCYOX1,PRG4,MST1,LMOD3,SERPINA1,TGFBI,CFB,FBN2,JCHAIN,APOA5,ATP1A1,VIM,TTN,CFD,PLCG2,KNG1,ACTB,B2M,FGA
DISEASES	DOID:2914	Immune system disease	33	675	0.62	3.51e-09	C5,TTR,APOE,APCS,CFHR5,C9,KLKB1,SAA4,SERPING1,A2ML1,LALBA,DST,F2,PLG,CFHR1,FN1,HP,COL18A1,CFHR3,CFH,ADAMTS13,CD99,CFI,C4A,C4B,CFB,JCHAIN,IGHV3-15,VEGFC,PLCG2,KNG1,ACTB,B2M
DISEASES	DOID:0080301	Atypical hemolytic-uremic syndrome	7	11	1.73	2.10e-07	CFHR5,CFHR1,CFHR3,CFH,ADAMTS13,CFI,CFB
DISEASES	DOID:9799	Eye degenerative disease	18	236	0.81	3.13e-07	CETP,APOB,APOE,CFHR5,C9,PCSK9,CFHR1,FILIP1L,F13B,CFHR2,CFHR4,CFHR3,CFH,HTRA1,EFEMP1,CFI,TGFBI,CFB
DISEASES	DOID:557	Kidney disease	19	275	0.77	4.73e-07	C5,TTR,APCS,CFHR5,SAA4,F2,CFHR1,APOL1,FN1,HP,APOA4,COL18A1,CFHR3,CFH,ADAMTS13,CFI,C4B,CFB,B2M
DISEASES	DOID:12554	Hemolytic-uremic syndrome	7	14	1.63	6.33e-07	CFHR5,CFHR1,CFHR3,CFH,ADAMTS13,CFI,CFB
DISEASES	DOID:1287	Cardiovascular system disease	24	493	0.61	2.20e-06	CETP,CHGA,APOB,APOA1,TTR,APOE,APCS,VWF,DNAH5,DSC2,PF4,PCSK9,F2,PLG,PAPPA,AKAP9,FLNA,ADAMTS13,F10,F7,CD99,C4A,FBN2,TTN
DISEASES	DOID:5614	Eye disease	30	749	0.53	2.20e-06	CETP,LTF,APOB,APOE,APCS,CFHR5,C9,LUM,SERPING1,PCSK9,PLG,CFHR1,CLU,FILIP1L,COL18A1,F13B,CFHR2,CFHR4,CFHR3,CFH,HTRA1,ADAMTSL4,EFEMP1,CFI,SERPINA1,TGFBI,CFB,FBN2,VIM,ACTB
DISEASES	DOID:37	Skin disease	24	518	0.59	5.06e-06	APOA1,CFP,APOE,APCS,C9,CSTA,KLKB1,SERPING1,DSC2,PIP,CAMP,A2ML1,SLC27A4,DST,EFEMP2,TUBB,BLM,HP,APOA4,COL18A1,C1S,SERPINA1,KNG1,ACTB
DISEASES	DOID:16	Integumentary system disease	25	575	0.57	8.00e-06	APOA1,CFP,APOE,APCS,C9,CSTA,KLKB1,AHSG,SERPING1,DSC2,PIP,CAMP,A2ML1,SLC27A4,DST,EFEMP2,TUBB,BLM,HP,APOA4,COL18A1,C1S,SERPINA1,KNG1,ACTB
DISEASES	DOID:0014667	Disease of metabolism	35	1076	0.44	1.49e-05	CETP,F9,MGP,APOB,APOA1,TTR,CAT,APOE,APCS,LYZ,C9,LCAT,CP,AHSG,SAA4,AZGP1,PCSK9,F2,CLU,NDUFS8,FN1,APOA4,CNDP1,ECM1,MCOLN2,GPX3,PROS1,PCYOX1,SERPINA1,TGFBI,SELENOP,APOA5,ACTB,B2M,FGA
DISEASES	DOID:0050155	Sensory system disease	32	929	0.46	1.59e-05	CETP,LTF,APOB,PROC,APOE,APCS,CFHR5,C9,LUM,SERPING1,A2ML1,PCSK9,PLG,CFHR1,CLU,FILIP1L,COL18A1,F13B,CFHR2,CFHR4,CFHR3,CFH,HTRA1,ADAMTSL4,EFEMP1,CFI,SERPINA1,TGFBI,CFB,FBN2,VIM,ACTB
DISEASES	DOID:178	Vascular disease	16	249	0.74	1.59e-05	CETP,APOB,APOA1,APOE,VWF,PF4,PCSK9,F2,PLG,PAPPA,ADAMTS13,F10,F7,CD99,C4A,FBN2

DISEASES	DOID:863	Nervous system disease	56	2275	0.32	1.59e-05	CETP,LTF,APOB,PROC,APOA1,TTR,APOE,APCS,CFHR5,VWF,C9,LCAT,CENPE,LUM,SERPING1,ANKRD44,CSF1R,SERPINI1,A2ML1,PCSK9,YWHAG,DST,F2,PLG,C1QB,CFHR1,CLU,SUZ12,ANG,FILIP1L,CNDP1,COL18A1,F13B,CFHR2,CFHR4,CFHR3,CFH,HTRA1,ADAMTSL4,FLNA,C1QA,CD99,EFEMP1,CFI,TNRC6A,YWHAZ,CCDC122,SERPINA1,TGFBI,CFB,FBN2,TSKU,ATP1A1,VIM,ACTB,TRIM66
DISEASES	DOID:0050117	Disease by infectious agent	19	368	0.64	2.46e-05	APOE,APCS,CP,AHSG,APEH,F2,PLG,PRG2,C1QB,APOL1,SLPI,HTRA1,C1QA,CA6,YWHAZ,C4A,CCDC122,PDGFC,HPR
DISEASES	DOID:612	Primary immunodeficiency disease	21	470	0.58	5.03e-05	C5,TTR,CFHR5,C9,KLKB1,SERPING1,A2ML1,DST,F2,CFHR1,HP,COL18A1,CFHR3,CFH,ADAMTS13,CFI,C4A,C4B,CFB,PLCG2,KNG1
DISEASES	DOID:11949	Creutzfeldt-Jakob disease	5	9	1.67	5.35e-05	APOE,APCS,C1QB,C1QA,YWHAZ
DISEASES	DOID:0060903	Thrombosis	6	21	1.38	7.79e-05	VWF,PF4,F2,PLG,F10,F7
DISEASES	DOID:2921	Glomerulonephritis	7	36	1.22	8.06e-05	CFHR5,APOL1,COL18A1,CFHR3,CFH,C4B,CFB
DISEASES	DOID:0060440	Epithelial and subepithelial dystrophy	5	11	1.58	0.00010	LTF,APOE,CLU,COL18A1,TGFBI
DISEASES	DOID:10772	Thrombotic thrombocytopenic purpura	4	4	1.93	0.00015	VWF,HP,CFH,ADAMTS13
DISEASES	DOID:0060449	Gelatinous drop-like corneal dystrophy	4	5	1.83	0.00026	LTF,APOE,CLU,TGFBI
DISEASES	DOID:65	Connective tissue disease	26	774	0.45	0.00030	F9,C5,MGP,SPARC,TTR,SERPINF1,TCIRG1,SEC24D,CSF1R,A2ML1,BMP1,DST,PLG,EFEMP2,P4HB,COL18A1,HTRA1,FLNA,F7,CD99,C1S,C4A,C4B,FBN2,JCHAIN,C1R
DISEASES	DOID:2218	Blood platelet disease	7	47	1.1	0.00034	VWF,PF4,F2,FLNA,ADAMTS13,F7,MMRN1
DISEASES	DOID:17	Musculoskeletal system disease	33	1154	0.38	0.00036	F9,C5,MGP,SPARC,TTR,APOE,SERPINF1,APCS,TCIRG1,SEC24D,CSF1R,A2ML1,BMP1,DST,PLG,EFEMP2,P4HB,COL18A1,HTRA1,FLNA,F7,CD99,C1S,C4A,LMOD3,C4B,FBN2,JCHAIN,C1R,ATP1A1,TTN,CFD,B2M
DISEASES	DOID:14735	Hereditary angioedema	4	6	1.75	0.00039	KLKB1,SERPING1,C1S,KNG1
DISEASES	DOID:3146	Lipid metabolism disorder	8	72	0.97	0.00044	CETP,APOB,APOA1,APOE,LCAT,PCSK9,ECM1,APOA5
DISEASES	DOID:10873	Kuhnt-Junius degeneration	4	7	1.68	0.00056	CETP,CFH,HTRA1,CFB
DISEASES	DOID:0050828	Artery disease	10	130	0.81	0.00057	CETP,APOB,APOA1,APOE,PCSK9,F2,PLG,PAPPA,ADAMTS13,FBN2
DISEASES	DOID:0050639	Primary cutaneous amyloidosis	5	19	1.35	0.00068	APOE,APCS,C9,SERPINA1,ACTB
DISEASES	DOID:0050637	Finnish type amyloidosis	4	9	1.57	0.0011	APOE,APCS,C9,SERPINA1
DISEASES	DOID:14250	Down syndrome	4	9	1.57	0.0011	APOE,C1QB,PAPPA,C1QA
DISEASES	DOID:10124	Corneal disease	8	85	0.9	0.0012	LTF,APOB,APOE,LUM,PCSK9,CLU,COL18A1,TGFBI
DISEASES	DOID:1387	Hypolipoproteinemia	4	11	1.49	0.0019	APOB,APOA1,LCAT,PCSK9
DISEASES	DOID:5113	Nutritional deficiency disease	6	43	1.07	0.0019	MGP,TTR,F2,GPX3,PROS1,SELENOP
DISEASES	DOID:1588	Thrombocytopenia	4	12	1.45	0.0024	VWF,PF4,F2,ADAMTS13
DISEASES	DOID:2222	Factor X deficiency	3	3	1.93	0.0025	F2,F10,F7
DISEASES	DOID:2236	Congenital afibrinogenemia	3	3	1.93	0.0025	FGB,FGG,FGA
DISEASES	DOID:409	Liver disease	8	97	0.84	0.0025	TTR,APCS,AHSG,SAA4,F2,SERPINA1,IGHV3-15,ACTB
DISEASES	DOID:0060002	C1 inhibitor deficiency	3	4	1.8	0.0041	KLKB1,SERPING1,KNG1
DISEASES	DOID:12134	Factor VIII deficiency	3	4	1.8	0.0041	F9,VWF,F7
DISEASES	DOID:8943	Lattice corneal dystrophy	3	4	1.8	0.0041	APOE,CLU,TGFBI
DISEASES	DOID:1168	Familial hyperlipidemia	4	15	1.35	0.0045	APOB,APOE,PCSK9,APOA5
DISEASES	DOID:0050737	Autosomal recessive disease	40	1785	0.28	0.0055	MGP,LTF,APOB,APOE,VWF,CP,KLKB1,F13A1,DNAH5,TCIRG1,SERPING1,MASP1,CAMP,PCSK9,FGB,DST,F2,PLG,EFEMP2,CLU,SERPINF2,FGG,BLM,F13B,F5,FLNA,F10,F7,IGFBP3,F11,C1S,PCYOX1,PRG4,LMOD3,TGFBI,APOA5,TTN,CFD,KNG1,FGA
DISEASES	DOID:655	Inherited metabolic disorder	26	949	0.36	0.0056	CETP,F9,APOB,APOA1,TTR,CAT,APOE,APCS,LYZ,C9,LCAT,CP,AZGP1,PCSK9,CLU,NDUFS8,APOA4,CNDP1,ECM1,MCOLN2,PCYOX1,SERPINA1,APOA5,ACTB,B2M,FGA
DISEASES	DOID:11249	Vitamin K deficiency bleeding	3	5	1.71	0.0059	MGP,F2,PROS1
DISEASES	DOID:12259	Hemophilia B	3	5	1.71	0.0059	F9,F7,F11
DISEASES	DOID:1237	Corneal degeneration	3	5	1.71	0.0059	APOB,PCSK9,TGFBI
DISEASES	DOID:2229	Factor XI deficiency	3	5	1.71	0.0059	F9,F7,F11
DISEASES	DOID:2451	Protein S deficiency	3	5	1.71	0.0059	F2,PROS1,C4B
DISEASES	DOID:12347	Osteogenesis imperfecta	5	35	1.08	0.0064	SPARC,SERPINF1,SEC24D,BMP1,P4HB
DISEASES	DOID:3393	Coronary artery disease	5	36	1.07	0.0071	APOB,APOA1,APOE,PCSK9,PLG
DISEASES	DOID:12531	Von Willebrands disease	3	6	1.63	0.0079	F9,VWF,ADAMTS13
DISEASES	DOID:170	Endocrine gland cancer	7	93	0.8	0.0106	CHGA,APOE,APCS,APOA4,CD99,IGHV3-15,ACTB
DISEASES	DOID:1936	Atherosclerosis	4	21	1.21	0.0113	CETP,APOB,APOA1,APOE
DISEASES	DOID:3118	Hepatobiliary disease	9	168	0.66	0.0141	TTR,APCS,AHSG,SAA4,F2,MST1,SERPINA1,IGHV3-15,ACTB
DISEASES	DOID:3973	Thyroid gland medullary carcinoma	3	8	1.5	0.0141	APOE,APCS,APOA4
DISEASES	DOID:0080001	Bone disease	17	540	0.43	0.0176	F9,C5,SPARC,TTR,SERPINF1,TCIRG1,SEC24D,CSF1R,BMP1,P4HB,COL18A1,HTRA1,FLNA,F7,CD99,C1S,JCHAIN
DISEASES	DOID:50	Thyroid gland disease	5	50	0.93	0.0247	TTR,APOE,APCS,SERPINA7,APOA4
DISEASES	DOID:5844	Myocardial infarction	4	27	1.1	0.0247	APOB,APOE,PCSK9,PLG
DISEASES	DOID:0070029	ITM2B-related cerebral amyloid angiopathy 1	3	12	1.33	0.0350	APOE,APCS,CLU
DISEASES	DOID:2213	Hemorrhagic disease	3	12	1.33	0.0350	F2,SERPINF2,ADAMTS13
DISEASES	DOID:0050638	Transthyretin amyloidosis	2	2	1.93	0.0417	TTR,APCS
DISEASES	DOID:0050831	Familial encephalopathy with neuroserpin inclusion bodies	2	2	1.93	0.0417	SERPINI1,SERPINA1
DISEASES	DOID:0070030	ITM2B-related cerebral amyloid angiopathy 2	3	13	1.29	0.0417	APOE,APCS,CLU
DISEASES	DOID:0111676	High molecular weight kininogen deficiency	2	2	1.93	0.0417	KLKB1,KNG1
DISEASES	DOID:10230	Aortic atherosclerosis	2	2	1.93	0.0417	APOB,APOE
DISEASES	DOID:1391	Norum disease	2	2	1.93	0.0417	APOA1,LCAT
DISEASES	DOID:2211	Factor XIII deficiency	2	2	1.93	0.0417	F13A1,F13B
DISEASES	DOID:0060158	Acquired metabolic disease	8	162	0.62	0.0428	MGP,TTR,CP,AHSG,F2,GPX3,PROS1,SELENOP
DISEASES	DOID:0080307	Myofibrillar myopathy	3	14	1.26	0.0450	FLNA,TTN,CFD
TISSUES	BTO:0004850	Bone marrow cell	66	198	1.45	9.52e-67	CPB2,CETP,C5,APOB,IGFBP5,TTR,LRP1,CFP,SERPINF1,APCS,THBS1,VWF,LGALS3BP,C9,CP,KLKB1,F13A1,AMBP,HPX,LUM,FCN3,AHSG,SAA4,SERPING1,DSC2,CSF1R,AZGP1,MASP1,FGB,F2,CSPG4,CFHR1,CLU,APOL1,SERPINF2,APOD,FN1,HP,CFH,F5,ECM1,ADAMTS13,C1QA,IGFBP3,SOD3,PROS1,CFI,MMRN1,F11,C1S,ORM2,C4A,PRG4,MST1,C4B,SERPINA1,CFB,HGFAC,SELENOP,JCHAIN,C1R,PAMR1,KNG1,CDH5,B2M,FGA
TISSUES	BTO:0000392	Plasma cell	60	171	1.47	1.47e-61	C5,APOB,IGFBP5,TTR,LRP1,CFP,SERPINF1,APCS,THBS1,VWF,LGALS3BP,C9,CP,KLKB1,F13A1,AMBP,HPX,LUM,FCN3,AHSG,SAA4,SERPING1,DSC2,CSF1R,AZGP1,MASP1,FGB,F2,CSPG4,CFHR1,CLU,APOL1,SERPINF2,APOD,FN1,HP,CFH,ECM1,ADAMTS13,C1QA,IGFBP3,SOD3,PROS1,CFI,MMRN1,C1S,ORM2,C4A,C4B,SERPINA1,CFB,HGFAC,SELENOP,JCHAIN,C1R,PAMR1,KNG1,CDH5,B2M,FGA
TISSUES	BTO:0000141	Bone marrow	76	528	1.09	1.75e-54	CPB2,CETP,APMAP,C5,LTF,APOB,IGFBP5,TTR,LRP1,CFP,SERPINF1,APCS,THBS1,VWF,LGALS3BP,C9,CSTA,CP,KLKB1,F13A1,AMBP,HPX,LUM,FCN3,AHSG,SAA4,SERPING1,DSC2,CSF1R,AZGP1,MASP1,CAMP,FGB,F2,PRG2,CSPG4,CFHR1,CLU,SUZ12,APOL1,SERPINF2,KDM3B,APOD,FN1,HP,CFH,F5,ECM1,ADAMTS13,C1QA,IGFBP3,SOD3,PROS1,CFI,MMRN1,YWHAZ,F11,C1S,BZW1,ORM2,C4A,PRG4,MST1,C4B,SERPINA1,CFB,HGFAC,SELENOP,JCHAIN,TUBA1C,C1R,PAMR1,KNG1,CDH5,B2M,FGA

TISSUES	BTO:0000759	Liver	118	2125	0.67	6.98e-48	SPP2,ST6GAL1,CPB2,CETP,CHGA,TPD52L2,APMAP,F9,GPLD1,LTF,APOB,PROC,APOA1,TTR,CAT,LRP1,APOE,SERPINF1,APCS,CFHR5,THBS1,VWF,PSMA6,SERPINA10,LGALS3BP,ATRN,C9,CP,KLKB1,AMBP,HPX,INHBE,LUM,FCN3,ITIH1,AHSG,IGFBP1,HABP2,SAA4,SERPING1,CSF1R,AZGP1,PGLYRP2,MASP1,APEH,PDIA3,FGB,YWHAG,DST,F2,INHBC,PLG,STAB1,C1QB,CFHR1,CLU,APOL1,SERPINF2,P4HB,SERPINA7,ANG,TUBA1B,FGG,TUBB,SLPI,FN1,ASGR2,HP,AKAP9,COL18A1,C8A,F13B,CFHR2,CFHR4,CFHR3,CFH,S100A9,ECM1,FLNA,SMARCA1,C8B,ADAMTS13,C1QA,F10,F7,APOM,IGFBP3,CD99,PROS1,CFI,YWHAZ,MASP2,F11,C1S,PCYOX1,BZW1,ORM2,C4A,MST1,ITIH3,C4B,SERPINA1,TGFBI,CFB,IGFALS,GC,HGFAC,JCHAIN,TUBA1C,C1R,APOA5,ATP1A1,VIM,APOC4,KNG1,ACTB,B2M,FGA
TISSUES	BTO:0001486	Skeletal system	97	1307	0.8	9.90e-48	SPP2,CPB2,CETP,CHGA,APMAP,C5,SPARC,LTF,APOB,IGFBP5,APOA1,TTR,LRP1,CFP,APOE,SERPINF1,APCS,THBS1,VWF,PSMA6,LGALS3BP,ATRN,C9,CSTA,CP,KLKB1,F13A1,AMBP,HPX,LUM,FCN3,AHSG,SAA4,SERPING1,DSC2,CSF1R,AZGP1,MASP1,CAMP,FGB,F2,UBE3C,PRG2,CSPG4,CFHR1,CLU,SUZ12,APOL1,SERPINF2,KDM3B,SERPINA7,TUBA1B,FGG,APOD,FILIP1L,FN1,HP,CFH,F5,HTRA1,ADAMTSL4,ECM1,CRTAC1,ADAMTS13,CCN5,C1QA,IGFBP3,SOD3,PROS1,CFI,MMRN1,YWHAZ,F11,C1S,PCYOX1,BZW1,ORM2,C4A,PRG4,MST1,LMOD3,C4B,SERPINA1,CFB,HGFAC,SELENOP,JCHAIN,TUBA1C,C1R,VIM,TTN,PAMR1,KNG1,ACTB,CDH5,B2M,FGA
TISSUES	BTO:0000345	Digestive gland	129	2881	0.58	7.78e-44	SPP2,ST6GAL1,CPB2,CETP,CHGA,TPD52L2,APMAP,F9,GPLD1,SPARC,LTF,APOB,PROC,APOA1,TTR,CAT,LRP1,APOE,SERPINF1,APCS,CFHR5,THBS1,VWF,PSMA6,SERPINA10,LGALS3BP,ATRN,C9,CP,KLKB1,F13A1,AMBP,TCIRG1,HPX,INHBE,LUM,FCN3,ITIH1,AHSG,IGFBP1,HABP2,SAA4,SERPING1,SEC24D,CSF1R,AZGP1,PGLYRP2,MASP1,APEH,PDIA3,ANGPTL4,FGB,YWHAG,DST,F2,INHBC,PLG,STAB1,C1QB,CFHR1,CLU,NDUFS8,APOL1,SERPINF2,P4HB,SERPINA7,ANG,TUBA1B,FGG,TUBB,SLPI,QPCT,APOD,FILIP1L,FN1,ASGR2,HP,AKAP9,COL18A1,C8A,F13B,CFHR2,CFHR4,CFHR3,CFH,S100A9,ECM1,FLNA,SMARCA1,C8B,ADAMTS13,C1QA,F10,F7,APOM,IGFBP3,CD99,GPX3,PROS1,CFI,YWHAZ,MASP2,F11,C1S,PCYOX1,BZW1,ORM2,C4A,MST1,ITIH3,C4B,SERPINA1,TGFBI,CFB,IGFALS,GC,HGFAC,JCHAIN,TUBA1C,C1R,APOA5,ATP1A1,VIM,RNASE4,APOC4,KNG1,ACTB,B2M,FGA
TISSUES	BTO:0001491	Viscus	151	5378	0.38	8.74e-30	SPP2,ST6GAL1,CPB2,CETP,CHGA,TPD52L2,APMAP,F9,GPLD1,SPARC,LTF,APOB,PROC,APOA1,TTR,CAT,LRP1,CFP,APOE,SERPINF1,APCS,CFHR5,THBS1,LYZ,VWF,PSMA6,SERPINA10,LGALS3BP,ATRN,C9,CSTA,CP,KLKB1,F13A1,CDH6,AMBP,TCIRG1,HPX,INHBE,LUM,FCN3,ITIH1,AHSG,IGFBP1,HABP2,SAA4,SERPING1,SEC24D,DSC2,CSF1R,AZGP1,PGLYRP2,MASP1,APEH,PDIA3,LALBA,ANGPTL4,FGB,YWHAG,DST,F2,INHBC,PLG,STAB1,C1QB,CFHR1,CLU,APOL1,SERPINF2,P4HB,SERPINA7,FBLN1,ANG,TUBA1B,FGG,TUBB,SLPI,QPCT,APOD,FILIP1L,FN1,ASGR2,HP,AKAP9,APOA4,COL18A1,C8A,F13B,CFHR2,CFHR4,CFHR3,CFH,S100A9,ECM1,FLNA,SMARCA1,C8B,ADAMTS13,C1QA,F10,F7,APOM,CALML5,IGFBP3,CD99,SOD3,GPX3,PROS1,EFEMP1,CFI,TNRC6A,YWHAZ,ILK,MASP2,F11,C1S,PCYOX1,BZW1,ORM2,C4A,ACSF2,CKMT1A,MST1,ITIH3,C4B,SERPINA1,TGFBI,CFB,IGFALS,RARRES2,GC,HGFAC,JCHAIN,TUBA1C,C1R,APOA5,ATP1A1,VIM,SERPINA4,RNASE4,APOC4,CFD,CCL18,VEGFC,PLCG2,KNG1,TPM4,ACTB,TRIM8,B2M,FGA
TISSUES	BTO:0000237	Cerebrospinal fluid	20	45	1.57	5.37e-21	CHGA,TTR,APOE,SERPINF1,CP,HPX,AHSG,SERPING1,F2,CLU,SERPINA7,APOD,HP,CFH,IGFBP3,CFI,ORM2,C4A,SERPINA1,KNG1
TISSUES	BTO:0001488	Endocrine gland	149	6403	0.29	4.11e-20	SPP2,ST6GAL1,CPB2,CETP,CHGA,TPD52L2,APMAP,F9,GPLD1,SPARC,LTF,APOB,PROC,APOA1,TTR,CAT,LRP1,APOE,SERPINF1,APCS,CFHR5,THBS1,VWF,PSMA6,SERPINA10,LGALS3BP,ATRN,C9,LCN1,CSTA,CP,KLKB1,F13A1,AMBP,TCIRG1,HPX,INHBE,LUM,FCN3,ITIH1,AHSG,IGFBP1,HABP2,SAA4,SERPING1,SEC24D,CSF1R,PIP,AZGP1,PGLYRP2,MASP1,CAMP,APEH,PDIA3,ANGPTL4,FGB,YWHAG,DST,F2,INHBC,PLG,STAB1,C1QB,CFHR1,CLU,NDUFS8,APOL1,SERPINF2,P4HB,SERPINA7,FBLN1,ANG,TUBA1B,FGG,ISYNA1,TUBB,SLPI,QPCT,APOD,FILIP1L,FN1,ASGR2,HP,AKAP9,COL18A1,C8A,F13B,CFHR2,CFHR4,CFHR3,CFH,S100A9,ECM1,FLNA,SMARCA1,C8B,ADAMTS13,CCN5,SVEP1,C1QA,F10,F7,APOM,CALML5,IGFBP3,CD99,SOD3,GPX3,PROS1,EFEMP1,CFI,EFCAB5,YWHAZ,MASP2,F11,C1S,PCYOX1,BZW1,ORM2,C4A,ATP9B,ACSF2,MST1,ITIH3,C4B,SERPINA1,TGFBI,CFB,IGFALS,RARRES2,GC,HGFAC,UIMC1,PDGFC,JCHAIN,TUBA1C,C1R,APOA5,ATP1A1,VIM,RNASE4,APOC4,CFD,CCL18,KNG1,TPM4,ACTB,B2M,FGA
TISSUES	BTO:0000522	Gland	155	7004	0.27	3.10e-19	SPP2,ST6GAL1,CPB2,CETP,CHGA,TPD52L2,APMAP,F9,C5,GPLD1,SPARC,LTF,APOB,PROC,APOA1,TTR,CAT,LRP1,APOE,BPIFB1,SERPINF1,APCS,CFHR5,THBS1,LYZ,VWF,PSMA6,SERPINA10,LGALS3BP,ATRN,C9,LCN1,CSTA,CP,KLKB1,F13A1,CDH6,AMBP,TCIRG1,HPX,INHBE,LUM,FCN3,ITIH1,AHSG,IGFBP1,HABP2,SAA4,SERPING1,SEC24D,CSF1R,PIP,AZGP1,PGLYRP2,MASP1,CAMP,APEH,PDIA3,ANGPTL4,FGB,YWHAG,DST,F2,INHBC,PLG,STAB1,C1QB,CFHR1,CLU,NDUFS8,APOL1,SERPINF2,P4HB,SERPINA7,FBLN1,ANG,TUBA1B,FGG,ISYNA1,TUBB,SLPI,QPCT,APOD,FILIP1L,FN1,ASGR2,HP,AKAP9,COL18A1,C8A,F13B,CFHR2,CFHR4,CFHR3,CFH,S100A9,ECM1,FLNA,SMARCA1,C8B,ADAMTS13,CCN5,SVEP1,C1QA,F10,F7,APOM,CA6,CALML5,IGFBP3,CD99,SOD3,GPX3,PROS1,EFEMP1,CFI,EFCAB5,TNRC6A,YWHAZ,MASP2,F11,C1S,PCYOX1,BZW1,ORM2,C4A,ATP9B,ACSF2,MST1,ITIH3,C4B,SERPINA1,TGFBI,CFB,IGFALS,RARRES2,GC,HGFAC,UIMC1,PDGFC,JCHAIN,TUBA1C,C1R,APOA5,ATP1A1,VIM,RNASE4,APOC4,CFD,CCL18,KNG1,TPM4,ACTB,B2M,FGA
TISSUES	BTO:0001489	Whole body	214	13099	0.14	1.41e-17	SPP2,ST6GAL1,CPB2,CETP,CHGA,TPD52L2,APMAP,F9,C5,MGP,GPLD1,SPARC,LTF,APOB,IGFBP5,PROC,APOA1,TTR,CAT,LRP1,CFP,APOE,BPIFB1,SERPINF1,APCS,CFHR5,THBS1,LYZ,VWF,PSMA6,SERPINA10,LGALS3BP,ATRN,C9,LCN1,LCAT,CSTA,CP,KLKB1,F13A1,DGKG,CDH6,DNAH5,AMBP,CENPE,TCIRG1,HPX,INHBE,LUM,FCN3,ITIH1,AHSG,IGFBP1,HABP2,SAA4,SERPING1,SEC24D,DSC2,CSF1R,PIP,PSG6,AZGP1,PGLYRP2,SCGB3A1,SERPINI1,PF4,MASP1,CAMP,APEH,A2ML1,PDIA3,SLC27A4,LALBA,ANGPTL4,PCSK9,BMP1,FGB,YWHAG,DST,F2,INHBC,PLG,PSG1,UBE3C,EFEMP2,PRG2,CSPG4,STAB1,C1QB,CFHR1,CLU,NDUFS8,SUZ12,APOL1,SERPINF2,C7,KDM3B,P4HB,SERPINA7,PAPPA,FBLN1,PSG3,DNAH8,ANG,TUBA1B,FGG,ISYNA1,TUBB,THBS4,SLPI,QPCT,APOD,FILIP1L,FN1,ASGR2,HP,AKAP9,APOA4,CNDP1,COL18A1,C8A,F13B,CFHR2,CFHR4,CFHR3,CFH,F5,ADAM12,S100A7,S100A9,HTRA1,ADAMTSL4,ECM1,FLNA,CRTAC1,SMARCA1,C8B,FCN1,ADAMTS13,CCN5,SVEP1,C1QA,F10,F7,APOM,CA6,ADGRG2,CALML5,IGFBP3,CD99,SOD3,GPX3,PROS1,EFEMP1,CFI,EFCAB5,MMRN1,TNRC6A,YWHAZ,NAV2,ILK,MAP3K7CL,MASP2,F11,C1S,PSG2,PCYOX1,BZW1,ORM2,C4A,ATP9B,PRG4,SLAIN1,ACSF2,CKMT1A,MST1,LMOD3,ITIH3,C4B,SERPINA1,TGFBI,CFB,IGFALS,PAEP,RARRES2,GC,HGFAC,UIMC1,PDGFC,FBN2,SELENOP,TSKU,JCHAIN,TUBA1C,C1R,APOA5,ATP1A1,VIM,SERPINA4,RNASE4,TTN,APOC4,CFD,CCL18,VEGFC,PLCG2,PAMR1,KNG1,TPM4,ACTB,TRIM8,CDH5,B2M,FGA
TISSUES	BTO:0001279	Spinal cord	24	233	0.94	7.50e-13	CHGA,TTR,APOE,SERPINF1,CP,HPX,AHSG,SERPING1,F2,CSPG4,CLU,SERPINA7,APOD,HP,CFH,FLNA,IGFBP3,CFI,ORM2,C4A,SERPINA1,VIM,KNG1,ACTB
TISSUES	BTO:0001239	Serum	12	25	1.61	1.13e-12	GPLD1,APOA1,LGALS3BP,ATRN,ITIH1,PGLYRP2,PRG2,CLU,PAPPA,HP,HGFAC,KNG1

TISSUES	BTO:0001078	Placenta	50	1244	0.53	6.16e-12	SPARC,IGFBP5,LRP1,THBS1,VWF,LGALS3BP,F13A1,LUM,IGFBP1,SERPING1,DSC2,CSF1R,PSG6,PDIA3,ANGPTL4,BMP1,DST,PSG1,EFEMP2,PRG2,APOL1,P4HB,PAPPA,FBLN1,PSG3,TUBA1B,FN1,COL18A1,F5,ADAM12,HTRA1,ADAMTSL4,SVEP1,IGFBP3,GPX3,YWHAZ,ILK,PSG2,PCYOX1,SERPINA1,TGFBI,FBN2,TUBA1C,ATP1A1,VIM,CFD,TPM4,ACTB,CDH5,B2M
TISSUES	BTO:0000449	Fetus	51	1318	0.51	1.27e-11	SPARC,IGFBP5,LRP1,THBS1,VWF,LGALS3BP,F13A1,LUM,IGFBP1,SERPING1,DSC2,CSF1R,PSG6,PDIA3,ANGPTL4,BMP1,DST,PSG1,EFEMP2,PRG2,APOL1,P4HB,PAPPA,FBLN1,PSG3,TUBA1B,TUBB,FN1,COL18A1,F5,ADAM12,HTRA1,ADAMTSL4,SVEP1,IGFBP3,GPX3,YWHAZ,ILK,PSG2,PCYOX1,SERPINA1,TGFBI,FBN2,TUBA1C,ATP1A1,VIM,CFD,TPM4,ACTB,CDH5,B2M
TISSUES	BTO:0000042	Animal	219	15148	0.09	2.92e-10	SPP2,ST6GAL1,CPB2,CETP,CHGA,TPD52L2,APMAP,F9,C5,MGP,GPLD1,SPARC,LTF,APOB,IGFBP5,PROC,APOA1,TTR,CAT,LRP1,CFP,APOE,ANGPTL6,BPIFB1,SERPINF1,APCS,CFHR5,THBS1,LYZ,VWF,PSMA6,SERPINA10,LGALS3BP,ATRN,C9,LCN1,LCAT,CSTA,CP,KLKB1,F13A1,PPP2R3A,DGKG,CDH6,DNAH5,AMBP,CENPE,TCIRG1,HPX,INHBE,LUM,FCN3,ITIH1,AHSG,IGFBP1,HABP2,SAA4,SERPING1,SEC24D,DSC2,CSF1R,PIP,PSG6,AZGP1,PGLYRP2,SCGB3A1,SERPINI1,PF4,MASP1,CAMP,APEH,A2ML1,PDIA3,SLC27A4,LALBA,ANGPTL4,PCSK9,BMP1,FGB,YWHAG,DST,F2,INHBC,PLG,PSG1,UBE3C,EFEMP2,PRG2,CSPG4,STAB1,C1QB,CFHR1,CLU,NDUFS8,SUZ12,APOL1,SERPINF2,C7,KDM3B,P4HB,SERPINA7,PAPPA,FBLN1,PSG3,DNAH8,ANG,TUBA1B,FGG,ISYNA1,TUBB,THBS4,SLPI,QPCT,APOD,FILIP1L,FN1,ASGR2,HP,AKAP9,APOA4,CNDP1,COL18A1,C8A,F13B,CFHR2,CFHR4,CFHR3,CFH,F5,ADAM12,S100A7,S100A9,HTRA1,ADAMTSL4,ECM1,FLNA,CRTAC1,SMARCA1,C8B,FCN1,ADAMTS13,CCN5,SVEP1,C1QA,F10,F7,APOM,CA6,ADGRG2,CALML5,IGFBP3,CD99,SOD3,GPX3,PROS1,EFEMP1,CFI,EFCAB5,MMRN1,TNRC6A,YWHAZ,NAV2,ILK,MAP3K7CL,MASP2,F11,C1S,PSG2,PCYOX1,BZW1,ORM2,C4A,ATP9B,PRG4,SLAIN1,ACSF2,CKMT1A,ABCA13,MST1,LMOD3,ITIH3,C4B,SERPINA1,TGFBI,CFB,IGFALS,PAEP,RARRES2,TCAF1,GC,HGFAC,UIMC1,PDGFC,FBN2,SELENOP,TSKU,JCHAIN,TUBA1C,C1R,APOA5,ATP1A1,VIM,SERPINA4,RNASE4,TTN,APOC4,CFD,CCL18,VEGFC,PLCG2,PAMR1,KNG1,TPM4,ACTB,TRIM66,TRIM8,CDH5,B2M,FGA
TISSUES	BTO:0000421	Connective tissue	38	954	0.53	1.17e-08	SPP2,APMAP,SPARC,APOA1,TTR,CAT,THBS1,LGALS3BP,LUM,AHSG,IGFBP1,PDIA3,ANGPTL4,YWHAG,EFEMP2,CLU,P4HB,PAPPA,TUBA1B,TUBB,THBS4,FN1,F5,HTRA1,ECM1,CRTAC1,EFEMP1,YWHAZ,BZW1,PRG4,ACSF2,SERPINA1,TUBA1C,VIM,CFD,TPM4,ACTB,B2M
TISSUES	BTO:0000284	Organism form	67	2542	0.35	2.09e-08	ST6GAL1,TPD52L2,SPARC,LTF,IGFBP5,LRP1,CFP,THBS1,VWF,PSMA6,LGALS3BP,F13A1,LUM,IGFBP1,SERPING1,DSC2,CSF1R,PSG6,CAMP,PDIA3,ANGPTL4,BMP1,DST,PSG1,UBE3C,EFEMP2,PRG2,APOL1,P4HB,PAPPA,FBLN1,PSG3,TUBA1B,ISYNA1,TUBB,FN1,AKAP9,COL18A1,F5,ADAM12,HTRA1,ADAMTSL4,FLNA,SVEP1,APOM,IGFBP3,CD99,GPX3,EFEMP1,YWHAZ,NAV2,ILK,PSG2,PCYOX1,SERPINA1,TGFBI,UIMC1,FBN2,TUBA1C,ATP1A1,VIM,CFD,VEGFC,TPM4,ACTB,CDH5,B2M
TISSUES	BTO:0003099	Internal female genital organ	71	2804	0.33	2.66e-08	TPD52L2,MGP,SPARC,LTF,IGFBP5,LRP1,THBS1,VWF,LGALS3BP,F13A1,LUM,IGFBP1,SERPING1,DSC2,CSF1R,PSG6,MASP1,A2ML1,PDIA3,ANGPTL4,BMP1,DST,PSG1,EFEMP2,PRG2,CLU,NDUFS8,SUZ12,APOL1,P4HB,PAPPA,FBLN1,PSG3,TUBA1B,ISYNA1,TUBB,SLPI,FN1,COL18A1,F5,ADAM12,S100A9,HTRA1,ADAMTSL4,FLNA,CCN5,SVEP1,F10,IGFBP3,CD99,GPX3,PROS1,YWHAZ,ILK,C1S,PSG2,PCYOX1,SERPINA1,TGFBI,PAEP,UIMC1,PDGFC,FBN2,TUBA1C,ATP1A1,VIM,CFD,TPM4,ACTB,CDH5,B2M
TISSUES	BTO:0000203	Respiratory system	52	1707	0.41	3.07e-08	ST6GAL1,SPARC,LTF,IGFBP5,APOE,BPIFB1,THBS1,LYZ,VWF,LGALS3BP,ATRN,LCN1,CP,DNAH5,TCIRG1,LUM,FCN3,SERPING1,PIP,PDIA3,P4HB,TUBA1B,TUBB,FN1,AKAP9,CFH,S100A9,FLNA,SMARCA1,FCN1,CCN5,IGFBP3,GPX3,PROS1,EFEMP1,MASP2,C1S,PCYOX1,C4B,SERPINA1,TGFBI,PDGFC,JCHAIN,TUBA1C,ATP1A1,VIM,CCL18,VEGFC,KNG1,ACTB,CDH5,B2M
TISSUES	BTO:0000511	Gastrointestinal tract	54	1816	0.4	3.07e-08	CHGA,SPARC,LTF,APOB,PROC,APOA1,TTR,CAT,LYZ,LGALS3BP,KLKB1,LUM,AHSG,HABP2,SERPING1,AZGP1,PDIA3,LALBA,YWHAG,DST,CLU,P4HB,FBLN1,ANG,TUBA1B,TUBB,SLPI,FN1,AKAP9,APOA4,FLNA,IGFBP3,CD99,SOD3,GPX3,TNRC6A,YWHAZ,ILK,C1S,CKMT1A,SERPINA1,TGFBI,CFB,JCHAIN,TUBA1C,C1R,ATP1A1,VIM,SERPINA4,CFD,TPM4,ACTB,TRIM8,B2M
TISSUES	BTO:0000570	Hematopoietic system	70	2755	0.33	3.07e-08	ST6GAL1,CETP,TPD52L2,GPLD1,LTF,APOB,PROC,APOA1,TTR,CAT,LRP1,CFP,APOE,THBS1,VWF,PSMA6,LGALS3BP,ATRN,C9,ITIH1,AHSG,SERPING1,AZGP1,PGLYRP2,PF4,PDIA3,ANGPTL4,FGB,YWHAG,F2,PRG2,CLU,P4HB,PAPPA,TUBA1B,FGG,FN1,HP,AKAP9,C8A,F5,S100A9,FLNA,C1QA,APOM,IGFBP3,CD99,SOD3,GPX3,MMRN1,YWHAZ,C1S,PRG4,C4B,SERPINA1,TGFBI,CFB,HGFAC,JCHAIN,TUBA1C,VIM,CFD,CCL18,VEGFC,PLCG2,KNG1,TPM4,ACTB,B2M,FGA
TISSUES	BTO:0001202	Saliva	11	57	1.21	3.50e-08	THBS1,LGALS3BP,HPX,AHSG,DSC2,PIP,AZGP1,CLU,HP,C4B,JCHAIN
TISSUES	BTO:0000132	Blood platelet	22	363	0.71	9.03e-08	APOA1,TTR,CAT,THBS1,VWF,C9,AHSG,PDIA3,FGB,YWHAG,CLU,P4HB,FGG,F5,FLNA,MMRN1,YWHAZ,PRG4,SERPINA1,TPM4,ACTB,FGA
TISSUES	BTO:0000058	Alimentary canal	56	2021	0.37	1.39e-07	CHGA,SPARC,LTF,APOB,PROC,APOA1,TTR,CAT,THBS1,LYZ,LGALS3BP,KLKB1,LUM,AHSG,HABP2,SERPING1,AZGP1,PDIA3,LALBA,YWHAG,DST,CLU,P4HB,FBLN1,ANG,TUBA1B,TUBB,SLPI,FN1,AKAP9,APOA4,FLNA,CALML5,IGFBP3,CD99,SOD3,GPX3,TNRC6A,YWHAZ,ILK,C1S,CKMT1A,SERPINA1,TGFBI,CFB,JCHAIN,TUBA1C,C1R,ATP1A1,VIM,SERPINA4,CFD,TPM4,ACTB,TRIM8,B2M
TISSUES	BTO:0000174	Embryonic structure	61	2369	0.34	3.24e-07	ST6GAL1,TPD52L2,SPARC,IGFBP5,LRP1,THBS1,VWF,PSMA6,LGALS3BP,F13A1,LUM,IGFBP1,SERPING1,DSC2,CSF1R,PSG6,PDIA3,ANGPTL4,BMP1,DST,PSG1,UBE3C,EFEMP2,PRG2,APOL1,P4HB,PAPPA,FBLN1,PSG3,TUBA1B,ISYNA1,TUBB,FN1,AKAP9,COL18A1,F5,ADAM12,HTRA1,ADAMTSL4,FLNA,SVEP1,APOM,IGFBP3,GPX3,YWHAZ,ILK,PSG2,PCYOX1,SERPINA1,TGFBI,UIMC1,FBN2,TUBA1C,ATP1A1,VIM,CFD,VEGFC,TPM4,ACTB,CDH5,B2M
TISSUES	BTO:0000088	Cardiovascular system	37	1057	0.47	4.31e-07	MGP,SPARC,APOB,APOA1,APOE,THBS1,VWF,LUM,SERPING1,PDIA3,SLC27A4,ANGPTL4,YWHAG,F2,P4HB,TUBA1B,THBS4,FN1,F13B,FLNA,GPX3,EFEMP1,MMRN1,PCYOX1,LMOD3,SERPINA1,TGFBI,FBN2,SELENOP,VIM,TTN,CCL18,VEGFC,ACTB,CDH5,B2M,FGA
TISSUES	BTO:0001419	Urine	8	28	1.38	5.52e-07	LYZ,AMBP,F2,FN1,MASP2,PRG4,SERPINA1,B2M
TISSUES	BTO:0000121	Bile	7	20	1.47	1.46e-06	LGALS3BP,CP,HPX,AZGP1,ORM2,SERPINA1,JCHAIN
TISSUES	BTO:0000634	Integument	55	2112	0.34	1.46e-06	ST6GAL1,CHGA,SPARC,LTF,LRP1,APOE,BPIFB1,THBS1,VWF,PSMA6,LGALS3BP,LCN1,CSTA,LUM,IGFBP1,DSC2,PIP,AZGP1,A2ML1,PDIA3,ANGPTL4,BMP1,DST,P4HB,TUBA1B,SLPI,APOD,FN1,AKAP9,S100A7,S100A9,ECM1,FLNA,CALML5,IGFBP3,CD99,EFEMP1,MMRN1,YWHAZ,ILK,TGFBI,PAEP,RARRES2,PDGFC,FBN2,TUBA1C,C1R,ATP1A1,VIM,CFD,VEGFC,TPM4,ACTB,CDH5,B2M
TISSUES	BTO:0000089	Blood	50	1824	0.37	1.49e-06	TPD52L2,LTF,APOB,PROC,APOA1,TTR,CAT,LRP1,APOE,THBS1,VWF,C9,AHSG,SERPING1,AZGP1,PF4,PDIA3,FGB,YWHAG,F2,CLU,P4HB,TUBA1B,FGG,FN1,HP,AKAP9,C8A,F5,S100A9,FLNA,C1QA,APOM,CD99,SOD3,GPX3,MMRN1,YWHAZ,C1S,PRG4,C4B,SERPINA1,CFB,VIM,CCL18,PLCG2,TPM4,ACTB,B2M,FGA
TISSUES	BTO:0003914	Interstitial cell of Cajal	15	193	0.82	1.63e-06	APOA1,TTR,CAT,AHSG,PDIA3,YWHAG,P4HB,TUBA1B,TUBB,YWHAZ,SERPINA1,TUBA1C,VIM,TPM4,ACTB

TISSUES	BTO:0001499	Tear	7	23	1.41	2.82e-06	LTF,LYZ,LCN1,PIP,SLPI,JCHAIN,B2M
TISSUES	BTO:0001253	Skin	37	1151	0.43	2.85e-06	ST6GAL1,SPARC,LRP1,APOE,PSMA6,LGALS3BP,LCN1,CSTA,DSC2,PDIA3,ANGPTL4,BMP1,DST,P4HB,TUBA1B,APOD,FN1,S100A7,S100A9,ECM1,FLNA,CALML5,IGFBP3,CD99,EFEMP1,YWHAZ,TGFBI,RARRES2,TUBA1C,C1R,ATP1A1,VIM,CFD,VEGFC,TPM4,ACTB,B2M
TISSUES	BTO:0000988	Pancreas	26	626	0.55	3.20e-06	CETP,CHGA,GPLD1,SPARC,TTR,F13A1,TCIRG1,SEC24D,PDIA3,ANGPTL4,CLU,APOL1,P4HB,TUBA1B,QPCT,FILIP1L,FN1,FLNA,IGFBP3,CD99,GPX3,TGFBI,ATP1A1,VIM,ACTB,B2M
TISSUES	BTO:0000255	Brain cell line	14	188	0.8	6.96e-06	APOA1,TTR,PDIA3,FGB,YWHAG,P4HB,TUBA1B,TUBB,YWHAZ,VIM,TTN,TPM4,ACTB,TRIM66
TISSUES	BTO:0001085	Vascular system	20	420	0.6	1.46e-05	MGP,APOB,APOE,THBS1,VWF,ANGPTL4,YWHAG,F2,P4HB,FN1,EFEMP1,MMRN1,TGFBI,FBN2,VIM,CCL18,VEGFC,ACTB,CDH5,B2M
TISSUES	BTO:0001307	Stomach	17	312	0.66	1.99e-05	CHGA,SPARC,LTF,LYZ,PDIA3,LALBA,CLU,P4HB,TUBA1B,FN1,AKAP9,IGFBP3,TNRC6A,TUBA1C,VIM,ACTB,B2M
TISSUES	BTO:0000763	Lung	39	1395	0.37	3.44e-05	SPARC,LTF,IGFBP5,APOE,BPIFB1,THBS1,VWF,LGALS3BP,LCN1,DNAH5,TCIRG1,LUM,FCN3,SERPING1,PDIA3,P4HB,TUBA1B,TUBB,FN1,AKAP9,S100A9,FLNA,SMARCA1,FCN1,CCN5,GPX3,EFEMP1,MASP2,C1S,C4B,SERPINA1,TGFBI,PDGFC,TUBA1C,VIM,CCL18,ACTB,CDH5,B2M
TISSUES	BTO:0000648	Intestine	38	1349	0.38	4.01e-05	APOB,PROC,LYZ,LGALS3BP,KLKB1,LUM,HABP2,AZGP1,PDIA3,DST,CLU,P4HB,ANG,TUBA1B,SLPI,FN1,APOA4,FLNA,IGFBP3,SOD3,GPX3,YWHAZ,ILK,C1S,CKMT1A,SERPINA1,TGFBI,CFB,JCHAIN,TUBA1C,C1R,ATP1A1,VIM,SERPINA4,CFD,ACTB,TRIM8,B2M
TISSUES	BTO:0001090	Mouth	21	532	0.52	0.00012	C5,LTF,BPIFB1,LYZ,LCN1,CSTA,CENPE,PIP,A2ML1,DST,C7,P4HB,SLPI,FN1,S100A9,CA6,YWHAZ,JCHAIN,TPM4,ACTB,B2M
TISSUES	BTO:0000439	Eye	27	835	0.44	0.00017	SPARC,LTF,IGFBP5,TTR,APOE,SERPINF1,LYZ,LCN1,LUM,SERPINI1,DST,CLU,TUBA1B,TUBB,APOD,FN1,HP,CFH,GPX3,YWHAZ,BZW1,TGFBI,FBN2,ATP1A1,VIM,ACTB,B2M
TISSUES	BTO:0000562	Heart	25	738	0.46	0.00018	APOA1,THBS1,VWF,LUM,SERPING1,PDIA3,SLC27A4,F2,TUBA1B,THBS4,FN1,F13B,FLNA,GPX3,MMRN1,PCYOX1,LMOD3,SERPINA1,SELENOP,VIM,TTN,VEGFC,ACTB,CDH5,FGA
TISSUES	BTO:0000431	Excretory gland	36	1385	0.34	0.00044	TPD52L2,SPARC,LTF,CAT,APOE,CDH6,HPX,AHSG,PIP,AZGP1,FGB,F2,PLG,CLU,P4HB,TUBA1B,APOD,FN1,HP,COL18A1,SMARCA1,SVEP1,IGFBP3,CD99,GPX3,TNRC6A,ACSF2,TGFBI,RARRES2,GC,TUBA1C,ATP1A1,VIM,KNG1,ACTB,B2M
TISSUES	BTO:0000202	Sense organ	31	1124	0.37	0.00064	SPARC,LTF,IGFBP5,TTR,APOE,BPIFB1,SERPINF1,LYZ,LCN1,CSTA,LUM,SERPING1,SERPINI1,DST,CLU,TUBA1B,TUBB,SLPI,APOD,FN1,HP,CFH,GPX3,YWHAZ,BZW1,TGFBI,FBN2,ATP1A1,VIM,ACTB,B2M
TISSUES	BTO:0000102	Blood clot	4	11	1.49	0.0010	VWF,F2,PLG,ADAMTS13
TISSUES	BTO:0001369	Vertebrate muscular system	24	780	0.42	0.0012	CPB2,APOA1,APOE,APCS,PSMA6,YWHAG,EFEMP2,P4HB,FGG,THBS4,FILIP1L,ECM1,CRTAC1,CCN5,GPX3,EFEMP1,BZW1,ORM2,PRG4,LMOD3,SERPINA1,TTN,KNG1,ACTB
TISSUES	BTO:0000083	Female reproductive system	101	6111	0.15	0.0025	ST6GAL1,TPD52L2,APMAP,MGP,SPARC,LTF,IGFBP5,APOA1,CAT,LRP1,APOE,SERPINF1,THBS1,VWF,PSMA6,LGALS3BP,ATRN,F13A1,HPX,LUM,AHSG,IGFBP1,SERPING1,DSC2,CSF1R,PSG6,AZGP1,PGLYRP2,MASP1,CAMP,APEH,A2ML1,PDIA3,LALBA,ANGPTL4,BMP1,FGB,DST,F2,PSG1,UBE3C,EFEMP2,PRG2,CLU,NDUFS8,SUZ12,APOL1,C7,P4HB,PAPPA,FBLN1,PSG3,DNAH8,TUBA1B,ISYNA1,TUBB,SLPI,FN1,HP,COL18A1,CFH,F5,ADAM12,S100A9,HTRA1,ADAMTSL4,ECM1,FLNA,ADAMTS13,CCN5,SVEP1,F10,IGFBP3,CD99,GPX3,PROS1,EFEMP1,EFCAB5,YWHAZ,NAV2,ILK,C1S,PSG2,PCYOX1,BZW1,SERPINA1,TGFBI,PAEP,UIMC1,PDGFC,FBN2,TSKU,TUBA1C,ATP1A1,VIM,CFD,PAMR1,TPM4,ACTB,CDH5,B2M
TISSUES	BTO:0000081	Reproductive system	105	6444	0.14	0.0027	ST6GAL1,TPD52L2,APMAP,MGP,SPARC,LTF,IGFBP5,APOA1,CAT,LRP1,APOE,SERPINF1,THBS1,VWF,PSMA6,LGALS3BP,ATRN,F13A1,HPX,LUM,AHSG,IGFBP1,SERPING1,DSC2,CSF1R,PIP,PSG6,AZGP1,PGLYRP2,MASP1,CAMP,APEH,A2ML1,PDIA3,LALBA,ANGPTL4,BMP1,FGB,DST,F2,PSG1,UBE3C,EFEMP2,PRG2,CLU,NDUFS8,SUZ12,APOL1,C7,P4HB,PAPPA,FBLN1,PSG3,DNAH8,TUBA1B,ISYNA1,TUBB,SLPI,FN1,HP,COL18A1,CFH,F5,ADAM12,S100A9,HTRA1,ADAMTSL4,ECM1,FLNA,ADAMTS13,CCN5,SVEP1,F10,ADGRG2,IGFBP3,CD99,GPX3,PROS1,EFEMP1,EFCAB5,YWHAZ,NAV2,ILK,C1S,PSG2,PCYOX1,BZW1,ATP9B,SERPINA1,TGFBI,PAEP,RARRES2,UIMC1,PDGFC,FBN2,TSKU,TUBA1C,ATP1A1,VIM,CFD,PAMR1,TPM4,ACTB,CDH5,B2M
TISSUES	BTO:0001485	Muscular system	31	1220	0.33	0.0027	CPB2,SPARC,APOA1,APOE,SERPINF1,APCS,PSMA6,INHBE,APEH,YWHAG,EFEMP2,P4HB,TUBA1B,FGG,TUBB,THBS4,FILIP1L,FN1,ECM1,CRTAC1,CCN5,GPX3,EFEMP1,BZW1,ORM2,PRG4,LMOD3,SERPINA1,TTN,KNG1,ACTB
TISSUES	BTO:0003091	Urogenital system	113	7090	0.13	0.0029	ST6GAL1,TPD52L2,APMAP,MGP,SPARC,LTF,IGFBP5,APOA1,CAT,LRP1,APOE,SERPINF1,THBS1,VWF,PSMA6,LGALS3BP,ATRN,F13A1,CDH6,HPX,LUM,AHSG,IGFBP1,SERPING1,DSC2,CSF1R,PIP,PSG6,AZGP1,PGLYRP2,MASP1,CAMP,APEH,A2ML1,PDIA3,LALBA,ANGPTL4,BMP1,FGB,DST,F2,PLG,PSG1,UBE3C,EFEMP2,PRG2,CLU,NDUFS8,SUZ12,APOL1,C7,P4HB,PAPPA,FBLN1,PSG3,DNAH8,TUBA1B,ISYNA1,TUBB,SLPI,FN1,HP,COL18A1,CFH,F5,ADAM12,S100A9,HTRA1,ADAMTSL4,ECM1,FLNA,SMARCA1,ADAMTS13,CCN5,SVEP1,F10,ADGRG2,IGFBP3,CD99,GPX3,PROS1,EFEMP1,EFCAB5,TNRC6A,YWHAZ,NAV2,ILK,C1S,PSG2,PCYOX1,BZW1,ATP9B,ACSF2,SERPINA1,TGFBI,PAEP,RARRES2,GC,UIMC1,PDGFC,FBN2,TSKU,TUBA1C,ATP1A1,VIM,CFD,VEGFC,PAMR1,KNG1,TPM4,ACTB,CDH5,B2M
TISSUES	BTO:0001356	Tendon	8	112	0.78	0.0035	EFEMP2,P4HB,THBS4,ECM1,CRTAC1,EFEMP1,PRG4,SERPINA1
TISSUES	BTO:0000667	Keratinocyte	8	113	0.78	0.0037	CSTA,DSC2,PDIA3,DST,TUBA1B,S100A7,S100A9,TPM4
TISSUES	BTO:0000765	Exocrine gland	9	150	0.71	0.0044	C5,BPIFB1,LYZ,LCN1,PIP,MASP1,SLPI,CA6,B2M
TISSUES	BTO:0001823	Synovium	7	88	0.83	0.0050	P4HB,THBS4,ECM1,CRTAC1,EFEMP1,PRG4,SERPINA1
TISSUES	BTO:0000091	Ascites	6	61	0.92	0.0051	LGALS3BP,TUBA1B,SERPINA1,KNG1,ACTB,B2M
TISSUES	BTO:0005288	CL-48 cell	10	200	0.63	0.0071	SERPINA10,AHSG,MASP1,CLU,SERPINA11,FGG,GPX3,PRG4,SERPINA1,SERPINA4
TISSUES	BTO:0000140	Bone	9	165	0.66	0.0078	SPP2,SPARC,THBS1,CLU,TUBA1B,FN1,VIM,ACTB,B2M
TISSUES	BTO:0001493	Trunk	26	1014	0.34	0.0078	ST6GAL1,CHGA,TPD52L2,LTF,APOE,LGALS3BP,HPX,AHSG,AZGP1,ANGPTL4,FGB,F2,CLU,P4HB,TUBA1B,FN1,HP,SVEP1,GPX3,SERPINA1,ATP1A1,VIM,TTN,KNG1,ACTB,B2M
TISSUES	BTO:0001028	Peripheral nervous system	9	169	0.65	0.0089	SPARC,THBS4,COL18A1,MMRN1,TNRC6A,YWHAZ,C1S,CKMT1A,SELENOP
TISSUES	BTO:0000393	Endothelium	9	172	0.65	0.0100	APOE,THBS1,VWF,ANGPTL4,FN1,MMRN1,FBN2,VEGFC,CDH5
TISSUES	BTO:0000706	Large intestine	21	750	0.37	0.0101	PROC,LYZ,LGALS3BP,KLKB1,HABP2,AZGP1,P4HB,APOA4,FLNA,SOD3,YWHAZ,ILK,CKMT1A,SERPINA1,CFB,JCHAIN,C1R,SERPINA4,ACTB,TRIM8,B2M
TISSUES	BTO:0000671	Kidney	26	1039	0.33	0.0107	TPD52L2,SPARC,CAT,APOE,CDH6,PLG,CLU,P4HB,TUBA1B,FN1,COL18A1,SMARCA1,IGFBP3,CD99,GPX3,TNRC6A,ACSF2,TGFBI,RARRES2,GC,TUBA1C,ATP1A1,VIM,KNG1,ACTB,B2M
TISSUES	BTO:0003120	Atherosclerotic plaque	3	9	1.45	0.0107	APOB,APOA1,APOE
TISSUES	BTO:0003872	Foam cell	3	9	1.45	0.0107	APOB,APOA1,APOE
TISSUES	BTO:0001102	Blood vessel	13	354	0.49	0.0131	APOB,APOE,THBS1,VWF,ANGPTL4,F2,FN1,MMRN1,FBN2,VIM,CCL18,VEGFC,CDH5
TISSUES	BTO:0001203	Salivary gland	7	111	0.73	0.0151	C5,BPIFB1,LYZ,PIP,SLPI,CA6,B2M
TISSUES	BTO:0001853	Vascular endothelium	7	111	0.73	0.0151	APOE,THBS1,VWF,ANGPTL4,FN1,FBN2,CDH5

TISSUES	BTO:0003092	Urinary system	29	1249	0.29	0.0151	TPD52L2,SPARC,CAT,APOE,CDH6,PLG,CLU,P4HB,TUBA1B,FN1,COL18A1,CFH,FLNA,SMARCA1,IGFBP3,CD99,GPX3,TNRC6A,ACSF2,TGFBI,RARRES2,GC,TUBA1C,ATP1A1,VIM,VEGFC,KNG1,ACTB,B2M
TISSUES	BTO:0000020	Abdomen	7	113	0.72	0.0163	LGALS3BP,F2,TUBA1B,SERPINA1,KNG1,ACTB,B2M
TISSUES	BTO:0001244	Urinary tract	29	1258	0.29	0.0163	TPD52L2,SPARC,CAT,APOE,CDH6,PLG,CLU,P4HB,TUBA1B,FN1,COL18A1,CFH,FLNA,SMARCA1,IGFBP3,CD99,GPX3,TNRC6A,ACSF2,TGFBI,RARRES2,GC,TUBA1C,ATP1A1,VIM,VEGFC,KNG1,ACTB,B2M
TISSUES	BTO:0001470	Epidermal cell	9	197	0.59	0.0221	CSTA,DSC2,PDIA3,ANGPTL4,DST,TUBA1B,S100A7,S100A9,TPM4
TISSUES	BTO:0000416	Epithelium	19	697	0.36	0.0233	LTF,APOE,BPIFB1,THBS1,VWF,LCN1,LUM,PIP,AZGP1,ANGPTL4,SLPI,FN1,MMRN1,RARRES2,PDGFC,FBN2,ATP1A1,VEGFC,CDH5
TISSUES	BTO:0003150	Pulmonary artery endothelial cell line	2	2	1.93	0.0235	KNG1,CDH5
TISSUES	BTO:0003632	Cervicovaginal fluid	2	2	1.93	0.0235	LTF,SLPI
TISSUES	BTO:0001388	Trachea	9	201	0.58	0.0241	ST6GAL1,LTF,LYZ,CP,P4HB,CFH,VEGFC,KNG1,B2M
TISSUES	BTO:0000394	Aorta endothelium	4	33	1.01	0.0250	THBS1,ANGPTL4,FN1,FBN2
TISSUES	BTO:0001067	Pineal gland	3	14	1.26	0.0265	TTR,DST,CLU
TISSUES	BTO:0001702	Left atrium	5	60	0.85	0.0269	APOA1,FLNA,SERPINA1,VIM,TTN
TISSUES	BTO:0001519	Endothelial cell line	3	16	1.2	0.0362	VEGFC,KNG1,CDH5
TISSUES	BTO:0003445	Carotid atherosclerotic plaque	2	3	1.75	0.0362	APOB,APOE
TISSUES	BTO:0000136	Bronchoalveolar system	5	65	0.81	0.0364	BPIFB1,LCN1,FN1,MASP2,CDH5
TISSUES	BTO:0000420	Neck	10	264	0.51	0.0395	CHGA,APOE,CLU,P4HB,FN1,GPX3,ATP1A1,VIM,ACTB,B2M
TISSUES	BTO:0000573	Artery	9	219	0.54	0.0395	APOB,APOE,THBS1,ANGPTL4,F2,FN1,FBN2,VIM,CCL18
TISSUES	BTO:0000243	Vagina	3	18	1.15	0.0458	LTF,SLPI,S100A9
TISSUES	BTO:0000269	Colon	17	639	0.35	0.0480	LYZ,LGALS3BP,KLKB1,HABP2,AZGP1,P4HB,APOA4,FLNA,SOD3,YWHAZ,ILK,CKMT1A,SERPINA1,CFB,C1R,SERPINA4,TRIM8
TISSUES	BTO:0001424	Uterus	25	1117	0.28	0.0482	MGP,SPARC,LTF,IGFBP5,THBS1,IGFBP1,SERPING1,A2ML1,NDUFS8,SUZ12,TUBA1B,ISYNA1,SLPI,FN1,CD99,PROS1,YWHAZ,ILK,C1S,PAEP,UIMC1,PDGFC,ATP1A1,VIM,ACTB
TISSUES	BTO:0001129	Prostate gland	14	476	0.4	0.0485	LTF,ATRN,LUM,PIP,AZGP1,P4HB,TUBA1B,FLNA,RARRES2,PDGFC,ATP1A1,VIM,ACTB,B2M
TISSUES	BTO:0002690	Biofilm	2	4	1.63	0.0485	LTF,CAMP
TISSUES	BTO:0002854	Corn silk	2	4	1.63	0.0485	MASP1,MASP2
COMPARTMENTS	GOCC:0005576	Extracellular region	158	2079	0.81	6.42e-91	SPP2,ST6GAL1,CPB2,CETP,CHGA,F9,C5,MGP,GPLD1,SPARC,LTF,APOB,IGFBP5,PROC,APOA1,TTR,CFP,APOE,ANGPTL6,BPIFB1,SERPINF1,APCS,CFHR5,THBS1,LYZ,VWF,SERPINA10,LGALS3BP,C9,LCN1,LCAT,CSTA,CP,KLKB1,F13A1,AMBP,CENPE,HPX,LUM,FCN3,ITIH1,AHSG,IGFBP1,HABP2,SAA4,SERPING1,PIP,PSG6,AZGP1,PGLYRP2,SCGB3A1,SERPINI1,PF4,MASP1,CAMP,A2ML1,PDIA3,LALBA,ANGPTL4,PCSK9,BMP1,FGB,DST,F2,INHBC,PLG,EFEMP2,PRG2,CSPG4,C1QB,CFHR1,CLU,APOL1,CPN2,SERPINF2,C7,P4HB,SERPINA7,PAPPA,FBLN1,ANG,FGG,TUBB,THBS4,SLPI,QPCT,APOD,FN1,HP,APOA4,COL18A1,C8A,F13B,CFHR2,CFHR4,CFHR3,CFH,F5,ADAM12,S100A7,S100A9,HTRA1,ECM1,FLNA,CRTAC1,C8B,FCN1,ADAMTS13,CCN5,C1QA,F10,F7,APOM,CA6,CALML5,IGFBP3,SOD3,GPX3,PROS1,EFEMP1,CFI,MMRN1,YWHAZ,MASP2,F11,C1S,PCYOX1,ORM2,C4A,MST1,ITIH3,C4B,SERPINA1,TGFBI,CFB,IGFALS,PAEP,RARRES2,GC,HGFAC,FBN2,TSKU,JCHAIN,HPR,C1R,APOA5,VIM,SERPINA4,RNASE4,APOC4,CFD,CCL18,VEGFC,PLCG2,KNG1,ACTB,B2M,FGA
COMPARTMENTS	GOCC:0005615	Extracellular space	112	1027	0.96	3.39e-73	CPB2,CETP,F9,C5,MGP,GPLD1,LTF,APOB,IGFBP5,PROC,APOA1,TTR,CFP,APOE,SERPINF1,APCS,THBS1,LYZ,VWF,LGALS3BP,C9,LCN1,LCAT,CSTA,CP,KLKB1,F13A1,AMBP,HPX,LUM,FCN3,ITIH1,AHSG,IGFBP1,SAA4,SERPING1,PIP,AZGP1,SCGB3A1,SERPINI1,MASP1,CAMP,A2ML1,PDIA3,ANGPTL4,PCSK9,FGB,F2,PLG,CSPG4,C1QB,CFHR1,CLU,APOL1,CPN2,SERPINF2,PAPPA,FBLN1,ANG,FGG,THBS4,SLPI,APOD,FN1,HP,APOA4,COL18A1,C8A,CFHR3,CFH,S100A7,S100A9,ECM1,FLNA,CCN5,C1QA,F7,APOM,CA6,IGFBP3,SOD3,GPX3,PROS1,EFEMP1,YWHAZ,MASP2,F11,C1S,PCYOX1,ORM2,C4A,MST1,C4B,SERPINA1,TGFBI,CFB,IGFALS,RARRES2,GC,FBN2,JCHAIN,HPR,C1R,APOA5,VIM,APOC4,CFD,PLCG2,KNG1,ACTB,B2M,FGA
COMPARTMENTS	GOCC:0072562	Blood microparticle	44	118	1.5	3.30e-45	APOA1,APOE,APCS,LGALS3BP,C9,CP,F13A1,AMBP,HPX,FCN3,ITIH1,AHSG,SERPING1,ANGPTL4,FGB,F2,PLG,C1QB,CFHR1,CLU,APOL1,CPN2,SERPINF2,FGG,FN1,HP,APOA4,C8A,CFHR3,CFH,PROS1,YWHAZ,C1S,ORM2,C4A,C4B,CFB,GC,JCHAIN,HPR,C1R,KNG1,ACTB,FGA
COMPARTMENTS	GOCC:0043230	Extracellular organelle	56	524	0.96	1.39e-32	CPB2,F9,C5,MGP,LTF,APOB,APOA1,TTR,APOE,SERPINF1,APCS,THBS1,VWF,LGALS3BP,CP,KLKB1,AMBP,HPX,LUM,AHSG,SERPING1,AZGP1,CAMP,FGB,F2,PLG,CSPG4,CLU,SERPINF2,FGG,SLPI,APOD,FN1,HP,COL18A1,CFH,S100A9,ECM1,FLNA,GPX3,PROS1,EFEMP1,YWHAZ,MASP2,F11,C4A,C4B,SERPINA1,TGFBI,GC,C1R,VIM,KNG1,ACTB,B2M,FGA
COMPARTMENTS	GOCC:0060205	Cytoplasmic vesicle lumen	43	245	1.17	2.18e-32	SPP2,SPARC,LTF,APOA1,TTR,CFP,THBS1,LYZ,VWF,LGALS3BP,F13A1,AHSG,SERPING1,SERPINI1,PF4,CAMP,FGB,PLG,CLU,SERPINF2,FGG,TUBB,SLPI,QPCT,FN1,HP,F5,S100A7,ECM1,FCN1,PROS1,MMRN1,ORM2,ITIH3,SERPINA1,RARRES2,SELENOP,SERPINA4,CFD,VEGFC,KNG1,B2M,FGA
COMPARTMENTS	GOCC:0034774	Secretory granule lumen	42	241	1.17	1.31e-31	SPP2,SPARC,LTF,APOA1,TTR,CFP,THBS1,LYZ,VWF,LGALS3BP,F13A1,AHSG,SERPING1,PF4,CAMP,FGB,PLG,CLU,SERPINF2,FGG,TUBB,SLPI,QPCT,FN1,HP,F5,S100A7,ECM1,FCN1,PROS1,MMRN1,ORM2,ITIH3,SERPINA1,RARRES2,SELENOP,SERPINA4,CFD,VEGFC,KNG1,B2M,FGA
COMPARTMENTS	GOCC:0065010	Extracellular membrane-bounded organelle	52	473	0.97	7.59e-31	CPB2,F9,MGP,LTF,APOB,APOA1,TTR,APOE,SERPINF1,APCS,THBS1,VWF,LGALS3BP,CP,KLKB1,AMBP,HPX,LUM,AHSG,SERPING1,AZGP1,CAMP,FGB,F2,PLG,CSPG4,CLU,SERPINF2,FGG,APOD,FN1,HP,COL18A1,CFH,S100A9,ECM1,FLNA,GPX3,PROS1,YWHAZ,F11,C4A,C4B,SERPINA1,TGFBI,GC,C1R,VIM,KNG1,ACTB,B2M,FGA
COMPARTMENTS	GOCC:1903561	Extracellular vesicle	53	500	0.95	7.92e-31	CPB2,F9,MGP,LTF,APOB,APOA1,TTR,APOE,SERPINF1,APCS,THBS1,VWF,LGALS3BP,CP,KLKB1,AMBP,HPX,LUM,AHSG,SERPING1,AZGP1,CAMP,FGB,F2,PLG,CSPG4,CLU,SERPINF2,FGG,APOD,FN1,HP,COL18A1,CFH,S100A9,ECM1,FLNA,GPX3,PROS1,EFEMP1,YWHAZ,F11,C4A,C4B,SERPINA1,TGFBI,GC,C1R,VIM,KNG1,ACTB,B2M,FGA
COMPARTMENTS	GOCC:0031982	Vesicle	91	2125	0.56	2.12e-26	SPP2,CPB2,CHGA,F9,MGP,SPARC,LTF,APOB,APOA1,TTR,CAT,LRP1,CFP,APOE,SERPINF1,APCS,THBS1,LYZ,VWF,LGALS3BP,CP,KLKB1,F13A1,AMBP,TCIRG1,HPX,LUM,ITIH1,AHSG,SERPING1,SEC24D,DSC2,AZGP1,SERPINI1,PF4,CAMP,APEH,PDIA3,PCSK9,FGB,YWHAG,DST,F2,PLG,PRG2,CSPG4,STAB1,CLU,SERPINF2,FGG,TUBB,SLPI,QPCT,APOD,FN1,HP,COL18A1,CFH,F5,S100A7,S100A9,ECM1,FLNA,FCN1,CALML5,GPX3,PROS1,EFEMP1,MMRN1,YWHAZ,F11,ORM2,C4A,ABCA13,ITIH3,C4B,SERPINA1,TGFBI,RARRES2,GC,SELENOP,C1R,VIM,SERPINA4,CFD,VEGFC,PLCG2,KNG1,ACTB,B2M,FGA
COMPARTMENTS	GOCC:0031093	Platelet alpha granule lumen	22	66	1.45	3.65e-21	SPARC,THBS1,VWF,F13A1,AHSG,SERPING1,PF4,FGB,PLG,CLU,SERPINF2,FGG,FN1,F5,PROS1,MMRN1,ORM2,SERPINA1,CFD,VEGFC,KNG1,FGA
COMPARTMENTS	GOCC:0070062	Extracellular exosome	40	428	0.9	6.87e-21	MGP,LTF,APOB,APOA1,TTR,APOE,SERPINF1,THBS1,VWF,LGALS3BP,CP,AMBP,HPX,AHSG,AZGP1,CAMP,FGB,PLG,CSPG4,CLU,FGG,APOD,FN1,HP,COL18A1,CFH,S100A9,ECM1,FLNA,GPX3,EFEMP1,YWHAZ,C4A,SERPINA1,TGFBI,VIM,KNG1,ACTB,B2M,FGA

COMPARTMENTS	GOCC:0030141	Secretory granule	49	719	0.76	1.82e-20	SPP2,CHGA,SPARC,LTF,APOA1,TTR,CAT,CFP,THBS1,LYZ,VWF,LGALS3BP,F13A1,TCIRG1,AHSG,SERPING1,PF4,CAMP,APEH,FGB,PLG,PRG2,CLU,SERPINF2,FGG,TUBB,SLPI,QPCT,FN1,HP,F5,S100A7,ECM1,FCN1,CALML5,PROS1,MMRN1,ORM2,ABCA13,ITIH3,SERPINA1,RARRES2,SELENOP,SERPINA4,CFD,VEGFC,KNG1,B2M,FGA
COMPARTMENTS	GOCC:0005788	Endoplasmic reticulum lumen	26	172	1.11	8.07e-18	SPP2,F9,APOB,APOA1,APOE,THBS1,AHSG,IGFBP1,PDIA3,PCSK9,CLU,APOL1,P4HB,FGG,FN1,APOA4,COL18A1,F5,F7,IGFBP3,C4A,SERPINA1,APOA5,KNG1,B2M,FGA
COMPARTMENTS	GOCC:0034358	Plasma lipoprotein particle	15	48	1.42	6.65e-14	CETP,APOB,APOA1,APOE,LCAT,SAA4,PCSK9,CLU,APOL1,APOA4,APOM,PCYOX1,HPR,APOA5,APOC4
COMPARTMENTS	GOCC:0034364	High-density lipoprotein particle	13	30	1.56	1.68e-13	CETP,APOB,APOA1,APOE,LCAT,SAA4,CLU,APOL1,APOA4,APOM,HPR,APOA5,APOC4
COMPARTMENTS	GOCC:0031012	Extracellular matrix	24	259	0.89	4.08e-12	CPB2,GPLD1,SPARC,APOE,THBS1,VWF,LUM,DST,PLG,EFEMP2,CLU,FBLN1,ANG,THBS4,FN1,COL18A1,ECM1,ADAMTS13,SOD3,EFEMP1,MMRN1,TGFBI,RARRES2,FBN2
COMPARTMENTS	GOCC:0031410	Cytoplasmic vesicle	60	1738	0.47	4.67e-12	SPP2,CHGA,SPARC,LTF,APOB,APOA1,TTR,CAT,LRP1,CFP,APOE,THBS1,LYZ,VWF,LGALS3BP,F13A1,TCIRG1,HPX,AHSG,SERPING1,SEC24D,DSC2,SERPINI1,PF4,CAMP,APEH,PDIA3,PCSK9,FGB,DST,PLG,PRG2,STAB1,CLU,SERPINF2,FGG,TUBB,SLPI,QPCT,FN1,HP,F5,S100A7,ECM1,FCN1,CALML5,PROS1,MMRN1,ORM2,ABCA13,ITIH3,SERPINA1,RARRES2,SELENOP,SERPINA4,CFD,VEGFC,KNG1,B2M,FGA
COMPARTMENTS	GOCC:0110165	Cellular anatomical entity	213	14060	0.11	5.61e-12	SPP2,ST6GAL1,CPB2,CETP,CHGA,TPD52L2,APMAP,F9,C5,MGP,GPLD1,SPARC,LTF,APOB,IGFBP5,PROC,APOA1,TTR,CAT,LRP1,CFP,APOE,ANGPTL6,BPIFB1,SERPINF1,APCS,CFHR5,THBS1,LYZ,VWF,PSMA6,SERPINA10,LGALS3BP,ATRN,C9,LCN1,LCAT,CSTA,CP,KLKB1,F13A1,DGKG,CDH6,DNAH5,AMBP,CENPE,TCIRG1,HPX,LUM,FCN3,ITIH1,AHSG,IGFBP1,HABP2,SAA4,SERPING1,SEC24D,DSC2,CSF1R,PIP,PSG6,AZGP1,PGLYRP2,SCGB3A1,SERPINI1,PF4,MASP1,CAMP,APEH,A2ML1,PDIA3,SLC27A4,LALBA,ANGPTL4,PCSK9,BMP1,FGB,YWHAG,DST,F2,INHBC,PLG,EFEMP2,PRG2,CSPG4,STAB1,C1QB,CFHR1,CLU,NDUFS8,SUZ12,APOL1,CPN2,SERPINF2,C7,KDM3B,P4HB,SERPINA7,PAPPA,FBLN1,DNAH8,ANG,TUBA1B,FGG,ISYNA1,TUBB,THBS4,SLPI,QPCT,APOD,FN1,ASGR2,BLM,HP,AKAP9,APOA4,CNDP1,COL18A1,C8A,F13B,CFHR2,CFHR4,CFHR3,CFH,F5,ADAM12,S100A7,S100A9,HTRA1,ECM1,FLNA,CRTAC1,MCOLN2,SMARCA1,C8B,FCN1,ADAMTS13,CCN5,SVEP1,C1QA,F10,F7,APOM,CA6,ADGRG2,CALML5,IGFBP3,CD99,SOD3,GPX3,PROS1,EFEMP1,CFI,MMRN1,TNRC6A,YWHAZ,NAV2,ILK,MAP3K7CL,MASP2,F11,C1S,PCYOX1,BZW1,ORM2,C4A,ATP9B,ACSF2,CKMT1A,ABCA13,MST1,LMOD3,ITIH3,C4B,SERPINA1,TGFBI,CFB,IGFALS,PAEP,RARRES2,TCAF1,GC,HGFAC,UIMC1,PDGFC,FBN2,SELENOP,TSKU,JCHAIN,HPR,TUBA1C,C1R,APOA5,ATP1A1,VIM,SERPINA4,RNASE4,TTN,APOC4,CFD,CCL18,VEGFC,SEMA3B,PLCG2,OR52B4,KNG1,TPM4,ACTB,TRIM66,TRIM8,CDH5,B2M,FGA
COMPARTMENTS	GOCC:0043233	Organelle lumen	80	2905	0.37	1.10e-11	SPP2,F9,SPARC,LTF,APOB,PROC,APOA1,TTR,CAT,CFP,APOE,THBS1,LYZ,VWF,LGALS3BP,F13A1,HPX,LUM,AHSG,IGFBP1,SERPING1,SERPINI1,PF4,CAMP,APEH,PDIA3,LALBA,PCSK9,FGB,F2,PLG,PRG2,CSPG4,CLU,NDUFS8,SUZ12,APOL1,SERPINF2,P4HB,ANG,FGG,TUBB,SLPI,QPCT,FN1,BLM,HP,APOA4,COL18A1,F5,S100A7,HTRA1,ECM1,SMARCA1,FCN1,F10,F7,CALML5,IGFBP3,SOD3,PROS1,MMRN1,ORM2,C4A,ITIH3,SERPINA1,RARRES2,GC,UIMC1,SELENOP,APOA5,SERPINA4,TTN,CFD,VEGFC,KNG1,ACTB,TRIM8,B2M,FGA
COMPARTMENTS	GOCC:0030312	External encapsulating structure	23	259	0.88	2.53e-11	GPLD1,SPARC,APOE,THBS1,VWF,LUM,DST,PLG,EFEMP2,CLU,PAPPA,FBLN1,ANG,THBS4,FN1,COL18A1,ECM1,ADAMTS13,SOD3,EFEMP1,MMRN1,RARRES2,FBN2
COMPARTMENTS	GOCC:0070013	Intracellular organelle lumen	79	2902	0.36	2.78e-11	SPP2,F9,SPARC,LTF,APOB,PROC,APOA1,TTR,CAT,CFP,APOE,THBS1,LYZ,VWF,LGALS3BP,F13A1,HPX,LUM,AHSG,IGFBP1,SERPING1,SERPINI1,PF4,CAMP,APEH,PDIA3,LALBA,PCSK9,FGB,F2,PLG,PRG2,CSPG4,CLU,NDUFS8,SUZ12,APOL1,SERPINF2,P4HB,ANG,FGG,TUBB,SLPI,QPCT,FN1,BLM,HP,APOA4,COL18A1,F5,S100A7,ECM1,SMARCA1,FCN1,F10,F7,CALML5,IGFBP3,SOD3,PROS1,MMRN1,ORM2,C4A,ITIH3,SERPINA1,RARRES2,GC,UIMC1,SELENOP,APOA5,SERPINA4,TTN,CFD,VEGFC,KNG1,ACTB,TRIM8,B2M,FGA
COMPARTMENTS	GOCC:0005577	Fibrinogen complex	10	20	1.63	7.63e-11	THBS1,VWF,FGB,F2,PLG,SERPINF2,FGG,FN1,F7,FGA
COMPARTMENTS	GOCC:0034361	Very-low-density lipoprotein particle	10	20	1.63	7.63e-11	CETP,APOB,APOA1,APOE,APOL1,APOA4,APOM,PCYOX1,APOA5,APOC4
COMPARTMENTS	GOCC:0012505	Endomembrane system	82	3156	0.34	8.46e-11	SPP2,ST6GAL1,CHGA,F9,SPARC,LTF,APOB,PROC,APOA1,TTR,CAT,LRP1,CFP,APOE,THBS1,LYZ,VWF,LGALS3BP,CP,F13A1,TCIRG1,LUM,AHSG,IGFBP1,SERPING1,SEC24D,PF4,CAMP,APEH,PDIA3,SLC27A4,LALBA,PCSK9,FGB,F2,PLG,PRG2,CSPG4,CLU,APOL1,SERPINF2,P4HB,FGG,TUBB,THBS4,SLPI,QPCT,FN1,ASGR2,HP,AKAP9,APOA4,COL18A1,F5,S100A7,ECM1,FCN1,F10,F7,CALML5,IGFBP3,SOD3,PROS1,MMRN1,TNRC6A,ORM2,C4A,ATP9B,ABCA13,ITIH3,SERPINA1,RARRES2,SELENOP,APOA5,SERPINA4,CFD,VEGFC,SEMA3B,KNG1,CDH5,B2M,FGA
COMPARTMENTS	GOCC:0062023	Collagen-containing extracellular matrix	19	198	0.91	7.57e-10	SPARC,APOE,THBS1,VWF,LUM,DST,PLG,EFEMP2,CLU,ANG,THBS4,FN1,COL18A1,ECM1,SOD3,MMRN1,TGFBI,RARRES2,FBN2
COMPARTMENTS	GOCC:0062167	Complement component C1q complex	7	10	1.77	3.25e-08	SERPING1,C1QB,C1QA,C1S,C4A,C4B,C1R
COMPARTMENTS	GOCC:1905286	Serine-type peptidase complex	7	11	1.73	5.11e-08	F9,FCN3,MASP1,FCN1,F7,MASP2,F11
COMPARTMENTS	GOCC:0031089	Platelet dense granule lumen	7	14	1.63	1.76e-07	SPP2,LGALS3BP,ECM1,ITIH3,RARRES2,SELENOP,SERPINA4
COMPARTMENTS	GOCC:0005579	Membrane attack complex	6	12	1.63	2.39e-06	C5,C9,C7,C8A,C8B,C4A
COMPARTMENTS	GOCC:0071682	Endocytic vesicle lumen	7	23	1.41	2.60e-06	SPARC,LTF,APOB,APOA1,APOE,HPX,HP
COMPARTMENTS	GOCC:0005783	Endoplasmic reticulum	35	1095	0.43	7.32e-06	SPP2,F9,APOB,PROC,APOA1,APOE,THBS1,VWF,CP,AHSG,IGFBP1,SEC24D,PDIA3,SLC27A4,PCSK9,CLU,APOL1,P4HB,FGG,THBS4,FN1,ASGR2,APOA4,COL18A1,F5,S100A7,F7,IGFBP3,C4A,SERPINA1,APOA5,SEMA3B,KNG1,B2M,FGA
COMPARTMENTS	GOCC:0035580	Specific granule lumen	9	62	1.09	7.78e-06	LTF,CFP,LYZ,CAMP,SLPI,QPCT,HP,ORM2,B2M
COMPARTMENTS	GOCC:1905370	Serine-type endopeptidase complex	5	7	1.78	8.49e-06	FCN3,MASP1,FCN1,MASP2,F11
COMPARTMENTS	GOCC:0032991	Protein-containing complex	99	5325	0.2	1.69e-05	CPB2,CETP,F9,C5,LTF,APOB,IGFBP5,APOA1,CAT,LRP1,APOE,THBS1,VWF,PSMA6,C9,LCAT,CSTA,PPP2R3A,CDH6,DNAH5,LUM,FCN3,IGFBP1,SAA4,SERPING1,SEC24D,CSF1R,MASP1,APEH,PDIA3,PCSK9,FGB,DST,F2,PLG,UBE3C,C1QB,CFHR1,CLU,NDUFS8,SUZ12,APOL1,SERPINF2,C7,P4HB,PAPPA,DNAH8,ANG,FGG,FN1,BLM,HP,AKAP9,APOA4,COL18A1,C8A,CFHR2,S100A7,S100A9,FLNA,SMARCA1,C8B,FCN1,ADAMTS13,SVEP1,C1QA,F7,APOM,CALML5,IGFBP3,GPX3,MMRN1,TNRC6A,MASP2,F11,C1S,PCYOX1,C4A,CKMT1A,C4B,SERPINA1,CFB,IGFALS,UIMC1,JCHAIN,HPR,C1R,APOA5,ATP1A1,VIM,TTN,APOC4,IGHV3-15,VEGFC,TPM4,ACTB,CDH5,B2M,FGA
COMPARTMENTS	GOCC:0062136	Low-density lipoprotein receptor complex	5	9	1.67	2.01e-05	APOB,APOA1,LRP1,APOE,PCSK9
COMPARTMENTS	GOCC:0034362	Low-density lipoprotein particle	6	22	1.36	3.64e-05	CETP,APOB,APOA1,APOE,PCSK9,APOM
COMPARTMENTS	GOCC:0005796	Golgi lumen	10	102	0.92	3.84e-05	F9,PROC,LUM,LALBA,F2,CSPG4,F10,F7,SOD3,PROS1
COMPARTMENTS	GOCC:0034366	Spherical high-density lipoprotein particle	5	11	1.58	3.94e-05	APOA1,LCAT,CLU,APOM,HPR
COMPARTMENTS	GOCC:0009986	Cell surface	19	438	0.56	9.23e-05	APMAP,SPARC,LTF,THBS1,CSF1R,PDIA3,PCSK9,FGB,PLG,CSPG4,CLU,SERPINF2,P4HB,FGG,FCN1,ADAMTS13,PDGFC,CDH5,FGA
COMPARTMENTS	GOCC:0042627	Chylomicron	5	14	1.48	9.79e-05	APOB,APOA1,APOE,APOA4,APOA5
COMPARTMENTS	GOCC:0005602	Complement component C1 complex	4	5	1.83	9.88e-05	C1QB,C1QA,C1S,C1R
COMPARTMENTS	GOCC:0034365	Discoidal high-density lipoprotein particle	4	6	1.75	0.00016	APOA1,APOE,LCAT,APOM

COMPARTMENTS	GOCC:0005925	Focal adhesion	14	269	0.64	0.00028	LRP1,PDIA3,YWHAG,DST,CSPG4,P4HB,S100A7,FLNA,YWHAZ,ILK,VIM,TPM4,ACTB,B2M
COMPARTMENTS	GOCC:1904724	Tertiary granule lumen	7	55	1.03	0.00032	LTF,CFP,LYZ,CAMP,QPCT,HP,B2M
COMPARTMENTS	GOCC:1905368	Peptidase complex	10	136	0.79	0.00036	F9,PSMA6,FCN3,MASP1,F2,UBE3C,FCN1,F7,MASP2,F11
COMPARTMENTS	GOCC:0005604	Basement membrane	7	61	0.99	0.00056	SPARC,DST,EFEMP2,ANG,THBS4,FN1,COL18A1
COMPARTMENTS	GOCC:0043227	Membrane-bounded organelle	140	9083	0.11	0.00056	SPP2,ST6GAL1,CPB2,CHGA,F9,MGP,GPLD1,SPARC,LTF,APOB,PROC,APOA1,TTR,CAT,LRP1,CFP,APOE,SERPINF1,APCS,THBS1,LYZ,VWF,PSMA6,LGALS3BP,LCAT,CSTA,CP,KLKB1,F13A1,DNAH5,AMBP,CENPE,TCIRG1,HPX,LUM,ITIH1,AHSG,IGFBP1,SERPING1,SEC24D,DSC2,PIP,AZGP1,SERPINI1,PF4,CAMP,APEH,PDIA3,SLC27A4,LALBA,PCSK9,FGB,YWHAG,DST,F2,PLG,PRG2,CSPG4,STAB1,CLU,NDUFS8,SUZ12,APOL1,SERPINF2,KDM3B,P4HB,DNAH8,ANG,FGG,TUBB,THBS4,SLPI,QPCT,APOD,FN1,ASGR2,BLM,HP,AKAP9,APOA4,COL18A1,CFH,F5,S100A7,S100A9,HTRA1,ECM1,FLNA,SMARCA1,FCN1,CCN5,SVEP1,F10,F7,CALML5,IGFBP3,CD99,SOD3,GPX3,PROS1,EFEMP1,MMRN1,TNRC6A,YWHAZ,NAV2,ILK,MASP2,F11,PCYOX1,ORM2,C4A,ATP9B,ACSF2,CKMT1A,ABCA13,ITIH3,C4B,SERPINA1,TGFBI,RARRES2,GC,UIMC1,SELENOP,TUBA1C,C1R,APOA5,VIM,SERPINA4,TTN,CFD,VEGFC,SEMA3B,PLCG2,KNG1,ACTB,TRIM66,TRIM8,CDH5,B2M,FGA
COMPARTMENTS	GOCC:0016942	Insulin-like growth factor binding protein complex	4	10	1.53	0.00064	IGFBP5,IGFBP1,IGFBP3,IGFALS
COMPARTMENTS	GOCC:0005601	Classical-complement-pathway C3/C5 convertase complex	3	4	1.8	0.0020	C4A,C4B,CFB
COMPARTMENTS	GOCC:0042567	Insulin-like growth factor ternary complex	3	4	1.8	0.0020	IGFBP5,IGFBP3,IGFALS
COMPARTMENTS	GOCC:0036454	Growth factor complex	5	31	1.13	0.0021	IGFBP5,IGFBP1,IGFBP3,IGFALS,VEGFC
COMPARTMENTS	GOCC:0098797	Plasma membrane protein complex	21	682	0.42	0.0030	C5,APOB,APOA1,LRP1,APOE,VWF,C9,CDH6,PCSK9,DST,C7,FN1,AKAP9,C8A,FLNA,C8B,SVEP1,C4A,ATP1A1,CDH5,B2M
COMPARTMENTS	GOCC:0030139	Endocytic vesicle	12	260	0.59	0.0032	SPARC,LTF,APOB,APOA1,LRP1,APOE,TCIRG1,HPX,PDIA3,STAB1,HP,B2M
COMPARTMENTS	GOCC:1905369	Endopeptidase complex	7	90	0.82	0.0050	PSMA6,FCN3,MASP1,UBE3C,FCN1,MASP2,F11
COMPARTMENTS	GOCC:1904813	ficolin-1-rich granule lumen	8	124	0.74	0.0056	CAT,APEH,PRG2,QPCT,FCN1,CALML5,SERPINA1,CFD
COMPARTMENTS	GOCC:0071944	Cell periphery	69	3860	0.18	0.0060	C5,GPLD1,SPARC,APOB,APOA1,LRP1,APOE,THBS1,VWF,ATRN,C9,CSTA,CDH6,AMBP,TCIRG1,LUM,DSC2,CSF1R,PCSK9,FGB,DST,PLG,EFEMP2,CSPG4,STAB1,CLU,C7,P4HB,PAPPA,FBLN1,ANG,FGG,THBS4,FN1,ASGR2,AKAP9,COL18A1,C8A,ADAM12,S100A9,ECM1,FLNA,MCOLN2,C8B,FCN1,ADAMTS13,SVEP1,ADGRG2,SOD3,GPX3,PROS1,EFEMP1,MMRN1,ILK,C4A,ABCA13,C4B,TGFBI,RARRES2,TCAF1,PDGFC,FBN2,ATP1A1,PLCG2,OR52B4,KNG1,CDH5,B2M,FGA
COMPARTMENTS	GOCC:0005775	Vacuolar lumen	9	168	0.66	0.0079	APOB,TTR,LYZ,LUM,CSPG4,TUBB,S100A7,ORM2,GC
COMPARTMENTS	GOCC:0034363	Intermediate-density lipoprotein particle	3	8	1.5	0.0079	APOB,APOA1,APOE
COMPARTMENTS	GOCC:0009897	External side of plasma membrane	7	102	0.76	0.0093	THBS1,FGB,PLG,P4HB,FGG,FCN1,FGA
COMPARTMENTS	GOCC:1990341	Thrombospondin complex	3	9	1.45	0.0100	THBS1,VWF,ADAMTS13
COMPARTMENTS	GOCC:0070161	Anchoring junction	17	553	0.41	0.0126	LRP1,DSC2,PDIA3,YWHAG,DST,CSPG4,P4HB,S100A7,FLNA,CD99,YWHAZ,ILK,VIM,TPM4,ACTB,CDH5,B2M
COMPARTMENTS	GOCC:0043226	Organelle	145	10113	0.08	0.0169	SPP2,ST6GAL1,CPB2,CHGA,F9,C5,MGP,GPLD1,SPARC,LTF,APOB,PROC,APOA1,TTR,CAT,LRP1,CFP,APOE,SERPINF1,APCS,THBS1,LYZ,VWF,PSMA6,LGALS3BP,LCAT,CSTA,CP,KLKB1,F13A1,DNAH5,AMBP,CENPE,TCIRG1,HPX,LUM,ITIH1,AHSG,IGFBP1,SERPING1,SEC24D,DSC2,PIP,AZGP1,SERPINI1,PF4,CAMP,APEH,PDIA3,SLC27A4,LALBA,PCSK9,FGB,YWHAG,DST,F2,PLG,PRG2,CSPG4,STAB1,CLU,NDUFS8,SUZ12,APOL1,SERPINF2,KDM3B,P4HB,DNAH8,ANG,TUBA1B,FGG,TUBB,THBS4,SLPI,QPCT,APOD,FN1,ASGR2,BLM,HP,AKAP9,APOA4,COL18A1,CFH,F5,S100A7,S100A9,HTRA1,ECM1,FLNA,SMARCA1,FCN1,CCN5,SVEP1,F10,F7,CALML5,IGFBP3,CD99,SOD3,GPX3,PROS1,EFEMP1,MMRN1,TNRC6A,YWHAZ,NAV2,ILK,MASP2,F11,PCYOX1,ORM2,C4A,ATP9B,ACSF2,CKMT1A,ABCA13,LMOD3,ITIH3,C4B,SERPINA1,TGFBI,RARRES2,GC,UIMC1,SELENOP,TUBA1C,C1R,APOA5,ATP1A1,VIM,SERPINA4,TTN,CFD,VEGFC,SEMA3B,PLCG2,KNG1,TPM4,ACTB,TRIM66,TRIM8,CDH5,B2M,FGA
COMPARTMENTS	GOCC:0005737	Cytoplasm	122	8195	0.1	0.0191	SPP2,ST6GAL1,CHGA,TPD52L2,F9,GPLD1,SPARC,LTF,APOB,PROC,APOA1,TTR,CAT,LRP1,CFP,APOE,THBS1,LYZ,VWF,PSMA6,LGALS3BP,CSTA,CP,F13A1,DGKG,DNAH5,AMBP,CENPE,TCIRG1,HPX,LUM,AHSG,IGFBP1,SERPING1,SEC24D,DSC2,SERPINI1,PF4,CAMP,APEH,PDIA3,SLC27A4,LALBA,PCSK9,FGB,YWHAG,DST,F2,PLG,PRG2,CSPG4,STAB1,CLU,NDUFS8,APOL1,SERPINF2,P4HB,DNAH8,FGG,ISYNA1,TUBB,THBS4,SLPI,QPCT,APOD,FN1,ASGR2,HP,AKAP9,APOA4,CNDP1,COL18A1,F5,S100A7,S100A9,ECM1,FLNA,FCN1,SVEP1,F10,F7,CALML5,IGFBP3,CD99,SOD3,GPX3,PROS1,MMRN1,TNRC6A,YWHAZ,ILK,MAP3K7CL,PCYOX1,BZW1,ORM2,C4A,ATP9B,ACSF2,CKMT1A,ABCA13,LMOD3,ITIH3,SERPINA1,TGFBI,RARRES2,GC,SELENOP,HPR,APOA5,VIM,SERPINA4,TTN,CFD,VEGFC,SEMA3B,PLCG2,KNG1,TPM4,ACTB,TRIM8,B2M,FGA
COMPARTMENTS	GOCC:0070820	Tertiary granule	8	164	0.62	0.0285	LTF,CFP,LYZ,TCIRG1,CAMP,QPCT,HP,B2M
COMPARTMENTS	GOCC:1904090	Peptidase inhibitor complex	3	17	1.17	0.0452	CSTA,SERPINA1,ACTB
UniProt Keywords	KW-0964	Secreted	158	1839	0.86	4.14e-99	SPP2,ST6GAL1,CPB2,CETP,CHGA,F9,C5,MGP,GPLD1,SPARC,LTF,APOB,IGFBP5,PROC,APOA1,TTR,CFP,APOE,ANGPTL6,BPIFB1,SERPINF1,APCS,CFHR5,THBS1,LYZ,VWF,SERPINA10,LGALS3BP,ATRN,C9,LCN1,LCAT,CP,KLKB1,F13A1,AMBP,HPX,INHBE,LUM,FCN3,ITIH1,AHSG,IGFBP1,HABP2,SAA4,SERPING1,PIP,PSG6,AZGP1,PGLYRP2,SCGB3A1,SERPINI1,PF4,CAMP,A2ML1,LALBA,ANGPTL4,PCSK9,BMP1,FGB,F2,INHBC,PLG,PSG1,EFEMP2,PRG2,C1QB,CFHR1,CLU,APOL1,CPN2,SERPINF2,C7,SERPINA7,PAPPA,FBLN1,PSG3,SERPINA11,ANG,FGG,THBS4,SLPI,QPCT,APOD,FN1,HP,APOA4,CNDP1,COL18A1,C8A,F13B,CFHR2,CFHR4,CFHR3,CFH,F5,ADAM12,S100A7,S100A9,HTRA1,ADAMTSL4,ECM1,CRTAC1,C8B,FCN1,ADAMTS13,CCN5,SVEP1,C1QA,F10,F7,APOM,CA6,IGFBP3,SOD3,GPX3,PROS1,EFEMP1,CFI,MMRN1,MASP2,F11,PSG2,ORM2,C4A,PRG4,MST1,ITIH3,C4B,SERPINA1,TGFBI,CFB,IGFALS,PAEP,RARRES2,GC,HGFAC,PDGFC,FBN2,SELENOP,TSKU,JCHAIN,HPR,C1R,APOA5,SERPINA4,RNASE4,APOC4,CFD,IGHV3-15,CCL18,IGHV3-72,VEGFC,SEMA3B,PAMR1,KNG1,B2M,FGA
UniProt Keywords	KW-0732	Signal	167	3277	0.63	4.17e-73	SPP2,CPB2,CETP,CHGA,F9,C5,MGP,GPLD1,SPARC,LTF,APOB,IGFBP5,PROC,APOA1,TTR,LRP1,CFP,APOE,ANGPTL6,BPIFB1,SERPINF1,APCS,CFHR5,THBS1,LYZ,VWF,LGALS3BP,ATRN,C9,LCN1,LCAT,CP,KLKB1,CDH6,AMBP,HPX,INHBE,LUM,FCN3,ITIH1,AHSG,IGFBP1,HABP2,SAA4,SERPING1,DSC2,CSF1R,PIP,PSG6,AZGP1,PGLYRP2,SCGB3A1,SERPINI1,PF4,CAMP,A2ML1,PDIA3,LALBA,ANGPTL4,PCSK9,BMP1,FGB,F2,INHBC,PLG,PSG1,EFEMP2,PRG2,CSPG4,STAB1,C1QB,CFHR1,CLU,APOL1,CPN2,SERPINF2,C7,P4HB,SERPINA7,PAPPA,FBLN1,PSG3,OIT3,SERPINA11,ANG,FGG,THBS4,SLPI,QPCT,APOD,FN1,HP,APOA4,CNDP1,COL18A1,C8A,F13B,CFHR2,CFHR4,CFHR3,CFH,F5,ADAM12,HTRA1,ADAMTSL4,ECM1,CRTAC1,C8B,FCN1,ADAMTS13,CCN5,SVEP1,C1QA,F10,F7,APOM,CA6,ADGRG2,IGFBP3,CD99,SOD3,GPX3,PROS1,EFEMP1,CFI,MMRN1,MASP2,F11,C1S,PSG2,PCYOX1,ORM2,C4A,PRG4,MST1,ITIH3,C4B,SERPINA1,TGFBI,CFB,IGFALS,PAEP,RARRES2,GC,HGFAC,PDGFC,FBN2,SELENOP,TSKU,JCHAIN,HPR,C1R,APOA5,SERPINA4,RNASE4,APOC4,CFD,IGHV3-15,CCL18,IGHV3-72,VEGFC,SEMA3B,PAMR1,KNG1,CDH5,B2M,FGA

UniProt Keywords	KW-1015	Disulfide bond	144	3338	0.56	1.22e-49	SPP2,ST6GAL1,CPB2,CETP,CHGA,F9,C5,MGP,SPARC,LTF,APOB,IGFBP5,PROC,LRP1,CFP,ANGPTL6,BPIFB1,APCS,CFHR5,THBS1,LYZ,VWF,LGALS3BP,ATRN,C9,LCN1,LCAT,CP,KLKB1,AMBP,HPX,INHBE,LUM,FCN3,ITIH1,AHSG,IGFBP1,HABP2,SERPING1,CSF1R,PIP,PSG6,AZGP1,PGLYRP2,SCGB3A1,PF4,MASP1,CAMP,A2ML1,PDIA3,LALBA,ANGPTL4,PCSK9,BMP1,FGB,F2,INHBC,PLG,PSG1,EFEMP2,PRG2,CSPG4,STAB1,C1QB,CFHR1,CLU,CPN2,SERPINF2,C7,P4HB,PAPPA,FBLN1,PSG3,OIT3,ANG,FGG,THBS4,SLPI,QPCT,APOD,FN1,ASGR2,HP,COL18A1,C8A,F13B,CFHR2,CFHR4,CFHR3,CFH,F5,ADAM12,S100A7,HTRA1,CRTAC1,MCOLN2,C8B,FCN1,ADAMTS13,SVEP1,C1QA,F10,F7,APOM,CA6,IGFBP3,SOD3,PROS1,EFEMP1,CFI,MMRN1,MASP2,F11,C1S,PSG2,ORM2,C4A,PRG4,MST1,C4B,TGFBI,CFB,PAEP,RARRES2,GC,HGFAC,PDGFC,FBN2,JCHAIN,HPR,C1R,RNASE4,TTN,CFD,IGHV3-15,CCL18,IGHV3-72,VEGFC,SEMA3B,PAMR1,OR52B4,KNG1,B2M,FGA
UniProt Keywords	KW-0325	Glycoprotein	149	4386	0.46	2.46e-39	ST6GAL1,CPB2,CETP,CHGA,APMAP,F9,C5,GPLD1,SPARC,LTF,APOB,IGFBP5,PROC,APOA1,TTR,LRP1,CFP,APOE,ANGPTL6,BPIFB1,SERPINF1,APCS,CFHR5,THBS1,VWF,PSMA6,SERPINA10,LGALS3BP,ATRN,C9,LCAT,CP,KLKB1,F13A1,CDH6,AMBP,HPX,INHBE,LUM,FCN3,ITIH1,AHSG,HABP2,SAA4,SERPING1,DSC2,CSF1R,PIP,PSG6,AZGP1,PGLYRP2,SERPINI1,A2ML1,LALBA,ANGPTL4,PCSK9,BMP1,FGB,F2,INHBC,PLG,PSG1,EFEMP2,PRG2,CSPG4,STAB1,C1QB,CFHR1,CLU,APOL1,CPN2,SERPINF2,C7,SERPINA7,PAPPA,FBLN1,PSG3,OIT3,SERPINA11,FGG,THBS4,QPCT,APOD,FN1,ASGR2,HP,CNDP1,COL18A1,C8A,F13B,CFHR2,CFHR4,CFHR3,CFH,F5,ADAM12,ADAMTSL4,ECM1,CRTAC1,C8B,FCN1,ADAMTS13,SVEP1,C1QA,F10,F7,APOM,CA6,ADGRG2,IGFBP3,CD99,SOD3,PROS1,EFEMP1,CFI,MMRN1,F11,C1S,PSG2,PCYOX1,ORM2,C4A,PRG4,MST1,ITIH3,C4B,SERPINA1,CFB,IGFALS,PAEP,GC,HGFAC,PDGFC,FBN2,SELENOP,TSKU,JCHAIN,C1R,VIM,SERPINA4,APOC4,VEGFC,SEMA3B,PAMR1,OR52B4,KNG1,CDH5,B2M,FGA
UniProt Keywords	KW-0094	Blood coagulation	22	46	1.61	3.80e-24	CPB2,F9,PROC,VWF,SERPINA10,KLKB1,F13A1,SERPING1,FGB,F2,PLG,FGG,F13B,F5,ADAMTS13,F10,F7,PROS1,F11,SERPINA1,KNG1,FGA
UniProt Keywords	KW-0245	EGF-like domain	31	232	1.05	3.10e-20	F9,PROC,LRP1,THBS1,ATRN,C9,HABP2,MASP1,BMP1,EFEMP2,STAB1,C7,FBLN1,OIT3,THBS4,C8A,ADAM12,CRTAC1,C8B,SVEP1,F10,F7,PROS1,EFEMP1,MMRN1,MASP2,C1S,HGFAC,FBN2,C1R,PAMR1
UniProt Keywords	KW-0768	Sushi	18	57	1.43	3.81e-17	CFHR5,MASP1,CFHR1,C7,PAPPA,HP,F13B,CFHR2,CFHR4,CFHR3,CFH,SVEP1,MASP2,C1S,CFB,HPR,C1R,PAMR1
UniProt Keywords	KW-0180	Complement pathway	15	30	1.63	1.25e-16	C5,C9,SERPING1,C1QB,CLU,C7,C8A,C8B,C1QA,CFI,MASP2,C1S,C4A,C4B,C1R
UniProt Keywords	KW-0106	Calcium	47	886	0.65	8.34e-16	CHGA,F9,SPARC,PROC,LRP1,APCS,THBS1,F13A1,PPP2R3A,DGKG,CDH6,FCN3,DSC2,LALBA,PCSK9,BMP1,DST,F2,EFEMP2,FBLN1,OIT3,SPATA21,FGG,THBS4,ASGR2,F5,S100A7,S100A9,MCOLN2,FCN1,ADAMTS13,SVEP1,F10,F7,CALML5,PROS1,EFEMP1,CFI,EFCAB5,MASP2,C1S,FBN2,TTN,PLCG2,TPM4,CDH5,FGA
UniProt Keywords	KW-0165	Cleavage on pair of basic residues	28	280	0.93	2.42e-15	CHGA,F9,C5,PROC,VWF,CDH6,AMBP,INHBE,HABP2,DSC2,CAMP,BMP1,F2,INHBC,PLG,C8A,ADAM12,ADAMTS13,F10,F7,PROS1,CFI,C4A,C4B,CFB,PDGFC,VEGFC,CDH5
UniProt Keywords	KW-0391	Immunity	36	537	0.75	8.25e-15	C5,LTF,CFP,BPIFB1,C9,FCN3,SERPING1,CSF1R,PGLYRP2,FGB,PRG2,C1QB,CLU,C7,SLPI,HP,C8A,CFH,S100A9,MCOLN2,C8B,FCN1,C1QA,CFI,MASP2,C1S,C4A,C4B,CFB,C1R,CFD,IGHV3-15,IGHV3-72,TRIM8,B2M,FGA
UniProt Keywords	KW-0399	Innate immunity	29	329	0.87	1.20e-14	C5,CFP,BPIFB1,C9,FCN3,SERPING1,CSF1R,FGB,C1QB,CLU,C7,SLPI,C8A,CFH,S100A9,MCOLN2,C8B,FCN1,C1QA,CFI,MASP2,C1S,C4A,C4B,CFB,C1R,CFD,TRIM8,FGA
UniProt Keywords	KW-0720	Serine protease	19	136	1.07	1.18e-12	F9,LTF,PROC,KLKB1,HABP2,PCSK9,F2,PLG,HTRA1,F10,F7,CFI,MASP2,F11,C1S,CFB,HGFAC,C1R,CFD
UniProt Keywords	KW-0873	Pyrrolidone carboxylic acid	14	67	1.25	2.46e-11	SERPINF1,LUM,PIP,AZGP1,FGB,C1QB,ANG,APOD,FN1,ORM2,JCHAIN,RNASE4,KNG1,B2M
UniProt Keywords	KW-0179	Complement alternate pathway	9	12	1.8	3.82e-11	C5,CFP,C9,C7,C8A,CFH,C8B,CFB,CFD
UniProt Keywords	KW-0677	Repeat	106	4794	0.27	9.12e-11	F9,GPLD1,LTF,PROC,APOA1,LRP1,CFP,APOE,CFHR5,THBS1,VWF,ATRN,CP,KLKB1,PPP2R3A,DGKG,CDH6,DNAH5,AMBP,HPX,LUM,FCN3,AHSG,ANKRD31,HABP2,SERPING1,DSC2,ANKRD44,CSF1R,PSG6,MASP1,PDIA3,BMP1,DST,F2,PLG,PSG1,EFEMP2,CSPG4,STAB1,C1QB,CFHR1,NDUFS8,CPN2,C7,P4HB,PAPPA,FBLN1,PSG3,DNAH8,THBS4,SLPI,FN1,HP,APOA4,COL18A1,C8A,F13B,CFHR2,CFHR4,CFHR3,CFH,F5,S100A7,S100A9,ADAMTSL4,ECM1,FLNA,CRTAC1,SMARCA1,C8B,FCN1,ADAMTS13,SVEP1,C1QA,F10,F7,CALML5,PROS1,EFEMP1,CFI,ILK,MASP2,F11,C1S,PSG2,PRG4,ABCA13,MST1,TGFBI,CFB,IGFALS,GC,HGFAC,UIMC1,FBN2,TSKU,C1R,TTN,VEGFC,PLCG2,PAMR1,KNG1,TRIM66,TRIM8,CDH5
UniProt Keywords	KW-0301	Gamma-carboxyglutamic acid	9	16	1.68	2.26e-10	F9,MGP,PROC,TTR,F2,F10,F7,PROS1,TGFBI
UniProt Keywords	KW-0865	Zymogen	19	210	0.88	9.85e-10	CPB2,F9,PROC,KLKB1,F13A1,PCSK9,BMP1,F2,PLG,PAPPA,F5,ADAM12,ADAMTS13,F10,F7,PROS1,CFB,HGFAC,CFD
UniProt Keywords	KW-0765	Sulfation	11	57	1.21	1.26e-08	CHGA,F9,LUM,PCSK9,SERPINF2,FGG,FN1,CFH,F5,C4A,C4B
UniProt Keywords	KW-0646	Protease inhibitor	14	117	1.01	1.41e-08	SERPINA10,CSTA,AMBP,ITIH1,SERPING1,SERPINI1,A2ML1,SERPINF2,SERPINA11,SLPI,ITIH3,SERPINA1,SERPINA4,KNG1
UniProt Keywords	KW-0722	Serine protease inhibitor	12	80	1.1	2.38e-08	SERPINA10,AMBP,ITIH1,SERPING1,SERPINI1,A2ML1,SERPINF2,SERPINA11,SLPI,ITIH3,SERPINA1,SERPINA4
UniProt Keywords	KW-0272	Extracellular matrix	19	267	0.78	3.51e-08	SPARC,APOE,THBS1,VWF,LGALS3BP,LUM,ANGPTL4,BMP1,FBLN1,THBS4,FN1,COL18A1,ADAMTSL4,ECM1,CRTAC1,EFEMP1,SERPINA1,TGFBI,FBN2
UniProt Keywords	KW-0645	Protease	26	516	0.63	3.51e-08	CPB2,F9,LTF,PROC,PSMA6,KLKB1,HABP2,PCSK9,BMP1,F2,PLG,PAPPA,CNDP1,ADAM12,HTRA1,ADAMTS13,F10,F7,CFI,MASP2,F11,C1S,CFB,HGFAC,C1R,CFD
UniProt Keywords	KW-0379	Hydroxylation	14	143	0.92	1.29e-07	F9,PROC,FCN3,C1QB,COL18A1,C1QA,F10,F7,PROS1,MASP2,C1S,C1R,KNG1,FGA
UniProt Keywords	KW-0345	HDL	7	16	1.57	1.47e-07	APOA1,APOE,SAA4,APOL1,APOA4,APOM,APOA5
UniProt Keywords	KW-0011	Acute phase	7	19	1.49	3.69e-07	SAA4,F2,SERPINF2,FN1,HP,ORM2,SERPINA1
UniProt Keywords	KW-0792	Thrombophilia	6	10	1.71	4.17e-07	F9,PROC,F2,PLG,F5,PROS1
UniProt Keywords	KW-1068	Hemolytic uremic syndrome	6	10	1.71	4.17e-07	CFHR5,CFHR1,CFHR3,CFH,CFI,CFB
UniProt Keywords	KW-0034	Amyloid	7	24	1.39	1.26e-06	APOA1,TTR,APCS,LYZ,TGFBI,B2M,FGA
UniProt Keywords	KW-0473	Membrane attack complex	5	7	1.78	3.36e-06	C5,C9,C7,C8A,C8B
UniProt Keywords	KW-0358	Heparin-binding	10	88	0.98	4.70e-06	LTF,APOB,APOE,THBS1,SERPINA10,PF4,PRG2,FN1,SOD3,F11
UniProt Keywords	KW-0280	Fibrinolysis	5	8	1.72	5.08e-06	CPB2,KLKB1,SERPING1,PLG,PROS1
UniProt Keywords	KW-1008	Amyloidosis	7	31	1.28	5.08e-06	APOA1,TTR,APOE,LYZ,TGFBI,B2M,FGA
UniProt Keywords	KW-0971	Glycation	5	12	1.55	2.22e-05	APOA1,APOE,SOD3,CFB,B2M
UniProt Keywords	KW-0445	Lipid transport	10	110	0.89	2.77e-05	CETP,APOB,APOA1,APOE,SLC27A4,APOL1,APOA4,APOM,APOA5,APOC4
UniProt Keywords	KW-0420	Kringle	5	16	1.42	6.46e-05	HABP2,F2,PLG,MST1,HGFAC
UniProt Keywords	KW-0913	Age-related macular degeneration	5	16	1.42	6.46e-05	C9,CFH,HTRA1,CFI,CFB
UniProt Keywords	KW-0395	Inflammatory response	11	163	0.76	0.00011	C5,ATRN,KLKB1,CSF1R,STAB1,S100A9,C4A,C4B,RARRES2,CCL18,KNG1
UniProt Keywords	KW-0654	Proteoglycan	7	55	1.03	0.00013	AMBP,LUM,ITIH1,PRG2,CSPG4,PRG4,ITIH3
UniProt Keywords	KW-0162	Chylomicron	4	9	1.57	0.00019	APOB,APOE,APOA4,APOA5
UniProt Keywords	KW-0721	Serine protease homolog	4	9	1.57	0.00019	HP,MST1,HPR,PAMR1
UniProt Keywords	KW-1065	Osteogenesis imperfecta	5	23	1.26	0.00025	SPARC,SERPINF1,SEC24D,BMP1,P4HB
UniProt Keywords	KW-0153	Cholesterol metabolism	7	65	0.96	0.00031	CETP,APOB,APOA1,APOE,LCAT,PCSK9,APOL1
UniProt Keywords	KW-0929	Antimicrobial	8	93	0.86	0.00036	CHGA,LTF,LYZ,CAMP,PRG2,SLPI,HP,S100A9

UniProt Keywords	KW-0797	Tissue remodeling	3	4	1.8	0.00075	PLG,CSPG4,THBS4
UniProt Keywords	KW-0558	Oxidation	5	32	1.12	0.00092	CHGA,APOA1,APOE,FN1,ACTB
UniProt Keywords	KW-0130	Cell adhesion	17	478	0.48	0.0010	THBS1,VWF,LGALS3BP,CSTA,CDH6,DSC2,DST,THBS4,FN1,COL18A1,ADAM12,CCN5,SVEP1,CD99,TGFBI,IGFALS,CDH5
UniProt Keywords	KW-0340	Growth factor binding	4	20	1.23	0.0021	IGFBP5,IGFBP1,HTRA1,IGFBP3
UniProt Keywords	KW-0378	Hydrolase	36	1612	0.28	0.0025	CPB2,F9,GPLD1,LTF,PROC,LYZ,PSMA6,KLKB1,HABP2,PGLYRP2,APEH,PCSK9,BMP1,F2,PLG,PAPPA,ANG,BLM,CNDP1,ADAM12,HTRA1,SMARCA1,ADAMTS13,F10,F7,CFI,NAV2,MASP2,F11,C1S,CFB,HGFAC,C1R,RNASE4,CFD,PLCG2
UniProt Keywords	KW-0065	Atherosclerosis	3	8	1.5	0.0030	CETP,APOB,APOA1
UniProt Keywords	KW-0882	Thioester bond	3	8	1.5	0.0030	A2ML1,C4A,C4B
UniProt Keywords	KW-0044	Antibiotic	6	77	0.82	0.0051	CHGA,LTF,CAMP,PRG2,SLPI,HP
UniProt Keywords	KW-0351	Hemoglobin-binding	2	2	1.93	0.0095	HP,HPR
UniProt Keywords	KW-0049	Antioxidant	3	14	1.26	0.0110	HP,S100A9,SOD3
UniProt Keywords	KW-0339	Growth factor	7	129	0.66	0.0127	INHBE,BMP1,INHBC,THBS4,EFEMP1,PDGFC,VEGFC
UniProt Keywords	KW-0037	Angiogenesis	7	131	0.65	0.0135	ANGPTL6,ANGPTL4,CSPG4,ANG,FN1,ECM1,VEGFC
UniProt Keywords	KW-0892	Osteogenesis	4	37	0.96	0.0145	MGP,LTF,BMP1,ECM1
UniProt Keywords	KW-0497	Mitogen	4	39	0.94	0.0170	CAT,THBS4,PDGFC,VEGFC
UniProt Keywords	KW-0495	Mineral balance	2	4	1.63	0.0211	AHSG,ECM1
UniProt Keywords	KW-0176	Collagen	5	83	0.71	0.0392	FCN3,C1QB,COL18A1,FCN1,C1QA
Pfam	PF01442	Apolipoprotein A1/A4/E domain	4	4	1.93	0.0028	APOA1,APOE,APOA4,APOA5
InterPro	IPR001881	EGF-like calcium-binding domain	24	124	1.21	7.04e-17	F9,PROC,LRP1,THBS1,HABP2,MASP1,BMP1,EFEMP2,STAB1,FBLN1,OIT3,THBS4,CRTAC1,SVEP1,F10,F7,PROS1,EFEMP1,MMRN1,MASP2,C1S,FBN2,C1R,PAMR1
InterPro	IPR035976	Sushi/SCR/CCP superfamily	18	58	1.42	2.11e-15	CFHR5,MASP1,CFHR1,C7,PAPPA,HP,F13B,CFHR2,CFHR4,CFHR3,CFH,SVEP1,MASP2,C1S,CFB,HPR,C1R,PAMR1
InterPro	IPR000436	Sushi/SCR/CCP domain	17	56	1.41	1.87e-14	CFHR5,MASP1,CFHR1,C7,PAPPA,HP,F13B,CFHR2,CFHR4,CFHR3,CFH,SVEP1,MASP2,C1S,CFB,C1R,PAMR1
InterPro	IPR001314	Peptidase S1A, chymotrypsin family	20	107	1.2	4.83e-14	F9,PROC,KLKB1,HABP2,F2,PLG,HP,F10,F7,CFI,MASP2,F11,C1S,MST1,CFB,HGFAC,HPR,C1R,CFD,PAMR1
InterPro	IPR009003	Peptidase S1, PA clan	21	123	1.16	4.83e-14	F9,PROC,KLKB1,HABP2,F2,PLG,HP,HTRA1,F10,F7,CFI,MASP2,F11,C1S,MST1,CFB,HGFAC,HPR,C1R,CFD,PAMR1
InterPro	IPR001254	Serine proteases, trypsin domain	20	115	1.17	1.38e-13	F9,PROC,KLKB1,HABP2,F2,PLG,HP,F10,F7,CFI,MASP2,F11,C1S,MST1,CFB,HGFAC,HPR,C1R,CFD,PAMR1
InterPro	IPR018097	EGF-like calcium-binding, conserved site	19	99	1.21	1.41e-13	F9,PROC,LRP1,MASP1,BMP1,EFEMP2,FBLN1,OIT3,THBS4,CRTAC1,SVEP1,F10,F7,PROS1,EFEMP1,MASP2,C1S,FBN2,C1R
InterPro	IPR000742	EGF-like domain	25	236	0.95	5.57e-13	F9,PROC,LRP1,THBS1,ATRN,HABP2,MASP1,BMP1,EFEMP2,STAB1,FBLN1,OIT3,THBS4,ADAM12,SVEP1,F10,F7,PROS1,EFEMP1,MMRN1,MASP2,HGFAC,FBN2,C1R,PAMR1
InterPro	IPR043504	Peptidase S1, PA clan, chymotrypsin-like fold	19	116	1.14	1.46e-12	F9,PROC,KLKB1,HABP2,F2,PLG,HP,F10,F7,CFI,MASP2,F11,C1S,MST1,HGFAC,HPR,C1R,CFD,PAMR1
InterPro	IPR033116	Serine proteases, trypsin family, serine active site	16	92	1.17	1.02e-10	F9,PROC,KLKB1,HABP2,F2,PLG,F10,F7,CFI,MASP2,F11,C1S,CFB,HGFAC,C1R,CFD
InterPro	IPR000152	EGF-type aspartate/asparagine hydroxylation site	15	98	1.11	2.84e-09	F9,PROC,LRP1,BMP1,EFEMP2,FBLN1,SVEP1,F10,F7,PROS1,EFEMP1,MMRN1,MASP2,C1S,FBN2
InterPro	IPR009030	Growth factor receptor cysteine-rich domain superfamily	15	127	1.0	7.29e-08	IGFBP5,LRP1,C9,IGFBP1,BMP1,EFEMP2,FBLN1,C8A,HTRA1,CCN5,SVEP1,IGFBP3,PROS1,EFEMP1,FBN2
InterPro	IPR018114	Serine proteases, trypsin family, histidine active site	13	100	1.04	4.11e-07	F9,PROC,KLKB1,HABP2,F2,PLG,F10,F7,CFI,F11,CFB,HGFAC,CFD
InterPro	IPR042185	Serpin superfamily, domain 2	9	35	1.34	8.90e-07	SERPINF1,SERPINA10,SERPING1,SERPINI1,SERPINF2,SERPINA7,SERPINA11,SERPINA1,SERPINA4
InterPro	IPR000215	Serpin family	9	36	1.33	1.03e-06	SERPINF1,SERPINA10,SERPING1,SERPINI1,SERPINF2,SERPINA7,SERPINA11,SERPINA1,SERPINA4
InterPro	IPR023796	Serpin domain	9	36	1.33	1.03e-06	SERPINF1,SERPINA10,SERPING1,SERPINI1,SERPINF2,SERPINA7,SERPINA11,SERPINA1,SERPINA4
InterPro	IPR036186	Serpin superfamily	9	36	1.33	1.03e-06	SERPINF1,SERPINA10,SERPING1,SERPINI1,SERPINF2,SERPINA7,SERPINA11,SERPINA1,SERPINA4
InterPro	IPR042178	Serpin superfamily, domain 1	9	36	1.33	1.03e-06	SERPINF1,SERPINA10,SERPING1,SERPINI1,SERPINF2,SERPINA7,SERPINA11,SERPINA1,SERPINA4
InterPro	IPR000294	Gamma-carboxyglutamic acid-rich (GLA) domain	7	15	1.6	1.51e-06	F9,MGP,PROC,F2,F10,F7,PROS1
InterPro	IPR035972	Gamma-carboxyglutamic acid-rich (GLA) domain superfamily	7	15	1.6	1.51e-06	F9,MGP,PROC,F2,F10,F7,PROS1
InterPro	IPR020837	Fibrinogen, conserved site	7	19	1.49	5.08e-06	ANGPTL6,FCN3,ANGPTL4,FGB,FGG,FCN1,FGA
InterPro	IPR035914	Spermadhesin, CUB domain superfamily	9	54	1.15	1.56e-05	ATRN,MASP1,BMP1,ADAMTS13,MASP2,C1S,PDGFC,C1R,PAMR1
InterPro	IPR014716	Fibrinogen, alpha/beta/gamma chain, C-terminal globular, subdomain 1	7	26	1.36	2.81e-05	ANGPTL6,FCN3,ANGPTL4,FGB,FGG,FCN1,FGA
InterPro	IPR036383	Thrombospondin type-1 (TSP1) repeat superfamily	9	64	1.08	5.30e-05	CFP,THBS1,C9,C7,C8A,ADAMTSL4,C8B,ADAMTS13,CCN5
InterPro	IPR000884	Thrombospondin type-1 (TSP1) repeat	9	65	1.07	5.73e-05	CFP,THBS1,C9,C7,C8A,ADAMTSL4,C8B,ADAMTS13,CCN5
InterPro	IPR002181	Fibrinogen, alpha/beta/gamma chain, C-terminal globular domain	7	32	1.27	8.42e-05	ANGPTL6,FCN3,ANGPTL4,FGB,FGG,FCN1,FGA
InterPro	IPR023795	Serpin, conserved site	7	32	1.27	8.42e-05	SERPINF1,SERPING1,SERPINI1,SERPINF2,SERPINA7,SERPINA1,SERPINA4
InterPro	IPR036056	Fibrinogen-like, C-terminal	7	32	1.27	8.42e-05	ANGPTL6,FCN3,ANGPTL4,FGB,FGG,FCN1,FGA
InterPro	IPR000859	CUB domain	8	51	1.12	0.00010	ATRN,MASP1,BMP1,MASP2,C1S,PDGFC,C1R,PAMR1
InterPro	IPR046350	Cystatin superfamily	6	23	1.34	0.00022	SPP2,CSTA,AHSG,CAMP,RARRES2,KNG1
InterPro	IPR017857	Coagulation factor-like, Gla domain superfamily	5	12	1.55	0.00027	F9,PROC,F10,F7,PROS1
InterPro	IPR000074	Apolipoprotein A/E	4	4	1.93	0.00028	APOA1,APOE,APOA4,APOA5
InterPro	IPR013806	Kringle-like fold	6	26	1.29	0.00037	HABP2,F2,PLG,FN1,MST1,HGFAC
InterPro	IPR001862	Membrane attack complex component/perforin/complement C9	4	5	1.83	0.00046	C9,C7,C8A,C8B
InterPro	IPR012224	Peptidase S1A, coagulation factor VII/IX/X/C/Z	4	5	1.83	0.00046	F9,PROC,F10,F7
InterPro	IPR000001	Kringle	5	16	1.42	0.00072	HABP2,F2,PLG,MST1,HGFAC
InterPro	IPR000020	Anaphylatoxin/fibulin	4	6	1.75	0.00072	C5,FBLN1,C4A,C4B
InterPro	IPR003609	PAN/Apple domain	4	6	1.75	0.00072	KLKB1,PLG,F11,MST1
InterPro	IPR018056	Kringle, conserved site	5	16	1.42	0.00072	HABP2,F2,PLG,MST1,HGFAC
InterPro	IPR020863	Membrane attack complex component/perforin domain, conserved site	4	6	1.75	0.00072	C9,C7,C8A,C8B
InterPro	IPR038178	Kringle superfamily	5	16	1.42	0.00072	HABP2,F2,PLG,MST1,HGFAC
InterPro	IPR040839	Macroglobulin domain MG4	4	8	1.63	0.0014	C5,A2ML1,C4A,C4B
InterPro	IPR000867	Insulin-like growth factor-binding protein, IGFBP	5	20	1.33	0.0015	IGFBP5,IGFBP1,HTRA1,CCN5,IGFBP3
InterPro	IPR001599	Alpha-2-macroglobulin	4	9	1.57	0.0019	C5,A2ML1,C4A,C4B
InterPro	IPR002890	Macroglobulin domain	4	9	1.57	0.0019	C5,A2ML1,C4A,C4B
InterPro	IPR009048	Alpha-macroglobulin, receptor-binding	4	9	1.57	0.0019	C5,A2ML1,C4A,C4B
InterPro	IPR011625	Alpha-2-macroglobulin, bait region domain	4	9	1.57	0.0019	C5,A2ML1,C4A,C4B
InterPro	IPR011626	Alpha-macroglobulin-like, TED domain	4	9	1.57	0.0019	C5,A2ML1,C4A,C4B

InterPro	IPR012674	Calycin	6	38	1.13	0.0019	LCN1,AMBP,APOD,APOM,ORM2,PAEP
InterPro	IPR036595	Alpha-macroglobulin, receptor-binding domain superfamily	4	9	1.57	0.0019	C5,A2ML1,C4A,C4B
InterPro	IPR041555	Macroglobulin domain MG3	4	9	1.57	0.0019	C5,A2ML1,C4A,C4B
InterPro	IPR023415	Low-density lipoprotein (LDL) receptor class A, conserved site	6	40	1.1	0.0021	LRP1,C9,C7,C8A,C8B,CFI
InterPro	IPR017891	Insulin-like growth factor binding protein, N-terminal, Cys-rich conserved site	4	11	1.49	0.0030	IGFBP5,IGFBP1,CCN5,IGFBP3
InterPro	IPR020864	Membrane attack complex component/perforin (MACPF) domain	4	12	1.45	0.0039	C9,C7,C8A,C8B
InterPro	IPR012290	Fibrinogen, alpha/beta/gamma chain, coiled coil domain	3	3	1.93	0.0040	FGB,FGG,FGA
InterPro	IPR024175	Peptidase S1A, complement C1r/C1S/mannan-binding	3	3	1.93	0.0040	MASP2,C1S,C1R
InterPro	IPR026823	Complement Clr-like EGF domain	5	27	1.19	0.0040	LRP1,EFEMP2,FBLN1,EFEMP1,FBN2
InterPro	IPR037579	Fibrinogen	3	3	1.93	0.0040	FGB,FGG,FGA
InterPro	IPR002172	Low-density lipoprotein (LDL) receptor class A repeat	6	47	1.03	0.0042	LRP1,C9,C7,C8A,C8B,CFI
InterPro	IPR036055	LDL receptor-like superfamily	6	47	1.03	0.0042	LRP1,C9,C7,C8A,C8B,CFI
InterPro	IPR022272	Lipocalin family conserved site	4	13	1.42	0.0045	AMBP,SLC27A4,APOD,PAEP
InterPro	IPR001840	Anaphylatoxin, complement system domain	3	4	1.8	0.0062	C5,C4A,C4B
InterPro	IPR018081	Anaphylatoxin, complement system	3	4	1.8	0.0062	C5,C4A,C4B
InterPro	IPR008930	Terpenoid cyclases/protein prenyltransferase alpha-alpha toroid	4	15	1.35	0.0069	C5,A2ML1,C4A,C4B
InterPro	IPR000566	Lipocalin/cytosolic fatty-acid binding domain	5	35	1.08	0.0109	LCN1,AMBP,APOD,ORM2,PAEP
InterPro	IPR022321	Insulin-like growth factor-binding protein family 1-6, chordata	3	6	1.63	0.0137	IGFBP5,IGFBP1,IGFBP3
InterPro	IPR019742	Alpha-2-macroglobulin, conserved site	3	8	1.5	0.0260	A2ML1,C4A,C4B
InterPro	IPR047565	Alpha-macroglobulin-like, thiol-ester bond-forming region	3	8	1.5	0.0260	A2ML1,C4A,C4B
InterPro	IPR018247	EF-Hand 1, calcium-binding site	9	176	0.64	0.0352	SPARC,PPP2R3A,DGKG,DST,SPATA21,S100A7,S100A9,CALML5,EFCAB5
SMART	SM00020	Trypsin-like serine protease	21	118	1.18	6.41e-15	F9,PROC,KLKB1,HABP2,MASP1,F2,PLG,HP,F10,F7,CFI,MASP2,F11,C1S,MST1,CFB,HGFAC,HPR,C1R,CFD,PAMR1
SMART	SM00032	Domain abundant in complement control proteins	17	56	1.41	6.41e-15	CFHR5,MASP1,CFHR1,C7,PAPPA,HP,F13B,CFHR2,CFHR4,CFHR3,CFH,SVEP1,MASP2,C1S,CFB,C1R,PAMR1
SMART	SM00179	Calcium-binding EGF-like domain	18	101	1.18	7.63e-13	F9,PROC,LRP1,MASP1,BMP1,EFEMP2,FBLN1,OIT3,THBS4,SVEP1,F10,F7,PROS1,EFEMP1,MASP2,C1S,FBN2,C1R
SMART	SM00181	Epidermal growth factor-like domain.	19	209	0.89	5.30e-09	F9,PROC,LRP1,THBS1,ATRN,HABP2,EFEMP2,STAB1,FBLN1,OIT3,THBS4,SVEP1,F10,F7,PROS1,MMRN1,HGFAC,FBN2,PAMR1
SMART	SM00093	SERine Proteinase INhibitors	9	35	1.34	2.85e-07	SERPINF1,SERPINA10,SERPING1,SERPINI1,SERPINF2,SERPINA7,SERPINA11,SERPINA1,SERPINA4
SMART	SM00069	Domain containing Gla (gamma-carboxyglutamate) residues.	7	15	1.6	5.48e-07	F9,MGP,PROC,F2,F10,F7,PROS1
SMART	SM00186	Fibrinogen-related domains (FReDs)	7	29	1.31	2.00e-05	ANGPTL6,FCN3,ANGPTL4,FGB,FGG,FCN1,FGA
SMART	SM00209	Thrombospondin type 1 repeats	9	64	1.08	2.00e-05	CFP,THBS1,C9,C7,C8A,ADAMTSL4,C8B,ADAMTS13,CCN5
SMART	SM00042	Domain first found in C1r, C1s, uEGF, and bone morphogenetic protein.	8	50	1.13	3.27e-05	ATRN,MASP1,BMP1,MASP2,C1S,PDGFC,C1R,PAMR1
SMART	SM00104	Anaphylatoxin homologous domain	4	6	1.75	0.00030	C5,FBLN1,C4A,C4B
SMART	SM00130	Kringle domain	5	16	1.42	0.00030	HABP2,F2,PLG,MST1,HGFAC
SMART	SM00121	Insulin growth factor-binding protein homologues	5	17	1.4	0.00032	IGFBP5,IGFBP1,HTRA1,CCN5,IGFBP3
SMART	SM01359	Alpha-2-Macroglobulin	4	9	1.57	0.00076	C5,A2ML1,C4A,C4B
SMART	SM01360	Alpha-2-macroglobulin family	4	9	1.57	0.00076	C5,A2ML1,C4A,C4B
SMART	SM01361	A-macroglobulin receptor	4	9	1.57	0.00076	C5,A2ML1,C4A,C4B
SMART	SM00457	Membrane-attack complex / perforin	4	12	1.45	0.0015	C9,C7,C8A,C8B
SMART	SM01212	Fibrinogen alpha/beta chain family	3	3	1.93	0.0015	FGB,FGG,FGA
SMART	SM00192	Low-density lipoprotein receptor domain class A	6	48	1.02	0.0018	LRP1,C9,C7,C8A,C8B,CFI
SMART	SM01419	Alpha-macro-globulin thiol-ester bond-forming region	3	8	1.5	0.0105	A2ML1,C4A,C4B
SMART	SM00001	EGF domain, unclasssified subfamily	6	70	0.86	0.0109	LRP1,ATRN,EFEMP2,STAB1,OIT3,EFEMP1
SMART	SM00223	APPLE domain	2	2	1.93	0.0314	KLKB1,F11
SMART	SM00643	Netrin C-terminal Domain	3	15	1.23	0.0422	C5,C4A,C4B

UC vs ACFET T3

#category	term ID	term description	observed gene count	background gene count	strength	false discovery rate	matching proteins in your network (labels)
GO Process	GO:0007596	Blood coagulation	36	173	1.27	2.98e-28	VCL,SERPIND1,MYH9,F9,TFPI2,PF4V1,HRG,PLEK,F12,VWF,SERPINA10,KLKB1,F13A1,FERMT3,PF4,FGB,F2,PLG,TLN1,FBLN1,FGG,F13B,F5,FLNA,ADAMTS13,F10,F7,PROS1,ILK,SAA1,F11,SERPINA1,HGFAC,PLCG2,ACTB,FGA
GO Process	GO:0006959	Humoral immune response	39	268	1.11	2.76e-26	ST6GAL1,C5,PF4V1,HRG,C3,CFHR5,LYZ,FCN3,FCN2,PPBP,PF4,MASP1,CAMP,FGB,F2,C1QB,CFHR1,COLEC10,ANG,SLPI,C8A,CFHR3,CFH,S100A7,C8B,FCN1,MBL2,C1QC,MASP2,C1S,C4A,COLEC11,C4B,CFB,JCHAIN,C1R,IGHV3-15,IGHV3-72,FGA
GO Process	GO:0006956	Complement activation	25	60	1.57	1.44e-25	C5,C3,CFHR5,FCN3,FCN2,MASP1,C1QB,CFHR1,COLEC10,C8A,CFHR3,CFH,C8B,FCN1,MBL2,C1QC,MASP2,C1S,C4A,COLEC11,C4B,CFB,C1R,IGHV3-15,IGHV3-72
GO Process	GO:0006950	Response to stress	110	3358	0.46	7.33e-25	VCL,SERPIND1,MYH9,F9,TFPI2,C5,PF4V1,HRG,PLEK,CAT,C3,APOE,PXDN,BPIFB1,F12,APCS,CFHR5,THBS1,LYZ,VWF,SERPINA10,ATRN,KLKB1,F13A1,AMBP,TCIRG1,HPX,ITIH4,FCN3,CRH,FERMT3,DSC2,CSF1R,FCN2,PPBP,PF4,MASP1,CAMP,LALBA,ANGPTL4,PCSK9,FGB,F2,PLG,PRG2,STAB1,C1QB,CFHR1,NDUFS8,TLN1,APOL1,SUMO4,SERPINF2,HBA1,P4HB,FBLN1,COLEC10,ANG,FGG,THBS4,SLPI,BLM,C8A,F13B,CFH,F5,S100A7,ECM1,FLNA,C8B,FCN1,ADAMTS13,MBL2,C1QC,F10,F7,SOD3,GPX3,IGF1,PROS1,TNRC6A,ILK,MASP2,SAA1,F11,C1S,CD14,ADIPOQ,C4A,CDK3,COLEC11,MST1,C4B,SERPINA1,CFB,HGFAC,UIMC1,SELENOP,TSKU,JCHAIN,HPR,C1R,IGHV3-15,IGHV3-72,VEGFC,PLCG2,HSPH1,ACTB,FGA,PDIA4
GO Process	GO:0042060	Wound healing	38	336	1.0	3.69e-22	VCL,SERPIND1,MYH9,F9,TFPI2,PF4V1,HRG,PLEK,F12,VWF,SERPINA10,KLKB1,F13A1,FERMT3,PF4,FGB,F2,PLG,TLN1,FBLN1,FGG,F13B,F5,FLNA,ADAMTS13,F10,F7,IGF1,PROS1,ILK,SAA1,F11,SERPINA1,HGFAC,TSKU,PLCG2,ACTB,FGA
GO Process	GO:0050878	Regulation of body fluid levels	39	371	0.97	8.24e-22	VCL,SERPIND1,MYH9,F9,TFPI2,PF4V1,HRG,PLEK,APOE,F12,THBS1,VWF,SERPINA10,KLKB1,F13A1,FERMT3,PF4,FGB,F2,PLG,TLN1,SERPINF2,FBLN1,FGG,F13B,F5,FLNA,ADAMTS13,F10,F7,PROS1,ILK,SAA1,F11,SERPINA1,HGFAC,PLCG2,ACTB,FGA

GO Process	GO:0006952	Defense response	61	1394	0.59	6.41e-17	C5,PF4V1,HRG,C3,BPIFB1,F12,APCS,CFHR5,THBS1,LYZ,ATRN,KLKB1,TCIRG1,HPX,ITIH4,FCN3,CRH,CSF1R,FCN2,PPBP,PF4,MASP1,CAMP,LALBA,FGB,F2,PRG2,STAB1,C1QB,CFHR1,APOL1,SERPINF2,COLEC10,ANG,SLPI,C8A,CFH,S100A7,ECM1,C8B,FCN1,ADAMTS13,MBL2,C1QC,F7,MASP2,SAA1,C1S,CD14,C4A,COLEC11,C4B,SERPINA1,CFB,JCHAIN,HPR,C1R,IGHV3-15,IGHV3-72,PLCG2,FGA
GO Process	GO:0072378	Blood coagulation, fibrin clot formation	15	24	1.74	8.92e-17	F9,F12,KLKB1,F13A1,FGB,F2,FBLN1,FGG,F13B,F5,FLNA,F10,F7,F11,FGA
GO Process	GO:0051346	Negative regulation of hydrolase activity	32	354	0.9	8.20e-16	SPP2,SERPIND1,TFPI2,C5,HRG,APOA1,C3,APCS,THBS1,SERPINA10,TIMP2,LCN1,CSTA,AMBP,ITIH4,ITIH1,SERPINI1,A2ML1,SLC27A4,ANGPTL4,SERPINF2,SERPINA11,SLPI,PAPLN,ITIH2,ECM1,PROS1,C4A,ITIH3,C4B,SERPINA1,APOC1
GO Process	GO:0006955	Immune response	57	1321	0.58	2.01e-15	ST6GAL1,LGALS1,C5,PF4V1,HRG,C3,PXDN,BPIFB1,F12,APCS,CFHR5,THBS1,LYZ,TCIRG1,HPX,PSG9,FCN3,CSF1R,FCN2,PPBP,PF4,MASP1,CAMP,FGB,F2,PRG2,C1QB,CFHR1,APOL1,COLEC10,ANG,SLPI,C8A,CFHR3,CFH,S100A7,C8B,FCN1,ADAMTS13,MBL2,C1QC,RAPGEF4,MASP2,C1S,CD14,C4A,PRG4,COLEC11,C4B,CFB,JCHAIN,C1R,IGKV2D-28,IGHV3-15,IGHV3-72,PLCG2,FGA
GO Process	GO:0098542	Defense response to other organism	49	989	0.64	4.03e-15	C5,PF4V1,HRG,C3,BPIFB1,F12,APCS,CFHR5,LYZ,HPX,FCN3,CSF1R,FCN2,PPBP,PF4,MASP1,CAMP,LALBA,FGB,F2,PRG2,STAB1,C1QB,CFHR1,APOL1,COLEC10,ANG,SLPI,C8A,CFH,S100A7,C8B,FCN1,ADAMTS13,MBL2,C1QC,MASP2,C1S,CD14,C4A,COLEC11,C4B,CFB,JCHAIN,C1R,IGHV3-15,IGHV3-72,PLCG2,FGA
GO Process	GO:0010466	Negative regulation of peptidase activity	27	249	0.98	6.58e-15	SPP2,SERPIND1,TFPI2,C5,HRG,C3,THBS1,SERPINA10,TIMP2,LCN1,CSTA,AMBP,ITIH4,ITIH1,SERPINI1,A2ML1,SERPINF2,SERPINA11,SLPI,PAPLN,ITIH2,ECM1,PROS1,C4A,ITIH3,C4B,SERPINA1
GO Process	GO:0050896	Response to stimulus	152	7835	0.24	7.65e-15	ST6GAL1,VCL,SERPIND1,LGALS1,MYH9,F9,TFPI2,C5,PF4V1,HRG,APOB,IGFBP5,PLEK,APOA1,TTR,CAT,C3,APOE,PXDN,BPIFB1,F12,APCS,CFHR5,THBS1,LYZ,VWF,SERPINA10,TIMP2,ATRN,LCN1,LCAT,KLKB1,F13A1,AMBP,TCIRG1,HPX,ITIH4,PSG9,FCN3,CRH,FERMT3,DSC2,CSF1R,PIP,FCN2,PSG6,SCGB3A1,PPBP,PF4,MASP1,CAMP,GUCY1A1,SLC27A4,LALBA,ANGPTL4,PCSK9,BMP1,FGB,F2,INHBC,PLG,PSG1,PRG2,STAB1,C1QB,CFHR1,NDUFS8,TLN1,APOL1,SUMO4,SERPINF2,HBA1,FBN1,P4HB,FBLN1,COLEC10,ANG,FGG,LIMS1,THBS4,SLPI,BLM,C8A,F13B,CFHR3,CFH,F5,ADAM12,S100A7,ECM1,FLNA,C8B,RBP4,FCN1,ADAMTS13,CCN5,MBL2,C1QC,F10,F7,APOM,CA6,ADGRG2,SOD3,GPX3,IGF1,PROS1,EFEMP1,TNRC6A,YWHAZ,ILK,RAPGEF4,MASP2,PSG4,SAA1,F11,C1S,CD14,LTBP1,ADIPOQ,C4A,PRG4,CDK3,COLEC11,MST1,C4B,SERPINA1,TGFBI,CFB,IGFALS,HGFAC,UIMC1,PDGFC,FBN2,SELENOP,TSKU,JCHAIN,HPR,C1R,ATP1A1,IGKV2D-28,TTN,IGHV3-15,IGHV3-72,VEGFC,SEMA3B,PLCG2,OR52B4,HSPH1,ACTB,FGA,PDIA4
GO Process	GO:0010951	Negative regulation of endopeptidase activity	26	240	0.98	2.53e-14	SPP2,SERPIND1,TFPI2,C5,HRG,C3,THBS1,SERPINA10,TIMP2,LCN1,CSTA,AMBP,ITIH4,ITIH1,SERPINI1,A2ML1,SERPINF2,SERPINA11,SLPI,PAPLN,ITIH2,PROS1,C4A,ITIH3,C4B,SERPINA1
GO Process	GO:0030168	Platelet activation	19	97	1.24	3.32e-14	VCL,MYH9,PF4V1,HRG,PLEK,VWF,FERMT3,PF4,FGB,F2,TLN1,FGG,FLNA,ADAMTS13,ILK,SAA1,PLCG2,ACTB,FGA
GO Process	GO:0002682	Regulation of immune system process	57	1438	0.55	5.55e-14	LGALS1,C5,HRG,APOA1,C3,APOE,BPIFB1,APCS,CFHR5,THBS1,AMBP,HPX,PSG9,FCN3,PLA2G7,CSF1R,PIP,FCN2,PSG6,PF4,MASP1,PSG1,PRG2,C1QB,CFHR1,FBN1,COLEC10,THBS4,C8A,CFHR3,CFH,S100A7,ECM1,C8B,RBP4,FCN1,MBL2,C1QC,F7,IGF1,MASP2,PSG4,C1S,CD14,ADIPOQ,C4A,COLEC11,MST1,C4B,CFB,C1R,IGHV3-15,IGHV3-72,VEGFC,PLCG2,HSPH1,ACTB
GO Process	GO:1903028	Positive regulation of opsonization	12	16	1.82	5.55e-14	C3,FCN3,FCN2,MASP1,COLEC10,FCN1,MBL2,MASP2,C4A,COLEC11,C4B,CFB
GO Process	GO:0032101	Regulation of response to external stimulus	46	964	0.63	1.38e-13	ST6GAL1,LGALS1,C5,HRG,APOA1,C3,APOE,F12,APCS,THBS1,PSMA6,KLKB1,HPX,FCN3,PLA2G7,CSF1R,FCN2,MASP1,FGB,F2,PLG,SERPINF2,FBN1,COLEC10,FGG,THBS4,CFH,S100A7,HTRA1,FLNA,FCN1,MBL2,F7,IGF1,PROS1,MASP2,SAA1,F11,CD14,ADIPOQ,COLEC11,MST1,VEGFC,SEMA3B,PLCG2,FGA
GO Process	GO:0002253	Activation of immune response	26	271	0.93	2.84e-13	C5,C3,CFHR5,FCN3,FCN2,MASP1,C1QB,CFHR1,COLEC10,C8A,CFHR3,CFH,C8B,FCN1,MBL2,C1QC,MASP2,C1S,C4A,COLEC11,C4B,CFB,C1R,IGHV3-15,IGHV3-72,PLCG2
GO Process	GO:0006958	Complement activation, classical pathway	14	40	1.49	4.14e-13	C5,C3,C1QB,C8A,C8B,MBL2,C1QC,MASP2,C1S,C4A,C4B,C1R,IGHV3-15,IGHV3-72
GO Process	GO:0045861	Negative regulation of proteolysis	28	339	0.87	6.31e-13	SPP2,SERPIND1,TFPI2,C5,HRG,C3,THBS1,SERPINA10,TIMP2,LCN1,CSTA,AMBP,ITIH4,ITIH1,SERPINI1,A2ML1,F2,SERPINF2,SERPINA11,SLPI,PAPLN,ITIH2,ECM1,PROS1,C4A,ITIH3,C4B,SERPINA1
GO Process	GO:0002252	Immune effector process	29	375	0.84	8.96e-13	LGALS1,C5,C3,CFHR5,TCIRG1,FCN3,FCN2,MASP1,F2,C1QB,CFHR1,COLEC10,C8A,CFHR3,CFH,C8B,FCN1,MBL2,C1QC,MASP2,C1S,C4A,COLEC11,C4B,CFB,C1R,IGHV3-15,IGHV3-72,PLCG2
GO Process	GO:0045087	Innate immune response	39	754	0.66	2.46e-12	C5,C3,BPIFB1,F12,APCS,CFHR5,HPX,FCN3,CSF1R,FCN2,MASP1,CAMP,FGB,C1QB,APOL1,COLEC10,ANG,SLPI,C8A,CFH,S100A7,C8B,FCN1,ADAMTS13,MBL2,C1QC,MASP2,C1S,CD14,C4A,COLEC11,C4B,CFB,JCHAIN,C1R,IGHV3-15,IGHV3-72,PLCG2,FGA
GO Process	GO:0051707	Response to other organism	51	1328	0.53	8.21e-12	C5,PF4V1,HRG,APOB,C3,BPIFB1,F12,APCS,CFHR5,LYZ,HPX,FCN3,CSF1R,FCN2,PPBP,PF4,MASP1,CAMP,LALBA,FGB,F2,PRG2,STAB1,C1QB,CFHR1,APOL1,COLEC10,ANG,SLPI,C8A,CFH,S100A7,C8B,FCN1,ADAMTS13,MBL2,C1QC,MASP2,C1S,CD14,ADIPOQ,C4A,COLEC11,C4B,CFB,JCHAIN,C1R,IGHV3-15,IGHV3-72,PLCG2,FGA
GO Process	GO:0002684	Positive regulation of immune system process	41	874	0.62	9.32e-12	LGALS1,C5,HRG,C3,CFHR5,THBS1,HPX,FCN3,PLA2G7,CSF1R,FCN2,PF4,MASP1,C1QB,CFHR1,COLEC10,THBS4,C8A,CFHR3,CFH,S100A7,C8B,RBP4,FCN1,MBL2,C1QC,F7,IGF1,MASP2,C1S,C4A,COLEC11,C4B,CFB,C1R,IGHV3-15,IGHV3-72,VEGFC,PLCG2,HSPH1,ACTB
GO Process	GO:0031638	Zymogen activation	14	59	1.32	2.88e-11	F9,F12,KLKB1,FGB,F2,FGG,F5,F10,F7,F11,HGFAC,HPR,C1R,FGA
GO Process	GO:0044419	Biological process involved in interspecies interaction between organisms	53	1490	0.5	3.78e-11	C5,PF4V1,HRG,APOB,C3,APOE,BPIFB1,F12,APCS,CFHR5,LYZ,HPX,FCN3,CSF1R,FCN2,PPBP,PF4,MASP1,CAMP,LALBA,FGB,F2,PLG,PRG2,STAB1,C1QB,CFHR1,APOL1,COLEC10,ANG,SLPI,C8A,CFH,S100A7,C8B,FCN1,ADAMTS13,MBL2,C1QC,MASP2,C1S,CD14,ADIPOQ,C4A,COLEC11,C4B,CFB,JCHAIN,C1R,IGHV3-15,IGHV3-72,PLCG2,FGA
GO Process	GO:0052547	Regulation of peptidase activity	29	446	0.76	4.36e-11	SPP2,SERPIND1,TFPI2,C5,HRG,C3,THBS1,SERPINA10,TIMP2,LCN1,CSTA,AMBP,ITIH4,ITIH1,SERPINI1,A2ML1,ANTXR1,SERPINF2,FBLN1,SERPINA11,SLPI,PAPLN,ITIH2,ECM1,PROS1,C4A,ITIH3,C4B,SERPINA1
GO Process	GO:0042730	Fibrinolysis	10	19	1.67	1.70e-10	HRG,F12,KLKB1,FGB,F2,PLG,SERPINF2,FGG,PROS1,FGA
GO Process	GO:0030193	Regulation of blood coagulation	14	70	1.25	1.99e-10	HRG,APOE,F12,THBS1,KLKB1,FGB,F2,PLG,SERPINF2,FGG,F7,PROS1,F11,FGA
GO Process	GO:0002376	Immune system process	63	2121	0.42	2.34e-10	ST6GAL1,LGALS1,MYH9,C5,PF4V1,HRG,PLEK,C3,PXDN,BPIFB1,F12,APCS,CFHR5,THBS1,LYZ,TCIRG1,HPX,PSG9,FCN3,CSF1R,FCN2,PPBP,PF4,MASP1,CAMP,FGB,F2,PLG,PRG2,C1QB,CFHR1,APOL1,COLEC10,ANG,SLPI,C8A,CFHR3,CFH,S100A7,FLNA,C8B,FCN1,ADAMTS13,MBL2,C1QC,ACTN1,RAPGEF4,MASP2,SAA1,C1S,CD14,C4A,PRG4,COLEC11,C4B,CFB,JCHAIN,C1R,IGKV2D-28,IGHV3-15,IGHV3-72,PLCG2,FGA

GO Process	GO:0009605	Response to external stimulus	67	2355	0.4	2.34e-10	SERPIND1,C5,PF4V1,HRG,APOB,APOA1,CAT,C3,APOE,BPIFB1,F12,APCS,CFHR5,THBS1,LYZ,HPX,FCN3,DSC2,CSF1R,FCN2,PPBP,PF4,MASP1,CAMP,SLC27A4,LALBA,PCSK9,FGB,F2,PRG2,STAB1,C1QB,CFHR1,APOL1,FBN1,COLEC10,ANG,SLPI,C8A,CFH,F5,S100A7,C8B,FCN1,ADAMTS13,MBL2,C1QC,F7,TNRC6A,MASP2,SAA1,C1S,CD14,ADIPOQ,C4A,COLEC11,C4B,CFB,JCHAIN,C1R,TTN,IGHV3-15,IGHV3-72,VEGFC,SEMA3B,PLCG2,FGA
GO Process	GO:0050766	Positive regulation of phagocytosis	14	75	1.22	4.12e-10	APOA1,C3,FCN3,FCN2,MASP1,COLEC10,FCN1,MBL2,MASP2,C4A,COLEC11,C4B,CFB,PLCG2
GO Process	GO:0016064	Immunoglobulin mediated immune response	15	95	1.15	5.20e-10	C5,C3,TCIRG1,C1QB,C8A,C8B,MBL2,C1QC,MASP2,C1S,C4A,C4B,C1R,IGHV3-15,IGHV3-72
GO Process	GO:0030162	Regulation of proteolysis	35	739	0.62	5.39e-10	SPP2,SERPIND1,MYH9,TFPI2,C5,HRG,C3,APOE,F12,THBS1,SERPINA10,TIMP2,LCN1,CSTA,KLKB1,AMBP,ITIH4,ITIH1,SERPINI1,A2ML1,ANTXR1,SH3D19,F2,SERPINF2,FBLN1,SERPINA11,SLPI,PAPLN,ITIH2,ECM1,PROS1,C4A,ITIH3,C4B,SERPINA1
GO Process	GO:0030195	Negative regulation of blood coagulation	12	46	1.36	5.39e-10	HRG,APOE,F12,KLKB1,FGB,F2,PLG,SERPINF2,FGG,PROS1,F11,FGA
GO Process	GO:0048584	Positive regulation of response to stimulus	62	2131	0.41	7.58e-10	LGALS1,C5,HRG,IGFBP5,APOA1,CAT,C3,APOE,F12,CFHR5,THBS1,VWF,HPX,FCN3,PLA2G7,CSF1R,FCN2,MASP1,GUCY1A1,FGB,F2,INHBC,PLG,C1QB,CFHR1,SERPINF2,FBN1,COLEC10,FGG,LIMS1,THBS4,C8A,CFHR3,CFH,S100A7,ECM1,FLNA,C8B,FCN1,MBL2,C1QC,F10,F7,IGF1,ILK,MASP2,C1S,CD14,ADIPOQ,C4A,COLEC11,C4B,CFB,UIMC1,PDGFC,C1R,IGHV3-15,IGHV3-72,VEGFC,PLCG2,ACTB,FGA
GO Process	GO:0048583	Regulation of response to stimulus	90	3931	0.31	8.12e-10	ST6GAL1,LGALS1,C5,HRG,IGFBP5,PLEK,APOA1,CAT,C3,APOE,PXDN,BPIFB1,F12,APCS,CFHR5,THBS1,VWF,PSMA6,KLKB1,AMBP,HPX,PSG9,FCN3,PLA2G7,CRH,CSF1R,FCN2,PF4,MASP1,GUCY1A1,SLC27A4,PCSK9,FGB,F2,INHBC,PLG,C1QB,CFHR1,SUMO4,SERPINF2,FBN1,P4HB,FBLN1,COLEC10,FGG,LIMS1,THBS4,FBLN5,C8A,CFHR3,CFH,S100A7,HTRA1,ECM1,FLNA,C8B,FCN1,MBL2,C1QC,F10,F7,IGF1,PROS1,YWHAZ,ILK,MASP2,SAA1,F11,C1S,CD14,LTBP1,ADIPOQ,C4A,CDK3,COLEC11,MST1,C4B,CFB,UIMC1,PDGFC,FBN2,TSKU,C1R,IGHV3-15,IGHV3-72,VEGFC,SEMA3B,PLCG2,ACTB,FGA
GO Process	GO:0002752	Cell surface pattern recognition receptor signaling pathway	8	9	1.9	1.38e-09	FCN3,FCN2,MASP1,COLEC10,FCN1,MBL2,MASP2,COLEC11
GO Process	GO:0050764	Regulation of phagocytosis	15	104	1.11	1.38e-09	APOA1,C3,FCN3,FCN2,MASP1,COLEC10,FCN1,MBL2,MASP2,ADIPOQ,C4A,COLEC11,C4B,CFB,PLCG2
GO Process	GO:0043086	Negative regulation of catalytic activity	35	771	0.61	1.39e-09	SPP2,SERPIND1,TFPI2,C5,HRG,APOA1,C3,APOE,APCS,THBS1,SERPINA10,TIMP2,LCN1,CSTA,AMBP,ITIH4,ITIH1,SERPINI1,A2ML1,SLC27A4,ANGPTL4,SERPINF2,SERPINA11,SLPI,PAPLN,ITIH2,ECM1,PROS1,ADIPOQ,C4A,ITIH3,C4B,SERPINA1,APOC1,APOC2
GO Process	GO:0044092	Negative regulation of molecular function	43	1143	0.52	1.47e-09	SPP2,SERPIND1,TFPI2,C5,HRG,APOA1,CAT,C3,APOE,PXDN,APCS,CFHR5,THBS1,SERPINA10,TIMP2,LCN1,CSTA,AMBP,ITIH4,ITIH1,SERPINI1,A2ML1,SLC27A4,ANGPTL4,PCSK9,CFHR1,SUMO4,SERPINF2,SERPINA11,SLPI,PAPLN,ITIH2,ECM1,FLNA,PROS1,ADIPOQ,C4A,ITIH3,C4B,SERPINA1,APOC1,APOC2,ACTB
GO Process	GO:0061045	Negative regulation of wound healing	13	69	1.22	1.79e-09	HRG,APOE,F12,APCS,KLKB1,FGB,F2,PLG,SERPINF2,FGG,PROS1,F11,FGA
GO Process	GO:0001867	Complement activation, lectin pathway	8	10	1.85	2.25e-09	FCN3,FCN2,MASP1,COLEC10,FCN1,MBL2,MASP2,COLEC11
GO Process	GO:0050778	Positive regulation of immune response	28	502	0.69	2.38e-09	C5,HRG,C3,CFHR5,HPX,FCN3,FCN2,MASP1,C1QB,CFHR1,COLEC10,C8A,CFHR3,CFH,C8B,FCN1,MBL2,C1QC,MASP2,C1S,C4A,COLEC11,C4B,CFB,C1R,IGHV3-15,IGHV3-72,PLCG2
GO Process	GO:0051241	Negative regulation of multicellular organismal process	40	1035	0.54	3.91e-09	SPARC,HRG,IGFBP5,APOA1,APOE,F12,APCS,THBS1,KLKB1,PSG9,CRH,PF4,GUCY1A1,PCSK9,FGB,F2,PLG,PRG2,STAB1,SERPINF2,FBN1,FBLN1,FGG,THBS4,ECM1,RBP4,C1QC,APOM,IGF1,PROS1,EFEMP1,F11,ADIPOQ,PAEP,TSKU,ATP1A1,APOC1,APOC2,SEMA3B,FGA
GO Process	GO:0070527	Platelet aggregation	11	43	1.36	4.15e-09	VCL,MYH9,PLEK,FERMT3,FGB,TLN1,FGG,FLNA,ILK,ACTB,FGA
GO Process	GO:0034109	Homotypic cell-cell adhesion	12	60	1.25	5.88e-09	VCL,MYH9,PLEK,FERMT3,DSC2,FGB,TLN1,FGG,FLNA,ILK,ACTB,FGA
GO Process	GO:0048518	Positive regulation of biological process	118	6207	0.23	6.65e-09	ST6GAL1,LGALS1,MYH9,C5,SPARC,HRG,APOB,IGFBP5,PLEK,APOA1,CAT,C3,DHX29,APOE,F12,CFHR5,THBS1,VWF,KLKB1,CENPE,TCIRG1,HPX,PSG9,FCN3,PLA2G7,CRH,FERMT3,CSF1R,PIP,FCN2,SCGB3A1,SERPINI1,PPBP,PF4,MASP1,CAMP,GUCY1A1,SLC27A4,ANGPTL4,ANTXR1,SH3D19,PCSK9,BMP1,FGB,F2,INHBC,PLG,PRG2,C1QB,CFHR1,SUZ12,SERPINF2,HBA1,FBN1,P4HB,FBLN1,COLEC10,ANG,FGG,LIMS1,THBS4,BLM,C8A,CFHR3,CFH,ADAM12,S100A7,HTRA1,ADAMTSL4,ECM1,FLNA,SMARCA1,C8B,RBP4,FCN1,MBL2,C1QC,F10,F7,IGF1,ACTN1,TNRC6A,ILK,MASP2,SAA1,F11,C1S,CD14,ADIPOQ,C4A,SLAIN1,COLEC11,MST1,C4B,CFB,PLTP,PAEP,TCAF1,UIMC1,PDGFC,FBN2,TSKU,JCHAIN,HPR,C1R,ATP1A1,APOC1,APOC2,TTN,APOC4,IGHV3-15,IGHV3-72,VEGFC,SEMA3B,PLCG2,HSPH1,ACTB,FGA
GO Process	GO:0032103	Positive regulation of response to external stimulus	26	453	0.71	7.10e-09	LGALS1,HRG,C3,THBS1,HPX,FCN3,PLA2G7,CSF1R,FCN2,MASP1,F2,PLG,SERPINF2,FBN1,COLEC10,THBS4,S100A7,FLNA,FCN1,MBL2,F7,MASP2,CD14,COLEC11,VEGFC,PLCG2
GO Process	GO:0051336	Regulation of hydrolase activity	39	1011	0.53	7.13e-09	SPP2,SERPIND1,TFPI2,C5,HRG,PLEK,APOA1,C3,APCS,THBS1,SERPINA10,TIMP2,LCN1,CSTA,AMBP,ITIH4,ITIH1,SERPINI1,A2ML1,SLC27A4,ANGPTL4,ANTXR1,SERPINF2,FBLN1,SERPINA11,ANG,LIMS1,SLPI,PAPLN,ITIH2,ECM1,PROS1,RAPGEF4,C4A,ITIH3,C4B,SERPINA1,APOC1,APOC2
GO Process	GO:0006954	Inflammatory response	28	538	0.66	9.93e-09	C5,PF4V1,C3,F12,APCS,THBS1,LYZ,ATRN,KLKB1,TCIRG1,ITIH4,CRH,CSF1R,PPBP,PF4,CAMP,F2,STAB1,SERPINF2,ECM1,MBL2,F7,SAA1,CD14,C4A,C4B,SERPINA1,HPR
GO Process	GO:0016485	Protein processing	18	202	0.9	1.26e-08	F9,F12,KLKB1,PLA2G7,PCSK9,BMP1,FGB,F2,FGG,F5,ADAMTS13,F10,F7,F11,HGFAC,HPR,C1R,FGA
GO Process	GO:0051248	Negative regulation of protein metabolic process	39	1038	0.52	1.45e-08	SPP2,SERPIND1,TFPI2,C5,HRG,IGFBP5,C3,APOE,APCS,THBS1,SERPINA10,TIMP2,LCN1,CSTA,AMBP,ITIH4,ITIH1,SERPINI1,A2ML1,F2,SERPINF2,FBLN1,SERPINA11,ANG,SLPI,PAPLN,ITIH2,ECM1,FLNA,IGF1,PROS1,TNRC6A,ADIPOQ,C4A,ITIH3,C4B,SERPINA1,ITM2B,FGA
GO Process	GO:0006508	Proteolysis	43	1247	0.49	1.76e-08	MYH9,F9,C3,F12,PSMA6,LCN1,KLKB1,FCN3,PLA2G7,HABP2,PIP,FCN2,MASP1,PCSK9,BMP1,FGB,F2,PLG,UBE3C,COLEC10,FGG,CNDP1,CFH,F5,ADAM12,HTRA1,FCN1,ADAMTS13,MBL2,F10,F7,MASP2,F11,C1S,PCYOX1,COLEC11,MST1,CFB,HGFAC,UIMC1,HPR,C1R,FGA
GO Process	GO:0061041	Regulation of wound healing	15	130	1.01	1.85e-08	HRG,APOE,F12,APCS,THBS1,KLKB1,FGB,F2,PLG,SERPINF2,FGG,F7,PROS1,F11,FGA
GO Process	GO:1903034	Regulation of response to wounding	16	162	0.94	3.53e-08	HRG,APOE,F12,APCS,THBS1,KLKB1,FGB,F2,PLG,SERPINF2,FGG,FLNA,F7,PROS1,F11,FGA

GO Process	GO:0065007	Biological regulation	183	12385	0.12	4.01e-08	SPP2,ST6GAL1,VCL,SERPIND1,LGALS1,MYH9,TPD52L2,F9,TFPI2,C5,PF4V1,MGP,SPARC,HRG,APOB,IGFBP5,PLEK,APOA1,TTR,CAT,C3,DHX29,APOE,PXDN,BPIFB1,F12,APCS,CFHR5,THBS1,LYZ,VWF,PSMA6,SERPINA10,TIMP2,ATRN,LCN1,LCAT,CSTA,CP,KLKB1,F13A1,AMBP,CENPE,TCIRG1,HPX,ITIH4,PSG9,FCN3,ITIH1,PLA2G7,CRH,FERMT3,DSC2,CSF1R,PIP,FCN2,PSG6,SCGB3A1,SERPINI1,PPBP,PF4,MASP1,CAMP,GUCY1A1,A2ML1,SLC27A4,LALBA,ANGPTL4,ANTXR1,SH3D19,PCSK9,BMP1,FGB,F2,INHBC,PLG,PSG1,PRG2,STAB1,C1QB,CFHR1,TLN1,SUZ12,SUMO4,SERPINF2,HBA1,FBN1,P4HB,FBLN1,COLEC10,OIT3,SERPINA11,ANG,FGG,LIMS1,THBS4,SLPI,FBLN5,PAPLN,BLM,ITIH2,CNDP1,COL18A1,C8A,F13B,CFHR3,CFH,F5,ADAM12,S100A7,HTRA1,ADAMTSL4,ECM1,FLNA,SMARCA1,C8B,RBP4,FCN1,ADAMTS13,CCN5,MBL2,C1QC,F10,F7,APOM,ADGRG2,SOD3,GOLM1,IGF1,PROS1,ACTN1,EFEMP1,TNRC6A,YWHAZ,ILK,RAPGEF4,MASP2,PSG4,SAA1,F11,C1S,CD14,LTBP1,ADIPOQ,C4A,ATP9B,SLAIN1,CDK3,COLEC11,MST1,ITIH3,C4B,SERPINA1,TGFBI,CFB,IGFALS,PLTP,PAEP,TCAF1,HGFAC,UIMC1,PDGFC,FBN2,SELENOP,TSKU,JCHAIN,HPR,C1R,ATP1A1,APOC1,APOC2,TTN,APOC4,IGHV3-15,IGHV3-72,VEGFC,SEMA3B,PLCG2,OR52B4,HSPH1,ACTB,ITM2B,FGA
GO Process	GO:0050776	Regulation of immune response	34	844	0.55	4.66e-08	C5,HRG,C3,APOE,BPIFB1,CFHR5,AMBP,HPX,PSG9,FCN3,FCN2,MASP1,C1QB,CFHR1,COLEC10,C8A,CFHR3,CFH,ECM1,C8B,FCN1,MBL2,C1QC,MASP2,C1S,CD14,C4A,COLEC11,C4B,CFB,C1R,IGHV3-15,IGHV3-72,PLCG2
GO Process	GO:0034369	Plasma lipoprotein particle remodeling	9	29	1.44	5.76e-08	APOB,APOA1,APOE,LCAT,PLA2G7,APOM,PLTP,APOC1,APOC2
GO Process	GO:0051917	Regulation of fibrinolysis	8	18	1.6	5.76e-08	HRG,F12,THBS1,KLKB1,F2,PLG,SERPINF2,F11
GO Process	GO:0002460	Adaptive immune response based on somatic recombination of immune receptors built from immunoglobulin superfamily domains	16	169	0.92	5.80e-08	C5,C3,TCIRG1,PSG9,C1QB,C8A,C8B,MBL2,C1QC,MASP2,C1S,C4A,C4B,C1R,IGHV3-15,IGHV3-72
GO Process	GO:0007155	Cell adhesion	36	965	0.52	9.06e-08	VCL,LGALS1,MYH9,PLEK,PXDN,THBS1,VWF,ATRN,CSTA,AMBP,HABP2,FERMT3,DSC2,ANTXR1,FGB,STAB1,TLN1,PSG11,FBN1,FGG,LIMS1,THBS4,FBLN5,COL18A1,PCDH18,ADAM12,FLNA,ADAMTS13,CCN5,ACTN1,ILK,PSG2,TGFBI,IGFALS,ACTB,FGA
GO Process	GO:0002220	Innate immune response activating cell surface receptor signaling pathway	9	32	1.4	1.12e-07	FCN3,FCN2,MASP1,COLEC10,FCN1,MBL2,MASP2,COLEC11,PLCG2
GO Process	GO:0048519	Negative regulation of biological process	103	5313	0.24	1.17e-07	SPP2,ST6GAL1,VCL,SERPIND1,MYH9,TFPI2,C5,SPARC,HRG,IGFBP5,PLEK,APOA1,CAT,C3,APOE,PXDN,BPIFB1,F12,APCS,THBS1,SERPINA10,TIMP2,LCN1,CSTA,KLKB1,AMBP,ITIH4,PSG9,FCN3,ITIH1,CRH,CSF1R,PIP,SCGB3A1,SERPINI1,PF4,MASP1,GUCY1A1,A2ML1,SLC27A4,ANGPTL4,ANTXR1,PCSK9,FGB,F2,PLG,PRG2,STAB1,SUZ12,SUMO4,SERPINF2,FBN1,FBLN1,SERPINA11,ANG,FGG,LIMS1,THBS4,SLPI,PAPLN,BLM,ITIH2,COL18A1,HTRA1,ECM1,FLNA,RBP4,FCN1,CCN5,MBL2,C1QC,APOM,IGF1,PROS1,EFEMP1,TNRC6A,YWHAZ,ILK,SAA1,F11,LTBP1,ADIPOQ,C4A,CDK3,MST1,ITIH3,C4B,SERPINA1,TGFBI,PAEP,TCAF1,UIMC1,FBN2,TSKU,ATP1A1,APOC1,APOC2,VEGFC,SEMA3B,PLCG2,ACTB,ITM2B,FGA
GO Process	GO:0032102	Negative regulation of response to external stimulus	22	387	0.7	2.31e-07	ST6GAL1,C5,HRG,APOA1,APOE,F12,APCS,THBS1,KLKB1,FGB,F2,PLG,SERPINF2,FGG,HTRA1,IGF1,PROS1,SAA1,F11,ADIPOQ,SEMA3B,FGA
GO Process	GO:0032501	Multicellular organismal process	117	6490	0.2	2.39e-07	SPP2,VCL,SERPIND1,LGALS1,MYH9,F9,TFPI2,C5,PF4V1,MGP,HRG,APOB,IGFBP5,PLEK,APOA1,CAT,C3,APOE,PXDN,ANGPTL6,F12,THBS1,LYZ,VWF,SERPINA10,ATRN,LCN1,LCAT,KLKB1,F13A1,DNAH5,AMBP,TCIRG1,PSG9,PLA2G7,CRH,FERMT3,SEC24D,DSC2,CSF1R,PIP,PSG6,SERPINI1,PF4,GUCY1A1,SLC27A4,ANGPTL4,ANTXR1,PCSK9,BMP1,FGB,F2,PLG,PSG1,TLN1,SUZ12,PSG11,SERPINF2,FBN1,FBLN1,ANG,FGG,THBS4,COL18A1,PCDH18,F13B,F5,S100A7,HTRA1,ECM1,FLNA,CRTAC1,SMARCA1,RBP4,ADAMTS13,F10,F7,APOM,CA6,ADGRG2,SOD3,IGF1,PROS1,ACTN1,EFEMP1,YWHAZ,ILK,PSG4,SAA1,F11,PSG2,ADIPOQ,FSIP2,MST1,SERPINA1,TGFBI,PLTP,HGFAC,PDGFC,FBN2,SELENOP,TSKU,JCHAIN,ATP1A1,MYL6,APOC1,APOC2,TTN,VEGFC,SEMA3B,PLCG2,OR52B4,TPM4,ACTB,ITM2B,FGA,TPM3
GO Process	GO:0002250	Adaptive immune response	21	359	0.72	3.38e-07	C5,C3,TCIRG1,PSG9,FGB,C1QB,C8A,C8B,MBL2,C1QC,RAPGEF4,MASP2,C1S,C4A,C4B,JCHAIN,C1R,IGKV2D-28,IGHV3-15,IGHV3-72,FGA
GO Process	GO:0097006	Regulation of plasma lipoprotein particle levels	10	54	1.22	3.85e-07	APOB,APOA1,APOE,LCAT,PLA2G7,APOM,ADIPOQ,PLTP,APOC1,APOC2
GO Process	GO:0051246	Regulation of protein metabolic process	63	2622	0.33	6.38e-07	SPP2,SERPIND1,MYH9,TFPI2,C5,HRG,IGFBP5,APOA1,C3,DHX29,APOE,F12,APCS,THBS1,SERPINA10,TIMP2,LCN1,CSTA,KLKB1,AMBP,CENPE,HPX,ITIH4,ITIH1,CSF1R,SERPINI1,CAMP,A2ML1,ANTXR1,SH3D19,PCSK9,F2,INHBC,SERPINF2,FBN1,FBLN1,SERPINA11,ANG,THBS4,SLPI,PAPLN,BLM,ITIH2,ECM1,FLNA,IGF1,PROS1,TNRC6A,ILK,ADIPOQ,C4A,MST1,ITIH3,C4B,SERPINA1,PAEP,PDGFC,TTN,VEGFC,PLCG2,ACTB,ITM2B,FGA
GO Process	GO:0006957	Complement activation, alternative pathway	7	16	1.59	7.13e-07	C5,C3,CFHR5,C8A,CFH,C8B,CFB
GO Process	GO:0019730	Antimicrobial humoral response	14	149	0.92	7.27e-07	PF4V1,HRG,LYZ,PPBP,PF4,CAMP,FGB,F2,ANG,SLPI,S100A7,COLEC11,JCHAIN,FGA
GO Process	GO:0002526	Acute inflammatory response	11	80	1.09	9.29e-07	F12,APCS,KLKB1,ITIH4,F2,SERPINF2,MBL2,F7,SAA1,SERPINA1,HPR
GO Process	GO:0002443	Leukocyte mediated immunity	16	211	0.83	9.45e-07	C5,C3,TCIRG1,F2,C1QB,C8A,C8B,MBL2,C1QC,MASP2,C1S,C4A,C4B,C1R,IGHV3-15,IGHV3-72
GO Process	GO:0051873	Killing by host of symbiont cells	8	29	1.39	1.00e-06	HRG,CFHR5,PF4,CAMP,F2,CFHR1,APOL1,MBL2
GO Process	GO:1900024	Regulation of substrate adhesion-dependent cell spreading	10	61	1.16	1.00e-06	ST6GAL1,APOA1,FGB,P4HB,FBLN1,FGG,LIMS1,FLNA,ILK,FGA
GO Process	GO:0050789	Regulation of biological process	172	11655	0.12	1.24e-06	SPP2,ST6GAL1,VCL,SERPIND1,LGALS1,MYH9,TPD52L2,TFPI2,C5,PF4V1,MGP,SPARC,HRG,APOB,IGFBP5,PLEK,APOA1,TTR,CAT,C3,DHX29,APOE,PXDN,BPIFB1,F12,APCS,CFHR5,THBS1,VWF,PSMA6,SERPINA10,TIMP2,ATRN,LCN1,LCAT,CSTA,KLKB1,AMBP,CENPE,TCIRG1,HPX,ITIH4,PSG9,FCN3,ITIH1,PLA2G7,CRH,FERMT3,DSC2,CSF1R,PIP,FCN2,PSG6,SCGB3A1,SERPINI1,PPBP,PF4,MASP1,CAMP,GUCY1A1,A2ML1,SLC27A4,LALBA,ANGPTL4,ANTXR1,SH3D19,PCSK9,BMP1,FGB,F2,INHBC,PLG,PSG1,PRG2,STAB1,C1QB,CFHR1,TLN1,SUZ12,SUMO4,SERPINF2,HBA1,FBN1,P4HB,FBLN1,COLEC10,SERPINA11,ANG,FGG,LIMS1,THBS4,SLPI,FBLN5,PAPLN,BLM,ITIH2,COL18A1,C8A,CFHR3,CFH,ADAM12,S100A7,HTRA1,ADAMTSL4,ECM1,FLNA,SMARCA1,C8B,RBP4,FCN1,ADAMTS13,CCN5,MBL2,C1QC,F10,F7,APOM,ADGRG2,GOLM1,IGF1,PROS1,ACTN1,EFEMP1,TNRC6A,YWHAZ,ILK,RAPGEF4,MASP2,PSG4,SAA1,F11,C1S,CD14,LTBP1,ADIPOQ,C4A,SLAIN1,CDK3,COLEC11,MST1,ITIH3,C4B,SERPINA1,TGFBI,CFB,IGFALS,PLTP,PAEP,TCAF1,UIMC1,PDGFC,FBN2,SELENOP,TSKU,JCHAIN,HPR,C1R,ATP1A1,APOC1,APOC2,TTN,APOC4,IGHV3-15,IGHV3-72,VEGFC,SEMA3B,PLCG2,OR52B4,HSPH1,ACTB,ITM2B,FGA
GO Process	GO:0051702	Biological process involved in interaction with symbiont	12	108	0.99	1.56e-06	HRG,APOE,APCS,CFHR5,CSF1R,PF4,CAMP,F2,PLG,CFHR1,APOL1,MBL2

GO Process	GO:0065008	Regulation of biological quality	77	3654	0.27	1.76e-06	VCL,SERPIND1,MYH9,F9,TFPI2,PF4V1,HRG,APOB,IGFBP5,PLEK,APOA1,TTR,APOE,F12,THBS1,LYZ,VWF,SERPINA10,LCN1,LCAT,CP,KLKB1,F13A1,TCIRG1,HPX,PLA2G7,CRH,FERMT3,DSC2,CSF1R,PIP,PF4,GUCY1A1,ANGPTL4,PCSK9,FGB,F2,PLG,TLN1,SERPINF2,FBN1,FBLN1,OIT3,ANG,FGG,F13B,F5,FLNA,RBP4,ADAMTS13,F10,F7,APOM,SOD3,IGF1,PROS1,YWHAZ,ILK,RAPGEF4,SAA1,F11,ADIPOQ,ATP9B,SERPINA1,HGFAC,PDGFC,FBN2,TSKU,JCHAIN,ATP1A1,APOC2,APOC4,VEGFC,SEMA3B,PLCG2,ACTB,FGA
GO Process	GO:0080134	Regulation of response to stress	41	1373	0.42	2.12e-06	LGALS1,HRG,APOA1,C3,APOE,F12,APCS,THBS1,PSMA6,KLKB1,AMBP,HPX,FCN3,PLA2G7,FCN2,MASP1,FGB,F2,PLG,SERPINF2,P4HB,COLEC10,FGG,FBLN5,CFH,HTRA1,FLNA,FCN1,MBL2,F7,IGF1,PROS1,MASP2,SAA1,F11,ADIPOQ,COLEC11,UIMC1,PLCG2,ACTB,FGA
GO Process	GO:0009617	Response to bacterium	27	663	0.56	2.33e-06	PF4V1,APOB,C3,LYZ,FCN2,PPBP,PF4,CAMP,LALBA,FGB,F2,PRG2,STAB1,ANG,SLPI,S100A7,ADAMTS13,MBL2,CD14,ADIPOQ,C4B,CFB,JCHAIN,IGHV3-15,IGHV3-72,PLCG2,FGA
GO Process	GO:0051050	Positive regulation of transport	32	915	0.49	3.19e-06	APOA1,C3,APOE,FCN3,CRH,FCN2,MASP1,PCSK9,FGB,F2,COLEC10,ANG,FGG,FLNA,RBP4,FCN1,MBL2,IGF1,MASP2,CD14,ADIPOQ,C4A,COLEC11,C4B,CFB,PLTP,TCAF1,TTN,VEGFC,PLCG2,ACTB,FGA
GO Process	GO:0051239	Regulation of multicellular organismal process	63	2749	0.31	3.26e-06	VCL,C5,MGP,SPARC,HRG,IGFBP5,APOA1,C3,APOE,F12,APCS,THBS1,ATRN,LCAT,KLKB1,PSG9,CRH,DSC2,CSF1R,PF4,CAMP,GUCY1A1,ANGPTL4,PCSK9,BMP1,FGB,F2,PLG,PRG2,STAB1,SERPINF2,FBN1,FBLN1,FGG,THBS4,ADAM12,ECM1,RBP4,FCN1,C1QC,F7,APOM,IGF1,PROS1,EFEMP1,SAA1,F11,CD14,LTBP1,ADIPOQ,MST1,PAEP,PDGFC,FBN2,TSKU,ATP1A1,APOC1,APOC2,VEGFC,SEMA3B,PLCG2,ACTB,FGA
GO Process	GO:0051838	Cytolysis by host of symbiont cells	6	12	1.65	4.40e-06	HRG,CFHR5,CAMP,F2,CFHR1,APOL1
GO Process	GO:0060627	Regulation of vesicle-mediated transport	24	551	0.59	4.49e-06	APOA1,C3,APOE,FCN3,FCN2,MASP1,PCSK9,FGB,COLEC10,FGG,FCN1,MBL2,RAPGEF4,MASP2,CD14,ADIPOQ,C4A,COLEC11,C4B,CFB,APOC1,APOC2,PLCG2,FGA
GO Process	GO:0034381	Plasma lipoprotein particle clearance	7	24	1.41	5.91e-06	APOB,APOA1,APOE,APOM,ADIPOQ,APOC1,APOC2
GO Process	GO:0007597	Blood coagulation, intrinsic pathway	6	13	1.61	6.19e-06	F9,F12,KLKB1,FLNA,F7,F11
GO Process	GO:0010810	Regulation of cell-substrate adhesion	15	217	0.79	7.82e-06	ST6GAL1,VCL,HRG,APOA1,THBS1,FGB,PLG,TLN1,P4HB,FBLN1,FGG,LIMS1,FLNA,ILK,FGA
GO Process	GO:0033344	Cholesterol efflux	7	27	1.36	1.14e-05	APOB,APOA1,APOE,APOM,TSKU,APOC1,APOC2
GO Process	GO:0030194	Positive regulation of blood coagulation	7	28	1.35	1.40e-05	HRG,F12,THBS1,F2,PLG,SERPINF2,F7
GO Process	GO:0034375	High-density lipoprotein particle remodeling	6	16	1.52	1.58e-05	APOA1,APOE,LCAT,APOM,PLTP,APOC1
GO Process	GO:1900026	Positive regulation of substrate adhesion-dependent cell spreading	8	45	1.2	1.65e-05	APOA1,FGB,P4HB,FGG,LIMS1,FLNA,ILK,FGA
GO Process	GO:0042742	Defense response to bacterium	17	306	0.69	1.88e-05	LYZ,FCN2,PPBP,CAMP,LALBA,FGB,F2,PRG2,STAB1,ANG,SLPI,S100A7,MBL2,JCHAIN,IGHV3-15,IGHV3-72,FGA
GO Process	GO:0031347	Regulation of defense response	24	638	0.52	5.38e-05	LGALS1,APOA1,C3,APOE,F12,APCS,PSMA6,KLKB1,HPX,FCN3,PLA2G7,FCN2,MASP1,COLEC10,CFH,HTRA1,FCN1,MBL2,IGF1,MASP2,SAA1,ADIPOQ,COLEC11,PLCG2
GO Process	GO:0002221	Pattern recognition receptor signaling pathway	10	101	0.94	6.04e-05	FCN3,FCN2,MASP1,COLEC10,FCN1,MBL2,MASP2,CD14,COLEC11,PLCG2
GO Process	GO:0002699	Positive regulation of immune effector process	15	264	0.7	7.66e-05	C3,HPX,FCN3,FCN2,MASP1,COLEC10,RBP4,FCN1,MBL2,MASP2,C4A,COLEC11,C4B,CFB,PLCG2
GO Process	GO:0065009	Regulation of molecular function	64	3085	0.27	7.83e-05	SPP2,SERPIND1,TFPI2,C5,HRG,PLEK,APOA1,CAT,C3,APOE,PXDN,APCS,CFHR5,THBS1,PSMA6,SERPINA10,TIMP2,LCN1,CSTA,AMBP,CENPE,ITIH4,ITIH1,CRH,CSF1R,SERPINI1,A2ML1,SLC27A4,ANGPTL4,ANTXR1,PCSK9,F2,CFHR1,SUZ12,SUMO4,SERPINF2,FBN1,FBLN1,SERPINA11,ANG,LIMS1,SLPI,PAPLN,BLM,ITIH2,ECM1,FLNA,IGF1,PROS1,RAPGEF4,ADIPOQ,C4A,MST1,ITIH3,C4B,SERPINA1,TCAF1,PDGFC,APOC1,APOC2,TTN,VEGFC,PLCG2,ACTB
GO Process	GO:0002697	Regulation of immune effector process	18	383	0.62	7.92e-05	APOA1,C3,HPX,FCN3,FCN2,MASP1,PRG2,COLEC10,CFH,RBP4,FCN1,MBL2,MASP2,C4A,COLEC11,C4B,CFB,PLCG2
GO Process	GO:0031639	Plasminogen activation	5	11	1.61	8.15e-05	KLKB1,FGB,FGG,F11,FGA
GO Process	GO:0015918	Sterol transport	9	82	0.99	9.96e-05	APOB,APOA1,APOE,LCAT,APOM,PLTP,TSKU,APOC1,APOC2
GO Process	GO:1905952	Regulation of lipid localization	12	167	0.8	0.00010	APOB,APOA1,C3,APOE,THBS1,CRH,PCSK9,ADIPOQ,PLTP,APOC1,APOC2,APOC4
GO Process	GO:0019538	Protein metabolic process	75	3910	0.23	0.00011	ST6GAL1,GNPTG,MYH9,F9,APOB,APOA1,CAT,C3,DHX29,APOE,F12,THBS1,PSMA6,LCN1,LCAT,CSTA,KLKB1,F13A1,AMBP,TCIRG1,HPX,FCN3,PLA2G7,HABP2,CSF1R,PIP,FCN2,MASP1,PCSK9,BMP1,FGB,F2,PLG,UBE3C,SUZ12,APOL1,SUMO4,P4HB,FBLN1,COLEC10,FGG,GANAB,CNDP1,F13B,CFH,F5,ADAM12,HTRA1,FLNA,FCN1,ADAMTS13,MBL2,F10,F7,APOM,IGF1,EFEMP1,YWHAZ,ILK,MASP2,F11,C1S,PCYOX1,CDK3,COLEC11,MST1,CFB,HGFAC,UIMC1,HPR,C1R,APOC1,TTN,ACTB,FGA
GO Process	GO:0050790	Regulation of catalytic activity	53	2370	0.3	0.00011	SPP2,SERPIND1,TFPI2,C5,HRG,PLEK,APOA1,C3,APOE,APCS,THBS1,SERPINA10,TIMP2,LCN1,CSTA,AMBP,CENPE,ITIH4,ITIH1,CSF1R,SERPINI1,A2ML1,SLC27A4,ANGPTL4,ANTXR1,F2,SUZ12,SERPINF2,FBN1,FBLN1,SERPINA11,ANG,LIMS1,SLPI,PAPLN,BLM,ITIH2,ECM1,IGF1,PROS1,RAPGEF4,ADIPOQ,C4A,MST1,ITIH3,C4B,SERPINA1,PDGFC,APOC1,APOC2,TTN,VEGFC,ACTB
GO Process	GO:0051919	Positive regulation of fibrinolysis	4	4	1.95	0.00012	F12,KLKB1,PLG,F11
GO Process	GO:0006953	Acute-phase response	7	42	1.17	0.00013	APCS,ITIH4,F2,SERPINF2,MBL2,SAA1,SERPINA1
GO Process	GO:0051918	Negative regulation of fibrinolysis	5	13	1.53	0.00015	HRG,THBS1,F2,PLG,SERPINF2
GO Process	GO:0030301	Cholesterol transport	8	65	1.04	0.00018	APOB,APOA1,APOE,LCAT,APOM,TSKU,APOC1,APOC2
GO Process	GO:0072377	Blood coagulation, common pathway	4	5	1.85	0.00021	F2,F5,F10,FGA
GO Process	GO:0002833	Positive regulation of response to biotic stimulus	12	187	0.76	0.00029	HRG,HPX,FCN3,FCN2,MASP1,COLEC10,FCN1,MBL2,MASP2,CD14,COLEC11,PLCG2
GO Process	GO:0022603	Regulation of anatomical structure morphogenesis	28	920	0.43	0.00029	ST6GAL1,MYH9,C5,SPARC,HRG,APOA1,C3,THBS1,CSF1R,PF4,CAMP,ANGPTL4,SH3D19,FGB,F2,STAB1,P4HB,FBLN1,FGG,LIMS1,THBS4,ADAM12,ECM1,FLNA,ILK,VEGFC,SEMA3B,FGA
GO Process	GO:1903036	Positive regulation of response to wounding	8	72	0.99	0.00035	HRG,F12,THBS1,F2,PLG,SERPINF2,FLNA,F7
GO Process	GO:0010811	Positive regulation of cell-substrate adhesion	10	128	0.84	0.00039	HRG,APOA1,FGB,P4HB,FBLN1,FGG,LIMS1,FLNA,ILK,FGA
GO Process	GO:0031589	Cell-substrate adhesion	12	195	0.74	0.00043	VCL,VWF,ATRN,FERMT3,ANTXR1,FGB,FGG,FBLN5,ADAMTS13,ACTN1,ILK,FGA
GO Process	GO:0050793	Regulation of developmental process	53	2492	0.28	0.00044	ST6GAL1,VCL,MYH9,C5,MGP,SPARC,HRG,APOB,IGFBP5,APOA1,C3,APOE,APCS,THBS1,ATRN,TCIRG1,PSG9,CSF1R,SCGB3A1,PF4,CAMP,ANGPTL4,SH3D19,BMP1,FGB,F2,STAB1,SUZ12,SERPINF2,FBN1,P4HB,FBLN1,FGG,LIMS1,THBS4,ADAM12,ECM1,FLNA,RBP4,C1QC,IGF1,EFEMP1,YWHAZ,ILK,ADIPOQ,MST1,PAEP,FBN2,TSKU,VEGFC,SEMA3B,ACTB,FGA
GO Process	GO:0022604	Regulation of cell morphogenesis	15	311	0.63	0.00046	ST6GAL1,MYH9,SPARC,APOA1,CSF1R,SH3D19,FGB,F2,P4HB,FBLN1,FGG,LIMS1,FLNA,ILK,FGA
GO Process	GO:0030155	Regulation of cell adhesion	25	784	0.45	0.00046	ST6GAL1,VCL,LGALS1,HRG,APOA1,THBS1,FERMT3,SERPINI1,FGB,PLG,TLN1,SERPINF2,P4HB,FBLN1,FGG,LIMS1,FLNA,IGF1,ILK,SAA1,ADIPOQ,TGFBI,HSPH1,ACTB,FGA
GO Process	GO:0034384	High-density lipoprotein particle clearance	4	7	1.71	0.00051	APOA1,APOE,APOM,APOC2
GO Process	GO:0043691	Reverse cholesterol transport	5	18	1.39	0.00051	APOA1,APOE,LCAT,APOM,APOC2
GO Process	GO:0045088	Regulation of innate immune response	13	238	0.69	0.00056	C3,APOE,HPX,FCN3,FCN2,MASP1,COLEC10,CFH,FCN1,MBL2,MASP2,COLEC11,PLCG2

GO Process	GO:0001775	Cell activation	23	693	0.47	0.00059	VCL,LGALS1,MYH9,PF4V1,HRG,PLEK,VWF,TCIRG1,FERMT3,PF4,CAMP,FGB,F2,TLN1,FGG,FLNA,ADAMTS13,IGF1,ILK,SAA1,PLCG2,ACTB,FGA
GO Process	GO:0002831	Regulation of response to biotic stimulus	16	361	0.59	0.00059	HRG,C3,APOE,HPX,FCN3,FCN2,MASP1,COLEC10,CFH,HTRA1,FCN1,MBL2,MASP2,CD14,COLEC11,PLCG2
GO Process	GO:0007565	Female pregnancy	11	170	0.76	0.00065	IGFBP5,AMBP,PSG9,CRH,PSG6,PSG1,PSG11,FBLN1,PSG4,PSG2,MST1
GO Process	GO:0001568	Blood vessel development	19	505	0.52	0.00074	MYH9,HRG,APOB,APOE,PXDN,ANGPTL6,THBS1,ANGPTL4,ANTXR1,PLG,SERPINF2,ANG,COL18A1,S100A7,ECM1,FLNA,YWHAZ,TGFBI,VEGFC
GO Process	GO:0051240	Positive regulation of multicellular organismal process	37	1505	0.34	0.00077	C5,HRG,C3,APOE,F12,THBS1,CRH,CSF1R,PF4,CAMP,ANGPTL4,BMP1,FGB,F2,PLG,PRG2,SERPINF2,FGG,ADAM12,ECM1,FCN1,F7,IGF1,SAA1,CD14,ADIPOQ,MST1,PAEP,PDGFC,FBN2,TSKU,ATP1A1,APOC2,VEGFC,PLCG2,ACTB,FGA
GO Process	GO:0030334	Regulation of cell migration	27	927	0.41	0.00088	VCL,C5,SPARC,HRG,IGFBP5,APOE,THBS1,PLA2G7,FERMT3,CSF1R,PLG,FBLN1,THBS4,S100A7,ECM1,FLNA,F10,F7,IGF1,ADIPOQ,MST1,TCAF1,PDGFC,FBN2,VEGFC,SEMA3B,PLCG2
GO Process	GO:0048514	Blood vessel morphogenesis	17	419	0.56	0.00088	MYH9,HRG,APOB,APOE,PXDN,ANGPTL6,THBS1,ANGPTL4,SERPINF2,ANG,COL18A1,S100A7,ECM1,FLNA,YWHAZ,TGFBI,VEGFC
GO Process	GO:0045089	Positive regulation of innate immune response	10	144	0.79	0.00093	HPX,FCN3,FCN2,MASP1,COLEC10,FCN1,MBL2,MASP2,COLEC11,PLCG2
GO Process	GO:0008228	Opsonization	4	9	1.6	0.00100	C3,FCN2,MBL2,C4B
GO Process	GO:0034372	Very-low-density lipoprotein particle remodeling	4	9	1.6	0.00100	APOA1,APOE,LCAT,APOC2
GO Process	GO:0035295	Tube development	26	880	0.42	0.00100	MYH9,HRG,APOB,IGFBP5,CAT,C3,APOE,PXDN,ANGPTL6,THBS1,CRH,CSF1R,ANGPTL4,SERPINF2,ANG,COL18A1,S100A7,ECM1,FLNA,RBP4,YWHAZ,ILK,MST1,TGFBI,PDGFC,VEGFC
GO Process	GO:0008203	Cholesterol metabolic process	9	119	0.83	0.0013	APOB,APOA1,CAT,APOE,LCAT,PCSK9,APOL1,TSKU,APOC1
GO Process	GO:0032374	Regulation of cholesterol transport	7	64	0.99	0.0013	APOA1,APOE,PCSK9,ADIPOQ,PLTP,APOC1,APOC2
GO Process	GO:0034377	Plasma lipoprotein particle assembly	5	23	1.29	0.0013	APOB,APOA1,APOE,APOM,APOC1
GO Process	GO:0042632	Cholesterol homeostasis	8	89	0.9	0.0013	APOB,APOA1,APOE,LCAT,PCSK9,APOM,TSKU,APOC2
GO Process	GO:0098869	Cellular oxidant detoxification	8	91	0.89	0.0014	CAT,APOE,PXDN,AMBP,HBA1,APOM,SOD3,GPX3
GO Process	GO:1901564	Organonitrogen compound metabolic process	85	4981	0.18	0.0014	ST6GAL1,GNPTG,MYH9,F9,APOB,APOA1,TTR,CAT,C3,DHX29,APOE,PXDN,F12,THBS1,PSMA6,BLVRB,LCN1,LCAT,CSTA,KLKB1,F13A1,AMBP,TCIRG1,HPX,ITIH4,FCN3,ITIH1,PLA2G7,HABP2,CSF1R,PIP,FCN2,MASP1,GUCY1A1,PCSK9,BMP1,FGB,F2,PLG,UBE3C,NDUFS8,SUZ12,APOL1,SUMO4,P4HB,FBLN1,COLEC10,FGG,GANAB,ITIH2,CNDP1,F13B,CFH,F5,ADAM12,HTRA1,FLNA,FCN1,ADAMTS13,MBL2,F10,F7,APOM,IGF1,EFEMP1,YWHAZ,ILK,MASP2,F11,C1S,PCYOX1,ACSF2,CDK3,COLEC11,MST1,ITIH3,CFB,HGFAC,UIMC1,HPR,C1R,APOC1,TTN,ACTB,FGA
GO Process	GO:0002768	Immune response-regulating cell surface receptor signaling pathway	12	228	0.67	0.0015	PSG9,FCN3,FCN2,MASP1,COLEC10,FCN1,MBL2,MASP2,COLEC11,IGHV3-15,IGHV3-72,PLCG2
GO Process	GO:0034114	Regulation of heterotypic cell-cell adhesion	5	24	1.27	0.0015	APOA1,FGB,FGG,ADIPOQ,FGA
GO Process	GO:0050920	Regulation of chemotaxis	12	227	0.67	0.0015	ST6GAL1,C5,HRG,THBS1,PLA2G7,CSF1R,THBS4,S100A7,F7,MST1,VEGFC,SEMA3B
GO Process	GO:0009892	Negative regulation of metabolic process	58	2982	0.24	0.0016	SPP2,SERPIND1,TFPI2,C5,HRG,IGFBP5,PLEK,APOA1,C3,APOE,APCS,THBS1,SERPINA10,TIMP2,LCN1,CSTA,AMBP,ITIH4,PSG9,FCN3,ITIH1,SERPINI1,PF4,A2ML1,SLC27A4,PCSK9,F2,PRG2,SUZ12,SUMO4,SERPINF2,FBLN1,SERPINA11,ANG,LIMS1,SLPI,PAPLN,BLM,ITIH2,ECM1,FLNA,IGF1,PROS1,TNRC6A,YWHAZ,ADIPOQ,C4A,MST1,ITIH3,C4B,SERPINA1,UIMC1,TSKU,ATP1A1,APOC1,APOC2,ITM2B,FGA
GO Process	GO:0098609	Cell-cell adhesion	19	542	0.49	0.0016	VCL,LGALS1,MYH9,PLEK,CSTA,FERMT3,DSC2,FGB,TLN1,PSG11,FGG,LIMS1,THBS4,PCDH18,FLNA,ILK,PSG2,ACTB,FGA
GO Process	GO:0040012	Regulation of locomotion	28	1032	0.38	0.0018	ST6GAL1,VCL,C5,SPARC,HRG,IGFBP5,APOE,THBS1,PLA2G7,FERMT3,CSF1R,PLG,FBLN1,THBS4,S100A7,ECM1,FLNA,F10,F7,IGF1,ADIPOQ,MST1,TCAF1,PDGFC,FBN2,VEGFC,SEMA3B,PLCG2
GO Process	GO:0002429	Immune response-activating cell surface receptor signaling pathway	11	197	0.7	0.0020	FCN3,FCN2,MASP1,COLEC10,FCN1,MBL2,MASP2,COLEC11,IGHV3-15,IGHV3-72,PLCG2
GO Process	GO:0002688	Regulation of leukocyte chemotaxis	9	128	0.8	0.0020	C5,THBS1,PLA2G7,CSF1R,THBS4,S100A7,F7,MST1,VEGFC
GO Process	GO:0006910	Phagocytosis, recognition	5	26	1.23	0.0020	FCN3,FCN2,FCN1,IGHV3-15,IGHV3-72
GO Process	GO:0055088	Lipid homeostasis	10	161	0.74	0.0020	APOB,APOA1,APOE,LCAT,ANGPTL4,PCSK9,APOM,TSKU,APOC2,APOC4
GO Process	GO:0015850	Organic hydroxy compound transport	10	163	0.74	0.0021	APOB,APOA1,APOE,LCAT,RBP4,APOM,PLTP,TSKU,APOC1,APOC2
GO Process	GO:0033700	Phospholipid efflux	4	12	1.47	0.0021	APOA1,APOE,APOC1,APOC2
GO Process	GO:0006066	Alcohol metabolic process	14	324	0.58	0.0023	APOB,APOA1,TTR,CAT,APOE,LCAT,PCSK9,APOL1,ISYNA1,RBP4,IGF1,TSKU,APOC1,PLCG2
GO Process	GO:0001525	Angiogenesis	14	325	0.58	0.0024	MYH9,HRG,PXDN,ANGPTL6,THBS1,ANGPTL4,ANG,COL18A1,S100A7,ECM1,FLNA,YWHAZ,TGFBI,VEGFC
GO Process	GO:0007229	Integrin-mediated signaling pathway	8	100	0.85	0.0024	MYH9,PLEK,APOA1,FERMT3,TLN1,ADAM12,ADAMTS13,ILK
GO Process	GO:0006869	Lipid transport	14	329	0.58	0.0027	APOB,APOA1,APOE,LCAT,SLC27A4,APOL1,RBP4,APOM,ATP9B,PLTP,TSKU,APOC1,APOC2,APOC4
GO Process	GO:0048585	Negative regulation of response to stimulus	37	1612	0.31	0.0027	ST6GAL1,C5,HRG,IGFBP5,PLEK,APOA1,APOE,PXDN,BPIFB1,F12,APCS,THBS1,KLKB1,AMBP,PF4,MASP1,SLC27A4,FGB,F2,PLG,SERPINF2,FBN1,FBLN1,FGG,HTRA1,ECM1,IGF1,PROS1,SAA1,F11,LTBP1,ADIPOQ,CDK3,FBN2,TSKU,SEMA3B,FGA
GO Process	GO:0045765	Regulation of angiogenesis	13	288	0.6	0.0029	C5,SPARC,HRG,C3,THBS1,PF4,CAMP,ANGPTL4,STAB1,THBS4,ADAM12,ECM1,VEGFC
GO Process	GO:0007160	Cell-matrix adhesion	9	136	0.77	0.0030	VCL,FERMT3,FGB,FGG,FBLN5,ADAMTS13,ACTN1,ILK,FGA
GO Process	GO:0032369	Negative regulation of lipid transport	5	30	1.17	0.0032	APOE,THBS1,PCSK9,APOC1,APOC2
GO Process	GO:0032375	Negative regulation of cholesterol transport	4	14	1.4	0.0032	APOE,PCSK9,APOC1,APOC2
GO Process	GO:0051049	Regulation of transport	39	1763	0.29	0.0033	APOA1,C3,APOE,THBS1,TCIRG1,FCN3,CRH,FCN2,MASP1,PCSK9,FGB,F2,COLEC10,ANG,FGG,FLNA,RBP4,FCN1,MBL2,IGF1,RAPGEF4,MASP2,SAA1,CD14,ADIPOQ,C4A,COLEC11,C4B,CFB,PLTP,TCAF1,ATP1A1,APOC1,APOC2,TTN,VEGFC,PLCG2,ACTB,FGA
GO Process	GO:0032368	Regulation of lipid transport	9	139	0.76	0.0034	APOA1,APOE,THBS1,CRH,PCSK9,ADIPOQ,PLTP,APOC1,APOC2
GO Process	GO:0031349	Positive regulation of defense response	13	296	0.59	0.0036	LGALS1,C3,HPX,FCN3,PLA2G7,FCN2,MASP1,COLEC10,FCN1,MBL2,MASP2,COLEC11,PLCG2
GO Process	GO:0035583	Sequestering of TGFbeta in extracellular matrix	3	4	1.82	0.0036	FBN1,LTBP1,FBN2
GO Process	GO:0010543	Regulation of platelet activation	6	53	1.0	0.0038	HRG,PLEK,APOE,F2,FGG,FLNA
GO Process	GO:1905954	Positive regulation of lipid localization	8	111	0.81	0.0043	APOB,APOA1,C3,APOE,CRH,ADIPOQ,PLTP,APOC4
GO Process	GO:0061844	Antimicrobial humoral immune response mediated by antimicrobial peptide	8	113	0.8	0.0049	PF4V1,HRG,PPBP,PF4,CAMP,F2,ANG,S100A7
GO Process	GO:0048522	Positive regulation of cellular process	90	5584	0.16	0.0053	ST6GAL1,LGALS1,SPARC,HRG,APOB,IGFBP5,PLEK,APOA1,CAT,C3,DHX29,APOE,THBS1,VWF,CENPE,TCIRG1,HPX,FCN3,PLA2G7,CRH,FERMT3,CSF1R,FCN2,SCGB3A1,SERPINI1,PPBP,PF4,MASP1,CAMP,GUCY1A1,SLC27A4,SH3D19,PCSK9,FGB,F2,INHBC,PLG,SUZ12,SERPINF2,HBA1,FBN1,P4HB,FBLN1,COLEC10,ANG,FGG,LIMS1,THBS4,BLM,S100A7,HTRA1,ADAMTSL4,ECM1,FLNA,SMARCA1,RBP4,FCN1,MBL2,F10,F7,IGF1,ACTN1,TNRC6A,ILK,MASP2,SAA1,CD14,ADIPOQ,C4A,SLAIN1,COLEC11,MST1,C4B,CFB,TCAF1,UIMC1,PDGFC,FBN2,HPR,APOC1,APOC2,TTN,IGHV3-15,IGHV3-72,VEGFC,SEMA3B,PLCG2,HSPH1,ACTB,FGA
GO Process	GO:0034382	Chylomicron remnant clearance	3	5	1.73	0.0055	APOE,APOC1,APOC2

GO Process	GO:0002764	Immune response-regulating signaling pathway	13	312	0.57	0.0057	PSG9,FCN3,FCN2,MASP1,COLEC10,FCN1,MBL2,MASP2,CD14,COLEC11,IGHV3-15,IGHV3-72,PLCG2
GO Process	GO:1902533	Positive regulation of intracellular signal transduction	26	997	0.36	0.0057	LGALS1,IGFBP5,APOA1,CAT,APOE,THBS1,VWF,CSF1R,GUCY1A1,FGB,F2,SERPINF2,FGG,LIMS1,S100A7,ECM1,FLNA,F10,F7,IGF1,ILK,CD14,ADIPOQ,PDGFC,PLCG2,FGA
GO Process	GO:0060284	Regulation of cell development	17	506	0.47	0.0062	VCL,APOA1,FGB,F2,FBN1,P4HB,FBLN1,FGG,LIMS1,FLNA,IGF1,ILK,ADIPOQ,PAEP,VEGFC,SEMA3B,FGA
GO Process	GO:0043903	Regulation of biological process involved in symbiotic interaction	6	60	0.95	0.0068	LGALS1,APCS,FCN3,P4HB,FBLN1,FCN1
GO Process	GO:0051004	Regulation of lipoprotein lipase activity	4	18	1.29	0.0068	APOA1,ANGPTL4,APOC1,APOC2
GO Process	GO:0035239	Tube morphogenesis	20	669	0.42	0.0069	MYH9,HRG,APOB,APOE,PXDN,ANGPTL6,THBS1,CSF1R,ANGPTL4,SERPINF2,ANG,COL18A1,S100A7,ECM1,FLNA,YWHAZ,ILK,MST1,TGFBI,VEGFC
GO Process	GO:0032879	Regulation of localization	43	2103	0.26	0.0070	VCL,APOB,APOA1,C3,APOE,THBS1,TCIRG1,FCN3,CRH,FCN2,MASP1,PCSK9,FGB,F2,SUMO4,COLEC10,ANG,FGG,FLNA,RBP4,FCN1,MBL2,IGF1,RAPGEF4,MASP2,SAA1,CD14,ADIPOQ,C4A,COLEC11,C4B,CFB,PLTP,TCAF1,ATP1A1,APOC1,APOC2,TTN,APOC4,VEGFC,PLCG2,ACTB,FGA
GO Process	GO:0006979	Response to oxidative stress	14	368	0.53	0.0074	CAT,APOE,PXDN,ATRN,AMBP,NDUFS8,SUMO4,HBA1,S100A7,MBL2,SOD3,GPX3,ADIPOQ,SELENOP
GO Process	GO:0072359	Circulatory system development	24	901	0.37	0.0077	MYH9,HRG,APOB,APOE,PXDN,ANGPTL6,THBS1,DNAH5,ANGPTL4,ANTXR1,PLG,SERPINF2,FBN1,ANG,COL18A1,S100A7,ECM1,FLNA,RBP4,YWHAZ,ILK,TGFBI,TTN,VEGFC
GO Process	GO:0042157	Lipoprotein metabolic process	8	123	0.76	0.0079	APOB,APOA1,APOE,LCAT,PCSK9,APOL1,APOM,APOC1
GO Process	GO:0070328	Triglyceride homeostasis	5	38	1.07	0.0079	APOA1,APOE,ANGPTL4,APOC2,APOC4
GO Process	GO:0010628	Positive regulation of gene expression	28	1146	0.34	0.0084	MYH9,C5,APOB,C3,DHX29,F12,THBS1,KLKB1,HPX,PSG9,CSF1R,PIP,PF4,CAMP,PRG2,SERPINF2,FBLN1,LIMS1,RBP4,FCN1,IGF1,SAA1,CD14,ADIPOQ,MST1,PAEP,TTN,PLCG2
GO Process	GO:0051172	Negative regulation of nitrogen compound metabolic process	47	2403	0.24	0.0086	SPP2,SERPIND1,TFPI2,C5,HRG,IGFBP5,C3,APOE,APCS,THBS1,SERPINA10,TIMP2,LCN1,CSTA,AMBP,ITIH4,FCN3,ITIH1,SERPINI1,A2ML1,F2,SUZ12,SUMO4,SERPINF2,FBLN1,SERPINA11,ANG,LIMS1,SLPI,PAPLN,BLM,ITIH2,ECM1,FLNA,IGF1,PROS1,TNRC6A,YWHAZ,ADIPOQ,C4A,ITIH3,C4B,SERPINA1,UIMC1,APOC1,ITM2B,FGA
GO Process	GO:0001817	Regulation of cytokine production	21	739	0.4	0.0087	C5,APOA1,C3,THBS1,PSG9,CSF1R,PF4,CAMP,F2,PRG2,SERPINF2,FBLN1,FCN1,IGF1,SAA1,CD14,LTBP1,ADIPOQ,PAEP,TSKU,PLCG2
GO Process	GO:0071634	Regulation of transforming growth factor beta production	5	39	1.06	0.0087	THBS1,SERPINF2,FBLN1,LTBP1,TSKU
GO Process	GO:0010605	Negative regulation of macromolecule metabolic process	52	2760	0.22	0.0088	SPP2,SERPIND1,TFPI2,C5,HRG,IGFBP5,APOA1,C3,APOE,APCS,THBS1,SERPINA10,TIMP2,LCN1,CSTA,AMBP,ITIH4,PSG9,FCN3,ITIH1,SERPINI1,PF4,A2ML1,PCSK9,F2,PRG2,SUZ12,SUMO4,SERPINF2,FBLN1,SERPINA11,ANG,LIMS1,SLPI,PAPLN,BLM,ITIH2,ECM1,FLNA,IGF1,PROS1,TNRC6A,YWHAZ,ADIPOQ,C4A,ITIH3,C4B,SERPINA1,UIMC1,TSKU,ITM2B,FGA
GO Process	GO:0019731	Antibacterial humoral response	6	64	0.92	0.0088	CAMP,FGB,ANG,SLPI,JCHAIN,FGA
GO Process	GO:0030335	Positive regulation of cell migration	17	529	0.46	0.0096	SPARC,IGFBP5,THBS1,PLA2G7,FERMT3,CSF1R,PLG,FBLN1,THBS4,S100A7,F10,F7,IGF1,PDGFC,VEGFC,SEMA3B,PLCG2
GO Process	GO:1901615	Organic hydroxy compound metabolic process	16	478	0.47	0.0096	APOB,APOA1,TTR,CAT,APOE,LCAT,PCSK9,APOL1,ISYNA1,RBP4,IGF1,PLTP,GC,TSKU,APOC1,PLCG2
GO Process	GO:0043654	Recognition of apoptotic cell	3	7	1.58	0.0104	FCN3,FCN2,FCN1
GO Process	GO:0045785	Positive regulation of cell adhesion	16	485	0.47	0.0111	LGALS1,HRG,APOA1,FGB,SERPINF2,P4HB,FBLN1,FGG,LIMS1,FLNA,IGF1,ILK,SAA1,HSPH1,ACTB,FGA
GO Process	GO:0050714	Positive regulation of protein secretion	8	131	0.73	0.0112	FGB,ANG,FGG,RBP4,IGF1,TTN,VEGFC,FGA
GO Process	GO:0050708	Regulation of protein secretion	11	250	0.59	0.0116	APOE,TCIRG1,FGB,ANG,FGG,RBP4,IGF1,SAA1,TTN,VEGFC,FGA
GO Process	GO:0051093	Negative regulation of developmental process	24	933	0.36	0.0119	SPARC,HRG,IGFBP5,APOE,APCS,THBS1,PF4,F2,STAB1,SUZ12,FBN1,FBLN1,THBS4,ECM1,RBP4,C1QC,IGF1,EFEMP1,ADIPOQ,PAEP,TSKU,VEGFC,SEMA3B,ACTB
GO Process	GO:0002690	Positive regulation of leukocyte chemotaxis	7	99	0.8	0.0121	THBS1,PLA2G7,CSF1R,THBS4,S100A7,F7,VEGFC
GO Process	GO:0070372	Regulation of ERK1 and ERK2 cascade	12	299	0.55	0.0132	APOE,CSF1R,FGB,SERPINF2,FBLN1,FGG,S100A7,IGF1,YWHAZ,ADIPOQ,PDGFC,FGA
GO Process	GO:0007166	Cell surface receptor signaling pathway	41	2040	0.25	0.0137	MYH9,C5,PF4V1,IGFBP5,PLEK,APOA1,C3,HPX,PSG9,FCN3,FERMT3,CSF1R,FCN2,PPBP,PF4,MASP1,F2,INHBC,TLN1,P4HB,COLEC10,LIMS1,ADAM12,FLNA,FCN1,ADAMTS13,MBL2,ADGRG2,IGF1,EFEMP1,ILK,MASP2,CD14,LTBP1,COLEC11,PDGFC,IGHV3-15,IGHV3-72,VEGFC,SEMA3B,PLCG2
GO Process	GO:2000427	Positive regulation of apoptotic cell clearance	3	8	1.52	0.0137	C3,C4A,C4B
GO Process	GO:0019835	Cytolysis	4	23	1.19	0.0138	C5,LYZ,C8A,C8B
GO Process	GO:0030212	Hyaluronan metabolic process	4	23	1.19	0.0138	ITIH4,ITIH1,ITIH2,ITIH3
GO Process	GO:0008202	Steroid metabolic process	11	258	0.58	0.0145	APOB,APOA1,CAT,APOE,LCAT,CRH,PCSK9,APOL1,GC,TSKU,APOC1
GO Process	GO:0046596	Regulation of viral entry into host cell	5	45	0.99	0.0147	LGALS1,APCS,FCN3,P4HB,FCN1
GO Process	GO:0042744	Hydrogen peroxide catabolic process	4	25	1.15	0.0180	CAT,PXDN,HBA1,GPX3
GO Process	GO:0010875	Positive regulation of cholesterol efflux	4	26	1.14	0.0205	APOA1,APOE,ADIPOQ,PLTP
GO Process	GO:0042221	Response to chemical	67	4010	0.17	0.0227	ST6GAL1,SERPIND1,C5,PF4V1,HRG,APOB,IGFBP5,APOA1,CAT,APOE,PXDN,F12,THBS1,TIMP2,LCAT,AMBP,TCIRG1,HPX,ITIH4,PSG9,CSF1R,PIP,PPBP,PF4,CAMP,SLC27A4,PCSK9,FGB,SUMO4,HBA1,FBN1,P4HB,COLEC10,ANG,FGG,LIMS1,THBS4,SLPI,BLM,F5,S100A7,FLNA,RBP4,ADAMTS13,F7,APOM,CA6,SOD3,GPX3,IGF1,ILK,SAA1,CD14,ADIPOQ,MST1,C4B,PDGFC,SELENOP,ATP1A1,TTN,VEGFC,SEMA3B,PLCG2,OR52B4,HSPH1,ACTB,FGA
GO Process	GO:0002685	Regulation of leukocyte migration	10	231	0.58	0.0238	C5,THBS1,PLA2G7,CSF1R,THBS4,S100A7,ECM1,F7,MST1,VEGFC
GO Process	GO:0050727	Regulation of inflammatory response	13	371	0.49	0.0241	LGALS1,APOA1,C3,APOE,F12,APCS,PSMA6,KLKB1,PLA2G7,IGF1,SAA1,ADIPOQ,PLCG2
GO Process	GO:2000351	Regulation of endothelial cell apoptotic process	5	51	0.94	0.0241	THBS1,ANGPTL4,FGB,FGG,FGA
GO Process	GO:0030198	Extracellular matrix organization	11	278	0.55	0.0253	PXDN,BMP1,PLG,SERPINF2,FBLN1,FBLN5,PAPLN,COL18A1,ADAMTSL4,ADAMTS13,TGFBI
GO Process	GO:0010035	Response to inorganic substance	16	532	0.43	0.0270	APOB,CAT,THBS1,LCAT,TCIRG1,FGB,HBA1,FGG,BLM,ADAMTS13,SOD3,CD14,SELENOP,TTN,PLCG2,FGA
GO Process	GO:0010985	Negative regulation of lipoprotein particle clearance	3	11	1.38	0.0271	PCSK9,APOC1,APOC2
GO Process	GO:0020027	Hemoglobin metabolic process	3	11	1.38	0.0271	CAT,AMBP,HPX
GO Process	GO:0098883	Synapse pruning	3	11	1.38	0.0271	C3,C1QB,C1QC
GO Process	GO:0010758	Regulation of macrophage chemotaxis	4	29	1.09	0.0280	C5,THBS1,CSF1R,MST1
GO Process	GO:0048261	Negative regulation of receptor-mediated endocytosis	4	30	1.07	0.0313	PCSK9,ADIPOQ,APOC1,APOC2
GO Process	GO:0034380	High-density lipoprotein particle assembly	3	12	1.35	0.0328	APOA1,APOE,APOM
GO Process	GO:2000352	Negative regulation of endothelial cell apoptotic process	4	31	1.06	0.0346	ANGPTL4,FGB,FGG,FGA
GO Process	GO:0050792	Regulation of viral process	8	161	0.64	0.0362	LGALS1,APCS,FCN3,P4HB,FBLN1,SLPI,FCN1,MBL2
GO Process	GO:0006629	Lipid metabolic process	27	1210	0.3	0.0367	APOB,PLEK,APOA1,TTR,CAT,C3,APOE,LCAT,PLA2G7,CRH,CSF1R,SLC27A4,ANGPTL4,PCSK9,APOL1,ANG,ISYNA1,RBP4,ADIPOQ,ACSF2,PLTP,GC,TSKU,APOC1,APOC2,APOC4,PLCG2
GO Process	GO:1901700	Response to oxygen-containing compound	32	1547	0.26	0.0407	ST6GAL1,PF4V1,APOB,IGFBP5,CAT,APOE,THBS1,AMBP,PPBP,PF4,CAMP,SLC27A4,PCSK9,HBA1,FBN1,SLPI,BLM,F5,S100A7,FLNA,RBP4,ADAMTS13,F7,SOD3,GPX3,IGF1,CD14,ADIPOQ,PDGFC,ATP1A1,PLCG2,ACTB

GO Process	GO:1904036	Negative regulation of epithelial cell apoptotic process	5	59	0.88	0.0417	ANGPTL4,FGB,FGG,MST1,FGA
GO Process	GO:0045766	Positive regulation of angiogenesis	8	166	0.63	0.0432	C5,C3,THBS1,CAMP,ANGPTL4,ADAM12,ECM1,VEGFC
GO Process	GO:0001878	Response to yeast	3	14	1.28	0.0463	CAMP,ANG,PLCG2
GO Process	GO:0034394	Protein localization to cell surface	4	34	1.02	0.0463	VCL,FBLN5,FLNA,FCN1
GO Process	GO:0050922	Negative regulation of chemotaxis	5	61	0.86	0.0470	ST6GAL1,C5,HRG,THBS1,SEMA3B
GO Process	GO:0001932	Regulation of protein phosphorylation	25	1108	0.3	0.0487	HRG,APOA1,C3,APOE,THBS1,CENPE,HPX,CSF1R,CAMP,F2,INHBC,FBN1,FBLN1,ANG,THBS4,BLM,IGF1,ILK,ADIPOQ,MST1,PDGFC,TTN,VEGFC,PLCG2,ACTB
GO Process	GO:0071692	Protein localization to extracellular region	7	130	0.68	0.0487	PLEK,FBN1,FGG,RAPGEF4,LTBP1,FBN2,PDIA4
GO Process	GO:0032091	Negative regulation of protein binding	6	94	0.75	0.0491	CFHR5,PCSK9,CFHR1,SLPI,ADIPOQ,ACTB
GO Process	GO:0048525	Negative regulation of viral process	6	94	0.75	0.0491	APCS,FCN3,FBLN1,SLPI,FCN1,MBL2
GO Process	GO:0070374	Positive regulation of ERK1 and ERK2 cascade	9	213	0.57	0.0491	APOE,CSF1R,FGB,SERPINF2,FGG,S100A7,IGF1,PDGFC,FGA
GO Function	GO:0004866	Endopeptidase inhibitor activity	25	177	1.1	1.99e-15	SPP2,SERPIND1,TFPI2,C5,HRG,C3,SERPINA10,TIMP2,LCN1,CSTA,AMBP,ITIH4,ITIH1,SERPINI1,A2ML1,SERPINF2,SERPINA11,SLPI,PAPLN,ITIH2,PROS1,C4A,ITIH3,C4B,SERPINA1
GO Function	GO:0005102	Signaling receptor binding	60	1499	0.55	6.97e-15	MYH9,C5,PF4V1,HRG,APOB,APOA1,TTR,C3,APOE,PXDN,ANGPTL6,THBS1,VWF,LCN1,CRH,FERMT3,FCN2,SCGB3A1,PPBP,PF4,ANGPTL4,PCSK9,BMP1,FGB,F2,INHBC,PLG,TLN1,HBA1,FBN1,P4HB,FBLN1,COLEC10,ANG,FGG,THBS4,FBLN5,S100A7,ECM1,FLNA,FCN1,ADAMTS13,CCN5,MBL2,F7,IGF1,ACTN1,EFEMP1,SAA1,ADIPOQ,MST1,TGFBI,PDGFC,FBN2,JCHAIN,IGHV3-15,IGHV3-72,VEGFC,SEMA3B,FGA
GO Function	GO:0061134	Peptidase regulator activity	26	227	1.01	8.26e-15	SPP2,SERPIND1,TFPI2,C5,HRG,C3,SERPINA10,TIMP2,LCN1,CSTA,AMBP,ITIH4,ITIH1,SERPINI1,A2ML1,SERPINF2,FBLN1,SERPINA11,SLPI,PAPLN,ITIH2,PROS1,C4A,ITIH3,C4B,SERPINA1
GO Function	GO:0005515	Protein binding	142	7242	0.24	2.44e-13	ST6GAL1,GNPTG,VCL,LGALS1,MYH9,TPD52L2,C5,PF4V1,HRG,APOB,IGFBP5,PLEK,APOA1,TTR,CAT,C3,DHX29,APOE,PXDN,ANGPTL6,F12,APCS,CFHR5,THBS1,LYZ,VWF,PSMA6,TIMP2,LCN1,LCAT,CSTA,CP,DNAH5,AMBP,CENPE,TCIRG1,PSG9,FCN3,CRH,FERMT3,SEC24D,DSC2,CSF1R,PIP,FCN2,PSG6,SCGB3A1,PPBP,PF4,MASP1,A2ML1,ANGPTL4,ANTXR1,SH3D19,PCSK9,BMP1,FGB,F2,INHBC,PLG,PSG1,C1QB,CFHR1,TLN1,SUZ12,SUMO4,SERPINF2,HBA1,FBN1,P4HB,FBLN1,COLEC10,DNAH8,ANG,FGG,LIMS1,THBS4,SLPI,FBLN5,BLM,COL18A1,C8A,CFHR3,CFH,ADAM12,S100A7,HTRA1,ADAMTSL4,ECM1,FLNA,SMARCA1,FCN1,ADAMTS13,CCN5,MBL2,F7,GPX3,IGF1,ACTN1,EFEMP1,YWHAZ,ILK,RAPGEF4,MASP2,PSG4,SAA1,F11,C1S,LTBP1,ADIPOQ,C4A,NBEAL1,COLEC11,MST1,C4B,SERPINA1,TGFBI,CFB,IGFALS,PLTP,TCAF1,GC,UIMC1,PDGFC,FBN2,TSKU,JCHAIN,HPR,C1R,ATP1A1,APOC2,TTN,IGHV3-15,IGHV3-72,VEGFC,SEMA3B,PLCG2,HSPH1,TPM4,ACTB,FGA,TPM3
GO Function	GO:0005509	Calcium ion binding	39	717	0.68	6.70e-13	F9,MGP,SPARC,F12,APCS,THBS1,ITIH1,HABP2,DSC2,MASP1,LALBA,BMP1,F2,STAB1,FBN1,FBLN1,OIT3,SPATA21,THBS4,FBLN5,PCDH18,S100A7,CRTAC1,ADAMTS13,F10,F7,PROS1,ACTN1,EFEMP1,EFCAB5,MASP2,C1S,LTBP1,COLEC11,FBN2,C1R,MYL6,TTN,TPM4
GO Function	GO:0004857	Enzyme inhibitor activity	28	396	0.8	2.23e-11	SPP2,SERPIND1,TFPI2,C5,HRG,C3,SERPINA10,TIMP2,LCN1,CSTA,AMBP,ITIH4,ITIH1,SERPINI1,A2ML1,ANGPTL4,SERPINF2,SERPINA11,SLPI,PAPLN,ITIH2,PROS1,C4A,ITIH3,C4B,SERPINA1,APOC1,APOC2
GO Function	GO:0005539	Glycosaminoglycan binding	23	245	0.92	2.23e-11	SERPIND1,PF4V1,HRG,APOB,APOE,THBS1,SERPINA10,AMBP,ITIH1,HABP2,PF4,F2,PRG2,STAB1,FBN1,ANG,THBS4,ITIH2,CFH,CCN5,SOD3,SAA1,F11
GO Function	GO:0005488	Binding	194	12838	0.13	3.07e-11	ST6GAL1,GNPTG,VCL,SERPIND1,LGALS1,MYH9,TPD52L2,F9,C5,PF4V1,MGP,SPARC,HRG,APOB,IGFBP5,PLEK,APOA1,TTR,CAT,C3,DHX29,APOE,PXDN,ANGPTL6,BPIFB1,F12,APCS,CFHR5,THBS1,LYZ,VWF,PSMA6,SERPINA10,TIMP2,ATRN,LCN1,LCAT,CSTA,CP,KLKB1,F13A1,DNAH5,AMBP,CENPE,TCIRG1,HPX,PSG9,FCN3,ITIH1,PLA2G7,CRH,HABP2,FERMT3,SEC24D,DSC2,CSF1R,PIP,FCN2,PSG6,SCGB3A1,PPBP,PF4,MASP1,CAMP,GUCY1A1,A2ML1,SLC27A4,LALBA,ANGPTL4,ANTXR1,SH3D19,PCSK9,BMP1,FGB,F2,INHBC,PLG,PSG1,PRG2,STAB1,C1QB,CFHR1,NDUFS8,TLN1,SUZ12,APOL1,SUMO4,SERPINF2,HBA1,FBN1,P4HB,FBLN1,COLEC10,DNAH8,OIT3,SPATA21,ANG,FGG,LIMS1,THBS4,GANAB,SLPI,FBLN5,BLM,ITIH2,CNDP1,COL18A1,C8A,PCDH18,CFHR3,CFH,F5,ADAM12,S100A7,HTRA1,ADAMTSL4,ECM1,FLNA,CRTAC1,SMARCA1,C8B,RBP4,FCN1,ADAMTS13,CCN5,MBL2,F10,F7,APOM,CA6,SOD3,GPX3,IGF1,PROS1,ACTN1,EFEMP1,EFCAB5,TNRC6A,YWHAZ,ILK,RAPGEF4,BPIFC,MASP2,PSG4,SAA1,F11,C1S,CD14,LTBP1,PCYOX1,ADIPOQ,C4A,ATP9B,PRG4,NBEAL1,ACSF2,CDK3,COLEC11,MST1,C4B,SERPINA1,TGFBI,CFB,IGFALS,PLTP,PAEP,TCAF1,GC,UIMC1,PDGFC,FBN2,SELENOP,TSKU,JCHAIN,HPR,C1R,ATP1A1,MYL6,IGKV2D-28,APOC1,APOC2,TTN,IGHV3-15,IGHV3-72,VEGFC,SEMA3B,PLCG2,HSPH1,TPM4,ACTB,ITM2B,FGA,TPM3,PDIA4
GO Function	GO:0004252	Serine-type endopeptidase activity	20	176	1.0	3.11e-11	F9,F12,KLKB1,HABP2,MASP1,PCSK9,BMP1,F2,PLG,HTRA1,F10,F7,MASP2,F11,C1S,MST1,CFB,HGFAC,HPR,C1R
GO Function	GO:0004867	Serine-type endopeptidase inhibitor activity	16	98	1.16	6.64e-11	SERPIND1,TFPI2,HRG,SERPINA10,AMBP,ITIH4,ITIH1,SERPINI1,A2ML1,SERPINF2,SERPINA11,SLPI,PAPLN,ITIH2,ITIH3,SERPINA1
GO Function	GO:0001848	Complement binding	10	26	1.53	1.56e-09	APCS,CFHR5,CFHR1,C8A,CFHR3,CFH,MASP2,C4A,C4B,CFB
GO Function	GO:0008201	Heparin binding	18	173	0.97	1.56e-09	SERPIND1,PF4V1,HRG,APOB,APOE,THBS1,SERPINA10,PF4,F2,PRG2,FBN1,ANG,THBS4,CFH,CCN5,SOD3,SAA1,F11
GO Function	GO:0004175	Endopeptidase activity	25	437	0.71	2.18e-08	F9,F12,PSMA6,KLKB1,HABP2,PIP,MASP1,PCSK9,BMP1,F2,PLG,PAPLN,ADAM12,HTRA1,ADAMTS13,F10,F7,MASP2,F11,C1S,MST1,CFB,HGFAC,HPR,C1R
GO Function	GO:0098772	Molecular function regulator activity	56	1960	0.4	2.18e-08	SPP2,SERPIND1,TFPI2,C5,PF4V1,HRG,APOA1,TTR,C3,APOE,PXDN,SERPINA10,TIMP2,LCN1,CSTA,AMBP,ITIH4,ITIH1,CRH,SCGB3A1,SERPINI1,PPBP,PF4,A2ML1,ANGPTL4,PCSK9,BMP1,F2,INHBC,SUZ12,SERPINF2,FBN1,FBLN1,COLEC10,SERPINA11,THBS4,SLPI,PAPLN,ITIH2,FLNA,IGF1,PROS1,EFEMP1,RAPGEF4,ADIPOQ,C4A,ITIH3,C4B,SERPINA1,PDGFC,FBN2,APOC1,APOC2,VEGFC,SEMA3B,HSPH1
GO Function	GO:0005201	Extracellular matrix structural constituent	15	131	1.01	2.40e-08	TFPI2,MGP,PXDN,THBS1,FGB,PRG2,FBN1,FBLN1,OIT3,FGG,COL18A1,LTBP1,TGFBI,FBN2,FGA
GO Function	GO:1901681	Sulfur compound binding	19	272	0.79	1.82e-07	SERPIND1,PF4V1,HRG,APOB,APOE,THBS1,SERPINA10,AMBP,PF4,F2,PRG2,FBN1,ANG,THBS4,CFH,CCN5,SOD3,SAA1,F11
GO Function	GO:0005178	Integrin binding	15	159	0.92	2.59e-07	MYH9,THBS1,VWF,FERMT3,TLN1,FBN1,P4HB,FBLN1,THBS4,FBLN5,ADAMTS13,CCN5,IGF1,ACTN1,TGFBI
GO Function	GO:0044877	Protein-containing complex binding	39	1261	0.44	2.96e-06	VCL,MYH9,SPARC,HRG,APOA1,TTR,DHX29,APOE,APCS,THBS1,VWF,AMBP,PSG9,FERMT3,PIP,ANTXR1,PCSK9,STAB1,TLN1,FBN1,P4HB,FBLN1,THBS4,FBLN5,C8A,FLNA,SMARCA1,C8B,ADAMTS13,CCN5,IGF1,ACTN1,C4A,TGFBI,PLTP,JCHAIN,TTN,TPM4,TPM3
GO Function	GO:0008233	Peptidase activity	26	617	0.57	3.15e-06	F9,F12,PSMA6,KLKB1,HABP2,PIP,MASP1,PCSK9,BMP1,F2,PLG,PAPLN,CNDP1,ADAM12,HTRA1,ADAMTS13,F10,F7,MASP2,F11,C1S,MST1,CFB,HGFAC,HPR,C1R

GO Function	GO:0042802	Identical protein binding	54	2144	0.35	3.15e-06	ST6GAL1,GNPTG,MYH9,TPD52L2,PLEK,APOA1,TTR,CAT,APOE,APCS,CFHR5,THBS1,LYZ,VWF,AMBP,FCN3,CSF1R,PIP,FCN2,MASP1,ANGPTL4,BMP1,C1QB,CFHR1,SERPINF2,FBN1,FBLN1,ANG,FGG,FBLN5,BLM,COL18A1,CFH,HTRA1,ADAMTSL4,FLNA,MBL2,GPX3,ACTN1,YWHAZ,MASP2,F11,C1S,ADIPOQ,COLEC11,SERPINA1,TGFBI,PDGFC,JCHAIN,C1R,APOC2,TTN,TPM4,ACTB
GO Function	GO:0001846	Opsonin binding	7	21	1.47	3.72e-06	APCS,CFHR5,CFHR1,CFHR3,CFH,MASP2,C4A
GO Function	GO:0030545	Signaling receptor regulator activity	23	552	0.57	2.47e-05	C5,PF4V1,APOA1,TTR,PXDN,CRH,SCGB3A1,PPBP,PF4,PCSK9,BMP1,F2,INHBC,FBN1,COLEC10,THBS4,IGF1,EFEMP1,ADIPOQ,PDGFC,FBN2,VEGFC,SEMA3B
GO Function	GO:0048018	Receptor ligand activity	21	499	0.57	7.37e-05	C5,PF4V1,APOA1,TTR,CRH,SCGB3A1,PPBP,PF4,BMP1,F2,INHBC,FBN1,COLEC10,THBS4,IGF1,EFEMP1,ADIPOQ,PDGFC,FBN2,VEGFC,SEMA3B
GO Function	GO:0071813	Lipoprotein particle binding	6	28	1.28	0.00032	APOA1,APOE,THBS1,PCSK9,STAB1,PLTP
GO Function	GO:0050839	Cell adhesion molecule binding	21	560	0.52	0.00038	VCL,MYH9,DHX29,THBS1,VWF,FERMT3,DSC2,TLN1,FBN1,P4HB,FBLN1,FGG,THBS4,FBLN5,FLNA,ADAMTS13,CCN5,IGF1,ACTN1,YWHAZ,TGFBI
GO Function	GO:0003823	Antigen binding	8	71	1.0	0.00043	FCN3,FCN2,PLG,FCN1,JCHAIN,IGKV2D-28,IGHV3-15,IGHV3-72
GO Function	GO:0030234	Enzyme regulator activity	33	1239	0.37	0.00074	SPP2,SERPIND1,TFPI2,C5,HRG,APOA1,C3,APOE,SERPINA10,TIMP2,LCN1,CSTA,AMBP,ITIH4,ITIH1,SERPINI1,A2ML1,ANGPTL4,SUZ12,SERPINF2,FBLN1,SERPINA11,SLPI,PAPLN,ITIH2,PROS1,RAPGEF4,C4A,ITIH3,C4B,SERPINA1,APOC1,APOC2
GO Function	GO:0019865	Immunoglobulin binding	5	23	1.29	0.0019	HRG,VWF,AMBP,PIP,JCHAIN
GO Function	GO:0097367	Carbohydrate derivative binding	48	2278	0.27	0.0023	SERPIND1,MYH9,PF4V1,HRG,APOB,DHX29,APOE,THBS1,SERPINA10,DNAH5,AMBP,CENPE,ITIH1,HABP2,CSF1R,FCN2,PF4,CAMP,GUCY1A1,F2,PRG2,STAB1,FBN1,DNAH8,ANG,THBS4,BLM,ITIH2,CFH,SMARCA1,FCN1,CCN5,SOD3,ILK,RAPGEF4,BPIFC,SAA1,F11,CD14,ADIPOQ,ATP9B,ACSF2,CDK3,ATP1A1,TTN,HSPH1,ACTB,ITM2B
GO Function	GO:0001851	Complement component C3b binding	4	11	1.51	0.0025	CFHR5,CFHR1,CFHR3,CFH
GO Function	GO:0030246	Carbohydrate binding	13	278	0.62	0.0033	LGALS1,APCS,ATRN,FCN3,FCN2,PRG2,COLEC10,GANAB,FCN1,MBL2,PRG4,COLEC11,C4B
GO Function	GO:0005198	Structural molecule activity	23	776	0.42	0.0041	VCL,TFPI2,MGP,APOE,PXDN,THBS1,FGB,PRG2,TLN1,FBN1,FBLN1,OIT3,FGG,COL18A1,ACTN1,LTBP1,TGFBI,FBN2,MYL6,TTN,TPM4,ACTB,FGA
GO Function	GO:0002020	Protease binding	9	135	0.77	0.0044	VWF,TIMP2,CSTA,A2ML1,SERPINF2,ADAMTSL4,ECM1,SERPINA1,TTN
GO Function	GO:0016209	Antioxidant activity	7	76	0.91	0.0048	CAT,APOE,PXDN,AMBP,APOM,SOD3,GPX3
GO Function	GO:0034987	Immunoglobulin receptor binding	4	16	1.35	0.0076	FLNA,JCHAIN,IGHV3-15,IGHV3-72
GO Function	GO:0050840	Extracellular matrix binding	6	56	0.98	0.0077	LGALS1,SPARC,PXDN,THBS1,ECM1,TGFBI
GO Function	GO:0042803	Protein homodimerization activity	21	709	0.42	0.0082	ST6GAL1,GNPTG,MYH9,TPD52L2,PLEK,APOA1,CAT,APOE,AMBP,CSF1R,MASP1,SERPINF2,ANG,FBLN5,BLM,FLNA,ACTN1,ADIPOQ,PDGFC,JCHAIN,TPM4
GO Function	GO:0030169	Low-density lipoprotein particle binding	4	17	1.32	0.0085	THBS1,PCSK9,STAB1,PLTP
GO Function	GO:0043169	Cation binding	74	4335	0.18	0.0085	F9,MGP,SPARC,HRG,APOA1,CAT,APOE,PXDN,F12,APCS,THBS1,TIMP2,LCN1,CP,KLKB1,F13A1,HPX,FCN3,ITIH1,HABP2,SEC24D,DSC2,FCN2,MASP1,LALBA,ANTXR1,BMP1,F2,STAB1,NDUFS8,SUZ12,HBA1,FBN1,FBLN1,OIT3,SPATA21,ANG,FGG,LIMS1,THBS4,FBLN5,BLM,CNDP1,COL18A1,PCDH18,F5,ADAM12,S100A7,CRTAC1,FCN1,ADAMTS13,F10,F7,CA6,SOD3,PROS1,ACTN1,EFEMP1,EFCAB5,MASP2,C1S,LTBP1,ATP9B,COLEC11,PLTP,UIMC1,FBN2,C1R,ATP1A1,MYL6,APOC1,TTN,TPM4,FGA
GO Function	GO:0043394	Proteoglycan binding	5	37	1.08	0.0107	HRG,APOE,THBS1,FCN2,CFH
GO Function	GO:0060228	Phosphatidylcholine-sterol O-acyltransferase activator activity	3	6	1.65	0.0115	APOA1,APOE,APOC1
GO Function	GO:0008083	Growth factor activity	9	162	0.69	0.0135	PPBP,BMP1,F2,INHBC,THBS4,IGF1,EFEMP1,PDGFC,VEGFC
GO Function	GO:0005319	Lipid transporter activity	9	163	0.69	0.0139	APOB,APOA1,APOE,SLC27A4,RBP4,APOM,ATP9B,PLTP,APOC4
GO Function	GO:0097493	Structural molecule activity conferring elasticity	3	8	1.52	0.0208	FBN1,FBN2,TTN
GO Function	GO:0120020	Cholesterol transfer activity	4	23	1.19	0.0208	APOB,APOA1,APOE,PLTP
GO Function	GO:0046872	Metal ion binding	71	4250	0.17	0.0214	F9,MGP,SPARC,HRG,CAT,APOE,PXDN,F12,APCS,THBS1,TIMP2,LCN1,CP,KLKB1,F13A1,HPX,FCN3,ITIH1,HABP2,SEC24D,DSC2,FCN2,MASP1,LALBA,ANTXR1,BMP1,F2,STAB1,NDUFS8,SUZ12,HBA1,FBN1,FBLN1,OIT3,SPATA21,ANG,FGG,LIMS1,THBS4,FBLN5,BLM,CNDP1,COL18A1,PCDH18,F5,ADAM12,S100A7,CRTAC1,FCN1,ADAMTS13,F10,F7,CA6,SOD3,PROS1,ACTN1,EFEMP1,EFCAB5,MASP2,C1S,LTBP1,ATP9B,COLEC11,UIMC1,FBN2,C1R,ATP1A1,MYL6,TTN,TPM4,FGA
GO Function	GO:0043167	Ion binding	93	6033	0.14	0.0248	MYH9,F9,MGP,SPARC,HRG,PLEK,APOA1,CAT,DHX29,APOE,PXDN,F12,APCS,THBS1,PSMA6,TIMP2,LCN1,CP,KLKB1,F13A1,DNAH5,CENPE,HPX,FCN3,ITIH1,HABP2,SEC24D,DSC2,CSF1R,FCN2,MASP1,GUCY1A1,LALBA,ANTXR1,BMP1,F2,STAB1,NDUFS8,SUZ12,HBA1,FBN1,FBLN1,DNAH8,OIT3,SPATA21,ANG,FGG,LIMS1,THBS4,FBLN5,BLM,CNDP1,COL18A1,PCDH18,F5,ADAM12,S100A7,CRTAC1,SMARCA1,FCN1,ADAMTS13,F10,F7,CA6,SOD3,PROS1,ACTN1,EFEMP1,EFCAB5,ILK,RAPGEF4,MASP2,C1S,LTBP1,PCYOX1,ADIPOQ,ATP9B,ACSF2,CDK3,COLEC11,PLTP,UIMC1,FBN2,C1R,ATP1A1,MYL6,APOC1,TTN,HSPH1,TPM4,ACTB,ITM2B,FGA
GO Function	GO:0043236	Laminin binding	4	28	1.1	0.0379	LGALS1,PXDN,THBS1,ECM1
GO Function	GO:0070325	Lipoprotein particle receptor binding	4	30	1.07	0.0472	APOB,APOA1,APOE,PCSK9
GO Component	GO:0005576	Extracellular region	183	4175	0.59	7.25e-80	SPP2,ST6GAL1,GNPTG,VCL,SERPIND1,LGALS1,MYH9,F9,TFPI2,C5,PF4V1,MGP,SPARC,HRG,APOB,IGFBP5,PLEK,APOA1,TTR,CAT,C3,APOE,PXDN,ANGPTL6,BPIFB1,F12,APCS,CFHR5,THBS1,LYZ,VWF,PSMA6,SERPINA10,TIMP2,ATRN,A1BG,BLVRB,LCN1,LCAT,CSTA,CP,KLKB1,F13A1,DNAH5,AMBP,CENPE,HPX,ITIH4,PSG9,FCN3,ITIH1,PLA2G7,CRH,HABP2,FERMT3,DSC2,PIP,FCN2,PSG6,SCGB3A1,SERPINI1,PPBP,PF4,MASP1,CAMP,A2ML1,LALBA,ANGPTL4,PCSK9,BMP1,FGB,F2,INHBC,PLG,PSG1,PRG2,C1QB,CFHR1,TLN1,APOL1,PSG11,SERPINF2,HBA1,FBN1,P4HB,VMO1,FBLN1,COLEC10,OIT3,SERPINA11,ANG,FGG,THBS4,GANAB,SLPI,FBLN5,PAPLN,ITIH2,CNDP1,COL18A1,C8A,F13B,CFHR3,CFH,F5,ADAM12,S100A7,HTRA1,ADAMTSL4,ECM1,FLNA,CRTAC1,C8B,RBP4,FCN1,ADAMTS13,CCN5,MBL2,C1QC,F10,F7,APOM,CA6,ADGRG2,SOD3,GOLM1,GPX3,IGF1,PROS1,ACTN1,EFEMP1,YWHAZ,BPIFC,MASP2,PSG4,SAA1,F11,C1S,CD14,PSG2,LTBP1,PCYOX1,ADIPOQ,C4A,PRG4,COLEC11,MST1,ITIH3,C4B,SERPINA1,TGFBI,CFB,IGFALS,PLTP,PAEP,GC,HGFAC,PDGFC,FBN2,SELENOP,TSKU,JCHAIN,HPR,C1R,ATP1A1,MYL6,IGKV2D-28,APOC1,APOC2,TTN,APOC4,IGHV3-15,IGHV3-72,VEGFC,SEMA3B,PLCG2,HSPH1,TPM4,ACTB,ITM2B,FGA,TPM3,PDIA4

GO Component	GO:0005615	Extracellular space	167	3247	0.66	1.24e-78	GNPTG,VCL,SERPIND1,LGALS1,MYH9,F9,TFPI2,C5,PF4V1,MGP,SPARC,HRG,APOB,IGFBP5,APOA1,TTR,CAT,C3,APOE,PXDN,ANGPTL6,BPIFB1,F12,APCS,CFHR5,THBS1,LYZ,VWF,PSMA6,SERPINA10,TIMP2,ATRN,A1BG,BLVRB,LCN1,LCAT,CSTA,CP,KLKB1,F13A1,AMBP,HPX,ITIH4,FCN3,ITIH1,PLA2G7,CRH,HABP2,FERMT3,DSC2,PIP,FCN2,SCGB3A1,SERPINI1,PPBP,PF4,MASP1,CAMP,A2ML1,LALBA,ANGPTL4,PCSK9,BMP1,FGB,F2,INHBC,PLG,PRG2,C1QB,CFHR1,TLN1,APOL1,SERPINF2,HBA1,FBN1,P4HB,VMO1,FBLN1,COLEC10,OIT3,SERPINA11,ANG,FGG,THBS4,GANAB,SLPI,FBLN5,ITIH2,COL18A1,C8A,F13B,CFHR3,CFH,F5,S100A7,HTRA1,ECM1,FLNA,CRTAC1,C8B,RBP4,FCN1,ADAMTS13,CCN5,MBL2,C1QC,F10,F7,APOM,CA6,ADGRG2,SOD3,GOLM1,GPX3,IGF1,PROS1,ACTN1,EFEMP1,YWHAZ,BPIFC,MASP2,SAA1,F11,C1S,CD14,PCYOX1,ADIPOQ,C4A,PRG4,COLEC11,MST1,ITIH3,C4B,SERPINA1,TGFBI,CFB,IGFALS,PLTP,PAEP,GC,HGFAC,PDGFC,FBN2,SELENOP,TSKU,JCHAIN,HPR,C1R,ATP1A1,MYL6,IGKV2D-28,APOC1,APOC2,TTN,APOC4,IGHV3-15,IGHV3-72,VEGFC,SEMA3B,PLCG2,HSPH1,TPM4,ACTB,ITM2B,FGA,TPM3,PDIA4
GO Component	GO:0062023	Collagen-containing extracellular matrix	60	407	1.12	8.99e-44	SPP2,LGALS1,F9,MGP,SPARC,HRG,APOA1,APOE,PXDN,ANGPTL6,F12,APCS,THBS1,VWF,TIMP2,ATRN,A1BG,F13A1,AMBP,HPX,ITIH4,FCN3,ITIH1,FCN2,PF4,ANGPTL4,FGB,F2,PLG,PRG2,C1QB,SERPINF2,FBN1,FBLN1,ANG,FGG,THBS4,SLPI,FBLN5,ITIH2,COL18A1,S100A7,HTRA1,ADAMTSL4,ECM1,FCN1,MBL2,C1QC,F7,SOD3,EFEMP1,LTBP1,ADIPOQ,PRG4,MST1,SERPINA1,TGFBI,FBN2,SEMA3B,FGA
GO Component	GO:0072562	Blood microparticle	42	118	1.5	2.00e-43	HRG,APOA1,C3,APOE,APCS,A1BG,CP,F13A1,AMBP,HPX,ITIH4,FCN3,ITIH1,FCN2,ANGPTL4,FGB,F2,PLG,C1QB,CFHR1,APOL1,SERPINF2,HBA1,FGG,ITIH2,C8A,CFHR3,CFH,C1QC,PROS1,YWHAZ,C1S,C4A,C4B,CFB,GC,JCHAIN,HPR,C1R,IGKV2D-28,ACTB,FGA
GO Component	GO:0031012	Extracellular matrix	65	552	1.02	1.65e-42	SPP2,LGALS1,F9,TFPI2,MGP,SPARC,HRG,APOA1,APOE,PXDN,ANGPTL6,F12,APCS,THBS1,VWF,TIMP2,ATRN,A1BG,F13A1,AMBP,HPX,ITIH4,FCN3,ITIH1,FCN2,PF4,ANGPTL4,FGB,F2,PLG,PRG2,C1QB,SERPINF2,FBN1,FBLN1,ANG,FGG,THBS4,SLPI,FBLN5,PAPLN,ITIH2,COL18A1,S100A7,HTRA1,ADAMTSL4,ECM1,FCN1,ADAMTS13,CCN5,MBL2,C1QC,F7,SOD3,EFEMP1,LTBP1,ADIPOQ,PRG4,MST1,SERPINA1,TGFBI,IGFALS,FBN2,SEMA3B,FGA
GO Component	GO:1903561	Extracellular vesicle	107	2120	0.65	3.39e-41	GNPTG,VCL,SERPIND1,LGALS1,MYH9,F9,C5,MGP,HRG,APOB,APOA1,TTR,CAT,C3,APOE,PXDN,ANGPTL6,BPIFB1,F12,APCS,THBS1,LYZ,VWF,PSMA6,SERPINA10,ATRN,A1BG,BLVRB,LCAT,CP,KLKB1,AMBP,HPX,ITIH4,ITIH1,FERMT3,DSC2,PIP,FCN2,SCGB3A1,SERPINI1,CAMP,A2ML1,FGB,F2,PLG,PRG2,TLN1,SERPINF2,HBA1,P4HB,VMO1,FBLN1,FGG,THBS4,GANAB,SLPI,FBLN5,ITIH2,COL18A1,C8A,CFHR3,CFH,F5,HTRA1,ECM1,FLNA,CRTAC1,C8B,RBP4,CA6,ADGRG2,SOD3,GPX3,PROS1,ACTN1,EFEMP1,YWHAZ,MASP2,SAA1,F11,CD14,PCYOX1,C4A,ITIH3,C4B,SERPINA1,TGFBI,CFB,IGFALS,GC,PDGFC,SELENOP,JCHAIN,HPR,C1R,ATP1A1,MYL6,IGKV2D-28,TTN,PLCG2,HSPH1,TPM4,ACTB,ITM2B,FGA,TPM3
GO Component	GO:0070062	Extracellular exosome	106	2096	0.65	6.29e-41	GNPTG,VCL,SERPIND1,LGALS1,MYH9,F9,C5,MGP,HRG,APOB,APOA1,TTR,CAT,C3,APOE,PXDN,ANGPTL6,BPIFB1,F12,APCS,THBS1,LYZ,VWF,PSMA6,SERPINA10,ATRN,A1BG,BLVRB,LCAT,CP,KLKB1,AMBP,HPX,ITIH4,ITIH1,FERMT3,DSC2,PIP,FCN2,SCGB3A1,SERPINI1,CAMP,A2ML1,FGB,F2,PLG,PRG2,TLN1,SERPINF2,HBA1,P4HB,VMO1,FBLN1,FGG,THBS4,GANAB,SLPI,FBLN5,ITIH2,COL18A1,C8A,CFHR3,CFH,HTRA1,ECM1,FLNA,CRTAC1,C8B,RBP4,CA6,ADGRG2,SOD3,GPX3,PROS1,ACTN1,EFEMP1,YWHAZ,MASP2,SAA1,F11,CD14,PCYOX1,C4A,ITIH3,C4B,SERPINA1,TGFBI,CFB,IGFALS,GC,PDGFC,SELENOP,JCHAIN,HPR,C1R,ATP1A1,MYL6,IGKV2D-28,TTN,PLCG2,HSPH1,TPM4,ACTB,ITM2B,FGA,TPM3
GO Component	GO:0031982	Vesicle	128	3957	0.46	7.58e-32	SPP2,GNPTG,VCL,SERPIND1,LGALS1,MYH9,F9,C5,MGP,SPARC,HRG,APOB,APOA1,TTR,CAT,C3,APOE,PXDN,ANGPTL6,BPIFB1,F12,APCS,THBS1,LYZ,VWF,PSMA6,SERPINA10,TIMP2,ATRN,A1BG,BLVRB,LCAT,CP,KLKB1,F13A1,AMBP,TCIRG1,HPX,ITIH4,ITIH1,FERMT3,SEC24D,DSC2,PIP,FCN2,SCGB3A1,SERPINI1,PPBP,PF4,CAMP,A2ML1,ANTXR1,PCSK9,BMP1,FGB,F2,PLG,PRG2,STAB1,TLN1,SERPINF2,HBA1,P4HB,VMO1,FBLN1,ANG,FGG,THBS4,GANAB,SLPI,FBLN5,ITIH2,COL18A1,C8A,CFHR3,CFH,F5,S100A7,HTRA1,ECM1,FLNA,CRTAC1,C8B,RBP4,FCN1,F7,CA6,ADGRG2,SOD3,GPX3,IGF1,PROS1,ACTN1,EFEMP1,YWHAZ,MASP2,SAA1,F11,CD14,PCYOX1,C4A,ATP9B,ITIH3,C4B,SERPINA1,TGFBI,CFB,IGFALS,GC,PDGFC,SELENOP,JCHAIN,HPR,C1R,ATP1A1,MYL6,IGKV2D-28,APOC2,TTN,VEGFC,PLCG2,HSPH1,TPM4,ACTB,ITM2B,FGA,TPM3,PDIA4
GO Component	GO:0031983	Vesicle lumen	38	326	1.01	1.04e-23	SPP2,VCL,SPARC,HRG,APOB,APOA1,TTR,CAT,C3,THBS1,LYZ,VWF,TIMP2,A1BG,F13A1,ITIH4,FERMT3,SERPINI1,PPBP,PF4,CAMP,FGB,PLG,SERPINF2,FGG,SLPI,F5,S100A7,ECM1,FCN1,IGF1,PROS1,ACTN1,ITIH3,SERPINA1,SELENOP,VEGFC,FGA
GO Component	GO:0034774	Secretory granule lumen	37	321	1.01	6.49e-23	SPP2,VCL,SPARC,HRG,APOA1,TTR,CAT,C3,THBS1,LYZ,VWF,TIMP2,A1BG,F13A1,ITIH4,FERMT3,SERPINI1,PPBP,PF4,CAMP,FGB,PLG,SERPINF2,FGG,SLPI,F5,S100A7,ECM1,FCN1,IGF1,PROS1,ACTN1,ITIH3,SERPINA1,SELENOP,VEGFC,FGA
GO Component	GO:1905370	Serine-type endopeptidase complex	18	26	1.79	3.80e-22	F9,C3,F12,KLKB1,FCN3,FCN2,MASP1,F2,COLEC10,CFH,F5,FCN1,MBL2,F10,F7,MASP2,F11,COLEC11
GO Component	GO:0005788	Endoplasmic reticulum lumen	33	312	0.97	2.61e-19	SPP2,SERPIND1,LGALS1,F9,APOB,IGFBP5,APOA1,C3,APOE,THBS1,SERPINA10,CP,PCSK9,F2,APOL1,FBN1,P4HB,FGG,GANAB,ITIH2,COL18A1,F5,ADAMTSL4,ADAMTS13,F10,F7,GOLM1,LTBP1,C4A,SERPINA1,PDGFC,FGA,PDIA4
GO Component	GO:0031093	Platelet alpha granule lumen	20	66	1.43	3.99e-19	SPARC,HRG,THBS1,VWF,A1BG,F13A1,FERMT3,PPBP,PF4,FGB,PLG,SERPINF2,FGG,F5,IGF1,PROS1,ACTN1,SERPINA1,VEGFC,FGA
GO Component	GO:1905369	Endopeptidase complex	20	104	1.23	8.16e-16	F9,C3,F12,PSMA6,KLKB1,FCN3,FCN2,MASP1,F2,UBE3C,COLEC10,CFH,F5,FCN1,MBL2,F10,F7,MASP2,F11,COLEC11
GO Component	GO:0071944	Cell periphery	128	6015	0.28	8.93e-15	SPP2,VCL,LGALS1,MYH9,F9,TFPI2,C5,MGP,SPARC,HRG,APOB,PLEK,APOA1,CAT,C3,APOE,PXDN,ANGPTL6,F12,APCS,THBS1,VWF,TIMP2,ATRN,A1BG,BLVRB,CSTA,CP,KLKB1,F13A1,AMBP,TCIRG1,HPX,ITIH4,PSG9,FCN3,ITIH1,DSC2,CSF1R,FCN2,PSG6,PF4,MASP1,SLC27A4,ANGPTL4,ANTXR1,SH3D19,PCSK9,FGB,F2,PLG,PSG1,PRG2,STAB1,C1QB,TLN1,SERPINF2,FBN1,P4HB,FBLN1,COLEC10,ANG,FGG,LIMS1,THBS4,SLPI,FBLN5,PAPLN,ITIH2,COL18A1,C8A,PCDH18,F5,ADAM12,S100A7,HTRA1,ADAMTSL4,ECM1,FLNA,C8B,FCN1,ADAMTS13,CCN5,MBL2,C1QC,F10,F7,ADGRG2,SOD3,GOLM1,IGF1,PROS1,ACTN1,EFEMP1,ILK,RAPGEF4,MASP2,PSG4,F11,CD14,LTBP1,PCYOX1,ADIPOQ,C4A,ATP9B,PRG4,COLEC11,MST1,C4B,SERPINA1,TGFBI,CFB,IGFALS,TCAF1,PDGFC,FBN2,ATP1A1,IGKV2D-28,TTN,IGHV3-15,IGHV3-72,SEMA3B,PLCG2,OR52B4,TPM4,ACTB,ITM2B,FGA
GO Component	GO:0034358	Plasma lipoprotein particle	14	38	1.51	3.42e-14	APOB,APOA1,APOE,LCAT,PLA2G7,APOL1,APOM,SAA1,PCYOX1,PLTP,HPR,APOC1,APOC2,APOC4
GO Component	GO:0034364	High-density lipoprotein particle	13	29	1.6	4.70e-14	APOB,APOA1,APOE,LCAT,PLA2G7,APOL1,APOM,SAA1,PLTP,HPR,APOC1,APOC2,APOC4

GO Component	GO:0030141	Secretory granule	42	873	0.63	2.86e-13	SPP2,VCL,MYH9,SPARC,HRG,APOA1,TTR,CAT,C3,ANGPTL6,THBS1,LYZ,VWF,TIMP2,A1BG,F13A1,TCIRG1,ITIH4,FERMT3,SERPINI1,PPBP,PF4,CAMP,FGB,PLG,PRG2,SERPINF2,FGG,SLPI,F5,S100A7,ECM1,FCN1,IGF1,PROS1,ACTN1,CD14,ITIH3,SERPINA1,SELENOP,VEGFC,FGA
GO Component	GO:0009986	Cell surface	41	894	0.61	2.86e-12	APMAP,SPARC,HRG,C3,PXDN,THBS1,AMBP,PSG9,FCN3,CSF1R,FCN2,PSG6,MASP1,ANTXR1,PCSK9,FGB,PLG,PSG1,TLN1,PSG11,SERPINF2,P4HB,COLEC10,OIT3,FGG,FCN1,ADAMTS13,MBL2,F10,ADGRG2,MASP2,PSG4,CD14,PSG2,ADIPOQ,COLEC11,PDGFC,IGHV3-15,IGHV3-72,FGA,PDIA4
GO Component	GO:0034361	Very-low-density lipoprotein particle	9	20	1.6	1.27e-09	APOB,APOA1,APOE,APOL1,APOM,PCYOX1,APOC1,APOC2,APOC4
GO Component	GO:0097708	Intracellular vesicle	63	2484	0.35	2.76e-08	SPP2,VCL,MYH9,SPARC,HRG,APOB,APOA1,TTR,CAT,C3,APOE,ANGPTL6,THBS1,LYZ,VWF,TIMP2,A1BG,F13A1,TCIRG1,HPX,ITIH4,FERMT3,SEC24D,DSC2,SERPINI1,PPBP,PF4,CAMP,ANTXR1,PCSK9,FGB,PLG,PRG2,STAB1,SERPINF2,HBA1,P4HB,ANG,FGG,GANAB,SLPI,F5,S100A7,ECM1,FCN1,IGF1,PROS1,ACTN1,YWHAZ,SAA1,CD14,ATP9B,ITIH3,SERPINA1,SELENOP,ATP1A1,APOC2,VEGFC,PLCG2,HSPH1,ITM2B,FGA,PDIA4
GO Component	GO:0031410	Cytoplasmic vesicle	62	2482	0.35	7.06e-08	SPP2,VCL,MYH9,SPARC,HRG,APOB,APOA1,TTR,CAT,C3,APOE,ANGPTL6,THBS1,LYZ,VWF,TIMP2,A1BG,F13A1,TCIRG1,HPX,ITIH4,FERMT3,SEC24D,DSC2,SERPINI1,PPBP,PF4,CAMP,ANTXR1,PCSK9,FGB,PLG,PRG2,STAB1,SERPINF2,HBA1,P4HB,ANG,FGG,GANAB,SLPI,F5,S100A7,ECM1,FCN1,IGF1,PROS1,ACTN1,YWHAZ,SAA1,CD14,ATP9B,ITIH3,SERPINA1,SELENOP,ATP1A1,APOC2,VEGFC,HSPH1,ITM2B,FGA,PDIA4
GO Component	GO:0071682	Endocytic vesicle lumen	8	23	1.49	7.99e-08	SPARC,APOB,APOA1,APOE,HPX,HBA1,SAA1,HSPH1
GO Component	GO:0012505	Endomembrane system	92	4721	0.24	5.08e-07	SPP2,ST6GAL1,GNPTG,VCL,SERPIND1,LGALS1,MYH9,F9,SPARC,HRG,APOB,IGFBP5,APOA1,TTR,CAT,C3,APOE,PXDN,ANGPTL6,F12,THBS1,LYZ,VWF,SERPINA10,TIMP2,A1BG,CP,F13A1,AMBP,TCIRG1,ITIH4,FERMT3,SEC24D,SERPINI1,PPBP,PF4,CAMP,SLC27A4,LALBA,ANTXR1,PCSK9,BMP1,FGB,F2,PLG,PRG2,APOL1,SERPINF2,FBN1,P4HB,COLEC10,OIT3,FGG,THBS4,GANAB,SLPI,ITIH2,COL18A1,F5,S100A7,ADAMTSL4,ECM1,FLNA,FCN1,ADAMTS13,F10,F7,SOD3,GOLM1,IGF1,PROS1,ACTN1,TNRC6A,CD14,LTBP1,ADIPOQ,C4A,ATP9B,ITIH3,SERPINA1,TGFBI,HGFAC,PDGFC,SELENOP,ATP1A1,APOC1,APOC2,VEGFC,SEMA3B,ITM2B,FGA,PDIA4
GO Component	GO:0032991	Protein-containing complex	101	5506	0.21	1.34e-06	GNPTG,VCL,LGALS1,MYH9,F9,C5,APOB,IGFBP5,APOA1,TTR,CAT,C3,DHX29,APOE,F12,CFHR5,THBS1,PSMA6,LCAT,CSTA,KLKB1,F13A1,DNAH5,TCIRG1,PSG9,FCN3,PLA2G7,SEC24D,CSF1R,FCN2,MASP1,GUCY1A1,LALBA,PCSK9,FGB,F2,UBE3C,C1QB,CFHR1,NDUFS8,SUZ12,APOL1,SUMO4,SERPINF2,HBA1,P4HB,COLEC10,DNAH8,ANG,FGG,GANAB,BLM,COL18A1,C8A,F13B,CFH,F5,FLNA,SMARCA1,C8B,FCN1,MBL2,C1QC,F10,F7,APOM,IGF1,TNRC6A,MASP2,SAA1,F11,C1S,CD14,LTBP1,PCYOX1,ADIPOQ,C4A,CDK3,COLEC11,C4B,CFB,IGFALS,PLTP,UIMC1,JCHAIN,HPR,C1R,ATP1A1,MYL6,IGKV2D-28,APOC1,APOC2,TTN,APOC4,IGHV3-15,IGHV3-72,HSPH1,TPM4,ACTB,FGA,TPM3
GO Component	GO:0009897	External side of plasma membrane	20	388	0.66	1.92e-06	THBS1,FCN3,FCN2,MASP1,ANTXR1,PCSK9,FGB,PLG,P4HB,COLEC10,FGG,FCN1,MBL2,F10,MASP2,CD14,COLEC11,IGHV3-15,IGHV3-72,FGA
GO Component	GO:0005783	Endoplasmic reticulum	50	2021	0.34	5.48e-06	SPP2,ST6GAL1,SERPIND1,LGALS1,F9,APOB,IGFBP5,APOA1,CAT,C3,APOE,PXDN,F12,THBS1,VWF,SERPINA10,CP,AMBP,SEC24D,SLC27A4,PCSK9,FGB,F2,APOL1,FBN1,P4HB,FGG,THBS4,GANAB,ITIH2,COL18A1,F5,S100A7,ADAMTSL4,ADAMTS13,F10,F7,GOLM1,PROS1,LTBP1,ADIPOQ,C4A,SERPINA1,HGFAC,PDGFC,ATP1A1,APOC1,SEMA3B,FGA,PDIA4
GO Component	GO:0005577	Fibrinogen complex	5	8	1.74	1.03e-05	THBS1,FGB,SERPINF2,FGG,FGA
GO Component	GO:0005581	Collagen trimer	10	95	0.97	1.41e-05	FCN3,FCN2,C1QB,COLEC10,COL18A1,FCN1,MBL2,C1QC,ADIPOQ,COLEC11
GO Component	GO:0034362	Low-density lipoprotein particle	5	12	1.57	4.55e-05	APOB,APOE,PLA2G7,APOM,APOC2
GO Component	GO:0042627	Chylomicron	5	13	1.53	6.10e-05	APOB,APOA1,APOE,APOC1,APOC2
GO Component	GO:0031089	Platelet dense granule lumen	5	14	1.5	8.01e-05	SPP2,ITIH4,ECM1,ITIH3,SELENOP
GO Component	GO:0005602	Complement component C1 complex	4	5	1.85	8.42e-05	C1QB,C1QC,C1S,C1R
GO Component	GO:0005604	Basement membrane	9	98	0.91	0.00014	SPARC,PXDN,ATRN,FBN1,FBLN1,ANG,THBS4,COL18A1,TGFBI
GO Component	GO:0034366	Spherical high-density lipoprotein particle	4	8	1.65	0.00031	APOA1,APOM,HPR,APOC2
GO Component	GO:0098552	Side of membrane	21	611	0.48	0.00038	MYH9,THBS1,FCN3,FCN2,MASP1,ANTXR1,PCSK9,FGB,PLG,P4HB,COLEC10,FGG,FCN1,MBL2,F10,MASP2,CD14,COLEC11,IGHV3-15,IGHV3-72,FGA
GO Component	GO:0070013	Intracellular organelle lumen	92	5660	0.16	0.0016	SPP2,VCL,SERPIND1,LGALS1,MYH9,F9,SPARC,HRG,APOB,IGFBP5,APOA1,TTR,CAT,C3,APOE,THBS1,LYZ,VWF,PSMA6,SERPINA10,TIMP2,A1BG,BLVRB,CSTA,CP,F13A1,HPX,ITIH4,FERMT3,CSF1R,SERPINI1,PPBP,PF4,MASP1,CAMP,C16orf46,LALBA,SH3D19,PCSK9,FGB,F2,PLG,PRG2,NDUFS8,SUZ12,APOL1,SERPINF2,HBA1,FBN1,P4HB,ANG,FGG,GANAB,SLPI,BLM,ITIH2,COL18A1,F5,ADAM12,S100A7,ADAMTSL4,ECM1,FLNA,SMARCA1,FCN1,ADAMTS13,F10,F7,SOD3,GOLM1,IGF1,PROS1,ACTN1,TNRC6A,YWHAZ,ILK,SAA1,LTBP1,C4A,ACSF2,ITIH3,SERPINA1,GC,UIMC1,PDGFC,SELENOP,TTN,VEGFC,HSPH1,ACTB,FGA,PDIA4
GO Component	GO:0005601	Classical-complement-pathway C3/C5 convertase complex	3	4	1.82	0.0018	C4A,C4B,CFB
GO Component	GO:0042567	Insulin-like growth factor ternary complex	3	4	1.82	0.0018	IGFBP5,IGF1,IGFALS
GO Component	GO:0034363	Intermediate-density lipoprotein particle	3	5	1.73	0.0028	APOB,APOE,APOC2
GO Component	GO:1902494	Catalytic complex	34	1539	0.29	0.0053	GNPTG,F9,CAT,C3,F12,PSMA6,KLKB1,F13A1,DNAH5,PSG9,FCN3,FCN2,MASP1,F2,UBE3C,NDUFS8,SUZ12,SUMO4,P4HB,COLEC10,DNAH8,F13B,CFH,F5,SMARCA1,FCN1,MBL2,F10,F7,MASP2,F11,CDK3,COLEC11,ACTB
GO Component	GO:0005579	Membrane attack complex	3	7	1.58	0.0056	C5,C8A,C8B
GO Component	GO:0005796	Golgi lumen	7	106	0.77	0.0096	F9,LALBA,F2,F10,F7,SOD3,PROS1
GO Component	GO:0019814	Immunoglobulin complex	4	25	1.15	0.0096	JCHAIN,IGKV2D-28,IGHV3-15,IGHV3-72
GO Component	GO:0031232	Extrinsic component of external side of plasma membrane	3	9	1.47	0.0096	PCSK9,PLG,FCN1
GO Component	GO:0030055	Cell-substrate junction	14	426	0.46	0.0146	VCL,MYH9,CAT,FERMT3,TLN1,P4HB,LIMS1,S100A7,FLNA,ACTN1,YWHAZ,ILK,TPM4,ACTB
GO Component	GO:0042571	Immunoglobulin complex, circulating	3	12	1.35	0.0183	JCHAIN,IGHV3-15,IGHV3-72
GO Component	GO:0001527	Microfibril	3	13	1.31	0.0216	FBN1,LTBP1,FBN2
GO Component	GO:1904813	ficolin-1-rich granule lumen	7	124	0.7	0.0216	VCL,CAT,TIMP2,A1BG,PRG2,FCN1,SERPINA1
GO Component	GO:0005886	Plasma membrane	85	5544	0.13	0.0227	VCL,MYH9,F9,C5,SPARC,HRG,APOB,PLEK,APOA1,CAT,C3,APOE,F12,THBS1,ATRN,BLVRB,CSTA,CP,KLKB1,AMBP,TCIRG1,ITIH4,PSG9,FCN3,DSC2,CSF1R,FCN2,PSG6,MASP1,SLC27A4,ANTXR1,SH3D19,PCSK9,FGB,F2,PLG,PSG1,STAB1,TLN1,P4HB,COLEC10,FGG,LIMS1,C8A,PCDH18,F5,ADAM12,HTRA1,FLNA,C8B,FCN1,MBL2,F10,F7,ADGRG2,GOLM1,IGF1,PROS1,ACTN1,ILK,RAPGEF4,MASP2,PSG4,F11,CD14,PCYOX1,C4A,ATP9B,COLEC11,C4B,TGFBI,CFB,TCAF1,PDGFC,ATP1A1,IGKV2D-28,TTN,IGHV3-15,IGHV3-72,SEMA3B,PLCG2,OR52B4,ACTB,ITM2B,FGA
GO Component	GO:0034365	Discoidal high-density lipoprotein particle	2	2	1.95	0.0227	APOE,APOM
GO Component	GO:0035580	Specific granule lumen	5	62	0.85	0.0272	VCL,LYZ,TIMP2,CAMP,SLPI
GO Component	GO:0005925	Focal adhesion	13	416	0.44	0.0326	VCL,MYH9,CAT,TLN1,P4HB,LIMS1,S100A7,FLNA,ACTN1,YWHAZ,ILK,TPM4,ACTB
GO Component	GO:0062167	Complement component C1q complex	2	3	1.77	0.0354	C1QB,C1QC
GO Component	GO:0101002	ficolin-1-rich granule	8	185	0.58	0.0428	VCL,CAT,TIMP2,A1BG,TCIRG1,PRG2,FCN1,SERPINA1
GO Component	GO:0005903	Brush border	6	106	0.7	0.0456	VCL,MYH9,SLC27A4,FLNA,ACTN1,MYL6

STRING clusters	CL:18723	Mixed, incl. Complement and coagulation cascades, and Protein-lipid complex	70	196	1.5	1.68e-74	SPP2,SERPIND1,F9,C5,HRG,APOA1,C3,APOE,F12,APCS,CFHR5,VWF,SERPINA10,LCAT,KLKB1,F13A1,AMBP,HPX,FCN3,ITIH1,HABP2,FCN2,MASP1,ANGPTL4,PCSK9,FGB,F2,PLG,STAB1,C1QB,CFHR1,APOL1,SERPINF2,COLEC10,SERPINA11,FGG,ITIH2,C8A,F13B,CFHR3,CFH,F5,ECM1,C8B,FCN1,ADAMTS13,MBL2,C1QC,F10,F7,APOM,PROS1,MASP2,SAA1,F11,C1S,PCYOX1,C4A,COLEC11,ITIH3,C4B,SERPINA1,CFB,PLTP,HPR,C1R,APOC1,APOC2,APOC4,FGA
STRING clusters	CL:18724	Mixed, incl. Complement and coagulation cascades, and Protein-lipid complex	68	172	1.55	1.68e-74	SPP2,SERPIND1,F9,C5,HRG,APOA1,C3,APOE,F12,APCS,CFHR5,VWF,SERPINA10,LCAT,KLKB1,F13A1,AMBP,HPX,FCN3,ITIH1,HABP2,FCN2,MASP1,ANGPTL4,PCSK9,FGB,F2,PLG,C1QB,CFHR1,APOL1,SERPINF2,COLEC10,SERPINA11,FGG,ITIH2,C8A,F13B,CFHR3,CFH,F5,ECM1,C8B,FCN1,ADAMTS13,MBL2,C1QC,F10,F7,APOM,MASP2,SAA1,F11,C1S,PCYOX1,C4A,COLEC11,ITIH3,C4B,SERPINA1,CFB,PLTP,HPR,C1R,APOC1,APOC2,APOC4,FGA
STRING clusters	CL:18725	Complement and coagulation cascades, and Protein-lipid complex	66	166	1.55	1.04e-72	SERPIND1,F9,C5,HRG,APOA1,C3,APOE,F12,APCS,CFHR5,VWF,SERPINA10,LCAT,KLKB1,F13A1,AMBP,HPX,FCN3,ITIH1,HABP2,FCN2,MASP1,ANGPTL4,PCSK9,FGB,F2,PLG,C1QB,CFHR1,APOL1,SERPINF2,COLEC10,SERPINA11,FGG,ITIH2,C8A,F13B,CFHR3,CFH,F5,C8B,FCN1,ADAMTS13,MBL2,C1QC,F10,F7,APOM,MASP2,SAA1,F11,C1S,PCYOX1,C4A,COLEC11,ITIH3,C4B,SERPINA1,CFB,PLTP,HPR,C1R,APOC1,APOC2,APOC4,FGA
STRING clusters	CL:18726	Complement and coagulation cascades, and Protein-lipid complex	65	161	1.55	5.46e-72	SERPIND1,F9,C5,HRG,APOA1,C3,APOE,F12,APCS,CFHR5,VWF,LCAT,KLKB1,F13A1,AMBP,HPX,FCN3,ITIH1,HABP2,FCN2,MASP1,ANGPTL4,PCSK9,FGB,F2,PLG,C1QB,CFHR1,APOL1,SERPINF2,COLEC10,SERPINA11,FGG,ITIH2,C8A,F13B,CFHR3,CFH,F5,C8B,FCN1,ADAMTS13,MBL2,C1QC,F10,F7,APOM,MASP2,SAA1,F11,C1S,PCYOX1,C4A,COLEC11,ITIH3,C4B,SERPINA1,CFB,PLTP,HPR,C1R,APOC1,APOC2,APOC4,FGA
STRING clusters	CL:18728	Complement and coagulation cascades, and Positive regulation of opsonization	51	109	1.62	3.99e-58	SERPIND1,F9,C5,HRG,C3,F12,APCS,CFHR5,VWF,KLKB1,F13A1,AMBP,HPX,FCN3,ITIH1,HABP2,FCN2,MASP1,FGB,F2,PLG,C1QB,CFHR1,SERPINF2,COLEC10,SERPINA11,FGG,ITIH2,C8A,F13B,CFHR3,CFH,F5,C8B,FCN1,ADAMTS13,MBL2,C1QC,F10,F7,MASP2,F11,C1S,C4A,COLEC11,ITIH3,C4B,SERPINA1,CFB,C1R,FGA
STRING clusters	CL:18731	Hemostasis, and Dissolution of Fibrin Clot	26	50	1.66	2.31e-29	SERPIND1,F9,HRG,F12,VWF,KLKB1,F13A1,AMBP,HPX,ITIH1,HABP2,FGB,F2,PLG,SERPINF2,FGG,ITIH2,F13B,F5,ADAMTS13,F10,F7,F11,ITIH3,SERPINA1,FGA
STRING clusters	CL:18846	Complement cascade	24	50	1.63	2.07e-26	C5,C3,APCS,CFHR5,FCN3,FCN2,MASP1,C1QB,CFHR1,COLEC10,C8A,CFHR3,CFH,C8B,FCN1,MBL2,C1QC,MASP2,C1S,C4A,COLEC11,C4B,CFB,C1R
STRING clusters	CL:18848	Complement cascade	20	40	1.65	4.74e-22	C5,C3,FCN3,FCN2,MASP1,C1QB,COLEC10,C8A,CFH,C8B,FCN1,MBL2,C1QC,MASP2,C1S,C4A,COLEC11,C4B,CFB,C1R
STRING clusters	CL:18849	Initial triggering of complement, and Regulation of complement activation	17	31	1.69	4.42e-19	C3,FCN3,FCN2,MASP1,C1QB,COLEC10,CFH,FCN1,MBL2,C1QC,MASP2,C1S,C4A,COLEC11,C4B,CFB,C1R
STRING clusters	CL:18732	Mixed, incl. COVID-19, thrombosis and anticoagulation, and Scavenging of heme from plasma	16	26	1.74	1.61e-18	SERPIND1,HRG,F13A1,AMBP,HPX,ITIH1,HABP2,FGB,PLG,SERPINF2,FGG,ITIH2,F13B,ITIH3,SERPINA1,FGA
STRING clusters	CL:18733	Mixed, incl. COVID-19, thrombosis and anticoagulation, and Inter-alpha-trypsin inhibitor heavy chain C-terminus	15	21	1.8	5.02e-18	SERPIND1,HRG,F13A1,AMBP,ITIH1,HABP2,FGB,PLG,SERPINF2,FGG,ITIH2,F13B,ITIH3,SERPINA1,FGA
STRING clusters	CL:18851	Initial triggering of complement, and Negative regulation of complement activation	15	24	1.74	2.04e-17	C3,FCN3,FCN2,MASP1,C1QB,COLEC10,FCN1,MBL2,C1QC,MASP2,C1S,C4A,COLEC11,C4B,C1R
STRING clusters	CL:18852	Creation of C4 and C2 activators	12	15	1.85	1.25e-14	FCN3,FCN2,MASP1,C1QB,COLEC10,FCN1,MBL2,C1QC,MASP2,C1S,COLEC11,C1R
STRING clusters	CL:18956	Lipoprotein particle, and Assembly of active LPL and LIPC lipase complexes	14	46	1.43	9.52e-13	APOA1,APOE,LCAT,ANGPTL4,PCSK9,APOL1,APOM,SAA1,PCYOX1,PLTP,HPR,APOC1,APOC2,APOC4
STRING clusters	CL:18734	COVID-19, thrombosis and anticoagulation, and Negative regulation of fibrinolysis	10	11	1.91	2.06e-12	SERPIND1,HRG,F13A1,HABP2,FGB,PLG,SERPINF2,FGG,F13B,FGA
STRING clusters	CL:18957	Lipoprotein particle	12	36	1.47	3.39e-11	APOA1,LCAT,ANGPTL4,APOL1,APOM,SAA1,PCYOX1,PLTP,HPR,APOC1,APOC2,APOC4
STRING clusters	CL:18960	High-density lipoprotein particle	11	26	1.57	4.10e-11	APOA1,LCAT,APOL1,APOM,SAA1,PCYOX1,PLTP,HPR,APOC1,APOC2,APOC4
STRING clusters	CL:18784	Formation of Fibrin Clot (Clotting Cascade)	10	18	1.69	6.07e-11	F9,F12,VWF,KLKB1,F2,F5,ADAMTS13,F10,F7,F11
STRING clusters	CL:18962	High-density lipoprotein particle	10	21	1.63	1.90e-10	APOA1,LCAT,APOL1,APOM,PCYOX1,PLTP,HPR,APOC1,APOC2,APOC4
STRING clusters	CL:18786	Hemophilia B, and Blood coagulation, common pathway	8	12	1.77	4.68e-09	F9,VWF,F2,F5,ADAMTS13,F10,F7,F11
STRING clusters	CL:18737	Fibrinogen, and Thrombophilia	6	6	1.95	2.98e-07	SERPIND1,HRG,FGB,PLG,FGG,FGA
STRING clusters	CL:18854	Cell surface pattern recognition receptor signaling pathway	6	6	1.95	2.98e-07	FCN3,FCN2,MASP1,FCN1,MBL2,MASP2
STRING clusters	CL:18966	High-density lipoprotein particle	7	16	1.59	7.20e-07	APOA1,LCAT,APOM,PLTP,APOC1,APOC2,APOC4
STRING clusters	CL:35044	Mixed, incl. Galactoside-binding lectin, and Transforming growth factor beta1 activation	6	16	1.52	1.95e-05	PSG9,PSG6,PSG1,PSG11,PSG4,PSG2
STRING clusters	CL:35049	Transforming growth factor beta1 activation, and Protein tyrosine kinase binding	5	7	1.8	2.00e-05	PSG9,PSG6,PSG1,PSG4,PSG2
STRING clusters	CL:19696	Elastic fibre formation, and Tolloid/BMP1 peptidase domain	7	34	1.26	4.99e-05	BMP1,FBN1,FBLN1,FBLN5,EFEMP1,LTBP1,FBN2
STRING clusters	CL:18755	Mixed, incl. Inter-alpha-trypsin inhibitor heavy chain C-terminus, and Alpha-1-acid glycoprotein	5	10	1.65	6.87e-05	AMBP,ITIH1,ITIH2,ITIH3,SERPINA1
STRING clusters	CL:19695	Elastic fibre formation, and Matrix metalloproteinases	8	61	1.07	0.00014	TIMP2,BMP1,FBN1,FBLN1,FBLN5,EFEMP1,LTBP1,FBN2
STRING clusters	CL:18122	Mixed, incl. Cell-extracellular matrix interactions, and Leukocyte adhesion deficiency 3	6	26	1.31	0.00018	VCL,FERMT3,TLN1,LIMS1,FLNA,ILK
STRING clusters	CL:19697	Elastic fibre formation, and Transforming growth factor beta receptor complex assembly	6	27	1.29	0.00021	FBN1,FBLN1,FBLN5,EFEMP1,LTBP1,FBN2
STRING clusters	CL:19457	Extracellular matrix organization	12	180	0.77	0.00022	THBS1,TIMP2,BMP1,FBN1,P4HB,FBLN1,THBS4,FBLN5,COL18A1,EFEMP1,LTBP1,FBN2
STRING clusters	CL:19699	Elastic fibre formation	5	14	1.5	0.00022	FBN1,FBLN1,FBLN5,EFEMP1,FBN2
STRING clusters	CL:18788	Extrinsic Pathway of Fibrin Clot Formation, and Factor V deficiency	4	6	1.77	0.00037	F2,F5,F10,F7
STRING clusters	CL:18869	Complement component C1 complex	4	6	1.77	0.00037	C1QB,C1QC,C1S,C1R
STRING clusters	CL:19115	Mixed, incl. Cystatin superfamily, and Specific granule lumen	7	60	1.02	0.0010	LGALS1,LYZ,A1BG,LCN1,CSTA,CAMP,SLPI
STRING clusters	CL:18970	Triglyceride-rich lipoprotein particle remodeling, and Spherical high-density lipoprotein particle	4	11	1.51	0.0020	APOA1,LCAT,APOC1,APOC2
STRING clusters	CL:19114	Mixed, incl. S100/CaBP-9k-type, calcium binding, subdomain, and Cystatin superfamily	8	103	0.84	0.0036	LGALS1,LYZ,A1BG,LCN1,CSTA,CAMP,SLPI,S100A7

STRING clusters	CL:24266	Insulin-like growth factor binding, and Pappalysin-1/2	4	16	1.35	0.0063	IGFBP5,PRG2,IGF1,IGFALS
STRING clusters	CL:18934	Hemolytic uremic syndrome	3	5	1.73	0.0070	CFHR5,CFHR1,CFHR3
STRING clusters	CL:18123	Cell-extracellular matrix interactions, and Alpha-catenin/vinculin-like superfamily	4	17	1.32	0.0072	VCL,LIMS1,FLNA,ILK
STRING clusters	CL:9658	Detoxification of Reactive Oxygen Species, and Copper transport	5	40	1.05	0.0128	CAT,BLVRB,CP,SOD3,GPX3
STRING clusters	CL:18911	Membrane attack complex	3	7	1.58	0.0139	C5,C8A,C8B
STRING clusters	CL:19701	Marfan syndrome, and Microfibril-associated glycoprotein	3	8	1.52	0.0182	FBN1,FBLN5,FBN2
STRING clusters	CL:24268	Insulin-like growth factor-binding protein family 1-6, chordata, and Insulin-like growth factor	3	10	1.43	0.0305	IGFBP5,IGF1,IGFALS
STRING clusters	CL:17209	Immunoglobulin complex, and DNA recombinase complex	4	27	1.12	0.0310	JCHAIN,IGKV2D-28,IGHV3-15,IGHV3-72
STRING clusters	CL:19181	Mixed, incl. Specific granule lumen, and Mononeuritis multiplex	4	27	1.12	0.0310	LYZ,A1BG,LCN1,CAMP
STRING clusters	CL:9660	Detoxification of Reactive Oxygen Species, and Heme catabolic process	4	30	1.07	0.0430	CAT,BLVRB,SOD3,GPX3
KEGG	hsa04610	Complement and coagulation cascades	36	82	1.59	1.06e-39	SERPIND1,F9,C5,C3,F12,CFHR5,VWF,KLKB1,F13A1,FGB,F2,PLG,C1QB,CFHR1,SERPINF2,FGG,C8A,F13B,CFHR3,CFH,F5,C8B,MBL2,C1QC,F10,F7,PROS1,MASP2,F11,C1S,C4A,C4B,SERPINA1,CFB,C1R,FGA
KEGG	hsa05150	Staphylococcus aureus infection	15	86	1.19	6.77e-11	C5,C3,CAMP,PLG,C1QB,FGG,CFH,MBL2,C1QC,MASP2,C1S,C4A,C4B,CFB,C1R
KEGG	hsa04979	Cholesterol metabolism	9	48	1.22	1.43e-06	APOB,APOA1,APOE,LCAT,ANGPTL4,PCSK9,PLTP,APOC1,APOC2
KEGG	hsa05322	Systemic lupus erythematosus	11	94	1.02	2.15e-06	C5,C3,C1QB,C8A,C8B,C1QC,ACTN1,C1S,C4A,C4B,C1R
KEGG	hsa05133	Pertussis	9	73	1.04	2.19e-05	C5,C3,C1QB,C1QC,C1S,CD14,C4A,C4B,C1R
KEGG	hsa04611	Platelet activation	10	122	0.86	0.00013	VWF,FERMT3,GUCY1A1,FGB,F2,TLN1,FGG,PLCG2,ACTB,FGA
KEGG	hsa04510	Focal adhesion	12	195	0.74	0.00018	VCL,THBS1,VWF,TLN1,THBS4,FLNA,IGF1,ACTN1,ILK,PDGFC,VEGFC,ACTB
KEGG	hsa04145	Phagosome	9	141	0.75	0.0020	C3,THBS1,TCIRG1,THBS4,MBL2,CD14,COLEC11,C1R,ACTB
KEGG	hsa03320	PPAR signaling pathway	6	75	0.85	0.0108	APOA1,SLC27A4,ANGPTL4,ILK,ADIPOQ,PLTP
Reactome	HSA-109582	Hemostasis	56	607	0.91	4.32e-30	SPP2,VCL,SERPIND1,F9,PF4V1,SPARC,HRG,APOB,PLEK,APOA1,F12,THBS1,VWF,A1BG,KLKB1,F13A1,CENPE,ITIH4,PSG9,FERMT3,PSG6,PPBP,PF4,GUCY1A1,FGB,F2,PLG,PSG1,TLN1,PSG11,SERPINF2,FGG,F13B,F5,ECM1,FLNA,F10,F7,IGF1,PROS1,ACTN1,YWHAZ,RAPGEF4,PSG4,F11,PSG2,ITIH3,SERPINA1,SELENOP,JCHAIN,IGKV2D-28,TTN,VEGFC,PLCG2,ACTB,FGA
Reactome	HSA-114608	Platelet degranulation	31	126	1.34	6.88e-27	SPP2,VCL,SPARC,HRG,PLEK,APOA1,THBS1,VWF,A1BG,F13A1,ITIH4,FERMT3,PPBP,PF4,FGB,PLG,TLN1,SERPINF2,FGG,F5,ECM1,FLNA,IGF1,PROS1,ACTN1,ITIH3,SERPINA1,SELENOP,TTN,VEGFC,FGA
Reactome	HSA-166658	Complement cascade	25	59	1.58	1.87e-26	C5,C3,CFHR5,FCN3,FCN2,F2,C1QB,CFHR1,COLEC10,C8A,CFHR3,CFH,C8B,FCN1,MBL2,C1QC,PROS1,MASP2,C1S,C4A,COLEC11,C4B,CFB,C1R,IGKV2D-28
Reactome	HSA-76002	Platelet activation, signaling and aggregation	35	260	1.08	3.67e-23	SPP2,VCL,SPARC,HRG,PLEK,APOA1,THBS1,VWF,A1BG,F13A1,ITIH4,FERMT3,PPBP,PF4,FGB,F2,PLG,TLN1,SERPINF2,FGG,F5,ECM1,FLNA,IGF1,PROS1,ACTN1,YWHAZ,RAPGEF4,ITIH3,SERPINA1,SELENOP,TTN,VEGFC,PLCG2,FGA
Reactome	HSA-381426	Regulation of Insulin-like Growth Factor (IGF) transport and uptake by Insulin-like Growth Factor Binding Proteins (IGFBPs)	26	124	1.27	4.05e-21	SPP2,SERPIND1,LGALS1,APOB,IGFBP5,APOA1,C3,APOE,SERPINA10,CP,PCSK9,F2,PLG,APOL1,FBN1,P4HB,FGG,ITIH2,F5,GOLM1,IGF1,LTBP1,C4A,SERPINA1,IGFALS,FGA
Reactome	HSA-140877	Formation of Fibrin Clot (Clotting Cascade)	18	39	1.61	2.41e-19	SERPIND1,F9,PF4V1,F12,VWF,KLKB1,F13A1,PF4,FGB,F2,FGG,F13B,F5,F10,F7,PROS1,F11,FGA
Reactome	HSA-166663	Initial triggering of complement	16	24	1.77	4.64e-19	C3,FCN3,FCN2,C1QB,COLEC10,FCN1,MBL2,C1QC,MASP2,C1S,C4A,COLEC11,C4B,CFB,C1R,IGKV2D-28
Reactome	HSA-977606	Regulation of Complement cascade	18	49	1.51	5.14e-18	C5,C3,CFHR5,F2,C1QB,CFHR1,C8A,CFHR3,CFH,C8B,C1QC,PROS1,C1S,C4A,C4B,CFB,C1R,IGKV2D-28
Reactome	HSA-8957275	Post-translational protein phosphorylation	22	107	1.26	1.09e-17	SPP2,SERPIND1,LGALS1,APOB,IGFBP5,APOA1,C3,APOE,SERPINA10,CP,PCSK9,APOL1,FBN1,P4HB,FGG,ITIH2,F5,GOLM1,LTBP1,C4A,SERPINA1,FGA
Reactome	HSA-168249	Innate Immune System	49	1041	0.62	5.81e-15	VCL,MYH9,C5,APOB,TTR,CAT,C3,BPIFB1,CFHR5,LYZ,PSMA6,TIMP2,A1BG,TCIRG1,FCN3,FCN2,PPBP,CAMP,FGB,F2,PRG2,C1QB,CFHR1,COLEC10,FGG,SLPI,C8A,CFHR3,CFH,S100A7,C8B,FCN1,MBL2,C1QC,PROS1,MASP2,SAA1,C1S,CD14,C4A,COLEC11,C4B,SERPINA1,CFB,C1R,IGKV2D-28,PLCG2,ACTB,FGA
Reactome	HSA-166786	Creation of C4 and C2 activators	12	16	1.82	1.35e-14	FCN3,FCN2,C1QB,COLEC10,FCN1,MBL2,C1QC,MASP2,C1S,COLEC11,C1R,IGKV2D-28
Reactome	HSA-140875	Common Pathway of Fibrin Clot Formation	12	22	1.68	2.11e-13	SERPIND1,PF4V1,F13A1,PF4,FGB,F2,FGG,F13B,F5,F10,PROS1,FGA
Reactome	HSA-2173782	Binding and Uptake of Ligands by Scavenger Receptors	14	43	1.46	2.48e-13	SPARC,APOB,APOA1,APOE,AMBP,HPX,STAB1,APOL1,SAA1,COLEC11,JCHAIN,HPR,IGKV2D-28,HSPH1
Reactome	HSA-1474244	Extracellular matrix organization	23	300	0.83	2.80e-10	SPARC,TTR,PXDN,THBS1,VWF,TIMP2,KLKB1,BMP1,FGB,PLG,FBN1,P4HB,FBLN1,FGG,FBLN5,COL18A1,ADAM12,HTRA1,ACTN1,EFEMP1,LTBP1,FBN2,FGA
Reactome	HSA-168256	Immune System	58	1979	0.42	1.62e-09	VCL,MYH9,C5,APOB,TTR,CAT,C3,BPIFB1,CFHR5,LYZ,PSMA6,TIMP2,A1BG,F13A1,CENPE,TCIRG1,FCN3,SEC24D,CSF1R,FCN2,PPBP,CAMP,FGB,F2,UBE3C,PRG2,C1QB,CFHR1,P4HB,COLEC10,FGG,SLPI,C8A,CFHR3,CFH,S100A7,FLNA,C8B,FCN1,MBL2,C1QC,PROS1,YWHAZ,RAPGEF4,MASP2,SAA1,C1S,CD14,C4A,COLEC11,C4B,SERPINA1,CFB,C1R,IGKV2D-28,PLCG2,ACTB,FGA
Reactome	HSA-140837	Intrinsic Pathway of Fibrin Clot Formation	9	23	1.54	6.80e-09	SERPIND1,F9,F12,VWF,KLKB1,F2,F10,PROS1,F11
Reactome	HSA-166662	Lectin pathway of complement activation	7	7	1.95	7.91e-09	FCN3,FCN2,COLEC10,FCN1,MBL2,MASP2,COLEC11
Reactome	HSA-174824	Plasma lipoprotein assembly, remodeling, and clearance	12	75	1.15	2.78e-08	APOB,APOA1,APOE,LCAT,ANGPTL4,PCSK9,BMP1,P4HB,PLTP,APOC1,APOC2,APOC4
Reactome	HSA-8963898	Plasma lipoprotein assembly	8	19	1.57	4.43e-08	APOB,APOA1,APOE,BMP1,P4HB,APOC1,APOC2,APOC4
Reactome	HSA-2168880	Scavenging of heme from plasma	7	15	1.62	3.12e-07	APOA1,AMBP,HPX,APOL1,JCHAIN,HPR,IGKV2D-28
Reactome	HSA-9651496	Defects of contact activation system (CAS) and kallikrein/kinin system (KKS)	7	16	1.59	4.25e-07	F9,F12,VWF,KLKB1,F2,F10,F11
Reactome	HSA-202733	Cell surface interactions at the vascular wall	13	139	0.92	1.53e-06	PF4V1,APOB,PSG9,PSG6,PF4,F2,PSG1,PSG11,PROS1,PSG4,PSG2,JCHAIN,IGKV2D-28
Reactome	HSA-8963899	Plasma lipoprotein remodeling	8	34	1.32	1.63e-06	APOB,APOA1,APOE,LCAT,ANGPTL4,P4HB,PLTP,APOC2
Reactome	HSA-392499	Metabolism of proteins	49	1917	0.36	4.64e-06	SPP2,ST6GAL1,SERPIND1,LGALS1,F9,APOB,IGFBP5,APOA1,TTR,C3,APOE,APCS,THBS1,LYZ,PSMA6,SERPINA10,CP,PLA2G7,SEC24D,PCSK9,F2,INHBC,PLG,SUZ12,APOL1,FBN1,P4HB,FGG,GANAB,BLM,ITIH2,F5,ADAMTSL4,ADAMTS13,F10,F7,GOLM1,IGF1,PROS1,SAA1,LTBP1,C4A,SERPINA1,TGFBI,IGFALS,UIMC1,ACTB,ITM2B,FGA
Reactome	HSA-174577	Activation of C3 and C5	5	7	1.8	1.03e-05	C5,C3,C4A,C4B,CFB
Reactome	HSA-159763	Transport of gamma-carboxylated protein precursors from the endoplasmic reticulum to the Golgi apparatus	5	9	1.69	2.47e-05	F9,F2,F10,F7,PROS1
Reactome	HSA-173623	Classical antibody-mediated complement activation	5	9	1.69	2.47e-05	C1QB,C1QC,C1S,C1R,IGKV2D-28
Reactome	HSA-159740	Gamma-carboxylation of protein precursors	5	10	1.65	3.39e-05	F9,F2,F10,F7,PROS1
Reactome	HSA-159782	Removal of aminoterminal propeptides from gamma-carboxylated proteins	5	10	1.65	3.39e-05	F9,F2,F10,F7,PROS1

Reactome	HSA-6802948	Signaling by high-kinase activity BRAF mutants	7	36	1.24	3.39e-05	VCL,VWF,FGB,TLN1,FGG,ACTB,FGA
Reactome	HSA-8963888	Chylomicron assembly	5	10	1.65	3.39e-05	APOB,APOA1,APOE,P4HB,APOC2
Reactome	HSA-8964058	HDL remodeling	5	10	1.65	3.39e-05	APOA1,APOE,LCAT,PLTP,APOC2
Reactome	HSA-597592	Post-translational protein modification	38	1405	0.38	3.84e-05	SPP2,ST6GAL1,SERPIND1,LGALS1,F9,APOB,IGFBP5,APOA1,C3,APOE,THBS1,PSMA6,SERPINA10,CP,SEC24D,PCSK9,F2,SUZ12,APOL1,FBN1,P4HB,FGG,GANAB,BLM,ITIH2,F5,ADAMTSL4,ADAMTS13,F10,F7,GOLM1,PROS1,LTBP1,C4A,SERPINA1,UIMC1,ACTB,FGA
Reactome	HSA-977225	Amyloid fiber formation	9	79	1.0	3.84e-05	APOA1,TTR,APOE,APCS,LYZ,SAA1,TGFBI,ITM2B,FGA
Reactome	HSA-76009	Platelet Aggregation (Plug Formation)	7	39	1.2	4.32e-05	VWF,FGB,F2,TLN1,FGG,RAPGEF4,FGA
Reactome	HSA-5674135	MAP2K and MAPK activation	7	40	1.19	4.90e-05	VCL,VWF,FGB,TLN1,FGG,ACTB,FGA
Reactome	HSA-9656223	Signaling by RAF1 mutants	7	41	1.18	5.54e-05	VCL,VWF,FGB,TLN1,FGG,ACTB,FGA
Reactome	HSA-2855086	Ficolins bind to repetitive carbohydrate structures on the target cell surface	4	4	1.95	5.65e-05	FCN3,FCN2,FCN1,MASP2
Reactome	HSA-975634	Retinoid metabolism and transport	7	44	1.15	8.06e-05	APOB,APOA1,TTR,APOE,RBP4,APOM,APOC2
Reactome	HSA-354192	Integrin signaling	6	27	1.29	8.19e-05	VWF,FGB,TLN1,FGG,RAPGEF4,FGA
Reactome	HSA-6802946	Signaling by moderate kinase activity BRAF mutants	7	45	1.14	8.80e-05	VCL,VWF,FGB,TLN1,FGG,ACTB,FGA
Reactome	HSA-6802955	Paradoxical activation of RAF signaling by kinase inactive BRAF	7	45	1.14	8.80e-05	VCL,VWF,FGB,TLN1,FGG,ACTB,FGA
Reactome	HSA-8866423	VLDL assembly	4	5	1.85	8.80e-05	APOB,P4HB,APOC1,APOC4
Reactome	HSA-9649948	Signaling downstream of RAS mutants	7	45	1.14	8.80e-05	VCL,VWF,FGB,TLN1,FGG,ACTB,FGA
Reactome	HSA-354194	GRB2:SOS provides linkage to MAPK signaling for Integrins	5	15	1.47	0.00010	VWF,FGB,TLN1,FGG,FGA
Reactome	HSA-372708	p130Cas linkage to MAPK signaling for integrins	5	15	1.47	0.00010	VWF,FGB,TLN1,FGG,FGA
Reactome	HSA-446353	Cell-extracellular matrix interactions	5	18	1.39	0.00020	LIMS1,FLNA,ACTN1,ILK,ACTB
Reactome	HSA-9662001	Defective factor VIII causes hemophilia A	4	7	1.71	0.00020	F9,VWF,F2,F10
Reactome	HSA-9029569	NR1H3 & NR1H2 regulate gene expression linked to cholesterol transport and efflux	6	36	1.17	0.00028	APOE,TNRC6A,PLTP,APOC1,APOC2,APOC4
Reactome	HSA-5686938	Regulation of TLR by endogenous ligand	5	20	1.35	0.00030	APOB,FGB,FGG,CD14,FGA
Reactome	HSA-2129379	Molecules associated with elastic fibres	6	37	1.16	0.00032	FBN1,FBLN1,FBLN5,EFEMP1,LTBP1,FBN2
Reactome	HSA-8964043	Plasma lipoprotein clearance	6	37	1.16	0.00032	APOB,APOA1,APOE,PCSK9,APOC1,APOC4
Reactome	HSA-5653656	Vesicle-mediated transport	22	666	0.47	0.00038	MYH9,SPARC,APOB,APOA1,APOE,AMBP,CENPE,HPX,SEC24D,SH3D19,STAB1,APOL1,F5,YWHAZ,SAA1,COLEC11,SERPINA1,JCHAIN,HPR,IGKV2D-28,HSPH1,ACTB
Reactome	HSA-8963901	Chylomicron remodeling	4	10	1.55	0.00053	APOB,APOA1,APOE,APOC2
Reactome	HSA-6802952	Signaling by BRAF and RAF1 fusions	7	65	0.98	0.00059	VCL,VWF,FGB,TLN1,FGG,ACTB,FGA
Reactome	HSA-445355	Smooth Muscle Contraction	6	43	1.09	0.00063	VCL,GUCY1A1,TLN1,MYL6,TPM4,TPM3
Reactome	HSA-1643685	Disease	39	1702	0.31	0.00072	ST6GAL1,VCL,MYH9,F9,APOA1,TTR,C3,F12,THBS1,VWF,PSMA6,CP,KLKB1,SEC24D,SLC27A4,ANTXR1,FGB,F2,TLN1,SUZ12,FGG,GANAB,BLM,ADAMTSL4,RBP4,ADAMTS13,MBL2,F10,YWHAZ,MASP2,F11,CD14,ATP1A1,IGKV2D-28,PLCG2,TPM4,ACTB,FGA,TPM3
Reactome	HSA-9657688	Defective factor XII causes hereditary angioedema	3	3	1.95	0.00094	F12,KLKB1,F2
Reactome	HSA-3000497	Scavenging by Class H Receptors	3	4	1.82	0.0016	SPARC,APOB,STAB1
Reactome	HSA-140834	Extrinsic Pathway of Fibrin Clot Formation	3	5	1.73	0.0025	F9,F10,F7
Reactome	HSA-216083	Integrin cell surface interactions	7	85	0.86	0.0025	THBS1,VWF,FGB,FBN1,FGG,COL18A1,FGA
Reactome	HSA-5602498	MyD88 deficiency (TLR2/4)	4	17	1.32	0.0025	FGB,FGG,CD14,FGA
Reactome	HSA-3000480	Scavenging by Class A Receptors	4	18	1.29	0.0030	APOB,APOA1,APOE,COLEC11
Reactome	HSA-5603041	IRAK4 deficiency (TLR2/4)	4	18	1.29	0.0030	FGB,FGG,CD14,FGA
Reactome	HSA-3000471	Scavenging by Class B Receptors	3	6	1.65	0.0034	APOB,APOA1,SAA1
Reactome	HSA-8964046	VLDL clearance	3	6	1.65	0.0034	APOB,APOC1,APOC4
Reactome	HSA-6798695	Neutrophil degranulation	16	476	0.47	0.0041	VCL,TTR,CAT,C3,LYZ,TIMP2,A1BG,TCIRG1,PPBP,CAMP,PRG2,SLPI,S100A7,FCN1,CD14,SERPINA1
Reactome	HSA-166665	Terminal pathway of complement	3	8	1.52	0.0062	C5,C8A,C8B
Reactome	HSA-1474228	Degradation of the extracellular matrix	8	140	0.71	0.0075	TIMP2,KLKB1,BMP1,PLG,FBN1,COL18A1,HTRA1,FBN2
Reactome	HSA-382551	Transport of small molecules	20	723	0.39	0.0075	APOB,APOA1,APOE,PSMA6,LCN1,LCAT,CP,TCIRG1,PIP,SLC27A4,ANGPTL4,PCSK9,BMP1,P4HB,ATP9B,PLTP,ATP1A1,APOC1,APOC2,APOC4
Reactome	HSA-9668250	Defective factor IX causes hemophilia B	3	9	1.47	0.0079	F9,F10,F11
Reactome	HSA-430116	GP1b-IX-V activation signalling	3	12	1.35	0.0157	VWF,FLNA,YWHAZ
Reactome	HSA-75205	Dissolution of Fibrin Clot	3	13	1.31	0.0184	HRG,PLG,SERPINF2
Reactome	HSA-1592389	Activation of Matrix Metalloproteinases	4	33	1.03	0.0197	TIMP2,KLKB1,PLG,COL18A1
Reactome	HSA-162582	Signal Transduction	46	2540	0.21	0.0211	VCL,MYH9,C5,SPARC,C3,APOE,THBS1,VWF,PSMA6,CENPE,TCIRG1,CRH,PPBP,PF4,FGB,F2,PLG,TLN1,SUZ12,FBN1,P4HB,FGG,THBS4,ADAM12,FLNA,IGF1,ACTN1,TNRC6A,YWHAZ,RAPGEF4,SAA1,LTBP1,MST1,PLTP,HGFAC,PDGFC,MYL6,APOC1,APOC2,APOC4,VEGFC,PLCG2,TPM4,ACTB,FGA,TPM3
Reactome	HSA-446728	Cell junction organization	6	92	0.76	0.0211	ANG,LIMS1,FLNA,ACTN1,ILK,ACTB
Reactome	HSA-3299685	Detoxification of Reactive Oxygen Species	4	36	0.99	0.0257	CAT,P4HB,SOD3,GPX3
Reactome	HSA-9657689	Defective SERPING1 causes hereditary angioedema	2	3	1.77	0.0307	F12,KLKB1
Reactome	HSA-9672383	Defective factor IX causes thrombophilia	2	3	1.77	0.0307	F9,F10
Reactome	HSA-9672391	Defective F8 cleavage by thrombin	2	3	1.77	0.0307	VWF,F2
Reactome	HSA-9672396	Defective cofactor function of FVIIIa variant	2	3	1.77	0.0307	F9,F10
Reactome	HSA-9673202	Defective F9 variant does not activate FX	2	3	1.77	0.0307	F9,F10
WikiPathways	WP558	Complement and coagulation cascades	25	58	1.58	1.81e-26	SERPIND1,F9,C3,F12,VWF,KLKB1,MASP1,FGB,F2,PLG,C1QB,SERPINF2,F13B,CFH,F5,C1QC,F10,F7,PROS1,MASP2,C1S,C4B,SERPINA1,CFB,C1R
WikiPathways	WP2806	Complement system	26	96	1.38	1.43e-23	C5,APOA1,C3,F12,APCS,THBS1,KLKB1,F13A1,FCN2,MASP1,FGB,PLG,FGG,C8A,CFH,FCN1,MBL2,F10,PROS1,MASP2,F11,C1S,ADIPOQ,C4A,CFB,FGA
WikiPathways	WP272	Blood clotting cascade	15	22	1.78	6.18e-18	F9,F12,VWF,KLKB1,FGB,F2,PLG,SERPINF2,FGG,F13B,F5,F10,F7,F11,FGA
WikiPathways	WP5090	Complement system in neuronal development and plasticity	21	105	1.25	1.12e-16	C5,C3,FCN3,FCN2,MASP1,C1QB,COLEC10,C8A,CFH,C8B,FCN1,MBL2,C1QC,PROS1,MASP2,C1S,C4A,COLEC11,C4B,CFB,C1R
WikiPathways	WP545	Complement activation	13	22	1.72	5.33e-15	C5,C3,MASP1,C1QB,C8A,C8B,C1QC,MASP2,C1S,C4A,C4B,CFB,C1R
WikiPathways	WP5115	Network map of SARS-CoV-2 signaling pathway	20	218	0.91	3.49e-10	HRG,APOA1,SERPINA10,ITIH4,PF4,FGB,APOL1,FGG,C8A,CFH,APOM,SAA1,C1S,CD14,ITIH3,CFB,C1R,APOC1,ACTB,FGA
WikiPathways	WP15	Selenium micronutrient network	14	84	1.17	4.81e-10	APOB,APOA1,CAT,FGB,F2,PLG,HBA1,FGG,F7,SOD3,GPX3,SAA1,SELENOP,FGA
WikiPathways	WP176	Folate metabolism	13	67	1.24	4.81e-10	APOB,APOA1,CAT,FGB,F2,PLG,HBA1,FGG,F7,SOD3,GPX3,SAA1,FGA
WikiPathways	WP4927	COVID-19, thrombosis and anticoagulation	7	7	1.95	5.41e-09	F13A1,FGB,F2,PLG,FGG,F13B,FGA
WikiPathways	WP1533	Vitamin B12 metabolism	9	50	1.2	1.36e-06	APOB,APOA1,APOE,F2,PLG,HBA1,F7,SOD3,SAA1
WikiPathways	WP430	Statin inhibition of cholesterol production	7	29	1.33	8.74e-06	APOB,APOA1,APOE,LCAT,PLTP,APOC1,APOC2
WikiPathways	WP4522	Metabolic pathway of LDL, HDL and TG, including diseases	6	17	1.5	1.00e-05	APOB,APOA1,APOE,LCAT,PCSK9,APOC2
WikiPathways	WP5304	Cholesterol metabolism	9	72	1.05	1.76e-05	APOB,APOA1,APOE,LCAT,SLC27A4,PCSK9,PLTP,APOC1,APOC2
WikiPathways	WP3601	Lipid particles composition	5	10	1.65	2.51e-05	APOB,APOA1,APOE,LCAT,APOC2
WikiPathways	WP5323	Fatty Acids and Lipoproteins Transport in Hepatocytes	18	380	0.62	2.92e-05	APOB,APOA1,APOE,LCAT,PLA2G7,SLC27A4,PCSK9,APOL1,APOM,SAA1,PCYOX1,ADIPOQ,ATP9B,PRG4,PLTP,APOC1,APOC2,APOC4
WikiPathways	WP5186	Vitamin K metabolism and activation of dependent proteins	5	12	1.57	4.45e-05	F9,F2,F10,F7,PROS1
WikiPathways	WP5109	Familial hyperlipidemia type 2	5	16	1.44	0.00013	APOB,APOA1,LCAT,PCSK9,PLTP

WikiPathways	WP306	Focal adhesion	12	195	0.74	0.00016	VCL,THBS1,VWF,TLN1,THBS4,FLNA,IGF1,ACTN1,ILK,PDGFC,VEGFC,ACTB
WikiPathways	WP5108	Familial hyperlipidemia type 1	5	18	1.39	0.00019	APOA1,LCAT,ANGPTL4,PLTP,APOC2
WikiPathways	WP3941	Oxidative damage response	6	39	1.14	0.00040	C5,C1QB,C1QC,C1S,C4B,C1R
WikiPathways	WP2328	Allograft rejection	8	88	0.91	0.00045	C5,C3,C1QB,C8A,C8B,C1QC,C4A,C4B
WikiPathways	WP5110	Familial hyperlipidemia type 3	4	13	1.44	0.0011	APOA1,APOE,LCAT,PLTP
WikiPathways	WP5112	Familial hyperlipidemia type 5	4	15	1.37	0.0017	APOA1,LCAT,PLTP,APOC2
WikiPathways	WP3942	PPAR signaling pathway	6	67	0.9	0.0053	APOA1,SLC27A4,ANGPTL4,ILK,ADIPOQ,PLTP
WikiPathways	WP4136	Fibrin complement receptor 3 signaling pathway	5	43	1.01	0.0058	FGB,PLG,FGG,CD14,FGA
WikiPathways	WP5055	Burn wound healing	7	100	0.79	0.0058	LGALS1,SPARC,TIMP2,F13A1,AMBP,FBN1,ADAM12
WikiPathways	WP5111	Familial hyperlipidemia type 4	4	22	1.21	0.0058	APOA1,LCAT,PLTP,APOC2
WikiPathways	WP3668	Hypothesized pathways in pathogenesis of cardiovascular disease	4	25	1.15	0.0080	FBN1,FLNA,LTBP1,FBN2
WikiPathways	WP5104	Acquired partial lipodystrophy / Barraquer-Simons syndrome	3	10	1.43	0.0097	C5,C3,CFB
WikiPathways	WP2572	Primary focal segmental glomerulosclerosis (FSGS)	5	72	0.79	0.0441	VCL,MYH9,TLN1,LIMS1,ILK
Monarch	EFO:0007937	Blood protein measurement	86	1810	0.62	9.41e-28	ST6GAL1,GNPTG,MYH9,C5,HRG,APOB,IGFBP5,CAT,C3,APOE,PXDN,BPIFB1,F12,CFHR5,LYZ,VWF,SERPINA10,TIMP2,LCAT,KLKB1,AMBP,HPX,ITIH4,PSG9,ITIH1,HABP2,DSC2,ANKRD44,CSF1R,FCN2,MASP1,ANTXR1,F2,PLG,PSG1,PRG2,APOL1,SERPINF2,OAF,COLEC10,SERPINA11,ANG,ITIH2,CNDP1,COL18A1,F13B,CFH,F5,ADAM12,ECM1,CRTAC1,C8B,RBP4,FCN1,ADAMTS13,CCN5,MBL2,C1QC,F10,F7,APOM,CA6,SOD3,PSG4,SAA1,F11,C1S,PSG2,PCYOX1,NBEAL1,COLEC11,MST1,C4B,SERPINA1,TGFBI,CFB,PLTP,GC,HGFAC,HPR,C1R,APOC1,APOC2,APOC4,VEGFC,PLCG2
Monarch	HP:0002597	Abnormality of the vasculature	63	1673	0.52	7.27e-14	SERPIND1,MYH9,F9,MGP,SPARC,APOB,APOA1,TTR,CAT,APOE,ANGPTL6,F12,LYZ,VWF,LCAT,CSTA,F13A1,DNAH5,TCIRG1,HABP2,FERMT3,SEC24D,MASP1,GUCY1A1,A2ML1,ANTXR1,PCSK9,BMP1,FGB,F2,PLG,SERPINF2,HBA1,FBN1,P4HB,FGG,GANAB,FBLN5,BLM,COL18A1,F13B,F5,HTRA1,FLNA,CRTAC1,RBP4,ADAMTS13,F10,F7,PROS1,EFEMP1,C1S,C4A,SERPINA1,TGFBI,FBN2,C1R,APOC2,VEGFC,PLCG2,ACTB,FGA,TPM3
Monarch	HP:0025015	Abnormal vascular morphology	48	1027	0.62	3.35e-13	SERPIND1,F9,MGP,SPARC,APOB,TTR,CAT,APOE,ANGPTL6,F12,VWF,LCAT,F13A1,DNAH5,TCIRG1,HABP2,SEC24D,MASP1,GUCY1A1,A2ML1,ANTXR1,PCSK9,FGB,F2,SERPINF2,FBN1,P4HB,FGG,GANAB,FBLN5,COL18A1,F13B,F5,HTRA1,FLNA,CRTAC1,RBP4,ADAMTS13,F10,F7,PROS1,EFEMP1,C4A,FBN2,VEGFC,ACTB,FGA,TPM3
Monarch	EFO:0004747	Protein measurement	125	5856	0.28	8.19e-13	ST6GAL1,GNPTG,MYH9,C5,HRG,APOB,IGFBP5,APOA1,CAT,C3,APOE,PXDN,ANGPTL6,BPIFB1,F12,APCS,CFHR5,LYZ,VWF,SERPINA10,TIMP2,LCAT,CP,KLKB1,DNAH5,AMBP,TCIRG1,HPX,ITIH4,PSG9,ITIH1,PLA2G7,HABP2,DSC2,ANKRD44,CSF1R,FCN2,MASP1,ANGPTL4,ANTXR1,PCSK9,FGB,F2,INHBC,PLG,PSG1,UBE3C,PRG2,STAB1,APOL1,SERPINF2,HBA1,FBLN1,OAF,COLEC10,DNAH8,OIT3,SERPINA11,ANG,FGG,LIMS1,THBS4,PAPLN,ITIH2,CNDP1,COL18A1,F13B,CFH,F5,ADAM12,ECM1,CRTAC1,C8B,RBP4,FCN1,ADAMTS13,CCN5,MBL2,C1QC,F10,F7,APOM,CA6,SOD3,IGF1,ACTN1,EFCAB5,TNRC6A,YWHAZ,RAPGEF4,PSG4,SAA1,F11,C1S,PSG2,LTBP1,PCYOX1,ADIPOQ,NBEAL1,COLEC11,MST1,ITIH3,C4B,SERPINA1,TGFBI,CFB,IGFALS,PLTP,GC,HGFAC,PDGFC,FBN2,SELENOP,TSKU,HPR,C1R,APOC1,APOC2,TTN,APOC4,VEGFC,PLCG2,ACTB,FGA,TPM3
Monarch	HP:0001892	Abnormal bleeding	31	411	0.83	8.19e-13	MYH9,F9,SPARC,CAT,APOE,ANGPTL6,F12,VWF,F13A1,TCIRG1,FERMT3,SEC24D,A2ML1,FGB,F2,SERPINF2,FBN1,P4HB,FGG,F13B,F5,FLNA,F10,F7,PROS1,ACTN1,F11,C1S,C4A,C1R,FGA
Monarch	HP:0000118	Phenotypic abnormality	115	5129	0.3	8.66e-13	ST6GAL1,GNPTG,VCL,SERPIND1,MYH9,F9,C5,MGP,SPARC,HRG,APOB,APOA1,TTR,CAT,C3,APOE,PXDN,ANGPTL6,F12,CFHR5,LYZ,VWF,PSMA6,LCAT,CSTA,CP,KLKB1,F13A1,DNAH5,CENPE,TCIRG1,FCN3,PLA2G7,CRH,HABP2,FERMT3,SEC24D,DSC2,CSF1R,SERPINI1,MASP1,GUCY1A1,A2ML1,SLC27A4,ANTXR1,PCSK9,BMP1,FGB,F2,PLG,STAB1,C1QB,CFHR1,NDUFS8,SUZ12,SERPINF2,HBA1,FBN1,P4HB,FBLN1,COLEC10,DNAH8,ANG,FGG,LIMS1,THBS4,GANAB,FBLN5,PAPLN,BLM,COL18A1,C8A,F13B,CFH,F5,HTRA1,ADAMTSL4,ECM1,FLNA,CRTAC1,C8B,RBP4,ADAMTS13,C1QC,F10,F7,ADGRG2,IGF1,PROS1,ACTN1,EFEMP1,MASP2,F11,C1S,C4A,PRG4,FSIP2,COLEC11,ITIH3,C4B,SERPINA1,TGFBI,IGFALS,GC,FBN2,C1R,ATP1A1,APOC2,TTN,VEGFC,PLCG2,ACTB,ITM2B,FGA,TPM3
Monarch	HP:0001871	Abnormality of blood and blood-forming tissues	51	1214	0.57	8.66e-13	VCL,SERPIND1,MYH9,F9,SPARC,HRG,APOB,APOA1,CAT,APOE,ANGPTL6,F12,VWF,LCAT,CP,KLKB1,F13A1,TCIRG1,FERMT3,SEC24D,GUCY1A1,A2ML1,SLC27A4,FGB,F2,PLG,NDUFS8,SERPINF2,HBA1,FBN1,P4HB,FGG,BLM,F13B,CFH,F5,FLNA,ADAMTS13,F10,F7,PROS1,ACTN1,F11,C1S,C4A,SERPINA1,FBN2,C1R,TTN,PLCG2,FGA
Monarch	HP:0001626	Abnormality of the cardiovascular system	74	2438	0.43	1.07e-12	GNPTG,VCL,SERPIND1,MYH9,F9,MGP,SPARC,APOB,APOA1,TTR,CAT,APOE,ANGPTL6,F12,LYZ,VWF,LCAT,CSTA,CP,KLKB1,F13A1,DNAH5,TCIRG1,HABP2,FERMT3,SEC24D,DSC2,MASP1,GUCY1A1,A2ML1,ANTXR1,PCSK9,BMP1,FGB,F2,PLG,NDUFS8,SUZ12,SERPINF2,HBA1,FBN1,P4HB,FGG,GANAB,FBLN5,BLM,COL18A1,F13B,F5,HTRA1,ADAMTSL4,FLNA,CRTAC1,RBP4,ADAMTS13,F10,F7,PROS1,EFEMP1,F11,C1S,C4A,PRG4,SERPINA1,TGFBI,FBN2,C1R,APOC2,TTN,VEGFC,PLCG2,ACTB,FGA,TPM3
Monarch	HP:0011025	Abnormal cardiovascular system physiology	48	1169	0.56	1.35e-11	GNPTG,VCL,MYH9,F9,MGP,SPARC,APOB,APOA1,TTR,APOE,ANGPTL6,LYZ,VWF,LCAT,CP,F13A1,TCIRG1,DSC2,GUCY1A1,A2ML1,PCSK9,BMP1,FGB,F2,SERPINF2,HBA1,FBN1,FGG,GANAB,FBLN5,F13B,F5,HTRA1,ADAMTSL4,FLNA,CRTAC1,ADAMTS13,F10,F7,PROS1,F11,SERPINA1,FBN2,TTN,PLCG2,ACTB,FGA,TPM3
Monarch	HP:0001928	Abnormality of coagulation	20	170	1.02	4.66e-11	F9,HRG,F12,VWF,KLKB1,F13A1,A2ML1,FGB,F2,PLG,SERPINF2,FGG,F13B,F5,FLNA,F10,F7,PROS1,F11,FGA
Monarch	HP:0000225	Gingival bleeding	13	45	1.41	6.46e-11	CAT,F13A1,FGB,F2,SERPINF2,FGG,F13B,F5,F10,F7,C1S,C1R,FGA
Monarch	HP:0005339	Abnormality of complement system	11	24	1.61	8.61e-11	C5,C3,C1QB,C8A,CFH,C8B,C1QC,MASP2,C1S,C4A,C4B
Monarch	HP:0000421	Epistaxis	15	78	1.23	1.05e-10	MYH9,F9,VWF,F13A1,FERMT3,FGB,F2,FGG,F13B,F5,F10,F7,ACTN1,F11,FGA
Monarch	HP:0100659	Abnormal cerebral vascular morphology	27	381	0.8	1.05e-10	F9,SPARC,APOB,TTR,APOE,ANGPTL6,F13A1,GUCY1A1,ANTXR1,PCSK9,FGB,F2,SERPINF2,FBN1,FGG,GANAB,F13B,F5,HTRA1,FLNA,CRTAC1,ADAMTS13,F10,F7,PROS1,ACTB,FGA
Monarch	HP:0012649	Increased inflammatory response	42	981	0.58	1.36e-10	MYH9,C5,MGP,APOA1,CAT,C3,APOE,CFHR5,LYZ,PSMA6,CSTA,F13A1,DNAH5,TCIRG1,FCN3,FERMT3,PLG,C1QB,CFHR1,HBA1,FBN1,GANAB,FBLN5,BLM,F13B,CFH,ECM1,FLNA,RBP4,C1QC,MASP2,C1S,C4A,PRG4,C4B,SERPINA1,C1R,APOC2,VEGFC,PLCG2,FGA,TPM3
Monarch	HP:0030680	Abnormality of cardiovascular system morphology	56	1680	0.47	1.36e-10	VCL,SERPIND1,F9,MGP,SPARC,APOB,TTR,CAT,APOE,ANGPTL6,F12,VWF,LCAT,F13A1,DNAH5,TCIRG1,HABP2,SEC24D,DSC2,MASP1,GUCY1A1,A2ML1,ANTXR1,PCSK9,FGB,F2,NDUFS8,SUZ12,SERPINF2,HBA1,FBN1,P4HB,FGG,GANAB,FBLN5,COL18A1,F13B,F5,HTRA1,FLNA,CRTAC1,RBP4,ADAMTS13,F10,F7,PROS1,EFEMP1,C4A,PRG4,SERPINA1,FBN2,TTN,VEGFC,ACTB,FGA,TPM3
Monarch	HP:0001933	Subcutaneous hemorrhage	20	189	0.97	1.60e-10	MYH9,APOE,VWF,F13A1,TCIRG1,FERMT3,SEC24D,F2,SERPINF2,FBN1,P4HB,F13B,F5,FLNA,F10,F7,PROS1,C1S,C4A,C1R

Monarch	HP:0011276	Vascular skin abnormality	28	433	0.76	2.01e-10	MYH9,APOE,F12,VWF,CSTA,F13A1,TCIRG1,FERMT3,SEC24D,MASP1,GUCY1A1,ANTXR1,F2,SERPINF2,FBN1,P4HB,BLM,F13B,F5,FLNA,F10,F7,PROS1,C1S,C4A,SERPINA1,C1R,PLCG2
Monarch	HP:0003256	Abnormality of the coagulation cascade	16	105	1.13	2.45e-10	F9,F12,VWF,F13A1,FGB,F2,PLG,FGG,F13B,F5,FLNA,F10,F7,PROS1,F11,FGA
Monarch	HP:0005261	Joint hemorrhage	10	19	1.67	2.45e-10	F9,VWF,F13A1,F2,SERPINF2,F13B,F5,F10,F7,F11
Monarch	HP:0002715	Abnormality of the immune system	55	1682	0.46	3.91e-10	VCL,MYH9,C5,MGP,APOA1,CAT,C3,APOE,CFHR5,LYZ,PSMA6,LCAT,CSTA,F13A1,DNAH5,TCIRG1,FCN3,HABP2,FERMT3,A2ML1,SLC27A4,FGB,F2,PLG,C1QB,CFHR1,HBA1,FBN1,FGG,GANAB,FBLN5,BLM,COL18A1,C8A,F13B,CFH,ECM1,FLNA,C8B,RBP4,C1QC,MASP2,C1S,C4A,PRG4,C4B,SERPINA1,C1R,APOC2,TTN,VEGFC,PLCG2,ACTB,FGA,TPM3
Monarch	HP:0004431	Complement deficiency	10	21	1.63	4.62e-10	C5,C3,C1QB,C8A,CFH,C8B,C1QC,MASP2,C4A,C4B
Monarch	HP:0000951	Abnormality of the skin	58	1888	0.44	8.85e-10	VCL,MYH9,F9,C5,MGP,SPARC,APOB,APOA1,CAT,APOE,F12,LYZ,VWF,PSMA6,CSTA,F13A1,CENPE,TCIRG1,FCN3,FERMT3,SEC24D,DSC2,MASP1,GUCY1A1,A2ML1,SLC27A4,ANTXR1,PCSK9,F2,PLG,SUZ12,SERPINF2,HBA1,FBN1,P4HB,COLEC10,FBLN5,BLM,F13B,F5,ECM1,FLNA,RBP4,ADAMTS13,F10,F7,IGF1,PROS1,C1S,C4A,SERPINA1,C1R,APOC2,TTN,VEGFC,PLCG2,ACTB,FGA
Monarch	HP:0001574	Abnormality of the integument	64	2266	0.4	1.58e-09	VCL,MYH9,F9,C5,MGP,SPARC,APOB,APOA1,CAT,APOE,F12,LYZ,VWF,PSMA6,CSTA,F13A1,CENPE,TCIRG1,FCN3,FERMT3,SEC24D,DSC2,MASP1,GUCY1A1,A2ML1,SLC27A4,ANTXR1,PCSK9,BMP1,F2,PLG,NDUFS8,SUZ12,SERPINF2,HBA1,FBN1,P4HB,COLEC10,LIMS1,FBLN5,BLM,COL18A1,F13B,F5,HTRA1,ECM1,FLNA,RBP4,ADAMTS13,F10,F7,IGF1,PROS1,C1S,C4A,COLEC11,SERPINA1,C1R,APOC2,TTN,VEGFC,PLCG2,ACTB,FGA
Monarch	HP:0000978	Bruising susceptibility	16	130	1.04	3.73e-09	MYH9,VWF,F13A1,TCIRG1,SEC24D,F2,SERPINF2,FBN1,P4HB,F13B,F5,FLNA,F10,F7,C1S,C1R
Monarch	EFO:0004634	Coagulation factor measurement	16	132	1.03	4.43e-09	CFHR5,VWF,KLKB1,F13A1,FGB,FGG,CFH,F5,ADAMTS13,F10,F7,F11,COLEC11,HGFAC,TSKU,FGA
Monarch	HP:0000123	Nephritis	12	59	1.26	8.52e-09	MYH9,C3,CFHR5,PLG,C1QB,CFHR1,GANAB,FBLN5,CFH,C1QC,C4A,C1R
Monarch	HP:0011121	Abnormality of skin morphology	51	1648	0.44	1.81e-08	VCL,MYH9,F9,MGP,SPARC,APOB,APOA1,CAT,APOE,F12,VWF,CSTA,F13A1,CENPE,TCIRG1,FERMT3,SEC24D,DSC2,MASP1,GUCY1A1,A2ML1,SLC27A4,ANTXR1,PCSK9,F2,SUZ12,SERPINF2,HBA1,FBN1,P4HB,COLEC10,FBLN5,BLM,F13B,F5,ECM1,FLNA,ADAMTS13,F10,F7,IGF1,PROS1,C1S,C4A,SERPINA1,C1R,APOC2,TTN,VEGFC,PLCG2,ACTB
Monarch	HP:0011890	Prolonged bleeding following procedure	9	23	1.54	2.06e-08	F9,VWF,F13A1,F2,F13B,F5,F10,F7,F11
Monarch	HP:0010978	Abnormality of immune system physiology	45	1347	0.47	2.81e-08	MYH9,C5,MGP,APOA1,CAT,C3,APOE,CFHR5,LYZ,PSMA6,CSTA,F13A1,DNAH5,TCIRG1,FCN3,FERMT3,PLG,C1QB,CFHR1,HBA1,FBN1,GANAB,FBLN5,BLM,C8A,F13B,CFH,ECM1,FLNA,C8B,RBP4,C1QC,MASP2,C1S,C4A,PRG4,C4B,SERPINA1,C1R,APOC2,VEGFC,PLCG2,ACTB,FGA,TPM3
Monarch	HP:0002170	Intracranial hemorrhage	15	130	1.01	3.19e-08	F9,SPARC,ANGPTL6,F13A1,FGB,F2,SERPINF2,FBN1,FGG,F13B,F5,F10,F7,PROS1,FGA
Monarch	HP:0010935	Abnormality of the upper urinary tract	40	1101	0.51	3.37e-08	MYH9,F9,APOB,APOA1,TTR,C3,APOE,CFHR5,LYZ,LCAT,HABP2,MASP1,ANTXR1,PCSK9,F2,PLG,C1QB,CFHR1,NDUFS8,SERPINF2,FBN1,COLEC10,GANAB,FBLN5,BLM,COL18A1,CFH,F5,FLNA,ADAMTS13,C1QC,F10,ADGRG2,C4A,COLEC11,SERPINA1,C1R,ATP1A1,ACTB,FGA
Monarch	HP:0006298	Prolonged bleeding after dental extraction	8	16	1.65	4.75e-08	F9,VWF,F13A1,F2,F13B,F5,F10,F11
Monarch	HP:0000077	Abnormality of the kidney	39	1077	0.51	6.22e-08	MYH9,F9,APOB,APOA1,TTR,C3,APOE,CFHR5,LYZ,LCAT,HABP2,MASP1,ANTXR1,PCSK9,F2,PLG,C1QB,CFHR1,NDUFS8,SERPINF2,FBN1,COLEC10,GANAB,FBLN5,BLM,CFH,F5,FLNA,ADAMTS13,C1QC,F10,ADGRG2,C4A,COLEC11,SERPINA1,C1R,ATP1A1,ACTB,FGA
Monarch	HP:0012233	Intramuscular hematoma	7	10	1.79	1.16e-07	F9,F13A1,F2,SERPINF2,F13B,F10,F7
Monarch	HP:0011354	Generalized abnormality of skin	33	821	0.55	1.46e-07	MYH9,F9,APOE,F12,VWF,CSTA,F13A1,CENPE,TCIRG1,FERMT3,SEC24D,MASP1,GUCY1A1,ANTXR1,F2,SERPINF2,HBA1,FBN1,P4HB,FBLN5,BLM,F13B,F5,FLNA,ADAMTS13,F10,F7,PROS1,C1S,C4A,SERPINA1,C1R,PLCG2
Monarch	HP:0012211	Abnormal renal physiology	27	560	0.63	1.55e-07	MYH9,F9,APOA1,TTR,C3,APOE,CFHR5,LYZ,LCAT,F2,PLG,C1QB,CFHR1,NDUFS8,SERPINF2,GANAB,FBLN5,CFH,F5,ADAMTS13,C1QC,F10,C4A,SERPINA1,C1R,ATP1A1,FGA
Monarch	HP:0000119	Abnormality of the genitourinary system	58	2189	0.37	1.71e-07	MYH9,F9,APOB,APOA1,TTR,C3,APOE,CFHR5,LYZ,VWF,LCAT,F13A1,DNAH5,CRH,HABP2,MASP1,GUCY1A1,A2ML1,ANTXR1,PCSK9,FGB,F2,PLG,C1QB,CFHR1,NDUFS8,SUZ12,SERPINF2,FBN1,FBLN1,COLEC10,DNAH8,FGG,GANAB,FBLN5,BLM,COL18A1,F13B,CFH,F5,HTRA1,FLNA,ADAMTS13,C1QC,F10,F7,ADGRG2,IGF1,F11,C4A,FSIP2,COLEC11,SERPINA1,C1R,ATP1A1,VEGFC,ACTB,FGA
Monarch	HP:0033353	Abnormal blood vessel morphology	32	792	0.55	2.26e-07	SERPIND1,MGP,APOB,CAT,APOE,ANGPTL6,F12,VWF,LCAT,DNAH5,TCIRG1,SEC24D,MASP1,GUCY1A1,A2ML1,ANTXR1,PCSK9,FBN1,P4HB,GANAB,FBLN5,COL18A1,HTRA1,FLNA,RBP4,PROS1,EFEMP1,C4A,FBN2,VEGFC,ACTB,TPM3
Monarch	HP:0000168	Abnormality of the gingiva	16	190	0.87	4.18e-07	CAT,F13A1,TCIRG1,FGB,F2,PLG,SERPINF2,FGG,F13B,F5,ECM1,F10,F7,C1S,C1R,FGA
Monarch	HP:0000132	Menorrhagia	9	38	1.32	6.18e-07	MYH9,VWF,F13A1,F2,F13B,F5,F10,F7,F11
Monarch	HP:0000790	Hematuria	13	118	0.99	7.44e-07	F9,APOA1,CFHR5,LYZ,F2,CFHR1,SERPINF2,GANAB,CFH,F5,ADAMTS13,F10,FGA
Monarch	HP:0002725	Systemic lupus erythematosus	8	26	1.44	8.04e-07	C3,C1QB,C8A,C1QC,MASP2,C1S,C4A,C1R
Monarch	HP:0011029	Internal hemorrhage	17	244	0.79	1.65e-06	F9,SPARC,ANGPTL6,VWF,F13A1,FGB,F2,SERPINF2,FBN1,FGG,F13B,F5,F10,F7,PROS1,F11,FGA
Monarch	HP:0004846	Prolonged bleeding after surgery	7	18	1.54	2.04e-06	F9,VWF,F13A1,F13B,F5,F10,F7
Monarch	HP:0011891	Post-partum hemorrhage	6	10	1.73	3.32e-06	F13A1,F2,F13B,F5,F10,F7
Monarch	HP:0002634	Arteriosclerosis	10	70	1.1	5.01e-06	SERPIND1,APOB,CAT,APOE,ANGPTL6,LCAT,ANTXR1,PCSK9,FBN1,HTRA1
Monarch	HP:0010990	Abnormality of the common coagulation pathway	8	35	1.31	5.31e-06	F13A1,FGB,PLG,FGG,F13B,F5,F10,FGA
Monarch	HP:0001977	Abnormal thrombosis	11	94	1.02	6.07e-06	SERPIND1,MYH9,F9,HRG,F12,FGB,F2,FGG,F5,PROS1,FGA
Monarch	HP:0001934	Persistent bleeding after trauma	6	12	1.65	6.84e-06	F9,VWF,F13A1,SERPINF2,F13B,F5
Monarch	HP:0025031	Abnormality of the digestive system	57	2389	0.33	7.61e-06	F9,C5,APOB,APOA1,TTR,APOE,F12,LYZ,VWF,LCAT,CP,F13A1,DNAH5,TCIRG1,FCN3,HABP2,FERMT3,MASP1,GUCY1A1,A2ML1,ANTXR1,PCSK9,BMP1,FGB,F2,PLG,NDUFS8,SUZ12,HBA1,FBN1,COLEC10,ANG,FGG,GANAB,FBLN5,BLM,COL18A1,F13B,F5,ECM1,FLNA,ADAMTS13,F10,F7,MASP2,F11,C1S,COLEC11,C4B,SERPINA1,FBN2,C1R,APOC2,PLCG2,ACTB,FGA,TPM3
Monarch	HP:0011884	Abnormal umbilical stump bleeding	5	5	1.95	8.78e-06	F13A1,F2,SERPINF2,F13B,F10
Monarch	HP:0003645	Prolonged partial thromboplastin time	7	24	1.41	8.86e-06	F9,F12,KLKB1,F2,F5,F10,F11
Monarch	HP:0000080	Abnormality of reproductive system physiology	26	659	0.54	1.13e-05	MYH9,F9,TTR,APOE,VWF,F13A1,DNAH5,GUCY1A1,A2ML1,ANTXR1,FGB,F2,DNAH8,FGG,GANAB,BLM,F13B,F5,F10,F7,ADGRG2,IGF1,F11,FSIP2,SERPINA1,FGA
Monarch	HP:0001367	Abnormal joint morphology	31	905	0.48	1.19e-05	GNPTG,F9,APOE,VWF,F13A1,MASP1,A2ML1,BMP1,FGB,F2,SUZ12,SERPINF2,FBN1,FBLN1,COLEC10,FGG,FBLN5,F13B,F5,FLNA,F10,F7,F11,PRG4,COLEC11,SERPINA1,FBN2,VEGFC,ACTB,FGA,TPM3
Monarch	HP:0000271	Abnormality of the face	60	2641	0.3	1.52e-05	GNPTG,MYH9,F9,MGP,SPARC,APOB,CAT,APOE,F12,LYZ,VWF,CP,F13A1,DNAH5,CENPE,TCIRG1,FERMT3,SEC24D,CSF1R,MASP1,A2ML1,ANTXR1,PCSK9,BMP1,FGB,F2,PLG,SUZ12,SERPINF2,FBN1,P4HB,FBLN1,COLEC10,ANG,FGG,FBLN5,BLM,COL18A1,F13B,F5,ADAMTSL4,ECM1,FLNA,CRTAC1,F10,F7,IGF1,ACTN1,F11,C1S,COLEC11,SERPINA1,FBN2,C1R,TTN,VEGFC,PLCG2,ACTB,FGA,TPM3
Monarch	EFO:0004310	Partial thromboplastin time	6	15	1.55	1.70e-05	HRG,F12,KLKB1,F5,F11,C1S

Monarch	HP:0000707	Abnormality of the nervous system	72	3471	0.26	1.70e-05	ST6GAL1,GNPTG,F9,MGP,SPARC,APOB,APOA1,TTR,CAT,APOE,ANGPTL6,CP,F13A1,DNAH5,CENPE,TCIRG1,CRH,HABP2,SEC24D,CSF1R,SERPINI1,MASP1,GUCY1A1,A2ML1,ANTXR1,PCSK9,BMP1,FGB,F2,PLG,STAB1,NDUFS8,SUZ12,SERPINF2,HBA1,FBN1,P4HB,FBLN1,COLEC10,ANG,FGG,GANAB,FBLN5,BLM,COL18A1,C8A,F13B,F5,HTRA1,ADAMTSL4,ECM1,FLNA,CRTAC1,C8B,ADAMTS13,F10,F7,IGF1,PROS1,COLEC11,ITIH3,C4B,SERPINA1,TGFBI,FBN2,ATP1A1,TTN,VEGFC,ACTB,ITM2B,FGA,TPM3
Monarch	HP:0000140	Abnormality of the menstrual cycle	15	228	0.77	1.98e-05	MYH9,F9,VWF,F13A1,ANTXR1,FGB,F2,FGG,F13B,F5,F10,F7,F11,SERPINA1,FGA
Monarch	HP:0000152	Abnormality of head or neck	63	2882	0.29	2.33e-05	GNPTG,MYH9,F9,MGP,SPARC,APOB,CAT,APOE,F12,LYZ,VWF,CP,F13A1,DNAH5,CENPE,TCIRG1,HABP2,FERMT3,SEC24D,CSF1R,MASP1,A2ML1,ANTXR1,PCSK9,BMP1,FGB,F2,PLG,NDUFS8,SUZ12,SERPINF2,FBN1,P4HB,FBLN1,COLEC10,ANG,FGG,FBLN5,BLM,COL18A1,F13B,F5,ADAMTSL4,ECM1,FLNA,CRTAC1,F10,F7,IGF1,ACTN1,F11,C1S,COLEC11,SERPINA1,IGFALS,FBN2,C1R,TTN,VEGFC,PLCG2,ACTB,FGA,TPM3
Monarch	HP:0002011	Morphological central nervous system abnormality	56	2416	0.31	2.33e-05	F9,MGP,SPARC,APOB,TTR,APOE,ANGPTL6,CP,F13A1,DNAH5,CENPE,TCIRG1,HABP2,SEC24D,CSF1R,SERPINI1,MASP1,GUCY1A1,ANTXR1,PCSK9,FGB,F2,PLG,NDUFS8,SUZ12,SERPINF2,HBA1,FBN1,P4HB,FBLN1,COLEC10,ANG,FGG,GANAB,FBLN5,BLM,COL18A1,C8A,F13B,F5,HTRA1,ECM1,FLNA,CRTAC1,C8B,ADAMTS13,F10,F7,IGF1,PROS1,COLEC11,C4B,ATP1A1,ACTB,ITM2B,FGA
Monarch	HP:0012639	Abnormal nervous system morphology	58	2546	0.31	2.33e-05	F9,MGP,SPARC,APOB,TTR,APOE,ANGPTL6,CP,F13A1,DNAH5,CENPE,TCIRG1,HABP2,SEC24D,CSF1R,SERPINI1,MASP1,GUCY1A1,ANTXR1,PCSK9,FGB,F2,PLG,NDUFS8,SUZ12,SERPINF2,HBA1,FBN1,P4HB,FBLN1,COLEC10,ANG,FGG,GANAB,FBLN5,BLM,COL18A1,C8A,F13B,F5,HTRA1,ECM1,FLNA,CRTAC1,C8B,ADAMTS13,F10,F7,IGF1,PROS1,COLEC11,C4B,ATP1A1,TTN,ACTB,ITM2B,FGA,TPM3
Monarch	HP:0011830	Abnormal oral mucosa morphology	17	305	0.69	2.48e-05	CAT,LYZ,F13A1,TCIRG1,FGB,F2,PLG,SERPINF2,FGG,F13B,F5,ECM1,F10,F7,C1S,C1R,FGA
Monarch	HP:0005368	Abnormality of humoral immunity	15	234	0.75	2.52e-05	C5,C3,C1QB,BLM,C8A,CFH,FLNA,C8B,C1QC,MASP2,C1S,C4A,C4B,SERPINA1,PLCG2
Monarch	HP:0030012	Abnormal female reproductive system physiology	16	273	0.72	3.00e-05	MYH9,F9,VWF,F13A1,ANTXR1,FGB,F2,FGG,BLM,F13B,F5,F10,F7,F11,SERPINA1,FGA
Monarch	EFO:0008390	Prothrombin time measurement	5	8	1.74	3.48e-05	F2,FGG,F5,F10,F7
Monarch	HP:0000234	Abnormality of the head	62	2865	0.28	4.04e-05	GNPTG,MYH9,F9,MGP,SPARC,APOB,CAT,APOE,F12,LYZ,VWF,CP,F13A1,DNAH5,CENPE,TCIRG1,FERMT3,SEC24D,CSF1R,MASP1,A2ML1,ANTXR1,PCSK9,BMP1,FGB,F2,PLG,NDUFS8,SUZ12,SERPINF2,FBN1,P4HB,FBLN1,COLEC10,ANG,FGG,FBLN5,BLM,COL18A1,F13B,F5,ADAMTSL4,ECM1,FLNA,CRTAC1,F10,F7,IGF1,ACTN1,F11,C1S,COLEC11,SERPINA1,IGFALS,FBN2,C1R,TTN,VEGFC,PLCG2,ACTB,FGA,TPM3
Monarch	HP:0011004	Abnormal systemic arterial morphology	19	398	0.63	4.13e-05	SERPIND1,APOB,CAT,APOE,ANGPTL6,F12,LCAT,SEC24D,GUCY1A1,ANTXR1,PCSK9,FBN1,P4HB,GANAB,FBLN5,HTRA1,FLNA,FBN2,TPM3
Monarch	HP:0000079	Abnormality of the urinary system	43	1657	0.36	4.75e-05	MYH9,F9,APOB,APOA1,TTR,C3,APOE,CFHR5,LYZ,LCAT,CRH,HABP2,MASP1,ANTXR1,PCSK9,F2,PLG,C1QB,CFHR1,NDUFS8,SERPINF2,FBN1,COLEC10,GANAB,FBLN5,PAPLN,BLM,COL18A1,CFH,F5,HTRA1,FLNA,ADAMTS13,C1QC,F10,ADGRG2,C4A,COLEC11,SERPINA1,C1R,ATP1A1,ACTB,FGA
Monarch	HP:0030140	Oral cavity bleeding	5	9	1.69	5.07e-05	F13A1,F2,F13B,F5,F10
Monarch	HP:0001939	Abnormality of metabolism/homeostasis	51	2168	0.32	5.63e-05	GNPTG,VCL,MYH9,F9,APOB,APOA1,TTR,CAT,APOE,F12,CFHR5,LYZ,LCAT,CP,TCIRG1,PLA2G7,CSF1R,A2ML1,ANTXR1,PCSK9,FGB,F2,PLG,CFHR1,NDUFS8,SERPINF2,HBA1,FBN1,FGG,GANAB,PAPLN,BLM,CFH,F5,FLNA,ADAMTS13,F10,IGF1,SERPINA1,TGFBI,IGFALS,GC,ATP1A1,APOC2,TTN,VEGFC,PLCG2,ACTB,ITM2B,FGA,TPM3
Monarch	HP:0011355	Localized skin lesion	26	738	0.5	7.17e-05	MGP,APOB,APOA1,APOE,F12,CSTA,TCIRG1,FERMT3,SEC24D,MASP1,A2ML1,ANTXR1,PCSK9,SUZ12,FBN1,P4HB,COLEC10,BLM,ECM1,FLNA,IGF1,PROS1,C1S,C1R,APOC2,ACTB
Monarch	HP:0000099	Glomerulonephritis	7	37	1.22	8.42e-05	C3,CFHR5,C1QB,CFHR1,CFH,C1QC,C4A
Monarch	HP:0000078	Abnormality of the genital system	37	1358	0.38	0.00011	MYH9,F9,TTR,APOE,VWF,F13A1,DNAH5,MASP1,GUCY1A1,A2ML1,ANTXR1,FGB,F2,PLG,SUZ12,FBN1,FBLN1,COLEC10,DNAH8,FGG,GANAB,FBLN5,BLM,F13B,F5,FLNA,F10,F7,ADGRG2,IGF1,F11,FSIP2,COLEC11,SERPINA1,VEGFC,ACTB,FGA
Monarch	HP:0033127	Abnormality of the musculoskeletal system	65	3173	0.26	0.00012	GNPTG,VCL,F9,MGP,SPARC,APOB,APOA1,TTR,APOE,VWF,CP,F13A1,DNAH5,CENPE,TCIRG1,HABP2,FERMT3,SEC24D,CSF1R,MASP1,A2ML1,ANTXR1,PCSK9,BMP1,FGB,F2,PLG,NDUFS8,SUZ12,SERPINF2,FBN1,P4HB,FBLN1,COLEC10,ANG,FGG,FBLN5,BLM,COL18A1,F13B,F5,HTRA1,ADAMTSL4,ECM1,FLNA,F10,F7,IGF1,F11,C1S,PRG4,COLEC11,SERPINA1,TGFBI,IGFALS,FBN2,C1R,ATP1A1,TTN,VEGFC,PLCG2,ACTB,ITM2B,FGA,TPM3
Monarch	HP:0000924	Abnormality of the skeletal system	58	2708	0.28	0.00014	GNPTG,F9,MGP,SPARC,APOB,TTR,APOE,VWF,F13A1,DNAH5,CENPE,TCIRG1,HABP2,FERMT3,SEC24D,CSF1R,MASP1,A2ML1,ANTXR1,PCSK9,BMP1,FGB,F2,PLG,NDUFS8,SUZ12,SERPINF2,FBN1,P4HB,FBLN1,COLEC10,FGG,FBLN5,BLM,COL18A1,F13B,F5,HTRA1,ADAMTSL4,ECM1,FLNA,F10,F7,IGF1,F11,C1S,PRG4,COLEC11,SERPINA1,IGFALS,FBN2,C1R,ATP1A1,TTN,VEGFC,ACTB,FGA,TPM3
Monarch	HP:0002239	Gastrointestinal hemorrhage	10	111	0.9	0.00016	F9,VWF,FGB,F2,FGG,F5,F10,F7,F11,FGA
Monarch	HP:0001634	Mitral valve prolapse	9	86	0.97	0.00017	VWF,SEC24D,FBN1,P4HB,GANAB,FLNA,FBN2,TTN,TPM3
Monarch	HP:0011277	Abnormality of the urinary system physiology	29	933	0.44	0.00017	MYH9,F9,APOA1,TTR,C3,APOE,CFHR5,LYZ,LCAT,F2,PLG,C1QB,CFHR1,NDUFS8,SERPINF2,GANAB,FBLN5,PAPLN,BLM,CFH,F5,ADAMTS13,C1QC,F10,C4A,SERPINA1,C1R,ATP1A1,FGA
Monarch	HP:0002621	Atherosclerosis	8	64	1.05	0.00020	SERPIND1,APOB,APOE,ANGPTL6,LCAT,ANTXR1,PCSK9,FBN1
Monarch	HP:0005692	Joint hyperflexibility	11	144	0.83	0.00020	CENPE,SEC24D,A2ML1,ANTXR1,SUZ12,P4HB,FBLN5,COL18A1,FLNA,C1S,C1R
Monarch	HP:0004936	Venous thrombosis	8	65	1.04	0.00021	SERPIND1,F9,FGB,F2,FGG,F5,PROS1,FGA
Monarch	HP:0005542	Prolonged whole-blood clotting time	4	5	1.85	0.00025	F9,F12,VWF,F5
Monarch	HP:0011842	Abnormal skeletal morphology	55	2573	0.28	0.00028	GNPTG,F9,MGP,SPARC,APOB,TTR,APOE,VWF,F13A1,DNAH5,CENPE,TCIRG1,FERMT3,SEC24D,CSF1R,MASP1,A2ML1,ANTXR1,PCSK9,BMP1,FGB,F2,PLG,NDUFS8,SUZ12,SERPINF2,FBN1,P4HB,FBLN1,COLEC10,FGG,FBLN5,BLM,COL18A1,F13B,F5,HTRA1,ADAMTSL4,FLNA,F10,F7,IGF1,F11,C1S,PRG4,COLEC11,SERPINA1,IGFALS,FBN2,C1R,TTN,VEGFC,ACTB,FGA,TPM3
Monarch	HP:0002960	Autoimmunity	10	122	0.86	0.00031	C3,TCIRG1,C1QB,C8A,C1QC,MASP2,C1S,C4A,C1R,PLCG2
Monarch	HP:0001382	Joint hypermobility	14	279	0.65	0.00075	CENPE,SEC24D,A2ML1,ANTXR1,BMP1,SUZ12,FBN1,P4HB,FBLN5,COL18A1,FLNA,C1S,COLEC11,C1R
Monarch	HP:0009145	Abnormal cerebral artery morphology	7	55	1.05	0.00075	APOB,ANGPTL6,GUCY1A1,PCSK9,FBN1,GANAB,HTRA1
Monarch	HP:0012443	Abnormality of brain morphology	49	2254	0.29	0.00075	F9,MGP,SPARC,APOB,TTR,APOE,ANGPTL6,CP,F13A1,DNAH5,CENPE,TCIRG1,SEC24D,CSF1R,SERPINI1,MASP1,GUCY1A1,ANTXR1,PCSK9,FGB,F2,PLG,NDUFS8,SUZ12,SERPINF2,HBA1,FBN1,P4HB,FBLN1,FGG,GANAB,FBLN5,BLM,COL18A1,F13B,F5,HTRA1,ECM1,FLNA,CRTAC1,ADAMTS13,F10,F7,IGF1,PROS1,ATP1A1,ACTB,ITM2B,FGA
Monarch	HP:0032101	Unusual infection	24	748	0.45	0.00077	C5,MGP,C3,DNAH5,TCIRG1,FCN3,FERMT3,PLG,C1QB,GANAB,FBLN5,BLM,C8A,CFH,ECM1,FLNA,C8B,C1QC,MASP2,C4B,SERPINA1,C1R,PLCG2,TPM3
Monarch	HP:0001633	Abnormal mitral valve morphology	9	109	0.86	0.00090	VWF,SEC24D,FBN1,P4HB,GANAB,FLNA,FBN2,TTN,TPM3
Monarch	HP:0002625	Deep venous thrombosis	5	20	1.35	0.00093	SERPIND1,F9,F2,F5,PROS1

Monarch	HP:0012372	Abnormal eye morphology	46	2077	0.29	0.00093	GNPTG,MYH9,MGP,SPARC,APOB,APOA1,TTR,APOE,PXDN,F12,LCAT,CP,DNAH5,TCIRG1,SEC24D,CSF1R,MASP1,A2ML1,ANTXR1,PCSK9,BMP1,PLG,NDUFS8,SUZ12,FBN1,P4HB,FBLN1,COLEC10,FBLN5,BLM,COL18A1,CFH,ADAMTSL4,FLNA,RBP4,PROS1,EFEMP1,COLEC11,TGFBI,FBN2,C1R,APOC2,PLCG2,ACTB,ITM2B,TPM3
Monarch	EFO:0005278	Cardiovascular disease biomarker measurement	48	2228	0.28	0.0011	ST6GAL1,F9,HRG,APOB,APOA1,APOE,F12,LCAT,KLKB1,DNAH5,ITIH1,PLA2G7,HABP2,SERPINI1,ANGPTL4,PCSK9,FGB,PLG,STAB1,FBN1,FBLN1,COLEC10,OIT3,LIMS1,FBLN5,COL18A1,CFH,F5,CRTAC1,ADAMTS13,EFEMP1,EFCAB5,TNRC6A,RAPGEF4,LTBP1,NBEAL1,ITIH3,SERPINA1,PLTP,HGFAC,FBN2,HPR,APOC1,APOC2,TTN,APOC4,PLCG2,ACTB
Monarch	HP:0030137	Prolonged bleeding following circumcision	4	9	1.6	0.0012	F13A1,F2,F13B,F5
Monarch	EFO:0004298	Cardiovascular measurement	50	2378	0.27	0.0013	ST6GAL1,VCL,F9,HRG,APOB,APOA1,APOE,F12,LCAT,KLKB1,DNAH5,ITIH1,PLA2G7,HABP2,SERPINI1,ANGPTL4,PCSK9,FGB,INHBC,PLG,STAB1,FBN1,FBLN1,COLEC10,OIT3,LIMS1,FBLN5,COL18A1,CFH,F5,CRTAC1,ADAMTS13,EFEMP1,EFCAB5,TNRC6A,RAPGEF4,LTBP1,NBEAL1,ITIH3,SERPINA1,PLTP,HGFAC,FBN2,HPR,APOC1,APOC2,TTN,APOC4,PLCG2,ACTB
Monarch	HP:0000991	Xanthomatosis	5	22	1.3	0.0013	APOB,APOA1,APOE,PCSK9,APOC2
Monarch	HP:0030962	Abnormal morphology of the great vessels	20	571	0.49	0.0013	MGP,APOB,APOE,ANGPTL6,DNAH5,TCIRG1,SEC24D,MASP1,A2ML1,PCSK9,FBN1,P4HB,GANAB,FBLN5,COL18A1,FLNA,RBP4,FBN2,ACTB,TPM3
Monarch	HP:0001654	Abnormal heart valve morphology	13	259	0.65	0.0014	VWF,TCIRG1,SEC24D,A2ML1,FBN1,P4HB,GANAB,FBLN5,FLNA,FBN2,TTN,ACTB,TPM3
Monarch	HP:0030163	Abnormal vascular physiology	15	340	0.59	0.0014	MGP,ANGPTL6,TCIRG1,GUCY1A1,BMP1,F2,FBN1,F5,HTRA1,FLNA,CRTAC1,ADAMTS13,PROS1,SERPINA1,ACTB
Monarch	HP:0001387	Joint stiffness	10	151	0.77	0.0016	GNPTG,F2,SUZ12,FBN1,ADAMTSL4,FLNA,PRG4,SERPINA1,FBN2,ACTB
Monarch	HP:0400008	Menometrorrhagia	4	10	1.55	0.0016	F9,FGB,FGG,FGA
Monarch	HP:0001000	Abnormality of skin pigmentation	17	439	0.54	0.0017	CAT,APOE,CSTA,A2ML1,ANTXR1,F2,SUZ12,FBN1,BLM,FLNA,IGF1,PROS1,C1S,SERPINA1,C1R,PLCG2,ACTB
Monarch	EFO:0004614	Apolipoprotein A 1 measurement	16	396	0.55	0.0019	APOB,APOA1,APOE,LCAT,ITIH1,ANGPTL4,PCSK9,PLG,STAB1,COL18A1,CRTAC1,HGFAC,HPR,APOC1,APOC2,APOC4
Monarch	HP:0012531	Pain	19	539	0.5	0.0019	GNPTG,APOB,CAT,F12,TCIRG1,HABP2,FERMT3,PCSK9,F2,SERPINF2,FBN1,ANG,GANAB,HTRA1,FLNA,C1S,SERPINA1,TGFBI,PLCG2
Monarch	HP:0011024	Abnormality of the gastrointestinal tract	34	1384	0.34	0.0020	F9,APOE,F12,VWF,F13A1,DNAH5,TCIRG1,FCN3,HABP2,GUCY1A1,FGB,F2,PLG,SUZ12,FBN1,FGG,FBLN5,BLM,COL18A1,F13B,F5,ECM1,FLNA,F10,F7,MASP2,F11,SERPINA1,FBN2,C1R,PLCG2,ACTB,FGA,TPM3
Monarch	HP:0011034	Amyloidosis	5	25	1.25	0.0020	APOA1,TTR,LYZ,ITM2B,FGA
Monarch	HP:0001376	Limitation of joint mobility	15	356	0.57	0.0021	GNPTG,MASP1,F2,SUZ12,FBN1,COLEC10,ADAMTSL4,FLNA,PRG4,COLEC11,SERPINA1,FBN2,TTN,ACTB,TPM3
Monarch	HP:0011843	Abnormal musculoskeletal physiology	33	1330	0.34	0.0022	GNPTG,SPARC,APOE,CENPE,TCIRG1,HABP2,SEC24D,MASP1,A2ML1,ANTXR1,BMP1,F2,SUZ12,SERPINF2,FBN1,P4HB,FBLN1,COLEC10,FBLN5,COL18A1,ADAMTSL4,FLNA,IGF1,C1S,PRG4,COLEC11,SERPINA1,IGFALS,FBN2,C1R,TTN,ACTB,TPM3
Monarch	HP:0000366	Abnormality of the nose	35	1470	0.32	0.0028	MYH9,F9,MGP,APOE,VWF,F13A1,DNAH5,CENPE,TCIRG1,FERMT3,MASP1,ANTXR1,FGB,F2,PLG,SUZ12,FBN1,COLEC10,FGG,BLM,COL18A1,F13B,F5,ECM1,FLNA,F10,F7,IGF1,ACTN1,F11,COLEC11,SERPINA1,PLCG2,ACTB,FGA
Monarch	HP:0010979	Abnormality of lipoprotein cholesterol concentration	6	47	1.05	0.0028	APOB,APOA1,APOE,LCAT,PCSK9,APOC2
Monarch	HP:0002719	Recurrent infections	22	721	0.43	0.0033	C5,MGP,C3,DNAH5,TCIRG1,FCN3,FERMT3,PLG,C1QB,GANAB,FBLN5,BLM,CFH,ECM1,FLNA,C8B,C1QC,MASP2,SERPINA1,C1R,PLCG2,TPM3
Monarch	HP:0004942	Aortic aneurysm	9	135	0.77	0.0036	ANGPTL6,SEC24D,FBN1,P4HB,GANAB,FBLN5,FLNA,FBN2,TPM3
Monarch	HP:0012719	Functional abnormality of the gastrointestinal tract	24	840	0.4	0.0038	F9,VWF,F13A1,TCIRG1,FCN3,GUCY1A1,FGB,F2,FBN1,FGG,BLM,F13B,F5,ECM1,FLNA,F10,F7,MASP2,F11,SERPINA1,PLCG2,ACTB,FGA,TPM3
Monarch	HP:0010647	Abnormal elasticity of skin	10	172	0.71	0.0040	MGP,SPARC,CSTA,ANTXR1,SUZ12,FBN1,FBLN5,FLNA,C1S,C1R
Monarch	HP:0011329	Abnormality of cranial sutures	13	292	0.6	0.0041	SPARC,CENPE,TCIRG1,SEC24D,MASP1,ANTXR1,BMP1,FBN1,P4HB,COLEC10,FLNA,COLEC11,ACTB
Monarch	HP:0025142	Constitutional symptom	25	906	0.39	0.0045	GNPTG,APOB,TTR,CAT,F12,TCIRG1,CRH,HABP2,FERMT3,DSC2,PCSK9,F2,SERPINF2,FBN1,ANG,GANAB,HTRA1,FLNA,PROS1,C1S,SERPINA1,TGFBI,TTN,PLCG2,TPM3
Monarch	HP:0007759	Opacification of the corneal stroma	7	78	0.9	0.0046	GNPTG,APOB,APOE,LCAT,PCSK9,COL18A1,TGFBI
Monarch	HP:0012223	Splenic rupture	3	4	1.82	0.0046	FGB,FGG,FGA
Monarch	HP:0040232	Delayed onset bleeding	3	4	1.82	0.0046	F9,F13A1,F13B
Monarch	HP:0000979	Purpura	7	79	0.9	0.0048	APOE,F13A1,FERMT3,F2,F13B,PROS1,C4A
Monarch	HP:0032263	Increased blood pressure	13	303	0.58	0.0056	MGP,APOB,APOA1,ANGPTL6,LYZ,GUCY1A1,PCSK9,F2,FBN1,GANAB,ADAMTSL4,SERPINA1,FGA
Monarch	HP:0000153	Abnormality of the mouth	42	1992	0.27	0.0058	MYH9,MGP,SPARC,CAT,APOE,LYZ,F13A1,CENPE,TCIRG1,SEC24D,MASP1,A2ML1,ANTXR1,BMP1,FGB,F2,PLG,SUZ12,SERPINF2,FBN1,P4HB,FBLN1,COLEC10,ANG,FGG,BLM,F13B,F5,ECM1,FLNA,CRTAC1,F10,F7,IGF1,C1S,COLEC11,FBN2,C1R,TTN,ACTB,FGA,TPM3
Monarch	HP:0005262	Abnormality of the synovia	13	305	0.58	0.0058	APOE,MASP1,BMP1,F2,FBN1,FBLN1,COLEC10,FBLN5,FLNA,PRG4,COLEC11,SERPINA1,TPM3
Monarch	HP:0010980	Hyperlipoproteinemia	5	33	1.13	0.0058	APOB,APOE,LCAT,PCSK9,APOC2
Monarch	HP:0031652	Abnormal aortic valve physiology	8	113	0.8	0.0060	GNPTG,APOB,VWF,PCSK9,FBN1,FBLN5,FLNA,ACTB
Monarch	HP:0000969	Edema	16	448	0.5	0.0064	APOA1,APOE,F12,LYZ,A2ML1,ANTXR1,FGB,F2,HBA1,FGG,SERPINA1,TGFBI,VEGFC,PLCG2,ACTB,FGA
Monarch	HP:0000235	Abnormality of the fontanelles or cranial sutures	14	355	0.54	0.0066	SPARC,APOE,CENPE,TCIRG1,SEC24D,MASP1,ANTXR1,BMP1,FBN1,P4HB,COLEC10,FLNA,COLEC11,ACTB
Monarch	HP:0003216	Generalized amyloid deposition	3	5	1.73	0.0067	APOA1,LYZ,FGA
Monarch	HP:0001363	Craniosynostosis	9	150	0.73	0.0068	CENPE,TCIRG1,SEC24D,MASP1,FBN1,P4HB,COLEC10,FLNA,COLEC11
Monarch	EFO:0001365	Age-related macular degeneration	5	35	1.1	0.0071	C3,APOE,CFHR5,CFH,CFB
Monarch	EFO:1001492	Atrophic macular degeneration	5	35	1.1	0.0071	C3,APOE,CFHR5,CFH,CFB
Monarch	HP:0000704	Periodontitis	5	35	1.1	0.0071	CAT,TCIRG1,PLG,C1S,C1R
Monarch	HP:0001386	Joint swelling	5	35	1.1	0.0071	FGB,F2,FGG,SERPINA1,FGA
Monarch	HP:0001537	Umbilical hernia	11	228	0.63	0.0071	APOE,MASP1,ANTXR1,BMP1,SUZ12,FBN1,COLEC10,FBLN5,FLNA,C1S,COLEC11
Monarch	HP:0011729	Abnormality of joint mobility	27	1063	0.35	0.0071	GNPTG,APOE,CENPE,SEC24D,MASP1,A2ML1,ANTXR1,BMP1,F2,SUZ12,FBN1,P4HB,FBLN1,COLEC10,FBLN5,COL18A1,ADAMTSL4,FLNA,C1S,PRG4,COLEC11,SERPINA1,FBN2,C1R,TTN,ACTB,TPM3
Monarch	HP:0410042	Abnormal liver morphology	21	715	0.42	0.0071	APOB,APOA1,APOE,LYZ,LCAT,CP,TCIRG1,FERMT3,A2ML1,ANTXR1,PCSK9,F2,NDUFS8,HBA1,FBN1,GANAB,C1S,C4B,SERPINA1,APOC2,FGA
Monarch	HP:0002644	Abnormal pelvic girdle bone morphology	17	512	0.47	0.0076	GNPTG,APOE,CENPE,TCIRG1,SEC24D,MASP1,ANTXR1,BMP1,SUZ12,FBN1,FBLN1,COLEC10,FBLN5,FLNA,COLEC11,TTN,TPM3
Monarch	HP:0003272	Abnormal hip bone morphology	16	461	0.49	0.0076	GNPTG,APOE,CENPE,TCIRG1,SEC24D,MASP1,BMP1,SUZ12,FBN1,FBLN1,COLEC10,FBLN5,FLNA,COLEC11,TTN,TPM3
Monarch	HP:0000163	Abnormal oral cavity morphology	40	1901	0.27	0.0077	MYH9,MGP,SPARC,CAT,APOE,LYZ,F13A1,CENPE,TCIRG1,SEC24D,MASP1,A2ML1,ANTXR1,BMP1,FGB,F2,PLG,SUZ12,SERPINF2,FBN1,P4HB,COLEC10,FGG,BLM,F13B,F5,ECM1,FLNA,CRTAC1,F10,F7,IGF1,C1S,COLEC11,FBN2,C1R,TTN,ACTB,FGA,TPM3

Monarch	HP:0000478	Abnormality of the eye	54	2876	0.22	0.0077	GNPTG,MYH9,MGP,SPARC,APOB,APOA1,TTR,APOE,PXDN,ANGPTL6,F12,LCAT,CSTA,CP,DNAH5,CENPE,TCIRG1,SEC24D,CSF1R,SERPINI1,MASP1,A2ML1,ANTXR1,PCSK9,BMP1,PLG,NDUFS8,SUZ12,FBN1,P4HB,FBLN1,COLEC10,THBS4,FBLN5,BLM,COL18A1,CFH,HTRA1,ADAMTSL4,FLNA,RBP4,IGF1,PROS1,EFEMP1,COLEC11,TGFBI,FBN2,C1R,APOC2,TTN,PLCG2,ACTB,ITM2B,TPM3
Monarch	HP:0001342	Cerebral hemorrhage	6	61	0.94	0.0078	F13A1,FGB,FGG,F13B,PROS1,FGA
Monarch	HP:0001551	Abnormal umbilicus morphology	11	235	0.62	0.0079	APOE,MASP1,ANTXR1,BMP1,SUZ12,FBN1,COLEC10,FBLN5,FLNA,C1S,COLEC11
Monarch	HP:0030972	Abnormal systemic blood pressure	14	369	0.53	0.0081	MGP,APOB,APOA1,TTR,ANGPTL6,LYZ,GUCY1A1,PCSK9,F2,FBN1,GANAB,ADAMTSL4,SERPINA1,FGA
Monarch	HP:0000793	Membranoproliferative glomerulonephritis	3	6	1.65	0.0082	C3,C1QB,C1QC
Monarch	HP:0002825	Caudal appendage	3	6	1.65	0.0082	MASP1,COLEC10,COLEC11
Monarch	HP:0033057	Decreased serum terminal complement component	3	6	1.65	0.0082	C5,C8A,C8B
Monarch	HP:0040016	Prominent coccyx	3	6	1.65	0.0082	MASP1,COLEC10,COLEC11
Monarch	HP:0100724	Hypercoagulability	4	19	1.27	0.0082	F9,HRG,F5,PROS1
Monarch	HP:0002829	Arthralgia	8	124	0.76	0.0086	GNPTG,APOB,PCSK9,F2,FBN1,C1S,SERPINA1,PLCG2
Monarch	HP:0100763	Abnormality of the lymphatic system	18	576	0.44	0.0091	APOA1,APOE,LYZ,LCAT,DNAH5,TCIRG1,HABP2,FERMT3,A2ML1,FGB,F2,HBA1,FGG,BLM,COL18A1,SERPINA1,APOC2,FGA
Monarch	HP:0011867	Abnormal iliac wing morphology	6	64	0.92	0.0093	GNPTG,APOE,TCIRG1,SEC24D,SUZ12,FLNA
Monarch	HP:0001058	Poor wound healing	4	20	1.25	0.0096	F9,F13A1,F13B,C1R
Monarch	HP:0007420	Spontaneous hematomas	4	20	1.25	0.0096	F13A1,F13B,F5,F10
Monarch	HP:0009004	Hypoplasia of the musculature	4	20	1.25	0.0096	FLNA,COLEC11,FBN2,TPM3
Monarch	HP:0003110	Abnormality of urine homeostasis	19	638	0.42	0.0105	MYH9,F9,APOA1,APOE,CFHR5,LYZ,LCAT,F2,CFHR1,SERPINF2,GANAB,PAPLN,CFH,F5,ADAMTS13,F10,SERPINA1,ATP1A1,FGA
Monarch	HP:0000799	Renal steatosis	3	7	1.58	0.0107	APOB,APOE,PCSK9
Monarch	HP:0008067	Abnormally lax or hyperextensible skin	8	129	0.74	0.0107	CSTA,ANTXR1,SUZ12,FBN1,FBLN5,FLNA,C1S,C1R
Monarch	HP:0012397	Aortic atherosclerotic lesion	3	7	1.58	0.0107	APOB,APOE,PCSK9
Monarch	HP:0001540	Diastasis recti	4	21	1.23	0.0108	MASP1,SUZ12,COLEC10,COLEC11
Monarch	HP:0001679	Abnormal aortic morphology	12	292	0.56	0.0108	APOB,APOE,ANGPTL6,SEC24D,PCSK9,FBN1,P4HB,GANAB,FBLN5,FLNA,FBN2,TPM3
Monarch	HP:0005268	Miscarriage	4	21	1.23	0.0108	MGP,FGB,FGG,FGA
Monarch	HP:0011420	Age of death	4	21	1.23	0.0108	MGP,FGB,FGG,FGA
Monarch	EFO:0000319	Cardiovascular disease	12	293	0.56	0.0110	VWF,SEC24D,FBN1,P4HB,GANAB,F5,FLNA,NBEAL1,FBN2,APOC1,TTN,TPM3
Monarch	HP:0001297	Stroke	11	249	0.59	0.0110	TTR,ANGPTL6,GUCY1A1,F2,FBN1,F5,HTRA1,FLNA,CRTAC1,ADAMTS13,ACTB
Monarch	HP:0003010	Prolonged bleeding time	5	42	1.02	0.0111	MYH9,F9,VWF,F2,F5
Monarch	HP:0008065	Aplasia/Hypoplasia of the skin	9	170	0.67	0.0119	MGP,TCIRG1,SEC24D,FBN1,P4HB,FLNA,PROS1,C1S,C1R
Monarch	EFO:0004574	Total cholesterol measurement	16	497	0.46	0.0134	APOB,APOA1,APOE,LCAT,PCSK9,FGB,OIT3,LIMS1,COL18A1,ADAMTS13,SERPINA1,HGFAC,HPR,APOC1,APOC2,APOC4
Monarch	HP:0000095	Abnormal renal glomerulus morphology	8	136	0.72	0.0134	C3,APOE,CFHR5,C1QB,CFHR1,CFH,C1QC,C4A
Monarch	HP:0000593	Abnormal anterior chamber morphology	5	44	1.0	0.0134	MASP1,FBN1,COLEC10,COL18A1,COLEC11
Monarch	HP:0000822	Hypertension	11	256	0.58	0.0134	MGP,APOB,APOA1,ANGPTL6,LYZ,GUCY1A1,PCSK9,FBN1,GANAB,ADAMTSL4,FGA
Monarch	HP:0001677	Coronary artery atherosclerosis	5	44	1.0	0.0134	SERPIND1,APOB,APOE,PCSK9,FBN1
Monarch	HP:0002086	Abnormality of the respiratory system	35	1636	0.28	0.0134	VCL,MGP,APOB,CAT,APOE,F12,DNAH5,TCIRG1,FCN3,CRH,HABP2,DSC2,A2ML1,SLC27A4,PCSK9,BMP1,F2,PLG,NDUFS8,SERPINF2,FBN1,ANG,FBLN5,BLM,F5,ECM1,FLNA,ADAMTS13,PROS1,MASP2,SERPINA1,FBN2,TTN,PLCG2,TPM3
Monarch	HP:0004381	Supravalvular aortic stenosis	3	8	1.52	0.0134	APOB,PCSK9,FBLN5
Monarch	HP:0006394	Limited pronation/supination of forearm	3	8	1.52	0.0134	MASP1,COLEC10,COLEC11
Monarch	HP:0010874	Tendon xanthomatosis	3	8	1.52	0.0134	APOB,APOE,PCSK9
Monarch	HP:0011506	Choroidal neovascularization	3	8	1.52	0.0134	FBLN1,FBLN5,FBN2
Monarch	HP:0000347	Micrognathia	19	659	0.41	0.0135	CENPE,TCIRG1,SEC24D,A2ML1,ANTXR1,BMP1,SUZ12,FBN1,P4HB,BLM,FLNA,IGF1,C1S,IGFALS,FBN2,C1R,TTN,ACTB,TPM3
Monarch	HP:0002012	Abnormality of the abdominal organs	26	1068	0.33	0.0135	APOB,APOA1,APOE,LYZ,LCAT,CP,DNAH5,TCIRG1,FERMT3,A2ML1,ANTXR1,PCSK9,FGB,F2,NDUFS8,HBA1,FBN1,FGG,GANAB,ADAMTS13,F10,C1S,C4B,SERPINA1,APOC2,FGA
Monarch	HP:0009118	Aplasia/Hypoplasia of the mandible	19	660	0.41	0.0136	CENPE,TCIRG1,SEC24D,A2ML1,ANTXR1,BMP1,SUZ12,FBN1,P4HB,BLM,FLNA,IGF1,C1S,IGFALS,FBN2,C1R,TTN,ACTB,TPM3
Monarch	HP:0001609	Hoarse voice	6	72	0.87	0.0140	SUZ12,FBN1,ECM1,FLNA,C1R,TPM3
Monarch	HP:0100545	Arterial stenosis	6	72	0.87	0.0140	SERPIND1,APOB,APOE,F12,PCSK9,FBN1
Monarch	HP:0001681	Angina pectoris	4	24	1.17	0.0147	APOB,APOE,LCAT,PCSK9
Monarch	HP:0200036	Skin nodule	6	74	0.86	0.0158	APOB,APOE,FERMT3,PCSK9,FBN1,ECM1
Monarch	HP:0001627	Abnormal heart morphology	28	1206	0.31	0.0159	VCL,MGP,APOB,TTR,APOE,VWF,DNAH5,TCIRG1,SEC24D,DSC2,MASP1,A2ML1,PCSK9,F2,NDUFS8,HBA1,FBN1,P4HB,GANAB,FBLN5,COL18A1,FLNA,PRG4,SERPINA1,FBN2,TTN,ACTB,TPM3
Monarch	HP:0000537	Epicanthus inversus	3	9	1.47	0.0161	MASP1,COLEC10,COLEC11
Monarch	HP:0001084	Corneal arcus	3	9	1.47	0.0161	APOB,APOE,PCSK9
Monarch	HP:0003141	Increased LDL cholesterol concentration	4	26	1.14	0.0187	APOB,APOE,LCAT,PCSK9
Monarch	HP:0007957	Corneal opacity	10	226	0.59	0.0187	GNPTG,APOB,APOA1,APOE,PXDN,LCAT,PCSK9,COLEC10,COL18A1,TGFBI
Monarch	HP:0008151	Prolonged prothrombin time	4	26	1.14	0.0187	F2,F5,F10,F7
Monarch	HP:0002996	Limited elbow movement	8	146	0.69	0.0191	MASP1,SUZ12,FBN1,COLEC10,FLNA,COLEC11,FBN2,TPM3
Monarch	EFO:0010071	Cardiac troponin I measurement	3	10	1.43	0.0200	F12,KLKB1,HABP2
Monarch	HP:0000926	Platyspondyly	7	111	0.75	0.0200	SPARC,TCIRG1,SEC24D,CSF1R,BMP1,P4HB,FLNA
Monarch	HP:0001114	Xanthelasma	3	10	1.43	0.0200	APOB,APOE,PCSK9
Monarch	HP:0200067	Recurrent spontaneous abortion	3	10	1.43	0.0200	F12,F13A1,F13B
Monarch	HP:0010981	Hypolipoproteinemia	4	27	1.12	0.0203	APOB,APOA1,APOE,LCAT
Monarch	HP:0003298	Spina bifida occulta	5	51	0.94	0.0213	MASP1,COLEC10,FLNA,COLEC11,ACTB
Monarch	EFO:0007986	Reticulocyte count	16	527	0.43	0.0219	ST6GAL1,MYH9,APOE,ATRN,BLVRB,TCIRG1,FERMT3,ANGPTL4,HBA1,F10,ACTN1,C4B,PDGFC,PLCG2,ACTB,TPM3
Monarch	HP:0012575	Abnormal nephron morphology	9	190	0.62	0.0219	C3,APOE,CFHR5,ANTXR1,C1QB,CFHR1,CFH,C1QC,C4A
Monarch	HP:0000703	Dentinogenesis imperfecta	4	28	1.1	0.0224	SPARC,SEC24D,BMP1,P4HB
Monarch	HP:0001547	Abnormal rib cage morphology	12	325	0.52	0.0224	GNPTG,MGP,TCIRG1,SEC24D,A2ML1,ANTXR1,BMP1,SUZ12,FBN1,P4HB,FLNA,ACTB
Monarch	HP:0012874	Abnormal male reproductive system physiology	10	234	0.58	0.0226	TTR,DNAH5,GUCY1A1,ANTXR1,F2,DNAH8,GANAB,BLM,ADGRG2,SERPINA1
Monarch	HP:0031365	Macular purpura	5	52	0.93	0.0226	APOE,F13A1,FERMT3,F2,F13B
Monarch	HP:0011821	Abnormal facial skeleton morphology	25	1056	0.32	0.0235	MGP,APOE,DNAH5,CENPE,TCIRG1,SEC24D,A2ML1,ANTXR1,BMP1,SUZ12,FBN1,P4HB,BLM,ADAMTSL4,FLNA,IGF1,C1S,COLEC11,SERPINA1,IGFALS,FBN2,C1R,TTN,ACTB,TPM3
Monarch	HP:0001635	Congestive heart failure	9	193	0.62	0.0236	VCL,CP,F2,HBA1,FBN1,FBLN5,FLNA,SERPINA1,TTN
Monarch	HP:0010991	Abnormal morphology of the abdominal musculature	5	53	0.92	0.0240	MASP1,A2ML1,SUZ12,COLEC10,COLEC11
Monarch	HP:0000592	Blue sclerae	6	83	0.81	0.0244	SPARC,SEC24D,BMP1,FBN1,P4HB,C1R
Monarch	HP:0004950	Peripheral arterial stenosis	4	29	1.09	0.0244	APOB,APOE,PCSK9,FBN1
Monarch	HP:0010989	Abnormality of the intrinsic pathway	4	29	1.09	0.0244	F9,F12,VWF,F11
Monarch	EFO:0004623	Fibrinogen measurement	5	54	0.91	0.0253	FGB,FGG,COLEC11,HGFAC,FGA

Monarch	HP:0002645	Wormian bones	5	54	0.91	0.0253	SPARC,SEC24D,BMP1,P4HB,FLNA
Monarch	HP:0000973	Cutis laxa	6	84	0.8	0.0254	ANTXR1,SUZ12,FBN1,FBLN5,FLNA,C1R
Monarch	EFO:0006794	Cerebrospinal fluid biomarker measurement	5	55	0.91	0.0269	HRG,APOE,CFHR5,PLA2G7,C1R
Monarch	HP:0002326	Transient ischemic attack	5	55	0.91	0.0269	ANGPTL6,FBN1,HTRA1,ADAMTS13,ACTB
Monarch	HP:0032180	Abnormal circulating metabolite concentration	24	1010	0.32	0.0272	GNPTG,VCL,APOB,APOA1,APOE,LCAT,CP,TCIRG1,PLA2G7,CSF1R,PCSK9,F2,PLG,NDUFS8,FBN1,GANAB,ADAMTS13,IGF1,SERPINA1,IGFALS,ATP1A1,APOC2,TTN,TPM3
Monarch	HP:0002265	Large fleshy ears	3	12	1.35	0.0275	MASP1,COLEC10,COLEC11
Monarch	HP:0002813	Abnormality of limb bone morphology	31	1459	0.28	0.0275	GNPTG,MGP,SPARC,APOE,VWF,DNAH5,CENPE,TCIRG1,SEC24D,CSF1R,MASP1,A2ML1,ANTXR1,BMP1,F2,SUZ12,FBN1,P4HB,FBLN1,COLEC10,FBLN5,BLM,FLNA,IGF1,PRG4,COLEC11,SERPINA1,FBN2,C1R,TTN,ACTB
Monarch	HP:0003321	Biconcave flattened vertebrae	3	12	1.35	0.0275	SPARC,SEC24D,P4HB
Monarch	HP:0002637	Cerebral ischemia	9	201	0.6	0.0287	ANGPTL6,GUCY1A1,F2,FBN1,F5,HTRA1,CRTAC1,ADAMTS13,ACTB
Monarch	HP:0033401	Tissue ischemia	9	201	0.6	0.0287	ANGPTL6,GUCY1A1,F2,FBN1,F5,HTRA1,CRTAC1,ADAMTS13,ACTB
Monarch	HP:0001392	Abnormality of the liver	22	895	0.34	0.0291	APOB,APOA1,APOE,LYZ,LCAT,CP,TCIRG1,FERMT3,A2ML1,ANTXR1,PCSK9,F2,NDUFS8,HBA1,FBN1,GANAB,ADAMTS13,C1S,C4B,SERPINA1,APOC2,FGA
Monarch	HP:0004329	Abnormal posterior eye segment morphology	26	1146	0.3	0.0301	MGP,APOB,TTR,APOE,F12,CP,DNAH5,TCIRG1,CSF1R,ANTXR1,PCSK9,NDUFS8,FBN1,FBLN1,FBLN5,BLM,COL18A1,CFH,ADAMTSL4,RBP4,PROS1,EFEMP1,FBN2,APOC2,ACTB,ITM2B
Monarch	HP:0100238	Synostosis involving bones of the upper limbs	6	90	0.77	0.0338	MASP1,A2ML1,FBLN1,COLEC10,FLNA,COLEC11
Monarch	EFO:0004983	Complement C3 measurement	2	2	1.95	0.0342	C3,CFH
Monarch	EFO:0008098	Complement factor H-related protein 5 measurement	2	2	1.95	0.0342	F13B,CFH
Monarch	HP:0001743	Abnormality of the spleen	15	503	0.42	0.0342	APOA1,APOE,LYZ,LCAT,DNAH5,TCIRG1,FERMT3,A2ML1,FGB,F2,HBA1,FGG,SERPINA1,APOC2,FGA
Monarch	HP:0004434	Decreased serum complement C8	2	2	1.95	0.0342	C8A,C8B
Monarch	HP:0004586	Biconcave vertebral bodies	4	33	1.03	0.0342	SPARC,SEC24D,BMP1,P4HB
Monarch	HP:0002823	Abnormality of femur morphology	10	253	0.54	0.0351	GNPTG,SPARC,TCIRG1,SEC24D,SUZ12,FBN1,P4HB,FLNA,PRG4,ACTB
Monarch	EFO:0004872	Inflammatory biomarker measurement	27	1226	0.29	0.0356	APOB,PLEK,APOA1,APOE,APCS,LYZ,LCAT,ITIH1,ANKRD44,ANGPTL4,PCSK9,PLG,STAB1,COL18A1,CFH,F5,CRTAC1,YWHAZ,COLEC11,SERPINA1,GC,HGFAC,HPR,APOC1,APOC2,APOC4,TPM3
Monarch	EFO:0000618	Nervous system disease	12	350	0.48	0.0359	C3,APOE,CFHR5,TIMP2,ITIH4,COL18A1,CFH,F5,NBEAL1,CFB,APOC1,PLCG2
Monarch	EFO:0004535	Serum albumin measurement	7	128	0.69	0.0359	TCIRG1,SERPINF2,SERPINA11,CFH,SERPINA1,HGFAC,HPR
Monarch	EFO:0009932	HMG CoA reductase inhibitor use measurement	5	60	0.87	0.0359	APOB,APOE,FERMT3,PCSK9,HPR
Monarch	HP:0002037	Inflammation of the large intestine	5	60	0.87	0.0359	F13A1,FCN3,F13B,MASP2,PLCG2
Monarch	HP:0003549	Abnormality of connective tissue	28	1294	0.28	0.0359	VCL,SPARC,APOE,CENPE,TCIRG1,SEC24D,MASP1,ANTXR1,BMP1,SUZ12,FBN1,P4HB,COLEC10,FBLN5,BLM,ECM1,FLNA,C1S,PRG4,COLEC11,TGFBI,FBN2,C1R,TTN,VEGFC,PLCG2,ACTB,TPM3
Monarch	HP:0011138	Abnormality of skin adnexa morphology	26	1165	0.3	0.0359	MGP,APOE,CSTA,CENPE,TCIRG1,DSC2,MASP1,A2ML1,ANTXR1,BMP1,F2,NDUFS8,SUZ12,FBN1,COLEC10,LIMS1,BLM,COL18A1,HTRA1,ECM1,FLNA,IGF1,COLEC11,SERPINA1,VEGFC,ACTB
Monarch	HP:0011314	Abnormal long bone morphology	18	675	0.37	0.0359	GNPTG,MGP,SPARC,CENPE,TCIRG1,SEC24D,CSF1R,MASP1,A2ML1,ANTXR1,BMP1,SUZ12,FBN1,P4HB,COLEC10,FLNA,COLEC11,FBN2
Monarch	HP:0011844	Abnormal appendicular skeleton morphology	32	1557	0.26	0.0359	GNPTG,MGP,SPARC,APOE,VWF,DNAH5,CENPE,TCIRG1,SEC24D,CSF1R,MASP1,A2ML1,ANTXR1,BMP1,F2,SUZ12,FBN1,P4HB,FBLN1,COLEC10,FBLN5,BLM,FLNA,IGF1,PRG4,COLEC11,SERPINA1,FBN2,C1R,TTN,ACTB,TPM3
Monarch	HP:0040070	Abnormal upper limb bone morphology	13	401	0.46	0.0359	SPARC,MASP1,A2ML1,BMP1,F2,SUZ12,FBN1,FBLN1,COLEC10,FLNA,PRG4,COLEC11,SERPINA1
Monarch	EFO:0006925	Lipoprotein A measurement	4	34	1.02	0.0363	APOE,PLG,APOC2,APOC4
Monarch	HP:0001595	Abnormal hair morphology	24	1040	0.31	0.0363	MGP,APOE,CENPE,TCIRG1,DSC2,MASP1,A2ML1,ANTXR1,BMP1,F2,NDUFS8,SUZ12,FBN1,COLEC10,LIMS1,BLM,COL18A1,HTRA1,ECM1,FLNA,IGF1,COLEC11,SERPINA1,ACTB
Monarch	HP:0002686	Prenatal maternal abnormality	7	129	0.68	0.0365	F12,F13A1,DNAH5,HBA1,FBN1,F13B,F5
Monarch	HP:0020129	Abnormal urine protein level	9	211	0.58	0.0365	MYH9,APOA1,APOE,LYZ,LCAT,GANAB,CFH,ADAMTS13,FGA
Monarch	HP:0031653	Abnormal heart valve physiology	10	256	0.54	0.0365	GNPTG,MGP,APOB,VWF,PCSK9,FBN1,FBLN5,FLNA,FBN2,ACTB
Monarch	HP:0002718	Recurrent bacterial infections	8	169	0.62	0.0369	C5,C3,DNAH5,TCIRG1,FCN3,FERMT3,CFH,C8B
Monarch	EFO:0000408	Disease	29	1373	0.27	0.0406	C3,APOE,CFHR5,VWF,TIMP2,ITIH4,SEC24D,FBN1,P4HB,COLEC10,GANAB,ITIH2,COL18A1,CFH,F5,ADAM12,FLNA,IGF1,EFEMP1,EFCAB5,LTBP1,NBEAL1,CFB,IGFALS,FBN2,APOC1,TTN,PLCG2,TPM3
Monarch	EFO:0004731	Eye measurement	20	804	0.34	0.0407	MYH9,APOE,SERPINI1,A2ML1,ANTXR1,F2,FBN1,LIMS1,THBS4,FBLN5,COL18A1,CFH,F5,ADAM12,RBP4,EFEMP1,TNRC6A,LTBP1,FBN2,PLCG2
Monarch	HP:0000974	Hyperextensible skin	5	63	0.85	0.0413	ANTXR1,FBLN5,FLNA,C1S,C1R
Monarch	HP:0012638	Abnormal nervous system physiology	54	3154	0.18	0.0413	ST6GAL1,GNPTG,MGP,SPARC,APOB,APOA1,TTR,CAT,APOE,ANGPTL6,CP,DNAH5,CENPE,TCIRG1,CRH,SEC24D,CSF1R,SERPINI1,MASP1,GUCY1A1,A2ML1,ANTXR1,PCSK9,BMP1,F2,STAB1,NDUFS8,SUZ12,HBA1,FBN1,P4HB,FBLN1,COLEC10,ANG,FBLN5,BLM,COL18A1,HTRA1,ADAMTSL4,ECM1,FLNA,ADAMTS13,IGF1,COLEC11,ITIH3,SERPINA1,TGFBI,FBN2,ATP1A1,TTN,VEGFC,ACTB,ITM2B,TPM3
Monarch	EFO:0004619	Factor VII measurement	3	15	1.25	0.0419	F10,F7,TSKU
Monarch	HP:0000362	Otosclerosis	3	15	1.25	0.0419	SPARC,SEC24D,P4HB
Monarch	HP:0001083	Ectopia lentis	4	36	0.99	0.0419	FBN1,COL18A1,ADAMTSL4,FBN2
Monarch	HP:0001166	Arachnodactyly	7	133	0.67	0.0419	BMP1,FBN1,FBLN5,FLNA,FBN2,C1R,TTN
Monarch	HP:0005181	Premature coronary artery atherosclerosis	3	15	1.25	0.0419	APOB,APOE,PCSK9
Monarch	HP:0012727	Thoracic aortic aneurysm	6	97	0.74	0.0431	ANGPTL6,FBN1,GANAB,FBLN5,FBN2,TPM3
Monarch	HP:0004298	Abnormality of the abdominal wall	13	415	0.44	0.0442	APOE,MASP1,A2ML1,ANTXR1,BMP1,SUZ12,FBN1,COLEC10,FBLN5,FLNA,C1S,COLEC11,C1R
Monarch	HP:0001620	High pitched voice	4	37	0.98	0.0449	SEC24D,P4HB,BLM,TTN
Monarch	HP:0007495	Prematurely aged appearance	7	136	0.66	0.0459	APOE,CSTA,CENPE,ANTXR1,FBN1,FBLN5,C1R
Monarch	HP:0030875	Abnormality of pulmonary circulation	7	136	0.66	0.0459	MGP,TCIRG1,BMP1,F2,FBN1,FLNA,PROS1
Monarch	EFO:0008142	Granulocyte colony-stimulating factor measurement	3	16	1.22	0.0469	F5,APOC2,APOC4
Monarch	EFO:0008432	Lipoprotein-associated phospholipase A(2) change measurement	2	3	1.77	0.0486	APOE,PLA2G7
Monarch	HP:0001658	Myocardial infarction	4	38	0.97	0.0486	MYH9,APOB,PCSK9,ADAMTS13
Monarch	HP:0008357	Reduced factor XIII activity	2	3	1.77	0.0486	F13A1,F13B
Monarch	HP:0025408	Abnormal spleen morphology	14	475	0.42	0.0486	APOA1,APOE,LYZ,LCAT,DNAH5,TCIRG1,FERMT3,FGB,F2,HBA1,FGG,SERPINA1,APOC2,FGA
Monarch	HP:0030657	Umbilical cord hematoma	2	3	1.77	0.0486	F13A1,F13B
Monarch	HP:0030791	Abnormal jaw morphology	21	883	0.32	0.0486	CENPE,TCIRG1,SEC24D,A2ML1,ANTXR1,BMP1,SUZ12,FBN1,P4HB,BLM,ADAMTSL4,FLNA,IGF1,C1S,COLEC11,IGFALS,FBN2,C1R,TTN,ACTB,TPM3
Monarch	HP:0002664	Neoplasm	19	763	0.34	0.0492	CAT,APOE,F13A1,TCIRG1,FCN3,HABP2,ANTXR1,F2,HBA1,GANAB,BLM,COL18A1,F13B,ECM1,FLNA,C1S,SERPINA1,VEGFC,ACTB
DISEASES	DOID:1247	Blood coagulation disease	26	89	1.41	3.20e-23	SERPIND1,MYH9,F9,PF4V1,HRG,F12,VWF,KLKB1,F13A1,PF4,FGB,F2,PLG,SERPINF2,FGG,F13B,CFH,F5,FLNA,ADAMTS13,F10,F7,PROS1,F11,C4B,FGA

DISEASES	DOID:74	Hematopoietic system disease	33	473	0.79	5.64e-13	SERPIND1,MYH9,F9,PF4V1,HRG,F12,VWF,KLKB1,F13A1,TCIRG1,FERMT3,PF4,CAMP,FGB,F2,PLG,SERPINF2,HBA1,FGG,F13B,CFH,F5,FLNA,ADAMTS13,F10,F7,PROS1,SAA1,F11,C4B,IGHV3-15,ACTB,FGA
DISEASES	DOID:4	Disease	130	6291	0.26	7.31e-13	GNPTG,VCL,SERPIND1,LGALS1,MYH9,F9,C5,PF4V1,MGP,SPARC,HRG,APOB,APOA1,TTR,CAT,C3,APOE,PXDN,F12,APCS,CFHR5,LYZ,VWF,LCAT,CSTA,CP,KLKB1,F13A1,DNAH5,CENPE,TCIRG1,ITIH4,PLA2G7,CRH,FERMT3,SEC24D,DSC2,ANKRD44,CSF1R,PIP,SCGB3A1,SERPINI1,PF4,MASP1,CAMP,A2ML1,SLC27A4,LALBA,ANTXR1,SH3D19,PCSK9,BMP1,FGB,F2,PLG,PRG2,C1QB,CFHR1,NDUFS8,SUZ12,APOL1,SUMO4,SERPINF2,HBA1,FBN1,P4HB,COLEC10,DNAH8,ANG,FGG,GANAB,SLPI,FBLN5,BLM,CNDP1,COL18A1,F13B,CFHR3,CFH,F5,HTRA1,ADAMTSL4,ECM1,FLNA,C8B,RBP4,ADAMTS13,C1QC,F10,F7,CA6,ADGRG2,GPX3,IGF1,PROS1,EFEMP1,TNRC6A,YWHAZ,SAA1,F11,C1S,PCYOX1,ADIPOQ,C4A,PRG4,COLEC11,MST1,C4B,SERPINA1,TGFBI,CFB,PDGFC,FBN2,SELENOP,TSKU,JCHAIN,HPR,C1R,ATP1A1,IGKV2D-28,APOC2,TTN,IGHV3-15,VEGFC,PLCG2,TPM4,ACTB,ITM2B,FGA,TPM3
DISEASES	DOID:7	Disease of anatomical entity	110	4798	0.31	8.34e-13	VCL,SERPIND1,LGALS1,MYH9,F9,C5,PF4V1,MGP,SPARC,HRG,APOB,APOA1,TTR,C3,APOE,PXDN,F12,APCS,CFHR5,VWF,LCAT,CSTA,KLKB1,F13A1,DNAH5,CENPE,TCIRG1,PLA2G7,CRH,FERMT3,SEC24D,DSC2,ANKRD44,CSF1R,PIP,SCGB3A1,SERPINI1,PF4,CAMP,A2ML1,SLC27A4,LALBA,ANTXR1,SH3D19,PCSK9,BMP1,FGB,F2,PLG,C1QB,CFHR1,SUZ12,APOL1,SUMO4,SERPINF2,HBA1,FBN1,P4HB,ANG,FGG,GANAB,SLPI,FBLN5,BLM,CNDP1,COL18A1,F13B,CFHR3,CFH,F5,HTRA1,ADAMTSL4,ECM1,FLNA,C8B,RBP4,ADAMTS13,C1QC,F10,F7,ADGRG2,IGF1,PROS1,EFEMP1,TNRC6A,YWHAZ,SAA1,F11,C1S,ADIPOQ,C4A,MST1,C4B,SERPINA1,TGFBI,CFB,FBN2,TSKU,JCHAIN,C1R,ATP1A1,IGKV2D-28,TTN,IGHV3-15,VEGFC,PLCG2,ACTB,ITM2B,FGA,TPM3
DISEASES	DOID:2452	Thrombophilia	11	21	1.67	2.48e-11	SERPIND1,F9,HRG,VWF,F2,PLG,CFH,F5,ADAMTS13,PROS1,C4B
DISEASES	DOID:9120	Amyloidosis	13	75	1.19	1.27e-08	APOA1,TTR,C3,APOE,APCS,LYZ,SAA1,SERPINA1,TGFBI,APOC2,ACTB,ITM2B,FGA
DISEASES	DOID:0050739	Autosomal genetic disease	71	2802	0.35	1.35e-08	GNPTG,SERPIND1,MYH9,F9,MGP,HRG,APOB,APOA1,TTR,C3,APOE,F12,APCS,CFHR5,LYZ,VWF,CP,KLKB1,F13A1,DNAH5,TCIRG1,FERMT3,ANKRD44,CSF1R,SERPINI1,MASP1,CAMP,ANTXR1,PCSK9,FGB,F2,PLG,CFHR1,SERPINF2,HBA1,FBN1,COLEC10,FGG,GANAB,FBLN5,BLM,F13B,CFHR3,CFH,F5,HTRA1,ADAMTSL4,FLNA,F10,F7,IGF1,EFEMP1,SAA1,F11,C1S,PCYOX1,PRG4,COLEC11,SERPINA1,TGFBI,CFB,FBN2,JCHAIN,ATP1A1,APOC2,TTN,PLCG2,ACTB,ITM2B,FGA,TPM3
DISEASES	DOID:0050636	Familial visceral amyloidosis	9	21	1.58	1.51e-08	APOA1,TTR,APOE,APCS,LYZ,SERPINA1,APOC2,ACTB,FGA
DISEASES	DOID:2007	Degeneration of macula and posterior pole	11	46	1.33	1.54e-08	C3,APOE,CFHR5,CFHR1,FBLN5,F13B,CFHR3,CFH,HTRA1,EFEMP1,CFB
DISEASES	DOID:630	Genetic disease	85	3778	0.3	1.85e-08	GNPTG,SERPIND1,MYH9,F9,MGP,HRG,APOB,APOA1,TTR,CAT,C3,APOE,F12,APCS,CFHR5,LYZ,VWF,LCAT,CP,KLKB1,F13A1,DNAH5,TCIRG1,FERMT3,SEC24D,ANKRD44,CSF1R,SERPINI1,MASP1,CAMP,A2ML1,ANTXR1,PCSK9,FGB,F2,PLG,C1QB,CFHR1,NDUFS8,SUMO4,SERPINF2,HBA1,FBN1,COLEC10,DNAH8,FGG,GANAB,FBLN5,BLM,CNDP1,F13B,CFHR3,CFH,F5,HTRA1,ADAMTSL4,ECM1,FLNA,F10,F7,ADGRG2,IGF1,PROS1,EFEMP1,SAA1,F11,C1S,PCYOX1,ADIPOQ,PRG4,COLEC11,MST1,SERPINA1,TGFBI,CFB,FBN2,JCHAIN,ATP1A1,APOC2,TTN,PLCG2,ACTB,ITM2B,FGA,TPM3
DISEASES	DOID:626	Complement deficiency	9	23	1.54	2.11e-08	C5,C3,CFHR5,KLKB1,CFHR1,CFHR3,CFH,ADAMTS13,CFB
DISEASES	DOID:0050177	Monogenic disease	77	3266	0.32	2.43e-08	GNPTG,SERPIND1,MYH9,F9,MGP,HRG,APOB,APOA1,TTR,C3,APOE,F12,APCS,CFHR5,LYZ,VWF,CP,KLKB1,F13A1,DNAH5,TCIRG1,FERMT3,SEC24D,ANKRD44,CSF1R,SERPINI1,MASP1,CAMP,A2ML1,ANTXR1,PCSK9,FGB,F2,PLG,CFHR1,SERPINF2,HBA1,FBN1,COLEC10,DNAH8,FGG,GANAB,FBLN5,BLM,F13B,CFHR3,CFH,F5,HTRA1,ADAMTSL4,FLNA,F10,F7,ADGRG2,IGF1,PROS1,EFEMP1,SAA1,F11,C1S,PCYOX1,PRG4,COLEC11,MST1,SERPINA1,TGFBI,CFB,FBN2,JCHAIN,ATP1A1,APOC2,TTN,PLCG2,ACTB,ITM2B,FGA,TPM3
DISEASES	DOID:2914	Immune system disease	30	675	0.6	1.02e-07	C5,TTR,C3,APOE,PXDN,APCS,CFHR5,KLKB1,FERMT3,A2ML1,LALBA,F2,PLG,CFHR1,SUMO4,HBA1,COL18A1,CFHR3,CFH,ADAMTS13,SAA1,C4A,C4B,CFB,JCHAIN,IGKV2D-28,IGHV3-15,VEGFC,PLCG2,ACTB
DISEASES	DOID:10871	Age related macular degeneration	10	44	1.3	1.15e-07	C3,APOE,CFHR5,CFHR1,FBLN5,F13B,CFHR3,CFH,HTRA1,CFB
DISEASES	DOID:0080301	Atypical hemolytic-uremic syndrome	7	11	1.75	1.60e-07	C3,CFHR5,CFHR1,CFHR3,CFH,ADAMTS13,CFB
DISEASES	DOID:0050736	Autosomal dominant disease	43	1386	0.44	5.05e-07	SERPIND1,MYH9,F9,HRG,APOA1,TTR,C3,APOE,F12,APCS,CFHR5,LYZ,VWF,KLKB1,ANKRD44,CSF1R,SERPINI1,F2,CFHR1,FBN1,GANAB,FBLN5,F13B,CFHR3,CFH,F5,HTRA1,FLNA,EFEMP1,SAA1,C1S,SERPINA1,TGFBI,CFB,FBN2,JCHAIN,ATP1A1,APOC2,TTN,PLCG2,ACTB,ITM2B,FGA
DISEASES	DOID:12554	Hemolytic-uremic syndrome	7	14	1.65	5.05e-07	C3,CFHR5,CFHR1,CFHR3,CFH,ADAMTS13,CFB
DISEASES	DOID:11949	Creutzfeldt-Jakob disease	6	9	1.77	1.99e-06	C3,APOE,APCS,C1QB,C1QC,YWHAZ
DISEASES	DOID:0050117	Disease by infectious agent	20	368	0.68	3.45e-06	C3,APOE,APCS,CP,ITIH4,ANTXR1,F2,PLG,PRG2,C1QB,APOL1,SLPI,HTRA1,C1QC,CA6,YWHAZ,SAA1,C4A,PDGFC,HPR
DISEASES	DOID:1287	Cardiovascular system disease	23	493	0.62	3.99e-06	VCL,APOB,APOA1,TTR,APOE,APCS,VWF,DNAH5,DSC2,PF4,PCSK9,F2,PLG,FBN1,FLNA,ADAMTS13,F10,F7,SAA1,ADIPOQ,C4A,FBN2,TTN
DISEASES	DOID:2921	Glomerulonephritis	8	36	1.29	5.50e-06	C3,CFHR5,APOL1,COL18A1,CFHR3,CFH,C4B,CFB
DISEASES	DOID:557	Kidney disease	17	275	0.74	6.53e-06	MYH9,C5,TTR,C3,APCS,CFHR5,F2,CFHR1,APOL1,GANAB,COL18A1,CFHR3,CFH,ADAMTS13,SAA1,C4B,CFB
DISEASES	DOID:178	Vascular disease	16	249	0.76	9.72e-06	APOB,APOA1,APOE,VWF,PF4,PCSK9,F2,PLG,FBN1,ADAMTS13,F10,F7,SAA1,ADIPOQ,C4A,FBN2
DISEASES	DOID:2218	Blood platelet disease	8	47	1.18	2.67e-05	MYH9,PF4V1,VWF,PF4,F2,FLNA,ADAMTS13,F7
DISEASES	DOID:612	Primary immunodeficiency disease	21	470	0.6	2.67e-05	C5,TTR,C3,PXDN,CFHR5,KLKB1,FERMT3,A2ML1,F2,CFHR1,SUMO4,HBA1,COL18A1,CFHR3,CFH,ADAMTS13,SAA1,C4A,C4B,CFB,PLCG2
DISEASES	DOID:37	Skin disease	22	518	0.58	2.87e-05	APOA1,C3,APOE,F12,APCS,CSTA,KLKB1,FERMT3,DSC2,PIP,CAMP,A2ML1,SLC27A4,ANTXR1,FBN1,FBLN5,BLM,COL18A1,IGF1,C1S,SERPINA1,ACTB
DISEASES	DOID:0014667	Disease of metabolism	33	1076	0.43	3.75e-05	GNPTG,F9,MGP,APOB,APOA1,TTR,CAT,C3,APOE,APCS,LYZ,LCAT,CP,PCSK9,F2,NDUFS8,SUMO4,HBA1,CNDP1,ECM1,GPX3,IGF1,PROS1,SAA1,PCYOX1,ADIPOQ,SERPINA1,TGFBI,SELENOP,APOC2,ACTB,ITM2B,FGA
DISEASES	DOID:0060903	Thrombosis	6	21	1.4	6.46e-05	VWF,PF4,F2,PLG,F10,F7
DISEASES	DOID:0050828	Artery disease	11	130	0.88	7.63e-05	APOB,APOA1,APOE,PCSK9,F2,PLG,FBN1,ADAMTS13,SAA1,ADIPOQ,FBN2
DISEASES	DOID:5614	Eye disease	26	749	0.49	8.05e-05	LGALS1,MYH9,APOB,C3,APOE,APCS,CFHR5,PCSK9,PLG,CFHR1,FBN1,FBLN5,COL18A1,F13B,CFHR3,CFH,HTRA1,ADAMTSL4,RBP4,IGF1,EFEMP1,SERPINA1,TGFBI,CFB,FBN2,ACTB
DISEASES	DOID:1588	Thrombocytopenia	5	12	1.57	0.00012	PF4V1,VWF,PF4,F2,ADAMTS13
DISEASES	DOID:9799	Eye degenerative disease	14	236	0.72	0.00012	APOB,C3,APOE,CFHR5,PCSK9,CFHR1,FBLN5,F13B,CFHR3,CFH,HTRA1,EFEMP1,TGFBI,CFB
DISEASES	DOID:0050737	Autosomal recessive disease	43	1785	0.33	0.00024	GNPTG,MYH9,MGP,APOB,APOE,F12,VWF,CP,KLKB1,F13A1,DNAH5,TCIRG1,FERMT3,MASP1,CAMP,ANTXR1,PCSK9,FGB,F2,PLG,SERPINF2,HBA1,COLEC10,FGG,FBLN5,BLM,F13B,F5,FLNA,F10,F7,IGF1,SAA1,F11,C1S,PCYOX1,PRG4,COLEC11,TGFBI,APOC2,TTN,FGA,TPM3
DISEASES	DOID:17	Musculoskeletal system disease	32	1154	0.39	0.00037	F9,C5,MGP,SPARC,TTR,APOE,F12,APCS,TCIRG1,SEC24D,CSF1R,A2ML1,ANTXR1,BMP1,PLG,FBN1,P4HB,FBLN5,COL18A1,HTRA1,FLNA,F7,SAA1,C1S,C4A,C4B,FBN2,JCHAIN,C1R,ATP1A1,TTN,TPM3
DISEASES	DOID:65	Connective tissue disease	25	774	0.46	0.00039	F9,C5,MGP,SPARC,TTR,TCIRG1,SEC24D,CSF1R,A2ML1,ANTXR1,BMP1,PLG,FBN1,P4HB,COL18A1,HTRA1,FLNA,F7,SAA1,C1S,C4A,C4B,FBN2,JCHAIN,C1R
DISEASES	DOID:0050639	Primary cutaneous amyloidosis	5	19	1.37	0.00066	C3,APOE,APCS,SERPINA1,ACTB

DISEASES	DOID:0050155	Sensory system disease	27	929	0.41	0.00096	LGALS1,MYH9,APOB,C3,APOE,APCS,CFHR5,A2ML1,PCSK9,PLG,CFHR1,FBN1,FBLN5,COL18A1,F13B,CFHR3,CFH,HTRA1,ADAMTSL4,RBP4,IGF1,EFEMP1,SERPINA1,TGFBI,CFB,FBN2,ACTB
DISEASES	DOID:0050637	Finnish type amyloidosis	4	9	1.6	0.0011	C3,APOE,APCS,SERPINA1
DISEASES	DOID:655	Inherited metabolic disorder	27	949	0.4	0.0013	GNPTG,F9,APOB,APOA1,TTR,CAT,C3,APOE,APCS,LYZ,LCAT,CP,PCSK9,NDUFS8,SUMO4,HBA1,CNDP1,ECM1,IGF1,SAA1,PCYOX1,ADIPOQ,SERPINA1,APOC2,ACTB,ITM2B,FGA
DISEASES	DOID:863	Nervous system disease	48	2275	0.27	0.0016	LGALS1,MYH9,APOB,APOA1,TTR,C3,APOE,APCS,CFHR5,VWF,LCAT,CENPE,ANKRD44,CSF1R,SERPINI1,A2ML1,SH3D19,PCSK9,F2,PLG,C1QB,CFHR1,SUZ12,FBN1,ANG,FBLN5,CNDP1,COL18A1,F13B,CFHR3,CFH,HTRA1,ADAMTSL4,FLNA,RBP4,C1QC,IGF1,EFEMP1,TNRC6A,YWHAZ,SERPINA1,TGFBI,CFB,FBN2,TSKU,ATP1A1,ACTB,ITM2B
DISEASES	DOID:5113	Nutritional deficiency disease	6	43	1.09	0.0017	MGP,TTR,F2,GPX3,PROS1,SELENOP
DISEASES	DOID:1387	Hypolipoproteinemia	4	11	1.51	0.0018	APOB,APOA1,LCAT,PCSK9
DISEASES	DOID:409	Liver disease	8	97	0.86	0.0022	TTR,APCS,F2,GANAB,SAA1,SERPINA1,IGHV3-15,ACTB
DISEASES	DOID:0060225	3MC syndrome	3	3	1.95	0.0025	MASP1,COLEC10,COLEC11
DISEASES	DOID:2222	Factor X deficiency	3	3	1.95	0.0025	F2,F10,F7
DISEASES	DOID:2236	Congenital afibrinogenemia	3	3	1.95	0.0025	FGB,FGG,FGA
DISEASES	DOID:3146	Lipid metabolism disorder	7	72	0.94	0.0025	APOB,APOA1,APOE,LCAT,PCSK9,ECM1,APOC2
DISEASES	DOID:10772	Thrombotic thrombocytopenic purpura	3	4	1.82	0.0040	VWF,CFH,ADAMTS13
DISEASES	DOID:12134	Factor VIII deficiency	3	4	1.82	0.0040	F9,VWF,F7
DISEASES	DOID:2569	Retinal drusen	3	4	1.82	0.0040	FBLN5,CFH,EFEMP1
DISEASES	DOID:2920	Membranoproliferative glomerulonephritis	3	4	1.82	0.0040	C3,CFHR5,CFH
DISEASES	DOID:1168	Familial hyperlipidemia	4	15	1.37	0.0041	APOB,APOE,PCSK9,APOC2
DISEASES	DOID:11249	Vitamin K deficiency bleeding	3	5	1.73	0.0058	MGP,F2,PROS1
DISEASES	DOID:12259	Hemophilia B	3	5	1.73	0.0058	F9,F7,F11
DISEASES	DOID:1237	Corneal degeneration	3	5	1.73	0.0058	APOB,PCSK9,TGFBI
DISEASES	DOID:2229	Factor XI deficiency	3	5	1.73	0.0058	F9,F7,F11
DISEASES	DOID:2451	Protein S deficiency	3	5	1.73	0.0058	F2,PROS1,C4B
DISEASES	DOID:3393	Coronary artery disease	5	36	1.09	0.0064	APOB,APOA1,APOE,PCSK9,PLG
DISEASES	DOID:12531	Von Willebrands disease	3	6	1.65	0.0077	F9,VWF,ADAMTS13
DISEASES	DOID:14735	Hereditary angioedema	3	6	1.65	0.0077	F12,KLKB1,C1S
DISEASES	DOID:10873	Kuhnt-Junius degeneration	3	7	1.58	0.0106	CFH,HTRA1,CFB
DISEASES	DOID:1936	Atherosclerosis	4	21	1.23	0.0106	APOB,APOA1,APOE,ADIPOQ
DISEASES	DOID:7693	Abdominal aortic aneurysm	3	7	1.58	0.0106	APOE,PLG,FBN1
DISEASES	DOID:3118	Hepatobiliary disease	9	168	0.68	0.0109	TTR,APCS,F2,GANAB,SAA1,MST1,SERPINA1,IGHV3-15,ACTB
DISEASES	DOID:0080001	Bone disease	17	540	0.45	0.0113	F9,C5,SPARC,TTR,TCIRG1,SEC24D,CSF1R,BMP1,FBN1,P4HB,COL18A1,HTRA1,FLNA,F7,SAA1,C1S,JCHAIN
DISEASES	DOID:5679	Retinal disease	12	309	0.54	0.0166	C3,APOE,CFHR5,CFHR1,FBLN5,F13B,CFHR3,CFH,HTRA1,IGF1,EFEMP1,CFB
DISEASES	DOID:9352	Type 2 diabetes mellitus	4	25	1.15	0.0173	APOE,APCS,IGF1,ADIPOQ
DISEASES	DOID:5844	Myocardial infarction	4	27	1.12	0.0223	APOB,APOE,PCSK9,PLG
DISEASES	DOID:0060440	Epithelial and subepithelial dystrophy	3	11	1.38	0.0267	APOE,COL18A1,TGFBI
DISEASES	DOID:374	Nutrition disease	7	116	0.73	0.0269	MGP,TTR,F2,GPX3,PROS1,ADIPOQ,SELENOP
DISEASES	DOID:10124	Corneal disease	6	85	0.8	0.0312	LGALS1,APOB,APOE,PCSK9,COL18A1,TGFBI
DISEASES	DOID:0070029	ITM2B-related cerebral amyloid angiopathy 1	3	12	1.35	0.0319	APOE,APCS,ITM2B
DISEASES	DOID:2213	Hemorrhagic disease	3	12	1.35	0.0319	F2,SERPINF2,ADAMTS13
DISEASES	DOID:0060158	Acquired metabolic disease	8	162	0.64	0.0363	MGP,TTR,CP,F2,GPX3,PROS1,ADIPOQ,SELENOP
DISEASES	DOID:0070030	ITM2B-related cerebral amyloid angiopathy 2	3	13	1.31	0.0376	APOE,APCS,ITM2B
DISEASES	DOID:0050638	Transthyretin amyloidosis	2	2	1.95	0.0391	TTR,APCS
DISEASES	DOID:0050831	Familial encephalopathy with neuroserpin inclusion bodies	2	2	1.95	0.0391	SERPINI1,SERPINA1
DISEASES	DOID:0060745	Doyne honeycomb retinal dystrophy	2	2	1.95	0.0391	FBLN5,EFEMP1
DISEASES	DOID:0111595	Congenital contractural arachnodactyly	2	2	1.95	0.0391	FBN1,FBN2
DISEASES	DOID:10230	Aortic atherosclerosis	2	2	1.95	0.0391	APOB,APOE
DISEASES	DOID:1391	Norum disease	2	2	1.95	0.0391	APOA1,LCAT
DISEASES	DOID:2211	Factor XIII deficiency	2	2	1.95	0.0391	F13A1,F13B
DISEASES	DOID:520	Aortic disease	4	34	1.02	0.0407	APOE,PLG,FBN1,FBN2
DISEASES	DOID:12347	Osteogenesis imperfecta	4	35	1.01	0.0445	SPARC,SEC24D,BMP1,P4HB
TISSUES	BTO:0004850	Bone marrow cell	48	198	1.33	1.20e-42	C5,APOB,IGFBP5,TTR,C3,APCS,THBS1,VWF,A1BG,CP,KLKB1,F13A1,AMBP,HPX,ITIH4,FCN3,DSC2,CSF1R,FCN2,MASP1,FGB,F2,CFHR1,APOL1,SERPINF2,ITIH2,CFH,F5,ECM1,ADAMTS13,MBL2,SOD3,PROS1,F11,C1S,CD14,C4A,PRG4,MST1,C4B,SERPINA1,CFB,PLTP,HGFAC,SELENOP,JCHAIN,C1R,FGA
TISSUES	BTO:0000759	Liver	105	2125	0.64	9.20e-39	SPP2,ST6GAL1,VCL,SERPIND1,LGALS1,MYH9,TPD52L2,APMAP,F9,HRG,APOB,APOA1,TTR,CAT,C3,APOE,PXDN,F12,APCS,CFHR5,THBS1,VWF,PSMA6,SERPINA10,ATRN,A1BG,BLVRB,CP,KLKB1,AMBP,HPX,FCN3,ITIH1,HABP2,CSF1R,FCN2,PPBP,MASP1,FGB,F2,INHBC,PLG,STAB1,C1QB,CFHR1,TLN1,APOL1,SERPINF2,HBA1,FBN1,P4HB,ANG,FGG,GANAB,SLPI,ITIH2,COL18A1,C8A,PCDH18,F13B,CFHR3,CFH,ECM1,FLNA,SMARCA1,C8B,RBP4,ADAMTS13,MBL2,F10,F7,APOM,GOLM1,IGF1,PROS1,ACTN1,YWHAZ,MASP2,SAA1,F11,C1S,CD14,PCYOX1,C4A,COLEC11,MST1,ITIH3,C4B,SERPINA1,TGFBI,CFB,IGFALS,PLTP,GC,HGFAC,JCHAIN,C1R,ATP1A1,MYL6,APOC1,APOC4,ACTB,FGA,TPM3,PDIA4
TISSUES	BTO:0000141	Bone marrow	60	528	1.0	9.23e-38	SERPIND1,APMAP,C5,APOB,IGFBP5,TTR,C3,PXDN,APCS,THBS1,VWF,A1BG,CSTA,CP,KLKB1,F13A1,AMBP,HPX,ITIH4,FCN3,DSC2,CSF1R,FCN2,MASP1,CAMP,FGB,F2,PRG2,CFHR1,SUZ12,APOL1,SERPINF2,HBA1,LIMS1,GANAB,ITIH2,CFH,F5,ECM1,ADAMTS13,MBL2,SOD3,PROS1,YWHAZ,F11,C1S,CD14,C4A,PRG4,MST1,C4B,SERPINA1,CFB,PLTP,HGFAC,SELENOP,JCHAIN,C1R,HSPH1,FGA
TISSUES	BTO:0000392	Plasma cell	42	171	1.34	1.19e-37	C5,APOB,IGFBP5,TTR,C3,APCS,THBS1,VWF,CP,KLKB1,F13A1,AMBP,HPX,ITIH4,FCN3,DSC2,CSF1R,FCN2,MASP1,FGB,F2,CFHR1,APOL1,SERPINF2,ITIH2,CFH,ECM1,ADAMTS13,MBL2,SOD3,PROS1,C1S,CD14,C4A,C4B,SERPINA1,CFB,HGFAC,SELENOP,JCHAIN,C1R,FGA
TISSUES	BTO:0000345	Digestive gland	114	2881	0.55	1.87e-34	SPP2,ST6GAL1,VCL,SERPIND1,LGALS1,MYH9,TPD52L2,APMAP,F9,SPARC,HRG,APOB,APOA1,TTR,CAT,C3,DHX29,APOE,PXDN,F12,APCS,CFHR5,THBS1,VWF,PSMA6,SERPINA10,ATRN,A1BG,BLVRB,CP,KLKB1,F13A1,AMBP,TCIRG1,HPX,FCN3,ITIH1,HABP2,SEC24D,CSF1R,FCN2,PPBP,MASP1,ANGPTL4,FGB,F2,INHBC,PLG,STAB1,C1QB,CFHR1,NDUFS8,TLN1,APOL1,SERPINF2,HBA1,FBN1,P4HB,ANG,FGG,GANAB,SLPI,ITIH2,COL18A1,C8A,PCDH18,F13B,CFHR3,CFH,ECM1,FLNA,SMARCA1,C8B,RBP4,ADAMTS13,MBL2,F10,F7,APOM,GOLM1,GPX3,IGF1,PROS1,ACTN1,YWHAZ,MASP2,SAA1,F11,C1S,CD14,PCYOX1,C4A,COLEC11,MST1,ITIH3,C4B,SERPINA1,TGFBI,CFB,IGFALS,PLTP,GC,HGFAC,JCHAIN,C1R,ATP1A1,MYL6,APOC1,APOC4,ACTB,ITM2B,FGA,TPM3,PDIA4

TISSUES	BTO:0001486	Skeletal system	77	1307	0.72	1.96e-31	SPP2,SERPIND1,APMAP,C5,SPARC,APOB,IGFBP5,APOA1,TTR,C3,APOE,PXDN,APCS,THBS1,VWF,PSMA6,ATRN,A1BG,CSTA,CP,KLKB1,F13A1,AMBP,HPX,ITIH4,FCN3,DSC2,CSF1R,FCN2,MASP1,CAMP,FGB,F2,UBE3C,PRG2,CFHR1,SUZ12,APOL1,SERPINF2,HBA1,FGG,LIMS1,GANAB,ITIH2,CFH,F5,HTRA1,ADAMTSL4,ECM1,CRTAC1,ADAMTS13,CCN5,MBL2,SOD3,PROS1,YWHAZ,F11,C1S,CD14,PCYOX1,C4A,PRG4,MST1,C4B,SERPINA1,CFB,PLTP,HGFAC,SELENOP,JCHAIN,C1R,APOC2,TTN,HSPH1,ACTB,FGA,TPM3
TISSUES	BTO:0001491	Viscus	133	5378	0.34	2.00e-21	SPP2,ST6GAL1,VCL,SERPIND1,LGALS1,MYH9,TPD52L2,APMAP,F9,SPARC,HRG,APOB,PLEK,APOA1,TTR,CAT,C3,DHX29,APOE,PXDN,F12,APCS,CFHR5,THBS1,LYZ,VWF,PSMA6,SERPINA10,ATRN,A1BG,BLVRB,CSTA,CP,KLKB1,F13A1,AMBP,TCIRG1,HPX,FCN3,ITIH1,PLA2G7,HABP2,SEC24D,DSC2,CSF1R,FCN2,PPBP,MASP1,GUCY1A1,LALBA,ANGPTL4,ANTXR1,SH3D19,FGB,F2,INHBC,PLG,STAB1,C1QB,CFHR1,TLN1,APOL1,SERPINF2,HBA1,FBN1,P4HB,FBLN1,ANG,FGG,GANAB,SLPI,FBLN5,ITIH2,COL18A1,C8A,PCDH18,F13B,CFHR3,CFH,ECM1,FLNA,SMARCA1,C8B,RBP4,ADAMTS13,MBL2,F10,F7,APOM,SOD3,GOLM1,GPX3,IGF1,PROS1,ACTN1,EFEMP1,TNRC6A,YWHAZ,ILK,MASP2,SAA1,F11,C1S,CD14,PCYOX1,C4A,ACSF2,COLEC11,MST1,ITIH3,C4B,SERPINA1,TGFBI,CFB,IGFALS,PLTP,GC,HGFAC,JCHAIN,C1R,ATP1A1,MYL6,APOC1,APOC4,VEGFC,PLCG2,HSPH1,TPM4,ACTB,ITM2B,FGA,TPM3,PDIA4
TISSUES	BTO:0001488	Endocrine gland	141	6403	0.29	1.67e-18	SPP2,ST6GAL1,VCL,SERPIND1,LGALS1,MYH9,TPD52L2,APMAP,F9,SPARC,HRG,APOB,APOA1,TTR,CAT,C3,DHX29,APOE,PXDN,F12,APCS,CFHR5,THBS1,VWF,PSMA6,SERPINA10,ATRN,A1BG,BLVRB,LCN1,CSTA,CP,KLKB1,F13A1,AMBP,TCIRG1,HPX,FCN3,ITIH1,PLA2G7,CRH,HABP2,FERMT3,SEC24D,CSF1R,PIP,FCN2,PPBP,MASP1,CAMP,ANGPTL4,ANTXR1,SH3D19,FGB,F2,INHBC,PLG,STAB1,C1QB,CFHR1,NDUFS8,TLN1,APOL1,SERPINF2,HBA1,FBN1,P4HB,FBLN1,ANG,FGG,LIMS1,ISYNA1,GANAB,SLPI,FBLN5,PAPLN,ITIH2,COL18A1,C8A,PCDH18,F13B,CFHR3,CFH,ECM1,FLNA,SMARCA1,C8B,RBP4,ADAMTS13,CCN5,MBL2,F10,F7,APOM,SOD3,GOLM1,GPX3,IGF1,PROS1,ACTN1,EFEMP1,EFCAB5,YWHAZ,RAPGEF4,MASP2,SAA1,F11,C1S,CD14,LTBP1,PCYOX1,C4A,ATP9B,FSIP2,ACSF2,COLEC11,MST1,ITIH3,C4B,SERPINA1,TGFBI,CFB,IGFALS,PLTP,GC,HGFAC,UIMC1,PDGFC,JCHAIN,C1R,ATP1A1,MYL6,APOC1,APOC4,HSPH1,TPM4,ACTB,ITM2B,FGA,TPM3,PDIA4
TISSUES	BTO:0000522	Gland	147	7004	0.27	7.66e-18	SPP2,ST6GAL1,VCL,SERPIND1,LGALS1,MYH9,TPD52L2,APMAP,F9,C5,SPARC,HRG,APOB,APOA1,TTR,CAT,C3,DHX29,APOE,PXDN,BPIFB1,F12,APCS,CFHR5,THBS1,LYZ,VWF,PSMA6,SERPINA10,ATRN,A1BG,BLVRB,LCN1,CSTA,CP,KLKB1,F13A1,AMBP,TCIRG1,HPX,FCN3,ITIH1,PLA2G7,CRH,HABP2,FERMT3,SEC24D,CSF1R,PIP,FCN2,PPBP,MASP1,CAMP,GUCY1A1,ANGPTL4,ANTXR1,SH3D19,FGB,F2,INHBC,PLG,STAB1,C1QB,CFHR1,NDUFS8,TLN1,APOL1,SERPINF2,HBA1,FBN1,P4HB,FBLN1,ANG,FGG,LIMS1,ISYNA1,GANAB,SLPI,FBLN5,PAPLN,ITIH2,COL18A1,C8A,PCDH18,F13B,CFHR3,CFH,ECM1,FLNA,SMARCA1,C8B,RBP4,ADAMTS13,CCN5,MBL2,F10,F7,APOM,CA6,SOD3,GOLM1,GPX3,IGF1,PROS1,ACTN1,EFEMP1,EFCAB5,TNRC6A,YWHAZ,RAPGEF4,MASP2,SAA1,F11,C1S,CD14,LTBP1,PCYOX1,C4A,ATP9B,FSIP2,ACSF2,COLEC11,MST1,ITIH3,C4B,SERPINA1,TGFBI,CFB,IGFALS,PLTP,GC,HGFAC,UIMC1,PDGFC,JCHAIN,C1R,ATP1A1,MYL6,APOC1,APOC4,HSPH1,TPM4,ACTB,ITM2B,FGA,TPM3,PDIA4
TISSUES	BTO:0001489	Whole body	201	13099	0.13	1.49e-14	SPP2,ST6GAL1,GNPTG,VCL,SERPIND1,LGALS1,MYH9,TPD52L2,APMAP,F9,TFPI2,C5,MGP,SPARC,HRG,APOB,IGFBP5,PLEK,APOA1,TTR,CAT,C3,DHX29,APOE,PXDN,BPIFB1,F12,APCS,CFHR5,THBS1,LYZ,VWF,PSMA6,SERPINA10,TIMP2,ATRN,A1BG,BLVRB,LCN1,LCAT,CSTA,CP,KLKB1,F13A1,DNAH5,AMBP,CENPE,TCIRG1,HPX,ITIH4,PSG9,FCN3,ITIH1,PLA2G7,CRH,HABP2,FERMT3,SEC24D,DSC2,CSF1R,PIP,FCN2,PSG6,SCGB3A1,SERPINI1,PPBP,PF4,MASP1,CAMP,GUCY1A1,A2ML1,SLC27A4,LALBA,ANGPTL4,ANTXR1,SH3D19,PCSK9,BMP1,FGB,F2,INHBC,PLG,PSG1,UBE3C,PRG2,STAB1,C1QB,CFHR1,NDUFS8,TLN1,SUZ12,APOL1,PSG11,SERPINF2,HBA1,FBN1,P4HB,VMO1,FBLN1,DNAH8,ANG,FGG,LIMS1,ISYNA1,THBS4,GANAB,SLPI,FBLN5,PAPLN,ITIH2,CNDP1,COL18A1,C8A,PCDH18,F13B,CFHR3,CFH,F5,ADAM12,S100A7,HTRA1,ADAMTSL4,ECM1,FLNA,CRTAC1,SMARCA1,C8B,RBP4,FCN1,ADAMTS13,CCN5,MBL2,C1QC,F10,F7,APOM,CA6,ADGRG2,SOD3,GOLM1,GPX3,IGF1,PROS1,ACTN1,EFEMP1,EFCAB5,TNRC6A,YWHAZ,ILK,RAPGEF4,MASP2,PSG4,SAA1,F11,C1S,CD14,PSG2,LTBP1,PCYOX1,ADIPOQ,C4A,ATP9B,PRG4,NBEAL1,SLAIN1,FSIP2,ACSF2,COLEC11,MST1,ITIH3,C4B,SERPINA1,TGFBI,CFB,IGFALS,PLTP,PAEP,GC,HGFAC,UIMC1,PDGFC,FBN2,SELENOP,TSKU,JCHAIN,C1R,ATP1A1,MYL6,APOC1,APOC2,TTN,APOC4,VEGFC,PLCG2,HSPH1,TPM4,ACTB,ITM2B,FGA,TPM3,PDIA4
TISSUES	BTO:0000132	Blood platelet	28	363	0.84	1.15e-12	VCL,LGALS1,MYH9,APOA1,TTR,CAT,C3,THBS1,VWF,FERMT3,PPBP,FGB,TLN1,HBA1,P4HB,FGG,LIMS1,F5,FLNA,ACTN1,YWHAZ,LTBP1,PRG4,SERPINA1,TPM4,ACTB,FGA,TPM3
TISSUES	BTO:0001078	Placenta	47	1244	0.53	6.99e-11	LGALS1,MYH9,TFPI2,SPARC,IGFBP5,THBS1,VWF,TIMP2,F13A1,PSG9,CRH,DSC2,CSF1R,PSG6,ANGPTL4,ANTXR1,BMP1,PSG1,PRG2,APOL1,PSG11,HBA1,FBN1,P4HB,FBLN1,GANAB,FBLN5,COL18A1,F5,ADAM12,HTRA1,ADAMTSL4,GPX3,ACTN1,YWHAZ,ILK,PSG4,PSG2,PCYOX1,SERPINA1,TGFBI,PLTP,FBN2,ATP1A1,MYL6,TPM4,ACTB
TISSUES	BTO:0000203	Respiratory system	53	1707	0.44	2.29e-09	ST6GAL1,LGALS1,MYH9,SPARC,IGFBP5,C3,APOE,PXDN,BPIFB1,THBS1,LYZ,VWF,ATRN,LCN1,CP,DNAH5,TCIRG1,FCN3,FERMT3,PIP,SH3D19,HBA1,P4HB,LIMS1,GANAB,FBLN5,PCDH18,CFH,FLNA,SMARCA1,FCN1,CCN5,GPX3,PROS1,ACTN1,EFEMP1,RAPGEF4,MASP2,C1S,LTBP1,PCYOX1,C4B,SERPINA1,TGFBI,PDGFC,JCHAIN,ATP1A1,MYL6,APOC1,VEGFC,ACTB,TPM3,PDIA4
TISSUES	BTO:0003099	Internal female genital organ	71	2804	0.35	3.22e-09	VCL,LGALS1,MYH9,TPD52L2,TFPI2,MGP,SPARC,IGFBP5,C3,THBS1,VWF,TIMP2,A1BG,F13A1,PSG9,CRH,DSC2,CSF1R,PSG6,MASP1,A2ML1,ANGPTL4,ANTXR1,BMP1,PSG1,PRG2,NDUFS8,SUZ12,APOL1,PSG11,HBA1,FBN1,P4HB,FBLN1,ISYNA1,GANAB,SLPI,FBLN5,PAPLN,COL18A1,PCDH18,F5,ADAM12,HTRA1,ADAMTSL4,FLNA,CCN5,F10,GPX3,PROS1,ACTN1,YWHAZ,ILK,PSG4,C1S,PSG2,PCYOX1,COLEC11,SERPINA1,TGFBI,PLTP,PAEP,UIMC1,PDGFC,FBN2,ATP1A1,MYL6,TPM4,ACTB,ITM2B,TPM3
TISSUES	BTO:0000284	Organism form	65	2542	0.36	2.06e-08	ST6GAL1,VCL,LGALS1,MYH9,TPD52L2,TFPI2,SPARC,IGFBP5,DHX29,THBS1,VWF,PSMA6,TIMP2,F13A1,ITIH4,PSG9,CRH,DSC2,CSF1R,PSG6,CAMP,ANGPTL4,ANTXR1,BMP1,PSG1,UBE3C,PRG2,TLN1,APOL1,PSG11,HBA1,FBN1,P4HB,FBLN1,ISYNA1,GANAB,FBLN5,COL18A1,PCDH18,F5,ADAM12,HTRA1,ADAMTSL4,FLNA,APOM,GPX3,IGF1,ACTN1,EFEMP1,YWHAZ,ILK,PSG4,PSG2,PCYOX1,SERPINA1,TGFBI,PLTP,UIMC1,FBN2,ATP1A1,MYL6,VEGFC,TPM4,ACTB,TPM3
TISSUES	BTO:0000237	Cerebrospinal fluid	10	45	1.29	6.14e-08	TTR,APOE,A1BG,CP,HPX,F2,CFH,CD14,C4A,SERPINA1
TISSUES	BTO:0000089	Blood	51	1824	0.39	1.69e-07	VCL,LGALS1,MYH9,TPD52L2,APOB,PLEK,APOA1,TTR,CAT,C3,APOE,F12,THBS1,VWF,BLVRB,PSG9,PLA2G7,FERMT3,PPBP,PF4,FGB,F2,TLN1,HBA1,P4HB,FGG,LIMS1,C8A,PCDH18,F5,FLNA,C1QC,APOM,SOD3,GPX3,ACTN1,YWHAZ,C1S,CD14,LTBP1,PRG4,C4B,SERPINA1,CFB,MYL6,PLCG2,HSPH1,TPM4,ACTB,FGA,TPM3

TISSUES	BTO:0000174	Embryonic structure	60	2369	0.35	1.69e-07	ST6GAL1,VCL,LGALS1,MYH9,TPD52L2,TFPI2,SPARC,IGFBP5,DHX29,THBS1,VWF,PSMA6,TIMP2,F13A1,PSG9,CRH,DSC2,CSF1R,PSG6,ANGPTL4,ANTXR1,BMP1,PSG1,UBE3C,PRG2,TLN1,APOL1,PSG11,HBA1,FBN1,P4HB,FBLN1,ISYNA1,GANAB,FBLN5,COL18A1,PCDH18,F5,ADAM12,HTRA1,ADAMTSL4,FLNA,APOM,GPX3,ACTN1,YWHAZ,ILK,PSG4,PSG2,PCYOX1,SERPINA1,TGFBI,PLTP,UIMC1,FBN2,ATP1A1,MYL6,VEGFC,TPM4,ACTB
TISSUES	BTO:0000042	Animal	204	15148	0.08	4.47e-07	SPP2,ST6GAL1,GNPTG,VCL,SERPIND1,LGALS1,MYH9,TPD52L2,APMAP,F9,TFPI2,C5,MGP,SPARC,HRG,APOB,IGFBP5,PLEK,APOA1,TTR,CAT,C3,DHX29,APOE,PXDN,ANGPTL6,BPIFB1,F12,APCS,CFHR5,THBS1,LYZ,VWF,PSMA6,SERPINA10,TIMP2,ATRN,A1BG,BLVRB,LCN1,LCAT,CSTA,CP,KLKB1,F13A1,DNAH5,AMBP,CENPE,TCIRG1,HPX,ITIH4,PSG9,FCN3,ITIH1,PLA2G7,CRH,HABP2,FERMT3,SEC24D,DSC2,CSF1R,PIP,FCN2,PSG6,SCGB3A1,SERPINI1,PPBP,PF4,MASP1,CAMP,GUCY1A1,A2ML1,SLC27A4,LALBA,ANGPTL4,ANTXR1,SH3D19,PCSK9,BMP1,FGB,F2,INHBC,PLG,PSG1,UBE3C,PRG2,STAB1,C1QB,CFHR1,NDUFS8,TLN1,SUZ12,APOL1,PSG11,SERPINF2,HBA1,FBN1,P4HB,VMO1,FBLN1,OAF,DNAH8,ANG,FGG,LIMS1,ISYNA1,THBS4,GANAB,SLPI,FBLN5,PAPLN,ITIH2,CNDP1,COL18A1,C8A,PCDH18,F13B,CFHR3,CFH,F5,ADAM12,S100A7,HTRA1,ADAMTSL4,ECM1,FLNA,CRTAC1,SMARCA1,C8B,RBP4,FCN1,ADAMTS13,CCN5,MBL2,C1QC,F10,F7,APOM,CA6,ADGRG2,SOD3,GOLM1,GPX3,IGF1,PROS1,ACTN1,EFEMP1,EFCAB5,TNRC6A,YWHAZ,ILK,RAPGEF4,MASP2,PSG4,SAA1,F11,C1S,CD14,PSG2,LTBP1,PCYOX1,ADIPOQ,C4A,ATP9B,PRG4,NBEAL1,SLAIN1,FSIP2,ACSF2,COLEC11,MST1,ITIH3,C4B,SERPINA1,TGFBI,CFB,IGFALS,PLTP,PAEP,TCAF1,GC,HGFAC,UIMC1,PDGFC,FBN2,SELENOP,TSKU,JCHAIN,C1R,ATP1A1,MYL6,APOC1,APOC2,TTN,APOC4,VEGFC,PLCG2,HSPH1,TPM4,ACTB,ITM2B,FGA,TPM3,PDIA4
TISSUES	BTO:0000570	Hematopoietic system	63	2755	0.31	2.30e-06	ST6GAL1,VCL,LGALS1,MYH9,TPD52L2,APOB,PLEK,APOA1,TTR,CAT,C3,APOE,F12,THBS1,VWF,PSMA6,ATRN,BLVRB,PSG9,ITIH1,PLA2G7,FERMT3,PPBP,PF4,ANGPTL4,FGB,F2,PRG2,TLN1,HBA1,P4HB,FGG,LIMS1,GANAB,ITIH2,C8A,PCDH18,F5,FLNA,C1QC,APOM,SOD3,GPX3,ACTN1,YWHAZ,C1S,CD14,LTBP1,PRG4,C4B,SERPINA1,TGFBI,CFB,HGFAC,JCHAIN,MYL6,VEGFC,PLCG2,HSPH1,TPM4,ACTB,FGA,TPM3
TISSUES	BTO:0000088	Cardiovascular system	34	1057	0.46	4.81e-06	VCL,TFPI2,MGP,SPARC,APOB,APOA1,C3,APOE,THBS1,VWF,TIMP2,SLC27A4,ANGPTL4,F2,HBA1,FBN1,P4HB,THBS4,PCDH18,F13B,FLNA,GPX3,EFEMP1,PCYOX1,SERPINA1,TGFBI,PLTP,FBN2,SELENOP,TTN,VEGFC,ACTB,FGA,TPM3
TISSUES	BTO:0001239	Serum	7	25	1.4	4.81e-06	APOA1,C3,ATRN,ITIH1,PRG2,ITIH2,HGFAC
TISSUES	BTO:0001702	Left atrium	9	60	1.12	6.20e-06	VCL,APOA1,C3,HBA1,FBN1,FLNA,SERPINA1,TTN,TPM3
TISSUES	BTO:0001419	Urine	7	28	1.35	8.38e-06	LYZ,AMBP,F2,FBLN5,MASP2,PRG4,SERPINA1
TISSUES	BTO:0000421	Connective tissue	31	954	0.46	1.42e-05	SPP2,LGALS1,APMAP,TFPI2,SPARC,APOA1,TTR,CAT,THBS1,BLVRB,ANGPTL4,FBN1,P4HB,THBS4,GANAB,PAPLN,F5,HTRA1,ECM1,CRTAC1,EFEMP1,YWHAZ,LTBP1,ADIPOQ,PRG4,ACSF2,SERPINA1,PLTP,TPM4,ACTB,TPM3
TISSUES	BTO:0000763	Lung	38	1395	0.38	3.88e-05	LGALS1,MYH9,SPARC,IGFBP5,C3,APOE,PXDN,BPIFB1,THBS1,VWF,LCN1,DNAH5,TCIRG1,FCN3,FERMT3,HBA1,P4HB,FBLN5,PCDH18,FLNA,SMARCA1,FCN1,CCN5,GPX3,ACTN1,EFEMP1,MASP2,C1S,LTBP1,C4B,SERPINA1,TGFBI,PDGFC,MYL6,APOC1,ACTB,TPM3,PDIA4
TISSUES	BTO:0001279	Spinal cord	14	233	0.73	5.60e-05	MYH9,TTR,APOE,A1BG,CP,HPX,F2,PCDH18,CFH,FLNA,CD14,C4A,SERPINA1,ACTB
TISSUES	BTO:0001703	Right atrium	8	61	1.07	6.98e-05	VCL,APOA1,C3,HBA1,FBN1,SERPINA1,TTN,TPM3
TISSUES	BTO:0001090	Mouth	20	532	0.52	0.00028	GNPTG,C5,BPIFB1,LYZ,LCN1,CSTA,CENPE,PIP,A2ML1,P4HB,LIMS1,GANAB,SLPI,CA6,ACTN1,YWHAZ,JCHAIN,MYL6,TPM4,ACTB
TISSUES	BTO:0003091	Urogenital system	113	7090	0.15	0.00030	ST6GAL1,GNPTG,VCL,LGALS1,MYH9,TPD52L2,APMAP,TFPI2,MGP,SPARC,HRG,IGFBP5,APOA1,CAT,C3,DHX29,APOE,PXDN,THBS1,VWF,PSMA6,TIMP2,ATRN,A1BG,F13A1,HPX,PSG9,CRH,DSC2,CSF1R,PIP,PSG6,MASP1,CAMP,GUCY1A1,A2ML1,LALBA,ANGPTL4,ANTXR1,SH3D19,BMP1,FGB,F2,PLG,PSG1,UBE3C,PRG2,NDUFS8,TLN1,SUZ12,APOL1,PSG11,HBA1,FBN1,P4HB,VMO1,FBLN1,DNAH8,LIMS1,ISYNA1,GANAB,SLPI,FBLN5,PAPLN,COL18A1,PCDH18,CFH,F5,ADAM12,HTRA1,ADAMTSL4,ECM1,FLNA,SMARCA1,ADAMTS13,CCN5,F10,ADGRG2,GPX3,PROS1,ACTN1,EFEMP1,EFCAB5,TNRC6A,YWHAZ,ILK,PSG4,C1S,CD14,PSG2,PCYOX1,ATP9B,FSIP2,ACSF2,COLEC11,SERPINA1,TGFBI,PLTP,PAEP,GC,UIMC1,PDGFC,FBN2,TSKU,ATP1A1,MYL6,VEGFC,HSPH1,TPM4,ACTB,ITM2B,TPM3,PDIA4
TISSUES	BTO:0000081	Reproductive system	105	6444	0.16	0.00032	ST6GAL1,GNPTG,VCL,LGALS1,MYH9,TPD52L2,APMAP,TFPI2,MGP,SPARC,IGFBP5,APOA1,CAT,C3,DHX29,APOE,PXDN,THBS1,VWF,PSMA6,TIMP2,ATRN,A1BG,F13A1,HPX,PSG9,CRH,DSC2,CSF1R,PIP,PSG6,MASP1,CAMP,A2ML1,LALBA,ANGPTL4,ANTXR1,SH3D19,BMP1,FGB,F2,PSG1,UBE3C,PRG2,NDUFS8,TLN1,SUZ12,APOL1,PSG11,HBA1,FBN1,P4HB,VMO1,FBLN1,DNAH8,LIMS1,ISYNA1,GANAB,SLPI,FBLN5,PAPLN,COL18A1,PCDH18,CFH,F5,ADAM12,HTRA1,ADAMTSL4,ECM1,FLNA,ADAMTS13,CCN5,F10,ADGRG2,GPX3,PROS1,ACTN1,EFEMP1,EFCAB5,YWHAZ,ILK,PSG4,C1S,CD14,PSG2,PCYOX1,ATP9B,FSIP2,COLEC11,SERPINA1,TGFBI,PLTP,PAEP,UIMC1,PDGFC,FBN2,TSKU,ATP1A1,MYL6,HSPH1,TPM4,ACTB,ITM2B,TPM3,PDIA4
TISSUES	BTO:0000083	Female reproductive system	101	6111	0.17	0.00032	ST6GAL1,GNPTG,VCL,LGALS1,MYH9,TPD52L2,APMAP,TFPI2,MGP,SPARC,IGFBP5,APOA1,CAT,C3,DHX29,APOE,PXDN,THBS1,VWF,PSMA6,TIMP2,ATRN,A1BG,F13A1,HPX,PSG9,CRH,DSC2,CSF1R,PSG6,MASP1,CAMP,A2ML1,LALBA,ANGPTL4,ANTXR1,SH3D19,BMP1,FGB,F2,PSG1,UBE3C,PRG2,NDUFS8,TLN1,SUZ12,APOL1,PSG11,HBA1,FBN1,P4HB,FBLN1,DNAH8,LIMS1,ISYNA1,GANAB,SLPI,FBLN5,PAPLN,COL18A1,PCDH18,CFH,F5,ADAM12,HTRA1,ADAMTSL4,ECM1,FLNA,ADAMTS13,CCN5,F10,GPX3,PROS1,ACTN1,EFEMP1,EFCAB5,YWHAZ,ILK,PSG4,C1S,CD14,PSG2,PCYOX1,FSIP2,COLEC11,SERPINA1,TGFBI,PLTP,PAEP,UIMC1,PDGFC,FBN2,TSKU,ATP1A1,MYL6,HSPH1,TPM4,ACTB,ITM2B,TPM3,PDIA4
TISSUES	BTO:0005288	CL-48 cell	12	200	0.73	0.00032	SERPINA10,ITIH4,PSG9,MASP1,SERPINA11,FGG,LIMS1,RBP4,GPX3,PSG4,PRG4,SERPINA1
TISSUES	BTO:0001202	Saliva	7	57	1.04	0.00043	THBS1,HPX,DSC2,PIP,GOLM1,C4B,JCHAIN
TISSUES	BTO:0001085	Vascular system	17	420	0.56	0.00052	VCL,TFPI2,MGP,APOB,APOE,THBS1,VWF,ANGPTL4,F2,FBN1,P4HB,EFEMP1,TGFBI,PLTP,FBN2,VEGFC,ACTB
TISSUES	BTO:0001823	Synovium	8	88	0.91	0.00069	P4HB,THBS4,PAPLN,ECM1,CRTAC1,EFEMP1,PRG4,SERPINA1
TISSUES	BTO:0001499	Tear	5	23	1.29	0.00072	LYZ,LCN1,PIP,SLPI,JCHAIN
TISSUES	BTO:0000255	Brain cell line	11	188	0.72	0.00080	VCL,LGALS1,APOA1,TTR,FGB,P4HB,YWHAZ,TTN,HSPH1,TPM4,ACTB
TISSUES	BTO:0000562	Heart	23	738	0.44	0.00080	VCL,APOA1,C3,THBS1,VWF,SLC27A4,F2,HBA1,FBN1,THBS4,PCDH18,F13B,FLNA,GPX3,PCYOX1,SERPINA1,PLTP,SELENOP,TTN,VEGFC,ACTB,FGA,TPM3
TISSUES	BTO:0000102	Blood clot	4	11	1.51	0.00096	VWF,F2,PLG,ADAMTS13
TISSUES	BTO:0003914	Interstitial cell of Cajal	11	193	0.7	0.00096	LGALS1,APOA1,TTR,CAT,BLVRB,P4HB,GANAB,YWHAZ,SERPINA1,TPM4,ACTB
TISSUES	BTO:0000511	Gastrointestinal tract	39	1816	0.28	0.0037	VCL,LGALS1,MYH9,SPARC,APOB,APOA1,TTR,CAT,C3,LYZ,BLVRB,KLKB1,HABP2,LALBA,SH3D19,TLN1,P4HB,FBLN1,ANG,GANAB,SLPI,PCDH18,FLNA,SOD3,GPX3,ACTN1,TNRC6A,YWHAZ,ILK,C1S,SERPINA1,TGFBI,CFB,JCHAIN,C1R,ATP1A1,MYL6,TPM4,ACTB
TISSUES	BTO:0000121	Bile	4	20	1.25	0.0061	CP,HPX,SERPINA1,JCHAIN

TISSUES	BTO:0003120	Atherosclerotic plaque	3	9	1.47	0.0125	APOB,APOA1,APOE
TISSUES	BTO:0003872	Foam cell	3	9	1.47	0.0125	APOB,APOA1,APOE
TISSUES	BTO:0000058	Alimentary canal	40	2021	0.24	0.0150	VCL,LGALS1,MYH9,SPARC,APOB,APOA1,TTR,CAT,C3,THBS1,LYZ,BLVRB,KLKB1,HABP2,LALBA,SH3D19,TLN1,P4HB,FBLN1,ANG,GANAB,SLPI,PCDH18,FLNA,SOD3,GPX3,ACTN1,TNRC6A,YWHAZ,ILK,C1S,SERPINA1,TGFBI,CFB,JCHAIN,C1R,ATP1A1,MYL6,TPM4,ACTB
TISSUES	BTO:0001629	Left ventricle	6	78	0.83	0.0150	VCL,APOA1,HBA1,SERPINA1,TTN,TPM3
TISSUES	BTO:0000765	Exocrine gland	8	150	0.68	0.0169	C5,BPIFB1,LYZ,LCN1,PIP,MASP1,SLPI,CA6
TISSUES	BTO:0000574	Hematopoietic cell	24	1019	0.32	0.0250	VCL,LGALS1,MYH9,TPD52L2,APOB,PLEK,APOA1,CAT,APOE,BLVRB,PPBP,PF4,PCDH18,FLNA,C1QC,GPX3,YWHAZ,C1S,CD14,SERPINA1,MYL6,PLCG2,HSPH1,ACTB
TISSUES	BTO:0001253	Skin	26	1151	0.3	0.0267	ST6GAL1,LGALS1,SPARC,APOE,PSMA6,TIMP2,LCN1,CSTA,DSC2,ANGPTL4,BMP1,P4HB,S100A7,ECM1,FLNA,ACTN1,EFEMP1,YWHAZ,ADIPOQ,TGFBI,C1R,ATP1A1,VEGFC,TPM4,ACTB,TPM3
TISSUES	BTO:0000648	Intestine	29	1349	0.28	0.0277	VCL,MYH9,APOB,C3,LYZ,KLKB1,HABP2,SH3D19,TLN1,P4HB,ANG,GANAB,SLPI,PCDH18,FLNA,SOD3,GPX3,ACTN1,YWHAZ,ILK,C1S,SERPINA1,TGFBI,CFB,JCHAIN,C1R,ATP1A1,MYL6,ACTB
TISSUES	BTO:0000634	Integument	40	2112	0.23	0.0311	ST6GAL1,VCL,LGALS1,SPARC,APOE,BPIFB1,THBS1,VWF,PSMA6,TIMP2,LCN1,CSTA,DSC2,PIP,A2ML1,ANGPTL4,ANTXR1,BMP1,P4HB,SLPI,S100A7,ECM1,FLNA,ACTN1,EFEMP1,YWHAZ,ILK,ADIPOQ,COLEC11,TGFBI,PLTP,PAEP,PDGFC,FBN2,C1R,ATP1A1,VEGFC,TPM4,ACTB,TPM3
TISSUES	BTO:0000393	Endothelium	8	172	0.62	0.0355	VCL,APOE,THBS1,VWF,ANGPTL4,PLTP,FBN2,VEGFC
TISSUES	BTO:0003445	Carotid atherosclerotic plaque	2	3	1.77	0.0444	APOB,APOE
TISSUES	BTO:0004695	Aortic root	2	3	1.77	0.0444	APOE,FBN1
TISSUES	BTO:0005739	Transfer cell	2	3	1.77	0.0444	PXDN,ACTB
TISSUES	BTO:0000545	Gut	5	69	0.81	0.0498	VCL,MYH9,TLN1,PCDH18,FLNA
COMPARTMENTS	GOCC:0005576	Extracellular region	149	2079	0.8	9.15e-85	SPP2,ST6GAL1,VCL,SERPIND1,LGALS1,MYH9,F9,TFPI2,C5,PF4V1,MGP,SPARC,HRG,APOB,IGFBP5,APOA1,TTR,C3,APOE,PXDN,ANGPTL6,BPIFB1,F12,APCS,CFHR5,THBS1,LYZ,VWF,SERPINA10,TIMP2,A1BG,LCN1,LCAT,CSTA,CP,KLKB1,F13A1,AMBP,CENPE,HPX,ITIH4,FCN3,ITIH1,PLA2G7,CRH,HABP2,PIP,FCN2,PSG6,SCGB3A1,SERPINI1,PPBP,PF4,MASP1,CAMP,A2ML1,LALBA,ANGPTL4,PCSK9,BMP1,FGB,F2,INHBC,PLG,PRG2,C1QB,CFHR1,TLN1,APOL1,SERPINF2,HBA1,FBN1,P4HB,VMO1,FBLN1,COLEC10,ANG,FGG,THBS4,SLPI,FBLN5,ITIH2,COL18A1,C8A,F13B,CFHR3,CFH,F5,ADAM12,S100A7,HTRA1,ECM1,FLNA,CRTAC1,C8B,RBP4,FCN1,ADAMTS13,CCN5,MBL2,C1QC,F10,F7,APOM,CA6,SOD3,GOLM1,GPX3,IGF1,PROS1,EFEMP1,YWHAZ,MASP2,SAA1,F11,C1S,CD14,LTBP1,PCYOX1,ADIPOQ,C4A,COLEC11,MST1,ITIH3,C4B,SERPINA1,TGFBI,CFB,IGFALS,PLTP,PAEP,GC,HGFAC,FBN2,TSKU,JCHAIN,HPR,C1R,IGKV2D-28,APOC1,APOC2,APOC4,VEGFC,PLCG2,ACTB,ITM2B,FGA,TPM3,PDIA4
COMPARTMENTS	GOCC:0005615	Extracellular space	107	1027	0.97	4.11e-70	VCL,SERPIND1,F9,C5,MGP,HRG,APOB,IGFBP5,APOA1,TTR,C3,APOE,PXDN,F12,APCS,THBS1,LYZ,VWF,A1BG,LCN1,LCAT,CSTA,CP,KLKB1,F13A1,AMBP,HPX,ITIH4,FCN3,ITIH1,PLA2G7,CRH,PIP,FCN2,SCGB3A1,SERPINI1,MASP1,CAMP,A2ML1,ANGPTL4,PCSK9,FGB,F2,PLG,C1QB,CFHR1,TLN1,APOL1,SERPINF2,HBA1,FBN1,FBLN1,COLEC10,ANG,FGG,THBS4,SLPI,ITIH2,COL18A1,C8A,CFHR3,CFH,S100A7,ECM1,FLNA,RBP4,CCN5,MBL2,C1QC,F7,APOM,CA6,SOD3,GPX3,IGF1,PROS1,EFEMP1,YWHAZ,MASP2,SAA1,F11,C1S,PCYOX1,ADIPOQ,C4A,COLEC11,MST1,C4B,SERPINA1,TGFBI,CFB,IGFALS,PLTP,GC,FBN2,JCHAIN,HPR,C1R,IGKV2D-28,APOC1,APOC2,APOC4,PLCG2,ACTB,ITM2B,FGA,PDIA4
COMPARTMENTS	GOCC:0072562	Blood microparticle	42	118	1.5	3.00e-43	HRG,APOA1,C3,APOE,APCS,A1BG,CP,F13A1,AMBP,HPX,ITIH4,FCN3,ITIH1,FCN2,ANGPTL4,FGB,F2,PLG,C1QB,CFHR1,APOL1,SERPINF2,HBA1,FGG,ITIH2,C8A,CFHR3,CFH,C1QC,PROS1,YWHAZ,C1S,C4A,C4B,CFB,GC,JCHAIN,HPR,C1R,IGKV2D-28,ACTB,FGA
COMPARTMENTS	GOCC:0060205	Cytoplasmic vesicle lumen	36	245	1.12	5.63e-25	SPP2,VCL,SPARC,HRG,APOA1,TTR,C3,THBS1,LYZ,VWF,TIMP2,A1BG,F13A1,ITIH4,FERMT3,SERPINI1,PPBP,PF4,CAMP,FGB,PLG,SERPINF2,FGG,SLPI,F5,S100A7,ECM1,FCN1,IGF1,PROS1,ACTN1,ITIH3,SERPINA1,SELENOP,VEGFC,FGA
COMPARTMENTS	GOCC:0043230	Extracellular organelle	47	524	0.9	7.04e-25	VCL,MYH9,F9,C5,MGP,APOB,APOA1,TTR,C3,APOE,PXDN,APCS,THBS1,VWF,CP,KLKB1,AMBP,HPX,ITIH4,FCN2,CAMP,FGB,F2,PLG,TLN1,SERPINF2,FGG,SLPI,COL18A1,CFH,ECM1,FLNA,GPX3,PROS1,EFEMP1,YWHAZ,MASP2,SAA1,F11,C4A,C4B,SERPINA1,TGFBI,GC,C1R,ACTB,FGA
COMPARTMENTS	GOCC:0034774	Secretory granule lumen	35	241	1.11	2.63e-24	SPP2,VCL,SPARC,HRG,APOA1,TTR,C3,THBS1,LYZ,VWF,TIMP2,A1BG,F13A1,ITIH4,FERMT3,PPBP,PF4,CAMP,FGB,PLG,SERPINF2,FGG,SLPI,F5,S100A7,ECM1,FCN1,IGF1,PROS1,ACTN1,ITIH3,SERPINA1,SELENOP,VEGFC,FGA
COMPARTMENTS	GOCC:1903561	Extracellular vesicle	43	500	0.88	5.52e-22	VCL,MYH9,F9,MGP,APOB,APOA1,TTR,C3,APOE,PXDN,APCS,THBS1,VWF,CP,KLKB1,AMBP,HPX,ITIH4,CAMP,FGB,F2,PLG,TLN1,SERPINF2,FGG,COL18A1,CFH,ECM1,FLNA,GPX3,PROS1,EFEMP1,YWHAZ,SAA1,F11,C4A,C4B,SERPINA1,TGFBI,GC,C1R,ACTB,FGA
COMPARTMENTS	GOCC:0065010	Extracellular membrane-bounded organelle	42	473	0.9	5.75e-22	VCL,MYH9,F9,MGP,APOB,APOA1,TTR,C3,APOE,PXDN,APCS,THBS1,VWF,CP,KLKB1,AMBP,HPX,ITIH4,CAMP,FGB,F2,PLG,TLN1,SERPINF2,FGG,COL18A1,CFH,ECM1,FLNA,GPX3,PROS1,YWHAZ,SAA1,F11,C4A,C4B,SERPINA1,TGFBI,GC,C1R,ACTB,FGA
COMPARTMENTS	GOCC:0031093	Platelet alpha granule lumen	20	66	1.43	8.08e-19	SPARC,HRG,THBS1,VWF,A1BG,F13A1,FERMT3,PPBP,PF4,FGB,PLG,SERPINF2,FGG,F5,IGF1,PROS1,ACTN1,SERPINA1,VEGFC,FGA
COMPARTMENTS	GOCC:0031982	Vesicle	76	2125	0.5	5.16e-18	SPP2,VCL,MYH9,F9,MGP,SPARC,HRG,APOB,APOA1,TTR,CAT,C3,APOE,PXDN,APCS,THBS1,LYZ,VWF,TIMP2,A1BG,CP,KLKB1,F13A1,AMBP,TCIRG1,HPX,ITIH4,ITIH1,FERMT3,SEC24D,DSC2,SERPINI1,PPBP,PF4,CAMP,ANTXR1,PCSK9,FGB,F2,PLG,PRG2,STAB1,TLN1,SERPINF2,HBA1,FGG,SLPI,COL18A1,CFH,F5,S100A7,ECM1,FLNA,FCN1,GPX3,IGF1,PROS1,ACTN1,EFEMP1,YWHAZ,SAA1,F11,C4A,ITIH3,C4B,SERPINA1,TGFBI,GC,SELENOP,C1R,VEGFC,PLCG2,HSPH1,ACTB,ITM2B,FGA
COMPARTMENTS	GOCC:0034358	Plasma lipoprotein particle	16	48	1.47	1.77e-15	APOB,APOA1,APOE,LCAT,PLA2G7,PCSK9,APOL1,APOM,SAA1,PCYOX1,ADIPOQ,PLTP,HPR,APOC1,APOC2,APOC4
COMPARTMENTS	GOCC:0070062	Extracellular exosome	32	428	0.82	1.65e-14	VCL,MGP,APOB,APOA1,TTR,C3,APOE,PXDN,THBS1,VWF,CP,AMBP,HPX,ITIH4,CAMP,FGB,PLG,TLN1,FGG,COL18A1,CFH,ECM1,FLNA,GPX3,EFEMP1,YWHAZ,SAA1,C4A,SERPINA1,TGFBI,ACTB,FGA
COMPARTMENTS	GOCC:0034364	High-density lipoprotein particle	13	30	1.58	1.06e-13	APOB,APOA1,APOE,LCAT,PLA2G7,APOL1,APOM,SAA1,PLTP,HPR,APOC1,APOC2,APOC4
COMPARTMENTS	GOCC:0030141	Secretory granule	39	719	0.68	1.33e-13	SPP2,VCL,MYH9,SPARC,HRG,APOA1,TTR,CAT,C3,THBS1,LYZ,VWF,TIMP2,A1BG,F13A1,TCIRG1,ITIH4,FERMT3,PPBP,PF4,CAMP,FGB,PLG,PRG2,SERPINF2,FGG,SLPI,F5,S100A7,ECM1,FCN1,IGF1,PROS1,ACTN1,ITIH3,SERPINA1,SELENOP,VEGFC,FGA
COMPARTMENTS	GOCC:0005788	Endoplasmic reticulum lumen	20	172	1.01	5.38e-12	SPP2,F9,APOB,APOA1,C3,APOE,THBS1,PCSK9,APOL1,FBN1,P4HB,FGG,GANAB,COL18A1,F5,F7,C4A,SERPINA1,FGA,PDIA4
COMPARTMENTS	GOCC:1905286	Serine-type peptidase complex	9	11	1.86	3.73e-11	F9,C3,FCN3,FCN2,MASP1,FCN1,F7,MASP2,F11
COMPARTMENTS	GOCC:0030312	External encapsulating structure	21	259	0.86	6.96e-10	TFPI2,SPARC,HRG,APOE,PXDN,THBS1,VWF,TIMP2,PLG,FBN1,FBLN1,ANG,THBS4,FBLN5,COL18A1,ECM1,ADAMTS13,SOD3,EFEMP1,LTBP1,FBN2
COMPARTMENTS	GOCC:0031012	Extracellular matrix	21	259	0.86	6.96e-10	TFPI2,SPARC,APOE,PXDN,THBS1,VWF,TIMP2,PLG,FBN1,FBLN1,ANG,THBS4,FBLN5,COL18A1,ECM1,ADAMTS13,SOD3,EFEMP1,LTBP1,TGFBI,FBN2

COMPARTMENTS	GOCC:0062167	Complement component C1q complex	8	10	1.85	7.99e-10	C3,C1QB,MBL2,C1QC,C1S,C4A,C4B,C1R
COMPARTMENTS	GOCC:0005577	Fibrinogen complex	9	20	1.6	1.71e-09	THBS1,VWF,FGB,F2,PLG,SERPINF2,FGG,F7,FGA
COMPARTMENTS	GOCC:0034361	Very-low-density lipoprotein particle	9	20	1.6	1.71e-09	APOB,APOA1,APOE,APOL1,APOM,PCYOX1,APOC1,APOC2,APOC4
COMPARTMENTS	GOCC:0110165	Cellular anatomical entity	199	14060	0.1	3.54e-09	SPP2,ST6GAL1,GNPTG,VCL,SERPIND1,LGALS1,MYH9,TPD52L2,APMAP,F9,TFPI2,C5,PF4V1,MGP,SPARC,HRG,APOB,IGFBP5,PLEK,APOA1,TTR,CAT,C3,DHX29,APOE,PXDN,ANGPTL6,BPIFB1,F12,APCS,CFHR5,THBS1,LYZ,VWF,PSMA6,SERPINA10,TIMP2,ATRN,A1BG,BLVRB,LCN1,LCAT,CSTA,CP,KLKB1,F13A1,DNAH5,AMBP,CENPE,TCIRG1,HPX,ITIH4,PSG9,FCN3,ITIH1,PLA2G7,CRH,HABP2,FERMT3,SEC24D,DSC2,CSF1R,PIP,FCN2,PSG6,SCGB3A1,SERPINI1,PPBP,PF4,MASP1,CAMP,GUCY1A1,C16orf46,A2ML1,SLC27A4,LALBA,ANGPTL4,ANTXR1,SH3D19,PCSK9,BMP1,FGB,F2,INHBC,PLG,PRG2,STAB1,C1QB,CFHR1,NDUFS8,TLN1,SUZ12,APOL1,SERPINF2,HBA1,FBN1,P4HB,VMO1,FBLN1,COLEC10,DNAH8,ANG,FGG,LIMS1,ISYNA1,THBS4,GANAB,SLPI,FBLN5,BLM,ITIH2,CNDP1,COL18A1,C8A,F13B,CFHR3,CFH,F5,ADAM12,S100A7,HTRA1,ECM1,FLNA,CRTAC1,SMARCA1,C8B,RBP4,FCN1,ADAMTS13,CCN5,MBL2,C1QC,F10,F7,APOM,CA6,ADGRG2,SOD3,GOLM1,GPX3,IGF1,PROS1,ACTN1,EFEMP1,TNRC6A,YWHAZ,ILK,RAPGEF4,MASP2,SAA1,F11,C1S,CD14,LTBP1,PCYOX1,ADIPOQ,C4A,ATP9B,FSIP2,ACSF2,COLEC11,MST1,ITIH3,C4B,SERPINA1,TGFBI,CFB,IGFALS,PLTP,PAEP,TCAF1,GC,HGFAC,UIMC1,PDGFC,FBN2,SELENOP,TSKU,JCHAIN,HPR,C1R,ATP1A1,MYL6,IGKV2D-28,APOC1,APOC2,TTN,APOC4,VEGFC,SEMA3B,PLCG2,OR52B4,HSPH1,TPM4,ACTB,ITM2B,FGA,TPM3,PDIA4
COMPARTMENTS	GOCC:1905370	Serine-type endopeptidase complex	7	7	1.95	4.95e-09	C3,FCN3,FCN2,MASP1,FCN1,MASP2,F11
COMPARTMENTS	GOCC:0031410	Cytoplasmic vesicle	52	1738	0.42	6.70e-09	SPP2,VCL,MYH9,SPARC,HRG,APOB,APOA1,TTR,CAT,C3,APOE,THBS1,LYZ,VWF,TIMP2,A1BG,F13A1,TCIRG1,HPX,ITIH4,FERMT3,SEC24D,DSC2,SERPINI1,PPBP,PF4,CAMP,ANTXR1,PCSK9,FGB,PLG,PRG2,STAB1,SERPINF2,HBA1,FGG,SLPI,F5,S100A7,ECM1,FCN1,IGF1,PROS1,ACTN1,SAA1,ITIH3,SERPINA1,SELENOP,VEGFC,HSPH1,ITM2B,FGA
COMPARTMENTS	GOCC:0062023	Collagen-containing extracellular matrix	17	198	0.88	2.31e-08	SPARC,APOE,PXDN,THBS1,VWF,TIMP2,PLG,FBN1,ANG,THBS4,FBLN5,COL18A1,ECM1,SOD3,LTBP1,TGFBI,FBN2
COMPARTMENTS	GOCC:0034362	Low-density lipoprotein particle	8	22	1.51	6.93e-08	APOB,APOA1,APOE,PLA2G7,PCSK9,APOM,ADIPOQ,APOC2
COMPARTMENTS	GOCC:0071682	Endocytic vesicle lumen	8	23	1.49	8.98e-08	SPARC,APOB,APOA1,APOE,HPX,HBA1,SAA1,HSPH1
COMPARTMENTS	GOCC:0043233	Organelle lumen	68	2905	0.32	1.27e-07	SPP2,VCL,F9,SPARC,HRG,APOB,APOA1,TTR,CAT,C3,APOE,THBS1,LYZ,VWF,TIMP2,A1BG,F13A1,HPX,ITIH4,FERMT3,SERPINI1,PPBP,PF4,CAMP,LALBA,PCSK9,FGB,F2,PLG,PRG2,NDUFS8,SUZ12,APOL1,SERPINF2,HBA1,FBN1,P4HB,ANG,FGG,GANAB,SLPI,BLM,COL18A1,F5,S100A7,HTRA1,ECM1,SMARCA1,FCN1,F10,F7,SOD3,IGF1,PROS1,ACTN1,SAA1,C4A,ITIH3,SERPINA1,GC,UIMC1,SELENOP,TTN,VEGFC,HSPH1,ACTB,FGA,PDIA4
COMPARTMENTS	GOCC:0070013	Intracellular organelle lumen	67	2902	0.31	2.92e-07	SPP2,VCL,F9,SPARC,HRG,APOB,APOA1,TTR,CAT,C3,APOE,THBS1,LYZ,VWF,TIMP2,A1BG,F13A1,HPX,ITIH4,FERMT3,SERPINI1,PPBP,PF4,CAMP,LALBA,PCSK9,FGB,F2,PLG,PRG2,NDUFS8,SUZ12,APOL1,SERPINF2,HBA1,FBN1,P4HB,ANG,FGG,GANAB,SLPI,BLM,COL18A1,F5,S100A7,ECM1,SMARCA1,FCN1,F10,F7,SOD3,IGF1,PROS1,ACTN1,SAA1,C4A,ITIH3,SERPINA1,GC,UIMC1,SELENOP,TTN,VEGFC,HSPH1,ACTB,FGA,PDIA4
COMPARTMENTS	GOCC:0012505	Endomembrane system	69	3156	0.29	1.39e-06	SPP2,ST6GAL1,VCL,MYH9,F9,SPARC,HRG,APOB,APOA1,TTR,CAT,C3,APOE,PXDN,THBS1,LYZ,VWF,TIMP2,A1BG,CP,F13A1,TCIRG1,ITIH4,FERMT3,SEC24D,PPBP,PF4,CAMP,SLC27A4,LALBA,ANTXR1,PCSK9,FGB,F2,PLG,PRG2,APOL1,SERPINF2,FBN1,P4HB,FGG,THBS4,GANAB,SLPI,COL18A1,F5,S100A7,ECM1,FCN1,F10,F7,SOD3,GOLM1,IGF1,PROS1,ACTN1,TNRC6A,CD14,ADIPOQ,C4A,ATP9B,ITIH3,SERPINA1,SELENOP,VEGFC,SEMA3B,ITM2B,FGA,PDIA4
COMPARTMENTS	GOCC:0005579	Membrane attack complex	6	12	1.65	1.75e-06	C5,C3,C8A,C8B,MBL2,C4A
COMPARTMENTS	GOCC:0009986	Cell surface	21	438	0.63	2.91e-06	APMAP,SPARC,HRG,PXDN,THBS1,CSF1R,ANTXR1,PCSK9,FGB,PLG,SERPINF2,P4HB,FGG,FCN1,ADAMTS13,MBL2,CD14,ADIPOQ,PDGFC,FGA,PDIA4
COMPARTMENTS	GOCC:1905368	Peptidase complex	12	136	0.89	5.89e-06	F9,C3,PSMA6,FCN3,FCN2,MASP1,F2,UBE3C,FCN1,F7,MASP2,F11
COMPARTMENTS	GOCC:0032991	Protein-containing complex	95	5325	0.2	1.98e-05	GNPTG,VCL,LGALS1,MYH9,F9,C5,APOB,IGFBP5,APOA1,CAT,C3,DHX29,APOE,PXDN,THBS1,VWF,PSMA6,LCAT,CSTA,DNAH5,PSG9,FCN3,PLA2G7,SEC24D,CSF1R,FCN2,MASP1,GUCY1A1,PCSK9,FGB,F2,PLG,UBE3C,C1QB,CFHR1,NDUFS8,SUZ12,APOL1,SUMO4,SERPINF2,HBA1,FBN1,P4HB,DNAH8,ANG,FGG,GANAB,BLM,COL18A1,C8A,S100A7,FLNA,SMARCA1,C8B,FCN1,ADAMTS13,MBL2,C1QC,F7,APOM,GPX3,IGF1,TNRC6A,MASP2,SAA1,F11,C1S,CD14,LTBP1,PCYOX1,ADIPOQ,C4A,CDK3,C4B,SERPINA1,CFB,IGFALS,PLTP,UIMC1,JCHAIN,HPR,C1R,ATP1A1,MYL6,APOC1,APOC2,TTN,APOC4,IGHV3-15,VEGFC,HSPH1,TPM4,ACTB,FGA,TPM3
COMPARTMENTS	GOCC:0034366	Spherical high-density lipoprotein particle	5	11	1.61	3.47e-05	APOA1,LCAT,APOM,HPR,APOC2
COMPARTMENTS	GOCC:1905369	Endopeptidase complex	9	90	0.95	8.56e-05	C3,PSMA6,FCN3,FCN2,MASP1,UBE3C,FCN1,MASP2,F11
COMPARTMENTS	GOCC:0031089	Platelet dense granule lumen	5	14	1.5	8.60e-05	SPP2,ITIH4,ECM1,ITIH3,SELENOP
COMPARTMENTS	GOCC:0042627	Chylomicron	5	14	1.5	8.60e-05	APOB,APOA1,APOE,APOC1,APOC2
COMPARTMENTS	GOCC:0005602	Complement component C1 complex	4	5	1.85	8.86e-05	C1QB,C1QC,C1S,C1R
COMPARTMENTS	GOCC:0005783	Endoplasmic reticulum	31	1095	0.4	0.00014	SPP2,F9,APOB,APOA1,C3,APOE,PXDN,THBS1,VWF,CP,SEC24D,SLC27A4,PCSK9,APOL1,FBN1,P4HB,FGG,THBS4,GANAB,COL18A1,F5,S100A7,F7,GOLM1,IGF1,ADIPOQ,C4A,SERPINA1,SEMA3B,FGA,PDIA4
COMPARTMENTS	GOCC:0034365	Discoidal high-density lipoprotein particle	4	6	1.77	0.00014	APOA1,APOE,LCAT,APOM
COMPARTMENTS	GOCC:0009897	External side of plasma membrane	9	102	0.89	0.00020	THBS1,ANTXR1,FGB,PLG,P4HB,FGG,FCN1,CD14,FGA
COMPARTMENTS	GOCC:0034363	Intermediate-density lipoprotein particle	4	8	1.65	0.00031	APOB,APOA1,APOE,APOC2
COMPARTMENTS	GOCC:0062136	Low-density lipoprotein receptor complex	4	9	1.6	0.00042	APOB,APOA1,APOE,PCSK9
COMPARTMENTS	GOCC:0005601	Classical-complement-pathway C3/C5 convertase complex	3	4	1.82	0.0019	C4A,C4B,CFB
COMPARTMENTS	GOCC:0042567	Insulin-like growth factor ternary complex	3	4	1.82	0.0019	IGFBP5,IGF1,IGFALS
COMPARTMENTS	GOCC:1904090	Peptidase inhibitor complex	4	17	1.32	0.0031	PXDN,CSTA,SERPINA1,ACTB
COMPARTMENTS	GOCC:0005604	Basement membrane	6	61	0.94	0.0040	SPARC,PXDN,FBN1,ANG,THBS4,COL18A1
COMPARTMENTS	GOCC:0071953	Elastic fiber	3	8	1.52	0.0081	FBN1,FBLN1,FBLN5
COMPARTMENTS	GOCC:0005796	Golgi lumen	7	102	0.78	0.0083	F9,LALBA,F2,F10,F7,SOD3,PROS1
COMPARTMENTS	GOCC:1990341	Thrombospondin complex	3	9	1.47	0.0104	THBS1,VWF,ADAMTS13
COMPARTMENTS	GOCC:0005925	Focal adhesion	11	269	0.56	0.0117	VCL,TLN1,P4HB,LIMS1,S100A7,FLNA,ACTN1,YWHAZ,ILK,TPM4,ACTB
COMPARTMENTS	GOCC:0071944	Cell periphery	65	3860	0.17	0.0134	VCL,MYH9,TFPI2,C5,SPARC,HRG,APOB,PLEK,APOA1,C3,APOE,PXDN,THBS1,VWF,TIMP2,ATRN,CSTA,AMBP,TCIRG1,PSG9,DSC2,CSF1R,ANTXR1,PCSK9,FGB,PLG,STAB1,FBN1,P4HB,FBLN1,ANG,FGG,LIMS1,THBS4,FBLN5,COL18A1,C8A,ADAM12,ECM1,FLNA,C8B,FCN1,ADAMTS13,MBL2,ADGRG2,SOD3,GPX3,IGF1,PROS1,ACTN1,EFEMP1,ILK,CD14,LTBP1,C4A,C4B,TGFBI,TCAF1,PDGFC,FBN2,ATP1A1,PLCG2,OR52B4,ITM2B,FGA
COMPARTMENTS	GOCC:0036454	Growth factor complex	4	31	1.06	0.0206	IGFBP5,IGF1,IGFALS,VEGFC
COMPARTMENTS	GOCC:1904813	ficolin-1-rich granule lumen	7	124	0.7	0.0228	VCL,CAT,TIMP2,A1BG,PRG2,FCN1,SERPINA1
COMPARTMENTS	GOCC:0001527	Microfibril	3	13	1.31	0.0229	FBN1,LTBP1,FBN2
COMPARTMENTS	GOCC:0097179	Protease inhibitor complex	3	14	1.28	0.0272	PXDN,SERPINA1,ACTB
COMPARTMENTS	GOCC:0035580	Specific granule lumen	5	62	0.85	0.0289	VCL,LYZ,TIMP2,CAMP,SLPI
COMPARTMENTS	GOCC:0030139	Endocytic vesicle	10	260	0.53	0.0295	SPARC,APOB,APOA1,APOE,TCIRG1,HPX,STAB1,HBA1,SAA1,HSPH1
COMPARTMENTS	GOCC:0098552	Side of membrane	10	264	0.53	0.0325	MYH9,THBS1,ANTXR1,FGB,PLG,P4HB,FGG,FCN1,CD14,FGA
COMPARTMENTS	GOCC:0001725	Stress fiber	4	39	0.96	0.0404	MYH9,ACTN1,TPM4,TPM3

UniProt Keywords	KW-0964	Secreted	149	1839	0.86	1.60e-92	SPP2,ST6GAL1,GNPTG,LGALS1,F9,TFPI2,C5,PF4V1,MGP,SPARC,HRG,APOB,IGFBP5,APOA1,TTR,C3,APOE,PXDN,ANGPTL6,BPIFB1,F12,APCS,CFHR5,THBS1,LYZ,VWF,SERPINA10,TIMP2,ATRN,A1BG,LCN1,LCAT,CP,KLKB1,F13A1,AMBP,HPX,ITIH4,PSG9,FCN3,ITIH1,PLA2G7,CRH,HABP2,PIP,FCN2,PSG6,SCGB3A1,SERPINI1,PPBP,PF4,CAMP,A2ML1,LALBA,ANGPTL4,PCSK9,BMP1,FGB,F2,INHBC,PLG,PSG1,PRG2,C1QB,CFHR1,APOL1,PSG11,SERPINF2,FBN1,VMO1,FBLN1,COLEC10,SERPINA11,ANG,FGG,THBS4,SLPI,FBLN5,PAPLN,ITIH2,CNDP1,COL18A1,C8A,F13B,CFHR3,CFH,F5,ADAM12,S100A7,HTRA1,ADAMTSL4,ECM1,CRTAC1,C8B,RBP4,FCN1,ADAMTS13,CCN5,MBL2,C1QC,F10,F7,APOM,CA6,SOD3,GPX3,IGF1,PROS1,EFEMP1,BPIFC,MASP2,PSG4,SAA1,F11,CD14,PSG2,LTBP1,ADIPOQ,C4A,PRG4,COLEC11,MST1,ITIH3,C4B,SERPINA1,TGFBI,CFB,IGFALS,PLTP,PAEP,GC,HGFAC,PDGFC,FBN2,SELENOP,TSKU,JCHAIN,HPR,C1R,IGKV2D-28,APOC1,APOC2,APOC4,IGHV3-15,IGHV3-72,VEGFC,SEMA3B,ITM2B,FGA
UniProt Keywords	KW-0732	Signal	157	3277	0.63	2.11e-67	SPP2,GNPTG,SERPIND1,F9,TFPI2,C5,PF4V1,MGP,SPARC,HRG,APOB,IGFBP5,APOA1,TTR,C3,APOE,PXDN,ANGPTL6,BPIFB1,F12,APCS,CFHR5,THBS1,LYZ,VWF,TIMP2,ATRN,A1BG,LCN1,LCAT,CP,KLKB1,AMBP,HPX,ITIH4,PSG9,FCN3,ITIH1,PLA2G7,CRH,HABP2,DSC2,CSF1R,PIP,FCN2,PSG6,SCGB3A1,SERPINI1,PPBP,PF4,CAMP,A2ML1,LALBA,ANGPTL4,ANTXR1,PCSK9,BMP1,FGB,F2,INHBC,PLG,PSG1,PRG2,STAB1,C1QB,CFHR1,APOL1,PSG11,SERPINF2,FBN1,P4HB,VMO1,FBLN1,OAF,COLEC10,OIT3,SERPINA11,ANG,FGG,THBS4,GANAB,SLPI,FBLN5,PAPLN,ITIH2,CNDP1,COL18A1,C8A,PCDH18,F13B,CFHR3,CFH,F5,ADAM12,HTRA1,ADAMTSL4,ECM1,CRTAC1,C8B,RBP4,FCN1,ADAMTS13,CCN5,MBL2,C1QC,F10,F7,APOM,CA6,ADGRG2,SOD3,GPX3,IGF1,PROS1,EFEMP1,BPIFC,MASP2,PSG4,SAA1,F11,C1S,CD14,PSG2,LTBP1,PCYOX1,ADIPOQ,C4A,PRG4,COLEC11,MST1,ITIH3,C4B,SERPINA1,TGFBI,CFB,IGFALS,PLTP,PAEP,GC,HGFAC,PDGFC,FBN2,SELENOP,TSKU,JCHAIN,HPR,C1R,IGKV2D-28,APOC1,APOC2,APOC4,IGHV3-15,IGHV3-72,VEGFC,SEMA3B,FGA,PDIA4
UniProt Keywords	KW-1015	Disulfide bond	135	3338	0.55	2.23e-45	SPP2,ST6GAL1,GNPTG,F9,TFPI2,C5,PF4V1,MGP,SPARC,HRG,APOB,IGFBP5,C3,PXDN,ANGPTL6,BPIFB1,F12,APCS,CFHR5,THBS1,LYZ,VWF,TIMP2,ATRN,A1BG,LCN1,LCAT,CP,KLKB1,AMBP,HPX,ITIH4,PSG9,FCN3,ITIH1,HABP2,CSF1R,PIP,FCN2,PSG6,SCGB3A1,PPBP,PF4,MASP1,CAMP,A2ML1,LALBA,ANGPTL4,ANTXR1,PCSK9,BMP1,FGB,F2,INHBC,PLG,PSG1,PRG2,STAB1,C1QB,CFHR1,PSG11,SERPINF2,FBN1,P4HB,VMO1,FBLN1,COLEC10,OIT3,ANG,FGG,THBS4,GANAB,SLPI,FBLN5,PAPLN,ITIH2,COL18A1,C8A,F13B,CFHR3,CFH,F5,ADAM12,S100A7,HTRA1,CRTAC1,C8B,RBP4,FCN1,ADAMTS13,MBL2,C1QC,F10,F7,APOM,CA6,SOD3,IGF1,PROS1,EFEMP1,BPIFC,MASP2,PSG4,F11,C1S,CD14,PSG2,LTBP1,ADIPOQ,C4A,PRG4,COLEC11,MST1,C4B,TGFBI,CFB,PLTP,PAEP,GC,HGFAC,PDGFC,FBN2,JCHAIN,HPR,C1R,IGKV2D-28,TTN,IGHV3-15,IGHV3-72,VEGFC,SEMA3B,OR52B4,ITM2B,FGA,PDIA4
UniProt Keywords	KW-0325	Glycoprotein	133	4386	0.43	8.28e-31	ST6GAL1,GNPTG,SERPIND1,APMAP,F9,TFPI2,C5,PF4V1,SPARC,HRG,APOB,IGFBP5,APOA1,TTR,C3,APOE,PXDN,ANGPTL6,BPIFB1,F12,APCS,CFHR5,THBS1,VWF,PSMA6,SERPINA10,ATRN,A1BG,LCAT,CP,KLKB1,F13A1,AMBP,HPX,ITIH4,PSG9,FCN3,ITIH1,PLA2G7,HABP2,DSC2,CSF1R,PIP,FCN2,PSG6,SERPINI1,A2ML1,LALBA,ANGPTL4,ANTXR1,PCSK9,BMP1,FGB,F2,INHBC,PLG,PSG1,PRG2,STAB1,C1QB,CFHR1,APOL1,PSG11,SERPINF2,FBN1,FBLN1,COLEC10,OIT3,SERPINA11,FGG,THBS4,GANAB,FBLN5,ITIH2,CNDP1,COL18A1,C8A,PCDH18,F13B,CFHR3,CFH,F5,ADAM12,ADAMTSL4,ECM1,CRTAC1,C8B,FCN1,ADAMTS13,C1QC,F10,F7,APOM,CA6,ADGRG2,SOD3,GOLM1,PROS1,EFEMP1,BPIFC,PSG4,F11,C1S,CD14,PSG2,LTBP1,PCYOX1,ADIPOQ,C4A,PRG4,MST1,ITIH3,C4B,SERPINA1,CFB,IGFALS,PLTP,PAEP,GC,HGFAC,PDGFC,FBN2,SELENOP,TSKU,JCHAIN,C1R,APOC2,APOC4,VEGFC,SEMA3B,OR52B4,ITM2B,FGA
UniProt Keywords	KW-0094	Blood coagulation	22	46	1.63	1.29e-24	SERPIND1,F9,TFPI2,HRG,F12,VWF,SERPINA10,KLKB1,F13A1,FGB,F2,PLG,FGG,F13B,F5,ADAMTS13,F10,F7,PROS1,F11,SERPINA1,FGA
UniProt Keywords	KW-0245	EGF-like domain	27	232	1.01	1.25e-16	F9,F12,THBS1,ATRN,HABP2,MASP1,BMP1,STAB1,FBN1,FBLN1,OIT3,THBS4,FBLN5,C8A,ADAM12,CRTAC1,C8B,F10,F7,PROS1,EFEMP1,MASP2,C1S,LTBP1,HGFAC,FBN2,C1R
UniProt Keywords	KW-0165	Cleavage on pair of basic residues	27	280	0.93	8.51e-15	F9,C5,HRG,C3,VWF,AMBP,CRH,HABP2,DSC2,PPBP,CAMP,BMP1,F2,INHBC,PLG,C8A,ADAM12,ADAMTS13,F10,F7,PROS1,C4A,C4B,CFB,PDGFC,VEGFC,ITM2B
UniProt Keywords	KW-0180	Complement pathway	12	30	1.55	1.67e-12	C5,C3,C1QB,C8A,C8B,MBL2,C1QC,MASP2,C1S,C4A,C4B,C1R
UniProt Keywords	KW-0106	Calcium	41	886	0.61	1.97e-12	F9,SPARC,PXDN,APCS,THBS1,F13A1,FCN3,DSC2,FCN2,LALBA,PCSK9,BMP1,F2,FBN1,FBLN1,COLEC10,OIT3,SPATA21,FGG,THBS4,FBLN5,PCDH18,F5,S100A7,FCN1,ADAMTS13,MBL2,F10,F7,PROS1,ACTN1,EFEMP1,EFCAB5,MASP2,C1S,COLEC11,FBN2,TTN,PLCG2,TPM4,FGA
UniProt Keywords	KW-0646	Protease inhibitor	17	117	1.11	9.30e-12	SERPIND1,TFPI2,SERPINA10,TIMP2,CSTA,AMBP,ITIH4,ITIH1,SERPINI1,A2ML1,SERPINF2,SERPINA11,SLPI,PAPLN,ITIH2,ITIH3,SERPINA1
UniProt Keywords	KW-0722	Serine protease inhibitor	15	80	1.22	9.30e-12	SERPIND1,TFPI2,SERPINA10,AMBP,ITIH4,ITIH1,SERPINI1,A2ML1,SERPINF2,SERPINA11,SLPI,PAPLN,ITIH2,ITIH3,SERPINA1
UniProt Keywords	KW-0399	Innate immunity	24	329	0.81	8.25e-11	C5,C3,BPIFB1,FCN3,CSF1R,FCN2,FGB,C1QB,SLPI,C8A,CFH,C8B,FCN1,MBL2,C1QC,MASP2,C1S,CD14,C4A,COLEC11,C4B,CFB,C1R,FGA
UniProt Keywords	KW-0301	Gamma-carboxyglutamic acid	9	16	1.7	1.89e-10	F9,MGP,TTR,F2,ITIH2,F10,F7,PROS1,TGFBI
UniProt Keywords	KW-0272	Extracellular matrix	21	267	0.84	5.09e-10	LGALS1,SPARC,APOE,PXDN,THBS1,VWF,ANGPTL4,BMP1,FBN1,FBLN1,THBS4,FBLN5,COL18A1,ADAMTSL4,ECM1,CRTAC1,EFEMP1,LTBP1,SERPINA1,TGFBI,FBN2
UniProt Keywords	KW-0720	Serine protease	16	136	1.02	6.21e-10	F9,F12,KLKB1,HABP2,PCSK9,F2,PLG,HTRA1,F10,F7,MASP2,F11,C1S,CFB,HGFAC,C1R
UniProt Keywords	KW-0379	Hydroxylation	16	143	1.0	1.16e-09	F9,FCN3,FCN2,C1QB,COL18A1,MBL2,C1QC,F10,F7,PROS1,MASP2,C1S,LTBP1,ADIPOQ,C1R,FGA
UniProt Keywords	KW-0391	Immunity	28	537	0.67	1.50e-09	C5,C3,BPIFB1,FCN3,CSF1R,FCN2,FGB,PRG2,C1QB,SLPI,C8A,CFH,C8B,FCN1,MBL2,C1QC,MASP2,C1S,CD14,C4A,COLEC11,C4B,CFB,C1R,IGKV2D-28,IGHV3-15,IGHV3-72,FGA
UniProt Keywords	KW-0677	Repeat	99	4794	0.26	1.68e-09	VCL,SERPIND1,F9,TFPI2,HRG,PLEK,APOA1,APOE,PXDN,F12,CFHR5,THBS1,VWF,ATRN,A1BG,CP,KLKB1,DNAH5,AMBP,HPX,PSG9,FCN3,HABP2,DSC2,ANKRD44,CSF1R,FCN2,PSG6,MASP1,SH3D19,BMP1,F2,PLG,PSG1,STAB1,C1QB,CFHR1,NDUFS8,PSG11,FBN1,P4HB,FBLN1,DNAH8,LIMS1,THBS4,SLPI,FBLN5,PAPLN,COL18A1,C8A,PCDH18,F13B,CFHR3,CFH,F5,S100A7,ADAMTSL4,ECM1,FLNA,CRTAC1,SMARCA1,C8B,FCN1,ADAMTS13,MBL2,C1QC,F10,F7,PROS1,ACTN1,EFEMP1,ILK,RAPGEF4,MASP2,PSG4,F11,C1S,CD14,PSG2,LTBP1,ADIPOQ,PRG4,NBEAL1,FSIP2,MST1,TGFBI,CFB,IGFALS,GC,HGFAC,UIMC1,FBN2,TSKU,C1R,MYL6,TTN,VEGFC,PLCG2,PDIA4
UniProt Keywords	KW-0358	Heparin-binding	13	88	1.12	3.32e-09	SERPIND1,PF4V1,HRG,APOB,APOE,THBS1,SERPINA10,PF4,PRG2,FBN1,SOD3,SAA1,F11
UniProt Keywords	KW-0768	Sushi	11	57	1.23	7.22e-09	CFHR5,MASP1,CFHR1,F13B,CFHR3,CFH,MASP2,C1S,CFB,HPR,C1R
UniProt Keywords	KW-0792	Thrombophilia	7	10	1.79	1.05e-08	SERPIND1,F9,HRG,F2,PLG,F5,PROS1
UniProt Keywords	KW-0034	Amyloid	8	24	1.47	5.12e-08	APOA1,TTR,APCS,LYZ,SAA1,TGFBI,ITM2B,FGA
UniProt Keywords	KW-0865	Zymogen	16	210	0.83	1.50e-07	F9,F12,KLKB1,F13A1,PCSK9,BMP1,F2,PLG,F5,ADAM12,ADAMTS13,F10,F7,PROS1,CFB,HGFAC

UniProt Keywords	KW-1008	Amyloidosis	8	31	1.36	2.58e-07	APOA1,TTR,APOE,LYZ,SAA1,TGFBI,ITM2B,FGA
UniProt Keywords	KW-1068	Hemolytic uremic syndrome	6	10	1.73	3.38e-07	C3,CFHR5,CFHR1,CFHR3,CFH,CFB
UniProt Keywords	KW-0179	Complement alternate pathway	6	12	1.65	7.40e-07	C5,C3,C8A,CFH,C8B,CFB
UniProt Keywords	KW-0765	Sulfation	9	57	1.15	1.15e-06	SERPIND1,F9,PCSK9,SERPINF2,FGG,CFH,F5,C4A,C4B
UniProt Keywords	KW-0176	Collagen	10	83	1.03	2.02e-06	FCN3,FCN2,C1QB,COLEC10,COL18A1,FCN1,MBL2,C1QC,ADIPOQ,COLEC11
UniProt Keywords	KW-0345	HDL	6	16	1.52	2.58e-06	APOA1,APOE,APOL1,APOM,SAA1,APOC2
UniProt Keywords	KW-0420	Kringle	6	16	1.52	2.58e-06	F12,HABP2,F2,PLG,MST1,HGFAC
UniProt Keywords	KW-0280	Fibrinolysis	5	8	1.74	4.13e-06	HRG,F12,KLKB1,PLG,PROS1
UniProt Keywords	KW-0645	Protease	21	516	0.56	1.39e-05	F9,F12,PSMA6,KLKB1,HABP2,PCSK9,BMP1,F2,PLG,CNDP1,ADAM12,HTRA1,ADAMTS13,F10,F7,MASP2,F11,C1S,CFB,HGFAC,C1R
UniProt Keywords	KW-0445	Lipid transport	10	110	0.91	1.90e-05	APOB,APOA1,APOE,SLC27A4,APOL1,APOM,PLTP,APOC1,APOC2,APOC4
UniProt Keywords	KW-0913	Age-related macular degeneration	5	16	1.44	5.55e-05	C3,FBLN5,CFH,HTRA1,CFB
UniProt Keywords	KW-0011	Acute phase	5	19	1.37	0.00011	ITIH4,F2,SERPINF2,SAA1,SERPINA1
UniProt Keywords	KW-0654	Proteoglycan	7	55	1.05	0.00011	PF4V1,AMBP,ITIH1,PRG2,ITIH2,PRG4,ITIH3
UniProt Keywords	KW-0882	Thioester bond	4	8	1.65	0.00012	C3,A2ML1,C4A,C4B
UniProt Keywords	KW-0430	Lectin	10	162	0.74	0.00041	LGALS1,APCS,ATRN,FCN3,FCN2,PRG2,COLEC10,FCN1,MBL2,COLEC11
UniProt Keywords	KW-0971	Glycation	4	12	1.47	0.00041	APOA1,APOE,SOD3,CFB
UniProt Keywords	KW-1018	Complement activation lectin pathway	3	3	1.95	0.00044	FCN3,FCN2,MBL2
UniProt Keywords	KW-0497	Mitogen	5	39	1.06	0.0020	CAT,PPBP,THBS4,PDGFC,VEGFC
UniProt Keywords	KW-0153	Cholesterol metabolism	6	65	0.91	0.0022	APOB,APOA1,APOE,LCAT,PCSK9,APOL1
UniProt Keywords	KW-0395	Inflammatory response	9	163	0.69	0.0022	C5,C3,ATRN,KLKB1,CSF1R,STAB1,CD14,C4A,C4B
UniProt Keywords	KW-0339	Growth factor	8	129	0.74	0.0023	PPBP,BMP1,INHBC,THBS4,IGF1,EFEMP1,PDGFC,VEGFC
UniProt Keywords	KW-0465	Mannose-binding	3	7	1.58	0.0023	COLEC10,MBL2,COLEC11
UniProt Keywords	KW-0473	Membrane attack complex	3	7	1.58	0.0023	C5,C8A,C8B
UniProt Keywords	KW-1065	Osteogenesis imperfecta	4	23	1.19	0.0030	SPARC,SEC24D,BMP1,P4HB
UniProt Keywords	KW-0162	Chylomicron	3	9	1.47	0.0037	APOB,APOE,APOC2
UniProt Keywords	KW-0393	Immunoglobulin domain	15	463	0.46	0.0042	PXDN,A1BG,PSG9,CSF1R,PSG6,PSG1,PSG11,PAPLN,PSG4,PSG2,IGKV2D-28,TTN,IGHV3-15,IGHV3-72,SEMA3B
UniProt Keywords	KW-0850	VLDL	3	10	1.43	0.0046	APOE,APOC1,APOC2
UniProt Keywords	KW-0130	Cell adhesion	15	478	0.44	0.0056	VCL,MYH9,THBS1,VWF,CSTA,FERMT3,DSC2,THBS4,FBLN5,COL18A1,PCDH18,ADAM12,CCN5,TGFBI,IGFALS
UniProt Keywords	KW-1280	Immunoglobulin	3	12	1.35	0.0070	IGKV2D-28,IGHV3-15,IGHV3-72
UniProt Keywords	KW-0186	Copper	5	65	0.83	0.0131	SPARC,HRG,CP,F5,SOD3
UniProt Keywords	KW-0873	Pyrrolidone carboxylic acid	5	67	0.82	0.0145	PIP,FGB,C1QB,ANG,JCHAIN
UniProt Keywords	KW-0443	Lipid metabolism	14	485	0.41	0.0169	APOB,APOA1,C3,APOE,LCAT,PLA2G7,SLC27A4,ANGPTL4,PCSK9,APOL1,ISYNA1,ACSF2,APOC2,PLCG2
UniProt Keywords	KW-0380	Hyperlipidemia	2	4	1.65	0.0202	APOE,APOC2
UniProt Keywords	KW-0797	Tissue remodeling	2	4	1.65	0.0202	PLG,THBS4
UniProt Keywords	KW-0340	Growth factor binding	3	20	1.12	0.0222	IGFBP5,HTRA1,LTBP1
UniProt Keywords	KW-0575	Peroxidase	3	25	1.03	0.0388	CAT,PXDN,GPX3
UniProt Keywords	KW-0037	Angiogenesis	6	131	0.61	0.0461	HRG,ANGPTL6,ANGPTL4,ANG,ECM1,VEGFC
UniProt Keywords	KW-0372	Hormone	5	92	0.68	0.0477	TTR,CRH,INHBC,FBN1,ADIPOQ
UniProt Keywords	KW-0929	Antimicrobial	5	93	0.68	0.0491	LYZ,PPBP,CAMP,PRG2,SLPI
Pfam	PF06668	Inter-alpha-trypsin inhibitor heavy chain C-terminus	4	6	1.77	0.0069	ITIH4,ITIH1,ITIH2,ITIH3
Pfam	PF01391	Collagen triple helix repeat (20 copies)	7	70	0.95	0.0279	FCN2,C1QB,COLEC10,COL18A1,FCN1,MBL2,COLEC11
InterPro	IPR001881	EGF-like calcium-binding domain	22	124	1.2	5.61e-15	F9,F12,THBS1,HABP2,MASP1,BMP1,STAB1,FBN1,FBLN1,OIT3,THBS4,FBLN5,CRTAC1,F10,F7,PROS1,EFEMP1,MASP2,C1S,LTBP1,FBN2,C1R
InterPro	IPR018097	EGF-like calcium-binding, conserved site	18	99	1.21	3.16e-12	F9,MASP1,BMP1,FBN1,FBLN1,OIT3,THBS4,FBLN5,CRTAC1,F10,F7,PROS1,EFEMP1,MASP2,C1S,LTBP1,FBN2,C1R
InterPro	IPR000742	EGF-like domain	23	236	0.94	4.17e-11	F9,F12,THBS1,ATRN,HABP2,MASP1,BMP1,STAB1,FBN1,FBLN1,OIT3,THBS4,FBLN5,ADAM12,F10,F7,PROS1,EFEMP1,MASP2,LTBP1,HGFAC,FBN2,C1R
InterPro	IPR009003	Peptidase S1, PA clan	17	123	1.09	5.69e-10	F9,F12,KLKB1,HABP2,F2,PLG,HTRA1,F10,F7,MASP2,F11,C1S,MST1,CFB,HGFAC,HPR,C1R
InterPro	IPR001314	Peptidase S1A, chymotrypsin family	16	107	1.12	7.80e-10	F9,F12,KLKB1,HABP2,F2,PLG,F10,F7,MASP2,F11,C1S,MST1,CFB,HGFAC,HPR,C1R
InterPro	IPR001254	Serine proteases, trypsin domain	16	115	1.09	1.76e-09	F9,F12,KLKB1,HABP2,F2,PLG,F10,F7,MASP2,F11,C1S,MST1,CFB,HGFAC,HPR,C1R
InterPro	IPR033116	Serine proteases, trypsin family, serine active site	14	92	1.13	1.33e-08	F9,F12,KLKB1,HABP2,F2,PLG,F10,F7,MASP2,F11,C1S,CFB,HGFAC,C1R
InterPro	IPR043504	Peptidase S1, PA clan, chymotrypsin-like fold	15	116	1.06	1.73e-08	F9,F12,KLKB1,HABP2,F2,PLG,F10,F7,MASP2,F11,C1S,MST1,HGFAC,HPR,C1R
InterPro	IPR035976	Sushi/SCR/CCP superfamily	11	58	1.23	2.12e-07	CFHR5,MASP1,CFHR1,F13B,CFHR3,CFH,MASP2,C1S,CFB,HPR,C1R
InterPro	IPR000152	EGF-type aspartate/asparagine hydroxylation site	13	98	1.07	2.37e-07	F9,BMP1,FBN1,FBLN1,FBLN5,F10,F7,PROS1,EFEMP1,MASP2,C1S,LTBP1,FBN2
InterPro	IPR020837	Fibrinogen, conserved site	8	19	1.57	2.53e-07	ANGPTL6,FCN3,FCN2,ANGPTL4,FGB,FGG,FCN1,FGA
InterPro	IPR000436	Sushi/SCR/CCP domain	10	56	1.2	1.77e-06	CFHR5,MASP1,CFHR1,F13B,CFHR3,CFH,MASP2,C1S,CFB,C1R
InterPro	IPR014716	Fibrinogen, alpha/beta/gamma chain, C-terminal globular, subdomain 1	8	26	1.44	1.77e-06	ANGPTL6,FCN3,FCN2,ANGPTL4,FGB,FGG,FCN1,FGA
InterPro	IPR002181	Fibrinogen, alpha/beta/gamma chain, C-terminal globular domain	8	32	1.35	6.09e-06	ANGPTL6,FCN3,FCN2,ANGPTL4,FGB,FGG,FCN1,FGA
InterPro	IPR036056	Fibrinogen-like, C-terminal	8	32	1.35	6.09e-06	ANGPTL6,FCN3,FCN2,ANGPTL4,FGB,FGG,FCN1,FGA
InterPro	IPR018114	Serine proteases, trypsin family, histidine active site	11	100	0.99	2.06e-05	F9,F12,KLKB1,HABP2,F2,PLG,F10,F7,F11,CFB,HGFAC
InterPro	IPR009030	Growth factor receptor cysteine-rich domain superfamily	12	127	0.92	2.21e-05	IGFBP5,BMP1,FBN1,FBLN1,FBLN5,C8A,HTRA1,CCN5,PROS1,EFEMP1,LTBP1,FBN2
InterPro	IPR000020	Anaphylatoxin/fibulin	5	6	1.87	2.87e-05	C5,C3,FBLN1,C4A,C4B
InterPro	IPR000294	Gamma-carboxyglutamic acid-rich (GLA) domain	6	15	1.55	3.19e-05	F9,MGP,F2,F10,F7,PROS1
InterPro	IPR035972	Gamma-carboxyglutamic acid-rich (GLA) domain superfamily	6	15	1.55	3.19e-05	F9,MGP,F2,F10,F7,PROS1
InterPro	IPR000001	Kringle	6	16	1.52	3.94e-05	F12,HABP2,F2,PLG,MST1,HGFAC
InterPro	IPR018056	Kringle, conserved site	6	16	1.52	3.94e-05	F12,HABP2,F2,PLG,MST1,HGFAC
InterPro	IPR038178	Kringle superfamily	6	16	1.52	3.94e-05	F12,HABP2,F2,PLG,MST1,HGFAC
InterPro	IPR040839	Macroglobulin domain MG4	5	8	1.74	5.88e-05	C5,C3,A2ML1,C4A,C4B
InterPro	IPR001599	Alpha-2-macroglobulin	5	9	1.69	8.71e-05	C5,C3,A2ML1,C4A,C4B
InterPro	IPR002890	Macroglobulin domain	5	9	1.69	8.71e-05	C5,C3,A2ML1,C4A,C4B
InterPro	IPR009048	Alpha-macroglobulin, receptor-binding	5	9	1.69	8.71e-05	C5,C3,A2ML1,C4A,C4B
InterPro	IPR011625	Alpha-2-macroglobulin, bait region domain	5	9	1.69	8.71e-05	C5,C3,A2ML1,C4A,C4B
InterPro	IPR011626	Alpha-macroglobulin-like, TED domain	5	9	1.69	8.71e-05	C5,C3,A2ML1,C4A,C4B
InterPro	IPR036595	Alpha-macroglobulin, receptor-binding domain superfamily	5	9	1.69	8.71e-05	C5,C3,A2ML1,C4A,C4B
InterPro	IPR041555	Macroglobulin domain MG3	5	9	1.69	8.71e-05	C5,C3,A2ML1,C4A,C4B
InterPro	IPR035914	Spermadhesin, CUB domain superfamily	8	54	1.12	9.49e-05	ATRN,MASP1,BMP1,ADAMTS13,MASP2,C1S,PDGFC,C1R
InterPro	IPR008160	Collagen triple helix repeat	9	77	1.02	0.00011	FCN2,C1QB,COLEC10,COL18A1,FCN1,MBL2,C1QC,ADIPOQ,COLEC11
InterPro	IPR001840	Anaphylatoxin, complement system domain	4	4	1.95	0.00021	C5,C3,C4A,C4B
InterPro	IPR018081	Anaphylatoxin, complement system	4	4	1.95	0.00021	C5,C3,C4A,C4B
InterPro	IPR013806	Kringle-like fold	6	26	1.31	0.00025	F12,HABP2,F2,PLG,MST1,HGFAC

InterPro	IPR026823	Complement Clr-like EGF domain	6	27	1.29	0.00030	FBN1,FBLN1,FBLN5,EFEMP1,LTBP1,FBN2
InterPro	IPR008930	Terpenoid cyclases/protein prenyltransferase alpha-alpha toroid	5	15	1.47	0.00042	C5,C3,A2ML1,C4A,C4B
InterPro	IPR003609	PAN/Apple domain	4	6	1.77	0.00055	KLKB1,PLG,F11,MST1
InterPro	IPR010600	Inter-alpha-trypsin inhibitor heavy chain, C-terminal	4	6	1.77	0.00055	ITIH4,ITIH1,ITIH2,ITIH3
InterPro	IPR000859	CUB domain	7	51	1.09	0.00063	ATRN,MASP1,BMP1,MASP2,C1S,PDGFC,C1R
InterPro	IPR042185	Serpin superfamily, domain 2	6	35	1.18	0.00099	SERPIND1,SERPINA10,SERPINI1,SERPINF2,SERPINA11,SERPINA1
InterPro	IPR000215	Serpin family	6	36	1.17	0.0011	SERPIND1,SERPINA10,SERPINI1,SERPINF2,SERPINA11,SERPINA1
InterPro	IPR019742	Alpha-2-macroglobulin, conserved site	4	8	1.65	0.0011	C3,A2ML1,C4A,C4B
InterPro	IPR023796	Serpin domain	6	36	1.17	0.0011	SERPIND1,SERPINA10,SERPINI1,SERPINF2,SERPINA11,SERPINA1
InterPro	IPR036186	Serpin superfamily	6	36	1.17	0.0011	SERPIND1,SERPINA10,SERPINI1,SERPINF2,SERPINA11,SERPINA1
InterPro	IPR042178	Serpin superfamily, domain 1	6	36	1.17	0.0011	SERPIND1,SERPINA10,SERPINI1,SERPINF2,SERPINA11,SERPINA1
InterPro	IPR047565	Alpha-macroglobulin-like, thiol-ester bond-forming region	4	8	1.65	0.0011	C3,A2ML1,C4A,C4B
InterPro	IPR013106	Immunoglobulin V-set domain	11	182	0.73	0.0017	PSG9,PSG6,PSG1,PSG11,PAPLN,PSG4,PSG2,IGKV2D-28,TTN,IGHV3-15,IGHV3-72
InterPro	IPR001134	Netrin domain	5	23	1.29	0.0019	C5,C3,TIMP2,C4A,C4B
InterPro	IPR013694	VIT domain	4	10	1.55	0.0019	ITIH4,ITIH1,ITIH2,ITIH3
InterPro	IPR013783	Immunoglobulin-like fold	23	732	0.45	0.0019	C5,C3,PXDN,A1BG,F13A1,PSG9,CSF1R,PIP,PSG6,A2ML1,PSG1,PSG11,PAPLN,FLNA,PSG4,PSG2,C4A,C4B,IGKV2D-28,TTN,IGHV3-15,IGHV3-72,SEMA3B
InterPro	IPR036383	Thrombospondin type-1 (TSP1) repeat superfamily	7	64	0.99	0.0019	THBS1,PAPLN,C8A,ADAMTSL4,C8B,ADAMTS13,CCN5
InterPro	IPR000884	Thrombospondin type-1 (TSP1) repeat	7	65	0.98	0.0021	THBS1,PAPLN,C8A,ADAMTSL4,C8B,ADAMTS13,CCN5
InterPro	IPR008993	Tissue inhibitor of metalloproteinases-like, OB-fold	5	24	1.27	0.0021	C5,C3,TIMP2,C4A,C4B
InterPro	IPR036465	Von Willebrand factor A-like domain superfamily	8	97	0.86	0.0030	VWF,ITIH4,ITIH1,SEC24D,ANTXR1,ITIH2,ITIH3,CFB
InterPro	IPR017857	Coagulation factor-like, Gla domain superfamily	4	12	1.47	0.0031	F9,F10,F7,PROS1
InterPro	IPR012290	Fibrinogen, alpha/beta/gamma chain, coiled coil domain	3	3	1.95	0.0033	FGB,FGG,FGA
InterPro	IPR024175	Peptidase S1A, complement C1r/C1S/mannan-binding	3	3	1.95	0.0033	MASP2,C1S,C1R
InterPro	IPR037579	Fibrinogen	3	3	1.95	0.0033	FGB,FGG,FGA
InterPro	IPR022272	Lipocalin family conserved site	4	13	1.44	0.0038	AMBP,SLC27A4,RBP4,PAEP
InterPro	IPR002035	Von Willebrand factor, type A	7	76	0.91	0.0046	VWF,ITIH4,ITIH1,ANTXR1,ITIH2,ITIH3,CFB
InterPro	IPR012224	Peptidase S1A, coagulation factor VII/IX/X/C/Z	3	5	1.73	0.0084	F9,F10,F7
InterPro	IPR033990	Collectin, C-type lectin-like domain	3	5	1.73	0.0084	COLEC10,MBL2,COLEC11
InterPro	IPR018933	Netrin module, non-TIMP type	4	17	1.32	0.0086	C5,C3,C4A,C4B
InterPro	IPR003599	Immunoglobulin subtype	15	411	0.51	0.0100	PXDN,A1BG,PSG9,CSF1R,PSG6,PSG1,PSG11,PAPLN,PSG4,PSG2,IGKV2D-28,TTN,IGHV3-15,IGHV3-72,SEMA3B
InterPro	IPR012674	Calycin	5	38	1.07	0.0120	LCN1,AMBP,RBP4,APOM,PAEP
InterPro	IPR003598	Immunoglobulin subtype 2	11	239	0.61	0.0127	PXDN,A1BG,PSG9,CSF1R,PSG6,PSG1,PSG11,PAPLN,PSG4,PSG2,TTN
InterPro	IPR016186	C-type lectin-like/link domain superfamily	7	98	0.8	0.0182	ATRN,PRG2,STAB1,COLEC10,COL18A1,MBL2,COLEC11
InterPro	IPR046350	Cystatin superfamily	4	23	1.19	0.0221	SPP2,HRG,CSTA,CAMP
InterPro	IPR016187	C-type lectin fold	7	106	0.77	0.0279	ATRN,PRG2,STAB1,COLEC10,COL18A1,MBL2,COLEC11
InterPro	IPR017878	TB domain	3	9	1.47	0.0279	FBN1,LTBP1,FBN2
InterPro	IPR036773	TGF-beta binding (TB) domain superfamily	3	9	1.47	0.0279	FBN1,LTBP1,FBN2
InterPro	IPR001124	Lipid-binding serum glycoprotein, C-terminal	3	10	1.43	0.0349	BPIFB1,BPIFC,PLTP
InterPro	IPR007110	Immunoglobulin-like domain	15	479	0.44	0.0434	PXDN,A1BG,PSG9,CSF1R,PSG6,PSG1,PSG11,PAPLN,PSG4,PSG2,IGKV2D-28,TTN,IGHV3-15,IGHV3-72,SEMA3B
SMART	SM00179	Calcium-binding EGF-like domain	17	101	1.17	1.45e-11	F9,MASP1,BMP1,FBN1,FBLN1,OIT3,THBS4,FBLN5,F10,F7,PROS1,EFEMP1,MASP2,C1S,LTBP1,FBN2,C1R
SMART	SM00020	Trypsin-like serine protease	17	118	1.11	7.08e-11	F9,F12,KLKB1,HABP2,MASP1,F2,PLG,F10,F7,MASP2,F11,C1S,MST1,CFB,HGFAC,HPR,C1R
SMART	SM00032	Domain abundant in complement control proteins	10	56	1.2	8.08e-07	CFHR5,MASP1,CFHR1,F13B,CFHR3,CFH,MASP2,C1S,CFB,C1R
SMART	SM00181	Epidermal growth factor-like domain.	16	209	0.83	1.03e-06	F9,F12,THBS1,ATRN,HABP2,STAB1,FBN1,FBLN1,OIT3,THBS4,F10,F7,PROS1,LTBP1,HGFAC,FBN2
SMART	SM00186	Fibrinogen-related domains (FReDs)	8	29	1.39	1.03e-06	ANGPTL6,FCN3,FCN2,ANGPTL4,FGB,FGG,FCN1,FGA
SMART	SM00104	Anaphylatoxin homologous domain	5	6	1.87	9.84e-06	C5,C3,FBLN1,C4A,C4B
SMART	SM00069	Domain containing Gla (gamma-carboxyglutamate) residues.	6	15	1.55	9.91e-06	F9,MGP,F2,F10,F7,PROS1
SMART	SM00130	Kringle domain	6	16	1.52	1.18e-05	F12,HABP2,F2,PLG,MST1,HGFAC
SMART	SM01359	Alpha-2-Macroglobulin	5	9	1.69	2.76e-05	C5,C3,A2ML1,C4A,C4B
SMART	SM01360	Alpha-2-macroglobulin family	5	9	1.69	2.76e-05	C5,C3,A2ML1,C4A,C4B
SMART	SM01361	A-macroglobulin receptor	5	9	1.69	2.76e-05	C5,C3,A2ML1,C4A,C4B
SMART	SM00042	Domain first found in C1r, C1s, uEGF, and bone morphogenetic protein.	7	50	1.09	0.00022	ATRN,MASP1,BMP1,MASP2,C1S,PDGFC,C1R
SMART	SM00609	Vault protein Inter-alpha-Trypsin domain	4	7	1.71	0.00029	ITIH4,ITIH1,ITIH2,ITIH3
SMART	SM00093	SERine Proteinase INhibitors	6	35	1.18	0.00034	SERPIND1,SERPINA10,SERPINI1,SERPINF2,SERPINA11,SERPINA1
SMART	SM01419	Alpha-macro-globulin thiol-ester bond-forming region	4	8	1.65	0.00038	C3,A2ML1,C4A,C4B
SMART	SM00209	Thrombospondin type 1 repeats	7	64	0.99	0.00072	THBS1,PAPLN,C8A,ADAMTSL4,C8B,ADAMTS13,CCN5
SMART	SM01212	Fibrinogen alpha/beta chain family	3	3	1.95	0.0013	FGB,FGG,FGA
SMART	SM00327	Von Willebrand factor (vWF) type A domain	7	77	0.91	0.0019	VWF,ITIH4,ITIH1,ANTXR1,ITIH2,ITIH3,CFB
SMART	SM00408	Immunoglobulin C-2 Type	11	212	0.66	0.0019	PXDN,A1BG,PSG9,CSF1R,PSG6,PSG1,PSG11,PAPLN,PSG4,PSG2,TTN
SMART	SM00643	Netrin C-terminal Domain	4	15	1.37	0.0021	C5,C3,C4A,C4B
SMART	SM00328	BPI/LBP/CETP N-terminal domain	3	9	1.47	0.0109	BPIFB1,BPIFC,PLTP
SMART	SM00223	APPLE domain	2	2	1.95	0.0273	KLKB1,F11
SMART	SM00214	Von Willebrand factor (vWF) type C domain	4	37	0.98	0.0393	PXDN,THBS1,VWF,CCN5
SMART	SM00121	Insulin growth factor-binding protein homologues	3	17	1.19	0.0463	IGFBP5,HTRA1,CCN5
SMART	SM00210	Thrombospondin N-terminal -like domains.	3	17	1.19	0.0463	THBS1,THBS4,COL18A1
SMART	SM00001	EGF domain, unclasssified subfamily	5	70	0.8	0.0475	ATRN,STAB1,OIT3,FBLN5,EFEMP1
SMART	SM00131	BPTI/Kunitz family of serine protease inhibitors.	3	18	1.17	0.0476	TFPI2,AMBP,PAPLN

UC vs NCFET T1

#category	term ID	term description	observed gene count	background gene count	strength	false discovery rate	matching proteins in your network (labels)
GO Process	GO:0006959	Humoral immune response	42	268	1.14	8.09e-29	ST6GAL1,EBI3,PF4V1,LTF,HRG,C4BPB,CFP,FCN3,SERPING1,FCN2,PF4,MASP1,CAMP,FGB,F2,C1QB,CFHR1,C7,ANG,C8A,CFHR3,CFH,S100A7,S100A9,C8B,C8G,C1QC,CFI,GAPDH,MASP2,C1S,C4A,COLEC11,C4B,CFB,RARRES2,C1R,CFD,IGHV3-15,IGHV3-72,KNG1,B2M

GO Process	GO:0006950	Response to stress	117	3358	0.48	2.59e-28	CPB2,SERPIND1,CHGA,F9,MMP2,COMP,MPO,PF4V1,LTF,HRG,PROC,PLEK,CAT,LRP1,C4BPB,CFP,APOE,PXDN,F12,APCS,ORM1,VWF,SERPINA10,ATRN,YWHAE,KLKB1,DGKG,TCIRG1,HPX,HYAL1,ITIH4,FCN3,AHSG,CRH,SAA4,SERPING1,CSF1R,FCN2,PGLYRP2,PF4,MASP1,CAMP,HSP90B1,LRG1,PCSK9,SHLD1,FGB,DST,F2,PLG,C1QB,CFHR1,APOL1,SUMO4,CALR,SERPINF2,C7,HBA1,HSPA5,P4HB,FBLN1,ANG,THBS4,PROZ,APOD,BLM,APOA4,C8A,F13B,CFH,F5,APOA2,CD5L,S100A7,S100A9,FLNA,MCOLN2,C8B,C8G,GSN,C1QC,F10,F7,TAF9,SOD3,GPX3,CFI,TNRC6A,ILK,GAPDH,RNASE1,CST3,MASP2,F11,C1S,ADIPOQ,ORM2,C4A,CDK3,COLEC11,MST1,C4B,CFB,RARRES2,UIMC1,SELENOP,C1R,SERPINA3,RNASE4,CFD,IGHV3-15,IGHV3-72,VEGFC,HYOU1,KNG1,B2M,PDIA4
GO Process	GO:0006956	Complement activation	26	60	1.58	1.27e-26	C4BPB,CFP,FCN3,SERPING1,FCN2,MASP1,C1QB,CFHR1,C7,C8A,CFHR3,CFH,C8B,C8G,C1QC,CFI,MASP2,C1S,C4A,COLEC11,C4B,CFB,C1R,CFD,IGHV3-15,IGHV3-72
GO Process	GO:0006952	Defense response	69	1394	0.63	8.50e-22	CHGA,MPO,PF4V1,LTF,HRG,LRP1,C4BPB,CFP,F12,APCS,ORM1,ATRN,KLKB1,TCIRG1,HPX,HYAL1,ITIH4,FCN3,AHSG,CRH,SAA4,SERPING1,CSF1R,FCN2,PGLYRP2,PF4,MASP1,CAMP,FGB,F2,C1QB,CFHR1,APOL1,SERPINF2,C7,ANG,APOA4,C8A,CFH,APOA2,CD5L,S100A7,S100A9,MCOLN2,C8B,C8G,GSN,C1QC,F7,CFI,GAPDH,RNASE1,CST3,MASP2,C1S,ORM2,C4A,COLEC11,C4B,CFB,RARRES2,C1R,SERPINA3,RNASE4,CFD,IGHV3-15,IGHV3-72,KNG1,B2M
GO Process	GO:0007596	Blood coagulation	29	173	1.16	3.49e-20	CPB2,SERPIND1,F9,COMP,PF4V1,HRG,PROC,PLEK,C4BPB,F12,VWF,SERPINA10,KLKB1,DGKG,SERPING1,PF4,FGB,F2,PLG,FBLN1,PROZ,F13B,F5,FLNA,F10,F7,ILK,F11,KNG1
GO Process	GO:0006955	Immune response	61	1321	0.6	1.62e-17	ST6GAL1,LGALS1,CHGA,EBI3,PF4V1,LTF,HRG,LRP1,C4BPB,CFP,PXDN,F12,APCS,TCIRG1,HPX,FCN3,SERPING1,CSF1R,FCN2,PGLYRP2,PF4,MASP1,CAMP,SHLD1,FGB,F2,C1QB,CFHR1,APOL1,C7,ANG,PIGR,APOA4,C8A,CFHR3,CFH,S100A7,S100A9,MCOLN2,C8B,C8G,GSN,C1QC,CFI,GAPDH,RAPGEF4,FGL1,MASP2,C1S,C4A,COLEC11,C4B,CFB,RARRES2,C1R,IGKV2D-28,CFD,IGHV3-15,IGHV3-72,KNG1,B2M
GO Process	GO:0050896	Response to stimulus	159	7835	0.25	4.03e-17	ST6GAL1,CPB2,SERPIND1,LGALS1,CHGA,F9,MMP2,EBI3,COMP,MPO,PF4V1,APOC3,GPLD1,LTF,HRG,APOB,IGFBP5,PROC,PLEK,APOA1,TTR,CAT,LRP1,C4BPB,CFP,APOE,PXDN,F12,APCS,ORM1,VWF,SERPINA10,ATRN,LCAT,YWHAE,KLKB1,DGKG,PON3,TCIRG1,HPX,HYAL1,ITIH4,FCN3,AHSG,CRH,SAA4,SERPING1,CSF1R,PIP,FCN2,PSG6,PGLYRP2,SCGB3A1,IHH,PF4,MASP1,CAMP,GUCY1A1,HSP90B1,LRG1,PCSK9,BMP1,SHLD1,FGB,DST,F2,INHBC,PLG,CSPG4,C1QB,CFHR1,APOL1,SUMO4,CALR,SERPINF2,C7,HBA1,ITLN1,HSPA5,FBN1,P4HB,FBLN1,ANG,LIMS1,THBS4,PROZ,APOD,ASGR2,BLM,AKAP9,PIGR,APOA4,C8A,CENPF,F13B,CFHR3,CFH,F5,APOA2,CD5L,S100A7,S100A9,FLNA,MCOLN2,C8B,C8G,CCN5,GSN,SVEP1,C1QC,F10,F7,APOM,CA6,CALML5,TAF9,IGFBP3,SOD3,GPX3,EFEMP1,CFI,TNRC6A,ILK,GAPDH,RAPGEF4,RNASE1,FGL1,CST3,MASP2,F11,C1S,NUCB1,ADIPOQ,IGF2,ORM2,C4A,CDK3,COLEC11,MST1,C4B,TGFBI,CFB,IGFALS,RARRES2,UIMC1,SELENOP,C1R,SERPINA3,RNASE4,IGKV2D-28,CFD,IGHV3-15,IGHV3-72,VEGFC,HYOU1,KNG1,CDH5,B2M,PDIA4
GO Process	GO:0098542	Defense response to other organism	52	989	0.66	8.87e-17	CHGA,MPO,PF4V1,LTF,HRG,C4BPB,CFP,F12,APCS,HPX,FCN3,SERPING1,CSF1R,FCN2,PGLYRP2,PF4,MASP1,CAMP,FGB,F2,C1QB,CFHR1,APOL1,C7,ANG,APOA4,C8A,CFH,S100A7,S100A9,MCOLN2,C8B,C8G,GSN,C1QC,CFI,GAPDH,RNASE1,MASP2,C1S,C4A,COLEC11,C4B,CFB,RARRES2,C1R,RNASE4,CFD,IGHV3-15,IGHV3-72,KNG1,B2M
GO Process	GO:0002252	Immune effector process	34	375	0.9	1.15e-16	LGALS1,CHGA,MPO,LRP1,C4BPB,CFP,TCIRG1,FCN3,SERPING1,FCN2,MASP1,SHLD1,F2,C1QB,CFHR1,C7,C8A,CFHR3,CFH,C8B,C8G,C1QC,CFI,MASP2,C1S,C4A,COLEC11,C4B,CFB,C1R,CFD,IGHV3-15,IGHV3-72,B2M
GO Process	GO:0010466	Negative regulation of peptidase activity	28	249	0.99	1.39e-15	SPP2,SERPIND1,LTF,HRG,LRP1,SERPINA10,CSTA,ITIH4,ITIH1,AHSG,SERPING1,SERPINI1,A2ML1,LPA,SERPINF2,SERPINA7,SERPINA5,SERPINA11,PAPLN,ITIH2,GAPDH,CST3,C4A,ITIH3,C4B,SERPINA3,SERPINA4,KNG1
GO Process	GO:0006958	Complement activation, classical pathway	16	40	1.54	1.41e-15	C4BPB,SERPING1,C1QB,C7,C8A,C8B,C8G,C1QC,CFI,MASP2,C1S,C4A,C4B,C1R,IGHV3-15,IGHV3-72
GO Process	GO:0009607	Response to biotic stimulus	59	1375	0.57	1.41e-15	CHGA,MPO,PF4V1,LTF,HRG,APOB,C4BPB,CFP,F12,APCS,HPX,HYAL1,FCN3,SERPING1,CSF1R,FCN2,PGLYRP2,PF4,MASP1,CAMP,LRG1,FGB,F2,C1QB,CFHR1,APOL1,CALR,C7,ITLN1,HSPA5,ANG,APOA4,C8A,CFH,S100A7,S100A9,MCOLN2,C8B,C8G,GSN,C1QC,CFI,GAPDH,RNASE1,MASP2,C1S,ADIPOQ,C4A,COLEC11,C4B,CFB,RARRES2,C1R,RNASE4,CFD,IGHV3-15,IGHV3-72,KNG1,B2M
GO Process	GO:0051346	Negative regulation of hydrolase activity	32	354	0.9	1.41e-15	SPP2,SERPIND1,APOC3,LTF,HRG,APOA1,LRP1,APCS,SERPINA10,CSTA,ITIH4,ITIH1,AHSG,SERPING1,SERPINI1,A2ML1,LPA,SERPINF2,SERPINA7,SERPINA5,SERPINA11,PAPLN,ITIH2,APOA2,GAPDH,CST3,C4A,ITIH3,C4B,SERPINA3,SERPINA4,KNG1
GO Process	GO:0051707	Response to other organism	58	1328	0.58	1.41e-15	CHGA,MPO,PF4V1,LTF,HRG,APOB,C4BPB,CFP,F12,APCS,HPX,HYAL1,FCN3,SERPING1,CSF1R,FCN2,PGLYRP2,PF4,MASP1,CAMP,LRG1,FGB,F2,C1QB,CFHR1,APOL1,CALR,C7,ITLN1,ANG,APOA4,C8A,CFH,S100A7,S100A9,MCOLN2,C8B,C8G,GSN,C1QC,CFI,GAPDH,RNASE1,MASP2,C1S,ADIPOQ,C4A,COLEC11,C4B,CFB,RARRES2,C1R,RNASE4,CFD,IGHV3-15,IGHV3-72,KNG1,B2M
GO Process	GO:0002682	Regulation of immune system process	60	1438	0.56	1.86e-15	LGALS1,EBI3,GPLD1,LTF,HRG,APOA1,C4BPB,CFP,APOE,APCS,ORM1,HPX,FCN3,SERPING1,CSF1R,PIP,FCN2,PSG6,PGLYRP2,IHH,PF4,MASP1,SHLD1,C1QB,CFHR1,CALR,C7,FBN1,THBS4,APOD,PIGR,C8A,CFHR3,CFH,APOA2,CD5L,S100A7,C8B,C8G,C1QC,F7,CFI,FGL1,MASP2,C1S,ADIPOQ,IGF2,ORM2,C4A,COLEC11,MST1,C4B,CFB,RARRES2,C1R,CFD,IGHV3-15,IGHV3-72,VEGFC,B2M
GO Process	GO:0042060	Wound healing	31	336	0.91	2.03e-15	CPB2,SERPIND1,F9,COMP,PF4V1,HRG,PROC,PLEK,C4BPB,F12,VWF,SERPINA10,KLKB1,DGKG,SERPING1,PF4,LRG1,FGB,DST,F2,PLG,FBLN1,PROZ,F13B,F5,FLNA,F10,F7,ILK,F11,KNG1
GO Process	GO:0045861	Negative regulation of proteolysis	31	339	0.9	2.44e-15	SPP2,CPB2,SERPIND1,LTF,HRG,LRP1,SERPINA10,CSTA,ITIH4,ITIH1,AHSG,SERPING1,SERPINI1,A2ML1,F2,LPA,SERPINF2,SERPINA7,SERPINA5,SERPINA11,PAPLN,ITIH2,TAF9,GAPDH,CST3,C4A,ITIH3,C4B,SERPINA3,SERPINA4,KNG1
GO Process	GO:0045087	Innate immune response	43	754	0.7	6.64e-15	CHGA,LTF,C4BPB,CFP,F12,APCS,HPX,FCN3,SERPING1,CSF1R,FCN2,PGLYRP2,MASP1,CAMP,FGB,C1QB,APOL1,C7,ANG,APOA4,C8A,CFH,S100A7,S100A9,MCOLN2,C8B,C8G,GSN,C1QC,CFI,GAPDH,MASP2,C1S,C4A,COLEC11,C4B,CFB,RARRES2,C1R,CFD,IGHV3-15,IGHV3-72,B2M
GO Process	GO:0009611	Response to wounding	34	444	0.82	6.72e-15	CPB2,SERPIND1,F9,MMP2,COMP,PF4V1,HRG,PROC,PLEK,C4BPB,F12,VWF,SERPINA10,KLKB1,DGKG,SERPING1,PF4,LRG1,FGB,DST,F2,PLG,FBLN1,PROZ,APOD,APOA4,F13B,F5,FLNA,F10,F7,ILK,F11,KNG1
GO Process	GO:0044419	Biological process involved in interspecies interaction between organisms	60	1490	0.55	6.80e-15	CHGA,MPO,PF4V1,LTF,HRG,APOB,C4BPB,CFP,APOE,F12,APCS,HPX,HYAL1,FCN3,SERPING1,CSF1R,FCN2,PGLYRP2,PF4,MASP1,CAMP,LRG1,FGB,F2,PLG,C1QB,CFHR1,APOL1,CALR,C7,ITLN1,ANG,APOA4,C8A,CFH,S100A7,S100A9,MCOLN2,C8B,C8G,GSN,C1QC,CFI,GAPDH,RNASE1,MASP2,C1S,ADIPOQ,C4A,COLEC11,C4B,CFB,RARRES2,C1R,RNASE4,CFD,IGHV3-15,IGHV3-72,KNG1,B2M

GO Process	GO:0050878	Regulation of body fluid levels	31	371	0.86	2.00e-14	CPB2,SERPIND1,F9,COMP,PF4V1,HRG,PROC,PLEK,C4BPB,APOE,F12,VWF,SERPINA10,KLKB1,DGKG,SERPING1,PF4,FGB,F2,PLG,SERPINF2,FBLN1,PROZ,F13B,F5,FLNA,F10,F7,ILK,F11,KNG1
GO Process	GO:0002253	Activation of immune response	27	271	0.94	4.36e-14	GPLD1,C4BPB,CFP,FCN3,SERPING1,FCN2,MASP1,C1QB,CFHR1,C7,C8A,CFHR3,CFH,C8B,C8G,C1QC,CFI,MASP2,C1S,C4A,COLEC11,C4B,CFB,C1R,CFD,IGHV3-15,IGHV3-72
GO Process	GO:0052547	Regulation of peptidase activity	33	446	0.81	4.69e-14	SPP2,SERPIND1,PCOLCE,LTF,HRG,LRP1,SERPINA10,CSTA,ITIH4,ITIH1,AHSG,SERPING1,SERPINI1,A2ML1,ANTXR1,LPA,SERPINF2,SERPINA7,FBLN1,SERPINA5,SERPINA11,PAPLN,ITIH2,S100A9,GSN,GAPDH,CST3,C4A,ITIH3,C4B,SERPINA3,SERPINA4,KNG1
GO Process	GO:0030162	Regulation of proteolysis	41	739	0.68	8.42e-14	SPP2,CPB2,SERPIND1,PCOLCE,GPLD1,LTF,HRG,LRP1,APOE,F12,SERPINA10,CSTA,KLKB1,ITIH4,ITIH1,AHSG,SERPING1,SERPINI1,A2ML1,ANTXR1,SH3D19,F2,LPA,SERPINF2,SERPINA7,FBLN1,SERPINA5,SERPINA11,PAPLN,ITIH2,S100A9,GSN,TAF9,GAPDH,CST3,C4A,ITIH3,C4B,SERPINA3,SERPINA4,KNG1
GO Process	GO:0048583	Regulation of response to stimulus	100	3931	0.35	1.05e-13	ST6GAL1,CPB2,LGALS1,CHGA,APOC3,GPLD1,LTF,HRG,IGFBP5,PROC,PLEK,APOA1,CAT,LRP1,C4BPB,CFP,APOE,PXDN,F12,APCS,VWF,PSMA6,KLKB1,DGKG,HPX,FCN3,AHSG,CRH,SERPING1,CSF1R,FCN2,PGLYRP2,IHH,PF4,MASP1,GUCY1A1,LRG1,PCSK9,SHLD1,FGB,F2,INHBC,PLG,CSPG4,C1QB,CFHR1,SUMO4,CALR,SERPINF2,C7,HSPA5,FBN1,P4HB,FBLN1,LIMS1,THBS4,APOD,GIT2,AKAP9,PIGR,C8A,CFHR3,CFH,CD5L,S100A7,S100A9,FLNA,C8B,C8G,C1QC,F10,F7,TAF9,IGFBP3,CFI,ILK,GAPDH,FGL1,MASP2,F11,C1S,ADIPOQ,IGF2,C4A,CDK3,COLEC11,MST1,C4B,CFB,RARRES2,UIMC1,C1R,CFD,IGHV3-15,IGHV3-72,VEGFC,HYOU1,KNG1,CDH5,B2M
GO Process	GO:0048584	Positive regulation of response to stimulus	70	2131	0.46	1.63e-13	CPB2,LGALS1,CHGA,GPLD1,LTF,HRG,IGFBP5,APOA1,CAT,C4BPB,CFP,APOE,F12,VWF,HPX,FCN3,SERPING1,CSF1R,FCN2,IHH,MASP1,GUCY1A1,LRG1,SHLD1,FGB,F2,INHBC,PLG,CSPG4,C1QB,CFHR1,CALR,SERPINF2,C7,FBN1,LIMS1,THBS4,C8A,CFHR3,CFH,S100A7,S100A9,FLNA,C8B,C8G,C1QC,F10,F7,TAF9,IGFBP3,CFI,ILK,GAPDH,MASP2,C1S,ADIPOQ,IGF2,C4A,COLEC11,C4B,CFB,RARRES2,UIMC1,C1R,CFD,IGHV3-15,IGHV3-72,VEGFC,CDH5,B2M
GO Process	GO:0016064	Immunoglobulin mediated immune response	18	95	1.22	3.12e-13	C4BPB,TCIRG1,SERPING1,SHLD1,C1QB,C7,C8A,C8B,C8G,C1QC,CFI,MASP2,C1S,C4A,C4B,C1R,IGHV3-15,IGHV3-72
GO Process	GO:0002684	Positive regulation of immune system process	43	874	0.63	7.08e-13	LGALS1,EBI3,GPLD1,HRG,C4BPB,CFP,HPX,FCN3,SERPING1,CSF1R,FCN2,IHH,PF4,MASP1,SHLD1,C1QB,CFHR1,CALR,C7,THBS4,C8A,CFHR3,CFH,S100A7,C8B,C8G,C1QC,F7,CFI,MASP2,C1S,IGF2,C4A,COLEC11,C4B,CFB,RARRES2,C1R,CFD,IGHV3-15,IGHV3-72,VEGFC,B2M
GO Process	GO:0002376	Immune system process	68	2121	0.45	1.48e-12	ST6GAL1,LGALS1,CHGA,EBI3,MPO,PF4V1,GPLD1,LTF,HRG,PLEK,LRP1,C4BPB,CFP,PXDN,F12,APCS,TCIRG1,HPX,FCN3,SERPING1,CSF1R,FCN2,PGLYRP2,PF4,MASP1,CAMP,SHLD1,FGB,F2,PLG,C1QB,CFHR1,APOL1,CALR,C7,ANG,PIGR,APOA4,C8A,CFHR3,CFH,CD5L,S100A7,S100A9,FLNA,MCOLN2,C8B,C8G,GSN,C1QC,CFI,GAPDH,RAPGEF4,FGL1,MASP2,C1S,C4A,COLEC11,C4B,CFB,RARRES2,C1R,IGKV2D-28,CFD,IGHV3-15,IGHV3-72,KNG1,B2M
GO Process	GO:0072378	Blood coagulation, fibrin clot formation	12	24	1.64	1.48e-12	F9,F12,KLKB1,FGB,F2,FBLN1,F13B,F5,FLNA,F10,F7,F11
GO Process	GO:1900047	Negative regulation of hemostasis	14	47	1.41	2.32e-12	CPB2,COMP,HRG,PROC,APOE,F12,KLKB1,SERPING1,FGB,F2,PLG,SERPINF2,F11,KNG1
GO Process	GO:0048518	Positive regulation of biological process	128	6207	0.25	3.47e-12	ST6GAL1,CPB2,CETP,LGALS1,CHGA,MMP2,EBI3,COMP,PCOLCE,GPLD1,LTF,HRG,APOB,IGFBP5,PROC,PLEK,APOA1,CAT,LRP1,C4BPB,CFP,APOE,F12,ORM1,VWF,YWHAE,KLKB1,CENPE,TCIRG1,HPX,HYAL1,FCN3,AHSG,ANKRD31,CRH,SERPING1,CSF1R,PIP,FCN2,SCGB3A1,IHH,SERPINI1,PF4,MASP1,CAMP,GUCY1A1,ANTXR1,LRG1,SH3D19,PCSK9,BMP1,SHLD1,FGB,F2,INHBC,PLG,EFEMP2,CSPG4,C1QB,CFHR1,CALR,SERPINF2,C7,HBA1,ITLN1,HSPA5,FBN1,P4HB,FBLN1,ANG,LIMS1,THBS4,BLM,AKAP9,APOA4,MTCL1,C8A,CFHR3,CFH,APOA2,CD5L,S100A7,S100A9,ADAMTSL4,FLNA,MCOLN2,SMARCA1,C8B,C8G,GSN,C1QC,F10,F7,TAF9,IGFBP3,CFI,TNRC6A,ILK,GAPDH,MASP2,F11,C1S,ADIPOQ,IGF2,ORM2,C4A,SLAIN1,ABCA13,COLEC11,MST1,LMOD3,C4B,CFB,PLTP,PAEP,RARRES2,UIMC1,C1R,APOA5,APOC4,CFD,IGHV3-15,IGHV3-72,VEGFC,KNG1,GRHL2,CDH5,B2M
GO Process	GO:0051336	Regulation of hydrolase activity	45	1011	0.59	3.90e-12	SPP2,SERPIND1,PCOLCE,APOC3,GPLD1,LTF,HRG,PLEK,APOA1,LRP1,APCS,SERPINA10,CSTA,ITIH4,ITIH1,AHSG,SERPING1,SERPINI1,A2ML1,HSP90B1,ANTXR1,LPA,SERPINF2,SERPINA7,FBLN1,SERPINA5,SERPINA11,ANG,LIMS1,PAPLN,APOA4,ITIH2,APOA2,S100A9,GSN,GAPDH,RAPGEF4,CST3,C4A,ITIH3,C4B,APOA5,SERPINA3,SERPINA4,KNG1
GO Process	GO:0009605	Response to external stimulus	71	2355	0.42	5.20e-12	SERPIND1,CHGA,MMP2,MPO,PF4V1,LTF,HRG,APOB,APOA1,CAT,C4BPB,CFP,APOE,F12,APCS,HPX,HYAL1,FCN3,SERPING1,CSF1R,FCN2,PGLYRP2,IHH,PF4,MASP1,CAMP,LRG1,PCSK9,FGB,F2,C1QB,CFHR1,APOL1,CALR,C7,ITLN1,HSPA5,FBN1,ANG,APOA4,C8A,CFH,F5,S100A7,S100A9,MCOLN2,C8B,C8G,GSN,C1QC,F7,CFI,TNRC6A,GAPDH,RNASE1,MASP2,C1S,ADIPOQ,C4A,COLEC11,C4B,CFB,RARRES2,C1R,RNASE4,CFD,IGHV3-15,IGHV3-72,VEGFC,KNG1,B2M
GO Process	GO:0034369	Plasma lipoprotein particle remodeling	12	29	1.56	7.47e-12	CETP,MPO,APOC3,APOB,APOA1,APOE,LCAT,APOA4,APOA2,APOM,PLTP,APOA5
GO Process	GO:0052548	Regulation of endopeptidase activity	29	414	0.79	9.59e-12	SPP2,SERPIND1,LTF,HRG,SERPINA10,CSTA,ITIH4,ITIH1,AHSG,SERPING1,SERPINI1,A2ML1,LPA,SERPINF2,SERPINA7,SERPINA5,SERPINA11,PAPLN,ITIH2,S100A9,GSN,GAPDH,CST3,C4A,ITIH3,C4B,SERPINA3,SERPINA4,KNG1
GO Process	GO:0002460	Adaptive immune response based on somatic recombination of immune receptors built from immunoglobulin superfamily domains	20	169	1.01	1.65e-11	EBI3,C4BPB,TCIRG1,SERPING1,SHLD1,C1QB,C7,C8A,C8B,C8G,C1QC,CFI,MASP2,C1S,C4A,C4B,C1R,IGHV3-15,IGHV3-72,B2M
GO Process	GO:1900046	Regulation of hemostasis	15	72	1.26	1.77e-11	CPB2,COMP,HRG,PROC,APOE,F12,KLKB1,SERPING1,FGB,F2,PLG,SERPINF2,F7,F11,KNG1
GO Process	GO:0050778	Positive regulation of immune response	31	502	0.73	2.57e-11	GPLD1,HRG,C4BPB,CFP,HPX,FCN3,SERPING1,FCN2,MASP1,SHLD1,C1QB,CFHR1,C7,C8A,CFHR3,CFH,C8B,C8G,C1QC,CFI,MASP2,C1S,C4A,COLEC11,C4B,CFB,C1R,CFD,IGHV3-15,IGHV3-72,B2M
GO Process	GO:0051246	Regulation of protein metabolic process	74	2622	0.39	2.69e-11	SPP2,CPB2,SERPIND1,PCOLCE,GPLD1,LTF,HRG,IGFBP5,APOA1,LRP1,C4BPB,APOE,F12,APCS,SERPINA10,YWHAE,CSTA,KLKB1,CENPE,HPX,ITIH4,ITIH1,AHSG,SERPING1,CSF1R,SERPINI1,CAMP,A2ML1,HSP90B1,ANTXR1,SH3D19,PCSK9,F2,INHBC,CSPG4,CALR,LPA,SERPINF2,ITLN1,HSPA5,FBN1,SERPINA7,FBLN1,SERPINA5,SERPINA11,ANG,THBS4,APOD,PAPLN,BLM,AKAP9,ITIH2,S100A9,FLNA,GSN,TAF9,IGFBP3,TNRC6A,ILK,GAPDH,CST3,ADIPOQ,IGF2,C4A,MST1,ITIH3,C4B,PAEP,RARRES2,SERPINA3,SERPINA4,VEGFC,KNG1,CDH5
GO Process	GO:0030195	Negative regulation of blood coagulation	13	46	1.39	2.91e-11	CPB2,HRG,PROC,APOE,F12,KLKB1,SERPING1,FGB,F2,PLG,SERPINF2,F11,KNG1
GO Process	GO:0002449	Lymphocyte mediated immunity	19	159	1.02	5.49e-11	C4BPB,TCIRG1,SERPING1,SHLD1,C1QB,C7,C8A,C8B,C8G,C1QC,CFI,MASP2,C1S,C4A,C4B,C1R,IGHV3-15,IGHV3-72,B2M
GO Process	GO:0032101	Regulation of response to external stimulus	42	964	0.58	5.49e-11	ST6GAL1,CPB2,LGALS1,LTF,HRG,PROC,APOA1,APOE,F12,APCS,PSMA6,KLKB1,HPX,FCN3,AHSG,SERPING1,CSF1R,FCN2,PGLYRP2,MASP1,FGB,F2,PLG,CALR,SERPINF2,FBN1,THBS4,CFH,S100A7,S100A9,FLNA,F7,CFI,MASP2,F11,ADIPOQ,COLEC11,MST1,RARRES2,VEGFC,KNG1,CDH5

GO Process	GO:0065007	Biological regulation	192	12385	0.13	6.91e-11	SPP2,ST6GAL1,CPB2,CETP,SERPIND1,LGALS1,CHGA,TPD52L2,F9,MMP2,EBI3,COMP,PCOLCE,MPO,PF4V1,APOC3,GPLD1,LTF,HRG,APOB,IGFBP5,PROC,PLEK,APOA1,TTR,CAT,LRP1,C4BPB,CFP,APOE,PXDN,F12,APCS,ORM1,VWF,PSMA6,SERPINA10,ATRN,LCAT,YWHAE,CSTA,CP,KLKB1,DGKG,CENPE,PON3,TCIRG1,HPX,HYAL1,ITIH4,FCN3,ITIH1,AHSG,ANKRD31,CRH,SERPING1,CSF1R,PIP,FCN2,PSG6,PGLYRP2,SCGB3A1,IHH,SERPINI1,PF4,MASP1,CAMP,GUCY1A1,A2ML1,HSP90B1,ANTXR1,LRG1,SH3D19,PCSK9,BMP1,SHLD1,FGB,DST,F2,INHBC,PLG,EFEMP2,CSPG4,C1QB,CFHR1,SUMO4,CPN2,CALR,LPA,SERPINF2,C7,HBA1,ITLN1,HSPA5,FBN1,P4HB,SERPINA7,FBLN1,SERPINA5,OIT3,SERPINA11,ANG,LIMS1,THBS4,PROZ,APOD,PAPLN,ASGR2,BLM,GIT2,AKAP9,PIGR,APOA4,ITIH2,CNDP1,MTCL1,C8A,CENPF,F13B,CFHR3,CFH,QSOX1,F5,APOA2,CD5L,S100A7,S100A9,ADAMTSL4,FLNA,MCOLN2,SMARCA1,C8B,C8G,CCN5,GSN,SVEP1,C1QC,F10,F7,APOM,CALML5,TAF9,IGFBP3,SOD3,EFEMP1,CFI,TNRC6A,NAV2,ILK,GAPDH,RAPGEF4,FGL1,CST3,MASP2,F11,C1S,NUCB1,ADIPOQ,IGF2,ORM2,C4A,ATP9B,SLAIN1,CDK3,ABCA13,COLEC11,MST1,LMOD3,ITIH3,C4B,TGFBI,CFB,IGFALS,PLTP,PAEP,RARRES2,UIMC1,SELENOP,C1R,APOA5,SERPINA3,SERPINA4,APOC4,CFD,IGHV3-15,IGHV3-72,VEGFC,HYOU1,KNG1,GRHL2,CDH5,B2M
GO Process	GO:0002443	Leukocyte mediated immunity	21	211	0.94	7.02e-11	CHGA,C4BPB,TCIRG1,SERPING1,SHLD1,F2,C1QB,C7,C8A,C8B,C8G,C1QC,CFI,MASP2,C1S,C4A,C4B,C1R,IGHV3-15,IGHV3-72,B2M
GO Process	GO:1903027	Regulation of opsonization	10	18	1.69	8.88e-11	C4BPB,CFP,FCN3,FCN2,MASP1,MASP2,C4A,COLEC11,C4B,CFB
GO Process	GO:0051248	Negative regulation of protein metabolic process	43	1038	0.56	1.24e-10	SPP2,CPB2,SERPIND1,LTF,HRG,IGFBP5,LRP1,APOE,APCS,SERPINA10,YWHAE,CSTA,ITIH4,ITIH1,AHSG,SERPING1,SERPINI1,A2ML1,F2,CALR,LPA,SERPINF2,SERPINA7,FBLN1,SERPINA5,SERPINA11,ANG,APOD,PAPLN,ITIH2,FLNA,TAF9,IGFBP3,TNRC6A,GAPDH,CST3,ADIPOQ,C4A,ITIH3,C4B,SERPINA3,SERPINA4,KNG1
GO Process	GO:0061045	Negative regulation of wound healing	14	69	1.25	1.39e-10	CPB2,HRG,PROC,APOE,F12,APCS,KLKB1,SERPING1,FGB,F2,PLG,SERPINF2,F11,KNG1
GO Process	GO:0097006	Regulation of plasma lipoprotein particle levels	13	54	1.32	1.43e-10	CETP,MPO,APOC3,APOB,APOA1,APOE,LCAT,APOA4,APOA2,APOM,ADIPOQ,PLTP,APOA5
GO Process	GO:0030193	Regulation of blood coagulation	14	70	1.24	1.60e-10	CPB2,HRG,PROC,APOE,F12,KLKB1,SERPING1,FGB,F2,PLG,SERPINF2,F7,F11,KNG1
GO Process	GO:0048519	Negative regulation of biological process	111	5313	0.26	4.06e-10	SPP2,ST6GAL1,CPB2,CETP,SERPIND1,CHGA,MMP2,COMP,MPO,APOC3,GPLD1,LTF,HRG,IGFBP5,PROC,PLEK,APOA1,CAT,LRP1,C4BPB,APOE,PXDN,F12,APCS,ORM1,SERPINA10,YWHAE,CSTA,KLKB1,DGKG,PON3,HYAL1,ITIH4,FCN3,ITIH1,AHSG,CRH,SERPING1,CSF1R,PIP,PGLYRP2,SCGB3A1,IHH,SERPINI1,PF4,MASP1,GUCY1A1,A2ML1,HSP90B1,ANTXR1,PCSK9,SHLD1,FGB,F2,PLG,EFEMP2,SUMO4,CALR,LPA,SERPINF2,HSPA5,FBN1,SERPINA7,FBLN1,SERPINA5,SERPINA11,ANG,LIMS1,THBS4,APOD,PAPLN,BLM,APOA4,ITIH2,CENPF,QSOX1,APOA2,FLNA,MCOLN2,CCN5,GSN,C1QC,APOM,TAF9,IGFBP3,EFEMP1,TNRC6A,ILK,GAPDH,FGL1,CST3,F11,ADIPOQ,IGF2,C4A,CDK3,MST1,LMOD3,ITIH3,C4B,TGFBI,PAEP,UIMC1,SERPINA3,SERPINA4,VEGFC,HYOU1,KNG1,GRHL2,CDH5,B2M
GO Process	GO:0051241	Negative regulation of multicellular organismal process	42	1035	0.55	4.08e-10	CPB2,MMP2,APOC3,LTF,HRG,IGFBP5,PROC,APOA1,APOE,F12,APCS,ORM1,KLKB1,AHSG,CRH,SERPING1,PGLYRP2,IHH,PF4,GUCY1A1,PCSK9,FGB,F2,PLG,CALR,SERPINF2,FBN1,FBLN1,THBS4,APOD,APOA4,APOA2,C1QC,APOM,EFEMP1,CST3,F11,ADIPOQ,PAEP,KNG1,GRHL2,B2M
GO Process	GO:0002250	Adaptive immune response	25	359	0.78	4.92e-10	EBI3,C4BPB,TCIRG1,SERPING1,SHLD1,FGB,C1QB,C7,C8A,MCOLN2,C8B,C8G,C1QC,CFI,RAPGEF4,FGL1,MASP2,C1S,C4A,C4B,C1R,IGKV2D-28,IGHV3-15,IGHV3-72,B2M
GO Process	GO:0034375	High-density lipoprotein particle remodeling	9	16	1.69	1.08e-09	CETP,APOC3,APOA1,APOE,LCAT,APOA4,APOA2,APOM,PLTP
GO Process	GO:1903028	Positive regulation of opsonization	9	16	1.69	1.08e-09	CFP,FCN3,FCN2,MASP1,MASP2,C4A,COLEC11,C4B,CFB
GO Process	GO:0050776	Regulation of immune response	37	844	0.58	1.16e-09	GPLD1,LTF,HRG,C4BPB,CFP,APOE,HPX,FCN3,SERPING1,FCN2,PGLYRP2,MASP1,SHLD1,C1QB,CFHR1,C7,PIGR,C8A,CFHR3,CFH,CD5L,C8B,C8G,C1QC,CFI,FGL1,MASP2,C1S,C4A,COLEC11,C4B,CFB,C1R,CFD,IGHV3-15,IGHV3-72,B2M
GO Process	GO:0043086	Negative regulation of catalytic activity	35	771	0.6	1.81e-09	SPP2,SERPIND1,APOC3,LTF,HRG,APOA1,LRP1,APOE,APCS,SERPINA10,CSTA,ITIH4,ITIH1,AHSG,SERPING1,SERPINI1,A2ML1,LPA,SERPINF2,SERPINA7,SERPINA5,SERPINA11,PAPLN,AKAP9,ITIH2,APOA2,GAPDH,CST3,ADIPOQ,C4A,ITIH3,C4B,SERPINA3,SERPINA4,KNG1
GO Process	GO:0044092	Negative regulation of molecular function	43	1143	0.52	2.11e-09	SPP2,SERPIND1,APOC3,LTF,HRG,APOA1,CAT,LRP1,APOE,PXDN,APCS,SERPINA10,YWHAE,CSTA,ITIH4,ITIH1,AHSG,SERPING1,SERPINI1,A2ML1,PCSK9,CFHR1,SUMO4,LPA,SERPINF2,SERPINA7,SERPINA5,SERPINA11,PAPLN,AKAP9,ITIH2,APOA2,FLNA,GAPDH,CST3,ADIPOQ,C4A,ITIH3,C4B,SERPINA3,SERPINA4,KNG1,B2M
GO Process	GO:0006508	Proteolysis	45	1247	0.5	2.42e-09	CPB2,F9,MMP2,COMP,PCOLCE,LTF,PROC,F12,PSMA6,KLKB1,FCN3,HABP2,PIP,FCN2,IHH,MASP1,HSP90B1,PCSK9,BMP1,FGB,F2,PLG,UBE3C,CALR,LPA,HSPA5,PROZ,CNDP1,CFH,F5,CD5L,F10,F7,TAF9,CFI,MASP2,F11,C1S,PCYOX1,COLEC11,MST1,CFB,UIMC1,C1R,CFD
GO Process	GO:0042730	Fibrinolysis	9	19	1.62	3.22e-09	CPB2,HRG,F12,KLKB1,SERPING1,FGB,F2,PLG,SERPINF2
GO Process	GO:0050766	Positive regulation of phagocytosis	13	75	1.18	4.31e-09	APOA1,CFP,FCN3,AHSG,FCN2,MASP1,CALR,APOA2,MASP2,C4A,COLEC11,C4B,CFB
GO Process	GO:0050789	Regulation of biological process	181	11655	0.13	4.60e-09	SPP2,ST6GAL1,CPB2,CETP,SERPIND1,LGALS1,CHGA,TPD52L2,MMP2,EBI3,COMP,PCOLCE,MPO,PF4V1,APOC3,GPLD1,LTF,HRG,APOB,IGFBP5,PROC,PLEK,APOA1,TTR,CAT,LRP1,C4BPB,CFP,APOE,PXDN,F12,APCS,ORM1,VWF,PSMA6,SERPINA10,ATRN,LCAT,YWHAE,CSTA,KLKB1,DGKG,CENPE,PON3,TCIRG1,HPX,HYAL1,ITIH4,FCN3,ITIH1,AHSG,ANKRD31,CRH,SERPING1,CSF1R,PIP,FCN2,PSG6,PGLYRP2,SCGB3A1,IHH,SERPINI1,PF4,MASP1,CAMP,GUCY1A1,A2ML1,HSP90B1,ANTXR1,LRG1,SH3D19,PCSK9,BMP1,SHLD1,FGB,DST,F2,INHBC,PLG,EFEMP2,CSPG4,C1QB,CFHR1,SUMO4,CALR,LPA,SERPINF2,C7,HBA1,ITLN1,HSPA5,FBN1,P4HB,SERPINA7,FBLN1,SERPINA5,SERPINA11,ANG,LIMS1,THBS4,APOD,PAPLN,ASGR2,BLM,GIT2,AKAP9,PIGR,APOA4,ITIH2,MTCL1,C8A,CENPF,CFHR3,CFH,QSOX1,APOA2,CD5L,S100A7,S100A9,ADAMTSL4,FLNA,MCOLN2,SMARCA1,C8B,C8G,CCN5,GSN,SVEP1,C1QC,F10,F7,APOM,CALML5,TAF9,IGFBP3,EFEMP1,CFI,TNRC6A,ILK,GAPDH,RAPGEF4,FGL1,CST3,MASP2,F11,C1S,NUCB1,ADIPOQ,IGF2,ORM2,C4A,SLAIN1,CDK3,ABCA13,COLEC11,MST1,LMOD3,ITIH3,C4B,TGFBI,CFB,IGFALS,PLTP,PAEP,RARRES2,UIMC1,SELENOP,C1R,APOA5,SERPINA3,SERPINA4,APOC4,CFD,IGHV3-15,IGHV3-72,VEGFC,HYOU1,KNG1,GRHL2,CDH5,B2M
GO Process	GO:0002526	Acute inflammatory response	13	80	1.15	8.64e-09	F12,APCS,ORM1,KLKB1,ITIH4,AHSG,SAA4,F2,SERPINF2,APOA2,F7,ORM2,SERPINA3
GO Process	GO:0032374	Regulation of cholesterol transport	12	64	1.21	1.11e-08	CETP,APOC3,APOA1,LRP1,APOE,PCSK9,APOA4,APOA2,ADIPOQ,ABCA13,PLTP,APOA5
GO Process	GO:0006954	Inflammatory response	28	538	0.66	1.25e-08	PF4V1,LRP1,F12,APCS,ORM1,ATRN,KLKB1,TCIRG1,HYAL1,ITIH4,AHSG,CRH,SAA4,CSF1R,PF4,CAMP,F2,SERPINF2,APOA2,CD5L,S100A9,F7,ORM2,C4A,C4B,RARRES2,SERPINA3,KNG1
GO Process	GO:0050764	Regulation of phagocytosis	14	104	1.07	1.39e-08	APOA1,CFP,FCN3,AHSG,FCN2,MASP1,CALR,APOA2,MASP2,ADIPOQ,C4A,COLEC11,C4B,CFB

GO Process	GO:0061041	Regulation of wound healing	15	130	1.0	2.06e-08	CPB2,HRG,PROC,APOE,F12,APCS,KLKB1,SERPING1,FGB,F2,PLG,SERPINF2,F7,F11,KNG1
GO Process	GO:0006957	Complement activation, alternative pathway	8	16	1.64	2.88e-08	CFP,C7,C8A,CFH,C8B,C8G,CFB,CFD
GO Process	GO:1903034	Regulation of response to wounding	16	162	0.93	3.94e-08	CPB2,HRG,PROC,APOE,F12,APCS,KLKB1,SERPING1,FGB,F2,PLG,SERPINF2,FLNA,F7,F11,KNG1
GO Process	GO:0043691	Reverse cholesterol transport	8	18	1.59	5.85e-08	CETP,APOC3,APOA1,APOE,LCAT,APOA4,APOA2,APOM
GO Process	GO:0051917	Regulation of fibrinolysis	8	18	1.59	5.85e-08	CPB2,HRG,F12,KLKB1,F2,PLG,SERPINF2,F11
GO Process	GO:0031638	Zymogen activation	11	59	1.21	6.79e-08	F9,F12,KLKB1,FGB,F2,F5,CD5L,F10,F7,F11,C1R
GO Process	GO:0034370	Triglyceride-rich lipoprotein particle remodeling	7	10	1.79	6.93e-08	CETP,APOA1,APOE,LCAT,APOA4,APOA2,APOA5
GO Process	GO:0050790	Regulation of catalytic activity	62	2370	0.36	7.12e-08	SPP2,SERPIND1,PCOLCE,APOC3,GPLD1,LTF,HRG,PLEK,APOA1,LRP1,APOE,APCS,SERPINA10,CSTA,CENPE,ITIH4,ITIH1,AHSG,SERPING1,CSF1R,SERPINI1,A2ML1,HSP90B1,ANTXR1,F2,CPN2,LPA,SERPINF2,FBN1,SERPINA7,FBLN1,SERPINA5,SERPINA11,ANG,LIMS1,PAPLN,BLM,GIT2,AKAP9,APOA4,ITIH2,APOA2,S100A9,GSN,CALML5,IGFBP3,GAPDH,RAPGEF4,CST3,NUCB1,ADIPOQ,IGF2,C4A,MST1,ITIH3,C4B,APOA5,SERPINA3,SERPINA4,VEGFC,KNG1,GRHL2
GO Process	GO:0019730	Antimicrobial humoral response	15	149	0.94	1.07e-07	PF4V1,LTF,HRG,PF4,CAMP,FGB,F2,ANG,S100A7,S100A9,GAPDH,COLEC11,RARRES2,KNG1,B2M
GO Process	GO:0065009	Regulation of molecular function	72	3085	0.31	2.38e-07	SPP2,SERPIND1,PCOLCE,APOC3,GPLD1,LTF,HRG,PLEK,APOA1,CAT,LRP1,APOE,PXDN,APCS,PSMA6,SERPINA10,YWHAE,CSTA,CENPE,ITIH4,ITIH1,AHSG,CRH,SERPING1,CSF1R,SERPINI1,A2ML1,HSP90B1,ANTXR1,PCSK9,F2,CFHR1,SUMO4,CPN2,LPA,SERPINF2,FBN1,SERPINA7,FBLN1,SERPINA5,SERPINA11,ANG,LIMS1,PAPLN,BLM,GIT2,AKAP9,APOA4,ITIH2,APOA2,S100A9,FLNA,GSN,CALML5,IGFBP3,GAPDH,RAPGEF4,CST3,NUCB1,ADIPOQ,IGF2,C4A,MST1,ITIH3,C4B,APOA5,SERPINA3,SERPINA4,VEGFC,KNG1,GRHL2,B2M
GO Process	GO:0034377	Plasma lipoprotein particle assembly	8	23	1.48	2.60e-07	APOC3,APOB,APOA1,APOE,APOA4,APOA2,APOM,APOA5
GO Process	GO:0065008	Regulation of biological quality	80	3654	0.28	3.53e-07	CPB2,CETP,SERPIND1,CHGA,F9,MMP2,COMP,PF4V1,APOC3,GPLD1,LTF,HRG,APOB,IGFBP5,PROC,PLEK,APOA1,TTR,C4BPB,APOE,F12,VWF,SERPINA10,LCAT,YWHAE,CP,KLKB1,DGKG,TCIRG1,HPX,CRH,SERPING1,CSF1R,PIP,IHH,PF4,GUCY1A1,HSP90B1,PCSK9,FGB,F2,PLG,CPN2,CALR,SERPINF2,FBN1,SERPINA7,FBLN1,OIT3,ANG,PROZ,AKAP9,PIGR,APOA4,F13B,F5,APOA2,S100A9,FLNA,GSN,F10,F7,APOM,TAF9,SOD3,NAV2,ILK,GAPDH,RAPGEF4,F11,ADIPOQ,ATP9B,LMOD3,APOA5,SERPINA3,APOC4,VEGFC,KNG1,CDH5,B2M
GO Process	GO:0051050	Positive regulation of transport	34	915	0.51	4.38e-07	CPB2,CETP,GPLD1,APOA1,LRP1,CFP,APOE,YWHAE,FCN3,AHSG,CRH,FCN2,MASP1,PCSK9,FGB,F2,CALR,ITLN1,ANG,AKAP9,MTCL1,APOA2,FLNA,MASP2,ADIPOQ,C4A,ABCA13,COLEC11,C4B,CFB,PLTP,APOA5,VEGFC,B2M
GO Process	GO:1905952	Regulation of lipid localization	15	167	0.89	4.38e-07	CETP,APOC3,APOB,APOA1,LRP1,APOE,CRH,PCSK9,APOA4,APOA2,ADIPOQ,ABCA13,PLTP,APOA5,APOC4
GO Process	GO:0051239	Regulation of multicellular organismal process	66	2749	0.32	4.87e-07	CPB2,CHGA,MMP2,EBI3,COMP,APOC3,GPLD1,LTF,HRG,IGFBP5,PROC,APOA1,LRP1,APOE,F12,APCS,ORM1,ATRN,LCAT,YWHAE,KLKB1,HYAL1,AHSG,CRH,SERPING1,CSF1R,PGLYRP2,IHH,PF4,CAMP,GUCY1A1,LRG1,PCSK9,BMP1,SHLD1,FGB,F2,PLG,CALR,SERPINF2,FBN1,FBLN1,THBS4,APOD,AKAP9,APOA4,APOA2,MCOLN2,C1QC,F7,APOM,EFEMP1,GAPDH,CST3,F11,ADIPOQ,IGF2,ORM2,MST1,PAEP,APOA5,VEGFC,KNG1,GRHL2,CDH5,B2M
GO Process	GO:0033344	Cholesterol efflux	8	27	1.41	7.00e-07	APOC3,APOB,APOA1,APOE,APOA4,APOA2,APOM,APOA5
GO Process	GO:0006953	Acute-phase response	9	42	1.27	8.44e-07	APCS,ORM1,ITIH4,AHSG,SAA4,F2,SERPINF2,ORM2,SERPINA3
GO Process	GO:0015918	Sterol transport	11	82	1.07	1.33e-06	CETP,APOC3,APOB,APOA1,APOE,LCAT,APOA4,APOA2,APOM,PLTP,APOA5
GO Process	GO:0034372	Very-low-density lipoprotein particle remodeling	6	9	1.76	1.42e-06	CETP,APOA1,APOE,LCAT,APOA4,APOA5
GO Process	GO:0051702	Biological process involved in interaction with symbiont	12	108	0.99	1.87e-06	LTF,HRG,APOE,APCS,CSF1R,PF4,CAMP,F2,PLG,CFHR1,APOL1,GAPDH
GO Process	GO:0030301	Cholesterol transport	10	65	1.13	1.91e-06	CETP,APOC3,APOB,APOA1,APOE,LCAT,APOA4,APOA2,APOM,APOA5
GO Process	GO:0042632	Cholesterol homeostasis	11	89	1.03	2.76e-06	CETP,APOC3,APOB,APOA1,APOE,LCAT,PCSK9,APOA4,APOA2,APOM,APOA5
GO Process	GO:0006869	Lipid transport	19	329	0.7	2.78e-06	CETP,APOC3,APOB,APOA1,APOE,LCAT,APOL1,LPA,SERPINA5,APOD,APOA4,APOA2,APOM,APOF,ATP9B,ABCA13,PLTP,APOA5,APOC4
GO Process	GO:0061844	Antimicrobial humoral immune response mediated by antimicrobial peptide	12	113	0.97	2.86e-06	PF4V1,LTF,HRG,PF4,CAMP,F2,ANG,S100A7,S100A9,GAPDH,KNG1,B2M
GO Process	GO:0032368	Regulation of lipid transport	13	139	0.91	2.91e-06	CETP,APOC3,APOA1,LRP1,APOE,CRH,PCSK9,APOA4,APOA2,ADIPOQ,ABCA13,PLTP,APOA5
GO Process	GO:0009617	Response to bacterium	27	663	0.55	3.21e-06	CHGA,MPO,PF4V1,LTF,APOB,C4BPB,CFP,FCN2,PGLYRP2,PF4,CAMP,LRG1,FGB,F2,ANG,S100A7,S100A9,RNASE1,ADIPOQ,C4B,CFB,RARRES2,RNASE4,CFD,IGHV3-15,IGHV3-72,B2M
GO Process	GO:0032103	Positive regulation of response to external stimulus	22	453	0.63	3.77e-06	CPB2,LGALS1,HRG,HPX,FCN3,CSF1R,FCN2,MASP1,F2,PLG,CALR,SERPINF2,FBN1,THBS4,S100A7,S100A9,FLNA,F7,MASP2,COLEC11,RARRES2,VEGFC
GO Process	GO:0016485	Protein processing	15	202	0.81	4.04e-06	F9,COMP,F12,KLKB1,IHH,PCSK9,BMP1,FGB,F2,F5,CD5L,F10,F7,F11,C1R
GO Process	GO:0008203	Cholesterol metabolic process	12	119	0.94	4.59e-06	CETP,APOB,APOA1,CAT,APOE,LCAT,PCSK9,APOL1,APOA4,APOA2,APOF,APOA5
GO Process	GO:0033700	Phospholipid efflux	6	12	1.64	4.59e-06	APOC3,APOA1,APOE,APOA4,APOA2,APOA5
GO Process	GO:0034380	High-density lipoprotein particle assembly	6	12	1.64	4.59e-06	APOA1,APOE,APOA4,APOA2,APOM,APOA5
GO Process	GO:0042742	Defense response to bacterium	18	306	0.71	4.68e-06	CHGA,MPO,LTF,CFP,FCN2,PGLYRP2,CAMP,FGB,F2,ANG,S100A7,S100A9,RNASE1,RARRES2,RNASE4,IGHV3-15,IGHV3-72,B2M
GO Process	GO:0002697	Regulation of immune effector process	20	383	0.66	5.13e-06	APOA1,C4BPB,CFP,HPX,FCN3,SERPING1,FCN2,PGLYRP2,MASP1,SHLD1,CFH,APOA2,CD5L,CFI,MASP2,C4A,COLEC11,C4B,CFB,B2M
GO Process	GO:0030168	Platelet activation	11	97	0.99	5.52e-06	COMP,PF4V1,HRG,PLEK,VWF,DGKG,PF4,FGB,F2,FLNA,ILK
GO Process	GO:0034381	Plasma lipoprotein particle clearance	7	24	1.41	6.10e-06	APOC3,APOB,APOA1,APOE,APOA2,APOM,ADIPOQ
GO Process	GO:0042157	Lipoprotein metabolic process	12	123	0.93	6.10e-06	APOC3,GPLD1,APOB,APOA1,APOE,LCAT,PCSK9,APOL1,APOA4,APOA2,APOM,APOA5
GO Process	GO:0007597	Blood coagulation, intrinsic pathway	6	13	1.6	6.25e-06	F9,F12,KLKB1,FLNA,F7,F11
GO Process	GO:0001944	Vasculature development	23	527	0.58	1.04e-05	MMP2,COMP,GPLD1,HRG,APOB,LRP1,APOE,PXDN,ANGPTL6,IHH,ANTXR1,PLG,EFEMP2,CSPG4,SERPINF2,ANG,APOD,S100A7,FLNA,SVEP1,TGFBI,VEGFC,CDH5
GO Process	GO:1900024	Regulation of substrate adhesion-dependent cell spreading	9	61	1.11	1.20e-05	ST6GAL1,APOA1,FGB,CALR,P4HB,FBLN1,LIMS1,FLNA,ILK
GO Process	GO:0019538	Protein metabolic process	79	3910	0.25	1.26e-05	ST6GAL1,CPB2,GNPTG,F9,MMP2,COMP,PCOLCE,APOC3,GPLD1,LTF,APOB,PROC,APOA1,CAT,APOE,F12,PSMA6,LCAT,CSTA,KLKB1,TCIRG1,HPX,HYAL1,FCN3,HABP2,CSF1R,PIP,FCN2,IHH,MASP1,HSP90B1,PCSK9,BMP1,FGB,F2,PLG,UBE3C,APOL1,SUMO4,CALR,LPA,HSPA5,P4HB,FBLN1,GANAB,PROZ,QPCT,APOA4,CNDP1,F13B,CFH,F5,APOA2,CD5L,S100A9,FLNA,GSN,F10,F7,APOM,TAF9,IGFBP3,EFEMP1,CFI,ILK,GAPDH,MASP2,F11,C1S,PCYOX1,CDK3,COLEC11,MST1,CFB,UIMC1,C1R,APOA5,CFD,B2M

GO Process	GO:0032501	Multicellular organismal process	113	6490	0.18	1.29e-05	SPP2,CPB2,CETP,SERPIND1,LGALS1,CHGA,F9,MMP2,COMP,MPO,PF4V1,APOC3,GPLD1,PCDH12,LTF,HRG,APOB,IGFBP5,PROC,PLEK,APOA1,CAT,LRP1,C4BPB,APOE,PXDN,ANGPTL6,F12,VWF,SERPINA10,ATRN,LCAT,YWHAE,KLKB1,DGKG,DNAH5,PON3,TCIRG1,HYAL1,AHSG,CRH,SERPING1,CSF1R,PIP,PSG6,IHH,SERPINI1,PF4,GUCY1A1,ANTXR1,LRG1,PCSK9,BMP1,SHLD1,FGB,F2,PLG,EFEMP2,CSPG4,CALR,LPA,SERPINF2,HSPA5,FBN1,FBLN1,SERPINA5,ANG,THBS4,PROZ,APOD,GIT2,AKAP9,PIGR,APOA4,PCDH18,CENPF,F13B,F5,APOA2,S100A7,S100A9,FLNA,CRTAC1,SMARCA1,GSN,SVEP1,F10,F7,APOM,CA6,IGFBP3,SOD3,EFEMP1,NAV2,ILK,FGL1,F11,ADIPOQ,IGF2,FSIP2,MST1,LMOD3,TGFBI,PLTP,RARRES2,SELENOP,APOA5,SERPINA3,VEGFC,KNG1,GRHL2,CDH5,B2M
GO Process	GO:0050832	Defense response to fungus	9	62	1.1	1.32e-05	CHGA,MPO,LTF,HRG,CAMP,ANG,S100A9,GAPDH,RARRES2
GO Process	GO:0030194	Positive regulation of blood coagulation	7	28	1.34	1.38e-05	CPB2,HRG,F12,F2,PLG,SERPINF2,F7
GO Process	GO:0051873	Killing by host of symbiont cells	7	29	1.32	1.66e-05	HRG,PF4,CAMP,F2,CFHR1,APOL1,GAPDH
GO Process	GO:0001568	Blood vessel development	22	505	0.58	1.93e-05	MMP2,COMP,GPLD1,HRG,APOB,LRP1,APOE,PXDN,ANGPTL6,IHH,ANTXR1,PLG,EFEMP2,CSPG4,SERPINF2,ANG,APOD,S100A7,FLNA,TGFBI,VEGFC,CDH5
GO Process	GO:0060627	Regulation of vesicle-mediated transport	23	551	0.56	2.04e-05	APOC3,APOA1,LRP1,CFP,APOE,FCN3,AHSG,FCN2,MASP1,PCSK9,FGB,CALR,APOA2,RAPGEF4,MASP2,ADIPOQ,C4A,ABCA13,COLEC11,C4B,CFB,APOA5,B2M
GO Process	GO:0032102	Negative regulation of response to external stimulus	19	387	0.63	2.44e-05	ST6GAL1,CPB2,LTF,HRG,PROC,APOA1,APOE,F12,APCS,KLKB1,SERPING1,FGB,F2,PLG,SERPINF2,F11,ADIPOQ,KNG1,CDH5
GO Process	GO:0098869	Cellular oxidant detoxification	10	91	0.98	2.60e-05	MPO,CAT,APOE,PXDN,HBA1,APOA4,S100A9,APOM,SOD3,GPX3
GO Process	GO:0051604	Protein maturation	16	275	0.71	2.67e-05	F9,COMP,F12,KLKB1,IHH,PCSK9,BMP1,FGB,F2,CALR,F5,CD5L,F10,F7,F11,C1R
GO Process	GO:0006639	Acylglycerol metabolic process	10	95	0.96	3.70e-05	CETP,APOC3,APOB,CAT,APOE,DGKG,PCSK9,ANG,APOA2,APOA5
GO Process	GO:0002752	Cell surface pattern recognition receptor signaling pathway	5	9	1.69	3.73e-05	FCN3,FCN2,MASP1,MASP2,COLEC11
GO Process	GO:0001867	Complement activation, lectin pathway	5	10	1.64	5.43e-05	FCN3,FCN2,MASP1,MASP2,COLEC11
GO Process	GO:0080134	Regulation of response to stress	38	1373	0.38	5.86e-05	CPB2,LGALS1,HRG,PROC,APOA1,APOE,F12,APCS,PSMA6,KLKB1,HPX,FCN3,AHSG,SERPING1,FCN2,PGLYRP2,MASP1,SHLD1,FGB,F2,PLG,SERPINF2,HSPA5,P4HB,CFH,S100A9,FLNA,F7,TAF9,CFI,MASP2,F11,ADIPOQ,COLEC11,UIMC1,HYOU1,KNG1,CDH5
GO Process	GO:0030449	Regulation of complement activation	6	22	1.38	6.64e-05	C4BPB,SERPING1,MASP1,CFH,CD5L,CFI
GO Process	GO:0048514	Blood vessel morphogenesis	19	419	0.6	7.19e-05	MMP2,COMP,GPLD1,HRG,APOB,LRP1,APOE,PXDN,ANGPTL6,IHH,EFEMP2,CSPG4,SERPINF2,ANG,APOD,S100A7,FLNA,TGFBI,VEGFC
GO Process	GO:0032376	Positive regulation of cholesterol transport	7	38	1.21	7.42e-05	CETP,APOA1,LRP1,APOE,ADIPOQ,ABCA13,PLTP
GO Process	GO:0055088	Lipid homeostasis	12	161	0.81	7.42e-05	CETP,APOC3,APOB,APOA1,APOE,LCAT,PCSK9,APOA4,APOA2,APOM,APOA5,APOC4
GO Process	GO:0070328	Triglyceride homeostasis	7	38	1.21	7.42e-05	CETP,APOC3,APOA1,APOE,APOA4,APOA5,APOC4
GO Process	GO:0009892	Negative regulation of metabolic process	63	2982	0.27	8.46e-05	SPP2,CPB2,SERPIND1,APOC3,GPLD1,LTF,HRG,IGFBP5,PLEK,APOA1,LRP1,APOE,APCS,ORM1,SERPINA10,YWHAE,CSTA,PON3,ITIH4,FCN3,ITIH1,AHSG,SERPING1,PGLYRP2,SERPINI1,PF4,A2ML1,PCSK9,SHLD1,F2,SUMO4,CALR,LPA,SERPINF2,SERPINA7,FBLN1,SERPINA5,SERPINA11,ANG,LIMS1,APOD,PAPLN,BLM,ITIH2,CENPF,QSOX1,APOA2,FLNA,TAF9,IGFBP3,TNRC6A,GAPDH,CST3,ADIPOQ,IGF2,C4A,MST1,ITIH3,C4B,UIMC1,SERPINA3,SERPINA4,KNG1
GO Process	GO:0051838	Cytolysis by host of symbiont cells	5	12	1.56	0.00010	HRG,CAMP,F2,CFHR1,APOL1
GO Process	GO:0051919	Positive regulation of fibrinolysis	4	4	1.94	0.00011	F12,KLKB1,PLG,F11
GO Process	GO:0042744	Hydrogen peroxide catabolic process	6	25	1.32	0.00012	MPO,CAT,PXDN,HBA1,APOA4,GPX3
GO Process	GO:1905954	Positive regulation of lipid localization	10	111	0.9	0.00012	CETP,APOB,APOA1,LRP1,APOE,CRH,ADIPOQ,ABCA13,PLTP,APOC4
GO Process	GO:0051918	Negative regulation of fibrinolysis	5	13	1.53	0.00014	CPB2,HRG,F2,PLG,SERPINF2
GO Process	GO:0010896	Regulation of triglyceride catabolic process	5	14	1.49	0.00018	APOC3,GPLD1,APOA1,APOA4,APOA5
GO Process	GO:0030155	Regulation of cell adhesion	26	784	0.46	0.00018	ST6GAL1,LGALS1,MMP2,EBI3,HRG,APOA1,HYAL1,IHH,SERPINI1,FGB,PLG,EFEMP2,CALR,SERPINF2,P4HB,FBLN1,LIMS1,APOD,FLNA,ILK,FGL1,ADIPOQ,IGF2,TGFBI,KNG1,B2M
GO Process	GO:1900026	Positive regulation of substrate adhesion-dependent cell spreading	7	45	1.13	0.00019	APOA1,FGB,CALR,P4HB,LIMS1,FLNA,ILK
GO Process	GO:0002920	Regulation of humoral immune response	7	46	1.12	0.00021	C4BPB,HPX,SERPING1,MASP1,CFH,CD5L,CFI
GO Process	GO:0006641	Triglyceride metabolic process	8	68	1.01	0.00022	CETP,APOC3,APOB,CAT,APOE,PCSK9,APOA2,APOA5
GO Process	GO:0048522	Positive regulation of cellular process	97	5584	0.18	0.00022	ST6GAL1,CPB2,LGALS1,CHGA,MMP2,EBI3,COMP,GPLD1,LTF,HRG,APOB,IGFBP5,PROC,PLEK,APOA1,CAT,LRP1,CFP,APOE,VWF,YWHAE,CENPE,TCIRG1,HPX,HYAL1,FCN3,ANKRD31,CRH,CSF1R,FCN2,SCGB3A1,IHH,SERPINI1,PF4,MASP1,CAMP,GUCY1A1,LRG1,SH3D19,PCSK9,SHLD1,FGB,F2,INHBC,PLG,EFEMP2,CSPG4,CALR,SERPINF2,HBA1,ITLN1,HSPA5,FBN1,P4HB,FBLN1,ANG,LIMS1,THBS4,BLM,AKAP9,APOA4,MTCL1,CD5L,S100A7,S100A9,ADAMTSL4,FLNA,SMARCA1,GSN,F10,F7,TAF9,IGFBP3,TNRC6A,ILK,GAPDH,MASP2,ADIPOQ,IGF2,C4A,SLAIN1,ABCA13,COLEC11,MST1,LMOD3,C4B,CFB,RARRES2,UIMC1,APOA5,IGHV3-15,IGHV3-72,VEGFC,KNG1,GRHL2,CDH5,B2M
GO Process	GO:0010810	Regulation of cell-substrate adhesion	13	217	0.72	0.00023	ST6GAL1,HRG,APOA1,FGB,PLG,EFEMP2,CALR,P4HB,FBLN1,LIMS1,APOD,FLNA,ILK
GO Process	GO:0072359	Circulatory system development	28	901	0.43	0.00023	MMP2,COMP,GPLD1,HRG,APOB,LRP1,APOE,PXDN,ANGPTL6,DNAH5,IHH,ANTXR1,PLG,EFEMP2,CSPG4,CALR,SERPINF2,FBN1,ANG,APOD,S100A7,FLNA,SVEP1,ILK,TGFBI,VEGFC,GRHL2,CDH5
GO Process	GO:0048878	Chemical homeostasis	28	904	0.43	0.00024	CPB2,CETP,APOC3,LTF,APOB,IGFBP5,APOA1,APOE,LCAT,YWHAE,CP,TCIRG1,HPX,HSP90B1,PCSK9,F2,CALR,FBN1,OIT3,APOA4,APOA2,S100A9,APOM,ADIPOQ,APOA5,APOC4,CDH5,B2M
GO Process	GO:0008202	Steroid metabolic process	14	258	0.67	0.00027	CETP,APOB,APOA1,CAT,APOE,LCAT,CRH,PCSK9,APOL1,APOA4,APOA2,APOF,GC,APOA5
GO Process	GO:0034374	Low-density lipoprotein particle remodeling	5	16	1.44	0.00029	CETP,MPO,APOB,APOE,APOA2
GO Process	GO:0010901	Regulation of very-low-density lipoprotein particle remodeling	4	6	1.76	0.00030	APOC3,APOA1,APOA2,APOA5
GO Process	GO:1903036	Positive regulation of response to wounding	8	72	0.99	0.00032	CPB2,HRG,F12,F2,PLG,SERPINF2,FLNA,F7
GO Process	GO:0010811	Positive regulation of cell-substrate adhesion	10	128	0.83	0.00036	HRG,APOA1,FGB,EFEMP2,CALR,P4HB,FBLN1,LIMS1,FLNA,ILK
GO Process	GO:0015914	Phospholipid transport	9	99	0.9	0.00036	CETP,APOC3,APOA1,APOE,APOA4,APOA2,ATP9B,PLTP,APOA5
GO Process	GO:0035239	Tube morphogenesis	23	669	0.48	0.00038	MMP2,COMP,GPLD1,HRG,APOB,LRP1,APOE,PXDN,ANGPTL6,CSF1R,IHH,EFEMP2,CSPG4,SERPINF2,ANG,APOD,S100A7,FLNA,ILK,MST1,TGFBI,VEGFC,GRHL2
GO Process	GO:0048771	Tissue remodeling	9	101	0.89	0.00040	SPP2,MMP2,IGFBP5,TCIRG1,IHH,PLG,CSPG4,THBS4,FLNA
GO Process	GO:0050830	Defense response to Gram-positive bacterium	10	130	0.83	0.00040	CHGA,LTF,FCN2,PGLYRP2,CAMP,ANG,RNASE1,RARRES2,RNASE4,B2M
GO Process	GO:0035295	Tube development	27	880	0.43	0.00041	MMP2,COMP,GPLD1,HRG,APOB,IGFBP5,CAT,LRP1,APOE,PXDN,ANGPTL6,CRH,CSF1R,IHH,EFEMP2,CSPG4,SERPINF2,ANG,APOD,S100A7,FLNA,ILK,MST1,TGFBI,RARRES2,VEGFC,GRHL2
GO Process	GO:0034384	High-density lipoprotein particle clearance	4	7	1.7	0.00043	APOA1,APOE,APOA2,APOM

GO Process	GO:1901564	Organonitrogen compound metabolic process	88	4981	0.19	0.00043	ST6GAL1,CPB2,CETP,GNPTG,F9,MMP2,COMP,PCOLCE,APOC3,GPLD1,LTF,APOB,PROC,APOA1,TTR,CAT,APOE,PXDN,F12,PSMA6,LCAT,CSTA,KLKB1,TCIRG1,HPX,HYAL1,ITIH4,FCN3,ITIH1,HABP2,CSF1R,PIP,FCN2,PGLYRP2,IHH,MASP1,GUCY1A1,HSP90B1,PCSK9,BMP1,FGB,F2,PLG,UBE3C,APOL1,SUMO4,CALR,LPA,HSPA5,P4HB,FBLN1,GANAB,PROZ,QPCT,APOA4,ITIH2,CNDP1,F13B,CFH,F5,APOA2,CD5L,S100A9,FLNA,GSN,F10,F7,APOM,TAF9,IGFBP3,EFEMP1,CFI,ILK,GAPDH,MASP2,F11,C1S,PCYOX1,CDK3,COLEC11,MST1,ITIH3,CFB,UIMC1,C1R,APOA5,CFD,B2M
GO Process	GO:0048662	Negative regulation of smooth muscle cell proliferation	7	53	1.06	0.00044	IGFBP5,APOE,EFEMP2,ANG,APOD,IGFBP3,ADIPOQ
GO Process	GO:0051235	Maintenance of location	11	166	0.76	0.00050	APOA1,APOE,HSP90B1,CALR,HSPA5,FBN1,AKAP9,S100A7,S100A9,FLNA,GSN
GO Process	GO:0046486	Glycerolipid metabolic process	16	357	0.59	0.00051	CETP,APOC3,GPLD1,APOB,PLEK,APOA1,CAT,APOE,LCAT,DGKG,CSF1R,PCSK9,ANG,APOA4,APOA2,APOA5
GO Process	GO:0003013	Circulatory system process	19	493	0.53	0.00055	CHGA,MMP2,COMP,LRP1,APOE,SERPING1,GUCY1A1,FGB,LPA,SERPINF2,F5,GSN,SVEP1,SOD3,NAV2,ADIPOQ,VEGFC,KNG1,CDH5
GO Process	GO:0008015	Blood circulation	17	403	0.57	0.00055	CHGA,MMP2,COMP,APOE,SERPING1,GUCY1A1,FGB,LPA,SERPINF2,F5,GSN,SOD3,NAV2,ADIPOQ,VEGFC,KNG1,CDH5
GO Process	GO:0010605	Negative regulation of macromolecule metabolic process	57	2760	0.26	0.00055	SPP2,CPB2,SERPIND1,LTF,HRG,IGFBP5,APOA1,LRP1,APOE,APCS,ORM1,SERPINA10,YWHAE,CSTA,ITIH4,FCN3,ITIH1,AHSG,SERPING1,PGLYRP2,SERPINI1,PF4,A2ML1,PCSK9,SHLD1,F2,SUMO4,CALR,LPA,SERPINF2,SERPINA7,FBLN1,SERPINA5,SERPINA11,ANG,LIMS1,APOD,PAPLN,BLM,ITIH2,CENPF,APOA2,FLNA,TAF9,IGFBP3,TNRC6A,GAPDH,CST3,ADIPOQ,IGF2,C4A,ITIH3,C4B,UIMC1,SERPINA3,SERPINA4,KNG1
GO Process	GO:0031347	Regulation of defense response	22	638	0.48	0.00055	LGALS1,PROC,APOA1,APOE,F12,APCS,PSMA6,KLKB1,HPX,FCN3,AHSG,SERPING1,FCN2,PGLYRP2,MASP1,CFH,S100A9,CFI,MASP2,ADIPOQ,COLEC11,CDH5
GO Process	GO:0050793	Regulation of developmental process	53	2492	0.27	0.00056	ST6GAL1,CETP,COMP,LTF,HRG,APOB,IGFBP5,PROC,APOA1,APOE,APCS,ATRN,DGKG,TCIRG1,HYAL1,AHSG,CSF1R,PGLYRP2,SCGB3A1,IHH,PF4,CAMP,LRG1,SH3D19,BMP1,SHLD1,FGB,F2,EFEMP2,CALR,SERPINF2,FBN1,P4HB,FBLN1,LIMS1,THBS4,CENPF,FLNA,C1QC,IGFBP3,EFEMP1,ILK,CST3,ADIPOQ,IGF2,MST1,LMOD3,PAEP,RARRES2,VEGFC,GRHL2,CDH5,B2M
GO Process	GO:1905920	Positive regulation of CoA-transferase activity	4	8	1.64	0.00061	APOA1,APOE,APOA4,APOA2
GO Process	GO:0006979	Response to oxidative stress	16	368	0.58	0.00069	MMP2,MPO,CAT,APOE,PXDN,ATRN,HYAL1,SUMO4,HBA1,APOD,APOA4,S100A7,SOD3,GPX3,ADIPOQ,SELENOP
GO Process	GO:0007155	Cell adhesion	28	965	0.4	0.00069	LGALS1,COMP,PCDH12,PLEK,PXDN,VWF,ATRN,CSTA,HABP2,IHH,ANTXR1,LRG1,FGB,DST,FBN1,LIMS1,THBS4,APOA4,PCDH18,S100A9,FLNA,CCN5,SVEP1,ILK,TGFBI,IGFALS,GRHL2,CDH5
GO Process	GO:0010874	Regulation of cholesterol efflux	6	37	1.15	0.00069	CETP,APOA1,LRP1,APOE,ADIPOQ,PLTP
GO Process	GO:1901700	Response to oxygen-containing compound	38	1547	0.33	0.00069	ST6GAL1,CPB2,MMP2,MPO,PF4V1,GPLD1,APOB,IGFBP5,CAT,LRP1,APOE,HYAL1,IHH,PF4,CAMP,HSP90B1,PCSK9,CALR,HBA1,HSPA5,FBN1,APOD,BLM,AKAP9,APOA4,F5,APOA2,S100A7,S100A9,FLNA,F7,TAF9,SOD3,GPX3,ADIPOQ,IGF2,RARRES2,B2M
GO Process	GO:0048585	Negative regulation of response to stimulus	39	1612	0.32	0.00071	ST6GAL1,CPB2,LTF,HRG,IGFBP5,PROC,PLEK,APOA1,LRP1,C4BPB,APOE,PXDN,F12,APCS,KLKB1,DGKG,AHSG,SERPING1,PGLYRP2,PF4,MASP1,SHLD1,FGB,F2,PLG,CALR,SERPINF2,HSPA5,FBN1,FBLN1,APOD,TAF9,IGFBP3,F11,ADIPOQ,CDK3,HYOU1,KNG1,CDH5
GO Process	GO:0043903	Regulation of biological process involved in symbiotic interaction	7	60	1.01	0.00084	LGALS1,LTF,APCS,FCN3,P4HB,FBLN1,GSN
GO Process	GO:0051172	Negative regulation of nitrogen compound metabolic process	51	2403	0.27	0.00088	SPP2,CPB2,SERPIND1,LTF,HRG,IGFBP5,LRP1,APOE,APCS,SERPINA10,YWHAE,CSTA,ITIH4,FCN3,ITIH1,AHSG,SERPING1,SERPINI1,A2ML1,SHLD1,F2,SUMO4,CALR,LPA,SERPINF2,SERPINA7,FBLN1,SERPINA5,SERPINA11,ANG,LIMS1,APOD,PAPLN,BLM,ITIH2,CENPF,FLNA,TAF9,IGFBP3,TNRC6A,GAPDH,CST3,ADIPOQ,IGF2,C4A,ITIH3,C4B,UIMC1,SERPINA3,SERPINA4,KNG1
GO Process	GO:0090207	Regulation of triglyceride metabolic process	6	40	1.12	0.00099	APOC3,GPLD1,APOA1,APOE,APOA4,APOA5
GO Process	GO:0044403	Biological process involved in symbiotic interaction	13	257	0.64	0.0010	LTF,HRG,APOE,APCS,CSF1R,PGLYRP2,PF4,CAMP,F2,PLG,CFHR1,APOL1,GAPDH
GO Process	GO:0050994	Regulation of lipid catabolic process	7	62	0.99	0.0010	APOC3,GPLD1,APOA1,APOA4,APOA2,RARRES2,APOA5
GO Process	GO:0030212	Hyaluronan metabolic process	5	23	1.28	0.0011	HYAL1,ITIH4,ITIH1,ITIH2,ITIH3
GO Process	GO:0030300	Regulation of intestinal cholesterol absorption	4	10	1.54	0.0011	APOA1,APOA4,APOA2,APOA5
GO Process	GO:0032370	Positive regulation of lipid transport	8	89	0.89	0.0011	CETP,APOA1,LRP1,APOE,CRH,ADIPOQ,ABCA13,PLTP
GO Process	GO:0042592	Homeostatic process	35	1406	0.34	0.0012	CPB2,CETP,COMP,APOC3,LTF,APOB,IGFBP5,APOA1,APOE,LCAT,YWHAE,CP,TCIRG1,HPX,PIP,IHH,HSP90B1,PCSK9,F2,PLG,CALR,FBN1,OIT3,ANG,PIGR,APOA4,APOA2,S100A9,APOM,ADIPOQ,APOA5,SERPINA3,APOC4,CDH5,B2M
GO Process	GO:0031099	Regeneration	10	154	0.75	0.0014	CPB2,MMP2,SERPINA10,IHH,PLG,APOD,APOA4,APOA2,F7,APOA5
GO Process	GO:0034975	Protein folding in endoplasmic reticulum	4	11	1.5	0.0015	HSP90B1,CALR,HSPA5,P4HB
GO Process	GO:0010875	Positive regulation of cholesterol efflux	5	26	1.22	0.0018	APOA1,LRP1,APOE,ADIPOQ,PLTP
GO Process	GO:0019732	Antifungal humoral response	4	12	1.46	0.0019	LTF,CAMP,ANG,RARRES2
GO Process	GO:0050829	Defense response to Gram-negative bacterium	8	97	0.86	0.0019	CHGA,LTF,FCN2,CAMP,F2,S100A7,RARRES2,B2M
GO Process	GO:0051240	Positive regulation of multicellular organismal process	36	1505	0.32	0.0019	CPB2,CHGA,EBI3,GPLD1,LTF,HRG,LRP1,APOE,F12,ORM1,HYAL1,CRH,CSF1R,IHH,PF4,CAMP,LRG1,BMP1,SHLD1,FGB,F2,PLG,SERPINF2,APOA2,MCOLN2,F7,GAPDH,ADIPOQ,IGF2,ORM2,MST1,PAEP,APOA5,VEGFC,CDH5,B2M
GO Process	GO:0030198	Extracellular matrix organization	13	278	0.61	0.0021	MMP2,COMP,PXDN,IHH,BMP1,PLG,EFEMP2,SERPINF2,FBLN1,PAPLN,QSOX1,ADAMTSL4,TGFBI
GO Process	GO:0046470	Phosphatidylcholine metabolic process	7	71	0.93	0.0021	CETP,GPLD1,APOA1,LCAT,APOA4,APOA2,APOA5
GO Process	GO:0048523	Negative regulation of cellular process	82	4736	0.18	0.0021	ST6GAL1,CPB2,CETP,CHGA,MMP2,COMP,MPO,APOC3,GPLD1,LTF,HRG,IGFBP5,PROC,PLEK,APOA1,CAT,LRP1,APOE,PXDN,APCS,YWHAE,DGKG,PON3,HYAL1,FCN3,AHSG,CRH,CSF1R,PIP,PGLYRP2,SCGB3A1,IHH,PF4,HSP90B1,ANTXR1,PCSK9,SHLD1,FGB,F2,PLG,EFEMP2,SUMO4,CALR,HSPA5,FBN1,FBLN1,ANG,LIMS1,APOD,BLM,APOA4,CENPF,QSOX1,APOA2,FLNA,MCOLN2,CCN5,GSN,C1QC,APOM,TAF9,IGFBP3,EFEMP1,TNRC6A,ILK,GAPDH,FGL1,CST3,ADIPOQ,IGF2,CDK3,MST1,LMOD3,TGFBI,PAEP,UIMC1,VEGFC,HYOU1,KNG1,GRHL2,CDH5,B2M
GO Process	GO:0051049	Regulation of transport	40	1763	0.3	0.0021	CPB2,CETP,CHGA,APOC3,GPLD1,APOA1,LRP1,CFP,APOE,YWHAE,TCIRG1,FCN3,AHSG,CRH,FCN2,MASP1,PCSK9,FGB,F2,CALR,ITLN1,ANG,APOD,AKAP9,APOA4,MTCL1,APOA2,FLNA,RAPGEF4,MASP2,ADIPOQ,C4A,ABCA13,COLEC11,C4B,CFB,PLTP,APOA5,VEGFC,B2M
GO Process	GO:0060284	Regulation of cell development	18	506	0.49	0.0023	LTF,PROC,APOA1,FGB,F2,CALR,FBN1,P4HB,FBLN1,LIMS1,FLNA,ILK,ADIPOQ,LMOD3,PAEP,VEGFC,CDH5,B2M
GO Process	GO:0032879	Regulation of localization	45	2103	0.27	0.0024	CPB2,CETP,CHGA,APOC3,GPLD1,APOB,APOA1,LRP1,CFP,APOE,YWHAE,TCIRG1,FCN3,AHSG,CRH,FCN2,MASP1,PCSK9,FGB,F2,SUMO4,CALR,ITLN1,ANG,APOD,AKAP9,APOA4,MTCL1,APOA2,FLNA,MCOLN2,GSN,RAPGEF4,MASP2,ADIPOQ,C4A,ABCA13,COLEC11,C4B,CFB,PLTP,APOA5,APOC4,VEGFC,B2M

GO Process	GO:0042221	Response to chemical	72	4010	0.19	0.0025	ST6GAL1,CPB2,SERPIND1,CHGA,MMP2,EBI3,COMP,MPO,PF4V1,GPLD1,HRG,APOB,IGFBP5,APOA1,CAT,LRP1,APOE,PXDN,F12,LCAT,PON3,TCIRG1,HPX,HYAL1,ITIH4,CSF1R,PIP,IHH,PF4,CAMP,HSP90B1,PCSK9,FGB,SUMO4,CALR,HBA1,HSPA5,FBN1,P4HB,ANG,LIMS1,THBS4,APOD,BLM,AKAP9,PIGR,APOA4,CENPF,F5,APOA2,S100A7,S100A9,FLNA,GSN,F7,APOM,CA6,TAF9,SOD3,GPX3,ILK,GAPDH,ADIPOQ,IGF2,MST1,C4B,RARRES2,SELENOP,VEGFC,HYOU1,CDH5,B2M
GO Process	GO:0000302	Response to reactive oxygen species	10	169	0.71	0.0027	MMP2,MPO,CAT,APOE,HYAL1,HBA1,APOD,APOA4,S100A7,SOD3
GO Process	GO:0032375	Negative regulation of cholesterol transport	4	14	1.4	0.0030	APOC3,APOE,PCSK9,APOA2
GO Process	GO:0010873	Positive regulation of cholesterol esterification	3	4	1.82	0.0033	APOA1,APOA4,APOA5
GO Process	GO:0010903	Negative regulation of very-low-density lipoprotein particle remodeling	3	4	1.82	0.0033	APOC3,APOA1,APOA2
GO Process	GO:0010033	Response to organic substance	53	2692	0.23	0.0038	ST6GAL1,CPB2,MMP2,EBI3,COMP,MPO,PF4V1,GPLD1,APOB,IGFBP5,CAT,LRP1,F12,LCAT,TCIRG1,HPX,HYAL1,ITIH4,CSF1R,IHH,PF4,CAMP,HSP90B1,PCSK9,FGB,CALR,HSPA5,FBN1,P4HB,ANG,LIMS1,THBS4,BLM,AKAP9,APOA4,F5,APOA2,S100A7,S100A9,FLNA,GSN,F7,TAF9,GPX3,ILK,GAPDH,ADIPOQ,IGF2,C4B,RARRES2,VEGFC,CDH5,B2M
GO Process	GO:0048660	Regulation of smooth muscle cell proliferation	9	142	0.74	0.0039	MMP2,IGFBP5,APOE,EFEMP2,SERPINF2,ANG,APOD,IGFBP3,ADIPOQ
GO Process	GO:0008152	Metabolic process	121	7988	0.12	0.0040	ST6GAL1,CPB2,CETP,GNPTG,TPD52L2,APMAP,F9,MMP2,COMP,PCOLCE,MPO,APOC3,GPLD1,PCDH12,LTF,APOB,PROC,PLEK,APOA1,TTR,CAT,LRP1,APOE,PXDN,F12,PSMA6,LCAT,CSTA,KLKB1,DGKG,PON3,TCIRG1,HPX,HYAL1,ITIH4,FCN3,ITIH1,ANKRD31,CRH,HABP2,CSF1R,PIP,FCN2,PGLYRP2,IHH,MASP1,GUCY1A1,HSP90B1,PCSK9,BMP1,SHLD1,FGB,F2,PLG,UBE3C,APOL1,SUMO4,CALR,LPA,HBA1,HSPA5,FBN1,P4HB,FBLN1,ANG,GANAB,PROZ,QPCT,APOD,BLM,GIT2,AKAP9,APOA4,ITIH2,CNDP1,CENPF,F13B,CFH,F5,APOA2,CD5L,S100A9,FLNA,GSN,SVEP1,F10,F7,APOM,CA6,TAF9,IGFBP3,SOD3,GPX3,EFEMP1,CFI,ILK,GAPDH,RNASE1,APOF,MASP2,F11,C1S,PCYOX1,ADIPOQ,IGF2,CDK3,COLEC11,MST1,ITIH3,CFB,PLTP,RARRES2,GC,UIMC1,SELENOP,C1R,APOA5,RNASE4,APOC4,CFD,B2M
GO Process	GO:0045785	Positive regulation of cell adhesion	17	485	0.49	0.0042	LGALS1,EBI3,HRG,APOA1,HYAL1,IHH,FGB,EFEMP2,CALR,SERPINF2,P4HB,FBLN1,LIMS1,FLNA,ILK,IGF2,B2M
GO Process	GO:0043170	Macromolecule metabolic process	94	5781	0.15	0.0043	ST6GAL1,CPB2,GNPTG,F9,MMP2,COMP,PCOLCE,APOC3,GPLD1,PCDH12,LTF,APOB,PROC,APOA1,CAT,APOE,F12,PSMA6,LCAT,CSTA,KLKB1,TCIRG1,HPX,HYAL1,ITIH4,FCN3,ITIH1,ANKRD31,HABP2,CSF1R,PIP,FCN2,PGLYRP2,IHH,MASP1,HSP90B1,PCSK9,BMP1,SHLD1,FGB,F2,PLG,UBE3C,APOL1,SUMO4,CALR,LPA,HSPA5,P4HB,FBLN1,ANG,GANAB,PROZ,QPCT,BLM,APOA4,ITIH2,CNDP1,CENPF,F13B,CFH,F5,APOA2,CD5L,S100A9,FLNA,GSN,SVEP1,F10,F7,APOM,TAF9,IGFBP3,EFEMP1,CFI,ILK,GAPDH,RNASE1,MASP2,F11,C1S,PCYOX1,ADIPOQ,CDK3,COLEC11,MST1,ITIH3,CFB,UIMC1,C1R,APOA5,RNASE4,CFD,B2M
GO Process	GO:0072377	Blood coagulation, common pathway	3	5	1.72	0.0050	F2,F5,F10
GO Process	GO:0002699	Positive regulation of immune effector process	12	264	0.6	0.0051	CFP,HPX,FCN3,FCN2,MASP1,SHLD1,MASP2,C4A,COLEC11,C4B,CFB,B2M
GO Process	GO:0019222	Regulation of metabolic process	106	6784	0.13	0.0052	SPP2,CPB2,SERPIND1,EBI3,COMP,PCOLCE,APOC3,GPLD1,LTF,HRG,APOB,IGFBP5,PLEK,APOA1,CAT,LRP1,C4BPB,APOE,F12,APCS,ORM1,PSMA6,SERPINA10,YWHAE,CSTA,KLKB1,CENPE,PON3,TCIRG1,HPX,ITIH4,FCN3,ITIH1,AHSG,ANKRD31,SERPING1,CSF1R,PIP,PGLYRP2,IHH,SERPINI1,PF4,CAMP,A2ML1,HSP90B1,ANTXR1,SH3D19,PCSK9,SHLD1,F2,INHBC,CSPG4,SUMO4,CALR,LPA,SERPINF2,ITLN1,HSPA5,FBN1,SERPINA7,FBLN1,SERPINA5,SERPINA11,ANG,LIMS1,THBS4,APOD,PAPLN,BLM,AKAP9,APOA4,ITIH2,MTCL1,CENPF,QSOX1,APOA2,S100A9,FLNA,MCOLN2,SMARCA1,GSN,TAF9,IGFBP3,EFEMP1,TNRC6A,ILK,GAPDH,CST3,ADIPOQ,IGF2,ORM2,C4A,MST1,ITIH3,C4B,PAEP,RARRES2,UIMC1,APOA5,SERPINA3,SERPINA4,VEGFC,KNG1,GRHL2,CDH5,B2M
GO Process	GO:0042127	Regulation of cell population proliferation	37	1669	0.29	0.0062	ST6GAL1,CPB2,TPD52L2,MMP2,EBI3,COMP,GPLD1,LTF,HRG,IGFBP5,APOE,TCIRG1,HYAL1,CSF1R,SCGB3A1,IHH,PF4,CAMP,LRG1,F2,PLG,EFEMP2,CALR,SERPINF2,FBLN1,ANG,THBS4,APOD,SMARCA1,IGFBP3,ILK,ADIPOQ,IGF2,MST1,VEGFC,CDH5,B2M
GO Process	GO:0009636	Response to toxic substance	11	229	0.62	0.0063	MPO,CAT,APOE,PXDN,PON3,HBA1,APOA4,S100A9,APOM,SOD3,GPX3
GO Process	GO:0051004	Regulation of lipoprotein lipase activity	4	18	1.29	0.0064	APOC3,APOA1,APOA4,APOA5
GO Process	GO:0060255	Regulation of macromolecule metabolic process	99	6249	0.14	0.0065	SPP2,CPB2,SERPIND1,EBI3,COMP,PCOLCE,GPLD1,LTF,HRG,APOB,IGFBP5,APOA1,CAT,LRP1,C4BPB,APOE,F12,APCS,ORM1,PSMA6,SERPINA10,YWHAE,CSTA,KLKB1,CENPE,TCIRG1,HPX,ITIH4,FCN3,ITIH1,AHSG,ANKRD31,SERPING1,CSF1R,PIP,PGLYRP2,IHH,SERPINI1,PF4,CAMP,A2ML1,HSP90B1,ANTXR1,SH3D19,PCSK9,SHLD1,F2,INHBC,CSPG4,SUMO4,CALR,LPA,SERPINF2,ITLN1,HSPA5,FBN1,SERPINA7,FBLN1,SERPINA5,SERPINA11,ANG,LIMS1,THBS4,APOD,PAPLN,BLM,AKAP9,ITIH2,CENPF,APOA2,S100A9,FLNA,MCOLN2,SMARCA1,GSN,TAF9,IGFBP3,EFEMP1,TNRC6A,ILK,GAPDH,CST3,ADIPOQ,IGF2,ORM2,C4A,MST1,ITIH3,C4B,PAEP,RARRES2,UIMC1,SERPINA3,SERPINA4,VEGFC,KNG1,GRHL2,CDH5,B2M
GO Process	GO:0060191	Regulation of lipase activity	7	88	0.84	0.0066	APOC3,APOA1,LRP1,ANG,APOA4,APOA2,APOA5
GO Process	GO:0006066	Alcohol metabolic process	13	324	0.54	0.0083	CETP,APOB,APOA1,TTR,CAT,APOE,LCAT,PCSK9,APOL1,APOA4,APOA2,APOF,APOA5
GO Process	GO:0001525	Angiogenesis	13	325	0.54	0.0085	MMP2,GPLD1,HRG,PXDN,ANGPTL6,IHH,CSPG4,ANG,APOD,S100A7,FLNA,TGFBI,VEGFC
GO Process	GO:0080090	Regulation of primary metabolic process	94	5899	0.14	0.0090	SPP2,CPB2,SERPIND1,PCOLCE,APOC3,GPLD1,LTF,HRG,APOB,IGFBP5,PLEK,APOA1,CAT,LRP1,C4BPB,APOE,F12,APCS,PSMA6,SERPINA10,YWHAE,CSTA,KLKB1,CENPE,HPX,ITIH4,FCN3,ITIH1,AHSG,ANKRD31,SERPING1,CSF1R,IHH,SERPINI1,PF4,CAMP,A2ML1,HSP90B1,ANTXR1,SH3D19,PCSK9,SHLD1,F2,INHBC,CSPG4,SUMO4,CALR,LPA,SERPINF2,ITLN1,HSPA5,FBN1,SERPINA7,FBLN1,SERPINA5,SERPINA11,ANG,LIMS1,THBS4,APOD,PAPLN,BLM,AKAP9,APOA4,ITIH2,CENPF,APOA2,S100A9,FLNA,SMARCA1,GSN,TAF9,IGFBP3,EFEMP1,TNRC6A,ILK,GAPDH,CST3,ADIPOQ,IGF2,C4A,MST1,ITIH3,C4B,PAEP,RARRES2,UIMC1,APOA5,SERPINA3,SERPINA4,VEGFC,KNG1,GRHL2,CDH5
GO Process	GO:0051247	Positive regulation of protein metabolic process	34	1512	0.29	0.0092	PCOLCE,GPLD1,LTF,LRP1,C4BPB,APOE,F12,KLKB1,CENPE,HPX,CSF1R,CAMP,ANTXR1,SH3D19,PCSK9,F2,INHBC,CSPG4,ITLN1,HSPA5,FBN1,FBLN1,ANG,THBS4,AKAP9,S100A9,GSN,ILK,ADIPOQ,IGF2,PAEP,RARRES2,VEGFC,CDH5
GO Process	GO:0045595	Regulation of cell differentiation	35	1582	0.29	0.0098	CETP,LTF,APOB,IGFBP5,PROC,APOA1,APCS,TCIRG1,PGLYRP2,IHH,PF4,LRG1,FGB,F2,EFEMP2,CALR,SERPINF2,FBN1,P4HB,FBLN1,LIMS1,FLNA,C1QC,IGFBP3,EFEMP1,ILK,ADIPOQ,IGF2,LMOD3,PAEP,RARRES2,VEGFC,GRHL2,CDH5,B2M
GO Process	GO:0014012	Peripheral nervous system axon regeneration	3	7	1.57	0.0100	MMP2,APOD,APOA4
GO Process	GO:0001503	Ossification	12	287	0.56	0.0101	MMP2,COMP,GPLD1,LTF,IGFBP5,CAT,TCIRG1,AHSG,IHH,BMP1,IGFBP3,IGF2
GO Process	GO:0002688	Regulation of leukocyte chemotaxis	8	128	0.74	0.0101	CSF1R,CALR,THBS4,S100A7,F7,MST1,RARRES2,VEGFC

GO Process	GO:0010941	Regulation of cell death	36	1651	0.28	0.0102	ST6GAL1,LGALS1,CHGA,MMP2,COMP,MPO,GPLD1,LTF,HRG,PROC,CAT,LRP1,APOE,CSF1R,PIP,IHH,PF4,HSP90B1,PCSK9,FGB,CALR,HBA1,HSPA5,P4HB,S100A9,ADAMTSL4,FLNA,CCN5,GSN,TAF9,IGFBP3,ADIPOQ,MST1,HYOU1,KNG1,CDH5
GO Process	GO:0051651	Maintenance of location in cell	7	97	0.8	0.0111	APOE,HSP90B1,CALR,HSPA5,AKAP9,S100A9,GSN
GO Process	GO:0070887	Cellular response to chemical stimulus	50	2609	0.22	0.0113	CPB2,CHGA,MMP2,EBI3,COMP,MPO,PF4V1,GPLD1,APOB,IGFBP5,CAT,LRP1,APOE,PXDN,PON3,TCIRG1,HPX,HYAL1,CSF1R,PF4,CAMP,HSP90B1,PCSK9,FGB,SUMO4,CALR,HBA1,HSPA5,FBN1,P4HB,LIMS1,BLM,AKAP9,APOA4,S100A9,FLNA,GSN,APOM,SOD3,GPX3,ILK,GAPDH,ADIPOQ,IGF2,MST1,RARRES2,VEGFC,HYOU1,CDH5,B2M
GO Process	GO:0002690	Positive regulation of leukocyte chemotaxis	7	99	0.79	0.0124	CSF1R,CALR,THBS4,S100A7,F7,RARRES2,VEGFC
GO Process	GO:0070527	Platelet aggregation	5	43	1.01	0.0124	COMP,PLEK,FGB,FLNA,ILK
GO Process	GO:0030334	Regulation of cell migration	24	927	0.35	0.0128	MMP2,GPLD1,HRG,IGFBP5,LRP1,APOE,HYAL1,CSF1R,PLG,CALR,HSPA5,FBLN1,THBS4,APOD,S100A7,FLNA,F10,F7,IGFBP3,ADIPOQ,MST1,RARRES2,VEGFC,CDH5
GO Process	GO:0071704	Organic substance metabolic process	113	7522	0.12	0.0129	ST6GAL1,CPB2,CETP,GNPTG,TPD52L2,F9,MMP2,COMP,PCOLCE,MPO,APOC3,GPLD1,PCDH12,LTF,APOB,PROC,PLEK,APOA1,TTR,CAT,LRP1,APOE,PXDN,F12,PSMA6,LCAT,CSTA,KLKB1,DGKG,PON3,TCIRG1,HPX,HYAL1,ITIH4,FCN3,ITIH1,ANKRD31,CRH,HABP2,CSF1R,PIP,FCN2,PGLYRP2,IHH,MASP1,GUCY1A1,HSP90B1,PCSK9,BMP1,SHLD1,FGB,F2,PLG,UBE3C,APOL1,SUMO4,CALR,LPA,HSPA5,FBN1,P4HB,FBLN1,ANG,GANAB,PROZ,QPCT,APOD,BLM,APOA4,ITIH2,CNDP1,CENPF,F13B,CFH,F5,APOA2,CD5L,S100A9,FLNA,GSN,SVEP1,F10,F7,APOM,TAF9,IGFBP3,EFEMP1,CFI,ILK,GAPDH,RNASE1,APOF,MASP2,F11,C1S,PCYOX1,ADIPOQ,IGF2,CDK3,COLEC11,MST1,ITIH3,CFB,PLTP,RARRES2,GC,UIMC1,C1R,APOA5,RNASE4,APOC4,CFD,B2M
GO Process	GO:0032489	Regulation of Cdc42 protein signal transduction	3	8	1.51	0.0131	APOC3,APOA1,APOE
GO Process	GO:0001932	Regulation of protein phosphorylation	27	1108	0.33	0.0132	LTF,HRG,APOA1,LRP1,APOE,CENPE,HPX,CSF1R,CAMP,F2,INHBC,CSPG4,ITLN1,FBN1,FBLN1,ANG,THBS4,BLM,AKAP9,IGFBP3,ILK,ADIPOQ,IGF2,MST1,RARRES2,VEGFC,CDH5
GO Process	GO:0019835	Cytolysis	4	23	1.18	0.0135	C7,C8A,C8B,C8G
GO Process	GO:0090208	Positive regulation of triglyceride metabolic process	4	23	1.18	0.0135	GPLD1,APOA1,APOA4,APOA5
GO Process	GO:0051093	Negative regulation of developmental process	24	933	0.35	0.0138	CETP,LTF,HRG,IGFBP5,APOE,APCS,AHSG,PGLYRP2,IHH,PF4,F2,CALR,FBN1,FBLN1,THBS4,C1QC,EFEMP1,CST3,ADIPOQ,IGF2,PAEP,VEGFC,GRHL2,B2M
GO Process	GO:0019216	Regulation of lipid metabolic process	13	346	0.52	0.0143	APOC3,GPLD1,APOB,APOA1,APOE,F2,APOD,APOA4,APOA2,ADIPOQ,RARRES2,APOA5,SERPINA3
GO Process	GO:0072593	Reactive oxygen species metabolic process	7	102	0.78	0.0143	MPO,CAT,PXDN,HBA1,APOA4,SOD3,GPX3
GO Process	GO:0046596	Regulation of viral entry into host cell	5	45	0.99	0.0145	LGALS1,APCS,FCN3,P4HB,GSN
GO Process	GO:1902533	Positive regulation of intracellular signal transduction	25	997	0.34	0.0146	LGALS1,LTF,IGFBP5,APOA1,CAT,APOE,VWF,CSF1R,GUCY1A1,FGB,F2,CSPG4,CALR,SERPINF2,LIMS1,S100A7,S100A9,FLNA,F10,F7,IGFBP3,ILK,GAPDH,ADIPOQ,IGF2
GO Process	GO:0010640	Regulation of platelet-derived growth factor receptor signaling pathway	4	24	1.16	0.0153	LRP1,APOD,F7,ADIPOQ
GO Process	GO:0048870	Cell motility	26	1061	0.33	0.0154	CHGA,MMP2,PF4V1,GPLD1,APOB,APOA1,ATRN,YWHAE,DNAH5,PF4,LRG1,DST,PLG,CSPG4,DNAH8,ANG,THBS4,S100A9,FLNA,MCOLN2,ILK,FSIP2,MST1,PLTP,VEGFC,CDH5
GO Process	GO:0010720	Positive regulation of cell development	12	304	0.54	0.0157	PROC,APOA1,FGB,CALR,P4HB,LIMS1,FLNA,ILK,ADIPOQ,LMOD3,VEGFC,CDH5
GO Process	GO:0010898	Positive regulation of triglyceride catabolic process	3	9	1.46	0.0166	APOA1,APOA4,APOA5
GO Process	GO:0051006	Positive regulation of lipoprotein lipase activity	3	9	1.46	0.0166	APOA1,APOA4,APOA5
GO Process	GO:0097746	Blood vessel diameter maintenance	8	140	0.7	0.0166	MMP2,COMP,APOE,GUCY1A1,FGB,SERPINF2,SOD3,KNG1
GO Process	GO:1902106	Negative regulation of leukocyte differentiation	7	107	0.76	0.0180	LTF,APCS,PGLYRP2,IHH,FBN1,C1QC,ADIPOQ
GO Process	GO:0022604	Regulation of cell morphogenesis	12	311	0.53	0.0187	ST6GAL1,APOA1,CSF1R,SH3D19,FGB,F2,CALR,P4HB,FBLN1,LIMS1,FLNA,ILK
GO Process	GO:0006910	Phagocytosis, recognition	4	26	1.13	0.0193	FCN3,FCN2,IGHV3-15,IGHV3-72
GO Process	GO:0014912	Negative regulation of smooth muscle cell migration	4	26	1.13	0.0193	IGFBP5,LRP1,IGFBP3,ADIPOQ
GO Process	GO:0042981	Regulation of apoptotic process	32	1462	0.28	0.0207	ST6GAL1,LGALS1,MMP2,COMP,MPO,GPLD1,LTF,HRG,PROC,CAT,APOE,CSF1R,PIP,IHH,PF4,HSP90B1,PCSK9,FGB,CALR,HSPA5,P4HB,S100A9,ADAMTSL4,FLNA,GSN,TAF9,IGFBP3,ADIPOQ,MST1,HYOU1,KNG1,CDH5
GO Process	GO:0090209	Negative regulation of triglyceride metabolic process	3	10	1.42	0.0207	APOC3,GPLD1,APOE
GO Process	GO:0006629	Lipid metabolic process	28	1210	0.3	0.0209	CETP,APOC3,GPLD1,APOB,PLEK,APOA1,TTR,CAT,LRP1,APOE,LCAT,DGKG,CRH,CSF1R,PCSK9,APOL1,LPA,ANG,APOD,APOA4,APOA2,APOF,ADIPOQ,PLTP,RARRES2,GC,APOA5,APOC4
GO Process	GO:0009966	Regulation of signal transduction	54	2978	0.2	0.0209	LGALS1,CHGA,APOC3,LTF,HRG,IGFBP5,PLEK,APOA1,CAT,LRP1,APOE,PXDN,VWF,DGKG,HPX,AHSG,CRH,CSF1R,IHH,PF4,GUCY1A1,LRG1,PCSK9,FGB,F2,INHBC,CSPG4,SUMO4,CALR,SERPINF2,HSPA5,FBN1,P4HB,FBLN1,LIMS1,APOD,GIT2,AKAP9,S100A7,S100A9,FLNA,F10,F7,TAF9,IGFBP3,ILK,GAPDH,ADIPOQ,IGF2,CDK3,MST1,VEGFC,HYOU1,CDH5
GO Process	GO:0048731	System development	66	3867	0.17	0.0211	SPP2,CPB2,LGALS1,MMP2,COMP,GPLD1,PCDH12,LTF,HRG,APOB,IGFBP5,PLEK,APOA1,CAT,LRP1,APOE,PXDN,ANGPTL6,SERPINA10,ATRN,YWHAE,DGKG,DNAH5,TCIRG1,HYAL1,AHSG,CRH,CSF1R,IHH,SERPINI1,ANTXR1,PCSK9,BMP1,SHLD1,PLG,EFEMP2,CSPG4,CALR,SERPINF2,HSPA5,FBN1,ANG,APOD,GIT2,APOA4,PCDH18,CENPF,S100A7,S100A9,FLNA,CRTAC1,SMARCA1,GSN,SVEP1,EFEMP1,NAV2,ILK,FGL1,IGF2,TGFBI,RARRES2,SELENOP,VEGFC,GRHL2,CDH5,B2M
GO Process	GO:0050920	Regulation of chemotaxis	10	227	0.58	0.0211	ST6GAL1,HRG,CSF1R,CALR,THBS4,S100A7,F7,MST1,RARRES2,VEGFC
GO Process	GO:0070482	Response to oxygen levels	12	318	0.52	0.0218	MMP2,CAT,GUCY1A1,HSP90B1,P4HB,ANG,F7,SOD3,ADIPOQ,MST1,VEGFC,HYOU1
GO Process	GO:0019220	Regulation of phosphate metabolic process	31	1405	0.28	0.0219	GPLD1,LTF,HRG,PLEK,APOA1,LRP1,APOE,YWHAE,CENPE,HPX,CSF1R,CAMP,HSP90B1,F2,INHBC,CSPG4,ITLN1,FBN1,FBLN1,ANG,THBS4,BLM,AKAP9,IGFBP3,ILK,ADIPOQ,IGF2,MST1,RARRES2,VEGFC,CDH5
GO Process	GO:0040012	Regulation of locomotion	25	1032	0.32	0.0222	ST6GAL1,MMP2,GPLD1,HRG,IGFBP5,LRP1,APOE,HYAL1,CSF1R,PLG,CALR,HSPA5,FBLN1,THBS4,APOD,S100A7,FLNA,F10,F7,IGFBP3,ADIPOQ,MST1,RARRES2,VEGFC,CDH5
GO Process	GO:0045596	Negative regulation of cell differentiation	19	685	0.38	0.0234	CETP,LTF,IGFBP5,APCS,PGLYRP2,IHH,PF4,F2,CALR,FBN1,FBLN1,C1QC,EFEMP1,ADIPOQ,IGF2,PAEP,VEGFC,GRHL2,B2M
GO Process	GO:0031529	Ruffle organization	4	28	1.1	0.0237	PLEK,TCIRG1,CSF1R,CSPG4
GO Process	GO:0050996	Positive regulation of lipid catabolic process	4	28	1.1	0.0237	APOA1,APOA4,APOA2,APOA5
GO Process	GO:0050727	Regulation of inflammatory response	13	371	0.48	0.0242	LGALS1,PROC,APOA1,APOE,F12,APCS,PSMA6,KLKB1,AHSG,PGLYRP2,S100A9,ADIPOQ,CDH5
GO Process	GO:0031639	Plasminogen activation	3	11	1.38	0.0247	KLKB1,FGB,F11
GO Process	GO:1900121	Negative regulation of receptor binding	3	11	1.38	0.0247	PCSK9,ADIPOQ,B2M
GO Process	GO:0034097	Response to cytokine	21	804	0.36	0.0248	MMP2,EBI3,PF4V1,APOB,TCIRG1,HPX,HYAL1,ITIH4,CSF1R,PF4,CAMP,FGB,HSPA5,P4HB,LIMS1,GSN,TAF9,ILK,GAPDH,ADIPOQ,VEGFC

GO Process	GO:0001666	Response to hypoxia	11	278	0.54	0.0249	MMP2,CAT,HSP90B1,P4HB,ANG,F7,SOD3,ADIPOQ,MST1,VEGFC,HYOU1
GO Process	GO:0051129	Negative regulation of cellular component organization	19	691	0.38	0.0252	APOC3,HRG,APOA1,APOE,ANTXR1,PCSK9,HSPA5,APOD,APOA4,CENPF,APOA2,FLNA,GSN,APOM,CST3,ADIPOQ,LMOD3,CDH5,B2M
GO Process	GO:0002762	Negative regulation of myeloid leukocyte differentiation	5	53	0.92	0.0257	LTF,APCS,FBN1,C1QC,ADIPOQ
GO Process	GO:0006826	Iron ion transport	5	53	0.92	0.0257	LTF,HRG,CP,HPX,B2M
GO Process	GO:0010543	Regulation of platelet activation	5	53	0.92	0.0257	HRG,PLEK,APOE,F2,FLNA
GO Process	GO:0046464	Acylglycerol catabolic process	4	29	1.08	0.0257	APOC3,APOB,APOA2,APOA5
GO Process	GO:0030335	Positive regulation of cell migration	16	529	0.42	0.0259	MMP2,GPLD1,IGFBP5,HYAL1,CSF1R,PLG,CALR,HSPA5,FBLN1,THBS4,S100A7,F10,F7,RARRES2,VEGFC,CDH5
GO Process	GO:1901615	Organic hydroxy compound metabolic process	15	478	0.44	0.0265	CETP,APOB,APOA1,TTR,CAT,APOE,LCAT,PCSK9,APOL1,APOA4,APOA2,APOF,PLTP,GC,APOA5
GO Process	GO:0010035	Response to inorganic substance	16	532	0.42	0.0271	MMP2,MPO,GPLD1,APOB,CAT,LCAT,TCIRG1,FGB,CALR,HBA1,HSPA5,BLM,APOA4,SOD3,SELENOP,B2M
GO Process	GO:0045088	Regulation of innate immune response	10	238	0.56	0.0275	APOE,HPX,FCN3,SERPING1,FCN2,MASP1,CFH,CFI,MASP2,COLEC11
GO Process	GO:0045834	Positive regulation of lipid metabolic process	8	155	0.65	0.0275	GPLD1,APOA1,APOE,F2,APOA4,APOA2,ADIPOQ,APOA5
GO Process	GO:0062012	Regulation of small molecule metabolic process	12	330	0.5	0.0275	APOC3,GPLD1,APOB,PLEK,APOA1,APOE,APOA4,IGFBP3,ADIPOQ,IGF2,MST1,APOA5
GO Process	GO:0045940	Positive regulation of steroid metabolic process	4	30	1.07	0.0280	APOA1,APOE,APOA4,APOA5
GO Process	GO:0002683	Negative regulation of immune system process	14	433	0.45	0.0299	LTF,APOA1,C4BPB,APCS,SERPING1,PGLYRP2,IHH,MASP1,FBN1,APOD,APOA2,C1QC,FGL1,ADIPOQ
GO Process	GO:0010743	Regulation of macrophage derived foam cell differentiation	4	31	1.05	0.0309	CETP,APOB,PF4,ADIPOQ
GO Process	GO:1903053	Regulation of extracellular matrix organization	5	56	0.89	0.0309	CPB2,LRP1,ANTXR1,EFEMP2,CST3
GO Process	GO:0007162	Negative regulation of cell adhesion	11	289	0.52	0.0318	MMP2,HRG,APOA1,IHH,PLG,FBLN1,APOD,FGL1,ADIPOQ,TGFBI,KNG1
GO Process	GO:0098609	Cell-cell adhesion	16	542	0.41	0.0319	LGALS1,COMP,PCDH12,PLEK,CSTA,IHH,LRG1,FGB,LIMS1,THBS4,APOA4,PCDH18,S100A9,FLNA,ILK,CDH5
GO Process	GO:0010642	Negative regulation of platelet-derived growth factor receptor signaling pathway	3	13	1.3	0.0345	LRP1,APOD,ADIPOQ
GO Process	GO:0034445	Negative regulation of plasma lipoprotein oxidation	2	2	1.94	0.0360	APOA4,APOM
GO Process	GO:0035606	Peptidyl-cysteine S-trans-nitrosylation	2	2	1.94	0.0360	S100A9,GAPDH
GO Process	GO:0060621	Negative regulation of cholesterol import	2	2	1.94	0.0360	APOC3,APOA2
GO Process	GO:0051094	Positive regulation of developmental process	29	1332	0.28	0.0369	LTF,APOB,PROC,APOA1,APOE,HYAL1,SCGB3A1,IHH,PF4,CAMP,LRG1,BMP1,SHLD1,FGB,EFEMP2,CALR,SERPINF2,P4HB,LIMS1,FLNA,IGFBP3,ILK,ADIPOQ,IGF2,MST1,LMOD3,RARRES2,VEGFC,CDH5
GO Process	GO:0001878	Response to yeast	3	14	1.27	0.0405	MPO,CAMP,ANG
GO Process	GO:0019430	Removal of superoxide radicals	3	14	1.27	0.0405	MPO,APOA4,SOD3
GO Process	GO:0045916	Negative regulation of complement activation	3	14	1.27	0.0405	C4BPB,SERPING1,MASP1
GO Process	GO:0001906	Cell killing	7	129	0.67	0.0430	CHGA,LTF,PF4,F2,GAPDH,KNG1,B2M
GO Process	GO:0085029	Extracellular matrix assembly	4	35	1.0	0.0447	PXDN,IHH,EFEMP2,QSOX1
GO Process	GO:0051171	Regulation of nitrogen compound metabolic process	88	5734	0.13	0.0469	SPP2,CPB2,SERPIND1,PCOLCE,GPLD1,LTF,HRG,IGFBP5,APOA1,CAT,LRP1,C4BPB,APOE,F12,APCS,PSMA6,SERPINA10,YWHAE,CSTA,KLKB1,CENPE,HPX,ITIH4,FCN3,ITIH1,AHSG,ANKRD31,SERPING1,CSF1R,IHH,SERPINI1,PF4,CAMP,A2ML1,HSP90B1,ANTXR1,SH3D19,PCSK9,SHLD1,F2,INHBC,CSPG4,SUMO4,CALR,LPA,SERPINF2,ITLN1,HSPA5,FBN1,SERPINA7,FBLN1,SERPINA5,SERPINA11,ANG,LIMS1,THBS4,APOD,PAPLN,BLM,AKAP9,ITIH2,CENPF,S100A9,FLNA,SMARCA1,GSN,TAF9,IGFBP3,EFEMP1,TNRC6A,ILK,GAPDH,CST3,ADIPOQ,IGF2,C4A,MST1,ITIH3,C4B,PAEP,RARRES2,UIMC1,SERPINA3,SERPINA4,VEGFC,KNG1,GRHL2,CDH5
GO Process	GO:2001140	Positive regulation of phospholipid transport	3	15	1.24	0.0474	CETP,APOA1,APOE
GO Process	GO:0001817	Regulation of cytokine production	19	739	0.35	0.0490	EBI3,LTF,APOA1,ORM1,CSF1R,PGLYRP2,PF4,CAMP,F2,SERPINF2,FBLN1,APOD,APOA2,MCOLN2,GAPDH,ADIPOQ,ORM2,PAEP,B2M
GO Process	GO:0006457	Protein folding	9	215	0.56	0.0490	APCS,HSP90B1,CALR,HSPA5,P4HB,QSOX1,HYOU1,B2M,PDIA4
GO Function	GO:0004866	Endopeptidase inhibitor activity	27	177	1.12	1.86e-17	SPP2,SERPIND1,LTF,HRG,SERPINA10,CSTA,ITIH4,ITIH1,AHSG,SERPING1,SERPINI1,A2ML1,LPA,SERPINF2,SERPINA7,SERPINA5,SERPINA11,PAPLN,ITIH2,GAPDH,CST3,C4A,ITIH3,C4B,SERPINA3,SERPINA4,KNG1
GO Function	GO:0061134	Peptidase regulator activity	29	227	1.05	2.02e-17	SPP2,SERPIND1,PCOLCE,LTF,HRG,SERPINA10,CSTA,ITIH4,ITIH1,AHSG,SERPING1,SERPINI1,A2ML1,LPA,SERPINF2,SERPINA7,FBLN1,SERPINA5,SERPINA11,PAPLN,ITIH2,GAPDH,CST3,C4A,ITIH3,C4B,SERPINA3,SERPINA4,KNG1
GO Function	GO:0005539	Glycosaminoglycan binding	29	245	1.01	6.72e-17	SERPIND1,COMP,PCOLCE,MPO,PF4V1,LTF,HRG,APOB,APOE,SERPINA10,ITIH1,HABP2,PGLYRP2,PF4,F2,EFEMP2,LPA,FBN1,SERPINA5,ANG,THBS4,ITIH2,CFH,CCN5,SOD3,NAV2,F11,APOA5,KNG1
GO Function	GO:0008201	Heparin binding	25	173	1.1	3.12e-16	SERPIND1,COMP,PCOLCE,MPO,PF4V1,LTF,HRG,APOB,APOE,SERPINA10,PF4,F2,EFEMP2,LPA,FBN1,SERPINA5,ANG,THBS4,CFH,CCN5,SOD3,NAV2,F11,APOA5,KNG1
GO Function	GO:0004252	Serine-type endopeptidase activity	25	176	1.09	3.88e-16	F9,MMP2,LTF,PROC,F12,KLKB1,HABP2,MASP1,PCSK9,BMP1,F2,PLG,LPA,PROZ,CD5L,F10,F7,CFI,MASP2,F11,C1S,MST1,CFB,C1R,CFD
GO Function	GO:0005509	Calcium ion binding	42	717	0.71	4.91e-15	F9,COMP,PCDH12,PROC,LRP1,F12,APCS,DGKG,ITIH1,HABP2,IHH,MASP1,HSP90B1,BMP1,DST,F2,EFEMP2,CALR,ITLN1,HSPA5,FBN1,FBLN1,OIT3,THBS4,PROZ,PCDH18,S100A7,S100A9,CRTAC1,GSN,SVEP1,F10,F7,CALML5,EFEMP1,EFCAB5,MASP2,C1S,NUCB1,COLEC11,C1R,CDH5
GO Function	GO:1901681	Sulfur compound binding	26	272	0.92	3.26e-13	SERPIND1,COMP,PCOLCE,MPO,PF4V1,LTF,HRG,APOB,LRP1,APOE,SERPINA10,PF4,F2,EFEMP2,LPA,FBN1,SERPINA5,ANG,THBS4,CFH,CCN5,SOD3,NAV2,F11,APOA5,KNG1
GO Function	GO:0005102	Signaling receptor binding	56	1499	0.51	1.69e-12	EBI3,COMP,PF4V1,APOC3,HRG,APOB,APOA1,TTR,LRP1,APOE,PXDN,ANGPTL6,VWF,CRH,FCN2,SCGB3A1,IHH,PF4,HSP90B1,LRG1,PCSK9,BMP1,FGB,DST,F2,INHBC,PLG,CALR,LPA,HBA1,FBN1,P4HB,FBLN1,ANG,THBS4,AKAP9,APOA2,S100A7,S100A9,FLNA,CCN5,F7,EFEMP1,FGL1,APOF,ADIPOQ,IGF2,MST1,TGFBI,RARRES2,APOA5,IGHV3-15,IGHV3-72,VEGFC,KNG1,CDH5
GO Function	GO:0004857	Enzyme inhibitor activity	29	396	0.81	3.51e-12	SPP2,SERPIND1,APOC3,LTF,HRG,SERPINA10,CSTA,ITIH4,ITIH1,AHSG,SERPING1,SERPINI1,A2ML1,LPA,SERPINF2,SERPINA7,SERPINA5,SERPINA11,PAPLN,ITIH2,APOA2,GAPDH,CST3,C4A,ITIH3,C4B,SERPINA3,SERPINA4,KNG1
GO Function	GO:0004867	Serine-type endopeptidase inhibitor activity	17	98	1.18	5.35e-12	SERPIND1,HRG,SERPINA10,ITIH4,ITIH1,SERPING1,SERPINI1,A2ML1,SERPINF2,SERPINA7,SERPINA5,SERPINA11,PAPLN,ITIH2,ITIH3,SERPINA3,SERPINA4
GO Function	GO:0098772	Molecular function regulator activity	62	1960	0.44	4.98e-11	SPP2,SERPIND1,EBI3,PCOLCE,PF4V1,APOC3,GPLD1,LTF,HRG,APOA1,TTR,APOE,PXDN,SERPINA10,YWHAE,CSTA,ITIH4,ITIH1,AHSG,CRH,SERPING1,SCGB3A1,SERPINI1,PF4,A2ML1,PCSK9,BMP1,F2,INHBC,CPN2,LPA,SERPINF2,FBN1,SERPINA7,FBLN1,SERPINA5,SERPINA11,THBS4,PAPLN,GIT2,AKAP9,APOA4,ITIH2,APOA2,FLNA,CALML5,IGFBP3,EFEMP1,GAPDH,RAPGEF4,CST3,NUCB1,ADIPOQ,IGF2,C4A,ITIH3,C4B,APOA5,SERPINA3,SERPINA4,VEGFC,KNG1
GO Function	GO:0001848	Complement binding	11	26	1.57	6.37e-11	APCS,CFHR1,CALR,C8A,CFHR3,CFH,C8G,MASP2,C4A,C4B,CFB

GO Function	GO:0004175	Endopeptidase activity	28	437	0.75	1.75e-10	F9,MMP2,LTF,PROC,F12,PSMA6,KLKB1,HABP2,PIP,MASP1,PCSK9,BMP1,F2,PLG,LPA,PROZ,PAPLN,CD5L,F10,F7,CFI,MASP2,F11,C1S,MST1,CFB,C1R,CFD
GO Function	GO:0008233	Peptidase activity	31	617	0.64	3.53e-09	CPB2,F9,MMP2,LTF,PROC,F12,PSMA6,KLKB1,HABP2,PIP,IHH,MASP1,PCSK9,BMP1,F2,PLG,LPA,PROZ,PAPLN,CNDP1,CD5L,F10,F7,CFI,MASP2,F11,C1S,MST1,CFB,C1R,CFD
GO Function	GO:0005515	Protein binding	131	7242	0.2	2.69e-08	ST6GAL1,GNPTG,LGALS1,TPD52L2,MMP2,EBI3,COMP,PF4V1,APOC3,HRG,APOB,IGFBP5,PLEK,APOA1,TTR,CAT,LRP1,APOE,PXDN,ANGPTL6,F12,APCS,VWF,PSMA6,LCAT,YWHAE,CSTA,CP,DNAH5,CENPE,PON3,TCIRG1,FCN3,CRH,CSF1R,PIP,FCN2,PSG6,SCGB3A1,IHH,PF4,MASP1,A2ML1,HSP90B1,ANTXR1,LRG1,SH3D19,PCSK9,BMP1,FGB,DST,F2,INHBC,PLG,EFEMP2,CSPG4,C1QB,CFHR1,SUMO4,CALR,LPA,SERPINF2,HBA1,ITLN1,HSPA5,FBN1,P4HB,FBLN1,SERPINA5,DNAH8,ANG,LIMS1,THBS4,BLM,GIT2,AKAP9,APOA4,MTCL1,C8A,CENPF,CFHR3,CFH,APOA2,S100A7,S100A9,ADAMTSL4,FLNA,MCOLN2,SMARCA1,C8G,CCN5,GSN,F7,TAF9,IGFBP3,GPX3,KIF19,EFEMP1,ILK,GAPDH,RAPGEF4,FGL1,APOF,CST3,MASP2,F11,C1S,NUCB1,ADIPOQ,IGF2,C4A,COLEC11,MST1,LMOD3,C4B,TGFBI,CFB,IGFALS,PLTP,RARRES2,GC,UIMC1,C1R,APOA5,IGHV3-15,IGHV3-72,VEGFC,HYOU1,KNG1,CDH5,B2M
GO Function	GO:0030234	Enzyme regulator activity	42	1239	0.47	9.90e-08	SPP2,SERPIND1,PCOLCE,APOC3,LTF,HRG,APOA1,APOE,SERPINA10,CSTA,ITIH4,ITIH1,AHSG,SERPING1,SERPINI1,A2ML1,CPN2,LPA,SERPINF2,SERPINA7,FBLN1,SERPINA5,SERPINA11,PAPLN,GIT2,APOA4,ITIH2,APOA2,CALML5,IGFBP3,GAPDH,RAPGEF4,CST3,NUCB1,IGF2,C4A,ITIH3,C4B,APOA5,SERPINA3,SERPINA4,KNG1
GO Function	GO:0070325	Lipoprotein particle receptor binding	9	30	1.42	9.90e-08	APOC3,APOB,APOA1,LRP1,APOE,HSP90B1,PCSK9,APOA2,APOA5
GO Function	GO:0005488	Binding	188	12838	0.11	3.74e-07	ST6GAL1,CPB2,CETP,GNPTG,SERPIND1,LGALS1,TPD52L2,F9,MMP2,EBI3,COMP,PCOLCE,MPO,PF4V1,APOC3,PCDH12,LTF,HRG,APOB,IGFBP5,PROC,PLEK,APOA1,TTR,CAT,LRP1,APOE,PXDN,ANGPTL6,F12,APCS,VWF,PSMA6,SERPINA10,ATRN,LCAT,YWHAE,CSTA,CP,KLKB1,DGKG,DNAH5,CENPE,PON3,TCIRG1,HPX,FCN3,ITIH1,CRH,HABP2,CSF1R,PIP,FCN2,PSG6,PGLYRP2,SCGB3A1,IHH,PF4,MASP1,CAMP,GUCY1A1,A2ML1,HSP90B1,ANTXR1,LRG1,SH3D19,PCSK9,BMP1,FGB,DST,F2,INHBC,PLG,EFEMP2,CSPG4,C1QB,CFHR1,APOL1,SUMO4,CALR,LPA,SERPINF2,HBA1,ITLN1,HSPA5,FBN1,P4HB,FBLN1,SERPINA5,DNAH8,OIT3,ANG,LIMS1,THBS4,GANAB,PROZ,QPCT,APOD,ASGR2,BLM,GIT2,AKAP9,APOA4,ITIH2,CNDP1,MTCL1,C8A,PCDH18,CENPF,CFHR3,CFH,QSOX1,F5,APOA2,S100A7,S100A9,ADAMTSL4,FLNA,CRTAC1,MCOLN2,SMARCA1,C8B,C8G,CCN5,GSN,SVEP1,F10,F7,APOM,CA6,CKAP4,CALML5,TAF9,IGFBP3,SOD3,GPX3,KIF19,EFEMP1,CFI,EFCAB5,TNRC6A,NAV2,ILK,GAPDH,RAPGEF4,RNASE1,FGL1,APOF,CST3,MASP2,F11,C1S,NUCB1,PCYOX1,ADIPOQ,IGF2,C4A,ATP9B,CDK3,ABCA13,COLEC11,MST1,LMOD3,C4B,TGFBI,CFB,IGFALS,PLTP,PAEP,RARRES2,GC,UIMC1,SELENOP,C1R,APOA5,SERPINA3,RNASE4,IGKV2D-28,IGHV3-15,IGHV3-72,VEGFC,HYOU1,KNG1,TRIM66,GRHL2,CDH5,B2M,PDIA4
GO Function	GO:0001846	Opsonin binding	7	21	1.46	4.56e-06	APCS,CFHR1,CALR,CFHR3,CFH,MASP2,C4A
GO Function	GO:0120020	Cholesterol transfer activity	7	23	1.42	7.38e-06	CETP,APOB,APOA1,APOE,APOA4,APOA2,PLTP
GO Function	GO:0060228	Phosphatidylcholine-sterol O-acyltransferase activator activity	5	6	1.86	1.49e-05	APOA1,APOE,APOA4,APOA2,APOA5
GO Function	GO:0005319	Lipid transporter activity	13	163	0.84	2.07e-05	CETP,APOB,APOA1,APOE,APOD,APOA4,APOA2,APOM,APOF,ATP9B,ABCA13,PLTP,APOC4
GO Function	GO:0031210	Phosphatidylcholine binding	7	29	1.32	2.45e-05	CETP,APOA1,SERPINA5,APOA4,APOA2,PLTP,APOA5
GO Function	GO:0015485	Cholesterol binding	8	52	1.13	6.34e-05	CETP,APOC3,APOA1,APOD,APOA4,APOA2,APOF,APOA5
GO Function	GO:0016209	Antioxidant activity	9	76	1.01	8.49e-05	MPO,CAT,APOE,PXDN,APOA4,S100A9,APOM,SOD3,GPX3
GO Function	GO:0043169	Cation binding	81	4335	0.21	0.00025	CPB2,CETP,F9,MMP2,COMP,MPO,PCDH12,LTF,HRG,PROC,APOA1,CAT,LRP1,APOE,PXDN,F12,APCS,CP,KLKB1,DGKG,PON3,HPX,FCN3,ITIH1,HABP2,FCN2,PGLYRP2,IHH,MASP1,HSP90B1,ANTXR1,BMP1,DST,F2,EFEMP2,CALR,HBA1,ITLN1,HSPA5,FBN1,FBLN1,SERPINA5,OIT3,ANG,LIMS1,THBS4,PROZ,QPCT,BLM,GIT2,APOA4,CNDP1,PCDH18,F5,APOA2,S100A7,S100A9,CRTAC1,GSN,SVEP1,F10,F7,CA6,CALML5,IGFBP3,SOD3,EFEMP1,CFI,EFCAB5,MASP2,C1S,NUCB1,ATP9B,COLEC11,PLTP,UIMC1,C1R,APOA5,KNG1,TRIM66,CDH5
GO Function	GO:0043178	Alcohol binding	9	91	0.94	0.00029	CETP,APOC3,APOA1,APOD,APOA4,APOA2,C8G,APOF,APOA5
GO Function	GO:0005178	Integrin binding	11	159	0.78	0.00053	COMP,VWF,DST,CALR,FBN1,P4HB,FBLN1,THBS4,CCN5,IGF2,TGFBI
GO Function	GO:0044877	Protein-containing complex binding	34	1261	0.37	0.00053	COMP,PCOLCE,HRG,APOA1,TTR,LRP1,APOE,APCS,VWF,YWHAE,PIP,ANTXR1,PCSK9,DST,CALR,HSPA5,FBN1,P4HB,FBLN1,THBS4,C8A,CENPF,APOA2,FLNA,SMARCA1,C8B,CCN5,GSN,IGF2,C4A,TGFBI,PLTP,CDH5,B2M
GO Function	GO:0005496	Steroid binding	9	101	0.89	0.00058	CETP,APOC3,APOA1,APOD,APOA4,APOA2,APOF,GC,APOA5
GO Function	GO:0097367	Carbohydrate derivative binding	50	2278	0.28	0.00070	SERPIND1,COMP,PCOLCE,MPO,PF4V1,LTF,HRG,APOB,LRP1,APOE,SERPINA10,DGKG,DNAH5,CENPE,ITIH1,HABP2,CSF1R,FCN2,PGLYRP2,PF4,CAMP,GUCY1A1,HSP90B1,F2,EFEMP2,LPA,HSPA5,FBN1,SERPINA5,DNAH8,ANG,THBS4,BLM,ITIH2,CFH,SMARCA1,CCN5,SOD3,KIF19,NAV2,ILK,RAPGEF4,F11,ADIPOQ,ATP9B,CDK3,ABCA13,APOA5,HYOU1,KNG1
GO Function	GO:0034185	Apolipoprotein binding	5	19	1.36	0.00077	LRP1,LCAT,PCSK9,PLG,LPA
GO Function	GO:0043395	Heparan sulfate proteoglycan binding	5	19	1.36	0.00077	COMP,HRG,LRP1,APOE,CFH
GO Function	GO:0043394	Proteoglycan binding	6	37	1.15	0.00098	COMP,HRG,LRP1,APOE,FCN2,CFH
GO Function	GO:0042802	Identical protein binding	47	2144	0.28	0.0014	ST6GAL1,GNPTG,TPD52L2,PLEK,APOA1,TTR,CAT,APOE,APCS,VWF,YWHAE,PON3,FCN3,CSF1R,PIP,FCN2,MASP1,BMP1,DST,EFEMP2,C1QB,CFHR1,SERPINF2,ITLN1,FBN1,FBLN1,ANG,BLM,APOA4,MTCL1,CENPF,CFH,APOA2,ADAMTSL4,FLNA,MCOLN2,GPX3,GAPDH,CST3,MASP2,F11,C1S,ADIPOQ,COLEC11,TGFBI,C1R,B2M
GO Function	GO:0050750	Low-density lipoprotein particle receptor binding	5	25	1.24	0.0022	APOB,APOE,HSP90B1,PCSK9,APOA5
GO Function	GO:0043167	Ion binding	99	6033	0.16	0.0024	CPB2,CETP,F9,MMP2,COMP,MPO,PCDH12,LTF,HRG,PROC,PLEK,APOA1,CAT,LRP1,APOE,PXDN,F12,APCS,PSMA6,CP,KLKB1,DGKG,DNAH5,CENPE,PON3,HPX,FCN3,ITIH1,HABP2,CSF1R,FCN2,PGLYRP2,IHH,MASP1,GUCY1A1,HSP90B1,ANTXR1,BMP1,DST,F2,EFEMP2,CALR,HBA1,ITLN1,HSPA5,FBN1,FBLN1,SERPINA5,DNAH8,OIT3,ANG,LIMS1,THBS4,PROZ,QPCT,BLM,GIT2,APOA4,CNDP1,PCDH18,QSOX1,F5,APOA2,S100A7,S100A9,CRTAC1,SMARCA1,GSN,SVEP1,F10,F7,CA6,CALML5,IGFBP3,SOD3,KIF19,EFEMP1,CFI,EFCAB5,NAV2,ILK,RAPGEF4,MASP2,C1S,NUCB1,PCYOX1,ADIPOQ,ATP9B,CDK3,ABCA13,COLEC11,PLTP,UIMC1,C1R,APOA5,HYOU1,KNG1,TRIM66,CDH5
GO Function	GO:0016787	Hydrolase activity	49	2347	0.26	0.0028	CPB2,APMAP,F9,MMP2,GPLD1,LTF,PROC,CAT,F12,PSMA6,LCAT,KLKB1,PON3,HYAL1,HABP2,PIP,PGLYRP2,IHH,MASP1,HSP90B1,PCSK9,BMP1,F2,PLG,LPA,HSPA5,ANG,GANAB,PROZ,PAPLN,BLM,CNDP1,CD5L,SMARCA1,F10,F7,KIF19,CFI,NAV2,RNASE1,MASP2,F11,C1S,ATP9B,MST1,CFB,C1R,RNASE4,CFD
GO Function	GO:0070653	High-density lipoprotein particle receptor binding	3	3	1.94	0.0029	APOC3,APOA1,APOA2
GO Function	GO:0042803	Protein homodimerization activity	22	709	0.43	0.0032	ST6GAL1,GNPTG,TPD52L2,PLEK,APOA1,CAT,APOE,PON3,CSF1R,MASP1,DST,EFEMP2,SERPINF2,ANG,BLM,APOA4,MTCL1,CENPF,APOA2,FLNA,ADIPOQ,B2M
GO Function	GO:0071813	Lipoprotein particle binding	5	28	1.19	0.0032	APOA1,APOE,PCSK9,APOA2,PLTP

GO Function	GO:0046872	Metal ion binding	75	4250	0.19	0.0039	CPB2,F9,MMP2,COMP,MPO,PCDH12,LTF,HRG,PROC,CAT,LRP1,APOE,PXDN,F12,APCS,CP,KLKB1,DGKG,PON3,HPX,FCN3,ITIH1,HABP2,FCN2,PGLYRP2,IHH,MASP1,HSP90B1,ANTXR1,BMP1,DST,F2,EFEMP2,CALR,HBA1,ITLN1,HSPA5,FBN1,FBLN1,OIT3,ANG,LIMS1,THBS4,PROZ,QPCT,BLM,GIT2,APOA4,CNDP1,PCDH18,F5,S100A7,S100A9,CRTAC1,GSN,SVEP1,F10,F7,CA6,CALML5,IGFBP3,SOD3,EFEMP1,CFI,EFCAB5,MASP2,C1S,NUCB1,ATP9B,COLEC11,UIMC1,C1R,KNG1,TRIM66,CDH5
GO Function	GO:0001968	Fibronectin binding	5	30	1.16	0.0041	MMP2,IGFBP5,LPA,FBLN1,IGFBP3
GO Function	GO:0004869	Cysteine-type endopeptidase inhibitor activity	6	54	0.99	0.0055	LTF,HRG,CSTA,AHSG,CST3,KNG1
GO Function	GO:0008289	Lipid binding	23	796	0.4	0.0055	CETP,APOC3,LTF,APOB,APOA1,APOE,DGKG,CAMP,F2,APOL1,SERPINA5,APOD,APOA4,APOA2,S100A9,C8G,GSN,F10,APOM,APOF,PLTP,GC,APOA5
GO Function	GO:0030545	Signaling receptor regulator activity	18	552	0.45	0.0083	EBI3,PF4V1,APOA1,TTR,PXDN,CRH,SCGB3A1,PF4,PCSK9,BMP1,F2,INHBC,FBN1,THBS4,EFEMP1,ADIPOQ,IGF2,VEGFC
GO Function	GO:0034190	Apolipoprotein receptor binding	3	6	1.64	0.0100	APOA1,PCSK9,APOA2
GO Function	GO:0005201	Extracellular matrix structural constituent	8	131	0.73	0.0162	COMP,PXDN,FGB,EFEMP2,FBN1,FBLN1,OIT3,TGFBI
GO Function	GO:0001849	Complement component C1q complex binding	3	8	1.51	0.0186	APCS,CALR,C4A
GO Function	GO:0002020	Protease binding	8	135	0.71	0.0190	COMP,VWF,CSTA,A2ML1,SERPINF2,SERPINA5,ADAMTSL4,CST3
GO Function	GO:0003823	Antigen binding	6	71	0.87	0.0195	FCN3,FCN2,PLG,IGKV2D-28,IGHV3-15,IGHV3-72
GO Function	GO:0048018	Receptor ligand activity	16	499	0.45	0.0221	EBI3,PF4V1,APOA1,TTR,CRH,SCGB3A1,PF4,BMP1,F2,INHBC,FBN1,THBS4,EFEMP1,ADIPOQ,IGF2,VEGFC
GO Function	GO:0051087	Chaperone binding	7	108	0.75	0.0263	VWF,CP,FGB,PLG,CALR,HSPA5,HYOU1
GO Function	GO:0008035	High-density lipoprotein particle binding	3	10	1.42	0.0287	APOA1,APOA2,PLTP
GO Function	GO:0001851	Complement component C3b binding	3	11	1.38	0.0357	CFHR1,CFHR3,CFH
GO Function	GO:0016670	Oxidoreductase activity, acting on a sulfur group of donors, oxygen as acceptor	3	11	1.38	0.0357	P4HB,QSOX1,PCYOX1
GO Function	GO:0030246	Carbohydrate binding	11	278	0.54	0.0357	LGALS1,APCS,ATRN,FCN3,FCN2,CALR,ITLN1,GANAB,ASGR2,COLEC11,C4B
GO Component	GO:0005615	Extracellular space	170	3247	0.66	9.71e-80	CPB2,CETP,GNPTG,SERPIND1,LGALS1,CHGA,F9,MMP2,EBI3,COMP,PCOLCE,MPO,PF4V1,APOC3,GPLD1,PCDH12,LTF,HRG,APOB,IGFBP5,PROC,APOA1,TTR,CAT,C4BPB,CFP,APOE,PXDN,ANGPTL6,F12,APCS,ORM1,VWF,PSMA6,SERPINA10,ATRN,LCAT,YWHAE,CSTA,CP,KLKB1,PON3,HPX,HYAL1,ITIH4,FCN3,ITIH1,AHSG,CRH,HABP2,SAA4,SERPING1,PIP,FCN2,PGLYRP2,SCGB3A1,IHH,SERPINI1,PF4,MASP1,CAMP,A2ML1,HSP90B1,LRG1,PCSK9,BMP1,FGB,F2,INHBC,PLG,EFEMP2,CSPG4,C1QB,CFHR1,APOL1,CPN2,CALR,LPA,SERPINF2,C7,HBA1,ITLN1,HSPA5,FBN1,P4HB,SERPINA7,FBLN1,SERPINA5,OIT3,SERPINA11,ANG,THBS4,GANAB,FAM184A,PROZ,QPCT,APOD,PIGR,APOA4,ITIH2,MTCL1,C8A,F13B,CFHR3,CFH,QSOX1,F5,APOA2,CD5L,S100A7,S100A9,FLNA,CRTAC1,C8B,C8G,CCN5,GSN,SVEP1,C1QC,F10,F7,APOM,CA6,CKAP4,IGFBP3,SOD3,GPX3,EFEMP1,CFI,GAPDH,RNASE1,FGL1,APOF,CST3,MASP2,F11,C1S,NUCB1,PCYOX1,ADIPOQ,IGF2,ORM2,C4A,COLEC11,MST1,ITIH3,C4B,TGFBI,CFB,IGFALS,PLTP,PAEP,RARRES2,GC,SELENOP,C1R,APOA5,SERPINA3,SERPINA4,RNASE4,IGKV2D-28,APOC4,CFD,IGHV3-15,IGHV3-72,VEGFC,HYOU1,KNG1,B2M,PDIA4
GO Component	GO:0005576	Extracellular region	180	4175	0.57	3.54e-74	SPP2,ST6GAL1,CPB2,CETP,GNPTG,SERPIND1,LGALS1,CHGA,F9,MMP2,EBI3,COMP,PCOLCE,MPO,PF4V1,APOC3,GPLD1,PCDH12,LTF,HRG,APOB,IGFBP5,PROC,PLEK,APOA1,TTR,CAT,C4BPB,CFP,APOE,PXDN,ANGPTL6,F12,APCS,ORM1,VWF,PSMA6,SERPINA10,ATRN,LCAT,YWHAE,CSTA,CP,KLKB1,DNAH5,CENPE,PON3,HPX,HYAL1,ITIH4,FCN3,ITIH1,AHSG,CRH,HABP2,SAA4,SERPING1,PIP,FCN2,PSG6,PGLYRP2,SCGB3A1,IHH,SERPINI1,PF4,MASP1,CAMP,A2ML1,HSP90B1,LRG1,PCSK9,BMP1,FGB,F2,INHBC,PLG,EFEMP2,CSPG4,C1QB,CFHR1,APOL1,CPN2,CALR,LPA,SERPINF2,C7,HBA1,ITLN1,HSPA5,FBN1,P4HB,SERPINA7,FBLN1,SERPINA5,OIT3,SERPINA11,ANG,THBS4,GANAB,FAM184A,PROZ,QPCT,APOD,PAPLN,PIGR,APOA4,ITIH2,CNDP1,MTCL1,C8A,F13B,CFHR3,CFH,QSOX1,F5,APOA2,CD5L,S100A7,S100A9,ADAMTSL4,FLNA,CRTAC1,C8B,C8G,CCN5,GSN,SVEP1,C1QC,F10,F7,APOM,CA6,CKAP4,CALML5,IGFBP3,SOD3,GPX3,EFEMP1,CFI,GAPDH,RNASE1,FGL1,APOF,CST3,MASP2,F11,C1S,NUCB1,PCYOX1,ADIPOQ,IGF2,ORM2,C4A,COLEC11,MST1,ITIH3,C4B,TGFBI,CFB,IGFALS,PLTP,PAEP,RARRES2,GC,SELENOP,C1R,APOA5,SERPINA3,SERPINA4,RNASE4,IGKV2D-28,APOC4,CFD,IGHV3-15,IGHV3-72,VEGFC,HYOU1,KNG1,B2M,PDIA4
GO Component	GO:1903561	Extracellular vesicle	115	2120	0.67	5.49e-47	CPB2,CETP,GNPTG,SERPIND1,LGALS1,F9,COMP,PCOLCE,MPO,APOC3,GPLD1,PCDH12,LTF,HRG,APOB,APOA1,TTR,CAT,APOE,PXDN,ANGPTL6,F12,APCS,ORM1,VWF,PSMA6,SERPINA10,ATRN,LCAT,YWHAE,CP,KLKB1,PON3,HPX,HYAL1,ITIH4,ITIH1,AHSG,SAA4,SERPING1,PIP,FCN2,PGLYRP2,SCGB3A1,SERPINI1,CAMP,A2ML1,HSP90B1,LRG1,FGB,F2,PLG,EFEMP2,CSPG4,CPN2,CALR,SERPINF2,C7,HBA1,ITLN1,HSPA5,P4HB,SERPINA7,FBLN1,SERPINA5,THBS4,GANAB,PROZ,QPCT,APOD,PIGR,APOA4,ITIH2,C8A,CFHR3,CFH,QSOX1,F5,APOA2,S100A9,FLNA,CRTAC1,C8B,C8G,GSN,CA6,CKAP4,SOD3,GPX3,EFEMP1,CFI,GAPDH,RNASE1,CST3,MASP2,F11,NUCB1,PCYOX1,ORM2,C4A,ITIH3,C4B,TGFBI,CFB,IGFALS,GC,SELENOP,C1R,SERPINA3,SERPINA4,IGKV2D-28,CFD,HYOU1,KNG1,B2M
GO Component	GO:0070062	Extracellular exosome	114	2096	0.68	7.35e-47	CPB2,CETP,GNPTG,SERPIND1,LGALS1,F9,COMP,PCOLCE,MPO,APOC3,GPLD1,PCDH12,LTF,HRG,APOB,APOA1,TTR,CAT,APOE,PXDN,ANGPTL6,F12,APCS,ORM1,VWF,PSMA6,SERPINA10,ATRN,LCAT,YWHAE,CP,KLKB1,PON3,HPX,HYAL1,ITIH4,ITIH1,AHSG,SAA4,SERPING1,PIP,FCN2,PGLYRP2,SCGB3A1,SERPINI1,CAMP,A2ML1,HSP90B1,LRG1,FGB,F2,PLG,EFEMP2,CSPG4,CPN2,CALR,SERPINF2,C7,HBA1,ITLN1,HSPA5,P4HB,SERPINA7,FBLN1,SERPINA5,THBS4,GANAB,PROZ,QPCT,APOD,PIGR,APOA4,ITIH2,C8A,CFHR3,CFH,QSOX1,APOA2,S100A9,FLNA,CRTAC1,C8B,C8G,GSN,CA6,CKAP4,SOD3,GPX3,EFEMP1,CFI,GAPDH,RNASE1,CST3,MASP2,F11,NUCB1,PCYOX1,ORM2,C4A,ITIH3,C4B,TGFBI,CFB,IGFALS,GC,SELENOP,C1R,SERPINA3,SERPINA4,IGKV2D-28,CFD,HYOU1,KNG1,B2M
GO Component	GO:0072562	Blood microparticle	42	118	1.49	2.54e-43	HRG,APOA1,APOE,APCS,ORM1,CP,HPX,ITIH4,FCN3,ITIH1,AHSG,SERPING1,FCN2,FGB,F2,PLG,C1QB,CFHR1,APOL1,CPN2,SERPINF2,HBA1,APOA4,ITIH2,C8A,CFHR3,CFH,APOA2,CD5L,C8G,GSN,C1QC,C1S,ORM2,C4A,C4B,CFB,GC,C1R,SERPINA3,IGKV2D-28,KNG1
GO Component	GO:0062023	Collagen-containing extracellular matrix	56	407	1.08	4.75e-39	SPP2,LGALS1,F9,MMP2,COMP,APOC3,HRG,APOA1,APOE,PXDN,ANGPTL6,F12,APCS,ORM1,VWF,ATRN,HPX,ITIH4,FCN3,ITIH1,AHSG,SERPING1,FCN2,PF4,HSP90B1,FGB,DST,F2,PLG,EFEMP2,CSPG4,C1QB,CALR,SERPINF2,FBN1,FBLN1,ANG,THBS4,APOA4,ITIH2,S100A7,S100A9,ADAMTSL4,C1QC,F7,SOD3,EFEMP1,NAV2,FGL1,ADIPOQ,ORM2,MST1,TGFBI,RARRES2,SERPINA3,KNG1
GO Component	GO:0031012	Extracellular matrix	62	552	0.99	5.95e-39	SPP2,LGALS1,F9,MMP2,COMP,APOC3,GPLD1,HRG,APOA1,APOE,PXDN,ANGPTL6,F12,APCS,ORM1,VWF,ATRN,HPX,ITIH4,FCN3,ITIH1,AHSG,SERPING1,FCN2,IHH,PF4,HSP90B1,FGB,DST,F2,PLG,EFEMP2,CSPG4,C1QB,CPN2,CALR,SERPINF2,FBN1,FBLN1,ANG,THBS4,PAPLN,APOA4,ITIH2,S100A7,S100A9,ADAMTSL4,CCN5,C1QC,F7,SOD3,EFEMP1,NAV2,FGL1,ADIPOQ,ORM2,MST1,TGFBI,IGFALS,RARRES2,SERPINA3,KNG1

GO Component	GO:0031982	Vesicle	137	3957	0.48	1.94e-37	SPP2,CPB2,CETP,GNPTG,SERPIND1,LGALS1,CHGA,F9,COMP,PCOLCE,MPO,APOC3,GPLD1,PCDH12,LTF,HRG,APOB,APOA1,TTR,CAT,LRP1,CFP,APOE,PXDN,ANGPTL6,F12,APCS,ORM1,VWF,PSMA6,SERPINA10,ATRN,LCAT,YWHAE,CP,KLKB1,PON3,TCIRG1,HPX,HYAL1,ITIH4,ITIH1,AHSG,SAA4,SERPING1,PIP,FCN2,PGLYRP2,SCGB3A1,SERPINI1,PF4,CAMP,A2ML1,HSP90B1,ANTXR1,LRG1,PCSK9,BMP1,FGB,DST,F2,PLG,EFEMP2,CSPG4,CPN2,CALR,SERPINF2,C7,HBA1,ITLN1,HSPA5,P4HB,SERPINA7,FBLN1,SERPINA5,ANG,THBS4,GANAB,PROZ,QPCT,APOD,PIGR,APOA4,ITIH2,C8A,CFHR3,CFH,QSOX1,F5,APOA2,S100A7,S100A9,FLNA,CRTAC1,MCOLN2,C8B,C8G,GSN,F7,CA6,CKAP4,CALML5,SOD3,GPX3,EFEMP1,CFI,GAPDH,RNASE1,CST3,MASP2,F11,NUCB1,PCYOX1,IGF2,ORM2,C4A,ATP9B,ABCA13,ITIH3,C4B,TGFBI,CFB,IGFALS,RARRES2,GC,SELENOP,C1R,APOA5,SERPINA3,SERPINA4,IGKV2D-28,CFD,VEGFC,HYOU1,KNG1,B2M,PDIA4
GO Component	GO:0005788	Endoplasmic reticulum lumen	43	312	1.08	1.75e-29	SPP2,SERPIND1,LGALS1,F9,EBI3,APOB,IGFBP5,PROC,APOA1,CFP,APOE,SERPINA10,CP,AHSG,SERPING1,HSP90B1,PCSK9,F2,APOL1,CALR,HSPA5,FBN1,P4HB,GANAB,PROZ,APOA4,ITIH2,QSOX1,F5,APOA2,ADAMTSL4,F10,F7,CKAP4,IGFBP3,CST3,NUCB1,C4A,APOA5,HYOU1,KNG1,B2M,PDIA4
GO Component	GO:0031983	Vesicle lumen	38	326	1.01	1.88e-23	SPP2,MPO,LTF,HRG,APOB,APOA1,TTR,CAT,CFP,ORM1,VWF,ITIH4,AHSG,SERPING1,SERPINI1,PF4,CAMP,LRG1,FGB,PLG,SERPINF2,QPCT,QSOX1,F5,S100A7,S100A9,GSN,IGF2,ORM2,ITIH3,RARRES2,SELENOP,SERPINA3,SERPINA4,CFD,VEGFC,KNG1,B2M
GO Component	GO:0034774	Secretory granule lumen	37	321	1.0	1.15e-22	SPP2,MPO,LTF,HRG,APOA1,TTR,CAT,CFP,ORM1,VWF,ITIH4,AHSG,SERPING1,SERPINI1,PF4,CAMP,LRG1,FGB,PLG,SERPINF2,QPCT,QSOX1,F5,S100A7,S100A9,GSN,IGF2,ORM2,ITIH3,RARRES2,SELENOP,SERPINA3,SERPINA4,CFD,VEGFC,KNG1,B2M
GO Component	GO:1905370	Serine-type endopeptidase complex	16	26	1.73	7.21e-19	F9,PROC,F12,KLKB1,FCN3,FCN2,MASP1,F2,CFH,F5,F10,F7,CFI,MASP2,F11,COLEC11
GO Component	GO:0034358	Plasma lipoprotein particle	17	38	1.59	2.46e-18	CETP,APOC3,APOB,APOA1,APOE,LCAT,SAA4,APOL1,LPA,APOA4,APOA2,APOM,APOF,PCYOX1,PLTP,APOA5,APOC4
GO Component	GO:0034364	High-density lipoprotein particle	15	29	1.65	6.94e-17	CETP,APOC3,APOB,APOA1,APOE,LCAT,SAA4,APOL1,APOA4,APOA2,APOM,APOF,PLTP,APOA5,APOC4
GO Component	GO:0030141	Secretory granule	47	873	0.67	1.85e-16	SPP2,CHGA,MPO,LTF,HRG,APOA1,TTR,CAT,CFP,ANGPTL6,ORM1,VWF,TCIRG1,ITIH4,AHSG,SERPING1,SERPINI1,PF4,CAMP,LRG1,FGB,PLG,CALR,SERPINF2,SERPINA5,QPCT,PIGR,QSOX1,F5,S100A7,S100A9,GSN,CKAP4,CALML5,CST3,IGF2,ORM2,ABCA13,ITIH3,RARRES2,SELENOP,SERPINA3,SERPINA4,CFD,VEGFC,KNG1,B2M
GO Component	GO:0031093	Platelet alpha granule lumen	17	66	1.35	4.34e-15	HRG,ORM1,VWF,AHSG,SERPING1,PF4,FGB,PLG,SERPINF2,QSOX1,F5,IGF2,ORM2,SERPINA3,CFD,VEGFC,KNG1
GO Component	GO:0031091	Platelet alpha granule	18	89	1.25	1.98e-14	HRG,ORM1,VWF,AHSG,SERPING1,PF4,FGB,PLG,SERPINF2,SERPINA5,QSOX1,F5,IGF2,ORM2,SERPINA3,CFD,VEGFC,KNG1
GO Component	GO:1905369	Endopeptidase complex	18	104	1.18	2.03e-13	F9,PROC,F12,PSMA6,KLKB1,FCN3,FCN2,MASP1,F2,UBE3C,CFH,F5,F10,F7,CFI,MASP2,F11,COLEC11
GO Component	GO:1905368	Peptidase complex	19	138	1.08	1.36e-12	F9,PROC,F12,PSMA6,KLKB1,FCN3,FCN2,MASP1,F2,UBE3C,CFH,F5,F10,F7,TAF9,CFI,MASP2,F11,COLEC11
GO Component	GO:0034361	Very-low-density lipoprotein particle	11	20	1.68	1.59e-12	APOC3,APOB,APOA1,APOE,APOL1,APOA4,APOA2,APOM,PCYOX1,APOA5,APOC4
GO Component	GO:0071944	Cell periphery	122	6015	0.25	2.07e-11	SPP2,LGALS1,F9,MMP2,EBI3,COMP,APOC3,GPLD1,PCDH12,LTF,HRG,APOB,PLEK,APOA1,CAT,LRP1,C4BPB,CFP,APOE,PXDN,ANGPTL6,F12,APCS,ORM1,VWF,ATRN,CSTA,CP,KLKB1,DGKG,TCIRG1,HPX,ITIH4,FCN3,ITIH1,AHSG,SERPING1,CSF1R,FCN2,PSG6,IHH,PF4,MASP1,HSP90B1,ANTXR1,SH3D19,PCSK9,FGB,DST,F2,PLG,EFEMP2,CSPG4,C1QB,CPN2,CALR,LPA,SERPINF2,C7,ITLN1,HSPA5,FBN1,P4HB,FBLN1,SERPINA5,ANG,LIMS1,THBS4,PAPLN,ASGR2,AKAP9,PIGR,APOA4,ITIH2,MTCL1,C8A,PCDH18,F5,CD5L,S100A7,S100A9,ADAMTSL4,FLNA,MCOLN2,C8B,C8G,CCN5,GSN,C1QC,F10,F7,CKAP4,SOD3,EFEMP1,NAV2,ILK,GAPDH,RAPGEF4,FGL1,CST3,MASP2,F11,PCYOX1,ADIPOQ,ORM2,C4A,ATP9B,ABCA13,COLEC11,MST1,C4B,TGFBI,CFB,IGFALS,RARRES2,SERPINA3,IGKV2D-28,IGHV3-15,IGHV3-72,KNG1,CDH5,B2M
GO Component	GO:0031410	Cytoplasmic vesicle	70	2482	0.39	3.65e-11	SPP2,CHGA,MPO,APOC3,LTF,HRG,APOB,APOA1,TTR,CAT,LRP1,CFP,APOE,ANGPTL6,ORM1,VWF,YWHAE,TCIRG1,HPX,HYAL1,ITIH4,AHSG,SERPING1,SERPINI1,PF4,CAMP,HSP90B1,ANTXR1,LRG1,PCSK9,FGB,DST,PLG,CALR,SERPINF2,HBA1,HSPA5,P4HB,SERPINA5,ANG,GANAB,QPCT,PIGR,APOA4,QSOX1,F5,APOA2,S100A7,S100A9,MCOLN2,GSN,CKAP4,CALML5,CST3,IGF2,ORM2,ATP9B,ABCA13,ITIH3,RARRES2,SELENOP,APOA5,SERPINA3,SERPINA4,CFD,VEGFC,HYOU1,KNG1,B2M,PDIA4
GO Component	GO:0012505	Endomembrane system	103	4721	0.28	9.44e-11	SPP2,ST6GAL1,GNPTG,SERPIND1,LGALS1,CHGA,F9,EBI3,MPO,APOC3,LTF,HRG,APOB,IGFBP5,PROC,APOA1,TTR,CAT,LRP1,CFP,APOE,PXDN,ANGPTL6,F12,ORM1,VWF,SERPINA10,CP,TCIRG1,ITIH4,AHSG,SERPING1,SERPINI1,PF4,CAMP,HSP90B1,ANTXR1,LRG1,PCSK9,BMP1,FGB,DST,F2,PLG,CSPG4,APOL1,CALR,SERPINF2,HSPA5,FBN1,P4HB,SERPINA5,OIT3,THBS4,GANAB,PROZ,QPCT,APOD,ASGR2,AKAP9,PIGR,APOA4,ITIH2,CENPF,QSOX1,F5,APOA2,S100A7,S100A9,ADAMTSL4,FLNA,MCOLN2,GSN,F10,F7,CKAP4,CALML5,IGFBP3,SOD3,TNRC6A,GAPDH,CST3,NUCB1,ADIPOQ,IGF2,ORM2,C4A,ATP9B,ABCA13,ITIH3,TGFBI,RARRES2,SELENOP,APOA5,SERPINA3,SERPINA4,CFD,VEGFC,HYOU1,KNG1,CDH5,B2M,PDIA4
GO Component	GO:0071682	Endocytic vesicle lumen	10	23	1.58	1.28e-10	MPO,LTF,APOB,APOA1,APOE,HPX,HSP90B1,CALR,HBA1,HYOU1
GO Component	GO:0005783	Endoplasmic reticulum	55	2021	0.38	8.04e-08	SPP2,ST6GAL1,SERPIND1,LGALS1,F9,EBI3,APOB,IGFBP5,PROC,APOA1,CAT,CFP,APOE,PXDN,F12,VWF,SERPINA10,CP,AHSG,SERPING1,HSP90B1,PCSK9,FGB,DST,F2,APOL1,CALR,HSPA5,FBN1,P4HB,THBS4,GANAB,PROZ,APOD,ASGR2,APOA4,ITIH2,QSOX1,F5,APOA2,S100A7,ADAMTSL4,F10,F7,CKAP4,IGFBP3,CST3,NUCB1,ADIPOQ,C4A,APOA5,HYOU1,KNG1,B2M,PDIA4
GO Component	GO:0042627	Chylomicron	7	13	1.67	8.56e-08	APOC3,APOB,APOA1,APOE,APOA4,APOA2,APOA5
GO Component	GO:0009986	Cell surface	32	894	0.49	1.06e-06	APMAP,EBI3,LTF,HRG,PXDN,FCN3,CSF1R,FCN2,PSG6,MASP1,ANTXR1,PCSK9,FGB,PLG,CSPG4,CALR,SERPINF2,HSPA5,P4HB,SERPINA5,OIT3,ASGR2,CD5L,F10,MASP2,ADIPOQ,COLEC11,IGHV3-15,IGHV3-72,CDH5,B2M,PDIA4
GO Component	GO:1904724	Tertiary granule lumen	9	55	1.15	2.29e-06	LTF,CFP,ORM1,CAMP,LRG1,QPCT,QSOX1,CST3,B2M
GO Component	GO:0031089	Platelet dense granule lumen	6	14	1.57	3.55e-06	SPP2,ITIH4,ITIH3,RARRES2,SELENOP,SERPINA4
GO Component	GO:0035580	Specific granule lumen	9	62	1.1	5.49e-06	LTF,CFP,ORM1,CAMP,LRG1,QPCT,QSOX1,ORM2,B2M
GO Component	GO:0009897	External side of plasma membrane	19	388	0.63	1.09e-05	EBI3,FCN3,FCN2,MASP1,ANTXR1,PCSK9,FGB,PLG,CALR,P4HB,SERPINA5,ASGR2,F10,MASP2,COLEC11,IGHV3-15,IGHV3-72,CDH5,B2M
GO Component	GO:0032991	Protein-containing complex	98	5506	0.19	2.87e-05	CETP,GNPTG,LGALS1,F9,EBI3,COMP,APOC3,LTF,APOB,IGFBP5,PROC,APOA1,TTR,CAT,LRP1,APOE,F12,PSMA6,LCAT,CSTA,KLKB1,DNAH5,TCIRG1,FCN3,SAA4,CSF1R,FCN2,MASP1,GUCY1A1,HSP90B1,PCSK9,FGB,F2,UBE3C,C1QB,CFHR1,APOL1,SUMO4,CALR,LPA,SERPINF2,C7,HBA1,ITLN1,HSPA5,P4HB,SERPINA5,DNAH8,ANG,GANAB,BLM,AKAP9,PIGR,APOA4,C8A,CENPF,F13B,CFH,F5,APOA2,FLNA,SMARCA1,C8B,C8G,C1QC,F10,F7,APOM,TAF9,IGFBP3,KIF19,CFI,TNRC6A,GAPDH,FGL1,APOF,MASP2,F11,C1S,PCYOX1,ADIPOQ,C4A,CDK3,COLEC11,C4B,CFB,IGFALS,PLTP,UIMC1,C1R,APOA5,IGKV2D-28,APOC4,IGHV3-15,IGHV3-72,HYOU1,CDH5,B2M

GO Component	GO:0070013	Intracellular organelle lumen	99	5660	0.18	5.47e-05	SPP2,SERPIND1,LGALS1,F9,EBI3,MPO,LTF,HRG,APOB,IGFBP5,PROC,APOA1,TTR,CAT,CFP,APOE,ORM1,VWF,PSMA6,SERPINA10,CSTA,CP,HPX,HYAL1,ITIH4,AHSG,SERPING1,CSF1R,SERPINI1,PF4,MASP1,CAMP,C16orf46,HSP90B1,LRG1,SH3D19,PCSK9,FGB,DST,F2,PLG,CSPG4,APOL1,CALR,SERPINF2,HBA1,HSPA5,FBN1,P4HB,ANG,GANAB,PROZ,QPCT,BLM,GIT2,APOA4,ITIH2,CENPF,QSOX1,F5,APOA2,S100A7,S100A9,ADAMTSL4,FLNA,SMARCA1,GSN,F10,F7,CKAP4,CALML5,TAF9,IGFBP3,SOD3,TNRC6A,NAV2,ILK,CST3,NUCB1,IGF2,ORM2,C4A,ITIH3,RARRES2,GC,UIMC1,SELENOP,APOA5,SERPINA3,SERPINA4,CFD,VEGFC,HYOU1,KNG1,TRIM66,GRHL2,CDH5,B2M,PDIA4
GO Component	GO:0005602	Complement component C1 complex	4	5	1.84	8.46e-05	C1QB,C1QC,C1S,C1R
GO Component	GO:0005604	Basement membrane	9	98	0.9	0.00015	PXDN,ATRN,DST,EFEMP2,FBN1,FBLN1,ANG,THBS4,TGFBI
GO Component	GO:0005579	Membrane attack complex	4	7	1.7	0.00021	C7,C8A,C8B,C8G
GO Component	GO:0034366	Spherical high-density lipoprotein particle	4	8	1.64	0.00030	APOC3,APOA1,APOA2,APOM
GO Component	GO:0043227	Membrane-bounded organelle	181	13188	0.08	0.00038	SPP2,ST6GAL1,CPB2,CETP,GNPTG,SERPIND1,LGALS1,CHGA,F9,MMP2,EBI3,COMP,PCOLCE,MPO,APOC3,GPLD1,PCDH12,LTF,HRG,APOB,IGFBP5,PROC,APOA1,TTR,CAT,LRP1,CFP,APOE,PXDN,ANGPTL6,F12,APCS,ORM1,VWF,PSMA6,SERPINA10,ATRN,LCAT,YWHAE,CSTA,CP,KLKB1,DNAH5,CENPE,PON3,TCIRG1,HPX,HYAL1,ITIH4,ITIH1,AHSG,ANKRD31,SAA4,SERPING1,CSF1R,PIP,FCN2,PGLYRP2,SCGB3A1,SERPINI1,PF4,MASP1,CAMP,C16orf46,A2ML1,HSP90B1,ANTXR1,LRG1,SH3D19,PCSK9,BMP1,FGB,DST,F2,PLG,EFEMP2,CSPG4,APOL1,SUMO4,CPN2,CALR,SERPINF2,C7,HBA1,ITLN1,HSPA5,FBN1,P4HB,SERPINA7,FBLN1,SERPINA5,DNAH8,OIT3,ANG,THBS4,GANAB,PROZ,QPCT,APOD,ASGR2,BLM,GIT2,AKAP9,PIGR,APOA4,ITIH2,C8A,CENPF,CFHR3,CFH,QSOX1,F5,APOA2,S100A7,S100A9,ADAMTSL4,FLNA,CRTAC1,MCOLN2,SMARCA1,C8B,C8G,CCN5,GSN,SVEP1,F10,F7,CA6,CKAP4,CALML5,TAF9,IGFBP3,SOD3,GPX3,KIF19,EFEMP1,CFI,TNRC6A,NAV2,ILK,GAPDH,RNASE1,CST3,MAP3K7CL,MASP2,F11,NUCB1,PCYOX1,ADIPOQ,IGF2,ORM2,C4A,ATP9B,FSIP2,CDK3,ABCA13,MST1,ITIH3,C4B,TGFBI,CFB,IGFALS,PLTP,RARRES2,GC,UIMC1,SELENOP,C1R,APOA5,SERPINA3,SERPINA4,IGKV2D-28,CFD,VEGFC,HYOU1,KNG1,TRIM66,GRHL2,CDH5,B2M,PDIA4
GO Component	GO:0005925	Focal adhesion	17	416	0.55	0.00039	CAT,LRP1,YWHAE,HSP90B1,DST,CSPG4,CALR,HSPA5,P4HB,LIMS1,GIT2,S100A7,FLNA,GSN,ILK,HYOU1,B2M
GO Component	GO:0005764	Lysosome	23	746	0.43	0.00088	GNPTG,MPO,GPLD1,HRG,APOB,TTR,CAT,LRP1,CP,TCIRG1,HYAL1,PCSK9,CSPG4,CALR,PIGR,S100A7,MCOLN2,CKAP4,PCYOX1,ORM2,ABCA13,GC,SERPINA3
GO Component	GO:0042581	Specific granule	10	159	0.74	0.00088	LTF,CFP,ORM1,CAMP,LRG1,QPCT,QSOX1,CKAP4,ORM2,B2M
GO Component	GO:0034362	Low-density lipoprotein particle	4	12	1.46	0.00095	APOB,APOE,APOM,APOF
GO Component	GO:0034663	Endoplasmic reticulum chaperone complex	4	12	1.46	0.00095	HSP90B1,HSPA5,P4HB,HYOU1
GO Component	GO:0070820	Tertiary granule	10	164	0.73	0.0011	LTF,CFP,ORM1,TCIRG1,CAMP,LRG1,QPCT,QSOX1,CST3,B2M
GO Component	GO:0005601	Classical-complement-pathway C3/C5 convertase complex	3	4	1.82	0.0016	C4A,C4B,CFB
GO Component	GO:0005773	Vacuole	24	839	0.4	0.0016	GNPTG,MPO,GPLD1,HRG,APOB,TTR,CAT,LRP1,CP,TCIRG1,HYAL1,PCSK9,CSPG4,CALR,PIGR,S100A7,MCOLN2,CKAP4,PCYOX1,ORM2,ABCA13,MST1,GC,SERPINA3
GO Component	GO:0005796	Golgi lumen	8	106	0.82	0.0016	F9,PROC,F2,CSPG4,PROZ,F10,F7,SOD3
GO Component	GO:0042567	Insulin-like growth factor ternary complex	3	4	1.82	0.0016	IGFBP5,IGFBP3,IGFALS
GO Component	GO:0030139	Endocytic vesicle	14	338	0.56	0.0017	MPO,LTF,APOB,APOA1,LRP1,APOE,TCIRG1,HPX,HSP90B1,CALR,HBA1,GSN,HYOU1,B2M
GO Component	GO:0034363	Intermediate-density lipoprotein particle	3	5	1.72	0.0024	APOC3,APOB,APOE
GO Component	GO:0043226	Organelle	186	14017	0.06	0.0025	SPP2,ST6GAL1,CPB2,CETP,GNPTG,SERPIND1,LGALS1,CHGA,F9,MMP2,EBI3,COMP,PCOLCE,MPO,APOC3,GPLD1,PCDH12,LTF,HRG,APOB,IGFBP5,PROC,APOA1,TTR,CAT,LRP1,CFP,APOE,PXDN,ANGPTL6,F12,APCS,ORM1,VWF,PSMA6,SERPINA10,ATRN,LCAT,YWHAE,CSTA,CP,KLKB1,DGKG,DNAH5,CENPE,PON3,TCIRG1,HPX,HYAL1,ITIH4,ITIH1,AHSG,ANKRD31,SAA4,SERPING1,CSF1R,PIP,FCN2,PGLYRP2,SCGB3A1,SERPINI1,PF4,MASP1,CAMP,C16orf46,A2ML1,HSP90B1,ANTXR1,LRG1,SH3D19,PCSK9,BMP1,SHLD1,FGB,DST,F2,PLG,EFEMP2,CSPG4,APOL1,SUMO4,CPN2,CALR,SERPINF2,C7,HBA1,ITLN1,HSPA5,FBN1,P4HB,SERPINA7,FBLN1,SERPINA5,DNAH8,OIT3,ANG,THBS4,GANAB,PROZ,QPCT,APOD,ASGR2,BLM,GIT2,AKAP9,PIGR,APOA4,ITIH2,MTCL1,C8A,CENPF,CFHR3,CFH,QSOX1,F5,APOA2,S100A7,S100A9,ADAMTSL4,FLNA,CRTAC1,MCOLN2,SMARCA1,C8B,C8G,CCN5,GSN,SVEP1,F10,F7,CA6,CKAP4,CALML5,TAF9,IGFBP3,SOD3,GPX3,KIF19,EFEMP1,CFI,TNRC6A,NAV2,ILK,GAPDH,RNASE1,CST3,MAP3K7CL,MASP2,F11,NUCB1,PCYOX1,ADIPOQ,IGF2,ORM2,C4A,ATP9B,SLAIN1,FSIP2,CDK3,ABCA13,MST1,LMOD3,ITIH3,C4B,TGFBI,CFB,IGFALS,PLTP,RARRES2,GC,UIMC1,SELENOP,C1R,APOA5,SERPINA3,SERPINA4,IGKV2D-28,CFD,VEGFC,HYOU1,KNG1,TRIM66,GRHL2,CDH5,B2M,PDIA4
GO Component	GO:0005577	Fibrinogen complex	3	8	1.51	0.0065	FGB,SERPINF2,FGL1
GO Component	GO:0005775	Vacuolar lumen	9	175	0.65	0.0075	MPO,APOB,TTR,HYAL1,CSPG4,S100A7,ORM2,GC,SERPINA3
GO Component	GO:0019814	Immunoglobulin complex	4	25	1.14	0.0087	PIGR,IGKV2D-28,IGHV3-15,IGHV3-72
GO Component	GO:0042470	Melanosome	7	109	0.75	0.0101	YWHAE,HSP90B1,HSPA5,P4HB,GANAB,GSN,PDIA4
GO Component	GO:0042582	Azurophil granule	8	154	0.66	0.0144	MPO,TTR,PIGR,S100A7,CKAP4,ORM2,ABCA13,SERPINA3
GO Component	GO:0005790	Smooth endoplasmic reticulum	4	30	1.07	0.0150	APOB,CALR,HSPA5,HYOU1
GO Component	GO:0042571	Immunoglobulin complex, circulating	3	12	1.34	0.0153	PIGR,IGHV3-15,IGHV3-72
GO Component	GO:0034365	Discoidal high-density lipoprotein particle	2	2	1.94	0.0191	APOE,APOM
GO Component	GO:1904813	ficolin-1-rich granule lumen	7	124	0.69	0.0191	CAT,LRG1,QPCT,GSN,CALML5,CST3,CFD
GO Component	GO:0140534	Endoplasmic reticulum protein-containing complex	7	129	0.67	0.0233	HSP90B1,CALR,HSPA5,P4HB,GANAB,HYOU1,B2M
GO Component	GO:0110165	Cellular anatomical entity	221	18293	0.02	0.0242	SPP2,ST6GAL1,CPB2,CETP,GNPTG,SERPIND1,LGALS1,CHGA,TPD52L2,APMAP,F9,MMP2,EBI3,COMP,PCOLCE,MPO,PF4V1,APOC3,GPLD1,PCDH12,LTF,HRG,APOB,IGFBP5,PROC,PLEK,APOA1,TTR,CAT,LRP1,C4BPB,CFP,APOE,PXDN,ANGPTL6,F12,APCS,ORM1,VWF,PSMA6,SERPINA10,ATRN,LCAT,YWHAE,CSTA,CP,KLKB1,DGKG,DNAH5,CENPE,PON3,TCIRG1,HPX,HYAL1,ITIH4,FCN3,ITIH1,AHSG,ANKRD31,CRH,HABP2,SAA4,SERPING1,CSF1R,PIP,FCN2,PSG6,PGLYRP2,SCGB3A1,IHH,SERPINI1,PF4,MASP1,CAMP,GUCY1A1,C16orf46,A2ML1,HSP90B1,ANTXR1,LRG1,SH3D19,PCSK9,BMP1,SHLD1,FGB,DST,F2,INHBC,PLG,EFEMP2,CSPG4,C1QB,CFHR1,APOL1,SUMO4,CPN2,CALR,LPA,SERPINF2,C7,HBA1,ITLN1,HSPA5,FBN1,P4HB,SERPINA7,FBLN1,SERPINA5,DNAH8,OIT3,SERPINA11,ANG,LIMS1,THBS4,GANAB,FAM184A,PROZ,QPCT,APOD,PAPLN,ASGR2,BLM,GIT2,AKAP9,PIGR,APOA4,ITIH2,CNDP1,MTCL1,C8A,PCDH18,CENPF,F13B,CFHR3,CFH,QSOX1,F5,APOA2,CD5L,S100A7,S100A9,ADAMTSL4,FLNA,CRTAC1,MCOLN2,SMARCA1,C8B,C8G,CCN5,GSN,SVEP1,C1QC,F10,F7,APOM,CA6,CKAP4,CALML5,TAF9,IGFBP3,SOD3,GPX3,KIF19,EFEMP1,CFI,TNRC6A,NAV2,ILK,GAPDH,RAPGEF4,RNASE1,FGL1,APOF,CST3,MAP3K7CL,MASP2,F11,C1S,NUCB1,PCYOX1,ADIPOQ,IGF2,ORM2,C4A,ATP9B,SLAIN1,FSIP2,CDK3,ABCA13,COLEC11,MST1,LMOD3,ITIH3,C4B,TGFBI,CFB,IGFALS,PLTP,PAEP,RARRES2,GC,UIMC1,SELENOP,C1R,APOA5,SERPINA3,SERPINA4,RNASE4,IGKV2D-28,APOC4,CFD,IGHV3-15,IGHV3-72,VEGFC,HYOU1,KNG1,TRIM66,GRHL2,CDH5,B2M,PDIA4
GO Component	GO:0005581	Collagen trimer	6	95	0.74	0.0251	FCN3,FCN2,C1QB,C1QC,ADIPOQ,COLEC11

GO Component	GO:0062167	Complement component C1q complex	2	3	1.76	0.0302	C1QB,C1QC
GO Component	GO:0101002	ficolin-1-rich granule	8	185	0.58	0.0395	CAT,TCIRG1,LRG1,QPCT,GSN,CALML5,CST3,CFD
GO Component	GO:0071953	Elastic fiber	2	4	1.64	0.0439	EFEMP2,FBLN1
STRING clusters	CL:18724	Mixed, incl. Complement and coagulation cascades, and Protein-lipid complex	77	172	1.59	4.04e-88	SPP2,CPB2,CETP,SERPIND1,F9,APOC3,HRG,PROC,APOA1,LRP1,C4BPB,CFP,APOE,F12,APCS,ORM1,VWF,SERPINA10,LCAT,KLKB1,HPX,FCN3,ITIH1,AHSG,HABP2,SAA4,SERPING1,FCN2,PGLYRP2,MASP1,PCSK9,FGB,F2,PLG,C1QB,CFHR1,APOL1,CPN2,LPA,SERPINF2,C7,SERPINA7,SERPINA5,SERPINA11,PROZ,APOA4,ITIH2,C8A,F13B,CFHR3,CFH,F5,APOA2,C8B,C8G,C1QC,F10,F7,APOM,CFI,APOF,MASP2,F11,C1S,PCYOX1,ORM2,C4A,COLEC11,ITIH3,C4B,CFB,PLTP,C1R,APOA5,SERPINA4,APOC4,CFD
STRING clusters	CL:18725	Complement and coagulation cascades, and Protein-lipid complex	76	166	1.6	9.29e-88	CPB2,CETP,SERPIND1,F9,APOC3,HRG,PROC,APOA1,LRP1,C4BPB,CFP,APOE,F12,APCS,ORM1,VWF,SERPINA10,LCAT,KLKB1,HPX,FCN3,ITIH1,AHSG,HABP2,SAA4,SERPING1,FCN2,PGLYRP2,MASP1,PCSK9,FGB,F2,PLG,C1QB,CFHR1,APOL1,CPN2,LPA,SERPINF2,C7,SERPINA7,SERPINA5,SERPINA11,PROZ,APOA4,ITIH2,C8A,F13B,CFHR3,CFH,F5,APOA2,C8B,C8G,C1QC,F10,F7,APOM,CFI,APOF,MASP2,F11,C1S,PCYOX1,ORM2,C4A,COLEC11,ITIH3,C4B,CFB,PLTP,C1R,APOA5,SERPINA4,APOC4,CFD
STRING clusters	CL:18723	Mixed, incl. Complement and coagulation cascades, and Protein-lipid complex	78	196	1.54	1.30e-86	SPP2,CPB2,CETP,SERPIND1,F9,APOC3,HRG,PROC,APOA1,LRP1,C4BPB,CFP,APOE,F12,APCS,ORM1,VWF,SERPINA10,LCAT,KLKB1,HPX,FCN3,ITIH1,AHSG,HABP2,SAA4,SERPING1,FCN2,PGLYRP2,MASP1,PCSK9,FGB,F2,PLG,C1QB,CFHR1,APOL1,CPN2,LPA,SERPINF2,C7,SERPINA7,SERPINA5,SERPINA11,PROZ,APOA4,ITIH2,C8A,F13B,CFHR3,CFH,F5,APOA2,CD5L,C8B,C8G,C1QC,F10,F7,APOM,CFI,APOF,MASP2,F11,C1S,PCYOX1,ORM2,C4A,COLEC11,ITIH3,C4B,CFB,PLTP,C1R,APOA5,SERPINA4,APOC4,CFD
STRING clusters	CL:18726	Complement and coagulation cascades, and Protein-lipid complex	74	161	1.6	1.24e-85	CPB2,CETP,SERPIND1,F9,APOC3,HRG,PROC,APOA1,LRP1,C4BPB,CFP,APOE,F12,APCS,ORM1,VWF,LCAT,KLKB1,HPX,FCN3,ITIH1,AHSG,HABP2,SAA4,SERPING1,FCN2,PGLYRP2,MASP1,PCSK9,FGB,F2,PLG,C1QB,CFHR1,APOL1,CPN2,LPA,SERPINF2,C7,SERPINA7,SERPINA5,SERPINA11,APOA4,ITIH2,C8A,F13B,CFHR3,CFH,F5,APOA2,C8B,C8G,C1QC,F10,F7,APOM,CFI,APOF,MASP2,F11,C1S,PCYOX1,ORM2,C4A,COLEC11,ITIH3,C4B,CFB,PLTP,C1R,APOA5,SERPINA4,APOC4,CFD
STRING clusters	CL:18727	Complement and coagulation cascades, and Positive regulation of opsonization	57	115	1.64	3.89e-66	CPB2,SERPIND1,F9,HRG,PROC,LRP1,C4BPB,CFP,F12,APCS,ORM1,VWF,KLKB1,HPX,FCN3,ITIH1,AHSG,HABP2,SERPING1,FCN2,PGLYRP2,MASP1,FGB,F2,PLG,C1QB,CFHR1,CPN2,SERPINF2,C7,SERPINA7,SERPINA5,SERPINA11,ITIH2,C8A,F13B,CFHR3,CFH,F5,C8B,C8G,C1QC,F10,F7,CFI,MASP2,F11,C1S,ORM2,C4A,COLEC11,ITIH3,C4B,CFB,C1R,SERPINA4,CFD
STRING clusters	CL:18728	Complement and coagulation cascades, and Positive regulation of opsonization	55	109	1.64	4.56e-64	CPB2,SERPIND1,F9,HRG,PROC,LRP1,C4BPB,CFP,F12,APCS,ORM1,VWF,KLKB1,HPX,FCN3,ITIH1,AHSG,HABP2,SERPING1,FCN2,MASP1,FGB,F2,PLG,C1QB,CFHR1,SERPINF2,C7,SERPINA7,SERPINA5,SERPINA11,ITIH2,C8A,F13B,CFHR3,CFH,F5,C8B,C8G,C1QC,F10,F7,CFI,MASP2,F11,C1S,ORM2,C4A,COLEC11,ITIH3,C4B,CFB,C1R,SERPINA4,CFD
STRING clusters	CL:18730	Hemostasis, and Dissolution of Fibrin Clot	28	55	1.65	2.37e-31	CPB2,SERPIND1,F9,HRG,PROC,LRP1,F12,ORM1,VWF,KLKB1,HPX,ITIH1,AHSG,HABP2,FGB,F2,PLG,SERPINF2,SERPINA5,ITIH2,F13B,F5,F10,F7,F11,ORM2,ITIH3,SERPINA4
STRING clusters	CL:18731	Hemostasis, and Dissolution of Fibrin Clot	26	50	1.66	3.26e-29	CPB2,SERPIND1,F9,HRG,PROC,LRP1,F12,ORM1,VWF,KLKB1,HPX,ITIH1,AHSG,HABP2,FGB,F2,PLG,SERPINF2,ITIH2,F13B,F5,F10,F7,F11,ORM2,ITIH3
STRING clusters	CL:18846	Complement cascade	25	50	1.64	9.79e-28	C4BPB,CFP,APCS,FCN3,SERPING1,FCN2,MASP1,C1QB,CFHR1,C7,C8A,CFHR3,CFH,C8B,C8G,C1QC,CFI,MASP2,C1S,C4A,COLEC11,C4B,CFB,C1R,CFD
STRING clusters	CL:18848	Complement cascade	22	40	1.68	5.95e-25	C4BPB,CFP,FCN3,SERPING1,FCN2,MASP1,C1QB,C7,C8A,CFH,C8B,C8G,C1QC,CFI,MASP2,C1S,C4A,COLEC11,C4B,CFB,C1R,CFD
STRING clusters	CL:18849	Initial triggering of complement, and Regulation of complement activation	17	31	1.68	5.99e-19	CFP,FCN3,SERPING1,FCN2,MASP1,C1QB,CFH,C1QC,CFI,MASP2,C1S,C4A,COLEC11,C4B,CFB,C1R,CFD
STRING clusters	CL:18732	Mixed, incl. COVID-19, thrombosis and anticoagulation, and Scavenging of heme from plasma	16	26	1.73	2.15e-18	CPB2,SERPIND1,HRG,LRP1,ORM1,HPX,ITIH1,AHSG,HABP2,FGB,PLG,SERPINF2,ITIH2,F13B,ORM2,ITIH3
STRING clusters	CL:18960	High-density lipoprotein particle	15	26	1.7	7.12e-17	CETP,APOC3,APOA1,LCAT,SAA4,APOL1,LPA,APOA4,APOA2,APOM,APOF,PCYOX1,PLTP,APOA5,APOC4
STRING clusters	CL:18956	Lipoprotein particle, and Assembly of active LPL and LIPC lipase complexes	17	46	1.51	9.68e-17	CETP,APOC3,APOA1,APOE,LCAT,SAA4,PCSK9,APOL1,LPA,APOA4,APOA2,APOM,APOF,PCYOX1,PLTP,APOA5,APOC4
STRING clusters	CL:18733	Mixed, incl. COVID-19, thrombosis and anticoagulation, and Inter-alpha-trypsin inhibitor heavy chain C-terminus	14	21	1.76	2.23e-16	CPB2,SERPIND1,HRG,ORM1,ITIH1,AHSG,HABP2,FGB,PLG,SERPINF2,ITIH2,F13B,ORM2,ITIH3
STRING clusters	CL:18962	High-density lipoprotein particle	14	21	1.76	2.23e-16	CETP,APOC3,APOA1,LCAT,APOL1,LPA,APOA4,APOA2,APOM,APOF,PCYOX1,PLTP,APOA5,APOC4
STRING clusters	CL:18851	Initial triggering of complement, and Negative regulation of complement activation	12	24	1.64	8.47e-13	FCN3,SERPING1,FCN2,MASP1,C1QB,C1QC,MASP2,C1S,C4A,COLEC11,C4B,C1R
STRING clusters	CL:18966	High-density lipoprotein particle	11	16	1.78	8.47e-13	CETP,APOC3,APOA1,LCAT,LPA,APOA4,APOA2,APOM,PLTP,APOA5,APOC4
STRING clusters	CL:18852	Creation of C4 and C2 activators	10	15	1.76	1.86e-11	FCN3,SERPING1,FCN2,MASP1,C1QB,C1QC,MASP2,C1S,COLEC11,C1R
STRING clusters	CL:18784	Formation of Fibrin Clot (Clotting Cascade)	10	18	1.69	6.90e-11	F9,PROC,F12,VWF,KLKB1,F2,F5,F10,F7,F11
STRING clusters	CL:18734	COVID-19, thrombosis and anticoagulation, and Negative regulation of fibrinolysis	8	11	1.8	3.27e-09	CPB2,SERPIND1,HRG,HABP2,FGB,PLG,SERPINF2,F13B
STRING clusters	CL:18970	Triglyceride-rich lipoprotein particle remodeling, and Spherical high-density lipoprotein particle	7	11	1.74	1.20e-07	CETP,APOC3,APOA1,LCAT,APOA4,APOA2,APOA5
STRING clusters	CL:18786	Hemophilia B, and Blood coagulation, common pathway	7	12	1.71	1.81e-07	F9,VWF,F2,F5,F10,F7,F11
STRING clusters	CL:18755	Mixed, incl. Inter-alpha-trypsin inhibitor heavy chain C-terminus, and Alpha-1-acid glycoprotein	6	10	1.72	2.55e-06	ORM1,ITIH1,AHSG,ITIH2,ORM2,ITIH3
STRING clusters	CL:18869	Complement component C1 complex	5	6	1.86	1.33e-05	SERPING1,C1QB,C1QC,C1S,C1R
STRING clusters	CL:18737	Fibrinogen, and Thrombophilia	4	6	1.76	0.00052	SERPIND1,HRG,FGB,PLG
STRING clusters	CL:18788	Extrinsic Pathway of Fibrin Clot Formation, and Factor V deficiency	4	6	1.76	0.00052	F2,F5,F10,F7
STRING clusters	CL:18854	Cell surface pattern recognition receptor signaling pathway	4	6	1.76	0.00052	FCN3,FCN2,MASP1,MASP2
STRING clusters	CL:18911	Membrane attack complex	4	7	1.7	0.00074	C7,C8A,C8B,C8G
STRING clusters	CL:19696	Elastic fibre formation, and Tolloid/BMP1 peptidase domain	6	34	1.19	0.00079	PCOLCE,BMP1,EFEMP2,FBN1,FBLN1,EFEMP1
STRING clusters	CL:19695	Elastic fibre formation, and Matrix metalloproteinases	7	61	1.0	0.0016	MMP2,PCOLCE,BMP1,EFEMP2,FBN1,FBLN1,EFEMP1
STRING clusters	CL:24268	Insulin-like growth factor-binding protein family 1-6, chordata, and Insulin-like growth factor	4	10	1.54	0.0020	IGFBP5,IGFBP3,IGF2,IGFALS
STRING clusters	CL:19699	Elastic fibre formation	4	14	1.4	0.0057	EFEMP2,FBN1,FBLN1,EFEMP1
STRING clusters	CL:19457	Extracellular matrix organization	10	180	0.69	0.0082	MMP2,PCOLCE,BMP1,DST,EFEMP2,FBN1,P4HB,FBLN1,THBS4,EFEMP1
STRING clusters	CL:18757	Alpha-1-acid glycoprotein, and Protein-losing enteropathy	3	5	1.72	0.0093	ORM1,AHSG,ORM2

STRING clusters	CL:18973	Regulation of very-low-density lipoprotein particle clearance, and Negative regulation of cholesterol import	3	5	1.72	0.0093	APOC3,APOA4,APOA2
STRING clusters	CL:1545	Photodynamic therapy-induced unfolded protein response, and IRE1-TRAF2-ASK1 complex	4	19	1.26	0.0138	HSP90B1,HSPA5,HYOU1,PDIA4
STRING clusters	CL:17205	Immunoglobulin complex, and IgG-binding protein	5	40	1.04	0.0169	PCDH12,PIGR,IGKV2D-28,IGHV3-15,IGHV3-72
STRING clusters	CL:19114	Mixed, incl. S100/CaBP-9k-type, calcium binding, subdomain, and Cystatin superfamily	7	103	0.77	0.0284	LGALS1,MPO,CSTA,CAMP,S100A7,S100A9,CST3
STRING clusters	CL:1546	Endoplasmic reticulum chaperone complex, and ARMET-like	3	10	1.42	0.0403	HSP90B1,HYOU1,PDIA4
STRING clusters	CL:17209	Immunoglobulin complex, and DNA recombinase complex	4	27	1.11	0.0415	PIGR,IGKV2D-28,IGHV3-15,IGHV3-72
KEGG	hsa04610	Complement and coagulation cascades	37	82	1.59	6.44e-41	CPB2,SERPIND1,F9,PROC,C4BPB,F12,VWF,KLKB1,SERPING1,FGB,F2,PLG,C1QB,CFHR1,SERPINF2,C7,SERPINA5,C8A,F13B,CFHR3,CFH,F5,C8B,C8G,C1QC,F10,F7,CFI,MASP2,F11,C1S,C4A,C4B,CFB,C1R,CFD,KNG1
KEGG	hsa04979	Cholesterol metabolism	12	48	1.34	4.92e-10	CETP,APOC3,APOB,APOA1,LRP1,APOE,LCAT,PCSK9,LPA,APOA4,APOA2,PLTP
KEGG	hsa05150	Staphylococcus aureus infection	13	86	1.12	1.07e-08	CAMP,PLG,C1QB,CFH,C1QC,CFI,MASP2,C1S,C4A,C4B,CFB,C1R,CFD
KEGG	hsa05322	Systemic lupus erythematosus	10	94	0.97	2.50e-05	C1QB,C7,C8A,C8B,C8G,C1QC,C1S,C4A,C4B,C1R
KEGG	hsa05133	Pertussis	9	73	1.03	2.54e-05	C4BPB,SERPING1,C1QB,C1QC,CALML5,C1S,C4A,C4B,C1R
KEGG	hsa04918	Thyroid hormone synthesis	7	73	0.92	0.0019	TTR,HSP90B1,HSPA5,SERPINA7,ASGR2,GPX3,PDIA4
KEGG	hsa03320	PPAR signaling pathway	7	75	0.91	0.0020	APOC3,APOA1,APOA2,ILK,ADIPOQ,PLTP,APOA5
KEGG	hsa04141	Protein processing in endoplasmic reticulum	8	163	0.63	0.0321	HSP90B1,CALR,HSPA5,P4HB,GANAB,CKAP4,HYOU1,PDIA4
Reactome	HSA-166658	Complement cascade	27	59	1.6	1.25e-28	CPB2,C4BPB,CFP,FCN3,SERPING1,FCN2,F2,C1QB,CFHR1,CPN2,C7,C8A,CFHR3,CFH,C8B,C8G,C1QC,CFI,MASP2,C1S,C4A,COLEC11,C4B,CFB,C1R,IGKV2D-28,CFD
Reactome	HSA-381426	Regulation of Insulin-like Growth Factor (IGF) transport and uptake by Insulin-like Growth Factor Binding Proteins (IGFBPs)	32	124	1.35	3.81e-28	SPP2,SERPIND1,LGALS1,MMP2,APOB,IGFBP5,PROC,APOA1,APOE,SERPINA10,CP,AHSG,HSP90B1,PCSK9,F2,PLG,APOL1,FBN1,P4HB,ITIH2,QSOX1,F5,APOA2,CKAP4,IGFBP3,CST3,NUCB1,IGF2,C4A,IGFALS,APOA5,KNG1
Reactome	HSA-8957275	Post-translational protein phosphorylation	27	107	1.34	2.43e-23	SPP2,SERPIND1,LGALS1,APOB,IGFBP5,PROC,APOA1,APOE,SERPINA10,CP,AHSG,HSP90B1,PCSK9,APOL1,FBN1,P4HB,ITIH2,QSOX1,F5,APOA2,CKAP4,IGFBP3,CST3,NUCB1,C4A,APOA5,KNG1
Reactome	HSA-977606	Regulation of Complement cascade	22	49	1.59	2.46e-23	CPB2,C4BPB,CFP,SERPING1,F2,C1QB,CFHR1,CPN2,C7,C8A,CFHR3,CFH,C8B,C8G,C1QC,CFI,C1S,C4A,C4B,CFB,C1R,IGKV2D-28
Reactome	HSA-109582	Hemostasis	47	607	0.83	6.32e-22	SPP2,SERPIND1,F9,PF4V1,HRG,APOB,PROC,PLEK,APOA1,F12,ORM1,VWF,KLKB1,DGKG,CENPE,ITIH4,AHSG,SERPING1,PSG6,PF4,GUCY1A1,FGB,F2,PLG,SERPINF2,HSPA5,SERPINA5,F13B,QSOX1,F5,FLNA,F10,F7,KIF19,RAPGEF4,F11,IGF2,ORM2,ITIH3,RARRES2,SELENOP,SERPINA3,SERPINA4,IGKV2D-28,CFD,VEGFC,KNG1
Reactome	HSA-114608	Platelet degranulation	27	126	1.27	6.32e-22	SPP2,HRG,PLEK,APOA1,ORM1,VWF,ITIH4,AHSG,SERPING1,PF4,FGB,PLG,SERPINF2,HSPA5,QSOX1,F5,FLNA,IGF2,ORM2,ITIH3,RARRES2,SELENOP,SERPINA3,SERPINA4,CFD,VEGFC,KNG1
Reactome	HSA-168249	Innate Immune System	56	1041	0.67	1.53e-19	CPB2,CHGA,MPO,LTF,APOB,TTR,CAT,C4BPB,CFP,ORM1,PSMA6,TCIRG1,FCN3,AHSG,SERPING1,FCN2,PGLYRP2,CAMP,HSP90B1,LRG1,FGB,F2,C1QB,CFHR1,CPN2,C7,ITLN1,QPCT,PIGR,C8A,CFHR3,CFH,QSOX1,S100A7,S100A9,C8B,C8G,GSN,C1QC,CKAP4,CALML5,CFI,CST3,MASP2,C1S,ORM2,C4A,ABCA13,COLEC11,C4B,CFB,C1R,SERPINA3,IGKV2D-28,CFD,B2M
Reactome	HSA-140877	Formation of Fibrin Clot (Clotting Cascade)	18	39	1.6	2.59e-19	SERPIND1,F9,PF4V1,PROC,F12,VWF,KLKB1,SERPING1,PF4,FGB,F2,SERPINA5,F13B,F5,F10,F7,F11,KNG1
Reactome	HSA-76002	Platelet activation, signaling and aggregation	30	260	1.0	6.62e-18	SPP2,HRG,PLEK,APOA1,ORM1,VWF,DGKG,ITIH4,AHSG,SERPING1,PF4,FGB,F2,PLG,SERPINF2,HSPA5,QSOX1,F5,FLNA,RAPGEF4,IGF2,ORM2,ITIH3,RARRES2,SELENOP,SERPINA3,SERPINA4,CFD,VEGFC,KNG1
Reactome	HSA-166663	Initial triggering of complement	14	24	1.71	6.14e-16	CFP,FCN3,FCN2,C1QB,C1QC,MASP2,C1S,C4A,COLEC11,C4B,CFB,C1R,IGKV2D-28,CFD
Reactome	HSA-168256	Immune System	66	1979	0.46	2.91e-13	CPB2,CHGA,MMP2,EBI3,MPO,LTF,APOB,TTR,CAT,C4BPB,CFP,ORM1,PSMA6,CENPE,TCIRG1,FCN3,AHSG,SERPING1,CSF1R,FCN2,PGLYRP2,CAMP,HSP90B1,LRG1,FGB,F2,UBE3C,C1QB,CFHR1,CPN2,CALR,C7,ITLN1,HSPA5,P4HB,QPCT,PIGR,C8A,CFHR3,CFH,QSOX1,S100A7,S100A9,FLNA,C8B,C8G,GSN,C1QC,CKAP4,CALML5,CFI,RAPGEF4,CST3,MASP2,C1S,ORM2,C4A,ABCA13,COLEC11,C4B,CFB,C1R,SERPINA3,IGKV2D-28,CFD,B2M
Reactome	HSA-140837	Intrinsic Pathway of Fibrin Clot Formation	12	23	1.66	3.94e-13	SERPIND1,F9,PROC,F12,VWF,KLKB1,SERPING1,F2,SERPINA5,F10,F11,KNG1
Reactome	HSA-8963899	Plasma lipoprotein remodeling	13	34	1.52	5.93e-13	CETP,APOC3,APOB,APOA1,APOE,LCAT,LPA,P4HB,APOA4,APOA2,APOF,PLTP,APOA5
Reactome	HSA-174824	Plasma lipoprotein assembly, remodeling, and clearance	16	75	1.27	8.22e-13	CETP,APOC3,APOB,APOA1,APOE,LCAT,PCSK9,BMP1,LPA,P4HB,APOA4,APOA2,APOF,PLTP,APOA5,APOC4
Reactome	HSA-140875	Common Pathway of Fibrin Clot Formation	10	22	1.6	2.14e-10	SERPIND1,PF4V1,PROC,PF4,FGB,F2,SERPINA5,F13B,F5,F10
Reactome	HSA-166786	Creation of C4 and C2 activators	9	16	1.69	6.21e-10	FCN3,FCN2,C1QB,C1QC,MASP2,C1S,COLEC11,C1R,IGKV2D-28
Reactome	HSA-8963898	Plasma lipoprotein assembly	9	19	1.62	1.92e-09	APOC3,APOB,APOA1,APOE,BMP1,P4HB,APOA4,APOA2,APOC4
Reactome	HSA-2173782	Binding and Uptake of Ligands by Scavenger Receptors	11	43	1.35	2.35e-09	APOB,APOA1,LRP1,APOE,HPX,HSP90B1,APOL1,CALR,COLEC11,IGKV2D-28,HYOU1
Reactome	HSA-9651496	Defects of contact activation system (CAS) and kallikrein/kinin system (KKS)	8	16	1.64	1.74e-08	F9,F12,VWF,KLKB1,SERPING1,F2,F10,F11
Reactome	HSA-392499	Metabolism of proteins	55	1917	0.4	2.14e-08	SPP2,ST6GAL1,CPB2,SERPIND1,LGALS1,F9,MMP2,GPLD1,LTF,APOB,IGFBP5,PROC,APOA1,TTR,CFP,APOE,APCS,PSMA6,SERPINA10,CP,AHSG,HSP90B1,PCSK9,F2,INHBC,PLG,APOL1,CALR,FBN1,P4HB,GANAB,PROZ,ASGR2,BLM,APOA4,ITIH2,QSOX1,F5,APOA2,ADAMTSL4,GSN,F10,F7,CKAP4,IGFBP3,CST3,NUCB1,IGF2,C4A,TGFBI,IGFALS,UIMC1,APOA5,KNG1,B2M
Reactome	HSA-8963888	Chylomicron assembly	7	10	1.79	3.86e-08	APOC3,APOB,APOA1,APOE,P4HB,APOA4,APOA2
Reactome	HSA-8963901	Chylomicron remodeling	7	10	1.79	3.86e-08	APOC3,APOB,APOA1,APOE,APOA4,APOA2,APOA5
Reactome	HSA-6798695	Neutrophil degranulation	24	476	0.64	2.69e-07	MPO,LTF,TTR,CAT,CFP,ORM1,TCIRG1,AHSG,CAMP,LRG1,QPCT,PIGR,QSOX1,S100A7,S100A9,GSN,CKAP4,CALML5,CST3,ORM2,ABCA13,SERPINA3,CFD,B2M
Reactome	HSA-975634	Retinoid metabolism and transport	9	44	1.25	6.69e-07	APOC3,APOB,APOA1,TTR,LRP1,APOE,APOA4,APOA2,APOM
Reactome	HSA-159763	Transport of gamma-carboxylated protein precursors from the endoplasmic reticulum to the Golgi apparatus	6	9	1.76	7.74e-07	F9,PROC,F2,PROZ,F10,F7
Reactome	HSA-159740	Gamma-carboxylation of protein precursors	6	10	1.72	1.18e-06	F9,PROC,F2,PROZ,F10,F7
Reactome	HSA-159782	Removal of aminoterminal propeptides from gamma-carboxylated proteins	6	10	1.72	1.18e-06	F9,PROC,F2,PROZ,F10,F7
Reactome	HSA-597592	Post-translational protein modification	42	1405	0.42	1.18e-06	SPP2,ST6GAL1,SERPIND1,LGALS1,F9,GPLD1,APOB,IGFBP5,PROC,APOA1,CFP,APOE,PSMA6,SERPINA10,CP,AHSG,HSP90B1,PCSK9,F2,APOL1,CALR,FBN1,P4HB,GANAB,PROZ,ASGR2,BLM,ITIH2,QSOX1,F5,APOA2,ADAMTSL4,F10,F7,CKAP4,IGFBP3,CST3,NUCB1,C4A,UIMC1,APOA5,KNG1
Reactome	HSA-8964058	HDL remodeling	6	10	1.72	1.18e-06	CETP,APOC3,APOA1,APOE,LCAT,PLTP

Reactome	HSA-977225	Amyloid fiber formation	10	79	1.04	4.51e-06	LTF,APOA1,TTR,APOE,APCS,APOA4,GSN,CST3,TGFBI,B2M
Reactome	HSA-8964041	LDL remodeling	5	6	1.86	5.12e-06	CETP,APOB,LPA,P4HB,APOF
Reactome	HSA-3000480	Scavenging by Class A Receptors	6	18	1.46	1.43e-05	APOB,APOA1,APOE,HSP90B1,CALR,COLEC11
Reactome	HSA-173623	Classical antibody-mediated complement activation	5	9	1.69	2.04e-05	C1QB,C1QC,C1S,C1R,IGKV2D-28
Reactome	HSA-1474244	Extracellular matrix organization	16	300	0.67	4.23e-05	MMP2,COMP,PCOLCE,TTR,PXDN,VWF,KLKB1,BMP1,FGB,DST,PLG,EFEMP2,FBN1,P4HB,FBLN1,EFEMP1
Reactome	HSA-2168880	Scavenging of heme from plasma	5	15	1.46	0.00014	APOA1,LRP1,HPX,APOL1,IGKV2D-28
Reactome	HSA-166662	Lectin pathway of complement activation	4	7	1.7	0.00028	FCN3,FCN2,MASP2,COLEC11
Reactome	HSA-174577	Activation of C3 and C5	4	7	1.7	0.00028	CFP,C4A,C4B,CFB
Reactome	HSA-9662001	Defective factor VIII causes hemophilia A	4	7	1.7	0.00028	F9,VWF,F2,F10
Reactome	HSA-166665	Terminal pathway of complement	4	8	1.64	0.00039	C7,C8A,C8B,C8G
Reactome	HSA-9029569	NR1H3 & NR1H2 regulate gene expression linked to cholesterol transport and efflux	6	36	1.16	0.00039	CETP,APOE,APOD,TNRC6A,PLTP,APOC4
Reactome	HSA-382551	Transport of small molecules	23	723	0.44	0.00071	CETP,APOC3,APOB,APOA1,APOE,PSMA6,LCAT,CP,TCIRG1,PIP,PCSK9,BMP1,LPA,P4HB,APOD,APOA4,APOA2,MCOLN2,APOF,ATP9B,PLTP,APOA5,APOC4
Reactome	HSA-9657688	Defective factor XII causes hereditary angioedema	3	3	1.94	0.0013	F12,KLKB1,F2
Reactome	HSA-9657689	Defective SERPING1 causes hereditary angioedema	3	3	1.94	0.0013	F12,KLKB1,SERPING1
Reactome	HSA-2855086	Ficolins bind to repetitive carbohydrate structures on the target cell surface	3	4	1.82	0.0022	FCN3,FCN2,MASP2
Reactome	HSA-140834	Extrinsic Pathway of Fibrin Clot Formation	3	5	1.72	0.0034	F9,F10,F7
Reactome	HSA-173736	Alternative complement activation	3	5	1.72	0.0034	CFP,CFB,CFD
Reactome	HSA-8866423	VLDL assembly	3	5	1.72	0.0034	APOB,P4HB,APOC4
Reactome	HSA-6803157	Antimicrobial peptides	7	87	0.85	0.0041	CHGA,LTF,PGLYRP2,CAMP,ITLN1,S100A7,S100A9
Reactome	HSA-8964043	Plasma lipoprotein clearance	5	37	1.07	0.0044	APOB,APOA1,APOE,PCSK9,APOC4
Reactome	HSA-3000484	Scavenging by Class F Receptors	3	6	1.64	0.0046	APOB,CALR,HYOU1
Reactome	HSA-6799990	Metal sequestration by antimicrobial proteins	3	6	1.64	0.0046	LTF,S100A7,S100A9
Reactome	HSA-9668250	Defective factor IX causes hemophilia B	3	9	1.46	0.0114	F9,F10,F11
Reactome	HSA-381183	ATF6 (ATF6-alpha) activates chaperone genes	3	10	1.42	0.0144	HSP90B1,CALR,HSPA5
Reactome	HSA-75205	Dissolution of Fibrin Clot	3	13	1.3	0.0262	HRG,PLG,SERPINF2
Reactome	HSA-446728	Cell junction organization	6	92	0.75	0.0314	DST,ANG,LIMS1,FLNA,ILK,CDH5
Reactome	HSA-3299685	Detoxification of Reactive Oxygen Species	4	36	0.99	0.0376	CAT,P4HB,SOD3,GPX3
Reactome	HSA-2129379	Molecules associated with elastic fibres	4	37	0.97	0.0406	EFEMP2,FBN1,FBLN1,EFEMP1
Reactome	HSA-9672383	Defective factor IX causes thrombophilia	2	3	1.76	0.0427	F9,F10
Reactome	HSA-9672391	Defective F8 cleavage by thrombin	2	3	1.76	0.0427	VWF,F2
Reactome	HSA-9672396	Defective cofactor function of FVIIIa variant	2	3	1.76	0.0427	F9,F10
Reactome	HSA-9673202	Defective F9 variant does not activate FX	2	3	1.76	0.0427	F9,F10
Reactome	HSA-202733	Cell surface interactions at the vascular wall	7	139	0.64	0.0452	PF4V1,APOB,PROC,PSG6,PF4,F2,IGKV2D-28
Reactome	HSA-76009	Platelet Aggregation (Plug Formation)	4	39	0.95	0.0452	VWF,FGB,F2,RAPGEF4
WikiPathways	WP558	Complement and coagulation cascades	31	58	1.67	2.25e-35	CPB2,SERPIND1,F9,PROC,F12,VWF,KLKB1,SERPING1,MASP1,FGB,F2,PLG,C1QB,SERPINF2,C7,SERPINA5,F13B,CFH,F5,C8G,C1QC,F10,F7,CFI,MASP2,C1S,C4B,CFB,C1R,CFD,KNG1
WikiPathways	WP2806	Complement system	23	96	1.32	1.90e-19	APOA1,CFP,F12,APCS,KLKB1,SERPING1,FCN2,MASP1,FGB,PLG,CALR,C7,C8A,CFH,F10,CFI,MASP2,F11,C1S,ADIPOQ,C4A,CFB,CFD
WikiPathways	WP545	Complement activation	15	22	1.77	8.10e-18	CFP,MASP1,C1QB,C7,C8A,C8B,C8G,C1QC,MASP2,C1S,C4A,C4B,CFB,C1R,CFD
WikiPathways	WP5090	Complement system in neuronal development and plasticity	22	105	1.26	9.66e-18	C4BPB,CFP,FCN3,SERPING1,FCN2,MASP1,C1QB,C7,C8A,CFH,C8B,C8G,C1QC,CFI,MASP2,C1S,C4A,COLEC11,C4B,CFB,C1R,CFD
WikiPathways	WP272	Blood clotting cascade	13	22	1.71	6.73e-15	F9,F12,VWF,KLKB1,FGB,F2,PLG,SERPINF2,F13B,F5,F10,F7,F11
WikiPathways	WP5115	Network map of SARS-CoV-2 signaling pathway	22	218	0.94	6.48e-12	HRG,APOA1,CFP,SERPINA10,ITIH4,PF4,LRG1,FGB,APOL1,SERPINA5,APOD,C8A,CFH,APOA2,GSN,APOM,IGFBP3,CFI,C1S,ITIH3,CFB,C1R
WikiPathways	WP430	Statin inhibition of cholesterol production	11	29	1.52	6.05e-11	CETP,APOC3,APOB,APOA1,LRP1,APOE,LCAT,APOA4,APOA2,PLTP,APOA5
WikiPathways	WP15	Selenium micronutrient network	14	84	1.16	5.35e-10	MPO,APOB,APOA1,CAT,SAA4,FGB,F2,PLG,HBA1,F7,SOD3,GPX3,SELENOP,SERPINA3
WikiPathways	WP176	Folate metabolism	13	67	1.23	5.35e-10	MPO,APOB,APOA1,CAT,SAA4,FGB,F2,PLG,HBA1,F7,SOD3,GPX3,SERPINA3
WikiPathways	WP5110	Familial hyperlipidemia type 3	8	13	1.73	3.12e-09	CETP,APOA1,LRP1,APOE,LCAT,APOA4,APOA2,PLTP
WikiPathways	WP5323	Fatty Acids and Lipoproteins Transport in Hepatocytes	24	380	0.74	3.12e-09	CETP,APOC3,APOB,APOA1,LRP1,APOE,LCAT,SAA4,PCSK9,APOL1,LPA,ASGR2,APOA4,APOA2,CD5L,APOM,CFI,APOF,PCYOX1,ADIPOQ,ATP9B,PLTP,APOA5,APOC4
WikiPathways	WP1533	Vitamin B12 metabolism	11	50	1.28	5.18e-09	MPO,APOB,APOA1,APOE,SAA4,F2,PLG,HBA1,F7,SOD3,SERPINA3
WikiPathways	WP5112	Familial hyperlipidemia type 5	8	15	1.67	6.16e-09	CETP,APOA1,LRP1,LCAT,APOA4,APOA2,PLTP,APOA5
WikiPathways	WP5109	Familial hyperlipidemia type 2	8	16	1.64	8.49e-09	CETP,APOB,APOA1,LCAT,PCSK9,APOA4,APOA2,PLTP
WikiPathways	WP5304	Cholesterol metabolism	12	72	1.16	9.70e-09	CETP,APOC3,APOB,APOA1,LRP1,APOE,LCAT,PCSK9,LPA,APOA4,APOA2,PLTP
WikiPathways	WP5108	Familial hyperlipidemia type 1	8	18	1.59	1.55e-08	CETP,APOA1,LRP1,LCAT,APOA4,APOA2,PLTP,APOA5
WikiPathways	WP3601	Lipid particles composition	7	10	1.79	1.79e-08	CETP,APOC3,APOB,APOA1,APOE,LCAT,APOA2
WikiPathways	WP5111	Familial hyperlipidemia type 4	8	22	1.5	4.96e-08	CETP,APOA1,LRP1,LCAT,APOA4,APOA2,PLTP,APOA5
WikiPathways	WP4522	Metabolic pathway of LDL, HDL and TG, including diseases	7	17	1.55	2.66e-07	CETP,APOB,APOA1,APOE,LCAT,PCSK9,APOA2
WikiPathways	WP5186	Vitamin K metabolism and activation of dependent proteins	6	12	1.64	1.29e-06	F9,PROC,F2,PROZ,F10,F7
WikiPathways	WP4927	COVID-19, thrombosis and anticoagulation	4	7	1.7	0.00019	FGB,F2,PLG,F13B
WikiPathways	WP5089	Kinin-Kallikrein pathway	4	7	1.7	0.00019	F12,KLKB1,SERPING1,KNG1
WikiPathways	WP3942	PPAR signaling pathway	7	67	0.96	0.00071	APOC3,APOA1,APOA2,ILK,ADIPOQ,PLTP,APOA5
WikiPathways	WP2878	PPAR-alpha pathway	5	26	1.22	0.00078	APOC3,APOA1,APOA2,PLTP,APOA5
WikiPathways	WP2328	Allograft rejection	7	88	0.84	0.0033	C1QB,C7,C8A,C8B,C1QC,C4A,C4B
WikiPathways	WP3941	Oxidative damage response	5	39	1.05	0.0041	C1QB,C1QC,C1S,C4B,C1R
WikiPathways	WP4969	RAS and bradykinin pathways in COVID-19	4	28	1.1	0.0131	F12,KLKB1,SERPING1,KNG1
WikiPathways	WP2877	Vitamin D receptor pathway	8	183	0.58	0.0434	IGFBP5,ORM1,CAMP,S100A9,IGFBP3,ORM2,COLEC11,KNG1
Monarch	EFO:0007937	Blood protein measurement	85	1810	0.61	2.55e-26	ST6GAL1,CPB2,GNPTG,CHGA,EBI3,MPO,APOC3,LTF,HRG,APOB,IGFBP5,PROC,CAT,APOE,PXDN,F12,VWF,SERPINA10,LCAT,KLKB1,HPX,ITIH4,ITIH1,AHSG,HABP2,SAA4,SERPING1,ANKRD44,CSF1R,FCN2,PGLYRP2,MASP1,HSP90B1,ANTXR1,F2,PLG,APOL1,CPN2,LPA,SERPINF2,C7,SERPINA5,SERPINA11,ANG,PROZ,ASGR2,PIGR,ITIH2,CNDP1,F13B,CFH,QSOX1,F5,S100A9,CRTAC1,C8B,C8G,CCN5,GSN,SVEP1,C1QC,F10,F7,APOM,CA6,IGFBP3,SOD3,FGL1,CST3,F11,C1S,PCYOX1,COLEC11,MST1,C4B,TGFBI,CFB,PLTP,GC,C1R,SERPINA4,RNASE4,APOC4,VEGFC,KNG1
Monarch	HP:0005339	Abnormality of complement system	13	24	1.67	6.21e-13	CFP,C1QB,C7,C8A,CFH,C8B,C1QC,CFI,MASP2,C1S,C4A,C4B,CFD

Monarch	EFO:0004747	Protein measurement	126	5856	0.27	2.36e-12	ST6GAL1,CPB2,CETP,GNPTG,CHGA,EBI3,MPO,APOC3,GPLD1,LTF,HRG,APOB,IGFBP5,PROC,APOA1,CAT,LRP1,C4BPB,APOE,PXDN,ANGPTL6,F12,APCS,ORM1,VWF,SERPINA10,LCAT,CP,KLKB1,DGKG,DNAH5,PON3,TCIRG1,HPX,ITIH4,ITIH1,AHSG,HABP2,SAA4,SERPING1,ANKRD44,CSF1R,FCN2,PGLYRP2,MASP1,HSP90B1,ANTXR1,PCSK9,FGB,F2,INHBC,PLG,UBE3C,APOL1,CPN2,LPA,SERPINF2,C7,HBA1,FBLN1,SERPINA5,DNAH8,OIT3,SERPINA11,ANG,LIMS1,THBS4,PROZ,APOD,PAPLN,ASGR2,GIT2,PIGR,APOA4,ITIH2,CNDP1,CENPF,F13B,CFH,QSOX1,F5,S100A9,CRTAC1,C8B,C8G,CCN5,GSN,SVEP1,C1QC,F10,F7,APOM,CA6,IGFBP3,SOD3,CFI,EFCAB5,TNRC6A,NAV2,RAPGEF4,FGL1,CST3,F11,C1S,PCYOX1,ADIPOQ,ABCA13,COLEC11,MST1,ITIH3,C4B,TGFBI,CFB,IGFALS,PLTP,GC,SELENOP,C1R,APOA5,SERPINA4,RNASE4,APOC4,VEGFC,KNG1,TRIM66,GRHL2
Monarch	HP:0004431	Complement deficiency	11	21	1.66	1.00e-10	C1QB,C7,C8A,CFH,C8B,C1QC,CFI,MASP2,C4A,C4B,CFD
Monarch	HP:0000118	Phenotypic abnormality	110	5129	0.27	9.53e-10	ST6GAL1,CETP,GNPTG,SERPIND1,F9,MMP2,COMP,MPO,APOC3,PCDH12,LTF,HRG,APOB,PROC,APOA1,TTR,CAT,LRP1,CFP,APOE,PXDN,ANGPTL6,F12,VWF,PSMA6,LCAT,CSTA,CP,KLKB1,DNAH5,CENPE,PON3,TCIRG1,HYAL1,FCN3,AHSG,CRH,HABP2,SERPING1,CSF1R,IHH,SERPINI1,MASP1,GUCY1A1,A2ML1,ANTXR1,PCSK9,BMP1,FGB,DST,F2,PLG,EFEMP2,C1QB,CFHR1,CALR,LPA,SERPINF2,C7,HBA1,FBN1,P4HB,FBLN1,DNAH8,ANG,LIMS1,THBS4,GANAB,PAPLN,BLM,AKAP9,C8A,CENPF,F13B,CFH,F5,ADAMTSL4,FLNA,CRTAC1,C8B,GSN,SVEP1,C1QC,F10,F7,EFEMP1,CFI,NAV2,CST3,MASP2,F11,C1S,IGF2,C4A,FSIP2,ABCA13,COLEC11,LMOD3,ITIH3,C4B,TGFBI,IGFALS,GC,C1R,APOA5,CFD,VEGFC,KNG1,GRHL2,B2M
Monarch	HP:0002597	Abnormality of the vasculature	54	1673	0.45	8.54e-09	SERPIND1,F9,MMP2,APOB,PROC,APOA1,TTR,CAT,APOE,ANGPTL6,F12,VWF,LCAT,CSTA,DNAH5,TCIRG1,HABP2,SERPING1,MASP1,GUCY1A1,A2ML1,ANTXR1,PCSK9,BMP1,FGB,F2,PLG,EFEMP2,CALR,SERPINF2,HBA1,FBN1,P4HB,GANAB,BLM,CENPF,F13B,F5,FLNA,CRTAC1,GSN,F10,F7,EFEMP1,CFI,CST3,C1S,IGF2,C4A,TGFBI,C1R,VEGFC,KNG1,B2M
Monarch	HP:0001892	Abnormal bleeding	26	411	0.74	9.03e-09	F9,PROC,CAT,APOE,ANGPTL6,F12,VWF,TCIRG1,A2ML1,FGB,F2,CALR,SERPINF2,FBN1,P4HB,F13B,F5,FLNA,GSN,F10,F7,CST3,F11,C1S,C4A,C1R
Monarch	HP:0001928	Abnormality of coagulation	18	170	0.97	9.03e-09	F9,HRG,PROC,F12,VWF,KLKB1,A2ML1,FGB,F2,PLG,SERPINF2,F13B,F5,FLNA,F10,F7,F11,KNG1
Monarch	HP:0025015	Abnormal vascular morphology	41	1027	0.54	9.03e-09	SERPIND1,F9,MMP2,APOB,PROC,TTR,CAT,APOE,ANGPTL6,F12,VWF,LCAT,DNAH5,TCIRG1,HABP2,MASP1,GUCY1A1,A2ML1,ANTXR1,PCSK9,FGB,F2,EFEMP2,CALR,SERPINF2,FBN1,P4HB,GANAB,F13B,F5,FLNA,CRTAC1,F10,F7,EFEMP1,CFI,CST3,IGF2,C4A,VEGFC,KNG1
Monarch	HP:0005261	Joint hemorrhage	9	19	1.62	1.85e-08	F9,VWF,F2,SERPINF2,F13B,F5,F10,F7,F11
Monarch	HP:0001933	Subcutaneous hemorrhage	18	189	0.92	2.91e-08	PROC,APOE,VWF,TCIRG1,F2,CALR,SERPINF2,FBN1,P4HB,F13B,F5,FLNA,GSN,F10,F7,C1S,C4A,C1R
Monarch	HP:0003256	Abnormality of the coagulation cascade	14	105	1.07	8.19e-08	F9,PROC,F12,VWF,FGB,F2,PLG,F13B,F5,FLNA,F10,F7,F11,KNG1
Monarch	HP:0011276	Vascular skin abnormality	25	433	0.7	8.74e-08	PROC,APOE,F12,VWF,CSTA,TCIRG1,SERPING1,MASP1,GUCY1A1,ANTXR1,F2,CALR,SERPINF2,FBN1,P4HB,BLM,F13B,F5,FLNA,GSN,F10,F7,C1S,C4A,C1R
Monarch	HP:0001871	Abnormality of blood and blood-forming tissues	42	1214	0.48	1.92e-07	SERPIND1,F9,MPO,HRG,APOB,PROC,APOA1,CAT,APOE,ANGPTL6,F12,VWF,LCAT,CP,KLKB1,TCIRG1,GUCY1A1,A2ML1,FGB,F2,PLG,CALR,SERPINF2,HBA1,FBN1,P4HB,BLM,F13B,CFH,F5,FLNA,GSN,SVEP1,F10,F7,CST3,F11,C1S,C4A,C1R,KNG1,B2M
Monarch	HP:0100659	Abnormal cerebral vascular morphology	23	381	0.72	1.97e-07	F9,APOB,PROC,TTR,APOE,ANGPTL6,GUCY1A1,ANTXR1,PCSK9,FGB,F2,CALR,SERPINF2,FBN1,GANAB,F13B,F5,FLNA,CRTAC1,F10,F7,CST3,KNG1
Monarch	HP:0000225	Gingival bleeding	10	45	1.29	4.12e-07	CAT,FGB,F2,SERPINF2,F13B,F5,F10,F7,C1S,C1R
Monarch	HP:0011025	Abnormal cardiovascular system physiology	40	1169	0.47	6.24e-07	GNPTG,F9,MMP2,APOB,PROC,APOA1,TTR,APOE,ANGPTL6,VWF,LCAT,CP,TCIRG1,SERPING1,GUCY1A1,A2ML1,PCSK9,BMP1,FGB,DST,F2,EFEMP2,CALR,SERPINF2,HBA1,FBN1,GANAB,AKAP9,F13B,F5,ADAMTSL4,FLNA,CRTAC1,GSN,F10,F7,CST3,F11,KNG1,B2M
Monarch	HP:0000978	Bruising susceptibility	14	130	0.97	7.08e-07	VWF,TCIRG1,F2,SERPINF2,FBN1,P4HB,F13B,F5,FLNA,GSN,F10,F7,C1S,C1R
Monarch	HP:0001626	Abnormality of the cardiovascular system	62	2438	0.35	7.85e-07	GNPTG,SERPIND1,F9,MMP2,APOB,PROC,APOA1,TTR,CAT,LRP1,APOE,ANGPTL6,F12,VWF,LCAT,CSTA,CP,KLKB1,DNAH5,TCIRG1,HABP2,SERPING1,MASP1,GUCY1A1,A2ML1,ANTXR1,PCSK9,BMP1,FGB,DST,F2,PLG,EFEMP2,CALR,SERPINF2,HBA1,FBN1,P4HB,GANAB,BLM,AKAP9,CENPF,F13B,F5,ADAMTSL4,FLNA,CRTAC1,GSN,F10,F7,EFEMP1,CFI,CST3,F11,C1S,IGF2,C4A,TGFBI,C1R,VEGFC,KNG1,B2M
Monarch	HP:0002715	Abnormality of the immune system	49	1682	0.4	9.30e-07	MMP2,MPO,APOA1,CAT,CFP,APOE,PSMA6,LCAT,CSTA,DNAH5,TCIRG1,HYAL1,FCN3,HABP2,SERPING1,A2ML1,FGB,F2,PLG,EFEMP2,C1QB,CFHR1,CALR,C7,HBA1,FBN1,GANAB,BLM,C8A,CENPF,F13B,CFH,FLNA,C8B,GSN,SVEP1,C1QC,CFI,NAV2,MASP2,C1S,IGF2,C4A,C4B,C1R,CFD,VEGFC,GRHL2,B2M
Monarch	HP:0011890	Prolonged bleeding following procedure	8	23	1.48	9.30e-07	F9,VWF,F2,F13B,F5,F10,F7,F11
Monarch	HP:0003645	Prolonged partial thromboplastin time	8	24	1.46	1.12e-06	F9,F12,KLKB1,F2,F5,F10,F11,KNG1
Monarch	EFO:0004310	Partial thromboplastin time	7	15	1.61	1.72e-06	HRG,F12,KLKB1,F5,F11,C1S,KNG1
Monarch	HP:0002725	Systemic lupus erythematosus	8	26	1.43	1.73e-06	SERPING1,C1QB,C8A,C1QC,MASP2,C1S,C4A,C1R
Monarch	HP:0006298	Prolonged bleeding after dental extraction	7	16	1.58	2.26e-06	F9,VWF,F2,F13B,F5,F10,F11
Monarch	HP:0000123	Nephritis	10	59	1.17	2.57e-06	PLG,EFEMP2,C1QB,CFHR1,GANAB,CFH,C1QC,CFI,C4A,C1R
Monarch	EFO:0004634	Coagulation factor measurement	13	132	0.93	5.08e-06	CPB2,PROC,ORM1,VWF,KLKB1,FGB,CFH,F5,F10,F7,F11,COLEC11,KNG1
Monarch	HP:0012233	Intramuscular hematoma	6	10	1.72	6.32e-06	F9,F2,SERPINF2,F13B,F10,F7
Monarch	HP:0005368	Abnormality of humoral immunity	16	234	0.78	1.21e-05	CFP,C1QB,C7,BLM,C8A,CFH,FLNA,C8B,C1QC,CFI,MASP2,C1S,C4A,C4B,CFD,B2M
Monarch	HP:0010978	Abnormality of immune system physiology	40	1347	0.41	1.68e-05	MMP2,APOA1,CAT,CFP,APOE,PSMA6,CSTA,DNAH5,TCIRG1,HYAL1,FCN3,SERPING1,PLG,EFEMP2,C1QB,CFHR1,C7,HBA1,FBN1,GANAB,BLM,C8A,F13B,CFH,FLNA,C8B,GSN,C1QC,CFI,NAV2,MASP2,C1S,IGF2,C4A,C4B,C1R,CFD,VEGFC,GRHL2,B2M
Monarch	HP:0010979	Abnormality of lipoprotein cholesterol concentration	8	47	1.17	7.33e-05	CETP,APOC3,APOB,APOA1,APOE,LCAT,PCSK9,APOA5
Monarch	HP:0004846	Prolonged bleeding after surgery	6	18	1.46	8.62e-05	F9,VWF,F13B,F5,F10,F7
Monarch	HP:0030680	Abnormality of cardiovascular system morphology	44	1680	0.36	9.57e-05	SERPIND1,F9,MMP2,APOB,PROC,TTR,CAT,APOE,ANGPTL6,F12,VWF,LCAT,DNAH5,TCIRG1,HABP2,MASP1,GUCY1A1,A2ML1,ANTXR1,PCSK9,FGB,F2,EFEMP2,CALR,SERPINF2,HBA1,FBN1,P4HB,GANAB,F13B,F5,FLNA,CRTAC1,GSN,F10,F7,EFEMP1,CFI,CST3,IGF2,C4A,VEGFC,KNG1,B2M
Monarch	HP:0002634	Arteriosclerosis	9	70	1.05	9.83e-05	SERPIND1,APOB,CAT,APOE,ANGPTL6,LCAT,ANTXR1,PCSK9,FBN1
Monarch	HP:0001977	Abnormal thrombosis	10	94	0.97	0.00010	SERPIND1,F9,HRG,PROC,F12,FGB,F2,CALR,F5,KNG1
Monarch	HP:0010980	Hyperlipoproteinemia	7	33	1.27	0.00010	CETP,APOC3,APOB,APOE,LCAT,PCSK9,APOA5
Monarch	HP:0000951	Abnormality of the skin	47	1888	0.34	0.00013	F9,MMP2,APOB,PROC,APOA1,CAT,LRP1,APOE,F12,VWF,PSMA6,CSTA,CENPE,TCIRG1,HYAL1,FCN3,AHSG,SERPING1,MASP1,GUCY1A1,A2ML1,ANTXR1,PCSK9,DST,F2,PLG,EFEMP2,CALR,SERPINF2,HBA1,FBN1,P4HB,BLM,F13B,F5,FLNA,GSN,F10,F7,CFI,C1S,IGF2,C4A,C1R,VEGFC,GRHL2,B2M
Monarch	HP:0011354	Generalized abnormality of skin	28	821	0.47	0.00013	F9,PROC,APOE,F12,VWF,CSTA,CENPE,TCIRG1,SERPING1,MASP1,GUCY1A1,ANTXR1,F2,CALR,SERPINF2,HBA1,FBN1,P4HB,BLM,F13B,F5,FLNA,GSN,F10,F7,C1S,C4A,C1R
Monarch	HP:0011891	Post-partum hemorrhage	5	10	1.64	0.00015	F2,F13B,F5,F10,F7
Monarch	HP:0012531	Pain	22	539	0.55	0.00015	GNPTG,MMP2,COMP,APOB,CAT,F12,PON3,TCIRG1,HABP2,SERPING1,PCSK9,F2,CALR,SERPINF2,FBN1,ANG,GANAB,FLNA,C1S,TGFBI,GRHL2,B2M

Monarch	HP:0012649	Increased inflammatory response	31	981	0.44	0.00015	APOA1,CAT,APOE,PSMA6,CSTA,DNAH5,TCIRG1,HYAL1,FCN3,PLG,EFEMP2,C1QB,CFHR1,HBA1,FBN1,GANAB,BLM,F13B,CFH,FLNA,GSN,C1QC,CFI,NAV2,MASP2,C1S,C4A,C4B,C1R,VEGFC,B2M
Monarch	HP:0031888	Abnormal HDL cholesterol concentration	6	21	1.4	0.00015	CETP,APOC3,APOA1,APOE,LCAT,APOA5
Monarch	HP:0000168	Abnormality of the gingiva	13	190	0.78	0.00016	MMP2,CAT,TCIRG1,FGB,F2,PLG,SERPINF2,F13B,F5,F10,F7,C1S,C1R
Monarch	HP:0001939	Abnormality of metabolism/homeostasis	51	2168	0.31	0.00016	CETP,GNPTG,F9,MMP2,MPO,APOC3,APOB,APOA1,TTR,CAT,CFP,APOE,F12,LCAT,CP,TCIRG1,SERPING1,CSF1R,A2ML1,ANTXR1,PCSK9,FGB,DST,F2,PLG,CFHR1,CALR,LPA,SERPINF2,HBA1,FBN1,GANAB,PAPLN,BLM,AKAP9,CFH,F5,FLNA,GSN,F10,CST3,IGF2,LMOD3,TGFBI,IGFALS,GC,APOA5,VEGFC,KNG1,GRHL2,B2M
Monarch	HP:0000421	Epistaxis	9	78	1.0	0.00017	F9,VWF,FGB,F2,F13B,F5,F10,F7,F11
Monarch	HP:0002170	Intracranial hemorrhage	11	130	0.87	0.00017	F9,ANGPTL6,FGB,F2,SERPINF2,FBN1,F13B,F5,F10,F7,CST3
Monarch	HP:0000132	Menorrhagia	7	38	1.21	0.00018	VWF,F2,F13B,F5,F10,F7,F11
Monarch	HP:0033353	Abnormal blood vessel morphology	27	792	0.47	0.00018	SERPIND1,MMP2,APOB,PROC,CAT,APOE,ANGPTL6,F12,VWF,LCAT,DNAH5,TCIRG1,MASP1,GUCY1A1,A2ML1,ANTXR1,PCSK9,EFEMP2,FBN1,P4HB,GANAB,FLNA,EFEMP1,CFI,IGF2,C4A,VEGFC
Monarch	HP:0001934	Persistent bleeding after trauma	5	12	1.56	0.00024	F9,VWF,SERPINF2,F13B,F5
Monarch	HP:0011121	Abnormality of skin morphology	42	1648	0.35	0.00025	F9,MMP2,APOB,PROC,APOA1,CAT,LRP1,APOE,F12,VWF,CSTA,CENPE,TCIRG1,AHSG,SERPING1,MASP1,GUCY1A1,A2ML1,ANTXR1,PCSK9,DST,F2,EFEMP2,CALR,SERPINF2,HBA1,FBN1,P4HB,BLM,F13B,F5,FLNA,GSN,F10,F7,C1S,IGF2,C4A,C1R,VEGFC,GRHL2,B2M
Monarch	HP:0000077	Abnormality of the kidney	32	1077	0.41	0.00026	F9,APOB,APOA1,TTR,APOE,LCAT,HABP2,MASP1,ANTXR1,PCSK9,F2,PLG,EFEMP2,C1QB,CFHR1,SERPINF2,FBN1,GANAB,BLM,CENPF,CFH,F5,FLNA,GSN,C1QC,F10,CFI,IGF2,C4A,COLEC11,C1R,B2M
Monarch	HP:0002621	Atherosclerosis	8	64	1.04	0.00036	SERPIND1,APOB,APOE,ANGPTL6,LCAT,ANTXR1,PCSK9,FBN1
Monarch	HP:0011029	Internal hemorrhage	14	244	0.7	0.00036	F9,PROC,ANGPTL6,VWF,FGB,F2,SERPINF2,FBN1,F13B,F5,F10,F7,CST3,F11
Monarch	HP:0000707	Abnormality of the nervous system	69	3471	0.24	0.00038	ST6GAL1,GNPTG,F9,MMP2,COMP,PCDH12,APOB,PROC,APOA1,TTR,CAT,LRP1,APOE,ANGPTL6,CP,DNAH5,CENPE,PON3,TCIRG1,AHSG,CRH,HABP2,SERPING1,CSF1R,SERPINI1,MASP1,GUCY1A1,A2ML1,ANTXR1,PCSK9,BMP1,FGB,DST,F2,PLG,EFEMP2,CALR,SERPINF2,HBA1,FBN1,P4HB,FBLN1,ANG,GANAB,BLM,AKAP9,C8A,CENPF,F13B,F5,ADAMTSL4,FLNA,CRTAC1,C8B,GSN,F10,F7,CFI,CST3,IGF2,COLEC11,LMOD3,ITIH3,C4B,TGFBI,VEGFC,KNG1,GRHL2,B2M
Monarch	HP:0004936	Venous thrombosis	8	65	1.03	0.00038	SERPIND1,F9,PROC,FGB,F2,CALR,F5,KNG1
Monarch	HP:0010935	Abnormality of the upper urinary tract	32	1101	0.4	0.00038	F9,APOB,APOA1,TTR,APOE,LCAT,HABP2,MASP1,ANTXR1,PCSK9,F2,PLG,EFEMP2,C1QB,CFHR1,SERPINF2,FBN1,GANAB,BLM,CENPF,CFH,F5,FLNA,GSN,C1QC,F10,CFI,IGF2,C4A,COLEC11,C1R,B2M
Monarch	HP:0010981	Hypolipoproteinemia	6	27	1.29	0.00038	APOC3,APOB,APOA1,APOE,LCAT,APOA5
Monarch	HP:0005542	Prolonged whole-blood clotting time	4	5	1.84	0.00040	F9,F12,VWF,F5
Monarch	HP:0011884	Abnormal umbilical stump bleeding	4	5	1.84	0.00040	F2,SERPINF2,F13B,F10
Monarch	HP:0001574	Abnormality of the integument	51	2266	0.29	0.00044	F9,MMP2,APOB,PROC,APOA1,CAT,LRP1,APOE,F12,VWF,PSMA6,CSTA,CENPE,TCIRG1,HYAL1,FCN3,AHSG,SERPING1,IHH,MASP1,GUCY1A1,A2ML1,ANTXR1,PCSK9,BMP1,DST,F2,PLG,EFEMP2,CALR,SERPINF2,HBA1,FBN1,P4HB,LIMS1,BLM,F13B,F5,FLNA,GSN,F10,F7,CFI,C1S,IGF2,C4A,COLEC11,C1R,VEGFC,GRHL2,B2M
Monarch	HP:0000152	Abnormality of head or neck	60	2882	0.26	0.00052	GNPTG,F9,MMP2,PCDH12,LTF,APOB,PROC,CAT,LRP1,APOE,F12,VWF,CP,DNAH5,CENPE,PON3,TCIRG1,HYAL1,AHSG,HABP2,SERPING1,CSF1R,IHH,MASP1,A2ML1,ANTXR1,PCSK9,BMP1,FGB,DST,F2,PLG,EFEMP2,SERPINF2,FBN1,P4HB,FBLN1,ANG,BLM,CENPF,F13B,F5,ADAMTSL4,FLNA,CRTAC1,GSN,F10,F7,CFI,F11,C1S,IGF2,ABCA13,COLEC11,LMOD3,IGFALS,C1R,VEGFC,GRHL2,B2M
Monarch	HP:0002960	Autoimmunity	10	122	0.85	0.00052	MMP2,TCIRG1,SERPING1,C1QB,C8A,C1QC,MASP2,C1S,C4A,C1R
Monarch	EFO:0005278	Cardiovascular disease biomarker measurement	50	2228	0.29	0.00059	ST6GAL1,CETP,F9,MPO,APOC3,HRG,APOB,APOA1,LRP1,C4BPB,APOE,F12,LCAT,YWHAE,KLKB1,DGKG,DNAH5,ITIH1,HABP2,SERPINI1,HSP90B1,PCSK9,SHLD1,FGB,PLG,LPA,FBN1,FBLN1,OIT3,LIMS1,FAM184A,APOA4,CFH,QSOX1,F5,CRTAC1,GSN,SVEP1,EFEMP1,EFCAB5,TNRC6A,NAV2,RAPGEF4,MAP3K7CL,ITIH3,PLTP,APOA5,APOC4,KNG1,GRHL2
Monarch	HP:0012211	Abnormal renal physiology	21	560	0.51	0.00059	F9,APOA1,TTR,APOE,LCAT,F2,PLG,EFEMP2,C1QB,CFHR1,SERPINF2,GANAB,CFH,F5,GSN,C1QC,F10,CFI,C4A,C1R,B2M
Monarch	HP:0000271	Abnormality of the face	56	2641	0.27	0.00069	GNPTG,F9,MMP2,LTF,APOB,PROC,CAT,LRP1,APOE,F12,VWF,CP,DNAH5,CENPE,PON3,TCIRG1,HYAL1,AHSG,SERPING1,CSF1R,MASP1,A2ML1,ANTXR1,PCSK9,BMP1,FGB,DST,F2,PLG,EFEMP2,SERPINF2,FBN1,P4HB,FBLN1,ANG,BLM,CENPF,F13B,F5,ADAMTSL4,FLNA,CRTAC1,GSN,F10,F7,CFI,F11,C1S,IGF2,ABCA13,COLEC11,LMOD3,C1R,VEGFC,GRHL2,B2M
Monarch	EFO:0004298	Cardiovascular measurement	52	2378	0.28	0.00071	ST6GAL1,CETP,F9,MPO,APOC3,GPLD1,HRG,APOB,APOA1,LRP1,C4BPB,APOE,F12,LCAT,YWHAE,KLKB1,DGKG,DNAH5,ITIH1,HABP2,SERPINI1,HSP90B1,PCSK9,SHLD1,FGB,INHBC,PLG,LPA,FBN1,FBLN1,OIT3,LIMS1,FAM184A,APOA4,CFH,QSOX1,F5,CRTAC1,GSN,SVEP1,EFEMP1,EFCAB5,TNRC6A,NAV2,RAPGEF4,MAP3K7CL,ITIH3,PLTP,APOA5,APOC4,KNG1,GRHL2
Monarch	HP:0000234	Abnormality of the head	59	2865	0.25	0.00084	GNPTG,F9,MMP2,PCDH12,LTF,APOB,PROC,CAT,LRP1,APOE,F12,VWF,CP,DNAH5,CENPE,PON3,TCIRG1,HYAL1,AHSG,SERPING1,CSF1R,IHH,MASP1,A2ML1,ANTXR1,PCSK9,BMP1,FGB,DST,F2,PLG,EFEMP2,SERPINF2,FBN1,P4HB,FBLN1,ANG,BLM,CENPF,F13B,F5,ADAMTSL4,FLNA,CRTAC1,GSN,F10,F7,CFI,F11,C1S,IGF2,ABCA13,COLEC11,LMOD3,IGFALS,C1R,VEGFC,GRHL2,B2M
Monarch	EFO:0008390	Prothrombin time measurement	4	8	1.64	0.0013	F2,F5,F10,F7
Monarch	HP:0001367	Abnormal joint morphology	27	905	0.42	0.0013	GNPTG,F9,MMP2,COMP,APOE,VWF,HYAL1,IHH,MASP1,A2ML1,BMP1,FGB,F2,EFEMP2,SERPINF2,FBN1,FBLN1,F13B,F5,FLNA,F10,F7,CFI,F11,COLEC11,LMOD3,VEGFC
Monarch	HP:0002625	Deep venous thrombosis	5	20	1.34	0.0013	SERPIND1,F9,PROC,F2,F5
Monarch	HP:0031886	Abnormal LDL cholesterol concentration	6	36	1.16	0.0013	APOC3,APOB,APOE,LCAT,PCSK9,APOA5
Monarch	HP:0000099	Glomerulonephritis	6	37	1.15	0.0014	C1QB,CFHR1,CFH,C1QC,CFI,C4A
Monarch	HP:0000119	Abnormality of the genitourinary system	48	2189	0.28	0.0015	F9,MMP2,APOB,APOA1,TTR,APOE,VWF,LCAT,DNAH5,AHSG,CRH,HABP2,MASP1,GUCY1A1,A2ML1,ANTXR1,PCSK9,FGB,F2,PLG,EFEMP2,C1QB,CFHR1,SERPINF2,FBN1,FBLN1,DNAH8,GANAB,BLM,CENPF,F13B,CFH,F5,FLNA,GSN,C1QC,F10,F7,CFI,F11,IGF2,C4A,FSIP2,COLEC11,LMOD3,C1R,VEGFC,B2M
Monarch	HP:0025031	Abnormality of the digestive system	51	2389	0.27	0.0015	F9,MMP2,APOB,APOA1,TTR,APOE,F12,VWF,LCAT,CP,DNAH5,PON3,TCIRG1,FCN3,HABP2,SERPING1,MASP1,GUCY1A1,A2ML1,ANTXR1,PCSK9,BMP1,FGB,DST,F2,PLG,EFEMP2,CALR,HBA1,FBN1,ANG,GANAB,BLM,CENPF,F13B,F5,FLNA,GSN,F10,F7,NAV2,MASP2,F11,C1S,IGF2,COLEC11,LMOD3,C4B,C1R,GRHL2,B2M
Monarch	HP:0030140	Oral cavity bleeding	4	9	1.59	0.0017	F2,F13B,F5,F10
Monarch	HP:0011492	Abnormal corneal stroma morphology	8	86	0.91	0.0018	GNPTG,MMP2,APOB,APOE,LCAT,PCSK9,TGFBI,GRHL2
Monarch	HP:0012639	Abnormal nervous system morphology	53	2546	0.26	0.0019	F9,COMP,PCDH12,APOB,PROC,TTR,APOE,ANGPTL6,CP,DNAH5,CENPE,PON3,TCIRG1,AHSG,HABP2,SERPING1,CSF1R,SERPINI1,MASP1,GUCY1A1,ANTXR1,PCSK9,FGB,F2,PLG,EFEMP2,CALR,SERPINF2,HBA1,FBN1,P4HB,FBLN1,ANG,GANAB,BLM,C8A,CENPF,F13B,F5,FLNA,CRTAC1,C8B,GSN,F10,F7,CFI,CST3,IGF2,COLEC11,LMOD3,C4B,KNG1,B2M
Monarch	HP:0000790	Hematuria	9	118	0.82	0.0022	F9,APOA1,F2,CFHR1,SERPINF2,GANAB,CFH,F5,F10

Monarch	HP:0008011	Peripheral opacification of the cornea	4	10	1.54	0.0022	MMP2,APOB,APOE,PCSK9
Monarch	HP:0012185	Constrictive median neuropathy	4	10	1.54	0.0022	COMP,TTR,GSN,B2M
Monarch	HP:0007957	Corneal opacity	12	226	0.67	0.0026	GNPTG,MMP2,APOB,APOA1,APOE,PXDN,LCAT,PCSK9,DST,CENPF,TGFBI,GRHL2
Monarch	HP:0011830	Abnormal oral mucosa morphology	14	305	0.6	0.0026	MMP2,LTF,CAT,TCIRG1,FGB,F2,PLG,SERPINF2,F13B,F5,F10,F7,C1S,C1R
Monarch	HP:0011034	Amyloidosis	5	25	1.24	0.0028	APOA1,TTR,GSN,CST3,B2M
Monarch	HP:0011355	Localized skin lesion	23	738	0.43	0.0028	MMP2,APOB,PROC,APOA1,LRP1,APOE,F12,CSTA,TCIRG1,MASP1,A2ML1,ANTXR1,PCSK9,DST,FBN1,P4HB,BLM,FLNA,GSN,C1S,IGF2,C1R,B2M
Monarch	HP:0011004	Abnormal systemic arterial morphology	16	398	0.54	0.0030	SERPIND1,MMP2,APOB,CAT,APOE,ANGPTL6,F12,LCAT,GUCY1A1,ANTXR1,PCSK9,EFEMP2,FBN1,P4HB,GANAB,FLNA
Monarch	HP:0025142	Constitutional symptom	26	906	0.4	0.0030	GNPTG,MMP2,COMP,APOB,PROC,TTR,CAT,F12,PON3,TCIRG1,CRH,HABP2,SERPING1,PCSK9,F2,CALR,SERPINF2,FBN1,ANG,GANAB,AKAP9,FLNA,C1S,TGFBI,GRHL2,B2M
Monarch	HP:0002011	Morphological central nervous system abnormality	50	2416	0.26	0.0037	F9,COMP,PCDH12,APOB,PROC,TTR,APOE,ANGPTL6,CP,DNAH5,CENPE,PON3,TCIRG1,AHSG,HABP2,CSF1R,SERPINI1,MASP1,GUCY1A1,ANTXR1,PCSK9,FGB,F2,PLG,EFEMP2,CALR,SERPINF2,HBA1,FBN1,P4HB,FBLN1,ANG,GANAB,BLM,C8A,CENPF,F13B,F5,FLNA,CRTAC1,C8B,F10,F7,CFI,CST3,IGF2,COLEC11,C4B,KNG1,B2M
Monarch	HP:0000080	Abnormality of reproductive system physiology	21	659	0.44	0.0044	F9,TTR,APOE,VWF,DNAH5,AHSG,GUCY1A1,A2ML1,ANTXR1,FGB,F2,DNAH8,GANAB,BLM,F13B,F5,F10,F7,F11,IGF2,FSIP2
Monarch	HP:0003272	Abnormal hip bone morphology	17	461	0.51	0.0045	GNPTG,MMP2,COMP,APOE,CENPE,TCIRG1,HYAL1,IHH,MASP1,BMP1,EFEMP2,FBN1,FBLN1,FLNA,COLEC11,LMOD3,B2M
Monarch	HP:0002644	Abnormal pelvic girdle bone morphology	18	512	0.49	0.0048	GNPTG,MMP2,COMP,APOE,CENPE,TCIRG1,HYAL1,IHH,MASP1,ANTXR1,BMP1,EFEMP2,FBN1,FBLN1,FLNA,COLEC11,LMOD3,B2M
Monarch	HP:0010989	Abnormality of the intrinsic pathway	5	29	1.18	0.0049	F9,F12,VWF,F11,KNG1
Monarch	EFO:0004694	Factor XI measurement	3	4	1.82	0.0061	KLKB1,F11,KNG1
Monarch	HP:0005356	Decreased serum complement factor I	3	4	1.82	0.0061	C1QB,C1QC,CFI
Monarch	HP:0007759	Opacification of the corneal stroma	7	78	0.89	0.0063	GNPTG,MMP2,APOB,APOE,LCAT,PCSK9,TGFBI
Monarch	HP:0002239	Gastrointestinal hemorrhage	8	111	0.8	0.0079	F9,VWF,FGB,F2,F5,F10,F7,F11
Monarch	HP:0009145	Abnormal cerebral artery morphology	6	55	0.98	0.0079	APOB,ANGPTL6,GUCY1A1,PCSK9,FBN1,GANAB
Monarch	EFO:0004614	Apolipoprotein A 1 measurement	15	396	0.52	0.0089	CETP,APOC3,APOB,APOA1,LRP1,APOE,LCAT,DGKG,ITIH1,PCSK9,PLG,LPA,APOA4,CRTAC1,APOC4
Monarch	EFO:0006925	Lipoprotein A measurement	5	34	1.11	0.0089	CETP,APOE,PLG,LPA,APOC4
Monarch	HP:0005262	Abnormality of the synovia	13	305	0.57	0.0089	MMP2,COMP,APOE,HYAL1,MASP1,BMP1,F2,EFEMP2,FBN1,FBLN1,FLNA,COLEC11,LMOD3
Monarch	HP:0003216	Generalized amyloid deposition	3	5	1.72	0.0090	APOA1,GSN,CST3
Monarch	HP:0005430	Recurrent Neisserial infections	3	5	1.72	0.0090	C7,C8B,CFI
Monarch	HP:0007937	Reticular pigmentary degeneration	3	5	1.72	0.0090	CFH,EFEMP1,CFI
Monarch	EFO:0001365	Age-related macular degeneration	5	35	1.1	0.0097	CETP,APOE,CFH,CFI,CFB
Monarch	EFO:1001492	Atrophic macular degeneration	5	35	1.1	0.0097	CETP,APOE,CFH,CFI,CFB
Monarch	HP:0000140	Abnormality of the menstrual cycle	11	228	0.62	0.0097	F9,VWF,ANTXR1,FGB,F2,F13B,F5,F10,F7,F11,IGF2
Monarch	HP:0000704	Periodontitis	5	35	1.1	0.0097	CAT,TCIRG1,PLG,C1S,C1R
Monarch	HP:0010990	Abnormality of the common coagulation pathway	5	35	1.1	0.0097	FGB,PLG,F13B,F5,F10
Monarch	EFO:0007804	LDL cholesterol change measurement	4	18	1.29	0.0101	APOB,APOE,PCSK9,LPA
Monarch	HP:0003233	Decreased HDL cholesterol concentration	4	18	1.29	0.0101	APOA1,APOE,LCAT,APOA5
Monarch	HP:0030012	Abnormal female reproductive system physiology	12	273	0.58	0.0105	F9,VWF,ANTXR1,FGB,F2,BLM,F13B,F5,F10,F7,F11,IGF2
Monarch	HP:0033057	Decreased serum terminal complement component	3	6	1.64	0.0113	C7,C8A,C8B
Monarch	HP:0100724	Hypercoagulability	4	19	1.26	0.0118	F9,HRG,PROC,F5
Monarch	EFO:0004713	FEV/FEC ratio	20	672	0.41	0.0124	MPO,LRP1,SERPINA10,DNAH5,SERPING1,ANTXR1,DST,C7,FBN1,FBLN1,FAM184A,MTCL1,CFH,EFEMP1,CFI,EFCAB5,NAV2,FGL1,VEGFC,HYOU1
Monarch	HP:0001382	Joint hypermobility	12	279	0.57	0.0124	COMP,CENPE,A2ML1,ANTXR1,BMP1,EFEMP2,FBN1,P4HB,FLNA,C1S,COLEC11,C1R
Monarch	HP:0030972	Abnormal systemic blood pressure	14	369	0.52	0.0124	MMP2,APOB,APOA1,TTR,ANGPTL6,SERPING1,GUCY1A1,PCSK9,F2,CALR,FBN1,GANAB,ADAMTSL4,GSN
Monarch	HP:0002829	Arthralgia	8	124	0.75	0.0126	GNPTG,MMP2,COMP,APOB,PCSK9,F2,FBN1,C1S
Monarch	HP:0000799	Renal steatosis	3	7	1.57	0.0153	APOB,APOE,PCSK9
Monarch	HP:0012397	Aortic atherosclerotic lesion	3	7	1.57	0.0153	APOB,APOE,PCSK9
Monarch	HP:0008067	Abnormally lax or hyperextensible skin	8	129	0.73	0.0160	CSTA,ANTXR1,EFEMP2,FBN1,FLNA,GSN,C1S,C1R
Monarch	HP:0033127	Abnormality of the musculoskeletal system	58	3173	0.2	0.0162	GNPTG,F9,MMP2,COMP,PCDH12,APOB,APOA1,TTR,LRP1,APOE,VWF,CP,DNAH5,CENPE,PON3,TCIRG1,HYAL1,AHSG,HABP2,CSF1R,IHH,MASP1,A2ML1,ANTXR1,PCSK9,BMP1,FGB,DST,F2,PLG,EFEMP2,CALR,SERPINF2,FBN1,P4HB,FBLN1,ANG,BLM,CENPF,F13B,F5,ADAMTSL4,FLNA,GSN,F10,F7,CFI,F11,C1S,IGF2,COLEC11,LMOD3,TGFBI,IGFALS,C1R,VEGFC,GRHL2,B2M
Monarch	HP:0000079	Abnormality of the urinary system	36	1657	0.28	0.0164	F9,APOB,APOA1,TTR,APOE,LCAT,CRH,HABP2,MASP1,ANTXR1,PCSK9,F2,PLG,EFEMP2,C1QB,CFHR1,LPA,SERPINF2,FBN1,GANAB,PAPLN,BLM,CENPF,CFH,F5,FLNA,GSN,C1QC,F10,CFI,IGF2,C4A,COLEC11,LMOD3,C1R,B2M
Monarch	HP:0003010	Prolonged bleeding time	5	42	1.02	0.0169	F9,VWF,F2,CALR,F5
Monarch	HP:0032101	Unusual infection	21	748	0.39	0.0170	DNAH5,TCIRG1,HYAL1,FCN3,PLG,EFEMP2,C1QB,C7,GANAB,BLM,C8A,CFH,FLNA,C8B,C1QC,CFI,MASP2,C4B,C1R,CFD,B2M
Monarch	HP:0001384	Abnormal hip joint morphology	12	292	0.55	0.0173	MMP2,COMP,APOE,HYAL1,MASP1,BMP1,EFEMP2,FBN1,FBLN1,FLNA,COLEC11,LMOD3
Monarch	HP:0000991	Xanthomatosis	4	22	1.2	0.0174	APOB,APOA1,APOE,PCSK9
Monarch	HP:0001297	Stroke	11	249	0.59	0.0174	TTR,ANGPTL6,GUCY1A1,F2,CALR,FBN1,F5,FLNA,CRTAC1,CST3,KNG1
Monarch	HP:0008065	Aplasia/Hypoplasia of the skin	9	170	0.66	0.0185	PROC,TCIRG1,DST,FBN1,P4HB,FLNA,GSN,C1S,C1R
Monarch	HP:0000078	Abnormality of the genital system	31	1358	0.3	0.0192	F9,MMP2,TTR,APOE,VWF,DNAH5,AHSG,MASP1,GUCY1A1,A2ML1,ANTXR1,FGB,F2,PLG,EFEMP2,FBN1,FBLN1,DNAH8,GANAB,BLM,F13B,F5,FLNA,F10,F7,F11,IGF2,FSIP2,COLEC11,LMOD3,VEGFC
Monarch	HP:0001000	Abnormality of skin pigmentation	15	439	0.47	0.0192	MMP2,PROC,CAT,APOE,CSTA,A2ML1,ANTXR1,DST,F2,FBN1,BLM,FLNA,C1S,IGF2,C1R
Monarch	HP:0001677	Coronary artery atherosclerosis	5	44	1.0	0.0192	SERPIND1,APOB,APOE,PCSK9,FBN1
Monarch	HP:0004381	Supravalvular aortic stenosis	3	8	1.51	0.0192	APOB,PCSK9,EFEMP2
Monarch	HP:0010874	Tendon xanthomatosis	3	8	1.51	0.0192	APOB,APOE,PCSK9
Monarch	HP:0011277	Abnormality of the urinary system physiology	24	933	0.35	0.0195	F9,APOA1,TTR,APOE,LCAT,F2,PLG,EFEMP2,C1QB,CFHR1,LPA,SERPINF2,GANAB,PAPLN,BLM,CFH,F5,GSN,C1QC,F10,CFI,C4A,C1R,B2M
Monarch	HP:0100545	Arterial stenosis	6	72	0.86	0.0212	SERPIND1,APOB,APOE,F12,PCSK9,FBN1
Monarch	HP:0001681	Angina pectoris	4	24	1.16	0.0217	APOB,APOE,LCAT,PCSK9
Monarch	EFO:0003907	Deep vein thrombosis	3	9	1.46	0.0237	F2,F5,KNG1
Monarch	EFO:0009925	Antithrombotic agent use measurement	3	9	1.46	0.0237	APOE,LPA,F5
Monarch	HP:0001084	Corneal arcus	3	9	1.46	0.0237	APOB,APOE,PCSK9
Monarch	HP:0030137	Prolonged bleeding following circumcision	3	9	1.46	0.0237	F2,F13B,F5
Monarch	HP:0003141	Increased LDL cholesterol concentration	4	26	1.13	0.0276	APOB,APOE,LCAT,PCSK9
Monarch	HP:0008151	Prolonged prothrombin time	4	26	1.13	0.0276	F2,F5,F10,F7
Monarch	HP:0000481	Abnormal cornea morphology	19	673	0.39	0.0280	GNPTG,MMP2,APOB,APOA1,APOE,PXDN,LCAT,DNAH5,SERPING1,ANTXR1,PCSK9,DST,FBN1,CENPF,FLNA,GSN,ABCA13,TGFBI,GRHL2
Monarch	EFO:0010071	Cardiac troponin I measurement	3	10	1.42	0.0292	F12,KLKB1,HABP2

Monarch	HP:0000153	Abnormality of the mouth	40	1992	0.24	0.0292	MMP2,LTF,CAT,APOE,CENPE,PON3,TCIRG1,HYAL1,SERPING1,MASP1,A2ML1,ANTXR1,BMP1,FGB,DST,F2,PLG,EFEMP2,SERPINF2,FBN1,P4HB,FBLN1,ANG,BLM,CENPF,F13B,F5,FLNA,CRTAC1,GSN,F10,F7,C1S,IGF2,ABCA13,COLEC11,LMOD3,C1R,GRHL2,B2M
Monarch	HP:0001114	Xanthelasma	3	10	1.42	0.0292	APOB,APOE,PCSK9
Monarch	HP:0000979	Purpura	6	79	0.82	0.0311	PROC,APOE,F2,CALR,F13B,C4A
Monarch	EFO:0004612	High density lipoprotein cholesterol measurement	20	740	0.37	0.0325	CETP,APOC3,APOB,APOA1,LRP1,APOE,LCAT,DGKG,PON3,ITIH1,F2,INHBC,LPA,APOD,GIT2,APOA4,CRTAC1,PLTP,APOA5,APOC4
Monarch	HP:0031652	Abnormal aortic valve physiology	7	113	0.73	0.0329	GNPTG,APOB,VWF,PCSK9,EFEMP2,FBN1,FLNA
Monarch	HP:0000924	Abnormality of the skeletal system	50	2708	0.21	0.0331	GNPTG,F9,MMP2,COMP,PCDH12,APOB,TTR,APOE,VWF,DNAH5,CENPE,TCIRG1,HYAL1,AHSG,HABP2,CSF1R,IHH,MASP1,A2ML1,ANTXR1,PCSK9,BMP1,FGB,DST,F2,PLG,EFEMP2,SERPINF2,FBN1,P4HB,FBLN1,BLM,CENPF,F13B,F5,ADAMTSL4,FLNA,F10,F7,CFI,F11,C1S,IGF2,COLEC11,LMOD3,IGFALS,C1R,VEGFC,GRHL2,B2M
Monarch	HP:0003170	Abnormal acetabulum morphology	6	81	0.81	0.0344	MMP2,COMP,APOE,HYAL1,BMP1,FBN1
Monarch	HP:0003563	Decreased LDL cholesterol concentration	3	11	1.38	0.0348	APOC3,APOB,APOA5
Monarch	HP:0011842	Abnormal skeletal morphology	48	2573	0.21	0.0348	GNPTG,F9,MMP2,COMP,PCDH12,APOB,TTR,APOE,VWF,DNAH5,CENPE,TCIRG1,HYAL1,AHSG,CSF1R,IHH,MASP1,A2ML1,ANTXR1,PCSK9,BMP1,FGB,DST,F2,PLG,EFEMP2,SERPINF2,FBN1,P4HB,FBLN1,BLM,CENPF,F13B,F5,ADAMTSL4,FLNA,F10,F7,CFI,F11,C1S,IGF2,COLEC11,LMOD3,IGFALS,C1R,VEGFC,B2M
Monarch	HP:0001547	Abnormal rib cage morphology	12	325	0.51	0.0351	GNPTG,COMP,TCIRG1,IHH,A2ML1,ANTXR1,BMP1,FBN1,P4HB,FLNA,IGF2,LMOD3
Monarch	HP:0040071	Abnormal morphology of ulna	7	115	0.72	0.0351	COMP,IHH,MASP1,A2ML1,FLNA,COLEC11,B2M
Monarch	HP:0004950	Peripheral arterial stenosis	4	29	1.08	0.0361	APOB,APOE,PCSK9,FBN1
Monarch	EFO:0004732	Lipoprotein measurement	31	1426	0.28	0.0368	CETP,APOC3,APOB,APOA1,LRP1,APOE,LCAT,KLKB1,DGKG,PON3,ITIH1,PCSK9,FGB,F2,INHBC,PLG,LPA,FBLN1,OIT3,LIMS1,THBS4,APOD,ASGR2,GIT2,APOA4,CRTAC1,ABCA13,PLTP,APOA5,APOC4,GRHL2
Monarch	HP:0003124	Hypercholesterolemia	5	54	0.91	0.0381	CETP,APOC3,APOB,APOE,PCSK9
Monarch	EFO:0003777	Heart disease	8	155	0.65	0.0385	MMP2,LRP1,VWF,FBN1,P4HB,GANAB,FLNA,MAP3K7CL
Monarch	HP:0000973	Cutis laxa	6	84	0.79	0.0385	ANTXR1,EFEMP2,FBN1,FLNA,GSN,C1R
Monarch	HP:0031815	Abnormal oral physiology	9	196	0.6	0.0391	PON3,SERPING1,F2,FBLN1,ANG,F13B,F5,GSN,F10
Monarch	EFO:0006794	Cerebrospinal fluid biomarker measurement	5	55	0.9	0.0400	LTF,HRG,APOE,C7,C1R
Monarch	HP:0001634	Mitral valve prolapse	6	86	0.78	0.0425	MMP2,VWF,FBN1,P4HB,GANAB,FLNA
Monarch	HP:0004334	Dermal atrophy	6	88	0.77	0.0469	TCIRG1,DST,FBN1,GSN,C1S,C1R
Monarch	HP:0011915	Cardiovascular calcification	4	32	1.04	0.0471	APOB,PCSK9,FBN1,B2M
Monarch	HP:0012330	Pyelonephritis	3	13	1.3	0.0474	EFEMP2,GANAB,CFI
Monarch	HP:0030163	Abnormal vascular physiology	12	340	0.49	0.0474	PROC,ANGPTL6,TCIRG1,GUCY1A1,BMP1,F2,CALR,FBN1,F5,FLNA,CRTAC1,KNG1
Monarch	HP:0033151	Abnormal pharynx morphology	8	162	0.63	0.0477	F12,DNAH5,TCIRG1,SERPING1,PLG,BLM,CFI,B2M
Monarch	EFO:0008022	Angiogenin measurement	2	2	1.94	0.0489	ANG,RNASE4
Monarch	EFO:0008098	Complement factor H-related protein 5 measurement	2	2	1.94	0.0489	F13B,CFH
Monarch	EFO:0008155	Histidine-rich glycoprotein measurement	2	2	1.94	0.0489	HRG,KNG1
Monarch	EFO:0008260	Plasma kallikrein measurement	2	2	1.94	0.0489	KLKB1,KNG1
Monarch	HP:0003119	Abnormal circulating lipid concentration	9	206	0.58	0.0489	CETP,APOC3,APOB,APOA1,APOE,LCAT,PCSK9,FBN1,APOA5
Monarch	HP:0004434	Decreased serum complement C8	2	2	1.94	0.0489	C8A,C8B
Monarch	HP:0005225	Intestinal edema	2	2	1.94	0.0489	F12,SERPING1
Monarch	HP:0005369	Decreased serum complement factor H	2	2	1.94	0.0489	CFH,CFI
Monarch	HP:0005482	Abnormality of the alternative complement pathway	2	2	1.94	0.0489	CFP,CFH
Monarch	HP:0011843	Abnormal musculoskeletal physiology	29	1330	0.28	0.0489	GNPTG,MMP2,COMP,APOE,CENPE,TCIRG1,AHSG,HABP2,IHH,MASP1,A2ML1,ANTXR1,BMP1,DST,F2,EFEMP2,SERPINF2,FBN1,P4HB,FBLN1,ADAMTSL4,FLNA,C1S,IGF2,COLEC11,LMOD3,IGFALS,C1R,B2M
Monarch	HP:0011855	Pharyngeal edema	2	2	1.94	0.0489	F12,SERPING1
Monarch	HP:0030843	Cardiac amyloidosis	2	2	1.94	0.0489	GSN,B2M
Monarch	HP:0100238	Synostosis involving bones of the upper limbs	6	90	0.76	0.0489	IHH,MASP1,A2ML1,FBLN1,FLNA,COLEC11
Monarch	HP:0002719	Recurrent infections	19	721	0.36	0.0490	DNAH5,TCIRG1,HYAL1,FCN3,PLG,EFEMP2,C1QB,C7,GANAB,BLM,CFH,FLNA,C8B,C1QC,CFI,MASP2,C1R,CFD,B2M
DISEASES	DOID:1247	Blood coagulation disease	23	89	1.35	5.11e-19	SERPIND1,F9,PF4V1,HRG,PROC,F12,VWF,KLKB1,PF4,FGB,F2,PLG,CALR,SERPINF2,F13B,CFH,F5,FLNA,F10,F7,F11,C4B,KNG1
DISEASES	DOID:4	Disease	128	6291	0.25	5.98e-11	CETP,GNPTG,SERPIND1,LGALS1,CHGA,F9,MMP2,COMP,MPO,PF4V1,APOC3,PCDH12,LTF,HRG,APOB,PROC,APOA1,TTR,CAT,CFP,APOE,PXDN,F12,APCS,VWF,LCAT,YWHAE,CSTA,CP,KLKB1,DNAH5,CENPE,TCIRG1,HYAL1,ITIH4,AHSG,CRH,SAA4,SERPING1,ANKRD44,CSF1R,PIP,SCGB3A1,IHH,SERPINI1,PF4,MASP1,CAMP,A2ML1,ANTXR1,LRG1,SH3D19,PCSK9,BMP1,FGB,DST,F2,PLG,EFEMP2,C1QB,CFHR1,APOL1,SUMO4,CALR,LPA,SERPINF2,HBA1,HSPA5,FBN1,P4HB,SERPINA7,DNAH8,ANG,GANAB,BLM,AKAP9,PIGR,APOA4,CNDP1,CENPF,F13B,CFHR3,CFH,F5,APOA2,ADAMTSL4,FLNA,MCOLN2,C8B,GSN,C1QC,F10,F7,CA6,IGFBP3,GPX3,EFEMP1,CFI,TNRC6A,GAPDH,CST3,F11,C1S,NUCB1,PCYOX1,ADIPOQ,IGF2,C4A,CCDC122,ABCA13,COLEC11,MST1,LMOD3,C4B,TGFBI,CFB,SELENOP,C1R,APOA5,SERPINA3,IGKV2D-28,CFD,IGHV3-15,VEGFC,KNG1,TRIM66,GRHL2,B2M
DISEASES	DOID:7	Disease of anatomical entity	108	4798	0.29	5.98e-11	CETP,SERPIND1,LGALS1,CHGA,F9,MMP2,COMP,MPO,PF4V1,PCDH12,LTF,HRG,APOB,PROC,APOA1,TTR,CFP,APOE,PXDN,F12,APCS,VWF,LCAT,YWHAE,CSTA,KLKB1,DNAH5,CENPE,TCIRG1,AHSG,CRH,SAA4,SERPING1,ANKRD44,CSF1R,PIP,SCGB3A1,IHH,SERPINI1,PF4,CAMP,A2ML1,ANTXR1,LRG1,SH3D19,PCSK9,BMP1,FGB,DST,F2,PLG,EFEMP2,C1QB,CFHR1,APOL1,SUMO4,CALR,LPA,SERPINF2,HBA1,FBN1,P4HB,SERPINA7,ANG,GANAB,BLM,AKAP9,PIGR,APOA4,CNDP1,F13B,CFHR3,CFH,F5,ADAMTSL4,FLNA,C8B,GSN,C1QC,F10,F7,EFEMP1,CFI,TNRC6A,GAPDH,CST3,F11,C1S,NUCB1,ADIPOQ,IGF2,C4A,CCDC122,MST1,LMOD3,C4B,TGFBI,CFB,C1R,SERPINA3,IGKV2D-28,CFD,IGHV3-15,VEGFC,KNG1,TRIM66,GRHL2,B2M
DISEASES	DOID:74	Hematopoietic system disease	30	473	0.74	1.23e-10	SERPIND1,F9,MPO,PF4V1,HRG,PROC,F12,VWF,KLKB1,TCIRG1,PF4,CAMP,FGB,F2,PLG,CALR,SERPINF2,HBA1,F13B,CFH,F5,FLNA,F10,F7,F11,C4B,SERPINA3,IGHV3-15,KNG1,B2M
DISEASES	DOID:2452	Thrombophilia	10	21	1.62	9.56e-10	SERPIND1,F9,HRG,PROC,VWF,F2,PLG,CFH,F5,C4B
DISEASES	DOID:0050739	Autosomal genetic disease	73	2802	0.36	4.95e-09	CETP,GNPTG,SERPIND1,F9,COMP,APOC3,LTF,HRG,APOB,PROC,APOA1,TTR,APOE,F12,APCS,VWF,YWHAE,CP,KLKB1,DNAH5,TCIRG1,SERPING1,ANKRD44,CSF1R,IHH,SERPINI1,MASP1,CAMP,ANTXR1,PCSK9,FGB,DST,F2,PLG,EFEMP2,CFHR1,CALR,SERPINF2,HBA1,FBN1,GANAB,BLM,AKAP9,PIGR,APOA4,F13B,CFHR3,CFH,F5,APOA2,ADAMTSL4,FLNA,GSN,F10,F7,IGFBP3,EFEMP1,CFI,CST3,F11,C1S,PCYOX1,IGF2,COLEC11,LMOD3,TGFBI,CFB,APOA5,SERPINA3,CFD,KNG1,GRHL2,B2M
DISEASES	DOID:9120	Amyloidosis	13	75	1.18	1.35e-08	APOC3,APOA1,TTR,APOE,APCS,SAA4,APOA4,APOA2,GSN,CST3,TGFBI,SERPINA3,B2M

DISEASES	DOID:0050736	Autosomal dominant disease	46	1386	0.46	5.58e-08	CETP,SERPIND1,F9,COMP,APOC3,HRG,PROC,APOA1,TTR,APOE,F12,APCS,VWF,YWHAE,KLKB1,SERPING1,ANKRD44,CSF1R,IHH,SERPINI1,F2,EFEMP2,CFHR1,CALR,FBN1,GANAB,AKAP9,APOA4,F13B,CFHR3,CFH,F5,APOA2,FLNA,GSN,EFEMP1,CFI,CST3,C1S,IGF2,TGFBI,CFB,SERPINA3,KNG1,GRHL2,B2M
DISEASES	DOID:630	Genetic disease	85	3778	0.29	5.91e-08	CETP,GNPTG,SERPIND1,F9,COMP,APOC3,LTF,HRG,APOB,PROC,APOA1,TTR,CAT,APOE,F12,APCS,VWF,LCAT,YWHAE,CP,KLKB1,DNAH5,TCIRG1,HYAL1,SERPING1,ANKRD44,CSF1R,IHH,SERPINI1,MASP1,CAMP,A2ML1,ANTXR1,PCSK9,FGB,DST,F2,PLG,EFEMP2,C1QB,CFHR1,SUMO4,CALR,SERPINF2,HBA1,FBN1,DNAH8,GANAB,BLM,AKAP9,PIGR,APOA4,CNDP1,CENPF,F13B,CFHR3,CFH,F5,APOA2,ADAMTSL4,FLNA,MCOLN2,GSN,F10,F7,IGFBP3,EFEMP1,CFI,CST3,F11,C1S,PCYOX1,ADIPOQ,IGF2,COLEC11,MST1,LMOD3,TGFBI,CFB,APOA5,SERPINA3,CFD,KNG1,GRHL2,B2M
DISEASES	DOID:0050177	Monogenic disease	77	3266	0.31	7.62e-08	CETP,GNPTG,SERPIND1,F9,COMP,APOC3,LTF,HRG,APOB,PROC,APOA1,TTR,APOE,F12,APCS,VWF,YWHAE,CP,KLKB1,DNAH5,TCIRG1,SERPING1,ANKRD44,CSF1R,IHH,SERPINI1,MASP1,CAMP,A2ML1,ANTXR1,PCSK9,FGB,DST,F2,PLG,EFEMP2,CFHR1,CALR,SERPINF2,HBA1,FBN1,DNAH8,GANAB,BLM,AKAP9,PIGR,APOA4,CENPF,F13B,CFHR3,CFH,F5,APOA2,ADAMTSL4,FLNA,GSN,F10,F7,IGFBP3,EFEMP1,CFI,CST3,F11,C1S,PCYOX1,IGF2,COLEC11,MST1,LMOD3,TGFBI,CFB,APOA5,SERPINA3,CFD,KNG1,GRHL2,B2M
DISEASES	DOID:2007	Degeneration of macula and posterior pole	10	46	1.28	2.72e-07	CETP,APOE,CFHR1,F13B,CFHR3,CFH,EFEMP1,CFI,CST3,CFB
DISEASES	DOID:0050636	Familial visceral amyloidosis	8	21	1.52	3.24e-07	APOC3,APOA1,TTR,APOE,APCS,APOA4,APOA2,B2M
DISEASES	DOID:626	Complement deficiency	8	23	1.48	5.39e-07	KLKB1,SERPING1,CFHR1,CFHR3,CFH,CFI,CFB,KNG1
DISEASES	DOID:10871	Age related macular degeneration	9	44	1.25	2.33e-06	CETP,APOE,CFHR1,F13B,CFHR3,CFH,CFI,CST3,CFB
DISEASES	DOID:2914	Immune system disease	27	675	0.54	9.14e-06	MPO,TTR,APOE,PXDN,APCS,KLKB1,SAA4,SERPING1,A2ML1,DST,F2,PLG,CFHR1,SUMO4,HBA1,PIGR,CFHR3,CFH,CFI,C4A,C4B,CFB,IGKV2D-28,IGHV3-15,VEGFC,KNG1,B2M
DISEASES	DOID:557	Kidney disease	17	275	0.73	1.15e-05	MPO,TTR,APCS,SAA4,F2,CFHR1,APOL1,GANAB,APOA4,CFHR3,CFH,CFI,CST3,IGF2,C4B,CFB,B2M
DISEASES	DOID:178	Vascular disease	16	249	0.75	1.66e-05	CETP,MPO,APOB,APOA1,APOE,VWF,PF4,PCSK9,F2,PLG,LPA,FBN1,F10,F7,ADIPOQ,C4A
DISEASES	DOID:14735	Hereditary angioedema	5	6	1.86	1.90e-05	F12,KLKB1,SERPING1,C1S,KNG1
DISEASES	DOID:1287	Cardiovascular system disease	22	493	0.59	2.51e-05	CETP,CHGA,MPO,APOB,APOA1,TTR,APOE,APCS,VWF,DNAH5,PF4,PCSK9,F2,PLG,LPA,FBN1,AKAP9,FLNA,F10,F7,ADIPOQ,C4A
DISEASES	DOID:37	Skin disease	22	518	0.57	5.15e-05	MPO,APOA1,CFP,APOE,F12,APCS,CSTA,KLKB1,SERPING1,PIP,CAMP,A2ML1,ANTXR1,DST,EFEMP2,FBN1,BLM,APOA4,GSN,CST3,C1S,KNG1
DISEASES	DOID:0014667	Disease of metabolism	33	1076	0.43	7.14e-05	CETP,GNPTG,F9,APOC3,APOB,APOA1,TTR,CAT,APOE,APCS,LCAT,CP,HYAL1,AHSG,SAA4,PCSK9,F2,SUMO4,HBA1,APOA4,CNDP1,APOA2,MCOLN2,GSN,GPX3,CST3,PCYOX1,ADIPOQ,TGFBI,SELENOP,APOA5,SERPINA3,B2M
DISEASES	DOID:16	Integumentary system disease	23	575	0.54	7.14e-05	MPO,APOA1,CFP,APOE,F12,APCS,CSTA,KLKB1,AHSG,SERPING1,PIP,CAMP,A2ML1,ANTXR1,DST,EFEMP2,FBN1,BLM,APOA4,GSN,CST3,C1S,KNG1
DISEASES	DOID:0060903	Thrombosis	6	21	1.4	8.88e-05	VWF,PF4,F2,PLG,F10,F7
DISEASES	DOID:1936	Atherosclerosis	6	21	1.4	8.88e-05	CETP,APOB,APOA1,APOE,LPA,ADIPOQ
DISEASES	DOID:0080301	Atypical hemolytic-uremic syndrome	5	11	1.6	0.00012	CFHR1,CFHR3,CFH,CFI,CFB
DISEASES	DOID:12554	Hemolytic-uremic syndrome	5	14	1.49	0.00027	CFHR1,CFHR3,CFH,CFI,CFB
DISEASES	DOID:0070028	APP-related cerebral amyloid angiopathy	4	5	1.84	0.00028	APOE,APCS,CST3,SERPINA3
DISEASES	DOID:1168	Familial hyperlipidemia	5	15	1.46	0.00034	APOC3,APOB,APOE,PCSK9,APOA5
DISEASES	DOID:2218	Blood platelet disease	7	47	1.11	0.00034	PF4V1,VWF,PF4,F2,CALR,FLNA,F7
DISEASES	DOID:649	Prion disease	5	16	1.44	0.00041	APOE,APCS,YWHAE,C1QB,C1QC
DISEASES	DOID:3146	Lipid metabolism disorder	8	72	0.99	0.00043	CETP,APOC3,APOB,APOA1,APOE,LCAT,PCSK9,APOA5
DISEASES	DOID:612	Primary immunodeficiency disease	19	470	0.55	0.00043	MPO,TTR,PXDN,KLKB1,SERPING1,A2ML1,DST,F2,CFHR1,SUMO4,HBA1,PIGR,CFHR3,CFH,CFI,C4A,C4B,CFB,KNG1
DISEASES	DOID:0050828	Artery disease	10	130	0.83	0.00055	CETP,APOB,APOA1,APOE,PCSK9,F2,PLG,LPA,FBN1,ADIPOQ
DISEASES	DOID:9799	Eye degenerative disease	13	236	0.68	0.00067	CETP,APOB,APOE,PCSK9,CFHR1,F13B,CFHR3,CFH,EFEMP1,CFI,CST3,TGFBI,CFB
DISEASES	DOID:2921	Glomerulonephritis	6	36	1.16	0.00085	MPO,APOL1,CFHR3,CFH,C4B,CFB
DISEASES	DOID:3393	Coronary artery disease	6	36	1.16	0.00085	APOB,APOA1,APOE,PCSK9,PLG,LPA
DISEASES	DOID:0050117	Disease by infectious agent	16	368	0.58	0.00095	APOE,APCS,YWHAE,CP,ITIH4,AHSG,ANTXR1,F2,PLG,C1QB,APOL1,HSPA5,C1QC,CA6,C4A,CCDC122
DISEASES	DOID:0050637	Finnish type amyloidosis	4	9	1.59	0.0011	APOE,APCS,GSN,CST3
DISEASES	DOID:11949	Creutzfeldt-Jakob disease	4	9	1.59	0.0011	APOE,APCS,C1QB,C1QC
DISEASES	DOID:0050737	Autosomal recessive disease	41	1785	0.3	0.0017	GNPTG,LTF,APOB,APOE,F12,VWF,CP,KLKB1,DNAH5,TCIRG1,SERPING1,IHH,MASP1,CAMP,ANTXR1,PCSK9,FGB,DST,F2,PLG,EFEMP2,SERPINF2,HBA1,BLM,PIGR,F13B,F5,FLNA,F10,F7,IGFBP3,F11,C1S,PCYOX1,IGF2,COLEC11,LMOD3,TGFBI,APOA5,CFD,KNG1
DISEASES	DOID:655	Inherited metabolic disorder	27	949	0.39	0.0018	CETP,GNPTG,F9,APOC3,APOB,APOA1,TTR,CAT,APOE,APCS,LCAT,CP,HYAL1,PCSK9,SUMO4,HBA1,APOA4,CNDP1,APOA2,MCOLN2,GSN,CST3,PCYOX1,ADIPOQ,APOA5,SERPINA3,B2M
DISEASES	DOID:1387	Hypolipoproteinemia	4	11	1.5	0.0019	APOB,APOA1,LCAT,PCSK9
DISEASES	DOID:170	Endocrine gland cancer	8	93	0.87	0.0019	CHGA,APOE,APCS,APOA4,GAPDH,IGF2,IGKV2D-28,IGHV3-15
DISEASES	DOID:863	Nervous system disease	48	2275	0.26	0.0023	CETP,LGALS1,MPO,PCDH12,LTF,APOB,PROC,APOA1,TTR,APOE,APCS,VWF,LCAT,YWHAE,CENPE,SERPING1,ANKRD44,CSF1R,SERPINI1,A2ML1,SH3D19,PCSK9,DST,F2,PLG,C1QB,CFHR1,FBN1,ANG,CNDP1,F13B,CFHR3,CFH,ADAMTSL4,FLNA,GSN,C1QC,EFEMP1,CFI,TNRC6A,CST3,IGF2,CCDC122,TGFBI,CFB,SERPINA3,TRIM66,GRHL2
DISEASES	DOID:0070029	ITM2B-related cerebral amyloid angiopathy 1	4	12	1.46	0.0024	APOE,APCS,CST3,SERPINA3
DISEASES	DOID:1588	Thrombocytopenia	4	12	1.46	0.0024	PF4V1,VWF,PF4,F2
DISEASES	DOID:2222	Factor X deficiency	3	3	1.94	0.0024	F2,F10,F7
DISEASES	DOID:409	Liver disease	8	97	0.86	0.0024	TTR,APCS,AHSG,SAA4,F2,GANAB,GAPDH,IGHV3-15
DISEASES	DOID:5844	Myocardial infarction	5	27	1.21	0.0024	APOB,APOE,PCSK9,PLG,LPA
DISEASES	DOID:0070030	ITM2B-related cerebral amyloid angiopathy 2	4	13	1.43	0.0028	APOE,APCS,CST3,SERPINA3
DISEASES	DOID:65	Connective tissue disease	23	774	0.41	0.0032	F9,MMP2,COMP,TTR,TCIRG1,CSF1R,IHH,A2ML1,ANTXR1,BMP1,DST,PLG,EFEMP2,FBN1,P4HB,FLNA,F7,C1S,IGF2,C4A,C4B,C1R,SERPINA3
DISEASES	DOID:0060002	C1 inhibitor deficiency	3	4	1.82	0.0038	KLKB1,SERPING1,KNG1
DISEASES	DOID:12134	Factor VIII deficiency	3	4	1.82	0.0038	F9,VWF,F7
DISEASES	DOID:5614	Eye disease	22	749	0.41	0.0053	CETP,LGALS1,LTF,APOB,APOE,APCS,SERPING1,PCSK9,PLG,CFHR1,FBN1,F13B,CFHR3,CFH,ADAMTSL4,GSN,EFEMP1,CFI,CST3,TGFBI,CFB,GRHL2
DISEASES	DOID:0060449	Gelatinous drop-like corneal dystrophy	3	5	1.72	0.0057	LTF,APOE,TGFBI
DISEASES	DOID:12259	Hemophilia B	3	5	1.72	0.0057	F9,F7,F11
DISEASES	DOID:1237	Corneal degeneration	3	5	1.72	0.0057	APOB,PCSK9,TGFBI
DISEASES	DOID:17	Musculoskeletal system disease	29	1154	0.34	0.0057	F9,MMP2,COMP,TTR,APOE,F12,APCS,TCIRG1,CSF1R,IHH,A2ML1,ANTXR1,BMP1,DST,PLG,EFEMP2,FBN1,P4HB,FLNA,F7,C1S,IGF2,C4A,LMOD3,C4B,C1R,SERPINA3,CFD,B2M
DISEASES	DOID:2229	Factor XI deficiency	3	5	1.72	0.0057	F9,F7,F11
DISEASES	DOID:10124	Corneal disease	7	85	0.86	0.0058	LGALS1,LTF,APOB,APOE,PCSK9,TGFBI,GRHL2
DISEASES	DOID:0050639	Primary cutaneous amyloidosis	4	19	1.26	0.0081	APOE,APCS,GSN,CST3
DISEASES	DOID:10873	Kuhnt-Junius degeneration	3	7	1.57	0.0109	CETP,CFH,CFB
DISEASES	DOID:7693	Abdominal aortic aneurysm	3	7	1.57	0.0109	APOE,PLG,FBN1

DISEASES	DOID:3118	Hepatobiliary disease	9	168	0.67	0.0126	TTR,APCS,AHSG,SAA4,F2,GANAB,GAPDH,MST1,IGHV3-15
DISEASES	DOID:0050155	Sensory system disease	24	929	0.35	0.0142	CETP,LGALS1,LTF,APOB,PROC,APOE,APCS,SERPING1,A2ML1,PCSK9,PLG,CFHR1,FBN1,F13B,CFHR3,CFH,ADAMTSL4,GSN,EFEMP1,CFI,CST3,TGFBI,CFB,GRHL2
DISEASES	DOID:3973	Thyroid gland medullary carcinoma	3	8	1.51	0.0142	APOE,APCS,APOA4
DISEASES	DOID:1188	Mononeuropathy	3	9	1.46	0.0182	MPO,F2,CCDC122
DISEASES	DOID:14250	Down syndrome	3	9	1.46	0.0182	APOE,C1QB,CST3
DISEASES	DOID:4079	Heart valve disease	4	26	1.13	0.0212	PLG,LPA,FBN1,FLNA
DISEASES	DOID:50	Thyroid gland disease	5	50	0.94	0.0234	TTR,APOE,APCS,SERPINA7,APOA4
DISEASES	DOID:77	Gastrointestinal system disease	17	576	0.41	0.0264	CHGA,LTF,TTR,APCS,AHSG,CRH,SAA4,PIP,ANTXR1,LRG1,F2,GANAB,FLNA,C8B,GAPDH,MST1,IGHV3-15
DISEASES	DOID:62	Aortic valve disease	3	13	1.3	0.0410	PLG,LPA,FBN1
DISEASES	DOID:0050638	Transthyretin amyloidosis	2	2	1.94	0.0419	TTR,APCS
DISEASES	DOID:0111676	High molecular weight kininogen deficiency	2	2	1.94	0.0419	KLKB1,KNG1
DISEASES	DOID:10230	Aortic atherosclerosis	2	2	1.94	0.0419	APOB,APOE
DISEASES	DOID:1391	Norum disease	2	2	1.94	0.0419	APOA1,LCAT
DISEASES	DOID:520	Aortic disease	4	34	1.01	0.0460	APOE,PLG,LPA,FBN1
TISSUES	BTO:0004850	Bone marrow cell	55	198	1.38	7.77e-52	CPB2,CETP,MPO,APOB,IGFBP5,TTR,LRP1,CFP,APCS,ORM1,VWF,CP,KLKB1,HPX,ITIH4,FCN3,AHSG,SAA4,SERPING1,CSF1R,FCN2,IHH,MASP1,LRG1,FGB,F2,CSPG4,CFHR1,APOL1,SERPINF2,SERPINA5,APOD,PIGR,ITIH2,CFH,F5,APOA2,IGFBP3,SOD3,CFI,F11,C1S,ORM2,C4A,MST1,C4B,CFB,PLTP,SELENOP,C1R,SERPINA3,HYOU1,KNG1,CDH5,B2M
TISSUES	BTO:0000759	Liver	119	2125	0.69	2.38e-50	SPP2,ST6GAL1,CPB2,CETP,SERPIND1,LGALS1,CHGA,TPD52L2,APMAP,F9,MPO,APOC3,GPLD1,LTF,HRG,APOB,PROC,APOA1,TTR,CAT,LRP1,C4BPB,APOE,PXDN,F12,APCS,ORM1,VWF,PSMA6,SERPINA10,ATRN,YWHAE,CP,KLKB1,PON3,HPX,FCN3,ITIH1,AHSG,HABP2,SAA4,SERPING1,CSF1R,FCN2,PGLYRP2,MASP1,HSP90B1,LRG1,FGB,DST,F2,INHBC,PLG,C1QB,CFHR1,APOL1,CALR,LPA,SERPINF2,HBA1,HSPA5,FBN1,P4HB,SERPINA7,SERPINA5,ANG,GANAB,PROZ,ASGR2,AKAP9,PIGR,ITIH2,C8A,PCDH18,F13B,CFHR3,CFH,APOA2,CD5L,S100A9,FLNA,SMARCA1,C8B,C8G,GSN,F10,F7,APOM,CKAP4,IGFBP3,CFI,GAPDH,FGL1,APOF,MASP2,F11,C1S,NUCB1,PCYOX1,IGF2,ORM2,C4A,COLEC11,MST1,ITIH3,C4B,TGFBI,CFB,IGFALS,PLTP,GC,C1R,APOA5,SERPINA3,APOC4,HYOU1,KNG1,B2M,PDIA4
TISSUES	BTO:0000392	Plasma cell	49	171	1.4	1.00e-46	MPO,APOB,IGFBP5,TTR,LRP1,CFP,APCS,ORM1,VWF,CP,KLKB1,HPX,ITIH4,FCN3,AHSG,SAA4,SERPING1,CSF1R,FCN2,IHH,MASP1,LRG1,FGB,F2,CSPG4,CFHR1,APOL1,SERPINF2,SERPINA5,APOD,PIGR,ITIH2,CFH,APOA2,IGFBP3,SOD3,CFI,C1S,ORM2,C4A,C4B,CFB,SELENOP,C1R,SERPINA3,HYOU1,KNG1,CDH5,B2M
TISSUES	BTO:0000141	Bone marrow	66	528	1.04	5.06e-44	CPB2,CETP,SERPIND1,APMAP,MPO,LTF,APOB,IGFBP5,TTR,LRP1,CFP,PXDN,APCS,ORM1,VWF,CSTA,CP,KLKB1,HPX,ITIH4,FCN3,AHSG,SAA4,SERPING1,CSF1R,FCN2,IHH,MASP1,CAMP,LRG1,FGB,F2,CSPG4,CFHR1,APOL1,SERPINF2,HBA1,SERPINA5,LIMS1,GANAB,APOD,GIT2,PIGR,ITIH2,CFH,QSOX1,F5,APOA2,IGFBP3,SOD3,CFI,F11,C1S,ORM2,C4A,MST1,C4B,CFB,PLTP,SELENOP,C1R,SERPINA3,HYOU1,KNG1,CDH5,B2M
TISSUES	BTO:0000345	Digestive gland	126	2881	0.58	3.54e-43	SPP2,ST6GAL1,CPB2,CETP,SERPIND1,LGALS1,CHGA,TPD52L2,APMAP,F9,MPO,APOC3,GPLD1,LTF,HRG,APOB,PROC,APOA1,TTR,CAT,LRP1,C4BPB,APOE,PXDN,F12,APCS,ORM1,VWF,PSMA6,SERPINA10,ATRN,YWHAE,CP,KLKB1,PON3,TCIRG1,HPX,HYAL1,FCN3,ITIH1,AHSG,HABP2,SAA4,SERPING1,CSF1R,FCN2,PGLYRP2,MASP1,HSP90B1,LRG1,FGB,DST,F2,INHBC,PLG,C1QB,CFHR1,APOL1,CALR,LPA,SERPINF2,HBA1,HSPA5,FBN1,P4HB,SERPINA7,SERPINA5,ANG,GANAB,PROZ,QPCT,APOD,ASGR2,AKAP9,PIGR,ITIH2,C8A,PCDH18,F13B,CFHR3,CFH,APOA2,CD5L,S100A9,FLNA,SMARCA1,C8B,C8G,GSN,F10,F7,APOM,CKAP4,IGFBP3,GPX3,CFI,GAPDH,RNASE1,FGL1,APOF,MASP2,F11,C1S,NUCB1,PCYOX1,IGF2,ORM2,C4A,COLEC11,MST1,ITIH3,C4B,TGFBI,CFB,IGFALS,PLTP,GC,C1R,APOA5,SERPINA3,RNASE4,APOC4,HYOU1,KNG1,B2M,PDIA4
TISSUES	BTO:0001486	Skeletal system	88	1307	0.77	1.40e-40	SPP2,CPB2,CETP,SERPIND1,CHGA,APMAP,MMP2,COMP,MPO,APOC3,LTF,APOB,IGFBP5,APOA1,TTR,LRP1,CFP,APOE,PXDN,APCS,ORM1,VWF,PSMA6,ATRN,CSTA,CP,KLKB1,HPX,HYAL1,ITIH4,FCN3,AHSG,SAA4,SERPING1,CSF1R,FCN2,IHH,MASP1,CAMP,HSP90B1,LRG1,FGB,F2,UBE3C,CSPG4,CFHR1,APOL1,SERPINF2,HBA1,HSPA5,SERPINA7,SERPINA5,LIMS1,GANAB,APOD,GIT2,PIGR,ITIH2,CFH,QSOX1,F5,APOA2,ADAMTSL4,CRTAC1,CCN5,IGFBP3,SOD3,CFI,GAPDH,CST3,F11,C1S,PCYOX1,IGF2,ORM2,C4A,MST1,LMOD3,C4B,CFB,PLTP,SELENOP,C1R,SERPINA3,HYOU1,KNG1,CDH5,B2M
TISSUES	BTO:0001491	Viscus	145	5378	0.37	6.92e-28	SPP2,ST6GAL1,CPB2,CETP,SERPIND1,LGALS1,CHGA,TPD52L2,APMAP,F9,MPO,APOC3,GPLD1,LTF,HRG,APOB,PROC,PLEK,APOA1,TTR,CAT,LRP1,C4BPB,CFP,APOE,PXDN,F12,APCS,ORM1,VWF,PSMA6,SERPINA10,ATRN,YWHAE,CSTA,CP,KLKB1,PON3,TCIRG1,HPX,FCN3,ITIH1,AHSG,HABP2,SAA4,SERPING1,CSF1R,FCN2,PGLYRP2,MASP1,GUCY1A1,HSP90B1,ANTXR1,LRG1,SH3D19,FGB,DST,F2,INHBC,PLG,C1QB,CFHR1,APOL1,CALR,LPA,SERPINF2,HBA1,ITLN1,HSPA5,FBN1,P4HB,SERPINA7,FBLN1,SERPINA5,ANG,GANAB,PROZ,QPCT,APOD,ASGR2,AKAP9,PIGR,APOA4,ITIH2,C8A,PCDH18,F13B,CFHR3,CFH,QSOX1,APOA2,CD5L,S100A9,FLNA,SMARCA1,C8B,C8G,GSN,F10,F7,APOM,CKAP4,CALML5,IGFBP3,SOD3,GPX3,EFEMP1,CFI,TNRC6A,ILK,GAPDH,RNASE1,FGL1,APOF,CST3,MASP2,F11,C1S,NUCB1,PCYOX1,IGF2,ORM2,C4A,COLEC11,MST1,ITIH3,C4B,TGFBI,CFB,IGFALS,PLTP,RARRES2,GC,C1R,APOA5,SERPINA3,SERPINA4,RNASE4,APOC4,CFD,VEGFC,HYOU1,KNG1,B2M,PDIA4
TISSUES	BTO:0001488	Endocrine gland	154	6403	0.32	4.37e-25	SPP2,ST6GAL1,CPB2,CETP,SERPIND1,LGALS1,CHGA,TPD52L2,APMAP,F9,MMP2,COMP,MPO,APOC3,GPLD1,LTF,HRG,APOB,PROC,APOA1,TTR,CAT,LRP1,C4BPB,APOE,PXDN,F12,APCS,ORM1,VWF,PSMA6,SERPINA10,ATRN,YWHAE,CSTA,CP,KLKB1,PON3,TCIRG1,HPX,HYAL1,FCN3,ITIH1,AHSG,CRH,HABP2,SAA4,SERPING1,CSF1R,PIP,FCN2,PGLYRP2,IHH,MASP1,CAMP,HSP90B1,ANTXR1,LRG1,SH3D19,FGB,DST,F2,INHBC,PLG,C1QB,CFHR1,APOL1,CALR,LPA,SERPINF2,HBA1,ITLN1,HSPA5,FBN1,P4HB,SERPINA7,FBLN1,SERPINA5,ANG,LIMS1,GANAB,PROZ,QPCT,APOD,PAPLN,ASGR2,GIT2,AKAP9,PIGR,ITIH2,C8A,PCDH18,F13B,CFHR3,CFH,QSOX1,APOA2,CD5L,S100A9,FLNA,SMARCA1,C8B,C8G,CCN5,GSN,SVEP1,F10,F7,APOM,CKAP4,CALML5,IGFBP3,SOD3,GPX3,EFEMP1,CFI,EFCAB5,GAPDH,RAPGEF4,RNASE1,FGL1,APOF,CST3,MASP2,F11,C1S,NUCB1,PCYOX1,IGF2,ORM2,C4A,ATP9B,FSIP2,COLEC11,MST1,ITIH3,C4B,TGFBI,CFB,IGFALS,PLTP,RARRES2,GC,UIMC1,C1R,APOA5,SERPINA3,RNASE4,APOC4,CFD,HYOU1,KNG1,B2M,PDIA4
TISSUES	BTO:0000237	Cerebrospinal fluid	21	45	1.61	8.40e-23	CHGA,APOC3,TTR,APOE,ORM1,CP,HPX,AHSG,SERPING1,LRG1,F2,SERPINA7,APOD,CFH,IGFBP3,CFI,CST3,IGF2,ORM2,C4A,KNG1

TISSUES	BTO:0000522	Gland	157	7004	0.29	1.67e-22	SPP2,ST6GAL1,CPB2,CETP,SERPIND1,LGALS1,CHGA,TPD52L2,APMAP,F9,MMP2,COMP,MPO,APOC3,GPLD1,LTF,HRG,APOB,PROC,APOA1,TTR,CAT,LRP1,C4BPB,APOE,PXDN,F12,APCS,ORM1,VWF,PSMA6,SERPINA10,ATRN,YWHAE,CSTA,CP,KLKB1,PON3,TCIRG1,HPX,HYAL1,FCN3,ITIH1,AHSG,CRH,HABP2,SAA4,SERPING1,CSF1R,PIP,FCN2,PGLYRP2,IHH,MASP1,CAMP,GUCY1A1,HSP90B1,ANTXR1,LRG1,SH3D19,FGB,DST,F2,INHBC,PLG,C1QB,CFHR1,APOL1,CALR,LPA,SERPINF2,HBA1,ITLN1,HSPA5,FBN1,P4HB,SERPINA7,FBLN1,SERPINA5,ANG,LIMS1,GANAB,PROZ,QPCT,APOD,PAPLN,ASGR2,GIT2,AKAP9,PIGR,ITIH2,C8A,PCDH18,F13B,CFHR3,CFH,QSOX1,APOA2,CD5L,S100A9,FLNA,SMARCA1,C8B,C8G,CCN5,GSN,SVEP1,F10,F7,APOM,CA6,CKAP4,CALML5,IGFBP3,SOD3,GPX3,EFEMP1,CFI,EFCAB5,TNRC6A,GAPDH,RAPGEF4,RNASE1,FGL1,APOF,CST3,MASP2,F11,C1S,NUCB1,PCYOX1,IGF2,ORM2,C4A,ATP9B,FSIP2,COLEC11,MST1,ITIH3,C4B,TGFBI,CFB,IGFALS,PLTP,RARRES2,GC,UIMC1,C1R,APOA5,SERPINA3,RNASE4,APOC4,CFD,HYOU1,KNG1,B2M,PDIA4
TISSUES	BTO:0001489	Whole body	204	13099	0.13	1.58e-14	SPP2,ST6GAL1,CPB2,CETP,GNPTG,SERPIND1,LGALS1,CHGA,TPD52L2,APMAP,F9,MMP2,EBI3,COMP,PCOLCE,MPO,APOC3,GPLD1,PCDH12,LTF,HRG,APOB,IGFBP5,PROC,PLEK,APOA1,TTR,CAT,LRP1,C4BPB,CFP,APOE,PXDN,F12,APCS,ORM1,VWF,PSMA6,SERPINA10,ATRN,LCAT,YWHAE,CSTA,CP,KLKB1,DGKG,DNAH5,CENPE,PON3,TCIRG1,HPX,HYAL1,ITIH4,FCN3,ITIH1,AHSG,CRH,HABP2,SAA4,SERPING1,CSF1R,PIP,FCN2,PSG6,PGLYRP2,SCGB3A1,IHH,SERPINI1,PF4,MASP1,CAMP,GUCY1A1,A2ML1,HSP90B1,ANTXR1,LRG1,SH3D19,PCSK9,BMP1,FGB,DST,F2,INHBC,PLG,UBE3C,EFEMP2,CSPG4,C1QB,CFHR1,APOL1,CALR,LPA,SERPINF2,C7,HBA1,ITLN1,HSPA5,FBN1,P4HB,SERPINA7,FBLN1,SERPINA5,DNAH8,ANG,LIMS1,THBS4,GANAB,FAM184A,PROZ,QPCT,APOD,PAPLN,ASGR2,GIT2,AKAP9,PIGR,APOA4,ITIH2,CNDP1,MTCL1,C8A,PCDH18,CENPF,F13B,CFHR3,CFH,QSOX1,F5,APOA2,CD5L,S100A7,S100A9,ADAMTSL4,FLNA,CRTAC1,SMARCA1,C8B,C8G,CCN5,GSN,SVEP1,C1QC,F10,F7,APOM,CA6,CKAP4,CALML5,IGFBP3,SOD3,GPX3,EFEMP1,CFI,EFCAB5,TNRC6A,NAV2,ILK,GAPDH,RAPGEF4,RNASE1,FGL1,APOF,CST3,MAP3K7CL,MASP2,F11,C1S,NUCB1,PCYOX1,ADIPOQ,IGF2,ORM2,C4A,ATP9B,SLAIN1,FSIP2,COLEC11,MST1,LMOD3,ITIH3,C4B,TGFBI,CFB,IGFALS,PLTP,PAEP,RARRES2,GC,UIMC1,SELENOP,C1R,APOA5,SERPINA3,SERPINA4,RNASE4,APOC4,CFD,VEGFC,HYOU1,KNG1,GRHL2,CDH5,B2M,PDIA4
TISSUES	BTO:0001279	Spinal cord	24	233	0.95	3.78e-13	CHGA,APOC3,TTR,APOE,ORM1,CP,HPX,AHSG,SERPING1,LRG1,F2,CSPG4,SERPINA7,APOD,PCDH18,CFH,FLNA,IGFBP3,CFI,CST3,IGF2,ORM2,C4A,KNG1
TISSUES	BTO:0000203	Respiratory system	52	1707	0.42	1.49e-08	ST6GAL1,LGALS1,MMP2,LTF,IGFBP5,APOE,PXDN,VWF,ATRN,YWHAE,CP,DNAH5,TCIRG1,FCN3,SERPING1,PIP,HSP90B1,SH3D19,CALR,HBA1,ITLN1,HSPA5,P4HB,LIMS1,GANAB,AKAP9,PIGR,PCDH18,CFH,QSOX1,S100A9,FLNA,SMARCA1,CCN5,CKAP4,IGFBP3,GPX3,EFEMP1,GAPDH,RAPGEF4,MASP2,C1S,NUCB1,PCYOX1,C4B,TGFBI,VEGFC,HYOU1,KNG1,CDH5,B2M,PDIA4
TISSUES	BTO:0001078	Placenta	42	1244	0.47	6.85e-08	LGALS1,EBI3,PCOLCE,PCDH12,IGFBP5,LRP1,VWF,YWHAE,CRH,SERPING1,CSF1R,PSG6,HSP90B1,ANTXR1,BMP1,DST,EFEMP2,APOL1,CALR,HBA1,FBN1,P4HB,FBLN1,GANAB,QSOX1,F5,ADAMTSL4,SVEP1,IGFBP3,GPX3,ILK,GAPDH,RNASE1,CST3,NUCB1,PCYOX1,IGF2,TGFBI,PLTP,CFD,CDH5,B2M
TISSUES	BTO:0000042	Animal	209	15148	0.08	8.00e-08	SPP2,ST6GAL1,CPB2,CETP,GNPTG,SERPIND1,LGALS1,CHGA,TPD52L2,APMAP,F9,MMP2,EBI3,COMP,PCOLCE,MPO,APOC3,GPLD1,PCDH12,LTF,HRG,APOB,IGFBP5,PROC,PLEK,APOA1,TTR,CAT,LRP1,C4BPB,CFP,APOE,PXDN,ANGPTL6,F12,APCS,ORM1,VWF,PSMA6,SERPINA10,ATRN,LCAT,YWHAE,CSTA,CP,KLKB1,DGKG,DNAH5,CENPE,PON3,TCIRG1,HPX,HYAL1,ITIH4,FCN3,ITIH1,AHSG,CRH,HABP2,SAA4,SERPING1,CSF1R,PIP,FCN2,PSG6,PGLYRP2,SCGB3A1,IHH,SERPINI1,PF4,MASP1,CAMP,GUCY1A1,A2ML1,HSP90B1,ANTXR1,LRG1,SH3D19,PCSK9,BMP1,SHLD1,FGB,DST,F2,INHBC,PLG,UBE3C,EFEMP2,CSPG4,C1QB,CFHR1,APOL1,CALR,LPA,SERPINF2,C7,HBA1,ITLN1,HSPA5,FBN1,P4HB,SERPINA7,FBLN1,SERPINA5,DNAH8,ANG,LIMS1,THBS4,GANAB,FAM184A,PROZ,QPCT,APOD,PAPLN,ASGR2,GIT2,AKAP9,PIGR,APOA4,ITIH2,CNDP1,MTCL1,C8A,PCDH18,CENPF,F13B,CFHR3,CFH,QSOX1,F5,APOA2,CD5L,S100A7,S100A9,ADAMTSL4,FLNA,CRTAC1,SMARCA1,C8B,C8G,CCN5,GSN,SVEP1,C1QC,F10,F7,APOM,CA6,CKAP4,CALML5,IGFBP3,SOD3,GPX3,KIF19,EFEMP1,CFI,EFCAB5,TNRC6A,NAV2,ILK,GAPDH,RAPGEF4,RNASE1,FGL1,APOF,CST3,MAP3K7CL,MASP2,F11,C1S,NUCB1,PCYOX1,ADIPOQ,IGF2,ORM2,C4A,ATP9B,SLAIN1,FSIP2,ABCA13,COLEC11,MST1,LMOD3,ITIH3,C4B,TGFBI,CFB,IGFALS,PLTP,PAEP,RARRES2,GC,UIMC1,SELENOP,C1R,APOA5,SERPINA3,SERPINA4,RNASE4,APOC4,CFD,VEGFC,HYOU1,KNG1,TRIM66,GRHL2,CDH5,B2M,PDIA4
TISSUES	BTO:0000449	Fetus	43	1318	0.45	1.01e-07	LGALS1,EBI3,PCOLCE,PCDH12,IGFBP5,LRP1,VWF,YWHAE,CRH,SERPING1,CSF1R,PSG6,HSP90B1,ANTXR1,BMP1,DST,EFEMP2,APOL1,CALR,HBA1,FBN1,P4HB,FBLN1,GANAB,QSOX1,F5,ADAMTSL4,SVEP1,CKAP4,IGFBP3,GPX3,ILK,GAPDH,RNASE1,CST3,NUCB1,PCYOX1,IGF2,TGFBI,PLTP,CFD,CDH5,B2M
TISSUES	BTO:0001239	Serum	8	25	1.45	3.19e-07	GPLD1,APOA1,ATRN,ITIH1,PGLYRP2,ITIH2,APOA2,KNG1
TISSUES	BTO:0000284	Organism form	62	2542	0.33	6.98e-07	ST6GAL1,LGALS1,TPD52L2,EBI3,PCOLCE,PCDH12,LTF,IGFBP5,LRP1,CFP,VWF,PSMA6,YWHAE,ITIH4,CRH,SERPING1,CSF1R,PSG6,CAMP,HSP90B1,ANTXR1,BMP1,DST,UBE3C,EFEMP2,APOL1,CALR,HBA1,HSPA5,FBN1,P4HB,FBLN1,GANAB,AKAP9,PCDH18,CENPF,QSOX1,F5,ADAMTSL4,FLNA,SVEP1,APOM,CKAP4,IGFBP3,GPX3,EFEMP1,NAV2,ILK,GAPDH,RNASE1,CST3,NUCB1,PCYOX1,IGF2,TGFBI,PLTP,UIMC1,CFD,VEGFC,GRHL2,CDH5,B2M
TISSUES	BTO:0000570	Hematopoietic system	64	2755	0.31	2.23e-06	ST6GAL1,CETP,LGALS1,TPD52L2,MPO,APOC3,GPLD1,LTF,APOB,PROC,PLEK,APOA1,TTR,CAT,LRP1,CFP,APOE,F12,VWF,PSMA6,ATRN,YWHAE,ITIH1,AHSG,SERPING1,PGLYRP2,PF4,HSP90B1,FGB,F2,LPA,HBA1,P4HB,LIMS1,GANAB,AKAP9,ITIH2,C8A,PCDH18,F5,APOA2,CD5L,S100A9,FLNA,GSN,C1QC,APOM,CKAP4,IGFBP3,SOD3,GPX3,GAPDH,CST3,C1S,NUCB1,IGF2,C4B,TGFBI,CFB,CFD,VEGFC,HYOU1,KNG1,B2M
TISSUES	BTO:0001202	Saliva	9	57	1.14	5.90e-06	MPO,ORM1,HPX,AHSG,PIP,LRG1,PIGR,CST3,C4B
TISSUES	BTO:0000174	Embryonic structure	55	2369	0.31	2.93e-05	ST6GAL1,LGALS1,TPD52L2,EBI3,PCOLCE,PCDH12,IGFBP5,LRP1,VWF,PSMA6,YWHAE,CRH,SERPING1,CSF1R,PSG6,HSP90B1,ANTXR1,BMP1,DST,UBE3C,EFEMP2,APOL1,CALR,HBA1,FBN1,P4HB,FBLN1,GANAB,AKAP9,PCDH18,CENPF,QSOX1,F5,ADAMTSL4,FLNA,SVEP1,APOM,CKAP4,IGFBP3,GPX3,ILK,GAPDH,RNASE1,CST3,NUCB1,PCYOX1,IGF2,TGFBI,PLTP,UIMC1,CFD,VEGFC,GRHL2,CDH5,B2M
TISSUES	BTO:0000511	Gastrointestinal tract	46	1816	0.34	3.23e-05	LGALS1,CHGA,LTF,APOB,PROC,APOA1,TTR,CAT,YWHAE,KLKB1,AHSG,HABP2,SERPING1,HSP90B1,LRG1,SH3D19,DST,CALR,ITLN1,HSPA5,P4HB,FBLN1,ANG,GANAB,AKAP9,PIGR,APOA4,PCDH18,QSOX1,FLNA,GSN,IGFBP3,SOD3,GPX3,TNRC6A,ILK,GAPDH,CST3,C1S,IGF2,TGFBI,CFB,C1R,SERPINA4,CFD,B2M
TISSUES	BTO:0000421	Connective tissue	30	954	0.44	7.57e-05	SPP2,LGALS1,APMAP,MMP2,COMP,APOA1,TTR,CAT,YWHAE,AHSG,IHH,HSP90B1,EFEMP2,CALR,ITLN1,HSPA5,FBN1,P4HB,THBS4,GANAB,PAPLN,F5,CRTAC1,EFEMP1,GAPDH,CST3,ADIPOQ,PLTP,CFD,B2M

TISSUES	BTO:0003099	Internal female genital organ	60	2804	0.27	9.80e-05	LGALS1,TPD52L2,EBI3,COMP,PCOLCE,PCDH12,LTF,IGFBP5,LRP1,VWF,YWHAE,CRH,SERPING1,CSF1R,PSG6,MASP1,A2ML1,HSP90B1,ANTXR1,BMP1,DST,EFEMP2,APOL1,CALR,HBA1,ITLN1,FBN1,P4HB,FBLN1,GANAB,PAPLN,PCDH18,QSOX1,F5,S100A9,ADAMTSL4,FLNA,CCN5,GSN,SVEP1,F10,CKAP4,IGFBP3,GPX3,ILK,GAPDH,RNASE1,CST3,C1S,NUCB1,PCYOX1,IGF2,COLEC11,TGFBI,PLTP,PAEP,UIMC1,CFD,CDH5,B2M
TISSUES	BTO:0000988	Pancreas	23	626	0.51	0.00013	CETP,CHGA,APOC3,GPLD1,TTR,TCIRG1,HSP90B1,APOL1,CALR,HSPA5,P4HB,QPCT,FLNA,GSN,CKAP4,IGFBP3,GPX3,GAPDH,RNASE1,IGF2,TGFBI,SERPINA3,B2M
TISSUES	BTO:0000132	Blood platelet	17	363	0.61	0.00014	LGALS1,APOA1,TTR,CAT,VWF,YWHAE,AHSG,HSP90B1,FGB,HBA1,P4HB,LIMS1,F5,FLNA,GSN,GAPDH,HYOU1
TISSUES	BTO:0000763	Lung	37	1395	0.36	0.00017	LGALS1,MMP2,LTF,IGFBP5,APOE,PXDN,VWF,YWHAE,DNAH5,TCIRG1,FCN3,SERPING1,HSP90B1,CALR,HBA1,ITLN1,HSPA5,P4HB,AKAP9,PCDH18,QSOX1,S100A9,FLNA,SMARCA1,CCN5,CKAP4,GPX3,EFEMP1,GAPDH,MASP2,C1S,NUCB1,C4B,TGFBI,CDH5,B2M,PDIA4
TISSUES	BTO:0000089	Blood	44	1824	0.32	0.00018	LGALS1,TPD52L2,MPO,LTF,APOB,PROC,PLEK,APOA1,TTR,CAT,LRP1,APOE,F12,VWF,YWHAE,AHSG,SERPING1,PF4,HSP90B1,FGB,F2,LPA,HBA1,P4HB,LIMS1,AKAP9,C8A,PCDH18,F5,S100A9,FLNA,GSN,C1QC,APOM,SOD3,GPX3,GAPDH,CST3,C1S,NUCB1,C4B,CFB,HYOU1,B2M
TISSUES	BTO:0000058	Alimentary canal	47	2021	0.31	0.00019	LGALS1,CHGA,LTF,APOB,PROC,APOA1,TTR,CAT,YWHAE,KLKB1,AHSG,HABP2,SERPING1,HSP90B1,LRG1,SH3D19,DST,CALR,ITLN1,HSPA5,P4HB,FBLN1,ANG,GANAB,AKAP9,PIGR,APOA4,PCDH18,QSOX1,FLNA,GSN,CALML5,IGFBP3,SOD3,GPX3,TNRC6A,ILK,GAPDH,CST3,C1S,IGF2,TGFBI,CFB,C1R,SERPINA4,CFD,B2M
TISSUES	BTO:0000088	Cardiovascular system	30	1057	0.39	0.00044	PCOLCE,APOB,APOA1,APOE,VWF,YWHAE,HYAL1,SERPING1,HSP90B1,F2,HBA1,FBN1,P4HB,THBS4,PCDH18,F13B,FLNA,GSN,GPX3,EFEMP1,GAPDH,PCYOX1,LMOD3,TGFBI,PLTP,SELENOP,VEGFC,HYOU1,CDH5,B2M
TISSUES	BTO:0000121	Bile	5	20	1.34	0.00057	ORM1,CP,HPX,PIGR,ORM2
TISSUES	BTO:0003914	Interstitial cell of Cajal	11	193	0.7	0.0015	LGALS1,APOA1,TTR,CAT,YWHAE,AHSG,CALR,HSPA5,P4HB,GANAB,GAPDH
TISSUES	BTO:0001388	Trachea	11	201	0.68	0.0021	ST6GAL1,LTF,CP,P4HB,LIMS1,CFH,RAPGEF4,VEGFC,HYOU1,KNG1,B2M
TISSUES	BTO:0000648	Intestine	33	1349	0.33	0.0024	APOB,PROC,KLKB1,HABP2,HSP90B1,LRG1,SH3D19,DST,CALR,ITLN1,HSPA5,P4HB,ANG,GANAB,PIGR,APOA4,PCDH18,FLNA,GSN,IGFBP3,SOD3,GPX3,ILK,GAPDH,CST3,C1S,IGF2,TGFBI,CFB,C1R,SERPINA4,CFD,B2M
TISSUES	BTO:0000562	Heart	22	738	0.41	0.0034	PCOLCE,APOA1,VWF,YWHAE,HYAL1,SERPING1,F2,HBA1,FBN1,THBS4,PCDH18,F13B,FLNA,GPX3,GAPDH,PCYOX1,LMOD3,PLTP,SELENOP,VEGFC,HYOU1,CDH5
TISSUES	BTO:0000255	Brain cell line	10	188	0.67	0.0053	LGALS1,APOA1,TTR,FGB,CALR,HSPA5,P4HB,GAPDH,HYOU1,TRIM66
TISSUES	BTO:0003092	Urinary system	30	1249	0.32	0.0071	TPD52L2,CAT,APOE,GUCY1A1,HSP90B1,ANTXR1,LRG1,PLG,P4HB,PCDH18,CFH,QSOX1,FLNA,SMARCA1,GSN,IGFBP3,GPX3,TNRC6A,GAPDH,FGL1,NUCB1,IGF2,COLEC11,TGFBI,RARRES2,GC,SERPINA3,VEGFC,KNG1,B2M
TISSUES	BTO:0001244	Urinary tract	30	1258	0.32	0.0078	TPD52L2,CAT,APOE,GUCY1A1,HSP90B1,ANTXR1,LRG1,PLG,P4HB,PCDH18,CFH,QSOX1,FLNA,SMARCA1,GSN,IGFBP3,GPX3,TNRC6A,GAPDH,FGL1,NUCB1,IGF2,COLEC11,TGFBI,RARRES2,GC,SERPINA3,VEGFC,KNG1,B2M
TISSUES	BTO:0000431	Excretory gland	32	1385	0.3	0.0079	TPD52L2,LTF,CAT,APOE,HPX,AHSG,PIP,GUCY1A1,HSP90B1,ANTXR1,LRG1,FGB,F2,PLG,P4HB,APOD,QSOX1,SMARCA1,GSN,SVEP1,IGFBP3,GPX3,TNRC6A,GAPDH,NUCB1,IGF2,COLEC11,TGFBI,RARRES2,GC,KNG1,B2M
TISSUES	BTO:0001253	Skin	28	1151	0.33	0.0092	ST6GAL1,LGALS1,LRP1,APOE,PSMA6,CSTA,HSP90B1,BMP1,DST,CALR,P4HB,APOD,S100A7,S100A9,FLNA,GSN,CALML5,IGFBP3,EFEMP1,GAPDH,ADIPOQ,TGFBI,RARRES2,C1R,SERPINA3,CFD,VEGFC,B2M
TISSUES	BTO:0001499	Tear	4	23	1.18	0.0119	LTF,PIP,PIGR,B2M
TISSUES	BTO:0003120	Atherosclerotic plaque	3	9	1.46	0.0148	APOB,APOA1,APOE
TISSUES	BTO:0003872	Foam cell	3	9	1.46	0.0148	APOB,APOA1,APOE
TISSUES	BTO:0000634	Integument	42	2112	0.24	0.0154	ST6GAL1,LGALS1,CHGA,LTF,LRP1,APOE,VWF,PSMA6,CSTA,PIP,A2ML1,HSP90B1,ANTXR1,BMP1,DST,CALR,HSPA5,P4HB,APOD,AKAP9,S100A7,S100A9,FLNA,GSN,CALML5,IGFBP3,EFEMP1,ILK,GAPDH,RNASE1,ADIPOQ,COLEC11,TGFBI,PLTP,PAEP,RARRES2,C1R,SERPINA3,CFD,VEGFC,CDH5,B2M
TISSUES	BTO:0000849	Melanoma cell line	9	180	0.64	0.0154	LGALS1,MMP2,YWHAE,HSP90B1,CSPG4,HSPA5,P4HB,GANAB,BLM
TISSUES	BTO:0001356	Tendon	7	112	0.74	0.0196	EFEMP2,P4HB,THBS4,PAPLN,CRTAC1,EFEMP1,CST3
TISSUES	BTO:0001419	Urine	4	28	1.1	0.0207	F2,RNASE1,MASP2,B2M
TISSUES	BTO:0000102	Blood clot	3	11	1.38	0.0215	VWF,F2,PLG
TISSUES	BTO:0003091	Urogenital system	106	7090	0.11	0.0232	ST6GAL1,GNPTG,LGALS1,TPD52L2,APMAP,MMP2,EBI3,COMP,PCOLCE,PCDH12,LTF,HRG,IGFBP5,APOA1,CAT,LRP1,APOE,PXDN,VWF,PSMA6,ATRN,YWHAE,HPX,AHSG,CRH,SERPING1,CSF1R,PIP,PSG6,PGLYRP2,IHH,MASP1,CAMP,GUCY1A1,A2ML1,HSP90B1,ANTXR1,LRG1,SH3D19,BMP1,FGB,DST,F2,PLG,UBE3C,EFEMP2,APOL1,CALR,C7,HBA1,ITLN1,HSPA5,FBN1,P4HB,FBLN1,SERPINA5,DNAH8,LIMS1,GANAB,PAPLN,PCDH18,CFH,QSOX1,F5,S100A9,ADAMTSL4,FLNA,SMARCA1,CCN5,GSN,SVEP1,F10,CKAP4,IGFBP3,GPX3,EFEMP1,EFCAB5,TNRC6A,NAV2,ILK,GAPDH,RNASE1,FGL1,CST3,C1S,NUCB1,PCYOX1,IGF2,ATP9B,FSIP2,COLEC11,TGFBI,PLTP,PAEP,RARRES2,GC,UIMC1,SERPINA3,CFD,VEGFC,HYOU1,KNG1,GRHL2,CDH5,B2M,PDIA4
TISSUES	BTO:0001823	Synovium	6	88	0.77	0.0306	P4HB,THBS4,PAPLN,CRTAC1,EFEMP1,CST3
TISSUES	BTO:0003150	Pulmonary artery endothelial cell line	2	2	1.94	0.0317	KNG1,CDH5
TISSUES	BTO:0001702	Left atrium	5	60	0.86	0.0351	APOA1,HBA1,FBN1,FLNA,GAPDH
TISSUES	BTO:0000083	Female reproductive system	93	6111	0.12	0.0353	ST6GAL1,GNPTG,LGALS1,TPD52L2,APMAP,MMP2,EBI3,COMP,PCOLCE,PCDH12,LTF,IGFBP5,APOA1,CAT,LRP1,APOE,PXDN,VWF,PSMA6,ATRN,YWHAE,HPX,AHSG,CRH,SERPING1,CSF1R,PSG6,PGLYRP2,IHH,MASP1,CAMP,A2ML1,HSP90B1,ANTXR1,SH3D19,BMP1,FGB,DST,F2,UBE3C,EFEMP2,APOL1,CALR,C7,HBA1,ITLN1,HSPA5,FBN1,P4HB,FBLN1,SERPINA5,DNAH8,LIMS1,GANAB,PAPLN,PCDH18,CFH,QSOX1,F5,S100A9,ADAMTSL4,FLNA,CCN5,GSN,SVEP1,F10,CKAP4,IGFBP3,GPX3,EFEMP1,EFCAB5,NAV2,ILK,GAPDH,RNASE1,CST3,C1S,NUCB1,PCYOX1,IGF2,FSIP2,COLEC11,TGFBI,PLTP,PAEP,UIMC1,SERPINA3,CFD,HYOU1,GRHL2,CDH5,B2M,PDIA4
TISSUES	BTO:0000166	Cecum	4	35	1.0	0.0386	PIGR,GAPDH,CST3,B2M
TISSUES	BTO:0000671	Kidney	24	1039	0.3	0.0403	TPD52L2,CAT,APOE,GUCY1A1,HSP90B1,ANTXR1,LRG1,PLG,P4HB,QSOX1,SMARCA1,GSN,IGFBP3,GPX3,TNRC6A,GAPDH,NUCB1,IGF2,COLEC11,TGFBI,RARRES2,GC,KNG1,B2M
TISSUES	BTO:0001519	Endothelial cell line	3	16	1.21	0.0462	VEGFC,KNG1,CDH5
TISSUES	BTO:0003445	Carotid atherosclerotic plaque	2	3	1.76	0.0467	APOB,APOE
TISSUES	BTO:0004695	Aortic root	2	3	1.76	0.0467	APOE,FBN1

COMPARTMENTS	GOCC:0005576	Extracellular region	159	2079	0.82	2.33e-95	SPP2,ST6GAL1,CPB2,CETP,SERPIND1,LGALS1,CHGA,F9,MMP2,EBI3,COMP,PCOLCE,MPO,PF4V1,APOC3,GPLD1,LTF,HRG,APOB,IGFBP5,PROC,APOA1,TTR,C4BPB,CFP,APOE,PXDN,ANGPTL6,F12,APCS,ORM1,VWF,SERPINA10,LCAT,YWHAE,CSTA,CP,KLKB1,CENPE,PON3,HPX,HYAL1,ITIH4,FCN3,ITIH1,AHSG,CRH,HABP2,SAA4,SERPING1,PIP,FCN2,PSG6,PGLYRP2,SCGB3A1,SERPINI1,PF4,MASP1,CAMP,A2ML1,HSP90B1,LRG1,PCSK9,BMP1,FGB,DST,F2,INHBC,PLG,EFEMP2,CSPG4,C1QB,CFHR1,APOL1,CPN2,CALR,LPA,SERPINF2,C7,HBA1,ITLN1,HSPA5,FBN1,P4HB,SERPINA7,FBLN1,SERPINA5,ANG,THBS4,PROZ,QPCT,APOD,PIGR,APOA4,ITIH2,C8A,F13B,CFHR3,CFH,QSOX1,F5,APOA2,CD5L,S100A7,S100A9,FLNA,CRTAC1,C8B,C8G,CCN5,GSN,C1QC,F10,F7,APOM,CA6,CALML5,IGFBP3,SOD3,GPX3,EFEMP1,CFI,GAPDH,RNASE1,FGL1,APOF,CST3,MASP2,F11,C1S,NUCB1,PCYOX1,ADIPOQ,IGF2,ORM2,C4A,COLEC11,MST1,ITIH3,C4B,TGFBI,CFB,IGFALS,PLTP,PAEP,RARRES2,GC,C1R,APOA5,SERPINA3,SERPINA4,RNASE4,IGKV2D-28,APOC4,CFD,VEGFC,KNG1,B2M,PDIA4
COMPARTMENTS	GOCC:0005615	Extracellular space	121	1027	1.01	3.13e-86	CPB2,CETP,SERPIND1,F9,MMP2,EBI3,MPO,APOC3,GPLD1,LTF,HRG,APOB,IGFBP5,PROC,APOA1,TTR,CFP,APOE,PXDN,F12,APCS,ORM1,VWF,LCAT,YWHAE,CSTA,CP,KLKB1,PON3,HPX,HYAL1,ITIH4,FCN3,ITIH1,AHSG,CRH,SAA4,SERPING1,PIP,FCN2,SCGB3A1,SERPINI1,MASP1,CAMP,A2ML1,HSP90B1,LRG1,PCSK9,FGB,F2,PLG,CSPG4,C1QB,CFHR1,APOL1,CPN2,CALR,LPA,SERPINF2,HBA1,ITLN1,HSPA5,FBN1,FBLN1,SERPINA5,ANG,THBS4,APOD,PIGR,APOA4,ITIH2,C8A,CFHR3,CFH,QSOX1,APOA2,CD5L,S100A7,S100A9,FLNA,C8G,CCN5,GSN,C1QC,F7,APOM,CA6,IGFBP3,SOD3,GPX3,EFEMP1,GAPDH,FGL1,APOF,CST3,MASP2,F11,C1S,PCYOX1,ADIPOQ,IGF2,ORM2,C4A,COLEC11,MST1,C4B,TGFBI,CFB,IGFALS,PLTP,RARRES2,GC,C1R,APOA5,SERPINA3,IGKV2D-28,APOC4,CFD,KNG1,B2M,PDIA4
COMPARTMENTS	GOCC:0072562	Blood microparticle	42	118	1.49	6.66e-43	HRG,APOA1,APOE,APCS,ORM1,CP,HPX,ITIH4,FCN3,ITIH1,AHSG,SERPING1,FCN2,FGB,F2,PLG,C1QB,CFHR1,APOL1,CPN2,SERPINF2,HBA1,APOA4,ITIH2,C8A,CFHR3,CFH,APOA2,CD5L,C8G,GSN,C1QC,C1S,ORM2,C4A,C4B,CFB,GC,C1R,SERPINA3,IGKV2D-28,KNG1
COMPARTMENTS	GOCC:0043230	Extracellular organelle	54	524	0.95	2.84e-31	CPB2,F9,MPO,LTF,APOB,APOA1,TTR,APOE,PXDN,APCS,ORM1,VWF,YWHAE,CP,KLKB1,HPX,ITIH4,AHSG,SERPING1,FCN2,CAMP,HSP90B1,LRG1,FGB,F2,PLG,CSPG4,CALR,SERPINF2,HSPA5,SERPINA5,APOD,PIGR,CFH,QSOX1,APOA2,S100A9,FLNA,GSN,GPX3,EFEMP1,GAPDH,RNASE1,CST3,MASP2,F11,C4A,C4B,TGFBI,GC,C1R,SERPINA3,KNG1,B2M
COMPARTMENTS	GOCC:1903561	Extracellular vesicle	52	500	0.96	2.96e-30	CPB2,F9,MPO,LTF,APOB,APOA1,TTR,APOE,PXDN,APCS,ORM1,VWF,YWHAE,CP,KLKB1,HPX,ITIH4,AHSG,SERPING1,CAMP,HSP90B1,LRG1,FGB,F2,PLG,CSPG4,CALR,SERPINF2,HSPA5,SERPINA5,APOD,PIGR,CFH,QSOX1,APOA2,S100A9,FLNA,GSN,GPX3,EFEMP1,GAPDH,RNASE1,CST3,F11,C4A,C4B,TGFBI,GC,C1R,SERPINA3,KNG1,B2M
COMPARTMENTS	GOCC:0065010	Extracellular membrane-bounded organelle	50	473	0.96	2.39e-29	CPB2,F9,MPO,LTF,APOB,APOA1,TTR,APOE,PXDN,APCS,ORM1,VWF,CP,KLKB1,HPX,ITIH4,AHSG,SERPING1,CAMP,HSP90B1,LRG1,FGB,F2,PLG,CSPG4,CALR,SERPINF2,HSPA5,SERPINA5,APOD,PIGR,CFH,QSOX1,APOA2,S100A9,FLNA,GSN,GPX3,GAPDH,RNASE1,CST3,F11,C4A,C4B,TGFBI,GC,C1R,SERPINA3,KNG1,B2M
COMPARTMENTS	GOCC:0060205	Cytoplasmic vesicle lumen	35	245	1.1	8.09e-24	SPP2,MPO,LTF,HRG,APOA1,TTR,CFP,ORM1,VWF,ITIH4,AHSG,SERPING1,SERPINI1,PF4,CAMP,LRG1,FGB,PLG,SERPINF2,QPCT,QSOX1,F5,S100A7,GSN,IGF2,ORM2,ITIH3,RARRES2,SELENOP,SERPINA3,SERPINA4,CFD,VEGFC,KNG1,B2M
COMPARTMENTS	GOCC:0034774	Secretory granule lumen	34	241	1.09	4.97e-23	SPP2,MPO,LTF,HRG,APOA1,TTR,CFP,ORM1,VWF,ITIH4,AHSG,SERPING1,PF4,CAMP,LRG1,FGB,PLG,SERPINF2,QPCT,QSOX1,F5,S100A7,GSN,IGF2,ORM2,ITIH3,RARRES2,SELENOP,SERPINA3,SERPINA4,CFD,VEGFC,KNG1,B2M
COMPARTMENTS	GOCC:0031982	Vesicle	82	2125	0.53	2.74e-21	SPP2,CPB2,CHGA,F9,MPO,LTF,HRG,APOB,APOA1,TTR,CAT,LRP1,CFP,APOE,PXDN,APCS,ORM1,VWF,YWHAE,CP,KLKB1,TCIRG1,HPX,HYAL1,ITIH4,ITIH1,AHSG,SERPING1,SERPINI1,PF4,CAMP,HSP90B1,ANTXR1,LRG1,PCSK9,FGB,DST,F2,PLG,CSPG4,CALR,SERPINF2,HBA1,HSPA5,SERPINA5,QPCT,APOD,PIGR,CFH,QSOX1,F5,APOA2,S100A7,S100A9,FLNA,GSN,CKAP4,CALML5,GPX3,EFEMP1,GAPDH,RNASE1,CST3,F11,IGF2,ORM2,C4A,ABCA13,ITIH3,C4B,TGFBI,RARRES2,GC,SELENOP,C1R,SERPINA3,SERPINA4,CFD,VEGFC,HYOU1,KNG1,B2M
COMPARTMENTS	GOCC:0034358	Plasma lipoprotein particle	19	48	1.54	1.59e-19	CETP,APOC3,APOB,APOA1,APOE,LCAT,SAA4,PCSK9,APOL1,LPA,APOA4,APOA2,APOM,APOF,PCYOX1,ADIPOQ,PLTP,APOA5,APOC4
COMPARTMENTS	GOCC:0070062	Extracellular exosome	38	428	0.89	1.59e-19	MPO,LTF,APOB,APOA1,TTR,APOE,PXDN,ORM1,VWF,YWHAE,CP,HPX,ITIH4,AHSG,CAMP,HSP90B1,LRG1,FGB,PLG,CSPG4,CALR,HSPA5,SERPINA5,APOD,CFH,QSOX1,APOA2,S100A9,FLNA,GSN,GPX3,EFEMP1,GAPDH,CST3,C4A,TGFBI,KNG1,B2M
COMPARTMENTS	GOCC:0005788	Endoplasmic reticulum lumen	27	172	1.14	3.17e-19	SPP2,F9,APOB,APOA1,APOE,AHSG,HSP90B1,PCSK9,APOL1,CALR,HSPA5,FBN1,P4HB,GANAB,APOA4,QSOX1,F5,F7,CKAP4,IGFBP3,NUCB1,C4A,APOA5,HYOU1,KNG1,B2M,PDIA4
COMPARTMENTS	GOCC:0030141	Secretory granule	44	719	0.73	4.39e-17	SPP2,CHGA,MPO,LTF,HRG,APOA1,TTR,CAT,CFP,ORM1,VWF,TCIRG1,ITIH4,AHSG,SERPING1,PF4,CAMP,LRG1,FGB,PLG,CALR,SERPINF2,SERPINA5,QPCT,PIGR,QSOX1,F5,S100A7,GSN,CKAP4,CALML5,CST3,IGF2,ORM2,ABCA13,ITIH3,RARRES2,SELENOP,SERPINA3,SERPINA4,CFD,VEGFC,KNG1,B2M
COMPARTMENTS	GOCC:0034364	High-density lipoprotein particle	15	30	1.64	1.43e-16	CETP,APOC3,APOB,APOA1,APOE,LCAT,SAA4,APOL1,APOA4,APOA2,APOM,APOF,PLTP,APOA5,APOC4
COMPARTMENTS	GOCC:0031093	Platelet alpha granule lumen	17	66	1.35	5.91e-15	HRG,ORM1,VWF,AHSG,SERPING1,PF4,FGB,PLG,SERPINF2,QSOX1,F5,IGF2,ORM2,SERPINA3,CFD,VEGFC,KNG1
COMPARTMENTS	GOCC:0031091	Platelet alpha granule	18	89	1.25	2.67e-14	HRG,ORM1,VWF,AHSG,SERPING1,PF4,FGB,PLG,SERPINF2,SERPINA5,QSOX1,F5,IGF2,ORM2,SERPINA3,CFD,VEGFC,KNG1
COMPARTMENTS	GOCC:0034361	Very-low-density lipoprotein particle	12	20	1.72	6.85e-14	CETP,APOC3,APOB,APOA1,APOE,APOL1,APOA4,APOA2,APOM,PCYOX1,APOA5,APOC4

COMPARTMENTS	GOCC:0110165	Cellular anatomical entity	209	14060	0.11	3.76e-13	SPP2,ST6GAL1,CPB2,CETP,GNPTG,SERPIND1,LGALS1,CHGA,TPD52L2,APMAP,F9,MMP2,EBI3,COMP,PCOLCE,MPO,PF4V1,APOC3,GPLD1,PCDH12,LTF,HRG,APOB,IGFBP5,PROC,PLEK,APOA1,TTR,CAT,LRP1,C4BPB,CFP,APOE,PXDN,ANGPTL6,F12,APCS,ORM1,VWF,PSMA6,SERPINA10,ATRN,LCAT,YWHAE,CSTA,CP,KLKB1,DGKG,DNAH5,CENPE,PON3,TCIRG1,HPX,HYAL1,ITIH4,FCN3,ITIH1,AHSG,CRH,HABP2,SAA4,SERPING1,CSF1R,PIP,FCN2,PSG6,PGLYRP2,SCGB3A1,IHH,SERPINI1,PF4,MASP1,CAMP,GUCY1A1,C16orf46,A2ML1,HSP90B1,ANTXR1,LRG1,SH3D19,PCSK9,BMP1,SHLD1,FGB,DST,F2,INHBC,PLG,EFEMP2,CSPG4,C1QB,CFHR1,APOL1,CPN2,CALR,LPA,SERPINF2,C7,HBA1,ITLN1,HSPA5,FBN1,P4HB,SERPINA7,FBLN1,SERPINA5,DNAH8,ANG,LIMS1,THBS4,GANAB,PROZ,QPCT,APOD,ASGR2,BLM,GIT2,AKAP9,PIGR,APOA4,ITIH2,CNDP1,MTCL1,C8A,CENPF,F13B,CFHR3,CFH,QSOX1,F5,APOA2,CD5L,S100A7,S100A9,FLNA,CRTAC1,MCOLN2,SMARCA1,C8B,C8G,CCN5,GSN,SVEP1,C1QC,F10,F7,APOM,CA6,CKAP4,CALML5,TAF9,IGFBP3,SOD3,GPX3,KIF19,EFEMP1,CFI,TNRC6A,NAV2,ILK,GAPDH,RAPGEF4,RNASE1,FGL1,APOF,CST3,MAP3K7CL,MASP2,F11,C1S,NUCB1,PCYOX1,ADIPOQ,IGF2,ORM2,C4A,ATP9B,FSIP2,ABCA13,COLEC11,MST1,LMOD3,ITIH3,C4B,TGFBI,CFB,IGFALS,PLTP,PAEP,RARRES2,GC,UIMC1,SELENOP,C1R,APOA5,SERPINA3,SERPINA4,RNASE4,IGKV2D-28,APOC4,CFD,VEGFC,HYOU1,KNG1,TRIM66,GRHL2,CDH5,B2M,PDIA4
COMPARTMENTS	GOCC:0012505	Endomembrane system	82	3156	0.36	1.69e-11	SPP2,ST6GAL1,CHGA,F9,MPO,LTF,HRG,APOB,PROC,APOA1,TTR,CAT,LRP1,CFP,APOE,PXDN,ORM1,VWF,CP,TCIRG1,ITIH4,AHSG,SERPING1,PF4,CAMP,HSP90B1,ANTXR1,LRG1,PCSK9,FGB,F2,PLG,CSPG4,APOL1,CALR,SERPINF2,HSPA5,FBN1,P4HB,SERPINA5,THBS4,GANAB,PROZ,QPCT,ASGR2,AKAP9,PIGR,APOA4,CENPF,QSOX1,F5,S100A7,GSN,F10,F7,CKAP4,CALML5,IGFBP3,SOD3,TNRC6A,GAPDH,CST3,NUCB1,ADIPOQ,IGF2,ORM2,C4A,ATP9B,ABCA13,ITIH3,RARRES2,SELENOP,APOA5,SERPINA3,SERPINA4,CFD,VEGFC,HYOU1,KNG1,CDH5,B2M,PDIA4
COMPARTMENTS	GOCC:0070013	Intracellular organelle lumen	76	2902	0.36	1.35e-10	SPP2,F9,MPO,LTF,HRG,APOB,PROC,APOA1,TTR,CAT,CFP,APOE,ORM1,VWF,HPX,HYAL1,ITIH4,AHSG,SERPING1,SERPINI1,PF4,CAMP,HSP90B1,LRG1,PCSK9,FGB,F2,PLG,CSPG4,APOL1,CALR,SERPINF2,HBA1,HSPA5,FBN1,P4HB,ANG,GANAB,PROZ,QPCT,BLM,GIT2,APOA4,CENPF,QSOX1,F5,S100A7,SMARCA1,GSN,F10,F7,CKAP4,CALML5,TAF9,IGFBP3,SOD3,GAPDH,CST3,NUCB1,IGF2,ORM2,C4A,ITIH3,RARRES2,GC,UIMC1,SELENOP,APOA5,SERPINA3,SERPINA4,CFD,VEGFC,HYOU1,KNG1,B2M,PDIA4
COMPARTMENTS	GOCC:0031410	Cytoplasmic vesicle	56	1738	0.45	1.37e-10	SPP2,CHGA,MPO,LTF,HRG,APOB,APOA1,TTR,CAT,LRP1,CFP,APOE,ORM1,VWF,TCIRG1,HPX,HYAL1,ITIH4,AHSG,SERPING1,SERPINI1,PF4,CAMP,HSP90B1,ANTXR1,LRG1,PCSK9,FGB,DST,PLG,CALR,SERPINF2,HBA1,SERPINA5,QPCT,PIGR,QSOX1,F5,S100A7,GSN,CKAP4,CALML5,CST3,IGF2,ORM2,ABCA13,ITIH3,RARRES2,SELENOP,SERPINA3,SERPINA4,CFD,VEGFC,HYOU1,KNG1,B2M
COMPARTMENTS	GOCC:0071682	Endocytic vesicle lumen	10	23	1.58	1.63e-10	MPO,LTF,APOB,APOA1,APOE,HPX,HSP90B1,CALR,HBA1,HYOU1
COMPARTMENTS	GOCC:0034362	Low-density lipoprotein particle	9	22	1.55	3.19e-09	CETP,APOB,APOA1,APOE,PCSK9,LPA,APOM,APOF,ADIPOQ
COMPARTMENTS	GOCC:0062167	Complement component C1q complex	7	10	1.79	2.79e-08	SERPING1,C1QB,C1QC,C1S,C4A,C4B,C1R
COMPARTMENTS	GOCC:0031012	Extracellular matrix	19	259	0.81	3.22e-08	CPB2,MMP2,COMP,GPLD1,APOE,PXDN,VWF,HSP90B1,DST,PLG,EFEMP2,FBN1,FBLN1,ANG,THBS4,SOD3,EFEMP1,TGFBI,RARRES2
COMPARTMENTS	GOCC:1905286	Serine-type peptidase complex	7	11	1.74	4.26e-08	F9,FCN3,FCN2,MASP1,F7,MASP2,F11
COMPARTMENTS	GOCC:0042627	Chylomicron	7	14	1.64	1.47e-07	APOC3,APOB,APOA1,APOE,APOA4,APOA2,APOA5
COMPARTMENTS	GOCC:0030312	External encapsulating structure	18	259	0.78	1.92e-07	MMP2,COMP,GPLD1,HRG,APOE,PXDN,VWF,HSP90B1,DST,PLG,EFEMP2,FBN1,FBLN1,ANG,THBS4,SOD3,EFEMP1,RARRES2
COMPARTMENTS	GOCC:0005577	Fibrinogen complex	7	20	1.48	1.00e-06	VWF,FGB,F2,PLG,SERPINF2,F7,FGL1
COMPARTMENTS	GOCC:0062023	Collagen-containing extracellular matrix	15	198	0.82	1.35e-06	MMP2,COMP,APOE,PXDN,VWF,HSP90B1,DST,PLG,EFEMP2,FBN1,ANG,THBS4,SOD3,TGFBI,RARRES2
COMPARTMENTS	GOCC:1904724	Tertiary granule lumen	9	55	1.15	2.51e-06	LTF,CFP,ORM1,CAMP,LRG1,QPCT,QSOX1,CST3,B2M
COMPARTMENTS	GOCC:0032991	Protein-containing complex	99	5325	0.21	3.10e-06	CPB2,CETP,GNPTG,LGALS1,F9,COMP,APOC3,LTF,APOB,IGFBP5,APOA1,CAT,LRP1,APOE,PXDN,VWF,PSMA6,LCAT,CSTA,DNAH5,FCN3,SAA4,SERPING1,CSF1R,FCN2,MASP1,GUCY1A1,HSP90B1,PCSK9,FGB,DST,F2,PLG,UBE3C,C1QB,CFHR1,APOL1,SUMO4,CALR,LPA,SERPINF2,C7,HBA1,ITLN1,HSPA5,FBN1,P4HB,SERPINA5,DNAH8,ANG,GANAB,BLM,AKAP9,PIGR,APOA4,C8A,CENPF,APOA2,S100A7,S100A9,FLNA,SMARCA1,C8B,C8G,SVEP1,C1QC,F7,APOM,CALML5,TAF9,IGFBP3,GPX3,KIF19,TNRC6A,GAPDH,FGL1,APOF,CST3,MASP2,F11,C1S,PCYOX1,ADIPOQ,IGF2,C4A,CDK3,C4B,CFB,IGFALS,PLTP,UIMC1,C1R,APOA5,APOC4,IGHV3-15,VEGFC,HYOU1,CDH5,B2M
COMPARTMENTS	GOCC:0005783	Endoplasmic reticulum	35	1095	0.44	3.33e-06	SPP2,F9,APOB,PROC,APOA1,APOE,PXDN,VWF,CP,AHSG,HSP90B1,PCSK9,APOL1,CALR,HSPA5,FBN1,P4HB,THBS4,GANAB,ASGR2,APOA4,QSOX1,F5,S100A7,F7,CKAP4,IGFBP3,NUCB1,ADIPOQ,C4A,APOA5,HYOU1,KNG1,B2M,PDIA4
COMPARTMENTS	GOCC:0031089	Platelet dense granule lumen	6	14	1.57	3.71e-06	SPP2,ITIH4,ITIH3,RARRES2,SELENOP,SERPINA4
COMPARTMENTS	GOCC:0035580	Specific granule lumen	9	62	1.1	5.74e-06	LTF,CFP,ORM1,CAMP,LRG1,QPCT,QSOX1,ORM2,B2M
COMPARTMENTS	GOCC:1905370	Serine-type endopeptidase complex	5	7	1.79	6.97e-06	FCN3,FCN2,MASP1,MASP2,F11
COMPARTMENTS	GOCC:0062136	Low-density lipoprotein receptor complex	5	9	1.69	1.69e-05	APOB,APOA1,LRP1,APOE,PCSK9
COMPARTMENTS	GOCC:0034366	Spherical high-density lipoprotein particle	5	11	1.6	3.40e-05	APOC3,APOA1,LCAT,APOA2,APOM
COMPARTMENTS	GOCC:0005925	Focal adhesion	15	269	0.69	4.38e-05	LRP1,YWHAE,HSP90B1,DST,CSPG4,CALR,HSPA5,P4HB,LIMS1,GIT2,S100A7,FLNA,GSN,ILK,B2M
COMPARTMENTS	GOCC:1905368	Peptidase complex	11	136	0.85	4.42e-05	F9,PSMA6,FCN3,FCN2,MASP1,F2,UBE3C,F7,TAF9,MASP2,F11
COMPARTMENTS	GOCC:0005579	Membrane attack complex	5	12	1.56	4.49e-05	C7,C8A,C8B,C8G,C4A
COMPARTMENTS	GOCC:0005602	Complement component C1 complex	4	5	1.84	8.43e-05	C1QB,C1QC,C1S,C1R
COMPARTMENTS	GOCC:0034365	Discoidal high-density lipoprotein particle	4	6	1.76	0.00014	APOA1,APOE,LCAT,APOM
COMPARTMENTS	GOCC:0009986	Cell surface	18	438	0.55	0.00021	APMAP,LTF,HRG,PXDN,CSF1R,ANTXR1,PCSK9,FGB,PLG,CSPG4,CALR,SERPINF2,P4HB,SERPINA5,CD5L,ADIPOQ,CDH5,PDIA4
COMPARTMENTS	GOCC:0034363	Intermediate-density lipoprotein particle	4	8	1.64	0.00031	APOC3,APOB,APOA1,APOE
COMPARTMENTS	GOCC:0016942	Insulin-like growth factor binding protein complex	4	10	1.54	0.00060	IGFBP5,IGFBP3,IGF2,IGFALS
COMPARTMENTS	GOCC:0030139	Endocytic vesicle	13	260	0.64	0.00061	MPO,LTF,APOB,APOA1,LRP1,APOE,TCIRG1,HPX,HSP90B1,CALR,HBA1,HYOU1,B2M
COMPARTMENTS	GOCC:0043227	Membrane-bounded organelle	136	9083	0.12	0.00065	SPP2,ST6GAL1,CPB2,GNPTG,LGALS1,CHGA,F9,MMP2,PCOLCE,MPO,GPLD1,LTF,HRG,APOB,PROC,APOA1,TTR,CAT,LRP1,CFP,APOE,PXDN,APCS,ORM1,VWF,PSMA6,LCAT,YWHAE,CSTA,CP,KLKB1,DNAH5,CENPE,PON3,TCIRG1,HPX,HYAL1,ITIH4,ITIH1,AHSG,SERPING1,PIP,SERPINI1,PF4,CAMP,C16orf46,HSP90B1,ANTXR1,LRG1,SH3D19,PCSK9,FGB,DST,F2,PLG,CSPG4,APOL1,CALR,SERPINF2,HBA1,HSPA5,FBN1,P4HB,SERPINA5,DNAH8,ANG,THBS4,GANAB,PROZ,QPCT,APOD,ASGR2,BLM,GIT2,AKAP9,PIGR,APOA4,CENPF,CFH,QSOX1,F5,APOA2,S100A7,S100A9,FLNA,SMARCA1,CCN5,GSN,SVEP1,F10,F7,CKAP4,CALML5,TAF9,IGFBP3,SOD3,GPX3,EFEMP1,TNRC6A,NAV2,ILK,GAPDH,RNASE1,CST3,MASP2,F11,NUCB1,PCYOX1,ADIPOQ,IGF2,ORM2,C4A,ATP9B,FSIP2,ABCA13,ITIH3,C4B,TGFBI,PLTP,RARRES2,GC,UIMC1,SELENOP,C1R,APOA5,SERPINA3,SERPINA4,CFD,VEGFC,HYOU1,KNG1,TRIM66,GRHL2,CDH5,B2M,PDIA4
COMPARTMENTS	GOCC:0034663	Endoplasmic reticulum chaperone complex	4	11	1.5	0.00076	HSP90B1,HSPA5,P4HB,HYOU1
COMPARTMENTS	GOCC:0042581	Specific granule	10	158	0.74	0.00086	LTF,CFP,ORM1,CAMP,LRG1,QPCT,QSOX1,CKAP4,ORM2,B2M

COMPARTMENTS	GOCC:0070820	Tertiary granule	10	164	0.73	0.0011	LTF,CFP,ORM1,TCIRG1,CAMP,LRG1,QPCT,QSOX1,CST3,B2M
COMPARTMENTS	GOCC:0005796	Golgi lumen	8	102	0.83	0.0013	F9,PROC,F2,CSPG4,PROZ,F10,F7,SOD3
COMPARTMENTS	GOCC:0005764	Lysosome	19	566	0.47	0.0015	GNPTG,MPO,APOB,TTR,LRP1,CP,TCIRG1,HYAL1,PCSK9,CSPG4,CALR,PIGR,S100A7,CKAP4,PCYOX1,ORM2,ABCA13,GC,SERPINA3
COMPARTMENTS	GOCC:0005601	Classical-complement-pathway C3/C5 convertase complex	3	4	1.82	0.0017	C4A,C4B,CFB
COMPARTMENTS	GOCC:0036454	Growth factor complex	5	31	1.15	0.0017	IGFBP5,IGFBP3,IGF2,IGFALS,VEGFC
COMPARTMENTS	GOCC:0042567	Insulin-like growth factor ternary complex	3	4	1.82	0.0017	IGFBP5,IGFBP3,IGFALS
COMPARTMENTS	GOCC:1904090	Peptidase inhibitor complex	4	17	1.31	0.0027	PXDN,CSTA,SERPINA5,GAPDH
COMPARTMENTS	GOCC:0005604	Basement membrane	6	61	0.93	0.0036	PXDN,DST,EFEMP2,FBN1,ANG,THBS4
COMPARTMENTS	GOCC:1905369	Endopeptidase complex	7	90	0.83	0.0038	PSMA6,FCN3,FCN2,MASP1,UBE3C,MASP2,F11
COMPARTMENTS	GOCC:0005775	Vacuolar lumen	9	168	0.67	0.0061	MPO,APOB,TTR,HYAL1,CSPG4,S100A7,ORM2,GC,SERPINA3
COMPARTMENTS	GOCC:0071953	Elastic fiber	3	8	1.51	0.0068	EFEMP2,FBN1,FBLN1
COMPARTMENTS	GOCC:0070161	Anchoring junction	17	553	0.43	0.0087	LRP1,YWHAE,HSP90B1,DST,CSPG4,CALR,HSPA5,P4HB,LIMS1,GIT2,S100A7,FLNA,GSN,ILK,GRHL2,CDH5,B2M
COMPARTMENTS	GOCC:0042582	Azurophil granule	8	154	0.66	0.0160	MPO,TTR,PIGR,S100A7,CKAP4,ORM2,ABCA13,SERPINA3
COMPARTMENTS	GOCC:0043226	Organelle	141	10113	0.08	0.0165	SPP2,ST6GAL1,CPB2,GNPTG,LGALS1,CHGA,F9,MMP2,PCOLCE,MPO,GPLD1,LTF,HRG,APOB,PROC,APOA1,TTR,CAT,LRP1,CFP,APOE,PXDN,APCS,ORM1,VWF,PSMA6,LCAT,YWHAE,CSTA,CP,KLKB1,DNAH5,CENPE,PON3,TCIRG1,HPX,HYAL1,ITIH4,ITIH1,AHSG,SERPING1,PIP,FCN2,SERPINI1,PF4,CAMP,C16orf46,HSP90B1,ANTXR1,LRG1,SH3D19,PCSK9,SHLD1,FGB,DST,F2,PLG,CSPG4,APOL1,CALR,SERPINF2,HBA1,HSPA5,FBN1,P4HB,SERPINA5,DNAH8,ANG,THBS4,GANAB,PROZ,QPCT,APOD,ASGR2,BLM,GIT2,AKAP9,PIGR,APOA4,MTCL1,CENPF,CFH,QSOX1,F5,APOA2,S100A7,S100A9,FLNA,SMARCA1,CCN5,GSN,SVEP1,F10,F7,CKAP4,CALML5,TAF9,IGFBP3,SOD3,GPX3,KIF19,EFEMP1,TNRC6A,NAV2,ILK,GAPDH,RNASE1,CST3,MASP2,F11,NUCB1,PCYOX1,ADIPOQ,IGF2,ORM2,C4A,ATP9B,FSIP2,ABCA13,LMOD3,ITIH3,C4B,TGFBI,PLTP,RARRES2,GC,UIMC1,SELENOP,C1R,APOA5,SERPINA3,SERPINA4,CFD,VEGFC,HYOU1,KNG1,TRIM66,GRHL2,CDH5,B2M,PDIA4
COMPARTMENTS	GOCC:1904813	ficolin-1-rich granule lumen	7	124	0.69	0.0215	CAT,LRG1,QPCT,GSN,CALML5,CST3,CFD
COMPARTMENTS	GOCC:0097179	Protease inhibitor complex	3	14	1.27	0.0247	PXDN,SERPINA5,GAPDH
UniProt Keywords	KW-0964	Secreted	152	1839	0.86	1.43e-94	SPP2,ST6GAL1,CPB2,CETP,GNPTG,LGALS1,CHGA,F9,MMP2,EBI3,COMP,PCOLCE,PF4V1,APOC3,GPLD1,PCDH12,LTF,HRG,APOB,IGFBP5,PROC,APOA1,TTR,C4BPB,CFP,APOE,PXDN,ANGPTL6,F12,APCS,ORM1,VWF,SERPINA10,ATRN,LCAT,CP,KLKB1,PON3,HPX,HYAL1,ITIH4,FCN3,ITIH1,AHSG,CRH,HABP2,SAA4,SERPING1,PIP,FCN2,PSG6,PGLYRP2,SCGB3A1,IHH,SERPINI1,PF4,CAMP,A2ML1,LRG1,PCSK9,BMP1,FGB,F2,INHBC,PLG,EFEMP2,C1QB,CFHR1,APOL1,CPN2,CALR,SERPINF2,C7,ITLN1,FBN1,SERPINA7,FBLN1,SERPINA5,SERPINA11,ANG,THBS4,PROZ,QPCT,APOD,PAPLN,PIGR,APOA4,ITIH2,CNDP1,C8A,F13B,CFHR3,CFH,QSOX1,F5,APOA2,CD5L,S100A7,S100A9,ADAMTSL4,CRTAC1,C8B,C8G,CCN5,GSN,SVEP1,C1QC,F10,F7,APOM,CA6,IGFBP3,SOD3,GPX3,EFEMP1,CFI,RNASE1,FGL1,APOF,CST3,MASP2,F11,NUCB1,ADIPOQ,IGF2,ORM2,C4A,COLEC11,MST1,ITIH3,C4B,TGFBI,CFB,IGFALS,PLTP,PAEP,RARRES2,GC,SELENOP,C1R,APOA5,SERPINA3,SERPINA4,RNASE4,IGKV2D-28,APOC4,CFD,IGHV3-15,IGHV3-72,VEGFC,KNG1,B2M
UniProt Keywords	KW-0732	Signal	165	3277	0.64	3.13e-74	SPP2,CPB2,CETP,GNPTG,SERPIND1,CHGA,F9,MMP2,EBI3,COMP,PCOLCE,MPO,PF4V1,APOC3,GPLD1,PCDH12,LTF,HRG,APOB,IGFBP5,PROC,APOA1,TTR,LRP1,C4BPB,CFP,APOE,PXDN,ANGPTL6,F12,APCS,ORM1,VWF,ATRN,LCAT,CP,KLKB1,PON3,HPX,HYAL1,ITIH4,FCN3,ITIH1,AHSG,CRH,HABP2,SAA4,SERPING1,CSF1R,PIP,FCN2,PSG6,PGLYRP2,SCGB3A1,IHH,SERPINI1,PF4,CAMP,A2ML1,HSP90B1,ANTXR1,LRG1,PCSK9,BMP1,FGB,F2,INHBC,PLG,EFEMP2,CSPG4,C1QB,CFHR1,APOL1,CPN2,CALR,LPA,SERPINF2,C7,ITLN1,HSPA5,FBN1,P4HB,SERPINA7,FBLN1,SERPINA5,OIT3,SERPINA11,ANG,THBS4,GANAB,PROZ,QPCT,APOD,PAPLN,PIGR,APOA4,ITIH2,CNDP1,C8A,PCDH18,F13B,CFHR3,CFH,QSOX1,F5,APOA2,CD5L,ADAMTSL4,CRTAC1,C8B,C8G,CCN5,GSN,SVEP1,C1QC,F10,F7,APOM,CA6,IGFBP3,SOD3,GPX3,EFEMP1,CFI,RNASE1,FGL1,APOF,CST3,MASP2,F11,C1S,NUCB1,PCYOX1,ADIPOQ,IGF2,ORM2,C4A,COLEC11,MST1,ITIH3,C4B,TGFBI,CFB,IGFALS,PLTP,PAEP,RARRES2,GC,SELENOP,C1R,APOA5,SERPINA3,SERPINA4,RNASE4,IGKV2D-28,APOC4,CFD,IGHV3-15,IGHV3-72,VEGFC,HYOU1,KNG1,CDH5,B2M,PDIA4
UniProt Keywords	KW-1015	Disulfide bond	135	3338	0.55	4.13e-44	SPP2,ST6GAL1,CPB2,CETP,GNPTG,CHGA,F9,MMP2,COMP,PCOLCE,MPO,PF4V1,LTF,HRG,APOB,IGFBP5,PROC,LRP1,C4BPB,CFP,PXDN,ANGPTL6,F12,APCS,ORM1,VWF,ATRN,LCAT,CP,KLKB1,PON3,HPX,HYAL1,ITIH4,FCN3,ITIH1,AHSG,HABP2,SERPING1,CSF1R,PIP,FCN2,PSG6,PGLYRP2,SCGB3A1,PF4,MASP1,CAMP,A2ML1,HSP90B1,ANTXR1,LRG1,PCSK9,BMP1,FGB,F2,INHBC,PLG,EFEMP2,CSPG4,C1QB,CFHR1,CPN2,CALR,LPA,SERPINF2,C7,ITLN1,FBN1,P4HB,FBLN1,OIT3,ANG,THBS4,GANAB,PROZ,QPCT,APOD,PAPLN,ASGR2,PIGR,ITIH2,C8A,F13B,CFHR3,CFH,QSOX1,F5,APOA2,CD5L,S100A7,CRTAC1,MCOLN2,C8B,C8G,GSN,SVEP1,C1QC,F10,F7,APOM,CA6,IGFBP3,SOD3,EFEMP1,CFI,RNASE1,FGL1,CST3,MASP2,F11,C1S,ADIPOQ,IGF2,ORM2,C4A,COLEC11,MST1,C4B,TGFBI,CFB,PLTP,PAEP,RARRES2,GC,C1R,RNASE4,IGKV2D-28,CFD,IGHV3-15,IGHV3-72,VEGFC,KNG1,B2M,PDIA4
UniProt Keywords	KW-0325	Glycoprotein	144	4386	0.46	9.52e-38	ST6GAL1,CPB2,CETP,GNPTG,SERPIND1,CHGA,APMAP,F9,MMP2,EBI3,COMP,PCOLCE,MPO,PF4V1,APOC3,GPLD1,PCDH12,LTF,HRG,APOB,IGFBP5,PROC,APOA1,TTR,LRP1,C4BPB,CFP,APOE,PXDN,ANGPTL6,F12,APCS,ORM1,VWF,PSMA6,SERPINA10,ATRN,LCAT,CP,KLKB1,PON3,HPX,HYAL1,ITIH4,FCN3,ITIH1,AHSG,HABP2,SAA4,SERPING1,CSF1R,PIP,FCN2,PSG6,PGLYRP2,IHH,SERPINI1,A2ML1,HSP90B1,ANTXR1,LRG1,PCSK9,BMP1,FGB,F2,INHBC,PLG,EFEMP2,CSPG4,C1QB,CFHR1,APOL1,CPN2,CALR,LPA,SERPINF2,C7,ITLN1,FBN1,SERPINA7,FBLN1,SERPINA5,OIT3,SERPINA11,THBS4,GANAB,PROZ,QPCT,APOD,ASGR2,PIGR,ITIH2,CNDP1,C8A,PCDH18,F13B,CFHR3,CFH,QSOX1,F5,APOA2,CD5L,ADAMTSL4,CRTAC1,C8B,SVEP1,C1QC,F10,F7,APOM,CA6,IGFBP3,SOD3,EFEMP1,CFI,RNASE1,APOF,CST3,F11,C1S,NUCB1,PCYOX1,ADIPOQ,IGF2,ORM2,C4A,MST1,ITIH3,C4B,CFB,IGFALS,PLTP,PAEP,GC,SELENOP,C1R,SERPINA3,SERPINA4,APOC4,VEGFC,HYOU1,KNG1,CDH5,B2M
UniProt Keywords	KW-0094	Blood coagulation	20	46	1.58	1.83e-21	CPB2,SERPIND1,F9,HRG,PROC,F12,VWF,SERPINA10,KLKB1,SERPING1,FGB,F2,PLG,PROZ,F13B,F5,F10,F7,F11,KNG1
UniProt Keywords	KW-0245	EGF-like domain	27	232	1.01	1.98e-16	F9,COMP,PROC,LRP1,F12,ATRN,HYAL1,HABP2,MASP1,BMP1,EFEMP2,C7,FBN1,FBLN1,OIT3,THBS4,PROZ,C8A,CRTAC1,C8B,SVEP1,F10,F7,EFEMP1,MASP2,C1S,C1R
UniProt Keywords	KW-0106	Calcium	47	886	0.66	2.92e-16	CHGA,F9,MMP2,COMP,MPO,PCDH12,PROC,LRP1,PXDN,APCS,DGKG,PON3,FCN3,FCN2,IHH,HSP90B1,PCSK9,BMP1,DST,F2,EFEMP2,CALR,ITLN1,FBN1,FBLN1,OIT3,THBS4,PROZ,ASGR2,PCDH18,F5,S100A7,S100A9,MCOLN2,GSN,SVEP1,F10,F7,CALML5,EFEMP1,CFI,EFCAB5,MASP2,C1S,NUCB1,COLEC11,CDH5
UniProt Keywords	KW-0180	Complement pathway	14	30	1.61	2.47e-15	C4BPB,SERPING1,C1QB,C7,C8A,C8B,C8G,C1QC,CFI,MASP2,C1S,C4A,C4B,C1R

UniProt Keywords	KW-0391	Immunity	33	537	0.73	8.75e-13	LTF,C4BPB,CFP,FCN3,SERPING1,CSF1R,FCN2,PGLYRP2,FGB,C1QB,C7,C8A,CFH,CD5L,S100A9,MCOLN2,C8B,C8G,C1QC,CFI,FGL1,MASP2,C1S,C4A,COLEC11,C4B,CFB,C1R,IGKV2D-28,CFD,IGHV3-15,IGHV3-72,B2M
UniProt Keywords	KW-0720	Serine protease	19	136	1.09	8.75e-13	F9,LTF,PROC,F12,KLKB1,HABP2,PCSK9,F2,PLG,LPA,F10,F7,CFI,MASP2,F11,C1S,CFB,C1R,CFD
UniProt Keywords	KW-0646	Protease inhibitor	18	117	1.13	8.98e-13	SERPIND1,SERPINA10,CSTA,ITIH4,ITIH1,SERPING1,SERPINI1,A2ML1,SERPINF2,SERPINA5,SERPINA11,PAPLN,ITIH2,CST3,ITIH3,SERPINA3,SERPINA4,KNG1
UniProt Keywords	KW-0165	Cleavage on pair of basic residues	24	280	0.87	4.77e-12	CHGA,F9,HRG,PROC,VWF,CRH,HABP2,CAMP,BMP1,F2,INHBC,PLG,PROZ,C8A,APOA2,F10,F7,CFI,IGF2,C4A,C4B,CFB,VEGFC,CDH5
UniProt Keywords	KW-0722	Serine protease inhibitor	15	80	1.21	9.86e-12	SERPIND1,SERPINA10,ITIH4,ITIH1,SERPING1,SERPINI1,A2ML1,SERPINF2,SERPINA5,SERPINA11,PAPLN,ITIH2,ITIH3,SERPINA3,SERPINA4
UniProt Keywords	KW-0399	Innate immunity	25	329	0.82	1.52e-11	C4BPB,CFP,FCN3,SERPING1,CSF1R,FCN2,FGB,C1QB,C7,C8A,CFH,S100A9,MCOLN2,C8B,C8G,C1QC,CFI,MASP2,C1S,C4A,COLEC11,C4B,CFB,C1R,CFD
UniProt Keywords	KW-0677	Repeat	105	4794	0.28	2.43e-11	SERPIND1,F9,MMP2,EBI3,COMP,PCOLCE,GPLD1,PCDH12,LTF,HRG,PROC,PLEK,APOA1,LRP1,C4BPB,CFP,APOE,PXDN,F12,VWF,ATRN,CP,KLKB1,DGKG,DNAH5,HPX,FCN3,AHSG,ANKRD31,HABP2,SERPING1,ANKRD44,CSF1R,FCN2,PSG6,MASP1,LRG1,SH3D19,BMP1,DST,F2,PLG,EFEMP2,CSPG4,C1QB,CFHR1,CPN2,CALR,LPA,C7,FBN1,P4HB,FBLN1,DNAH8,LIMS1,THBS4,PROZ,PAPLN,GIT2,PIGR,APOA4,C8A,PCDH18,CENPF,F13B,CFHR3,CFH,F5,CD5L,S100A7,S100A9,ADAMTSL4,FLNA,CRTAC1,SMARCA1,C8B,GSN,SVEP1,C1QC,F10,F7,CALML5,EFEMP1,CFI,ILK,RAPGEF4,MASP2,F11,C1S,NUCB1,ADIPOQ,FSIP2,ABCA13,MST1,TGFBI,CFB,IGFALS,GC,UIMC1,C1R,VEGFC,KNG1,TRIM66,CDH5,PDIA4
UniProt Keywords	KW-0301	Gamma-carboxyglutamic acid	9	16	1.69	1.83e-10	F9,PROC,TTR,F2,PROZ,ITIH2,F10,F7,TGFBI
UniProt Keywords	KW-0345	HDL	9	16	1.69	1.83e-10	APOA1,APOE,SAA4,APOL1,APOA4,APOA2,APOM,APOF,APOA5
UniProt Keywords	KW-0445	Lipid transport	15	110	1.08	4.42e-10	CETP,APOC3,APOB,APOA1,APOE,APOL1,LPA,SERPINA5,APOA4,APOA2,APOM,APOF,PLTP,APOA5,APOC4
UniProt Keywords	KW-0768	Sushi	12	57	1.26	5.67e-10	C4BPB,MASP1,CFHR1,C7,F13B,CFHR3,CFH,SVEP1,MASP2,C1S,CFB,C1R
UniProt Keywords	KW-0179	Complement alternate pathway	8	12	1.76	8.68e-10	CFP,C7,C8A,CFH,C8B,C8G,CFB,CFD
UniProt Keywords	KW-0873	Pyrrolidone carboxylic acid	12	67	1.19	2.71e-09	ORM1,PIP,FGB,C1QB,ANG,APOD,APOA2,C8G,ORM2,RNASE4,KNG1,B2M
UniProt Keywords	KW-0358	Heparin-binding	13	88	1.11	3.60e-09	SERPIND1,COMP,PF4V1,LTF,HRG,APOB,APOE,SERPINA10,PF4,FBN1,SERPINA5,SOD3,F11
UniProt Keywords	KW-0792	Thrombophilia	7	10	1.79	1.09e-08	SERPIND1,F9,HRG,PROC,F2,PLG,F5
UniProt Keywords	KW-0645	Protease	25	516	0.63	7.85e-08	CPB2,F9,MMP2,LTF,PROC,F12,PSMA6,KLKB1,HABP2,IHH,PCSK9,BMP1,F2,PLG,LPA,CNDP1,F10,F7,CFI,MASP2,F11,C1S,CFB,C1R,CFD
UniProt Keywords	KW-0379	Hydroxylation	14	143	0.93	8.41e-08	F9,PROC,FCN3,FCN2,C1QB,PROZ,C1QC,F10,F7,MASP2,C1S,ADIPOQ,C1R,KNG1
UniProt Keywords	KW-0280	Fibrinolysis	6	8	1.82	1.38e-07	CPB2,HRG,F12,KLKB1,SERPING1,PLG
UniProt Keywords	KW-0011	Acute phase	7	19	1.51	2.89e-07	ORM1,ITIH4,SAA4,F2,SERPINF2,ORM2,SERPINA3
UniProt Keywords	KW-0034	Amyloid	7	24	1.41	1.06e-06	APOA1,TTR,APCS,GSN,CST3,TGFBI,B2M
UniProt Keywords	KW-0865	Zymogen	15	210	0.79	1.06e-06	CPB2,F9,MMP2,PROC,F12,KLKB1,PCSK9,BMP1,F2,PLG,F5,F10,F7,CFB,CFD
UniProt Keywords	KW-0765	Sulfation	9	57	1.14	1.21e-06	SERPIND1,CHGA,F9,PCSK9,SERPINF2,CFH,F5,C4A,C4B
UniProt Keywords	KW-0420	Kringle	6	16	1.51	2.68e-06	F12,HABP2,F2,PLG,LPA,MST1
UniProt Keywords	KW-1008	Amyloidosis	7	31	1.29	4.19e-06	APOA1,TTR,APOE,GSN,CST3,TGFBI,B2M
UniProt Keywords	KW-0162	Chylomicron	5	9	1.69	6.54e-06	APOC3,APOB,APOE,APOA4,APOA5
UniProt Keywords	KW-1068	Hemolytic uremic syndrome	5	10	1.64	9.44e-06	CFHR1,CFHR3,CFH,CFI,CFB
UniProt Keywords	KW-0272	Extracellular matrix	15	267	0.69	1.59e-05	LGALS1,MMP2,COMP,APOE,PXDN,VWF,BMP1,CALR,FBN1,FBLN1,THBS4,ADAMTSL4,CRTAC1,EFEMP1,TGFBI
UniProt Keywords	KW-0971	Glycation	5	12	1.56	1.81e-05	APOA1,APOE,SOD3,CFB,B2M
UniProt Keywords	KW-0153	Cholesterol metabolism	8	65	1.03	3.05e-05	CETP,APOB,APOA1,APOE,LCAT,PCSK9,APOL1,APOF
UniProt Keywords	KW-0558	Oxidation	6	32	1.21	7.00e-05	CHGA,MPO,APOA1,APOE,APOA2,GAPDH
UniProt Keywords	KW-0473	Membrane attack complex	4	7	1.7	8.19e-05	C7,C8A,C8B,C8G
UniProt Keywords	KW-0065	Atherosclerosis	4	8	1.64	0.00012	CETP,APOB,APOA1,LPA
UniProt Keywords	KW-0378	Hydrolase	37	1612	0.3	0.00071	CPB2,F9,MMP2,GPLD1,LTF,PROC,F12,PSMA6,KLKB1,PON3,HYAL1,HABP2,PGLYRP2,IHH,PCSK9,BMP1,F2,PLG,LPA,HSPA5,ANG,GANAB,BLM,CNDP1,SMARCA1,F10,F7,CFI,NAV2,RNASE1,MASP2,F11,C1S,CFB,C1R,RNASE4,CFD
UniProt Keywords	KW-0797	Tissue remodeling	3	4	1.82	0.00071	PLG,CSPG4,THBS4
UniProt Keywords	KW-0913	Age-related macular degeneration	4	16	1.34	0.00097	CFH,CFI,CST3,CFB
UniProt Keywords	KW-1212	Corneal dystrophy	4	20	1.24	0.0020	LCAT,GSN,TGFBI,GRHL2
UniProt Keywords	KW-0395	Inflammatory response	9	163	0.68	0.0021	ATRN,KLKB1,CSF1R,CD5L,S100A9,C4A,C4B,RARRES2,KNG1
UniProt Keywords	KW-0430	Lectin	9	162	0.69	0.0021	LGALS1,APCS,ATRN,FCN3,FCN2,CALR,ITLN1,ASGR2,COLEC11
UniProt Keywords	KW-0882	Thioester bond	3	8	1.51	0.0029	A2ML1,C4A,C4B
UniProt Keywords	KW-0068	Autocatalytic cleavage	5	46	0.98	0.0036	MMP2,IHH,PCSK9,LPA,MASP2
UniProt Keywords	KW-0376	Hydrogen peroxide	3	9	1.46	0.0037	MPO,CAT,PXDN
UniProt Keywords	KW-0575	Peroxidase	4	25	1.14	0.0038	MPO,CAT,PXDN,GPX3
UniProt Keywords	KW-0850	VLDL	3	10	1.42	0.0046	APOC3,APOE,APOA5
UniProt Keywords	KW-0176	Collagen	6	83	0.8	0.0063	FCN3,FCN2,C1QB,C1QC,ADIPOQ,COLEC11
UniProt Keywords	KW-0654	Proteoglycan	5	55	0.9	0.0070	PF4V1,ITIH1,CSPG4,ITIH2,ITIH3
UniProt Keywords	KW-1280	Immunoglobulin	3	12	1.34	0.0070	IGKV2D-28,IGHV3-15,IGHV3-72
UniProt Keywords	KW-0372	Hormone	6	92	0.75	0.0100	TTR,CRH,INHBC,FBN1,ADIPOQ,IGF2
UniProt Keywords	KW-1018	Complement activation lectin pathway	2	3	1.76	0.0140	FCN3,FCN2
UniProt Keywords	KW-0497	Mitogen	4	39	0.95	0.0155	CAT,THBS4,IGF2,VEGFC
UniProt Keywords	KW-0789	Thiol protease inhibitor	3	25	1.02	0.0415	CSTA,CST3,KNG1
UniProt Keywords	KW-0339	Growth factor	6	129	0.61	0.0475	BMP1,INHBC,THBS4,EFEMP1,IGF2,VEGFC
Pfam	PF01442	Apolipoprotein A1/A4/E domain	4	4	1.94	0.0025	APOA1,APOE,APOA4,APOA5
Pfam	PF06668	Inter-alpha-trypsin inhibitor heavy chain C-terminus	4	6	1.76	0.0037	ITIH4,ITIH1,ITIH2,ITIH3
InterPro	IPR001881	EGF-like calcium-binding domain	22	124	1.19	8.24e-15	F9,COMP,PROC,LRP1,F12,HABP2,MASP1,BMP1,EFEMP2,FBN1,FBLN1,OIT3,THBS4,PROZ,CRTAC1,SVEP1,F10,F7,EFEMP1,MASP2,C1S,C1R
InterPro	IPR018097	EGF-like calcium-binding, conserved site	19	99	1.22	2.81e-13	F9,COMP,PROC,LRP1,MASP1,BMP1,EFEMP2,FBN1,FBLN1,OIT3,THBS4,CRTAC1,SVEP1,F10,F7,EFEMP1,MASP2,C1S,C1R
InterPro	IPR001314	Peptidase S1A, chymotrypsin family	19	107	1.19	6.67e-13	F9,PROC,F12,KLKB1,HABP2,F2,PLG,LPA,PROZ,F10,F7,CFI,MASP2,F11,C1S,MST1,CFB,C1R,CFD
InterPro	IPR001254	Serine proteases, trypsin domain	19	115	1.16	1.63e-12	F9,PROC,F12,KLKB1,HABP2,F2,PLG,LPA,PROZ,F10,F7,CFI,MASP2,F11,C1S,MST1,CFB,C1R,CFD
InterPro	IPR009003	Peptidase S1, PA clan	19	123	1.13	3.91e-12	F9,PROC,F12,KLKB1,HABP2,F2,PLG,LPA,PROZ,F10,F7,CFI,MASP2,F11,C1S,MST1,CFB,C1R,CFD
InterPro	IPR033116	Serine proteases, trypsin family, serine active site	17	92	1.21	7.22e-12	F9,PROC,F12,KLKB1,HABP2,F2,PLG,LPA,F10,F7,CFI,MASP2,F11,C1S,CFB,C1R,CFD
InterPro	IPR043504	Peptidase S1, PA clan, chymotrypsin-like fold	18	116	1.13	1.44e-11	F9,PROC,F12,KLKB1,HABP2,F2,PLG,LPA,PROZ,F10,F7,CFI,MASP2,F11,C1S,MST1,C1R,CFD
InterPro	IPR000742	EGF-like domain	21	236	0.89	1.62e-09	F9,COMP,PROC,LRP1,F12,ATRN,HABP2,MASP1,BMP1,EFEMP2,FBN1,FBLN1,OIT3,THBS4,PROZ,SVEP1,F10,F7,EFEMP1,MASP2,C1R
InterPro	IPR000436	Sushi/SCR/CCP domain	12	56	1.27	1.12e-08	C4BPB,MASP1,CFHR1,C7,F13B,CFHR3,CFH,SVEP1,MASP2,C1S,CFB,C1R
InterPro	IPR035976	Sushi/SCR/CCP superfamily	12	58	1.26	1.45e-08	C4BPB,MASP1,CFHR1,C7,F13B,CFHR3,CFH,SVEP1,MASP2,C1S,CFB,C1R
InterPro	IPR000152	EGF-type aspartate/asparagine hydroxylation site	14	98	1.1	2.29e-08	F9,PROC,LRP1,BMP1,EFEMP2,FBN1,FBLN1,PROZ,SVEP1,F10,F7,EFEMP1,MASP2,C1S
InterPro	IPR018114	Serine proteases, trypsin family, histidine active site	14	100	1.09	2.68e-08	F9,PROC,F12,KLKB1,HABP2,F2,PLG,LPA,F10,F7,CFI,F11,CFB,CFD

InterPro	IPR042185	Serpin superfamily, domain 2	10	35	1.4	3.60e-08	SERPIND1,SERPINA10,SERPING1,SERPINI1,SERPINF2,SERPINA7,SERPINA5,SERPINA11,SERPINA3,SERPINA4
InterPro	IPR000215	Serpin family	10	36	1.38	4.23e-08	SERPIND1,SERPINA10,SERPING1,SERPINI1,SERPINF2,SERPINA7,SERPINA5,SERPINA11,SERPINA3,SERPINA4
InterPro	IPR023796	Serpin domain	10	36	1.38	4.23e-08	SERPIND1,SERPINA10,SERPING1,SERPINI1,SERPINF2,SERPINA7,SERPINA5,SERPINA11,SERPINA3,SERPINA4
InterPro	IPR036186	Serpin superfamily	10	36	1.38	4.23e-08	SERPIND1,SERPINA10,SERPING1,SERPINI1,SERPINF2,SERPINA7,SERPINA5,SERPINA11,SERPINA3,SERPINA4
InterPro	IPR042178	Serpin superfamily, domain 1	10	36	1.38	4.23e-08	SERPIND1,SERPINA10,SERPING1,SERPINI1,SERPINF2,SERPINA7,SERPINA5,SERPINA11,SERPINA3,SERPINA4
InterPro	IPR046350	Cystatin superfamily	8	23	1.48	6.09e-07	SPP2,HRG,CSTA,AHSG,CAMP,CST3,RARRES2,KNG1
InterPro	IPR023795	Serpin, conserved site	8	32	1.34	5.15e-06	SERPIND1,SERPING1,SERPINI1,SERPINF2,SERPINA7,SERPINA5,SERPINA3,SERPINA4
InterPro	IPR012224	Peptidase S1A, coagulation factor VII/IX/X/C/Z	5	5	1.94	1.55e-05	F9,PROC,PROZ,F10,F7
InterPro	IPR009030	Growth factor receptor cysteine-rich domain superfamily	12	127	0.92	2.17e-05	COMP,IGFBP5,LRP1,BMP1,EFEMP2,FBN1,FBLN1,C8A,CCN5,SVEP1,IGFBP3,EFEMP1
InterPro	IPR013806	Kringle-like fold	7	26	1.37	2.39e-05	MMP2,F12,HABP2,F2,PLG,LPA,MST1
InterPro	IPR000294	Gamma-carboxyglutamic acid-rich (GLA) domain	6	15	1.54	2.93e-05	F9,PROC,F2,PROZ,F10,F7
InterPro	IPR035972	Gamma-carboxyglutamic acid-rich (GLA) domain superfamily	6	15	1.54	2.93e-05	F9,PROC,F2,PROZ,F10,F7
InterPro	IPR000001	Kringle	6	16	1.51	3.67e-05	F12,HABP2,F2,PLG,LPA,MST1
InterPro	IPR018056	Kringle, conserved site	6	16	1.51	3.67e-05	F12,HABP2,F2,PLG,LPA,MST1
InterPro	IPR038178	Kringle superfamily	6	16	1.51	3.67e-05	F12,HABP2,F2,PLG,LPA,MST1
InterPro	IPR017857	Coagulation factor-like, Gla domain superfamily	5	12	1.56	0.00026	F9,PROC,PROZ,F10,F7
InterPro	IPR000074	Apolipoprotein A/E	4	4	1.94	0.00027	APOA1,APOE,APOA4,APOA5
InterPro	IPR014716	Fibrinogen, alpha/beta/gamma chain, C-terminal globular, subdomain 1	6	26	1.3	0.00034	ANGPTL6,FCN3,FCN2,FGB,ITLN1,FGL1
InterPro	IPR003609	PAN/Apple domain	4	6	1.76	0.00074	KLKB1,PLG,F11,MST1
InterPro	IPR010600	Inter-alpha-trypsin inhibitor heavy chain, C-terminal	4	6	1.76	0.00074	ITIH4,ITIH1,ITIH2,ITIH3
InterPro	IPR000010	Cystatin domain	5	17	1.41	0.00088	HRG,CSTA,AHSG,CST3,KNG1
InterPro	IPR000859	CUB domain	7	51	1.08	0.00088	PCOLCE,ATRN,MASP1,BMP1,MASP2,C1S,C1R
InterPro	IPR002181	Fibrinogen, alpha/beta/gamma chain, C-terminal globular domain	6	32	1.21	0.00088	ANGPTL6,FCN3,FCN2,FGB,ITLN1,FGL1
InterPro	IPR036056	Fibrinogen-like, C-terminal	6	32	1.21	0.00088	ANGPTL6,FCN3,FCN2,FGB,ITLN1,FGL1
InterPro	IPR002035	Von Willebrand factor, type A	8	76	0.96	0.00098	VWF,ITIH4,ITIH1,ANTXR1,ITIH2,SVEP1,ITIH3,CFB
InterPro	IPR035914	Spermadhesin, CUB domain superfamily	7	54	1.05	0.0011	PCOLCE,ATRN,MASP1,BMP1,MASP2,C1S,C1R
InterPro	IPR020837	Fibrinogen, conserved site	5	19	1.36	0.0012	ANGPTL6,FCN3,FCN2,FGB,FGL1
InterPro	IPR012674	Calycin	6	38	1.14	0.0018	ORM1,APOD,C8G,APOM,ORM2,PAEP
InterPro	IPR013694	VIT domain	4	10	1.54	0.0026	ITIH4,ITIH1,ITIH2,ITIH3
InterPro	IPR036383	Thrombospondin type-1 (TSP1) repeat superfamily	7	64	0.98	0.0027	CFP,C7,PAPLN,C8A,ADAMTSL4,C8B,CCN5
InterPro	IPR000884	Thrombospondin type-1 (TSP1) repeat	7	65	0.97	0.0029	CFP,C7,PAPLN,C8A,ADAMTSL4,C8B,CCN5
InterPro	IPR036465	Von Willebrand factor A-like domain superfamily	8	97	0.86	0.0043	VWF,ITIH4,ITIH1,ANTXR1,ITIH2,SVEP1,ITIH3,CFB
InterPro	IPR024175	Peptidase S1A, complement C1r/C1S/mannan-binding	3	3	1.94	0.0045	MASP2,C1S,C1R
InterPro	IPR026823	Complement Clr-like EGF domain	5	27	1.21	0.0045	LRP1,EFEMP2,FBN1,FBLN1,EFEMP1
InterPro	IPR001862	Membrane attack complex component/perforin/complement C9	3	5	1.72	0.0118	C7,C8A,C8B
InterPro	IPR000566	Lipocalin/cytosolic fatty-acid binding domain	5	35	1.1	0.0128	ORM1,APOD,C8G,ORM2,PAEP
InterPro	IPR000020	Anaphylatoxin/fibulin	3	6	1.64	0.0169	FBLN1,C4A,C4B
InterPro	IPR020863	Membrane attack complex component/perforin domain, conserved site	3	6	1.64	0.0169	C7,C8A,C8B
InterPro	IPR023415	Low-density lipoprotein (LDL) receptor class A, conserved site	5	40	1.04	0.0214	LRP1,C7,C8A,C8B,CFI
InterPro	IPR019742	Alpha-2-macroglobulin, conserved site	3	8	1.51	0.0307	A2ML1,C4A,C4B
InterPro	IPR023411	Ribonuclease A, active site	3	8	1.51	0.0307	ANG,RNASE1,RNASE4
InterPro	IPR040839	Macroglobulin domain MG4	3	8	1.51	0.0307	A2ML1,C4A,C4B
InterPro	IPR047565	Alpha-macroglobulin-like, thiol-ester bond-forming region	3	8	1.51	0.0307	A2ML1,C4A,C4B
InterPro	IPR001599	Alpha-2-macroglobulin	3	9	1.46	0.0377	A2ML1,C4A,C4B
InterPro	IPR002172	Low-density lipoprotein (LDL) receptor class A repeat	5	47	0.97	0.0377	LRP1,C7,C8A,C8B,CFI
InterPro	IPR002890	Macroglobulin domain	3	9	1.46	0.0377	A2ML1,C4A,C4B
InterPro	IPR009048	Alpha-macroglobulin, receptor-binding	3	9	1.46	0.0377	A2ML1,C4A,C4B
InterPro	IPR011625	Alpha-2-macroglobulin, bait region domain	3	9	1.46	0.0377	A2ML1,C4A,C4B
InterPro	IPR011626	Alpha-macroglobulin-like, TED domain	3	9	1.46	0.0377	A2ML1,C4A,C4B
InterPro	IPR018073	Proteinase inhibitor I25, cystatin, conserved site	3	9	1.46	0.0377	CSTA,CST3,KNG1
InterPro	IPR036055	LDL receptor-like superfamily	5	47	0.97	0.0377	LRP1,C7,C8A,C8B,CFI
InterPro	IPR036595	Alpha-macroglobulin, receptor-binding domain superfamily	3	9	1.46	0.0377	A2ML1,C4A,C4B
InterPro	IPR041555	Macroglobulin domain MG3	3	9	1.46	0.0377	A2ML1,C4A,C4B
SMART	SM00020	Trypsin-like serine protease	20	118	1.17	8.16e-14	F9,PROC,F12,KLKB1,HABP2,MASP1,F2,PLG,LPA,PROZ,F10,F7,CFI,MASP2,F11,C1S,MST1,CFB,C1R,CFD
SMART	SM00179	Calcium-binding EGF-like domain	18	101	1.19	6.73e-13	F9,COMP,PROC,LRP1,MASP1,BMP1,EFEMP2,FBN1,FBLN1,OIT3,THBS4,SVEP1,F10,F7,EFEMP1,MASP2,C1S,C1R
SMART	SM00032	Domain abundant in complement control proteins	12	56	1.27	3.86e-09	C4BPB,MASP1,CFHR1,C7,F13B,CFHR3,CFH,SVEP1,MASP2,C1S,CFB,C1R
SMART	SM00093	SERine Proteinase INhibitors	10	35	1.4	1.34e-08	SERPIND1,SERPINA10,SERPING1,SERPINI1,SERPINF2,SERPINA7,SERPINA5,SERPINA11,SERPINA3,SERPINA4
SMART	SM00181	Epidermal growth factor-like domain.	17	209	0.85	1.50e-07	F9,COMP,PROC,LRP1,F12,ATRN,HYAL1,HABP2,EFEMP2,FBN1,FBLN1,OIT3,THBS4,PROZ,SVEP1,F10,F7
SMART	SM00069	Domain containing Gla (gamma-carboxyglutamate) residues.	6	15	1.54	1.28e-05	F9,PROC,F2,PROZ,F10,F7
SMART	SM00130	Kringle domain	6	16	1.51	1.50e-05	F12,HABP2,F2,PLG,LPA,MST1
SMART	SM00186	Fibrinogen-related domains (FReDs)	6	29	1.26	0.00025	ANGPTL6,FCN3,FCN2,FGB,ITLN1,FGL1
SMART	SM00042	Domain first found in C1r, C1s, uEGF, and bone morphogenetic protein.	7	50	1.09	0.00033	PCOLCE,ATRN,MASP1,BMP1,MASP2,C1S,C1R
SMART	SM00043	Cystatin-like domain	5	17	1.41	0.00033	HRG,CSTA,AHSG,CST3,KNG1
SMART	SM00609	Vault protein Inter-alpha-Trypsin domain	4	7	1.7	0.00037	ITIH4,ITIH1,ITIH2,ITIH3
SMART	SM00327	Von Willebrand factor (vWF) type A domain	8	77	0.96	0.00038	VWF,ITIH4,ITIH1,ANTXR1,ITIH2,SVEP1,ITIH3,CFB
SMART	SM00209	Thrombospondin type 1 repeats	7	64	0.98	0.00100	CFP,C7,PAPLN,C8A,ADAMTSL4,C8B,CCN5
SMART	SM00104	Anaphylatoxin homologous domain	3	6	1.64	0.0067	FBLN1,C4A,C4B

SMART	SM00092	Pancreatic ribonuclease	3	8	1.51	0.0122	ANG,RNASE1,RNASE4
SMART	SM01419	Alpha-macro-globulin thiol-ester bond-forming region	3	8	1.51	0.0122	A2ML1,C4A,C4B
SMART	SM00192	Low-density lipoprotein receptor domain class A	5	48	0.96	0.0142	LRP1,C7,C8A,C8B,CFI
SMART	SM01359	Alpha-2-Macroglobulin	3	9	1.46	0.0142	A2ML1,C4A,C4B
SMART	SM01360	Alpha-2-macroglobulin family	3	9	1.46	0.0142	A2ML1,C4A,C4B
SMART	SM01361	A-macroglobulin receptor	3	9	1.46	0.0142	A2ML1,C4A,C4B
SMART	SM00457	Membrane-attack complex / perforin	3	12	1.34	0.0232	C7,C8A,C8B
SMART	SM00223	APPLE domain	2	2	1.94	0.0283	KLKB1,F11
SMART	SM00643	Netrin C-terminal Domain	3	15	1.24	0.0370	PCOLCE,C4A,C4B
SMART	SM00121	Insulin growth factor-binding protein homologues	3	17	1.19	0.0487	IGFBP5,CCN5,IGFBP3

UC vs NCFET T2

#category	term ID	term description	observed gene count	background gene count	strength	false discovery rate	matching proteins in your network (labels)
GO Process	GO:0006959	Humoral immune response	39	268	1.12	4.49e-26	C5,LTF,CFHR5,LYZ,FCN3,SERPING1,FCN2,PPBP,PF4,MASP1,CAMP,FGB,F2,C1QB,CFHR1,SLPI,C8A,CFHR2,CFHR4,CFHR3,CFH,S100A7,C8B,FCN1,C1QC,C1QA,MASP2,C1S,C4B,CFB,RARRES2,JCHAIN,C1R,CFD,IGHV3-15,IGHV3-72,KNG1,B2M,FGA
GO Process	GO:0006956	Complement activation	25	60	1.58	2.01e-25	C5,CFHR5,FCN3,SERPING1,FCN2,MASP1,C1QB,CFHR1,C8A,CFHR2,CFHR4,CFHR3,CFH,C8B,FCN1,C1QC,C1QA,MASP2,C1S,C4B,CFB,C1R,CFD,IGHV3-15,IGHV3-72
GO Process	GO:0007596	Blood coagulation	31	173	1.21	7.24e-23	CPB2,APOH,SERPIND1,F9,PROC,F12,VWF,SERPINA10,KLKB1,F13A1,SERPING1,PF4,FGB,F2,FBLN1,FGG,F8,F13B,SERPINC1,F5,FLNA,ADAMTS13,F7,PROS1,MMRN1,ILK,F11,PLCG2,KNG1,ACTB,FGA
GO Process	GO:0006950	Response to stress	104	3358	0.45	5.89e-22	CPB2,APOH,SERPIND1,F9,MMP2,C5,LTF,PROC,CAT,APOE,F12,APCS,CFHR5,LYZ,VWF,SERPINA10,ATRN,KLKB1,F13A1,TCIRG1,HPX,IGFBP4,FCN3,AHSG,SAA4,SERPING1,CSF1R,IGF2BP1,FCN2,PGLYRP2,PPBP,PF4,MASP1,CAMP,PDIA3,LALBA,PCSK9,FGB,DST,F2,PRG2,STAB1,C1QB,CFHR1,NDUFS8,APOL1,SERPINF2,P4HB,FBLN1,HSP90AA1,FGG,TUBB,THBS4,PRDX6,SLPI,APOD,BLM,APOA4,F8,C8A,F13B,CFHR2,CFH,SERPINC1,F5,S100A7,ECM1,FLNA,MCOLN2,C8B,FCN1,ADAMTS13,C1QC,C1QA,F7,SOD3,GPX3,PROS1,MMRN1,ILK,MASP2,F11,C1S,ADIPOQ,MST1,C4B,CFB,RARRES2,UIMC1,TSKU,JCHAIN,HPR,C1R,VIM,RNASE4,CFD,IGHV3-15,IGHV3-72,PLCG2,KNG1,ACTB,TRIM8,B2M,FGA
GO Process	GO:0006952	Defense response	65	1394	0.63	3.50e-20	C5,LTF,F12,APCS,CFHR5,LYZ,ATRN,KLKB1,TCIRG1,HPX,IGFBP4,FCN3,AHSG,SAA4,SERPING1,CSF1R,FCN2,PGLYRP2,PPBP,PF4,MASP1,CAMP,LALBA,FGB,F2,PRG2,STAB1,C1QB,CFHR1,APOL1,SERPINF2,TUBB,SLPI,APOA4,F8,C8A,CFHR2,CFH,S100A7,ECM1,MCOLN2,C8B,FCN1,ADAMTS13,C1QC,C1QA,F7,MASP2,C1S,C4B,CFB,RARRES2,JCHAIN,HPR,C1R,VIM,RNASE4,CFD,IGHV3-15,IGHV3-72,PLCG2,KNG1,TRIM8,B2M,FGA
GO Process	GO:0098542	Defense response to other organism	54	989	0.7	4.60e-19	C5,LTF,F12,APCS,CFHR5,LYZ,HPX,FCN3,SERPING1,CSF1R,FCN2,PGLYRP2,PPBP,PF4,MASP1,CAMP,LALBA,FGB,F2,PRG2,STAB1,C1QB,CFHR1,APOL1,TUBB,SLPI,APOA4,C8A,CFHR2,CFH,S100A7,MCOLN2,C8B,FCN1,ADAMTS13,C1QC,C1QA,MASP2,C1S,C4B,CFB,RARRES2,JCHAIN,C1R,VIM,RNASE4,CFD,IGHV3-15,IGHV3-72,PLCG2,KNG1,TRIM8,B2M,FGA
GO Process	GO:0072378	Blood coagulation, fibrin clot formation	16	24	1.78	1.96e-18	APOH,F9,F12,KLKB1,F13A1,FGB,F2,FBLN1,FGG,F8,F13B,F5,FLNA,F7,F11,FGA
GO Process	GO:0042060	Wound healing	33	336	0.95	1.23e-17	CPB2,APOH,SERPIND1,F9,PROC,F12,VWF,SERPINA10,KLKB1,F13A1,SERPING1,PF4,FGB,DST,F2,FBLN1,FGG,F8,F13B,SERPINC1,F5,FLNA,ADAMTS13,F7,PROS1,MMRN1,ILK,F11,TSKU,PLCG2,KNG1,ACTB,FGA
GO Process	GO:0009611	Response to wounding	36	444	0.87	5.93e-17	CPB2,APOH,SERPIND1,F9,MMP2,PROC,F12,VWF,SERPINA10,KLKB1,F13A1,SERPING1,PF4,FGB,DST,F2,FBLN1,FGG,APOD,APOA4,F8,F13B,SERPINC1,F5,FLNA,ADAMTS13,F7,PROS1,MMRN1,ILK,F11,TSKU,PLCG2,KNG1,ACTB,FGA
GO Process	GO:0050878	Regulation of body fluid levels	33	371	0.91	1.71e-16	CPB2,APOH,SERPIND1,F9,PROC,APOE,F12,VWF,SERPINA10,KLKB1,F13A1,SERPING1,PF4,FGB,F2,SERPINF2,FBLN1,FGG,F8,F13B,SERPINC1,F5,FLNA,ADAMTS13,F7,PROS1,MMRN1,ILK,F11,PLCG2,KNG1,ACTB,FGA
GO Process	GO:0051707	Response to other organism	57	1328	0.59	8.08e-16	C5,LTF,APOB,F12,APCS,CFHR5,LYZ,HPX,FCN3,SERPING1,CSF1R,FCN2,PGLYRP2,PPBP,PF4,MASP1,CAMP,LALBA,FGB,F2,PRG2,STAB1,C1QB,CFHR1,APOL1,HSP90AA1,TUBB,SLPI,APOA4,C8A,CFHR2,CFH,S100A7,MCOLN2,C8B,FCN1,ADAMTS13,C1QC,C1QA,MASP2,C1S,ADIPOQ,C4B,CFB,RARRES2,JCHAIN,C1R,VIM,RNASE4,CFD,IGHV3-15,IGHV3-72,PLCG2,KNG1,TRIM8,B2M,FGA
GO Process	GO:0045087	Innate immune response	43	754	0.71	1.96e-15	C5,LTF,F12,APCS,CFHR5,HPX,FCN3,SERPING1,CSF1R,FCN2,PGLYRP2,MASP1,CAMP,FGB,C1QB,APOL1,TUBB,SLPI,APOA4,C8A,CFH,S100A7,MCOLN2,C8B,FCN1,ADAMTS13,C1QC,C1QA,MASP2,C1S,C4B,CFB,RARRES2,JCHAIN,C1R,VIM,CFD,IGHV3-15,IGHV3-72,PLCG2,TRIM8,B2M,FGA
GO Process	GO:0002253	Activation of immune response	28	271	0.97	2.53e-15	C5,GPLD1,CFHR5,FCN3,SERPING1,FCN2,MASP1,C1QB,CFHR1,HSP90AA1,C8A,CFHR2,CFHR4,CFHR3,CFH,C8B,FCN1,C1QC,C1QA,MASP2,C1S,C4B,CFB,C1R,CFD,IGHV3-15,IGHV3-72,PLCG2
GO Process	GO:0050896	Response to stimulus	150	7835	0.24	2.94e-15	CPB2,APOH,SERPIND1,F9,MMP2,C5,HSD17B1,GPLD1,LTF,APOB,IGFBP5,PROC,APOA1,TTR,CAT,APOE,F12,APCS,CFHR5,LYZ,VWF,SERPINA10,ATRN,LCN1,LCAT,KLKB1,F13A1,PPP2R3A,PON3,TCIRG1,HPX,IGFBP4,FCN3,AHSG,IGFBP1,SAA4,SERPING1,CSF1R,IGF2BP1,PIP,FCN2,PGLYRP2,SCGB3A1,PPBP,PF4,MASP1,CAMP,GUCY1A1,PDIA3,SLC27A4,LALBA,PCSK9,BMP1,FGB,DST,F2,INHBC,PSG1,PRG2,CSPG4,STAB1,C1QB,CFHR1,NDUFS8,APOL1,SERPINF2,FBN1,P4HB,PAPPA,FBLN1,HSP90AA1,TUBA1B,FGG,TUBB,THBS4,PRDX6,SLPI,APOD,ASGR2,BLM,APOA4,F8,C8A,CENPF,F13B,CFHR2,CFHR4,CFHR3,CFH,SERPINC1,F5,ADAM12,S100A7,ECM1,FLNA,MCOLN2,C8B,FCN1,ADAMTS13,CCN5,SVEP1,C1QC,C1QA,F7,APOM,CA6,ADGRG2,CALML5,SOD3,GPX3,PROS1,EFEMP1,MMRN1,YWHAZ,ILK,MASP2,PSG4,F11,C1S,ADIPOQ,PRG4,MST1,SPARCL1,C4B,TGFBI,CFB,IGFALS,RARRES2,UIMC1,FBN2,TSKU,JCHAIN,HPR,C1R,ATP1A1,VIM,RNASE4,TTN,CFD,IGHV3-15,IGHV3-72,SEMA3B,PLCG2,OR52B4,KNG1,ACTB,TRIM8,CDH5,B2M,FGA
GO Process	GO:0006955	Immune response	55	1321	0.58	1.00e-14	C5,LTF,F12,APCS,CFHR5,LYZ,TCIRG1,HPX,FCN3,SERPING1,CSF1R,FCN2,PGLYRP2,PPBP,PF4,MASP1,CAMP,FGB,F2,PRG2,C1QB,CFHR1,APOL1,TUBB,SLPI,APOA4,C8A,CFHR2,CFHR4,CFHR3,CFH,S100A7,MCOLN2,C8B,FCN1,ADAMTS13,C1QC,C1QA,MASP2,C1S,PRG4,C4B,CFB,RARRES2,JCHAIN,C1R,VIM,CFD,IGHV3-15,IGHV3-72,PLCG2,KNG1,TRIM8,B2M,FGA
GO Process	GO:0044419	Biological process involved in interspecies interaction between organisms	58	1490	0.55	1.94e-14	C5,LTF,APOB,APOE,F12,APCS,CFHR5,LYZ,HPX,FCN3,SERPING1,CSF1R,FCN2,PGLYRP2,PPBP,PF4,MASP1,CAMP,LALBA,FGB,F2,PRG2,STAB1,C1QB,CFHR1,APOL1,HSP90AA1,TUBB,SLPI,APOA4,C8A,CFHR2,CFH,S100A7,MCOLN2,C8B,FCN1,ADAMTS13,C1QC,C1QA,MASP2,C1S,ADIPOQ,C4B,CFB,RARRES2,JCHAIN,C1R,VIM,RNASE4,CFD,IGHV3-15,IGHV3-72,PLCG2,KNG1,TRIM8,B2M,FGA

GO Process	GO:0061045	Negative regulation of wound healing	17	69	1.35	3.34e-14	CPB2,APOH,PROC,APOE,F12,APCS,KLKB1,SERPING1,FGB,F2,SERPINF2,FGG,PROS1,MMRN1,F11,KNG1,FGA
GO Process	GO:0030193	Regulation of blood coagulation	17	70	1.34	3.93e-14	CPB2,APOH,PROC,APOE,F12,KLKB1,SERPING1,FGB,F2,SERPINF2,FGG,SERPINC1,F7,PROS1,F11,KNG1,FGA
GO Process	GO:0030195	Negative regulation of blood coagulation	15	46	1.47	6.32e-14	CPB2,APOH,PROC,APOE,F12,KLKB1,SERPING1,FGB,F2,SERPINF2,FGG,PROS1,F11,KNG1,FGA
GO Process	GO:0002252	Immune effector process	30	375	0.86	6.56e-14	C5,CFHR5,TCIRG1,FCN3,SERPING1,FCN2,MASP1,F2,C1QB,CFHR1,TUBB,C8A,CFHR2,CFHR4,CFHR3,CFH,C8B,FCN1,C1QC,C1QA,MASP2,C1S,C4B,CFB,C1R,CFD,IGHV3-15,IGHV3-72,PLCG2,B2M
GO Process	GO:0032101	Regulation of response to external stimulus	45	964	0.63	2.04e-13	CPB2,APOH,C5,LTF,PROC,APOA1,APOE,F12,APCS,PSMA6,KLKB1,HPX,FCN3,AHSG,SERPING1,CSF1R,FCN2,PGLYRP2,MASP1,FGB,F2,SERPINF2,FBN1,HSP90AA1,FGG,THBS4,CFH,SERPINC1,S100A7,HTRA1,FLNA,FCN1,F7,PROS1,MMRN1,MASP2,F11,ADIPOQ,MST1,RARRES2,SEMA3B,PLCG2,KNG1,CDH5,FGA
GO Process	GO:0061041	Regulation of wound healing	19	130	1.12	1.58e-12	CPB2,APOH,PROC,APOE,F12,APCS,KLKB1,SERPING1,FGB,F2,SERPINF2,FGG,SERPINC1,F7,PROS1,MMRN1,F11,KNG1,FGA
GO Process	GO:1903034	Regulation of response to wounding	20	162	1.05	5.14e-12	CPB2,APOH,PROC,APOE,F12,APCS,KLKB1,SERPING1,FGB,F2,SERPINF2,FGG,SERPINC1,FLNA,F7,PROS1,MMRN1,F11,KNG1,FGA
GO Process	GO:0006958	Complement activation, classical pathway	13	40	1.47	5.62e-12	C5,SERPING1,C1QB,C8A,C8B,C1QC,C1QA,MASP2,C1S,C4B,C1R,IGHV3-15,IGHV3-72
GO Process	GO:0009605	Response to external stimulus	69	2355	0.42	7.20e-12	SERPIND1,MMP2,C5,LTF,APOB,APOA1,CAT,APOE,F12,APCS,CFHR5,LYZ,HPX,FCN3,SERPING1,CSF1R,FCN2,PGLYRP2,PPBP,PF4,MASP1,CAMP,SLC27A4,LALBA,PCSK9,FGB,F2,PRG2,STAB1,C1QB,CFHR1,APOL1,FBN1,HSP90AA1,TUBB,SLPI,APOA4,C8A,CFHR2,CFH,F5,S100A7,MCOLN2,C8B,FCN1,ADAMTS13,C1QC,C1QA,F7,MASP2,C1S,ADIPOQ,C4B,CFB,RARRES2,JCHAIN,C1R,VIM,RNASE4,TTN,CFD,IGHV3-15,IGHV3-72,SEMA3B,PLCG2,KNG1,TRIM8,B2M,FGA
GO Process	GO:0048584	Positive regulation of response to stimulus	65	2131	0.44	8.80e-12	CPB2,APOH,C5,GPLD1,LTF,IGFBP5,APOA1,CAT,APOE,F12,CFHR5,VWF,NID1,PPP2R3A,HPX,IGFBP4,FCN3,SERPING1,CSF1R,FCN2,MASP1,GUCY1A1,PDIA3,FGB,F2,INHBC,CSPG4,C1QB,CFHR1,SERPINF2,FBN1,HSP90AA1,FGG,THBS4,C8A,CFHR2,CFHR4,CFHR3,CFH,S100A7,ECM1,FLNA,C8B,FCN1,C1QC,C1QA,F7,ILK,MASP2,C1S,ADIPOQ,C4B,CFB,RARRES2,UIMC1,C1R,CFD,IGHV3-15,IGHV3-72,PLCG2,ACTB,TRIM8,CDH5,B2M,FGA
GO Process	GO:0002682	Regulation of immune system process	52	1438	0.52	1.32e-11	C5,GPLD1,LTF,APOA1,APOE,APCS,CFHR5,HPX,FCN3,SERPING1,CSF1R,PIP,FCN2,PGLYRP2,PF4,MASP1,PSG1,PRG2,C1QB,CFHR1,FBN1,HSP90AA1,THBS4,APOD,C8A,CFHR2,CFHR4,CFHR3,CFH,S100A7,ECM1,C8B,FCN1,C1QC,C1QA,F7,CD99,MASP2,PSG4,C1S,ADIPOQ,MST1,C4B,CFB,RARRES2,C1R,CFD,IGHV3-15,IGHV3-72,PLCG2,ACTB,B2M
GO Process	GO:0031638	Zymogen activation	14	59	1.33	1.84e-11	APOH,F9,F12,KLKB1,FGB,F2,FGG,F8,F5,F7,F11,HPR,C1R,FGA
GO Process	GO:0050778	Positive regulation of immune response	30	502	0.73	6.12e-11	C5,GPLD1,CFHR5,HPX,FCN3,SERPING1,FCN2,MASP1,C1QB,CFHR1,HSP90AA1,C8A,CFHR2,CFHR4,CFHR3,CFH,C8B,FCN1,C1QC,C1QA,MASP2,C1S,C4B,CFB,C1R,CFD,IGHV3-15,IGHV3-72,PLCG2,B2M
GO Process	GO:0042730	Fibrinolysis	10	19	1.68	1.22e-10	CPB2,F12,KLKB1,SERPING1,FGB,F2,SERPINF2,FGG,PROS1,FGA
GO Process	GO:0051241	Negative regulation of multicellular organismal process	42	1035	0.57	1.55e-10	CPB2,APOH,MMP2,LTF,IGFBP5,PROC,APOA1,APOE,F12,APCS,KLKB1,AHSG,SERPING1,PGLYRP2,PF4,GUCY1A1,PCSK9,FGB,F2,PRG2,STAB1,SERPINF2,FBN1,FBLN1,FGG,THBS4,APOD,APOA4,ECM1,C1QC,APOM,PROS1,EFEMP1,F11,ADIPOQ,PAEP,TSKU,ATP1A1,SEMA3B,KNG1,B2M,FGA
GO Process	GO:0048518	Positive regulation of biological process	120	6207	0.24	1.97e-10	CPB2,CETP,APOH,MMP2,C5,GPLD1,LTF,APOB,IGFBP5,PROC,APOA1,CAT,DHX29,APOE,F12,CFHR5,VWF,NID1,KLKB1,PPP2R3A,CENPE,TCIRG1,HPX,LUM,IGFBP4,FCN3,AHSG,ANKRD31,IGFBP1,SERPING1,CSF1R,IGF2BP1,PIP,FCN2,SCGB3A1,SERPINI1,PPBP,PF4,MASP1,CAMP,GUCY1A1,PDIA3,SLC27A4,PCSK9,BMP1,FGB,F2,INHBC,EFEMP2,PRG2,CSPG4,C1QB,CFHR1,SUZ12,SERPINF2,FBN1,P4HB,FBLN1,HSP90AA1,FGG,THBS4,PRDX6,BLM,APOA4,MTCL1,C8A,CFHR2,CFHR4,CFHR3,CFH,ADAM12,S100A7,HTRA1,ADAMTSL4,ECM1,FLNA,MCOLN2,SMARCA1,C8B,FCN1,C1QC,C1QA,F7,CD99,MMRN1,ILK,MASP2,F11,C1S,ADIPOQ,SLAIN1,ABCA13,MST1,C4B,CFB,PAEP,RARRES2,TCAF1,UIMC1,FBN2,TSKU,JCHAIN,HPR,C1R,APOA5,ATP1A1,VIM,TTN,APOC4,CFD,IGHV3-15,IGHV3-72,SEMA3B,PLCG2,KNG1,ACTB,TRIM8,CDH5,B2M,FGA
GO Process	GO:0048583	Regulation of response to stimulus	90	3931	0.32	2.01e-10	CPB2,APOH,C5,GPLD1,LTF,IGFBP5,PROC,APOA1,CAT,APOE,F12,APCS,CFHR5,VWF,PSMA6,NID1,KLKB1,PPP2R3A,HPX,IGFBP4,FCN3,AHSG,IGFBP1,SERPING1,CSF1R,FCN2,PGLYRP2,PF4,MASP1,GUCY1A1,PDIA3,SLC27A4,PCSK9,FGB,F2,INHBC,CSPG4,C1QB,CFHR1,SERPINF2,FBN1,P4HB,FBLN1,HSP90AA1,FGG,THBS4,APOD,GIT2,C8A,CFHR2,CFHR4,CFHR3,CFH,SERPINC1,S100A7,HTRA1,ECM1,FLNA,C8B,FCN1,C1QC,C1QA,F7,PROS1,MMRN1,YWHAZ,ILK,MASP2,F11,C1S,ADIPOQ,MST1,C4B,CFB,RARRES2,UIMC1,FBN2,TSKU,C1R,CFD,IGHV3-15,IGHV3-72,SEMA3B,PLCG2,KNG1,ACTB,TRIM8,CDH5,B2M,FGA
GO Process	GO:0002684	Positive regulation of immune system process	38	874	0.6	3.10e-10	C5,GPLD1,CFHR5,HPX,FCN3,SERPING1,CSF1R,FCN2,PF4,MASP1,C1QB,CFHR1,HSP90AA1,THBS4,C8A,CFHR2,CFHR4,CFHR3,CFH,S100A7,C8B,FCN1,C1QC,C1QA,F7,CD99,MASP2,C1S,C4B,CFB,RARRES2,C1R,CFD,IGHV3-15,IGHV3-72,PLCG2,ACTB,B2M
GO Process	GO:0010466	Negative regulation of peptidase activity	21	249	0.88	7.31e-10	SPP2,SERPIND1,C5,LTF,SERPINA10,LCN1,CSTA,ITIH1,AHSG,SERPING1,SERPINI1,A2ML1,SERPINF2,SLPI,PAPLN,SERPINC1,ECM1,PROS1,ITIH3,C4B,KNG1
GO Process	GO:0065007	Biological regulation	183	12385	0.13	1.14e-09	SPP2,CPB2,CETP,APOH,SERPIND1,TPD52L2,F9,MMP2,C5,HSD17B1,AFM,MGP,GPLD1,LTF,APOB,IGFBP5,PROC,APOA1,TTR,CAT,DHX29,APOE,F12,APCS,CFHR5,LYZ,VWF,PSMA6,SERPINA10,ATRN,LCN1,LCAT,NID1,CSTA,CP,KLKB1,F13A1,PPP2R3A,CENPE,PON3,TCIRG1,HPX,LUM,CDH11,IGFBP4,FCN3,ITIH1,AHSG,ANKRD31,IGFBP1,SERPING1,CSF1R,IGF2BP1,PIP,FCN2,PGLYRP2,SCGB3A1,SERPINI1,PPBP,PF4,MASP1,CAMP,GUCY1A1,A2ML1,PDIA3,SLC27A4,LALBA,PCSK9,BMP1,FGB,DST,F2,INHBC,PSG1,EFEMP2,PRG2,CSPG4,STAB1,C1QB,CFHR1,SUZ12,SERPINF2,FBN1,KDM3B,P4HB,PAPPA,FBLN1,OIT3,HSP90AA1,FGG,TUBB,THBS4,PRDX6,SLPI,APOD,PAPLN,ASGR2,BLM,GIT2,APOA4,CNDP1,COL18A1,MTCL1,F8,C8A,CENPF,F13B,CFHR2,CFHR4,CFHR3,CFH,QSOX1,SERPINC1,F5,ADAM12,S100A7,HTRA1,ADAMTSL4,ECM1,FLNA,MCOLN2,SMARCA1,C8B,FCN1,ADAMTS13,CCN5,CNTRL,SVEP1,C1QC,C1QA,F7,APOM,ADGRG2,CALML5,CD99,SOD3,PROS1,EFEMP1,MMRN1,YWHAZ,NAV2,ILK,MASP2,PSG4,F11,C1S,ADIPOQ,ATP9B,SLAIN1,ABCA13,MST1,SPARCL1,ITIH3,C4B,TGFBI,CFB,IGFALS,PAEP,RARRES2,TCAF1,UIMC1,FBN2,TSKU,JCHAIN,HPR,C1R,APOA5,ATP1A1,VIM,TTN,APOC4,CFD,IGHV3-15,IGHV3-72,SEMA3B,PLCG2,OR52B4,KNG1,ACTB,TRIM8,CDH5,B2M,FGA
GO Process	GO:0051346	Negative regulation of hydrolase activity	24	354	0.79	1.37e-09	SPP2,SERPIND1,C5,LTF,APOA1,APCS,SERPINA10,LCN1,CSTA,ITIH1,AHSG,SERPING1,SERPINI1,A2ML1,SLC27A4,SERPINF2,SLPI,PAPLN,SERPINC1,ECM1,PROS1,ITIH3,C4B,KNG1

GO Process	GO:0002376	Immune system process	60	2121	0.41	2.13e-09	C5,GPLD1,LTF,F12,APCS,CFHR5,LYZ,TCIRG1,HPX,FCN3,SERPING1,CSF1R,FCN2,PGLYRP2,PPBP,PF4,MASP1,CAMP,PDIA3,FGB,F2,PRG2,C1QB,CFHR1,APOL1,HSP90AA1,TUBB,SLPI,APOA4,C8A,CFHR2,CFHR4,CFHR3,CFH,S100A7,FLNA,MCOLN2,C8B,FCN1,ADAMTS13,C1QC,C1QA,CD99,MASP2,C1S,PRG4,C4B,CFB,RARRES2,JCHAIN,C1R,VIM,CFD,IGHV3-15,IGHV3-72,PLCG2,KNG1,TRIM8,B2M,FGA
GO Process	GO:0006508	Proteolysis	44	1247	0.51	3.06e-09	CPB2,APOH,F9,MMP2,LTF,PROC,F12,PSMA6,LCN1,KLKB1,FCN3,HABP2,PIP,FCN2,MASP1,APEH,PCSK9,BMP1,FGB,F2,UBE3C,PAPPA,FGG,CNDP1,F8,CFH,F5,ADAM12,HTRA1,FCN1,ADAMTS13,F7,MASP2,F11,C1S,PCYOX1,MST1,CFB,UIMC1,HPR,C1R,CFD,PAMR1,FGA
GO Process	GO:0045861	Negative regulation of proteolysis	23	339	0.79	3.59e-09	SPP2,CPB2,SERPIND1,C5,LTF,SERPINA10,LCN1,CSTA,ITIH1,AHSG,SERPING1,SERPINI1,A2ML1,F2,SERPINF2,SLPI,PAPLN,SERPINC1,ECM1,PROS1,ITIH3,C4B,KNG1
GO Process	GO:0016064	Immunoglobulin mediated immune response	14	95	1.13	4.16e-09	C5,TCIRG1,SERPING1,C1QB,C8A,C8B,C1QC,C1QA,MASP2,C1S,C4B,C1R,IGHV3-15,IGHV3-72
GO Process	GO:0032102	Negative regulation of response to external stimulus	24	387	0.75	6.94e-09	CPB2,APOH,C5,LTF,PROC,APOA1,APOE,F12,APCS,KLKB1,SERPING1,FGB,F2,SERPINF2,FGG,HTRA1,PROS1,MMRN1,F11,ADIPOQ,SEMA3B,KNG1,CDH5,FGA
GO Process	GO:0007597	Blood coagulation, intrinsic pathway	8	13	1.75	8.81e-09	APOH,F9,F12,KLKB1,F8,FLNA,F7,F11
GO Process	GO:0002449	Lymphocyte mediated immunity	16	159	0.96	2.47e-08	C5,TCIRG1,SERPING1,C1QB,TUBB,C8A,C8B,C1QC,C1QA,MASP2,C1S,C4B,C1R,IGHV3-15,IGHV3-72,B2M
GO Process	GO:0050776	Regulation of immune response	34	844	0.56	3.19e-08	C5,GPLD1,LTF,APOE,CFHR5,HPX,FCN3,SERPING1,FCN2,PGLYRP2,MASP1,C1QB,CFHR1,HSP90AA1,C8A,CFHR2,CFHR4,CFHR3,CFH,ECM1,C8B,FCN1,C1QC,C1QA,MASP2,C1S,C4B,CFB,C1R,CFD,IGHV3-15,IGHV3-72,PLCG2,B2M
GO Process	GO:0016485	Protein processing	17	202	0.88	8.00e-08	APOH,F9,F12,KLKB1,PCSK9,BMP1,FGB,F2,FGG,F8,F5,ADAMTS13,F7,F11,HPR,C1R,FGA
GO Process	GO:0032501	Multicellular organismal process	116	6490	0.21	1.32e-07	SPP2,CPB2,CETP,APOH,SERPIND1,F9,MMP2,C5,HSD17B1,MGP,GPLD1,LTF,APOB,IGFBP5,PROC,APOA1,CAT,APOE,ANGPTL6,F12,LYZ,VWF,SERPINA10,ATRN,LCN1,LCAT,NID1,KLKB1,F13A1,PPP2R3A,DNAH5,PON3,TCIRG1,LUM,CDH11,AHSG,SERPING1,SEC24D,CSF1R,IGF2BP1,PIP,SERPINI1,PF4,GUCY1A1,SLC27A4,PCSK9,BMP1,FGB,F2,PSG1,EFEMP2,CSPG4,SUZ12,PSG11,SERPINF2,FBN1,PAPPA,FBLN1,HSP90AA1,FGG,THBS4,APOD,GIT2,APOA4,COL18A1,F8,PCDH18,CENPF,F13B,SERPINC1,F5,S100A7,HTRA1,ECM1,FLNA,CRTAC1,SMARCA1,ADAMTS13,CNTRL,SVEP1,C1QA,F7,APOM,CA6,ADGRG2,SOD3,PROS1,EFEMP1,MMRN1,YWHAZ,NAV2,ILK,PSG4,F11,PSG2,ADIPOQ,MST1,TGFBI,RARRES2,FBN2,TSKU,JCHAIN,APOA5,ATP1A1,VIM,TTN,SEMA3B,PLCG2,OR52B4,KNG1,ACTB,TRIM8,CDH5,B2M,FGA,TPM3
GO Process	GO:0009617	Response to bacterium	29	663	0.6	1.33e-07	LTF,APOB,LYZ,FCN2,PGLYRP2,PPBP,PF4,CAMP,LALBA,FGB,F2,PRG2,STAB1,SLPI,S100A7,ADAMTS13,ADIPOQ,C4B,CFB,RARRES2,JCHAIN,VIM,RNASE4,CFD,IGHV3-15,IGHV3-72,PLCG2,B2M,FGA
GO Process	GO:0042742	Defense response to bacterium	20	306	0.77	1.33e-07	LTF,LYZ,FCN2,PGLYRP2,PPBP,CAMP,LALBA,FGB,F2,PRG2,STAB1,SLPI,S100A7,RARRES2,JCHAIN,RNASE4,IGHV3-15,IGHV3-72,B2M,FGA
GO Process	GO:0002443	Leukocyte mediated immunity	17	211	0.86	1.40e-07	C5,TCIRG1,SERPING1,F2,C1QB,TUBB,C8A,C8B,C1QC,C1QA,MASP2,C1S,C4B,C1R,IGHV3-15,IGHV3-72,B2M
GO Process	GO:0002460	Adaptive immune response based on somatic recombination of immune receptors built from immunoglobulin superfamily domains	15	169	0.91	4.31e-07	C5,TCIRG1,SERPING1,C1QB,C8A,C8B,C1QC,C1QA,MASP2,C1S,C4B,C1R,IGHV3-15,IGHV3-72,B2M
GO Process	GO:0006954	Inflammatory response	25	538	0.63	6.85e-07	C5,F12,APCS,LYZ,ATRN,KLKB1,TCIRG1,IGFBP4,AHSG,SAA4,CSF1R,PPBP,PF4,CAMP,F2,STAB1,SERPINF2,F8,ECM1,C1QA,F7,C4B,RARRES2,HPR,KNG1
GO Process	GO:0019730	Antimicrobial humoral response	14	149	0.93	7.50e-07	LTF,LYZ,PPBP,PF4,CAMP,FGB,F2,SLPI,S100A7,RARRES2,JCHAIN,KNG1,B2M,FGA
GO Process	GO:0051239	Regulation of multicellular organismal process	64	2749	0.32	7.75e-07	CPB2,APOH,MMP2,C5,MGP,GPLD1,LTF,IGFBP5,PROC,APOA1,APOE,F12,APCS,ATRN,LCAT,KLKB1,LUM,AHSG,SERPING1,CSF1R,IGF2BP1,PGLYRP2,PF4,CAMP,GUCY1A1,PCSK9,BMP1,FGB,F2,PRG2,STAB1,SERPINF2,FBN1,FBLN1,HSP90AA1,FGG,THBS4,APOD,APOA4,SERPINC1,ADAM12,ECM1,MCOLN2,FCN1,C1QC,F7,APOM,PROS1,EFEMP1,F11,ADIPOQ,MST1,PAEP,FBN2,TSKU,APOA5,ATP1A1,SEMA3B,PLCG2,KNG1,ACTB,CDH5,B2M,FGA
GO Process	GO:0006957	Complement activation, alternative pathway	7	16	1.6	8.07e-07	C5,CFHR5,C8A,CFH,C8B,CFB,CFD
GO Process	GO:1903028	Positive regulation of opsonization	7	16	1.6	8.07e-07	FCN3,FCN2,MASP1,FCN1,MASP2,C4B,CFB
GO Process	GO:0034369	Plasma lipoprotein particle remodeling	8	29	1.4	1.15e-06	CETP,APOB,APOA1,APOE,LCAT,APOA4,APOM,APOA5
GO Process	GO:0050789	Regulation of biological process	169	11655	0.12	1.15e-06	SPP2,CPB2,CETP,APOH,SERPIND1,TPD52L2,MMP2,C5,MGP,GPLD1,LTF,APOB,IGFBP5,PROC,APOA1,TTR,CAT,DHX29,APOE,F12,APCS,CFHR5,VWF,PSMA6,SERPINA10,ATRN,LCN1,LCAT,NID1,CSTA,KLKB1,PPP2R3A,CENPE,PON3,TCIRG1,HPX,LUM,CDH11,IGFBP4,FCN3,ITIH1,AHSG,ANKRD31,IGFBP1,SERPING1,CSF1R,IGF2BP1,PIP,FCN2,PGLYRP2,SCGB3A1,SERPINI1,PPBP,PF4,MASP1,CAMP,GUCY1A1,A2ML1,PDIA3,SLC27A4,LALBA,PCSK9,BMP1,FGB,DST,F2,INHBC,PSG1,EFEMP2,PRG2,CSPG4,STAB1,C1QB,CFHR1,SUZ12,SERPINF2,FBN1,KDM3B,P4HB,PAPPA,FBLN1,HSP90AA1,FGG,TUBB,THBS4,PRDX6,SLPI,APOD,PAPLN,ASGR2,BLM,GIT2,APOA4,COL18A1,MTCL1,C8A,CENPF,CFHR2,CFHR4,CFHR3,CFH,QSOX1,SERPINC1,ADAM12,S100A7,HTRA1,ADAMTSL4,ECM1,FLNA,MCOLN2,SMARCA1,C8B,FCN1,ADAMTS13,CCN5,CNTRL,SVEP1,C1QC,C1QA,F7,APOM,ADGRG2,CALML5,CD99,PROS1,EFEMP1,MMRN1,YWHAZ,ILK,MASP2,PSG4,F11,C1S,ADIPOQ,SLAIN1,ABCA13,MST1,SPARCL1,ITIH3,C4B,TGFBI,CFB,IGFALS,PAEP,RARRES2,TCAF1,UIMC1,FBN2,TSKU,JCHAIN,HPR,C1R,APOA5,ATP1A1,VIM,TTN,APOC4,CFD,IGHV3-15,IGHV3-72,SEMA3B,PLCG2,OR52B4,KNG1,ACTB,TRIM8,CDH5,B2M,FGA
GO Process	GO:0051917	Regulation of fibrinolysis	7	18	1.55	1.45e-06	CPB2,APOH,F12,KLKB1,F2,SERPINF2,F11
GO Process	GO:0034372	Very-low-density lipoprotein particle remodeling	6	9	1.78	1.51e-06	CETP,APOA1,APOE,LCAT,APOA4,APOA5
GO Process	GO:0065008	Regulation of biological quality	76	3654	0.28	1.77e-06	CPB2,CETP,APOH,SERPIND1,F9,MMP2,HSD17B1,AFM,GPLD1,LTF,APOB,IGFBP5,PROC,APOA1,TTR,APOE,F12,LYZ,VWF,SERPINA10,LCN1,LCAT,CP,KLKB1,F13A1,TCIRG1,HPX,SERPING1,CSF1R,IGF2BP1,PIP,PF4,GUCY1A1,PCSK9,FGB,F2,SERPINF2,FBN1,FBLN1,OIT3,HSP90AA1,FGG,TUBB,PRDX6,APOA4,F8,F13B,SERPINC1,F5,FLNA,ADAMTS13,F7,APOM,SOD3,PROS1,MMRN1,YWHAZ,NAV2,ILK,F11,ADIPOQ,ATP9B,FBN2,TSKU,JCHAIN,APOA5,ATP1A1,VIM,APOC4,SEMA3B,PLCG2,KNG1,ACTB,CDH5,B2M,FGA
GO Process	GO:0052547	Regulation of peptidase activity	22	446	0.65	1.95e-06	SPP2,SERPIND1,C5,LTF,SERPINA10,LCN1,CSTA,ITIH1,AHSG,SERPING1,SERPINI1,A2ML1,SERPINF2,FBLN1,SLPI,PAPLN,SERPINC1,ECM1,PROS1,ITIH3,C4B,KNG1
GO Process	GO:0031639	Plasminogen activation	6	11	1.69	3.43e-06	APOH,KLKB1,FGB,FGG,F11,FGA
GO Process	GO:0030162	Regulation of proteolysis	28	739	0.54	4.20e-06	SPP2,CPB2,SERPIND1,C5,GPLD1,LTF,APOE,F12,SERPINA10,LCN1,CSTA,KLKB1,ITIH1,AHSG,SERPING1,SERPINI1,A2ML1,F2,SERPINF2,FBLN1,SLPI,PAPLN,SERPINC1,ECM1,PROS1,ITIH3,C4B,KNG1
GO Process	GO:0051838	Cytolysis by host of symbiont cells	6	12	1.66	4.91e-06	CFHR5,CAMP,F2,CFHR1,APOL1,CFHR2
GO Process	GO:0097006	Regulation of plasma lipoprotein particle levels	9	54	1.18	4.91e-06	CETP,APOB,APOA1,APOE,LCAT,APOA4,APOM,ADIPOQ,APOA5
GO Process	GO:0030168	Platelet activation	11	97	1.01	5.03e-06	VWF,PF4,FGB,F2,FGG,FLNA,ADAMTS13,ILK,PLCG2,ACTB,FGA
GO Process	GO:0050766	Positive regulation of phagocytosis	10	75	1.08	5.36e-06	APOA1,FCN3,AHSG,FCN2,MASP1,FCN1,MASP2,C4B,CFB,PLCG2

GO Process	GO:0002250	Adaptive immune response	19	359	0.68	6.55e-06	C5,TCIRG1,SERPING1,FGB,C1QB,C8A,MCOLN2,C8B,C1QC,C1QA,MASP2,C1S,C4B,JCHAIN,C1R,IGHV3-15,IGHV3-72,B2M,FGA
GO Process	GO:0002526	Acute inflammatory response	10	80	1.05	9.14e-06	F12,APCS,KLKB1,AHSG,SAA4,F2,SERPINF2,F8,F7,HPR
GO Process	GO:0050764	Regulation of phagocytosis	11	104	0.98	9.28e-06	APOA1,FCN3,AHSG,FCN2,MASP1,FCN1,MASP2,ADIPOQ,C4B,CFB,PLCG2
GO Process	GO:0080134	Regulation of response to stress	39	1373	0.41	1.11e-05	CPB2,APOH,PROC,APOA1,APOE,F12,APCS,PSMA6,KLKB1,HPX,FCN3,AHSG,SERPING1,FCN2,PGLYRP2,MASP1,FGB,F2,SERPINF2,P4HB,HSP90AA1,FGG,CFH,SERPINC1,HTRA1,FLNA,FCN1,F7,PROS1,MMRN1,MASP2,F11,ADIPOQ,UIMC1,PLCG2,KNG1,ACTB,CDH5,FGA
GO Process	GO:0033344	Cholesterol efflux	7	27	1.37	1.17e-05	APOB,APOA1,APOE,APOA4,APOM,TSKU,APOA5
GO Process	GO:0051702	Biological process involved in interaction with symbiont	11	108	0.97	1.25e-05	LTF,APOE,APCS,CFHR5,CSF1R,PF4,CAMP,F2,CFHR1,APOL1,CFHR2
GO Process	GO:0034375	High-density lipoprotein particle remodeling	6	16	1.53	1.66e-05	CETP,APOA1,APOE,LCAT,APOA4,APOM
GO Process	GO:0051873	Killing by host of symbiont cells	7	29	1.34	1.71e-05	CFHR5,PF4,CAMP,F2,CFHR1,APOL1,CFHR2
GO Process	GO:0030301	Cholesterol transport	9	65	1.1	1.76e-05	CETP,APOB,APOA1,APOE,LCAT,APOA4,APOM,TSKU,APOA5
GO Process	GO:0042632	Cholesterol homeostasis	10	89	1.01	2.03e-05	CETP,APOB,APOA1,APOE,LCAT,PCSK9,APOA4,APOM,TSKU,APOA5
GO Process	GO:0007155	Cell adhesion	31	965	0.46	2.10e-05	VWF,ATRN,NID1,CSTA,CDH11,HABP2,FGB,DST,STAB1,PSG11,FBN1,FGG,THBS4,APOA4,COL18A1,PCDH18,ADAM12,FLNA,ADAMTS13,CCN5,SVEP1,CD99,MMRN1,ILK,PSG2,SPARCL1,TGFBI,IGFALS,ACTB,CDH5,FGA
GO Process	GO:0051246	Regulation of protein metabolic process	58	2622	0.3	2.18e-05	SPP2,CPB2,SERPIND1,C5,GPLD1,LTF,IGFBP5,APOA1,DHX29,APOE,F12,APCS,SERPINA10,LCN1,CSTA,KLKB1,PPP2R3A,CENPE,HPX,ITIH1,AHSG,SERPING1,CSF1R,IGF2BP1,SERPINI1,CAMP,A2ML1,PCSK9,F2,INHBC,CSPG4,SERPINF2,FBN1,FBLN1,HSP90AA1,THBS4,SLPI,APOD,PAPLN,BLM,SERPINC1,ECM1,FLNA,PROS1,ILK,ADIPOQ,MST1,ITIH3,C4B,PAEP,RARRES2,VIM,TTN,PLCG2,KNG1,ACTB,CDH5,FGA
GO Process	GO:0019538	Protein metabolic process	76	3910	0.25	2.40e-05	CPB2,APOH,F9,MMP2,GPLD1,LTF,APOB,PROC,APOA1,CAT,DHX29,APOE,F12,PSMA6,LCN1,LCAT,CSTA,KLKB1,F13A1,PPP2R3A,TCIRG1,HPX,FCN3,HABP2,CSF1R,PIP,FCN2,MASP1,APEH,PCSK9,BMP1,FGB,F2,UBE3C,SUZ12,APOL1,KDM3B,P4HB,PAPPA,FBLN1,HSP90AA1,FGG,QPCT,APOA4,CNDP1,F8,F13B,CFH,F5,ADAM12,HTRA1,FLNA,FCN1,ADAMTS13,F7,APOM,EFEMP1,YWHAZ,ILK,MASP2,F11,C1S,PCYOX1,MST1,CFB,UIMC1,HPR,C1R,APOA5,TTN,CFD,PAMR1,ACTB,TRIM8,B2M,FGA
GO Process	GO:0043691	Reverse cholesterol transport	6	18	1.48	2.69e-05	CETP,APOA1,APOE,LCAT,APOA4,APOM
GO Process	GO:0044092	Negative regulation of molecular function	34	1143	0.43	2.71e-05	SPP2,SERPIND1,C5,LTF,APOA1,CAT,APOE,APCS,CFHR5,SERPINA10,LCN1,CSTA,ITIH1,AHSG,SERPING1,SERPINI1,A2ML1,SLC27A4,PCSK9,CFHR1,SERPINF2,SLPI,PAPLN,CFHR2,SERPINC1,ECM1,FLNA,PROS1,ADIPOQ,ITIH3,C4B,KNG1,ACTB,B2M
GO Process	GO:0008203	Cholesterol metabolic process	11	119	0.92	2.76e-05	CETP,APOB,APOA1,CAT,APOE,LCAT,PCSK9,APOL1,APOA4,TSKU,APOA5
GO Process	GO:0048519	Negative regulation of biological process	94	5313	0.21	2.79e-05	SPP2,CPB2,CETP,APOH,SERPIND1,MMP2,C5,GPLD1,LTF,IGFBP5,PROC,APOA1,CAT,APOE,F12,APCS,SERPINA10,LCN1,CSTA,KLKB1,PPP2R3A,PON3,IGFBP4,FCN3,ITIH1,AHSG,IGFBP1,SERPING1,CSF1R,IGF2BP1,PIP,PGLYRP2,SCGB3A1,SERPINI1,PF4,MASP1,GUCY1A1,A2ML1,SLC27A4,PCSK9,FGB,F2,EFEMP2,PRG2,STAB1,SUZ12,SERPINF2,FBN1,FBLN1,FGG,THBS4,SLPI,APOD,PAPLN,BLM,APOA4,COL18A1,CENPF,QSOX1,SERPINC1,HTRA1,ECM1,FLNA,MCOLN2,FCN1,CCN5,C1QC,APOM,PROS1,EFEMP1,MMRN1,YWHAZ,ILK,F11,ADIPOQ,MST1,ITIH3,C4B,TGFBI,PAEP,TCAF1,UIMC1,FBN2,TSKU,ATP1A1,VIM,SEMA3B,PLCG2,KNG1,ACTB,TRIM8,CDH5,B2M,FGA
GO Process	GO:0051248	Negative regulation of protein metabolic process	32	1038	0.45	2.94e-05	SPP2,CPB2,SERPIND1,C5,LTF,IGFBP5,APOE,APCS,SERPINA10,LCN1,CSTA,ITIH1,AHSG,SERPING1,IGF2BP1,SERPINI1,A2ML1,F2,SERPINF2,FBLN1,SLPI,APOD,PAPLN,SERPINC1,ECM1,FLNA,PROS1,ADIPOQ,ITIH3,C4B,KNG1,FGA
GO Process	GO:0002752	Cell surface pattern recognition receptor signaling pathway	5	9	1.7	3.88e-05	FCN3,FCN2,MASP1,FCN1,MASP2
GO Process	GO:0006869	Lipid transport	17	329	0.67	3.88e-05	CETP,APOH,APOB,APOA1,APOE,LCAT,SLC27A4,APOL1,APOD,APOA4,CFHR4,APOM,ATP9B,ABCA13,TSKU,APOA5,APOC4
GO Process	GO:0001867	Complement activation, lectin pathway	5	10	1.66	5.64e-05	FCN3,FCN2,MASP1,FCN1,MASP2
GO Process	GO:0051050	Positive regulation of transport	29	915	0.46	6.47e-05	CPB2,CETP,GPLD1,APOA1,APOE,FCN3,AHSG,FCN2,MASP1,PCSK9,FGB,F2,HSP90AA1,FGG,MTCL1,FLNA,FCN1,MASP2,ADIPOQ,ABCA13,C4B,CFB,TCAF1,APOA5,TTN,PLCG2,ACTB,B2M,FGA
GO Process	GO:0034377	Plasma lipoprotein particle assembly	6	23	1.37	8.28e-05	APOB,APOA1,APOE,APOA4,APOM,APOA5
GO Process	GO:0043086	Negative regulation of catalytic activity	26	771	0.49	8.55e-05	SPP2,SERPIND1,C5,LTF,APOA1,APOE,APCS,SERPINA10,LCN1,CSTA,ITIH1,AHSG,SERPING1,SERPINI1,A2ML1,SLC27A4,SERPINF2,SLPI,PAPLN,SERPINC1,ECM1,PROS1,ADIPOQ,ITIH3,C4B,KNG1
GO Process	GO:0034380	High-density lipoprotein particle assembly	5	12	1.58	0.00011	APOA1,APOE,APOA4,APOM,APOA5
GO Process	GO:1900024	Regulation of substrate adhesion-dependent cell spreading	8	61	1.08	0.00011	APOA1,FGB,P4HB,FBLN1,FGG,FLNA,ILK,FGA
GO Process	GO:0032103	Positive regulation of response to external stimulus	19	453	0.58	0.00014	CPB2,APOH,HPX,FCN3,CSF1R,FCN2,MASP1,F2,SERPINF2,FBN1,HSP90AA1,THBS4,S100A7,FLNA,FCN1,F7,MASP2,RARRES2,PLCG2
GO Process	GO:0032374	Regulation of cholesterol transport	8	64	1.05	0.00015	CETP,APOA1,APOE,PCSK9,APOA4,ADIPOQ,ABCA13,APOA5
GO Process	GO:1900026	Positive regulation of substrate adhesion-dependent cell spreading	7	45	1.15	0.00018	APOA1,FGB,P4HB,FGG,FLNA,ILK,FGA
GO Process	GO:0030194	Positive regulation of blood coagulation	6	28	1.29	0.00020	CPB2,APOH,F12,F2,SERPINF2,F7
GO Process	GO:0042157	Lipoprotein metabolic process	10	123	0.87	0.00025	GPLD1,APOB,APOA1,APOE,LCAT,PCSK9,APOL1,APOA4,APOM,APOA5
GO Process	GO:0010811	Positive regulation of cell-substrate adhesion	10	128	0.85	0.00034	APOA1,NID1,FGB,EFEMP2,P4HB,FBLN1,FGG,FLNA,ILK,FGA
GO Process	GO:0055088	Lipid homeostasis	11	161	0.79	0.00036	CETP,APOB,APOA1,APOE,LCAT,PCSK9,APOA4,APOM,TSKU,APOA5,APOC4
GO Process	GO:0002220	Innate immune response activating cell surface receptor signaling pathway	6	32	1.23	0.00038	FCN3,FCN2,MASP1,FCN1,MASP2,PLCG2
GO Process	GO:0051336	Regulation of hydrolase activity	29	1011	0.42	0.00038	SPP2,APOH,SERPIND1,C5,GPLD1,LTF,APOA1,APCS,SERPINA10,LCN1,CSTA,ITIH1,AHSG,SERPING1,SERPINI1,A2ML1,SLC27A4,SERPINF2,FBLN1,SLPI,PAPLN,APOA4,SERPINC1,ECM1,PROS1,ITIH3,C4B,APOA5,KNG1
GO Process	GO:0048585	Negative regulation of response to stimulus	39	1612	0.34	0.00039	CPB2,APOH,C5,LTF,IGFBP5,PROC,APOA1,APOE,F12,APCS,KLKB1,PPP2R3A,IGFBP4,AHSG,IGFBP1,SERPING1,PGLYRP2,PF4,MASP1,SLC27A4,FGB,F2,SERPINF2,FBN1,FBLN1,FGG,APOD,HTRA1,ECM1,PROS1,MMRN1,F11,ADIPOQ,FBN2,TSKU,SEMA3B,KNG1,CDH5,FGA
GO Process	GO:0031347	Regulation of defense response	22	638	0.5	0.00040	PROC,APOA1,APOE,F12,APCS,PSMA6,KLKB1,HPX,FCN3,AHSG,SERPING1,FCN2,PGLYRP2,MASP1,HSP90AA1,CFH,HTRA1,FCN1,MASP2,ADIPOQ,PLCG2,CDH5
GO Process	GO:0070328	Triglyceride homeostasis	6	38	1.16	0.00089	CETP,APOA1,APOE,APOA4,APOA5,APOC4
GO Process	GO:0034109	Homotypic cell-cell adhesion	7	60	1.02	0.00093	FGB,FGG,FLNA,CD99,ILK,ACTB,FGA
GO Process	GO:0043903	Regulation of biological process involved in symbiotic interaction	7	60	1.02	0.00093	LTF,APCS,FCN3,P4HB,FBLN1,FCN1,TRIM8
GO Process	GO:0008202	Steroid metabolic process	13	258	0.66	0.0010	CETP,HSD17B1,APOB,APOA1,CAT,APOE,LCAT,PCSK9,APOL1,APOA4,GC,TSKU,APOA5
GO Process	GO:0050793	Regulation of developmental process	51	2492	0.27	0.0010	CETP,APOH,C5,MGP,LTF,APOB,IGFBP5,PROC,APOA1,APOE,APCS,ATRN,NID1,TCIRG1,AHSG,CSF1R,PGLYRP2,SCGB3A1,PF4,CAMP,BMP1,FGB,F2,EFEMP2,STAB1,SUZ12,SERPINF2,FBN1,P4HB,FBLN1,FGG,THBS4,CENPF,ADAM12,ECM1,FLNA,C1QC,EFEMP1,YWHAZ,ILK,ADIPOQ,MST1,PAEP,RARRES2,FBN2,TSKU,SEMA3B,ACTB,CDH5,B2M,FGA
GO Process	GO:0051006	Positive regulation of lipoprotein lipase activity	4	9	1.61	0.0010	APOH,APOA1,APOA4,APOA5

GO Process	GO:1901564	Organonitrogen compound metabolic process	84	4981	0.18	0.0011	CPB2,CETP,APOH,F9,MMP2,GPLD1,LTF,APOB,PROC,APOA1,TTR,CAT,DHX29,APOE,F12,PSMA6,LCN1,LCAT,CSTA,KLKB1,F13A1,PPP2R3A,TCIRG1,HPX,FCN3,ITIH1,HABP2,CSF1R,PIP,FCN2,PGLYRP2,MASP1,APEH,GUCY1A1,PCSK9,BMP1,FGB,F2,UBE3C,NDUFS8,SUZ12,APOL1,KDM3B,P4HB,PAPPA,FBLN1,HSP90AA1,FGG,QPCT,APOA4,CNDP1,F8,F13B,CFH,F5,ADAM12,HTRA1,FLNA,FCN1,ADAMTS13,F7,APOM,EFEMP1,YWHAZ,ILK,MASP2,F11,C1S,PCYOX1,CKMT1A,MST1,ITIH3,CFB,UIMC1,HPR,C1R,APOA5,TTN,CFD,PAMR1,ACTB,TRIM8,B2M,FGA
GO Process	GO:0019731	Antibacterial humoral response	7	64	1.0	0.0013	LTF,CAMP,FGB,SLPI,JCHAIN,B2M,FGA
GO Process	GO:0002218	Activation of innate immune response	7	65	0.99	0.0014	FCN3,FCN2,MASP1,HSP90AA1,FCN1,MASP2,PLCG2
GO Process	GO:0003013	Circulatory system process	18	493	0.52	0.0014	MMP2,APOE,SERPING1,GUCY1A1,SLC27A4,FGB,SERPINF2,FGG,F5,SVEP1,SOD3,NAV2,ADIPOQ,ATP1A1,TTN,KNG1,CDH5,FGA
GO Process	GO:0006953	Acute-phase response	6	42	1.11	0.0014	APCS,AHSG,SAA4,F2,SERPINF2,F8
GO Process	GO:0034114	Regulation of heterotypic cell-cell adhesion	5	24	1.28	0.0015	APOA1,FGB,FGG,ADIPOQ,FGA
GO Process	GO:0034381	Plasma lipoprotein particle clearance	5	24	1.28	0.0015	APOB,APOA1,APOE,APOM,ADIPOQ
GO Process	GO:0070527	Platelet aggregation	6	43	1.1	0.0015	FGB,FGG,FLNA,ILK,ACTB,FGA
GO Process	GO:0008015	Blood circulation	16	403	0.56	0.0016	MMP2,APOE,SERPING1,GUCY1A1,FGB,SERPINF2,FGG,F5,SOD3,NAV2,ADIPOQ,ATP1A1,TTN,KNG1,CDH5,FGA
GO Process	GO:0032091	Negative regulation of protein binding	8	94	0.89	0.0016	CFHR5,PCSK9,CFHR1,SLPI,CFHR2,ADIPOQ,ACTB,B2M
GO Process	GO:0060627	Regulation of vesicle-mediated transport	19	551	0.5	0.0017	APOA1,APOE,FCN3,AHSG,FCN2,MASP1,PCSK9,FGB,FGG,FCN1,MASP2,ADIPOQ,ABCA13,C4B,CFB,APOA5,PLCG2,B2M,FGA
GO Process	GO:0006641	Triglyceride metabolic process	7	68	0.97	0.0018	CETP,APOH,APOB,CAT,APOE,PCSK9,APOA5
GO Process	GO:0006910	Phagocytosis, recognition	5	26	1.24	0.0020	FCN3,FCN2,FCN1,IGHV3-15,IGHV3-72
GO Process	GO:0030198	Extracellular matrix organization	13	278	0.63	0.0020	MMP2,NID1,LUM,BMP1,EFEMP2,SERPINF2,FBLN1,PAPLN,COL18A1,QSOX1,ADAMTSL4,ADAMTS13,TGFBI
GO Process	GO:0050829	Defense response to Gram-negative bacterium	8	97	0.87	0.0020	LTF,LYZ,FCN2,CAMP,F2,S100A7,RARRES2,B2M
GO Process	GO:0033700	Phospholipid efflux	4	12	1.48	0.0022	APOA1,APOE,APOA4,APOA5
GO Process	GO:1903036	Positive regulation of response to wounding	7	72	0.95	0.0024	CPB2,APOH,F12,F2,SERPINF2,FLNA,F7
GO Process	GO:1905952	Regulation of lipid localization	10	167	0.74	0.0024	CETP,APOB,APOA1,APOE,PCSK9,APOA4,ADIPOQ,ABCA13,APOA5,APOC4
GO Process	GO:0051240	Positive regulation of multicellular organismal process	35	1505	0.32	0.0025	CPB2,APOH,C5,GPLD1,LTF,APOE,F12,LUM,CSF1R,PF4,CAMP,BMP1,FGB,F2,PRG2,SERPINF2,HSP90AA1,FGG,ADAM12,ECM1,MCOLN2,FCN1,F7,ADIPOQ,MST1,PAEP,FBN2,TSKU,APOA5,ATP1A1,PLCG2,ACTB,CDH5,B2M,FGA
GO Process	GO:0051918	Negative regulation of fibrinolysis	4	13	1.45	0.0027	CPB2,APOH,F2,SERPINF2
GO Process	GO:0048878	Chemical homeostasis	25	904	0.4	0.0028	CPB2,CETP,LTF,APOB,IGFBP5,APOA1,APOE,LCAT,CP,TCIRG1,HPX,PCSK9,F2,FBN1,OIT3,APOA4,APOM,ADIPOQ,FBN2,TSKU,APOA5,ATP1A1,APOC4,CDH5,B2M
GO Process	GO:0001817	Regulation of cytokine production	22	739	0.43	0.0029	C5,LTF,APOA1,LUM,CSF1R,IGF2BP1,PGLYRP2,PF4,CAMP,F2,PRG2,SERPINF2,FBLN1,HSP90AA1,APOD,MCOLN2,FCN1,ADIPOQ,PAEP,TSKU,PLCG2,B2M
GO Process	GO:0001944	Vasculature development	18	527	0.49	0.0029	MMP2,GPLD1,APOB,APOE,ANGPTL6,EFEMP2,CSPG4,SERPINF2,APOD,COL18A1,S100A7,ECM1,FLNA,CNTRL,SVEP1,YWHAZ,TGFBI,CDH5
GO Process	GO:0002697	Regulation of immune effector process	15	383	0.55	0.0030	APOA1,HPX,FCN3,SERPING1,FCN2,PGLYRP2,MASP1,PRG2,CFH,FCN1,MASP2,C4B,CFB,PLCG2,B2M
GO Process	GO:0010896	Regulation of triglyceride catabolic process	4	14	1.41	0.0034	GPLD1,APOA1,APOA4,APOA5
GO Process	GO:0097746	Blood vessel diameter maintenance	9	140	0.77	0.0035	MMP2,APOE,GUCY1A1,FGB,SERPINF2,FGG,SOD3,KNG1,FGA
GO Process	GO:0001895	Retina homeostasis	7	78	0.91	0.0036	LTF,LYZ,LCN1,PIP,JCHAIN,ACTB,B2M
GO Process	GO:0044403	Biological process involved in symbiotic interaction	12	257	0.63	0.0037	LTF,APOE,APCS,CFHR5,CSF1R,PGLYRP2,PF4,CAMP,F2,CFHR1,APOL1,CFHR2
GO Process	GO:0010810	Regulation of cell-substrate adhesion	11	217	0.66	0.0038	APOA1,NID1,FGB,EFEMP2,P4HB,FBLN1,FGG,APOD,FLNA,ILK,FGA
GO Process	GO:0010873	Positive regulation of cholesterol esterification	3	4	1.83	0.0038	APOA1,APOA4,APOA5
GO Process	GO:0051919	Positive regulation of fibrinolysis	3	4	1.83	0.0038	F12,KLKB1,F11
GO Process	GO:0048522	Positive regulation of cellular process	89	5584	0.16	0.0040	CPB2,MMP2,GPLD1,LTF,APOB,IGFBP5,PROC,APOA1,CAT,DHX29,APOE,VWF,NID1,PPP2R3A,CENPE,TCIRG1,HPX,LUM,IGFBP4,FCN3,ANKRD31,IGFBP1,CSF1R,IGF2BP1,FCN2,SCGB3A1,SERPINI1,PPBP,PF4,MASP1,CAMP,GUCY1A1,PDIA3,SLC27A4,PCSK9,FGB,F2,INHBC,EFEMP2,CSPG4,SUZ12,SERPINF2,FBN1,P4HB,FBLN1,HSP90AA1,FGG,THBS4,PRDX6,BLM,APOA4,MTCL1,S100A7,HTRA1,ADAMTSL4,ECM1,FLNA,SMARCA1,FCN1,C1QA,F7,CD99,MMRN1,ILK,MASP2,ADIPOQ,SLAIN1,ABCA13,MST1,C4B,CFB,RARRES2,TCAF1,UIMC1,FBN2,HPR,APOA5,VIM,TTN,IGHV3-15,IGHV3-72,SEMA3B,PLCG2,KNG1,ACTB,TRIM8,CDH5,B2M,FGA
GO Process	GO:0061844	Antimicrobial humoral immune response mediated by antimicrobial peptide	8	113	0.81	0.0047	LTF,PPBP,PF4,CAMP,F2,S100A7,KNG1,B2M
GO Process	GO:0046486	Glycerolipid metabolic process	14	357	0.55	0.0051	CETP,APOH,GPLD1,APOB,APOA1,CAT,APOE,LCAT,CSF1R,PCSK9,PRDX6,APOA4,APOA5,PLCG2
GO Process	GO:0001568	Blood vessel development	17	505	0.49	0.0053	MMP2,GPLD1,APOB,APOE,ANGPTL6,EFEMP2,CSPG4,SERPINF2,APOD,COL18A1,S100A7,ECM1,FLNA,CNTRL,YWHAZ,TGFBI,CDH5
GO Process	GO:0060284	Regulation of cell development	17	506	0.48	0.0054	LTF,PROC,APOA1,FGB,F2,FBN1,P4HB,FBLN1,FGG,FLNA,ILK,ADIPOQ,PAEP,SEMA3B,CDH5,B2M,FGA
GO Process	GO:0072377	Blood coagulation, common pathway	3	5	1.74	0.0058	F2,F5,FGA
GO Process	GO:0006066	Alcohol metabolic process	13	324	0.56	0.0073	CETP,APOB,APOA1,TTR,CAT,APOE,LCAT,PCSK9,APOL1,APOA4,TSKU,APOA5,PLCG2
GO Process	GO:0048514	Blood vessel morphogenesis	15	419	0.51	0.0073	MMP2,GPLD1,APOB,APOE,ANGPTL6,EFEMP2,CSPG4,SERPINF2,APOD,COL18A1,S100A7,ECM1,FLNA,YWHAZ,TGFBI
GO Process	GO:0045088	Regulation of innate immune response	11	238	0.62	0.0077	APOE,HPX,FCN3,SERPING1,FCN2,MASP1,HSP90AA1,CFH,FCN1,MASP2,PLCG2
GO Process	GO:0032376	Positive regulation of cholesterol transport	5	38	1.08	0.0081	CETP,APOA1,APOE,ADIPOQ,ABCA13
GO Process	GO:1901700	Response to oxygen-containing compound	34	1547	0.3	0.0082	CPB2,MMP2,GPLD1,APOB,IGFBP5,CAT,APOE,IGFBP1,PPBP,PF4,CAMP,SLC27A4,PCSK9,FBN1,PAPPA,HSP90AA1,SLPI,APOD,BLM,APOA4,F5,S100A7,FLNA,ADAMTS13,F7,SOD3,GPX3,ADIPOQ,RARRES2,ATP1A1,VIM,PLCG2,ACTB,B2M
GO Process	GO:0010035	Response to inorganic substance	17	532	0.46	0.0091	MMP2,HSD17B1,GPLD1,APOB,CAT,LCAT,TCIRG1,FGB,FGG,BLM,APOA4,ADAMTS13,SOD3,TTN,PLCG2,B2M,FGA
GO Process	GO:0030334	Regulation of cell migration	24	927	0.37	0.0092	APOH,MMP2,C5,GPLD1,IGFBP5,APOE,PPP2R3A,CSF1R,FBLN1,THBS4,APOD,S100A7,ECM1,FLNA,F7,CD99,ADIPOQ,MST1,RARRES2,TCAF1,FBN2,SEMA3B,PLCG2,CDH5
GO Process	GO:0048525	Negative regulation of viral process	7	94	0.83	0.0093	LTF,APCS,FCN3,FBLN1,SLPI,FCN1,TRIM8
GO Process	GO:0051093	Negative regulation of developmental process	24	933	0.37	0.0098	CETP,APOH,LTF,IGFBP5,APOE,APCS,AHSG,PGLYRP2,PF4,F2,STAB1,SUZ12,FBN1,FBLN1,THBS4,ECM1,C1QC,EFEMP1,ADIPOQ,PAEP,TSKU,SEMA3B,ACTB,B2M
GO Process	GO:0090207	Regulation of triglyceride metabolic process	5	40	1.05	0.0098	GPLD1,APOA1,APOE,APOA4,APOA5
GO Process	GO:0001906	Cell killing	8	129	0.75	0.0100	LTF,LYZ,PPBP,PF4,F2,TUBB,KNG1,B2M
GO Process	GO:0050830	Defense response to Gram-positive bacterium	8	130	0.75	0.0104	LTF,LYZ,FCN2,PGLYRP2,CAMP,RARRES2,RNASE4,B2M
GO Process	GO:0014012	Peripheral nervous system axon regeneration	3	7	1.59	0.0109	MMP2,APOD,APOA4
GO Process	GO:0034384	High-density lipoprotein particle clearance	3	7	1.59	0.0109	APOA1,APOE,APOM
GO Process	GO:0043654	Recognition of apoptotic cell	3	7	1.59	0.0109	FCN3,FCN2,FCN1

GO Process	GO:0007565	Female pregnancy	9	170	0.68	0.0116	MMP2,IGFBP5,PSG1,PSG11,PAPPA,FBLN1,PSG4,PSG2,MST1
GO Process	GO:0045595	Regulation of cell differentiation	34	1582	0.29	0.0116	CETP,LTF,APOB,IGFBP5,PROC,APOA1,APCS,NID1,TCIRG1,PGLYRP2,PF4,FGB,F2,EFEMP2,SUZ12,SERPINF2,FBN1,P4HB,FBLN1,FGG,FLNA,C1QC,EFEMP1,ILK,ADIPOQ,PAEP,RARRES2,FBN2,TSKU,SEMA3B,ACTB,CDH5,B2M,FGA
GO Process	GO:0065009	Regulation of molecular function	55	3085	0.21	0.0127	SPP2,APOH,SERPIND1,C5,GPLD1,LTF,APOA1,CAT,APOE,APCS,CFHR5,PSMA6,SERPINA10,LCN1,CSTA,PPP2R3A,CENPE,ITIH1,AHSG,SERPING1,CSF1R,SERPINI1,A2ML1,SLC27A4,PCSK9,F2,CFHR1,SUZ12,SERPINF2,FBN1,FBLN1,HSP90AA1,SLPI,PAPLN,BLM,GIT2,APOA4,CFHR2,SERPINC1,ECM1,FLNA,CALML5,PROS1,ADIPOQ,MST1,ITIH3,C4B,TCAF1,APOA5,TTN,PLCG2,KNG1,ACTB,TRIM8,B2M
GO Process	GO:0048771	Tissue remodeling	7	101	0.8	0.0133	SPP2,MMP2,IGFBP5,TCIRG1,CSPG4,THBS4,FLNA
GO Process	GO:0010628	Positive regulation of gene expression	27	1146	0.33	0.0142	C5,APOB,DHX29,F12,KLKB1,HPX,LUM,CSF1R,IGF2BP1,PIP,PF4,CAMP,PRG2,SERPINF2,FBLN1,HSP90AA1,PRDX6,MCOLN2,FCN1,ADIPOQ,MST1,PAEP,VIM,TTN,PLCG2,CDH5,B2M
GO Process	GO:0046470	Phosphatidylcholine metabolic process	6	71	0.88	0.0142	CETP,GPLD1,APOA1,LCAT,APOA4,APOA5
GO Process	GO:0019835	Cytolysis	4	23	1.2	0.0143	C5,LYZ,C8A,C8B
GO Process	GO:0046597	Negative regulation of viral entry into host cell	4	23	1.2	0.0143	APCS,FCN3,FCN1,TRIM8
GO Process	GO:0090208	Positive regulation of triglyceride metabolic process	4	23	1.2	0.0143	GPLD1,APOA1,APOA4,APOA5
GO Process	GO:1905920	Positive regulation of CoA-transferase activity	3	8	1.53	0.0143	APOA1,APOE,APOA4
GO Process	GO:0042592	Homeostatic process	31	1406	0.3	0.0144	CPB2,CETP,LTF,APOB,IGFBP5,APOA1,APOE,LYZ,LCN1,LCAT,CP,TCIRG1,HPX,PIP,PCSK9,F2,FBN1,OIT3,PRDX6,APOA4,APOM,ADIPOQ,FBN2,TSKU,JCHAIN,APOA5,ATP1A1,APOC4,ACTB,CDH5,B2M
GO Process	GO:0046596	Regulation of viral entry into host cell	5	45	1.0	0.0147	APCS,FCN3,P4HB,FCN1,TRIM8
GO Process	GO:0003018	Vascular process in circulatory system	11	262	0.58	0.0149	MMP2,APOE,GUCY1A1,SLC27A4,FGB,SERPINF2,FGG,SOD3,KNG1,CDH5,FGA
GO Process	GO:0002831	Regulation of response to biotic stimulus	13	361	0.51	0.0170	LTF,APOE,HPX,FCN3,SERPING1,FCN2,MASP1,HSP90AA1,CFH,HTRA1,FCN1,MASP2,PLCG2
GO Process	GO:0010038	Response to metal ion	13	362	0.51	0.0174	HSD17B1,GPLD1,CAT,LCAT,TCIRG1,FGB,FGG,ADAMTS13,SOD3,TTN,PLCG2,B2M,FGA
GO Process	GO:0010898	Positive regulation of triglyceride catabolic process	3	9	1.48	0.0179	APOA1,APOA4,APOA5
GO Process	GO:0045089	Positive regulation of innate immune response	8	144	0.7	0.0182	HPX,FCN3,FCN2,MASP1,HSP90AA1,FCN1,MASP2,PLCG2
GO Process	GO:1905954	Positive regulation of lipid localization	7	111	0.76	0.0212	CETP,APOB,APOA1,APOE,ADIPOQ,ABCA13,APOC4
GO Process	GO:0002685	Regulation of leukocyte migration	10	231	0.59	0.0219	C5,CSF1R,THBS4,APOD,S100A7,ECM1,F7,CD99,MST1,RARRES2
GO Process	GO:1902533	Positive regulation of intracellular signal transduction	24	997	0.34	0.0222	LTF,IGFBP5,APOA1,CAT,APOE,VWF,IGFBP4,CSF1R,GUCY1A1,FGB,F2,CSPG4,SERPINF2,HSP90AA1,FGG,S100A7,ECM1,FLNA,F7,ILK,ADIPOQ,PLCG2,TRIM8,FGA
GO Process	GO:0030300	Regulation of intestinal cholesterol absorption	3	10	1.44	0.0224	APOA1,APOA4,APOA5
GO Process	GO:1901615	Organic hydroxy compound metabolic process	15	478	0.45	0.0229	CETP,HSD17B1,APOB,APOA1,TTR,CAT,APOE,LCAT,PCSK9,APOL1,APOA4,GC,TSKU,APOA5,PLCG2
GO Process	GO:0050790	Regulation of catalytic activity	44	2370	0.23	0.0255	SPP2,APOH,SERPIND1,C5,GPLD1,LTF,APOA1,APOE,APCS,SERPINA10,LCN1,CSTA,PPP2R3A,CENPE,ITIH1,AHSG,SERPING1,CSF1R,SERPINI1,A2ML1,SLC27A4,F2,SUZ12,SERPINF2,FBN1,FBLN1,HSP90AA1,SLPI,PAPLN,BLM,GIT2,APOA4,SERPINC1,ECM1,CALML5,PROS1,ADIPOQ,MST1,ITIH3,C4B,APOA5,TTN,KNG1,ACTB
GO Process	GO:0031099	Regeneration	8	154	0.67	0.0265	CPB2,MMP2,SERPINA10,IGFBP1,APOD,APOA4,F7,APOA5
GO Process	GO:0002762	Negative regulation of myeloid leukocyte differentiation	5	53	0.93	0.0270	LTF,APCS,FBN1,C1QC,ADIPOQ
GO Process	GO:0010543	Regulation of platelet activation	5	53	0.93	0.0270	APOE,F2,FGG,FLNA,MMRN1
GO Process	GO:0048662	Negative regulation of smooth muscle cell proliferation	5	53	0.93	0.0270	IGFBP5,APOE,EFEMP2,APOD,ADIPOQ
GO Process	GO:0098609	Cell-cell adhesion	16	542	0.43	0.0270	CSTA,CDH11,FGB,PSG11,FGG,THBS4,APOA4,PCDH18,FLNA,CD99,ILK,PSG2,SPARCL1,ACTB,CDH5,FGA
GO Process	GO:0098883	Synapse pruning	3	11	1.39	0.0270	C1QB,C1QC,C1QA
GO Process	GO:1900121	Negative regulation of receptor binding	3	11	1.39	0.0270	PCSK9,ADIPOQ,B2M
GO Process	GO:0010758	Regulation of macrophage chemotaxis	4	29	1.1	0.0274	C5,CSF1R,MST1,RARRES2
GO Process	GO:0001503	Ossification	11	287	0.54	0.0277	MMP2,MGP,GPLD1,LTF,IGFBP5,CAT,TCIRG1,CDH11,AHSG,BMP1,ECM1
GO Process	GO:0002429	Immune response-activating cell surface receptor signaling pathway	9	197	0.62	0.0277	GPLD1,FCN3,FCN2,MASP1,FCN1,MASP2,IGHV3-15,IGHV3-72,PLCG2
GO Process	GO:0045940	Positive regulation of steroid metabolic process	4	30	1.08	0.0303	APOA1,APOE,APOA4,APOA5
GO Process	GO:0072359	Circulatory system development	22	901	0.35	0.0303	MMP2,GPLD1,APOB,APOE,ANGPTL6,DNAH5,EFEMP2,CSPG4,SERPINF2,FBN1,APOD,COL18A1,S100A7,ECM1,FLNA,CNTRL,SVEP1,YWHAZ,ILK,TGFBI,TTN,CDH5
GO Process	GO:0043393	Regulation of protein binding	9	201	0.61	0.0310	APOE,CFHR5,PCSK9,CFHR1,SLPI,CFHR2,ADIPOQ,ACTB,B2M
GO Process	GO:0007166	Cell surface receptor signaling pathway	39	2040	0.24	0.0311	MMP2,C5,GPLD1,IGFBP5,APOA1,PPP2R3A,HPX,FCN3,IGFBP1,CSF1R,FCN2,PPBP,PF4,MASP1,PDIA3,DST,F2,INHBC,CSPG4,P4HB,PAPPA,ASGR2,ADAM12,FLNA,FCN1,ADAMTS13,SVEP1,ADGRG2,EFEMP1,ILK,MASP2,RARRES2,VIM,CFD,IGHV3-15,IGHV3-72,SEMA3B,PLCG2,CDH5
GO Process	GO:0019732	Antifungal humoral response	3	12	1.36	0.0318	LTF,CAMP,RARRES2
GO Process	GO:0030155	Regulation of cell adhesion	20	784	0.36	0.0318	MMP2,APOA1,NID1,SERPINI1,FGB,EFEMP2,SERPINF2,P4HB,FBLN1,FGG,APOD,FLNA,MMRN1,ILK,ADIPOQ,TGFBI,KNG1,ACTB,B2M,FGA
GO Process	GO:0050792	Regulation of viral process	8	161	0.65	0.0324	LTF,APCS,FCN3,P4HB,FBLN1,SLPI,FCN1,TRIM8
GO Process	GO:0010743	Regulation of macrophage derived foam cell differentiation	4	31	1.07	0.0327	CETP,APOB,PF4,ADIPOQ
GO Process	GO:0035239	Tube morphogenesis	18	669	0.39	0.0327	MMP2,GPLD1,APOB,APOE,ANGPTL6,CSF1R,EFEMP2,CSPG4,SERPINF2,APOD,COL18A1,S100A7,ECM1,FLNA,YWHAZ,ILK,MST1,TGFBI
GO Process	GO:2000352	Negative regulation of endothelial cell apoptotic process	4	31	1.07	0.0327	FGB,FGG,CDH5,FGA
GO Process	GO:0019216	Regulation of lipid metabolic process	12	346	0.5	0.0345	GPLD1,APOB,APOA1,APOE,F2,APOD,APOA4,ADIPOQ,RARRES2,APOA5,ATP1A1,PLCG2
GO Process	GO:1904036	Negative regulation of epithelial cell apoptotic process	5	59	0.89	0.0385	FGB,FGG,MST1,CDH5,FGA
GO Process	GO:0034445	Negative regulation of plasma lipoprotein oxidation	2	2	1.96	0.0404	APOA4,APOM
GO Process	GO:0002688	Regulation of leukocyte chemotaxis	7	128	0.7	0.0405	C5,CSF1R,THBS4,S100A7,F7,MST1,RARRES2
GO Process	GO:0001501	Skeletal system development	15	513	0.42	0.0407	SPP2,MMP2,HSD17B1,MGP,GPLD1,LTF,CDH11,AHSG,BMP1,FBN1,ECM1,FLNA,TGFBI,FBN2,TSKU
GO Process	GO:0045596	Negative regulation of cell differentiation	18	685	0.38	0.0411	CETP,LTF,IGFBP5,APCS,PGLYRP2,PF4,F2,SUZ12,FBN1,FBLN1,C1QC,EFEMP1,ADIPOQ,PAEP,TSKU,SEMA3B,ACTB,B2M
GO Process	GO:0048870	Cell motility	24	1061	0.31	0.0447	MMP2,C5,GPLD1,APOB,APOA1,ATRN,DNAH5,PPBP,PF4,DST,CSPG4,DNAH8,HSP90AA1,THBS4,CFAP46,FLNA,MCOLN2,CD99,ILK,PSG2,MST1,SEMA3B,ACTB,CDH5

GO Process	GO:0048856	Anatomical structure development	78	5117	0.14	0.0456	SPP2,CPB2,MMP2,C5,HSD17B1,MGP,GPLD1,LTF,APOB,IGFBP5,APOA1,CAT,APOE,ANGPTL6,SERPINA10,ATRN,NID1,CSTA,PPP2R3A,DNAH5,TCIRG1,CDH11,AHSG,IGFBP1,SEC24D,CSF1R,IGF2BP1,SERPINI1,SLC27A4,PCSK9,BMP1,EFEMP2,CSPG4,C1QB,SUZ12,SERPINF2,FBN1,FBLN1,HSP90AA1,FGG,TUBB,APOD,GIT2,APOA4,COL18A1,PCDH18,CENPF,ADAM12,S100A7,HTRA1,ADAMTSL4,ECM1,FLNA,CRTAC1,SMARCA1,CNTRL,SVEP1,C1QA,F7,CALML5,EFEMP1,YWHAZ,NAV2,ILK,MST1,SPARCL1,TGFBI,RARRES2,FBN2,TSKU,APOA5,VIM,TTN,SEMA3B,PLCG2,ACTB,CDH5,B2M
GO Process	GO:0050994	Regulation of lipid catabolic process	5	62	0.86	0.0460	GPLD1,APOA1,APOA4,RARRES2,APOA5
GO Process	GO:0002699	Positive regulation of immune effector process	10	264	0.54	0.0492	HPX,FCN3,FCN2,MASP1,FCN1,MASP2,C4B,CFB,PLCG2,B2M
GO Process	GO:0035295	Tube development	21	880	0.34	0.0492	MMP2,GPLD1,APOB,IGFBP5,CAT,APOE,ANGPTL6,CSF1R,EFEMP2,CSPG4,SERPINF2,APOD,COL18A1,S100A7,ECM1,FLNA,YWHAZ,ILK,MST1,TGFBI,RARRES2
GO Function	GO:0004252	Serine-type endopeptidase activity	23	176	1.07	1.58e-13	F9,MMP2,LTF,PROC,F12,KLKB1,HABP2,MASP1,APEH,PCSK9,BMP1,F2,HTRA1,F7,MASP2,F11,C1S,MST1,CFB,HPR,C1R,CFD,PAMR1
GO Function	GO:0005509	Calcium ion binding	40	717	0.7	1.86e-13	F9,MGP,PROC,F12,APCS,NID1,PPP2R3A,CDH11,ITIH1,HABP2,MASP1,LALBA,BMP1,DST,F2,EFEMP2,STAB1,FBN1,FBLN1,OIT3,THBS4,PCDH18,S100A7,CRTAC1,ADAMTS13,SVEP1,F7,CALML5,PROS1,EFEMP1,EFCAB5,MMRN1,MASP2,C1S,SPARCL1,FBN2,C1R,TTN,PAMR1,CDH5
GO Function	GO:0005539	Glycosaminoglycan binding	24	245	0.95	2.42e-12	APOH,SERPIND1,LTF,APOB,APOE,SERPINA10,ITIH1,HABP2,PGLYRP2,PF4,F2,EFEMP2,PRG2,STAB1,FBN1,THBS4,CFH,SERPINC1,CCN5,SOD3,NAV2,F11,APOA5,KNG1
GO Function	GO:0004175	Endopeptidase activity	30	437	0.79	4.19e-12	F9,MMP2,LTF,PROC,F12,PSMA6,KLKB1,HABP2,PIP,MASP1,APEH,PDIA3,PCSK9,BMP1,F2,PAPPA,PAPLN,ADAM12,HTRA1,ADAMTS13,F7,MASP2,F11,C1S,MST1,CFB,HPR,C1R,CFD,PAMR1
GO Function	GO:0008201	Heparin binding	20	173	1.02	2.37e-11	APOH,SERPIND1,LTF,APOB,APOE,SERPINA10,PF4,F2,EFEMP2,PRG2,FBN1,THBS4,CFH,SERPINC1,CCN5,SOD3,NAV2,F11,APOA5,KNG1
GO Function	GO:0004866	Endopeptidase inhibitor activity	20	177	1.01	3.07e-11	SPP2,SERPIND1,C5,LTF,SERPINA10,LCN1,CSTA,ITIH1,AHSG,SERPING1,SERPINI1,A2ML1,SERPINF2,SLPI,PAPLN,SERPINC1,PROS1,ITIH3,C4B,KNG1
GO Function	GO:0005488	Binding	190	12838	0.13	4.00e-11	CPB2,CETP,APOH,SERPIND1,TPD52L2,F9,MMP2,C5,HSD17B1,AFM,MGP,LTF,APOB,IGFBP5,PROC,APOA1,TTR,CAT,DHX29,APOE,ANGPTL6,F12,APCS,CFHR5,LYZ,VWF,PSMA6,SERPINA10,ATRN,LCN1,LCAT,NID1,CSTA,CP,KLKB1,F13A1,PPP2R3A,DNAH5,CENPE,PON3,TCIRG1,HPX,LUM,CDH11,IGFBP4,FCN3,ITIH1,IGFBP1,HABP2,SEC24D,CSF1R,IGF2BP1,PIP,FCN2,PGLYRP2,SCGB3A1,PPBP,PF4,MASP1,CAMP,APEH,GUCY1A1,A2ML1,PDIA3,SLC27A4,LALBA,PCSK9,BMP1,FGB,DST,F2,INHBC,PSG1,EFEMP2,PRG2,CSPG4,STAB1,C1QB,CFHR1,NDUFS8,SUZ12,APOL1,SERPINF2,FBN1,KDM3B,P4HB,PAPPA,FBLN1,DNAH8,OIT3,HSP90AA1,TUBA1B,FGG,TUBB,THBS4,PRDX6,SLPI,QPCT,APOD,ASGR2,BLM,GIT2,APOA4,CNDP1,COL18A1,MTCL1,F8,C8A,PCDH18,CENPF,CFHR2,CFHR4,CFHR3,CFH,QSOX1,SERPINC1,F5,ADAM12,S100A7,HTRA1,ADAMTSL4,ECM1,FLNA,CRTAC1,MCOLN2,SMARCA1,C8B,FCN1,ADAMTS13,CCN5,CNTRL,SVEP1,C1QA,F7,APOM,CA6,CALML5,SOD3,GPX3,PROS1,EFEMP1,EFCAB5,MMRN1,YWHAZ,NAV2,ILK,MASP2,PSG4,F11,C1S,PCYOX1,ADIPOQ,ATP9B,PRG4,CKMT1A,ABCA13,MST1,SPARCL1,C4B,TGFBI,CFB,IGFALS,PAEP,RARRES2,TCAF1,GC,UIMC1,FBN2,TSKU,JCHAIN,HPR,C1R,APOA5,ATP1A1,VIM,RNASE4,TTN,IGHV3-15,IGHV3-72,SEMA3B,PLCG2,PAMR1,KNG1,ACTB,TRIM66,TRIM8,CDH5,B2M,FGA,TPM3
GO Function	GO:0001848	Complement binding	11	26	1.58	5.96e-11	APCS,CFHR5,CFHR1,C8A,CFHR2,CFHR4,CFHR3,CFH,MASP2,C4B,CFB
GO Function	GO:0061134	Peptidase regulator activity	21	227	0.92	1.56e-10	SPP2,SERPIND1,C5,LTF,SERPINA10,LCN1,CSTA,ITIH1,AHSG,SERPING1,SERPINI1,A2ML1,SERPINF2,FBLN1,SLPI,PAPLN,SERPINC1,PROS1,ITIH3,C4B,KNG1
GO Function	GO:0008233	Peptidase activity	32	617	0.67	3.03e-10	CPB2,F9,MMP2,LTF,PROC,F12,PSMA6,KLKB1,HABP2,PIP,MASP1,APEH,PDIA3,PCSK9,BMP1,F2,PAPPA,PAPLN,CNDP1,ADAM12,HTRA1,ADAMTS13,F7,MASP2,F11,C1S,MST1,CFB,HPR,C1R,CFD,PAMR1
GO Function	GO:0005515	Protein binding	130	7242	0.21	1.73e-09	APOH,TPD52L2,MMP2,C5,HSD17B1,APOB,IGFBP5,APOA1,TTR,CAT,DHX29,APOE,ANGPTL6,F12,APCS,CFHR5,LYZ,VWF,PSMA6,LCN1,LCAT,NID1,CSTA,CP,DNAH5,CENPE,PON3,TCIRG1,CDH11,IGFBP4,FCN3,IGFBP1,SEC24D,CSF1R,PIP,FCN2,SCGB3A1,PPBP,PF4,MASP1,APEH,A2ML1,PDIA3,PCSK9,BMP1,FGB,DST,F2,INHBC,PSG1,EFEMP2,CSPG4,C1QB,CFHR1,SUZ12,SERPINF2,FBN1,P4HB,FBLN1,DNAH8,HSP90AA1,TUBA1B,FGG,TUBB,THBS4,PRDX6,SLPI,BLM,GIT2,APOA4,COL18A1,MTCL1,C8A,CENPF,CFHR2,CFHR4,CFHR3,CFH,SERPINC1,ADAM12,S100A7,HTRA1,ADAMTSL4,ECM1,FLNA,MCOLN2,SMARCA1,FCN1,ADAMTS13,CCN5,CNTRL,F7,GPX3,EFEMP1,YWHAZ,ILK,MASP2,PSG4,F11,C1S,ADIPOQ,MST1,C4B,TGFBI,CFB,IGFALS,RARRES2,TCAF1,GC,UIMC1,FBN2,TSKU,JCHAIN,HPR,C1R,APOA5,ATP1A1,VIM,TTN,IGHV3-15,IGHV3-72,SEMA3B,PLCG2,KNG1,ACTB,TRIM8,CDH5,B2M,FGA,TPM3
GO Function	GO:0005102	Signaling receptor binding	48	1499	0.46	7.96e-09	C5,APOB,APOA1,TTR,APOE,ANGPTL6,VWF,LCN1,IGFBP4,IGFBP1,FCN2,SCGB3A1,PPBP,PF4,PCSK9,BMP1,FGB,DST,F2,INHBC,FBN1,P4HB,FBLN1,HSP90AA1,FGG,TUBB,THBS4,S100A7,ECM1,FLNA,FCN1,ADAMTS13,CCN5,F7,EFEMP1,ADIPOQ,MST1,TGFBI,RARRES2,FBN2,JCHAIN,APOA5,IGHV3-15,IGHV3-72,SEMA3B,KNG1,CDH5,FGA
GO Function	GO:0001846	Opsonin binding	8	21	1.54	1.69e-07	APCS,CFHR5,CFHR1,CFHR2,CFHR4,CFHR3,CFH,MASP2
GO Function	GO:0005201	Extracellular matrix structural constituent	14	131	0.99	1.76e-07	MGP,NID1,LUM,FGB,EFEMP2,PRG2,FBN1,FBLN1,OIT3,FGG,COL18A1,TGFBI,FBN2,FGA
GO Function	GO:0042802	Identical protein binding	56	2144	0.37	2.01e-07	APOH,TPD52L2,HSD17B1,APOA1,TTR,CAT,APOE,APCS,CFHR5,LYZ,VWF,PON3,FCN3,CSF1R,PIP,FCN2,MASP1,APEH,PDIA3,BMP1,DST,EFEMP2,C1QB,CFHR1,SERPINF2,FBN1,FBLN1,HSP90AA1,FGG,PRDX6,BLM,APOA4,COL18A1,MTCL1,CENPF,CFHR2,CFH,SERPINC1,HTRA1,ADAMTSL4,FLNA,MCOLN2,GPX3,YWHAZ,MASP2,F11,C1S,ADIPOQ,TGFBI,JCHAIN,C1R,VIM,TTN,ACTB,TRIM8,B2M
GO Function	GO:0001851	Complement component C3b binding	6	11	1.69	4.11e-06	CFHR5,CFHR1,CFHR2,CFHR4,CFHR3,CFH
GO Function	GO:0004867	Serine-type endopeptidase inhibitor activity	11	98	1.01	6.74e-06	SERPIND1,SERPINA10,ITIH1,SERPING1,SERPINI1,A2ML1,SERPINF2,SLPI,PAPLN,SERPINC1,ITIH3
GO Function	GO:0043169	Cation binding	83	4335	0.24	1.14e-05	CPB2,CETP,F9,MMP2,AFM,MGP,LTF,PROC,APOA1,CAT,APOE,F12,APCS,LCN1,NID1,CP,KLKB1,F13A1,PPP2R3A,PON3,HPX,CDH11,FCN3,ITIH1,HABP2,SEC24D,FCN2,PGLYRP2,MASP1,LALBA,BMP1,DST,F2,EFEMP2,STAB1,NDUFS8,SUZ12,FBN1,KDM3B,PAPPA,FBLN1,OIT3,FGG,THBS4,QPCT,BLM,GIT2,APOA4,CNDP1,COL18A1,F8,PCDH18,F5,ADAM12,S100A7,CRTAC1,FCN1,ADAMTS13,SVEP1,F7,CA6,CALML5,SOD3,PROS1,EFEMP1,EFCAB5,MMRN1,MASP2,C1S,ATP9B,SPARCL1,UIMC1,FBN2,C1R,APOA5,ATP1A1,TTN,PAMR1,KNG1,TRIM66,TRIM8,CDH5,FGA

GO Function	GO:0043167	Ion binding	103	6033	0.19	3.53e-05	CPB2,CETP,F9,MMP2,HSD17B1,AFM,MGP,LTF,PROC,APOA1,CAT,DHX29,APOE,F12,APCS,PSMA6,LCN1,NID1,CP,KLKB1,F13A1,PPP2R3A,DNAH5,CENPE,PON3,HPX,CDH11,FCN3,ITIH1,HABP2,SEC24D,CSF1R,FCN2,PGLYRP2,MASP1,GUCY1A1,LALBA,BMP1,DST,F2,EFEMP2,STAB1,NDUFS8,SUZ12,FBN1,KDM3B,PAPPA,FBLN1,DNAH8,OIT3,HSP90AA1,TUBA1B,FGG,TUBB,THBS4,QPCT,BLM,GIT2,APOA4,CNDP1,COL18A1,F8,PCDH18,QSOX1,F5,ADAM12,S100A7,CRTAC1,SMARCA1,FCN1,ADAMTS13,SVEP1,F7,CA6,CALML5,SOD3,PROS1,EFEMP1,EFCAB5,MMRN1,NAV2,ILK,MASP2,C1S,PCYOX1,ADIPOQ,ATP9B,CKMT1A,ABCA13,SPARCL1,UIMC1,FBN2,C1R,APOA5,ATP1A1,TTN,PAMR1,KNG1,ACTB,TRIM66,TRIM8,CDH5,FGA
GO Function	GO:0046872	Metal ion binding	80	4250	0.23	4.54e-05	CPB2,F9,MMP2,AFM,MGP,LTF,PROC,CAT,APOE,F12,APCS,LCN1,NID1,CP,KLKB1,F13A1,PPP2R3A,PON3,HPX,CDH11,FCN3,ITIH1,HABP2,SEC24D,FCN2,PGLYRP2,MASP1,LALBA,BMP1,DST,F2,EFEMP2,STAB1,NDUFS8,SUZ12,FBN1,KDM3B,PAPPA,FBLN1,OIT3,FGG,THBS4,QPCT,BLM,GIT2,APOA4,CNDP1,COL18A1,F8,PCDH18,F5,ADAM12,S100A7,CRTAC1,FCN1,ADAMTS13,SVEP1,F7,CA6,CALML5,SOD3,PROS1,EFEMP1,EFCAB5,MMRN1,MASP2,C1S,ATP9B,SPARCL1,UIMC1,FBN2,C1R,ATP1A1,TTN,PAMR1,KNG1,TRIM66,TRIM8,CDH5,FGA
GO Function	GO:0098772	Molecular function regulator activity	47	1960	0.34	6.02e-05	SPP2,APOH,SERPIND1,C5,GPLD1,LTF,APOA1,TTR,APOE,SERPINA10,LCN1,CSTA,PPP2R3A,ITIH1,AHSG,SERPING1,SCGB3A1,SERPINI1,PPBP,PF4,A2ML1,PCSK9,BMP1,F2,INHBC,SUZ12,SERPINF2,FBN1,FBLN1,HSP90AA1,THBS4,SLPI,PAPLN,GIT2,APOA4,SERPINC1,FLNA,CALML5,PROS1,EFEMP1,ADIPOQ,ITIH3,C4B,FBN2,APOA5,SEMA3B,KNG1
GO Function	GO:0005319	Lipid transporter activity	12	163	0.82	8.98e-05	CETP,APOB,APOA1,APOE,SLC27A4,APOD,APOA4,CFHR4,APOM,ATP9B,ABCA13,APOC4
GO Function	GO:0060228	Phosphatidylcholine-sterol O-acyltransferase activator activity	4	6	1.78	0.00047	APOA1,APOE,APOA4,APOA5
GO Function	GO:0016787	Hydrolase activity	50	2347	0.29	0.00064	CPB2,APMAP,F9,MMP2,GPLD1,LTF,PROC,CAT,DHX29,F12,LYZ,PSMA6,LCAT,KLKB1,PON3,HABP2,PIP,PGLYRP2,MASP1,APEH,PDIA3,LALBA,PCSK9,BMP1,F2,PAPPA,HSP90AA1,PRDX6,PAPLN,BLM,CNDP1,ADAM12,HTRA1,SMARCA1,ADAMTS13,F7,NAV2,MASP2,F11,C1S,ATP9B,MST1,CFB,HPR,C1R,ATP1A1,RNASE4,CFD,PLCG2,PAMR1
GO Function	GO:0044877	Protein-containing complex binding	33	1261	0.38	0.00069	APOA1,TTR,DHX29,APOE,APCS,VWF,NID1,LUM,PIP,PCSK9,DST,STAB1,FBN1,P4HB,FBLN1,HSP90AA1,TUBB,THBS4,C8A,CENPF,FLNA,SMARCA1,C8B,ADAMTS13,CCN5,SPARCL1,TGFBI,JCHAIN,VIM,TTN,CDH5,B2M,TPM3
GO Function	GO:0120020	Cholesterol transfer activity	5	23	1.3	0.0018	CETP,APOB,APOA1,APOE,APOA4
GO Function	GO:0097367	Carbohydrate derivative binding	47	2278	0.27	0.0027	APOH,SERPIND1,LTF,APOB,DHX29,APOE,SERPINA10,NID1,DNAH5,CENPE,ITIH1,HABP2,CSF1R,FCN2,PGLYRP2,PF4,CAMP,GUCY1A1,F2,EFEMP2,PRG2,STAB1,FBN1,DNAH8,HSP90AA1,TUBA1B,TUBB,THBS4,BLM,CFH,SERPINC1,SMARCA1,FCN1,CCN5,SOD3,NAV2,ILK,F11,ADIPOQ,ATP9B,CKMT1A,ABCA13,APOA5,ATP1A1,TTN,KNG1,ACTB
GO Function	GO:0030234	Enzyme regulator activity	31	1239	0.36	0.0029	SPP2,APOH,SERPIND1,C5,LTF,APOA1,APOE,SERPINA10,LCN1,CSTA,PPP2R3A,ITIH1,AHSG,SERPING1,SERPINI1,A2ML1,SUZ12,SERPINF2,FBLN1,HSP90AA1,SLPI,PAPLN,GIT2,APOA4,SERPINC1,CALML5,PROS1,ITIH3,C4B,APOA5,KNG1
GO Function	GO:0005496	Steroid binding	8	101	0.86	0.0035	CETP,HSD17B1,APOA1,APOD,APOA4,GC,APOA5,ATP1A1
GO Function	GO:0016209	Antioxidant activity	7	76	0.92	0.0045	CAT,APOE,PRDX6,APOA4,APOM,SOD3,GPX3
GO Function	GO:0070325	Lipoprotein particle receptor binding	5	30	1.18	0.0047	APOB,APOA1,APOE,PCSK9,APOA5
GO Function	GO:0042803	Protein homodimerization activity	21	709	0.43	0.0066	TPD52L2,HSD17B1,APOA1,CAT,APOE,PON3,CSF1R,MASP1,DST,EFEMP2,SERPINF2,HSP90AA1,BLM,APOA4,MTCL1,CENPF,FLNA,ADIPOQ,JCHAIN,TRIM8,B2M
GO Function	GO:0034987	Immunoglobulin receptor binding	4	16	1.36	0.0071	FLNA,JCHAIN,IGHV3-15,IGHV3-72
GO Function	GO:0005520	Insulin-like growth factor binding	4	17	1.33	0.0085	IGFBP5,IGFBP4,IGFBP1,IGFALS
GO Function	GO:0005178	Integrin binding	9	159	0.71	0.0111	VWF,DST,FBN1,P4HB,FBLN1,THBS4,ADAMTS13,CCN5,TGFBI
GO Function	GO:0140096	Catalytic activity, acting on a protein	44	2279	0.24	0.0189	CPB2,F9,MMP2,LTF,PROC,F12,PSMA6,KLKB1,F13A1,HABP2,CSF1R,PIP,MASP1,APEH,PDIA3,PCSK9,BMP1,F2,UBE3C,STAB1,SUZ12,KDM3B,P4HB,PAPPA,QPCT,PAPLN,CNDP1,QSOX1,ADAM12,HTRA1,ADAMTS13,F7,EFEMP1,ILK,MASP2,F11,C1S,MST1,CFB,HPR,C1R,TTN,CFD,PAMR1
GO Function	GO:0002020	Protease binding	8	135	0.73	0.0193	VWF,CSTA,A2ML1,SERPINF2,SERPINC1,ADAMTSL4,ECM1,TTN
GO Function	GO:0005198	Structural molecule activity	21	776	0.39	0.0193	MGP,APOE,NID1,LUM,FGB,DST,EFEMP2,PRG2,FBN1,FBLN1,OIT3,TUBA1B,FGG,TUBB,COL18A1,TGFBI,FBN2,VIM,TTN,ACTB,FGA
GO Function	GO:0003823	Antigen binding	6	71	0.88	0.0204	FCN3,FCN2,FCN1,JCHAIN,IGHV3-15,IGHV3-72
GO Function	GO:0031995	Insulin-like growth factor II binding	3	8	1.53	0.0204	IGFBP5,IGFBP4,IGFBP1
GO Function	GO:0097493	Structural molecule activity conferring elasticity	3	8	1.53	0.0204	FBN1,FBN2,TTN
GO Function	GO:0050750	Low-density lipoprotein particle receptor binding	4	25	1.16	0.0259	APOB,APOE,PCSK9,APOA5
GO Function	GO:0031994	Insulin-like growth factor I binding	3	10	1.44	0.0319	IGFBP5,IGFBP4,IGFBP1
GO Function	GO:0015485	Cholesterol binding	5	52	0.94	0.0360	CETP,APOA1,APOD,APOA4,APOA5
GO Function	GO:0071813	Lipoprotein particle binding	4	28	1.11	0.0360	APOA1,APOE,PCSK9,STAB1
GO Function	GO:0016670	Oxidoreductase activity, acting on a sulfur group of donors, oxygen as acceptor	3	11	1.39	0.0374	P4HB,QSOX1,PCYOX1
GO Function	GO:0031210	Phosphatidylcholine binding	4	29	1.1	0.0379	CETP,APOA1,APOA4,APOA5
GO Function	GO:0004869	Cysteine-type endopeptidase inhibitor activity	5	54	0.92	0.0387	LTF,LCN1,CSTA,AHSG,KNG1
GO Function	GO:0030545	Signaling receptor regulator activity	16	552	0.42	0.0434	C5,APOA1,TTR,SCGB3A1,PPBP,PF4,PCSK9,BMP1,F2,INHBC,FBN1,THBS4,EFEMP1,ADIPOQ,FBN2,SEMA3B
GO Function	GO:0048018	Receptor ligand activity	15	499	0.44	0.0444	C5,APOA1,TTR,SCGB3A1,PPBP,PF4,BMP1,F2,INHBC,FBN1,THBS4,EFEMP1,ADIPOQ,FBN2,SEMA3B
GO Function	GO:0050839	Cell adhesion molecule binding	16	560	0.41	0.0488	DHX29,VWF,CDH11,DST,FBN1,P4HB,FBLN1,FGG,THBS4,PRDX6,FLNA,ADAMTS13,CCN5,YWHAZ,TGFBI,CDH5
GO Component	GO:0005615	Extracellular space	155	3247	0.64	2.63e-67	CPB2,CETP,APOH,SERPIND1,F9,MMP2,C5,AFM,MGP,GPLD1,LTF,APOB,IGFBP5,PROC,APOA1,TTR,CAT,APOE,ANGPTL6,F12,APCS,CFHR5,LYZ,VWF,PSMA6,SERPINA10,ATRN,LCN1,LCAT,NID1,CSTA,CP,KLKB1,F13A1,PON3,HPX,LUM,CDH11,IGFBP4,FCN3,ITIH1,AHSG,IGFBP1,HABP2,SAA4,SERPING1,PIP,FCN2,PGLYRP2,SCGB3A1,SERPINI1,PPBP,PF4,MASP1,CAMP,APEH,A2ML1,PDIA3,LALBA,PCSK9,BMP1,FGB,F2,INHBC,EFEMP2,PRG2,CSPG4,C1QB,CFHR1,APOL1,SERPINF2,FBN1,P4HB,PAPPA,FBLN1,OIT3,HSP90AA1,FGG,TUBB,THBS4,PRDX6,SLPI,QPCT,APOD,APOA4,COL18A1,MTCL1,F8,C8A,F13B,CFHR2,CFHR4,CFHR3,CFH,QSOX1,SERPINC1,F5,S100A7,HTRA1,ECM1,FLNA,CRTAC1,C8B,FCN1,ADAMTS13,CCN5,SVEP1,C1QC,C1QA,F7,APOM,CA6,ADGRG2,SOD3,GPX3,PROS1,EFEMP1,YWHAZ,MASP2,F11,C1S,PCYOX1,ADIPOQ,PRG4,MST1,SPARCL1,ITIH3,C4B,TGFBI,CFB,IGFALS,PAEP,RARRES2,GC,FBN2,TSKU,JCHAIN,HPR,C1R,APOA5,ATP1A1,VIM,RNASE4,TTN,APOC4,CFD,IGHV3-15,IGHV3-72,SEMA3B,PLCG2,KNG1,ACTB,B2M,FGA,TPM3

GO Component	GO:0005576	Extracellular region	169	4175	0.57	6.46e-67	SPP2,CPB2,CETP,APOH,SERPIND1,F9,MMP2,C5,AFM,MGP,GPLD1,LTF,APOB,IGFBP5,PROC,APOA1,TTR,CAT,APOE,ANGPTL6,F12,APCS,CFHR5,LYZ,VWF,PSMA6,SERPINA10,ATRN,LCN1,LCAT,NID1,CSTA,CP,KLKB1,F13A1,DNAH5,CENPE,PON3,HPX,LUM,CDH11,IGFBP4,FCN3,ITIH1,AHSG,IGFBP1,HABP2,SAA4,SERPING1,PIP,FCN2,PGLYRP2,SCGB3A1,SERPINI1,PPBP,PF4,MASP1,CAMP,APEH,A2ML1,PDIA3,LALBA,PCSK9,BMP1,FGB,F2,INHBC,PSG1,EFEMP2,PRG2,CSPG4,C1QB,CFHR1,APOL1,PSG11,SERPINF2,FBN1,P4HB,PAPPA,FBLN1,OIT3,HSP90AA1,FGG,TUBB,THBS4,PRDX6,SLPI,QPCT,APOD,PAPLN,APOA4,CNDP1,COL18A1,MTCL1,F8,C8A,F13B,CFHR2,CFHR4,CFHR3,CFH,QSOX1,SERPINC1,F5,ADAM12,S100A7,HTRA1,ADAMTSL4,ECM1,FLNA,CRTAC1,C8B,FCN1,ADAMTS13,CCN5,SVEP1,C1QC,C1QA,F7,APOM,CA6,ADGRG2,CALML5,SOD3,GPX3,PROS1,EFEMP1,MMRN1,YWHAZ,MASP2,PSG4,F11,C1S,PSG2,PCYOX1,ADIPOQ,PRG4,MST1,SPARCL1,ITIH3,C4B,TGFBI,CFB,IGFALS,PAEP,RARRES2,GC,FBN2,TSKU,JCHAIN,HPR,C1R,APOA5,ATP1A1,VIM,RNASE4,TTN,APOC4,CFD,IGHV3-15,IGHV3-72,SEMA3B,PLCG2,PAMR1,KNG1,ACTB,B2M,FGA,TPM3
GO Component	GO:0062023	Collagen-containing extracellular matrix	61	407	1.13	1.27e-45	SPP2,APOH,F9,MMP2,MGP,APOA1,APOE,ANGPTL6,F12,APCS,VWF,ATRN,NID1,F13A1,HPX,LUM,FCN3,ITIH1,AHSG,SERPING1,FCN2,PF4,FGB,DST,F2,EFEMP2,PRG2,CSPG4,C1QB,SERPINF2,FBN1,FBLN1,HSP90AA1,FGG,THBS4,SLPI,APOA4,COL18A1,SERPINC1,S100A7,HTRA1,ADAMTSL4,ECM1,FCN1,C1QC,C1QA,F7,SOD3,EFEMP1,MMRN1,NAV2,ADIPOQ,PRG4,MST1,SPARCL1,TGFBI,RARRES2,FBN2,SEMA3B,KNG1,FGA
GO Component	GO:0031012	Extracellular matrix	66	552	1.04	3.08e-44	SPP2,APOH,F9,MMP2,MGP,GPLD1,APOA1,APOE,ANGPTL6,F12,APCS,VWF,ATRN,NID1,F13A1,HPX,LUM,FCN3,ITIH1,AHSG,SERPING1,FCN2,PF4,FGB,DST,F2,EFEMP2,PRG2,CSPG4,C1QB,SERPINF2,FBN1,FBLN1,HSP90AA1,FGG,THBS4,SLPI,PAPLN,APOA4,COL18A1,SERPINC1,S100A7,HTRA1,ADAMTSL4,ECM1,FCN1,ADAMTS13,CCN5,C1QC,C1QA,F7,SOD3,EFEMP1,MMRN1,NAV2,ADIPOQ,PRG4,MST1,SPARCL1,TGFBI,IGFALS,RARRES2,FBN2,SEMA3B,KNG1,FGA
GO Component	GO:1903561	Extracellular vesicle	102	2120	0.64	5.68e-38	CPB2,CETP,APOH,SERPIND1,F9,C5,AFM,MGP,GPLD1,LTF,APOB,APOA1,TTR,CAT,APOE,ANGPTL6,F12,APCS,LYZ,VWF,PSMA6,SERPINA10,ATRN,LCAT,NID1,CP,KLKB1,PON3,HPX,LUM,CDH11,ITIH1,AHSG,SAA4,SERPING1,PIP,FCN2,PGLYRP2,SCGB3A1,SERPINI1,CAMP,APEH,A2ML1,PDIA3,FGB,F2,EFEMP2,PRG2,CSPG4,SERPINF2,P4HB,FBLN1,HSP90AA1,FGG,TUBB,THBS4,PRDX6,SLPI,QPCT,APOD,APOA4,COL18A1,C8A,CFHR3,CFH,QSOX1,SERPINC1,F5,HTRA1,ECM1,FLNA,CRTAC1,C8B,CA6,ADGRG2,SOD3,GPX3,PROS1,EFEMP1,YWHAZ,MASP2,F11,PCYOX1,ITIH3,C4B,TGFBI,CFB,IGFALS,GC,JCHAIN,HPR,C1R,ATP1A1,VIM,TTN,CFD,PLCG2,KNG1,ACTB,B2M,FGA,TPM3
GO Component	GO:0070062	Extracellular exosome	101	2096	0.64	1.01e-37	CPB2,CETP,APOH,SERPIND1,F9,C5,AFM,MGP,GPLD1,LTF,APOB,APOA1,TTR,CAT,APOE,ANGPTL6,F12,APCS,LYZ,VWF,PSMA6,SERPINA10,ATRN,LCAT,NID1,CP,KLKB1,PON3,HPX,LUM,CDH11,ITIH1,AHSG,SAA4,SERPING1,PIP,FCN2,PGLYRP2,SCGB3A1,SERPINI1,CAMP,APEH,A2ML1,PDIA3,FGB,F2,EFEMP2,PRG2,CSPG4,SERPINF2,P4HB,FBLN1,HSP90AA1,FGG,TUBB,THBS4,PRDX6,SLPI,QPCT,APOD,APOA4,COL18A1,C8A,CFHR3,CFH,QSOX1,SERPINC1,HTRA1,ECM1,FLNA,CRTAC1,C8B,CA6,ADGRG2,SOD3,GPX3,PROS1,EFEMP1,YWHAZ,MASP2,F11,PCYOX1,ITIH3,C4B,TGFBI,CFB,IGFALS,GC,JCHAIN,HPR,C1R,ATP1A1,VIM,TTN,CFD,PLCG2,KNG1,ACTB,B2M,FGA,TPM3
GO Component	GO:0072562	Blood microparticle	37	118	1.45	5.08e-37	AFM,APOA1,APOE,APCS,CP,F13A1,HPX,FCN3,ITIH1,AHSG,SERPING1,FCN2,FGB,F2,C1QB,CFHR1,APOL1,SERPINF2,FGG,APOA4,C8A,CFHR3,CFH,SERPINC1,C1QC,PROS1,YWHAZ,C1S,C4B,CFB,GC,JCHAIN,HPR,C1R,KNG1,ACTB,FGA
GO Component	GO:0031982	Vesicle	123	3957	0.45	1.30e-29	SPP2,CPB2,CETP,APOH,SERPIND1,F9,C5,AFM,MGP,GPLD1,LTF,APOB,APOA1,TTR,CAT,APOE,ANGPTL6,F12,APCS,LYZ,VWF,PSMA6,SERPINA10,ATRN,LCAT,NID1,CP,KLKB1,F13A1,PON3,TCIRG1,HPX,LUM,CDH11,ITIH1,AHSG,SAA4,SERPING1,SEC24D,PIP,FCN2,PGLYRP2,SCGB3A1,SERPINI1,PPBP,PF4,CAMP,APEH,A2ML1,PDIA3,PCSK9,BMP1,FGB,DST,F2,EFEMP2,PRG2,CSPG4,STAB1,SERPINF2,P4HB,FBLN1,HSP90AA1,FGG,TUBB,THBS4,PRDX6,SLPI,QPCT,APOD,APOA4,COL18A1,F8,C8A,CFHR3,CFH,QSOX1,SERPINC1,F5,S100A7,HTRA1,ECM1,FLNA,CRTAC1,MCOLN2,C8B,FCN1,F7,CA6,ADGRG2,CALML5,SOD3,GPX3,PROS1,EFEMP1,MMRN1,YWHAZ,MASP2,F11,PCYOX1,ATP9B,ABCA13,ITIH3,C4B,TGFBI,CFB,IGFALS,RARRES2,GC,JCHAIN,HPR,C1R,APOA5,ATP1A1,VIM,TTN,CFD,PLCG2,KNG1,ACTB,B2M,FGA,TPM3
GO Component	GO:0031983	Vesicle lumen	38	326	1.02	4.41e-24	SPP2,APOH,LTF,APOB,APOA1,TTR,CAT,LYZ,VWF,F13A1,AHSG,SERPING1,SERPINI1,PPBP,PF4,CAMP,FGB,SERPINF2,HSP90AA1,FGG,TUBB,PRDX6,SLPI,QPCT,F8,QSOX1,F5,S100A7,ECM1,FCN1,PROS1,MMRN1,ITIH3,RARRES2,CFD,KNG1,B2M,FGA
GO Component	GO:0034774	Secretory granule lumen	37	321	1.02	2.82e-23	SPP2,APOH,LTF,APOA1,TTR,CAT,LYZ,VWF,F13A1,AHSG,SERPING1,SERPINI1,PPBP,PF4,CAMP,FGB,SERPINF2,HSP90AA1,FGG,TUBB,PRDX6,SLPI,QPCT,F8,QSOX1,F5,S100A7,ECM1,FCN1,PROS1,MMRN1,ITIH3,RARRES2,CFD,KNG1,B2M,FGA
GO Component	GO:0005788	Endoplasmic reticulum lumen	35	312	1.01	1.26e-21	SPP2,SERPIND1,F9,APOB,IGFBP5,PROC,APOA1,APOE,SERPINA10,CP,IGFBP4,AHSG,IGFBP1,SERPING1,PDIA3,PCSK9,F2,APOL1,FBN1,P4HB,FGG,APOA4,COL18A1,F8,QSOX1,SERPINC1,F5,ADAMTSL4,ADAMTS13,F7,SPARCL1,APOA5,KNG1,B2M,FGA
GO Component	GO:1905370	Serine-type endopeptidase complex	15	26	1.72	1.40e-17	F9,PROC,F12,KLKB1,FCN3,FCN2,MASP1,F2,F8,CFH,F5,FCN1,F7,MASP2,F11
GO Component	GO:0031093	Platelet alpha granule lumen	17	66	1.37	2.81e-15	VWF,F13A1,AHSG,SERPING1,PPBP,PF4,FGB,SERPINF2,FGG,F8,QSOX1,F5,PROS1,MMRN1,CFD,KNG1,FGA
GO Component	GO:0034358	Plasma lipoprotein particle	14	38	1.52	2.88e-14	CETP,APOH,APOB,APOA1,APOE,LCAT,SAA4,APOL1,APOA4,APOM,PCYOX1,HPR,APOA5,APOC4
GO Component	GO:0030141	Secretory granule	43	873	0.65	3.13e-14	SPP2,APOH,LTF,APOA1,TTR,CAT,ANGPTL6,LYZ,VWF,F13A1,TCIRG1,AHSG,SERPING1,SERPINI1,PPBP,PF4,CAMP,APEH,FGB,PRG2,SERPINF2,HSP90AA1,FGG,TUBB,PRDX6,SLPI,QPCT,F8,QSOX1,F5,S100A7,ECM1,FCN1,CALML5,PROS1,MMRN1,ABCA13,ITIH3,RARRES2,CFD,KNG1,B2M,FGA
GO Component	GO:0034364	High-density lipoprotein particle	13	29	1.61	3.82e-14	CETP,APOH,APOB,APOA1,APOE,LCAT,SAA4,APOL1,APOA4,APOM,HPR,APOA5,APOC4
GO Component	GO:1905369	Endopeptidase complex	17	104	1.17	1.53e-12	F9,PROC,F12,PSMA6,KLKB1,FCN3,FCN2,MASP1,F2,UBE3C,F8,CFH,F5,FCN1,F7,MASP2,F11
GO Component	GO:0034361	Very-low-density lipoprotein particle	10	20	1.66	3.61e-11	APOH,APOB,APOA1,APOE,APOL1,APOA4,APOM,PCYOX1,APOA5,APOC4
GO Component	GO:0071944	Cell periphery	115	6015	0.24	4.66e-10	SPP2,APOH,F9,MMP2,C5,MGP,GPLD1,LTF,APOB,APOA1,CAT,APOE,ANGPTL6,F12,APCS,VWF,ATRN,NID1,CSTA,CP,KLKB1,F13A1,TCIRG1,HPX,LUM,CDH11,FCN3,ITIH1,AHSG,SERPING1,CSF1R,FCN2,PF4,MASP1,SLC27A4,PCSK9,FGB,DST,F2,PSG1,EFEMP2,PRG2,CSPG4,STAB1,C1QB,SERPINF2,FBN1,P4HB,FBLN1,HSP90AA1,FGG,THBS4,SLPI,PAPLN,ASGR2,APOA4,COL18A1,MTCL1,F8,C8A,PCDH18,SERPINC1,F5,ADAM12,S100A7,HTRA1,ADAMTSL4,ECM1,FLNA,MCOLN2,C8B,FCN1,ADAMTS13,CCN5,C1QC,C1QA,F7,ADGRG2,CD99,SOD3,PROS1,EFEMP1,MMRN1,NAV2,ILK,MASP2,PSG4,F11,PCYOX1,ADIPOQ,ATP9B,PRG4,ABCA13,MST1,SPARCL1,C4B,TGFBI,CFB,IGFALS,RARRES2,TCAF1,FBN2,ATP1A1,VIM,TTN,IGHV3-15,IGHV3-72,SEMA3B,PLCG2,OR52B4,KNG1,ACTB,CDH5,B2M,FGA

GO Component	GO:0097708	Intracellular vesicle	60	2484	0.34	2.13e-07	SPP2,APOH,LTF,APOB,APOA1,TTR,CAT,APOE,ANGPTL6,LYZ,VWF,F13A1,TCIRG1,HPX,AHSG,SERPING1,SEC24D,SERPINI1,PPBP,PF4,CAMP,APEH,PDIA3,PCSK9,FGB,DST,PRG2,STAB1,SERPINF2,P4HB,HSP90AA1,FGG,TUBB,PRDX6,SLPI,QPCT,APOA4,F8,QSOX1,F5,S100A7,ECM1,MCOLN2,FCN1,CALML5,PROS1,MMRN1,YWHAZ,ATP9B,ABCA13,ITIH3,RARRES2,APOA5,ATP1A1,VIM,CFD,PLCG2,KNG1,B2M,FGA
GO Component	GO:0031410	Cytoplasmic vesicle	59	2482	0.33	5.26e-07	SPP2,APOH,LTF,APOB,APOA1,TTR,CAT,APOE,ANGPTL6,LYZ,VWF,F13A1,TCIRG1,HPX,AHSG,SERPING1,SEC24D,SERPINI1,PPBP,PF4,CAMP,APEH,PDIA3,PCSK9,FGB,DST,PRG2,STAB1,SERPINF2,P4HB,HSP90AA1,FGG,TUBB,PRDX6,SLPI,QPCT,APOA4,F8,QSOX1,F5,S100A7,ECM1,MCOLN2,FCN1,CALML5,PROS1,MMRN1,YWHAZ,ATP9B,ABCA13,ITIH3,RARRES2,APOA5,ATP1A1,VIM,CFD,KNG1,B2M,FGA
GO Component	GO:0009986	Cell surface	31	894	0.5	1.58e-06	APOH,APMAP,LTF,FCN3,CSF1R,FCN2,MASP1,PDIA3,PCSK9,FGB,PSG1,CSPG4,PSG11,SERPINF2,P4HB,OIT3,HSP90AA1,FGG,ASGR2,FCN1,ADAMTS13,ADGRG2,MASP2,PSG4,PSG2,ADIPOQ,IGHV3-15,IGHV3-72,CDH5,B2M,FGA
GO Component	GO:0012505	Endomembrane system	89	4721	0.23	1.60e-06	SPP2,APOH,SERPIND1,F9,LTF,APOB,IGFBP5,PROC,APOA1,TTR,CAT,APOE,ANGPTL6,F12,LYZ,VWF,SERPINA10,CP,F13A1,TCIRG1,LUM,IGFBP4,AHSG,IGFBP1,SERPING1,SEC24D,SERPINI1,PPBP,PF4,CAMP,APEH,PDIA3,SLC27A4,LALBA,PCSK9,BMP1,FGB,DST,F2,PRG2,CSPG4,APOL1,SERPINF2,FBN1,P4HB,OIT3,HSP90AA1,FGG,TUBB,THBS4,PRDX6,SLPI,QPCT,APOD,ASGR2,APOA4,COL18A1,F8,CENPF,QSOX1,SERPINC1,F5,S100A7,ADAMTSL4,ECM1,FLNA,MCOLN2,FCN1,ADAMTS13,F7,CALML5,SOD3,PROS1,MMRN1,ADIPOQ,ATP9B,ABCA13,SPARCL1,ITIH3,TGFBI,RARRES2,APOA5,ATP1A1,CFD,SEMA3B,KNG1,CDH5,B2M,FGA
GO Component	GO:0005602	Complement component C1 complex	5	5	1.96	2.07e-06	C1QB,C1QC,C1QA,C1S,C1R
GO Component	GO:0042627	Chylomicron	6	13	1.62	2.14e-06	APOH,APOB,APOA1,APOE,APOA4,APOA5
GO Component	GO:0005783	Endoplasmic reticulum	50	2021	0.35	2.64e-06	SPP2,SERPIND1,F9,APOB,IGFBP5,PROC,APOA1,CAT,APOE,F12,VWF,SERPINA10,CP,IGFBP4,AHSG,IGFBP1,SERPING1,SEC24D,PDIA3,SLC27A4,PCSK9,FGB,DST,F2,APOL1,FBN1,P4HB,FGG,THBS4,APOD,ASGR2,APOA4,COL18A1,F8,QSOX1,SERPINC1,F5,S100A7,ADAMTSL4,ADAMTS13,F7,PROS1,ADIPOQ,SPARCL1,APOA5,ATP1A1,SEMA3B,KNG1,B2M,FGA
GO Component	GO:0032991	Protein-containing complex	96	5506	0.2	1.39e-05	CETP,APOH,F9,C5,LTF,APOB,IGFBP5,PROC,APOA1,TTR,CAT,DHX29,APOE,F12,CFHR5,PSMA6,LCAT,NID1,CSTA,KLKB1,F13A1,PPP2R3A,DNAH5,TCIRG1,LUM,CDH11,FCN3,SAA4,SEC24D,CSF1R,IGF2BP1,FCN2,MASP1,GUCY1A1,PDIA3,LALBA,PCSK9,FGB,F2,UBE3C,C1QB,CFHR1,NDUFS8,SUZ12,APOL1,SERPINF2,KDM3B,P4HB,DNAH8,HSP90AA1,FGG,TUBB,BLM,APOA4,COL18A1,F8,C8A,CENPF,F13B,CFHR2,CFH,F5,FLNA,SMARCA1,C8B,FCN1,CNTRL,C1QC,C1QA,F7,APOM,MMRN1,MASP2,F11,C1S,PCYOX1,ADIPOQ,C4B,CFB,IGFALS,UIMC1,JCHAIN,HPR,C1R,APOA5,ATP1A1,VIM,TTN,APOC4,IGHV3-15,IGHV3-72,ACTB,CDH5,B2M,FGA,TPM3
GO Component	GO:0005796	Golgi lumen	10	106	0.93	3.00e-05	F9,PROC,LUM,LALBA,F2,CSPG4,F8,F7,SOD3,PROS1
GO Component	GO:0071682	Endocytic vesicle lumen	6	23	1.37	3.08e-05	LTF,APOB,APOA1,APOE,HPX,HSP90AA1
GO Component	GO:0031089	Platelet dense granule lumen	5	14	1.51	7.52e-05	SPP2,APOH,ECM1,ITIH3,RARRES2
GO Component	GO:0005581	Collagen trimer	9	95	0.93	9.51e-05	LUM,FCN3,FCN2,C1QB,COL18A1,FCN1,C1QC,C1QA,ADIPOQ
GO Component	GO:0070013	Intracellular organelle lumen	95	5660	0.18	9.51e-05	SPP2,APOH,SERPIND1,F9,LTF,APOB,IGFBP5,PROC,APOA1,TTR,CAT,APOE,LYZ,VWF,PSMA6,SERPINA10,CSTA,CP,F13A1,HPX,LUM,IGFBP4,AHSG,IGFBP1,SERPING1,CSF1R,IGF2BP1,SERPINI1,PPBP,PF4,MASP1,CAMP,APEH,PDIA3,LALBA,PCSK9,FGB,DST,F2,PRG2,CSPG4,NDUFS8,SUZ12,APOL1,SERPINF2,FBN1,KDM3B,P4HB,HSP90AA1,FGG,TUBB,PRDX6,SLPI,QPCT,BLM,GIT2,APOA4,COL18A1,F8,CENPF,QSOX1,SERPINC1,F5,ADAM12,S100A7,ADAMTSL4,ECM1,FLNA,SMARCA1,FCN1,ADAMTS13,F7,CALML5,SOD3,PROS1,MMRN1,YWHAZ,NAV2,ILK,SPARCL1,ITIH3,RARRES2,GC,UIMC1,APOA5,VIM,TTN,CFD,KNG1,ACTB,TRIM66,TRIM8,CDH5,B2M,FGA
GO Component	GO:0005604	Basement membrane	9	98	0.92	0.00012	ATRN,NID1,DST,EFEMP2,FBN1,FBLN1,THBS4,COL18A1,TGFBI
GO Component	GO:1904724	Tertiary granule lumen	7	55	1.06	0.00021	LTF,LYZ,PPBP,CAMP,QPCT,QSOX1,B2M
GO Component	GO:0005577	Fibrinogen complex	4	8	1.66	0.00027	FGB,SERPINF2,FGG,FGA
GO Component	GO:0035580	Specific granule lumen	7	62	1.01	0.00041	LTF,LYZ,CAMP,SLPI,QPCT,QSOX1,B2M
GO Component	GO:0062167	Complement component C1q complex	3	3	1.96	0.00100	C1QB,C1QC,C1QA
GO Component	GO:0005775	Vacuolar lumen	10	175	0.71	0.0015	APOB,TTR,LYZ,LUM,CSPG4,HSP90AA1,TUBB,PRDX6,S100A7,GC
GO Component	GO:0009897	External side of plasma membrane	15	388	0.55	0.0015	FCN3,FCN2,MASP1,PCSK9,FGB,P4HB,FGG,ASGR2,FCN1,MASP2,IGHV3-15,IGHV3-72,CDH5,B2M,FGA
GO Component	GO:1904813	ficolin-1-rich granule lumen	8	124	0.77	0.0038	CAT,APEH,PRG2,HSP90AA1,QPCT,FCN1,CALML5,CFD
GO Component	GO:0005579	Membrane attack complex	3	7	1.59	0.0054	C5,C8A,C8B
GO Component	GO:0034366	Spherical high-density lipoprotein particle	3	8	1.53	0.0072	APOA1,APOM,HPR
GO Component	GO:0005925	Focal adhesion	14	416	0.49	0.0100	CAT,PDIA3,DST,CSPG4,P4HB,GIT2,S100A7,FLNA,CD99,YWHAZ,ILK,VIM,ACTB,B2M
GO Component	GO:0043227	Membrane-bounded organelle	169	13188	0.07	0.0101	SPP2,CPB2,CETP,APOH,SERPIND1,F9,MMP2,C5,AFM,MGP,GPLD1,LTF,APOB,IGFBP5,PROC,APOA1,TTR,CAT,APOE,ANGPTL6,F12,APCS,LYZ,VWF,PSMA6,SERPINA10,ATRN,LCAT,NID1,CSTA,CP,KLKB1,F13A1,DNAH5,CENPE,PON3,TCIRG1,HPX,LUM,CDH11,IGFBP4,ITIH1,AHSG,ANKRD31,IGFBP1,SAA4,SERPING1,SEC24D,CSF1R,IGF2BP1,PIP,FCN2,PGLYRP2,SCGB3A1,SERPINI1,PPBP,PF4,MASP1,CAMP,APEH,A2ML1,PDIA3,SLC27A4,LALBA,PCSK9,BMP1,FGB,DST,F2,EFEMP2,PRG2,CSPG4,STAB1,NDUFS8,SUZ12,APOL1,SERPINF2,FBN1,KDM3B,P4HB,FBLN1,DNAH8,OIT3,HSP90AA1,FGG,TUBB,THBS4,PRDX6,SLPI,QPCT,APOD,FILIP1L,ASGR2,BLM,GIT2,APOA4,COL18A1,F8,C8A,CENPF,CFHR3,CFH,QSOX1,SERPINC1,F5,CFAP46,ADAM12,S100A7,HTRA1,ADAMTSL4,ECM1,FLNA,CRTAC1,MCOLN2,SMARCA1,C8B,FCN1,ADAMTS13,CCN5,CNTRL,SVEP1,F7,CA6,ADGRG2,CALML5,SOD3,GPX3,PROS1,EFEMP1,MMRN1,YWHAZ,NAV2,ILK,MAP3K7CL,MASP2,F11,PCYOX1,ADIPOQ,ATP9B,CKMT1A,ABCA13,MST1,SPARCL1,ITIH3,C4B,TGFBI,CFB,IGFALS,RARRES2,GC,UIMC1,JCHAIN,HPR,C1R,APOA5,ATP1A1,VIM,TTN,CFD,SEMA3B,PLCG2,KNG1,ACTB,TRIM66,TRIM8,CDH5,B2M,FGA,TPM3
GO Component	GO:0101002	ficolin-1-rich granule	9	185	0.65	0.0101	CAT,TCIRG1,APEH,PRG2,HSP90AA1,QPCT,FCN1,CALML5,CFD
GO Component	GO:0034362	Low-density lipoprotein particle	3	12	1.36	0.0177	APOB,APOE,APOM
GO Component	GO:0042571	Immunoglobulin complex, circulating	3	12	1.36	0.0177	JCHAIN,IGHV3-15,IGHV3-72
GO Component	GO:0070820	Tertiary granule	8	164	0.65	0.0198	LTF,LYZ,TCIRG1,PPBP,CAMP,QPCT,QSOX1,B2M
GO Component	GO:0001527	Microfibril	3	13	1.32	0.0206	EFEMP2,FBN1,FBN2
GO Component	GO:0034365	Discoidal high-density lipoprotein particle	2	2	1.96	0.0220	APOE,APOM
STRING clusters	CL:18723	Mixed, incl. Complement and coagulation cascades, and Protein-lipid complex	70	196	1.51	2.82e-75	SPP2,CPB2,CETP,APOH,SERPIND1,F9,C5,AFM,PROC,APOA1,APOE,F12,APCS,CFHR5,VWF,SERPINA10,LCAT,KLKB1,F13A1,HPX,FCN3,ITIH1,AHSG,HABP2,SAA4,SERPING1,FCN2,PGLYRP2,MASP1,PCSK9,FGB,F2,STAB1,C1QB,CFHR1,APOL1,SERPINF2,FGG,APOA4,F8,C8A,F13B,CFHR2,CFHR4,CFHR3,CFH,SERPINC1,F5,ECM1,C8B,FCN1,ADAMTS13,C1QC,C1QA,F7,APOM,PROS1,MASP2,F11,C1S,PCYOX1,ITIH3,C4B,CFB,HPR,C1R,APOA5,APOC4,CFD,FGA

STRING clusters	CL:18724	Mixed, incl. Complement and coagulation cascades, and Protein-lipid complex	68	172	1.55	2.82e-75	SPP2,CPB2,CETP,APOH,SERPIND1,F9,C5,AFM,PROC,APOA1,APOE,F12,APCS,CFHR5,VWF,SERPINA10,LCAT,KLKB1,F13A1,HPX,FCN3,ITIH1,AHSG,HABP2,SAA4,SERPING1,FCN2,PGLYRP2,MASP1,PCSK9,FGB,F2,C1QB,CFHR1,APOL1,SERPINF2,FGG,APOA4,F8,C8A,F13B,CFHR2,CFHR4,CFHR3,CFH,SERPINC1,F5,ECM1,C8B,FCN1,ADAMTS13,C1QC,C1QA,F7,APOM,MASP2,F11,C1S,PCYOX1,ITIH3,C4B,CFB,HPR,C1R,APOA5,APOC4,CFD,FGA
STRING clusters	CL:18725	Complement and coagulation cascades, and Protein-lipid complex	66	166	1.56	1.86e-73	CPB2,CETP,APOH,SERPIND1,F9,C5,AFM,PROC,APOA1,APOE,F12,APCS,CFHR5,VWF,SERPINA10,LCAT,KLKB1,F13A1,HPX,FCN3,ITIH1,AHSG,HABP2,SAA4,SERPING1,FCN2,PGLYRP2,MASP1,PCSK9,FGB,F2,C1QB,CFHR1,APOL1,SERPINF2,FGG,APOA4,F8,C8A,F13B,CFHR2,CFHR4,CFHR3,CFH,SERPINC1,F5,C8B,FCN1,ADAMTS13,C1QC,C1QA,F7,APOM,MASP2,F11,C1S,PCYOX1,ITIH3,C4B,CFB,HPR,C1R,APOA5,APOC4,CFD,FGA
STRING clusters	CL:18726	Complement and coagulation cascades, and Protein-lipid complex	65	161	1.56	1.00e-72	CPB2,CETP,APOH,SERPIND1,F9,C5,AFM,PROC,APOA1,APOE,F12,APCS,CFHR5,VWF,LCAT,KLKB1,F13A1,HPX,FCN3,ITIH1,AHSG,HABP2,SAA4,SERPING1,FCN2,PGLYRP2,MASP1,PCSK9,FGB,F2,C1QB,CFHR1,APOL1,SERPINF2,FGG,APOA4,F8,C8A,F13B,CFHR2,CFHR4,CFHR3,CFH,SERPINC1,F5,C8B,FCN1,ADAMTS13,C1QC,C1QA,F7,APOM,MASP2,F11,C1S,PCYOX1,ITIH3,C4B,CFB,HPR,C1R,APOA5,APOC4,CFD,FGA
STRING clusters	CL:18727	Complement and coagulation cascades, and Positive regulation of opsonization	51	115	1.6	9.96e-58	CPB2,SERPIND1,F9,C5,AFM,PROC,F12,APCS,CFHR5,VWF,KLKB1,F13A1,HPX,FCN3,ITIH1,AHSG,HABP2,SERPING1,FCN2,PGLYRP2,MASP1,FGB,F2,C1QB,CFHR1,SERPINF2,FGG,F8,C8A,F13B,CFHR2,CFHR4,CFHR3,CFH,SERPINC1,F5,C8B,FCN1,ADAMTS13,C1QC,C1QA,F7,MASP2,F11,C1S,ITIH3,C4B,CFB,C1R,CFD,FGA
STRING clusters	CL:18728	Complement and coagulation cascades, and Positive regulation of opsonization	49	109	1.61	1.29e-55	CPB2,SERPIND1,F9,C5,PROC,F12,APCS,CFHR5,VWF,KLKB1,F13A1,HPX,FCN3,ITIH1,AHSG,HABP2,SERPING1,FCN2,MASP1,FGB,F2,C1QB,CFHR1,SERPINF2,FGG,F8,C8A,F13B,CFHR2,CFHR4,CFHR3,CFH,SERPINC1,F5,C8B,FCN1,ADAMTS13,C1QC,C1QA,F7,MASP2,F11,C1S,ITIH3,C4B,CFB,C1R,CFD,FGA
STRING clusters	CL:18731	Hemostasis, and Dissolution of Fibrin Clot	25	50	1.66	4.44e-28	CPB2,SERPIND1,F9,PROC,F12,VWF,KLKB1,F13A1,HPX,ITIH1,AHSG,HABP2,FGB,F2,SERPINF2,FGG,F8,F13B,SERPINC1,F5,ADAMTS13,F7,F11,ITIH3,FGA
STRING clusters	CL:18846	Complement cascade	24	50	1.64	1.18e-26	C5,APCS,CFHR5,FCN3,SERPING1,FCN2,MASP1,C1QB,CFHR1,C8A,CFHR2,CFHR4,CFHR3,CFH,C8B,FCN1,C1QC,C1QA,MASP2,C1S,C4B,CFB,C1R,CFD
STRING clusters	CL:18848	Complement cascade	18	40	1.61	3.22e-19	C5,FCN3,SERPING1,FCN2,MASP1,C1QB,C8A,CFH,C8B,FCN1,C1QC,C1QA,MASP2,C1S,C4B,CFB,C1R,CFD
STRING clusters	CL:18849	Initial triggering of complement, and Regulation of complement activation	15	31	1.64	3.51e-16	FCN3,SERPING1,FCN2,MASP1,C1QB,CFH,FCN1,C1QC,C1QA,MASP2,C1S,C4B,CFB,C1R,CFD
STRING clusters	CL:18732	Mixed, incl. COVID-19, thrombosis and anticoagulation, and Scavenging of heme from plasma	13	26	1.66	5.13e-14	CPB2,SERPIND1,F13A1,HPX,ITIH1,AHSG,HABP2,FGB,SERPINF2,FGG,F13B,ITIH3,FGA
STRING clusters	CL:18784	Formation of Fibrin Clot (Clotting Cascade)	12	18	1.78	5.28e-14	F9,PROC,F12,VWF,KLKB1,F2,F8,SERPINC1,F5,ADAMTS13,F7,F11
STRING clusters	CL:18733	Mixed, incl. COVID-19, thrombosis and anticoagulation, and Inter-alpha-trypsin inhibitor heavy chain C-terminus	12	21	1.71	1.96e-13	CPB2,SERPIND1,F13A1,ITIH1,AHSG,HABP2,FGB,SERPINF2,FGG,F13B,ITIH3,FGA
STRING clusters	CL:18852	Creation of C4 and C2 activators	11	15	1.82	4.07e-13	FCN3,SERPING1,FCN2,MASP1,C1QB,FCN1,C1QC,C1QA,MASP2,C1S,C1R
STRING clusters	CL:18851	Initial triggering of complement, and Negative regulation of complement activation	12	24	1.66	5.86e-13	FCN3,SERPING1,FCN2,MASP1,C1QB,FCN1,C1QC,C1QA,MASP2,C1S,C4B,C1R
STRING clusters	CL:18956	Lipoprotein particle, and Assembly of active LPL and LIPC lipase complexes	14	46	1.44	6.18e-13	CETP,APOH,APOA1,APOE,LCAT,SAA4,PCSK9,APOL1,APOA4,APOM,PCYOX1,HPR,APOA5,APOC4
STRING clusters	CL:18960	High-density lipoprotein particle	12	26	1.62	1.05e-12	CETP,APOH,APOA1,LCAT,SAA4,APOL1,APOA4,APOM,PCYOX1,HPR,APOA5,APOC4
STRING clusters	CL:18962	High-density lipoprotein particle	11	21	1.68	4.81e-12	CETP,APOH,APOA1,LCAT,APOL1,APOA4,APOM,PCYOX1,HPR,APOA5,APOC4
STRING clusters	CL:18734	COVID-19, thrombosis and anticoagulation, and Negative regulation of fibrinolysis	9	11	1.87	5.77e-11	CPB2,SERPIND1,F13A1,HABP2,FGB,SERPINF2,FGG,F13B,FGA
STRING clusters	CL:18786	Hemophilia B, and Blood coagulation, common pathway	9	12	1.83	9.57e-11	F9,VWF,F2,F8,SERPINC1,F5,ADAMTS13,F7,F11
STRING clusters	CL:18966	High-density lipoprotein particle	8	16	1.66	2.11e-08	CETP,APOH,APOA1,LCAT,APOA4,APOM,APOA5,APOC4
STRING clusters	CL:18869	Complement component C1 complex	6	6	1.96	2.50e-07	SERPING1,C1QB,C1QC,C1QA,C1S,C1R
STRING clusters	CL:18854	Cell surface pattern recognition receptor signaling pathway	5	6	1.88	1.13e-05	FCN3,FCN2,MASP1,FCN1,MASP2
STRING clusters	CL:24266	Insulin-like growth factor binding, and Pappalysin-1/2	6	16	1.53	1.71e-05	IGFBP5,IGFBP4,IGFBP1,PRG2,PAPPA,IGFALS
STRING clusters	CL:19457	Extracellular matrix organization	13	180	0.82	3.40e-05	MMP2,NID1,LUM,BMP1,DST,EFEMP2,FBN1,P4HB,FBLN1,THBS4,COL18A1,EFEMP1,FBN2
STRING clusters	CL:18970	Triglyceride-rich lipoprotein particle remodeling, and Spherical high-density lipoprotein particle	5	11	1.62	9.16e-05	CETP,APOA1,LCAT,APOA4,APOA5
STRING clusters	CL:19699	Elastic fibre formation	5	14	1.51	0.00023	EFEMP2,FBN1,FBLN1,EFEMP1,FBN2
STRING clusters	CL:18934	Hemolytic uremic syndrome	4	5	1.86	0.00024	CFHR5,CFHR1,CFHR4,CFHR3
STRING clusters	CL:18737	Fibrinogen, and Thrombophilia	4	6	1.78	0.00039	SERPIND1,FGB,FGG,FGA
STRING clusters	CL:19696	Elastic fibre formation, and Tolloid/BMP1 peptidase domain	6	34	1.2	0.00061	BMP1,EFEMP2,FBN1,FBLN1,EFEMP1,FBN2
STRING clusters	CL:19695	Elastic fibre formation, and Matrix metalloproteinases	7	61	1.02	0.0012	MMP2,BMP1,EFEMP2,FBN1,FBLN1,EFEMP1,FBN2
STRING clusters	CL:24268	Insulin-like growth factor-binding protein family 1-6, chordata, and Insulin-like growth factor	4	10	1.56	0.0016	IGFBP5,IGFBP4,IGFBP1,IGFALS
STRING clusters	CL:35044	Mixed, incl. Galactoside-binding lectin, and Transforming growth factor beta1 activation	4	16	1.36	0.0070	PSG1,PSG11,PSG4,PSG2
STRING clusters	CL:18788	Extrinsic Pathway of Fibrin Clot Formation, and Factor V deficiency	3	6	1.66	0.0120	F2,F5,F7
STRING clusters	CL:9658	Detoxification of Reactive Oxygen Species, and Copper transport	5	40	1.05	0.0144	CAT,CP,PRDX6,SOD3,GPX3
STRING clusters	CL:18911	Membrane attack complex	3	7	1.59	0.0161	C5,C8A,C8B
STRING clusters	CL:35049	Transforming growth factor beta1 activation, and Protein tyrosine kinase binding	3	7	1.59	0.0161	PSG1,PSG4,PSG2
STRING clusters	CL:19701	Marfan syndrome, and Microfibril-associated glycoprotein	3	8	1.53	0.0205	EFEMP2,FBN1,FBN2
STRING clusters	CL:9662	Detoxification of Reactive Oxygen Species, and mRNA, protein, and metabolite inducation pathway by cyclosporin A	4	24	1.18	0.0232	CAT,PRDX6,SOD3,GPX3
STRING clusters	CL:18755	Mixed, incl. Inter-alpha-trypsin inhibitor heavy chain C-terminus, and Alpha-1-acid glycoprotein	3	10	1.44	0.0335	ITIH1,AHSG,ITIH3
KEGG	hsa04610	Complement and coagulation cascades	40	82	1.65	2.90e-46	CPB2,SERPIND1,F9,C5,PROC,F12,CFHR5,VWF,KLKB1,F13A1,SERPING1,FGB,F2,C1QB,CFHR1,SERPINF2,FGG,F8,C8A,F13B,CFHR2,CFHR4,CFHR3,CFH,SERPINC1,F5,C8B,C1QC,C1QA,F7,PROS1,MASP2,F11,C1S,C4B,CFB,C1R,CFD,KNG1,FGA

KEGG	hsa05150	Staphylococcus aureus infection	13	86	1.14	9.69e-09	C5,CAMP,C1QB,FGG,CFH,C1QC,C1QA,MASP2,C1S,C4B,CFB,C1R,CFD
KEGG	hsa04979	Cholesterol metabolism	8	48	1.18	1.77e-05	CETP,APOH,APOB,APOA1,APOE,LCAT,PCSK9,APOA4
KEGG	hsa05133	Pertussis	9	73	1.05	2.27e-05	C5,SERPING1,C1QB,C1QC,C1QA,CALML5,C1S,C4B,C1R
KEGG	hsa05322	Systemic lupus erythematosus	9	94	0.94	0.00013	C5,C1QB,C8A,C8B,C1QC,C1QA,C1S,C4B,C1R
KEGG	hsa04611	Platelet activation	8	122	0.77	0.0051	VWF,GUCY1A1,FGB,F2,FGG,PLCG2,ACTB,FGA
KEGG	hsa05020	Prion disease	10	263	0.54	0.0412	C5,PSMA6,C1QB,NDUFS8,TUBA1B,TUBB,C8A,C8B,C1QC,C1QA
KEGG	hsa05143	African trypanosomiasis	4	36	1.0	0.0412	APOA1,APOL1,HPR,KNG1
Reactome	HSA-166658	Complement cascade	25	59	1.59	4.18e-26	CPB2,C5,CFHR5,FCN3,SERPING1,FCN2,F2,C1QB,CFHR1,C8A,CFHR2,CFHR4,CFHR3,CFH,C8B,FCN1,C1QC,C1QA,PROS1,MASP2,C1S,C4B,CFB,C1R,CFD
Reactome	HSA-140877	Formation of Fibrin Clot (Clotting Cascade)	21	39	1.69	1.42e-23	SERPIND1,F9,PROC,F12,VWF,KLKB1,F13A1,SERPING1,PF4,FGB,F2,FGG,F8,F13B,SERPINC1,F5,F7,PROS1,F11,KNG1,FGA
Reactome	HSA-381426	Regulation of Insulin-like Growth Factor (IGF) transport and uptake by Insulin-like Growth Factor Binding Proteins (IGFBPs)	28	124	1.31	1.42e-23	SPP2,SERPIND1,MMP2,APOB,IGFBP5,PROC,APOA1,APOE,SERPINA10,CP,IGFBP4,AHSG,IGFBP1,PCSK9,F2,APOL1,FBN1,P4HB,PAPPA,FGG,QSOX1,SERPINC1,F5,SPARCL1,IGFALS,APOA5,KNG1,FGA
Reactome	HSA-109582	Hemostasis	48	607	0.86	1.62e-23	SPP2,APOH,SERPIND1,F9,APOB,PROC,APOA1,F12,VWF,KLKB1,F13A1,CENPE,AHSG,SERPING1,PPBP,PF4,GUCY1A1,FGB,F2,PSG1,PSG11,SERPINF2,TUBA1B,FGG,F8,F13B,QSOX1,SERPINC1,F5,ECM1,FLNA,F7,CD99,PROS1,MMRN1,YWHAZ,PSG4,F11,PSG2,ITIH3,RARRES2,JCHAIN,TTN,CFD,PLCG2,KNG1,ACTB,FGA
Reactome	HSA-977606	Regulation of Complement cascade	20	49	1.57	7.55e-21	CPB2,C5,CFHR5,SERPING1,F2,C1QB,CFHR1,C8A,CFHR2,CFHR4,CFHR3,CFH,C8B,C1QC,C1QA,PROS1,C1S,C4B,CFB,C1R
Reactome	HSA-8957275	Post-translational protein phosphorylation	24	107	1.31	3.30e-20	SPP2,SERPIND1,APOB,IGFBP5,PROC,APOA1,APOE,SERPINA10,CP,IGFBP4,AHSG,IGFBP1,PCSK9,APOL1,FBN1,P4HB,FGG,QSOX1,SERPINC1,F5,SPARCL1,APOA5,KNG1,FGA
Reactome	HSA-114608	Platelet degranulation	25	126	1.26	4.85e-20	SPP2,APOH,APOA1,VWF,F13A1,AHSG,SERPING1,PPBP,PF4,FGB,SERPINF2,FGG,F8,QSOX1,F5,ECM1,FLNA,PROS1,MMRN1,ITIH3,RARRES2,TTN,CFD,KNG1,FGA
Reactome	HSA-168249	Innate Immune System	53	1041	0.66	3.22e-18	CPB2,C5,LTF,APOB,TTR,CAT,CFHR5,LYZ,PSMA6,TCIRG1,FCN3,AHSG,SERPING1,FCN2,PGLYRP2,PPBP,CAMP,APEH,FGB,F2,PRG2,C1QB,CFHR1,HSP90AA1,FGG,TUBB,PRDX6,SLPI,QPCT,C8A,CFHR2,CFHR4,CFHR3,CFH,QSOX1,S100A7,C8B,FCN1,C1QC,C1QA,CALML5,PROS1,MASP2,C1S,ABCA13,C4B,CFB,C1R,CFD,PLCG2,ACTB,B2M,FGA
Reactome	HSA-76002	Platelet activation, signaling and aggregation	28	260	0.99	2.31e-16	SPP2,APOH,APOA1,VWF,F13A1,AHSG,SERPING1,PPBP,PF4,FGB,F2,SERPINF2,FGG,F8,QSOX1,F5,ECM1,FLNA,PROS1,MMRN1,YWHAZ,ITIH3,RARRES2,TTN,CFD,PLCG2,KNG1,FGA
Reactome	HSA-140875	Common Pathway of Fibrin Clot Formation	13	22	1.73	5.27e-15	SERPIND1,PROC,F13A1,PF4,FGB,F2,FGG,F8,F13B,SERPINC1,F5,PROS1,FGA
Reactome	HSA-140837	Intrinsic Pathway of Fibrin Clot Formation	13	23	1.71	7.49e-15	SERPIND1,F9,PROC,F12,VWF,KLKB1,SERPING1,F2,F8,SERPINC1,PROS1,F11,KNG1
Reactome	HSA-168256	Immune System	67	1979	0.49	7.49e-15	CPB2,MMP2,C5,LTF,APOB,TTR,CAT,CFHR5,LYZ,PSMA6,F13A1,CENPE,TCIRG1,FCN3,AHSG,SERPING1,SEC24D,CSF1R,FCN2,PGLYRP2,PPBP,CAMP,APEH,PDIA3,FGB,F2,UBE3C,PRG2,C1QB,CFHR1,P4HB,HSP90AA1,TUBA1B,FGG,TUBB,PRDX6,SLPI,QPCT,C8A,CFHR2,CFHR4,CFHR3,CFH,QSOX1,S100A7,FLNA,C8B,FCN1,C1QC,C1QA,CALML5,CD99,PROS1,YWHAZ,MASP2,C1S,ABCA13,C4B,CFB,C1R,VIM,CFD,PLCG2,ACTB,TRIM8,B2M,FGA
Reactome	HSA-166663	Initial triggering of complement	12	24	1.66	3.34e-13	FCN3,FCN2,C1QB,FCN1,C1QC,C1QA,MASP2,C1S,C4B,CFB,C1R,CFD
Reactome	HSA-166786	Creation of C4 and C2 activators	9	16	1.71	4.90e-10	FCN3,FCN2,C1QB,FCN1,C1QC,C1QA,MASP2,C1S,C1R
Reactome	HSA-392499	Metabolism of proteins	54	1917	0.41	1.58e-08	SPP2,CPB2,SERPIND1,F9,MMP2,GPLD1,LTF,APOB,IGFBP5,PROC,APOA1,TTR,APOE,APCS,LYZ,PSMA6,SERPINA10,CP,IGFBP4,AHSG,IGFBP1,SEC24D,APEH,PDIA3,PCSK9,F2,INHBC,SUZ12,APOL1,FBN1,P4HB,PAPPA,TUBA1B,FGG,ASGR2,BLM,APOA4,F8,QSOX1,SERPINC1,F5,ADAMTSL4,ADAMTS13,F7,PROS1,SPARCL1,TGFBI,IGFALS,UIMC1,APOA5,KNG1,ACTB,B2M,FGA
Reactome	HSA-9651496	Defects of contact activation system (CAS) and kallikrein/kinin system (KKS)	8	16	1.66	1.58e-08	F9,F12,VWF,KLKB1,SERPING1,F2,F8,F11
Reactome	HSA-1474244	Extracellular matrix organization	20	300	0.78	4.48e-08	MMP2,TTR,VWF,NID1,KLKB1,LUM,BMP1,FGB,DST,EFEMP2,FBN1,P4HB,FBLN1,FGG,COL18A1,ADAM12,HTRA1,EFEMP1,FBN2,FGA
Reactome	HSA-174824	Plasma lipoprotein assembly, remodeling, and clearance	11	75	1.12	2.72e-07	CETP,APOB,APOA1,APOE,LCAT,PCSK9,BMP1,P4HB,APOA4,APOA5,APOC4
Reactome	HSA-2173782	Binding and Uptake of Ligands by Scavenger Receptors	9	43	1.28	4.89e-07	APOB,APOA1,APOE,HPX,STAB1,APOL1,HSP90AA1,JCHAIN,HPR
Reactome	HSA-8963898	Plasma lipoprotein assembly	7	19	1.52	9.47e-07	APOB,APOA1,APOE,BMP1,P4HB,APOA4,APOC4
Reactome	HSA-597592	Post-translational protein modification	41	1405	0.42	1.31e-06	SPP2,SERPIND1,F9,GPLD1,APOB,IGFBP5,PROC,APOA1,APOE,PSMA6,SERPINA10,CP,IGFBP4,AHSG,IGFBP1,SEC24D,PDIA3,PCSK9,F2,SUZ12,APOL1,FBN1,P4HB,TUBA1B,FGG,ASGR2,BLM,F8,QSOX1,SERPINC1,F5,ADAMTSL4,ADAMTS13,F7,PROS1,SPARCL1,UIMC1,APOA5,KNG1,ACTB,FGA
Reactome	HSA-8963899	Plasma lipoprotein remodeling	8	34	1.33	1.43e-06	CETP,APOB,APOA1,APOE,LCAT,P4HB,APOA4,APOA5
Reactome	HSA-6798695	Neutrophil degranulation	22	476	0.62	2.69e-06	LTF,TTR,CAT,LYZ,TCIRG1,AHSG,PPBP,CAMP,APEH,PRG2,HSP90AA1,TUBB,PRDX6,SLPI,QPCT,QSOX1,S100A7,FCN1,CALML5,ABCA13,CFD,B2M
Reactome	HSA-977225	Amyloid fiber formation	10	79	1.06	4.04e-06	LTF,APOA1,TTR,APOE,APCS,LYZ,APOA4,TGFBI,B2M,FGA
Reactome	HSA-159763	Transport of gamma-carboxylated protein precursors from the endoplasmic reticulum to the Golgi apparatus	5	9	1.7	2.29e-05	F9,PROC,F2,F7,PROS1
Reactome	HSA-173623	Classical antibody-mediated complement activation	5	9	1.7	2.29e-05	C1QB,C1QC,C1QA,C1S,C1R
Reactome	HSA-159740	Gamma-carboxylation of protein precursors	5	10	1.66	3.17e-05	F9,PROC,F2,F7,PROS1
Reactome	HSA-159782	Removal of aminoterminal propeptides from gamma-carboxylated proteins	5	10	1.66	3.17e-05	F9,PROC,F2,F7,PROS1
Reactome	HSA-8963888	Chylomicron assembly	5	10	1.66	3.17e-05	APOB,APOA1,APOE,P4HB,APOA4
Reactome	HSA-8963901	Chylomicron remodeling	5	10	1.66	3.17e-05	APOB,APOA1,APOE,APOA4,APOA5
Reactome	HSA-202733	Cell surface interactions at the vascular wall	11	139	0.86	5.30e-05	APOB,PROC,PF4,F2,PSG1,PSG11,CD99,PROS1,PSG4,PSG2,JCHAIN
Reactome	HSA-2855086	Ficolins bind to repetitive carbohydrate structures on the target cell surface	4	4	1.96	6.05e-05	FCN3,FCN2,FCN1,MASP2
Reactome	HSA-2168880	Scavenging of heme from plasma	5	15	1.48	0.00013	APOA1,HPX,APOL1,JCHAIN,HPR
Reactome	HSA-9662001	Defective factor VIII causes hemophilia A	4	7	1.71	0.00026	F9,VWF,F2,F8
Reactome	HSA-8964058	HDL remodeling	4	10	1.56	0.00074	CETP,APOA1,APOE,LCAT
Reactome	HSA-163841	Gamma carboxylation, hypusine formation and arylsulfatase activation	6	42	1.11	0.00075	F9,PROC,F2,F8,F7,PROS1
Reactome	HSA-975634	Retinoid metabolism and transport	6	44	1.09	0.00093	APOB,APOA1,TTR,APOE,APOA4,APOM
Reactome	HSA-9657688	Defective factor XII causes hereditary angioedema	3	3	1.96	0.0014	F12,KLKB1,F2
Reactome	HSA-9657689	Defective SERPING1 causes hereditary angioedema	3	3	1.96	0.0014	F12,KLKB1,SERPING1
Reactome	HSA-9672391	Defective F8 cleavage by thrombin	3	3	1.96	0.0014	VWF,F2,F8
Reactome	HSA-354194	GRB2:SOS provides linkage to MAPK signaling for Integrins	4	15	1.38	0.0023	VWF,FGB,FGG,FGA
Reactome	HSA-372708	p130Cas linkage to MAPK signaling for integrins	4	15	1.38	0.0023	VWF,FGB,FGG,FGA
Reactome	HSA-216083	Integrin cell surface interactions	7	85	0.87	0.0031	VWF,LUM,FGB,FBN1,FGG,COL18A1,FGA
Reactome	HSA-382551	Transport of small molecules	21	723	0.42	0.0032	CETP,APOB,APOA1,APOE,PSMA6,LCN1,LCAT,CP,TCIRG1,PIP,SLC27A4,PCSK9,BMP1,P4HB,APOD,APOA4,MCOLN2,ATP9B,APOA5,ATP1A1,APOC4

Reactome	HSA-8866423	VLDL assembly	3	5	1.74	0.0032	APOB,P4HB,APOC4
Reactome	HSA-3299685	Detoxification of Reactive Oxygen Species	5	36	1.1	0.0036	CAT,P4HB,PRDX6,SOD3,GPX3
Reactome	HSA-6802948	Signaling by high-kinase activity BRAF mutants	5	36	1.1	0.0036	VWF,FGB,FGG,ACTB,FGA
Reactome	HSA-2129379	Molecules associated with elastic fibres	5	37	1.09	0.0039	EFEMP2,FBN1,FBLN1,EFEMP1,FBN2
Reactome	HSA-8964043	Plasma lipoprotein clearance	5	37	1.09	0.0039	APOB,APOA1,APOE,PCSK9,APOC4
Reactome	HSA-8964041	LDL remodeling	3	6	1.66	0.0042	CETP,APOB,P4HB
Reactome	HSA-76009	Platelet Aggregation (Plug Formation)	5	39	1.07	0.0046	VWF,FGB,F2,FGG,FGA
Reactome	HSA-5674135	MAP2K and MAPK activation	5	40	1.05	0.0049	VWF,FGB,FGG,ACTB,FGA
Reactome	HSA-5686938	Regulation of TLR by endogenous ligand	4	20	1.26	0.0049	APOB,FGB,FGG,FGA
Reactome	HSA-9656223	Signaling by RAF1 mutants	5	41	1.04	0.0054	VWF,FGB,FGG,ACTB,FGA
Reactome	HSA-174577	Activation of C3 and C5	3	7	1.59	0.0055	C5,C4B,CFB
Reactome	HSA-2187338	Visual phototransduction	7	99	0.81	0.0061	HSD17B1,APOB,APOA1,TTR,APOE,APOA4,APOM
Reactome	HSA-166665	Terminal pathway of complement	3	8	1.53	0.0072	C5,C8A,C8B
Reactome	HSA-804914	Transport of fatty acids	3	8	1.53	0.0072	LCN1,SLC27A4,APOD
Reactome	HSA-6802946	Signaling by moderate kinase activity BRAF mutants	5	45	1.0	0.0073	VWF,FGB,FGG,ACTB,FGA
Reactome	HSA-6802955	Paradoxical activation of RAF signaling by kinase inactive BRAF	5	45	1.0	0.0073	VWF,FGB,FGG,ACTB,FGA
Reactome	HSA-9649948	Signaling downstream of RAS mutants	5	45	1.0	0.0073	VWF,FGB,FGG,ACTB,FGA
Reactome	HSA-1474228	Degradation of the extracellular matrix	8	140	0.71	0.0074	MMP2,NID1,KLKB1,BMP1,FBN1,COL18A1,HTRA1,FBN2
Reactome	HSA-9668250	Defective factor IX causes hemophilia B	3	9	1.48	0.0085	F9,F8,F11
Reactome	HSA-1643685	Disease	35	1702	0.27	0.0091	F9,LTF,APOA1,TTR,F12,VWF,PSMA6,CP,KLKB1,LUM,SERPING1,SEC24D,SLC27A4,FGB,F2,CSPG4,SUZ12,HSP90AA1,TUBA1B,FGG,TUBB,BLM,F8,ADAMTSL4,ADAMTS13,CNTRL,YWHAZ,MASP2,F11,ATP1A1,PLCG2,ACTB,B2M,FGA,TPM3
Reactome	HSA-430116	GP1b-IX-V activation signalling	3	12	1.36	0.0165	VWF,FLNA,YWHAZ
Reactome	HSA-1236974	ER-Phagosome pathway	6	89	0.79	0.0187	PSMA6,PDIA3,FGB,FGG,B2M,FGA
Reactome	HSA-9672393	Defective F8 binding to von Willebrand factor	2	2	1.96	0.0213	VWF,F8
Reactome	HSA-446728	Cell junction organization	6	92	0.77	0.0215	CDH11,DST,FLNA,ILK,ACTB,CDH5
Reactome	HSA-9029569	NR1H3 & NR1H2 regulate gene expression linked to cholesterol transport and efflux	4	36	1.0	0.0273	CETP,APOE,APOD,APOC4
Reactome	HSA-6802952	Signaling by BRAF and RAF1 fusions	5	65	0.84	0.0292	VWF,FGB,FGG,ACTB,FGA
Reactome	HSA-9672383	Defective factor IX causes thrombophilia	2	3	1.78	0.0334	F9,F8
Reactome	HSA-9672396	Defective cofactor function of FVIIIa variant	2	3	1.78	0.0334	F9,F8
Reactome	HSA-9673202	Defective F9 variant does not activate FX	2	3	1.78	0.0334	F9,F8
Reactome	HSA-5602498	MyD88 deficiency (TLR2/4)	3	17	1.2	0.0353	FGB,FGG,FGA
Reactome	HSA-3000480	Scavenging by Class A Receptors	3	18	1.18	0.0399	APOB,APOA1,APOE
Reactome	HSA-446353	Cell-extracellular matrix interactions	3	18	1.18	0.0399	FLNA,ILK,ACTB
Reactome	HSA-5603041	IRAK4 deficiency (TLR2/4)	3	18	1.18	0.0399	FGB,FGG,FGA
Reactome	HSA-3000497	Scavenging by Class H Receptors	2	4	1.66	0.0452	APOB,STAB1
WikiPathways	WP558	Complement and coagulation cascades	29	58	1.66	8.05e-33	CPB2,SERPIND1,F9,PROC,F12,VWF,KLKB1,SERPING1,MASP1,FGB,F2,C1QB,SERPINF2,F8,F13B,CFH,SERPINC1,F5,C1QC,C1QA,F7,PROS1,MASP2,C1S,C4B,CFB,C1R,CFD,KNG1
WikiPathways	WP2806	Complement system	24	96	1.36	3.66e-21	C5,APOA1,F12,APCS,KLKB1,F13A1,SERPING1,FCN2,MASP1,FGB,FGG,C8A,CFHR2,CFHR4,CFH,FCN1,PROS1,MASP2,F11,C1S,ADIPOQ,CFB,CFD,FGA
WikiPathways	WP272	Blood clotting cascade	14	22	1.76	1.74e-16	F9,F12,VWF,KLKB1,FGB,F2,SERPINF2,FGG,F8,F13B,F5,F7,F11,FGA
WikiPathways	WP545	Complement activation	13	22	1.73	4.96e-15	C5,MASP1,C1QB,C8A,C8B,C1QC,C1QA,MASP2,C1S,C4B,CFB,C1R,CFD
WikiPathways	WP5090	Complement system in neuronal development and plasticity	19	105	1.22	1.51e-14	C5,FCN3,SERPING1,FCN2,MASP1,C1QB,C8A,CFH,C8B,FCN1,C1QC,C1QA,PROS1,MASP2,C1S,C4B,CFB,C1R,CFD
WikiPathways	WP176	Folate metabolism	11	67	1.17	1.07e-07	APOB,APOA1,CAT,SAA4,FGB,F2,FGG,F7,SOD3,GPX3,FGA
WikiPathways	WP5115	Network map of SARS-CoV-2 signaling pathway	17	218	0.85	1.07e-07	APOH,APOA1,SERPINA10,PF4,FGB,APOL1,FGG,APOD,C8A,CFH,APOM,C1S,ITIH3,CFB,C1R,ACTB,FGA
WikiPathways	WP4927	COVID-19, thrombosis and anticoagulation	6	7	1.89	2.45e-07	F13A1,FGB,F2,FGG,F13B,FGA
WikiPathways	WP15	Selenium micronutrient network	11	84	1.08	6.06e-07	APOB,APOA1,CAT,SAA4,FGB,F2,FGG,F7,SOD3,GPX3,FGA
WikiPathways	WP5323	Fatty Acids and Lipoproteins Transport in Hepatocytes	19	380	0.66	6.64e-06	CETP,APOH,APOB,APOA1,APOE,LCAT,SAA4,SLC27A4,PCSK9,APOL1,ASGR2,APOA4,APOM,PCYOX1,ADIPOQ,ATP9B,PRG4,APOA5,APOC4
WikiPathways	WP430	Statin inhibition of cholesterol production	7	29	1.34	7.15e-06	CETP,APOB,APOA1,APOE,LCAT,APOA4,APOA5
WikiPathways	WP5109	Familial hyperlipidemia type 2	6	16	1.53	7.15e-06	CETP,APOB,APOA1,LCAT,PCSK9,APOA4
WikiPathways	WP4522	Metabolic pathway of LDL, HDL and TG, including diseases	6	17	1.51	8.11e-06	CETP,APOB,APOA1,APOE,LCAT,PCSK9
WikiPathways	WP5304	Cholesterol metabolism	9	72	1.05	1.35e-05	CETP,APOH,APOB,APOA1,APOE,LCAT,SLC27A4,PCSK9,APOA4
WikiPathways	WP3601	Lipid particles composition	5	10	1.66	2.10e-05	CETP,APOB,APOA1,APOE,LCAT
WikiPathways	WP3941	Oxidative damage response	7	39	1.21	3.01e-05	C5,C1QB,C1QC,C1QA,C1S,C4B,C1R
WikiPathways	WP5186	Vitamin K metabolism and activation of dependent proteins	5	12	1.58	3.75e-05	F9,PROC,F2,F7,PROS1
WikiPathways	WP2328	Allograft rejection	9	88	0.97	4.86e-05	C5,C1QB,TUBA1B,C8A,C8B,C1QC,C1QA,C4B,VIM
WikiPathways	WP5110	Familial hyperlipidemia type 3	5	13	1.54	4.86e-05	CETP,APOA1,APOE,LCAT,APOA4
WikiPathways	WP5112	Familial hyperlipidemia type 5	5	15	1.48	7.77e-05	CETP,APOA1,LCAT,APOA4,APOA5
WikiPathways	WP1533	Vitamin B12 metabolism	7	50	1.1	0.00010	APOB,APOA1,APOE,SAA4,F2,F7,SOD3
WikiPathways	WP5089	Kinin-Kallikrein pathway	4	7	1.71	0.00015	F12,KLKB1,SERPING1,KNG1
WikiPathways	WP5108	Familial hyperlipidemia type 1	5	18	1.4	0.00015	CETP,APOA1,LCAT,APOA4,APOA5
WikiPathways	WP5111	Familial hyperlipidemia type 4	5	22	1.31	0.00032	CETP,APOA1,LCAT,APOA4,APOA5
WikiPathways	WP5104	Acquired partial lipodystrophy / Barraquer-Simons syndrome	3	10	1.44	0.0105	C5,CFB,CFD
WikiPathways	WP4969	RAS and bradykinin pathways in COVID-19	4	28	1.11	0.0117	F12,KLKB1,SERPING1,KNG1
WikiPathways	WP5333	Cholesterol metabolism	4	36	1.0	0.0266	APOB,APOA1,SLC27A4,APOA4
WikiPathways	WP3942	PPAR signaling pathway	5	67	0.83	0.0316	APOA1,SLC27A4,ILK,ADIPOQ,APOA5
WikiPathways	WP3937	Microglia pathogen phagocytosis pathway	4	40	0.96	0.0356	C1QB,C1QC,C1QA,PLCG2
Monarch	EFO:0007937	Blood protein measurement	76	1810	0.58	4.76e-21	CPB2,APOH,C5,AFM,LTF,APOB,IGFBP5,PROC,CAT,APOE,F12,CFHR5,LYZ,VWF,SERPINA10,LCAT,NID1,KLKB1,HPX,CDH11,ITIH1,AHSG,HABP2,SAA4,SERPING1,ANKRD44,CSF1R,FCN2,PGLYRP2,MASP1,F2,PSG1,PRG2,APOL1,SERPINF2,PAPPA,HSP90AA1,PRDX6,ASGR2,CNDP1,COL18A1,F13B,CFHR4,CFH,QSOX1,F5,ADAM12,ECM1,CRTAC1,C8B,FCN1,ADAMTS13,CCN5,SVEP1,C1QC,F7,APOM,CA6,SOD3,PSG4,F11,C1S,PSG2,PCYOX1,MST1,SPARCL1,C4B,TGFBI,CFB,GC,HPR,C1R,RNASE4,APOC4,PLCG2,KNG1
Monarch	HP:0000118	Phenotypic abnormality	115	5129	0.31	2.90e-13	CETP,SERPIND1,F9,MMP2,C5,HSD17B1,MGP,LTF,APOB,PROC,APOA1,TTR,CAT,APOE,ANGPTL6,F12,CFHR5,LYZ,VWF,PSMA6,LCAT,NID1,CSTA,CP,KLKB1,F13A1,DNAH5,CENPE,PON3,TCIRG1,CDH11,FCN3,AHSG,HABP2,SERPING1,SEC24D,CSF1R,SERPINI1,MASP1,GUCY1A1,A2ML1,SLC27A4,PCSK9,BMP1,FGB,DST,F2,EFEMP2,STAB1,C1QB,CFHR1,NDUFS8,SUZ12,SERPINF2,FBN1,KDM3B,P4HB,PAPPA,FBLN1,DNAH8,FGG,TUBB,THBS4,PAPLN,FILIP1L,BLM,COL18A1,F8,C8A,CENPF,F13B,CFHR4,CFH,SERPINC1,F5,HTRA1,ADAMTSL4,ECM1,FLNA,CRTAC1,C8B,ADAMTS13,SVEP1,C1QC,C1QA,F7,ADGRG2,PROS1,EFEMP1,NAV2,MASP2,F11,C1S,PRG4,ABCA13,ITIH3,C4B,TGFBI,IGFALS,GC,FBN2,C1R,APOA5,ATP1A1,VIM,TTN,CFD,PLCG2,PAMR1,KNG1,ACTB,TRIM8,B2M,FGA,TPM3

Monarch	HP:0025015	Abnormal vascular morphology	47	1027	0.62	6.39e-13	SERPIND1,F9,MMP2,MGP,APOB,PROC,TTR,CAT,APOE,ANGPTL6,F12,VWF,LCAT,NID1,F13A1,DNAH5,TCIRG1,HABP2,SEC24D,MASP1,GUCY1A1,A2ML1,PCSK9,FGB,F2,EFEMP2,SERPINF2,FBN1,KDM3B,P4HB,FGG,COL18A1,F8,F13B,F5,HTRA1,FLNA,CRTAC1,ADAMTS13,F7,PROS1,EFEMP1,FBN2,KNG1,ACTB,FGA,TPM3
Monarch	HP:0002597	Abnormality of the vasculature	60	1673	0.51	7.75e-13	SERPIND1,F9,MMP2,MGP,APOB,PROC,APOA1,TTR,CAT,APOE,ANGPTL6,F12,LYZ,VWF,LCAT,NID1,CSTA,F13A1,DNAH5,TCIRG1,HABP2,SERPING1,SEC24D,MASP1,GUCY1A1,A2ML1,PCSK9,BMP1,FGB,F2,EFEMP2,SERPINF2,FBN1,KDM3B,P4HB,FGG,BLM,COL18A1,F8,CENPF,F13B,SERPINC1,F5,HTRA1,FLNA,CRTAC1,ADAMTS13,F7,PROS1,EFEMP1,C1S,TGFBI,FBN2,C1R,PLCG2,KNG1,ACTB,B2M,FGA,TPM3
Monarch	HP:0003256	Abnormality of the coagulation cascade	18	105	1.19	3.60e-12	F9,PROC,F12,VWF,F13A1,FGB,F2,FGG,F8,F13B,SERPINC1,F5,FLNA,F7,PROS1,F11,KNG1,FGA
Monarch	EFO:0004747	Protein measurement	121	5856	0.27	4.25e-12	CPB2,CETP,APOH,C5,AFM,GPLD1,LTF,APOB,IGFBP5,PROC,APOA1,CAT,APOE,ANGPTL6,F12,APCS,CFHR5,LYZ,VWF,SERPINA10,LCAT,NID1,CP,KLKB1,PPP2R3A,DNAH5,PON3,TCIRG1,HPX,CDH11,ITIH1,AHSG,HABP2,SAA4,SERPING1,ANKRD44,CSF1R,IGF2BP1,FCN2,PGLYRP2,MASP1,APEH,PCSK9,FGB,F2,INHBC,PSG1,UBE3C,PRG2,STAB1,APOL1,SERPINF2,PAPPA,FBLN1,DNAH8,OIT3,HSP90AA1,FGG,THBS4,PRDX6,APOD,PAPLN,ASGR2,GIT2,APOA4,CNDP1,COL18A1,CENPF,F13B,CFHR4,CFH,QSOX1,F5,CFAP46,ADAM12,ECM1,CRTAC1,C8B,FCN1,ADAMTS13,CCN5,CNTRL,SVEP1,C1QC,F7,APOM,CA6,SOD3,EFCAB5,YWHAZ,NAV2,PSG4,F11,C1S,PSG2,PCYOX1,ADIPOQ,ABCA13,MST1,SPARCL1,ITIH3,C4B,TGFBI,CFB,IGFALS,GC,FBN2,TSKU,HPR,C1R,APOA5,RNASE4,TTN,APOC4,PLCG2,KNG1,ACTB,TRIM66,TRIM8,FGA,TPM3
Monarch	HP:0001928	Abnormality of coagulation	21	170	1.05	4.25e-12	F9,PROC,F12,VWF,KLKB1,F13A1,A2ML1,FGB,F2,SERPINF2,FGG,F8,F13B,SERPINC1,F5,FLNA,F7,PROS1,F11,KNG1,FGA
Monarch	HP:0011025	Abnormal cardiovascular system physiology	48	1169	0.57	6.04e-12	F9,MMP2,MGP,APOB,PROC,APOA1,TTR,APOE,ANGPTL6,LYZ,VWF,LCAT,CP,F13A1,TCIRG1,SERPING1,GUCY1A1,A2ML1,PCSK9,BMP1,FGB,DST,F2,EFEMP2,SERPINF2,FBN1,FGG,TUBB,F8,F13B,SERPINC1,F5,HTRA1,ADAMTSL4,FLNA,CRTAC1,ADAMTS13,F7,PROS1,F11,FBN2,TTN,PLCG2,KNG1,ACTB,B2M,FGA,TPM3
Monarch	HP:0001626	Abnormality of the cardiovascular system	71	2438	0.42	1.03e-11	SERPIND1,F9,MMP2,MGP,APOB,PROC,APOA1,TTR,CAT,APOE,ANGPTL6,F12,LYZ,VWF,LCAT,NID1,CSTA,CP,KLKB1,F13A1,DNAH5,TCIRG1,HABP2,SERPING1,SEC24D,MASP1,GUCY1A1,A2ML1,PCSK9,BMP1,FGB,DST,F2,EFEMP2,NDUFS8,SUZ12,SERPINF2,FBN1,KDM3B,P4HB,FGG,TUBB,BLM,COL18A1,F8,CENPF,F13B,SERPINC1,F5,HTRA1,ADAMTSL4,FLNA,CRTAC1,ADAMTS13,F7,PROS1,EFEMP1,F11,C1S,PRG4,TGFBI,FBN2,C1R,TTN,PLCG2,KNG1,ACTB,TRIM8,B2M,FGA,TPM3
Monarch	HP:0000225	Gingival bleeding	13	45	1.42	5.31e-11	CAT,F13A1,FGB,F2,SERPINF2,FGG,F8,F13B,F5,F7,C1S,C1R,FGA
Monarch	EFO:0004634	Coagulation factor measurement	18	132	1.09	5.75e-11	CPB2,PROC,CFHR5,VWF,KLKB1,F13A1,PPP2R3A,FGB,FGG,CFHR4,CFH,F5,ADAMTS13,F7,F11,TSKU,KNG1,FGA
Monarch	HP:0005339	Abnormality of complement system	11	24	1.62	6.72e-11	C5,C1QB,C8A,CFH,C8B,C1QC,C1QA,MASP2,C1S,C4B,CFD
Monarch	HP:0001871	Abnormality of blood and blood-forming tissues	46	1214	0.54	2.54e-10	SERPIND1,F9,APOB,PROC,APOA1,CAT,APOE,ANGPTL6,F12,VWF,LCAT,CP,KLKB1,F13A1,TCIRG1,SEC24D,GUCY1A1,A2ML1,SLC27A4,FGB,F2,NDUFS8,SERPINF2,FBN1,P4HB,FGG,BLM,F8,F13B,CFH,SERPINC1,F5,FLNA,ADAMTS13,SVEP1,F7,PROS1,F11,C1S,FBN2,C1R,TTN,PLCG2,KNG1,B2M,FGA
Monarch	HP:0005261	Joint hemorrhage	10	19	1.68	2.79e-10	F9,VWF,F13A1,F2,SERPINF2,F8,F13B,F5,F7,F11
Monarch	HP:0001892	Abnormal bleeding	27	411	0.78	2.85e-10	F9,PROC,CAT,APOE,ANGPTL6,F12,VWF,F13A1,TCIRG1,SEC24D,A2ML1,FGB,F2,SERPINF2,FBN1,P4HB,FGG,F8,F13B,F5,FLNA,F7,PROS1,F11,C1S,C1R,FGA
Monarch	HP:0100659	Abnormal cerebral vascular morphology	26	381	0.79	3.33e-10	F9,APOB,PROC,TTR,APOE,ANGPTL6,F13A1,GUCY1A1,PCSK9,FGB,F2,SERPINF2,FBN1,FGG,F8,F13B,F5,HTRA1,FLNA,CRTAC1,ADAMTS13,F7,PROS1,KNG1,ACTB,FGA
Monarch	HP:0004431	Complement deficiency	10	21	1.64	4.99e-10	C5,C1QB,C8A,CFH,C8B,C1QC,C1QA,MASP2,C4B,CFD
Monarch	HP:0002715	Abnormality of the immune system	54	1682	0.46	6.03e-10	MMP2,C5,HSD17B1,MGP,APOA1,CAT,APOE,CFHR5,LYZ,PSMA6,LCAT,CSTA,F13A1,DNAH5,TCIRG1,FCN3,HABP2,SERPING1,A2ML1,SLC27A4,FGB,F2,EFEMP2,C1QB,CFHR1,FBN1,FGG,BLM,COL18A1,F8,C8A,CENPF,F13B,CFHR4,CFH,ECM1,FLNA,C8B,SVEP1,C1QC,C1QA,NAV2,MASP2,C1S,PRG4,C4B,C1R,TTN,CFD,PLCG2,ACTB,B2M,FGA,TPM3
Monarch	HP:0030680	Abnormality of cardiovascular system morphology	54	1680	0.47	6.03e-10	SERPIND1,F9,MMP2,MGP,APOB,PROC,TTR,CAT,APOE,ANGPTL6,F12,VWF,LCAT,NID1,F13A1,DNAH5,TCIRG1,HABP2,SEC24D,MASP1,GUCY1A1,A2ML1,PCSK9,FGB,F2,EFEMP2,NDUFS8,SUZ12,SERPINF2,FBN1,KDM3B,P4HB,FGG,TUBB,COL18A1,F8,F13B,F5,HTRA1,FLNA,CRTAC1,ADAMTS13,F7,PROS1,EFEMP1,PRG4,FBN2,TTN,KNG1,ACTB,TRIM8,B2M,FGA,TPM3
Monarch	HP:0001933	Subcutaneous hemorrhage	18	189	0.94	8.17e-09	PROC,APOE,VWF,F13A1,TCIRG1,SEC24D,F2,SERPINF2,FBN1,P4HB,F8,F13B,F5,FLNA,F7,PROS1,C1S,C1R
Monarch	HP:0011276	Vascular skin abnormality	25	433	0.72	2.25e-08	PROC,APOE,F12,VWF,CSTA,F13A1,TCIRG1,SERPING1,SEC24D,MASP1,GUCY1A1,F2,SERPINF2,FBN1,P4HB,BLM,F8,F13B,F5,FLNA,F7,PROS1,C1S,C1R,PLCG2
Monarch	HP:0011890	Prolonged bleeding following procedure	9	23	1.55	2.29e-08	F9,VWF,F13A1,F2,F8,F13B,F5,F7,F11
Monarch	HP:0000978	Bruising susceptibility	15	130	1.02	3.23e-08	VWF,F13A1,TCIRG1,SEC24D,F2,SERPINF2,FBN1,P4HB,F8,F13B,F5,FLNA,F7,C1S,C1R
Monarch	HP:0006298	Prolonged bleeding after dental extraction	8	16	1.66	5.62e-08	F9,VWF,F13A1,F2,F8,F13B,F5,F11
Monarch	HP:0001977	Abnormal thrombosis	13	94	1.1	7.64e-08	SERPIND1,F9,PROC,F12,FGB,F2,FGG,F8,SERPINC1,F5,PROS1,KNG1,FGA
Monarch	HP:0000951	Abnormality of the skin	53	1888	0.41	1.02e-07	F9,MMP2,C5,HSD17B1,MGP,APOB,PROC,APOA1,CAT,APOE,F12,LYZ,VWF,PSMA6,CSTA,F13A1,CENPE,TCIRG1,CDH11,FCN3,AHSG,SERPING1,SEC24D,MASP1,GUCY1A1,A2ML1,SLC27A4,PCSK9,DST,F2,EFEMP2,SUZ12,SERPINF2,FBN1,P4HB,TUBB,BLM,F8,F13B,CFHR4,F5,ECM1,FLNA,ADAMTS13,F7,PROS1,C1S,C1R,TTN,PLCG2,ACTB,B2M,FGA
Monarch	HP:0000421	Epistaxis	12	78	1.15	1.21e-07	F9,VWF,F13A1,FGB,F2,FGG,F8,F13B,F5,F7,F11,FGA
Monarch	HP:0012233	Intramuscular hematoma	7	10	1.8	1.28e-07	F9,F13A1,F2,SERPINF2,F8,F13B,F7
Monarch	HP:0012649	Increased inflammatory response	36	981	0.52	1.56e-07	C5,HSD17B1,MGP,APOA1,CAT,APOE,CFHR5,LYZ,PSMA6,CSTA,F13A1,DNAH5,TCIRG1,FCN3,EFEMP2,C1QB,CFHR1,FBN1,BLM,F13B,CFHR4,CFH,ECM1,FLNA,C1QC,C1QA,NAV2,MASP2,C1S,PRG4,C4B,C1R,PLCG2,B2M,FGA,TPM3
Monarch	HP:0002170	Intracranial hemorrhage	14	130	0.99	2.42e-07	F9,ANGPTL6,F13A1,FGB,F2,SERPINF2,FBN1,FGG,F8,F13B,F5,F7,PROS1,FGA
Monarch	HP:0004936	Venous thrombosis	11	65	1.19	2.42e-07	SERPIND1,F9,PROC,FGB,F2,FGG,SERPINC1,F5,PROS1,KNG1,FGA
Monarch	HP:0000271	Abnormality of the face	64	2641	0.34	2.49e-07	F9,MMP2,MGP,LTF,APOB,PROC,CAT,APOE,F12,LYZ,VWF,NID1,CP,F13A1,DNAH5,CENPE,PON3,TCIRG1,CDH11,AHSG,SERPING1,SEC24D,CSF1R,MASP1,A2ML1,PCSK9,BMP1,FGB,DST,F2,EFEMP2,SUZ12,SERPINF2,FBN1,KDM3B,P4HB,PAPPA,FBLN1,FGG,TUBB,FILIP1L,BLM,COL18A1,F8,CENPF,F13B,F5,ADAMTSL4,ECM1,FLNA,CRTAC1,F7,F11,C1S,ABCA13,FBN2,C1R,TTN,PLCG2,ACTB,TRIM8,B2M,FGA,TPM3
Monarch	HP:0001934	Persistent bleeding after trauma	7	12	1.72	2.68e-07	F9,VWF,F13A1,SERPINF2,F8,F13B,F5

Monarch	HP:0001574	Abnormality of the integument	58	2266	0.37	2.72e-07	F9,MMP2,C5,HSD17B1,MGP,APOB,PROC,APOA1,CAT,APOE,F12,LYZ,VWF,PSMA6,CSTA,F13A1,CENPE,TCIRG1,CDH11,FCN3,AHSG,SERPING1,SEC24D,MASP1,GUCY1A1,A2ML1,SLC27A4,PCSK9,BMP1,DST,F2,EFEMP2,NDUFS8,SUZ12,SERPINF2,FBN1,P4HB,TUBB,BLM,COL18A1,F8,F13B,CFHR4,F5,HTRA1,ECM1,FLNA,ADAMTS13,F7,PROS1,C1S,C1R,TTN,PLCG2,ACTB,TRIM8,B2M,FGA
Monarch	HP:0000168	Abnormality of the gingiva	16	190	0.88	3.43e-07	MMP2,CAT,F13A1,TCIRG1,FGB,F2,SERPINF2,FGG,F8,F13B,F5,ECM1,F7,C1S,C1R,FGA
Monarch	HP:0000152	Abnormality of head or neck	67	2882	0.32	4.17e-07	F9,MMP2,MGP,LTF,APOB,PROC,CAT,APOE,F12,LYZ,VWF,NID1,CP,F13A1,DNAH5,CENPE,PON3,TCIRG1,CDH11,AHSG,HABP2,SERPING1,SEC24D,CSF1R,MASP1,A2ML1,PCSK9,BMP1,FGB,DST,F2,EFEMP2,NDUFS8,SUZ12,SERPINF2,FBN1,KDM3B,P4HB,PAPPA,FBLN1,FGG,TUBB,FILIP1L,BLM,COL18A1,F8,CENPF,F13B,F5,ADAMTSL4,ECM1,FLNA,CRTAC1,F7,F11,C1S,ABCA13,IGFALS,FBN2,C1R,TTN,PLCG2,ACTB,TRIM8,B2M,FGA,TPM3
Monarch	HP:0010978	Abnormality of immune system physiology	42	1347	0.45	4.17e-07	MMP2,C5,HSD17B1,MGP,APOA1,CAT,APOE,CFHR5,LYZ,PSMA6,CSTA,F13A1,DNAH5,TCIRG1,FCN3,SERPING1,EFEMP2,C1QB,CFHR1,FBN1,BLM,C8A,F13B,CFHR4,CFH,ECM1,FLNA,C8B,C1QC,C1QA,NAV2,MASP2,C1S,PRG4,C4B,C1R,CFD,PLCG2,ACTB,B2M,FGA,TPM3
Monarch	HP:0003645	Prolonged partial thromboplastin time	8	24	1.48	4.59e-07	F9,F12,KLKB1,F2,F8,F5,F11,KNG1
Monarch	HP:0033353	Abnormal blood vessel morphology	31	792	0.55	5.09e-07	SERPIND1,MMP2,MGP,APOB,PROC,CAT,APOE,ANGPTL6,F12,VWF,LCAT,NID1,DNAH5,TCIRG1,SEC24D,MASP1,GUCY1A1,A2ML1,PCSK9,EFEMP2,FBN1,KDM3B,P4HB,COL18A1,HTRA1,FLNA,PROS1,EFEMP1,FBN2,ACTB,TPM3
Monarch	EFO:0004310	Partial thromboplastin time	7	15	1.63	7.31e-07	APOH,F12,KLKB1,F5,F11,C1S,KNG1
Monarch	HP:0000234	Abnormality of the head	66	2865	0.32	7.31e-07	F9,MMP2,MGP,LTF,APOB,PROC,CAT,APOE,F12,LYZ,VWF,NID1,CP,F13A1,DNAH5,CENPE,PON3,TCIRG1,CDH11,AHSG,SERPING1,SEC24D,CSF1R,MASP1,A2ML1,PCSK9,BMP1,FGB,DST,F2,EFEMP2,NDUFS8,SUZ12,SERPINF2,FBN1,KDM3B,P4HB,PAPPA,FBLN1,FGG,TUBB,FILIP1L,BLM,COL18A1,F8,CENPF,F13B,F5,ADAMTSL4,ECM1,FLNA,CRTAC1,F7,F11,C1S,ABCA13,IGFALS,FBN2,C1R,TTN,PLCG2,ACTB,TRIM8,B2M,FGA,TPM3
Monarch	HP:0002725	Systemic lupus erythematosus	8	26	1.45	7.31e-07	SERPING1,C1QB,C8A,C1QC,C1QA,MASP2,C1S,C1R
Monarch	HP:0011029	Internal hemorrhage	17	244	0.8	1.22e-06	F9,PROC,ANGPTL6,VWF,F13A1,FGB,F2,SERPINF2,FBN1,FGG,F8,F13B,F5,F7,PROS1,F11,FGA
Monarch	HP:0011121	Abnormality of skin morphology	46	1648	0.4	1.47e-06	F9,MMP2,MGP,APOB,PROC,APOA1,CAT,APOE,F12,VWF,CSTA,F13A1,CENPE,TCIRG1,CDH11,AHSG,SERPING1,SEC24D,MASP1,GUCY1A1,A2ML1,SLC27A4,PCSK9,DST,F2,EFEMP2,SUZ12,SERPINF2,FBN1,P4HB,TUBB,BLM,F8,F13B,F5,ECM1,FLNA,ADAMTS13,F7,PROS1,C1S,C1R,TTN,PLCG2,ACTB,B2M
Monarch	HP:0025031	Abnormality of the digestive system	58	2389	0.34	1.47e-06	F9,MMP2,C5,APOB,APOA1,TTR,APOE,F12,LYZ,VWF,LCAT,CP,F13A1,DNAH5,PON3,TCIRG1,CDH11,FCN3,HABP2,SERPING1,MASP1,GUCY1A1,A2ML1,PCSK9,BMP1,FGB,DST,F2,EFEMP2,NDUFS8,SUZ12,FBN1,KDM3B,FGG,TUBB,BLM,COL18A1,F8,CENPF,F13B,F5,ECM1,FLNA,ADAMTS13,F7,NAV2,MASP2,F11,C1S,C4B,FBN2,C1R,PLCG2,ACTB,TRIM8,B2M,FGA,TPM3
Monarch	HP:0004846	Prolonged bleeding after surgery	7	18	1.55	1.75e-06	F9,VWF,F13A1,F8,F13B,F5,F7
Monarch	HP:0010935	Abnormality of the upper urinary tract	36	1101	0.47	1.84e-06	F9,APOB,APOA1,TTR,APOE,CFHR5,LYZ,LCAT,CDH11,HABP2,MASP1,PCSK9,F2,EFEMP2,C1QB,CFHR1,NDUFS8,SERPINF2,FBN1,BLM,COL18A1,F8,CENPF,CFH,F5,FLNA,ADAMTS13,C1QC,C1QA,ADGRG2,C1R,ATP1A1,ACTB,TRIM8,B2M,FGA
Monarch	HP:0012639	Abnormal nervous system morphology	60	2546	0.33	2.00e-06	F9,MGP,APOB,PROC,TTR,APOE,ANGPTL6,NID1,CP,F13A1,DNAH5,CENPE,PON3,TCIRG1,CDH11,AHSG,HABP2,SERPING1,SEC24D,CSF1R,SERPINI1,MASP1,GUCY1A1,PCSK9,FGB,F2,EFEMP2,NDUFS8,SUZ12,SERPINF2,FBN1,KDM3B,P4HB,FBLN1,FGG,TUBB,BLM,COL18A1,F8,C8A,CENPF,F13B,F5,HTRA1,ECM1,FLNA,CRTAC1,C8B,ADAMTS13,F7,PROS1,C4B,ATP1A1,TTN,KNG1,ACTB,TRIM8,B2M,FGA,TPM3
Monarch	HP:0011891	Post-partum hemorrhage	6	10	1.74	2.79e-06	F13A1,F2,F8,F13B,F5,F7
Monarch	HP:0002625	Deep venous thrombosis	7	20	1.5	2.92e-06	SERPIND1,F9,PROC,F2,SERPINC1,F5,PROS1
Monarch	HP:0000119	Abnormality of the genitourinary system	54	2189	0.35	2.98e-06	F9,MMP2,APOB,APOA1,TTR,APOE,CFHR5,LYZ,VWF,LCAT,F13A1,DNAH5,CDH11,AHSG,HABP2,MASP1,GUCY1A1,A2ML1,PCSK9,FGB,F2,EFEMP2,C1QB,CFHR1,NDUFS8,SUZ12,SERPINF2,FBN1,KDM3B,FBLN1,DNAH8,FGG,TUBB,BLM,COL18A1,F8,CENPF,F13B,CFH,F5,HTRA1,FLNA,ADAMTS13,C1QC,C1QA,F7,ADGRG2,F11,C1R,ATP1A1,ACTB,TRIM8,B2M,FGA
Monarch	HP:0000707	Abnormality of the nervous system	73	3471	0.28	2.98e-06	F9,MMP2,MGP,APOB,PROC,APOA1,TTR,CAT,APOE,ANGPTL6,NID1,CP,F13A1,DNAH5,CENPE,PON3,TCIRG1,CDH11,AHSG,HABP2,SERPING1,SEC24D,CSF1R,SERPINI1,MASP1,GUCY1A1,A2ML1,PCSK9,BMP1,FGB,DST,F2,EFEMP2,STAB1,NDUFS8,SUZ12,SERPINF2,FBN1,KDM3B,P4HB,FBLN1,FGG,TUBB,BLM,COL18A1,F8,C8A,CENPF,F13B,F5,HTRA1,ADAMTSL4,ECM1,FLNA,CRTAC1,C8B,ADAMTS13,F7,PROS1,ITIH3,C4B,TGFBI,FBN2,ATP1A1,VIM,TTN,PAMR1,KNG1,ACTB,TRIM8,B2M,FGA,TPM3
Monarch	HP:0011830	Abnormal oral mucosa morphology	18	305	0.73	3.97e-06	MMP2,LTF,CAT,LYZ,F13A1,TCIRG1,FGB,F2,SERPINF2,FGG,F8,F13B,F5,ECM1,F7,C1S,C1R,FGA
Monarch	HP:0000790	Hematuria	12	118	0.97	4.20e-06	F9,APOA1,CFHR5,LYZ,F2,CFHR1,SERPINF2,F8,CFH,F5,ADAMTS13,FGA
Monarch	HP:0002011	Morphological central nervous system abnormality	57	2416	0.33	4.43e-06	F9,MGP,APOB,PROC,TTR,APOE,ANGPTL6,NID1,CP,F13A1,DNAH5,CENPE,PON3,TCIRG1,CDH11,AHSG,HABP2,SEC24D,CSF1R,SERPINI1,MASP1,GUCY1A1,PCSK9,FGB,F2,EFEMP2,NDUFS8,SUZ12,SERPINF2,FBN1,KDM3B,P4HB,FBLN1,FGG,TUBB,BLM,COL18A1,F8,C8A,CENPF,F13B,F5,HTRA1,ECM1,FLNA,CRTAC1,C8B,ADAMTS13,F7,PROS1,C4B,ATP1A1,KNG1,ACTB,TRIM8,B2M,FGA
Monarch	HP:0000153	Abnormality of the mouth	50	1992	0.36	6.28e-06	MMP2,MGP,LTF,CAT,APOE,LYZ,NID1,F13A1,CENPE,PON3,TCIRG1,CDH11,SERPING1,SEC24D,MASP1,A2ML1,BMP1,FGB,DST,F2,EFEMP2,SUZ12,SERPINF2,FBN1,KDM3B,P4HB,PAPPA,FBLN1,FGG,TUBB,FILIP1L,BLM,F8,CENPF,F13B,F5,ECM1,FLNA,CRTAC1,F7,C1S,ABCA13,FBN2,C1R,TTN,ACTB,TRIM8,B2M,FGA,TPM3
Monarch	HP:0000132	Menorrhagia	8	38	1.28	6.49e-06	VWF,F13A1,F2,F8,F13B,F5,F7,F11
Monarch	HP:0000077	Abnormality of the kidney	34	1077	0.46	9.20e-06	F9,APOB,APOA1,TTR,APOE,CFHR5,LYZ,LCAT,HABP2,MASP1,PCSK9,F2,EFEMP2,C1QB,CFHR1,NDUFS8,SERPINF2,FBN1,BLM,F8,CENPF,CFH,F5,FLNA,ADAMTS13,C1QC,C1QA,ADGRG2,C1R,ATP1A1,ACTB,TRIM8,B2M,FGA
Monarch	HP:0000163	Abnormal oral cavity morphology	48	1901	0.36	1.02e-05	MMP2,MGP,LTF,CAT,APOE,LYZ,NID1,F13A1,CENPE,TCIRG1,CDH11,SERPING1,SEC24D,MASP1,A2ML1,BMP1,FGB,DST,F2,EFEMP2,SUZ12,SERPINF2,FBN1,KDM3B,P4HB,PAPPA,FGG,TUBB,FILIP1L,BLM,F8,CENPF,F13B,F5,ECM1,FLNA,CRTAC1,F7,C1S,ABCA13,FBN2,C1R,TTN,ACTB,TRIM8,B2M,FGA,TPM3
Monarch	HP:0011354	Generalized abnormality of skin	29	821	0.51	1.02e-05	F9,PROC,APOE,F12,VWF,CSTA,F13A1,CENPE,TCIRG1,CDH11,SERPING1,SEC24D,MASP1,GUCY1A1,F2,SERPINF2,FBN1,P4HB,BLM,F8,F13B,F5,FLNA,ADAMTS13,F7,PROS1,C1S,C1R,PLCG2
Monarch	HP:0005368	Abnormality of humoral immunity	15	234	0.76	1.94e-05	C5,C1QB,BLM,C8A,CFH,FLNA,C8B,C1QC,C1QA,MASP2,C1S,C4B,CFD,PLCG2,B2M
Monarch	HP:0010989	Abnormality of the intrinsic pathway	7	29	1.34	1.95e-05	F9,F12,VWF,F8,SERPINC1,F11,KNG1
Monarch	HP:0012211	Abnormal renal physiology	23	560	0.57	2.01e-05	F9,APOA1,TTR,APOE,CFHR5,LYZ,LCAT,F2,EFEMP2,C1QB,CFHR1,NDUFS8,SERPINF2,F8,CFH,F5,ADAMTS13,C1QC,C1QA,C1R,ATP1A1,B2M,FGA
Monarch	HP:0002634	Arteriosclerosis	9	70	1.07	3.50e-05	SERPIND1,APOB,CAT,APOE,ANGPTL6,LCAT,PCSK9,FBN1,HTRA1
Monarch	HP:0002960	Autoimmunity	11	122	0.91	4.16e-05	MMP2,TCIRG1,SERPING1,C1QB,C8A,C1QC,C1QA,MASP2,C1S,C1R,PLCG2
Monarch	HP:0030137	Prolonged bleeding following circumcision	5	9	1.7	4.72e-05	F13A1,F2,F8,F13B,F5

Monarch	HP:0030140	Oral cavity bleeding	5	9	1.7	4.72e-05	F13A1,F2,F8,F13B,F5
Monarch	HP:0010990	Abnormality of the common coagulation pathway	7	35	1.26	5.47e-05	F13A1,FGB,FGG,F13B,SERPINC1,F5,FGA
Monarch	HP:0000924	Abnormality of the skeletal system	58	2708	0.29	6.91e-05	F9,MMP2,MGP,APOB,TTR,APOE,VWF,NID1,F13A1,DNAH5,CENPE,TCIRG1,CDH11,AHSG,HABP2,SEC24D,CSF1R,MASP1,A2ML1,PCSK9,BMP1,FGB,DST,F2,EFEMP2,NDUFS8,SUZ12,SERPINF2,FBN1,KDM3B,P4HB,FBLN1,FGG,TUBB,BLM,COL18A1,F8,CENPF,F13B,F5,HTRA1,ADAMTSL4,ECM1,FLNA,F7,F11,C1S,PRG4,IGFALS,FBN2,C1R,ATP1A1,TTN,ACTB,TRIM8,B2M,FGA,TPM3
Monarch	HP:0030163	Abnormal vascular physiology	17	340	0.66	6.91e-05	MGP,PROC,ANGPTL6,TCIRG1,GUCY1A1,BMP1,F2,FBN1,SERPINC1,F5,HTRA1,FLNA,CRTAC1,ADAMTS13,PROS1,KNG1,ACTB
Monarch	HP:0012443	Abnormality of brain morphology	51	2254	0.31	8.54e-05	F9,MGP,APOB,PROC,TTR,APOE,ANGPTL6,NID1,CP,F13A1,DNAH5,CENPE,TCIRG1,CDH11,AHSG,SEC24D,CSF1R,SERPINI1,MASP1,GUCY1A1,PCSK9,FGB,F2,EFEMP2,NDUFS8,SUZ12,SERPINF2,FBN1,KDM3B,P4HB,FBLN1,FGG,TUBB,BLM,COL18A1,F8,CENPF,F13B,F5,HTRA1,ECM1,FLNA,CRTAC1,ADAMTS13,F7,PROS1,ATP1A1,KNG1,ACTB,TRIM8,FGA
Monarch	HP:0000123	Nephritis	8	59	1.09	0.00010	CFHR5,EFEMP2,C1QB,CFHR1,CFH,C1QC,C1QA,C1R
Monarch	HP:0011024	Abnormality of the gastrointestinal tract	37	1384	0.39	0.00010	F9,APOE,F12,VWF,F13A1,DNAH5,TCIRG1,CDH11,FCN3,HABP2,SERPING1,GUCY1A1,FGB,F2,EFEMP2,SUZ12,FBN1,KDM3B,FGG,BLM,COL18A1,F8,CENPF,F13B,F5,ECM1,FLNA,F7,MASP2,F11,FBN2,C1R,PLCG2,ACTB,B2M,FGA,TPM3
Monarch	HP:0011004	Abnormal systemic arterial morphology	18	398	0.61	0.00012	SERPIND1,MMP2,APOB,CAT,APOE,ANGPTL6,F12,LCAT,SEC24D,GUCY1A1,PCSK9,EFEMP2,FBN1,P4HB,HTRA1,FLNA,FBN2,TPM3
Monarch	HP:0002239	Gastrointestinal hemorrhage	10	111	0.91	0.00013	F9,VWF,FGB,F2,FGG,F8,F5,F7,F11,FGA
Monarch	HP:0001634	Mitral valve prolapse	9	86	0.98	0.00014	MMP2,VWF,SEC24D,FBN1,P4HB,FLNA,FBN2,TTN,TPM3
Monarch	HP:0011355	Localized skin lesion	25	738	0.49	0.00014	MMP2,MGP,APOB,PROC,APOA1,APOE,F12,CSTA,TCIRG1,SEC24D,MASP1,A2ML1,PCSK9,DST,SUZ12,FBN1,P4HB,BLM,ECM1,FLNA,PROS1,C1S,C1R,ACTB,B2M
Monarch	HP:0011842	Abnormal skeletal morphology	55	2573	0.29	0.00014	F9,MMP2,MGP,APOB,TTR,APOE,VWF,NID1,F13A1,DNAH5,CENPE,TCIRG1,CDH11,AHSG,SEC24D,CSF1R,MASP1,A2ML1,PCSK9,BMP1,FGB,DST,F2,EFEMP2,NDUFS8,SUZ12,SERPINF2,FBN1,KDM3B,P4HB,FBLN1,FGG,TUBB,BLM,COL18A1,F8,CENPF,F13B,F5,HTRA1,ADAMTSL4,FLNA,F7,F11,C1S,PRG4,IGFALS,FBN2,C1R,TTN,ACTB,TRIM8,B2M,FGA,TPM3
Monarch	HP:0012223	Splenic rupture	4	4	1.96	0.00014	FGB,FGG,F8,FGA
Monarch	HP:0033127	Abnormality of the musculoskeletal system	63	3173	0.26	0.00021	F9,MMP2,MGP,APOB,APOA1,TTR,APOE,VWF,NID1,CP,F13A1,DNAH5,CENPE,PON3,TCIRG1,CDH11,AHSG,HABP2,SEC24D,CSF1R,MASP1,A2ML1,PCSK9,BMP1,FGB,DST,F2,EFEMP2,NDUFS8,SUZ12,SERPINF2,FBN1,KDM3B,P4HB,FBLN1,FGG,TUBB,BLM,COL18A1,F8,CENPF,F13B,F5,HTRA1,ADAMTSL4,ECM1,FLNA,F7,F11,C1S,PRG4,TGFBI,IGFALS,FBN2,C1R,ATP1A1,TTN,PLCG2,ACTB,TRIM8,B2M,FGA,TPM3
Monarch	HP:0005542	Prolonged whole-blood clotting time	4	5	1.86	0.00023	F9,F12,VWF,F5
Monarch	HP:0011884	Abnormal umbilical stump bleeding	4	5	1.86	0.00023	F13A1,F2,SERPINF2,F13B
Monarch	HP:0010979	Abnormality of lipoprotein cholesterol concentration	7	47	1.13	0.00026	CETP,APOB,APOA1,APOE,LCAT,PCSK9,APOA5
Monarch	HP:0030962	Abnormal morphology of the great vessels	21	571	0.52	0.00030	MMP2,MGP,APOB,APOE,ANGPTL6,NID1,DNAH5,TCIRG1,SEC24D,MASP1,A2ML1,PCSK9,EFEMP2,FBN1,KDM3B,P4HB,COL18A1,FLNA,FBN2,ACTB,TPM3
Monarch	HP:0000079	Abnormality of the urinary system	40	1657	0.34	0.00034	F9,APOB,APOA1,TTR,APOE,CFHR5,LYZ,LCAT,CDH11,HABP2,MASP1,PCSK9,F2,EFEMP2,C1QB,CFHR1,NDUFS8,SERPINF2,FBN1,KDM3B,TUBB,PAPLN,BLM,COL18A1,F8,CENPF,CFH,F5,HTRA1,FLNA,ADAMTS13,C1QC,C1QA,ADGRG2,C1R,ATP1A1,ACTB,TRIM8,B2M,FGA
Monarch	HP:0001000	Abnormality of skin pigmentation	18	439	0.57	0.00038	MMP2,PROC,CAT,APOE,CSTA,A2ML1,DST,F2,SUZ12,FBN1,TUBB,BLM,FLNA,PROS1,C1S,C1R,PLCG2,ACTB
Monarch	HP:0001367	Abnormal joint morphology	27	905	0.43	0.00048	F9,MMP2,APOE,VWF,F13A1,MASP1,A2ML1,BMP1,FGB,F2,EFEMP2,SUZ12,SERPINF2,FBN1,FBLN1,FGG,F8,F13B,F5,FLNA,F7,F11,PRG4,FBN2,ACTB,FGA,TPM3
Monarch	HP:0010980	Hyperlipoproteinemia	6	33	1.22	0.00049	CETP,APOB,APOE,LCAT,PCSK9,APOA5
Monarch	HP:0012372	Abnormal eye morphology	46	2077	0.3	0.00050	MMP2,MGP,APOB,PROC,APOA1,TTR,APOE,F12,LCAT,CP,DNAH5,TCIRG1,CDH11,SERPING1,SEC24D,CSF1R,MASP1,A2ML1,PCSK9,BMP1,DST,EFEMP2,NDUFS8,SUZ12,FBN1,P4HB,FBLN1,TUBB,BLM,COL18A1,CENPF,CFH,SERPINC1,ADAMTSL4,FLNA,PROS1,EFEMP1,ABCA13,TGFBI,FBN2,C1R,VIM,PLCG2,ACTB,B2M,TPM3
Monarch	HP:0004418	Thrombophlebitis	4	7	1.71	0.00054	PROC,F2,SERPINC1,PROS1
Monarch	HP:0005305	Cerebral venous thrombosis	4	7	1.71	0.00054	PROC,F2,SERPINC1,PROS1
Monarch	HP:0008065	Aplasia/Hypoplasia of the skin	11	170	0.77	0.00062	MGP,PROC,TCIRG1,SEC24D,DST,FBN1,P4HB,FLNA,PROS1,C1S,C1R
Monarch	HP:0001939	Abnormality of metabolism/homeostasis	47	2168	0.29	0.00065	CETP,F9,MMP2,APOB,APOA1,TTR,CAT,APOE,F12,CFHR5,LYZ,LCAT,CP,TCIRG1,CDH11,SERPING1,CSF1R,A2ML1,PCSK9,FGB,DST,F2,CFHR1,NDUFS8,SERPINF2,FBN1,FGG,TUBB,PAPLN,BLM,F8,CFH,F5,FLNA,ADAMTS13,TGFBI,IGFALS,GC,APOA5,ATP1A1,TTN,PLCG2,KNG1,ACTB,B2M,FGA,TPM3
Monarch	HP:0001633	Abnormal mitral valve morphology	9	109	0.87	0.00072	MMP2,VWF,SEC24D,FBN1,P4HB,FLNA,FBN2,TTN,TPM3
Monarch	EFO:0008390	Prothrombin time measurement	4	8	1.66	0.00077	F2,FGG,F5,F7
Monarch	HP:0001058	Poor wound healing	5	20	1.36	0.00078	F9,F13A1,F8,F13B,C1R
Monarch	HP:0000099	Glomerulonephritis	6	37	1.17	0.00081	CFHR5,C1QB,CFHR1,CFH,C1QC,C1QA
Monarch	HP:0005268	Miscarriage	5	21	1.33	0.00094	MGP,FGB,FGG,SERPINC1,FGA
Monarch	HP:0011420	Age of death	5	21	1.33	0.00094	MGP,FGB,FGG,SERPINC1,FGA
Monarch	HP:0031888	Abnormal HDL cholesterol concentration	5	21	1.33	0.00094	CETP,APOA1,APOE,LCAT,APOA5
Monarch	HP:0012719	Functional abnormality of the gastrointestinal tract	25	840	0.43	0.00097	F9,VWF,F13A1,TCIRG1,FCN3,SERPING1,GUCY1A1,FGB,F2,FBN1,FGG,BLM,F8,F13B,F5,ECM1,FLNA,F7,MASP2,F11,PLCG2,ACTB,B2M,FGA,TPM3
Monarch	HP:0000478	Abnormality of the eye	56	2876	0.25	0.0013	MMP2,MGP,APOB,PROC,APOA1,TTR,APOE,ANGPTL6,F12,LCAT,NID1,CSTA,CP,DNAH5,CENPE,TCIRG1,CDH11,AHSG,SERPING1,SEC24D,CSF1R,SERPINI1,MASP1,A2ML1,PCSK9,BMP1,DST,EFEMP2,NDUFS8,SUZ12,FBN1,P4HB,FBLN1,TUBB,THBS4,BLM,COL18A1,CENPF,CFH,SERPINC1,HTRA1,ADAMTSL4,FLNA,PROS1,EFEMP1,ABCA13,TGFBI,FBN2,C1R,VIM,TTN,PLCG2,ACTB,TRIM8,B2M,TPM3
Monarch	HP:0002621	Atherosclerosis	7	64	1.0	0.0013	SERPIND1,APOB,APOE,ANGPTL6,LCAT,PCSK9,FBN1
Monarch	HP:0008011	Peripheral opacification of the cornea	4	10	1.56	0.0013	MMP2,APOB,APOE,PCSK9
Monarch	HP:0012531	Pain	19	539	0.51	0.0013	MMP2,APOB,CAT,F12,PON3,TCIRG1,HABP2,SERPING1,PCSK9,F2,SERPINF2,FBN1,F8,HTRA1,FLNA,C1S,TGFBI,PLCG2,B2M
Monarch	HP:0200067	Recurrent spontaneous abortion	4	10	1.56	0.0013	F12,F13A1,F13B,SERPINC1
Monarch	HP:0400008	Menometrorrhagia	4	10	1.56	0.0013	F9,FGB,FGG,FGA
Monarch	EFO:0003777	Heart disease	10	155	0.77	0.0014	MMP2,VWF,SEC24D,FBN1,P4HB,FLNA,MAP3K7CL,FBN2,TTN,TPM3
Monarch	HP:0000140	Abnormality of the menstrual cycle	12	228	0.68	0.0014	F9,VWF,F13A1,FGB,F2,FGG,F8,F13B,F5,F7,F11,FGA
Monarch	HP:0000366	Abnormality of the nose	35	1470	0.33	0.0016	F9,MMP2,MGP,APOE,VWF,F13A1,DNAH5,CENPE,TCIRG1,CDH11,AHSG,MASP1,FGB,F2,EFEMP2,SUZ12,FBN1,KDM3B,FGG,TUBB,BLM,COL18A1,F8,CENPF,F13B,F5,ECM1,FLNA,F7,F11,PLCG2,ACTB,TRIM8,B2M,FGA
Monarch	HP:0009115	Aplasia/hypoplasia involving the skeleton	27	985	0.4	0.0016	MMP2,MGP,APOE,CENPE,TCIRG1,CDH11,SEC24D,MASP1,A2ML1,BMP1,EFEMP2,SUZ12,FBN1,P4HB,TUBB,BLM,CENPF,FLNA,C1S,IGFALS,FBN2,C1R,TTN,ACTB,TRIM8,B2M,TPM3
Monarch	HP:0011034	Amyloidosis	5	25	1.26	0.0016	APOA1,TTR,LYZ,B2M,FGA
Monarch	HP:0030012	Abnormal female reproductive system physiology	13	273	0.64	0.0016	F9,VWF,F13A1,FGB,F2,FGG,BLM,F8,F13B,F5,F7,F11,FGA

Monarch	HP:0000078	Abnormality of the genital system	33	1358	0.34	0.0018	F9,MMP2,TTR,APOE,VWF,F13A1,DNAH5,CDH11,AHSG,MASP1,GUCY1A1,A2ML1,FGB,F2,EFEMP2,SUZ12,FBN1,KDM3B,FBLN1,DNAH8,FGG,TUBB,BLM,F8,F13B,F5,FLNA,F7,ADGRG2,F11,ACTB,TRIM8,FGA
Monarch	HP:0000080	Abnormality of reproductive system physiology	21	659	0.46	0.0018	F9,TTR,APOE,VWF,F13A1,DNAH5,AHSG,GUCY1A1,A2ML1,FGB,F2,DNAH8,FGG,BLM,F8,F13B,F5,F7,ADGRG2,F11,FGA
Monarch	HP:0000347	Micrognathia	21	659	0.46	0.0018	MMP2,CENPE,TCIRG1,SEC24D,A2ML1,BMP1,EFEMP2,SUZ12,FBN1,P4HB,TUBB,BLM,CENPF,FLNA,C1S,IGFALS,FBN2,C1R,TTN,ACTB,TPM3
Monarch	HP:0009118	Aplasia/Hypoplasia of the mandible	21	660	0.46	0.0018	MMP2,CENPE,TCIRG1,SEC24D,A2ML1,BMP1,EFEMP2,SUZ12,FBN1,P4HB,TUBB,BLM,CENPF,FLNA,C1S,IGFALS,FBN2,C1R,TTN,ACTB,TPM3
Monarch	HP:0001382	Joint hypermobility	13	279	0.63	0.0019	CENPE,SEC24D,A2ML1,BMP1,EFEMP2,SUZ12,FBN1,KDM3B,P4HB,COL18A1,FLNA,C1S,C1R
Monarch	HP:0010981	Hypolipoproteinemia	5	27	1.23	0.0021	APOB,APOA1,APOE,LCAT,APOA5
Monarch	HP:0001609	Hoarse voice	7	72	0.95	0.0023	SERPING1,SUZ12,FBN1,ECM1,FLNA,C1R,TPM3
Monarch	HP:0002638	Superficial thrombophlebitis	3	3	1.96	0.0023	PROC,SERPINC1,PROS1
Monarch	HP:0001297	Stroke	12	249	0.64	0.0028	TTR,ANGPTL6,GUCY1A1,F2,FBN1,F5,HTRA1,FLNA,CRTAC1,ADAMTS13,KNG1,ACTB
Monarch	HP:0011843	Abnormal musculoskeletal physiology	32	1330	0.34	0.0028	MMP2,APOE,CENPE,TCIRG1,AHSG,HABP2,SEC24D,MASP1,A2ML1,BMP1,DST,F2,EFEMP2,SUZ12,SERPINF2,FBN1,KDM3B,P4HB,FBLN1,COL18A1,F8,ADAMTSL4,FLNA,C1S,PRG4,IGFALS,FBN2,C1R,TTN,ACTB,B2M,TPM3
Monarch	HP:0030875	Abnormality of pulmonary circulation	9	136	0.78	0.0028	MGP,PROC,TCIRG1,BMP1,F2,FBN1,SERPINC1,FLNA,PROS1
Monarch	EFO:0000319	Cardiovascular disease	13	293	0.61	0.0029	MMP2,VWF,SEC24D,FBN1,P4HB,F8,F5,FLNA,MAP3K7CL,FBN2,TTN,KNG1,TPM3
Monarch	HP:0032101	Unusual infection	22	748	0.43	0.0033	C5,MGP,DNAH5,TCIRG1,FCN3,EFEMP2,C1QB,BLM,C8A,CFH,ECM1,FLNA,C8B,C1QC,C1QA,MASP2,C4B,C1R,CFD,PLCG2,B2M,TPM3
Monarch	HP:0007759	Opacification of the corneal stroma	7	78	0.91	0.0035	MMP2,APOB,APOE,LCAT,PCSK9,COL18A1,TGFBI
Monarch	EFO:0004694	Factor XI measurement	3	4	1.83	0.0037	KLKB1,F11,KNG1
Monarch	HP:0005356	Decreased serum complement factor I	3	4	1.83	0.0037	C1QB,C1QC,C1QA
Monarch	HP:0012541	Cephalohematoma	3	4	1.83	0.0037	F9,F2,F8
Monarch	HP:0040232	Delayed onset bleeding	3	4	1.83	0.0037	F9,F13A1,F13B
Monarch	HP:0001654	Abnormal heart valve morphology	12	259	0.62	0.0038	MMP2,VWF,TCIRG1,SEC24D,A2ML1,FBN1,P4HB,FLNA,FBN2,TTN,ACTB,TPM3
Monarch	HP:0005692	Joint hyperflexibility	9	144	0.75	0.0039	CENPE,SEC24D,A2ML1,SUZ12,P4HB,COL18A1,FLNA,C1S,C1R
Monarch	HP:0011277	Abnormality of the urinary system physiology	25	933	0.39	0.0040	F9,APOA1,TTR,APOE,CFHR5,LYZ,LCAT,F2,EFEMP2,C1QB,CFHR1,NDUFS8,SERPINF2,PAPLN,BLM,F8,CFH,F5,ADAMTS13,C1QC,C1QA,C1R,ATP1A1,B2M,FGA
Monarch	HP:0000969	Edema	16	448	0.51	0.0043	APOA1,APOE,F12,LYZ,CDH11,SERPING1,A2ML1,FGB,F2,FGG,TUBB,F8,TGFBI,PLCG2,ACTB,FGA
Monarch	HP:0009145	Abnormal cerebral artery morphology	6	55	1.0	0.0043	APOB,ANGPTL6,GUCY1A1,PCSK9,FBN1,HTRA1
Monarch	HP:0011821	Abnormal facial skeleton morphology	27	1056	0.37	0.0043	MMP2,MGP,APOE,DNAH5,CENPE,TCIRG1,CDH11,SEC24D,A2ML1,BMP1,EFEMP2,SUZ12,FBN1,P4HB,TUBB,BLM,CENPF,ADAMTSL4,FLNA,C1S,IGFALS,FBN2,C1R,TTN,ACTB,B2M,TPM3
Monarch	EFO:0000408	Disease	32	1373	0.33	0.0046	CETP,MMP2,APOE,CFHR5,VWF,CDH11,IGFBP4,SEC24D,EFEMP2,FBN1,KDM3B,P4HB,TUBB,COL18A1,F8,CFHR4,CFH,F5,ADAM12,FLNA,EFEMP1,EFCAB5,NAV2,MAP3K7CL,CFB,IGFALS,FBN2,TTN,PLCG2,PAMR1,KNG1,TPM3
Monarch	HP:0001376	Limitation of joint mobility	14	356	0.55	0.0046	MMP2,MASP1,DST,F2,SUZ12,FBN1,F8,ADAMTSL4,FLNA,PRG4,FBN2,TTN,ACTB,TPM3
Monarch	HP:0011729	Abnormality of joint mobility	27	1063	0.36	0.0046	MMP2,APOE,CENPE,AHSG,SEC24D,MASP1,A2ML1,BMP1,DST,F2,EFEMP2,SUZ12,FBN1,KDM3B,P4HB,FBLN1,COL18A1,F8,ADAMTSL4,FLNA,C1S,PRG4,FBN2,C1R,TTN,ACTB,TPM3
Monarch	HP:0025323	Abnormal arterial physiology	11	224	0.65	0.0046	ANGPTL6,GUCY1A1,F2,FBN1,SERPINC1,F5,HTRA1,CRTAC1,ADAMTS13,KNG1,ACTB
Monarch	HP:0001537	Umbilical hernia	11	228	0.64	0.0051	APOE,CDH11,MASP1,BMP1,SUZ12,FBN1,KDM3B,TUBB,FLNA,C1S,TRIM8
Monarch	HP:0003216	Generalized amyloid deposition	3	5	1.74	0.0053	APOA1,LYZ,FGA
Monarch	EFO:0001365	Age-related macular degeneration	5	35	1.11	0.0055	CETP,APOE,CFHR5,CFH,CFB
Monarch	EFO:1001492	Atrophic macular degeneration	5	35	1.11	0.0055	CETP,APOE,CFHR5,CFH,CFB
Monarch	HP:0000277	Abnormal mandible morphology	23	841	0.39	0.0055	MMP2,CENPE,TCIRG1,CDH11,SEC24D,A2ML1,BMP1,EFEMP2,SUZ12,FBN1,P4HB,TUBB,BLM,CENPF,ADAMTSL4,FLNA,C1S,IGFALS,FBN2,C1R,TTN,ACTB,TPM3
Monarch	HP:0000481	Abnormal cornea morphology	20	673	0.43	0.0055	MMP2,APOB,APOA1,APOE,LCAT,DNAH5,CDH11,SERPING1,PCSK9,DST,FBN1,TUBB,COL18A1,CENPF,FLNA,ABCA13,TGFBI,VIM,PLCG2,ACTB
Monarch	HP:0001386	Joint swelling	5	35	1.11	0.0055	FGB,F2,FGG,F8,FGA
Monarch	HP:0003272	Abnormal hip bone morphology	16	461	0.5	0.0055	MMP2,APOE,CENPE,TCIRG1,SEC24D,MASP1,BMP1,EFEMP2,SUZ12,FBN1,KDM3B,FBLN1,FLNA,TTN,B2M,TPM3
Monarch	HP:0001083	Ectopia lentis	5	36	1.1	0.0056	FBN1,COL18A1,ADAMTSL4,FBN2,B2M
Monarch	HP:0004298	Abnormality of the abdominal wall	15	415	0.52	0.0056	MMP2,APOE,CDH11,MASP1,A2ML1,BMP1,EFEMP2,SUZ12,FBN1,KDM3B,TUBB,FLNA,C1S,C1R,TRIM8
Monarch	HP:0031886	Abnormal LDL cholesterol concentration	5	36	1.1	0.0056	APOB,APOE,LCAT,PCSK9,APOA5
Monarch	HP:0025142	Constitutional symptom	24	906	0.38	0.0057	MMP2,APOB,PROC,TTR,CAT,F12,PON3,TCIRG1,HABP2,SERPING1,PCSK9,F2,SERPINF2,FBN1,F8,HTRA1,FLNA,PROS1,C1S,TGFBI,TTN,PLCG2,B2M,TPM3
Monarch	HP:0001551	Abnormal umbilicus morphology	11	235	0.63	0.0058	APOE,CDH11,MASP1,BMP1,SUZ12,FBN1,KDM3B,TUBB,FLNA,C1S,TRIM8
Monarch	HP:0030972	Abnormal systemic blood pressure	14	369	0.54	0.0058	MMP2,MGP,APOB,APOA1,TTR,ANGPTL6,LYZ,SERPING1,GUCY1A1,PCSK9,F2,FBN1,ADAMTSL4,FGA
Monarch	HP:0003233	Decreased HDL cholesterol concentration	4	18	1.3	0.0059	APOA1,APOE,LCAT,APOA5
Monarch	HP:0001342	Cerebral hemorrhage	6	61	0.95	0.0060	F13A1,FGB,FGG,F13B,PROS1,FGA
Monarch	HP:0004299	Hernia of the abdominal wall	14	371	0.53	0.0060	MMP2,APOE,CDH11,MASP1,BMP1,EFEMP2,SUZ12,FBN1,KDM3B,TUBB,FLNA,C1S,C1R,TRIM8
Monarch	HP:0002829	Arthralgia	8	124	0.77	0.0067	MMP2,APOB,PCSK9,F2,FBN1,F8,C1S,PLCG2
Monarch	HP:0010866	Abdominal wall defect	14	375	0.53	0.0067	MMP2,APOE,CDH11,MASP1,BMP1,EFEMP2,SUZ12,FBN1,KDM3B,TUBB,FLNA,C1S,C1R,TRIM8
Monarch	HP:0000793	Membranoproliferative glomerulonephritis	3	6	1.66	0.0068	C1QB,C1QC,C1QA
Monarch	HP:0011889	Bleeding with minor or no trauma	3	6	1.66	0.0068	F13A1,F8,F13B
Monarch	HP:0030780	Abnormality of the protein C anticoagulant pathway	3	6	1.66	0.0068	PROC,F5,PROS1
Monarch	HP:0033057	Decreased serum terminal complement component	3	6	1.66	0.0068	C5,C8A,C8B
Monarch	HP:0100724	Hypercoagulability	4	19	1.28	0.0068	F9,PROC,F5,PROS1
Monarch	HP:0002637	Cerebral ischemia	10	201	0.65	0.0071	ANGPTL6,GUCY1A1,F2,FBN1,F5,HTRA1,CRTAC1,ADAMTS13,KNG1,ACTB
Monarch	HP:0003549	Abnormality of connective tissue	30	1294	0.32	0.0071	MMP2,APOE,CENPE,TCIRG1,CDH11,AHSG,SEC24D,MASP1,BMP1,DST,EFEMP2,SUZ12,FBN1,KDM3B,P4HB,TUBB,BLM,F8,ECM1,FLNA,C1S,PRG4,TGFBI,FBN2,C1R,TTN,PLCG2,ACTB,TRIM8,TPM3
Monarch	HP:0033401	Tissue ischemia	10	201	0.65	0.0071	ANGPTL6,GUCY1A1,F2,FBN1,F5,HTRA1,CRTAC1,ADAMTS13,KNG1,ACTB
Monarch	HP:0007420	Spontaneous hematomas	4	20	1.26	0.0077	F13A1,F8,F13B,F5
Monarch	HP:0008067	Abnormally lax or hyperextensible skin	8	129	0.75	0.0082	CSTA,EFEMP2,SUZ12,FBN1,TUBB,FLNA,C1S,C1R
Monarch	HP:0001679	Abnormal aortic morphology	12	292	0.57	0.0083	MMP2,APOB,APOE,ANGPTL6,SEC24D,PCSK9,EFEMP2,FBN1,P4HB,FLNA,FBN2,TPM3
Monarch	HP:0002086	Abnormality of the respiratory system	35	1636	0.29	0.0083	MGP,APOB,PROC,CAT,APOE,F12,DNAH5,PON3,TCIRG1,FCN3,HABP2,SERPING1,A2ML1,SLC27A4,PCSK9,BMP1,DST,F2,EFEMP2,NDUFS8,SERPINF2,FBN1,BLM,SERPINC1,F5,ECM1,FLNA,ADAMTS13,PROS1,MASP2,FBN2,TTN,PLCG2,B2M,TPM3

Monarch	HP:0000799	Renal steatosis	3	7	1.59	0.0089	APOB,APOE,PCSK9
Monarch	HP:0008800	Limited hip movement	6	67	0.91	0.0089	MMP2,DST,F8,FBN2,TTN,TPM3
Monarch	HP:0012397	Aortic atherosclerotic lesion	3	7	1.59	0.0089	APOB,APOE,PCSK9
Monarch	HP:0001166	Arachnodactyly	8	133	0.74	0.0096	BMP1,EFEMP2,FBN1,TUBB,FLNA,FBN2,C1R,TTN
Monarch	EFO:0004574	Total cholesterol measurement	16	497	0.47	0.0100	CETP,APOH,APOB,APOA1,APOE,LCAT,PCSK9,FGB,OIT3,ASGR2,APOA4,COL18A1,ADAMTS13,HPR,APOA5,APOC4
Monarch	EFO:0004614	Apolipoprotein A 1 measurement	14	396	0.51	0.0101	CETP,APOH,APOB,APOA1,APOE,LCAT,ITIH1,PCSK9,STAB1,APOA4,COL18A1,CRTAC1,HPR,APOC4
Monarch	HP:0000991	Xanthomatosis	4	22	1.22	0.0101	APOB,APOA1,APOE,PCSK9
Monarch	HP:0001787	Abnormal delivery	5	43	1.02	0.0101	F9,F2,KDM3B,F8,TPM3
Monarch	HP:0010647	Abnormal elasticity of skin	9	172	0.68	0.0101	MGP,CSTA,EFEMP2,SUZ12,FBN1,TUBB,FLNA,C1S,C1R
Monarch	HP:0000822	Hypertension	11	256	0.59	0.0102	MMP2,MGP,APOB,APOA1,ANGPTL6,LYZ,GUCY1A1,PCSK9,FBN1,ADAMTSL4,FGA
Monarch	HP:0004942	Aortic aneurysm	8	135	0.73	0.0102	ANGPTL6,SEC24D,EFEMP2,FBN1,P4HB,FLNA,FBN2,TPM3
Monarch	HP:0002719	Recurrent infections	20	721	0.4	0.0106	C5,MGP,DNAH5,TCIRG1,FCN3,EFEMP2,C1QB,BLM,CFH,ECM1,FLNA,C8B,C1QC,C1QA,MASP2,C1R,CFD,PLCG2,B2M,TPM3
Monarch	HP:0032263	Increased blood pressure	12	303	0.56	0.0106	MMP2,MGP,APOB,APOA1,ANGPTL6,LYZ,GUCY1A1,PCSK9,F2,FBN1,ADAMTSL4,FGA
Monarch	HP:0001627	Abnormal heart morphology	28	1206	0.32	0.0107	MMP2,MGP,APOB,TTR,APOE,VWF,NID1,DNAH5,TCIRG1,SEC24D,MASP1,A2ML1,PCSK9,F2,EFEMP2,NDUFS8,FBN1,KDM3B,P4HB,COL18A1,FLNA,PRG4,FBN2,TTN,ACTB,TRIM8,B2M,TPM3
Monarch	HP:0001677	Coronary artery atherosclerosis	5	44	1.01	0.0107	SERPIND1,APOB,APOE,PCSK9,FBN1
Monarch	HP:0002204	Pulmonary embolism	4	23	1.2	0.0111	PROC,F2,SERPINC1,PROS1
Monarch	HP:0004381	Supravalvular aortic stenosis	3	8	1.53	0.0111	APOB,PCSK9,EFEMP2
Monarch	HP:0005262	Abnormality of the synovia	12	305	0.55	0.0111	MMP2,APOE,MASP1,BMP1,F2,EFEMP2,FBN1,FBLN1,F8,FLNA,PRG4,TPM3
Monarch	HP:0010874	Tendon xanthomatosis	3	8	1.53	0.0111	APOB,APOE,PCSK9
Monarch	HP:0100665	Angioedema	3	8	1.53	0.0111	F12,SERPING1,PLCG2
Monarch	EFO:0005278	Cardiovascular disease biomarker measurement	43	2228	0.24	0.0113	CETP,APOH,F9,APOB,APOA1,APOE,F12,LCAT,KLKB1,DNAH5,CDH11,ITIH1,HABP2,SERPINI1,PCSK9,FGB,STAB1,FBN1,FBLN1,OIT3,FILIP1L,APOA4,COL18A1,CFH,QSOX1,F5,CRTAC1,ADAMTS13,CNTRL,SVEP1,EFEMP1,EFCAB5,NAV2,MAP3K7CL,ITIH3,FBN2,HPR,APOA5,TTN,APOC4,PLCG2,KNG1,ACTB
Monarch	HP:0012718	Morphological abnormality of the gastrointestinal tract	22	845	0.37	0.0115	APOE,F12,VWF,F13A1,DNAH5,CDH11,FCN3,HABP2,SERPING1,EFEMP2,SUZ12,KDM3B,BLM,COL18A1,CENPF,F13B,FLNA,MASP2,FBN2,C1R,PLCG2,B2M
Monarch	HP:0100545	Arterial stenosis	6	72	0.88	0.0115	SERPIND1,APOB,APOE,F12,PCSK9,FBN1
Monarch	EFO:0004298	Cardiovascular measurement	45	2378	0.23	0.0120	CETP,APOH,F9,GPLD1,APOB,APOA1,APOE,F12,LCAT,KLKB1,DNAH5,CDH11,ITIH1,HABP2,SERPINI1,PCSK9,FGB,INHBC,STAB1,FBN1,FBLN1,OIT3,FILIP1L,APOA4,COL18A1,CFH,QSOX1,F5,CRTAC1,ADAMTS13,CNTRL,SVEP1,EFEMP1,EFCAB5,NAV2,MAP3K7CL,ITIH3,FBN2,HPR,APOA5,TTN,APOC4,PLCG2,KNG1,ACTB
Monarch	HP:0025033	Abnormality of digestive system morphology	22	849	0.37	0.0121	APOE,F12,VWF,F13A1,DNAH5,CDH11,FCN3,HABP2,SERPING1,EFEMP2,SUZ12,KDM3B,BLM,COL18A1,CENPF,F13B,FLNA,MASP2,FBN2,C1R,PLCG2,B2M
Monarch	HP:0001239	Wrist flexion contracture	4	24	1.18	0.0123	MMP2,FLNA,PRG4,FBN2
Monarch	HP:0001681	Angina pectoris	4	24	1.18	0.0123	APOB,APOE,LCAT,PCSK9
Monarch	HP:0200036	Skin nodule	6	74	0.87	0.0128	MMP2,APOB,APOE,PCSK9,FBN1,ECM1
Monarch	HP:0006480	Premature loss of teeth	5	47	0.98	0.0133	CAT,TCIRG1,CDH11,C1S,C1R
Monarch	HP:0011844	Abnormal appendicular skeleton morphology	33	1557	0.28	0.0134	MMP2,MGP,APOE,VWF,DNAH5,CENPE,TCIRG1,CDH11,AHSG,SEC24D,CSF1R,MASP1,A2ML1,BMP1,F2,EFEMP2,SUZ12,FBN1,KDM3B,P4HB,FBLN1,TUBB,BLM,CENPF,FLNA,PRG4,FBN2,C1R,TTN,ACTB,TRIM8,B2M,TPM3
Monarch	EFO:0003907	Deep vein thrombosis	3	9	1.48	0.0137	F2,F5,KNG1
Monarch	HP:0001084	Corneal arcus	3	9	1.48	0.0137	APOB,APOE,PCSK9
Monarch	HP:0007957	Corneal opacity	10	226	0.6	0.0144	MMP2,APOB,APOA1,APOE,LCAT,PCSK9,DST,COL18A1,CENPF,TGFBI
Monarch	HP:0003141	Increased LDL cholesterol concentration	4	26	1.15	0.0156	APOB,APOE,LCAT,PCSK9
Monarch	HP:0012638	Abnormal nervous system physiology	55	3154	0.2	0.0157	MMP2,MGP,APOB,PROC,APOA1,TTR,CAT,APOE,ANGPTL6,NID1,CP,DNAH5,CENPE,PON3,TCIRG1,CDH11,AHSG,SERPING1,SEC24D,CSF1R,SERPINI1,MASP1,GUCY1A1,A2ML1,PCSK9,BMP1,DST,F2,EFEMP2,STAB1,NDUFS8,SUZ12,FBN1,KDM3B,P4HB,FBLN1,TUBB,BLM,COL18A1,F8,HTRA1,ADAMTSL4,ECM1,FLNA,ADAMTS13,ITIH3,TGFBI,FBN2,ATP1A1,VIM,TTN,ACTB,TRIM8,B2M,TPM3
Monarch	HP:0000979	Purpura	6	79	0.84	0.0172	PROC,APOE,F13A1,F2,F13B,PROS1
Monarch	EFO:0010071	Cardiac troponin I measurement	3	10	1.44	0.0173	F12,KLKB1,HABP2
Monarch	HP:0001114	Xanthelasma	3	10	1.44	0.0173	APOB,APOE,PCSK9
Monarch	HP:0001363	Craniosynostosis	8	150	0.68	0.0177	CENPE,TCIRG1,CDH11,SEC24D,MASP1,FBN1,P4HB,FLNA
Monarch	HP:0031652	Abnormal aortic valve physiology	7	113	0.75	0.0177	APOB,VWF,PCSK9,EFEMP2,FBN1,FLNA,ACTB
Monarch	HP:0000963	Thin skin	6	80	0.83	0.0178	PROC,SEC24D,P4HB,FLNA,PROS1,C1R
Monarch	HP:0003273	Hip contracture	5	51	0.95	0.0178	MMP2,F8,FBN2,TTN,TPM3
Monarch	HP:0001387	Joint stiffness	8	151	0.68	0.0180	F2,SUZ12,FBN1,ADAMTSL4,FLNA,PRG4,FBN2,ACTB
Monarch	HP:0002813	Abnormality of limb bone morphology	31	1459	0.29	0.0189	MMP2,MGP,APOE,VWF,DNAH5,CENPE,TCIRG1,CDH11,AHSG,SEC24D,CSF1R,MASP1,A2ML1,BMP1,F2,EFEMP2,SUZ12,FBN1,P4HB,FBLN1,TUBB,BLM,CENPF,FLNA,PRG4,FBN2,C1R,TTN,ACTB,TRIM8,B2M
Monarch	HP:0031815	Abnormal oral physiology	9	196	0.62	0.0210	F13A1,PON3,SERPING1,F2,FBLN1,F8,F13B,F5,TPM3
Monarch	HP:0004950	Peripheral arterial stenosis	4	29	1.1	0.0213	APOB,APOE,PCSK9,FBN1
Monarch	HP:0010299	Abnormal dentin morphology	4	29	1.1	0.0213	CDH11,SEC24D,BMP1,P4HB
Monarch	HP:0004329	Abnormal posterior eye segment morphology	26	1146	0.31	0.0219	MMP2,MGP,APOB,PROC,TTR,APOE,F12,CP,DNAH5,TCIRG1,CSF1R,PCSK9,NDUFS8,FBN1,FBLN1,TUBB,BLM,COL18A1,CENPF,CFH,SERPINC1,ADAMTSL4,PROS1,EFEMP1,FBN2,ACTB
Monarch	HP:0002326	Transient ischemic attack	5	55	0.92	0.0235	ANGPTL6,FBN1,HTRA1,ADAMTS13,ACTB
Monarch	HP:0011329	Abnormality of cranial sutures	11	292	0.53	0.0245	MMP2,CENPE,TCIRG1,CDH11,SEC24D,MASP1,BMP1,FBN1,P4HB,FLNA,ACTB
Monarch	EFO:0004732	Lipoprotein measurement	30	1426	0.28	0.0266	CETP,APOH,APOB,APOA1,APOE,LCAT,KLKB1,PPP2R3A,PON3,ITIH1,IGF2BP1,PCSK9,FGB,F2,INHBC,STAB1,FBLN1,OIT3,THBS4,APOD,ASGR2,GIT2,APOA4,COL18A1,CRTAC1,ADAMTS13,ABCA13,HPR,APOA5,APOC4
Monarch	HP:0000174	Abnormal palate morphology	24	1035	0.32	0.0266	APOE,NID1,TCIRG1,CDH11,SERPING1,SEC24D,MASP1,A2ML1,DST,EFEMP2,FBN1,PAPPA,TUBB,FILIP1L,CENPF,ECM1,FLNA,CRTAC1,ABCA13,FBN2,TTN,ACTB,TRIM8,TPM3
Monarch	HP:0004334	Dermal atrophy	6	88	0.79	0.0270	MGP,TCIRG1,DST,FBN1,C1S,C1R
Monarch	HP:0002659	Increased susceptibility to fractures	9	205	0.6	0.0275	MMP2,TCIRG1,HABP2,SEC24D,BMP1,EFEMP2,P4HB,B2M,TPM3
Monarch	HP:0002242	Abnormal intestine morphology	18	673	0.39	0.0276	APOE,F12,F13A1,DNAH5,FCN3,HABP2,SERPING1,EFEMP2,KDM3B,BLM,CENPF,F13B,FLNA,MASP2,FBN2,C1R,PLCG2,B2M
Monarch	HP:0011915	Cardiovascular calcification	4	32	1.05	0.0286	APOB,PCSK9,FBN1,B2M
Monarch	HP:0030872	Abnormal cardiac ventricular function	6	90	0.78	0.0297	APOB,PCSK9,EFEMP2,FBN1,TTN,B2M
Monarch	EFO:0000618	Nervous system disease	12	350	0.49	0.0299	CETP,APOE,CFHR5,EFEMP2,KDM3B,COL18A1,F8,CFH,F5,CFB,PLCG2,KNG1
Monarch	EFO:0004612	High density lipoprotein cholesterol measurement	19	740	0.37	0.0310	CETP,APOB,APOA1,APOE,LCAT,PPP2R3A,PON3,ITIH1,IGF2BP1,F2,INHBC,STAB1,APOD,GIT2,APOA4,COL18A1,CRTAC1,APOA5,APOC4
Monarch	HP:0001832	Abnormal metatarsal morphology	7	127	0.7	0.0310	MMP2,SUZ12,FBN1,FBLN1,FLNA,PRG4,FBN2
Monarch	HP:0001907	Thromboembolism	4	33	1.04	0.0312	F12,F2,F8,SERPINC1
Monarch	HP:0002751	Kyphoscoliosis	8	167	0.64	0.0312	MMP2,CDH11,BMP1,FBN1,FLNA,FBN2,ACTB,TPM3
Monarch	EFO:0003966	Eye disease	8	168	0.64	0.0317	CETP,APOE,CFHR5,EFEMP2,CFH,CFB,FBN2,PAMR1

Monarch	EFO:0008091	Complement C1s subcomponent measurement	2	2	1.96	0.0317	CFHR4,CFH
Monarch	EFO:0008098	Complement factor H-related protein 5 measurement	2	2	1.96	0.0317	F13B,CFH
Monarch	EFO:0008260	Plasma kallikrein measurement	2	2	1.96	0.0317	KLKB1,KNG1
Monarch	EFO:0008543	c3d:C3 ratio	2	2	1.96	0.0317	CFHR4,CFH
Monarch	HP:0000235	Abnormality of the fontanelles or cranial sutures	12	355	0.49	0.0317	MMP2,APOE,CENPE,TCIRG1,CDH11,SEC24D,MASP1,BMP1,FBN1,P4HB,FLNA,ACTB
Monarch	HP:0001038	Warfarin-induced skin necrosis	2	2	1.96	0.0317	PROC,PROS1
Monarch	HP:0002037	Inflammation of the large intestine	5	60	0.88	0.0317	F13A1,FCN3,F13B,MASP2,PLCG2
Monarch	HP:0004434	Decreased serum complement C8	2	2	1.96	0.0317	C8A,C8B
Monarch	HP:0005225	Intestinal edema	2	2	1.96	0.0317	F12,SERPING1
Monarch	HP:0011855	Pharyngeal edema	2	2	1.96	0.0317	F12,SERPING1
Monarch	HP:0011971	Dermatographic urticaria	2	2	1.96	0.0317	SERPING1,PLCG2
Monarch	HP:0002686	Prenatal maternal abnormality	7	129	0.69	0.0318	F12,F13A1,DNAH5,FBN1,F13B,SERPINC1,F5
Monarch	EFO:0006925	Lipoprotein A measurement	4	34	1.03	0.0324	CETP,APOH,APOE,APOC4
Monarch	HP:0100763	Abnormality of the lymphatic system	16	576	0.4	0.0345	APOA1,APOE,LYZ,LCAT,DNAH5,TCIRG1,HABP2,A2ML1,FGB,F2,FGG,BLM,COL18A1,F8,CENPF,FGA
Monarch	HP:0000704	Periodontitis	4	35	1.02	0.0356	CAT,TCIRG1,C1S,C1R
Monarch	HP:0000766	Abnormal sternum morphology	12	362	0.48	0.0365	TCIRG1,CDH11,SEC24D,A2ML1,BMP1,EFEMP2,FBN1,TUBB,FLNA,FBN2,ACTB,TPM3
Monarch	HP:0011805	Abnormal skeletal muscle morphology	31	1537	0.26	0.0377	F9,MMP2,APOE,CP,F13A1,PON3,TCIRG1,AHSG,MASP1,A2ML1,BMP1,DST,F2,EFEMP2,NDUFS8,SUZ12,SERPINF2,FBN1,KDM3B,F8,CENPF,F13B,FLNA,F7,PRG4,FBN2,ATP1A1,TTN,ACTB,B2M,TPM3
Monarch	HP:0005181	Premature coronary artery atherosclerosis	3	15	1.26	0.0388	APOB,APOE,PCSK9
Monarch	HP:0011867	Abnormal iliac wing morphology	5	64	0.85	0.0392	APOE,TCIRG1,SEC24D,SUZ12,FLNA
Monarch	EFO:0003839	Retinopathy	6	98	0.74	0.0408	CETP,APOE,CFHR5,EFEMP2,CFH,CFB
Monarch	HP:0012337	Abnormal homeostasis	27	1275	0.28	0.0408	MMP2,APOA1,CAT,APOE,F12,LYZ,CP,TCIRG1,CDH11,SERPING1,A2ML1,FGB,DST,F2,NDUFS8,FBN1,FGG,TUBB,BLM,F8,ADAMTS13,TGFBI,IGFALS,APOA5,PLCG2,ACTB,FGA
Monarch	HP:0000095	Abnormal renal glomerulus morphology	7	136	0.67	0.0411	APOE,CFHR5,C1QB,CFHR1,CFH,C1QC,C1QA
Monarch	HP:0001620	High pitched voice	4	37	0.99	0.0417	SEC24D,P4HB,BLM,TTN
Monarch	HP:0025032	Abnormality of digestive system physiology	34	1763	0.24	0.0435	F9,C5,TTR,F12,VWF,F13A1,PON3,TCIRG1,FCN3,SERPING1,GUCY1A1,A2ML1,FGB,DST,F2,NDUFS8,SUZ12,FBN1,KDM3B,FGG,BLM,F8,F13B,F5,ECM1,FLNA,F7,MASP2,F11,PLCG2,ACTB,B2M,FGA,TPM3
Monarch	HP:0002795	Abnormal respiratory system physiology	25	1151	0.29	0.0439	MGP,APOB,PROC,F12,DNAH5,PON3,TCIRG1,HABP2,SERPING1,SLC27A4,PCSK9,BMP1,DST,F2,EFEMP2,NDUFS8,FBN1,BLM,SERPINC1,FLNA,ADAMTS13,PROS1,TTN,PLCG2,TPM3
Monarch	HP:0002817	Abnormality of the upper limb	29	1426	0.27	0.0470	MMP2,MGP,APOE,VWF,CSTA,CENPE,CDH11,AHSG,MASP1,A2ML1,BMP1,DST,F2,EFEMP2,SUZ12,FBN1,FBLN1,TUBB,BLM,F8,FLNA,PRG4,FBN2,C1R,TTN,ACTB,TRIM8,B2M,TPM3
Monarch	HP:0003312	Abnormal form of the vertebral bodies	10	274	0.52	0.0470	MMP2,TCIRG1,CDH11,SEC24D,CSF1R,BMP1,FBN1,P4HB,FLNA,ACTB
Monarch	HP:0004419	Recurrent thrombophlebitis	2	3	1.78	0.0470	F2,SERPINC1
Monarch	HP:0008330	Reduced von Willebrand factor activity	2	3	1.78	0.0470	VWF,F8
Monarch	HP:0008357	Reduced factor XIII activity	2	3	1.78	0.0470	F13A1,F13B
Monarch	HP:0012636	Retinal vein occlusion	2	3	1.78	0.0470	F12,SERPINC1
Monarch	HP:0030657	Umbilical cord hematoma	2	3	1.78	0.0470	F13A1,F13B
Monarch	HP:0003040	Arthropathy	4	39	0.97	0.0480	MMP2,F2,F8,PRG4
DISEASES	DOID:1247	Blood coagulation disease	26	89	1.42	1.75e-23	SERPIND1,F9,PROC,F12,VWF,KLKB1,F13A1,PF4,FGB,F2,SERPINF2,FGG,F8,F13B,CFH,SERPINC1,F5,FLNA,ADAMTS13,F7,PROS1,MMRN1,F11,C4B,KNG1,FGA
DISEASES	DOID:7	Disease of anatomical entity	111	4798	0.32	6.56e-14	CETP,APOH,SERPIND1,F9,MMP2,C5,AFM,MGP,LTF,APOB,PROC,APOA1,TTR,APOE,F12,APCS,CFHR5,VWF,LCAT,CSTA,KLKB1,F13A1,DNAH5,CENPE,TCIRG1,LUM,AHSG,SAA4,SERPING1,SEC24D,ANKRD44,CSF1R,PIP,SCGB3A1,SERPINI1,PF4,CAMP,APEH,A2ML1,SLC27A4,LALBA,PCSK9,BMP1,FGB,DST,F2,EFEMP2,C1QB,CFHR1,SUZ12,APOL1,SERPINF2,FBN1,P4HB,PAPPA,FGG,TUBB,SLPI,FILIP1L,BLM,APOA4,CNDP1,COL18A1,F8,F13B,CFHR2,CFHR4,CFHR3,CFH,SERPINC1,F5,CFAP46,HTRA1,ADAMTSL4,ECM1,FLNA,C8B,ADAMTS13,C1QC,C1QA,F7,ADGRG2,CD99,PROS1,EFEMP1,MMRN1,YWHAZ,F11,C1S,ADIPOQ,CCDC122,MST1,C4B,TGFBI,CFB,FBN2,TSKU,JCHAIN,C1R,ATP1A1,VIM,TTN,CFD,IGHV3-15,PLCG2,KNG1,ACTB,TRIM66,B2M,FGA,TPM3
DISEASES	DOID:4	Disease	129	6291	0.27	1.79e-13	CETP,APOH,SERPIND1,F9,MMP2,C5,AFM,MGP,LTF,APOB,PROC,APOA1,TTR,CAT,APOE,F12,APCS,CFHR5,LYZ,VWF,LCAT,CSTA,CP,KLKB1,F13A1,DNAH5,CENPE,TCIRG1,LUM,CDH11,AHSG,SAA4,SERPING1,SEC24D,ANKRD44,CSF1R,PIP,SCGB3A1,SERPINI1,PF4,MASP1,CAMP,APEH,A2ML1,SLC27A4,LALBA,PCSK9,BMP1,FGB,DST,F2,EFEMP2,PRG2,C1QB,CFHR1,NDUFS8,SUZ12,APOL1,SERPINF2,FBN1,P4HB,PAPPA,DNAH8,FGG,TUBB,SLPI,FILIP1L,BLM,APOA4,CNDP1,COL18A1,F8,CENPF,F13B,CFHR2,CFHR4,CFHR3,CFH,SERPINC1,F5,CFAP46,HTRA1,ADAMTSL4,ECM1,FLNA,MCOLN2,C8B,ADAMTS13,CNTRL,C1QC,C1QA,F7,CA6,ADGRG2,CD99,GPX3,PROS1,EFEMP1,MMRN1,YWHAZ,F11,C1S,PCYOX1,ADIPOQ,PRG4,CCDC122,ABCA13,MST1,C4B,TGFBI,CFB,FBN2,TSKU,JCHAIN,HPR,C1R,APOA5,ATP1A1,VIM,TTN,CFD,IGHV3-15,PLCG2,KNG1,ACTB,TRIM66,B2M,FGA,TPM3
DISEASES	DOID:74	Hematopoietic system disease	32	473	0.79	9.75e-13	SERPIND1,F9,PROC,F12,VWF,KLKB1,F13A1,TCIRG1,PF4,CAMP,FGB,F2,SERPINF2,FGG,F8,F13B,CFH,SERPINC1,F5,FLNA,ADAMTS13,F7,CD99,PROS1,MMRN1,F11,C4B,IGHV3-15,KNG1,ACTB,B2M,FGA
DISEASES	DOID:2452	Thrombophilia	11	21	1.68	1.93e-11	SERPIND1,F9,PROC,VWF,F2,CFH,SERPINC1,F5,ADAMTS13,PROS1,C4B
DISEASES	DOID:2007	Degeneration of macula and posterior pole	13	46	1.41	4.24e-11	CETP,APOE,CFHR5,CFHR1,FILIP1L,F13B,CFHR2,CFHR4,CFHR3,CFH,HTRA1,EFEMP1,CFB
DISEASES	DOID:630	Genetic disease	88	3778	0.33	2.67e-10	CETP,SERPIND1,F9,MGP,LTF,APOB,PROC,APOA1,TTR,CAT,APOE,F12,APCS,CFHR5,LYZ,VWF,LCAT,CP,KLKB1,F13A1,DNAH5,TCIRG1,SERPING1,SEC24D,ANKRD44,CSF1R,SERPINI1,MASP1,CAMP,A2ML1,PCSK9,FGB,DST,F2,EFEMP2,C1QB,CFHR1,NDUFS8,SERPINF2,FBN1,PAPPA,DNAH8,FGG,FILIP1L,BLM,APOA4,CNDP1,F8,CENPF,F13B,CFHR2,CFHR4,CFHR3,CFH,SERPINC1,F5,CFAP46,HTRA1,ADAMTSL4,ECM1,FLNA,MCOLN2,C1QA,F7,ADGRG2,PROS1,EFEMP1,F11,C1S,PCYOX1,ADIPOQ,PRG4,MST1,TGFBI,CFB,FBN2,JCHAIN,APOA5,ATP1A1,VIM,TTN,CFD,PLCG2,KNG1,ACTB,B2M,FGA,TPM3
DISEASES	DOID:10871	Age related macular degeneration	12	44	1.39	3.86e-10	CETP,APOE,CFHR5,CFHR1,FILIP1L,F13B,CFHR2,CFHR4,CFHR3,CFH,HTRA1,CFB
DISEASES	DOID:626	Complement deficiency	10	23	1.6	6.55e-10	C5,CFHR5,KLKB1,SERPING1,CFHR1,CFHR3,CFH,ADAMTS13,CFB,KNG1
DISEASES	DOID:0050736	Autosomal dominant disease	48	1386	0.5	7.33e-10	CETP,SERPIND1,F9,PROC,APOA1,TTR,APOE,F12,APCS,CFHR5,LYZ,VWF,KLKB1,SERPING1,ANKRD44,CSF1R,SERPINI1,F2,EFEMP2,CFHR1,FBN1,FILIP1L,APOA4,F8,F13B,CFHR2,CFHR4,CFHR3,CFH,SERPINC1,F5,CFAP46,HTRA1,FLNA,EFEMP1,C1S,TGFBI,CFB,FBN2,JCHAIN,ATP1A1,VIM,TTN,PLCG2,KNG1,ACTB,B2M,FGA

DISEASES	DOID:0050177	Monogenic disease	78	3266	0.34	2.40e-09	CETP,SERPIND1,F9,MGP,LTF,APOB,PROC,APOA1,TTR,APOE,F12,APCS,CFHR5,LYZ,VWF,CP,KLKB1,F13A1,DNAH5,TCIRG1,SERPING1,SEC24D,ANKRD44,CSF1R,SERPINI1,MASP1,CAMP,A2ML1,PCSK9,FGB,DST,F2,EFEMP2,CFHR1,SERPINF2,FBN1,DNAH8,FGG,FILIP1L,BLM,APOA4,F8,CENPF,F13B,CFHR2,CFHR4,CFHR3,CFH,SERPINC1,F5,CFAP46,HTRA1,ADAMTSL4,FLNA,F7,ADGRG2,PROS1,EFEMP1,F11,C1S,PCYOX1,PRG4,MST1,TGFBI,CFB,FBN2,JCHAIN,APOA5,ATP1A1,VIM,TTN,CFD,PLCG2,KNG1,ACTB,B2M,FGA,TPM3
DISEASES	DOID:0050739	Autosomal genetic disease	71	2802	0.36	2.40e-09	CETP,SERPIND1,F9,MGP,LTF,APOB,PROC,APOA1,TTR,APOE,F12,APCS,CFHR5,LYZ,VWF,CP,KLKB1,F13A1,DNAH5,TCIRG1,SERPING1,ANKRD44,CSF1R,SERPINI1,MASP1,CAMP,PCSK9,FGB,DST,F2,EFEMP2,CFHR1,SERPINF2,FBN1,FGG,FILIP1L,BLM,APOA4,F8,F13B,CFHR2,CFHR4,CFHR3,CFH,SERPINC1,F5,CFAP46,HTRA1,ADAMTSL4,FLNA,F7,EFEMP1,F11,C1S,PCYOX1,PRG4,TGFBI,CFB,FBN2,JCHAIN,APOA5,ATP1A1,VIM,TTN,CFD,PLCG2,KNG1,ACTB,B2M,FGA,TPM3
DISEASES	DOID:0050636	Familial visceral amyloidosis	9	21	1.59	7.06e-09	APOA1,TTR,APOE,APCS,LYZ,APOA4,ACTB,B2M,FGA
DISEASES	DOID:9120	Amyloidosis	11	75	1.12	7.31e-07	APOA1,TTR,APOE,APCS,LYZ,SAA4,APOA4,TGFBI,ACTB,B2M,FGA
DISEASES	DOID:9799	Eye degenerative disease	17	236	0.82	7.93e-07	CETP,APOB,APOE,CFHR5,PCSK9,CFHR1,FILIP1L,F13B,CFHR2,CFHR4,CFHR3,CFH,CFAP46,HTRA1,EFEMP1,TGFBI,CFB
DISEASES	DOID:2914	Immune system disease	28	675	0.58	8.94e-07	APOH,C5,TTR,APOE,APCS,CFHR5,KLKB1,SAA4,SERPING1,A2ML1,LALBA,DST,F2,CFHR1,COL18A1,CFHR3,CFH,SERPINC1,ADAMTS13,CD99,C4B,CFB,JCHAIN,IGHV3-15,PLCG2,KNG1,ACTB,B2M
DISEASES	DOID:178	Vascular disease	17	249	0.79	1.50e-06	CETP,APOH,APOB,APOA1,APOE,VWF,PF4,PCSK9,F2,FBN1,PAPPA,SERPINC1,ADAMTS13,F7,CD99,ADIPOQ,FBN2
DISEASES	DOID:11949	Creutzfeldt-Jakob disease	6	9	1.78	1.74e-06	APOE,APCS,C1QB,C1QC,C1QA,YWHAZ
DISEASES	DOID:0080301	Atypical hemolytic-uremic syndrome	6	11	1.69	4.02e-06	CFHR5,CFHR1,CFHR3,CFH,ADAMTS13,CFB
DISEASES	DOID:0050828	Artery disease	12	130	0.92	1.13e-05	CETP,APOB,APOA1,APOE,PCSK9,F2,FBN1,PAPPA,SERPINC1,ADAMTS13,ADIPOQ,FBN2
DISEASES	DOID:12554	Hemolytic-uremic syndrome	6	14	1.59	1.13e-05	CFHR5,CFHR1,CFHR3,CFH,ADAMTS13,CFB
DISEASES	DOID:1287	Cardiovascular system disease	22	493	0.61	1.13e-05	CETP,APOH,APOB,APOA1,TTR,APOE,APCS,VWF,DNAH5,PF4,PCSK9,F2,FBN1,PAPPA,SERPINC1,FLNA,ADAMTS13,F7,CD99,ADIPOQ,FBN2,TTN
DISEASES	DOID:14735	Hereditary angioedema	5	6	1.88	1.23e-05	F12,KLKB1,SERPING1,C1S,KNG1
DISEASES	DOID:5614	Eye disease	27	749	0.51	1.86e-05	CETP,LTF,APOB,APOE,APCS,CFHR5,LUM,SERPING1,PCSK9,CFHR1,FBN1,FILIP1L,COL18A1,F13B,CFHR2,CFHR4,CFHR3,CFH,CFAP46,HTRA1,ADAMTSL4,EFEMP1,TGFBI,CFB,FBN2,VIM,ACTB
DISEASES	DOID:557	Kidney disease	16	275	0.72	2.21e-05	C5,TTR,APCS,CFHR5,SAA4,F2,CFHR1,APOL1,APOA4,COL18A1,CFHR3,CFH,ADAMTS13,C4B,CFB,B2M
DISEASES	DOID:863	Nervous system disease	52	2275	0.32	4.55e-05	CETP,LTF,APOB,PROC,APOA1,TTR,APOE,APCS,CFHR5,VWF,LCAT,CENPE,LUM,SERPING1,ANKRD44,CSF1R,SERPINI1,A2ML1,PCSK9,DST,F2,C1QB,CFHR1,SUZ12,FBN1,FILIP1L,CNDP1,COL18A1,F13B,CFHR2,CFHR4,CFHR3,CFH,SERPINC1,CFAP46,HTRA1,ADAMTSL4,FLNA,C1QC,C1QA,CD99,EFEMP1,YWHAZ,CCDC122,TGFBI,CFB,FBN2,TSKU,ATP1A1,VIM,ACTB,TRIM66
DISEASES	DOID:0060903	Thrombosis	6	21	1.41	5.85e-05	APOH,VWF,PF4,F2,SERPINC1,F7
DISEASES	DOID:2921	Glomerulonephritis	7	36	1.25	5.85e-05	CFHR5,APOL1,COL18A1,CFHR3,CFH,C4B,CFB
DISEASES	DOID:8466	Retinal degeneration	14	227	0.75	6.20e-05	CETP,APOE,CFHR5,CFHR1,FILIP1L,F13B,CFHR2,CFHR4,CFHR3,CFH,CFAP46,HTRA1,EFEMP1,CFB
DISEASES	DOID:37	Skin disease	21	518	0.57	6.93e-05	APOA1,APOE,F12,APCS,CSTA,KLKB1,SERPING1,PIP,CAMP,A2ML1,SLC27A4,DST,EFEMP2,FBN1,TUBB,BLM,APOA4,COL18A1,C1S,KNG1,ACTB
DISEASES	DOID:0050155	Sensory system disease	29	929	0.45	8.51e-05	CETP,LTF,APOB,PROC,APOE,APCS,CFHR5,LUM,SERPING1,A2ML1,PCSK9,CFHR1,FBN1,FILIP1L,COL18A1,F13B,CFHR2,CFHR4,CFHR3,CFH,CFAP46,HTRA1,ADAMTSL4,EFEMP1,TGFBI,CFB,FBN2,VIM,ACTB
DISEASES	DOID:16	Integumentary system disease	22	575	0.54	8.76e-05	APOA1,APOE,F12,APCS,CSTA,KLKB1,AHSG,SERPING1,PIP,CAMP,A2ML1,SLC27A4,DST,EFEMP2,FBN1,TUBB,BLM,APOA4,COL18A1,C1S,KNG1,ACTB
DISEASES	DOID:17	Musculoskeletal system disease	33	1154	0.41	8.76e-05	F9,MMP2,C5,MGP,TTR,APOE,F12,APCS,TCIRG1,SEC24D,CSF1R,A2ML1,BMP1,DST,EFEMP2,FBN1,P4HB,COL18A1,F8,HTRA1,FLNA,F7,CD99,C1S,C4B,FBN2,JCHAIN,C1R,ATP1A1,TTN,CFD,B2M,TPM3
DISEASES	DOID:12134	Factor VIII deficiency	4	4	1.96	0.00012	F9,VWF,F8,F7
DISEASES	DOID:0050117	Disease by infectious agent	17	368	0.62	0.00014	APOE,APCS,CP,AHSG,APEH,F2,PRG2,C1QB,APOL1,SLPI,HTRA1,C1QC,C1QA,CA6,YWHAZ,CCDC122,HPR
DISEASES	DOID:12259	Hemophilia B	4	5	1.86	0.00019	F9,F8,F7,F11
DISEASES	DOID:2229	Factor XI deficiency	4	5	1.86	0.00019	F9,F8,F7,F11
DISEASES	DOID:2451	Protein S deficiency	4	5	1.86	0.00019	F2,SERPINC1,PROS1,C4B
DISEASES	DOID:2218	Blood platelet disease	7	47	1.13	0.00020	VWF,PF4,F2,FLNA,ADAMTS13,F7,MMRN1
DISEASES	DOID:612	Primary immunodeficiency disease	19	470	0.56	0.00020	APOH,C5,TTR,CFHR5,KLKB1,SERPING1,A2ML1,DST,F2,CFHR1,COL18A1,CFHR3,CFH,SERPINC1,ADAMTS13,C4B,CFB,PLCG2,KNG1
DISEASES	DOID:65	Connective tissue disease	25	774	0.47	0.00023	F9,MMP2,C5,MGP,TTR,TCIRG1,SEC24D,CSF1R,A2ML1,BMP1,DST,EFEMP2,FBN1,P4HB,COL18A1,F8,HTRA1,FLNA,F7,CD99,C1S,C4B,FBN2,JCHAIN,C1R
DISEASES	DOID:3146	Lipid metabolism disorder	8	72	1.0	0.00026	CETP,APOB,APOA1,APOE,LCAT,PCSK9,ECM1,APOA5
DISEASES	DOID:12531	Von Willebrands disease	4	6	1.78	0.00027	F9,VWF,F8,ADAMTS13
DISEASES	DOID:0014667	Disease of metabolism	30	1076	0.4	0.00035	CETP,F9,MGP,APOB,APOA1,TTR,CAT,APOE,APCS,LYZ,LCAT,CP,AHSG,SAA4,PCSK9,F2,NDUFS8,APOA4,CNDP1,ECM1,MCOLN2,GPX3,PROS1,PCYOX1,ADIPOQ,TGFBI,APOA5,ACTB,B2M,FGA
DISEASES	DOID:10873	Kuhnt-Junius degeneration	4	7	1.71	0.00039	CETP,CFH,HTRA1,CFB
DISEASES	DOID:1936	Atherosclerosis	5	21	1.33	0.00072	CETP,APOB,APOA1,APOE,ADIPOQ
DISEASES	DOID:14250	Down syndrome	4	9	1.61	0.00079	APOE,C1QB,PAPPA,C1QA
DISEASES	DOID:0060440	Epithelial and subepithelial dystrophy	4	11	1.52	0.0014	LTF,APOE,COL18A1,TGFBI
DISEASES	DOID:1387	Hypolipoproteinemia	4	11	1.52	0.0014	APOB,APOA1,LCAT,PCSK9
DISEASES	DOID:409	Liver disease	8	97	0.87	0.0016	TTR,APCS,AHSG,SAA4,F2,SERPINC1,IGHV3-15,ACTB
DISEASES	DOID:1588	Thrombocytopenia	4	12	1.48	0.0018	VWF,PF4,F2,ADAMTS13
DISEASES	DOID:0111146	Acquired von Willebrand syndrome	3	3	1.96	0.0019	VWF,F8,ADAMTS13
DISEASES	DOID:2236	Congenital afibrinogenemia	3	3	1.96	0.0019	FGB,FGG,FGA
DISEASES	DOID:801	Hemarthrosis	3	3	1.96	0.0019	F9,F8,F7
DISEASES	DOID:0060002	C1 inhibitor deficiency	3	4	1.83	0.0031	KLKB1,SERPING1,KNG1
DISEASES	DOID:0080001	Bone disease	18	540	0.48	0.0031	F9,MMP2,C5,TTR,TCIRG1,SEC24D,CSF1R,BMP1,FBN1,P4HB,COL18A1,F8,HTRA1,FLNA,F7,CD99,C1S,JCHAIN
DISEASES	DOID:10772	Thrombotic thrombocytopenic purpura	3	4	1.83	0.0031	VWF,CFH,ADAMTS13
DISEASES	DOID:2988	Antiphospholipid syndrome	3	4	1.83	0.0031	APOH,F2,SERPINC1
DISEASES	DOID:3756	Protein C deficiency	3	4	1.83	0.0031	PROC,F2,SERPINC1
DISEASES	DOID:1168	Familial hyperlipidemia	4	15	1.38	0.0032	APOB,APOE,PCSK9,APOA5
DISEASES	DOID:10124	Corneal disease	7	85	0.87	0.0044	LTF,APOB,APOE,LUM,PCSK9,COL18A1,TGFBI
DISEASES	DOID:0060449	Gelatinous drop-like corneal dystrophy	3	5	1.74	0.0045	LTF,APOE,TGFBI
DISEASES	DOID:0111902	Thrombophilia due to activated protein C resistance	3	5	1.74	0.0045	F2,SERPINC1,F5
DISEASES	DOID:11249	Vitamin K deficiency bleeding	3	5	1.74	0.0045	MGP,F2,PROS1
DISEASES	DOID:1237	Corneal degeneration	3	5	1.74	0.0045	APOB,PCSK9,TGFBI
DISEASES	DOID:0050737	Autosomal recessive disease	38	1785	0.29	0.0046	MGP,LTF,APOB,APOE,F12,VWF,CP,KLKB1,F13A1,DNAH5,TCIRG1,SERPING1,MASP1,CAMP,PCSK9,FGB,DST,F2,EFEMP2,SERPINF2,FGG,BLM,F13B,SERPINC1,F5,FLNA,F7,F11,C1S,PCYOX1,PRG4,TGFBI,APOA5,TTN,CFD,KNG1,FGA,TPM3
DISEASES	DOID:11247	Disseminated intravascular coagulation	3	7	1.59	0.0088	VWF,F2,SERPINC1

DISEASES	DOID:3118	Hepatobiliary disease	9	168	0.69	0.0088	TTR,APCS,AHSG,SAA4,F2,SERPINC1,MST1,IGHV3-15,ACTB
DISEASES	DOID:5113	Nutritional deficiency disease	5	43	1.02	0.0102	MGP,TTR,F2,GPX3,PROS1
DISEASES	DOID:3973	Thyroid gland medullary carcinoma	3	8	1.53	0.0116	APOE,APCS,APOA4
DISEASES	DOID:0080010	Bone structure disease	4	24	1.18	0.0133	MMP2,TTR,FBN1,HTRA1
DISEASES	DOID:655	Inherited metabolic disorder	23	949	0.34	0.0233	CETP,F9,APOB,APOA1,TTR,CAT,APOE,APCS,LYZ,LCAT,CP,PCSK9,NDUFS8,APOA4,CNDP1,ECM1,MCOLN2,PCYOX1,ADIPOQ,APOA5,ACTB,B2M,FGA
DISEASES	DOID:2213	Hemorrhagic disease	3	12	1.36	0.0299	F2,SERPINF2,ADAMTS13
DISEASES	DOID:0060158	Acquired metabolic disease	8	162	0.65	0.0317	MGP,TTR,CP,AHSG,F2,GPX3,PROS1,ADIPOQ
DISEASES	DOID:0050638	Transthyretin amyloidosis	2	2	1.96	0.0382	TTR,APCS
DISEASES	DOID:0060573	Von Willebrands disease 1	2	2	1.96	0.0382	VWF,F8
DISEASES	DOID:0060574	Von Willebrands disease 2	2	2	1.96	0.0382	VWF,F8
DISEASES	DOID:0080307	Myofibrillar myopathy	3	14	1.29	0.0382	FLNA,TTN,CFD
DISEASES	DOID:0111054	Von Willebrands disease 3	2	2	1.96	0.0382	VWF,F8
DISEASES	DOID:0111595	Congenital contractural arachnodactyly	2	2	1.96	0.0382	FBN1,FBN2
DISEASES	DOID:0111676	High molecular weight kininogen deficiency	2	2	1.96	0.0382	KLKB1,KNG1
DISEASES	DOID:0111901	Heparin cofactor II deficiency	2	2	1.96	0.0382	SERPIND1,SERPINC1
DISEASES	DOID:10230	Aortic atherosclerosis	2	2	1.96	0.0382	APOB,APOE
DISEASES	DOID:1391	Norum disease	2	2	1.96	0.0382	APOA1,LCAT
DISEASES	DOID:170	Endocrine gland cancer	6	93	0.77	0.0382	APOE,APCS,APOA4,CD99,IGHV3-15,ACTB
DISEASES	DOID:2211	Factor XIII deficiency	2	2	1.96	0.0382	F13A1,F13B
DISEASES	DOID:3576	Sagittal sinus thrombosis	2	2	1.96	0.0382	F2,SERPINC1
DISEASES	DOID:3393	Coronary artery disease	4	36	1.0	0.0432	APOB,APOA1,APOE,PCSK9
TISSUES	BTO:0004850	Bone marrow cell	53	198	1.39	4.53e-50	CPB2,CETP,APOH,C5,AFM,APOB,IGFBP5,TTR,APCS,VWF,CP,KLKB1,F13A1,HPX,LUM,FCN3,AHSG,SAA4,SERPING1,CSF1R,FCN2,MASP1,FGB,F2,CSPG4,CFHR1,APOL1,SERPINF2,APOD,F8,CFH,SERPINC1,F5,ECM1,ADAMTS13,C1QA,SOD3,PROS1,MMRN1,F11,C1S,PRG4,MST1,SPARCL1,C4B,CFB,JCHAIN,C1R,PAMR1,KNG1,CDH5,B2M,FGA
TISSUES	BTO:0000141	Bone marrow	65	528	1.05	7.82e-44	CPB2,CETP,APOH,SERPIND1,APMAP,C5,AFM,LTF,APOB,IGFBP5,TTR,APCS,VWF,CSTA,CP,KLKB1,F13A1,HPX,LUM,FCN3,AHSG,SAA4,SERPING1,CSF1R,FCN2,MASP1,CAMP,FGB,F2,PRG2,CSPG4,CFHR1,SUZ12,APOL1,SERPINF2,KDM3B,PRDX6,APOD,GIT2,F8,CFH,QSOX1,SERPINC1,F5,ECM1,ADAMTS13,C1QA,SOD3,PROS1,MMRN1,YWHAZ,F11,C1S,PRG4,MST1,SPARCL1,C4B,CFB,JCHAIN,C1R,PAMR1,KNG1,CDH5,B2M,FGA
TISSUES	BTO:0000392	Plasma cell	46	171	1.39	1.92e-43	APOH,C5,APOB,IGFBP5,TTR,APCS,VWF,CP,KLKB1,F13A1,HPX,LUM,FCN3,AHSG,SAA4,SERPING1,CSF1R,FCN2,MASP1,FGB,F2,CSPG4,CFHR1,APOL1,SERPINF2,APOD,F8,CFH,SERPINC1,ECM1,ADAMTS13,C1QA,SOD3,PROS1,MMRN1,C1S,SPARCL1,C4B,CFB,JCHAIN,C1R,PAMR1,KNG1,CDH5,B2M,FGA
TISSUES	BTO:0000759	Liver	105	2125	0.65	3.23e-40	SPP2,CPB2,CETP,APOH,SERPIND1,TPD52L2,APMAP,F9,AFM,GPLD1,LTF,APOB,PROC,APOA1,TTR,CAT,APOE,F12,APCS,CFHR5,VWF,PSMA6,SERPINA10,ATRN,NID1,CP,KLKB1,PON3,HPX,LUM,IGFBP4,FCN3,ITIH1,AHSG,IGFBP1,HABP2,SAA4,SERPING1,CSF1R,FCN2,PGLYRP2,PPBP,MASP1,APEH,PDIA3,FGB,DST,F2,INHBC,STAB1,C1QB,CFHR1,APOL1,SERPINF2,FBN1,P4HB,HSP90AA1,TUBA1B,FGG,TUBB,PRDX6,SLPI,ASGR2,COL18A1,C8A,PCDH18,F13B,CFHR2,CFHR4,CFHR3,CFH,SERPINC1,ECM1,FLNA,SMARCA1,C8B,ADAMTS13,C1QA,F7,APOM,CD99,PROS1,YWHAZ,MASP2,F11,C1S,PCYOX1,MST1,ITIH3,C4B,TGFBI,CFB,IGFALS,GC,JCHAIN,C1R,APOA5,ATP1A1,VIM,APOC4,KNG1,ACTB,B2M,FGA,TPM3
TISSUES	BTO:0001486	Skeletal system	85	1307	0.77	2.35e-39	SPP2,CPB2,CETP,APOH,SERPIND1,APMAP,MMP2,C5,AFM,LTF,APOB,IGFBP5,APOA1,TTR,APOE,APCS,VWF,PSMA6,ATRN,CSTA,CP,KLKB1,F13A1,HPX,LUM,FCN3,AHSG,SAA4,SERPING1,CSF1R,FCN2,MASP1,CAMP,FGB,F2,UBE3C,PRG2,CSPG4,CFHR1,SUZ12,APOL1,SERPINF2,KDM3B,HSP90AA1,TUBA1B,FGG,PRDX6,APOD,FILIP1L,GIT2,F8,CFH,QSOX1,SERPINC1,F5,HTRA1,ADAMTSL4,ECM1,CRTAC1,ADAMTS13,CCN5,C1QA,SOD3,PROS1,MMRN1,YWHAZ,F11,C1S,PCYOX1,PRG4,MST1,SPARCL1,C4B,CFB,JCHAIN,C1R,VIM,TTN,PAMR1,KNG1,ACTB,CDH5,B2M,FGA,TPM3
TISSUES	BTO:0000345	Digestive gland	115	2881	0.56	1.43e-36	SPP2,CPB2,CETP,APOH,SERPIND1,TPD52L2,APMAP,F9,AFM,GPLD1,LTF,APOB,PROC,APOA1,TTR,CAT,DHX29,APOE,F12,APCS,CFHR5,VWF,PSMA6,SERPINA10,ATRN,NID1,CP,KLKB1,F13A1,PON3,TCIRG1,HPX,LUM,IGFBP4,FCN3,ITIH1,AHSG,IGFBP1,HABP2,SAA4,SERPING1,SEC24D,CSF1R,FCN2,PGLYRP2,PPBP,MASP1,APEH,PDIA3,FGB,DST,F2,INHBC,STAB1,C1QB,CFHR1,NDUFS8,APOL1,SERPINF2,FBN1,P4HB,HSP90AA1,TUBA1B,FGG,TUBB,PRDX6,SLPI,QPCT,APOD,FILIP1L,ASGR2,COL18A1,C8A,PCDH18,F13B,CFHR2,CFHR4,CFHR3,CFH,SERPINC1,ECM1,FLNA,SMARCA1,C8B,ADAMTS13,C1QA,F7,APOM,CD99,GPX3,PROS1,YWHAZ,MASP2,F11,C1S,PCYOX1,MST1,ITIH3,C4B,TGFBI,CFB,IGFALS,GC,JCHAIN,C1R,APOA5,ATP1A1,VIM,RNASE4,APOC4,KNG1,ACTB,B2M,FGA,TPM3
TISSUES	BTO:0001491	Viscus	133	5378	0.35	9.60e-23	SPP2,CPB2,CETP,APOH,SERPIND1,TPD52L2,APMAP,F9,AFM,GPLD1,LTF,APOB,PROC,APOA1,TTR,CAT,DHX29,APOE,F12,APCS,CFHR5,LYZ,VWF,PSMA6,SERPINA10,ATRN,NID1,CSTA,CP,KLKB1,F13A1,PON3,TCIRG1,HPX,LUM,IGFBP4,FCN3,ITIH1,AHSG,IGFBP1,HABP2,SAA4,SERPING1,SEC24D,CSF1R,FCN2,PGLYRP2,PPBP,MASP1,APEH,GUCY1A1,PDIA3,LALBA,FGB,DST,F2,INHBC,STAB1,C1QB,CFHR1,APOL1,SERPINF2,FBN1,P4HB,FBLN1,HSP90AA1,TUBA1B,FGG,TUBB,PRDX6,SLPI,QPCT,APOD,FILIP1L,ASGR2,APOA4,COL18A1,F8,C8A,PCDH18,F13B,CFHR2,CFHR4,CFHR3,CFH,QSOX1,SERPINC1,ECM1,FLNA,SMARCA1,C8B,ADAMTS13,CNTRL,C1QA,F7,APOM,CALML5,CD99,SOD3,GPX3,PROS1,EFEMP1,YWHAZ,ILK,MASP2,F11,C1S,PCYOX1,CKMT1A,MST1,SPARCL1,ITIH3,C4B,TGFBI,CFB,IGFALS,RARRES2,GC,JCHAIN,C1R,APOA5,ATP1A1,VIM,RNASE4,APOC4,CFD,PLCG2,KNG1,ACTB,TRIM8,B2M,FGA,TPM3
TISSUES	BTO:0001488	Endocrine gland	134	6403	0.28	5.10e-16	SPP2,CPB2,CETP,APOH,SERPIND1,TPD52L2,APMAP,F9,MMP2,AFM,GPLD1,LTF,APOB,PROC,APOA1,TTR,CAT,DHX29,APOE,F12,APCS,CFHR5,VWF,PSMA6,SERPINA10,ATRN,LCN1,NID1,CSTA,CP,KLKB1,F13A1,PON3,TCIRG1,HPX,LUM,IGFBP4,FCN3,ITIH1,AHSG,IGFBP1,HABP2,SAA4,SERPING1,SEC24D,CSF1R,PIP,FCN2,PGLYRP2,PPBP,MASP1,CAMP,APEH,PDIA3,FGB,DST,F2,INHBC,STAB1,C1QB,CFHR1,NDUFS8,APOL1,SERPINF2,FBN1,P4HB,FBLN1,HSP90AA1,TUBA1B,FGG,TUBB,PRDX6,SLPI,QPCT,APOD,PAPLN,FILIP1L,ASGR2,GIT2,COL18A1,C8A,PCDH18,F13B,CFHR2,CFHR4,CFHR3,CFH,QSOX1,SERPINC1,ECM1,FLNA,SMARCA1,C8B,ADAMTS13,CCN5,SVEP1,C1QA,F7,APOM,CALML5,CD99,SOD3,GPX3,PROS1,EFEMP1,EFCAB5,YWHAZ,MASP2,F11,C1S,PCYOX1,ATP9B,MST1,ITIH3,C4B,TGFBI,CFB,IGFALS,RARRES2,GC,UIMC1,JCHAIN,C1R,APOA5,ATP1A1,VIM,RNASE4,APOC4,CFD,KNG1,ACTB,B2M,FGA,TPM3

TISSUES	BTO:0001489	Whole body	198	13099	0.14	3.23e-15	SPP2,CPB2,CETP,APOH,SERPIND1,TPD52L2,APMAP,F9,MMP2,C5,HSD17B1,AFM,MGP,GPLD1,LTF,APOB,IGFBP5,PROC,APOA1,TTR,CAT,DHX29,APOE,F12,APCS,CFHR5,LYZ,VWF,PSMA6,SERPINA10,ATRN,LCN1,LCAT,NID1,CSTA,CP,KLKB1,F13A1,DNAH5,CENPE,PON3,TCIRG1,HPX,LUM,CDH11,IGFBP4,FCN3,ITIH1,AHSG,IGFBP1,HABP2,SAA4,SERPING1,SEC24D,CSF1R,PIP,FCN2,PGLYRP2,SCGB3A1,SERPINI1,PPBP,PF4,MASP1,CAMP,APEH,GUCY1A1,A2ML1,PDIA3,SLC27A4,LALBA,PCSK9,BMP1,FGB,DST,F2,INHBC,PSG1,UBE3C,EFEMP2,PRG2,CSPG4,STAB1,C1QB,CFHR1,NDUFS8,SUZ12,APOL1,PSG11,SERPINF2,FBN1,KDM3B,P4HB,PAPPA,FBLN1,DNAH8,HSP90AA1,TUBA1B,FGG,TUBB,THBS4,PRDX6,SLPI,QPCT,APOD,PAPLN,FILIP1L,ASGR2,GIT2,APOA4,CNDP1,COL18A1,MTCL1,F8,C8A,PCDH18,CENPF,F13B,CFHR2,CFHR4,CFHR3,CFH,QSOX1,SERPINC1,F5,ADAM12,S100A7,HTRA1,ADAMTSL4,ECM1,FLNA,CRTAC1,SMARCA1,C8B,FCN1,ADAMTS13,CCN5,CNTRL,SVEP1,C1QC,C1QA,F7,APOM,CA6,ADGRG2,CALML5,CD99,SOD3,GPX3,PROS1,EFEMP1,EFCAB5,MMRN1,YWHAZ,NAV2,ILK,MAP3K7CL,MASP2,PSG4,F11,C1S,PSG2,PCYOX1,ADIPOQ,ATP9B,PRG4,SLAIN1,CKMT1A,MST1,SPARCL1,ITIH3,C4B,TGFBI,CFB,IGFALS,PAEP,RARRES2,GC,UIMC1,FBN2,TSKU,JCHAIN,C1R,APOA5,ATP1A1,VIM,RNASE4,TTN,APOC4,CFD,PLCG2,PAMR1,KNG1,ACTB,TRIM8,CDH5,B2M,FGA,TPM3
TISSUES	BTO:0000522	Gland	139	7004	0.26	5.25e-15	SPP2,CPB2,CETP,APOH,SERPIND1,TPD52L2,APMAP,F9,MMP2,C5,AFM,GPLD1,LTF,APOB,PROC,APOA1,TTR,CAT,DHX29,APOE,F12,APCS,CFHR5,LYZ,VWF,PSMA6,SERPINA10,ATRN,LCN1,NID1,CSTA,CP,KLKB1,F13A1,PON3,TCIRG1,HPX,LUM,IGFBP4,FCN3,ITIH1,AHSG,IGFBP1,HABP2,SAA4,SERPING1,SEC24D,CSF1R,PIP,FCN2,PGLYRP2,PPBP,MASP1,CAMP,APEH,GUCY1A1,PDIA3,FGB,DST,F2,INHBC,STAB1,C1QB,CFHR1,NDUFS8,APOL1,SERPINF2,FBN1,P4HB,FBLN1,HSP90AA1,TUBA1B,FGG,TUBB,PRDX6,SLPI,QPCT,APOD,PAPLN,FILIP1L,ASGR2,GIT2,COL18A1,F8,C8A,PCDH18,F13B,CFHR2,CFHR4,CFHR3,CFH,QSOX1,SERPINC1,ECM1,FLNA,SMARCA1,C8B,ADAMTS13,CCN5,SVEP1,C1QA,F7,APOM,CA6,CALML5,CD99,SOD3,GPX3,PROS1,EFEMP1,EFCAB5,YWHAZ,MASP2,F11,C1S,PCYOX1,ATP9B,MST1,ITIH3,C4B,TGFBI,CFB,IGFALS,RARRES2,GC,UIMC1,JCHAIN,C1R,APOA5,ATP1A1,VIM,RNASE4,APOC4,CFD,KNG1,ACTB,B2M,FGA,TPM3
TISSUES	BTO:0000237	Cerebrospinal fluid	14	45	1.45	4.15e-13	APOH,AFM,TTR,APOE,CP,HPX,AHSG,SERPING1,F2,APOD,CFH,SERPINC1,SPARCL1,KNG1
TISSUES	BTO:0001078	Placenta	45	1244	0.52	4.21e-10	HSD17B1,IGFBP5,VWF,NID1,F13A1,LUM,IGFBP4,IGFBP1,SERPING1,CSF1R,PDIA3,BMP1,DST,PSG1,EFEMP2,PRG2,APOL1,PSG11,FBN1,P4HB,PAPPA,FBLN1,HSP90AA1,TUBA1B,COL18A1,QSOX1,F5,ADAM12,HTRA1,ADAMTSL4,SVEP1,GPX3,YWHAZ,ILK,PSG4,PSG2,PCYOX1,TGFBI,FBN2,ATP1A1,VIM,CFD,ACTB,CDH5,B2M
TISSUES	BTO:0000449	Fetus	46	1318	0.5	7.07e-10	HSD17B1,IGFBP5,VWF,NID1,F13A1,LUM,IGFBP4,IGFBP1,SERPING1,CSF1R,PDIA3,BMP1,DST,PSG1,EFEMP2,PRG2,APOL1,PSG11,FBN1,P4HB,PAPPA,FBLN1,HSP90AA1,TUBA1B,TUBB,COL18A1,QSOX1,F5,ADAM12,HTRA1,ADAMTSL4,SVEP1,GPX3,YWHAZ,ILK,PSG4,PSG2,PCYOX1,TGFBI,FBN2,ATP1A1,VIM,CFD,ACTB,CDH5,B2M
TISSUES	BTO:0000042	Animal	204	15148	0.09	1.74e-09	SPP2,CPB2,CETP,APOH,SERPIND1,TPD52L2,APMAP,F9,MMP2,C5,HSD17B1,AFM,MGP,GPLD1,LTF,APOB,IGFBP5,PROC,APOA1,TTR,CAT,DHX29,APOE,ANGPTL6,F12,APCS,CFHR5,LYZ,VWF,PSMA6,SERPINA10,ATRN,LCN1,LCAT,NID1,CSTA,CP,KLKB1,F13A1,PPP2R3A,DNAH5,CENPE,PON3,TCIRG1,HPX,LUM,CDH11,IGFBP4,FCN3,ITIH1,AHSG,IGFBP1,HABP2,SAA4,SERPING1,SEC24D,CSF1R,IGF2BP1,PIP,FCN2,PGLYRP2,SCGB3A1,SERPINI1,PPBP,PF4,MASP1,CAMP,APEH,GUCY1A1,A2ML1,PDIA3,SLC27A4,LALBA,PCSK9,BMP1,FGB,DST,F2,INHBC,PSG1,UBE3C,EFEMP2,PRG2,CSPG4,STAB1,C1QB,CFHR1,NDUFS8,SUZ12,APOL1,PSG11,SERPINF2,FBN1,KDM3B,P4HB,PAPPA,FBLN1,DNAH8,HSP90AA1,TUBA1B,FGG,TUBB,THBS4,PRDX6,SLPI,QPCT,APOD,PAPLN,FILIP1L,ASGR2,GIT2,APOA4,CNDP1,COL18A1,MTCL1,F8,C8A,PCDH18,CENPF,F13B,CFHR2,CFHR4,CFHR3,CFH,QSOX1,SERPINC1,F5,ADAM12,S100A7,HTRA1,ADAMTSL4,ECM1,FLNA,CRTAC1,SMARCA1,C8B,FCN1,ADAMTS13,CCN5,CNTRL,SVEP1,C1QC,C1QA,F7,APOM,CA6,ADGRG2,CALML5,CD99,SOD3,GPX3,PROS1,EFEMP1,EFCAB5,MMRN1,YWHAZ,NAV2,ILK,MAP3K7CL,MASP2,PSG4,F11,C1S,PSG2,PCYOX1,ADIPOQ,ATP9B,PRG4,SLAIN1,CKMT1A,ABCA13,MST1,SPARCL1,ITIH3,C4B,TGFBI,CFB,IGFALS,PAEP,RARRES2,TCAF1,GC,UIMC1,FBN2,TSKU,JCHAIN,C1R,APOA5,ATP1A1,VIM,RNASE4,TTN,APOC4,CFD,PLCG2,PAMR1,KNG1,ACTB,TRIM66,TRIM8,CDH5,B2M,FGA,TPM3
TISSUES	BTO:0001279	Spinal cord	19	233	0.87	5.81e-09	APOH,AFM,TTR,APOE,CP,HPX,AHSG,SERPING1,F2,CSPG4,APOD,PCDH18,CFH,SERPINC1,FLNA,SPARCL1,VIM,KNG1,ACTB
TISSUES	BTO:0003099	Internal female genital organ	66	2804	0.33	1.37e-07	APOH,TPD52L2,HSD17B1,MGP,LTF,IGFBP5,VWF,NID1,F13A1,LUM,IGFBP4,IGFBP1,SERPING1,CSF1R,MASP1,A2ML1,PDIA3,BMP1,DST,PSG1,EFEMP2,PRG2,NDUFS8,SUZ12,APOL1,PSG11,FBN1,P4HB,PAPPA,FBLN1,HSP90AA1,TUBA1B,TUBB,SLPI,PAPLN,COL18A1,PCDH18,QSOX1,F5,ADAM12,HTRA1,ADAMTSL4,FLNA,CCN5,SVEP1,CD99,GPX3,PROS1,YWHAZ,ILK,PSG4,C1S,PSG2,PCYOX1,SPARCL1,TGFBI,PAEP,UIMC1,FBN2,ATP1A1,VIM,CFD,ACTB,CDH5,B2M,TPM3
TISSUES	BTO:0000132	Blood platelet	21	363	0.72	1.82e-07	APOA1,TTR,CAT,VWF,AHSG,PPBP,PDIA3,FGB,P4HB,HSP90AA1,FGG,PRDX6,SERPINC1,F5,FLNA,MMRN1,YWHAZ,PRG4,ACTB,FGA,TPM3
TISSUES	BTO:0001239	Serum	8	25	1.46	2.22e-07	GPLD1,APOA1,ATRN,ITIH1,PGLYRP2,PRG2,PAPPA,KNG1
TISSUES	BTO:0000284	Organism form	61	2542	0.34	3.19e-07	TPD52L2,HSD17B1,LTF,IGFBP5,DHX29,VWF,PSMA6,NID1,F13A1,LUM,IGFBP4,IGFBP1,SERPING1,CSF1R,CAMP,PDIA3,BMP1,DST,PSG1,UBE3C,EFEMP2,PRG2,APOL1,PSG11,FBN1,P4HB,PAPPA,FBLN1,HSP90AA1,TUBA1B,TUBB,COL18A1,PCDH18,CENPF,QSOX1,F5,ADAM12,HTRA1,ADAMTSL4,FLNA,SVEP1,APOM,CD99,GPX3,EFEMP1,YWHAZ,NAV2,ILK,PSG4,PSG2,PCYOX1,TGFBI,UIMC1,FBN2,ATP1A1,VIM,CFD,ACTB,CDH5,B2M,TPM3
TISSUES	BTO:0000421	Connective tissue	33	954	0.5	9.53e-07	SPP2,APMAP,MMP2,APOA1,TTR,CAT,LUM,AHSG,IGFBP1,PDIA3,EFEMP2,FBN1,P4HB,PAPPA,HSP90AA1,TUBA1B,TUBB,THBS4,PRDX6,PAPLN,F5,HTRA1,ECM1,CRTAC1,EFEMP1,YWHAZ,ADIPOQ,PRG4,VIM,CFD,ACTB,B2M,TPM3
TISSUES	BTO:0000203	Respiratory system	46	1707	0.39	1.59e-06	MMP2,LTF,IGFBP5,APOE,LYZ,VWF,ATRN,LCN1,CP,DNAH5,TCIRG1,LUM,IGFBP4,FCN3,SERPING1,PIP,PDIA3,P4HB,HSP90AA1,TUBA1B,TUBB,PRDX6,PCDH18,CFH,QSOX1,FLNA,SMARCA1,FCN1,CCN5,GPX3,PROS1,EFEMP1,MASP2,C1S,PCYOX1,SPARCL1,C4B,TGFBI,JCHAIN,ATP1A1,VIM,KNG1,ACTB,CDH5,B2M,TPM3
TISSUES	BTO:0001499	Tear	7	23	1.44	2.54e-06	LTF,LYZ,LCN1,PIP,SLPI,JCHAIN,B2M
TISSUES	BTO:0000088	Cardiovascular system	34	1057	0.47	2.66e-06	AFM,MGP,APOB,APOA1,APOE,VWF,LUM,SERPING1,PDIA3,SLC27A4,F2,FBN1,P4HB,HSP90AA1,TUBA1B,THBS4,PCDH18,F13B,SERPINC1,FLNA,GPX3,EFEMP1,MMRN1,PCYOX1,SPARCL1,TGFBI,FBN2,VIM,TTN,ACTB,CDH5,B2M,FGA,TPM3
TISSUES	BTO:0000255	Brain cell line	14	188	0.83	4.25e-06	APOA1,TTR,PDIA3,FGB,P4HB,HSP90AA1,TUBA1B,TUBB,PRDX6,YWHAZ,VIM,TTN,ACTB,TRIM66

TISSUES	BTO:0000174	Embryonic structure	55	2369	0.32	5.80e-06	TPD52L2,HSD17B1,IGFBP5,DHX29,VWF,PSMA6,NID1,F13A1,LUM,IGFBP4,IGFBP1,SERPING1,CSF1R,PDIA3,BMP1,DST,PSG1,UBE3C,EFEMP2,PRG2,APOL1,PSG11,FBN1,P4HB,PAPPA,FBLN1,HSP90AA1,TUBA1B,TUBB,COL18A1,PCDH18,CENPF,QSOX1,F5,ADAM12,HTRA1,ADAMTSL4,FLNA,SVEP1,APOM,GPX3,YWHAZ,ILK,PSG4,PSG2,PCYOX1,TGFBI,UIMC1,FBN2,ATP1A1,VIM,CFD,ACTB,CDH5,B2M
TISSUES	BTO:0000089	Blood	46	1824	0.36	8.85e-06	TPD52L2,LTF,APOB,PROC,APOA1,TTR,CAT,APOE,F12,VWF,AHSG,SERPING1,PPBP,PF4,PDIA3,FGB,F2,P4HB,HSP90AA1,TUBA1B,FGG,PRDX6,C8A,PCDH18,SERPINC1,F5,FLNA,CNTRL,C1QC,C1QA,APOM,CD99,SOD3,GPX3,MMRN1,YWHAZ,C1S,PRG4,C4B,CFB,VIM,PLCG2,ACTB,B2M,FGA,TPM3
TISSUES	BTO:0000511	Gastrointestinal tract	45	1816	0.35	1.99e-05	LTF,APOB,PROC,APOA1,TTR,CAT,LYZ,KLKB1,LUM,IGFBP4,AHSG,HABP2,SERPING1,PDIA3,LALBA,DST,P4HB,FBLN1,HSP90AA1,TUBA1B,TUBB,PRDX6,SLPI,APOA4,PCDH18,QSOX1,FLNA,CD99,SOD3,GPX3,YWHAZ,ILK,C1S,CKMT1A,SPARCL1,TGFBI,CFB,JCHAIN,C1R,ATP1A1,VIM,CFD,ACTB,TRIM8,B2M
TISSUES	BTO:0000570	Hematopoietic system	59	2755	0.29	2.40e-05	CETP,TPD52L2,GPLD1,LTF,APOB,PROC,APOA1,TTR,CAT,APOE,F12,VWF,PSMA6,ATRN,ITIH1,AHSG,SERPING1,PGLYRP2,PPBP,PF4,PDIA3,FGB,F2,PRG2,P4HB,PAPPA,HSP90AA1,TUBA1B,FGG,PRDX6,C8A,PCDH18,SERPINC1,F5,FLNA,CNTRL,C1QC,C1QA,APOM,CD99,SOD3,GPX3,MMRN1,YWHAZ,C1S,PRG4,SPARCL1,C4B,TGFBI,CFB,JCHAIN,VIM,CFD,PLCG2,KNG1,ACTB,B2M,FGA,TPM3
TISSUES	BTO:0003914	Interstitial cell of Cajal	13	193	0.79	3.24e-05	APOA1,TTR,CAT,AHSG,PDIA3,P4HB,HSP90AA1,TUBA1B,TUBB,PRDX6,YWHAZ,VIM,ACTB
TISSUES	BTO:0000562	Heart	25	738	0.49	6.77e-05	AFM,APOA1,VWF,LUM,SERPING1,PDIA3,SLC27A4,F2,FBN1,HSP90AA1,TUBA1B,THBS4,PCDH18,F13B,FLNA,GPX3,MMRN1,PCYOX1,SPARCL1,VIM,TTN,ACTB,CDH5,FGA,TPM3
TISSUES	BTO:0000058	Alimentary canal	46	2021	0.32	0.00013	LTF,APOB,PROC,APOA1,TTR,CAT,LYZ,KLKB1,LUM,IGFBP4,AHSG,HABP2,SERPING1,PDIA3,LALBA,DST,P4HB,FBLN1,HSP90AA1,TUBA1B,TUBB,PRDX6,SLPI,APOA4,PCDH18,QSOX1,FLNA,CALML5,CD99,SOD3,GPX3,YWHAZ,ILK,C1S,CKMT1A,SPARCL1,TGFBI,CFB,JCHAIN,C1R,ATP1A1,VIM,CFD,ACTB,TRIM8,B2M
TISSUES	BTO:0000763	Lung	36	1395	0.37	0.00013	MMP2,LTF,IGFBP5,APOE,VWF,LCN1,DNAH5,TCIRG1,LUM,IGFBP4,FCN3,SERPING1,PDIA3,P4HB,HSP90AA1,TUBA1B,TUBB,PRDX6,PCDH18,QSOX1,FLNA,SMARCA1,FCN1,CCN5,GPX3,EFEMP1,MASP2,C1S,SPARCL1,C4B,TGFBI,VIM,ACTB,CDH5,B2M,TPM3
TISSUES	BTO:0000988	Pancreas	22	626	0.5	0.00016	CETP,GPLD1,TTR,NID1,F13A1,TCIRG1,SEC24D,PDIA3,APOL1,P4HB,HSP90AA1,TUBA1B,QPCT,FILIP1L,FLNA,CD99,GPX3,TGFBI,ATP1A1,VIM,ACTB,B2M
TISSUES	BTO:0001202	Saliva	7	57	1.05	0.00038	APOH,AFM,HPX,AHSG,PIP,C4B,JCHAIN
TISSUES	BTO:0000121	Bile	5	20	1.36	0.00039	APOH,CP,HPX,SERPINC1,JCHAIN
TISSUES	BTO:0001085	Vascular system	17	420	0.57	0.00039	MGP,APOB,APOE,VWF,F2,FBN1,P4HB,HSP90AA1,SERPINC1,EFEMP1,MMRN1,TGFBI,FBN2,VIM,ACTB,CDH5,B2M
TISSUES	BTO:0000102	Blood clot	4	11	1.52	0.00098	VWF,F2,SERPINC1,ADAMTS13
TISSUES	BTO:0001419	Urine	5	28	1.21	0.0015	LYZ,F2,MASP2,PRG4,B2M
TISSUES	BTO:0000648	Intestine	32	1349	0.33	0.0022	APOB,PROC,LYZ,KLKB1,LUM,IGFBP4,HABP2,PDIA3,DST,P4HB,HSP90AA1,TUBA1B,SLPI,APOA4,PCDH18,FLNA,SOD3,GPX3,YWHAZ,ILK,C1S,CKMT1A,TGFBI,CFB,JCHAIN,C1R,ATP1A1,VIM,CFD,ACTB,TRIM8,B2M
TISSUES	BTO:0001356	Tendon	8	112	0.81	0.0027	EFEMP2,P4HB,THBS4,PAPLN,ECM1,CRTAC1,EFEMP1,PRG4
TISSUES	BTO:0000020	Abdomen	8	113	0.81	0.0028	F2,HSP90AA1,TUBA1B,SERPINC1,ADIPOQ,KNG1,ACTB,B2M
TISSUES	BTO:0000081	Reproductive system	99	6444	0.14	0.0028	APOH,TPD52L2,APMAP,MMP2,HSD17B1,MGP,LTF,IGFBP5,APOA1,CAT,DHX29,APOE,VWF,PSMA6,ATRN,NID1,F13A1,HPX,LUM,CDH11,IGFBP4,AHSG,IGFBP1,SERPING1,CSF1R,PIP,PGLYRP2,MASP1,CAMP,APEH,A2ML1,PDIA3,LALBA,BMP1,FGB,DST,F2,PSG1,UBE3C,EFEMP2,PRG2,NDUFS8,SUZ12,APOL1,PSG11,FBN1,P4HB,PAPPA,FBLN1,DNAH8,HSP90AA1,TUBA1B,TUBB,PRDX6,SLPI,PAPLN,COL18A1,PCDH18,CFH,QSOX1,F5,ADAM12,HTRA1,ADAMTSL4,ECM1,FLNA,ADAMTS13,CCN5,CNTRL,SVEP1,ADGRG2,CD99,GPX3,PROS1,EFEMP1,EFCAB5,YWHAZ,NAV2,ILK,PSG4,C1S,PSG2,PCYOX1,ATP9B,SPARCL1,TGFBI,PAEP,RARRES2,UIMC1,FBN2,TSKU,ATP1A1,VIM,CFD,PAMR1,ACTB,CDH5,B2M,TPM3
TISSUES	BTO:0000083	Female reproductive system	95	6111	0.15	0.0028	APOH,TPD52L2,APMAP,MMP2,HSD17B1,MGP,LTF,IGFBP5,APOA1,CAT,DHX29,APOE,VWF,PSMA6,ATRN,NID1,F13A1,HPX,LUM,CDH11,IGFBP4,AHSG,IGFBP1,SERPING1,CSF1R,PGLYRP2,MASP1,CAMP,APEH,A2ML1,PDIA3,LALBA,BMP1,FGB,DST,F2,PSG1,UBE3C,EFEMP2,PRG2,NDUFS8,SUZ12,APOL1,PSG11,FBN1,P4HB,PAPPA,FBLN1,DNAH8,HSP90AA1,TUBA1B,TUBB,PRDX6,SLPI,PAPLN,COL18A1,PCDH18,CFH,QSOX1,F5,ADAM12,HTRA1,ADAMTSL4,ECM1,FLNA,ADAMTS13,CCN5,CNTRL,SVEP1,CD99,GPX3,PROS1,EFEMP1,EFCAB5,YWHAZ,NAV2,ILK,PSG4,C1S,PSG2,PCYOX1,SPARCL1,TGFBI,PAEP,UIMC1,FBN2,TSKU,ATP1A1,VIM,CFD,PAMR1,ACTB,CDH5,B2M,TPM3
TISSUES	BTO:0001702	Left atrium	6	60	0.96	0.0039	APOA1,FBN1,FLNA,VIM,TTN,TPM3
TISSUES	BTO:0001823	Synovium	7	88	0.86	0.0039	P4HB,THBS4,PAPLN,ECM1,CRTAC1,EFEMP1,PRG4
TISSUES	BTO:0001253	Skin	28	1151	0.34	0.0040	APOE,PSMA6,LCN1,CSTA,PDIA3,BMP1,DST,P4HB,HSP90AA1,TUBA1B,APOD,S100A7,ECM1,FLNA,CALML5,CD99,EFEMP1,YWHAZ,ADIPOQ,TGFBI,RARRES2,C1R,ATP1A1,VIM,CFD,ACTB,B2M,TPM3
TISSUES	BTO:0001090	Mouth	17	532	0.46	0.0052	C5,LTF,LYZ,LCN1,CSTA,CENPE,PIP,A2ML1,DST,P4HB,HSP90AA1,SLPI,CA6,YWHAZ,JCHAIN,ACTB,B2M
TISSUES	BTO:0000439	Eye	22	835	0.38	0.0077	LTF,IGFBP5,TTR,APOE,LYZ,LCN1,LUM,SERPINI1,DST,HSP90AA1,TUBA1B,TUBB,APOD,CFH,GPX3,YWHAZ,TGFBI,FBN2,ATP1A1,VIM,ACTB,B2M
TISSUES	BTO:0001369	Vertebrate muscular system	21	780	0.39	0.0080	CPB2,APOA1,APOE,APCS,PSMA6,EFEMP2,P4HB,FGG,THBS4,PAPLN,FILIP1L,ECM1,CRTAC1,CCN5,GPX3,EFEMP1,PRG4,TTN,KNG1,ACTB,TPM3
TISSUES	BTO:0000970	Osteosarcoma cell	5	45	1.0	0.0087	IGFBP5,CDH11,IGFBP4,VIM,TPM3
TISSUES	BTO:0003091	Urogenital system	104	7090	0.12	0.0092	APOH,TPD52L2,APMAP,MMP2,HSD17B1,MGP,LTF,IGFBP5,APOA1,CAT,DHX29,APOE,VWF,PSMA6,ATRN,NID1,F13A1,HPX,LUM,CDH11,IGFBP4,AHSG,IGFBP1,SERPING1,CSF1R,PIP,PGLYRP2,MASP1,CAMP,APEH,GUCY1A1,A2ML1,PDIA3,LALBA,BMP1,FGB,DST,F2,PSG1,UBE3C,EFEMP2,PRG2,NDUFS8,SUZ12,APOL1,PSG11,FBN1,P4HB,PAPPA,FBLN1,DNAH8,HSP90AA1,TUBA1B,TUBB,PRDX6,SLPI,PAPLN,COL18A1,F8,PCDH18,CFH,QSOX1,F5,ADAM12,HTRA1,ADAMTSL4,ECM1,FLNA,SMARCA1,ADAMTS13,CCN5,CNTRL,SVEP1,ADGRG2,CD99,GPX3,PROS1,EFEMP1,EFCAB5,YWHAZ,NAV2,ILK,PSG4,C1S,PSG2,PCYOX1,ATP9B,SPARCL1,TGFBI,PAEP,RARRES2,GC,UIMC1,FBN2,TSKU,ATP1A1,VIM,CFD,PAMR1,KNG1,ACTB,CDH5,B2M,TPM3
TISSUES	BTO:0000372	Bone cancer cell	9	183	0.65	0.0104	IGFBP5,TCIRG1,CDH11,IGFBP4,QSOX1,VIM,TTN,PAMR1,TPM3
TISSUES	BTO:0001049	Pharynx	6	75	0.86	0.0104	LTF,LYZ,HSP90AA1,JCHAIN,ACTB,B2M
TISSUES	BTO:0003120	Atherosclerotic plaque	3	9	1.48	0.0104	APOB,APOA1,APOE
TISSUES	BTO:0003872	Foam cell	3	9	1.48	0.0104	APOB,APOA1,APOE
TISSUES	BTO:0001129	Prostate gland	15	476	0.46	0.0125	LTF,ATRN,LUM,PIP,P4HB,HSP90AA1,TUBA1B,PCDH18,FLNA,RARRES2,ATP1A1,VIM,ACTB,B2M,TPM3
TISSUES	BTO:0000431	Excretory gland	30	1385	0.29	0.0128	TPD52L2,LTF,CAT,APOE,HPX,AHSG,PIP,GUCY1A1,FGB,F2,P4HB,HSP90AA1,TUBA1B,APOD,COL18A1,F8,QSOX1,SMARCA1,SVEP1,CD99,GPX3,TGFBI,RARRES2,GC,ATP1A1,VIM,KNG1,ACTB,B2M,TPM3
TISSUES	BTO:0000765	Exocrine gland	8	150	0.68	0.0128	C5,LYZ,LCN1,PIP,MASP1,SLPI,CA6,B2M

TISSUES	BTO:0000634	Integument	40	2112	0.24	0.0185	LTF,APOE,VWF,PSMA6,LCN1,CSTA,LUM,IGFBP1,PIP,A2ML1,PDIA3,BMP1,DST,P4HB,HSP90AA1,TUBA1B,SLPI,APOD,S100A7,ECM1,FLNA,CALML5,CD99,EFEMP1,MMRN1,YWHAZ,ILK,ADIPOQ,TGFBI,PAEP,RARRES2,FBN2,C1R,ATP1A1,VIM,CFD,ACTB,CDH5,B2M,TPM3
TISSUES	BTO:0000140	Bone	8	165	0.64	0.0223	SPP2,MMP2,HSP90AA1,TUBA1B,VIM,ACTB,B2M,TPM3
TISSUES	BTO:0002783	Superior mesenteric vein	2	2	1.96	0.0242	F2,SERPINC1
TISSUES	BTO:0003150	Pulmonary artery endothelial cell line	2	2	1.96	0.0242	KNG1,CDH5
TISSUES	BTO:0003632	Cervicovaginal fluid	2	2	1.96	0.0242	LTF,SLPI
TISSUES	BTO:0000091	Ascites	5	61	0.87	0.0254	HSP90AA1,TUBA1B,KNG1,ACTB,B2M
TISSUES	BTO:0000202	Sense organ	25	1124	0.31	0.0254	LTF,IGFBP5,TTR,APOE,LYZ,LCN1,CSTA,LUM,SERPING1,SERPINI1,DST,HSP90AA1,TUBA1B,TUBB,SLPI,APOD,CFH,GPX3,YWHAZ,TGFBI,FBN2,ATP1A1,VIM,ACTB,B2M
TISSUES	BTO:0000706	Large intestine	19	750	0.36	0.0254	PROC,LYZ,KLKB1,IGFBP4,HABP2,P4HB,APOA4,PCDH18,FLNA,SOD3,YWHAZ,ILK,CKMT1A,CFB,JCHAIN,C1R,ACTB,TRIM8,B2M
TISSUES	BTO:0001207	Sarcoma cell	8	171	0.63	0.0254	IGFBP5,CDH11,IGFBP4,QSOX1,VIM,TTN,PAMR1,TPM3
TISSUES	BTO:0001703	Right atrium	5	61	0.87	0.0254	APOA1,FBN1,VIM,TTN,TPM3
TISSUES	BTO:0001307	Stomach	11	312	0.51	0.0267	LTF,LYZ,IGFBP4,PDIA3,LALBA,P4HB,HSP90AA1,TUBA1B,VIM,ACTB,B2M
TISSUES	BTO:0000849	Melanoma cell line	8	180	0.61	0.0330	MMP2,PDIA3,CSPG4,P4HB,HSP90AA1,BLM,YWHAZ,ATP1A1
TISSUES	BTO:0003445	Carotid atherosclerotic plaque	2	3	1.78	0.0346	APOB,APOE
TISSUES	BTO:0004695	Aortic root	2	3	1.78	0.0346	APOE,FBN1
TISSUES	BTO:0002690	Biofilm	2	4	1.66	0.0497	LTF,CAMP
TISSUES	BTO:0002854	Corn silk	2	4	1.66	0.0497	MASP1,MASP2
COMPARTMENTS	GOCC:0005576	Extracellular region	146	2079	0.8	2.74e-83	SPP2,CPB2,CETP,APOH,SERPIND1,F9,MMP2,C5,AFM,MGP,GPLD1,LTF,APOB,IGFBP5,PROC,APOA1,TTR,APOE,ANGPTL6,F12,APCS,CFHR5,LYZ,VWF,SERPINA10,LCN1,LCAT,NID1,CSTA,CP,KLKB1,F13A1,CENPE,PON3,HPX,LUM,CDH11,IGFBP4,FCN3,ITIH1,AHSG,IGFBP1,HABP2,SAA4,SERPING1,PIP,FCN2,PGLYRP2,SCGB3A1,SERPINI1,PPBP,PF4,MASP1,CAMP,A2ML1,PDIA3,LALBA,PCSK9,BMP1,FGB,DST,F2,INHBC,EFEMP2,PRG2,CSPG4,C1QB,CFHR1,APOL1,SERPINF2,FBN1,P4HB,PAPPA,FBLN1,HSP90AA1,FGG,TUBB,THBS4,SLPI,QPCT,APOD,APOA4,COL18A1,F8,C8A,F13B,CFHR2,CFHR4,CFHR3,CFH,QSOX1,SERPINC1,F5,ADAM12,S100A7,HTRA1,ECM1,FLNA,CRTAC1,C8B,FCN1,ADAMTS13,CCN5,C1QC,C1QA,F7,APOM,CA6,CALML5,SOD3,GPX3,PROS1,EFEMP1,MMRN1,YWHAZ,MASP2,F11,C1S,PCYOX1,ADIPOQ,MST1,SPARCL1,ITIH3,C4B,TGFBI,CFB,IGFALS,PAEP,RARRES2,GC,FBN2,TSKU,JCHAIN,HPR,C1R,APOA5,VIM,RNASE4,APOC4,CFD,PLCG2,KNG1,ACTB,B2M,FGA,TPM3
COMPARTMENTS	GOCC:0005615	Extracellular space	109	1027	0.98	7.29e-74	CPB2,CETP,APOH,SERPIND1,F9,MMP2,C5,AFM,MGP,GPLD1,LTF,APOB,IGFBP5,PROC,APOA1,TTR,APOE,F12,APCS,LYZ,VWF,LCN1,LCAT,CSTA,CP,KLKB1,F13A1,PON3,HPX,LUM,CDH11,IGFBP4,FCN3,ITIH1,AHSG,IGFBP1,SAA4,SERPING1,PIP,FCN2,SCGB3A1,SERPINI1,MASP1,CAMP,A2ML1,PDIA3,PCSK9,FGB,F2,CSPG4,C1QB,CFHR1,APOL1,SERPINF2,FBN1,PAPPA,FBLN1,HSP90AA1,FGG,THBS4,SLPI,APOD,APOA4,COL18A1,F8,C8A,CFHR3,CFH,QSOX1,SERPINC1,S100A7,ECM1,FLNA,CCN5,C1QC,C1QA,F7,APOM,CA6,SOD3,GPX3,PROS1,EFEMP1,YWHAZ,MASP2,F11,C1S,PCYOX1,ADIPOQ,MST1,C4B,TGFBI,CFB,IGFALS,RARRES2,GC,FBN2,JCHAIN,HPR,C1R,APOA5,VIM,APOC4,CFD,PLCG2,KNG1,ACTB,B2M,FGA
COMPARTMENTS	GOCC:0072562	Blood microparticle	37	118	1.45	1.90e-36	AFM,APOA1,APOE,APCS,CP,F13A1,HPX,FCN3,ITIH1,AHSG,SERPING1,FCN2,FGB,F2,C1QB,CFHR1,APOL1,SERPINF2,FGG,APOA4,C8A,CFHR3,CFH,SERPINC1,C1QC,PROS1,YWHAZ,C1S,C4B,CFB,GC,JCHAIN,HPR,C1R,KNG1,ACTB,FGA
COMPARTMENTS	GOCC:0043230	Extracellular organelle	51	524	0.95	3.76e-29	CPB2,APOH,F9,C5,AFM,MGP,LTF,APOB,APOA1,TTR,APOE,APCS,VWF,CP,KLKB1,HPX,LUM,CDH11,AHSG,SERPING1,FCN2,CAMP,FGB,F2,CSPG4,SERPINF2,HSP90AA1,FGG,SLPI,APOD,COL18A1,CFH,QSOX1,SERPINC1,ECM1,FLNA,GPX3,PROS1,EFEMP1,YWHAZ,MASP2,F11,C4B,TGFBI,GC,C1R,VIM,KNG1,ACTB,B2M,FGA
COMPARTMENTS	GOCC:1903561	Extracellular vesicle	47	500	0.93	4.38e-26	CPB2,APOH,F9,AFM,MGP,LTF,APOB,APOA1,TTR,APOE,APCS,VWF,CP,KLKB1,HPX,LUM,CDH11,AHSG,SERPING1,CAMP,FGB,F2,CSPG4,SERPINF2,HSP90AA1,FGG,APOD,COL18A1,CFH,QSOX1,SERPINC1,ECM1,FLNA,GPX3,PROS1,EFEMP1,YWHAZ,F11,C4B,TGFBI,GC,C1R,VIM,KNG1,ACTB,B2M,FGA
COMPARTMENTS	GOCC:0060205	Cytoplasmic vesicle lumen	36	245	1.13	1.65e-25	SPP2,APOH,LTF,APOA1,TTR,LYZ,VWF,F13A1,AHSG,SERPING1,SERPINI1,PPBP,PF4,CAMP,FGB,SERPINF2,HSP90AA1,FGG,TUBB,PRDX6,SLPI,QPCT,F8,QSOX1,F5,S100A7,ECM1,FCN1,PROS1,MMRN1,ITIH3,RARRES2,CFD,KNG1,B2M,FGA
COMPARTMENTS	GOCC:0034774	Secretory granule lumen	35	241	1.12	1.04e-24	SPP2,APOH,LTF,APOA1,TTR,LYZ,VWF,F13A1,AHSG,SERPING1,PPBP,PF4,CAMP,FGB,SERPINF2,HSP90AA1,FGG,TUBB,PRDX6,SLPI,QPCT,F8,QSOX1,F5,S100A7,ECM1,FCN1,PROS1,MMRN1,ITIH3,RARRES2,CFD,KNG1,B2M,FGA
COMPARTMENTS	GOCC:0065010	Extracellular membrane-bounded organelle	44	473	0.93	2.57e-24	CPB2,APOH,F9,MGP,LTF,APOB,APOA1,TTR,APOE,APCS,VWF,CP,KLKB1,HPX,LUM,AHSG,SERPING1,CAMP,FGB,F2,CSPG4,SERPINF2,HSP90AA1,FGG,APOD,COL18A1,CFH,QSOX1,SERPINC1,ECM1,FLNA,GPX3,PROS1,YWHAZ,F11,C4B,TGFBI,GC,C1R,VIM,KNG1,ACTB,B2M,FGA
COMPARTMENTS	GOCC:0031982	Vesicle	78	2125	0.52	6.09e-20	SPP2,CPB2,APOH,F9,AFM,MGP,LTF,APOB,APOA1,TTR,CAT,APOE,APCS,LYZ,VWF,CP,KLKB1,F13A1,TCIRG1,HPX,LUM,CDH11,ITIH1,AHSG,SERPING1,SEC24D,SERPINI1,PPBP,PF4,CAMP,APEH,PDIA3,PCSK9,FGB,DST,F2,PRG2,CSPG4,STAB1,SERPINF2,HSP90AA1,FGG,TUBB,PRDX6,SLPI,QPCT,APOD,COL18A1,F8,CFH,QSOX1,SERPINC1,F5,S100A7,ECM1,FLNA,FCN1,CALML5,GPX3,PROS1,EFEMP1,MMRN1,YWHAZ,F11,ABCA13,ITIH3,C4B,TGFBI,RARRES2,GC,C1R,VIM,CFD,PLCG2,KNG1,ACTB,B2M,FGA
COMPARTMENTS	GOCC:0034358	Plasma lipoprotein particle	16	48	1.48	1.46e-15	CETP,APOH,APOB,APOA1,APOE,LCAT,SAA4,PCSK9,APOL1,APOA4,APOM,PCYOX1,ADIPOQ,HPR,APOA5,APOC4
COMPARTMENTS	GOCC:0030141	Secretory granule	41	719	0.71	2.49e-15	SPP2,APOH,LTF,APOA1,TTR,CAT,LYZ,VWF,F13A1,TCIRG1,AHSG,SERPING1,PPBP,PF4,CAMP,APEH,FGB,PRG2,SERPINF2,HSP90AA1,FGG,TUBB,PRDX6,SLPI,QPCT,F8,QSOX1,F5,S100A7,ECM1,FCN1,CALML5,PROS1,MMRN1,ABCA13,ITIH3,RARRES2,CFD,KNG1,B2M,FGA
COMPARTMENTS	GOCC:0005788	Endoplasmic reticulum lumen	23	172	1.08	3.31e-15	SPP2,F9,APOB,APOA1,APOE,AHSG,IGFBP1,PDIA3,PCSK9,APOL1,FBN1,P4HB,FGG,APOA4,COL18A1,F8,QSOX1,F5,F7,APOA5,KNG1,B2M,FGA
COMPARTMENTS	GOCC:0031093	Platelet alpha granule lumen	17	66	1.37	3.55e-15	VWF,F13A1,AHSG,SERPING1,PPBP,PF4,FGB,SERPINF2,FGG,F8,QSOX1,F5,PROS1,MMRN1,CFD,KNG1,FGA
COMPARTMENTS	GOCC:0070062	Extracellular exosome	32	428	0.83	7.94e-15	AFM,MGP,LTF,APOB,APOA1,TTR,APOE,VWF,CP,HPX,CDH11,AHSG,CAMP,FGB,CSPG4,HSP90AA1,FGG,APOD,COL18A1,CFH,QSOX1,ECM1,FLNA,GPX3,EFEMP1,YWHAZ,TGFBI,VIM,KNG1,ACTB,B2M,FGA
COMPARTMENTS	GOCC:0034364	High-density lipoprotein particle	13	30	1.59	7.06e-14	CETP,APOH,APOB,APOA1,APOE,LCAT,SAA4,APOL1,APOA4,APOM,HPR,APOA5,APOC4
COMPARTMENTS	GOCC:0034361	Very-low-density lipoprotein particle	11	20	1.7	1.53e-12	CETP,APOH,APOB,APOA1,APOE,APOL1,APOA4,APOM,PCYOX1,APOA5,APOC4
COMPARTMENTS	GOCC:0031012	Extracellular matrix	21	259	0.87	4.31e-10	CPB2,MMP2,GPLD1,APOE,VWF,NID1,LUM,DST,EFEMP2,FBN1,FBLN1,THBS4,COL18A1,ECM1,ADAMTS13,SOD3,EFEMP1,MMRN1,TGFBI,RARRES2,FBN2

COMPARTMENTS	GOCC:0110165	Cellular anatomical entity	196	14060	0.1	1.03e-09	SPP2,CPB2,CETP,APOH,SERPIND1,TPD52L2,APMAP,F9,MMP2,C5,HSD17B1,AFM,MGP,GPLD1,LTF,APOB,IGFBP5,PROC,APOA1,TTR,CAT,DHX29,APOE,ANGPTL6,F12,APCS,CFHR5,LYZ,VWF,PSMA6,SERPINA10,ATRN,LCN1,LCAT,NID1,CSTA,CP,KLKB1,F13A1,DNAH5,CENPE,PON3,TCIRG1,HPX,LUM,CDH11,IGFBP4,FCN3,ITIH1,AHSG,IGFBP1,HABP2,SAA4,SERPING1,SEC24D,CSF1R,IGF2BP1,PIP,FCN2,PGLYRP2,SCGB3A1,SERPINI1,PPBP,PF4,MASP1,CAMP,APEH,GUCY1A1,A2ML1,PDIA3,SLC27A4,LALBA,PCSK9,BMP1,FGB,DST,F2,INHBC,EFEMP2,PRG2,CSPG4,STAB1,C1QB,CFHR1,NDUFS8,SUZ12,APOL1,SERPINF2,FBN1,KDM3B,P4HB,PAPPA,FBLN1,DNAH8,HSP90AA1,TUBA1B,FGG,TUBB,THBS4,PRDX6,SLPI,QPCT,APOD,ASGR2,BLM,GIT2,APOA4,CNDP1,COL18A1,MTCL1,F8,C8A,CENPF,F13B,CFHR2,CFHR4,CFHR3,CFH,QSOX1,SERPINC1,F5,CFAP46,ADAM12,S100A7,HTRA1,ECM1,FLNA,CRTAC1,MCOLN2,SMARCA1,C8B,FCN1,ADAMTS13,CCN5,CNTRL,SVEP1,C1QC,C1QA,F7,APOM,CA6,ADGRG2,CALML5,CD99,SOD3,GPX3,PROS1,EFEMP1,MMRN1,YWHAZ,NAV2,ILK,MAP3K7CL,MASP2,F11,C1S,PCYOX1,ADIPOQ,ATP9B,CKMT1A,ABCA13,MST1,SPARCL1,ITIH3,C4B,TGFBI,CFB,IGFALS,PAEP,RARRES2,TCAF1,GC,UIMC1,FBN2,TSKU,JCHAIN,HPR,C1R,APOA5,ATP1A1,VIM,RNASE4,TTN,APOC4,CFD,SEMA3B,PLCG2,OR52B4,KNG1,ACTB,TRIM66,TRIM8,CDH5,B2M,FGA,TPM3
COMPARTMENTS	GOCC:0043233	Organelle lumen	72	2905	0.35	1.08e-09	SPP2,APOH,F9,LTF,APOB,PROC,APOA1,TTR,CAT,APOE,LYZ,VWF,F13A1,HPX,LUM,AHSG,IGFBP1,SERPING1,SERPINI1,PPBP,PF4,CAMP,APEH,PDIA3,LALBA,PCSK9,FGB,F2,PRG2,CSPG4,NDUFS8,SUZ12,APOL1,SERPINF2,FBN1,P4HB,HSP90AA1,FGG,TUBB,PRDX6,SLPI,QPCT,BLM,GIT2,APOA4,COL18A1,F8,CENPF,QSOX1,F5,S100A7,HTRA1,ECM1,SMARCA1,FCN1,F7,CALML5,SOD3,PROS1,MMRN1,ITIH3,RARRES2,GC,UIMC1,APOA5,TTN,CFD,KNG1,ACTB,TRIM8,B2M,FGA
COMPARTMENTS	GOCC:1905286	Serine-type peptidase complex	8	11	1.82	1.08e-09	F9,FCN3,FCN2,MASP1,FCN1,F7,MASP2,F11
COMPARTMENTS	GOCC:0030312	External encapsulating structure	20	259	0.85	2.60e-09	MMP2,GPLD1,APOE,VWF,NID1,LUM,DST,EFEMP2,FBN1,PAPPA,FBLN1,THBS4,COL18A1,ECM1,ADAMTS13,SOD3,EFEMP1,MMRN1,RARRES2,FBN2
COMPARTMENTS	GOCC:0070013	Intracellular organelle lumen	71	2902	0.35	2.60e-09	SPP2,APOH,F9,LTF,APOB,PROC,APOA1,TTR,CAT,APOE,LYZ,VWF,F13A1,HPX,LUM,AHSG,IGFBP1,SERPING1,SERPINI1,PPBP,PF4,CAMP,APEH,PDIA3,LALBA,PCSK9,FGB,F2,PRG2,CSPG4,NDUFS8,SUZ12,APOL1,SERPINF2,FBN1,P4HB,HSP90AA1,FGG,TUBB,PRDX6,SLPI,QPCT,BLM,GIT2,APOA4,COL18A1,F8,CENPF,QSOX1,F5,S100A7,ECM1,SMARCA1,FCN1,F7,CALML5,SOD3,PROS1,MMRN1,ITIH3,RARRES2,GC,UIMC1,APOA5,TTN,CFD,KNG1,ACTB,TRIM8,B2M,FGA
COMPARTMENTS	GOCC:0062167	Complement component C1q complex	7	10	1.8	2.25e-08	SERPING1,C1QB,C1QC,C1QA,C1S,C4B,C1R
COMPARTMENTS	GOCC:0031410	Cytoplasmic vesicle	50	1738	0.42	2.67e-08	SPP2,APOH,LTF,APOB,APOA1,TTR,CAT,APOE,LYZ,VWF,F13A1,TCIRG1,HPX,AHSG,SERPING1,SEC24D,SERPINI1,PPBP,PF4,CAMP,APEH,PDIA3,PCSK9,FGB,DST,PRG2,STAB1,SERPINF2,HSP90AA1,FGG,TUBB,PRDX6,SLPI,QPCT,F8,QSOX1,F5,S100A7,ECM1,FCN1,CALML5,PROS1,MMRN1,ABCA13,ITIH3,RARRES2,CFD,KNG1,B2M,FGA
COMPARTMENTS	GOCC:0005577	Fibrinogen complex	8	20	1.56	3.14e-08	VWF,FGB,F2,SERPINF2,FGG,SERPINC1,F7,FGA
COMPARTMENTS	GOCC:0062023	Collagen-containing extracellular matrix	16	198	0.87	1.19e-07	MMP2,APOE,VWF,NID1,LUM,DST,EFEMP2,FBN1,THBS4,COL18A1,ECM1,SOD3,MMRN1,TGFBI,RARRES2,FBN2
COMPARTMENTS	GOCC:1905370	Serine-type endopeptidase complex	6	7	1.89	1.65e-07	FCN3,FCN2,MASP1,FCN1,MASP2,F11
COMPARTMENTS	GOCC:0012505	Endomembrane system	70	3156	0.3	2.13e-07	SPP2,APOH,F9,LTF,APOB,PROC,APOA1,TTR,CAT,APOE,LYZ,VWF,CP,F13A1,TCIRG1,LUM,AHSG,IGFBP1,SERPING1,SEC24D,PPBP,PF4,CAMP,APEH,PDIA3,SLC27A4,LALBA,PCSK9,FGB,F2,PRG2,CSPG4,APOL1,SERPINF2,FBN1,P4HB,HSP90AA1,FGG,TUBB,THBS4,PRDX6,SLPI,QPCT,ASGR2,APOA4,COL18A1,F8,CENPF,QSOX1,F5,S100A7,ECM1,FCN1,F7,CALML5,SOD3,PROS1,MMRN1,ADIPOQ,ATP9B,ABCA13,ITIH3,RARRES2,APOA5,CFD,SEMA3B,KNG1,CDH5,B2M,FGA
COMPARTMENTS	GOCC:0034362	Low-density lipoprotein particle	7	22	1.46	1.31e-06	CETP,APOB,APOA1,APOE,PCSK9,APOM,ADIPOQ
COMPARTMENTS	GOCC:0032991	Protein-containing complex	97	5325	0.22	1.32e-06	CPB2,CETP,APOH,F9,C5,LTF,APOB,IGFBP5,APOA1,CAT,DHX29,APOE,VWF,PSMA6,LCAT,NID1,CSTA,PPP2R3A,DNAH5,LUM,CDH11,IGFBP4,FCN3,IGFBP1,SAA4,SERPING1,SEC24D,CSF1R,IGF2BP1,FCN2,MASP1,APEH,GUCY1A1,PDIA3,PCSK9,FGB,DST,F2,UBE3C,C1QB,CFHR1,NDUFS8,SUZ12,APOL1,SERPINF2,FBN1,P4HB,PAPPA,DNAH8,HSP90AA1,FGG,BLM,APOA4,COL18A1,C8A,CENPF,CFHR2,SERPINC1,S100A7,FLNA,SMARCA1,C8B,FCN1,ADAMTS13,CNTRL,SVEP1,C1QC,C1QA,F7,APOM,CALML5,GPX3,MMRN1,MASP2,F11,C1S,PCYOX1,ADIPOQ,CKMT1A,C4B,CFB,IGFALS,UIMC1,JCHAIN,HPR,C1R,APOA5,ATP1A1,VIM,TTN,APOC4,IGHV3-15,ACTB,CDH5,B2M,FGA,TPM3
COMPARTMENTS	GOCC:0005602	Complement component C1 complex	5	5	1.96	2.09e-06	C1QB,C1QC,C1QA,C1S,C1R
COMPARTMENTS	GOCC:0042627	Chylomicron	6	14	1.59	3.06e-06	APOH,APOB,APOA1,APOE,APOA4,APOA5
COMPARTMENTS	GOCC:0005783	Endoplasmic reticulum	33	1095	0.44	1.19e-05	SPP2,F9,APOB,PROC,APOA1,APOE,VWF,CP,AHSG,IGFBP1,SEC24D,PDIA3,SLC27A4,PCSK9,APOL1,FBN1,P4HB,FGG,THBS4,ASGR2,APOA4,COL18A1,F8,QSOX1,F5,S100A7,F7,ADIPOQ,APOA5,SEMA3B,KNG1,B2M,FGA
COMPARTMENTS	GOCC:0005796	Golgi lumen	10	102	0.95	2.26e-05	F9,PROC,LUM,LALBA,F2,CSPG4,F8,F7,SOD3,PROS1
COMPARTMENTS	GOCC:0071682	Endocytic vesicle lumen	6	23	1.37	3.22e-05	LTF,APOB,APOA1,APOE,HPX,HSP90AA1
COMPARTMENTS	GOCC:1905368	Peptidase complex	11	136	0.87	3.38e-05	F9,PSMA6,FCN3,FCN2,MASP1,F2,UBE3C,FCN1,F7,MASP2,F11
COMPARTMENTS	GOCC:0031089	Platelet dense granule lumen	5	14	1.51	7.70e-05	SPP2,APOH,ECM1,ITIH3,RARRES2
COMPARTMENTS	GOCC:0034365	Discoidal high-density lipoprotein particle	4	6	1.78	0.00014	APOA1,APOE,LCAT,APOM
COMPARTMENTS	GOCC:1904724	Tertiary granule lumen	7	55	1.06	0.00024	LTF,LYZ,PPBP,CAMP,QPCT,QSOX1,B2M
COMPARTMENTS	GOCC:0062136	Low-density lipoprotein receptor complex	4	9	1.61	0.00043	APOB,APOA1,APOE,PCSK9
COMPARTMENTS	GOCC:0035580	Specific granule lumen	7	62	1.01	0.00045	LTF,LYZ,CAMP,SLPI,QPCT,QSOX1,B2M
COMPARTMENTS	GOCC:1905369	Endopeptidase complex	8	90	0.91	0.00052	PSMA6,FCN3,FCN2,MASP1,UBE3C,FCN1,MASP2,F11
COMPARTMENTS	GOCC:0016942	Insulin-like growth factor binding protein complex	4	10	1.56	0.00055	IGFBP5,IGFBP4,IGFBP1,IGFALS
COMPARTMENTS	GOCC:0034366	Spherical high-density lipoprotein particle	4	11	1.52	0.00073	APOA1,LCAT,APOM,HPR
COMPARTMENTS	GOCC:0005775	Vacuolar lumen	10	168	0.73	0.0011	APOB,TTR,LYZ,LUM,CSPG4,HSP90AA1,TUBB,PRDX6,S100A7,GC
COMPARTMENTS	GOCC:0009986	Cell surface	16	438	0.52	0.0017	APOH,APMAP,LTF,CSF1R,PDIA3,PCSK9,FGB,CSPG4,SERPINF2,P4HB,FGG,FCN1,ADAMTS13,ADIPOQ,CDH5,FGA
COMPARTMENTS	GOCC:0005925	Focal adhesion	12	269	0.61	0.0026	PDIA3,DST,CSPG4,P4HB,GIT2,S100A7,FLNA,YWHAZ,ILK,VIM,ACTB,B2M
COMPARTMENTS	GOCC:0005604	Basement membrane	6	61	0.95	0.0035	NID1,DST,EFEMP2,FBN1,THBS4,COL18A1
COMPARTMENTS	GOCC:1904813	ficolin-1-rich granule lumen	8	124	0.77	0.0039	CAT,APEH,PRG2,HSP90AA1,QPCT,FCN1,CALML5,CFD
COMPARTMENTS	GOCC:0034363	Intermediate-density lipoprotein particle	3	8	1.53	0.0073	APOB,APOA1,APOE
COMPARTMENTS	GOCC:0071953	Elastic fiber	3	8	1.53	0.0073	EFEMP2,FBN1,FBLN1
COMPARTMENTS	GOCC:0043227	Membrane-bounded organelle	126	9083	0.1	0.0104	SPP2,CPB2,APOH,F9,MMP2,AFM,MGP,GPLD1,LTF,APOB,PROC,APOA1,TTR,CAT,APOE,APCS,LYZ,VWF,PSMA6,LCAT,CSTA,CP,KLKB1,F13A1,DNAH5,CENPE,PON3,TCIRG1,HPX,LUM,CDH11,ITIH1,AHSG,IGFBP1,SERPING1,SEC24D,IGF2BP1,PIP,SERPINI1,PPBP,PF4,CAMP,APEH,PDIA3,SLC27A4,LALBA,PCSK9,FGB,DST,F2,PRG2,CSPG4,STAB1,NDUFS8,SUZ12,APOL1,SERPINF2,FBN1,KDM3B,P4HB,DNAH8,HSP90AA1,FGG,TUBB,THBS4,PRDX6,SLPI,QPCT,APOD,ASGR2,BLM,GIT2,APOA4,COL18A1,F8,CENPF,CFH,QSOX1,SERPINC1,F5,S100A7,HTRA1,ECM1,FLNA,SMARCA1,FCN1,CCN5,SVEP1,F7,CALML5,CD99,SOD3,GPX3,PROS1,EFEMP1,MMRN1,YWHAZ,NAV2,ILK,MASP2,F11,PCYOX1,ADIPOQ,ATP9B,CKMT1A,ABCA13,ITIH3,C4B,TGFBI,RARRES2,GC,UIMC1,C1R,APOA5,VIM,TTN,CFD,SEMA3B,PLCG2,KNG1,ACTB,TRIM66,TRIM8,CDH5,B2M,FGA

COMPARTMENTS	GOCC:0005579	Membrane attack complex	3	12	1.36	0.0187	C5,C8A,C8B
COMPARTMENTS	GOCC:0070820	Tertiary granule	8	164	0.65	0.0210	LTF,LYZ,TCIRG1,PPBP,CAMP,QPCT,QSOX1,B2M
COMPARTMENTS	GOCC:0001527	Microfibril	3	13	1.32	0.0218	EFEMP2,FBN1,FBN2
COMPARTMENTS	GOCC:0030139	Endocytic vesicle	10	260	0.54	0.0263	LTF,APOB,APOA1,APOE,TCIRG1,HPX,PDIA3,STAB1,HSP90AA1,B2M
COMPARTMENTS	GOCC:0043226	Organelle	135	10113	0.08	0.0282	SPP2,CPB2,APOH,F9,MMP2,C5,AFM,MGP,GPLD1,LTF,APOB,PROC,APOA1,TTR,CAT,DHX29,APOE,APCS,LYZ,VWF,PSMA6,LCAT,CSTA,CP,KLKB1,F13A1,DNAH5,CENPE,PON3,TCIRG1,HPX,LUM,CDH11,ITIH1,AHSG,IGFBP1,SERPING1,SEC24D,IGF2BP1,PIP,FCN2,SERPINI1,PPBP,PF4,CAMP,APEH,PDIA3,SLC27A4,LALBA,PCSK9,FGB,DST,F2,PRG2,CSPG4,STAB1,NDUFS8,SUZ12,APOL1,SERPINF2,FBN1,KDM3B,P4HB,DNAH8,HSP90AA1,TUBA1B,FGG,TUBB,THBS4,PRDX6,SLPI,QPCT,APOD,ASGR2,BLM,GIT2,APOA4,COL18A1,MTCL1,F8,CENPF,CFH,QSOX1,SERPINC1,F5,CFAP46,S100A7,HTRA1,ECM1,FLNA,SMARCA1,FCN1,CCN5,CNTRL,SVEP1,F7,CALML5,CD99,SOD3,GPX3,PROS1,EFEMP1,MMRN1,YWHAZ,NAV2,ILK,MASP2,F11,PCYOX1,ADIPOQ,ATP9B,CKMT1A,ABCA13,ITIH3,C4B,TGFBI,RARRES2,GC,UIMC1,C1R,APOA5,ATP1A1,VIM,TTN,CFD,SEMA3B,PLCG2,KNG1,ACTB,TRIM66,TRIM8,CDH5,B2M,FGA,TPM3
UniProt Keywords	KW-0964	Secreted	140	1839	0.84	1.85e-83	SPP2,CPB2,CETP,APOH,F9,MMP2,C5,AFM,MGP,GPLD1,LTF,APOB,IGFBP5,PROC,APOA1,TTR,APOE,ANGPTL6,F12,APCS,CFHR5,LYZ,VWF,SERPINA10,ATRN,LCN1,LCAT,NID1,CP,KLKB1,F13A1,PON3,HPX,LUM,IGFBP4,FCN3,ITIH1,AHSG,IGFBP1,HABP2,SAA4,SERPING1,PIP,FCN2,PGLYRP2,SCGB3A1,SERPINI1,PPBP,PF4,CAMP,A2ML1,LALBA,PCSK9,BMP1,FGB,F2,INHBC,PSG1,EFEMP2,PRG2,C1QB,CFHR1,APOL1,PSG11,SERPINF2,FBN1,PAPPA,FBLN1,FGG,THBS4,SLPI,QPCT,APOD,PAPLN,APOA4,CNDP1,COL18A1,F8,C8A,F13B,CFHR2,CFHR4,CFHR3,CFH,QSOX1,SERPINC1,F5,ADAM12,S100A7,HTRA1,ADAMTSL4,ECM1,CRTAC1,C8B,FCN1,ADAMTS13,CCN5,SVEP1,C1QC,C1QA,F7,APOM,CA6,SOD3,GPX3,PROS1,EFEMP1,MMRN1,MASP2,PSG4,F11,PSG2,ADIPOQ,PRG4,MST1,SPARCL1,ITIH3,C4B,TGFBI,CFB,IGFALS,PAEP,RARRES2,GC,FBN2,TSKU,JCHAIN,HPR,C1R,APOA5,RNASE4,APOC4,CFD,IGHV3-15,IGHV3-72,SEMA3B,PAMR1,KNG1,B2M,FGA
UniProt Keywords	KW-0732	Signal	151	3277	0.62	2.68e-63	SPP2,CPB2,CETP,APOH,SERPIND1,F9,MMP2,C5,AFM,MGP,GPLD1,LTF,APOB,IGFBP5,PROC,APOA1,TTR,APOE,ANGPTL6,F12,APCS,CFHR5,LYZ,VWF,ATRN,LCN1,LCAT,NID1,CP,KLKB1,PON3,HPX,LUM,CDH11,IGFBP4,FCN3,ITIH1,AHSG,IGFBP1,HABP2,SAA4,SERPING1,CSF1R,PIP,FCN2,PGLYRP2,SCGB3A1,SERPINI1,PPBP,PF4,CAMP,A2ML1,PDIA3,LALBA,PCSK9,BMP1,FGB,F2,INHBC,PSG1,EFEMP2,PRG2,CSPG4,STAB1,C1QB,CFHR1,APOL1,PSG11,SERPINF2,FBN1,P4HB,PAPPA,FBLN1,OIT3,FGG,THBS4,SLPI,QPCT,APOD,PAPLN,APOA4,CNDP1,COL18A1,F8,C8A,PCDH18,F13B,CFHR2,CFHR4,CFHR3,CFH,QSOX1,SERPINC1,F5,ADAM12,HTRA1,ADAMTSL4,ECM1,CRTAC1,C8B,FCN1,ADAMTS13,CCN5,SVEP1,C1QC,C1QA,F7,APOM,CA6,ADGRG2,CD99,SOD3,GPX3,PROS1,EFEMP1,MMRN1,MASP2,PSG4,F11,C1S,PSG2,PCYOX1,ADIPOQ,PRG4,MST1,SPARCL1,ITIH3,C4B,TGFBI,CFB,IGFALS,PAEP,RARRES2,GC,FBN2,TSKU,JCHAIN,HPR,C1R,APOA5,RNASE4,APOC4,CFD,IGHV3-15,IGHV3-72,SEMA3B,PAMR1,KNG1,CDH5,B2M,FGA
UniProt Keywords	KW-1015	Disulfide bond	132	3338	0.56	2.16e-44	SPP2,CPB2,CETP,APOH,F9,MMP2,C5,AFM,MGP,LTF,APOB,IGFBP5,PROC,ANGPTL6,F12,APCS,CFHR5,LYZ,VWF,ATRN,LCN1,LCAT,NID1,CP,KLKB1,PON3,HPX,LUM,IGFBP4,FCN3,ITIH1,AHSG,IGFBP1,HABP2,SERPING1,CSF1R,PIP,FCN2,PGLYRP2,SCGB3A1,PPBP,PF4,MASP1,CAMP,A2ML1,PDIA3,LALBA,PCSK9,BMP1,FGB,F2,INHBC,PSG1,EFEMP2,PRG2,CSPG4,STAB1,C1QB,CFHR1,PSG11,SERPINF2,FBN1,P4HB,PAPPA,FBLN1,OIT3,FGG,THBS4,SLPI,QPCT,APOD,PAPLN,ASGR2,COL18A1,F8,C8A,F13B,CFHR2,CFHR4,CFHR3,CFH,QSOX1,SERPINC1,F5,ADAM12,S100A7,HTRA1,CRTAC1,MCOLN2,C8B,FCN1,ADAMTS13,SVEP1,C1QC,C1QA,F7,APOM,CA6,SOD3,PROS1,EFEMP1,MMRN1,MASP2,PSG4,F11,C1S,PSG2,ADIPOQ,PRG4,MST1,SPARCL1,C4B,TGFBI,CFB,PAEP,RARRES2,GC,FBN2,JCHAIN,HPR,C1R,RNASE4,TTN,CFD,IGHV3-15,IGHV3-72,SEMA3B,PAMR1,OR52B4,KNG1,B2M,FGA
UniProt Keywords	KW-0325	Glycoprotein	132	4386	0.44	1.53e-31	CPB2,CETP,APOH,SERPIND1,APMAP,F9,MMP2,C5,AFM,GPLD1,LTF,APOB,IGFBP5,PROC,APOA1,TTR,APOE,ANGPTL6,F12,APCS,CFHR5,VWF,PSMA6,SERPINA10,ATRN,LCAT,NID1,CP,KLKB1,F13A1,PON3,HPX,LUM,CDH11,IGFBP4,FCN3,ITIH1,AHSG,HABP2,SAA4,SERPING1,CSF1R,PIP,FCN2,PGLYRP2,SERPINI1,A2ML1,LALBA,PCSK9,BMP1,FGB,F2,INHBC,PSG1,EFEMP2,PRG2,CSPG4,STAB1,C1QB,CFHR1,APOL1,PSG11,SERPINF2,FBN1,PAPPA,FBLN1,OIT3,FGG,THBS4,QPCT,APOD,ASGR2,CNDP1,COL18A1,F8,C8A,PCDH18,F13B,CFHR2,CFHR4,CFHR3,CFH,QSOX1,SERPINC1,F5,ADAM12,ADAMTSL4,ECM1,CRTAC1,C8B,FCN1,ADAMTS13,SVEP1,C1QC,C1QA,F7,APOM,CA6,ADGRG2,CD99,SOD3,PROS1,EFEMP1,MMRN1,PSG4,F11,C1S,PSG2,PCYOX1,ADIPOQ,PRG4,MST1,SPARCL1,ITIH3,C4B,CFB,IGFALS,PAEP,GC,FBN2,TSKU,JCHAIN,C1R,VIM,APOC4,SEMA3B,PAMR1,OR52B4,KNG1,CDH5,B2M,FGA
UniProt Keywords	KW-0094	Blood coagulation	23	46	1.66	2.28e-26	CPB2,SERPIND1,F9,PROC,F12,VWF,SERPINA10,KLKB1,F13A1,SERPING1,FGB,F2,FGG,F8,F13B,SERPINC1,F5,ADAMTS13,F7,PROS1,F11,KNG1,FGA
UniProt Keywords	KW-0245	EGF-like domain	28	232	1.04	6.17e-18	F9,PROC,F12,ATRN,NID1,HABP2,MASP1,BMP1,EFEMP2,STAB1,FBN1,FBLN1,OIT3,THBS4,C8A,ADAM12,CRTAC1,C8B,SVEP1,F7,PROS1,EFEMP1,MMRN1,MASP2,C1S,FBN2,C1R,PAMR1
UniProt Keywords	KW-0768	Sushi	17	57	1.43	2.55e-16	APOH,CFHR5,MASP1,CFHR1,PAPPA,F13B,CFHR2,CFHR4,CFHR3,CFH,SVEP1,MASP2,C1S,CFB,HPR,C1R,PAMR1
UniProt Keywords	KW-0106	Calcium	44	886	0.65	7.63e-15	F9,MMP2,PROC,APCS,NID1,F13A1,PPP2R3A,PON3,CDH11,FCN3,FCN2,LALBA,PCSK9,BMP1,DST,F2,EFEMP2,FBN1,FBLN1,OIT3,FGG,THBS4,ASGR2,F8,PCDH18,F5,S100A7,MCOLN2,FCN1,ADAMTS13,SVEP1,F7,CALML5,PROS1,EFEMP1,EFCAB5,MASP2,C1S,SPARCL1,FBN2,TTN,PLCG2,CDH5,FGA
UniProt Keywords	KW-0677	Repeat	108	4794	0.31	5.60e-14	APOH,SERPIND1,F9,MMP2,AFM,GPLD1,LTF,PROC,APOA1,APOE,F12,CFHR5,VWF,ATRN,NID1,CP,KLKB1,PPP2R3A,DNAH5,HPX,LUM,CDH11,FCN3,AHSG,ANKRD31,HABP2,SERPING1,ANKRD44,CSF1R,IGF2BP1,FCN2,MASP1,PDIA3,BMP1,DST,F2,PSG1,EFEMP2,CSPG4,STAB1,C1QB,CFHR1,NDUFS8,PSG11,FBN1,P4HB,PAPPA,FBLN1,DNAH8,THBS4,SLPI,PAPLN,GIT2,APOA4,COL18A1,F8,C8A,PCDH18,CENPF,F13B,CFHR2,CFHR4,CFHR3,CFH,F5,CFAP46,S100A7,ADAMTSL4,ECM1,FLNA,CRTAC1,SMARCA1,C8B,FCN1,ADAMTS13,CNTRL,SVEP1,C1QC,C1QA,F7,CALML5,PROS1,EFEMP1,ILK,MASP2,PSG4,F11,C1S,PSG2,ADIPOQ,PRG4,ABCA13,MST1,TGFBI,CFB,IGFALS,GC,UIMC1,FBN2,TSKU,C1R,TTN,PLCG2,PAMR1,KNG1,TRIM66,TRIM8,CDH5
UniProt Keywords	KW-0180	Complement pathway	11	30	1.52	2.89e-11	C5,SERPING1,C1QB,C8A,C8B,C1QC,C1QA,MASP2,C1S,C4B,C1R
UniProt Keywords	KW-0391	Immunity	29	537	0.69	2.47e-10	C5,LTF,FCN3,SERPING1,CSF1R,FCN2,PGLYRP2,FGB,PRG2,C1QB,SLPI,C8A,CFH,MCOLN2,C8B,FCN1,C1QC,C1QA,MASP2,C1S,C4B,CFB,C1R,CFD,IGHV3-15,IGHV3-72,TRIM8,B2M,FGA
UniProt Keywords	KW-0399	Innate immunity	23	329	0.8	3.48e-10	C5,FCN3,SERPING1,CSF1R,FCN2,FGB,C1QB,SLPI,C8A,CFH,MCOLN2,C8B,FCN1,C1QC,C1QA,MASP2,C1S,C4B,CFB,C1R,CFD,TRIM8,FGA
UniProt Keywords	KW-0720	Serine protease	16	136	1.03	5.05e-10	F9,LTF,PROC,F12,KLKB1,HABP2,PCSK9,F2,HTRA1,F7,MASP2,F11,C1S,CFB,C1R,CFD
UniProt Keywords	KW-0379	Hydroxylation	16	143	1.01	9.40e-10	F9,PROC,FCN3,FCN2,C1QB,COL18A1,C1QC,C1QA,F7,PROS1,MASP2,C1S,ADIPOQ,C1R,KNG1,FGA

UniProt Keywords	KW-0865	Zymogen	18	210	0.89	2.82e-09	CPB2,F9,MMP2,PROC,F12,KLKB1,F13A1,PCSK9,BMP1,F2,PAPPA,F5,ADAM12,ADAMTS13,F7,PROS1,CFB,CFD
UniProt Keywords	KW-0301	Gamma-carboxyglutamic acid	8	16	1.66	4.37e-09	F9,MGP,PROC,TTR,F2,F7,PROS1,TGFBI
UniProt Keywords	KW-0765	Sulfation	11	57	1.24	6.62e-09	SERPIND1,F9,NID1,LUM,PCSK9,SERPINF2,FGG,F8,CFH,F5,C4B
UniProt Keywords	KW-0792	Thrombophilia	7	10	1.8	1.04e-08	SERPIND1,F9,PROC,F2,SERPINC1,F5,PROS1
UniProt Keywords	KW-0165	Cleavage on pair of basic residues	19	280	0.79	2.67e-08	F9,C5,PROC,VWF,CDH11,HABP2,PPBP,CAMP,BMP1,F2,INHBC,C8A,ADAM12,ADAMTS13,F7,PROS1,C4B,CFB,CDH5
UniProt Keywords	KW-0358	Heparin-binding	12	88	1.09	2.99e-08	APOH,SERPIND1,LTF,APOB,APOE,SERPINA10,PF4,PRG2,FBN1,SERPINC1,SOD3,F11
UniProt Keywords	KW-0646	Protease inhibitor	13	117	1.0	5.54e-08	SERPIND1,SERPINA10,CSTA,ITIH1,SERPING1,SERPINI1,A2ML1,SERPINF2,SLPI,PAPLN,SERPINC1,ITIH3,KNG1
UniProt Keywords	KW-0345	HDL	7	16	1.6	1.02e-07	APOA1,APOE,SAA4,APOL1,APOA4,APOM,APOA5
UniProt Keywords	KW-0722	Serine protease inhibitor	11	80	1.1	1.23e-07	SERPIND1,SERPINA10,ITIH1,SERPING1,SERPINI1,A2ML1,SERPINF2,SLPI,PAPLN,SERPINC1,ITIH3
UniProt Keywords	KW-0645	Protease	24	516	0.63	1.59e-07	CPB2,F9,MMP2,LTF,PROC,F12,PSMA6,KLKB1,HABP2,PCSK9,BMP1,F2,PAPPA,CNDP1,ADAM12,HTRA1,ADAMTS13,F7,MASP2,F11,C1S,CFB,C1R,CFD
UniProt Keywords	KW-0272	Extracellular matrix	17	267	0.76	4.02e-07	MMP2,APOE,VWF,NID1,LUM,BMP1,FBN1,FBLN1,THBS4,COL18A1,ADAMTSL4,ECM1,CRTAC1,EFEMP1,SPARCL1,TGFBI,FBN2
UniProt Keywords	KW-0179	Complement alternate pathway	6	12	1.66	6.47e-07	C5,C8A,CFH,C8B,CFB,CFD
UniProt Keywords	KW-0034	Amyloid	7	24	1.42	8.36e-07	APOA1,TTR,APCS,LYZ,TGFBI,B2M,FGA
UniProt Keywords	KW-0873	Pyrrolidone carboxylic acid	9	67	1.09	3.22e-06	LUM,PIP,FGB,C1QB,APOD,JCHAIN,RNASE4,KNG1,B2M
UniProt Keywords	KW-1008	Amyloidosis	7	31	1.31	3.51e-06	APOA1,TTR,APOE,LYZ,TGFBI,B2M,FGA
UniProt Keywords	KW-0280	Fibrinolysis	5	8	1.75	3.81e-06	CPB2,F12,KLKB1,SERPING1,PROS1
UniProt Keywords	KW-1068	Hemolytic uremic syndrome	5	10	1.66	8.46e-06	CFHR5,CFHR1,CFHR3,CFH,CFB
UniProt Keywords	KW-0445	Lipid transport	10	110	0.92	1.60e-05	CETP,APOB,APOA1,APOE,SLC27A4,APOL1,APOA4,APOM,APOA5,APOC4
UniProt Keywords	KW-0971	Glycation	5	12	1.58	1.61e-05	APOA1,APOE,SOD3,CFB,B2M
UniProt Keywords	KW-0176	Collagen	8	83	0.94	0.00013	FCN3,FCN2,C1QB,COL18A1,FCN1,C1QC,C1QA,ADIPOQ
UniProt Keywords	KW-0162	Chylomicron	4	9	1.61	0.00017	APOB,APOE,APOA4,APOA5
UniProt Keywords	KW-0153	Cholesterol metabolism	7	65	0.99	0.00024	CETP,APOB,APOA1,APOE,LCAT,PCSK9,APOL1
UniProt Keywords	KW-0378	Hydrolase	36	1612	0.31	0.00078	CPB2,F9,MMP2,GPLD1,LTF,PROC,DHX29,F12,LYZ,PSMA6,KLKB1,PON3,HABP2,PGLYRP2,APEH,PCSK9,BMP1,F2,PAPPA,PRDX6,BLM,CNDP1,ADAM12,HTRA1,SMARCA1,ADAMTS13,F7,NAV2,MASP2,F11,C1S,CFB,C1R,RNASE4,CFD,PLCG2
UniProt Keywords	KW-0654	Proteoglycan	6	55	1.0	0.00088	LUM,ITIH1,PRG2,CSPG4,PRG4,ITIH3
UniProt Keywords	KW-0420	Kringle	4	16	1.36	0.00095	F12,HABP2,F2,MST1
UniProt Keywords	KW-0011	Acute phase	4	19	1.28	0.0017	SAA4,F2,SERPINF2,F8
UniProt Keywords	KW-0340	Growth factor binding	4	20	1.26	0.0019	IGFBP5,IGFBP4,IGFBP1,HTRA1
UniProt Keywords	KW-0473	Membrane attack complex	3	7	1.59	0.0022	C5,C8A,C8B
UniProt Keywords	KW-0065	Atherosclerosis	3	8	1.53	0.0029	CETP,APOB,APOA1
UniProt Keywords	KW-0721	Serine protease homolog	3	9	1.48	0.0037	MST1,HPR,PAMR1
UniProt Keywords	KW-0130	Cell adhesion	15	478	0.45	0.0049	VWF,NID1,CSTA,CDH11,DST,THBS4,COL18A1,PCDH18,ADAM12,CCN5,SVEP1,CD99,TGFBI,IGFALS,CDH5
UniProt Keywords	KW-0395	Inflammatory response	8	163	0.65	0.0081	C5,ATRN,KLKB1,CSF1R,STAB1,C4B,RARRES2,KNG1
UniProt Keywords	KW-0355	Hemophilia	2	2	1.96	0.0094	F9,F8
UniProt Keywords	KW-0929	Antimicrobial	6	93	0.77	0.0099	LTF,LYZ,PPBP,CAMP,PRG2,SLPI
UniProt Keywords	KW-0892	Osteogenesis	4	37	0.99	0.0128	MGP,LTF,BMP1,ECM1
UniProt Keywords	KW-0913	Age-related macular degeneration	3	16	1.23	0.0135	CFH,HTRA1,CFB
UniProt Keywords	KW-1018	Complement activation lectin pathway	2	3	1.78	0.0141	FCN3,FCN2
UniProt Keywords	KW-0495	Mineral balance	2	4	1.66	0.0207	AHSG,ECM1
UniProt Keywords	KW-0797	Tissue remodeling	2	4	1.66	0.0207	CSPG4,THBS4
UniProt Keywords	KW-0482	Metalloprotease	7	152	0.62	0.0211	CPB2,MMP2,BMP1,PAPPA,CNDP1,ADAM12,ADAMTS13
UniProt Keywords	KW-0044	Antibiotic	5	77	0.77	0.0231	LTF,PPBP,CAMP,PRG2,SLPI
UniProt Keywords	KW-0430	Lectin	7	162	0.59	0.0288	APCS,ATRN,FCN3,FCN2,PRG2,ASGR2,FCN1
UniProt Keywords	KW-1065	Osteogenesis imperfecta	3	23	1.07	0.0304	SEC24D,BMP1,P4HB
UniProt Keywords	KW-0575	Peroxidase	3	25	1.04	0.0371	CAT,PRDX6,GPX3
Pfam	PF01442	Apolipoprotein A1/A4/E domain	4	4	1.96	0.0021	APOA1,APOE,APOA4,APOA5
InterPro	IPR001881	EGF-like calcium-binding domain	24	124	1.24	1.30e-17	F9,PROC,F12,NID1,HABP2,MASP1,BMP1,EFEMP2,STAB1,FBN1,FBLN1,OIT3,THBS4,CRTAC1,SVEP1,F7,PROS1,EFEMP1,MMRN1,MASP2,C1S,FBN2,C1R,PAMR1
InterPro	IPR035976	Sushi/SCR/CCP superfamily	17	58	1.43	1.39e-14	APOH,CFHR5,MASP1,CFHR1,PAPPA,F13B,CFHR2,CFHR4,CFHR3,CFH,SVEP1,MASP2,C1S,CFB,HPR,C1R,PAMR1
InterPro	IPR018097	EGF-like calcium-binding, conserved site	19	99	1.24	8.81e-14	F9,PROC,NID1,MASP1,BMP1,EFEMP2,FBN1,FBLN1,OIT3,THBS4,CRTAC1,SVEP1,F7,PROS1,EFEMP1,MASP2,C1S,FBN2,C1R
InterPro	IPR000436	Sushi/SCR/CCP domain	16	56	1.41	9.75e-14	APOH,CFHR5,MASP1,CFHR1,PAPPA,F13B,CFHR2,CFHR4,CFHR3,CFH,SVEP1,MASP2,C1S,CFB,C1R,PAMR1
InterPro	IPR000742	EGF-like domain	24	236	0.97	1.63e-12	F9,PROC,F12,ATRN,NID1,HABP2,MASP1,BMP1,EFEMP2,STAB1,FBN1,FBLN1,OIT3,THBS4,ADAM12,SVEP1,F7,PROS1,EFEMP1,MMRN1,MASP2,FBN2,C1R,PAMR1
InterPro	IPR009003	Peptidase S1, PA clan	17	123	1.1	2.62e-10	F9,PROC,F12,KLKB1,HABP2,F2,HTRA1,F7,MASP2,F11,C1S,MST1,CFB,HPR,C1R,CFD,PAMR1
InterPro	IPR001314	Peptidase S1A, chymotrypsin family	16	107	1.13	3.93e-10	F9,PROC,F12,KLKB1,HABP2,F2,F7,MASP2,F11,C1S,MST1,CFB,HPR,C1R,CFD,PAMR1
InterPro	IPR001254	Serine proteases, trypsin domain	16	115	1.1	9.32e-10	F9,PROC,F12,KLKB1,HABP2,F2,F7,MASP2,F11,C1S,MST1,CFB,HPR,C1R,CFD,PAMR1
InterPro	IPR000152	EGF-type aspartate/asparagine hydroxylation site	15	98	1.14	1.26e-09	F9,PROC,NID1,BMP1,EFEMP2,FBN1,FBLN1,SVEP1,F7,PROS1,EFEMP1,MMRN1,MASP2,C1S,FBN2
InterPro	IPR043504	Peptidase S1, PA clan, chymotrypsin-like fold	15	116	1.07	1.00e-08	F9,PROC,F12,KLKB1,HABP2,F2,F7,MASP2,F11,C1S,MST1,HPR,C1R,CFD,PAMR1
InterPro	IPR009030	Growth factor receptor cysteine-rich domain superfamily	15	127	1.03	2.94e-08	IGFBP5,NID1,IGFBP4,IGFBP1,BMP1,EFEMP2,FBN1,FBLN1,C8A,HTRA1,CCN5,SVEP1,PROS1,EFEMP1,FBN2
InterPro	IPR033116	Serine proteases, trypsin family, serine active site	13	92	1.11	7.36e-08	F9,PROC,F12,KLKB1,HABP2,F2,F7,MASP2,F11,C1S,CFB,C1R,CFD
InterPro	IPR020837	Fibrinogen, conserved site	7	19	1.52	5.04e-06	ANGPTL6,FCN3,FCN2,FGB,FGG,FCN1,FGA
InterPro	IPR014716	Fibrinogen, alpha/beta/gamma chain, C-terminal globular, subdomain 1	7	26	1.39	2.85e-05	ANGPTL6,FCN3,FCN2,FGB,FGG,FCN1,FGA
InterPro	IPR000294	Gamma-carboxyglutamic acid-rich (GLA) domain	6	15	1.56	3.54e-05	F9,MGP,PROC,F2,F7,PROS1
InterPro	IPR035972	Gamma-carboxyglutamic acid-rich (GLA) domain superfamily	6	15	1.56	3.54e-05	F9,MGP,PROC,F2,F7,PROS1
InterPro	IPR002181	Fibrinogen, alpha/beta/gamma chain, C-terminal globular domain	7	32	1.3	8.00e-05	ANGPTL6,FCN3,FCN2,FGB,FGG,FCN1,FGA
InterPro	IPR036056	Fibrinogen-like, C-terminal	7	32	1.3	8.00e-05	ANGPTL6,FCN3,FCN2,FGB,FGG,FCN1,FGA
InterPro	IPR018114	Serine proteases, trypsin family, histidine active site	10	100	0.96	0.00013	F9,PROC,F12,KLKB1,HABP2,F2,F7,F11,CFB,CFD
InterPro	IPR035914	Spermadhesin, CUB domain superfamily	8	54	1.13	0.00013	ATRN,MASP1,BMP1,ADAMTS13,MASP2,C1S,C1R,PAMR1
InterPro	IPR046350	Cystatin superfamily	6	23	1.37	0.00021	SPP2,CSTA,AHSG,CAMP,RARRES2,KNG1
InterPro	IPR000074	Apolipoprotein A/E	4	4	1.96	0.00030	APOA1,APOE,APOA4,APOA5
InterPro	IPR026823	Complement Clr-like EGF domain	6	27	1.3	0.00042	NID1,EFEMP2,FBN1,FBLN1,EFEMP1,FBN2
InterPro	IPR000859	CUB domain	7	51	1.1	0.00093	ATRN,MASP1,BMP1,MASP2,C1S,C1R,PAMR1
InterPro	IPR042185	Serpin superfamily, domain 2	6	35	1.19	0.0015	SERPIND1,SERPINA10,SERPING1,SERPINI1,SERPINF2,SERPINC1

InterPro	IPR000215	Serpin family	6	36	1.18	0.0016	SERPIND1,SERPINA10,SERPING1,SERPINI1,SERPINF2,SERPINC1
InterPro	IPR000867	Insulin-like growth factor-binding protein, IGFBP	5	20	1.36	0.0016	IGFBP5,IGFBP4,IGFBP1,HTRA1,CCN5
InterPro	IPR023796	Serpin domain	6	36	1.18	0.0016	SERPIND1,SERPINA10,SERPING1,SERPINI1,SERPINF2,SERPINC1
InterPro	IPR036186	Serpin superfamily	6	36	1.18	0.0016	SERPIND1,SERPINA10,SERPING1,SERPINI1,SERPINF2,SERPINC1
InterPro	IPR042178	Serpin superfamily, domain 1	6	36	1.18	0.0016	SERPIND1,SERPINA10,SERPING1,SERPINI1,SERPINF2,SERPINC1
InterPro	IPR017891	Insulin-like growth factor binding protein, N-terminal, Cys-rich conserved site	4	11	1.52	0.0039	IGFBP5,IGFBP4,IGFBP1,CCN5
InterPro	IPR013806	Kringle-like fold	5	26	1.24	0.0044	MMP2,F12,HABP2,F2,MST1
InterPro	IPR017857	Coagulation factor-like, Gla domain superfamily	4	12	1.48	0.0049	F9,PROC,F7,PROS1
InterPro	IPR012290	Fibrinogen, alpha/beta/gamma chain, coiled coil domain	3	3	1.96	0.0053	FGB,FGG,FGA
InterPro	IPR024175	Peptidase S1A, complement C1r/C1S/mannan-binding	3	3	1.96	0.0053	MASP2,C1S,C1R
InterPro	IPR037579	Fibrinogen	3	3	1.96	0.0053	FGB,FGG,FGA
InterPro	IPR008160	Collagen triple helix repeat	7	77	0.92	0.0071	FCN2,C1QB,COL18A1,FCN1,C1QC,C1QA,ADIPOQ
InterPro	IPR023795	Serpin, conserved site	5	32	1.15	0.0091	SERPIND1,SERPING1,SERPINI1,SERPINF2,SERPINC1
InterPro	IPR001073	C1q domain	5	33	1.14	0.0101	C1QB,C1QC,C1QA,MMRN1,ADIPOQ
InterPro	IPR000001	Kringle	4	16	1.36	0.0104	F12,HABP2,F2,MST1
InterPro	IPR018056	Kringle, conserved site	4	16	1.36	0.0104	F12,HABP2,F2,MST1
InterPro	IPR024731	EGF domain	4	16	1.36	0.0104	NID1,STAB1,FBN1,FBN2
InterPro	IPR038178	Kringle superfamily	4	16	1.36	0.0104	F12,HABP2,F2,MST1
InterPro	IPR000716	Thyroglobulin type-1	4	17	1.33	0.0112	IGFBP5,NID1,IGFBP4,IGFBP1
InterPro	IPR011707	Multicopper oxidase, N-terminal	3	5	1.74	0.0112	CP,F8,F5
InterPro	IPR012224	Peptidase S1A, coagulation factor VII/IX/X/C/Z	3	5	1.74	0.0112	F9,PROC,F7
InterPro	IPR020858	Serum albumin-like	3	5	1.74	0.0112	AFM,ECM1,GC
InterPro	IPR033138	Multicopper oxidases, conserved site	3	5	1.74	0.0112	CP,F8,F5
InterPro	IPR036857	Thyroglobulin type-1 superfamily	4	17	1.33	0.0112	IGFBP5,NID1,IGFBP4,IGFBP1
InterPro	IPR000020	Anaphylatoxin/fibulin	3	6	1.66	0.0147	C5,FBLN1,C4B
InterPro	IPR003609	PAN/Apple domain	3	6	1.66	0.0147	KLKB1,F11,MST1
InterPro	IPR022321	Insulin-like growth factor-binding protein family 1-6, chordata	3	6	1.66	0.0147	IGFBP5,IGFBP4,IGFBP1
InterPro	IPR036383	Thrombospondin type-1 (TSP1) repeat superfamily	6	64	0.93	0.0155	PAPLN,C8A,ADAMTSL4,C8B,ADAMTS13,CCN5
InterPro	IPR000884	Thrombospondin type-1 (TSP1) repeat	6	65	0.92	0.0165	PAPLN,C8A,ADAMTSL4,C8B,ADAMTS13,CCN5
InterPro	IPR040839	Macroglobulin domain MG4	3	8	1.53	0.0258	C5,A2ML1,C4B
InterPro	IPR001599	Alpha-2-macroglobulin	3	9	1.48	0.0335	C5,A2ML1,C4B
InterPro	IPR002890	Macroglobulin domain	3	9	1.48	0.0335	C5,A2ML1,C4B
InterPro	IPR009048	Alpha-macroglobulin, receptor-binding	3	9	1.48	0.0335	C5,A2ML1,C4B
InterPro	IPR011625	Alpha-2-macroglobulin, bait region domain	3	9	1.48	0.0335	C5,A2ML1,C4B
InterPro	IPR011626	Alpha-macroglobulin-like, TED domain	3	9	1.48	0.0335	C5,A2ML1,C4B
InterPro	IPR036595	Alpha-macroglobulin, receptor-binding domain superfamily	3	9	1.48	0.0335	C5,A2ML1,C4B
InterPro	IPR041555	Macroglobulin domain MG3	3	9	1.48	0.0335	C5,A2ML1,C4B
InterPro	IPR008983	Tumour necrosis factor-like domain superfamily	5	53	0.93	0.0479	C1QB,C1QC,C1QA,MMRN1,ADIPOQ
SMART	SM00032	Domain abundant in complement control proteins	16	56	1.41	4.46e-14	APOH,CFHR5,MASP1,CFHR1,PAPPA,F13B,CFHR2,CFHR4,CFHR3,CFH,SVEP1,MASP2,C1S,CFB,C1R,PAMR1
SMART	SM00179	Calcium-binding EGF-like domain	18	101	1.21	3.31e-13	F9,PROC,NID1,MASP1,BMP1,EFEMP2,FBN1,FBLN1,OIT3,THBS4,SVEP1,F7,PROS1,EFEMP1,MASP2,C1S,FBN2,C1R
SMART	SM00020	Trypsin-like serine protease	17	118	1.12	3.26e-11	F9,PROC,F12,KLKB1,HABP2,MASP1,F2,F7,MASP2,F11,C1S,MST1,CFB,HPR,C1R,CFD,PAMR1
SMART	SM00181	Epidermal growth factor-like domain.	18	209	0.89	1.28e-08	F9,PROC,F12,ATRN,NID1,HABP2,EFEMP2,STAB1,FBN1,FBLN1,OIT3,THBS4,SVEP1,F7,PROS1,MMRN1,FBN2,PAMR1
SMART	SM00069	Domain containing Gla (gamma-carboxyglutamate) residues.	6	15	1.56	1.21e-05	F9,MGP,PROC,F2,F7,PROS1
SMART	SM00186	Fibrinogen-related domains (FReDs)	7	29	1.34	1.45e-05	ANGPTL6,FCN3,FCN2,FGB,FGG,FCN1,FGA
SMART	SM00042	Domain first found in C1r, C1s, uEGF, and bone morphogenetic protein.	7	50	1.1	0.00032	ATRN,MASP1,BMP1,MASP2,C1S,C1R,PAMR1
SMART	SM00121	Insulin growth factor-binding protein homologues	5	17	1.43	0.00034	IGFBP5,IGFBP4,IGFBP1,HTRA1,CCN5
SMART	SM00093	SERine Proteinase INhibitors	6	35	1.19	0.00047	SERPIND1,SERPINA10,SERPING1,SERPINI1,SERPINF2,SERPINC1
SMART	SM00110	Complement component C1q domain.	5	26	1.24	0.0016	C1QB,C1QC,C1QA,MMRN1,ADIPOQ
SMART	SM01212	Fibrinogen alpha/beta chain family	3	3	1.96	0.0019	FGB,FGG,FGA
SMART	SM00130	Kringle domain	4	16	1.36	0.0040	F12,HABP2,F2,MST1
SMART	SM00211	Thyroglobulin type I repeats.	4	17	1.33	0.0045	IGFBP5,NID1,IGFBP4,IGFBP1
SMART	SM00104	Anaphylatoxin homologous domain	3	6	1.66	0.0060	C5,FBLN1,C4B
SMART	SM00209	Thrombospondin type 1 repeats	6	64	0.93	0.0063	PAPLN,C8A,ADAMTSL4,C8B,ADAMTS13,CCN5
SMART	SM01359	Alpha-2-Macroglobulin	3	9	1.48	0.0134	C5,A2ML1,C4B
SMART	SM01360	Alpha-2-macroglobulin family	3	9	1.48	0.0134	C5,A2ML1,C4B
SMART	SM01361	A-macroglobulin receptor	3	9	1.48	0.0134	C5,A2ML1,C4B
SMART	SM00223	APPLE domain	2	2	1.96	0.0302	KLKB1,F11

UC vs NCFET T3

#category	term ID	term description	observed gene count	background gene count	strength	false discovery rate	matching proteins in your network (labels)
GO Process	GO:0006950	Response to stress	119	3358	0.46	1.54e-25	CPB2,SERPIND1,MYH9,F9,MMP2,TFPI2,C5,MPO,PF4V1,PROC,PLEK,CAT,LRP1,APOE,PXDN,F12,CFHR5,THBS1,LYZ,VWF,SERPINA10,ATRN,KLKB1,F13A1,AMBP,TCIRG1,HPX,ITIH4,IGFBP4,FCN3,CRH,SAA4,SERPING1,FERMT3,DSC2,CSF1R,FCN2,PGLYRP2,PPBP,PF4,MASP1,LALBA,LRG1,PCSK9,FGB,DST,F2,PRG2,STAB1,C1QB,CFHR1,NDUFS8,TLN1,APOL1,SUMO4,C7,HBA1,A2M,HSPA5,P4HB,FBLN1,PSG3,HSP90AA1,ANG,FGG,THBS4,APOD,FN1,BLM,APOA4,C8A,F13B,CFHR2,CFH,PAPPA2,F5,S100A7,S100A8,ECM1,FLNA,C8B,GSN,C1QC,F10,F7,SOD3,GPX3,IGF1,PROS1,TNRC6A,ILK,GAPDH,MASP2,F11,C1S,TF,ADIPOQ,C4A,CDK3,MST1,C4B,CFB,UIMC1,SELENOP,TSKU,SAA2,HPR,C1R,RNASE4,CFD,IGHV3-72,VEGFC,PLCG2,HSPH1,KNG1,HBB,ACTB,FGA,PDIA4
GO Process	GO:0007596	Blood coagulation	35	173	1.21	1.54e-25	CPB2,SERPIND1,MYH9,F9,TFPI2,PF4V1,PROC,PLEK,F12,VWF,SERPINA10,KLKB1,F13A1,SERPING1,FERMT3,PF4,FGB,F2,TLN1,FBLN1,FGG,FN1,F13B,F5,FLNA,F10,F7,PROS1,ILK,F11,PLCG2,KNG1,HBB,ACTB,FGA
GO Process	GO:0042060	Wound healing	40	336	0.98	1.71e-22	CPB2,SERPIND1,MYH9,F9,TFPI2,PF4V1,PROC,PLEK,F12,VWF,SERPINA10,KLKB1,F13A1,SERPING1,FERMT3,PF4,LRG1,FGB,DST,F2,TLN1,FBLN1,FGG,FN1,F13B,F5,S100A8,FLNA,F10,F7,IGF1,PROS1,ILK,F11,TSKU,PLCG2,KNG1,HBB,ACTB,FGA
GO Process	GO:0006956	Complement activation	23	60	1.49	9.07e-22	C5,CFHR5,FCN3,SERPING1,FCN2,MASP1,C1QB,CFHR1,C7,C8A,CFHR2,CFHR3,CFH,C8B,C1QC,MASP2,C1S,C4A,C4B,CFB,C1R,CFD,IGHV3-72

GO Process	GO:0006959	Humoral immune response	36	268	1.04	1.04e-21	ST6GAL1,C5,PF4V1,CFHR5,LYZ,FCN3,SERPING1,FCN2,PPBP,PF4,MASP1,FGB,F2,C1QB,CFHR1,C7,ANG,C8A,CFHR2,CFHR3,CFH,S100A7,C8B,C1QC,GAPDH,MASP2,C1S,TF,C4A,C4B,CFB,C1R,CFD,IGHV3-72,KNG1,FGA
GO Process	GO:0009611	Response to wounding	43	444	0.89	2.46e-21	CPB2,SERPIND1,MYH9,F9,MMP2,TFPI2,PF4V1,PROC,PLEK,F12,VWF,SERPINA10,KLKB1,F13A1,SERPING1,FERMT3,PF4,LRG1,FGB,DST,F2,TLN1,FBLN1,FGG,APOD,FN1,APOA4,F13B,F5,S100A8,FLNA,F10,F7,IGF1,PROS1,ILK,F11,TSKU,PLCG2,KNG1,HBB,ACTB,FGA
GO Process	GO:0050878	Regulation of body fluid levels	38	371	0.92	2.37e-19	CPB2,SERPIND1,MYH9,F9,TFPI2,VTN,PF4V1,PROC,PLEK,APOE,F12,THBS1,VWF,SERPINA10,KLKB1,F13A1,SERPING1,FERMT3,PF4,FGB,F2,TLN1,FBLN1,FGG,FN1,F13B,F5,FLNA,F10,F7,PROS1,ILK,F11,PLCG2,KNG1,HBB,ACTB,FGA
GO Process	GO:0050896	Response to stimulus	171	7835	0.25	2.81e-18	ST6GAL1,CPB2,SERPIND1,MYH9,F9,MMP2,TFPI2,C5,MPO,HSD17B1,VTN,PF4V1,GPLD1,IGFBP5,PROC,PLEK,APOA1,TTR,CAT,LRP1,APOE,PXDN,F12,CFHR5,THBS1,LYZ,VWF,SERPINA10,ITGA2B,TIMP2,ATRN,NOTCH3,LCAT,KLKB1,F13A1,AMBP,TCIRG1,HPX,ITIH4,IGFBP4,PSG9,FCN3,CRH,SAA4,SERPING1,FERMT3,DSC2,CSF1R,FCN2,PSG6,PGLYRP2,SCGB3A1,PPBP,PF4,MASP1,GUCY1A1,LALBA,LRG1,PCSK9,FGB,DST,F2,INHBC,PSG1,PRG2,CSPG4,STAB1,C1QB,CFHR1,NDUFS8,TLN1,APOL1,SUMO4,PKM,C7,HBA1,A2M,HSPA5,FBN1,P4HB,PAPPA,FBLN1,PSG3,HSP90AA1,ANG,TUBA1B,FGG,LIMS1,THBS4,APOD,FN1,BLM,APOA4,C8A,CENPF,F13B,CFHR2,CFHR3,CFH,PAPPA2,F5,ADAM12,S100A7,S100A8,ECM1,FLNA,ANGPTL3,C8B,RBP4,CCN5,GSN,C1QC,F10,F7,APOM,CA6,ADGRG2,CALML5,IGFBP3,SOD3,GPX3,IGF1,PROS1,EFEMP1,TNRC6A,YWHAZ,ILK,GAPDH,RAPGEF4,ROCK1,MASP2,F11,C1S,TF,DCDC1,LTBP1,KSR2,ADIPOQ,IGF2,C4A,PRG4,CDK3,MST1,C4B,TGFBI,CFB,IGFALS,UIMC1,SELENOP,TSKU,SAA2,HPR,C1R,ATP1A1,RNASE4,IGKV2D-28,TTN,CFD,IGHV3-72,VEGFC,SEMA3B,PLCG2,OR52B4,HSPH1,IGLV10-54,KNG1,HBB,ACTB,ENO1,FGA,PDIA4
GO Process	GO:0072378	Blood coagulation, fibrin clot formation	16	24	1.73	1.06e-17	F9,F12,KLKB1,F13A1,FGB,F2,FBLN1,FGG,FN1,F13B,F5,FLNA,F10,F7,F11,FGA
GO Process	GO:0006952	Defense response	64	1394	0.57	8.78e-17	C5,MPO,PF4V1,LRP1,F12,CFHR5,THBS1,LYZ,ATRN,KLKB1,TCIRG1,HPX,ITIH4,IGFBP4,FCN3,CRH,SAA4,SERPING1,CSF1R,FCN2,PGLYRP2,PPBP,PF4,MASP1,LALBA,FGB,F2,PRG2,STAB1,C1QB,CFHR1,APOL1,C7,A2M,PSG3,ANG,FN1,APOA4,C8A,CFHR2,CFH,S100A7,S100A8,ECM1,C8B,GSN,C1QC,F7,GAPDH,MASP2,C1S,TF,C4A,C4B,CFB,SAA2,HPR,C1R,RNASE4,CFD,IGHV3-72,PLCG2,KNG1,FGA
GO Process	GO:0010466	Negative regulation of peptidase activity	27	249	0.94	8.38e-14	SPP2,SERPIND1,TFPI2,C5,VTN,LRP1,THBS1,SERPINA10,TIMP2,CSTA,AMBP,ITIH4,SERPING1,A2ML1,A2M,SERPINA7,SERPINA11,PAPLN,ITIH2,ECM1,PROS1,GAPDH,ROCK1,C4A,ITIH3,C4B,KNG1
GO Process	GO:0006955	Immune response	57	1321	0.54	1.91e-13	ST6GAL1,C5,VTN,PF4V1,LRP1,PXDN,F12,CFHR5,THBS1,LYZ,TCIRG1,HPX,PSG9,FCN3,SERPING1,CSF1R,FCN2,PGLYRP2,PPBP,PF4,MASP1,FGB,F2,PRG2,C1QB,CFHR1,APOL1,C7,A2M,ANG,APOA4,C8A,CFHR2,CFHR3,CFH,S100A7,S100A8,C8B,GSN,C1QC,GAPDH,RAPGEF4,MASP2,C1S,TF,C4A,PRG4,C4B,CFB,C1R,IGKV2D-28,CFD,IGHV3-72,PLCG2,IGLV10-54,KNG1,FGA
GO Process	GO:0030162	Regulation of proteolysis	42	739	0.66	4.66e-13	SPP2,CPB2,SERPIND1,MYH9,TFPI2,PCOLCE,C5,VTN,GPLD1,LRP1,APOE,F12,THBS1,SERPINA10,TIMP2,CSTA,KLKB1,AMBP,ITIH4,SERPING1,A2ML1,ANTXR1,SH3D19,F2,A2M,SERPINA7,FBLN1,SERPINA11,PAPLN,FN1,ITIH2,S100A8,ECM1,GSN,PROS1,GAPDH,ROCK1,C4A,ITIH3,C4B,KNG1,ENO1
GO Process	GO:0052547	Regulation of peptidase activity	33	446	0.78	7.67e-13	SPP2,SERPIND1,TFPI2,PCOLCE,C5,VTN,LRP1,THBS1,SERPINA10,TIMP2,CSTA,AMBP,ITIH4,SERPING1,A2ML1,ANTXR1,A2M,SERPINA7,FBLN1,SERPINA11,PAPLN,FN1,ITIH2,S100A8,ECM1,GSN,PROS1,GAPDH,ROCK1,C4A,ITIH3,C4B,KNG1
GO Process	GO:0098542	Defense response to other organism	48	989	0.59	8.73e-13	C5,MPO,PF4V1,F12,CFHR5,LYZ,HPX,FCN3,SERPING1,CSF1R,FCN2,PGLYRP2,PPBP,PF4,MASP1,LALBA,FGB,F2,PRG2,STAB1,C1QB,CFHR1,APOL1,C7,ANG,APOA4,C8A,CFHR2,CFH,S100A7,S100A8,C8B,GSN,C1QC,GAPDH,MASP2,C1S,TF,C4A,C4B,CFB,C1R,RNASE4,CFD,IGHV3-72,PLCG2,KNG1,FGA
GO Process	GO:0045861	Negative regulation of proteolysis	29	339	0.84	1.35e-12	SPP2,CPB2,SERPIND1,TFPI2,C5,VTN,LRP1,THBS1,SERPINA10,TIMP2,CSTA,AMBP,ITIH4,SERPING1,A2ML1,F2,A2M,SERPINA7,SERPINA11,PAPLN,ITIH2,ECM1,PROS1,GAPDH,ROCK1,C4A,ITIH3,C4B,KNG1
GO Process	GO:0010951	Negative regulation of endopeptidase activity	25	240	0.92	2.04e-12	SPP2,SERPIND1,TFPI2,C5,VTN,THBS1,SERPINA10,TIMP2,CSTA,AMBP,ITIH4,SERPING1,A2ML1,A2M,SERPINA7,SERPINA11,PAPLN,ITIH2,PROS1,GAPDH,ROCK1,C4A,ITIH3,C4B,KNG1
GO Process	GO:0002253	Activation of immune response	26	271	0.89	3.18e-12	C5,GPLD1,CFHR5,FCN3,SERPING1,FCN2,MASP1,C1QB,CFHR1,C7,HSP90AA1,C8A,CFHR2,CFHR3,CFH,C8B,C1QC,MASP2,C1S,C4A,C4B,CFB,C1R,CFD,IGHV3-72,PLCG2
GO Process	GO:0051346	Negative regulation of hydrolase activity	29	354	0.82	3.31e-12	SPP2,SERPIND1,TFPI2,C5,VTN,APOA1,LRP1,THBS1,SERPINA10,TIMP2,CSTA,AMBP,ITIH4,SERPING1,A2ML1,A2M,SERPINA7,SERPINA11,PAPLN,ITIH2,ECM1,ANGPTL3,PROS1,GAPDH,ROCK1,C4A,ITIH3,C4B,KNG1
GO Process	GO:0002682	Regulation of immune system process	57	1438	0.51	4.46e-12	C5,GPLD1,APOA1,APOE,CFHR5,THBS1,AMBP,HPX,PSG9,FCN3,PLA2G7,SERPING1,CSF1R,FCN2,PSG6,PGLYRP2,PF4,MASP1,PSG1,PRG2,C1QB,CFHR1,C7,A2M,FBN1,PSG3,HSP90AA1,THBS4,APOD,FN1,C8A,CFHR2,CFHR3,CFH,S100A7,ECM1,C8B,RBP4,C1QC,F7,CD99,IGF1,MASP2,C1S,ADIPOQ,IGF2,C4A,MST1,C4B,CFB,C1R,CFD,IGHV3-72,VEGFC,PLCG2,HSPH1,ACTB
GO Process	GO:0030193	Regulation of blood coagulation	16	70	1.27	4.54e-12	CPB2,VTN,PROC,APOE,F12,THBS1,KLKB1,SERPING1,FGB,F2,FGG,F7,PROS1,F11,KNG1,FGA
GO Process	GO:0030195	Negative regulation of blood coagulation	14	46	1.39	7.36e-12	CPB2,VTN,PROC,APOE,F12,KLKB1,SERPING1,FGB,F2,FGG,PROS1,F11,KNG1,FGA
GO Process	GO:0030168	Platelet activation	17	97	1.15	2.39e-11	MYH9,PF4V1,PLEK,VWF,FERMT3,PF4,FGB,F2,TLN1,FGG,FN1,FLNA,ILK,PLCG2,HBB,ACTB,FGA
GO Process	GO:0006958	Complement activation, classical pathway	13	40	1.42	2.76e-11	C5,SERPING1,C1QB,C7,C8A,C8B,C1QC,MASP2,C1S,C4A,C4B,C1R,IGHV3-72
GO Process	GO:0002252	Immune effector process	28	375	0.78	5.93e-11	C5,MPO,LRP1,CFHR5,TCIRG1,FCN3,SERPING1,FCN2,MASP1,F2,C1QB,CFHR1,C7,C8A,CFHR2,CFHR3,CFH,C8B,C1QC,MASP2,C1S,C4A,C4B,CFB,C1R,CFD,IGHV3-72,PLCG2
GO Process	GO:0007155	Cell adhesion	44	965	0.57	7.22e-11	MYH9,VTN,PLEK,PXDN,THBS1,VWF,ITGA2B,ATRN,CSTA,AMBP,CDH11,HABP2,FERMT3,DSC2,PCDH1,ANTXR1,LRG1,FGB,DST,STAB1,TLN1,PSG11,FBN1,FGG,LIMS1,THBS4,FN1,APOA4,COL18A1,ADAM12,S100A8,FLNA,ANGPTL3,CCN5,CD99,ACTN1,ILK,ROCK1,PSG2,TGFBI,IGFALS,HBB,ACTB,FGA
GO Process	GO:0009607	Response to biotic stimulus	53	1375	0.49	9.41e-11	C5,MPO,PF4V1,F12,CFHR5,LYZ,HPX,FCN3,SERPING1,CSF1R,FCN2,PGLYRP2,PPBP,PF4,MASP1,LALBA,LRG1,FGB,F2,PRG2,STAB1,C1QB,CFHR1,APOL1,C7,HSPA5,HSP90AA1,ANG,APOA4,C8A,CFHR2,CFH,S100A7,S100A8,C8B,GSN,C1QC,GAPDH,MASP2,C1S,TF,ADIPOQ,C4A,C4B,CFB,C1R,RNASE4,CFD,IGHV3-72,PLCG2,KNG1,ENO1,FGA
GO Process	GO:0051707	Response to other organism	52	1328	0.5	9.41e-11	C5,MPO,PF4V1,F12,CFHR5,LYZ,HPX,FCN3,SERPING1,CSF1R,FCN2,PGLYRP2,PPBP,PF4,MASP1,LALBA,LRG1,FGB,F2,PRG2,STAB1,C1QB,CFHR1,APOL1,C7,HSP90AA1,ANG,APOA4,C8A,CFHR2,CFH,S100A7,S100A8,C8B,GSN,C1QC,GAPDH,MASP2,C1S,TF,ADIPOQ,C4A,C4B,CFB,C1R,RNASE4,CFD,IGHV3-72,PLCG2,KNG1,ENO1,FGA
GO Process	GO:0034109	Homotypic cell-cell adhesion	14	60	1.28	1.14e-10	MYH9,PLEK,FERMT3,DSC2,FGB,TLN1,FGG,FN1,FLNA,CD99,ILK,HBB,ACTB,FGA

GO Process	GO:0051241	Negative regulation of multicellular organismal process	45	1035	0.55	1.54e-10	CPB2,MMP2,VTN,SPARC,IGFBP5,PROC,APOA1,APOE,F12,THBS1,KLKB1,PSG9,CRH,SERPING1,PGLYRP2,PF4,GUCY1A1,PCSK9,FGB,F2,PRG2,STAB1,FBN1,FBLN1,FGG,THBS4,APOD,FN1,APOA4,ECM1,RBP4,C1QC,APOM,IGF1,PROS1,EFEMP1,ROCK1,F11,ADIPOQ,PAEP,TSKU,ATP1A1,SEMA3B,KNG1,FGA
GO Process	GO:0002684	Positive regulation of immune system process	41	874	0.58	1.92e-10	C5,GPLD1,CFHR5,THBS1,HPX,FCN3,PLA2G7,SERPING1,CSF1R,FCN2,PF4,MASP1,C1QB,CFHR1,C7,HSP90AA1,THBS4,C8A,CFHR2,CFHR3,CFH,S100A7,C8B,RBP4,C1QC,F7,CD99,IGF1,MASP2,C1S,IGF2,C4A,C4B,CFB,C1R,CFD,IGHV3-72,VEGFC,PLCG2,HSPH1,ACTB
GO Process	GO:0002376	Immune system process	67	2121	0.41	2.35e-10	ST6GAL1,MYH9,C5,MPO,VTN,PF4V1,GPLD1,PLEK,LRP1,PXDN,F12,CFHR5,THBS1,LYZ,TCIRG1,HPX,PSG9,FCN3,SERPING1,CSF1R,FCN2,PGLYRP2,PPBP,PF4,MASP1,FGB,F2,PRG2,C1QB,CFHR1,APOL1,C7,A2M,HSP90AA1,ANG,APOA4,MFAP5,C8A,CFHR2,CFHR3,CFH,S100A7,S100A8,FLNA,C8B,GSN,C1QC,CD99,ACTN1,GAPDH,RAPGEF4,ROCK1,MASP2,C1S,TF,C4A,PRG4,C4B,CFB,C1R,IGKV2D-28,CFD,IGHV3-72,PLCG2,IGLV10-54,KNG1,FGA
GO Process	GO:0032101	Regulation of response to external stimulus	43	964	0.56	2.35e-10	ST6GAL1,CPB2,C5,VTN,PROC,APOA1,APOE,F12,THBS1,PSMA6,KLKB1,HPX,FCN3,PLA2G7,SERPING1,CSF1R,FCN2,PGLYRP2,MASP1,FGB,F2,A2M,FBN1,HSP90AA1,FGG,THBS4,CFH,S100A7,S100A8,HTRA1,FLNA,F7,IGF1,PROS1,MASP2,F11,ADIPOQ,MST1,VEGFC,SEMA3B,PLCG2,KNG1,FGA
GO Process	GO:0048584	Positive regulation of response to stimulus	67	2131	0.4	2.78e-10	CPB2,C5,VTN,GPLD1,IGFBP5,APOA1,CAT,APOE,F12,CFHR5,THBS1,VWF,HPX,IGFBP4,FCN3,PLA2G7,SERPING1,CSF1R,FCN2,MASP1,GUCY1A1,LRG1,FGB,F2,INHBC,CSPG4,C1QB,CFHR1,C7,FBN1,HSP90AA1,FGG,LIMS1,THBS4,FN1,C8A,CFHR2,CFHR3,CFH,S100A7,S100A8,ECM1,FLNA,C8B,C1QC,F10,F7,IGFBP3,IGF1,ILK,GAPDH,ROCK1,MASP2,C1S,ADIPOQ,IGF2,C4A,C4B,CFB,UIMC1,C1R,CFD,IGHV3-72,VEGFC,PLCG2,ACTB,FGA
GO Process	GO:0044419	Biological process involved in interspecies interaction between organisms	54	1490	0.47	4.55e-10	C5,MPO,PF4V1,APOE,F12,CFHR5,LYZ,HPX,FCN3,SERPING1,CSF1R,FCN2,PGLYRP2,PPBP,PF4,MASP1,LALBA,LRG1,FGB,F2,PRG2,STAB1,C1QB,CFHR1,APOL1,C7,HSP90AA1,ANG,FN1,APOA4,C8A,CFHR2,CFH,S100A7,S100A8,C8B,GSN,C1QC,GAPDH,MASP2,C1S,TF,ADIPOQ,C4A,C4B,CFB,C1R,RNASE4,CFD,IGHV3-72,PLCG2,KNG1,ENO1,FGA
GO Process	GO:0048583	Regulation of response to stimulus	97	3931	0.3	5.25e-10	ST6GAL1,CPB2,C5,VTN,GPLD1,IGFBP5,PROC,PLEK,APOA1,CAT,LRP1,APOE,PXDN,F12,CFHR5,THBS1,VWF,PSMA6,KLKB1,AMBP,HPX,IGFBP4,PSG9,FCN3,PLA2G7,CRH,SERPING1,CSF1R,FCN2,PGLYRP2,PF4,MASP1,GUCY1A1,LRG1,PCSK9,FGB,F2,INHBC,CSPG4,C1QB,CFHR1,SUMO4,C7,A2M,HSPA5,FBN1,P4HB,FBLN1,HSP90AA1,FGG,LIMS1,THBS4,APOD,FN1,C8A,CFHR2,CFHR3,CFH,S100A7,S100A8,HTRA1,ECM1,FLNA,C8B,C1QC,F10,F7,IGFBP3,IGF1,PROS1,YWHAZ,ILK,GAPDH,ROCK1,MASP2,F11,C1S,LTBP1,ADIPOQ,IGF2,C4A,CDK3,MST1,C4B,CFB,UIMC1,TSKU,C1R,CFD,IGHV3-72,VEGFC,SEMA3B,PLCG2,KNG1,ACTB,ENO1,FGA
GO Process	GO:0048518	Positive regulation of biological process	130	6207	0.23	7.57e-10	ST6GAL1,CPB2,CETP,MYH9,MMP2,PCOLCE,C5,VTN,GPLD1,SPARC,IGFBP5,PROC,PLEK,APOA1,CAT,LRP1,APOE,F12,CFHR5,THBS1,VWF,NOTCH3,KLKB1,CENPE,TCIRG1,HPX,IGFBP4,PSG9,FCN3,PLA2G7,CRH,SERPING1,FERMT3,CSF1R,FCN2,SCGB3A1,PPBP,PF4,FYCO1,MASP1,GUCY1A1,ANTXR1,LRG1,SH3D19,PCSK9,FGB,F2,INHBC,PRG2,CSPG4,C1QB,CFHR1,SUZ12,PKM,STXBP5,C7,HBA1,HSPA5,FBN1,P4HB,FBLN1,HSP90AA1,ANG,FGG,LIMS1,THBS4,FN1,BLM,APOA4,C8A,CFHR2,CFHR3,CFH,ADAM12,S100A7,S100A8,HTRA1,ADAMTSL4,ECM1,FLNA,SMARCA1,ANGPTL3,C8B,RBP4,GSN,C1QC,F10,F7,IGFBP3,CD99,IGF1,ACTN1,TNRC6A,ILK,GAPDH,ROCK1,MASP2,F11,C1S,TF,KSR2,ADIPOQ,IGF2,C4A,SLAIN1,MST1,LMOD3,C4B,CFB,PAEP,TCAF1,UIMC1,TSKU,HPR,C1R,APOA5,ATP1A1,TTN,APOC4,CFD,IGHV3-72,VEGFC,SEMA3B,PLCG2,HSPH1,KNG1,HBB,ACTB,ENO1,FGA
GO Process	GO:0070527	Platelet aggregation	12	43	1.35	7.73e-10	MYH9,PLEK,FERMT3,FGB,TLN1,FGG,FN1,FLNA,ILK,HBB,ACTB,FGA
GO Process	GO:0009605	Response to external stimulus	70	2355	0.38	8.42e-10	SERPIND1,MMP2,C5,MPO,PF4V1,APOA1,CAT,APOE,F12,CFHR5,THBS1,LYZ,NOTCH3,HPX,FCN3,SERPING1,DSC2,CSF1R,FCN2,PGLYRP2,PPBP,PF4,MASP1,LALBA,LRG1,PCSK9,FGB,F2,PRG2,STAB1,C1QB,CFHR1,APOL1,C7,A2M,HSPA5,FBN1,HSP90AA1,ANG,APOA4,C8A,CFHR2,CFH,F5,S100A7,S100A8,C8B,GSN,C1QC,F7,TNRC6A,GAPDH,MASP2,C1S,TF,ADIPOQ,C4A,C4B,CFB,C1R,RNASE4,TTN,CFD,IGHV3-72,VEGFC,SEMA3B,PLCG2,KNG1,ENO1,FGA
GO Process	GO:0031638	Zymogen activation	13	59	1.25	1.17e-09	F9,F12,KLKB1,FGB,F2,FGG,F5,F10,F7,F11,HPR,C1R,FGA
GO Process	GO:0052548	Regulation of endopeptidase activity	27	414	0.72	2.04e-09	SPP2,SERPIND1,TFPI2,C5,VTN,THBS1,SERPINA10,TIMP2,CSTA,AMBP,ITIH4,SERPING1,A2ML1,A2M,SERPINA7,SERPINA11,PAPLN,ITIH2,S100A8,GSN,PROS1,GAPDH,ROCK1,C4A,ITIH3,C4B,KNG1
GO Process	GO:0051336	Regulation of hydrolase activity	42	1011	0.53	3.25e-09	SPP2,SERPIND1,TFPI2,PCOLCE,C5,VTN,GPLD1,PLEK,APOA1,LRP1,THBS1,SERPINA10,TIMP2,CSTA,AMBP,ITIH4,SERPING1,A2ML1,ANTXR1,A2M,SERPINA7,FBLN1,SERPINA11,ANG,LIMS1,PAPLN,FN1,APOA4,ITIH2,S100A8,ECM1,ANGPTL3,GSN,PROS1,GAPDH,RAPGEF4,ROCK1,C4A,ITIH3,C4B,APOA5,KNG1
GO Process	GO:0051246	Regulation of protein metabolic process	73	2622	0.35	4.80e-09	SPP2,CPB2,SERPIND1,MYH9,TFPI2,PCOLCE,C5,VTN,GPLD1,IGFBP5,APOA1,LRP1,APOE,F12,THBS1,SERPINA10,TIMP2,CSTA,KLKB1,AMBP,CENPE,HPX,ITIH4,SERPING1,CSF1R,A2ML1,ANTXR1,SH3D19,PCSK9,F2,INHBC,CSPG4,PKM,A2M,HSPA5,FBN1,SERPINA7,FBLN1,SERPINA11,HSP90AA1,ANG,THBS4,APOD,PAPLN,FN1,BLM,ITIH2,S100A8,ECM1,FLNA,GSN,IGFBP3,IGF1,PROS1,TNRC6A,ILK,GAPDH,ROCK1,ADIPOQ,IGF2,BZW1,C4A,MST1,ITIH3,C4B,PAEP,TTN,VEGFC,PLCG2,KNG1,ACTB,ENO1,FGA
GO Process	GO:0042730	Fibrinolysis	9	19	1.58	8.53e-09	CPB2,F12,KLKB1,SERPING1,FGB,F2,FGG,PROS1,FGA
GO Process	GO:0016064	Immunoglobulin mediated immune response	14	95	1.08	1.82e-08	C5,TCIRG1,SERPING1,C1QB,C7,C8A,C8B,C1QC,MASP2,C1S,C4A,C4B,C1R,IGHV3-72
GO Process	GO:1903034	Regulation of response to wounding	17	162	0.93	2.10e-08	CPB2,VTN,PROC,APOE,F12,THBS1,KLKB1,SERPING1,FGB,F2,FGG,FLNA,F7,PROS1,F11,KNG1,FGA
GO Process	GO:0045087	Innate immune response	34	754	0.56	4.32e-08	C5,F12,CFHR5,HPX,FCN3,SERPING1,CSF1R,FCN2,PGLYRP2,MASP1,FGB,C1QB,APOL1,C7,ANG,APOA4,C8A,CFH,S100A7,S100A8,C8B,GSN,C1QC,GAPDH,MASP2,C1S,C4A,C4B,CFB,C1R,CFD,IGHV3-72,PLCG2,FGA

GO Process	GO:0065007	Biological regulation	199	12385	0.11	5.21e-08	SPP2,ST6GAL1,CPB2,CETP,SERPIND1,MYH9,TPD52L2,F9,MMP2,TFPI2,PCOLCE,C5,MPO,HSD17B1,VTN,PF4V1,MGP,GPLD1,SPARC,IGFBP5,PROC,PLEK,APOA1,TTR,CAT,LRP1,APOE,PXDN,F12,CFHR5,THBS1,LYZ,VWF,PSMA6,SERPINA10,ITGA2B,TIMP2,ATRN,NOTCH3,LCAT,CSTA,CP,KLKB1,F13A1,AMBP,CENPE,TCIRG1,HPX,ITIH4,CDH11,IGFBP4,PSG9,FCN3,PLA2G7,CRH,SERPING1,FERMT3,DSC2,CSF1R,FCN2,PSG6,PGLYRP2,SCGB3A1,PPBP,PF4,FYCO1,MASP1,GUCY1A1,A2ML1,LALBA,ANTXR1,LRG1,SH3D19,PCSK9,FGB,DST,F2,INHBC,PSG1,PRG2,CSPG4,STAB1,C1QB,CFHR1,TLN1,SUZ12,SUMO4,PKM,STXBP5,C7,HBA1,A2M,HSPA5,FBN1,P4HB,SERPINA7,PAPPA,FBLN1,PSG3,OIT3,SERPINA11,HSP90AA1,ANG,FGG,LIMS1,THBS4,APOD,PAPLN,FN1,BLM,APOA4,ITIH2,COL18A1,C8A,CENPF,F13B,CFHR2,CFHR3,CFH,PAPPA2,F5,MKI67,ADAM12,S100A7,S100A8,HTRA1,ADAMTSL4,ECM1,FLNA,SMARCA1,ANGPTL3,C8B,RBP4,CCN5,GSN,C1QC,F10,F7,APOM,ADGRG2,CALML5,IGFBP3,CD99,SOD3,GOLM1,IGF1,PROS1,ACTN1,EFEMP1,TNRC6A,YWHAZ,ILK,GAPDH,RAPGEF4,ROCK1,MASP2,F11,C1S,TF,DCDC1,LTBP1,KSR2,ADIPOQ,IGF2,BZW1,C4A,ATP9B,SLAIN1,CDK3,MST1,LMOD3,ITIH3,C4B,TGFBI,CFB,IGFALS,PAEP,TCAF1,UIMC1,SELENOP,TSKU,HPR,C1R,APOA5,ATP1A1,TTN,APOC4,CFD,IGHV3-72,VEGFC,SEMA3B,PLCG2,OR52B4,HSPH1,KNG1,HBB,ACTB,ENO1,FGA
GO Process	GO:0006957	Complement activation, alternative pathway	8	16	1.61	7.03e-08	C5,CFHR5,C7,C8A,CFH,C8B,CFB,CFD
GO Process	GO:0051248	Negative regulation of protein metabolic process	40	1038	0.49	7.77e-08	SPP2,CPB2,SERPIND1,TFPI2,C5,VTN,IGFBP5,LRP1,APOE,THBS1,SERPINA10,TIMP2,CSTA,AMBP,ITIH4,SERPING1,A2ML1,F2,A2M,SERPINA7,FBLN1,SERPINA11,ANG,APOD,PAPLN,ITIH2,ECM1,FLNA,IGFBP3,IGF1,PROS1,TNRC6A,GAPDH,ROCK1,ADIPOQ,C4A,ITIH3,C4B,KNG1,FGA
GO Process	GO:0006954	Inflammatory response	28	538	0.62	9.29e-08	C5,PF4V1,LRP1,F12,THBS1,LYZ,ATRN,KLKB1,TCIRG1,ITIH4,IGFBP4,CRH,SAA4,CSF1R,PPBP,PF4,F2,STAB1,A2M,FN1,S100A8,ECM1,F7,C4A,C4B,SAA2,HPR,KNG1
GO Process	GO:0050778	Positive regulation of immune response	27	502	0.64	9.73e-08	C5,GPLD1,CFHR5,HPX,FCN3,SERPING1,FCN2,MASP1,C1QB,CFHR1,C7,HSP90AA1,C8A,CFHR2,CFHR3,CFH,C8B,C1QC,MASP2,C1S,C4A,C4B,CFB,C1R,CFD,IGHV3-72,PLCG2
GO Process	GO:0034369	Plasma lipoprotein particle remodeling	9	29	1.4	1.51e-07	CETP,MPO,APOA1,APOE,LCAT,PLA2G7,APOA4,APOM,APOA5
GO Process	GO:0032102	Negative regulation of response to external stimulus	23	387	0.68	3.27e-07	ST6GAL1,CPB2,C5,VTN,PROC,APOA1,APOE,F12,THBS1,KLKB1,SERPING1,FGB,F2,A2M,FGG,HTRA1,IGF1,PROS1,F11,ADIPOQ,SEMA3B,KNG1,FGA
GO Process	GO:0050789	Regulation of biological process	189	11655	0.12	3.45e-07	SPP2,ST6GAL1,CPB2,CETP,SERPIND1,MYH9,TPD52L2,MMP2,TFPI2,PCOLCE,C5,MPO,VTN,PF4V1,MGP,GPLD1,SPARC,IGFBP5,PROC,PLEK,APOA1,TTR,CAT,LRP1,APOE,PXDN,F12,CFHR5,THBS1,VWF,PSMA6,SERPINA10,ITGA2B,TIMP2,ATRN,NOTCH3,LCAT,CSTA,KLKB1,AMBP,CENPE,TCIRG1,HPX,ITIH4,CDH11,IGFBP4,PSG9,FCN3,PLA2G7,CRH,SERPING1,FERMT3,DSC2,CSF1R,FCN2,PSG6,PGLYRP2,SCGB3A1,PPBP,PF4,FYCO1,MASP1,GUCY1A1,A2ML1,LALBA,ANTXR1,LRG1,SH3D19,PCSK9,FGB,DST,F2,INHBC,PSG1,PRG2,CSPG4,STAB1,C1QB,CFHR1,TLN1,SUZ12,SUMO4,PKM,STXBP5,C7,HBA1,A2M,HSPA5,FBN1,P4HB,SERPINA7,PAPPA,FBLN1,PSG3,SERPINA11,HSP90AA1,ANG,FGG,LIMS1,THBS4,APOD,PAPLN,FN1,BLM,APOA4,ITIH2,COL18A1,C8A,CENPF,CFHR2,CFHR3,CFH,PAPPA2,MKI67,ADAM12,S100A7,S100A8,HTRA1,ADAMTSL4,ECM1,FLNA,SMARCA1,ANGPTL3,C8B,RBP4,CCN5,GSN,C1QC,F10,F7,APOM,ADGRG2,CALML5,IGFBP3,CD99,GOLM1,IGF1,PROS1,ACTN1,EFEMP1,TNRC6A,YWHAZ,ILK,GAPDH,RAPGEF4,ROCK1,MASP2,F11,C1S,TF,DCDC1,LTBP1,KSR2,ADIPOQ,IGF2,BZW1,C4A,SLAIN1,CDK3,MST1,LMOD3,ITIH3,C4B,TGFBI,CFB,IGFALS,PAEP,TCAF1,UIMC1,SELENOP,TSKU,HPR,C1R,APOA5,ATP1A1,TTN,APOC4,CFD,IGHV3-72,VEGFC,SEMA3B,PLCG2,OR52B4,HSPH1,KNG1,HBB,ACTB,ENO1,FGA
GO Process	GO:0065008	Regulation of biological quality	85	3654	0.27	3.67e-07	CPB2,CETP,SERPIND1,MYH9,F9,MMP2,TFPI2,HSD17B1,VTN,PF4V1,GPLD1,IGFBP5,PROC,PLEK,APOA1,TTR,APOE,F12,THBS1,LYZ,VWF,SERPINA10,LCAT,CP,KLKB1,F13A1,TCIRG1,HPX,PLA2G7,CRH,SERPING1,FERMT3,DSC2,CSF1R,PF4,GUCY1A1,PCSK9,FGB,F2,TLN1,STXBP5,FBN1,SERPINA7,FBLN1,OIT3,HSP90AA1,ANG,FGG,FN1,APOA4,F13B,F5,S100A8,FLNA,ANGPTL3,RBP4,GSN,F10,F7,APOM,SOD3,IGF1,PROS1,YWHAZ,ILK,GAPDH,RAPGEF4,ROCK1,F11,TF,KSR2,ADIPOQ,ATP9B,LMOD3,TSKU,APOA5,ATP1A1,APOC4,VEGFC,SEMA3B,PLCG2,KNG1,HBB,ACTB,FGA
GO Process	GO:0051239	Regulation of multicellular organismal process	70	2749	0.31	5.15e-07	CPB2,MMP2,C5,VTN,MGP,GPLD1,SPARC,IGFBP5,PROC,APOA1,LRP1,APOE,F12,THBS1,ATRN,LCAT,KLKB1,PSG9,CRH,SERPING1,DSC2,CSF1R,PGLYRP2,PF4,GUCY1A1,LRG1,PCSK9,FGB,F2,PRG2,STAB1,PKM,FBN1,FBLN1,HSP90AA1,FGG,THBS4,APOD,FN1,APOA4,ADAM12,ECM1,ANGPTL3,RBP4,C1QC,F7,APOM,IGF1,PROS1,EFEMP1,GAPDH,ROCK1,F11,TF,LTBP1,KSR2,ADIPOQ,IGF2,MST1,PAEP,TSKU,APOA5,ATP1A1,VEGFC,SEMA3B,PLCG2,KNG1,ACTB,ENO1,FGA
GO Process	GO:0032501	Multicellular organismal process	125	6490	0.19	7.88e-07	SPP2,CPB2,CETP,SERPIND1,MYH9,F9,MMP2,TFPI2,C5,MPO,HSD17B1,VTN,PF4V1,MGP,GPLD1,IGFBP5,PROC,PLEK,APOA1,CAT,LRP1,APOE,PXDN,ANGPTL6,F12,THBS1,LYZ,VWF,SERPINA10,ITGA2B,ATRN,NOTCH3,LCAT,KLKB1,F13A1,DNAH5,AMBP,TCIRG1,CDH11,PSG9,PLA2G7,CRH,SERPING1,FERMT3,SEC24D,DSC2,CSF1R,PCDH1,PSG6,PF4,GUCY1A1,ANTXR1,LRG1,PCSK9,FGB,F2,PSG1,CSPG4,TLN1,SUZ12,PSG11,A2M,HSPA5,FBN1,PAPPA,FBLN1,PSG3,HSP90AA1,ANG,FGG,THBS4,APOD,FN1,APOA4,MFAP5,COL18A1,CENPF,F13B,PAPPA2,F5,S100A7,S100A8,HTRA1,ECM1,FLNA,SMARCA1,ANGPTL3,RBP4,GSN,F10,F7,APOM,CA6,ADGRG2,IGFBP3,SOD3,IGF1,PROS1,ACTN1,EFEMP1,YWHAZ,ILK,ROCK1,F11,PSG2,TF,ADIPOQ,IGF2,MST1,LMOD3,TGFBI,SELENOP,TSKU,APOA5,ATP1A1,TTN,VEGFC,SEMA3B,PLCG2,OR52B4,KNG1,HBB,ACTB,FGA,TPM3
GO Process	GO:0044092	Negative regulation of molecular function	40	1143	0.45	9.65e-07	SPP2,SERPIND1,TFPI2,C5,VTN,APOA1,CAT,LRP1,APOE,PXDN,CFHR5,THBS1,SERPINA10,TIMP2,CSTA,AMBP,ITIH4,SERPING1,A2ML1,PCSK9,CFHR1,SUMO4,A2M,SERPINA7,SERPINA11,PAPLN,ITIH2,CFHR2,ECM1,FLNA,ANGPTL3,PROS1,GAPDH,ROCK1,ADIPOQ,C4A,ITIH3,C4B,KNG1,ACTB
GO Process	GO:0097006	Regulation of plasma lipoprotein particle levels	10	54	1.17	1.11e-06	CETP,MPO,APOA1,APOE,LCAT,PLA2G7,APOA4,APOM,ADIPOQ,APOA5
GO Process	GO:0051050	Positive regulation of transport	35	915	0.49	1.15e-06	CPB2,CETP,VTN,GPLD1,APOA1,LRP1,APOE,FCN3,CRH,FCN2,MASP1,PCSK9,FGB,F2,STXBP5,HSP90AA1,ANG,FGG,S100A8,FLNA,RBP4,IGF1,MASP2,TF,ADIPOQ,C4A,C4B,CFB,TCAF1,APOA5,TTN,VEGFC,PLCG2,ACTB,FGA
GO Process	GO:0048519	Negative regulation of biological process	108	5313	0.22	1.29e-06	SPP2,ST6GAL1,CPB2,CETP,SERPIND1,MYH9,MMP2,TFPI2,C5,MPO,VTN,GPLD1,SPARC,IGFBP5,PROC,PLEK,APOA1,CAT,LRP1,APOE,PXDN,F12,THBS1,SERPINA10,TIMP2,NOTCH3,CSTA,KLKB1,AMBP,ITIH4,IGFBP4,PSG9,FCN3,CRH,SERPING1,CSF1R,PGLYRP2,SCGB3A1,PF4,MASP1,GUCY1A1,A2ML1,ANTXR1,PCSK9,FGB,F2,PRG2,STAB1,SUZ12,SUMO4,A2M,HSPA5,FBN1,SERPINA7,FBLN1,SERPINA11,ANG,FGG,LIMS1,THBS4,APOD,PAPLN,FN1,BLM,APOA4,ITIH2,COL18A1,CENPF,HTRA1,ECM1,FLNA,RBP4,CCN5,GSN,C1QC,APOM,IGFBP3,IGF1,PROS1,EFEMP1,TNRC6A,YWHAZ,ILK,GAPDH,ROCK1,F11,LTBP1,ADIPOQ,IGF2,C4A,CDK3,MST1,LMOD3,ITIH3,C4B,TGFBI,PAEP,TCAF1,UIMC1,TSKU,ATP1A1,VEGFC,SEMA3B,PLCG2,KNG1,ACTB,ENO1,FGA
GO Process	GO:1903028	Positive regulation of opsonization	7	16	1.55	1.57e-06	FCN3,FCN2,MASP1,MASP2,C4A,C4B,CFB

GO Process	GO:0002460	Adaptive immune response based on somatic recombination of immune receptors built from immunoglobulin superfamily domains	15	169	0.86	1.67e-06	C5,TCIRG1,PSG9,SERPING1,C1QB,C7,C8A,C8B,C1QC,MASP2,C1S,C4A,C4B,C1R,IGHV3-72
GO Process	GO:0098609	Cell-cell adhesion	26	542	0.59	1.67e-06	MYH9,PLEK,ITGA2B,CSTA,CDH11,FERMT3,DSC2,PCDH1,LRG1,FGB,TLN1,PSG11,FGG,LIMS1,THBS4,FN1,APOA4,S100A8,FLNA,CD99,ILK,ROCK1,PSG2,HBB,ACTB,FGA
GO Process	GO:0050776	Regulation of immune response	33	844	0.5	1.86e-06	C5,GPLD1,APOE,CFHR5,AMBP,HPX,PSG9,FCN3,SERPING1,FCN2,PGLYRP2,MASP1,C1QB,CFHR1,C7,A2M,HSP90AA1,C8A,CFHR2,CFHR3,CFH,ECM1,C8B,C1QC,MASP2,C1S,C4A,C4B,CFB,C1R,CFD,IGHV3-72,PLCG2
GO Process	GO:0001525	Angiogenesis	20	325	0.7	1.96e-06	MYH9,MMP2,GPLD1,PXDN,ANGPTL6,THBS1,ITGA2B,NOTCH3,CSPG4,ANG,APOD,FN1,COL18A1,S100A7,ECM1,FLNA,ANGPTL3,YWHAZ,TGFBI,VEGFC
GO Process	GO:0034372	Very-low-density lipoprotein particle remodeling	6	9	1.73	2.69e-06	CETP,APOA1,APOE,LCAT,APOA4,APOA5
GO Process	GO:0002526	Acute inflammatory response	11	80	1.05	2.71e-06	F12,KLKB1,ITIH4,SAA4,F2,A2M,FN1,S100A8,F7,SAA2,HPR
GO Process	GO:0043086	Negative regulation of catalytic activity	31	771	0.51	2.74e-06	SPP2,SERPIND1,TFPI2,C5,VTN,APOA1,LRP1,APOE,THBS1,SERPINA10,TIMP2,CSTA,AMBP,ITIH4,SERPING1,A2ML1,A2M,SERPINA7,SERPINA11,PAPLN,ITIH2,ECM1,ANGPTL3,PROS1,GAPDH,ROCK1,ADIPOQ,C4A,ITIH3,C4B,KNG1
GO Process	GO:0051917	Regulation of fibrinolysis	7	18	1.5	2.74e-06	CPB2,VTN,F12,THBS1,KLKB1,F2,F11
GO Process	GO:1900024	Regulation of substrate adhesion-dependent cell spreading	10	61	1.12	2.74e-06	ST6GAL1,APOA1,FGB,P4HB,FBLN1,FGG,LIMS1,FLNA,ILK,FGA
GO Process	GO:0048514	Blood vessel morphogenesis	22	419	0.63	4.74e-06	MYH9,MMP2,GPLD1,LRP1,APOE,PXDN,ANGPTL6,THBS1,ITGA2B,NOTCH3,CSPG4,ANG,APOD,FN1,COL18A1,S100A7,ECM1,FLNA,ANGPTL3,YWHAZ,TGFBI,VEGFC
GO Process	GO:0010810	Regulation of cell-substrate adhesion	16	217	0.77	5.19e-06	ST6GAL1,VTN,APOA1,THBS1,FGB,TLN1,P4HB,FBLN1,FGG,LIMS1,APOD,FN1,FLNA,ILK,ROCK1,FGA
GO Process	GO:0006508	Proteolysis	40	1247	0.41	7.70e-06	CPB2,MYH9,F9,MMP2,PCOLCE,PROC,F12,PSMA6,KLKB1,FCN3,PLA2G7,HABP2,FCN2,MASP1,APEH,PCSK9,FGB,F2,UBE3C,HSPA5,PAPPA,FGG,CFH,PAPPA2,F5,ADAM12,HTRA1,F10,F7,MASP2,F11,C1S,PCYOX1,MST1,CFB,UIMC1,HPR,C1R,CFD,FGA
GO Process	GO:0065009	Regulation of molecular function	72	3085	0.28	7.80e-06	SPP2,SERPIND1,TFPI2,PCOLCE,C5,VTN,GPLD1,PLEK,APOA1,CAT,LRP1,APOE,PXDN,CFHR5,THBS1,PSMA6,SERPINA10,TIMP2,CSTA,AMBP,CENPE,ITIH4,CRH,SERPING1,CSF1R,A2ML1,ANTXR1,PCSK9,F2,CFHR1,SUZ12,SUMO4,STXBP5,A2M,FBN1,SERPINA7,FBLN1,SERPINA11,HSP90AA1,ANG,LIMS1,PAPLN,FN1,BLM,APOA4,ITIH2,CFHR2,S100A8,ECM1,FLNA,ANGPTL3,GSN,CALML5,IGFBP3,IGF1,PROS1,GAPDH,RAPGEF4,ROCK1,ADIPOQ,IGF2,C4A,MST1,ITIH3,C4B,TCAF1,APOA5,TTN,VEGFC,PLCG2,KNG1,ACTB
GO Process	GO:0002250	Adaptive immune response	20	359	0.65	7.90e-06	C5,TCIRG1,PSG9,SERPING1,FGB,C1QB,C7,C8A,C8B,C1QC,RAPGEF4,MASP2,C1S,C4A,C4B,C1R,IGKV2D-28,IGHV3-72,IGLV10-54,FGA
GO Process	GO:0098869	Cellular oxidant detoxification	11	91	0.99	7.90e-06	MPO,CAT,APOE,PXDN,AMBP,HBA1,APOA4,APOM,SOD3,GPX3,HBB
GO Process	GO:0050790	Regulation of catalytic activity	60	2370	0.31	9.61e-06	SPP2,SERPIND1,TFPI2,PCOLCE,C5,VTN,GPLD1,PLEK,APOA1,LRP1,APOE,THBS1,SERPINA10,TIMP2,CSTA,AMBP,CENPE,ITIH4,SERPING1,CSF1R,A2ML1,ANTXR1,F2,SUZ12,STXBP5,A2M,FBN1,SERPINA7,FBLN1,SERPINA11,HSP90AA1,ANG,LIMS1,PAPLN,FN1,BLM,APOA4,ITIH2,S100A8,ECM1,ANGPTL3,GSN,CALML5,IGFBP3,IGF1,PROS1,GAPDH,RAPGEF4,ROCK1,ADIPOQ,IGF2,C4A,MST1,ITIH3,C4B,APOA5,TTN,VEGFC,KNG1,ACTB
GO Process	GO:0007597	Blood coagulation, intrinsic pathway	6	13	1.57	1.17e-05	F9,F12,KLKB1,FLNA,F7,F11
GO Process	GO:0016485	Protein processing	15	202	0.78	1.21e-05	F9,F12,KLKB1,PLA2G7,PCSK9,FGB,F2,FGG,F5,F10,F7,F11,HPR,C1R,FGA
GO Process	GO:0030334	Regulation of cell migration	33	927	0.46	1.27e-05	MMP2,C5,VTN,GPLD1,SPARC,IGFBP5,LRP1,APOE,THBS1,PLA2G7,FERMT3,CSF1R,HSPA5,FBLN1,THBS4,APOD,FN1,S100A7,ECM1,FLNA,ANGPTL3,F10,F7,IGFBP3,CD99,IGF1,ROCK1,ADIPOQ,MST1,TCAF1,VEGFC,SEMA3B,PLCG2
GO Process	GO:0042744	Hydrogen peroxide catabolic process	7	25	1.35	1.49e-05	MPO,CAT,PXDN,HBA1,APOA4,GPX3,HBB
GO Process	GO:0040012	Regulation of locomotion	35	1032	0.44	1.51e-05	ST6GAL1,MMP2,C5,VTN,GPLD1,SPARC,IGFBP5,LRP1,APOE,THBS1,PLA2G7,FERMT3,CSF1R,HSPA5,FBLN1,THBS4,APOD,FN1,S100A7,ECM1,FLNA,ANGPTL3,F10,F7,IGFBP3,CD99,IGF1,ROCK1,TF,ADIPOQ,MST1,TCAF1,VEGFC,SEMA3B,PLCG2
GO Process	GO:2000145	Regulation of cell motility	34	988	0.44	1.67e-05	MMP2,C5,VTN,GPLD1,SPARC,IGFBP5,LRP1,APOE,THBS1,PLA2G7,FERMT3,CSF1R,HSPA5,FBLN1,THBS4,APOD,FN1,S100A7,ECM1,FLNA,ANGPTL3,F10,F7,IGFBP3,CD99,IGF1,ROCK1,TF,ADIPOQ,MST1,TCAF1,VEGFC,SEMA3B,PLCG2
GO Process	GO:0007229	Integrin-mediated signaling pathway	11	100	0.95	1.75e-05	MYH9,PLEK,APOA1,ITGA2B,FERMT3,DST,TLN1,FN1,ADAM12,ANGPTL3,ILK
GO Process	GO:0002443	Leukocyte mediated immunity	15	211	0.76	1.94e-05	C5,TCIRG1,SERPING1,F2,C1QB,C7,C8A,C8B,C1QC,MASP2,C1S,C4A,C4B,C1R,IGHV3-72
GO Process	GO:0001568	Blood vessel development	23	505	0.57	2.26e-05	MYH9,MMP2,GPLD1,LRP1,APOE,PXDN,ANGPTL6,THBS1,ITGA2B,NOTCH3,ANTXR1,CSPG4,ANG,APOD,FN1,COL18A1,S100A7,ECM1,FLNA,ANGPTL3,YWHAZ,TGFBI,VEGFC
GO Process	GO:0010811	Positive regulation of cell-substrate adhesion	12	128	0.88	2.26e-05	VTN,APOA1,FGB,P4HB,FBLN1,FGG,LIMS1,FN1,FLNA,ILK,ROCK1,FGA
GO Process	GO:0034375	High-density lipoprotein particle remodeling	6	16	1.48	2.85e-05	CETP,APOA1,APOE,LCAT,APOA4,APOM
GO Process	GO:0051873	Killing by host of symbiont cells	7	29	1.29	3.25e-05	CFHR5,PF4,F2,CFHR1,APOL1,CFHR2,GAPDH
GO Process	GO:1900026	Positive regulation of substrate adhesion-dependent cell spreading	8	45	1.16	3.43e-05	APOA1,FGB,P4HB,FGG,LIMS1,FLNA,ILK,FGA
GO Process	GO:0043691	Reverse cholesterol transport	6	18	1.43	4.81e-05	CETP,APOA1,APOE,LCAT,APOA4,APOM
GO Process	GO:0042632	Cholesterol homeostasis	10	89	0.96	5.08e-05	CETP,APOA1,APOE,LCAT,PCSK9,APOA4,ANGPTL3,APOM,TSKU,APOA5
GO Process	GO:0007565	Female pregnancy	13	170	0.79	5.61e-05	MMP2,IGFBP5,AMBP,PSG9,CRH,PSG6,PSG1,PSG11,PAPPA,FBLN1,PSG3,PSG2,MST1
GO Process	GO:0042742	Defense response to bacterium	17	306	0.65	6.92e-05	MPO,LYZ,FCN2,PGLYRP2,PPBP,LALBA,FGB,F2,PRG2,STAB1,ANG,S100A7,S100A8,TF,RNASE4,IGHV3-72,FGA
GO Process	GO:0080134	Regulation of response to stress	40	1373	0.37	7.04e-05	CPB2,VTN,PROC,APOA1,APOE,F12,THBS1,PSMA6,KLKB1,AMBP,HPX,FCN3,PLA2G7,SERPING1,FCN2,PGLYRP2,MASP1,FGB,F2,A2M,HSPA5,P4HB,HSP90AA1,FGG,CFH,S100A8,HTRA1,FLNA,F7,IGF1,PROS1,MASP2,F11,ADIPOQ,UIMC1,PLCG2,KNG1,ACTB,ENO1,FGA
GO Process	GO:0008203	Cholesterol metabolic process	11	119	0.87	7.62e-05	CETP,APOA1,CAT,APOE,LCAT,PCSK9,APOL1,APOA4,ANGPTL3,TSKU,APOA5
GO Process	GO:0019730	Antimicrobial humoral response	12	149	0.81	9.12e-05	PF4V1,LYZ,PPBP,PF4,FGB,F2,ANG,S100A7,GAPDH,TF,KNG1,FGA
GO Process	GO:2000147	Positive regulation of cell motility	23	555	0.52	9.59e-05	MMP2,VTN,GPLD1,SPARC,IGFBP5,THBS1,PLA2G7,FERMT3,CSF1R,HSPA5,FBLN1,THBS4,FN1,S100A7,ANGPTL3,F10,F7,CD99,IGF1,TF,VEGFC,SEMA3B,PLCG2
GO Process	GO:0019538	Protein metabolic process	81	3910	0.22	9.98e-05	ST6GAL1,CPB2,GNPTG,MYH9,F9,MMP2,PCOLCE,GPLD1,PROC,APOA1,CAT,APOE,F12,THBS1,PSMA6,LCAT,CSTA,KLKB1,F13A1,AMBP,TCIRG1,HPX,FCN3,PLA2G7,HABP2,CSF1R,FCN2,MASP1,APEH,PCSK9,FGB,F2,UBE3C,SUZ12,APOL1,SUMO4,PKM,HSPA5,P4HB,PAPPA,FBLN1,HSP90AA1,FGG,GANAB,FN1,APOA4,F13B,CFH,PAPPA2,F5,ADAM12,S100A8,HTRA1,FLNA,GSN,F10,F7,APOM,IGFBP3,IGF1,EFEMP1,YWHAZ,ILK,GAPDH,ROCK1,MASP2,F11,C1S,PCYOX1,KSR2,CDK3,MST1,CFB,UIMC1,HPR,C1R,APOA5,TTN,CFD,ACTB,FGA

GO Process	GO:0050766	Positive regulation of phagocytosis	9	75	0.99	0.00011	APOA1,FCN3,FCN2,MASP1,MASP2,C4A,C4B,CFB,PLCG2
GO Process	GO:0031639	Plasminogen activation	5	11	1.56	0.00013	KLKB1,FGB,FGG,F11,FGA
GO Process	GO:0070328	Triglyceride homeostasis	7	38	1.17	0.00014	CETP,APOA1,APOE,APOA4,ANGPTL3,APOA5,APOC4
GO Process	GO:0009617	Response to bacterium	25	663	0.48	0.00015	MPO,PF4V1,LYZ,FCN2,PGLYRP2,PPBP,PF4,LALBA,LRG1,FGB,F2,PRG2,STAB1,ANG,S100A7,S100A8,TF,ADIPOQ,C4B,CFB,RNASE4,CFD,IGHV3-72,PLCG2,FGA
GO Process	GO:0030335	Positive regulation of cell migration	22	529	0.53	0.00015	MMP2,VTN,GPLD1,SPARC,IGFBP5,THBS1,PLA2G7,FERMT3,CSF1R,HSPA5,FBLN1,THBS4,FN1,S100A7,ANGPTL3,F10,F7,CD99,IGF1,VEGFC,SEMA3B,PLCG2
GO Process	GO:0006979	Response to oxidative stress	18	368	0.6	0.00016	MMP2,MPO,CAT,APOE,PXDN,ATRN,AMBP,NDUFS8,SUMO4,HBA1,APOD,APOA4,S100A7,SOD3,GPX3,ADIPOQ,SELENOP,HBB
GO Process	GO:0010035	Response to inorganic substance	22	532	0.52	0.00016	MMP2,MPO,HSD17B1,GPLD1,CAT,THBS1,LCAT,TCIRG1,FGB,HBA1,HSPA5,FGG,BLM,APOA4,S100A8,SOD3,TF,SELENOP,TTN,PLCG2,HBB,FGA
GO Process	GO:0034380	High-density lipoprotein particle assembly	5	12	1.53	0.00016	APOA1,APOE,APOA4,APOM,APOA5
GO Process	GO:0050764	Regulation of phagocytosis	10	104	0.89	0.00016	APOA1,FCN3,FCN2,MASP1,MASP2,ADIPOQ,C4A,C4B,CFB,PLCG2
GO Process	GO:0051838	Cytolysis by host of symbiont cells	5	12	1.53	0.00016	CFHR5,F2,CFHR1,APOL1,CFHR2
GO Process	GO:0035239	Tube morphogenesis	25	669	0.48	0.00017	MYH9,MMP2,GPLD1,LRP1,APOE,PXDN,ANGPTL6,THBS1,ITGA2B,NOTCH3,CSF1R,CSPG4,ANG,APOD,FN1,COL18A1,S100A7,ECM1,FLNA,ANGPTL3,YWHAZ,ILK,MST1,TGFBI,VEGFC
GO Process	GO:0032103	Positive regulation of response to external stimulus	20	453	0.55	0.00018	CPB2,VTN,THBS1,HPX,FCN3,PLA2G7,CSF1R,FCN2,MASP1,F2,FBN1,HSP90AA1,THBS4,S100A7,S100A8,FLNA,F7,MASP2,VEGFC,PLCG2
GO Process	GO:0031589	Cell-substrate adhesion	13	195	0.73	0.00020	VTN,VWF,ITGA2B,ATRN,FERMT3,ANTXR1,FGB,FGG,FN1,ANGPTL3,ACTN1,ILK,FGA
GO Process	GO:0051702	Biological process involved in interaction with symbiont	10	108	0.87	0.00020	APOE,CFHR5,CSF1R,PF4,F2,CFHR1,APOL1,FN1,CFHR2,GAPDH
GO Process	GO:0035295	Tube development	29	880	0.42	0.00025	MYH9,MMP2,GPLD1,IGFBP5,CAT,LRP1,APOE,PXDN,ANGPTL6,THBS1,ITGA2B,NOTCH3,CRH,CSF1R,CSPG4,ANG,APOD,FN1,COL18A1,S100A7,ECM1,FLNA,ANGPTL3,RBP4,YWHAZ,ILK,MST1,TGFBI,VEGFC
GO Process	GO:0060627	Regulation of vesicle-mediated transport	22	551	0.51	0.00025	VTN,APOA1,LRP1,APOE,FCN3,FCN2,MASP1,PCSK9,FGB,STXBP5,FGG,RAPGEF4,ROCK1,MASP2,TF,ADIPOQ,C4A,C4B,CFB,APOA5,PLCG2,FGA
GO Process	GO:0033344	Cholesterol efflux	6	27	1.25	0.00027	APOA1,APOE,APOA4,APOM,TSKU,APOA5
GO Process	GO:0072377	Blood coagulation, common pathway	4	5	1.81	0.00027	F2,F5,F10,FGA
GO Process	GO:0032374	Regulation of cholesterol transport	8	64	1.0	0.00028	CETP,APOA1,LRP1,APOE,PCSK9,APOA4,ADIPOQ,APOA5
GO Process	GO:0022604	Regulation of cell morphogenesis	16	311	0.62	0.00030	ST6GAL1,MYH9,SPARC,APOA1,CSF1R,SH3D19,FGB,F2,P4HB,FBLN1,FGG,LIMS1,FN1,FLNA,ILK,FGA
GO Process	GO:0030301	Cholesterol transport	8	65	1.0	0.00030	CETP,APOA1,APOE,LCAT,APOA4,APOM,TSKU,APOA5
GO Process	GO:0030194	Positive regulation of blood coagulation	6	28	1.24	0.00032	CPB2,VTN,F12,THBS1,F2,F7
GO Process	GO:1902533	Positive regulation of intracellular signal transduction	31	997	0.4	0.00033	IGFBP5,APOA1,CAT,APOE,THBS1,VWF,IGFBP4,CSF1R,GUCY1A1,FGB,F2,CSPG4,HSP90AA1,FGG,LIMS1,FN1,S100A7,S100A8,ECM1,FLNA,F10,F7,IGFBP3,IGF1,ILK,GAPDH,ROCK1,ADIPOQ,IGF2,PLCG2,FGA
GO Process	GO:0072359	Circulatory system development	29	901	0.41	0.00037	MYH9,MMP2,GPLD1,LRP1,APOE,PXDN,ANGPTL6,THBS1,ITGA2B,NOTCH3,DNAH5,ANTXR1,CSPG4,FBN1,ANG,APOD,FN1,COL18A1,S100A7,ECM1,FLNA,ANGPTL3,RBP4,YWHAZ,ILK,ROCK1,TGFBI,TTN,VEGFC
GO Process	GO:1901700	Response to oxygen-containing compound	41	1547	0.33	0.00038	ST6GAL1,CPB2,MMP2,MPO,PF4V1,GPLD1,IGFBP5,CAT,LRP1,APOE,THBS1,AMBP,PPBP,PF4,PCSK9,PKM,HBA1,A2M,HSPA5,FBN1,PAPPA,HSP90AA1,APOD,BLM,APOA4,F5,S100A7,S100A8,FLNA,RBP4,F7,SOD3,GPX3,IGF1,ROCK1,ADIPOQ,IGF2,ATP1A1,PLCG2,HBB,ACTB
GO Process	GO:1901564	Organonitrogen compound metabolic process	94	4981	0.18	0.00040	ST6GAL1,CPB2,CETP,GNPTG,MYH9,F9,MMP2,PCOLCE,GPLD1,PROC,APOA1,TTR,CAT,APOE,PXDN,F12,THBS1,PSMA6,BLVRB,LCAT,CSTA,KLKB1,F13A1,AMBP,TCIRG1,HPX,ITIH4,FCN3,PLA2G7,HABP2,CSF1R,FCN2,PGLYRP2,MASP1,APEH,GUCY1A1,PCSK9,FGB,F2,UBE3C,NDUFS8,SUZ12,APOL1,SUMO4,PKM,HSPA5,P4HB,PAPPA,FBLN1,HSP90AA1,FGG,GANAB,FN1,APOA4,ITIH2,F13B,CFH,PAPPA2,F5,ADAM12,S100A8,HTRA1,FLNA,GSN,F10,F7,APOM,IGFBP3,IGF1,EFEMP1,YWHAZ,ILK,GAPDH,ROCK1,MASP2,F11,C1S,PCYOX1,KSR2,ACSF2,CKMT1A,CDK3,MST1,ITIH3,CFB,UIMC1,HPR,C1R,APOA5,TTN,CFD,ACTB,ENO1,FGA
GO Process	GO:0042221	Response to chemical	80	4010	0.21	0.00041	ST6GAL1,CPB2,SERPIND1,MMP2,C5,MPO,HSD17B1,PF4V1,GPLD1,IGFBP5,APOA1,CAT,LRP1,APOE,PXDN,F12,THBS1,TIMP2,NOTCH3,LCAT,AMBP,TCIRG1,HPX,ITIH4,PSG9,CSF1R,PPBP,PF4,PCSK9,FGB,SUMO4,PKM,HBA1,A2M,HSPA5,FBN1,P4HB,PAPPA,HSP90AA1,ANG,TUBA1B,FGG,LIMS1,THBS4,APOD,BLM,APOA4,CENPF,F5,S100A7,S100A8,FLNA,ANGPTL3,RBP4,GSN,F7,APOM,CA6,SOD3,GPX3,IGF1,ILK,GAPDH,ROCK1,TF,ADIPOQ,IGF2,MST1,C4B,SELENOP,ATP1A1,TTN,VEGFC,SEMA3B,PLCG2,OR52B4,HSPH1,HBB,ACTB,FGA
GO Process	GO:0048585	Negative regulation of response to stimulus	42	1612	0.32	0.00043	ST6GAL1,CPB2,C5,VTN,IGFBP5,PROC,PLEK,APOA1,LRP1,APOE,PXDN,F12,THBS1,KLKB1,AMBP,IGFBP4,SERPING1,PGLYRP2,PF4,MASP1,FGB,F2,A2M,HSPA5,FBN1,FBLN1,FGG,APOD,HTRA1,ECM1,IGFBP3,IGF1,PROS1,F11,LTBP1,ADIPOQ,CDK3,TSKU,SEMA3B,KNG1,ENO1,FGA
GO Process	GO:0022603	Regulation of anatomical structure morphogenesis	29	920	0.41	0.00052	ST6GAL1,MYH9,C5,SPARC,APOA1,THBS1,CSF1R,PF4,LRG1,SH3D19,FGB,F2,STAB1,PKM,P4HB,FBLN1,FGG,LIMS1,THBS4,FN1,ADAM12,ECM1,FLNA,ANGPTL3,ILK,ROCK1,VEGFC,SEMA3B,FGA
GO Process	GO:0008202	Steroid metabolic process	14	258	0.64	0.00066	CETP,HSD17B1,APOA1,CAT,APOE,LCAT,CRH,PCSK9,APOL1,APOA4,ANGPTL3,GC,TSKU,APOA5
GO Process	GO:0050793	Regulation of developmental process	56	2492	0.26	0.00071	ST6GAL1,CETP,MYH9,C5,MGP,SPARC,IGFBP5,PROC,APOA1,APOE,THBS1,ATRN,NOTCH3,TCIRG1,PSG9,CSF1R,PGLYRP2,SCGB3A1,PF4,LRG1,SH3D19,FGB,F2,STAB1,SUZ12,PKM,FBN1,P4HB,FBLN1,FGG,LIMS1,THBS4,FN1,CENPF,ADAM12,ECM1,FLNA,ANGPTL3,RBP4,C1QC,IGFBP3,IGF1,EFEMP1,YWHAZ,ILK,ROCK1,ADIPOQ,IGF2,MST1,LMOD3,PAEP,TSKU,VEGFC,SEMA3B,ACTB,FGA
GO Process	GO:0060284	Regulation of cell development	20	506	0.5	0.00074	PROC,APOA1,FGB,F2,FBN1,P4HB,FBLN1,FGG,LIMS1,FN1,FLNA,IGF1,ILK,ROCK1,ADIPOQ,LMOD3,PAEP,VEGFC,SEMA3B,FGA
GO Process	GO:0055088	Lipid homeostasis	11	161	0.74	0.00084	CETP,APOA1,APOE,LCAT,PCSK9,APOA4,ANGPTL3,APOM,TSKU,APOA5,APOC4
GO Process	GO:0009892	Negative regulation of metabolic process	63	2982	0.23	0.0011	SPP2,CPB2,SERPIND1,TFPI2,C5,VTN,GPLD1,IGFBP5,PLEK,APOA1,LRP1,APOE,THBS1,SERPINA10,TIMP2,NOTCH3,CSTA,AMBP,ITIH4,PSG9,FCN3,SERPING1,PGLYRP2,PF4,A2ML1,PCSK9,F2,PRG2,SUZ12,SUMO4,A2M,SERPINA7,FBLN1,SERPINA11,ANG,LIMS1,APOD,PAPLN,FN1,BLM,ITIH2,CENPF,ECM1,FLNA,IGFBP3,IGF1,PROS1,TNRC6A,YWHAZ,GAPDH,ROCK1,ADIPOQ,IGF2,C4A,MST1,ITIH3,C4B,UIMC1,TSKU,ATP1A1,KNG1,ENO1,FGA
GO Process	GO:0051240	Positive regulation of multicellular organismal process	39	1505	0.32	0.0011	CPB2,C5,VTN,GPLD1,LRP1,APOE,F12,THBS1,CRH,CSF1R,PF4,LRG1,FGB,F2,PRG2,PKM,HSP90AA1,FGG,FN1,ADAM12,ECM1,ANGPTL3,F7,IGF1,GAPDH,ROCK1,KSR2,ADIPOQ,IGF2,MST1,PAEP,TSKU,APOA5,ATP1A1,VEGFC,PLCG2,ACTB,ENO1,FGA
GO Process	GO:1905952	Regulation of lipid localization	11	167	0.73	0.0011	CETP,APOA1,LRP1,APOE,THBS1,CRH,PCSK9,APOA4,ADIPOQ,APOA5,APOC4
GO Process	GO:0000302	Response to reactive oxygen species	11	169	0.72	0.0012	MMP2,MPO,CAT,APOE,AMBP,HBA1,APOD,APOA4,S100A7,SOD3,HBB
GO Process	GO:0007160	Cell-matrix adhesion	10	136	0.77	0.0012	VTN,ITGA2B,FERMT3,FGB,FGG,FN1,ANGPTL3,ACTN1,ILK,FGA

GO Process	GO:0009653	Anatomical structure morphogenesis	51	2229	0.27	0.0012	MYH9,MMP2,VTN,MGP,GPLD1,IGFBP5,LRP1,APOE,PXDN,ANGPTL6,THBS1,ITGA2B,ATRN,NOTCH3,TCIRG1,CDH11,FERMT3,CSF1R,ANTXR1,LRG1,CSPG4,HSPA5,FBN1,HSP90AA1,ANG,APOD,FN1,MFAP5,COL18A1,PAPPA2,ADAM12,S100A7,HTRA1,ECM1,FLNA,ANGPTL3,RBP4,ACTN1,EFEMP1,YWHAZ,ILK,ROCK1,IGF2,MST1,LMOD3,TGFBI,TSKU,TTN,VEGFC,SEMA3B,ACTB
GO Process	GO:0002752	Cell surface pattern recognition receptor signaling pathway	4	9	1.55	0.0013	FCN3,FCN2,MASP1,MASP2
GO Process	GO:0032368	Regulation of lipid transport	10	139	0.76	0.0013	CETP,APOA1,LRP1,APOE,THBS1,CRH,PCSK9,APOA4,ADIPOQ,APOA5
GO Process	GO:0008015	Blood circulation	17	403	0.53	0.0015	MMP2,APOE,SERPING1,GUCY1A1,FGB,FGG,F5,GSN,SOD3,ROCK1,ADIPOQ,ATP1A1,TTN,VEGFC,KNG1,HBB,FGA
GO Process	GO:0010605	Negative regulation of macromolecule metabolic process	59	2760	0.24	0.0015	SPP2,CPB2,SERPIND1,TFPI2,C5,VTN,IGFBP5,APOA1,LRP1,APOE,THBS1,SERPINA10,TIMP2,NOTCH3,CSTA,AMBP,ITIH4,PSG9,FCN3,SERPING1,PGLYRP2,PF4,A2ML1,PCSK9,F2,PRG2,SUZ12,SUMO4,A2M,SERPINA7,FBLN1,SERPINA11,ANG,LIMS1,APOD,PAPLN,FN1,BLM,ITIH2,CENPF,ECM1,FLNA,IGFBP3,IGF1,PROS1,TNRC6A,YWHAZ,GAPDH,ROCK1,ADIPOQ,IGF2,C4A,ITIH3,C4B,UIMC1,TSKU,KNG1,ENO1,FGA
GO Process	GO:0048522	Positive regulation of cellular process	100	5584	0.16	0.0015	ST6GAL1,CPB2,MMP2,VTN,GPLD1,SPARC,IGFBP5,PROC,PLEK,APOA1,CAT,LRP1,APOE,THBS1,VWF,NOTCH3,CENPE,TCIRG1,HPX,IGFBP4,FCN3,PLA2G7,CRH,FERMT3,CSF1R,FCN2,SCGB3A1,PPBP,PF4,FYCO1,MASP1,GUCY1A1,LRG1,SH3D19,PCSK9,FGB,F2,INHBC,CSPG4,SUZ12,PKM,STXBP5,HBA1,HSPA5,FBN1,P4HB,FBLN1,HSP90AA1,ANG,FGG,LIMS1,THBS4,FN1,BLM,APOA4,S100A7,S100A8,HTRA1,ADAMTSL4,ECM1,FLNA,SMARCA1,ANGPTL3,RBP4,GSN,F10,F7,IGFBP3,CD99,IGF1,ACTN1,TNRC6A,ILK,GAPDH,ROCK1,MASP2,TF,ADIPOQ,IGF2,C4A,SLAIN1,MST1,LMOD3,C4B,CFB,TCAF1,UIMC1,HPR,APOA5,TTN,IGHV3-72,VEGFC,SEMA3B,PLCG2,HSPH1,KNG1,HBB,ACTB,ENO1,FGA
GO Process	GO:0048660	Regulation of smooth muscle cell proliferation	10	142	0.75	0.0016	MMP2,IGFBP5,APOE,THBS1,NOTCH3,ANG,APOD,IGFBP3,IGF1,ADIPOQ
GO Process	GO:0001867	Complement activation, lectin pathway	4	10	1.51	0.0017	FCN3,FCN2,MASP1,MASP2
GO Process	GO:0006066	Alcohol metabolic process	15	324	0.57	0.0017	CETP,APOA1,TTR,CAT,APOE,LCAT,PCSK9,APOL1,APOA4,ANGPTL3,RBP4,IGF1,TSKU,APOA5,PLCG2
GO Process	GO:0019835	Cytolysis	5	23	1.24	0.0017	C5,LYZ,C7,C8A,C8B
GO Process	GO:0030155	Regulation of cell adhesion	25	784	0.41	0.0018	ST6GAL1,MMP2,VTN,APOA1,THBS1,FERMT3,FGB,TLN1,P4HB,FBLN1,FGG,LIMS1,APOD,FN1,FLNA,IGF1,ILK,ROCK1,ADIPOQ,IGF2,TGFBI,HSPH1,KNG1,ACTB,FGA
GO Process	GO:0031347	Regulation of defense response	22	638	0.44	0.0018	PROC,APOA1,APOE,F12,PSMA6,KLKB1,HPX,FCN3,PLA2G7,SERPING1,FCN2,PGLYRP2,MASP1,A2M,HSP90AA1,CFH,S100A8,HTRA1,IGF1,MASP2,ADIPOQ,PLCG2
GO Process	GO:0048646	Anatomical structure formation involved in morphogenesis	28	938	0.38	0.0018	MYH9,MMP2,VTN,GPLD1,PXDN,ANGPTL6,THBS1,ITGA2B,NOTCH3,TCIRG1,CSPG4,ANG,APOD,FN1,COL18A1,ADAM12,S100A7,HTRA1,ECM1,FLNA,ANGPTL3,RBP4,ACTN1,YWHAZ,LMOD3,TGFBI,TTN,VEGFC
GO Process	GO:0006953	Acute-phase response	6	42	1.06	0.0020	ITIH4,SAA4,F2,A2M,FN1,SAA2
GO Process	GO:0034114	Regulation of heterotypic cell-cell adhesion	5	24	1.23	0.0020	APOA1,FGB,FGG,ADIPOQ,FGA
GO Process	GO:0048870	Cell motility	30	1061	0.36	0.0023	MYH9,MMP2,C5,VTN,PF4V1,GPLD1,APOA1,THBS1,ATRN,DNAH5,PPBP,PF4,LRG1,DST,CSPG4,DNAH8,HSP90AA1,ANG,THBS4,FN1,S100A8,FLNA,CD99,ILK,ROCK1,PSG2,MST1,VEGFC,SEMA3B,ACTB
GO Process	GO:0051043	Regulation of membrane protein ectodomain proteolysis	5	25	1.21	0.0023	GPLD1,APOE,TIMP2,SH3D19,ROCK1
GO Process	GO:0010033	Response to organic substance	57	2692	0.23	0.0027	ST6GAL1,CPB2,MMP2,MPO,PF4V1,GPLD1,IGFBP5,CAT,LRP1,F12,THBS1,TIMP2,LCAT,TCIRG1,HPX,ITIH4,PSG9,CSF1R,PPBP,PF4,PCSK9,FGB,PKM,A2M,HSPA5,FBN1,P4HB,PAPPA,HSP90AA1,ANG,TUBA1B,FGG,LIMS1,THBS4,BLM,APOA4,F5,S100A7,S100A8,FLNA,ANGPTL3,RBP4,GSN,F7,GPX3,IGF1,ILK,GAPDH,ROCK1,ADIPOQ,IGF2,C4B,ATP1A1,VEGFC,PLCG2,HSPH1,ACTB
GO Process	GO:0002697	Regulation of immune effector process	16	383	0.53	0.0028	APOA1,HPX,FCN3,SERPING1,FCN2,PGLYRP2,MASP1,PRG2,A2M,CFH,RBP4,MASP2,C4A,C4B,CFB,PLCG2
GO Process	GO:0033700	Phospholipid efflux	4	12	1.43	0.0028	APOA1,APOE,APOA4,APOA5
GO Process	GO:0042157	Lipoprotein metabolic process	9	123	0.77	0.0028	GPLD1,APOA1,APOE,LCAT,PCSK9,APOL1,APOA4,APOM,APOA5
GO Process	GO:0051049	Regulation of transport	42	1763	0.28	0.0029	CPB2,CETP,VTN,GPLD1,APOA1,LRP1,APOE,THBS1,TCIRG1,FCN3,CRH,FCN2,MASP1,PCSK9,FGB,F2,STXBP5,HSP90AA1,ANG,FGG,APOD,APOA4,S100A8,FLNA,RBP4,IGF1,RAPGEF4,ROCK1,MASP2,TF,ADIPOQ,C4A,C4B,CFB,TCAF1,APOA5,ATP1A1,TTN,VEGFC,PLCG2,ACTB,FGA
GO Process	GO:0010954	Positive regulation of protein processing	5	27	1.17	0.0031	MYH9,F12,KLKB1,GSN,ENO1
GO Process	GO:0070613	Regulation of protein processing	7	69	0.91	0.0031	CPB2,MYH9,F12,THBS1,KLKB1,GSN,ENO1
GO Process	GO:0043568	Positive regulation of insulin-like growth factor receptor signaling pathway	4	13	1.4	0.0035	IGFBP5,IGFBP4,IGFBP3,IGF1
GO Process	GO:0051918	Negative regulation of fibrinolysis	4	13	1.4	0.0035	CPB2,VTN,THBS1,F2
GO Process	GO:0002685	Regulation of leukocyte migration	12	231	0.62	0.0036	C5,THBS1,PLA2G7,CSF1R,THBS4,APOD,S100A7,ECM1,F7,CD99,MST1,VEGFC
GO Process	GO:0046470	Phosphatidylcholine metabolic process	7	71	0.9	0.0036	CETP,GPLD1,APOA1,LCAT,PLA2G7,APOA4,APOA5
GO Process	GO:0002688	Regulation of leukocyte chemotaxis	9	128	0.75	0.0037	C5,THBS1,PLA2G7,CSF1R,THBS4,S100A7,F7,MST1,VEGFC
GO Process	GO:0051093	Negative regulation of developmental process	27	933	0.37	0.0038	CETP,SPARC,IGFBP5,APOE,THBS1,NOTCH3,PGLYRP2,PF4,F2,STAB1,SUZ12,FBN1,FBLN1,THBS4,ECM1,RBP4,C1QC,IGF1,EFEMP1,ROCK1,ADIPOQ,IGF2,PAEP,TSKU,VEGFC,SEMA3B,ACTB
GO Process	GO:1903036	Positive regulation of response to wounding	7	72	0.89	0.0038	CPB2,VTN,F12,THBS1,F2,FLNA,F7
GO Process	GO:0007166	Cell surface receptor signaling pathway	46	2040	0.26	0.0041	MYH9,MMP2,C5,PF4V1,GPLD1,IGFBP5,PLEK,APOA1,LRP1,ITGA2B,NOTCH3,HPX,PSG9,FCN3,FERMT3,CSF1R,FCN2,PPBP,PF4,MASP1,DST,F2,INHBC,CSPG4,TLN1,P4HB,PAPPA,LIMS1,FN1,PAPPA2,ADAM12,FLNA,ANGPTL3,ADGRG2,IGF1,EFEMP1,ILK,MASP2,TF,LTBP1,IGF2,CFD,IGHV3-72,VEGFC,SEMA3B,PLCG2
GO Process	GO:0010896	Regulation of triglyceride catabolic process	4	14	1.36	0.0042	GPLD1,APOA1,APOA4,APOA5
GO Process	GO:0050714	Positive regulation of protein secretion	9	131	0.74	0.0042	GPLD1,FGB,ANG,FGG,RBP4,IGF1,TTN,VEGFC,FGA
GO Process	GO:0010873	Positive regulation of cholesterol esterification	3	4	1.78	0.0044	APOA1,APOA4,APOA5
GO Process	GO:0051919	Positive regulation of fibrinolysis	3	4	1.78	0.0044	F12,KLKB1,F11
GO Process	GO:0003013	Circulatory system process	18	493	0.47	0.0045	MMP2,LRP1,APOE,SERPING1,GUCY1A1,FGB,FGG,F5,GSN,SOD3,ROCK1,ADIPOQ,ATP1A1,TTN,VEGFC,KNG1,HBB,FGA
GO Process	GO:0072593	Reactive oxygen species metabolic process	8	102	0.8	0.0045	MPO,CAT,PXDN,HBA1,APOA4,SOD3,GPX3,HBB
GO Process	GO:0051235	Maintenance of location	10	166	0.69	0.0046	APOA1,APOE,HSPA5,FBN1,S100A7,S100A8,FLNA,ANGPTL3,GSN,LTBP1
GO Process	GO:0051716	Cellular response to stimulus	108	6357	0.14	0.0047	CPB2,MYH9,MMP2,TFPI2,C5,MPO,HSD17B1,PF4V1,GPLD1,IGFBP5,PLEK,APOA1,TTR,CAT,LRP1,APOE,PXDN,THBS1,ITGA2B,NOTCH3,AMBP,TCIRG1,HPX,IGFBP4,PSG9,FCN3,CRH,FERMT3,DSC2,CSF1R,FCN2,PSG6,SCGB3A1,PPBP,PF4,MASP1,GUCY1A1,LALBA,PCSK9,FGB,DST,F2,INHBC,PSG1,CSPG4,TLN1,SUMO4,PKM,HBA1,HSPA5,FBN1,P4HB,PAPPA,PSG3,HSP90AA1,TUBA1B,FGG,LIMS1,THBS4,APOD,FN1,BLM,APOA4,CENPF,PAPPA2,ADAM12,S100A8,ECM1,FLNA,ANGPTL3,CCN5,GSN,APOM,ADGRG2,CALML5,IGFBP3,SOD3,GPX3,IGF1,EFEMP1,TNRC6A,YWHAZ,ILK,GAPDH,RAPGEF4,ROCK1,MASP2,TF,DCDC1,LTBP1,KSR2,ADIPOQ,IGF2,CDK3,MST1,IGFALS,UIMC1,ATP1A1,TTN,CFD,IGHV3-72,VEGFC,SEMA3B,PLCG2,OR52B4,HBB,ACTB,PDIA4

GO Process	GO:0010038	Response to metal ion	15	362	0.52	0.0048	HSD17B1,GPLD1,CAT,THBS1,LCAT,TCIRG1,FGB,HSPA5,FGG,S100A8,SOD3,TF,TTN,PLCG2,FGA
GO Process	GO:0002220	Innate immune response activating cell surface receptor signaling pathway	5	32	1.1	0.0058	FCN3,FCN2,MASP1,MASP2,PLCG2
GO Process	GO:0048662	Negative regulation of smooth muscle cell proliferation	6	53	0.96	0.0058	IGFBP5,APOE,ANG,APOD,IGFBP3,ADIPOQ
GO Process	GO:0001936	Regulation of endothelial cell proliferation	9	138	0.72	0.0059	SPARC,APOE,THBS1,LRG1,ANG,THBS4,ECM1,IGF2,VEGFC
GO Process	GO:0034374	Low-density lipoprotein particle remodeling	4	16	1.3	0.0063	CETP,MPO,APOE,PLA2G7
GO Process	GO:0045765	Regulation of angiogenesis	13	288	0.56	0.0063	C5,SPARC,THBS1,PF4,LRG1,STAB1,PKM,THBS4,ADAM12,ECM1,ANGPTL3,ROCK1,VEGFC
GO Process	GO:0051172	Negative regulation of nitrogen compound metabolic process	51	2403	0.23	0.0064	SPP2,CPB2,SERPIND1,TFPI2,C5,VTN,IGFBP5,LRP1,APOE,THBS1,SERPINA10,TIMP2,NOTCH3,CSTA,AMBP,ITIH4,FCN3,SERPING1,A2ML1,F2,SUZ12,SUMO4,A2M,SERPINA7,FBLN1,SERPINA11,ANG,LIMS1,APOD,PAPLN,BLM,ITIH2,CENPF,ECM1,FLNA,IGFBP3,IGF1,PROS1,TNRC6A,YWHAZ,GAPDH,ROCK1,ADIPOQ,IGF2,C4A,ITIH3,C4B,UIMC1,KNG1,ENO1,FGA
GO Process	GO:0097746	Blood vessel diameter maintenance	9	140	0.72	0.0064	MMP2,APOE,GUCY1A1,FGB,FGG,SOD3,ROCK1,KNG1,FGA
GO Process	GO:0050708	Regulation of protein secretion	12	250	0.59	0.0066	GPLD1,APOE,TCIRG1,FGB,STXBP5,ANG,FGG,RBP4,IGF1,TTN,VEGFC,FGA
GO Process	GO:0061844	Antimicrobial humoral immune response mediated by antimicrobial peptide	8	113	0.76	0.0082	PF4V1,PPBP,PF4,F2,ANG,S100A7,GAPDH,KNG1
GO Process	GO:0062013	Positive regulation of small molecule metabolic process	9	147	0.69	0.0088	GPLD1,APOA1,APOE,APOA4,IGF1,ADIPOQ,IGF2,APOA5,ENO1
GO Process	GO:0010755	Regulation of plasminogen activation	4	18	1.25	0.0089	CPB2,F12,THBS1,ENO1
GO Process	GO:0051004	Regulation of lipoprotein lipase activity	4	18	1.25	0.0089	APOA1,APOA4,ANGPTL3,APOA5
GO Process	GO:1901615	Organic hydroxy compound metabolic process	17	478	0.46	0.0089	CETP,HSD17B1,APOA1,TTR,CAT,APOE,LCAT,PCSK9,APOL1,APOA4,ANGPTL3,RBP4,IGF1,GC,TSKU,APOA5,PLCG2
GO Process	GO:0006006	Glucose metabolic process	8	116	0.75	0.0094	PKM,FBN1,APOD,RBP4,GAPDH,ADIPOQ,IGF2,ENO1
GO Process	GO:0051247	Positive regulation of protein metabolic process	36	1512	0.28	0.0094	MYH9,PCOLCE,VTN,GPLD1,LRP1,APOE,F12,THBS1,KLKB1,CENPE,HPX,CSF1R,ANTXR1,SH3D19,PCSK9,F2,INHBC,CSPG4,PKM,HSPA5,FBN1,FBLN1,HSP90AA1,ANG,THBS4,FN1,S100A8,GSN,IGF1,ILK,ADIPOQ,IGF2,PAEP,VEGFC,PLCG2,ENO1
GO Process	GO:0010720	Positive regulation of cell development	13	304	0.54	0.0099	PROC,APOA1,FGB,P4HB,FGG,LIMS1,FN1,FLNA,ILK,ADIPOQ,LMOD3,VEGFC,FGA
GO Process	GO:0010874	Regulation of cholesterol efflux	5	37	1.04	0.0099	CETP,APOA1,LRP1,APOE,ADIPOQ
GO Process	GO:0018149	Peptide cross-linking	5	37	1.04	0.0099	THBS1,CSTA,F13A1,FN1,F13B
GO Process	GO:0032376	Positive regulation of cholesterol transport	5	38	1.03	0.0110	CETP,APOA1,LRP1,APOE,ADIPOQ
GO Process	GO:0050920	Regulation of chemotaxis	11	227	0.59	0.0110	ST6GAL1,C5,THBS1,PLA2G7,CSF1R,THBS4,S100A7,F7,MST1,VEGFC,SEMA3B
GO Process	GO:0051047	Positive regulation of secretion	13	308	0.53	0.0110	CPB2,GPLD1,CRH,FGB,STXBP5,ANG,FGG,S100A8,RBP4,IGF1,TTN,VEGFC,FGA
GO Process	GO:0010941	Regulation of cell death	38	1651	0.27	0.0113	ST6GAL1,MMP2,MPO,GPLD1,PROC,CAT,LRP1,APOE,THBS1,CSF1R,PF4,PCSK9,FGB,HBA1,HSPA5,P4HB,HSP90AA1,FGG,S100A8,HTRA1,ADAMTSL4,FLNA,CCN5,GSN,IGFBP3,IGF1,ACTN1,YWHAZ,ROCK1,ADIPOQ,MST1,HPR,PLCG2,KNG1,HBB,ACTB,ENO1,FGA
GO Process	GO:0031099	Regeneration	9	154	0.67	0.0115	CPB2,MMP2,VTN,SERPINA10,APOD,APOA4,F7,IGF1,APOA5
GO Process	GO:0016477	Cell migration	25	903	0.35	0.0117	MYH9,MMP2,C5,VTN,PF4V1,GPLD1,APOA1,THBS1,ATRN,PPBP,PF4,LRG1,CSPG4,HSP90AA1,ANG,THBS4,FN1,S100A8,FLNA,CD99,ILK,ROCK1,PSG2,VEGFC,SEMA3B
GO Process	GO:0071634	Regulation of transforming growth factor beta production	5	39	1.01	0.0119	THBS1,FBLN1,FN1,LTBP1,TSKU
GO Process	GO:0014012	Peripheral nervous system axon regeneration	3	7	1.54	0.0124	MMP2,APOD,APOA4
GO Process	GO:0034384	High-density lipoprotein particle clearance	3	7	1.54	0.0124	APOA1,APOE,APOM
GO Process	GO:0046486	Glycerolipid metabolic process	14	357	0.5	0.0124	CETP,GPLD1,PLEK,APOA1,CAT,APOE,LCAT,PLA2G7,CSF1R,PCSK9,ANG,APOA4,APOA5,PLCG2
GO Process	GO:0090207	Regulation of triglyceride metabolic process	5	40	1.0	0.0130	GPLD1,APOA1,APOE,APOA4,APOA5
GO Process	GO:0032879	Regulation of localization	45	2103	0.24	0.0133	CPB2,CETP,VTN,GPLD1,APOA1,LRP1,APOE,THBS1,TCIRG1,FCN3,CRH,FCN2,MASP1,PCSK9,FGB,F2,SUMO4,STXBP5,HSP90AA1,ANG,FGG,APOD,APOA4,S100A8,FLNA,RBP4,GSN,IGF1,RAPGEF4,ROCK1,MASP2,TF,ADIPOQ,C4A,C4B,CFB,TCAF1,APOA5,ATP1A1,TTN,APOC4,VEGFC,PLCG2,ACTB,FGA
GO Process	GO:0002218	Activation of innate immune response	6	65	0.87	0.0139	FCN3,FCN2,MASP1,HSP90AA1,MASP2,PLCG2
GO Process	GO:0014910	Regulation of smooth muscle cell migration	6	65	0.87	0.0139	VTN,IGFBP5,LRP1,IGFBP3,IGF1,ADIPOQ
GO Process	GO:0032091	Negative regulation of protein binding	7	94	0.78	0.0144	CFHR5,PCSK9,CFHR1,CFHR2,ROCK1,ADIPOQ,ACTB
GO Process	GO:0051223	Regulation of protein transport	17	503	0.44	0.0144	GPLD1,APOE,TCIRG1,FGB,STXBP5,HSP90AA1,ANG,FGG,APOD,FLNA,RBP4,IGF1,ADIPOQ,TCAF1,TTN,VEGFC,FGA
GO Process	GO:0010876	Lipid localization	14	365	0.49	0.0150	CETP,APOA1,APOE,LCAT,APOL1,APOD,APOA4,ANGPTL3,RBP4,APOM,ATP9B,TSKU,APOA5,APOC4
GO Process	GO:0045088	Regulation of innate immune response	11	238	0.57	0.0153	APOE,HPX,FCN3,SERPING1,FCN2,MASP1,A2M,HSP90AA1,CFH,MASP2,PLCG2
GO Process	GO:0030449	Regulation of complement activation	4	22	1.17	0.0155	SERPING1,MASP1,A2M,CFH
GO Process	GO:0015850	Organic hydroxy compound transport	9	163	0.65	0.0161	CETP,APOA1,APOE,LCAT,APOA4,RBP4,APOM,TSKU,APOA5
GO Process	GO:1900223	Positive regulation of amyloid-beta clearance	3	8	1.48	0.0161	LRP1,APOE,ROCK1
GO Process	GO:1905920	Positive regulation of CoA-transferase activity	3	8	1.48	0.0161	APOA1,APOE,APOA4
GO Process	GO:0043393	Regulation of protein binding	10	201	0.6	0.0163	VTN,LRP1,APOE,CFHR5,PCSK9,CFHR1,CFHR2,ROCK1,ADIPOQ,ACTB
GO Process	GO:1903532	Positive regulation of secretion by cell	12	282	0.54	0.0164	CPB2,GPLD1,CRH,FGB,STXBP5,ANG,FGG,RBP4,IGF1,TTN,VEGFC,FGA
GO Process	GO:0010647	Positive regulation of cell communication	38	1693	0.26	0.0170	VTN,GPLD1,IGFBP5,APOA1,CAT,APOE,THBS1,VWF,HPX,IGFBP4,CRH,CSF1R,GUCY1A1,LRG1,FGB,F2,INHBC,CSPG4,HSP90AA1,FGG,LIMS1,FN1,S100A7,S100A8,ECM1,FLNA,RBP4,F10,F7,IGFBP3,IGF1,ILK,GAPDH,ROCK1,ADIPOQ,IGF2,PLCG2,FGA
GO Process	GO:0001818	Negative regulation of cytokine production	12	284	0.53	0.0173	APOA1,THBS1,PSG9,PGLYRP2,F2,PRG2,FBLN1,APOD,FN1,IGF1,ADIPOQ,TSKU
GO Process	GO:0002793	Positive regulation of peptide secretion	7	98	0.76	0.0174	GPLD1,CRH,FGB,FGG,S100A8,RBP4,FGA
GO Process	GO:0090208	Positive regulation of triglyceride metabolic process	4	23	1.15	0.0174	GPLD1,APOA1,APOA4,APOA5
GO Process	GO:0023056	Positive regulation of signaling	38	1698	0.26	0.0176	VTN,GPLD1,IGFBP5,APOA1,CAT,APOE,THBS1,VWF,HPX,IGFBP4,CRH,CSF1R,GUCY1A1,LRG1,FGB,F2,INHBC,CSPG4,HSP90AA1,FGG,LIMS1,FN1,S100A7,S100A8,ECM1,FLNA,RBP4,F10,F7,IGFBP3,IGF1,ILK,GAPDH,ROCK1,ADIPOQ,IGF2,PLCG2,FGA
GO Process	GO:0006869	Lipid transport	13	329	0.5	0.0179	CETP,APOA1,APOE,LCAT,APOL1,APOD,APOA4,RBP4,APOM,ATP9B,TSKU,APOA5,APOC4
GO Process	GO:0002690	Positive regulation of leukocyte chemotaxis	7	99	0.76	0.0183	THBS1,PLA2G7,CSF1R,THBS4,S100A7,F7,VEGFC
GO Process	GO:0044057	Regulation of system process	18	567	0.41	0.0183	MMP2,IGFBP5,APOA1,APOE,CRH,DSC2,GUCY1A1,FGB,HSP90AA1,FGG,APOA4,IGF1,ROCK1,ADIPOQ,APOA5,ATP1A1,ENO1,FGA
GO Process	GO:0045807	Positive regulation of endocytosis	7	99	0.76	0.0183	VTN,LRP1,APOE,PCSK9,TF,APOA5,PLCG2
GO Process	GO:0062012	Regulation of small molecule metabolic process	13	330	0.5	0.0183	GPLD1,PLEK,APOA1,APOE,IGFBP4,APOA4,IGFBP3,IGF1,ADIPOQ,IGF2,MST1,APOA5,ENO1
GO Process	GO:0001932	Regulation of protein phosphorylation	28	1108	0.31	0.0185	VTN,APOA1,LRP1,APOE,THBS1,CENPE,HPX,CSF1R,F2,INHBC,CSPG4,FBN1,FBLN1,HSP90AA1,ANG,THBS4,FN1,BLM,IGFBP3,IGF1,ILK,ADIPOQ,IGF2,MST1,TTN,VEGFC,PLCG2,ACTB

GO Process	GO:0009967	Positive regulation of signal transduction	35	1525	0.27	0.0194	VTN,IGFBP5,APOA1,CAT,APOE,THBS1,VWF,HPX,IGFBP4,CSF1R,GUCY1A1,LRG1,FGB,F2,INHBC,CSPG4,HSP90AA1,FGG,LIMS1,FN1,S100A7,S100A8,ECM1,FLNA,F10,F7,IGFBP3,IGF1,ILK,GAPDH,ROCK1,ADIPOQ,IGF2,PLCG2,FGA
GO Process	GO:0010640	Regulation of platelet-derived growth factor receptor signaling pathway	4	24	1.13	0.0194	LRP1,APOD,F7,ADIPOQ
GO Process	GO:0019220	Regulation of phosphate metabolic process	33	1405	0.28	0.0194	VTN,GPLD1,PLEK,APOA1,LRP1,APOE,THBS1,CENPE,HPX,CSF1R,F2,INHBC,CSPG4,FBN1,FBLN1,HSP90AA1,ANG,THBS4,FN1,BLM,IGFBP3,IGF1,ILK,ROCK1,TF,ADIPOQ,IGF2,MST1,TTN,VEGFC,PLCG2,ACTB,ENO1
GO Process	GO:0034381	Plasma lipoprotein particle clearance	4	24	1.13	0.0194	APOA1,APOE,APOM,ADIPOQ
GO Process	GO:0010898	Positive regulation of triglyceride catabolic process	3	9	1.43	0.0197	APOA1,APOA4,APOA5
GO Process	GO:0048771	Tissue remodeling	7	101	0.75	0.0197	SPP2,MMP2,IGFBP5,TCIRG1,CSPG4,THBS4,FLNA
GO Process	GO:0051006	Positive regulation of lipoprotein lipase activity	3	9	1.43	0.0197	APOA1,APOA4,APOA5
GO Process	GO:0051258	Protein polymerization	7	101	0.75	0.0197	VTN,FGB,ANG,FGG,GSN,SLAIN1,FGA
GO Process	GO:0070887	Cellular response to chemical stimulus	52	2609	0.21	0.0197	CPB2,MMP2,C5,MPO,HSD17B1,PF4V1,GPLD1,IGFBP5,CAT,LRP1,APOE,PXDN,THBS1,AMBP,TCIRG1,HPX,PSG9,CSF1R,PPBP,PF4,PCSK9,FGB,SUMO4,PKM,HBA1,HSPA5,FBN1,P4HB,TUBA1B,FGG,LIMS1,BLM,APOA4,S100A8,FLNA,GSN,APOM,SOD3,GPX3,IGF1,ILK,GAPDH,TF,ADIPOQ,IGF2,MST1,ATP1A1,VEGFC,SEMA3B,PLCG2,HBB,ACTB
GO Process	GO:0002920	Regulation of humoral immune response	5	46	0.94	0.0206	HPX,SERPING1,MASP1,A2M,CFH
GO Process	GO:1902531	Regulation of intracellular signal transduction	38	1726	0.25	0.0226	IGFBP5,PLEK,APOA1,CAT,APOE,THBS1,VWF,AMBP,IGFBP4,CSF1R,GUCY1A1,FGB,F2,CSPG4,SUMO4,P4HB,FBLN1,HSP90AA1,FGG,LIMS1,FN1,S100A7,S100A8,ECM1,FLNA,F10,F7,IGFBP3,IGF1,YWHAZ,ILK,GAPDH,ROCK1,ADIPOQ,IGF2,PLCG2,ENO1,FGA
GO Process	GO:0010906	Regulation of glucose metabolic process	7	104	0.74	0.0227	GPLD1,IGFBP4,IGFBP3,IGF1,ADIPOQ,IGF2,MST1
GO Process	GO:0051128	Regulation of cellular component organization	48	2365	0.21	0.0228	CPB2,MYH9,VTN,IGFBP5,PLEK,APOA1,LRP1,APOE,THBS1,LCAT,CENPE,IGFBP4,CSF1R,SCGB3A1,FYCO1,ANTXR1,PCSK9,F2,TLN1,STXBP5,HSPA5,HSP90AA1,LIMS1,APOD,FN1,APOA4,CENPF,PAPPA2,MKI67,S100A8,FLNA,GSN,APOM,IGFBP3,IGF1,YWHAZ,ROCK1,TF,ADIPOQ,IGF2,SLAIN1,LMOD3,TSKU,APOA5,SEMA3B,PLCG2,ACTB,ENO1
GO Process	GO:0010875	Positive regulation of cholesterol efflux	4	26	1.09	0.0241	APOA1,LRP1,APOE,ADIPOQ
GO Process	GO:0014912	Negative regulation of smooth muscle cell migration	4	26	1.09	0.0241	IGFBP5,LRP1,IGFBP3,ADIPOQ
GO Process	GO:0048878	Chemical homeostasis	24	904	0.33	0.0241	CPB2,CETP,IGFBP5,APOA1,APOE,LCAT,CP,TCIRG1,HPX,PCSK9,F2,FBN1,OIT3,APOA4,S100A8,ANGPTL3,RBP4,APOM,TF,ADIPOQ,TSKU,APOA5,ATP1A1,APOC4
GO Process	GO:0030300	Regulation of intestinal cholesterol absorption	3	10	1.38	0.0243	APOA1,APOA4,APOA5
GO Process	GO:0033622	Integrin activation	3	10	1.38	0.0243	FERMT3,TLN1,FN1
GO Process	GO:0045785	Positive regulation of cell adhesion	16	485	0.43	0.0243	VTN,APOA1,FGB,P4HB,FBLN1,FGG,LIMS1,FN1,FLNA,IGF1,ILK,ROCK1,IGF2,HSPH1,ACTB,FGA
GO Process	GO:0042325	Regulation of phosphorylation	30	1251	0.29	0.0245	VTN,APOA1,LRP1,APOE,THBS1,CENPE,HPX,CSF1R,F2,INHBC,CSPG4,FBN1,FBLN1,HSP90AA1,ANG,THBS4,FN1,BLM,IGFBP3,IGF1,ILK,ROCK1,TF,ADIPOQ,IGF2,MST1,TTN,VEGFC,PLCG2,ACTB
GO Process	GO:0044403	Biological process involved in symbiotic interaction	11	257	0.54	0.0246	APOE,CFHR5,CSF1R,PGLYRP2,PF4,F2,CFHR1,APOL1,FN1,CFHR2,GAPDH
GO Process	GO:0001775	Cell activation	20	693	0.37	0.0258	MYH9,PF4V1,PLEK,LRP1,VWF,TCIRG1,FERMT3,PF4,FGB,F2,TLN1,FGG,FN1,FLNA,IGF1,ILK,PLCG2,HBB,ACTB,FGA
GO Process	GO:0051222	Positive regulation of protein transport	12	303	0.5	0.0267	GPLD1,FGB,HSP90AA1,ANG,FGG,FLNA,RBP4,IGF1,TCAF1,TTN,VEGFC,FGA
GO Process	GO:0020027	Hemoglobin metabolic process	3	11	1.34	0.0299	CAT,AMBP,HPX
GO Process	GO:0031529	Ruffle organization	4	28	1.06	0.0299	PLEK,TCIRG1,CSF1R,CSPG4
GO Process	GO:0050996	Positive regulation of lipid catabolic process	4	28	1.06	0.0299	APOA1,APOA4,ANGPTL3,APOA5
GO Process	GO:1905954	Positive regulation of lipid localization	7	111	0.71	0.0310	CETP,APOA1,LRP1,APOE,CRH,ADIPOQ,APOC4
GO Process	GO:0009725	Response to hormone	21	762	0.35	0.0328	MMP2,GPLD1,IGFBP5,CAT,THBS1,TIMP2,LCAT,PCSK9,FGB,PKM,A2M,FBN1,PAPPA,HSP90AA1,ANG,ANGPTL3,F7,ROCK1,ADIPOQ,IGF2,ATP1A1
GO Process	GO:0010758	Regulation of macrophage chemotaxis	4	29	1.05	0.0333	C5,THBS1,CSF1R,MST1
GO Process	GO:0045595	Regulation of cell differentiation	35	1582	0.25	0.0334	CETP,IGFBP5,PROC,APOA1,NOTCH3,TCIRG1,PSG9,PGLYRP2,PF4,LRG1,FGB,F2,SUZ12,FBN1,P4HB,FBLN1,FGG,LIMS1,FN1,FLNA,C1QC,IGFBP3,IGF1,EFEMP1,ILK,ROCK1,ADIPOQ,IGF2,LMOD3,PAEP,TSKU,VEGFC,SEMA3B,ACTB,FGA
GO Process	GO:0010543	Regulation of platelet activation	5	53	0.88	0.0347	PLEK,APOE,F2,FGG,FLNA
GO Process	GO:0051592	Response to calcium ion	8	149	0.64	0.0348	GPLD1,THBS1,FGB,HSPA5,FGG,TTN,PLCG2,FGA
GO Process	GO:0045940	Positive regulation of steroid metabolic process	4	30	1.03	0.0369	APOA1,APOE,APOA4,APOA5
GO Process	GO:0002687	Positive regulation of leukocyte migration	8	151	0.63	0.0376	THBS1,PLA2G7,CSF1R,THBS4,S100A7,F7,CD99,VEGFC
GO Process	GO:0048731	System development	69	3867	0.16	0.0377	SPP2,CPB2,MYH9,MMP2,HSD17B1,VTN,MGP,GPLD1,IGFBP5,PLEK,APOA1,CAT,LRP1,APOE,PXDN,ANGPTL6,THBS1,SERPINA10,ITGA2B,ATRN,NOTCH3,DNAH5,TCIRG1,CDH11,PSG9,CRH,CSF1R,PCDH1,ANTXR1,PCSK9,CSPG4,SUZ12,A2M,HSPA5,FBN1,HSP90AA1,ANG,APOD,FN1,APOA4,MFAP5,COL18A1,CENPF,PAPPA2,S100A7,S100A8,HTRA1,ECM1,FLNA,SMARCA1,ANGPTL3,RBP4,GSN,IGF1,ACTN1,EFEMP1,YWHAZ,ILK,ROCK1,TF,IGF2,TGFBI,SELENOP,TSKU,TTN,VEGFC,SEMA3B,PLCG2,ACTB
GO Process	GO:0008152	Metabolic process	124	7988	0.1	0.0387	ST6GAL1,CPB2,CETP,GNPTG,MYH9,TPD52L2,APMAP,F9,MMP2,PCOLCE,MPO,HSD17B1,GPLD1,PROC,PLEK,APOA1,TTR,CAT,LRP1,APOE,PXDN,F12,THBS1,LYZ,PSMA6,BLVRB,LCAT,CSTA,KLKB1,F13A1,AMBP,TCIRG1,HPX,ITIH4,PSG9,FCN3,PLA2G7,CRH,HABP2,CSF1R,FCN2,PGLYRP2,MASP1,APEH,GUCY1A1,LALBA,PCSK9,FGB,F2,UBE3C,NDUFS8,SUZ12,APOL1,SUMO4,PKM,HBA1,HSPA5,FBN1,P4HB,PAPPA,FBLN1,HSP90AA1,ANG,FGG,GANAB,APOD,FN1,BLM,APOA4,ITIH2,CENPF,F13B,CFH,PAPPA2,F5,ADAM12,S100A8,HTRA1,FLNA,ANGPTL3,RBP4,GSN,F10,F7,APOM,CA6,IGFBP3,SOD3,GPX3,IGF1,EFEMP1,YWHAZ,ILK,GAPDH,ROCK1,MASP2,F11,C1S,PCYOX1,KSR2,ADIPOQ,IGF2,ACSF2,CKMT1A,CDK3,MST1,ITIH3,CFB,GC,UIMC1,SELENOP,TSKU,HPR,C1R,APOA5,RNASE4,TTN,APOC4,CFD,PLCG2,HBB,ACTB,ENO1,FGA
GO Process	GO:0048260	Positive regulation of receptor-mediated endocytosis	5	55	0.87	0.0397	VTN,PCSK9,TF,APOA5,PLCG2
GO Process	GO:0043408	Regulation of MAPK cascade	19	668	0.36	0.0398	APOE,THBS1,AMBP,IGFBP4,CSF1R,FGB,CSPG4,FBLN1,FGG,FN1,S100A7,IGFBP3,IGF1,YWHAZ,ROCK1,ADIPOQ,IGF2,PLCG2,FGA
GO Process	GO:0050678	Regulation of epithelial cell proliferation	13	366	0.46	0.0398	CPB2,SPARC,APOE,THBS1,LRG1,ANG,THBS4,HTRA1,ECM1,IGF1,IGF2,MST1,VEGFC
GO Process	GO:0009966	Regulation of signal transduction	56	2978	0.18	0.0410	VTN,IGFBP5,PLEK,APOA1,CAT,LRP1,APOE,PXDN,THBS1,VWF,AMBP,HPX,IGFBP4,CRH,CSF1R,PF4,GUCY1A1,LRG1,PCSK9,FGB,F2,INHBC,CSPG4,SUMO4,HSPA5,FBN1,P4HB,FBLN1,HSP90AA1,FGG,LIMS1,APOD,FN1,S100A7,S100A8,HTRA1,ECM1,FLNA,F10,F7,IGFBP3,IGF1,YWHAZ,ILK,GAPDH,ROCK1,LTBP1,ADIPOQ,IGF2,CDK3,MST1,TSKU,VEGFC,PLCG2,ENO1,FGA
GO Process	GO:0001869	Negative regulation of complement activation, lectin pathway	2	2	1.91	0.0416	SERPING1,A2M
GO Process	GO:0007159	Leukocyte cell-cell adhesion	5	56	0.86	0.0416	FERMT3,LRG1,APOA4,S100A8,ROCK1
GO Process	GO:0010037	Response to carbon dioxide	2	2	1.91	0.0416	A2M,F7

GO Process	GO:0010642	Negative regulation of platelet-derived growth factor receptor signaling pathway	3	13	1.27	0.0416	LRP1,APOD,ADIPOQ
GO Process	GO:0034445	Negative regulation of plasma lipoprotein oxidation	2	2	1.91	0.0416	APOA4,APOM
GO Process	GO:0071635	Negative regulation of transforming growth factor beta production	3	13	1.27	0.0416	FBLN1,FN1,TSKU
GO Process	GO:1903923	Positive regulation of protein processing in phagocytic vesicle	2	2	1.91	0.0416	MYH9,GSN
GO Process	GO:0045834	Positive regulation of lipid metabolic process	8	155	0.62	0.0418	GPLD1,APOA1,APOE,F2,APOA4,ANGPTL3,ADIPOQ,APOA5
GO Process	GO:0050731	Positive regulation of peptidyl-tyrosine phosphorylation	9	194	0.57	0.0418	VTN,HPX,CSF1R,CSPG4,THBS4,IGF1,ADIPOQ,IGF2,PLCG2
GO Process	GO:0043067	Regulation of programmed cell death	33	1492	0.25	0.0448	ST6GAL1,MMP2,MPO,GPLD1,PROC,CAT,APOE,THBS1,CSF1R,PF4,PCSK9,FGB,HSPA5,P4HB,HSP90AA1,FGG,S100A8,HTRA1,ADAMTSL4,FLNA,GSN,IGFBP3,IGF1,ACTN1,YWHAZ,ROCK1,ADIPOQ,MST1,PLCG2,KNG1,ACTB,ENO1,FGA
GO Process	GO:0010675	Regulation of cellular carbohydrate metabolic process	8	158	0.61	0.0466	GPLD1,PLEK,IGFBP4,IGFBP3,IGF1,ADIPOQ,IGF2,MST1
GO Process	GO:0034446	Substrate adhesion-dependent cell spreading	5	58	0.84	0.0469	ATRN,FERMT3,ANTXR1,FN1,ILK
GO Process	GO:0045862	Positive regulation of proteolysis	13	375	0.45	0.0469	MYH9,PCOLCE,GPLD1,APOE,F12,KLKB1,ANTXR1,SH3D19,FBLN1,FN1,S100A8,GSN,ENO1
GO Process	GO:0060191	Regulation of lipase activity	6	88	0.74	0.0470	APOA1,LRP1,ANG,APOA4,ANGPTL3,APOA5
GO Process	GO:0001878	Response to yeast	3	14	1.24	0.0484	MPO,ANG,PLCG2
GO Process	GO:0019430	Removal of superoxide radicals	3	14	1.24	0.0484	MPO,APOA4,SOD3
GO Process	GO:0044342	Type B pancreatic cell proliferation	3	14	1.24	0.0484	IGFBP5,IGFBP4,IGFBP3
GO Process	GO:0045916	Negative regulation of complement activation	3	14	1.24	0.0484	SERPING1,MASP1,A2M
GO Process	GO:0001817	Regulation of cytokine production	20	739	0.34	0.0488	C5,APOA1,THBS1,PSG9,CSF1R,PGLYRP2,PF4,F2,PRG2,FBLN1,HSP90AA1,APOD,FN1,IGF1,GAPDH,LTBP1,ADIPOQ,PAEP,TSKU,PLCG2
GO Process	GO:0010646	Regulation of cell communication	61	3355	0.17	0.0488	VTN,GPLD1,IGFBP5,PLEK,APOA1,CAT,LRP1,APOE,PXDN,THBS1,VWF,AMBP,TCIRG1,HPX,CDH11,IGFBP4,CRH,CSF1R,PF4,GUCY1A1,LRG1,PCSK9,FGB,F2,INHBC,CSPG4,SUMO4,STXBP5,HSPA5,FBN1,P4HB,FBLN1,HSP90AA1,FGG,LIMS1,APOD,FN1,S100A7,S100A8,HTRA1,ECM1,FLNA,RBP4,F10,F7,IGFBP3,IGF1,YWHAZ,ILK,GAPDH,ROCK1,LTBP1,ADIPOQ,IGF2,CDK3,MST1,TSKU,VEGFC,PLCG2,ENO1,FGA
GO Process	GO:0032370	Positive regulation of lipid transport	6	89	0.74	0.0488	CETP,APOA1,LRP1,APOE,CRH,ADIPOQ
GO Process	GO:0045596	Negative regulation of cell differentiation	19	685	0.35	0.0488	CETP,IGFBP5,NOTCH3,PGLYRP2,PF4,F2,SUZ12,FBN1,FBLN1,C1QC,IGF1,EFEMP1,ADIPOQ,IGF2,PAEP,TSKU,VEGFC,SEMA3B,ACTB
GO Process	GO:0032880	Regulation of protein localization	22	852	0.32	0.0496	GPLD1,LRP1,APOE,TCIRG1,FGB,F2,SUMO4,STXBP5,HSP90AA1,ANG,FGG,APOD,FLNA,RBP4,GSN,IGF1,ADIPOQ,TCAF1,TTN,VEGFC,ACTB,FGA
GO Function	GO:0005539	Glycosaminoglycan binding	27	245	0.95	2.54e-13	SERPIND1,PCOLCE,MPO,VTN,PF4V1,APOE,THBS1,SERPINA10,AMBP,HABP2,PGLYRP2,PF4,F2,PRG2,STAB1,FBN1,ANG,THBS4,FN1,ITIH2,CFH,ANGPTL3,CCN5,SOD3,F11,APOA5,KNG1
GO Function	GO:0061134	Peptidase regulator activity	26	227	0.97	2.54e-13	SPP2,SERPIND1,TFPI2,PCOLCE,C5,SERPINA10,TIMP2,CSTA,AMBP,ITIH4,SERPING1,A2ML1,A2M,SERPINA7,FBLN1,SERPINA11,PAPLN,FN1,ITIH2,PROS1,GAPDH,ROCK1,C4A,ITIH3,C4B,KNG1
GO Function	GO:0004866	Endopeptidase inhibitor activity	23	177	1.02	7.72e-13	SPP2,SERPIND1,TFPI2,C5,SERPINA10,TIMP2,CSTA,AMBP,ITIH4,SERPING1,A2ML1,A2M,SERPINA7,SERPINA11,PAPLN,ITIH2,PROS1,GAPDH,ROCK1,C4A,ITIH3,C4B,KNG1
GO Function	GO:0005515	Protein binding	151	7242	0.23	2.07e-12	ST6GAL1,GNPTG,MYH9,TPD52L2,MMP2,C5,HSD17B1,VTN,PF4V1,IGFBP5,PLEK,APOA1,TTR,CAT,LRP1,APOE,PXDN,ANGPTL6,F12,CFHR5,THBS1,LYZ,VWF,PSMA6,ITGA2B,TIMP2,NOTCH3,LCAT,CSTA,CP,DNAH5,AMBP,CENPE,TCIRG1,CDH11,IGFBP4,PSG9,FCN3,CRH,FERMT3,SEC24D,DSC2,CSF1R,FCN2,PSG6,SCGB3A1,PPBP,PF4,MASP1,APEH,A2ML1,ANTXR1,LRG1,SH3D19,PCSK9,FGB,DST,F2,INHBC,PSG1,CSPG4,C1QB,CFHR1,TLN1,SUZ12,SUMO4,PKM,STXBP5,HBA1,A2M,HSPA5,FBN1,P4HB,FBLN1,PSG3,DNAH8,HSP90AA1,ANG,TUBA1B,FGG,LIMS1,THBS4,FN1,BLM,APOA4,COL18A1,C8A,CENPF,CFHR2,CFHR3,CFH,MKI67,ADAM12,S100A7,S100A8,HTRA1,ADAMTSL4,ECM1,FLNA,SMARCA1,ANGPTL3,CCN5,GSN,F7,IGFBP3,GPX3,IGF1,ACTN1,EFEMP1,YWHAZ,ILK,GAPDH,RAPGEF4,ROCK1,MASP2,F11,C1S,TF,DCDC1,LTBP1,ADIPOQ,IGF2,BZW1,C4A,NBEAL1,MST1,LMOD3,C4B,TGFBI,CFB,IGFALS,TCAF1,GC,UIMC1,TSKU,HPR,C1R,APOA5,ATP1A1,TTN,IGHV3-72,VEGFC,SEMA3B,PLCG2,HSPH1,KNG1,HBB,ACTB,ENO1,FGA,TPM3
GO Function	GO:0008201	Heparin binding	22	173	1.01	2.21e-12	SERPIND1,PCOLCE,MPO,VTN,PF4V1,APOE,THBS1,SERPINA10,PF4,F2,PRG2,FBN1,ANG,THBS4,FN1,CFH,ANGPTL3,CCN5,SOD3,F11,APOA5,KNG1
GO Function	GO:0005102	Signaling receptor binding	58	1499	0.49	6.08e-12	MYH9,C5,VTN,PF4V1,APOA1,TTR,LRP1,APOE,PXDN,ANGPTL6,THBS1,VWF,ITGA2B,IGFBP4,CRH,FERMT3,FCN2,SCGB3A1,PPBP,PF4,LRG1,PCSK9,FGB,DST,F2,INHBC,TLN1,HBA1,A2M,FBN1,P4HB,FBLN1,HSP90AA1,ANG,FGG,THBS4,FN1,S100A7,S100A8,ECM1,FLNA,ANGPTL3,CCN5,F7,IGF1,ACTN1,EFEMP1,TF,ADIPOQ,IGF2,MST1,TGFBI,APOA5,IGHV3-72,VEGFC,SEMA3B,KNG1,FGA
GO Function	GO:0004252	Serine-type endopeptidase activity	21	176	0.98	2.35e-11	F9,MMP2,PROC,F12,KLKB1,HABP2,MASP1,APEH,PCSK9,F2,HTRA1,F10,F7,MASP2,F11,C1S,MST1,CFB,HPR,C1R,CFD
GO Function	GO:0005509	Calcium ion binding	38	717	0.63	5.56e-11	F9,MGP,SPARC,PROC,LRP1,F12,THBS1,NOTCH3,CDH11,HABP2,DSC2,PCDH1,MASP1,LALBA,DST,F2,STAB1,HSPA5,FBN1,FBLN1,OIT3,SPATA21,THBS4,S100A7,S100A8,GSN,F10,F7,CALML5,PROS1,ACTN1,EFEMP1,EFCAB5,MASP2,C1S,LTBP1,C1R,TTN
GO Function	GO:1901681	Sulfur compound binding	24	272	0.85	1.13e-10	SERPIND1,PCOLCE,MPO,VTN,PF4V1,LRP1,APOE,THBS1,SERPINA10,AMBP,PF4,F2,PRG2,FBN1,ANG,THBS4,FN1,CFH,ANGPTL3,CCN5,SOD3,F11,APOA5,KNG1

GO Function	GO:0005488	Binding	209	12838	0.12	2.32e-10	ST6GAL1,CPB2,CETP,GNPTG,SERPIND1,MYH9,TPD52L2,F9,MMP2,PCOLCE,C5,MPO,HSD17B1,VTN,PF4V1,MGP,SPARC,IGFBP5,PROC,PLEK,APOA1,TTR,CAT,LRP1,APOE,PXDN,ANGPTL6,F12,CFHR5,THBS1,LYZ,VWF,PSMA6,SERPINA10,ITGA2B,TIMP2,ATRN,NOTCH3,LCAT,CSTA,CP,KLKB1,F13A1,DNAH5,AMBP,CENPE,TCIRG1,HPX,CDH11,IGFBP4,PSG9,FCN3,PLA2G7,CRH,HABP2,FERMT3,SEC24D,DSC2,CSF1R,PCDH1,FCN2,PSG6,PGLYRP2,SCGB3A1,PPBP,PF4,FYCO1,MASP1,APEH,GUCY1A1,A2ML1,LALBA,ANTXR1,LRG1,SH3D19,PCSK9,FGB,DST,F2,INHBC,PSG1,PRG2,CSPG4,STAB1,C1QB,CFHR1,NDUFS8,TLN1,SUZ12,APOL1,SUMO4,PKM,STXBP5,HBA1,A2M,HSPA5,FBN1,P4HB,PAPPA,FBLN1,PSG3,DNAH8,OIT3,HSP90AA1,SPATA21,ANG,TUBA1B,FGG,LIMS1,THBS4,GANAB,APOD,FN1,BLM,APOA4,ITIH2,COL18A1,C8A,CENPF,CFHR2,CFHR3,CFH,PAPPA2,F5,MKI67,ADAM12,S100A7,S100A8,HTRA1,ADAMTSL4,ECM1,FLNA,SMARCA1,ANGPTL3,C8B,RBP4,CCN5,GSN,F10,F7,APOM,CA6,CALML5,IGFBP3,SOD3,GPX3,IGF1,PROS1,ACTN1,EFEMP1,EFCAB5,TNRC6A,YWHAZ,ILK,GAPDH,RAPGEF4,BPIFC,ROCK1,MASP2,F11,C1S,TF,DCDC1,LTBP1,PCYOX1,KSR2,ADIPOQ,IGF2,BZW1,C4A,ATP9B,PRG4,NBEAL1,ACSF2,CKMT1A,CDK3,MST1,LMOD3,C4B,TGFBI,CFB,IGFALS,PAEP,TCAF1,GC,UIMC1,SELENOP,TSKU,HPR,C1R,APOA5,ATP1A1,RNASE4,IGKV2D-28,NBPF10,TTN,IGHV3-72,VEGFC,SEMA3B,PLCG2,HSPH1,KNG1,HBB,ACTB,TRIM66,ENO1,FGA,TPM3,PDIA4
GO Function	GO:0005178	Integrin binding	19	159	0.98	2.33e-10	MYH9,VTN,THBS1,VWF,ITGA2B,FERMT3,DST,TLN1,FBN1,P4HB,FBLN1,THBS4,FN1,ANGPTL3,CCN5,IGF1,ACTN1,IGF2,TGFBI
GO Function	GO:0005201	Extracellular matrix structural constituent	17	131	1.02	1.11e-09	TFPI2,VTN,MGP,PXDN,THBS1,FGB,PRG2,FBN1,FBLN1,OIT3,FGG,FN1,MFAP5,COL18A1,LTBP1,TGFBI,FGA
GO Function	GO:0001848	Complement binding	10	26	1.49	3.49e-09	CFHR5,CFHR1,C8A,CFHR2,CFHR3,CFH,MASP2,C4A,C4B,CFB
GO Function	GO:0004175	Endopeptidase activity	26	437	0.68	3.07e-08	F9,MMP2,PROC,F12,PSMA6,KLKB1,HABP2,MASP1,APEH,PCSK9,F2,PAPPA,PAPLN,PAPPA2,ADAM12,HTRA1,F10,F7,MASP2,F11,C1S,MST1,CFB,HPR,C1R,CFD
GO Function	GO:0050839	Cell adhesion molecule binding	29	560	0.62	5.21e-08	MYH9,VTN,THBS1,VWF,ITGA2B,NOTCH3,CDH11,FERMT3,DSC2,DST,TLN1,PKM,HSPA5,FBN1,P4HB,FBLN1,FGG,THBS4,FN1,FLNA,ANGPTL3,CCN5,IGF1,ACTN1,YWHAZ,IGF2,BZW1,TGFBI,ENO1
GO Function	GO:0004857	Enzyme inhibitor activity	24	396	0.69	1.04e-07	SPP2,SERPIND1,TFPI2,C5,SERPINA10,TIMP2,CSTA,AMBP,ITIH4,SERPING1,A2ML1,A2M,SERPINA7,SERPINA11,PAPLN,ITIH2,ANGPTL3,PROS1,GAPDH,ROCK1,C4A,ITIH3,C4B,KNG1
GO Function	GO:0098772	Molecular function regulator activity	58	1960	0.38	1.04e-07	SPP2,SERPIND1,TFPI2,PCOLCE,C5,PF4V1,GPLD1,APOA1,TTR,APOE,PXDN,SERPINA10,TIMP2,CSTA,AMBP,ITIH4,CRH,SERPING1,SCGB3A1,PPBP,PF4,A2ML1,PCSK9,F2,INHBC,SUZ12,STXBP5,A2M,FBN1,SERPINA7,FBLN1,SERPINA11,HSP90AA1,THBS4,PAPLN,FN1,APOA4,ITIH2,FLNA,ANGPTL3,CALML5,IGFBP3,IGF1,PROS1,EFEMP1,GAPDH,RAPGEF4,ROCK1,ADIPOQ,IGF2,C4A,ITIH3,C4B,APOA5,VEGFC,SEMA3B,HSPH1,KNG1
GO Function	GO:0004867	Serine-type endopeptidase inhibitor activity	13	98	1.03	2.33e-07	SERPIND1,TFPI2,SERPINA10,AMBP,ITIH4,SERPING1,A2ML1,A2M,SERPINA7,SERPINA11,PAPLN,ITIH2,ITIH3
GO Function	GO:0044877	Protein-containing complex binding	42	1261	0.43	1.53e-06	MYH9,PCOLCE,VTN,SPARC,APOA1,TTR,LRP1,APOE,THBS1,VWF,ITGA2B,AMBP,PSG9,FERMT3,ANTXR1,PCSK9,DST,STAB1,TLN1,PKM,HSPA5,FBN1,P4HB,FBLN1,HSP90AA1,THBS4,FN1,C8A,CENPF,FLNA,SMARCA1,ANGPTL3,C8B,CCN5,GSN,IGF1,ACTN1,IGF2,C4A,TGFBI,TTN,TPM3
GO Function	GO:0008233	Peptidase activity	27	617	0.55	5.51e-06	CPB2,F9,MMP2,PROC,F12,PSMA6,KLKB1,HABP2,MASP1,APEH,PCSK9,F2,PAPPA,PAPLN,PAPPA2,ADAM12,HTRA1,F10,F7,MASP2,F11,C1S,MST1,CFB,HPR,C1R,CFD
GO Function	GO:0001846	Opsonin binding	7	21	1.43	7.07e-06	CFHR5,CFHR1,CFHR2,CFHR3,CFH,MASP2,C4A
GO Function	GO:0042802	Identical protein binding	56	2144	0.32	1.31e-05	ST6GAL1,GNPTG,MYH9,TPD52L2,HSD17B1,VTN,PLEK,APOA1,TTR,CAT,APOE,CFHR5,THBS1,LYZ,VWF,ITGA2B,NOTCH3,AMBP,FCN3,CSF1R,FCN2,MASP1,APEH,DST,C1QB,CFHR1,PKM,A2M,FBN1,FBLN1,HSP90AA1,ANG,FGG,FN1,BLM,APOA4,COL18A1,CENPF,CFHR2,CFH,HTRA1,ADAMTSL4,FLNA,GPX3,ACTN1,YWHAZ,GAPDH,MASP2,F11,C1S,ADIPOQ,TGFBI,C1R,TTN,ACTB,ENO1
GO Function	GO:0030234	Enzyme regulator activity	38	1239	0.39	5.84e-05	SPP2,SERPIND1,TFPI2,PCOLCE,C5,APOA1,APOE,SERPINA10,TIMP2,CSTA,AMBP,ITIH4,SERPING1,A2ML1,SUZ12,STXBP5,A2M,SERPINA7,FBLN1,SERPINA11,HSP90AA1,PAPLN,FN1,APOA4,ITIH2,ANGPTL3,CALML5,IGFBP3,PROS1,GAPDH,RAPGEF4,ROCK1,IGF2,C4A,ITIH3,C4B,APOA5,KNG1
GO Function	GO:0097367	Carbohydrate derivative binding	56	2278	0.3	8.68e-05	SERPIND1,MYH9,PCOLCE,MPO,VTN,PF4V1,LRP1,APOE,THBS1,SERPINA10,DNAH5,AMBP,CENPE,HABP2,CSF1R,FCN2,PGLYRP2,PF4,GUCY1A1,F2,PRG2,STAB1,PKM,HSPA5,FBN1,DNAH8,HSP90AA1,ANG,TUBA1B,THBS4,FN1,BLM,ITIH2,CFH,MKI67,SMARCA1,ANGPTL3,CCN5,SOD3,ILK,RAPGEF4,BPIFC,ROCK1,F11,KSR2,ADIPOQ,ATP9B,ACSF2,CKMT1A,CDK3,APOA5,ATP1A1,TTN,HSPH1,KNG1,ACTB
GO Function	GO:0001851	Complement component C3b binding	5	11	1.56	0.00016	CFHR5,CFHR1,CFHR2,CFHR3,CFH
GO Function	GO:0016209	Antioxidant activity	9	76	0.98	0.00016	MPO,CAT,APOE,PXDN,AMBP,APOA4,APOM,SOD3,GPX3
GO Function	GO:0043169	Cation binding	87	4335	0.21	0.00016	CPB2,CETP,F9,MMP2,MPO,MGP,SPARC,PROC,APOA1,CAT,LRP1,APOE,PXDN,F12,THBS1,ITGA2B,TIMP2,NOTCH3,CP,KLKB1,F13A1,HPX,CDH11,FCN3,HABP2,SEC24D,DSC2,PCDH1,FCN2,PGLYRP2,FYCO1,MASP1,LALBA,ANTXR1,DST,F2,STAB1,NDUFS8,SUZ12,PKM,HBA1,HSPA5,FBN1,PAPPA,FBLN1,OIT3,SPATA21,ANG,FGG,LIMS1,THBS4,BLM,APOA4,COL18A1,PAPPA2,F5,ADAM12,S100A7,S100A8,GSN,F10,F7,CA6,CALML5,IGFBP3,SOD3,PROS1,ACTN1,EFEMP1,EFCAB5,ROCK1,MASP2,C1S,TF,LTBP1,KSR2,ATP9B,UIMC1,C1R,APOA5,ATP1A1,TTN,KNG1,HBB,TRIM66,ENO1,FGA
GO Function	GO:0043167	Ion binding	109	6033	0.16	0.00048	CPB2,CETP,MYH9,F9,MMP2,MPO,HSD17B1,MGP,SPARC,PROC,PLEK,APOA1,CAT,LRP1,APOE,PXDN,F12,THBS1,PSMA6,ITGA2B,TIMP2,NOTCH3,CP,KLKB1,F13A1,DNAH5,CENPE,HPX,CDH11,FCN3,HABP2,SEC24D,DSC2,CSF1R,PCDH1,FCN2,PGLYRP2,FYCO1,MASP1,GUCY1A1,LALBA,ANTXR1,DST,F2,STAB1,NDUFS8,SUZ12,PKM,HBA1,HSPA5,FBN1,PAPPA,FBLN1,DNAH8,OIT3,HSP90AA1,SPATA21,ANG,TUBA1B,FGG,LIMS1,THBS4,BLM,APOA4,COL18A1,PAPPA2,F5,MKI67,ADAM12,S100A7,S100A8,SMARCA1,GSN,F10,F7,CA6,CALML5,IGFBP3,SOD3,PROS1,ACTN1,EFEMP1,EFCAB5,ILK,RAPGEF4,ROCK1,MASP2,C1S,TF,LTBP1,PCYOX1,KSR2,ADIPOQ,ATP9B,ACSF2,CKMT1A,CDK3,UIMC1,C1R,APOA5,ATP1A1,TTN,HSPH1,KNG1,HBB,ACTB,TRIM66,ENO1,FGA
GO Function	GO:0046872	Metal ion binding	84	4250	0.2	0.00048	CPB2,F9,MMP2,MPO,MGP,SPARC,PROC,CAT,LRP1,APOE,PXDN,F12,THBS1,ITGA2B,TIMP2,NOTCH3,CP,KLKB1,F13A1,HPX,CDH11,FCN3,HABP2,SEC24D,DSC2,PCDH1,FCN2,PGLYRP2,FYCO1,MASP1,LALBA,ANTXR1,DST,F2,STAB1,NDUFS8,SUZ12,PKM,HBA1,HSPA5,FBN1,PAPPA,FBLN1,OIT3,SPATA21,ANG,FGG,LIMS1,THBS4,BLM,APOA4,COL18A1,PAPPA2,F5,ADAM12,S100A7,S100A8,GSN,F10,F7,CA6,CALML5,IGFBP3,SOD3,PROS1,ACTN1,EFEMP1,EFCAB5,ROCK1,MASP2,C1S,TF,LTBP1,KSR2,ATP9B,UIMC1,C1R,ATP1A1,TTN,KNG1,HBB,TRIM66,ENO1,FGA
GO Function	GO:0005518	Collagen binding	8	66	0.99	0.00049	PCOLCE,VTN,SPARC,THBS1,VWF,ANTXR1,FN1,TGFBI
GO Function	GO:0060228	Phosphatidylcholine-sterol O-acyltransferase activator activity	4	6	1.73	0.00061	APOA1,APOE,APOA4,APOA5
GO Function	GO:0019838	Growth factor binding	10	127	0.8	0.0010	IGFBP5,PXDN,THBS1,IGFBP4,A2M,HTRA1,IGFBP3,LTBP1,IGFALS,TSKU

GO Function	GO:0030545	Signaling receptor regulator activity	21	552	0.49	0.0011	C5,PF4V1,APOA1,TTR,PXDN,CRH,SCGB3A1,PPBP,PF4,PCSK9,F2,INHBC,FBN1,THBS4,ANGPTL3,IGF1,EFEMP1,ADIPOQ,IGF2,VEGFC,SEMA3B
GO Function	GO:0050840	Extracellular matrix binding	7	56	1.0	0.0015	VTN,SPARC,PXDN,THBS1,ITGA2B,ECM1,TGFBI
GO Function	GO:0043394	Proteoglycan binding	6	37	1.12	0.0017	LRP1,APOE,THBS1,FCN2,FN1,CFH
GO Function	GO:0048018	Receptor ligand activity	19	499	0.49	0.0028	C5,PF4V1,APOA1,TTR,CRH,SCGB3A1,PPBP,PF4,F2,INHBC,FBN1,THBS4,ANGPTL3,IGF1,EFEMP1,ADIPOQ,IGF2,VEGFC,SEMA3B
GO Function	GO:0042803	Protein homodimerization activity	23	709	0.42	0.0042	ST6GAL1,GNPTG,MYH9,TPD52L2,HSD17B1,PLEK,APOA1,CAT,APOE,AMBP,CSF1R,MASP1,DST,PKM,HSP90AA1,ANG,BLM,APOA4,CENPF,FLNA,ACTN1,ADIPOQ,ENO1
GO Function	GO:0071813	Lipoprotein particle binding	5	28	1.16	0.0054	APOA1,APOE,THBS1,PCSK9,STAB1
GO Function	GO:0005198	Structural molecule activity	24	776	0.4	0.0055	TFPI2,VTN,MGP,APOE,PXDN,THBS1,FGB,DST,PRG2,TLN1,FBN1,FBLN1,OIT3,TUBA1B,FGG,FN1,MFAP5,COL18A1,ACTN1,LTBP1,TGFBI,TTN,ACTB,FGA
GO Function	GO:0005496	Steroid binding	8	101	0.81	0.0063	CETP,HSD17B1,APOA1,APOD,APOA4,GC,APOA5,ATP1A1
GO Function	GO:0001968	Fibronectin binding	5	30	1.13	0.0066	MMP2,IGFBP5,THBS1,FBLN1,IGFBP3
GO Function	GO:0070325	Lipoprotein particle receptor binding	5	30	1.13	0.0066	APOA1,LRP1,APOE,PCSK9,APOA5
GO Function	GO:0002020	Protease binding	9	135	0.73	0.0072	VWF,TIMP2,CSTA,A2ML1,A2M,FN1,ADAMTSL4,ECM1,TTN
GO Function	GO:0005520	Insulin-like growth factor binding	4	17	1.28	0.0110	IGFBP5,IGFBP4,IGFBP3,IGFALS
GO Function	GO:0008083	Growth factor activity	9	162	0.65	0.0256	PPBP,F2,INHBC,THBS4,ANGPTL3,IGF1,EFEMP1,IGF2,VEGFC
GO Function	GO:0005319	Lipid transporter activity	9	163	0.65	0.0262	CETP,APOA1,APOE,APOD,APOA4,RBP4,APOM,ATP9B,APOC4
GO Function	GO:0031995	Insulin-like growth factor II binding	3	8	1.48	0.0262	IGFBP5,IGFBP4,IGFBP3
GO Function	GO:0120020	Cholesterol transfer activity	4	23	1.15	0.0281	CETP,APOA1,APOE,APOA4
GO Function	GO:0031994	Insulin-like growth factor I binding	3	10	1.38	0.0416	IGFBP5,IGFBP4,IGFBP3
GO Function	GO:0020037	Heme binding	8	140	0.66	0.0419	MPO,CAT,PXDN,KLKB1,AMBP,GUCY1A1,HBA1,HBB
GO Component	GO:0005576	Extracellular region	192	4175	0.57	3.71e-77	SPP2,ST6GAL1,CPB2,CETP,GNPTG,SERPIND1,MYH9,F9,MMP2,TFPI2,PCOLCE,C5,MPO,VTN,PF4V1,MGP,GPLD1,SPARC,IGFBP5,PROC,PLEK,APOA1,TTR,CAT,APOE,PXDN,ANGPTL6,F12,CFHR5,THBS1,LYZ,VWF,PSMA6,SERPINA10,ITGA2B,TIMP2,ATRN,A1BG,BLVRB,NOTCH3,LCAT,CSTA,CP,KLKB1,F13A1,DNAH5,AMBP,CENPE,HPX,ITIH4,CDH11,IGFBP4,PSG9,FCN3,PLA2G7,CRH,HABP2,SAA4,SERPING1,FERMT3,DSC2,FCN2,PSG6,PGLYRP2,SCGB3A1,PPBP,PF4,MASP1,APEH,A2ML1,LALBA,LRG1,PCSK9,FGB,F2,INHBC,PSG1,PRG2,CSPG4,C1QB,CFHR1,TLN1,APOL1,PSG11,PKM,C7,HBA1,A2M,HSPA5,FBN1,P4HB,VMO1,SERPINA7,PAPPA,FBLN1,PSG3,OIT3,SERPINA11,HSP90AA1,ANG,FGG,THBS4,GANAB,APOD,PAPLN,FN1,APOA4,ITIH2,MFAP5,COL18A1,C8A,F13B,CFHR2,CFHR3,CFH,PAPPA2,F5,ADAM12,S100A7,S100A8,HTRA1,ADAMTSL4,ECM1,FLNA,ANGPTL3,C8B,RBP4,CCN5,GSN,C1QC,F10,F7,APOM,CA6,ADGRG2,CALML5,IGFBP3,SOD3,GOLM1,GPX3,IGF1,PROS1,ACTN1,EFEMP1,YWHAZ,GAPDH,BPIFC,ROCK1,MASP2,F11,C1S,PSG2,TF,LTBP1,PCYOX1,ADIPOQ,IGF2,C4A,PRG4,MST1,ITIH3,C4B,TGFBI,CFB,IGFALS,PAEP,GC,SELENOP,TSKU,SAA2,HPR,C1R,APOA5,ATP1A1,RNASE4,IGKV2D-28,TTN,APOC4,CFD,IGHV3-72,VEGFC,SEMA3B,PLCG2,HSPH1,IGLV10-54,KNG1,HBB,ACTB,ENO1,FGA,TPM3,PDIA4
GO Component	GO:0005615	Extracellular space	173	3247	0.63	1.20e-74	CPB2,CETP,GNPTG,SERPIND1,MYH9,F9,MMP2,TFPI2,PCOLCE,C5,MPO,VTN,PF4V1,MGP,GPLD1,SPARC,IGFBP5,PROC,APOA1,TTR,CAT,APOE,PXDN,ANGPTL6,F12,CFHR5,THBS1,LYZ,VWF,PSMA6,SERPINA10,ITGA2B,TIMP2,ATRN,A1BG,BLVRB,LCAT,CSTA,CP,KLKB1,F13A1,AMBP,HPX,ITIH4,CDH11,IGFBP4,FCN3,PLA2G7,CRH,HABP2,SAA4,SERPING1,FERMT3,DSC2,FCN2,PGLYRP2,SCGB3A1,PPBP,PF4,MASP1,APEH,A2ML1,LALBA,LRG1,PCSK9,FGB,F2,INHBC,PRG2,CSPG4,C1QB,CFHR1,TLN1,APOL1,PKM,C7,HBA1,A2M,HSPA5,FBN1,P4HB,VMO1,SERPINA7,PAPPA,FBLN1,OIT3,SERPINA11,HSP90AA1,ANG,FGG,THBS4,GANAB,APOD,FN1,APOA4,ITIH2,COL18A1,C8A,F13B,CFHR2,CFHR3,CFH,PAPPA2,F5,S100A7,S100A8,HTRA1,ECM1,FLNA,ANGPTL3,C8B,RBP4,CCN5,GSN,C1QC,F10,F7,APOM,CA6,ADGRG2,IGFBP3,SOD3,GOLM1,GPX3,IGF1,PROS1,ACTN1,EFEMP1,YWHAZ,GAPDH,BPIFC,MASP2,F11,C1S,TF,PCYOX1,ADIPOQ,IGF2,C4A,PRG4,MST1,ITIH3,C4B,TGFBI,CFB,IGFALS,PAEP,GC,SELENOP,TSKU,SAA2,HPR,C1R,APOA5,ATP1A1,RNASE4,IGKV2D-28,TTN,APOC4,CFD,IGHV3-72,VEGFC,SEMA3B,PLCG2,HSPH1,IGLV10-54,KNG1,HBB,ACTB,ENO1,FGA,TPM3,PDIA4
GO Component	GO:0072562	Blood microparticle	44	118	1.48	2.52e-44	VTN,APOA1,APOE,ITGA2B,A1BG,CP,F13A1,AMBP,HPX,ITIH4,FCN3,SERPING1,FCN2,FGB,F2,C1QB,CFHR1,APOL1,HBA1,A2M,FGG,FN1,APOA4,ITIH2,C8A,CFHR3,CFH,GSN,C1QC,PROS1,YWHAZ,C1S,TF,C4A,C4B,CFB,GC,HPR,C1R,IGKV2D-28,KNG1,HBB,ACTB,FGA
GO Component	GO:1903561	Extracellular vesicle	116	2120	0.65	1.21e-43	CPB2,CETP,GNPTG,SERPIND1,MYH9,F9,PCOLCE,C5,MPO,VTN,MGP,GPLD1,APOA1,TTR,CAT,APOE,PXDN,ANGPTL6,F12,THBS1,LYZ,VWF,PSMA6,SERPINA10,ITGA2B,ATRN,A1BG,BLVRB,LCAT,CP,KLKB1,AMBP,HPX,ITIH4,CDH11,SAA4,SERPING1,FERMT3,DSC2,FCN2,PGLYRP2,SCGB3A1,APEH,A2ML1,LRG1,FGB,F2,PRG2,CSPG4,TLN1,PKM,C7,HBA1,A2M,HSPA5,P4HB,VMO1,SERPINA7,FBLN1,HSP90AA1,FGG,THBS4,GANAB,APOD,FN1,APOA4,ITIH2,COL18A1,C8A,CFHR3,CFH,PAPPA2,F5,S100A8,HTRA1,ECM1,FLNA,C8B,RBP4,GSN,CA6,ADGRG2,SOD3,GPX3,PROS1,ACTN1,EFEMP1,YWHAZ,GAPDH,MASP2,F11,TF,PCYOX1,C4A,ITIH3,C4B,TGFBI,CFB,IGFALS,GC,SELENOP,SAA2,HPR,C1R,ATP1A1,IGKV2D-28,TTN,CFD,PLCG2,HSPH1,KNG1,HBB,ACTB,ENO1,FGA,TPM3
GO Component	GO:0070062	Extracellular exosome	115	2096	0.65	1.65e-43	CPB2,CETP,GNPTG,SERPIND1,MYH9,F9,PCOLCE,C5,MPO,VTN,MGP,GPLD1,APOA1,TTR,CAT,APOE,PXDN,ANGPTL6,F12,THBS1,LYZ,VWF,PSMA6,SERPINA10,ITGA2B,ATRN,A1BG,BLVRB,LCAT,CP,KLKB1,AMBP,HPX,ITIH4,CDH11,SAA4,SERPING1,FERMT3,DSC2,FCN2,PGLYRP2,SCGB3A1,APEH,A2ML1,LRG1,FGB,F2,PRG2,CSPG4,TLN1,PKM,C7,HBA1,A2M,HSPA5,P4HB,VMO1,SERPINA7,FBLN1,HSP90AA1,FGG,THBS4,GANAB,APOD,FN1,APOA4,ITIH2,COL18A1,C8A,CFHR3,CFH,PAPPA2,S100A8,HTRA1,ECM1,FLNA,C8B,RBP4,GSN,CA6,ADGRG2,SOD3,GPX3,PROS1,ACTN1,EFEMP1,YWHAZ,GAPDH,MASP2,F11,TF,PCYOX1,C4A,ITIH3,C4B,TGFBI,CFB,IGFALS,GC,SELENOP,SAA2,HPR,C1R,ATP1A1,IGKV2D-28,TTN,CFD,PLCG2,HSPH1,KNG1,HBB,ACTB,ENO1,FGA,TPM3
GO Component	GO:0062023	Collagen-containing extracellular matrix	61	407	1.08	1.01e-42	SPP2,F9,MMP2,VTN,MGP,SPARC,APOA1,APOE,PXDN,ANGPTL6,F12,THBS1,VWF,TIMP2,ATRN,A1BG,F13A1,AMBP,HPX,ITIH4,FCN3,SERPING1,FCN2,PF4,FGB,DST,F2,PRG2,CSPG4,C1QB,PKM,A2M,FBN1,FBLN1,HSP90AA1,ANG,FGG,THBS4,FN1,APOA4,ITIH2,MFAP5,COL18A1,S100A7,S100A8,HTRA1,ADAMTSL4,ECM1,ANGPTL3,C1QC,F7,SOD3,EFEMP1,LTBP1,ADIPOQ,PRG4,MST1,TGFBI,SEMA3B,KNG1,FGA
GO Component	GO:0031012	Extracellular matrix	66	552	0.98	5.30e-41	SPP2,F9,MMP2,TFPI2,VTN,MGP,GPLD1,SPARC,APOA1,APOE,PXDN,ANGPTL6,F12,THBS1,VWF,TIMP2,ATRN,A1BG,F13A1,AMBP,HPX,ITIH4,FCN3,SERPING1,FCN2,PF4,FGB,DST,F2,PRG2,CSPG4,C1QB,PKM,A2M,FBN1,FBLN1,HSP90AA1,ANG,FGG,THBS4,PAPLN,FN1,APOA4,ITIH2,MFAP5,COL18A1,S100A7,S100A8,HTRA1,ADAMTSL4,ECM1,ANGPTL3,CCN5,C1QC,F7,SOD3,EFEMP1,LTBP1,ADIPOQ,PRG4,MST1,TGFBI,IGFALS,SEMA3B,KNG1,FGA

GO Component	GO:0031982	Vesicle	143	3957	0.47	1.06e-36	SPP2,CPB2,CETP,GNPTG,SERPIND1,MYH9,F9,PCOLCE,C5,MPO,VTN,MGP,GPLD1,SPARC,APOA1,TTR,CAT,LRP1,APOE,PXDN,ANGPTL6,F12,THBS1,LYZ,VWF,PSMA6,SERPINA10,ITGA2B,TIMP2,ATRN,A1BG,BLVRB,LCAT,CP,KLKB1,F13A1,AMBP,TCIRG1,HPX,ITIH4,CDH11,SAA4,SERPING1,FERMT3,SEC24D,DSC2,FCN2,PGLYRP2,SCGB3A1,PPBP,PF4,FYCO1,APEH,A2ML1,ANTXR1,LRG1,PCSK9,FGB,DST,F2,PRG2,CSPG4,STAB1,TLN1,PKM,STXBP5,C7,HBA1,A2M,HSPA5,P4HB,VMO1,SERPINA7,FBLN1,HSP90AA1,ANG,FGG,THBS4,GANAB,APOD,FN1,APOA4,ITIH2,COL18A1,C8A,CFHR3,CFH,PAPPA2,F5,S100A7,S100A8,HTRA1,ECM1,FLNA,ANGPTL3,C8B,RBP4,GSN,F7,CA6,ADGRG2,CALML5,SOD3,GPX3,IGF1,PROS1,ACTN1,EFEMP1,YWHAZ,GAPDH,ROCK1,MASP2,F11,TF,PCYOX1,IGF2,C4A,ATP9B,ITIH3,C4B,TGFBI,CFB,IGFALS,GC,SELENOP,SAA2,HPR,C1R,APOA5,ATP1A1,IGKV2D-28,TTN,CFD,VEGFC,PLCG2,HSPH1,KNG1,HBB,ACTB,ENO1,FGA,TPM3,PDIA4
GO Component	GO:0034774	Secretory granule lumen	41	321	1.01	1.77e-25	SPP2,MPO,SPARC,APOA1,TTR,CAT,THBS1,LYZ,VWF,TIMP2,A1BG,F13A1,ITIH4,SERPING1,FERMT3,PPBP,PF4,LRG1,FGB,PKM,A2M,HSP90AA1,FGG,FN1,F5,S100A7,S100A8,ECM1,GSN,IGF1,PROS1,ACTN1,ROCK1,TF,IGF2,ITIH3,SELENOP,CFD,VEGFC,KNG1,FGA
GO Component	GO:0005788	Endoplasmic reticulum lumen	38	312	0.99	6.71e-23	SPP2,SERPIND1,F9,VTN,IGFBP5,PROC,APOA1,APOE,THBS1,SERPINA10,CP,IGFBP4,SERPING1,PCSK9,F2,APOL1,HSPA5,FBN1,P4HB,FGG,GANAB,FN1,APOA4,ITIH2,COL18A1,F5,ADAMTSL4,F10,F7,IGFBP3,GOLM1,TF,LTBP1,C4A,APOA5,KNG1,FGA,PDIA4
GO Component	GO:0031093	Platelet alpha granule lumen	22	66	1.43	6.16e-21	SPARC,THBS1,VWF,A1BG,F13A1,SERPING1,FERMT3,PPBP,PF4,FGB,A2M,FGG,FN1,F5,IGF1,PROS1,ACTN1,IGF2,CFD,VEGFC,KNG1,FGA
GO Component	GO:0031091	Platelet alpha granule	23	89	1.32	7.72e-20	SPARC,THBS1,VWF,ITGA2B,A1BG,F13A1,SERPING1,FERMT3,PPBP,PF4,FGB,A2M,FGG,FN1,F5,IGF1,PROS1,ACTN1,IGF2,CFD,VEGFC,KNG1,FGA
GO Component	GO:0030141	Secretory granule	50	873	0.67	3.90e-17	SPP2,MYH9,MPO,SPARC,APOA1,TTR,CAT,ANGPTL6,THBS1,LYZ,VWF,ITGA2B,TIMP2,A1BG,F13A1,TCIRG1,ITIH4,SERPING1,FERMT3,PPBP,PF4,APEH,LRG1,FGB,PRG2,PKM,STXBP5,A2M,HSP90AA1,FGG,FN1,F5,S100A7,S100A8,ECM1,GSN,CALML5,IGF1,PROS1,ACTN1,ROCK1,TF,IGF2,ITIH3,SELENOP,CFD,VEGFC,KNG1,HBB,FGA
GO Component	GO:1905370	Serine-type endopeptidase complex	14	26	1.64	2.18e-15	F9,PROC,F12,KLKB1,FCN3,FCN2,MASP1,F2,CFH,F5,F10,F7,MASP2,F11
GO Component	GO:0034358	Plasma lipoprotein particle	14	38	1.47	1.26e-13	CETP,APOA1,APOE,LCAT,PLA2G7,SAA4,APOL1,APOA4,APOM,PCYOX1,SAA2,HPR,APOA5,APOC4
GO Component	GO:0034364	High-density lipoprotein particle	13	29	1.56	1.59e-13	CETP,APOA1,APOE,LCAT,PLA2G7,SAA4,APOL1,APOA4,APOM,SAA2,HPR,APOA5,APOC4
GO Component	GO:0071944	Cell periphery	134	6015	0.25	2.99e-13	SPP2,MYH9,F9,MMP2,TFPI2,C5,VTN,MGP,GPLD1,SPARC,PLEK,APOA1,CAT,LRP1,APOE,PXDN,ANGPTL6,F12,THBS1,VWF,ITGA2B,TIMP2,ATRN,A1BG,BLVRB,NOTCH3,CSTA,CP,KLKB1,F13A1,AMBP,TCIRG1,HPX,ITIH4,CDH11,PSG9,FCN3,SERPING1,DSC2,CSF1R,PCDH1,FCN2,PSG6,PF4,MASP1,ANTXR1,SH3D19,PCSK9,FGB,DST,F2,PSG1,PRG2,CSPG4,STAB1,C1QB,TLN1,PKM,STXBP5,C7,A2M,HSPA5,FBN1,P4HB,FBLN1,PSG3,HSP90AA1,ANG,FGG,LIMS1,THBS4,PAPLN,FN1,APOA4,ITIH2,MFAP5,COL18A1,C8A,PAPPA2,F5,ADAM12,S100A7,S100A8,HTRA1,ADAMTSL4,ECM1,FLNA,ANGPTL3,C8B,CCN5,GSN,C1QC,F10,F7,ADGRG2,CD99,SOD3,GOLM1,IGF1,PROS1,ACTN1,EFEMP1,ILK,GAPDH,RAPGEF4,ROCK1,MASP2,F11,TF,LTBP1,PCYOX1,KSR2,ADIPOQ,C4A,ATP9B,PRG4,MST1,C4B,TGFBI,CFB,IGFALS,TCAF1,ATP1A1,IGKV2D-28,TTN,IGHV3-72,SEMA3B,PLCG2,OR52B4,IGLV10-54,KNG1,ACTB,ENO1,FGA
GO Component	GO:0097708	Intracellular vesicle	74	2484	0.38	3.22e-11	SPP2,MYH9,MPO,SPARC,APOA1,TTR,CAT,LRP1,APOE,ANGPTL6,THBS1,LYZ,VWF,ITGA2B,TIMP2,A1BG,F13A1,TCIRG1,HPX,ITIH4,SERPING1,FERMT3,SEC24D,DSC2,PPBP,PF4,FYCO1,APEH,ANTXR1,LRG1,PCSK9,FGB,DST,PRG2,STAB1,PKM,STXBP5,HBA1,A2M,HSPA5,P4HB,HSP90AA1,ANG,FGG,GANAB,FN1,APOA4,F5,S100A7,S100A8,ECM1,ANGPTL3,GSN,CALML5,IGF1,PROS1,ACTN1,YWHAZ,ROCK1,TF,IGF2,ATP9B,ITIH3,SELENOP,APOA5,ATP1A1,CFD,VEGFC,PLCG2,HSPH1,KNG1,HBB,FGA,PDIA4
GO Component	GO:0031410	Cytoplasmic vesicle	73	2482	0.38	8.88e-11	SPP2,MYH9,MPO,SPARC,APOA1,TTR,CAT,LRP1,APOE,ANGPTL6,THBS1,LYZ,VWF,ITGA2B,TIMP2,A1BG,F13A1,TCIRG1,HPX,ITIH4,SERPING1,FERMT3,SEC24D,DSC2,PPBP,PF4,FYCO1,APEH,ANTXR1,LRG1,PCSK9,FGB,DST,PRG2,STAB1,PKM,STXBP5,HBA1,A2M,HSPA5,P4HB,HSP90AA1,ANG,FGG,GANAB,FN1,APOA4,F5,S100A7,S100A8,ECM1,ANGPTL3,GSN,CALML5,IGF1,PROS1,ACTN1,YWHAZ,ROCK1,TF,IGF2,ATP9B,ITIH3,SELENOP,APOA5,ATP1A1,CFD,VEGFC,HSPH1,KNG1,HBB,FGA,PDIA4
GO Component	GO:1905369	Endopeptidase complex	16	104	1.09	1.09e-10	F9,PROC,F12,PSMA6,KLKB1,FCN3,FCN2,MASP1,F2,UBE3C,CFH,F5,F10,F7,MASP2,F11
GO Component	GO:0012505	Endomembrane system	106	4721	0.26	1.82e-09	SPP2,ST6GAL1,GNPTG,SERPIND1,MYH9,F9,MPO,VTN,SPARC,IGFBP5,PROC,APOA1,TTR,CAT,LRP1,APOE,PXDN,ANGPTL6,F12,THBS1,LYZ,VWF,SERPINA10,ITGA2B,TIMP2,A1BG,NOTCH3,CP,F13A1,AMBP,TCIRG1,ITIH4,IGFBP4,SERPING1,FERMT3,SEC24D,PPBP,PF4,FYCO1,APEH,LALBA,ANTXR1,LRG1,PCSK9,FGB,DST,F2,PRG2,CSPG4,APOL1,PKM,STXBP5,A2M,HSPA5,FBN1,P4HB,OIT3,HSP90AA1,FGG,THBS4,GANAB,APOD,FN1,APOA4,ITIH2,COL18A1,CENPF,F5,S100A7,S100A8,ADAMTSL4,ECM1,FLNA,ANGPTL3,GSN,F10,F7,CALML5,IGFBP3,SOD3,GOLM1,IGF1,PROS1,ACTN1,TNRC6A,GAPDH,ROCK1,TF,LTBP1,ADIPOQ,IGF2,C4A,ATP9B,ITIH3,TGFBI,SELENOP,APOA5,ATP1A1,CFD,VEGFC,SEMA3B,KNG1,HBB,ENO1,FGA,PDIA4
GO Component	GO:0071682	Endocytic vesicle lumen	9	23	1.5	7.44e-09	MPO,SPARC,APOA1,APOE,HPX,HBA1,HSP90AA1,HSPH1,HBB
GO Component	GO:0009986	Cell surface	37	894	0.52	1.49e-08	APMAP,SPARC,PXDN,THBS1,ITGA2B,NOTCH3,AMBP,PSG9,FCN3,CSF1R,FCN2,PSG6,MASP1,ANTXR1,PCSK9,FGB,PSG1,CSPG4,TLN1,PSG11,HSPA5,P4HB,PSG3,OIT3,HSP90AA1,FGG,ANGPTL3,F10,ADGRG2,MASP2,PSG2,TF,ADIPOQ,IGHV3-72,ENO1,FGA,PDIA4
GO Component	GO:0034361	Very-low-density lipoprotein particle	8	20	1.51	6.78e-08	APOA1,APOE,APOL1,APOA4,APOM,PCYOX1,APOA5,APOC4
GO Component	GO:0005783	Endoplasmic reticulum	56	2021	0.35	5.32e-07	SPP2,ST6GAL1,SERPIND1,F9,VTN,IGFBP5,PROC,APOA1,CAT,APOE,PXDN,F12,THBS1,VWF,SERPINA10,NOTCH3,CP,AMBP,IGFBP4,SERPING1,SEC24D,PCSK9,FGB,DST,F2,APOL1,PKM,HSPA5,FBN1,P4HB,FGG,THBS4,GANAB,APOD,FN1,APOA4,ITIH2,COL18A1,F5,S100A7,ADAMTSL4,F10,F7,IGFBP3,GOLM1,PROS1,TF,LTBP1,ADIPOQ,C4A,APOA5,ATP1A1,SEMA3B,KNG1,FGA,PDIA4
GO Component	GO:0005604	Basement membrane	12	98	1.0	5.80e-07	VTN,SPARC,PXDN,ATRN,DST,FBN1,FBLN1,ANG,THBS4,FN1,COL18A1,TGFBI
GO Component	GO:1904813	ficolin-1-rich granule lumen	12	124	0.89	6.15e-06	CAT,TIMP2,A1BG,APEH,LRG1,PRG2,PKM,HSP90AA1,GSN,CALML5,CFD,HBB

GO Component	GO:0032991	Protein-containing complex	106	5506	0.19	1.03e-05	CETP,GNPTG,MYH9,F9,C5,VTN,IGFBP5,PROC,APOA1,TTR,CAT,LRP1,APOE,F12,CFHR5,THBS1,PSMA6,ITGA2B,NOTCH3,LCAT,CSTA,KLKB1,F13A1,DNAH5,TCIRG1,CDH11,PSG9,FCN3,PLA2G7,SAA4,SEC24D,CSF1R,FCN2,MASP1,GUCY1A1,LALBA,PCSK9,FGB,F2,UBE3C,C1QB,CFHR1,NDUFS8,SUZ12,APOL1,SUMO4,STXBP5,C7,HBA1,HSPA5,P4HB,DNAH8,HSP90AA1,ANG,FGG,GANAB,FN1,BLM,APOA4,COL18A1,C8A,CENPF,F13B,CFHR2,CFH,F5,FLNA,SMARCA1,C8B,C1QC,F10,F7,APOM,IGFBP3,IGF1,TNRC6A,GAPDH,MASP2,F11,C1S,TF,LTBP1,PCYOX1,ADIPOQ,C4A,CDK3,C4B,CFB,IGFALS,UIMC1,SAA2,HPR,C1R,APOA5,ATP1A1,IGKV2D-28,TTN,APOC4,IGHV3-72,HSPH1,IGLV10-54,HBB,ACTB,ENO1,FGA,TPM3
GO Component	GO:0070013	Intracellular organelle lumen	108	5660	0.19	1.11e-05	SPP2,SERPIND1,MYH9,F9,MPO,VTN,SPARC,IGFBP5,PROC,APOA1,TTR,CAT,APOE,THBS1,LYZ,VWF,PSMA6,SERPINA10,TIMP2,A1BG,BLVRB,NOTCH3,CSTA,CP,F13A1,HPX,ITIH4,IGFBP4,SERPING1,FERMT3,CSF1R,PCDH1,PPBP,PF4,MASP1,APEH,C16orf46,LALBA,LRG1,SH3D19,PCSK9,FGB,DST,F2,PRG2,CSPG4,NDUFS8,SUZ12,APOL1,PKM,HBA1,A2M,HSPA5,FBN1,P4HB,HSP90AA1,ANG,FGG,GANAB,FN1,BLM,APOA4,ITIH2,COL18A1,CENPF,F5,MKI67,ADAM12,S100A7,S100A8,ADAMTSL4,ECM1,FLNA,SMARCA1,GSN,F10,F7,CALML5,IGFBP3,SOD3,GOLM1,IGF1,PROS1,ACTN1,TNRC6A,YWHAZ,ILK,ROCK1,TF,LTBP1,IGF2,C4A,ACSF2,ITIH3,GC,UIMC1,SELENOP,APOA5,TTN,CFD,VEGFC,HSPH1,KNG1,HBB,ACTB,TRIM66,FGA,PDIA4
GO Component	GO:0005577	Fibrinogen complex	5	8	1.7	1.49e-05	THBS1,FGB,FGG,FN1,FGA
GO Component	GO:0101002	ficolin-1-rich granule	13	185	0.75	4.86e-05	CAT,TIMP2,A1BG,TCIRG1,APEH,LRG1,PRG2,PKM,HSP90AA1,GSN,CALML5,CFD,HBB
GO Component	GO:0042567	Insulin-like growth factor ternary complex	4	4	1.91	6.70e-05	IGFBP5,IGFBP3,IGF1,IGFALS
GO Component	GO:0005796	Golgi lumen	10	106	0.88	7.30e-05	F9,VTN,PROC,LALBA,F2,CSPG4,F10,F7,SOD3,PROS1
GO Component	GO:0005602	Complement component C1 complex	4	5	1.81	0.00011	C1QB,C1QC,C1S,C1R
GO Component	GO:0030055	Cell-substrate junction	19	426	0.56	0.00011	MYH9,CAT,LRP1,ITGA2B,FERMT3,DST,CSPG4,TLN1,HSPA5,P4HB,LIMS1,S100A7,FLNA,GSN,CD99,ACTN1,YWHAZ,ILK,ACTB
GO Component	GO:0031089	Platelet dense granule lumen	5	14	1.46	0.00011	SPP2,ITIH4,ECM1,ITIH3,SELENOP
GO Component	GO:0005579	Membrane attack complex	4	7	1.66	0.00027	C5,C7,C8A,C8B
GO Component	GO:0005925	Focal adhesion	18	416	0.54	0.00028	MYH9,CAT,LRP1,ITGA2B,DST,CSPG4,TLN1,HSPA5,P4HB,LIMS1,S100A7,FLNA,GSN,CD99,ACTN1,YWHAZ,ILK,ACTB
GO Component	GO:0042627	Chylomicron	4	13	1.4	0.0017	APOA1,APOE,APOA4,APOA5
GO Component	GO:0005601	Classical-complement-pathway C3/C5 convertase complex	3	4	1.78	0.0022	C4A,C4B,CFB
GO Component	GO:0042470	Melanosome	8	109	0.77	0.0035	HSPA5,P4HB,HSP90AA1,GANAB,GSN,YWHAZ,ATP1A1,PDIA4
GO Component	GO:0030139	Endocytic vesicle	14	338	0.52	0.0041	MPO,SPARC,APOA1,LRP1,APOE,TCIRG1,HPX,STAB1,HBA1,HSP90AA1,GSN,TF,HSPH1,HBB
GO Component	GO:0009897	External side of plasma membrane	15	388	0.49	0.0048	THBS1,ITGA2B,FCN3,FCN2,MASP1,ANTXR1,PCSK9,FGB,P4HB,FGG,F10,MASP2,TF,IGHV3-72,FGA
GO Component	GO:0005886	Plasma membrane	95	5544	0.14	0.0065	MYH9,F9,MMP2,C5,VTN,SPARC,PLEK,APOA1,CAT,LRP1,APOE,F12,THBS1,ITGA2B,ATRN,BLVRB,NOTCH3,CSTA,CP,KLKB1,AMBP,TCIRG1,ITIH4,CDH11,PSG9,FCN3,DSC2,CSF1R,PCDH1,FCN2,PSG6,MASP1,ANTXR1,SH3D19,PCSK9,FGB,DST,F2,PSG1,CSPG4,STAB1,TLN1,STXBP5,C7,HSPA5,P4HB,PSG3,HSP90AA1,FGG,LIMS1,FN1,C8A,PAPPA2,F5,ADAM12,S100A8,HTRA1,FLNA,C8B,GSN,F10,F7,ADGRG2,CD99,GOLM1,IGF1,PROS1,ACTN1,ILK,GAPDH,RAPGEF4,ROCK1,MASP2,F11,TF,PCYOX1,KSR2,C4A,ATP9B,C4B,TGFBI,CFB,TCAF1,ATP1A1,IGKV2D-28,TTN,IGHV3-72,SEMA3B,PLCG2,OR52B4,IGLV10-54,KNG1,ACTB,ENO1,FGA
GO Component	GO:0034366	Spherical high-density lipoprotein particle	3	8	1.48	0.0092	APOA1,APOM,HPR
GO Component	GO:0110165	Cellular anatomical entity	239	18293	0.02	0.0122	SPP2,ST6GAL1,CPB2,CETP,GNPTG,SERPIND1,MYH9,TPD52L2,APMAP,F9,MMP2,TFPI2,PCOLCE,C5,MPO,HSD17B1,VTN,PF4V1,MGP,GPLD1,SPARC,IGFBP5,PROC,PLEK,APOA1,TTR,CAT,LRP1,APOE,PXDN,ANGPTL6,F12,CFHR5,THBS1,LYZ,VWF,PSMA6,SERPINA10,ITGA2B,TIMP2,ATRN,A1BG,BLVRB,NOTCH3,LCAT,CSTA,CP,KLKB1,F13A1,DNAH5,AMBP,CENPE,TCIRG1,HPX,ITIH4,CDH11,IGFBP4,PSG9,FCN3,PLA2G7,CRH,HABP2,SAA4,SERPING1,FERMT3,SEC24D,DSC2,CSF1R,PCDH1,FCN2,PSG6,PGLYRP2,SCGB3A1,PPBP,PF4,FYCO1,MASP1,APEH,GUCY1A1,C16orf46,A2ML1,LALBA,ANTXR1,LRG1,SH3D19,PCSK9,FGB,DST,F2,INHBC,PSG1,PRG2,CSPG4,STAB1,C1QB,CFHR1,NDUFS8,TLN1,SUZ12,APOL1,SUMO4,PSG11,PKM,STXBP5,C7,HBA1,A2M,HSPA5,FBN1,P4HB,VMO1,SERPINA7,PAPPA,FBLN1,PSG3,DNAH8,OIT3,SERPINA11,HSP90AA1,ANG,TUBA1B,FGG,LIMS1,THBS4,GANAB,APOD,PAPLN,FN1,BLM,APOA4,ITIH2,MFAP5,COL18A1,C8A,KIAA0753,CENPF,F13B,CFHR2,CFHR3,CFH,PAPPA2,F5,MKI67,ADAM12,S100A7,S100A8,HTRA1,ADAMTSL4,ECM1,FLNA,SMARCA1,ANGPTL3,C8B,RBP4,CCN5,GSN,C1QC,F10,F7,APOM,CA6,ADGRG2,CALML5,IGFBP3,CD99,SOD3,GOLM1,GPX3,IGF1,PROS1,ACTN1,EFEMP1,TNRC6A,YWHAZ,ILK,GAPDH,RAPGEF4,BPIFC,ROCK1,MAP3K7CL,MASP2,F11,C1S,PSG2,TF,DCDC1,LTBP1,PCYOX1,KSR2,ADIPOQ,IGF2,BZW1,C4A,ATP9B,PRG4,NBEAL1,SLAIN1,ACSF2,CKMT1A,CDK3,MST1,LMOD3,ITIH3,C4B,TGFBI,CFB,IGFALS,PAEP,TCAF1,GC,UIMC1,SELENOP,TSKU,SAA2,HPR,C1R,APOA5,ATP1A1,RNASE4,IGKV2D-28,NBPF10,TTN,APOC4,CFD,IGHV3-72,VEGFC,SEMA3B,PLCG2,OR52B4,HSPH1,IGLV10-54,KNG1,HBB,ACTB,TRIM66,ENO1,FGA,TPM3,PDIA4
GO Component	GO:0030017	Sarcomere	10	217	0.57	0.0162	MMP2,PSMA6,DST,FLNA,ACTN1,ILK,LMOD3,TTN,ENO1,TPM3
GO Component	GO:0043227	Membrane-bounded organelle	187	13188	0.06	0.0202	SPP2,ST6GAL1,CPB2,CETP,GNPTG,SERPIND1,MYH9,F9,MMP2,PCOLCE,C5,MPO,VTN,MGP,GPLD1,SPARC,IGFBP5,PROC,APOA1,TTR,CAT,LRP1,APOE,PXDN,ANGPTL6,F12,THBS1,LYZ,VWF,PSMA6,SERPINA10,ITGA2B,TIMP2,ATRN,A1BG,BLVRB,NOTCH3,LCAT,CSTA,CP,KLKB1,F13A1,DNAH5,AMBP,CENPE,TCIRG1,HPX,ITIH4,CDH11,IGFBP4,SAA4,SERPING1,FERMT3,SEC24D,DSC2,CSF1R,PCDH1,FCN2,PGLYRP2,SCGB3A1,PPBP,PF4,FYCO1,MASP1,APEH,C16orf46,A2ML1,LALBA,ANTXR1,LRG1,SH3D19,PCSK9,FGB,DST,F2,PRG2,CSPG4,STAB1,NDUFS8,TLN1,SUZ12,APOL1,SUMO4,PKM,STXBP5,C7,HBA1,A2M,HSPA5,FBN1,P4HB,VMO1,SERPINA7,FBLN1,DNAH8,OIT3,HSP90AA1,ANG,FGG,THBS4,GANAB,APOD,FN1,BLM,APOA4,ITIH2,COL18A1,C8A,CENPF,CFHR3,CFH,PAPPA2,F5,MKI67,ADAM12,S100A7,S100A8,HTRA1,ADAMTSL4,ECM1,FLNA,SMARCA1,ANGPTL3,C8B,RBP4,CCN5,GSN,F10,F7,CA6,ADGRG2,CALML5,IGFBP3,SOD3,GOLM1,GPX3,IGF1,PROS1,ACTN1,EFEMP1,TNRC6A,YWHAZ,ILK,GAPDH,ROCK1,MAP3K7CL,MASP2,F11,TF,LTBP1,PCYOX1,ADIPOQ,IGF2,C4A,ATP9B,ACSF2,CKMT1A,CDK3,MST1,ITIH3,C4B,TGFBI,CFB,IGFALS,GC,UIMC1,SELENOP,SAA2,HPR,C1R,APOA5,ATP1A1,IGKV2D-28,TTN,CFD,VEGFC,SEMA3B,PLCG2,HSPH1,KNG1,HBB,ACTB,TRIM66,ENO1,FGA,TPM3,PDIA4
GO Component	GO:0034362	Low-density lipoprotein particle	3	12	1.3	0.0226	APOE,PLA2G7,APOM
GO Component	GO:1904724	Tertiary granule lumen	5	55	0.87	0.0250	LYZ,TIMP2,PPBP,LRG1,HBB
GO Component	GO:0034365	Discoidal high-density lipoprotein particle	2	2	1.91	0.0261	APOE,APOM
GO Component	GO:0001527	Microfibril	3	13	1.27	0.0263	FBN1,MFAP5,LTBP1
GO Component	GO:0031252	Cell leading edge	14	422	0.43	0.0287	MYH9,PLEK,ANTXR1,DST,CSPG4,TLN1,P4HB,ANGPTL3,GSN,ACTN1,ILK,ROCK1,PLCG2,ACTB
GO Component	GO:0062167	Complement component C1q complex	2	3	1.73	0.0402	C1QB,C1QC

GO Component	GO:0005581	Collagen trimer	6	95	0.71	0.0414	FCN3,FCN2,C1QB,COL18A1,C1QC,ADIPOQ
GO Component	GO:0031672	A band	4	38	0.93	0.0458	DST,LMOD3,TTN,ENO1
STRING clusters	CL:18723	Mixed, incl. Complement and coagulation cascades, and Protein-lipid complex	70	196	1.46	2.54e-71	SPP2,CPB2,CETP,SERPIND1,F9,C5,PROC,APOA1,LRP1,APOE,F12,CFHR5,VWF,SERPINA10,LCAT,KLKB1,F13A1,AMBP,HPX,FCN3,HABP2,SAA4,SERPING1,FCN2,PGLYRP2,MASP1,PCSK9,FGB,F2,STAB1,C1QB,CFHR1,APOL1,C7,A2M,SERPINA7,SERPINA11,FGG,APOA4,ITIH2,C8A,F13B,CFHR2,CFHR3,CFH,F5,ECM1,ANGPTL3,C8B,C1QC,F10,F7,APOM,PROS1,MASP2,F11,C1S,TF,PCYOX1,C4A,ITIH3,C4B,CFB,SAA2,HPR,C1R,APOA5,APOC4,CFD,FGA
STRING clusters	CL:18724	Mixed, incl. Complement and coagulation cascades, and Protein-lipid complex	68	172	1.5	2.54e-71	SPP2,CPB2,CETP,SERPIND1,F9,C5,PROC,APOA1,LRP1,APOE,F12,CFHR5,VWF,SERPINA10,LCAT,KLKB1,F13A1,AMBP,HPX,FCN3,HABP2,SAA4,SERPING1,FCN2,PGLYRP2,MASP1,PCSK9,FGB,F2,C1QB,CFHR1,APOL1,C7,A2M,SERPINA7,SERPINA11,FGG,APOA4,ITIH2,C8A,F13B,CFHR2,CFHR3,CFH,F5,ECM1,ANGPTL3,C8B,C1QC,F10,F7,APOM,MASP2,F11,C1S,TF,PCYOX1,C4A,ITIH3,C4B,CFB,SAA2,HPR,C1R,APOA5,APOC4,CFD,FGA
STRING clusters	CL:18725	Complement and coagulation cascades, and Protein-lipid complex	66	166	1.51	1.21e-69	CPB2,CETP,SERPIND1,F9,C5,PROC,APOA1,LRP1,APOE,F12,CFHR5,VWF,SERPINA10,LCAT,KLKB1,F13A1,AMBP,HPX,FCN3,HABP2,SAA4,SERPING1,FCN2,PGLYRP2,MASP1,PCSK9,FGB,F2,C1QB,CFHR1,APOL1,C7,A2M,SERPINA7,SERPINA11,FGG,APOA4,ITIH2,C8A,F13B,CFHR2,CFHR3,CFH,F5,ANGPTL3,C8B,C1QC,F10,F7,APOM,MASP2,F11,C1S,TF,PCYOX1,C4A,ITIH3,C4B,CFB,SAA2,HPR,C1R,APOA5,APOC4,CFD,FGA
STRING clusters	CL:18726	Complement and coagulation cascades, and Protein-lipid complex	65	161	1.51	5.63e-69	CPB2,CETP,SERPIND1,F9,C5,PROC,APOA1,LRP1,APOE,F12,CFHR5,VWF,LCAT,KLKB1,F13A1,AMBP,HPX,FCN3,HABP2,SAA4,SERPING1,FCN2,PGLYRP2,MASP1,PCSK9,FGB,F2,C1QB,CFHR1,APOL1,C7,A2M,SERPINA7,SERPINA11,FGG,APOA4,ITIH2,C8A,F13B,CFHR2,CFHR3,CFH,F5,ANGPTL3,C8B,C1QC,F10,F7,APOM,MASP2,F11,C1S,TF,PCYOX1,C4A,ITIH3,C4B,CFB,SAA2,HPR,C1R,APOA5,APOC4,CFD,FGA
STRING clusters	CL:18727	Complement and coagulation cascades, and Positive regulation of opsonization	50	115	1.55	2.12e-53	CPB2,SERPIND1,F9,C5,PROC,LRP1,F12,CFHR5,VWF,KLKB1,F13A1,AMBP,HPX,FCN3,HABP2,SERPING1,FCN2,PGLYRP2,MASP1,FGB,F2,C1QB,CFHR1,C7,A2M,SERPINA7,SERPINA11,FGG,ITIH2,C8A,F13B,CFHR2,CFHR3,CFH,F5,C8B,C1QC,F10,F7,MASP2,F11,C1S,TF,C4A,ITIH3,C4B,CFB,C1R,CFD,FGA
STRING clusters	CL:18728	Complement and coagulation cascades, and Positive regulation of opsonization	49	109	1.56	6.59e-53	CPB2,SERPIND1,F9,C5,PROC,LRP1,F12,CFHR5,VWF,KLKB1,F13A1,AMBP,HPX,FCN3,HABP2,SERPING1,FCN2,MASP1,FGB,F2,C1QB,CFHR1,C7,A2M,SERPINA7,SERPINA11,FGG,ITIH2,C8A,F13B,CFHR2,CFHR3,CFH,F5,C8B,C1QC,F10,F7,MASP2,F11,C1S,TF,C4A,ITIH3,C4B,CFB,C1R,CFD,FGA
STRING clusters	CL:18731	Hemostasis, and Dissolution of Fibrin Clot	25	50	1.61	8.89e-27	CPB2,SERPIND1,F9,PROC,LRP1,F12,VWF,KLKB1,F13A1,AMBP,HPX,HABP2,FGB,F2,A2M,FGG,ITIH2,F13B,F5,F10,F7,F11,TF,ITIH3,FGA
STRING clusters	CL:18846	Complement cascade	22	50	1.55	1.72e-22	C5,CFHR5,FCN3,SERPING1,FCN2,MASP1,C1QB,CFHR1,C7,C8A,CFHR2,CFHR3,CFH,C8B,C1QC,MASP2,C1S,C4A,C4B,CFB,C1R,CFD
STRING clusters	CL:18848	Complement cascade	18	40	1.56	2.67e-18	C5,FCN3,SERPING1,FCN2,MASP1,C1QB,C7,C8A,CFH,C8B,C1QC,MASP2,C1S,C4A,C4B,CFB,C1R,CFD
STRING clusters	CL:18732	Mixed, incl. COVID-19, thrombosis and anticoagulation, and Scavenging of heme from plasma	15	26	1.67	2.66e-16	CPB2,SERPIND1,LRP1,F13A1,AMBP,HPX,HABP2,FGB,A2M,FGG,ITIH2,F13B,TF,ITIH3,FGA
STRING clusters	CL:18849	Initial triggering of complement, and Regulation of complement activation	14	31	1.56	5.26e-14	FCN3,SERPING1,FCN2,MASP1,C1QB,CFH,C1QC,MASP2,C1S,C4A,C4B,CFB,C1R,CFD
STRING clusters	CL:18956	Lipoprotein particle, and Assembly of active LPL and LIPC lipase complexes	15	46	1.42	2.01e-13	CETP,APOA1,APOE,LCAT,SAA4,PCSK9,APOL1,APOA4,ANGPTL3,APOM,PCYOX1,SAA2,HPR,APOA5,APOC4
STRING clusters	CL:18733	Mixed, incl. COVID-19, thrombosis and anticoagulation, and Inter-alpha-trypsin inhibitor heavy chain C-terminus	12	21	1.66	7.93e-13	CPB2,SERPIND1,F13A1,AMBP,HABP2,FGB,FGG,ITIH2,F13B,TF,ITIH3,FGA
STRING clusters	CL:18957	Lipoprotein particle	13	36	1.46	5.35e-12	CETP,APOA1,LCAT,SAA4,APOL1,APOA4,ANGPTL3,APOM,PCYOX1,SAA2,HPR,APOA5,APOC4
STRING clusters	CL:18960	High-density lipoprotein particle	12	26	1.57	5.35e-12	CETP,APOA1,LCAT,SAA4,APOL1,APOA4,APOM,PCYOX1,SAA2,HPR,APOA5,APOC4
STRING clusters	CL:18851	Initial triggering of complement, and Negative regulation of complement activation	11	24	1.57	6.36e-11	FCN3,SERPING1,FCN2,MASP1,C1QB,C1QC,MASP2,C1S,C4A,C4B,C1R
STRING clusters	CL:18784	Formation of Fibrin Clot (Clotting Cascade)	10	18	1.65	1.72e-10	F9,PROC,F12,VWF,KLKB1,F2,F5,F10,F7,F11
STRING clusters	CL:18962	High-density lipoprotein particle	10	21	1.58	5.33e-10	CETP,APOA1,LCAT,APOL1,APOA4,APOM,PCYOX1,HPR,APOA5,APOC4
STRING clusters	CL:18852	Creation of C4 and C2 activators	9	15	1.69	1.35e-09	FCN3,SERPING1,FCN2,MASP1,C1QB,C1QC,MASP2,C1S,C1R
STRING clusters	CL:24266	Insulin-like growth factor binding, and Pappalysin-1/2	9	16	1.66	1.90e-09	IGFBP5,IGFBP4,PRG2,PAPPA,PAPPA2,IGFBP3,IGF1,IGF2,IGFALS
STRING clusters	CL:18734	COVID-19, thrombosis and anticoagulation, and Negative regulation of fibrinolysis	8	11	1.77	5.99e-09	CPB2,SERPIND1,F13A1,HABP2,FGB,FGG,F13B,FGA
STRING clusters	CL:24268	Insulin-like growth factor-binding protein family 1-6, chordata, and Insulin-like growth factor	7	10	1.75	1.26e-07	IGFBP5,IGFBP4,PAPPA2,IGFBP3,IGF1,IGF2,IGFALS
STRING clusters	CL:18786	Hemophilia B, and Blood coagulation, common pathway	7	12	1.67	3.06e-07	F9,VWF,F2,F5,F10,F7,F11
STRING clusters	CL:19457	Extracellular matrix organization	16	180	0.86	4.64e-07	MMP2,PCOLCE,VTN,THBS1,ITGA2B,TIMP2,DST,FBN1,P4HB,FBLN1,THBS4,FN1,MFAP5,COL18A1,EFEMP1,LTBP1
STRING clusters	CL:18966	High-density lipoprotein particle	7	16	1.55	1.28e-06	CETP,APOA1,LCAT,APOA4,APOM,APOA5,APOC4
STRING clusters	CL:18869	Complement component C1 complex	5	6	1.83	1.81e-05	SERPING1,C1QB,C1QC,C1S,C1R
STRING clusters	CL:35044	Mixed, incl. Galactoside-binding lectin, and Transforming growth factor beta1 activation	6	16	1.48	2.96e-05	PSG9,PSG6,PSG1,PSG11,PSG3,PSG2
STRING clusters	CL:35049	Transforming growth factor beta1 activation, and Protein tyrosine kinase binding	5	7	1.76	2.96e-05	PSG9,PSG6,PSG1,PSG3,PSG2
STRING clusters	CL:18970	Triglyceride-rich lipoprotein particle remodeling, and Spherical high-density lipoprotein particle	5	11	1.56	0.00014	CETP,APOA1,LCAT,APOA4,APOA5
STRING clusters	CL:19695	Elastic fibre formation, and Matrix metalloproteinases	8	61	1.02	0.00026	MMP2,PCOLCE,TIMP2,FBN1,FBLN1,MFAP5,EFEMP1,LTBP1
STRING clusters	CL:18737	Fibrinogen, and Thrombophilia	4	6	1.73	0.00057	SERPIND1,FGB,FGG,FGA
STRING clusters	CL:18788	Extrinsic Pathway of Fibrin Clot Formation, and Factor V deficiency	4	6	1.73	0.00057	F2,F5,F10,F7
STRING clusters	CL:18854	Cell surface pattern recognition receptor signaling pathway	4	6	1.73	0.00057	FCN3,FCN2,MASP1,MASP2
STRING clusters	CL:18911	Membrane attack complex	4	7	1.66	0.00081	C5,C7,C8A,C8B
STRING clusters	CL:19696	Elastic fibre formation, and Tolloid/BMP1 peptidase domain	6	34	1.15	0.0010	PCOLCE,FBN1,FBLN1,MFAP5,EFEMP1,LTBP1
STRING clusters	CL:18755	Mixed, incl. Inter-alpha-trypsin inhibitor heavy chain C-terminus, and Alpha-1-acid glycoprotein	4	10	1.51	0.0022	AMBP,ITIH2,TF,ITIH3
STRING clusters	CL:18122	Mixed, incl. Cell-extracellular matrix interactions, and Leukocyte adhesion deficiency 3	5	26	1.19	0.0038	FERMT3,TLN1,LIMS1,FLNA,ILK

STRING clusters	CL:19697	Elastic fibre formation, and Transforming growth factor beta receptor complex assembly	5	27	1.17	0.0043	FBN1,FBLN1,MFAP5,EFEMP1,LTBP1
STRING clusters	CL:19699	Elastic fibre formation	4	14	1.36	0.0061	FBN1,FBLN1,MFAP5,EFEMP1
STRING clusters	CL:19587	Protein complex involved in cell adhesion, and Junctional epidermolysis bullosa	6	53	0.96	0.0082	VTN,THBS1,ITGA2B,DST,THBS4,FN1
STRING clusters	CL:19588	Integrin domain superfamily, and Cerebral malaria	5	32	1.1	0.0082	VTN,THBS1,ITGA2B,THBS4,FN1
STRING clusters	CL:18774	Scavenging of heme from plasma	3	5	1.69	0.0090	LRP1,HPX,A2M
STRING clusters	CL:18934	Hemolytic uremic syndrome	3	5	1.69	0.0090	CFHR5,CFHR1,CFHR3
STRING clusters	CL:24270	Insulin-like growth factor, and Insulin-like growth factor-binding protein family 1-6, chordata	3	5	1.69	0.0090	IGFBP4,IGF1,IGF2
STRING clusters	CL:19458	Collagen formation, and Protein complex involved in cell adhesion	8	119	0.73	0.0148	VTN,THBS1,ITGA2B,DST,P4HB,THBS4,FN1,COL18A1
STRING clusters	CL:9658	Detoxification of Reactive Oxygen Species, and Copper transport	5	40	1.0	0.0179	CAT,BLVRB,CP,SOD3,GPX3
KEGG	hsa04610	Complement and coagulation cascades	40	82	1.6	4.21e-44	CPB2,SERPIND1,F9,C5,VTN,PROC,F12,CFHR5,VWF,KLKB1,F13A1,SERPING1,FGB,F2,C1QB,CFHR1,C7,A2M,FGG,C8A,F13B,CFHR2,CFHR3,CFH,F5,C8B,C1QC,F10,F7,PROS1,MASP2,F11,C1S,C4A,C4B,CFB,C1R,CFD,KNG1,FGA
KEGG	hsa05150	Staphylococcus aureus infection	12	86	1.05	4.63e-07	C5,C1QB,FGG,CFH,C1QC,MASP2,C1S,C4A,C4B,CFB,C1R,CFD
KEGG	hsa05322	Systemic lupus erythematosus	11	94	0.98	7.45e-06	C5,C1QB,C7,C8A,C8B,C1QC,ACTN1,C1S,C4A,C4B,C1R
KEGG	hsa04611	Platelet activation	12	122	0.9	8.14e-06	VWF,ITGA2B,FERMT3,GUCY1A1,FGB,F2,TLN1,FGG,ROCK1,PLCG2,ACTB,FGA
KEGG	hsa04510	Focal adhesion	14	195	0.76	2.10e-05	VTN,THBS1,VWF,ITGA2B,TLN1,THBS4,FN1,FLNA,IGF1,ACTN1,ILK,ROCK1,VEGFC,ACTB
KEGG	hsa04979	Cholesterol metabolism	8	48	1.13	2.15e-05	CETP,APOA1,LRP1,APOE,LCAT,PCSK9,APOA4,ANGPTL3
KEGG	hsa05133	Pertussis	9	73	1.0	3.43e-05	C5,SERPING1,C1QB,C1QC,CALML5,C1S,C4A,C4B,C1R
KEGG	hsa05143	African trypanosomiasis	5	36	1.05	0.0058	APOA1,APOL1,HPR,KNG1,HBB
KEGG	hsa05205	Proteoglycans in cancer	10	194	0.62	0.0081	MMP2,VTN,THBS1,FN1,FLNA,IGF1,ROCK1,IGF2,PLCG2,ACTB
KEGG	hsa04918	Thyroid hormone synthesis	6	73	0.82	0.0140	TTR,HSPA5,SERPINA7,GPX3,ATP1A1,PDIA4
KEGG	hsa04670	Leukocyte transendothelial migration	7	111	0.71	0.0192	MMP2,CD99,ACTN1,RAPGEF4,ROCK1,PLCG2,ACTB
KEGG	hsa04512	ECM-receptor interaction	6	88	0.74	0.0293	VTN,THBS1,VWF,ITGA2B,THBS4,FN1
KEGG	hsa04015	Rap1 signaling pathway	9	201	0.56	0.0300	THBS1,ITGA2B,CSF1R,TLN1,CALML5,IGF1,RAPGEF4,VEGFC,ACTB
KEGG	hsa04810	Regulation of actin cytoskeleton	9	209	0.54	0.0361	MYH9,ITGA2B,F2,FN1,GSN,ACTN1,ROCK1,KNG1,ACTB
KEGG	hsa04151	PI3K-Akt signaling pathway	12	349	0.44	0.0384	VTN,THBS1,VWF,ITGA2B,CSF1R,HSP90AA1,THBS4,FN1,IGF1,YWHAZ,IGF2,VEGFC
KEGG	hsa05020	Prion disease	10	263	0.49	0.0428	C5,PSMA6,C1QB,NDUFS8,C7,HSPA5,TUBA1B,C8A,C8B,C1QC
KEGG	hsa04145	Phagosome	7	141	0.6	0.0462	MPO,THBS1,TCIRG1,TUBA1B,THBS4,C1R,ACTB
KEGG	hsa05200	Pathways in cancer	15	515	0.37	0.0462	MMP2,ITGA2B,NOTCH3,CSF1R,F2,HSP90AA1,FN1,CALML5,IGF1,ROCK1,IGF2,VEGFC,PLCG2,KNG1,TPM3
Reactome	HSA-109582	Hemostasis	62	607	0.92	1.35e-33	SPP2,SERPIND1,F9,PF4V1,SPARC,PROC,PLEK,APOA1,F12,THBS1,VWF,ITGA2B,A1BG,KLKB1,F13A1,CENPE,ITIH4,PSG9,SERPING1,FERMT3,PSG6,PPBP,PF4,GUCY1A1,FGB,F2,PSG1,TLN1,PSG11,A2M,HSPA5,PSG3,TUBA1B,FGG,FN1,F13B,F5,ECM1,FLNA,F10,F7,CD99,IGF1,PROS1,ACTN1,YWHAZ,RAPGEF4,F11,PSG2,TF,IGF2,ITIH3,SELENOP,IGKV2D-28,TTN,CFD,VEGFC,PLCG2,KNG1,HBB,ACTB,FGA
Reactome	HSA-114608	Platelet degranulation	35	126	1.35	8.95e-31	SPP2,SPARC,PLEK,APOA1,THBS1,VWF,ITGA2B,A1BG,F13A1,ITIH4,SERPING1,FERMT3,PPBP,PF4,FGB,TLN1,A2M,HSPA5,FGG,FN1,F5,ECM1,FLNA,IGF1,PROS1,ACTN1,TF,IGF2,ITIH3,SELENOP,TTN,CFD,VEGFC,KNG1,FGA
Reactome	HSA-381426	Regulation of Insulin-like Growth Factor (IGF) transport and uptake by Insulin-like Growth Factor Binding Proteins (IGFBPs)	32	124	1.32	2.30e-27	SPP2,SERPIND1,MMP2,IGFBP5,PROC,APOA1,APOE,SERPINA10,CP,IGFBP4,PCSK9,F2,APOL1,FBN1,P4HB,PAPPA,FGG,FN1,ITIH2,PAPPA2,F5,IGFBP3,GOLM1,IGF1,TF,LTBP1,IGF2,C4A,IGFALS,APOA5,KNG1,FGA
Reactome	HSA-166658	Complement cascade	26	59	1.55	5.95e-27	CPB2,C5,VTN,CFHR5,FCN3,SERPING1,FCN2,F2,C1QB,CFHR1,C7,C8A,CFHR2,CFHR3,CFH,C8B,C1QC,PROS1,MASP2,C1S,C4A,C4B,CFB,C1R,IGKV2D-28,CFD
Reactome	HSA-76002	Platelet activation, signaling and aggregation	39	260	1.08	3.88e-26	SPP2,SPARC,PLEK,APOA1,THBS1,VWF,ITGA2B,A1BG,F13A1,ITIH4,SERPING1,FERMT3,PPBP,PF4,FGB,F2,TLN1,A2M,HSPA5,FGG,FN1,F5,ECM1,FLNA,IGF1,PROS1,ACTN1,YWHAZ,RAPGEF4,TF,IGF2,ITIH3,SELENOP,TTN,CFD,VEGFC,PLCG2,KNG1,FGA
Reactome	HSA-140877	Formation of Fibrin Clot (Clotting Cascade)	22	39	1.66	1.53e-24	SERPIND1,F9,PF4V1,PROC,F12,VWF,KLKB1,F13A1,SERPING1,PF4,FGB,F2,A2M,FGG,F13B,F5,F10,F7,PROS1,F11,KNG1,FGA
Reactome	HSA-977606	Regulation of Complement cascade	22	49	1.56	6.77e-23	CPB2,C5,VTN,CFHR5,SERPING1,F2,C1QB,CFHR1,C7,C8A,CFHR2,CFHR3,CFH,C8B,C1QC,PROS1,C1S,C4A,C4B,CFB,C1R,IGKV2D-28
Reactome	HSA-8957275	Post-translational protein phosphorylation	25	107	1.28	2.10e-20	SPP2,SERPIND1,IGFBP5,PROC,APOA1,APOE,SERPINA10,CP,IGFBP4,PCSK9,APOL1,FBN1,P4HB,FGG,FN1,ITIH2,F5,IGFBP3,GOLM1,TF,LTBP1,C4A,APOA5,KNG1,FGA
Reactome	HSA-168249	Innate Immune System	53	1041	0.61	7.51e-16	CPB2,MYH9,C5,MPO,VTN,TTR,CAT,CFHR5,LYZ,PSMA6,TIMP2,A1BG,TCIRG1,FCN3,SERPING1,FCN2,PGLYRP2,PPBP,APEH,LRG1,FGB,F2,PRG2,C1QB,CFHR1,PKM,C7,HSP90AA1,FGG,C8A,CFHR2,CFHR3,CFH,S100A7,S100A8,C8B,GSN,C1QC,CALML5,PROS1,ROCK1,MASP2,C1S,C4A,C4B,CFB,C1R,IGKV2D-28,CFD,PLCG2,HBB,ACTB,FGA
Reactome	HSA-140875	Common Pathway of Fibrin Clot Formation	13	22	1.68	2.41e-14	SERPIND1,PF4V1,PROC,F13A1,PF4,FGB,F2,FGG,F13B,F5,F10,PROS1,FGA
Reactome	HSA-140837	Intrinsic Pathway of Fibrin Clot Formation	13	23	1.66	3.42e-14	SERPIND1,F9,PROC,F12,VWF,KLKB1,SERPING1,F2,A2M,F10,PROS1,F11,KNG1
Reactome	HSA-1474244	Extracellular matrix organization	27	300	0.86	9.16e-13	MMP2,PCOLCE,VTN,SPARC,TTR,PXDN,THBS1,VWF,ITGA2B,TIMP2,KLKB1,FGB,DST,A2M,FBN1,P4HB,FBLN1,FGG,FN1,MFAP5,COL18A1,ADAM12,HTRA1,ACTN1,EFEMP1,LTBP1,FGA
Reactome	HSA-166663	Initial triggering of complement	12	24	1.61	1.35e-12	FCN3,FCN2,C1QB,C1QC,MASP2,C1S,C4A,C4B,CFB,C1R,IGKV2D-28,CFD
Reactome	HSA-168256	Immune System	67	1979	0.44	3.89e-12	CPB2,MYH9,MMP2,C5,MPO,VTN,TTR,CAT,CFHR5,LYZ,PSMA6,TIMP2,A1BG,F13A1,CENPE,TCIRG1,FCN3,SERPING1,SEC24D,CSF1R,FCN2,PGLYRP2,PPBP,APEH,LRG1,FGB,F2,UBE3C,PRG2,C1QB,CFHR1,PKM,C7,HSPA5,P4HB,HSP90AA1,TUBA1B,FGG,FN1,C8A,CFHR2,CFHR3,CFH,S100A7,S100A8,FLNA,C8B,GSN,C1QC,CALML5,CD99,PROS1,YWHAZ,RAPGEF4,ROCK1,MASP2,C1S,C4A,C4B,CFB,C1R,IGKV2D-28,CFD,PLCG2,HBB,ACTB,FGA
Reactome	HSA-2173782	Binding and Uptake of Ligands by Scavenger Receptors	13	43	1.39	1.69e-11	SPARC,APOA1,LRP1,APOE,AMBP,HPX,STAB1,APOL1,HSP90AA1,HPR,IGKV2D-28,HSPH1,HBB
Reactome	HSA-2168880	Scavenging of heme from plasma	8	15	1.63	2.52e-08	APOA1,LRP1,AMBP,HPX,APOL1,HPR,IGKV2D-28,HBB
Reactome	HSA-166786	Creation of C4 and C2 activators	8	16	1.61	3.53e-08	FCN3,FCN2,C1QB,C1QC,MASP2,C1S,C1R,IGKV2D-28
Reactome	HSA-9651496	Defects of contact activation system (CAS) and kallikrein/kinin system (KKS)	8	16	1.61	3.53e-08	F9,F12,VWF,KLKB1,SERPING1,F2,F10,F11
Reactome	HSA-392499	Metabolism of proteins	57	1917	0.38	5.71e-08	SPP2,ST6GAL1,CPB2,SERPIND1,F9,MMP2,GPLD1,IGFBP5,PROC,APOA1,TTR,APOE,THBS1,LYZ,PSMA6,SERPINA10,CP,IGFBP4,PLA2G7,SEC24D,APEH,PCSK9,F2,INHBC,SUZ12,APOL1,FBN1,P4HB,PAPPA,TUBA1B,FGG,GANAB,FN1,BLM,APOA4,ITIH2,PAPPA2,F5,ADAMTSL4,GSN,F10,F7,IGFBP3,GOLM1,IGF1,PROS1,TF,LTBP1,IGF2,C4A,TGFBI,IGFALS,UIMC1,APOA5,KNG1,ACTB,FGA
Reactome	HSA-6802948	Signaling by high-kinase activity BRAF mutants	9	36	1.3	3.32e-07	VWF,ITGA2B,FGB,TLN1,FGG,FN1,KSR2,ACTB,FGA

Reactome	HSA-202733	Cell surface interactions at the vascular wall	14	139	0.91	5.45e-07	PF4V1,PROC,PSG9,PSG6,PF4,F2,PSG1,PSG11,PSG3,FN1,CD99,PROS1,PSG2,IGKV2D-28
Reactome	HSA-354194	GRB2:SOS provides linkage to MAPK signaling for Integrins	7	15	1.58	5.45e-07	VWF,ITGA2B,FGB,TLN1,FGG,FN1,FGA
Reactome	HSA-372708	p130Cas linkage to MAPK signaling for integrins	7	15	1.58	5.45e-07	VWF,ITGA2B,FGB,TLN1,FGG,FN1,FGA
Reactome	HSA-76009	Platelet Aggregation (Plug Formation)	9	39	1.27	5.45e-07	VWF,ITGA2B,FGB,F2,TLN1,FGG,FN1,RAPGEF4,FGA
Reactome	HSA-5674135	MAP2K and MAPK activation	9	40	1.26	6.01e-07	VWF,ITGA2B,FGB,TLN1,FGG,FN1,KSR2,ACTB,FGA
Reactome	HSA-174824	Plasma lipoprotein assembly, remodeling, and clearance	11	75	1.07	6.46e-07	CETP,APOA1,APOE,LCAT,PCSK9,A2M,P4HB,APOA4,ANGPTL3,APOA5,APOC4
Reactome	HSA-354192	Integrin signaling	8	27	1.38	6.46e-07	VWF,ITGA2B,FGB,TLN1,FGG,FN1,RAPGEF4,FGA
Reactome	HSA-9656223	Signaling by RAF1 mutants	9	41	1.25	6.53e-07	VWF,ITGA2B,FGB,TLN1,FGG,FN1,KSR2,ACTB,FGA
Reactome	HSA-159763	Transport of gamma-carboxylated protein precursors from the endoplasmic reticulum to the Golgi apparatus	6	9	1.73	1.06e-06	F9,PROC,F2,F10,F7,PROS1
Reactome	HSA-6802946	Signaling by moderate kinase activity BRAF mutants	9	45	1.21	1.25e-06	VWF,ITGA2B,FGB,TLN1,FGG,FN1,KSR2,ACTB,FGA
Reactome	HSA-6802955	Paradoxical activation of RAF signaling by kinase inactive BRAF	9	45	1.21	1.25e-06	VWF,ITGA2B,FGB,TLN1,FGG,FN1,KSR2,ACTB,FGA
Reactome	HSA-9649948	Signaling downstream of RAS mutants	9	45	1.21	1.25e-06	VWF,ITGA2B,FGB,TLN1,FGG,FN1,KSR2,ACTB,FGA
Reactome	HSA-159740	Gamma-carboxylation of protein precursors	6	10	1.69	1.45e-06	F9,PROC,F2,F10,F7,PROS1
Reactome	HSA-159782	Removal of aminoterminal propeptides from gamma-carboxylated proteins	6	10	1.69	1.45e-06	F9,PROC,F2,F10,F7,PROS1
Reactome	HSA-8963899	Plasma lipoprotein remodeling	8	34	1.28	2.16e-06	CETP,APOA1,APOE,LCAT,P4HB,APOA4,ANGPTL3,APOA5
Reactome	HSA-597592	Post-translational protein modification	42	1405	0.38	7.79e-06	SPP2,ST6GAL1,SERPIND1,F9,GPLD1,IGFBP5,PROC,APOA1,APOE,THBS1,PSMA6,SERPINA10,CP,IGFBP4,SEC24D,PCSK9,F2,SUZ12,APOL1,FBN1,P4HB,TUBA1B,FGG,GANAB,FN1,BLM,ITIH2,F5,ADAMTSL4,F10,F7,IGFBP3,GOLM1,PROS1,TF,LTBP1,C4A,UIMC1,APOA5,KNG1,ACTB,FGA
Reactome	HSA-216083	Integrin cell surface interactions	10	85	0.98	1.42e-05	VTN,THBS1,VWF,ITGA2B,FGB,FBN1,FGG,FN1,COL18A1,FGA
Reactome	HSA-6802952	Signaling by BRAF and RAF1 fusions	9	65	1.05	1.59e-05	VWF,ITGA2B,FGB,TLN1,FGG,FN1,KSR2,ACTB,FGA
Reactome	HSA-8963898	Plasma lipoprotein assembly	6	19	1.41	2.44e-05	APOA1,APOE,A2M,P4HB,APOA4,APOC4
Reactome	HSA-173623	Classical antibody-mediated complement activation	5	9	1.65	2.50e-05	C1QB,C1QC,C1S,C1R,IGKV2D-28
Reactome	HSA-2129379	Molecules associated with elastic fibres	7	37	1.18	4.98e-05	VTN,FBN1,FBLN1,FN1,MFAP5,EFEMP1,LTBP1
Reactome	HSA-975634	Retinoid metabolism and transport	7	44	1.11	0.00013	APOA1,TTR,LRP1,APOE,APOA4,RBP4,APOM
Reactome	HSA-6798695	Neutrophil degranulation	20	476	0.53	0.00016	MPO,TTR,CAT,LYZ,TIMP2,A1BG,TCIRG1,PPBP,APEH,LRG1,PRG2,PKM,HSP90AA1,S100A7,S100A8,GSN,CALML5,ROCK1,CFD,HBB
Reactome	HSA-174577	Activation of C3 and C5	4	7	1.66	0.00030	C5,C4A,C4B,CFB
Reactome	HSA-9662001	Defective factor VIII causes hemophilia A	4	7	1.66	0.00030	F9,VWF,F2,F10
Reactome	HSA-1643685	Disease	43	1702	0.31	0.00031	ST6GAL1,MYH9,F9,APOA1,TTR,F12,THBS1,VWF,PSMA6,ITGA2B,NOTCH3,CP,KLKB1,SERPING1,SEC24D,ANTXR1,FGB,F2,CSPG4,TLN1,SUZ12,HSP90AA1,TUBA1B,FGG,GANAB,FN1,BLM,S100A8,ADAMTSL4,RBP4,F10,YWHAZ,ROCK1,MASP2,F11,KSR2,ATP1A1,IGKV2D-28,PLCG2,ACTB,ENO1,FGA,TPM3
Reactome	HSA-446353	Cell-extracellular matrix interactions	5	18	1.35	0.00031	LIMS1,FLNA,ACTN1,ILK,ACTB
Reactome	HSA-166665	Terminal pathway of complement	4	8	1.61	0.00042	C5,C7,C8A,C8B
Reactome	HSA-977225	Amyloid fiber formation	8	79	0.91	0.00046	APOA1,TTR,APOE,LYZ,APOA4,GSN,TGFBI,FGA
Reactome	HSA-8963888	Chylomicron assembly	4	10	1.51	0.00080	APOA1,APOE,P4HB,APOA4
Reactome	HSA-8963901	Chylomicron remodeling	4	10	1.51	0.00080	APOA1,APOE,APOA4,APOA5
Reactome	HSA-8964058	HDL remodeling	4	10	1.51	0.00080	CETP,APOA1,APOE,LCAT
Reactome	HSA-446728	Cell junction organization	8	92	0.85	0.0012	CDH11,DST,ANG,LIMS1,FLNA,ACTN1,ILK,ACTB
Reactome	HSA-9657688	Defective factor XII causes hereditary angioedema	3	3	1.91	0.0013	F12,KLKB1,F2
Reactome	HSA-9657689	Defective SERPING1 causes hereditary angioedema	3	3	1.91	0.0013	F12,KLKB1,SERPING1
Reactome	HSA-5653656	Vesicle-mediated transport	22	666	0.43	0.0014	MYH9,SPARC,APOA1,LRP1,APOE,AMBP,CENPE,HPX,SEC24D,SH3D19,STAB1,APOL1,HSP90AA1,TUBA1B,F5,YWHAZ,TF,HPR,IGKV2D-28,HSPH1,HBB,ACTB
Reactome	HSA-9024446	NR1H2 and NR1H3-mediated signaling	6	46	1.02	0.0014	CETP,APOE,APOD,ANGPTL3,TNRC6A,APOC4
Reactome	HSA-2187338	Visual phototransduction	8	99	0.81	0.0018	HSD17B1,APOA1,TTR,LRP1,APOE,APOA4,RBP4,APOM
Reactome	HSA-2855086	Ficolins bind to repetitive carbohydrate structures on the target cell surface	3	4	1.78	0.0021	FCN3,FCN2,MASP2
Reactome	HSA-140834	Extrinsic Pathway of Fibrin Clot Formation	3	5	1.69	0.0033	F9,F10,F7
Reactome	HSA-5602498	MyD88 deficiency (TLR2/4)	4	17	1.28	0.0037	FGB,FGG,S100A8,FGA
Reactome	HSA-5603041	IRAK4 deficiency (TLR2/4)	4	18	1.25	0.0044	FGB,FGG,S100A8,FGA
Reactome	HSA-9029569	NR1H3 & NR1H2 regulate gene expression linked to cholesterol transport and efflux	5	36	1.05	0.0044	CETP,APOE,APOD,TNRC6A,APOC4
Reactome	HSA-5686938	Regulation of TLR by endogenous ligand	4	20	1.21	0.0061	FGB,FGG,S100A8,FGA
Reactome	HSA-162582	Signal Transduction	51	2540	0.21	0.0109	CETP,MYH9,MMP2,C5,SPARC,APOE,THBS1,VWF,PSMA6,ITGA2B,NOTCH3,CENPE,TCIRG1,CRH,PPBP,PF4,FGB,DST,F2,TLN1,SUZ12,FBN1,P4HB,HSP90AA1,TUBA1B,FGG,THBS4,APOD,FN1,CENPF,ADAM12,S100A8,FLNA,ANGPTL3,IGF1,ACTN1,TNRC6A,YWHAZ,RAPGEF4,ROCK1,LTBP1,KSR2,IGF2,MST1,APOC4,VEGFC,PLCG2,KNG1,ACTB,FGA,TPM3
Reactome	HSA-9668250	Defective factor IX causes hemophilia B	3	9	1.43	0.0110	F9,F10,F11
Reactome	HSA-1474228	Degradation of the extracellular matrix	8	140	0.66	0.0144	MMP2,TIMP2,KLKB1,A2M,FBN1,FN1,COL18A1,HTRA1
Reactome	HSA-3000170	Syndecan interactions	4	27	1.08	0.0155	VTN,THBS1,FN1,ACTN1
Reactome	HSA-430116	GP1b-IX-V activation signalling	3	12	1.3	0.0213	VWF,FLNA,YWHAZ
Reactome	HSA-1592389	Activation of Matrix Metalloproteinases	4	33	0.99	0.0293	MMP2,TIMP2,KLKB1,COL18A1
Reactome	HSA-3000171	Non-integrin membrane-ECM interactions	5	59	0.84	0.0307	VTN,TTR,THBS1,FN1,ACTN1
Reactome	HSA-3299685	Detoxification of Reactive Oxygen Species	4	36	0.95	0.0384	CAT,P4HB,SOD3,GPX3
Reactome	HSA-9672383	Defective factor IX causes thrombophilia	2	3	1.73	0.0391	F9,F10
Reactome	HSA-9672391	Defective F8 cleavage by thrombin	2	3	1.73	0.0391	VWF,F2
Reactome	HSA-9672396	Defective cofactor function of FVIIIa variant	2	3	1.73	0.0391	F9,F10
Reactome	HSA-9673202	Defective F9 variant does not activate FX	2	3	1.73	0.0391	F9,F10
Reactome	HSA-8964043	Plasma lipoprotein clearance	4	37	0.94	0.0398	APOA1,APOE,PCSK9,APOC4
WikiPathways	WP558	Complement and coagulation cascades	27	58	1.57	2.47e-28	CPB2,SERPIND1,F9,PROC,F12,VWF,KLKB1,SERPING1,MASP1,FGB,F2,C1QB,C7,F13B,CFH,F5,C1QC,F10,F7,PROS1,MASP2,C1S,C4B,CFB,C1R,CFD,KNG1
WikiPathways	WP2806	Complement system	27	96	1.36	8.43e-24	C5,VTN,APOA1,F12,THBS1,ITGA2B,KLKB1,F13A1,SERPING1,FCN2,MASP1,FGB,C7,FGG,C8A,CFHR2,CFH,F10,PROS1,MASP2,F11,C1S,ADIPOQ,C4A,CFB,CFD,FGA
WikiPathways	WP545	Complement activation	14	22	1.71	9.01e-16	C5,MASP1,C1QB,C7,C8A,C8B,C1QC,MASP2,C1S,C4A,C4B,CFB,C1R,CFD
WikiPathways	WP5090	Complement system in neuronal development and plasticity	20	105	1.19	1.15e-14	C5,VTN,FCN3,SERPING1,FCN2,MASP1,C1QB,C7,C8A,CFH,C8B,C1QC,PROS1,MASP2,C1S,C4A,C4B,CFB,C1R,CFD
WikiPathways	WP272	Blood clotting cascade	13	22	1.68	1.81e-14	F9,F12,VWF,KLKB1,FGB,F2,FGG,F13B,F5,F10,F7,F11,FGA
WikiPathways	WP5115	Network map of SARS-CoV-2 signaling pathway	24	218	0.95	4.07e-13	APOA1,SERPINA10,ITIH4,PF4,LRG1,FGB,APOL1,FGG,APOD,FN1,C8A,CFH,GSN,APOM,IGFBP3,C1S,TF,ITIH3,CFB,SAA2,C1R,HBB,ACTB,FGA
WikiPathways	WP15	Selenium micronutrient network	15	84	1.16	1.14e-10	MPO,APOA1,CAT,SAA4,FGB,F2,HBA1,FGG,F7,SOD3,GPX3,SELENOP,SAA2,HBB,FGA
WikiPathways	WP176	Folate metabolism	14	67	1.23	1.14e-10	MPO,APOA1,CAT,SAA4,FGB,F2,HBA1,FGG,F7,SOD3,GPX3,SAA2,HBB,FGA

WikiPathways	WP1533	Vitamin B12 metabolism	10	50	1.21	2.39e-07	MPO,APOA1,APOE,SAA4,F2,HBA1,F7,SOD3,SAA2,HBB
WikiPathways	WP4927	COVID-19, thrombosis and anticoagulation	6	7	1.84	3.93e-07	F13A1,FGB,F2,FGG,F13B,FGA
WikiPathways	WP5108	Familial hyperlipidemia type 1	7	18	1.5	1.07e-06	CETP,APOA1,LRP1,LCAT,APOA4,ANGPTL3,APOA5
WikiPathways	WP5186	Vitamin K metabolism and activation of dependent proteins	6	12	1.61	3.37e-06	F9,PROC,F2,F10,F7,PROS1
WikiPathways	WP5110	Familial hyperlipidemia type 3	6	13	1.57	4.50e-06	CETP,APOA1,LRP1,APOE,LCAT,APOA4
WikiPathways	WP5112	Familial hyperlipidemia type 5	6	15	1.51	8.19e-06	CETP,APOA1,LRP1,LCAT,APOA4,APOA5
WikiPathways	WP430	Statin inhibition of cholesterol production	7	29	1.29	1.21e-05	CETP,APOA1,LRP1,APOE,LCAT,APOA4,APOA5
WikiPathways	WP306	Focal adhesion	14	195	0.76	1.53e-05	VTN,THBS1,VWF,ITGA2B,TLN1,THBS4,FN1,FLNA,IGF1,ACTN1,ILK,ROCK1,VEGFC,ACTB
WikiPathways	WP5323	Fatty Acids and Lipoproteins Transport in Hepatocytes	19	380	0.61	2.38e-05	CETP,VTN,APOA1,LRP1,APOE,LCAT,PLA2G7,SAA4,PCSK9,APOL1,APOA4,APOM,PCYOX1,ADIPOQ,ATP9B,PRG4,SAA2,APOA5,APOC4
WikiPathways	WP5111	Familial hyperlipidemia type 4	6	22	1.34	4.11e-05	CETP,APOA1,LRP1,LCAT,APOA4,APOA5
WikiPathways	WP2328	Allograft rejection	9	88	0.92	0.00012	C5,C1QB,C7,TUBA1B,C8A,C8B,C1QC,C4A,C4B
WikiPathways	WP5109	Familial hyperlipidemia type 2	5	16	1.4	0.00018	CETP,APOA1,LCAT,PCSK9,APOA4
WikiPathways	WP4522	Metabolic pathway of LDL, HDL and TG, including diseases	5	17	1.38	0.00022	CETP,APOA1,APOE,LCAT,PCSK9
WikiPathways	WP5304	Cholesterol metabolism	8	72	0.95	0.00022	CETP,APOA1,LRP1,APOE,LCAT,PCSK9,APOA4,ANGPTL3
WikiPathways	WP5089	Kinin-Kallikrein pathway	4	7	1.66	0.00023	F12,KLKB1,SERPING1,KNG1
WikiPathways	WP3941	Oxidative damage response	6	39	1.09	0.00056	C5,C1QB,C1QC,C1S,C4B,C1R
WikiPathways	WP3601	Lipid particles composition	4	10	1.51	0.00062	CETP,APOA1,APOE,LCAT
WikiPathways	WP4969	RAS and bradykinin pathways in COVID-19	5	28	1.16	0.0014	F12,KLKB1,SERPING1,ROCK1,KNG1
WikiPathways	WP5055	Burn wound healing	8	100	0.81	0.0016	MMP2,SPARC,TIMP2,F13A1,AMBP,FBN1,FN1,ADAM12
WikiPathways	WP2572	Primary focal segmental glomerulosclerosis (FSGS)	6	72	0.83	0.0108	MYH9,VTN,TLN1,LIMS1,MKI67,ILK
WikiPathways	WP5104	Acquired partial lipodystrophy / Barraquer-Simons syndrome	3	10	1.38	0.0127	C5,CFB,CFD
WikiPathways	WP5049	Glycolysis in senescence	3	11	1.34	0.0155	PKM,GAPDH,ENO1
WikiPathways	WP4629	Aerobic glycolysis	3	12	1.3	0.0186	PKM,GAPDH,ENO1
WikiPathways	WP289	Myometrial relaxation and contraction pathways	8	156	0.62	0.0232	IGFBP5,IGFBP4,CRH,GUCY1A1,IGFBP3,YWHAZ,PLCG2,ACTB
WikiPathways	WP4493	Cells and molecules involved in local acute inflammatory response	3	17	1.15	0.0418	C5,C7,KNG1
Monarch	EFO:0007937	Blood protein measurement	83	1810	0.57	3.50e-22	ST6GAL1,CPB2,GNPTG,MYH9,C5,MPO,VTN,IGFBP5,PROC,CAT,APOE,PXDN,F12,CFHR5,LYZ,VWF,SERPINA10,TIMP2,LCAT,KLKB1,AMBP,HPX,ITIH4,CDH11,PSG9,HABP2,SAA4,SERPING1,DSC2,ANKRD44,CSF1R,FCN2,PGLYRP2,FYCO1,MASP1,ANTXR1,F2,PSG1,PRG2,APOL1,C7,PAPPA,SERPINA11,HSP90AA1,ANG,FN1,ITIH2,COL18A1,F13B,CFH,F5,ADAM12,ECM1,C8B,RBP4,CCN5,GSN,C1QC,F10,F7,APOM,CA6,IGFBP3,SOD3,F11,C1S,PSG2,TF,PCYOX1,NBEAL1,MST1,C4B,TGFBI,CFB,GC,HPR,C1R,RNASE4,APOC4,VEGFC,PLCG2,KNG1,ENO1
Monarch	HP:0001892	Abnormal bleeding	36	411	0.85	1.40e-15	MYH9,F9,SPARC,PROC,CAT,APOE,ANGPTL6,F12,VWF,ITGA2B,NOTCH3,F13A1,TCIRG1,FERMT3,SEC24D,A2ML1,FGB,F2,FBN1,P4HB,FGG,FN1,MFAP5,F13B,F5,FLNA,GSN,F10,F7,PROS1,ACTN1,F11,C1S,C4A,C1R,FGA
Monarch	HP:0025015	Abnormal vascular morphology	51	1027	0.6	1.76e-13	SERPIND1,F9,MMP2,MGP,SPARC,PROC,TTR,CAT,APOE,ANGPTL6,F12,VWF,ITGA2B,NOTCH3,LCAT,F13A1,DNAH5,TCIRG1,HABP2,SEC24D,MASP1,GUCY1A1,A2ML1,ANTXR1,PCSK9,FGB,F2,FBN1,P4HB,FGG,GANAB,FN1,MFAP5,COL18A1,F13B,F5,HTRA1,FLNA,RBP4,F10,F7,PROS1,EFEMP1,IGF2,C4A,VEGFC,KNG1,HBB,ACTB,FGA,TPM3
Monarch	HP:0002597	Abnormality of the vasculature	65	1673	0.5	3.65e-13	SERPIND1,MYH9,F9,MMP2,MGP,SPARC,PROC,APOA1,TTR,CAT,APOE,ANGPTL6,F12,LYZ,VWF,ITGA2B,NOTCH3,LCAT,CSTA,F13A1,DNAH5,TCIRG1,HABP2,SERPING1,FERMT3,SEC24D,MASP1,GUCY1A1,A2ML1,ANTXR1,PCSK9,FGB,F2,HBA1,FBN1,P4HB,FGG,GANAB,FN1,BLM,MFAP5,COL18A1,CENPF,F13B,F5,HTRA1,FLNA,RBP4,GSN,F10,F7,PROS1,EFEMP1,C1S,IGF2,C4A,TGFBI,C1R,VEGFC,PLCG2,KNG1,HBB,ACTB,FGA,TPM3
Monarch	HP:0000118	Phenotypic abnormality	124	5129	0.29	5.43e-13	ST6GAL1,CETP,GNPTG,SERPIND1,MYH9,F9,MMP2,C5,MPO,HSD17B1,MGP,SPARC,PROC,APOA1,TTR,CAT,LRP1,APOE,PXDN,ANGPTL6,F12,CFHR5,LYZ,VWF,PSMA6,ITGA2B,NOTCH3,LCAT,CSTA,CP,KLKB1,F13A1,DNAH5,CENPE,TCIRG1,CDH11,FCN3,PLA2G7,CRH,HABP2,SERPING1,FERMT3,SEC24D,DSC2,CSF1R,FYCO1,MASP1,GUCY1A1,A2ML1,ANTXR1,PCSK9,FGB,DST,F2,STAB1,C1QB,CFHR1,NDUFS8,SUZ12,C7,HBA1,FBN1,P4HB,PAPPA,FBLN1,DNAH8,ANG,FGG,LIMS1,THBS4,GANAB,PAPLN,FN1,BLM,MFAP5,COL18A1,C8A,KIAA0753,CENPF,F13B,CFH,F5,HTRA1,ADAMTSL4,ECM1,FLNA,ANGPTL3,C8B,RBP4,GSN,C1QC,F10,F7,ADGRG2,IGF1,PROS1,ACTN1,EFEMP1,MASP2,F11,C1S,TF,KSR2,IGF2,C4A,PRG4,LMOD3,ITIH3,C4B,TGFBI,IGFALS,GC,C1R,APOA5,ATP1A1,TTN,CFD,VEGFC,PLCG2,KNG1,HBB,ACTB,FGA,TPM3
Monarch	EFO:0004747	Protein measurement	134	5856	0.27	7.72e-13	ST6GAL1,CPB2,CETP,GNPTG,MYH9,C5,MPO,VTN,GPLD1,IGFBP5,PROC,APOA1,CAT,LRP1,APOE,PXDN,ANGPTL6,F12,CFHR5,LYZ,VWF,SERPINA10,TIMP2,LCAT,CP,KLKB1,DNAH5,AMBP,TCIRG1,HPX,ITIH4,CDH11,PSG9,PLA2G7,HABP2,SAA4,SERPING1,DSC2,ANKRD44,CSF1R,FCN2,PGLYRP2,FYCO1,MASP1,APEH,ANTXR1,PCSK9,FGB,F2,INHBC,PSG1,UBE3C,PRG2,STAB1,APOL1,PKM,STXBP5,C7,HBA1,PAPPA,FBLN1,DNAH8,OIT3,SERPINA11,HSP90AA1,ANG,FGG,LIMS1,THBS4,APOD,PAPLN,FN1,APOA4,ITIH2,COL18A1,KIAA0753,CENPF,F13B,CFH,PAPPA2,F5,ADAM12,ECM1,C8B,RBP4,CCN5,GSN,C1QC,F10,F7,APOM,CA6,IGFBP3,SOD3,IGF1,ACTN1,EFCAB5,TNRC6A,YWHAZ,RAPGEF4,F11,C1S,PSG2,TF,DCDC1,LTBP1,PCYOX1,KSR2,ADIPOQ,NBEAL1,MST1,ITIH3,C4B,TGFBI,CFB,IGFALS,GC,SELENOP,TSKU,HPR,C1R,APOA5,RNASE4,TTN,APOC4,VEGFC,PLCG2,KNG1,HBB,ACTB,TRIM66,ENO1,FGA,TPM3
Monarch	HP:0001626	Abnormality of the cardiovascular system	79	2438	0.42	8.77e-13	GNPTG,SERPIND1,MYH9,F9,MMP2,MGP,SPARC,PROC,APOA1,TTR,CAT,LRP1,APOE,ANGPTL6,F12,LYZ,VWF,ITGA2B,NOTCH3,LCAT,CSTA,CP,KLKB1,F13A1,DNAH5,TCIRG1,HABP2,SERPING1,FERMT3,SEC24D,DSC2,MASP1,GUCY1A1,A2ML1,ANTXR1,PCSK9,FGB,DST,F2,NDUFS8,SUZ12,HBA1,FBN1,P4HB,FGG,GANAB,FN1,BLM,MFAP5,COL18A1,KIAA0753,CENPF,F13B,F5,HTRA1,ADAMTSL4,FLNA,RBP4,GSN,F10,F7,PROS1,EFEMP1,F11,C1S,TF,IGF2,C4A,PRG4,TGFBI,C1R,TTN,VEGFC,PLCG2,KNG1,HBB,ACTB,FGA,TPM3
Monarch	HP:0001933	Subcutaneous hemorrhage	23	189	0.99	2.60e-12	MYH9,PROC,APOE,VWF,ITGA2B,F13A1,TCIRG1,FERMT3,SEC24D,F2,FBN1,P4HB,MFAP5,F13B,F5,FLNA,GSN,F10,F7,PROS1,C1S,C4A,C1R
Monarch	HP:0100659	Abnormal cerebral vascular morphology	30	381	0.8	5.50e-12	F9,SPARC,PROC,TTR,APOE,ANGPTL6,ITGA2B,NOTCH3,F13A1,GUCY1A1,ANTXR1,PCSK9,FGB,F2,FBN1,FGG,GANAB,FN1,MFAP5,F13B,F5,HTRA1,FLNA,F10,F7,PROS1,KNG1,HBB,ACTB,FGA
Monarch	HP:0001871	Abnormality of blood and blood-forming tissues	52	1214	0.54	8.01e-12	SERPIND1,MYH9,F9,MPO,SPARC,PROC,APOA1,CAT,APOE,ANGPTL6,F12,VWF,ITGA2B,NOTCH3,LCAT,CP,KLKB1,F13A1,TCIRG1,FERMT3,SEC24D,GUCY1A1,A2ML1,FGB,F2,NDUFS8,HBA1,FBN1,P4HB,FGG,FN1,BLM,MFAP5,F13B,CFH,F5,FLNA,GSN,F10,F7,PROS1,ACTN1,F11,C1S,TF,C4A,C1R,TTN,PLCG2,KNG1,HBB,FGA

Monarch	HP:0011025	Abnormal cardiovascular system physiology	51	1169	0.55	8.01e-12	GNPTG,MYH9,F9,MMP2,MGP,SPARC,PROC,APOA1,TTR,APOE,ANGPTL6,LYZ,VWF,ITGA2B,NOTCH3,LCAT,CP,F13A1,TCIRG1,SERPING1,DSC2,GUCY1A1,A2ML1,PCSK9,FGB,DST,F2,HBA1,FBN1,FGG,GANAB,FN1,MFAP5,F13B,F5,HTRA1,ADAMTSL4,FLNA,GSN,F10,F7,PROS1,F11,TF,TTN,PLCG2,KNG1,HBB,ACTB,FGA,TPM3
Monarch	HP:0005339	Abnormality of complement system	12	24	1.61	8.42e-12	C5,C1QB,C7,C8A,CFH,C8B,C1QC,MASP2,C1S,C4A,C4B,CFD
Monarch	HP:0003256	Abnormality of the coagulation cascade	18	105	1.14	1.06e-11	F9,PROC,F12,VWF,ITGA2B,F13A1,FGB,F2,FGG,F13B,F5,FLNA,F10,F7,PROS1,F11,KNG1,FGA
Monarch	HP:0011276	Vascular skin abnormality	31	433	0.76	1.34e-11	MYH9,PROC,APOE,F12,VWF,ITGA2B,CSTA,F13A1,TCIRG1,SERPING1,FERMT3,SEC24D,MASP1,GUCY1A1,ANTXR1,F2,FBN1,P4HB,BLM,MFAP5,F13B,F5,FLNA,GSN,F10,F7,PROS1,C1S,C4A,C1R,PLCG2
Monarch	HP:0001928	Abnormality of coagulation	21	170	1.0	1.87e-11	F9,PROC,F12,VWF,ITGA2B,KLKB1,F13A1,A2ML1,FGB,F2,FGG,F13B,F5,FLNA,F10,F7,PROS1,F11,KNG1,HBB,FGA
Monarch	HP:0000421	Epistaxis	16	78	1.22	2.31e-11	MYH9,F9,VWF,ITGA2B,F13A1,FERMT3,FGB,F2,FGG,F13B,F5,F10,F7,ACTN1,F11,FGA
Monarch	HP:0004431	Complement deficiency	11	21	1.63	5.42e-11	C5,C1QB,C7,C8A,CFH,C8B,C1QC,MASP2,C4A,C4B,CFD
Monarch	HP:0000225	Gingival bleeding	13	45	1.37	1.31e-10	CAT,ITGA2B,F13A1,FGB,F2,FGG,F13B,F5,F10,F7,C1S,C1R,FGA
Monarch	HP:0000978	Bruising susceptibility	18	130	1.05	1.94e-10	MYH9,VWF,ITGA2B,F13A1,TCIRG1,SEC24D,F2,FBN1,P4HB,MFAP5,F13B,F5,FLNA,GSN,F10,F7,C1S,C1R
Monarch	HP:0002170	Intracranial hemorrhage	18	130	1.05	1.94e-10	F9,SPARC,ANGPTL6,ITGA2B,NOTCH3,F13A1,FGB,F2,FBN1,FGG,FN1,MFAP5,F13B,F5,F10,F7,PROS1,FGA
Monarch	HP:0002715	Abnormality of the immune system	59	1682	0.45	1.94e-10	MYH9,MMP2,C5,MPO,HSD17B1,MGP,APOA1,CAT,APOE,CFHR5,LYZ,PSMA6,LCAT,CSTA,F13A1,DNAH5,TCIRG1,FCN3,HABP2,SERPING1,FERMT3,A2ML1,FGB,F2,C1QB,CFHR1,C7,HBA1,FBN1,FGG,GANAB,BLM,COL18A1,C8A,CENPF,F13B,CFH,ECM1,FLNA,C8B,RBP4,GSN,C1QC,MASP2,C1S,TF,IGF2,C4A,PRG4,C4B,C1R,TTN,CFD,VEGFC,PLCG2,HBB,ACTB,FGA,TPM3
Monarch	HP:0030680	Abnormality of cardiovascular system morphology	59	1680	0.45	1.94e-10	SERPIND1,F9,MMP2,MGP,SPARC,PROC,TTR,CAT,APOE,ANGPTL6,F12,VWF,ITGA2B,NOTCH3,LCAT,F13A1,DNAH5,TCIRG1,HABP2,SEC24D,DSC2,MASP1,GUCY1A1,A2ML1,ANTXR1,PCSK9,FGB,F2,NDUFS8,SUZ12,HBA1,FBN1,P4HB,FGG,GANAB,FN1,MFAP5,COL18A1,KIAA0753,F13B,F5,HTRA1,FLNA,RBP4,GSN,F10,F7,PROS1,EFEMP1,IGF2,C4A,PRG4,TTN,VEGFC,KNG1,HBB,ACTB,FGA,TPM3
Monarch	HP:0000951	Abnormality of the skin	61	1888	0.42	1.98e-09	MYH9,F9,MMP2,C5,HSD17B1,MGP,SPARC,PROC,APOA1,CAT,LRP1,APOE,F12,LYZ,VWF,PSMA6,ITGA2B,NOTCH3,CSTA,F13A1,CENPE,TCIRG1,CDH11,FCN3,SERPING1,FERMT3,SEC24D,DSC2,MASP1,GUCY1A1,A2ML1,ANTXR1,PCSK9,DST,F2,SUZ12,HBA1,FBN1,P4HB,BLM,MFAP5,F13B,F5,ECM1,FLNA,RBP4,GSN,F10,F7,IGF1,PROS1,C1S,IGF2,C4A,C1R,TTN,VEGFC,PLCG2,HBB,ACTB,FGA
Monarch	EFO:0004634	Coagulation factor measurement	17	132	1.02	2.05e-09	CPB2,PROC,CFHR5,VWF,KLKB1,F13A1,FGB,STXBP5,FGG,CFH,F5,F10,F7,F11,TSKU,KNG1,FGA
Monarch	HP:0011890	Prolonged bleeding following procedure	10	23	1.55	2.21e-09	F9,VWF,ITGA2B,F13A1,F2,F13B,F5,F10,F7,F11
Monarch	HP:0011029	Internal hemorrhage	21	244	0.84	6.54e-09	F9,SPARC,PROC,ANGPTL6,VWF,ITGA2B,NOTCH3,F13A1,FGB,F2,FBN1,FGG,FN1,MFAP5,F13B,F5,F10,F7,PROS1,F11,FGA
Monarch	HP:0010978	Abnormality of immune system physiology	49	1347	0.47	7.04e-09	MYH9,MMP2,C5,HSD17B1,MGP,APOA1,CAT,APOE,CFHR5,LYZ,PSMA6,CSTA,F13A1,DNAH5,TCIRG1,FCN3,SERPING1,FERMT3,C1QB,CFHR1,C7,HBA1,FBN1,GANAB,BLM,C8A,F13B,CFH,ECM1,FLNA,C8B,RBP4,GSN,C1QC,MASP2,C1S,TF,IGF2,C4A,PRG4,C4B,C1R,CFD,VEGFC,PLCG2,HBB,ACTB,FGA,TPM3
Monarch	HP:0005261	Joint hemorrhage	9	19	1.58	1.30e-08	F9,VWF,F13A1,F2,F13B,F5,F10,F7,F11
Monarch	HP:0001574	Abnormality of the integument	66	2266	0.37	1.41e-08	MYH9,F9,MMP2,C5,HSD17B1,MGP,SPARC,PROC,APOA1,CAT,LRP1,APOE,F12,LYZ,VWF,PSMA6,ITGA2B,NOTCH3,CSTA,F13A1,CENPE,TCIRG1,CDH11,FCN3,SERPING1,FERMT3,SEC24D,DSC2,MASP1,GUCY1A1,A2ML1,ANTXR1,PCSK9,DST,F2,NDUFS8,SUZ12,HBA1,FBN1,P4HB,LIMS1,BLM,MFAP5,COL18A1,KIAA0753,F13B,F5,HTRA1,ECM1,FLNA,RBP4,GSN,F10,F7,IGF1,PROS1,C1S,IGF2,C4A,C1R,TTN,VEGFC,PLCG2,HBB,ACTB,FGA
Monarch	HP:0011121	Abnormality of skin morphology	54	1648	0.42	2.20e-08	MYH9,F9,MMP2,MGP,SPARC,PROC,APOA1,CAT,LRP1,APOE,F12,VWF,ITGA2B,NOTCH3,CSTA,F13A1,CENPE,TCIRG1,CDH11,SERPING1,FERMT3,SEC24D,DSC2,MASP1,GUCY1A1,A2ML1,ANTXR1,PCSK9,DST,F2,SUZ12,HBA1,FBN1,P4HB,BLM,MFAP5,F13B,F5,ECM1,FLNA,GSN,F10,F7,IGF1,PROS1,C1S,IGF2,C4A,C1R,TTN,VEGFC,PLCG2,HBB,ACTB
Monarch	HP:0012649	Increased inflammatory response	40	981	0.52	2.44e-08	MYH9,C5,HSD17B1,MGP,APOA1,CAT,APOE,CFHR5,LYZ,PSMA6,CSTA,F13A1,DNAH5,TCIRG1,FCN3,FERMT3,C1QB,CFHR1,HBA1,FBN1,GANAB,BLM,F13B,CFH,ECM1,FLNA,RBP4,GSN,C1QC,MASP2,C1S,C4A,PRG4,C4B,C1R,VEGFC,PLCG2,HBB,FGA,TPM3
Monarch	HP:0011354	Generalized abnormality of skin	36	821	0.55	3.39e-08	MYH9,F9,PROC,APOE,F12,VWF,ITGA2B,CSTA,F13A1,CENPE,TCIRG1,CDH11,SERPING1,FERMT3,SEC24D,MASP1,GUCY1A1,ANTXR1,F2,HBA1,FBN1,P4HB,BLM,MFAP5,F13B,F5,FLNA,GSN,F10,F7,PROS1,C1S,C4A,C1R,PLCG2,HBB
Monarch	HP:0033353	Abnormal blood vessel morphology	35	792	0.55	5.07e-08	SERPIND1,MMP2,MGP,PROC,CAT,APOE,ANGPTL6,F12,VWF,NOTCH3,LCAT,DNAH5,TCIRG1,SEC24D,MASP1,GUCY1A1,A2ML1,ANTXR1,PCSK9,FBN1,P4HB,GANAB,FN1,MFAP5,COL18A1,HTRA1,FLNA,RBP4,PROS1,EFEMP1,IGF2,C4A,VEGFC,ACTB,TPM3
Monarch	HP:0000132	Menorrhagia	10	38	1.33	9.22e-08	MYH9,VWF,ITGA2B,F13A1,F2,F13B,F5,F10,F7,F11
Monarch	HP:0006298	Prolonged bleeding after dental extraction	8	16	1.61	9.22e-08	F9,VWF,F13A1,F2,F13B,F5,F10,F11
Monarch	HP:0004846	Prolonged bleeding after surgery	8	18	1.55	1.86e-07	F9,VWF,ITGA2B,F13A1,F13B,F5,F10,F7
Monarch	HP:0001977	Abnormal thrombosis	13	94	1.05	2.05e-07	SERPIND1,MYH9,F9,PROC,F12,FGB,F2,FGG,F5,PROS1,KNG1,HBB,FGA
Monarch	HP:0000168	Abnormality of the gingiva	17	190	0.86	2.30e-07	MMP2,CAT,ITGA2B,NOTCH3,F13A1,TCIRG1,FGB,F2,FGG,F13B,F5,ECM1,F10,F7,C1S,C1R,FGA
Monarch	HP:0000790	Hematuria	14	118	0.98	2.63e-07	F9,APOA1,CFHR5,LYZ,ITGA2B,F2,CFHR1,GANAB,FN1,CFH,F5,F10,HBB,FGA
Monarch	HP:0010935	Abnormality of the upper urinary tract	40	1101	0.47	4.62e-07	MYH9,F9,APOA1,TTR,APOE,CFHR5,LYZ,ITGA2B,NOTCH3,LCAT,CDH11,HABP2,MASP1,ANTXR1,PCSK9,F2,C1QB,CFHR1,NDUFS8,FBN1,GANAB,FN1,BLM,COL18A1,KIAA0753,CENPF,CFH,F5,FLNA,GSN,C1QC,F10,ADGRG2,IGF2,C4A,C1R,ATP1A1,HBB,ACTB,FGA
Monarch	HP:0004936	Venous thrombosis	11	65	1.14	5.79e-07	SERPIND1,F9,PROC,FGB,F2,FGG,F5,PROS1,KNG1,HBB,FGA
Monarch	HP:0001939	Abnormality of metabolism/homeostasis	60	2168	0.35	6.60e-07	CETP,GNPTG,MYH9,F9,MMP2,MPO,APOA1,TTR,CAT,APOE,F12,CFHR5,LYZ,ITGA2B,NOTCH3,LCAT,CP,TCIRG1,CDH11,PLA2G7,SERPING1,CSF1R,A2ML1,ANTXR1,PCSK9,FGB,DST,F2,CFHR1,NDUFS8,HBA1,FBN1,FGG,GANAB,PAPLN,FN1,BLM,CFH,F5,FLNA,ANGPTL3,GSN,F10,IGF1,TF,IGF2,LMOD3,TGFBI,IGFALS,GC,APOA5,ATP1A1,TTN,VEGFC,PLCG2,KNG1,HBB,ACTB,FGA,TPM3
Monarch	HP:0003645	Prolonged partial thromboplastin time	8	24	1.43	9.69e-07	F9,F12,KLKB1,F2,F5,F10,F11,KNG1
Monarch	HP:0011355	Localized skin lesion	31	738	0.53	1.49e-06	MMP2,MGP,PROC,APOA1,LRP1,APOE,F12,NOTCH3,CSTA,TCIRG1,FERMT3,SEC24D,MASP1,A2ML1,ANTXR1,PCSK9,DST,SUZ12,FBN1,P4HB,BLM,ECM1,FLNA,GSN,IGF1,PROS1,C1S,IGF2,C1R,HBB,ACTB
Monarch	HP:0002725	Systemic lupus erythematosus	8	26	1.4	1.57e-06	SERPING1,C1QB,C8A,C1QC,MASP2,C1S,C4A,C1R

Monarch	HP:0000119	Abnormality of the genitourinary system	59	2189	0.34	2.24e-06	MYH9,F9,MMP2,APOA1,TTR,APOE,CFHR5,LYZ,VWF,ITGA2B,NOTCH3,LCAT,F13A1,DNAH5,CDH11,CRH,HABP2,MASP1,GUCY1A1,A2ML1,ANTXR1,PCSK9,FGB,F2,C1QB,CFHR1,NDUFS8,SUZ12,FBN1,FBLN1,DNAH8,FGG,GANAB,FN1,BLM,COL18A1,KIAA0753,CENPF,F13B,CFH,F5,HTRA1,FLNA,GSN,C1QC,F10,F7,ADGRG2,IGF1,F11,IGF2,C4A,LMOD3,C1R,ATP1A1,VEGFC,HBB,ACTB,FGA
Monarch	HP:0000077	Abnormality of the kidney	38	1077	0.45	2.31e-06	MYH9,F9,APOA1,TTR,APOE,CFHR5,LYZ,ITGA2B,NOTCH3,LCAT,HABP2,MASP1,ANTXR1,PCSK9,F2,C1QB,CFHR1,NDUFS8,FBN1,GANAB,FN1,BLM,KIAA0753,CENPF,CFH,F5,FLNA,GSN,C1QC,F10,ADGRG2,IGF2,C4A,C1R,ATP1A1,HBB,ACTB,FGA
Monarch	HP:0000271	Abnormality of the face	66	2641	0.3	4.04e-06	GNPTG,MYH9,F9,MMP2,MGP,SPARC,PROC,CAT,LRP1,APOE,F12,LYZ,VWF,ITGA2B,NOTCH3,CP,F13A1,DNAH5,CENPE,TCIRG1,CDH11,SERPING1,FERMT3,SEC24D,CSF1R,MASP1,A2ML1,ANTXR1,PCSK9,FGB,DST,F2,SUZ12,FBN1,P4HB,PAPPA,FBLN1,ANG,FGG,BLM,MFAP5,COL18A1,KIAA0753,CENPF,F13B,F5,ADAMTSL4,ECM1,FLNA,GSN,F10,F7,IGF1,ACTN1,F11,C1S,IGF2,LMOD3,C1R,TTN,VEGFC,PLCG2,HBB,ACTB,FGA,TPM3
Monarch	HP:0011891	Post-partum hemorrhage	6	10	1.69	5.35e-06	F13A1,F2,F13B,F5,F10,F7
Monarch	HP:0012233	Intramuscular hematoma	6	10	1.69	5.35e-06	F9,F13A1,F2,F13B,F10,F7
Monarch	HP:0025031	Abnormality of the digestive system	61	2389	0.31	7.54e-06	F9,MMP2,C5,APOA1,TTR,APOE,F12,LYZ,VWF,ITGA2B,NOTCH3,LCAT,CP,F13A1,DNAH5,TCIRG1,CDH11,FCN3,HABP2,SERPING1,FERMT3,MASP1,GUCY1A1,A2ML1,ANTXR1,PCSK9,FGB,DST,F2,NDUFS8,SUZ12,HBA1,FBN1,ANG,FGG,GANAB,BLM,MFAP5,COL18A1,KIAA0753,CENPF,F13B,F5,ECM1,FLNA,GSN,F10,F7,MASP2,F11,C1S,TF,IGF2,LMOD3,C4B,C1R,PLCG2,HBB,ACTB,FGA,TPM3
Monarch	HP:0000152	Abnormality of head or neck	69	2882	0.29	8.51e-06	GNPTG,MYH9,F9,MMP2,MGP,SPARC,PROC,CAT,LRP1,APOE,F12,LYZ,VWF,ITGA2B,NOTCH3,CP,F13A1,DNAH5,CENPE,TCIRG1,CDH11,HABP2,SERPING1,FERMT3,SEC24D,CSF1R,MASP1,A2ML1,ANTXR1,PCSK9,FGB,DST,F2,NDUFS8,SUZ12,FBN1,P4HB,PAPPA,FBLN1,ANG,FGG,BLM,MFAP5,COL18A1,KIAA0753,CENPF,F13B,F5,ADAMTSL4,ECM1,FLNA,GSN,F10,F7,IGF1,ACTN1,F11,C1S,IGF2,LMOD3,IGFALS,C1R,TTN,VEGFC,PLCG2,HBB,ACTB,FGA,TPM3
Monarch	HP:0002634	Arteriosclerosis	10	70	1.06	1.06e-05	SERPIND1,CAT,APOE,ANGPTL6,LCAT,ANTXR1,PCSK9,FBN1,MFAP5,HTRA1
Monarch	HP:0000140	Abnormality of the menstrual cycle	16	228	0.75	1.27e-05	MYH9,F9,VWF,ITGA2B,F13A1,ANTXR1,FGB,F2,FGG,F13B,F5,F10,F7,F11,IGF2,FGA
Monarch	HP:0001000	Abnormality of skin pigmentation	22	439	0.61	1.28e-05	MMP2,PROC,CAT,APOE,NOTCH3,CSTA,A2ML1,ANTXR1,DST,F2,SUZ12,FBN1,BLM,FLNA,IGF1,PROS1,C1S,IGF2,C1R,PLCG2,HBB,ACTB
Monarch	HP:0012211	Abnormal renal physiology	25	560	0.56	1.30e-05	MYH9,F9,APOA1,TTR,APOE,CFHR5,LYZ,ITGA2B,LCAT,F2,C1QB,CFHR1,NDUFS8,GANAB,FN1,CFH,F5,GSN,C1QC,F10,C4A,C1R,ATP1A1,HBB,FGA
Monarch	HP:0000234	Abnormality of the head	68	2865	0.28	1.43e-05	GNPTG,MYH9,F9,MMP2,MGP,SPARC,PROC,CAT,LRP1,APOE,F12,LYZ,VWF,ITGA2B,NOTCH3,CP,F13A1,DNAH5,CENPE,TCIRG1,CDH11,SERPING1,FERMT3,SEC24D,CSF1R,MASP1,A2ML1,ANTXR1,PCSK9,FGB,DST,F2,NDUFS8,SUZ12,FBN1,P4HB,PAPPA,FBLN1,ANG,FGG,BLM,MFAP5,COL18A1,KIAA0753,CENPF,F13B,F5,ADAMTSL4,ECM1,FLNA,GSN,F10,F7,IGF1,ACTN1,F11,C1S,IGF2,LMOD3,IGFALS,C1R,TTN,VEGFC,PLCG2,HBB,ACTB,FGA,TPM3
Monarch	HP:0000080	Abnormality of reproductive system physiology	27	659	0.52	1.94e-05	MYH9,F9,TTR,APOE,VWF,ITGA2B,F13A1,DNAH5,GUCY1A1,A2ML1,ANTXR1,FGB,F2,DNAH8,FGG,GANAB,BLM,F13B,F5,F10,F7,ADGRG2,IGF1,F11,IGF2,HBB,FGA
Monarch	HP:0011830	Abnormal oral mucosa morphology	18	305	0.68	2.05e-05	MMP2,CAT,LYZ,ITGA2B,NOTCH3,F13A1,TCIRG1,FGB,F2,FGG,F13B,F5,ECM1,F10,F7,C1S,C1R,FGA
Monarch	HP:0030012	Abnormal female reproductive system physiology	17	273	0.7	2.24e-05	MYH9,F9,VWF,ITGA2B,F13A1,ANTXR1,FGB,F2,FGG,BLM,F13B,F5,F10,F7,F11,IGF2,FGA
Monarch	HP:0012639	Abnormal nervous system morphology	62	2546	0.29	2.58e-05	F9,MGP,SPARC,PROC,TTR,APOE,ANGPTL6,ITGA2B,NOTCH3,CP,F13A1,DNAH5,CENPE,TCIRG1,CDH11,HABP2,SERPING1,SEC24D,CSF1R,MASP1,GUCY1A1,ANTXR1,PCSK9,FGB,F2,NDUFS8,SUZ12,HBA1,FBN1,P4HB,FBLN1,ANG,FGG,GANAB,FN1,BLM,MFAP5,COL18A1,C8A,KIAA0753,CENPF,F13B,F5,HTRA1,ECM1,FLNA,C8B,GSN,F10,F7,IGF1,PROS1,IGF2,LMOD3,C4B,ATP1A1,TTN,KNG1,HBB,ACTB,FGA,TPM3
Monarch	HP:0000123	Nephritis	9	59	1.09	2.62e-05	MYH9,CFHR5,C1QB,CFHR1,GANAB,CFH,C1QC,C4A,C1R
Monarch	EFO:0004310	Partial thromboplastin time	6	15	1.51	2.71e-05	F12,KLKB1,F5,F11,C1S,KNG1
Monarch	HP:0000707	Abnormality of the nervous system	76	3471	0.25	4.35e-05	ST6GAL1,GNPTG,F9,MMP2,MGP,SPARC,PROC,APOA1,TTR,CAT,LRP1,APOE,ANGPTL6,ITGA2B,NOTCH3,CP,F13A1,DNAH5,CENPE,TCIRG1,CDH11,CRH,HABP2,SERPING1,SEC24D,CSF1R,MASP1,GUCY1A1,A2ML1,ANTXR1,PCSK9,FGB,DST,F2,STAB1,NDUFS8,SUZ12,HBA1,FBN1,P4HB,FBLN1,ANG,FGG,GANAB,FN1,BLM,MFAP5,COL18A1,C8A,KIAA0753,CENPF,F13B,F5,HTRA1,ADAMTSL4,ECM1,FLNA,C8B,GSN,F10,F7,IGF1,PROS1,IGF2,LMOD3,ITIH3,C4B,TGFBI,ATP1A1,TTN,VEGFC,KNG1,HBB,ACTB,FGA,TPM3
Monarch	HP:0001634	Mitral valve prolapse	10	86	0.97	5.04e-05	MMP2,VWF,SEC24D,FBN1,P4HB,GANAB,MFAP5,FLNA,TTN,TPM3
Monarch	EFO:0008390	Prothrombin time measurement	5	8	1.7	5.36e-05	F2,FGG,F5,F10,F7
Monarch	HP:0002239	Gastrointestinal hemorrhage	11	111	0.9	5.36e-05	F9,VWF,ITGA2B,FGB,F2,FGG,F5,F10,F7,F11,FGA
Monarch	HP:0030972	Abnormal systemic blood pressure	19	369	0.62	5.67e-05	MMP2,MGP,APOA1,TTR,ANGPTL6,LYZ,NOTCH3,SERPING1,GUCY1A1,PCSK9,F2,FBN1,GANAB,FN1,MFAP5,ADAMTSL4,GSN,HBB,FGA
Monarch	HP:0005368	Abnormality of humoral immunity	15	234	0.71	7.62e-05	C5,C1QB,C7,BLM,C8A,CFH,FLNA,C8B,C1QC,MASP2,C1S,C4A,C4B,CFD,PLCG2
Monarch	HP:0030137	Prolonged bleeding following circumcision	5	9	1.65	7.83e-05	ITGA2B,F13A1,F2,F13B,F5
Monarch	HP:0030140	Oral cavity bleeding	5	9	1.65	7.83e-05	F13A1,F2,F13B,F5,F10
Monarch	HP:0002625	Deep venous thrombosis	6	20	1.38	9.37e-05	SERPIND1,F9,PROC,F2,F5,PROS1
Monarch	HP:0002960	Autoimmunity	11	122	0.86	0.00011	MMP2,TCIRG1,SERPING1,C1QB,C8A,C1QC,MASP2,C1S,C4A,C1R,PLCG2
Monarch	HP:0010990	Abnormality of the common coagulation pathway	7	35	1.21	0.00011	F13A1,FGB,FGG,F13B,F5,F10,FGA
Monarch	HP:0400008	Menometrorrhagia	5	10	1.61	0.00011	F9,ITGA2B,FGB,FGG,FGA
Monarch	HP:0011004	Abnormal systemic arterial morphology	19	398	0.59	0.00015	SERPIND1,MMP2,CAT,APOE,ANGPTL6,F12,NOTCH3,LCAT,SEC24D,GUCY1A1,ANTXR1,PCSK9,FBN1,P4HB,GANAB,MFAP5,HTRA1,FLNA,TPM3
Monarch	EFO:0003777	Heart disease	12	155	0.8	0.00016	MMP2,LRP1,VWF,SEC24D,FBN1,P4HB,GANAB,MFAP5,FLNA,MAP3K7CL,TTN,TPM3
Monarch	HP:0000078	Abnormality of the genital system	39	1358	0.37	0.00016	MYH9,F9,MMP2,TTR,APOE,VWF,ITGA2B,NOTCH3,F13A1,DNAH5,CDH11,MASP1,GUCY1A1,A2ML1,ANTXR1,FGB,F2,SUZ12,FBN1,FBLN1,DNAH8,FGG,GANAB,BLM,KIAA0753,F13B,F5,FLNA,F10,F7,ADGRG2,IGF1,F11,IGF2,LMOD3,VEGFC,HBB,ACTB,FGA
Monarch	HP:0000822	Hypertension	15	256	0.67	0.00019	MMP2,MGP,APOA1,ANGPTL6,LYZ,NOTCH3,GUCY1A1,PCSK9,FBN1,GANAB,FN1,MFAP5,ADAMTSL4,HBB,FGA
Monarch	HP:0000979	Purpura	9	79	0.96	0.00019	PROC,APOE,ITGA2B,F13A1,FERMT3,F2,F13B,PROS1,C4A
Monarch	HP:0000969	Edema	20	448	0.56	0.00020	APOA1,APOE,F12,LYZ,CDH11,SERPING1,A2ML1,ANTXR1,FGB,F2,HBA1,FGG,FN1,GSN,LMOD3,TGFBI,VEGFC,PLCG2,ACTB,FGA
Monarch	HP:0001367	Abnormal joint morphology	30	905	0.43	0.00020	GNPTG,F9,MMP2,APOE,VWF,NOTCH3,F13A1,MASP1,A2ML1,FGB,F2,SUZ12,FBN1,FBLN1,FGG,FN1,F13B,F5,FLNA,F10,F7,F11,TF,PRG4,LMOD3,VEGFC,HBB,ACTB,FGA,TPM3
Monarch	HP:0001934	Persistent bleeding after trauma	5	12	1.53	0.00020	F9,VWF,F13A1,F13B,F5

Monarch	HP:0002011	Morphological central nervous system abnormality	57	2416	0.28	0.00020	F9,MGP,SPARC,PROC,TTR,APOE,ANGPTL6,ITGA2B,NOTCH3,CP,F13A1,DNAH5,CENPE,TCIRG1,CDH11,HABP2,SEC24D,CSF1R,MASP1,GUCY1A1,ANTXR1,PCSK9,FGB,F2,NDUFS8,SUZ12,HBA1,FBN1,P4HB,FBLN1,ANG,FGG,GANAB,FN1,BLM,MFAP5,COL18A1,C8A,KIAA0753,CENPF,F13B,F5,HTRA1,ECM1,FLNA,C8B,F10,F7,IGF1,PROS1,IGF2,C4B,ATP1A1,KNG1,HBB,ACTB,FGA
Monarch	HP:0000079	Abnormality of the urinary system	44	1657	0.33	0.00021	MYH9,F9,APOA1,TTR,APOE,CFHR5,LYZ,ITGA2B,NOTCH3,LCAT,CDH11,CRH,HABP2,MASP1,ANTXR1,PCSK9,F2,C1QB,CFHR1,NDUFS8,FBN1,GANAB,PAPLN,FN1,BLM,COL18A1,KIAA0753,CENPF,CFH,F5,HTRA1,FLNA,GSN,C1QC,F10,ADGRG2,IGF2,C4A,LMOD3,C1R,ATP1A1,HBB,ACTB,FGA
Monarch	HP:0001342	Cerebral hemorrhage	8	61	1.02	0.00026	NOTCH3,F13A1,FGB,FGG,FN1,F13B,PROS1,FGA
Monarch	HP:0001633	Abnormal mitral valve morphology	10	109	0.87	0.00027	MMP2,VWF,SEC24D,FBN1,P4HB,GANAB,MFAP5,FLNA,TTN,TPM3
Monarch	HP:0032263	Increased blood pressure	16	303	0.63	0.00028	MMP2,MGP,APOA1,ANGPTL6,LYZ,NOTCH3,GUCY1A1,PCSK9,F2,FBN1,GANAB,FN1,MFAP5,ADAMTSL4,HBB,FGA
Monarch	HP:0000924	Abnormality of the skeletal system	61	2708	0.26	0.00031	GNPTG,F9,MMP2,MGP,SPARC,TTR,APOE,VWF,NOTCH3,F13A1,DNAH5,CENPE,TCIRG1,CDH11,HABP2,FERMT3,SEC24D,CSF1R,MASP1,A2ML1,ANTXR1,PCSK9,FGB,DST,F2,NDUFS8,SUZ12,FBN1,P4HB,FBLN1,FGG,FN1,BLM,MFAP5,COL18A1,KIAA0753,CENPF,F13B,F5,HTRA1,ADAMTSL4,ECM1,FLNA,F10,F7,IGF1,F11,C1S,TF,IGF2,PRG4,LMOD3,IGFALS,C1R,ATP1A1,TTN,VEGFC,HBB,ACTB,FGA,TPM3
Monarch	HP:0002621	Atherosclerosis	8	64	1.0	0.00034	SERPIND1,APOE,ANGPTL6,LCAT,ANTXR1,PCSK9,FBN1,MFAP5
Monarch	HP:0003216	Generalized amyloid deposition	4	5	1.81	0.00034	APOA1,LYZ,GSN,FGA
Monarch	HP:0005542	Prolonged whole-blood clotting time	4	5	1.81	0.00034	F9,F12,VWF,F5
Monarch	HP:0008065	Aplasia/Hypoplasia of the skin	12	170	0.76	0.00034	MGP,PROC,TCIRG1,SEC24D,DST,FBN1,P4HB,FLNA,GSN,PROS1,C1S,C1R
Monarch	HP:0011884	Abnormal umbilical stump bleeding	4	5	1.81	0.00034	F13A1,F2,F13B,F10
Monarch	HP:0000153	Abnormality of the mouth	49	1992	0.3	0.00036	MYH9,MMP2,MGP,SPARC,CAT,APOE,LYZ,ITGA2B,NOTCH3,F13A1,CENPE,TCIRG1,CDH11,SERPING1,SEC24D,MASP1,A2ML1,ANTXR1,FGB,DST,F2,SUZ12,FBN1,P4HB,PAPPA,FBLN1,ANG,FGG,BLM,MFAP5,KIAA0753,CENPF,F13B,F5,ECM1,FLNA,GSN,F10,F7,IGF1,C1S,IGF2,LMOD3,C1R,TTN,HBB,ACTB,FGA,TPM3
Monarch	HP:0033127	Abnormality of the musculoskeletal system	68	3173	0.24	0.00036	GNPTG,F9,MMP2,MGP,SPARC,APOA1,TTR,LRP1,APOE,VWF,NOTCH3,CP,F13A1,DNAH5,CENPE,TCIRG1,CDH11,HABP2,FERMT3,SEC24D,CSF1R,MASP1,A2ML1,ANTXR1,PCSK9,FGB,DST,F2,NDUFS8,SUZ12,FBN1,P4HB,FBLN1,ANG,FGG,FN1,BLM,MFAP5,COL18A1,KIAA0753,CENPF,F13B,F5,HTRA1,ADAMTSL4,ECM1,FLNA,GSN,F10,F7,IGF1,F11,C1S,TF,IGF2,PRG4,LMOD3,TGFBI,IGFALS,C1R,ATP1A1,TTN,VEGFC,PLCG2,HBB,ACTB,FGA,TPM3
Monarch	HP:0005692	Joint hyperflexibility	11	144	0.79	0.00040	NOTCH3,CENPE,SEC24D,A2ML1,ANTXR1,SUZ12,P4HB,COL18A1,FLNA,C1S,C1R
Monarch	HP:0010979	Abnormality of lipoprotein cholesterol concentration	7	47	1.08	0.00048	CETP,APOA1,APOE,LCAT,PCSK9,ANGPTL3,APOA5
Monarch	HP:0030962	Abnormal morphology of the great vessels	22	571	0.49	0.00049	MMP2,MGP,APOE,ANGPTL6,NOTCH3,DNAH5,TCIRG1,SEC24D,MASP1,A2ML1,PCSK9,FBN1,P4HB,GANAB,FN1,MFAP5,COL18A1,FLNA,RBP4,IGF2,ACTB,TPM3
Monarch	HP:0033057	Decreased serum terminal complement component	4	6	1.73	0.00050	C5,C7,C8A,C8B
Monarch	HP:0000163	Abnormal oral cavity morphology	47	1901	0.3	0.00051	MYH9,MMP2,MGP,SPARC,CAT,APOE,LYZ,ITGA2B,NOTCH3,F13A1,CENPE,TCIRG1,CDH11,SERPING1,SEC24D,MASP1,A2ML1,ANTXR1,FGB,DST,F2,SUZ12,FBN1,P4HB,PAPPA,FGG,BLM,MFAP5,KIAA0753,CENPF,F13B,F5,ECM1,FLNA,GSN,F10,F7,IGF1,C1S,IGF2,LMOD3,C1R,TTN,HBB,ACTB,FGA,TPM3
Monarch	HP:0011842	Abnormal skeletal morphology	58	2573	0.26	0.00051	GNPTG,F9,MMP2,MGP,SPARC,TTR,APOE,VWF,NOTCH3,F13A1,DNAH5,CENPE,TCIRG1,CDH11,FERMT3,SEC24D,CSF1R,MASP1,A2ML1,ANTXR1,PCSK9,FGB,DST,F2,NDUFS8,SUZ12,FBN1,P4HB,FBLN1,FGG,FN1,BLM,MFAP5,COL18A1,KIAA0753,CENPF,F13B,F5,HTRA1,ADAMTSL4,FLNA,F10,F7,IGF1,F11,C1S,TF,IGF2,PRG4,LMOD3,IGFALS,C1R,TTN,VEGFC,HBB,ACTB,FGA,TPM3
Monarch	EFO:0005278	Cardiovascular disease biomarker measurement	52	2228	0.28	0.00069	ST6GAL1,CETP,F9,MPO,VTN,APOA1,LRP1,APOE,F12,NOTCH3,LCAT,KLKB1,DNAH5,CDH11,PLA2G7,HABP2,PCSK9,FGB,STAB1,STXBP5,FBN1,FBLN1,OIT3,LIMS1,FN1,APOA4,COL18A1,KIAA0753,CFH,PAPPA2,F5,GSN,EFEMP1,EFCAB5,TNRC6A,RAPGEF4,ROCK1,MAP3K7CL,TF,DCDC1,LTBP1,KSR2,NBEAL1,ITIH3,HPR,APOA5,TTN,APOC4,PLCG2,KNG1,HBB,ACTB
Monarch	EFO:0000319	Cardiovascular disease	15	293	0.62	0.00071	MMP2,LRP1,VWF,SEC24D,FBN1,P4HB,GANAB,MFAP5,F5,FLNA,MAP3K7CL,NBEAL1,TTN,KNG1,TPM3
Monarch	HP:0001654	Abnormal heart valve morphology	14	259	0.64	0.00080	MMP2,VWF,NOTCH3,TCIRG1,SEC24D,A2ML1,FBN1,P4HB,GANAB,MFAP5,FLNA,TTN,ACTB,TPM3
Monarch	HP:0012719	Functional abnormality of the gastrointestinal tract	27	840	0.41	0.00092	F9,VWF,ITGA2B,NOTCH3,F13A1,TCIRG1,FCN3,SERPING1,GUCY1A1,FGB,F2,FBN1,FGG,BLM,F13B,F5,ECM1,FLNA,F10,F7,MASP2,F11,LMOD3,PLCG2,ACTB,FGA,TPM3
Monarch	EFO:0004298	Cardiovascular measurement	54	2378	0.26	0.00093	ST6GAL1,CETP,F9,MPO,VTN,GPLD1,APOA1,LRP1,APOE,F12,NOTCH3,LCAT,KLKB1,DNAH5,CDH11,PLA2G7,HABP2,PCSK9,FGB,INHBC,STAB1,STXBP5,FBN1,FBLN1,OIT3,LIMS1,FN1,APOA4,COL18A1,KIAA0753,CFH,PAPPA2,F5,GSN,EFEMP1,EFCAB5,TNRC6A,RAPGEF4,ROCK1,MAP3K7CL,TF,DCDC1,LTBP1,KSR2,NBEAL1,ITIH3,HPR,APOA5,TTN,APOC4,PLCG2,KNG1,HBB,ACTB
Monarch	HP:0030163	Abnormal vascular physiology	16	340	0.58	0.00093	MGP,PROC,ANGPTL6,NOTCH3,TCIRG1,GUCY1A1,F2,FBN1,MFAP5,F5,HTRA1,FLNA,PROS1,KNG1,HBB,ACTB
Monarch	HP:0012372	Abnormal eye morphology	49	2077	0.28	0.00097	GNPTG,MYH9,MMP2,MGP,SPARC,PROC,APOA1,TTR,APOE,PXDN,F12,NOTCH3,LCAT,CP,DNAH5,TCIRG1,CDH11,SERPING1,SEC24D,CSF1R,FYCO1,MASP1,A2ML1,ANTXR1,PCSK9,DST,NDUFS8,SUZ12,FBN1,P4HB,FBLN1,BLM,MFAP5,COL18A1,KIAA0753,CENPF,CFH,ADAMTSL4,FLNA,RBP4,GSN,PROS1,EFEMP1,TGFBI,C1R,PLCG2,HBB,ACTB,TPM3
Monarch	HP:0100724	Hypercoagulability	5	19	1.33	0.00097	F9,PROC,F5,PROS1,HBB
Monarch	HP:0009145	Abnormal cerebral artery morphology	7	55	1.01	0.0011	ANGPTL6,GUCY1A1,PCSK9,FBN1,GANAB,MFAP5,HTRA1
Monarch	HP:0011024	Abnormality of the gastrointestinal tract	37	1384	0.33	0.0011	F9,APOE,F12,VWF,ITGA2B,NOTCH3,F13A1,DNAH5,TCIRG1,CDH11,FCN3,HABP2,SERPING1,GUCY1A1,FGB,F2,SUZ12,FBN1,FGG,BLM,COL18A1,CENPF,F13B,F5,ECM1,FLNA,F10,F7,MASP2,F11,LMOD3,C1R,PLCG2,HBB,ACTB,FGA,TPM3
Monarch	HP:0000347	Micrognathia	23	659	0.45	0.0012	MMP2,NOTCH3,CENPE,TCIRG1,SEC24D,A2ML1,ANTXR1,SUZ12,FBN1,P4HB,BLM,KIAA0753,CENPF,FLNA,IGF1,C1S,IGF2,LMOD3,IGFALS,C1R,TTN,ACTB,TPM3
Monarch	HP:0007420	Spontaneous hematomas	5	20	1.3	0.0012	ITGA2B,F13A1,F13B,F5,F10
Monarch	HP:0009118	Aplasia/Hypoplasia of the mandible	23	660	0.45	0.0012	MMP2,NOTCH3,CENPE,TCIRG1,SEC24D,A2ML1,ANTXR1,SUZ12,FBN1,P4HB,BLM,KIAA0753,CENPF,FLNA,IGF1,C1S,IGF2,LMOD3,IGFALS,C1R,TTN,ACTB,TPM3
Monarch	HP:0025142	Constitutional symptom	28	906	0.4	0.0012	GNPTG,MMP2,PROC,TTR,CAT,F12,NOTCH3,TCIRG1,CRH,HABP2,SERPING1,FERMT3,DSC2,PCSK9,F2,FBN1,ANG,GANAB,MFAP5,HTRA1,FLNA,PROS1,C1S,TGFBI,TTN,PLCG2,HBB,TPM3
Monarch	HP:0000099	Glomerulonephritis	6	37	1.12	0.0013	CFHR5,C1QB,CFHR1,CFH,C1QC,C4A
Monarch	HP:0031888	Abnormal HDL cholesterol concentration	5	21	1.28	0.0014	CETP,APOA1,APOE,LCAT,APOA5
Monarch	HP:0000366	Abnormality of the nose	38	1470	0.32	0.0016	MYH9,F9,MMP2,MGP,APOE,VWF,ITGA2B,NOTCH3,F13A1,DNAH5,CENPE,TCIRG1,CDH11,FERMT3,MASP1,ANTXR1,FGB,F2,SUZ12,FBN1,FGG,BLM,COL18A1,KIAA0753,CENPF,F13B,F5,ECM1,FLNA,F10,F7,IGF1,ACTN1,F11,PLCG2,HBB,ACTB,FGA

Monarch	HP:0012443	Abnormality of brain morphology	51	2254	0.26	0.0017	F9,MGP,SPARC,PROC,TTR,APOE,ANGPTL6,ITGA2B,NOTCH3,CP,F13A1,DNAH5,CENPE,TCIRG1,CDH11,SEC24D,CSF1R,MASP1,GUCY1A1,ANTXR1,PCSK9,FGB,F2,NDUFS8,SUZ12,HBA1,FBN1,P4HB,FBLN1,FGG,GANAB,FN1,BLM,MFAP5,COL18A1,KIAA0753,CENPF,F13B,F5,HTRA1,ECM1,FLNA,F10,F7,IGF1,PROS1,ATP1A1,KNG1,HBB,ACTB,FGA
Monarch	HP:0012531	Pain	20	539	0.48	0.0018	GNPTG,MMP2,CAT,F12,TCIRG1,HABP2,SERPING1,FERMT3,PCSK9,F2,FBN1,ANG,GANAB,MFAP5,HTRA1,FLNA,C1S,TGFBI,PLCG2,HBB
Monarch	HP:0030791	Abnormal jaw morphology	27	883	0.39	0.0019	MMP2,NOTCH3,CENPE,TCIRG1,CDH11,SEC24D,A2ML1,ANTXR1,SUZ12,FBN1,P4HB,BLM,MFAP5,KIAA0753,CENPF,ADAMTSL4,FLNA,IGF1,C1S,IGF2,LMOD3,IGFALS,C1R,TTN,HBB,ACTB,TPM3
Monarch	HP:0001297	Stroke	13	249	0.62	0.0020	TTR,ANGPTL6,NOTCH3,GUCY1A1,F2,FBN1,MFAP5,F5,HTRA1,FLNA,KNG1,HBB,ACTB
Monarch	HP:0000277	Abnormal mandible morphology	26	841	0.4	0.0022	MMP2,NOTCH3,CENPE,TCIRG1,CDH11,SEC24D,A2ML1,ANTXR1,SUZ12,FBN1,P4HB,BLM,MFAP5,KIAA0753,CENPF,ADAMTSL4,FLNA,IGF1,C1S,IGF2,LMOD3,IGFALS,C1R,TTN,ACTB,TPM3
Monarch	HP:0003010	Prolonged bleeding time	6	42	1.06	0.0023	MYH9,F9,VWF,ITGA2B,F2,F5
Monarch	HP:0011821	Abnormal facial skeleton morphology	30	1056	0.36	0.0024	MMP2,MGP,APOE,NOTCH3,DNAH5,CENPE,TCIRG1,CDH11,SEC24D,A2ML1,ANTXR1,SUZ12,FBN1,P4HB,BLM,MFAP5,KIAA0753,CENPF,ADAMTSL4,FLNA,IGF1,C1S,IGF2,LMOD3,IGFALS,C1R,TTN,HBB,ACTB,TPM3
Monarch	HP:0012337	Abnormal homeostasis	34	1275	0.33	0.0024	MMP2,APOA1,CAT,APOE,F12,LYZ,NOTCH3,CP,TCIRG1,CDH11,SERPING1,A2ML1,ANTXR1,FGB,DST,F2,NDUFS8,HBA1,FBN1,FGG,FN1,BLM,GSN,IGF1,IGF2,LMOD3,TGFBI,IGFALS,APOA5,VEGFC,PLCG2,HBB,ACTB,FGA
Monarch	HP:0032101	Unusual infection	24	748	0.41	0.0025	C5,MGP,DNAH5,TCIRG1,FCN3,FERMT3,C1QB,C7,GANAB,BLM,C8A,CFH,ECM1,FLNA,C8B,C1QC,MASP2,TF,C4B,C1R,CFD,PLCG2,HBB,TPM3
Monarch	HP:0011034	Amyloidosis	5	25	1.21	0.0026	APOA1,TTR,LYZ,GSN,FGA
Monarch	HP:0009122	Aplasia/hypoplasia affecting bones of the axial skeleton	24	751	0.41	0.0027	MMP2,NOTCH3,CENPE,TCIRG1,SEC24D,A2ML1,ANTXR1,SUZ12,FBN1,P4HB,FN1,BLM,KIAA0753,CENPF,FLNA,IGF1,C1S,IGF2,LMOD3,IGFALS,C1R,TTN,ACTB,TPM3
Monarch	HP:0010981	Hypolipoproteinemia	5	27	1.17	0.0035	APOA1,APOE,LCAT,ANGPTL3,APOA5
Monarch	EFO:0000408	Disease	35	1373	0.31	0.0042	CETP,MMP2,LRP1,APOE,CFHR5,VWF,TIMP2,ITIH4,CDH11,IGFBP4,SEC24D,FBN1,P4HB,GANAB,ITIH2,MFAP5,COL18A1,CFH,F5,ADAM12,FLNA,IGF1,EFEMP1,EFCAB5,MAP3K7CL,LTBP1,KSR2,BZW1,NBEAL1,CFB,IGFALS,TTN,PLCG2,KNG1,TPM3
Monarch	HP:0009115	Aplasia/hypoplasia involving the skeleton	28	985	0.36	0.0043	MMP2,MGP,APOE,NOTCH3,CENPE,TCIRG1,CDH11,SEC24D,MASP1,A2ML1,ANTXR1,SUZ12,FBN1,P4HB,FN1,BLM,KIAA0753,CENPF,FLNA,IGF1,C1S,IGF2,LMOD3,IGFALS,C1R,TTN,ACTB,TPM3
Monarch	HP:0011277	Abnormality of the urinary system physiology	27	933	0.37	0.0043	MYH9,F9,APOA1,TTR,APOE,CFHR5,LYZ,ITGA2B,LCAT,F2,C1QB,CFHR1,NDUFS8,GANAB,PAPLN,FN1,BLM,CFH,F5,GSN,C1QC,F10,C4A,C1R,ATP1A1,HBB,FGA
Monarch	HP:0001609	Hoarse voice	7	72	0.89	0.0044	SERPING1,SUZ12,FBN1,ECM1,FLNA,C1R,TPM3
Monarch	HP:0100545	Arterial stenosis	7	72	0.89	0.0044	SERPIND1,APOE,F12,NOTCH3,PCSK9,FBN1,MFAP5
Monarch	HP:0000235	Abnormality of the fontanelles or cranial sutures	15	355	0.53	0.0046	MMP2,SPARC,APOE,NOTCH3,CENPE,TCIRG1,CDH11,SEC24D,MASP1,ANTXR1,FBN1,P4HB,FLNA,LMOD3,ACTB
Monarch	HP:0010989	Abnormality of the intrinsic pathway	5	29	1.14	0.0046	F9,F12,VWF,F11,KNG1
Monarch	HP:0003312	Abnormal form of the vertebral bodies	13	274	0.58	0.0047	MMP2,SPARC,NOTCH3,TCIRG1,CDH11,SEC24D,CSF1R,ANTXR1,FBN1,P4HB,FN1,FLNA,ACTB
Monarch	EFO:0004694	Factor XI measurement	3	4	1.78	0.0050	KLKB1,F11,KNG1
Monarch	HP:0012223	Splenic rupture	3	4	1.78	0.0050	FGB,FGG,FGA
Monarch	HP:0040232	Delayed onset bleeding	3	4	1.78	0.0050	F9,F13A1,F13B
Monarch	HP:0200036	Skin nodule	7	74	0.88	0.0050	MMP2,APOE,NOTCH3,FERMT3,PCSK9,FBN1,ECM1
Monarch	HP:0002718	Recurrent bacterial infections	10	169	0.68	0.0059	C5,DNAH5,TCIRG1,FCN3,FERMT3,C7,CFH,C8B,CFD,HBB
Monarch	HP:0031365	Macular purpura	6	52	0.97	0.0059	APOE,ITGA2B,F13A1,FERMT3,F2,F13B
Monarch	HP:0001547	Abnormal rib cage morphology	14	325	0.54	0.0063	GNPTG,MGP,NOTCH3,TCIRG1,SEC24D,A2ML1,ANTXR1,SUZ12,FBN1,P4HB,FLNA,IGF2,LMOD3,ACTB
Monarch	HP:0007759	Opacification of the corneal stroma	7	78	0.86	0.0065	GNPTG,MMP2,APOE,LCAT,PCSK9,COL18A1,TGFBI
Monarch	HP:0010647	Abnormal elasticity of skin	10	172	0.67	0.0066	MGP,SPARC,CSTA,ANTXR1,SUZ12,FBN1,FLNA,GSN,C1S,C1R
Monarch	HP:0005293	Venous insufficiency	6	54	0.95	0.0070	PROC,VWF,NOTCH3,PROS1,EFEMP1,VEGFC
Monarch	HP:0010980	Hyperlipoproteinemia	5	33	1.09	0.0073	CETP,APOE,LCAT,PCSK9,APOA5
Monarch	HP:0005430	Recurrent Neisserial infections	3	5	1.69	0.0074	C5,C7,C8B
Monarch	HP:0002326	Transient ischemic attack	6	55	0.94	0.0076	ANGPTL6,NOTCH3,FBN1,MFAP5,HTRA1,ACTB
Monarch	HP:0002664	Neoplasm	23	763	0.39	0.0079	CAT,APOE,NOTCH3,F13A1,TCIRG1,FCN3,HABP2,ANTXR1,F2,HBA1,GANAB,FN1,BLM,COL18A1,KIAA0753,F13B,ECM1,FLNA,C1S,IGF2,VEGFC,HBB,ACTB
Monarch	HP:0011329	Abnormality of cranial sutures	13	292	0.56	0.0079	MMP2,SPARC,NOTCH3,CENPE,TCIRG1,CDH11,SEC24D,MASP1,ANTXR1,FBN1,P4HB,FLNA,ACTB
Monarch	HP:0000478	Abnormality of the eye	58	2876	0.21	0.0083	GNPTG,MYH9,MMP2,MGP,SPARC,PROC,APOA1,TTR,APOE,PXDN,ANGPTL6,F12,NOTCH3,LCAT,CSTA,CP,DNAH5,CENPE,TCIRG1,CDH11,SERPING1,SEC24D,CSF1R,FYCO1,MASP1,A2ML1,ANTXR1,PCSK9,DST,NDUFS8,SUZ12,FBN1,P4HB,FBLN1,THBS4,BLM,MFAP5,COL18A1,KIAA0753,CENPF,CFH,HTRA1,ADAMTSL4,FLNA,RBP4,GSN,IGF1,PROS1,EFEMP1,KSR2,LMOD3,TGFBI,C1R,TTN,PLCG2,HBB,ACTB,TPM3
Monarch	EFO:0001365	Age-related macular degeneration	5	35	1.06	0.0090	CETP,APOE,CFHR5,CFH,CFB
Monarch	EFO:1001492	Atrophic macular degeneration	5	35	1.06	0.0090	CETP,APOE,CFHR5,CFH,CFB
Monarch	HP:0002719	Recurrent infections	22	721	0.39	0.0090	C5,MGP,DNAH5,TCIRG1,FCN3,FERMT3,C1QB,C7,GANAB,BLM,CFH,ECM1,FLNA,C8B,C1QC,MASP2,TF,C1R,CFD,PLCG2,HBB,TPM3
Monarch	HP:0000481	Abnormal cornea morphology	21	673	0.4	0.0093	GNPTG,MMP2,APOA1,APOE,PXDN,LCAT,DNAH5,CDH11,SERPING1,ANTXR1,PCSK9,DST,FBN1,COL18A1,CENPF,FLNA,RBP4,GSN,TGFBI,PLCG2,ACTB
Monarch	HP:0003233	Decreased HDL cholesterol concentration	4	18	1.25	0.0093	APOA1,APOE,LCAT,APOA5
Monarch	HP:0011843	Abnormal musculoskeletal physiology	33	1330	0.3	0.0093	GNPTG,MMP2,SPARC,APOE,NOTCH3,CENPE,TCIRG1,HABP2,SEC24D,MASP1,A2ML1,ANTXR1,DST,F2,SUZ12,FBN1,P4HB,FBLN1,FN1,COL18A1,ADAMTSL4,FLNA,IGF1,C1S,IGF2,PRG4,LMOD3,IGFALS,C1R,TTN,HBB,ACTB,TPM3
Monarch	HP:0031886	Abnormal LDL cholesterol concentration	5	36	1.05	0.0094	APOE,LCAT,PCSK9,ANGPTL3,APOA5
Monarch	HP:0100763	Abnormality of the lymphatic system	19	576	0.43	0.0096	APOA1,APOE,LYZ,LCAT,DNAH5,TCIRG1,HABP2,FERMT3,A2ML1,FGB,F2,HBA1,FGG,BLM,COL18A1,CENPF,GSN,HBB,FGA
Monarch	HP:0030780	Abnormality of the protein C anticoagulant pathway	3	6	1.61	0.0097	PROC,F5,PROS1
Monarch	HP:0011138	Abnormality of skin adnexa morphology	30	1165	0.32	0.0099	MMP2,MGP,LRP1,APOE,NOTCH3,CSTA,CENPE,TCIRG1,CDH11,DSC2,MASP1,A2ML1,ANTXR1,DST,F2,NDUFS8,SUZ12,FBN1,LIMS1,BLM,COL18A1,KIAA0753,HTRA1,ECM1,FLNA,GSN,IGF1,IGF2,VEGFC,ACTB
Monarch	HP:0002813	Abnormality of limb bone morphology	35	1459	0.29	0.0103	GNPTG,MMP2,MGP,SPARC,APOE,VWF,NOTCH3,DNAH5,CENPE,TCIRG1,CDH11,SEC24D,CSF1R,MASP1,A2ML1,ANTXR1,F2,SUZ12,FBN1,P4HB,FBLN1,FN1,BLM,MFAP5,KIAA0753,CENPF,FLNA,IGF1,IGF2,PRG4,LMOD3,C1R,TTN,HBB,ACTB
Monarch	HP:0004334	Dermal atrophy	7	88	0.81	0.0109	MGP,TCIRG1,DST,FBN1,GSN,C1S,C1R
Monarch	HP:0001482	Subcutaneous nodule	6	61	0.9	0.0110	MMP2,APOE,NOTCH3,FERMT3,FBN1,ECM1
Monarch	HP:0007957	Corneal opacity	11	226	0.59	0.0110	GNPTG,MMP2,APOA1,APOE,PXDN,LCAT,PCSK9,DST,COL18A1,CENPF,TGFBI
Monarch	HP:0000282	Facial edema	6	62	0.89	0.0117	F12,CDH11,SERPING1,ANTXR1,GSN,ACTB
Monarch	HP:0003110	Abnormality of urine homeostasis	20	638	0.4	0.0117	MYH9,F9,APOA1,APOE,CFHR5,LYZ,ITGA2B,LCAT,F2,CFHR1,GANAB,PAPLN,FN1,CFH,F5,GSN,F10,ATP1A1,HBB,FGA

Monarch	HP:0032180	Abnormal circulating metabolite concentration	27	1010	0.33	0.0117	CETP,GNPTG,APOA1,APOE,NOTCH3,LCAT,CP,TCIRG1,PLA2G7,CSF1R,PCSK9,F2,NDUFS8,FBN1,GANAB,FN1,ANGPTL3,IGF1,TF,LMOD3,IGFALS,APOA5,ATP1A1,TTN,KNG1,HBB,TPM3
Monarch	HP:0001058	Poor wound healing	4	20	1.21	0.0121	F9,F13A1,F13B,C1R
Monarch	HP:0011877	Increased mean platelet volume	4	20	1.21	0.0121	MYH9,ITGA2B,FLNA,ACTN1
Monarch	HP:0001376	Limitation of joint mobility	14	356	0.5	0.0124	GNPTG,MMP2,NOTCH3,MASP1,DST,F2,SUZ12,FBN1,ADAMTSL4,FLNA,PRG4,TTN,ACTB,TPM3
Monarch	HP:0004418	Thrombophlebitis	3	7	1.54	0.0127	PROC,F2,PROS1
Monarch	HP:0005305	Cerebral venous thrombosis	3	7	1.54	0.0127	PROC,F2,PROS1
Monarch	HP:0011867	Abnormal iliac wing morphology	6	64	0.88	0.0132	GNPTG,APOE,TCIRG1,SEC24D,SUZ12,FLNA
Monarch	HP:0002808	Kyphosis	15	405	0.48	0.0136	GNPTG,MMP2,SPARC,NOTCH3,CDH11,SEC24D,CSF1R,SUZ12,FBN1,P4HB,FN1,FLNA,LMOD3,ACTB,TPM3
Monarch	HP:0002829	Arthralgia	8	124	0.72	0.0137	GNPTG,MMP2,PCSK9,F2,FBN1,C1S,PLCG2,HBB
Monarch	HP:0005268	Miscarriage	4	21	1.19	0.0137	MGP,FGB,FGG,FGA
Monarch	HP:0011420	Age of death	4	21	1.19	0.0137	MGP,FGB,FGG,FGA
Monarch	HP:0031815	Abnormal oral physiology	10	196	0.61	0.0138	F13A1,SERPING1,F2,FBLN1,ANG,F13B,F5,GSN,F10,TPM3
Monarch	HP:0011844	Abnormal appendicular skeleton morphology	36	1557	0.27	0.0147	GNPTG,MMP2,MGP,SPARC,APOE,VWF,NOTCH3,DNAH5,CENPE,TCIRG1,CDH11,SEC24D,CSF1R,MASP1,A2ML1,ANTXR1,F2,SUZ12,FBN1,P4HB,FBLN1,FN1,BLM,MFAP5,KIAA0753,CENPF,FLNA,IGF1,IGF2,PRG4,LMOD3,C1R,TTN,HBB,ACTB,TPM3
Monarch	HP:0001382	Joint hypermobility	12	279	0.54	0.0151	NOTCH3,CENPE,SEC24D,A2ML1,ANTXR1,SUZ12,FBN1,P4HB,COL18A1,FLNA,C1S,C1R
Monarch	HP:0001627	Abnormal heart morphology	30	1206	0.3	0.0154	MMP2,MGP,TTR,APOE,VWF,NOTCH3,DNAH5,TCIRG1,SEC24D,DSC2,MASP1,A2ML1,PCSK9,F2,NDUFS8,HBA1,FBN1,P4HB,GANAB,FN1,MFAP5,COL18A1,KIAA0753,FLNA,GSN,PRG4,TTN,HBB,ACTB,TPM3
Monarch	HP:0100665	Angioedema	3	8	1.48	0.0160	F12,SERPING1,PLCG2
Monarch	HP:0002637	Cerebral ischemia	10	201	0.6	0.0163	ANGPTL6,NOTCH3,GUCY1A1,F2,FBN1,MFAP5,F5,HTRA1,KNG1,ACTB
Monarch	HP:0002686	Prenatal maternal abnormality	8	129	0.7	0.0163	F12,F13A1,DNAH5,HBA1,FBN1,MFAP5,F13B,F5
Monarch	HP:0008067	Abnormally lax or hyperextensible skin	8	129	0.7	0.0163	CSTA,ANTXR1,SUZ12,FBN1,FLNA,GSN,C1S,C1R
Monarch	HP:0033401	Tissue ischemia	10	201	0.6	0.0163	ANGPTL6,NOTCH3,GUCY1A1,F2,FBN1,MFAP5,F5,HTRA1,KNG1,ACTB
Monarch	HP:0000944	Abnormal metaphysis morphology	10	204	0.6	0.0177	MMP2,NOTCH3,TCIRG1,SEC24D,CSF1R,ANTXR1,SUZ12,P4HB,FN1,FLNA
Monarch	HP:0001677	Coronary artery atherosclerosis	5	44	0.96	0.0179	SERPIND1,APOE,PCSK9,FBN1,MFAP5
Monarch	HP:0025032	Abnormality of digestive system physiology	39	1763	0.25	0.0180	F9,C5,TTR,F12,VWF,ITGA2B,NOTCH3,F13A1,TCIRG1,FCN3,SERPING1,GUCY1A1,A2ML1,FGB,DST,F2,NDUFS8,SUZ12,FBN1,ANG,FGG,GANAB,BLM,KIAA0753,F13B,F5,ECM1,FLNA,F10,F7,MASP2,F11,IGF2,LMOD3,PLCG2,HBB,ACTB,FGA,TPM3
Monarch	HP:0002659	Increased susceptibility to fractures	10	205	0.6	0.0181	MMP2,SPARC,TCIRG1,HABP2,SEC24D,P4HB,FN1,LMOD3,HBB,TPM3
Monarch	EFO:0004713	FEV/FEC ratio	20	672	0.38	0.0192	MYH9,MPO,LRP1,SERPINA10,TIMP2,DNAH5,SERPING1,ANTXR1,DST,C7,FBN1,FBLN1,CFH,ADAM12,HTRA1,EFEMP1,EFCAB5,LTBP1,KSR2,VEGFC
Monarch	HP:0002138	Subarachnoid hemorrhage	4	24	1.13	0.0194	ANGPTL6,FBN1,MFAP5,F10
Monarch	HP:0001647	Bicuspid aortic valve	6	71	0.83	0.0196	MMP2,NOTCH3,FBN1,MFAP5,FLNA,ACTB
Monarch	EFO:0003907	Deep vein thrombosis	3	9	1.43	0.0198	F2,F5,KNG1
Monarch	HP:0004942	Aortic aneurysm	8	135	0.68	0.0207	ANGPTL6,SEC24D,FBN1,P4HB,GANAB,MFAP5,FLNA,TPM3
Monarch	HP:0031567	Abnormal aortic valve cusp morphology	6	72	0.83	0.0208	MMP2,NOTCH3,FBN1,MFAP5,FLNA,ACTB
Monarch	HP:0000095	Abnormal renal glomerulus morphology	8	136	0.68	0.0215	APOE,CFHR5,C1QB,CFHR1,FN1,CFH,C1QC,C4A
Monarch	HP:0004452	Abnormality of the middle ear ossicles	4	25	1.11	0.0215	SPARC,NOTCH3,SEC24D,P4HB
Monarch	HP:0020129	Abnormal urine protein level	10	211	0.58	0.0215	MYH9,APOA1,APOE,LYZ,LCAT,GANAB,FN1,CFH,GSN,FGA
Monarch	HP:0030875	Abnormality of pulmonary circulation	8	136	0.68	0.0215	MGP,PROC,TCIRG1,F2,FBN1,FLNA,PROS1,HBB
Monarch	HP:0002012	Abnormality of the abdominal organs	27	1068	0.31	0.0222	APOA1,APOE,LYZ,NOTCH3,LCAT,CP,DNAH5,TCIRG1,FERMT3,A2ML1,ANTXR1,PCSK9,FGB,F2,NDUFS8,HBA1,FBN1,FGG,GANAB,CENPF,GSN,F10,C1S,TF,C4B,HBB,FGA
Monarch	HP:0002823	Abnormality of femur morphology	11	253	0.55	0.0222	GNPTG,SPARC,TCIRG1,SEC24D,SUZ12,FBN1,P4HB,FN1,FLNA,PRG4,ACTB
Monarch	HP:0006480	Premature loss of teeth	5	47	0.93	0.0222	CAT,TCIRG1,CDH11,C1S,C1R
Monarch	HP:0000953	Hyperpigmentation of the skin	12	297	0.51	0.0230	MMP2,APOE,NOTCH3,CSTA,F2,BLM,IGF1,C1S,IGF2,C1R,HBB,ACTB
Monarch	HP:0008151	Prolonged prothrombin time	4	26	1.09	0.0238	F2,F5,F10,F7
Monarch	EFO:0010071	Cardiac troponin I measurement	3	10	1.38	0.0240	F12,KLKB1,HABP2
Monarch	HP:0008011	Peripheral opacification of the cornea	3	10	1.38	0.0240	MMP2,APOE,PCSK9
Monarch	HP:0200067	Recurrent spontaneous abortion	3	10	1.38	0.0240	F12,F13A1,F13B
Monarch	EFO:0004614	Apolipoprotein A 1 measurement	14	396	0.46	0.0279	CETP,VTN,APOA1,LRP1,APOE,LCAT,PCSK9,STAB1,APOA4,COL18A1,PAPPA2,TF,HPR,APOC4
Monarch	EFO:0000618	Nervous system disease	13	350	0.48	0.0280	CETP,APOE,CFHR5,TIMP2,ITIH4,COL18A1,CFH,F5,KSR2,NBEAL1,CFB,PLCG2,KNG1
Monarch	HP:0002624	Abnormal venous morphology	8	145	0.65	0.0300	PROC,VWF,NOTCH3,DNAH5,ANTXR1,PROS1,EFEMP1,VEGFC
Monarch	EFO:0004574	Total cholesterol measurement	16	497	0.41	0.0303	CETP,APOA1,APOE,LCAT,PCSK9,FGB,OIT3,LIMS1,FN1,APOA4,COL18A1,KSR2,HPR,APOA5,APOC4,HBB
Monarch	HP:0000963	Thin skin	6	80	0.78	0.0321	PROC,SEC24D,P4HB,FLNA,PROS1,C1R
Monarch	HP:0000093	Proteinuria	10	225	0.55	0.0323	MYH9,APOA1,APOE,LYZ,LCAT,PAPLN,FN1,CFH,GSN,FGA
Monarch	HP:0002143	Abnormality of the spinal cord	10	225	0.55	0.0323	APOE,NOTCH3,HABP2,MASP1,FBN1,FBLN1,MFAP5,FLNA,HBB,ACTB
Monarch	HP:0004950	Peripheral arterial stenosis	4	29	1.05	0.0328	APOE,PCSK9,FBN1,MFAP5
Monarch	HP:0010299	Abnormal dentin morphology	4	29	1.05	0.0328	SPARC,CDH11,SEC24D,P4HB
Monarch	HP:0001743	Abnormality of the spleen	16	503	0.41	0.0332	APOA1,APOE,LYZ,LCAT,DNAH5,TCIRG1,FERMT3,A2ML1,FGB,F2,HBA1,FGG,CENPF,GSN,HBB,FGA
Monarch	HP:0031652	Abnormal aortic valve physiology	7	113	0.7	0.0332	GNPTG,VWF,PCSK9,FBN1,MFAP5,FLNA,ACTB
Monarch	HP:0004328	Abnormal anterior eye segment morphology	27	1106	0.29	0.0343	GNPTG,MYH9,MMP2,APOA1,APOE,PXDN,NOTCH3,LCAT,DNAH5,CDH11,SERPING1,FYCO1,MASP1,ANTXR1,PCSK9,DST,FBN1,MFAP5,COL18A1,CENPF,ADAMTSL4,FLNA,RBP4,GSN,TGFBI,PLCG2,ACTB
Monarch	HP:0003321	Biconcave flattened vertebrae	3	12	1.3	0.0349	SPARC,SEC24D,P4HB
Monarch	HP:0004840	Hypochromic microcytic anemia	3	12	1.3	0.0349	CP,HBA1,HBB
Monarch	HP:0001363	Craniosynostosis	8	150	0.63	0.0350	CENPE,TCIRG1,CDH11,SEC24D,MASP1,FBN1,P4HB,FLNA
Monarch	HP:0000766	Abnormal sternum morphology	13	362	0.46	0.0356	GNPTG,NOTCH3,TCIRG1,CDH11,SEC24D,A2ML1,FBN1,FN1,MFAP5,FLNA,LMOD3,ACTB,TPM3
Monarch	HP:0002645	Wormian bones	5	54	0.87	0.0358	SPARC,NOTCH3,SEC24D,P4HB,FLNA
Monarch	HP:0001387	Joint stiffness	8	151	0.63	0.0359	GNPTG,F2,SUZ12,FBN1,ADAMTSL4,FLNA,PRG4,ACTB
Monarch	HP:0012575	Abnormal nephron morphology	9	190	0.58	0.0366	APOE,CFHR5,ANTXR1,C1QB,CFHR1,FN1,CFH,C1QC,C4A
Monarch	HP:0003549	Abnormality of connective tissue	30	1294	0.27	0.0378	MMP2,SPARC,LRP1,APOE,NOTCH3,CENPE,TCIRG1,CDH11,SEC24D,MASP1,ANTXR1,DST,SUZ12,FBN1,P4HB,BLM,MFAP5,ECM1,FLNA,C1S,IGF2,PRG4,LMOD3,TGFBI,C1R,TTN,VEGFC,PLCG2,ACTB,TPM3
Monarch	EFO:0006794	Cerebrospinal fluid biomarker measurement	5	55	0.87	0.0380	APOE,CFHR5,PLA2G7,C7,C1R
Monarch	HP:0000973	Cutis laxa	6	84	0.76	0.0380	ANTXR1,SUZ12,FBN1,FLNA,GSN,C1R
Monarch	HP:0002644	Abnormal pelvic girdle bone morphology	16	512	0.4	0.0380	GNPTG,MMP2,APOE,CENPE,TCIRG1,SEC24D,MASP1,ANTXR1,SUZ12,FBN1,FBLN1,FN1,FLNA,LMOD3,TTN,TPM3
Monarch	HP:0031364	Ecchymosis	4	31	1.02	0.0384	ITGA2B,F13A1,F2,F13B
Monarch	HP:0004298	Abnormality of the abdominal wall	14	415	0.44	0.0390	MMP2,APOE,NOTCH3,CDH11,MASP1,A2ML1,ANTXR1,SUZ12,FBN1,MFAP5,FLNA,C1S,IGF2,C1R
Monarch	EFO:0008022	Angiogenin measurement	2	2	1.91	0.0395	ANG,RNASE4
Monarch	EFO:0008098	Complement factor H-related protein 5 measurement	2	2	1.91	0.0395	F13B,CFH
Monarch	EFO:0008260	Plasma kallikrein measurement	2	2	1.91	0.0395	KLKB1,KNG1
Monarch	HP:0001038	Warfarin-induced skin necrosis	2	2	1.91	0.0395	PROC,PROS1

Monarch	HP:0002694	Sclerosis of skull base	3	13	1.27	0.0395	NOTCH3,TCIRG1,FLNA
Monarch	HP:0004434	Decreased serum complement C8	2	2	1.91	0.0395	C8A,C8B
Monarch	HP:0005225	Intestinal edema	2	2	1.91	0.0395	F12,SERPING1
Monarch	HP:0005511	Heinz body anemia	2	2	1.91	0.0395	HBA1,HBB
Monarch	HP:0006086	Thin metacarpal cortices	2	2	1.91	0.0395	MMP2,SPARC
Monarch	HP:0011855	Pharyngeal edema	2	2	1.91	0.0395	F12,SERPING1
Monarch	HP:0011971	Dermatographic urticaria	2	2	1.91	0.0395	SERPING1,PLCG2
Monarch	HP:0012874	Abnormal male reproductive system physiology	10	234	0.54	0.0395	TTR,DNAH5,GUCY1A1,ANTXR1,F2,DNAH8,GANAB,BLM,ADGRG2,HBB
Monarch	HP:0004299	Hernia of the abdominal wall	13	371	0.45	0.0404	MMP2,APOE,NOTCH3,CDH11,MASP1,ANTXR1,SUZ12,FBN1,MFAP5,FLNA,C1S,IGF2,C1R
Monarch	HP:0011314	Abnormal long bone morphology	19	675	0.36	0.0404	GNPTG,MMP2,MGP,SPARC,NOTCH3,CENPE,TCIRG1,SEC24D,CSF1R,MASP1,A2ML1,ANTXR1,SUZ12,FBN1,P4HB,FN1,FLNA,LMOD3,HBB
Monarch	HP:0100540	Palpebral edema	4	32	1.0	0.0404	CDH11,ANTXR1,GSN,ACTB
Monarch	HP:0010866	Abdominal wall defect	13	375	0.45	0.0441	MMP2,APOE,NOTCH3,CDH11,MASP1,ANTXR1,SUZ12,FBN1,MFAP5,FLNA,C1S,IGF2,C1R
Monarch	HP:0004586	Biconcave vertebral bodies	4	33	0.99	0.0445	SPARC,NOTCH3,SEC24D,P4HB
Monarch	HP:0000767	Pectus excavatum	10	241	0.53	0.0462	NOTCH3,CDH11,SEC24D,A2ML1,FBN1,MFAP5,FLNA,LMOD3,ACTB,TPM3
Monarch	HP:0002650	Scoliosis	23	908	0.31	0.0474	GNPTG,MMP2,SPARC,NOTCH3,CENPE,CDH11,SEC24D,MASP1,A2ML1,ANTXR1,SUZ12,FBN1,P4HB,FN1,MFAP5,FLNA,C1S,IGF2,LMOD3,C1R,TTN,ACTB,TPM3
Monarch	HP:0011800	Midface retrusion	10	243	0.52	0.0487	MGP,CDH11,SEC24D,A2ML1,ANTXR1,FBN1,P4HB,COL18A1,FLNA,ACTB
Monarch	HP:0000765	Abnormal thorax morphology	20	743	0.34	0.0490	GNPTG,MMP2,MGP,NOTCH3,TCIRG1,CDH11,SEC24D,A2ML1,ANTXR1,SUZ12,FBN1,P4HB,FN1,MFAP5,FLNA,IGF2,LMOD3,TTN,ACTB,TPM3
Monarch	HP:0002037	Inflammation of the large intestine	5	60	0.83	0.0496	F13A1,FCN3,F13B,MASP2,PLCG2
Monarch	HP:0040064	Abnormality of limbs	41	2018	0.21	0.0496	GNPTG,MMP2,MGP,SPARC,APOE,VWF,NOTCH3,CSTA,DNAH5,CENPE,TCIRG1,CDH11,SEC24D,DSC2,CSF1R,MASP1,A2ML1,ANTXR1,DST,F2,SUZ12,FBN1,P4HB,FBLN1,FN1,BLM,MFAP5,KIAA0753,CENPF,FLNA,IGF1,IGF2,PRG4,LMOD3,C1R,ATP1A1,TTN,VEGFC,HBB,ACTB,TPM3
Monarch	HP:0002086	Abnormality of the respiratory system	35	1636	0.24	0.0500	MGP,PROC,CAT,APOE,F12,NOTCH3,DNAH5,TCIRG1,FCN3,CRH,HABP2,SERPING1,PCSK9,DST,F2,NDUFS8,FBN1,ANG,BLM,MFAP5,KIAA0753,F5,ECM1,FLNA,GSN,PROS1,MASP2,IGF2,LMOD3,TTN,PLCG2,HBB,TPM3
DISEASES	DOID:1247	Blood coagulation disease	25	89	1.36	7.79e-21	SERPIND1,MYH9,F9,PF4V1,PROC,F12,VWF,ITGA2B,KLKB1,F13A1,PF4,FGB,F2,FGG,F13B,CFH,F5,FLNA,F10,F7,PROS1,F11,C4B,KNG1,FGA
DISEASES	DOID:7	Disease of anatomical entity	120	4798	0.31	1.82e-13	CETP,SERPIND1,MYH9,F9,MMP2,C5,MPO,PF4V1,MGP,SPARC,PROC,APOA1,TTR,APOE,PXDN,F12,CFHR5,VWF,ITGA2B,NOTCH3,LCAT,CSTA,KLKB1,F13A1,DNAH5,CENPE,TCIRG1,PLA2G7,CRH,SAA4,SERPING1,FERMT3,SEC24D,DSC2,ANKRD44,CSF1R,PCDH1,SCGB3A1,PF4,FYCO1,APEH,A2ML1,LALBA,ANTXR1,LRG1,SH3D19,PCSK9,FGB,DST,F2,C1QB,CFHR1,SUZ12,APOL1,SUMO4,HBA1,A2M,FBN1,P4HB,SERPINA7,PAPPA,ANG,FGG,GANAB,FN1,BLM,APOA4,MFAP5,COL18A1,KIAA0753,F13B,CFHR2,CFHR3,CFH,F5,S100A8,HTRA1,ADAMTSL4,ECM1,FLNA,C8B,RBP4,GSN,C1QC,F10,F7,ADGRG2,CD99,IGF1,PROS1,EFEMP1,TNRC6A,YWHAZ,GAPDH,F11,C1S,ADIPOQ,IGF2,C4A,MST1,LMOD3,C4B,TGFBI,CFB,TSKU,SAA2,C1R,ATP1A1,IGKV2D-28,NBPF10,TTN,CFD,VEGFC,PLCG2,KNG1,HBB,ACTB,TRIM66,FGA,TPM3
DISEASES	DOID:4	Disease	141	6291	0.26	2.16e-13	CETP,GNPTG,SERPIND1,MYH9,F9,MMP2,C5,MPO,PF4V1,MGP,SPARC,PROC,APOA1,TTR,CAT,APOE,PXDN,F12,CFHR5,LYZ,VWF,ITGA2B,NOTCH3,LCAT,CSTA,CP,KLKB1,F13A1,DNAH5,CENPE,TCIRG1,ITIH4,CDH11,PLA2G7,CRH,SAA4,SERPING1,FERMT3,SEC24D,DSC2,ANKRD44,CSF1R,PCDH1,SCGB3A1,PF4,FYCO1,MASP1,APEH,A2ML1,LALBA,ANTXR1,LRG1,SH3D19,PCSK9,FGB,DST,F2,PRG2,C1QB,CFHR1,NDUFS8,SUZ12,APOL1,SUMO4,HBA1,A2M,HSPA5,FBN1,P4HB,SERPINA7,PAPPA,DNAH8,ANG,FGG,GANAB,FN1,BLM,APOA4,MFAP5,COL18A1,KIAA0753,CENPF,F13B,CFHR2,CFHR3,CFH,F5,S100A8,HTRA1,ADAMTSL4,ECM1,FLNA,ANGPTL3,C8B,RBP4,GSN,C1QC,F10,F7,CA6,ADGRG2,IGFBP3,CD99,GPX3,IGF1,PROS1,EFEMP1,TNRC6A,YWHAZ,GAPDH,F11,C1S,PCYOX1,ADIPOQ,IGF2,C4A,PRG4,MST1,LMOD3,C4B,TGFBI,CFB,SELENOP,TSKU,SAA2,HPR,C1R,APOA5,ATP1A1,IGKV2D-28,NBPF10,TTN,CFD,VEGFC,PLCG2,KNG1,HBB,ACTB,TRIM66,FGA,TPM3
DISEASES	DOID:74	Hematopoietic system disease	33	473	0.75	4.67e-12	SERPIND1,MYH9,F9,MPO,PF4V1,PROC,F12,VWF,ITGA2B,KLKB1,F13A1,TCIRG1,FERMT3,PF4,FGB,F2,HBA1,FGG,F13B,CFH,F5,FLNA,F10,F7,CD99,PROS1,F11,C4B,SAA2,KNG1,HBB,ACTB,FGA
DISEASES	DOID:9120	Amyloidosis	14	75	1.18	3.60e-09	APOA1,TTR,APOE,LYZ,SAA4,A2M,FN1,APOA4,GSN,TGFBI,SAA2,HBB,ACTB,FGA
DISEASES	DOID:2452	Thrombophilia	9	21	1.54	4.64e-08	SERPIND1,F9,PROC,VWF,F2,CFH,F5,PROS1,C4B
DISEASES	DOID:2007	Degeneration of macula and posterior pole	11	46	1.29	5.41e-08	CETP,APOE,CFHR5,CFHR1,F13B,CFHR2,CFHR3,CFH,HTRA1,EFEMP1,CFB
DISEASES	DOID:0050739	Autosomal genetic disease	74	2802	0.33	6.68e-08	CETP,GNPTG,SERPIND1,MYH9,F9,MGP,PROC,APOA1,TTR,APOE,F12,CFHR5,LYZ,VWF,ITGA2B,NOTCH3,CP,KLKB1,F13A1,DNAH5,TCIRG1,SERPING1,FERMT3,ANKRD44,CSF1R,FYCO1,MASP1,ANTXR1,PCSK9,FGB,DST,F2,CFHR1,HBA1,FBN1,FGG,GANAB,BLM,APOA4,KIAA0753,F13B,CFHR2,CFHR3,CFH,F5,HTRA1,ADAMTSL4,FLNA,ANGPTL3,GSN,F10,F7,IGFBP3,IGF1,EFEMP1,F11,C1S,PCYOX1,IGF2,PRG4,LMOD3,TGFBI,CFB,SAA2,APOA5,ATP1A1,TTN,CFD,PLCG2,KNG1,HBB,ACTB,FGA,TPM3
DISEASES	DOID:626	Complement deficiency	9	23	1.5	6.68e-08	C5,CFHR5,KLKB1,SERPING1,CFHR1,CFHR3,CFH,CFB,KNG1
DISEASES	DOID:2914	Immune system disease	32	675	0.58	8.38e-08	C5,MPO,TTR,APOE,PXDN,CFHR5,ITGA2B,KLKB1,SAA4,SERPING1,FERMT3,A2ML1,LALBA,DST,F2,CFHR1,SUMO4,HBA1,FN1,COL18A1,CFHR3,CFH,CD99,C4A,C4B,CFB,SAA2,IGKV2D-28,VEGFC,PLCG2,KNG1,ACTB
DISEASES	DOID:0050177	Monogenic disease	81	3266	0.3	1.01e-07	CETP,GNPTG,SERPIND1,MYH9,F9,MGP,PROC,APOA1,TTR,APOE,F12,CFHR5,LYZ,VWF,ITGA2B,NOTCH3,CP,KLKB1,F13A1,DNAH5,TCIRG1,SERPING1,FERMT3,SEC24D,ANKRD44,CSF1R,FYCO1,MASP1,A2ML1,ANTXR1,PCSK9,FGB,DST,F2,CFHR1,HBA1,FBN1,DNAH8,FGG,GANAB,BLM,APOA4,KIAA0753,CENPF,F13B,CFHR2,CFHR3,CFH,F5,HTRA1,ADAMTSL4,FLNA,ANGPTL3,GSN,F10,F7,ADGRG2,IGFBP3,IGF1,PROS1,EFEMP1,F11,C1S,PCYOX1,IGF2,PRG4,MST1,LMOD3,TGFBI,CFB,SAA2,APOA5,ATP1A1,TTN,CFD,PLCG2,KNG1,HBB,ACTB,FGA,TPM3
DISEASES	DOID:630	Genetic disease	89	3778	0.28	1.06e-07	CETP,GNPTG,SERPIND1,MYH9,F9,MGP,PROC,APOA1,TTR,CAT,APOE,F12,CFHR5,LYZ,VWF,ITGA2B,NOTCH3,LCAT,CP,KLKB1,F13A1,DNAH5,TCIRG1,SERPING1,FERMT3,SEC24D,ANKRD44,CSF1R,FYCO1,MASP1,A2ML1,ANTXR1,PCSK9,FGB,DST,F2,C1QB,CFHR1,NDUFS8,SUMO4,HBA1,FBN1,PAPPA,DNAH8,FGG,GANAB,BLM,APOA4,KIAA0753,CENPF,F13B,CFHR2,CFHR3,CFH,F5,HTRA1,ADAMTSL4,ECM1,FLNA,ANGPTL3,GSN,F10,F7,ADGRG2,IGFBP3,IGF1,PROS1,EFEMP1,F11,C1S,PCYOX1,ADIPOQ,IGF2,PRG4,MST1,LMOD3,TGFBI,CFB,SAA2,APOA5,ATP1A1,TTN,CFD,PLCG2,KNG1,HBB,ACTB,FGA,TPM3
DISEASES	DOID:10871	Age related macular degeneration	10	44	1.26	3.06e-07	CETP,APOE,CFHR5,CFHR1,F13B,CFHR2,CFHR3,CFH,HTRA1,CFB
DISEASES	DOID:0050636	Familial visceral amyloidosis	8	21	1.49	4.72e-07	APOA1,TTR,APOE,LYZ,APOA4,HBB,ACTB,FGA
DISEASES	DOID:557	Kidney disease	19	275	0.75	1.20e-06	MYH9,C5,MPO,TTR,CFHR5,SAA4,F2,CFHR1,APOL1,GANAB,FN1,APOA4,COL18A1,CFHR3,CFH,IGF2,C4B,CFB,SAA2
DISEASES	DOID:178	Vascular disease	18	249	0.77	1.51e-06	CETP,MPO,APOA1,APOE,VWF,ITGA2B,PF4,PCSK9,F2,FBN1,PAPPA,MFAP5,F10,F7,CD99,ADIPOQ,C4A,SAA2

DISEASES	DOID:612	Primary immunodeficiency disease	24	470	0.62	2.67e-06	C5,MPO,TTR,PXDN,CFHR5,ITGA2B,KLKB1,SERPING1,FERMT3,A2ML1,DST,F2,CFHR1,SUMO4,HBA1,COL18A1,CFHR3,CFH,C4A,C4B,CFB,SAA2,PLCG2,KNG1
DISEASES	DOID:0050736	Autosomal dominant disease	43	1386	0.4	8.21e-06	CETP,SERPIND1,MYH9,F9,PROC,APOA1,TTR,APOE,F12,CFHR5,LYZ,VWF,NOTCH3,KLKB1,SERPING1,ANKRD44,CSF1R,F2,CFHR1,FBN1,GANAB,APOA4,F13B,CFHR2,CFHR3,CFH,F5,HTRA1,FLNA,GSN,EFEMP1,C1S,IGF2,TGFBI,CFB,SAA2,ATP1A1,TTN,PLCG2,KNG1,HBB,ACTB,FGA
DISEASES	DOID:2921	Glomerulonephritis	8	36	1.25	1.25e-05	MPO,CFHR5,APOL1,COL18A1,CFHR3,CFH,C4B,CFB
DISEASES	DOID:1287	Cardiovascular system disease	23	493	0.58	2.26e-05	CETP,MPO,APOA1,TTR,APOE,VWF,ITGA2B,DNAH5,DSC2,PF4,PCSK9,F2,FBN1,PAPPA,MFAP5,FLNA,F10,F7,CD99,ADIPOQ,C4A,SAA2,TTN
DISEASES	DOID:14735	Hereditary angioedema	5	6	1.83	2.39e-05	F12,KLKB1,SERPING1,C1S,KNG1
DISEASES	DOID:0014667	Disease of metabolism	35	1076	0.42	4.65e-05	CETP,GNPTG,F9,MGP,APOA1,TTR,CAT,APOE,LYZ,LCAT,CP,SAA4,PCSK9,F2,NDUFS8,SUMO4,HBA1,A2M,FN1,APOA4,ECM1,ANGPTL3,GSN,GPX3,IGF1,PROS1,PCYOX1,ADIPOQ,TGFBI,SELENOP,SAA2,APOA5,HBB,ACTB,FGA
DISEASES	DOID:0050737	Autosomal recessive disease	48	1785	0.34	5.61e-05	GNPTG,MYH9,MGP,APOE,F12,VWF,ITGA2B,CP,KLKB1,F13A1,DNAH5,TCIRG1,SERPING1,FERMT3,FYCO1,MASP1,ANTXR1,PCSK9,FGB,DST,F2,HBA1,FGG,BLM,KIAA0753,F13B,F5,FLNA,ANGPTL3,F10,F7,IGFBP3,IGF1,F11,C1S,PCYOX1,IGF2,PRG4,LMOD3,TGFBI,SAA2,APOA5,TTN,CFD,KNG1,HBB,FGA,TPM3
DISEASES	DOID:2218	Blood platelet disease	8	47	1.14	5.61e-05	MYH9,PF4V1,VWF,ITGA2B,PF4,F2,FLNA,F7
DISEASES	DOID:0060903	Thrombosis	6	21	1.36	0.00012	VWF,ITGA2B,PF4,F2,F10,F7
DISEASES	DOID:0080301	Atypical hemolytic-uremic syndrome	5	11	1.56	0.00016	CFHR5,CFHR1,CFHR3,CFH,CFB
DISEASES	DOID:0050117	Disease by infectious agent	18	368	0.6	0.00020	APOE,CP,ITIH4,PCDH1,APEH,ANTXR1,F2,PRG2,C1QB,APOL1,HSPA5,HTRA1,C1QC,CA6,YWHAZ,C4A,SAA2,HPR
DISEASES	DOID:0050828	Artery disease	11	130	0.83	0.00020	CETP,APOA1,APOE,ITGA2B,PCSK9,F2,FBN1,PAPPA,MFAP5,ADIPOQ,SAA2
DISEASES	DOID:1588	Thrombocytopenia	5	12	1.53	0.00020	PF4V1,VWF,ITGA2B,PF4,F2
DISEASES	DOID:12554	Hemolytic-uremic syndrome	5	14	1.46	0.00036	CFHR5,CFHR1,CFHR3,CFH,CFB
DISEASES	DOID:17	Musculoskeletal system disease	34	1154	0.38	0.00043	F9,MMP2,C5,MGP,SPARC,TTR,APOE,F12,TCIRG1,SEC24D,CSF1R,A2ML1,ANTXR1,DST,A2M,FBN1,P4HB,COL18A1,KIAA0753,HTRA1,FLNA,F7,CD99,C1S,IGF2,C4A,LMOD3,C4B,SAA2,C1R,ATP1A1,TTN,CFD,TPM3
DISEASES	DOID:37	Skin disease	21	518	0.51	0.00043	MPO,APOA1,APOE,F12,CSTA,KLKB1,SERPING1,FERMT3,DSC2,A2ML1,ANTXR1,DST,FBN1,BLM,APOA4,COL18A1,GSN,IGF1,C1S,KNG1,ACTB
DISEASES	DOID:3146	Lipid metabolism disorder	8	72	0.95	0.00074	CETP,APOA1,APOE,LCAT,PCSK9,ECM1,ANGPTL3,APOA5
DISEASES	DOID:65	Connective tissue disease	26	774	0.43	0.00074	F9,MMP2,C5,MGP,SPARC,TTR,TCIRG1,SEC24D,CSF1R,A2ML1,ANTXR1,DST,FBN1,P4HB,COL18A1,KIAA0753,HTRA1,FLNA,F7,CD99,C1S,IGF2,C4A,C4B,SAA2,C1R
DISEASES	DOID:10873	Kuhnt-Junius degeneration	4	7	1.66	0.00079	CETP,CFH,HTRA1,CFB
DISEASES	DOID:9799	Eye degenerative disease	13	236	0.65	0.0015	CETP,APOE,CFHR5,PCSK9,CFHR1,F13B,CFHR2,CFHR3,CFH,HTRA1,EFEMP1,TGFBI,CFB
DISEASES	DOID:11949	Creutzfeldt-Jakob disease	4	9	1.55	0.0016	APOE,C1QB,C1QC,YWHAZ
DISEASES	DOID:1387	Hypolipoproteinemia	4	11	1.47	0.0029	APOA1,LCAT,PCSK9,ANGPTL3
DISEASES	DOID:5113	Nutritional deficiency disease	6	43	1.05	0.0030	MGP,TTR,F2,GPX3,PROS1,SELENOP
DISEASES	DOID:2222	Factor X deficiency	3	3	1.91	0.0036	F2,F10,F7
DISEASES	DOID:2236	Congenital afibrinogenemia	3	3	1.91	0.0036	FGB,FGG,FGA
DISEASES	DOID:863	Nervous system disease	50	2275	0.25	0.0046	CETP,MYH9,MPO,PROC,APOA1,TTR,APOE,CFHR5,VWF,NOTCH3,LCAT,CENPE,SERPING1,ANKRD44,CSF1R,FYCO1,A2ML1,SH3D19,PCSK9,DST,F2,C1QB,CFHR1,SUZ12,A2M,FBN1,ANG,COL18A1,F13B,CFHR2,CFHR3,CFH,HTRA1,ADAMTSL4,FLNA,RBP4,GSN,C1QC,CD99,IGF1,EFEMP1,TNRC6A,YWHAZ,IGF2,TGFBI,CFB,TSKU,ATP1A1,ACTB,TRIM66
DISEASES	DOID:0060002	C1 inhibitor deficiency	3	4	1.78	0.0058	KLKB1,SERPING1,KNG1
DISEASES	DOID:0080001	Bone disease	19	540	0.45	0.0058	F9,MMP2,C5,SPARC,TTR,TCIRG1,SEC24D,CSF1R,FBN1,P4HB,COL18A1,KIAA0753,HTRA1,FLNA,F7,CD99,C1S,IGF2,SAA2
DISEASES	DOID:12134	Factor VIII deficiency	3	4	1.78	0.0058	F9,VWF,F7
DISEASES	DOID:417	Autoimmune disease	14	317	0.55	0.0058	C5,MPO,TTR,PXDN,ITGA2B,A2ML1,DST,F2,SUMO4,HBA1,COL18A1,C4A,C4B,SAA2
DISEASES	DOID:5614	Eye disease	23	749	0.39	0.0071	CETP,MYH9,APOE,CFHR5,SERPING1,FYCO1,PCSK9,CFHR1,FBN1,COL18A1,F13B,CFHR2,CFHR3,CFH,HTRA1,ADAMTSL4,RBP4,GSN,IGF1,EFEMP1,TGFBI,CFB,ACTB
DISEASES	DOID:11249	Vitamin K deficiency bleeding	3	5	1.69	0.0082	MGP,F2,PROS1
DISEASES	DOID:12259	Hemophilia B	3	5	1.69	0.0082	F9,F7,F11
DISEASES	DOID:2219	Glanzmanns thrombasthenia	3	5	1.69	0.0082	VWF,ITGA2B,F7
DISEASES	DOID:2229	Factor XI deficiency	3	5	1.69	0.0082	F9,F7,F11
DISEASES	DOID:2451	Protein S deficiency	3	5	1.69	0.0082	F2,PROS1,C4B
DISEASES	DOID:655	Inherited metabolic disorder	26	949	0.34	0.0129	CETP,GNPTG,F9,APOA1,TTR,CAT,APOE,LYZ,LCAT,CP,PCSK9,NDUFS8,SUMO4,HBA1,APOA4,ECM1,ANGPTL3,GSN,IGF1,PCYOX1,ADIPOQ,SAA2,APOA5,HBB,ACTB,FGA
DISEASES	DOID:2217	Bernard-Soulier syndrome	3	7	1.54	0.0153	MYH9,VWF,ITGA2B
DISEASES	DOID:170	Endocrine gland cancer	7	93	0.78	0.0167	APOE,APOA4,CD99,GAPDH,IGF2,IGKV2D-28,ACTB
DISEASES	DOID:1936	Atherosclerosis	4	21	1.19	0.0167	CETP,APOA1,APOE,ADIPOQ
DISEASES	DOID:0050155	Sensory system disease	25	929	0.34	0.0205	CETP,MYH9,PROC,APOE,CFHR5,SERPING1,FYCO1,A2ML1,PCSK9,CFHR1,FBN1,COL18A1,F13B,CFHR2,CFHR3,CFH,HTRA1,ADAMTSL4,RBP4,GSN,IGF1,EFEMP1,TGFBI,CFB,ACTB
DISEASES	DOID:409	Liver disease	7	97	0.77	0.0205	TTR,SAA4,F2,GANAB,GAPDH,SAA2,ACTB
DISEASES	DOID:0080010	Bone structure disease	4	24	1.13	0.0240	MMP2,TTR,FBN1,HTRA1
DISEASES	DOID:14250	Down syndrome	3	9	1.43	0.0244	APOE,C1QB,PAPPA
DISEASES	DOID:0060440	Epithelial and subepithelial dystrophy	3	11	1.34	0.0391	APOE,COL18A1,TGFBI
DISEASES	DOID:5679	Retinal disease	12	309	0.5	0.0406	CETP,APOE,CFHR5,CFHR1,F13B,CFHR2,CFHR3,CFH,HTRA1,IGF1,EFEMP1,CFB
TISSUES	BTO:0004850	Bone marrow cell	52	198	1.33	8.07e-46	CPB2,CETP,C5,MPO,VTN,IGFBP5,TTR,LRP1,THBS1,VWF,ITGA2B,A1BG,CP,KLKB1,F13A1,AMBP,HPX,ITIH4,FCN3,SAA4,SERPING1,DSC2,CSF1R,FCN2,MASP1,LRG1,FGB,F2,CSPG4,CFHR1,APOL1,A2M,APOD,FN1,ITIH2,CFH,F5,ECM1,IGFBP3,SOD3,PROS1,F11,C1S,C4A,PRG4,MST1,C4B,CFB,SELENOP,C1R,KNG1,FGA
TISSUES	BTO:0000759	Liver	119	2125	0.66	9.12e-46	SPP2,ST6GAL1,CPB2,CETP,SERPIND1,MYH9,TPD52L2,APMAP,F9,MPO,GPLD1,PROC,APOA1,TTR,CAT,LRP1,APOE,PXDN,F12,CFHR5,THBS1,VWF,PSMA6,SERPINA10,ATRN,A1BG,BLVRB,CP,KLKB1,AMBP,HPX,IGFBP4,FCN3,HABP2,SAA4,SERPING1,CSF1R,FCN2,PGLYRP2,PPBP,MASP1,APEH,LRG1,FGB,DST,F2,INHBC,STAB1,C1QB,CFHR1,TLN1,APOL1,PKM,HBA1,A2M,HSPA5,FBN1,P4HB,SERPINA7,HSP90AA1,ANG,TUBA1B,FGG,GANAB,FN1,ITIH2,COL18A1,C8A,F13B,CFHR2,CFHR3,CFH,S100A8,ECM1,FLNA,SMARCA1,ANGPTL3,C8B,RBP4,GSN,F10,F7,APOM,IGFBP3,CD99,GOLM1,IGF1,PROS1,ACTN1,YWHAZ,GAPDH,ROCK1,MASP2,F11,C1S,TF,PCYOX1,IGF2,BZW1,C4A,MST1,ITIH3,C4B,TGFBI,CFB,IGFALS,GC,SAA2,C1R,APOA5,ATP1A1,APOC4,KNG1,HBB,ACTB,ENO1,FGA,TPM3,PDIA4

TISSUES	BTO:0001486	Skeletal system	95	1307	0.77	1.70e-43	SPP2,CPB2,CETP,SERPIND1,APMAP,MMP2,C5,MPO,VTN,SPARC,IGFBP5,APOA1,TTR,LRP1,APOE,PXDN,THBS1,VWF,PSMA6,ITGA2B,ATRN,A1BG,CSTA,CP,KLKB1,F13A1,AMBP,HPX,ITIH4,FCN3,SAA4,SERPING1,DSC2,CSF1R,FCN2,FYCO1,MASP1,LRG1,FGB,F2,UBE3C,PRG2,CSPG4,CFHR1,SUZ12,APOL1,PKM,HBA1,A2M,HSPA5,SERPINA7,HSP90AA1,TUBA1B,FGG,LIMS1,GANAB,APOD,FN1,ITIH2,MFAP5,CFH,F5,S100A8,HTRA1,ADAMTSL4,ECM1,ANGPTL3,CCN5,IGFBP3,SOD3,PROS1,YWHAZ,GAPDH,F11,C1S,TF,PCYOX1,IGF2,BZW1,C4A,PRG4,MST1,LMOD3,C4B,CFB,SELENOP,C1R,TTN,HSPH1,KNG1,HBB,ACTB,ENO1,FGA,TPM3
TISSUES	BTO:0000141	Bone marrow	65	528	1.0	1.33e-40	CPB2,CETP,SERPIND1,APMAP,C5,MPO,VTN,IGFBP5,TTR,LRP1,PXDN,THBS1,VWF,ITGA2B,A1BG,CSTA,CP,KLKB1,F13A1,AMBP,HPX,ITIH4,FCN3,SAA4,SERPING1,DSC2,CSF1R,FCN2,MASP1,LRG1,FGB,F2,PRG2,CSPG4,CFHR1,SUZ12,APOL1,HBA1,A2M,LIMS1,GANAB,APOD,FN1,ITIH2,CFH,F5,ECM1,IGFBP3,SOD3,PROS1,YWHAZ,F11,C1S,BZW1,C4A,PRG4,MST1,C4B,CFB,SELENOP,C1R,HSPH1,KNG1,HBB,FGA
TISSUES	BTO:0000345	Digestive gland	128	2881	0.55	5.03e-40	SPP2,ST6GAL1,CPB2,CETP,SERPIND1,MYH9,TPD52L2,APMAP,F9,MPO,GPLD1,SPARC,PROC,APOA1,TTR,CAT,LRP1,APOE,PXDN,F12,CFHR5,THBS1,VWF,PSMA6,SERPINA10,ATRN,A1BG,BLVRB,CP,KLKB1,F13A1,AMBP,TCIRG1,HPX,IGFBP4,FCN3,HABP2,SAA4,SERPING1,SEC24D,CSF1R,FCN2,PGLYRP2,PPBP,MASP1,APEH,LRG1,FGB,DST,F2,INHBC,STAB1,C1QB,CFHR1,NDUFS8,TLN1,APOL1,PKM,STXBP5,HBA1,A2M,HSPA5,FBN1,P4HB,SERPINA7,HSP90AA1,ANG,TUBA1B,FGG,GANAB,APOD,FN1,ITIH2,COL18A1,C8A,F13B,CFHR2,CFHR3,CFH,S100A8,ECM1,FLNA,SMARCA1,ANGPTL3,C8B,RBP4,GSN,F10,F7,APOM,IGFBP3,CD99,GOLM1,GPX3,IGF1,PROS1,ACTN1,YWHAZ,GAPDH,ROCK1,MASP2,F11,C1S,TF,PCYOX1,IGF2,BZW1,C4A,MST1,ITIH3,C4B,TGFBI,CFB,IGFALS,GC,SAA2,C1R,APOA5,ATP1A1,RNASE4,APOC4,KNG1,HBB,ACTB,ENO1,FGA,TPM3,PDIA4
TISSUES	BTO:0000392	Plasma cell	45	171	1.33	6.12e-40	C5,MPO,VTN,IGFBP5,TTR,LRP1,THBS1,VWF,ITGA2B,CP,KLKB1,F13A1,AMBP,HPX,ITIH4,FCN3,SAA4,SERPING1,DSC2,CSF1R,FCN2,MASP1,LRG1,FGB,F2,CSPG4,CFHR1,APOL1,A2M,APOD,FN1,ITIH2,CFH,ECM1,IGFBP3,SOD3,PROS1,C1S,C4A,C4B,CFB,SELENOP,C1R,KNG1,FGA
TISSUES	BTO:0001491	Viscus	150	5378	0.35	6.38e-26	SPP2,ST6GAL1,CPB2,CETP,SERPIND1,MYH9,TPD52L2,APMAP,F9,MPO,GPLD1,SPARC,PROC,PLEK,APOA1,TTR,CAT,LRP1,APOE,PXDN,F12,CFHR5,THBS1,LYZ,VWF,PSMA6,SERPINA10,ITGA2B,ATRN,A1BG,BLVRB,CSTA,CP,KLKB1,F13A1,AMBP,TCIRG1,HPX,IGFBP4,FCN3,PLA2G7,HABP2,SAA4,SERPING1,SEC24D,DSC2,CSF1R,FCN2,PGLYRP2,PPBP,FYCO1,MASP1,APEH,GUCY1A1,LALBA,ANTXR1,LRG1,SH3D19,FGB,DST,F2,INHBC,STAB1,C1QB,CFHR1,TLN1,APOL1,PKM,HBA1,A2M,HSPA5,FBN1,P4HB,SERPINA7,FBLN1,HSP90AA1,ANG,TUBA1B,FGG,GANAB,APOD,FN1,APOA4,ITIH2,COL18A1,C8A,F13B,CFHR2,CFHR3,CFH,S100A8,ECM1,FLNA,SMARCA1,ANGPTL3,C8B,RBP4,GSN,F10,F7,APOM,CALML5,IGFBP3,CD99,SOD3,GOLM1,GPX3,IGF1,PROS1,ACTN1,EFEMP1,TNRC6A,YWHAZ,ILK,GAPDH,ROCK1,MASP2,F11,C1S,TF,PCYOX1,IGF2,BZW1,C4A,ACSF2,CKMT1A,MST1,ITIH3,C4B,TGFBI,CFB,IGFALS,GC,SAA2,C1R,APOA5,ATP1A1,RNASE4,APOC4,CFD,VEGFC,PLCG2,HSPH1,KNG1,HBB,ACTB,ENO1,FGA,TPM3,PDIA4
TISSUES	BTO:0001488	Endocrine gland	153	6403	0.29	2.54e-19	SPP2,ST6GAL1,CPB2,CETP,SERPIND1,MYH9,TPD52L2,APMAP,F9,MMP2,MPO,VTN,GPLD1,SPARC,PROC,APOA1,TTR,CAT,LRP1,APOE,PXDN,F12,CFHR5,THBS1,VWF,PSMA6,SERPINA10,ATRN,A1BG,BLVRB,CSTA,CP,KLKB1,F13A1,AMBP,TCIRG1,HPX,IGFBP4,FCN3,PLA2G7,CRH,HABP2,SAA4,SERPING1,FERMT3,SEC24D,CSF1R,FCN2,PGLYRP2,PPBP,FYCO1,MASP1,APEH,ANTXR1,LRG1,SH3D19,FGB,DST,F2,INHBC,STAB1,C1QB,CFHR1,NDUFS8,TLN1,APOL1,PKM,STXBP5,HBA1,A2M,HSPA5,FBN1,P4HB,SERPINA7,FBLN1,HSP90AA1,ANG,TUBA1B,FGG,LIMS1,GANAB,APOD,PAPLN,FN1,ITIH2,COL18A1,C8A,KIAA0753,F13B,CFHR2,CFHR3,CFH,S100A8,ECM1,FLNA,SMARCA1,ANGPTL3,C8B,RBP4,CCN5,GSN,F10,F7,APOM,CALML5,IGFBP3,CD99,SOD3,GOLM1,GPX3,IGF1,PROS1,ACTN1,EFEMP1,EFCAB5,YWHAZ,GAPDH,RAPGEF4,ROCK1,MASP2,F11,C1S,TF,LTBP1,PCYOX1,IGF2,BZW1,C4A,ATP9B,ACSF2,MST1,ITIH3,C4B,TGFBI,CFB,IGFALS,GC,UIMC1,SAA2,C1R,APOA5,ATP1A1,RNASE4,APOC4,CFD,HSPH1,KNG1,HBB,ACTB,ENO1,FGA,TPM3,PDIA4
TISSUES	BTO:0000522	Gland	158	7004	0.26	9.04e-18	SPP2,ST6GAL1,CPB2,CETP,SERPIND1,MYH9,TPD52L2,APMAP,F9,MMP2,C5,MPO,VTN,GPLD1,SPARC,PROC,APOA1,TTR,CAT,LRP1,APOE,PXDN,F12,CFHR5,THBS1,LYZ,VWF,PSMA6,SERPINA10,ATRN,A1BG,BLVRB,CSTA,CP,KLKB1,F13A1,AMBP,TCIRG1,HPX,IGFBP4,FCN3,PLA2G7,CRH,HABP2,SAA4,SERPING1,FERMT3,SEC24D,CSF1R,FCN2,PGLYRP2,PPBP,FYCO1,MASP1,APEH,GUCY1A1,ANTXR1,LRG1,SH3D19,FGB,DST,F2,INHBC,STAB1,C1QB,CFHR1,NDUFS8,TLN1,APOL1,PKM,STXBP5,HBA1,A2M,HSPA5,FBN1,P4HB,SERPINA7,FBLN1,HSP90AA1,ANG,TUBA1B,FGG,LIMS1,GANAB,APOD,PAPLN,FN1,ITIH2,COL18A1,C8A,KIAA0753,F13B,CFHR2,CFHR3,CFH,S100A8,ECM1,FLNA,SMARCA1,ANGPTL3,C8B,RBP4,CCN5,GSN,F10,F7,APOM,CA6,CALML5,IGFBP3,CD99,SOD3,GOLM1,GPX3,IGF1,PROS1,ACTN1,EFEMP1,EFCAB5,TNRC6A,YWHAZ,GAPDH,RAPGEF4,ROCK1,MASP2,F11,C1S,TF,LTBP1,PCYOX1,IGF2,BZW1,C4A,ATP9B,ACSF2,MST1,ITIH3,C4B,TGFBI,CFB,IGFALS,GC,UIMC1,SAA2,C1R,APOA5,ATP1A1,RNASE4,APOC4,CFD,HSPH1,KNG1,HBB,ACTB,ENO1,FGA,TPM3,PDIA4
TISSUES	BTO:0001078	Placenta	57	1244	0.57	3.33e-15	MYH9,TFPI2,PCOLCE,HSD17B1,SPARC,IGFBP5,LRP1,THBS1,VWF,TIMP2,F13A1,IGFBP4,PSG9,CRH,SERPING1,DSC2,CSF1R,PSG6,ANTXR1,DST,PSG1,PRG2,APOL1,PSG11,PKM,HBA1,A2M,FBN1,P4HB,PAPPA,FBLN1,PSG3,HSP90AA1,TUBA1B,GANAB,FN1,COL18A1,PAPPA2,F5,ADAM12,HTRA1,ADAMTSL4,IGFBP3,GPX3,ACTN1,YWHAZ,ILK,GAPDH,PSG2,PCYOX1,IGF2,TGFBI,ATP1A1,CFD,HBB,ACTB,ENO1
TISSUES	BTO:0000237	Cerebrospinal fluid	16	45	1.46	4.22e-15	TTR,APOE,A1BG,CP,HPX,SERPING1,LRG1,F2,SERPINA7,APOD,CFH,IGFBP3,TF,IGF2,C4A,KNG1

TISSUES	BTO:0001489	Whole body	218	13099	0.13	2.68e-14	SPP2,ST6GAL1,CPB2,CETP,GNPTG,SERPIND1,MYH9,TPD52L2,APMAP,F9,MMP2,TFPI2,PCOLCE,C5,MPO,HSD17B1,VTN,MGP,GPLD1,SPARC,IGFBP5,PROC,PLEK,APOA1,TTR,CAT,LRP1,APOE,PXDN,F12,CFHR5,THBS1,LYZ,VWF,PSMA6,SERPINA10,ITGA2B,TIMP2,ATRN,A1BG,BLVRB,LCAT,CSTA,CP,KLKB1,F13A1,DNAH5,AMBP,CENPE,TCIRG1,HPX,ITIH4,CDH11,IGFBP4,PSG9,FCN3,PLA2G7,CRH,HABP2,SAA4,SERPING1,FERMT3,SEC24D,DSC2,CSF1R,FCN2,PSG6,PGLYRP2,SCGB3A1,PPBP,PF4,FYCO1,MASP1,APEH,GUCY1A1,A2ML1,LALBA,ANTXR1,LRG1,SH3D19,PCSK9,FGB,DST,F2,INHBC,PSG1,UBE3C,PRG2,CSPG4,STAB1,C1QB,CFHR1,NDUFS8,TLN1,SUZ12,APOL1,PSG11,PKM,STXBP5,C7,HBA1,A2M,HSPA5,FBN1,P4HB,VMO1,SERPINA7,PAPPA,FBLN1,PSG3,DNAH8,HSP90AA1,ANG,TUBA1B,FGG,LIMS1,THBS4,GANAB,APOD,PAPLN,FN1,APOA4,ITIH2,MFAP5,COL18A1,C8A,KIAA0753,CENPF,F13B,CFHR2,CFHR3,CFH,PAPPA2,F5,ADAM12,S100A7,S100A8,HTRA1,ADAMTSL4,ECM1,FLNA,SMARCA1,ANGPTL3,C8B,RBP4,CCN5,GSN,C1QC,F10,F7,APOM,CA6,ADGRG2,CALML5,IGFBP3,CD99,SOD3,GOLM1,GPX3,IGF1,PROS1,ACTN1,EFEMP1,EFCAB5,TNRC6A,YWHAZ,ILK,GAPDH,RAPGEF4,ROCK1,MAP3K7CL,MASP2,F11,C1S,PSG2,TF,LTBP1,PCYOX1,ADIPOQ,IGF2,BZW1,C4A,ATP9B,PRG4,NBEAL1,SLAIN1,ACSF2,CKMT1A,MST1,LMOD3,ITIH3,C4B,TGFBI,CFB,IGFALS,PAEP,GC,UIMC1,SELENOP,TSKU,SAA2,C1R,APOA5,ATP1A1,RNASE4,TTN,APOC4,CFD,VEGFC,PLCG2,HSPH1,KNG1,HBB,ACTB,ENO1,FGA,TPM3,PDIA4
TISSUES	BTO:0000132	Blood platelet	30	363	0.82	1.97e-13	MYH9,VTN,APOA1,TTR,CAT,THBS1,VWF,ITGA2B,FERMT3,PPBP,FGB,TLN1,PKM,STXBP5,HBA1,P4HB,HSP90AA1,FGG,LIMS1,F5,FLNA,GSN,ACTN1,YWHAZ,GAPDH,LTBP1,PRG4,ACTB,FGA,TPM3
TISSUES	BTO:0000284	Organism form	75	2542	0.38	5.99e-11	ST6GAL1,MYH9,TPD52L2,TFPI2,PCOLCE,HSD17B1,SPARC,IGFBP5,LRP1,THBS1,VWF,PSMA6,TIMP2,F13A1,ITIH4,IGFBP4,PSG9,CRH,SERPING1,DSC2,CSF1R,PSG6,FYCO1,ANTXR1,DST,PSG1,UBE3C,PRG2,TLN1,APOL1,PSG11,PKM,HBA1,A2M,HSPA5,FBN1,P4HB,PAPPA,FBLN1,PSG3,HSP90AA1,TUBA1B,GANAB,FN1,COL18A1,CENPF,PAPPA2,F5,ADAM12,HTRA1,ADAMTSL4,FLNA,APOM,IGFBP3,CD99,GPX3,IGF1,ACTN1,EFEMP1,YWHAZ,ILK,GAPDH,ROCK1,PSG2,PCYOX1,IGF2,TGFBI,UIMC1,ATP1A1,CFD,VEGFC,HBB,ACTB,ENO1,TPM3
TISSUES	BTO:0000570	Hematopoietic system	77	2755	0.35	3.64e-10	ST6GAL1,CETP,MYH9,TPD52L2,MPO,VTN,GPLD1,PROC,PLEK,APOA1,TTR,CAT,LRP1,APOE,F12,THBS1,VWF,PSMA6,ITGA2B,ATRN,BLVRB,PSG9,PLA2G7,SERPING1,FERMT3,PGLYRP2,PPBP,PF4,FYCO1,FGB,F2,PRG2,TLN1,PKM,STXBP5,HBA1,A2M,P4HB,PAPPA,HSP90AA1,TUBA1B,FGG,LIMS1,GANAB,FN1,ITIH2,C8A,F5,S100A8,FLNA,GSN,C1QC,APOM,IGFBP3,CD99,SOD3,GPX3,ACTN1,YWHAZ,GAPDH,C1S,LTBP1,IGF2,PRG4,C4B,TGFBI,CFB,CFD,VEGFC,PLCG2,HSPH1,KNG1,HBB,ACTB,ENO1,FGA,TPM3
TISSUES	BTO:0001279	Spinal cord	21	233	0.86	7.54e-10	MYH9,TTR,APOE,A1BG,CP,HPX,SERPING1,LRG1,F2,CSPG4,PKM,SERPINA7,APOD,CFH,FLNA,IGFBP3,TF,IGF2,C4A,KNG1,ACTB
TISSUES	BTO:0003099	Internal female genital organ	77	2804	0.35	7.84e-10	MYH9,TPD52L2,TFPI2,PCOLCE,HSD17B1,MGP,SPARC,IGFBP5,LRP1,THBS1,VWF,TIMP2,A1BG,F13A1,IGFBP4,PSG9,CRH,SERPING1,DSC2,CSF1R,PSG6,FYCO1,MASP1,A2ML1,ANTXR1,DST,PSG1,PRG2,NDUFS8,SUZ12,APOL1,PSG11,PKM,HBA1,A2M,FBN1,P4HB,PAPPA,FBLN1,PSG3,HSP90AA1,TUBA1B,GANAB,PAPLN,FN1,COL18A1,KIAA0753,PAPPA2,F5,ADAM12,HTRA1,ADAMTSL4,FLNA,CCN5,GSN,F10,IGFBP3,CD99,GPX3,PROS1,ACTN1,YWHAZ,ILK,GAPDH,C1S,PSG2,PCYOX1,IGF2,TGFBI,PAEP,UIMC1,ATP1A1,CFD,HBB,ACTB,ENO1,TPM3
TISSUES	BTO:0000089	Blood	59	1824	0.42	9.87e-10	MYH9,TPD52L2,MPO,VTN,PROC,PLEK,APOA1,TTR,CAT,LRP1,APOE,F12,THBS1,VWF,ITGA2B,BLVRB,PSG9,PLA2G7,SERPING1,FERMT3,PPBP,PF4,FGB,F2,TLN1,PKM,STXBP5,HBA1,P4HB,HSP90AA1,TUBA1B,FGG,LIMS1,FN1,C8A,F5,S100A8,FLNA,GSN,C1QC,APOM,CD99,SOD3,GPX3,ACTN1,YWHAZ,GAPDH,C1S,LTBP1,PRG4,C4B,CFB,PLCG2,HSPH1,HBB,ACTB,ENO1,FGA,TPM3
TISSUES	BTO:0000174	Embryonic structure	69	2369	0.37	1.06e-09	ST6GAL1,MYH9,TPD52L2,TFPI2,PCOLCE,HSD17B1,SPARC,IGFBP5,LRP1,THBS1,VWF,PSMA6,TIMP2,F13A1,IGFBP4,PSG9,CRH,SERPING1,DSC2,CSF1R,PSG6,FYCO1,ANTXR1,DST,PSG1,UBE3C,PRG2,TLN1,APOL1,PSG11,PKM,HBA1,A2M,FBN1,P4HB,PAPPA,FBLN1,PSG3,HSP90AA1,TUBA1B,GANAB,FN1,COL18A1,CENPF,PAPPA2,F5,ADAM12,HTRA1,ADAMTSL4,FLNA,APOM,IGFBP3,GPX3,ACTN1,YWHAZ,ILK,GAPDH,ROCK1,PSG2,PCYOX1,IGF2,TGFBI,UIMC1,ATP1A1,CFD,VEGFC,HBB,ACTB,ENO1
TISSUES	BTO:0000203	Respiratory system	56	1707	0.42	2.09e-09	ST6GAL1,MYH9,MMP2,VTN,SPARC,IGFBP5,APOE,PXDN,THBS1,LYZ,VWF,ITGA2B,ATRN,CP,DNAH5,TCIRG1,IGFBP4,FCN3,SERPING1,FERMT3,SH3D19,PKM,HBA1,A2M,HSPA5,P4HB,HSP90AA1,TUBA1B,LIMS1,GANAB,FN1,CFH,FLNA,SMARCA1,CCN5,IGFBP3,GPX3,PROS1,ACTN1,EFEMP1,GAPDH,RAPGEF4,MASP2,C1S,LTBP1,PCYOX1,C4B,TGFBI,ATP1A1,VEGFC,KNG1,HBB,ACTB,ENO1,TPM3,PDIA4
TISSUES	BTO:0000042	Animal	224	15148	0.08	8.90e-08	SPP2,ST6GAL1,CPB2,CETP,GNPTG,SERPIND1,MYH9,TPD52L2,APMAP,F9,MMP2,TFPI2,PCOLCE,C5,MPO,HSD17B1,VTN,MGP,GPLD1,SPARC,IGFBP5,PROC,PLEK,APOA1,TTR,CAT,LRP1,APOE,PXDN,ANGPTL6,F12,CFHR5,THBS1,LYZ,VWF,PSMA6,SERPINA10,ITGA2B,TIMP2,ATRN,A1BG,BLVRB,NOTCH3,LCAT,CSTA,CP,KLKB1,F13A1,DNAH5,AMBP,CENPE,TCIRG1,HPX,ITIH4,CDH11,IGFBP4,PSG9,FCN3,PLA2G7,CRH,HABP2,SAA4,SERPING1,FERMT3,SEC24D,DSC2,CSF1R,PCDH1,FCN2,PSG6,PGLYRP2,SCGB3A1,PPBP,PF4,FYCO1,MASP1,APEH,GUCY1A1,A2ML1,LALBA,ANTXR1,LRG1,SH3D19,PCSK9,FGB,DST,F2,INHBC,PSG1,UBE3C,PRG2,CSPG4,STAB1,C1QB,CFHR1,NDUFS8,TLN1,SUZ12,APOL1,PSG11,PKM,STXBP5,C7,HBA1,A2M,HSPA5,FBN1,P4HB,VMO1,SERPINA7,PAPPA,FBLN1,PSG3,DNAH8,HSP90AA1,ANG,TUBA1B,FGG,LIMS1,THBS4,GANAB,APOD,PAPLN,FN1,APOA4,ITIH2,MFAP5,COL18A1,C8A,KIAA0753,CENPF,F13B,CFHR2,CFHR3,CFH,PAPPA2,F5,MKI67,ADAM12,S100A7,S100A8,HTRA1,ADAMTSL4,ECM1,FLNA,SMARCA1,ANGPTL3,C8B,RBP4,CCN5,GSN,C1QC,F10,F7,APOM,CA6,ADGRG2,CALML5,IGFBP3,CD99,SOD3,GOLM1,GPX3,IGF1,PROS1,ACTN1,EFEMP1,EFCAB5,TNRC6A,YWHAZ,ILK,GAPDH,RAPGEF4,ROCK1,MAP3K7CL,MASP2,F11,C1S,PSG2,TF,LTBP1,PCYOX1,ADIPOQ,IGF2,BZW1,C4A,ATP9B,PRG4,NBEAL1,SLAIN1,ACSF2,CKMT1A,MST1,LMOD3,ITIH3,C4B,TGFBI,CFB,IGFALS,PAEP,TCAF1,GC,UIMC1,SELENOP,TSKU,SAA2,C1R,APOA5,ATP1A1,RNASE4,TTN,APOC4,CFD,VEGFC,PLCG2,HSPH1,KNG1,HBB,ACTB,TRIM66,ENO1,FGA,TPM3,PDIA4
TISSUES	BTO:0000421	Connective tissue	37	954	0.5	1.25e-07	SPP2,APMAP,MMP2,TFPI2,SPARC,APOA1,TTR,CAT,THBS1,BLVRB,PKM,HSPA5,FBN1,P4HB,PAPPA,HSP90AA1,TUBA1B,THBS4,GANAB,PAPLN,FN1,F5,HTRA1,ECM1,EFEMP1,YWHAZ,GAPDH,TF,LTBP1,ADIPOQ,BZW1,PRG4,ACSF2,CFD,ACTB,ENO1,TPM3
TISSUES	BTO:0000088	Cardiovascular system	39	1057	0.47	1.53e-07	TFPI2,PCOLCE,MGP,SPARC,APOA1,APOE,THBS1,VWF,TIMP2,SERPING1,FYCO1,F2,PKM,HBA1,A2M,FBN1,P4HB,HSP90AA1,TUBA1B,THBS4,FN1,F13B,FLNA,GSN,GPX3,EFEMP1,GAPDH,TF,PCYOX1,LMOD3,TGFBI,SELENOP,TTN,VEGFC,HBB,ACTB,ENO1,FGA,TPM3
TISSUES	BTO:0001239	Serum	8	25	1.41	3.99e-07	GPLD1,APOA1,ATRN,PGLYRP2,PRG2,PAPPA,ITIH2,KNG1
TISSUES	BTO:0001702	Left atrium	10	60	1.13	1.05e-06	APOA1,PKM,HBA1,FBN1,FLNA,GAPDH,TF,TTN,HBB,TPM3
TISSUES	BTO:0000255	Brain cell line	15	188	0.81	2.51e-06	APOA1,TTR,FGB,HSPA5,P4HB,HSP90AA1,TUBA1B,YWHAZ,GAPDH,TF,TTN,HSPH1,ACTB,TRIM66,ENO1

TISSUES	BTO:0000511	Gastrointestinal tract	51	1816	0.36	2.55e-06	MYH9,SPARC,PROC,APOA1,TTR,CAT,LYZ,BLVRB,KLKB1,IGFBP4,HABP2,SERPING1,LALBA,LRG1,SH3D19,DST,TLN1,PKM,A2M,HSPA5,P4HB,FBLN1,HSP90AA1,ANG,TUBA1B,GANAB,FN1,APOA4,S100A8,FLNA,GSN,IGFBP3,CD99,SOD3,GPX3,ACTN1,TNRC6A,YWHAZ,ILK,GAPDH,C1S,TF,IGF2,CKMT1A,TGFBI,CFB,C1R,ATP1A1,CFD,ACTB,ENO1
TISSUES	BTO:0003914	Interstitial cell of Cajal	15	193	0.8	3.27e-06	APOA1,TTR,CAT,BLVRB,PKM,HSPA5,P4HB,HSP90AA1,TUBA1B,GANAB,YWHAZ,GAPDH,TF,ACTB,ENO1
TISSUES	BTO:0000988	Pancreas	26	626	0.53	8.91e-06	CETP,GPLD1,SPARC,TTR,BLVRB,F13A1,TCIRG1,SEC24D,APOL1,PKM,HSPA5,P4HB,HSP90AA1,TUBA1B,FN1,FLNA,GSN,IGFBP3,CD99,GPX3,GAPDH,IGF2,TGFBI,ATP1A1,ACTB,ENO1
TISSUES	BTO:0000058	Alimentary canal	53	2021	0.33	9.89e-06	MYH9,SPARC,PROC,APOA1,TTR,CAT,THBS1,LYZ,BLVRB,KLKB1,IGFBP4,HABP2,SERPING1,LALBA,LRG1,SH3D19,DST,TLN1,PKM,A2M,HSPA5,P4HB,FBLN1,HSP90AA1,ANG,TUBA1B,GANAB,FN1,APOA4,S100A8,FLNA,GSN,CALML5,IGFBP3,CD99,SOD3,GPX3,ACTN1,TNRC6A,YWHAZ,ILK,GAPDH,C1S,TF,IGF2,CKMT1A,TGFBI,CFB,C1R,ATP1A1,CFD,ACTB,ENO1
TISSUES	BTO:0001253	Skin	37	1151	0.41	9.89e-06	ST6GAL1,SPARC,LRP1,APOE,PSMA6,TIMP2,CSTA,DSC2,DST,PKM,A2M,P4HB,HSP90AA1,TUBA1B,APOD,FN1,S100A7,S100A8,ECM1,FLNA,GSN,CALML5,IGFBP3,CD99,ACTN1,EFEMP1,YWHAZ,GAPDH,ADIPOQ,TGFBI,C1R,ATP1A1,CFD,VEGFC,ACTB,ENO1,TPM3
TISSUES	BTO:0001703	Right atrium	9	61	1.08	1.12e-05	APOA1,PKM,HBA1,FBN1,GAPDH,TF,TTN,HBB,TPM3
TISSUES	BTO:0000562	Heart	28	738	0.49	1.53e-05	PCOLCE,APOA1,THBS1,VWF,SERPING1,FYCO1,F2,PKM,HBA1,FBN1,HSP90AA1,TUBA1B,THBS4,FN1,F13B,FLNA,GPX3,GAPDH,TF,PCYOX1,LMOD3,SELENOP,TTN,VEGFC,HBB,ACTB,FGA,TPM3
TISSUES	BTO:0000763	Lung	41	1395	0.38	1.81e-05	MYH9,MMP2,VTN,SPARC,IGFBP5,APOE,PXDN,THBS1,VWF,ITGA2B,DNAH5,TCIRG1,IGFBP4,FCN3,SERPING1,FERMT3,PKM,HBA1,A2M,HSPA5,P4HB,HSP90AA1,TUBA1B,FN1,FLNA,SMARCA1,CCN5,GPX3,ACTN1,EFEMP1,GAPDH,MASP2,C1S,LTBP1,C4B,TGFBI,HBB,ACTB,ENO1,TPM3,PDIA4
TISSUES	BTO:0000648	Intestine	39	1349	0.37	5.35e-05	MYH9,PROC,LYZ,KLKB1,IGFBP4,HABP2,LRG1,SH3D19,DST,TLN1,PKM,A2M,HSPA5,P4HB,HSP90AA1,ANG,TUBA1B,GANAB,FN1,APOA4,FLNA,GSN,IGFBP3,SOD3,GPX3,ACTN1,YWHAZ,ILK,GAPDH,C1S,IGF2,CKMT1A,TGFBI,CFB,C1R,ATP1A1,CFD,ACTB,ENO1
TISSUES	BTO:0001202	Saliva	8	57	1.05	6.99e-05	MPO,THBS1,HPX,DSC2,LRG1,GOLM1,TF,C4B
TISSUES	BTO:0001090	Mouth	22	532	0.52	7.61e-05	GNPTG,C5,LYZ,CSTA,CENPE,A2ML1,DST,C7,P4HB,HSP90AA1,LIMS1,GANAB,FN1,MFAP5,KIAA0753,S100A8,GSN,CA6,ACTN1,YWHAZ,ACTB,ENO1
TISSUES	BTO:0000091	Ascites	8	61	1.02	0.00011	PKM,HSP90AA1,TUBA1B,S100A8,GAPDH,KNG1,ACTB,ENO1
TISSUES	BTO:0000140	Bone	12	165	0.77	0.00011	SPP2,MMP2,SPARC,THBS1,PKM,HSP90AA1,TUBA1B,FN1,GAPDH,ACTB,ENO1,TPM3
TISSUES	BTO:0000020	Abdomen	10	113	0.85	0.00014	F2,PKM,HSP90AA1,TUBA1B,S100A8,GAPDH,ADIPOQ,KNG1,ACTB,ENO1
TISSUES	BTO:0000083	Female reproductive system	110	6111	0.16	0.00016	ST6GAL1,GNPTG,MYH9,TPD52L2,APMAP,MMP2,TFPI2,PCOLCE,HSD17B1,VTN,MGP,SPARC,IGFBP5,APOA1,CAT,LRP1,APOE,PXDN,THBS1,VWF,PSMA6,TIMP2,ATRN,A1BG,F13A1,HPX,CDH11,IGFBP4,PSG9,CRH,SERPING1,DSC2,CSF1R,PSG6,PGLYRP2,FYCO1,MASP1,APEH,A2ML1,LALBA,ANTXR1,SH3D19,FGB,DST,F2,PSG1,UBE3C,PRG2,NDUFS8,TLN1,SUZ12,APOL1,PSG11,PKM,C7,HBA1,A2M,HSPA5,FBN1,P4HB,PAPPA,FBLN1,PSG3,DNAH8,HSP90AA1,TUBA1B,LIMS1,GANAB,PAPLN,FN1,COL18A1,KIAA0753,CFH,PAPPA2,F5,ADAM12,HTRA1,ADAMTSL4,ECM1,FLNA,CCN5,GSN,F10,IGFBP3,CD99,GPX3,PROS1,ACTN1,EFEMP1,EFCAB5,YWHAZ,ILK,GAPDH,C1S,PSG2,PCYOX1,IGF2,BZW1,TGFBI,PAEP,UIMC1,TSKU,ATP1A1,CFD,HSPH1,HBB,ACTB,ENO1,TPM3,PDIA4
TISSUES	BTO:0001419	Urine	6	28	1.24	0.00016	LYZ,AMBP,F2,FN1,MASP2,PRG4
TISSUES	BTO:0003091	Urogenital system	122	7090	0.14	0.00029	ST6GAL1,GNPTG,MYH9,TPD52L2,APMAP,MMP2,TFPI2,PCOLCE,HSD17B1,VTN,MGP,SPARC,IGFBP5,APOA1,CAT,LRP1,APOE,PXDN,THBS1,VWF,PSMA6,TIMP2,ATRN,A1BG,F13A1,HPX,CDH11,IGFBP4,PSG9,CRH,SERPING1,DSC2,CSF1R,PSG6,PGLYRP2,FYCO1,MASP1,APEH,GUCY1A1,A2ML1,LALBA,ANTXR1,LRG1,SH3D19,FGB,DST,F2,PSG1,UBE3C,PRG2,NDUFS8,TLN1,SUZ12,APOL1,PSG11,PKM,C7,HBA1,A2M,HSPA5,FBN1,P4HB,VMO1,PAPPA,FBLN1,PSG3,DNAH8,HSP90AA1,TUBA1B,LIMS1,GANAB,PAPLN,FN1,COL18A1,KIAA0753,CFH,PAPPA2,F5,ADAM12,HTRA1,ADAMTSL4,ECM1,FLNA,SMARCA1,CCN5,GSN,F10,ADGRG2,IGFBP3,CD99,GPX3,PROS1,ACTN1,EFEMP1,EFCAB5,TNRC6A,YWHAZ,ILK,GAPDH,C1S,PSG2,TF,PCYOX1,IGF2,BZW1,ATP9B,ACSF2,TGFBI,PAEP,GC,UIMC1,TSKU,ATP1A1,CFD,VEGFC,HSPH1,KNG1,HBB,ACTB,ENO1,TPM3,PDIA4
TISSUES	BTO:0000081	Reproductive system	113	6444	0.15	0.00037	ST6GAL1,GNPTG,MYH9,TPD52L2,APMAP,MMP2,TFPI2,PCOLCE,HSD17B1,VTN,MGP,SPARC,IGFBP5,APOA1,CAT,LRP1,APOE,PXDN,THBS1,VWF,PSMA6,TIMP2,ATRN,A1BG,F13A1,HPX,CDH11,IGFBP4,PSG9,CRH,SERPING1,DSC2,CSF1R,PSG6,PGLYRP2,FYCO1,MASP1,APEH,A2ML1,LALBA,ANTXR1,SH3D19,FGB,DST,F2,PSG1,UBE3C,PRG2,NDUFS8,TLN1,SUZ12,APOL1,PSG11,PKM,C7,HBA1,A2M,HSPA5,FBN1,P4HB,VMO1,PAPPA,FBLN1,PSG3,DNAH8,HSP90AA1,TUBA1B,LIMS1,GANAB,PAPLN,FN1,COL18A1,KIAA0753,CFH,PAPPA2,F5,ADAM12,HTRA1,ADAMTSL4,ECM1,FLNA,CCN5,GSN,F10,ADGRG2,IGFBP3,CD99,GPX3,PROS1,ACTN1,EFEMP1,EFCAB5,YWHAZ,ILK,GAPDH,C1S,PSG2,PCYOX1,IGF2,BZW1,ATP9B,TGFBI,PAEP,UIMC1,TSKU,ATP1A1,CFD,HSPH1,HBB,ACTB,ENO1,TPM3,PDIA4
TISSUES	BTO:0001485	Muscular system	34	1220	0.35	0.00049	CPB2,SPARC,APOA1,APOE,PSMA6,FYCO1,APEH,PKM,HSPA5,P4HB,TUBA1B,FGG,THBS4,PAPLN,FN1,MFAP5,S100A8,ECM1,ANGPTL3,CCN5,GPX3,EFEMP1,GAPDH,IGF2,BZW1,PRG4,LMOD3,TTN,KNG1,HBB,ACTB,ENO1,TPM3,PDIA4
TISSUES	BTO:0001629	Left ventricle	8	78	0.92	0.00049	APOA1,PKM,HBA1,GAPDH,TF,TTN,HBB,TPM3
TISSUES	BTO:0005288	CL-48 cell	12	200	0.69	0.00056	SERPINA10,ITGA2B,ITIH4,PSG9,MASP1,SERPINA11,FGG,LIMS1,RBP4,GPX3,TF,PRG4
TISSUES	BTO:0001307	Stomach	15	312	0.59	0.00058	SPARC,LYZ,IGFBP4,LALBA,PKM,P4HB,HSP90AA1,TUBA1B,FN1,S100A8,IGFBP3,TNRC6A,GAPDH,ACTB,ENO1
TISSUES	BTO:0000671	Kidney	30	1039	0.37	0.00085	TPD52L2,SPARC,CAT,APOE,GUCY1A1,ANTXR1,LRG1,PKM,P4HB,HSP90AA1,TUBA1B,FN1,COL18A1,SMARCA1,GSN,IGFBP3,CD99,GPX3,TNRC6A,GAPDH,TF,IGF2,ACSF2,TGFBI,GC,ATP1A1,KNG1,ACTB,ENO1,TPM3
TISSUES	BTO:0001369	Vertebrate muscular system	25	780	0.41	0.00085	CPB2,APOA1,APOE,PSMA6,FYCO1,P4HB,FGG,THBS4,PAPLN,MFAP5,S100A8,ECM1,ANGPTL3,CCN5,GPX3,EFEMP1,GAPDH,BZW1,PRG4,LMOD3,TTN,KNG1,HBB,ACTB,TPM3
TISSUES	BTO:0000431	Excretory gland	36	1385	0.32	0.0010	TPD52L2,VTN,SPARC,CAT,APOE,A1BG,HPX,GUCY1A1,ANTXR1,LRG1,FGB,F2,PKM,P4HB,HSP90AA1,TUBA1B,APOD,FN1,COL18A1,SMARCA1,GSN,IGFBP3,CD99,GPX3,TNRC6A,GAPDH,TF,IGF2,ACSF2,TGFBI,GC,ATP1A1,KNG1,ACTB,ENO1,TPM3
TISSUES	BTO:0001085	Vascular system	17	420	0.51	0.0012	TFPI2,MGP,APOE,THBS1,VWF,F2,PKM,A2M,FBN1,P4HB,HSP90AA1,FN1,EFEMP1,TGFBI,VEGFC,ACTB,ENO1
TISSUES	BTO:0000420	Neck	13	264	0.6	0.0016	MYH9,APOE,PKM,HSPA5,P4HB,GANAB,FN1,GPX3,GAPDH,SAA2,ATP1A1,ACTB,ENO1
TISSUES	BTO:0003092	Urinary system	33	1249	0.33	0.0016	TPD52L2,SPARC,CAT,APOE,GUCY1A1,ANTXR1,LRG1,PKM,P4HB,HSP90AA1,TUBA1B,FN1,COL18A1,CFH,FLNA,SMARCA1,GSN,IGFBP3,CD99,GPX3,TNRC6A,GAPDH,TF,IGF2,ACSF2,TGFBI,GC,ATP1A1,VEGFC,KNG1,ACTB,ENO1,TPM3

TISSUES	BTO:0001244	Urinary tract	33	1258	0.33	0.0018	TPD52L2,SPARC,CAT,APOE,GUCY1A1,ANTXR1,LRG1,PKM,P4HB,HSP90AA1,TUBA1B,FN1,COL18A1,CFH,FLNA,SMARCA1,GSN,IGFBP3,CD99,GPX3,TNRC6A,GAPDH,TF,IGF2,ACSF2,TGFBI,GC,ATP1A1,VEGFC,KNG1,ACTB,ENO1,TPM3
TISSUES	BTO:0000828	Throat	12	234	0.62	0.0020	MYH9,PKM,HSPA5,P4HB,GANAB,FN1,GPX3,GAPDH,SAA2,ATP1A1,ACTB,ENO1
TISSUES	BTO:0001493	Trunk	28	1014	0.35	0.0030	ST6GAL1,MYH9,TPD52L2,VTN,APOE,A1BG,HPX,FERMT3,FGB,F2,PKM,HBA1,HSPA5,P4HB,HSP90AA1,TUBA1B,GANAB,FN1,S100A8,GPX3,GAPDH,ADIPOQ,SAA2,ATP1A1,TTN,KNG1,ACTB,ENO1
TISSUES	BTO:0000007	HEK-293 cell	3	5	1.69	0.0036	PXDN,GAPDH,ACTB
TISSUES	BTO:0000855	Lymph	10	177	0.66	0.0039	ST6GAL1,PLEK,FERMT3,PKM,TUBA1B,GANAB,GAPDH,PLCG2,ACTB,ENO1
TISSUES	BTO:0000202	Sense organ	29	1124	0.32	0.0066	GNPTG,SPARC,IGFBP5,TTR,APOE,LYZ,TIMP2,CSTA,SERPING1,DST,PKM,HSP90AA1,TUBA1B,APOD,FN1,MFAP5,CFH,S100A8,GSN,GPX3,ACTN1,YWHAZ,GAPDH,TF,BZW1,TGFBI,ATP1A1,ACTB,ENO1
TISSUES	BTO:0000917	Needle	3	7	1.54	0.0071	PXDN,GAPDH,ACTB
TISSUES	BTO:0000574	Hematopoietic cell	27	1019	0.33	0.0072	MYH9,TPD52L2,MPO,PLEK,APOA1,CAT,APOE,ITGA2B,BLVRB,PPBP,PF4,PKM,HSP90AA1,FN1,S100A8,FLNA,C1QC,CD99,GPX3,YWHAZ,GAPDH,C1S,PLCG2,HSPH1,HBB,ACTB,ENO1
TISSUES	BTO:0001388	Trachea	10	201	0.6	0.0096	ST6GAL1,LYZ,CP,A2M,P4HB,LIMS1,CFH,RAPGEF4,VEGFC,KNG1
TISSUES	BTO:0000744	Lymphocytic leukemia cell	15	419	0.46	0.0104	ST6GAL1,MYH9,TLN1,SUZ12,PKM,HSP90AA1,CENPF,MKI67,FLNA,YWHAZ,GAPDH,ROCK1,PLCG2,ACTB,ENO1
TISSUES	BTO:0001424	Uterus	28	1117	0.31	0.0125	MGP,SPARC,IGFBP5,THBS1,IGFBP4,SERPING1,A2ML1,NDUFS8,SUZ12,PKM,A2M,TUBA1B,GANAB,FN1,GSN,CD99,PROS1,YWHAZ,ILK,GAPDH,C1S,IGF2,PAEP,UIMC1,ATP1A1,ACTB,ENO1,TPM3
TISSUES	BTO:0000975	Ovary	17	528	0.41	0.0134	A1BG,SERPING1,FYCO1,PKM,HSP90AA1,TUBA1B,PAPLN,KIAA0753,FLNA,CCN5,F10,GAPDH,IGF2,UIMC1,ATP1A1,ACTB,ENO1
TISSUES	BTO:0000887	Muscle	27	1070	0.31	0.0141	SPARC,APOA1,APOE,PSMA6,FYCO1,APEH,PKM,HSPA5,TUBA1B,FGG,FN1,MFAP5,S100A8,ANGPTL3,CCN5,GPX3,GAPDH,IGF2,PRG4,LMOD3,TTN,KNG1,HBB,ACTB,ENO1,TPM3,PDIA4
TISSUES	BTO:0000634	Integument	44	2112	0.23	0.0146	ST6GAL1,SPARC,LRP1,APOE,THBS1,VWF,PSMA6,TIMP2,CSTA,DSC2,A2ML1,ANTXR1,DST,PKM,A2M,HSPA5,P4HB,HSP90AA1,TUBA1B,APOD,FN1,S100A7,S100A8,ECM1,FLNA,GSN,CALML5,IGFBP3,CD99,ACTN1,EFEMP1,YWHAZ,ILK,GAPDH,ADIPOQ,TGFBI,PAEP,C1R,ATP1A1,CFD,VEGFC,ACTB,ENO1,TPM3
TISSUES	BTO:0001281	Spleen	17	540	0.41	0.0165	ST6GAL1,CETP,PLEK,ITGA2B,PLA2G7,FYCO1,PKM,HBA1,A2M,P4HB,FLNA,IGFBP3,C1S,CFD,PLCG2,HBB,ACTB
TISSUES	BTO:0000849	Melanoma cell line	9	180	0.61	0.0169	MMP2,CSPG4,HSPA5,P4HB,HSP90AA1,GANAB,BLM,YWHAZ,ATP1A1
TISSUES	BTO:0000102	Blood clot	3	11	1.34	0.0181	VWF,ITGA2B,F2
TISSUES	BTO:0000372	Bone cancer cell	9	183	0.6	0.0185	IGFBP5,PXDN,TCIRG1,CDH11,IGFBP4,SH3D19,PKM,TTN,TPM3
TISSUES	BTO:0000667	Keratinocyte	7	113	0.7	0.0205	CSTA,DSC2,DST,TUBA1B,S100A7,S100A8,TPM3
TISSUES	BTO:0004078	Non-small cell lung adenocarcinoma cell line	3	12	1.3	0.0216	PCSK9,GAPDH,ACTB
TISSUES	BTO:0000439	Eye	22	835	0.33	0.0254	SPARC,IGFBP5,TTR,APOE,LYZ,DST,PKM,HSP90AA1,TUBA1B,APOD,FN1,CFH,GSN,GPX3,YWHAZ,GAPDH,TF,BZW1,TGFBI,ATP1A1,ACTB,ENO1
TISSUES	BTO:0005692	Column	2	2	1.91	0.0254	GAPDH,ACTB
TISSUES	BTO:0001823	Synovium	6	88	0.74	0.0292	P4HB,THBS4,PAPLN,ECM1,EFEMP1,PRG4
TISSUES	BTO:0001385	Tongue	8	161	0.6	0.0314	GNPTG,CSTA,DST,MFAP5,S100A8,GSN,ACTN1,YWHAZ
TISSUES	BTO:0000706	Large intestine	20	750	0.33	0.0354	MYH9,PROC,LYZ,KLKB1,IGFBP4,HABP2,LRG1,P4HB,APOA4,FLNA,GSN,SOD3,ACTN1,YWHAZ,ILK,GAPDH,CKMT1A,CFB,C1R,ACTB
TISSUES	BTO:0004695	Aortic root	2	3	1.73	0.0388	APOE,FBN1
TISSUES	BTO:0005739	Transfer cell	2	3	1.73	0.0388	PXDN,ACTB
TISSUES	BTO:0001028	Peripheral nervous system	8	169	0.58	0.0400	SPARC,THBS4,COL18A1,TNRC6A,YWHAZ,C1S,CKMT1A,SELENOP
TISSUES	BTO:0001379	Thyroid gland	8	169	0.58	0.0400	PKM,HSPA5,P4HB,FN1,GPX3,SAA2,ACTB,ENO1
TISSUES	BTO:0001207	Sarcoma cell	8	171	0.58	0.0420	IGFBP5,PXDN,CDH11,IGFBP4,SH3D19,PKM,TTN,TPM3
COMPARTMENTS	GOCC:0005576	Extracellular region	160	2079	0.79	1.19e-88	SPP2,ST6GAL1,CPB2,CETP,SERPIND1,MYH9,F9,MMP2,TFPI2,PCOLCE,C5,MPO,VTN,PF4V1,MGP,GPLD1,SPARC,IGFBP5,PROC,APOA1,TTR,APOE,PXDN,ANGPTL6,F12,CFHR5,THBS1,LYZ,VWF,SERPINA10,ITGA2B,TIMP2,A1BG,LCAT,CSTA,CP,KLKB1,F13A1,AMBP,CENPE,HPX,ITIH4,CDH11,IGFBP4,FCN3,PLA2G7,CRH,HABP2,SAA4,SERPING1,FCN2,PSG6,PGLYRP2,SCGB3A1,PPBP,PF4,MASP1,A2ML1,LALBA,LRG1,PCSK9,FGB,DST,F2,INHBC,PRG2,CSPG4,C1QB,CFHR1,TLN1,APOL1,PKM,C7,HBA1,A2M,HSPA5,FBN1,P4HB,VMO1,SERPINA7,PAPPA,FBLN1,HSP90AA1,ANG,FGG,THBS4,APOD,FN1,APOA4,ITIH2,MFAP5,COL18A1,C8A,F13B,CFHR2,CFHR3,CFH,PAPPA2,F5,ADAM12,S100A7,S100A8,HTRA1,ECM1,FLNA,ANGPTL3,C8B,RBP4,CCN5,GSN,C1QC,F10,F7,APOM,CA6,CALML5,IGFBP3,SOD3,GOLM1,GPX3,IGF1,PROS1,EFEMP1,YWHAZ,GAPDH,MASP2,F11,C1S,TF,LTBP1,PCYOX1,ADIPOQ,IGF2,C4A,MST1,ITIH3,C4B,TGFBI,CFB,IGFALS,PAEP,GC,TSKU,SAA2,HPR,C1R,APOA5,RNASE4,IGKV2D-28,APOC4,CFD,VEGFC,PLCG2,KNG1,HBB,ACTB,ENO1,FGA,TPM3,PDIA4
COMPARTMENTS	GOCC:0005615	Extracellular space	117	1027	0.96	2.85e-76	CPB2,CETP,SERPIND1,F9,MMP2,C5,MPO,VTN,MGP,GPLD1,IGFBP5,PROC,APOA1,TTR,APOE,PXDN,F12,THBS1,LYZ,VWF,ITGA2B,A1BG,LCAT,CSTA,CP,KLKB1,F13A1,AMBP,HPX,ITIH4,CDH11,IGFBP4,FCN3,PLA2G7,CRH,SAA4,SERPING1,FCN2,SCGB3A1,MASP1,A2ML1,LRG1,PCSK9,FGB,F2,CSPG4,C1QB,CFHR1,TLN1,APOL1,PKM,HBA1,A2M,HSPA5,FBN1,PAPPA,FBLN1,HSP90AA1,ANG,FGG,THBS4,APOD,FN1,APOA4,ITIH2,COL18A1,C8A,CFHR3,CFH,S100A7,S100A8,ECM1,FLNA,ANGPTL3,RBP4,CCN5,GSN,C1QC,F7,APOM,CA6,IGFBP3,SOD3,GPX3,IGF1,PROS1,EFEMP1,YWHAZ,GAPDH,MASP2,F11,C1S,TF,PCYOX1,ADIPOQ,IGF2,C4A,MST1,C4B,TGFBI,CFB,IGFALS,GC,SAA2,HPR,C1R,APOA5,IGKV2D-28,APOC4,CFD,PLCG2,KNG1,HBB,ACTB,ENO1,FGA,PDIA4
COMPARTMENTS	GOCC:0072562	Blood microparticle	44	118	1.48	2.84e-44	VTN,APOA1,APOE,ITGA2B,A1BG,CP,F13A1,AMBP,HPX,ITIH4,FCN3,SERPING1,FCN2,FGB,F2,C1QB,CFHR1,APOL1,HBA1,A2M,FGG,FN1,APOA4,ITIH2,C8A,CFHR3,CFH,GSN,C1QC,PROS1,YWHAZ,C1S,TF,C4A,C4B,CFB,GC,HPR,C1R,IGKV2D-28,KNG1,HBB,ACTB,FGA
COMPARTMENTS	GOCC:0043230	Extracellular organelle	59	524	0.96	1.73e-34	CPB2,MYH9,F9,C5,MPO,VTN,MGP,APOA1,TTR,APOE,PXDN,THBS1,VWF,ITGA2B,CP,KLKB1,AMBP,HPX,ITIH4,CDH11,SERPING1,FCN2,LRG1,FGB,F2,CSPG4,TLN1,PKM,A2M,HSPA5,HSP90AA1,FGG,APOD,FN1,COL18A1,CFH,S100A8,ECM1,FLNA,GSN,GPX3,PROS1,EFEMP1,YWHAZ,GAPDH,MASP2,F11,TF,C4A,C4B,TGFBI,GC,SAA2,C1R,KNG1,HBB,ACTB,ENO1,FGA
COMPARTMENTS	GOCC:1903561	Extracellular vesicle	56	500	0.96	1.60e-32	CPB2,MYH9,F9,MPO,VTN,MGP,APOA1,TTR,APOE,PXDN,THBS1,VWF,ITGA2B,CP,KLKB1,AMBP,HPX,ITIH4,CDH11,SERPING1,LRG1,FGB,F2,CSPG4,TLN1,PKM,A2M,HSPA5,HSP90AA1,FGG,APOD,FN1,COL18A1,CFH,S100A8,ECM1,FLNA,GSN,GPX3,PROS1,EFEMP1,YWHAZ,GAPDH,F11,TF,C4A,C4B,TGFBI,GC,SAA2,C1R,KNG1,HBB,ACTB,ENO1,FGA
COMPARTMENTS	GOCC:0065010	Extracellular membrane-bounded organelle	53	473	0.96	1.10e-30	CPB2,MYH9,F9,MPO,VTN,MGP,APOA1,TTR,APOE,PXDN,THBS1,VWF,ITGA2B,CP,KLKB1,AMBP,HPX,ITIH4,SERPING1,LRG1,FGB,F2,CSPG4,TLN1,PKM,A2M,HSPA5,HSP90AA1,FGG,APOD,FN1,COL18A1,CFH,S100A8,ECM1,FLNA,GSN,GPX3,PROS1,YWHAZ,GAPDH,F11,C4A,C4B,TGFBI,GC,SAA2,C1R,KNG1,HBB,ACTB,ENO1,FGA

COMPARTMENTS	GOCC:0034774	Secretory granule lumen	37	241	1.09	4.51e-25	SPP2,MPO,SPARC,APOA1,TTR,THBS1,LYZ,VWF,TIMP2,A1BG,F13A1,ITIH4,SERPING1,FERMT3,PPBP,PF4,LRG1,FGB,A2M,HSP90AA1,FGG,FN1,F5,S100A7,ECM1,GSN,IGF1,PROS1,ACTN1,TF,IGF2,ITIH3,SELENOP,CFD,VEGFC,KNG1,FGA
COMPARTMENTS	GOCC:0031982	Vesicle	91	2125	0.54	1.15e-24	SPP2,CPB2,MYH9,F9,MPO,VTN,MGP,SPARC,APOA1,TTR,CAT,LRP1,APOE,PXDN,THBS1,LYZ,VWF,ITGA2B,TIMP2,A1BG,CP,KLKB1,F13A1,AMBP,TCIRG1,HPX,ITIH4,CDH11,SERPING1,FERMT3,SEC24D,DSC2,PPBP,PF4,FYCO1,APEH,ANTXR1,LRG1,PCSK9,FGB,DST,F2,PRG2,CSPG4,STAB1,TLN1,PKM,STXBP5,HBA1,A2M,HSPA5,HSP90AA1,FGG,APOD,FN1,COL18A1,CFH,F5,S100A7,S100A8,ECM1,FLNA,GSN,CALML5,GPX3,IGF1,PROS1,ACTN1,EFEMP1,YWHAZ,GAPDH,F11,TF,IGF2,C4A,ITIH3,C4B,TGFBI,GC,SELENOP,SAA2,C1R,CFD,VEGFC,PLCG2,HSPH1,KNG1,HBB,ACTB,ENO1,FGA
COMPARTMENTS	GOCC:0070062	Extracellular exosome	43	428	0.91	6.85e-23	MPO,VTN,MGP,APOA1,TTR,APOE,PXDN,THBS1,VWF,ITGA2B,CP,AMBP,HPX,ITIH4,CDH11,LRG1,FGB,CSPG4,TLN1,PKM,A2M,HSPA5,HSP90AA1,FGG,APOD,FN1,COL18A1,CFH,ECM1,FLNA,GSN,GPX3,EFEMP1,YWHAZ,GAPDH,TF,C4A,TGFBI,KNG1,HBB,ACTB,ENO1,FGA
COMPARTMENTS	GOCC:0031093	Platelet alpha granule lumen	22	66	1.43	9.23e-21	SPARC,THBS1,VWF,A1BG,F13A1,SERPING1,FERMT3,PPBP,PF4,FGB,A2M,FGG,FN1,F5,IGF1,PROS1,ACTN1,IGF2,CFD,VEGFC,KNG1,FGA
COMPARTMENTS	GOCC:0031091	Platelet alpha granule	23	89	1.32	1.14e-19	SPARC,THBS1,VWF,ITGA2B,A1BG,F13A1,SERPING1,FERMT3,PPBP,PF4,FGB,A2M,FGG,FN1,F5,IGF1,PROS1,ACTN1,IGF2,CFD,VEGFC,KNG1,FGA
COMPARTMENTS	GOCC:0030141	Secretory granule	46	719	0.71	3.17e-17	SPP2,MYH9,MPO,SPARC,APOA1,TTR,CAT,THBS1,LYZ,VWF,ITGA2B,TIMP2,A1BG,F13A1,TCIRG1,ITIH4,SERPING1,FERMT3,PPBP,PF4,APEH,LRG1,FGB,PRG2,PKM,A2M,HSP90AA1,FGG,FN1,F5,S100A7,ECM1,GSN,CALML5,IGF1,PROS1,ACTN1,TF,IGF2,ITIH3,SELENOP,CFD,VEGFC,KNG1,HBB,FGA
COMPARTMENTS	GOCC:0099503	Secretory vesicle	47	797	0.68	2.47e-16	SPP2,MYH9,MPO,SPARC,APOA1,TTR,CAT,THBS1,LYZ,VWF,ITGA2B,TIMP2,A1BG,F13A1,TCIRG1,ITIH4,SERPING1,FERMT3,PPBP,PF4,APEH,LRG1,FGB,PRG2,PKM,STXBP5,A2M,HSP90AA1,FGG,FN1,F5,S100A7,ECM1,GSN,CALML5,IGF1,PROS1,ACTN1,TF,IGF2,ITIH3,SELENOP,CFD,VEGFC,KNG1,HBB,FGA
COMPARTMENTS	GOCC:0034358	Plasma lipoprotein particle	16	48	1.43	6.41e-15	CETP,APOA1,APOE,LCAT,PLA2G7,SAA4,PCSK9,APOL1,APOA4,APOM,PCYOX1,ADIPOQ,SAA2,HPR,APOA5,APOC4
COMPARTMENTS	GOCC:0005788	Endoplasmic reticulum lumen	23	172	1.03	3.24e-14	SPP2,F9,APOA1,APOE,THBS1,PCSK9,APOL1,HSPA5,FBN1,P4HB,FGG,GANAB,FN1,APOA4,COL18A1,F5,F7,IGFBP3,C4A,APOA5,KNG1,FGA,PDIA4
COMPARTMENTS	GOCC:0034364	High-density lipoprotein particle	13	30	1.54	3.04e-13	CETP,APOA1,APOE,LCAT,PLA2G7,SAA4,APOL1,APOA4,APOM,SAA2,HPR,APOA5,APOC4
COMPARTMENTS	GOCC:0031012	Extracellular matrix	25	259	0.89	1.42e-12	CPB2,MMP2,TFPI2,VTN,GPLD1,SPARC,APOE,PXDN,THBS1,VWF,TIMP2,DST,PKM,FBN1,FBLN1,ANG,THBS4,FN1,MFAP5,COL18A1,ECM1,SOD3,EFEMP1,LTBP1,TGFBI
COMPARTMENTS	GOCC:0030312	External encapsulating structure	24	259	0.87	1.07e-11	MMP2,TFPI2,VTN,GPLD1,SPARC,APOE,PXDN,THBS1,VWF,TIMP2,DST,PKM,FBN1,PAPPA,FBLN1,ANG,THBS4,FN1,MFAP5,COL18A1,ECM1,SOD3,EFEMP1,LTBP1
COMPARTMENTS	GOCC:0110165	Cellular anatomical entity	221	14060	0.1	2.61e-11	SPP2,ST6GAL1,CPB2,CETP,GNPTG,SERPIND1,MYH9,TPD52L2,APMAP,F9,MMP2,TFPI2,PCOLCE,C5,MPO,HSD17B1,VTN,PF4V1,MGP,GPLD1,SPARC,IGFBP5,PROC,PLEK,APOA1,TTR,CAT,LRP1,APOE,PXDN,ANGPTL6,F12,CFHR5,THBS1,LYZ,VWF,PSMA6,SERPINA10,ITGA2B,TIMP2,ATRN,A1BG,BLVRB,NOTCH3,LCAT,CSTA,CP,KLKB1,F13A1,DNAH5,AMBP,CENPE,TCIRG1,HPX,ITIH4,CDH11,IGFBP4,PSG9,FCN3,PLA2G7,CRH,HABP2,SAA4,SERPING1,FERMT3,SEC24D,DSC2,CSF1R,PCDH1,FCN2,PSG6,PGLYRP2,SCGB3A1,PPBP,PF4,FYCO1,MASP1,APEH,GUCY1A1,C16orf46,A2ML1,LALBA,ANTXR1,LRG1,SH3D19,PCSK9,FGB,DST,F2,INHBC,PRG2,CSPG4,STAB1,C1QB,CFHR1,NDUFS8,TLN1,SUZ12,APOL1,PKM,STXBP5,C7,HBA1,A2M,HSPA5,FBN1,P4HB,VMO1,SERPINA7,PAPPA,FBLN1,DNAH8,HSP90AA1,ANG,TUBA1B,FGG,LIMS1,THBS4,GANAB,APOD,FN1,BLM,APOA4,ITIH2,MFAP5,COL18A1,C8A,KIAA0753,CENPF,F13B,CFHR2,CFHR3,CFH,PAPPA2,F5,MKI67,ADAM12,S100A7,S100A8,HTRA1,ECM1,FLNA,SMARCA1,ANGPTL3,C8B,RBP4,CCN5,GSN,C1QC,F10,F7,APOM,CA6,ADGRG2,CALML5,IGFBP3,CD99,SOD3,GOLM1,GPX3,IGF1,PROS1,ACTN1,EFEMP1,TNRC6A,YWHAZ,ILK,GAPDH,RAPGEF4,ROCK1,MAP3K7CL,MASP2,F11,C1S,TF,DCDC1,LTBP1,PCYOX1,ADIPOQ,IGF2,BZW1,C4A,ATP9B,ACSF2,CKMT1A,MST1,LMOD3,ITIH3,C4B,TGFBI,CFB,IGFALS,PAEP,TCAF1,GC,UIMC1,SELENOP,TSKU,SAA2,HPR,C1R,APOA5,ATP1A1,RNASE4,IGKV2D-28,TTN,APOC4,CFD,VEGFC,SEMA3B,PLCG2,OR52B4,HSPH1,KNG1,HBB,ACTB,TRIM66,ENO1,FGA,TPM3,PDIA4
COMPARTMENTS	GOCC:0031410	Cytoplasmic vesicle	59	1738	0.44	1.23e-10	SPP2,MYH9,MPO,SPARC,APOA1,TTR,CAT,LRP1,APOE,THBS1,LYZ,VWF,ITGA2B,TIMP2,A1BG,F13A1,TCIRG1,HPX,ITIH4,SERPING1,FERMT3,SEC24D,DSC2,PPBP,PF4,FYCO1,APEH,ANTXR1,LRG1,PCSK9,FGB,DST,PRG2,STAB1,PKM,STXBP5,HBA1,A2M,HSP90AA1,FGG,FN1,F5,S100A7,ECM1,GSN,CALML5,IGF1,PROS1,ACTN1,TF,IGF2,ITIH3,SELENOP,CFD,VEGFC,HSPH1,KNG1,HBB,FGA
COMPARTMENTS	GOCC:0043233	Organelle lumen	80	2905	0.35	1.64e-10	SPP2,F9,MPO,SPARC,PROC,APOA1,TTR,CAT,APOE,THBS1,LYZ,VWF,TIMP2,A1BG,F13A1,HPX,ITIH4,SERPING1,FERMT3,PPBP,PF4,APEH,LALBA,LRG1,PCSK9,FGB,F2,PRG2,CSPG4,NDUFS8,SUZ12,APOL1,PKM,HBA1,A2M,HSPA5,FBN1,P4HB,HSP90AA1,ANG,FGG,GANAB,FN1,BLM,APOA4,COL18A1,CENPF,F5,MKI67,S100A7,HTRA1,ECM1,SMARCA1,GSN,F10,F7,CALML5,IGFBP3,SOD3,IGF1,PROS1,ACTN1,GAPDH,TF,IGF2,C4A,ITIH3,GC,UIMC1,SELENOP,APOA5,TTN,CFD,VEGFC,HSPH1,KNG1,HBB,ACTB,FGA,PDIA4
COMPARTMENTS	GOCC:0012505	Endomembrane system	84	3156	0.33	1.95e-10	SPP2,ST6GAL1,MYH9,F9,MPO,SPARC,PROC,APOA1,TTR,CAT,LRP1,APOE,PXDN,THBS1,LYZ,VWF,ITGA2B,TIMP2,A1BG,CP,F13A1,TCIRG1,ITIH4,SERPING1,FERMT3,SEC24D,PPBP,PF4,FYCO1,APEH,LALBA,ANTXR1,LRG1,PCSK9,FGB,F2,PRG2,CSPG4,APOL1,PKM,STXBP5,A2M,HSPA5,FBN1,P4HB,HSP90AA1,FGG,THBS4,GANAB,FN1,APOA4,COL18A1,CENPF,F5,S100A7,ECM1,GSN,F10,F7,CALML5,IGFBP3,SOD3,GOLM1,IGF1,PROS1,ACTN1,TNRC6A,GAPDH,TF,ADIPOQ,IGF2,C4A,ATP9B,ITIH3,SELENOP,APOA5,CFD,VEGFC,SEMA3B,KNG1,HBB,ENO1,FGA,PDIA4
COMPARTMENTS	GOCC:0070013	Intracellular organelle lumen	79	2902	0.34	3.87e-10	SPP2,F9,MPO,SPARC,PROC,APOA1,TTR,CAT,APOE,THBS1,LYZ,VWF,TIMP2,A1BG,F13A1,HPX,ITIH4,SERPING1,FERMT3,PPBP,PF4,APEH,LALBA,LRG1,PCSK9,FGB,F2,PRG2,CSPG4,NDUFS8,SUZ12,APOL1,PKM,HBA1,A2M,HSPA5,FBN1,P4HB,HSP90AA1,ANG,FGG,GANAB,FN1,BLM,APOA4,COL18A1,CENPF,F5,MKI67,S100A7,ECM1,SMARCA1,GSN,F10,F7,CALML5,IGFBP3,SOD3,IGF1,PROS1,ACTN1,GAPDH,TF,IGF2,C4A,ITIH3,GC,UIMC1,SELENOP,APOA5,TTN,CFD,VEGFC,HSPH1,KNG1,HBB,ACTB,FGA,PDIA4
COMPARTMENTS	GOCC:0062023	Collagen-containing extracellular matrix	19	198	0.89	1.85e-09	MMP2,VTN,SPARC,APOE,PXDN,THBS1,VWF,TIMP2,DST,PKM,FBN1,ANG,THBS4,FN1,COL18A1,ECM1,SOD3,LTBP1,TGFBI
COMPARTMENTS	GOCC:0005577	Fibrinogen complex	9	20	1.56	3.18e-09	THBS1,VWF,ITGA2B,FGB,F2,FGG,FN1,F7,FGA
COMPARTMENTS	GOCC:0034361	Very-low-density lipoprotein particle	9	20	1.56	3.18e-09	CETP,APOA1,APOE,APOL1,APOA4,APOM,PCYOX1,APOA5,APOC4
COMPARTMENTS	GOCC:0071682	Endocytic vesicle lumen	9	23	1.5	7.87e-09	MPO,SPARC,APOA1,APOE,HPX,HBA1,HSP90AA1,HSPH1,HBB
COMPARTMENTS	GOCC:0062167	Complement component C1q complex	7	10	1.75	4.34e-08	SERPING1,C1QB,C1QC,C1S,C4A,C4B,C1R
COMPARTMENTS	GOCC:1905286	Serine-type peptidase complex	7	11	1.71	6.83e-08	F9,FCN3,FCN2,MASP1,F7,MASP2,F11

COMPARTMENTS	GOCC:0032991	Protein-containing complex	108	5325	0.21	4.47e-07	CPB2,CETP,GNPTG,MYH9,F9,C5,VTN,IGFBP5,APOA1,CAT,LRP1,APOE,PXDN,THBS1,VWF,PSMA6,ITGA2B,NOTCH3,LCAT,CSTA,DNAH5,CDH11,IGFBP4,PSG9,FCN3,PLA2G7,SAA4,SERPING1,SEC24D,CSF1R,FCN2,MASP1,APEH,GUCY1A1,PCSK9,FGB,DST,F2,UBE3C,C1QB,CFHR1,NDUFS8,SUZ12,APOL1,SUMO4,PKM,STXBP5,C7,HBA1,HSPA5,FBN1,P4HB,PAPPA,DNAH8,HSP90AA1,ANG,FGG,GANAB,FN1,BLM,APOA4,COL18A1,C8A,CENPF,CFHR2,S100A7,S100A8,FLNA,SMARCA1,C8B,C1QC,F7,APOM,CALML5,IGFBP3,GPX3,IGF1,TNRC6A,GAPDH,MASP2,F11,C1S,TF,LTBP1,PCYOX1,ADIPOQ,IGF2,C4A,CKMT1A,CDK3,C4B,CFB,IGFALS,UIMC1,SAA2,HPR,C1R,APOA5,ATP1A1,TTN,APOC4,VEGFC,HSPH1,HBB,ACTB,ENO1,FGA,TPM3
COMPARTMENTS	GOCC:0016942	Insulin-like growth factor binding protein complex	6	10	1.69	1.38e-06	IGFBP5,IGFBP4,IGFBP3,IGF1,IGF2,IGFALS
COMPARTMENTS	GOCC:0034362	Low-density lipoprotein particle	7	22	1.41	2.76e-06	CETP,APOA1,APOE,PLA2G7,PCSK9,APOM,ADIPOQ
COMPARTMENTS	GOCC:1904813	ficolin-1-rich granule lumen	12	124	0.89	6.57e-06	CAT,TIMP2,A1BG,APEH,LRG1,PRG2,PKM,HSP90AA1,GSN,CALML5,CFD,HBB
COMPARTMENTS	GOCC:0005604	Basement membrane	9	61	1.08	1.01e-05	VTN,SPARC,PXDN,DST,FBN1,ANG,THBS4,FN1,COL18A1
COMPARTMENTS	GOCC:1905370	Serine-type endopeptidase complex	5	7	1.76	1.04e-05	FCN3,FCN2,MASP1,MASP2,F11
COMPARTMENTS	GOCC:0036454	Growth factor complex	7	31	1.26	1.80e-05	IGFBP5,IGFBP4,IGFBP3,IGF1,IGF2,IGFALS,VEGFC
COMPARTMENTS	GOCC:0005783	Endoplasmic reticulum	35	1095	0.41	2.01e-05	SPP2,F9,PROC,APOA1,APOE,PXDN,THBS1,VWF,CP,SEC24D,PCSK9,APOL1,PKM,HSPA5,FBN1,P4HB,FGG,THBS4,GANAB,FN1,APOA4,COL18A1,F5,S100A7,F7,IGFBP3,GOLM1,IGF1,ADIPOQ,C4A,APOA5,SEMA3B,KNG1,FGA,PDIA4
COMPARTMENTS	GOCC:0005579	Membrane attack complex	5	12	1.53	7.22e-05	C5,C7,C8A,C8B,C4A
COMPARTMENTS	GOCC:0042567	Insulin-like growth factor ternary complex	4	4	1.91	7.22e-05	IGFBP5,IGFBP3,IGF1,IGFALS
COMPARTMENTS	GOCC:0005602	Complement component C1 complex	4	5	1.81	0.00013	C1QB,C1QC,C1S,C1R
COMPARTMENTS	GOCC:0031089	Platelet dense granule lumen	5	14	1.46	0.00013	SPP2,ITIH4,ECM1,ITIH3,SELENOP
COMPARTMENTS	GOCC:0034365	Discoidal high-density lipoprotein particle	4	6	1.73	0.00020	APOA1,APOE,LCAT,APOM
COMPARTMENTS	GOCC:1904090	Peptidase inhibitor complex	5	17	1.38	0.00026	VTN,PXDN,CSTA,GAPDH,ACTB
COMPARTMENTS	GOCC:0005796	Golgi lumen	9	102	0.85	0.00040	F9,PROC,LALBA,F2,CSPG4,F10,F7,SOD3,PROS1
COMPARTMENTS	GOCC:0005925	Focal adhesion	14	269	0.62	0.00048	LRP1,DST,CSPG4,TLN1,HSPA5,P4HB,LIMS1,S100A7,FLNA,GSN,ACTN1,YWHAZ,ILK,ACTB
COMPARTMENTS	GOCC:1905368	Peptidase complex	10	136	0.77	0.00055	F9,PSMA6,FCN3,FCN2,MASP1,F2,UBE3C,F7,MASP2,F11
COMPARTMENTS	GOCC:0062136	Low-density lipoprotein receptor complex	4	9	1.55	0.00059	APOA1,LRP1,APOE,PCSK9
COMPARTMENTS	GOCC:0034366	Spherical high-density lipoprotein particle	4	11	1.47	0.0010	APOA1,LCAT,APOM,HPR
COMPARTMENTS	GOCC:0030139	Endocytic vesicle	13	260	0.61	0.0013	MPO,SPARC,APOA1,LRP1,APOE,TCIRG1,HPX,STAB1,HBA1,HSP90AA1,TF,HSPH1,HBB
COMPARTMENTS	GOCC:0043227	Membrane-bounded organelle	144	9083	0.11	0.0015	SPP2,ST6GAL1,CPB2,GNPTG,MYH9,F9,MMP2,PCOLCE,MPO,VTN,MGP,GPLD1,SPARC,PROC,APOA1,TTR,CAT,LRP1,APOE,PXDN,THBS1,LYZ,VWF,PSMA6,ITGA2B,TIMP2,A1BG,BLVRB,NOTCH3,LCAT,CSTA,CP,KLKB1,F13A1,DNAH5,AMBP,CENPE,TCIRG1,HPX,ITIH4,CDH11,SERPING1,FERMT3,SEC24D,DSC2,PPBP,PF4,FYCO1,APEH,C16orf46,LALBA,ANTXR1,LRG1,SH3D19,PCSK9,FGB,DST,F2,PRG2,CSPG4,STAB1,NDUFS8,TLN1,SUZ12,APOL1,PKM,STXBP5,HBA1,A2M,HSPA5,FBN1,P4HB,DNAH8,HSP90AA1,ANG,FGG,THBS4,GANAB,APOD,FN1,BLM,APOA4,COL18A1,CENPF,CFH,F5,MKI67,S100A7,S100A8,HTRA1,ECM1,FLNA,SMARCA1,CCN5,GSN,F10,F7,CALML5,IGFBP3,CD99,SOD3,GOLM1,GPX3,IGF1,PROS1,ACTN1,EFEMP1,TNRC6A,YWHAZ,ILK,GAPDH,ROCK1,MASP2,F11,TF,PCYOX1,ADIPOQ,IGF2,C4A,ATP9B,ACSF2,CKMT1A,ITIH3,C4B,TGFBI,GC,UIMC1,SELENOP,SAA2,C1R,APOA5,TTN,CFD,VEGFC,SEMA3B,PLCG2,HSPH1,KNG1,HBB,ACTB,TRIM66,ENO1,FGA,PDIA4
COMPARTMENTS	GOCC:0009986	Cell surface	17	438	0.5	0.0018	APMAP,SPARC,PXDN,THBS1,ITGA2B,CSF1R,ANTXR1,PCSK9,FGB,CSPG4,P4HB,FGG,TF,ADIPOQ,ENO1,FGA,PDIA4
COMPARTMENTS	GOCC:0042627	Chylomicron	4	14	1.36	0.0021	APOA1,APOE,APOA4,APOA5
COMPARTMENTS	GOCC:0005601	Classical-complement-pathway C3/C5 convertase complex	3	4	1.78	0.0022	C4A,C4B,CFB
COMPARTMENTS	GOCC:0071944	Cell periphery	73	3860	0.18	0.0030	MYH9,MMP2,TFPI2,C5,VTN,GPLD1,SPARC,PLEK,APOA1,LRP1,APOE,PXDN,THBS1,VWF,ITGA2B,TIMP2,ATRN,NOTCH3,CSTA,AMBP,TCIRG1,CDH11,PSG9,DSC2,CSF1R,PCDH1,ANTXR1,PCSK9,FGB,DST,CSPG4,STAB1,PKM,C7,FBN1,P4HB,PAPPA,FBLN1,ANG,FGG,LIMS1,THBS4,FN1,MFAP5,COL18A1,C8A,ADAM12,S100A8,ECM1,FLNA,C8B,GSN,ADGRG2,SOD3,GPX3,IGF1,PROS1,ACTN1,EFEMP1,ILK,GAPDH,TF,LTBP1,C4A,C4B,TGFBI,TCAF1,ATP1A1,PLCG2,OR52B4,KNG1,ENO1,FGA
COMPARTMENTS	GOCC:0005737	Cytoplasm	131	8195	0.11	0.0036	SPP2,ST6GAL1,GNPTG,MYH9,TPD52L2,F9,MMP2,MPO,HSD17B1,GPLD1,SPARC,PROC,PLEK,APOA1,TTR,CAT,LRP1,APOE,PXDN,THBS1,LYZ,VWF,PSMA6,ITGA2B,TIMP2,A1BG,BLVRB,NOTCH3,CSTA,CP,F13A1,DNAH5,AMBP,CENPE,TCIRG1,HPX,ITIH4,SERPING1,FERMT3,SEC24D,DSC2,PPBP,PF4,FYCO1,APEH,GUCY1A1,C16orf46,LALBA,ANTXR1,LRG1,SH3D19,PCSK9,FGB,DST,F2,PRG2,CSPG4,STAB1,NDUFS8,TLN1,APOL1,PKM,STXBP5,HBA1,A2M,HSPA5,FBN1,P4HB,DNAH8,HSP90AA1,FGG,LIMS1,THBS4,GANAB,APOD,FN1,APOA4,COL18A1,CENPF,F5,S100A7,S100A8,ECM1,FLNA,GSN,F10,F7,CALML5,IGFBP3,CD99,SOD3,GOLM1,GPX3,IGF1,PROS1,ACTN1,TNRC6A,YWHAZ,ILK,GAPDH,ROCK1,MAP3K7CL,TF,PCYOX1,ADIPOQ,IGF2,BZW1,C4A,ATP9B,ACSF2,CKMT1A,LMOD3,ITIH3,TGFBI,GC,SELENOP,HPR,APOA5,TTN,CFD,VEGFC,SEMA3B,PLCG2,HSPH1,KNG1,HBB,ACTB,ENO1,FGA,TPM3,PDIA4
COMPARTMENTS	GOCC:1905369	Endopeptidase complex	7	90	0.8	0.0064	PSMA6,FCN3,FCN2,MASP1,UBE3C,MASP2,F11
COMPARTMENTS	GOCC:1990779	Glycoprotein Ib-IX-V complex	3	8	1.48	0.0093	VWF,ITGA2B,FLNA
COMPARTMENTS	GOCC:0043226	Organelle	152	10113	0.08	0.0129	SPP2,ST6GAL1,CPB2,GNPTG,MYH9,F9,MMP2,PCOLCE,C5,MPO,VTN,MGP,GPLD1,SPARC,PROC,APOA1,TTR,CAT,LRP1,APOE,PXDN,THBS1,LYZ,VWF,PSMA6,ITGA2B,TIMP2,A1BG,BLVRB,NOTCH3,LCAT,CSTA,CP,KLKB1,F13A1,DNAH5,AMBP,CENPE,TCIRG1,HPX,ITIH4,CDH11,SERPING1,FERMT3,SEC24D,DSC2,FCN2,PPBP,PF4,FYCO1,APEH,C16orf46,LALBA,ANTXR1,LRG1,SH3D19,PCSK9,FGB,DST,F2,PRG2,CSPG4,STAB1,NDUFS8,TLN1,SUZ12,APOL1,PKM,STXBP5,HBA1,A2M,HSPA5,FBN1,P4HB,DNAH8,HSP90AA1,ANG,TUBA1B,FGG,THBS4,GANAB,APOD,FN1,BLM,APOA4,COL18A1,KIAA0753,CENPF,CFH,F5,MKI67,S100A7,S100A8,HTRA1,ECM1,FLNA,SMARCA1,CCN5,GSN,F10,F7,CALML5,IGFBP3,CD99,SOD3,GOLM1,GPX3,IGF1,PROS1,ACTN1,EFEMP1,TNRC6A,YWHAZ,ILK,GAPDH,ROCK1,MASP2,F11,TF,DCDC1,PCYOX1,ADIPOQ,IGF2,C4A,ATP9B,ACSF2,CKMT1A,LMOD3,ITIH3,C4B,TGFBI,GC,UIMC1,SELENOP,SAA2,C1R,APOA5,ATP1A1,TTN,CFD,VEGFC,SEMA3B,PLCG2,HSPH1,KNG1,HBB,ACTB,TRIM66,ENO1,FGA,TPM3,PDIA4
COMPARTMENTS	GOCC:0098797	Plasma membrane protein complex	20	682	0.37	0.0137	C5,VTN,APOA1,LRP1,APOE,VWF,ITGA2B,CDH11,PSG9,PCSK9,DST,C7,FN1,C8A,FLNA,C8B,IGF1,TF,C4A,ATP1A1
COMPARTMENTS	GOCC:0045298	Tubulin complex	3	10	1.38	0.0151	PXDN,GAPDH,ACTB
COMPARTMENTS	GOCC:0008305	Integrin complex	5	50	0.91	0.0175	VTN,VWF,ITGA2B,DST,FN1
COMPARTMENTS	GOCC:0070161	Anchoring junction	17	553	0.39	0.0214	LRP1,DSC2,PCDH1,DST,CSPG4,TLN1,HSPA5,P4HB,LIMS1,S100A7,FLNA,GSN,CD99,ACTN1,YWHAZ,ILK,ACTB
COMPARTMENTS	GOCC:1904724	Tertiary granule lumen	5	55	0.87	0.0255	LYZ,TIMP2,PPBP,LRG1,HBB
COMPARTMENTS	GOCC:0001527	Microfibril	3	13	1.27	0.0273	FBN1,MFAP5,LTBP1
COMPARTMENTS	GOCC:0097179	Protease inhibitor complex	3	14	1.24	0.0324	PXDN,GAPDH,ACTB
COMPARTMENTS	GOCC:0140224	SLAC complex	2	3	1.73	0.0429	GAPDH,ACTB

UniProt Keywords	KW-0964	Secreted	146	1839	0.81	2.30e-80	SPP2,ST6GAL1,CPB2,CETP,GNPTG,F9,MMP2,TFPI2,PCOLCE,C5,VTN,PF4V1,MGP,GPLD1,SPARC,IGFBP5,PROC,APOA1,TTR,APOE,PXDN,ANGPTL6,F12,CFHR5,THBS1,LYZ,VWF,SERPINA10,TIMP2,ATRN,A1BG,LCAT,CP,KLKB1,F13A1,AMBP,HPX,ITIH4,IGFBP4,PSG9,FCN3,PLA2G7,CRH,HABP2,SAA4,SERPING1,FCN2,PSG6,PGLYRP2,SCGB3A1,PPBP,PF4,A2ML1,LALBA,LRG1,PCSK9,FGB,F2,INHBC,PSG1,PRG2,C1QB,CFHR1,APOL1,PSG11,C7,A2M,FBN1,VMO1,SERPINA7,PAPPA,FBLN1,PSG3,SERPINA11,ANG,FGG,THBS4,APOD,PAPLN,FN1,APOA4,ITIH2,MFAP5,COL18A1,C8A,F13B,CFHR2,CFHR3,CFH,PAPPA2,F5,ADAM12,S100A7,S100A8,HTRA1,ADAMTSL4,ECM1,ANGPTL3,C8B,RBP4,CCN5,GSN,C1QC,F10,F7,APOM,CA6,IGFBP3,SOD3,GPX3,IGF1,PROS1,EFEMP1,BPIFC,MASP2,F11,PSG2,TF,LTBP1,ADIPOQ,IGF2,C4A,PRG4,MST1,ITIH3,C4B,TGFBI,CFB,IGFALS,PAEP,GC,SELENOP,TSKU,SAA2,HPR,C1R,APOA5,RNASE4,IGKV2D-28,APOC4,CFD,IGHV3-72,VEGFC,SEMA3B,KNG1,FGA
UniProt Keywords	KW-0732	Signal	163	3277	0.6	2.86e-64	SPP2,CPB2,CETP,GNPTG,SERPIND1,F9,MMP2,TFPI2,PCOLCE,C5,MPO,VTN,PF4V1,MGP,GPLD1,SPARC,IGFBP5,PROC,APOA1,TTR,LRP1,APOE,PXDN,ANGPTL6,F12,CFHR5,THBS1,LYZ,VWF,ITGA2B,TIMP2,ATRN,A1BG,NOTCH3,LCAT,CP,KLKB1,AMBP,HPX,ITIH4,CDH11,IGFBP4,PSG9,FCN3,PLA2G7,CRH,HABP2,SAA4,SERPING1,DSC2,CSF1R,PCDH1,FCN2,PSG6,PGLYRP2,SCGB3A1,PPBP,PF4,A2ML1,LALBA,ANTXR1,LRG1,PCSK9,FGB,F2,INHBC,PSG1,PRG2,CSPG4,STAB1,C1QB,CFHR1,APOL1,PSG11,C7,A2M,HSPA5,FBN1,P4HB,VMO1,SERPINA7,PAPPA,FBLN1,PSG3,OIT3,SERPINA11,ANG,FGG,THBS4,GANAB,APOD,PAPLN,FN1,APOA4,ITIH2,MFAP5,COL18A1,C8A,F13B,CFHR2,CFHR3,CFH,PAPPA2,F5,ADAM12,HTRA1,ADAMTSL4,ECM1,ANGPTL3,C8B,RBP4,CCN5,GSN,C1QC,F10,F7,APOM,CA6,ADGRG2,IGFBP3,CD99,SOD3,GPX3,IGF1,PROS1,EFEMP1,BPIFC,MASP2,F11,C1S,PSG2,TF,LTBP1,PCYOX1,ADIPOQ,IGF2,C4A,PRG4,MST1,ITIH3,C4B,TGFBI,CFB,IGFALS,PAEP,GC,SELENOP,TSKU,SAA2,HPR,C1R,APOA5,RNASE4,IGKV2D-28,APOC4,CFD,IGHV3-72,VEGFC,SEMA3B,IGLV10-54,KNG1,FGA,PDIA4
UniProt Keywords	KW-1015	Disulfide bond	137	3338	0.52	1.76e-40	SPP2,ST6GAL1,CPB2,CETP,GNPTG,F9,MMP2,TFPI2,PCOLCE,C5,MPO,VTN,PF4V1,MGP,SPARC,IGFBP5,PROC,LRP1,PXDN,ANGPTL6,F12,CFHR5,THBS1,LYZ,VWF,ITGA2B,TIMP2,ATRN,A1BG,NOTCH3,LCAT,CP,KLKB1,AMBP,HPX,ITIH4,IGFBP4,PSG9,FCN3,HABP2,SERPING1,CSF1R,FCN2,PSG6,PGLYRP2,SCGB3A1,PPBP,PF4,MASP1,A2ML1,LALBA,ANTXR1,LRG1,PCSK9,FGB,F2,INHBC,PSG1,PRG2,CSPG4,STAB1,C1QB,CFHR1,PSG11,C7,A2M,FBN1,P4HB,VMO1,PAPPA,FBLN1,PSG3,OIT3,ANG,FGG,THBS4,GANAB,APOD,PAPLN,FN1,ITIH2,MFAP5,COL18A1,C8A,F13B,CFHR2,CFHR3,CFH,PAPPA2,F5,ADAM12,S100A7,HTRA1,ANGPTL3,C8B,RBP4,GSN,C1QC,F10,F7,APOM,CA6,IGFBP3,SOD3,IGF1,PROS1,EFEMP1,BPIFC,MASP2,F11,C1S,PSG2,TF,LTBP1,ADIPOQ,IGF2,C4A,PRG4,MST1,C4B,TGFBI,CFB,PAEP,GC,HPR,C1R,RNASE4,IGKV2D-28,TTN,CFD,IGHV3-72,VEGFC,SEMA3B,OR52B4,KNG1,FGA,PDIA4
UniProt Keywords	KW-0325	Glycoprotein	140	4386	0.41	1.23e-29	ST6GAL1,CPB2,CETP,GNPTG,SERPIND1,APMAP,F9,MMP2,TFPI2,PCOLCE,C5,MPO,VTN,PF4V1,GPLD1,SPARC,IGFBP5,PROC,APOA1,TTR,LRP1,APOE,PXDN,ANGPTL6,F12,CFHR5,THBS1,VWF,PSMA6,SERPINA10,ITGA2B,ATRN,A1BG,NOTCH3,LCAT,CP,KLKB1,F13A1,AMBP,HPX,ITIH4,CDH11,IGFBP4,PSG9,FCN3,PLA2G7,HABP2,SAA4,SERPING1,DSC2,CSF1R,PCDH1,FCN2,PSG6,PGLYRP2,A2ML1,LALBA,ANTXR1,LRG1,PCSK9,FGB,F2,INHBC,PSG1,PRG2,CSPG4,STAB1,C1QB,CFHR1,APOL1,PSG11,C7,A2M,FBN1,SERPINA7,PAPPA,FBLN1,PSG3,OIT3,SERPINA11,FGG,THBS4,GANAB,APOD,FN1,ITIH2,MFAP5,COL18A1,C8A,F13B,CFHR2,CFHR3,CFH,PAPPA2,F5,ADAM12,ADAMTSL4,ECM1,ANGPTL3,C8B,C1QC,F10,F7,APOM,CA6,ADGRG2,IGFBP3,CD99,SOD3,GOLM1,PROS1,EFEMP1,BPIFC,F11,C1S,PSG2,TF,LTBP1,PCYOX1,ADIPOQ,IGF2,C4A,PRG4,MST1,ITIH3,C4B,CFB,IGFALS,PAEP,GC,SELENOP,TSKU,C1R,APOC4,VEGFC,SEMA3B,OR52B4,KNG1,HBB,FGA
UniProt Keywords	KW-0094	Blood coagulation	22	46	1.59	1.06e-23	CPB2,SERPIND1,F9,TFPI2,PROC,F12,VWF,SERPINA10,KLKB1,F13A1,SERPING1,FGB,F2,FGG,F13B,F5,F10,F7,PROS1,F11,KNG1,FGA
UniProt Keywords	KW-0245	EGF-like domain	26	232	0.96	1.34e-14	F9,PROC,LRP1,F12,THBS1,ATRN,NOTCH3,HABP2,MASP1,STAB1,C7,FBN1,FBLN1,OIT3,THBS4,C8A,ADAM12,C8B,F10,F7,PROS1,EFEMP1,MASP2,C1S,LTBP1,C1R
UniProt Keywords	KW-0768	Sushi	15	57	1.33	6.99e-13	CFHR5,MASP1,CFHR1,C7,PAPPA,F13B,CFHR2,CFHR3,CFH,PAPPA2,MASP2,C1S,CFB,HPR,C1R
UniProt Keywords	KW-0180	Complement pathway	12	30	1.51	5.12e-12	C5,SERPING1,C1QB,C7,C8A,C8B,C1QC,MASP2,C1S,C4A,C4B,C1R
UniProt Keywords	KW-0677	Repeat	113	4794	0.28	7.11e-12	SERPIND1,F9,MMP2,TFPI2,PCOLCE,VTN,GPLD1,PROC,PLEK,APOA1,LRP1,APOE,PXDN,F12,CFHR5,THBS1,VWF,ITGA2B,ATRN,A1BG,NOTCH3,CP,KLKB1,DNAH5,AMBP,HPX,CDH11,PSG9,FCN3,HABP2,SERPING1,DSC2,ANKRD44,CSF1R,PCDH1,FCN2,PSG6,MASP1,LRG1,SH3D19,DST,F2,PSG1,CSPG4,STAB1,C1QB,CFHR1,NDUFS8,PSG11,STXBP5,C7,FBN1,P4HB,PAPPA,FBLN1,PSG3,DNAH8,LIMS1,THBS4,PAPLN,FN1,APOA4,COL18A1,C8A,CENPF,F13B,CFHR2,CFHR3,CFH,PAPPA2,F5,MKI67,S100A7,S100A8,ADAMTSL4,ECM1,FLNA,SMARCA1,C8B,GSN,C1QC,F10,F7,CALML5,PROS1,ACTN1,EFEMP1,ILK,RAPGEF4,MASP2,F11,C1S,PSG2,TF,DCDC1,LTBP1,ADIPOQ,PRG4,NBEAL1,MST1,TGFBI,CFB,IGFALS,GC,UIMC1,TSKU,C1R,TTN,VEGFC,PLCG2,KNG1,TRIM66,PDIA4
UniProt Keywords	KW-0379	Hydroxylation	19	143	1.03	7.76e-12	F9,PROC,FCN3,FCN2,C1QB,PKM,COL18A1,C1QC,F10,F7,PROS1,MASP2,C1S,LTBP1,ADIPOQ,C1R,KNG1,ENO1,FGA
UniProt Keywords	KW-0301	Gamma-carboxyglutamic acid	10	16	1.7	1.57e-11	F9,MGP,PROC,TTR,F2,ITIH2,F10,F7,PROS1,TGFBI
UniProt Keywords	KW-0106	Calcium	41	886	0.57	3.86e-11	F9,MMP2,MPO,SPARC,PROC,LRP1,PXDN,THBS1,ITGA2B,F13A1,CDH11,FCN3,DSC2,PCDH1,FCN2,LALBA,PCSK9,DST,F2,FBN1,FBLN1,OIT3,SPATA21,FGG,THBS4,F5,S100A7,S100A8,GSN,F10,F7,CALML5,PROS1,ACTN1,EFEMP1,EFCAB5,MASP2,C1S,TTN,PLCG2,FGA
UniProt Keywords	KW-0165	Cleavage on pair of basic residues	23	280	0.82	1.66e-10	F9,C5,PROC,VWF,ITGA2B,AMBP,CDH11,CRH,HABP2,DSC2,PPBP,F2,INHBC,C8A,ADAM12,F10,F7,PROS1,IGF2,C4A,C4B,CFB,VEGFC
UniProt Keywords	KW-0720	Serine protease	16	136	0.98	2.70e-09	F9,PROC,F12,KLKB1,HABP2,PCSK9,F2,HTRA1,F10,F7,MASP2,F11,C1S,CFB,C1R,CFD
UniProt Keywords	KW-0646	Protease inhibitor	15	117	1.01	3.40e-09	SERPIND1,TFPI2,SERPINA10,TIMP2,CSTA,AMBP,ITIH4,SERPING1,A2ML1,A2M,SERPINA11,PAPLN,ITIH2,ITIH3,KNG1
UniProt Keywords	KW-0345	HDL	8	16	1.61	1.10e-08	APOA1,APOE,SAA4,APOL1,APOA4,APOM,SAA2,APOA5
UniProt Keywords	KW-0358	Heparin-binding	13	88	1.08	1.17e-08	SERPIND1,VTN,PF4V1,APOE,THBS1,SERPINA10,PF4,PRG2,FBN1,FN1,ANGPTL3,SOD3,F11
UniProt Keywords	KW-0865	Zymogen	18	210	0.84	1.59e-08	CPB2,F9,MMP2,PROC,F12,KLKB1,F13A1,PCSK9,F2,PAPPA,PAPPA2,F5,ADAM12,F10,F7,PROS1,CFB,CFD
UniProt Keywords	KW-0722	Serine protease inhibitor	12	80	1.08	4.41e-08	SERPIND1,TFPI2,SERPINA10,AMBP,ITIH4,SERPING1,A2ML1,A2M,SERPINA11,PAPLN,ITIH2,ITIH3
UniProt Keywords	KW-0179	Complement alternate pathway	7	12	1.67	5.36e-08	C5,C7,C8A,CFH,C8B,CFB,CFD
UniProt Keywords	KW-0399	Innate immunity	21	329	0.71	7.16e-08	C5,FCN3,SERPING1,CSF1R,FCN2,FGB,C1QB,C7,C8A,CFH,S100A8,C8B,C1QC,MASP2,C1S,C4A,C4B,CFB,C1R,CFD,FGA
UniProt Keywords	KW-0765	Sulfation	10	57	1.15	2.46e-07	SERPIND1,F9,VTN,PCSK9,FGG,FN1,CFH,F5,C4A,C4B
UniProt Keywords	KW-1008	Amyloidosis	8	31	1.32	5.56e-07	APOA1,TTR,APOE,LYZ,GSN,TGFBI,SAA2,FGA
UniProt Keywords	KW-0792	Thrombophilia	6	10	1.69	6.13e-07	SERPIND1,F9,PROC,F2,F5,PROS1

UniProt Keywords	KW-0391	Immunity	25	537	0.58	7.50e-07	C5,FCN3,SERPING1,CSF1R,FCN2,PGLYRP2,FGB,PRG2,C1QB,C7,C8A,CFH,S100A8,C8B,C1QC,MASP2,C1S,C4A,C4B,CFB,C1R,IGKV2D-28,CFD,IGHV3-72,FGA
UniProt Keywords	KW-0034	Amyloid	7	24	1.37	1.92e-06	APOA1,TTR,LYZ,GSN,TGFBI,SAA2,FGA
UniProt Keywords	KW-0272	Extracellular matrix	17	267	0.71	2.05e-06	MMP2,SPARC,APOE,PXDN,THBS1,VWF,FBN1,FBLN1,THBS4,FN1,MFAP5,COL18A1,ADAMTSL4,ECM1,EFEMP1,LTBP1,TGFBI
UniProt Keywords	KW-0280	Fibrinolysis	5	8	1.7	7.24e-06	CPB2,F12,KLKB1,SERPING1,PROS1
UniProt Keywords	KW-1068	Hemolytic uremic syndrome	5	10	1.61	1.60e-05	CFHR5,CFHR1,CFHR3,CFH,CFB
UniProt Keywords	KW-0645	Protease	22	516	0.54	1.84e-05	CPB2,F9,MMP2,PROC,F12,PSMA6,KLKB1,HABP2,PCSK9,F2,PAPPA,PAPPA2,ADAM12,HTRA1,F10,F7,MASP2,F11,C1S,CFB,C1R,CFD
UniProt Keywords	KW-0971	Glycation	5	12	1.53	3.03e-05	APOA1,APOE,SOD3,CFB,HBB
UniProt Keywords	KW-0130	Cell adhesion	20	478	0.53	7.30e-05	MYH9,VTN,THBS1,VWF,ITGA2B,CSTA,CDH11,FERMT3,DSC2,PCDH1,DST,THBS4,FN1,COL18A1,ADAM12,ANGPTL3,CCN5,CD99,TGFBI,IGFALS
UniProt Keywords	KW-0873	Pyrrolidone carboxylic acid	8	67	0.98	7.37e-05	ITGA2B,FGB,C1QB,ANG,APOD,FN1,RNASE4,KNG1
UniProt Keywords	KW-0558	Oxidation	6	32	1.18	0.00012	MPO,APOA1,APOE,FN1,GAPDH,ACTB
UniProt Keywords	KW-0473	Membrane attack complex	4	7	1.66	0.00013	C5,C7,C8A,C8B
UniProt Keywords	KW-0011	Acute phase	5	19	1.33	0.00017	ITIH4,SAA4,F2,FN1,SAA2
UniProt Keywords	KW-0654	Proteoglycan	7	55	1.01	0.00018	PF4V1,AMBP,PRG2,CSPG4,ITIH2,PRG4,ITIH3
UniProt Keywords	KW-0882	Thioester bond	4	8	1.61	0.00018	A2ML1,A2M,C4A,C4B
UniProt Keywords	KW-0340	Growth factor binding	5	20	1.3	0.00019	IGFBP5,IGFBP4,HTRA1,IGFBP3,LTBP1
UniProt Keywords	KW-0420	Kringle	4	16	1.3	0.0015	F12,HABP2,F2,MST1
UniProt Keywords	KW-0445	Lipid transport	8	110	0.77	0.0016	CETP,APOA1,APOE,APOL1,APOA4,APOM,APOA5,APOC4
UniProt Keywords	KW-0497	Mitogen	5	39	1.01	0.0030	CAT,PPBP,THBS4,IGF2,VEGFC
UniProt Keywords	KW-0153	Cholesterol metabolism	6	65	0.87	0.0035	CETP,APOA1,APOE,LCAT,PCSK9,APOL1
UniProt Keywords	KW-0395	Inflammatory response	9	163	0.65	0.0040	C5,ATRN,KLKB1,CSF1R,STAB1,S100A8,C4A,C4B,KNG1
UniProt Keywords	KW-0037	Angiogenesis	8	131	0.69	0.0045	MMP2,ANGPTL6,CSPG4,ANG,FN1,ECM1,ANGPTL3,VEGFC
UniProt Keywords	KW-0162	Chylomicron	3	9	1.43	0.0051	APOE,APOA4,APOA5
UniProt Keywords	KW-0376	Hydrogen peroxide	3	9	1.43	0.0051	MPO,CAT,PXDN
UniProt Keywords	KW-0575	Peroxidase	4	25	1.11	0.0055	MPO,CAT,PXDN,GPX3
UniProt Keywords	KW-0176	Collagen	6	83	0.77	0.0102	FCN3,FCN2,C1QB,COL18A1,C1QC,ADIPOQ
UniProt Keywords	KW-0372	Hormone	6	92	0.72	0.0165	TTR,CRH,INHBC,FBN1,ADIPOQ,IGF2
UniProt Keywords	KW-0339	Growth factor	7	129	0.64	0.0180	PPBP,INHBC,THBS4,IGF1,EFEMP1,IGF2,VEGFC
UniProt Keywords	KW-1018	Complement activation lectin pathway	2	3	1.73	0.0180	FCN3,FCN2
UniProt Keywords	KW-0913	Age-related macular degeneration	3	16	1.18	0.0182	CFH,HTRA1,CFB
UniProt Keywords	KW-0378	Hydrolase	34	1612	0.23	0.0209	CPB2,F9,MMP2,GPLD1,PROC,F12,LYZ,PSMA6,KLKB1,PLA2G7,HABP2,PGLYRP2,APEH,PCSK9,F2,HSPA5,PAPPA,ANG,GANAB,BLM,PAPPA2,ADAM12,HTRA1,SMARCA1,F10,F7,MASP2,F11,C1S,CFB,C1R,RNASE4,CFD,PLCG2
UniProt Keywords	KW-0082	Bait region	2	4	1.61	0.0247	A2ML1,A2M
UniProt Keywords	KW-0797	Tissue remodeling	2	4	1.61	0.0247	CSPG4,THBS4
UniProt Keywords	KW-0393	Immunoglobulin domain	14	463	0.39	0.0257	PXDN,A1BG,PSG9,CSF1R,PSG6,PSG1,PSG11,PSG3,PAPLN,PSG2,IGKV2D-28,TTN,IGHV3-72,SEMA3B
UniProt Keywords	KW-1212	Corneal dystrophy	3	20	1.08	0.0290	LCAT,GSN,TGFBI
UniProt Keywords	KW-1065	Osteogenesis imperfecta	3	23	1.02	0.0408	SPARC,SEC24D,P4HB
Pfam	PF01442	Apolipoprotein A1/A4/E domain	4	4	1.91	0.0034	APOA1,APOE,APOA4,APOA5
InterPro	IPR001881	EGF-like calcium-binding domain	21	124	1.14	5.70e-13	F9,PROC,LRP1,F12,THBS1,NOTCH3,HABP2,MASP1,STAB1,FBN1,FBLN1,OIT3,THBS4,F10,F7,PROS1,EFEMP1,MASP2,C1S,LTBP1,C1R
InterPro	IPR035976	Sushi/SCR/CCP superfamily	15	58	1.32	3.72e-11	CFHR5,MASP1,CFHR1,C7,PAPPA,F13B,CFHR2,CFHR3,CFH,PAPPA2,MASP2,C1S,CFB,HPR,C1R
InterPro	IPR018097	EGF-like calcium-binding, conserved site	17	99	1.14	1.47e-10	F9,PROC,LRP1,NOTCH3,MASP1,FBN1,FBLN1,OIT3,THBS4,F10,F7,PROS1,EFEMP1,MASP2,C1S,LTBP1,C1R
InterPro	IPR000436	Sushi/SCR/CCP domain	14	56	1.3	2.19e-10	CFHR5,MASP1,CFHR1,C7,PAPPA,F13B,CFHR2,CFHR3,CFH,PAPPA2,MASP2,C1S,CFB,C1R
InterPro	IPR000742	EGF-like domain	22	236	0.88	1.47e-09	F9,PROC,LRP1,F12,THBS1,ATRN,NOTCH3,HABP2,MASP1,STAB1,FBN1,FBLN1,OIT3,THBS4,ADAM12,F10,F7,PROS1,EFEMP1,MASP2,LTBP1,C1R
InterPro	IPR009003	Peptidase S1, PA clan	17	123	1.05	1.73e-09	F9,PROC,F12,KLKB1,HABP2,F2,HTRA1,F10,F7,MASP2,F11,C1S,MST1,CFB,HPR,C1R,CFD
InterPro	IPR001314	Peptidase S1A, chymotrypsin family	16	107	1.08	2.34e-09	F9,PROC,F12,KLKB1,HABP2,F2,F10,F7,MASP2,F11,C1S,MST1,CFB,HPR,C1R,CFD
InterPro	IPR001254	Serine proteases, trypsin domain	16	115	1.05	5.49e-09	F9,PROC,F12,KLKB1,HABP2,F2,F10,F7,MASP2,F11,C1S,MST1,CFB,HPR,C1R,CFD
InterPro	IPR033116	Serine proteases, trypsin family, serine active site	14	92	1.09	3.60e-08	F9,PROC,F12,KLKB1,HABP2,F2,F10,F7,MASP2,F11,C1S,CFB,C1R,CFD
InterPro	IPR043504	Peptidase S1, PA clan, chymotrypsin-like fold	15	116	1.02	5.21e-08	F9,PROC,F12,KLKB1,HABP2,F2,F10,F7,MASP2,F11,C1S,MST1,HPR,C1R,CFD
InterPro	IPR000152	EGF-type aspartate/asparagine hydroxylation site	13	98	1.03	6.78e-07	F9,PROC,LRP1,NOTCH3,FBN1,FBLN1,F10,F7,PROS1,EFEMP1,MASP2,C1S,LTBP1
InterPro	IPR000294	Gamma-carboxyglutamic acid-rich (GLA) domain	7	15	1.58	3.29e-06	F9,MGP,PROC,F2,F10,F7,PROS1
InterPro	IPR035972	Gamma-carboxyglutamic acid-rich (GLA) domain superfamily	7	15	1.58	3.29e-06	F9,MGP,PROC,F2,F10,F7,PROS1
InterPro	IPR009030	Growth factor receptor cysteine-rich domain superfamily	13	127	0.92	9.31e-06	IGFBP5,LRP1,NOTCH3,IGFBP4,FBN1,FBLN1,C8A,HTRA1,CCN5,IGFBP3,PROS1,EFEMP1,LTBP1
InterPro	IPR014716	Fibrinogen, alpha/beta/gamma chain, C-terminal globular, subdomain 1	7	26	1.34	5.67e-05	ANGPTL6,FCN3,FCN2,FGB,FGG,ANGPTL3,FGA
InterPro	IPR018114	Serine proteases, trypsin family, histidine active site	11	100	0.95	5.67e-05	F9,PROC,F12,KLKB1,HABP2,F2,F10,F7,F11,CFB,CFD
InterPro	IPR040839	Macroglobulin domain MG4	5	8	1.7	0.00013	C5,A2ML1,A2M,C4A,C4B
InterPro	IPR001599	Alpha-2-macroglobulin	5	9	1.65	0.00017	C5,A2ML1,A2M,C4A,C4B
InterPro	IPR002181	Fibrinogen, alpha/beta/gamma chain, C-terminal globular domain	7	32	1.25	0.00017	ANGPTL6,FCN3,FCN2,FGB,FGG,ANGPTL3,FGA
InterPro	IPR002890	Macroglobulin domain	5	9	1.65	0.00017	C5,A2ML1,A2M,C4A,C4B
InterPro	IPR009048	Alpha-macroglobulin, receptor-binding	5	9	1.65	0.00017	C5,A2ML1,A2M,C4A,C4B
InterPro	IPR011625	Alpha-2-macroglobulin, bait region domain	5	9	1.65	0.00017	C5,A2ML1,A2M,C4A,C4B
InterPro	IPR011626	Alpha-macroglobulin-like, TED domain	5	9	1.65	0.00017	C5,A2ML1,A2M,C4A,C4B
InterPro	IPR020837	Fibrinogen, conserved site	6	19	1.41	0.00017	ANGPTL6,FCN3,FCN2,FGB,FGG,FGA
InterPro	IPR036056	Fibrinogen-like, C-terminal	7	32	1.25	0.00017	ANGPTL6,FCN3,FCN2,FGB,FGG,ANGPTL3,FGA
InterPro	IPR036595	Alpha-macroglobulin, receptor-binding domain superfamily	5	9	1.65	0.00017	C5,A2ML1,A2M,C4A,C4B
InterPro	IPR041555	Macroglobulin domain MG3	5	9	1.65	0.00017	C5,A2ML1,A2M,C4A,C4B
InterPro	IPR000074	Apolipoprotein A/E	4	4	1.91	0.00037	APOA1,APOE,APOA4,APOA5
InterPro	IPR017857	Coagulation factor-like, Gla domain superfamily	5	12	1.53	0.00037	F9,PROC,F10,F7,PROS1
InterPro	IPR013806	Kringle-like fold	6	26	1.27	0.00052	MMP2,F12,HABP2,F2,FN1,MST1
InterPro	IPR012224	Peptidase S1A, coagulation factor VII/IX/X/C/Z	4	5	1.81	0.00061	F9,PROC,F10,F7
InterPro	IPR008930	Terpenoid cyclases/protein prenyltransferase alpha-alpha toroid	5	15	1.43	0.00079	C5,A2ML1,A2M,C4A,C4B
InterPro	IPR000020	Anaphylatoxin/fibulin	4	6	1.73	0.00094	C5,FBLN1,C4A,C4B
InterPro	IPR019742	Alpha-2-macroglobulin, conserved site	4	8	1.61	0.0021	A2ML1,A2M,C4A,C4B

InterPro	IPR047565	Alpha-macroglobulin-like, thiol-ester bond-forming region	4	8	1.61	0.0021	A2ML1,A2M,C4A,C4B
InterPro	IPR000867	Insulin-like growth factor-binding protein, IGFBP	5	20	1.3	0.0023	IGFBP5,IGFBP4,HTRA1,CCN5,IGFBP3
InterPro	IPR001134	Netrin domain	5	23	1.24	0.0040	PCOLCE,C5,TIMP2,C4A,C4B
InterPro	IPR008993	Tissue inhibitor of metalloproteinases-like, OB-fold	5	24	1.23	0.0046	PCOLCE,C5,TIMP2,C4A,C4B
InterPro	IPR036383	Thrombospondin type-1 (TSP1) repeat superfamily	7	64	0.95	0.0047	THBS1,C7,PAPLN,C8A,ADAMTSL4,C8B,CCN5
InterPro	IPR017891	Insulin-like growth factor binding protein, N-terminal, Cys-rich conserved site	4	11	1.47	0.0048	IGFBP5,IGFBP4,CCN5,IGFBP3
InterPro	IPR000884	Thrombospondin type-1 (TSP1) repeat	7	65	0.94	0.0049	THBS1,C7,PAPLN,C8A,ADAMTSL4,C8B,CCN5
InterPro	IPR012290	Fibrinogen, alpha/beta/gamma chain, coiled coil domain	3	3	1.91	0.0060	FGB,FGG,FGA
InterPro	IPR024175	Peptidase S1A, complement C1r/C1S/mannan-binding	3	3	1.91	0.0060	MASP2,C1S,C1R
InterPro	IPR037579	Fibrinogen	3	3	1.91	0.0060	FGB,FGG,FGA
InterPro	IPR026823	Complement Clr-like EGF domain	5	27	1.17	0.0065	LRP1,FBN1,FBLN1,EFEMP1,LTBP1
InterPro	IPR022272	Lipocalin family conserved site	4	13	1.4	0.0072	AMBP,APOD,RBP4,PAEP
InterPro	IPR001840	Anaphylatoxin, complement system domain	3	4	1.78	0.0093	C5,C4A,C4B
InterPro	IPR018081	Anaphylatoxin, complement system	3	4	1.78	0.0093	C5,C4A,C4B
InterPro	IPR000859	CUB domain	6	51	0.98	0.0100	PCOLCE,ATRN,MASP1,MASP2,C1S,C1R
InterPro	IPR000001	Kringle	4	16	1.3	0.0130	F12,HABP2,F2,MST1
InterPro	IPR001862	Membrane attack complex component/perforin/complement C9	3	5	1.69	0.0130	C7,C8A,C8B
InterPro	IPR018056	Kringle, conserved site	4	16	1.3	0.0130	F12,HABP2,F2,MST1
InterPro	IPR035914	Spermadhesin, CUB domain superfamily	6	54	0.95	0.0130	PCOLCE,ATRN,MASP1,MASP2,C1S,C1R
InterPro	IPR038178	Kringle superfamily	4	16	1.3	0.0130	F12,HABP2,F2,MST1
InterPro	IPR018486	Hemopexin, conserved site	4	17	1.28	0.0145	MMP2,VTN,HPX,PRG4
InterPro	IPR018933	Netrin module, non-TIMP type	4	17	1.28	0.0145	PCOLCE,C5,C4A,C4B
InterPro	IPR042185	Serpin superfamily, domain 2	5	35	1.06	0.0154	SERPIND1,SERPINA10,SERPING1,SERPINA7,SERPINA11
InterPro	IPR013106	Immunoglobulin V-set domain	10	182	0.65	0.0164	PSG9,PSG6,PSG1,PSG11,PSG3,PAPLN,PSG2,IGKV2D-28,TTN,IGHV3-72
InterPro	IPR000215	Serpin family	5	36	1.05	0.0168	SERPIND1,SERPINA10,SERPING1,SERPINA7,SERPINA11
InterPro	IPR003609	PAN/Apple domain	3	6	1.61	0.0168	KLKB1,F11,MST1
InterPro	IPR010600	Inter-alpha-trypsin inhibitor heavy chain, C-terminal	3	6	1.61	0.0168	ITIH4,ITIH2,ITIH3
InterPro	IPR013783	Immunoglobulin-like fold	22	732	0.38	0.0168	C5,PXDN,A1BG,F13A1,PSG9,CSF1R,PSG6,A2ML1,PSG1,PSG11,A2M,PSG3,PAPLN,FN1,FLNA,PSG2,C4A,C4B,IGKV2D-28,TTN,IGHV3-72,SEMA3B
InterPro	IPR020863	Membrane attack complex component/perforin domain, conserved site	3	6	1.61	0.0168	C7,C8A,C8B
InterPro	IPR022321	Insulin-like growth factor-binding protein family 1-6, chordata	3	6	1.61	0.0168	IGFBP5,IGFBP4,IGFBP3
InterPro	IPR023796	Serpin domain	5	36	1.05	0.0168	SERPIND1,SERPINA10,SERPING1,SERPINA7,SERPINA11
InterPro	IPR036186	Serpin superfamily	5	36	1.05	0.0168	SERPIND1,SERPINA10,SERPING1,SERPINA7,SERPINA11
InterPro	IPR042178	Serpin superfamily, domain 1	5	36	1.05	0.0168	SERPIND1,SERPINA10,SERPING1,SERPINA7,SERPINA11
InterPro	IPR012674	Calycin	5	38	1.03	0.0183	AMBP,APOD,RBP4,APOM,PAEP
InterPro	IPR000800	Notch domain	3	7	1.54	0.0212	NOTCH3,PAPPA,PAPPA2
InterPro	IPR003598	Immunoglobulin subtype 2	11	239	0.57	0.0277	PXDN,A1BG,PSG9,CSF1R,PSG6,PSG1,PSG11,PSG3,PAPLN,PSG2,TTN
InterPro	IPR036465	Von Willebrand factor A-like domain superfamily	7	97	0.77	0.0295	VWF,ITIH4,SEC24D,ANTXR1,ITIH2,ITIH3,CFB
InterPro	IPR000585	Hemopexin-like domain	4	23	1.15	0.0307	MMP2,VTN,HPX,PRG4
InterPro	IPR018487	Hemopexin-like repeats	4	24	1.13	0.0350	MMP2,VTN,HPX,PRG4
InterPro	IPR036375	Hemopexin-like domain superfamily	4	24	1.13	0.0350	MMP2,VTN,HPX,PRG4
InterPro	IPR013694	VIT domain	3	10	1.38	0.0452	ITIH4,ITIH2,ITIH3
InterPro	IPR002035	Von Willebrand factor, type A	6	76	0.8	0.0480	VWF,ITIH4,ANTXR1,ITIH2,ITIH3,CFB
SMART	SM00032	Domain abundant in complement control proteins	14	56	1.3	6.75e-11	CFHR5,MASP1,CFHR1,C7,PAPPA,F13B,CFHR2,CFHR3,CFH,PAPPA2,MASP2,C1S,CFB,C1R
SMART	SM00179	Calcium-binding EGF-like domain	17	101	1.13	6.75e-11	F9,PROC,LRP1,NOTCH3,MASP1,FBN1,FBLN1,OIT3,THBS4,F10,F7,PROS1,EFEMP1,MASP2,C1S,LTBP1,C1R
SMART	SM00020	Trypsin-like serine protease	17	118	1.07	2.16e-10	F9,PROC,F12,KLKB1,HABP2,MASP1,F2,F10,F7,MASP2,F11,C1S,MST1,CFB,HPR,C1R,CFD
SMART	SM00181	Epidermal growth factor-like domain.	17	209	0.82	5.95e-07	F9,PROC,LRP1,F12,THBS1,ATRN,NOTCH3,HABP2,STAB1,FBN1,FBLN1,OIT3,THBS4,F10,F7,PROS1,LTBP1
SMART	SM00069	Domain containing Gla (gamma-carboxyglutamate) residues.	7	15	1.58	9.02e-07	F9,MGP,PROC,F2,F10,F7,PROS1
SMART	SM00186	Fibrinogen-related domains (FReDs)	7	29	1.29	3.18e-05	ANGPTL6,FCN3,FCN2,FGB,FGG,ANGPTL3,FGA
SMART	SM01359	Alpha-2-Macroglobulin	5	9	1.65	5.65e-05	C5,A2ML1,A2M,C4A,C4B
SMART	SM01360	Alpha-2-macroglobulin family	5	9	1.65	5.65e-05	C5,A2ML1,A2M,C4A,C4B
SMART	SM01361	A-macroglobulin receptor	5	9	1.65	5.65e-05	C5,A2ML1,A2M,C4A,C4B
SMART	SM00104	Anaphylatoxin homologous domain	4	6	1.73	0.00036	C5,FBLN1,C4A,C4B
SMART	SM00121	Insulin growth factor-binding protein homologues	5	17	1.38	0.00044	IGFBP5,IGFBP4,HTRA1,CCN5,IGFBP3
SMART	SM01419	Alpha-macro-globulin thiol-ester bond-forming region	4	8	1.61	0.00069	A2ML1,A2M,C4A,C4B
SMART	SM00209	Thrombospondin type 1 repeats	7	64	0.95	0.0016	THBS1,C7,PAPLN,C8A,ADAMTSL4,C8B,CCN5
SMART	SM01212	Fibrinogen alpha/beta chain family	3	3	1.91	0.0021	FGB,FGG,FGA
SMART	SM00042	Domain first found in C1r, C1s, uEGF, and bone morphogenetic protein.	6	50	0.99	0.0034	PCOLCE,ATRN,MASP1,MASP2,C1S,C1R
SMART	SM00643	Netrin C-terminal Domain	4	15	1.33	0.0038	PCOLCE,C5,C4A,C4B
SMART	SM00130	Kringle domain	4	16	1.3	0.0044	F12,HABP2,F2,MST1
SMART	SM00408	Immunoglobulin C-2 Type	11	212	0.62	0.0046	PXDN,A1BG,PSG9,CSF1R,PSG6,PSG1,PSG11,PSG3,PAPLN,PSG2,TTN
SMART	SM00093	SERine Proteinase INhibitors	5	35	1.06	0.0053	SERPIND1,SERPINA10,SERPING1,SERPINA7,SERPINA11
SMART	SM00004	Domain found in Notch and Lin-12	3	7	1.54	0.0084	NOTCH3,PAPPA,PAPPA2
SMART	SM00609	Vault protein Inter-alpha-Trypsin domain	3	7	1.54	0.0084	ITIH4,ITIH2,ITIH3
SMART	SM00120	Hemopexin-like repeats.	4	23	1.15	0.0115	MMP2,VTN,HPX,PRG4
SMART	SM00327	Von Willebrand factor (vWF) type A domain	6	77	0.8	0.0194	VWF,ITIH4,ANTXR1,ITIH2,ITIH3,CFB
SMART	SM00457	Membrane-attack complex / perforin	3	12	1.3	0.0253	C7,C8A,C8B
SMART	SM00223	APPLE domain	2	2	1.91	0.0289	KLKB1,F11
SMART	SM00059	Fibronectin type 2 domain	3	14	1.24	0.0342	MMP2,F12,FN1
SMART	SM00214	Von Willebrand factor (vWF) type C domain	4	37	0.94	0.0472	PXDN,THBS1,VWF,CCN5

UC vs AC+NCFET T1

#category	term ID	term description	observed gene count	background gene count	strength	false discovery rate	matching proteins in your network (labels)

GO Process	GO:0006959	Humoral immune response	53	268	1.17	2.62e-38	ST6GAL1,EBI3,C5,PF4V1,LTF,HRG,C4BPB,CFP,CFHR5,LYZ,C6,FCN3,SERPING1,FCN2,PPBP,PF4,MASP1,CAMP,FGB,F2,C1QB,CFHR1,C7,ANG,C8A,C4BPA,CFHR2,CFHR4,CFHR3,CFH,S100A7,S100A9,C8B,C8G,MBL2,C1QC,CFI,GAPDH,MASP2,C1S,C4A,COLEC11,C4B,CFB,RARRES2,JCHAIN,C1R,CFD,IGHV3-15,IGHV3-72,KNG1,B2M,FGA
GO Process	GO:0006956	Complement activation	33	60	1.61	1.06e-34	C5,C4BPB,CFP,CFHR5,C6,FCN3,SERPING1,FCN2,MASP1,C1QB,CFHR1,C7,C8A,C4BPA,CFHR2,CFHR4,CFHR3,CFH,C8B,C8G,MBL2,C1QC,CFI,MASP2,C1S,C4A,COLEC11,C4B,CFB,C1R,CFD,IGHV3-15,IGHV3-72
GO Process	GO:0006950	Response to stress	134	3358	0.47	3.22e-31	CPB2,APOH,VCL,SERPIND1,CHGA,LBP,F9,MMP2,COMP,C5,MPO,PF4V1,LTF,HRG,PROC,PLEK,CAT,LRP1,C4BPB,CFP,APOE,PXDN,F12,APCS,CFHR5,LYZ,VWF,SERPINA10,LGALS3BP,ATRN,C6,YWHAE,DGKG,AMBP,TCIRG1,HYAL1,ITIH4,FCN3,AHSG,CRH,SAA4,SERPING1,CSF1R,FCN2,PGLYRP2,PPBP,PF4,MASP1,CAMP,HSP90B1,LRG1,PCSK9,SHLD1,FGB,DST,F2,PLG,C1QB,CFHR1,APOL1,SUMO4,CALR,SERPINF2,C7,HBA1,HSPA5,P4HB,FBLN1,ANG,THBS4,PRDX6,PROZ,APOD,BLM,APOA4,F8,C8A,C4BPA,F13B,CFHR2,CFH,F5,CD5L,S100A7,S100A9,FLNA,MCOLN2,C8B,C8G,ADAMTS13,GSN,MBL2,C1QC,F10,TAF9,SOD3,GPX3,PROS1,CFI,TNRC6A,ILK,GAPDH,RNASE1,CST3,MASP2,SAA1,F11,C1S,ADIPOQ,ORM2,C4A,CDK3,COLEC11,MST1,C4B,CFB,RARRES2,HGFAC,UIMC1,SELENOP,JCHAIN,HPR,C1R,SERPINA3,RNASE4,CFD,IGHV3-15,IGHV3-72,VEGFC,HYOU1,KNG1,B2M,FGA,PDIA4
GO Process	GO:0006952	Defense response	80	1394	0.63	5.30e-25	CHGA,LBP,C5,MPO,PF4V1,LTF,HRG,LRP1,C4BPB,CFP,F12,APCS,CFHR5,LYZ,LGALS3BP,ATRN,C6,TCIRG1,HYAL1,ITIH4,FCN3,AHSG,CRH,SAA4,SERPING1,CSF1R,FCN2,PGLYRP2,PPBP,PF4,MASP1,CAMP,FGB,F2,C1QB,CFHR1,APOL1,SERPINF2,C7,ANG,APOA4,F8,C8A,C4BPA,CFHR2,CFH,CD5L,S100A7,S100A9,MCOLN2,C8B,C8G,ADAMTS13,GSN,MBL2,C1QC,CFI,GAPDH,RNASE1,CST3,MASP2,SAA1,C1S,ORM2,C4A,COLEC11,C4B,CFB,RARRES2,JCHAIN,HPR,C1R,SERPINA3,RNASE4,CFD,IGHV3-15,IGHV3-72,KNG1,B2M,FGA
GO Process	GO:0007596	Blood coagulation	35	173	1.18	6.32e-25	CPB2,APOH,VCL,SERPIND1,F9,COMP,PF4V1,HRG,PROC,PLEK,C4BPB,F12,VWF,SERPINA10,DGKG,SERPING1,PF4,FGB,F2,PLG,FBLN1,PROZ,F8,F13B,F5,FLNA,ADAMTS13,F10,PROS1,ILK,SAA1,F11,HGFAC,KNG1,FGA
GO Process	GO:0098542	Defense response to other organism	63	989	0.67	3.48e-21	CHGA,LBP,C5,MPO,PF4V1,LTF,HRG,C4BPB,CFP,F12,APCS,CFHR5,LYZ,C6,FCN3,SERPING1,CSF1R,FCN2,PGLYRP2,PPBP,PF4,MASP1,CAMP,FGB,F2,C1QB,CFHR1,APOL1,C7,ANG,APOA4,C8A,C4BPA,CFHR2,CFH,S100A7,S100A9,MCOLN2,C8B,C8G,ADAMTS13,GSN,MBL2,C1QC,CFI,GAPDH,RNASE1,MASP2,C1S,C4A,COLEC11,C4B,CFB,RARRES2,JCHAIN,C1R,RNASE4,CFD,IGHV3-15,IGHV3-72,KNG1,B2M,FGA
GO Process	GO:0006955	Immune response	72	1321	0.61	5.03e-21	ST6GAL1,CHGA,LBP,EBI3,C5,VTN,PF4V1,LTF,HRG,LRP1,C4BPB,CFP,PXDN,F12,APCS,CFHR5,LYZ,C6,TCIRG1,FCN3,SERPING1,CSF1R,FCN2,PGLYRP2,PPBP,PF4,MASP1,CAMP,SHLD1,FGB,F2,C1QB,CFHR1,APOL1,C7,ANG,PIGR,APOA4,C8A,C4BPA,CFHR2,CFHR4,CFHR3,CFH,S100A7,S100A9,MCOLN2,C8B,C8G,ADAMTS13,GSN,MBL2,C1QC,CFI,GAPDH,FGL1,MASP2,C1S,C4A,COLEC11,C4B,CFB,RARRES2,JCHAIN,C1R,IGKV2D-28,CFD,IGHV3-15,IGHV3-72,KNG1,B2M,FGA
GO Process	GO:0002252	Immune effector process	41	375	0.91	1.16e-20	CHGA,LBP,C5,MPO,LRP1,C4BPB,CFP,CFHR5,C6,TCIRG1,FCN3,SERPING1,FCN2,MASP1,SHLD1,F2,C1QB,CFHR1,C7,C8A,C4BPA,CFHR2,CFHR4,CFHR3,CFH,C8B,C8G,MBL2,C1QC,CFI,MASP2,C1S,C4A,COLEC11,C4B,CFB,C1R,CFD,IGHV3-15,IGHV3-72,B2M
GO Process	GO:0006958	Complement activation, classical pathway	20	40	1.57	5.62e-20	C5,C4BPB,C6,SERPING1,C1QB,C7,C8A,C4BPA,C8B,C8G,MBL2,C1QC,CFI,MASP2,C1S,C4A,C4B,C1R,IGHV3-15,IGHV3-72
GO Process	GO:0051707	Response to other organism	69	1328	0.59	5.28e-19	CHGA,LBP,C5,MPO,PF4V1,LTF,HRG,APOB,C4BPB,CFP,F12,APCS,CFHR5,LYZ,C6,HYAL1,FCN3,SERPING1,CSF1R,FCN2,PGLYRP2,PPBP,PF4,MASP1,CAMP,LRG1,FGB,F2,C1QB,CFHR1,APOL1,CALR,C7,ITLN1,ANG,APOA4,C8A,C4BPA,CFHR2,CFH,S100A7,S100A9,MCOLN2,C8B,C8G,ADAMTS13,GSN,MBL2,C1QC,CFI,GAPDH,RNASE1,MASP2,C1S,ADIPOQ,C4A,COLEC11,C4B,CFB,RARRES2,JCHAIN,C1R,RNASE4,CFD,IGHV3-15,IGHV3-72,KNG1,B2M,FGA
GO Process	GO:0009607	Response to biotic stimulus	70	1375	0.58	6.35e-19	CHGA,LBP,C5,MPO,PF4V1,LTF,HRG,APOB,C4BPB,CFP,F12,APCS,CFHR5,LYZ,C6,HYAL1,FCN3,SERPING1,CSF1R,FCN2,PGLYRP2,PPBP,PF4,MASP1,CAMP,LRG1,FGB,F2,C1QB,CFHR1,APOL1,CALR,C7,ITLN1,HSPA5,ANG,APOA4,C8A,C4BPA,CFHR2,CFH,S100A7,S100A9,MCOLN2,C8B,C8G,ADAMTS13,GSN,MBL2,C1QC,CFI,GAPDH,RNASE1,MASP2,C1S,ADIPOQ,C4A,COLEC11,C4B,CFB,RARRES2,JCHAIN,C1R,RNASE4,CFD,IGHV3-15,IGHV3-72,KNG1,B2M,FGA
GO Process	GO:0010466	Negative regulation of peptidase activity	33	249	0.99	1.08e-18	SPP2,SERPIND1,C5,VTN,LTF,HRG,LRP1,SERPINF1,SERPINA10,LCN1,CSTA,AMBP,ITIH4,ITIH1,AHSG,SERPING1,SERPINI1,A2ML1,LPA,SERPINF2,SERPINA7,SERPINA5,SERPINA11,PAPLN,ITIH2,PROS1,GAPDH,CST3,C4A,ITIH3,C4B,SERPINA3,KNG1
GO Process	GO:0042060	Wound healing	37	336	0.91	1.08e-18	CPB2,APOH,VCL,SERPIND1,F9,COMP,PF4V1,HRG,PROC,PLEK,C4BPB,F12,VWF,SERPINA10,DGKG,SERPING1,PF4,LRG1,FGB,DST,F2,PLG,FBLN1,PROZ,F8,F13B,F5,FLNA,ADAMTS13,F10,PROS1,ILK,SAA1,F11,HGFAC,KNG1,FGA
GO Process	GO:0002253	Activation of immune response	34	271	0.97	1.13e-18	C5,GPLD1,C4BPB,CFP,CFHR5,C6,FCN3,SERPING1,FCN2,MASP1,C1QB,CFHR1,C7,C8A,C4BPA,CFHR2,CFHR4,CFHR3,CFH,C8B,C8G,MBL2,C1QC,CFI,MASP2,C1S,C4A,COLEC11,C4B,CFB,C1R,CFD,IGHV3-15,IGHV3-72
GO Process	GO:0050878	Regulation of body fluid levels	38	371	0.88	2.50e-18	CPB2,APOH,VCL,SERPIND1,F9,COMP,VTN,PF4V1,HRG,PROC,PLEK,C4BPB,APOE,F12,VWF,SERPINA10,DGKG,SERPING1,PF4,FGB,F2,PLG,SERPINF2,FBLN1,PROZ,F8,F13B,F5,FLNA,ADAMTS13,F10,PROS1,ILK,SAA1,F11,HGFAC,KNG1,FGA
GO Process	GO:0010951	Negative regulation of endopeptidase activity	32	240	0.99	3.25e-18	SPP2,SERPIND1,C5,VTN,LTF,HRG,SERPINF1,SERPINA10,LCN1,CSTA,AMBP,ITIH4,ITIH1,AHSG,SERPING1,SERPINI1,A2ML1,LPA,SERPINF2,SERPINA7,SERPINA5,SERPINA11,PAPLN,ITIH2,PROS1,GAPDH,CST3,C4A,ITIH3,C4B,SERPINA3,KNG1
GO Process	GO:0050896	Response to stimulus	182	7835	0.24	4.91e-18	ST6GAL1,CPB2,APOH,VCL,SERPIND1,CHGA,LBP,F9,MMP2,EBI3,COMP,PON1,C5,MPO,VTN,PF4V1,APOC3,GPLD1,LTF,HRG,APOB,IGFBP5,PROC,PLEK,APOA1,TTR,CAT,LRP1,C4BPB,CFP,APOE,PXDN,F12,SERPINF1,APCS,CFHR5,LYZ,VWF,SERPINA10,LGALS3BP,ATRN,C6,LCN1,LCAT,YWHAE,DGKG,CDH6,AMBP,PON3,TCIRG1,HYAL1,ITIH4,FCN3,AHSG,IGFBP1,CRH,SAA4,SERPING1,CSF1R,FCN2,PGLYRP2,SCGB3A1,IHH,PPBP,PF4,MASP1,CAMP,GUCY1A1,HSP90B1,LRG1,PCSK9,BMP1,SHLD1,FGB,DST,F2,INHBC,PLG,CSPG4,C1QB,CFHR1,APOL1,SUMO4,CALR,SERPINF2,C7,HBA1,ITLN1,HSPA5,FBN1,P4HB,FBLN1,ANG,LIMS1,THBS4,PRDX6,PROZ,APOD,ASGR2,BLM,AKAP9,PIGR,APOA4,F8,C8A,C4BPA,F13B,CFHR2,CFHR4,CFHR3,CFH,F5,CD5L,S100A7,S100A9,FLNA,MCOLN2,C8B,C8G,ADAMTS13,GSN,MBL2,SVEP1,C1QC,F10,APOM,CA6,CALML5,TAF9,IGFBP3,SOD3,GPX3,PROS1,EFEMP1,CFI,TNRC6A,ILK,GAPDH,RNASE1,FGL1,CST3,MASP2,PSG4,SAA1,F11,C1S,NUCB1,KSR2,ADIPOQ,IGF2,ORM2,C4A,CDK3,COLEC11,MST1,SPARCL1,C4B,TGFBI,CFB,IGFALS,RARRES2,HGFAC,UIMC1,SELENOP,JCHAIN,HPR,C1R,ATP1A1,SERPINA3,RNASE4,IGKV2D-28,CFD,IGHV3-15,IGHV3-72,VEGFC,HYOU1,KNG1,CDH5,B2M,FGA,PDIA4,SDF4

GO Process	GO:0045087	Innate immune response	51	754	0.7	5.81e-18	CHGA,LBP,C5,LTF,C4BPB,CFP,F12,APCS,CFHR5,C6,FCN3,SERPING1,CSF1R,FCN2,PGLYRP2,MASP1,CAMP,FGB,C1QB,APOL1,C7,ANG,APOA4,C8A,C4BPA,CFH,S100A7,S100A9,MCOLN2,C8B,C8G,ADAMTS13,GSN,MBL2,C1QC,CFI,GAPDH,MASP2,C1S,C4A,COLEC11,C4B,CFB,RARRES2,JCHAIN,C1R,CFD,IGHV3-15,IGHV3-72,B2M,FGA
GO Process	GO:0044419	Biological process involved in interspecies interaction between organisms	71	1490	0.55	6.99e-18	CHGA,LBP,C5,MPO,PF4V1,LTF,HRG,APOB,C4BPB,CFP,APOE,F12,APCS,CFHR5,LYZ,C6,HYAL1,FCN3,SERPING1,CSF1R,FCN2,PGLYRP2,PPBP,PF4,MASP1,CAMP,LRG1,FGB,F2,PLG,C1QB,CFHR1,APOL1,CALR,C7,ITLN1,ANG,APOA4,C8A,C4BPA,CFHR2,CFH,S100A7,S100A9,MCOLN2,C8B,C8G,ADAMTS13,GSN,MBL2,C1QC,CFI,GAPDH,RNASE1,MASP2,C1S,ADIPOQ,C4A,COLEC11,C4B,CFB,RARRES2,JCHAIN,C1R,RNASE4,CFD,IGHV3-15,IGHV3-72,KNG1,B2M,FGA
GO Process	GO:0045861	Negative regulation of proteolysis	36	339	0.9	8.02e-18	SPP2,CPB2,SERPIND1,C5,VTN,LTF,HRG,LRP1,SERPINF1,SERPINA10,LCN1,CSTA,AMBP,ITIH4,ITIH1,AHSG,SERPING1,SERPINI1,A2ML1,F2,LPA,SERPINF2,SERPINA7,SERPINA5,SERPINA11,PAPLN,ITIH2,TAF9,PROS1,GAPDH,CST3,C4A,ITIH3,C4B,SERPINA3,KNG1
GO Process	GO:0009611	Response to wounding	40	444	0.82	1.13e-17	CPB2,APOH,VCL,SERPIND1,F9,MMP2,COMP,PF4V1,HRG,PROC,PLEK,C4BPB,F12,VWF,SERPINA10,DGKG,SERPING1,PF4,LRG1,FGB,DST,F2,PLG,FBLN1,PROZ,APOD,APOA4,F8,F13B,F5,FLNA,ADAMTS13,F10,PROS1,ILK,SAA1,F11,HGFAC,KNG1,FGA
GO Process	GO:0051346	Negative regulation of hydrolase activity	36	354	0.88	2.74e-17	SPP2,SERPIND1,C5,VTN,APOC3,LTF,HRG,APOA1,LRP1,SERPINF1,APCS,SERPINA10,LCN1,CSTA,AMBP,ITIH4,ITIH1,AHSG,SERPING1,SERPINI1,A2ML1,LPA,SERPINF2,SERPINA7,SERPINA5,SERPINA11,PAPLN,ITIH2,PROS1,GAPDH,CST3,C4A,ITIH3,C4B,SERPINA3,KNG1
GO Process	GO:0016064	Immunoglobulin mediated immune response	22	95	1.23	1.48e-16	C5,C4BPB,C6,TCIRG1,SERPING1,SHLD1,C1QB,C7,C8A,C4BPA,C8B,C8G,MBL2,C1QC,CFI,MASP2,C1S,C4A,C4B,C1R,IGHV3-15,IGHV3-72
GO Process	GO:0052547	Regulation of peptidase activity	38	446	0.8	5.55e-16	SPP2,SERPIND1,PCOLCE,C5,VTN,LTF,HRG,LRP1,SERPINF1,SERPINA10,LCN1,CSTA,AMBP,ITIH4,ITIH1,AHSG,SERPING1,SERPINI1,A2ML1,ANTXR1,LPA,SERPINF2,SERPINA7,FBLN1,SERPINA5,SERPINA11,PAPLN,ITIH2,S100A9,GSN,PROS1,GAPDH,CST3,C4A,ITIH3,C4B,SERPINA3,KNG1
GO Process	GO:0002376	Immune system process	81	2121	0.45	2.23e-15	ST6GAL1,CHGA,LBP,EBI3,C5,MPO,VTN,PF4V1,GPLD1,LTF,HRG,PLEK,LRP1,C4BPB,CFP,PXDN,F12,APCS,CFHR5,LYZ,C6,TCIRG1,FCN3,SERPING1,CSF1R,FCN2,PGLYRP2,PPBP,PF4,MASP1,CAMP,SHLD1,FGB,F2,PLG,C1QB,CFHR1,APOL1,CALR,C7,ANG,PIGR,APOA4,C8A,C4BPA,CFHR2,CFHR4,CFHR3,CFH,CD5L,S100A7,S100A9,FLNA,MCOLN2,C8B,C8G,ADAMTS13,GSN,MBL2,C1QC,CD99,CFI,GAPDH,FGL1,MASP2,SAA1,C1S,C4A,COLEC11,C4B,CFB,RARRES2,JCHAIN,C1R,IGKV2D-28,CFD,IGHV3-15,IGHV3-72,KNG1,B2M,FGA
GO Process	GO:1900047	Negative regulation of hemostasis	17	47	1.43	3.13e-15	CPB2,APOH,COMP,VTN,HRG,PROC,APOE,F12,SERPING1,FGB,F2,PLG,SERPINF2,PROS1,F11,KNG1,FGA
GO Process	GO:0002682	Regulation of immune system process	64	1438	0.52	1.73e-14	LBP,EBI3,C5,GPLD1,LTF,HRG,APOA1,C4BPB,CFP,APOE,APCS,CFHR5,C6,AMBP,FCN3,SERPING1,CSF1R,FCN2,PGLYRP2,IHH,PF4,MASP1,SHLD1,C1QB,CFHR1,CALR,C7,FBN1,THBS4,APOD,PIGR,C8A,C4BPA,CFHR2,CFHR4,CFHR3,CFH,CD5L,S100A7,C8B,C8G,MBL2,C1QC,CD99,CFI,FGL1,MASP2,PSG4,C1S,ADIPOQ,IGF2,ORM2,C4A,COLEC11,MST1,C4B,CFB,RARRES2,C1R,CFD,IGHV3-15,IGHV3-72,VEGFC,B2M
GO Process	GO:0050778	Positive regulation of immune response	38	502	0.75	1.93e-14	LBP,C5,GPLD1,HRG,C4BPB,CFP,CFHR5,C6,FCN3,SERPING1,FCN2,MASP1,SHLD1,C1QB,CFHR1,C7,C8A,C4BPA,CFHR2,CFHR4,CFHR3,CFH,C8B,C8G,MBL2,C1QC,CFI,MASP2,C1S,C4A,COLEC11,C4B,CFB,C1R,CFD,IGHV3-15,IGHV3-72,B2M
GO Process	GO:0048584	Positive regulation of response to stimulus	79	2131	0.44	3.13e-14	CPB2,APOH,CHGA,LBP,C5,VTN,GPLD1,LTF,HRG,IGFBP5,APOA1,CAT,C4BPB,CFP,APOE,F12,CFHR5,VWF,C6,NID1,FCN3,SERPING1,CSF1R,FCN2,IHH,MASP1,GUCY1A1,LRG1,SHLD1,FGB,F2,INHBC,PLG,CSPG4,C1QB,CFHR1,CALR,SERPINF2,C7,FBN1,LIMS1,THBS4,C8A,C4BPA,CFHR2,CFHR4,CFHR3,CFH,S100A7,S100A9,FLNA,C8B,C8G,MBL2,C1QC,F10,TAF9,IGFBP3,CFI,ILK,GAPDH,MASP2,C1S,ADIPOQ,IGF2,C4A,COLEC11,C4B,CFB,RARRES2,UIMC1,C1R,CFD,IGHV3-15,IGHV3-72,VEGFC,CDH5,B2M,FGA
GO Process	GO:0002684	Positive regulation of immune system process	49	874	0.62	3.36e-14	LBP,EBI3,C5,GPLD1,HRG,C4BPB,CFP,CFHR5,C6,FCN3,SERPING1,CSF1R,FCN2,IHH,PF4,MASP1,SHLD1,C1QB,CFHR1,CALR,C7,THBS4,C8A,C4BPA,CFHR2,CFHR4,CFHR3,CFH,S100A7,C8B,C8G,MBL2,C1QC,CD99,CFI,MASP2,C1S,IGF2,C4A,COLEC11,C4B,CFB,RARRES2,C1R,CFD,IGHV3-15,IGHV3-72,VEGFC,B2M
GO Process	GO:0030162	Regulation of proteolysis	45	739	0.65	3.99e-14	SPP2,CPB2,SERPIND1,PCOLCE,C5,VTN,GPLD1,LTF,HRG,LRP1,APOE,F12,SERPINF1,SERPINA10,LCN1,CSTA,AMBP,ITIH4,ITIH1,AHSG,SERPING1,SERPINI1,A2ML1,ANTXR1,SH3D19,F2,LPA,SERPINF2,SERPINA7,FBLN1,SERPINA5,SERPINA11,PAPLN,ITIH2,S100A9,GSN,TAF9,PROS1,GAPDH,CST3,C4A,ITIH3,C4B,SERPINA3,KNG1
GO Process	GO:0030195	Negative regulation of blood coagulation	16	46	1.41	4.20e-14	CPB2,APOH,VTN,HRG,PROC,APOE,F12,SERPING1,FGB,F2,PLG,SERPINF2,PROS1,F11,KNG1,FGA
GO Process	GO:0002460	Adaptive immune response based on somatic recombination of immune receptors built from immunoglobulin superfamily domains	24	169	1.02	4.99e-14	EBI3,C5,C4BPB,C6,TCIRG1,SERPING1,SHLD1,C1QB,C7,C8A,C4BPA,C8B,C8G,MBL2,C1QC,CFI,MASP2,C1S,C4A,C4B,C1R,IGHV3-15,IGHV3-72,B2M
GO Process	GO:0009605	Response to external stimulus	83	2355	0.42	5.99e-14	SERPIND1,CHGA,LBP,MMP2,PON1,C5,MPO,PF4V1,LTF,HRG,APOB,APOA1,CAT,C4BPB,CFP,APOE,F12,APCS,CFHR5,LYZ,C6,HYAL1,FCN3,SERPING1,CSF1R,FCN2,PGLYRP2,IHH,PPBP,PF4,MASP1,CAMP,LRG1,PCSK9,FGB,F2,C1QB,CFHR1,APOL1,CALR,C7,ITLN1,HSPA5,FBN1,ANG,APOA4,C8A,C4BPA,CFHR2,CFH,F5,S100A7,S100A9,MCOLN2,C8B,C8G,ADAMTS13,GSN,MBL2,C1QC,CFI,TNRC6A,GAPDH,RNASE1,MASP2,SAA1,C1S,ADIPOQ,C4A,COLEC11,C4B,CFB,RARRES2,JCHAIN,C1R,RNASE4,CFD,IGHV3-15,IGHV3-72,VEGFC,KNG1,B2M,FGA
GO Process	GO:0052548	Regulation of endopeptidase activity	34	414	0.78	8.70e-14	SPP2,SERPIND1,C5,VTN,LTF,HRG,SERPINF1,SERPINA10,LCN1,CSTA,AMBP,ITIH4,ITIH1,AHSG,SERPING1,SERPINI1,A2ML1,LPA,SERPINF2,SERPINA7,SERPINA5,SERPINA11,PAPLN,ITIH2,S100A9,GSN,PROS1,GAPDH,CST3,C4A,ITIH3,C4B,SERPINA3,KNG1
GO Process	GO:0048518	Positive regulation of biological process	149	6207	0.25	9.04e-14	ST6GAL1,CPB2,CETP,APOH,CHGA,LBP,MMP2,EBI3,COMP,PON1,PCOLCE,C5,VTN,GPLD1,SPARC,LTF,HRG,APOB,IGFBP5,PROC,PLEK,APOA1,CAT,LRP1,C4BPB,CFP,DHX29,APOE,F12,SERPINF1,CFHR5,VWF,C6,NID1,YWHAE,CENPE,TCIRG1,HYAL1,LUM,FCN3,AHSG,ANKRD31,IGFBP1,CRH,SERPING1,CSF1R,FCN2,SCGB3A1,IHH,SERPINI1,PPBP,PF4,MASP1,CAMP,GUCY1A1,ANTXR1,LRG1,SH3D19,PCSK9,BMP1,SHLD1,FGB,F2,INHBC,PLG,EFEMP2,CSPG4,C1QB,CFHR1,SUZ12,CALR,SERPINF2,C7,HBA1,ITLN1,HSPA5,FBN1,P4HB,FBLN1,ANG,LIMS1,THBS4,PRDX6,BLM,AKAP9,APOA4,MTCL1,C8A,C4BPA,CFHR2,CFHR4,CFHR3,CFH,CD5L,S100A7,S100A9,ADAMTSL4,FLNA,MCOLN2,SMARCA1,C8B,C8G,GSN,MBL2,C1QC,F10,TAF9,IGFBP3,CD99,CFI,TNRC6A,ILK,GAPDH,MASP2,FBLN2,SAA1,F11,C1S,KSR2,ADIPOQ,IGF2,ORM2,C4A,SLAIN1,ABCA13,COLEC11,MST1,LMOD3,C4B,CFB,PLTP,PAEP,RARRES2,UIMC1,JCHAIN,HPR,C1R,APOA5,ATP1A1,APOC4,CFD,IGHV3-15,IGHV3-72,VEGFC,KNG1,GRHL2,CDH5,B2M,FGA

GO Process	GO:0048583	Regulation of response to stimulus	112	3931	0.32	1.08e-13	ST6GAL1,CPB2,APOH,CHGA,LBP,C5,VTN,APOC3,GPLD1,LTF,HRG,IGFBP5,PROC,PLEK,APOA1,CAT,LRP1,C4BPB,CFP,APOE,PXDN,F12,SERPINF1,APCS,CFHR5,VWF,PSMA6,C6,NID1,DGKG,AMBP,FCN3,AHSG,IGFBP1,CRH,SERPING1,CSF1R,FCN2,PGLYRP2,IHH,PF4,MASP1,GUCY1A1,LRG1,PCSK9,SHLD1,FGB,F2,INHBC,PLG,CSPG4,C1QB,CFHR1,SUMO4,CALR,SERPINF2,C7,HSPA5,FBN1,P4HB,FBLN1,LIMS1,THBS4,APOD,AKAP9,PIGR,C8A,C4BPA,CFHR2,CFHR4,CFHR3,CFH,CD5L,S100A7,S100A9,FLNA,C8B,C8G,MBL2,C1QC,F10,TAF9,IGFBP3,PROS1,CFI,ILK,GAPDH,FGL1,MASP2,SAA1,F11,C1S,ADIPOQ,IGF2,C4A,CDK3,COLEC11,MST1,C4B,CFB,RARRES2,UIMC1,C1R,CFD,IGHV3-15,IGHV3-72,VEGFC,HYOU1,KNG1,CDH5,B2M,FGA
GO Process	GO:0002449	Lymphocyte mediated immunity	23	159	1.03	1.33e-13	C5,C4BPB,C6,TCIRG1,SERPING1,SHLD1,C1QB,C7,C8A,C4BPA,C8B,C8G,MBL2,C1QC,CFI,MASP2,C1S,C4A,C4B,C1R,IGHV3-15,IGHV3-72,B2M
GO Process	GO:0072378	Blood coagulation, fibrin clot formation	13	24	1.6	2.90e-13	APOH,F9,F12,FGB,F2,FBLN1,F8,F13B,F5,FLNA,F10,F11,FGA
GO Process	GO:0002443	Leukocyte mediated immunity	25	211	0.94	4.31e-13	CHGA,C5,C4BPB,C6,TCIRG1,SERPING1,SHLD1,F2,C1QB,C7,C8A,C4BPA,C8B,C8G,MBL2,C1QC,CFI,MASP2,C1S,C4A,C4B,C1R,IGHV3-15,IGHV3-72,B2M
GO Process	GO:0061045	Negative regulation of wound healing	17	69	1.26	4.60e-13	CPB2,APOH,VTN,HRG,PROC,APOE,F12,APCS,SERPING1,FGB,F2,PLG,SERPINF2,PROS1,F11,KNG1,FGA
GO Process	GO:1903027	Regulation of opsonization	12	18	1.69	5.57e-13	C4BPB,CFP,FCN3,FCN2,MASP1,C4BPA,MBL2,MASP2,C4A,COLEC11,C4B,CFB
GO Process	GO:0002250	Adaptive immune response	30	359	0.79	2.87e-12	EBI3,C5,C4BPB,C6,TCIRG1,SERPING1,SHLD1,FGB,C1QB,C7,C8A,C4BPA,MCOLN2,C8B,C8G,MBL2,C1QC,CFI,FGL1,MASP2,C1S,C4A,C4B,JCHAIN,C1R,IGKV2D-28,IGHV3-15,IGHV3-72,B2M,FGA
GO Process	GO:0050776	Regulation of immune response	45	844	0.6	2.87e-12	LBP,C5,GPLD1,LTF,HRG,C4BPB,CFP,APOE,CFHR5,C6,AMBP,FCN3,SERPING1,FCN2,PGLYRP2,MASP1,SHLD1,C1QB,CFHR1,C7,PIGR,C8A,C4BPA,CFHR2,CFHR4,CFHR3,CFH,CD5L,C8B,C8G,MBL2,C1QC,CFI,FGL1,MASP2,C1S,C4A,COLEC11,C4B,CFB,C1R,CFD,IGHV3-15,IGHV3-72,B2M
GO Process	GO:0051336	Regulation of hydrolase activity	49	1011	0.56	5.00e-12	SPP2,APOH,SERPIND1,PCOLCE,C5,VTN,APOC3,GPLD1,LTF,HRG,PLEK,APOA1,LRP1,SERPINF1,APCS,SERPINA10,LCN1,CSTA,AMBP,ITIH4,ITIH1,AHSG,SERPING1,SERPINI1,A2ML1,HSP90B1,ANTXR1,LPA,SERPINF2,SERPINA7,FBLN1,SERPINA5,SERPINA11,ANG,LIMS1,PAPLN,APOA4,ITIH2,S100A9,GSN,PROS1,GAPDH,CST3,C4A,ITIH3,C4B,APOA5,SERPINA3,KNG1
GO Process	GO:0051248	Negative regulation of protein metabolic process	49	1038	0.54	1.23e-11	SPP2,CPB2,SERPIND1,C5,VTN,LTF,HRG,IGFBP5,LRP1,APOE,SERPINF1,APCS,SERPINA10,LCN1,YWHAE,CSTA,AMBP,ITIH4,ITIH1,AHSG,SERPING1,SERPINI1,A2ML1,F2,CALR,LPA,SERPINF2,SERPINA7,FBLN1,SERPINA5,SERPINA11,ANG,APOD,PAPLN,ITIH2,FLNA,TAF9,IGFBP3,PROS1,TNRC6A,GAPDH,CST3,ADIPOQ,C4A,ITIH3,C4B,SERPINA3,KNG1,FGA
GO Process	GO:0032101	Regulation of response to external stimulus	47	964	0.56	1.42e-11	ST6GAL1,CPB2,APOH,LBP,C5,VTN,LTF,HRG,PROC,APOA1,APOE,F12,SERPINF1,APCS,PSMA6,FCN3,AHSG,SERPING1,CSF1R,FCN2,PGLYRP2,MASP1,FGB,F2,PLG,CALR,SERPINF2,FBN1,THBS4,CFH,S100A7,S100A9,FLNA,MBL2,PROS1,CFI,MASP2,SAA1,F11,ADIPOQ,COLEC11,MST1,RARRES2,VEGFC,KNG1,CDH5,FGA
GO Process	GO:0065007	Biological regulation	222	12385	0.12	4.99e-11	SPP2,ST6GAL1,CPB2,CETP,APOH,VCL,SERPIND1,CHGA,TPD52L2,LBP,F9,MMP2,EBI3,COMP,PON1,PCOLCE,C5,MPO,VTN,AFM,PF4V1,APOC3,GPLD1,SPARC,LTF,HRG,APOB,IGFBP5,PROC,PLEK,APOA1,TTR,CAT,LRP1,C4BPB,CFP,DHX29,APOE,PXDN,F12,SERPINF1,APCS,CFHR5,LYZ,VWF,PSMA6,DSG2,SERPINA10,LGALS3BP,ATRN,C6,LCN1,LCAT,NID1,YWHAE,CSTA,CP,DGKG,CDH6,AMBP,CENPE,PON3,TCIRG1,HYAL1,ITIH4,LUM,FCN3,ITIH1,AHSG,ANKRD31,IGFBP1,CRH,SERPING1,CSF1R,FCN2,PGLYRP2,SCGB3A1,IHH,SERPINI1,PPBP,PF4,MASP1,CAMP,GUCY1A1,A2ML1,HSP90B1,ANTXR1,LRG1,SH3D19,PCSK9,BMP1,SHLD1,FGB,DST,F2,INHBC,PLG,EFEMP2,CSPG4,C1QB,CFHR1,SUZ12,SUMO4,CPN2,CALR,LPA,SERPINF2,C7,HBA1,ITLN1,HSPA5,FBN1,P4HB,SERPINA7,FBLN1,SERPINA5,OIT3,SERPINA11,ANG,LIMS1,THBS4,PRDX6,PROZ,APOD,PAPLN,ASGR2,BLM,AKAP9,PIGR,APOA4,ITIH2,CNDP1,COL18A1,MTCL1,F8,C8A,C4BPA,F13B,CFHR2,CFHR4,CFHR3,CFH,QSOX1,F5,CD5L,S100A7,S100A9,ADAMTSL4,FLNA,MCOLN2,SMARCA1,C8B,C8G,ADAMTS13,GSN,MBL2,SVEP1,C1QC,F10,APOM,CALML5,TAF9,IGFBP3,CD99,SOD3,PROS1,EFEMP1,CFI,TNRC6A,NAV2,ILK,GAPDH,FGL1,CST3,MASP2,FBLN2,PSG4,SAA1,F11,C1S,NUCB1,KSR2,ADIPOQ,IGF2,ORM2,C4A,SLAIN1,CDK3,ABCA13,COLEC11,MST1,LMOD3,SPARCL1,ITIH3,C4B,TGFBI,CFB,IGFALS,PLTP,PAEP,RARRES2,HGFAC,UIMC1,SELENOP,JCHAIN,HPR,C1R,APOA5,ATP1A1,SERPINA3,APOC4,CFD,CLTC,IGHV3-15,IGHV3-72,VEGFC,HYOU1,KNG1,GRHL2,CDH5,B2M,FGA
GO Process	GO:0051241	Negative regulation of multicellular organismal process	47	1035	0.53	1.57e-10	CPB2,APOH,LBP,MMP2,VTN,APOC3,SPARC,LTF,HRG,IGFBP5,PROC,APOA1,APOE,F12,SERPINF1,APCS,AHSG,CRH,SERPING1,PGLYRP2,IHH,PF4,GUCY1A1,PCSK9,FGB,F2,PLG,CALR,SERPINF2,FBN1,FBLN1,THBS4,APOD,APOA4,C1QC,APOM,PROS1,EFEMP1,CST3,F11,ADIPOQ,PAEP,ATP1A1,KNG1,GRHL2,B2M,FGA
GO Process	GO:0006957	Complement activation, alternative pathway	10	16	1.67	1.68e-10	C5,CFP,CFHR5,C7,C8A,CFH,C8B,C8G,CFB,CFD
GO Process	GO:1903028	Positive regulation of opsonization	10	16	1.67	1.68e-10	CFP,FCN3,FCN2,MASP1,MBL2,MASP2,C4A,COLEC11,C4B,CFB
GO Process	GO:0031638	Zymogen activation	14	59	1.24	1.80e-10	APOH,F9,F12,FGB,F2,F8,F5,CD5L,F10,F11,HGFAC,HPR,C1R,FGA
GO Process	GO:0019730	Antimicrobial humoral response	19	149	0.98	2.78e-10	PF4V1,LTF,HRG,LYZ,PPBP,PF4,CAMP,FGB,F2,ANG,S100A7,S100A9,GAPDH,COLEC11,RARRES2,JCHAIN,KNG1,B2M,FGA
GO Process	GO:0044092	Negative regulation of molecular function	49	1143	0.5	3.29e-10	SPP2,SERPIND1,C5,VTN,APOC3,LTF,HRG,APOA1,CAT,LRP1,APOE,PXDN,SERPINF1,APCS,CFHR5,SERPINA10,LCN1,YWHAE,CSTA,AMBP,ITIH4,ITIH1,AHSG,SERPING1,SERPINI1,A2ML1,PCSK9,CFHR1,SUMO4,LPA,SERPINF2,SERPINA7,SERPINA5,SERPINA11,PAPLN,AKAP9,ITIH2,CFHR2,FLNA,PROS1,GAPDH,CST3,ADIPOQ,C4A,ITIH3,C4B,SERPINA3,KNG1,B2M
GO Process	GO:0051246	Regulation of protein metabolic process	80	2622	0.35	3.31e-10	SPP2,CPB2,SERPIND1,PCOLCE,C5,VTN,GPLD1,LTF,HRG,IGFBP5,APOA1,LRP1,C4BPB,DHX29,APOE,F12,SERPINF1,APCS,SERPINA10,LCN1,YWHAE,CSTA,AMBP,CENPE,ITIH4,ITIH1,AHSG,SERPING1,CSF1R,SERPINI1,CAMP,A2ML1,HSP90B1,ANTXR1,SH3D19,PCSK9,F2,INHBC,CSPG4,CALR,LPA,SERPINF2,ITLN1,HSPA5,FBN1,SERPINA7,FBLN1,SERPINA5,SERPINA11,ANG,THBS4,APOD,PAPLN,BLM,AKAP9,ITIH2,C4BPA,S100A9,FLNA,GSN,TAF9,IGFBP3,PROS1,TNRC6A,ILK,GAPDH,CST3,ADIPOQ,IGF2,C4A,MST1,ITIH3,C4B,PAEP,RARRES2,SERPINA3,VEGFC,KNG1,CDH5,FGA
GO Process	GO:0006508	Proteolysis	51	1247	0.48	5.58e-10	CPB2,APOH,F9,MMP2,COMP,PCOLCE,LTF,PROC,F12,PSMA6,LCN1,FCN3,HABP2,FCN2,IHH,MASP1,APEH,HSP90B1,PCSK9,BMP1,FGB,F2,PLG,UBE3C,CALR,LPA,HSPA5,PROZ,CNDP1,F8,CFH,F5,CD5L,ADAMTS13,MBL2,F10,TAF9,CFI,MASP2,F11,C1S,PCYOX1,COLEC11,MST1,CFB,HGFAC,UIMC1,HPR,C1R,CFD,FGA
GO Process	GO:0042730	Fibrinolysis	10	19	1.59	5.58e-10	CPB2,HRG,F12,SERPING1,FGB,F2,PLG,SERPINF2,PROS1,FGA
GO Process	GO:0034369	Plasma lipoprotein particle remodeling	11	29	1.45	7.13e-10	CETP,MPO,APOC3,APOB,APOA1,APOE,LCAT,APOA4,APOM,PLTP,APOA5
GO Process	GO:0043086	Negative regulation of catalytic activity	39	771	0.57	7.33e-10	SPP2,SERPIND1,C5,VTN,APOC3,LTF,HRG,APOA1,LRP1,APOE,SERPINF1,APCS,SERPINA10,LCN1,CSTA,AMBP,ITIH4,ITIH1,AHSG,SERPING1,SERPINI1,A2ML1,LPA,SERPINF2,SERPINA7,SERPINA5,SERPINA11,PAPLN,AKAP9,ITIH2,PROS1,GAPDH,CST3,ADIPOQ,C4A,ITIH3,C4B,SERPINA3,KNG1
GO Process	GO:0006953	Acute-phase response	12	42	1.33	1.11e-09	LBP,APCS,ITIH4,AHSG,SAA4,F2,SERPINF2,F8,MBL2,SAA1,ORM2,SERPINA3

GO Process	GO:0006954	Inflammatory response	32	538	0.64	1.45e-09	LBP,C5,PF4V1,LRP1,F12,APCS,LYZ,ATRN,TCIRG1,HYAL1,ITIH4,AHSG,CRH,SAA4,CSF1R,PPBP,PF4,CAMP,F2,SERPINF2,F8,CD5L,S100A9,MBL2,SAA1,ORM2,C4A,C4B,RARRES2,HPR,SERPINA3,KNG1
GO Process	GO:0009617	Response to bacterium	35	663	0.59	3.25e-09	CHGA,LBP,MPO,PF4V1,LTF,APOB,C4BPB,CFP,LYZ,FCN2,PGLYRP2,PPBP,PF4,CAMP,LRG1,FGB,F2,ANG,C4BPA,S100A7,S100A9,ADAMTS13,MBL2,RNASE1,ADIPOQ,C4B,CFB,RARRES2,JCHAIN,RNASE4,CFD,IGHV3-15,IGHV3-72,B2M,FGA
GO Process	GO:0042742	Defense response to bacterium	24	306	0.76	3.60e-09	CHGA,LBP,MPO,LTF,CFP,LYZ,FCN2,PGLYRP2,PPBP,CAMP,FGB,F2,ANG,S100A7,S100A9,MBL2,RNASE1,RARRES2,JCHAIN,RNASE4,IGHV3-15,IGHV3-72,B2M,FGA
GO Process	GO:0048519	Negative regulation of biological process	123	5313	0.23	4.05e-09	SPP2,ST6GAL1,CPB2,CETP,APOH,VCL,SERPIND1,CHGA,LBP,MMP2,COMP,C5,MPO,VTN,APOC3,GPLD1,SPARC,LTF,HRG,IGFBP5,PROC,PLEK,APOA1,CAT,LRP1,C4BPB,APOE,PXDN,F12,SERPINF1,APCS,SERPINA10,LCN1,YWHAE,CSTA,DGKG,AMBP,PON3,HYAL1,ITIH4,FCN3,ITIH1,AHSG,IGFBP1,CRH,SERPING1,CSF1R,PGLYRP2,SCGB3A1,IHH,SERPINI1,PF4,MASP1,GUCY1A1,A2ML1,HSP90B1,ANTXR1,PCSK9,SHLD1,FGB,F2,PLG,EFEMP2,SUZ12,SUMO4,CALR,LPA,SERPINF2,HSPA5,FBN1,SERPINA7,FBLN1,SERPINA5,SERPINA11,ANG,LIMS1,THBS4,APOD,PAPLN,BLM,APOA4,ITIH2,COL18A1,C4BPA,QSOX1,FLNA,MCOLN2,GSN,MBL2,C1QC,APOM,TAF9,IGFBP3,PROS1,EFEMP1,TNRC6A,ILK,GAPDH,FGL1,CST3,SAA1,F11,ADIPOQ,IGF2,C4A,CDK3,MST1,LMOD3,ITIH3,C4B,TGFBI,PAEP,UIMC1,ATP1A1,SERPINA3,CLTC,VEGFC,HYOU1,KNG1,GRHL2,CDH5,B2M,FGA
GO Process	GO:0030168	Platelet activation	15	97	1.06	4.91e-09	VCL,COMP,PF4V1,HRG,PLEK,VWF,DGKG,PF4,FGB,F2,FLNA,ADAMTS13,ILK,SAA1,FGA
GO Process	GO:0002526	Acute inflammatory response	14	80	1.11	5.21e-09	LBP,F12,APCS,ITIH4,AHSG,SAA4,F2,SERPINF2,F8,MBL2,SAA1,ORM2,HPR,SERPINA3
GO Process	GO:1903034	Regulation of response to wounding	18	162	0.92	7.08e-09	CPB2,APOH,VTN,HRG,PROC,APOE,F12,APCS,SERPING1,FGB,F2,PLG,SERPINF2,FLNA,PROS1,F11,KNG1,FGA
GO Process	GO:0051917	Regulation of fibrinolysis	9	18	1.57	8.40e-09	CPB2,APOH,VTN,HRG,F12,F2,PLG,SERPINF2,F11
GO Process	GO:0050789	Regulation of biological process	208	11655	0.12	9.95e-09	SPP2,ST6GAL1,CPB2,CETP,APOH,VCL,SERPIND1,CHGA,TPD52L2,LBP,MMP2,EBI3,COMP,PON1,PCOLCE,C5,MPO,VTN,PF4V1,APOC3,GPLD1,SPARC,LTF,HRG,APOB,IGFBP5,PROC,PLEK,APOA1,TTR,CAT,LRP1,C4BPB,CFP,DHX29,APOE,PXDN,F12,SERPINF1,APCS,CFHR5,VWF,PSMA6,DSG2,SERPINA10,LGALS3BP,ATRN,C6,LCN1,LCAT,NID1,YWHAE,CSTA,DGKG,CDH6,AMBP,CENPE,PON3,TCIRG1,HYAL1,ITIH4,LUM,FCN3,ITIH1,AHSG,ANKRD31,IGFBP1,CRH,SERPING1,CSF1R,FCN2,PGLYRP2,SCGB3A1,IHH,SERPINI1,PPBP,PF4,MASP1,CAMP,GUCY1A1,A2ML1,HSP90B1,ANTXR1,LRG1,SH3D19,PCSK9,BMP1,SHLD1,FGB,DST,F2,INHBC,PLG,EFEMP2,CSPG4,C1QB,CFHR1,SUZ12,SUMO4,CALR,LPA,SERPINF2,C7,HBA1,ITLN1,HSPA5,FBN1,P4HB,SERPINA7,FBLN1,SERPINA5,SERPINA11,ANG,LIMS1,THBS4,PRDX6,APOD,PAPLN,ASGR2,BLM,AKAP9,PIGR,APOA4,ITIH2,COL18A1,MTCL1,C8A,C4BPA,CFHR2,CFHR4,CFHR3,CFH,QSOX1,CD5L,S100A7,S100A9,ADAMTSL4,FLNA,MCOLN2,SMARCA1,C8B,C8G,ADAMTS13,GSN,MBL2,SVEP1,C1QC,F10,APOM,CALML5,TAF9,IGFBP3,CD99,PROS1,EFEMP1,CFI,TNRC6A,ILK,GAPDH,FGL1,CST3,MASP2,FBLN2,PSG4,SAA1,F11,C1S,NUCB1,KSR2,ADIPOQ,IGF2,ORM2,C4A,SLAIN1,CDK3,ABCA13,COLEC11,MST1,LMOD3,SPARCL1,ITIH3,C4B,TGFBI,CFB,IGFALS,PLTP,PAEP,RARRES2,UIMC1,SELENOP,JCHAIN,HPR,C1R,APOA5,ATP1A1,SERPINA3,APOC4,CFD,CLTC,IGHV3-15,IGHV3-72,VEGFC,HYOU1,KNG1,GRHL2,CDH5,B2M,FGA
GO Process	GO:0097006	Regulation of plasma lipoprotein particle levels	12	54	1.22	1.19e-08	CETP,MPO,APOC3,APOB,APOA1,APOE,LCAT,APOA4,APOM,ADIPOQ,PLTP,APOA5
GO Process	GO:0051873	Killing by host of symbiont cells	10	29	1.41	1.24e-08	HRG,CFHR5,PF4,CAMP,F2,CFHR1,APOL1,CFHR2,MBL2,GAPDH
GO Process	GO:0051702	Biological process involved in interaction with symbiont	15	108	1.01	1.75e-08	LTF,HRG,APOE,APCS,CFHR5,CSF1R,PF4,CAMP,F2,PLG,CFHR1,APOL1,CFHR2,MBL2,GAPDH
GO Process	GO:0016485	Protein processing	19	202	0.84	2.57e-08	APOH,F9,COMP,F12,IHH,PCSK9,BMP1,FGB,F2,F8,F5,CD5L,ADAMTS13,F10,F11,HGFAC,HPR,C1R,FGA
GO Process	GO:0050766	Positive regulation of phagocytosis	13	75	1.11	2.70e-08	APOA1,CFP,FCN3,AHSG,FCN2,MASP1,CALR,MBL2,MASP2,C4A,COLEC11,C4B,CFB
GO Process	GO:0007155	Cell adhesion	41	965	0.5	2.74e-08	VCL,COMP,VTN,PCDH12,PLEK,PXDN,VWF,DSG2,LGALS3BP,ATRN,NID1,CSTA,CDH6,AMBP,HABP2,PCDH1,IHH,ANTXR1,LRG1,FGB,DST,PSG11,FBN1,LIMS1,THBS4,APOA4,COL18A1,PCDH18,S100A9,FLNA,ADAMTS13,SVEP1,CD99,ILK,PSG2,SPARCL1,TGFBI,IGFALS,GRHL2,CDH5,FGA
GO Process	GO:0032102	Negative regulation of response to external stimulus	25	387	0.68	5.39e-08	ST6GAL1,CPB2,APOH,C5,VTN,LTF,HRG,PROC,APOA1,APOE,F12,SERPINF1,APCS,SERPING1,FGB,F2,PLG,SERPINF2,PROS1,SAA1,F11,ADIPOQ,KNG1,CDH5,FGA
GO Process	GO:0032374	Regulation of cholesterol transport	12	64	1.14	6.00e-08	CETP,PON1,APOC3,APOA1,LRP1,APOE,PCSK9,APOA4,ADIPOQ,ABCA13,PLTP,APOA5
GO Process	GO:0034375	High-density lipoprotein particle remodeling	8	16	1.57	9.02e-08	CETP,APOC3,APOA1,APOE,LCAT,APOA4,APOM,PLTP
GO Process	GO:0050764	Regulation of phagocytosis	14	104	1.0	9.79e-08	APOA1,CFP,FCN3,AHSG,FCN2,MASP1,CALR,MBL2,MASP2,ADIPOQ,C4A,COLEC11,C4B,CFB
GO Process	GO:0051239	Regulation of multicellular organismal process	76	2749	0.31	1.03e-07	CPB2,APOH,VCL,CHGA,LBP,MMP2,EBI3,COMP,C5,VTN,APOC3,GPLD1,SPARC,LTF,HRG,IGFBP5,PROC,APOA1,LRP1,APOE,F12,SERPINF1,APCS,DSG2,ATRN,LCAT,YWHAE,HYAL1,LUM,AHSG,CRH,SERPING1,CSF1R,PGLYRP2,IHH,PF4,CAMP,GUCY1A1,LRG1,PCSK9,BMP1,SHLD1,FGB,F2,PLG,CALR,SERPINF2,FBN1,FBLN1,THBS4,APOD,AKAP9,APOA4,MCOLN2,C1QC,APOM,PROS1,EFEMP1,GAPDH,CST3,SAA1,F11,KSR2,ADIPOQ,IGF2,ORM2,MST1,PAEP,APOA5,ATP1A1,VEGFC,KNG1,GRHL2,CDH5,B2M,FGA
GO Process	GO:0065008	Regulation of biological quality	91	3654	0.27	2.12e-07	CPB2,CETP,APOH,VCL,SERPIND1,CHGA,F9,MMP2,COMP,VTN,AFM,PF4V1,APOC3,GPLD1,LTF,HRG,APOB,IGFBP5,PROC,PLEK,APOA1,TTR,C4BPB,APOE,F12,SERPINF1,LYZ,VWF,DSG2,SERPINA10,LCN1,LCAT,YWHAE,CP,DGKG,TCIRG1,CRH,SERPING1,CSF1R,IHH,PF4,GUCY1A1,HSP90B1,PCSK9,FGB,F2,PLG,CPN2,CALR,SERPINF2,FBN1,SERPINA7,FBLN1,OIT3,ANG,PRDX6,PROZ,AKAP9,PIGR,APOA4,F8,F13B,F5,S100A9,FLNA,ADAMTS13,GSN,F10,APOM,TAF9,SOD3,PROS1,NAV2,ILK,GAPDH,SAA1,F11,KSR2,ADIPOQ,LMOD3,HGFAC,JCHAIN,APOA5,ATP1A1,SERPINA3,APOC4,VEGFC,KNG1,CDH5,B2M,FGA
GO Process	GO:0032501	Multicellular organismal process	135	6490	0.19	3.18e-07	RCN1,SPP2,CPB2,CETP,APOH,VCL,SERPIND1,CHGA,F9,MMP2,COMP,C5,MPO,VTN,PF4V1,APOC3,GPLD1,PCDH12,LTF,HRG,APOB,IGFBP5,PROC,PLEK,APOA1,CAT,LRP1,C4BPB,APOE,PXDN,ANGPTL6,F12,SERPINF1,LYZ,VWF,DSG2,SERPINA10,ATRN,C6,LCN1,LCAT,NID1,YWHAE,DGKG,DNAH5,AMBP,PON3,TCIRG1,HYAL1,LUM,AHSG,CRH,SERPING1,CSF1R,PCDH1,IHH,SERPINI1,PF4,GUCY1A1,ANTXR1,LRG1,PCSK9,BMP1,SHLD1,FGB,F2,PLG,EFEMP2,CSPG4,SUZ12,PSG11,CALR,LPA,SERPINF2,HSPA5,FBN1,FBLN1,SERPINA5,ANG,THBS4,PROZ,APOD,AKAP9,PIGR,APOA4,COL18A1,F8,PCDH18,F13B,F5,S100A7,S100A9,FLNA,CRTAC1,SMARCA1,ADAMTS13,MINPP1,GSN,SVEP1,F10,APOM,CA6,IGFBP3,SOD3,PROS1,EFEMP1,NAV2,ILK,FGL1,PSG4,SAA1,F11,PSG2,ADIPOQ,IGF2,FSIP2,MST1,LMOD3,TGFBI,PLTP,RARRES2,HGFAC,SELENOP,JCHAIN,APOA5,ATP1A1,SERPINA3,CLTC,VEGFC,KNG1,GRHL2,CDH5,B2M,FGA,SDF4

GO Process	GO:0010810	Regulation of cell-substrate adhesion	18	217	0.79	4.48e-07	ST6GAL1,VCL,VTN,HRG,APOA1,NID1,FGB,PLG,EFEMP2,CALR,P4HB,FBLN1,LIMS1,APOD,FLNA,ILK,FBLN2,FGA
GO Process	GO:0051838	Cytolysis by host of symbiont cells	7	12	1.64	4.70e-07	HRG,CFHR5,CAMP,F2,CFHR1,APOL1,CFHR2
GO Process	GO:0051604	Protein maturation	20	275	0.73	4.73e-07	APOH,F9,COMP,F12,IHH,PCSK9,BMP1,FGB,F2,CALR,F8,F5,CD5L,ADAMTS13,F10,F11,HGFAC,HPR,C1R,FGA
GO Process	GO:0051918	Negative regulation of fibrinolysis	7	13	1.6	7.01e-07	CPB2,APOH,VTN,HRG,F2,PLG,SERPINF2
GO Process	GO:0051050	Positive regulation of transport	37	915	0.48	7.45e-07	CPB2,CETP,PON1,VTN,GPLD1,APOA1,LRP1,CFP,APOE,YWHAE,FCN3,AHSG,CRH,FCN2,MASP1,PCSK9,FGB,F2,CALR,ITLN1,ANG,AKAP9,MTCL1,FLNA,MBL2,MASP2,ADIPOQ,C4A,ABCA13,COLEC11,C4B,CFB,PLTP,APOA5,VEGFC,B2M,FGA
GO Process	GO:0010811	Positive regulation of cell-substrate adhesion	14	128	0.91	1.03e-06	VTN,HRG,APOA1,NID1,FGB,EFEMP2,CALR,P4HB,FBLN1,LIMS1,FLNA,ILK,FBLN2,FGA
GO Process	GO:0065009	Regulation of molecular function	79	3085	0.28	1.23e-06	SPP2,APOH,SERPIND1,PON1,PCOLCE,C5,VTN,APOC3,GPLD1,LTF,HRG,PLEK,APOA1,CAT,LRP1,APOE,PXDN,SERPINF1,APCS,CFHR5,PSMA6,SERPINA10,LCN1,YWHAE,CSTA,AMBP,CENPE,ITIH4,ITIH1,AHSG,CRH,SERPING1,CSF1R,SERPINI1,A2ML1,HSP90B1,ANTXR1,PCSK9,F2,CFHR1,SUZ12,SUMO4,CPN2,LPA,SERPINF2,FBN1,SERPINA7,FBLN1,SERPINA5,SERPINA11,ANG,LIMS1,PAPLN,BLM,AKAP9,APOA4,ITIH2,CFHR2,S100A9,FLNA,GSN,CALML5,IGFBP3,PROS1,GAPDH,CST3,NUCB1,ADIPOQ,IGF2,C4A,MST1,ITIH3,C4B,APOA5,SERPINA3,VEGFC,KNG1,GRHL2,B2M
GO Process	GO:0050790	Regulation of catalytic activity	66	2370	0.31	1.43e-06	SPP2,APOH,SERPIND1,PCOLCE,C5,VTN,APOC3,GPLD1,LTF,HRG,PLEK,APOA1,LRP1,APOE,SERPINF1,APCS,SERPINA10,LCN1,CSTA,AMBP,CENPE,ITIH4,ITIH1,AHSG,SERPING1,CSF1R,SERPINI1,A2ML1,HSP90B1,ANTXR1,F2,SUZ12,CPN2,LPA,SERPINF2,FBN1,SERPINA7,FBLN1,SERPINA5,SERPINA11,ANG,LIMS1,PAPLN,BLM,AKAP9,APOA4,ITIH2,S100A9,GSN,CALML5,IGFBP3,PROS1,GAPDH,CST3,NUCB1,ADIPOQ,IGF2,C4A,MST1,ITIH3,C4B,APOA5,SERPINA3,VEGFC,KNG1,GRHL2
GO Process	GO:0098869	Cellular oxidant detoxification	12	91	0.99	1.85e-06	MPO,CAT,APOE,PXDN,AMBP,HBA1,PRDX6,APOA4,S100A9,APOM,SOD3,GPX3
GO Process	GO:0061844	Antimicrobial humoral immune response mediated by antimicrobial peptide	13	113	0.93	2.00e-06	PF4V1,LTF,HRG,PPBP,PF4,CAMP,F2,ANG,S100A7,S100A9,GAPDH,KNG1,B2M
GO Process	GO:0030194	Positive regulation of blood coagulation	8	28	1.33	2.79e-06	CPB2,APOH,VTN,HRG,F12,F2,PLG,SERPINF2
GO Process	GO:0030198	Extracellular matrix organization	19	278	0.7	2.79e-06	MMP2,COMP,VTN,PXDN,NID1,LUM,IHH,BMP1,PLG,EFEMP2,SERPINF2,FBLN1,PAPLN,COL18A1,QSOX1,ADAMTSL4,ADAMTS13,FBLN2,TGFBI
GO Process	GO:1905952	Regulation of lipid localization	15	167	0.82	3.18e-06	CETP,PON1,APOC3,APOB,APOA1,LRP1,APOE,CRH,PCSK9,APOA4,ADIPOQ,ABCA13,PLTP,APOA5,APOC4
GO Process	GO:0002752	Cell surface pattern recognition receptor signaling pathway	6	9	1.69	3.23e-06	FCN3,FCN2,MASP1,MBL2,MASP2,COLEC11
GO Process	GO:0034372	Very-low-density lipoprotein particle remodeling	6	9	1.69	3.23e-06	CETP,APOA1,APOE,LCAT,APOA4,APOA5
GO Process	GO:0043691	Reverse cholesterol transport	7	18	1.46	3.58e-06	CETP,APOC3,APOA1,APOE,LCAT,APOA4,APOM
GO Process	GO:1900024	Regulation of substrate adhesion-dependent cell spreading	10	61	1.08	4.40e-06	ST6GAL1,APOA1,FGB,CALR,P4HB,FBLN1,LIMS1,FLNA,ILK,FGA
GO Process	GO:0001867	Complement activation, lectin pathway	6	10	1.65	4.91e-06	FCN3,FCN2,MASP1,MBL2,MASP2,COLEC11
GO Process	GO:0006869	Lipid transport	20	329	0.65	6.61e-06	CETP,APOH,LBP,APOC3,APOB,APOA1,APOE,LCAT,APOL1,LPA,SERPINA5,APOD,APOA4,CFHR4,APOM,APOF,ABCA13,PLTP,APOA5,APOC4
GO Process	GO:0050830	Defense response to Gram-positive bacterium	13	130	0.87	8.01e-06	CHGA,LBP,LTF,LYZ,FCN2,PGLYRP2,CAMP,ANG,MBL2,RNASE1,RARRES2,RNASE4,B2M
GO Process	GO:0030449	Regulation of complement activation	7	22	1.37	1.04e-05	C4BPB,SERPING1,MASP1,C4BPA,CFH,CD5L,CFI
GO Process	GO:0019835	Cytolysis	7	23	1.35	1.33e-05	C5,LYZ,C6,C7,C8A,C8B,C8G
GO Process	GO:0034377	Plasma lipoprotein particle assembly	7	23	1.35	1.33e-05	APOC3,APOB,APOA1,APOE,APOA4,APOM,APOA5
GO Process	GO:0032103	Positive regulation of response to external stimulus	23	453	0.58	1.40e-05	CPB2,APOH,LBP,VTN,HRG,FCN3,CSF1R,FCN2,MASP1,F2,PLG,CALR,SERPINF2,FBN1,THBS4,S100A7,S100A9,FLNA,MBL2,MASP2,COLEC11,RARRES2,VEGFC
GO Process	GO:0002697	Regulation of immune effector process	21	383	0.61	1.49e-05	LBP,APOA1,C4BPB,CFP,FCN3,SERPING1,FCN2,PGLYRP2,MASP1,SHLD1,C4BPA,CFH,CD5L,MBL2,CFI,MASP2,C4A,COLEC11,C4B,CFB,B2M
GO Process	GO:0007597	Blood coagulation, intrinsic pathway	6	13	1.53	1.49e-05	APOH,F9,F12,F8,FLNA,F11
GO Process	GO:0032368	Regulation of lipid transport	13	139	0.84	1.55e-05	CETP,PON1,APOC3,APOA1,LRP1,APOE,CRH,PCSK9,APOA4,ADIPOQ,ABCA13,PLTP,APOA5
GO Process	GO:0032376	Positive regulation of cholesterol transport	8	38	1.19	1.76e-05	CETP,PON1,APOA1,LRP1,APOE,ADIPOQ,ABCA13,PLTP
GO Process	GO:0030155	Regulation of cell adhesion	31	784	0.47	1.82e-05	ST6GAL1,VCL,MMP2,EBI3,VTN,HRG,APOA1,NID1,HYAL1,IHH,SERPINI1,FGB,PLG,EFEMP2,CALR,SERPINF2,P4HB,FBLN1,LIMS1,APOD,FLNA,ILK,FGL1,FBLN2,SAA1,ADIPOQ,IGF2,TGFBI,KNG1,B2M,FGA
GO Process	GO:0008203	Cholesterol metabolic process	12	119	0.87	2.18e-05	CETP,PON1,APOB,APOA1,CAT,APOE,LCAT,PCSK9,APOL1,APOA4,APOF,APOA5
GO Process	GO:0048585	Negative regulation of response to stimulus	48	1612	0.34	2.81e-05	ST6GAL1,CPB2,APOH,C5,VTN,LTF,HRG,IGFBP5,PROC,PLEK,APOA1,LRP1,C4BPB,APOE,PXDN,F12,SERPINF1,APCS,DGKG,AMBP,AHSG,IGFBP1,SERPING1,PGLYRP2,PF4,MASP1,SHLD1,FGB,F2,PLG,CALR,SERPINF2,HSPA5,FBN1,FBLN1,APOD,C4BPA,TAF9,IGFBP3,PROS1,SAA1,F11,ADIPOQ,CDK3,HYOU1,KNG1,CDH5,FGA
GO Process	GO:0033344	Cholesterol efflux	7	27	1.28	3.03e-05	APOC3,APOB,APOA1,APOE,APOA4,APOM,APOA5
GO Process	GO:0080134	Regulation of response to stress	43	1373	0.37	3.49e-05	CPB2,APOH,LBP,VTN,HRG,PROC,APOA1,APOE,F12,SERPINF1,APCS,PSMA6,AMBP,FCN3,AHSG,SERPING1,FCN2,PGLYRP2,MASP1,SHLD1,FGB,F2,PLG,SERPINF2,HSPA5,P4HB,CFH,S100A9,FLNA,MBL2,TAF9,PROS1,CFI,MASP2,SAA1,F11,ADIPOQ,COLEC11,UIMC1,HYOU1,KNG1,CDH5,FGA
GO Process	GO:0034109	Homotypic cell-cell adhesion	9	60	1.05	3.57e-05	VCL,COMP,PLEK,DSG2,FGB,FLNA,CD99,ILK,FGA
GO Process	GO:0019538	Protein metabolic process	88	3910	0.22	3.61e-05	ST6GAL1,CPB2,GNPTG,APOH,F9,MMP2,COMP,PCOLCE,APOC3,GPLD1,LTF,APOB,PROC,APOA1,CAT,DHX29,APOE,F12,PSMA6,LCN1,LCAT,CSTA,AMBP,TCIRG1,HYAL1,FCN3,HABP2,CSF1R,FCN2,IHH,MASP1,APEH,HSP90B1,PCSK9,BMP1,FGB,F2,PLG,UBE3C,SUZ12,APOL1,SUMO4,CALR,LPA,HSPA5,P4HB,FBLN1,GANAB,PROZ,QPCT,APOA4,CNDP1,F8,CDC42BPA,F13B,CFH,F5,CD5L,S100A9,FLNA,ADAMTS13,GSN,MBL2,F10,APOM,TAF9,IGFBP3,EFEMP1,CFI,ILK,GAPDH,MASP2,F11,C1S,PCYOX1,KSR2,CDK3,COLEC11,MST1,CFB,HGFAC,UIMC1,HPR,C1R,APOA5,CFD,B2M,FGA
GO Process	GO:0001944	Vasculature development	24	527	0.53	4.37e-05	MMP2,COMP,GPLD1,HRG,APOB,LRP1,APOE,PXDN,ANGPTL6,IHH,ANTXR1,PLG,EFEMP2,CSPG4,SERPINF2,ANG,APOD,COL18A1,S100A7,FLNA,SVEP1,TGFBI,VEGFC,CDH5
GO Process	GO:0015918	Sterol transport	10	82	0.96	4.42e-05	CETP,APOC3,APOB,APOA1,APOE,LCAT,APOA4,APOM,PLTP,APOA5
GO Process	GO:0050832	Defense response to fungus	9	62	1.03	4.47e-05	CHGA,MPO,LTF,HRG,CAMP,ANG,S100A9,GAPDH,RARRES2
GO Process	GO:1900026	Positive regulation of substrate adhesion-dependent cell spreading	8	45	1.12	4.96e-05	APOA1,FGB,CALR,P4HB,LIMS1,FLNA,ILK,FGA
GO Process	GO:0030301	Cholesterol transport	9	65	1.01	6.23e-05	CETP,APOC3,APOB,APOA1,APOE,LCAT,APOA4,APOM,APOA5
GO Process	GO:0098609	Cell-cell adhesion	24	542	0.52	6.73e-05	VCL,COMP,PCDH12,PLEK,DSG2,CSTA,CDH6,PCDH1,IHH,LRG1,FGB,PSG11,LIMS1,THBS4,APOA4,PCDH18,S100A9,FLNA,CD99,ILK,PSG2,SPARCL1,CDH5,FGA
GO Process	GO:0001568	Blood vessel development	23	505	0.53	7.24e-05	MMP2,COMP,GPLD1,HRG,APOB,LRP1,APOE,PXDN,ANGPTL6,IHH,ANTXR1,PLG,EFEMP2,CSPG4,SERPINF2,ANG,APOD,COL18A1,S100A7,FLNA,TGFBI,VEGFC,CDH5
GO Process	GO:1905954	Positive regulation of lipid localization	11	111	0.87	7.35e-05	CETP,PON1,APOB,APOA1,LRP1,APOE,CRH,ADIPOQ,ABCA13,PLTP,APOC4
GO Process	GO:0042632	Cholesterol homeostasis	10	89	0.92	8.23e-05	CETP,APOC3,APOB,APOA1,APOE,LCAT,PCSK9,APOA4,APOM,APOA5

GO Process	GO:0044403	Biological process involved in symbiotic interaction	16	257	0.66	8.61e-05	LTF,HRG,APOE,APCS,CFHR5,CSF1R,PGLYRP2,PF4,CAMP,F2,PLG,CFHR1,APOL1,CFHR2,MBL2,GAPDH
GO Process	GO:0060627	Regulation of vesicle-mediated transport	24	551	0.51	8.61e-05	VTN,APOC3,APOA1,LRP1,CFP,APOE,FCN3,AHSG,FCN2,MASP1,PCSK9,FGB,CALR,MBL2,MASP2,ADIPOQ,C4A,ABCA13,COLEC11,C4B,CFB,APOA5,B2M,FGA
GO Process	GO:0009636	Response to toxic substance	15	229	0.69	0.00010	PON1,MPO,CAT,APOE,PXDN,AMBP,PON3,HBA1,PRDX6,APOA4,S100A9,ADAMTS13,APOM,SOD3,GPX3
GO Process	GO:1901700	Response to oxygen-containing compound	45	1547	0.33	0.00011	ST6GAL1,CPB2,LBP,MMP2,PON1,MPO,PF4V1,GPLD1,APOB,IGFBP5,CAT,LRP1,APOE,SERPINF1,AMBP,HYAL1,IGFBP1,IHH,PPBP,PF4,CAMP,HSP90B1,PCSK9,CALR,HBA1,HSPA5,FBN1,APOD,BLM,AKAP9,APOA4,F5,S100A7,S100A9,FLNA,ADAMTS13,TAF9,SOD3,GPX3,ADIPOQ,IGF2,RARRES2,ATP1A1,B2M,SDF4
GO Process	GO:0006979	Response to oxidative stress	19	368	0.58	0.00012	MMP2,MPO,CAT,APOE,PXDN,ATRN,AMBP,HYAL1,SUMO4,HBA1,PRDX6,APOD,APOA4,S100A7,MBL2,SOD3,GPX3,ADIPOQ,SELENOP
GO Process	GO:1903036	Positive regulation of response to wounding	9	72	0.97	0.00013	CPB2,APOH,VTN,HRG,F12,F2,PLG,SERPINF2,FLNA
GO Process	GO:0006639	Acylglycerol metabolic process	10	95	0.89	0.00014	CETP,APOH,APOC3,APOB,CAT,APOE,DGKG,PCSK9,ANG,APOA5
GO Process	GO:0010874	Regulation of cholesterol efflux	7	37	1.15	0.00016	CETP,PON1,APOA1,LRP1,APOE,ADIPOQ,PLTP
GO Process	GO:0050829	Defense response to Gram-negative bacterium	10	97	0.88	0.00016	CHGA,LBP,LTF,LYZ,FCN2,CAMP,F2,S100A7,RARRES2,B2M
GO Process	GO:0042157	Lipoprotein metabolic process	11	123	0.82	0.00017	APOC3,GPLD1,APOB,APOA1,APOE,LCAT,PCSK9,APOL1,APOA4,APOM,APOA5
GO Process	GO:0048514	Blood vessel morphogenesis	20	419	0.55	0.00018	MMP2,COMP,GPLD1,HRG,APOB,LRP1,APOE,PXDN,ANGPTL6,IHH,EFEMP2,CSPG4,SERPINF2,ANG,APOD,COL18A1,S100A7,FLNA,TGFBI,VEGFC
GO Process	GO:0070328	Triglyceride homeostasis	7	38	1.13	0.00018	CETP,APOC3,APOA1,APOE,APOA4,APOA5,APOC4
GO Process	GO:0009892	Negative regulation of metabolic process	70	2982	0.24	0.00019	SPP2,CPB2,SERPIND1,LBP,C5,VTN,APOC3,GPLD1,LTF,HRG,IGFBP5,PLEK,APOA1,LRP1,APOE,SERPINF1,APCS,SERPINA10,LCN1,YWHAE,CSTA,AMBP,PON3,ITIH4,FCN3,ITIH1,AHSG,SERPING1,PGLYRP2,SERPINI1,PF4,A2ML1,PCSK9,SHLD1,F2,SUZ12,SUMO4,CALR,LPA,SERPINF2,SERPINA7,FBLN1,SERPINA5,SERPINA11,ANG,LIMS1,APOD,PAPLN,BLM,ITIH2,QSOX1,FLNA,TAF9,IGFBP3,PROS1,TNRC6A,GAPDH,CST3,ADIPOQ,IGF2,C4A,MST1,ITIH3,C4B,UIMC1,ATP1A1,SERPINA3,CLTC,KNG1,FGA
GO Process	GO:0033700	Phospholipid efflux	5	12	1.49	0.00020	APOC3,APOA1,APOE,APOA4,APOA5
GO Process	GO:0034380	High-density lipoprotein particle assembly	5	12	1.49	0.00020	APOA1,APOE,APOA4,APOM,APOA5
GO Process	GO:0034381	Plasma lipoprotein particle clearance	6	24	1.27	0.00021	APOC3,APOB,APOA1,APOE,APOM,ADIPOQ
GO Process	GO:0048522	Positive regulation of cellular process	111	5584	0.17	0.00021	ST6GAL1,CPB2,CHGA,LBP,MMP2,EBI3,COMP,VTN,GPLD1,SPARC,LTF,HRG,APOB,IGFBP5,PROC,PLEK,APOA1,CAT,LRP1,CFP,DHX29,APOE,SERPINF1,VWF,NID1,YWHAE,CENPE,TCIRG1,HYAL1,LUM,FCN3,ANKRD31,IGFBP1,CRH,CSF1R,FCN2,SCGB3A1,IHH,SERPINI1,PPBP,PF4,MASP1,CAMP,GUCY1A1,LRG1,SH3D19,PCSK9,SHLD1,FGB,F2,INHBC,PLG,EFEMP2,CSPG4,SUZ12,CALR,SERPINF2,HBA1,ITLN1,HSPA5,FBN1,P4HB,FBLN1,ANG,LIMS1,THBS4,PRDX6,BLM,AKAP9,APOA4,MTCL1,CD5L,S100A7,S100A9,ADAMTSL4,FLNA,SMARCA1,GSN,MBL2,F10,TAF9,IGFBP3,CD99,TNRC6A,ILK,GAPDH,MASP2,FBLN2,SAA1,ADIPOQ,IGF2,C4A,SLAIN1,ABCA13,COLEC11,MST1,LMOD3,C4B,CFB,RARRES2,UIMC1,HPR,APOA5,IGHV3-15,IGHV3-72,VEGFC,KNG1,GRHL2,CDH5,B2M,FGA
GO Process	GO:0042744	Hydrogen peroxide catabolic process	6	25	1.25	0.00026	MPO,CAT,PXDN,HBA1,APOA4,GPX3
GO Process	GO:0046486	Glycerolipid metabolic process	18	357	0.57	0.00027	CETP,APOH,PON1,APOC3,GPLD1,APOB,PLEK,APOA1,CAT,APOE,LCAT,DGKG,CSF1R,PCSK9,ANG,PRDX6,APOA4,APOA5
GO Process	GO:0051240	Positive regulation of multicellular organismal process	43	1505	0.33	0.00029	CPB2,APOH,CHGA,LBP,EBI3,C5,VTN,GPLD1,LTF,HRG,LRP1,APOE,F12,SERPINF1,HYAL1,LUM,CRH,CSF1R,IHH,PF4,CAMP,LRG1,BMP1,SHLD1,FGB,F2,PLG,SERPINF2,MCOLN2,GAPDH,SAA1,KSR2,ADIPOQ,IGF2,ORM2,MST1,PAEP,APOA5,ATP1A1,VEGFC,CDH5,B2M,FGA
GO Process	GO:0010875	Positive regulation of cholesterol efflux	6	26	1.23	0.00031	PON1,APOA1,LRP1,APOE,ADIPOQ,PLTP
GO Process	GO:0072377	Blood coagulation, common pathway	4	5	1.77	0.00031	F2,F5,F10,FGA
GO Process	GO:0048870	Cell motility	34	1061	0.38	0.00033	CHGA,LBP,MMP2,C5,VTN,PF4V1,GPLD1,APOB,APOA1,ATRN,YWHAE,DNAH5,PPBP,PF4,LRG1,DST,PLG,CSPG4,DNAH8,ANG,THBS4,CDC42BPA,S100A9,FLNA,MCOLN2,CD99,ILK,SAA1,PSG2,FSIP2,MST1,PLTP,VEGFC,CDH5
GO Process	GO:0010896	Regulation of triglyceride catabolic process	5	14	1.42	0.00034	APOC3,GPLD1,APOA1,APOA4,APOA5
GO Process	GO:0008015	Blood circulation	19	403	0.54	0.00035	CHGA,MMP2,COMP,APOE,SERPING1,GUCY1A1,FGB,LPA,SERPINF2,F5,GSN,SOD3,NAV2,ADIPOQ,ATP1A1,VEGFC,KNG1,CDH5,FGA
GO Process	GO:0070527	Platelet aggregation	7	43	1.08	0.00035	VCL,COMP,PLEK,FGB,FLNA,ILK,FGA
GO Process	GO:0030334	Regulation of cell migration	31	927	0.39	0.00039	APOH,VCL,LBP,MMP2,C5,VTN,GPLD1,SPARC,HRG,IGFBP5,LRP1,APOE,SERPINF1,HYAL1,CSF1R,PLG,CALR,HSPA5,FBLN1,THBS4,APOD,S100A7,FLNA,F10,IGFBP3,CD99,ADIPOQ,MST1,RARRES2,VEGFC,CDH5
GO Process	GO:0045785	Positive regulation of cell adhesion	21	485	0.51	0.00039	EBI3,VTN,HRG,APOA1,NID1,HYAL1,IHH,FGB,EFEMP2,CALR,SERPINF2,P4HB,FBLN1,LIMS1,FLNA,ILK,FBLN2,SAA1,IGF2,B2M,FGA
GO Process	GO:0003013	Circulatory system process	21	493	0.5	0.00049	CHGA,MMP2,COMP,LRP1,APOE,SERPING1,GUCY1A1,FGB,LPA,SERPINF2,F5,GSN,SVEP1,SOD3,NAV2,ADIPOQ,ATP1A1,VEGFC,KNG1,CDH5,FGA
GO Process	GO:0032370	Positive regulation of lipid transport	9	89	0.87	0.00053	CETP,PON1,APOA1,LRP1,APOE,CRH,ADIPOQ,ABCA13,PLTP
GO Process	GO:0050793	Regulation of developmental process	60	2492	0.25	0.00054	ST6GAL1,CETP,APOH,VCL,COMP,C5,SPARC,LTF,HRG,APOB,IGFBP5,PROC,APOA1,APOE,SERPINF1,APCS,ATRN,NID1,DGKG,TCIRG1,HYAL1,AHSG,CSF1R,PGLYRP2,SCGB3A1,IHH,PF4,CAMP,LRG1,SH3D19,BMP1,SHLD1,FGB,F2,EFEMP2,SUZ12,CALR,SERPINF2,FBN1,P4HB,FBLN1,LIMS1,THBS4,FLNA,C1QC,IGFBP3,EFEMP1,ILK,CST3,ADIPOQ,IGF2,MST1,LMOD3,PAEP,RARRES2,VEGFC,GRHL2,CDH5,B2M,FGA
GO Process	GO:0072359	Circulatory system development	30	901	0.39	0.00060	MMP2,COMP,GPLD1,HRG,APOB,LRP1,APOE,PXDN,ANGPTL6,DSG2,DNAH5,IHH,ANTXR1,PLG,EFEMP2,CSPG4,CALR,SERPINF2,FBN1,ANG,APOD,COL18A1,S100A7,FLNA,SVEP1,ILK,TGFBI,VEGFC,GRHL2,CDH5
GO Process	GO:0006641	Triglyceride metabolic process	8	68	0.94	0.00062	CETP,APOH,APOC3,APOB,CAT,APOE,PCSK9,APOA5
GO Process	GO:0060284	Regulation of cell development	21	506	0.49	0.00069	VCL,LTF,PROC,APOA1,SERPINF1,FGB,F2,CALR,FBN1,P4HB,FBLN1,LIMS1,FLNA,ILK,ADIPOQ,LMOD3,PAEP,VEGFC,CDH5,B2M,FGA
GO Process	GO:0051172	Negative regulation of nitrogen compound metabolic process	58	2403	0.25	0.00073	SPP2,CPB2,SERPIND1,C5,VTN,LTF,HRG,IGFBP5,LRP1,APOE,SERPINF1,APCS,SERPINA10,LCN1,YWHAE,CSTA,AMBP,ITIH4,FCN3,ITIH1,AHSG,SERPING1,SERPINI1,A2ML1,SHLD1,F2,SUZ12,SUMO4,CALR,LPA,SERPINF2,SERPINA7,FBLN1,SERPINA5,SERPINA11,ANG,LIMS1,APOD,PAPLN,BLM,ITIH2,FLNA,TAF9,IGFBP3,PROS1,TNRC6A,GAPDH,CST3,ADIPOQ,IGF2,C4A,ITIH3,C4B,UIMC1,SERPINA3,CLTC,KNG1,FGA
GO Process	GO:0051004	Regulation of lipoprotein lipase activity	5	18	1.31	0.00088	APOH,APOC3,APOA1,APOA4,APOA5
GO Process	GO:1901564	Organonitrogen compound metabolic process	99	4981	0.17	0.0010	ST6GAL1,CPB2,CETP,GNPTG,APOH,F9,MMP2,COMP,PON1,PCOLCE,APOC3,GPLD1,LTF,APOB,PROC,APOA1,TTR,CAT,DHX29,APOE,PXDN,F12,PSMA6,LCN1,LCAT,CSTA,AMBP,TCIRG1,HYAL1,ITIH4,FCN3,ITIH1,HABP2,CSF1R,FCN2,PGLYRP2,IHH,MASP1,APEH,GUCY1A1,HSP90B1,PCSK9,BMP1,FGB,F2,PLG,UBE3C,SUZ12,APOL1,SUMO4,CALR,LPA,HSPA5,P4HB,FBLN1,GANAB,PROZ,QPCT,APOA4,ITIH2,CNDP1,F8,CDC42BPA,F13B,CFH,F5,CD5L,S100A9,FLNA,ADAMTS13,GSN,MBL2,F10,APOM,TAF9,IGFBP3,EFEMP1,CFI,ILK,GAPDH,MASP2,F11,C1S,PCYOX1,KSR2,ACSF2,CDK3,COLEC11,MST1,ITIH3,CFB,HGFAC,UIMC1,HPR,C1R,APOA5,CFD,B2M,FGA

GO Process	GO:0040012	Regulation of locomotion	32	1032	0.36	0.0011	ST6GAL1,APOH,VCL,LBP,MMP2,C5,VTN,GPLD1,SPARC,HRG,IGFBP5,LRP1,APOE,SERPINF1,HYAL1,CSF1R,PLG,CALR,HSPA5,FBLN1,THBS4,APOD,S100A7,FLNA,F10,IGFBP3,CD99,ADIPOQ,MST1,RARRES2,VEGFC,CDH5
GO Process	GO:0048662	Negative regulation of smooth muscle cell proliferation	7	53	0.99	0.0011	IGFBP5,APOE,EFEMP2,ANG,APOD,IGFBP3,ADIPOQ
GO Process	GO:0048771	Tissue remodeling	9	101	0.82	0.0013	SPP2,MMP2,IGFBP5,TCIRG1,IHH,PLG,CSPG4,THBS4,FLNA
GO Process	GO:0008202	Steroid metabolic process	14	258	0.6	0.0014	CETP,PON1,APOB,APOA1,CAT,APOE,LCAT,CRH,PCSK9,APOL1,APOA4,APOF,GC,APOA5
GO Process	GO:0008228	Opsonization	4	9	1.52	0.0015	LBP,FCN2,MBL2,C4B
GO Process	GO:0010605	Negative regulation of macromolecule metabolic process	63	2760	0.23	0.0015	SPP2,CPB2,SERPIND1,LBP,C5,VTN,LTF,HRG,IGFBP5,APOA1,LRP1,APOE,SERPINF1,APCS,SERPINA10,LCN1,YWHAE,CSTA,AMBP,ITIH4,FCN3,ITIH1,AHSG,SERPING1,PGLYRP2,SERPINI1,PF4,A2ML1,PCSK9,SHLD1,F2,SUZ12,SUMO4,CALR,LPA,SERPINF2,SERPINA7,FBLN1,SERPINA5,SERPINA11,ANG,LIMS1,APOD,PAPLN,BLM,ITIH2,FLNA,TAF9,IGFBP3,PROS1,TNRC6A,GAPDH,CST3,ADIPOQ,IGF2,C4A,ITIH3,C4B,UIMC1,SERPINA3,CLTC,KNG1,FGA
GO Process	GO:0035239	Tube morphogenesis	24	669	0.42	0.0015	MMP2,COMP,GPLD1,HRG,APOB,LRP1,APOE,PXDN,ANGPTL6,CSF1R,IHH,EFEMP2,CSPG4,SERPINF2,ANG,APOD,COL18A1,S100A7,FLNA,ILK,MST1,TGFBI,VEGFC,GRHL2
GO Process	GO:0042592	Homeostatic process	39	1406	0.31	0.0015	CPB2,CETP,VCL,COMP,APOC3,LTF,APOB,IGFBP5,APOA1,APOE,SERPINF1,LYZ,LCN1,LCAT,YWHAE,CP,TCIRG1,IHH,HSP90B1,PCSK9,F2,PLG,CALR,FBN1,OIT3,ANG,PRDX6,PIGR,APOA4,S100A9,APOM,ADIPOQ,JCHAIN,APOA5,ATP1A1,SERPINA3,APOC4,CDH5,B2M
GO Process	GO:0051006	Positive regulation of lipoprotein lipase activity	4	9	1.52	0.0015	APOH,APOA1,APOA4,APOA5
GO Process	GO:0055088	Lipid homeostasis	11	161	0.7	0.0015	CETP,APOC3,APOB,APOA1,APOE,LCAT,PCSK9,APOA4,APOM,APOA5,APOC4
GO Process	GO:0051235	Maintenance of location	11	166	0.69	0.0019	APOA1,APOE,HSP90B1,CALR,HSPA5,FBN1,AKAP9,S100A7,S100A9,FLNA,GSN
GO Process	GO:0031347	Regulation of defense response	23	638	0.43	0.0020	LBP,PROC,APOA1,APOE,F12,SERPINF1,APCS,PSMA6,FCN3,AHSG,SERPING1,FCN2,PGLYRP2,MASP1,CFH,S100A9,MBL2,CFI,MASP2,SAA1,ADIPOQ,COLEC11,CDH5
GO Process	GO:0000302	Response to reactive oxygen species	11	169	0.68	0.0022	MMP2,MPO,CAT,APOE,AMBP,HYAL1,HBA1,APOD,APOA4,S100A7,SOD3
GO Process	GO:0030212	Hyaluronan metabolic process	5	23	1.21	0.0022	HYAL1,ITIH4,ITIH1,ITIH2,ITIH3
GO Process	GO:0090207	Regulation of triglyceride metabolic process	6	40	1.05	0.0022	APOC3,GPLD1,APOA1,APOE,APOA4,APOA5
GO Process	GO:0032879	Regulation of localization	51	2103	0.25	0.0023	CPB2,CETP,VCL,CHGA,PON1,VTN,APOC3,GPLD1,APOB,APOA1,LRP1,CFP,APOE,YWHAE,TCIRG1,FCN3,AHSG,CRH,FCN2,MASP1,PCSK9,FGB,F2,SUMO4,CALR,ITLN1,ANG,APOD,AKAP9,APOA4,MTCL1,FLNA,MCOLN2,GSN,MBL2,MASP2,SAA1,ADIPOQ,C4A,ABCA13,COLEC11,C4B,CFB,PLTP,APOA5,ATP1A1,APOC4,CLTC,VEGFC,B2M,FGA
GO Process	GO:0035295	Tube development	28	880	0.37	0.0024	MMP2,COMP,GPLD1,HRG,APOB,IGFBP5,CAT,LRP1,APOE,PXDN,ANGPTL6,CRH,CSF1R,IHH,EFEMP2,CSPG4,SERPINF2,ANG,APOD,COL18A1,S100A7,FLNA,ILK,MST1,TGFBI,RARRES2,VEGFC,GRHL2
GO Process	GO:0031639	Plasminogen activation	4	11	1.43	0.0026	APOH,FGB,F11,FGA
GO Process	GO:0034975	Protein folding in endoplasmic reticulum	4	11	1.43	0.0026	HSP90B1,CALR,HSPA5,P4HB
GO Process	GO:0019731	Antibacterial humoral response	7	64	0.91	0.0031	LTF,CAMP,FGB,ANG,JCHAIN,B2M,FGA
GO Process	GO:0019732	Antifungal humoral response	4	12	1.39	0.0034	LTF,CAMP,ANG,RARRES2
GO Process	GO:0070887	Cellular response to chemical stimulus	59	2609	0.22	0.0035	CPB2,CHGA,LBP,MMP2,EBI3,COMP,C5,MPO,PF4V1,GPLD1,APOB,IGFBP5,CAT,LRP1,APOE,PXDN,SERPINF1,AMBP,PON3,TCIRG1,HYAL1,IGFBP1,CSF1R,PPBP,PF4,CAMP,HSP90B1,PCSK9,FGB,SUMO4,CALR,HBA1,HSPA5,FBN1,P4HB,LIMS1,PRDX6,BLM,AKAP9,APOA4,S100A9,FLNA,ADAMTS13,GSN,APOM,SOD3,GPX3,ILK,GAPDH,SAA1,ADIPOQ,IGF2,MST1,RARRES2,ATP1A1,VEGFC,HYOU1,CDH5,B2M
GO Process	GO:0048878	Chemical homeostasis	28	904	0.36	0.0036	CPB2,CETP,APOC3,LTF,APOB,IGFBP5,APOA1,APOE,SERPINF1,LCAT,YWHAE,CP,TCIRG1,HSP90B1,PCSK9,F2,CALR,FBN1,OIT3,APOA4,S100A9,APOM,ADIPOQ,APOA5,ATP1A1,APOC4,CDH5,B2M
GO Process	GO:0010035	Response to inorganic substance	20	532	0.44	0.0037	MMP2,PON1,MPO,GPLD1,APOB,CAT,SERPINF1,LCAT,TCIRG1,FGB,CALR,HBA1,HSPA5,BLM,APOA4,ADAMTS13,SOD3,SELENOP,B2M,FGA
GO Process	GO:0001503	Ossification	14	287	0.56	0.0038	MMP2,COMP,GPLD1,LTF,IGFBP5,CAT,TCIRG1,AHSG,IHH,BMP1,MINPP1,IGFBP3,IGF2,CLTC
GO Process	GO:0051049	Regulation of transport	44	1763	0.27	0.0044	CPB2,CETP,CHGA,PON1,VTN,APOC3,GPLD1,APOA1,LRP1,CFP,APOE,YWHAE,TCIRG1,FCN3,AHSG,CRH,FCN2,MASP1,PCSK9,FGB,F2,CALR,ITLN1,ANG,APOD,AKAP9,APOA4,MTCL1,FLNA,MBL2,MASP2,SAA1,ADIPOQ,C4A,ABCA13,COLEC11,C4B,CFB,PLTP,APOA5,ATP1A1,VEGFC,B2M,FGA
GO Process	GO:0031099	Regeneration	10	154	0.68	0.0049	CPB2,MMP2,VTN,SERPINA10,IGFBP1,IHH,PLG,APOD,APOA4,APOA5
GO Process	GO:0010873	Positive regulation of cholesterol esterification	3	4	1.74	0.0053	APOA1,APOA4,APOA5
GO Process	GO:0051919	Positive regulation of fibrinolysis	3	4	1.74	0.0053	F12,PLG,F11
GO Process	GO:0031640	Killing of cells of another organism	7	71	0.86	0.0054	CHGA,LTF,LYZ,PPBP,PF4,GAPDH,KNG1
GO Process	GO:0042127	Regulation of cell population proliferation	42	1669	0.27	0.0054	ST6GAL1,CPB2,APOH,TPD52L2,MMP2,EBI3,COMP,GPLD1,SPARC,LTF,HRG,IGFBP5,APOE,SERPINF1,TCIRG1,HYAL1,CSF1R,SCGB3A1,IHH,PF4,CAMP,LRG1,F2,PLG,EFEMP2,SUZ12,CALR,SERPINF2,FBLN1,ANG,THBS4,APOD,COL18A1,SMARCA1,IGFBP3,ILK,ADIPOQ,IGF2,MST1,VEGFC,CDH5,B2M
GO Process	GO:0045916	Negative regulation of complement activation	4	14	1.33	0.0054	C4BPB,SERPING1,MASP1,C4BPA
GO Process	GO:0046470	Phosphatidylcholine metabolic process	7	71	0.86	0.0054	CETP,PON1,GPLD1,APOA1,LCAT,APOA4,APOA5
GO Process	GO:0051093	Negative regulation of developmental process	28	933	0.35	0.0059	CETP,APOH,SPARC,LTF,HRG,IGFBP5,APOE,SERPINF1,APCS,AHSG,PGLYRP2,IHH,PF4,F2,SUZ12,CALR,FBN1,FBLN1,THBS4,C1QC,EFEMP1,CST3,ADIPOQ,IGF2,PAEP,VEGFC,GRHL2,B2M
GO Process	GO:0002688	Regulation of leukocyte chemotaxis	9	128	0.72	0.0061	LBP,C5,CSF1R,CALR,THBS4,S100A7,MST1,RARRES2,VEGFC
GO Process	GO:0002699	Positive regulation of immune effector process	13	264	0.56	0.0061	LBP,CFP,FCN3,FCN2,MASP1,SHLD1,MBL2,MASP2,C4A,COLEC11,C4B,CFB,B2M
GO Process	GO:0001906	Cell killing	9	129	0.71	0.0064	CHGA,LTF,LYZ,PPBP,PF4,F2,GAPDH,KNG1,B2M
GO Process	GO:0010720	Positive regulation of cell development	14	304	0.53	0.0064	PROC,APOA1,SERPINF1,FGB,CALR,P4HB,LIMS1,FLNA,ILK,ADIPOQ,LMOD3,VEGFC,CDH5,FGA
GO Process	GO:0045595	Regulation of cell differentiation	40	1582	0.27	0.0071	CETP,VCL,LTF,APOB,IGFBP5,PROC,APOA1,SERPINF1,APCS,NID1,TCIRG1,PGLYRP2,IHH,PF4,LRG1,FGB,F2,EFEMP2,SUZ12,CALR,SERPINF2,FBN1,P4HB,FBLN1,LIMS1,FLNA,C1QC,IGFBP3,EFEMP1,ILK,ADIPOQ,IGF2,LMOD3,PAEP,RARRES2,VEGFC,GRHL2,CDH5,B2M,FGA
GO Process	GO:0022604	Regulation of cell morphogenesis	14	311	0.52	0.0079	ST6GAL1,SPARC,APOA1,CSF1R,SH3D19,FGB,F2,CALR,P4HB,FBLN1,LIMS1,FLNA,ILK,FGA
GO Process	GO:0034374	Low-density lipoprotein particle remodeling	4	16	1.27	0.0079	CETP,MPO,APOB,APOE
GO Process	GO:0042221	Response to chemical	80	4010	0.17	0.0079	ST6GAL1,CPB2,SERPIND1,CHGA,LBP,MMP2,EBI3,COMP,PON1,C5,MPO,PF4V1,GPLD1,HRG,APOB,IGFBP5,APOA1,CAT,LRP1,APOE,PXDN,F12,SERPINF1,LCAT,AMBP,PON3,TCIRG1,HYAL1,ITIH4,IGFBP1,CSF1R,IHH,PPBP,PF4,CAMP,HSP90B1,PCSK9,FGB,SUMO4,CALR,HBA1,HSPA5,FBN1,P4HB,ANG,LIMS1,THBS4,PRDX6,APOD,BLM,AKAP9,PIGR,APOA4,F5,S100A7,S100A9,FLNA,ADAMTS13,GSN,APOM,CA6,TAF9,SOD3,GPX3,ILK,GAPDH,SAA1,ADIPOQ,IGF2,MST1,C4B,RARRES2,SELENOP,ATP1A1,VEGFC,HYOU1,CDH5,B2M,FGA,SDF4

GO Process	GO:0016477	Cell migration	27	903	0.35	0.0080	CHGA,LBP,MMP2,C5,VTN,PF4V1,GPLD1,APOA1,ATRN,YWHAE,PPBP,PF4,LRG1,PLG,CSPG4,ANG,THBS4,CDC42BPA,S100A9,FLNA,MCOLN2,CD99,ILK,SAA1,PSG2,VEGFC,CDH5
GO Process	GO:0001894	Tissue homeostasis	12	238	0.57	0.0088	VCL,COMP,LTF,LYZ,LCN1,TCIRG1,IHH,PIGR,JCHAIN,SERPINA3,CDH5,B2M
GO Process	GO:0030335	Positive regulation of cell migration	19	529	0.42	0.0093	LBP,MMP2,VTN,GPLD1,SPARC,IGFBP5,HYAL1,CSF1R,PLG,CALR,HSPA5,FBLN1,THBS4,S100A7,F10,CD99,RARRES2,VEGFC,CDH5
GO Process	GO:0007565	Female pregnancy	10	170	0.64	0.0096	MMP2,IGFBP5,AMBP,CRH,IHH,PSG11,FBLN1,PSG4,PSG2,MST1
GO Process	GO:1903053	Regulation of extracellular matrix organization	6	56	0.9	0.0107	CPB2,LRP1,NID1,ANTXR1,EFEMP2,CST3
GO Process	GO:0097746	Blood vessel diameter maintenance	9	140	0.68	0.0109	MMP2,COMP,APOE,GUCY1A1,FGB,SERPINF2,SOD3,KNG1,FGA
GO Process	GO:0006066	Alcohol metabolic process	14	324	0.51	0.0113	CETP,PON1,APOB,APOA1,TTR,CAT,APOE,LCAT,PCSK9,APOL1,APOA4,MINPP1,APOF,APOA5
GO Process	GO:0010901	Regulation of very-low-density lipoprotein particle remodeling	3	6	1.57	0.0113	APOC3,APOA1,APOA5
GO Process	GO:0001525	Angiogenesis	14	325	0.5	0.0116	MMP2,GPLD1,HRG,PXDN,ANGPTL6,IHH,CSPG4,ANG,APOD,COL18A1,S100A7,FLNA,TGFBI,VEGFC
GO Process	GO:0048660	Regulation of smooth muscle cell proliferation	9	142	0.67	0.0117	MMP2,IGFBP5,APOE,EFEMP2,SERPINF2,ANG,APOD,IGFBP3,ADIPOQ
GO Process	GO:0010033	Response to organic substance	58	2692	0.2	0.0128	ST6GAL1,CPB2,LBP,MMP2,EBI3,COMP,PON1,MPO,PF4V1,GPLD1,APOB,IGFBP5,CAT,LRP1,F12,SERPINF1,LCAT,TCIRG1,HYAL1,ITIH4,IGFBP1,CSF1R,IHH,PPBP,PF4,CAMP,HSP90B1,PCSK9,FGB,CALR,HSPA5,FBN1,P4HB,ANG,LIMS1,THBS4,BLM,AKAP9,APOA4,F5,S100A7,S100A9,FLNA,ADAMTS13,GSN,TAF9,GPX3,ILK,GAPDH,ADIPOQ,IGF2,C4B,RARRES2,ATP1A1,VEGFC,CDH5,B2M,SDF4
GO Process	GO:0048523	Negative regulation of cellular process	90	4736	0.15	0.0131	ST6GAL1,CPB2,CETP,APOH,VCL,CHGA,MMP2,COMP,C5,MPO,APOC3,GPLD1,SPARC,LTF,HRG,IGFBP5,PROC,PLEK,APOA1,CAT,LRP1,APOE,PXDN,SERPINF1,APCS,YWHAE,DGKG,AMBP,PON3,HYAL1,FCN3,AHSG,IGFBP1,CRH,CSF1R,PGLYRP2,SCGB3A1,IHH,PF4,HSP90B1,ANTXR1,PCSK9,SHLD1,FGB,F2,PLG,EFEMP2,SUZ12,SUMO4,CALR,HSPA5,FBN1,FBLN1,ANG,LIMS1,APOD,BLM,APOA4,COL18A1,QSOX1,FLNA,MCOLN2,GSN,C1QC,APOM,TAF9,IGFBP3,EFEMP1,TNRC6A,ILK,GAPDH,FGL1,CST3,ADIPOQ,IGF2,CDK3,MST1,LMOD3,TGFBI,PAEP,UIMC1,ATP1A1,CLTC,VEGFC,HYOU1,KNG1,GRHL2,CDH5,B2M,FGA
GO Process	GO:0043903	Regulation of biological process involved in symbiotic interaction	6	60	0.87	0.0144	LTF,APCS,FCN3,P4HB,FBLN1,GSN
GO Process	GO:0001775	Cell activation	22	693	0.37	0.0145	VCL,CHGA,LBP,EBI3,COMP,PF4V1,HRG,PLEK,LRP1,VWF,DGKG,TCIRG1,PF4,CAMP,FGB,F2,FLNA,ADAMTS13,ILK,SAA1,B2M,FGA
GO Process	GO:0014012	Peripheral nervous system axon regeneration	3	7	1.5	0.0153	MMP2,APOD,APOA4
GO Process	GO:0034384	High-density lipoprotein particle clearance	3	7	1.5	0.0153	APOA1,APOE,APOM
GO Process	GO:0060249	Anatomical structure homeostasis	13	296	0.51	0.0159	VCL,COMP,LTF,LYZ,LCN1,TCIRG1,IHH,PLG,PIGR,JCHAIN,SERPINA3,CDH5,B2M
GO Process	GO:0060191	Regulation of lipase activity	7	88	0.77	0.0161	APOH,APOC3,APOA1,LRP1,ANG,APOA4,APOA5
GO Process	GO:0050994	Regulation of lipid catabolic process	6	62	0.86	0.0166	APOC3,GPLD1,APOA1,APOA4,RARRES2,APOA5
GO Process	GO:1901701	Cellular response to oxygen-containing compound	29	1057	0.31	0.0169	CPB2,LBP,MMP2,MPO,PF4V1,GPLD1,IGFBP5,CAT,LRP1,SERPINF1,AMBP,IGFBP1,PPBP,PF4,CAMP,HSP90B1,PCSK9,HSPA5,FBN1,BLM,AKAP9,APOA4,FLNA,ADAMTS13,SOD3,ADIPOQ,IGF2,RARRES2,B2M
GO Process	GO:0040013	Negative regulation of locomotion	14	340	0.48	0.0172	ST6GAL1,APOH,VCL,C5,HRG,IGFBP5,LRP1,APOE,SERPINF1,CALR,FBLN1,APOD,IGFBP3,ADIPOQ
GO Process	GO:2000146	Negative regulation of cell motility	13	304	0.5	0.0199	APOH,VCL,C5,HRG,IGFBP5,LRP1,APOE,SERPINF1,CALR,FBLN1,APOD,IGFBP3,ADIPOQ
GO Process	GO:0032489	Regulation of Cdc42 protein signal transduction	3	8	1.44	0.0201	APOC3,APOA1,APOE
GO Process	GO:1905920	Positive regulation of CoA-transferase activity	3	8	1.44	0.0201	APOA1,APOE,APOA4
GO Process	GO:1990266	Neutrophil migration	7	92	0.75	0.0202	LBP,PF4V1,PPBP,PF4,S100A9,MCOLN2,SAA1
GO Process	GO:0002683	Negative regulation of immune system process	16	433	0.44	0.0203	C5,LTF,APOA1,C4BPB,APCS,AMBP,SERPING1,PGLYRP2,IHH,MASP1,FBN1,APOD,C4BPA,C1QC,FGL1,ADIPOQ
GO Process	GO:0050920	Regulation of chemotaxis	11	227	0.55	0.0203	ST6GAL1,LBP,C5,HRG,CSF1R,CALR,THBS4,S100A7,MST1,RARRES2,VEGFC
GO Process	GO:0022603	Regulation of anatomical structure morphogenesis	26	920	0.32	0.0218	ST6GAL1,APOH,C5,SPARC,HRG,APOA1,SERPINF1,HYAL1,CSF1R,PF4,CAMP,LRG1,SH3D19,FGB,F2,CALR,P4HB,FBLN1,LIMS1,THBS4,FLNA,ILK,CST3,VEGFC,CDH5,FGA
GO Process	GO:0002685	Regulation of leukocyte migration	11	231	0.55	0.0230	LBP,C5,CSF1R,CALR,THBS4,APOD,S100A7,CD99,MST1,RARRES2,VEGFC
GO Process	GO:0090208	Positive regulation of triglyceride metabolic process	4	23	1.11	0.0230	GPLD1,APOA1,APOA4,APOA5
GO Process	GO:0031589	Cell-substrate adhesion	10	195	0.58	0.0240	VCL,VTN,VWF,ATRN,NID1,ANTXR1,FGB,ADAMTS13,ILK,FGA
GO Process	GO:0010898	Positive regulation of triglyceride catabolic process	3	9	1.39	0.0254	APOA1,APOA4,APOA5
GO Process	GO:0034114	Regulation of heterotypic cell-cell adhesion	4	24	1.09	0.0262	APOA1,FGB,ADIPOQ,FGA
GO Process	GO:0051651	Maintenance of location in cell	7	97	0.73	0.0262	APOE,HSP90B1,CALR,HSPA5,AKAP9,S100A9,GSN
GO Process	GO:0090075	Relaxation of muscle	4	24	1.09	0.0262	CHGA,GUCY1A1,GSN,ATP1A1
GO Process	GO:0045088	Regulation of innate immune response	11	238	0.53	0.0284	LBP,APOE,FCN3,SERPING1,FCN2,MASP1,CFH,MBL2,CFI,MASP2,COLEC11
GO Process	GO:0002690	Positive regulation of leukocyte chemotaxis	7	99	0.72	0.0289	LBP,CSF1R,CALR,THBS4,S100A7,RARRES2,VEGFC
GO Process	GO:0015914	Phospholipid transport	7	99	0.72	0.0289	CETP,APOC3,APOA1,APOE,APOA4,PLTP,APOA5
GO Process	GO:0045807	Positive regulation of endocytosis	7	99	0.72	0.0289	VTN,LRP1,APOE,PCSK9,ABCA13,APOA5,B2M
GO Process	GO:0006629	Lipid metabolic process	31	1210	0.28	0.0297	CETP,APOH,PON1,APOC3,GPLD1,APOB,PLEK,APOA1,TTR,CAT,LRP1,APOE,LCAT,DGKG,CRH,CSF1R,PCSK9,APOL1,LPA,ANG,PRDX6,APOD,APOA4,APOF,ADIPOQ,ACSF2,PLTP,RARRES2,GC,APOA5,APOC4
GO Process	GO:0030300	Regulation of intestinal cholesterol absorption	3	10	1.35	0.0316	APOA1,APOA4,APOA5
GO Process	GO:0090209	Negative regulation of triglyceride metabolic process	3	10	1.35	0.0316	APOC3,GPLD1,APOE
GO Process	GO:0050678	Regulation of epithelial cell proliferation	14	366	0.45	0.0317	CPB2,APOH,SPARC,APOE,SERPINF1,HYAL1,IHH,LRG1,ANG,THBS4,IGF2,MST1,VEGFC,B2M
GO Process	GO:0051258	Protein polymerization	7	101	0.71	0.0317	VTN,FGB,ANG,AKAP9,GSN,SLAIN1,FGA
GO Process	GO:0006910	Phagocytosis, recognition	4	26	1.06	0.0329	FCN3,FCN2,IGHV3-15,IGHV3-72
GO Process	GO:0014912	Negative regulation of smooth muscle cell migration	4	26	1.06	0.0329	IGFBP5,LRP1,IGFBP3,ADIPOQ
GO Process	GO:0072593	Reactive oxygen species metabolic process	7	102	0.71	0.0329	MPO,CAT,PXDN,HBA1,APOA4,SOD3,GPX3
GO Process	GO:0010594	Regulation of endothelial cell migration	9	169	0.6	0.0334	APOH,GPLD1,SPARC,HRG,APOE,SERPINF1,PLG,CALR,VEGFC
GO Process	GO:0051094	Positive regulation of developmental process	33	1332	0.26	0.0335	C5,LTF,APOB,PROC,APOA1,APOE,SERPINF1,NID1,HYAL1,SCGB3A1,IHH,PF4,CAMP,LRG1,BMP1,SHLD1,FGB,EFEMP2,CALR,SERPINF2,P4HB,LIMS1,FLNA,IGFBP3,ILK,ADIPOQ,IGF2,MST1,LMOD3,RARRES2,VEGFC,CDH5,FGA
GO Process	GO:0050727	Regulation of inflammatory response	14	371	0.45	0.0351	LBP,PROC,APOA1,APOE,F12,SERPINF1,APCS,PSMA6,AHSG,PGLYRP2,S100A9,SAA1,ADIPOQ,CDH5
GO Process	GO:0030336	Negative regulation of cell migration	12	288	0.49	0.0369	APOH,VCL,C5,HRG,IGFBP5,LRP1,APOE,SERPINF1,CALR,APOD,IGFBP3,ADIPOQ
GO Process	GO:0045765	Regulation of angiogenesis	12	288	0.49	0.0369	APOH,C5,SPARC,HRG,SERPINF1,HYAL1,PF4,CAMP,LRG1,THBS4,VEGFC,CDH5
GO Process	GO:0007568	Aging	9	172	0.59	0.0370	MMP2,COMP,MPO,IGFBP5,CAT,SERPINF1,IGFBP1,SERPING1,APOD

GO Process	GO:1900121	Negative regulation of receptor binding	3	11	1.31	0.0380	PCSK9,ADIPOQ,B2M
GO Process	GO:0001936	Regulation of endothelial cell proliferation	8	138	0.63	0.0383	APOH,SPARC,APOE,LRG1,ANG,THBS4,IGF2,VEGFC
GO Process	GO:0097529	Myeloid leukocyte migration	8	138	0.63	0.0383	CHGA,LBP,PF4V1,PPBP,PF4,S100A9,MCOLN2,SAA1
GO Process	GO:0045216	Cell-cell junction organization	9	174	0.58	0.0394	VCL,DSG2,CDH6,CSF1R,LIMS1,FLNA,SVEP1,GRHL2,CDH5
GO Process	GO:0008152	Metabolic process	134	7988	0.09	0.0397	ST6GAL1,CPB2,CETP,GNPTG,APOH,TPD52L2,APMAP,F9,MMP2,COMP,PON1,PCOLCE,MPO,APOC3,GPLD1,PCDH12,LTF,APOB,PROC,PLEK,APOA1,TTR,CAT,LRP1,DHX29,APOE,PXDN,F12,LYZ,PSMA6,LCN1,LCAT,CSTA,DGKG,AMBP,PON3,TCIRG1,HYAL1,ITIH4,FCN3,ITIH1,ANKRD31,CRH,HABP2,CSF1R,FCN2,PGLYRP2,IHH,MASP1,APEH,GUCY1A1,HSP90B1,PCSK9,BMP1,SHLD1,FGB,F2,PLG,UBE3C,SUZ12,APOL1,SUMO4,CALR,LPA,HBA1,HSPA5,FBN1,P4HB,FBLN1,ANG,GANAB,PRDX6,PROZ,QPCT,APOD,BLM,AKAP9,APOA4,ITIH2,CNDP1,F8,CDC42BPA,F13B,CFH,F5,CD5L,S100A9,FLNA,ADAMTS13,MINPP1,GSN,MBL2,SVEP1,F10,APOM,CA6,TAF9,IGFBP3,SOD3,GPX3,EFEMP1,CFI,ILK,GAPDH,RNASE1,APOF,MASP2,F11,C1S,PCYOX1,KSR2,ADIPOQ,IGF2,ACSF2,CDK3,COLEC11,MST1,ITIH3,CFB,PLTP,RARRES2,GC,HGFAC,UIMC1,SELENOP,HPR,C1R,APOA5,RNASE4,APOC4,CFD,CLTC,B2M,FGA
GO Process	GO:0031529	Ruffle organization	4	28	1.02	0.0402	PLEK,TCIRG1,CSF1R,CSPG4
GO Process	GO:1902106	Negative regulation of leukocyte differentiation	7	107	0.69	0.0410	LTF,APCS,PGLYRP2,IHH,FBN1,C1QC,ADIPOQ
GO Process	GO:0019222	Regulation of metabolic process	117	6784	0.11	0.0411	SPP2,CPB2,SERPIND1,LBP,EBI3,COMP,PCOLCE,C5,VTN,APOC3,GPLD1,LTF,HRG,APOB,IGFBP5,PLEK,APOA1,CAT,LRP1,C4BPB,DHX29,APOE,F12,SERPINF1,APCS,PSMA6,SERPINA10,LCN1,YWHAE,CSTA,AMBP,CENPE,PON3,TCIRG1,ITIH4,LUM,FCN3,ITIH1,AHSG,ANKRD31,SERPING1,CSF1R,PGLYRP2,IHH,SERPINI1,PF4,CAMP,A2ML1,HSP90B1,ANTXR1,SH3D19,PCSK9,SHLD1,F2,INHBC,CSPG4,SUZ12,SUMO4,CALR,LPA,SERPINF2,ITLN1,HSPA5,FBN1,SERPINA7,FBLN1,SERPINA5,SERPINA11,ANG,LIMS1,THBS4,PRDX6,APOD,PAPLN,BLM,AKAP9,APOA4,ITIH2,MTCL1,C4BPA,QSOX1,S100A9,FLNA,MCOLN2,SMARCA1,GSN,TAF9,IGFBP3,PROS1,EFEMP1,TNRC6A,ILK,GAPDH,CST3,SAA1,KSR2,ADIPOQ,IGF2,ORM2,C4A,MST1,ITIH3,C4B,PAEP,RARRES2,UIMC1,JCHAIN,APOA5,ATP1A1,SERPINA3,CLTC,VEGFC,KNG1,GRHL2,CDH5,B2M,FGA
GO Process	GO:0008285	Negative regulation of cell population proliferation	21	713	0.34	0.0423	CPB2,APOH,GPLD1,SPARC,HRG,IGFBP5,APOE,SERPINF1,CSF1R,IHH,PLG,EFEMP2,FBLN1,ANG,APOD,COL18A1,IGFBP3,ILK,ADIPOQ,CDH5,B2M
GO Process	GO:0001895	Retina homeostasis	6	78	0.76	0.0434	LTF,LYZ,LCN1,PIGR,JCHAIN,B2M
GO Process	GO:0010758	Regulation of macrophage chemotaxis	4	29	1.01	0.0446	C5,CSF1R,MST1,RARRES2
GO Process	GO:0071622	Regulation of granulocyte chemotaxis	5	52	0.85	0.0460	LBP,CSF1R,THBS4,S100A7,RARRES2
GO Process	GO:0045597	Positive regulation of cell differentiation	24	876	0.31	0.0483	LTF,APOB,PROC,APOA1,SERPINF1,NID1,IHH,PF4,LRG1,FGB,EFEMP2,CALR,SERPINF2,P4HB,LIMS1,FLNA,IGFBP3,ILK,ADIPOQ,LMOD3,RARRES2,VEGFC,CDH5,FGA
GO Process	GO:0030593	Neutrophil chemotaxis	6	80	0.74	0.0485	LBP,PF4V1,PPBP,PF4,S100A9,SAA1
GO Process	GO:1901615	Organic hydroxy compound metabolic process	16	478	0.39	0.0485	CETP,PON1,APOB,APOA1,TTR,CAT,APOE,LCAT,PCSK9,APOL1,APOA4,MINPP1,APOF,PLTP,GC,APOA5
GO Process	GO:0002762	Negative regulation of myeloid leukocyte differentiation	5	53	0.84	0.0490	LTF,APCS,FBN1,C1QC,ADIPOQ
GO Process	GO:0006826	Iron ion transport	5	53	0.84	0.0490	LTF,HRG,CP,CLTC,B2M
GO Process	GO:0010543	Regulation of platelet activation	5	53	0.84	0.0490	HRG,PLEK,APOE,F2,FLNA
GO Process	GO:0033993	Response to lipid	23	827	0.31	0.0490	LBP,MMP2,PON1,MPO,PF4V1,GPLD1,APOB,CAT,SERPINF1,LCAT,IHH,PPBP,PF4,CAMP,CALR,APOA4,S100A7,S100A9,ADAMTS13,GPX3,ADIPOQ,ATP1A1,B2M
GO Process	GO:0034445	Negative regulation of plasma lipoprotein oxidation	2	2	1.87	0.0490	APOA4,APOM
GO Process	GO:0035606	Peptidyl-cysteine S-trans-nitrosylation	2	2	1.87	0.0490	S100A9,GAPDH
GO Process	GO:0045940	Positive regulation of steroid metabolic process	4	30	0.99	0.0490	APOA1,APOE,APOA4,APOA5
GO Process	GO:0097421	Liver regeneration	4	30	0.99	0.0490	CPB2,VTN,SERPINA10,IHH
GO Function	GO:0004866	Endopeptidase inhibitor activity	31	177	1.11	6.50e-20	SPP2,SERPIND1,C5,LTF,HRG,SERPINF1,SERPINA10,LCN1,CSTA,AMBP,ITIH4,ITIH1,AHSG,SERPING1,SERPINI1,A2ML1,LPA,SERPINF2,SERPINA7,SERPINA5,SERPINA11,PAPLN,ITIH2,PROS1,GAPDH,CST3,C4A,ITIH3,C4B,SERPINA3,KNG1
GO Function	GO:0005509	Calcium ion binding	53	717	0.74	6.74e-20	RCN1,F9,COMP,PON1,SPARC,PCDH12,PROC,LRP1,F12,APCS,DSG2,NID1,DGKG,CDH6,ITIH1,HABP2,PCDH1,IHH,MASP1,HSP90B1,BMP1,DST,F2,EFEMP2,CALR,ITLN1,HSPA5,FBN1,FBLN1,OIT3,THBS4,PROZ,PCDH18,S100A7,S100A9,CRTAC1,ADAMTS13,GSN,SVEP1,F10,CALML5,PROS1,EFEMP1,EFCAB5,MASP2,FBLN2,C1S,NUCB1,COLEC11,SPARCL1,C1R,CDH5,SDF4
GO Function	GO:0061134	Peptidase regulator activity	33	227	1.03	1.05e-19	SPP2,SERPIND1,PCOLCE,C5,LTF,HRG,SERPINF1,SERPINA10,LCN1,CSTA,AMBP,ITIH4,ITIH1,AHSG,SERPING1,SERPINI1,A2ML1,LPA,SERPINF2,SERPINA7,FBLN1,SERPINA5,SERPINA11,PAPLN,ITIH2,PROS1,GAPDH,CST3,C4A,ITIH3,C4B,SERPINA3,KNG1
GO Function	GO:0005539	Glycosaminoglycan binding	32	245	0.99	5.97e-18	APOH,SERPIND1,COMP,PCOLCE,MPO,VTN,PF4V1,LTF,HRG,APOB,APOE,SERPINA10,AMBP,ITIH1,HABP2,PGLYRP2,PF4,F2,EFEMP2,LPA,FBN1,SERPINA5,ANG,THBS4,ITIH2,CFH,SOD3,NAV2,SAA1,F11,APOA5,KNG1
GO Function	GO:0008201	Heparin binding	27	173	1.06	1.11e-16	APOH,SERPIND1,COMP,PCOLCE,MPO,VTN,PF4V1,LTF,HRG,APOB,APOE,SERPINA10,PF4,F2,EFEMP2,LPA,FBN1,SERPINA5,ANG,THBS4,CFH,SOD3,NAV2,SAA1,F11,APOA5,KNG1
GO Function	GO:0004252	Serine-type endopeptidase activity	26	176	1.04	1.58e-15	F9,MMP2,LTF,PROC,F12,HABP2,MASP1,APEH,PCSK9,BMP1,F2,PLG,LPA,PROZ,CD5L,F10,CFI,MASP2,F11,C1S,MST1,CFB,HGFAC,HPR,C1R,CFD
GO Function	GO:0001848	Complement binding	14	26	1.6	2.90e-14	APCS,CFHR5,CFHR1,CALR,C8A,CFHR2,CFHR4,CFHR3,CFH,C8G,MASP2,C4A,C4B,CFB
GO Function	GO:0005102	Signaling receptor binding	65	1499	0.51	2.90e-14	LBP,EBI3,COMP,C5,VTN,PF4V1,APOC3,HRG,APOB,APOA1,TTR,LRP1,APOE,PXDN,ANGPTL6,VWF,LCN1,IGFBP1,CRH,FCN2,SCGB3A1,IHH,PPBP,PF4,HSP90B1,LRG1,PCSK9,BMP1,FGB,DST,F2,INHBC,PLG,CALR,LPA,HBA1,FBN1,P4HB,FBLN1,ANG,THBS4,AKAP9,S100A7,S100A9,FLNA,ADAMTS13,MBL2,EFEMP1,FGL1,APOF,SAA1,ADIPOQ,IGF2,MST1,TGFBI,RARRES2,JCHAIN,APOA5,CLTC,IGHV3-15,IGHV3-72,VEGFC,KNG1,CDH5,FGA
GO Function	GO:1901681	Sulfur compound binding	29	272	0.9	2.94e-14	APOH,SERPIND1,COMP,PCOLCE,MPO,VTN,PF4V1,LTF,HRG,APOB,LRP1,APOE,SERPINA10,AMBP,PF4,F2,EFEMP2,LPA,FBN1,SERPINA5,ANG,THBS4,CFH,SOD3,NAV2,SAA1,F11,APOA5,KNG1
GO Function	GO:0004857	Enzyme inhibitor activity	32	396	0.78	9.72e-13	SPP2,SERPIND1,C5,APOC3,LTF,HRG,SERPINF1,SERPINA10,LCN1,CSTA,AMBP,ITIH4,ITIH1,AHSG,SERPING1,SERPINI1,A2ML1,LPA,SERPINF2,SERPINA7,SERPINA5,SERPINA11,PAPLN,ITIH2,PROS1,GAPDH,CST3,C4A,ITIH3,C4B,SERPINA3,KNG1
GO Function	GO:0004867	Serine-type endopeptidase inhibitor activity	18	98	1.13	5.66e-12	SERPIND1,HRG,SERPINF1,SERPINA10,AMBP,ITIH4,ITIH1,SERPING1,SERPINI1,A2ML1,SERPINF2,SERPINA7,SERPINA5,SERPINA11,PAPLN,ITIH2,ITIH3,SERPINA3

GO Function	GO:0005515	Protein binding	155	7242	0.2	4.00e-10	ST6GAL1,GNPTG,APOH,VCL,TPD52L2,LBP,MMP2,EBI3,COMP,PON1,C5,VTN,PF4V1,APOC3,HRG,APOB,IGFBP5,PLEK,APOA1,TTR,CAT,LRP1,DHX29,APOE,PXDN,ANGPTL6,F12,APCS,CFHR5,LYZ,VWF,PSMA6,DSG2,LCN1,LCAT,NID1,YWHAE,CSTA,CP,CDH6,DNAH5,AMBP,CENPE,PON3,TCIRG1,FCN3,IGFBP1,CRH,CSF1R,FCN2,SCGB3A1,IHH,PPBP,PF4,MASP1,APEH,A2ML1,HSP90B1,ANTXR1,LRG1,SH3D19,PCSK9,BMP1,FGB,DST,F2,INHBC,PLG,EFEMP2,CSPG4,C1QB,CFHR1,SUZ12,SUMO4,CALR,LPA,SERPINF2,HBA1,ITLN1,HSPA5,FBN1,P4HB,FBLN1,SERPINA5,DNAH8,ANG,LIMS1,THBS4,PRDX6,BLM,AKAP9,APOA4,COL18A1,MTCL1,C8A,CDC42BPA,CFHR2,CFHR4,CFHR3,CFH,S100A7,S100A9,ADAMTSL4,FLNA,MCOLN2,SMARCA1,C8G,ADAMTS13,GSN,MBL2,TAF9,IGFBP3,GPX3,KIF19,EFEMP1,ILK,GAPDH,FGL1,APOF,CST3,MASP2,PSG4,SAA1,F11,C1S,NUCB1,ADIPOQ,IGF2,C4A,COLEC11,MST1,LMOD3,C4B,TGFBI,CFB,IGFALS,PLTP,RARRES2,GC,UIMC1,JCHAIN,HPR,C1R,APOA5,ATP1A1,CLTC,IGHV3-15,IGHV3-72,VEGFC,HYOU1,KNG1,CDH5,B2M,FGA,SDF4
GO Function	GO:0098772	Molecular function regulator activity	66	1960	0.4	1.14e-09	SPP2,APOH,SERPIND1,EBI3,PCOLCE,C5,PF4V1,APOC3,GPLD1,LTF,HRG,APOA1,TTR,APOE,PXDN,SERPINF1,SERPINA10,LCN1,YWHAE,CSTA,AMBP,ITIH4,ITIH1,AHSG,CRH,SERPING1,SCGB3A1,SERPINI1,PPBP,PF4,A2ML1,PCSK9,BMP1,F2,INHBC,SUZ12,CPN2,LPA,SERPINF2,FBN1,SERPINA7,FBLN1,SERPINA5,SERPINA11,THBS4,PAPLN,AKAP9,APOA4,ITIH2,FLNA,CALML5,IGFBP3,PROS1,EFEMP1,GAPDH,CST3,NUCB1,ADIPOQ,IGF2,C4A,ITIH3,C4B,APOA5,SERPINA3,VEGFC,KNG1
GO Function	GO:0001846	Opsonin binding	10	21	1.55	1.41e-09	APCS,CFHR5,CFHR1,CALR,CFHR2,CFHR4,CFHR3,CFH,MASP2,C4A
GO Function	GO:0004175	Endopeptidase activity	29	437	0.69	1.55e-09	F9,MMP2,LTF,PROC,F12,PSMA6,HABP2,MASP1,APEH,PCSK9,BMP1,F2,PLG,LPA,PROZ,PAPLN,CD5L,ADAMTS13,F10,CFI,MASP2,F11,C1S,MST1,CFB,HGFAC,HPR,C1R,CFD
GO Function	GO:0005488	Binding	221	12838	0.11	2.12e-08	RCN1,ST6GAL1,CPB2,CETP,GNPTG,APOH,VCL,SERPIND1,TPD52L2,LBP,F9,MMP2,EBI3,COMP,PON1,PCOLCE,C5,MPO,VTN,AFM,PF4V1,APOC3,SPARC,PCDH12,LTF,HRG,APOB,IGFBP5,PROC,PLEK,APOA1,TTR,CAT,LRP1,DHX29,APOE,PXDN,ANGPTL6,F12,APCS,CFHR5,LYZ,VWF,PSMA6,DSG2,SERPINA10,ATRN,LCN1,LCAT,NID1,YWHAE,CSTA,CP,DGKG,CDH6,DNAH5,AMBP,CENPE,PON3,TCIRG1,LUM,FCN3,ITIH1,IGFBP1,CRH,HABP2,CSF1R,PCDH1,FCN2,PGLYRP2,SCGB3A1,IHH,PPBP,PF4,MASP1,CAMP,APEH,GUCY1A1,A2ML1,HSP90B1,ANTXR1,LRG1,SH3D19,PCSK9,BMP1,FGB,DST,F2,INHBC,PLG,EFEMP2,CSPG4,C1QB,CFHR1,SUZ12,APOL1,SUMO4,CALR,LPA,SERPINF2,HBA1,ITLN1,HSPA5,FBN1,P4HB,FBLN1,SERPINA5,DNAH8,OIT3,ANG,LIMS1,THBS4,GANAB,PRDX6,PROZ,QPCT,APOD,ASGR2,BLM,AKAP9,APOA4,ITIH2,CNDP1,COL18A1,MTCL1,F8,C8A,PCDH18,CDC42BPA,C4BPA,CFHR2,CFHR4,CFHR3,CFH,QSOX1,F5,S100A7,S100A9,ADAMTSL4,FLNA,CRTAC1,MCOLN2,SMARCA1,C8B,C8G,ADAMTS13,GSN,MBL2,SVEP1,F10,APOM,CA6,CKAP4,CALML5,TAF9,IGFBP3,SOD3,GPX3,KIF19,PROS1,EFEMP1,CFI,EFCAB5,TNRC6A,NAV2,ILK,GAPDH,RNASE1,FGL1,APOF,CST3,MASP2,FBLN2,PSG4,SAA1,F11,C1S,NUCB1,PCYOX1,KSR2,ADIPOQ,IGF2,C4A,ACSF2,CDK3,ABCA13,COLEC11,MST1,LMOD3,SPARCL1,C4B,TGFBI,CFB,IGFALS,PLTP,PAEP,RARRES2,GC,UIMC1,SELENOP,JCHAIN,HPR,C1R,APOA5,ATP1A1,SERPINA3,RNASE4,IGKV2D-28,CLTC,IGHV3-15,IGHV3-72,VEGFC,HYOU1,KNG1,TRIM66,GRHL2,CDH5,B2M,FGA,PDIA4,SDF4
GO Function	GO:0008233	Peptidase activity	32	617	0.58	4.54e-08	CPB2,F9,MMP2,LTF,PROC,F12,PSMA6,HABP2,IHH,MASP1,APEH,PCSK9,BMP1,F2,PLG,LPA,PROZ,PAPLN,CNDP1,CD5L,ADAMTS13,F10,CFI,MASP2,F11,C1S,MST1,CFB,HGFAC,HPR,C1R,CFD
GO Function	GO:0070325	Lipoprotein particle receptor binding	9	30	1.35	3.87e-07	APOC3,APOB,APOA1,LRP1,APOE,HSP90B1,PCSK9,APOA5,CLTC
GO Function	GO:0030234	Enzyme regulator activity	45	1239	0.43	4.81e-07	SPP2,APOH,SERPIND1,PCOLCE,C5,APOC3,LTF,HRG,APOA1,APOE,SERPINF1,SERPINA10,LCN1,CSTA,AMBP,ITIH4,ITIH1,AHSG,SERPING1,SERPINI1,A2ML1,SUZ12,CPN2,LPA,SERPINF2,SERPINA7,FBLN1,SERPINA5,SERPINA11,PAPLN,APOA4,ITIH2,CALML5,IGFBP3,PROS1,GAPDH,CST3,NUCB1,IGF2,C4A,ITIH3,C4B,APOA5,SERPINA3,KNG1
GO Function	GO:0005201	Extracellular matrix structural constituent	14	131	0.9	1.87e-06	COMP,VTN,PXDN,NID1,LUM,FGB,EFEMP2,FBN1,FBLN1,OIT3,COL18A1,FBLN2,TGFBI,FGA
GO Function	GO:0016209	Antioxidant activity	11	76	1.03	4.31e-06	MPO,CAT,APOE,PXDN,AMBP,PRDX6,APOA4,S100A9,APOM,SOD3,GPX3
GO Function	GO:0001851	Complement component C3b binding	6	11	1.61	1.10e-05	CFHR5,CFHR1,CFHR2,CFHR4,CFHR3,CFH
GO Function	GO:0005518	Collagen binding	10	66	1.05	1.20e-05	COMP,PCOLCE,VTN,SPARC,VWF,NID1,LUM,ANTXR1,SPARCL1,TGFBI
GO Function	GO:0043169	Cation binding	96	4335	0.21	2.59e-05	RCN1,CPB2,CETP,F9,MMP2,COMP,PON1,MPO,AFM,SPARC,PCDH12,LTF,HRG,PROC,APOA1,CAT,LRP1,APOE,PXDN,F12,APCS,DSG2,LCN1,NID1,CP,DGKG,CDH6,PON3,FCN3,ITIH1,HABP2,PCDH1,FCN2,PGLYRP2,IHH,MASP1,HSP90B1,ANTXR1,BMP1,DST,F2,EFEMP2,SUZ12,CALR,HBA1,ITLN1,HSPA5,FBN1,FBLN1,SERPINA5,OIT3,ANG,LIMS1,THBS4,PROZ,QPCT,BLM,APOA4,CNDP1,COL18A1,F8,PCDH18,CDC42BPA,F5,S100A7,S100A9,CRTAC1,ADAMTS13,GSN,SVEP1,F10,CA6,CALML5,IGFBP3,SOD3,PROS1,EFEMP1,CFI,EFCAB5,MASP2,FBLN2,C1S,NUCB1,KSR2,COLEC11,SPARCL1,PLTP,UIMC1,C1R,APOA5,ATP1A1,KNG1,TRIM66,CDH5,FGA,SDF4
GO Function	GO:0042802	Identical protein binding	58	2144	0.3	4.45e-05	ST6GAL1,GNPTG,APOH,TPD52L2,PON1,VTN,PLEK,APOA1,TTR,CAT,APOE,APCS,CFHR5,LYZ,VWF,YWHAE,AMBP,PON3,FCN3,CSF1R,FCN2,MASP1,APEH,BMP1,DST,EFEMP2,C1QB,CFHR1,SERPINF2,ITLN1,FBN1,FBLN1,ANG,PRDX6,BLM,APOA4,COL18A1,MTCL1,CDC42BPA,CFHR2,CFH,ADAMTSL4,FLNA,MCOLN2,MBL2,GPX3,GAPDH,CST3,MASP2,F11,C1S,ADIPOQ,COLEC11,TGFBI,JCHAIN,C1R,B2M,SDF4
GO Function	GO:0044877	Protein-containing complex binding	40	1261	0.37	0.00010	VCL,COMP,PCOLCE,VTN,SPARC,HRG,APOA1,TTR,LRP1,DHX29,APOE,APCS,VWF,NID1,YWHAE,AMBP,LUM,ANTXR1,PCSK9,DST,CALR,HSPA5,FBN1,P4HB,FBLN1,THBS4,C8A,FLNA,SMARCA1,C8B,ADAMTS13,GSN,IGF2,C4A,SPARCL1,TGFBI,PLTP,JCHAIN,CDH5,B2M
GO Function	GO:0043394	Proteoglycan binding	7	37	1.15	0.00027	COMP,HRG,LRP1,APOE,NID1,FCN2,CFH
GO Function	GO:0046872	Metal ion binding	91	4250	0.2	0.00027	RCN1,CPB2,F9,MMP2,COMP,PON1,MPO,AFM,SPARC,PCDH12,LTF,HRG,PROC,CAT,LRP1,APOE,PXDN,F12,APCS,DSG2,LCN1,NID1,CP,DGKG,CDH6,PON3,FCN3,ITIH1,HABP2,PCDH1,FCN2,PGLYRP2,IHH,MASP1,HSP90B1,ANTXR1,BMP1,DST,F2,EFEMP2,SUZ12,CALR,HBA1,ITLN1,HSPA5,FBN1,FBLN1,OIT3,ANG,LIMS1,THBS4,PROZ,QPCT,BLM,APOA4,CNDP1,COL18A1,F8,PCDH18,CDC42BPA,F5,S100A7,S100A9,CRTAC1,ADAMTS13,GSN,SVEP1,F10,CA6,CALML5,IGFBP3,SOD3,PROS1,EFEMP1,CFI,EFCAB5,MASP2,FBLN2,C1S,NUCB1,KSR2,COLEC11,SPARCL1,UIMC1,C1R,ATP1A1,KNG1,TRIM66,CDH5,FGA,SDF4
GO Function	GO:0097367	Carbohydrate derivative binding	58	2278	0.28	0.00027	APOH,SERPIND1,LBP,COMP,PCOLCE,MPO,VTN,PF4V1,LTF,HRG,APOB,LRP1,DHX29,APOE,SERPINA10,NID1,DGKG,DNAH5,AMBP,CENPE,ITIH1,HABP2,CSF1R,FCN2,PGLYRP2,PF4,CAMP,GUCY1A1,HSP90B1,F2,EFEMP2,LPA,HSPA5,FBN1,SERPINA5,DNAH8,ANG,THBS4,BLM,ITIH2,CDC42BPA,CFH,SMARCA1,SOD3,KIF19,NAV2,ILK,SAA1,F11,KSR2,ADIPOQ,ACSF2,CDK3,ABCA13,APOA5,ATP1A1,HYOU1,KNG1
GO Function	GO:0120020	Cholesterol transfer activity	6	23	1.29	0.00028	CETP,APOB,APOA1,APOE,APOA4,PLTP
GO Function	GO:0050750	Low-density lipoprotein particle receptor binding	6	25	1.25	0.00040	APOB,APOE,HSP90B1,PCSK9,APOA5,CLTC

GO Function	GO:0005178	Integrin binding	12	159	0.75	0.00044	COMP,VTN,VWF,DST,CALR,FBN1,P4HB,FBLN1,THBS4,ADAMTS13,IGF2,TGFBI
GO Function	GO:0005319	Lipid transporter activity	12	163	0.74	0.00054	CETP,APOB,APOA1,APOE,APOD,APOA4,CFHR4,APOM,APOF,ABCA13,PLTP,APOC4
GO Function	GO:0031210	Phosphatidylcholine binding	6	29	1.19	0.00077	CETP,APOA1,SERPINA5,APOA4,PLTP,APOA5
GO Function	GO:0060228	Phosphatidylcholine-sterol O-acyltransferase activator activity	4	6	1.69	0.00077	APOA1,APOE,APOA4,APOA5
GO Function	GO:0043167	Ion binding	115	6033	0.15	0.0013	RCN1,CPB2,CETP,F9,MMP2,COMP,PON1,MPO,AFM,SPARC,PCDH12,LTF,HRG,PROC,PLEK,APOA1,CAT,LRP1,DHX29,APOE,PXDN,F12,APCS,PSMA6,DSG2,LCN1,NID1,CP,DGKG,CDH6,DNAH5,CENPE,PON3,FCN3,ITIH1,HABP2,CSF1R,PCDH1,FCN2,PGLYRP2,IHH,MASP1,GUCY1A1,HSP90B1,ANTXR1,BMP1,DST,F2,EFEMP2,SUZ12,CALR,HBA1,ITLN1,HSPA5,FBN1,FBLN1,SERPINA5,DNAH8,OIT3,ANG,LIMS1,THBS4,PROZ,QPCT,BLM,APOA4,CNDP1,COL18A1,F8,PCDH18,CDC42BPA,QSOX1,F5,S100A7,S100A9,CRTAC1,SMARCA1,ADAMTS13,GSN,SVEP1,F10,CA6,CALML5,IGFBP3,SOD3,KIF19,PROS1,EFEMP1,CFI,EFCAB5,NAV2,ILK,MASP2,FBLN2,C1S,NUCB1,PCYOX1,KSR2,ADIPOQ,ACSF2,CDK3,ABCA13,COLEC11,SPARCL1,PLTP,UIMC1,C1R,APOA5,ATP1A1,HYOU1,KNG1,TRIM66,CDH5,FGA,SDF4
GO Function	GO:0015485	Cholesterol binding	7	52	1.0	0.0015	CETP,APOC3,APOA1,APOD,APOA4,APOF,APOA5
GO Function	GO:0034185	Apolipoprotein binding	5	19	1.29	0.0016	LRP1,LCAT,PCSK9,PLG,LPA
GO Function	GO:0043395	Heparan sulfate proteoglycan binding	5	19	1.29	0.0016	COMP,HRG,LRP1,APOE,CFH
GO Function	GO:0004869	Cysteine-type endopeptidase inhibitor activity	7	54	0.98	0.0018	LTF,HRG,LCN1,CSTA,AHSG,CST3,KNG1
GO Function	GO:0005496	Steroid binding	9	101	0.82	0.0018	CETP,APOC3,APOA1,APOD,APOA4,APOF,GC,APOA5,ATP1A1
GO Function	GO:0008289	Lipid binding	27	796	0.4	0.0018	CETP,APOH,LBP,PON1,AFM,APOC3,LTF,APOB,APOA1,APOE,DGKG,CAMP,F2,APOL1,SERPINA5,APOD,APOA4,S100A9,C8G,GSN,F10,APOM,APOF,PLTP,GC,APOA5,ATP1A1
GO Function	GO:0050840	Extracellular matrix binding	7	56	0.97	0.0021	VTN,SPARC,PXDN,NID1,FBLN2,SPARCL1,TGFBI
GO Function	GO:0016787	Hydrolase activity	55	2347	0.24	0.0036	CPB2,APMAP,F9,MMP2,PON1,GPLD1,LTF,PROC,CAT,DHX29,F12,LYZ,PSMA6,LCAT,PON3,HYAL1,HABP2,PGLYRP2,IHH,MASP1,APEH,HSP90B1,PCSK9,BMP1,F2,PLG,LPA,HSPA5,ANG,GANAB,PRDX6,PROZ,PAPLN,BLM,CNDP1,CD5L,SMARCA1,ADAMTS13,MINPP1,F10,KIF19,CFI,NAV2,RNASE1,MASP2,F11,C1S,MST1,CFB,HGFAC,HPR,C1R,ATP1A1,RNASE4,CFD
GO Function	GO:0050839	Cell adhesion molecule binding	21	560	0.44	0.0036	VCL,COMP,VTN,DHX29,VWF,DSG2,YWHAE,CDH6,DST,CALR,HSPA5,FBN1,P4HB,FBLN1,THBS4,PRDX6,FLNA,ADAMTS13,IGF2,TGFBI,CDH5
GO Function	GO:0043178	Alcohol binding	8	91	0.81	0.0049	CETP,APOC3,APOA1,APOD,APOA4,C8G,APOF,APOA5
GO Function	GO:0003823	Antigen binding	7	71	0.86	0.0072	FCN3,FCN2,PLG,JCHAIN,IGKV2D-28,IGHV3-15,IGHV3-72
GO Function	GO:0030545	Signaling receptor regulator activity	20	552	0.43	0.0077	EBI3,C5,PF4V1,APOA1,TTR,PXDN,CRH,SCGB3A1,PPBP,PF4,PCSK9,BMP1,F2,INHBC,FBN1,THBS4,EFEMP1,ADIPOQ,IGF2,VEGFC
GO Function	GO:0001968	Fibronectin binding	5	30	1.09	0.0080	MMP2,IGFBP5,LPA,FBLN1,IGFBP3
GO Function	GO:0034987	Immunoglobulin receptor binding	4	16	1.27	0.0105	FLNA,JCHAIN,IGHV3-15,IGHV3-72
GO Function	GO:0042803	Protein homodimerization activity	23	709	0.38	0.0109	ST6GAL1,GNPTG,TPD52L2,PON1,PLEK,APOA1,CAT,APOE,AMBP,PON3,CSF1R,MASP1,DST,EFEMP2,SERPINF2,ANG,BLM,APOA4,MTCL1,FLNA,ADIPOQ,JCHAIN,B2M
GO Function	GO:0002020	Protease binding	9	135	0.69	0.0111	COMP,VWF,CSTA,A2ML1,SERPINF2,SERPINA5,ADAMTSL4,CST3,CLTC
GO Function	GO:0005520	Insulin-like growth factor binding	4	17	1.24	0.0123	IGFBP5,IGFBP1,IGFBP3,IGFALS
GO Function	GO:0051087	Chaperone binding	8	108	0.74	0.0129	VWF,CP,FGB,PLG,CALR,HSPA5,ATP1A1,HYOU1
GO Function	GO:0004064	Arylesterase activity	3	6	1.57	0.0143	APMAP,PON1,PON3
GO Function	GO:0048018	Receptor ligand activity	18	499	0.43	0.0161	EBI3,C5,PF4V1,APOA1,TTR,CRH,SCGB3A1,PPBP,PF4,BMP1,F2,INHBC,FBN1,THBS4,EFEMP1,ADIPOQ,IGF2,VEGFC
GO Function	GO:0005507	Copper ion binding	6	60	0.87	0.0184	CP,ANG,APOA4,F8,F5,SOD3
GO Function	GO:0001849	Complement component C1q complex binding	3	8	1.44	0.0257	APCS,CALR,C4A
GO Function	GO:0004601	Peroxidase activity	5	41	0.96	0.0257	MPO,CAT,PXDN,PRDX6,GPX3
GO Function	GO:0031995	Insulin-like growth factor II binding	3	8	1.44	0.0257	IGFBP5,IGFBP1,IGFBP3
GO Function	GO:0019865	Immunoglobulin binding	4	23	1.11	0.0295	HRG,VWF,AMBP,JCHAIN
GO Function	GO:0030246	Carbohydrate binding	12	278	0.5	0.0368	VTN,APCS,ATRN,FCN3,FCN2,CALR,ITLN1,GANAB,ASGR2,MBL2,COLEC11,C4B
GO Function	GO:0031994	Insulin-like growth factor I binding	3	10	1.35	0.0404	IGFBP5,IGFBP1,IGFBP3
GO Function	GO:0005044	Scavenger receptor activity	5	48	0.89	0.0453	VTN,LRP1,LGALS3BP,CD5L,CFI
GO Function	GO:0016670	Oxidoreductase activity, acting on a sulfur group of donors, oxygen as acceptor	3	11	1.31	0.0495	P4HB,QSOX1,PCYOX1
GO Component	GO:0005615	Extracellular space	202	3247	0.66	4.32e-96	CPB2,CETP,GNPTG,APOH,VCL,SERPIND1,CHGA,LBP,F9,MMP2,EBI3,COMP,PON1,PCOLCE,C5,MPO,VTN,AFM,PF4V1,APOC3,GPLD1,SPARC,PCDH12,LTF,HRG,APOB,IGFBP5,PROC,APOA1,TTR,CAT,C4BPB,CFP,APOE,PXDN,ANGPTL6,F12,SERPINF1,APCS,CFHR5,LYZ,VWF,PSMA6,DSG2,SERPINA10,LGALS3BP,ATRN,C6,LCN1,LCAT,NID1,YWHAE,CSTA,CP,AMBP,PON3,HYAL1,ITIH4,LUM,FCN3,ITIH1,AHSG,IGFBP1,CRH,HABP2,SAA4,SERPING1,FCN2,PGLYRP2,SCGB3A1,IHH,SERPINI1,PPBP,PF4,MASP1,CAMP,APEH,A2ML1,HSP90B1,LRG1,PCSK9,BMP1,FGB,F2,INHBC,PLG,EFEMP2,CSPG4,C1QB,CFHR1,APOL1,CPN2,CALR,LPA,SERPINF2,C7,HBA1,ITLN1,HSPA5,FBN1,P4HB,SERPINA7,FBLN1,SERPINA5,OIT3,SERPINA11,ANG,THBS4,GANAB,PRDX6,FAM184A,PROZ,QPCT,APOD,PIGR,APOA4,ITIH2,COL18A1,MTCL1,F8,C8A,CDC42BPA,C4BPA,F13B,CFHR2,CFHR4,CFHR3,CFH,QSOX1,F5,CD5L,S100A7,S100A9,FLNA,CRTAC1,C8B,C8G,ADAMTS13,MINPP1,GSN,MBL2,SVEP1,C1QC,F10,APOM,CA6,CKAP4,IGFBP3,SOD3,GPX3,PROS1,EFEMP1,CFI,GAPDH,RNASE1,FGL1,APOF,CST3,MASP2,SAA1,F11,C1S,NUCB1,PCYOX1,ADIPOQ,IGF2,ORM2,C4A,COLEC11,MST1,SPARCL1,ITIH3,C4B,TGFBI,CFB,IGFALS,PLTP,PAEP,RARRES2,GC,HGFAC,SELENOP,JCHAIN,HPR,C1R,APOA5,ATP1A1,SERPINA3,RNASE4,IGKV2D-28,APOC4,CFD,CLTC,IGHV3-15,IGHV3-72,VEGFC,HYOU1,KNG1,B2M,FGA,PDIA4,SDF4

GO Component	GO:0005576	Extracellular region	215	4175	0.58	6.05e-91	SPP2,ST6GAL1,CPB2,CETP,GNPTG,APOH,VCL,SERPIND1,CHGA,LBP,F9,MMP2,EBI3,COMP,PON1,PCOLCE,C5,MPO,VTN,AFM,PF4V1,APOC3,GPLD1,SPARC,PCDH12,LTF,HRG,APOB,IGFBP5,PROC,PLEK,APOA1,TTR,CAT,C4BPB,CFP,APOE,PXDN,ANGPTL6,F12,SERPINF1,APCS,CFHR5,LYZ,VWF,PSMA6,DSG2,SERPINA10,LGALS3BP,ATRN,C6,LCN1,LCAT,NID1,YWHAE,CSTA,CP,DNAH5,AMBP,CENPE,PON3,HYAL1,ITIH4,LUM,FCN3,ITIH1,AHSG,IGFBP1,CRH,HABP2,SAA4,SERPING1,FCN2,PGLYRP2,SCGB3A1,IHH,SERPINI1,PPBP,PF4,MASP1,CAMP,APEH,A2ML1,HSP90B1,LRG1,PCSK9,BMP1,FGB,F2,INHBC,PLG,EFEMP2,CSPG4,C1QB,CFHR1,APOL1,PSG11,CPN2,CALR,LPA,SERPINF2,C7,HBA1,ITLN1,HSPA5,FBN1,P4HB,SERPINA7,FBLN1,SERPINA5,OIT3,SERPINA11,ANG,THBS4,GANAB,PRDX6,FAM184A,PROZ,QPCT,APOD,PAPLN,PIGR,APOA4,ITIH2,CNDP1,COL18A1,MTCL1,F8,C8A,CDC42BPA,C4BPA,F13B,CFHR2,CFHR4,CFHR3,CFH,QSOX1,F5,CD5L,S100A7,S100A9,ADAMTSL4,FLNA,CRTAC1,C8B,C8G,ADAMTS13,MINPP1,GSN,MBL2,SVEP1,C1QC,F10,APOM,CA6,CKAP4,CALML5,IGFBP3,SOD3,GPX3,PROS1,EFEMP1,CFI,GAPDH,RNASE1,FGL1,APOF,CST3,MASP2,FBLN2,PSG4,SAA1,F11,C1S,PSG2,NUCB1,PCYOX1,ADIPOQ,IGF2,ORM2,C4A,COLEC11,MST1,SPARCL1,ITIH3,C4B,TGFBI,CFB,IGFALS,PLTP,PAEP,RARRES2,GC,HGFAC,SELENOP,JCHAIN,HPR,C1R,APOA5,ATP1A1,SERPINA3,RNASE4,IGKV2D-28,APOC4,CFD,CLTC,IGHV3-15,IGHV3-72,VEGFC,HYOU1,KNG1,B2M,FGA,PDIA4,SDF4
GO Component	GO:1903561	Extracellular vesicle	137	2120	0.68	8.13e-57	CPB2,CETP,GNPTG,APOH,VCL,SERPIND1,LBP,F9,COMP,PON1,PCOLCE,C5,MPO,VTN,AFM,APOC3,GPLD1,PCDH12,LTF,HRG,APOB,APOA1,TTR,CAT,APOE,PXDN,ANGPTL6,F12,SERPINF1,APCS,LYZ,VWF,PSMA6,DSG2,SERPINA10,LGALS3BP,ATRN,C6,LCAT,NID1,YWHAE,CP,AMBP,PON3,HYAL1,ITIH4,LUM,ITIH1,AHSG,SAA4,SERPING1,FCN2,PGLYRP2,SCGB3A1,SERPINI1,CAMP,APEH,A2ML1,HSP90B1,LRG1,FGB,F2,PLG,EFEMP2,CSPG4,CPN2,CALR,SERPINF2,C7,HBA1,ITLN1,HSPA5,P4HB,SERPINA7,FBLN1,SERPINA5,THBS4,GANAB,PRDX6,PROZ,QPCT,APOD,PIGR,APOA4,ITIH2,COL18A1,C8A,CDC42BPA,CFHR3,CFH,QSOX1,F5,S100A9,FLNA,CRTAC1,C8B,C8G,MINPP1,GSN,CA6,CKAP4,SOD3,GPX3,PROS1,EFEMP1,CFI,GAPDH,RNASE1,CST3,MASP2,FBLN2,SAA1,F11,NUCB1,PCYOX1,ORM2,C4A,ITIH3,C4B,TGFBI,CFB,IGFALS,GC,SELENOP,JCHAIN,HPR,C1R,ATP1A1,SERPINA3,IGKV2D-28,CFD,CLTC,HYOU1,KNG1,B2M,FGA,SDF4
GO Component	GO:0070062	Extracellular exosome	135	2096	0.68	7.40e-56	CPB2,CETP,GNPTG,APOH,VCL,SERPIND1,LBP,F9,COMP,PON1,PCOLCE,C5,MPO,VTN,AFM,APOC3,GPLD1,PCDH12,LTF,HRG,APOB,APOA1,TTR,CAT,APOE,PXDN,ANGPTL6,F12,SERPINF1,APCS,LYZ,VWF,PSMA6,DSG2,SERPINA10,LGALS3BP,ATRN,C6,LCAT,NID1,YWHAE,CP,AMBP,PON3,HYAL1,ITIH4,LUM,ITIH1,AHSG,SAA4,SERPING1,FCN2,PGLYRP2,SCGB3A1,SERPINI1,CAMP,APEH,A2ML1,HSP90B1,LRG1,FGB,F2,PLG,EFEMP2,CSPG4,CPN2,CALR,SERPINF2,C7,HBA1,ITLN1,HSPA5,P4HB,SERPINA7,FBLN1,SERPINA5,THBS4,GANAB,PRDX6,PROZ,QPCT,APOD,PIGR,APOA4,ITIH2,COL18A1,C8A,CDC42BPA,CFHR3,CFH,QSOX1,S100A9,FLNA,CRTAC1,C8B,C8G,MINPP1,GSN,CA6,CKAP4,SOD3,GPX3,PROS1,EFEMP1,CFI,GAPDH,RNASE1,CST3,MASP2,SAA1,F11,NUCB1,PCYOX1,ORM2,C4A,ITIH3,C4B,TGFBI,CFB,IGFALS,GC,SELENOP,JCHAIN,HPR,C1R,ATP1A1,SERPINA3,IGKV2D-28,CFD,CLTC,HYOU1,KNG1,B2M,FGA,SDF4
GO Component	GO:0072562	Blood microparticle	49	118	1.49	5.24e-50	PON1,VTN,AFM,HRG,APOA1,APOE,APCS,LGALS3BP,CP,AMBP,ITIH4,FCN3,ITIH1,AHSG,SERPING1,FCN2,FGB,F2,PLG,C1QB,CFHR1,APOL1,CPN2,SERPINF2,HBA1,APOA4,ITIH2,C8A,C4BPA,CFHR3,CFH,CD5L,C8G,GSN,C1QC,PROS1,C1S,ORM2,C4A,C4B,CFB,GC,JCHAIN,HPR,C1R,SERPINA3,IGKV2D-28,KNG1,FGA
GO Component	GO:0062023	Collagen-containing extracellular matrix	65	407	1.07	8.74e-45	SPP2,APOH,F9,MMP2,COMP,VTN,APOC3,SPARC,HRG,APOA1,APOE,PXDN,ANGPTL6,F12,SERPINF1,APCS,VWF,LGALS3BP,ATRN,NID1,AMBP,ITIH4,LUM,FCN3,ITIH1,AHSG,SERPING1,FCN2,PF4,HSP90B1,FGB,DST,F2,PLG,EFEMP2,CSPG4,C1QB,CALR,SERPINF2,FBN1,FBLN1,ANG,THBS4,APOA4,ITIH2,COL18A1,S100A7,S100A9,ADAMTSL4,MBL2,C1QC,SOD3,EFEMP1,NAV2,FGL1,FBLN2,ADIPOQ,ORM2,MST1,SPARCL1,TGFBI,RARRES2,SERPINA3,KNG1,FGA
GO Component	GO:0031012	Extracellular matrix	71	552	0.98	1.27e-43	SPP2,APOH,F9,MMP2,COMP,VTN,APOC3,GPLD1,SPARC,HRG,APOA1,APOE,PXDN,ANGPTL6,F12,SERPINF1,APCS,VWF,LGALS3BP,ATRN,NID1,AMBP,ITIH4,LUM,FCN3,ITIH1,AHSG,SERPING1,FCN2,IHH,PF4,HSP90B1,FGB,DST,F2,PLG,EFEMP2,CSPG4,C1QB,CPN2,CALR,SERPINF2,FBN1,FBLN1,ANG,THBS4,PAPLN,APOA4,ITIH2,COL18A1,S100A7,S100A9,ADAMTSL4,ADAMTS13,MBL2,C1QC,SOD3,EFEMP1,NAV2,FGL1,FBLN2,ADIPOQ,ORM2,MST1,SPARCL1,TGFBI,IGFALS,RARRES2,SERPINA3,KNG1,FGA
GO Component	GO:0031982	Vesicle	160	3957	0.48	4.36e-43	SPP2,CPB2,CETP,GNPTG,APOH,VCL,SERPIND1,CHGA,LBP,F9,COMP,PON1,PCOLCE,C5,MPO,VTN,AFM,APOC3,GPLD1,SPARC,PCDH12,LTF,HRG,APOB,APOA1,TTR,CAT,LRP1,CFP,APOE,PXDN,ANGPTL6,F12,SERPINF1,APCS,LYZ,VWF,PSMA6,DSG2,SERPINA10,LGALS3BP,ATRN,C6,LCAT,NID1,YWHAE,CP,AMBP,PON3,TCIRG1,HYAL1,ITIH4,LUM,ITIH1,AHSG,SAA4,SERPING1,FCN2,PGLYRP2,SCGB3A1,SERPINI1,PPBP,PF4,CAMP,APEH,A2ML1,HSP90B1,ANTXR1,LRG1,PCSK9,BMP1,FGB,DST,F2,PLG,EFEMP2,CSPG4,CPN2,CALR,SERPINF2,C7,HBA1,ITLN1,HSPA5,P4HB,SERPINA7,FBLN1,SERPINA5,ANG,THBS4,GANAB,PRDX6,PROZ,QPCT,APOD,PIGR,APOA4,ITIH2,COL18A1,F8,C8A,CDC42BPA,CFHR3,CFH,QSOX1,F5,S100A7,S100A9,FLNA,CRTAC1,MCOLN2,C8B,C8G,MINPP1,GSN,CA6,CKAP4,CALML5,SOD3,GPX3,PROS1,EFEMP1,CFI,GAPDH,RNASE1,CST3,MASP2,FBLN2,SAA1,F11,NUCB1,PCYOX1,IGF2,ORM2,C4A,ABCA13,ITIH3,C4B,TGFBI,CFB,IGFALS,RARRES2,GC,SELENOP,JCHAIN,HPR,C1R,APOA5,ATP1A1,SERPINA3,IGKV2D-28,CFD,CLTC,VEGFC,HYOU1,KNG1,B2M,FGA,PDIA4,SDF4
GO Component	GO:0005788	Endoplasmic reticulum lumen	49	312	1.07	7.96e-33	RCN1,SPP2,SERPIND1,F9,EBI3,VTN,APOB,IGFBP5,PROC,APOA1,CFP,APOE,SERPINA10,CP,AHSG,IGFBP1,SERPING1,HSP90B1,PCSK9,F2,APOL1,CALR,HSPA5,FBN1,P4HB,GANAB,PROZ,APOA4,ITIH2,COL18A1,F8,QSOX1,F5,ADAMTSL4,ADAMTS13,MINPP1,F10,CKAP4,IGFBP3,CST3,NUCB1,C4A,SPARCL1,APOA5,HYOU1,KNG1,B2M,FGA,PDIA4
GO Component	GO:0031983	Vesicle lumen	46	326	1.02	7.08e-29	SPP2,APOH,VCL,MPO,SPARC,LTF,HRG,APOB,APOA1,TTR,CAT,CFP,LYZ,VWF,LGALS3BP,ITIH4,AHSG,SERPING1,SERPINI1,PPBP,PF4,CAMP,LRG1,FGB,PLG,SERPINF2,PRDX6,QPCT,F8,QSOX1,F5,S100A7,S100A9,GSN,PROS1,IGF2,ORM2,ITIH3,RARRES2,SELENOP,SERPINA3,CFD,VEGFC,KNG1,B2M,FGA
GO Component	GO:0034774	Secretory granule lumen	45	321	1.02	3.99e-28	SPP2,APOH,VCL,MPO,SPARC,LTF,HRG,APOA1,TTR,CAT,CFP,LYZ,VWF,LGALS3BP,ITIH4,AHSG,SERPING1,SERPINI1,PPBP,PF4,CAMP,LRG1,FGB,PLG,SERPINF2,PRDX6,QPCT,F8,QSOX1,F5,S100A7,S100A9,GSN,PROS1,IGF2,ORM2,ITIH3,RARRES2,SELENOP,SERPINA3,CFD,VEGFC,KNG1,B2M,FGA
GO Component	GO:0034358	Plasma lipoprotein particle	20	38	1.59	2.27e-21	CETP,APOH,PON1,APOC3,APOB,APOA1,APOE,LCAT,SAA4,APOL1,LPA,APOA4,APOM,APOF,SAA1,PCYOX1,PLTP,HPR,APOA5,APOC4
GO Component	GO:0034364	High-density lipoprotein particle	18	29	1.66	3.42e-20	CETP,APOH,PON1,APOC3,APOB,APOA1,APOE,LCAT,SAA4,APOL1,APOA4,APOM,APOF,SAA1,PLTP,HPR,APOA5,APOC4

GO Component	GO:0030141	Secretory granule	56	873	0.68	8.01e-20	SPP2,APOH,VCL,CHGA,MPO,SPARC,LTF,HRG,APOA1,TTR,CAT,CFP,ANGPTL6,LYZ,VWF,LGALS3BP,TCIRG1,ITIH4,AHSG,SERPING1,SERPINI1,PPBP,PF4,CAMP,APEH,LRG1,FGB,PLG,CALR,SERPINF2,SERPINA5,PRDX6,QPCT,PIGR,F8,QSOX1,F5,S100A7,S100A9,GSN,CKAP4,CALML5,PROS1,CST3,IGF2,ORM2,ABCA13,ITIH3,RARRES2,SELENOP,SERPINA3,CFD,VEGFC,KNG1,B2M,FGA
GO Component	GO:0031093	Platelet alpha granule lumen	21	66	1.37	6.30e-19	SPARC,HRG,VWF,AHSG,SERPING1,PPBP,PF4,FGB,PLG,SERPINF2,F8,QSOX1,F5,PROS1,IGF2,ORM2,SERPINA3,CFD,VEGFC,KNG1,FGA
GO Component	GO:0031091	Platelet alpha granule	22	89	1.26	7.07e-18	SPARC,HRG,VWF,AHSG,SERPING1,PPBP,PF4,FGB,PLG,SERPINF2,SERPINA5,F8,QSOX1,F5,PROS1,IGF2,ORM2,SERPINA3,CFD,VEGFC,KNG1,FGA
GO Component	GO:1905370	Serine-type endopeptidase complex	16	26	1.66	7.07e-18	F9,PROC,F12,FCN3,FCN2,MASP1,F2,F8,CFH,F5,MBL2,F10,CFI,MASP2,F11,COLEC11
GO Component	GO:0071944	Cell periphery	142	6015	0.24	1.03e-12	SPP2,APOH,VCL,F9,MMP2,EBI3,COMP,C5,VTN,APOC3,GPLD1,SPARC,PCDH12,LTF,HRG,APOB,PLEK,APOA1,CAT,LRP1,C4BPB,CFP,APOE,PXDN,ANGPTL6,F12,SERPINF1,APCS,VWF,DSG2,LGALS3BP,ATRN,C6,NID1,CSTA,CP,DGKG,CDH6,AMBP,TCIRG1,ITIH4,LUM,FCN3,ITIH1,AHSG,SERPING1,CSF1R,PCDH1,FCN2,IHH,PF4,MASP1,HSP90B1,ANTXR1,SH3D19,PCSK9,FGB,DST,F2,PLG,EFEMP2,CSPG4,C1QB,CPN2,CALR,LPA,SERPINF2,C7,ITLN1,HSPA5,FBN1,P4HB,FBLN1,SERPINA5,ANG,LIMS1,THBS4,PAPLN,ASGR2,AKAP9,PIGR,APOA4,ITIH2,COL18A1,MTCL1,F8,C8A,PCDH18,C4BPA,F5,CD5L,S100A7,S100A9,ADAMTSL4,FLNA,MCOLN2,C8B,C8G,ADAMTS13,GSN,MBL2,C1QC,F10,CKAP4,CD99,SOD3,PROS1,EFEMP1,NAV2,ILK,GAPDH,FGL1,CST3,MASP2,FBLN2,PSG4,F11,PCYOX1,KSR2,ADIPOQ,ORM2,C4A,ABCA13,COLEC11,MST1,SPARCL1,C4B,TGFBI,CFB,IGFALS,RARRES2,ATP1A1,SERPINA3,IGKV2D-28,CLTC,IGHV3-15,IGHV3-72,KNG1,CDH5,B2M,FGA,SDF4
GO Component	GO:0031410	Cytoplasmic vesicle	81	2482	0.38	2.07e-12	SPP2,APOH,VCL,CHGA,MPO,APOC3,SPARC,LTF,HRG,APOB,APOA1,TTR,CAT,LRP1,CFP,APOE,ANGPTL6,SERPINF1,LYZ,VWF,LGALS3BP,YWHAE,TCIRG1,HYAL1,ITIH4,AHSG,SERPING1,SERPINI1,PPBP,PF4,CAMP,APEH,HSP90B1,ANTXR1,LRG1,PCSK9,FGB,DST,PLG,CALR,SERPINF2,HBA1,HSPA5,P4HB,SERPINA5,ANG,GANAB,PRDX6,QPCT,PIGR,APOA4,F8,QSOX1,F5,S100A7,S100A9,MCOLN2,GSN,CKAP4,CALML5,PROS1,CST3,SAA1,IGF2,ORM2,ABCA13,ITIH3,RARRES2,SELENOP,APOA5,ATP1A1,SERPINA3,CFD,CLTC,VEGFC,HYOU1,KNG1,B2M,FGA,PDIA4,SDF4
GO Component	GO:1905369	Endopeptidase complex	18	104	1.11	3.02e-12	F9,PROC,F12,PSMA6,FCN3,FCN2,MASP1,F2,UBE3C,F8,CFH,F5,MBL2,F10,CFI,MASP2,F11,COLEC11
GO Component	GO:0012505	Endomembrane system	120	4721	0.27	4.20e-12	RCN1,SPP2,ST6GAL1,GNPTG,APOH,VCL,SERPIND1,CHGA,F9,EBI3,MPO,VTN,APOC3,SPARC,LTF,HRG,APOB,IGFBP5,PROC,APOA1,TTR,CAT,LRP1,CFP,APOE,PXDN,ANGPTL6,F12,LYZ,VWF,SERPINA10,LGALS3BP,CP,AMBP,TCIRG1,ITIH4,LUM,AHSG,IGFBP1,SERPING1,SERPINI1,PPBP,PF4,CAMP,APEH,HSP90B1,ANTXR1,LRG1,PCSK9,BMP1,FGB,DST,F2,PLG,CSPG4,APOL1,CALR,SERPINF2,HSPA5,FBN1,P4HB,SERPINA5,OIT3,THBS4,GANAB,PRDX6,PROZ,QPCT,APOD,ASGR2,AKAP9,PIGR,APOA4,ITIH2,COL18A1,F8,QSOX1,F5,S100A7,S100A9,ADAMTSL4,FLNA,MCOLN2,ADAMTS13,MINPP1,GSN,F10,CKAP4,CALML5,IGFBP3,SOD3,PROS1,TNRC6A,GAPDH,CST3,NUCB1,ADIPOQ,IGF2,ORM2,C4A,ABCA13,SPARCL1,ITIH3,TGFBI,RARRES2,HGFAC,SELENOP,APOA5,ATP1A1,SERPINA3,CFD,CLTC,VEGFC,HYOU1,KNG1,CDH5,B2M,FGA,PDIA4,SDF4
GO Component	GO:0034361	Very-low-density lipoprotein particle	11	20	1.61	8.44e-12	APOH,APOC3,APOB,APOA1,APOE,APOL1,APOA4,APOM,PCYOX1,APOA5,APOC4
GO Component	GO:1905368	Peptidase complex	19	138	1.01	2.05e-11	F9,PROC,F12,PSMA6,FCN3,FCN2,MASP1,F2,UBE3C,F8,CFH,F5,MBL2,F10,TAF9,CFI,MASP2,F11,COLEC11
GO Component	GO:0071682	Endocytic vesicle lumen	11	23	1.55	2.51e-11	MPO,SPARC,LTF,APOB,APOA1,APOE,HSP90B1,CALR,HBA1,SAA1,HYOU1
GO Component	GO:0009986	Cell surface	42	894	0.54	2.54e-10	APOH,LBP,APMAP,EBI3,SPARC,LTF,HRG,PXDN,DSG2,AMBP,FCN3,CSF1R,FCN2,MASP1,ANTXR1,PCSK9,FGB,PLG,CSPG4,PSG11,CALR,SERPINF2,HSPA5,P4HB,SERPINA5,OIT3,ASGR2,CD5L,ADAMTS13,MBL2,F10,MASP2,PSG4,PSG2,ADIPOQ,COLEC11,IGHV3-15,IGHV3-72,CDH5,B2M,FGA,PDIA4
GO Component	GO:0005783	Endoplasmic reticulum	66	2021	0.38	8.38e-10	RCN1,SPP2,ST6GAL1,SERPIND1,F9,EBI3,VTN,APOB,IGFBP5,PROC,APOA1,CAT,CFP,APOE,PXDN,F12,VWF,SERPINA10,CP,AMBP,AHSG,IGFBP1,SERPING1,HSP90B1,PCSK9,FGB,DST,F2,APOL1,CALR,HSPA5,FBN1,P4HB,THBS4,GANAB,PROZ,APOD,ASGR2,APOA4,ITIH2,COL18A1,F8,QSOX1,F5,S100A7,ADAMTSL4,ADAMTS13,MINPP1,F10,CKAP4,IGFBP3,PROS1,CST3,NUCB1,ADIPOQ,C4A,SPARCL1,HGFAC,APOA5,ATP1A1,HYOU1,KNG1,B2M,FGA,PDIA4,SDF4
GO Component	GO:0005604	Basement membrane	14	98	1.02	1.64e-08	VTN,SPARC,PXDN,SERPINF1,ATRN,NID1,DST,EFEMP2,FBN1,FBLN1,ANG,THBS4,COL18A1,TGFBI
GO Component	GO:0042627	Chylomicron	7	13	1.6	2.48e-07	APOH,APOC3,APOB,APOA1,APOE,APOA4,APOA5
GO Component	GO:0031089	Platelet dense granule lumen	7	14	1.57	3.58e-07	SPP2,APOH,LGALS3BP,ITIH4,ITIH3,RARRES2,SELENOP
GO Component	GO:0005579	Membrane attack complex	6	7	1.8	4.29e-07	C5,C6,C7,C8A,C8B,C8G
GO Component	GO:1904724	Tertiary granule lumen	10	55	1.13	6.99e-07	LTF,CFP,LYZ,PPBP,CAMP,LRG1,QPCT,QSOX1,CST3,B2M
GO Component	GO:0035580	Specific granule lumen	10	62	1.08	1.88e-06	VCL,LTF,CFP,LYZ,CAMP,LRG1,QPCT,QSOX1,ORM2,B2M
GO Component	GO:0005796	Golgi lumen	12	106	0.92	2.84e-06	F9,VTN,PROC,LUM,F2,CSPG4,PROZ,F8,F10,SOD3,PROS1,SDF4
GO Component	GO:0032991	Protein-containing complex	115	5506	0.19	3.92e-06	CETP,GNPTG,APOH,VCL,F9,EBI3,COMP,PON1,C5,VTN,APOC3,LTF,APOB,IGFBP5,PROC,APOA1,TTR,CAT,LRP1,DHX29,APOE,F12,CFHR5,PSMA6,C6,LCAT,NID1,CSTA,CDH6,DNAH5,TCIRG1,LUM,FCN3,SAA4,CSF1R,FCN2,MASP1,GUCY1A1,HSP90B1,PCSK9,FGB,F2,UBE3C,C1QB,CFHR1,SUZ12,APOL1,SUMO4,CALR,LPA,SERPINF2,C7,HBA1,ITLN1,HSPA5,P4HB,SERPINA5,DNAH8,ANG,GANAB,BLM,AKAP9,PIGR,APOA4,COL18A1,F8,C8A,F13B,CFHR2,CFH,F5,FLNA,SMARCA1,C8B,C8G,MBL2,C1QC,F10,APOM,TAF9,IGFBP3,KIF19,CFI,TNRC6A,GAPDH,FGL1,APOF,MASP2,SAA1,F11,C1S,PCYOX1,ADIPOQ,C4A,CDK3,COLEC11,C4B,CFB,IGFALS,PLTP,UIMC1,JCHAIN,HPR,C1R,APOA5,ATP1A1,IGKV2D-28,APOC4,CLTC,IGHV3-15,IGHV3-72,HYOU1,CDH5,B2M,FGA
GO Component	GO:0070013	Intracellular organelle lumen	117	5660	0.18	4.79e-06	RCN1,SPP2,APOH,VCL,SERPIND1,F9,EBI3,MPO,VTN,SPARC,LTF,HRG,APOB,IGFBP5,PROC,APOA1,TTR,CAT,CFP,APOE,LYZ,VWF,PSMA6,SERPINA10,LGALS3BP,CSTA,CP,CDH6,HYAL1,ITIH4,LUM,AHSG,IGFBP1,SERPING1,CSF1R,PCDH1,SERPINI1,PPBP,PF4,MASP1,CAMP,APEH,C16orf46,HSP90B1,LRG1,SH3D19,PCSK9,FGB,DST,F2,PLG,CSPG4,SUZ12,APOL1,CALR,SERPINF2,HBA1,HSPA5,FBN1,P4HB,ANG,GANAB,PRDX6,PROZ,QPCT,BLM,APOA4,ITIH2,COL18A1,F8,QSOX1,F5,S100A7,S100A9,ADAMTSL4,FLNA,SMARCA1,ADAMTS13,MINPP1,GSN,F10,CKAP4,CALML5,TAF9,IGFBP3,SOD3,PROS1,TNRC6A,NAV2,ILK,CST3,ANKRD28,SAA1,NUCB1,IGF2,ORM2,C4A,ACSF2,SPARCL1,ITIH3,RARRES2,GC,UIMC1,SELENOP,APOA5,SERPINA3,CFD,VEGFC,HYOU1,KNG1,TRIM66,GRHL2,CDH5,B2M,FGA,PDIA4,SDF4
GO Component	GO:0009897	External side of plasma membrane	21	388	0.6	6.40e-06	EBI3,FCN3,FCN2,MASP1,ANTXR1,PCSK9,FGB,PLG,CALR,P4HB,SERPINA5,ASGR2,MBL2,F10,MASP2,COLEC11,IGHV3-15,IGHV3-72,CDH5,B2M,FGA
GO Component	GO:0034366	Spherical high-density lipoprotein particle	5	8	1.67	1.95e-05	PON1,APOC3,APOA1,APOM,HPR
GO Component	GO:0005602	Complement component C1 complex	4	5	1.77	0.00015	C1QB,C1QC,C1S,C1R
GO Component	GO:0042470	Melanosome	10	109	0.83	0.00017	SERPINF1,YWHAE,HSP90B1,HSPA5,P4HB,GANAB,GSN,ATP1A1,CLTC,PDIA4

GO Component	GO:0005764	Lysosome	27	746	0.43	0.00019	GNPTG,MPO,GPLD1,HRG,APOB,TTR,CAT,LRP1,LYZ,CP,TCIRG1,HYAL1,LUM,PCSK9,CSPG4,CALR,PRDX6,PIGR,S100A7,MCOLN2,CKAP4,PCYOX1,ORM2,ABCA13,GC,SERPINA3,CLTC
GO Component	GO:0005925	Focal adhesion	19	416	0.53	0.00023	VCL,CAT,LRP1,YWHAE,HSP90B1,DST,CSPG4,CALR,HSPA5,P4HB,LIMS1,S100A7,FLNA,GSN,CD99,ILK,CLTC,HYOU1,B2M
GO Component	GO:0043227	Membrane-bounded organelle	211	13188	0.07	0.00023	RCN1,SPP2,ST6GAL1,CPB2,CETP,GNPTG,APOH,VCL,SERPIND1,CHGA,LBP,F9,MMP2,EBI3,COMP,PON1,PCOLCE,C5,MPO,VTN,AFM,APOC3,GPLD1,SPARC,PCDH12,LTF,HRG,APOB,IGFBP5,PROC,APOA1,TTR,CAT,LRP1,CFP,APOE,PXDN,ANGPTL6,F12,SERPINF1,APCS,LYZ,VWF,PSMA6,DSG2,SERPINA10,LGALS3BP,ATRN,C6,LCAT,NID1,YWHAE,CSTA,CP,CDH6,DNAH5,AMBP,CENPE,PON3,TCIRG1,HYAL1,ITIH4,LUM,ITIH1,AHSG,ANKRD31,IGFBP1,SAA4,SERPING1,CSF1R,PCDH1,FCN2,PGLYRP2,SCGB3A1,SERPINI1,PPBP,PF4,MASP1,CAMP,APEH,C16orf46,A2ML1,HSP90B1,ANTXR1,LRG1,SH3D19,PCSK9,BMP1,FGB,DST,F2,PLG,EFEMP2,CSPG4,SUZ12,APOL1,SUMO4,CPN2,CALR,SERPINF2,C7,HBA1,ITLN1,HSPA5,FBN1,P4HB,SERPINA7,FBLN1,SERPINA5,DNAH8,OIT3,ANG,THBS4,GANAB,PRDX6,PROZ,QPCT,APOD,ASGR2,BLM,AKAP9,PIGR,APOA4,ITIH2,COL18A1,F8,C8A,CDC42BPA,CFHR3,CFH,QSOX1,F5,S100A7,S100A9,ADAMTSL4,FLNA,CRTAC1,MCOLN2,SMARCA1,C8B,C8G,ADAMTS13,MINPP1,GSN,SVEP1,F10,CA6,CKAP4,CALML5,TAF9,IGFBP3,SOD3,GPX3,KIF19,PROS1,EFEMP1,CFI,TNRC6A,NAV2,ILK,GAPDH,RNASE1,CST3,ANKRD28,MAP3K7CL,MASP2,FBLN2,SAA1,F11,NUCB1,PCYOX1,ADIPOQ,IGF2,ORM2,C4A,FSIP2,ACSF2,CDK3,ABCA13,MST1,SPARCL1,ITIH3,C4B,TGFBI,CFB,IGFALS,PLTP,RARRES2,GC,HGFAC,UIMC1,SELENOP,JCHAIN,HPR,C1R,APOA5,ATP1A1,SERPINA3,IGKV2D-28,CFD,CLTC,VEGFC,HYOU1,KNG1,TRIM66,GRHL2,CDH5,B2M,FGA,PDIA4,SDF4
GO Component	GO:0005775	Vacuolar lumen	12	175	0.71	0.00029	MPO,APOB,TTR,LYZ,HYAL1,LUM,CSPG4,PRDX6,S100A7,ORM2,GC,SERPINA3
GO Component	GO:0005581	Collagen trimer	9	95	0.85	0.00035	LUM,FCN3,FCN2,C1QB,COL18A1,MBL2,C1QC,ADIPOQ,COLEC11
GO Component	GO:0005577	Fibrinogen complex	4	8	1.57	0.00046	FGB,SERPINF2,FGL1,FGA
GO Component	GO:0005773	Vacuole	28	839	0.39	0.00048	GNPTG,MPO,GPLD1,HRG,APOB,TTR,CAT,LRP1,LYZ,CP,TCIRG1,HYAL1,LUM,PCSK9,CSPG4,CALR,PRDX6,PIGR,S100A7,MCOLN2,CKAP4,PCYOX1,ORM2,ABCA13,MST1,GC,SERPINA3,CLTC
GO Component	GO:0042581	Specific granule	11	159	0.71	0.00058	VCL,LTF,CFP,LYZ,CAMP,LRG1,QPCT,QSOX1,CKAP4,ORM2,B2M
GO Component	GO:0030139	Endocytic vesicle	16	338	0.54	0.00074	MPO,SPARC,LTF,APOB,APOA1,LRP1,APOE,TCIRG1,HSP90B1,CALR,HBA1,GSN,SAA1,CLTC,HYOU1,B2M
GO Component	GO:0070820	Tertiary granule	11	164	0.7	0.00074	LTF,CFP,LYZ,TCIRG1,PPBP,CAMP,LRG1,QPCT,QSOX1,CST3,B2M
GO Component	GO:0019814	Immunoglobulin complex	5	25	1.17	0.0013	PIGR,JCHAIN,IGKV2D-28,IGHV3-15,IGHV3-72
GO Component	GO:0034362	Low-density lipoprotein particle	4	12	1.39	0.0015	APOB,APOE,APOM,APOF
GO Component	GO:0034663	Endoplasmic reticulum chaperone complex	4	12	1.39	0.0015	HSP90B1,HSPA5,P4HB,HYOU1
GO Component	GO:0042571	Immunoglobulin complex, circulating	4	12	1.39	0.0015	PIGR,JCHAIN,IGHV3-15,IGHV3-72
GO Component	GO:0043226	Organelle	217	14017	0.06	0.0020	RCN1,SPP2,ST6GAL1,CPB2,CETP,GNPTG,APOH,VCL,SERPIND1,CHGA,LBP,F9,MMP2,EBI3,COMP,PON1,PCOLCE,C5,MPO,VTN,AFM,APOC3,GPLD1,SPARC,PCDH12,LTF,HRG,APOB,IGFBP5,PROC,APOA1,TTR,CAT,LRP1,CFP,DHX29,APOE,PXDN,ANGPTL6,F12,SERPINF1,APCS,LYZ,VWF,PSMA6,DSG2,SERPINA10,LGALS3BP,ATRN,C6,LCAT,NID1,YWHAE,CSTA,CP,DGKG,CDH6,DNAH5,AMBP,CENPE,PON3,TCIRG1,HYAL1,ITIH4,LUM,ITIH1,AHSG,ANKRD31,IGFBP1,SAA4,SERPING1,CSF1R,PCDH1,FCN2,PGLYRP2,SCGB3A1,SERPINI1,PPBP,PF4,MASP1,CAMP,APEH,C16orf46,A2ML1,HSP90B1,ANTXR1,LRG1,SH3D19,PCSK9,BMP1,SHLD1,FGB,DST,F2,PLG,EFEMP2,CSPG4,SUZ12,APOL1,SUMO4,CPN2,CALR,SERPINF2,C7,HBA1,ITLN1,HSPA5,FBN1,P4HB,SERPINA7,FBLN1,SERPINA5,DNAH8,OIT3,ANG,THBS4,GANAB,PRDX6,PROZ,QPCT,APOD,ASGR2,BLM,AKAP9,PIGR,APOA4,ITIH2,COL18A1,MTCL1,F8,C8A,CDC42BPA,CFHR3,CFH,QSOX1,F5,S100A7,S100A9,ADAMTSL4,FLNA,CRTAC1,MCOLN2,SMARCA1,C8B,C8G,ADAMTS13,MINPP1,GSN,SVEP1,F10,CA6,CKAP4,CALML5,TAF9,IGFBP3,SOD3,GPX3,KIF19,PROS1,EFEMP1,CFI,TNRC6A,NAV2,ILK,GAPDH,RNASE1,CST3,ANKRD28,MAP3K7CL,MASP2,FBLN2,SAA1,F11,NUCB1,PCYOX1,ADIPOQ,IGF2,ORM2,C4A,SLAIN1,FSIP2,ACSF2,CDK3,ABCA13,MST1,LMOD3,SPARCL1,ITIH3,C4B,TGFBI,CFB,IGFALS,PLTP,RARRES2,GC,HGFAC,UIMC1,SELENOP,JCHAIN,HPR,C1R,APOA5,ATP1A1,SERPINA3,IGKV2D-28,CFD,CLTC,VEGFC,HYOU1,KNG1,TRIM66,GRHL2,CDH5,B2M,FGA,PDIA4,SDF4
GO Component	GO:0005601	Classical-complement-pathway C3/C5 convertase complex	3	4	1.74	0.0021	C4A,C4B,CFB
GO Component	GO:0042567	Insulin-like growth factor ternary complex	3	4	1.74	0.0021	IGFBP5,IGFBP3,IGFALS
GO Component	GO:0042582	Azurophil granule	10	154	0.68	0.0021	MPO,TTR,LYZ,PRDX6,PIGR,S100A7,CKAP4,ORM2,ABCA13,SERPINA3
GO Component	GO:1904813	ficolin-1-rich granule lumen	9	124	0.73	0.0021	VCL,CAT,APEH,LRG1,QPCT,GSN,CALML5,CST3,CFD
GO Component	GO:0110165	Cellular anatomical entity	261	18293	0.02	0.0030	RCN1,SPP2,ST6GAL1,CPB2,CETP,GNPTG,APOH,VCL,SERPIND1,CHGA,TPD52L2,LBP,APMAP,F9,MMP2,EBI3,COMP,PON1,PCOLCE,C5,MPO,VTN,AFM,PF4V1,APOC3,GPLD1,SPARC,PCDH12,LTF,HRG,APOB,IGFBP5,PROC,PLEK,APOA1,TTR,CAT,LRP1,C4BPB,CFP,DHX29,APOE,PXDN,ANGPTL6,F12,SERPINF1,APCS,CFHR5,LYZ,VWF,PSMA6,DSG2,SERPINA10,LGALS3BP,ATRN,C6,LCN1,LCAT,NID1,YWHAE,CSTA,CP,DGKG,CDH6,DNAH5,AMBP,CENPE,PON3,TCIRG1,HYAL1,ITIH4,LUM,FCN3,ITIH1,AHSG,ANKRD31,IGFBP1,CRH,HABP2,SAA4,SERPING1,CSF1R,PCDH1,FCN2,PGLYRP2,SCGB3A1,IHH,SERPINI1,PPBP,PF4,MASP1,CAMP,APEH,GUCY1A1,C16orf46,A2ML1,HSP90B1,ANTXR1,LRG1,SH3D19,PCSK9,BMP1,SHLD1,FGB,DST,F2,INHBC,PLG,EFEMP2,CSPG4,C1QB,CFHR1,SUZ12,APOL1,SUMO4,PSG11,CPN2,CALR,LPA,SERPINF2,C7,HBA1,ITLN1,HSPA5,FBN1,P4HB,SERPINA7,FBLN1,SERPINA5,DNAH8,OIT3,SERPINA11,ANG,LIMS1,THBS4,GANAB,PRDX6,FAM184A,PROZ,QPCT,APOD,PAPLN,ASGR2,BLM,AKAP9,PIGR,APOA4,ITIH2,CNDP1,COL18A1,MTCL1,F8,C8A,PCDH18,CDC42BPA,C4BPA,F13B,CFHR2,CFHR4,CFHR3,CFH,QSOX1,F5,CD5L,S100A7,S100A9,ADAMTSL4,FLNA,CRTAC1,MCOLN2,SMARCA1,C8B,C8G,ADAMTS13,MINPP1,GSN,MBL2,SVEP1,C1QC,F10,APOM,CA6,CKAP4,CALML5,TAF9,IGFBP3,CD99,SOD3,GPX3,KIF19,PROS1,EFEMP1,CFI,TNRC6A,NAV2,ILK,GAPDH,RNASE1,FGL1,APOF,CST3,ANKRD28,MAP3K7CL,MASP2,FBLN2,PSG4,SAA1,F11,C1S,PSG2,NUCB1,PCYOX1,KSR2,ADIPOQ,IGF2,ORM2,C4A,SLAIN1,FSIP2,ACSF2,CDK3,ABCA13,COLEC11,MST1,LMOD3,SPARCL1,ITIH3,C4B,TGFBI,CFB,IGFALS,PLTP,PAEP,RARRES2,GC,HGFAC,UIMC1,SELENOP,JCHAIN,HPR,C1R,APOA5,ATP1A1,SERPINA3,RNASE4,IGKV2D-28,APOC4,CFD,CLTC,IGHV3-15,IGHV3-72,VEGFC,HYOU1,KNG1,TRIM66,GRHL2,CDH5,B2M,FGA,PDIA4,SDF4
GO Component	GO:0034363	Intermediate-density lipoprotein particle	3	5	1.65	0.0032	APOC3,APOB,APOE
GO Component	GO:0101002	ficolin-1-rich granule	10	185	0.6	0.0074	VCL,CAT,TCIRG1,APEH,LRG1,QPCT,GSN,CALML5,CST3,CFD
GO Component	GO:0035578	Azurophil granule lumen	7	91	0.76	0.0084	MPO,TTR,LYZ,PRDX6,S100A7,ORM2,SERPINA3
GO Component	GO:1904090	Peptidase inhibitor complex	3	11	1.31	0.0186	VTN,CSTA,SERPINA5
GO Component	GO:0005790	Smooth endoplasmic reticulum	4	30	0.99	0.0251	APOB,CALR,HSPA5,HYOU1
GO Component	GO:0034365	Discoidal high-density lipoprotein particle	2	2	1.87	0.0251	APOE,APOM
GO Component	GO:0071751	Secretory IgA immunoglobulin complex	2	2	1.87	0.0251	PIGR,JCHAIN

GO Component	GO:0005886	Plasma membrane	98	5544	0.12	0.0311	VCL,F9,MMP2,EBI3,C5,VTN,SPARC,PCDH12,LTF,HRG,APOB,PLEK,APOA1,CAT,LRP1,C4BPB,CFP,APOE,F12,DSG2,ATRN,C6,NID1,CSTA,CP,DGKG,CDH6,AMBP,TCIRG1,ITIH4,FCN3,CSF1R,PCDH1,FCN2,IHH,MASP1,HSP90B1,ANTXR1,SH3D19,PCSK9,FGB,DST,F2,PLG,CSPG4,CALR,LPA,C7,ITLN1,HSPA5,P4HB,SERPINA5,LIMS1,ASGR2,AKAP9,PIGR,MTCL1,F8,C8A,PCDH18,C4BPA,F5,CD5L,S100A9,FLNA,MCOLN2,C8B,C8G,GSN,MBL2,F10,CKAP4,CD99,PROS1,ILK,GAPDH,CST3,MASP2,PSG4,F11,PCYOX1,KSR2,C4A,ABCA13,COLEC11,C4B,TGFBI,CFB,ATP1A1,IGKV2D-28,CLTC,IGHV3-15,IGHV3-72,KNG1,CDH5,B2M,FGA,SDF4
GO Component	GO:0062167	Complement component C1q complex	2	3	1.69	0.0383	C1QB,C1QC
STRING clusters	CL:18724	Mixed, incl. Complement and coagulation cascades, and Protein-lipid complex	87	172	1.57	3.33e-97	SPP2,CPB2,CETP,APOH,SERPIND1,F9,PON1,C5,AFM,APOC3,HRG,PROC,APOA1,LRP1,C4BPB,CFP,APOE,F12,APCS,CFHR5,VWF,SERPINA10,C6,LCAT,AMBP,FCN3,ITIH1,AHSG,HABP2,SAA4,SERPING1,FCN2,PGLYRP2,MASP1,PCSK9,FGB,F2,PLG,C1QB,CFHR1,APOL1,CPN2,LPA,SERPINF2,C7,SERPINA7,SERPINA5,SERPINA11,PROZ,APOA4,ITIH2,F8,C8A,C4BPA,F13B,CFHR2,CFHR4,CFHR3,CFH,F5,C8B,C8G,ADAMTS13,MBL2,C1QC,F10,APOM,CFI,APOF,MASP2,SAA1,F11,C1S,PCYOX1,ORM2,C4A,COLEC11,ITIH3,C4B,CFB,PLTP,HPR,C1R,APOA5,APOC4,CFD,FGA
STRING clusters	CL:18725	Complement and coagulation cascades, and Protein-lipid complex	86	166	1.58	5.52e-97	CPB2,CETP,APOH,SERPIND1,F9,PON1,C5,AFM,APOC3,HRG,PROC,APOA1,LRP1,C4BPB,CFP,APOE,F12,APCS,CFHR5,VWF,SERPINA10,C6,LCAT,AMBP,FCN3,ITIH1,AHSG,HABP2,SAA4,SERPING1,FCN2,PGLYRP2,MASP1,PCSK9,FGB,F2,PLG,C1QB,CFHR1,APOL1,CPN2,LPA,SERPINF2,C7,SERPINA7,SERPINA5,SERPINA11,PROZ,APOA4,ITIH2,F8,C8A,C4BPA,F13B,CFHR2,CFHR4,CFHR3,CFH,F5,C8B,C8G,ADAMTS13,MBL2,C1QC,F10,APOM,CFI,APOF,MASP2,SAA1,F11,C1S,PCYOX1,ORM2,C4A,COLEC11,ITIH3,C4B,CFB,PLTP,HPR,C1R,APOA5,APOC4,CFD,FGA
STRING clusters	CL:18723	Mixed, incl. Complement and coagulation cascades, and Protein-lipid complex	89	196	1.53	8.16e-97	SPP2,CPB2,CETP,APOH,SERPIND1,F9,PON1,C5,AFM,APOC3,HRG,PROC,APOA1,LRP1,C4BPB,CFP,APOE,F12,APCS,CFHR5,VWF,SERPINA10,C6,LCAT,AMBP,FCN3,ITIH1,AHSG,HABP2,SAA4,SERPING1,FCN2,PGLYRP2,MASP1,PCSK9,FGB,F2,PLG,C1QB,CFHR1,APOL1,CPN2,LPA,SERPINF2,C7,SERPINA7,SERPINA5,SERPINA11,PROZ,APOA4,ITIH2,F8,C8A,C4BPA,F13B,CFHR2,CFHR4,CFHR3,CFH,F5,CD5L,C8B,C8G,ADAMTS13,MBL2,C1QC,F10,APOM,PROS1,CFI,APOF,MASP2,SAA1,F11,C1S,PCYOX1,ORM2,C4A,COLEC11,ITIH3,C4B,CFB,PLTP,HPR,C1R,APOA5,APOC4,CFD,FGA
STRING clusters	CL:18726	Complement and coagulation cascades, and Protein-lipid complex	84	161	1.59	4.98e-95	CPB2,CETP,APOH,SERPIND1,F9,PON1,C5,AFM,APOC3,HRG,PROC,APOA1,LRP1,C4BPB,CFP,APOE,F12,APCS,CFHR5,VWF,C6,LCAT,AMBP,FCN3,ITIH1,AHSG,HABP2,SAA4,SERPING1,FCN2,PGLYRP2,MASP1,PCSK9,FGB,F2,PLG,C1QB,CFHR1,APOL1,CPN2,LPA,SERPINF2,C7,SERPINA7,SERPINA5,SERPINA11,APOA4,ITIH2,F8,C8A,C4BPA,F13B,CFHR2,CFHR4,CFHR3,CFH,F5,C8B,C8G,ADAMTS13,MBL2,C1QC,F10,APOM,CFI,APOF,MASP2,SAA1,F11,C1S,PCYOX1,ORM2,C4A,COLEC11,ITIH3,C4B,CFB,PLTP,HPR,C1R,APOA5,APOC4,CFD,FGA
STRING clusters	CL:18727	Complement and coagulation cascades, and Positive regulation of opsonization	64	115	1.62	2.32e-72	CPB2,SERPIND1,F9,C5,AFM,HRG,PROC,LRP1,C4BPB,CFP,F12,APCS,CFHR5,VWF,C6,AMBP,FCN3,ITIH1,AHSG,HABP2,SERPING1,FCN2,PGLYRP2,MASP1,FGB,F2,PLG,C1QB,CFHR1,CPN2,SERPINF2,C7,SERPINA7,SERPINA5,SERPINA11,ITIH2,F8,C8A,C4BPA,F13B,CFHR2,CFHR4,CFHR3,CFH,F5,C8B,C8G,ADAMTS13,MBL2,C1QC,F10,CFI,MASP2,F11,C1S,ORM2,C4A,COLEC11,ITIH3,C4B,CFB,C1R,CFD,FGA
STRING clusters	CL:18728	Complement and coagulation cascades, and Positive regulation of opsonization	61	109	1.62	6.19e-69	CPB2,SERPIND1,F9,C5,HRG,PROC,LRP1,C4BPB,CFP,F12,APCS,CFHR5,VWF,C6,AMBP,FCN3,ITIH1,AHSG,HABP2,SERPING1,FCN2,MASP1,FGB,F2,PLG,C1QB,CFHR1,SERPINF2,C7,SERPINA7,SERPINA5,SERPINA11,ITIH2,F8,C8A,C4BPA,F13B,CFHR2,CFHR4,CFHR3,CFH,F5,C8B,C8G,ADAMTS13,MBL2,C1QC,F10,CFI,MASP2,F11,C1S,ORM2,C4A,COLEC11,ITIH3,C4B,CFB,C1R,CFD,FGA
STRING clusters	CL:18846	Complement cascade	32	50	1.68	3.12e-36	C5,C4BPB,CFP,APCS,CFHR5,C6,FCN3,SERPING1,FCN2,MASP1,C1QB,CFHR1,C7,C8A,C4BPA,CFHR2,CFHR4,CFHR3,CFH,C8B,C8G,MBL2,C1QC,CFI,MASP2,C1S,C4A,COLEC11,C4B,CFB,C1R,CFD
STRING clusters	CL:18848	Complement cascade	26	40	1.68	2.96e-29	C5,C4BPB,CFP,C6,FCN3,SERPING1,FCN2,MASP1,C1QB,C7,C8A,C4BPA,CFH,C8B,C8G,MBL2,C1QC,CFI,MASP2,C1S,C4A,COLEC11,C4B,CFB,C1R,CFD
STRING clusters	CL:18730	Hemostasis, and Dissolution of Fibrin Clot	27	55	1.56	5.54e-28	CPB2,SERPIND1,F9,HRG,PROC,LRP1,F12,VWF,AMBP,ITIH1,AHSG,HABP2,FGB,F2,PLG,SERPINF2,SERPINA5,ITIH2,F8,F13B,F5,ADAMTS13,F10,F11,ORM2,ITIH3,FGA
STRING clusters	CL:18731	Hemostasis, and Dissolution of Fibrin Clot	26	50	1.59	1.96e-27	CPB2,SERPIND1,F9,HRG,PROC,LRP1,F12,VWF,AMBP,ITIH1,AHSG,HABP2,FGB,F2,PLG,SERPINF2,ITIH2,F8,F13B,F5,ADAMTS13,F10,F11,ORM2,ITIH3,FGA
STRING clusters	CL:18960	High-density lipoprotein particle	18	26	1.71	3.08e-20	CETP,APOH,PON1,APOC3,APOA1,LCAT,SAA4,APOL1,LPA,APOA4,APOM,APOF,SAA1,PCYOX1,PLTP,HPR,APOA5,APOC4
STRING clusters	CL:18956	Lipoprotein particle, and Assembly of active LPL and LIPC lipase complexes	20	46	1.51	1.36e-19	CETP,APOH,PON1,APOC3,APOA1,APOE,LCAT,SAA4,PCSK9,APOL1,LPA,APOA4,APOM,APOF,SAA1,PCYOX1,PLTP,HPR,APOA5,APOC4
STRING clusters	CL:18849	Initial triggering of complement, and Regulation of complement activation	18	31	1.63	2.76e-19	CFP,FCN3,SERPING1,FCN2,MASP1,C1QB,CFH,MBL2,C1QC,CFI,MASP2,C1S,C4A,COLEC11,C4B,CFB,C1R,CFD
STRING clusters	CL:18962	High-density lipoprotein particle	16	21	1.75	2.02e-18	CETP,APOH,PON1,APOC3,APOA1,LCAT,APOL1,LPA,APOA4,APOM,APOF,PCYOX1,PLTP,HPR,APOA5,APOC4
STRING clusters	CL:18732	Mixed, incl. COVID-19, thrombosis and anticoagulation, and Scavenging of heme from plasma	16	26	1.66	2.19e-17	CPB2,SERPIND1,HRG,LRP1,AMBP,ITIH1,AHSG,HABP2,FGB,PLG,SERPINF2,ITIH2,F13B,ORM2,ITIH3,FGA
STRING clusters	CL:18733	Mixed, incl. COVID-19, thrombosis and anticoagulation, and Inter-alpha-trypsin inhibitor heavy chain C-terminus	15	21	1.72	5.81e-17	CPB2,SERPIND1,HRG,AMBP,ITIH1,AHSG,HABP2,FGB,PLG,SERPINF2,ITIH2,F13B,ORM2,ITIH3,FGA
STRING clusters	CL:18966	High-density lipoprotein particle	12	16	1.74	1.56e-13	CETP,APOH,PON1,APOC3,APOA1,LCAT,LPA,APOA4,APOM,PLTP,APOA5,APOC4
STRING clusters	CL:18851	Initial triggering of complement, and Negative regulation of complement activation	13	24	1.6	2.01e-13	FCN3,SERPING1,FCN2,MASP1,C1QB,MBL2,C1QC,MASP2,C1S,C4A,COLEC11,C4B,C1R
STRING clusters	CL:18852	Creation of C4 and C2 activators	11	15	1.73	2.83e-12	FCN3,SERPING1,FCN2,MASP1,C1QB,MBL2,C1QC,MASP2,C1S,COLEC11,C1R
STRING clusters	CL:18784	Formation of Fibrin Clot (Clotting Cascade)	10	18	1.61	3.45e-10	F9,PROC,F12,VWF,F2,F8,F5,ADAMTS13,F10,F11
STRING clusters	CL:18734	COVID-19, thrombosis and anticoagulation, and Negative regulation of fibrinolysis	9	11	1.78	3.55e-10	CPB2,SERPIND1,HRG,HABP2,FGB,PLG,SERPINF2,F13B,FGA
STRING clusters	CL:18786	Hemophilia B, and Blood coagulation, common pathway	8	12	1.69	1.87e-08	F9,VWF,F2,F8,F5,ADAMTS13,F10,F11
STRING clusters	CL:18970	Triglyceride-rich lipoprotein particle remodeling, and Spherical high-density lipoprotein particle	7	11	1.67	3.54e-07	CETP,PON1,APOC3,APOA1,LCAT,APOA4,APOA5
STRING clusters	CL:18911	Membrane attack complex	6	7	1.8	1.48e-06	C5,C6,C7,C8A,C8B,C8G
STRING clusters	CL:19457	Extracellular matrix organization	16	180	0.82	1.51e-06	MMP2,PCOLCE,VTN,SERPINF1,NID1,LUM,BMP1,DST,EFEMP2,FBN1,P4HB,FBLN1,THBS4,COL18A1,EFEMP1,FBLN2
STRING clusters	CL:18755	Mixed, incl. Inter-alpha-trypsin inhibitor heavy chain C-terminus, and Alpha-1-acid glycoprotein	6	10	1.65	6.19e-06	AMBP,ITIH1,AHSG,ITIH2,ORM2,ITIH3

STRING clusters	CL:18737	Fibrinogen, and Thrombophilia	5	6	1.79	2.76e-05	SERPIND1,HRG,FGB,PLG,FGA
STRING clusters	CL:18854	Cell surface pattern recognition receptor signaling pathway	5	6	1.79	2.76e-05	FCN3,FCN2,MASP1,MBL2,MASP2
STRING clusters	CL:18869	Complement component C1 complex	5	6	1.79	2.76e-05	SERPING1,C1QB,C1QC,C1S,C1R
STRING clusters	CL:19696	Elastic fibre formation, and Tolloid/BMP1 peptidase domain	7	34	1.18	0.00015	PCOLCE,BMP1,EFEMP2,FBN1,FBLN1,EFEMP1,FBLN2
STRING clusters	CL:24268	Insulin-like growth factor-binding protein family 1-6, chordata, and Insulin-like growth factor	5	10	1.57	0.00015	IGFBP5,IGFBP1,IGFBP3,IGF2,IGFALS
STRING clusters	CL:18934	Hemolytic uremic syndrome	4	5	1.77	0.00050	CFHR5,CFHR1,CFHR4,CFHR3
STRING clusters	CL:19695	Elastic fibre formation, and Matrix metalloproteinases	8	61	0.99	0.00050	MMP2,PCOLCE,BMP1,EFEMP2,FBN1,FBLN1,EFEMP1,FBLN2
STRING clusters	CL:19699	Elastic fibre formation	5	14	1.42	0.00051	EFEMP2,FBN1,FBLN1,EFEMP1,FBLN2
STRING clusters	CL:19114	Mixed, incl. S100/CaBP-9k-type, calcium binding, subdomain, and Cystatin superfamily	9	103	0.81	0.0023	MPO,LYZ,LGALS3BP,LCN1,CSTA,CAMP,S100A7,S100A9,CST3
STRING clusters	CL:17205	Immunoglobulin complex, and IgG-binding protein	6	40	1.05	0.0036	PCDH12,PIGR,JCHAIN,IGKV2D-28,IGHV3-15,IGHV3-72
STRING clusters	CL:19115	Mixed, incl. Cystatin superfamily, and Specific granule lumen	7	60	0.94	0.0036	MPO,LYZ,LGALS3BP,LCN1,CSTA,CAMP,CST3
STRING clusters	CL:17209	Immunoglobulin complex, and DNA recombinase complex	5	27	1.14	0.0068	PIGR,JCHAIN,IGKV2D-28,IGHV3-15,IGHV3-72
STRING clusters	CL:18123	Cell-extracellular matrix interactions, and Alpha-catenin/vinculin-like superfamily	4	17	1.24	0.0160	VCL,LIMS1,FLNA,ILK
STRING clusters	CL:18788	Extrinsic Pathway of Fibrin Clot Formation, and Factor V deficiency	3	6	1.57	0.0188	F2,F5,F10
STRING clusters	CL:18945	Mixed, incl. Peptide amidation, and TonB box, conserved site	3	6	1.57	0.0188	AFM,PGLYRP2,CPN2
STRING clusters	CL:1545	Photodynamic therapy-induced unfolded protein response, and IRE1-TRAF2-ASK1 complex	4	19	1.19	0.0209	HSP90B1,HSPA5,HYOU1,PDIA4
STRING clusters	CL:19458	Collagen formation, and Protein complex involved in cell adhesion	8	119	0.7	0.0280	VTN,SERPINF1,NID1,LUM,DST,P4HB,THBS4,COL18A1
STRING clusters	CL:9658	Detoxification of Reactive Oxygen Species, and Copper transport	5	40	0.97	0.0287	CAT,CP,PRDX6,SOD3,GPX3
STRING clusters	CL:9662	Detoxification of Reactive Oxygen Species, and mRNA, protein, and metabolite inducation pathway by cyclosporin A	4	24	1.09	0.0431	CAT,PRDX6,SOD3,GPX3
KEGG	hsa04610	Complement and coagulation cascades	46	82	1.62	1.85e-51	CPB2,SERPIND1,F9,C5,VTN,PROC,C4BPB,F12,CFHR5,VWF,C6,SERPING1,FGB,F2,PLG,C1QB,CFHR1,SERPINF2,C7,SERPINA5,F8,C8A,C4BPA,F13B,CFHR2,CFHR4,CFHR3,CFH,F5,C8B,C8G,MBL2,C1QC,F10,PROS1,CFI,MASP2,F11,C1S,C4A,C4B,CFB,C1R,CFD,KNG1,FGA
KEGG	hsa05150	Staphylococcus aureus infection	15	86	1.11	8.96e-10	C5,CAMP,PLG,C1QB,CFH,MBL2,C1QC,CFI,MASP2,C1S,C4A,C4B,CFB,C1R,CFD
KEGG	hsa04979	Cholesterol metabolism	12	48	1.27	2.17e-09	CETP,APOH,APOC3,APOB,APOA1,LRP1,APOE,LCAT,PCSK9,LPA,APOA4,PLTP
KEGG	hsa05133	Pertussis	11	73	1.05	1.26e-06	C5,C4BPB,SERPING1,C1QB,C4BPA,C1QC,CALML5,C1S,C4A,C4B,C1R
KEGG	hsa05322	Systemic lupus erythematosus	12	94	0.98	1.26e-06	C5,C6,C1QB,C7,C8A,C8B,C8G,C1QC,C1S,C4A,C4B,C1R
KEGG	hsa04918	Thyroid hormone synthesis	8	73	0.91	0.00069	TTR,HSP90B1,HSPA5,SERPINA7,ASGR2,GPX3,ATP1A1,PDIA4
KEGG	hsa03320	PPAR signaling pathway	6	75	0.77	0.0357	APOC3,APOA1,ILK,ADIPOQ,PLTP,APOA5
KEGG	hsa04145	Phagosome	8	141	0.62	0.0371	COMP,MPO,TCIRG1,CALR,THBS4,MBL2,COLEC11,C1R
Reactome	HSA-166658	Complement cascade	36	59	1.66	6.20e-40	CPB2,C5,VTN,C4BPB,CFP,CFHR5,C6,FCN3,SERPING1,FCN2,F2,C1QB,CFHR1,CPN2,C7,C8A,C4BPA,CFHR2,CFHR4,CFHR3,CFH,C8B,C8G,MBL2,C1QC,PROS1,CFI,MASP2,C1S,C4A,COLEC11,C4B,CFB,C1R,IGKV2D-28,CFD
Reactome	HSA-977606	Regulation of Complement cascade	30	49	1.66	3.68e-33	CPB2,C5,VTN,C4BPB,CFP,CFHR5,C6,SERPING1,F2,C1QB,CFHR1,CPN2,C7,C8A,C4BPA,CFHR2,CFHR4,CFHR3,CFH,C8B,C8G,C1QC,PROS1,CFI,C1S,C4A,C4B,CFB,C1R,IGKV2D-28
Reactome	HSA-381426	Regulation of Insulin-like Growth Factor (IGF) transport and uptake by Insulin-like Growth Factor Binding Proteins (IGFBPs)	34	124	1.31	1.51e-28	RCN1,SPP2,SERPIND1,MMP2,APOB,IGFBP5,PROC,APOA1,APOE,SERPINA10,CP,AHSG,IGFBP1,HSP90B1,PCSK9,F2,PLG,APOL1,FBN1,P4HB,ITIH2,QSOX1,F5,CKAP4,IGFBP3,CST3,NUCB1,IGF2,C4A,SPARCL1,IGFALS,APOA5,KNG1,FGA
Reactome	HSA-168249	Innate Immune System	72	1041	0.71	1.72e-27	CPB2,VCL,CHGA,LBP,C5,MPO,VTN,LTF,APOB,TTR,CAT,C4BPB,CFP,CFHR5,LYZ,PSMA6,C6,TCIRG1,FCN3,AHSG,SERPING1,FCN2,PGLYRP2,PPBP,CAMP,APEH,HSP90B1,LRG1,FGB,F2,C1QB,CFHR1,CPN2,C7,ITLN1,PRDX6,QPCT,PIGR,C8A,C4BPA,CFHR2,CFHR4,CFHR3,CFH,QSOX1,S100A7,S100A9,C8B,C8G,GSN,MBL2,C1QC,CKAP4,CALML5,PROS1,CFI,CST3,MASP2,SAA1,C1S,ORM2,C4A,ABCA13,COLEC11,C4B,CFB,C1R,SERPINA3,IGKV2D-28,CFD,B2M,FGA
Reactome	HSA-114608	Platelet degranulation	33	126	1.29	2.59e-27	SPP2,APOH,VCL,SPARC,HRG,PLEK,APOA1,VWF,LGALS3BP,ITIH4,AHSG,SERPING1,PPBP,PF4,FGB,PLG,SERPINF2,HSPA5,F8,QSOX1,F5,FLNA,PROS1,IGF2,ORM2,ITIH3,RARRES2,SELENOP,SERPINA3,CFD,VEGFC,KNG1,FGA
Reactome	HSA-109582	Hemostasis	54	607	0.82	8.21e-25	SPP2,APOH,VCL,SERPIND1,F9,PF4V1,SPARC,HRG,APOB,PROC,PLEK,APOA1,F12,VWF,LGALS3BP,DGKG,CENPE,ITIH4,AHSG,SERPING1,PPBP,PF4,GUCY1A1,FGB,F2,PLG,PSG11,SERPINF2,HSPA5,SERPINA5,F8,F13B,QSOX1,F5,FLNA,F10,CD99,KIF19,PROS1,PSG4,F11,PSG2,IGF2,ORM2,ITIH3,RARRES2,SELENOP,JCHAIN,SERPINA3,IGKV2D-28,CFD,VEGFC,KNG1,FGA
Reactome	HSA-8957275	Post-translational protein phosphorylation	29	107	1.3	2.40e-24	RCN1,SPP2,SERPIND1,APOB,IGFBP5,PROC,APOA1,APOE,SERPINA10,CP,AHSG,IGFBP1,HSP90B1,PCSK9,APOL1,FBN1,P4HB,ITIH2,QSOX1,F5,CKAP4,IGFBP3,CST3,NUCB1,C4A,SPARCL1,APOA5,KNG1,FGA
Reactome	HSA-76002	Platelet activation, signaling and aggregation	35	260	1.0	9.85e-21	SPP2,APOH,VCL,SPARC,HRG,PLEK,APOA1,VWF,LGALS3BP,DGKG,ITIH4,AHSG,SERPING1,PPBP,PF4,FGB,F2,PLG,SERPINF2,HSPA5,F8,QSOX1,F5,FLNA,PROS1,IGF2,ORM2,ITIH3,RARRES2,SELENOP,SERPINA3,CFD,VEGFC,KNG1,FGA
Reactome	HSA-140877	Formation of Fibrin Clot (Clotting Cascade)	19	39	1.56	1.66e-19	SERPIND1,F9,PF4V1,PROC,F12,VWF,SERPING1,PF4,FGB,F2,SERPINA5,F8,F13B,F5,F10,PROS1,F11,KNG1,FGA
Reactome	HSA-168256	Immune System	83	1979	0.49	1.01e-18	CPB2,VCL,CHGA,LBP,MMP2,EBI3,C5,MPO,VTN,LTF,APOB,TTR,CAT,C4BPB,CFP,CFHR5,LYZ,PSMA6,C6,CENPE,TCIRG1,FCN3,AHSG,SERPING1,CSF1R,FCN2,PGLYRP2,PPBP,CAMP,APEH,HSP90B1,LRG1,FGB,F2,UBE3C,C1QB,CFHR1,CPN2,CALR,C7,ITLN1,HSPA5,P4HB,PRDX6,QPCT,PIGR,C8A,C4BPA,CFHR2,CFHR4,CFHR3,CFH,QSOX1,S100A7,S100A9,FLNA,C8B,C8G,GSN,MBL2,C1QC,CKAP4,CALML5,CD99,PROS1,CFI,CST3,MASP2,SAA1,C1S,ORM2,C4A,ABCA13,COLEC11,C4B,CFB,C1R,SERPINA3,IGKV2D-28,CFD,CLTC,B2M,FGA
Reactome	HSA-166663	Initial triggering of complement	15	24	1.67	1.79e-16	CFP,FCN3,FCN2,C1QB,MBL2,C1QC,MASP2,C1S,C4A,COLEC11,C4B,CFB,C1R,IGKV2D-28,CFD
Reactome	HSA-140875	Common Pathway of Fibrin Clot Formation	13	22	1.64	6.21e-14	SERPIND1,PF4V1,PROC,PF4,FGB,F2,SERPINA5,F8,F13B,F5,F10,PROS1,FGA
Reactome	HSA-140837	Intrinsic Pathway of Fibrin Clot Formation	13	23	1.62	8.91e-14	SERPIND1,F9,PROC,F12,VWF,SERPING1,F2,SERPINA5,F8,F10,PROS1,F11,KNG1
Reactome	HSA-2173782	Binding and Uptake of Ligands by Scavenger Receptors	15	43	1.41	1.35e-13	SPARC,APOB,APOA1,LRP1,APOE,AMBP,HSP90B1,APOL1,CALR,SAA1,COLEC11,JCHAIN,HPR,IGKV2D-28,HYOU1

Reactome	HSA-174824	Plasma lipoprotein assembly, remodeling, and clearance	16	75	1.2	9.55e-12	CETP,APOC3,APOB,APOA1,APOE,LCAT,PCSK9,BMP1,LPA,P4HB,APOA4,APOF,PLTP,APOA5,APOC4,CLTC
Reactome	HSA-166786	Creation of C4 and C2 activators	10	16	1.67	8.53e-11	FCN3,FCN2,C1QB,MBL2,C1QC,MASP2,C1S,COLEC11,C1R,IGKV2D-28
Reactome	HSA-8963899	Plasma lipoprotein remodeling	12	34	1.42	8.53e-11	CETP,APOC3,APOB,APOA1,APOE,LCAT,LPA,P4HB,APOA4,APOF,PLTP,APOA5
Reactome	HSA-392499	Metabolism of proteins	64	1917	0.39	1.59e-09	RCN1,SPP2,ST6GAL1,CPB2,SERPIND1,F9,MMP2,GPLD1,LTF,APOB,IGFBP5,PROC,APOA1,TTR,CFP,APOE,APCS,LYZ,PSMA6,SERPINA10,CP,AHSG,IGFBP1,APEH,HSP90B1,PCSK9,F2,INHBC,PLG,SUZ12,APOL1,CALR,FBN1,P4HB,GANAB,PROZ,ASGR2,BLM,APOA4,ITIH2,F8,QSOX1,F5,ADAMTSL4,ADAMTS13,GSN,F10,CKAP4,IGFBP3,PROS1,CST3,ANKRD28,SAA1,NUCB1,IGF2,C4A,SPARCL1,TGFBI,IGFALS,UIMC1,APOA5,KNG1,B2M,FGA
Reactome	HSA-6798695	Neutrophil degranulation	28	476	0.64	2.25e-08	VCL,MPO,LTF,TTR,CAT,CFP,LYZ,TCIRG1,AHSG,PPBP,CAMP,APEH,LRG1,PRDX6,QPCT,PIGR,QSOX1,S100A7,S100A9,GSN,CKAP4,CALML5,CST3,ORM2,ABCA13,SERPINA3,CFD,B2M
Reactome	HSA-977225	Amyloid fiber formation	13	79	1.09	2.96e-08	LTF,APOA1,TTR,APOE,APCS,LYZ,APOA4,GSN,CST3,SAA1,TGFBI,B2M,FGA
Reactome	HSA-1474244	Extracellular matrix organization	22	300	0.73	4.36e-08	MMP2,COMP,PCOLCE,VTN,SPARC,TTR,PXDN,VWF,NID1,LUM,BMP1,FGB,DST,PLG,EFEMP2,FBN1,P4HB,FBLN1,COL18A1,EFEMP1,FBLN2,FGA
Reactome	HSA-9651496	Defects of contact activation system (CAS) and kallikrein/kinin system (KKS)	8	16	1.57	5.43e-08	F9,F12,VWF,SERPING1,F2,F8,F10,F11
Reactome	HSA-8963898	Plasma lipoprotein assembly	8	19	1.49	1.46e-07	APOC3,APOB,APOA1,APOE,BMP1,P4HB,APOA4,APOC4
Reactome	HSA-597592	Post-translational protein modification	48	1405	0.4	3.59e-07	RCN1,SPP2,ST6GAL1,SERPIND1,F9,GPLD1,APOB,IGFBP5,PROC,APOA1,CFP,APOE,PSMA6,SERPINA10,CP,AHSG,IGFBP1,HSP90B1,PCSK9,F2,SUZ12,APOL1,CALR,FBN1,P4HB,GANAB,PROZ,ASGR2,BLM,ITIH2,F8,QSOX1,F5,ADAMTSL4,ADAMTS13,F10,CKAP4,IGFBP3,PROS1,CST3,ANKRD28,NUCB1,C4A,SPARCL1,UIMC1,APOA5,KNG1,FGA
Reactome	HSA-2168880	Scavenging of heme from plasma	7	15	1.54	8.37e-07	APOA1,LRP1,AMBP,APOL1,JCHAIN,HPR,IGKV2D-28
Reactome	HSA-166665	Terminal pathway of complement	6	8	1.74	1.18e-06	C5,C6,C7,C8A,C8B,C8G
Reactome	HSA-159763	Transport of gamma-carboxylated protein precursors from the endoplasmic reticulum to the Golgi apparatus	6	9	1.69	1.88e-06	F9,PROC,F2,PROZ,F10,PROS1
Reactome	HSA-159740	Gamma-carboxylation of protein precursors	6	10	1.65	2.88e-06	F9,PROC,F2,PROZ,F10,PROS1
Reactome	HSA-159782	Removal of aminoterminal propeptides from gamma-carboxylated proteins	6	10	1.65	2.88e-06	F9,PROC,F2,PROZ,F10,PROS1
Reactome	HSA-8963888	Chylomicron assembly	6	10	1.65	2.88e-06	APOC3,APOB,APOA1,APOE,P4HB,APOA4
Reactome	HSA-8963901	Chylomicron remodeling	6	10	1.65	2.88e-06	APOC3,APOB,APOA1,APOE,APOA4,APOA5
Reactome	HSA-8964058	HDL remodeling	6	10	1.65	2.88e-06	CETP,APOC3,APOA1,APOE,LCAT,PLTP
Reactome	HSA-8964041	LDL remodeling	5	6	1.79	1.11e-05	CETP,APOB,LPA,P4HB,APOF
Reactome	HSA-166662	Lectin pathway of complement activation	5	7	1.72	1.83e-05	FCN3,FCN2,MBL2,MASP2,COLEC11
Reactome	HSA-174577	Activation of C3 and C5	5	7	1.72	1.83e-05	C5,CFP,C4A,C4B,CFB
Reactome	HSA-9662001	Defective factor VIII causes hemophilia A	5	7	1.72	1.83e-05	F9,VWF,F2,F8,F10
Reactome	HSA-975634	Retinoid metabolism and transport	8	44	1.13	2.31e-05	APOC3,APOB,APOA1,TTR,LRP1,APOE,APOA4,APOM
Reactome	HSA-3000480	Scavenging by Class A Receptors	6	18	1.39	3.24e-05	APOB,APOA1,APOE,HSP90B1,CALR,COLEC11
Reactome	HSA-173623	Classical antibody-mediated complement activation	5	9	1.61	3.97e-05	C1QB,C1QC,C1S,C1R,IGKV2D-28
Reactome	HSA-202733	Cell surface interactions at the vascular wall	12	139	0.81	4.60e-05	PF4V1,APOB,PROC,PF4,F2,PSG11,CD99,PROS1,PSG4,PSG2,JCHAIN,IGKV2D-28
Reactome	HSA-163841	Gamma carboxylation, hypusine formation and arylsulfatase activation	7	42	1.09	0.00019	F9,PROC,F2,PROZ,F8,F10,PROS1
Reactome	HSA-5686938	Regulation of TLR by endogenous ligand	5	20	1.27	0.00086	LBP,APOB,FGB,S100A9,FGA
Reactome	HSA-9029569	NR1H3 & NR1H2 regulate gene expression linked to cholesterol transport and efflux	6	36	1.09	0.00090	CETP,APOE,APOD,TNRC6A,PLTP,APOC4
Reactome	HSA-9668250	Defective factor IX causes hemophilia B	4	9	1.52	0.00095	F9,F8,F10,F11
Reactome	HSA-2129379	Molecules associated with elastic fibres	6	37	1.08	0.00099	VTN,EFEMP2,FBN1,FBLN1,EFEMP1,FBLN2
Reactome	HSA-8964043	Plasma lipoprotein clearance	6	37	1.08	0.00099	APOB,APOA1,APOE,PCSK9,APOC4,CLTC
Reactome	HSA-216083	Integrin cell surface interactions	8	85	0.84	0.0015	COMP,VTN,VWF,LUM,FGB,FBN1,COL18A1,FGA
Reactome	HSA-6803157	Antimicrobial peptides	8	87	0.83	0.0018	CHGA,LTF,LYZ,PGLYRP2,CAMP,ITLN1,S100A7,S100A9
Reactome	HSA-9672383	Defective factor IX causes thrombophilia	3	3	1.87	0.0020	F9,F8,F10
Reactome	HSA-9672391	Defective F8 cleavage by thrombin	3	3	1.87	0.0020	VWF,F2,F8
Reactome	HSA-9672396	Defective cofactor function of FVIIIa variant	3	3	1.87	0.0020	F9,F8,F10
Reactome	HSA-9673202	Defective F9 variant does not activate FX	3	3	1.87	0.0020	F9,F8,F10
Reactome	HSA-5653656	Vesicle-mediated transport	23	666	0.41	0.0021	SPARC,APOB,APOA1,LRP1,APOE,YWHAE,AMBP,CENPE,HSP90B1,SH3D19,APOL1,CALR,F8,F5,KIF19,ANKRD28,SAA1,COLEC11,JCHAIN,HPR,IGKV2D-28,CLTC,HYOU1
Reactome	HSA-2855086	Ficolins bind to repetitive carbohydrate structures on the target cell surface	3	4	1.74	0.0030	FCN3,FCN2,MASP2
Reactome	HSA-173736	Alternative complement activation	3	5	1.65	0.0046	CFP,CFB,CFD
Reactome	HSA-8866423	VLDL assembly	3	5	1.65	0.0046	APOB,P4HB,APOC4
Reactome	HSA-382551	Transport of small molecules	23	723	0.37	0.0062	CETP,APOC3,APOB,APOA1,APOE,PSMA6,LCN1,LCAT,CP,TCIRG1,PCSK9,BMP1,LPA,P4HB,APOD,APOA4,MCOLN2,APOF,PLTP,APOA5,ATP1A1,APOC4,CLTC
Reactome	HSA-3000471	Scavenging by Class B Receptors	3	6	1.57	0.0065	APOB,APOA1,SAA1
Reactome	HSA-3000484	Scavenging by Class F Receptors	3	6	1.57	0.0065	APOB,CALR,HYOU1
Reactome	HSA-6799990	Metal sequestration by antimicrobial proteins	3	6	1.57	0.0065	LTF,S100A7,S100A9
Reactome	HSA-3299685	Detoxification of Reactive Oxygen Species	5	36	1.01	0.0069	CAT,P4HB,PRDX6,SOD3,GPX3
Reactome	HSA-6802948	Signaling by high-kinase activity BRAF mutants	5	36	1.01	0.0069	VCL,VWF,FGB,KSR2,FGA
Reactome	HSA-5674135	MAP2K and MAPK activation	5	40	0.97	0.0104	VCL,VWF,FGB,KSR2,FGA
Reactome	HSA-9656223	Signaling by RAF1 mutants	5	41	0.96	0.0114	VCL,VWF,FGB,KSR2,FGA
Reactome	HSA-446728	Cell junction organization	7	92	0.75	0.0115	CDH6,DST,ANG,LIMS1,FLNA,ILK,CDH5
Reactome	HSA-6802952	Signaling by BRAF and RAF1 fusions	6	65	0.83	0.0115	VCL,VWF,FGB,AKAP9,KSR2,FGA
Reactome	HSA-1643685	Disease	40	1702	0.24	0.0149	ST6GAL1,VCL,F9,LTF,APOA1,TTR,CFP,F12,VWF,PSMA6,YWHAE,CP,HYAL1,LUM,SERPING1,IHH,ANTXR1,FGB,F2,CSPG4,SUZ12,CALR,GANAB,BLM,AKAP9,F8,S100A9,ADAMTSL4,ADAMTS13,MBL2,F10,TAF9,MASP2,F11,KSR2,ATP1A1,IGKV2D-28,CLTC,B2M,FGA
Reactome	HSA-6802946	Signaling by moderate kinase activity BRAF mutants	5	45	0.92	0.0160	VCL,VWF,FGB,KSR2,FGA
Reactome	HSA-6802955	Paradoxical activation of RAF signaling by kinase inactive BRAF	5	45	0.92	0.0160	VCL,VWF,FGB,KSR2,FGA
Reactome	HSA-9649948	Signaling downstream of RAS mutants	5	45	0.92	0.0160	VCL,VWF,FGB,KSR2,FGA
Reactome	HSA-381183	ATF6 (ATF6-alpha) activates chaperone genes	3	10	1.35	0.0175	HSP90B1,CALR,HSPA5
Reactome	HSA-9672393	Defective F8 binding to von Willebrand factor	2	2	1.87	0.0294	VWF,F8
Reactome	HSA-75205	Dissolution of Fibrin Clot	3	13	1.23	0.0320	HRG,PLG,SERPINF2
Reactome	HSA-354194	GRB2:SOS provides linkage to MAPK signaling for Integrins	3	15	1.17	0.0447	VWF,FGB,FGA
Reactome	HSA-372708	p130Cas linkage to MAPK signaling for integrins	3	15	1.17	0.0447	VWF,FGB,FGA

Reactome	HSA-1236974	ER-Phagosome pathway	6	89	0.7	0.0453	PSMA6,FGB,CALR,S100A9,B2M,FGA
Reactome	HSA-9657688	Defective factor XII causes hereditary angioedema	2	3	1.69	0.0453	F12,F2
Reactome	HSA-9657689	Defective SERPING1 causes hereditary angioedema	2	3	1.69	0.0453	F12,SERPING1
Reactome	HSA-1474290	Collagen formation	6	90	0.69	0.0462	PCOLCE,PXDN,BMP1,DST,P4HB,COL18A1
Reactome	HSA-2022090	Assembly of collagen fibrils and other multimeric structures	5	61	0.78	0.0473	PCOLCE,PXDN,BMP1,DST,COL18A1
WikiPathways	WP558	Complement and coagulation cascades	33	58	1.62	4.47e-36	CPB2,SERPIND1,F9,PROC,F12,VWF,C6,SERPING1,MASP1,FGB,F2,PLG,C1QB,SERPINF2,C7,SERPINA5,F8,F13B,CFH,F5,C8G,C1QC,F10,PROS1,CFI,MASP2,C1S,C4B,CFB,C1R,CFD,CLTC,KNG1
WikiPathways	WP2806	Complement system	31	96	1.38	5.79e-28	C5,VTN,APOA1,CFP,F12,APCS,C6,SERPING1,FCN2,MASP1,FGB,PLG,CALR,C7,C8A,C4BPA,CFHR2,CFHR4,CFH,MBL2,F10,PROS1,CFI,MASP2,F11,C1S,ADIPOQ,C4A,CFB,CFD,FGA
WikiPathways	WP5090	Complement system in neuronal development and plasticity	28	105	1.3	2.50e-23	C5,VTN,C4BPB,CFP,C6,FCN3,SERPING1,FCN2,MASP1,C1QB,C7,C8A,C4BPA,CFH,C8B,C8G,MBL2,C1QC,PROS1,CFI,MASP2,C1S,C4A,COLEC11,C4B,CFB,C1R,CFD
WikiPathways	WP545	Complement activation	17	22	1.76	6.02e-20	C5,CFP,C6,MASP1,C1QB,C7,C8A,C8B,C8G,C1QC,MASP2,C1S,C4A,C4B,CFB,C1R,CFD
WikiPathways	WP5115	Network map of SARS-CoV-2 signaling pathway	26	218	0.95	4.48e-14	APOH,LBP,HRG,APOA1,CFP,SERPINA10,LGALS3BP,ITIH4,PF4,LRG1,FGB,APOL1,SERPINA5,APOD,C8A,CFH,GSN,APOM,IGFBP3,CFI,SAA1,C1S,ITIH3,CFB,C1R,FGA
WikiPathways	WP272	Blood clotting cascade	13	22	1.64	4.60e-14	F9,F12,VWF,FGB,F2,PLG,SERPINF2,F8,F13B,F5,F10,F11,FGA
WikiPathways	WP176	Folate metabolism	14	67	1.19	3.62e-10	MPO,APOB,APOA1,CAT,SAA4,FGB,F2,PLG,HBA1,SOD3,GPX3,SAA1,SERPINA3,FGA
WikiPathways	WP15	Selenium micronutrient network	15	84	1.12	3.86e-10	MPO,APOB,APOA1,CAT,SAA4,FGB,F2,PLG,HBA1,SOD3,GPX3,SAA1,SELENOP,SERPINA3,FGA
WikiPathways	WP5323	Fatty Acids and Lipoproteins Transport in Hepatocytes	27	380	0.72	6.70e-10	CETP,APOH,PON1,VTN,APOC3,APOB,APOA1,LRP1,APOE,LGALS3BP,LCAT,SAA4,PCSK9,APOL1,LPA,ASGR2,APOA4,CD5L,APOM,CFI,APOF,SAA1,PCYOX1,ADIPOQ,PLTP,APOA5,APOC4
WikiPathways	WP430	Statin inhibition of cholesterol production	10	29	1.41	5.26e-09	CETP,APOC3,APOB,APOA1,LRP1,APOE,LCAT,APOA4,PLTP,APOA5
WikiPathways	WP1533	Vitamin B12 metabolism	11	50	1.21	3.15e-08	MPO,APOB,APOA1,APOE,SAA4,F2,PLG,HBA1,SOD3,SAA1,SERPINA3
WikiPathways	WP5304	Cholesterol metabolism	12	72	1.09	7.67e-08	CETP,APOH,APOC3,APOB,APOA1,LRP1,APOE,LCAT,PCSK9,LPA,APOA4,PLTP
WikiPathways	WP5110	Familial hyperlipidemia type 3	7	13	1.6	2.77e-07	CETP,APOA1,LRP1,APOE,LCAT,APOA4,PLTP
WikiPathways	WP5112	Familial hyperlipidemia type 5	7	15	1.54	5.52e-07	CETP,APOA1,LRP1,LCAT,APOA4,PLTP,APOA5
WikiPathways	WP5109	Familial hyperlipidemia type 2	7	16	1.51	7.33e-07	CETP,APOB,APOA1,LCAT,PCSK9,APOA4,PLTP
WikiPathways	WP5108	Familial hyperlipidemia type 1	7	18	1.46	1.32e-06	CETP,APOA1,LRP1,LCAT,APOA4,PLTP,APOA5
WikiPathways	WP3601	Lipid particles composition	6	10	1.65	1.74e-06	CETP,APOC3,APOB,APOA1,APOE,LCAT
WikiPathways	WP5111	Familial hyperlipidemia type 4	7	22	1.37	3.63e-06	CETP,APOA1,LRP1,LCAT,APOA4,PLTP,APOA5
WikiPathways	WP5186	Vitamin K metabolism and activation of dependent proteins	6	12	1.57	3.63e-06	F9,PROC,F2,PROZ,F10,PROS1
WikiPathways	WP4927	COVID-19, thrombosis and anticoagulation	5	7	1.72	1.16e-05	FGB,F2,PLG,F13B,FGA
WikiPathways	WP4522	Metabolic pathway of LDL, HDL and TG, including diseases	6	17	1.42	1.64e-05	CETP,APOB,APOA1,APOE,LCAT,PCSK9
WikiPathways	WP2328	Allograft rejection	9	88	0.88	0.00021	C5,C6,C1QB,C7,C8A,C8B,C1QC,C4A,C4B
WikiPathways	WP3941	Oxidative damage response	6	39	1.06	0.00095	C5,C1QB,C1QC,C1S,C4B,C1R
WikiPathways	WP4493	Cells and molecules involved in local acute inflammatory response	4	17	1.24	0.0050	C5,C6,C7,KNG1
WikiPathways	WP5089	Kinin-Kallikrein pathway	3	7	1.5	0.0082	F12,SERPING1,KNG1
WikiPathways	WP3942	PPAR signaling pathway	6	67	0.82	0.0128	APOC3,APOA1,ILK,ADIPOQ,PLTP,APOA5
WikiPathways	WP5104	Acquired partial lipodystrophy / Barraquer-Simons syndrome	3	10	1.35	0.0175	C5,CFB,CFD
WikiPathways	WP2878	PPAR-alpha pathway	4	26	1.06	0.0179	APOC3,APOA1,PLTP,APOA5
WikiPathways	WP3888	VEGFA-VEGFR2 signaling	15	428	0.41	0.0253	RCN1,VCL,MMP2,DHX29,YWHAE,FGB,CALR,P4HB,PRDX6,IGFBP3,GAPDH,CLTC,HYOU1,CDH5,FGA
Monarch	EFO:0007937	Blood protein measurement	106	1810	0.64	6.39e-36	ST6GAL1,CPB2,GNPTG,APOH,CHGA,LBP,EBI3,PON1,C5,MPO,VTN,AFM,APOC3,LTF,HRG,APOB,IGFBP5,PROC,CAT,APOE,PXDN,F12,SERPINF1,CFHR5,LYZ,VWF,DSG2,SERPINA10,LCAT,NID1,AMBP,ITIH4,ITIH1,AHSG,HABP2,SAA4,SERPING1,ANKRD44,CSF1R,FCN2,PGLYRP2,MASP1,HSP90B1,ANTXR1,F2,PLG,APOL1,CPN2,LPA,SERPINF2,C7,SERPINA5,SERPINA11,ANG,PRDX6,PROZ,ASGR2,PIGR,ITIH2,CNDP1,COL18A1,C4BPA,F13B,CFHR4,CFH,QSOX1,F5,S100A9,CRTAC1,C8B,C8G,ADAMTS13,MINPP1,GSN,MBL2,SVEP1,C1QC,F10,APOM,CA6,IGFBP3,SOD3,FGL1,CST3,PSG4,SAA1,F11,C1S,PSG2,PCYOX1,COLEC11,MST1,SPARCL1,C4B,TGFBI,CFB,PLTP,GC,HGFAC,HPR,C1R,RNASE4,APOC4,VEGFC,KNG1,SDF4
Monarch	EFO:0004747	Protein measurement	149	5856	0.28	5.23e-15	RCN1,ST6GAL1,CPB2,CETP,GNPTG,APOH,CHGA,LBP,EBI3,PON1,C5,MPO,VTN,AFM,APOC3,GPLD1,LTF,HRG,APOB,IGFBP5,PROC,APOA1,CAT,LRP1,C4BPB,APOE,PXDN,ANGPTL6,F12,SERPINF1,APCS,CFHR5,LYZ,VWF,DSG2,SERPINA10,LCAT,NID1,CP,DGKG,DNAH5,AMBP,PON3,TCIRG1,ITIH4,ITIH1,AHSG,HABP2,SAA4,SERPING1,ANKRD44,CSF1R,FCN2,PGLYRP2,MASP1,APEH,HSP90B1,ANTXR1,PCSK9,FGB,F2,INHBC,PLG,UBE3C,APOL1,CPN2,LPA,SERPINF2,C7,HBA1,FBLN1,SERPINA5,DNAH8,OIT3,SERPINA11,ANG,LIMS1,THBS4,PRDX6,PROZ,APOD,PAPLN,ASGR2,PIGR,APOA4,ITIH2,CNDP1,COL18A1,CDC42BPA,C4BPA,F13B,CFHR4,CFH,QSOX1,F5,S100A9,CRTAC1,C8B,C8G,ADAMTS13,MINPP1,GSN,MBL2,SVEP1,C1QC,F10,APOM,CA6,IGFBP3,SOD3,CFI,EFCAB5,TNRC6A,NAV2,FGL1,CST3,ANKRD28,PSG4,SAA1,F11,C1S,PSG2,PCYOX1,KSR2,ADIPOQ,ABCA13,COLEC11,MST1,SPARCL1,ITIH3,C4B,TGFBI,CFB,IGFALS,PLTP,GC,HGFAC,SELENOP,HPR,C1R,APOA5,RNASE4,APOC4,VEGFC,KNG1,TRIM66,GRHL2,FGA,SDF4
Monarch	HP:0005339	Abnormality of complement system	15	24	1.67	5.23e-15	C5,CFP,C6,C1QB,C7,C8A,CFH,C8B,C1QC,CFI,MASP2,C1S,C4A,C4B,CFD
Monarch	HP:0004431	Complement deficiency	13	21	1.66	6.86e-13	C5,C6,C1QB,C7,C8A,CFH,C8B,C1QC,CFI,MASP2,C4A,C4B,CFD
Monarch	HP:0000118	Phenotypic abnormality	127	5129	0.26	1.01e-10	ST6GAL1,CETP,GNPTG,VCL,SERPIND1,F9,MMP2,COMP,PON1,C5,MPO,APOC3,SPARC,PCDH12,LTF,HRG,APOB,PROC,APOA1,TTR,CAT,LRP1,CFP,APOE,PXDN,ANGPTL6,F12,SERPINF1,CFHR5,LYZ,VWF,PSMA6,DSG2,C6,LCAT,NID1,CSTA,CP,DNAH5,CENPE,PON3,TCIRG1,HYAL1,FCN3,AHSG,CRH,HABP2,SERPING1,CSF1R,IHH,SERPINI1,MASP1,GUCY1A1,A2ML1,ANTXR1,PCSK9,BMP1,FGB,DST,F2,PLG,EFEMP2,C1QB,CFHR1,SUZ12,CALR,LPA,SERPINF2,C7,HBA1,FBN1,P4HB,FBLN1,DNAH8,ANG,LIMS1,THBS4,GANAB,PAPLN,BLM,AKAP9,COL18A1,F8,C8A,F13B,CFHR4,CFH,F5,ADAMTSL4,FLNA,CRTAC1,C8B,ADAMTS13,GSN,SVEP1,C1QC,F10,PROS1,EFEMP1,CFI,NAV2,CST3,MASP2,F11,C1S,KSR2,IGF2,C4A,FSIP2,ABCA13,COLEC11,LMOD3,ITIH3,C4B,TGFBI,IGFALS,GC,C1R,APOA5,ATP1A1,CFD,CLTC,VEGFC,KNG1,GRHL2,B2M,FGA
Monarch	HP:0025015	Abnormal vascular morphology	47	1027	0.53	9.39e-10	SERPIND1,F9,MMP2,SPARC,APOB,PROC,TTR,CAT,APOE,ANGPTL6,F12,VWF,LCAT,NID1,DNAH5,TCIRG1,HABP2,MASP1,GUCY1A1,A2ML1,ANTXR1,PCSK9,FGB,F2,EFEMP2,CALR,SERPINF2,FBN1,P4HB,GANAB,COL18A1,F8,F13B,F5,FLNA,CRTAC1,ADAMTS13,F10,PROS1,EFEMP1,CFI,CST3,IGF2,C4A,VEGFC,KNG1,FGA

Monarch	HP:0001892	Abnormal bleeding	29	411	0.72	2.24e-09	F9,SPARC,PROC,CAT,APOE,ANGPTL6,F12,VWF,TCIRG1,A2ML1,FGB,F2,CALR,SERPINF2,FBN1,P4HB,F8,F13B,F5,FLNA,GSN,F10,PROS1,CST3,F11,C1S,C4A,C1R,FGA
Monarch	HP:0002597	Abnormality of the vasculature	61	1673	0.43	2.24e-09	SERPIND1,F9,MMP2,SPARC,APOB,PROC,APOA1,TTR,CAT,APOE,ANGPTL6,F12,SERPINF1,LYZ,VWF,LCAT,NID1,CSTA,DNAH5,TCIRG1,HABP2,SERPING1,MASP1,GUCY1A1,A2ML1,ANTXR1,PCSK9,BMP1,FGB,F2,PLG,EFEMP2,CALR,SERPINF2,HBA1,FBN1,P4HB,GANAB,BLM,COL18A1,F8,F13B,F5,FLNA,CRTAC1,ADAMTS13,GSN,F10,PROS1,EFEMP1,CFI,CST3,C1S,IGF2,C4A,TGFBI,C1R,VEGFC,KNG1,B2M,FGA
Monarch	HP:0003256	Abnormality of the coagulation cascade	16	105	1.05	8.73e-09	F9,PROC,F12,VWF,FGB,F2,PLG,F8,F13B,F5,FLNA,F10,PROS1,F11,KNG1,FGA
Monarch	HP:0100659	Abnormal cerebral vascular morphology	27	381	0.72	9.97e-09	F9,SPARC,APOB,PROC,TTR,APOE,ANGPTL6,GUCY1A1,ANTXR1,PCSK9,FGB,F2,CALR,SERPINF2,FBN1,GANAB,F8,F13B,F5,FLNA,CRTAC1,ADAMTS13,F10,PROS1,CST3,KNG1,FGA
Monarch	HP:0001928	Abnormality of coagulation	19	170	0.92	1.04e-08	F9,HRG,PROC,F12,VWF,A2ML1,FGB,F2,PLG,SERPINF2,F8,F13B,F5,FLNA,F10,PROS1,F11,KNG1,FGA
Monarch	HP:0011025	Abnormal cardiovascular system physiology	48	1169	0.48	1.06e-08	GNPTG,VCL,F9,MMP2,SPARC,APOB,PROC,APOA1,TTR,APOE,ANGPTL6,SERPINF1,LYZ,VWF,DSG2,LCAT,CP,TCIRG1,SERPING1,GUCY1A1,A2ML1,PCSK9,BMP1,FGB,DST,F2,EFEMP2,CALR,SERPINF2,HBA1,FBN1,GANAB,AKAP9,F8,F13B,F5,ADAMTSL4,FLNA,CRTAC1,ADAMTS13,GSN,F10,PROS1,CST3,F11,KNG1,B2M,FGA
Monarch	HP:0002715	Abnormality of the immune system	58	1682	0.41	4.32e-08	VCL,MMP2,C5,MPO,APOA1,CAT,CFP,APOE,CFHR5,LYZ,PSMA6,DSG2,C6,LCAT,CSTA,DNAH5,TCIRG1,HYAL1,FCN3,HABP2,SERPING1,A2ML1,FGB,F2,PLG,EFEMP2,C1QB,CFHR1,CALR,C7,HBA1,FBN1,GANAB,BLM,COL18A1,F8,C8A,F13B,CFHR4,CFH,FLNA,C8B,GSN,SVEP1,C1QC,CFI,NAV2,MASP2,C1S,IGF2,C4A,C4B,C1R,CFD,VEGFC,GRHL2,B2M,FGA
Monarch	HP:0001933	Subcutaneous hemorrhage	19	189	0.87	4.40e-08	PROC,APOE,VWF,TCIRG1,F2,CALR,SERPINF2,FBN1,P4HB,F8,F13B,F5,FLNA,GSN,F10,PROS1,C1S,C4A,C1R
Monarch	HP:0005261	Joint hemorrhage	9	19	1.55	5.20e-08	F9,VWF,F2,SERPINF2,F8,F13B,F5,F10,F11
Monarch	HP:0001871	Abnormality of blood and blood-forming tissues	47	1214	0.46	9.25e-08	VCL,SERPIND1,F9,MPO,SPARC,HRG,APOB,PROC,APOA1,CAT,APOE,ANGPTL6,F12,VWF,DSG2,LCAT,CP,TCIRG1,GUCY1A1,A2ML1,FGB,F2,PLG,CALR,SERPINF2,HBA1,FBN1,P4HB,BLM,F8,F13B,CFH,F5,FLNA,ADAMTS13,GSN,SVEP1,F10,PROS1,CST3,F11,C1S,C4A,C1R,KNG1,B2M,FGA
Monarch	HP:0000225	Gingival bleeding	11	45	1.26	1.20e-07	CAT,FGB,F2,SERPINF2,F8,F13B,F5,F10,C1S,C1R,FGA
Monarch	HP:0001626	Abnormality of the cardiovascular system	71	2438	0.33	2.18e-07	GNPTG,VCL,SERPIND1,F9,MMP2,SPARC,APOB,PROC,APOA1,TTR,CAT,LRP1,APOE,ANGPTL6,F12,SERPINF1,LYZ,VWF,DSG2,LCAT,NID1,CSTA,CP,DNAH5,TCIRG1,HABP2,SERPING1,MASP1,GUCY1A1,A2ML1,ANTXR1,PCSK9,BMP1,FGB,DST,F2,PLG,EFEMP2,SUZ12,CALR,SERPINF2,HBA1,FBN1,P4HB,GANAB,BLM,AKAP9,COL18A1,F8,F13B,F5,ADAMTSL4,FLNA,CRTAC1,ADAMTS13,GSN,F10,PROS1,EFEMP1,CFI,CST3,F11,C1S,IGF2,C4A,TGFBI,C1R,VEGFC,KNG1,B2M,FGA
Monarch	HP:0006298	Prolonged bleeding after dental extraction	8	16	1.57	3.51e-07	F9,VWF,F2,F8,F13B,F5,F10,F11
Monarch	HP:0011276	Vascular skin abnormality	26	433	0.65	3.68e-07	PROC,APOE,F12,VWF,CSTA,TCIRG1,SERPING1,MASP1,GUCY1A1,ANTXR1,F2,CALR,SERPINF2,FBN1,P4HB,BLM,F8,F13B,F5,FLNA,GSN,F10,PROS1,C1S,C4A,C1R
Monarch	EFO:0004634	Coagulation factor measurement	15	132	0.93	7.13e-07	CPB2,PROC,CFHR5,VWF,FGB,CFHR4,CFH,F5,ADAMTS13,F10,F11,COLEC11,HGFAC,KNG1,FGA
Monarch	HP:0001977	Abnormal thrombosis	13	94	1.01	1.03e-06	SERPIND1,F9,HRG,PROC,F12,FGB,F2,CALR,F8,F5,PROS1,KNG1,FGA
Monarch	HP:0000123	Nephritis	11	59	1.14	1.09e-06	CFHR5,PLG,EFEMP2,C1QB,CFHR1,GANAB,CFH,C1QC,CFI,C4A,C1R
Monarch	HP:0000790	Hematuria	14	118	0.94	1.35e-06	F9,APOA1,CFHR5,LYZ,F2,CFHR1,SERPINF2,GANAB,F8,CFH,F5,ADAMTS13,F10,FGA
Monarch	HP:0003645	Prolonged partial thromboplastin time	8	24	1.39	3.22e-06	F9,F12,F2,F8,F5,F10,F11,KNG1
Monarch	HP:0030680	Abnormality of cardiovascular system morphology	53	1680	0.37	3.41e-06	VCL,SERPIND1,F9,MMP2,SPARC,APOB,PROC,TTR,CAT,APOE,ANGPTL6,F12,VWF,DSG2,LCAT,NID1,DNAH5,TCIRG1,HABP2,MASP1,GUCY1A1,A2ML1,ANTXR1,PCSK9,FGB,F2,EFEMP2,SUZ12,CALR,SERPINF2,HBA1,FBN1,P4HB,GANAB,COL18A1,F8,F13B,F5,FLNA,CRTAC1,ADAMTS13,GSN,F10,PROS1,EFEMP1,CFI,CST3,IGF2,C4A,VEGFC,KNG1,B2M,FGA
Monarch	HP:0000951	Abnormality of the skin	57	1888	0.35	3.50e-06	VCL,F9,MMP2,C5,SPARC,APOB,PROC,APOA1,CAT,LRP1,APOE,F12,LYZ,VWF,PSMA6,DSG2,CSTA,CENPE,TCIRG1,HYAL1,FCN3,AHSG,SERPING1,MASP1,GUCY1A1,A2ML1,ANTXR1,PCSK9,DST,F2,PLG,EFEMP2,SUZ12,CALR,SERPINF2,HBA1,FBN1,P4HB,BLM,F8,F13B,CFHR4,F5,FLNA,ADAMTS13,GSN,F10,PROS1,CFI,C1S,IGF2,C4A,C1R,VEGFC,GRHL2,B2M,FGA
Monarch	HP:0000978	Bruising susceptibility	14	130	0.9	3.50e-06	VWF,TCIRG1,F2,SERPINF2,FBN1,P4HB,F8,F13B,F5,FLNA,GSN,F10,C1S,C1R
Monarch	HP:0002170	Intracranial hemorrhage	14	130	0.9	3.50e-06	F9,SPARC,ANGPTL6,FGB,F2,SERPINF2,FBN1,F8,F13B,F5,F10,PROS1,CST3,FGA
Monarch	HP:0005368	Abnormality of humoral immunity	18	234	0.76	3.50e-06	C5,CFP,C6,C1QB,C7,BLM,C8A,CFH,FLNA,C8B,C1QC,CFI,MASP2,C1S,C4A,C4B,CFD,B2M
Monarch	HP:0010978	Abnormality of immune system physiology	46	1347	0.4	3.50e-06	MMP2,C5,APOA1,CAT,CFP,APOE,CFHR5,LYZ,PSMA6,C6,CSTA,DNAH5,TCIRG1,HYAL1,FCN3,SERPING1,PLG,EFEMP2,C1QB,CFHR1,C7,HBA1,FBN1,GANAB,BLM,C8A,F13B,CFHR4,CFH,FLNA,C8B,GSN,C1QC,CFI,NAV2,MASP2,C1S,IGF2,C4A,C4B,C1R,CFD,VEGFC,GRHL2,B2M,FGA
Monarch	EFO:0004310	Partial thromboplastin time	7	15	1.54	3.55e-06	APOH,HRG,F12,F5,F11,C1S,KNG1
Monarch	HP:0002725	Systemic lupus erythematosus	8	26	1.36	4.19e-06	SERPING1,C1QB,C8A,C1QC,MASP2,C1S,C4A,C1R
Monarch	HP:0012211	Abnormal renal physiology	27	560	0.55	8.12e-06	F9,APOA1,TTR,APOE,CFHR5,LYZ,LCAT,F2,PLG,EFEMP2,C1QB,CFHR1,SERPINF2,GANAB,F8,CFH,F5,ADAMTS13,GSN,C1QC,F10,CFI,C4A,C1R,ATP1A1,B2M,FGA
Monarch	HP:0012233	Intramuscular hematoma	6	10	1.65	1.25e-05	F9,F2,SERPINF2,F8,F13B,F10
Monarch	HP:0004936	Venous thrombosis	10	65	1.06	1.85e-05	SERPIND1,F9,PROC,FGB,F2,CALR,F5,PROS1,KNG1,FGA
Monarch	HP:0001574	Abnormality of the integument	62	2266	0.31	1.86e-05	VCL,F9,MMP2,C5,SPARC,APOB,PROC,APOA1,CAT,LRP1,APOE,F12,LYZ,VWF,PSMA6,DSG2,CSTA,CENPE,TCIRG1,HYAL1,FCN3,AHSG,SERPING1,IHH,MASP1,GUCY1A1,A2ML1,ANTXR1,PCSK9,BMP1,DST,F2,PLG,EFEMP2,SUZ12,CALR,SERPINF2,HBA1,FBN1,P4HB,LIMS1,BLM,COL18A1,F8,F13B,CFHR4,F5,FLNA,ADAMTS13,GSN,F10,PROS1,CFI,C1S,IGF2,C4A,COLEC11,C1R,VEGFC,GRHL2,B2M,FGA
Monarch	HP:0001939	Abnormality of metabolism/homeostasis	60	2168	0.31	2.14e-05	CETP,GNPTG,VCL,F9,MMP2,PON1,MPO,APOC3,APOB,APOA1,TTR,CAT,CFP,APOE,F12,CFHR5,LYZ,DSG2,LCAT,CP,TCIRG1,SERPING1,CSF1R,A2ML1,ANTXR1,PCSK9,FGB,DST,F2,PLG,CFHR1,CALR,LPA,SERPINF2,HBA1,FBN1,GANAB,PAPLN,BLM,AKAP9,F8,CFH,F5,FLNA,ADAMTS13,GSN,F10,CST3,IGF2,LMOD3,TGFBI,IGFALS,GC,APOA5,ATP1A1,VEGFC,KNG1,GRHL2,B2M,FGA
Monarch	HP:0001934	Persistent bleeding after trauma	6	12	1.57	2.55e-05	F9,VWF,SERPINF2,F8,F13B,F5
Monarch	HP:0011029	Internal hemorrhage	17	244	0.71	2.55e-05	F9,SPARC,PROC,ANGPTL6,VWF,FGB,F2,SERPINF2,FBN1,F8,F13B,F5,F10,PROS1,CST3,F11,FGA
Monarch	HP:0012649	Increased inflammatory response	36	981	0.43	2.55e-05	C5,APOA1,CAT,APOE,CFHR5,LYZ,PSMA6,CSTA,DNAH5,TCIRG1,HYAL1,FCN3,PLG,EFEMP2,C1QB,CFHR1,HBA1,FBN1,GANAB,BLM,F13B,CFHR4,CFH,FLNA,GSN,C1QC,CFI,NAV2,MASP2,C1S,C4A,C4B,C1R,VEGFC,B2M,FGA
Monarch	HP:0003216	Generalized amyloid deposition	5	5	1.87	2.62e-05	APOA1,LYZ,GSN,CST3,FGA
Monarch	HP:0005430	Recurrent Neisserial infections	5	5	1.87	2.62e-05	C5,C6,C7,C8B,CFI
Monarch	HP:0010935	Abnormality of the upper urinary tract	38	1101	0.41	4.34e-05	F9,APOB,APOA1,TTR,APOE,CFHR5,LYZ,LCAT,HABP2,MASP1,ANTXR1,PCSK9,F2,PLG,EFEMP2,C1QB,CFHR1,SERPINF2,FBN1,GANAB,BLM,COL18A1,F8,CFH,F5,FLNA,ADAMTS13,GSN,C1QC,F10,CFI,IGF2,C4A,COLEC11,C1R,ATP1A1,B2M,FGA

Monarch	HP:0011034	Amyloidosis	7	25	1.32	4.43e-05	APOA1,TTR,LYZ,GSN,CST3,B2M,FGA
Monarch	HP:0033057	Decreased serum terminal complement component	5	6	1.79	4.43e-05	C5,C6,C7,C8A,C8B
Monarch	EFO:0004614	Apolipoprotein A 1 measurement	21	396	0.59	5.16e-05	CETP,APOH,VTN,APOC3,APOB,APOA1,LRP1,APOE,LCAT,DGKG,ITIH1,PCSK9,PLG,LPA,APOA4,COL18A1,CRTAC1,ANKRD28,HGFAC,HPR,APOC4
Monarch	HP:0000421	Epistaxis	10	78	0.98	6.45e-05	F9,VWF,FGB,F2,F8,F13B,F5,F10,F11,FGA
Monarch	HP:0000077	Abnormality of the kidney	37	1077	0.41	6.49e-05	F9,APOB,APOA1,TTR,APOE,CFHR5,LYZ,LCAT,HABP2,MASP1,ANTXR1,PCSK9,F2,PLG,EFEMP2,C1QB,CFHR1,SERPINF2,FBN1,GANAB,BLM,F8,CFH,F5,FLNA,ADAMTS13,GSN,C1QC,F10,CFI,IGF2,C4A,COLEC11,C1R,ATP1A1,B2M,FGA
Monarch	HP:0011121	Abnormality of skin morphology	48	1648	0.33	0.00011	VCL,F9,MMP2,SPARC,APOB,PROC,APOA1,CAT,LRP1,APOE,F12,VWF,DSG2,CSTA,CENPE,TCIRG1,AHSG,SERPING1,MASP1,GUCY1A1,A2ML1,ANTXR1,PCSK9,DST,F2,EFEMP2,SUZ12,CALR,SERPINF2,HBA1,FBN1,P4HB,BLM,F8,F13B,F5,FLNA,ADAMTS13,GSN,F10,PROS1,C1S,IGF2,C4A,C1R,VEGFC,GRHL2,B2M
Monarch	HP:0000168	Abnormality of the gingiva	14	190	0.74	0.00013	MMP2,CAT,TCIRG1,FGB,F2,PLG,SERPINF2,F8,F13B,F5,F10,C1S,C1R,FGA
Monarch	HP:0004846	Prolonged bleeding after surgery	6	18	1.39	0.00013	F9,VWF,F8,F13B,F5,F10
Monarch	HP:0010979	Abnormality of lipoprotein cholesterol concentration	8	47	1.1	0.00013	CETP,APOC3,APOB,APOA1,APOE,LCAT,PCSK9,APOA5
Monarch	HP:0012531	Pain	24	539	0.52	0.00013	GNPTG,MMP2,COMP,PON1,APOB,CAT,F12,PON3,TCIRG1,HABP2,SERPING1,PCSK9,F2,CALR,SERPINF2,FBN1,ANG,GANAB,F8,FLNA,C1S,TGFBI,GRHL2,B2M
Monarch	HP:0033353	Abnormal blood vessel morphology	30	792	0.45	0.00013	SERPIND1,MMP2,APOB,PROC,CAT,APOE,ANGPTL6,F12,VWF,LCAT,NID1,DNAH5,TCIRG1,MASP1,GUCY1A1,A2ML1,ANTXR1,PCSK9,EFEMP2,FBN1,P4HB,GANAB,COL18A1,FLNA,PROS1,EFEMP1,CFI,IGF2,C4A,VEGFC
Monarch	EFO:0004298	Cardiovascular measurement	61	2378	0.28	0.00014	ST6GAL1,CETP,APOH,VCL,F9,PON1,MPO,VTN,APOC3,GPLD1,HRG,APOB,APOA1,LRP1,C4BPB,APOE,F12,LCAT,YWHAE,DGKG,DNAH5,ITIH1,HABP2,SERPINI1,HSP90B1,PCSK9,SHLD1,FGB,INHBC,PLG,LPA,FBN1,FBLN1,OIT3,LIMS1,FAM184A,APOA4,COL18A1,CDC42BPA,CFH,QSOX1,F5,CRTAC1,ADAMTS13,GSN,SVEP1,EFEMP1,EFCAB5,TNRC6A,NAV2,ANKRD28,MAP3K7CL,KSR2,ITIH3,PLTP,HGFAC,HPR,APOA5,APOC4,KNG1,GRHL2
Monarch	HP:0030140	Oral cavity bleeding	5	9	1.61	0.00015	F2,F8,F13B,F5,F10
Monarch	HP:0010980	Hyperlipoproteinemia	7	33	1.2	0.00017	CETP,APOC3,APOB,APOE,LCAT,PCSK9,APOA5
Monarch	EFO:0005278	Cardiovascular disease biomarker measurement	58	2228	0.29	0.00018	ST6GAL1,CETP,APOH,F9,PON1,MPO,VTN,APOC3,HRG,APOB,APOA1,LRP1,C4BPB,APOE,F12,LCAT,YWHAE,DGKG,DNAH5,ITIH1,HABP2,SERPINI1,HSP90B1,PCSK9,SHLD1,FGB,PLG,LPA,FBN1,FBLN1,OIT3,LIMS1,FAM184A,APOA4,COL18A1,CDC42BPA,CFH,QSOX1,F5,CRTAC1,ADAMTS13,GSN,SVEP1,EFEMP1,EFCAB5,TNRC6A,NAV2,ANKRD28,MAP3K7CL,KSR2,ITIH3,PLTP,HGFAC,HPR,APOA5,APOC4,KNG1,GRHL2
Monarch	HP:0002625	Deep venous thrombosis	6	20	1.35	0.00019	SERPIND1,F9,PROC,F2,F5,PROS1
Monarch	HP:0002634	Arteriosclerosis	9	70	0.98	0.00020	SERPIND1,APOB,CAT,APOE,ANGPTL6,LCAT,ANTXR1,PCSK9,FBN1
Monarch	HP:0011891	Post-partum hemorrhage	5	10	1.57	0.00020	F2,F8,F13B,F5,F10
Monarch	HP:0011354	Generalized abnormality of skin	30	821	0.43	0.00023	F9,PROC,APOE,F12,VWF,CSTA,CENPE,TCIRG1,SERPING1,MASP1,GUCY1A1,ANTXR1,F2,CALR,SERPINF2,HBA1,FBN1,P4HB,BLM,F8,F13B,F5,FLNA,ADAMTS13,GSN,F10,PROS1,C1S,C4A,C1R
Monarch	HP:0031888	Abnormal HDL cholesterol concentration	6	21	1.33	0.00023	CETP,APOC3,APOA1,APOE,LCAT,APOA5
Monarch	HP:0000099	Glomerulonephritis	7	37	1.15	0.00031	CFHR5,C1QB,CFHR1,CFH,C1QC,CFI,C4A
Monarch	HP:0000132	Menorrhagia	7	38	1.13	0.00035	VWF,F2,F8,F13B,F5,F10,F11
Monarch	HP:0005381	Recurrent meningococcal disease	4	4	1.87	0.00036	C5,C6,C7,CFI
Monarch	HP:0000707	Abnormality of the nervous system	78	3471	0.22	0.00039	ST6GAL1,GNPTG,F9,MMP2,COMP,PON1,SPARC,PCDH12,APOB,PROC,APOA1,TTR,CAT,LRP1,APOE,ANGPTL6,NID1,CP,DNAH5,CENPE,PON3,TCIRG1,AHSG,CRH,HABP2,SERPING1,CSF1R,SERPINI1,MASP1,GUCY1A1,A2ML1,ANTXR1,PCSK9,BMP1,FGB,DST,F2,PLG,EFEMP2,SUZ12,CALR,SERPINF2,HBA1,FBN1,P4HB,FBLN1,ANG,GANAB,BLM,AKAP9,COL18A1,F8,C8A,F13B,F5,ADAMTSL4,FLNA,CRTAC1,C8B,ADAMTS13,GSN,F10,PROS1,CFI,CST3,IGF2,COLEC11,LMOD3,ITIH3,C4B,TGFBI,ATP1A1,CLTC,VEGFC,KNG1,GRHL2,B2M,FGA
Monarch	HP:0000271	Abnormality of the face	64	2641	0.25	0.00040	GNPTG,F9,MMP2,PON1,SPARC,LTF,APOB,PROC,CAT,LRP1,APOE,F12,SERPINF1,LYZ,VWF,NID1,CP,DNAH5,CENPE,PON3,TCIRG1,HYAL1,AHSG,SERPING1,CSF1R,MASP1,A2ML1,ANTXR1,PCSK9,BMP1,FGB,DST,F2,PLG,EFEMP2,SUZ12,SERPINF2,FBN1,P4HB,FBLN1,ANG,BLM,COL18A1,F8,F13B,F5,ADAMTSL4,FLNA,CRTAC1,GSN,F10,CFI,F11,C1S,IGF2,ABCA13,COLEC11,LMOD3,C1R,CLTC,VEGFC,GRHL2,B2M,FGA
Monarch	HP:0000152	Abnormality of head or neck	68	2882	0.24	0.00043	GNPTG,F9,MMP2,PON1,SPARC,PCDH12,LTF,APOB,PROC,CAT,LRP1,APOE,F12,SERPINF1,LYZ,VWF,NID1,CP,DNAH5,CENPE,PON3,TCIRG1,HYAL1,AHSG,HABP2,SERPING1,CSF1R,IHH,MASP1,A2ML1,ANTXR1,PCSK9,BMP1,FGB,DST,F2,PLG,EFEMP2,SUZ12,SERPINF2,FBN1,P4HB,FBLN1,ANG,BLM,COL18A1,F8,F13B,F5,ADAMTSL4,FLNA,CRTAC1,GSN,F10,CFI,F11,C1S,IGF2,ABCA13,COLEC11,LMOD3,IGFALS,C1R,CLTC,VEGFC,GRHL2,B2M,FGA
Monarch	HP:0012639	Abnormal nervous system morphology	62	2546	0.26	0.00050	F9,COMP,PON1,SPARC,PCDH12,APOB,PROC,TTR,APOE,ANGPTL6,NID1,CP,DNAH5,CENPE,PON3,TCIRG1,AHSG,HABP2,SERPING1,CSF1R,SERPINI1,MASP1,GUCY1A1,ANTXR1,PCSK9,FGB,F2,PLG,EFEMP2,SUZ12,CALR,SERPINF2,HBA1,FBN1,P4HB,FBLN1,ANG,GANAB,BLM,COL18A1,F8,C8A,F13B,F5,FLNA,CRTAC1,C8B,ADAMTS13,GSN,F10,PROS1,CFI,CST3,IGF2,COLEC11,LMOD3,C4B,ATP1A1,CLTC,KNG1,B2M,FGA
Monarch	HP:0005542	Prolonged whole-blood clotting time	4	5	1.77	0.00059	F9,F12,VWF,F5
Monarch	HP:0011884	Abnormal umbilical stump bleeding	4	5	1.77	0.00059	F2,SERPINF2,F13B,F10
Monarch	HP:0000234	Abnormality of the head	67	2865	0.24	0.00067	GNPTG,F9,MMP2,PON1,SPARC,PCDH12,LTF,APOB,PROC,CAT,LRP1,APOE,F12,SERPINF1,LYZ,VWF,NID1,CP,DNAH5,CENPE,PON3,TCIRG1,HYAL1,AHSG,SERPING1,CSF1R,IHH,MASP1,A2ML1,ANTXR1,PCSK9,BMP1,FGB,DST,F2,PLG,EFEMP2,SUZ12,SERPINF2,FBN1,P4HB,FBLN1,ANG,BLM,COL18A1,F8,F13B,F5,ADAMTSL4,FLNA,CRTAC1,GSN,F10,CFI,F11,C1S,IGF2,ABCA13,COLEC11,LMOD3,IGFALS,C1R,CLTC,VEGFC,GRHL2,B2M,FGA
Monarch	HP:0010981	Hypolipoproteinemia	6	27	1.22	0.00067	APOC3,APOB,APOA1,APOE,LCAT,APOA5
Monarch	HP:0011492	Abnormal corneal stroma morphology	9	86	0.89	0.00076	GNPTG,MMP2,APOB,APOE,LCAT,PCSK9,COL18A1,TGFBI,GRHL2
Monarch	HP:0002621	Atherosclerosis	8	64	0.97	0.00077	SERPIND1,APOB,APOE,ANGPTL6,LCAT,ANTXR1,PCSK9,FBN1
Monarch	HP:0002011	Morphological central nervous system abnormality	59	2416	0.26	0.00079	F9,COMP,PON1,SPARC,PCDH12,APOB,PROC,TTR,APOE,ANGPTL6,NID1,CP,DNAH5,CENPE,PON3,TCIRG1,AHSG,HABP2,CSF1R,SERPINI1,MASP1,GUCY1A1,ANTXR1,PCSK9,FGB,F2,PLG,EFEMP2,SUZ12,CALR,SERPINF2,HBA1,FBN1,P4HB,FBLN1,ANG,GANAB,BLM,COL18A1,F8,C8A,F13B,F5,FLNA,CRTAC1,C8B,ADAMTS13,F10,PROS1,CFI,CST3,IGF2,COLEC11,C4B,ATP1A1,CLTC,KNG1,B2M,FGA
Monarch	HP:0010989	Abnormality of the intrinsic pathway	6	29	1.19	0.00091	F9,F12,VWF,F8,F11,KNG1
Monarch	HP:0011830	Abnormal oral mucosa morphology	16	305	0.59	0.0010	MMP2,LTF,CAT,LYZ,TCIRG1,FGB,F2,PLG,SERPINF2,F8,F13B,F5,F10,C1S,C1R,FGA
Monarch	HP:0001367	Abnormal joint morphology	30	905	0.39	0.0011	GNPTG,F9,MMP2,COMP,APOE,SERPINF1,VWF,HYAL1,IHH,MASP1,A2ML1,BMP1,FGB,F2,EFEMP2,SUZ12,SERPINF2,FBN1,FBLN1,F8,F13B,F5,FLNA,F10,CFI,F11,COLEC11,LMOD3,VEGFC,FGA

Monarch	HP:0025031	Abnormality of the digestive system	58	2389	0.25	0.0011	F9,MMP2,PON1,C5,APOB,APOA1,TTR,APOE,F12,LYZ,VWF,LCAT,CP,DNAH5,PON3,TCIRG1,FCN3,HABP2,SERPING1,MASP1,GUCY1A1,A2ML1,ANTXR1,PCSK9,BMP1,FGB,DST,F2,PLG,EFEMP2,SUZ12,CALR,HBA1,FBN1,ANG,GANAB,BLM,COL18A1,F8,F13B,F5,FLNA,ADAMTS13,GSN,F10,NAV2,MASP2,F11,C1S,IGF2,COLEC11,LMOD3,C4B,C1R,CLTC,GRHL2,B2M,FGA
Monarch	HP:0025142	Constitutional symptom	30	906	0.39	0.0011	GNPTG,MMP2,COMP,PON1,APOB,PROC,TTR,CAT,F12,DSG2,PON3,TCIRG1,CRH,HABP2,SERPING1,PCSK9,F2,CALR,SERPINF2,FBN1,ANG,GANAB,AKAP9,F8,FLNA,PROS1,C1S,TGFBI,GRHL2,B2M
Monarch	HP:0000119	Abnormality of the genitourinary system	54	2189	0.26	0.0015	F9,MMP2,APOB,APOA1,TTR,APOE,CFHR5,LYZ,VWF,LCAT,DNAH5,AHSG,CRH,HABP2,MASP1,GUCY1A1,A2ML1,ANTXR1,PCSK9,FGB,F2,PLG,EFEMP2,C1QB,CFHR1,SUZ12,SERPINF2,FBN1,FBLN1,DNAH8,GANAB,BLM,COL18A1,F8,F13B,CFH,F5,FLNA,ADAMTS13,GSN,C1QC,F10,CFI,F11,IGF2,C4A,FSIP2,COLEC11,LMOD3,C1R,ATP1A1,VEGFC,B2M,FGA
Monarch	HP:0002960	Autoimmunity	10	122	0.78	0.0015	MMP2,TCIRG1,SERPING1,C1QB,C8A,C1QC,MASP2,C1S,C4A,C1R
Monarch	EFO:0006925	Lipoprotein A measurement	6	34	1.12	0.0019	CETP,APOH,APOE,PLG,LPA,APOC4
Monarch	HP:0011277	Abnormality of the urinary system physiology	30	933	0.38	0.0019	F9,APOA1,TTR,APOE,CFHR5,LYZ,LCAT,F2,PLG,EFEMP2,C1QB,CFHR1,LPA,SERPINF2,GANAB,PAPLN,BLM,F8,CFH,F5,ADAMTS13,GSN,C1QC,F10,CFI,C4A,C1R,ATP1A1,B2M,FGA
Monarch	HP:0100724	Hypercoagulability	5	19	1.29	0.0019	F9,HRG,PROC,F5,PROS1
Monarch	EFO:0001365	Age-related macular degeneration	6	35	1.1	0.0021	CETP,APOE,CFHR5,CFH,CFI,CFB
Monarch	EFO:1001492	Atrophic macular degeneration	6	35	1.1	0.0021	CETP,APOE,CFHR5,CFH,CFI,CFB
Monarch	HP:0010990	Abnormality of the common coagulation pathway	6	35	1.1	0.0021	FGB,PLG,F13B,F5,F10,FGA
Monarch	HP:0007759	Opacification of the corneal stroma	8	78	0.88	0.0022	GNPTG,MMP2,APOB,APOE,LCAT,PCSK9,COL18A1,TGFBI
Monarch	HP:0031886	Abnormal LDL cholesterol concentration	6	36	1.09	0.0022	APOC3,APOB,APOE,LCAT,PCSK9,APOA5
Monarch	HP:0030137	Prolonged bleeding following circumcision	4	9	1.52	0.0023	F2,F8,F13B,F5
Monarch	EFO:0004574	Total cholesterol measurement	20	497	0.47	0.0025	CETP,APOH,APOB,APOA1,APOE,LCAT,PCSK9,FGB,LPA,OIT3,LIMS1,ASGR2,APOA4,COL18A1,ADAMTS13,KSR2,HGFAC,HPR,APOA5,APOC4
Monarch	HP:0003272	Abnormal hip bone morphology	19	461	0.48	0.0029	GNPTG,MMP2,COMP,APOE,SERPINF1,CENPE,TCIRG1,HYAL1,IHH,MASP1,BMP1,EFEMP2,SUZ12,FBN1,FBLN1,FLNA,COLEC11,LMOD3,B2M
Monarch	HP:0005262	Abnormality of the synovia	15	305	0.56	0.0031	MMP2,COMP,APOE,SERPINF1,HYAL1,MASP1,BMP1,F2,EFEMP2,FBN1,FBLN1,F8,FLNA,COLEC11,LMOD3
Monarch	HP:0008011	Peripheral opacification of the cornea	4	10	1.47	0.0031	MMP2,APOB,APOE,PCSK9
Monarch	HP:0012185	Constrictive median neuropathy	4	10	1.47	0.0031	COMP,TTR,GSN,B2M
Monarch	HP:0002239	Gastrointestinal hemorrhage	9	111	0.78	0.0035	F9,VWF,FGB,F2,F8,F5,F10,F11,FGA
Monarch	HP:0002644	Abnormal pelvic girdle bone morphology	20	512	0.46	0.0035	GNPTG,MMP2,COMP,APOE,SERPINF1,CENPE,TCIRG1,HYAL1,IHH,MASP1,ANTXR1,BMP1,EFEMP2,SUZ12,FBN1,FBLN1,FLNA,COLEC11,LMOD3,B2M
Monarch	HP:0011355	Localized skin lesion	25	738	0.4	0.0036	MMP2,APOB,PROC,APOA1,LRP1,APOE,F12,CSTA,TCIRG1,MASP1,A2ML1,ANTXR1,PCSK9,DST,SUZ12,FBN1,P4HB,BLM,FLNA,GSN,PROS1,C1S,IGF2,C1R,B2M
Monarch	HP:0001382	Joint hypermobility	14	279	0.57	0.0043	COMP,CENPE,A2ML1,ANTXR1,BMP1,EFEMP2,SUZ12,FBN1,P4HB,COL18A1,FLNA,C1S,COLEC11,C1R
Monarch	EFO:0004732	Lipoprotein measurement	38	1426	0.3	0.0060	CETP,APOH,VTN,APOC3,APOB,APOA1,LRP1,APOE,LCAT,DGKG,PON3,ITIH1,PCSK9,FGB,F2,INHBC,PLG,LPA,FBLN1,OIT3,LIMS1,THBS4,APOD,ASGR2,APOA4,COL18A1,CDC42BPA,CRTAC1,ADAMTS13,ANKRD28,KSR2,ABCA13,PLTP,HGFAC,HPR,APOA5,APOC4,GRHL2
Monarch	HP:0033127	Abnormality of the musculoskeletal system	68	3173	0.2	0.0062	GNPTG,VCL,F9,MMP2,COMP,PON1,SPARC,PCDH12,APOB,APOA1,TTR,LRP1,APOE,SERPINF1,VWF,DSG2,NID1,CP,DNAH5,CENPE,PON3,TCIRG1,HYAL1,AHSG,HABP2,CSF1R,IHH,MASP1,A2ML1,ANTXR1,PCSK9,BMP1,FGB,DST,F2,PLG,EFEMP2,SUZ12,CALR,SERPINF2,FBN1,P4HB,FBLN1,ANG,BLM,COL18A1,F8,F13B,F5,ADAMTSL4,FLNA,GSN,F10,CFI,F11,C1S,IGF2,COLEC11,LMOD3,TGFBI,IGFALS,C1R,ATP1A1,CLTC,VEGFC,GRHL2,B2M,FGA
Monarch	HP:0030972	Abnormal systemic blood pressure	16	369	0.51	0.0065	MMP2,APOB,APOA1,TTR,ANGPTL6,LYZ,SERPING1,GUCY1A1,PCSK9,F2,CALR,FBN1,GANAB,ADAMTSL4,GSN,FGA
Monarch	HP:0000079	Abnormality of the urinary system	42	1657	0.27	0.0070	F9,APOB,APOA1,TTR,APOE,CFHR5,LYZ,LCAT,CRH,HABP2,MASP1,ANTXR1,PCSK9,F2,PLG,EFEMP2,C1QB,CFHR1,LPA,SERPINF2,FBN1,GANAB,PAPLN,BLM,COL18A1,F8,CFH,F5,FLNA,ADAMTS13,GSN,C1QC,F10,CFI,IGF2,C4A,COLEC11,LMOD3,C1R,ATP1A1,B2M,FGA
Monarch	HP:0002829	Arthralgia	9	124	0.73	0.0075	GNPTG,MMP2,COMP,APOB,PCSK9,F2,FBN1,F8,C1S
Monarch	HP:0005356	Decreased serum complement factor I	3	4	1.74	0.0078	C1QB,C1QC,CFI
Monarch	HP:0012223	Splenic rupture	3	4	1.74	0.0078	FGB,F8,FGA
Monarch	HP:0012541	Cephalohematoma	3	4	1.74	0.0078	F9,F2,F8
Monarch	HP:0007957	Corneal opacity	12	226	0.59	0.0085	GNPTG,MMP2,APOB,APOA1,APOE,PXDN,LCAT,PCSK9,DST,COL18A1,TGFBI,GRHL2
Monarch	HP:0030163	Abnormal vascular physiology	15	340	0.51	0.0086	PROC,ANGPTL6,SERPINF1,TCIRG1,GUCY1A1,BMP1,F2,CALR,FBN1,F5,FLNA,CRTAC1,ADAMTS13,PROS1,KNG1
Monarch	EFO:0004535	Serum albumin measurement	9	128	0.72	0.0089	TCIRG1,LPA,SERPINF2,SERPINA5,SERPINA11,CFH,SVEP1,HGFAC,HPR
Monarch	HP:0000140	Abnormality of the menstrual cycle	12	228	0.59	0.0090	F9,VWF,ANTXR1,FGB,F2,F8,F13B,F5,F10,F11,IGF2,FGA
Monarch	HP:0008067	Abnormally lax or hyperextensible skin	9	129	0.71	0.0092	CSTA,ANTXR1,EFEMP2,SUZ12,FBN1,FLNA,GSN,C1S,C1R
Monarch	HP:0031815	Abnormal oral physiology	11	196	0.62	0.0101	PON1,PON3,SERPING1,F2,FBLN1,ANG,F8,F13B,F5,GSN,F10
Monarch	HP:0000080	Abnormality of reproductive system physiology	22	659	0.39	0.0114	F9,TTR,APOE,VWF,DNAH5,AHSG,GUCY1A1,A2ML1,ANTXR1,FGB,F2,DNAH8,GANAB,BLM,F8,F13B,F5,F10,F11,IGF2,FSIP2,FGA
Monarch	HP:0007937	Reticular pigmentary degeneration	3	5	1.65	0.0114	CFH,EFEMP1,CFI
Monarch	HP:0030012	Abnormal female reproductive system physiology	13	273	0.55	0.0117	F9,VWF,ANTXR1,FGB,F2,BLM,F8,F13B,F5,F10,F11,IGF2,FGA
Monarch	HP:0000153	Abnormality of the mouth	47	1992	0.24	0.0119	MMP2,PON1,SPARC,LTF,CAT,APOE,SERPINF1,LYZ,NID1,CENPE,PON3,TCIRG1,HYAL1,SERPING1,MASP1,A2ML1,ANTXR1,BMP1,FGB,DST,F2,PLG,EFEMP2,SUZ12,SERPINF2,FBN1,P4HB,FBLN1,ANG,BLM,F8,F13B,F5,FLNA,CRTAC1,GSN,F10,C1S,IGF2,ABCA13,COLEC11,LMOD3,C1R,CLTC,GRHL2,B2M,FGA
Monarch	HP:0001000	Abnormality of skin pigmentation	17	439	0.46	0.0127	MMP2,PROC,CAT,APOE,CSTA,A2ML1,ANTXR1,DST,F2,SUZ12,FBN1,BLM,FLNA,PROS1,C1S,IGF2,C1R
Monarch	HP:0002718	Recurrent bacterial infections	10	169	0.64	0.0131	C5,C6,DNAH5,TCIRG1,FCN3,C7,CFH,C8B,CFI,CFD
Monarch	HP:0011004	Abnormal systemic arterial morphology	16	398	0.47	0.0131	SERPIND1,MMP2,APOB,CAT,APOE,ANGPTL6,F12,LCAT,GUCY1A1,ANTXR1,PCSK9,EFEMP2,FBN1,P4HB,GANAB,FLNA
Monarch	HP:0000979	Purpura	7	79	0.82	0.0132	PROC,APOE,F2,CALR,F13B,PROS1,C4A
Monarch	EFO:0004872	Inflammatory biomarker measurement	33	1226	0.3	0.0135	CETP,APOH,MPO,VTN,APOC3,APOB,PLEK,APOA1,LRP1,APOE,APCS,LYZ,LCAT,DGKG,ITIH1,ANKRD44,PCSK9,PLG,EFEMP2,LPA,APOA4,COL18A1,CFH,F5,CRTAC1,CKAP4,ANKRD28,KSR2,COLEC11,GC,HGFAC,HPR,APOC4
Monarch	HP:0008065	Aplasia/Hypoplasia of the skin	10	170	0.64	0.0135	PROC,TCIRG1,DST,FBN1,P4HB,FLNA,GSN,PROS1,C1S,C1R
Monarch	EFO:0004611	Low density lipoprotein cholesterol measurement	21	624	0.4	0.0136	CETP,APOH,VTN,APOB,APOE,PCSK9,FGB,LPA,FBLN1,OIT3,LIMS1,APOA4,CDC42BPA,ADAMTS13,KSR2,PLTP,HGFAC,HPR,APOA5,APOC4,GRHL2
Monarch	EFO:0006794	Cerebrospinal fluid biomarker measurement	6	55	0.91	0.0136	LTF,HRG,APOE,CFHR5,C7,C1R
Monarch	HP:0009145	Abnormal cerebral artery morphology	6	55	0.91	0.0136	APOB,ANGPTL6,GUCY1A1,PCSK9,FBN1,GANAB
Monarch	HP:0010647	Abnormal elasticity of skin	10	172	0.63	0.0143	SPARC,CSTA,ANTXR1,EFEMP2,SUZ12,FBN1,FLNA,GSN,C1S,C1R
Monarch	HP:0003170	Abnormal acetabulum morphology	7	81	0.81	0.0145	MMP2,COMP,APOE,SERPINF1,HYAL1,BMP1,FBN1
Monarch	HP:0000969	Edema	17	448	0.45	0.0148	APOA1,APOE,F12,LYZ,SERPING1,A2ML1,ANTXR1,FGB,F2,HBA1,F8,GSN,LMOD3,TGFBI,VEGFC,GRHL2,FGA
Monarch	HP:0000704	Periodontitis	5	35	1.02	0.0149	CAT,TCIRG1,PLG,C1S,C1R

Monarch	EFO:0007804	LDL cholesterol change measurement	4	18	1.22	0.0151	APOB,APOE,PCSK9,LPA
Monarch	HP:0003233	Decreased HDL cholesterol concentration	4	18	1.22	0.0151	APOA1,APOE,LCAT,APOA5
Monarch	HP:0030780	Abnormality of the protein C anticoagulant pathway	3	6	1.57	0.0151	PROC,F5,PROS1
Monarch	HP:0000926	Platyspondyly	8	111	0.73	0.0156	COMP,SPARC,SERPINF1,TCIRG1,CSF1R,BMP1,P4HB,FLNA
Monarch	HP:0001547	Abnormal rib cage morphology	14	325	0.5	0.0156	GNPTG,COMP,SERPINF1,TCIRG1,IHH,A2ML1,ANTXR1,BMP1,SUZ12,FBN1,P4HB,FLNA,IGF2,LMOD3
Monarch	HP:0001297	Stroke	12	249	0.55	0.0166	TTR,ANGPTL6,GUCY1A1,F2,CALR,FBN1,F5,FLNA,CRTAC1,ADAMTS13,CST3,KNG1
Monarch	HP:0000973	Cutis laxa	7	84	0.79	0.0168	ANTXR1,EFEMP2,SUZ12,FBN1,FLNA,GSN,C1R
Monarch	HP:0005692	Joint hyperflexibility	9	144	0.67	0.0169	CENPE,A2ML1,ANTXR1,SUZ12,P4HB,COL18A1,FLNA,C1S,C1R
Monarch	HP:0001384	Abnormal hip joint morphology	13	292	0.52	0.0187	MMP2,COMP,APOE,SERPINF1,HYAL1,MASP1,BMP1,EFEMP2,FBN1,FBLN1,FLNA,COLEC11,LMOD3
Monarch	HP:0000799	Renal steatosis	3	7	1.5	0.0201	APOB,APOE,PCSK9
Monarch	HP:0001058	Poor wound healing	4	20	1.17	0.0201	F9,F8,F13B,C1R
Monarch	HP:0004418	Thrombophlebitis	3	7	1.5	0.0201	PROC,F2,PROS1
Monarch	HP:0005305	Cerebral venous thrombosis	3	7	1.5	0.0201	PROC,F2,PROS1
Monarch	HP:0007420	Spontaneous hematomas	4	20	1.17	0.0201	F8,F13B,F5,F10
Monarch	HP:0012397	Aortic atherosclerotic lesion	3	7	1.5	0.0201	APOB,APOE,PCSK9
Monarch	HP:0032101	Unusual infection	23	748	0.36	0.0207	C5,C6,DNAH5,TCIRG1,HYAL1,FCN3,PLG,EFEMP2,C1QB,C7,GANAB,BLM,C8A,CFH,FLNA,C8B,C1QC,CFI,MASP2,C4B,C1R,CFD,B2M
Monarch	HP:0003179	Protrusio acetabuli	4	21	1.15	0.0230	MMP2,SERPINF1,BMP1,FBN1
Monarch	HP:0011843	Abnormal musculoskeletal physiology	34	1330	0.28	0.0230	GNPTG,MMP2,COMP,SPARC,APOE,SERPINF1,CENPE,TCIRG1,AHSG,HABP2,IHH,MASP1,A2ML1,ANTXR1,BMP1,DST,F2,EFEMP2,SUZ12,SERPINF2,FBN1,P4HB,FBLN1,COL18A1,F8,ADAMTSL4,FLNA,C1S,IGF2,COLEC11,LMOD3,IGFALS,C1R,B2M
Monarch	HP:0006487	Bowing of the long bones	11	224	0.56	0.0240	GNPTG,COMP,SPARC,SERPINF1,TCIRG1,IHH,BMP1,P4HB,FLNA,LMOD3,B2M
Monarch	HP:0011867	Abnormal iliac wing morphology	6	64	0.84	0.0245	GNPTG,APOE,TCIRG1,IHH,SUZ12,FLNA
Monarch	HP:0032263	Increased blood pressure	13	303	0.5	0.0245	MMP2,APOB,APOA1,ANGPTL6,LYZ,GUCY1A1,PCSK9,F2,CALR,FBN1,GANAB,ADAMTSL4,FGA
Monarch	EFO:0008390	Prothrombin time measurement	3	8	1.44	0.0254	F2,F5,F10
Monarch	HP:0000991	Xanthomatosis	4	22	1.13	0.0254	APOB,APOA1,APOE,PCSK9
Monarch	HP:0004381	Supravalvular aortic stenosis	3	8	1.44	0.0254	APOB,PCSK9,EFEMP2
Monarch	HP:0010874	Tendon xanthomatosis	3	8	1.44	0.0254	APOB,APOE,PCSK9
Monarch	HP:0003010	Prolonged bleeding time	5	42	0.95	0.0271	F9,VWF,F2,CALR,F5
Monarch	EFO:0004713	FEV/FEC ratio	21	672	0.36	0.0288	MPO,LRP1,SERPINA10,DNAH5,SERPING1,ANTXR1,DST,C7,FBN1,FBLN1,FAM184A,MTCL1,CFH,EFEMP1,CFI,EFCAB5,NAV2,FGL1,KSR2,VEGFC,HYOU1
Monarch	HP:0000924	Abnormality of the skeletal system	57	2708	0.19	0.0305	GNPTG,F9,MMP2,COMP,SPARC,PCDH12,APOB,TTR,APOE,SERPINF1,VWF,NID1,DNAH5,CENPE,TCIRG1,HYAL1,AHSG,HABP2,CSF1R,IHH,MASP1,A2ML1,ANTXR1,PCSK9,BMP1,FGB,DST,F2,PLG,EFEMP2,SUZ12,SERPINF2,FBN1,P4HB,FBLN1,BLM,COL18A1,F8,F13B,F5,ADAMTSL4,FLNA,F10,CFI,F11,C1S,IGF2,COLEC11,LMOD3,IGFALS,C1R,ATP1A1,CLTC,VEGFC,GRHL2,B2M,FGA
Monarch	HP:0000235	Abnormality of the fontanelles or cranial sutures	14	355	0.47	0.0311	MMP2,SPARC,APOE,SERPINF1,CENPE,TCIRG1,MASP1,ANTXR1,BMP1,FBN1,P4HB,FLNA,COLEC11,LMOD3
Monarch	EFO:0003907	Deep vein thrombosis	3	9	1.39	0.0317	F2,F5,KNG1
Monarch	EFO:0009925	Antithrombotic agent use measurement	3	9	1.39	0.0317	APOE,LPA,F5
Monarch	HP:0000593	Abnormal anterior chamber morphology	5	44	0.93	0.0317	MASP1,FBN1,COL18A1,COLEC11,GRHL2
Monarch	HP:0001084	Corneal arcus	3	9	1.39	0.0317	APOB,APOE,PCSK9
Monarch	HP:0001677	Coronary artery atherosclerosis	5	44	0.93	0.0317	SERPIND1,APOB,APOE,PCSK9,FBN1
Monarch	HP:0003312	Abnormal form of the vertebral bodies	12	274	0.51	0.0317	MMP2,COMP,SPARC,SERPINF1,TCIRG1,CSF1R,IHH,ANTXR1,BMP1,FBN1,P4HB,FLNA
Monarch	HP:0001681	Angina pectoris	4	24	1.09	0.0320	APOB,APOE,LCAT,PCSK9
Monarch	EFO:0003839	Retinopathy	7	98	0.72	0.0335	CETP,APOE,CFHR5,EFEMP2,CFH,CFI,CFB
Monarch	HP:0003110	Abnormality of urine homeostasis	20	638	0.37	0.0357	F9,APOA1,APOE,CFHR5,LYZ,LCAT,F2,CFHR1,LPA,SERPINF2,GANAB,PAPLN,F8,CFH,F5,ADAMTS13,GSN,F10,ATP1A1,FGA
Monarch	EFO:0004612	High density lipoprotein cholesterol measurement	22	740	0.34	0.0378	CETP,VTN,APOC3,APOB,APOA1,LRP1,APOE,LCAT,DGKG,PON3,ITIH1,F2,INHBC,LPA,APOD,APOA4,COL18A1,CRTAC1,ANKRD28,PLTP,APOA5,APOC4
Monarch	HP:0001114	Xanthelasma	3	10	1.35	0.0387	APOB,APOE,PCSK9
Monarch	HP:0100545	Arterial stenosis	6	72	0.79	0.0387	SERPIND1,APOB,APOE,F12,PCSK9,FBN1
Monarch	HP:0400008	Menometrorrhagia	3	10	1.35	0.0387	F9,FGB,FGA
Monarch	HP:0000163	Abnormal oral cavity morphology	43	1901	0.22	0.0399	MMP2,SPARC,LTF,CAT,APOE,SERPINF1,LYZ,NID1,CENPE,TCIRG1,HYAL1,SERPING1,MASP1,A2ML1,ANTXR1,BMP1,FGB,DST,F2,PLG,EFEMP2,SUZ12,SERPINF2,FBN1,P4HB,BLM,F8,F13B,F5,FLNA,CRTAC1,GSN,F10,C1S,IGF2,ABCA13,COLEC11,LMOD3,C1R,CLTC,GRHL2,B2M,FGA
Monarch	HP:0000236	Abnormality of the anterior fontanelle	7	102	0.71	0.0400	MMP2,SERPINF1,TCIRG1,MASP1,ANTXR1,BMP1,FLNA
Monarch	HP:0002659	Increased susceptibility to fractures	10	205	0.56	0.0400	MMP2,SPARC,SERPINF1,TCIRG1,HABP2,BMP1,EFEMP2,P4HB,LMOD3,B2M
Monarch	HP:0003141	Increased LDL cholesterol concentration	4	26	1.06	0.0400	APOB,APOE,LCAT,PCSK9
Monarch	HP:0011842	Abnormal skeletal morphology	54	2573	0.19	0.0430	GNPTG,F9,MMP2,COMP,SPARC,PCDH12,APOB,TTR,APOE,SERPINF1,VWF,NID1,DNAH5,CENPE,TCIRG1,HYAL1,AHSG,CSF1R,IHH,MASP1,A2ML1,ANTXR1,PCSK9,BMP1,FGB,DST,F2,PLG,EFEMP2,SUZ12,SERPINF2,FBN1,P4HB,FBLN1,BLM,COL18A1,F8,F13B,F5,ADAMTSL4,FLNA,F10,CFI,F11,C1S,IGF2,COLEC11,LMOD3,IGFALS,C1R,CLTC,VEGFC,B2M,FGA
Monarch	HP:0000095	Abnormal renal glomerulus morphology	8	136	0.64	0.0431	APOE,CFHR5,C1QB,CFHR1,CFH,C1QC,CFI,C4A
Monarch	HP:0030875	Abnormality of pulmonary circulation	8	136	0.64	0.0431	PROC,SERPINF1,TCIRG1,BMP1,F2,FBN1,FLNA,PROS1
Monarch	HP:0002953	Vertebral compression fracture	4	27	1.04	0.0439	MMP2,SPARC,SERPINF1,P4HB
Monarch	HP:0000940	Abnormal diaphysis morphology	11	249	0.51	0.0457	GNPTG,COMP,SPARC,SERPINF1,TCIRG1,IHH,BMP1,P4HB,FLNA,LMOD3,B2M
Monarch	HP:0003563	Decreased LDL cholesterol concentration	3	11	1.31	0.0457	APOC3,APOB,APOA5
Monarch	HP:0000703	Dentinogenesis imperfecta	4	28	1.02	0.0485	SPARC,SERPINF1,BMP1,P4HB
Monarch	HP:0011329	Abnormality of cranial sutures	12	292	0.48	0.0485	MMP2,SPARC,SERPINF1,CENPE,TCIRG1,MASP1,ANTXR1,BMP1,FBN1,P4HB,FLNA,COLEC11
DISEASES	DOID:1247	Blood coagulation disease	25	89	1.32	6.66e-20	SERPIND1,F9,PF4V1,HRG,PROC,F12,VWF,PF4,FGB,F2,PLG,CALR,SERPINF2,F8,F13B,CFH,F5,FLNA,ADAMTS13,F10,PROS1,F11,C4B,KNG1,FGA
DISEASES	DOID:7	Disease of anatomical entity	129	4798	0.3	5.80e-14	CETP,APOH,VCL,SERPIND1,CHGA,LBP,F9,MMP2,COMP,C5,MPO,AFM,PF4V1,SPARC,PCDH12,LTF,HRG,APOB,PROC,APOA1,TTR,CFP,APOE,PXDN,F12,SERPINF1,APCS,CFHR5,VWF,DSG2,LCAT,YWHAE,CSTA,DNAH5,CENPE,TCIRG1,LUM,AHSG,CRH,SAA4,SERPING1,ANKRD44,CSF1R,PCDH1,SCGB3A1,IHH,SERPINI1,PF4,CAMP,APEH,A2ML1,ANTXR1,LRG1,SH3D19,PCSK9,BMP1,FGB,DST,F2,PLG,EFEMP2,C1QB,CFHR1,SUZ12,APOL1,SUMO4,CALR,LPA,SERPINF2,HBA1,FBN1,P4HB,SERPINA7,ANG,GANAB,BLM,AKAP9,PIGR,APOA4,CNDP1,COL18A1,F8,F13B,CFHR2,CFHR4,CFHR3,CFH,F5,ADAMTSL4,FLNA,C8B,ADAMTS13,GSN,C1QC,F10,CD99,PROS1,EFEMP1,CFI,TNRC6A,GAPDH,CST3,ANKRD28,SAA1,F11,C1S,NUCB1,ADIPOQ,IGF2,C4A,CCDC122,MST1,LMOD3,C4B,TGFBI,CFB,JCHAIN,C1R,ATP1A1,SERPINA3,IGKV2D-28,CFD,IGHV3-15,VEGFC,KNG1,TRIM66,GRHL2,B2M,FGA

DISEASES	DOID:4	Disease	152	6291	0.25	6.11e-14	CETP,GNPTG,APOH,VCL,SERPIND1,CHGA,LBP,F9,MMP2,COMP,PON1,C5,MPO,AFM,PF4V1,APOC3,SPARC,PCDH12,LTF,HRG,APOB,PROC,APOA1,TTR,CAT,CFP,APOE,PXDN,F12,SERPINF1,APCS,CFHR5,LYZ,VWF,DSG2,LCAT,YWHAE,CSTA,CP,CDH6,DNAH5,CENPE,TCIRG1,HYAL1,ITIH4,LUM,AHSG,CRH,SAA4,SERPING1,ANKRD44,CSF1R,PCDH1,SCGB3A1,IHH,SERPINI1,PF4,MASP1,CAMP,APEH,A2ML1,ANTXR1,LRG1,SH3D19,PCSK9,BMP1,FGB,DST,F2,PLG,EFEMP2,C1QB,CFHR1,SUZ12,APOL1,SUMO4,CALR,LPA,SERPINF2,HBA1,HSPA5,FBN1,P4HB,SERPINA7,DNAH8,ANG,GANAB,BLM,AKAP9,PIGR,APOA4,CNDP1,COL18A1,F8,F13B,CFHR2,CFHR4,CFHR3,CFH,F5,ADAMTSL4,FLNA,MCOLN2,C8B,ADAMTS13,GSN,C1QC,F10,CA6,IGFBP3,CD99,GPX3,PROS1,EFEMP1,CFI,TNRC6A,GAPDH,CST3,ANKRD28,SAA1,F11,C1S,NUCB1,PCYOX1,ADIPOQ,IGF2,C4A,CCDC122,ABCA13,COLEC11,MST1,LMOD3,C4B,TGFBI,CFB,SELENOP,JCHAIN,HPR,C1R,APOA5,ATP1A1,SERPINA3,IGKV2D-28,CFD,CLTC,IGHV3-15,VEGFC,KNG1,TRIM66,GRHL2,B2M,FGA
DISEASES	DOID:2452	Thrombophilia	12	21	1.63	7.76e-12	SERPIND1,F9,HRG,PROC,VWF,F2,PLG,CFH,F5,ADAMTS13,PROS1,C4B
DISEASES	DOID:74	Hematopoietic system disease	34	473	0.73	8.06e-12	SERPIND1,F9,MPO,PF4V1,HRG,PROC,F12,VWF,TCIRG1,PF4,CAMP,FGB,F2,PLG,CALR,SERPINF2,HBA1,F8,F13B,CFH,F5,FLNA,ADAMTS13,F10,CD99,PROS1,SAA1,F11,C4B,SERPINA3,IGHV3-15,KNG1,B2M,FGA
DISEASES	DOID:2007	Degeneration of macula and posterior pole	13	46	1.32	5.59e-10	CETP,APOE,CFHR5,CFHR1,F13B,CFHR2,CFHR4,CFHR3,CFH,EFEMP1,CFI,CST3,CFB
DISEASES	DOID:0050736	Autosomal dominant disease	55	1386	0.47	5.74e-10	CETP,SERPIND1,F9,COMP,APOC3,HRG,PROC,APOA1,TTR,APOE,F12,APCS,CFHR5,LYZ,VWF,YWHAE,CDH6,SERPING1,ANKRD44,CSF1R,IHH,SERPINI1,F2,EFEMP2,CFHR1,CALR,FBN1,GANAB,AKAP9,APOA4,F8,F13B,CFHR2,CFHR4,CFHR3,CFH,F5,FLNA,GSN,EFEMP1,CFI,CST3,SAA1,C1S,IGF2,TGFBI,CFB,JCHAIN,ATP1A1,SERPINA3,CLTC,KNG1,GRHL2,B2M,FGA
DISEASES	DOID:9120	Amyloidosis	15	75	1.17	5.74e-10	APOC3,APOA1,TTR,APOE,APCS,LYZ,SAA4,APOA4,GSN,CST3,SAA1,TGFBI,SERPINA3,B2M,FGA
DISEASES	DOID:10871	Age related macular degeneration	12	44	1.31	3.72e-09	CETP,APOE,CFHR5,CFHR1,F13B,CFHR2,CFHR4,CFHR3,CFH,CFI,CST3,CFB
DISEASES	DOID:626	Complement deficiency	10	23	1.51	4.39e-09	C5,CFHR5,SERPING1,CFHR1,CFHR3,CFH,ADAMTS13,CFI,CFB,KNG1
DISEASES	DOID:0050739	Autosomal genetic disease	81	2802	0.33	5.65e-09	CETP,GNPTG,SERPIND1,F9,COMP,APOC3,LTF,HRG,APOB,PROC,APOA1,TTR,APOE,F12,APCS,CFHR5,LYZ,VWF,YWHAE,CP,CDH6,DNAH5,TCIRG1,SERPING1,ANKRD44,CSF1R,IHH,SERPINI1,MASP1,CAMP,ANTXR1,PCSK9,FGB,DST,F2,PLG,EFEMP2,CFHR1,CALR,SERPINF2,HBA1,FBN1,GANAB,BLM,AKAP9,PIGR,APOA4,F8,F13B,CFHR2,CFHR4,CFHR3,CFH,F5,ADAMTSL4,FLNA,GSN,F10,IGFBP3,EFEMP1,CFI,CST3,SAA1,F11,C1S,PCYOX1,IGF2,COLEC11,LMOD3,TGFBI,CFB,JCHAIN,APOA5,ATP1A1,SERPINA3,CFD,CLTC,KNG1,GRHL2,B2M,FGA
DISEASES	DOID:557	Kidney disease	22	275	0.77	3.16e-08	C5,MPO,TTR,APCS,CFHR5,SAA4,F2,CFHR1,APOL1,GANAB,APOA4,COL18A1,CFHR3,CFH,ADAMTS13,CFI,CST3,SAA1,IGF2,C4B,CFB,B2M
DISEASES	DOID:2914	Immune system disease	34	675	0.57	3.42e-08	APOH,C5,MPO,TTR,APOE,PXDN,APCS,CFHR5,SAA4,SERPING1,A2ML1,DST,F2,PLG,CFHR1,SUMO4,HBA1,PIGR,COL18A1,CFHR3,CFH,ADAMTS13,CD99,CFI,SAA1,C4A,C4B,CFB,JCHAIN,IGKV2D-28,IGHV3-15,VEGFC,KNG1,B2M
DISEASES	DOID:0050636	Familial visceral amyloidosis	9	21	1.5	3.97e-08	APOC3,APOA1,TTR,APOE,APCS,LYZ,APOA4,B2M,FGA
DISEASES	DOID:0050177	Monogenic disease	86	3266	0.29	9.13e-08	CETP,GNPTG,SERPIND1,F9,COMP,APOC3,LTF,HRG,APOB,PROC,APOA1,TTR,APOE,F12,SERPINF1,APCS,CFHR5,LYZ,VWF,YWHAE,CP,CDH6,DNAH5,TCIRG1,SERPING1,ANKRD44,CSF1R,IHH,SERPINI1,MASP1,CAMP,A2ML1,ANTXR1,PCSK9,FGB,DST,F2,PLG,EFEMP2,CFHR1,CALR,SERPINF2,HBA1,FBN1,DNAH8,GANAB,BLM,AKAP9,PIGR,APOA4,F8,F13B,CFHR2,CFHR4,CFHR3,CFH,F5,ADAMTSL4,FLNA,GSN,F10,IGFBP3,PROS1,EFEMP1,CFI,CST3,SAA1,F11,C1S,PCYOX1,IGF2,COLEC11,MST1,LMOD3,TGFBI,CFB,JCHAIN,APOA5,ATP1A1,SERPINA3,CFD,CLTC,KNG1,GRHL2,B2M,FGA
DISEASES	DOID:630	Genetic disease	94	3778	0.27	1.69e-07	CETP,GNPTG,SERPIND1,F9,COMP,APOC3,LTF,HRG,APOB,PROC,APOA1,TTR,CAT,APOE,F12,SERPINF1,APCS,CFHR5,LYZ,VWF,LCAT,YWHAE,CP,CDH6,DNAH5,TCIRG1,HYAL1,SERPING1,ANKRD44,CSF1R,IHH,SERPINI1,MASP1,CAMP,A2ML1,ANTXR1,PCSK9,FGB,DST,F2,PLG,EFEMP2,C1QB,CFHR1,SUMO4,CALR,SERPINF2,HBA1,FBN1,DNAH8,GANAB,BLM,AKAP9,PIGR,APOA4,CNDP1,F8,F13B,CFHR2,CFHR4,CFHR3,CFH,F5,ADAMTSL4,FLNA,MCOLN2,GSN,F10,IGFBP3,PROS1,EFEMP1,CFI,CST3,SAA1,F11,C1S,PCYOX1,ADIPOQ,IGF2,COLEC11,MST1,LMOD3,TGFBI,CFB,JCHAIN,APOA5,ATP1A1,SERPINA3,CFD,CLTC,KNG1,GRHL2,B2M,FGA
DISEASES	DOID:18	Urinary system disease	23	350	0.69	3.13e-07	C5,MPO,TTR,APCS,CFHR5,SAA4,F2,CFHR1,APOL1,GANAB,APOA4,COL18A1,CFHR3,CFH,ADAMTS13,CFI,CST3,ANKRD28,SAA1,IGF2,C4B,CFB,B2M
DISEASES	DOID:0080301	Atypical hemolytic-uremic syndrome	7	11	1.67	4.48e-07	CFHR5,CFHR1,CFHR3,CFH,ADAMTS13,CFI,CFB
DISEASES	DOID:1287	Cardiovascular system disease	27	493	0.61	4.48e-07	CETP,APOH,VCL,CHGA,MPO,APOB,APOA1,TTR,APOE,APCS,VWF,DSG2,DNAH5,PF4,PCSK9,F2,PLG,LPA,FBN1,AKAP9,FLNA,ADAMTS13,F10,CD99,SAA1,ADIPOQ,C4A
DISEASES	DOID:178	Vascular disease	19	249	0.75	8.11e-07	CETP,APOH,MPO,APOB,APOA1,APOE,VWF,PF4,PCSK9,F2,PLG,LPA,FBN1,ADAMTS13,F10,CD99,SAA1,ADIPOQ,C4A
DISEASES	DOID:12554	Hemolytic-uremic syndrome	7	14	1.57	1.43e-06	CFHR5,CFHR1,CFHR3,CFH,ADAMTS13,CFI,CFB
DISEASES	DOID:612	Primary immunodeficiency disease	24	470	0.58	1.09e-05	APOH,C5,MPO,TTR,PXDN,CFHR5,SERPING1,A2ML1,DST,F2,CFHR1,SUMO4,HBA1,PIGR,COL18A1,CFHR3,CFH,ADAMTS13,CFI,SAA1,C4A,C4B,CFB,KNG1
DISEASES	DOID:2921	Glomerulonephritis	8	36	1.22	2.00e-05	MPO,CFHR5,APOL1,COL18A1,CFHR3,CFH,C4B,CFB
DISEASES	DOID:0050117	Disease by infectious agent	20	368	0.6	5.28e-05	APOE,APCS,YWHAE,CP,ITIH4,AHSG,PCDH1,APEH,ANTXR1,F2,PLG,C1QB,APOL1,HSPA5,C1QC,CA6,SAA1,C4A,CCDC122,HPR
DISEASES	DOID:9799	Eye degenerative disease	16	236	0.7	5.28e-05	CETP,APOB,APOE,CFHR5,PCSK9,CFHR1,F13B,CFHR2,CFHR4,CFHR3,CFH,EFEMP1,CFI,CST3,TGFBI,CFB
DISEASES	DOID:65	Connective tissue disease	30	774	0.46	6.49e-05	F9,MMP2,COMP,C5,SPARC,TTR,SERPINF1,TCIRG1,CSF1R,IHH,A2ML1,ANTXR1,BMP1,DST,PLG,EFEMP2,FBN1,P4HB,COL18A1,F8,FLNA,CD99,SAA1,C1S,IGF2,C4A,C4B,JCHAIN,C1R,SERPINA3
DISEASES	DOID:0050828	Artery disease	12	130	0.83	7.26e-05	CETP,APOB,APOA1,APOE,PCSK9,F2,PLG,LPA,FBN1,ADAMTS13,SAA1,ADIPOQ
DISEASES	DOID:0014667	Disease of metabolism	36	1076	0.39	0.00011	CETP,GNPTG,F9,APOC3,APOB,APOA1,TTR,CAT,APOE,APCS,LYZ,LCAT,CP,HYAL1,AHSG,SAA4,PCSK9,F2,SUMO4,HBA1,APOA4,CNDP1,MCOLN2,GSN,GPX3,PROS1,CST3,SAA1,PCYOX1,ADIPOQ,TGFBI,SELENOP,APOA5,SERPINA3,B2M,FGA
DISEASES	DOID:0060903	Thrombosis	6	21	1.33	0.00018	APOH,VWF,PF4,F2,PLG,F10
DISEASES	DOID:1936	Atherosclerosis	6	21	1.33	0.00018	CETP,APOB,APOA1,APOE,LPA,ADIPOQ
DISEASES	DOID:17	Musculoskeletal system disease	37	1154	0.38	0.00019	F9,MMP2,COMP,C5,SPARC,TTR,APOE,F12,SERPINF1,APCS,TCIRG1,CSF1R,IHH,A2ML1,ANTXR1,BMP1,DST,PLG,EFEMP2,FBN1,P4HB,COL18A1,F8,FLNA,CD99,SAA1,C1S,IGF2,C4A,LMOD3,C4B,JCHAIN,C1R,ATP1A1,SERPINA3,CFD,B2M
DISEASES	DOID:1588	Thrombocytopenia	5	12	1.49	0.00028	PF4V1,VWF,PF4,F2,ADAMTS13
DISEASES	DOID:0070028	APP-related cerebral amyloid angiopathy	4	5	1.77	0.00046	APOE,APCS,CST3,SERPINA3
DISEASES	DOID:37	Skin disease	22	518	0.5	0.00046	MPO,APOA1,CFP,APOE,F12,APCS,DSG2,CSTA,SERPING1,CAMP,A2ML1,ANTXR1,DST,EFEMP2,FBN1,BLM,APOA4,COL18A1,GSN,CST3,C1S,KNG1
DISEASES	DOID:1168	Familial hyperlipidemia	5	15	1.39	0.00063	APOC3,APOB,APOE,PCSK9,APOA5
DISEASES	DOID:16	Integumentary system disease	23	575	0.47	0.00066	MPO,APOA1,CFP,APOE,F12,APCS,DSG2,CSTA,AHSG,SERPING1,CAMP,A2ML1,ANTXR1,DST,EFEMP2,FBN1,BLM,APOA4,COL18A1,GSN,CST3,C1S,KNG1

DISEASES	DOID:12531	Von Willebrands disease	4	6	1.69	0.00068	F9,VWF,F8,ADAMTS13
DISEASES	DOID:14735	Hereditary angioedema	4	6	1.69	0.00068	F12,SERPING1,C1S,KNG1
DISEASES	DOID:0080001	Bone disease	22	540	0.48	0.00074	F9,MMP2,COMP,C5,SPARC,TTR,SERPINF1,TCIRG1,CSF1R,IHH,BMP1,FBN1,P4HB,COL18A1,F8,FLNA,CD99,SAA1,C1S,IGF2,JCHAIN,SERPINA3
DISEASES	DOID:2218	Blood platelet disease	7	47	1.04	0.00074	PF4V1,VWF,PF4,F2,CALR,FLNA,ADAMTS13
DISEASES	DOID:649	Prion disease	5	16	1.36	0.00074	APOE,APCS,YWHAE,C1QB,C1QC
DISEASES	DOID:170	Endocrine gland cancer	9	93	0.86	0.00090	CHGA,APOE,APCS,APOA4,CD99,GAPDH,IGF2,IGKV2D-28,IGHV3-15
DISEASES	DOID:3146	Lipid metabolism disorder	8	72	0.92	0.0011	CETP,APOC3,APOB,APOA1,APOE,LCAT,PCSK9,APOA5
DISEASES	DOID:409	Liver disease	9	97	0.84	0.0012	TTR,APCS,AHSG,SAA4,F2,GANAB,GAPDH,SAA1,IGHV3-15
DISEASES	DOID:5614	Eye disease	26	749	0.41	0.0015	CETP,LTF,APOB,APOE,APCS,CFHR5,LUM,SERPING1,PCSK9,PLG,CFHR1,FBN1,COL18A1,F13B,CFHR2,CFHR4,CFHR3,CFH,ADAMTSL4,GSN,EFEMP1,CFI,CST3,TGFBI,CFB,GRHL2
DISEASES	DOID:863	Nervous system disease	55	2275	0.25	0.0015	CETP,MPO,PCDH12,LTF,APOB,PROC,APOA1,TTR,APOE,APCS,CFHR5,VWF,LCAT,YWHAE,CENPE,LUM,SERPING1,ANKRD44,CSF1R,SERPINI1,A2ML1,SH3D19,PCSK9,DST,F2,PLG,C1QB,CFHR1,SUZ12,FBN1,ANG,CNDP1,COL18A1,F13B,CFHR2,CFHR4,CFHR3,CFH,ADAMTSL4,FLNA,GSN,C1QC,CD99,EFEMP1,CFI,TNRC6A,CST3,IGF2,CCDC122,TGFBI,CFB,ATP1A1,SERPINA3,TRIM66,GRHL2
DISEASES	DOID:3393	Coronary artery disease	6	36	1.09	0.0016	APOB,APOA1,APOE,PCSK9,PLG,LPA
DISEASES	DOID:0050637	Finnish type amyloidosis	4	9	1.52	0.0017	APOE,APCS,GSN,CST3
DISEASES	DOID:11949	Creutzfeldt-Jakob disease	4	9	1.52	0.0017	APOE,APCS,C1QB,C1QC
DISEASES	DOID:10124	Corneal disease	8	85	0.84	0.0028	LTF,APOB,APOE,LUM,PCSK9,COL18A1,TGFBI,GRHL2
DISEASES	DOID:0060440	Epithelial and subepithelial dystrophy	4	11	1.43	0.0030	LTF,APOE,COL18A1,TGFBI
DISEASES	DOID:1387	Hypolipoproteinemia	4	11	1.43	0.0030	APOB,APOA1,LCAT,PCSK9
DISEASES	DOID:0111146	Acquired von Willebrand syndrome	3	3	1.87	0.0035	VWF,F8,ADAMTS13
DISEASES	DOID:0070029	ITM2B-related cerebral amyloid angiopathy 1	4	12	1.39	0.0038	APOE,APCS,CST3,SERPINA3
DISEASES	DOID:655	Inherited metabolic disorder	29	949	0.35	0.0038	CETP,GNPTG,F9,APOC3,APOB,APOA1,TTR,CAT,APOE,APCS,LYZ,LCAT,CP,HYAL1,PCSK9,SUMO4,HBA1,APOA4,CNDP1,MCOLN2,GSN,CST3,SAA1,PCYOX1,ADIPOQ,APOA5,SERPINA3,B2M,FGA
DISEASES	DOID:5844	Myocardial infarction	5	27	1.14	0.0045	APOB,APOE,PCSK9,PLG,LPA
DISEASES	DOID:0070030	ITM2B-related cerebral amyloid angiopathy 2	4	13	1.36	0.0047	APOE,APCS,CST3,SERPINA3
DISEASES	DOID:10772	Thrombotic thrombocytopenic purpura	3	4	1.74	0.0056	VWF,CFH,ADAMTS13
DISEASES	DOID:12134	Factor VIII deficiency	3	4	1.74	0.0056	F9,VWF,F8
DISEASES	DOID:0050155	Sensory system disease	28	929	0.35	0.0057	CETP,LTF,APOB,PROC,APOE,APCS,CFHR5,LUM,SERPING1,A2ML1,PCSK9,PLG,CFHR1,FBN1,COL18A1,F13B,CFHR2,CFHR4,CFHR3,CFH,ADAMTSL4,GSN,EFEMP1,CFI,CST3,TGFBI,CFB,GRHL2
DISEASES	DOID:0060449	Gelatinous drop-like corneal dystrophy	3	5	1.65	0.0084	LTF,APOE,TGFBI
DISEASES	DOID:12259	Hemophilia B	3	5	1.65	0.0084	F9,F8,F11
DISEASES	DOID:1237	Corneal degeneration	3	5	1.65	0.0084	APOB,PCSK9,TGFBI
DISEASES	DOID:2229	Factor XI deficiency	3	5	1.65	0.0084	F9,F8,F11
DISEASES	DOID:2451	Protein S deficiency	3	5	1.65	0.0084	F2,PROS1,C4B
DISEASES	DOID:3118	Hepatobiliary disease	10	168	0.64	0.0090	TTR,APCS,AHSG,SAA4,F2,GANAB,GAPDH,SAA1,MST1,IGHV3-15
DISEASES	DOID:417	Autoimmune disease	14	317	0.51	0.0095	APOH,C5,MPO,TTR,PXDN,A2ML1,DST,F2,SUMO4,HBA1,COL18A1,SAA1,C4A,C4B
DISEASES	DOID:0050639	Primary cutaneous amyloidosis	4	19	1.19	0.0137	APOE,APCS,GSN,CST3
DISEASES	DOID:10873	Kuhnt-Junius degeneration	3	7	1.5	0.0156	CETP,CFH,CFB
DISEASES	DOID:2566	Corneal dystrophy	5	38	0.99	0.0156	LTF,APOE,COL18A1,TGFBI,GRHL2
DISEASES	DOID:7693	Abdominal aortic aneurysm	3	7	1.5	0.0156	APOE,PLG,FBN1
DISEASES	DOID:3973	Thyroid gland medullary carcinoma	3	8	1.44	0.0207	APOE,APCS,APOA4
DISEASES	DOID:0080006	Bone development disease	12	269	0.52	0.0234	COMP,SPARC,SERPINF1,IHH,BMP1,FBN1,P4HB,COL18A1,F8,FLNA,C1S,JCHAIN
DISEASES	DOID:114	Heart disease	12	270	0.52	0.0235	APOH,VCL,TTR,APCS,DSG2,DNAH5,F2,PLG,LPA,FBN1,AKAP9,FLNA
DISEASES	DOID:77	Gastrointestinal system disease	19	576	0.39	0.0235	CHGA,AFM,LTF,TTR,APCS,AHSG,CRH,SAA4,APEH,ANTXR1,LRG1,F2,GANAB,FLNA,C8B,GAPDH,SAA1,MST1,IGHV3-15
DISEASES	DOID:5113	Nutritional deficiency disease	5	43	0.94	0.0237	TTR,F2,GPX3,PROS1,SELENOP
DISEASES	DOID:1188	Mononeuropathy	3	9	1.39	0.0254	MPO,F2,CCDC122
DISEASES	DOID:14250	Down syndrome	3	9	1.39	0.0254	APOE,C1QB,CST3
DISEASES	DOID:0050737	Autosomal recessive disease	41	1785	0.23	0.0338	GNPTG,LTF,APOB,APOE,F12,VWF,CP,DNAH5,TCIRG1,SERPING1,IHH,MASP1,CAMP,ANTXR1,PCSK9,FGB,DST,F2,PLG,EFEMP2,SERPINF2,HBA1,BLM,PIGR,F13B,F5,FLNA,F10,IGFBP3,SAA1,F11,C1S,PCYOX1,IGF2,COLEC11,LMOD3,TGFBI,APOA5,CFD,KNG1,FGA
DISEASES	DOID:4079	Heart valve disease	4	26	1.06	0.0338	PLG,LPA,FBN1,FLNA
DISEASES	DOID:75	Lymphatic system disease	9	174	0.58	0.0414	SAA4,F2,PLG,CD99,SAA1,IGKV2D-28,IGHV3-15,VEGFC,B2M
DISEASES	DOID:50	Thyroid gland disease	5	50	0.87	0.0417	TTR,APOE,APCS,SERPINA7,APOA4
DISEASES	DOID:2213	Hemorrhagic disease	3	12	1.27	0.0477	F2,SERPINF2,ADAMTS13
TISSUES	BTO:0004850	Bone marrow cell	70	198	1.42	3.67e-68	CPB2,CETP,APOH,PON1,C5,MPO,VTN,AFM,APOB,IGFBP5,TTR,LRP1,CFP,SERPINF1,APCS,VWF,DSG2,LGALS3BP,CP,AMBP,ITIH4,LUM,FCN3,AHSG,SAA4,SERPING1,CSF1R,FCN2,IHH,MASP1,LRG1,FGB,F2,CSPG4,CFHR1,APOL1,SERPINF2,SERPINA5,APOD,PIGR,ITIH2,F8,C4BPA,CFH,F5,ADAMTS13,MBL2,IGFBP3,SOD3,PROS1,CFI,F11,C1S,ORM2,C4A,MST1,SPARCL1,C4B,CFB,PLTP,HGFAC,SELENOP,JCHAIN,C1R,SERPINA3,HYOU1,KNG1,CDH5,B2M,FGA
TISSUES	BTO:0000759	Liver	145	2125	0.7	5.20e-64	RCN1,SPP2,ST6GAL1,CPB2,CETP,APOH,VCL,SERPIND1,CHGA,TPD52L2,LBP,APMAP,F9,PON1,MPO,AFM,APOC3,GPLD1,LTF,HRG,APOB,PROC,APOA1,TTR,CAT,LRP1,C4BPB,APOE,PXDN,F12,SERPINF1,APCS,CFHR5,VWF,PSMA6,SERPINA10,LGALS3BP,ATRN,C6,NID1,YWHAE,CP,AMBP,PON3,LUM,FCN3,ITIH1,AHSG,IGFBP1,HABP2,SAA4,SERPING1,CSF1R,FCN2,PGLYRP2,PPBP,MASP1,APEH,HSP90B1,LRG1,FGB,DST,F2,INHBC,PLG,C1QB,CFHR1,APOL1,CALR,LPA,SERPINF2,HBA1,HSPA5,FBN1,P4HB,SERPINA7,SERPINA5,ANG,GANAB,PRDX6,PROZ,ASGR2,AKAP9,PIGR,ITIH2,COL18A1,C8A,PCDH18,C4BPA,F13B,CFHR2,CFHR4,CFHR3,CFH,CD5L,S100A9,FLNA,SMARCA1,C8B,C8G,ADAMTS13,GSN,MBL2,F10,APOM,CKAP4,IGFBP3,CD99,PROS1,CFI,GAPDH,FGL1,APOF,MASP2,SAA1,F11,C1S,NUCB1,PCYOX1,IGF2,ORM2,C4A,COLEC11,MST1,ITIH3,C4B,TGFBI,CFB,IGFALS,PLTP,GC,HGFAC,JCHAIN,C1R,APOA5,ATP1A1,SERPINA3,APOC4,CLTC,HYOU1,KNG1,B2M,FGA,PDIA4,SDF4
TISSUES	BTO:0000392	Plasma cell	63	171	1.44	3.47e-62	APOH,PON1,C5,MPO,VTN,APOB,IGFBP5,TTR,LRP1,CFP,SERPINF1,APCS,VWF,DSG2,LGALS3BP,CP,AMBP,ITIH4,LUM,FCN3,AHSG,SAA4,SERPING1,CSF1R,FCN2,IHH,MASP1,LRG1,FGB,F2,CSPG4,CFHR1,APOL1,SERPINF2,SERPINA5,APOD,PIGR,ITIH2,F8,C4BPA,CFH,ADAMTS13,MBL2,IGFBP3,SOD3,PROS1,CFI,C1S,ORM2,C4A,SPARCL1,C4B,CFB,HGFAC,SELENOP,JCHAIN,C1R,SERPINA3,HYOU1,KNG1,CDH5,B2M,FGA
TISSUES	BTO:0000141	Bone marrow	83	528	1.07	8.84e-58	CPB2,CETP,APOH,SERPIND1,APMAP,PON1,C5,MPO,VTN,AFM,LTF,APOB,IGFBP5,TTR,LRP1,CFP,PXDN,SERPINF1,APCS,VWF,DSG2,LGALS3BP,CSTA,CP,AMBP,ITIH4,LUM,FCN3,AHSG,SAA4,SERPING1,CSF1R,FCN2,IHH,MASP1,CAMP,LRG1,FGB,F2,CSPG4,CFHR1,SUZ12,APOL1,SERPINF2,HBA1,SERPINA5,LIMS1,GANAB,PRDX6,APOD,PIGR,ITIH2,F8,C4BPA,CFH,QSOX1,F5,ADAMTS13,MBL2,IGFBP3,SOD3,PROS1,CFI,F11,C1S,ORM2,C4A,MST1,SPARCL1,C4B,CFB,PLTP,HGFAC,SELENOP,JCHAIN,C1R,SERPINA3,CLTC,HYOU1,KNG1,CDH5,B2M,FGA

TISSUES	BTO:0000345	Digestive gland	155	2881	0.6	1.16e-56	RCN1,SPP2,ST6GAL1,CPB2,CETP,APOH,VCL,SERPIND1,CHGA,TPD52L2,LBP,APMAP,F9,PON1,MPO,AFM,APOC3,GPLD1,SPARC,LTF,HRG,APOB,PROC,APOA1,TTR,CAT,LRP1,C4BPB,DHX29,APOE,PXDN,F12,SERPINF1,APCS,CFHR5,VWF,PSMA6,SERPINA10,LGALS3BP,ATRN,C6,NID1,YWHAE,CP,AMBP,PON3,TCIRG1,HYAL1,LUM,FCN3,ITIH1,AHSG,IGFBP1,HABP2,SAA4,SERPING1,CSF1R,FCN2,PGLYRP2,PPBP,MASP1,APEH,HSP90B1,LRG1,FGB,DST,F2,INHBC,PLG,C1QB,CFHR1,APOL1,CALR,LPA,SERPINF2,HBA1,HSPA5,FBN1,P4HB,SERPINA7,SERPINA5,ANG,GANAB,PRDX6,PROZ,QPCT,APOD,ASGR2,AKAP9,PIGR,ITIH2,COL18A1,C8A,PCDH18,CDC42BPA,C4BPA,F13B,CFHR2,CFHR4,CFHR3,CFH,CD5L,S100A9,FLNA,SMARCA1,C8B,C8G,ADAMTS13,GSN,MBL2,F10,APOM,CKAP4,IGFBP3,CD99,GPX3,PROS1,CFI,GAPDH,RNASE1,FGL1,APOF,MASP2,SAA1,F11,C1S,NUCB1,PCYOX1,IGF2,ORM2,C4A,COLEC11,MST1,ITIH3,C4B,TGFBI,CFB,IGFALS,PLTP,GC,HGFAC,JCHAIN,C1R,APOA5,ATP1A1,SERPINA3,RNASE4,APOC4,CLTC,HYOU1,KNG1,B2M,FGA,PDIA4,SDF4
TISSUES	BTO:0001486	Skeletal system	106	1307	0.78	5.41e-50	SPP2,CPB2,CETP,APOH,SERPIND1,CHGA,APMAP,MMP2,COMP,PON1,C5,MPO,VTN,AFM,APOC3,SPARC,LTF,APOB,IGFBP5,APOA1,TTR,LRP1,CFP,APOE,PXDN,SERPINF1,APCS,VWF,PSMA6,DSG2,LGALS3BP,ATRN,CSTA,CP,AMBP,HYAL1,ITIH4,LUM,FCN3,AHSG,SAA4,SERPING1,CSF1R,FCN2,IHH,MASP1,CAMP,HSP90B1,LRG1,FGB,F2,UBE3C,CSPG4,CFHR1,SUZ12,APOL1,SERPINF2,HBA1,HSPA5,SERPINA7,SERPINA5,LIMS1,GANAB,PRDX6,APOD,PIGR,ITIH2,F8,C4BPA,CFH,QSOX1,F5,ADAMTSL4,CRTAC1,ADAMTS13,MBL2,IGFBP3,SOD3,PROS1,CFI,GAPDH,CST3,F11,C1S,PCYOX1,IGF2,ORM2,C4A,MST1,LMOD3,SPARCL1,C4B,CFB,PLTP,HGFAC,SELENOP,JCHAIN,C1R,SERPINA3,CLTC,HYOU1,KNG1,CDH5,B2M,FGA,SDF4
TISSUES	BTO:0001491	Viscus	178	5378	0.39	5.47e-38	RCN1,SPP2,ST6GAL1,CPB2,CETP,APOH,VCL,SERPIND1,CHGA,TPD52L2,LBP,APMAP,F9,PON1,MPO,AFM,APOC3,GPLD1,SPARC,LTF,HRG,APOB,PROC,PLEK,APOA1,TTR,CAT,LRP1,C4BPB,CFP,DHX29,APOE,PXDN,F12,SERPINF1,APCS,CFHR5,LYZ,VWF,PSMA6,DSG2,SERPINA10,LGALS3BP,ATRN,C6,NID1,YWHAE,CSTA,CP,CDH6,AMBP,PON3,TCIRG1,LUM,FCN3,ITIH1,AHSG,IGFBP1,HABP2,SAA4,SERPING1,CSF1R,FCN2,PGLYRP2,PPBP,MASP1,APEH,GUCY1A1,HSP90B1,ANTXR1,LRG1,SH3D19,FGB,DST,F2,INHBC,PLG,C1QB,CFHR1,APOL1,CALR,LPA,SERPINF2,HBA1,ITLN1,HSPA5,FBN1,P4HB,SERPINA7,FBLN1,SERPINA5,ANG,GANAB,PRDX6,PROZ,QPCT,APOD,ASGR2,AKAP9,PIGR,APOA4,ITIH2,COL18A1,F8,C8A,PCDH18,C4BPA,F13B,CFHR2,CFHR4,CFHR3,CFH,QSOX1,CD5L,S100A9,FLNA,SMARCA1,C8B,C8G,ADAMTS13,GSN,MBL2,F10,APOM,CKAP4,CALML5,IGFBP3,CD99,SOD3,GPX3,PROS1,EFEMP1,CFI,TNRC6A,ILK,GAPDH,RNASE1,FGL1,APOF,CST3,MASP2,SAA1,F11,C1S,NUCB1,PCYOX1,IGF2,ORM2,C4A,ACSF2,COLEC11,MST1,SPARCL1,ITIH3,C4B,TGFBI,CFB,IGFALS,PLTP,RARRES2,GC,HGFAC,JCHAIN,C1R,APOA5,ATP1A1,SERPINA3,RNASE4,APOC4,CFD,CLTC,VEGFC,HYOU1,KNG1,B2M,FGA,PDIA4,SDF4
TISSUES	BTO:0001488	Endocrine gland	183	6403	0.33	9.92e-31	RCN1,SPP2,ST6GAL1,CPB2,CETP,APOH,VCL,SERPIND1,CHGA,TPD52L2,LBP,APMAP,F9,MMP2,COMP,PON1,MPO,VTN,AFM,APOC3,GPLD1,SPARC,LTF,HRG,APOB,PROC,APOA1,TTR,CAT,LRP1,C4BPB,DHX29,APOE,PXDN,F12,SERPINF1,APCS,CFHR5,VWF,PSMA6,SERPINA10,LGALS3BP,ATRN,C6,LCN1,NID1,YWHAE,CSTA,CP,AMBP,PON3,TCIRG1,HYAL1,LUM,FCN3,ITIH1,AHSG,IGFBP1,CRH,HABP2,SAA4,SERPING1,CSF1R,FCN2,PGLYRP2,IHH,PPBP,MASP1,CAMP,APEH,HSP90B1,ANTXR1,LRG1,SH3D19,FGB,DST,F2,INHBC,PLG,C1QB,CFHR1,APOL1,CALR,LPA,SERPINF2,HBA1,ITLN1,HSPA5,FBN1,P4HB,SERPINA7,FBLN1,SERPINA5,ANG,LIMS1,GANAB,PRDX6,PROZ,QPCT,APOD,PAPLN,ASGR2,AKAP9,PIGR,ITIH2,COL18A1,C8A,PCDH18,CDC42BPA,C4BPA,F13B,CFHR2,CFHR4,CFHR3,CFH,QSOX1,CD5L,S100A9,FLNA,SMARCA1,C8B,C8G,ADAMTS13,GSN,MBL2,SVEP1,F10,APOM,CKAP4,CALML5,IGFBP3,CD99,SOD3,GPX3,PROS1,EFEMP1,CFI,EFCAB5,GAPDH,RNASE1,FGL1,APOF,CST3,ANKRD28,MASP2,FBLN2,SAA1,F11,C1S,NUCB1,PCYOX1,IGF2,ORM2,C4A,FSIP2,ACSF2,COLEC11,MST1,ITIH3,C4B,TGFBI,CFB,IGFALS,PLTP,RARRES2,GC,HGFAC,UIMC1,JCHAIN,C1R,APOA5,ATP1A1,SERPINA3,RNASE4,APOC4,CFD,CLTC,HYOU1,KNG1,B2M,FGA,PDIA4,SDF4
TISSUES	BTO:0000522	Gland	190	7004	0.3	9.76e-30	RCN1,SPP2,ST6GAL1,CPB2,CETP,APOH,VCL,SERPIND1,CHGA,TPD52L2,LBP,APMAP,F9,MMP2,COMP,PON1,C5,MPO,VTN,AFM,APOC3,GPLD1,SPARC,LTF,HRG,APOB,PROC,APOA1,TTR,CAT,LRP1,C4BPB,DHX29,APOE,PXDN,F12,SERPINF1,APCS,CFHR5,LYZ,VWF,PSMA6,SERPINA10,LGALS3BP,ATRN,C6,LCN1,NID1,YWHAE,CSTA,CP,CDH6,AMBP,PON3,TCIRG1,HYAL1,LUM,FCN3,ITIH1,AHSG,IGFBP1,CRH,HABP2,SAA4,SERPING1,CSF1R,FCN2,PGLYRP2,IHH,PPBP,MASP1,CAMP,APEH,GUCY1A1,HSP90B1,ANTXR1,LRG1,SH3D19,FGB,DST,F2,INHBC,PLG,C1QB,CFHR1,APOL1,CALR,LPA,SERPINF2,HBA1,ITLN1,HSPA5,FBN1,P4HB,SERPINA7,FBLN1,SERPINA5,ANG,LIMS1,GANAB,PRDX6,PROZ,QPCT,APOD,PAPLN,ASGR2,AKAP9,PIGR,ITIH2,COL18A1,F8,C8A,PCDH18,CDC42BPA,C4BPA,F13B,CFHR2,CFHR4,CFHR3,CFH,QSOX1,CD5L,S100A9,FLNA,SMARCA1,C8B,C8G,ADAMTS13,GSN,MBL2,SVEP1,F10,APOM,CA6,CKAP4,CALML5,IGFBP3,CD99,SOD3,GPX3,PROS1,EFEMP1,CFI,EFCAB5,TNRC6A,GAPDH,RNASE1,FGL1,APOF,CST3,ANKRD28,MASP2,FBLN2,SAA1,F11,C1S,NUCB1,PCYOX1,IGF2,ORM2,C4A,FSIP2,ACSF2,COLEC11,MST1,ITIH3,C4B,TGFBI,CFB,IGFALS,PLTP,RARRES2,GC,HGFAC,UIMC1,JCHAIN,C1R,APOA5,ATP1A1,SERPINA3,RNASE4,APOC4,CFD,CLTC,HYOU1,KNG1,B2M,FGA,PDIA4,SDF4
TISSUES	BTO:0000237	Cerebrospinal fluid	23	45	1.58	3.20e-24	APOH,CHGA,AFM,APOC3,TTR,APOE,SERPINF1,CP,AHSG,SERPING1,LRG1,F2,SERPINA7,APOD,CFH,IGFBP3,CFI,CST3,IGF2,ORM2,C4A,SPARCL1,KNG1
TISSUES	BTO:0001489	Whole body	240	13099	0.13	3.86e-17	RCN1,SPP2,ST6GAL1,CPB2,CETP,GNPTG,APOH,VCL,SERPIND1,CHGA,TPD52L2,LBP,APMAP,F9,MMP2,EBI3,COMP,PON1,PCOLCE,C5,MPO,VTN,AFM,APOC3,GPLD1,SPARC,PCDH12,LTF,HRG,APOB,IGFBP5,PROC,PLEK,APOA1,TTR,CAT,LRP1,C4BPB,CFP,DHX29,APOE,PXDN,F12,SERPINF1,APCS,CFHR5,LYZ,VWF,PSMA6,DSG2,SERPINA10,LGALS3BP,ATRN,C6,LCN1,LCAT,NID1,YWHAE,CSTA,CP,DGKG,CDH6,DNAH5,AMBP,CENPE,PON3,TCIRG1,HYAL1,ITIH4,LUM,FCN3,ITIH1,AHSG,IGFBP1,CRH,HABP2,SAA4,SERPING1,CSF1R,FCN2,PGLYRP2,SCGB3A1,IHH,SERPINI1,PPBP,PF4,MASP1,CAMP,APEH,GUCY1A1,A2ML1,HSP90B1,ANTXR1,LRG1,SH3D19,PCSK9,BMP1,FGB,DST,F2,INHBC,PLG,UBE3C,EFEMP2,CSPG4,C1QB,CFHR1,SUZ12,APOL1,PSG11,CALR,LPA,SERPINF2,C7,HBA1,ITLN1,HSPA5,FBN1,P4HB,SERPINA7,FBLN1,SERPINA5,DNAH8,ANG,LIMS1,THBS4,GANAB,PRDX6,FAM184A,PROZ,QPCT,APOD,PAPLN,ASGR2,AKAP9,PIGR,APOA4,ITIH2,CNDP1,COL18A1,MTCL1,F8,C8A,PCDH18,CDC42BPA,C4BPA,F13B,CFHR2,CFHR4,CFHR3,CFH,QSOX1,F5,CD5L,S100A7,S100A9,ADAMTSL4,FLNA,CRTAC1,SMARCA1,C8B,C8G,ADAMTS13,GSN,MBL2,SVEP1,C1QC,F10,APOM,CA6,CKAP4,CALML5,IGFBP3,CD99,SOD3,GPX3,PROS1,EFEMP1,CFI,EFCAB5,TNRC6A,NAV2,ILK,GAPDH,RNASE1,FGL1,APOF,CST3,ANKRD28,MAP3K7CL,MASP2,FBLN2,PSG4,SAA1,F11,C1S,PSG2,NUCB1,PCYOX1,ADIPOQ,IGF2,ORM2,C4A,SLAIN1,FSIP2,ACSF2,COLEC11,MST1,LMOD3,SPARCL1,ITIH3,C4B,TGFBI,CFB,IGFALS,PLTP,PAEP,RARRES2,GC,HGFAC,UIMC1,SELENOP,JCHAIN,C1R,APOA5,ATP1A1,SERPINA3,RNASE4,APOC4,CFD,CLTC,VEGFC,HYOU1,KNG1,GRHL2,CDH5,B2M,FGA,PDIA4,SDF4

TISSUES	BTO:0001279	Spinal cord	27	233	0.93	2.49e-14	APOH,CHGA,AFM,APOC3,TTR,APOE,SERPINF1,CP,AHSG,SERPING1,LRG1,F2,CSPG4,SERPINA7,APOD,PCDH18,CFH,FLNA,IGFBP3,CFI,CST3,IGF2,ORM2,C4A,SPARCL1,CLTC,KNG1
TISSUES	BTO:0000042	Animal	247	15148	0.08	7.54e-10	RCN1,SPP2,ST6GAL1,CPB2,CETP,GNPTG,APOH,VCL,SERPIND1,CHGA,TPD52L2,LBP,APMAP,F9,MMP2,EBI3,COMP,PON1,PCOLCE,C5,MPO,VTN,AFM,APOC3,GPLD1,SPARC,PCDH12,LTF,HRG,APOB,IGFBP5,PROC,PLEK,APOA1,TTR,CAT,LRP1,C4BPB,CFP,DHX29,APOE,PXDN,ANGPTL6,F12,SERPINF1,APCS,CFHR5,LYZ,VWF,PSMA6,DSG2,SERPINA10,LGALS3BP,ATRN,C6,LCN1,LCAT,NID1,YWHAE,CSTA,CP,DGKG,CDH6,DNAH5,AMBP,CENPE,PON3,TCIRG1,HYAL1,ITIH4,LUM,FCN3,ITIH1,AHSG,IGFBP1,CRH,HABP2,SAA4,SERPING1,CSF1R,PCDH1,FCN2,PGLYRP2,SCGB3A1,IHH,SERPINI1,PPBP,PF4,MASP1,CAMP,APEH,GUCY1A1,A2ML1,HSP90B1,ANTXR1,LRG1,SH3D19,PCSK9,BMP1,SHLD1,FGB,DST,F2,INHBC,PLG,UBE3C,EFEMP2,CSPG4,C1QB,CFHR1,SUZ12,APOL1,PSG11,CALR,LPA,SERPINF2,C7,HBA1,ITLN1,HSPA5,FBN1,P4HB,SERPINA7,FBLN1,SERPINA5,DNAH8,ANG,LIMS1,THBS4,GANAB,PRDX6,FAM184A,PROZ,QPCT,APOD,PAPLN,ASGR2,AKAP9,PIGR,APOA4,ITIH2,CNDP1,COL18A1,MTCL1,F8,C8A,PCDH18,CDC42BPA,C4BPA,F13B,CFHR2,CFHR4,CFHR3,CFH,QSOX1,F5,CD5L,S100A7,S100A9,ADAMTSL4,FLNA,CRTAC1,SMARCA1,C8B,C8G,ADAMTS13,MINPP1,GSN,MBL2,SVEP1,C1QC,F10,APOM,CA6,CKAP4,CALML5,IGFBP3,CD99,SOD3,GPX3,KIF19,PROS1,EFEMP1,CFI,EFCAB5,TNRC6A,NAV2,ILK,GAPDH,RNASE1,FGL1,APOF,CST3,ANKRD28,MAP3K7CL,MASP2,FBLN2,PSG4,SAA1,F11,C1S,PSG2,NUCB1,PCYOX1,ADIPOQ,IGF2,ORM2,C4A,SLAIN1,FSIP2,ACSF2,ABCA13,COLEC11,MST1,LMOD3,SPARCL1,ITIH3,C4B,TGFBI,CFB,IGFALS,PLTP,PAEP,RARRES2,GC,HGFAC,UIMC1,SELENOP,JCHAIN,C1R,APOA5,ATP1A1,SERPINA3,RNASE4,APOC4,CFD,CLTC,VEGFC,HYOU1,KNG1,TRIM66,GRHL2,CDH5,B2M,FGA,PDIA4,SDF4
TISSUES	BTO:0001078	Placenta	50	1244	0.47	1.14e-09	EBI3,PCOLCE,SPARC,PCDH12,IGFBP5,LRP1,VWF,LGALS3BP,NID1,YWHAE,LUM,IGFBP1,CRH,SERPING1,CSF1R,HSP90B1,ANTXR1,BMP1,DST,EFEMP2,APOL1,PSG11,CALR,HBA1,FBN1,P4HB,FBLN1,GANAB,COL18A1,QSOX1,F5,ADAMTSL4,SVEP1,IGFBP3,GPX3,ILK,GAPDH,RNASE1,CST3,PSG4,PSG2,NUCB1,PCYOX1,IGF2,TGFBI,PLTP,ATP1A1,CFD,CDH5,B2M
TISSUES	BTO:0000203	Respiratory system	60	1707	0.42	1.23e-09	ST6GAL1,MMP2,VTN,SPARC,LTF,IGFBP5,APOE,PXDN,LYZ,VWF,LGALS3BP,ATRN,LCN1,YWHAE,CP,DNAH5,TCIRG1,LUM,FCN3,SERPING1,HSP90B1,SH3D19,CALR,HBA1,ITLN1,HSPA5,P4HB,LIMS1,GANAB,PRDX6,AKAP9,PIGR,PCDH18,C4BPA,CFH,QSOX1,S100A9,FLNA,SMARCA1,CKAP4,IGFBP3,GPX3,PROS1,EFEMP1,GAPDH,MASP2,C1S,NUCB1,PCYOX1,SPARCL1,C4B,TGFBI,JCHAIN,ATP1A1,VEGFC,HYOU1,KNG1,CDH5,B2M,PDIA4
TISSUES	BTO:0000449	Fetus	51	1318	0.46	2.18e-09	EBI3,PCOLCE,SPARC,PCDH12,IGFBP5,LRP1,VWF,LGALS3BP,NID1,YWHAE,LUM,IGFBP1,CRH,SERPING1,CSF1R,HSP90B1,ANTXR1,BMP1,DST,EFEMP2,APOL1,PSG11,CALR,HBA1,FBN1,P4HB,FBLN1,GANAB,COL18A1,QSOX1,F5,ADAMTSL4,SVEP1,CKAP4,IGFBP3,GPX3,ILK,GAPDH,RNASE1,CST3,PSG4,PSG2,NUCB1,PCYOX1,IGF2,TGFBI,PLTP,ATP1A1,CFD,CDH5,B2M
TISSUES	BTO:0000284	Organism form	74	2542	0.33	1.44e-08	ST6GAL1,VCL,TPD52L2,EBI3,PCOLCE,SPARC,PCDH12,LTF,IGFBP5,LRP1,CFP,DHX29,VWF,PSMA6,LGALS3BP,NID1,YWHAE,ITIH4,LUM,IGFBP1,CRH,SERPING1,CSF1R,CAMP,HSP90B1,ANTXR1,BMP1,DST,UBE3C,EFEMP2,APOL1,PSG11,CALR,HBA1,HSPA5,FBN1,P4HB,FBLN1,GANAB,AKAP9,COL18A1,PCDH18,QSOX1,F5,ADAMTSL4,FLNA,SVEP1,APOM,CKAP4,IGFBP3,CD99,GPX3,EFEMP1,NAV2,ILK,GAPDH,RNASE1,CST3,PSG4,PSG2,NUCB1,PCYOX1,IGF2,TGFBI,PLTP,UIMC1,ATP1A1,CFD,CLTC,VEGFC,GRHL2,CDH5,B2M,SDF4
TISSUES	BTO:0001239	Serum	9	25	1.43	5.23e-08	GPLD1,APOA1,LGALS3BP,ATRN,ITIH1,PGLYRP2,ITIH2,HGFAC,KNG1
TISSUES	BTO:0003099	Internal female genital organ	77	2804	0.31	7.01e-08	RCN1,APOH,VCL,TPD52L2,EBI3,COMP,PCOLCE,SPARC,PCDH12,LTF,IGFBP5,LRP1,VWF,LGALS3BP,C6,NID1,YWHAE,LUM,IGFBP1,CRH,SERPING1,CSF1R,MASP1,A2ML1,HSP90B1,ANTXR1,BMP1,DST,EFEMP2,SUZ12,APOL1,PSG11,CALR,HBA1,ITLN1,FBN1,P4HB,FBLN1,GANAB,PAPLN,COL18A1,PCDH18,QSOX1,F5,S100A9,ADAMTSL4,FLNA,GSN,SVEP1,F10,CKAP4,IGFBP3,CD99,GPX3,PROS1,ILK,GAPDH,RNASE1,CST3,FBLN2,PSG4,C1S,PSG2,NUCB1,PCYOX1,IGF2,COLEC11,SPARCL1,TGFBI,PLTP,PAEP,UIMC1,ATP1A1,CFD,CLTC,CDH5,B2M
TISSUES	BTO:0000570	Hematopoietic system	74	2755	0.3	4.33e-07	ST6GAL1,CETP,VCL,TPD52L2,PON1,MPO,VTN,APOC3,GPLD1,LTF,APOB,PROC,PLEK,APOA1,TTR,CAT,LRP1,CFP,APOE,F12,VWF,PSMA6,LGALS3BP,ATRN,YWHAE,ITIH1,AHSG,SERPING1,PGLYRP2,PPBP,PF4,HSP90B1,FGB,F2,LPA,HBA1,P4HB,LIMS1,GANAB,PRDX6,AKAP9,ITIH2,C8A,PCDH18,F5,CD5L,S100A9,FLNA,GSN,C1QC,APOM,CKAP4,IGFBP3,CD99,SOD3,GPX3,GAPDH,CST3,C1S,NUCB1,IGF2,SPARCL1,C4B,TGFBI,CFB,HGFAC,JCHAIN,CFD,CLTC,VEGFC,HYOU1,KNG1,B2M,FGA
TISSUES	BTO:0000132	Blood platelet	22	363	0.65	1.16e-06	VCL,VTN,APOA1,TTR,CAT,VWF,YWHAE,AHSG,PPBP,HSP90B1,FGB,HBA1,P4HB,LIMS1,PRDX6,F5,FLNA,GSN,GAPDH,CLTC,HYOU1,FGA
TISSUES	BTO:0000421	Connective tissue	37	954	0.46	1.38e-06	SPP2,APMAP,MMP2,COMP,SPARC,APOA1,TTR,CAT,LGALS3BP,YWHAE,LUM,AHSG,IGFBP1,IHH,HSP90B1,EFEMP2,CALR,ITLN1,HSPA5,FBN1,P4HB,THBS4,GANAB,PRDX6,PAPLN,F5,CRTAC1,EFEMP1,GAPDH,CST3,FBLN2,ADIPOQ,ACSF2,PLTP,CFD,B2M,SDF4
TISSUES	BTO:0001202	Saliva	10	57	1.11	1.77e-06	APOH,MPO,AFM,LGALS3BP,AHSG,LRG1,PIGR,CST3,C4B,JCHAIN
TISSUES	BTO:0000174	Embryonic structure	65	2369	0.31	2.06e-06	ST6GAL1,VCL,TPD52L2,EBI3,PCOLCE,SPARC,PCDH12,IGFBP5,LRP1,DHX29,VWF,PSMA6,LGALS3BP,NID1,YWHAE,LUM,IGFBP1,CRH,SERPING1,CSF1R,HSP90B1,ANTXR1,BMP1,DST,UBE3C,EFEMP2,APOL1,PSG11,CALR,HBA1,FBN1,P4HB,FBLN1,GANAB,AKAP9,COL18A1,PCDH18,QSOX1,F5,ADAMTSL4,FLNA,SVEP1,APOM,CKAP4,IGFBP3,GPX3,ILK,GAPDH,RNASE1,CST3,PSG4,PSG2,NUCB1,PCYOX1,IGF2,TGFBI,PLTP,UIMC1,ATP1A1,CFD,CLTC,VEGFC,GRHL2,CDH5,B2M
TISSUES	BTO:0000511	Gastrointestinal tract	54	1816	0.34	3.35e-06	VCL,CHGA,SPARC,LTF,APOB,PROC,APOA1,TTR,CAT,LYZ,LGALS3BP,YWHAE,LUM,AHSG,HABP2,SERPING1,HSP90B1,LRG1,SH3D19,DST,CALR,ITLN1,HSPA5,P4HB,FBLN1,ANG,GANAB,PRDX6,AKAP9,PIGR,APOA4,PCDH18,QSOX1,FLNA,GSN,IGFBP3,CD99,SOD3,GPX3,TNRC6A,ILK,GAPDH,CST3,C1S,IGF2,SPARCL1,TGFBI,CFB,JCHAIN,C1R,ATP1A1,CFD,CLTC,B2M
TISSUES	BTO:0000988	Pancreas	28	626	0.52	4.95e-06	CETP,CHGA,APOC3,GPLD1,SPARC,TTR,NID1,TCIRG1,HSP90B1,APOL1,CALR,HSPA5,P4HB,QPCT,FLNA,GSN,CKAP4,IGFBP3,CD99,GPX3,GAPDH,RNASE1,IGF2,TGFBI,ATP1A1,SERPINA3,B2M,SDF4
TISSUES	BTO:0000088	Cardiovascular system	37	1057	0.41	1.38e-05	VCL,PCOLCE,AFM,SPARC,APOB,APOA1,APOE,VWF,YWHAE,HYAL1,LUM,SERPING1,HSP90B1,F2,HBA1,FBN1,P4HB,THBS4,PCDH18,F13B,FLNA,GSN,GPX3,EFEMP1,GAPDH,PCYOX1,LMOD3,SPARCL1,TGFBI,PLTP,SELENOP,CLTC,VEGFC,HYOU1,CDH5,B2M,FGA
TISSUES	BTO:0000058	Alimentary canal	55	2021	0.3	3.64e-05	VCL,CHGA,SPARC,LTF,APOB,PROC,APOA1,TTR,CAT,LYZ,LGALS3BP,YWHAE,LUM,AHSG,HABP2,SERPING1,HSP90B1,LRG1,SH3D19,DST,CALR,ITLN1,HSPA5,P4HB,FBLN1,ANG,GANAB,PRDX6,AKAP9,PIGR,APOA4,PCDH18,QSOX1,FLNA,GSN,CALML5,IGFBP3,CD99,SOD3,GPX3,TNRC6A,ILK,GAPDH,CST3,C1S,IGF2,SPARCL1,TGFBI,CFB,JCHAIN,C1R,ATP1A1,CFD,CLTC,B2M

TISSUES	BTO:0000763	Lung	43	1395	0.36	3.64e-05	MMP2,VTN,SPARC,LTF,IGFBP5,APOE,PXDN,VWF,LGALS3BP,LCN1,YWHAE,DNAH5,TCIRG1,LUM,FCN3,SERPING1,HSP90B1,CALR,HBA1,ITLN1,HSPA5,P4HB,PRDX6,AKAP9,PCDH18,C4BPA,QSOX1,S100A9,FLNA,SMARCA1,CKAP4,GPX3,EFEMP1,GAPDH,MASP2,C1S,NUCB1,SPARCL1,C4B,TGFBI,CDH5,B2M,PDIA4
TISSUES	BTO:0000089	Blood	51	1824	0.32	4.52e-05	VCL,TPD52L2,PON1,MPO,VTN,LTF,APOB,PROC,PLEK,APOA1,TTR,CAT,LRP1,APOE,F12,VWF,YWHAE,AHSG,SERPING1,PPBP,PF4,HSP90B1,FGB,F2,LPA,HBA1,P4HB,LIMS1,PRDX6,AKAP9,C8A,PCDH18,F5,S100A9,FLNA,GSN,C1QC,APOM,CD99,SOD3,GPX3,GAPDH,CST3,C1S,NUCB1,C4B,CFB,CLTC,HYOU1,B2M,FGA
TISSUES	BTO:0000121	Bile	6	20	1.35	6.74e-05	APOH,LGALS3BP,CP,PIGR,ORM2,JCHAIN
TISSUES	BTO:0000562	Heart	28	738	0.45	9.66e-05	VCL,PCOLCE,AFM,APOA1,VWF,YWHAE,HYAL1,LUM,SERPING1,F2,HBA1,FBN1,THBS4,PCDH18,F13B,FLNA,GPX3,GAPDH,PCYOX1,LMOD3,SPARCL1,PLTP,SELENOP,CLTC,VEGFC,HYOU1,CDH5,FGA
TISSUES	BTO:0001499	Tear	6	23	1.29	0.00013	LTF,LYZ,LCN1,PIGR,JCHAIN,B2M
TISSUES	BTO:0003092	Urinary system	38	1249	0.35	0.00021	VCL,TPD52L2,SPARC,CAT,APOE,CDH6,GUCY1A1,HSP90B1,ANTXR1,LRG1,PLG,P4HB,COL18A1,F8,PCDH18,CFH,QSOX1,FLNA,SMARCA1,GSN,IGFBP3,CD99,GPX3,TNRC6A,GAPDH,FGL1,NUCB1,IGF2,ACSF2,COLEC11,TGFBI,RARRES2,GC,ATP1A1,SERPINA3,VEGFC,KNG1,B2M
TISSUES	BTO:0000083	Female reproductive system	118	6111	0.16	0.00024	RCN1,ST6GAL1,GNPTG,APOH,VCL,TPD52L2,APMAP,MMP2,EBI3,COMP,PCOLCE,VTN,SPARC,PCDH12,LTF,IGFBP5,APOA1,CAT,LRP1,DHX29,APOE,PXDN,SERPINF1,VWF,PSMA6,LGALS3BP,ATRN,C6,NID1,YWHAE,LUM,AHSG,IGFBP1,CRH,SERPING1,CSF1R,PGLYRP2,IHH,MASP1,CAMP,APEH,A2ML1,HSP90B1,ANTXR1,SH3D19,BMP1,FGB,DST,F2,UBE3C,EFEMP2,SUZ12,APOL1,PSG11,CALR,C7,HBA1,ITLN1,HSPA5,FBN1,P4HB,FBLN1,SERPINA5,DNAH8,LIMS1,GANAB,PRDX6,PAPLN,COL18A1,PCDH18,CDC42BPA,CFH,QSOX1,F5,S100A9,ADAMTSL4,FLNA,ADAMTS13,GSN,SVEP1,F10,CKAP4,IGFBP3,CD99,GPX3,PROS1,EFEMP1,EFCAB5,NAV2,ILK,GAPDH,RNASE1,CST3,ANKRD28,FBLN2,PSG4,C1S,PSG2,NUCB1,PCYOX1,IGF2,FSIP2,COLEC11,SPARCL1,TGFBI,PLTP,PAEP,UIMC1,ATP1A1,SERPINA3,CFD,CLTC,HYOU1,GRHL2,CDH5,B2M,PDIA4,SDF4
TISSUES	BTO:0001244	Urinary tract	38	1258	0.35	0.00024	VCL,TPD52L2,SPARC,CAT,APOE,CDH6,GUCY1A1,HSP90B1,ANTXR1,LRG1,PLG,P4HB,COL18A1,F8,PCDH18,CFH,QSOX1,FLNA,SMARCA1,GSN,IGFBP3,CD99,GPX3,TNRC6A,GAPDH,FGL1,NUCB1,IGF2,ACSF2,COLEC11,TGFBI,RARRES2,GC,ATP1A1,SERPINA3,VEGFC,KNG1,B2M
TISSUES	BTO:0003091	Urogenital system	132	7090	0.14	0.00025	RCN1,ST6GAL1,GNPTG,APOH,VCL,TPD52L2,APMAP,MMP2,EBI3,COMP,PCOLCE,VTN,SPARC,PCDH12,LTF,HRG,IGFBP5,APOA1,CAT,LRP1,DHX29,APOE,PXDN,SERPINF1,VWF,PSMA6,LGALS3BP,ATRN,C6,NID1,YWHAE,CDH6,LUM,AHSG,IGFBP1,CRH,SERPING1,CSF1R,PGLYRP2,IHH,MASP1,CAMP,APEH,GUCY1A1,A2ML1,HSP90B1,ANTXR1,LRG1,SH3D19,BMP1,FGB,DST,F2,PLG,UBE3C,EFEMP2,SUZ12,APOL1,PSG11,CALR,C7,HBA1,ITLN1,HSPA5,FBN1,P4HB,FBLN1,SERPINA5,DNAH8,LIMS1,GANAB,PRDX6,PAPLN,COL18A1,F8,PCDH18,CDC42BPA,CFH,QSOX1,F5,S100A9,ADAMTSL4,FLNA,SMARCA1,ADAMTS13,GSN,SVEP1,F10,CKAP4,IGFBP3,CD99,GPX3,PROS1,EFEMP1,EFCAB5,TNRC6A,NAV2,ILK,GAPDH,RNASE1,FGL1,CST3,ANKRD28,FBLN2,PSG4,C1S,PSG2,NUCB1,PCYOX1,IGF2,FSIP2,ACSF2,COLEC11,SPARCL1,TGFBI,PLTP,PAEP,RARRES2,GC,UIMC1,ATP1A1,SERPINA3,CFD,CLTC,VEGFC,HYOU1,KNG1,GRHL2,CDH5,B2M,PDIA4,SDF4
TISSUES	BTO:0001419	Urine	6	28	1.2	0.00030	LYZ,AMBP,F2,RNASE1,MASP2,B2M
TISSUES	BTO:0000648	Intestine	38	1349	0.32	0.00100	VCL,APOB,PROC,LYZ,LGALS3BP,LUM,HABP2,HSP90B1,LRG1,SH3D19,DST,CALR,ITLN1,HSPA5,P4HB,ANG,GANAB,PIGR,APOA4,PCDH18,FLNA,GSN,IGFBP3,SOD3,GPX3,ILK,GAPDH,CST3,C1S,IGF2,TGFBI,CFB,JCHAIN,C1R,ATP1A1,CFD,CLTC,B2M
TISSUES	BTO:0000081	Reproductive system	119	6444	0.14	0.0017	RCN1,ST6GAL1,GNPTG,APOH,VCL,TPD52L2,APMAP,MMP2,EBI3,COMP,PCOLCE,VTN,SPARC,PCDH12,LTF,IGFBP5,APOA1,CAT,LRP1,DHX29,APOE,PXDN,SERPINF1,VWF,PSMA6,LGALS3BP,ATRN,C6,NID1,YWHAE,LUM,AHSG,IGFBP1,CRH,SERPING1,CSF1R,PGLYRP2,IHH,MASP1,CAMP,APEH,A2ML1,HSP90B1,ANTXR1,SH3D19,BMP1,FGB,DST,F2,UBE3C,EFEMP2,SUZ12,APOL1,PSG11,CALR,C7,HBA1,ITLN1,HSPA5,FBN1,P4HB,FBLN1,SERPINA5,DNAH8,LIMS1,GANAB,PRDX6,PAPLN,COL18A1,PCDH18,CDC42BPA,CFH,QSOX1,F5,S100A9,ADAMTSL4,FLNA,ADAMTS13,GSN,SVEP1,F10,CKAP4,IGFBP3,CD99,GPX3,PROS1,EFEMP1,EFCAB5,NAV2,ILK,GAPDH,RNASE1,CST3,ANKRD28,FBLN2,PSG4,C1S,PSG2,NUCB1,PCYOX1,IGF2,FSIP2,COLEC11,SPARCL1,TGFBI,PLTP,PAEP,RARRES2,UIMC1,ATP1A1,SERPINA3,CFD,CLTC,HYOU1,GRHL2,CDH5,B2M,PDIA4,SDF4
TISSUES	BTO:0000431	Excretory gland	38	1385	0.31	0.0017	TPD52L2,VTN,SPARC,LTF,CAT,APOE,CDH6,AHSG,GUCY1A1,HSP90B1,ANTXR1,LRG1,FGB,F2,PLG,P4HB,APOD,COL18A1,F8,QSOX1,SMARCA1,GSN,SVEP1,IGFBP3,CD99,GPX3,TNRC6A,GAPDH,NUCB1,IGF2,ACSF2,COLEC11,TGFBI,RARRES2,GC,ATP1A1,KNG1,B2M
TISSUES	BTO:0000102	Blood clot	4	11	1.43	0.0019	VWF,F2,PLG,ADAMTS13
TISSUES	BTO:0000671	Kidney	31	1039	0.34	0.0021	TPD52L2,SPARC,CAT,APOE,CDH6,GUCY1A1,HSP90B1,ANTXR1,LRG1,PLG,P4HB,COL18A1,F8,QSOX1,SMARCA1,GSN,IGFBP3,CD99,GPX3,TNRC6A,GAPDH,NUCB1,IGF2,ACSF2,COLEC11,TGFBI,RARRES2,GC,ATP1A1,KNG1,B2M
TISSUES	BTO:0001253	Skin	33	1151	0.33	0.0024	ST6GAL1,SPARC,LRP1,APOE,PSMA6,LGALS3BP,LCN1,CSTA,HSP90B1,BMP1,DST,CALR,P4HB,APOD,S100A7,S100A9,FLNA,GSN,CALML5,IGFBP3,CD99,EFEMP1,GAPDH,ADIPOQ,TGFBI,RARRES2,C1R,ATP1A1,SERPINA3,CFD,VEGFC,B2M,SDF4
TISSUES	BTO:0000849	Melanoma cell line	11	180	0.66	0.0026	MMP2,SERPINF1,YWHAE,HSP90B1,CSPG4,HSPA5,P4HB,GANAB,BLM,ATP1A1,CLTC
TISSUES	BTO:0000255	Brain cell line	11	188	0.64	0.0037	VCL,APOA1,TTR,FGB,CALR,HSPA5,P4HB,PRDX6,GAPDH,HYOU1,TRIM66
TISSUES	BTO:0003914	Interstitial cell of Cajal	11	193	0.63	0.0045	APOA1,TTR,CAT,YWHAE,AHSG,CALR,HSPA5,P4HB,GANAB,PRDX6,GAPDH
TISSUES	BTO:0000634	Integument	49	2112	0.24	0.0062	ST6GAL1,VCL,CHGA,SPARC,LTF,LRP1,APOE,VWF,PSMA6,LGALS3BP,LCN1,CSTA,LUM,IGFBP1,A2ML1,HSP90B1,ANTXR1,BMP1,DST,CALR,HSPA5,P4HB,APOD,AKAP9,S100A7,S100A9,FLNA,GSN,CALML5,IGFBP3,CD99,EFEMP1,ILK,GAPDH,RNASE1,ADIPOQ,COLEC11,TGFBI,PLTP,PAEP,RARRES2,C1R,ATP1A1,SERPINA3,CFD,VEGFC,CDH5,B2M,SDF4
TISSUES	BTO:0001388	Trachea	11	201	0.61	0.0062	ST6GAL1,LTF,LYZ,CP,P4HB,LIMS1,CFH,VEGFC,HYOU1,KNG1,B2M
TISSUES	BTO:0000254	Female reproductive gland	64	3017	0.2	0.0063	RCN1,APOH,VCL,TPD52L2,APMAP,MMP2,COMP,VTN,SPARC,LTF,APOA1,DHX29,APOE,PSMA6,LGALS3BP,ATRN,C6,NID1,YWHAE,AHSG,SERPING1,PGLYRP2,IHH,CAMP,HSP90B1,SH3D19,FGB,F2,CALR,ITLN1,HSPA5,P4HB,SERPINA5,LIMS1,GANAB,PRDX6,PAPLN,PCDH18,CDC42BPA,CFH,QSOX1,FLNA,ADAMTS13,GSN,SVEP1,F10,IGFBP3,CD99,EFCAB5,GAPDH,RNASE1,CST3,ANKRD28,FBLN2,C1S,IGF2,FSIP2,COLEC11,UIMC1,ATP1A1,CFD,CLTC,HYOU1,B2M
TISSUES	BTO:0000975	Ovary	19	528	0.43	0.0063	RCN1,APOH,VCL,COMP,LGALS3BP,C6,SERPING1,ITLN1,PAPLN,PCDH18,FLNA,F10,GAPDH,IGF2,COLEC11,UIMC1,ATP1A1,CLTC,B2M
TISSUES	BTO:0001090	Mouth	19	532	0.42	0.0067	GNPTG,C5,LTF,LYZ,LCN1,CSTA,CENPE,A2ML1,DST,C7,P4HB,LIMS1,GANAB,S100A9,GSN,CA6,JCHAIN,B2M,SDF4
TISSUES	BTO:0000166	Cecum	5	35	1.02	0.0075	PIGR,GAPDH,CST3,JCHAIN,B2M
TISSUES	BTO:0002979	Secretion	3	6	1.57	0.0075	LTF,PIGR,JCHAIN

TISSUES	BTO:0001702	Left atrium	6	60	0.87	0.0098	VCL,APOA1,HBA1,FBN1,FLNA,GAPDH
TISSUES	BTO:0000672	Hindbrain	19	565	0.4	0.0130	RCN1,CHGA,PON1,VTN,APOE,PSMA6,LCN1,PON3,CRH,PCSK9,QPCT,APOD,MTCL1,C1QC,ILK,IGF2,ATP1A1,HYOU1,FGA
TISSUES	BTO:0001129	Prostate gland	17	476	0.42	0.0136	VCL,LTF,ATRN,LUM,HSP90B1,ANTXR1,CALR,P4HB,PCDH18,FLNA,GAPDH,RNASE1,NUCB1,RARRES2,ATP1A1,B2M,SDF4
TISSUES	BTO:0000673	Metencephalon	18	528	0.4	0.0154	RCN1,CHGA,PON1,VTN,APOE,LCN1,PON3,CRH,PCSK9,QPCT,APOD,MTCL1,C1QC,ILK,IGF2,ATP1A1,HYOU1,FGA
TISSUES	BTO:0003120	Atherosclerotic plaque	3	9	1.39	0.0176	APOB,APOA1,APOE
TISSUES	BTO:0003872	Foam cell	3	9	1.39	0.0176	APOB,APOA1,APOE
TISSUES	BTO:0000140	Bone	9	165	0.61	0.0202	SPP2,MMP2,COMP,SPARC,IHH,HSP90B1,GAPDH,B2M,SDF4
TISSUES	BTO:0003150	Pulmonary artery endothelial cell line	2	2	1.87	0.0367	KNG1,CDH5
TISSUES	BTO:0001356	Tendon	7	112	0.67	0.0376	EFEMP2,P4HB,THBS4,PAPLN,CRTAC1,EFEMP1,CST3
TISSUES	BTO:0000484	Frontal lobe	7	113	0.66	0.0390	APOE,APOD,PCDH18,GAPDH,CST3,CLTC,SDF4
TISSUES	BTO:0000439	Eye	23	835	0.31	0.0405	VCL,SPARC,LTF,IGFBP5,TTR,APOE,SERPINF1,LYZ,LCN1,LUM,SERPINI1,DST,CALR,FAM184A,APOD,CFH,GSN,GPX3,GAPDH,TGFBI,ATP1A1,CLTC,B2M
TISSUES	BTO:0001484	Nervous system	105	6016	0.11	0.0430	RCN1,GNPTG,APOH,CHGA,TPD52L2,APMAP,MMP2,PON1,PCOLCE,VTN,AFM,APOC3,SPARC,IGFBP5,TTR,CAT,LRP1,DHX29,APOE,SERPINF1,PSMA6,LGALS3BP,ATRN,LCN1,NID1,YWHAE,CP,DGKG,PON3,AHSG,CRH,SERPING1,CSF1R,SERPINI1,MASP1,GUCY1A1,HSP90B1,ANTXR1,LRG1,SH3D19,PCSK9,BMP1,DST,F2,EFEMP2,CSPG4,C1QB,CALR,HBA1,HSPA5,P4HB,SERPINA7,FBLN1,THBS4,GANAB,PRDX6,QPCT,APOD,AKAP9,CNDP1,COL18A1,MTCL1,F8,PCDH18,CDC42BPA,CFH,QSOX1,S100A9,FLNA,CRTAC1,GSN,C1QC,IGFBP3,CD99,GPX3,EFEMP1,CFI,TNRC6A,NAV2,ILK,GAPDH,CST3,MAP3K7CL,C1S,NUCB1,PCYOX1,IGF2,ORM2,C4A,SLAIN1,ACSF2,SPARCL1,TGFBI,PLTP,UIMC1,SELENOP,ATP1A1,SERPINA3,CLTC,HYOU1,KNG1,CDH5,B2M,FGA,SDF4
TISSUES	BTO:0000227	Central nervous system	102	5825	0.11	0.0469	RCN1,GNPTG,APOH,CHGA,TPD52L2,APMAP,MMP2,PON1,PCOLCE,VTN,AFM,APOC3,SPARC,IGFBP5,TTR,CAT,LRP1,DHX29,APOE,SERPINF1,PSMA6,LGALS3BP,ATRN,LCN1,NID1,YWHAE,CP,DGKG,PON3,AHSG,CRH,SERPING1,CSF1R,SERPINI1,MASP1,GUCY1A1,HSP90B1,ANTXR1,LRG1,SH3D19,PCSK9,BMP1,DST,F2,EFEMP2,CSPG4,C1QB,CALR,HBA1,HSPA5,P4HB,SERPINA7,FBLN1,GANAB,PRDX6,QPCT,APOD,AKAP9,CNDP1,MTCL1,F8,PCDH18,CDC42BPA,CFH,QSOX1,S100A9,FLNA,CRTAC1,GSN,C1QC,IGFBP3,CD99,GPX3,EFEMP1,CFI,TNRC6A,NAV2,ILK,GAPDH,CST3,MAP3K7CL,C1S,NUCB1,PCYOX1,IGF2,ORM2,C4A,SLAIN1,ACSF2,SPARCL1,TGFBI,PLTP,UIMC1,ATP1A1,SERPINA3,CLTC,HYOU1,KNG1,CDH5,B2M,FGA,SDF4
COMPARTMENTS	GOCC:0005576	Extracellular region	185	2079	0.82	2.07e-109	SPP2,ST6GAL1,CPB2,CETP,APOH,VCL,SERPIND1,CHGA,LBP,F9,MMP2,EBI3,COMP,PON1,PCOLCE,C5,MPO,VTN,AFM,PF4V1,APOC3,GPLD1,SPARC,LTF,HRG,APOB,IGFBP5,PROC,APOA1,TTR,C4BPB,CFP,APOE,PXDN,ANGPTL6,F12,SERPINF1,APCS,CFHR5,LYZ,VWF,SERPINA10,LGALS3BP,C6,LCN1,LCAT,NID1,YWHAE,CSTA,CP,AMBP,CENPE,PON3,HYAL1,ITIH4,LUM,FCN3,ITIH1,AHSG,IGFBP1,CRH,HABP2,SAA4,SERPING1,FCN2,PGLYRP2,SCGB3A1,SERPINI1,PPBP,PF4,MASP1,CAMP,A2ML1,HSP90B1,LRG1,PCSK9,BMP1,FGB,DST,F2,INHBC,PLG,EFEMP2,CSPG4,C1QB,CFHR1,APOL1,CPN2,CALR,LPA,SERPINF2,C7,HBA1,ITLN1,HSPA5,FBN1,P4HB,SERPINA7,FBLN1,SERPINA5,ANG,THBS4,PROZ,QPCT,APOD,PIGR,APOA4,ITIH2,COL18A1,F8,C8A,C4BPA,F13B,CFHR2,CFHR4,CFHR3,CFH,QSOX1,F5,CD5L,S100A7,S100A9,FLNA,CRTAC1,C8B,C8G,ADAMTS13,GSN,MBL2,C1QC,F10,APOM,CA6,CALML5,IGFBP3,SOD3,GPX3,PROS1,EFEMP1,CFI,GAPDH,RNASE1,FGL1,APOF,CST3,MASP2,FBLN2,SAA1,F11,C1S,NUCB1,PCYOX1,ADIPOQ,IGF2,ORM2,C4A,COLEC11,MST1,SPARCL1,ITIH3,C4B,TGFBI,CFB,IGFALS,PLTP,PAEP,RARRES2,GC,HGFAC,JCHAIN,HPR,C1R,APOA5,SERPINA3,RNASE4,IGKV2D-28,APOC4,CFD,CLTC,VEGFC,KNG1,B2M,FGA,PDIA4,SDF4
COMPARTMENTS	GOCC:0005615	Extracellular space	138	1027	1.0	1.12e-95	CPB2,CETP,APOH,VCL,SERPIND1,LBP,F9,MMP2,EBI3,PON1,C5,MPO,VTN,AFM,APOC3,GPLD1,LTF,HRG,APOB,IGFBP5,PROC,APOA1,TTR,CFP,APOE,PXDN,F12,SERPINF1,APCS,LYZ,VWF,LGALS3BP,LCN1,LCAT,YWHAE,CSTA,CP,AMBP,PON3,HYAL1,ITIH4,LUM,FCN3,ITIH1,AHSG,IGFBP1,CRH,SAA4,SERPING1,FCN2,SCGB3A1,SERPINI1,MASP1,CAMP,A2ML1,HSP90B1,LRG1,PCSK9,FGB,F2,PLG,CSPG4,C1QB,CFHR1,APOL1,CPN2,CALR,LPA,SERPINF2,HBA1,ITLN1,HSPA5,FBN1,FBLN1,SERPINA5,ANG,THBS4,APOD,PIGR,APOA4,ITIH2,COL18A1,F8,C8A,C4BPA,CFHR3,CFH,QSOX1,CD5L,S100A7,S100A9,FLNA,C8G,GSN,MBL2,C1QC,APOM,CA6,IGFBP3,SOD3,GPX3,PROS1,EFEMP1,GAPDH,FGL1,APOF,CST3,MASP2,SAA1,F11,C1S,PCYOX1,ADIPOQ,IGF2,ORM2,C4A,COLEC11,MST1,C4B,TGFBI,CFB,IGFALS,PLTP,RARRES2,GC,JCHAIN,HPR,C1R,APOA5,SERPINA3,IGKV2D-28,APOC4,CFD,CLTC,KNG1,B2M,FGA,PDIA4
COMPARTMENTS	GOCC:0072562	Blood microparticle	49	118	1.49	1.38e-49	PON1,VTN,AFM,HRG,APOA1,APOE,APCS,LGALS3BP,CP,AMBP,ITIH4,FCN3,ITIH1,AHSG,SERPING1,FCN2,FGB,F2,PLG,C1QB,CFHR1,APOL1,CPN2,SERPINF2,HBA1,APOA4,ITIH2,C8A,C4BPA,CFHR3,CFH,CD5L,C8G,GSN,C1QC,PROS1,C1S,ORM2,C4A,C4B,CFB,GC,JCHAIN,HPR,C1R,SERPINA3,IGKV2D-28,KNG1,FGA
COMPARTMENTS	GOCC:0043230	Extracellular organelle	65	524	0.96	2.75e-38	CPB2,APOH,VCL,LBP,F9,C5,MPO,VTN,AFM,LTF,APOB,APOA1,TTR,APOE,PXDN,SERPINF1,APCS,VWF,LGALS3BP,YWHAE,CP,AMBP,ITIH4,LUM,AHSG,SERPING1,FCN2,CAMP,HSP90B1,LRG1,FGB,F2,PLG,CSPG4,CALR,SERPINF2,HSPA5,SERPINA5,APOD,PIGR,COL18A1,CFH,QSOX1,S100A9,FLNA,GSN,GPX3,PROS1,EFEMP1,GAPDH,RNASE1,CST3,MASP2,SAA1,F11,C4A,C4B,TGFBI,GC,C1R,SERPINA3,CLTC,KNG1,B2M,FGA
COMPARTMENTS	GOCC:1903561	Extracellular vesicle	61	500	0.96	2.15e-35	CPB2,APOH,VCL,F9,MPO,VTN,AFM,LTF,APOB,APOA1,TTR,APOE,PXDN,SERPINF1,APCS,VWF,LGALS3BP,YWHAE,CP,AMBP,ITIH4,LUM,AHSG,SERPING1,CAMP,HSP90B1,LRG1,FGB,F2,PLG,CSPG4,CALR,SERPINF2,HSPA5,SERPINA5,APOD,PIGR,COL18A1,CFH,QSOX1,S100A9,FLNA,GSN,GPX3,PROS1,EFEMP1,GAPDH,RNASE1,CST3,SAA1,F11,C4A,C4B,TGFBI,GC,C1R,SERPINA3,CLTC,KNG1,B2M,FGA
COMPARTMENTS	GOCC:0065010	Extracellular membrane-bounded organelle	58	473	0.96	1.16e-33	CPB2,APOH,VCL,F9,MPO,VTN,LTF,APOB,APOA1,TTR,APOE,PXDN,SERPINF1,APCS,VWF,LGALS3BP,CP,AMBP,ITIH4,LUM,AHSG,SERPING1,CAMP,HSP90B1,LRG1,FGB,F2,PLG,CSPG4,CALR,SERPINF2,HSPA5,SERPINA5,APOD,PIGR,COL18A1,CFH,QSOX1,S100A9,FLNA,GSN,GPX3,PROS1,GAPDH,RNASE1,CST3,SAA1,F11,C4A,C4B,TGFBI,GC,C1R,SERPINA3,CLTC,KNG1,B2M,FGA
COMPARTMENTS	GOCC:0060205	Cytoplasmic vesicle lumen	43	245	1.11	4.98e-30	SPP2,APOH,VCL,MPO,SPARC,LTF,HRG,APOA1,TTR,CFP,LYZ,VWF,LGALS3BP,ITIH4,AHSG,SERPING1,SERPINI1,PPBP,PF4,CAMP,LRG1,FGB,PLG,SERPINF2,PRDX6,QPCT,F8,QSOX1,F5,S100A7,GSN,PROS1,IGF2,ORM2,ITIH3,RARRES2,SELENOP,SERPINA3,CFD,VEGFC,KNG1,B2M,FGA
COMPARTMENTS	GOCC:0034774	Secretory granule lumen	42	241	1.11	2.82e-29	SPP2,APOH,VCL,MPO,SPARC,LTF,HRG,APOA1,TTR,CFP,LYZ,VWF,LGALS3BP,ITIH4,AHSG,SERPING1,PPBP,PF4,CAMP,LRG1,FGB,PLG,SERPINF2,PRDX6,QPCT,F8,QSOX1,F5,S100A7,GSN,PROS1,IGF2,ORM2,ITIH3,RARRES2,SELENOP,SERPINA3,CFD,VEGFC,KNG1,B2M,FGA

COMPARTMENTS	GOCC:0031982	Vesicle	98	2125	0.53	4.33e-26	SPP2,CPB2,APOH,VCL,CHGA,LBP,F9,MPO,VTN,AFM,SPARC,LTF,HRG,APOB,APOA1,TTR,CAT,LRP1,CFP,APOE,PXDN,SERPINF1,APCS,LYZ,VWF,LGALS3BP,YWHAE,CP,AMBP,TCIRG1,HYAL1,ITIH4,LUM,ITIH1,AHSG,SERPING1,SERPINI1,PPBP,PF4,CAMP,APEH,HSP90B1,ANTXR1,LRG1,PCSK9,FGB,DST,F2,PLG,CSPG4,CALR,SERPINF2,HBA1,HSPA5,SERPINA5,PRDX6,QPCT,APOD,PIGR,COL18A1,F8,CDC42BPA,CFH,QSOX1,F5,S100A7,S100A9,FLNA,GSN,CKAP4,CALML5,GPX3,PROS1,EFEMP1,GAPDH,RNASE1,CST3,SAA1,F11,IGF2,ORM2,C4A,ABCA13,ITIH3,C4B,TGFBI,RARRES2,GC,SELENOP,C1R,SERPINA3,CFD,CLTC,VEGFC,HYOU1,KNG1,B2M,FGA
COMPARTMENTS	GOCC:0070062	Extracellular exosome	45	428	0.89	3.46e-23	VCL,MPO,VTN,AFM,LTF,APOB,APOA1,TTR,APOE,PXDN,SERPINF1,VWF,LGALS3BP,YWHAE,CP,AMBP,ITIH4,AHSG,CAMP,HSP90B1,LRG1,FGB,PLG,CSPG4,CALR,HSPA5,SERPINA5,APOD,COL18A1,CFH,QSOX1,S100A9,FLNA,GSN,GPX3,EFEMP1,GAPDH,CST3,SAA1,C4A,TGFBI,CLTC,KNG1,B2M,FGA
COMPARTMENTS	GOCC:0034358	Plasma lipoprotein particle	22	48	1.53	2.14e-22	CETP,APOH,PON1,APOC3,APOB,APOA1,APOE,LCAT,SAA4,PCSK9,APOL1,LPA,APOA4,APOM,APOF,SAA1,PCYOX1,ADIPOQ,PLTP,HPR,APOA5,APOC4
COMPARTMENTS	GOCC:0005788	Endoplasmic reticulum lumen	31	172	1.13	9.48e-22	RCN1,SPP2,F9,APOB,APOA1,APOE,AHSG,IGFBP1,HSP90B1,PCSK9,APOL1,CALR,HSPA5,FBN1,P4HB,GANAB,APOA4,COL18A1,F8,QSOX1,F5,CKAP4,IGFBP3,NUCB1,C4A,APOA5,HYOU1,KNG1,B2M,FGA,PDIA4
COMPARTMENTS	GOCC:0030141	Secretory granule	53	719	0.74	4.43e-21	SPP2,APOH,VCL,CHGA,MPO,SPARC,LTF,HRG,APOA1,TTR,CAT,CFP,LYZ,VWF,LGALS3BP,TCIRG1,ITIH4,AHSG,SERPING1,PPBP,PF4,CAMP,APEH,LRG1,FGB,PLG,CALR,SERPINF2,SERPINA5,PRDX6,QPCT,PIGR,F8,QSOX1,F5,S100A7,GSN,CKAP4,CALML5,PROS1,CST3,IGF2,ORM2,ABCA13,ITIH3,RARRES2,SELENOP,SERPINA3,CFD,VEGFC,KNG1,B2M,FGA
COMPARTMENTS	GOCC:0034364	High-density lipoprotein particle	18	30	1.65	6.79e-20	CETP,APOH,PON1,APOC3,APOB,APOA1,APOE,LCAT,SAA4,APOL1,APOA4,APOM,APOF,SAA1,PLTP,HPR,APOA5,APOC4
COMPARTMENTS	GOCC:0031093	Platelet alpha granule lumen	21	66	1.37	7.83e-19	SPARC,HRG,VWF,AHSG,SERPING1,PPBP,PF4,FGB,PLG,SERPINF2,F8,QSOX1,F5,PROS1,IGF2,ORM2,SERPINA3,CFD,VEGFC,KNG1,FGA
COMPARTMENTS	GOCC:0031091	Platelet alpha granule	22	89	1.26	8.75e-18	SPARC,HRG,VWF,AHSG,SERPING1,PPBP,PF4,FGB,PLG,SERPINF2,SERPINA5,F8,QSOX1,F5,PROS1,IGF2,ORM2,SERPINA3,CFD,VEGFC,KNG1,FGA
COMPARTMENTS	GOCC:0110165	Cellular anatomical entity	244	14060	0.11	3.37e-14	RCN1,SPP2,ST6GAL1,CPB2,CETP,GNPTG,APOH,VCL,SERPIND1,CHGA,TPD52L2,LBP,APMAP,F9,MMP2,EBI3,COMP,PON1,PCOLCE,C5,MPO,VTN,AFM,PF4V1,APOC3,GPLD1,SPARC,PCDH12,LTF,HRG,APOB,IGFBP5,PROC,PLEK,APOA1,TTR,CAT,LRP1,C4BPB,CFP,DHX29,APOE,PXDN,ANGPTL6,F12,SERPINF1,APCS,CFHR5,LYZ,VWF,PSMA6,DSG2,SERPINA10,LGALS3BP,ATRN,C6,LCN1,LCAT,NID1,YWHAE,CSTA,CP,DGKG,CDH6,DNAH5,AMBP,CENPE,PON3,TCIRG1,HYAL1,ITIH4,LUM,FCN3,ITIH1,AHSG,IGFBP1,CRH,HABP2,SAA4,SERPING1,CSF1R,PCDH1,FCN2,PGLYRP2,SCGB3A1,IHH,SERPINI1,PPBP,PF4,MASP1,CAMP,APEH,GUCY1A1,C16orf46,A2ML1,HSP90B1,ANTXR1,LRG1,SH3D19,PCSK9,BMP1,SHLD1,FGB,DST,F2,INHBC,PLG,EFEMP2,CSPG4,C1QB,CFHR1,SUZ12,APOL1,CPN2,CALR,LPA,SERPINF2,C7,HBA1,ITLN1,HSPA5,FBN1,P4HB,SERPINA7,FBLN1,SERPINA5,DNAH8,ANG,LIMS1,THBS4,GANAB,PRDX6,PROZ,QPCT,APOD,ASGR2,BLM,AKAP9,PIGR,APOA4,ITIH2,CNDP1,COL18A1,MTCL1,F8,C8A,CDC42BPA,C4BPA,F13B,CFHR2,CFHR4,CFHR3,CFH,QSOX1,F5,CD5L,S100A7,S100A9,FLNA,CRTAC1,MCOLN2,SMARCA1,C8B,C8G,ADAMTS13,MINPP1,GSN,MBL2,SVEP1,C1QC,F10,APOM,CA6,CKAP4,CALML5,TAF9,IGFBP3,CD99,SOD3,GPX3,KIF19,PROS1,EFEMP1,CFI,TNRC6A,NAV2,ILK,GAPDH,RNASE1,FGL1,APOF,CST3,MAP3K7CL,MASP2,FBLN2,SAA1,F11,C1S,NUCB1,PCYOX1,ADIPOQ,IGF2,ORM2,C4A,FSIP2,ACSF2,ABCA13,COLEC11,MST1,LMOD3,SPARCL1,ITIH3,C4B,TGFBI,CFB,IGFALS,PLTP,PAEP,RARRES2,GC,HGFAC,UIMC1,SELENOP,JCHAIN,HPR,C1R,APOA5,ATP1A1,SERPINA3,RNASE4,IGKV2D-28,APOC4,CFD,CLTC,VEGFC,HYOU1,KNG1,TRIM66,GRHL2,CDH5,B2M,FGA,PDIA4,SDF4
COMPARTMENTS	GOCC:0034361	Very-low-density lipoprotein particle	12	20	1.65	4.55e-13	CETP,APOH,APOC3,APOB,APOA1,APOE,APOL1,APOA4,APOM,PCYOX1,APOA5,APOC4
COMPARTMENTS	GOCC:0031012	Extracellular matrix	26	259	0.87	1.18e-12	CPB2,MMP2,COMP,VTN,GPLD1,SPARC,APOE,PXDN,VWF,NID1,LUM,HSP90B1,DST,PLG,EFEMP2,FBN1,FBLN1,ANG,THBS4,COL18A1,ADAMTS13,SOD3,EFEMP1,FBLN2,TGFBI,RARRES2
COMPARTMENTS	GOCC:0031410	Cytoplasmic vesicle	66	1738	0.45	1.67e-12	SPP2,APOH,VCL,CHGA,MPO,SPARC,LTF,HRG,APOB,APOA1,TTR,CAT,LRP1,CFP,APOE,LYZ,VWF,LGALS3BP,TCIRG1,HYAL1,ITIH4,AHSG,SERPING1,SERPINI1,PPBP,PF4,CAMP,APEH,HSP90B1,ANTXR1,LRG1,PCSK9,FGB,DST,PLG,CALR,SERPINF2,HBA1,SERPINA5,PRDX6,QPCT,PIGR,F8,QSOX1,F5,S100A7,GSN,CKAP4,CALML5,PROS1,CST3,SAA1,IGF2,ORM2,ABCA13,ITIH3,RARRES2,SELENOP,SERPINA3,CFD,CLTC,VEGFC,HYOU1,KNG1,B2M,FGA
COMPARTMENTS	GOCC:0012505	Endomembrane system	94	3156	0.34	1.87e-12	RCN1,SPP2,ST6GAL1,APOH,VCL,CHGA,F9,MPO,SPARC,LTF,HRG,APOB,PROC,APOA1,TTR,CAT,LRP1,CFP,APOE,PXDN,LYZ,VWF,LGALS3BP,CP,TCIRG1,ITIH4,LUM,AHSG,IGFBP1,SERPING1,PPBP,PF4,CAMP,APEH,HSP90B1,ANTXR1,LRG1,PCSK9,FGB,F2,PLG,CSPG4,APOL1,CALR,SERPINF2,HSPA5,FBN1,P4HB,SERPINA5,THBS4,GANAB,PRDX6,PROZ,QPCT,ASGR2,AKAP9,PIGR,APOA4,COL18A1,F8,QSOX1,F5,S100A7,GSN,F10,CKAP4,CALML5,IGFBP3,SOD3,PROS1,TNRC6A,GAPDH,CST3,NUCB1,ADIPOQ,IGF2,ORM2,C4A,ABCA13,ITIH3,RARRES2,SELENOP,APOA5,SERPINA3,CFD,CLTC,VEGFC,HYOU1,KNG1,CDH5,B2M,FGA,PDIA4,SDF4
COMPARTMENTS	GOCC:0030312	External encapsulating structure	25	259	0.85	7.60e-12	MMP2,COMP,VTN,GPLD1,SPARC,HRG,APOE,PXDN,VWF,NID1,LUM,HSP90B1,DST,PLG,EFEMP2,FBN1,FBLN1,ANG,THBS4,COL18A1,ADAMTS13,SOD3,EFEMP1,FBLN2,RARRES2
COMPARTMENTS	GOCC:0070013	Intracellular organelle lumen	87	2902	0.35	1.88e-11	RCN1,SPP2,APOH,VCL,F9,MPO,SPARC,LTF,HRG,APOB,PROC,APOA1,TTR,CAT,CFP,APOE,LYZ,VWF,LGALS3BP,HYAL1,ITIH4,LUM,AHSG,IGFBP1,SERPING1,SERPINI1,PPBP,PF4,CAMP,APEH,HSP90B1,LRG1,PCSK9,FGB,F2,PLG,CSPG4,SUZ12,APOL1,CALR,SERPINF2,HBA1,HSPA5,FBN1,P4HB,ANG,GANAB,PRDX6,PROZ,QPCT,BLM,APOA4,COL18A1,F8,QSOX1,F5,S100A7,SMARCA1,GSN,F10,CKAP4,CALML5,TAF9,IGFBP3,SOD3,PROS1,GAPDH,CST3,SAA1,NUCB1,IGF2,ORM2,C4A,ITIH3,RARRES2,GC,UIMC1,SELENOP,APOA5,SERPINA3,CFD,VEGFC,HYOU1,KNG1,B2M,FGA,PDIA4
COMPARTMENTS	GOCC:0071682	Endocytic vesicle lumen	11	23	1.55	3.00e-11	MPO,SPARC,LTF,APOB,APOA1,APOE,HSP90B1,CALR,HBA1,SAA1,HYOU1
COMPARTMENTS	GOCC:0062023	Collagen-containing extracellular matrix	21	198	0.9	1.28e-10	MMP2,COMP,VTN,SPARC,APOE,PXDN,VWF,NID1,LUM,HSP90B1,DST,PLG,EFEMP2,FBN1,ANG,THBS4,COL18A1,SOD3,FBLN2,TGFBI,RARRES2
COMPARTMENTS	GOCC:0062167	Complement component C1q complex	8	10	1.77	2.36e-09	SERPING1,C1QB,MBL2,C1QC,C1S,C4A,C4B,C1R
COMPARTMENTS	GOCC:0005579	Membrane attack complex	8	12	1.69	6.45e-09	C5,C6,C7,C8A,C8B,C8G,MBL2,C4A
COMPARTMENTS	GOCC:0034362	Low-density lipoprotein particle	9	22	1.48	1.15e-08	CETP,APOB,APOA1,APOE,PCSK9,LPA,APOM,APOF,ADIPOQ

COMPARTMENTS	GOCC:0032991	Protein-containing complex	118	5325	0.22	8.18e-08	CPB2,CETP,GNPTG,APOH,VCL,F9,COMP,PON1,C5,VTN,APOC3,LTF,APOB,IGFBP5,APOA1,CAT,LRP1,DHX29,APOE,PXDN,VWF,PSMA6,C6,LCAT,NID1,CSTA,CDH6,DNAH5,LUM,FCN3,IGFBP1,SAA4,SERPING1,CSF1R,FCN2,MASP1,APEH,GUCY1A1,HSP90B1,PCSK9,FGB,DST,F2,PLG,UBE3C,C1QB,CFHR1,SUZ12,APOL1,SUMO4,CALR,LPA,SERPINF2,C7,HBA1,ITLN1,HSPA5,FBN1,P4HB,SERPINA5,DNAH8,ANG,GANAB,BLM,AKAP9,PIGR,APOA4,COL18A1,C8A,CFHR2,S100A7,S100A9,FLNA,SMARCA1,C8B,C8G,ADAMTS13,MBL2,SVEP1,C1QC,APOM,CALML5,TAF9,IGFBP3,GPX3,KIF19,TNRC6A,GAPDH,FGL1,APOF,CST3,MASP2,SAA1,F11,C1S,PCYOX1,ADIPOQ,IGF2,C4A,CDK3,C4B,CFB,IGFALS,PLTP,UIMC1,JCHAIN,HPR,C1R,APOA5,ATP1A1,APOC4,CLTC,IGHV3-15,VEGFC,HYOU1,CDH5,B2M,FGA
COMPARTMENTS	GOCC:0009986	Cell surface	25	438	0.63	2.11e-07	APOH,LBP,APMAP,SPARC,LTF,HRG,PXDN,DSG2,CSF1R,ANTXR1,PCSK9,FGB,PLG,CSPG4,CALR,SERPINF2,P4HB,SERPINA5,CD5L,ADAMTS13,MBL2,ADIPOQ,CDH5,FGA,PDIA4
COMPARTMENTS	GOCC:0031089	Platelet dense granule lumen	7	14	1.57	4.03e-07	SPP2,APOH,LGALS3BP,ITIH4,ITIH3,RARRES2,SELENOP
COMPARTMENTS	GOCC:0042627	Chylomicron	7	14	1.57	4.03e-07	APOH,APOC3,APOB,APOA1,APOE,APOA4,APOA5
COMPARTMENTS	GOCC:1904724	Tertiary granule lumen	10	55	1.13	7.85e-07	LTF,CFP,LYZ,PPBP,CAMP,LRG1,QPCT,QSOX1,CST3,B2M
COMPARTMENTS	GOCC:0005604	Basement membrane	10	61	1.08	1.84e-06	VTN,SPARC,PXDN,NID1,DST,EFEMP2,FBN1,ANG,THBS4,COL18A1
COMPARTMENTS	GOCC:0035580	Specific granule lumen	10	62	1.08	2.07e-06	VCL,LTF,CFP,LYZ,CAMP,LRG1,QPCT,QSOX1,ORM2,B2M
COMPARTMENTS	GOCC:0005577	Fibrinogen complex	7	20	1.41	2.55e-06	VWF,FGB,F2,PLG,SERPINF2,FGL1,FGA
COMPARTMENTS	GOCC:0005783	Endoplasmic reticulum	39	1095	0.42	2.59e-06	RCN1,SPP2,F9,APOB,PROC,APOA1,APOE,PXDN,VWF,CP,AHSG,IGFBP1,HSP90B1,PCSK9,APOL1,CALR,HSPA5,FBN1,P4HB,THBS4,GANAB,ASGR2,APOA4,COL18A1,F8,QSOX1,F5,S100A7,CKAP4,IGFBP3,NUCB1,ADIPOQ,C4A,APOA5,HYOU1,KNG1,B2M,FGA,PDIA4
COMPARTMENTS	GOCC:0034366	Spherical high-density lipoprotein particle	6	11	1.61	2.89e-06	PON1,APOC3,APOA1,LCAT,APOM,HPR
COMPARTMENTS	GOCC:1905286	Serine-type peptidase complex	6	11	1.61	2.89e-06	F9,FCN3,FCN2,MASP1,MASP2,F11
COMPARTMENTS	GOCC:1905370	Serine-type endopeptidase complex	5	7	1.72	1.37e-05	FCN3,FCN2,MASP1,MASP2,F11
COMPARTMENTS	GOCC:0062136	Low-density lipoprotein receptor complex	5	9	1.61	3.31e-05	APOB,APOA1,LRP1,APOE,PCSK9
COMPARTMENTS	GOCC:0016942	Insulin-like growth factor binding protein complex	5	10	1.57	4.73e-05	IGFBP5,IGFBP1,IGFBP3,IGF2,IGFALS
COMPARTMENTS	GOCC:0005925	Focal adhesion	16	269	0.64	6.46e-05	VCL,LRP1,YWHAE,HSP90B1,DST,CSPG4,CALR,HSPA5,P4HB,LIMS1,S100A7,FLNA,GSN,ILK,CLTC,B2M
COMPARTMENTS	GOCC:0005796	Golgi lumen	10	102	0.86	0.00010	F9,PROC,LUM,F2,CSPG4,PROZ,F8,F10,SOD3,PROS1
COMPARTMENTS	GOCC:0005602	Complement component C1 complex	4	5	1.77	0.00015	C1QB,C1QC,C1S,C1R
COMPARTMENTS	GOCC:0005764	Lysosome	23	566	0.48	0.00018	GNPTG,MPO,APOB,TTR,LRP1,LYZ,CP,TCIRG1,HYAL1,LUM,PCSK9,CSPG4,CALR,PRDX6,PIGR,S100A7,CKAP4,PCYOX1,ORM2,ABCA13,GC,SERPINA3,CLTC
COMPARTMENTS	GOCC:0030139	Endocytic vesicle	15	260	0.63	0.00018	MPO,SPARC,LTF,APOB,APOA1,LRP1,APOE,TCIRG1,HSP90B1,CALR,HBA1,SAA1,CLTC,HYOU1,B2M
COMPARTMENTS	GOCC:0005775	Vacuolar lumen	12	168	0.72	0.00021	MPO,APOB,TTR,LYZ,HYAL1,LUM,CSPG4,PRDX6,S100A7,ORM2,GC,SERPINA3
COMPARTMENTS	GOCC:0034365	Discoidal high-density lipoprotein particle	4	6	1.69	0.00023	APOA1,APOE,LCAT,APOM
COMPARTMENTS	GOCC:0036454	Growth factor complex	6	31	1.16	0.00032	IGFBP5,IGFBP1,IGFBP3,IGF2,IGFALS,VEGFC
COMPARTMENTS	GOCC:1904090	Peptidase inhibitor complex	5	17	1.34	0.00032	VTN,PXDN,CSTA,SERPINA5,GAPDH
COMPARTMENTS	GOCC:0005773	Vacuole	24	640	0.44	0.00035	GNPTG,MPO,APOB,TTR,LRP1,LYZ,CP,TCIRG1,HYAL1,LUM,PCSK9,CSPG4,CALR,PRDX6,PIGR,S100A7,MINPP1,CKAP4,PCYOX1,ORM2,ABCA13,GC,SERPINA3,CLTC
COMPARTMENTS	GOCC:0070161	Anchoring junction	22	553	0.47	0.00035	VCL,LRP1,DSG2,YWHAE,PCDH1,HSP90B1,DST,CSPG4,CALR,HSPA5,P4HB,LIMS1,CDC42BPA,S100A7,FLNA,GSN,CD99,ILK,CLTC,GRHL2,CDH5,B2M
COMPARTMENTS	GOCC:0034363	Intermediate-density lipoprotein particle	4	8	1.57	0.00048	APOC3,APOB,APOA1,APOE
COMPARTMENTS	GOCC:0042581	Specific granule	11	158	0.71	0.00058	VCL,LTF,CFP,LYZ,CAMP,LRG1,QPCT,QSOX1,CKAP4,ORM2,B2M
COMPARTMENTS	GOCC:0070820	Tertiary granule	11	164	0.7	0.00080	LTF,CFP,LYZ,TCIRG1,PPBP,CAMP,LRG1,QPCT,QSOX1,CST3,B2M
COMPARTMENTS	GOCC:1905368	Peptidase complex	10	136	0.74	0.00088	F9,PSMA6,FCN3,FCN2,MASP1,F2,UBE3C,TAF9,MASP2,F11
COMPARTMENTS	GOCC:0098797	Plasma membrane protein complex	24	682	0.42	0.00089	C5,VTN,APOB,APOA1,LRP1,APOE,VWF,C6,CDH6,PCSK9,DST,C7,AKAP9,C8A,FLNA,C8B,C8G,MBL2,SVEP1,C4A,ATP1A1,CLTC,CDH5,B2M
COMPARTMENTS	GOCC:0034663	Endoplasmic reticulum chaperone complex	4	11	1.43	0.0012	HSP90B1,HSPA5,P4HB,HYOU1
COMPARTMENTS	GOCC:0071944	Cell periphery	79	3860	0.18	0.0019	VCL,MMP2,COMP,C5,VTN,GPLD1,SPARC,PCDH12,HRG,APOB,PLEK,APOA1,LRP1,APOE,PXDN,VWF,DSG2,ATRN,C6,NID1,CSTA,CDH6,AMBP,TCIRG1,LUM,CSF1R,PCDH1,IHH,HSP90B1,ANTXR1,PCSK9,FGB,DST,PLG,EFEMP2,CSPG4,C7,ITLN1,FBN1,P4HB,FBLN1,SERPINA5,ANG,LIMS1,THBS4,ASGR2,AKAP9,PIGR,COL18A1,MTCL1,C8A,C4BPA,S100A9,FLNA,MCOLN2,C8B,C8G,ADAMTS13,GSN,MBL2,SVEP1,SOD3,GPX3,PROS1,EFEMP1,ILK,GAPDH,FBLN2,C4A,ABCA13,C4B,TGFBI,RARRES2,ATP1A1,CLTC,KNG1,CDH5,B2M,FGA
COMPARTMENTS	GOCC:0042582	Azurophil granule	10	154	0.68	0.0022	MPO,TTR,LYZ,PRDX6,PIGR,S100A7,CKAP4,ORM2,ABCA13,SERPINA3
COMPARTMENTS	GOCC:1904813	ficolin-1-rich granule lumen	9	124	0.73	0.0023	VCL,CAT,APEH,LRG1,QPCT,GSN,CALML5,CST3,CFD
COMPARTMENTS	GOCC:0005601	Classical-complement-pathway C3/C5 convertase complex	3	4	1.74	0.0024	C4A,C4B,CFB
COMPARTMENTS	GOCC:0042567	Insulin-like growth factor ternary complex	3	4	1.74	0.0024	IGFBP5,IGFBP3,IGFALS
COMPARTMENTS	GOCC:0043227	Membrane-bounded organelle	153	9083	0.1	0.0039	RCN1,SPP2,ST6GAL1,CPB2,GNPTG,APOH,VCL,CHGA,LBP,F9,MMP2,PON1,PCOLCE,MPO,VTN,AFM,GPLD1,SPARC,LTF,HRG,APOB,PROC,APOA1,TTR,CAT,LRP1,CFP,APOE,PXDN,SERPINF1,APCS,LYZ,VWF,PSMA6,LGALS3BP,LCAT,YWHAE,CSTA,CP,DNAH5,AMBP,CENPE,PON3,TCIRG1,HYAL1,ITIH4,LUM,ITIH1,AHSG,IGFBP1,SERPING1,SERPINI1,PPBP,PF4,CAMP,APEH,C16orf46,HSP90B1,ANTXR1,LRG1,SH3D19,PCSK9,FGB,DST,F2,PLG,CSPG4,SUZ12,APOL1,CALR,SERPINF2,HBA1,HSPA5,FBN1,P4HB,SERPINA5,DNAH8,ANG,THBS4,GANAB,PRDX6,PROZ,QPCT,APOD,ASGR2,BLM,AKAP9,PIGR,APOA4,COL18A1,F8,CDC42BPA,CFH,QSOX1,F5,S100A7,S100A9,FLNA,SMARCA1,MINPP1,GSN,SVEP1,F10,CKAP4,CALML5,TAF9,IGFBP3,CD99,SOD3,GPX3,PROS1,EFEMP1,TNRC6A,NAV2,ILK,GAPDH,RNASE1,CST3,MASP2,SAA1,F11,NUCB1,PCYOX1,ADIPOQ,IGF2,ORM2,C4A,FSIP2,ACSF2,ABCA13,ITIH3,C4B,TGFBI,PLTP,RARRES2,GC,UIMC1,SELENOP,C1R,APOA5,SERPINA3,CFD,CLTC,VEGFC,HYOU1,KNG1,TRIM66,GRHL2,CDH5,B2M,FGA,PDIA4,SDF4
COMPARTMENTS	GOCC:1905369	Endopeptidase complex	7	90	0.76	0.0091	PSMA6,FCN3,FCN2,MASP1,UBE3C,MASP2,F11
COMPARTMENTS	GOCC:0035578	Azurophil granule lumen	7	91	0.76	0.0095	MPO,TTR,LYZ,PRDX6,S100A7,ORM2,SERPINA3
COMPARTMENTS	GOCC:0071953	Elastic fiber	3	8	1.44	0.0099	EFEMP2,FBN1,FBLN1
COMPARTMENTS	GOCC:0071751	Secretory IgA immunoglobulin complex	2	2	1.87	0.0286	PIGR,JCHAIN
COMPARTMENTS	GOCC:0030054	Cell junction	27	1053	0.28	0.0340	VCL,LRP1,APOE,DSG2,YWHAE,CDH6,PCDH1,HSP90B1,DST,CSPG4,CALR,HSPA5,P4HB,LIMS1,AKAP9,CDC42BPA,S100A7,FLNA,GSN,CD99,GPX3,ILK,C4A,CLTC,GRHL2,CDH5,B2M
COMPARTMENTS	GOCC:0097179	Protease inhibitor complex	3	14	1.2	0.0362	PXDN,SERPINA5,GAPDH

COMPARTMENTS	GOCC:0043226	Organelle	161	10113	0.07	0.0441	RCN1,SPP2,ST6GAL1,CPB2,GNPTG,APOH,VCL,CHGA,LBP,F9,MMP2,PON1,PCOLCE,C5,MPO,VTN,AFM,GPLD1,SPARC,LTF,HRG,APOB,PROC,APOA1,TTR,CAT,LRP1,CFP,DHX29,APOE,PXDN,SERPINF1,APCS,LYZ,VWF,PSMA6,LGALS3BP,LCAT,YWHAE,CSTA,CP,DNAH5,AMBP,CENPE,PON3,TCIRG1,HYAL1,ITIH4,LUM,ITIH1,AHSG,IGFBP1,SERPING1,FCN2,SERPINI1,PPBP,PF4,CAMP,APEH,C16orf46,HSP90B1,ANTXR1,LRG1,SH3D19,PCSK9,SHLD1,FGB,DST,F2,PLG,CSPG4,SUZ12,APOL1,CALR,SERPINF2,HBA1,HSPA5,FBN1,P4HB,SERPINA5,DNAH8,ANG,THBS4,GANAB,PRDX6,PROZ,QPCT,APOD,ASGR2,BLM,AKAP9,PIGR,APOA4,COL18A1,MTCL1,F8,CDC42BPA,CFH,QSOX1,F5,S100A7,S100A9,FLNA,SMARCA1,MINPP1,GSN,SVEP1,F10,CKAP4,CALML5,TAF9,IGFBP3,CD99,SOD3,GPX3,KIF19,PROS1,EFEMP1,TNRC6A,NAV2,ILK,GAPDH,RNASE1,CST3,MASP2,SAA1,F11,NUCB1,PCYOX1,ADIPOQ,IGF2,ORM2,C4A,FSIP2,ACSF2,ABCA13,LMOD3,ITIH3,C4B,TGFBI,PLTP,RARRES2,GC,UIMC1,SELENOP,C1R,APOA5,ATP1A1,SERPINA3,CFD,CLTC,VEGFC,HYOU1,KNG1,TRIM66,GRHL2,CDH5,B2M,FGA,PDIA4,SDF4
UniProt Keywords	KW-0964	Secreted	178	1839	0.86	6.24e-110	SPP2,ST6GAL1,CPB2,CETP,GNPTG,APOH,CHGA,LBP,F9,MMP2,EBI3,COMP,PON1,PCOLCE,C5,VTN,AFM,PF4V1,APOC3,GPLD1,SPARC,PCDH12,LTF,HRG,APOB,IGFBP5,PROC,APOA1,TTR,C4BPB,CFP,APOE,PXDN,ANGPTL6,F12,SERPINF1,APCS,CFHR5,LYZ,VWF,SERPINA10,LGALS3BP,ATRN,C6,LCN1,LCAT,NID1,CP,AMBP,PON3,HYAL1,ITIH4,LUM,FCN3,ITIH1,AHSG,IGFBP1,CRH,HABP2,SAA4,SERPING1,FCN2,PGLYRP2,SCGB3A1,IHH,SERPINI1,PPBP,PF4,CAMP,A2ML1,LRG1,PCSK9,BMP1,FGB,F2,INHBC,PLG,EFEMP2,C1QB,CFHR1,APOL1,PSG11,CPN2,CALR,SERPINF2,C7,ITLN1,FBN1,SERPINA7,FBLN1,SERPINA5,SERPINA11,ANG,THBS4,PROZ,QPCT,APOD,PAPLN,PIGR,APOA4,ITIH2,CNDP1,COL18A1,F8,C8A,C4BPA,F13B,CFHR2,CFHR4,CFHR3,CFH,QSOX1,F5,CD5L,S100A7,S100A9,ADAMTSL4,CRTAC1,C8B,C8G,ADAMTS13,GSN,MBL2,SVEP1,C1QC,F10,APOM,CA6,IGFBP3,SOD3,GPX3,PROS1,EFEMP1,CFI,RNASE1,FGL1,APOF,CST3,MASP2,FBLN2,PSG4,SAA1,F11,PSG2,NUCB1,ADIPOQ,IGF2,ORM2,C4A,COLEC11,MST1,SPARCL1,ITIH3,C4B,TGFBI,CFB,IGFALS,PLTP,PAEP,RARRES2,GC,HGFAC,SELENOP,JCHAIN,HPR,C1R,APOA5,SERPINA3,RNASE4,IGKV2D-28,APOC4,CFD,IGHV3-15,IGHV3-72,VEGFC,KNG1,B2M,FGA
UniProt Keywords	KW-0732	Signal	199	3277	0.65	1.16e-92	RCN1,SPP2,CPB2,CETP,GNPTG,APOH,SERPIND1,CHGA,LBP,F9,MMP2,EBI3,COMP,PON1,PCOLCE,C5,MPO,VTN,AFM,PF4V1,APOC3,GPLD1,SPARC,PCDH12,LTF,HRG,APOB,IGFBP5,PROC,APOA1,TTR,LRP1,C4BPB,CFP,APOE,PXDN,ANGPTL6,F12,SERPINF1,APCS,CFHR5,LYZ,VWF,DSG2,LGALS3BP,ATRN,C6,LCN1,LCAT,NID1,CP,CDH6,AMBP,PON3,HYAL1,ITIH4,LUM,FCN3,ITIH1,AHSG,IGFBP1,CRH,HABP2,SAA4,SERPING1,CSF1R,PCDH1,FCN2,PGLYRP2,SCGB3A1,IHH,SERPINI1,PPBP,PF4,CAMP,A2ML1,HSP90B1,ANTXR1,LRG1,PCSK9,BMP1,FGB,F2,INHBC,PLG,EFEMP2,CSPG4,C1QB,CFHR1,APOL1,PSG11,CPN2,CALR,LPA,SERPINF2,C7,ITLN1,HSPA5,FBN1,P4HB,SERPINA7,FBLN1,SERPINA5,OIT3,SERPINA11,ANG,THBS4,GANAB,PROZ,QPCT,APOD,PAPLN,PIGR,APOA4,ITIH2,CNDP1,COL18A1,F8,C8A,PCDH18,C4BPA,F13B,CFHR2,CFHR4,CFHR3,CFH,QSOX1,F5,CD5L,ADAMTSL4,CRTAC1,C8B,C8G,ADAMTS13,MINPP1,GSN,MBL2,SVEP1,C1QC,F10,APOM,CA6,IGFBP3,CD99,SOD3,GPX3,PROS1,EFEMP1,CFI,RNASE1,FGL1,APOF,CST3,MASP2,FBLN2,PSG4,SAA1,F11,C1S,PSG2,NUCB1,PCYOX1,ADIPOQ,IGF2,ORM2,C4A,COLEC11,MST1,SPARCL1,ITIH3,C4B,TGFBI,CFB,IGFALS,PLTP,PAEP,RARRES2,GC,HGFAC,SELENOP,JCHAIN,HPR,C1R,APOA5,SERPINA3,RNASE4,IGKV2D-28,APOC4,CFD,IGHV3-15,IGHV3-72,VEGFC,HYOU1,KNG1,CDH5,B2M,FGA,PDIA4,SDF4
UniProt Keywords	KW-1015	Disulfide bond	162	3338	0.56	1.55e-54	SPP2,ST6GAL1,CPB2,CETP,GNPTG,APOH,CHGA,LBP,F9,MMP2,COMP,PON1,PCOLCE,C5,MPO,VTN,AFM,PF4V1,SPARC,LTF,HRG,APOB,IGFBP5,PROC,LRP1,C4BPB,CFP,PXDN,ANGPTL6,F12,APCS,CFHR5,LYZ,VWF,LGALS3BP,ATRN,C6,LCN1,LCAT,NID1,CP,AMBP,PON3,HYAL1,ITIH4,LUM,FCN3,ITIH1,AHSG,IGFBP1,HABP2,SERPING1,CSF1R,FCN2,PGLYRP2,SCGB3A1,PPBP,PF4,MASP1,CAMP,A2ML1,HSP90B1,ANTXR1,LRG1,PCSK9,BMP1,FGB,F2,INHBC,PLG,EFEMP2,CSPG4,C1QB,CFHR1,PSG11,CPN2,CALR,LPA,SERPINF2,C7,ITLN1,FBN1,P4HB,FBLN1,OIT3,ANG,THBS4,GANAB,PROZ,QPCT,APOD,PAPLN,ASGR2,PIGR,ITIH2,COL18A1,F8,C8A,C4BPA,F13B,CFHR2,CFHR4,CFHR3,CFH,QSOX1,F5,CD5L,S100A7,CRTAC1,MCOLN2,C8B,C8G,ADAMTS13,GSN,MBL2,SVEP1,C1QC,F10,APOM,CA6,IGFBP3,SOD3,PROS1,EFEMP1,CFI,RNASE1,FGL1,CST3,MASP2,FBLN2,PSG4,F11,C1S,PSG2,ADIPOQ,IGF2,ORM2,C4A,COLEC11,MST1,SPARCL1,C4B,TGFBI,CFB,PLTP,PAEP,RARRES2,GC,HGFAC,JCHAIN,HPR,C1R,RNASE4,IGKV2D-28,CFD,IGHV3-15,IGHV3-72,VEGFC,KNG1,B2M,FGA,PDIA4
UniProt Keywords	KW-0325	Glycoprotein	172	4386	0.46	2.25e-46	RCN1,ST6GAL1,CPB2,CETP,GNPTG,APOH,SERPIND1,CHGA,LBP,APMAP,F9,MMP2,EBI3,COMP,PON1,PCOLCE,C5,MPO,VTN,AFM,PF4V1,APOC3,GPLD1,SPARC,PCDH12,LTF,HRG,APOB,IGFBP5,PROC,APOA1,TTR,LRP1,C4BPB,CFP,APOE,PXDN,ANGPTL6,F12,SERPINF1,APCS,CFHR5,VWF,PSMA6,DSG2,SERPINA10,LGALS3BP,ATRN,C6,LCAT,NID1,CP,CDH6,AMBP,PON3,HYAL1,ITIH4,LUM,FCN3,ITIH1,AHSG,HABP2,SAA4,SERPING1,CSF1R,PCDH1,FCN2,PGLYRP2,IHH,SERPINI1,A2ML1,HSP90B1,ANTXR1,LRG1,PCSK9,BMP1,FGB,F2,INHBC,PLG,EFEMP2,CSPG4,C1QB,CFHR1,APOL1,PSG11,CPN2,CALR,LPA,SERPINF2,C7,ITLN1,FBN1,SERPINA7,FBLN1,SERPINA5,OIT3,SERPINA11,THBS4,GANAB,PROZ,QPCT,APOD,ASGR2,PIGR,ITIH2,CNDP1,COL18A1,F8,C8A,PCDH18,C4BPA,F13B,CFHR2,CFHR4,CFHR3,CFH,QSOX1,F5,CD5L,ADAMTSL4,CRTAC1,C8B,ADAMTS13,MINPP1,SVEP1,C1QC,F10,APOM,CA6,IGFBP3,CD99,SOD3,PROS1,EFEMP1,CFI,RNASE1,APOF,CST3,FBLN2,PSG4,F11,C1S,PSG2,NUCB1,PCYOX1,ADIPOQ,IGF2,ORM2,C4A,MST1,SPARCL1,ITIH3,C4B,CFB,IGFALS,PLTP,PAEP,GC,HGFAC,SELENOP,JCHAIN,C1R,SERPINA3,APOC4,VEGFC,HYOU1,KNG1,CDH5,B2M,FGA,SDF4
UniProt Keywords	KW-0106	Calcium	61	886	0.71	3.29e-23	RCN1,CHGA,F9,MMP2,COMP,PON1,MPO,SPARC,PCDH12,PROC,LRP1,PXDN,APCS,DSG2,NID1,DGKG,CDH6,PON3,FCN3,PCDH1,FCN2,IHH,HSP90B1,PCSK9,BMP1,DST,F2,EFEMP2,CALR,ITLN1,FBN1,FBLN1,OIT3,THBS4,PROZ,ASGR2,F8,PCDH18,F5,S100A7,S100A9,MCOLN2,ADAMTS13,GSN,MBL2,SVEP1,F10,CALML5,PROS1,EFEMP1,CFI,EFCAB5,MASP2,FBLN2,C1S,NUCB1,COLEC11,SPARCL1,CDH5,FGA,SDF4
UniProt Keywords	KW-0094	Blood coagulation	22	46	1.55	5.97e-23	CPB2,SERPIND1,F9,HRG,PROC,F12,VWF,SERPINA10,SERPING1,FGB,F2,PLG,PROZ,F8,F13B,F5,ADAMTS13,F10,PROS1,F11,KNG1,FGA
UniProt Keywords	KW-0180	Complement pathway	18	30	1.65	4.21e-20	C5,C4BPB,C6,SERPING1,C1QB,C7,C8A,C4BPA,C8B,C8G,MBL2,C1QC,CFI,MASP2,C1S,C4A,C4B,C1R
UniProt Keywords	KW-0245	EGF-like domain	31	232	1.0	1.25e-18	F9,COMP,PROC,LRP1,F12,ATRN,C6,NID1,HYAL1,HABP2,MASP1,BMP1,EFEMP2,C7,FBN1,FBLN1,OIT3,THBS4,PROZ,C8A,CRTAC1,C8B,SVEP1,F10,PROS1,EFEMP1,MASP2,FBLN2,C1S,HGFAC,C1R
UniProt Keywords	KW-0768	Sushi	19	57	1.39	1.59e-17	APOH,C4BPB,CFHR5,C6,MASP1,CFHR1,C7,C4BPA,F13B,CFHR2,CFHR4,CFHR3,CFH,SVEP1,MASP2,C1S,CFB,HPR,C1R

UniProt Keywords	KW-0391	Immunity	39	537	0.73	3.39e-15	LBP,C5,LTF,C4BPB,CFP,C6,FCN3,SERPING1,CSF1R,FCN2,PGLYRP2,FGB,C1QB,C7,C8A,C4BPA,CFH,CD5L,S100A9,MCOLN2,C8B,C8G,MBL2,C1QC,CFI,FGL1,MASP2,C1S,C4A,COLEC11,C4B,CFB,C1R,IGKV2D-28,CFD,IGHV3-15,IGHV3-72,B2M,FGA
UniProt Keywords	KW-0165	Cleavage on pair of basic residues	29	280	0.88	8.57e-15	CHGA,F9,C5,HRG,PROC,VWF,DSG2,CDH6,AMBP,CRH,HABP2,PPBP,CAMP,BMP1,F2,INHBC,PLG,PROZ,C8A,ADAMTS13,F10,PROS1,CFI,IGF2,C4A,C4B,CFB,VEGFC,CDH5
UniProt Keywords	KW-0399	Innate immunity	31	329	0.84	8.57e-15	LBP,C5,C4BPB,CFP,C6,FCN3,SERPING1,CSF1R,FCN2,FGB,C1QB,C7,C8A,C4BPA,CFH,S100A9,MCOLN2,C8B,C8G,MBL2,C1QC,CFI,MASP2,C1S,C4A,COLEC11,C4B,CFB,C1R,CFD,FGA
UniProt Keywords	KW-0677	Repeat	127	4794	0.29	9.22e-15	RCN1,APOH,VCL,SERPIND1,F9,MMP2,EBI3,COMP,PCOLCE,VTN,AFM,GPLD1,PCDH12,LTF,HRG,PROC,PLEK,APOA1,LRP1,C4BPB,CFP,APOE,PXDN,F12,CFHR5,VWF,DSG2,ATRN,C6,NID1,CP,DGKG,CDH6,DNAH5,AMBP,LUM,FCN3,AHSG,ANKRD31,HABP2,SERPING1,ANKRD44,CSF1R,PCDH1,FCN2,MASP1,LRG1,SH3D19,BMP1,DST,F2,PLG,EFEMP2,CSPG4,C1QB,CFHR1,PSG11,CPN2,CALR,LPA,C7,FBN1,P4HB,FBLN1,DNAH8,LIMS1,THBS4,PROZ,PAPLN,PIGR,APOA4,COL18A1,F8,C8A,PCDH18,C4BPA,F13B,CFHR2,CFHR4,CFHR3,CFH,F5,CD5L,S100A7,S100A9,ADAMTSL4,FLNA,CRTAC1,SMARCA1,C8B,ADAMTS13,GSN,MBL2,SVEP1,C1QC,F10,CALML5,PROS1,EFEMP1,CFI,ILK,ANKRD28,MASP2,FBLN2,PSG4,F11,C1S,PSG2,NUCB1,ADIPOQ,FSIP2,ABCA13,MST1,TGFBI,CFB,IGFALS,GC,HGFAC,UIMC1,C1R,CLTC,VEGFC,KNG1,TRIM66,CDH5,PDIA4,SDF4
UniProt Keywords	KW-0646	Protease inhibitor	18	117	1.06	1.16e-11	SERPIND1,SERPINA10,CSTA,AMBP,ITIH4,ITIH1,SERPING1,SERPINI1,A2ML1,SERPINF2,SERPINA5,SERPINA11,PAPLN,ITIH2,CST3,ITIH3,SERPINA3,KNG1
UniProt Keywords	KW-0358	Heparin-binding	16	88	1.13	2.46e-11	APOH,SERPIND1,COMP,VTN,PF4V1,LTF,HRG,APOB,APOE,SERPINA10,PF4,FBN1,SERPINA5,SOD3,SAA1,F11
UniProt Keywords	KW-0345	HDL	10	16	1.67	2.59e-11	PON1,APOA1,APOE,SAA4,APOL1,APOA4,APOM,APOF,SAA1,APOA5
UniProt Keywords	KW-0722	Serine protease inhibitor	15	80	1.14	7.94e-11	SERPIND1,SERPINA10,AMBP,ITIH4,ITIH1,SERPING1,SERPINI1,A2ML1,SERPINF2,SERPINA5,SERPINA11,PAPLN,ITIH2,ITIH3,SERPINA3
UniProt Keywords	KW-0720	Serine protease	18	136	0.99	9.11e-11	F9,LTF,PROC,F12,HABP2,PCSK9,F2,PLG,LPA,F10,CFI,MASP2,F11,C1S,CFB,HGFAC,C1R,CFD
UniProt Keywords	KW-0179	Complement alternate pathway	9	12	1.74	9.93e-11	C5,CFP,C7,C8A,CFH,C8B,C8G,CFB,CFD
UniProt Keywords	KW-0765	Sulfation	13	57	1.23	2.40e-10	SERPIND1,CHGA,F9,VTN,NID1,LUM,PCSK9,SERPINF2,F8,CFH,F5,C4A,C4B
UniProt Keywords	KW-0034	Amyloid	10	24	1.49	4.50e-10	APOA1,TTR,APCS,LYZ,GSN,CST3,SAA1,TGFBI,B2M,FGA
UniProt Keywords	KW-0301	Gamma-carboxyglutamic acid	9	16	1.62	5.73e-10	F9,PROC,TTR,F2,PROZ,ITIH2,F10,PROS1,TGFBI
UniProt Keywords	KW-0792	Thrombophilia	8	10	1.77	9.76e-10	SERPIND1,F9,HRG,PROC,F2,PLG,F5,PROS1
UniProt Keywords	KW-0379	Hydroxylation	17	143	0.94	1.33e-09	F9,PROC,FCN3,FCN2,C1QB,PROZ,COL18A1,MBL2,C1QC,F10,PROS1,MASP2,C1S,ADIPOQ,C1R,KNG1,FGA
UniProt Keywords	KW-1008	Amyloidosis	10	31	1.38	3.00e-09	APOA1,TTR,APOE,LYZ,GSN,CST3,SAA1,TGFBI,B2M,FGA
UniProt Keywords	KW-0445	Lipid transport	15	110	1.0	3.04e-09	CETP,LBP,APOC3,APOB,APOA1,APOE,APOL1,LPA,SERPINA5,APOA4,APOM,APOF,PLTP,APOA5,APOC4
UniProt Keywords	KW-0272	Extracellular matrix	21	267	0.77	7.81e-09	MMP2,COMP,SPARC,APOE,PXDN,VWF,LGALS3BP,NID1,LUM,BMP1,CALR,FBN1,FBLN1,THBS4,COL18A1,ADAMTSL4,CRTAC1,EFEMP1,FBLN2,SPARCL1,TGFBI
UniProt Keywords	KW-0873	Pyrrolidone carboxylic acid	12	67	1.12	1.31e-08	SERPINF1,LUM,FGB,C1QB,ANG,APOD,C8G,ORM2,JCHAIN,RNASE4,KNG1,B2M
UniProt Keywords	KW-0011	Acute phase	8	19	1.49	3.57e-08	ITIH4,SAA4,F2,SERPINF2,F8,SAA1,ORM2,SERPINA3
UniProt Keywords	KW-0473	Membrane attack complex	6	7	1.8	1.82e-07	C5,C6,C7,C8A,C8B,C8G
UniProt Keywords	KW-0280	Fibrinolysis	6	8	1.74	2.96e-07	CPB2,HRG,F12,SERPING1,PLG,PROS1
UniProt Keywords	KW-0420	Kringle	7	16	1.51	2.96e-07	F12,HABP2,F2,PLG,LPA,MST1,HGFAC
UniProt Keywords	KW-1068	Hemolytic uremic syndrome	6	10	1.65	7.48e-07	CFHR5,CFHR1,CFHR3,CFH,CFI,CFB
UniProt Keywords	KW-0865	Zymogen	16	210	0.75	1.28e-06	CPB2,F9,MMP2,PROC,F12,PCSK9,BMP1,F2,PLG,F5,ADAMTS13,F10,PROS1,CFB,HGFAC,CFD
UniProt Keywords	KW-0645	Protease	25	516	0.55	1.39e-06	CPB2,F9,MMP2,LTF,PROC,F12,PSMA6,HABP2,IHH,PCSK9,BMP1,F2,PLG,LPA,CNDP1,ADAMTS13,F10,CFI,MASP2,F11,C1S,CFB,HGFAC,C1R,CFD
UniProt Keywords	KW-0162	Chylomicron	5	9	1.61	1.30e-05	APOC3,APOB,APOE,APOA4,APOA5
UniProt Keywords	KW-0971	Glycation	5	12	1.49	3.78e-05	APOA1,APOE,SOD3,CFB,B2M
UniProt Keywords	KW-0153	Cholesterol metabolism	8	65	0.96	9.55e-05	CETP,APOB,APOA1,APOE,LCAT,PCSK9,APOL1,APOF
UniProt Keywords	KW-0130	Cell adhesion	20	478	0.49	0.00021	VCL,COMP,VTN,PCDH12,VWF,DSG2,LGALS3BP,NID1,CSTA,CDH6,PCDH1,DST,THBS4,COL18A1,PCDH18,SVEP1,CD99,TGFBI,IGFALS,CDH5
UniProt Keywords	KW-0065	Atherosclerosis	4	8	1.57	0.00022	CETP,APOB,APOA1,LPA
UniProt Keywords	KW-0378	Hydrolase	43	1612	0.3	0.00027	CPB2,F9,MMP2,PON1,GPLD1,LTF,PROC,DHX29,F12,LYZ,PSMA6,PON3,HYAL1,HABP2,PGLYRP2,IHH,APEH,PCSK9,BMP1,F2,PLG,LPA,HSPA5,ANG,GANAB,PRDX6,BLM,CNDP1,SMARCA1,ADAMTS13,MINPP1,F10,CFI,NAV2,RNASE1,MASP2,F11,C1S,CFB,HGFAC,C1R,RNASE4,CFD
UniProt Keywords	KW-0654	Proteoglycan	7	55	0.97	0.00027	PF4V1,AMBP,LUM,ITIH1,CSPG4,ITIH2,ITIH3
UniProt Keywords	KW-0176	Collagen	8	83	0.85	0.00043	FCN3,FCN2,C1QB,COL18A1,MBL2,C1QC,ADIPOQ,COLEC11
UniProt Keywords	KW-0575	Peroxidase	5	25	1.17	0.00063	MPO,CAT,PXDN,PRDX6,GPX3
UniProt Keywords	KW-1018	Complement activation lectin pathway	3	3	1.87	0.00064	FCN3,FCN2,MBL2
UniProt Keywords	KW-0797	Tissue remodeling	3	4	1.74	0.0011	PLG,CSPG4,THBS4
UniProt Keywords	KW-0558	Oxidation	5	32	1.06	0.0016	CHGA,MPO,APOA1,APOE,GAPDH
UniProt Keywords	KW-0913	Age-related macular degeneration	4	16	1.27	0.0016	CFH,CFI,CST3,CFB
UniProt Keywords	KW-1212	Corneal dystrophy	4	20	1.17	0.0034	LCAT,GSN,TGFBI,GRHL2
UniProt Keywords	KW-0497	Mitogen	5	39	0.98	0.0036	CAT,PPBP,THBS4,IGF2,VEGFC
UniProt Keywords	KW-0882	Thioester bond	3	8	1.44	0.0044	A2ML1,C4A,C4B
UniProt Keywords	KW-0929	Antimicrobial	7	93	0.75	0.0046	CHGA,LBP,LTF,LYZ,PPBP,CAMP,S100A9
UniProt Keywords	KW-1065	Osteogenesis imperfecta	4	23	1.11	0.0050	SPARC,SERPINF1,BMP1,P4HB
UniProt Keywords	KW-0376	Hydrogen peroxide	3	9	1.39	0.0055	MPO,CAT,PXDN
UniProt Keywords	KW-0430	Lectin	9	162	0.61	0.0055	APCS,ATRN,FCN3,FCN2,CALR,ITLN1,ASGR2,MBL2,COLEC11
UniProt Keywords	KW-0721	Serine protease homolog	3	9	1.39	0.0055	PROZ,MST1,HPR
UniProt Keywords	KW-0395	Inflammatory response	9	163	0.61	0.0057	C5,ATRN,CSF1R,CD5L,S100A9,C4A,C4B,RARRES2,KNG1
UniProt Keywords	KW-0068	Autocatalytic cleavage	5	46	0.91	0.0062	MMP2,IHH,PCSK9,LPA,MASP2
UniProt Keywords	KW-0850	VLDL	3	10	1.35	0.0065	APOC3,APOE,APOA5
UniProt Keywords	KW-1280	Immunoglobulin	3	12	1.27	0.0099	IGKV2D-28,IGHV3-15,IGHV3-72
UniProt Keywords	KW-0355	Hemophilia	2	2	1.87	0.0108	F9,F8
UniProt Keywords	KW-0049	Antioxidant	3	14	1.2	0.0141	PRDX6,S100A9,SOD3
UniProt Keywords	KW-0372	Hormone	6	92	0.68	0.0201	TTR,CRH,INHBC,FBN1,ADIPOQ,IGF2
UniProt Keywords	KW-0145	Chemotaxis	6	94	0.67	0.0219	SERPIND1,HRG,PPBP,PF4,S100A9,RARRES2
UniProt Keywords	KW-0339	Growth factor	7	129	0.6	0.0228	PPBP,BMP1,INHBC,THBS4,EFEMP1,IGF2,VEGFC
UniProt Keywords	KW-0186	Copper	5	65	0.76	0.0232	SPARC,HRG,CP,F5,SOD3
UniProt Keywords	KW-0340	Growth factor binding	3	20	1.05	0.0322	IGFBP5,IGFBP1,IGFBP3
UniProt Keywords	KW-0044	Antibiotic	5	77	0.68	0.0447	CHGA,LBP,LTF,PPBP,CAMP
Pfam	PF01442	Apolipoprotein A1/A4/E domain	4	4	1.87	0.0048	APOA1,APOE,APOA4,APOA5
Pfam	PF06668	Inter-alpha-trypsin inhibitor heavy chain C-terminus	4	6	1.69	0.0070	ITIH4,ITIH1,ITIH2,ITIH3
InterPro	IPR001881	EGF-like calcium-binding domain	24	124	1.16	1.56e-15	F9,COMP,PROC,LRP1,F12,NID1,HABP2,MASP1,BMP1,EFEMP2,FBN1,FBLN1,OIT3,THBS4,PROZ,CRTAC1,SVEP1,F10,PROS1,EFEMP1,MASP2,FBLN2,C1S,C1R

InterPro	IPR035976	Sushi/SCR/CCP superfamily	19	58	1.38	1.56e-15	APOH,C4BPB,CFHR5,C6,MASP1,CFHR1,C7,C4BPA,F13B,CFHR2,CFHR4,CFHR3,CFH,SVEP1,MASP2,C1S,CFB,HPR,C1R
InterPro	IPR000436	Sushi/SCR/CCP domain	18	56	1.38	8.77e-15	APOH,C4BPB,CFHR5,C6,MASP1,CFHR1,C7,C4BPA,F13B,CFHR2,CFHR4,CFHR3,CFH,SVEP1,MASP2,C1S,CFB,C1R
InterPro	IPR018097	EGF-like calcium-binding, conserved site	21	99	1.2	1.46e-14	F9,COMP,PROC,LRP1,NID1,MASP1,BMP1,EFEMP2,FBN1,FBLN1,OIT3,THBS4,CRTAC1,SVEP1,F10,PROS1,EFEMP1,MASP2,FBLN2,C1S,C1R
InterPro	IPR001314	Peptidase S1A, chymotrypsin family	19	107	1.12	7.83e-12	F9,PROC,F12,HABP2,F2,PLG,LPA,PROZ,F10,CFI,MASP2,F11,C1S,MST1,CFB,HGFAC,HPR,C1R,CFD
InterPro	IPR001254	Serine proteases, trypsin domain	19	115	1.09	2.09e-11	F9,PROC,F12,HABP2,F2,PLG,LPA,PROZ,F10,CFI,MASP2,F11,C1S,MST1,CFB,HGFAC,HPR,C1R,CFD
InterPro	IPR009003	Peptidase S1, PA clan	19	123	1.06	5.31e-11	F9,PROC,F12,HABP2,F2,PLG,LPA,PROZ,F10,CFI,MASP2,F11,C1S,MST1,CFB,HGFAC,HPR,C1R,CFD
InterPro	IPR000742	EGF-like domain	24	236	0.88	9.36e-11	F9,COMP,PROC,LRP1,F12,ATRN,NID1,HABP2,MASP1,BMP1,EFEMP2,FBN1,FBLN1,OIT3,THBS4,PROZ,SVEP1,F10,PROS1,EFEMP1,MASP2,FBLN2,HGFAC,C1R
InterPro	IPR043504	Peptidase S1, PA clan, chymotrypsin-like fold	18	116	1.06	1.81e-10	F9,PROC,F12,HABP2,F2,PLG,LPA,PROZ,F10,CFI,MASP2,F11,C1S,MST1,HGFAC,HPR,C1R,CFD
InterPro	IPR033116	Serine proteases, trypsin family, serine active site	16	92	1.11	7.68e-10	F9,PROC,F12,HABP2,F2,PLG,LPA,F10,CFI,MASP2,F11,C1S,CFB,HGFAC,C1R,CFD
InterPro	IPR000152	EGF-type aspartate/asparagine hydroxylation site	16	98	1.08	1.65e-09	F9,PROC,LRP1,NID1,BMP1,EFEMP2,FBN1,FBLN1,PROZ,SVEP1,F10,PROS1,EFEMP1,MASP2,FBLN2,C1S
InterPro	IPR042185	Serpin superfamily, domain 2	10	35	1.33	1.89e-07	SERPIND1,SERPINF1,SERPINA10,SERPING1,SERPINI1,SERPINF2,SERPINA7,SERPINA5,SERPINA11,SERPINA3
InterPro	IPR000215	Serpin family	10	36	1.31	2.20e-07	SERPIND1,SERPINF1,SERPINA10,SERPING1,SERPINI1,SERPINF2,SERPINA7,SERPINA5,SERPINA11,SERPINA3
InterPro	IPR023796	Serpin domain	10	36	1.31	2.20e-07	SERPIND1,SERPINF1,SERPINA10,SERPING1,SERPINI1,SERPINF2,SERPINA7,SERPINA5,SERPINA11,SERPINA3
InterPro	IPR036186	Serpin superfamily	10	36	1.31	2.20e-07	SERPIND1,SERPINF1,SERPINA10,SERPING1,SERPINI1,SERPINF2,SERPINA7,SERPINA5,SERPINA11,SERPINA3
InterPro	IPR042178	Serpin superfamily, domain 1	10	36	1.31	2.20e-07	SERPIND1,SERPINF1,SERPINA10,SERPING1,SERPINI1,SERPINF2,SERPINA7,SERPINA5,SERPINA11,SERPINA3
InterPro	IPR009030	Growth factor receptor cysteine-rich domain superfamily	15	127	0.94	3.19e-07	COMP,IGFBP5,LRP1,NID1,IGFBP1,BMP1,EFEMP2,FBN1,FBLN1,C8A,SVEP1,IGFBP3,PROS1,EFEMP1,FBLN2
InterPro	IPR018114	Serine proteases, trypsin family, histidine active site	13	100	0.98	1.46e-06	F9,PROC,F12,HABP2,F2,PLG,LPA,F10,CFI,F11,CFB,HGFAC,CFD
InterPro	IPR046350	Cystatin superfamily	8	23	1.41	2.04e-06	SPP2,HRG,CSTA,AHSG,CAMP,CST3,RARRES2,KNG1
InterPro	IPR013806	Kringle-like fold	8	26	1.36	4.32e-06	MMP2,F12,HABP2,F2,PLG,LPA,MST1,HGFAC
InterPro	IPR000001	Kringle	7	16	1.51	4.81e-06	F12,HABP2,F2,PLG,LPA,MST1,HGFAC
InterPro	IPR018056	Kringle, conserved site	7	16	1.51	4.81e-06	F12,HABP2,F2,PLG,LPA,MST1,HGFAC
InterPro	IPR038178	Kringle superfamily	7	16	1.51	4.81e-06	F12,HABP2,F2,PLG,LPA,MST1,HGFAC
InterPro	IPR023795	Serpin, conserved site	8	32	1.27	1.42e-05	SERPIND1,SERPINF1,SERPING1,SERPINI1,SERPINF2,SERPINA7,SERPINA5,SERPINA3
InterPro	IPR000020	Anaphylatoxin/fibulin	5	6	1.79	5.03e-05	C5,FBLN1,FBLN2,C4A,C4B
InterPro	IPR014716	Fibrinogen, alpha/beta/gamma chain, C-terminal globular, subdomain 1	7	26	1.3	6.04e-05	ANGPTL6,FCN3,FCN2,FGB,ITLN1,FGL1,FGA
InterPro	IPR000294	Gamma-carboxyglutamic acid-rich (GLA) domain	6	15	1.47	6.45e-05	F9,PROC,F2,PROZ,F10,PROS1
InterPro	IPR035972	Gamma-carboxyglutamic acid-rich (GLA) domain superfamily	6	15	1.47	6.45e-05	F9,PROC,F2,PROZ,F10,PROS1
InterPro	IPR026823	Complement Clr-like EGF domain	7	27	1.28	6.75e-05	LRP1,NID1,EFEMP2,FBN1,FBLN1,EFEMP1,FBLN2
InterPro	IPR002181	Fibrinogen, alpha/beta/gamma chain, C-terminal globular domain	7	32	1.21	0.00018	ANGPTL6,FCN3,FCN2,FGB,ITLN1,FGL1,FGA
InterPro	IPR020837	Fibrinogen, conserved site	6	19	1.37	0.00018	ANGPTL6,FCN3,FCN2,FGB,FGL1,FGA
InterPro	IPR036056	Fibrinogen-like, C-terminal	7	32	1.21	0.00018	ANGPTL6,FCN3,FCN2,FGB,ITLN1,FGL1,FGA
InterPro	IPR035914	Spermadhesin, CUB domain superfamily	8	54	1.04	0.00035	PCOLCE,ATRN,MASP1,BMP1,ADAMTS13,MASP2,C1S,C1R
InterPro	IPR000074	Apolipoprotein A/E	4	4	1.87	0.00043	APOA1,APOE,APOA4,APOA5
InterPro	IPR012674	Calycin	7	38	1.13	0.00043	LCN1,AMBP,APOD,C8G,APOM,ORM2,PAEP
InterPro	IPR017857	Coagulation factor-like, Gla domain superfamily	5	12	1.49	0.00044	F9,PROC,PROZ,F10,PROS1
InterPro	IPR001862	Membrane attack complex component/perforin/complement C9	4	5	1.77	0.00071	C6,C7,C8A,C8B
InterPro	IPR012224	Peptidase S1A, coagulation factor VII/IX/X/C/Z	4	5	1.77	0.00071	F9,PROC,PROZ,F10
InterPro	IPR036383	Thrombospondin type-1 (TSP1) repeat superfamily	8	64	0.97	0.00094	CFP,C6,C7,PAPLN,C8A,ADAMTSL4,C8B,ADAMTS13
InterPro	IPR000884	Thrombospondin type-1 (TSP1) repeat	8	65	0.96	0.0010	CFP,C6,C7,PAPLN,C8A,ADAMTSL4,C8B,ADAMTS13
InterPro	IPR010600	Inter-alpha-trypsin inhibitor heavy chain, C-terminal	4	6	1.69	0.0011	ITIH4,ITIH1,ITIH2,ITIH3
InterPro	IPR020863	Membrane attack complex component/perforin domain, conserved site	4	6	1.69	0.0011	C6,C7,C8A,C8B
InterPro	IPR000010	Cystatin domain	5	17	1.34	0.0015	HRG,CSTA,AHSG,CST3,KNG1
InterPro	IPR000859	CUB domain	7	51	1.01	0.0019	PCOLCE,ATRN,MASP1,BMP1,MASP2,C1S,C1R
InterPro	IPR040839	Macroglobulin domain MG4	4	8	1.57	0.0022	C5,A2ML1,C4A,C4B
InterPro	IPR000566	Lipocalin/cytosolic fatty-acid binding domain	6	35	1.1	0.0025	LCN1,AMBP,APOD,C8G,ORM2,PAEP
InterPro	IPR002035	Von Willebrand factor, type A	8	76	0.89	0.0025	VWF,ITIH4,ITIH1,ANTXR1,ITIH2,SVEP1,ITIH3,CFB
InterPro	IPR001599	Alpha-2-macroglobulin	4	9	1.52	0.0030	C5,A2ML1,C4A,C4B
InterPro	IPR002890	Macroglobulin domain	4	9	1.52	0.0030	C5,A2ML1,C4A,C4B
InterPro	IPR009048	Alpha-macroglobulin, receptor-binding	4	9	1.52	0.0030	C5,A2ML1,C4A,C4B
InterPro	IPR011625	Alpha-2-macroglobulin, bait region domain	4	9	1.52	0.0030	C5,A2ML1,C4A,C4B
InterPro	IPR011626	Alpha-macroglobulin-like, TED domain	4	9	1.52	0.0030	C5,A2ML1,C4A,C4B
InterPro	IPR036595	Alpha-macroglobulin, receptor-binding domain superfamily	4	9	1.52	0.0030	C5,A2ML1,C4A,C4B
InterPro	IPR041555	Macroglobulin domain MG3	4	9	1.52	0.0030	C5,A2ML1,C4A,C4B
InterPro	IPR011042	Six-bladed beta-propeller, TolB-like	6	38	1.07	0.0032	APMAP,PON1,LRP1,NID1,PON3,APEH
InterPro	IPR013694	VIT domain	4	10	1.47	0.0036	ITIH4,ITIH1,ITIH2,ITIH3
InterPro	IPR023415	Low-density lipoprotein (LDL) receptor class A, conserved site	6	40	1.05	0.0040	LRP1,C6,C7,C8A,C8B,CFI
InterPro	IPR018247	EF-Hand 1, calcium-binding site	11	176	0.67	0.0054	RCN1,SPARC,DGKG,DST,S100A7,S100A9,CALML5,EFCAB5,NUCB1,SPARCL1,SDF4
InterPro	IPR024175	Peptidase S1A, complement C1r/C1S/mannan-binding	3	3	1.87	0.0055	MASP2,C1S,C1R
InterPro	IPR020864	Membrane attack complex component/perforin (MACPF) domain	4	12	1.39	0.0059	C6,C7,C8A,C8B
InterPro	IPR022272	Lipocalin family conserved site	4	13	1.36	0.0075	AMBP,APOD,C8G,PAEP
InterPro	IPR002172	Low-density lipoprotein (LDL) receptor class A repeat	6	47	0.98	0.0083	LRP1,C6,C7,C8A,C8B,CFI
InterPro	IPR036055	LDL receptor-like superfamily	6	47	0.98	0.0083	LRP1,C6,C7,C8A,C8B,CFI

InterPro	IPR001840	Anaphylatoxin, complement system domain	3	4	1.74	0.0088	C5,C4A,C4B
InterPro	IPR018081	Anaphylatoxin, complement system	3	4	1.74	0.0088	C5,C4A,C4B
InterPro	IPR036465	Von Willebrand factor A-like domain superfamily	8	97	0.79	0.0089	VWF,ITIH4,ITIH1,ANTXR1,ITIH2,SVEP1,ITIH3,CFB
InterPro	IPR008930	Terpenoid cyclases/protein prenyltransferase alpha-alpha toroid	4	15	1.3	0.0109	C5,A2ML1,C4A,C4B
InterPro	IPR003884	Factor I / membrane attack complex	3	5	1.65	0.0132	C6,C7,CFI
InterPro	IPR008160	Collagen triple helix repeat	7	77	0.83	0.0132	FCN2,C1QB,COL18A1,MBL2,C1QC,ADIPOQ,COLEC11
InterPro	IPR011707	Multicopper oxidase, N-terminal	3	5	1.65	0.0132	CP,F8,F5
InterPro	IPR017954	Lipid-binding serum glycoprotein, conserved site	3	5	1.65	0.0132	CETP,LBP,PLTP
InterPro	IPR033138	Multicopper oxidases, conserved site	3	5	1.65	0.0132	CP,F8,F5
InterPro	IPR000716	Thyroglobulin type-1	4	17	1.24	0.0152	IGFBP5,NID1,IGFBP1,IGFBP3
InterPro	IPR018933	Netrin module, non-TIMP type	4	17	1.24	0.0152	PCOLCE,C5,C4A,C4B
InterPro	IPR036857	Thyroglobulin type-1 superfamily	4	17	1.24	0.0152	IGFBP5,NID1,IGFBP1,IGFBP3
InterPro	IPR003609	PAN/Apple domain	3	6	1.57	0.0175	PLG,F11,MST1
InterPro	IPR022321	Insulin-like growth factor-binding protein family 1-6, chordata	3	6	1.57	0.0175	IGFBP5,IGFBP1,IGFBP3
InterPro	IPR019742	Alpha-2-macroglobulin, conserved site	3	8	1.44	0.0328	A2ML1,C4A,C4B
InterPro	IPR023411	Ribonuclease A, active site	3	8	1.44	0.0328	ANG,RNASE1,RNASE4
InterPro	IPR047565	Alpha-macroglobulin-like, thiol-ester bond-forming region	3	8	1.44	0.0328	A2ML1,C4A,C4B
InterPro	IPR001134	Netrin domain	4	23	1.11	0.0377	PCOLCE,C5,C4A,C4B
InterPro	IPR018073	Proteinase inhibitor I25, cystatin, conserved site	3	9	1.39	0.0412	CSTA,CST3,KNG1
InterPro	IPR008993	Tissue inhibitor of metalloproteinases-like, OB-fold	4	24	1.09	0.0424	PCOLCE,C5,C4A,C4B
SMART	SM00032	Domain abundant in complement control proteins	18	56	1.38	3.01e-15	APOH,C4BPB,CFHR5,C6,MASP1,CFHR1,C7,C4BPA,F13B,CFHR2,CFHR4,CFHR3,CFH,SVEP1,MASP2,C1S,CFB,C1R
SMART	SM00179	Calcium-binding EGF-like domain	20	101	1.17	6.61e-14	F9,COMP,PROC,LRP1,NID1,MASP1,BMP1,EFEMP2,FBN1,FBLN1,OIT3,THBS4,SVEP1,F10,PROS1,EFEMP1,MASP2,FBLN2,C1S,C1R
SMART	SM00020	Trypsin-like serine protease	20	118	1.1	6.20e-13	F9,PROC,F12,HABP2,MASP1,F2,PLG,LPA,PROZ,F10,CFI,MASP2,F11,C1S,MST1,CFB,HGFAC,HPR,C1R,CFD
SMART	SM00181	Epidermal growth factor-like domain.	20	209	0.85	7.17e-09	F9,COMP,PROC,LRP1,F12,ATRN,NID1,HYAL1,HABP2,EFEMP2,FBN1,FBLN1,OIT3,THBS4,PROZ,SVEP1,F10,PROS1,FBLN2,HGFAC
SMART	SM00093	SERine Proteinase INhibitors	10	35	1.33	5.17e-08	SERPIND1,SERPINF1,SERPINA10,SERPING1,SERPINI1,SERPINF2,SERPINA7,SERPINA5,SERPINA11,SERPINA3
SMART	SM00130	Kringle domain	7	16	1.51	1.93e-06	F12,HABP2,F2,PLG,LPA,MST1,HGFAC
SMART	SM00104	Anaphylatoxin homologous domain	5	6	1.79	2.05e-05	C5,FBLN1,FBLN2,C4A,C4B
SMART	SM00069	Domain containing Gla (gamma-carboxyglutamate) residues.	6	15	1.47	2.49e-05	F9,PROC,F2,PROZ,F10,PROS1
SMART	SM00186	Fibrinogen-related domains (FReDs)	7	29	1.25	3.77e-05	ANGPTL6,FCN3,FCN2,FGB,ITLN1,FGL1,FGA
SMART	SM00209	Thrombospondin type 1 repeats	8	64	0.97	0.00042	CFP,C6,C7,PAPLN,C8A,ADAMTSL4,C8B,ADAMTS13
SMART	SM00042	Domain first found in C1r, C1s, uEGF, and bone morphogenetic protein.	7	50	1.02	0.00066	PCOLCE,ATRN,MASP1,BMP1,MASP2,C1S,C1R
SMART	SM00043	Cystatin-like domain	5	17	1.34	0.00066	HRG,CSTA,AHSG,CST3,KNG1
SMART	SM00609	Vault protein Inter-alpha-Trypsin domain	4	7	1.63	0.00066	ITIH4,ITIH1,ITIH2,ITIH3
SMART	SM00327	Von Willebrand factor (vWF) type A domain	8	77	0.89	0.0010	VWF,ITIH4,ITIH1,ANTXR1,ITIH2,SVEP1,ITIH3,CFB
SMART	SM01359	Alpha-2-Macroglobulin	4	9	1.52	0.0011	C5,A2ML1,C4A,C4B
SMART	SM01360	Alpha-2-macroglobulin family	4	9	1.52	0.0011	C5,A2ML1,C4A,C4B
SMART	SM01361	A-macroglobulin receptor	4	9	1.52	0.0011	C5,A2ML1,C4A,C4B
SMART	SM00457	Membrane-attack complex / perforin	4	12	1.39	0.0023	C6,C7,C8A,C8B
SMART	SM00057	Factor I membrane attack complex	3	4	1.74	0.0034	C6,C7,CFI
SMART	SM00192	Low-density lipoprotein receptor domain class A	6	48	0.97	0.0034	LRP1,C6,C7,C8A,C8B,CFI
SMART	SM00643	Netrin C-terminal Domain	4	15	1.3	0.0040	PCOLCE,C5,C4A,C4B
SMART	SM00211	Thyroglobulin type I repeats.	4	17	1.24	0.0058	IGFBP5,NID1,IGFBP1,IGFBP3
SMART	SM00092	Pancreatic ribonuclease	3	8	1.44	0.0127	ANG,RNASE1,RNASE4
SMART	SM01419	Alpha-macro-globulin thiol-ester bond-forming region	3	8	1.44	0.0127	A2ML1,C4A,C4B
SMART	SM00328	BPI/LBP/CETP N-terminal domain	3	9	1.39	0.0155	CETP,LBP,PLTP
SMART	SM00329	BPI/LBP/CETP C-terminal domain	3	9	1.39	0.0155	CETP,LBP,PLTP
SMART	SM00059	Fibronectin type 2 domain	3	14	1.2	0.0421	MMP2,F12,HGFAC

UC vs AC+NCFET T2

#category	term ID	term description	observed gene count	background gene count	strength	false discovery rate	matching proteins in your network (labels)
GO Process	GO:0006959	Humoral immune response	46	268	1.13	1.19e-31	ST6GAL1,LTF,CFP,CFHR5,LYZ,C9,FCN3,SERPING1,FCN2,PF4,MASP1,CAMP,FGB,F2,C1QB,CLU,C7,ANG,SLPI,C8A,C4BPA,CFHR2,CFHR4,CFHR3,CFH,S100A7,C8B,FCN1,C1QC,C1QA,CFI,MASP2,C1S,COLEC11,C4B,CFB,RARRES2,JCHAIN,C1R,CFD,IGHV3-15,CCL18,IGHV3-72,KNG1,B2M,FGA
GO Process	GO:0006956	Complement activation	30	60	1.6	3.14e-31	CFP,CFHR5,C9,FCN3,SERPING1,FCN2,MASP1,C1QB,CLU,C7,C8A,C4BPA,CFHR2,CFHR4,CFHR3,CFH,C8B,FCN1,C1QC,C1QA,CFI,MASP2,C1S,COLEC11,C4B,CFB,C1R,CFD,IGHV3-15,IGHV3-72
GO Process	GO:0006950	Response to stress	121	3358	0.46	2.25e-26	CPB2,SERPIND1,CHGA,F9,MMP2,LTF,PROC,CAT,LRP1,CFP,APOE,F12,CFHR5,THBS1,LYZ,VWF,SERPINA10,ATRN,C9,KLKB1,F13A1,DGKG,TCIRG1,HPX,IGFBP4,FCN3,AHSG,SAA4,SERPING1,CSF1R,IGF2BP1,FCN2,PGLYRP2,PF4,MASP1,CAMP,PDIA3,LALBA,LRG1,PCSK9,FGB,DST,F2,PLG,PRG2,STAB1,C1QB,CLU,NDUFS8,APOL1,SERPINF2,C7,P4HB,FBLN1,PSG3,HSP90AA1,ANG,FGG,TUBB,THBS4,PRDX6,SLPI,APOD,FN1,BLM,HP,APOA4,F8,C8A,C4BPA,F13B,CFHR2,CFH,SERPINC1,F5,S100A7,ECM1,FLNA,MCOLN2,C8B,FCN1,ADAMTS13,C1QC,C1QA,F10,F7,GPX3,PROS1,CFI,MMRN1,TNRC6A,ILK,MASP2,F11,C1S,ADIPOQ,ORM2,COLEC11,MST1,C4B,SERPINA1,CFB,RARRES2,UIMC1,SELENOP,TSKU,JCHAIN,C1R,VIM,RNASE4,CFD,IGHV3-15,CCL18,IGHV3-72,VEGFC,PLCG2,KNG1,ACTB,TRIM8,B2M,FGA
GO Process	GO:0007596	Blood coagulation	35	173	1.21	6.84e-26	CPB2,SERPIND1,F9,PROC,F12,VWF,SERPINA10,KLKB1,F13A1,DGKG,SERPING1,PF4,FGB,F2,PLG,FBLN1,FGG,FN1,F8,F13B,SERPINC1,F5,FLNA,ADAMTS13,F10,F7,PROS1,MMRN1,ILK,F11,SERPINA1,PLCG2,KNG1,ACTB,FGA
GO Process	GO:0006952	Defense response	77	1394	0.64	2.91e-25	CHGA,LTF,LRP1,CFP,F12,CFHR5,THBS1,LYZ,ATRN,C9,KLKB1,TCIRG1,HPX,IGFBP4,FCN3,AHSG,SAA4,SERPING1,CSF1R,FCN2,PGLYRP2,PF4,MASP1,CAMP,LALBA,FGB,F2,PRG2,STAB1,C1QB,CLU,APOL1,SERPINF2,C7,PSG3,ANG,TUBB,SLPI,FN1,HP,APOA4,F8,C8A,C4BPA,CFHR2,CFH,S100A7,ECM1,MCOLN2,C8B,FCN1,ADAMTS13,C1QC,C1QA,F7,CFI,MASP2,C1S,ORM2,COLEC11,C4B,SERPINA1,CFB,RARRES2,JCHAIN,C1R,VIM,RNASE4,CFD,IGHV3-15,CCL18,IGHV3-72,PLCG2,KNG1,TRIM8,B2M,FGA

GO Process	GO:0098542	Defense response to other organism	61	989	0.69	2.12e-21	CHGA,LTF,CFP,F12,CFHR5,LYZ,C9,HPX,FCN3,SERPING1,CSF1R,FCN2,PGLYRP2,PF4,MASP1,CAMP,LALBA,FGB,F2,PRG2,STAB1,C1QB,CLU,APOL1,C7,ANG,TUBB,SLPI,HP,APOA4,C8A,C4BPA,CFHR2,CFH,S100A7,MCOLN2,C8B,FCN1,ADAMTS13,C1QC,C1QA,CFI,MASP2,C1S,COLEC11,C4B,CFB,RARRES2,JCHAIN,C1R,VIM,RNASE4,CFD,IGHV3-15,CCL18,IGHV3-72,PLCG2,KNG1,TRIM8,B2M,FGA
GO Process	GO:0042060	Wound healing	38	336	0.95	1.74e-20	CPB2,SERPIND1,F9,PROC,F12,VWF,SERPINA10,KLKB1,F13A1,DGKG,SERPING1,PF4,LRG1,FGB,DST,F2,PLG,FBLN1,FGG,FN1,F8,F13B,SERPINC1,F5,FLNA,ADAMTS13,F10,F7,PROS1,MMRN1,ILK,F11,SERPINA1,TSKU,PLCG2,KNG1,ACTB,FGA
GO Process	GO:0009611	Response to wounding	41	444	0.87	2.62e-19	CPB2,SERPIND1,F9,MMP2,PROC,F12,VWF,SERPINA10,KLKB1,F13A1,DGKG,SERPING1,PF4,LRG1,FGB,DST,F2,PLG,FBLN1,FGG,APOD,FN1,APOA4,F8,F13B,SERPINC1,F5,FLNA,ADAMTS13,F10,F7,PROS1,MMRN1,ILK,F11,SERPINA1,TSKU,PLCG2,KNG1,ACTB,FGA
GO Process	GO:0050878	Regulation of body fluid levels	38	371	0.91	3.50e-19	CPB2,SERPIND1,F9,PROC,APOE,F12,THBS1,VWF,SERPINA10,KLKB1,F13A1,DGKG,SERPING1,PF4,FGB,F2,PLG,SERPINF2,FBLN1,FGG,FN1,F8,F13B,SERPINC1,F5,FLNA,ADAMTS13,F10,F7,PROS1,MMRN1,ILK,F11,SERPINA1,PLCG2,KNG1,ACTB,FGA
GO Process	GO:0072378	Blood coagulation, fibrin clot formation	17	24	1.75	3.54e-19	F9,F12,KLKB1,F13A1,FGB,F2,FBLN1,FGG,FN1,F8,F13B,F5,FLNA,F10,F7,F11,FGA
GO Process	GO:0045087	Innate immune response	51	754	0.73	3.81e-19	CHGA,LTF,CFP,F12,CFHR5,C9,HPX,FCN3,SERPING1,CSF1R,FCN2,PGLYRP2,MASP1,CAMP,FGB,C1QB,CLU,APOL1,C7,ANG,TUBB,SLPI,APOA4,C8A,C4BPA,CFH,S100A7,MCOLN2,C8B,FCN1,ADAMTS13,C1QC,C1QA,CFI,MASP2,C1S,COLEC11,C4B,CFB,RARRES2,JCHAIN,C1R,VIM,CFD,IGHV3-15,CCL18,IGHV3-72,PLCG2,TRIM8,B2M,FGA
GO Process	GO:0002253	Activation of immune response	33	271	0.99	1.42e-18	GPLD1,CFP,CFHR5,C9,FCN3,SERPING1,FCN2,MASP1,C1QB,CLU,C7,HSP90AA1,C8A,C4BPA,CFHR2,CFHR4,CFHR3,CFH,C8B,FCN1,C1QC,C1QA,CFI,MASP2,C1S,COLEC11,C4B,CFB,C1R,CFD,IGHV3-15,IGHV3-72,PLCG2
GO Process	GO:0006955	Immune response	65	1321	0.59	2.62e-18	ST6GAL1,CHGA,LTF,LRP1,CFP,F12,CFHR5,THBS1,LYZ,C9,TCIRG1,HPX,FCN3,SERPING1,CSF1R,FCN2,PGLYRP2,PF4,MASP1,CAMP,FGB,F2,PRG2,C1QB,CLU,APOL1,C7,ANG,TUBB,SLPI,APOA4,C8A,C4BPA,CFHR2,CFHR4,CFHR3,CFH,S100A7,MCOLN2,C8B,FCN1,ADAMTS13,C1QC,C1QA,CFI,MASP2,C1S,PRG4,COLEC11,C4B,CFB,RARRES2,JCHAIN,C1R,VIM,IGKV2D-28,CFD,IGHV3-15,CCL18,IGHV3-72,PLCG2,KNG1,TRIM8,B2M,FGA
GO Process	GO:0002252	Immune effector process	37	375	0.89	2.73e-18	CHGA,LRP1,CFP,CFHR5,C9,TCIRG1,FCN3,SERPING1,FCN2,MASP1,F2,C1QB,CLU,C7,TUBB,C8A,C4BPA,CFHR2,CFHR4,CFHR3,CFH,C8B,FCN1,C1QC,C1QA,CFI,MASP2,C1S,COLEC11,C4B,CFB,C1R,CFD,IGHV3-15,IGHV3-72,PLCG2,B2M
GO Process	GO:0051707	Response to other organism	65	1328	0.59	3.05e-18	CHGA,LTF,APOB,CFP,F12,CFHR5,LYZ,C9,HPX,FCN3,SERPING1,CSF1R,FCN2,PGLYRP2,PF4,MASP1,CAMP,LALBA,LRG1,FGB,F2,PRG2,STAB1,C1QB,CLU,APOL1,C7,HSP90AA1,ANG,TUBB,SLPI,HP,APOA4,C8A,C4BPA,CFHR2,CFH,S100A7,MCOLN2,C8B,FCN1,ADAMTS13,C1QC,C1QA,CFI,MASP2,C1S,ADIPOQ,COLEC11,C4B,CFB,RARRES2,JCHAIN,C1R,VIM,RNASE4,CFD,IGHV3-15,CCL18,IGHV3-72,PLCG2,KNG1,TRIM8,B2M,FGA
GO Process	GO:0044419	Biological process involved in interspecies interaction between organisms	68	1490	0.56	1.01e-17	CHGA,LTF,APOB,CFP,APOE,F12,CFHR5,LYZ,C9,HPX,FCN3,SERPING1,CSF1R,FCN2,PGLYRP2,PF4,MASP1,CAMP,LALBA,LRG1,FGB,F2,PLG,PRG2,STAB1,C1QB,CLU,APOL1,C7,HSP90AA1,ANG,TUBB,SLPI,FN1,HP,APOA4,C8A,C4BPA,CFHR2,CFH,S100A7,MCOLN2,C8B,FCN1,ADAMTS13,C1QC,C1QA,CFI,MASP2,C1S,ADIPOQ,COLEC11,C4B,CFB,RARRES2,JCHAIN,C1R,VIM,RNASE4,CFD,IGHV3-15,CCL18,IGHV3-72,PLCG2,KNG1,TRIM8,B2M,FGA
GO Process	GO:0050896	Response to stimulus	170	7835	0.24	6.26e-17	ST6GAL1,CPB2,SERPIND1,CHGA,F9,MMP2,GPLD1,LTF,APOB,IGFBP5,PROC,APOA1,TTR,CAT,LRP1,CFP,APOE,F12,SERPINF1,CFHR5,THBS1,LYZ,VWF,SERPINA10,ATRN,C9,LCN1,LCAT,KLKB1,F13A1,PPP2R3A,DGKG,PON3,TCIRG1,HPX,INHBE,IGFBP4,FCN3,AHSG,IGFBP1,SAA4,SERPING1,CSF1R,IGF2BP1,PIP,FCN2,PSG6,PGLYRP2,SCGB3A1,PF4,MASP1,CAMP,PDIA3,SLC27A4,LALBA,LRG1,PCSK9,BMP1,FGB,DST,F2,INHBC,PLG,PSG1,PRG2,STAB1,C1QB,CLU,NDUFS8,APOL1,SERPINF2,C7,FBN1,P4HB,PAPPA,FBLN1,PSG3,HSP90AA1,ANG,TUBA1B,FGG,TUBB,THBS4,PRDX6,SLPI,APOD,FN1,ASGR2,BLM,HP,APOA4,F8,C8A,CENPF,C4BPA,F13B,CFHR2,CFHR4,CFHR3,CFH,SERPINC1,F5,ADAM12,S100A7,ECM1,FLNA,MCOLN2,C8B,FCN1,ADAMTS13,CCN5,SVEP1,C1QC,C1QA,F10,F7,APOM,CA6,ADGRG2,CALML5,IGFBP3,GPX3,PROS1,EFEMP1,CFI,MMRN1,TNRC6A,YWHAZ,ILK,MASP2,PSG4,F11,C1S,ADIPOQ,ORM2,PRG4,COLEC11,MST1,SPARCL1,C4B,SERPINA1,TGFBI,CFB,IGFALS,RARRES2,UIMC1,FBN2,SELENOP,TSKU,JCHAIN,C1R,ATP1A1,VIM,RNASE4,IGKV2D-28,TTN,CFD,IGHV3-15,CCL18,IGHV3-72,VEGFC,SEMA3B,PLCG2,OR52B4,KNG1,ACTB,TRIM8,CDH5,B2M,FGA
GO Process	GO:0006958	Complement activation, classical pathway	17	40	1.53	1.43e-16	C9,SERPING1,C1QB,CLU,C7,C8A,C4BPA,C8B,C1QC,C1QA,CFI,MASP2,C1S,C4B,C1R,IGHV3-15,IGHV3-72
GO Process	GO:0048584	Positive regulation of response to stimulus	77	2131	0.46	6.40e-15	CPB2,CHGA,GPLD1,LTF,IGFBP5,APOA1,CAT,CFP,APOE,F12,CFHR5,THBS1,VWF,C9,NID1,PPP2R3A,HPX,INHBE,IGFBP4,FCN3,SERPING1,CSF1R,FCN2,MASP1,PDIA3,LRG1,FGB,F2,INHBC,PLG,C1QB,CLU,SERPINF2,C7,FBN1,HSP90AA1,FGG,THBS4,FN1,C8A,C4BPA,CFHR2,CFHR4,CFHR3,CFH,S100A7,ECM1,FLNA,C8B,FCN1,C1QC,C1QA,F10,F7,IGFBP3,CFI,ILK,MASP2,C1S,ADIPOQ,COLEC11,C4B,CFB,RARRES2,UIMC1,C1R,CFD,IGHV3-15,CCL18,IGHV3-72,VEGFC,PLCG2,ACTB,TRIM8,CDH5,B2M,FGA
GO Process	GO:0002682	Regulation of immune system process	62	1438	0.53	8.82e-15	GPLD1,LTF,APOA1,CFP,APOE,CFHR5,THBS1,C9,HPX,FCN3,SERPING1,CSF1R,PIP,FCN2,PSG6,PGLYRP2,PF4,MASP1,PSG1,PRG2,C1QB,CLU,C7,FBN1,PSG3,HSP90AA1,THBS4,APOD,FN1,C8A,C4BPA,CFHR2,CFHR4,CFHR3,CFH,S100A7,ECM1,C8B,FCN1,C1QC,C1QA,F7,CD99,CFI,MASP2,PSG4,C1S,ADIPOQ,ORM2,COLEC11,MST1,C4B,CFB,RARRES2,C1R,CFD,IGHV3-15,IGHV3-72,VEGFC,PLCG2,ACTB,B2M
GO Process	GO:0030193	Regulation of blood coagulation	18	70	1.31	1.76e-14	CPB2,PROC,APOE,F12,THBS1,KLKB1,SERPING1,FGB,F2,PLG,SERPINF2,FGG,SERPINC1,F7,PROS1,F11,KNG1,FGA
GO Process	GO:0009605	Response to external stimulus	80	2355	0.43	3.27e-14	SERPIND1,CHGA,MMP2,LTF,APOB,APOA1,CAT,CFP,APOE,F12,CFHR5,THBS1,LYZ,C9,HPX,FCN3,SERPING1,CSF1R,FCN2,PGLYRP2,PF4,MASP1,CAMP,SLC27A4,LALBA,LRG1,PCSK9,FGB,F2,PRG2,STAB1,C1QB,CLU,APOL1,C7,FBN1,HSP90AA1,ANG,TUBB,SLPI,HP,APOA4,C8A,C4BPA,CFHR2,CFH,F5,S100A7,MCOLN2,C8B,FCN1,ADAMTS13,C1QC,C1QA,F7,CFI,TNRC6A,MASP2,C1S,ADIPOQ,COLEC11,C4B,CFB,RARRES2,JCHAIN,C1R,VIM,RNASE4,TTN,CFD,IGHV3-15,CCL18,IGHV3-72,VEGFC,SEMA3B,PLCG2,KNG1,TRIM8,B2M,FGA
GO Process	GO:0032101	Regulation of response to external stimulus	49	964	0.61	8.29e-14	ST6GAL1,CPB2,LTF,PROC,APOA1,APOE,F12,SERPINF1,THBS1,PSMA6,KLKB1,HPX,FCN3,AHSG,SERPING1,CSF1R,FCN2,PGLYRP2,MASP1,FGB,F2,PLG,SERPINF2,FBN1,HSP90AA1,FGG,THBS4,CFH,SERPINC1,S100A7,HTRA1,FLNA,FCN1,F7,PROS1,CFI,MMRN1,MASP2,F11,ADIPOQ,COLEC11,MST1,RARRES2,VEGFC,SEMA3B,PLCG2,KNG1,CDH5,FGA
GO Process	GO:0030195	Negative regulation of blood coagulation	15	46	1.41	3.56e-13	CPB2,PROC,APOE,F12,KLKB1,SERPING1,FGB,F2,PLG,SERPINF2,FGG,PROS1,F11,KNG1,FGA

[truncated: 887,611 more chars]
