# Supplementary figures and images for: Exploring the plasma proteome linked to corpus luteum presence and conception mode across pregnancy stages and postpartum
Source: J Assist Reprod Genet. 2025 Sep 20;42(10):3275–89. doi: 10.1007/s10815-025-03632-0 (PMC12602745; doi:10.1007/s10815-025-03632-0)

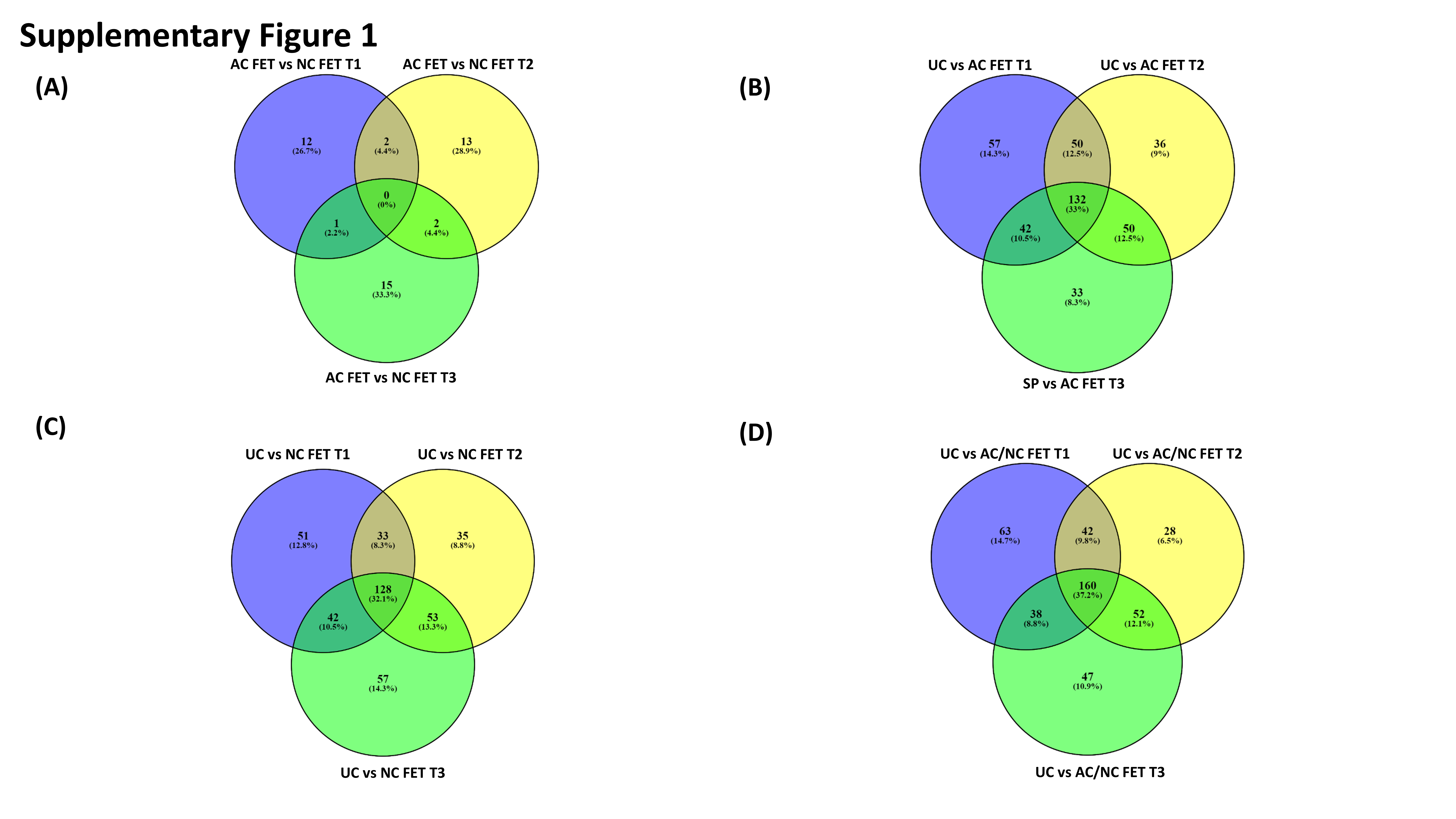

Supplement: Supplementary file 4 — (3.28 MB TIF) [file 10815_2025_3632_MOESM4_ESM.tif]

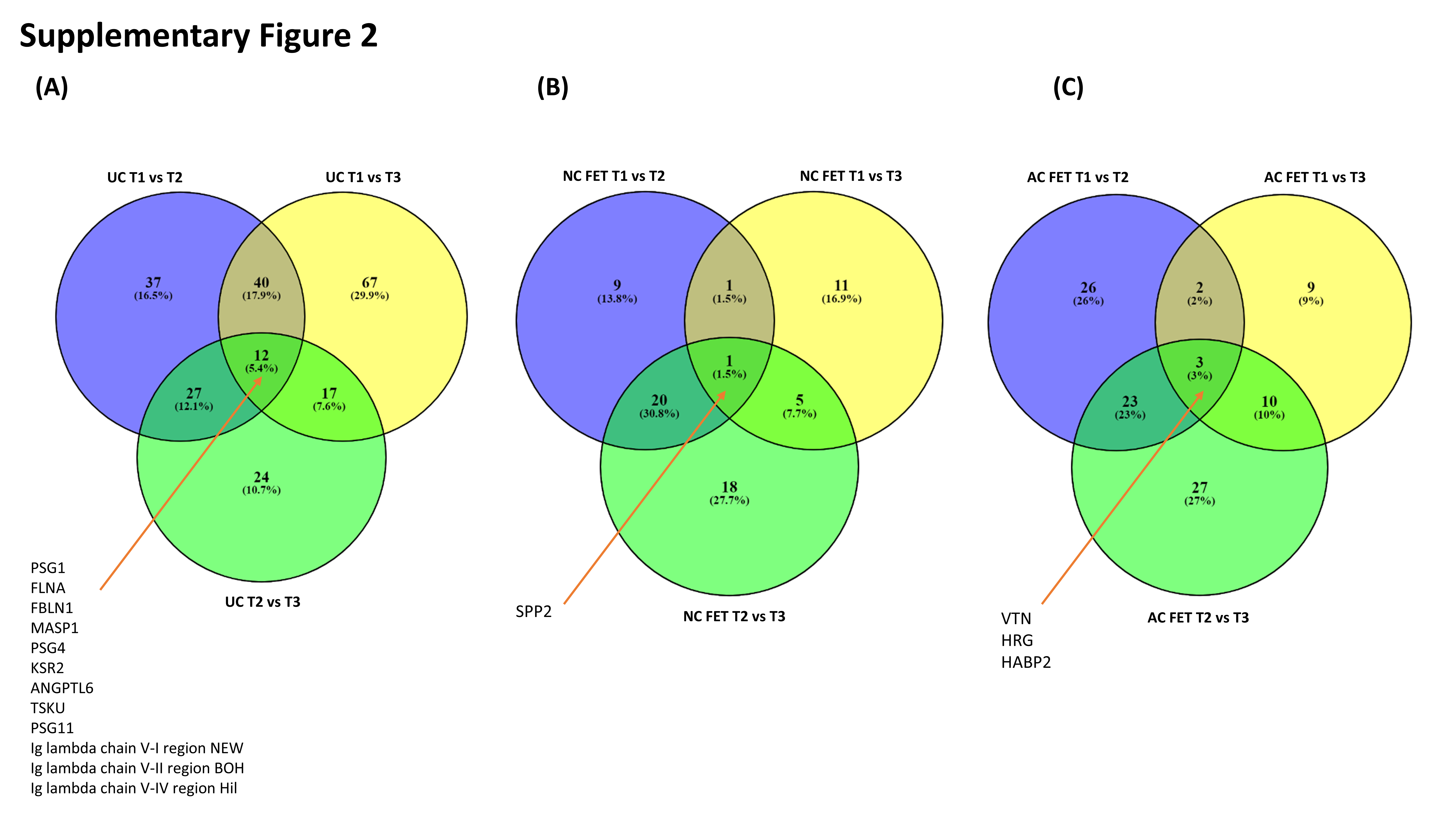

Supplement: Supplementary file 5 — (3.78 MB TIF) [file 10815_2025_3632_MOESM5_ESM.tif]

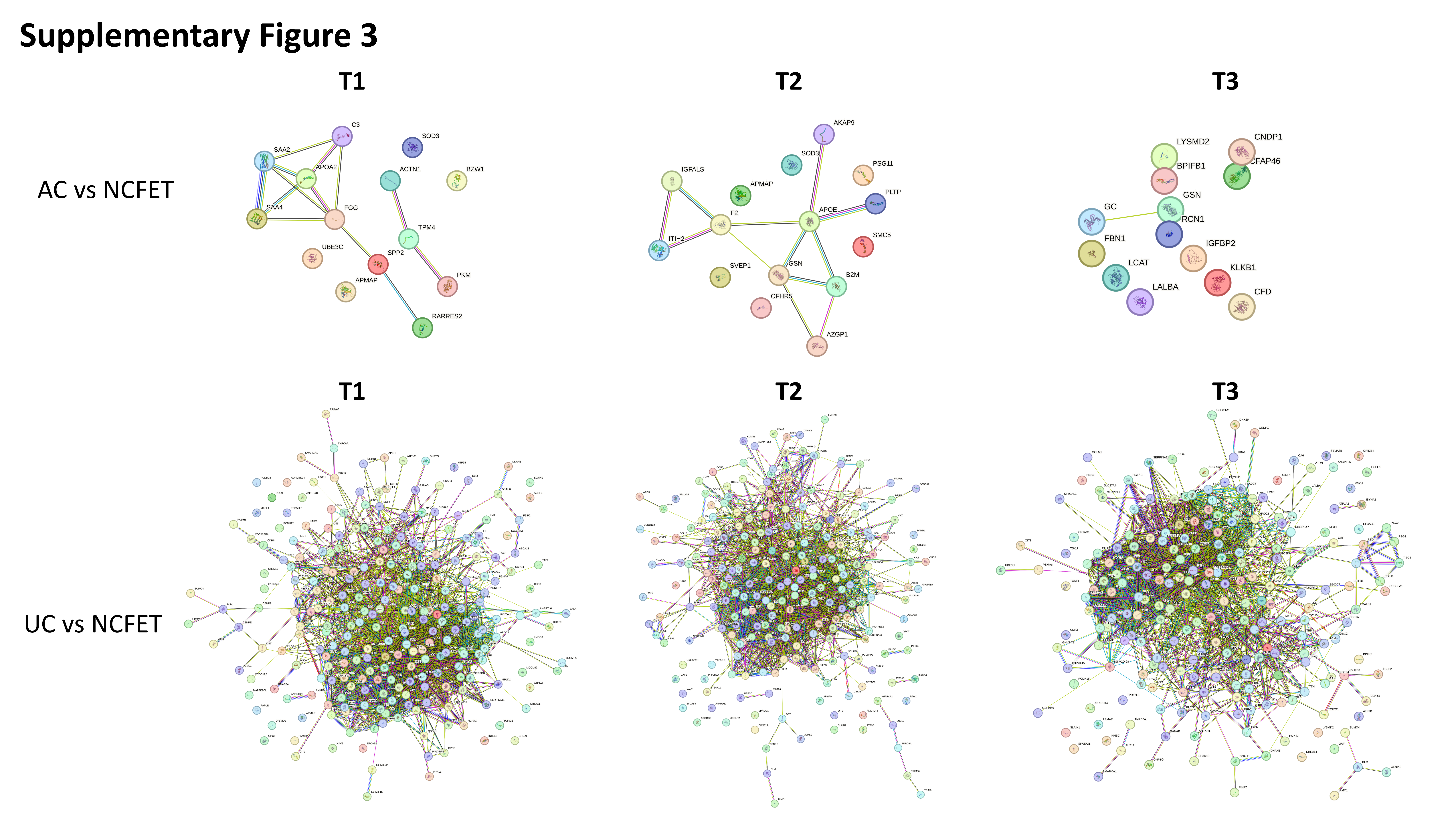

Supplement: Supplementary file 6 — (14.8 MB TIF) [file 10815_2025_3632_MOESM6_ESM.tif]

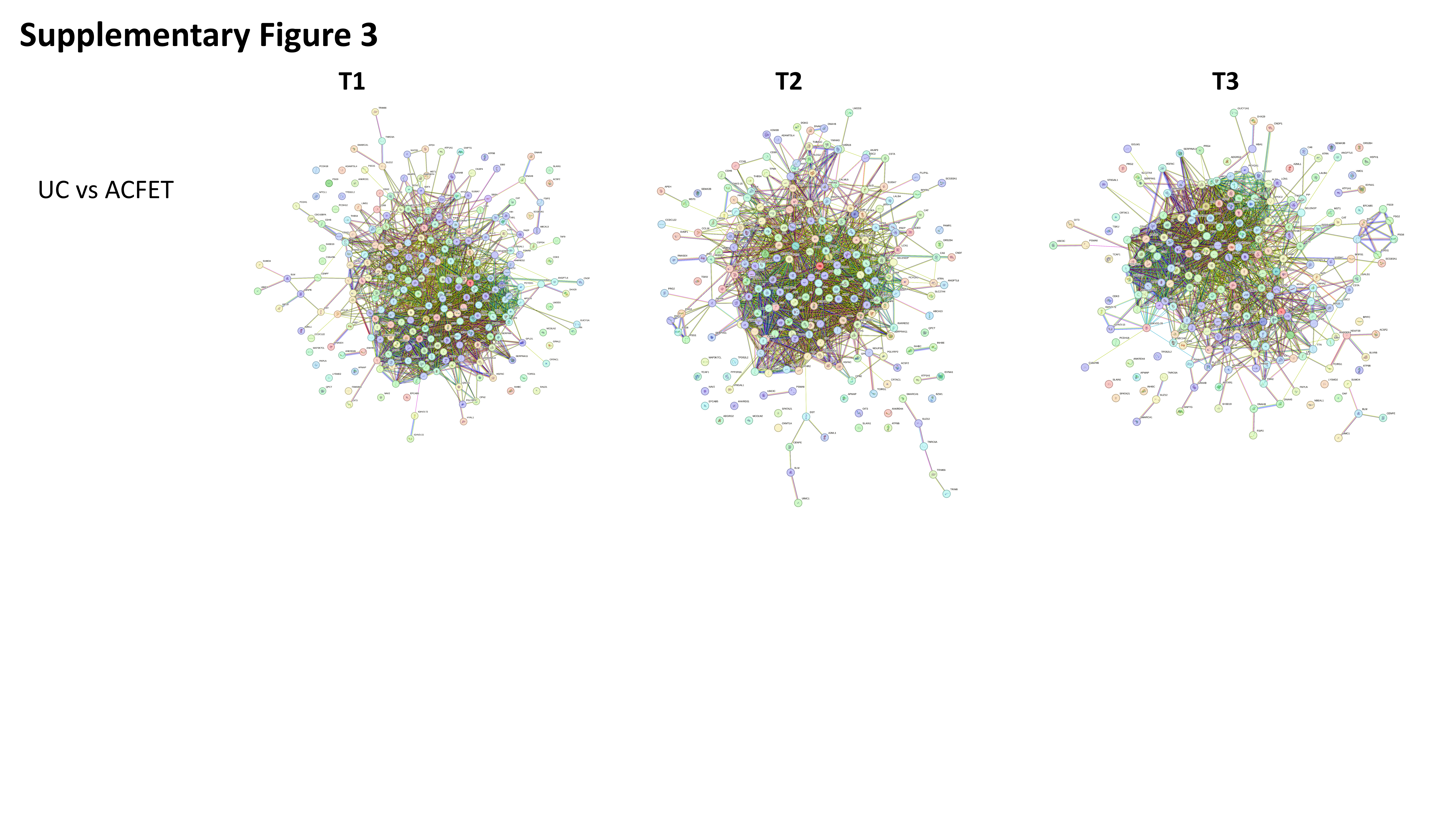

Supplement: Supplementary file 7 — (11.5 MB TIF) [file 10815_2025_3632_MOESM7_ESM.tif]

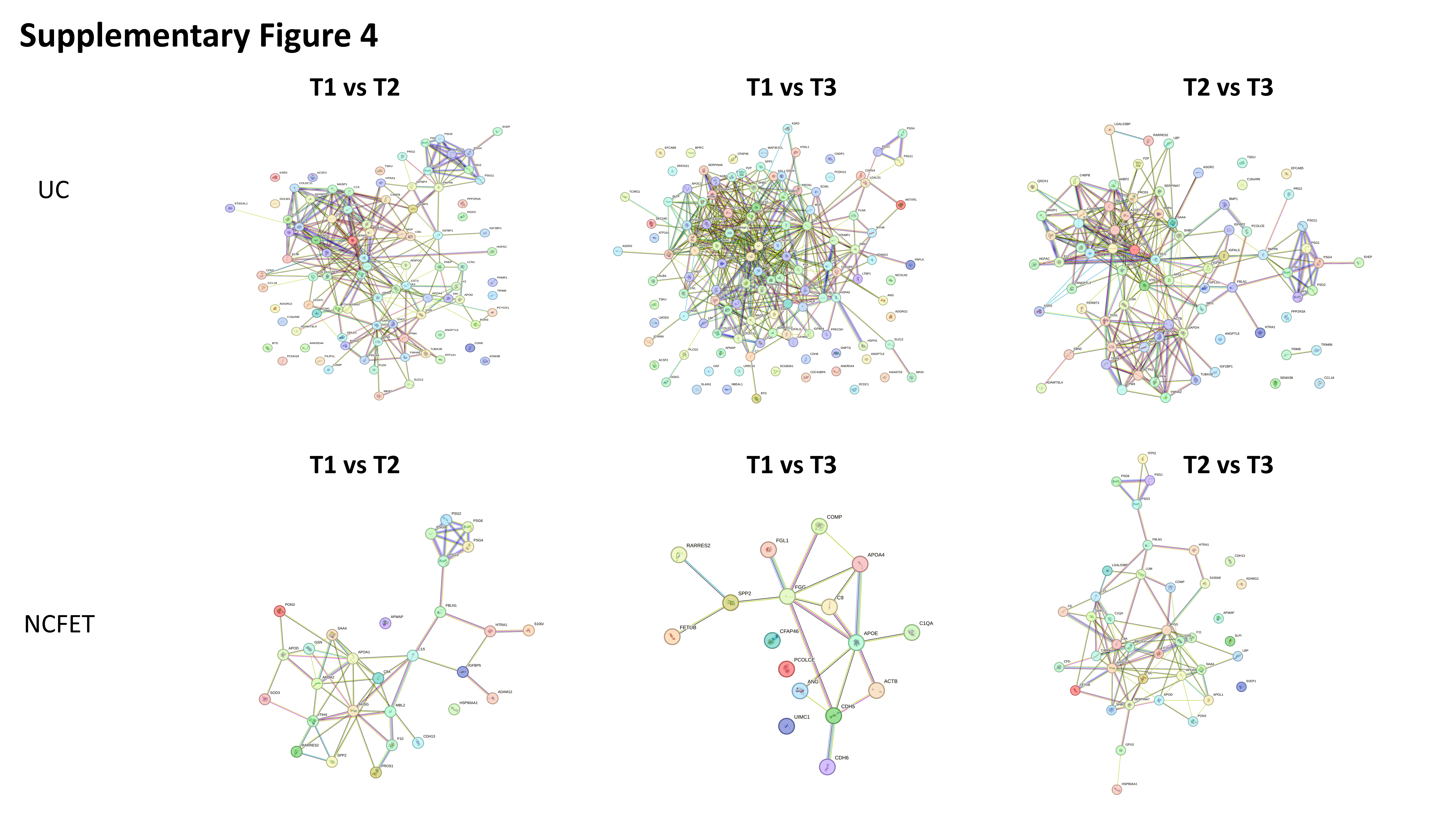

Supplement: Supplementary file 8 — (9.31 MB TIF) [file 10815_2025_3632_MOESM8_ESM.tif]

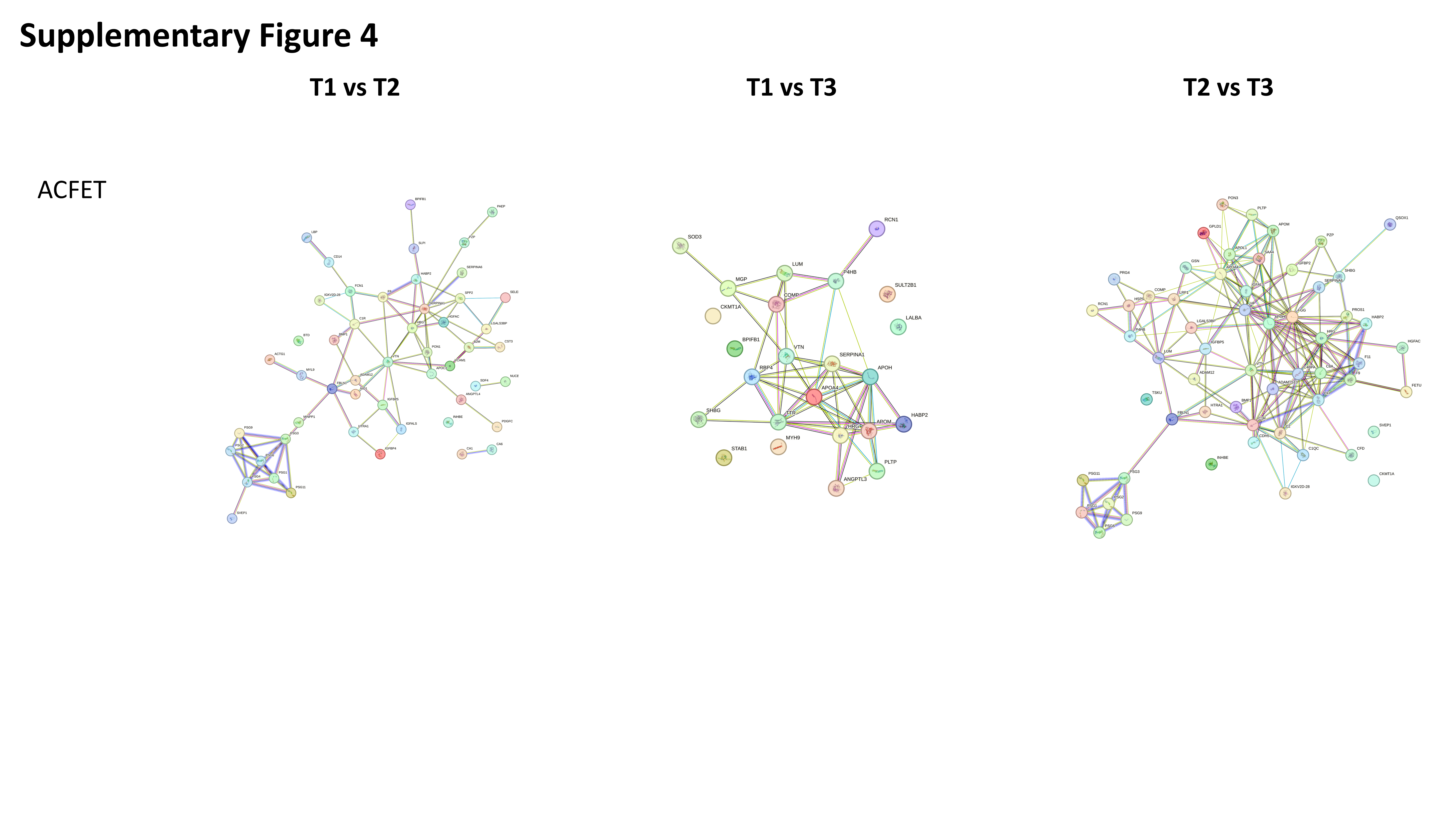

Supplement: Supplementary file 9 — (4.75 MB TIF) [file 10815_2025_3632_MOESM9_ESM.tif]
